# *Aspergillus fumigatus* versus Genus *Aspergillus*: Conservation, Adaptive Evolution and Specific Virulence Genes

**DOI:** 10.3390/microorganisms9102014

**Published:** 2021-09-23

**Authors:** Shishir K. Gupta, Mugdha Srivastava, Özge Osmanoglu, Zhuofei Xu, Axel A. Brakhage, Thomas Dandekar

**Affiliations:** 1 Functional Genomics & Systems Biology Group, Department of Bioinformatics, Biocenter, Am Hubland, University of Wuerzburg, D-97074 Wuerzburg, Germany; shishir.gupta@uni-wuerzburg.de (S.K.G.); mugdha.srivastava@uni-wuerzburg.de (M.S.); oezge.osmanoglu@uni-wuerzburg.de (Ö.O.); 2 Core Unit Systems Medicine, University of Wuerzburg, D-97080 Wuerzburg, Germany; 3 State Key Laboratory of Agricultural Microbiology, College of Veterinary Medicine, Huazhong Agricultural University, No. 1, Shizishan Street, Hongshan District, Wuhan 430070, China; zhuofei.xu@gmail.com; 4 Microbiology and Molecular Biology, Institute for Microbiology, Friedrich Schiller University Jena, Neugasse 23, 07745 Jena, Germany; axel.brakhage@hki-jena.de; 5 Molecular and Applied Microbiology, Leibniz Institute for Natural Product Research and Infection Biology (Leibniz-HKI), Adolf-Reichwein-Str. 23, 07745 Jena, Germany; 6 BioComputing Unit, EMBL Heidelberg, Meyerhofstraße 1, D-69117 Heidelberg, Germany

**Keywords:** molecular evolution, phylogenetic analysis, adaptation, recombination, positive selection, human pathogenic fungi, genus *Aspergillus*, *Aspergillus fumigatus*

## Abstract

*Aspergillus* is an important fungal genus containing economically important species, as well as pathogenic species of animals and plants. Using eighteen fungal species of the genus *Aspergillus*, we conducted a comprehensive investigation of conserved genes and their evolution. This also allows us to investigate the selection pressure driving the adaptive evolution in the pathogenic species *A. fumigatus.* Among single-copy orthologs (SCOs) for *A. fumigatus* and the closely related species *A. fischeri*, we identified 122 versus 50 positively selected genes (PSGs), respectively. Moreover, twenty conserved genes of unknown function were established to be positively selected and thus important for adaption. *A. fumigatus* PSGs interacting with human host proteins show over-representation of adaptive, symbiosis-related, immunomodulatory and virulence-related pathways, such as the TGF-β pathway, insulin receptor signaling, IL1 pathway and interfering with phagosomal GTPase signaling. Additionally, among the virulence factor coding genes, secretory and membrane protein-coding genes in multi-copy gene families, 212 genes underwent positive selection and also suggest increased adaptation, such as fungal immune evasion mechanisms (*aspf2*), siderophore biosynthesis (*sidD*), fumarylalanine production (*sidE*), stress tolerance (*atfA*) and thermotolerance (*sodA*). These genes presumably contribute to host adaptation strategies. Genes for the biosynthesis of gliotoxin are shared among all the close relatives of *A. fumigatus* as an ancient defense mechanism. Positive selection plays a crucial role in the adaptive evolution of *A. fumigatus*. The genome-wide profile of PSGs provides valuable targets for further research on the mechanisms of immune evasion, antimycotic targeting and understanding fundamental virulence processes.

## 1. Introduction

Fungal diseases and, specifically, invasive fungal infections lead to an estimated 1.5 to 2 million deaths each year, which exceeds the global mortality estimates for both malaria and tuberculosis [1]. During 200 million years of evolution, Aspergilli developed as a group of ubiquitous fungi [2]. Among the known Aspergilli, the environmentally acquired pathogen *Aspergillus fumigatus* is the predominant, ubiquitous, opportunistic pathogenic species causing life-threatening invasive aspergillosis (IA) and chronic pulmonary aspergillosis (CPA) in immunodeficient patients, as well as allergic disease in immunoreactive patients. *A. fumigatus* has a superior ability to survive and grow in a wide range of environmental conditions. The conidia of *A. fumigatus* released from the conidiophores are dispersed in the environment and remain dormant until encountering the environmental conditions that allow metabolic activation. Once metabolically active, conidia swell and germinate into hyphal filaments that form mycelia and produce conidiophores [3]. The inhalation of conidia, fungal growth and tissue invasion in immunocompromised patients can result in the establishment of invasive disease and represents a major cause of morbidity and mortality.

The availability of the sequenced genomes from several species of the genus *Aspergillus* opens the possibility of examining the demarcation of fungal species at the whole-genome level. These include economically important fungi (*A. oryzae*, *A. niger*), plant pathogens (*A. zonatus*), animal pathogens (*A. fumigatus*, *A. clavatus*, *A. fischeri*, *A. brasiliensis*, *A. sydowii*, *A. tubingenesis* and *A. terreus*), plant and animal dual pathogens (*A. glaucus*, *A. versicolor*, *A. niger*, *A. flavus* and *A. wentii*) and non-pathogens (*A. nidulans*, *A. kawachii*, *A. oryzae*, *A. carbonarius* and *A. luchuensis*). Among these, *A. fischeri*, a close homothallic sexual relative to *A. fumigatus,* can also cause keratitis and possibly pulmonary aspergillosis in transplant patients but is an extremely rare invasive pathogen [4,5].

In experimental models of IA, although less virulent than *A. fumigatus*, *A. fischeri* can cause IA; however, in clinical models, it rarely causes human disease—and is thus considered nonpathogenic—and responds differently to antifungal drugs [6,7]. It shows reduced viability in host conditions, such as hypoxia and temperature, and has reduced metabolic flexibility compared to *A. fumigatus* [6]. Although the two species genomes are very similar, their secondary metabolism differs a lot (very low level of biosynthetic gene clusters (BGCs) conservancy (share ~30%, [6]). Thus, the molecular differences between the two organisms can reveal the mechanisms of high pathogenesis of *A. fumigatus*. Furthermore, an evolutionary genomic comparison of *A. fumigatus* and *A. fischeri* with other pathogenic and nonpathogenic Aspergilli holds great potential of illuminating the evolution of their pathogenesis.

A recent comparative study emphasizes the high genomic and functional diversity within the genus *Aspergillus* [8]. In the study, strong conservation was observed for central biological functions while high diversity was observed for many other physiological traits, such as secondary metabolism, carbon utilization and stress response [8]. However, a genome-wide scan indicating evolutionary forces operating on *Aspergillus* genes has not been performed yet. The analysis we performed here addresses this and provides a general resource to study gene evolution in the genus *Aspergillus*. Our analysis also gives an accurate analysis of gene families (number of genes as well as the difference in sequences) with positive selection as an important characteristic of the Aspergillus genome (an overview of all families is given in [8]).

Positive selection and homologous recombination are major forces for the evolution of microorganisms that drive adaptation to new hosts, antimycotics and promote survival of pathogens in hostile environments, including the human host [9]. Evolution experiments showed that recombination could accelerate species adaptation in stressful environments [10] by combining advantageous mutations and thereby assisting in their fixation [11]. Additionally, natural selection is the central force shaping the diversity of genotypes by acting on resulting phenotypes. The study of natural selection provides significant insights into the possible functional alterations during gene evolution and important nucleotide substitutions involved in adaptation. Adaptive changes of the protein-coding genes are ultimately responsible for evolutionary innovations that can have a significant impact on the adaptation of species to their environment and generate an overview of diversity [12]. Fungal gene products that play a significant role in environmental adaptation, including survival in the human body, are more likely to be positively selected.

Aspergilli has a wide diversity of lifestyles and resulting roles in current medicine, agriculture and biotechnology. This diversity comes not only from the phylogenetic relationships between species but also from the saprobic conditions that are selected for features that have contributed to the current harmful lifestyles [13]. In other words, two Aspergillus species can be similar in their pathogenicity independent from their phylogenetic distance. The transition from a saprobic lifestyle to a harmful lifestyle is thus highly interesting, and we want to investigate here in silico traces of this transition in the genomes of different Aspergilli. These are no substitutes for experiments, but we provide a basis on which one can plan such experiments. We sought out to look for a positive selection of traits in single-copy genes in the genus of Aspergillus as clear markers of evolutionary change from saprobic to harmful lifestyle, but, surprisingly, the matter is much more complex; some of the more critical virulence factors, such as genes involved in gliotoxin biosynthesis, show no positive selection, while some genes are under positive selection but show no obvious function. As a genomic view on this transition, we present here an informatics-guided comparison of genus Aspergillus.

The evolutionary origin of virulence of *A. fumigatus* is not well known. Nevertheless, a well-accepted hypothesis suggests that the interaction with free-living predator amoebae during its saprophytic existence may have posed a selection pressure on *A. fumigatus* to optimize survival, which later endorsed accidental virulence in the human host [14,15]. Thus, we investigated whether evolutionary driving forces are visible for virulence-related genes and how far, in general, *A. fumigatus* protein-coding genes are associated with a genome-wide signature of positive selection. Since genetic similarity between two very close species, such as *A. fumigatus* and *A. fischeri,* does not necessarily mean similar virulence mechanisms or the same level of pathogenicity, we further analyzed the positive selection in *A. fischeri* single-copy orthologs (SCOs) to identify virulence mechanisms that may be shared between the two or specific to one of the species [16]. We hypothesize positive selection’s possible role in enhancing survival in different predator-related or environmental stress conditions that later translated into pathogenicity in the host, although a big portion of virulence genes are conserved [13].

Therefore, we first compare the genomic features and then the conserved gene families of Aspergilli in detail, conduct a species tree estimation, followed by recombination and evolutionary analysis of SCOs. Next, we show the results indicating positively selected genes (PSGs) involved in host–pathogen interactions and environmental adaptations. Besides analyzing the well-annotated virulence genes, the screen identified a further twenty conserved genes of unknown function, which are clearly positively selected and hence conveying important uncharacterized adaptive functions. However, a more complete picture of virulence evolution in multi-gene families must be included, and we point out the positive selection in several virulence factors from these multi-gene families. Hence, we finally considered analysis of multi-gene families containing annotated virulence factor coding genes, but apart from virulence genes, we also analyzed secretory and membrane protein-coding genes in *A. fumigatus*. However, for each of these categories, we establish here clear virulence, infection or growth-mediating factors with positive selection and single them out as those which are positively selected. These PSGs illuminate virulence for *A. fumigatus* with genes for conidia germination, signal transduction, metabolism, mitochondrial activity and transcriptional regulation. Note that the singletons and species-specific duplicated genes in *A. fumigatus,* which could be associated with the increased virulence of the species (Table S1; all Supplemental Tables S1–S15 are collected in excel format in Additional File S2), were not analyzed for the species-level selection signature. Finally, we show that genes involved in the biosynthesis of a very old and strong virulence factor, gliotoxin, are essential inventory, conserved among most Aspergilli for a long time and hence do not share a species-resolved selection.

## 2. Materials and Methods

### 2.1. Initial Dataset, Quality Assessment and Filtering

**Figure 1:**
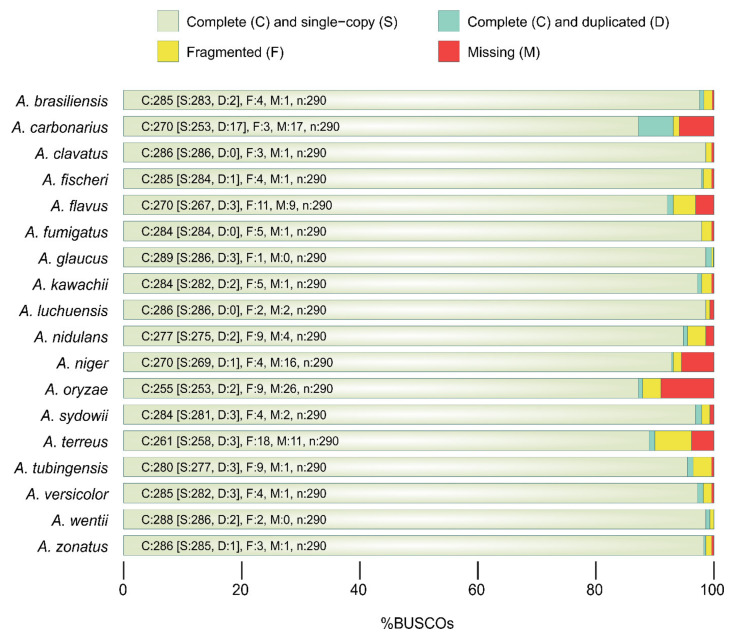
Genome completeness in the Aspergilli compared. Statistics and assessment of Aspergilli protein-coding gene sets and their completeness in BUSCO notation (C: complete (S: single copy) and (D: duplicated); F: fragmented; M: missing; n: gene number).

The complete set of protein-coding gene sequences and corresponding amino acid sequences of the eighteen *Aspergillus* genomes *(A. brasiliensis* (GCA_001889945.1), *A. carbonarius* (GCA_001990825.1), *A. clavatus* (GCA_000002715.1), *A. fischeri* (GCA_000149645.2), *A. flavus* (GCA_000006275.2), *A. fumigatus* (GCA_000002655.1), *A. glaucus* (GCA_001890805.1), *A. kawachii* (GCA_000239835.2), *A. luchuensis* (GCA_001890685.1), *A. nidulans* (GCA_000011425.1), *A. niger* (GCA_000230395.2), *A. oryzae* (GCA_000269785.2), *A. sydowii* (GCA_001890705.1), *A. terreus* (GCA_000149615.1), *A. tubingensis* (GCA_001890745.1), *A. versicolor* (GCA_001890125.1), *A. wentii* (GCA_001890725.1) and *A. zonatus* (GCA_001890105.1)) were retrieved from the NCBI GenBank database [17]. The full genome dataset has a size of 591 mb. Table S2 lists the details of the used fungal genomic assembly. BUSCO [18] was used to assess the completeness of proteomes based on the presence of a benchmarking set of universal fungal single-copy orthologs. With this helpful benchmarking set, we could compare the different genomes and test how complete the proteomes of the individual fungal proteomes were. BUSCO (Benchmarking Universal Single-Copy Orthologs) was furthermore used to assess genome quality, including gene prediction and applying phylogenomics. In particular, we hypothesized that if the genes are well demarcated in the sequenced genome, they should have an ortholog to the corresponding BUSCO set [18]. Nearly all Aspergilli genomes could be selected for the analysis with acceptable quality and completeness (Figure 1). Basic sequence quality features were carefully controlled, as described in [19]. CDSs (coding sequences) whose length was not a multiple of 3, or did not correspond to the length of the predicted protein, or that contained an internal stop codon were eliminated; CDSs shorter than 90 nt were eliminated; similarly, short peptides with an unsuitable length (<30 aa) for orthology analysis were eliminated. After the quality filtering, 208,055 protein-coding gene sequences and the same number of amino acid sequences (available at https://github.com/ShishirGupta-Wu/aspergillus_ps) were used in the further steps.

### 2.2. Orthologs and Unique Genes

The OrthoMCL algorithm, which uses multiple steps including BLASTP [20] and Markov clustering [21] to group proteins into likely orthologous clusters, was used for identifying the orthologs between eighteen Aspergilli proteomes. Additionally, the proteins that are not shared with any other species but contain intra-species very close homologs were extracted from the orthologous clusters. We also found single species clusters for the analyzed Aspergilli. Sequences in such clusters could be the consequence of gene duplications and, together with singletons, represent the species-specific proteins. This should be interpreted with caution, as an increase in the number of species in the analysis can change the number of proposed species-specific sequences. Although the evolutionary information seems to be lost for species-specific sequences, these sequences can define the specific character of each species.

### 2.3. Reconciling of Gene Trees and Species Trees

Sequences of single-copy ortholog clusters were further used for estimating the species tree using the super-matrix method [22]. The filtered alignments were concatenated, and the species tree was constructed using RaxML [23] under the probabilistic maximum-likelihood (ML) framework. We ran RaxML analyses to find the optimal ML estimate with 1000 bootstrap replicates. The resulting topologies of the species trees were applied to the selection analyses [24]. For this purpose, we first produced the multiple sequence alignment (MSA) of 3175 SCOs clusters shared by all eighteen Aspergilli and concatenated them into a single alignment. The alignment quality was improved first by selecting conserved blocks, then by removing sequence parts with multiple substitutions. This allowed us to reduce their potentially misleading effects due to alignment error. We started with the 2,112,647 columns in the alignment and ended up with 1,246,185 columns to produce a giant phylogenetic matrix. Such extensive data removal is often impractical in single-gene analyses because too few positions remain available and produce a poorly resolved tree [25]. Further, using the evidence from all characters in the derived phylogenetic matrix, the best probabilistic ML species tree was estimated under the assumption that each character provides independent evidence of relationships.

### 2.4. Single-Copy Orthologs Gene Families Data Set

Gene families were obtained from a custom run of the OrthoMCL pipeline [26], which implements Reverse Best Hit (RBH) Blast [27] and MCL clustering algorithm [28] to identify the ortholog clusters based on all-against-all Smith–Waterman protein sequence comparisons with an e-value cut off of 1 × 10^−5^. Gene families including strictly one ortholog in each of the 18 species were selected (3175 gene families).

### 2.5. Multiple Sequence Alignment (MSA)

Nucleotide and amino acid sequences of all orthologous gene groups were extracted from the initial sequence dataset. To reduce the effect of incorrect insertions/deletions (indels) on the codon alignments, MSA was initially performed for amino acid sequences of each ortholog group using T-Coffee [29], which combines the output of different aligners. The aligned amino acid sequences together with the corresponding nucleotide sequences of each ortholog group were converted into DNA alignments at the codon level using the program PAL2NAL [30]. The misalignment errors can be an important source of false positives in genome-wide scans for positive selection in coding sequences [31], and the removal of unreliable regions increases the power to detect positive selection [32]. Therefore, carefully filtered the potentially problematic sites in the alignments. To further improve alignment quality, we used stringent Gblocks filtering (type = codons; minimum length of a block = 4; no gaps allowed) [33] to remove gap-rich regions from the alignments, as these are problematic for positive selection inference [34].

### 2.6. Test for Recombination 

We filtered out genes with any evidence for recombination signals to decrease the rate of false positives in positive selection analysis (see Supplemental Methods in Additional File S3 for details).

### 2.7. Positive Selection in A. fumigatus Protein-Coding Genes

To explicitly test for positive selection in *A. fumigatus* among the orthologous clusters (alignment data available at https://funginet.hki-jena.de/data_files/76?version=1), we used a branch-site model implemented in PAML [35], which allows four classes of codons: a strictly conserved class (ω < 1), a class that is conserved in the ‘background’ lineages but under positive selection in the ‘foreground’ lineage of interest, a class that is strictly neutral (ω = 1) and a class that is neutral on the ‘background’ lineages but under positive selection in the ‘foreground’ lineage of interest. When compared to the null model, which does not allow positive selection in the foreground lineage, this model provides a robust test for positive selection in a subset of codons on a particular lineage [36]. We applied this branch-site model to two sets of foreground lineages; the *A. fumigatus* terminal branch and *A. fischeri*. The significance of the test was assessed using standard asymptotic assumptions [37]. The calculations were performed using the codeml program from the PAML 4.2b package [35]. The Bayes empirical Bayes approach was employed to estimate the probabilities of positive selection for specific codons under the likelihood framework [38].

To check whether the selection worked on the branch containing *A. fischeri,* we similarly designated the corresponding branch as foreground and the rest as background. The log-likelihoods from each test were compared in a likelihood ratio test assuming a χ2 distribution of the test statistic. Bonferroni correction [39] and FDR of 5% [40] were used to correct for multiple testing.

### 2.8. Tests of Functional Category Enrichment 

We identified significantly over-represented gene ontology (GO) annotations of *A. fumigatus* single copy orthologs (see Supplemental Methods in Additional File S3 for details). 

### 2.9. Interolog Network

The experimentally derived dataset of host–pathogen protein–protein interaction from the HPIDB [41] and PHISTO database [42] was used as a template to reconstruct interologs [43] based PPIs network of PSGs coding proteins and human proteins. To determine the orthologs, we used the Inparanoid algorithm [44], which integrates all-versus-all Blast similarity results and Markov single linkage binding to construct putative orthologous groups in two proteomes. The Blast e-value was set to 1e-05 for clustering. Only the seed orthologs were considered to increase the prediction confidence. For the pathogenic genes, we also calculated interactions based on the interacting domain profile pairs between host and pathogen, as mentioned in [45].

### 2.10. Inference of Positive Selection in A. fumigatus Virulence Genes 

We analyzed the positive selection in 1498 virulence genes from multi-gene famililes (see Supplemental Methods in Additional File S3).

### 2.11. Statistical Analysis of PSGs

Multiple testing correction was performed to control for Type I errors according to the approach presented by Benjamini and Hochberg [39]. For the analyses of homologous recombination, recombination breakpoints were inferred by the Shimodaira–Hasegawa test [46] with Bonferroni-corrected p-value, and the threshold for significance was set at 0.05. For all genes tested for positive selection, the false discovery rate (FDR) was controlled by using the Benjamini–Yekutieli (BY) method [40] and the significance level was set to 10%. For all genes tested for recombination and positive selection, q-values were calculated from p-values using the R package q-value with the proportion of true null hypothesis set to 1 (π0 = 1) [47]. Where appropriate, a chi-square test and Fisher-exact test were used to assess associations for the gene count data in the individual KOG categories; Bonferroni corrections for multiple tests were applied. All statistical analyses were carried out using R 3.4.4 [48].

## 3. Results

### 3.1. Genomic Features and Quality Assessment

The general and genomic features of Aspergilli analyzed in this study are listed in Table S2. The size and G+C content of the genomes ranged from 26.09 to 37.45 Mb and 48% to 52.8%, respectively. *A. luchuensis* represented the largest *Aspergillus* genome in the analyzed genus. Since many of the fungal genomes are poorly annotated [49], we assessed the quality of the genome annotation by testing for the presence of universally conserved fungal SCOs before performing the sequence comparisons (see Section 2). Almost all Aspergilli genomes for the analysis had acceptable quality and completeness (Figure 1). This approach here is superior to large-scale genome analysis where the nonappearance of a gene in a particular genome might result from a poor-quality gene structure annotation rather than its true absence.

### 3.2. Orthologs and Unique Genes

Genome-wise comparisons revealed the similarity and differences among the different organisms (see Section 2 for details). The clustering of proteomes yields 18,474 groups from 189,010 protein sequences (Additional Data File S1). The core genome spans ~23–35% of the total proteins encoded by each genome, revealing that the core genome has a rather homogeneous gene content (Additional Data File S2 containing Tables S1–S12). We found that 3175 protein-coding genes are present as single copies in each genome or 17% of the total ortholog clusters. These clusters comprise proteins involved in core biological processes, such as cell growth, cell division, protein folding and translation. Interestingly, sporocarp development involved in sexual reproduction was among the top five statistically over-represented (GO:0000909, FDR 4.79 × 10^−5^) terms, indicating that Aspergilli have the potential of undergoing sexual reproduction, which seems to be a conserved process. The full list of over-represented Gene Ontology (GO) terms in the three GO categories (i.e., ‘Biological Processes’, BP; ‘Molecular Function’, MF; ‘Cellular Component’, CC) is listed in Table S3.

**Figure 2:**
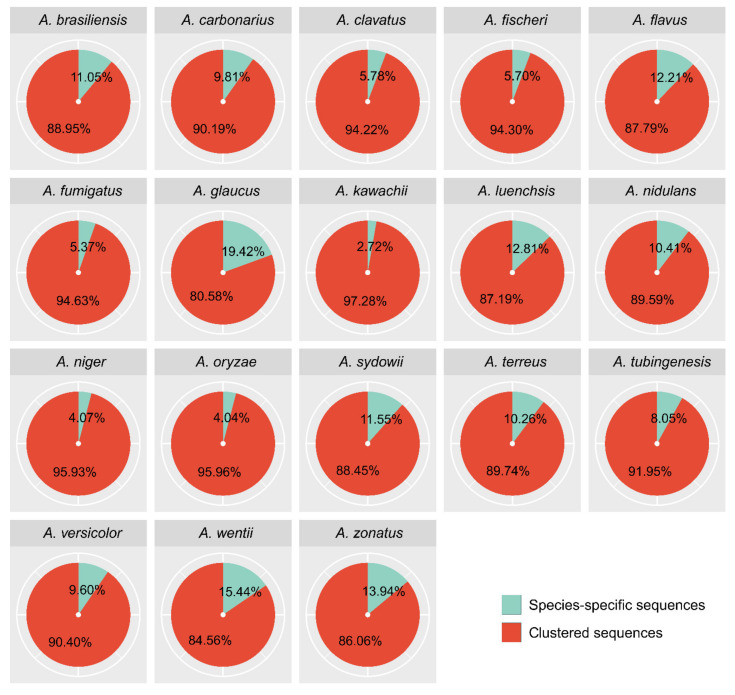
Distribution of Aspergilli proteins as unique or shared relative to the entire set of proteins. Partitioning Aspergilli gene sets by their traceable orthology reveals a spectrum of conservation from widespread orthologues found across the Aspergilli to species-specific genes with no recognizable homologues. The pie chart shows the percentage of unique and conserved proteins coded among all eighteen sequenced Aspergilli identified by OrthoMCL analysis. Species-specific sequences include both the singletons and the same species’ inparalog clusters.

Our comparative analysis also revealed that each genome also encodes species-specific proteins that have no homologues in other species. A total of ~2.5–17% of the sequences in each *Aspergillus* genome were estimated to be so-called singletons at a conservative clustering threshold of OrthoMCL using a 1.5 inflation index. The largest number of singletons was present in *A. glaucus,* followed by *A. wentii*. Interestingly, out of a total of 517 species-specific sequences found in *A. fumigatus*, 29 contain secretion signals as detected by SignalP [50] (Table S1). Figure 2 depicts the percentage of clustered and species-specific proteins in each analyzed proteome. A quantitative summary of orthology assignments is given in Table S4.

### 3.3. Reconciling of Species Tree and Gene Trees

**Figure 3:**
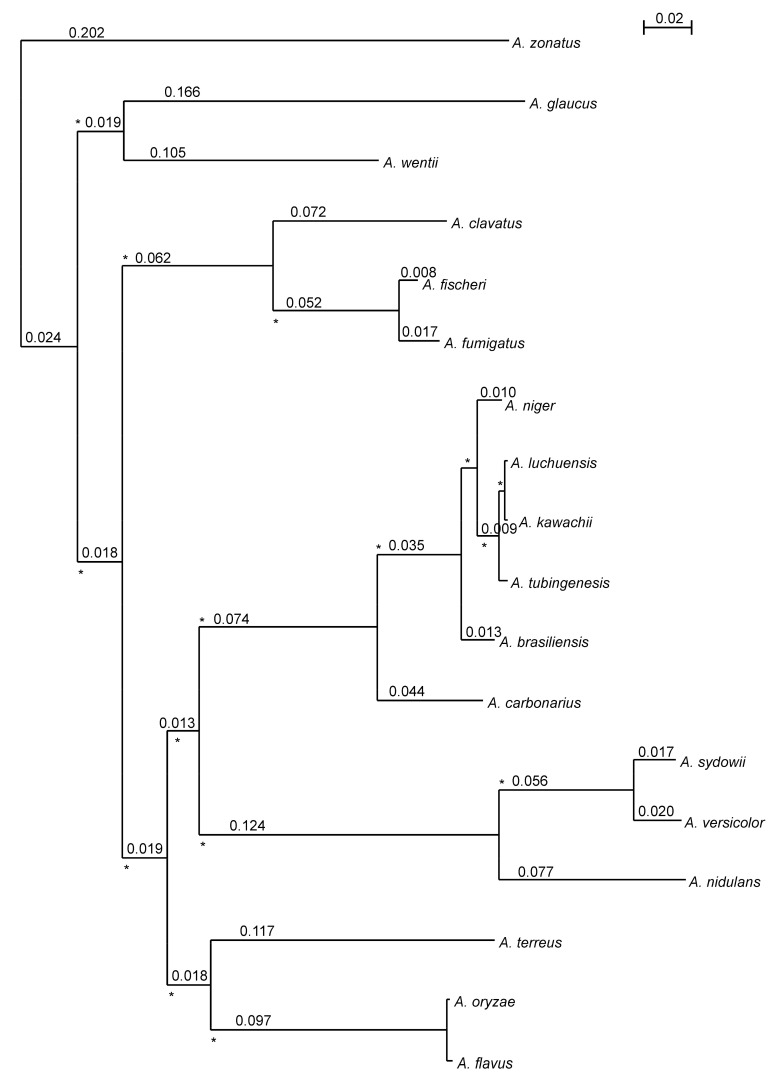
Aspergillus species tree. The maximum likelihood tree was obtained after concatenating the 3175 congruent orthologous proteins or protein fragments shared by all 18 genomes. On the branches of the tree are reported the branch length (black). The branches of the tree with 100% bootstrap support are shown by asterisks. The scale bar (s/s) indicates the number of substitutions per site.

Molecular phylogenies based on single genes often yield apparently conflicting tree structures and often produce incongruences [51]. To overcome this problem, genome-scale methods for phylogenetic inference by combining multiple genes are well established to resolve incongruences [52]. Here, we reconstructed a species tree using a supermatrix approach (Figure 3). We see strong bootstrap values that support the relationships among analyzed species. The proposed relationships agree with those recently reported by de Vries et al. (2017) [8]. As expected, *A. fumigatus* and *A. fischeri* represented sister groups. Moreover, *A. clavatus* was found as the closest ancestor of these two Aspergilli, but human infections due to *A. clavatus,* as for *A. fischeri* [6], are extremely rare case reports [53]. Finally, the three species, *A. zonatus*, *A. glaucus* and *A. wentii,* that are phylogenetically distant from *A. fumigatus*, also displayed the highest number of species-specific proteins (Figure 2).

### 3.4. Positive Selection in A. fumigatus Single-Copy Ortholog Protein-Coding Genes

**Figure 4:**
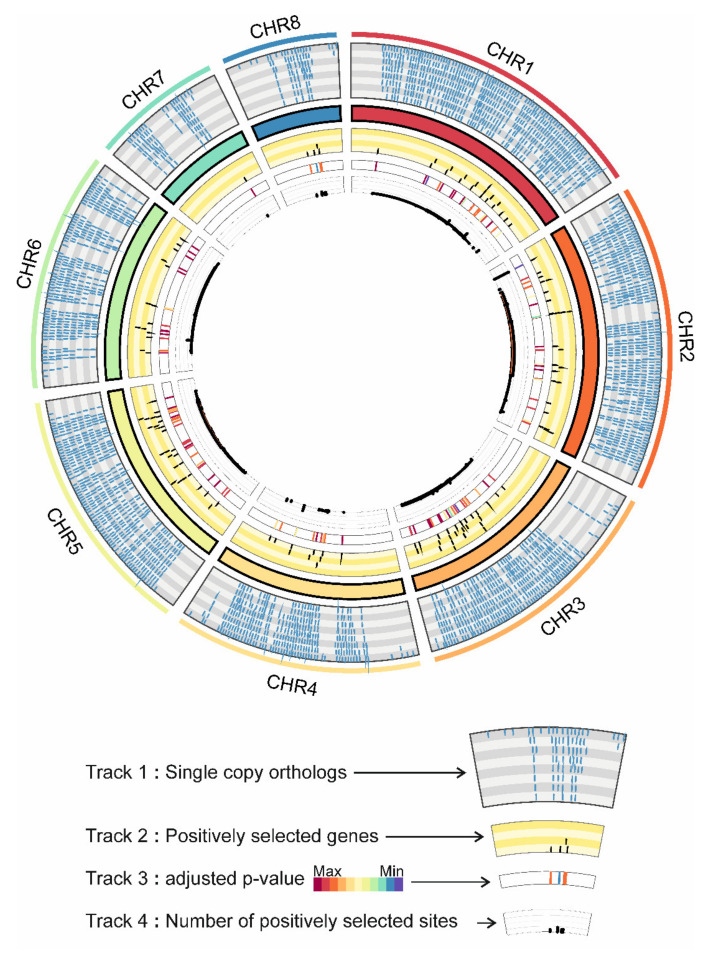
Chromosomal distribution of single-copy orthologs (SCOs) and positively selected genes (PSGs) in *A. fumigatus*. The circus plot shows the distribution of SCOs and PSGs over the different *A. fumigatus* chromosomes. Each different colored track shows different information. Track 1: SCOs in eight gray-shaded subtracks; Track 2: PSGs in four yellow shaded subtracks; Track 3: heatmap of the q-values of PSGs; Track 4: number of sites under selection in corresponding PSGs.

In our analysis, recombination events were excluded to avoid spurious signals by these (see Section 2). Here, for *A. fumigatus,* we identified 122 PSGs and, for *A. fischeri,* 50 PSGs (see below). The distribution of SCOs and *A. fumigatus* PSGs over the chromosomes is shown in Figure 4.

### 3.5. Host-Interacting Single-Copy Ortholog PSGs

**Figure 5:**
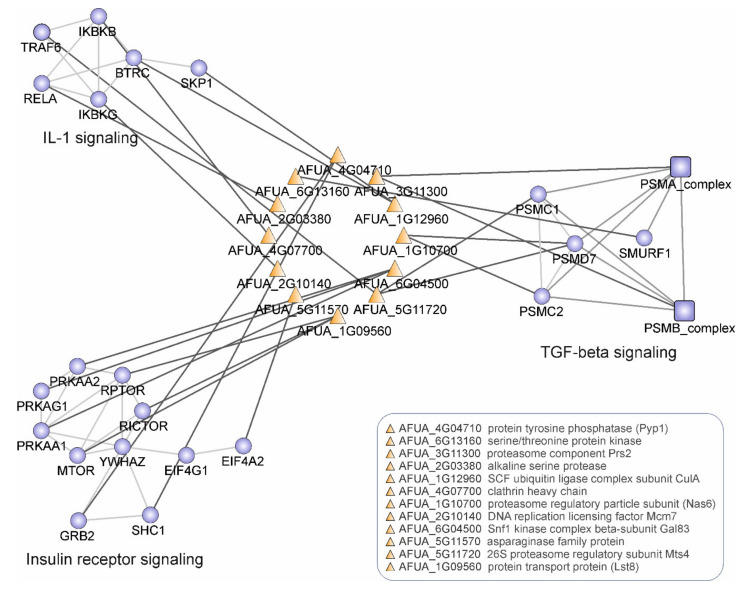
*A. fumigatus*–human host proteins–protein interactions (PPIs) map. PPIs between *A. fumigatus* proteins coded by positively selected genes (PSGs) and human proteins involved in three pathways are illustrated: IL1-signaling, Insulin receptor signaling and TGF-beta signaling. Orange—*A. fumigatus* proteins and blue—human proteins.

The encounter between host and pathogen is mediated by host–pathogen protein–protein interactions (HP-PPI), which play crucial roles in infections, as they define the balance either in favor of the spread of the pathogen or their clearance [54]. Genes involved in host–pathogen interactions are a frequent substrate of positive selection. We addressed the question of whether the identified PSGs interact with host proteins and interfere with host defense processes. To answer this, we verified the interacting partners of *A. fumigatus* protein products of PSGs in a previously established interspecies-interolog-based *A. fumigatus*–host interaction network [55] and the interspecies-interolog-based *A. fumigatus*–human interaction network reconstructed in this study. In total, 36% of SCO PSGs were identified to be involved in host–pathogen interactions (chi-squared = 306.18, *p*-value < 0.01). The resulting host–pathogen protein–protein interaction network consists of 44 *A. fumigatus* proteins coded by PSGs and 228 human proteins (Figure S1). The 40S ribosomal protein S9 Afua_3g06970 (Rps9A) was identified as the top pathogen hub with 35 interactions, followed by putative DEAD/DEAH box helicase Afua_5g02410 (Fal1) and putative proteasome component protein Afua_3g11300 (Prs2) (Table S5). Of note, such pathogenicity-related hub proteins with a high number of host interactions are involved in increasing the fitness of the pathogen in the host. We further performed a pathway and over-representation analysis (ORA) of targeted host proteins, which showed the over-representation of several immune-related pathways (Table S6 in Additional File S2; see Additional File S3 for Supplemental methods). Signaling proteins are preferentially targeted by pathogens because they globally regulate many cellular processes. The pathway over-representation analysis of PSGs interacting with human proteins resulted in significant over-representation of several signaling pathways (Table S6; Figure 5).

Highly significant over-representation was observed for the transforming growth factor beta (TGF-β) pathway (pathway ORA corrected *p*-value 3.95 × 10^−12^). Other top over-represented pathways include the insulin signaling pathway and the IL-1 signaling pathway. Notably, other potent immune pathways against *A. fumigatus,* such as the Toll-like receptor (TLR) pathway and the T-cell and B-cell pathway [56], were also significantly enriched. The adaptive evolution of several host-interacting *A. fumigatus* genes might be due to environmental pressure, and these could contribute towards the various fungal strategies to adapt and evade recognition by amoeba as well as in the rare event of infection of the human host’s immune system. Table S7 summarizes the significantly enriched GO terms for PSGs interacting host proteins.

### 3.6. Functional Classification of Single-Copy Ortholog PSGs

**Figure 6:**
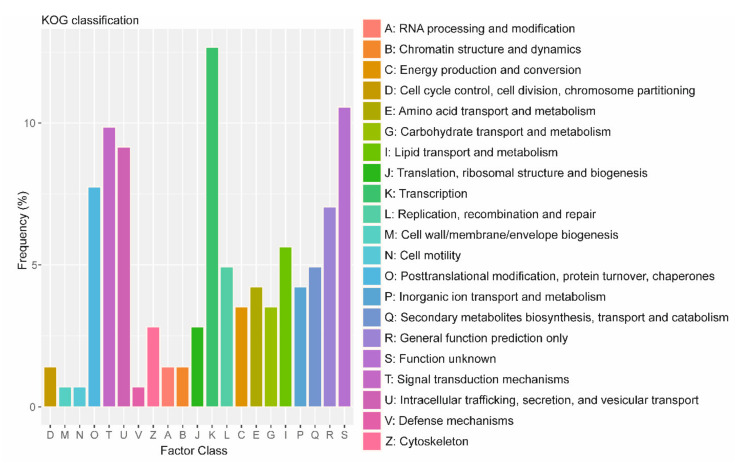
Functional categorization of positively selected genes (PSGs) in *A. fumigatus*. Distribution of the KOG function classification of PSGs. The categories of the KOG are shown on the horizontal axis, and frequencies are plotted on the vertical axis. The number of PSGs involved in transcription 18; in signal transduction mechanisms is 14; in intracellular trafficking, secretion, and vesicular transport is 13; in posttranslational modification, protein turnover, and chaperones is 11; in general function, prediction is 10; in lipid transport and metabolism, it is 8; in replication, recombination and repair, it is 7; in secondary metabolites biosynthesis, transport and catabolism, it is 7; in inorganic ion transport and metabolism, it is 6; in amino acid transport and metabolism, it is 6; and in unknown function, it is 15. The number of PSGs in other subgroups is ≤5.

We further categorized the *A. fumigatus* PSGs in euKaryotic Orthologous Groups (KOG) categories based on a blast-based sequence similarity search. The most suitable categories were identified, and multiple categories were used if genes were significantly similar to multiple query sequences with different KOG categories associated with them. If no annotation was retrieved for any of the genes, we scanned sequences against EGGNOGs [57] HHM profiles for classification. In total, we were able to assign functional KOG categories to 107 PSGs (Figure 6). These genes are our prime candidates for having been shaped by positive selection during evolution and adaptation of *A. fumigatus* and were assigned to three large functional categories (as well as to the corresponding functional classes) according to the KOG database: (i) metabolism, (ii) cellular processes and signaling and (iii) information storage and processing. In each category, we studied the most interesting genes in greater detail to further investigate the probable causes of positive selection (see Additional File S1 Supplemental Note “Positive selection in genes classified under KOG functional categories”). Table S8 contains a full list of the *A. fumigatus* genes in single-copy orthogroups evolved under positive selection.

### 3.7. Positive Selection in A. fumigatus in Multi-Gene Families

Our analysis above focused only on SCOs; therefore, many secretory, membrane protein-coding genes and virulence-associated genes were involuntarily excluded by this criterion. To overcome this limitation, we collected multi-gene families from the literature [58,59] and analyzed the positive selection for these genes. We focused on virulence-associated genes in multi-gene families, i.e., instead of single-copy genes, now we selected orthogroups in which any of the eighteen Aspergilli (Table S2) can have more than one paralog for a given gene. Out of 1498 non-SCOs genes analyzed, we found 212 genes that underwent positive selection in *A. fumigatus* (Table S9). Major virulence-related genes under positive selection included genes involved in immunoreactivity, nutrient uptake, resistance to immune response, signaling and toxin and secondary metabolite biosynthesis and metabolism. Key examples are given in Table 1.

Among 212 PSGs, 41 encoded proteins were predicted to interact with human proteins. This was established by the analysis of an interolog and domain–domain interactions-based host–pathogen interaction network. Enrichment analysis revealed the significant over-representation of pathogen interaction relevant GO terms, such as GTPase mediated signaling, regulatory activity and response to stimuli (Figure S2; FDR ≤ 0.05).

Studies have suggested that pathogens can exploit membrane trafficking events and a variety of host signaling cascades to facilitate invasion, colonization, and proliferation by targeting host GTPases [60]. Since during the saprophytic lifestyle, *A. fumigatus* can interact with free-living amoeba, we speculate that selection in host GTPases interacting genes might be the result of conflict between *A. fumigatus* and amoeba. To investigate this, we have collected the unique genes belonging to the most enriched three categories (regulation of GTPases) and analyzed their orthology relations with two amoeba species: *Dictyostelium discoideum* and *Acanthamoeba castellanii*. Not to our surprise, we found that 45 of the GTPase-related genes had orthologs in one or both of the Amoeba species (Table S10), including homologs from direct BLAST [20] hits between human and *A. castellanii* and *D. Discoideum*; and orthologs from Orthofinder [61] orthogroups of human, mouse, *A. castellanii* and *D. Discoideum*. Supported by further functional similarity, the evidence for the human vs. amoeba orthology relationship of these genes, which play a prominent role in host–pathogen interactions in humans, may point to the similarity between mechanisms employed by *A. fumigatus* in human and amoeba hosts. These mechanisms may have evolved under the predation pressure from amoeba and can now be utilized to survive in the human host.

### 3.8. Ancient Defenses: Example Gliotoxin Biosynthesis Cluster

Environmental virulence theories suggest that virulence traits of *A. fumigatus* have evolved as an adaptation to natural predators in soil, such as free-living amoeba [15,62]. The similarities between mechanisms used by *A. fumigatus*, especially at the conidial stage, to counteract against phagocytes both in the host and the environment points to the hypothesis that selective pressure exerted by amoeba predation caused the evolution of virulence strategies in the mammalian host [63]. Furthermore, *A. fumigatus* was also shown to decrease viability in amoeba not only by germinating in the cell after its uptake but also via secretion of mainly gliotoxin [15].

To investigate this example, we collected thirteen genes from the gliotoxin gene cluster, including biosynthesis genes, regulatory genes and transport genes [64], and studied their conservation in sequenced *A. fumigatus* strains and eighteen Aspergilli. We found that all the thirteen gliotoxin gene cluster genes are conserved in all six sequenced *A. fumigatus* strains and are single-copy orthologs except *gliA*, which had two paralogs in each strain (Table 2).

**Figure 7:**
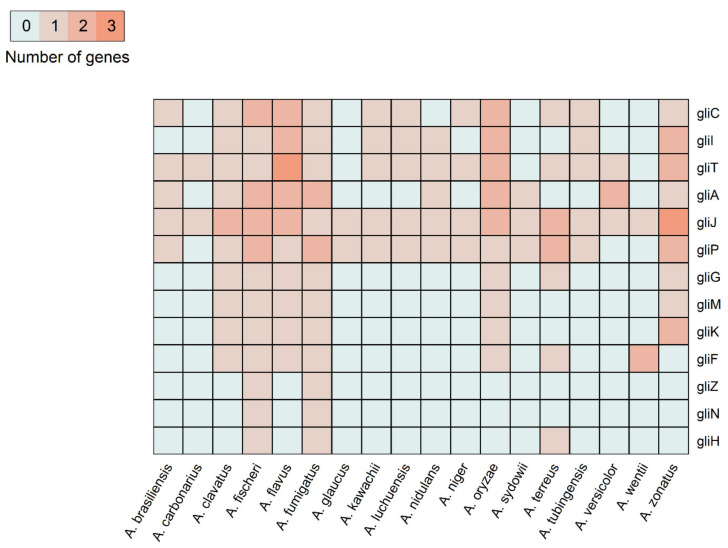
Conservation of gliotoxin biosynthesis gene cluster in Aspergillus species. Counts for each Aspergillus species in gliotoxin biosynthesis orthogroups are shown. Species that do not have orthologs in each orthogroup are represented gray.

Comparison of eighteen Aspergillus species, on the other hand, showed full conservation of the gliotoxin gene cluster among *A. fumigatus* and its close relative *A. fischeri*. Furthermore, another close relative, *A. clavatus,* as well as the more distant species, *A. flavus*, *A. oryzae* and *A. zonatus,* had orthologs for more than 80% of the gliotoxin cluster genes. Indeed, *A. fischeri*, a ‘very close nonpathogenic relative of *A. fumigatus*’, is the only species that has been also previously shown to have orthologs of all the gliotoxin biosynthesis gene cluster (Figure 7) and to biosynthesize gliotoxin [64]. Nevertheless, gliotoxin biosynthesis and secondary metabolite production, in general, was not as important for the virulence of *A. fischeri,* and the loss of *laeA*, a major regulator of secondary metabolism, did not affect its virulence [64]. We have also analyzed the traces of positive selection in gliotoxin biosynthesis genes of *A. fumigatus,* and none of the genes showed signs of positive selection (Table 2). Gliotoxin was found to be the major amoebicidal factor in *A. fumigatus* [15,64], which explains the lack of positive selection in gliotoxin biosynthesis pathway genes. Since gliotoxin evolved a long time ago against amoeba predation and now serves in “accidental virulence” against a mammalian host, it is plausible that there is no recent positive selection on those genes in an analysis comparing selection in current Aspergilli species.

## 4. Discussion

The effectiveness of natural selection acting on an advantageous mutation and the likelihood of its long-term fixation is proportional to its fitness effect [65]. Fighting an infection in immunocompromised patients has to cope with the versatile adaptation strategies of *A. fumigatus* [66], most likely resulting from its saprophytic life-style and its interaction with soil microorganisms including amoeba.

Previous work in this field includes the description of a toxin gene cluster evolved under positive selection in *A. parasiticus* [67]. Yang et al. (2012) identified that the duplicated gene pairs in *A. fumigatus* evolved under positive selection [68]. Species-wide comparisons of the genus Aspergillus provide a broad view of the biological diversity of the Aspergilli and highlighted protein versatility and conservation [8]. However, these studies did not offer the genome-wide signatures of recent selection in *A. fumigatus*, which was investigated here. Indeed, positive selection is the evolutionary force to drive divergence of species from its close relatives [69]. To examine how positive selection may have operated during *A. fumigatus’* evolution, we took advantage of the branch-site model’s ability to detect selection on the branch of interest. Maximum likelihood (ML) codon-specific estimation of the d_N_/d_S_ ratio may highlight other fungal adaptation strategies. Only with such an analysis can functional questions, such as positive selection of virulence genes, be answered, and hence, this is an important step forward to better understand the hidden evolutionary features behind Aspergilli and *A. fumigatus* in particular. The data provided here further emphasize the functional importance of PSGs in multiple functional categories.

### 4.1. Species-Specific A. fumigatus Proteins

Out of a total of 517 species-specific sequences found in *A. fumigatus*, 29 contain secretion signals, as detected by SignalP [50] (Table S1). It is likely that such proteins may also participate in some biological processes unique for *A. fumigatus* or play a role in the survival and propagation of this pathogen. However, in this regard, more in silico and in vitro functional characterization is required. For instance, an analysis of lineage-specific genes involved in the adaptation of a species to a particular environment suggests that such genes evolved rapidly since their deduced proteins have a substrate to act on, and therefore, a number of such genes have been postulated to play a role and confer an adaptive advantage on a particular species [70]. In contrast to this hypothesis, studies on *Drosophila* suggested that, instead of being fast-evolving genes, singletons seem to have largely originated by de novo synthesis from non-coding regions, such as intergenic sequences [71]. A further analysis of the species-specific proteins is beyond the scope of this manuscript but will be the subject of future studies.

### 4.2. Pathway and GO Over-Representation of A. fumigatus SCO Proteins Interacting with Human Host Proteins

Our analysis indicates that the over-representation of SCO PSGs interacting with host proteins downregulates the TGF-β [72], insulin receptor and IL-1 signaling pathways [73]. Pro-inflammatory cytokines are crucial for stimulating an effective immune response to *A. fumigatus* infection, which includes the recruitment of neutrophils to the alveolar spaces, where they constitute more than 90% of the phagocytic cells [74]. The role of these pathways in *A. fumigatus* infection have already been published [75,76,77,78], and we hope that our bioinformatics analysis will be an incentive to probe some of our predicted interactions by experiments. (The GO terms are given in Table S7.) The GO term ‘symbiosis, encompassing mutualism through parasitism’, was significantly enriched for PSGs-targeted human proteins. This finding is consistent with earlier data showing the significant enrichment of this category in *A. fumigatus*-targeted human proteins [55]. The GO term ‘viral process’ and parent GO term ‘symbiont process’ were also over-represented. Together with other GO terms, this indicates that in *A. fumigatus,* positive selection occurs mostly on genes involved in the initial stage of infection, where the host attempts to avoid pathogen establishment.

Among the multi-gene family PSGs interacting with the host, we identified that many genes involved in the regulation of the GTPase cascade were targeted by *A. fumigatus*. Notably, the *D. discoideum* genome contains an unexpectedly large number of GTPases, which involves complex signaling mechanisms in a wide range of biological processes [79]. Rho subfamily GTPases are also involved in controlling the reorganization of actin, which is essential for both phagocytosis [80] and the subsequent maturation of phagosomes [81]. The kinetics and regulation of phagosomal maturation are very similar in *D. discoideum* and mammals [82]. Once the pathogen is internalized in the host cell, fission and fusion events occur in the phagosomal membrane, and the phagosomal membrane interacts with elements of the endolysosomal system to obtain proteases and lysosomal enzymes, which is regulated by GTPases. The natural selection in GTPase regulator-interacting genes is likely the consequence of the conflict between fungi and amoeba.

We suggest that these host–pathogen interactions help *A. fumigatus* to survive also in the human host (see also [15]). As a further example, host–pathogen interactions critical for the survival of *Cryptococcus neoformans* in *A. castellanii* were shown to contribute to the survival of the fungus inside macrophages [83]. A recent review of such conserved virulence determinants focuses on this dual use of fungal adaptations beneficial to survive innate immune cells but also soil amoebae as the natural phagocytes [63].

### 4.3. Positive Selection in Other Functionally Annotated Categories

All PSGs classified under KOG categories are discussed in the Additional File S1 Supplemental Note “Positive selection in genes classified under KOG functional categories”.

#### 4.3.1. Conserved Hypothetical Protein-Coding Genes

Certain exceptions notwithstanding, conserved hypothetical proteins present in several sufficiently different genomes are not truly ‘hypothetical’ anymore [84]. Twenty SCO PSGs code for conserved hypothetical proteins, expressed to survive hypoxic conditions (Afua_2g09800, Afua_3g13930, Afua_8g05450 and Afua_5g07480) or in early conidial development (Afua_6g13670, Afua_3g13930, Afua_5g06310, Afua_4g07030) in [85]. Two are differentially expressed during dendritic cell infection (Afua_5g02110 and Afua_5g08770), and one is differentially expressed during neutrophil infection (Afua_4g07030). Moreover, two conserved hypothetical PSGs were annotated to be involved in signal transduction mechanisms (Afua_4g09890 and Afua_4g09910) by KOG categorization. These previously ignored conserved hypothetical genes are important as a general resource to understand the complex biology of Aspergillus better. Conserved hypothetical PSGs could be targets for novel and broad antimycotics as their positive selection and broad conservation suggests important functions for Aspergilli.

#### 4.3.2. Hypoxia-Responsive Genes

Hypoxia tolerance is necessary for *A. fumigatus* to survive in host tissue but also during its saprophytic lifestyle. Oxygen levels drop from 21% in the atmosphere to 14% in the lung alveoli [86], and in surrounding tissue, oxygen availability is further reduced to 2 to 4% and in inflamed tissue to less than 1% [87]. A broad range of metabolic pathways maintains energy levels under oxygen-limiting conditions. Compared with the gene expression profile of hypoxia-inducible target genes of *A. fumigatus* [85], 27% of the PSGs identified in our study significantly change their expression behavior during low-oxygen conditions (Table S11).

#### 4.3.3. Genes Involved in Early Development of *A. fumigatus*

Conidia of *A. fumigatus* ingested or inhaled by immunocompromised patients can germinate in the lung and produce hyphae within 6–8 h to trigger infection [88] and adaptation to aerobic metabolism and growth [89]. Among the PSGs we identified, ~25% of the genes were significantly expressed at 8 h, and 18% were significantly expressed at 16 h of growth. Highly downregulated PSGs at 8 h were the bZIP transcription factor Afua_3g11330 (*atfA*), followed by the ammonium transporter Afua_1g10930 (*mep2*), while highly upregulated PSGs were 40S ribosomal protein S9-coding gene Afua_3g06970 (*rps9a*), followed by GDP-mannose pyrophosphorylase A (GMPP)-coding gene Afua_6g07620 (*srb1*). In *A. fumigatus,* the expression of 63% of the conidia-associated genes is controlled by *atfA,* which is an essential gene for the viability of this fungus in various environmental conditions [90]. It positively regulates conidial stress-related genes and negatively regulates the genes for germination, suggesting a role for *atfA* in conidial dormancy [91]. *atfA* was also found to be pivotal for heat and oxidative stress tolerance in conidia by regulating the conidia-related genes responsible for stress protection [90]. *mep2* coded proteins function as ammonium sensors in fungal development [92] and the induction of invasive filamentous growth [93]. *srb1* gene encodes the GMPP in *A. fumigatus* and is essential for its viability. A highly downregulated PSG at 16 h was acyl-CoA thioesterase II-coding gene Afua_1g15170 (*acot2*), followed by *atfA*, while highly upregulated PSG was meiosis induction protein kinase Ime2, followed by an integral membrane protein 25D9-6 coding gene. *acot2* catalyzes the hydrolysis of acyl-CoAs to the free fatty acids and coenzyme A (CoASH), providing the potential to regulate intracellular levels of acyl-CoAs, free fatty acids and CoASH and has important functions in lipid metabolism and other cellular processes [94]. Overexpression of *acot2* can significantly modulate mitochondrial fatty acid oxidation [95]. Positive selection in *acot2* suggests respiratory activity was improved during the evolution of *A. fumigatus*. Other important genes in this category included serine/threonine-protein kinase Afua_2g13140 (*ime2*), which is critical for proper initiation of meiotic progression and sporulation. In *A. nidulans*, *ime2* is required for the light-dependent control of mycotoxin production [96]. The integral membrane protein 25D9-6 coding gene Afua_2g17080, which is a major component of peroxisomal membranes, was also found to be evolved under positive selection.

#### 4.3.4. Essential Protein-Coding Genes

The essential genes of an organism constitute the minimal gene set required for the survival and growth of an organism, in particular defined as to sustain a functioning cellular life under the most favorable culture conditions [97]. Typically, such genes are more evolutionarily conserved than the non-essential genes [98], and rapid evolution is usually not expected among essential genes [99] with few exceptions [100]. Several reports identified the essential genes in *A. fumigatus* using both the functional experimental methods and bioinformatics approaches [101,102,103,104,105]. By comparing these with our results, we noticed that among the experimentally identified essential genes, only the spliceosomal protein-coding gene, Afua_3g12290 (*dib1*) [105], and the mitochondrial import receptor subunit, Afua_3g11860 (*tom22*) [104], evolved under positive selection, while among computationally identified essential genes by sequence similarity against essential eukaryotic genes [102], four genes (transcription initiation factor TFIID subunit 2 Afua_8g04950, DNA-directed RNA polymerase III subunit 22.9 kDa Afua_8g04350, DNA replication licensing factor Afua_2g10140 (*mcm7*) and Ccr4-Not transcription complex subunit Afua_3g10240 (*not1*)) evolved under positive selection.

Likewise, positive selection is often more prevalent in peripheral proteins, while purifying selection is more stringent among central network proteins (hubs) in the interactome of organisms [106,107]. To identify whether the PSGs follow the same criteria, we analyzed the *A. fumigatus* interactome [108] and compared the top 20% hubs with PSGs. In contrast to the general tendency, fifteen *A. fumigatus* hub proteins (≥5 interaction partners) were found to have evolved under adaptive evolution (Table S12). Notably, twelve of these proteins are also involved in host interactions (Table S13; e.g., asparagine synthase Afua_4g06900 (*asn2*), Ion protease homolog Afua_2g11740 (*pim1*)), as detailed in the result section “Host-interacting PSGs” (see above). The importance of proteins during the infection is directly related to their number of interactions with the host [109]. This suggests that the observed positive selection at these hubs may be due, first, to environmentally imposed pressure to affect the pathogen fitness during amoeba attack and, second, for survival in macrophages.

### 4.4. Positive Selection in A. fischeri Protein-Coding Genes

*A. fischeri* is a sister-species to *A. fumigatus* but generally is saprophytic and a pathogen only under rare circumstances [4,5]. The species tree confirms the similarity of the *A. fischeri* genome with *A. fumigatus* (Figure 3). Both species share 8536 one-to-one orthologs (Additional Data File S3). To assess the extent of genome rearrangements more closely, the complete chromosome sequences of *A. fumigatus* and *A. fischeri* were aligned using the Mauve genome aligner [110]. The GC (guanine–cytosine) content was seen to be highly homogenous (49.2%), and genome sequences were relatively well-conserved. The alignment consisted of 318 Local Collinear Blocks (LCBs) with a minimum weight of 999. A genome synteny map is shown in Figure S3. We find several instances of genome rearrangements (reverse complement of the reference sequence) since *A. fumigatus* and *A. fischeri* separated from each other. The genome rearrangements possibly occurred because the recombination can be advantageous for fungal pathogens in stressful environments, such as inside a human host [111]. Furthermore, using the BS model (see methods), we identified 50 SCO PSGs in *A. fischeri* (Table S14). Among the orthologs of these genes in *A. fumigatus,* only eight genes evolved under positive selection, although the sites where selection occurs were not the same for these genes. Taken together, this analysis shows clear differences regarding PSGs between both organisms and the adaptation of both Aspergilli. In particular, *A. fischeri* has less than half as many PSGs and shares only eight PSGs with *A. fumigatus.* This difference suggests much less potential of *A. fischeri* to adapt even to a human host and is in line with only rarely observed clinical infections.

### 4.5. A. fumigatus Multi-Copy Virulence Genes with Positive Selection

The analysis of only SCOs excluded many virulence-related genes that have more than one copy in AAspergilli genomes. Therefore, it is important and necessary to analyze positive selection in virulence genes that do not belong to SCOs. Our analysis of these orthogroups identified 212 virulence genes that showed signatures of positive selection in *A. fumigatus* (Table S9). Some of these genes, such as the major allergen Afua_4g09580, genetic name *aspf2,* the non-ribosomal peptide synthases and several ABC transporters were involved in processes, such as immunoreactivity, nutrient uptake, resistance to immune response, signaling and toxin and secondary metabolite biosynthesis/metabolism (Table 1). In particular, *aspf2* is found in eleven Aspergilli species from the 18 compared (not found in *A. brasiliensis, A. carbonarius, A. kawachii, A. luchuensis, A. niger, A. tubingensis* and *A. zonatus*). Positive selection of *aspf2* in *A. fumigatus* and its conservation in so many *Aspergilli* may point to the evolution of an anti-amoeba strategy employed by these fungal species based on inhibiting phagocytosis by protein binding. Thus, during human infection, the conidia of *A. fumigatus* start germinating and activate human immune responses from the complement system. However, *A. fumigatus* evades this immune response by regulating the complement system. For immune evasion, the allergen *aspf2* recruits immune regulators, such as Factor-H, which is normally a factor to oppose complement activation. Furthermore, *aspf2* was shown to bind plasminogen, and its activated version of plasmin cleaves fibrinogen, and this process aids the damage of lung epithelial cells and helps with early infection. More important to confirming a more generally conserved evasion strategy against phagocytosis, conidia with mutant *aspf2* were shown to be more effectively phagocytosed and killed by neutrophils [112].

Despite the insufficient nutrients in the host phagolysosome environment, *A. fumigatus* can swell up and initiate growth by utilizing their siderophore machinery to overcome the limited iron supply [113]. This process is also well documented when *A. fumigatus* interacts with *A. castellanii* and *D. discoideum* [15,114]. The nonribosomal peptide synthases Afua_3g03420 (*sidD*) involved in siderophore biosynthesis that facilitates survival in iron-scarce environments, complemented by other nutrient transporters (Table 1), was found to be positively selected. Decreasing iron content during infection is a strategy employed by the host during infections, and pathogens evolve mechanisms to “obtain iron from the host”. Therefore, *A. fumigatus* uses two iron-uptake methods; one is via siderophores. Mutants of *sidD,* along with other enzymes of the siderophore biosynthesis, showed decreased conidiation and attenuated virulence in iron-scarcity, indicating the induction of a siderophore biosynthesis pathway during infection [115,116]. Of note, we also observed positive selection in non-ribosomal peptide synthetases Afua_3g03350 (*sidE*). Although *sidE* does not contribute to siderophore biosynthesis, it is involved in the production of fumaryl-alanine [117]. Fumaryl-alanine acts as an immunomodulatory metabolite, and *sidE* highly upregulates in murine lung infection [115,117].

Another result that came out from our study is that multifunctional Cu/Zn superoxide dismutase Afua_5g09240 *sodA* evolved under positive selection. During iron deplete conditions and host colonization, high expression of *sodA* in *A. fumigatus* provides self-protection against own fungal oxidants and gliotoxin levels [118,119,120,121]. *sodA* is also recognized by sera from patients with confirmed *A. fumigatus* infections [122]. Due to the high expression of *sodA* during pathogenic growth, it represents a valuable immunodiagnostic marker for *A. fumigatus* infections [122]. Compared to the wild-type, high susceptibility to reactive oxygen species (ROS) and high temperatures has also been observed in *sodA* mutants [120].

Moreover, three ABC transporters, Afua_1g17440 *abcA*, Afua_1g10390 *abcB* and Afua_7g00480 *abcE,* were found to be under positive selection in *A. fumigatus*. It was shown that all five ABC transporters (abcA-E) are induced by voriconazole and take part in azole resistance [123]. In particular, *abcB* was found to be related to azole resistance, and its loss contributed relatively more to increased susceptibility to azole, indicating that *abcB* is essential for virulence and azole resistance, while *abcA,* although not required, may increase resistance in infection [124,125]. The annotated virulence genes and their function constitute highly sought out data to understand pathogenic fungi.

### 4.6. Evolutionary Overview

Positive selection on *A. fumigatus* genes that are conserved among eighteen Aspergilli species can underline the *A. fumigatus*-specific functions of their protein products and consequently may be important in the pathogenicity of *A. fumigatus*. The same applies to *A. fumigatus*-specific genes; however, it is not possible to run a positive selection analysis on these genes. Nevertheless, although not covered in the study, the next step may involve analyzing positive selection in the genes compared with other sequenced *A. fumigatus* strains since we have shown that some of these genes are conserved in all strains and related to virulence (Table S1).

PSGs identified both in SCOs and multi-gene families can be related to recent virulence mechanisms evolved in *A. fumigatus* and show its unique virulence strategies against the host.

Since the human host niche is rarely used by *A. fumigatus* in comparison to its global abundance in saprophytic niches, the selection pressure on *A. fumigatus* should be rather imposed by free-living amoebae and other microorganisms (e.g., [126]) during their co-survival in the soil niche. *A. fumigatus* possesses a molecular arsenal to survive in most environments, including the human host [127]. This requires powerful strategies for initial colonization [128] and biofilm formation [129], which, in the human host, then permits to establish infection.

Especially the virulence mechanisms involving products of secondary metabolism such as gliotoxin, fumagillin, fumarylalanine, fumitremorgin, verruculogen, fumigaclavine, helvolic acid, sphingofungins and DHN-melanin may have evolved against their competitors and their predators in soil rather than host selection [64].

Here, we show that one of the major virulence mechanisms of *A. fumigatus*, gliotoxin biosynthesis pathway, does not have signs of positive selection in our analysis set. This finding demonstrates that old and settled virulence mechanisms of *A. fumigatus,* such as gliotoxin toxicity, have in fact evolved under long term, global and continuous predation pressure as survival tricks and not as more recent and more species-specific virulence strategies.

Biosynthetic gene clusters responsible for secondary metabolite production are less conserved than the rest of the genome. Although *A. fumigatus* share 80% of its genome with *A. fischeri* and *A. clavatus*, only 30% of the secondary metabolite biosynthesis pathways are conserved among the three [13]. Moreover, our criteria (orthology as defined by respective sequence identity, see Section 2) is not sufficiently accurate for judging secondary metabolism specificity. Even in the presence of a very high sequence identity (e.g., as observed between *A. oryzae* and *A. flavus*), the overall metabolite profiles may differ strongly [130]. Thus, conserved genes (and protein structures) in the well-conserved gliotoxin cluster do not necessarily imply that gliotoxin is synthesized in these species.

A typical discussion point for evolutionary studies is that these genes have already been reported as virulence genes, and hence, it may be questionable how novel our findings are. The answer to this question is that we bring the vast amounts of genes already described in an evolutionary context. This can then even help to improve their annotation and better understand their function, as well as whether they are important:

First of all, virulence genes, defined by an interaction with the host such that the infection process is enhanced and improved, are partly labeled as such if directly recognized (experimental data) or if recognized by sequence comparison and sequence similarity to other virulence genes from other species. This then leads to their annotation, for instance, if we think of fungal toxins. However, there are other processes that act similarly as an enhancement of the infection process. In our study, we found, in particular, among the 212 secretory, membrane and virulence related multi-gene families, a number of genes were involved in immune evasion mechanisms. These are not readily apparent from their annotation (often no mention of the term “virulence” in their description), but as we found specific genes in this category with positive selection, we can be sure that the genes with positive selection enhance the survival chances for fungi overall and in general. This non-trivial result hence singles out these genes as special from other immune evasion genes without positive selection. This is also important to recognize as the natural major environment of *Aspergilli* is saprophytic, and here, the positive selection points again to an evolutionary advantage of these specific genes. Similarly, non-obvious virulence factors (not apparent from annotation) concern several metabolic genes (siderophore biosynthesis, three ABC transporters, stress-tolerance). Again, the advantage is that we see which among these gene families are important as being selected by positive selection pressure. A case in point is the positively selected multifunctional Cu/Zn superoxide dismutase Afua_5g09240 *sodA*, which turns out to be an immunodiagnostic marker for Aspergillus infection.

Moreover, in hypothetical genes, it is notoriously difficult to get any clue on annotation. Here, our evolutionary study took expression data to single out and identify genes expressed to survive hypoxic conditions (Afua_2g09800, Afua_3g13930, Afua_8g05450 and Afua_5g07480) and important in early conidial development (Afua_6g13670, Afua_3g13930, Afua_5g06310 and Afua_4g07030). Without our test for positive selection, these genes would not be singled out from the many other hypothetical conserved genes for fungi already described.

Taken together, our work provides a detailed evolutionary perspective on *A. fumigatus* biology. For the first time, the currently available extensive genome information (18 genomes) is not just analyzed in terms of annotation or phylogenetic comparison, but rather, we show how positively selected genes in the pathogen *A. fumigatus* are identified, analyze their connection to virulence and add detailed interactome analysis to reveal interactions with the host. Blind spots in Aspergillus biology are illuminated, revealing highly conserved genes in the genus from our detailed positive selection analysis. Finally, we provide all data and scripts of the whole analysis as a resource to the community.

## 5. Conclusions

Selection plays an important role for Aspergilli in general and specifically for the adaptive evolution of *A. fumigatus* involving different environments from saprophytic lifestyle and soil microorganism to human opportunistic pathogen. Eighteen fungal species of the genus *Aspergillus* were compared and analyzed to identify PSGs. This large-scale analysis, including all genes and analysis data, is fully made available here. We identified 122 well-annotated genes of *A. fumigatus* that evolved under positive selection pressure, including signal transduction, metabolism, mitochondrial activity, regulation of transcription and conidia growth-related genes. Moreover, in conserved genes of unknown function, we establish several to be clearly positively selected. Finally, we detected positive selection signals in a total of 212 secretory or membrane related multi-gene families, and besides these two categories, we also found them in virulence-related multi-gene families. The genes analyzed here also point to immune evasion mechanisms (*aspf2*, one of the central immune evasion genes, was identified to evolve under positive selection). Further positive selection was also identified in genes involved in siderophore biosynthesis (*sidD*), metabolite fumarylalanine production (*sidE*), stress tolerance controlling transcription factor (*atfA*) and multifunctional thermotolerance and self-protection-regulating gene (*sodA,* immunodiagnostic marker for *A. fumigatus* infections). We found many PSGs interact with host GTPase regulation mechanisms and adaptive and immunomodulatory pathways. These are also medically relevant as they mitigate human host defenses. Moreover, proteins coded by PSGs may provide targets for new antifungals. Taken together, our results identify the *A. fumigatus* genes that are strong candidates for also having functional effects in the human host environment and provide an evolutionary overview on positively selected genes in the genus *Aspergillus*.

## Figures and Tables

**Table 1:** Positive selection in *A. fumigatus* virulence genes ^1^.

Gene	Gene Name	Annotation	Category
Afua_4g09580	*aspf2*	Allergen Asp f2	Allergens
Afua_5g03520		Immunoreactive secreted protein	
Afua_3g03420	*sidD*	Nonribosomal peptide synthetase 4	Nutrient uptake
Afua_1g10390	*abcB*	Putative ABC multidrug transporter	Resistance to immune response
Afua_1g17440	*abcA*	ABC drug exporter	
Afua_5g09240	*sodA*	Cu/Zn superoxide dismutase	
Afua_7g00480	*abcE*	Putative ABC transporter	
Afua_2g00660	*tcsB*	Putative sensor histidine kinase/response regulator	Signaling and regulation
Afua_2g18060	*fgaMT*	4-dimethylallyltryptophan N-methyltransferase	Toxins and secondary metabolites
Afua_4g14490	*tpcJ*	Putative dihydrogeodin oxidase	
Afua_8g00370	*fma-PKS*	Fumagillin biosynthesis polyketide synthase	
Afua_8g00440		Dual-functional monooxygenase/methyltransferase	
Afua_8g00460		Methionine aminopeptidase type I, putative	

^1^ See Table S9 for the complete list of PSGs in multi-gene family secretory, membrane protein-coding genes and virulence-associated genes (as defined by [58]).

**Table 2:** Gliotoxin biosynthesis genes ^1^.

Gene	Gene Name	Annotation	*q*-Value (BY)
Afua_6g09660	*gliP*	cyclo (L-Phe-L-Ser) synthetase	1
Afua_6g09670	*gliC*	cyclo(L-Phe-L-Ser) hydroxylase	1
Afua_6g09690	*gliG*	glutathione S-transferase	1
Afua_6g09700	*gliK*	gamma-glutamylcyclotransferase	0.16
Afua_6g09650	*gliJ*	3-benzyl-3,6-bis(cysteinylglycine)-6-(hydroxymethyl)-diketopiperazine dipeptidase	0.42
Afua_6g09640	*glil*	3-benzyl-3,6-bis(cysteinyl)-6-(hydroxymethyl)-diketopiperazine lyase	0.16
Afua_6g09740	*gliT*	3-benzyl-3,6-dithio-6-(hydroxymethyl)-diketopiperazine oxidase	1
Afua_6g09720	*gliN*	N-desmethyl-gliotoxin N-methyltransferase	-- *
Afua_6g09630	*gliZ*	Zn2Cys6 binuclear transcription factor	-- *
Afua_6g09680	*gliM*	Predicted O-methyltransferase	1
Afua_6g09710	*gliA*	Predicted major facilitator type glioxin transporter	1
Afua_6g09730	*gliF*	Predicted cytochrome P450 monooxygenase	0.53
Afua_6g09750	*gliH*	conserved hypothetical protein	1

* PS analysis for the two genes could not be performed since the clusters contained only two genes. ^1^ All but gliA are SCOs and conserved in all 6 strains, and gliA has duplicates in all strains (12 genes in one orthogroups in total).

## Data Availability

All results are contained in the manuscript and its supplementary files. The tools used for the analysis of evolution on gene families in the genus *Aspergillus* with a focus on *A. fumigatus* are made fully available by us, including all codes and steps used. These analysis tools and methods can now be applied to any genus and phenotype of interest. To enable full reproducibility of the data, the dataset and codes (1 Bash and 14 Perl scripts) underlying our positive selection pipeline, as well as other additional data files with all accession numbers, are available at https://github.com/ShishirGupta-Wu/aspergillus_ps.

## Supplementary Materials

The following are available online at www.mdpi.com/article/10.3390/microorganisms9102014/s1. Additional File S1: Supplemental notes, Supplementary Figures S1–S3, large supporting additional data files 1–3. Additional File S2: Supplementary Tables S1–S15. Additional File S3: Supplemental methods.
