# Supplementary material for: Aspergillus fumigatus versus Genus Aspergillus: Conservation, Adaptive Evolution and Specific Virulence Genes
Source: Microorganisms. 2021 Sep 23;9(10):2014. doi: 10.3390/microorganisms9102014 (PMC8539515; doi:10.3390/microorganisms9102014)
Supplement: Supplementary file 1 [file Additional file S1.pdf › Additional file S1.pdf]

Additional file 1

This PDF file includes:

1. Supplemental note

(i) Recombination breakpoints
(ii) Positive selection in genes classified under KOG functional categories
- Metabolism
- Cellular processes and signaling
- Information storage and processing
References

2. Supplementary figures
- Figs. S1, S2 and S3

3. Additional data file 1
4. Additional data file 2
5. Additional data file 3

Other Additional file for this manuscript includes the following:

Additional file 2

- **Captions for Tables S1 to S15 (Sheet in excel file)**
- **Tables S1 to S15 (Sheets in excel file)**

* Corresponding author
Address correspondence to Prof. Dr. Thomas Dandekar, dandekar@biozentrum.uni-wuerzburg.de

Note: We recommend to use bookmarks for navigation.

Supplemental Note

Recombination breakpoints

Recombination is a phenomenon that occurs in the genomes of many organisms through the exchange or transfer of genomic fragments demarcated by recombination breakpoints. In the evolution of fungi, recombination is documented as one of the major forces driving diversity and adaptation [1, 2] subsequently resulting in the creation of mosaic sequences where the evolutionary history at each site may be different. With recombination, nucleotide sites in a sequence do not evolve along a single tree, but instead along a set of correlated trees [3]. Violating this tree-like assumption of evolution can lead to serious consequences when performing positive selection analysis [4]. Thus, the evolution of homologous recombinant sequences must be modelled by several phylogenies, one for each nonrecombinant fragment in the alignment [5]. The widely used likelihood methods for quantifying selection pressure on codon alignments [6] may suffer from high rates of false positives when the sequences being analyzed have undergone recombination [4, 7]. Therefore, we analyze the presence of recombination breakpoints in SCOs clusters. We used the Genetic Algorithm Recombination Detection (GARD) tool that implements the likelihood-based model selection procedure and uses the genetic algorithm to screen MSA for recombination breakpoints and quantifies the strength of support for their location [5, 8]. We implemented the Shimodaira-Hasegawa (SH) test to reject the potentially recombinant areas which might be due to substitution rate variation and not the recombination, since only a subset of recombination breakpoints convey any selective advantage, and the majority may be selectively neutral with limited biological significance [9]. We found that significant recombination events occurred in 879 single copy ortholog clusters (Additional file 2: Table S15).

We further conducted GO over-representation analysis of proteins that possibly experienced recombination. In the BP terms, the top two enriched GO terms were response to stress and purine ribonucleotide biosynthetic process, the MF terms were structural constituents of ribosome and phospholipase D (PLD) activity, and the CC terms were related to organelle and mitochondria. Here we have highlighted the role of proteins under BP and MF categories. Detailed alignments are listed in (Additional file 2: Table S5) including the low-level over-representation of recombination breakpoints. Studies have shown the positive correlation between stress and recombination activity [10, 11]. In *Drosophila melanogaster*, it has also been suggested that stress-induced recombination might be a mechanism of evolvability as

certain types of stress, for instance heat shock stress, have the capacity to stimulate evolutionary changes [12]. Several stress-related proteins of *A. fumigatus* and *A. nidulans* have previously shown to be involved in recombination [13-16]. Highly significant over-representation of the GO category, cellular response to stress (GO:0033554, FDR 1.44E-03), suggest recombination might be a general stress response in aspergilli. The increased genetic flexibility by recombination might facilitate evolutionary adaptation of fungi to stressful environments.

Purines are essential nutrients required for functions as diverse as signal transduction, energy metabolism, and DNA synthesis. They are also required by the pathogenic fungi to enable growth and dissemination in the host. Due to varying concentrations of purines in their environmental niches and the host, most of the fungal pathogens can degrade, salvage, and synthesize de novo purines through several conserved enzymatic steps [17]. The purine nucleotide biosynthesis pathway is conserved between yeast and humans and is critically important for the normal functioning of cells. Additionally, dysregulation of this pathway can result in cytotoxicity [18]. High-frequency genetic variation in two purine biosynthesis genes influences survival of bacterial pathogen *Campylobacter jejuni* under stress and promotes its success as a pathogen [19]. We found that the over-representation of the GO category, purine ribonucleotide biosynthetic process (GO:0009152, FDR 1.82E-03), suggests the recombination in these genes could be the adaptive strategy of aspergilli.

Highly transcribed genes, such as the ribosomal genes, are more susceptible to recombination [20]. In agreement with this, we also found over-representation of several ribosome-related GO terms such as ribosome biogenesis (GO:0042254, FDR 2.54E-02), ribosome (GO:0005840, FDR 2.83E-02) and structural constituent of ribosome (GO:0003735, 2.77E-02) (Additional file 1: Table S5). Other than their primary functions in protein translation, ribosomal proteins (RPs) are found to have extra-ribosomal functions that are related to regulation of apoptosis, cell cycle arrest, cell proliferation, cell migration and invasion, and DNA damage repair [21, 22]. In response to DNA damage, RPs can help to maintain genomic stability by regulating the activity of the relevant key transcription factors [23]. To support the hyphal growth, the number of ribosomes in an ungerminated spore is insufficient, therefore, ribosome production is a major synthetic process during the early minutes of germination in filamentous fungi [24]. This process results in a 10-fold increase in the number of ribosomes actively engaged in translation within the first few minutes [25-27]. The degree of transcription involved enhances the opportunity for recombination. Thus, the over-representation of ribosome related GO terms in

recombination breakpoints containing proteins is not surprising as there the transcription is strong.

Phospholipases are ubiquitous enzymes that participate in multiple cellular functions such as vesicular trafficking, receptor signaling and signal transduction. Phospholipase D (PLD) catalyzes the hydrolysis of phosphatidylcholine into phosphatidic acid (PA). The PLD encoding gene modulates the internalization of this pathogen into epithelial cells and represents a virulence factor of *A. fumigatus* in IA under immunosuppression by corticosteroids [28]. PLD activity and its presence in the nucleus are necessary for signaling and the completion of meiosis [29, 30]. Therefore, over-representation of phospholipase D activity is not surprising.

Positive selection in genes classified under KOG functional categories

Metabolism

Inhaled resting conidia in immunocompromised individuals become metabolically active within 30 min of ingestion. Cellular energy is essential for the multiplication and stress tolerance. Aspergillus species have an remarkable capacity for energy generation, and high efficiency to acquire and utilize a wide range of resources and can store considerable quantities of nutrients within the cell; all traits which contribute to their energy-generating capacity and competitive ability [31]. *A. fumigatus* and some other aspergilli possess and express multiple genes for the enzymes that regulate pathways which allow fungi to adapt their primary carbon metabolism requirement in the niche they inhabit and confer a selective advantage [32]. Also, the possibilities of positive selection signatures in other metabolic genes can be expected. Our scan revealed that several genes involved in metabolic processes evolve under positive selection pressure. This also includes eleven differentially expressed metabolic genes during conidial/hyphae growth. Positive selection pressure was observed in five genes (Afua_2g15730, Afua_2g17080, Afua_6g04500, Afua_1g07220 and Afua_3g08480) in KOG class carbohydrate transport and metabolism. Interestingly, a highly significant positive selection signal (Benjamini-Yekutieli, q-value 0) was observed in a key regulatory glycolytic enzyme 6-phosphofructo-2-kinase 1 coding gene (Afua_1g07220) which catalyzes the conversion of fructose 6-phosphate and ATP to fructose 1,6-bisphosphate and ADP. In response to the cells energy requirements, this enzyme can increase or decrease the rate of glycolysis through allosteric inhibition.

Active fatty acid and lipid metabolism ensure the capability of *A. fumigatus* for using alternative carbon sources in the stressful host environment in infected tissue during invasive

aspergillosis [33, 34]. Additionally, also serve as the energy source for intracellular growth of *A. fumigatus* conidia [35]. Triacylglycerides (TAGs) are a class of neutral lipids that represent the most important primary storage form of energy for eukaryotic cells, which is often synthesized via multiple pathways. Phospholipid:DAG acyltransferase (PDAT) that transfer a fatty acyl moiety from a phospholipid (PL) to diacylglycerol (DAG) to form TAG represents one such acyl-CoA–independent TAG synthesis pathway. This membrane-localized enzyme coding gene Afua_3g09950 was identified as PSG by our analysis. The artificial microRNA silencing of PDAT suggests that membrane lipid turnover and TAG synthesis mediated by PDAT is essential for vigorous growth of alga *Chlamydomonas reinhardtii* under favorable culture conditions and for membrane lipid degradation with concomitant production of TAG for survival under stress [36]. BlastP based similarity search shows *A. fumigatus* PDAT shares 43% sequence similarity with *C. reinhardtii* PDAT. Further, having similar domains and similar GO annotations of both the proteins indicate their similar functions. Five genes involved in oxidative stress response (Afua_4g04710, Afua_3g11330, Afua_1g10930, Afua_3g09950 and Afua_2g11510) were found to be positively selected. We also found the signal of positive selection in acyl-CoA thioesterase II coding gene Afua_1g15170 which highly downregulates during 16 h of conidia growth. The enzyme is involved in lipid metabolism [37] as mentioned in later in the manuscript however, its role in *A. fumigatus* is not yet characterized. The Afua_5g08930 *ivdA* gene codes for the isovaleryl-CoA dehydrogenase enzyme, which participates in branched-chain amino acid leucine catabolic process and fatty acid beta-oxidation, (and) was found to be evolved under positive selection. The distant bacterial homolog *Mycobacterium tuberculosis* acyl-CoA dehydrogenase *mbtN*, which shows about 25% sequence identity to *A. fumigatus* Isovaleryl-CoA dehydrogenase and contains similar domain composition and GO annotation, is considered a virulence factor because of its involvement in the production of mycobactins and possibly vital for the iron acquisition within the macrophage [38, 39]. Another mitochondrial enzyme 3-methylcrotonyl-CoA carboxylase subunit alpha coding gene Afua_5g08910 *mccA*, involved in leucine degradation, was evolved under positive selection. This enzyme showed differential expression during conidia growth [40], oxygen-limitation [41], and host neutrophil interaction [42]. In oxygen-limiting conditions many pathogens are able to use leucine as the sole carbon or nitrogen source through the leucine degradation pathway leading to acetyl-CoA and acetoacetate, an important end-product of energy metabolism [43].

In fact, metabolism cannot proceed without energy, and thus adaptive evolution is expected in genes involved in the energy production and conversion subcategory, if the host encounters

pathogen adaptation. *A. fumigatus* generate energy in vivo during fungal pathogenesis to drive the metabolic pathways required for fungal growth, host tissue invasion, and disease development using different mechanisms [44]. Mitochondrial respiration is active in hypoxia and critical for fungal pathogenesis [45, 46]. Interruption of mitochondrial respiration in *A. fumigatus* significantly impaired growth in normoxia and hypoxia, conidial germination, and displayed attenuated virulence [47]. Positive selection was observed for electron transfer flavoprotein-ubiquinone oxidoreductase (ETF:QO) coding gene Afua_3g10110, which is critical for linking central metabolism with the respiratory chain [48]. The Afua_3g08770 *grim-19* gene codes for the component of mitochondrial respiratory chain NADH:ubiquinone oxidoreductase complex (complex I), which catalyzes the first step of electron transfer in the oxidative phosphorylation system (OXPHOS), was positively selected. Elimination of *grim-19* destroys the assembly and electron transfer activity of complex I and influences the other complexes in the mitochondrial respiratory chain [49, 50]. It also influences the Warburg effect, favors cell protection and/or prevent aberrant cell growth, apoptosis and also appears to regulate innate and acquired immune responses in mouse models [50-52]. The subunit of mitochondrial matrix pyruvate dehydrogenase (PDH) complex, E1 component subunit alpha coding gene Afua_1g06960, was found to be rapidly evolved. The PDH complex catalyzes the overall conversion of pyruvate into acetyl-CoA and CO2, and thereby provides the primary link between the glycolytic pathway to the tricarboxylic acid (TCA) cycle. The product of PSG acetate kinase Afua_3g10750 *ack* gene converts acetyl phosphate to acetate along with the phosphorylation of ADP and is one of the most prominent ATP-generating reactions in anaerobic respiration. Therefore, *ack* is involved in glucose fermentation to acetate. The growth of a bacterial mutant lacking *ack* has been shown to be inhibited by glucose, suggesting that *ack* is involved in excretion of excess carbohydrate [53]. Positive selection of *A. fumigatus Ack* may therefore be the pathogens strategy to escape with the host interference during anaerobic respiration.

The amino acid asparagine is required for the synthesis of many important cellular proteins in normal cells. Differential expression of asparagine synthetase Afua_4g06900 *asn2* has been observed during early developmental stages of *A. fumigatus* conidia [40]. *asn2* and an another asparaginase family protein Afua_5g11570 showed the signature of positive selection. The positive selection was seen for the gene Afua_5g11450 which encodes for the regulatory protein of arginine metabolism. Arginine can be used as a nitrogen compound to repress the secretion of the alkaline protease that *A. fumigatus* uses to degrade and destroy the host complement system ability to make fungal hyphae susceptible to phagocytosis [54]. The

biosynthesis of arginine is important for pathogenicity, hyphal growth, and siderophore biosynthesis, which is coordinated by the supply of ornithine that is produced either by mitochondrial glutamate or in the cytosol by hydrolysis of arginine [55].

Cellular processes and signaling

A high number of PSGs annotated are involved in cellular processes and signaling (Additional file 2: Table S8). Majorly involved functional subcategories were signal transduction mechanisms; intracellular trafficking, secretion, and vesicular transport, posttranslational modification, protein turnover, chaperones, and others (Fig. 6). During the conidial growth, like other filamentous fungi, a germ tube apical extension of *A. fumigatus*, a mode of growth that involves the establishment of a stable axis of polarity, followed by the maintenance of growth in the same direction, is regulated by several polarity maintenance genes [56]. The ability to sustain polarization requires a constant stream of new cell wall and plasma membrane material to the hyphal apex [57]. The Centrin-binding protein coding gene Afua_1g13700 *sfi1*, essential for the initiation of spindle pole body duplication [58] and critical in the cell division cycle, was found to be positively selected. PSG Afua_3g14340 *mad2* which codes for the evolutionarily conserved central component of the mitotic checkpoint complex, is essential for the spindle assembly checkpoint mechanism [59]. PSG Afua_2g06110 *arp4* codes for a cytoskeletal protein involved in histone acetylation, histone exchange, and regulation of nucleosome density. In *C. albicans*, a mutant phenotype of *arp4* resulted in a decrease of cell size [60]. Several genes related to vesicles were positively selected (KOG class - 'U', Additional file 2: Table S8). Membrane-enclosed vesicles of the secretory system transport here components. The latter are required for membrane and cell wall biogenesis to the growing tip cell [61]. Besides involvement in growth, vesicles participate in importing nutrients from the host and exporting virulence factors to extracellular space. Differentially expressed PSGs during the conidia growth include the clathrin heavy chain, mitochondrial import receptor subunit Afua_1g11860 *tom22*, and Rab small monomeric GTPase Afua_5g12130 *rab7*. The clathrin heavy chain is involved in the formation of coated vesicles and clathrin-dependent endocytosis. *tom22* mediates protein import into the mitochondrial matrix and mitochondrial outer membrane [62]. *rab7* plays a central role in the transport steps connecting late endosomes to upstream and downstream endocytic compartments and possibly to the phagocytic route [63]. The components of the cytoplasmic dynein complex, dynein family protein coding gene Afua_5g08510 *nudN*, showed the signature of positive selection. The cytoplasmic dynein complex play roles in mitotic regulation and intracellular transport of vesicles and organelles

[64]. Selection pressure was also observed on several genes involved in signal transduction mechanisms (KOG class - 'T', Additional file 2: Table S8). For instance, Afua_4g04710 *pyp1*, which has an ortholog in *Schizosaccharomyces pombe,* is involved in MAP kinase tyrosine phosphatase activity, negative regulation of G2/M transition of mitotic cell cycle, stress response, and osmo-sensory signaling pathway [65] and was found to be evolved under positive selection. Since *pyp1* is annotated as negative regulator of stress-activated MAPK cascade, it probably can control the functional consequence of MAPK activity in *A. fumigatus,* such as cell wall integrity, fungal morphology, and oxidative stress [66]. Protein phosphatase 2C (Afua_1g15800) mutants of *A. fumigatus* grow better than wild type in iron excess conditions [67]. Besides serine/threonine phosphatase activity, protein phosphatase 2C orthologs in fungi are involved in diverse functions (reviewed in [68]), including the virulence [69] in human fungal pathogen *Candida albicans*. Another important gene that evolved under adaptive evolution includes Afua_4g08050 *sac1*, which is required for virulence of *C. albicans* and its mutants. This gene also has induces attenuated filamentous growth and biofilm formation, hypersensitivity to cell wall stresses, and changed content and distribution of chitin [70]. Furthermore, several genes coding for proteins involved in posttranslational modification, protein turnover, and chaperone activity were positively selected (KOG class - 'O', Additional file 1: Table S8). This subcategory also includes Afua_3g12290 *dim1*, which is an essential gene for the viability of *A. fumigatus* [71]. The ortholog of *dim1* is involved in mRNA splicing, regulation of DNA methylation, mitotic spindle formation, and chromosome segregation [72].

Information storage and processing

In this category 33 PSGs were identified and among them most participate in transcription and related processes (Additional file 1: Table S8). PSG Afua_3g10240 *not1* codes a scaffold protein which is a critical component of Ccr4-Not complex regulating gene expression, mRNA turnover, translational repression, and transcription [73]. Not protein subunits have been found to repress transcription of promoters without a canonical TATA sequence and among the different components of Ccr4-Not complex, only *not1* is known to be essential for yeast viability [73] and growth [74]. The complex in general is regulatory and it regulates cellular processes upon sensing the nutrient levels and other signals and it is thought to be required to keep cells in the appropriate state and to respond to extracellular signals [75]. The ortholog of Afua_2g05020 *dopA* codes a regulatory protein which has been discovered to play a role in several processes, including transport from Golgi to endosome, hyphal growth, cell morphogenesis, spatiotemporal organization of multicellular structures, conidium formation

and endoplasmic reticulum organization and viability [76]. PSG Afua_5g04200 *rpc82* codes for the RNA polymerase C subunit protein, which has been identified to be essential for yeast cell viability [77]. Additionally, this protein was also annotated with the GO term 'pathogenesis' (GO:0009405). The ortholog of C6 transcription factor (Afua_6g11110) regulates cell wall architecture, hyphal morphogenesis, virulence, and drug sensitivity in *C. albicans* [78]. In yeast, this protein participates in the regulation of glucose metabolism and fermentation [79]. Afua_5g12190 *taf30* codes a hub protein in the *A. fumigatus* interactome [80], which interacts with many complexes, such as general transcription factors that make up the RNA polymerase II preinitiation complex, chromatin remodeling complexes, and the histone modification enzymes. The highly conserved domain "Yeats" containing the *taf30* ortholog of yeast was found to affect the cell growth in a negative way, in which cells grow better than wild-type in stress conditions when this domain is absent [81]. Some genes involved in the initiation of transcription for instance, Afua_8g04950 *tsm1* and Afua_8g04350, were also found to be evolved under positive selection. Moreover, positive selected genes are involved in DNA replication, recombination, and repair. For instance, gene Afua_2g10140 *mcm7* that codes a part of MCM complex, which plays a role in initiation and regulation of DNA replication, was found to be positively selected. Higher amounts of evolutionarily conserved MCM proteins has been observed in proliferating cells [82]. Afua_5g03320 *ssl2* provides DNA helicase functions and participates in transcription and DNA repair. Furthermore, the ortholog of *ssl2* leads to growth defects when mutated in yeast [83] and is also essential for cell viability [84]. Under the subcategory RNA processing and modification, Afua_1g14310 *pan3*, the required subunit of the PAN enzyme the function of which is to shorten mRNA poly(A) tails [85] and regulate the activity of post replication repair complexes [86], was identified.

References

1. Arenas M: **The importance and application of the ancestral recombination graph.** *Front Genet* 2013, **4**:206.
2. Awadalla P: **The evolutionary genomics of pathogen recombination.** *Nat Rev Genet* 2003, **4**:50-60.
3. Hudson RR: **Properties of a neutral allele model with intragenic recombination.** *Theor Popul Biol* 1983, **23**:183-201.
4. Shriner D, Nickle DC, Jensen MA, Mullins JI: **Potential impact of recombination on sitewise approaches for detecting positive natural selection.** *Genet Res* 2003, **81**:115-121.

5. Kosakovsky Pond SL, Posada D, Gravenor MB, Woelk CH, Frost SD: **Automated phylogenetic detection of recombination using a genetic algorithm.** *Mol Biol Evol* 2006, **23**:1891-1901.
6. Nielsen R, Yang Z: **Likelihood models for detecting positively selected amino acid sites and applications to the HIV-1 envelope gene.** *Genetics* 1998, **148**:929-936.
7. Anisimova M, Nielsen R, Yang Z: **Effect of recombination on the accuracy of the likelihood method for detecting positive selection at amino acid sites.** *Genetics* 2003, **164**:1229-1236.
8. Kosakovsky Pond SL, Posada D, Gravenor MB, Woelk CH, Frost SD: **GARD: a genetic algorithm for recombination detection.** *Bioinformatics* 2006, **22**:3096-3098.
9. Archer J, Pinney JW, Fan J, Simon-Loriere E, Arts EJ, Negroni M, Robertson DL: **Identifying the important HIV-1 recombination breakpoints.** *PLoS Comput Biol* 2008, **4**:e1000178.
10. Parsons PA: **Evolutionary Rates - Effects of Stress Upon Recombination.** *Biological Journal of the Linnean Society* 1988, **35**:49-68.
11. Bellini A, Girard PM, Lambert S, Tessier L, Sage E, Francesconi S: **Stress activated protein kinase pathway modulates homologous recombination in fission yeast.** *PLoS One* 2012, **7**:e47987.
12. Zhong WH, Priest NK: **Stress-induced recombination and the mechanism of evolvability.** *Behavioral Ecology and Sociobiology* 2011, **65**:493-502.
13. Semighini CP, von Zeska Kress Fagundes MR, Ferreira JC, Pascon RC, de Souza Goldman MH, Goldman GH: **Different roles of the Mre11 complex in the DNA damage response in Aspergillus nidulans.** *Mol Microbiol* 2003, **48**:1693-1709.
14. Malavazi I, Lima JF, von Zeska Kress Fagundes MR, Efimov VP, de Souza Goldman MH, Goldman GH: **The Aspergillus nidulans sldI(RAD50) gene interacts with bimE(APC1), a homologue of an anaphase-promoting complex subunit.** *Mol Microbiol* 2005, **57**:222-237.
15. Krappmann S, Sasse C, Braus GH: **Gene targeting in Aspergillus fumigatus by homologous recombination is facilitated in a nonhomologous end- joining-deficient genetic background.** *Eukaryot Cell* 2006, **5**:212-215.
16. Karanyi Z, Holb I, Hornok L, Pocsi I, Miskei M: **FSRD: fungal stress response database.** *Database (Oxford)* 2013, **2013**:bat037.
17. Chitty JL, Fraser JA: **Purine Acquisition and Synthesis by Human Fungal Pathogens.** *Microorganisms* 2017, **5**.
18. Kowalski D, Pendyala L, Daignan-Fornier B, Howell SB, Huang RY: **Dysregulation of purine nucleotide biosynthesis pathways modulates cisplatin cytotoxicity in Saccharomyces cerevisiae.** *Mol Pharmacol* 2008, **74**:1092-1100.
19. Cameron A, Huynh S, Scott NE, Frirdich E, Apel D, Foster LJ, Parker CT, Gaynor EC: **High-Frequency Variation of Purine Biosynthesis Genes Is a Mechanism of Success in Campylobacter jejuni.** *MBio* 2015, **6**:e00612-00615.
20. Lisby M, Rothstein R: **Cell biology of mitotic recombination.** *Cold Spring Harb Perspect Biol* 2015, **7**:a016535.
21. Xu X, Xiong X, Sun Y: **The role of ribosomal proteins in the regulation of cell proliferation, tumorigenesis, and genomic integrity.** *Sci China Life Sci* 2016, **59**:656-672.
22. Nakamura S, Sato H, Tanaka R, Yaguchi T: **Verification of Ribosomal Proteins of Aspergillus fumigatus for Use as Biomarkers in MALDI-TOF MS Identification.** *Mass Spectrom (Tokyo)* 2016, **5**:A0049.
23. Kim TH, Leslie P, Zhang Y: **Ribosomal proteins as unrevealed caretakers for cellular stress and genomic instability.** *Oncotarget* 2014, **5**:860-871.
24. Bhabhra R, Askew DS: **Thermotolerance and virulence of Aspergillus fumigatus: role of the fungal nucleolus.** *Med Mycol* 2005, **43 Suppl 1**:S87-93.
25. Winther MD, Stevens L: **RNA synthesis during the germination of conidia of Aspergillus nidulans.** *Microbios* 1981, **30**:153-162.
26. Mirkes PE: **Polysomes, ribonucleic acid, and protein synthesis during germination of Neurospora crassa conidia.** *J Bacteriol* 1974, **117**:196-202.

27. Brogden KA, Phillips M, Thurston JR, Richard JL: **Electron microscopic examination of ribosome preparations from germinated spores of Aspergillus fumigatus.** *Mycopathologia* 1984, **86:**59-64.
28. Li X, Gao M, Han X, Tao S, Zheng D, Cheng Y, Yu R, Han G, Schmidt M, Han L: **Disruption of the phospholipase D gene attenuates the virulence of Aspergillus fumigatus.** *Infect Immun* 2012, **80:**429-440.
29. Sung TC, Roper RL, Zhang Y, Rudge SA, Temel R, Hammond SM, Morris AJ, Moss B, Engebrecht J, Frohman MA: **Mutagenesis of phospholipase D defines a superfamily including a trans-Golgi viral protein required for poxvirus pathogenicity.** *EMBO J* 1997, **16:**4519-4530.
30. Rose K, Rudge SA, Frohman MA, Morris AJ, Engebrecht J: **Phospholipase D signaling is essential for meiosis.** *Proc Natl Acad Sci U S A* 1995, **92:**12151-12155.
31. Cray JA, Bell AN, Bhaganna P, Mswaka AY, Timson DJ, Hallsworth JE: **The biology of habitat dominance; can microbes behave as weeds?** *Microb Biotechnol* 2013, **6:**453-492.
32. Flipphi M, Sun J, Robellet X, Karaffa L, Fekete E, Zeng AP, Kubicek CP: **Biodiversity and evolution of primary carbon metabolism in Aspergillus nidulans and other Aspergillus spp.** *Fungal Genet Biol* 2009, **46 Suppl 1:**S19-S44.
33. Ebel F, Schwienbacher M, Beyer J, Heesemann J, Brakhage AA, Brock M: **Analysis of the regulation, expression, and localisation of the isocitrate lyase from Aspergillus fumigatus, a potential target for antifungal drug development.** *Fungal Genet Biol* 2006, **43:**476-489.
34. Bishai W: **Lipid lunch for persistent pathogen.** *Nature* 2000, **406:**683-685.
35. Olivas I, Royuela M, Romero B, Monteiro MC, Minguez JM, Laborda F, De Lucas JR: **Ability to grow on lipids accounts for the fully virulent phenotype in neutropenic mice of Aspergillus fumigatus null mutants in the key glyoxylate cycle enzymes.** *Fungal Genet Biol* 2008, **45:**45-60.
36. Moore TS, Du Z, Chen Z: **Membrane lipid biosynthesis in Chlamydomonas reinhardtii. In vitro biosynthesis of diacylglyceryltrimethylhomoserine.** *Plant Physiol* 2001, **125:**423-429.
37. Hunt MC, Siponen MI, Alexson SE: **The emerging role of acyl-CoA thioesterases and acyltransferases in regulating peroxisomal lipid metabolism.** *Biochim Biophys Acta* 2012, **1822:**1397-1410.
38. Madigan CA, Martinot AJ, Wei JR, Madduri A, Cheng TY, Young DC, Layre E, Murry JP, Rubin EJ, Moody DB: **Lipidomic analysis links mycobactin synthase K to iron uptake and virulence in M. tuberculosis.** *PLoS Pathog* 2015, **11:**e1004792.
39. De Voss JJ, Rutter K, Schroeder BG, Su H, Zhu Y, Barry CE, 3rd: **The salicylate-derived mycobactin siderophores of Mycobacterium tuberculosis are essential for growth in macrophages.** *Proc Natl Acad Sci U S A* 2000, **97:**1252-1257.
40. Cagas SE, Jain MR, Li H, Perlin DS: **The proteomic signature of Aspergillus fumigatus during early development.** *Mol Cell Proteomics* 2011, **10:**M111 010108.
41. Kroll K, Pahtz V, Hillmann F, Vaknin Y, Schmidt-Heck W, Roth M, Jacobsen ID, Osherov N, Brakhage AA, Kniemeyer O: **Identification of hypoxia-inducible target genes of Aspergillus fumigatus by transcriptome analysis reveals cellular respiration as an important contributor to hypoxic survival.** *Eukaryot Cell* 2014, **13:**1241-1253.
42. McDonagh A, Fedorova ND, Crabtree J, Yu Y, Kim S, Chen D, Loss O, Cairns T, Goldman G, Armstrong-James D, et al: **Sub-telomere directed gene expression during initiation of invasive aspergillosis.** *PLoS Pathog* 2008, **4:**e1000154.
43. Tielens AG, van Grinsven KW, Henze K, van Hellemond JJ, Martin W: **Acetate formation in the energy metabolism of parasitic helminths and protists.** *Int J Parasitol* 2010, **40:**387-397.
44. Willger SD, Grahl N, Cramer RA, Jr.: **Aspergillus fumigatus metabolism: clues to mechanisms of in vivo fungal growth and virulence.** *Med Mycol* 2009, **47 Suppl 1:**S72-79.

45. Poyton RO, Castello PR, Ball KA, Woo DK, Pan N: **Mitochondria and hypoxic signaling: a new view.** *Ann N Y Acad Sci* 2009, **1177:**48-56.
46. Kwast KE, Burke PV, Staahl BT, Poyton RO: **Oxygen sensing in yeast: evidence for the involvement of the respiratory chain in regulating the transcription of a subset of hypoxic genes.** *Proc Natl Acad Sci U S A* 1999, **96:**5446-5451.
47. Grahl N, Dinamarco TM, Willger SD, Goldman GH, Cramer RA: **Aspergillus fumigatus mitochondrial electron transport chain mediates oxidative stress homeostasis, hypoxia responses and fungal pathogenesis.** *Mol Microbiol* 2012, **84:**383-399.
48. Watmough NJ, Frerman FE: **The electron transfer flavoprotein: ubiquinone oxidoreductases.** *Biochim Biophys Acta* 2010, **1797:**1910-1916.
49. Lu H, Cao X: **GRIM-19 is essential for maintenance of mitochondrial membrane potential.** *Mol Biol Cell* 2008, **19:**1893-1902.
50. Huang G, Lu H, Hao A, Ng DC, Ponniah S, Guo K, Lufei C, Zeng Q, Cao X: **GRIM-19, a cell death regulatory protein, is essential for assembly and function of mitochondrial complex I.** *Mol Cell Biol* 2004, **24:**8447-8456.
51. Nallar SC, Kalvakolanu DV: **GRIM-19: A master regulator of cytokine induced tumor suppression, metastasis and energy metabolism.** *Cytokine Growth Factor Rev* 2017, **33:**1-18.
52. Maximo V, Botelho T, Capela J, Soares P, Lima J, Taveira A, Amaro T, Barbosa AP, Preto A, Harach HR, et al: **Somatic and germline mutation in GRIM-19, a dual function gene involved in mitochondrial metabolism and cell death, is linked to mitochondrion-rich (Hurthle cell) tumours of the thyroid.** *Br J Cancer* 2005, **92:**1892-1898.
53. Grundy FJ, Waters DA, Allen SH, Henkin TM: **Regulation of the Bacillus subtilis acetate kinase gene by CcpA.** *J Bacteriol* 1993, **175:**7348-7355.
54. Rambach G, Dum D, Mohsenipour I, Hagleitner M, Wurzner R, Lass-Florl C, Speth C: **Secretion of a fungal protease represents a complement evasion mechanism in cerebral aspergillosis.** *Mol Immunol* 2010, **47:**1438-1449.
55. Schafferer L, Beckmann N, Binder U, Brosch G, Haas H: **AmcA-a putative mitochondrial ornithine transporter supporting fungal siderophore biosynthesis.** *Front Microbiol* 2015, **6:**252.
56. Momany M: **Polarity in filamentous fungi: establishment, maintenance and new axes.** *Curr Opin Microbiol* 2002, **5:**580-585.
57. Steinberg G: **Hyphal growth: a tale of motors, lipids, and the Spitzenkorper.** *Eukaryot Cell* 2007, **6:**351-360.
58. Lee IJ, Wang N, Hu W, Schott K, Bahler J, Giddings TH, Jr., Pringle JR, Du LL, Wu JQ: **Regulation of spindle pole body assembly and cytokinesis by the centrin-binding protein Sfi1 in fission yeast.** *Mol Biol Cell* 2014, **25:**2735-2749.
59. Nezi L, Rancati G, De Antoni A, Pasqualato S, Piatti S, Musacchio A: **Accumulation of Mad2-Cdc20 complex during spindle checkpoint activation requires binding of open and closed conformers of Mad2 in Saccharomyces cerevisiae.** *J Cell Biol* 2006, **174:**39-51.
60. Lu Y, Su C, Mao X, Raniga PP, Liu H, Chen J: **Efg1-mediated recruitment of NuA4 to promoters is required for hypha-specific Swi/Snf binding and activation in Candida albicans.** *Mol Biol Cell* 2008, **19:**4260-4272.
61. Riquelme M, Yarden O, Bartnicki-Garcia S, Bowman B, Castro-Longoria E, Free SJ, Fleissner A, Freitag M, Lew RR, Mourino-Perez R, et al: **Architecture and development of the Neurospora crassa hypha -- a model cell for polarized growth.** *Fungal Biol* 2011, **115:**446-474.
62. Wiedemann N, Frazier AE, Pfanner N: **The protein import machinery of mitochondria.** *J Biol Chem* 2004, **279:**14473-14476.
63. Novick P, Zerial M: **The diversity of Rab proteins in vesicle transport.** *Curr Opin Cell Biol* 1997, **9:**496-504.
64. Zhang J, Li S, Musa S, Zhou H, Xiang X: **Dynein light intermediate chain in Aspergillus nidulans is essential for the interaction between heavy and intermediate chains.** *J Biol Chem* 2009, **284:**34760-34768.

65. Santo PD, Blanchard B, Hoffman CS: **The Schizosaccharomyces pombe pyp1 protein tyrosine phosphatase negatively regulates nutrient monitoring pathways.** *J Cell Sci* 1996, **109 (Pt 7)**:1919-1925.
66. Valiante V, Heinekamp T, Jain R, Hartl A, Brakhage AA: **The mitogen-activated protein kinase MpkA of Aspergillus fumigatus regulates cell wall signaling and oxidative stress response.** *Fungal Genet Biol* 2008, **45**:618-627.
67. Winkelstroter LK, Dolan SK, Fernanda Dos Reis T, Bom VL, Alves de Castro P, Hagiwara D, Alowni R, Jones GW, Doyle S, Brown NA, Goldman GH: **Systematic Global Analysis of Genes Encoding Protein Phosphatases in Aspergillus fumigatus.** *G3 (Bethesda)* 2015, **5**:1525-1539.
68. Arino J, Casamayor A, Gonzalez A: **Type 2C protein phosphatases in fungi.** *Eukaryot Cell* 2011, **10**:21-33.
69. Feng J, Zhao J, Li J, Zhang L, Jiang L: **Functional characterization of the PP2C phosphatase CaPtc2p in the human fungal pathogen Candida albicans.** *Yeast* 2010, **27**:753-764.
70. Zhang B, Yu Q, Jia C, Wang Y, Xiao C, Dong Y, Xu N, Wang L, Li M: **The actin-related protein Sac1 is required for morphogenesis and cell wall integrity in Candida albicans.** *Fungal Genet Biol* 2015, **81**:261-270.
71. Carr PD, Tuckwell D, Hey PM, Simon L, d'Enfert C, Birch M, Oliver JD, Bromley MJ: **The transposon impala is activated by low temperatures: use of a controlled transposition system to identify genes critical for viability of Aspergillus fumigatus.** *Eukaryot Cell* 2010, **9**:438-448.
72. Berry LD, Gould KL: **Fission yeast dim1(+) encodes a functionally conserved polypeptide essential for mitosis.** *J Cell Biol* 1997, **137**:1337-1354.
73. Bhaskar V, Basquin J, Conti E: **Architecture of the ubiquitylation module of the yeast Ccr4-Not complex.** *Structure* 2015, **23**:921-928.
74. Maillet L, Tu C, Hong YK, Shuster EO, Collart MA: **The essential function of Not1 lies within the Ccr4-Not complex.** *J Mol Biol* 2000, **303**:131-143.
75. Collart MA: **Global control of gene expression in yeast by the Ccr4-Not complex.** *Gene* 2003, **313**:1-16.
76. Pascon RC, Miller BL: **Morphogenesis in Aspergillus nidulans requires Dopey (DopA), a member of a novel family of leucine zipper-like proteins conserved from yeast to humans.** *Mol Microbiol* 2000, **36**:1250-1264.
77. Chiannilkulchai N, Stalder R, Riva M, Carles C, Werner M, Sentenac A: **RPC82 encodes the highly conserved, third-largest subunit of RNA polymerase C (III) from Saccharomyces cerevisiae.** *Mol Cell Biol* 1992, **12**:4433-4440.
78. Moreno I, Martinez-Esparza M, Laforet LC, Sentandreu R, Ernst JF, Valentin E: **Dosage-dependent roles of the Cwt1 transcription factor for cell wall architecture, morphogenesis, drug sensitivity and virulence in Candida albicans.** *Yeast* 2010, **27**:77-87.
79. Gasmi N, Jacques PE, Klimova N, Guo X, Ricciardi A, Robert F, Turcotte B: **The switch from fermentation to respiration in Saccharomyces cerevisiae is regulated by the Ert1 transcriptional activator/repressor.** *Genetics* 2014, **198**:547-560.
80. Kaltdorf M, Srivastava M, Gupta SK, Liang C, Binder J, Dietl AM, Meir Z, Haas H, Osherov N, Krappmann S, Dandekar T: **Systematic Identification of Anti-Fungal Drug Targets by a Metabolic Network Approach.** *Front Mol Biosci* 2016, **3**:22.
81. Schulze JM, Kane CM, Ruiz-Manzano A: **The YEATS domain of Taf14 in Saccharomyces cerevisiae has a negative impact on cell growth.** *Mol Genet Genomics* 2010, **283**:365-380.
82. Tye BK: **MCM proteins in DNA replication.** *Annu Rev Biochem* 1999, **68**:649-686.
83. Flom G, Weekes J, Johnson JL: **Novel interaction of the Hsp90 chaperone machine with Ssl2, an essential DNA helicase in Saccharomyces cerevisiae.** *Curr Genet* 2005, **47**:368-380.
84. Qiu H, Park E, Prakash L, Prakash S: **The Saccharomyces cerevisiae DNA repair gene RAD25 is required for transcription by RNA polymerase II.** *Genes Dev* 1993, **7**:2161-2171.

85. Brown CE, Tarun SZ, Jr., Boeck R, Sachs AB: **PAN3 encodes a subunit of the Pab1p-dependent poly(A) nuclease in Saccharomyces cerevisiae.** *Mol Cell Biol* 1996, **16**:5744-5753.

86. Hammet A, Pike BL, Heierhorst J: **Posttranscriptional regulation of the RAD5 DNA repair gene by the Dun1 kinase and the Pan2-Pan3 poly(A)-nuclease complex contributes to survival of replication blocks.** *J Biol Chem* 2002, **277**:22469-22474.

Supplementary figures

Figure S1: Proteins-protein interactions (PPIs) map. PPIs between *A. fumigatus* proteins coded by positively selected genes (PSGs) and human proteins. Orange - *A. fumigatus* proteins and blue - human proteins.

Figure S2: Gene Ontology (GO) overrepresentation analysis. Biological process (GO) significantly overrepresented in human proteins targeted by *A. fumigatus* virulence related proteins coded by multi-copy category positively selected genes (PSGs).

Figure S3: *A. fumigatus* and *A. fischeri* genome synteny map. Image was generated using the Mauve genome alignment tool. Upper and lower lines of the genomes correspond to *A. fumigatus* and *A. fischeri* respectively. Red vertical bars indicate concatenated chromosomal boundaries. Color-coded syntenic blocks indicate conserved segments (LCBs; Locally Collinear Blocks) identified by Mauve (minimum LCB weight = 999). Plots of sequence similarity are shown within each syntenic block. Regions with no color indicate no detectable homology between the two genomes with the settings used in Mauve. Unmatched regions (white area) within an LCBs indicate the presence of strain-specific sequence. The connecting lines between blocks indicate the location of each block in two genomes. Homologous regions with possible rearrangements are shown in first row.

Figure S1

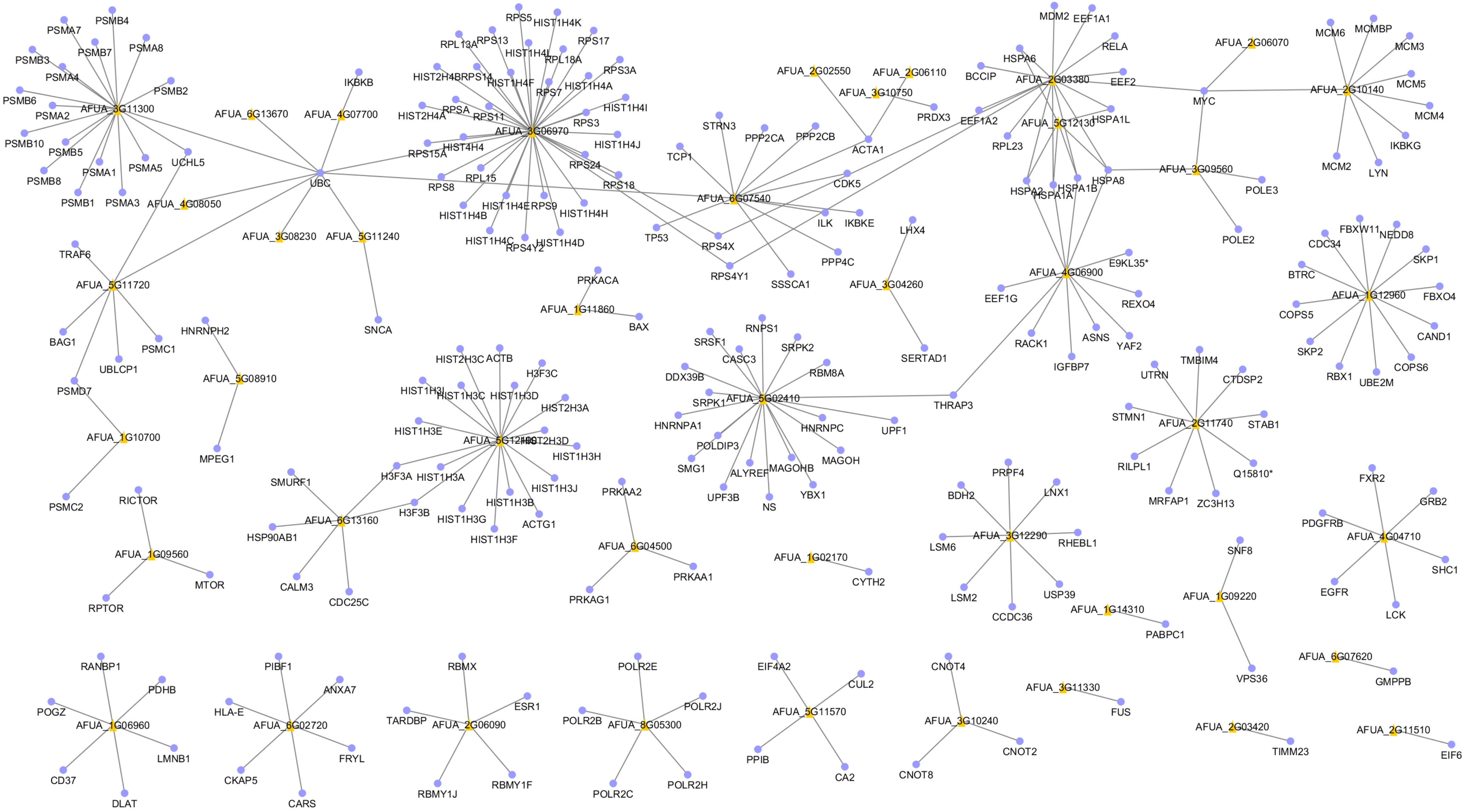

PSMB4
PSMA7
PSMB7
PSMA8
PSMB3
PSMA4
PSMB2
PSMB6
PSMA2
AFUA_3G11300
PSMB10
PSMB5
UCHL5
PSMA5
PSMA1
PSMB8
PSMB1
PSMA3
AFUA_4G08050
IKBKB
AFUA_6G13670
AFUA_4G07700
UBC
AFUA_3G08230
AFUA_5G11240
SNCA
TRAF6
AFUA_5G11720
BAG1
PSMC1
UBLCP1
PSMD7
HNRNPH2
AFUA_5G08910
MPEG1
AFUA_1G10700
PSMC2
RICTOR
AFUA_1G09560
MTOR
RPTOR
RPS5
HIST1H4K
RPL13A
RPS13
RPS17
HIST1H4L
RPL18A
RPS3A
HIST2H4B
RPS14
HIST1H4F
RPS7
HIST1H4A
HIST2H4A
RPSA
RPS11
HIST1H4I
AFUA_3G06970
RPS3
RPS15A
HIST4H4
HIST1H4J
RPS24
RPL15
RPS8
RPS18
HIST1H4E
RPS9
HIST1H4H
HIST1H4B
HIST1H4C
RPS4Y2
HIST1H4D
TCP1
STRN3
PPP2CA
PPP2CB
AFUA_2G02550
AFUA_2G06110
AFUA_3G10750
PRDX3
ACTA1
CDK5
AFUA_6G07540
ILK
IKBKE
TP53
RPS4X
RPS4Y1
SSSCA1
PPP4C
MDM2
EEF1A1
RELA
HSPA6
BCCIP
AFUA_2G03380
EEF2
EEF1A2
AFUA_5G12130
HSPA1L
RPL23
HSPA2
HSPA1B
HSPA1A
HSPA8
AFUA_2G06070
MYC
AFUA_3G09560
POLE3
POLE2
MCM6
MCMBP
MCM3
AFUA_2G10140
MCM5
MCM4
IKBKG
MCM2
LYN
LHX4
AFUA_3G04260
SERTAD1
E9KL35*
AFUA_4G06900
EEF1G
REXO4
RACK1
ASNS
YAF2
IGFBP7
FBXW11
NEDD8
CDC34
SKP1
BTRC
AFUA_1G12960
FBXO4
COPS5
CAND1
SKP2
RBX1
UBE2M
COPS6
PRKACA
AFUA_1G11860
BAX
RNPS1
SRSF1
SRPK2
CASC3
RBM8A
DDX39B
SRPK1
AFUA_5G02410
THRAP3
HNRNPA1
HNRNPC
UPF1
POLDIP3
MAGOH
SMG1
ALYREF
MAGOHB
UPF3B
NS
YBX1
HIST2H3C
ACTB
H3F3C
HIST1H3I
HIST1H3C
HIST1H3D
HIST2H3A
HIST1H3E
AFUA_5G12180
HIST2H3D
HIST1H3H
H3F3A
HIST1H3A
HIST1H3J
SMURF1
H3F3B
HIST1H3B
HIST1H3G
ACTG1
HIST1H3F
AFUA_6G13160
HSP90AB1
CALM3
CDC25C
PRKAA2
AFUA_6G04500
PRKAA1
PRKAG1
AFUA_1G02170
CYTH2
TMBIM4
UTRN
CTDSP2
STMN1
AFUA_2G11740
STAB1
RILPL1
Q15810*
MRFAP1
ZC3H13
PRPF4
BDH2
LNX1
AFUA_3G12290
RHEBL1
LSM6
LSM2
USP39
CCDC36
AFUA_1G14310
PABPC1
SNF8
AFUA_1G09220
VPS36
FXR2
GRB2
PDGFRB
AFUA_4G04710
SHC1
EGFR
LCK
AFUA_6G07620
GMPPB
RANBP1
PDHB
POGZ
AFUA_1G06960
LMNB1
CD37
DLAT
PIBF1
ANXA7
HLA-E
AFUA_6G02720
FRYL
CKAP5
CARS
RBMX
ESR1
TARDBP
AFUA_2G06090
RBMY1J
RBMY1F
POLR2E
POLR2J
POLR2B
AFUA_8G05300
POLR2C
POLR2H
EIF4A2
CUL2
AFUA_5G11570
PPIB
CA2
CNOT4
AFUA_3G10240
CNOT2
CNOT8
AFUA_3G11330
FUS
AFUA_2G03420
TIMM23
AFUA_2G11510
EIF6

Figure S2

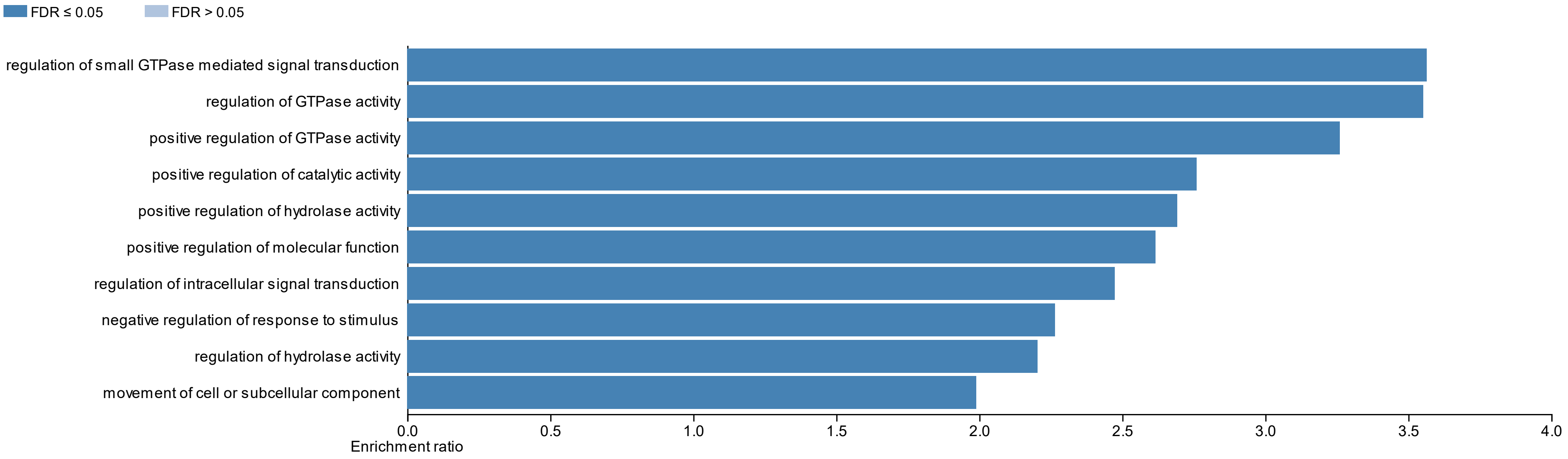
FDR ≤ 0.05
FDR > 0.05
regulation of small GTPase mediated signal transduction
regulation of GTPase activity
positive regulation of GTPase activity
positive regulation of catalytic activity
positive regulation of hydrolase activity
positive regulation of molecular function
regulation of intracellular signal transduction
negative regulation of response to stimulus
regulation of hydrolase activity
movement of cell or subcellular component
0.0
0.5
1.0
1.5
2.0
2.5
3.0
3.5
4.0
Enrichment ratio

Figure S3

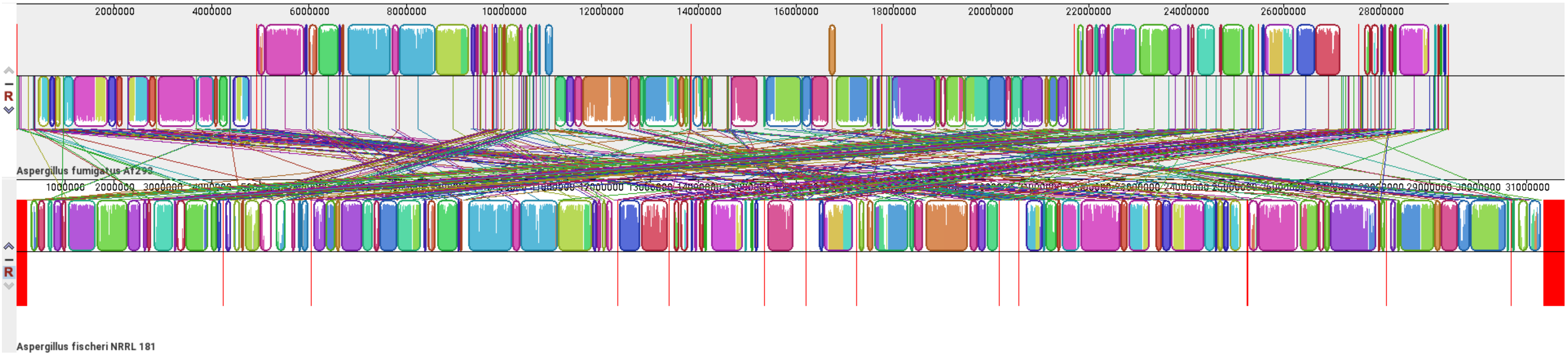

2000000
4000000
6000000
8000000
10000000
12000000
14000000
16000000
18000000
20000000
22000000
24000000
26000000
28000000
Aspergillus fumigatus Af293
Aspergillus fischeri NRRL 181

Additional data file 1: Clusters of orthologues identified by OrthoMCL (inflation index: 1.5). Abbreviations - abra : *Aspergillus brasiliensis*, acar : *A. carbonarius*, acla : *A. clavatus*, afis : *A. fischeri*, afla : *A. flavus*, afum : *A. fumigatus*, agla : *A. glaucus*, akaw : *A. kawachii*, alue : *A. luchuensis*, anid : *A. nidulans*, anig : *A. niger*, aora : *A. oryzae*, asyd : *A. sydowii*, ater : *A. terreus*, atub : *A. tubingenesis*, aver : *A. versicolor*, awen : *A. wentii*, azon : *A. zonatus*.

Group1: abra|OJJ66207.1 abra|OJJ67944.1 abra|OJJ69734.1 abra|OJJ73921.1 abra|OJJ75305.1 abra|OJJ75840.1 abra|OJJ73699.1 abra|OJJ69537.1 abra|OJJ78094.1 acar|OOF90653.1 acar|OOF94187.1 acar|OOG00776.1 acar|OOG01052.1 acar|OOF92566.1 acar|OOF93189.1 acar|OOF97512.1 acar|OOF98777.1 acla|EAW07798.1 acla|EAW08899.1 acla|EAW11681.1 akaw|GAA89528.1 akaw|GAA90187.1 akaw|GAA91252.1 aluc|OJZ80511.1 aluc|OJZ82816.1 aluc|OJZ88125.1 aluc|OJZ89282.1 aluc|OJZ83162.1 anid|CBF73441.1 anid|CBF90374.1 anid|CBF87872.1 anig|EHA17891.1 anig|EHA21256.1 anig|EHA21434.1 anig|EHA25711.1 anig|EHA28226.1 anig|EHA18088.1 anig|EHA19858.1 anig|EHA25622.1 anig|EHA25682.1 anig|EHA23283.1 aory|EIT74664.1 aory|EIT83097.1 aory|EIT80579.1 aory|EIT81467.1 aory|EIT83286.1 asyd|OJJ56148.1 asyd|OJJ63431.1 atub|OJI87775.1 atub|OJI89047.1 atub|OJI90732.1 atub|OJI88419.1 aver|OJI97506.1 aver|OJI99587.1 aver|OJJ03355.1 aver|OJJ05088.1 aver|OJJ08114.1 aver|OJJ03487.1 awen|OJJ29812.1 awen|OJJ35864.1 awen|OJJ38889.1 azon|OJJ42489.1 azon|OJJ44494.1 azon|OJJ46205.1 azon|OJJ47369.1 azon|OJJ45854.1 afis|EAW22835.1 afla|EED50110.1 afla|EED51745.1 afum|EAL90367.2 agla|OJJ78560.1 akaw|GAA89080.1 aory|EIT81736.1 ater|EAU35730.1 abra|OJJ67593.1 akaw|GAA89112.1 atub|OJI88453.1 acar|OOF91540.1 abra|OJJ70223.1 afla|EED52040.1 afum|EAL91343.1 akaw|GAA85904.1 aluc|OJZ91163.1 anid|CBF80212.1 atub|OJI83179.1 acar|OOF96620.1 akaw|GAA84550.1 atub|OJI85517.1 afla|EED52433.1 acla|EAW10758.1 afis|EAW16932.1 afla|EED48405.1 afla|EED51820.1 afla|EED51822.1 afla|EED52154.1 aory|EIT74440.1 anid|CBF73982.1 ater|EAU33387.1

Group2: abra|OJJ67588.1 abra|OJJ73922.1 abra|OJJ75304.1 acar|OOF99263.1 acar|OOG01053.1 acar|OOF99291.1 acar|OOF99559.1 acla|EAW07086.1 acla|EAW08665.1 acla|EAW08900.1 acla|EAW11679.1 acla|EAW15286.1 acla|EAW07799.1 afis|EAW16180.1 afis|EAW16446.1 afis|EAW20684.1 afla|EED44993.1 afla|EED47804.1 afla|EED49850.1 afla|EED50111.1 afla|EED52159.1 afum|EAL89049.1 afum|EAL91342.1 akaw|GAA82422.1 akaw|GAA89107.1 akaw|GAA89529.1 anid|CBF73453.1 anid|CBF87069.1 anid|CBF76036.1 anid|CBF87869.1 anid|CBF90372.1 anig|EHA17890.1 anig|EHA25707.1 anig|EHA20896.1 anig|EHA21433.1 anig|EHA24938.1 aory|EIT73739.1 aory|EIT74412.1 aory|EIT74615.1 aory|EIT80956.1 asyd|OJJ56147.1 asyd|OJJ63429.1 ater|EAU29302.1 ater|EAU30156.1 ater|EAU37759.1 ater|EAU30201.1 atub|OJI86150.1 atub|OJI88448.1 atub|OJI88518.1 atub|OJI90731.1 aver|OJI97508.1 aver|OJI99595.1 aver|OJJ03909.1 aver|OJJ05087.1 aver|OJI99581.1 aver|OJI99596.1 aver|OJJ07364.1 aver|OJJ08120.1 aver|OJJ08464.1 aver|OJI99625.1 aver|OJJ07311.1 aver|OJJ08781.1 awen|OJJ36076.1 awen|OJJ38362.1 awen|OJJ32929.1 awen|OJJ38888.1 azon|OJJ42030.1 azon|OJJ42483.1 azon|OJJ46579.1 azon|OJJ48580.1 afis|EAW22836.1 afum|EAL90366.1 agla|OJJ80563.1 aluc|OJZ85583.1 aluc|OJZ88124.1 asyd|OJJ61536.1 ater|EAU38874.1 awen|OJJ32292.1 abra|OJJ67652.1 afis|EAW16453.1 afis|EAW25605.1 agla|OJJ79232.1 akaw|GAA89168.1 aluc|OJZ81665.1 anig|EHA25778.1 ater|EAU33874.1 aver|OJJ07366.1 azon|OJJ50363.1 acla|EAW14636.1 ater|EAU36802.1 awen|OJJ34576.1 afum|EAL88812.1 aver|OJI99592.1 asyd|OJJ52456.1 aver|OJJ03957.1 awen|OJJ34650.1

Group3: abra|OJJ67584.1 abra|OJJ67975.1 abra|OJJ75778.1 abra|OJJ77754.1 acar|OOF92816.1 acar|OOF96723.1 acla|EAW08895.1 acla|EAW11760.1 acla|EAW12170.1 acla|EAW12336.1 acla|EAW14856.1 afla|EED47354.1 afla|EED52019.1 afla|EED47890.1 afla|EED48583.1 agla|OJJ79933.1 agla|OJJ80111.1 agla|OJJ83619.1 agla|OJJ86349.1 akaw|GAA84491.1 akaw|GAA85345.1 akaw|GAA87318.1 akaw|GAA91228.1 akaw|GAA92525.1 akaw|GAA90346.1 akaw|GAA85925.1 aluc|OJZ82842.1 aluc|OJZ85886.1 aluc|OJZ89214.1 aluc|OJZ90707.1 aluc|OJZ91539.1 aluc|OJZ91139.1 anid|CBF71467.1 anid|CBF83055.1 anid|CBF86052.1 anid|CBF87072.1 anid|CBF88289.1 anig|EHA19567.1 anig|EHA20193.1 anig|EHA21280.1 anig|EHA22576.1 anig|EHA24574.1 anig|EHA24700.1 anig|EHA28244.1 aory|EIT73118.1 aory|EIT76300.1 aory|EIT77964.1 aory|EIT81441.1 aory|EIT82867.1 ater|EAU29417.1 ater|EAU31921.1 ater|EAU32663.1 ater|EAU36720.1 atub|OJI79672.1 atub|OJI83150.1 atub|OJI85993.1 atub|OJI91114.1 atub|OJI82134.1 atub|OJI85578.1 atub|OJI89072.1 atub|OJI82802.1 aver|OJI96730.1 aver|OJJ05906.1 awen|OJJ29758.1 awen|OJJ34705.1 awen|OJJ40111.1 awen|OJJ29917.1 awen|OJJ30910.1 awen|OJJ38044.1 awen|OJJ39589.1 awen|OJJ29944.1 afis|EAW23793.1 afum|EAL87227.2 asyd|OJJ62861.1 aver|OJI96914.1 asyd|OJJ61528.1 abra|OJJ70374.1 acar|OOF91895.1 afis|EAW16886.1 afum|EAL86424.2 ater|EAU30225.1 azon|OJJ48577.1 acar|OOF93599.1 azon|OJJ49991.1 afla|EED46560.1 akaw|GAA87467.1 aluc|OJZ88529.1

Group4: abra|OJJ67662.1 abra|OJJ77815.1 abra|OJJ78181.1 abra|OJJ74373.1 abra|OJJ78098.1 abra|OJJ76619.1 acar|OOF91828.1 acar|OOF91847.1 acar|OOF92934.1 acar|OOF94993.1 acar|OOF97632.1 acar|OOF95055.1 acar|OOF95122.1 acla|EAW07064.1 afis|EAW19759.1 afis|EAW23638.1 akaw|GAA83602.1 akaw|GAA84851.1 akaw|GAA92094.1 akaw|GAA83831.1 akaw|GAA85928.1 akaw|GAA89177.1 akaw|GAA86655.1 akaw|GAA85575.1 akaw|GAA85859.1 aluc|OJZ81656.1 aluc|OJZ83631.1 aluc|OJZ90887.1 aluc|OJZ91221.1 aluc|OJZ81972.1 aluc|OJZ86820.1 aluc|OJZ89749.1 aluc|OJZ92392.1 aluc|OJZ83511.1 aluc|OJZ89332.1 aluc|OJZ91137.1 anid|CBF80211.1 anid|CBF82304.1 anig|EHA19289.1 anig|EHA24757.1 anig|EHA19606.1 anig|EHA25789.1 anig|EHA26583.1 aory|EIT74370.1 aory|EIT76972.1 ater|EAU35165.1 ater|EAU36856.1 atub|OJI79585.1 atub|OJI79696.1 atub|OJI83697.1 atub|OJI85053.1 atub|OJI86822.1 atub|OJI82881.1 atub|OJI88527.1 atub|OJI90000.1 atub|OJI83148.1 aver|OJJ04870.1 aver|OJJ06306.1

aver|OJJ07785.1 awen|OJJ29662.1 awen|OJJ29874.1 awen|OJJ33672.1 agla|OJJ83634.1 asyd|OJJ52727.1 aver|OJJ02001.1 azon|OJJ47400.1 afla|EED51023.1 abra|OJJ74811.1 acar|OOF95802.1 afla|EED52130.1 afum|EAL85129.1 atub|OJI85468.1 abra|OJJ77455.1 afla|EED49595.1 anig|EHA19288.1 asyd|OJJ56406.1 ater|EAU32666.1 ater|EAU38928.1 ater|EAU39559.1 anid|CBF79115.1 afis|EAW19771.1 akaw|GAA84588.1 akaw|GAA84589.1 aluc|OJZ89334.1 atub|OJI82404.1

Group5: abra|OJJ67238.1 abra|OJJ68732.1 acar|OOF92227.1 acar|OOF98313.1 acla|EAW12167.1 acla|EAW12516.1 afis|EAW15603.1 afis|EAW17072.1 afis|EAW19893.1 afis|EAW21005.1 afis|EAW24131.1 afis|EAW24651.1 afis|EAW22083.1 afis|EAW24734.1 afla|EED46089.1 afla|EED46801.1 afla|EED48098.1 afla|EED52234.1 afla|EED48355.1 afla|EED49615.1 afla|EED55083.1 afla|EED56403.1 afum|EAL85692.1 afum|EAL89381.1 afum|EAL91987.1 afum|EAL85730.1 afum|EAL86560.1 agla|OJJ79085.1 agla|OJJ82566.1 agla|OJJ86784.1 agla|OJJ87207.1 akaw|GAA89881.1 akaw|GAA91663.1 aluc|OJZ81798.1 aluc|OJZ87737.1 anid|CBF75787.1 anid|CBF86562.1 anid|CBF86663.1 anig|EHA20229.1 anig|EHA22418.1 aory|EIT72725.1 aory|EIT74466.1 aory|EIT76413.1 aory|EIT80974.1 aory|EIT81644.1 aory|EIT83015.1 asyd|OJJ59270.1 asyd|OJJ64851.1 asyd|OJJ60947.1 asyd|OJJ64782.1 asyd|OJJ65051.1 ater|EAU30561.1 ater|EAU31344.1 ater|EAU38103.1 atub|OJI80903.1 atub|OJI84369.1 aver|OJI98893.1 aver|OJI99174.1 aver|OJJ00274.1 aver|OJJ08481.1 aver|OJI98963.1 aver|OJJ01883.1 aver|OJJ07136.1 awen|OJJ30103.1 awen|OJJ40222.1 awen|OJJ41358.1 awen|OJJ31256.1 awen|OJJ37981.1 azon|OJJ46971.1 azon|OJJ48822.1 abra|OJJ73660.1 acar|OOG00838.1 akaw|GAA88498.1 aluc|OJZ88393.1 anig|EHA18121.1 aory|EIT72296.1 atub|OJI90988.1 awen|OJJ38335.1 anid|CBF74724.1 aluc|OJZ80164.1 aory|EIT76217.1 ater|EAU32520.1

Group6: abra|OJJ65900.1 abra|OJJ66009.1 abra|OJJ67667.1 abra|OJJ67833.1 abra|OJJ70432.1 abra|OJJ73134.1 abra|OJJ74399.1 abra|OJJ77760.1 abra|OJJ66336.1 abra|OJJ75222.1 abra|OJJ75882.1 acla|EAW13531.1 acla|EAW13625.1 afis|EAW16387.1 afis|EAW17052.1 afla|EED45665.1 afla|EED48448.1 afla|EED47332.1 afla|EED53171.1 afla|EED57354.1 akaw|GAA83600.1 akaw|GAA86003.1 akaw|GAA89183.1 akaw|GAA90148.1 akaw|GAA92662.1 akaw|GAA84585.1 akaw|GAA90211.1 akaw|GAA90881.1 aluc|OJZ81649.1 aluc|OJZ82246.1 aluc|OJZ82952.1 aluc|OJZ90228.1 aluc|OJZ92394.1 aluc|OJZ89329.1 aluc|OJZ83585.1 aluc|OJZ83788.1 aluc|OJZ90116.1 anig|EHA19545.1 anig|EHA19698.1 anig|EHA26581.1 anig|EHA21367.1 anig|EHA23246.1 anig|EHA25794.1 anig|EHA28496.1 aory|EIT80163.1 aory|EIT82886.1 asyd|OJJ53336.1 asyd|OJJ54299.1 ater|EAU30237.1 ater|EAU33224.1 atub|OJI80755.1 atub|OJI84681.1 atub|OJI86824.1 atub|OJI87738.1 atub|OJI88533.1 atub|OJI89152.1 atub|OJI89184.1 atub|OJI85471.1 atub|OJI88192.1 atub|OJI87802.1 aver|OJI96712.1 aver|OJJ08408.1 awen|OJJ30102.1 awen|OJJ41501.1 awen|OJJ41603.1 awen|OJJ31974.1 acar|OOF99302.1 afis|EAW21094.1 aory|EIT75368.1 ater|EAU31345.1 acar|OOF92089.1 aver|OJJ07905.1 aluc|OJZ83134.1 awen|OJJ35031.1 acar|OOF98127.1 afum|EAL87813.1 afum|EAL92117.1 anid|CBF75778.1 afis|EAW17483.1 anid|CBF84683.1 akaw|GAA83278.1

Group7: abra|OJJ65820.1 abra|OJJ66803.1 acar|OOF90643.1 acar|OOF94182.1 afis|EAW16457.1 afis|EAW21767.1 afla|EED55145.1 afla|EED55917.1 akaw|GAA92417.1 akaw|GAA93242.1 anid|CBF75176.1 anid|CBF84668.1 anid|CBF85153.1 anid|CBF88209.1 anid|CBF75912.1 anid|CBF80593.1 anid|CBF80303.1 anid|CBF80770.1 anig|EHA18419.1 anig|EHA24965.1 anig|EHA21352.1 aory|EIT76564.1 aory|EIT81636.1 aory|EIT81823.1 asyd|OJJ52880.1 asyd|OJJ54446.1 asyd|OJJ60235.1 asyd|OJJ61610.1 ater|EAU32131.1 ater|EAU35090.1 ater|EAU37273.1 aver|OJJ04030.1 aver|OJJ08401.1 aver|OJJ08851.1 aver|OJJ07396.1 azon|OJJ42892.1 azon|OJJ45918.1 azon|OJJ48221.1 azon|OJJ48680.1 azon|OJJ49289.1 agla|OJJ83550.1 anig|EHA26118.1 awen|OJJ41193.1 azon|OJJ42522.1 abra|OJJ67032.1 acla|EAW08788.1 afis|EAW19897.1 afis|EAW23754.1 agla|OJJ82646.1 akaw|GAA92151.1 aluc|OJZ85558.1 ater|EAU30865.1 atub|OJI79347.1 abra|OJJ76132.1 acar|OOF99791.1 acla|EAW09686.1 afis|EAW21453.1 afla|EED55883.1 afum|EAL92472.1 agla|OJJ85599.1 akaw|GAA88835.1 aluc|OJZ89593.1 anid|CBF76549.1 anig|EHA23018.1 aory|EIT76608.1 asyd|OJJ61156.1 atub|OJI85209.1 aver|OJJ00478.1 awen|OJJ39247.1 azon|OJJ51608.1 abra|OJJ77926.1 acar|OOF89902.1 afum|EAL87190.2 akaw|GAA83738.1 aluc|OJZ90994.1 atub|OJI82984.1 aver|OJJ07910.1 aver|OJJ00933.1

Group8: abra|OJJ66275.1 abra|OJJ77982.1 abra|OJJ70821.1 abra|OJJ71091.1 abra|OJJ75537.1 abra|OJJ78258.1 acar|OOF97571.1 acar|OOF98050.1 acar|OOG00735.1 acar|OOF98604.1 acla|EAW07624.1 acla|EAW08950.1 acla|EAW09019.1 acla|EAW09117.1 acla|EAW13520.1 acla|EAW15062.1 afla|EED45116.1 afla|EED47649.1 afla|EED49862.1 afla|EED51183.1 akaw|GAA82870.1 akaw|GAA91957.1 akaw|GAA85998.1 aluc|OJZ82661.1 aluc|OJZ84344.1 aluc|OJZ87437.1 aluc|OJZ91057.1 aluc|OJZ91332.1 anig|EHA18001.1 anig|EHA18935.1 anig|EHA20663.1 anig|EHA27435.1 anig|EHA27680.1 anig|EHA19760.1 anig|EHA23147.1 anig|EHA23656.1 anig|EHA20014.1 aory|EIT73707.1 aory|EIT80187.1 aory|EIT83384.1 asyd|OJJ57246.1 asyd|OJJ60063.1 asyd|OJJ63687.1 atub|OJI81211.1 atub|OJI83130.1 atub|OJI83325.1 atub|OJI88893.1 atub|OJI89724.1 atub|OJI83053.1 atub|OJI89947.1 aver|OJI99011.1 aver|OJJ08178.1 awen|OJJ29725.1 awen|OJJ32235.1 awen|OJJ34622.1 afla|EED49876.1 afum|EAL85113.2 akaw|GAA87094.1 aory|EIT83412.1 ater|EAU38971.1 afis|EAW16804.1 agla|OJJ85462.1 anid|CBF80487.1 ater|EAU29410.1 azon|OJJ42361.1 acla|EAW10019.1 afis|EAW20415.1 afum|EAL89230.2 anid|CBF85555.1 ater|EAU29808.1 azon|OJJ43495.1 aver|OJI97248.1 afum|EAL91103.2 akaw|GAA83290.1 akaw|GAA83291.1 afla|EED45117.1 aver|OJJ08105.1

Group9: abra|OJJ68001.1 abra|OJJ69377.1 abra|OJJ77432.1 abra|OJJ78055.1 acar|OOF92044.1 acar|OOF94347.1 afis|EAW19338.1 afis|EAW24682.1 afis|EAW20700.1 afis|EAW24697.1 afis|EAW25724.1 afla|EED45393.1 afla|EED47975.1 afla|EED51175.1 afla|EED57518.1 afum|EAL84397.1 afum|EAL84875.1 afum|EAL89339.1 afum|EAL94057.1 agla|OJJ83163.1 agla|OJJ85718.1 agla|OJJ88362.1 akaw|GAA85937.1 akaw|GAA88581.1 akaw|GAA88246.1 akaw|GAA90949.1 aluc|OJZ82213.1 aluc|OJZ82875.1 aluc|OJZ88485.1

aluc|OJZ91125.1 anid|CBF70387.1 anid|CBF79143.1 anid|CBF90097.1 anid|CBF74114.1 anid|CBF80184.1 anig|EHA21301.1 anig|EHA28527.1 aory|EIT75060.1 aory|EIT80443.1 aory|EIT79828.1 aory|EIT81356.1 aory|EIT81872.1 aory|EIT82404.1 asyd|OJJ53903.1 asyd|OJJ56275.1 asyd|OJJ57401.1 asyd|OJJ57276.1 asyd|OJJ58638.1 asyd|OJJ62031.1 ater|EAU31624.1 ater|EAU37396.1 ater|EAU31762.1 ater|EAU38791.1 atub|OJI79831.1 atub|OJI83136.1 atub|OJI88222.1 atub|OJI89099.1 aver|OJI96122.1 aver|OJJ01245.1 aver|OJI99530.1 aver|OJJ08000.1 aver|OJJ08490.1 awen|OJJ32995.1 awen|OJJ37839.1 awen|OJJ40791.1 azon|OJJ48191.1 azon|OJJ48520.1 acla|EAW14609.1 azon|OJJ43922.1 acla|EAW13612.1 afla|EED53479.1 anig|EHA20150.1 anig|EHA19826.1 afla|EED51738.1 afla|EED51739.1 ater|EAU33967.1

Group10: abra|OJJ73857.1 abra|OJJ76179.1 acar|OOF94216.1 acar|OOF99761.1 acar|OOG01005.1 acla|EAW07747.1 acla|EAW09647.1 afis|EAW17299.1 afis|EAW22784.1 afis|EAW20842.1 afis|EAW21493.1 afla|EED48870.1 afla|EED50046.1 afum|EAL84790.1 afum|EAL92512.1 afum|EAL90415.1 agla|OJJ83596.1 agla|OJJ85655.1 akaw|GAA82124.1 akaw|GAA89472.1 akaw|GAA92487.1 aluc|OJZ80154.1 aluc|OJZ88191.1 aluc|OJZ92051.1 aluc|OJZ91499.1 anid|CBF76107.1 anid|CBF87969.1 anid|CBF76478.1 anid|CBF80461.1 anid|CBF83927.1 anig|EHA17943.1 anig|EHA22980.1 anig|EHA22539.1 aory|EIT74611.1 aory|EIT76730.1 aory|EIT80155.1 aory|EIT81260.1 asyd|OJJ53335.1 asyd|OJJ61118.1 asyd|OJJ55439.1 asyd|OJJ60355.1 asyd|OJJ63488.1 ater|EAU29437.1 ater|EAU35045.1 ater|EAU38925.1 ater|EAU29951.1 atub|OJI80378.1 atub|OJI90790.1 atub|OJI85952.1 atub|OJI86482.1 aver|OJI97441.1 aver|OJJ06514.1 aver|OJI99693.1 aver|OJJ08409.1 aver|OJJ00436.1 awen|OJJ36113.1 awen|OJJ38951.1 awen|OJJ39202.1 awen|OJJ40168.1 azon|OJJ49724.1 azon|OJJ51661.1 abra|OJJ70289.1 acar|OOF91742.1 akaw|GAA92859.1 aluc|OJZ82554.1 anig|EHA22518.1 atub|OJI88795.1 awen|OJJ38795.1 afla|EED57355.1 afla|EED55928.1

Group11: abra|OJJ66566.1 abra|OJJ74913.1 acar|OOF91007.1 acar|OOF95904.1 acla|EAW08458.1 acla|EAW10929.1 afis|EAW20932.1 afis|EAW23503.1 afla|EED50286.1 afla|EED57404.1 afum|EAL84702.1 afum|EAL87958.1 agla|OJJ83956.1 agla|OJJ86749.1 akaw|GAA83509.1 akaw|GAA91151.1 aluc|OJZ81479.1 aluc|OJZ92486.1 anid|CBF82508.1 anid|CBF89725.1 anig|EHA18724.1 anig|EHA18871.1 anig|EHA25948.1 anig|EHA26487.1 aory|EIT77296.1 aory|EIT80109.1 asyd|OJJ54080.1 asyd|OJJ62235.1 ater|EAU35292.1 ater|EAU38577.1 atub|OJI86917.1 atub|OJI88701.1 aver|OJI96344.1 aver|OJJ08658.1 awen|OJJ34386.1 awen|OJJ39829.1 azon|OJJ47952.1 azon|OJJ48118.1 acar|OOF91006.1 acla|EAW10930.1 afis|EAW20931.1 afla|EED50285.1 afum|EAL84703.2 agla|OJJ83957.1 akaw|GAA91152.1 anid|CBF82510.1 anig|EHA25947.1 aory|EIT77323.1 asyd|OJJ54081.1 ater|EAU38576.1 aver|OJJ08657.1 awen|OJJ34387.1 azon|OJJ48119.1 abra|OJJ68224.1 acar|OOF95905.1 acla|EAW08457.1 afis|EAW23498.1 afla|EED57403.1 afum|EAL87965.1 akaw|GAA83219.1 aluc|OJZ83721.1 anig|EHA18870.1 aory|EIT80136.1 ater|EAU35291.1 atub|OJI90095.1 awen|OJJ39827.1 anig|EHA26486.1 atub|OJI86918.1 agla|OJJ83079.1

Group12: abra|OJJ67365.1 abra|OJJ70373.1 abra|OJJ72695.1 abra|OJJ77105.1 abra|OJJ78039.1 abra|OJJ77002.1 acar|OOF90082.1 acar|OOF91896.1 acar|OOF92990.1 acar|OOF91448.1 acla|EAW08775.1 acla|EAW12337.1 afis|EAW16885.1 afis|EAW20732.1 afis|EAW25626.1 afla|EED47350.1 afla|EED50231.1 afla|EED54427.1 afum|EAL84314.2 afum|EAL86423.2 agla|OJJ78903.1 agla|OJJ79932.1 agla|OJJ85297.1 akaw|GAA86951.1 akaw|GAA91977.1 aluc|OJZ80600.1 aluc|OJZ84401.1 aluc|OJZ82637.1 aluc|OJZ84514.1 anid|CBF80656.1 anid|CBF82934.1 anid|CBF83105.1 anig|EHA27442.1 anig|EHA27812.1 aory|EIT75862.1 aory|EIT77345.1 aory|EIT81172.1 aory|EIT81924.1 aory|EIT82860.1 asyd|OJJ53449.1 asyd|OJJ54612.1 asyd|OJJ61456.1 ater|EAU30228.1 ater|EAU38239.1 atub|OJI88868.1 atub|OJI89567.1 atub|OJI89671.1 aver|OJJ00792.1 aver|OJJ05324.1 aver|OJJ07546.1 aver|OJJ08303.1 awen|OJJ29598.1 awen|OJJ29759.1 awen|OJJ31482.1 awen|OJJ34518.1 awen|OJJ36047.1 azon|OJJ42524.1 afla|EED55182.1 akaw|GAA87046.1 anig|EHA27731.1 aory|EIT81588.1 asyd|OJJ55521.1 ater|EAU32705.1 aver|OJJ06599.1 azon|OJJ50398.1 afla|EED48862.1 ater|EAU32222.1

Group13: abra|OJJ74143.1 abra|OJJ77919.1 acar|OOF95048.1 acar|OOF95115.1 acar|OOF95586.1 acar|OOF98862.1 acar|OOF99364.1 afla|EED47372.1 afla|EED48594.1 akaw|GAA83744.1 akaw|GAA89741.1 akaw|GAA92822.1 akaw|GAA92977.1 aluc|OJZ80240.1 aluc|OJZ91576.1 aluc|OJZ80643.1 aluc|OJZ90987.1 anid|CBF75107.1 anid|CBF76688.1 anid|CBF85354.1 anid|CBF86773.1 anig|EHA19692.1 anig|EHA27189.1 aory|EIT73093.1 aory|EIT82880.1 asyd|OJJ59996.1 asyd|OJJ61226.1 asyd|OJJ64036.1 asyd|OJJ64686.1 ater|EAU32382.1 ater|EAU33556.1 atub|OJI82978.1 atub|OJI87498.1 aver|OJI98121.1 aver|OJJ00549.1 aver|OJJ02295.1 aver|OJI98789.1 abra|OJJ69652.1 acar|OOF98861.1 acla|EAW06691.1 afis|EAW16074.1 afla|EED48593.1 afum|EAL88706.2 agla|OJJ86094.1 akaw|GAA91631.1 aluc|OJZ82458.1 anid|CBF75109.1 anig|EHA28317.1 aory|EIT73047.1 ater|EAU33557.1 atub|OJI87980.1 awen|OJJ30359.1 azon|OJJ45380.1 acla|EAW06690.1 afis|EAW16075.1 afum|EAL88707.1 awen|OJJ30358.1 abra|OJJ76634.1 afla|EED53145.1 agla|OJJ82986.1 anig|EHA22603.1 aory|EIT75089.1 ater|EAU37809.1 atub|OJI86025.1 awen|OJJ31622.1 azon|OJJ47320.1

Group14: abra|OJJ65763.1 abra|OJJ65764.1 abra|OJJ68458.1 abra|OJJ70187.1 acar|OOF91603.1 acar|OOF93288.1 acar|OOF99308.1 acar|OOF99313.1 afis|EAW16734.1 afis|EAW19114.1 afla|EED45012.1 afla|EED57380.1 afla|EED57383.1 afum|EAL86261.1 afum|EAL86308.1 afum|EAL86264.1 afum|EAL93846.1 akaw|GAA83845.1 akaw|GAA86588.1 akaw|GAA92381.1 aluc|OJZ83240.1 aluc|OJZ85135.1 aluc|OJZ90870.1 anid|CBF74944.1 anid|CBF83824.1 anid|CBF84265.1 anig|EHA19590.1 anig|EHA25367.1 anig|EHA21095.1 anig|EHA22863.1 aory|EIT73582.1 aory|EIT75916.1 aory|EIT80067.1 aory|EIT80069.1 asyd|OJJ57214.1 asyd|OJJ58070.1 asyd|OJJ63941.1 asyd|OJJ60903.1 asyd|OJJ63609.1 asyd|OJJ63938.1 atub|OJI80457.1 atub|OJI82868.1 atub|OJI87697.1 aver|OJI97001.1 aver|OJJ00229.1 aver|OJJ03075.1 aver|OJJ04337.1 aver|OJJ07009.1 awen|OJJ30374.1 awen|OJJ30814.1 awen|OJJ32530.1 acla|EAW14406.1 agla|OJJ89198.1

azon|OJJ47391.1 aver|OJI97004.1 aver|OJI97328.1 awen|OJJ40814.1 azon|OJJ50090.1 ater|EAU37377.1 azon|OJJ42904.1 ater|EAU38814.1 asyd|OJJ55207.1

Group15: abra|OJJ69361.1 abra|OJJ71787.1 abra|OJJ75622.1 acar|OOF94364.1 acar|OOF96466.1 acar|OOG00218.1 acla|EAW08453.1 acla|EAW09357.1 acla|EAW14751.1 afis|EAW21780.1 afis|EAW23502.1 afis|EAW24282.1 afla|EED46347.1 afla|EED47994.1 afum|EAL85951.1 afum|EAL87959.1 afum|EAL92809.1 agla|OJJ80637.1 agla|OJJ84344.1 akaw|GAA84369.1 akaw|GAA85124.1 akaw|GAA88230.1 aluc|OJZ82194.1 aluc|OJZ86517.1 aluc|OJZ89073.1 anig|EHA22129.1 anig|EHA23464.1 anig|EHA28543.1 aory|EIT79847.1 aory|EIT81244.1 asyd|OJJ53917.1 asyd|OJJ56885.1 asyd|OJJ61185.1 asyd|OJJ62326.1 ater|EAU31230.1 ater|EAU38460.1 atub|OJI84007.1 atub|OJI85713.1 atub|OJI88241.1 aver|OJI96431.1 aver|OJJ00509.1 aver|OJJ08015.1 awen|OJJ33015.1 awen|OJJ34069.1 awen|OJJ39823.1 azon|OJJ47947.1 azon|OJJ48162.1 azon|OJJ48502.1 afla|EED57398.1 anid|CBF76607.1 agla|OJJ86745.1 abra|OJJ74918.1 acar|OOF95909.1 akaw|GAA83505.1 aluc|OJZ92489.1 anid|CBF89735.1 anig|EHA26483.1 aory|EIT80098.1 asyd|OJJ62232.1 ater|EAU35287.1 atub|OJI86922.1 aver|OJI96341.1

Group16: abra|OJJ69532.1 abra|OJJ73245.1 abra|OJJ75746.1 abra|OJJ71793.1 acar|OOF90431.1 acar|OOF92663.1 acar|OOG00484.1 acar|OOF96473.1 acla|EAW06919.1 acla|EAW08051.1 afis|EAW16536.1 afis|EAW23087.1 afla|EED48561.1 afla|EED48728.1 afla|EED56133.1 afum|EAL88392.1 afum|EAL89133.1 agla|OJJ82786.1 agla|OJJ89597.1 akaw|GAA84479.1 akaw|GAA85117.1 akaw|GAA87935.1 akaw|GAA88383.1 aluc|OJZ81182.1 aluc|OJZ82371.1 aluc|OJZ86523.1 aluc|OJZ89199.1 anid|CBF75152.1 anid|CBF81741.1 anid|CBF82143.1 anig|EHA18500.1 anig|EHA23358.1 anig|EHA28393.1 anig|EHA22123.1 aory|EIT73145.1 aory|EIT73234.1 aory|EIT76572.1 aory|EIT79614.1 asyd|OJJ54322.1 asyd|OJJ59597.1 asyd|OJJ59975.1 asyd|OJJ56163.1 atub|OJI83999.1 atub|OJI91379.1 atub|OJI85591.1 aver|OJJ02316.1 aver|OJJ05102.1 aver|OJJ05276.1 aver|OJJ02671.1 awen|OJJ30245.1 awen|OJJ39992.1 awen|OJJ40286.1 ater|EAU36211.1 ater|EAU36599.1 atub|OJI88071.1 awen|OJJ36697.1 azon|OJJ47500.1 azon|OJJ49147.1 afla|EED56590.1 agla|OJJ79942.1 anig|EHA23359.1

Group17: abra|OJJ67548.1 abra|OJJ67738.1 abra|OJJ69732.1 abra|OJJ75389.1 abra|OJJ67871.1 acar|OOF90253.1 acar|OOF90937.1 acar|OOF94189.1 acar|OOF94221.1 acar|OOF94457.1 acla|EAW11677.1 acla|EAW12103.1 afla|EED45500.1 afla|EED48836.1 afla|EED51171.1 afla|EED52156.1 akaw|GAA88112.1 akaw|GAA90185.1 akaw|GAA92429.1 akaw|GAA92752.1 akaw|GAA91880.1 aluc|OJZ81382.1 aluc|OJZ81593.1 aluc|OJZ83164.1 aluc|OJZ92960.1 aluc|OJZ82019.1 anid|CBF73431.1 anid|CBF87554.1 anig|EHA18677.1 anig|EHA25848.1 anig|EHA26015.1 anig|EHA28228.1 anig|EHA22237.1 aory|EIT74393.1 aory|EIT75554.1 aory|EIT81271.1 aory|EIT82420.1 aory|EIT81347.1 ater|EAU30799.1 ater|EAU34705.1 atub|OJI84570.1 atub|OJI87418.1 atub|OJI87773.1 atub|OJI88590.1 atub|OJI91588.1 aver|OJJ08108.1 aver|OJJ08968.1 azon|OJJ42487.1 azon|OJJ45532.1 afis|EAW17283.1 afum|EAL86701.1 agla|OJJ81086.1 anid|CBF82821.1 asyd|OJJ52999.1 asyd|OJJ61577.1 aver|OJJ00899.1 awen|OJJ35752.1 afla|EED45454.1 anid|CBF80162.1 aver|OJI99518.1

Group18: abra|OJJ67797.1 abra|OJJ73515.1 abra|OJJ68679.1 abra|OJJ70328.1 abra|OJJ76402.1 acar|OOF91783.1 acar|OOF92181.1 acar|OOG00703.1 acla|EAW07449.1 acla|EAW12568.1 afis|EAW18727.1 afis|EAW22482.1 afis|EAW24082.1 afla|EED45270.1 afla|EED51391.1 afum|EAL90712.1 afum|EAL91937.1 agla|OJJ79953.1 agla|OJJ82286.1 agla|OJJ87548.1 akaw|GAA82322.1 akaw|GAA89832.1 akaw|GAA92583.1 akaw|GAA87464.1 aluc|OJZ81855.1 aluc|OJZ82597.1 aluc|OJZ88532.1 aluc|OJZ91810.1 anig|EHA18233.1 anig|EHA22369.1 anig|EHA27480.1 anig|EHA22803.1 aory|EIT73742.1 aory|EIT78822.1 aory|EIT80707.1 asyd|OJJ53887.1 asyd|OJJ62829.1 ater|EAU30215.1 ater|EAU30505.1 ater|EAU39231.1 ater|EAU38177.1 atub|OJI84423.1 atub|OJI88833.1 atub|OJI91117.1 atub|OJI86266.1 aver|OJI96872.1 aver|OJJ07985.1 awen|OJJ29718.1 awen|OJJ32268.1 awen|OJJ35101.1 acla|EAW14021.1 aluc|OJZ90857.1 anid|CBF88889.1 atub|OJI82853.1 azon|OJJ50007.1 afla|EED50684.1 afla|EED54508.1 acar|OOF99644.1 afum|EAL93452.1 aory|EIT78049.1

Group19: abra|OJJ67556.1 abra|OJJ67739.1 abra|OJJ69721.1 abra|OJJ75388.1 abra|OJJ67870.1 acar|OOF90043.1 acar|OOF90938.1 acar|OOF94195.1 acar|OOF94224.1 acar|OOF94458.1 acla|EAW11676.1 acla|EAW12104.1 afla|EED45513.1 afla|EED48837.1 afla|EED51172.1 afla|EED52163.1 akaw|GAA88108.1 akaw|GAA90174.1 akaw|GAA92430.1 akaw|GAA92751.1 akaw|GAA91879.1 aluc|OJZ81378.1 aluc|OJZ81592.1 aluc|OJZ92959.1 aluc|OJZ82020.1 aluc|OJZ83176.1 anid|CBF73444.1 anid|CBF82822.1 anid|CBF87553.1 anig|EHA18673.1 anig|EHA25849.1 anig|EHA26016.1 anig|EHA28250.1 anig|EHA22236.1 aory|EIT74418.1 aory|EIT75385.1 aory|EIT81285.1 aory|EIT82421.1 aory|EIT81323.1 asyd|OJJ52998.1 asyd|OJJ61578.1 atub|OJI84572.1 atub|OJI87417.1 atub|OJI87761.1 atub|OJI91584.1 atub|OJI88591.1 aver|OJJ00900.1 aver|OJJ08115.1 aver|OJJ08967.1 azon|OJJ42478.1 azon|OJJ45531.1 afis|EAW17282.1 afum|EAL86700.1 agla|OJJ81085.1 ater|EAU30800.1 ater|EAU34706.1 awen|OJJ35753.1 anid|CBF80164.1 aver|OJI99519.1

Group20: abra|OJJ67608.1 abra|OJJ70431.1 acar|OOF89840.1 acar|OOF91955.1 acla|EAW11023.1 acla|EAW14999.1 afis|EAW18722.1 afis|EAW19401.1 afis|EAW20837.1 afis|EAW24523.1 afla|EED45470.1 afla|EED46489.1 afla|EED50679.1 afum|EAL84797.1 afum|EAL86188.1 afum|EAL93446.1 agla|OJJ79209.1 agla|OJJ83714.1 akaw|GAA89124.1 akaw|GAA90149.1 aluc|OJZ81714.1 aluc|OJZ83199.1 anid|CBF74338.1 anid|CBF82877.1 aory|EIT72354.1 aory|EIT75736.1 asyd|OJJ57954.1 asyd|OJJ59255.1 asyd|OJJ61569.1 ater|EAU30785.1 ater|EAU36144.1 ater|EAU36801.1 atub|OJI87739.1 atub|OJI88468.1 aver|OJJ00889.1 aver|OJJ01866.1 awen|OJJ32698.1 awen|OJJ34547.1 azon|OJJ42767.1 azon|OJJ43003.1 anig|EHA25725.1 agla|OJJ78774.1 anig|EHA21062.1 abra|OJJ76408.1 acar|OOF99650.1 acla|EAW14020.1 agla|OJJ80462.1

akaw|GAA82327.1 aluc|OJZ91804.1 anid|CBF70690.1 anig|EHA22798.1 asyd|OJJ58469.1 ater|EAU38182.1 atub|OJI86258.1 aver|OJJ01070.1 awen|OJJ32266.1 azon|OJJ46779.1 azon|OJJ51803.1 asyd|OJJ51967.1

Group21: abra|OJJ67735.1 abra|OJJ68593.1 abra|OJJ73309.1 abra|OJJ77765.1 acar|OOF90932.1 acar|OOF97505.1 acla|EAW12049.1 acla|EAW12177.1 afis|EAW17018.1 afis|EAW19983.1 afla|EED45460.1 afla|EED45667.1 afla|EED48486.1 afla|EED52023.1 afum|EAL84933.1 afum|EAL86540.1 akaw|GAA89817.1 akaw|GAA92425.1 aluc|OJZ81597.1 aluc|OJZ81873.1 anid|CBF69451.1 anid|CBF80428.1 anid|CBF82812.1 anid|CBF83139.1 anid|CBF88295.1 anid|CBF89312.1 anid|CBF86046.1 anig|EHA20911.1 anig|EHA25844.1 anig|EHA28237.1 aory|EIT73076.1 aory|EIT75517.1 aory|EIT81451.1 aory|EIT82410.1 aory|EIT79328.1 asyd|OJJ61573.1 asyd|OJJ62373.1 ater|EAU29529.1 ater|EAU30794.1 ater|EAU31923.1 ater|EAU35431.1 ater|EAU36706.1 atub|OJI80342.1 atub|OJI87755.1 aver|OJI96507.1 aver|OJJ00893.1 aver|OJJ07788.1 aver|OJJ06454.1 agla|OJJ79464.1 atub|OJI88585.1 awen|OJJ38079.1 azon|OJJ46787.1 azon|OJJ47411.1 akaw|GAA90162.1 aluc|OJZ83185.1 abra|OJJ70061.1 anig|EHA21035.1 acar|OOF91380.1 anig|EHA28257.1

Group22: abra|OJJ67988.1 abra|OJJ68663.1 abra|OJJ71084.1 abra|OJJ76673.1 acar|OOF92030.1 acar|OOF92164.1 acar|OOF98052.1 acar|OOF99336.1 akaw|GAA82877.1 akaw|GAA83886.1 akaw|GAA89737.1 akaw|GAA89804.1 akaw|GAA90960.1 akaw|GAA92519.1 akaw|GAA92933.1 aluc|OJZ80647.1 aluc|OJZ81887.1 aluc|OJZ82526.1 aluc|OJZ82862.1 aluc|OJZ87444.1 aluc|OJZ91142.1 aluc|OJZ91532.1 anig|EHA18444.1 anig|EHA19537.1 anig|EHA19580.1 anig|EHA20668.1 anig|EHA21292.1 anig|EHA22353.1 anig|EHA22571.1 anig|EHA27186.1 anig|EHA27490.1 anig|EHA22492.1 atub|OJI81204.1 atub|OJI82798.1 atub|OJI83155.1 atub|OJI84440.1 atub|OJI85986.1 atub|OJI87502.1 atub|OJI89087.1 atub|OJI87377.1 atub|OJI87376.1 awen|OJJ30975.1 awen|OJJ34464.1 azon|OJJ42197.1 azon|OJJ43917.1 azon|OJJ50236.1 azon|OJJ45149.1 azon|OJJ45207.1 afis|EAW19725.1 afla|EED53110.1 afum|EAL86801.1 agla|OJJ84005.1 aory|EIT74790.1 ater|EAU37613.1 abra|OJJ77748.1 aluc|OJZ90809.1 agla|OJJ88178.1 azon|OJJ46566.1 azon|OJJ42198.1

Group23: abra|OJJ70376.1 abra|OJJ76944.1 acar|OOF91394.1 acar|OOF91893.1 acar|OOF94281.1 acla|EAW09924.1 acla|EAW12333.1 acla|EAW12657.1 acla|EAW13468.1 afis|EAW16889.1 afis|EAW18023.1 afis|EAW20581.1 afis|EAW21172.1 afla|EED47357.1 afla|EED52279.1 afla|EED54755.1 afum|EAL84483.1 afum|EAL86427.2 afum|EAL92192.1 afum|EAL91835.1 agla|OJJ79937.1 agla|OJJ80007.1 agla|OJJ88693.1 akaw|GAA87096.1 akaw|GAA88352.1 akaw|GAA91976.1 aluc|OJZ82303.1 aluc|OJZ84342.1 aluc|OJZ82638.1 aory|EIT79353.1 aory|EIT82885.1 asyd|OJJ54585.1 asyd|OJJ55452.1 ater|EAU35839.1 ater|EAU37103.1 atub|OJI88134.1 atub|OJI89726.1 atub|OJI88871.1 aver|OJJ06525.1 aver|OJJ07569.1 awen|OJJ29755.1 awen|OJJ33285.1 awen|OJJ36510.1 azon|OJJ42518.1 azon|OJJ46073.1 azon|OJJ50660.1 abra|OJJ69460.1 anid|CBF83321.1 anig|EHA28452.1 asyd|OJJ60544.1 aver|OJI99870.1 azon|OJJ44385.1 anid|CBF76070.1 anid|CBF80669.1 anig|EHA27432.1 ater|EAU29931.1 ater|EAU30227.1 anig|EHA27678.1 aory|EIT74323.1

Group24: abra|OJJ69144.1 abra|OJJ70352.1 abra|OJJ77612.1 acar|OOF91158.1 acar|OOF99116.1 acla|EAW06570.1 acla|EAW08667.1 acla|EAW13589.1 afis|EAW15744.1 afis|EAW16026.1 afis|EAW20014.1 afum|EAL84904.1 afum|EAL85604.1 afum|EAL88655.1 agla|OJJ81906.1 agla|OJJ86054.1 agla|OJJ87984.1 akaw|GAA84019.1 akaw|GAA86313.1 aluc|OJZ80922.1 aluc|OJZ86130.1 anid|CBF71034.1 anid|CBF77968.1 anid|CBF84719.1 anig|EHA19425.1 anig|EHA24491.1 aory|EIT75778.1 aory|EIT81521.1 aory|EIT83013.1 asyd|OJJ52664.1 asyd|OJJ53328.1 asyd|OJJ55266.1 asyd|OJJ60058.1 ater|EAU29366.1 ater|EAU30124.1 ater|EAU32401.1 atub|OJI79926.1 atub|OJI82658.1 aver|OJJ02231.1 aver|OJJ04691.1 aver|OJJ06960.1 aver|OJJ08416.1 awen|OJJ30028.1 awen|OJJ38444.1 azon|OJJ43750.1 azon|OJJ45430.1 azon|OJJ48427.1 ater|EAU33615.1 awen|OJJ30431.1 awen|OJJ41731.1 afla|EED46021.1 afla|EED56407.1 anid|CBF75022.1 acla|EAW12683.1 afis|EAW17846.1 afum|EAL91655.1 ater|EAU33048.1 afla|EED51306.1

Group25: abra|OJJ70961.1 abra|OJJ76031.1 acar|OOF98169.1 acar|OOF99897.1 acla|EAW09101.1 acla|EAW09782.1 acla|EAW10522.1 afis|EAW21356.1 afis|EAW25044.1 afis|EAW25699.1 afis|EAW25729.1 afla|EED45847.1 afla|EED55407.1 afla|EED55771.1 afum|EAL84400.2 afum|EAL89712.1 afum|EAL92371.2 agla|OJJ78663.1 agla|OJJ87377.1 agla|OJJ89150.1 agla|OJJ88275.1 akaw|GAA82986.1 akaw|GAA91201.1 aluc|OJZ81536.1 aluc|OJZ87571.1 aluc|OJZ89483.1 anid|CBF76249.1 anid|CBF85912.1 anig|EHA20773.1 anig|EHA23111.1 anig|EHA25900.1 aory|EIT75461.1 aory|EIT80822.1 aory|EIT75500.1 aory|EIT76538.1 asyd|OJJ59042.1 asyd|OJJ60969.1 asyd|OJJ64398.1 ater|EAU35202.1 ater|EAU35794.1 ater|EAU36445.1 ater|EAU37742.1 atub|OJI81078.1 atub|OJI85318.1 aver|OJI98494.1 aver|OJJ00294.1 aver|OJJ01637.1 awen|OJJ37057.1 awen|OJJ39342.1 awen|OJJ40655.1 azon|OJJ51688.1 ater|EAU36998.1 afla|EED54858.1 anid|CBF69709.1 atub|OJI88650.1 aory|EIT79380.1

Group26: abra|OJJ71513.1 abra|OJJ73019.1 abra|OJJ77809.1 acar|OOF96188.1 acar|OOF96228.1 acar|OOF96266.1 acar|OOF98958.1 acla|EAW07995.1 acla|EAW11308.1 acla|EAW15070.1 afis|EAW18195.1 afis|EAW23030.1 afis|EAW20042.1 afla|EED45337.1 afla|EED54986.1 afla|EED56290.1 afum|EAL85162.1 afum|EAL88452.1 afum|EAL87556.1 agla|OJJ85896.1 agla|OJJ89448.1 agla|OJJ88920.1 akaw|GAA83840.1 akaw|GAA85596.1 akaw|GAA89403.1 aluc|OJZ83533.1 aluc|OJZ86251.1 aluc|OJZ90876.1 anid|CBF80914.1 anid|CBF86102.1 anig|EHA19596.1 anig|EHA24355.1 anig|EHA21614.1 aory|EIT73286.1 aory|EIT80288.1 asyd|OJJ56326.1 asyd|OJJ64481.1 ater|EAU29627.1 ater|EAU33801.1 atub|OJI82424.1 atub|OJI82873.1 atub|OJI84291.1 aver|OJI98582.1 aver|OJJ06387.1 awen|OJJ30557.1 awen|OJJ35716.1 azon|OJJ44174.1 azon|OJJ45510.1 azon|OJJ46319.1 anid|CBF84784.1 anid|CBF84786.1 aory|EIT73629.1 asyd|OJJ63232.1 ater|EAU38649.1 aver|OJI97724.1 awen|OJJ31798.1

Group27: abra|OJJ67095.1 abra|OJJ67686.1 abra|OJJ69631.1 abra|OJJ76432.1 acar|OOF92679.1 acar|OOF94074.1 acar|OOF94144.1 acar|OOF99676.1 acla|EAW10021.1 acla|EAW12377.1 acla|EAW13992.1 afis|EAW16823.1 afis|EAW18700.1 afis|EAW22316.1 afla|EED50648.1 afla|EED55075.1 afum|EAL86374.1 afum|EAL90874.1 afum|EAL93422.1 agla|OJJ79102.1 agla|OJJ82361.1 akaw|GAA82347.1 akaw|GAA89198.1 akaw|GAA91066.1 akaw|GAA91611.1 aluc|OJZ81632.1 aluc|OJZ82438.1 aluc|OJZ87880.1 aluc|OJZ91780.1 anid|CBF70637.1 anid|CBF82989.1 anig|EHA20183.1 anig|EHA25811.1 anig|EHA28335.1 aory|EIT78077.1 aory|EIT81953.1 aory|EIT82431.1 asyd|OJJ52074.1 asyd|OJJ54406.1 asyd|OJJ58493.1 asyd|OJJ64734.1 ater|EAU29368.1 ater|EAU35424.1 ater|EAU38206.1 atub|OJI80771.1 atub|OJI86236.1 atub|OJI88003.1 atub|OJI88548.1 aver|OJI98838.1 aver|OJJ01096.1 aver|OJJ03029.1 awen|OJJ32183.1 awen|OJJ40928.1 azon|OJJ46764.1 anig|EHA22777.1

Group28: abra|OJJ68117.1 abra|OJJ77078.1 abra|OJJ68815.1 acar|OOF90616.1 acar|OOF92960.1 acar|OOF93911.1 afis|EAW17741.1 afis|EAW17974.1 afla|EED45883.1 afla|EED55321.1 afum|EAL91555.1 afum|EAL91783.1 agla|OJJ81756.1 agla|OJJ85465.1 agla|OJJ89268.1 akaw|GAA86898.1 akaw|GAA86975.1 akaw|GAA90440.1 aluc|OJZ84488.1 aluc|OJZ85787.1 aluc|OJZ88708.1 aluc|OJZ90021.1 anid|CBF71442.1 anid|CBF84572.1 anig|EHA24771.1 anig|EHA27397.1 anig|EHA27792.1 aory|EIT75581.1 aory|EIT76204.1 asyd|OJJ53389.1 asyd|OJJ54954.1 ater|EAU30960.1 ater|EAU35114.1 ater|EAU36891.1 atub|OJI79571.1 atub|OJI84786.1 atub|OJI89592.1 atub|OJI91296.1 aver|OJI99661.1 aver|OJJ03907.1 aver|OJJ07242.1 aver|OJJ08354.1 awen|OJJ32944.1 awen|OJJ39601.1 azon|OJJ42107.1 azon|OJJ42757.1 azon|OJJ42786.1 azon|OJJ44596.1 azon|OJJ47560.1 acla|EAW11105.1 afla|EED52131.1 acla|EAW12809.1 ater|EAU36942.1 awen|OJJ32177.1 aory|EIT74465.1

Group29: anid|CBF71484.1 anid|CBF71852.1 anid|CBF76028.1 anid|CBF80836.1 anid|CBF81153.1 anid|CBF81850.1 anid|CBF84162.1 anid|CBF84238.1 anid|CBF86037.1 anid|CBF89606.1 anid|CBF80194.1 anid|CBF80847.1 anid|CBF84163.1 anid|CBF76085.1 anid|CBF71848.1 anid|CBF80228.1 anid|CBF81852.1 anid|CBF80226.1 anid|CBF82237.1 anid|CBF81848.1 anid|CBF86356.1 anid|CBF80843.1 anid|CBF80845.1 anid|CBF76165.1 anid|CBF80196.1 anid|CBF76161.1 anid|CBF80272.1 anid|CBF80253.1 anid|CBF80230.1 anid|CBF82238.1 anid|CBF80252.1 anid|CBF80219.1 anid|CBF89288.1 anid|CBF81155.1 anid|CBF76163.1 anid|CBF84359.1 anid|CBF89285.1 anid|CBF80218.1 anid|CBF82246.1 anid|CBF87221.1 anid|CBF81844.1 anid|CBF89286.1 anid|CBF81853.1 anid|CBF71850.1 anid|CBF74006.1 anid|CBF78409.1 anid|CBF87223.1 anid|CBF81157.1 anid|CBF79347.1 acar|OOF89759.1 afis|EAW17204.1 afis|EAW17205.1 afis|EAW22029.1 agla|OJJ82974.1 aory|EIT76232.1

Group30: abra|OJJ68096.1 abra|OJJ70248.1 abra|OJJ73110.1 abra|OJJ74007.1 acar|OOF92551.1 acar|OOF93899.1 acar|OOG00337.1 acar|OOG01129.1 afla|EED44907.1 afla|EED44932.1 afla|EED56562.1 agla|OJJ78703.1 agla|OJJ88418.1 akaw|GAA86542.1 akaw|GAA86880.1 akaw|GAA88056.1 akaw|GAA92925.1 akaw|GAA87488.1 aluc|OJZ81318.1 aluc|OJZ82516.1 aluc|OJZ85090.1 aluc|OJZ88037.1 aluc|OJZ90004.1 anid|CBF74975.1 anid|CBF78035.1 anid|CBF81473.1 anig|EHA17810.1 anig|EHA18624.1 anig|EHA25311.1 anig|EHA27379.1 anig|EHA20903.1 aory|EIT73500.1 aory|EIT73507.1 asyd|OJJ55224.1 asyd|OJJ59448.1 asyd|OJJ60089.1 asyd|OJJ61384.1 asyd|OJJ57439.1 ater|EAU39548.1 ater|EAU39587.1 atub|OJI80509.1 atub|OJI84804.1 atub|OJI91521.1 atub|OJI90644.1 aver|OJJ00711.1 aver|OJJ04176.1 aver|OJJ05739.1 aver|OJJ02824.1 awen|OJJ33639.1 awen|OJJ40047.1 acla|EAW07039.1 afis|EAW16665.1 afum|EAL89269.1 azon|OJJ50123.1

Group31: abra|OJJ69067.1 abra|OJJ70519.1 abra|OJJ77965.1 acar|OOF91229.1 acar|OOF97409.1 acar|OOF98850.1 acla|EAW08156.1 acla|EAW08763.1 afis|EAW15666.1 afis|EAW16089.1 afis|EAW23194.1 afla|EED46889.1 afla|EED47060.1 afla|EED48625.1 afum|EAL85682.1 afum|EAL88289.1 afum|EAL88721.1 agla|OJJ81804.1 agla|OJJ86130.1 agla|OJJ86345.1 akaw|GAA83366.1 akaw|GAA83698.1 akaw|GAA90597.1 aluc|OJZ83864.1 aluc|OJZ91035.1 aluc|OJZ86055.1 anid|CBF71178.1 anid|CBF74704.1 anid|CBF75129.1 anig|EHA19005.1 anig|EHA19734.1 anig|EHA24556.1 aory|EIT72496.1 aory|EIT73097.1 asyd|OJJ55863.1 asyd|OJJ60218.1 asyd|OJJ59982.1 ater|EAU32505.1 ater|EAU33161.1 ater|EAU36321.1 atub|OJI79852.1 atub|OJI83031.1 atub|OJI90232.1 aver|OJJ02309.1 aver|OJJ04050.1 aver|OJJ04804.1 awen|OJJ30341.1 awen|OJJ38341.1 awen|OJJ36585.1 azon|OJJ42517.1 azon|OJJ43041.1 aory|EIT72708.1 acla|EAW06676.1 azon|OJJ47622.1

Group32: abra|OJJ66128.1 abra|OJJ70165.1 abra|OJJ77944.1 acar|OOF90574.1 acar|OOF94003.1 afis|EAW24242.1 afis|EAW25195.1 afum|EAL85910.2 afum|EAL89210.1 anid|CBF71201.1 anid|CBF74964.1 asyd|OJJ52805.1 asyd|OJJ59802.1 ater|EAU29607.1 ater|EAU33975.1 ater|EAU34693.1 atub|OJI87381.1 atub|OJI87443.1 aver|OJI96126.1 aver|OJJ03002.1 aver|OJI98905.1 aver|OJI99605.1 aver|OJJ03852.1 afla|EED47392.1 akaw|GAA83719.1 aluc|OJZ91014.1 anid|CBF89341.1 aory|EIT73813.1 aory|EIT76002.1 atub|OJI83005.1 aver|OJJ05262.1 awen|OJJ36264.1 azon|OJJ48048.1 agla|OJJ85483.1 akaw|GAA86612.1 akaw|GAA92274.1 aluc|OJZ85163.1 aluc|OJZ90168.1 atub|OJI80439.1 atub|OJI81596.1 anig|EHA19716.1 ater|EAU36085.1 aver|OJJ08785.1 afum|EAL91911.1 acar|OOF93333.1 acar|OOF98718.1 asyd|OJJ55517.1 aver|OJJ06595.1 afla|EED45160.1 afla|EED54076.1 anid|CBF82991.1 azon|OJJ46519.1 asyd|OJJ52549.1

Group33: abra|OJJ66203.1 abra|OJJ68990.1 abra|OJJ74346.1 acar|OOF91561.1 acar|OOF91930.1 acar|OOF95383.1 acla|EAW07400.1 acla|EAW14202.1 afis|EAW19080.1 afis|EAW22426.1 afla|EED50168.1 afla|EED52504.1 afum|EAL91073.1 afum|EAL90765.1 afum|EAL93809.1 agla|OJJ84526.1 agla|OJJ87597.1 agla|OJJ84718.1 akaw|GAA86684.1 akaw|GAA89082.1 aluc|OJZ80508.1 aluc|OJZ85975.1 aluc|OJZ89784.1 anid|CBF76870.1 anid|CBF79672.1 anid|CBF88817.1 anig|EHA24631.1 anig|EHA26999.1 anig|EHA25684.1

aory|EIT76001.1 aory|EIT82781.1 asyd|OJJ52200.1 asyd|OJJ58034.1 asyd|OJJ61321.1 asyd|OJJ62887.1 ater|EAU32126.1 ater|EAU32863.1 ater|EAU32522.1 ater|EAU39281.1 atub|OJI85021.1 atub|OJI88421.1 aver|OJI96939.1 aver|OJJ00646.1 aver|OJJ04365.1 aver|OJJ05950.1 aver|OJJ07745.1 awen|OJJ32472.1 awen|OJJ35005.1 awen|OJJ34090.1 azon|OJJ45647.1 azon|OJJ46232.1 akaw|GAA90510.1 atub|OJI79765.1

Group34: abra|OJJ71614.1 abra|OJJ77069.1 acar|OOF92952.1 acar|OOF96330.1 acla|EAW13507.1 acla|EAW15156.1 afis|EAW20123.1 afis|EAW21377.1 afis|EAW20124.1 afis|EAW20605.1 afla|EED47726.1 afla|EED57252.1 afum|EAL84455.1 afum|EAL85242.1 agla|OJJ88636.1 agla|OJJ88823.1 akaw|GAA86984.1 akaw|GAA89313.1 aluc|OJZ84477.1 aluc|OJZ86355.1 anid|CBF84947.1 anid|CBF86944.1 anig|EHA21535.1 anig|EHA27785.1 aory|EIT73980.1 aory|EIT77148.1 asyd|OJJ55590.1 asyd|OJJ63148.1 ater|EAU35261.1 ater|EAU38740.1 atub|OJI84179.1 atub|OJI89602.1 aver|OJI97806.1 aver|OJJ06669.1 awen|OJJ31893.1 awen|OJJ33448.1 azon|OJJ46243.1 azon|OJJ50705.1 abra|OJJ71611.1 acar|OOF96327.1 acla|EAW15155.1 afla|EED57253.1 afum|EAL85241.2 agla|OJJ88828.1 akaw|GAA89316.1 anid|CBF84945.1 aory|EIT74161.1 asyd|OJJ63149.1 atub|OJI84182.1 aver|OJI97805.1 awen|OJJ31892.1 azon|OJJ42421.1 azon|OJJ46244.1

Group35: afis|EAW16487.1 afis|EAW19960.1 afla|EED48721.1 afla|EED49671.1 afum|EAL84955.1 afum|EAL89081.1 agla|OJJ79324.1 agla|OJJ82857.1 agla|OJJ88164.1 aory|EIT73140.1 aory|EIT80950.1 aory|EIT75645.1 aory|EIT79202.1 ater|EAU35270.1 ater|EAU36551.1 abra|OJJ68065.1 acla|EAW06870.1 afla|EED45908.1 akaw|GAA90894.1 aluc|OJZ82935.1 anid|CBF75512.1 anid|CBF84598.1 anig|EHA21355.1 anig|EHA22760.1 asyd|OJJ53379.1 atub|OJI89164.1 aver|OJJ02547.1 awen|OJJ29993.1 azon|OJJ49197.1 abra|OJJ71770.1 akaw|GAA85137.1 aluc|OJZ86500.1 anig|EHA22144.1 atub|OJI84026.1 azon|OJJ48613.1 abra|OJJ78207.1 acar|OOF98647.1 acla|EAW11241.1 afis|EAW18262.1 afum|EAL87626.1 akaw|GAA85819.1 aluc|OJZ91280.1 anid|CBF79531.1 anig|EHA19965.1 asyd|OJJ57895.1 ater|EAU33270.1 atub|OJI83270.1 aver|OJJ04500.1 awen|OJJ30636.1 afla|EED47227.1 asyd|OJJ59741.1 awen|OJJ40346.1 aver|OJJ08364.1

Group36: abra|OJJ72217.1 abra|OJJ77905.1 abra|OJJ77236.1 abra|OJJ77528.1 acar|OOF93116.1 acar|OOF98878.1 acar|OOF99197.1 acar|OOF96839.1 acla|EAW06700.1 acla|EAW13243.1 afis|EAW16233.1 afis|EAW16245.1 afis|EAW17393.1 afla|EED49461.1 afla|EED53306.1 afla|EED53348.1 afum|EAL88856.1 afum|EAL88873.1 afum|EAL91231.1 akaw|GAA83757.1 akaw|GAA84092.1 akaw|GAA90707.1 aluc|OJZ81007.1 aluc|OJZ84646.1 aluc|OJZ90974.1 anig|EHA19353.1 anig|EHA19676.1 anig|EHA27930.1 aory|EIT74908.1 aory|EIT75241.1 aory|EIT79470.1 atub|OJI82578.1 atub|OJI82965.1 atub|OJI89434.1 awen|OJJ30211.1 awen|OJJ30227.1 awen|OJJ37422.1 afla|EED48174.1 agla|OJJ80414.1 akaw|GAA84718.1 aluc|OJZ86969.1 anig|EHA21681.1 aory|EIT83042.1 atub|OJI83552.1 awen|OJJ31302.1 agla|OJJ86254.1 anid|CBF78900.1 asyd|OJJ64603.1 ater|EAU37455.1 aver|OJI98700.1 acla|EAW06515.1 ater|EAU33711.1

Group37: abra|OJJ72940.1 abra|OJJ77077.1 acar|OOF92763.1 acar|OOF92959.1 acar|OOF97146.1 afis|EAW18980.1 afis|EAW21099.1 afis|EAW24628.1 afla|EED53187.1 afla|EED54887.1 agla|OJJ80287.1 agla|OJJ82940.1 akaw|GAA86976.1 akaw|GAA91285.1 aluc|OJZ82784.1 aluc|OJZ84487.1 anig|EHA18922.1 anig|EHA21226.1 aory|EIT75171.1 aory|EIT79411.1 asyd|OJJ58358.1 asyd|OJJ60157.1 ater|EAU34862.1 ater|EAU37837.1 atub|OJI89016.1 atub|OJI89593.1 aver|OJJ02049.1 aver|OJJ04107.1 awen|OJJ31588.1 awen|OJJ37322.1 azon|OJJ47304.1 azon|OJJ47677.1 abra|OJJ70575.1 acla|EAW08374.1 afis|EAW23403.1 afla|EED47010.1 afum|EAL88067.1 agla|OJJ86391.1 akaw|GAA83419.1 aluc|OJZ83919.1 anid|CBF74791.1 anig|EHA19052.1 aory|EIT72601.1 ater|EAU36372.1 atub|OJI90290.1 awen|OJJ39474.1 acla|EAW14304.1 afum|EAL93716.2 anid|CBF69653.1 asyd|OJJ59075.1 aver|OJJ01668.1 anig|EHA27791.1

Group38: abra|OJJ65887.1 abra|OJJ73728.1 abra|OJJ73840.1 abra|OJJ75062.1 acar|OOF94948.1 acar|OOF97272.1 acar|OOG00894.1 acar|OOG00991.1 acla|EAW07735.1 acla|EAW09321.1 afis|EAW21824.1 afis|EAW24409.1 afla|EED45566.1 afla|EED50029.1 afum|EAL86078.1 afum|EAL92840.1 agla|OJJ83601.1 agla|OJJ85455.1 agla|OJJ89020.1 akaw|GAA86017.1 akaw|GAA88434.1 akaw|GAA88674.1 akaw|GAA89458.1 aluc|OJZ88209.1 aluc|OJZ88322.1 aluc|OJZ90245.1 aluc|OJZ92628.1 anid|CBF84638.1 anid|CBF87998.1 anig|EHA17956.1 anig|EHA18058.1 anig|EHA26362.1 aory|EIT74733.1 aory|EIT75750.1 asyd|OJJ53366.1 asyd|OJJ63506.1 ater|EAU38301.1 ater|EAU38950.1 atub|OJI81673.1 atub|OJI87058.1 atub|OJI90810.1 atub|OJI90917.1 aver|OJI97424.1 aver|OJJ08381.1 awen|OJJ32729.1 awen|OJJ37942.1 awen|OJJ38966.1 awen|OJJ41210.1 azon|OJJ46022.1 azon|OJJ48676.1 aory|EIT76918.1

Group39: abra|OJJ66342.1 abra|OJJ71162.1 abra|OJJ75572.1 acar|OOF89825.1 acar|OOF94094.1 acar|OOF94165.1 acar|OOF97998.1 acla|EAW09994.1 acla|EAW10337.1 afis|EAW21096.1 afis|EAW25252.1 afla|EED51678.1 afla|EED53719.1 afla|EED54679.1 afum|EAL86170.1 afum|EAL89914.1 afum|EAL92119.1 agla|OJJ80114.1 agla|OJJ89225.1 akaw|GAA82817.1 akaw|GAA84323.1 akaw|GAA90216.1 aluc|OJZ83127.1 aluc|OJZ87378.1 aluc|OJZ89020.1 anid|CBF80117.1 anid|CBF85345.1 anig|EHA20610.1 anig|EHA23510.1 anig|EHA28188.1 aory|EIT75350.1 aory|EIT78900.1 aory|EIT80684.1 asyd|OJJ54726.1 asyd|OJJ64027.1 atub|OJI81268.1 atub|OJI85769.1 atub|OJI87806.1 aver|OJI98113.1 aver|OJJ07441.1 awen|OJJ36352.1 awen|OJJ40966.1 awen|OJJ38784.1 ater|EAU34396.1 azon|OJJ44550.1 abra|OJJ67575.1 acar|OOF90092.1 akaw|GAA89104.1 anig|EHA25706.1 atub|OJI88443.1 afis|EAW24500.1

Group40: abra|OJJ73400.1 abra|OJJ73868.1 acar|OOG00598.1 acar|OOG01011.1 acla|EAW07007.1 acla|EAW07756.1 afis|EAW16623.1 afis|EAW19494.1 afis|EAW22793.1 afla|EED44972.1 afla|EED48978.1 afla|EED50061.1 afla|EED56714.1 afum|EAL87000.1 afum|EAL89214.1 afum|EAL90406.1 agla|OJJ83569.1 agla|OJJ85298.1 akaw|GAA83092.1 akaw|GAA89474.1 akaw|GAA89692.1 aluc|OJZ80692.1 aluc|OJZ88189.1 aluc|OJZ88639.1 anid|CBF81984.1 anid|CBF87954.1 anig|EHA17940.1 anig|EHA18328.1 anig|EHA27323.1

aory|EIT73515.1 aory|EIT73856.1 aory|EIT74647.1 aory|EIT79983.1 asyd|OJJ56154.1 asyd|OJJ59777.1 asyd|OJJ63479.1 ater|EAU34806.1 ater|EAU36732.1 ater|EAU38918.1 atub|OJI87552.1 atub|OJI90788.1 atub|OJI91225.1 aver|OJI97451.1 aver|OJJ02509.1 aver|OJJ05094.1 awen|OJJ36912.1 awen|OJJ38940.1 azon|OJJ45543.1 azon|OJJ49714.1 anid|CBF75442.1

Group41: abra|OJJ65606.1 abra|OJJ68131.1 abra|OJJ68372.1 abra|OJJ69834.1 acar|OOF90085.1 acar|OOF93362.1 acar|OOG00535.1 afla|EED45615.1 afla|EED45682.1 afla|EED54532.1 agla|OJJ80470.1 agla|OJJ80842.1 agla|OJJ83418.1 akaw|GAA86911.1 akaw|GAA87826.1 aluc|OJZ85462.1 aluc|OJZ90034.1 anig|EHA25004.1 anig|EHA25073.1 anig|EHA27410.1 aory|EIT75511.1 aory|EIT75540.1 aory|EIT78869.1 aory|EIT81830.1 asyd|OJJ52349.1 asyd|OJJ56735.1 asyd|OJJ60489.1 atub|OJI80101.1 atub|OJI84772.1 aver|OJI99815.1 aver|OJJ05882.1 aver|OJJ05988.1 awen|OJJ32535.1 awen|OJJ33535.1 awen|OJJ34624.1 awen|OJJ35129.1 awen|OJJ40106.1 acla|EAW07472.1 afis|EAW17054.1 afum|EAL86551.2 akaw|GAA83834.1 aluc|OJZ90883.1 anid|CBF86106.1 ater|EAU30279.1 atub|OJI82467.1 anig|EHA19603.1 atub|OJI82878.1 azon|OJJ47403.1 afla|EED57508.1

Group42: abra|OJJ65781.1 abra|OJJ69519.1 abra|OJJ69805.1 acar|OOF93570.1 acar|OOF94236.1 acla|EAW09965.1 acla|EAW15041.1 afis|EAW17149.1 afis|EAW21123.1 afis|EAW24564.1 afla|EED50957.1 afla|EED51796.1 afla|EED54117.1 afla|EED57909.1 afum|EAL86225.1 afum|EAL92146.1 agla|OJJ78698.1 agla|OJJ79809.1 agla|OJJ80088.1 akaw|GAA87854.1 akaw|GAA88307.1 akaw|GAA90882.1 aluc|OJZ82358.1 aluc|OJZ82950.1 aluc|OJZ85496.1 anid|CBF77670.1 anid|CBF87020.1 anig|EHA21430.1 anig|EHA25029.1 anig|EHA28405.1 aory|EIT76140.1 aory|EIT77042.1 aory|EIT81685.1 aory|EIT82334.1 asyd|OJJ53106.1 asyd|OJJ60460.1 ater|EAU32188.1 ater|EAU36777.1 atub|OJI80069.1 atub|OJI88083.1 atub|OJI89182.1 aver|OJI99780.1 aver|OJJ03746.1 awen|OJJ36453.1 awen|OJJ37008.1 awen|OJJ40041.1 azon|OJJ44424.1 azon|OJJ50127.1 aver|OJI99293.1

Group43: abra|OJJ67343.1 abra|OJJ74161.1 abra|OJJ74904.1 acar|OOF92471.1 acar|OOF95566.1 acar|OOF95902.1 afis|EAW18497.1 afis|EAW23515.1 afis|EAW24769.1 afum|EAL89410.1 afum|EAL93187.1 agla|OJJ83565.1 agla|OJJ85096.1 agla|OJJ86763.1 agla|OJJ88214.1 akaw|GAA83518.1 akaw|GAA86474.1 akaw|GAA89723.1 aluc|OJZ80660.1 aluc|OJZ85015.1 aluc|OJZ92477.1 anid|CBF70261.1 anid|CBF89705.1 anig|EHA25236.1 anig|EHA26493.1 anig|EHA27172.1 aory|EIT77595.1 aory|EIT78639.1 aory|EIT80122.1 asyd|OJJ58753.1 asyd|OJJ62252.1 asyd|OJJ62467.1 atub|OJI80582.1 atub|OJI86908.1 atub|OJI87516.1 aver|OJI96362.1 aver|OJJ01361.1 awen|OJJ31051.1 awen|OJJ37928.1 awen|OJJ39840.1 acla|EAW08468.1 afla|EED49731.1 afum|EAL87948.1 anid|CBF89382.1 ater|EAU35304.1 azon|OJJ43252.1 ater|EAU34034.1 aver|OJI96602.1 afla|EED57415.1

Group44: abra|OJJ68123.1 abra|OJJ69603.1 abra|OJJ72218.1 acar|OOF92701.1 acar|OOF93917.1 acar|OOF96841.1 acla|EAW12866.1 acla|EAW14975.1 afis|EAW17815.1 afis|EAW24499.1 afla|EED45599.1 afla|EED51849.1 afla|EED54703.1 afum|EAL86169.1 afum|EAL91624.1 agla|OJJ78971.1 agla|OJJ85550.1 agla|OJJ81686.1 akaw|GAA84717.1 akaw|GAA86904.1 akaw|GAA91591.1 aluc|OJZ82414.1 aluc|OJZ86970.1 aluc|OJZ90027.1 anig|EHA21680.1 anig|EHA27403.1 anig|EHA28353.1 aory|EIT75721.1 aory|EIT78771.1 aory|EIT81733.1 asyd|OJJ54895.1 asyd|OJJ58303.1 asyd|OJJ56516.1 asyd|OJJ56725.1 atub|OJI83551.1 atub|OJI84780.1 atub|OJI88027.1 aver|OJJ02109.1 aver|OJJ05998.1 aver|OJJ06206.1 awen|OJJ32626.1 awen|OJJ36932.1 anid|CBF73850.1 ater|EAU30287.1 azon|OJJ50345.1 anid|CBF80089.1 ater|EAU31562.1 awen|OJJ33657.1 ater|EAU29952.1

Group45: abra|OJJ68536.1 abra|OJJ72481.1 acar|OOF90150.1 acar|OOF94753.1 acla|EAW10385.1 acla|EAW12826.1 afis|EAW17874.1 afis|EAW17991.1 afis|EAW25211.1 afla|EED45114.1 afla|EED51912.1 afla|EED46241.1 afum|EAL89872.1 afum|EAL91801.1 afum|EAL91682.1 agla|OJJ80251.1 agla|OJJ83074.1 akaw|GAA86216.1 akaw|GAA91315.1 aluc|OJZ82750.1 aluc|OJZ90468.1 anid|CBF79986.1 anid|CBF89933.1 anig|EHA21199.1 anig|EHA23927.1 aory|EIT73554.1 aory|EIT76181.1 aory|EIT76362.1 ater|EAU31453.1 ater|EAU37217.1 ater|EAU31502.1 ater|EAU33093.1 atub|OJI81892.1 atub|OJI88986.1 aver|OJI96221.1 aver|OJJ02185.1 awen|OJJ41004.1 awen|OJJ41694.1 azon|OJJ45116.1 azon|OJJ46814.1 azon|OJJ48270.1 azon|OJJ48393.1 acla|EAW12711.1 asyd|OJJ58240.1 asyd|OJJ62114.1 awen|OJJ41555.1 anid|CBF69461.1 aver|OJJ01892.1 asyd|OJJ59277.1

Group46: abra|OJJ72575.1 abra|OJJ77969.1 acar|OOF94684.1 acar|OOF98846.1 akaw|GAA82423.1 akaw|GAA87621.1 akaw|GAA83693.1 akaw|GAA85201.1 aluc|OJZ90556.1 aluc|OJZ91039.1 anig|EHA19738.1 anig|EHA24003.1 asyd|OJJ54419.1 asyd|OJJ59096.1 atub|OJI81979.1 atub|OJI83035.1 acla|EAW06671.1 afis|EAW16094.1 afla|EED48630.1 afla|EED56583.1 afum|EAL88726.1 agla|OJJ88578.1 anid|CBF85458.1 aory|EIT73102.1 aory|EIT79674.1 asyd|OJJ58704.1 asyd|OJJ64100.1 ater|EAU30075.1 ater|EAU33170.1 aver|OJI98183.1 aver|OJJ01316.1 awen|OJJ30336.1 awen|OJJ40588.1 azon|OJJ42310.1 azon|OJJ45364.1 abra|OJJ76510.1 acar|OOF99461.1 afla|EED54610.1 agla|OJJ87529.1 aluc|OJZ91695.1 anig|EHA22708.1 aory|EIT78921.1 ater|EAU38073.1 atub|OJI86149.1 aver|OJJ01687.1 aver|OJJ06547.1 awen|OJJ36612.1 agla|OJJ86127.1 anid|CBF70342.1

Group47: acar|OOF91492.1 acar|OOF98333.1 acar|OOF92665.1 acar|OOF93877.1 afis|EAW15510.1 afis|EAW17137.1 afis|EAW16415.1 afis|EAW16568.1 afis|EAW17138.1 afis|EAW25618.1 afum|EAL89039.1 afum|EAL89165.1 anid|CBF80635.1 anid|CBF89254.1 ater|EAU31707.1 ater|EAU36836.1 abra|OJJ74339.1 acla|EAW07389.1 afis|EAW22415.1 afla|EED52493.1 afum|EAL90777.1 agla|OJJ87679.1 akaw|GAA86691.1 aluc|OJZ89792.1 anid|CBF88800.1 anig|EHA27005.1 aory|EIT82748.1 asyd|OJJ62896.1 ater|EAU39292.1

atub|OJI85014.1 aver|OJI96948.1 awen|OJJ34994.1 azon|OJJ45637.1 agla|OJJ82753.1 anig|EHA22499.1 atub|OJI84627.1 afis|EAW19287.1 acla|EAW06953.1 awen|OJJ40251.1 azon|OJJ49111.1 acar|OOG00127.1 akaw|GAA86019.1 aluc|OJZ90247.1 asyd|OJJ61228.1 aver|OJJ00551.1 anid|CBF76692.1 akaw|GAA91465.1 aluc|OJZ82070.1 afum|EAL84308.2

Group48: abra|OJJ66388.1 abra|OJJ69074.1 acla|EAW08755.1 acla|EAW11763.1 afis|EAW15677.1 afis|EAW23796.1 afis|EAW25170.1 afla|EED46854.1 afla|EED51603.1 afla|EED53628.1 afum|EAL85670.1 afum|EAL87230.1 afum|EAL89843.1 akaw|GAA90254.1 akaw|GAA90605.1 aluc|OJZ83086.1 aluc|OJZ86063.1 anid|CBF71157.1 anid|CBF79858.1 anid|CBF85251.1 aory|EIT72587.1 aory|EIT74962.1 aory|EIT80702.1 asyd|OJJ51987.1 asyd|OJJ54461.1 asyd|OJJ54551.1 asyd|OJJ55842.1 asyd|OJJ58159.1 asyd|OJJ59172.1 asyd|OJJ62925.1 asyd|OJJ64763.1 ater|EAU31334.1 ater|EAU32484.1 ater|EAU34334.1 atub|OJI79860.1 atub|OJI87858.1 aver|OJI98064.1 aver|OJJ04243.1 aver|OJJ04785.1 aver|OJJ07708.1 awen|OJJ30141.1 awen|OJJ33753.1 awen|OJJ38354.1 azon|OJJ42861.1 azon|OJJ43857.1 acar|OOF91216.1 agla|OJJ81826.1 anig|EHA24550.1

Group49: abra|OJJ66054.1 abra|OJJ67991.1 abra|OJJ68633.1 acar|OOF92033.1 acar|OOF92137.1 acla|EAW07055.1 acla|EAW12619.1 afis|EAW16409.1 afis|EAW16681.1 afis|EAW18065.1 afla|EED45379.1 afla|EED47440.1 afla|EED51440.1 afum|EAL84867.1 afum|EAL89033.1 afum|EAL89285.1 afum|EAL91877.1 akaw|GAA89772.1 akaw|GAA90957.1 aluc|OJZ81922.1 aluc|OJZ82865.1 anid|CBF73413.1 anid|CBF86422.1 anid|CBF87201.1 anig|EHA21295.1 anig|EHA22325.1 aory|EIT80529.1 aory|EIT81893.1 aory|EIT82380.1 asyd|OJJ59980.1 asyd|OJJ64938.1 ater|EAU30466.1 ater|EAU35871.1 atub|OJI84474.1 atub|OJI89090.1 aver|OJI99059.1 aver|OJJ02311.1 awen|OJJ36169.1 awen|OJJ41452.1 azon|OJJ47178.1 azon|OJJ48910.1 agla|OJJ87352.1 anid|CBF75131.1 aver|OJJ01785.1 asyd|OJJ59979.1 anid|CBF75132.1 aver|OJJ02312.1

Group50: abra|OJJ69511.1 abra|OJJ74314.1 acar|OOF94242.1 acar|OOF95413.1 acla|EAW07365.1 acla|EAW09962.1 afis|EAW21133.1 afis|EAW22392.1 afla|EED46140.1 afla|EED56055.1 afum|EAL90799.1 afum|EAL92151.1 agla|OJJ80083.1 agla|OJJ87654.1 akaw|GAA86711.1 akaw|GAA88313.1 aluc|OJZ82349.1 aluc|OJZ89812.1 anid|CBF87003.1 anid|CBF88741.1 anig|EHA27022.1 anig|EHA28411.1 aory|EIT76474.1 aory|EIT76698.1 asyd|OJJ60468.1 asyd|OJJ63953.1 ater|EAU36765.1 ater|EAU39326.1 atub|OJI84993.1 atub|OJI88091.1 aver|OJI96988.1 aver|OJI99788.1 awen|OJJ34969.1 awen|OJJ36463.1 acar|OOF94243.1 acla|EAW09961.1 afis|EAW21134.1 afum|EAL92153.1 agla|OJJ80077.1 anid|CBF87002.1 aory|EIT76417.1 ater|EAU36766.1 awen|OJJ36462.1 azon|OJJ44415.1 azon|OJJ44422.1 azon|OJJ45618.1 afla|EED46139.1

Group51: abra|OJJ66047.1 abra|OJJ76934.1 abra|OJJ77452.1 acar|OOF91381.1 acar|OOF99274.1 afis|EAW19956.1 afis|EAW22115.1 afla|EED52041.1 afla|EED54167.1 afla|EED55064.1 afum|EAL84958.1 afum|EAL91070.1 agla|OJJ80818.1 agla|OJJ80927.1 akaw|GAA84156.1 akaw|GAA87105.1 aluc|OJZ81077.1 aluc|OJZ84333.1 anig|EHA19285.1 anig|EHA27667.1 aory|EIT73694.1 aory|EIT75905.1 aory|EIT81463.1 aory|EIT81952.1 asyd|OJJ52197.1 asyd|OJJ52737.1 asyd|OJJ53817.1 asyd|OJJ54624.1 ater|EAU29271.1 ater|EAU29317.1 ater|EAU36080.1 atub|OJI82508.1 atub|OJI89736.1 aver|OJI95757.1 aver|OJI99556.1 aver|OJJ02993.1 aver|OJJ07236.1 aver|OJJ07532.1 aver|OJJ07918.1 awen|OJJ35197.1 awen|OJJ41937.1 anid|CBF78465.1 asyd|OJJ54956.1 azon|OJJ42548.1 afla|EED45216.1 agla|OJJ85358.1

Group52: abra|OJJ67587.1 abra|OJJ72232.1 abra|OJJ68022.1 abra|OJJ71087.1 acar|OOF96856.1 acar|OOF98832.1 afis|EAW19900.1 afis|EAW25740.1 afla|EED45923.1 afla|EED46506.1 afla|EED55031.1 afla|EED50270.1 afla|EED55600.1 akaw|GAA82874.1 akaw|GAA83260.1 akaw|GAA90929.1 aluc|OJZ80366.1 aluc|OJZ82896.1 aluc|OJZ83766.1 anig|EHA20401.1 anig|EHA20666.1 anig|EHA21320.1 aory|EIT72370.1 aory|EIT77313.1 aory|EIT81962.1 aory|EIT82672.1 ater|EAU30170.1 ater|EAU30810.1 ater|EAU34044.1 atub|OJI81207.1 atub|OJI89121.1 atub|OJI90137.1 afum|EAL85004.1 agla|OJJ80468.1 akaw|GAA84705.1 aluc|OJZ86982.1 anid|CBF73897.1 anig|EHA21665.1 aory|EIT75502.1 atub|OJI83537.1 acar|OOF92065.1 afis|EAW17166.1 awen|OJJ30813.1 aluc|OJZ87441.1 afla|EED45753.1 ater|EAU38566.1

Group53: acla|EAW06287.1 acla|EAW08342.1 acla|EAW12933.1 acla|EAW13032.1 afis|EAW17635.1 afis|EAW17710.1 afis|EAW18392.1 afis|EAW23377.1 afla|EED45712.1 afla|EED50529.1 afla|EED53015.1 afla|EED55580.1 afum|EAL88094.1 afum|EAL91525.1 afum|EAL93068.1 afum|EAL91450.1 akaw|GAA83332.1 akaw|GAA86352.1 akaw|GAA90807.1 akaw|GAA83394.1 anid|CBF70766.1 anid|CBF74845.1 anid|CBF76926.1 anid|CBF90134.1 aory|EIT75805.1 aory|EIT77402.1 aory|EIT82175.1 aory|EIT82691.1 ater|EAU31902.1 ater|EAU32280.1 ater|EAU35631.1 ater|EAU37071.1 awen|OJJ37126.1 awen|OJJ37209.1 acar|OOF97170.1 agla|OJJ86446.1 azon|OJJ50825.1 agla|OJJ81264.1 aluc|OJZ84881.1 anig|EHA27829.1 asyd|OJJ57069.1 atub|OJI90195.1 aver|OJJ03229.1 azon|OJJ50909.1 aluc|OJZ83828.1 asyd|OJJ62014.1

Group54: abra|OJJ65575.1 abra|OJJ65577.1 abra|OJJ66394.1 abra|OJJ68072.1 abra|OJJ78158.1 acar|OOF90635.1 acar|OOF94051.1 acar|OOF94121.1 agla|OJJ81248.1 agla|OJJ85122.1 agla|OJJ89317.1 akaw|GAA90259.1 akaw|GAA92419.1 aluc|OJZ80816.1 aluc|OJZ83081.1 aluc|OJZ87399.1 anid|CBF80614.1 anid|CBF80695.1 anig|EHA19835.1 anig|EHA28144.1 anig|EHA19917.1 anig|EHA20628.1 anig|EHA26671.1 ater|EAU29371.1 ater|EAU30114.1 atub|OJI81247.1 atub|OJI87864.1 aver|OJI97590.1 aver|OJJ07256.1 awen|OJJ29741.1 awen|OJJ32237.1 awen|OJJ32260.1 afis|EAW17155.1 afum|EAL86727.1 aory|EIT76930.1 azon|OJJ42341.1 afla|EED50167.1 asyd|OJJ52055.1 atub|OJI88902.1 abra|OJJ66294.1 abra|OJJ71136.1 akaw|GAA82838.1 azon|OJJ45212.1 awen|OJJ36390.1 azon|OJJ42821.1

Group55: abra|OJJ66953.1 abra|OJJ70025.1 acar|OOF93535.1 acar|OOF93655.1 agla|OJJ79210.1 agla|OJJ85149.1 akaw|GAA87635.1 akaw|GAA92208.1 aluc|OJZ85251.1 aluc|OJZ85625.1 anig|EHA24903.1 anig|EHA25451.1 aory|EIT80438.1 aory|EIT82112.1 asyd|OJJ55882.1 asyd|OJJ65094.1 ater|EAU32531.1 ater|EAU37243.1 atub|OJI79418.1 atub|OJI80318.1 aver|OJI99218.1 aver|OJJ04827.1 awen|OJJ30173.1 awen|OJJ38273.1 awen|OJJ41598.1 acla|EAW08800.1 afis|EAW15597.1 afla|EED50419.1 afum|EBA27239.1 anid|CBF78088.1 azon|OJJ43909.1 asyd|OJJ55868.1 acar|OOF93534.1 acla|EAW08799.1 afis|EAW15598.1 afum|EAL85734.1 agla|OJJ88612.1 akaw|GAA87636.1 anid|CBF78056.1 anig|EHA25452.1 aory|EIT82031.1 ater|EAU32530.1 atub|OJI80317.1 aver|OJJ04811.1 azon|OJJ43905.1

Group56: agla|OJJ81571.1 agla|OJJ85255.1 asyd|OJJ54206.1 asyd|OJJ59064.1 asyd|OJJ54427.1 ater|EAU31423.1 ater|EAU38101.1 aver|OJJ01658.1 aver|OJJ01906.1 awen|OJJ30145.1 awen|OJJ31520.1 abra|OJJ76240.1 acar|OOF99485.1 acla|EAW14198.1 afis|EAW18923.1 afla|EED50910.1 afum|EAL93655.1 agla|OJJ85250.1 akaw|GAA82174.1 aluc|OJZ91991.1 anid|CBF69674.1 anig|EHA22937.1 aory|EIT79045.1 asyd|OJJ59065.1 ater|EAU38098.1 atub|OJI86426.1 awen|OJJ31518.1 azon|OJJ47234.1 abra|OJJ76243.1 acar|OOF99488.1 acla|EAW06837.1 afis|EAW18926.1 afla|EED50916.1 afum|EAL93657.1 akaw|GAA82176.1 aluc|OJZ91988.1 anid|CBF69676.1 anig|EHA22935.1 aory|EIT78980.1 atub|OJI86423.1 azon|OJJ47236.1 afis|EAW16385.1 afla|EED48287.1 afum|EAL89013.2 aory|EIT72910.1

Group57: abra|OJJ69646.1 abra|OJJ77015.1 abra|OJJ77136.1 acar|OOF90285.1 acar|OOF91423.1 acar|OOF91458.1 acla|EAW09001.1 acla|EAW10860.1 acla|EAW12947.1 acla|EAW13503.1 afis|EAW17722.1 afis|EAW20602.1 afis|EAW20998.1 afum|EAL84459.1 afum|EAL91537.1 agla|OJJ78915.1 agla|OJJ79354.1 agla|OJJ88647.1 akaw|GAA87035.1 akaw|GAA90793.1 akaw|GAA91624.1 aluc|OJZ82450.1 aluc|OJZ84416.1 aluc|OJZ84546.1 anig|EHA27739.1 anig|EHA27843.1 anig|EHA28324.1 aory|EIT75439.1 aory|EIT77113.1 asyd|OJJ52539.1 asyd|OJJ55599.1 atub|OJI87989.1 atub|OJI89534.1 atub|OJI89658.1 aver|OJJ03865.1 aver|OJJ06679.1 awen|OJJ33394.1 awen|OJJ35790.1 afla|EED45724.1 afla|EED47731.1 anid|CBF86932.1 ater|EAU35247.1 azon|OJJ43330.1 agla|OJJ84075.1

Group58: acar|OOF92599.1 acar|OOF92620.1 abra|OJJ72332.1 acar|OOF94882.1 acla|EAW11990.1 afis|EAW19630.1 afla|EED56982.1 afum|EAL86892.1 agla|OJJ80775.1 akaw|GAA86084.1 aluc|OJZ90316.1 anid|CBF71323.1 anig|EHA23803.1 aory|EIT77586.1 asyd|OJJ55109.1 ater|EAU32896.1 atub|OJI81744.1 aver|OJJ07097.1 awen|OJJ41874.1 azon|OJJ47072.1 abra|OJJ72333.1 acar|OOF94880.1 acla|EAW11989.1 afis|EAW19629.1 afla|EED56981.1 afum|EAL86893.1 akaw|GAA86085.1 anid|CBF71321.1 aory|EIT77447.1 ater|EAU32897.1 azon|OJJ47071.1 abra|OJJ72335.1 acla|EAW11987.1 afis|EAW19627.1 afla|EED56979.1 afum|EAL86894.1 agla|OJJ80773.1 akaw|GAA86087.1 anid|CBF71319.1 aory|EIT77480.1 asyd|OJJ55111.1 ater|EAU32899.1 aver|OJJ07095.1 azon|OJJ47069.1

Group59: acar|OOF92600.1 acar|OOF92621.1 abra|OJJ76130.1 acar|OOF99793.1 acla|EAW09689.1 afis|EAW21450.1 afla|EED55879.1 afum|EAL92469.1 agla|OJJ85598.1 akaw|GAA88837.1 aluc|OJZ89591.1 anid|CBF76555.1 anig|EHA23020.1 aory|EIT76712.1 asyd|OJJ61157.1 ater|EAU35092.1 atub|OJI85211.1 aver|OJJ00479.1 awen|OJJ39248.1 azon|OJJ51607.1 abra|OJJ76131.1 acar|OOF99792.1 acla|EAW09687.1 afis|EAW21452.1 afla|EED55881.1 afum|EAL92471.1 akaw|GAA88836.1 aluc|OJZ89592.1 anid|CBF76551.1 anig|EHA23019.1 aory|EIT76739.1 ater|EAU35091.1 atub|OJI85210.1 abra|OJJ77110.1 acla|EAW09688.1 afis|EAW21451.1 afla|EED55880.1 afum|EAL92470.1 akaw|GAA90818.1 aluc|OJZ84519.1 anid|CBF76553.1 anig|EHA27817.1 aory|EIT76614.1 atub|OJI89561.1

Group60: afis|EAW15375.1 afis|EAW19388.1 afis|EAW19745.1 afis|EAW19746.1 afis|EAW20418.1 afis|EAW20688.1 afis|EAW21002.1 afis|EAW24636.1 afis|EAW25758.1 afis|EAW25761.1 afis|EAW17293.1 afis|EAW15376.1 afis|EAW17294.1 afis|EAW24637.1 afis|EAW15377.1 afis|EAW24638.1 afis|EAW15378.1 afis|EAW19381.1 afis|EAW22065.1 afis|EAW22066.1 afis|EAW24635.1 afis|EAW24639.1 afis|EAW24640.1 afis|EAW25759.1 afis|EAW22048.1 afis|EAW22062.1 afis|EAW23656.1 afis|EAW23657.1 afis|EAW25774.1 afis|EAW25760.1 anid|CBF76095.1 anid|CBF82248.1 ater|EAU29403.1 ater|EAU37740.1 acar|OOF89756.1 afum|EAL89306.1 asyd|OJJ55339.1 aver|OJJ07727.1 ater|EAU31660.1 aory|EIT81932.1 asyd|OJJ56289.1 afla|EED45889.1 aory|EIT75613.1 aluc|OJZ79928.1

Group61: abra|OJJ65992.1 abra|OJJ66994.1 abra|OJJ68819.1 acar|OOF90628.1 acar|OOF92880.1 afis|EAW20003.1 afis|EAW20740.1 afla|EED46008.1 afla|EED47482.1 afla|EED49530.1 agla|OJJ87844.1 agla|OJJ87962.1 akaw|GAA90432.1 akaw|GAA90866.1 akaw|GAA92170.1 aluc|OJZ82967.1 aluc|OJZ85578.1 aluc|OJZ85795.1 anig|EHA18425.1 anig|EHA21383.1 anig|EHA24761.1 anig|EHA24941.1 aory|EIT75392.1 aory|EIT79965.1 aory|EIT82595.1 asyd|OJJ52756.1 asyd|OJJ53312.1 asyd|OJJ54834.1 atub|OJI79374.1 atub|OJI79581.1 atub|OJI89200.1 atub|OJI91621.1 aver|OJJ07327.1 aver|OJJ08431.1 aver|OJJ08738.1 azon|OJJ43639.1 azon|OJJ50209.1 acla|EAW13602.1 afum|EAL84916.1 anid|CBF80344.1 anid|CBF84693.1 ater|EAU37349.1 awen|OJJ30010.1

Group62: abra|OJJ68708.1 abra|OJJ69824.1 acar|OOF92210.1 acar|OOF93339.1 acla|EAW08971.1 acla|EAW12537.1 afis|EAW15450.1 afis|EAW24112.1 afla|EED46821.1 afla|EED56823.1 akaw|GAA87836.1 akaw|GAA89862.1 aluc|OJZ81821.1 aluc|OJZ85472.1 anig|EHA22396.1 anig|EHA25013.1 atub|OJI80091.1 atub|OJI84392.1 awen|OJJ41377.1 awen|OJJ41767.1 abra|OJJ68707.1 acar|OOF92209.1 acla|EAW12538.1 afis|EAW24111.1 afum|EAL91965.1 agla|OJJ87188.1 akaw|GAA89861.1 aluc|OJZ81823.1 anid|CBF86525.1 anig|EHA22395.1 aory|EIT72582.1 asyd|OJJ64873.1 ater|EAU30539.1 atub|OJI84393.1 aver|OJI98986.1

azon|OJJ48844.1 afum|EAL91966.1 agla|OJJ87189.1 anid|CBF86527.1 aory|EIT72529.1 asyd|OJJ64872.1 ater|EAU30540.1 aver|OJI98984.1

Group63: agla|OJJ80048.1 agla|OJJ86306.1 agla|OJJ89670.1 agla|OJJ81524.1 agla|OJJ84920.1 agla|OJJ81876.1 agla|OJJ88808.1 akaw|GAA92616.1 akaw|GAA92655.1 akaw|GAA93101.1 akaw|GAA93218.1 akaw|GAA93326.1 akaw|GAA93341.1 akaw|GAA93349.1 akaw|GAA92654.1 anid|CBF73635.1 anid|CBF78260.1 anid|CBF84144.1 anid|CBF84167.1 anid|CBF74958.1 anid|CBF73637.1 anid|CBF75922.1 anid|CBF82905.1 anid|CBF83997.1 anid|CBF84165.1 anid|CBF89628.1 anid|CBF84258.1 anid|CBF83995.1 anid|CBF89115.1 anid|CBF79097.1 anid|CBF80200.1 anid|CBF79095.1 anid|CBF77664.1 anid|CBF80044.1 anid|CBF78884.1 anid|CBF78717.1 anid|CBF74110.1 anid|CBF74956.1 anid|CBF83687.1 acar|OOG00276.1 aluc|OJZ79659.1 afum|EAL90122.1 akaw|GAA93209.1

Group64: agla|OJJ84606.1 agla|OJJ86279.1 anid|CBF78809.1 anid|CBF85739.1 asyd|OJJ64288.1 asyd|OJJ65260.1 aver|OJI98381.1 aver|OJI99411.1 awen|OJJ30086.1 awen|OJJ30833.1 awen|OJJ38668.1 azon|OJJ44554.1 azon|OJJ44649.1 abra|OJJ67129.1 acar|OOF98424.1 afis|EAW19329.1 afla|EED47551.1 afum|EAL94046.1 akaw|GAA91039.1 aluc|OJZ87849.1 anig|EHA20152.1 aory|EIT74544.1 ater|EAU29453.1 atub|OJI80799.1 abra|OJJ67137.1 acar|OOF98421.1 acla|EAW14610.1 afis|EAW19339.1 afla|EED47549.1 afum|EAL94058.1 akaw|GAA91032.1 aluc|OJZ87840.1 anid|CBF78807.1 anig|EHA20314.1 aory|EIT74530.1 asyd|OJJ65259.1 ater|EAU29452.1 atub|OJI80806.1 aver|OJI99410.1 agla|OJJ83890.1 awen|OJJ41067.1 agla|OJJ89228.1 awen|OJJ40968.1

Group65: abra|OJJ65919.1 abra|OJJ71178.1 acar|OOF94968.1 acar|OOF97988.1 acla|EAW10328.1 acla|EAW13616.1 afis|EAW19973.1 afis|EAW25266.1 afla|EED50456.1 afla|EED53730.1 afum|EAL84937.1 afum|EAL89929.1 agla|OJJ80057.1 agla|OJJ89242.1 akaw|GAA82807.1 akaw|GAA92313.1 aluc|OJZ87365.1 aluc|OJZ90211.1 anid|CBF84421.1 anid|CBF85372.1 anig|EHA20600.1 anig|EHA23709.1 aory|EIT75304.1 aory|EIT82028.1 asyd|OJJ53545.1 asyd|OJJ57927.1 asyd|OJJ64047.1 ater|EAU31473.1 ater|EAU34407.1 atub|OJI81279.1 atub|OJI81642.1 aver|OJI97591.1 aver|OJI98132.1 aver|OJI98886.1 aver|OJJ08208.1 awen|OJJ36011.1 awen|OJJ40979.1 azon|OJJ42397.1 azon|OJJ44566.1 azon|OJJ45171.1 azon|OJJ50333.1 asyd|OJJ54388.1

Group66: abra|OJJ66757.1 abra|OJJ68421.1 abra|OJJ70242.1 acar|OOF92554.1 acar|OOF93838.1 afis|EAW19495.1 afis|EAW21208.1 afum|EAL86999.1 afum|EAL92219.1 agla|OJJ78585.1 agla|OJJ81224.1 agla|OJJ86343.1 agla|OJJ87022.1 akaw|GAA86545.1 akaw|GAA89691.1 akaw|GAA92527.1 aluc|OJZ80693.1 aluc|OJZ85093.1 aluc|OJZ90076.1 anig|EHA20997.1 anig|EHA27322.1 anig|EHA25314.1 aory|EIT73935.1 aory|EIT77370.1 asyd|OJJ59201.1 asyd|OJJ59780.1 ater|EAU34261.1 ater|EAU34807.1 atub|OJI80506.1 atub|OJI87553.1 atub|OJI84632.1 atub|OJI84730.1 aver|OJJ01814.1 aver|OJJ02507.1 awen|OJJ36122.1 awen|OJJ36911.1 azon|OJJ42905.1 azon|OJJ45981.1 acla|EAW11883.1 afla|EED50220.1 afla|EED56713.1 anid|CBF75444.1

Group67: abra|OJJ67357.1 abra|OJJ69620.1 abra|OJJ69925.1 acar|OOF92464.1 acar|OOF92686.1 acar|OOF93436.1 acla|EAW06385.1 acla|EAW08905.1 afis|EAW15491.1 afis|EAW18491.1 afla|EED49005.1 afla|EED52913.1 afum|EAL85832.1 afum|EAL93181.1 agla|OJJ80168.1 agla|OJJ85086.1 akaw|GAA86464.1 akaw|GAA87732.1 akaw|GAA91604.1 aluc|OJZ82429.1 aluc|OJZ85004.1 aluc|OJZ85363.1 anig|EHA25223.1 anig|EHA25546.1 anig|EHA28342.1 aory|EIT78593.1 aory|EIT82534.1 asyd|OJJ53723.1 asyd|OJJ56020.1 asyd|OJJ58801.1 ater|EAU29917.1 ater|EAU32628.1 atub|OJI80207.1 atub|OJI80594.1 atub|OJI88012.1 awen|OJJ31044.1 awen|OJJ38147.1 azon|OJJ43140.1 azon|OJJ50555.1 anid|CBF70161.1 aver|OJJ01405.1 aver|OJJ04967.1

Group68: abra|OJJ69659.1 abra|OJJ71032.1 acar|OOF92597.1 acar|OOF92618.1 acar|OOF92644.1 acar|OOF98104.1 aluc|OJZ82466.1 aluc|OJZ87497.1 anig|EHA20713.1 anig|EHA28309.1 atub|OJI81151.1 atub|OJI87972.1 acla|EAW08698.1 acla|EAW12766.1 afis|EAW15737.1 afis|EAW17928.1 afla|EED49112.1 afla|EED51315.1 afum|EAL85611.1 afum|EAL91739.1 agla|OJJ81894.1 agla|OJJ83005.1 akaw|GAA91638.1 akaw|GAA91639.1 aluc|OJZ82465.1 anid|CBF71049.1 anid|CBF80811.1 anig|EHA28310.1 aory|EIT78548.1 aory|EIT81553.1 asyd|OJJ52673.1 asyd|OJJ54450.1 ater|EAU32405.1 ater|EAU33543.1 atub|OJI87973.1 aver|OJJ04699.1 aver|OJJ07704.1 awen|OJJ38433.1 awen|OJJ41618.1 azon|OJJ43810.1 azon|OJJ48339.1 akaw|GAA82922.1

Group69: abra|OJJ70622.1 abra|OJJ71755.1 abra|OJJ72061.1 abra|OJJ76523.1 acar|OOF96715.1 acar|OOF96808.1 acar|OOF99444.1 acla|EAW13671.1 acla|EAW14172.1 afis|EAW15618.1 afis|EAW18886.1 afla|EED50879.1 afla|EED53904.1 afla|EED57853.1 agla|OJJ80215.1 agla|OJJ80629.1 akaw|GAA82436.1 akaw|GAA84874.1 aluc|OJZ84834.1 aluc|OJZ86797.1 aluc|OJZ91685.1 anig|EHA21886.1 anig|EHA22697.1 anig|EHA25744.1 aory|EIT78491.1 aory|EIT78970.1 aory|EIT81115.1 ater|EAU31982.1 ater|EAU32969.1 ater|EAU38058.1 atub|OJI83721.1 atub|OJI86137.1 aver|OJJ01614.1 aver|OJJ02222.1 awen|OJJ32444.1 awen|OJJ38463.1 azon|OJJ47202.1 azon|OJJ47982.1 afum|EAL93619.1 anid|CBF69896.1 asyd|OJJ59001.1

Group70: agla|OJJ81088.1 agla|OJJ81098.1 agla|OJJ84079.1 agla|OJJ81610.1 agla|OJJ85958.1 agla|OJJ84407.1 anid|CBF70684.1 anid|CBF83505.1 anid|CBF86544.1 anid|CBF70985.1 anid|CBF73878.1 anid|CBF76072.1 anid|CBF76571.1 anid|CBF77622.1 anid|CBF78018.1 anid|CBF78361.1 anid|CBF79546.1 anid|CBF82083.1 anid|CBF84126.1 anid|CBF88413.1 anid|CBF88708.1 anid|CBF89022.1 anid|CBF89295.1 ater|EAU29721.1 ater|EAU32284.1 ater|EAU32866.1 ater|EAU33186.1 ater|EAU30981.1 ater|EAU30143.1

ater|EAU35995.1 abra|OJJ69716.1 aluc|OJZ83181.1 anig|EHA28255.1 aory|EIT76332.1 atub|OJI87756.1 awen|OJJ29588.1 acar|OOF89784.1 aluc|OJZ82089.1 asyd|OJJ55193.1 agla|OJJ85314.1 agla|OJJ88024.1

Group71: abra|OJJ65673.1 abra|OJJ68238.1 abra|OJJ66038.1 abra|OJJ68515.1 abra|OJJ74212.1 abra|OJJ74240.1 acar|OOF92683.1 acar|OOF95490.1 acar|OOF95508.1 afla|EED47448.1 afla|EED57720.1 akaw|GAA86780.1 akaw|GAA86806.1 akaw|GAA91332.1 aluc|OJZ79540.1 aluc|OJZ89922.1 aluc|OJZ82729.1 aluc|OJZ89892.1 anig|EHA21182.1 anig|EHA27091.1 anig|EHA27122.1 aory|EIT79930.1 aory|EIT81920.1 atub|OJI84883.1 atub|OJI84915.1 atub|OJI88962.1 aver|OJI97104.1 aver|OJJ03419.1 acla|EAW07265.1 afum|EAL90895.1 ater|EAU34298.1 agla|OJJ87719.1 anid|CBF88605.1 awen|OJJ34900.1 azon|OJJ50067.1 afis|EAW22286.1 anid|CBF88560.1 asyd|OJJ63833.1 ater|EAU34323.1 asyd|OJJ63857.1

Group72: abra|OJJ66697.1 abra|OJJ68381.1 acar|OOF93705.1 acar|OOF93791.1 acar|OOF93987.1 acla|EAW11832.1 acla|EAW12357.1 afis|EAW16780.1 afis|EAW19443.1 afla|EED56190.1 afla|EED56651.1 afum|EAL86351.1 afum|EAL87052.1 agla|OJJ79770.1 agla|OJJ81157.1 akaw|GAA89641.1 akaw|GAA92564.1 aluc|OJZ80748.1 aluc|OJZ90112.1 anid|CBF80588.1 anid|CBF81398.1 anig|EHA20960.1 anig|EHA27274.1 aory|EIT73439.1 aory|EIT79687.1 asyd|OJJ54689.1 asyd|OJJ59403.1 ater|EAU30272.1 ater|EAU34745.1 atub|OJI84692.1 atub|OJI87197.1 atub|OJI87605.1 aver|OJI99850.1 aver|OJJ02870.1 aver|OJJ07480.1 aver|OJJ07987.1 awen|OJJ29847.1 awen|OJJ36844.1 azon|OJJ42956.1 azon|OJJ50350.1

Group73: abra|OJJ68502.1 abra|OJJ73653.1 acar|OOF98748.1 acar|OOG00831.1 acla|EAW07563.1 acla|EAW12139.1 acla|EAW15367.1 afis|EAW17197.1 afis|EAW17227.1 afis|EAW22597.1 afla|EED45179.1 afla|EED51197.1 afla|EED51520.1 afla|EED54722.1 afum|EAL86625.1 afum|EAL86649.1 afum|EAL90601.1 agla|OJJ80961.1 agla|OJJ86657.1 akaw|GAA88505.1 akaw|GAA92769.1 aluc|OJZ82715.1 aluc|OJZ88400.1 anid|CBF69884.1 anid|CBF89553.1 anig|EHA18128.1 anig|EHA21170.1 aory|EIT73539.1 aory|EIT78945.1 aory|EIT80690.1 asyd|OJJ59011.1 asyd|OJJ65377.1 ater|EAU31751.1 ater|EAU39106.1 atub|OJI88949.1 atub|OJI90995.1 aver|OJI99545.1 aver|OJJ01604.1 awen|OJJ35225.1 azon|OJJ51827.1

Group74: abra|OJJ66378.1 abra|OJJ68574.1 abra|OJJ75363.1 acar|OOF89872.1 acar|OOF92870.1 akaw|GAA90247.1 akaw|GAA91914.1 aluc|OJZ81966.1 aluc|OJZ83093.1 atub|OJI84517.1 atub|OJI87392.1 atub|OJI87843.1 acla|EAW08967.1 agla|OJJ89584.1 anid|CBF87061.1 anig|EHA22281.1 anig|EHA28155.1 asyd|OJJ60430.1 ater|EAU36814.1 aver|OJI99753.1 afla|EED48897.1 afum|EAL93812.1 akaw|GAA92059.1 aluc|OJZ92932.1 azon|OJJ46231.1 afis|EAW19083.1 agla|OJJ81069.1 anig|EHA26039.1 aory|EIT81242.1 ater|EAU36156.1 awen|OJJ35776.1 azon|OJJ45936.1 acla|EAW14375.1 afla|EED54315.1 aory|EIT76109.1 ater|EAU32130.1 awen|OJJ32478.1 afla|EED46053.1 aory|EIT83489.1

Group75: abra|OJJ66645.1 abra|OJJ77622.1 acar|OOF93665.1 acar|OOF93749.1 acar|OOF99113.1 afis|EAW19398.1 afis|EAW25441.1 afla|EED46395.1 afla|EED56341.1 afum|EAL87096.1 afum|EAL90099.1 agla|OJJ81109.1 agla|OJJ84218.1 akaw|GAA84009.1 akaw|GAA89598.1 aluc|OJZ80804.1 aluc|OJZ80911.1 anid|CBF74274.1 anid|CBF85786.1 anig|EHA19435.1 anig|EHA27230.1 aory|EIT72345.1 aory|EIT73378.1 aory|EIT83124.1 ater|EAU33678.1 ater|EAU36124.1 atub|OJI82668.1 atub|OJI87652.1 acla|EAW10153.1 acla|EAW11788.1 afla|EED50354.1 asyd|OJJ51908.1 asyd|OJJ64317.1 aver|OJJ05362.1 awen|OJJ36793.1 azon|OJJ43006.1 azon|OJJ43662.1 aver|OJI98410.1 awen|OJJ30031.1

Group76: abra|OJJ68339.1 abra|OJJ77792.1 acar|OOF89870.1 acar|OOF90688.1 acar|OOF90718.1 agla|OJJ81064.1 agla|OJJ85571.1 aluc|OJZ80535.1 aluc|OJZ83619.1 anig|EHA18777.1 anig|EHA25660.1 asyd|OJJ53057.1 asyd|OJJ61781.1 atub|OJI88396.1 atub|OJI89992.1 aver|OJI95881.1 aver|OJJ03787.1 awen|OJJ29921.1 awen|OJJ33519.1 awen|OJJ38011.1 abra|OJJ66230.1 afis|EAW16867.1 afla|EED45515.1 afla|EED46127.1 afum|EAL90432.1 akaw|GAA89059.1 akaw|GAA92106.1 anid|CBF80612.1 aory|EIT76508.1 asyd|OJJ64304.1 ater|EAU31983.1 aver|OJI98397.1 awen|OJJ35964.1 azon|OJJ42115.1 ater|EAU33486.1 aory|EIT73494.1 acar|OOF90354.1 afla|EED44960.1 awen|OJJ35329.1

Group77: abra|OJJ68971.1 abra|OJJ74920.1 acar|OOF91548.1 acar|OOF95911.1 acla|EAW08451.1 acla|EAW14216.1 afis|EAW19065.1 afis|EAW23496.1 afla|EED54431.1 afla|EED57396.1 afum|EAL87967.1 afum|EAL93794.1 agla|OJJ83427.1 agla|OJJ84542.1 agla|OJJ86743.1 akaw|GAA83503.1 akaw|GAA90498.1 aluc|OJZ85958.1 aluc|OJZ92491.1 anid|CBF79648.1 anid|CBF89739.1 anig|EHA24643.1 anig|EHA26481.1 aory|EIT75884.1 aory|EIT80127.1 asyd|OJJ52407.1 asyd|OJJ58022.1 asyd|OJJ62230.1 ater|EAU32108.1 ater|EAU35285.1 atub|OJI79746.1 atub|OJI86924.1 aver|OJI96339.1 aver|OJJ04377.1 awen|OJJ31497.1 awen|OJJ39821.1 azon|OJJ46209.1 azon|OJJ47945.1 anid|CBF76133.1

Group78: abra|OJJ72527.1 abra|OJJ73074.1 abra|OJJ74512.1 acar|OOF95231.1 acar|OOG00307.1 afis|EAW15886.1 afis|EAW22192.1 afla|EED49069.1 afla|EED49846.1 afla|EED56486.1 afum|EAL88520.1 afum|EAL90999.1 akaw|GAA82087.1 akaw|GAA86250.1 akaw|GAA88087.1 aluc|OJZ81351.1 aluc|OJZ90507.1 aluc|OJZ92093.1 anig|EHA18653.1 anig|EHA23964.1 anig|EHA26848.1 aory|EIT72810.1 aory|EIT82565.1 ater|EAU31708.1 ater|EAU34200.1 atub|OJI81931.1 atub|OJI86526.1 atub|OJI91555.1 aver|OJI97191.1 aver|OJJ02116.1 aver|OJJ08595.1 awen|OJJ34773.1 awen|OJJ36745.1 azon|OJJ45756.1 azon|OJJ45757.1 acla|EAW11155.1 anid|CBF88404.1 asyd|OJJ63749.1 aory|EIT83409.1

Group79: abra|OJJ69282.1 abra|OJJ71129.1 acar|OOF90163.1 acar|OOF98023.1 acar|OOF94440.1 acla|EAW10369.1 acla|EAW14671.1 afis|EAW24202.1 afis|EAW25223.1 afla|EED48070.1 afla|EED53683.1 afum|EAL85872.1 afum|EAL89884.1 agla|OJJ79190.1 agla|OJJ79675.1 akaw|GAA82845.1 akaw|GAA88154.1 aluc|OJZ82110.1 aluc|OJZ87406.1 anid|CBF74328.1 anid|CBF85271.1 anig|EHA20636.1 anig|EHA28620.1

aory|EIT75297.1 aory|EIT83329.1 asyd|OJJ53993.1 asyd|OJJ62910.1 ater|EAU31153.1 ater|EAU34382.1 atub|OJI81240.1 atub|OJI88326.1 aver|OJI98079.1 aver|OJJ08091.1 awen|OJJ33096.1 awen|OJJ40889.1 azon|OJJ42425.1 azon|OJJ44518.1 azon|OJJ44851.1

Group80: abra|OJJ69429.1 abra|OJJ76880.1 abra|OJJ74397.1 abra|OJJ75836.1 acar|OOF94306.1 acar|OOF95338.1 akaw|GAA84546.1 akaw|GAA87295.1 aluc|OJZ89279.1 aluc|OJZ89723.1 asyd|OJJ56900.1 asyd|OJJ58390.1 ater|EAU31031.1 ater|EAU36089.1 ater|EAU36364.1 ater|EAU36837.1 ater|EAU36966.1 ater|EAU39346.1 atub|OJI85077.1 atub|OJI85520.1 aver|OJJ02028.1 aver|OJJ03392.1 agla|OJJ83397.1 akaw|GAA88295.1 aluc|OJZ82266.1 anig|EHA28477.1 atub|OJI88172.1 anid|CBF82791.1 anig|EHA23290.1 anig|EHA26951.1 aory|EIT80997.1 aver|OJI99456.1 acla|EAW13699.1 afum|EAL86287.1 anid|CBF80711.1 awen|OJJ32049.1 afla|EED52718.1 afla|EED52719.1

Group81: abra|OJJ72988.1 abra|OJJ77279.1 acar|OOF93153.1 acar|OOF96164.1 acla|EAW08023.1 acla|EAW13278.1 afis|EAW17339.1 afis|EAW23057.1 afum|EAL88423.2 afum|EAL91183.1 akaw|GAA85562.1 akaw|GAA90670.1 aluc|OJZ83496.1 aluc|OJZ84689.1 anig|EHA24331.1 anig|EHA27964.1 asyd|OJJ54336.1 asyd|OJJ56391.1 ater|EAU33849.1 ater|EAU37607.1 atub|OJI82390.1 atub|OJI89393.1 aver|OJJ05288.1 aver|OJJ06322.1 awen|OJJ35679.1 awen|OJJ40816.1 afla|EED51042.1 afla|EED54960.1 agla|OJJ79345.1 agla|OJJ89418.1 anid|CBF81037.1 aory|EIT80299.1 azon|OJJ44147.1 azon|OJJ50224.1 anid|CBF84215.1 aory|EIT77041.1 asyd|OJJ57175.1 aver|OJJ03139.1

Group82: abra|OJJ73798.1 abra|OJJ76038.1 acar|OOF99891.1 acar|OOG00949.1 acla|EAW07105.1 acla|EAW07691.1 afla|EED45540.1 afla|EED49992.1 agla|OJJ86348.1 agla|OJJ87936.1 akaw|GAA88927.1 akaw|GAA89416.1 aluc|OJZ88257.1 aluc|OJZ89493.1 anid|CBF71497.1 anid|CBF88069.1 anig|EHA18002.1 anig|EHA23106.1 aory|EIT74740.1 aory|EIT75450.1 asyd|OJJ54934.1 asyd|OJJ60320.1 asyd|OJJ63552.1 ater|EAU32047.1 ater|EAU38995.1 atub|OJI85309.1 atub|OJI90856.1 aver|OJI97382.1 aver|OJI99669.1 aver|OJJ07264.1 awen|OJJ34497.1 awen|OJJ34697.1 awen|OJJ35343.1 azon|OJJ45277.1 azon|OJJ49485.1 azon|OJJ49775.1 afis|EAW22706.1 afum|EAL90486.1

Group83: abra|OJJ74861.1 abra|OJJ78108.1 aluc|OJZ91173.1 aluc|OJZ92439.1 anig|EHA19866.1 anig|EHA26528.1 atub|OJI83187.1 atub|OJI86868.1 acar|OOF95861.1 agla|OJJ86823.1 akaw|GAA83559.1 akaw|GAA85898.1 awen|OJJ39889.1 azon|OJJ43220.1 acar|OOF98757.1 acla|EAW06608.1 afis|EAW16215.1 afla|EED48709.1 afum|EAL88842.1 agla|OJJ86220.1 anid|CBF79542.1 aory|EIT73181.1 asyd|OJJ57900.1 ater|EAU33259.1 aver|OJJ04494.1 awen|OJJ30257.1 azon|OJJ43953.1 abra|OJJ78109.1 acla|EAW06607.1 afis|EAW16216.1 afla|EED48710.1 afum|EAL88843.1 akaw|GAA85897.1 anid|CBF79540.1 aory|EIT73231.1 ater|EAU33260.1 awen|OJJ30256.1 azon|OJJ43954.1

Group84: afla|EED45777.1 afla|EED56579.1 afla|EED46479.1 afla|EED47420.1 aory|EIT72361.1 aory|EIT81890.1 aory|EIT75763.1 aory|EIT79626.1 asyd|OJJ54274.1 asyd|OJJ58712.1 asyd|OJJ61516.1 asyd|OJJ61954.1 aver|OJI96045.1 aver|OJJ00852.1 aver|OJJ01325.1 aver|OJJ05230.1 abra|OJJ67908.1 afum|EAL86295.1 agla|OJJ85785.1 aluc|OJZ81978.1 anig|EHA22277.1 atub|OJI84530.1 awen|OJJ29991.1 azon|OJJ42128.1 abra|OJJ70236.1 akaw|GAA86549.1 aluc|OJZ85097.1 anid|CBF73730.1 anig|EHA25319.1 asyd|OJJ53339.1 ater|EAU37949.1 atub|OJI80501.1 aver|OJJ08406.1 awen|OJJ31368.1 awen|OJJ35920.1 asyd|OJJ65167.1 aver|OJI99319.1 awen|OJJ39978.1

Group85: abra|OJJ69585.1 acar|OOF91569.1 acla|EAW14381.1 acla|EAW14383.1 acla|EAW14384.1 afis|EAW19087.1 afis|EAW19089.1 afis|EAW19090.1 afla|EED54319.1 afla|EED54321.1 afla|EED54322.1 afum|EAL93817.1 afum|EAL93819.1 afum|EAL93820.1 agla|OJJ84514.1 agla|OJJ84515.1 agla|OJJ84517.1 akaw|GAA91580.1 akaw|GAA91581.1 akaw|GAA91583.1 aluc|OJZ82402.1 anid|CBF79686.1 anid|CBF79690.1 anid|CBF79692.1 anig|EHA28363.1 aory|EIT76048.1 aory|EIT76070.1 aory|EIT76088.1 asyd|OJJ58048.1 ater|EAU32135.1 ater|EAU32138.1 ater|EAU32139.1 atub|OJI88039.1 aver|OJJ04355.1 awen|OJJ32484.1 azon|OJJ46222.1 azon|OJJ46228.1 azon|OJJ46230.1

Group86: abra|OJJ65665.1 abra|OJJ65727.1 abra|OJJ70427.1 abra|OJJ73414.1 acar|OOG00335.1 acar|OOG00616.1 acla|EAW07048.1 acla|EAW10826.1 agla|OJJ78693.1 agla|OJJ85646.1 aluc|OJZ81154.1 aluc|OJZ88623.1 anid|CBF79793.1 anid|CBF82339.1 asyd|OJJ56350.1 asyd|OJJ56512.1 asyd|OJJ58126.1 aver|OJJ04280.1 aver|OJJ06363.1 awen|OJJ30203.1 awen|OJJ36125.1 azon|OJJ43588.1 azon|OJJ45284.1 afis|EAW21006.1 afum|EAL84365.1 agla|OJJ85438.1 akaw|GAA93193.1 atub|OJI88767.1 afla|EED56769.1 akaw|GAA83077.1 akaw|GAA93192.1 aory|EIT73951.1 atub|OJI91208.1 ater|EAU37240.1 afla|EED48948.1 aory|EIT79979.1 aory|EIT74379.1

Group87: abra|OJJ73172.1 abra|OJJ75139.1 acar|OOF97197.1 acar|OOG00411.1 acla|EAW06855.1 acla|EAW08318.1 afis|EAW16472.1 afis|EAW23353.1 afla|EED47207.1 afla|EED55602.1 afum|EAL88123.1 afum|EAL89066.1 agla|OJJ82874.1 agla|OJJ86473.1 akaw|GAA87999.1 akaw|GAA88746.1 aluc|OJZ81255.1 aluc|OJZ92706.1 anid|CBF74897.1 anid|CBF81437.1 anig|EHA18563.1 anig|EHA26294.1 aory|EIT79222.1 aory|EIT82665.1 asyd|OJJ59426.1 asyd|OJJ60103.1 ater|EAU35596.1 ater|EAU36570.1 atub|OJI87132.1 atub|OJI91451.1 aver|OJJ02845.1 aver|OJJ04159.1 awen|OJJ39536.1 awen|OJJ40364.1 azon|OJJ47731.1 azon|OJJ49213.1 anid|CBF74899.1

Group88: afis|EAW16767.1 afis|EAW20787.1 afis|EAW20816.1 aluc|OJZ89742.1 aluc|OJZ90793.1 atub|OJI82782.1 atub|OJI85059.1 aver|OJJ04748.1 aver|OJJ08594.1 awen|OJJ33411.1 awen|OJJ35564.1 afla|EED55613.1 anig|EHA22174.1 afum|EAL84837.1 afum|EAL86763.1 anid|CBF71491.1 asyd|OJJ52867.1

asyd|OJJ53645.1 asyd|OJJ54618.1 ater|EAU29406.1 awen|OJJ33444.1 azon|OJJ43208.1 afla|EED51750.1 agla|OJJ87003.1 akaw|GAA87278.1 anig|EHA19527.1 aory|EIT80503.1 asyd|OJJ56543.1 azon|OJJ47397.1 awen|OJJ33735.1 anid|CBF80305.1 aver|OJJ07540.1 awen|OJJ40176.1 agla|OJJ79599.1 akaw|GAA83902.1 anid|CBF86026.1 anid|CBF80765.1

Group89: aory|EIT73009.1 aory|EIT82639.1 abra|OJJ75430.1 acar|OOF90776.1 acla|EAW13800.1 acla|EAW13801.1 acla|EAW13802.1 afis|EAW18330.1 afis|EAW18331.1 afis|EAW18332.1 afla|EED48794.1 afla|EED48795.1 afla|EED49574.1 afum|EAL87695.1 afum|EAL87696.1 afum|EAL87697.1 agla|OJJ88054.1 akaw|GAA84206.1 akaw|GAA84207.1 aluc|OJZ88883.1 aluc|OJZ88884.1 anid|CBF79398.1 anid|CBF79400.1 anid|CBF79402.1 anig|EHA23623.1 aory|EIT72994.1 aory|EIT73029.1 asyd|OJJ57827.1 ater|EAU33343.1 ater|EAU33344.1 atub|OJI85907.1 atub|OJI85908.1 atub|OJI85909.1 aver|OJJ04565.1 awen|OJJ30712.1 azon|OJJ43305.1 afla|EED48796.1

Group90: aory|EIT73779.1 aory|EIT77467.1 awen|OJJ29827.1 awen|OJJ36939.1 abra|OJJ65853.1 acar|OOF95014.1 acla|EAW11610.1 afis|EAW23843.1 afla|EED56987.1 afum|EAL93778.1 agla|OJJ78690.1 akaw|GAA92291.1 aluc|OJZ90188.1 anig|EHA23687.1 ater|EAU31468.1 atub|OJI81615.1 aver|OJI99116.1 awen|OJJ29704.1 azon|OJJ46986.1 abra|OJJ77924.1 acar|OOF90880.1 afla|EED45127.1 akaw|GAA83740.1 aluc|OJZ90991.1 anid|CBF86319.1 anig|EHA19699.1 asyd|OJJ64996.1 atub|OJI82982.1 acar|OOG00327.1 asyd|OJJ55713.1 atub|OJI87196.1 aver|OJJ06799.1 afum|EAL87274.1 agla|OJJ86442.1 asyd|OJJ62873.1 ater|EAU31778.1 aver|OJI96926.1

Group91: abra|OJJ67337.1 acar|OOF92476.1 acla|EAW06397.1 afis|EAW18501.1 afla|EED52900.1 afum|EAL93192.1 agla|OJJ85126.1 akaw|GAA86479.1 aluc|OJZ85020.1 anid|CBF75182.1 anig|EHA25241.1 aory|EIT78666.1 asyd|OJJ59951.1 ater|EAU30070.1 atub|OJI80577.1 aver|OJJ02338.1 awen|OJJ31055.1 azon|OJJ50565.1 abra|OJJ69524.1 acar|OOF94230.1 acla|EAW11627.1 afis|EAW23829.1 afla|EED54410.1 afum|EAL87261.1 agla|OJJ82483.1 akaw|GAA88301.1 aluc|OJZ82364.1 anid|CBF75186.1 anig|EHA28401.1 aory|EIT75866.1 ater|EAU31384.1 atub|OJI88078.1 awen|OJJ38733.1 azon|OJJ50221.1 afla|EED51976.1 aory|EIT81396.1 agla|OJJ78561.1

Group92: abra|OJJ66053.1 abra|OJJ66142.1 abra|OJJ74823.1 abra|OJJ77498.1 acar|OOF98679.1 acar|OOF99228.1 afla|EED49691.1 afla|EED54455.1 afla|EED57013.1 agla|OJJ78526.1 agla|OJJ80805.1 agla|OJJ83730.1 akaw|GAA84123.1 akaw|GAA88361.1 aluc|OJZ81040.1 aluc|OJZ82294.1 anig|EHA18383.1 anig|EHA19327.1 anig|EHA25403.1 aory|EIT81151.1 aory|EIT82306.1 ater|EAU29270.1 ater|EAU38253.1 atub|OJI82544.1 atub|OJI88145.1 aver|OJJ01748.1 aver|OJJ01775.1 aver|OJJ08353.1 awen|OJJ34671.1 awen|OJJ41918.1 afis|EAW19512.1 afum|EAL86984.1 anid|CBF89390.1 asyd|OJJ59158.1 azon|OJJ42220.1 aory|EIT77599.1

Group93: abra|OJJ67791.1 abra|OJJ73324.1 abra|OJJ75373.1 acar|OOF94472.1 acar|OOF95395.1 afis|EAW17263.1 afis|EAW17790.1 afla|EED48868.1 afla|EED50759.1 afum|EAL86680.1 afum|EAL91599.1 akaw|GAA92735.1 akaw|GAA92794.1 aluc|OJZ88714.1 aluc|OJZ92943.1 aluc|OJZ88755.1 anig|EHA18417.1 anig|EHA26030.1 atub|OJI87402.1 atub|OJI91301.1 atub|OJI91325.1 awen|OJJ32920.1 awen|OJJ35770.1 acla|EAW12886.1 agla|OJJ84166.1 akaw|GAA92711.1 anid|CBF75220.1 aory|EIT78062.1 asyd|OJJ53461.1 ater|EAU29261.1 aver|OJJ08294.1 abra|OJJ67793.1 anid|CBF75222.1 aory|EIT78107.1 ater|EAU29259.1 awen|OJJ32922.1

Group94: abra|OJJ68844.1 abra|OJJ73357.1 acar|OOF90545.1 acar|OOG00565.1 acla|EAW06977.1 acla|EAW15277.1 afis|EAW16591.1 afis|EAW20248.1 afla|EED45005.1 afla|EED57097.1 afum|EAL85366.1 afum|EAL89187.1 akaw|GAA83127.1 akaw|GAA90404.1 aluc|OJZ85824.1 aluc|OJZ88683.1 anid|CBF78712.1 anid|CBF81894.1 anig|EHA18363.1 anig|EHA24737.1 aory|EIT73755.1 aory|EIT82298.1 asyd|OJJ59680.1 asyd|OJJ63029.1 ater|EAU32039.1 ater|EAU36671.1 atub|OJI79611.1 atub|OJI91263.1 aver|OJI97939.1 aver|OJJ02608.1 awen|OJJ31246.1 awen|OJJ40224.1 azon|OJJ48439.1 azon|OJJ49082.1 agla|OJJ82668.1 azon|OJJ45914.1

Group95: abra|OJJ69553.1 abra|OJJ70394.1 aory|EIT78629.1 aory|EIT82274.1 acar|OOF91880.1 afla|EED57090.1 agla|OJJ82606.1 akaw|GAA91965.1 aluc|OJZ82652.1 anid|CBF75146.1 anig|EHA27420.1 asyd|OJJ59972.1 ater|EAU30264.1 atub|OJI88884.1 aver|OJJ02319.1 awen|OJJ41493.1 abra|OJJ71088.1 acar|OOF98047.1 acla|EAW06408.1 afis|EAW18512.1 afla|EED52888.1 afum|EAL93207.1 agla|OJJ85018.1 akaw|GAA82873.1 aluc|OJZ87440.1 anid|CBF70237.1 anig|EHA20665.1 asyd|OJJ58768.1 ater|EAU29455.1 atub|OJI81208.1 aver|OJJ01377.1 awen|OJJ31066.1 azon|OJJ46021.1 acar|OOF98419.1 acar|OOF99514.1 azon|OJJ42344.1

Group96: abra|OJJ71749.1 abra|OJJ72735.1 acar|OOF90204.1 acar|OOF95966.1 afla|EED52022.1 afla|EED52611.1 afum|EAL85108.1 afum|EAL87310.1 agla|OJJ78437.1 agla|OJJ82556.1 akaw|GAA85163.1 akaw|GAA85341.1 aluc|OJZ86473.1 aluc|OJZ90703.1 anig|EHA22158.1 anig|EHA24129.1 aory|EIT73811.1 aory|EIT74178.1 aory|EIT81402.1 asyd|OJJ54106.1 asyd|OJJ56840.1 aver|OJJ03498.1 aver|OJJ08629.1 awen|OJJ31677.1 awen|OJJ33864.1 afis|EAW23882.1 agla|OJJ80999.1 anid|CBF77189.1 ater|EAU33464.1 atub|OJI82130.1 atub|OJI84056.1 azon|OJJ42521.1 azon|OJJ48689.1 acla|EAW11571.1 afis|EAW19784.1 afla|EED45206.1

Group97: abra|OJJ71850.1 abra|OJJ73479.1 acar|OOF96520.1 acar|OOG00674.1 acla|EAW06803.1 acla|EAW13882.1 afis|EAW16352.1 afis|EAW18594.1 afla|EED52753.1 afla|EED53519.1 afum|EAL88977.1

afum|EAL93302.1 agla|OJJ81646.1 agla|OJJ88248.1 akaw|GAA85067.1 akaw|GAA87429.1 aluc|OJZ86584.1 aluc|OJZ88568.1 anid|CBF70389.1 anid|CBF89223.1 anig|EHA18265.1 anig|EHA22076.1 aory|EIT75113.1 aory|EIT78157.1 asyd|OJJ58637.1 asyd|OJJ62519.1 ater|EAU34109.1 ater|EAU34888.1 atub|OJI83939.1 atub|OJI91154.1 aver|OJI96638.1 aver|OJJ01243.1 awen|OJJ31157.1 awen|OJJ37755.1 azon|OJJ45016.1 azon|OJJ46632.1

Group98: abra|OJJ72143.1 abra|OJJ77114.1 acar|OOF93003.1 acar|OOF96781.1 acla|EAW12957.1 acla|EAW14937.1 afis|EAW17702.1 afis|EAW24453.1 afum|EAL86122.1 afum|EAL91517.1 agla|OJJ79467.1 agla|OJJ80300.1 akaw|GAA84780.1 akaw|GAA90815.1 aluc|OJZ84523.1 aluc|OJZ86900.1 anid|CBF73820.1 anid|CBF89927.1 anig|EHA21742.1 anig|EHA27821.1 aory|EIT75637.1 aory|EIT83612.1 asyd|OJJ56569.1 asyd|OJJ62120.1 atub|OJI83619.1 atub|OJI89556.1 aver|OJI96228.1 aver|OJJ06153.1 awen|OJJ32744.1 awen|OJJ37133.1 azon|OJJ45294.1 azon|OJJ50831.1 afla|EED55221.1 ater|EAU29992.1 ater|EAU31909.1 afla|EED45698.1

Group99: abra|OJJ72622.1 abra|OJJ76815.1 acar|OOF91310.1 acar|OOF94641.1 acla|EAW11602.1 acla|EAW13375.1 afis|EAW20489.1 afis|EAW23856.1 afum|EAL84566.1 afum|EAL87284.1 agla|OJJ82510.1 agla|OJJ84827.1 akaw|GAA85243.1 akaw|GAA87206.1 aluc|OJZ84225.1 aluc|OJZ90600.1 anid|CBF77244.1 anid|CBF79220.1 anig|EHA24038.1 anig|EHA27574.1 aory|EIT74289.1 aory|EIT77786.1 asyd|OJJ55735.1 asyd|OJJ56812.1 ater|EAU29567.1 ater|EAU33499.1 atub|OJI82024.1 atub|OJI89859.1 aver|OJJ03523.1 aver|OJJ06821.1 awen|OJJ33189.1 awen|OJJ33817.1 azon|OJJ46391.1 azon|OJJ48719.1 afla|EED52576.1 afla|EED44774.1

Group100: abra|OJJ73827.1 abra|OJJ74268.1 acar|OOF95463.1 acar|OOG00979.1 acla|EAW07312.1 acla|EAW07722.1 afis|EAW22334.1 afis|EAW22754.1 afla|EED50015.1 afla|EED57748.1 afum|EAL90445.1 afum|EAL90858.1 agla|OJJ83671.1 agla|OJJ87714.1 akaw|GAA86755.1 akaw|GAA89445.1 aluc|OJZ88222.1 aluc|OJZ89865.1 anid|CBF88019.1 anid|CBF88645.1 anig|EHA17969.1 anig|EHA27065.1 aory|EIT74682.1 aory|EIT81059.1 asyd|OJJ63522.1 asyd|OJJ63881.1 ater|EAU38966.1 ater|EAU39471.1 atub|OJI84943.1 atub|OJI90822.1 aver|OJI97058.1 aver|OJI97408.1 awen|OJJ34912.1 awen|OJJ35426.1 azon|OJJ45572.1 azon|OJJ49750.1

Group101: abra|OJJ76503.1 abra|OJJ76504.1 acar|OOF99462.1 acar|OOF99465.1 afis|EAW18906.1 afis|EAW18907.1 afla|EED50840.1 afla|EED50841.1 afum|EAL93638.1 afum|EAL93639.1 agla|OJJ81511.1 agla|OJJ81512.1 akaw|GAA82419.1 akaw|GAA82420.1 aluc|OJZ91697.1 aluc|OJZ91699.1 anid|CBF69713.1 anid|CBF69715.1 anig|EHA22712.1 anig|EHA22713.1 aory|EIT78986.1 aory|EIT79030.1 asyd|OJJ59043.1 asyd|OJJ59044.1 ater|EAU38075.1 ater|EAU38076.1 atub|OJI86152.1 atub|OJI86154.1 aver|OJJ01638.1 aver|OJJ01639.1 awen|OJJ32463.1 awen|OJJ32464.1 azon|OJJ47220.1 azon|OJJ47221.1 acla|EAW14191.1 acla|EAW14192.1

Group102: afis|EAW17317.1 afis|EAW24567.1 afla|EED50210.1 afla|EED51099.1 afla|EED56609.1 afla|EED57630.1 anid|CBF82898.1 anid|CBF82901.1 aory|EIT75441.1 aory|EIT77262.1 aory|EIT79641.1 aory|EIT81362.1 aory|EIT82960.1 aory|EIT82255.1 aory|EIT81401.1 ater|EAU30305.1 ater|EAU39298.1 awen|OJJ35064.1 awen|OJJ38659.1 abra|OJJ69840.1 acar|OOF93363.1 acla|EAW13292.1 afla|EED57061.1 afum|EAL91153.1 akaw|GAA87820.1 aluc|OJZ85456.1 anig|EHA24998.1 asyd|OJJ62103.1 asyd|OJJ63346.1 atub|OJI80107.1 aver|OJI96209.1 aver|OJI97614.1 afla|EED52057.1 afum|EAL86230.1 acla|EAW15044.1 azon|OJJ48710.1

Group103: afla|EED46026.1 afla|EED49987.1 akaw|GAA89413.1 akaw|GAA90839.1 aluc|OJZ82994.1 aluc|OJZ88260.1 aory|EIT74653.1 aory|EIT75776.1 atub|OJI89228.1 atub|OJI90859.1 awen|OJJ30039.1 awen|OJJ35338.1 abra|OJJ65964.1 acar|OOF92910.1 acla|EAW13583.1 afis|EAW20018.1 afis|EAW22701.1 afum|EAL84899.2 afum|EAL90491.1 agla|OJJ87998.1 anid|CBF84731.1 anig|EHA21408.1 asyd|OJJ53303.1 ater|EAU29378.1 ater|EAU39001.1 aver|OJJ08438.1 azon|OJJ43685.1 abra|OJJ73794.1 acar|OOG00953.1 acla|EAW07686.1 agla|OJJ79008.1 anid|CBF88080.1 anig|EHA18005.1 asyd|OJJ63557.1 aver|OJI97375.1 azon|OJJ49780.1

Group104: agla|OJJ79992.1 agla|OJJ79999.1 azon|OJJ42536.1 azon|OJJ46701.1 abra|OJJ72154.1 acar|OOF95729.1 afis|EAW21487.1 afla|EED50612.1 agla|OJJ88432.1 anid|CBF86065.1 anig|EHA21734.1 aory|EIT78148.1 asyd|OJJ57414.1 ater|EAU29971.1 awen|OJJ31235.1 abra|OJJ77874.1 agla|OJJ85397.1 akaw|GAA83778.1 aluc|OJZ90949.1 anig|EHA19656.1 atub|OJI82940.1 azon|OJJ42437.1 afis|EAW18426.1 aluc|OJZ92434.1 awen|OJJ38296.1 akaw|GAA83564.1 ater|EAU38826.1 afla|EED48407.1 afla|EED49503.1 aory|EIT82647.1 awen|OJJ38298.1 agla|OJJ81249.1 aver|OJJ05768.1 asyd|OJJ52949.1 aver|OJI97602.1 azon|OJJ46505.1

Group105: abra|OJJ76497.1 acar|OOF99470.1 acla|EAW14363.1 afis|EAW18913.1 afla|EED50836.1 afum|EAL93644.1 agla|OJJ81517.1 akaw|GAA82414.1 aluc|OJZ91703.1 anid|CBF69695.1 anig|EHA22717.1 aory|EIT79010.1 asyd|OJJ59053.1 ater|EAU38080.1 atub|OJI86163.1 aver|OJJ01647.1 awen|OJJ31506.1 azon|OJJ41989.1 azon|OJJ47222.1 abra|OJJ76500.1 acar|OOF99467.1 acla|EAW14196.1 afis|EAW18912.1 afla|EED50837.1 afum|EAL93643.1 akaw|GAA82416.1 aluc|OJZ91702.1 anid|CBF69697.1 anig|EHA22716.1 aory|EIT79064.1 asyd|OJJ59051.1 ater|EAU38079.1 atub|OJI86161.1 awen|OJJ31505.1 azon|OJJ47223.1 azon|OJJ41991.1

Group106: abra|OJJ72950.1 abra|OJJ74416.1 acla|EAW06831.1 acla|EAW11196.1 afis|EAW15926.1 afis|EAW16381.1 afis|EAW19938.1 afum|EAL88562.1 afum|EAL89006.1 akaw|GAA85531.1 akaw|GAA87307.1 aluc|OJZ83460.1 aluc|OJZ89710.1 anid|CBF81021.1 anid|CBF89371.1 atub|OJI82352.1 atub|OJI85092.1 aver|OJI96612.1 aver|OJJ06329.1 awen|OJJ35654.1 awen|OJJ37914.1 acar|OOF96132.1 afla|EED54935.1 agla|OJJ89379.1 anig|EHA24304.1 aory|EIT80345.1 asyd|OJJ56383.1 ater|EAU35529.1 azon|OJJ48008.1 acar|OOF95327.1 afla|EED49933.1 anig|EHA26941.1 aory|EIT83444.1 asyd|OJJ62476.1 ater|EAU34071.1

Group107: abra|OJJ68266.1 acar|OOF97546.1 acla|EAW06826.1 afis|EAW16377.1 afla|EED53460.1 afum|EAL89002.1 agla|OJJ78595.1 akaw|GAA91930.1 aluc|OJZ82692.1 anid|CBF74547.1 anig|EHA27369.1 aory|EIT75115.1 asyd|OJJ57687.1 ater|EAU34075.1 atub|OJI88924.1 aver|OJJ05493.1 awen|OJJ37911.1 azon|OJJ45090.1 abra|OJJ68268.1 acar|OOF97548.1 acla|EAW07026.1 afis|EAW16652.1 afla|EED44948.1 afum|EAL89256.1 agla|OJJ78685.1 akaw|GAA91932.1 aluc|OJZ82690.1 anid|CBF74545.1 anig|EHA27367.1 aory|EIT73496.1 ater|EAU39576.1 atub|OJI88922.1 aver|OJJ05494.1 awen|OJJ40062.1 azon|OJJ45565.1

Group108: abra|OJJ69218.1 acar|OOF91100.1 acla|EAW08627.1 afis|EAW15804.1 afla|EED51241.1 afum|EAL85546.1 agla|OJJ81971.1 akaw|GAA92227.1 aluc|OJZ86200.1 anid|CBF70912.1 anig|EHA24423.1 aory|EIT83194.1 asyd|OJJ52602.1 ater|EAU33444.1 atub|OJI79993.1 aver|OJJ04628.1 awen|OJJ38514.1 azon|OJJ43738.1 abra|OJJ69219.1 acar|OOF91099.1 acla|EAW08626.1 afis|EAW15805.1 afla|EED51240.1 afum|EAL85545.1 akaw|GAA92228.1 aluc|OJZ86201.1 anid|CBF70910.1 anig|EHA24422.1 aory|EIT83177.1 asyd|OJJ52601.1 ater|EAU33443.1 atub|OJI79994.1 aver|OJJ04627.1 awen|OJJ38516.1 azon|OJJ43737.1

Group109: abra|OJJ73774.1 acar|OOG00931.1 acla|EAW07658.1 afis|EAW22676.1 afla|EED49957.1 afum|EAL90516.1 agla|OJJ79041.1 akaw|GAA88402.1 aluc|OJZ88280.1 anid|CBF88133.1 anig|EHA18025.1 aory|EIT83452.1 asyd|OJJ63590.1 ater|EAU39033.1 atub|OJI90880.1 aver|OJI97343.1 awen|OJJ35305.1 azon|OJJ49811.1 abra|OJJ73775.1 acar|OOG00932.1 acla|EAW07660.1 afis|EAW22677.1 afla|EED49958.1 afum|EAL90515.1 agla|OJJ79040.1 akaw|GAA88401.1 aluc|OJZ88279.1 anid|CBF88131.1 anig|EHA18024.1 aory|EIT83440.1 asyd|OJJ63589.1 ater|EAU39032.1 atub|OJI90879.1 aver|OJI97344.1 azon|OJJ49810.1

Group110: abra|OJJ74284.1 acar|OOF89817.1 acla|EAW07328.1 afis|EAW22360.1 afla|EED57768.1 afum|EAL90831.1 agla|OJJ87609.1 akaw|GAA86740.1 aluc|OJZ89849.1 anid|CBF88679.1 anig|EHA27050.1 aory|EIT81097.1 asyd|OJJ63900.1 ater|EAU39494.1 atub|OJI84957.1 aver|OJI97040.1 awen|OJJ34934.1 azon|OJJ45587.1 abra|OJJ74285.1 acar|OOF95446.1 acla|EAW07329.1 afis|EAW22361.1 afla|EED57769.1 afum|EAL90830.1 agla|OJJ87610.1 akaw|GAA86739.1 aluc|OJZ89848.1 anid|CBF88686.1 anig|EHA27049.1 aory|EIT81110.1 asyd|OJJ63902.1 ater|EAU39495.1 atub|OJI84958.1 aver|OJI97038.1 awen|OJJ34935.1

Group111: abra|OJJ74383.1 acar|OOF95354.1 acla|EAW07424.1 afis|EAW22450.1 afla|EED54465.1 afum|EAL90741.1 agla|OJJ87572.1 akaw|GAA87284.1 aluc|OJZ89735.1 anid|CBF88925.1 anig|EHA26961.1 aory|EIT81125.1 asyd|OJJ62853.1 ater|EAU39257.1 atub|OJI85066.1 aver|OJI96906.1 awen|OJJ35075.1 azon|OJJ50032.1 abra|OJJ74384.1 acar|OOF95353.1 acla|EAW07423.1 afis|EAW22449.1 afla|EED54467.1 afum|EAL90742.1 akaw|GAA87285.1 aluc|OJZ89734.1 anid|CBF88927.1 anig|EHA26960.1 aory|EIT81127.1 asyd|OJJ62852.1 ater|EAU39255.1 atub|OJI85067.1 aver|OJI96905.1 awen|OJJ35076.1 azon|OJJ50031.1

Group112: abra|OJJ75811.1 acar|OOG00066.1 acla|EAW14862.1 afis|EAW24390.1 afla|EED47820.1 afum|EAL86062.1 agla|OJJ78870.1 akaw|GAA84525.1 aluc|OJZ89258.1 anid|CBF73709.1 anig|EHA23310.1 aory|EIT77118.1 asyd|OJJ56644.1 ater|EAU30344.1 atub|OJI85541.1 aver|OJJ06082.1 awen|OJJ32814.1 azon|OJJ49018.1 abra|OJJ75812.1 acar|OOG00063.1 acla|EAW14861.1 afis|EAW24389.1 afum|EAL86061.1 agla|OJJ83285.1 akaw|GAA84526.1 aluc|OJZ89259.1 anid|CBF73707.1 anig|EHA23309.1 aory|EIT77172.1 asyd|OJJ56645.1 ater|EAU30345.1 atub|OJI85540.1 aver|OJJ06081.1 awen|OJJ32815.1 azon|OJJ49017.1

Group113: abra|OJJ77674.1 acar|OOF99062.1 acla|EAW06583.1 afis|EAW16009.1 afla|EED56386.1 afum|EAL88639.1 agla|OJJ86034.1 akaw|GAA83958.1 aluc|OJZ80845.1 anid|CBF74571.1 anig|EHA19478.1 aory|EIT83011.1 asyd|OJJ57651.1 ater|EAU33634.1 atub|OJI82722.1 aver|OJJ05511.1 awen|OJJ30456.1 azon|OJJ45447.1 abra|OJJ77675.1 acar|OOF99061.1 acla|EAW06587.1 afis|EAW16010.1 afla|EED56387.1 afum|EAL88640.1 agla|OJJ86035.1 akaw|GAA83957.1 aluc|OJZ80844.1 anid|CBF74569.1 anig|EHA19479.1 aory|EIT82998.1 ater|EAU33633.1 atub|OJI82723.1 aver|OJJ05510.1 awen|OJJ30455.1 azon|OJJ45446.1

Group114: abra|OJJ65981.1 abra|OJJ73719.1 acar|OOF92892.1 acar|OOG00881.1 acla|EAW07608.1 acla|EAW13600.1 afis|EAW20005.1 afis|EAW22638.1 afla|EED46007.1 afla|EED49889.1 afum|EAL84913.1 afum|EAL90554.2 akaw|GAA88444.1 akaw|GAA90854.1 aluc|OJZ82979.1 aluc|OJZ88332.1 anid|CBF84697.1 anid|CBF88205.1 anig|EHA18067.1 anig|EHA21394.1 asyd|OJJ53314.1 asyd|OJJ63639.1 atub|OJI89212.1 atub|OJI90927.1 aver|OJI97297.1 aver|OJJ08429.1 awen|OJJ30019.1 awen|OJJ35261.1 agla|OJJ80980.1 aory|EIT72460.1 aory|EIT75522.1 ater|EAU29347.1 ater|EAU39068.1 azon|OJJ44553.1

Group115: abra|OJJ66667.1 abra|OJJ72896.1 acar|OOF93682.1 acar|OOF93767.1 acar|OOF96087.1 acla|EAW11355.1 acla|EAW11808.1 afis|EAW18139.1 afis|EAW19418.1 afla|EED50375.1 afla|EED58065.1 afum|EAL87076.1 afum|EAL87496.1 agla|OJJ81131.1 agla|OJJ83822.1 akaw|GAA85483.1 akaw|GAA89616.1 aluc|OJZ80780.1 aluc|OJZ83405.1 anig|EHA24256.1 anig|EHA27249.1 aory|EIT79750.1 aory|EIT83148.1 atub|OJI82294.1 atub|OJI87632.1 awen|OJJ35581.1 awen|OJJ36816.1 azon|OJJ42986.1 azon|OJJ46941.1 anid|CBF74365.1 asyd|OJJ57784.1 ater|EAU34724.1 aver|OJJ05380.1 ater|EAU35462.1

Group116: abra|OJJ69228.1 abra|OJJ71754.1 asyd|OJJ52597.1 asyd|OJJ53400.1 aver|OJJ04201.1 aver|OJJ04624.1 abra|OJJ69224.1 acar|OOF91096.1 acla|EAW08622.1 afis|EAW15810.1 afla|EED51237.1 afum|EAL85541.1 agla|OJJ81976.1 akaw|GAA92232.1 aluc|OJZ86204.1 anid|CBF70905.1 anig|EHA24418.1 aory|EIT83188.1 ater|EAU33440.1 atub|OJI79998.1 awen|OJJ38521.1 azon|OJJ43734.1 acar|OOF91092.1 acla|EAW08620.1 afis|EAW15813.1 afla|EED51233.1 afum|EAL85538.1 akaw|GAA92236.1 aluc|OJZ86208.1 anid|CBF70903.1 aory|EIT83195.1 ater|EAU33435.1 atub|OJI80002.1 awen|OJJ38526.1

Group117: abra|OJJ69289.1 abra|OJJ70324.1 acar|OOF91780.1 acar|OOF94433.1 acla|EAW12318.1 acla|EAW14678.1 afis|EAW16903.1 afis|EAW24209.1 afla|EED45264.1 afla|EED48062.1 afum|EAL85879.1 afum|EAL86439.1 akaw|GAA88161.1 akaw|GAA92580.1 aluc|OJZ82118.1 aluc|OJZ82593.1 anig|EHA27482.1 anig|EHA28613.1 asyd|OJJ53986.1 asyd|OJJ54375.1 ater|EAU30213.1 ater|EAU31159.1 atub|OJI88319.1 atub|OJI88828.1 aver|OJJ05329.1 aver|OJJ08084.1 awen|OJJ29716.1 awen|OJJ33087.1 azon|OJJ42612.1 azon|OJJ44859.1 agla|OJJ79662.1 anid|CBF74267.1 aory|EIT83358.1 aory|EIT73698.1

Group118: abra|OJJ69846.1 abra|OJJ72321.1 acar|OOF94891.1 acar|OOF97566.1 afis|EAW19582.1 afis|EAW19642.1 afla|EED47805.1 afla|EED56993.1 agla|OJJ79220.1 agla|OJJ80784.1 akaw|GAA86072.1 akaw|GAA87813.1 aluc|OJZ85448.1 aluc|OJZ90307.1 anig|EHA23794.1 anig|EHA24991.1 aory|EIT77130.1 aory|EIT77504.1 atub|OJI80119.1 atub|OJI81731.1 aver|OJJ05597.1 aver|OJJ07110.1 acla|EAW12002.1 afum|EAL86880.1 anid|CBF71346.1 asyd|OJJ55096.1 ater|EAU32886.1 awen|OJJ41887.1 azon|OJJ42708.1 azon|OJJ46844.1 acla|EAW07815.1 anid|CBF74628.1 asyd|OJJ57585.1 ater|EAU30349.1

Group119: abra|OJJ70036.1 abra|OJJ72033.1 acla|EAW08504.1 acla|EAW09509.1 afis|EAW17096.1 afis|EAW21624.1 afla|EED48527.1 afla|EED57027.1 afla|EED57851.1 akaw|GAA84899.1 akaw|GAA88365.1 aluc|OJZ82290.1 aluc|OJZ86772.1 anig|EHA18733.1 anig|EHA21909.1 anig|EHA26569.1 aory|EIT72294.1 aory|EIT73160.1 aory|EIT81085.1 ater|EAU38312.1 ater|EAU38711.1 atub|OJI83756.1 atub|OJI88149.1 aver|OJJ03867.1 aver|OJJ08171.1 acar|OOF96691.1 afum|EAL92660.1 agla|OJJ79695.1 anid|CBF71556.1 asyd|OJJ52536.1 awen|OJJ33874.1 azon|OJJ48675.1 afla|EED46381.1 afla|EED46382.1

Group120: abra|OJJ72283.1 abra|OJJ78033.1 acar|OOF92932.1 acar|OOF94938.1 afla|EED49548.1 afla|EED50919.1 aluc|OJZ90262.1 aluc|OJZ91590.1 aory|EIT79028.1 aory|EIT82573.1 asyd|OJJ53419.1 asyd|OJJ54983.1 asyd|OJJ55080.1 asyd|OJJ65156.1 aver|OJJ07128.1 aver|OJJ08332.1 awen|OJJ32213.1 awen|OJJ32251.1 awen|OJJ41909.1 afis|EAW20797.1 afum|EAL84832.1 agla|OJJ85015.1 akaw|GAA86035.1 anid|CBF71371.1 anig|EHA23758.1 ater|EAU32868.1 atub|OJI81688.1 azon|OJJ47360.1 abra|OJJ76621.1 afla|EED51770.1 akaw|GAA92835.1 anig|EHA22614.1 aory|EIT81741.1 atub|OJI86039.1

Group121: abra|OJJ74623.1 abra|OJJ74837.1 acar|OOF95085.1 acar|OOF95151.1 acar|OOF95841.1 acla|EAW10761.1 acla|EAW14613.1 afis|EAW19343.1 afis|EAW24815.1 afla|EED47168.1 afla|EED50136.1 afla|EED56891.1 afum|EAL89459.1 afum|EAL94062.1 akaw|GAA81986.1 akaw|GAA83583.1 aluc|OJZ92204.1 aluc|OJZ92414.1 anig|EHA26551.1 anig|EHA26754.1 aory|EIT74736.1 aory|EIT79324.1 aory|EIT77583.1 ater|EAU36492.1 ater|EAU39073.1 atub|OJI86632.1 atub|OJI86844.1 azon|OJJ48665.1 azon|OJJ49253.1 agla|OJJ82911.1 anid|CBF85881.1 asyd|OJJ64378.1 aver|OJI98474.1 awen|OJJ40401.1

Group122: agla|OJJ84376.1 agla|OJJ85709.1 asyd|OJJ57959.1 asyd|OJJ61061.1 abra|OJJ71937.1 acar|OOF96594.1 acar|OOF96595.1 acla|EAW09592.1 acla|EAW09593.1 afis|EAW21545.1 afis|EAW21546.1 afla|EED55996.1 afla|EED55997.1 afum|EAL92565.1 afum|EAL92566.1 agla|OJJ85708.1 akaw|GAA84987.1 aluc|OJZ86674.1 anid|CBF76366.1 anid|CBF76368.1 anig|EHA21999.1 anig|EHA22000.1 aory|EIT76685.1 aory|EIT76776.1 asyd|OJJ61062.1 ater|EAU34993.1 ater|EAU34994.1 atub|OJI83854.1 aver|OJJ00378.1 aver|OJJ00379.1 awen|OJJ39144.1 awen|OJJ39146.1 azon|OJJ51476.1 azon|OJJ51477.1

Group123: abra|OJJ67331.1 acar|OOF92482.1 acla|EAW06403.1 afis|EAW18507.1 afla|EED52893.1 afum|EAL93201.1 agla|OJJ85114.1 akaw|GAA86485.1 aluc|OJZ85026.1 anid|CBF70247.1 anig|EHA25247.1 aory|EIT78591.1 asyd|OJJ58761.1 ater|EAU30064.1 atub|OJI80571.1 aver|OJJ01371.1 awen|OJJ31060.1 azon|OJJ50571.1 abra|OJJ67332.1 acar|OOF92481.1 acla|EAW06402.1 afis|EAW18506.1 afla|EED52895.1 afum|EAL93200.1 akaw|GAA86484.1 aluc|OJZ85025.1 anig|EHA25246.1 aory|EIT78664.1 asyd|OJJ58760.1 ater|EAU30065.1 atub|OJI80572.1 aver|OJJ01368.1 awen|OJJ31059.1 azon|OJJ50569.1

Group124: abra|OJJ68518.1 acar|OOF92724.1 acla|EAW12845.1 afis|EAW18008.1 afla|EED51887.1 afum|EAL91820.1 agla|OJJ80274.1 akaw|GAA91330.1 aluc|OJZ82732.1 anid|CBF80034.1 anig|EHA21184.1 aory|EIT81755.1 asyd|OJJ58272.1 ater|EAU31524.1 atub|OJI88965.1 aver|OJJ02152.1 awen|OJJ41532.1 azon|OJJ48252.1 atub|OJI79785.1 abra|OJJ69012.1 acar|OOF92723.1 acla|EAW12846.1 afis|EAW18009.1 afla|EED51886.1 afum|EAL91821.1 agla|OJJ80275.1 akaw|GAA90529.1 aluc|OJZ85998.1 anid|CBF80035.1 anig|EHA24612.1 aory|EIT81715.1 ater|EAU31525.1 awen|OJJ41531.1 azon|OJJ48251.1

Group125: abra|OJJ69958.1 acar|OOF93468.1 acla|EAW08829.1 afis|EAW15568.1 afla|EED49078.1 afum|EAL85765.1 agla|OJJ85182.1 akaw|GAA87702.1 aluc|OJZ85324.1 anid|CBF78110.1 anig|EHA25514.1 aory|EIT82568.1 ater|EAU32562.1 atub|OJI80248.1 abra|OJJ69959.1 acla|EAW08833.1 afis|EAW15567.1 afla|EED49076.1 afla|EED49077.1 afum|EAL85766.1 agla|OJJ85184.1 akaw|GAA87701.1 anid|CBF78112.1 anid|CBF78114.1 aory|EIT82498.1 aory|EIT82525.1 asyd|OJJ55899.1 ater|EAU32566.1 atub|OJI80249.1 aver|OJJ04844.1 awen|OJJ38227.1 azon|OJJ43068.1 acla|EAW08832.1 azon|OJJ47853.1

Group126: abra|OJJ71382.1 acar|OOF97787.1 acla|EAW10157.1 afis|EAW25437.1 afla|EED53925.1 afum|EAL90095.1 agla|OJJ87082.1 akaw|GAA82612.1 aluc|OJZ87158.1 anid|CBF77596.1 anig|EHA20417.1 aory|EIT78347.1 asyd|OJJ53142.1 ater|EAU34609.1 atub|OJI81482.1 aver|OJJ03710.1 awen|OJJ41165.1 azon|OJJ44743.1 abra|OJJ71383.1 acar|OOF97786.1 acla|EAW10158.1 afis|EAW25436.1 afla|EED53924.1 afum|EAL90094.1 agla|OJJ87081.1 akaw|GAA82611.1 aluc|OJZ87157.1 anid|CBF77594.1 anig|EHA20416.1 aory|EIT78262.1 asyd|OJJ53143.1 ater|EAU34608.1 atub|OJI81483.1 aver|OJJ03709.1

Group127: abra|OJJ72469.1 acar|OOF94768.1 acla|EAW12702.1 afis|EAW17865.1 afla|EED46230.1 afum|EAL91673.1 agla|OJJ83099.1 akaw|GAA86207.1 aluc|OJZ90456.1 anid|CBF76910.1 anig|EHA23918.1 aory|EIT76409.1 asyd|OJJ57089.1 ater|EAU33080.1 atub|OJI81883.1 aver|OJJ03211.1 awen|OJJ41706.1 azon|OJJ48402.1 abra|OJJ72470.1 acar|OOF94765.1 acla|EAW12703.1 afis|EAW17866.1 afla|EED46231.1 afum|EAL91674.1 agla|OJJ83086.1 akaw|GAA86208.1 aluc|OJZ90457.1 anid|CBF76912.1 anig|EHA23919.1 aory|EIT76527.1 ater|EAU33081.1 atub|OJI81884.1 awen|OJJ41705.1 azon|OJJ48401.1

Group128: abra|OJJ72932.1 acar|OOF96112.1 acla|EAW06458.1 afis|EAW15954.1 afla|EED58104.1 afum|EAL88587.1 agla|OJJ89359.1 akaw|GAA85514.1 aluc|OJZ83442.1 anid|CBF81063.1 anig|EHA24288.1 aory|EIT79164.1 asyd|OJJ56456.1 ater|EAU35503.1 atub|OJI82333.1 aver|OJJ06266.1 awen|OJJ35622.1 azon|OJJ46902.1 abra|OJJ72934.1 acar|OOF96115.1 acla|EAW06457.1 afis|EAW15953.1 afla|EED58105.1 afum|EAL88586.1 akaw|GAA85515.1 aluc|OJZ83443.1 anid|CBF81061.1 anig|EHA24289.1 aory|EIT79130.1 asyd|OJJ56455.1 ater|EAU35504.1 atub|OJI82334.1 aver|OJJ06267.1 awen|OJJ35623.1

Group129: abra|OJJ76465.1 acar|OOF99703.1 acla|EAW13966.1 afis|EAW18675.1 afla|EED50624.1 afum|EAL93395.2 agla|OJJ82401.1 aluc|OJZ91745.1 anid|CBF70588.1 anig|EHA22748.1 aory|EIT78145.1 asyd|OJJ58523.1 ater|EAU38235.1 atub|OJI86203.1 aver|OJJ01124.1 awen|OJJ32147.1 azon|OJJ46731.1 abra|OJJ76466.1 acar|OOF99704.1 acla|EAW13965.1 afis|EAW18674.1 afla|EED50625.1 afum|EAL93394.1 akaw|GAA82377.1 aluc|OJZ91744.1 anid|CBF70586.1 anig|EHA22747.1 aory|EIT77989.1 asyd|OJJ58524.1 ater|EAU38236.1 atub|OJI86202.1 aver|OJJ01125.1 awen|OJJ32146.1 azon|OJJ46730.1

Group130: abra|OJJ66702.1 abra|OJJ76322.1 acar|OOF93706.1 acar|OOF93792.1 afla|EED45227.1 afla|EED56652.1 anid|CBF78509.1 anid|CBF81402.1 anig|EHA22872.1 anig|EHA27277.1 aory|EIT73751.1 aory|EIT79654.1 asyd|OJJ59404.1 asyd|OJJ65313.1 ater|EAU29738.1 ater|EAU34746.1 atub|OJI86345.1 atub|OJI87602.1 acla|EAW11833.1 afis|EAW19444.1 afum|EAL87051.1 agla|OJJ81158.1 akaw|GAA89644.1 aluc|OJZ80745.1 aver|OJJ02869.1 awen|OJJ36845.1 azon|OJJ42319.1 azon|OJJ42955.1 acar|OOF97765.1 akaw|GAA82251.1 aluc|OJZ91893.1 aver|OJI99451.1 awen|OJJ40922.1

Group131: abra|OJJ68714.1 abra|OJJ76939.1 acar|OOF91389.1 acar|OOF92214.1 afum|EAL84486.2 afum|EAL91972.2 aluc|OJZ81814.1 aluc|OJZ84338.1 anig|EHA22401.1 anig|EHA27672.1 atub|OJI84386.1 atub|OJI89730.1 awen|OJJ33278.1 awen|OJJ41371.1 azon|OJJ48840.1 azon|OJJ50652.1 acla|EAW12532.1 acla|EAW13466.1 afis|EAW20578.1 afis|EAW24117.1 afla|EED46815.1 afla|EED52313.1 agla|OJJ88717.1 akaw|GAA87100.1 akaw|GAA89867.1 anid|CBF86537.1 aory|EIT74384.1 asyd|OJJ64866.1 ater|EAU37106.1 aver|OJI98979.1 anid|CBF78940.1 asyd|OJJ55646.1 aver|OJJ06731.1

Group132: abra|OJJ69799.1 abra|OJJ75720.1 acar|OOF90260.1 acar|OOF93578.1 afis|EAW21759.1 afis|EAW24741.1 afum|EAL89387.1 afum|EAL92789.1 akaw|GAA84456.1 akaw|GAA87860.1 aluc|OJZ85501.1 aluc|OJZ89172.1 anid|CBF80400.1 anid|CBF83678.1 anig|EHA23383.1 anig|EHA25034.1 aory|EIT75373.1 aory|EIT79769.1 asyd|OJJ54761.1 asyd|OJJ55920.1 asyd|OJJ60823.1 atub|OJI80064.1 atub|OJI85618.1 aver|OJJ00149.1 aver|OJJ04873.1 aver|OJJ07409.1 azon|OJJ45021.1 azon|OJJ48200.1 acla|EAW09376.1 afla|EED45538.1 agla|OJJ84391.1 ater|EAU38431.1 awen|OJJ34043.1

Group133: abra|OJJ71453.1 abra|OJJ72857.1 acar|OOF96054.1 acar|OOF97693.1 acar|OOF97744.1 acar|OOF98763.1 afis|EAW22749.1 afis|EAW24033.1 afla|EED45033.1 afla|EED49120.1 afum|EAL87464.1 afum|EAL90450.1 akaw|GAA82558.1 akaw|GAA85456.1 aluc|OJZ83364.1 aluc|OJZ87090.1 anig|EHA20361.1 anig|EHA24229.1 aory|EIT73568.1 aory|EIT78580.1 asyd|OJJ59869.1 asyd|OJJ65349.1 ater|EAU29414.1 ater|EAU29711.1 atub|OJI81548.1 atub|OJI82255.1 aver|OJI99497.1 aver|OJJ02418.1 acla|EAW11389.1 agla|OJJ86999.1 anid|CBF75320.1 awen|OJJ35505.1 azon|OJJ46979.1

Group134: abra|OJJ72895.1 abra|OJJ73247.1 acar|OOF96086.1 acar|OOG00487.1 acla|EAW06921.1 acla|EAW11356.1 afis|EAW16538.1 afis|EAW18138.1 afla|EED56131.1 afla|EED58064.1 afum|EAL87495.1 afum|EAL89135.1 agla|OJJ82784.1 agla|OJJ83821.1 akaw|GAA85482.1 akaw|GAA87933.1 aluc|OJZ81180.1 aluc|OJZ83404.1 anig|EHA18498.1 anig|EHA24255.1 aory|EIT76761.1 aory|EIT79757.1 ater|EAU35461.1 ater|EAU36601.1 atub|OJI82293.1 atub|OJI91377.1 awen|OJJ35580.1 awen|OJJ40283.1 azon|OJJ46943.1 azon|OJJ49145.1 anid|CBF81745.1 asyd|OJJ59599.1 aver|OJJ02669.1

Group135: anid|CBF74543.1 anid|CBF80800.1 asyd|OJJ54487.1 asyd|OJJ57688.1 aver|OJJ05495.1 aver|OJJ07692.1 awen|OJJ30771.1 awen|OJJ31710.1 abra|OJJ67440.1 acar|OOF92387.1 acar|OOF92388.1 acla|EAW06324.1 afis|EAW18438.1 afla|EED52977.1 afum|EAL93120.1 agla|OJJ85004.1 akaw|GAA86390.1 aluc|OJZ84924.1 anid|CBF70039.1 anig|EHA25142.1 anig|EHA25143.1 aory|EIT78691.1 aory|EIT78713.1 asyd|OJJ58866.1 ater|EAU29862.1 atub|OJI80675.1 aver|OJJ01467.1 azon|OJJ50475.1 acla|EAW06323.1 afis|EAW18437.1 afum|EAL93119.1 ater|EAU29854.1 afla|EED52975.1

Group136: aory|EIT76219.1 abra|OJJ66160.1 acla|EAW07091.1 ater|EAU30184.1 abra|OJJ76943.1 acar|OOF91392.1 acla|EAW14645.1 afis|EAW23759.1 afla|EED45469.1 afum|EAL87193.2 agla|OJJ78794.1 akaw|GAA87097.1 aluc|OJZ84341.1 anid|CBF86208.1 anig|EHA27675.1 anig|EHA27676.1 aory|EIT75611.1 asyd|OJJ64548.1 atub|OJI89727.1 aver|OJI98644.1 awen|OJJ40873.1 afis|EAW21192.1 anid|CBF78488.1 asyd|OJJ58352.1 ater|EAU36866.1 aver|OJJ02055.1 awen|OJJ31298.1 afis|EAW22111.1 afla|EED48116.1 aver|OJJ07112.1 azon|OJJ50585.1 awen|OJJ29841.1 awen|OJJ30060.1

Group137: abra|OJJ68073.1 acar|OOF92659.1 acla|EAW14013.1 afis|EAW18719.1 afla|EED50674.1 afum|EAL93443.2 agla|OJJ82339.1 akaw|GAA92398.1 aluc|OJZ83259.1 anid|CBF70677.1 anig|EHA21113.1 aory|EIT78139.1 asyd|OJJ58472.1 ater|EAU38185.1 atub|OJI87680.1 aver|OJJ01073.1 awen|OJJ32224.1 azon|OJJ46775.1 abra|OJJ68477.1 acar|OOF92676.1 acla|EAW07215.1 afis|EAW22228.1 afla|EED57619.1 afum|EAL90966.1 agla|OJJ87821.1 anid|CBF88467.1 anig|EHA21112.1 aory|EIT82940.1 asyd|OJJ63779.1 ater|EAU34233.1 aver|OJI97158.1 awen|OJJ34810.1 azon|OJJ45740.1

Group138: abra|OJJ71306.1 acar|OOF97864.1 acla|EAW10234.1 afis|EAW25358.1 afla|EED53840.1 afum|EAL90020.1 agla|OJJ83908.1 akaw|GAA82685.1 aluc|OJZ87238.1 anid|CBF77463.1 anig|EHA20484.1 aory|EIT78472.1 asyd|OJJ53218.1 ater|EAU34506.1 atub|OJI81404.1 aver|OJJ03634.1 awen|OJJ41084.1 azon|OJJ44664.1 abra|OJJ71307.1 acla|EAW10233.1 afis|EAW25359.1 afla|EED53841.1 afum|EAL90021.1 agla|OJJ83909.1 akaw|GAA82684.1 aluc|OJZ87237.1 anid|CBF77461.1 aory|EIT78435.1 asyd|OJJ53217.1 ater|EAU34507.1 atub|OJI81405.1 awen|OJJ41085.1 azon|OJJ44665.1

Group139: abra|OJJ71587.1 acar|OOF96317.1 acla|EAW15133.1 afis|EAW20105.1 afla|EED53369.1 afum|EAL85222.1 agla|OJJ88850.1 akaw|GAA89336.1 aluc|OJZ86327.1 anid|CBF84906.1 anig|EHA21551.1 aory|EIT75191.1 asyd|OJJ63169.1 ater|EAU38724.1 atub|OJI84207.1 aver|OJI97785.1 awen|OJJ31876.1 azon|OJJ46259.1 abra|OJJ71588.1 acla|EAW15132.1 afis|EAW20104.1 afum|EAL85221.1 agla|OJJ88849.1 akaw|GAA89335.1 aluc|OJZ86328.1 anid|CBF84908.1 anig|EHA21550.1 aory|EIT75224.1 asyd|OJJ63168.1 atub|OJI84206.1 aver|OJI97786.1 awen|OJJ31877.1 azon|OJJ46257.1

Group140: abra|OJJ72666.1 acar|OOF94609.1 acla|EAW11529.1 afis|EAW23925.1 afla|EED50596.1 afum|EAL87350.1 agla|OJJ79403.1 akaw|GAA85287.1 aluc|OJZ90645.1 anid|CBF77250.1 anig|EHA24080.1 aory|EIT82072.1 asyd|OJJ56809.1 ater|EAU32310.1 atub|OJI82075.1 aver|OJJ03525.1 awen|OJJ39029.1 azon|OJJ51358.1 abra|OJJ72668.1 acla|EAW11530.1 afis|EAW23924.1 afla|EED50597.1 afum|EAL87349.1 agla|OJJ79402.1 akaw|GAA85288.1 aluc|OJZ90646.1 anid|CBF77248.1 anig|EHA24081.1 aory|EIT78140.1 asyd|OJJ56810.1 ater|EAU32312.1 awen|OJJ39031.1 azon|OJJ51359.1

Group141: abra|OJJ74273.1 acar|OOF95457.1 acla|EAW07318.1 afis|EAW22350.1 afla|EED57754.1 afum|EAL90842.1 agla|OJJ87709.1 akaw|GAA86750.1 aluc|OJZ89860.1 anid|CBF88653.1 anig|EHA27060.1 aory|EIT81054.1 ater|EAU39489.1 aver|OJI97054.1 awen|OJJ34904.1 azon|OJJ45578.1 abra|OJJ74279.1 acar|OOF95452.1 acla|EAW07323.1 afis|EAW22355.1 afla|EED57761.1 afum|EAL90836.1 agla|OJJ87608.1 akaw|GAA86745.1 aluc|OJZ89854.1 anid|CBF88666.1 anig|EHA27055.1 aory|EIT81031.1 asyd|OJJ63892.1 atub|OJI84952.1 aver|OJI97047.1 awen|OJJ34929.1 azon|OJJ45582.1

Group142: abra|OJJ68218.1 abra|OJJ69789.1 acar|OOF93590.1 acar|OOF97507.1 agla|OJJ78902.1 agla|OJJ86249.1 akaw|GAA83228.1 akaw|GAA87869.1 aluc|OJZ83728.1 aluc|OJZ85510.1 anid|CBF84393.1 anid|CBF86693.1 atub|OJI80056.1 atub|OJI90104.1 acla|EAW09054.1 acla|EAW12123.1 afis|EAW25629.1 afla|EED55183.1 afum|EAL84317.1 anig|EHA18876.1 aory|EIT80665.1 aory|EIT81643.1 asyd|OJJ55507.1 ater|EAU32706.1 aver|OJJ06584.1 aver|OJJ08175.1 awen|OJJ36048.1 azon|OJJ43184.1 afla|EED51722.1 anig|EHA25042.1 asyd|OJJ53581.1 anid|CBF83169.1

Group143: abra|OJJ68305.1 abra|OJJ68976.1 afla|EED55146.1 afla|EED57528.1 agla|OJJ80112.1 agla|OJJ84537.1 akaw|GAA83169.1 akaw|GAA90501.1 aluc|OJZ83668.1 aluc|OJZ85964.1 aory|EIT81602.1 aory|EIT81847.1 asyd|OJJ59831.1 asyd|OJJ65307.1 atub|OJI79752.1 atub|OJI90039.1 awen|OJJ31501.1 awen|OJJ36340.1 azon|OJJ46215.1 azon|OJJ48729.1 acar|OOF91551.1 acar|OOF96683.1 acla|EAW14213.1 afis|EAW19068.1 afum|EAL93797.1 anid|CBF78527.1 anig|EHA18823.1 anig|EHA24640.1 aver|OJI99445.1 afla|EED54434.1 aory|EIT75875.1 ater|EAU32117.1

Group144: abra|OJJ69780.1 abra|OJJ73124.1 acar|OOF90902.1 acar|OOF91910.1 afis|EAW16809.1 afis|EAW20667.1 afis|EAW16810.1 atub|OJI88609.1 atub|OJI91509.1 awen|OJJ29822.1 awen|OJJ37778.1 azon|OJJ42117.1 azon|OJJ42607.1 afis|EAW17206.1 afis|EAW23422.1 afla|EED49098.1 afum|EAL85399.1 afum|EAL86742.1 afum|EBA27333.1 agla|OJJ84598.1 akaw|GAA88041.1 akaw|GAA92683.1 aluc|OJZ81300.1 aluc|OJZ85605.1 anig|EHA18609.1 ater|EAU32778.1 anig|EHA25864.1 afum|EAL86743.1 afis|EAW20625.1 afla|EED49487.1 atub|OJI83712.1 aluc|OJZ90138.1

Group145: abra|OJJ70543.1 abra|OJJ73773.1 acar|OOF94608.1 acar|OOG00929.1 afis|EAW15411.1 afis|EAW22675.1 afum|EAL84235.1 afum|EAL90517.1 akaw|GAA83389.1 akaw|GAA88403.1 aluc|OJZ83888.1 aluc|OJZ88281.1 anid|CBF74491.1 anid|CBF88137.1 anig|EHA18026.1 anig|EHA19025.1 aory|EIT72732.1 aory|EIT83438.1 asyd|OJJ57716.1 asyd|OJJ63598.1 ater|EAU36301.1 ater|EAU39034.1 atub|OJI90255.1 atub|OJI90881.1 azon|OJJ45068.1 azon|OJJ49812.1 acla|EAW07657.1 afla|EED49956.1 agla|OJJ79042.1 aver|OJI97338.1 aver|OJJ05451.1 awen|OJJ35304.1

Group146: abra|OJJ70875.1 abra|OJJ77724.1 acar|OOF98245.1 acar|OOF99016.1 acla|EAW10463.1 acla|EAW10819.1 acla|EAW11708.1 afis|EAW23731.1 afis|EAW25104.1 afum|EAL87170.1 afum|EAL89775.1 akaw|GAA83911.1 akaw|GAA91738.1 akaw|GAA93340.1 aluc|OJZ87647.1 aluc|OJZ90786.1 ater|EAU29552.1 ater|EAU31452.1 atub|OJI80998.1 atub|OJI82773.1 afla|EED55342.1 anid|CBF82779.1 anid|CBF86021.1 anig|EHA20840.1 asyd|OJJ55916.1 asyd|OJJ64453.1 ater|EAU39370.1 aver|OJI98556.1 aver|OJJ04856.1 awen|OJJ40718.1 anig|EHA19519.1 azon|OJJ48020.1

Group147: abra|OJJ73071.1 abra|OJJ77012.1 acla|EAW09005.1 acla|EAW15322.1 afis|EAW20323.1 afis|EAW25545.1 akaw|GAA87037.1 akaw|GAA88090.1 aluc|OJZ81354.1 aluc|OJZ84412.1 asyd|OJJ54270.1 asyd|OJJ55537.1 ater|EAU32181.1 ater|EAU39595.1 atub|OJI89661.1 atub|OJI91558.1 aver|OJJ05226.1 aver|OJJ06615.1 acar|OOG00304.1 afla|EED44875.1 afum|EAL85448.1 agla|OJJ78910.1 anid|CBF82101.1 anid|CBF83180.1 anig|EHA18655.1 aory|EIT83261.1 awen|OJJ36038.1 azon|OJJ51343.1 afla|EED54130.1 afum|EAL84263.1 anig|EHA27738.1 aory|EIT76050.1

Group148: abra|OJJ76652.1 abra|OJJ77533.1 acar|OOF99194.1 acar|OOF99350.1 afla|EED53116.1 afla|EED53300.1 akaw|GAA84088.1 akaw|GAA92992.1 aluc|OJZ81003.1 aluc|OJZ91553.1 anid|CBF82367.1 anid|CBF86914.1 anig|EHA19357.1 anig|EHA22588.1 asyd|OJJ54198.1 asyd|OJJ64607.1 ater|EAU30240.1 ater|EAU37451.1 ater|EAU37789.1 atub|OJI82582.1 atub|OJI86006.1 aver|OJI96893.1 aver|OJJ08526.1 aver|OJI98704.1 awen|OJJ31647.1 awen|OJJ37655.1 acla|EAW06705.1 agla|OJJ88350.1 aory|EIT74767.1 aory|EIT74899.1 azon|OJJ44918.1 asyd|OJJ53794.1

Group149: acar|OOF94510.1 acar|OOG00617.1 agla|OJJ78704.1 agla|OJJ82589.1 agla|OJJ88673.1 awen|OJJ33431.1 awen|OJJ40048.1 abra|OJJ73366.1 acar|OOG00573.1 acla|EAW12876.1 afis|EAW17800.1 afum|EAL91608.1 akaw|GAA83118.1 aluc|OJZ88674.1 anid|CBF87487.1 anig|EHA18356.1 asyd|OJJ52958.1 ater|EAU31398.1 atub|OJI91255.1 aver|OJJ08926.1 abra|OJJ73415.1 afla|EED44896.1 akaw|GAA83076.1 aluc|OJZ88622.1 anig|EHA18315.1 aory|EIT83248.1 asyd|OJJ62646.1 atub|OJI91207.1 aver|OJJ07782.1 azon|OJJ50122.1 afis|EAW20384.1 asyd|OJJ54467.1

Group150: acar|OOF95065.1 acar|OOF95131.1 acar|OOF95066.1 acar|OOF95132.1 abra|OJJ74648.1 acla|EAW10738.1 acla|EAW10739.1 afis|EAW24835.1 afis|EAW24836.1 afla|EED47139.1 afla|EED47140.1 afum|EAL89480.1 afum|EAL89481.2 agla|OJJ82028.1 akaw|GAA81962.1 akaw|GAA81963.1 aluc|OJZ92226.1 anid|CBF74584.1 anid|CBF74586.1 anig|EHA26730.1 aory|EIT79207.1 aory|EIT79226.1 asyd|OJJ57656.1 ater|EAU36469.1 ater|EAU36470.1 atub|OJI86656.1 aver|OJJ05516.1 aver|OJJ05517.1 awen|OJJ40422.1 awen|OJJ40423.1 azon|OJJ49280.1 azon|OJJ49281.1

Group151: acar|OOF95215.1 acar|OOG00549.1 afla|EED45224.1 afla|EED52255.1 afla|EED57542.1 afum|EAL86491.1 afum|EAL91022.1 aory|EIT73609.1 aory|EIT74331.1 aory|EIT81831.1 asyd|OJJ52204.1 asyd|OJJ54774.1 asyd|OJJ64753.1 ater|EAU30189.1 ater|EAU32767.1 ater|EAU34175.1 aver|OJI98855.1 aver|OJJ07392.1 aver|OJJ07305.1 abra|OJJ74541.1 acla|EAW07155.1 acla|EAW12276.1 afis|EAW16964.1 agla|OJJ87885.1 akaw|GAA82061.1 aluc|OJZ92121.1 anid|CBF86686.1 anig|EHA26824.1 atub|OJI86554.1 awen|OJJ34744.1 azon|OJJ42779.1 anid|CBF80465.1

Group152: anig|EHA18119.1 anig|EHA26153.1 awen|OJJ35539.1 awen|OJJ38334.1 acla|EAW13628.1 afis|EAW24672.1 acar|OOG00839.1 afla|EED48354.1 afum|EAL92114.1 akaw|GAA88497.1 aluc|OJZ88392.1 anid|CBF87369.1 anig|EHA18120.1 aory|EIT72297.1 asyd|OJJ53356.1 atub|OJI90987.1 aver|OJJ08385.1 acar|OOG00840.1 acla|EAW07713.1 afis|EAW20752.1 afla|EED48353.1 afum|EAL84865.1 agla|OJJ88184.1 akaw|GAA88496.1 anid|CBF82358.1 aory|EIT72300.1 asyd|OJJ54748.1 azon|OJJ43889.1 afum|EAL86291.2 afis|EAW21091.1 aver|OJJ08174.1 asyd|OJJ54203.1

Group153: asyd|OJJ60702.1 asyd|OJJ64821.1 aver|OJI98934.1 aver|OJJ00028.1 abra|OJJ72028.1 acar|OOF96677.1 afis|EAW21619.1 afla|EED57848.1 akaw|GAA84904.1 aluc|OJZ86765.1 anid|CBF83493.1 anig|EHA21914.1 aory|EIT81067.1 ater|EAU38309.1 atub|OJI83762.1 awen|OJJ37096.1 azon|OJJ48684.1 abra|OJJ77154.1 acar|OOF93040.1 acla|EAW12917.1 afis|EAW17740.1 afla|EED45741.1 afum|EAL91554.1 agla|OJJ81711.1 akaw|GAA90776.1 aluc|OJZ84568.1 anid|CBF86610.1 anig|EHA27862.1 aory|EIT75552.1 ater|EAU31867.1 atub|OJI89514.1 azon|OJJ50798.1

Group154: atub|OJI81651.1 atub|OJI86062.1 abra|OJJ65911.1 acar|OOF94961.1 acla|EAW12054.1 afis|EAW19697.1 afla|EED57032.1 afum|EAL86830.1 agla|OJJ80819.1 akaw|GAA92321.1 aluc|OJZ90219.1 anid|CBF71392.1 anig|EHA23717.1 aory|EIT82283.1 asyd|OJJ55041.1 ater|EAU33691.1 aver|OJI98387.1 awen|OJJ41939.1 azon|OJJ50749.1 abra|OJJ76597.1 akaw|GAA82501.1 aluc|OJZ91610.1 anig|EHA22639.1 acla|EAW11329.1 afis|EAW18166.1 afla|EED56329.1 afum|EAL87526.1 agla|OJJ85952.1 anid|CBF85749.1 aory|EIT73387.1 asyd|OJJ64295.1 aver|OJJ07167.1

Group155: abra|OJJ66622.1 acar|OOF91045.1 acla|EAW10875.1 afis|EAW20983.1 afla|EED50338.1 afum|EAL84647.2 agla|OJJ84060.1 akaw|GAA91103.1 aluc|OJZ81427.1 anid|CBF82618.1 anig|EHA25995.1 aory|EIT77260.1 asyd|OJJ54020.1 ater|EAU38633.1 atub|OJI88753.1 aver|OJJ08720.1 awen|OJJ34328.1 azon|OJJ48073.1 afla|EED50340.1 akaw|GAA91102.1 aluc|OJZ81426.1 acar|OOF91046.1 acla|EAW10874.1 afis|EAW20984.1 afla|EED50339.1 afum|EAL84646.1 agla|OJJ84063.1 anid|CBF82622.1 aory|EIT77350.1 atub|OJI88754.1 awen|OJJ34327.1 azon|OJJ48069.1

Group156: abra|OJJ68143.1 acar|OOF97481.1 acla|EAW13069.1 afis|EAW17595.1 afla|EED49225.1 afum|EAL91408.1 agla|OJJ81333.1 akaw|GAA92375.1 aluc|OJZ83234.1 anid|CBF90206.1 anig|EHA21091.1 aory|EIT83153.1 asyd|OJJ61974.1 ater|EAU37033.1 atub|OJI87702.1 aver|OJI96067.1 awen|OJJ37251.1 azon|OJJ50946.1 abra|OJJ68185.1 acla|EAW13066.1 afis|EAW17598.1 afla|EED49223.1 afum|EAL91411.1 agla|OJJ81335.1 akaw|GAA83261.1 aluc|OJZ83767.1 anid|CBF90203.1 asyd|OJJ61976.1 ater|EAU37035.1 atub|OJI90138.1 aver|OJI96069.1 awen|OJJ37248.1

Group157: abra|OJJ70602.1 acar|OOF97117.1 acla|EAW08180.1 afis|EAW23215.1 afla|EED47037.1 afum|EAL88269.1 akaw|GAA89942.1 aluc|OJZ83947.1 anig|EHA19076.1 aory|EIT72544.1 asyd|OJJ60180.1 ater|EAU36345.1 atub|OJI90318.1 awen|OJJ39449.1 abra|OJJ70604.1 acar|OOF97116.1 acla|EAW08178.1 afis|EAW23213.1 afum|EBA27340.1 agla|OJJ78731.1 akaw|GAA89944.1 aluc|OJZ83949.1 anid|CBF74742.1 anig|EHA19078.1 aory|EIT72666.1 asyd|OJJ60182.1 ater|EAU36343.1 atub|OJI90320.1 aver|OJJ04082.1 awen|OJJ39447.1 azon|OJJ47649.1 anid|CBF74745.1

Group158: abra|OJJ71268.1 acar|OOF97905.1 acar|OOF97907.1 acla|EAW10269.1 acla|EAW10270.1 afis|EAW25324.1 afis|EAW25325.1 afla|EED53799.1 afla|EED53800.1 afum|EAL89985.1 afum|EAL89986.1 agla|OJJ83862.1 agla|OJJ83863.1 akaw|GAA82722.1 aluc|OJZ87276.1 anid|CBF75258.1 anid|CBF75260.1 anig|EHA20517.1 anig|EHA20518.1 aory|EIT78367.1 aory|EIT78397.1 asyd|OJJ59900.1 asyd|OJJ59901.1 ater|EAU34470.1 atub|OJI81365.1 aver|OJJ02385.1 aver|OJJ02386.1 awen|OJJ41038.1 awen|OJJ41039.1 azon|OJJ44627.1 ater|EAU34472.1 ater|EAU34471.1

Group159: abra|OJJ72382.1 acar|OOF94837.1 acla|EAW11953.1 acla|EAW11954.1 afis|EAW19590.1 afis|EAW19591.1 afla|EED56945.1 afum|EAL86927.1 afum|EAL86928.1 agla|OJJ80730.1 agla|OJJ80732.1 akaw|GAA86129.1 akaw|GAA86131.1 aluc|OJZ90369.1 anid|CBF71264.1 anid|CBF71266.1 anig|EHA23843.1 anig|EHA23844.1 aory|EIT77519.1 aory|EIT77568.1 asyd|OJJ55144.1 asyd|OJJ55146.1 ater|EAU32934.1 ater|EAU32936.1 atub|OJI81795.1 aver|OJJ07061.1 aver|OJJ07063.1 awen|OJJ41805.1 awen|OJJ41808.1 azon|OJJ47024.1 azon|OJJ47026.1 acar|OOF94836.1

Group160: abra|OJJ72953.1 acar|OOF96134.1 acla|EAW11198.1 afis|EAW15928.1 afla|EED54933.1 afum|EAL88564.1 agla|OJJ89377.1 akaw|GAA85534.1 aluc|OJZ83463.1 anid|CBF81024.1 anig|EHA24306.1 aory|EIT80331.1 asyd|OJJ56385.1 ater|EAU35524.1 atub|OJI82355.1 aver|OJJ06327.1 awen|OJJ35652.1 azon|OJJ46886.1 abra|OJJ72954.1 acar|OOF96135.1 acla|EAW11199.1 afis|EAW15929.1 afla|EED54932.1 afum|EAL88565.1 akaw|GAA85535.1 anid|CBF81026.1 anig|EHA24307.1 aory|EIT80358.1 asyd|OJJ56386.1 ater|EAU35523.1 awen|OJJ35651.1 azon|OJJ46887.1

Group161: abra|OJJ74876.1 acar|OOF95878.1 acar|OOF95879.1 acla|EAW08494.1 acla|EAW08495.1 afis|EAW23540.1 afis|EAW23541.1 afla|EED57438.1 afla|EED57439.1 afum|EAL87925.1 afum|EAL87926.1 agla|OJJ86795.1 akaw|GAA83544.1 akaw|GAA83545.1 aluc|OJZ92453.1 anid|CBF89661.1 anid|CBF89663.1 anig|EHA26514.1 anig|EHA26515.1 aory|EIT80116.1 aory|EIT80129.1 asyd|OJJ62276.1 asyd|OJJ62277.1 ater|EAU35326.1 ater|EAU35327.1 atub|OJI86881.1 atub|OJI86882.1 aver|OJI96385.1 aver|OJI96386.1 awen|OJJ39871.1 awen|OJJ39872.1 azon|OJJ43230.1

Group162: abra|OJJ75341.1 acar|OOF89856.1 acla|EAW09853.1 afis|EAW21282.1 afla|EED54833.1 afum|EAL92299.1 akaw|GAA92034.1 aluc|OJZ92904.1 anid|CBF83359.1 anig|EHA26059.1 aory|EIT79370.1 ater|EAU35911.1 atub|OJI87363.1 awen|OJJ39417.1 abra|OJJ75940.1 acar|OOF99970.1 acla|EAW09854.1 afis|EAW21281.1 afla|EED54832.1 afum|EAL92298.1 agla|OJJ87478.1 akaw|GAA84642.1 aluc|OJZ89387.1 anid|CBF83358.1 anig|EHA23197.1 aory|EIT79445.1 asyd|OJJ60618.1 ater|EAU35910.1 atub|OJI85410.1 aver|OJI99942.1 awen|OJJ39418.1 azon|OJJ44300.1

Group163: abra|OJJ75791.1 acar|OOG00087.1 acar|OOG00097.1 acla|EAW14890.1 acla|EAW14891.1 afis|EAW24412.1 afis|EAW24413.1 afla|EED47763.1 afla|EED47764.1 afum|EAL86081.1 afum|EAL86082.1 agla|OJJ79215.1 agla|OJJ79216.1 akaw|GAA84506.1 akaw|GAA84510.1 aluc|OJZ89235.1 aluc|OJZ89239.1 anid|CBF73743.1 anid|CBF73745.1 anig|EHA23328.1 aory|EIT77117.1 aory|EIT77194.1 asyd|OJJ56623.1 ater|EAU30316.1 ater|EAU30317.1 atub|OJI85559.1 aver|OJJ06102.1 aver|OJJ06103.1 awen|OJJ32796.1 awen|OJJ32798.1 azon|OJJ49036.1 azon|OJJ49037.1

Group164: abra|OJJ76816.1 acar|OOF91311.1 acla|EAW13376.1 afis|EAW20490.1 afla|EED44773.1 afum|EAL84564.1 agla|OJJ84837.1 akaw|GAA87205.1 aluc|OJZ84226.1 anig|EHA27575.1 aory|EIT77777.1 asyd|OJJ55730.1 atub|OJI89858.1 aver|OJJ06816.1 awen|OJJ33190.1 azon|OJJ46392.1 abra|OJJ76822.1 acar|OOF91317.1 acla|EAW13382.1 afis|EAW20496.1 afla|EED44772.1 afum|EAL84558.1 agla|OJJ84806.1 akaw|GAA87200.1 aluc|OJZ84233.1 anid|CBF79209.1 anig|EHA27580.1 aory|EIT77769.1 ater|EAU37190.1 atub|OJI89852.1 awen|OJJ33196.1 azon|OJJ46400.1

Group165: abra|OJJ78214.1 acla|EAW11236.1 afis|EAW18269.1 afla|EED48735.1 afum|EAL87634.1 agla|OJJ88158.1 akaw|GAA85813.1 aluc|OJZ91286.1 anid|CBF79514.1 anig|EHA19971.1 aory|EIT73143.1 asyd|OJJ57887.1 ater|EAU33276.1 atub|OJI83276.1 aver|OJJ04507.1 awen|OJJ30643.1 azon|OJJ47972.1 abra|OJJ78215.1 acar|OOF98641.1 acla|EAW11235.1 afis|EAW18270.1 afla|EED48736.1 afum|EAL87635.1 agla|OJJ88157.1 akaw|GAA85812.1 aluc|OJZ91287.1 anid|CBF79512.1 anig|EHA19972.1 aory|EIT73147.1 asyd|OJJ57886.1 atub|OJI83277.1 aver|OJJ04508.1

Group166: abra|OJJ65897.1 abra|OJJ72196.1 acar|OOF94957.1 acar|OOF96818.1 agla|OJJ80319.1 agla|OJJ83741.1 agla|OJJ82619.1 akaw|GAA84738.1 akaw|GAA86008.1 aluc|OJZ86948.1 aluc|OJZ90234.1 anig|EHA21698.1 anig|EHA23734.1 asyd|OJJ53466.1 asyd|OJJ56558.1 ater|EAU29975.1 ater|EAU30764.1 atub|OJI81664.1 atub|OJI83575.1 aver|OJJ06162.1 aver|OJJ08289.1 azon|OJJ44535.1 azon|OJJ49495.1 acla|EAW07004.1 afis|EAW16621.1 afis|EAW23621.1 afla|EED55210.1 afum|EAL89216.2 anid|CBF73835.1 awen|OJJ32732.1 awen|OJJ40080.1

Group167: abra|OJJ66384.1 abra|OJJ76979.1 acar|OOF91425.1 acar|OOF94058.1 acar|OOF94128.1 acla|EAW11869.1 acla|EAW15042.1 afis|EAW19480.1 afis|EAW24565.1 afla|EED48343.1 afla|EED56690.1 afum|EAL86228.1 afum|EAL87015.1 akaw|GAA87066.1 akaw|GAA90250.1 aluc|OJZ83090.1 aluc|OJZ84377.1 anig|EHA27710.1 anig|EHA28152.1 aory|EIT73915.1 aory|EIT76297.1 atub|OJI87853.1 atub|OJI89692.1 azon|OJJ42424.1 azon|OJJ42921.1 asyd|OJJ56521.1 ater|EAU34790.1 aver|OJJ06201.1 awen|OJJ36886.1 anid|CBF84609.1 ater|EAU37362.1

Group168: abra|OJJ66675.1 abra|OJJ75504.1 acar|OOF90835.1 acar|OOF93687.1 acar|OOF93772.1 afis|EAW16470.1 afis|EAW19425.1 afum|EAL87069.1 afum|EAL89064.1 akaw|GAA84268.1 akaw|GAA89623.1 aluc|OJZ80773.1 aluc|OJZ88960.1 anig|EHA23560.1 anig|EHA27257.1 atub|OJI85829.1 atub|OJI87625.1 acla|EAW06851.1 afla|EED50383.1 agla|OJJ81137.1 anid|CBF74378.1 aory|EIT83131.1 asyd|OJJ57778.1 ater|EAU34729.1 aver|OJJ05387.1 awen|OJJ36823.1 azon|OJJ42980.1 afla|EED49104.1 asyd|OJJ57938.1 aver|OJJ04456.1 aory|EIT82490.1

Group169: abra|OJJ67685.1 abra|OJJ69630.1 acar|OOF92680.1 acar|OOF94075.1 acar|OOF94145.1 acar|OOF96015.1 afum|EAL86373.1 afum|EAL87420.1 akaw|GAA89197.1 akaw|GAA91610.1 aluc|OJZ81633.1 aluc|OJZ82435.1 anig|EHA25810.1 anig|EHA28336.1 atub|OJI88006.1 atub|OJI88547.1 afla|EED55077.1 aory|EIT81950.1 acla|EAW12376.1 afis|EAW16822.1 agla|OJJ86924.1 anid|CBF82987.1 asyd|OJJ64732.1 ater|EAU35425.1 aver|OJI98836.1 awen|OJJ35458.1 azon|OJJ51285.1 anid|CBF77369.1 asyd|OJJ53260.1 aver|OJJ03591.1 acla|EAW11432.1

Group170: acar|OOF91973.1 acar|OOF94079.1 acar|OOF94149.1 acar|OOF99292.1 afla|EED45949.1 afla|EED47803.1 aluc|OJZ79501.1 aluc|OJZ79636.1 aluc|OJZ81691.1 aluc|OJZ80076.1 aory|EIT75701.1 aory|EIT77131.1 asyd|OJJ52720.1 asyd|OJJ61396.1 asyd|OJJ61537.1 ater|EAU29987.1 ater|EAU30155.1 aver|OJJ00725.1 aver|OJJ04752.1 awen|OJJ32293.1 awen|OJJ35892.1 abra|OJJ67625.1 acla|EAW15287.1 afis|EAW20776.1 afum|EAL84844.1 agla|OJJ85083.1 akaw|GAA89145.1 anid|CBF87439.1 anig|EHA25753.1 atub|OJI88489.1 azon|OJJ42146.1

Group171: acar|OOF92319.1 acar|OOF92361.1 abra|OJJ67475.1 acla|EAW06297.1 afis|EAW18404.1 afla|EED53004.1 afum|EAL93084.1 agla|OJJ84961.1 akaw|GAA86362.1 aluc|OJZ84889.1 anid|CBF70745.1 anig|EHA25118.1 aory|EIT77437.1 asyd|OJJ58438.1 ater|EAU29821.1 atub|OJI80708.1 aver|OJJ01038.1 awen|OJJ31746.1 abra|OJJ67478.1 acla|EAW06294.1 afis|EAW18400.1 afla|EED53006.1 afum|EAL93078.1 agla|OJJ84971.1 akaw|GAA86360.1 anid|CBF70749.1 aory|EIT77424.1 ater|EAU29816.1 atub|OJI80711.1 awen|OJJ31749.1 azon|OJJ50474.1

Group172: acar|OOF98484.1 acar|OOF98497.1 abra|OJJ78390.1 acar|OOF98498.1 acla|EAW09142.1 acla|EAW09143.1 afis|EAW22004.1 afis|EAW22005.1 afla|EED46729.1 afum|EAL93019.1 afum|EAL93020.1 agla|OJJ84093.1 agla|OJJ84094.1 akaw|GAA85641.1 akaw|GAA85642.1 aluc|OJZ91469.1 anid|CBF82660.1 anid|CBF82662.1 anig|EHA20131.1 aory|EIT77865.1 aory|EIT77967.1 asyd|OJJ61666.1 asyd|OJJ61667.1 ater|EAU31123.1 ater|EAU31125.1 atub|OJI83462.1 aver|OJJ00987.1 awen|OJJ34299.1 awen|OJJ34300.1 azon|OJJ43338.1 azon|OJJ43339.1

Group173: afis|EAW21024.1 afis|EAW22408.1 afum|EAL90784.1 afum|EAL92063.1 awen|OJJ31391.1 abra|OJJ74333.1 acar|OOF95396.1 acla|EAW07383.1 afla|EED56072.1 akaw|GAA86696.1 aluc|OJZ89796.1 anid|CBF88792.1 anig|EHA27010.1 aory|EIT76782.1 asyd|OJJ62898.1 ater|EAU39300.1 atub|OJI85009.1 aver|OJI96952.1 awen|OJJ34988.1 acar|OOF95397.1 acla|EAW07385.1 afis|EAW22410.1 afla|EED52490.1 afum|EAL90782.1 akaw|GAA86694.1 anid|CBF88794.1 aory|EIT82779.1 ater|EAU39295.1 azon|OJJ50553.1 anig|EHA27008.1 aver|OJI96951.1

Group174: afla|EED46122.1 afla|EED53117.1 afla|EED48931.1 afla|EED51100.1 akaw|GAA86153.1 akaw|GAA92990.1 aory|EIT74792.1 aory|EIT79985.1 aory|EIT81307.1 atub|OJI81824.1 atub|OJI86011.1 abra|OJJ72410.1 acar|OOF94819.1 acla|EAW14266.1 afis|EAW19012.1 afum|EAL93746.1 agla|OJJ81770.1 aluc|OJZ90394.1 anig|EHA23863.1 aory|EIT76367.1 ater|EAU37790.1 awen|OJJ31644.1 abra|OJJ76649.1 acar|OOF99352.1 aluc|OJZ91559.1 anid|CBF86747.1 anig|EHA22591.1 asyd|OJJ64703.1 aver|OJI98807.1 azon|OJJ47337.1 afis|EAW16798.1

Group175: afum|EAL87729.1 afum|EAL88789.1 abra|OJJ65746.1 afis|EAW16109.1 afla|EED49345.1 agla|OJJ83935.1 akaw|GAA93207.1 aory|EIT79717.1 asyd|OJJ57625.1 ater|EAU33192.1 awen|OJJ41813.1 azon|OJJ42378.1 abra|OJJ77889.1 acar|OOF98892.1 afla|EED48269.1 akaw|GAA83769.1 aluc|OJZ90959.1 anig|EHA19664.1 asyd|OJJ64307.1 atub|OJI82950.1 aver|OJJ00097.1 aory|EIT72931.1 ater|EAU33625.1 acla|EAW06656.1 aluc|OJZ79714.1 asyd|OJJ60763.1 afla|EED57955.1 agla|OJJ86139.1 awen|OJJ38786.1 aory|EIT79719.1 aver|OJJ05561.1

Group176: ater|EAU33722.1 ater|EAU38738.1 abra|OJJ71609.1 acar|OOF96326.1 acla|EAW15150.1 acla|EAW15152.1 afis|EAW20118.1 afis|EAW20120.1 afla|EED53353.1 afla|EED57256.1 afum|EAL85235.1 afum|EBA27224.1 agla|OJJ88826.1 agla|OJJ88831.1 akaw|GAA89318.1 akaw|GAA89319.1 aluc|OJZ86351.1 aluc|OJZ86353.1 anid|CBF84937.1 anid|CBF84939.1 anig|EHA21536.1 aory|EIT74846.1 asyd|OJJ63156.1 ater|EAU38737.1 atub|OJI84184.1 aver|OJI97800.1 aver|OJI97801.1 awen|OJJ31888.1 azon|OJJ46246.1 aory|EIT74143.1 asyd|OJJ63154.1

Group177: awen|OJJ33301.1 awen|OJJ37090.1 abra|OJJ76956.1 acar|OOF91397.1 acla|EAW13475.1 afla|EED52244.1 agla|OJJ88687.1 akaw|GAA87087.1 aluc|OJZ84351.1 anid|CBF78932.1 anig|EHA27688.1 aory|EIT74404.1 asyd|OJJ62348.1 ater|EAU37096.1 atub|OJI89715.1 azon|OJJ50794.1 abra|OJJ77159.1 acar|OOF93045.1 acla|EAW12913.1 afis|EAW17747.1 afla|EED45747.1 afum|EAL91559.1 agla|OJJ81707.1 akaw|GAA90772.1 aluc|OJZ84572.1 anid|CBF86617.1 aory|EIT75770.1 asyd|OJJ64815.1 ater|EAU31860.1 atub|OJI89510.1 aver|OJI98928.1

Group178: abra|OJJ65971.1 acar|OOF92902.1 acla|EAW13592.1 afis|EAW20012.1 afla|EED46018.1 afum|EAL84906.1 akaw|GAA90846.1 aluc|OJZ82987.1 anid|CBF84721.1 anig|EHA21402.1 aory|EIT75680.1 asyd|OJJ53329.1 ater|EAU29354.1 atub|OJI89221.1 aver|OJJ08415.1 awen|OJJ30025.1 abra|OJJ65972.1 acar|OOF92900.1 acla|EAW13593.1 afis|EAW20011.1 afla|EED46017.1 afum|EAL84907.1 agla|OJJ87976.1 anid|CBF83963.1 anig|EHA21401.1 aory|EIT75560.1 asyd|OJJ61425.1 ater|EAU29353.1 aver|OJJ00759.1 awen|OJJ30024.1 azon|OJJ43649.1

Group179: abra|OJJ66528.1 acar|OOF90981.1 acla|EAW10962.1 afis|EAW20897.1 afla|EED50247.1 afum|EAL84735.1 agla|OJJ84007.1 akaw|GAA91185.1 aluc|OJZ81518.1 anid|CBF82460.1 anig|EHA25916.1 aory|EIT77290.1 ater|EAU38531.1 atub|OJI88667.1 awen|OJJ34450.1 azon|OJJ51738.1 abra|OJJ66529.1 acar|OOF90980.1 acla|EAW10961.1 afis|EAW20898.1 afla|EED50248.1 afum|EAL84734.2 agla|OJJ84008.1 akaw|GAA91184.1 aluc|OJZ81519.1 anid|CBF82458.1 aory|EIT77356.1 asyd|OJJ54115.1 atub|OJI88668.1 aver|OJJ08620.1 azon|OJJ51737.1

Group180: abra|OJJ66543.1 acar|OOF90995.1 afla|EED50933.1 agla|OJJ78717.1 akaw|GAA91168.1 aluc|OJZ81496.1 anid|CBF71517.1 anig|EHA25934.1 aory|EIT79048.1 asyd|OJJ54917.1 ater|EAU29874.1 atub|OJI88685.1 aver|OJJ07286.1 awen|OJJ30168.1 abra|OJJ67418.1 acar|OOF92403.1 acla|EAW06346.1 afis|EAW18457.1 afla|EED52951.1 afum|EAL93142.1 agla|OJJ85040.1 akaw|GAA86410.1 aluc|OJZ84950.1 anid|CBF70055.1 anig|EHA25165.1 aory|EIT78701.1 asyd|OJJ58858.1 atub|OJI80652.1 aver|OJJ01459.1 awen|OJJ31009.1 azon|OJJ50523.1

Group181: abra|OJJ67409.1 acar|OOF92406.1 acla|EAW06349.1 afis|EAW18461.1 afla|EED52947.1 afum|EAL93147.1 agla|OJJ85045.1 akaw|GAA86418.1 aluc|OJZ84959.1 anid|CBF70089.1 anig|EHA25173.1 aory|EIT78700.1 asyd|OJJ58841.1 ater|EAU29879.1 atub|OJI80641.1 aver|OJJ01443.1 awen|OJJ31013.1 azon|OJJ50526.1 abra|OJJ67410.1 acar|OOF92404.1 afis|EAW18460.1 afla|EED52948.1 afum|EAL93146.1 akaw|GAA86417.1 aluc|OJZ84958.1 anid|CBF70087.1 anig|EHA25172.1 aory|EIT78733.1 ater|EAU29878.1 atub|OJI80642.1 awen|OJJ31012.1

Group182: abra|OJJ67954.1 acar|OOF92799.1 acla|EAW11644.1 afis|EAW23813.1 afis|EAW23814.1 afla|EED51991.1 afla|EED52002.1 afum|EAL87248.1 agla|OJJ82453.1 akaw|GAA91242.1 akaw|GAA91243.1 aluc|OJZ82827.1 anid|CBF79881.1 anig|EHA21264.1 anig|EHA21265.1 aory|EIT81469.1 aory|EIT81487.1 asyd|OJJ58172.1 ater|EAU31366.1 atub|OJI89057.1 aver|OJJ04231.1 awen|OJJ33769.1 azon|OJJ46009.1 anid|CBF79883.1 asyd|OJJ58173.1 acla|EAW11643.1 afum|EAL87249.1 agla|OJJ82454.1 ater|EAU31369.1 awen|OJJ33770.1 azon|OJJ42873.1

Group183: abra|OJJ68614.1 acar|OOF92124.1 acar|OOF92125.1 acla|EAW12605.1 acla|EAW12606.1 afis|EAW18078.1 afis|EAW18079.1 afla|EED51472.1 afla|EED51473.1 afum|EAL91890.1 afum|EAL91891.1 agla|OJJ87372.1 akaw|GAA92907.1 akaw|GAA92908.1 aluc|OJZ81940.1 aluc|OJZ81941.1 anid|CBF86395.1 anid|CBF86397.1 anig|EHA22308.1 aory|EIT80638.1 aory|EIT80738.1 asyd|OJJ64952.1 asyd|OJJ64954.1 ater|EAU30443.1 ater|EAU30445.1 atub|OJI84493.1 atub|OJI84494.1 aver|OJI99074.1 aver|OJI99076.1 awen|OJJ41467.1 azon|OJJ48924.1

Group184: abra|OJJ71429.1 acar|OOF90402.1 acla|EAW10109.1 afis|EAW25478.1 afla|EED53973.1 afum|EAL90141.1 akaw|GAA82574.1 aluc|OJZ87109.1 anid|CBF77705.1 anig|EHA20376.1 asyd|OJJ53075.1 ater|EAU34650.1 atub|OJI81530.1 aver|OJJ03772.1 awen|OJJ41218.1 azon|OJJ44787.1 abra|OJJ71430.1 acla|EAW10108.1 afis|EAW25479.1 afla|EED53974.1 afum|EAL90144.1 akaw|GAA82573.1 aluc|OJZ87108.1 anid|CBF77703.1 anig|EHA20375.1 aory|EIT78327.1 asyd|OJJ53074.1 ater|EAU34651.1 atub|OJI81531.1 awen|OJJ41220.1 azon|OJJ44786.1

Group185: abra|OJJ73132.1 acar|OOF90742.1 acla|EAW13627.1 afis|EAW21092.1 afla|EED48284.1 afum|EAL92115.1 akaw|GAA92664.1 aluc|OJZ90118.1 anid|CBF83072.1 anig|EHA19695.1 anig|EHA19696.1 asyd|OJJ52355.1 ater|EAU32835.1 atub|OJI84679.1 aver|OJJ00828.1 awen|OJJ31224.1 azon|OJJ46149.1 acar|OOF90935.1 anid|CBF82817.1 aver|OJJ00896.1 acar|OOF90741.1 afis|EAW15628.1 afum|EAL85706.1 aory|EIT80551.1 asyd|OJJ61575.1 acar|OOF98130.1 acar|OOG00091.1 acla|EAW08502.1 afla|EED51590.1 asyd|OJJ61486.1 aory|EIT72957.1

Group186: abra|OJJ74487.1 acar|OOF95262.1 acla|EAW07208.1 afis|EAW22220.1 afla|EED57613.1 afum|EAL90974.1 agla|OJJ87824.1 akaw|GAA87381.1 aluc|OJZ89631.1 anig|EHA26874.1 ater|EAU34230.1 atub|OJI85171.1 aver|OJI97165.1 awen|OJJ34805.1 abra|OJJ74488.1 acar|OOF95261.1 acla|EAW07207.1 afis|EAW22219.1 afla|EED57612.1 afum|EAL90975.1 agla|OJJ87825.1 akaw|GAA87382.1 aluc|OJZ89630.1 anid|CBF88457.1 anig|EHA26873.1 aory|EIT82983.1 asyd|OJJ63772.1 ater|EAU34229.1 atub|OJI85172.1 aver|OJI97166.1 azon|OJJ45743.1

Group187: abra|OJJ74835.1 acar|OOF95828.1 acla|EAW09102.1 afis|EAW25706.1 afla|EED55155.1 afum|EAL84379.1 akaw|GAA83585.1 aluc|OJZ92412.1 anid|CBF83935.1 anig|EHA26553.1 aory|EIT81620.1 asyd|OJJ55434.1 ater|EAU32723.1 atub|OJI86842.1 aver|OJJ06511.1 awen|OJJ36213.1 azon|OJJ43614.1 abra|OJJ76623.1 acar|OOF99375.1 acla|EAW10020.1 afis|EAW22744.1 afla|EED50498.1 afum|EAL90452.1 akaw|GAA92834.1 aluc|OJZ91589.1 anig|EHA22613.1 aory|EIT82109.1 ater|EAU34860.1 atub|OJI86038.1 awen|OJJ40151.1 azon|OJJ48726.1

Group188: abra|OJJ74864.1 acar|OOF95864.1 acar|OOF95866.1 acla|EAW08515.1 acla|EAW08517.1 afis|EAW23553.1 afis|EAW23555.1 afla|EED57448.1 afla|EED57450.1 afum|EAL87911.1 afum|EAL87913.1 agla|OJJ86813.1 agla|OJJ86815.1 akaw|GAA83554.1 akaw|GAA83556.1 aluc|OJZ92442.1 anid|CBF89641.1 anid|CBF89643.1 anig|EHA26525.1 aory|EIT80074.1 aory|EIT80092.1 asyd|OJJ62288.1 asyd|OJJ62290.1 ater|EAU35336.1 ater|EAU35338.1 atub|OJI86871.1 aver|OJI96396.1 aver|OJI96398.1 awen|OJJ39884.1 awen|OJJ39886.1 azon|OJJ43221.1

Group189: abra|OJJ75158.1 acar|OOF97180.1 acar|OOF97181.1 acla|EAW08333.1 acla|EAW08334.1 afis|EAW23368.1 afis|EAW23369.1 afla|EED55588.1 afla|EED55589.1 afum|EAL88104.2 afum|EAL88105.1 agla|OJJ86455.1 agla|OJJ86456.1 akaw|GAA88764.1 akaw|GAA88769.1 aluc|OJZ92728.1 anid|CBF74819.1 anid|CBF74821.1 anig|EHA26273.1 aory|EIT82662.1 aory|EIT82702.1 asyd|OJJ60141.1 asyd|OJJ60142.1 ater|EAU35619.1 ater|EAU35620.1 atub|OJI87152.1 aver|OJJ04121.1 awen|OJJ39518.1 awen|OJJ39519.1 azon|OJJ47713.1 azon|OJJ47715.1

Group190: abra|OJJ75500.1 acar|OOF90833.1 acla|EAW09294.1 afis|EAW21853.1 afla|EED46457.1 afum|EAL92866.1 agla|OJJ84283.1 akaw|GAA84266.1 aluc|OJZ88959.1 anid|CBF76739.1 anig|EHA23561.1 aory|EIT72412.1 asyd|OJJ61254.1 ater|EAU30852.1 atub|OJI85830.1 aver|OJJ00577.1 awen|OJJ34137.1 azon|OJJ44207.1 abra|OJJ75502.1 acar|OOF90834.1 acla|EAW09295.1 afis|EAW21852.1 afla|EED46458.1 afum|EAL92865.1 akaw|GAA84267.1 anid|CBF76737.1 aory|EIT72344.1 asyd|OJJ61253.1 ater|EAU30851.1 awen|OJJ34135.1 azon|OJJ44209.1

Group191: abra|OJJ76457.1 acar|OOF99697.1 acla|EAW13972.1 acla|EAW13973.1 afis|EAW18681.1 afis|EAW18682.1 afla|EED50628.1 afum|EAL93402.1 afum|EAL93403.1 agla|OJJ82393.1 agla|OJJ82394.1 akaw|GAA82369.1 akaw|GAA82370.1 aluc|OJZ91752.1 aluc|OJZ91753.1 anid|CBF70601.1 anid|CBF70603.1 anig|EHA22755.1 anig|EHA22756.1 aory|EIT78067.1 asyd|OJJ58516.1 asyd|OJJ58517.1 ater|EAU38228.1 atub|OJI86210.1 atub|OJI86211.1 aver|OJJ01117.1 aver|OJJ01118.1 awen|OJJ32153.1 azon|OJJ46737.1 afla|EED50629.1 aory|EIT78047.1

Group192: abra|OJJ77384.1 acla|EAW09036.1 afla|EED56897.1 akaw|GAA91377.1 aluc|OJZ84792.1 anig|EHA28060.1 aory|EIT77450.1 asyd|OJJ57351.1 ater|EAU38783.1 atub|OJI89290.1 aver|OJJ05814.1 awen|OJJ35827.1 abra|OJJ78137.1 acar|OOF99382.1 acla|EAW14634.1 afis|EAW22732.1 afla|EED55765.1 afum|EAL90463.1 agla|OJJ78522.1 aluc|OJZ82078.1 anid|CBF78393.1 ater|EAU30739.1 awen|OJJ32040.1 azon|OJJ47350.1 acar|OOF96813.1 anig|EHA25049.1 aluc|OJZ82079.1 atub|OJI88447.1 akaw|GAA87875.1 aluc|OJZ85516.1 atub|OJI80050.1

Group193: abra|OJJ67081.1 abra|OJJ68837.1 abra|OJJ69761.1 agla|OJJ84620.1 agla|OJJ89301.1 aory|EIT74438.1 aory|EIT79603.1 asyd|OJJ52546.1 asyd|OJJ57148.1 asyd|OJJ63033.1 ater|EAU29247.1 ater|EAU29286.1 ater|EAU32050.1 ater|EAU35700.1 aver|OJI97935.1 aver|OJJ03858.1 awen|OJJ30061.1 awen|OJJ31262.1 acar|OOF90552.1 acla|EAW15294.1 afis|EAW20307.1 afla|EED52202.1 afla|EED56612.1 afum|EAL85435.2 akaw|GAA90413.1 aluc|OJZ85816.1 anid|CBF85212.1 atub|OJI79602.1 awen|OJJ29673.1 azon|OJJ45897.1

Group194: abra|OJJ67792.1 abra|OJJ73325.1 anig|EHA18415.1 anig|EHA18416.1 acla|EAW12887.1 afis|EAW17789.1 afla|EED50756.1 afum|EAL91598.1 agla|OJJ84165.1 akaw|GAA92793.1 aluc|OJZ88713.1 anid|CBF75218.1 aory|EIT78111.1 asyd|OJJ53462.1 ater|EAU29260.1 atub|OJI91300.1 aver|OJJ08293.1 awen|OJJ32921.1 acar|OOF95394.1 abra|OJJ75374.1 acar|OOF94473.1 afis|EAW17262.1 afla|EED48869.1 afum|EAL86679.1 akaw|GAA92734.1 aluc|OJZ92942.1 anig|EHA26031.1 aory|EIT81203.1 atub|OJI87401.1 awen|OJJ35771.1

Group195: abra|OJJ68217.1 abra|OJJ69796.1 acar|OOF92535.1 acar|OOF97501.1 akaw|GAA83229.1 akaw|GAA87863.1 aluc|OJZ83729.1 aluc|OJZ85504.1 anig|EHA18877.1 anig|EHA25036.1 aory|EIT80435.1 aory|EIT81864.1 atub|OJI80061.1 atub|OJI90106.1 awen|OJJ31685.1 awen|OJJ32019.1 azon|OJJ42231.1 azon|OJJ46071.1 acla|EAW15175.1 afis|EAW20145.1 afla|EED51644.1 afum|EAL85260.1 agla|OJJ89015.1 anid|CBF81813.1 asyd|OJJ59648.1 ater|EAU39446.1 aver|OJJ02634.1 asyd|OJJ58357.1 ater|EAU33077.1 aver|OJJ02050.1

Group196: abra|OJJ68439.1 abra|OJJ71007.1 acar|OOF93955.1 acar|OOF98135.1 afis|EAW19801.1 afis|EAW25026.1 agla|OJJ79158.1 agla|OJJ87950.1 akaw|GAA82946.1 akaw|GAA86933.1 aluc|OJZ87524.1 aluc|OJZ90058.1 anid|CBF81549.1 anid|CBF82379.1 anig|EHA20734.1 anig|EHA21013.1 asyd|OJJ54138.1 asyd|OJJ59494.1 ater|EAU30748.1 ater|EAU35761.1 atub|OJI81124.1 atub|OJI84749.1 aver|OJJ02780.1 aver|OJJ08598.1 acla|EAW10559.1 afla|EED55448.1 afum|EAL89669.1 aory|EIT80786.1 awen|OJJ40613.1 azon|OJJ49462.1

Group197: abra|OJJ69676.1 abra|OJJ72100.1 acar|OOF90056.1 acar|OOF96745.1 aluc|OJZ82484.1 aluc|OJZ86855.1 anig|EHA21784.1 anig|EHA28290.1 asyd|OJJ60799.1 asyd|OJJ62562.1 atub|OJI83664.1 atub|OJI87948.1 awen|OJJ31984.1 awen|OJJ34027.1 afis|EAW21744.1 afum|EAL92777.1 agla|OJJ81779.1 agla|OJJ84428.1 akaw|GAA84821.1 akaw|GAA88801.1 ater|EAU32512.1 aver|OJI96686.1 azon|OJJ42257.1 acla|EAW09389.1 afla|EED58033.1 anid|CBF83660.1 aory|EIT79754.1 ater|EAU38444.1 aver|OJJ00132.1 acar|OOF95939.1

Group198: abra|OJJ72836.1 abra|OJJ73712.1 agla|OJJ78798.1 agla|OJJ86951.1 akaw|GAA85433.1 akaw|GAA88451.1 aluc|OJZ83336.1 aluc|OJZ88338.1 anig|EHA18073.1 anig|EHA24212.1 aory|EIT75956.1 aory|EIT78575.1 asyd|OJJ53233.1 asyd|OJJ54785.1 ater|EAU29729.1 ater|EAU32826.1 atub|OJI82233.1 atub|OJI90934.1 aver|OJJ03618.1 aver|OJJ07380.1 awen|OJJ35489.1 awen|OJJ38098.1 acar|OOF96038.1 acla|EAW11404.1 afis|EAW24017.1 afla|EED49142.1 afum|EAL87447.1 anid|CBF77418.1 azon|OJJ47000.1 afla|EED54085.1

Group199: acar|OOF90457.1 acar|OOF90464.1 abra|OJJ71887.1 acla|EAW09633.1 afis|EAW21507.1 afla|EED55944.1 afum|EAL92527.1 agla|OJJ85669.1 akaw|GAA85029.1 aluc|OJZ86625.1 anid|CBF76445.1 anig|EHA22041.1 asyd|OJJ61103.1 ater|EAU35031.1 atub|OJI83900.1 aver|OJJ00420.1 awen|OJJ39188.1 azon|OJJ51505.1 acla|EAW09634.1 afis|EAW21506.1 afla|EED55943.1 afum|EAL92526.1 anid|CBF76447.1 asyd|OJJ61104.1 ater|EAU35032.1 aver|OJJ00421.1 awen|OJJ39189.1 acar|OOF96550.1 anig|EHA22040.1 afum|EAL92525.1

Group200: acar|OOF98095.1 acar|OOF99166.1 acla|EAW06529.1 acla|EAW10590.1 afis|EAW16066.1 afis|EAW24978.1 afla|EED48587.1 afla|EED55481.1 afum|EAL88698.1 afum|EAL89638.1 anid|CBF75098.1 anid|CBF81606.1 asyd|OJJ59522.1 asyd|OJJ60007.1 ater|EAU33563.1 ater|EAU35727.1 aver|OJJ02278.1 aver|OJJ02750.1 awen|OJJ30369.1 awen|OJJ40575.1 azon|OJJ45388.1 azon|OJJ49435.1 abra|OJJ71044.1 agla|OJJ79139.1 akaw|GAA82912.1 aluc|OJZ87482.1 anig|EHA20704.1 aory|EIT73215.1 aory|EIT80785.1 atub|OJI81166.1

Group201: afla|EED48129.1 afla|EED48474.1 anid|CBF75189.1 anid|CBF83027.1 aory|EIT73105.1 aory|EIT76193.1 aory|EIT79635.1 asyd|OJJ55485.1 asyd|OJJ56197.1 asyd|OJJ59940.1 ater|EAU31592.1 ater|EAU31786.1 aver|OJJ02347.1 aver|OJJ05124.1 aver|OJJ06564.1 aver|OJJ08128.1 azon|OJJ42131.1 azon|OJJ45782.1 azon|OJJ42807.1 abra|OJJ70953.1 acar|OOF98179.1 asyd|OJJ60083.1 asyd|OJJ64199.1 aver|OJI98288.1 aver|OJJ07880.1 afla|EED56568.1 aver|OJJ04169.1 anid|CBF87321.1 asyd|OJJ59110.1 aver|OJJ01702.1

Group202: agla|OJJ79264.1 agla|OJJ81588.1 asyd|OJJ56968.1 asyd|OJJ61442.1 aver|OJJ00778.1 aver|OJJ03319.1 azon|OJJ43207.1 azon|OJJ51818.1 abra|OJJ67615.1 acar|OOF91964.1 afla|EED44878.1 akaw|GAA89133.1 aluc|OJZ81703.1 anig|EHA25732.1 aory|EIT83259.1 atub|OJI88479.1 awen|OJJ36276.1 abra|OJJ68338.1 acar|OOF97638.1 akaw|GAA92100.1 aluc|OJZ83625.1 anid|CBF83952.1 anig|EHA18778.1 atub|OJI89994.1 awen|OJJ35809.1 azon|OJJ47565.1 akaw|GAA92300.1 aluc|OJZ90197.1 anig|EHA23695.1 atub|OJI81624.1

Group203: asyd|OJJ55488.1 asyd|OJJ61510.1 asyd|OJJ61953.1 asyd|OJJ60082.1 aver|OJI96044.1 aver|OJJ00847.1 aver|OJJ06567.1 awen|OJJ33588.1 awen|OJJ35927.1 abra|OJJ70268.1 afla|EED46478.1 agla|OJJ78786.1 akaw|GAA86529.1 aluc|OJZ85077.1 anid|CBF83020.1 anig|EHA25292.1 aory|EIT72376.1 aory|EIT82615.1 ater|EAU38779.1 atub|OJI80521.1 azon|OJJ42129.1 abra|OJJ70950.1 acar|OOF98180.1 akaw|GAA82994.1 aluc|OJZ87579.1 anig|EHA20781.1 atub|OJI81070.1 afla|EED49569.1 aver|OJJ04168.1 azon|OJJ42134.1

Group204: asyd|OJJ62594.1 awen|OJJ38006.1 awen|OJJ39405.1 abra|OJJ75729.1 acar|OOF90233.1 afis|EAW24067.1 afla|EED54453.1 afum|EAL91923.1 agla|OJJ87782.1 akaw|GAA84464.1 aluc|OJZ89181.1 anid|CBF83672.1 anig|EHA23374.1 aory|EIT81134.1 asyd|OJJ60812.1 ater|EAU32054.1 atub|OJI85608.1 aver|OJJ00139.1 abra|OJJ75956.1 acar|OOF99957.1 acla|EAW09841.1 akaw|GAA84658.1 aluc|OJZ89405.1 anid|CBF83380.1 anig|EHA23182.1 aory|EIT79378.1 ater|EAU35927.1 atub|OJI85392.1 afis|EAW21294.1 afum|EAL92311.1

Group205: awen|OJJ32368.1 awen|OJJ32676.1 azon|OJJ42336.1 azon|OJJ46795.1 abra|OJJ68299.1 acar|OOF95350.1 acar|OOF97574.1 acla|EAW14263.1 afla|EED54478.1 akaw|GAA83175.1 aluc|OJZ83673.1 anid|CBF85201.1 anig|EHA18829.1 aory|EIT80470.1 asyd|OJJ63024.1 ater|EAU32059.1 atub|OJI90044.1 aver|OJJ00341.1 awen|OJJ32193.1 abra|OJJ74389.1 akaw|GAA87289.1 aluc|OJZ89730.1 aory|EIT81129.1 atub|OJI85071.1 aory|EIT79607.1 asyd|OJJ55922.1 afla|EED56619.1 aver|OJI97944.1 afla|EED51613.1 agla|OJJ81483.1

Group206: abra|OJJ66942.1 acar|OOF91706.1 acar|OOF91708.1 acla|EAW14500.1 acla|EAW14501.1 afis|EAW19214.1 afis|EAW19215.1 afla|EED47685.1 afla|EED47686.1 afum|EAL93947.1 afum|EAL93948.1 agla|OJJ88482.1 akaw|GAA91478.1 akaw|GAA91479.1 aluc|OJZ85640.1 anid|CBF78591.1 anig|EHA24894.1 aory|EIT80213.1 aory|EIT80244.1 asyd|OJJ65083.1 ater|EAU37253.1 atub|OJI79431.1 aver|OJI99209.1 awen|OJJ37572.1 azon|OJJ51231.1 anid|CBF78589.1 ater|EAU37254.1 aver|OJI99208.1 awen|OJJ37571.1 asyd|OJJ65084.1

Group207: abra|OJJ67140.1 acar|OOF98417.1 acla|EAW14599.1 afis|EAW19328.1 afum|EAL94045.1 agla|OJJ86277.1 akaw|GAA91029.1 aluc|OJZ87832.1 anid|CBF83940.1 anig|EHA20311.1 aory|EIT74592.1 asyd|OJJ53609.1 ater|EAU29456.1 atub|OJI80809.1 aver|OJJ08139.1 awen|OJJ30097.1 azon|OJJ44450.1 abra|OJJ67993.1 acar|OOF92035.1 acla|EAW13634.1 afis|EAW23673.1 afla|EED47394.1 afum|EAL87116.1 akaw|GAA90955.1 aluc|OJZ82867.1 anig|EHA21297.1 aory|EIT81925.1 atub|OJI89092.1 azon|OJJ45993.1 afla|EED47553.1

Group208: abra|OJJ70660.1 acar|OOF97061.1 acla|EAW15046.1 afis|EAW23152.1 afla|EED48515.1 afum|EAL86237.1 agla|OJJ89650.1 akaw|GAA89996.1 aluc|OJZ84010.1 anid|CBF74423.1 anig|EHA19132.1 aory|EIT73250.1 asyd|OJJ57754.1 ater|EAU36259.1 atub|OJI90379.1 aver|OJJ05413.1 awen|OJJ36648.1 azon|OJJ47567.1 acla|EAW08098.1 afum|EAL88329.1 agla|OJJ89649.1 anig|EHA19133.1 azon|OJJ47566.1 acar|OOF97062.1 afis|EAW24607.1 anid|CBF87133.1 aory|EIT73278.1 asyd|OJJ60373.1 ater|EAU36260.1 aver|OJI99708.1

Group209: abra|OJJ71126.1 acar|OOF98025.1 acar|OOF98026.1 acla|EAW10372.1 acla|EAW10373.1 afis|EAW25219.1 afis|EAW25220.1 afla|EED53686.1 afla|EED53687.1 afum|EAL89880.1 afum|EAL89881.1 agla|OJJ79187.1 akaw|GAA82848.1 akaw|GAA82849.1 aluc|OJZ87409.1 anid|CBF85301.1 anig|EHA20639.1 aory|EIT75287.1 aory|EIT75345.1 asyd|OJJ62915.1 asyd|OJJ62916.1 ater|EAU34378.1 ater|EAU34379.1 atub|OJI81236.1 atub|OJI81237.1 aver|OJI98074.1 awen|OJJ40885.1 awen|OJJ40886.1 azon|OJJ44513.1 azon|OJJ44514.1

Group210: abra|OJJ73704.1 afis|EAW17806.1 afla|EED53530.1 afum|EBA27423.1 agla|OJJ78725.1 akaw|GAA88459.1 aluc|OJZ88348.1 ater|EAU30902.1 aver|OJI99268.1 awen|OJJ40823.1 afis|EAW16815.1 afis|EAW17324.1 afla|EED44724.1 afla|EED45247.1 akaw|GAA89045.1 aory|EIT72906.1 aory|EIT73594.1 asyd|OJJ53750.1 ater|EAU36770.1 azon|OJJ45102.1 aver|OJI98047.1 agla|OJJ86073.1 aory|EIT75237.1 asyd|OJJ57660.1 atub|OJI88380.1 aver|OJJ05522.1 asyd|OJJ62943.1 agla|OJJ80314.1 aluc|OJZ80551.1 anig|EHA27689.1

Group211: abra|OJJ74957.1 acar|OOF97356.1 acla|EAW08414.1 afis|EAW23455.1 afla|EED57346.1 afum|EAL88011.1 agla|OJJ86695.1 akaw|GAA83465.1 akaw|GAA83466.1 aluc|OJZ92529.1 anid|CBF89806.1 anig|EHA26445.1 anig|EHA26446.1 aory|EIT80091.1 asyd|OJJ62193.1 ater|EAU36921.1 atub|OJI86965.1 atub|OJI86966.1 aver|OJI96302.1 awen|OJJ39774.1 azon|OJJ47901.1 acla|EAW08415.1 afis|EAW23454.1 afla|EED57349.1 afum|EAL88012.1 agla|OJJ86696.1 anid|CBF89804.1 ater|EAU36920.1 aver|OJI96305.1 awen|OJJ39775.1

Group212: abra|OJJ75967.1 acar|OOF99952.1 acla|EAW09831.1 afis|EAW21302.1 afla|EED57262.1 afum|EAL92319.1 agla|OJJ87435.1 akaw|GAA88989.1 aluc|OJZ89414.1 anid|CBF83395.1 anig|EHA23173.1 aory|EIT74086.1 asyd|OJJ60638.1 ater|EAU35935.1 atub|OJI85381.1 aver|OJI99960.1 awen|OJJ39396.1 azon|OJJ44280.1 acla|EAW09826.1 afis|EAW21307.1 afum|EAL92323.1 anid|CBF83397.1 asyd|OJJ60645.1 ater|EAU35937.1 aver|OJI99963.1 afis|EAW21308.1 afum|EAL92324.1 anid|CBF83399.1 asyd|OJJ60644.1 ater|EAU35938.1

Group213: abra|OJJ76386.1 acar|OOF99627.1 acla|EAW14037.1 acla|EAW14038.1 afis|EAW18743.1 afis|EAW18744.1 afla|EED50699.1 afla|EED50700.1 afum|EAL93469.1 afum|EAL93470.1 agla|OJJ82309.1 akaw|GAA82305.1 akaw|GAA82306.1 aluc|OJZ91827.1 aluc|OJZ91828.1 anid|CBF69994.1 anid|CBF69996.1 anig|EHA22816.1 aory|EIT78103.1 aory|EIT78138.1 asyd|OJJ58888.1 asyd|OJJ58889.1 ater|EAU37870.1 ater|EAU37873.1 atub|OJI86282.1 atub|OJI86283.1 aver|OJJ01489.1 awen|OJJ32308.1 awen|OJJ32309.1 azon|OJJ42718.1

Group214: abra|OJJ65770.1 abra|OJJ69062.1 acar|OOF91221.1 acar|OOG00531.1 afla|EED46866.1 afla|EED56884.1 akaw|GAA90592.1 akaw|GAA90890.1 aluc|OJZ82939.1 aluc|OJZ86050.1 anig|EHA21359.1 anig|EHA24561.1 aory|EIT72522.1 aory|EIT77460.1 atub|OJI79847.1 atub|OJI89171.1 aver|OJJ03011.1 aver|OJJ07939.1 afis|EAW15670.1 afum|EAL85677.1 anid|CBF81578.1 asyd|OJJ53834.1 ater|EAU32494.1 awen|OJJ38346.1 acla|EAW11671.1 agla|OJJ81808.1 agla|OJJ85313.1 awen|OJJ38760.1 azon|OJJ42640.1

Group215: abra|OJJ66046.1 abra|OJJ74366.1 abra|OJJ77282.1 acar|OOF95364.1 acar|OOG00557.1 afis|EAW16747.1 afis|EAW22442.1 afla|EED54458.1 afla|EED54746.1 afum|EAL86322.1 afum|EAL90749.1 akaw|GAA86664.1 akaw|GAA90667.1 aluc|OJZ84692.1 aluc|OJZ89761.1 anig|EHA26981.1 anig|EHA27968.1 aory|EIT81130.1 aory|EIT81408.1 atub|OJI85041.1 atub|OJI89390.1 awen|OJJ35068.1 awen|OJJ37925.1 acla|EAW07417.1 agla|OJJ87580.1 anid|CBF88847.1 asyd|OJJ62866.1 ater|EAU39262.1 aver|OJI96919.1

Group216: abra|OJJ67846.1 abra|OJJ69869.1 acar|OOF90337.1 acar|OOF92827.1 acla|EAW11753.1 acla|EAW15317.1 akaw|GAA87787.1 akaw|GAA91854.1 aluc|OJZ82045.1 aluc|OJZ85422.1 anig|EHA22212.1 anig|EHA25601.1 asyd|OJJ52765.1 asyd|OJJ58155.1 ater|EAU31327.1 ater|EAU32679.1 aver|OJJ04248.1

aver|OJJ08748.1 awen|OJJ31347.1 awen|OJJ33731.1 afis|EAW23786.1 afla|EED51595.1 afum|EAL87220.2 agla|OJJ78939.1 anid|CBF79847.1 atub|OJI84604.1 azon|OJJ42847.1 atub|OJI80153.1 aory|EIT80419.1

Group217: abra|OJJ69627.1 abra|OJJ76818.1 acar|OOF91313.1 acar|OOF92684.1 acla|EAW08738.1 acla|EAW13378.1 agla|OJJ84834.1 agla|OJJ86329.1 akaw|GAA87204.1 akaw|GAA91607.1 anig|EHA27577.1 anig|EHA28339.1 atub|OJI88009.1 atub|OJI89856.1 awen|OJJ33192.1 awen|OJJ38390.1 azon|OJJ43848.1 azon|OJJ46477.1 afis|EAW15698.1 afla|EED51354.1 afum|EAL85650.1 aluc|OJZ82432.1 anid|CBF78282.1 aory|EIT80486.1 asyd|OJJ56072.1 ater|EAU39440.1 aver|OJJ05015.1 aluc|OJZ84228.1 asyd|OJJ52533.1

Group218: abra|OJJ69629.1 abra|OJJ74245.1 acar|OOF92681.1 acar|OOF95485.1 afla|EED55078.1 afla|EED57736.1 aluc|OJZ82434.1 aluc|OJZ89887.1 asyd|OJJ63867.1 asyd|OJJ64731.1 acla|EAW07299.1 agla|OJJ87688.1 akaw|GAA91609.1 anig|EHA28337.1 aory|EIT81976.1 atub|OJI84920.1 atub|OJI88007.1 aver|OJI98835.1 awen|OJJ34926.1 afis|EAW22346.1 afum|EAL90846.1 akaw|GAA86775.1 anid|CBF82985.1 anig|EHA27086.1 ater|EAU39484.1 aver|OJI97072.1 azon|OJJ50069.1 ater|EAU35426.1 aory|EIT79895.1

Group219: abra|OJJ69794.1 abra|OJJ76964.1 acar|OOF93584.1 acar|OOG00383.1 agla|OJJ81809.1 agla|OJJ84793.1 agla|OJJ85103.1 aluc|OJZ84359.1 aluc|OJZ85505.1 anig|EHA25037.1 anig|EHA27695.1 atub|OJI80060.1 atub|OJI89709.1 awen|OJJ30169.1 awen|OJJ37984.1 azon|OJJ46123.1 azon|OJJ46412.1 acla|EAW11252.1 afis|EAW18245.1 afla|EED44938.1 afum|EAL87610.1 akaw|GAA87864.1 aory|EIT73480.1 asyd|OJJ60485.1 ater|EAU30027.1 aver|OJI99811.1 acar|OOF91408.1 ater|EAU29360.1 akaw|GAA87080.1

Group220: abra|OJJ72131.1 abra|OJJ76584.1 acar|OOF96771.1 acar|OOF99402.1 agla|OJJ80546.1 agla|OJJ86323.1 agla|OJJ89016.1 akaw|GAA82487.1 akaw|GAA84791.1 aluc|OJZ86889.1 aluc|OJZ91622.1 anig|EHA21754.1 anig|EHA22647.1 asyd|OJJ57662.1 asyd|OJJ58966.1 atub|OJI83630.1 atub|OJI86075.1 aver|OJJ01560.1 aver|OJJ05524.1 awen|OJJ32389.1 awen|OJJ32572.1 awen|OJJ33544.1 acla|EAW14111.1 afis|EAW18813.1 afum|EAL93549.1 anid|CBF69799.1 aory|EIT77991.1 ater|EAU37980.1 azon|OJJ47150.1

Group221: acar|OOF92608.1 acar|OOF92629.1 abra|OJJ67199.1 acar|OOF98365.1 acla|EAW14525.1 acla|EAW14526.1 afis|EAW19243.1 afis|EAW19244.1 afla|EED47633.1 afla|EED47634.1 afum|EAL93972.1 afum|EAL93973.1 agla|OJJ88454.1 akaw|GAA90979.1 akaw|GAA90980.1 aluc|OJZ87775.1 anid|CBF78652.1 anig|EHA20259.1 aory|EIT80236.1 asyd|OJJ65195.1 ater|EAU29500.1 atub|OJI80870.1 aver|OJI99344.1 awen|OJJ37599.1 azon|OJJ51254.1 agla|OJJ88453.1 anid|CBF78654.1 azon|OJJ51255.1 ater|EAU29499.1

Group222: afis|EAW21841.1 afis|EAW25659.1 agla|OJJ84294.1 agla|OJJ87901.1 aory|EIT72356.1 aory|EIT79167.1 asyd|OJJ52337.1 asyd|OJJ61239.1 asyd|OJJ54547.1 aver|OJI96725.1 aver|OJI96959.1 aver|OJJ07630.1 aver|OJJ00562.1 abra|OJJ68267.1 acar|OOF97547.1 acla|EAW09304.1 afla|EED46529.1 afum|EAL92853.1 akaw|GAA91931.1 aluc|OJZ82691.1 anid|CBF76711.1 anig|EHA27368.1 ater|EAU38518.1 atub|OJI88923.1 awen|OJJ34124.1 azon|OJJ44222.1 aluc|OJZ80200.1 afla|EED47266.1 anid|CBF85164.1

Group223: afla|EED48230.1 afla|EED58028.1 anid|CBF83667.1 anid|CBF86363.1 aory|EIT72902.1 aory|EIT79778.1 asyd|OJJ60808.1 asyd|OJJ64969.1 aver|OJI99093.1 aver|OJJ00136.1 awen|OJJ30134.1 awen|OJJ35830.1 abra|OJJ72106.1 acar|OOF96749.1 acla|EAW09385.1 afis|EAW21750.1 afum|EAL92781.2 agla|OJJ84424.1 akaw|GAA84816.1 aluc|OJZ86861.1 anig|EHA21779.1 ater|EAU38440.1 atub|OJI83659.1 awen|OJJ34031.1 azon|OJJ48204.1 anid|CBF83471.1 ater|EAU34242.1 aver|OJJ00014.1 asyd|OJJ60689.1

Group224: azon|OJJ42769.1 azon|OJJ50372.1 abra|OJJ71617.1 acar|OOF96333.1 afla|EED57118.1 agla|OJJ80138.1 akaw|GAA89310.1 aluc|OJZ86358.1 anig|EHA21532.1 aory|EIT73985.1 atub|OJI84176.1 awen|OJJ40249.1 abra|OJJ77100.1 acar|OOF92984.1 acla|EAW12282.1 afis|EAW16959.1 afla|EED45239.1 afum|EAL86486.1 akaw|GAA86956.1 aluc|OJZ84508.1 anid|CBF80512.1 anig|EHA27807.1 aory|EIT73744.1 asyd|OJJ54736.1 ater|EAU30185.1 atub|OJI89572.1 aver|OJJ07435.1 agla|OJJ85349.1 awen|OJJ30386.1

Group225: azon|OJJ46793.1 azon|OJJ51796.1 abra|OJJ77127.1 acar|OOF93013.1 acla|EAW12902.1 afis|EAW16407.1 afla|EED51674.1 agla|OJJ81607.1 akaw|GAA90801.1 aluc|OJZ84537.1 anid|CBF82038.1 anig|EHA27835.1 aory|EIT79575.1 asyd|OJJ59200.1 ater|EAU36805.1 atub|OJI89543.1 aver|OJJ01812.1 awen|OJJ36343.1 acar|OOF93300.1 afla|EED49754.1 aory|EIT81671.1 asyd|OJJ54221.1 acar|OOF99299.1 aory|EIT77631.1 afla|EED48439.1 awen|OJJ38768.1 aory|EIT80498.1 afum|EAL91133.1 aver|OJJ05173.1

Group226: abra|OJJ67152.1 acar|OOF90071.1 acar|OOF98406.1 acla|EAW14572.1 acla|EAW14573.1 afis|EAW19295.1 afis|EAW19296.1 afla|EED47590.1 afla|EED47591.1 afum|EAL94014.1 afum|EAL94015.1 agla|OJJ88385.1 akaw|GAA91020.1 akaw|GAA91021.1 aluc|OJZ87823.1 aluc|OJZ87824.1 anid|CBF78768.1 anid|CBF78770.1 anig|EHA20303.1 aory|EIT74518.1 aory|EIT74526.1 asyd|OJJ65238.1 ater|EAU29476.1 ater|EAU29477.1 atub|OJI80820.1 atub|OJI80821.1 aver|OJI99388.1 awen|OJJ37646.1 azon|OJJ44911.1

Group227: abra|OJJ67842.1 acar|OOF92019.1 afis|EAW19539.1 afla|EED54092.1 afum|EAL86968.1 akaw|GAA91848.1 aluc|OJZ82050.1 anid|CBF75400.1 anig|EHA22208.1 aory|EIT75915.1 asyd|OJJ63641.1 ater|EAU35153.1 atub|OJI84608.1 aver|OJI97295.1 azon|OJJ48646.1 abra|OJJ76922.1 acar|OOF91369.1 afla|EED54074.1 akaw|GAA87116.1 aluc|OJZ84322.1 anid|CBF71754.1 anig|EHA27657.1 asyd|OJJ61735.1 atub|OJI89746.1 aver|OJI95828.1 awen|OJJ33258.1 anid|CBF71752.1 azon|OJJ44017.1 aory|EIT75930.1

Group228: abra|OJJ68668.1 acar|OOF92168.1 acla|EAW12580.1 afis|EAW24068.1 afla|EED51407.1 afum|EAL91924.1 agla|OJJ87316.1 aluc|OJZ81882.1 anid|CBF86457.1 anig|EHA22358.1 aory|EIT80674.1

asyd|OJJ64915.1 ater|EAU30490.1 atub|OJI84435.1 aver|OJI99035.1 awen|OJJ41422.1 azon|OJJ48887.1 acla|EAW12579.1 afis|EAW24069.1 afla|EED51406.1 afum|EAL91925.1 agla|OJJ87315.1 akaw|GAA89809.1 aluc|OJZ81881.1 anid|CBF86458.1 aory|EIT80695.1 asyd|OJJ64914.1 awen|OJJ41421.1 azon|OJJ48886.1

Group229: abra|OJJ68715.1 acar|OOF92215.1 acar|OOF92216.1 acla|EAW12530.1 acla|EAW12531.1 afis|EAW24118.1 afis|EAW24119.1 afla|EED46814.1 afum|EAL91973.1 afum|EAL91974.1 agla|OJJ87193.1 akaw|GAA89868.1 aluc|OJZ81813.1 anid|CBF86539.1 anid|CBF86540.1 anig|EHA22402.1 anig|EHA22403.1 aory|EIT72603.1 aory|EIT72683.1 asyd|OJJ64863.1 asyd|OJJ64865.1 ater|EAU30546.1 ater|EAU30547.1 atub|OJI84385.1 aver|OJI98974.1 aver|OJI98978.1 awen|OJJ41370.1 azon|OJJ48839.1 afla|EED46813.1

Group230: abra|OJJ68757.1 acar|OOF92245.1 acla|EAW12494.1 afis|EAW24151.1 afla|EED46780.1 afum|EAL92006.1 akaw|GAA89901.1 aluc|OJZ81770.1 anid|CBF77913.1 anig|EHA22446.1 aory|EIT72705.1 asyd|OJJ55298.1 ater|EAU30583.1 atub|OJI84342.1 aver|OJJ06928.1 awen|OJJ41327.1 abra|OJJ68758.1 acla|EAW12493.1 afis|EAW24152.1 afla|EED46779.1 afum|EAL92007.1 agla|OJJ89543.1 akaw|GAA89902.1 aluc|OJZ81769.1 anid|CBF77911.1 aory|EIT72741.1 atub|OJI84341.1 awen|OJJ41326.1 azon|OJJ48800.1

Group231: abra|OJJ70392.1 acar|OOF90061.1 acla|EAW12387.1 acla|EAW12388.1 afis|EAW16834.1 afis|EAW16835.1 afla|EED47328.1 afum|EAL86381.1 afum|EAL86382.2 agla|OJJ81545.1 agla|OJJ89426.1 akaw|GAA91967.1 aluc|OJZ82649.1 anid|CBF74687.1 anid|CBF74689.1 anig|EHA27422.1 anig|EHA27423.1 aory|EIT82824.1 asyd|OJJ60210.1 ater|EAU30239.1 atub|OJI88881.1 aver|OJJ04058.1 awen|OJJ35685.1 azon|OJJ44153.1 acar|OOF90060.1 afla|EED47327.1 aory|EIT82846.1 ater|EAU30242.1 acar|OOF91882.1

Group232: abra|OJJ70540.1 acar|OOF97393.1 acla|EAW08138.1 acla|EAW08139.1 afis|EAW23174.1 afis|EAW23175.1 afla|EED46944.1 afla|EED46945.1 afum|EAL88308.1 afum|EAL88309.1 agla|OJJ89686.1 agla|OJJ89687.1 akaw|GAA83385.1 akaw|GAA83386.1 aluc|OJZ83885.1 anid|CBF74496.1 anid|CBF74498.1 anig|EHA19022.1 aory|EIT72667.1 aory|EIT72762.1 asyd|OJJ57719.1 asyd|OJJ57720.1 ater|EAU36303.1 ater|EAU36304.1 atub|OJI90252.1 aver|OJJ05448.1 awen|OJJ36603.1 azon|OJJ47603.1 azon|OJJ47604.1

Group233: abra|OJJ73887.1 acar|OOG01025.1 acar|OOG01026.1 acla|EAW07769.1 acla|EAW07771.1 afis|EAW22805.1 afis|EAW22807.1 afla|EED50075.1 afum|EAL90394.1 afum|EBA27407.1 agla|OJJ83582.1 akaw|GAA89498.1 akaw|GAA89499.1 aluc|OJZ88162.1 anid|CBF87924.1 anig|EHA17916.1 anig|EHA17917.1 aory|EIT74704.1 asyd|OJJ63459.1 asyd|OJJ63460.1 ater|EAU38903.1 ater|EAU38904.1 atub|OJI90761.1 aver|OJI97472.1 aver|OJI97473.1 awen|OJJ38923.1 azon|OJJ49700.1 aory|EIT74626.1 afla|EED50076.1

Group234: abra|OJJ74769.1 acar|OOF95760.1 acla|EAW10642.1 afis|EAW24924.1 afla|EED55549.1 afum|EAL89579.1 agla|OJJ82153.1 akaw|GAA83643.1 aluc|OJZ92352.1 anid|CBF75699.1 anig|EHA26622.1 aory|EIT82671.1 asyd|OJJ57496.1 atub|OJI86780.1 aver|OJJ05684.1 awen|OJJ40521.1 azon|OJJ49380.1 abra|OJJ74770.1 acla|EAW10641.1 afis|EAW24925.1 afla|EED55548.1 afum|EAL89580.1 akaw|GAA83642.1 anid|CBF75701.1 aory|EIT82683.1 ater|EAU35655.1 atub|OJI86781.1 aver|OJJ05683.1 azon|OJJ49381.1

Group235: abra|OJJ74915.1 acar|OOF95906.1 acar|OOF95907.1 acla|EAW08455.1 acla|EAW08456.1 afis|EAW23499.1 afis|EAW23500.1 afla|EED57400.1 afla|EED57401.1 afum|EAL87962.2 afum|EAL87963.1 agla|OJJ86747.1 agla|OJJ86748.1 akaw|GAA83507.1 akaw|GAA83508.1 aluc|OJZ92487.1 anid|CBF89730.1 anid|CBF89731.1 anig|EHA26485.1 aory|EIT80151.1 aory|EIT80164.1 asyd|OJJ62234.1 ater|EAU35289.1 ater|EAU35290.1 atub|OJI86920.1 aver|OJI96343.1 awen|OJJ39825.1 azon|OJJ47949.1 azon|OJJ47950.1

Group236: abra|OJJ75075.1 acar|OOF97258.1 acar|OOF97259.1 acla|EAW08256.1 afis|EAW23290.1 afla|EED55682.1 afum|EAL88189.1 agla|OJJ86548.1 akaw|GAA88687.1 akaw|GAA88688.1 aluc|OJZ92641.1 anid|CBF89509.1 anig|EHA26351.1 aory|EIT77074.1 asyd|OJJ62397.1 ater|EAU37583.1 atub|OJI87072.1 aver|OJI96531.1 awen|OJJ39667.1 azon|OJJ47797.1 aory|EIT77075.1 acla|EAW08258.1 afis|EAW23291.1 afla|EED55680.1 afum|EAL88188.1 anid|CBF89508.1 asyd|OJJ62398.1 ater|EAU37582.1 azon|OJJ47796.1

Group237: abra|OJJ75137.1 acar|OOF97199.1 acar|OOF97200.1 afis|EAW23348.1 afis|EAW23350.1 afla|EED55617.1 afum|EAL88126.1 afum|EAL88128.1 agla|OJJ86478.1 akaw|GAA88743.1 aluc|OJZ92703.1 anid|CBF74910.1 anig|EHA26296.1 anig|EHA26297.1 aory|EIT81793.1 asyd|OJJ60100.1 ater|EAU35593.1 atub|OJI87130.1 aver|OJJ04162.1 awen|OJJ39539.1 awen|OJJ39543.1 azon|OJJ47741.1 agla|OJJ80588.1 aver|OJJ04161.1 anid|CBF74914.1 aory|EIT81802.1 ater|EAU35592.1 azon|OJJ47736.1 afla|EED55618.1

Group238: abra|OJJ75492.1 acar|OOF90824.1 acar|OOF90825.1 acla|EAW11224.1 acla|EAW11225.1 afis|EAW18278.1 afis|EAW18279.1 afla|EED48745.1 afla|EED48746.1 afum|EAL87644.1 afum|EAL87645.1 agla|OJJ88147.1 agla|OJJ88148.1 akaw|GAA84257.1 akaw|GAA84258.1 aluc|OJZ88951.1 anid|CBF79492.1 anid|CBF79494.1 anig|EHA23569.1 anig|EHA23570.1 aory|EIT73072.1 aory|EIT73086.1 asyd|OJJ57877.1 ater|EAU33284.1 ater|EAU33285.1 atub|OJI85838.1 aver|OJJ04517.1 awen|OJJ30651.1 azon|OJJ47964.1

Group239: abra|OJJ75717.1 acar|OOG00139.1 acar|OOG00141.1 acla|EAW09392.1 acla|EAW09394.1 afis|EAW21739.1 afis|EAW21741.1 afla|EED58014.1 afla|EED58016.1 afum|EAL92773.1 afum|EAL92774.1 agla|OJJ84432.1 agla|OJJ84449.1 akaw|GAA84452.1 akaw|GAA84453.1 aluc|OJZ89169.1 anid|CBF83696.1 anid|CBF83699.1 anig|EHA23386.1 aory|EIT79752.1 aory|EIT79775.1 asyd|OJJ60825.1 ater|EAU38420.1 ater|EAU38429.1 atub|OJI85621.1 aver|OJJ00151.1 awen|OJJ34021.1 awen|OJJ34023.1 azon|OJJ48535.1

Group240: abra|OJJ77333.1 acar|OOF93207.1 acla|EAW13139.1 afis|EAW17511.1 afla|EED49310.1 afum|EAL91331.1 agla|OJJ81447.1 akaw|GAA91425.1 aluc|OJZ84741.1 anid|CBF71568.1 anig|EHA28015.1 aory|EIT82226.1 asyd|OJJ61881.1 ater|EAU36952.1 atub|OJI89340.1 aver|OJI95979.1 awen|OJJ37333.1 azon|OJJ51021.1 afla|EED49307.1 afum|EAL91330.1 anid|CBF71531.1 asyd|OJJ61893.1 aver|OJI95989.1 azon|OJJ51020.1 acla|EAW13140.1 afis|EAW17512.1 anig|EHA28014.1 aory|EIT82236.1 awen|OJJ37334.1

Group241: abra|OJJ65980.1 abra|OJJ66239.1 acar|OOF90701.1 acar|OOF92893.1 acla|EAW11609.1 acla|EAW15289.1 afis|EAW23842.1 afis|EAW24576.1 akaw|GAA89050.1 akaw|GAA90853.1 aluc|OJZ80544.1 aluc|OJZ82980.1 anig|EHA21395.1 anig|EHA25651.1 asyd|OJJ54925.1 asyd|OJJ65267.1 ater|EAU32172.1 ater|EAU33781.1 atub|OJI88387.1 atub|OJI89213.1 aver|OJI99419.1 aver|OJJ07270.1 afla|EED45390.1 afum|EAL87273.1 agla|OJJ87625.1 anid|CBF78824.1 aory|EIT82439.1 azon|OJJ46551.1

Group242: abra|OJJ66056.1 abra|OJJ77098.1 afla|EED49909.1 afla|EED53379.1 akaw|GAA86958.1 akaw|GAA93126.1 aluc|OJZ81108.1 aluc|OJZ84506.1 anid|CBF80783.1 anid|CBF84621.1 anig|EHA19261.1 anig|EHA27805.1 aory|EIT72449.1 aory|EIT75126.1 asyd|OJJ54497.1 asyd|OJJ55641.1 ater|EAU30218.1 ater|EAU30988.1 atub|OJI82471.1 atub|OJI89574.1 aver|OJJ06724.1 aver|OJJ07683.1 awen|OJJ29737.1 awen|OJJ33568.1 acar|OOF92980.1 afis|EAW17111.1 afum|EAL86575.1 agla|OJJ79951.1

Group243: abra|OJJ66383.1 abra|OJJ71952.1 acar|OOF94059.1 acar|OOF94129.1 acar|OOF96603.1 afla|EED51643.1 afla|EED53089.1 akaw|GAA84976.1 akaw|GAA90249.1 aluc|OJZ83091.1 aluc|OJZ86686.1 aory|EIT74787.1 aory|EIT80741.1 atub|OJI83839.1 atub|OJI87845.1 azon|OJJ48234.1 azon|OJJ51069.1 acla|EAW12654.1 afis|EAW18031.1 afum|EAL91842.1 agla|OJJ85401.1 anid|CBF76050.1 anig|EHA21987.1 asyd|OJJ52485.1 ater|EAU36811.1 aver|OJJ03921.1 awen|OJJ41506.1 ater|EAU35537.1

Group244: abra|OJJ67133.1 abra|OJJ72705.1 akaw|GAA85309.1 akaw|GAA91036.1 aluc|OJZ87846.1 aluc|OJZ90669.1 atub|OJI80802.1 atub|OJI82099.1 aver|OJI96726.1 aver|OJJ07629.1 acar|OOF95940.1 anig|EHA24103.1 asyd|OJJ64079.1 azon|OJJ42255.1 afis|EAW22517.1 afum|EAL90676.1 aory|EIT73492.1 azon|OJJ49956.1 abra|OJJ75758.1 afla|EED44952.1 akaw|GAA84490.1 aluc|OJZ89211.1 anid|CBF85166.1 anig|EHA23347.1 asyd|OJJ54548.1 ater|EAU33498.1 atub|OJI85581.1 awen|OJJ40261.1

Group245: abra|OJJ68302.1 abra|OJJ74425.1 acar|OOF90436.1 acar|OOF90443.1 acar|OOF96685.1 afis|EAW24469.1 afis|EAW25516.1 aluc|OJZ80145.1 aluc|OJZ83671.1 aluc|OJZ89698.1 atub|OJI85105.1 atub|OJI90042.1 awen|OJJ34908.1 awen|OJJ41259.1 acla|EAW14952.1 afla|EED55056.1 afum|EAL86141.1 afum|EAL90181.1 agla|OJJ87287.1 akaw|GAA83172.1 anid|CBF77786.1 anig|EHA18826.1 aory|EIT81972.1 asyd|OJJ56269.1 ater|EAU33362.1 aver|OJJ08496.1 azon|OJJ42850.1 asyd|OJJ54439.1

Group246: abra|OJJ68357.1 abra|OJJ70141.1 afla|EED46535.1 afla|EED53487.1 akaw|GAA86624.1 akaw|GAA92129.1 aluc|OJZ83593.1 aluc|OJZ85179.1 anig|EHA18753.1 anig|EHA25391.1 aory|EIT72381.1 aory|EIT74971.1 asyd|OJJ56981.1 asyd|OJJ60301.1 ater|EAU38514.1 ater|EAU39003.1 atub|OJI80418.1 atub|OJI89965.1 awen|OJJ34116.1 awen|OJJ34635.1 acar|OOF93592.1 afis|EAW19844.1 afum|EAL85050.1 agla|OJJ84299.1 anid|CBF87147.1 aver|OJI99641.1 aver|OJJ03306.1 azon|OJJ50352.1

Group247: abra|OJJ71231.1 abra|OJJ72448.1 acar|OOF94788.1 acar|OOF97944.1 afla|EED46190.1 afla|EED56273.1 akaw|GAA82757.1 akaw|GAA86186.1 aluc|OJZ87316.1 aluc|OJZ90432.1 ater|EAU33047.1 ater|EAU33789.1 atub|OJI81329.1 atub|OJI81864.1 awen|OJJ31638.1 awen|OJJ41732.1 acla|EAW12682.1 afis|EAW17845.1 afum|EAL91654.1 agla|OJJ81772.1 agla|OJJ83123.1 anig|EHA20550.1 asyd|OJJ55268.1 aver|OJJ06959.1 azon|OJJ48428.1 anid|CBF77966.1 anig|EHA23901.1 aory|EIT76458.1

Group248: acar|OOF95098.1 acar|OOF95164.1 acar|OOF96577.1 afis|EAW21529.1 afis|EAW24614.1 agla|OJJ80793.1 agla|OJJ81846.1 agla|OJJ85693.1 agla|OJJ84478.1 aory|EIT76721.1 aory|EIT79947.1 asyd|OJJ52471.1 asyd|OJJ61079.1 ater|EAU31519.1 ater|EAU35012.1 abra|OJJ71920.1 acla|EAW09610.1 afla|EED47452.1 afla|EED55978.1 afum|EAL92550.1 akaw|GAA85002.1 aluc|OJZ86657.1 anid|CBF76400.1 anig|EHA22014.1 atub|OJI83870.1 aver|OJJ00395.1 awen|OJJ39164.1 azon|OJJ51493.1

Group249: afis|EAW16443.1 afis|EAW16700.1 afla|EED45811.1 afla|EED46054.1 anid|CBF78974.1 anid|CBF82132.1 anid|CBF88938.1 aory|EIT75703.1 aory|EIT80899.1 asyd|OJJ54312.1 asyd|OJJ55669.1 asyd|OJJ62858.1 aver|OJI95760.1 aver|OJI96910.1 aver|OJJ05266.1 aver|OJJ06750.1 abra|OJJ67169.1 acar|OOF97010.1 afla|EED55529.1 afum|EAL89297.1 akaw|GAA91005.1 aluc|OJZ87806.1 anig|EHA20288.1 ater|EAU36196.1 atub|OJI80839.1 azon|OJJ50602.1 afla|EED49535.1 aory|EIT82649.1

Group250: afis|EAW18309.1 afis|EAW25696.1 awen|OJJ30683.1 awen|OJJ32214.1 abra|OJJ75441.1 acar|OOF90786.1 acla|EAW13822.1 afla|EED53243.1 afum|EAL87675.1 agla|OJJ88078.1 akaw|GAA84216.1 aluc|OJZ88894.1 anid|CBF79436.1 anig|EHA23612.1 aory|EIT72989.1 asyd|OJJ54980.1 asyd|OJJ57846.1 ater|EAU33319.1 atub|OJI85895.1 aver|OJJ04318.1 aver|OJJ04546.1 awen|OJJ32538.1 azon|OJJ43103.1 afla|EED50918.1 aory|EIT74822.1 aory|EIT78997.1 asyd|OJJ52725.1 ater|EAU32817.1

Group251: afla|EED51654.1 afla|EED52464.1 aluc|OJZ84281.1 aluc|OJZ87033.1 anig|EHA25215.1 anig|EHA26913.1 anig|EHA27626.1 aory|EIT80434.1 aory|EIT82812.1 asyd|OJJ54529.1 asyd|OJJ62992.1 atub|OJI83482.1 atub|OJI89800.1 aver|OJI97976.1 aver|OJJ07658.1 abra|OJJ76870.1 acla|EAW10087.1 afis|EAW16918.1 akaw|GAA87153.1 anid|CBF80752.1 awen|OJJ30986.1 afis|EAW25500.1 afla|EED50854.1 afum|EAL90166.1 agla|OJJ87304.1 aory|EIT79059.1 awen|OJJ41242.1 azon|OJJ44808.1

Group252: agla|OJJ82640.1 agla|OJJ85276.1 asyd|OJJ57347.1 asyd|OJJ65114.1 aver|OJI99240.1 aver|OJJ05818.1 aver|OJJ00352.1 aver|OJJ05340.1 awen|OJJ30078.1 awen|OJJ32593.1 abra|OJJ78114.1 acar|OOF98754.1 acla|EAW07003.1 afis|EAW16620.1 afla|EED48645.1 afum|EAL89217.1 agla|OJJ84764.1 akaw|GAA85893.1 aluc|OJZ91179.1 anid|CBF81971.1 anig|EHA19870.1 aory|EIT73182.1 ater|EAU36705.1 atub|OJI83191.1 azon|OJJ42184.1 aory|EIT73798.1 asyd|OJJ61034.1 ater|EAU37620.1

Group253: asyd|OJJ62591.1 asyd|OJJ64803.1 abra|OJJ77288.1 acar|OOF93165.1 acla|EAW12880.1 afis|EAW17797.1 afla|EED45790.1 afum|EAL91605.2 agla|OJJ88361.1 akaw|GAA90661.1 aluc|OJZ84699.1 anid|CBF86643.1 anig|EHA27974.1 aory|EIT75720.1 ater|EAU31827.1 atub|OJI89383.1 aver|OJI98916.1 awen|OJJ37068.1 acar|OOF93164.1 afis|EAW17799.1 afla|EED45791.1 afum|EAL91607.1 ater|EAU31828.1 anid|CBF86641.1 aver|OJI98917.1 agla|OJJ78851.1 awen|OJJ37061.1 aory|EIT75595.1

Group254: abra|OJJ67087.1 afum|EAL86535.1 agla|OJJ83616.1 anid|CBF75171.1 anig|EHA20191.1 abra|OJJ67973.1 acar|OOF92814.1 acla|EAW11761.1 afis|EAW23794.1 afla|EED48581.1 akaw|GAA91229.1 aluc|OJZ82841.1 anig|EHA21278.1 aory|EIT73168.1 asyd|OJJ62859.1 atub|OJI89071.1 aver|OJI96912.1 awen|OJJ38042.1 azon|OJJ44002.1 akaw|GAA90147.1 aluc|OJZ91537.1 anig|EHA22574.1 atub|OJI87737.1 akaw|GAA92523.1 asyd|OJJ60253.1 atub|OJI85991.1 azon|OJJ46498.1 azon|OJJ45663.1

Group255: abra|OJJ67268.1 acar|OOF98298.1 acar|OOF98299.1 afis|EAW17109.1 afla|EED55276.1 afum|EAL86573.1 agla|OJJ88998.1 akaw|GAA91683.1 aluc|OJZ87711.1 anid|CBF85644.1 anig|EHA20205.1 anig|EHA20206.1 aory|EIT83585.1 asyd|OJJ64238.1 asyd|OJJ64239.1 ater|EAU29281.1 ater|EAU39434.1 atub|OJI80931.1 aver|OJI98331.1 aver|OJI98332.1 awen|OJJ40796.1 ater|EAU39435.1 afis|EAW17112.1 afum|EAL86576.1 anid|CBF85642.1 aory|EIT83532.1 afla|EED55275.1 aory|EIT83583.1

Group256: abra|OJJ67322.1 acar|OOF92491.1 acar|OOF92492.1 acla|EAW06413.1 acla|EAW06414.1 afis|EAW18517.1 afis|EAW18518.1 afla|EED52880.1 afla|EED52881.1 afum|EAL93212.1 afum|EAL93213.1 agla|OJJ85130.1 akaw|GAA86495.1 akaw|GAA86496.1 aluc|OJZ85037.1 aluc|OJZ85038.1 anid|CBF70228.1 anid|CBF70230.1 anig|EHA25256.1 aory|EIT78607.1 aory|EIT78620.1 asyd|OJJ58772.1 ater|EAU30052.1 ater|EAU30053.1 atub|OJI80559.1 aver|OJJ01381.1 awen|OJJ31070.1 azon|OJJ50580.1

Group257: abra|OJJ67358.1 acar|OOF92463.1 acla|EAW06384.1 afis|EAW18490.1 afla|EED52916.1 afum|EAL93180.1 agla|OJJ85085.1 akaw|GAA86463.1 aluc|OJZ85003.1 anid|CBF70157.1 anig|EHA25222.1 aory|EIT78645.1 asyd|OJJ58803.1 ater|EAU29914.1 atub|OJI80595.1 aver|OJJ01407.1 awen|OJJ31043.1 azon|OJJ50554.1 abra|OJJ67360.1 acla|EAW06383.1 afis|EAW18489.1 afla|EED52917.1 afum|EAL93179.1 akaw|GAA86461.1 anid|CBF70155.1 anig|EHA25220.1 aory|EIT78598.1 asyd|OJJ58804.1

Group258: abra|OJJ68582.1 atub|OJI91460.1 azon|OJJ47723.1 abra|OJJ75562.1 acar|OOF94716.1 acla|EAW13645.1 asyd|OJJ52397.1 ater|EAU35611.1 aver|OJJ01926.1 awen|OJJ38360.1 azon|OJJ47155.1 abra|OJJ76231.1 afis|EAW24533.1 afla|EED47408.1 afum|EAL86199.1 anid|CBF69447.1 anig|EHA18750.1 acla|EAW08325.1 afis|EAW23360.1 afla|EED55598.1 afum|EBA27335.1 agla|OJJ86466.1 aluc|OJZ92717.1 anig|EHA26282.1 aory|EIT82668.1 asyd|OJJ60112.1 ater|EAU35434.1 anig|EHA22166.1

Group259: abra|OJJ68687.1 acar|OOF92189.1 acar|OOF92190.1 acla|EAW12558.1 acla|EAW12560.1 afis|EAW24090.1 afis|EAW24092.1 afla|EED46847.1 afla|EED46849.1 afum|EAL91945.1 afum|EAL91947.2 agla|OJJ87160.1 agla|OJJ87166.1 akaw|GAA89840.1 akaw|GAA89842.1 aluc|OJZ81846.1 anid|CBF86494.1 anig|EHA22376.1 aory|EIT72742.1 asyd|OJJ64893.1 ater|EAU30514.1 ater|EAU30516.1 atub|OJI84415.1 aver|OJI99014.1 awen|OJJ41397.1 awen|OJJ41399.1 azon|OJJ48865.1 aory|EIT72593.1

Group260: abra|OJJ68761.1 acar|OOF92248.1 acla|EAW12490.1 afis|EAW24155.1 afla|EED46777.1 afum|EAL92010.1 akaw|GAA89905.1 aluc|OJZ81765.1 anid|CBF77904.1 anig|EHA22449.1 asyd|OJJ55301.1 atub|OJI84337.1 abra|OJJ68762.1 acla|EAW12489.1 afis|EAW24156.1 afum|EAL92011.1 agla|OJJ89525.1 akaw|GAA89906.1 aluc|OJZ81764.1 anid|CBF77902.1 anig|EHA22450.1 aory|EIT72652.1 asyd|OJJ55302.1 ater|EAU30587.1 atub|OJI84336.1 aver|OJJ06924.1 awen|OJJ41322.1 azon|OJJ48795.1

Group261: abra|OJJ69707.1 acar|OOF94204.1 acla|EAW14911.1 afla|EED55248.1 agla|OJJ79247.1 akaw|GAA90156.1 aluc|OJZ83191.1 anid|CBF73771.1 anig|EHA28261.1 aory|EIT77697.1 asyd|OJJ56592.1 atub|OJI87748.1 awen|OJJ35897.1 azon|OJJ50407.1 abra|OJJ72111.1 acar|OOF90025.1 afis|EAW24432.1 afum|EAL86100.1 akaw|GAA84811.1 aluc|OJZ86866.1 anig|EHA21775.1 ater|EAU30013.1 atub|OJI83654.1 aver|OJJ06133.1 awen|OJJ32770.1 aory|EIT83627.1 azon|OJJ44000.1 afla|EED49732.1

Group262: abra|OJJ69819.1 acar|OOF93344.1 afis|EAW15617.1 afla|EED52777.1 afum|EAL85718.1 agla|OJJ79351.1 akaw|GAA87841.1 aluc|OJZ85477.1 anid|CBF76275.1 anig|EHA25018.1 aory|EIT78176.1 asyd|OJJ60985.1 ater|EAU37197.1 atub|OJI80086.1 aver|OJJ00304.1 abra|OJJ74486.1 acar|OOF95263.1 acla|EAW08782.1 afla|EED57614.1 akaw|GAA87379.1 aluc|OJZ89633.1 anig|EHA26875.1 aory|EIT82993.1 atub|OJI85170.1 awen|OJJ40133.1 ater|EAU36083.1 asyd|OJJ52418.1 aver|OJJ03363.1

Group263: abra|OJJ70208.1 acar|OOF90365.1 acla|EAW13177.1 afis|EAW22620.1 afla|EED51827.1 afum|EAL87187.2 agla|OJJ85385.1 akaw|GAA86568.1 aluc|OJZ85119.1 anid|CBF84539.1 anig|EHA25339.1 aory|EIT81723.1 asyd|OJJ53483.1 ater|EAU32773.1 atub|OJI80474.1 aver|OJJ08276.1 awen|OJJ33335.1 azon|OJJ51798.1 acla|EAW11725.1 afis|EAW23750.1 asyd|OJJ64104.1 aver|OJI98187.1 azon|OJJ51793.1 afum|EAL90576.1 afla|EED53104.1 anid|CBF85467.1 agla|OJJ87544.1 aory|EIT74796.1

Group264: anig|EHA20613.1 atub|OJI81264.1 abra|OJJ71157.1 acar|OOF98002.1 acla|EAW10341.1 afis|EAW25250.1 afla|EED53714.1 afum|EAL89912.1 agla|OJJ89220.1 akaw|GAA82821.1 aluc|OJZ87382.1 anid|CBF85305.1 aory|EIT74933.1 asyd|OJJ63998.1 ater|EAU34356.1 aver|OJI98087.1 awen|OJJ40962.1 azon|OJJ44543.1 acla|EAW10342.1 afis|EAW25247.1 afla|EED53713.1 afum|EAL89909.1 agla|OJJ89214.1 akaw|GAA82822.1 aluc|OJZ87383.1 anid|CBF85306.1 awen|OJJ40961.1 azon|OJJ44529.1

Group265: abra|OJJ71667.1 acar|OOF96377.1 acar|OOF96378.1 acla|EAW15205.1 acla|EAW15206.1 afis|EAW20170.1 afis|EAW20171.1 afla|EED57177.1 afla|EED57179.1 afum|EAL85288.1 afum|EAL85289.1 agla|OJJ88759.1 akaw|GAA89264.1 akaw|GAA89265.1 aluc|OJZ86409.1 anid|CBF85026.1 anid|CBF85028.1 anig|EHA21490.1 aory|EIT73988.1 aory|EIT74058.1 asyd|OJJ63103.1 ater|EAU35205.1 ater|EAU35206.1 atub|OJI84122.1 aver|OJI97858.1 aver|OJI97859.1 awen|OJJ32067.1 azon|OJJ46099.1

Group266: abra|OJJ74363.1 acar|OOF95367.1 agla|OJJ88403.1 akaw|GAA86667.1 aluc|OJZ89764.1 anid|CBF83899.1 anig|EHA26984.1 asyd|OJJ55412.1 atub|OJI85038.1 aver|OJJ06490.1 awen|OJJ32658.1 abra|OJJ75265.1 acar|OOF94538.1 afis|EAW20807.1 afla|EED46108.1 afum|EAL84821.1 akaw|GAA91801.1 aluc|OJZ92823.1 anid|CBF87420.1 anig|EHA26186.1 aory|EIT76498.1 atub|OJI87279.1 atub|OJI84647.1 afla|EED56188.1 anig|EHA20917.1 aory|EIT73391.1 akaw|GAA92694.1 aluc|OJZ90152.1

Group267: abra|OJJ75148.1 acar|OOF97189.1 acar|OOF97190.1 acla|EAW08321.1 acla|EAW08322.1 afis|EAW23356.1 afis|EAW23357.1 afla|EED55606.1 afla|EED55607.1 afum|EAL88119.2 afum|EAL88120.2 agla|OJJ86469.1 akaw|GAA88753.1 akaw|GAA88754.1 aluc|OJZ92713.1 aluc|OJZ92714.1 anid|CBF74884.1 anig|EHA26286.1 aory|EIT81807.1 asyd|OJJ60109.1 ater|EAU35607.1 ater|EAU35608.1 atub|OJI87139.1 atub|OJI87140.1 aver|OJJ04153.1 awen|OJJ39530.1 azon|OJJ47726.1 azon|OJJ47728.1

Group268: abra|OJJ75712.1 acar|OOG00147.1 acla|EAW09400.1 afis|EAW21736.1 afla|EED58008.1 afum|EAL92769.1 agla|OJJ79847.1 akaw|GAA84446.1 aluc|OJZ89163.1 anig|EHA23391.1 aory|EIT79742.1 ater|EAU38417.1 atub|OJI85627.1 awen|OJJ34009.1 abra|OJJ75713.1 acar|OOG00146.1 acla|EAW09399.1 afla|EED58009.1 afum|EAL92770.1 agla|OJJ88777.1 akaw|GAA84447.1 aluc|OJZ89164.1 anig|EHA23390.1 aory|EIT79726.1 asyd|OJJ65106.1 atub|OJI85626.1 aver|OJI99233.1 azon|OJJ48541.1

Group269: abra|OJJ76491.1 acar|OOF99722.1 acar|OOF99723.1 acla|EAW13941.1 acla|EAW13944.1 afis|EAW18652.1 afis|EAW18653.1 afla|EED52680.1 afla|EED52681.1 afum|EAL93368.1 afum|EAL93369.1 agla|OJJ82422.1 akaw|GAA82406.1 akaw|GAA82407.1 aluc|OJZ91713.1 aluc|OJZ91714.1 anid|CBF70539.1 anid|CBF70540.1 anig|EHA22724.1 aory|EIT74312.1 aory|EIT74318.1 asyd|OJJ58549.1 ater|EAU38259.1 ater|EAU38260.1 atub|OJI86172.1 aver|OJJ01147.1 awen|OJJ32120.1 azon|OJJ46706.1

Group270: abra|OJJ76517.1 acar|OOF99456.1 acla|EAW14184.1 acla|EAW14185.1 afis|EAW18899.1 afis|EAW18900.1 afla|EED50848.1 afla|EED50853.1 afum|EAL93631.1 afum|EAL93632.1 agla|OJJ81505.1 akaw|GAA82429.1 akaw|GAA82430.1 aluc|OJZ91689.1 anid|CBF69917.1 anid|CBF69919.1 anig|EHA22702.1 aory|EIT78983.1 aory|EIT79046.1 asyd|OJJ58989.1 ater|EAU38067.1 ater|EAU38068.1 atub|OJI86142.1 aver|OJJ01626.1 aver|OJJ01627.1 awen|OJJ32457.1 azon|OJJ47214.1 azon|OJJ47215.1

Group271: abra|OJJ76807.1 acar|OOF91299.1 acla|EAW13366.1 afis|EAW20480.1 afla|EED44787.1 afum|EAL84574.1 agla|OJJ84832.1 akaw|GAA87216.1 aluc|OJZ84214.1 anid|CBF79227.1 anig|EHA27567.1 aory|EIT77778.1 asyd|OJJ55739.1 ater|EAU29559.1 atub|OJI89869.1 aver|OJJ06825.1 awen|OJJ33179.1 azon|OJJ46382.1 afla|EED44788.1 aory|EIT77780.1 awen|OJJ33175.1 azon|OJJ46828.1 acar|OOF91301.1 acla|EAW13367.1 afis|EAW20481.1 afum|EAL84573.1 akaw|GAA87215.1 aluc|OJZ84215.1

Group272: abra|OJJ77199.1 acar|OOF93082.1 acar|OOF93087.1 acla|EAW13188.1 acla|EAW13189.1 afis|EAW17452.1 afis|EAW17453.1 afla|EED49369.1 afla|EED49370.1 afum|EAL91280.1 afum|EAL91281.2 agla|OJJ80350.1 agla|OJJ80360.1 akaw|GAA90742.1 aluc|OJZ84606.1 anid|CBF71675.1 anid|CBF71677.1 anig|EHA27895.1 anig|EHA27896.1 aory|EIT79469.1 aory|EIT79540.1 asyd|OJJ61825.1 ater|EAU33932.1 ater|EAU33933.1 atub|OJI89473.1 aver|OJI95925.1 awen|OJJ37394.1 azon|OJJ51067.1

Group273: abra|OJJ77369.1 acar|OOF93239.1 acla|EAW13106.1 acla|EAW13107.1 afis|EAW17558.1 afla|EED49266.1 afla|EED49267.1 afum|EAL91372.1 agla|OJJ81410.1 akaw|GAA91392.1 akaw|GAA91393.1 aluc|OJZ84778.1 aluc|OJZ84779.1 anid|CBF90279.1 anig|EHA28046.1 aory|EIT82182.1 aory|EIT82245.1 asyd|OJJ61916.1 ater|EAU36989.1 atub|OJI89303.1 aver|OJI96009.1 awen|OJJ37296.1 azon|OJJ50989.1 afis|EAW17557.1 afum|EAL91371.1 agla|OJJ81411.1 anid|CBF90281.1 asyd|OJJ61917.1

Group274: abra|OJJ66221.1 abra|OJJ69595.1 aluc|OJZ80527.1 aluc|OJZ90304.1 anig|EHA19754.1 anig|EHA25667.1 aver|OJI98854.1 aver|OJJ06288.1 acar|OOF90660.1 acar|OOF90661.1 acla|EAW08925.1 afis|EAW17322.1 afum|EAL91158.1 agla|OJJ87386.1 akaw|GAA89065.1 anig|EHA25668.1 asyd|OJJ56432.1 ater|EAU32950.1 atub|OJI88403.1 awen|OJJ39606.1 azon|OJJ46461.1 akaw|GAA86069.1 awen|OJJ39353.1 acla|EAW09791.1 afis|EAW21345.1 afum|EAL92362.1 azon|OJJ51694.1

Group275: abra|OJJ68039.1 abra|OJJ73342.1 acar|OOF91804.1 acar|OOF94627.1 akaw|GAA83140.1 akaw|GAA90913.1 aluc|OJZ82913.1 aluc|OJZ88697.1 anig|EHA18377.1 anig|EHA21336.1 asyd|OJJ53721.1 asyd|OJJ60902.1 atub|OJI89139.1 atub|OJI91278.1 aver|OJJ00228.1 aver|OJJ07846.1 awen|OJJ29994.1 awen|OJJ33889.1 azon|OJJ42783.1 azon|OJJ50111.1 acla|EAW06969.1 afis|EAW24632.1 afla|EED55052.1 afum|EAL86254.1 anid|CBF83822.1 aory|EIT81944.1 ater|EAU36659.1

Group276: acar|OOF92615.1 acar|OOF92636.1 abra|OJJ69082.1 acar|OOF91209.1 acla|EAW08752.1 afis|EAW15684.1 afla|EED51370.1 afum|EAL85664.1 agla|OJJ81833.1 akaw|GAA90611.1 akaw|GAA90612.1 aluc|OJZ86070.1 anid|CBF71148.1 anig|EHA24544.1 aory|EIT80661.1 asyd|OJJ55837.1 ater|EAU32473.1 atub|OJI79866.1 atub|OJI79867.1 aver|OJJ04780.1 awen|OJJ38376.1 azon|OJJ43860.1 acla|EAW08751.1 afis|EAW15685.1 afla|EED51369.1 afum|EAL85663.1 anid|CBF71146.1

Group277: acar|OOF93141.1 acar|OOF96894.1 asyd|OJJ55471.1 asyd|OJJ61030.1 asyd|OJJ57185.1 abra|OJJ72261.1 acla|EAW13265.1 afis|EAW16849.1 afla|EED49789.1 afum|EAL86395.1 agla|OJJ88181.1 akaw|GAA84669.1 aluc|OJZ87020.1 anid|CBF84231.1 anig|EHA21635.1 aory|EIT77692.1 ater|EAU33865.1 atub|OJI83498.1 aver|OJJ00350.1 aver|OJJ03105.1 awen|OJJ36910.1 azon|OJJ50296.1 acla|EAW12401.1 afla|EED51180.1 aory|EIT81355.1 anid|CBF89260.1 azon|OJJ50917.1

Group278: acar|OOF94414.1 acar|OOF97591.1 afis|EAW24229.1 afis|EAW24933.1 afla|EED46551.1 afla|EED48042.1 afum|EAL85898.1 afum|EAL89589.1 aory|EIT77904.1 aory|EIT83324.1 asyd|OJJ53967.1 asyd|OJJ64678.1 ater|EAU29958.1 ater|EAU31178.1 aver|OJI98780.1 aver|OJJ08063.1 anig|EHA28592.1 abra|OJJ69311.1 acla|EAW14698.1 agla|OJJ79642.1 akaw|GAA88180.1 aluc|OJZ82140.1 anid|CBF74228.1 anig|EHA24084.1 atub|OJI88297.1 awen|OJJ33068.1 azon|OJJ48455.1

Group279: acla|EAW07586.1 acla|EAW11103.1 afis|EAW17139.1 afis|EAW25619.1 anid|CBF80637.1 anid|CBF89256.1 acar|OOF93375.1 abra|OJJ69171.1 acar|OOF91133.1 afla|EED55556.1 agla|OJJ80465.1 akaw|GAA86292.1 aluc|OJZ86158.1 anig|EHA24466.1 asyd|OJJ61355.1 ater|EAU32675.1 atub|OJI79950.1 aver|OJJ00682.1 awen|OJJ38095.1 azon|OJJ42539.1 aluc|OJZ82069.1 anid|CBF87524.1 ater|EAU31706.1 atub|OJI84626.1 akaw|GAA91464.1 anig|EHA22500.1 aory|EIT82677.1

Group280: afis|EAW18048.1 afis|EAW24488.1 afum|EAL86157.1 afum|EAL86730.1 afum|EAL91860.1 abra|OJJ68602.1 acar|OOF92112.1 acla|EAW12637.1 afla|EED51498.1 agla|OJJ85419.1 akaw|GAA91926.1 aluc|OJZ81954.1 anid|CBF79800.1 anig|EHA22293.1 aory|EIT80704.1 asyd|OJJ58129.1 ater|EAU31275.1 atub|OJI84505.1 aver|OJJ04276.1 awen|OJJ32709.1 awen|OJJ41486.1 azon|OJJ48216.1 afla|EED48171.1 agla|OJJ83081.1 aory|EIT83032.1 ater|EAU38954.1 aver|OJI98885.1

Group281: afla|EED45594.1 afla|EED53079.1 aory|EIT74816.1 aory|EIT75766.1 abra|OJJ66499.1 asyd|OJJ54572.1 aver|OJJ07582.1 azon|OJJ50366.1 abra|OJJ72643.1 acar|OOF94628.1 akaw|GAA85264.1 aluc|OJZ90622.1 anid|CBF69513.1 anig|EHA24059.1 asyd|OJJ56879.1 ater|EAU39542.1 atub|OJI82048.1 aver|OJJ08508.1 afla|EED47413.1 aory|EIT81918.1 aver|OJI99560.1 afla|EED52187.1 aory|EIT74424.1 ater|EAU31590.1 awen|OJJ31457.1 asyd|OJJ56253.1 ater|EAU31815.1

Group282: afla|EED51568.1 afla|EED51716.1 afla|EED56540.1 agla|OJJ78952.1 agla|OJJ80278.1 aory|EIT79700.1 aory|EIT80654.1 aory|EIT80755.1 asyd|OJJ57607.1 asyd|OJJ58114.1 aver|OJJ04291.1 aver|OJJ05577.1 awen|OJJ33707.1 awen|OJJ34440.1 abra|OJJ67863.1 acar|OOF92843.1 acla|EAW11735.1 afis|EAW23768.1 afum|EAL87202.1 agla|OJJ85386.1 akaw|GAA91870.1 aluc|OJZ82029.1 anid|CBF79783.1 anig|EHA22227.1 ater|EAU31312.1 atub|OJI84583.1 azon|OJJ42833.1

Group283: afum|EAL85403.1 afum|EAL87814.1 aory|EIT74045.1 aory|EIT79062.1 ater|EAU31644.1 ater|EAU36768.1 atub|OJI87665.1 atub|OJI90002.1 abra|OJJ65728.1 acar|OOF93412.1 afis|EAW19529.1 afla|EED50913.1 akaw|GAA93255.1 aluc|OJZ83633.1 asyd|OJJ62934.1 azon|OJJ46829.1 awen|OJJ30425.1 afis|EAW16772.1 akaw|GAA92091.1 anid|CBF73717.1 anig|EHA18786.1 asyd|OJJ64570.1 aver|OJI97354.1 aver|OJI98056.1 aver|OJI98667.1 afla|EED57223.1 asyd|OJJ63582.1

Group284: afum|EAL86514.1 afum|EAL91321.1 asyd|OJJ52759.1 asyd|OJJ54478.1 asyd|OJJ61874.1 azon|OJJ42558.1 azon|OJJ51475.1 azon|OJJ51028.1 abra|OJJ74834.1 acar|OOF95824.1 acla|EAW13149.1 afis|EAW17498.1 afla|EED49318.1 agla|OJJ81459.1 akaw|GAA83586.1 aluc|OJZ92411.1 anid|CBF71582.1 anig|EHA26554.1 aory|EIT82205.1 ater|EAU34444.1 ater|EAU36943.1 atub|OJI86841.1 aver|OJI95972.1 awen|OJJ37343.1 acla|EAW12237.1 afis|EAW16990.1 aver|OJJ08741.1

Group285: agla|OJJ79575.1 agla|OJJ86866.1 agla|OJJ88176.1 agla|OJJ89239.1 asyd|OJJ62473.1 asyd|OJJ64049.1 ater|EAU34049.1 ater|EAU34409.1 aver|OJI96608.1 aver|OJI98135.1 awen|OJJ38034.1 awen|OJJ40680.1 awen|OJJ40981.1 abra|OJJ74157.1 acar|OOF95570.1 acla|EAW12279.1 afis|EAW16961.1 afla|EED49740.1 afum|EAL86487.2 akaw|GAA89727.1 aluc|OJZ80656.1 anid|CBF89363.1 anig|EHA27176.1 aory|EIT75292.1 atub|OJI87511.1 azon|OJJ44568.1 anid|CBF85376.1

Group286: akaw|GAA84498.1 akaw|GAA89765.1 anig|EHA23338.1 anig|EHA27214.1 aver|OJJ00853.1 aver|OJJ07847.1 abra|OJJ75781.1 acar|OOF91480.1 acla|EAW10808.1 afis|EAW24770.1 afla|EED55185.1 afum|EAL89411.1 agla|OJJ83340.1 aluc|OJZ80618.1 aluc|OJZ89223.1 anid|CBF86704.1 aory|EIT83621.1 asyd|OJJ64740.1 ater|EAU37121.1 atub|OJI85569.1 atub|OJI87472.1 aver|OJI98844.1 awen|OJJ29862.1 anid|CBF83000.1 afla|EED53623.1 aory|EIT75056.1 asyd|OJJ61517.1

Group287: asyd|OJJ54631.1 asyd|OJJ65150.1 asyd|OJJ58326.1 abra|OJJ68110.1 afla|EED53398.1 akaw|GAA86893.1 aluc|OJZ90016.1 anid|CBF80628.1 anig|EHA27391.1 aory|EIT74923.1 ater|EAU36928.1 atub|OJI84791.1 aver|OJJ07525.1 awen|OJJ33176.1 abra|OJJ71207.1 acar|OOF97964.1 afla|EED54683.1 akaw|GAA82777.1 aluc|OJZ87336.1 anid|CBF80110.1 anig|EHA20573.1 aory|EIT78795.1 atub|OJI81308.1 aver|OJJ02085.1 afla|EED46389.1 aory|EIT72418.1 atub|OJI84522.1

Group288: awen|OJJ30753.1 awen|OJJ31728.1 awen|OJJ30755.1 awen|OJJ31726.1 abra|OJJ67455.1 acar|OOF92379.1 afis|EAW18419.1 afum|EAL93103.1 akaw|GAA86376.1 aluc|OJZ84910.1 anid|CBF70711.1 asyd|OJJ58453.1 ater|EAU29836.1 atub|OJI80688.1 aver|OJJ01052.1 azon|OJJ50489.1 acar|OOF92377.1 asyd|OJJ58451.1 acla|EAW06310.1 afla|EED52989.1 agla|OJJ84987.1 anid|CBF70709.1 aory|EIT78704.1 ater|EAU29837.1 aver|OJJ01053.1 ater|EAU29835.1 asyd|OJJ58452.1

Group289: azon|OJJ42747.1 azon|OJJ51180.1 abra|OJJ76401.1 acar|OOF99642.1 acla|EAW14023.1 afis|EAW18729.1 afla|EED50685.1 afum|EAL93454.1 agla|OJJ82287.1 akaw|GAA82321.1 aluc|OJZ91811.1 anid|CBF70016.1 anig|EHA22804.1 aory|EIT78004.1 asyd|OJJ58877.1 ater|EAU38175.1 atub|OJI86267.1 aver|OJJ01479.1 awen|OJJ32270.1 akaw|GAA82320.1 aluc|OJZ91812.1 acar|OOF99643.1 acla|EAW14022.1 afis|EAW18728.1 afum|EAL93453.1 anid|CBF70018.1 ater|EAU38176.1

Group290: abra|OJJ65930.1 acar|OOF94991.1 acla|EAW12067.1 afis|EAW19710.1 afla|EED57049.1 afum|EAL86817.2 agla|OJJ82841.1 akaw|GAA92305.1 akaw|GAA92306.1 aluc|OJZ90202.1 anid|CBF84278.1 anig|EHA23699.1 aory|EIT82284.1 asyd|OJJ57230.1 ater|EAU32834.1 atub|OJI81631.1 aver|OJJ03059.1 awen|OJJ32205.1 azon|OJJ42677.1 afla|EED57054.1 acar|OOF94992.1 acla|EAW12066.1 afis|EAW19709.1 afum|EAL86818.1 aory|EIT82258.1 ater|EAU32836.1 azon|OJJ42678.1

Group291: abra|OJJ66585.1 acar|OOF91022.1 acla|EAW10906.1 acla|EAW10907.1 afis|EAW20951.1 afis|EAW20952.1 afla|EED50307.1 afla|EED50308.1 afum|EAL84682.1 afum|EAL84683.2 agla|OJJ83929.1 akaw|GAA91132.1 akaw|GAA91134.1 aluc|OJZ81461.1 anid|CBF82552.1 anid|CBF82554.1 anig|EHA25968.1 aory|EIT77335.1 aory|EIT77337.1 asyd|OJJ54058.1 ater|EAU38602.1 ater|EAU38603.1 atub|OJI88718.1 aver|OJJ08683.1 awen|OJJ34364.1 awen|OJJ34365.1 azon|OJJ48100.1

Group292: abra|OJJ66932.1 acar|OOF91699.1 acla|EAW14495.1 afis|EAW19209.1 afla|EED52263.1 afum|EAL93939.2 agla|OJJ85720.1 akaw|GAA91486.1 aluc|OJZ85648.1 anid|CBF69569.1 anig|EHA24885.1 asyd|OJJ59150.1 ater|EAU34159.1 atub|OJI79439.1 aver|OJJ02018.1 awen|OJJ34677.1 azon|OJJ45456.1 acar|OOF91700.1 agla|OJJ88500.1 anid|CBF78572.1 anig|EHA24886.1 asyd|OJJ65074.1 ater|EAU37259.1 aver|OJI99199.1 awen|OJJ37563.1 azon|OJJ51226.1 aver|OJJ01740.1

Group293: abra|OJJ67205.1 acar|OOF98357.1 acar|OOF98358.1 acla|EAW14519.1 afis|EAW19237.1 afla|EED47640.1 afum|EAL93965.2 agla|OJJ88462.1 akaw|GAA90972.1 akaw|GAA90973.1 aluc|OJZ87769.1 anid|CBF78640.1 anig|EHA20253.1 aory|EIT80182.1 asyd|OJJ65189.1 ater|EAU37226.1 atub|OJI80876.1 aver|OJI99338.1 awen|OJJ37593.1 azon|OJJ51248.1 acla|EAW14518.1 afis|EAW19236.1 afla|EED47646.1 afum|EAL93964.1 anid|CBF78638.1 aory|EIT80263.1 ater|EAU37227.1

Group294: abra|OJJ67284.1 afla|EED54338.1 afum|EAL87110.1 agla|OJJ82024.1 akaw|GAA86525.1 aluc|OJZ85073.1 anid|CBF71655.1 anig|EHA25287.1 aory|EIT76146.1 asyd|OJJ61837.1 ater|EAU32860.1 atub|OJI80525.1 aver|OJI95936.1 awen|OJJ36119.1 abra|OJJ68987.1 acar|OOF91558.1 afla|EED48927.1 akaw|GAA90508.1 aluc|OJZ85973.1 anid|CBF87485.1 anig|EHA24632.1 aory|EIT79999.1 ater|EAU31715.1 atub|OJI79761.1 asyd|OJJ52961.1 aver|OJJ08927.1 awen|OJJ41925.1

Group295: abra|OJJ68390.1 afla|EED49558.1 agla|OJJ87147.1 aory|EIT82645.1 atub|OJI84701.1 abra|OJJ77648.1 acar|OOF99088.1 acla|EAW12091.1 afis|EAW19985.1 afum|EBA27209.1 akaw|GAA83987.1 aluc|OJZ80881.1 anid|CBF87267.1 anig|EHA19455.1 asyd|OJJ62371.1 ater|EAU33659.1 atub|OJI82696.1 aver|OJI98437.1 awen|OJJ38077.1 azon|OJJ42589.1 anid|CBF73965.1 aver|OJI96505.1 afla|EED52196.1 aory|EIT74391.1 ater|EAU29811.1 aluc|OJZ83787.1 anid|CBF85559.1

Group296: abra|OJJ69188.1 acar|OOF91124.1 acla|EAW08657.1 afis|EAW15774.1 afla|EED51274.1 afum|EAL85575.1 agla|OJJ81937.1 akaw|GAA86276.1 akaw|GAA86277.1 aluc|OJZ86174.1 anid|CBF70974.1 anig|EHA24450.1 anig|EHA24451.1 aory|EIT81499.1 asyd|OJJ52632.1 ater|EAU32367.1 atub|OJI79966.1 aver|OJJ04660.1 awen|OJJ38481.1 azon|OJJ43782.1 acla|EAW08658.1 afis|EAW15773.1 afla|EED49465.1 anid|CBF70972.1 ater|EAU32368.1 azon|OJJ43781.1 aory|EIT79571.1

Group297: abra|OJJ69236.1 acar|OOF91082.1 acar|OOF91084.1 acla|EAW08611.1 acla|EAW08613.1 afis|EAW15821.1 afis|EAW15822.1 afla|EED51224.1 afla|EED51226.1 afum|EAL85529.1 afum|EAL85530.1 agla|OJJ81985.1 akaw|GAA92244.1 akaw|GAA92246.1 aluc|OJZ86216.1 anid|CBF70886.1 anid|CBF70887.1 anig|EHA24410.1 aory|EIT83181.1 aory|EIT83189.1 asyd|OJJ52589.1 ater|EAU33426.1 ater|EAU33427.1 atub|OJI80011.1 aver|OJJ04616.1 awen|OJJ38535.1 azon|OJJ43727.1

Group298: abra|OJJ70462.1 acar|OOF97462.1 acar|OOF97465.1 acla|EAW13051.1 acla|EAW13053.1 afis|EAW17613.1 afis|EAW17616.1 afla|EED49204.1 afla|EED49207.1 afum|EAL91426.1 afum|EAL91429.1 agla|OJJ81324.1 akaw|GAA83308.1 akaw|GAA83311.1 aluc|OJZ83805.1 anid|CBF90169.1 anid|CBF90172.1 anig|EHA18952.1 aory|EIT78583.1 aory|EIT83172.1 asyd|OJJ61991.1 ater|EAU37048.1 ater|EAU37050.1 atub|OJI90172.1 aver|OJI96083.1 awen|OJJ37229.1 azon|OJJ50932.1

Group299: abra|OJJ71130.1 acar|OOF98022.1 acla|EAW10367.1 acla|EAW10368.1 afis|EAW25224.1 afis|EAW25225.1 afla|EED53669.1 afla|EED53682.1 afum|EAL89885.1 afum|EAL89886.1 akaw|GAA82844.1 aluc|OJZ87405.1 anid|CBF85264.1 anid|CBF85265.1 anig|EHA20634.1 anig|EHA20635.1 aory|EIT75307.1 aory|EIT75353.1 ater|EAU34383.1 ater|EAU34384.1 atub|OJI81241.1 aver|OJI98082.1 aver|OJI98083.1 agla|OJJ79183.1 asyd|OJJ62906.1 awen|OJJ40890.1 azon|OJJ44519.1

Group300: abra|OJJ72036.1 acar|OOF94174.1 acla|EAW09507.1 afla|EED57855.1 agla|OJJ82582.1 akaw|GAA84897.1 aluc|OJZ86774.1 anid|CBF83512.1 anig|EHA21907.1 aory|EIT81060.1 asyd|OJJ53748.1 asyd|OJJ60708.1 ater|EAU38317.1 atub|OJI83747.1 aver|OJJ00034.1 awen|OJJ33880.1 abra|OJJ72037.1 afla|EED56917.1 akaw|GAA84896.1 aluc|OJZ86775.1 anid|CBF73981.1 anig|EHA21906.1 aory|EIT77541.1 asyd|OJJ55360.1 atub|OJI83746.1 aver|OJJ06443.1 aver|OJJ07863.1

Group301: abra|OJJ72938.1 acar|OOF96120.1 acar|OOF96121.1 acla|EAW06452.1 acla|EAW06453.1 afis|EAW15949.1 afla|EED58109.1 afla|EED58110.1 afum|EAL88582.1 agla|OJJ89365.1 akaw|GAA85519.1 aluc|OJZ83447.1 anid|CBF81052.1 anig|EHA24293.1 anig|EHA24294.1 aory|EIT79139.1 aory|EIT79141.1 asyd|OJJ56450.1 ater|EAU35509.1 atub|OJI82338.1 atub|OJI82339.1 aver|OJJ06272.1 awen|OJJ35627.1 azon|OJJ46898.1 anid|CBF81054.1 ater|EAU35508.1 aver|OJJ06271.1

Group302: abra|OJJ73010.1 acar|OOF96182.1 acar|OOF96183.1 acla|EAW08002.1 acla|EAW08003.1 afis|EAW23037.1 afis|EAW23038.1 afla|EED54980.1 afum|EAL88443.1 afum|EAL88444.1 agla|OJJ89440.1 akaw|GAA85588.1 akaw|GAA85589.1 aluc|OJZ83525.1 anid|CBF80929.1 anid|CBF80931.1 anig|EHA24348.1 aory|EIT80318.1 aory|EIT80383.1 asyd|OJJ56316.1 ater|EAU29618.1 ater|EAU29621.1 atub|OJI82414.1 aver|OJJ06398.1 awen|OJJ35704.1 azon|OJJ44166.1 afla|EED54979.1

Group303: abra|OJJ73242.1 acar|OOG00479.1 acar|OOG00481.1 acla|EAW06915.1 acla|EAW06916.1 afis|EAW16532.1 afis|EAW16533.1 afla|EED56136.1 afla|EED56140.1 afum|EAL89129.1 afum|EAL89130.2 agla|OJJ82788.1 akaw|GAA87938.1 akaw|GAA87939.1 aluc|OJZ81186.1 anid|CBF81734.1 anid|CBF81736.1 anig|EHA18503.1 aory|EIT76580.1 aory|EIT76630.1 asyd|OJJ59594.1 ater|EAU36595.1 ater|EAU36596.1 atub|OJI91382.1 aver|OJJ02674.1 awen|OJJ40289.1 azon|OJJ49150.1

Group304: abra|OJJ73503.1 acar|OOG00688.1 acla|EAW07435.1 acla|EAW07436.1 afis|EAW22461.1 afis|EAW22462.1 afla|EED54482.1 afla|EED54483.1 afum|EAL90729.1 afum|EAL90730.1 agla|OJJ87561.1 akaw|GAA87450.1 akaw|GAA87451.1 aluc|OJZ88545.1 anid|CBF88911.1 anid|CBF88912.1 anig|EHA18245.1 aory|EIT81150.1 asyd|OJJ62841.1 ater|EAU39244.1 atub|OJI91130.1 aver|OJI96884.1 aver|OJI96885.1 awen|OJJ35087.1 awen|OJJ35088.1 azon|OJJ50020.1 aory|EIT81157.1

Group305: abra|OJJ74418.1 acar|OOF95322.1 acar|OOF95325.1 acla|EAW06834.1 afla|EED53447.1 afla|EED53451.1 agla|OJJ88208.1 akaw|GAA87309.1 akaw|GAA87312.1 aluc|OJZ89705.1 aluc|OJZ89708.1 anid|CBF89366.1 anid|CBF89368.1 anig|EHA26939.1 aory|EIT75073.1 asyd|OJJ62474.1 ater|EAU34066.1 ater|EAU34069.1 atub|OJI85094.1 aver|OJI96610.1 awen|OJJ37916.1 awen|OJJ37920.1 azon|OJJ45806.1 acar|OOF90541.1 acla|EAW11778.1 azon|OJJ50188.1 aory|EIT75099.1

Group306: abra|OJJ76286.1 acar|OOF99527.1 acla|EAW14330.1 afis|EAW18958.1 afum|EAL93691.1 akaw|GAA82215.1 aory|EIT76999.1 asyd|OJJ59136.1 awen|OJJ31557.1 azon|OJJ47274.1 abra|OJJ76287.1 acar|OOF99530.1 acla|EAW14329.1 afis|EAW18959.1 afla|EED51005.1 afum|EAL93692.1 agla|OJJ85336.1 akaw|GAA82216.1 aluc|OJZ91935.1 anid|CBF69598.1 anig|EHA22895.1 aory|EIT76941.1 ater|EAU38141.1 atub|OJI86377.1 aver|OJJ01725.1 awen|OJJ31558.1 azon|OJJ47275.1

Group307: abra|OJJ77576.1 acar|OOF99151.1 acla|EAW06541.1 acla|EAW06542.1 afis|EAW16053.1 afis|EAW16054.1 afla|EED56444.1 afla|EED56445.1 afum|EAL88685.1 afum|EAL88686.1 agla|OJJ86087.1 akaw|GAA84049.1 akaw|GAA84050.1 aluc|OJZ80961.1 anid|CBF75070.1 anid|CBF75072.1 anig|EHA19396.1 aory|EIT72818.1 asyd|OJJ60021.1 ater|EAU33580.1 ater|EAU33584.1 atub|OJI82625.1 aver|OJJ02264.1 awen|OJJ30385.1 awen|OJJ30389.1 azon|OJJ45401.1 aory|EIT72866.1

Group308: abra|OJJ77839.1 acar|OOF98932.1 acla|EAW11284.1 afis|EAW18216.1 afla|EED56254.1 afum|EAL87582.1 akaw|GAA83808.1 aory|EIT73366.1 ater|EAU33774.1 azon|OJJ50174.1 abra|OJJ77840.1 acar|OOF98931.1 acla|EAW11283.1 afis|EAW18217.1 afla|EED56253.1 afum|EAL87583.1 agla|OJJ85865.1 akaw|GAA83807.1 aluc|OJZ90915.1 anid|CBF86146.1 anig|EHA19627.1 aory|EIT73438.1 asyd|OJJ64511.1 atub|OJI82907.1 aver|OJI98609.1 awen|OJJ30595.1 azon|OJJ50175.1

Group309: abra|OJJ65742.1 abra|OJJ77048.1 acar|OOF91730.1 acar|OOF92937.1 aluc|OJZ84452.1 aluc|OJZ86322.1 atub|OJI84212.1 atub|OJI89624.1 acla|EAW13550.1 afis|EAW20627.1 afla|EED49777.1 afum|EAL84441.1 agla|OJJ86946.1 akaw|GAA87005.1 anid|CBF83228.1 anig|EHA27767.1 aory|EIT77606.1 asyd|OJJ55581.1 ater|EAU32164.1 aver|OJJ06660.1 awen|OJJ33462.1 azon|OJJ50718.1 afla|EED52520.1 anig|EHA19838.1 aory|EIT82787.1 awen|OJJ33741.1

Group310: abra|OJJ66443.1 abra|OJJ68021.1 abra|OJJ68923.1 acar|OOF91508.1 acar|OOF92064.1 agla|OJJ78513.1 agla|OJJ81294.1 agla|OJJ85809.1 akaw|GAA90325.1 akaw|GAA90930.1 aluc|OJZ82895.1 aluc|OJZ85908.1 anig|EHA21319.1 anig|EHA24684.1 aory|EIT75910.1 aory|EIT81247.1 asyd|OJJ59089.1 asyd|OJJ63774.1 atub|OJI79692.1 atub|OJI89120.1 aver|OJI97163.1 aver|OJJ01679.1 acla|EAW09049.1 afla|EED46360.1 ater|EAU34525.1 awen|OJJ36242.1

Group311: abra|OJJ68133.1 abra|OJJ69675.1 acar|OOF90055.1 acar|OOF90087.1 acla|EAW12871.1 afis|EAW17819.1 afla|EED45605.1 afum|EAL91629.1 agla|OJJ81780.1 akaw|GAA86913.1 akaw|GAA88800.1 aluc|OJZ90036.1 anid|CBF71190.1 anig|EHA27411.1 aory|EIT75710.1 asyd|OJJ55855.1 atub|OJI84770.1 atub|OJI87949.1 aver|OJJ04798.1 awen|OJJ38287.1 acla|EAW08770.1 afis|EAW15658.1 afum|EAL85688.2 aluc|OJZ82483.1 anig|EHA28291.1 ater|EAU32513.1

Group312: abra|OJJ68841.1 abra|OJJ73289.1 afis|EAW20222.1 afis|EAW20406.1 afla|EED48455.1 afla|EED50145.1 anig|EHA24185.1 anig|EHA24740.1 aory|EIT74688.1 aory|EIT83472.1 asyd|OJJ57669.1 asyd|OJJ58613.1 asyd|OJJ59804.1 asyd|OJJ64211.1 aver|OJI97339.1 aver|OJJ01220.1 aver|OJJ02487.1 acar|OOF90549.1 agla|OJJ82202.1 akaw|GAA90408.1 aluc|OJZ85820.1 anid|CBF70434.1 ater|EAU37675.1 atub|OJI79606.1 awen|OJJ30933.1 acar|OOG00326.1

Group313: abra|OJJ71059.1 abra|OJJ73085.1 akaw|GAA82900.1 akaw|GAA88075.1 anig|EHA18644.1 anig|EHA20691.1 ater|EAU29720.1 ater|EAU35713.1 atub|OJI81179.1 atub|OJI91543.1 aver|OJJ02730.1 aver|OJJ06544.1 awen|OJJ34513.1 awen|OJJ40561.1 acar|OOF98083.1 acla|EAW10600.1 afis|EAW24968.1 afum|EAL89627.1 agla|OJJ82234.1 aluc|OJZ87469.1 anid|CBF81643.1 aory|EIT80808.1 asyd|OJJ59538.1 azon|OJJ49422.1 afla|EED57716.1 aory|EIT79920.1

Group314: abra|OJJ77512.1 abra|OJJ78209.1 acar|OOF98645.1 acar|OOF99212.1 agla|OJJ84617.1 agla|OJJ88162.1 akaw|GAA84108.1 akaw|GAA85817.1 aluc|OJZ81024.1 aluc|OJZ91282.1 atub|OJI82559.1 atub|OJI83272.1 awen|OJJ30638.1 awen|OJJ31266.1 afis|EAW18264.1 anid|CBF79534.1 anig|EHA19340.1 aory|EIT75007.1 asyd|OJJ57897.1 aver|OJJ04498.1 azon|OJJ47978.1 afla|EED48723.1 afum|EAL87628.1 anig|EHA19967.1 ater|EAU33271.1 aory|EIT73106.1

Group315: acar|OOF95616.1 acar|OOF95668.1 afum|EAL86712.1 afum|EAL89365.2 aluc|OJZ83797.1 aluc|OJZ91020.1 atub|OJI83012.1 atub|OJI90166.1 abra|OJJ70451.1 afis|EAW19928.1 afis|EAW24742.1 agla|OJJ88966.1 akaw|GAA83713.1 anid|CBF78706.1 anig|EHA18945.1 asyd|OJJ64219.1 ater|EAU37496.1 aver|OJI98040.1 aver|OJI98302.1 awen|OJJ33410.1 azon|OJJ45929.1 afla|EED48357.1 afis|EAW16196.1 afum|EAL89388.1 asyd|OJJ61748.1 aver|OJI95845.1

Group316: acla|EAW06814.1 acla|EAW11026.1 afis|EAW16363.1 afis|EAW20840.1 afum|EAL84793.1 afum|EAL88988.1 agla|OJJ78575.1 agla|OJJ80753.1 aver|OJI96276.1 aver|OJI96627.1 awen|OJJ37768.1 awen|OJJ40172.1 abra|OJJ73491.1 acar|OOG00684.1 afla|EED53503.1 agla|OJJ79965.1 akaw|GAA87440.1 aluc|OJZ88557.1 anid|CBF89245.1 anig|EHA18255.1 aory|EIT74859.1 asyd|OJJ62507.1 ater|EAU34089.1 atub|OJI91142.1 azon|OJJ45066.1 asyd|OJJ62165.1

Group317: acla|EAW11040.1 acla|EAW11477.1 afla|EED47466.1 afla|EED49623.1 afla|EED50532.1 agla|OJJ82839.1 agla|OJJ86906.1 aory|EIT79957.1 aory|EIT81005.1 aory|EIT82111.1 awen|OJJ31954.1 awen|OJJ35441.1 azon|OJJ45954.1 azon|OJJ51301.1 abra|OJJ72795.1 acar|OOF89880.1 afis|EAW23976.1 afum|EAL87406.1 akaw|GAA85398.1 aluc|OJZ83292.1 anid|CBF77345.1 anig|EHA24176.1 asyd|OJJ53272.1 ater|EAU29776.1 atub|OJI82193.1 aver|OJJ03577.1

Group318: afla|EED44930.1 afla|EED51540.1 ater|EAU31301.1 ater|EAU38559.1 awen|OJJ33684.1 awen|OJJ36221.1 abra|OJJ67873.1 acar|OOF92853.1 acla|EAW11724.1 afis|EAW23749.1 afum|EAL87183.1 agla|OJJ78962.1 agla|OJJ89691.1 akaw|GAA91882.1 aluc|OJZ82015.1 anid|CBF79817.1 anig|EHA22241.1 aory|EIT80515.1 asyd|OJJ58137.1 atub|OJI84564.1 aver|OJJ04268.1 azon|OJJ42827.1 aver|OJJ03949.1 afis|EAW24762.1 afum|EAL89404.1 asyd|OJJ52466.1

Group319: afla|EED50195.1 afla|EED57633.1 anid|CBF78576.1 anid|CBF84581.1 anid|CBF88483.1 aory|EIT77388.1 aory|EIT82958.1 asyd|OJJ59798.1 asyd|OJJ63787.1 asyd|OJJ65070.1 aver|OJI97151.1 aver|OJI99195.1 aver|OJJ02491.1 abra|OJJ74472.1 acar|OOF95272.1 acla|EAW07225.1 afis|EAW22239.1 afum|EAL90953.1 agla|OJJ87812.1 akaw|GAA87366.1 aluc|OJZ89647.1 anig|EHA26885.1 ater|EAU34246.1 atub|OJI85157.1 awen|OJJ34817.1 azon|OJJ45732.1

Group320: aluc|OJZ79781.1 aluc|OJZ80029.1 aluc|OJZ80242.1 aluc|OJZ79859.1 aluc|OJZ80430.1 abra|OJJ65440.1 acla|EAW11311.1 afis|EAW16412.1 afla|EED56291.1 afum|EAL87548.1 akaw|GAA92640.1 anid|CBF73419.1 anig|EHA17691.1 asyd|OJJ52220.1 ater|EAU33807.1 aver|OJI98367.1 awen|OJJ29583.1 afis|EAW18185.1 akaw|GAA92960.1 anid|CBF85719.1 aory|EIT73430.1 asyd|OJJ64275.1 awen|OJJ30543.1 akaw|GAA89012.1 akaw|GAA93064.1 akaw|GAA93159.1

Group321: aluc|OJZ81129.1 aluc|OJZ82063.1 awen|OJJ30180.1 awen|OJJ35549.1 awen|OJJ30187.1 abra|OJJ65739.1 acar|OOF99878.1 acla|EAW14576.1 afla|EED53062.1 agla|OJJ86268.1 akaw|GAA91460.1 anid|CBF78777.1 aory|EIT74772.1 asyd|OJJ65241.1 atub|OJI84621.1 aver|OJI99392.1 azon|OJJ44977.1 abra|OJJ72613.1 afla|EED51141.1 anig|EHA24034.1 atub|OJI82018.1 awen|OJJ38779.1 aory|EIT81339.1 aory|EIT74785.1 akaw|GAA85238.1 aluc|OJZ90594.1

Group322: asyd|OJJ56859.1 abra|OJJ67208.1 acar|OOF98353.1 acla|EAW14728.1 afla|EED53515.1 akaw|GAA90969.1 aluc|OJZ87766.1 anid|CBF73997.1 anig|EHA20250.1 asyd|OJJ51948.1 ater|EAU33288.1 atub|OJI80879.1 aver|OJI96457.1 awen|OJJ36200.1 azon|OJJ50142.1 azon|OJJ46441.1 acar|OOF99565.1 afla|EED49927.1 agla|OJJ84266.1 akaw|GAA82243.1 aluc|OJZ91902.1 ater|EAU35687.1 acla|EAW09973.1 acla|EAW10724.1 agla|OJJ84410.1 agla|OJJ79135.1

Group323: abra|OJJ66489.1 acar|OOF90965.1 acar|OOF90966.1 acla|EAW10979.1 acla|EAW10980.1 afis|EAW20879.1 afis|EAW20880.1 afla|EED45565.1 afum|EAL84752.1 afum|EAL84753.1 agla|OJJ83989.1 akaw|GAA92472.1 akaw|GAA92473.1 aluc|OJZ81546.1 anid|CBF82862.1 anid|CBF82864.1 anig|EHA25889.1 aory|EIT75388.1 aory|EIT75432.1 asyd|OJJ61598.1 ater|EAU30811.1 atub|OJI88638.1 aver|OJJ00922.1 awen|OJJ34480.1 azon|OJJ51757.1 afla|EED45564.1

Group324: abra|OJJ67172.1 acar|OOF98391.1 acla|EAW14551.1 afis|EAW19268.1 afla|EED47606.1 afum|EAL93995.1 agla|OJJ88427.1 akaw|GAA91002.1 aluc|OJZ87803.1 anid|CBF78699.1 anig|EHA20284.1 aory|EIT80198.1 asyd|OJJ65220.1 ater|EAU29549.1 atub|OJI80844.1 aver|OJI99369.1 awen|OJJ37629.1 azon|OJJ47017.1 afla|EED47607.1 anid|CBF78697.1 aory|EIT80260.1 acar|OOF98392.1 acla|EAW14552.1 afis|EAW19269.1 afum|EAL93996.1 anig|EHA20285.1

Group325: abra|OJJ67303.1 acar|OOF92510.1 acla|EAW06432.1 afis|EAW18538.1 afla|EED52858.1 afum|EAL93236.1 agla|OJJ84448.1 akaw|GAA86513.1 aluc|OJZ85058.1 anig|EHA25273.1 aory|EIT78604.1 asyd|OJJ52110.1 atub|OJI80542.1 aver|OJJ05256.1 awen|OJJ36329.1 azon|OJJ42393.1 agla|OJJ82374.1 asyd|OJJ52384.1 aver|OJI96494.1 afis|EAW24603.1 afla|EED52462.1 aory|EIT82755.1 atub|OJI80421.1 aver|OJJ01939.1 asyd|OJJ53464.1 aver|OJJ08291.1

Group326: abra|OJJ68235.1 acar|OOF93581.1 acla|EAW10392.1 afis|EAW25199.1 afum|EBA27389.1 agla|OJJ85249.1 anid|CBF85213.1 asyd|OJJ54665.1 ater|EAU34341.1 atub|OJI83115.1 aver|OJI97964.1 awen|OJJ33408.1 abra|OJJ71100.1 afla|EED53646.1 akaw|GAA82868.1 aluc|OJZ87435.1 anig|EHA20657.1 aory|EIT75355.1 atub|OJI81213.1 azon|OJJ47678.1 aver|OJJ02024.1 awen|OJJ31517.1 asyd|OJJ63002.1 agla|OJJ86390.1 awen|OJJ39475.1 agla|OJJ89313.1

Group327: abra|OJJ68548.1 acar|OOF92744.1 acla|EAW12819.1 acla|EAW12820.1 afis|EAW17984.1 afis|EAW17985.1 afla|EED51920.1 afla|EED51921.1 afum|EAL91793.1 afum|EAL91794.1 agla|OJJ80241.1 akaw|GAA91305.1 akaw|GAA91306.1 aluc|OJZ82765.1 anid|CBF79988.1 anid|CBF79990.1 anig|EHA21208.1 aory|EIT81392.1 aory|EIT81447.1 asyd|OJJ58239.1 ater|EAU31494.1 ater|EAU31495.1 atub|OJI88997.1 aver|OJJ02186.1 awen|OJJ41561.1 azon|OJJ48276.1

Group328: abra|OJJ68713.1 acar|OOF92213.1 acla|EAW11027.1 afis|EAW20841.1 afla|EED46816.1 afum|EAL84792.1 agla|OJJ80754.1 akaw|GAA89866.1 aluc|OJZ81815.1 anid|CBF86535.1 anig|EHA22400.1 aory|EIT72698.1 asyd|OJJ62166.1 ater|EAU30544.1 atub|OJI84387.1 aver|OJI96277.1 awen|OJJ40171.1 azon|OJJ50392.1 afis|EAW24116.1 anid|CBF84526.1 acla|EAW12533.1 agla|OJJ88735.1 afum|EAL91971.1 awen|OJJ41372.1 aver|OJI98980.1 asyd|OJJ64867.1

Group329: abra|OJJ70297.1 acar|OOF91751.1 afis|EAW16949.1 afla|EED54756.1 afum|EAL86476.1 agla|OJJ79025.1 akaw|GAA93023.1 aluc|OJZ82562.1 anid|CBF73556.1 anig|EHA27157.1 aory|EIT79421.1 asyd|OJJ56717.1 atub|OJI88804.1 aver|OJJ06008.1 awen|OJJ32083.1 azon|OJJ45848.1 acar|OOF95544.1 agla|OJJ86358.1 akaw|GAA86838.1 aluc|OJZ89957.1 atub|OJI84849.1 agla|OJJ82216.1 ater|EAU31076.1 azon|OJJ46662.1 ater|EAU29923.1 awen|OJJ38719.1

Group330: abra|OJJ70396.1 acar|OOG01144.1 acla|EAW11244.1 afis|EAW19549.1 afla|EED49895.1 afum|EAL89219.1 agla|OJJ88525.1 akaw|GAA91962.1 aluc|OJZ82655.1 anig|EHA24571.1 asyd|OJJ65105.1 ater|EAU33680.1 atub|OJI88886.1 aver|OJI99232.1 awen|OJJ31981.1 azon|OJJ47790.1 afis|EAW18105.1 akaw|GAA92884.1 aory|EIT77372.1 afis|EAW16617.1 afum|EAL86960.1 anig|EHA27418.1 afla|EED50268.1 anid|CBF84502.1 asyd|OJJ53512.1 aver|OJI96187.1

Group331: abra|OJJ71980.1 acar|OOF96631.1 acla|EAW09559.1 acla|EAW09561.1 afis|EAW21576.1 afis|EAW21578.1 afla|EED57795.1 afum|EAL92595.1 afum|EAL92596.1 agla|OJJ85761.1 akaw|GAA84949.1 akaw|GAA84950.1 aluc|OJZ86717.1 anid|CBF76292.1 anid|CBF76294.1 anig|EHA21962.1 aory|EIT81026.1 aory|EIT81117.1 asyd|OJJ61004.1 ater|EAU34955.1 ater|EAU34956.1 atub|OJI83812.1 aver|OJJ00322.1 awen|OJJ39103.1 azon|OJJ51431.1 afla|EED57794.1

Group332: abra|OJJ75736.1 acar|OOF89930.1 acla|EAW14905.1 afis|EAW24426.1 afla|EED55252.1 afum|EAL86094.1 agla|OJJ79240.1 akaw|GAA84469.1 aluc|OJZ89188.1 anid|CBF73780.1 anig|EHA23369.1 aory|EIT83550.1 asyd|OJJ56598.1 ater|EAU30018.1 atub|OJI85602.1 aver|OJJ06127.1 awen|OJJ32778.1 azon|OJJ44006.1 acla|EAW14906.1 afis|EAW24427.1 afla|EED55251.1 afum|EAL86095.1 aluc|OJZ89187.1 anig|EHA23370.1 ater|EAU30019.1 awen|OJJ32779.1

Group333: abra|OJJ75938.1 acar|OOF99972.1 acar|OOF99973.1 acla|EAW09855.1 acla|EAW09856.1 afis|EAW21271.1 afis|EAW21280.1 afla|EED54831.1 afum|EAL92286.1 afum|EAL92296.1 agla|OJJ87477.1 akaw|GAA84639.1 akaw|GAA84640.1 aluc|OJZ89385.1 anid|CBF83352.1 anid|CBF83354.1 anig|EHA23199.1 aory|EIT79381.1 aory|EIT79404.1 asyd|OJJ60616.1 ater|EAU35907.1 ater|EAU35908.1 atub|OJI85413.1 aver|OJI99940.1 awen|OJJ39421.1 azon|OJJ44303.1

Group334: abra|OJJ76077.1 acar|OOF99848.1 acar|OOF99849.1 acla|EAW09743.1 acla|EAW09744.1 afis|EAW21398.1 afis|EAW21399.1 afla|EED55817.1 afla|EED55818.1 afum|EAL92412.1 afum|EAL92413.1 agla|OJJ85524.1 akaw|GAA88889.1 akaw|GAA88890.1 aluc|OJZ89533.1 anid|CBF83833.1 anig|EHA23070.1 aory|EIT76532.1 aory|EIT76875.1 asyd|OJJ60909.1 ater|EAU35159.1 atub|OJI85272.1 aver|OJJ00236.1 awen|OJJ39304.1 azon|OJJ51548.1 ater|EAU35160.1

Group335: abra|OJJ78321.1 acar|OOF98553.1 acla|EAW09204.1 acla|EAW09205.1 afis|EAW21942.1 afis|EAW21943.1 afla|EED46659.1 afla|EED46660.1 afum|EAL92956.1 afum|EAL92957.1 agla|OJJ84181.1 akaw|GAA85707.1 akaw|GAA85708.1 aluc|OJZ91396.1 anid|CBF84053.1 anid|CBF84055.1 anig|EHA20073.1 aory|EIT77914.1 aory|EIT77936.1 asyd|OJJ61344.1 ater|EAU30947.1 ater|EAU30991.1 atub|OJI83388.1 aver|OJJ00669.1 awen|OJJ34234.1 azon|OJJ43402.1

Group336: abra|OJJ65726.1 abra|OJJ73290.1 abra|OJJ77740.1 akaw|GAA83889.1 akaw|GAA92713.1 aluc|OJZ88758.1 aluc|OJZ90806.1 acar|OOF98988.1 acla|EAW12295.1 agla|OJJ85902.1 anig|EHA19533.1 aory|EIT79001.1 asyd|OJJ59393.1 ater|EAU30964.1 atub|OJI82795.1 atub|OJI91329.1 awen|OJJ36235.1 afis|EAW16926.1 afla|EED50932.1 anid|CBF79557.1 anig|EHA18455.1 aver|OJJ02879.1 afum|EAL87839.1 afis|EAW17484.1 agla|OJJ87319.1

Group337: abra|OJJ66243.1 abra|OJJ72838.1 afla|EED45789.1 afla|EED49140.1 aluc|OJZ80548.1 aluc|OJZ83338.1 anig|EHA24214.1 anig|EHA25648.1 aory|EIT75668.1 aory|EIT78573.1 atub|OJI82235.1 atub|OJI88383.1 acar|OOF96040.1 acla|EAW11403.1 afis|EAW24018.1 afum|EAL87448.1 akaw|GAA85435.1 akaw|GAA89048.1 anid|CBF87091.1 asyd|OJJ55451.1 ater|EAU29728.1 awen|OJJ35491.1 azon|OJJ46999.1 aver|OJJ06524.1 agla|OJJ78562.1

Group338: abra|OJJ66379.1 abra|OJJ67896.1 acar|OOF92868.1 acar|OOF94061.1 acar|OOF94131.1 akaw|GAA90248.1 akaw|GAA91900.1 aluc|OJZ81994.1 aluc|OJZ83092.1 anid|CBF82331.1 anid|CBF84191.1 anig|EHA22262.1 anig|EHA28154.1 atub|OJI84544.1 atub|OJI87844.1 afis|EAW20565.1 afla|EED45271.1 afum|EAL84496.1 agla|OJJ81768.1 aory|EIT73641.1 asyd|OJJ57136.1 ater|EAU32199.1 aver|OJJ03167.1 awen|OJJ36239.1 azon|OJJ45336.1

Group339: abra|OJJ66548.1 abra|OJJ77152.1 aluc|OJZ81501.1 aluc|OJZ84566.1 anig|EHA25929.1 anig|EHA27860.1 atub|OJI88680.1 atub|OJI89516.1 awen|OJJ34406.1 awen|OJJ37097.1 acar|OOF89954.1 afis|EAW17738.1 afla|EED50272.1 afum|EAL91553.1 agla|OJJ84037.1 akaw|GAA90778.1 akaw|GAA91173.1 anid|CBF82482.1 aory|EIT77312.1 asyd|OJJ54103.1 ater|EAU31869.1 aver|OJJ08632.1 azon|OJJ48148.1 acar|OOF93038.1 aory|EIT75734.1

Group340: abra|OJJ69049.1 abra|OJJ77527.1 akaw|GAA84093.1 akaw|GAA90580.1 aluc|OJZ81008.1 aluc|OJZ86038.1 anig|EHA19352.1 anig|EHA24573.1 asyd|OJJ51977.1 asyd|OJJ54328.1 atub|OJI79825.1 atub|OJI82576.1 acar|OOF99199.1 acla|EAW06702.1 afis|EAW16247.1 afum|EAL88875.1 agla|OJJ86256.1 anid|CBF82150.1 aory|EIT75005.1 ater|EAU37457.1 aver|OJJ05282.1 awen|OJJ30209.1 azon|OJJ43933.1 afla|EED53308.1 asyd|OJJ57961.1

Group341: abra|OJJ69066.1 abra|OJJ69440.1 acar|OOF91217.1 acar|OOF94300.1 afla|EED46891.1 afla|EED47923.1 akaw|GAA88378.1 akaw|GAA90596.1 aluc|OJZ82276.1 aluc|OJZ86054.1 anig|EHA24557.1 anig|EHA28470.1 aory|EIT72607.1 aory|EIT83373.1 atub|OJI79851.1 atub|OJI88163.1 awen|OJJ32949.1 awen|OJJ38342.1 acla|EAW08762.1 afis|EAW24340.1 afum|EAL86011.1 agla|OJJ83225.1 ater|EAU30407.1 aver|OJI96736.1 azon|OJJ48971.1

Group342: abra|OJJ70211.1 abra|OJJ76495.1 acar|OOF92571.1 acar|OOF99727.1 akaw|GAA82411.1 akaw|GAA86565.1 aluc|OJZ85115.1 aluc|OJZ91709.1 azon|OJJ46702.1 azon|OJJ50101.1 acla|EAW13937.1 afis|EAW18648.1 afla|EED52691.1 afum|EAL93364.1 agla|OJJ82426.1 anid|CBF70533.1 anig|EHA22720.1 anig|EHA25335.1 aory|EIT74236.1 asyd|OJJ58553.1 atub|OJI80479.1 atub|OJI86168.1 aver|OJJ01151.1 awen|OJJ32116.1 ater|EAU38263.1

Group343: abra|OJJ74685.1 abra|OJJ77443.1 acar|OOF95646.1 acar|OOF95697.1 asyd|OJJ52766.1 asyd|OJJ57547.1 asyd|OJJ56548.1 aver|OJJ05631.1 aver|OJJ06174.1 aver|OJJ08749.1 awen|OJJ30371.1 awen|OJJ40449.1 acla|EAW10711.1 afis|EAW24859.1 afla|EED47099.1 afum|EAL89507.1 agla|OJJ82077.1 akaw|GAA81929.1 aluc|OJZ92262.1 anid|CBF75821.1 anig|EHA26697.1 aory|EIT72503.1 ater|EAU36441.1 atub|OJI86696.1 azon|OJJ49305.1

Group344: abra|OJJ75156.1 abra|OJJ77714.1 acar|OOF97183.1 acar|OOF99026.1 akaw|GAA83918.1 akaw|GAA88762.1 aluc|OJZ90779.1 aluc|OJZ92725.1 anig|EHA19512.1 anig|EHA26276.1 ater|EAU35617.1 ater|EAU38821.1 atub|OJI82766.1 atub|OJI87150.1 acla|EAW08331.1 afis|EAW23366.1 afla|EED55592.1 afum|EAL88107.1 agla|OJJ86458.1 anid|CBF74825.1 aory|EIT82696.1 asyd|OJJ60139.1 aver|OJJ04123.1 awen|OJJ39521.1 azon|OJJ47717.1

Group345: acar|OOF90695.1 acar|OOF91261.1 afla|EED44834.1 afla|EED46120.1 agla|OJJ84890.1 agla|OJJ88358.1 anid|CBF79319.1 anid|CBF85127.1 asyd|OJJ55782.1 asyd|OJJ63353.1 aver|OJI97608.1 aver|OJJ06871.1 aver|OJJ07891.1 abra|OJJ76767.1 acla|EAW13321.1 afis|EAW20436.1 afum|EAL84616.1 akaw|GAA87259.1 aluc|OJZ84170.1 anig|EHA27531.1 aory|EIT77716.1 ater|EAU37722.1 atub|OJI89914.1 awen|OJJ33127.1 azon|OJJ46343.1

Group346: acar|OOG00252.1 acar|OOG00270.1 abra|OJJ66461.1 afis|EAW24599.1 agla|OJJ80217.1 aver|OJI96133.1 abra|OJJ67546.1 akaw|GAA88114.1 aluc|OJZ81386.1 anig|EHA18679.1 atub|OJI91591.1 abra|OJJ70572.1 acar|OOF97149.1 afla|EED47007.1 akaw|GAA83416.1 aluc|OJZ83916.1 anid|CBF74799.1 anig|EHA19049.1 aory|EIT72694.1 asyd|OJJ60153.1 ater|EAU36375.1 atub|OJI90286.1 aver|OJJ04111.1 awen|OJJ39478.1 awen|OJJ33480.1

Group347: acla|EAW06619.1 acla|EAW13553.1 awen|OJJ30278.1 awen|OJJ33599.1 azon|OJJ42199.1 azon|OJJ50720.1 abra|OJJ77044.1 acar|OOF90272.1 afis|EAW16146.1 afis|EAW20629.1 afla|EED49763.1 afum|EAL84438.1 afum|EAL88775.1 agla|OJJ80445.1 agla|OJJ86199.1 akaw|GAA87009.1 aluc|OJZ84447.1 anid|CBF84471.1 anig|EHA27764.1 aory|EIT77658.1 asyd|OJJ53537.1 ater|EAU32165.1 atub|OJI89628.1 aver|OJJ08214.1 azon|OJJ43971.1

Group348: acla|EAW07744.1 acla|EAW10138.1 afis|EAW20748.1 afis|EAW22781.1 afla|EED47130.1 afla|EED50040.1 afum|EAL84866.1 afum|EAL90418.1 anid|CBF71564.1 anid|CBF87981.1 ater|EAU33992.1 ater|EAU38933.1 awen|OJJ36890.1 awen|OJJ38956.1 abra|OJJ73849.1 acar|OOG00999.1 agla|OJJ83643.1 akaw|GAA89466.1 aluc|OJZ88200.1 anig|EHA17948.1 aory|EIT79245.1 asyd|OJJ63496.1 atub|OJI90798.1 aver|OJI97433.1 azon|OJJ49728.1

Group349: afis|EAW15424.1 afis|EAW16124.1 afum|EAL84220.1 afum|EAL88754.1 aory|EIT73054.1 aory|EIT80955.1 abra|OJJ69804.1 acar|OOF93572.1 acla|EAW06641.1 afla|EED48664.1 agla|OJJ86166.1 akaw|GAA87855.1 aluc|OJZ85497.1 anid|CBF85532.1 anig|EHA25030.1 asyd|OJJ64135.1 ater|EAU37174.1 atub|OJI80068.1 aver|OJI98218.1 awen|OJJ30306.1 azon|OJJ48681.1 acar|OOF91913.1 afla|EED47503.1 aory|EIT74578.1 ater|EAU33207.1

Group350: afis|EAW20695.1 afis|EAW25202.1 afum|EAL86271.1 afum|EAL89865.1 anid|CBF80363.1 anid|CBF85220.1 asyd|OJJ54819.1 asyd|OJJ60277.1 asyd|OJJ62998.1 ater|EAU34342.1 ater|EAU35428.1 aver|OJI97968.1 aver|OJJ07342.1 abra|OJJ71102.1 acar|OOF98041.1 acla|EAW10389.1 afla|EED53649.1 agla|OJJ88973.1 akaw|GAA82866.1 aluc|OJZ87433.1 anig|EHA20655.1 aory|EIT75301.1 atub|OJI81215.1 awen|OJJ40837.1 azon|OJJ46857.1

Group351: afla|EED45001.1 afla|EED45531.1 akaw|GAA83108.1 akaw|GAA90569.1 anig|EHA18348.1 anig|EHA22621.1 aory|EIT73636.1 aory|EIT75377.1 abra|OJJ68866.1 akaw|GAA90387.1 aluc|OJZ85844.1 anig|EHA24720.1 asyd|OJJ56361.1 ater|EAU36829.1 atub|OJI79632.1 aver|OJJ06353.1 abra|OJJ73381.1 acar|OOF90417.1 agla|OJJ82635.1 aluc|OJZ88657.1 anid|CBF90081.1 atub|OJI91243.1 awen|OJJ40195.1 azon|OJJ49062.1 awen|OJJ33525.1

Group352: afla|EED45010.1 afla|EED45410.1 anig|EHA21017.1 anig|EHA22867.1 aory|EIT73809.1 aory|EIT82470.1 aver|OJI96998.1 aver|OJJ03416.1 azon|OJJ43608.1 azon|OJJ47388.1 abra|OJJ68444.1 acar|OOF93950.1 afis|EAW19806.1 afla|EED50514.1 afum|EAL85087.1 akaw|GAA86929.1 aluc|OJZ90053.1 anid|CBF86126.1 asyd|OJJ63944.1 ater|EAU30743.1 atub|OJI84753.1 aver|OJJ08602.1 awen|OJJ34562.1 anid|CBF82389.1 asyd|OJJ54133.1

Group353: afla|EED45238.1 afla|EED49404.1 aory|EIT73665.1 aory|EIT79465.1 asyd|OJJ56263.1 asyd|OJJ61698.1 asyd|OJJ56732.1 aver|OJI95784.1 aver|OJJ05991.1 aver|OJJ08503.1 awen|OJJ33602.1 awen|OJJ36942.1 abra|OJJ77040.1 acar|OOF90076.1 acla|EAW13556.1 afis|EAW24047.1 agla|OJJ78446.1 akaw|GAA87012.1 aluc|OJZ84442.1 anid|CBF83192.1 anig|EHA27761.1 ater|EAU32167.1 atub|OJI89631.1 azon|OJJ50725.1 agla|OJJ78918.1

Group354: afla|EED45547.1 afla|EED48860.1 afum|EAL84770.1 afum|EAL86315.1 aory|EIT75539.1 aory|EIT81273.1 awen|OJJ29954.1 awen|OJJ34505.1 abra|OJJ67746.1 acar|OOF90945.1 acla|EAW12433.1 afis|EAW20865.1 agla|OJJ83914.1 akaw|GAA92437.1 aluc|OJZ81583.1 anid|CBF80387.1 anig|EHA25856.1 asyd|OJJ61074.1 ater|EAU30808.1 atub|OJI88598.1 aver|OJJ00391.1 azon|OJJ51781.1 acla|EAW10996.1 agla|OJJ79837.1 afis|EAW16740.1

Group355: afla|EED45554.1 afla|EED57298.1 aory|EIT74121.1 aory|EIT75629.1 asyd|OJJ56283.1 asyd|OJJ61592.1 asyd|OJJ61824.1 ater|EAU30081.1 ater|EAU30816.1 aver|OJI95924.1 aver|OJJ00914.1 aver|OJJ08478.1 abra|OJJ67752.1 acar|OOF90956.1 acla|EAW10988.1 afis|EAW20872.1 afum|EAL84762.1 agla|OJJ83979.1 akaw|GAA92442.1 aluc|OJZ81577.1 anid|CBF82847.1 anig|EHA25861.1 atub|OJI88603.1 awen|OJJ34489.1 azon|OJJ51766.1

Group356: afla|EED55154.1 afla|EED56856.1 aory|EIT77459.1 aory|EIT81607.1 asyd|OJJ52781.1 asyd|OJJ53407.1 asyd|OJJ55050.1 aver|OJI99562.1 aver|OJJ04194.1 aver|OJJ08759.1 abra|OJJ76674.1 acar|OOF99335.1 acla|EAW09104.1 afis|EAW25708.1 afum|EAL84381.1 agla|OJJ85490.1 akaw|GAA92518.1 aluc|OJZ91531.1 anid|CBF83922.1 anig|EHA22570.1 ater|EAU32722.1 atub|OJI85985.1 anig|EHA19936.1 asyd|OJJ55429.1 aver|OJJ06508.1

Group357: afum|EAL90938.1 afum|EAL91118.1 abra|OJJ70518.1 acar|OOF97412.1 acla|EAW08157.1 afis|EAW23195.1 afla|EED57873.1 afum|EAL88288.2 agla|OJJ86332.1 akaw|GAA83365.1 aluc|OJZ83863.1 anid|CBF74706.1 anig|EHA19003.1 aory|EIT83278.1 asyd|OJJ60219.1 ater|EAU36323.1 atub|OJI90231.1 aver|OJJ04049.1 awen|OJJ39425.1 azon|OJJ47626.1 afis|EAW20299.1 agla|OJJ84725.1 asyd|OJJ62722.1 aver|OJJ01792.1 azon|OJJ49994.1

Group358: agla|OJJ79814.1 agla|OJJ81857.1 agla|OJJ83394.1 asyd|OJJ52362.1 asyd|OJJ57936.1 asyd|OJJ60297.1 aver|OJI99638.1 aver|OJJ01984.1 aver|OJJ04458.1 awen|OJJ30856.1 awen|OJJ34435.1 awen|OJJ40033.1 acla|EAW09090.1 afis|EAW25682.1 afla|EED48118.1 afum|EAL84352.1 anid|CBF71525.1 aory|EIT76242.1 acla|EAW09112.1 afis|EAW25714.1 afum|EAL84386.2 anid|CBF74681.1 anid|CBF87142.1 ater|EAU32480.1 azon|OJJ46476.1

Group359: agla|OJJ83698.1 agla|OJJ89439.1 akaw|GAA85587.1 akaw|GAA85825.1 aluc|OJZ83524.1 aluc|OJZ91276.1 anig|EHA19962.1 anig|EHA24347.1 asyd|OJJ56315.1 asyd|OJJ58149.1 atub|OJI82413.1 atub|OJI83265.1 abra|OJJ73009.1 acar|OOF96181.1 acla|EAW08004.1 afis|EAW23039.1 afla|EED45399.1 afum|EAL88442.1 anid|CBF80933.1 aory|EIT82471.1 ater|EAU29619.1 aver|OJJ06399.1 awen|OJJ35703.1 azon|OJJ44165.1 aver|OJJ04255.1

Group360: aluc|OJZ83601.1 aluc|OJZ88595.1 anig|EHA18289.1 anig|EHA18760.1 asyd|OJJ56054.1 asyd|OJJ61370.1 atub|OJI89973.1 atub|OJI91180.1 aver|OJJ00697.1 aver|OJJ04995.1 awen|OJJ35022.1 awen|OJJ37732.1 abra|OJJ73445.1 acar|OOG00650.1 acla|EAW06781.1 afis|EAW16331.1 afla|EED53547.1 afum|EAL88956.1 agla|OJJ88261.1 akaw|GAA83049.1 anid|CBF84027.1 aory|EIT75029.1 ater|EAU34127.1 azon|OJJ45215.1 akaw|GAA92121.1

Group361: anid|CBF79016.1 anid|CBF89281.1 anid|CBF89401.1 anid|CBF82943.1 abra|OJJ69545.1 acla|EAW15297.1 afis|EAW17240.1 afla|EED45268.1 agla|OJJ88965.1 akaw|GAA91212.1 aluc|OJZ82859.1 aory|EIT72628.1 asyd|OJJ65384.1 ater|EAU37653.1 aver|OJI95878.1 awen|OJJ31342.1 atub|OJI81111.1 anig|EHA23888.1 aory|EIT73575.1 asyd|OJJ53722.1 anig|EHA19683.1 awen|OJJ36938.1 anid|CBF75970.1 ater|EAU39534.1 ater|EAU39535.1

Group362: anid|CBF80391.1 anid|CBF83286.1 asyd|OJJ54788.1 asyd|OJJ57255.1 aver|OJJ03039.1 aver|OJJ07376.1 abra|OJJ68496.1 acar|OOF98751.1 acla|EAW15364.1 afis|EAW17224.1 afla|EED51521.1 afum|EAL86646.1 agla|OJJ86656.1 akaw|GAA92774.1 aluc|OJZ82709.1 anid|CBF80759.1 anig|EHA21164.1 aory|EIT80558.1 ater|EAU37673.1 atub|OJI88942.1 azon|OJJ42623.1 azon|OJJ42665.1 asyd|OJJ57905.1 aver|OJJ04489.1 azon|OJJ45203.1

Group363: asyd|OJJ54205.1 asyd|OJJ55560.1 ater|EAU32170.1 ater|EAU32943.1 abra|OJJ75335.1 acar|OOF94500.1 acla|EAW13560.1 afis|EAW20637.1 afla|EED49750.1 afum|EAL84431.1 akaw|GAA92029.1 aluc|OJZ92899.1 anid|CBF83194.1 anig|EHA26064.1 aory|EIT77711.1 atub|OJI87357.1 awen|OJJ33608.1 abra|OJJ77031.1 acar|OOF91467.1 agla|OJJ78916.1 akaw|GAA87020.1 aluc|OJZ84434.1 anig|EHA27753.1 atub|OJI89639.1 aver|OJJ06641.1

Group364: atub|OJI82939.1 atub|OJI88437.1 abra|OJJ67568.1 afla|EED50521.1 akaw|GAA89098.1 anig|EHA25700.1 ater|EAU33843.1 abra|OJJ72071.1 acla|EAW14867.1 afis|EAW19913.1 afum|EAL84993.2 agla|OJJ81787.1 aory|EIT77284.1 ater|EAU32297.1 abra|OJJ77873.1 acar|OOF98898.1 aluc|OJZ90946.1 anig|EHA19655.1 aory|EIT82039.1 acar|OOG00619.1 afla|EED50223.1 anig|EHA19654.1 afla|EED50520.1 awen|OJJ34149.1 ater|EAU39457.1

Group365: awen|OJJ31642.1 awen|OJJ39457.1 abra|OJJ70593.1 acar|OOF97125.1 acar|OOF97948.1 acla|EAW08187.1 afis|EAW23221.1 afla|EED47029.1 afum|EAL88263.1 agla|OJJ86378.1 akaw|GAA83438.1 aluc|OJZ83939.1 anid|CBF76684.1 anig|EHA19069.1 ater|EAU36352.1 atub|OJI90312.1 abra|OJJ71237.1 afla|EED56272.1 akaw|GAA82751.1 aluc|OJZ87308.1 anig|EHA20544.1 aory|EIT73409.1 asyd|OJJ56499.1 atub|OJI81337.1 aver|OJJ06220.1

Group366: abra|OJJ66082.1 acar|OOF99287.1 acla|EAW06431.1 afis|EAW24735.1 afla|EED52785.1 afum|EAL89382.1 akaw|GAA84164.1 aluc|OJZ81089.1 anid|CBF79598.1 anig|EHA19281.1 aory|EIT78193.1 asyd|OJJ57988.1 ater|EAU31606.1 atub|OJI82495.1 aver|OJJ04414.1 awen|OJJ40223.1 afis|EAW18537.1 afla|EED56576.1 afum|EAL93235.1 anid|CBF82125.1 aory|EIT79676.1 awen|OJJ36227.1 ater|EAU32072.1 asyd|OJJ54295.1 aver|OJJ05250.1

Group367: abra|OJJ67359.1 acar|OOF93335.1 acla|EAW10444.1 afla|EED55318.1 agla|OJJ89047.1 akaw|GAA86462.1 anid|CBF87506.1 anig|EHA25221.1 aory|EIT76259.1 asyd|OJJ58362.1 ater|EAU39395.1 aver|OJJ02044.1 awen|OJJ40742.1 abra|OJJ70853.1 acar|OOF98263.1 afis|EAW25123.1 afum|EAL89794.1 akaw|GAA91720.1 aluc|OJZ87668.1 anid|CBF85688.1 anig|EHA20859.1 asyd|OJJ64259.1 atub|OJI80975.1 aver|OJI98351.1 azon|OJJ44113.1

Group368: abra|OJJ67850.1 acar|OOF92831.1 acla|EAW11749.1 afis|EAW23782.1 afum|EAL87216.1 agla|OJJ78934.1 akaw|GAA91858.1 aluc|OJZ82041.1 anig|EHA22215.1 atub|OJI84600.1 awen|OJJ33726.1 abra|OJJ75896.1 acar|OOG00008.1 afla|EED49453.1 agla|OJJ80043.1 akaw|GAA84602.1 aluc|OJZ89346.1 anig|EHA23235.1 aory|EIT79472.1 asyd|OJJ57259.1 ater|EAU39366.1 atub|OJI85452.1 aver|OJJ03037.1 awen|OJJ36056.1 azon|OJJ50429.1

Group369: abra|OJJ68472.1 acar|OOF94211.1 acla|EAW13768.1 afis|EAW19790.1 afla|EED45871.1 afum|EAL94073.1 akaw|GAA92393.1 aluc|OJZ83253.1 anid|CBF79916.1 anig|EHA21107.1 asyd|OJJ58193.1 ater|EAU36161.1 atub|OJI87684.1 aver|OJJ04212.1 awen|OJJ31450.1 acla|EAW14615.1 afis|EAW19353.1 afla|EED46572.1 anid|CBF76648.1 asyd|OJJ61206.1 aver|OJJ00530.1 acar|OOF94212.1 ater|EAU38489.1 aory|EIT77883.1 afum|EAL85101.1

Group370: abra|OJJ68801.1 acar|OOF91599.1 acla|EAW13559.1 afis|EAW20636.1 afum|EAL84432.1 akaw|GAA90452.1 aluc|OJZ85772.1 anig|EHA24781.1 aory|EIT77665.1 ater|EAU37652.1 atub|OJI79557.1 awen|OJJ33440.1 azon|OJJ50788.1 abra|OJJ70265.1 afla|EED54696.1 akaw|GAA86532.1 aluc|OJZ85080.1 anid|CBF85668.1 anig|EHA25295.1 aory|EIT78807.1 asyd|OJJ64247.1 aver|OJJ04749.1 afla|EED50779.1 aver|OJI98338.1 aory|EIT77987.1

Group371: abra|OJJ68927.1 acar|OOF90675.1 agla|OJJ86552.1 akaw|GAA90318.1 aluc|OJZ85915.1 anig|EHA24679.1 atub|OJI79705.1 aver|OJJ00751.1 abra|OJJ69600.1 acar|OOF93267.1 afis|EAW19224.1 afla|EED48204.1 anid|CBF83972.1 asyd|OJJ61415.1 awen|OJJ38318.1 abra|OJJ77028.1 afum|EAL93955.1 akaw|GAA87023.1 aluc|OJZ84430.1 anig|EHA27749.1 aory|EIT72916.1 atub|OJI89644.1 aver|OJJ08327.1 aver|OJJ02995.1 awen|OJJ29960.1

Group372: abra|OJJ70648.1 acar|OOF97073.1 acla|EAW08111.1 acla|EAW08112.1 afis|EAW23139.1 afis|EAW23140.1 afla|EED48502.1 afum|EAL88341.1 afum|EAL88342.1 agla|OJJ89661.1 akaw|GAA89984.1 aluc|OJZ83998.1 anid|CBF74444.1 anid|CBF74446.1 anig|EHA19121.1 aory|EIT73212.1 aory|EIT73270.1 asyd|OJJ57742.1 ater|EAU36277.1 atub|OJI90367.1 aver|OJJ05425.1 awen|OJJ36636.1 azon|OJJ47580.1 afla|EED48503.1 ater|EAU36278.1

Group373: abra|OJJ70697.1 acar|OOF90045.1 acla|EAW08067.1 afis|EAW23103.1 afla|EED48544.1 afum|EAL88376.1 agla|OJJ89639.1 akaw|GAA90028.1 akaw|GAA90029.1 aluc|OJZ84044.1 anid|CBF81363.1 anig|EHA19166.1 aory|EIT73061.1 asyd|OJJ59383.1 ater|EAU36232.1 atub|OJI90416.1 aver|OJJ02890.1 awen|OJJ36677.1 azon|OJJ47521.1 afla|EED48545.1 acla|EAW08068.1 afis|EAW23104.1 afum|EAL88375.1 anid|CBF81365.1 aory|EIT73082.1

Group374: abra|OJJ71208.1 acar|OOF97959.1 afla|EED54682.1 akaw|GAA82776.1 aluc|OJZ87335.1 anid|CBF80112.1 anig|EHA20571.1 anig|EHA20572.1 aory|EIT78810.1 asyd|OJJ65149.1 ater|EAU36930.1 atub|OJI81309.1 aver|OJJ02086.1 azon|OJJ44458.1 acar|OOF96688.1 afla|EED54681.1 asyd|OJJ65153.1 ater|EAU36931.1 acar|OOF97963.1 aory|EIT78922.1 afla|EED46387.1 afla|EED46388.1 aory|EIT72360.1 asyd|OJJ58325.1 atub|OJI84521.1

Group375: abra|OJJ72278.1 acar|OOF94942.1 afis|EAW20802.1 afla|EED56693.1 afum|EBA27208.1 agla|OJJ85014.1 akaw|GAA86030.1 aluc|OJZ90257.1 anid|CBF71378.1 anig|EHA23752.1 aory|EIT73887.1 asyd|OJJ55075.1 ater|EAU32872.1 atub|OJI81683.1 aver|OJJ07133.1 awen|OJJ41914.1 azon|OJJ47367.1 afis|EAW22078.1 afum|EAL91092.1 anid|CBF82802.1 asyd|OJJ57118.1 ater|EAU30146.1 afla|EED49551.1 aory|EIT82585.1 aory|EIT82590.1

Group376: abra|OJJ72657.1 acar|OOF94617.1 acla|EAW11521.1 afis|EAW23933.1 afla|EED50585.1 afum|EAL87357.1 agla|OJJ79413.1 akaw|GAA85279.1 akaw|GAA85280.1 aluc|OJZ90638.1 anid|CBF77273.1 anig|EHA24073.1 aory|EIT82169.1 asyd|OJJ56797.1 ater|EAU32304.1 atub|OJI82066.1 aver|OJJ03537.1 awen|OJJ39022.1 azon|OJJ51347.1 acla|EAW11520.1 afum|EAL87358.1 aory|EIT82139.1 afis|EAW23932.1 afla|EED50586.1 azon|OJJ51348.1

Group377: abra|OJJ72828.1 acar|OOF93602.1 acla|EAW14973.1 afis|EAW24498.1 afla|EED56620.1 afum|EAL86168.1 agla|OJJ79958.1 anid|CBF85575.1 aory|EIT79659.1 asyd|OJJ64180.1 ater|EAU31051.1 aver|OJJ07247.1 awen|OJJ34897.1 abra|OJJ75484.1 akaw|GAA84250.1 aluc|OJZ88942.1 anid|CBF80279.1 anig|EHA23578.1 atub|OJI85847.1 ater|EAU34059.1 anid|CBF71459.1 asyd|OJJ54948.1 afla|EED52220.1 aory|EIT74386.1 aver|OJI98257.1

Group378: abra|OJJ74406.1 acar|OOF95334.1 acla|EAW06822.1 afis|EAW16185.1 afla|EED53465.1 afum|EAL88816.2 agla|OJJ78591.1 akaw|GAA87300.1 aluc|OJZ89718.1 anid|CBF89935.1 anig|EHA26946.1 aory|EIT74868.1 asyd|OJJ62121.1 ater|EAU34079.1 atub|OJI85082.1 aver|OJI96229.1 awen|OJJ37907.1 azon|OJJ45085.1 afis|EAW16370.1 acla|EAW11051.1 afla|EED49634.1 aory|EIT80960.1 azon|OJJ45955.1 ater|EAU36747.1 azon|OJJ45949.1

Group379: abra|OJJ76313.1 acar|OOF99561.1 acar|OOF99562.1 acla|EAW14106.1 afis|EAW18806.1 afis|EAW18807.1 afla|EED50770.1 afum|EAL93542.1 afum|EAL93543.1 agla|OJJ80536.1 akaw|GAA82241.1 aluc|OJZ91904.1 anid|CBF69790.1 anig|EHA22880.1 aory|EIT78083.1 asyd|OJJ58961.1 ater|EAU37958.1 atub|OJI86354.1 aver|OJJ01555.1 awen|OJJ32382.1 azon|OJJ47143.1 agla|OJJ80538.1 acla|EAW14105.1 anid|CBF69788.1 awen|OJJ32381.1

Group380: abra|OJJ76772.1 acar|OOF91266.1 acla|EAW13327.1 afis|EAW20442.1 afla|EED44828.1 afum|EAL84610.2 agla|OJJ84883.1 akaw|GAA87253.1 akaw|GAA87254.1 aluc|OJZ84176.1 anid|CBF79308.1 anig|EHA27535.1 aory|EIT77723.1 asyd|OJJ55777.1 ater|EAU37717.1 atub|OJI89908.1 atub|OJI89909.1 aver|OJJ06866.1 awen|OJJ33132.1 azon|OJJ46347.1 acla|EAW13326.1 afis|EAW20441.1 afla|EED44829.1 afum|EAL84611.1 anid|CBF79310.1

Group381: abra|OJJ77345.1 acar|OOF93215.1 acla|EAW13129.1 acla|EAW13130.1 afis|EAW17533.1 afis|EAW17534.1 afla|EED49295.1 afla|EED49296.1 afum|EAL91347.1 afum|EAL91348.1 agla|OJJ81437.1 akaw|GAA91415.1 akaw|GAA91416.1 aluc|OJZ84756.1 anid|CBF90328.1 anid|CBF90329.1 anig|EHA28025.1 aory|EIT82179.1 asyd|OJJ61906.1 ater|EAU36962.1 ater|EAU36963.1 atub|OJI89328.1 aver|OJI95999.1 awen|OJJ37319.1 azon|OJJ51011.1

Group382: abra|OJJ66008.1 abra|OJJ66036.1 abra|OJJ72701.1 agla|OJJ79097.1 agla|OJJ82950.1 asyd|OJJ54673.1 asyd|OJJ57511.1 aver|OJJ05668.1 aver|OJJ07496.1 acar|OOF92090.1 akaw|GAA90880.1 aluc|OJZ82953.1 anig|EHA21368.1 aory|EIT75821.1 atub|OJI89185.1 awen|OJJ30435.1 atub|OJI87431.1 abra|OJJ67817.1 afum|EAL86776.1 anid|CBF79156.1 ater|EAU38014.1 afla|EED45927.1 afla|EED45926.1 anid|CBF75733.1

Group383: abra|OJJ66089.1 abra|OJJ76716.1 acar|OOF95538.1 afis|EAW21215.1 afla|EED53625.1 afum|EAL92226.1 aluc|OJZ91496.1 anig|EHA22536.1 aory|EIT75031.1 ater|EAU38549.1 atub|OJI85949.1 aver|OJI98307.1 aver|OJI99282.1 awen|OJJ30167.1 abra|OJJ66099.1 acla|EAW14986.1 afis|EAW24508.1 afum|EBA27258.1 agla|OJJ86285.1 anid|CBF78328.1 asyd|OJJ56085.1 aver|OJJ05028.1 akaw|GAA92484.1 awen|OJJ36979.1

Group384: abra|OJJ66370.1 abra|OJJ77693.1 acar|OOF98983.1 acar|OOF99046.1 akaw|GAA83937.1 akaw|GAA90239.1 atub|OJI82743.1 atub|OJI87834.1 acla|EAW06572.1 afis|EAW16024.1 afla|EED56405.1 afum|EAL88653.1 agla|OJJ81572.1 aluc|OJZ83101.1 anid|CBF75012.1 anig|EHA19493.1 anig|EHA28166.1 aory|EIT83000.1 asyd|OJJ60056.1 ater|EAU33617.1 aver|OJJ02233.1 awen|OJJ30433.1 azon|OJJ45432.1 aluc|OJZ80819.1

Group385: abra|OJJ67097.1 abra|OJJ77658.1 aluc|OJZ80870.1 aluc|OJZ87876.1 anig|EHA19465.1 anig|EHA20181.1 atub|OJI80773.1 atub|OJI82707.1 acar|OOF98457.1 acar|OOF99078.1 acla|EAW06500.1 afis|EAW15996.1 afla|EED56370.1 afum|EAL88628.2 agla|OJJ86011.1 akaw|GAA83977.1 akaw|GAA91064.1 anid|CBF81691.1 aory|EIT73327.1 asyd|OJJ59562.1 ater|EAU33651.1 aver|OJJ02706.1 awen|OJJ30473.1 azon|OJJ45465.1

Group386: abra|OJJ67844.1 abra|OJJ76158.1 acar|OOF92021.1 acar|OOF99774.1 akaw|GAA82106.1 akaw|GAA91850.1 aluc|OJZ82048.1 aluc|OJZ92070.1 anig|EHA22210.1 anig|EHA22999.1 atub|OJI84606.1 atub|OJI86502.1 awen|OJJ37808.1 awen|OJJ39219.1 acla|EAW09660.1 afis|EAW21479.1 afla|EED55911.1 afum|EAL92499.1 agla|OJJ85632.1 anid|CBF76506.1 aory|EIT76810.1 asyd|OJJ61132.1 aver|OJJ00453.1 azon|OJJ51636.1

Group387: abra|OJJ68864.1 abra|OJJ75043.1 acar|OOF90525.1 acar|OOF97290.1 akaw|GAA88660.1 akaw|GAA90389.1 aluc|OJZ85842.1 aluc|OJZ92609.1 atub|OJI79630.1 atub|OJI87043.1 awen|OJJ31662.1 awen|OJJ39696.1 acla|EAW08232.1 afis|EAW23266.1 afla|EED55747.1 afum|EAL88213.1 agla|OJJ86586.1 anid|CBF84130.1 anig|EHA24722.1 aory|EIT83228.1 asyd|OJJ57114.1 ater|EAU37532.1 aver|OJJ03186.1 azon|OJJ47828.1

Group388: abra|OJJ70275.1 abra|OJJ75558.1 acar|OOF90876.1 acar|OOF98673.1 agla|OJJ82823.1 agla|OJJ88521.1 akaw|GAA84313.1 akaw|GAA92874.1 aluc|OJZ82537.1 aluc|OJZ89008.1 anig|EHA22505.1 anig|EHA23520.1 asyd|OJJ55019.1 asyd|OJJ59700.1 atub|OJI85781.1 atub|OJI88781.1 aver|OJJ02587.1 aver|OJJ07186.1 awen|OJJ32269.1 awen|OJJ32616.1 anid|CBF78793.1 afla|EED45595.1 aory|EIT75779.1 aory|EIT75483.1

Group389: abra|OJJ70579.1 abra|OJJ75835.1 akaw|GAA83423.1 akaw|GAA84545.1 aluc|OJZ83923.1 aluc|OJZ89278.1 anig|EHA19056.1 anig|EHA23291.1 atub|OJI85521.1 atub|OJI90295.1 awen|OJJ32048.1 awen|OJJ39470.1 acar|OOF97142.1 acla|EAW08201.1 afis|EAW23235.1 afla|EED47014.1 afum|EAL88247.1 agla|OJJ86395.1 anid|CBF75150.1 aory|EIT72630.1 asyd|OJJ59974.1 ater|EAU36368.1 aver|OJJ02317.1 azon|OJJ47672.1

Group390: abra|OJJ70867.1 abra|OJJ72108.1 acar|OOF90121.1 acar|OOF96751.1 aluc|OJZ86863.1 aluc|OJZ87652.1 anig|EHA20846.1 anig|EHA21778.1 aory|EIT75800.1 aory|EIT76249.1 atub|OJI80991.1 atub|OJI83657.1 acla|EAW10459.1 afis|EAW25108.1 afla|EED55334.1 afum|EAL89779.1 anid|CBF86068.1 asyd|OJJ64458.1 ater|EAU39378.1 aver|OJI98561.1 awen|OJJ37055.1 azon|OJJ44547.1 akaw|GAA84814.1 afla|EED45959.1

Group391: acar|OOF90694.1 acar|OOF95969.1 afla|EED46110.1 afla|EED52607.1 agla|OJJ82549.1 agla|OJJ84416.1 awen|OJJ31372.1 awen|OJJ33859.1 abra|OJJ72741.1 acla|EAW11574.1 afis|EAW23880.1 afum|EAL87308.1 akaw|GAA85352.1 aluc|OJZ90714.1 anid|CBF77193.1 anig|EHA24133.1 aory|EIT74176.1 asyd|OJJ56836.1 ater|EAU33467.1 atub|OJI82140.1 aver|OJJ03501.1 azon|OJJ48693.1 asyd|OJJ58816.1 aory|EIT76373.1

Group392: acar|OOF92653.1 acar|OOG00348.1 abra|OJJ69030.1 afla|EED56357.1 akaw|GAA90546.1 aluc|OJZ86016.1 anig|EHA24595.1 aory|EIT73420.1 ater|EAU31400.1 atub|OJI79805.1 awen|OJJ34779.1 abra|OJJ73125.1 acla|EAW07183.1 afis|EAW22196.1 afum|EAL90995.1 agla|OJJ87849.1 akaw|GAA88040.1 aluc|OJZ81299.1 anid|CBF88408.1 anig|EHA18608.1 asyd|OJJ63753.1 atub|OJI91508.1 aver|OJI97188.1 azon|OJJ45760.1

Group393: acar|OOF94038.1 acar|OOF94108.1 abra|OJJ77702.1 acar|OOF99038.1 acla|EAW12405.1 afis|EAW16864.1 afla|EED51093.1 afum|EAL86403.2 agla|OJJ78684.1 akaw|GAA83928.1 aluc|OJZ90767.1 anig|EHA19502.1 aory|EIT81997.1 asyd|OJJ62647.1 ater|EAU37649.1 atub|OJI82755.1 awen|OJJ36987.1 azon|OJJ45261.1 acla|EAW15311.1 akaw|GAA90280.1 aluc|OJZ83060.1 anig|EHA28126.1 atub|OJI87886.1 awen|OJJ38066.1

Group394: acar|OOF95062.1 acar|OOF95129.1 afla|EED47135.1 afla|EED49651.1 anid|CBF74606.1 anid|CBF84541.1 asyd|OJJ53476.1 asyd|OJJ57659.1 aver|OJJ05520.1 aver|OJJ08280.1 abra|OJJ74650.1 acla|EAW10735.1 afis|EAW24839.1 afum|EAL89485.1 agla|OJJ82030.1 akaw|GAA81960.1 aluc|OJZ92229.1 anig|EHA26728.1 aory|EIT79283.1 ater|EAU36464.1 atub|OJI86659.1 awen|OJJ40425.1 azon|OJJ49283.1 ater|EAU29279.1

Group395: afis|EAW18417.1 afis|EAW22052.1 afum|EAL86752.2 afum|EAL93101.1 aory|EIT78709.1 aory|EIT83477.1 asyd|OJJ51851.1 asyd|OJJ58449.1 awen|OJJ30750.1 awen|OJJ31731.1 abra|OJJ65787.1 acar|OOF92376.1 acla|EAW06308.1 agla|OJJ84984.1 akaw|GAA86375.1 anid|CBF70714.1 anig|EHA25131.1 ater|EAU29833.1 atub|OJI80690.1 aver|OJJ01050.1 azon|OJJ50491.1 abra|OJJ67456.1 aluc|OJZ84908.1 akaw|GAA92636.1

Group396: afis|EAW19022.1 afis|EAW24624.1 afis|EAW25668.1 afum|EAL84338.1 afum|EAL93753.1 ater|EAU32064.1 ater|EAU33941.1 ater|EAU38525.1 abra|OJJ68917.1 acar|OOF91505.1 acla|EAW14258.1 afla|EED56624.1 agla|OJJ82736.1 akaw|GAA90332.1 aluc|OJZ85900.1 anid|CBF79090.1 anid|CBF85580.1 anig|EHA24692.1 aory|EIT79597.1 asyd|OJJ64176.1 atub|OJI79685.1 aver|OJI98251.1 awen|OJJ31400.1 aver|OJJ08543.1

Group397: afla|EED46864.1 afla|EED51110.1 aory|EIT72646.1 aory|EIT81348.1 ater|EAU32491.1 ater|EAU37681.1 abra|OJJ65858.1 acar|OOF95019.1 acla|EAW14585.1 agla|OJJ81814.1 akaw|GAA92287.1 aluc|OJZ90184.1 anig|EHA23683.1 atub|OJI81611.1 awen|OJJ38347.1 abra|OJJ76688.1 acar|OOF99324.1 akaw|GAA92506.1 aluc|OJZ91519.1 anid|CBF73586.1 anig|EHA22557.1 asyd|OJJ56216.1 atub|OJI85974.1 aver|OJJ05148.1

Group398: afla|EED49844.1 afla|EED50421.1 anid|CBF71215.1 anid|CBF78131.1 aory|EIT82058.1 aory|EIT83396.1 asyd|OJJ55867.1 asyd|OJJ57404.1 azon|OJJ43885.1 azon|OJJ46240.1 abra|OJJ70022.1 acar|OOF93528.1 acla|EAW08798.1 afis|EAW15601.1 afum|EAL85732.1 akaw|GAA87639.1 aluc|OJZ85254.1 anig|EHA25455.1 ater|EAU32528.1 atub|OJI80314.1 aver|OJJ02118.1 aver|OJJ04809.1 awen|OJJ38276.1 asyd|OJJ57345.1

Group399: agla|OJJ82200.1 agla|OJJ84366.1 abra|OJJ67531.1 afis|EAW24553.1 afla|EED51537.1 afum|EAL86218.1 aluc|OJZ87028.1 anid|CBF78469.1 anig|EHA21629.1 asyd|OJJ57285.1 ater|EAU37648.1 atub|OJI83486.1 aver|OJJ05871.1 awen|OJJ38297.1 abra|OJJ68399.1 acar|OOF93974.1 akaw|GAA92548.1 aluc|OJZ90097.1 anig|EHA20979.1 aory|EIT74555.1 atub|OJI84709.1 awen|OJJ37870.1 awen|OJJ40901.1 ater|EAU36047.1

Group400: agla|OJJ84117.1 agla|OJJ84488.1 ater|EAU31647.1 ater|EAU32102.1 ater|EAU32790.1 awen|OJJ34277.1 awen|OJJ34655.1 awen|OJJ38014.1 awen|OJJ38039.1 abra|OJJ74152.1 acar|OOF95573.1 afla|EED54552.1 akaw|GAA89731.1 aluc|OJZ80652.1 anig|EHA27180.1 aory|EIT78872.1 asyd|OJJ61410.1 atub|OJI87507.1 aver|OJJ00740.1 akaw|GAA89130.1 anig|EHA25730.1 atub|OJI88476.1 anid|CBF73465.1 aluc|OJZ81708.1

Group401: akaw|GAA85083.1 akaw|GAA91235.1 aluc|OJZ82835.1 aluc|OJZ86559.1 atub|OJI83963.1 atub|OJI89065.1 abra|OJJ67962.1 acar|OOF92808.1 acla|EAW11652.1 afis|EAW23801.1 afla|EED51619.1 afum|EAL87235.2 agla|OJJ78848.1 anig|EHA21273.1 aory|EIT80604.1 asyd|OJJ61019.1 ater|EAU31355.1 awen|OJJ33760.1 azon|OJJ42864.1 abra|OJJ71827.1 acar|OOF96504.1 anid|CBF85398.1 anig|EHA22090.1 ater|EAU39385.1

Group402: akaw|GAA91381.1 akaw|GAA92466.1 aluc|OJZ81553.1 aluc|OJZ84788.1 anig|EHA25881.1 anig|EHA28056.1 atub|OJI88632.1 atub|OJI89294.1 abra|OJJ66481.1 acar|OOF93248.1 afla|EED46099.1 anid|CBF82158.1 aory|EIT76444.1 asyd|OJJ54333.1 ater|EAU37661.1 aver|OJJ05287.1 awen|OJJ41952.1 abra|OJJ77379.1 afla|EED46096.1 anid|CBF82155.1 afla|EED46097.1 akaw|GAA92464.1 anid|CBF82156.1 aory|EIT76468.1

Group403: aory|EIT74183.1 aory|EIT82897.1 ater|EAU31229.1 ater|EAU37935.1 aver|OJJ03325.1 aver|OJJ08019.1 abra|OJJ69359.1 acar|OOF94365.1 acla|EAW14750.1 afis|EAW24281.1 afla|EED47995.1 afum|EAL85950.1 agla|OJJ80635.1 akaw|GAA88229.1 aluc|OJZ82191.1 anid|CBF74148.1 anig|EHA28544.1 asyd|OJJ53921.1 asyd|OJJ56960.1 atub|OJI88244.1 awen|OJJ33016.1 azon|OJJ48501.1 acar|OOF90185.1 afla|EED52650.1

Group404: awen|OJJ30773.1 awen|OJJ31708.1 abra|OJJ68030.1 acar|OOF92075.1 afla|EED45369.1 agla|OJJ84153.1 akaw|GAA90923.1 aluc|OJZ82903.1 aory|EIT82429.1 ater|EAU36888.1 atub|OJI89129.1 abra|OJJ70336.1 acla|EAW11117.1 afla|EED51703.1 aluc|OJZ82606.1 anig|EHA27474.1 aory|EIT81577.1 asyd|OJJ64590.1 ater|EAU34064.1 atub|OJI88841.1 aver|OJI98688.1 azon|OJJ45907.1 aver|OJI98018.1 afla|EED55092.1

Group405: abra|OJJ66091.1 acla|EAW07082.1 afla|EED53627.1 aory|EIT75030.1 asyd|OJJ64735.1 ater|EAU38556.1 aver|OJJ06182.1 awen|OJJ30165.1 abra|OJJ77781.1 afis|EAW19895.1 afum|EAL92224.2 agla|OJJ86287.1 akaw|GAA83866.1 aluc|OJZ90834.1 anid|CBF78324.1 anig|EHA19557.1 atub|OJI82820.1 aver|OJI98311.1 awen|OJJ36980.1 afis|EAW21213.1 asyd|OJJ56540.1 asyd|OJJ56087.1 aver|OJJ05030.1 awen|OJJ33354.1

Group406: abra|OJJ66593.1 acar|OOF91028.1 acla|EAW10898.1 afis|EAW20960.1 afum|EAL84673.1 agla|OJJ83918.1 akaw|GAA91124.1 aluc|OJZ81454.1 anid|CBF82567.1 anig|EHA25974.1 aory|EIT77369.1 asyd|OJJ54050.1 ater|EAU38610.1 atub|OJI88727.1 aver|OJJ08692.1 awen|OJJ34357.1 azon|OJJ48093.1 acla|EAW10899.1 afis|EAW20959.1 afla|EED50315.1 afum|EAL84674.1 akaw|GAA91125.1 aluc|OJZ81455.1 aory|EIT77298.1

Group407: anid|CBF79149.1 abra|OJJ68851.1 acar|OOF93604.1 afis|EAW23435.1 afla|EED49617.1 akaw|GAA87401.1 aluc|OJZ87639.1 anid|CBF79147.1 anig|EHA25624.1 aory|EIT80987.1 ater|EAU36062.1 atub|OJI79619.1 abra|OJJ76884.1 afla|EED53582.1 anid|CBF85241.1 asyd|OJJ56896.1 afis|EAW23434.1 aory|EIT80692.1 ater|EAU31633.1 atub|OJI88357.1 afla|EED51736.1 aory|EIT80667.1 aory|EIT75082.1 atub|OJI81007.1

Group408: abra|OJJ66957.1 acar|OOF93651.1 acla|EAW14508.1 afis|EAW19221.1 afla|EED47668.1 agla|OJJ88465.1 akaw|GAA92204.1 aluc|OJZ85619.1 anid|CBF78606.1 anig|EHA24907.1 azon|OJJ51238.1 abra|OJJ69809.1 acar|OOF93568.1 akaw|GAA87851.1 aluc|OJZ85491.1 aory|EIT82845.1 atub|OJI80074.1 awen|OJJ37584.1 azon|OJJ46584.1 afla|EED52824.1 asyd|OJJ65099.1 aver|OJI99225.1 afla|EED47373.1 atub|OJI79412.1

Group409: abra|OJJ66987.1 acar|OOF93625.1 acla|EAW08983.1 afis|EAW24689.1 afla|EED48347.1 afum|EAL89330.1 agla|OJJ83266.1 akaw|GAA92176.1 aluc|OJZ85586.1 anid|CBF73671.1 anig|EHA24935.1 aory|EIT76285.1 asyd|OJJ56664.1 ater|EAU31416.1 atub|OJI79381.1 aver|OJJ06059.1 awen|OJJ32833.1 azon|OJJ49002.1 acla|EAW14852.1 afis|EAW24379.1 afla|EED47832.1 afum|EAL86051.1 aory|EIT77109.1 ater|EAU30357.1

Group410: abra|OJJ67104.1 acar|OOF95836.1 afla|EED47889.1 aluc|OJZ82016.1 anig|EHA22240.1 aory|EIT77218.1 atub|OJI84565.1 abra|OJJ75997.1 acar|OOF99928.1 acla|EAW09808.1 afis|EAW21328.1 afla|EED57286.1 afum|EAL92346.2 agla|OJJ87411.1 akaw|GAA88962.1 aluc|OJZ89443.1 anid|CBF83430.1 anig|EHA23143.1 aory|EIT74039.1 asyd|OJJ60660.1 ater|EAU35953.1 aver|OJI99983.1 awen|OJJ39370.1 azon|OJJ51710.1

Group411: abra|OJJ67606.1 acar|OOF93337.1 acla|EAW11030.1 afis|EAW20824.1 afla|EED45471.1 afum|EAL84805.1 akaw|GAA89123.1 aluc|OJZ81715.1 anid|CBF77690.1 anig|EHA25724.1 aory|EIT75480.1 asyd|OJJ60009.1 ater|EAU37676.1 atub|OJI88467.1 aver|OJJ04442.1 awen|OJJ36217.1 afla|EED52784.1 aory|EIT78156.1 atub|OJI87922.1 acar|OOF98999.1 akaw|GAA83906.1 anig|EHA19524.1 aver|OJJ02276.1 azon|OJJ50341.1

Group412: afis|EAW15422.1 afum|EAL84222.1 abra|OJJ74863.1 acar|OOF95863.1 acla|EAW08764.1 afla|EED57451.1 agla|OJJ81802.1 akaw|GAA83557.1 aluc|OJZ92441.1 anid|CBF89639.1 anig|EHA26526.1 aory|EIT80148.1 asyd|OJJ62291.1 ater|EAU32509.1 atub|OJI86870.1 aver|OJI96399.1 awen|OJJ39887.1 azon|OJJ50235.1 afum|EAL85683.1 afis|EAW15665.1 afla|EED46890.1 awen|OJJ38340.1 acla|EAW08989.1 aory|EIT72560.1

Group413: abra|OJJ67909.1 acar|OOF98177.1 afla|EED46476.1 agla|OJJ79399.1 anig|EHA22276.1 aory|EIT72342.1 asyd|OJJ61955.1 ater|EAU37942.1 aver|OJI96047.1 awen|OJJ33590.1 azon|OJJ42127.1 abra|OJJ70267.1 akaw|GAA86530.1 aluc|OJZ85078.1 anid|CBF83033.1 anig|EHA25293.1 asyd|OJJ61489.1 atub|OJI80520.1 aver|OJJ00830.1 aory|EIT73700.1 asyd|OJJ55487.1 aver|OJJ06566.1 afla|EED45145.1 aory|EIT82578.1

Group414: abra|OJJ68074.1 acar|OOF96032.1 acla|EAW11411.1 afis|EAW24010.1 afla|EED49151.1 afum|EAL87440.1 agla|OJJ86942.1 akaw|GAA92399.1 aluc|OJZ83260.1 anid|CBF77404.1 anig|EHA21114.1 aory|EIT79051.1 asyd|OJJ53240.1 ater|EAU29741.1 atub|OJI87679.1 aver|OJJ03610.1 awen|OJJ35482.1 azon|OJJ51266.1 aory|EIT78571.1 afla|EED50884.1 ater|EAU38050.1 asyd|OJJ59006.1 aver|OJJ01610.1 awen|OJJ37601.1

Group415: abra|OJJ68223.1 acla|EAW07100.1 afis|EAW20575.1 afum|EAL84490.1 akaw|GAA83221.1 aluc|OJZ83722.1 anig|EHA18872.1 atub|OJI90097.1 azon|OJJ50650.1 abra|OJJ76992.1 acar|OOF91437.1 afla|EED52310.1 agla|OJJ88718.1 akaw|GAA87054.1 aluc|OJZ84392.1 anid|CBF78937.1 anig|EHA27721.1 aory|EIT74487.1 asyd|OJJ55645.1 ater|EAU37109.1 atub|OJI89679.1 aver|OJJ06730.1 awen|OJJ33275.1 acla|EAW13463.1

Group416: abra|OJJ68471.1 acar|OOF94213.1 acla|EAW13767.1 afis|EAW19791.1 afis|EAW22422.1 afla|EED45872.1 afum|EAL85100.2 afum|EAL90770.1 agla|OJJ87604.1 akaw|GAA92392.1 aluc|OJZ83252.1 anid|CBF88815.1 anig|EHA21105.1 aory|EIT75744.1 asyd|OJJ62890.1 ater|EAU36158.1 atub|OJI87685.1 awen|OJJ35001.1 azon|OJJ50741.1 acla|EAW07396.1 ater|EAU39285.1 anig|EHA21106.1 afla|EED52500.1 aory|EIT82777.1

Group417: abra|OJJ69765.1 afis|EAW19682.1 afla|EED54789.1 afum|EAL86840.1 agla|OJJ80273.1 akaw|GAA87888.1 aluc|OJZ85533.1 anid|CBF74487.1 anig|EHA25064.1 aory|EIT79365.1 asyd|OJJ57713.1 awen|OJJ37833.1 abra|OJJ73953.1 acar|OOG01081.1 akaw|GAA89558.1 aluc|OJZ88091.1 anig|EHA17861.1 asyd|OJJ58685.1 ater|EAU36023.1 atub|OJI90699.1 anid|CBF87757.1 aver|OJI97605.1 aory|EIT73667.1 asyd|OJJ63356.1

Group418: abra|OJJ70549.1 afla|EED53198.1 agla|OJJ89090.1 akaw|GAA83393.1 aluc|OJZ83893.1 asyd|OJJ55203.1 atub|OJI90260.1 aver|OJJ07013.1 abra|OJJ70550.1 acar|OOF97171.1 acla|EAW08341.1 afis|EAW23376.1 afla|EED55581.1 afum|EAL88095.1 agla|OJJ86447.1 anid|CBF74843.1 anig|EHA19029.1 asyd|OJJ60130.1 ater|EAU35630.1 atub|OJI90261.1 aver|OJJ04131.1 awen|OJJ39507.1 azon|OJJ47706.1 aory|EIT74938.1

Group419: agla|OJJ85379.1 asyd|OJJ59944.1 aver|OJJ02344.1 abra|OJJ77167.1 acar|OOF93057.1 acla|EAW12893.1 afis|EAW17769.1 afla|EED45780.1 afum|EAL91577.1 agla|OJJ81693.1 akaw|GAA90765.1 aluc|OJZ84580.1 anid|CBF86634.1 anig|EHA27873.1 asyd|OJJ64807.1 ater|EAU31848.1 atub|OJI89500.1 aver|OJI98920.1 awen|OJJ37072.1 acar|OOF91588.1 afla|EED45781.1 aory|EIT75674.1 afla|EED45782.1 aory|EIT75774.1

Group420: acar|OOF98107.1 akaw|GAA82924.1 anig|EHA20714.1 atub|OJI81150.1 abra|OJJ71031.1 acar|OOF98105.1 afis|EAW24984.1 afla|EED55473.1 afum|EBA27385.1 agla|OJJ82277.1 akaw|GAA82923.1 anid|CBF81625.1 aory|EIT80888.1 asyd|OJJ59528.1 ater|EAU35736.1 aver|OJJ02742.1 awen|OJJ40581.1 azon|OJJ49444.1 acla|EAW10584.1 afis|EAW24985.1 afum|EBA27386.1 anid|CBF81605.1 aory|EIT80797.1 aver|OJJ02751.1

Group421: abra|OJJ71639.1 acar|OOF96356.1 acla|EAW15180.1 afis|EAW20150.1 afla|EED57200.1 afum|EAL85264.1 agla|OJJ88784.1 akaw|GAA89286.1 akaw|GAA89287.1 aluc|OJZ86382.1 anid|CBF84986.1 anig|EHA21512.1 aory|EIT73993.1 asyd|OJJ63125.1 ater|EAU38765.1 atub|OJI84151.1 aver|OJI97830.1 awen|OJJ32023.1 azon|OJJ46067.1 acla|EAW15179.1 afis|EAW20149.1 anid|CBF84984.1 aory|EIT74054.1 afum|EAL85265.1

Group422: abra|OJJ71666.1 acar|OOF96376.1 acla|EAW15203.1 acla|EAW15204.1 afis|EAW20168.1 afis|EAW20169.1 afla|EED57180.1 afum|EAL85286.1 afum|EAL85287.1 agla|OJJ88761.1 akaw|GAA89266.1 akaw|GAA89267.1 aluc|OJZ86408.1 anid|CBF85015.1 anid|CBF85017.1 anig|EHA21491.1 aory|EIT74089.1 asyd|OJJ63104.1 ater|EAU38800.1 atub|OJI84123.1 aver|OJI97857.1 awen|OJJ32065.1 awen|OJJ32066.1 azon|OJJ46098.1

Group423: abra|OJJ72573.1 acar|OOF94686.1 akaw|GAA85199.1 aluc|OJZ90554.1 anig|EHA24001.1 atub|OJI81977.1 abra|OJJ77405.1 acar|OOF93271.1 acla|EAW13764.1 afis|EAW19795.1 afla|EED52177.1 afum|EAL85095.2 agla|OJJ83624.1 akaw|GAA91360.1 aluc|OJZ84812.1 anig|EHA28076.1 aory|EIT74472.1 asyd|OJJ54440.1 asyd|OJJ56546.1 ater|EAU36883.1 atub|OJI89273.1 aver|OJJ06176.1 awen|OJJ36480.1 azon|OJJ42299.1

Group424: abra|OJJ73313.1 acar|OOG00380.1 afis|EAW20348.1 afla|EED51166.1 agla|OJJ89330.1 akaw|GAA92810.1 aluc|OJZ88733.1 anid|CBF85228.1 anig|EHA18436.1 aory|EIT82315.1 asyd|OJJ62986.1 atub|OJI91309.1 aver|OJI97982.1 awen|OJJ29882.1 afis|EAW17164.1 anid|CBF76678.1 asyd|OJJ61222.1 aver|OJI96139.1 anid|CBF80144.1 aory|EIT81349.1 aver|OJI99535.1 afla|EED57011.1 asyd|OJJ61869.1 aver|OJI95967.1

Group425: abra|OJJ73412.1 acar|OOG00609.1 akaw|GAA83079.1 aluc|OJZ88625.1 anid|CBF80318.1 anig|EHA18317.1 asyd|OJJ57587.1 atub|OJI91210.1 aver|OJJ05595.1 awen|OJJ38061.1 abra|OJJ76670.1 acar|OOF99340.1 acla|EAW09094.1 afis|EAW25688.1 afla|EED55156.1 afum|EAL84357.1 agla|OJJ78885.1 akaw|GAA92522.1 aluc|OJZ91536.1 anig|EHA22573.1 aory|EIT81660.1 ater|EAU32946.1 atub|OJI85990.1 awen|OJJ41728.1

Group426: abra|OJJ74271.1 acar|OOF95459.1 acla|EAW07315.1 afis|EAW22331.1 afla|EED57752.1 afum|EAL90861.1 agla|OJJ87711.1 akaw|GAA86752.1 aluc|OJZ89862.1 anid|CBF88649.1 anig|EHA27062.1 aory|EIT81123.1 asyd|OJJ63884.1 ater|EAU39460.1 atub|OJI84946.1 aver|OJI97056.1 awen|OJJ34909.1 azon|OJJ45576.1 abra|OJJ74564.1 acar|OOF95196.1 akaw|GAA82037.1 aluc|OJZ92145.1 anig|EHA26804.1 atub|OJI86574.1

Group427: abra|OJJ74337.1 acar|OOF95390.1 acla|EAW07388.1 afis|EAW22412.1 afla|EED52492.1 afum|EAL90780.1 agla|OJJ80127.1 akaw|GAA86692.1 aluc|OJZ89793.1 anid|CBF88798.1 anig|EHA27006.1 aory|EIT82746.1 asyd|OJJ64791.1 ater|EAU39293.1 atub|OJI85013.1 aver|OJI98903.1 awen|OJJ34993.1 azon|OJJ45636.1 afis|EAW15417.1 afla|EED50613.1 afum|EAL84228.1 aory|EIT78052.1 ater|EAU37467.1 awen|OJJ32510.1

Group428: abra|OJJ76931.1 acar|OOF90090.1 acla|EAW13434.1 afis|EAW20550.1 afla|EED45551.1 afum|EAL84510.1 akaw|GAA87109.1 aluc|OJZ84329.1 anid|CBF73888.1 anig|EHA27663.1 anig|EHA27664.1 aory|EIT75407.1 asyd|OJJ53735.1 ater|EAU31070.1 atub|OJI89739.1 aver|OJJ07850.1 awen|OJJ33265.1 azon|OJJ50631.1 acar|OOF99064.1 acla|EAW11193.1 afis|EAW15923.1 afum|EAL88560.2 ater|EAU32036.1 awen|OJJ35657.1

Group429: abra|OJJ77163.1 acar|OOF93049.1 acla|EAW11721.1 afis|EAW23742.1 afla|EED45436.1 afum|EAL87179.1 agla|OJJ81702.1 akaw|GAA90769.1 aluc|OJZ84576.1 anid|CBF86622.1 anig|EHA27869.1 aory|EIT82382.1 asyd|OJJ65088.1 ater|EAU37973.1 atub|OJI89506.1 aver|OJI99214.1 awen|OJJ37083.1 azon|OJJ42895.1 anid|CBF87116.1 awen|OJJ35821.1 ater|EAU31855.1 asyd|OJJ64812.1 aver|OJI98925.1 anid|CBF71823.1

Group430: abra|OJJ65741.1 abra|OJJ71669.1 asyd|OJJ61538.1 asyd|OJJ63100.1 aver|OJI97862.1 aver|OJI98822.1 aver|OJJ00865.1 acar|OOF96381.1 acla|EAW15209.1 afis|EAW20174.1 afla|EED57174.1 afum|EAL85292.1 agla|OJJ88756.1 akaw|GAA89262.1 aluc|OJZ86412.1 anid|CBF85033.1 anig|EHA21487.1 aory|EIT74095.1 ater|EAU35210.1 atub|OJI84119.1 awen|OJJ41764.1 azon|OJJ46103.1 afla|EED54878.1

Group431: abra|OJJ65836.1 abra|OJJ67758.1 abra|OJJ72366.1 afum|EAL87737.1 afum|EAL88791.1 agla|OJJ79294.1 agla|OJJ80455.1 akaw|GAA87903.1 akaw|GAA92853.1 akaw|GAA92883.1 akaw|GAA93148.1 anig|EHA21873.1 anig|EHA26120.1 azon|OJJ46212.1 azon|OJJ47661.1 acar|OOF97545.1 aluc|OJZ80010.1 aluc|OJZ90353.1 anid|CBF79035.1 asyd|OJJ52047.1 atub|OJI81780.1 agla|OJJ84748.1 akaw|GAA86116.1

Group432: abra|OJJ65984.1 abra|OJJ67112.1 abra|OJJ73081.1 acar|OOF92889.1 acar|OOF98440.1 akaw|GAA88080.1 akaw|GAA90857.1 akaw|GAA91052.1 aluc|OJZ81343.1 aluc|OJZ82976.1 aluc|OJZ87863.1 anig|EHA18648.1 anig|EHA20169.1 anig|EHA21392.1 atub|OJI80785.1 atub|OJI89209.1 atub|OJI91548.1 azon|OJJ42157.1 azon|OJJ42333.1 azon|OJJ42440.1 azon|OJJ42744.1 anid|CBF71426.1 awen|OJJ31434.1

Group433: abra|OJJ66045.1 abra|OJJ73392.1 abra|OJJ77643.1 afla|EED46392.1 afla|EED49513.1 atub|OJI82691.1 atub|OJI87256.1 atub|OJI87448.1 atub|OJI91231.1 acar|OOF99092.1 agla|OJJ85351.1 akaw|GAA83097.1 akaw|GAA83991.1 aluc|OJZ80886.1 aluc|OJZ88644.1 anig|EHA18335.1 anig|EHA19451.1 aory|EIT72357.1 asyd|OJJ54995.1 aver|OJJ07206.1 awen|OJJ40082.1 afis|EAW17049.1 aver|OJI95758.1

Group434: abra|OJJ67714.1 abra|OJJ68753.1 akaw|GAA89213.1 akaw|GAA89897.1 aluc|OJZ81616.1 aluc|OJZ81774.1 anig|EHA22442.1 anig|EHA25827.1 atub|OJI84347.1 atub|OJI88567.1 acar|OOF92242.1 acla|EAW12498.1 afis|EAW24147.1 afla|EED46784.1 afum|EAL92002.1 agla|OJJ87227.1 anid|CBF77921.1 aory|EIT72760.1 asyd|OJJ55295.1 ater|EAU30579.1 aver|OJJ06931.1 awen|OJJ41332.1 azon|OJJ48802.1

Group435: abra|OJJ70999.1 abra|OJJ75834.1 acar|OOF98141.1 acla|EAW10556.1 afis|EAW25023.1 afla|EED55445.1 afum|EAL89672.1 agla|OJJ89191.1 akaw|GAA82949.1 aluc|OJZ87528.1 anid|CBF81543.1 anig|EHA20738.1 aory|EIT80817.1 asyd|OJJ59491.1 ater|EAU35764.1 atub|OJI81120.1 aver|OJJ02783.1 awen|OJJ40617.1 azon|OJJ49465.1 akaw|GAA84544.1 aluc|OJZ89277.1 anig|EHA23292.1 atub|OJI85522.1

Group436: abra|OJJ73227.1 abra|OJJ77180.1 acar|OOF91798.1 acar|OOG00465.1 agla|OJJ82802.1 agla|OJJ84567.1 asyd|OJJ52864.1 asyd|OJJ59702.1 acla|EAW06901.1 afis|EAW16518.1 afla|EED56153.1 afum|EAL89113.1 akaw|GAA87953.1 aluc|OJZ81201.1 anid|CBF75551.1 anig|EHA18516.1 aory|EIT76710.1 ater|EAU36584.1 atub|OJI91396.1 aver|OJJ02585.1 aver|OJJ08843.1 awen|OJJ40305.1 azon|OJJ49166.1

Group437: abra|OJJ75244.1 abra|OJJ75269.1 acar|OOF94534.1 acar|OOF94549.1 akaw|GAA91787.1 akaw|GAA91807.1 aluc|OJZ92806.1 aluc|OJZ92829.1 anig|EHA26180.1 anig|EHA26203.1 atub|OJI87249.1 atub|OJI87286.1 acla|EAW12239.1 afis|EAW16988.1 afla|EED48988.1 afum|EAL86512.1 agla|OJJ79828.1 anid|CBF87433.1 aory|EIT80045.1 ater|EAU31739.1 aver|OJJ08882.1 awen|OJJ35990.1 asyd|OJJ52913.1

Group438: abra|OJJ75609.1 abra|OJJ76068.1 akaw|GAA84359.1 akaw|GAA88899.1 aluc|OJZ89061.1 aluc|OJZ89522.1 anig|EHA23079.1 anig|EHA23477.1 atub|OJI85281.1 atub|OJI85725.1 acar|OOG00229.1 acla|EAW09371.1 afis|EAW21764.1 afla|EED46335.1 afum|EAL92794.1 agla|OJJ84387.1 anid|CBF83753.1 aory|EIT81195.1 asyd|OJJ60858.1 ater|EAU38451.1 aver|OJJ00182.1 awen|OJJ34047.1 azon|OJJ48184.1

Group439: acar|OOF90104.1 acar|OOF90211.1 acar|OOF90646.1 akaw|GAA86640.1 akaw|GAA87321.1 akaw|GAA88463.1 akaw|GAA90279.1 aluc|OJZ83061.1 aluc|OJZ88352.1 aluc|OJZ85197.1 anig|EHA18084.1 anig|EHA28127.1 atub|OJI80404.1 atub|OJI87885.1 atub|OJI90947.1 azon|OJJ42159.1 azon|OJJ48921.1 azon|OJJ43897.1 azon|OJJ46414.1 abra|OJJ73701.1 afla|EED57243.1 aory|EIT74140.1 awen|OJJ30907.1

Group440: acar|OOF90258.1 acar|OOF90411.1 acar|OOF94537.1 akaw|GAA90238.1 akaw|GAA91802.1 aluc|OJZ83102.1 aluc|OJZ92824.1 atub|OJI87281.1 atub|OJI87833.1 awen|OJJ33606.1 awen|OJJ39914.1 abra|OJJ75266.1 acla|EAW08995.1 afla|EED48390.1 afum|EAL91138.1 anig|EHA26183.1 aory|EIT83109.1 ater|EAU30261.1 ater|EAU37025.1 azon|OJJ45138.1 anig|EHA27462.1 ater|EAU34045.1 acar|OOF95569.1

Group441: acar|OOF94062.1 acar|OOF94132.1 abra|OJJ66364.1 acla|EAW08975.1 afis|EAW15454.1 afla|EED54752.1 afum|EAL84191.1 agla|OJJ80556.1 akaw|GAA90237.1 aluc|OJZ83103.1 anid|CBF80456.1 anig|EHA28167.1 aory|EIT78886.1 asyd|OJJ54768.1 ater|EAU32265.1 atub|OJI87832.1 aver|OJJ07399.1 awen|OJJ36431.1 azon|OJJ43598.1 atub|OJI80330.1 afla|EED48876.1 aory|EIT81202.1 awen|OJJ31672.1

Group442: acla|EAW10867.1 acla|EAW11076.1 afla|EED50345.1 afla|EED55539.1 afum|EAL84638.1 afum|EAL87765.1 aory|EIT77302.1 aory|EIT80884.1 asyd|OJJ54018.1 asyd|OJJ56874.1 abra|OJJ66629.1 acar|OOF91053.1 afis|EAW20991.1 agla|OJJ84069.1 akaw|GAA91096.1 aluc|OJZ81419.1 anid|CBF82616.1 anig|EHA26002.1 ater|EAU38640.1 atub|OJI88762.1 aver|OJJ08722.1 awen|OJJ34319.1 azon|OJJ48065.1

Group443: afis|EAW17232.1 afis|EAW21390.1 afum|EAL86652.1 afum|EAL92404.2 aory|EIT72927.1 aory|EIT76529.1 azon|OJJ48879.1 azon|OJJ50259.1 azon|OJJ51536.1 abra|OJJ65877.1 abra|OJJ76067.1 acar|OOF99863.1 acla|EAW09752.1 afla|EED48188.1 akaw|GAA88900.1 aluc|OJZ89521.1 anid|CBF83840.1 anig|EHA23080.1 asyd|OJJ60914.1 ater|EAU35174.1 atub|OJI85282.1 aver|OJJ00241.1 awen|OJJ30796.1

Group444: afis|EAW19359.1 afis|EAW20492.1 aver|OJJ07259.1 aver|OJJ07872.1 awen|OJJ33502.1 awen|OJJ34946.1 abra|OJJ66132.1 acar|OOF94018.1 acla|EAW14627.1 afla|EED56212.1 afum|EAL84562.1 agla|OJJ80511.1 akaw|GAA92689.1 aluc|OJZ90147.1 anid|CBF73880.1 anig|EHA20923.1 aory|EIT73321.1 asyd|OJJ64747.1 ater|EAU37645.1 atub|OJI84652.1 awen|OJJ38031.1 asyd|OJJ53765.1 asyd|OJJ54937.1

Group445: afis|EAW20796.1 afis|EAW22075.1 afum|EAL84833.1 afum|EAL91095.1 anid|CBF71369.1 anid|CBF82800.1 ater|EAU30149.1 ater|EAU32867.1 abra|OJJ72284.1 acar|OOF94937.1 afla|EED49547.1 agla|OJJ85016.1 akaw|GAA86036.1 aluc|OJZ90263.1 anig|EHA23759.1 aory|EIT73956.1 asyd|OJJ55081.1 asyd|OJJ57119.1 atub|OJI81689.1 aver|OJJ07127.1 awen|OJJ41908.1 azon|OJJ47364.1 afla|EED56697.1

Group446: afla|EED44969.1 afla|EED46569.1 afla|EED51147.1 aory|EIT73475.1 aory|EIT77864.1 aory|EIT81352.1 asyd|OJJ56870.1 asyd|OJJ61208.1 abra|OJJ75570.1 acar|OOF90888.1 acla|EAW09352.1

afis|EAW21817.1 afum|EAL92833.1 agla|OJJ84325.1 akaw|GAA84321.1 aluc|OJZ89018.1 anid|CBF76652.1 anig|EHA23512.1 ater|EAU38486.1 atub|OJI85771.1 aver|OJJ00532.1 awen|OJJ34091.1 azon|OJJ44255.1

Group447: afla|EED47292.1 afla|EED54844.1 agla|OJJ87461.1 agla|OJJ89110.1 aory|EIT79178.1 aory|EIT79336.1 asyd|OJJ60317.1 asyd|OJJ60627.1 asyd|OJJ62712.1 abra|OJJ75953.1 acar|OOF99959.1 acla|EAW09843.1 afis|EAW21292.1 afum|EAL92309.1 akaw|GAA84656.1 aluc|OJZ89402.1 anid|CBF83376.1 anig|EHA23185.1 ater|EAU35925.1 atub|OJI85395.1 aver|OJI99951.1 awen|OJJ39407.1 azon|OJJ44290.1

Group448: afla|EED48892.1 afla|EED55381.1 aory|EIT80798.1 aory|EIT81174.1 asyd|OJJ56534.1 asyd|OJJ64421.1 aver|OJI98520.1 aver|OJJ06188.1 abra|OJJ70928.1 acar|OOF98198.1 acla|EAW10494.1 afis|EAW25073.1 afum|EAL89742.1 agla|OJJ89119.1 akaw|GAA83014.1 aluc|OJZ87599.1 anid|CBF85962.1 anig|EHA20799.1 ater|EAU39552.1 atub|OJI81048.1 awen|OJJ40684.1 azon|OJJ44061.1 awen|OJJ34526.1

Group449: afla|EED54572.1 afla|EED56566.1 asyd|OJJ53770.1 asyd|OJJ62761.1 ater|EAU30266.1 ater|EAU39167.1 aver|OJI96804.1 aver|OJJ07878.1 abra|OJJ73591.1 acar|OOG00775.1 acla|EAW07510.1 afis|EAW22541.1 afum|EAL90653.1 agla|OJJ80900.1 akaw|GAA88561.1 aluc|OJZ88459.1 anid|CBF89038.1 anig|EHA18179.1 aory|EIT78798.1 aory|EIT79646.1 atub|OJI91057.1 awen|OJJ35170.1 azon|OJJ49937.1

Group450: agla|OJJ78857.1 agla|OJJ87323.1 abra|OJJ66001.1 acar|OOF92098.1 acla|EAW11077.1 afla|EED45932.1 akaw|GAA90873.1 aluc|OJZ82959.1 anid|CBF84691.1 anig|EHA21375.1 aory|EIT75815.1 asyd|OJJ53330.1 ater|EAU32160.1 atub|OJI89191.1 aver|OJJ08414.1 azon|OJJ46594.1 afla|EED53485.1 aory|EIT75146.1 asyd|OJJ61788.1 aver|OJI95887.1 ater|EAU32697.1 asyd|OJJ55442.1 ater|EAU35122.1

Group451: agla|OJJ81417.1 agla|OJJ84332.1 abra|OJJ77363.1 acar|OOF93232.1 acla|EAW13113.1 afis|EAW17551.1 afla|EED49274.1 afum|EAL91365.1 akaw|GAA91399.1 aluc|OJZ84772.1 anid|CBF82159.1 anig|EHA28040.1 aory|EIT82213.1 asyd|OJJ54334.1 ater|EAU36983.1 atub|OJI89309.1 awen|OJJ32551.1 azon|OJJ50995.1 afis|EAW17400.1 afla|EED46104.1 afum|EAL91238.1 anid|CBF79798.1 aory|EIT76423.1

Group452: akaw|GAA83965.1 akaw|GAA90739.1 aluc|OJZ80854.1 aluc|OJZ84609.1 abra|OJJ77200.1 acar|OOF93088.1 acla|EAW13191.1 afis|EAW17450.1 afla|EED49372.1 afum|EAL91278.2 agla|OJJ80362.1 akaw|GAA90738.1 aluc|OJZ84610.1 anid|CBF71683.1 anig|EHA27899.1 aory|EIT79587.1 asyd|OJJ61822.1 ater|EAU33930.1 atub|OJI89471.1 aver|OJI95922.1 awen|OJJ37396.1 azon|OJJ51068.1 anig|EHA27898.1

Group453: anid|CBF69431.1 anid|CBF73986.1 abra|OJJ66331.1 acar|OOG00530.1 acla|EAW09349.1 afis|EAW21804.1 afla|EED56853.1 afum|EAL92830.1 agla|OJJ79164.1 akaw|GAA90208.1 aluc|OJZ83137.1 anig|EHA28196.1 aory|EIT77503.1 asyd|OJJ65096.1 ater|EAU38484.1 atub|OJI87797.1 aver|OJI99221.1 azon|OJJ47555.1 aver|OJJ01938.1 afla|EED46583.1 aory|EIT77946.1 asyd|OJJ52385.1 agla|OJJ83529.1

Group454: anid|CBF73961.1 anid|CBF84419.1 asyd|OJJ53557.1 asyd|OJJ53743.1 asyd|OJJ57304.1 ater|EAU32191.1 ater|EAU37274.1 aver|OJJ05855.1 aver|OJJ07856.1 aver|OJJ08197.1 abra|OJJ66428.1 acla|EAW13742.1 afis|EAW19838.1 afla|EED45944.1 afum|EAL85055.2 agla|OJJ84458.1 akaw|GAA90293.1 aluc|OJZ83045.1 anig|EHA28114.1 aory|EIT75577.1 atub|OJI87903.1 awen|OJJ29654.1 azon|OJJ42315.1

Group455: aory|EIT75378.1 aory|EIT78539.1 awen|OJJ34056.1 awen|OJJ35494.1 azon|OJJ46995.1 azon|OJJ50589.1 abra|OJJ72842.1 acar|OOF96043.1 acla|EAW11400.1 afis|EAW24021.1 afla|EED45752.1 afla|EED49134.1 afla|EED49135.1 afum|EAL87451.1 agla|OJJ86954.1 akaw|GAA85444.1 aluc|OJZ83350.1 anid|CBF77424.1 anig|EHA24217.1 asyd|OJJ53230.1 ater|EAU29725.1 atub|OJI82243.1 aver|OJJ03622.1

Group456: asyd|OJJ51978.1 asyd|OJJ52250.1 asyd|OJJ56074.1 abra|OJJ76969.1 acar|OOF91414.1 acla|EAW13273.1 acla|EAW13494.1 afis|EAW20594.1 afla|EED47642.1 afum|EAL84467.1 agla|OJJ88662.1 akaw|GAA87075.1 aluc|OJZ84365.1 anid|CBF78279.1 anig|EHA27700.1 aory|EIT80239.1 ater|EAU35240.1 atub|OJI89703.1 aver|OJJ05017.1 awen|OJJ33318.1 azon|OJJ50690.1 afis|EAW17345.1 afum|EAL91189.2

Group457: asyd|OJJ52031.1 asyd|OJJ52140.1 abra|OJJ68385.1 acar|OOF93982.1 acla|EAW12147.1 afla|EED51420.1 afum|EAL86589.1 anid|CBF81959.1 aory|EIT80646.1 aver|OJJ05804.1 abra|OJJ68386.1 afis|EAW17129.1 akaw|GAA92560.1 aluc|OJZ90106.1 anig|EHA20964.1 ater|EAU31612.1 atub|OJI84696.1 awen|OJJ32473.1 azon|OJJ46830.1 afis|EAW25756.1 akaw|GAA93234.1 aluc|OJZ79642.1 asyd|OJJ57363.1

Group458: asyd|OJJ57402.1 asyd|OJJ58377.1 abra|OJJ75419.1 acar|OOF90764.1 acla|EAW13791.1 afis|EAW18341.1 afla|EED54057.1 afum|EAL87704.1 agla|OJJ88043.1 akaw|GAA84194.1 aluc|OJZ88871.1 anid|CBF79375.1 anig|EHA23633.1 asyd|OJJ57817.1 ater|EAU34676.1 atub|OJI85920.1 aver|OJJ04178.1 awen|OJJ35725.1 azon|OJJ42531.1 acar|OOF90590.1 ater|EAU35690.1 awen|OJJ36256.1 asyd|OJJ60091.1

Group459: asyd|OJJ59828.1 asyd|OJJ60302.1 asyd|OJJ60331.1 aver|OJI99642.1 aver|OJJ02459.1 aver|OJI99677.1 awen|OJJ29685.1 awen|OJJ30621.1 awen|OJJ36172.1 abra|OJJ77900.1 acar|OOF98884.1 acla|EAW11251.1 afis|EAW18246.1 afla|EED56217.1 afum|EAL87611.1 agla|OJJ85824.1 akaw|GAA83762.1 aluc|OJZ90969.1 anid|CBF87149.1 anig|EHA19671.1 aory|EIT73372.1 ater|EAU33739.1 atub|OJI82960.1

Group460: asyd|OJJ60480.1 asyd|OJJ61028.1 awen|OJJ32562.1 awen|OJJ36305.1 azon|OJJ43188.1 azon|OJJ47398.1 afum|EAL86366.1 abra|OJJ75145.1 akaw|GAA88750.1 aluc|OJZ92710.1 anig|EHA26289.1 acar|OOF98762.1 ater|EAU36669.1 acla|EAW09016.1 aver|OJI99800.1 afla|EED54373.1 aluc|OJZ81261.1 aory|EIT75853.1 ater|EAU36009.1 aver|OJJ07827.1 atub|OJI87136.1 ater|EAU33600.1 asyd|OJJ53703.1

Group461: ater|EAU29737.1 ater|EAU36828.1 abra|OJJ68505.1 acar|OOF90180.1 acar|OOF91789.1 acla|EAW11100.1 afis|EAW20763.1 afla|EED53200.1 afum|EAL84856.1 agla|OJJ84006.1 akaw|GAA92768.1 aluc|OJZ82717.1 anid|CBF80354.1 anig|EHA21171.1 aory|EIT75122.1 asyd|OJJ54828.1 atub|OJI88951.1 aver|OJJ07333.1 awen|OJJ29787.1 azon|OJJ42797.1 asyd|OJJ58563.1 aver|OJJ01162.1 azon|OJJ42773.1

Group462: atub|OJI83219.1 atub|OJI83220.1 abra|OJJ73330.1 afla|EED57225.1 agla|OJJ88240.1 akaw|GAA92790.1 anid|CBF74610.1 anig|EHA18401.1 aory|EIT73990.1 ater|EAU30210.1 atub|OJI91293.1 aver|OJJ05551.1 awen|OJJ36971.1 abra|OJJ78150.1 acar|OOF91819.1 agla|OJJ79110.1 akaw|GAA85875.1 aluc|OJZ91202.1 anig|EHA19907.1 asyd|OJJ55391.1 aver|OJJ08266.1 agla|OJJ78827.1 azon|OJJ42621.1

Group463: azon|OJJ45795.1 azon|OJJ48029.1 abra|OJJ67774.1 acla|EAW12350.1 afis|EAW16775.1 afla|EED45004.1 afum|EAL86344.1 agla|OJJ83713.1 aory|EIT73669.1 asyd|OJJ60273.1 ater|EAU32446.1 atub|OJI80491.1 aver|OJJ07654.1 awen|OJJ34537.1 acla|EAW13294.1 afla|EED47565.1 afum|EAL86272.1 aory|EIT74524.1 awen|OJJ35954.1 ater|EAU33716.1 aver|OJJ06720.1 afis|EAW20694.1 ater|EAU36512.1

Group464: abra|OJJ65849.1 acla|EAW08786.1 afis|EAW15938.1 aluc|OJZ80301.1 ater|EAU31058.1 atub|OJI87454.1 aver|OJI96479.1 awen|OJJ39979.1 abra|OJJ70264.1 agla|OJJ88746.1 akaw|GAA86533.1 aluc|OJZ85081.1 anig|EHA25296.1 aory|EIT73096.1 asyd|OJJ55484.1 atub|OJI80518.1 aver|OJJ06563.1 abra|OJJ70920.1 acar|OOF98204.1 akaw|GAA83020.1 aluc|OJZ87606.1 atub|OJI81042.1 asyd|OJJ52755.1

Group465: abra|OJJ66209.1 acar|OOF90651.1 acla|EAW06843.1 afis|EAW20271.1 afum|EAL85405.2 akaw|GAA89078.1 aluc|OJZ80513.1 anig|EHA25680.1 aory|EIT83312.1 asyd|OJJ60801.1 ater|EAU35068.1 atub|OJI88417.1 abra|OJJ67048.1 afla|EED52068.1 anig|EHA18595.1 azon|OJJ42510.1 afla|EED52437.1 aluc|OJZ90229.1 anig|EHA23731.1 ater|EAU37218.1 afla|EED52417.1 akaw|GAA86004.1 azon|OJJ50736.1

Group466: anid|CBF73652.1 abra|OJJ78129.1 acar|OOF90670.1 acla|EAW14637.1 afla|EED45059.1 akaw|GAA85878.1 aluc|OJZ91191.1 anid|CBF78296.1 anig|EHA19887.1 asyd|OJJ56106.1 atub|OJI83210.1 aver|OJJ05048.1 azon|OJJ45964.1 acla|EAW11632.1 asyd|OJJ52073.1 ater|EAU31025.1 atub|OJI91230.1 aver|OJJ03030.1 afla|EED45199.1 aory|EIT73607.1 asyd|OJJ59264.1 awen|OJJ35370.1 aver|OJI99658.1

Group467: abra|OJJ66772.1 acar|OOF93853.1 acla|EAW08992.1 afis|EAW15416.1 afla|EED48724.1 afum|EAL84229.2 agla|OJJ79941.1 anid|CBF81992.1 anig|EHA27337.1 aory|EIT73206.1 asyd|OJJ56162.1 ater|EAU32712.1 aver|OJJ05101.1 awen|OJJ39993.1 azon|OJJ42917.1 abra|OJJ75745.1 akaw|GAA84478.1 aluc|OJZ89198.1 anig|EHA23360.1 atub|OJI85592.1 afla|EED55174.1 aory|EIT81585.1 awen|OJJ36897.1

Group468: abra|OJJ67101.1 acar|OOF98451.1 afis|EAW20274.1 afla|EED57102.1 afum|EAL85408.1 agla|OJJ87503.1 akaw|GAA91060.1 aluc|OJZ87872.1 anid|CBF78242.1 anig|EHA20178.1 aory|EIT82822.1 asyd|OJJ56196.1 ater|EAU31785.1 atub|OJI80777.1 aver|OJJ05123.1 awen|OJJ35879.1 afla|EED48128.1 aory|EIT76261.1 agla|OJJ85549.1 asyd|OJJ61684.1 ater|EAU38792.1 aver|OJJ03092.1 awen|OJJ29992.1

Group469: abra|OJJ67813.1 acar|OOF89857.1 afis|EAW19368.1 afla|EED48200.1 afum|EAL94087.1 akaw|GAA87912.1 aluc|OJZ85623.1 anid|CBF87299.1 anig|EHA18476.1 aory|EIT72963.1 asyd|OJJ57229.1 ater|EAU30775.1 atub|OJI79416.1 aver|OJJ03060.1 awen|OJJ32619.1 agla|OJJ83617.1 afis|EAW24515.1 afla|EED47664.1 afum|EAL86181.1 aluc|OJZ88782.1 anid|CBF84276.1 aory|EIT80178.1 atub|OJI91354.1

Group470: abra|OJJ67869.1 acar|OOF92849.1 afla|EED51544.1 agla|OJJ78960.1 akaw|GAA91876.1 aluc|OJZ82023.1 anid|CBF79791.1 anig|EHA22232.1 aory|EIT80600.1 asyd|OJJ58125.1 ater|EAU31305.1 atub|OJI84576.1 aver|OJJ04281.1 awen|OJJ33687.1 abra|OJJ68892.1 acla|EAW11730.1 afis|EAW23762.1 afum|EAL87195.1 agla|OJJ82460.1 akaw|GAA90357.1 aluc|OJZ85875.1 anig|EHA24715.1 atub|OJI79661.1

Group471: abra|OJJ67925.1 acar|OOF92776.1 afis|EAW17948.1 afum|EAL91758.1 agla|OJJ89340.1 akaw|GAA91272.1 aluc|OJZ82797.1 anid|CBF80065.1 anig|EHA21238.1 ater|EAU31422.1 atub|OJI89028.1 aver|OJJ02091.1 awen|OJJ41595.1 azon|OJJ48310.1 abra|OJJ74125.1 acla|EAW14963.1 afla|EED48217.1 akaw|GAA89760.1 aluc|OJZ80623.1 anig|EHA27208.1 aory|EIT72959.1 asyd|OJJ58320.1 awen|OJJ31633.1

Group472: abra|OJJ67981.1 acla|EAW10293.1 afis|EAW16666.1 afla|EED50863.1 afum|EAL89270.1 agla|OJJ79452.1 akaw|GAA91221.1 aluc|OJZ82849.1 anid|CBF83064.1 anig|EHA21286.1 aory|EIT78981.1 asyd|OJJ53493.1 ater|EAU38052.1 atub|OJI89079.1 aver|OJJ08255.1 awen|OJJ41013.1 abra|OJJ74173.1 acar|OOF95553.1 akaw|GAA86843.1 aluc|OJZ89962.1 anig|EHA27162.1 atub|OJI84843.1 agla|OJJ89278.1

Group473: awen|OJJ41504.1 abra|OJJ71797.1 acar|OOF96477.1 acla|EAW13728.1 afis|EAW18033.1 afum|EAL91844.1 agla|OJJ85408.1 akaw|GAA85113.1 aluc|OJZ86527.1 anid|CBF81681.1 anig|EHA22119.1 aory|EIT81720.1 asyd|OJJ53625.1 asyd|OJJ58292.1 atub|OJI83995.1 aver|OJJ05942.1 awen|OJJ31297.1 azon|OJJ50337.1 acar|OOF92709.1 afla|EED51862.1 anid|CBF80076.1 ater|EAU31550.1 aver|OJJ02132.1

Group474: abra|OJJ68397.1 acar|OOF94205.1 acla|EAW12189.1 afis|EAW23755.1 afla|EED52086.1 afum|EAL87191.1 akaw|GAA92551.1 aluc|OJZ90099.1 anid|CBF84318.1 anig|EHA20976.1 aory|EIT76905.1 asyd|OJJ55649.1 ater|EAU29520.1 atub|OJI84707.1 aver|OJJ06734.1 awen|OJJ34639.1 acla|EAW14123.1 afla|EED45259.1 aory|EIT73569.1 ater|EAU38832.1 afis|EAW18834.1 afum|EAL93570.1 afis|EAW20404.1

Group475: abra|OJJ68432.1 acar|OOF93958.1 afis|EAW25721.1 afla|EED52101.1 afum|EAL84395.2 agla|OJJ87948.1 akaw|GAA86939.1 akaw|GAA86940.1 aluc|OJZ90065.1 anid|CBF82387.1 anig|EHA21009.1

aory|EIT76909.1 asyd|OJJ54134.1 ater|EAU30751.1 atub|OJI84742.1 aver|OJJ08601.1 awen|OJJ29682.1 afla|EED52100.1 anid|CBF82386.1 aory|EIT76914.1 afla|EED45119.1 aory|EIT73766.1 ater|EAU30752.1

Group476: abra|OJJ68512.1 akaw|GAA91334.1 aluc|OJZ82727.1 anig|EHA21180.1 atub|OJI88960.1 abra|OJJ69632.1 acar|OOF92677.1 acla|EAW10562.1 afis|EAW25029.1 afla|EED55452.1 afum|EAL89666.1 agla|OJJ79155.1 akaw|GAA91612.1 aluc|OJZ82439.1 anid|CBF81553.1 anig|EHA28334.1 aory|EIT80876.1 asyd|OJJ59496.1 ater|EAU35758.1 atub|OJI88002.1 aver|OJJ02778.1 awen|OJJ40610.1 azon|OJJ49459.1

Group477: afum|EAL94100.1 abra|OJJ73872.1 afla|EED45842.1 akaw|GAA89484.1 aluc|OJZ88178.1 anid|CBF80737.1 anig|EHA17930.1 aory|EIT75520.1 asyd|OJJ55544.1 ater|EAU35671.1 atub|OJI90777.1 aver|OJJ06622.1 awen|OJJ38075.1 abra|OJJ78163.1 akaw|GAA85862.1 aluc|OJZ91217.1 anig|EHA19923.1 asyd|OJJ61397.1 atub|OJI83235.1 afis|EAW20414.1 afum|EBA27371.1 ater|EAU29532.1 aver|OJJ00726.1

Group478: abra|OJJ72302.1 acar|OOF94922.1 acla|EAW10039.1 afis|EAW24476.1 afla|EED56937.1 afum|EAL86149.1 agla|OJJ86412.1 akaw|GAA86053.1 aluc|OJZ90283.1 anid|CBF78363.1 anig|EHA23773.1 aory|EIT77524.1 asyd|OJJ57323.1 ater|EAU29927.1 atub|OJI81707.1 aver|OJJ05837.1 awen|OJJ40788.1 acla|EAW12030.1 afis|EAW24671.1 afum|EAL89324.1 anig|EHA28157.1 azon|OJJ48190.1 awen|OJJ34571.1

Group479: abra|OJJ72891.1 acar|OOF96081.1 acla|EAW11359.1 afis|EAW18135.1 afla|EED58060.1 afum|EAL87492.1 akaw|GAA85478.1 aluc|OJZ83399.1 anid|CBF75378.1 anid|CBF84404.1 aory|EIT72894.1 aory|EIT79796.1 asyd|OJJ53570.1 asyd|OJJ59841.1 ater|EAU35458.1 atub|OJI82285.1 aver|OJJ02448.1 aver|OJJ08185.1 awen|OJJ35577.1 afla|EED56220.1 aory|EIT73370.1 afla|EED48228.1 ater|EAU33738.1

Group480: abra|OJJ73797.1 acar|OOG00952.1 acla|EAW07688.1 afis|EAW22703.1 afla|EED49989.1 afum|EAL90489.1 agla|OJJ79006.1 akaw|GAA89415.1 aluc|OJZ88258.1 anid|CBF88074.1 anig|EHA18003.1 aory|EIT73485.1 asyd|OJJ63555.1 ater|EAU38999.1 atub|OJI90857.1 aver|OJI97379.1 awen|OJJ35340.1 azon|OJJ49778.1 aory|EIT74604.1 afis|EAW21081.1 afum|EAL92102.1 agla|OJJ79284.1 afla|EED44920.1

Group481: abra|OJJ74536.1 acar|OOF96722.1 afla|EED46724.1 akaw|GAA82065.1 aluc|OJZ92117.1 anig|EHA26827.1 aory|EIT77932.1 asyd|OJJ61662.1 ater|EAU31119.1 atub|OJI86550.1 abra|OJJ78387.1 acar|OOF98501.1 acla|EAW09146.1 afis|EAW22000.1 afum|EAL93015.1 agla|OJJ84097.1 akaw|GAA85645.1 aluc|OJZ91466.1 anid|CBF82670.1 atub|OJI83459.1 aver|OJJ00983.1 awen|OJJ34296.1 azon|OJJ43342.1

Group482: abra|OJJ76072.1 acar|OOF99854.1 afis|EAW16839.1 afla|EED55812.1 afum|EAL86386.1 agla|OJJ80806.1 akaw|GAA88895.1 aluc|OJZ89526.1 anid|CBF73895.1 anig|EHA23075.1 aory|EIT76740.1 asyd|OJJ53659.1 ater|EAU36679.1 atub|OJI85277.1 aver|OJJ07767.1 abra|OJJ76505.1 akaw|GAA92900.1 anig|EHA22711.1 acar|OOF99003.1 agla|OJJ79991.1 awen|OJJ36307.1 acla|EAW12392.1 anig|EHA25400.1

Group483: abra|OJJ76831.1 acar|OOF91325.1 acla|EAW13393.1 afis|EAW16462.1 afla|EED45207.1 afum|EAL89058.1 agla|OJJ84801.1 akaw|GAA87191.1 aluc|OJZ84243.1 anid|CBF75808.1 anig|EHA27588.1 aory|EIT73549.1 asyd|OJJ55721.1 atub|OJI89840.1 aver|OJJ06807.1 awen|OJJ33206.1 azon|OJJ46480.1 awen|OJJ33434.1 acla|EAW06841.1 asyd|OJJ57540.1 aver|OJJ05638.1 afis|EAW20505.1 afum|EAL84551.1

Group484: abra|OJJ76940.1 acar|OOF91390.1 acla|EAW09287.1 afis|EAW21860.1 afla|EED45974.1 afum|EAL92873.1 agla|OJJ79533.1 akaw|GAA87099.1 aluc|OJZ84339.1 anid|CBF89674.1 anig|EHA27673.1 aory|EIT75604.1 asyd|OJJ62270.1 ater|EAU29774.1 atub|OJI89729.1 aver|OJI96379.1 awen|OJJ34144.1 azon|OJJ50245.1 afla|EED46450.1 agla|OJJ84275.1 aory|EIT72411.1 ater|EAU30859.1 azon|OJJ43482.1

Group485: abra|OJJ77386.1 acar|OOF93255.1 acla|EAW11088.1 afis|EAW20767.1 afla|EED50191.1 afum|EAL84852.1 akaw|GAA91375.1 aluc|OJZ84794.1 anid|CBF73862.1 anig|EHA28062.1 aory|EIT77384.1 asyd|OJJ56520.1 ater|EAU31812.1 atub|OJI89288.1 aver|OJJ06202.1 awen|OJJ33710.1 azon|OJJ50068.1 awen|OJJ34924.1 acla|EAW07300.1 aory|EIT79889.1 ater|EAU39485.1 asyd|OJJ57568.1 aver|OJJ05611.1

Group486: abra|OJJ78343.1 acar|OOF98537.1 acla|EAW13688.1 afis|EAW19912.1 afla|EED47242.1 afum|EAL84995.1 agla|OJJ84607.1 akaw|GAA85690.1 aluc|OJZ91415.1 anid|CBF87843.1 anig|EHA20088.1 aory|EIT79169.1 asyd|OJJ61361.1 ater|EAU31064.1 atub|OJI83410.1 aver|OJJ00688.1 awen|OJJ33622.1 azon|OJJ43388.1 afla|EED46677.1 anid|CBF84039.1 aory|EIT77968.1 awen|OJJ34250.1 agla|OJJ88201.1

Group487: abra|OJJ65482.1 abra|OJJ70272.1 awen|OJJ34689.1 awen|OJJ34690.1 acar|OOF93269.1 afum|EAL92101.1 agla|OJJ82948.1 aluc|OJZ82533.1 abra|OJJ68088.1 acla|EAW15262.1 afla|EED50507.1 akaw|GAA86873.1 aluc|OJZ89997.1 anig|EHA27372.1 ater|EAU33813.1 atub|OJI84811.1 afis|EAW21079.1 aory|EIT82101.1 acar|OOF90259.1 agla|OJJ86366.1 awen|OJJ32169.1 anig|EHA17942.1

Group488: abra|OJJ67142.1 abra|OJJ74374.1 acar|OOF95362.1 acar|OOF98414.1 akaw|GAA87274.1 akaw|GAA91027.1 aluc|OJZ87830.1 aluc|OJZ89747.1 atub|OJI80811.1 atub|OJI85055.1 acla|EAW13670.1 afis|EAW19949.1 afla|EED45542.1 afum|EAL84966.1 anid|CBF80394.1 anig|EHA20309.1 aory|EIT75592.1 asyd|OJJ54728.1 ater|EAU31211.1 aver|OJJ07440.1 azon|OJJ45120.1 anig|EHA26970.1

Group489: abra|OJJ68479.1 abra|OJJ69702.1 akaw|GAA88821.1 akaw|GAA92402.1 aluc|OJZ82505.1 aluc|OJZ83263.1 atub|OJI87674.1 atub|OJI87926.1 acar|OOF92670.1 acla|EAW15184.1 afis|EAW20154.1 afla|EED57195.1 afum|EAL85269.1 agla|OJJ88775.1 anid|CBF84996.1 anig|EHA28265.1 aory|EIT74166.1 asyd|OJJ63119.1 ater|EAU38769.1 aver|OJI97839.1 awen|OJJ32030.1 azon|OJJ46082.1

Group490: abra|OJJ69764.1 abra|OJJ76049.1 acla|EAW08772.1 acla|EAW09780.1 aluc|OJZ85534.1 aluc|OJZ89505.1 ater|EAU29246.1 ater|EAU35201.1 acar|OOF99880.1 afis|EAW21358.1 afla|EED55773.1 afum|EAL92373.1 agla|OJJ85477.1 akaw|GAA88916.1 anid|CBF76263.1 anig|EHA23095.1 aory|EIT76750.1 asyd|OJJ60971.1 atub|OJI85298.1 aver|OJJ00296.1 awen|OJJ39340.1 azon|OJJ51686.1

Group491: abra|OJJ74560.1 abra|OJJ78116.1 asyd|OJJ52502.1 asyd|OJJ54384.1 atub|OJI83194.1 atub|OJI86158.1 acla|EAW12888.1 afis|EAW17788.1 afum|EAL91597.1 acar|OOF98752.1 afla|EED48713.1 agla|OJJ82644.1 akaw|GAA85891.1 aluc|OJZ91181.1 aluc|OJZ92144.1 anid|CBF84655.1 anig|EHA26805.1 aory|EIT73117.1 ater|EAU33264.1 awen|OJJ36279.1 anig|EHA19872.1 anig|EHA19902.1

Group492: acar|OOF93735.1 acar|OOF93823.1 abra|OJJ66740.1 acla|EAW07107.1 afis|EAW19478.1 afla|EED56686.1 afum|EAL87018.1 agla|OJJ78581.1 akaw|GAA89678.1 aluc|OJZ80708.1 anid|CBF69805.1 anig|EHA27309.1 aory|EIT73937.1 asyd|OJJ58969.1 ater|EAU30765.1 atub|OJI87566.1 aver|OJJ01564.1 awen|OJJ40135.1 azon|OJJ45831.1 afis|EAW22119.1 agla|OJJ81206.1 afum|EAL91067.1

Group493: acar|OOF95045.1 acar|OOF95112.1 abra|OJJ73294.1 acla|EAW14608.1 afis|EAW19337.1 afum|EAL94055.1 agla|OJJ79423.1 akaw|GAA92709.1 aluc|OJZ88753.1 anid|CBF90107.1 anig|EHA18454.1 asyd|OJJ59230.1 ater|EAU30249.1 atub|OJI91323.1 aver|OJJ07884.1 awen|OJJ40776.1 afis|EAW24675.1 afla|EED54362.1 asyd|OJJ57396.1 ater|EAU31824.1 aver|OJJ01840.1 asyd|OJJ53780.1

Group494: acar|OOF95079.1 acar|OOF95144.1 afla|EED48018.1 afla|EED48886.1 anig|EHA24784.1 anig|EHA26744.1 aory|EIT81173.1 aory|EIT82914.1 awen|OJJ31657.1 awen|OJJ37608.1 abra|OJJ74633.1 acla|EAW14723.1 afis|EAW24259.1 afum|EAL85927.2 akaw|GAA81976.1 aluc|OJZ92213.1 asyd|OJJ62240.1 ater|EAU31205.1 atub|OJI86644.1 aver|OJI96349.1 awen|OJJ34660.1 azon|OJJ51810.1

Group495: acar|OOG00243.1 acar|OOG00261.1 acar|OOG00244.1 acar|OOG00262.1 abra|OJJ67538.1 acla|EAW06974.1 afis|EAW16588.1 afla|EED56077.1 afum|EAL89183.1 agla|OJJ78537.1 akaw|GAA88121.1 aluc|OJZ81394.1 anid|CBF78530.1 anig|EHA18687.1 aory|EIT76791.1 asyd|OJJ65303.1 atub|OJI91599.1 aver|OJI99441.1 awen|OJJ37035.1 azon|OJJ49092.1 awen|OJJ37036.1 awen|OJJ40227.1

Group496: acla|EAW12269.1 acla|EAW15163.1 agla|OJJ78609.1 agla|OJJ88803.1 ater|EAU31506.1 ater|EAU38748.1 aver|OJI97813.1 aver|OJI98043.1 abra|OJJ71622.1 acar|OOF96339.1 afis|EAW20132.1 afla|EED57220.1 afum|EAL85248.1 akaw|GAA89303.1 aluc|OJZ86365.1 anid|CBF84960.1 anig|EHA21527.1 aory|EIT74164.1 asyd|OJJ63141.1 atub|OJI84169.1 awen|OJJ31998.1 azon|OJJ50773.1

Group497: afis|EAW15552.1 afis|EAW16853.1 anid|CBF75924.1 anid|CBF80262.1 anid|CBF83068.1 abra|OJJ72655.1 akaw|GAA83700.1 aluc|OJZ91032.1 aory|EIT81911.1 ater|EAU36759.1 atub|OJI83029.1 aver|OJJ04021.1 awen|OJJ30091.1 ater|EAU37833.1 aluc|OJZ90302.1 agla|OJJ89297.1 afla|EED47474.1 awen|OJJ38684.1 agla|OJJ80033.1 asyd|OJJ61018.1 ater|EAU29857.1 atub|OJI81728.1

Group498: afis|EAW16688.1 afis|EAW25102.1 ater|EAU31451.1 ater|EAU31645.1 ater|EAU39369.1 awen|OJJ29684.1 awen|OJJ39500.1 awen|OJJ40716.1 abra|OJJ70876.1 acar|OOF98244.1 acla|EAW10465.1 afla|EED55343.1 afum|EAL89773.1 agla|OJJ85448.1 akaw|GAA91739.1 aluc|OJZ87646.1 anid|CBF86019.1 anig|EHA20839.1 asyd|OJJ64452.1 atub|OJI80999.1 aver|OJI98555.1 azon|OJJ42298.1

Group499: afis|EAW17401.1 afis|EAW23311.1 agla|OJJ82761.1 agla|OJJ86536.1 asyd|OJJ52975.1 asyd|OJJ54697.1 asyd|OJJ62410.1 aver|OJI96543.1 aver|OJJ08941.1 abra|OJJ75086.1 acar|OOF97247.1 acla|EAW08278.1 afla|EED55666.1 afum|EAL88166.1 akaw|GAA88698.1 aluc|OJZ92652.1 anid|CBF89498.1 aory|EIT81808.1 ater|EAU35550.1 atub|OJI87083.1 awen|OJJ39656.1 azon|OJJ47784.1

Group500: afis|EAW18163.1 afis|EAW21088.1 afum|EAL87523.1 afum|EAL92111.1 abra|OJJ77694.1 acar|OOF99045.1 acla|EAW11332.1 afla|EED56330.1 agla|OJJ85962.1 akaw|GAA83936.1 aluc|OJZ90757.1 anid|CBF85753.1 anig|EHA19494.1 aory|EIT73367.1 asyd|OJJ64297.1 ater|EAU33690.1 atub|OJI82744.1 aver|OJI98390.1 awen|OJJ30508.1 azon|OJJ50317.1 acla|EAW10845.1 acla|EAW13632.1

Group501: afla|EED45701.1 afla|EED55177.1 aory|EIT75733.1 aory|EIT81570.1 ater|EAU31908.1 ater|EAU32716.1 azon|OJJ50388.1 azon|OJJ50758.1 abra|OJJ68251.1 acar|OOF97530.1 acla|EAW09063.1 afis|EAW25635.1 afum|EAL84323.1 agla|OJJ78897.1 akaw|GAA83210.1 aluc|OJZ83712.1 anid|CBF83153.1 anig|EHA18863.1 asyd|OJJ55515.1 atub|OJI90087.1 aver|OJJ06592.1 awen|OJJ36092.1

Group502: afla|EED49422.1 afla|EED56716.1 afla|EED53576.1 aory|EIT73869.1 aory|EIT74843.1 aory|EIT79474.1 abra|OJJ66759.1 acar|OOF93842.1 acla|EAW11885.1 afis|EAW19497.1 afum|EAL86996.1 agla|OJJ81661.1 akaw|GAA89696.1 aluc|OJZ80689.1 anid|CBF75434.1 anig|EHA27326.1 asyd|OJJ59785.1 ater|EAU34809.1 atub|OJI87548.1 aver|OJJ02503.1 awen|OJJ36914.1 azon|OJJ42897.1

Group503: afla|EED49854.1 afla|EED57953.1 asyd|OJJ60093.1 asyd|OJJ60346.1 ater|EAU29423.1 ater|EAU34802.1 aver|OJI97568.1 aver|OJI99687.1 aver|OJJ03906.1 aver|OJJ04180.1 aver|OJI98275.1 aver|OJJ03018.1 acla|EAW11879.1 afis|EAW19490.1 afla|EED45383.1 afum|EAL87004.1 anid|CBF87414.1 aory|EIT82446.1 aory|EIT83413.1 asyd|OJJ65387.1 awen|OJJ36906.1 azon|OJJ47353.1

Group504: afla|EED52027.1 afla|EED55465.1 aory|EIT80878.1 aory|EIT81484.1 awen|OJJ35115.1 awen|OJJ40599.1 abra|OJJ77085.1 acar|OOF92969.1 acla|EAW10573.1 afis|EAW24994.1 afum|EAL89655.1

agla|OJJ82261.1 akaw|GAA86970.1 aluc|OJZ84494.1 anid|CBF81588.1 anig|EHA27797.1 asyd|OJJ59513.1 ater|EAU35745.1 atub|OJI89586.1 aver|OJJ02760.1 azon|OJJ49449.1 azon|OJJ49978.1

Group505: afla|EED54894.1 afla|EED55162.1 aory|EIT79416.1 aory|EIT81659.1 abra|OJJ69688.1 acar|OOF93996.1 afis|EAW25764.1 akaw|GAA88812.1 aluc|OJZ82495.1 anig|EHA28277.1 aory|EIT79352.1 asyd|OJJ53577.1 atub|OJI87936.1 aver|OJJ08180.1 awen|OJJ29839.1 azon|OJJ45270.1 acla|EAW12187.1 afis|EAW17124.1 afum|EAL86585.1 anid|CBF83965.1 asyd|OJJ61426.1 aver|OJJ00760.1

Group506: afum|EAL86746.1 afum|EAL86767.1 afum|EAL90935.1 afum|EAL91121.1 awen|OJJ29553.1 awen|OJJ29590.1 awen|OJJ29640.1 abra|OJJ65612.1 afis|EAW15508.1 agla|OJJ81552.1 aver|OJJ03119.1 abra|OJJ66326.1 afis|EAW20713.1 afla|EED52357.1 agla|OJJ80478.1 akaw|GAA87890.1 anid|CBF84445.1 anig|EHA21856.1 asyd|OJJ52037.1 azon|OJJ42065.1 asyd|OJJ62703.1 asyd|OJJ51872.1

Group507: agla|OJJ78579.1 agla|OJJ86328.1 agla|OJJ87482.1 agla|OJJ87483.1 azon|OJJ42717.1 azon|OJJ45989.1 abra|OJJ77701.1 acar|OOF99039.1 acla|EAW09904.1 afis|EAW17120.1 afla|EED48004.1 akaw|GAA83929.1 aluc|OJZ90766.1 anig|EHA19500.1 aory|EIT82898.1 asyd|OJJ57236.1 ater|EAU33755.1 atub|OJI82754.1 aver|OJJ00941.1 awen|OJJ35399.1 anid|CBF87717.1 aver|OJJ03049.1

Group508: agla|OJJ79099.1 agla|OJJ80685.1 abra|OJJ65682.1 afis|EAW18187.1 afum|EAL87550.1 abra|OJJ67677.1 acar|OOF92161.1 afla|EED45129.1 agla|OJJ82590.1 akaw|GAA83877.1 aluc|OJZ81891.1 anig|EHA22351.1 ater|EAU32846.1 atub|OJI84444.1 afis|EAW17780.1 akaw|GAA89800.1 aver|OJJ08350.1 awen|OJJ31361.1 acla|EAW13858.1 anid|CBF83961.1 aory|EIT73733.1 ater|EAU34039.1

Group509: agla|OJJ79128.1 agla|OJJ84540.1 asyd|OJJ53810.1 asyd|OJJ58024.1 aver|OJJ04376.1 aver|OJJ07911.1 awen|OJJ31498.1 awen|OJJ36278.1 abra|OJJ68972.1 acar|OOF91549.1 acla|EAW14215.1 afis|EAW19066.1 afla|EED54432.1 afum|EAL93795.1 akaw|GAA90499.1 aluc|OJZ85961.1 anid|CBF79650.1 anig|EHA24642.1 aory|EIT75890.1 ater|EAU32114.1 atub|OJI79748.1 azon|OJJ46211.1

Group510: agla|OJJ81570.1 agla|OJJ84260.1 asyd|OJJ54207.1 asyd|OJJ61270.1 awen|OJJ30146.1 awen|OJJ34161.1 abra|OJJ78235.1 acar|OOF98625.1 acla|EAW09275.1 afis|EAW21872.1 afla|EED46436.1 afum|EAL92886.1 akaw|GAA85790.1 aluc|OJZ91309.1 anid|CBF76772.1 anig|EHA19993.1 aory|EIT72312.1 ater|EAU30870.1 atub|OJI83302.1 aver|OJJ00599.1 aver|OJJ01907.1 azon|OJJ43468.1

Group511: agla|OJJ82328.1 agla|OJJ89180.1 ater|EAU35772.1 ater|EAU39458.1 atub|OJI81101.1 atub|OJI87185.1 awen|OJJ30919.1 awen|OJJ40626.1 abra|OJJ70986.1 acar|OOF98145.1 acla|EAW10546.1 afis|EAW25014.1 afla|EED55435.1 afum|EAL89682.1 akaw|GAA82963.1 aluc|OJZ87544.1 anid|CBF81525.1 anig|EHA20748.1 aory|EIT80926.1 asyd|OJJ59480.1 aver|OJJ02794.1 azon|OJJ49057.1

Group512: agla|OJJ83608.1 agla|OJJ87442.1 anig|EHA21812.1 anig|EHA23178.1 ater|EAU35930.1 ater|EAU39415.1 awen|OJJ36034.1 awen|OJJ39401.1 abra|OJJ75961.1 acar|OOF99954.1 acla|EAW09837.1 afis|EAW21297.1 afla|EED54848.1 afum|EAL92314.1 akaw|GAA84661.1 aluc|OJZ89409.1 anid|CBF83385.1 aory|EIT79420.1 asyd|OJJ60632.1 atub|OJI85387.1 aver|OJI99955.1 azon|OJJ44285.1

Group513: akaw|GAA83114.1 akaw|GAA92362.1 aluc|OJZ79905.1 aluc|OJZ81127.1 aluc|OJZ88669.1 aluc|OJZ88850.1 abra|OJJ73371.1 acar|OOG00577.1 acla|EAW06992.1 afis|EAW16606.1 afla|EED45025.1 afum|EAL89203.1 agla|OJJ82647.1 anid|CBF81913.1 anig|EHA18352.1 aory|EIT73821.1 asyd|OJJ59694.1 ater|EAU36688.1 atub|OJI91251.1 aver|OJJ02593.1 awen|OJJ40200.1 azon|OJJ49067.1

Group514: akaw|GAA89013.1 akaw|GAA93063.1 akaw|GAA93173.1 aluc|OJZ79783.1 aluc|OJZ79855.1 aluc|OJZ80033.1 aluc|OJZ80379.1 acar|OOF93307.1 acla|EAW11112.1 afis|EAW25575.1 afla|EED45095.1 afum|EAL84285.2 agla|OJJ78554.1 anid|CBF80123.1 anig|EHA17692.1 aory|EIT73824.1 asyd|OJJ58342.1 ater|EAU31353.1 aver|OJJ02069.1 awen|OJJ38810.1 agla|OJJ85585.1 awen|OJJ40900.1

Group515: asyd|OJJ54189.1 asyd|OJJ55024.1 ater|EAU32854.1 ater|EAU36887.1 aver|OJJ07182.1 aver|OJJ08550.1 abra|OJJ69557.1 acar|OOF91593.1 acla|EAW12881.1 afis|EAW17796.1 afla|EED57033.1 afum|EAL91604.2 agla|OJJ89112.1 akaw|GAA91566.1 aluc|OJZ82384.1 anid|CBF71395.1 anig|EHA28379.1 aory|EIT82249.1 atub|OJI88056.1 awen|OJJ41608.1 azon|OJJ48327.1 agla|OJJ84736.1

Group516: asyd|OJJ54603.1 asyd|OJJ56125.1 asyd|OJJ60498.1 aver|OJI99822.1 aver|OJJ05067.1 aver|OJJ07552.1 abra|OJJ67246.1 acar|OOF98311.1 acla|EAW08524.1 afis|EAW23563.1 afum|EAL87905.1 agla|OJJ80849.1 akaw|GAA91666.1 aluc|OJZ87731.1 anid|CBF69379.1 anig|EHA20223.1 aory|EIT74378.1 ater|EAU37656.1 atub|OJI80909.1 awen|OJJ30069.1 awen|OJJ37809.1 anid|CBF78333.1

Group517: asyd|OJJ55069.1 asyd|OJJ55250.1 ater|EAU31464.1 ater|EAU32149.1 aver|OJJ06973.1 aver|OJJ07142.1 abra|OJJ77233.1 acar|OOF93115.1 acla|EAW12666.1 afis|EAW17830.1 afla|EED46172.1 afum|EAL91640.1 agla|OJJ80651.1 akaw|GAA90709.1 aluc|OJZ84643.1 anid|CBF77996.1 anig|EHA27929.1 aory|EIT74935.1 atub|OJI89438.1 awen|OJJ41759.1 azon|OJJ48451.1 afla|EED53219.1

Group518: aver|OJJ00309.1 aver|OJJ08547.1 abra|OJJ66043.1 afis|EAW24627.1 afum|EAL86253.1 abra|OJJ69961.1 acar|OOF99004.1 acla|EAW11755.1 afla|EED51597.1 agla|OJJ78815.1 akaw|GAA87698.1 aluc|OJZ85322.1 anid|CBF76281.1 anig|EHA25512.1 aory|EIT80452.1 asyd|OJJ60989.1 ater|EAU31328.1 atub|OJI80251.1 awen|OJJ31328.1 afis|EAW23788.1 afum|EAL87222.1 awen|OJJ33732.1

Group519: awen|OJJ30937.1 awen|OJJ35649.1 acar|OOF98703.1 afla|EED50782.1 aory|EIT78014.1 abra|OJJ75482.1 afis|EAW24629.1 agla|OJJ82330.1 anid|CBF87140.1 asyd|OJJ53426.1 ater|EAU36583.1 aver|OJJ08319.1 acar|OOF93299.1 anid|CBF82039.1 asyd|OJJ54222.1 aver|OJJ05174.1 anig|EHA20951.1 atub|OJI84677.1 acar|OOF99300.1 ater|EAU34863.1 ater|EAU32944.1 awen|OJJ32536.1

Group520: azon|OJJ42806.1 azon|OJJ43585.1 asyd|OJJ55440.1 abra|OJJ73119.1 acla|EAW13712.1 afum|EAL91905.2 aluc|OJZ80612.1 asyd|OJJ57580.1 aver|OJJ05600.1 abra|OJJ75285.1 acar|OOF91874.1 afla|EED47775.1 akaw|GAA91823.1 aluc|OJZ92846.1 anig|EHA26164.1 atub|OJI87302.1 awen|OJJ38175.1 akaw|GAA85218.1 aver|OJJ06515.1 afis|EAW18097.1 aory|EIT77197.1 aory|EIT77132.1

Group521: abra|OJJ65490.1 anig|EHA18426.1 atub|OJI91620.1 anid|CBF78136.1 abra|OJJ70409.1 acla|EAW12074.1 afis|EAW19715.1 afla|EED47481.1 afum|EAL86812.1 agla|OJJ83008.1 akaw|GAA91946.1 aluc|OJZ82672.1 anid|CBF82263.1 anig|EHA21039.1 aory|EIT79956.1 asyd|OJJ52475.1 atub|OJI88908.1 aver|OJJ03940.1 awen|OJJ33572.1 acar|OOF90658.1 afla|EED47480.1 aory|EIT79950.1

Group522: abra|OJJ65676.1 acar|OOF97626.1 afla|EED58063.1 agla|OJJ89300.1 aluc|OJZ79543.1 aory|EIT79786.1 ater|EAU33067.1 aver|OJJ03420.1 awen|OJJ35949.1 azon|OJJ46841.1 abra|OJJ72223.1 acar|OOF96846.1 afum|EAL86781.1 agla|OJJ85961.1 akaw|GAA84714.1 aluc|OJZ86973.1 anid|CBF77158.1 anig|EHA21675.1 asyd|OJJ56903.1 atub|OJI83548.1 aver|OJJ03390.1 awen|OJJ36080.1

Group523: abra|OJJ65756.1 acla|EAW07338.1 afis|EAW19764.1 afla|EED46307.1 afum|EAL84477.1 agla|OJJ89164.1 anid|CBF85553.1 aory|EIT76353.1 asyd|OJJ64152.1 ater|EAU36860.1 aver|OJI99267.1 abra|OJJ66793.1 acar|OOF93870.1 akaw|GAA86858.1 aluc|OJZ89981.1 anig|EHA27353.1 aory|EIT73825.1 atub|OJI84829.1 azon|OJJ50413.1 afum|EAL85138.1 aver|OJI98232.1 afla|EED44981.1

Group524: abra|OJJ65922.1 acar|OOF94983.1 afla|EED52093.1 aluc|OJZ90209.1 anig|EHA23706.1 atub|OJI81639.1 aver|OJJ07181.1 awen|OJJ29695.1 abra|OJJ68440.1 acar|OOF93953.1 afis|EAW19803.1 afla|EED52094.1 agla|OJJ87952.1 akaw|GAA86932.1 aluc|OJZ90057.1 anid|CBF82416.1 anig|EHA21014.1 aory|EIT76923.1 asyd|OJJ54150.1 ater|EAU30746.1 atub|OJI84750.1 aver|OJJ08591.1

Group525: asyd|OJJ65354.1 ater|EAU38218.1 awen|OJJ32091.1 azon|OJJ46668.1 abra|OJJ67699.1 afis|EAW16912.1 afla|EED51126.1 agla|OJJ81314.1 akaw|GAA89208.1 aluc|OJZ81621.1 anid|CBF84302.1 anig|EHA25822.1 aory|EIT81344.1 asyd|OJJ55578.1 atub|OJI88559.1 aver|OJJ06657.1 akaw|GAA90566.1 anid|CBF90346.1 ater|EAU37575.1 asyd|OJJ62616.1 aver|OJJ03451.1 aver|OJI99503.1

Group526: acla|EAW15302.1 anid|CBF84723.1 abra|OJJ76196.1 acar|OOF99748.1 afla|EED52713.1 agla|OJJ81243.1 akaw|GAA82138.1 aluc|OJZ92033.1 anig|EHA22966.1 aory|EIT78196.1 ater|EAU38283.1 atub|OJI86466.1 awen|OJJ32095.1 azon|OJJ46673.1 afis|EAW20227.1 afum|EAL85344.1 aver|OJJ08433.1 afis|EAW25742.1 afla|EED46022.1 aory|EIT75659.1 asyd|OJJ53309.1 awen|OJJ30032.1

Group527: abra|OJJ66248.1 acar|OOF90703.1 afis|EAW15427.1 afla|EED56771.1 afum|EAL84216.1 agla|OJJ83506.1 akaw|GAA89041.1 aluc|OJZ80554.1 anid|CBF78298.1 anig|EHA25643.1 aory|EIT73857.1 asyd|OJJ61726.1 ater|EAU33504.1 atub|OJI88377.1 aver|OJJ05045.1 awen|OJJ38047.1 atub|OJI82848.1 aver|OJJ05231.1 afla|EED52547.1 aory|EIT74224.1 asyd|OJJ56102.1 aluc|OJZ90847.1

Group528: abra|OJJ67067.1 acar|OOF90566.1 afis|EAW22299.1 afla|EED53134.1 afum|EAL91113.1 agla|OJJ82720.1 anid|CBF85181.1 anig|EHA18939.1 aory|EIT75127.1 ater|EAU32823.1 atub|OJI88353.1 aver|OJJ05787.1 awen|OJJ31414.1 abra|OJJ77976.1 akaw|GAA83682.1 aluc|OJZ91051.1 anig|EHA19748.1 atub|OJI83047.1 azon|OJJ42193.1 anid|CBF87385.1 akaw|GAA89027.1 aluc|OJZ80577.1

Group529: abra|OJJ67286.1 afla|EED54791.1 anid|CBF87758.1 anig|EHA25285.1 aory|EIT79425.1 asyd|OJJ63355.1 atub|OJI80526.1 aver|OJI97606.1 abra|OJJ70023.1 acar|OOF93529.1 afis|EAW15602.1 afla|EED50420.1 afum|EAL85731.1 agla|OJJ81554.1 akaw|GAA87638.1 aluc|OJZ85253.1 anid|CBF78955.1 anig|EHA25454.1 aory|EIT82173.1 ater|EAU32529.1 atub|OJI80315.1 awen|OJJ38274.1

Group530: abra|OJJ67324.1 acar|OOF92489.1 acla|EAW06411.1 afis|EAW18515.1 afla|EED52883.1 afum|EAL93210.1 agla|OJJ85106.1 akaw|GAA86492.1 aluc|OJZ85034.1 anid|CBF84636.1 anig|EHA25254.1 aory|EIT78633.1 atub|OJI80563.1 aver|OJJ08377.1 awen|OJJ31069.1 azon|OJJ50578.1 acar|OOF91935.1 afla|EED47190.1 akaw|GAA89092.1 anig|EHA25694.1 aory|EIT79183.1 atub|OJI88431.1

Group531: abra|OJJ67569.1 akaw|GAA89099.1 anig|EHA25701.1 aver|OJJ07705.1 abra|OJJ70824.1 acar|OOF91940.1 acla|EAW09022.1 afis|EAW17020.1 afla|EED57105.1 agla|OJJ89322.1 akaw|GAA89419.1 aluc|OJZ88254.1 anid|CBF86050.1 anig|EHA17999.1 aory|EIT74056.1 atub|OJI90852.1 awen|OJJ32256.1 azon|OJJ49710.1 acla|EAW10946.1 akaw|GAA89819.1 aluc|OJZ81871.1 aver|OJJ07786.1

Group532: abra|OJJ67571.1 acar|OOF91945.1 acla|EAW07104.1 afis|EAW17308.1 afla|EED57039.1 afum|EAL91143.1 agla|OJJ83702.1 akaw|GAA84673.1 aluc|OJZ87016.1 anid|CBF79774.1 aory|EIT77654.1 asyd|OJJ58117.1 atub|OJI88439.1 aver|OJJ04288.1 awen|OJJ30697.1 acar|OOG00400.1 afla|EED49713.1 agla|OJJ88113.1 asyd|OJJ58093.1 atub|OJI83503.1 asyd|OJJ53589.1 aver|OJJ08159.1

Group533: abra|OJJ67572.1 acar|OOF91946.1 afla|EED57712.1 agla|OJJ83703.1 akaw|GAA89101.1 anid|CBF79773.1 anig|EHA25703.1 aory|EIT79942.1 asyd|OJJ58116.1 ater|EAU33168.1 atub|OJI88440.1

aver|OJJ04289.1 awen|OJJ30698.1 abra|OJJ77771.1 acar|OOF93071.1 agla|OJJ86555.1 akaw|GAA83875.1 aluc|OJZ90821.1 anig|EHA19547.1 atub|OJI82809.1 azon|OJJ50365.1 azon|OJJ42498.1

Group534: abra|OJJ67769.1 acla|EAW13437.1 afis|EAW17522.1 agla|OJJ79313.1 aory|EIT83023.1 ater|EAU39522.1 awen|OJJ32751.1 azon|OJJ42601.1 abra|OJJ74180.1 acar|OOF95540.1 akaw|GAA86835.1 aluc|OJZ89954.1 anig|EHA27155.1 atub|OJI84852.1 abra|OJJ78121.1 akaw|GAA89008.1 aluc|OJZ80000.1 anig|EHA19877.1 atub|OJI83202.1 aver|OJI97603.1 azon|OJJ43572.1 awen|OJJ34683.1

Group535: abra|OJJ67872.1 acla|EAW11678.1 akaw|GAA91881.1 aluc|OJZ82018.1 anid|CBF73446.1 anig|EHA22238.1 atub|OJI84569.1 aver|OJJ08116.1 abra|OJJ69722.1 acar|OOF94194.1 afla|EED52162.1 akaw|GAA90175.1 aluc|OJZ83175.1 anig|EHA28249.1 aory|EIT74416.1 asyd|OJJ54298.1 atub|OJI87762.1 awen|OJJ40352.1 azon|OJJ42479.1 acar|OOF99262.1 acar|OOF99303.1 atub|OJI80754.1

Group536: abra|OJJ68322.1 acar|OOF97579.1 acla|EAW08933.1 afis|EAW15466.1 afla|EED51782.1 afum|EAL85856.1 akaw|GAA92073.1 aluc|OJZ83651.1 anid|CBF83126.1 anig|EHA18802.1 aory|EIT81734.1 asyd|OJJ55850.1 ater|EAU34052.1 atub|OJI90020.1 awen|OJJ29732.1 azon|OJJ48706.1 anig|EHA24765.1 afla|EED45870.1 aory|EIT75363.1 aver|OJJ04793.1 awen|OJJ38111.1 akaw|GAA83679.1

Group537: abra|OJJ68433.1 acar|OOF93957.1 afla|EED52099.1 agla|OJJ87949.1 akaw|GAA86938.1 aluc|OJZ90064.1 anid|CBF82384.1 anig|EHA21010.1 aory|EIT76917.1 asyd|OJJ54135.1 ater|EAU30750.1 atub|OJI84743.1 aver|OJJ08600.1 abra|OJJ68434.1 afla|EED52098.1 akaw|GAA86937.1 aluc|OJZ90063.1 anid|CBF82382.1 asyd|OJJ54136.1 atub|OJI84744.1 awen|OJJ29691.1 azon|OJJ48597.1

Group538: abra|OJJ68774.1 acar|OOF92259.1 acla|EAW12478.1 afla|EED46766.1 afum|EAL92024.1 agla|OJJ89513.1 akaw|GAA89917.1 aluc|OJZ81752.1 anid|CBF77878.1 anig|EHA22460.1 asyd|OJJ55313.1 ater|EAU30598.1 atub|OJI84323.1 aver|OJJ06913.1 awen|OJJ41310.1 azon|OJJ48783.1 azon|OJJ48784.1 acar|OOF92261.1 acla|EAW12477.1 agla|OJJ89512.1 akaw|GAA89918.1 ater|EAU30599.1

Group539: abra|OJJ69330.1 acar|OOF94397.1 acla|EAW14710.1 afis|EAW24245.1 afla|EED48029.1 afum|EAL85913.2 agla|OJJ79629.1 akaw|GAA88201.1 aluc|OJZ82162.1 anid|CBF86924.1 anig|EHA28570.1 aory|EIT83325.1 asyd|OJJ55608.1 ater|EAU32738.1 atub|OJI88275.1 aver|OJJ06683.1 awen|OJJ38017.1 azon|OJJ51787.1 asyd|OJJ53731.1 afis|EAW23670.1 afum|EAL87115.1 agla|OJJ81340.1

Group540: abra|OJJ70397.1 acar|OOF91879.1 acla|EAW07052.1 afis|EAW25614.1 afla|EED45236.1 agla|OJJ79504.1 akaw|GAA91961.1 aluc|OJZ82656.1 anig|EHA27417.1 aory|EIT73635.1 ater|EAU31421.1 atub|OJI88887.1 awen|OJJ37774.1 azon|OJJ44467.1 acar|OOF98993.1 agla|OJJ88672.1 asyd|OJJ54458.1 ater|EAU30186.1 aver|OJJ07698.1 awen|OJJ33156.1 afla|EED53590.1 aory|EIT75094.1

Group541: abra|OJJ70452.1 acar|OOF95031.1 acla|EAW08562.1 afis|EAW23604.1 afla|EED52317.1 afum|EAL86541.1 agla|OJJ83593.1 akaw|GAA90131.1 aluc|OJZ83218.1 anid|CBF81471.1 anig|EHA21076.1 aory|EIT74491.1 asyd|OJJ52470.1 atub|OJI87719.1 aver|OJJ03017.1 awen|OJJ36161.1 azon|OJJ45785.1 aver|OJJ03945.1 agla|OJJ79566.1 awen|OJJ39928.1 asyd|OJJ59447.1 aver|OJJ02825.1

Group542: abra|OJJ70674.1 acar|OOF97053.1 acla|EAW08090.1 afis|EAW23125.1 afla|EED48525.1 afum|EAL88355.1 agla|OJJ87945.1 akaw|GAA90006.1 aluc|OJZ84022.1 anid|CBF74410.1 anig|EHA19143.1 aory|EIT73109.1 asyd|OJJ57761.1 ater|EAU36253.1 atub|OJI90393.1 aver|OJJ05405.1 awen|OJJ36655.1 azon|OJJ47551.1 awen|OJJ33435.1 afla|EED47672.1 aory|EIT80214.1 ater|EAU37239.1

Group543: abra|OJJ71147.1 acar|OOF98008.1 acla|EAW10357.1 afis|EAW25237.1 afla|EED53704.1 afum|EAL89898.1 agla|OJJ89205.1 akaw|GAA82830.1 aluc|OJZ87391.1 anid|CBF85312.1 anig|EHA20620.1 aory|EIT75340.1 asyd|OJJ64005.1 ater|EAU34361.1 atub|OJI81256.1 aver|OJI98093.1 awen|OJJ40948.1 azon|OJJ44541.1 afis|EAW25236.1 afum|EAL89897.1 awen|OJJ40953.1 azon|OJJ44540.1

Group544: abra|OJJ72457.1 acar|OOF94781.1 acla|EAW12689.1 afis|EAW17853.1 afla|EED46207.1 afum|EAL91661.1 agla|OJJ83117.1 akaw|GAA86193.1 aluc|OJZ90443.1 anid|CBF77956.1 anig|EHA23907.1 aory|EIT76431.1 asyd|OJJ61702.1 ater|EAU33055.1 atub|OJI81872.1 aver|OJI95791.1 awen|OJJ41720.1 azon|OJJ48418.1 afla|EED45133.1 aory|EIT73615.1 awen|OJJ30412.1 agla|OJJ86835.1

Group545: abra|OJJ73099.1 acar|OOG00332.1 acla|EAW07049.1 afis|EAW16675.1 afla|EED49659.1 afum|EBA27373.1 agla|OJJ78688.1 akaw|GAA88064.1 aluc|OJZ81327.1 anid|CBF89880.1 anig|EHA18631.1 aory|EIT80967.1 asyd|OJJ54564.1 ater|EAU33734.1 atub|OJI91530.1 aver|OJJ07590.1 awen|OJJ40031.1 azon|OJJ50640.1 acla|EAW08376.1 afis|EAW23405.1 afum|EAL88065.2 ater|EAU37518.1

Group546: abra|OJJ74582.1 acar|OOF90133.1 acla|EAW13424.1 afis|EAW16727.1 afla|EED45977.1 afum|EAL86301.1 agla|OJJ78669.1 akaw|GAA82023.1 aluc|OJZ92160.1 anid|CBF85294.1 anig|EHA26789.1 aory|EIT75394.1 asyd|OJJ58681.1 ater|EAU38428.1 atub|OJI86588.1 aver|OJJ01293.1 azon|OJJ50620.1 acar|OOF98979.1 afla|EED51702.1 aory|EIT80478.1 aver|OJI99084.1 anid|CBF71192.1

Group547: abra|OJJ76094.1 acar|OOF99830.1 acar|OOF99831.1 acla|EAW09725.1 afis|EAW21416.1 afla|EED55842.1 afum|EAL92431.1 agla|OJJ85546.1 akaw|GAA88872.1 akaw|GAA88873.1 aluc|OJZ89550.1 anid|CBF83794.1 anig|EHA23056.1 aory|EIT76615.1 asyd|OJJ60885.1 ater|EAU35132.1 atub|OJI85255.1 atub|OJI85256.1 aver|OJJ00209.1 awen|OJJ39286.1 azon|OJJ51568.1 aory|EIT76662.1

Group548: abra|OJJ76170.1 acar|OOF99769.1 acla|EAW13698.1 afis|EAW21484.1 afla|EED55916.1 afum|EAL92504.1 agla|OJJ85638.1 akaw|GAA82116.1 aluc|OJZ92060.1 anid|CBF76494.1 anig|EHA22989.1 aory|EIT76674.1 asyd|OJJ61126.1 ater|EAU35054.1 atub|OJI86492.1 aver|OJJ00447.1 awen|OJJ39213.1 azon|OJJ51641.1 acla|EAW09655.1 afla|EED49764.1 aory|EIT77609.1 awen|OJJ29776.1

Group549: abra|OJJ66058.1 abra|OJJ74755.1 akaw|GAA83656.1 akaw|GAA93128.1 aluc|OJZ81105.1 aluc|OJZ92336.1 atub|OJI82473.1 atub|OJI86765.1 awen|OJJ33564.1 awen|OJJ36363.1 afla|EED47092.1 agla|OJJ80118.1 anig|EHA19264.1 ater|EAU30986.1 acar|OOF95748.1 anid|CBF84619.1 anig|EHA26635.1 aory|EIT72514.1 asyd|OJJ55640.1 aver|OJJ06723.1 aory|EIT72452.1

Group550: abra|OJJ68047.1 abra|OJJ70802.1 acar|OOF96908.1 acar|OOF96943.1 aory|EIT72863.1 aory|EIT81579.1 acla|EAW11122.1 afis|EAW15851.1 afla|EED56522.1 afum|EAL88487.1 agla|OJJ87257.1 akaw|GAA90118.1 aluc|OJZ84148.1 anid|CBF81176.1 anig|EHA21341.1 asyd|OJJ59292.1 ater|EAU31666.1 atub|OJI90520.1 aver|OJJ02983.1 awen|OJJ36776.1 azon|OJJ47422.1

Group551: abra|OJJ69621.1 abra|OJJ74815.1 abra|OJJ78091.1 afis|EAW21065.1 afis|EAW22323.1 afla|EED44992.1 afla|EED49898.1 akaw|GAA85057.1 akaw|GAA85907.1 aluc|OJZ86595.1 aluc|OJZ91160.1 anig|EHA19855.1 anig|EHA26580.1 aory|EIT72455.1 aory|EIT73686.1 atub|OJI83175.1 atub|OJI83928.1 acar|OOF98779.1 afum|EAL86268.1 aver|OJJ04473.1 awen|OJJ40160.1

Group552: abra|OJJ72113.1 abra|OJJ72712.1 anig|EHA21773.1 anig|EHA24109.1 atub|OJI82107.1 atub|OJI83652.1 acar|OOF96753.1 acla|EAW14914.1 afis|EAW24435.1 afla|EED55246.1 afum|EAL86103.1 agla|OJJ79250.1 akaw|GAA84809.1 aluc|OJZ86868.1 anid|CBF73765.1 aory|EIT83588.1 asyd|OJJ56590.1 ater|EAU30011.1 aver|OJJ06135.1 awen|OJJ32764.1 azon|OJJ43998.1

Group553: abra|OJJ73065.1 abra|OJJ73716.1 acar|OOG00294.1 acar|OOG00876.1 akaw|GAA88095.1 akaw|GAA88447.1 aluc|OJZ81361.1 aluc|OJZ88334.1 anig|EHA18070.1 anig|EHA18661.1 atub|OJI90930.1 atub|OJI91565.1 azon|OJJ45231.1 azon|OJJ46835.1 azon|OJJ51792.1 acla|EAW10825.1 afis|EAW24747.1 afla|EED47445.1 afum|EAL89394.1 aory|EIT81906.1 awen|OJJ29808.1

Group554: acar|OOF91686.1 acar|OOF91797.1 ater|EAU30834.1 ater|EAU37279.1 awen|OJJ37554.1 awen|OJJ37864.1 abra|OJJ66921.1 acla|EAW14487.1 afis|EAW19196.1 afla|EED47712.1 afum|EAL93928.1 agla|OJJ88511.1 akaw|GAA91498.1 aluc|OJZ85659.1 anid|CBF78550.1 anig|EHA24874.1 aory|EIT80197.1 asyd|OJJ65057.1 atub|OJI79452.1 aver|OJI99182.1 azon|OJJ51215.1

Group555: acar|OOF93691.1 acar|OOF93775.1 abra|OJJ66681.1 acla|EAW11818.1 afis|EAW19429.1 afla|EED56633.1 afum|EAL87065.1 agla|OJJ81141.1 akaw|GAA89626.1 aluc|OJZ80768.1 anid|CBF74384.1 anig|EHA27260.1 aory|EIT79639.1 asyd|OJJ57774.1 ater|EAU34732.1 atub|OJI87621.1 awen|OJJ36828.1 azon|OJJ42976.1 aver|OJJ05392.1 aver|OJJ05391.1 awen|OJJ36827.1

Group556: acar|OOF94034.1 acar|OOF94104.1 afis|EAW20233.1 afis|EAW24546.1 afum|EAL85350.1 afum|EAL86210.1 ater|EAU29965.1 ater|EAU37764.1 abra|OJJ66417.1 afla|EED55131.1 akaw|GAA90283.1 aluc|OJZ83057.1 anig|EHA28123.1 aory|EIT81603.1 asyd|OJJ56039.1 atub|OJI87890.1 aver|OJJ04985.1 awen|OJJ41447.1 azon|OJJ47374.1 abra|OJJ67779.1 ater|EAU37374.1

Group557: acar|OOG00254.1 acar|OOG01226.1 acar|OOG01227.1 abra|OJJ70128.1 acla|EAW13482.1 afis|EAW20590.1 afla|EED52238.1 afum|EAL84471.1 agla|OJJ88668.1 akaw|GAA86642.1 aluc|OJZ85199.1 anid|CBF85436.1 anig|EHA25406.1 aory|EIT74451.1 asyd|OJJ64088.1 ater|EAU35229.1 atub|OJI80402.1 aver|OJI98172.1 awen|OJJ36225.1 acar|OOF91412.1 awen|OJJ33308.1

Group558: acla|EAW09303.1 acla|EAW15329.1 afum|EAL85377.2 afum|EAL92855.1 asyd|OJJ53789.1 asyd|OJJ61241.1 abra|OJJ75519.1 acar|OOF90846.1 afis|EAW21843.1 afla|EED46527.1 agla|OJJ84291.1 akaw|GAA84283.1 aluc|OJZ88976.1 anid|CBF76715.1 anig|EHA23549.1 aory|EIT72431.1 ater|EAU38520.1 atub|OJI85813.1 awen|OJJ34127.1 azon|OJJ44216.1 azon|OJJ51717.1

Group559: acla|EAW09341.1 acla|EAW11685.1 afis|EAW21796.1 afis|EAW23700.1 afum|EAL87141.1 afum|EAL92821.1 abra|OJJ75576.1 acar|OOF90894.1 afla|EED46599.1 agla|OJJ84330.1 akaw|GAA84328.1 aluc|OJZ89025.1 anid|CBF76627.1 anig|EHA23507.1 aory|EIT77837.1 asyd|OJJ61195.1 ater|EAU38478.1 atub|OJI85765.1 aver|OJJ00519.1 awen|OJJ34081.1 azon|OJJ44259.1

Group560: afis|EAW16155.1 afis|EAW23200.1 afum|EAL88027.1 afum|EAL88283.1 ater|EAU36328.1 ater|EAU36668.1 abra|OJJ70513.1 acar|OOF97416.1 acla|EAW08162.1 afla|EED47054.1 agla|OJJ78748.1 akaw|GAA83360.1 aluc|OJZ83858.1 anid|CBF74719.1 anig|EHA18999.1 aory|EIT72491.1 asyd|OJJ60202.1 atub|OJI90225.1 aver|OJJ04064.1 awen|OJJ39430.1 azon|OJJ47634.1

Group561: afis|EAW17104.1 afis|EAW25308.1 agla|OJJ88961.1 agla|OJJ89287.1 abra|OJJ71251.1 acar|OOF97919.1 acla|EAW10284.1 afum|EAL89971.1 akaw|GAA82736.1 aluc|OJZ87291.1 anid|CBF75291.1 anig|EHA20532.1 aory|EIT78474.1 asyd|OJJ59887.1 ater|EAU34456.1 atub|OJI81350.1 aver|OJJ02399.1 awen|OJJ41022.1 azon|OJJ44609.1 asyd|OJJ65171.1 afla|EED53785.1

Group562: afis|EAW19712.1 afis|EAW25702.1 afum|EAL84375.1 afum|EAL86815.1 abra|OJJ77041.1 acar|OOF90274.1 afla|EED50904.1 akaw|GAA87011.1 aluc|OJZ84444.1 anig|EHA27762.1 aory|EIT79058.1

asyd|OJJ63378.1 ater|EAU32932.1 atub|OJI89630.1 aver|OJJ05795.1 awen|OJJ39868.1 anid|CBF81946.1 agla|OJJ84461.1 aver|OJI97557.1 ater|EAU34785.1 asyd|OJJ57373.1

Group563: afis|EAW22109.1 afis|EAW22582.1 afum|EAL90617.1 afum|EAL91075.1 abra|OJJ73636.1 acar|OOG00816.1 acla|EAW07548.1 afla|EED54627.1 agla|OJJ80942.1 akaw|GAA88519.1 aluc|OJZ88414.1 anid|CBF88345.1 anig|EHA18141.1 aory|EIT78908.1 asyd|OJJ63718.1 ater|EAU39122.1 atub|OJI91010.1 aver|OJI97222.1 awen|OJJ35210.1 azon|OJJ49899.1 anid|CBF89563.1

Group564: afla|EED44994.1 afla|EED49089.1 asyd|OJJ52855.1 asyd|OJJ55885.1 aver|OJJ04830.1 aver|OJJ08832.1 awen|OJJ36406.1 awen|OJJ39156.1 abra|OJJ70006.1 acar|OOF93512.1 acla|EAW15361.1 agla|OJJ83218.1 akaw|GAA87655.1 aluc|OJZ85273.1 anid|CBF78094.1 anig|EHA25471.1 aory|EIT82516.1 ater|EAU34442.1 atub|OJI80297.1 aory|EIT73678.1 aver|OJJ01999.1

Group565: afla|EED45878.1 afla|EED45984.1 afla|EED47653.1 afum|EAL84420.1 afum|EAL86318.1 aory|EIT75536.1 aory|EIT75748.1 aory|EIT80201.1 acar|OOF99716.1 acla|EAW12434.1 afis|EAW16743.1 akaw|GAA82399.1 aluc|OJZ91723.1 anid|CBF89574.1 anig|EHA22730.1 asyd|OJJ62337.1 ater|EAU30784.1 atub|OJI86180.1 aver|OJI96442.1 awen|OJJ29697.1 anid|CBF83502.1

Group566: afla|EED45981.1 afla|EED55382.1 aory|EIT75435.1 aory|EIT80896.1 ater|EAU33990.1 ater|EAU39553.1 abra|OJJ70929.1 acar|OOF98196.1 acla|EAW10495.1 afis|EAW25072.1 afum|EAL89741.1 agla|OJJ89122.1 akaw|GAA83013.1 aluc|OJZ87598.1 anid|CBF85960.1 anig|EHA20798.1 asyd|OJJ64420.1 atub|OJI81049.1 aver|OJI98519.1 awen|OJJ40683.1 azon|OJJ44060.1

Group567: afla|EED48911.1 afla|EED51260.1 aory|EIT81176.1 aory|EIT81506.1 awen|OJJ29671.1 awen|OJJ38491.1 abra|OJJ69199.1 acar|OOF91116.1 acla|EAW08647.1 afis|EAW15784.1 afum|EAL85566.1 agla|OJJ81947.1 akaw|GAA92212.1 aluc|OJZ86184.1 anid|CBF70950.1 anig|EHA24440.1 asyd|OJJ52622.1 ater|EAU32357.1 atub|OJI79976.1 aver|OJJ04649.1 azon|OJJ43792.1

Group568: afla|EED51201.1 afla|EED53566.1 asyd|OJJ62648.1 asyd|OJJ65376.1 aver|OJI96748.1 aver|OJI99544.1 abra|OJJ73167.1 acar|OOG00407.1 acla|EAW06285.1 afis|EAW22140.1 afum|EAL91050.1 agla|OJJ87935.1 akaw|GAA88004.1 aluc|OJZ81260.1 anid|CBF89140.1 anig|EHA18567.1 aory|EIT75205.1 ater|EAU34142.1 atub|OJI91456.1 awen|OJJ34714.1 azon|OJJ50465.1

Group569: afum|EAL87769.1 afum|EAL87803.1 akaw|GAA92345.1 akaw|GAA93055.1 akaw|GAA92650.1 akaw|GAA93090.1 awen|OJJ29538.1 awen|OJJ29541.1 abra|OJJ65531.1 acla|EAW13472.1 aluc|OJZ79518.1 anid|CBF73631.1 anig|EHA21825.1 abra|OJJ65574.1 agla|OJJ85769.1 abra|OJJ65598.1 ater|EAU30115.1 acar|OOF90498.1 ater|EAU30117.1 acla|EAW11437.1 anig|EHA21861.1

Group570: agla|OJJ78645.1 agla|OJJ81233.1 agla|OJJ83403.1 abra|OJJ71870.1 acar|OOF96539.1 acla|EAW13900.1 afis|EAW18612.1 afla|EED52733.1 afum|EAL93322.1 akaw|GAA85045.1 aluc|OJZ86609.1 anid|CBF70422.1 anig|EHA22057.1 aory|EIT78158.1 asyd|OJJ58619.1 ater|EAU34909.1 atub|OJI83917.1 aver|OJJ01226.1 awen|OJJ32073.1 azon|OJJ46654.1 agla|OJJ81232.1

Group571: agla|OJJ82854.1 agla|OJJ88960.1 abra|OJJ71795.1 acar|OOF96475.1 acla|EAW12077.1 afis|EAW19718.1 afla|EED52815.1 afum|EAL86808.1 akaw|GAA85115.1 aluc|OJZ86525.1 anid|CBF78801.1 anig|EHA22121.1 aory|EIT78250.1 asyd|OJJ55181.1 ater|EAU34844.1 atub|OJI83997.1 aver|OJJ07026.1 acar|OOG00120.1 anid|CBF84151.1 asyd|OJJ65073.1 aver|OJI99198.1

Group572: agla|OJJ83728.1 agla|OJJ88980.1 abra|OJJ65910.1 acar|OOF90095.1 afla|EED53605.1 afum|EAL88809.2 akaw|GAA92322.1 aluc|OJZ90220.1 anid|CBF70180.1 anig|EHA23718.1 aory|EIT75102.1 atub|OJI81652.1 aver|OJJ03066.1 abra|OJJ66145.1 afis|EAW16403.1 anid|CBF86674.1 aver|OJJ05884.1 awen|OJJ29890.1 afis|EAW16176.1 aver|OJJ01772.1 asyd|OJJ52559.1

Group573: akaw|GAA82012.1 akaw|GAA83300.1 anig|EHA23471.1 anig|EHA26778.1 azon|OJJ46821.1 azon|OJJ49225.1 abra|OJJ74596.1 acar|OOF95173.1 acla|EAW10785.1 afis|EAW24793.1 afla|EED47193.1 afum|EAL89435.1 agla|OJJ82887.1 aluc|OJZ92171.1 anid|CBF81462.1 aory|EIT79250.1 asyd|OJJ59441.1 ater|EAU36515.1 atub|OJI86603.1 aver|OJJ02832.1 awen|OJJ40374.1

Group574: aluc|OJZ79634.1 aluc|OJZ79637.1 aluc|OJZ79681.1 aluc|OJZ90921.1 abra|OJJ77846.1 acar|OOF98926.1 acla|EAW11277.1 afis|EAW18223.1 afla|EED56246.1 afum|EAL87589.2 agla|OJJ85859.1 akaw|GAA83801.1 anid|CBF86151.1 anig|EHA19633.1 aory|EIT73368.1 asyd|OJJ64517.1 ater|EAU33768.1 atub|OJI82913.1 aver|OJI98615.1 awen|OJJ30601.1 azon|OJJ50167.1

Group575: aluc|OJZ79645.1 aluc|OJZ80467.1 anid|CBF81159.1 anid|CBF87224.1 anid|CBF82244.1 agla|OJJ86031.1 akaw|GAA88990.1 awen|OJJ36065.1 abra|OJJ70212.1 afla|EED52528.1 akaw|GAA86564.1 anig|EHA25334.1 aory|EIT82764.1 atub|OJI80480.1 acar|OOG00877.1 asyd|OJJ57261.1 aver|OJJ03035.1 azon|OJJ47412.1 awen|OJJ30459.1 azon|OJJ45449.1 aluc|OJZ85114.1

Group576: aluc|OJZ80315.1 aluc|OJZ86416.1 anid|CBF76119.1 anid|CBF85040.1 atub|OJI80382.1 atub|OJI84115.1 abra|OJJ71674.1 acar|OOF96385.1 acla|EAW15213.1 afis|EAW20178.1 afla|EED57170.1 afum|EAL85296.1 agla|OJJ84688.1 akaw|GAA89258.1 anig|EHA21483.1 aory|EIT74051.1 asyd|OJJ63096.1 ater|EAU35214.1 aver|OJI97866.1 awen|OJJ31177.1 azon|OJJ46108.1

Group577: aluc|OJZ87121.1 aluc|OJZ90552.1 anig|EHA20385.1 anig|EHA23999.1 atub|OJI81520.1 atub|OJI81975.1 awen|OJJ29935.1 awen|OJJ40118.1 awen|OJJ31429.1 awen|OJJ33906.1 abra|OJJ70173.1 acar|OOF90667.1 afla|EED52274.1 akaw|GAA82584.1 asyd|OJJ53431.1 aver|OJJ04439.1 azon|OJJ42631.1 abra|OJJ72571.1 aory|EIT80573.1 afla|EED51481.1 aory|EIT83049.1

Group578: aluc|OJZ89485.1 aluc|OJZ91533.1 asyd|OJJ52882.1 asyd|OJJ54759.1 atub|OJI85315.1 atub|OJI85987.1 abra|OJJ76672.1 acar|OOF99337.1 afla|EED47468.1 akaw|GAA92520.1 anid|CBF87401.1 anig|EHA18623.1 aory|EIT79967.1 ater|EAU31454.1 aver|OJJ08854.1 awen|OJJ38083.1 agla|OJJ86847.1 ater|EAU36924.1 awen|OJJ41649.1 aver|OJJ00789.1 aver|OJJ07411.1

Group579: anid|CBF70118.1 anid|CBF76209.1 awen|OJJ38751.1 awen|OJJ40905.1 abra|OJJ66448.1 acla|EAW11664.1 afis|EAW15397.1 agla|OJJ79144.1 asyd|OJJ56278.1 ater|EAU32821.1 aver|OJJ08485.1 awen|OJJ37492.1 abra|OJJ67824.1 acar|OOF93612.1 akaw|GAA91839.1 anig|EHA22193.1 atub|OJI84618.1 acla|EAW11665.1 awen|OJJ38753.1 aver|OJJ05914.1 aluc|OJZ82059.1

Group580: anid|CBF71344.1 anid|CBF71770.1 asyd|OJJ55097.1 asyd|OJJ55529.1 aver|OJJ07109.1 aver|OJJ07313.1 abra|OJJ72319.1 acar|OOF94892.1 acla|EAW12001.1 afis|EAW19641.1 afla|EED56992.1 afum|EAL86881.1 agla|OJJ80783.1 akaw|GAA86071.1 aluc|OJZ90306.1 anig|EHA23792.1 aory|EIT77578.1 ater|EAU32888.1 atub|OJI81730.1 awen|OJJ41886.1 azon|OJJ50727.1

Group581: anid|CBF80560.1 anid|CBF82261.1 abra|OJJ66944.1 akaw|GAA91476.1 aluc|OJZ85637.1 atub|OJI79427.1 abra|OJJ71064.1 acar|OOF90585.1 acla|EAW07015.1 afis|EAW25726.1 afla|EED53906.1 aory|EIT78492.1 asyd|OJJ52818.1 ater|EAU32822.1 aver|OJJ07278.1 awen|OJJ38756.1 azon|OJJ42176.1 aver|OJJ08797.1 awen|OJJ32537.1 afum|EAL87822.1 acla|EAW11668.1

Group582: asyd|OJJ52091.1 asyd|OJJ52556.1 asyd|OJJ55512.1 asyd|OJJ65388.1 abra|OJJ68248.1 acar|OOF97527.1 acla|EAW09059.1 afis|EAW25632.1 afla|EED55180.1 afum|EAL84320.1 agla|OJJ78899.1 akaw|GAA83213.1 aluc|OJZ83715.1 anid|CBF83158.1 anig|EHA18866.1 aory|EIT81642.1 ater|EAU32718.1 atub|OJI90091.1 aver|OJJ06589.1 awen|OJJ36052.1 aver|OJJ03845.1

Group583: asyd|OJJ52420.1 asyd|OJJ54955.1 abra|OJJ76861.1 acar|OOF91348.1 acla|EAW13422.1 afis|EAW20534.1 afla|EED52483.1 afum|EAL84524.1 agla|OJJ84698.1 akaw|GAA87161.1 aluc|OJZ84273.1 anid|CBF71440.1 anig|EHA27615.1 aory|EIT82754.1 asyd|OJJ55687.1 ater|EAU37151.1 atub|OJI89810.1 aver|OJJ06771.1 aver|OJJ07241.1 awen|OJJ33240.1 anid|CBF78998.1

Group584: asyd|OJJ53054.1 asyd|OJJ62331.1 aver|OJI96437.1 aver|OJJ03790.1 abra|OJJ67212.1 acar|OOF98347.1 acla|EAW08554.1 afis|EAW23596.1 afla|EED55272.1 afum|EAL87871.1 agla|OJJ86856.1 akaw|GAA90965.1 aluc|OJZ87761.1 anid|CBF89583.1 anig|EHA20246.1 aory|EIT83575.1 ater|EAU35356.1 atub|OJI80883.1 awen|OJJ39918.1 azon|OJJ45892.1 anid|CBF77738.1

Group585: asyd|OJJ53773.1 asyd|OJJ64196.1 aver|OJI98285.1 aver|OJJ01986.1 abra|OJJ71111.1 acar|OOF98036.1 acla|EAW10381.1 afis|EAW25212.1 afla|EED53666.1 afum|EAL89873.1 agla|OJJ88949.1 akaw|GAA82858.1 aluc|OJZ87423.1 anid|CBF85604.1 anig|EHA19933.1 anig|EHA20646.1 aory|EIT75278.1 ater|EAU34369.1 atub|OJI81225.1 awen|OJJ40874.1 azon|OJJ46813.1

Group586: asyd|OJJ54182.1 asyd|OJJ55740.1 aver|OJJ06826.1 aver|OJJ08558.1 abra|OJJ76808.1 acar|OOF91302.1 acla|EAW13368.1 afis|EAW20482.1 afla|EED44786.1 afum|EAL84572.1 akaw|GAA87214.1 aluc|OJZ84216.1 anid|CBF79229.1 anig|EHA27568.1 aory|EIT73548.1 aory|EIT77772.1 ater|EAU29560.1 atub|OJI89868.1 awen|OJJ33180.1 azon|OJJ46383.1 afla|EED45102.1

Group587: asyd|OJJ54581.1 asyd|OJJ54583.1 asyd|OJJ59186.1 awen|OJJ29889.1 awen|OJJ35053.1 awen|OJJ38450.1 azon|OJJ44478.1 azon|OJJ45681.1 abra|OJJ67703.1 acar|OOF97596.1 agla|OJJ81404.1 akaw|GAA89211.1 aluc|OJZ81618.1 anig|EHA25825.1 ater|EAU38935.1 atub|OJI88562.1 acar|OOG00088.1 afla|EED51583.1 aory|EIT80517.1 afis|EAW20205.1 afla|EED51443.1

Group588: asyd|OJJ60296.1 asyd|OJJ62116.1 aver|OJI96223.1 aver|OJI99634.1 aver|OJJ02653.1 aver|OJJ06318.1 abra|OJJ68285.1 acar|OOF90110.1 afis|EAW22106.1 afla|EED55144.1 afum|EAL91085.1 agla|OJJ82696.1 akaw|GAA83187.1 aluc|OJZ83686.1 anid|CBF87172.1 anig|EHA18840.1 aory|EIT81583.1 asyd|OJJ56395.1 ater|EAU32730.1 atub|OJI90057.1 awen|OJJ40235.1

Group589: ater|EAU35203.1 ater|EAU36444.1 abra|OJJ76030.1 acar|OOF99899.1 acla|EAW09783.1 afis|EAW21355.1 afla|EED55770.1 afum|EAL92370.2 agla|OJJ87376.1 aluc|OJZ89481.1 anid|CBF76251.1 anig|EHA23112.1 aory|EIT76609.1 asyd|OJJ60968.1 atub|OJI85319.1 aver|OJJ00292.1 awen|OJJ39343.1 azon|OJJ51689.1 afla|EED53075.1 aory|EIT74793.1 asyd|OJJ59168.1

Group590: azon|OJJ46530.1 azon|OJJ50393.1 acla|EAW13391.1 afla|EED49406.1 agla|OJJ78968.1 aory|EIT79482.1 asyd|OJJ52710.1 aver|OJJ04739.1 awen|OJJ35362.1 acar|OOF92857.1 afis|EAW24526.1 agla|OJJ81446.1 akaw|GAA82748.1 afum|EAL86190.1 ater|EAU37241.1 atub|OJI83744.1 afis|EAW22564.1 afum|EAL94067.1 agla|OJJ81434.1 aluc|OJZ87304.1 ater|EAU36467.1

Group591: abra|OJJ65586.1 acla|EAW06849.1 afum|EAL86639.1 anid|CBF87353.1 asyd|OJJ53531.1 ater|EAU31407.1 aver|OJJ08219.1 abra|OJJ68254.1 acar|OOF97533.1 afla|EED51066.1 akaw|GAA83207.1

aluc|OJZ83709.1 anig|EHA18860.1 aory|EIT77028.1 ater|EAU32153.1 atub|OJI90081.1 aver|OJJ00862.1 awen|OJJ38280.1 azon|OJJ43291.1 asyd|OJJ61534.1 azon|OJJ43503.1

Group592: abra|OJJ65854.1 acar|OOF95015.1 acla|EAW13528.1 afis|EAW20729.1 afla|EED48906.1 afum|EAL84879.1 agla|OJJ84616.1 akaw|GAA92290.1 aluc|OJZ90187.1 anid|CBF85584.1 anig|EHA23686.1 aory|EIT81194.1 asyd|OJJ64174.1 atub|OJI81614.1 aver|OJJ06463.1 awen|OJJ29911.1 azon|OJJ42620.1 afla|EED55134.1 aory|EIT81587.1 ater|EAU32788.1 aver|OJI98249.1

Group593: abra|OJJ66409.1 acar|OOF97558.1 acla|EAW15053.1 afla|EED47219.1 aluc|OJZ81110.1 anid|CBF76141.1 aory|EIT79310.1 ater|EAU32457.1 aver|OJJ03023.1 awen|OJJ37014.1 abra|OJJ67968.1 afis|EAW17012.1 afla|EED55205.1 agla|OJJ86640.1 aory|EIT83525.1 azon|OJJ42140.1 aory|EIT79570.1 akaw|GAA86290.1 awen|OJJ29897.1 aver|OJJ06536.1 asyd|OJJ54884.1

Group594: abra|OJJ66784.1 afla|EED47234.1 anid|CBF87107.1 aory|EIT79229.1 abra|OJJ75747.1 acar|OOG00125.1 acla|EAW09092.1 afla|EED52418.1 agla|OJJ82218.1 akaw|GAA84480.1 aluc|OJZ89200.1 anig|EHA23357.1 aory|EIT83316.1 asyd|OJJ56738.1 ater|EAU31807.1 atub|OJI85590.1 aver|OJJ05987.1 awen|OJJ41471.1 azon|OJJ44445.1 afum|EAL84355.1 afis|EAW25686.1

Group595: abra|OJJ67354.1 akaw|GAA86466.1 aluc|OJZ85007.1 asyd|OJJ58136.1 ater|EAU35299.1 atub|OJI80590.1 aver|OJJ04269.1 awen|OJJ33479.1 abra|OJJ69839.1 akaw|GAA87821.1 aluc|OJZ85457.1 anid|CBF84465.1 anig|EHA24999.1 atub|OJI80106.1 azon|OJJ47408.1 afla|EED57088.1 aory|EIT82252.1 awen|OJJ29832.1 asyd|OJJ53544.1 aver|OJJ08209.1 awen|OJJ37797.1

Group596: abra|OJJ67576.1 acla|EAW07712.1 afis|EAW19707.1 afla|EED52185.1 afum|EAL86292.2 agla|OJJ80363.1 akaw|GAA89105.1 anid|CBF82328.1 aory|EIT74319.1 asyd|OJJ59047.1 ater|EAU32212.1 atub|OJI88444.1 aver|OJJ01643.1 awen|OJJ35443.1 azon|OJJ49488.1 asyd|OJJ52892.1 aver|OJJ08863.1 afum|EBA27291.1 afis|EAW25591.1 awen|OJJ40142.1 agla|OJJ81186.1

Group597: abra|OJJ67718.1 afis|EAW16742.1 afla|EED49725.1 afum|EAL86317.2 aory|EIT77616.1 abra|OJJ76618.1 akaw|GAA86494.1 aluc|OJZ85036.1 anig|EHA22616.1 aory|EIT76392.1 asyd|OJJ54900.1 ater|EAU30402.1 atub|OJI86041.1 awen|OJJ32259.1 asyd|OJJ60225.1 atub|OJI80561.1 aver|OJJ04042.1 afla|EED46080.1 afla|EED46081.1 agla|OJJ80285.1 awen|OJJ32907.1

Group598: abra|OJJ67990.1 acar|OOF92032.1 afis|EAW22103.1 afla|EED45380.1 akaw|GAA90958.1 aluc|OJZ82864.1 anig|EHA21294.1 aory|EIT82399.1 atub|OJI89089.1 awen|OJJ36170.1 abra|OJJ71233.1 akaw|GAA82755.1 aluc|OJZ87312.1 anid|CBF84724.1 anig|EHA20548.1 asyd|OJJ53307.1 atub|OJI81333.1 aver|OJJ08434.1 azon|OJJ43637.1 abra|OJJ76888.1 afis|EAW16705.1

Group599: abra|OJJ68494.1 acar|OOF98749.1 acla|EAW12142.1 afis|EAW17194.1 afla|EED45176.1 afum|EAL86622.1 agla|OJJ86660.1 akaw|GAA92776.1 aluc|OJZ82707.1 anid|CBF69890.1 anig|EHA21162.1 aory|EIT73731.1 asyd|OJJ59007.1 ater|EAU34144.1 atub|OJI88940.1 aver|OJJ01608.1 awen|OJJ34716.1 agla|OJJ87933.1 anid|CBF89139.1 asyd|OJJ62650.1 aver|OJI96750.1

Group600: abra|OJJ69337.1 acar|OOF94389.1 acla|EAW06601.1 afis|EAW16003.1 afla|EED48032.1 afum|EAL88635.2 agla|OJJ79631.1 akaw|GAA88207.1 aluc|OJZ82169.1 anid|CBF85846.1 anig|EHA28563.1 aory|EIT83338.1 asyd|OJJ53958.1 ater|EAU31185.1 atub|OJI88268.1 aver|OJJ08054.1 awen|OJJ33053.1 azon|OJJ48464.1 asyd|OJJ64357.1 ater|EAU33640.1 aver|OJI98453.1

Group601: abra|OJJ69426.1 acar|OOF94310.1 acla|EAW08550.1 afis|EAW23591.1 afum|EAL87875.1 akaw|GAA88292.1 aluc|OJZ82263.1 anid|CBF73805.1 anig|EHA28480.1 asyd|OJJ56617.1 aver|OJJ06107.1 awen|OJJ35870.1 abra|OJJ69775.1 acar|OOF93598.1 afla|EED45633.1 akaw|GAA87878.1 aluc|OJZ85519.1 anig|EHA25052.1 aory|EIT75817.1 atub|OJI80047.1 azon|OJJ47676.1

Group602: abra|OJJ69887.1 acar|OOF93848.1 acla|EAW11893.1 afis|EAW19504.1 afla|EED46651.1 agla|OJJ81680.1 akaw|GAA87765.1 aluc|OJZ85400.1 anid|CBF87939.1 anig|EHA25582.1 aory|EIT77814.1 asyd|OJJ63469.1 ater|EAU38715.1 atub|OJI80171.1 aver|OJI97463.1 awen|OJJ36926.1 azon|OJJ45056.1 akaw|GAA89704.1 aluc|OJZ80680.1 anig|EHA27332.1 atub|OJI87539.1

Group603: abra|OJJ70214.1 acar|OOF92565.1 afla|EED46373.1 akaw|GAA86562.1 aluc|OJZ85112.1 anid|CBF78840.1 anig|EHA25332.1 aory|EIT77546.1 ater|EAU29773.1 atub|OJI80482.1 azon|OJJ45196.1 afis|EAW23745.1 anid|CBF78842.1 afla|EED52200.1 asyd|OJJ65270.1 aver|OJI99422.1 awen|OJJ33680.1 akaw|GAA93170.1 aluc|OJZ83029.1 aory|EIT74347.1 afla|EED56929.1

Group604: abra|OJJ70243.1 anig|EHA25313.1 abra|OJJ71272.1 acar|OOF92553.1 afis|EAW17415.1 afla|EED48947.1 agla|OJJ88526.1 akaw|GAA82718.1 aluc|OJZ87272.1 anid|CBF75478.1 anig|EHA20513.1 asyd|OJJ63978.1 ater|EAU39296.1 atub|OJI81370.1 aver|OJI96975.1 awen|OJJ31635.1 azon|OJJ46586.1 afla|EED48946.1 agla|OJJ87753.1 aory|EIT79982.1 aory|EIT80005.1

Group605: abra|OJJ70312.1 acar|OOF93014.1 acla|EAW13439.1 afis|EAW20553.1 afla|EED45906.1 afum|EAL84508.1 agla|OJJ80823.1 akaw|GAA93011.1 aluc|OJZ82574.1 anid|CBF78981.1 anig|EHA27499.1 aory|EIT75600.1 asyd|OJJ55678.1 ater|EAU37124.1 atub|OJI88818.1 aver|OJJ06761.1 awen|OJJ41921.1 azon|OJJ42685.1 anig|EHA23882.1 afla|EED52345.1 aory|EIT74410.1

Group606: abra|OJJ70650.1 acar|OOF97072.1 acla|EAW08109.1 afis|EAW23142.1 afla|EED47570.1 afum|EAL88339.1 agla|OJJ89659.1 akaw|GAA89986.1 aluc|OJZ84000.1 anid|CBF74440.1 anig|EHA19122.1 aory|EIT73130.1 asyd|OJJ57744.1 ater|EAU36275.1 atub|OJI90369.1 aver|OJJ05423.1 awen|OJJ36638.1 azon|OJJ47578.1 afla|EED44702.1 aluc|OJZ80351.1 aory|EIT79586.1

Group607: abra|OJJ70731.1 acar|OOF97007.1 acla|EAW11182.1 afis|EAW15913.1 afla|EED57767.1 afum|EAL88548.2 agla|OJJ89577.1 akaw|GAA90059.1 aluc|OJZ84076.1 anid|CBF81305.1 anig|EHA19195.1 aory|EIT81027.1 asyd|OJJ59352.1 ater|EAU36192.1 atub|OJI90448.1 aver|OJJ02922.1 awen|OJJ36717.1 azon|OJJ47476.1 acla|EAW10824.1 afum|EAL89393.1 awen|OJJ36142.1

Group608: abra|OJJ70975.1 acar|OOF98156.1 acla|EAW10534.1 afis|EAW25001.1 afla|EED55421.1 afum|EAL89697.1 agla|OJJ89163.1 akaw|GAA82974.1 aluc|OJZ87557.1 anid|CBF81488.1 anig|EHA20760.1 atub|OJI81090.1 awen|OJJ40638.1 abra|OJJ71753.1 aluc|OJZ84833.1 anig|EHA25743.1 asyd|OJJ53401.1 ater|EAU35784.1 aver|OJJ04200.1 asyd|OJJ61749.1 aver|OJI95846.1

Group609: abra|OJJ71034.1 acar|OOF98103.1 acla|EAW10585.1 afla|EED55474.1 afum|EAL89644.1 agla|OJJ82278.1 akaw|GAA93319.1 aluc|OJZ87495.1 anid|CBF81619.1 anig|EHA20712.1 asyd|OJJ59527.1 ater|EAU35735.1 atub|OJI81154.1 aver|OJJ02744.1 awen|OJJ40580.1 azon|OJJ49440.1 afis|EAW24983.1 akaw|GAA82920.1 acla|EAW12399.1 afis|EAW16847.1 afum|EAL86393.1

Group610: abra|OJJ71163.1 acar|OOF97997.1 acla|EAW14151.1 afis|EAW18864.1 afla|EED53721.1 afum|EAL93596.1 agla|OJJ85239.1 akaw|GAA82816.1 aluc|OJZ87377.1 anid|CBF85347.1 anig|EHA20609.1 aory|EIT75273.1 asyd|OJJ64028.1 ater|EAU34397.1 atub|OJI81269.1 aver|OJI98114.1 awen|OJJ32721.1 azon|OJJ50355.1 afla|EED45395.1 aory|EIT82387.1 awen|OJJ39952.1

Group611: abra|OJJ71567.1 acar|OOF96301.1 acla|EAW12384.1 afis|EAW16831.1 afla|EED47160.1 afum|EAL86379.1 akaw|GAA89357.1 aluc|OJZ86304.1 anid|CBF69502.1 anig|EHA21568.1 aory|EIT79185.1 asyd|OJJ63349.1 ater|EAU36074.1 atub|OJI84232.1 aver|OJI97611.1 awen|OJJ31351.1 azon|OJJ50260.1 asyd|OJJ59227.1 aver|OJJ01837.1 azon|OJJ48154.1 awen|OJJ35570.1

Group612: abra|OJJ71741.1 acar|OOF96432.1 acla|EAW06445.1 afis|EAW18550.1 afla|EED52825.1 afum|EAL93250.1 agla|OJJ81594.1 akaw|GAA85155.1 aluc|OJZ86482.1 anid|CBF70273.1 anig|EHA22165.1 aory|EIT78166.1 asyd|OJJ58746.1 ater|EAU33222.1 atub|OJI84048.1 aver|OJJ01356.1 awen|OJJ31108.1 azon|OJJ46554.1 ater|EAU31802.1 afla|EED56761.1 aory|EIT73941.1

Group613: abra|OJJ72042.1 acar|OOF96701.1 acla|EAW09503.1 afis|EAW21633.1 afla|EED57861.1 afum|EAL92666.2 agla|OJJ79697.1 akaw|GAA84891.1 aluc|OJZ86780.1 anid|CBF83510.1 anig|EHA21902.1 aory|EIT81075.1 asyd|OJJ60709.1 ater|EAU38319.1 atub|OJI83740.1 aver|OJJ00035.1 awen|OJJ33882.1 azon|OJJ48672.1 afum|EAL91062.1 acla|EAW07114.1 afis|EAW22126.1

Group614: abra|OJJ72229.1 acar|OOF96852.1 acla|EAW12118.1 afis|EAW17264.1 afla|EED48866.1 afum|EAL86681.1 agla|OJJ84958.1 akaw|GAA84708.1 aluc|OJZ86979.1 anig|EHA21670.1 aory|EIT81253.1 atub|OJI83541.1 azon|OJJ50249.1 abra|OJJ75321.1 acar|OOF94506.1 akaw|GAA92016.1 aluc|OJZ92882.1 anig|EHA26078.1 atub|OJI87341.1 acar|OOF94471.1 ater|EAU36154.1

Group615: abra|OJJ72267.1 acar|OOG00128.1 acla|EAW10037.1 afis|EAW23420.1 afla|EED49100.1 afum|EAL84260.1 akaw|GAA86022.1 anig|EHA23745.1 ater|EAU37850.1 atub|OJI81675.1 abra|OJJ73122.1 acar|OOF92500.1 afis|EAW22027.1 akaw|GAA88043.1 aluc|OJZ81302.1 anig|EHA18611.1 aory|EIT82499.1 atub|OJI91511.1 afum|EAL88049.1 acla|EAW12426.1 afis|EAW15611.1

Group616: abra|OJJ72269.1 acar|OOF90351.1 afis|EAW19947.1 afla|EED46130.1 afum|EAL84968.2 agla|OJJ85569.1 akaw|GAA86024.1 aluc|OJZ90251.1 anid|CBF84249.1 anig|EHA23747.1 aory|EIT76460.1 asyd|OJJ57200.1 ater|EAU30029.1 atub|OJI81677.1 aver|OJJ03090.1 awen|OJJ32569.1 afum|EAL90430.1 akaw|GAA84794.1 aluc|OJZ86886.1 atub|OJI83633.1 awen|OJJ31250.1

Group617: abra|OJJ72859.1 acar|OOF96056.1 acla|EAW11387.1 afis|EAW18109.1 afla|EED49118.1 afum|EAL87466.1 agla|OJJ86997.1 akaw|GAA85458.1 aluc|OJZ83366.1 anid|CBF75326.1 anig|EHA24231.1 aory|EIT78544.1 asyd|OJJ59867.1 ater|EAU29709.1 atub|OJI82257.1 aver|OJJ02421.1 awen|OJJ35507.1 azon|OJJ46977.1 afla|EED55499.1 agla|OJJ87955.1 aory|EIT80816.1

Group618: abra|OJJ72915.1 acar|OOF96097.1 acla|EAW11688.1 afis|EAW15970.1 afla|EED58087.1 afum|EAL87149.1 agla|OJJ83846.1 aluc|OJZ83425.1 anig|EHA24277.1 aory|EIT79158.1 asyd|OJJ56475.1 ater|EAU35478.1 atub|OJI82311.1 aver|OJJ06243.1 awen|OJJ35603.1 azon|OJJ46922.1 anid|CBF69504.1 asyd|OJJ59223.1 aver|OJJ01833.1 acla|EAW11689.1 afis|EAW23705.1

Group619: abra|OJJ73104.1 acar|OOG00342.1 acla|EAW12431.1 afis|EAW16738.1 afla|EED44914.1 afum|EAL86313.2 agla|OJJ82713.1 akaw|GAA88061.1 aluc|OJZ81323.1 anid|CBF82088.1 anig|EHA18629.1 aory|EIT73472.1 asyd|OJJ54256.1 ater|EAU39589.1 atub|OJI91526.1 aver|OJJ05209.1 awen|OJJ39338.1 anid|CBF75878.1 asyd|OJJ60230.1 aver|OJJ04036.1 azon|OJJ51650.1

Group620: abra|OJJ73308.1 acar|OOG00533.1 acla|EAW12048.1 afis|EAW17017.1 afla|EED45666.1 akaw|GAA89816.1 aluc|OJZ81874.1 anig|EHA21034.1 aory|EIT81376.1 asyd|OJJ54465.1 ater|EAU36711.1

aver|OJJ07789.1 awen|OJJ31272.1 azon|OJJ47410.1 afla|EED48480.1 anid|CBF86045.1 anig|EHA28230.1 afla|EED52021.1 anid|CBF73486.1 aver|OJJ06468.1 anid|CBF89301.1

Group621: abra|OJJ74126.1 acar|OOF95610.1 afis|EAW24721.1 afla|EED53234.1 afum|EAL89370.1 agla|OJJ87996.1 akaw|GAA89758.1 aluc|OJZ80625.1 anid|CBF82175.1 anig|EHA27207.1 aory|EIT74852.1 asyd|OJJ54960.1 atub|OJI87480.1 aver|OJJ08805.1 awen|OJJ30037.1 azon|OJJ43678.1 asyd|OJJ64485.1 atub|OJI88492.1 aver|OJI98586.1 aver|OJJ07231.1 asyd|OJJ52829.1

Group622: abra|OJJ74136.1 acar|OOF95594.1 acla|EAW07094.1 afis|EAW22104.1 afla|EED54116.1 afum|EAL91087.1 akaw|GAA89750.1 aluc|OJZ80634.1 anid|CBF79913.1 anig|EHA27198.1 aory|EIT76054.1 asyd|OJJ58191.1 ater|EAU32259.1 atub|OJI87489.1 aver|OJJ04214.1 awen|OJJ34666.1 azon|OJJ50218.1 acla|EAW11902.1 afis|EAW19515.1 afum|EAL86981.2 anid|CBF86632.1

Group623: abra|OJJ74191.1 acar|OOF95530.1 acla|EAW07243.1 afis|EAW22264.1 afla|EED57656.1 afum|EAL90916.1 agla|OJJ87791.1 akaw|GAA86826.1 aluc|OJZ89944.1 anid|CBF88515.1 anig|EHA27143.1 aory|EIT80760.1 asyd|OJJ63805.1 ater|EAU34274.1 atub|OJI84862.1 aver|OJI97133.1 awen|OJJ34837.1 azon|OJJ45710.1 afla|EED51484.1 asyd|OJJ58154.1 aver|OJJ04249.1

Group624: abra|OJJ74556.1 acar|OOF95199.1 acla|EAW12292.1 afis|EAW16945.1 afla|EED46082.1 afum|EAL86472.2 agla|OJJ89555.1 akaw|GAA82040.1 aluc|OJZ92139.1 anid|CBF88435.1 anig|EHA26810.1 aory|EIT76401.1 asyd|OJJ63762.1 ater|EAU35227.1 atub|OJI86571.1 aver|OJI97178.1 awen|OJJ36332.1 azon|OJJ46487.1 acla|EAW11680.1 afis|EAW23697.1 anid|CBF76117.1

Group625: abra|OJJ74819.1 acar|OOF95807.1 acla|EAW08501.1 afis|EAW16872.1 afum|EAL86410.2 akaw|GAA83596.1 aluc|OJZ92398.1 anid|CBF86626.1 anig|EHA26567.1 asyd|OJJ64810.1 ater|EAU32516.1 atub|OJI86828.1 aver|OJI98923.1 awen|OJJ32502.1 azon|OJJ43488.1 acar|OOF90627.1 afis|EAW17135.1 afla|EED45618.1 anig|EHA24760.1 aory|EIT75444.1 ater|EAU38152.1

Group626: abra|OJJ76271.1 acar|OOF99519.1 afis|EAW24673.1 akaw|GAA82204.1 aluc|OJZ91949.1 anid|CBF90103.1 anig|EHA22908.1 atub|OJI86394.1 aver|OJI96119.1 abra|OJJ77743.1 afis|EAW19876.1 afla|EED56872.1 agla|OJJ82021.1 anid|CBF74093.1 asyd|OJJ65119.1 ater|EAU36111.1 aver|OJI99248.1 awen|OJJ34656.1 awen|OJJ38125.1 aory|EIT77510.1 asyd|OJJ62028.1

Group627: abra|OJJ76451.1 acar|OOF99694.1 acla|EAW13977.1 afis|EAW18686.1 afla|EED53481.1 afum|EAL89344.2 akaw|GAA82363.1 aluc|OJZ91759.1 anig|EHA22761.1 aory|EIT75172.1 asyd|OJJ58512.1 ater|EAU38222.1 atub|OJI86217.1 aver|OJJ01113.1 awen|OJJ32157.1 azon|OJJ46741.1 acla|EAW13614.1 afis|EAW16454.1 anig|EHA20148.1 afum|EAL93407.1 afis|EAW24702.1

Group628: abra|OJJ76531.1 acar|OOF99442.1 acla|EAW12929.1 afis|EAW17727.1 afla|EED48895.1 afum|EAL91542.1 agla|OJJ87872.1 akaw|GAA82443.1 aluc|OJZ91673.1 anid|CBF90286.1 anig|EHA22691.1 aory|EIT81184.1 asyd|OJJ61920.1 ater|EAU36831.1 atub|OJI86128.1 aver|OJI96012.1 awen|OJJ39163.1 azon|OJJ44435.1 acar|OOF93188.1 acla|EAW12526.1 awen|OJJ40114.1

Group629: abra|OJJ76817.1 acar|OOF91312.1 acla|EAW13377.1 afis|EAW19711.1 afla|EED47757.1 afum|EAL86816.2 agla|OJJ84836.1 aluc|OJZ84227.1 anid|CBF79211.1 anig|EHA27576.1 aory|EIT77795.1 asyd|OJJ55731.1 ater|EAU30448.1 atub|OJI89857.1 aver|OJJ06817.1 awen|OJJ33191.1 azon|OJJ46393.1 aory|EIT77115.1 afla|EED44738.1 asyd|OJJ54596.1 aver|OJJ07556.1

Group630: abra|OJJ76842.1 acar|OOF89970.1 acla|EAW13405.1 afis|EAW20516.1 afla|EED52466.1 afum|EAL84541.1 agla|OJJ84774.1 akaw|GAA87180.1 aluc|OJZ84253.1 anid|CBF79059.1 anig|EHA27598.1 aory|EIT82819.1 asyd|OJJ55708.1 ater|EAU37185.1 atub|OJI89830.1 aver|OJJ06794.1 awen|OJJ33222.1 azon|OJJ45121.1 aluc|OJZ87023.1 anig|EHA21634.1 atub|OJI83495.1

Group631: abra|OJJ77007.1 acar|OOF91453.1 acla|EAW15047.1 afis|EAW24611.1 afla|EED47377.1 afum|EAL86241.1 agla|OJJ78906.1 akaw|GAA87041.1 aluc|OJZ84406.1 anid|CBF83177.1 anig|EHA27736.1 aory|EIT75955.1 asyd|OJJ55523.1 ater|EAU32183.1 atub|OJI89666.1 aver|OJJ06601.1 awen|OJJ36042.1 azon|OJJ45129.1 acla|EAW15048.1 aory|EIT82833.1 afla|EED54123.1

Group632: abra|OJJ77033.1 acar|OOF91472.1 acla|EAW07271.1 afis|EAW22292.1 afla|EED57692.1 afum|EAL90889.1 agla|OJJ87756.1 akaw|GAA87018.1 aluc|OJZ84436.1 anid|CBF88573.1 anig|EHA27755.1 aory|EIT79900.1 asyd|OJJ63839.1 ater|EAU34305.1 atub|OJI89637.1 aver|OJI97098.1 awen|OJJ34866.1 azon|OJJ45662.1 afla|EED51864.1 aory|EIT81771.1 awen|OJJ30800.1

Group633: abra|OJJ77037.1 acar|OOF90079.1 acla|EAW13558.1 afis|EAW20634.1 afla|EED49758.1 afum|EAL84434.1 agla|OJJ86283.1 akaw|GAA87014.1 aluc|OJZ84440.1 anid|CBF83213.1 anig|EHA27759.1 aory|EIT77698.1 asyd|OJJ65203.1 ater|EAU32169.1 atub|OJI89633.1 aver|OJJ06646.1 awen|OJJ33605.1 azon|OJJ50726.1 asyd|OJJ55565.1 azon|OJJ42610.1 aver|OJI99352.1

Group634: abra|OJJ77109.1 acar|OOF92994.1 afla|EED51851.1 agla|OJJ86977.1 akaw|GAA90819.1 aluc|OJZ84518.1 anid|CBF89943.1 anig|EHA27816.1 aory|EIT81728.1 asyd|OJJ62111.1 ater|EAU31034.1 atub|OJI89562.1 aver|OJJ03877.1 awen|OJJ33658.1 acla|EAW14977.1 aver|OJI96219.1 asyd|OJJ57090.1 ater|EAU34328.1 awen|OJJ33488.1 aver|OJJ03210.1 asyd|OJJ62627.1

Group635: abra|OJJ77191.1 acar|OOF93077.1 acla|EAW13182.1 afis|EAW17458.1 afla|EED49364.1 afum|EAL91286.1 agla|OJJ80049.1 akaw|GAA90749.1 aluc|OJZ84599.1 anid|CBF71666.1 anig|EHA27889.1 aory|EIT79494.1 asyd|OJJ59263.1 ater|EAU33943.1 atub|OJI89480.1 aver|OJJ01874.1 awen|OJJ37389.1 azon|OJJ51062.1 agla|OJJ80359.1 asyd|OJJ61831.1 aver|OJI95930.1

Group636: abra|OJJ77325.1 acar|OOF93201.1 acla|EAW13147.1 afis|EAW17500.1 afla|EED45479.1 afum|EAL91323.1 agla|OJJ81453.1 akaw|GAA91431.1 aluc|OJZ84735.1 anid|CBF71578.1 anig|EHA28008.1 aory|EIT82181.1 asyd|OJJ61876.1 ater|EAU36946.1 atub|OJI89346.1 aver|OJI95974.1 awen|OJJ37341.1 azon|OJJ51026.1 agla|OJJ78829.1 aory|EIT75365.1 asyd|OJJ62947.1

Group637: abra|OJJ77475.1 acar|OOF99250.1 acla|EAW06757.1 afis|EAW16307.1 afla|EED53242.1 afum|EAL88929.1 agla|OJJ88269.1 akaw|GAA84143.1 aluc|OJZ81063.1 anid|CBF69567.1 anig|EHA19305.1 aory|EIT75142.1 asyd|OJJ59151.1 ater|EAU29335.1 atub|OJI82522.1 aver|OJJ08866.1 awen|OJJ34880.1 azon|OJJ44967.1 aver|OJJ01741.1 awen|OJJ36323.1 asyd|OJJ52895.1

Group638: abra|OJJ78236.1 acar|OOF98624.1 acla|EAW09274.1 afis|EAW21874.1 afla|EED46435.1 afum|EAL92888.1 agla|OJJ84259.1 akaw|GAA85789.1 aluc|OJZ91310.1 anid|CBF76774.1 anig|EHA19994.1 aory|EIT72385.1 asyd|OJJ61272.1 ater|EAU30873.1 atub|OJI83303.1 aver|OJJ00600.1 awen|OJJ34162.1 azon|OJJ43467.1 afla|EED46434.1 awen|OJJ34163.1 asyd|OJJ61271.1

Group639: abra|OJJ65541.1 abra|OJJ65833.1 abra|OJJ67278.1 akaw|GAA92069.1 akaw|GAA92846.1 asyd|OJJ52003.1 asyd|OJJ62485.1 acar|OOF95207.1 afla|EED55152.1 agla|OJJ86434.1 anid|CBF76638.1 anig|EHA18761.1 aory|EIT81617.1 aver|OJJ07599.1 awen|OJJ31646.1 aluc|OJZ88789.1 acar|OOG00873.1 agla|OJJ79830.1 asyd|OJJ54555.1 awen|OJJ30844.1

Group640: abra|OJJ65699.1 abra|OJJ77440.1 aluc|OJZ81081.1 aluc|OJZ88716.1 asyd|OJJ59917.1 asyd|OJJ61765.1 aver|OJI96472.1 aver|OJJ02369.1 afla|EED56833.1 afum|EAL84269.2 agla|OJJ80658.1 akaw|GAA84158.1 anid|CBF85281.1 anig|EHA18818.1 aory|EIT73911.1 ater|EAU34168.1 atub|OJI82505.1 awen|OJJ30259.1 akaw|GAA92795.1 anig|EHA28199.1

Group641: abra|OJJ66191.1 abra|OJJ68084.1 acar|OOF91939.1 acar|OOF93882.1 akaw|GAA86869.1 akaw|GAA89096.1 anig|EHA25698.1 anig|EHA27364.1 atub|OJI84816.1 atub|OJI88435.1 acla|EAW09010.1 afis|EAW25589.1 afum|EAL84297.2 aluc|OJZ89993.1 asyd|OJJ61550.1 aver|OJJ00875.1 awen|OJJ41959.1 afla|EED48865.1 anid|CBF82995.1 aory|EIT81254.1

Group642: abra|OJJ68152.1 abra|OJJ75347.1 aluc|OJZ83228.1 aluc|OJZ92909.1 atub|OJI87369.1 atub|OJI87708.1 acar|OOF94489.1 acla|EAW08546.1 afis|EAW23583.1 afla|EED48907.1 afum|EAL87881.1 akaw|GAA92040.1 anid|CBF87513.1 anig|EHA21084.1 anig|EHA26055.1 aory|EIT81206.1 ater|EAU36167.1 awen|OJJ30073.1 asyd|OJJ52977.1 aver|OJJ08943.1

Group643: abra|OJJ70063.1 abra|OJJ75532.1 akaw|GAA84171.1 akaw|GAA87613.1 akaw|GAA91952.1 aluc|OJZ81096.1 aluc|OJZ82666.1 aluc|OJZ85234.1 anig|EHA19275.1 anig|EHA25438.1 anig|EHA27440.1 atub|OJI80344.1 atub|OJI82486.1 atub|OJI88899.1 acar|OOF93551.1 afis|EAW25548.1 afla|EED45174.1 afla|EED46370.1 anid|CBF82283.1 aory|EIT81213.1

Group644: abra|OJJ74604.1 abra|OJJ77441.1 acar|OOF95100.1 acar|OOF95166.1 acla|EAW10775.1 afis|EAW24801.1 afla|EED47186.1 afum|EAL89442.1 agla|OJJ82895.1 akaw|GAA82004.1 aluc|OJZ92183.1 anid|CBF81510.1 anig|EHA26770.1 aory|EIT79225.1 asyd|OJJ59469.1 ater|EAU36506.1 atub|OJI86611.1 aver|OJJ02805.1 awen|OJJ40384.1 azon|OJJ49233.1

Group645: acar|OOF90070.1 acar|OOF90633.1 akaw|GAA85210.1 akaw|GAA92948.1 abra|OJJ65532.1 anig|EHA21123.1 abra|OJJ67562.1 afla|EED57361.1 akaw|GAA84330.1 aluc|OJZ89029.1 aory|EIT78652.1 ater|EAU36945.1 atub|OJI85762.1 awen|OJJ30886.1 abra|OJJ72584.1 afla|EED44973.1 aluc|OJZ90565.1 anig|EHA24011.1 atub|OJI81988.1 afla|EED52856.1

Group646: acar|OOF90425.1 acar|OOF90429.1 acar|OOF93386.1 abra|OJJ69872.1 acla|EAW08934.1 afis|EAW15465.1 afla|EED46918.1 afum|EAL85857.1 agla|OJJ78859.1 akaw|GAA87784.1 aluc|OJZ85419.1 anid|CBF71773.1 anig|EHA25598.1 aory|EIT72659.1 asyd|OJJ61719.1 ater|EAU32668.1 atub|OJI80156.1 aver|OJI95813.1 awen|OJJ38110.1 azon|OJJ43167.1

Group647: acar|OOF90437.1 acar|OOF90444.1 abra|OJJ68818.1 acla|EAW13660.1 afis|EAW19540.1 agla|OJJ84857.1 akaw|GAA90433.1 aluc|OJZ85794.1 anid|CBF73461.1 anig|EHA24767.1 aory|EIT74867.1 asyd|OJJ55924.1 ater|EAU34189.1 atub|OJI79580.1 azon|OJJ42666.1 afum|EAL84970.1 azon|OJJ45282.1 afis|EAW19943.1 agla|OJJ79424.1 agla|OJJ89038.1

Group648: acar|OOF90442.1 acar|OOF90448.1 abra|OJJ78364.1 acar|OOF98523.1 acla|EAW09171.1 afis|EAW21975.1 afla|EED46698.1 afum|EAL92990.1 agla|OJJ84127.1 akaw|GAA85669.1 aluc|OJZ91440.1 anid|CBF82718.1 anig|EHA20104.1 aory|EIT77954.1 asyd|OJJ61636.1 ater|EAU31093.1 atub|OJI83434.1 aver|OJJ00959.1 awen|OJJ34268.1 azon|OJJ43367.1

Group649: acar|OOF90479.1 acar|OOF90486.1 acar|OOF96185.1 abra|OJJ73013.1 acla|EAW07999.1 afis|EAW23034.1 afla|EED54982.1 afum|EAL88447.1 agla|OJJ89443.1 akaw|GAA85592.1 aluc|OJZ83528.1

anid|CBF80924.1 anig|EHA24351.1 aory|EIT80284.1 asyd|OJJ56320.1 ater|EAU29623.1 atub|OJI82417.1 aver|OJJ06394.1 awen|OJJ35708.1 azon|OJJ44168.1

Group650: acar|OOF90594.1 acar|OOG00347.1 abra|OJJ73121.1 afis|EAW19817.1 afla|EED53226.1 afum|EAL85074.1 akaw|GAA88044.1 aluc|OJZ81304.1 anid|CBF82022.1 anig|EHA18612.1 aory|EIT75068.1 asyd|OJJ56203.1 ater|EAU29416.1 atub|OJI91512.1 aver|OJJ05130.1 awen|OJJ36147.1 azon|OJJ42172.1 azon|OJJ50764.1 aver|OJJ06466.1 ater|EAU34240.1

Group651: acar|OOF90625.1 acar|OOF90863.1 acar|OOF92572.1 acar|OOF94857.1 akaw|GAA86114.1 akaw|GAA86569.1 akaw|GAA90436.1 aluc|OJZ85120.1 aluc|OJZ85791.1 aluc|OJZ90350.1 atub|OJI79576.1 atub|OJI80473.1 atub|OJI81777.1 azon|OJJ43546.1 abra|OJJ70235.1 afla|EED57683.1 akaw|GAA86570.1 anig|EHA25341.1 aory|EIT79899.1 anig|EHA23836.1

Group652: acar|OOF92603.1 acar|OOF92624.1 asyd|OJJ51990.1 asyd|OJJ56630.1 abra|OJJ68460.1 acla|EAW14885.1 afis|EAW24404.1 afla|EED47809.1 afum|EAL86075.1 agla|OJJ89341.1 akaw|GAA92382.1 aluc|OJZ83241.1 anid|CBF73735.1 anig|EHA21096.1 aory|EIT77126.1 ater|EAU30327.1 atub|OJI87696.1 aver|OJJ06096.1 awen|OJJ32804.1 azon|OJJ49024.1

Group653: acar|OOF92614.1 acar|OOF92635.1 awen|OJJ30756.1 awen|OJJ31725.1 abra|OJJ67454.1 acla|EAW06311.1 afis|EAW18420.1 afla|EED52988.1 afum|EAL93104.1 agla|OJJ84988.1 akaw|GAA86377.1 aluc|OJZ84911.1 anid|CBF70700.1 anig|EHA25132.1 aory|EIT78732.1 asyd|OJJ58461.1 ater|EAU29838.1 atub|OJI80687.1 aver|OJJ01061.1 azon|OJJ50488.1

Group654: acar|OOF93661.1 acar|OOF93745.1 abra|OJJ66641.1 acla|EAW11784.1 afis|EAW19394.1 afum|EAL87100.1 agla|OJJ81105.1 akaw|GAA89594.1 aluc|OJZ80808.1 anid|CBF74281.1 anig|EHA27226.1 aory|EIT83121.1 asyd|OJJ56700.1 ater|EAU36057.1 atub|OJI87656.1 aver|OJJ05358.1 awen|OJJ36788.1 azon|OJJ43011.1 asyd|OJJ51861.1 aver|OJJ06025.1

Group655: acar|OOF93670.1 acar|OOF93754.1 abra|OJJ66651.1 acla|EAW11794.1 afis|EAW19404.1 afla|EED50359.1 afum|EAL87091.1 agla|OJJ81118.1 akaw|GAA89603.1 aluc|OJZ80795.1 anid|CBF74343.1 anig|EHA27236.1 aory|EIT83145.1 asyd|OJJ51900.1 asyd|OJJ57797.1 ater|EAU36141.1 atub|OJI87646.1 aver|OJJ05367.1 awen|OJJ36800.1 azon|OJJ42998.1

Group656: acar|OOF93714.1 acar|OOF93801.1 agla|OJJ81170.1 agla|OJJ82654.1 abra|OJJ66712.1 acla|EAW11843.1 afis|EAW19454.1 afla|EED56662.1 afum|EAL87042.1 akaw|GAA89654.1 aluc|OJZ80735.1 anid|CBF81419.1 anig|EHA27286.1 aory|EIT79629.1 asyd|OJJ59414.1 ater|EAU34757.1 atub|OJI87592.1 aver|OJJ02859.1 awen|OJJ36855.1 azon|OJJ42945.1

Group657: acar|OOF94063.1 acar|OOF94133.1 abra|OJJ66361.1 acla|EAW10010.1 afis|EAW21046.1 afla|EED51624.1 afum|EAL92082.1 agla|OJJ82364.1 akaw|GAA90234.1 aluc|OJZ83107.1 anid|CBF87132.1 anig|EHA28170.1 aory|EIT80496.1 asyd|OJJ60365.1 atub|OJI87827.1 aver|OJI99702.1 awen|OJJ32176.1 afla|EED48902.1 aory|EIT81167.1 awen|OJJ36966.1

Group658: acar|OOF94066.1 acar|OOF94136.1 abra|OJJ66356.1 acla|EAW10006.1 afis|EAW21050.1 afla|EED51620.1 afum|EAL92086.1 akaw|GAA90230.1 aluc|OJZ83111.1 anid|CBF87104.1 anig|EHA28174.1 aory|EIT76079.1 asyd|OJJ60388.1 ater|EAU36822.1 atub|OJI87823.1 aver|OJI99717.1 awen|OJJ32173.1 afla|EED54348.1 asyd|OJJ53360.1 aver|OJJ07646.1

Group659: acar|OOF95099.1 acar|OOF95165.1 asyd|OJJ59458.1 asyd|OJJ62484.1 abra|OJJ74605.1 acla|EAW10774.1 afis|EAW24802.1 afla|EED47184.1 afum|EAL89443.1 agla|OJJ82896.1 akaw|GAA82003.1 aluc|OJZ92184.1 anid|CBF81490.1 anig|EHA26769.1 aory|EIT79286.1 ater|EAU36505.1 atub|OJI86612.1 aver|OJJ02815.1 awen|OJJ40385.1 azon|OJJ49235.1

Group660: acar|OOF95626.1 acar|OOF95677.1 abra|OJJ74662.1 acla|EAW10965.1 afis|EAW20769.1 afla|EED50244.1 afum|EAL84738.2 agla|OJJ84002.1 akaw|GAA81949.1 aluc|OJZ92239.1 anig|EHA26715.1 asyd|OJJ54118.1 ater|EAU32725.1 atub|OJI86673.1 aver|OJJ08618.1 awen|OJJ34453.1 acar|OOF90978.1 afis|EAW20895.1 aory|EIT77360.1 ater|EAU38529.1

Group661: acar|OOF95637.1 acar|OOF95688.1 aver|OJJ05622.1 aver|OJJ07803.1 abra|OJJ74674.1 acla|EAW10723.1 afis|EAW24850.1 afla|EED47121.1 afum|EAL89496.1 agla|OJJ82064.1 akaw|GAA81939.1 aluc|OJZ92251.1 anid|CBF75837.1 anig|EHA26705.1 aory|EIT79282.1 asyd|OJJ57556.1 ater|EAU36453.1 atub|OJI86684.1 awen|OJJ40435.1 azon|OJJ49296.1

Group662: acar|OOF96241.1 acar|OOF96278.1 afum|EAL85174.2 afum|EAL87772.2 abra|OJJ71525.1 acla|EAW15082.1 afis|EAW20054.1 afla|EED45326.1 agla|OJJ88906.1 akaw|GAA89392.1 aluc|OJZ86265.1 anid|CBF84810.1 anig|EHA21603.1 aory|EIT73801.1 asyd|OJJ63220.1 ater|EAU38663.1 atub|OJI84278.1 aver|OJI97735.1 awen|OJJ31812.1 azon|OJJ46310.1

Group663: acar|OOF96244.1 acar|OOF96281.1 afum|EAL85177.1 afum|EAL87761.1 abra|OJJ71529.1 acla|EAW15085.1 afis|EAW20057.1 afla|EED45323.1 agla|OJJ88903.1 akaw|GAA89389.1 aluc|OJZ86269.1 anid|CBF84842.1 anig|EHA21601.1 aory|EIT73791.1 asyd|OJJ63216.1 ater|EAU38666.1 atub|OJI84273.1 aver|OJI97739.1 awen|OJJ31816.1 azon|OJJ46307.1

Group664: acar|OOF96246.1 acar|OOF96283.1 afum|EAL85179.1 afum|EAL87759.1 abra|OJJ71533.1 acla|EAW15087.1 afis|EAW20059.1 afla|EED45321.1 agla|OJJ88901.1 akaw|GAA89387.1 aluc|OJZ86271.1 anid|CBF84838.1 anig|EHA21598.1 aory|EIT73620.1 asyd|OJJ63214.1 ater|EAU38668.1 atub|OJI84270.1 aver|OJI97741.1 awen|OJJ31819.1 azon|OJJ46305.1

Group665: acar|OOF96247.1 acar|OOF96284.1 afum|EAL85180.1 afum|EAL87758.1 abra|OJJ71534.1 acla|EAW15088.1 afis|EAW20060.1 afla|EED45319.1 agla|OJJ88899.1 akaw|GAA89386.1 aluc|OJZ86272.1 anid|CBF84836.1 anig|EHA21597.1 aory|EIT73579.1 asyd|OJJ63213.1 ater|EAU38669.1 atub|OJI84268.1 aver|OJI97742.1 awen|OJJ31820.1 azon|OJJ46304.1

Group666: acar|OOF97654.1 acar|OOF97701.1 abra|OJJ71503.1 acla|EAW10045.1 afis|EAW25539.1 afla|EED44716.1 afum|EAL90202.1 agla|OJJ87266.1 akaw|GAA82517.1 aluc|OJZ87044.1 anid|CBF77824.1 anig|EHA20317.1 aory|EIT83461.1 asyd|OJJ53006.1 ater|EAU33380.1 atub|OJI81591.1 aver|OJJ03837.1 awen|OJJ41281.1 azon|OJJ49499.1 afla|EED44715.1

Group667: acar|OOF97687.1 acar|OOF97737.1 abra|OJJ71461.1 acla|EAW10090.1 afis|EAW25497.1 afla|EED53997.1 afum|EAL90163.1 agla|OJJ87307.1 akaw|GAA82552.1 aluc|OJZ87083.1 anid|CBF77751.1 anig|EHA20354.1 aory|EIT78321.1 asyd|OJJ53046.1 asyd|OJJ54800.1 ater|EAU34668.1 atub|OJI81554.1 aver|OJI95782.1 aver|OJJ03799.1 awen|OJJ41240.1

Group668: acar|OOF99309.1 acar|OOF99314.1 abra|OJJ74376.1 akaw|GAA87275.1 aluc|OJZ89746.1 anig|EHA26968.1 atub|OJI85056.1 abra|OJJ78032.1 akaw|GAA85950.1 aluc|OJZ91112.1 anig|EHA19809.1 aory|EIT82873.1 asyd|OJJ64719.1 ater|EAU30104.1 atub|OJI83109.1 aver|OJJ05112.1 awen|OJJ33401.1 azon|OJJ45152.1 acla|EAW11016.1 awen|OJJ38250.1

Group669: acla|EAW06566.1 acla|EAW13723.1 afis|EAW16030.1 afis|EAW19868.1 afum|EAL85029.1 afum|EAL88661.1 agla|OJJ86058.1 agla|OJJ87705.1 awen|OJJ30418.1 awen|OJJ39976.1 abra|OJJ67634.1 acar|OOF91979.1 afla|EED48196.1 akaw|GAA89153.1 aluc|OJZ81683.1 anig|EHA25759.1 aory|EIT72945.1 asyd|OJJ52747.1 atub|OJI88498.1 azon|OJJ45425.1

Group670: acla|EAW07085.1 acla|EAW08664.1 abra|OJJ67836.1 afum|EAL87810.1 agla|OJJ83639.1 akaw|GAA84845.1 aluc|OJZ86826.1 anig|EHA28243.1 asyd|OJJ62960.1 ater|EAU36707.1 atub|OJI89149.1 aver|OJJ06460.1 aver|OJJ03911.1 anig|EHA28234.1 asyd|OJJ64572.1 atub|OJI83692.1 aver|OJI98007.1 anid|CBF78723.1 aver|OJI98669.1 aver|OJJ06464.1

Group671: acla|EAW10425.1 acla|EAW11189.1 afum|EAL88556.2 afum|EAL89817.2 abra|OJJ70818.1 acar|OOF98284.1 afis|EAW25144.1 afla|EED55301.1 agla|OJJ89017.1 akaw|GAA91697.1 aluc|OJZ87695.1 anid|CBF85681.1 anig|EHA20883.1 aory|EIT83608.1 asyd|OJJ64255.1 ater|EAU39416.1 atub|OJI80947.1 aver|OJI98346.1 awen|OJJ40769.1 azon|OJJ46879.1

Group672: afis|EAW16434.1 afis|EAW20510.1 agla|OJJ84787.1 agla|OJJ85600.1 abra|OJJ76837.1 acar|OOF91329.1 acla|EAW13399.1 afla|EED52451.1 afum|EAL84546.1 akaw|GAA87186.1 aluc|OJZ84248.1 anid|CBF79187.1 anig|EHA27593.1 aory|EIT82791.1 asyd|OJJ55716.1 ater|EAU37204.1 atub|OJI89835.1 aver|OJJ06802.1 awen|OJJ33211.1 azon|OJJ46484.1

Group673: afis|EAW17153.1 afis|EAW21838.1 afum|EAL87745.2 afum|EBA27475.1 abra|OJJ75520.1 acar|OOF90847.1 acla|EAW09307.1 afla|EED46537.1 agla|OJJ84300.1 akaw|GAA84284.1 aluc|OJZ88977.1 anid|CBF76703.1 anig|EHA23548.1 aory|EIT72350.1 asyd|OJJ61234.1 ater|EAU38513.1 atub|OJI85812.1 aver|OJJ00557.1 awen|OJJ34114.1 azon|OJJ44226.1

Group674: afis|EAW17990.1 afis|EAW20711.1 afum|EAL91127.1 afum|EAL91800.1 abra|OJJ68535.1 acar|OOF92737.1 acla|EAW12825.1 afla|EED51913.1 agla|OJJ80250.1 akaw|GAA91316.1 aluc|OJZ82748.1 anid|CBF79983.1 anig|EHA21198.1 aory|EIT81369.1 asyd|OJJ58245.1 ater|EAU31501.1 atub|OJI88983.1 aver|OJJ02181.1 awen|OJJ41556.1 azon|OJJ48271.1

Group675: afis|EAW19531.1 afis|EAW22425.1 afum|EAL86975.1 afum|EAL90766.1 abra|OJJ74345.1 acar|OOF95384.1 acla|EAW07399.1 afla|EED52503.1 agla|OJJ87607.1 akaw|GAA86685.1 aluc|OJZ89785.1 anid|CBF88811.1 anig|EHA27000.1 asyd|OJJ62888.1 ater|EAU39282.1 atub|OJI85020.1 aver|OJI96940.1 awen|OJJ35004.1 azon|OJJ42263.1 azon|OJJ45640.1

Group676: afis|EAW23730.1 afis|EAW25103.1 awen|OJJ30012.1 awen|OJJ40717.1 abra|OJJ65991.1 acar|OOF92881.1 acla|EAW10464.1 acla|EAW11709.1 afla|EED46010.1 afum|EAL87168.1 akaw|GAA90865.1 aluc|OJZ82968.1 anid|CBF78451.1 anid|CBF84695.1 anig|EHA21384.1 aory|EIT75669.1 ater|EAU34822.1 atub|OJI89201.1 aver|OJJ08430.1 azon|OJJ48019.1

Group677: afla|EED45834.1 afla|EED51243.1 aory|EIT75702.1 aory|EIT83187.1 abra|OJJ69216.1 acar|OOF91102.1 acla|EAW08629.1 afis|EAW15802.1 afum|EAL85548.1 agla|OJJ81969.1 akaw|GAA92225.1 aluc|OJZ86198.1 anid|CBF70916.1 anig|EHA24425.1 asyd|OJJ52604.1 ater|EAU33446.1 atub|OJI79990.1 aver|OJJ04630.1 awen|OJJ38512.1 azon|OJJ43740.1

Group678: afla|EED46513.1 afla|EED55226.1 aory|EIT72395.1 aory|EIT83617.1 abra|OJJ72126.1 acar|OOF96766.1 acla|EAW14932.1 afis|EAW24447.1 afum|EAL86116.1 agla|OJJ80293.1 akaw|GAA84797.1

aluc|OJZ86883.1 anid|CBF73809.1 anig|EHA21761.1 asyd|OJJ56577.1 ater|EAU29999.1 atub|OJI83636.1 aver|OJJ06146.1 awen|OJJ32750.1 azon|OJJ43988.1

Group679: afla|EED46574.1 afla|EED49749.1 afla|EED52838.1 akaw|GAA87766.1 akaw|GAA91614.1 aluc|OJZ82440.1 aluc|OJZ85401.1 aluc|OJZ82904.1 anig|EHA21327.1 anig|EHA28333.1 aory|EIT77940.1 aory|EIT78630.1 abra|OJJ69634.1 agla|OJJ80224.1 asyd|OJJ52421.1 ater|EAU37501.1 atub|OJI88000.1 awen|OJJ31941.1 atub|OJI89130.1 akaw|GAA90922.1

Group680: afla|EED47735.1 afla|EED54121.1 abra|OJJ66976.1 acar|OOF93637.1 acla|EAW08563.1 afis|EAW23605.1 afum|EAL87861.1 agla|OJJ86863.1 akaw|GAA92189.1 aluc|OJZ85601.1 anid|CBF89577.1 anig|EHA24923.1 aory|EIT75914.1 aory|EIT77123.1 asyd|OJJ62333.1 ater|EAU35372.1 atub|OJI79396.1 aver|OJI96439.1 awen|OJJ39929.1 ater|EAU39302.1

Group681: agla|OJJ78415.1 agla|OJJ84293.1 abra|OJJ69363.1 acar|OOF94362.1 acla|EAW14753.1 afis|EAW24284.1 afla|EED47992.1 afum|EAL85953.1 akaw|GAA88232.1 aluc|OJZ82196.1 anid|CBF74135.1 anig|EHA28541.1 aory|EIT79812.1 asyd|OJJ53915.1 ater|EAU31232.1 atub|OJI88239.1 aver|OJJ08013.1 awen|OJJ33012.1 azon|OJJ48503.1 agla|OJJ83146.1

Group682: agla|OJJ78543.1 agla|OJJ79518.1 anid|CBF74064.1 anid|CBF77736.1 anid|CBF83503.1 anid|CBF87508.1 anid|CBF88991.1 anid|CBF89857.1 abra|OJJ69705.1 acla|EAW09195.1 afis|EAW18410.1 afla|EED46570.1 afum|EAL93090.1 aluc|OJZ80203.1 aory|EIT77911.1 asyd|OJJ53853.1 awen|OJJ41393.1 asyd|OJJ62070.1 anid|CBF73616.1 asyd|OJJ56787.1

Group683: agla|OJJ79096.1 agla|OJJ82949.1 abra|OJJ65777.1 acar|OOF92088.1 afla|EED45916.1 akaw|GAA90885.1 aluc|OJZ82946.1 anid|CBF84425.1 anig|EHA21364.1 aory|EIT75550.1 atub|OJI89178.1 abra|OJJ72700.1 afis|EAW19995.1 afum|EAL84921.1 asyd|OJJ54671.1 aver|OJJ07499.1 awen|OJJ30436.1 afla|EED49831.1 asyd|OJJ53641.1 aver|OJJ01165.1

Group684: agla|OJJ79930.1 agla|OJJ82748.1 awen|OJJ29762.1 awen|OJJ33576.1 abra|OJJ70370.1 acar|OOF91900.1 acla|EAW12338.1 afis|EAW16884.1 afla|EED47358.1 afum|EAL86420.1 akaw|GAA91980.1 aluc|OJZ82634.1 anid|CBF80682.1 anig|EHA27445.1 aory|EIT82887.1 asyd|OJJ54592.1 ater|EAU30230.1 atub|OJI88865.1 aver|OJJ07560.1 azon|OJJ50386.1

Group685: akaw|GAA85714.1 akaw|GAA87141.1 aluc|OJZ84296.1 aluc|OJZ91389.1 aory|EIT81041.1 aory|EIT81749.1 atub|OJI83382.1 atub|OJI89773.1 abra|OJJ78316.1 acar|OOF90169.1 acla|EAW09510.1 afis|EAW21622.1 afla|EED51839.1 afum|EAL92658.1 anig|EHA20067.1 asyd|OJJ65401.1 ater|EAU38311.1 aver|OJI95769.1 awen|OJJ38680.1 azon|OJJ47988.1

Group686: aluc|OJZ79488.1 aluc|OJZ79629.1 abra|OJJ68492.1 acar|OOF90053.1 acla|EAW12140.1 afis|EAW17196.1 afla|EED45178.1 afum|EAL86624.1 agla|OJJ86658.1 akaw|GAA92778.1 aluc|OJZ82705.1 anid|CBF69887.1 anig|EHA21160.1 aory|EIT73771.1 asyd|OJJ59009.1 ater|EAU31750.1 atub|OJI88938.1 aver|OJJ01606.1 azon|OJJ51828.1 asyd|OJJ53646.1

Group687: anid|CBF84338.1 anid|CBF85236.1 abra|OJJ66254.1 acar|OOF90725.1 acla|EAW13654.1 afis|EAW19951.1 afum|EAL84964.1 aluc|OJZ80562.1 atub|OJI88369.1 aver|OJI97993.1 abra|OJJ68406.1 afla|EED50983.1 akaw|GAA92542.1 aluc|OJZ90090.1 anig|EHA20984.1 aory|EIT75403.1 asyd|OJJ62976.1 atub|OJI84715.1 afla|EED45866.1 aory|EIT77017.1

Group688: aory|EIT72414.1 aory|EIT75970.1 abra|OJJ66829.1 acar|OOF91607.1 acla|EAW14412.1 afis|EAW19120.1 afla|EED46512.1 afla|EED54294.1 afum|EAL93852.1 agla|OJJ80435.1 akaw|GAA90466.1 aluc|OJZ85758.1 anid|CBF75403.1 anig|EHA24798.1 asyd|OJJ59823.1 ater|EAU37383.1 atub|OJI79543.1 aver|OJJ02464.1 awen|OJJ37451.1 azon|OJJ51128.1

Group689: aory|EIT73542.1 aory|EIT78881.1 awen|OJJ31263.1 awen|OJJ35154.1 awen|OJJ38678.1 abra|OJJ73576.1 acar|OOG00757.1 acla|EAW07494.1 afis|EAW22527.1 afla|EED54555.1 afum|EAL90667.1 agla|OJJ88671.1 akaw|GAA88574.1 aluc|OJZ88476.1 anig|EHA18193.1 asyd|OJJ54459.1 ater|EAU39184.1 atub|OJI91071.1 aver|OJJ07697.1 azon|OJJ49947.1

Group690: asyd|OJJ52010.1 asyd|OJJ52511.1 asyd|OJJ58584.1 abra|OJJ76189.1 acar|OOF99753.1 acla|EAW13913.1 afis|EAW18624.1 afum|EAL93335.1 agla|OJJ81236.1 akaw|GAA82133.1 aluc|OJZ92039.1 anid|CBF70490.1 anig|EHA22972.1 aory|EIT78164.1 ater|EAU34924.1 atub|OJI86473.1 aver|OJJ01188.1 awen|OJJ32087.1 azon|OJJ46666.1 afla|EED52723.1

Group691: asyd|OJJ52365.1 asyd|OJJ62740.1 aver|OJI96783.1 aver|OJJ01982.1 abra|OJJ74531.1 acar|OOF95221.1 acla|EAW07161.1 afis|EAW22176.1 afla|EED57555.1 afum|EAL91013.1 agla|OJJ87878.1 akaw|GAA82069.1 aluc|OJZ92113.1 anid|CBF89075.1 anig|EHA26832.1 aory|EIT81844.1 ater|EAU34181.1 atub|OJI86546.1 awen|OJJ34753.1 azon|OJJ45807.1

Group692: asyd|OJJ52442.1 asyd|OJJ59129.1 ater|EAU33481.1 ater|EAU38150.1 abra|OJJ76293.1 acar|OOF99538.1 acla|EAW14323.1 afis|EAW18965.1 afla|EED51014.1 afum|EAL93697.2 agla|OJJ85347.1 akaw|GAA82221.1 aluc|OJZ91929.1 anid|CBF69609.1 anig|EHA22891.1 aory|EIT76953.1 atub|OJI86372.1 aver|OJJ01717.1 awen|OJJ31565.1 azon|OJJ46798.1

Group693: asyd|OJJ52683.1 asyd|OJJ58527.1 aver|OJJ01128.1 aver|OJJ08669.1 abra|OJJ76471.1 acar|OOF99708.1 acla|EAW13962.1 afis|EAW18671.1 afla|EED50620.1 afum|EAL93390.1 agla|OJJ82404.1 akaw|GAA82383.1 aluc|OJZ91738.1 anid|CBF70581.1 anig|EHA22744.1 aory|EIT78086.1 ater|EAU38240.1 atub|OJI86196.1 awen|OJJ32143.1 azon|OJJ46724.1

Group694: asyd|OJJ52787.1 asyd|OJJ58807.1 aver|OJJ01410.1 aver|OJJ08762.1 abra|OJJ67374.1 acar|OOF92437.1 acla|EAW06381.1 afis|EAW18487.1 afla|EED52921.1 afum|EAL93176.1 agla|OJJ85082.1 akaw|GAA86448.1 aluc|OJZ84991.1 anid|CBF70149.1 anig|EHA25203.1 aory|EIT78597.1 ater|EAU29912.1 atub|OJI80609.1 awen|OJJ31041.1 azon|OJJ48423.1

Group695: asyd|OJJ54188.1 asyd|OJJ58628.1 aver|OJJ01234.1 aver|OJJ08551.1 abra|OJJ71860.1 acar|OOF96532.1 acla|EAW13891.1 afis|EAW18603.1 afla|EED52744.1 afum|EAL93311.1 agla|OJJ81655.1 akaw|GAA85055.1 aluc|OJZ86597.1 anid|CBF70407.1 anig|EHA22065.1 aory|EIT78216.1 ater|EAU34898.1 atub|OJI83926.1 awen|OJJ31169.1 azon|OJJ46646.1

Group696: asyd|OJJ54443.1 asyd|OJJ57339.1 awen|OJJ33856.1 awen|OJJ40919.1 abra|OJJ66256.1 acar|OOF90727.1 aluc|OJZ80564.1 anig|EHA25634.1 atub|OJI88367.1 awen|OJJ38260.1 abra|OJJ77747.1 akaw|GAA83887.1 aluc|OJZ90808.1 anid|CBF81976.1 anig|EHA19536.1 aory|EIT82874.1 atub|OJI82797.1 aver|OJJ05823.1 azon|OJJ50277.1 afla|EED47338.1

Group697: asyd|OJJ55918.1 asyd|OJJ60822.1 aver|OJJ00148.1 aver|OJJ04871.1 abra|OJJ75721.1 acar|OOF90261.1 acla|EAW09377.1 afis|EAW21758.1 afla|EED58019.1 afum|EAL92788.2 agla|OJJ84392.1 akaw|GAA84457.1 aluc|OJZ89173.1 anid|CBF83680.1 anig|EHA23382.1 aory|EIT79759.1 ater|EAU38432.1 atub|OJI85617.1 awen|OJJ34042.1 azon|OJJ48199.1

Group698: asyd|OJJ57403.1 asyd|OJJ62790.1 aver|OJI96837.1 aver|OJJ07752.1 abra|OJJ73561.1 acar|OOG00743.1 acla|EAW07478.1 afis|EAW22510.1 afla|EED54538.1 afum|EAL90683.1 agla|OJJ80868.1 akaw|GAA88593.1 aluc|OJZ88500.1 anid|CBF88979.1 anig|EHA18209.1 aory|EIT78737.1 ater|EAU39204.1 atub|OJI91087.1 awen|OJJ35136.1 azon|OJJ49963.1

Group699: asyd|OJJ61696.1 asyd|OJJ63657.1 aver|OJI95780.1 aver|OJI97277.1 abra|OJJ73686.1 acar|OOG00858.1 acla|EAW07601.1 afis|EAW22632.1 afla|EED49882.1 afum|EAL90561.1 agla|OJJ80987.1 akaw|GAA88473.1 aluc|OJZ88363.1 anid|CBF88232.1 anig|EHA18095.1 aory|EIT72462.1 ater|EAU39076.1 atub|OJI90959.1 awen|OJJ35254.1 azon|OJJ49852.1

Group700: asyd|OJJ63591.1 asyd|OJJ65132.1 abra|OJJ65990.1 acar|OOF92883.1 acla|EAW13601.1 afis|EAW20004.1 afla|EED46009.1 afum|EAL84915.1 agla|OJJ87963.1 akaw|GAA90864.1 aluc|OJZ82969.1 anig|EHA21385.1 aory|EIT75787.1 ater|EAU29342.1 atub|OJI89202.1 aver|OJJ07612.1 awen|OJJ30013.1 azon|OJJ43642.1 aver|OJI97342.1 ater|EAU30135.1

Group701: ater|EAU29422.1 ater|EAU29976.1 awen|OJJ32730.1 awen|OJJ40859.1 abra|OJJ72200.1 acar|OOF96820.1 acla|EAW14947.1 afis|EAW24462.1 afla|EED55204.1 afum|EAL86133.1 agla|OJJ80320.1 akaw|GAA84736.1 aluc|OJZ86950.1 anid|CBF73838.1 anig|EHA21697.1 aory|EIT83625.1 asyd|OJJ56557.1 atub|OJI83573.1 aver|OJJ06163.1 azon|OJJ45283.1

Group702: aver|OJI99622.1 aver|OJJ08782.1 abra|OJJ69941.1 acar|OOF93451.1 acla|EAW08881.1 afis|EAW15514.1 afla|EED49021.1 agla|OJJ80185.1 akaw|GAA87718.1 aluc|OJZ85345.1 anid|CBF87265.1 anig|EHA25533.1 asyd|OJJ60386.1 ater|EAU32610.1 atub|OJI80225.1 aver|OJI99715.1 awen|OJJ38168.1 azon|OJJ43128.1 afum|EAL85817.1 aory|EIT82522.1

Group703: aver|OJJ00302.1 aver|OJJ08608.1 abra|OJJ68423.1 acar|OOF93930.1 acla|EAW11595.1 afis|EAW23861.1 afla|EED51802.1 afum|EAL87289.1 agla|OJJ87920.1 akaw|GAA86949.1 aluc|OJZ90074.1 anid|CBF82425.1 anig|EHA20999.1 aory|EIT81707.1 asyd|OJJ54128.1 asyd|OJJ60984.1 ater|EAU30753.1 atub|OJI84732.1 awen|OJJ36149.1 azon|OJJ43896.1

Group704: aver|OJJ03177.1 aver|OJJ07034.1 abra|OJJ72236.1 acar|OOF96858.1 acla|EAW14993.1 afis|EAW24519.1 afla|EED47314.1 afum|EAL86185.1 akaw|GAA84703.1 aluc|OJZ86984.1 anid|CBF71222.1 anig|EHA21663.1 aory|EIT82888.1 asyd|OJJ55173.1 ater|EAU29959.1 atub|OJI83535.1 awen|OJJ32602.1 afla|EED57070.1 aory|EIT82265.1 azon|OJJ48731.1

Group705: azon|OJJ42149.1 azon|OJJ43551.1 abra|OJJ67976.1 acar|OOF92817.1 acla|EAW11759.1 afis|EAW23792.1 afla|EED51600.1 afum|EAL87226.1 agla|OJJ78821.1 akaw|GAA91227.1 aluc|OJZ82843.1 anid|CBF79854.1 anig|EHA21281.1 aory|EIT80489.1 asyd|OJJ58158.1 ater|EAU31332.1 atub|OJI89073.1 aver|OJJ04245.1 awen|OJJ33749.1 awen|OJJ34607.1

Group706: azon|OJJ42457.1 azon|OJJ44531.1 abra|OJJ77870.1 acar|OOF98903.1 afla|EED56222.1 akaw|GAA83780.1 aluc|OJZ90944.1 anid|CBF86194.1 anig|EHA19652.1 aory|EIT73290.1 asyd|OJJ64539.1 ater|EAU33742.1 atub|OJI82936.1 aver|OJI98635.1 awen|OJJ38717.1 acla|EAW11254.1 afis|EAW19361.1 afum|EAL94081.1 afla|EED49734.1 aory|EIT77649.1

Group707: azon|OJJ43566.1 azon|OJJ47404.1 azon|OJJ43567.1 abra|OJJ73130.1 acla|EAW12351.1 afis|EAW16776.1 afla|EED50851.1 afum|EAL86345.1 agla|OJJ79835.1 anig|EHA18704.1 aory|EIT78959.1

ater|EAU34771.1 azon|OJJ42286.1 akaw|GAA89139.1 aluc|OJZ81697.1 aory|EIT81792.1 atub|OJI88483.1 awen|OJJ29815.1 afla|EED55657.1 awen|OJJ34470.1

Group708: abra|OJJ65712.1 acla|EAW11438.1 afis|EAW24094.1 awen|OJJ32718.1 azon|OJJ51154.1 abra|OJJ68624.1 acla|EAW12144.1 aluc|OJZ81931.1 aory|EIT78678.1 asyd|OJJ58603.1 atub|OJI84484.1 abra|OJJ70046.1 acar|OOF98851.1 acla|EAW07758.1 akaw|GAA93052.1 anig|EHA25445.1 atub|OJI80326.1 ater|EAU38181.1 azon|OJJ51651.1 awen|OJJ36405.1

Group709: abra|OJJ65771.1 acar|OOG00532.1 afis|EAW15669.1 afum|EAL85679.1 agla|OJJ81807.1 akaw|GAA90889.1 aluc|OJZ82940.1 anig|EHA21360.1 asyd|OJJ53833.1 ater|EAU32495.1 atub|OJI89172.1 aver|OJJ07938.1 awen|OJJ38345.1 abra|OJJ69063.1 acar|OOF91220.1 akaw|GAA90593.1 aluc|OJZ86051.1 anig|EHA24560.1 atub|OJI79848.1 aver|OJJ03010.1

Group710: abra|OJJ65912.1 acar|OOF94962.1 afla|EED52047.1 agla|OJJ80820.1 akaw|GAA92320.1 aluc|OJZ90218.1 anig|EHA23716.1 aory|EIT81383.1 atub|OJI81650.1 abra|OJJ78044.1 agla|OJJ88747.1 aluc|OJZ80304.1 anid|CBF82048.1 asyd|OJJ54231.1 ater|EAU30133.1 atub|OJI87457.1 aver|OJJ05184.1 awen|OJJ39981.1 awen|OJJ41941.1 ater|EAU32853.1

Group711: abra|OJJ66027.1 acar|OOF98054.1 akaw|GAA84509.1 aluc|OJZ89238.1 atub|OJI87439.1 awen|OJJ39904.1 azon|OJJ45880.1 abra|OJJ68242.1 acar|OOG00392.1 acla|EAW08538.1 afis|EAW23576.1 afla|EED48210.1 afum|EAL87889.1 anid|CBF86753.1 aory|EIT72922.1 asyd|OJJ64708.1 ater|EAU32766.1 aver|OJI98810.1 awen|OJJ29877.1 acar|OOG00089.1

Group712: abra|OJJ66125.1 acar|OOF94014.1 acla|EAW12396.1 afis|EAW16844.1 afla|EED54593.1 afum|EAL86390.1 akaw|GAA92685.1 aluc|OJZ90143.1 anid|CBF82122.1 anig|EHA20926.1 aory|EIT78860.1 asyd|OJJ54293.1 ater|EAU29537.1 ater|EAU37948.1 atub|OJI84656.1 aver|OJJ08125.1 awen|OJJ41474.1 anid|CBF87727.1 awen|OJJ36145.1 aver|OJJ05248.1

Group713: abra|OJJ66251.1 acar|OOF90722.1 acla|EAW13684.1 afis|EAW19922.1 afla|EED50973.1 afum|EAL84987.2 agla|OJJ79334.1 akaw|GAA89036.1 aluc|OJZ80559.1 anid|CBF78449.1 anig|EHA25638.1 aory|EIT77050.1 asyd|OJJ52716.1 ater|EAU39414.1 atub|OJI88372.1 aver|OJJ04745.1 awen|OJJ29958.1 ater|EAU38132.1 awen|OJJ36275.1 anid|CBF71116.1

Group714: abra|OJJ66260.1 acar|OOF90729.1 afla|EED53425.1 agla|OJJ88623.1 aluc|OJZ80567.1 anid|CBF87285.1 anig|EHA25631.1 aory|EIT75214.1 asyd|OJJ56365.1 atub|OJI88364.1 aver|OJJ06349.1 awen|OJJ40804.1 azon|OJJ42356.1 abra|OJJ68264.1 acar|OOF97543.1 akaw|GAA83201.1 aluc|OJZ83702.1 anig|EHA18853.1 atub|OJI90074.1 acla|EAW12046.1

Group715: abra|OJJ66444.1 acar|OOG00548.1 acla|EAW08961.1 afis|EAW15440.1 afla|EED49520.1 afum|EAL84206.1 agla|OJJ89304.1 akaw|GAA86649.1 aluc|OJZ85207.1 anid|CBF80104.1 anig|EHA26091.1 aory|EIT82592.1 asyd|OJJ58321.1 aver|OJI98310.1 awen|OJJ32245.1 azon|OJJ50232.1 asyd|OJJ56181.1 aver|OJJ02088.1 asyd|OJJ52211.1 awen|OJJ36078.1

Group716: abra|OJJ66628.1 acar|OOF91051.1 acla|EAW10868.1 afis|EAW20990.1 afla|EED46119.1 afum|EAL84639.1 agla|OJJ84068.1 akaw|GAA91097.1 aluc|OJZ81420.1 anid|CBF82620.1 anig|EHA26001.1 aory|EIT77263.1 asyd|OJJ54019.1 ater|EAU38639.1 atub|OJI88761.1 aver|OJJ08721.1 awen|OJJ34320.1 azon|OJJ48067.1 aory|EIT76479.1 afla|EED50344.1

Group717: abra|OJJ66789.1 acar|OOF93863.1 afla|EED49543.1 akaw|GAA86855.1 aluc|OJZ89976.1 anig|EHA27350.1 aory|EIT82644.1 atub|OJI84832.1 acla|EAW08552.1 afis|EAW23594.1 afum|EAL87873.1 agla|OJJ86851.1 aver|OJJ05126.1 awen|OJJ39916.1 azon|OJJ45890.1 afla|EED49567.1 aory|EIT82571.1 aver|OJI96425.1 anid|CBF82020.1 asyd|OJJ56199.1

Group718: abra|OJJ66926.1 acar|OOF91694.1 akaw|GAA91491.1 anig|EHA24880.1 atub|OJI79444.1 abra|OJJ67524.1 acar|OOG00111.1 agla|OJJ87236.1 awen|OJJ29656.1 aluc|OJZ85159.1 anid|CBF84480.1 aver|OJI96719.1 awen|OJJ39541.1 afis|EAW23743.1 asyd|OJJ53652.1 aver|OJI98697.1 asyd|OJJ64600.1 agla|OJJ88829.1 aver|OJJ04862.1 ater|EAU31016.1

Group719: abra|OJJ66927.1 acar|OOF91695.1 acla|EAW14492.1 afis|EAW19206.1 afla|EED47706.1 afum|EAL93936.1 agla|OJJ88505.1 akaw|GAA91490.1 aluc|OJZ85652.1 anid|CBF78561.1 anig|EHA24881.1 aory|EIT80267.1 asyd|OJJ65063.1 ater|EAU37266.1 atub|OJI79443.1 aver|OJI99187.1 awen|OJJ37559.1 azon|OJJ51221.1 ater|EAU31748.1 awen|OJJ37930.1

Group720: abra|OJJ66958.1 acar|OOF93650.1 acla|EAW14962.1 afis|EAW24485.1 afla|EED45449.1 afum|EAL86154.1 agla|OJJ88956.1 akaw|GAA92203.1 aluc|OJZ85618.1 anid|CBF82826.1 anig|EHA24908.1 aory|EIT82465.1 asyd|OJJ55929.1 ater|EAU37956.1 atub|OJI79411.1 aver|OJJ04879.1 awen|OJJ34538.1 azon|OJJ51794.1 agla|OJJ83743.1 aver|OJJ00902.1

Group721: abra|OJJ67036.1 acla|EAW07070.1 afis|EAW20317.1 afum|EAL85445.1 agla|OJJ78448.1 akaw|GAA93114.1 abra|OJJ72609.1 acar|OOF94646.1 akaw|GAA85235.1 aluc|OJZ90591.1 anig|EHA24030.1 atub|OJI82014.1 azon|OJJ44436.1 aver|OJJ06054.1 acla|EAW08310.1 asyd|OJJ57393.1 azon|OJJ45053.1 acla|EAW15313.1 afla|EED55819.1 aory|EIT76576.1

Group722: abra|OJJ67530.1 afis|EAW20793.1 anig|EHA18697.1 ater|EAU31981.1 atub|OJI91610.1 aver|OJJ05285.1 abra|OJJ74028.1 acar|OOG01145.1 afla|EED54908.1 agla|OJJ83374.1 akaw|GAA87511.1 aluc|OJZ88009.1 anid|CBF87701.1 anig|EHA17789.1 aory|EIT80304.1 asyd|OJJ63330.1 atub|OJI90618.1 awen|OJJ38650.1 aver|OJI97632.1 anid|CBF84596.1

Group723: abra|OJJ67629.1 acar|OOF91975.1 acla|EAW08578.1 afis|EAW19829.1 afla|EED55476.1 afum|EAL85063.1 akaw|GAA89149.1 aluc|OJZ81687.1 anid|CBF81789.1 anig|EHA25756.1 aory|EIT80791.1 asyd|OJJ59633.1 ater|EAU38817.1 atub|OJI88494.1 aver|OJJ08870.1 azon|OJJ44622.1 acar|OOF94209.1 anid|CBF87415.1 asyd|OJJ52901.1 acla|EAW12655.1

Group724: abra|OJJ67755.1 agla|OJJ81288.1 aver|OJJ06529.1 awen|OJJ36303.1 abra|OJJ68240.1 acar|OOF91925.1 afis|EAW22251.1 afla|EED45387.1 anig|EHA27220.1 aory|EIT82443.1 asyd|OJJ64210.1 atub|OJI82856.1 awen|OJJ29879.1 afla|EED49833.1 agla|OJJ89342.1 aory|EIT83402.1 awen|OJJ29870.1 anid|CBF88940.1 aver|OJI99604.1 asyd|OJJ56544.1

Group725: abra|OJJ68054.1 acla|EAW07812.1 afla|EED48275.1 agla|OJJ87900.1 akaw|GAA90900.1 aluc|OJZ82926.1 anid|CBF87851.1 anig|EHA21348.1 aory|EIT72960.1 asyd|OJJ56525.1 ater|EAU29450.1 atub|OJI89156.1 aver|OJJ06198.1 awen|OJJ31590.1 aluc|OJZ80199.1 asyd|OJJ63418.1 ater|EAU38861.1 aver|OJI97520.1 awen|OJJ38874.1 awen|OJJ39633.1

Group726: abra|OJJ68111.1 acla|EAW15307.1 afla|EED55277.1 agla|OJJ83148.1 akaw|GAA92450.1 aluc|OJZ81568.1 anig|EHA27392.1 aory|EIT75017.1 asyd|OJJ62945.1 ater|EAU32219.1 atub|OJI88617.1 aver|OJI98045.1 awen|OJJ35781.1 afla|EED53156.1 awen|OJJ29938.1 ater|EAU30130.1 asyd|OJJ62946.1 aver|OJI98044.1 aver|OJJ07921.1 aver|OJJ03358.1

Group727: abra|OJJ68833.1 acar|OOF90556.1 acla|EAW11325.1 afis|EAW18170.1 afla|EED56323.1 afum|EAL87532.1 agla|OJJ85940.1 akaw|GAA90418.1 aluc|OJZ85812.1 anid|CBF82352.1 anig|EHA24747.1 aory|EIT73358.1 asyd|OJJ54213.1 ater|EAU33984.1 atub|OJI79598.1 aver|OJJ08518.1 awen|OJJ31290.1 ater|EAU33834.1 awen|OJJ30517.1 aluc|OJZ80361.1

Group728: abra|OJJ68942.1 acar|OOF91523.1 acla|EAW14240.1 afis|EAW19040.1 afla|EED50395.1 afum|EAL93769.1 agla|OJJ84572.1 akaw|GAA90311.1 aluc|OJZ85928.1 anig|EHA24668.1 aory|EIT82123.1 asyd|OJJ65324.1 ater|EAU32079.1 atub|OJI79718.1 aver|OJI99469.1 azon|OJJ46176.1 afla|EED56613.1 agla|OJJ84621.1 aory|EIT79679.1 awen|OJJ31261.1

Group729: abra|OJJ69052.1 acar|OOF91238.1 afis|EAW20344.1 afla|EED45064.1 afum|EAL85464.1 agla|OJJ85556.1 akaw|GAA90584.1 aluc|OJZ86042.1 anid|CBF85628.1 anig|EHA24569.1 aory|EIT73572.1 asyd|OJJ57132.1 ater|EAU37144.1 atub|OJI79829.1 aver|OJJ03172.1 awen|OJJ38366.1 azon|OJJ45225.1 ater|EAU37679.1 afla|EED56599.1 aory|EIT79596.1

Group730: abra|OJJ69229.1 acar|OOF91091.1 acla|EAW12717.1 afis|EAW15814.1 afla|EED52103.1 afum|EAL85537.1 agla|OJJ83067.1 akaw|GAA92237.1 aluc|OJZ86209.1 anid|CBF70901.1 anig|EHA24417.1 asyd|OJJ52596.1 ater|EAU33434.1 atub|OJI80003.1 aver|OJJ04623.1 awen|OJJ38527.1 afis|EAW17879.1 afum|EAL91687.1 aver|OJJ05547.1 awen|OJJ41685.1

Group731: abra|OJJ69242.1 acar|OOF91077.1 acla|EAW08606.1 afis|EAW15827.1 afla|EED51218.1 afum|EAL85524.1 agla|OJJ81990.1 akaw|GAA92251.1 aluc|OJZ86222.1 anid|CBF70872.1 anig|EHA24405.1 aory|EIT80412.1 asyd|OJJ52582.1 ater|EAU33421.1 atub|OJI80016.1 aver|OJJ04610.1 awen|OJJ38541.1 azon|OJJ43722.1 asyd|OJJ54750.1 aver|OJJ07417.1

Group732: abra|OJJ69380.1 acar|OOF94345.1 acla|EAW08419.1 afis|EAW23450.1 afla|EED57353.1 afum|EBA27331.1 agla|OJJ86147.1 akaw|GAA88248.1 aluc|OJZ82215.1 anid|CBF89797.1 anig|EHA28525.1 aory|EIT80130.1 asyd|OJJ53729.1 atub|OJI88220.1 aver|OJI98284.1 awen|OJJ31384.1 azon|OJJ47909.1 asyd|OJJ62197.1 aver|OJI96309.1 awen|OJJ39779.1

Group733: abra|OJJ69833.1 acar|OOF93358.1 afla|EED54260.1 agla|OJJ79197.1 akaw|GAA87827.1 aluc|OJZ85463.1 anid|CBF88684.1 anig|EHA25005.1 aory|EIT76164.1 asyd|OJJ56863.1 atub|OJI80100.1 aver|OJJ03462.1 awen|OJJ32851.1 abra|OJJ70031.1 acla|EAW15278.1 afis|EAW16787.1 afum|EAL86357.1 anig|EHA18716.1 ater|EAU36812.1 atub|OJI79320.1

Group734: abra|OJJ69864.1 acar|OOF90332.1 afis|EAW22301.1 afla|EED45628.1 agla|OJJ82723.1 akaw|GAA87792.1 aluc|OJZ85427.1 anid|CBF85184.1 anig|EHA25606.1 aory|EIT75771.1 ater|EAU32684.1 atub|OJI80148.1 aver|OJI96605.1 awen|OJJ31411.1 azon|OJJ42404.1 acar|OOF91868.1 afla|EED49499.1 aory|EIT82625.1 asyd|OJJ62470.1 ater|EAU38702.1

Group735: abra|OJJ70266.1 afla|EED54695.1 akaw|GAA86531.1 aluc|OJZ85079.1 anig|EHA25294.1 aory|EIT78911.1 atub|OJI80519.1 abra|OJJ78041.1 acla|EAW08785.1 afis|EAW15939.1 afla|EED54424.1 afum|EAL88574.1 agla|OJJ80367.1 aluc|OJZ80302.1 asyd|OJJ52753.1 ater|EAU31057.1 atub|OJI87455.1 aver|OJI96478.1 awen|OJJ39980.1 ater|EAU37127.1

Group736: abra|OJJ70395.1 acar|OOF90083.1 afla|EED52271.1 agla|OJJ85325.1 akaw|GAA91963.1 aluc|OJZ82654.1 anig|EHA27419.1 aory|EIT74349.1 asyd|OJJ61023.1 atub|OJI88885.1 aver|OJJ00344.1

awen|OJJ33584.1 afla|EED48143.1 aory|EIT74445.1 asyd|OJJ58659.1 afla|EED52287.1 afla|EED55148.1 aory|EIT81619.1 awen|OJJ34668.1 awen|OJJ37795.1

Group737: abra|OJJ70402.1 acar|OOF93249.1 acla|EAW14348.1 afis|EAW20782.1 afla|EED52329.1 afum|EAL84841.1 agla|OJJ85851.1 aory|EIT74419.1 asyd|OJJ62498.1 ater|EAU32955.1 aver|OJI96618.1 azon|OJJ44872.1 abra|OJJ77381.1 akaw|GAA91380.1 aluc|OJZ84789.1 anig|EHA28057.1 atub|OJI89293.1 aory|EIT74407.1 ater|EAU31843.1 azon|OJJ50673.1

Group738: acla|EAW07697.1 afis|EAW22714.1 afum|EAL90480.1 agla|OJJ78998.1 azon|OJJ49768.1 abra|OJJ76709.1 acar|OOF98067.1 akaw|GAA92489.1 aluc|OJZ91501.1 anid|CBF75580.1 anig|EHA22541.1 atub|OJI85954.1 awen|OJJ37863.1 afla|EED45147.1 aory|EIT73529.1 asyd|OJJ55486.1 aver|OJJ06565.1 awen|OJJ35349.1 awen|OJJ33591.1 azon|OJJ42132.1

Group739: abra|OJJ70566.1 acla|EAW08365.1 afis|EAW23395.1 afla|EED55562.1 afum|EAL88075.1 agla|OJJ86422.1 akaw|GAA83410.1 aluc|OJZ83909.1 anid|CBF74809.1 anig|EHA19043.1 aory|EIT82686.1 asyd|OJJ60147.1 ater|EAU35648.1 atub|OJI90280.1 aver|OJJ04116.1 awen|OJJ39484.1 azon|OJJ47686.1 acla|EAW12884.1 afis|EAW17793.1 afum|EAL91602.1

Group740: abra|OJJ70960.1 acar|OOF98170.1 acla|EAW10521.1 afis|EAW25045.1 afla|EED48351.1 afum|EAL89713.1 agla|OJJ89149.1 akaw|GAA82987.1 aluc|OJZ87572.1 anid|CBF85914.1 anig|EHA20774.1 aory|EIT80893.1 asyd|OJJ64399.1 ater|EAU35795.1 atub|OJI81077.1 aver|OJI98495.1 awen|OJJ40656.1 azon|OJJ44034.1 aory|EIT72298.1 afla|EED55406.1

Group741: abra|OJJ71146.1 acar|OOF98009.1 acar|OOF98010.1 acla|EAW10360.1 afis|EAW25233.1 afla|EED53679.1 afum|EAL89894.1 agla|OJJ79174.1 akaw|GAA82831.1 aluc|OJZ87392.1 anid|CBF85327.1 anig|EHA20621.1 anig|EHA20622.1 aory|EIT75303.1 asyd|OJJ64017.1 ater|EAU34392.1 atub|OJI81254.1 aver|OJI98103.1 awen|OJJ40899.1 azon|OJJ44526.1

Group742: abra|OJJ71723.1 acar|OOF96427.1 acla|EAW10027.1 afis|EAW15606.1 afum|EAL85727.1 akaw|GAA85167.1 aluc|OJZ86469.1 anig|EHA21441.1 atub|OJI84060.1 azon|OJJ45251.1 afla|EED55033.1 agla|OJJ82925.1 aory|EIT76156.1 asyd|OJJ53836.1 afla|EED48194.1 aory|EIT72936.1 azon|OJJ45977.1 afla|EED54138.1 aory|EIT81964.1 asyd|OJJ56435.1

Group743: abra|OJJ71853.1 acar|OOF96523.1 afla|EED52750.1 afum|EAL93305.1 agla|OJJ81650.1 akaw|GAA85064.1 aluc|OJZ86587.1 anid|CBF70395.1 anig|EHA22073.1 aory|EIT78183.1 asyd|OJJ58634.1 atub|OJI83936.1 aver|OJJ01240.1 awen|OJJ31160.1 acla|EAW13885.1 afis|EAW18597.1 ater|EAU34891.1 azon|OJJ46636.1 asyd|OJJ54226.1 aver|OJJ05179.1

Group744: abra|OJJ72081.1 acar|OOF90349.1 afla|EED45358.1 agla|OJJ82915.1 aluc|OJZ86835.1 aory|EIT82374.1 asyd|OJJ56205.1 atub|OJI83683.1 aver|OJJ05132.1 awen|OJJ35919.1 azon|OJJ42174.1 afla|EED45415.1 akaw|GAA92568.1 aluc|OJZ82578.1 anig|EHA22490.1 aory|EIT73709.1 asyd|OJJ65181.1 aver|OJI99335.1 aory|EIT82376.1 awen|OJJ39611.1

Group745: abra|OJJ72207.1 acar|OOF96828.1 acla|EAW14969.1 afis|EAW24492.1 afla|EED54697.1 afum|EAL86161.1 agla|OJJ80329.1 akaw|GAA84727.1 aluc|OJZ86959.1 anid|CBF71478.1 anig|EHA21689.1 aory|EIT78783.1 asyd|OJJ54943.1 ater|EAU29944.1 atub|OJI83564.1 aver|OJJ07252.1 awen|OJJ33962.1 azon|OJJ42613.1 azon|OJJ48606.1 awen|OJJ32703.1

Group746: abra|OJJ72535.1 acar|OOF94717.1 acla|EAW12742.1 afis|EAW17905.1 afla|EED46297.1 afum|EAL91714.1 agla|OJJ83037.1 akaw|GAA86257.1 aluc|OJZ90515.1 anid|CBF76985.1 anig|EHA23970.1 aory|EIT76354.1 asyd|OJJ57035.1 ater|EAU33123.1 atub|OJI81939.1 aver|OJJ03257.1 awen|OJJ41645.1 azon|OJJ48367.1 aluc|OJZ90514.1 aory|EIT76342.1

Group747: abra|OJJ72848.1 acar|OOF96049.1 acla|EAW11395.1 afis|EAW24026.1 afla|EED49128.1 afum|EAL87457.1 agla|OJJ86959.1 akaw|GAA85449.1 aluc|OJZ83357.1 anid|CBF84482.1 anig|EHA24222.1 aory|EIT78568.1 asyd|OJJ53530.1 ater|EAU29718.1 atub|OJI82248.1 aver|OJJ08220.1 awen|OJJ35499.1 azon|OJJ43175.1 acla|EAW12378.1 awen|OJJ40008.1

Group748: abra|OJJ73145.1 acar|OOG00357.1 acla|EAW07021.1 afis|EAW16647.1 afla|EED45442.1 afum|EAL89251.1 agla|OJJ82595.1 akaw|GAA88027.1 aluc|OJZ81286.1 anid|CBF82058.1 anig|EHA18585.1 aory|EIT82414.1 asyd|OJJ54238.1 ater|EAU39572.1 atub|OJI91493.1 aver|OJJ05190.1 awen|OJJ40067.1 azon|OJJ45557.1 aver|OJJ01178.1 afla|EED50972.1

Group749: abra|OJJ73181.1 acar|OOG00417.1 afla|EED47213.1 afum|EAL89070.1 akaw|GAA87994.1 aluc|OJZ81249.1 anig|EHA18557.1 ater|EAU36561.1 awen|OJJ40360.1 azon|OJJ49208.1 acla|EAW06859.1 afis|EAW16476.1 agla|OJJ82869.1 anid|CBF75495.1 aory|EIT79277.1 asyd|OJJ59750.1 atub|OJI91445.1 aver|OJJ02537.1 acar|OOG00418.1 atub|OJI91444.1

Group750: abra|OJJ73280.1 acar|OOG00516.1 acla|EAW06955.1 afis|EAW16570.1 afla|EED56094.1 afum|EAL89167.1 agla|OJJ82745.1 akaw|GAA92725.1 aluc|OJZ88769.1 anid|CBF81824.1 anig|EHA18465.1 aory|EIT76687.1 asyd|OJJ59656.1 ater|EAU36638.1 atub|OJI91342.1 aver|OJJ02626.1 awen|OJJ40248.1 azon|OJJ49110.1 afum|EAL87777.1 aory|EIT72969.1

Group751: abra|OJJ73388.1 acar|OOG00590.1 acla|EAW07000.1 afis|EAW16615.1 afla|EED44990.1 afum|EAL89221.1 agla|OJJ82629.1 akaw|GAA83101.1 aluc|OJZ88649.1 anid|CBF82980.1 anig|EHA18340.1 aory|EIT73600.1 asyd|OJJ53668.1 ater|EAU36700.1 atub|OJI91236.1 aver|OJJ07774.1 awen|OJJ40187.1 azon|OJJ49474.1 anig|EHA18404.1 ater|EAU37639.1

Group752: abra|OJJ73407.1 acar|OOG00604.1 acla|EAW07012.1 afis|EAW16632.1 afla|EED48324.1 afum|EAL89228.1 agla|OJJ82617.1 akaw|GAA83084.1 aluc|OJZ88631.1 anid|CBF82011.1 anig|EHA18321.1 aory|EIT73498.1 asyd|OJJ56190.1 ater|EAU36752.1 atub|OJI91215.1 aver|OJJ05118.1 awen|OJJ40077.1 azon|OJJ43490.1 aory|EIT76284.1 afla|EED48295.1

Group753: abra|OJJ73423.1 acar|OOG00627.1 acla|EAW07036.1 afis|EAW16662.1 afla|EED44895.1 afum|EAL89266.1 agla|OJJ78707.1 akaw|GAA83069.1 aluc|OJZ88620.1 anid|CBF84550.1 anig|EHA18311.1 aory|EIT83238.1 asyd|OJJ53473.1 ater|EAU31782.1 atub|OJI91205.1 aver|OJJ08283.1 awen|OJJ40051.1 azon|OJJ47485.1 afla|EED56569.1 aory|EIT79633.1

Group754: abra|OJJ73685.1 acar|OOG00855.1 acar|OOG00856.1 acla|EAW07600.1 afis|EAW22631.1 afla|EED49880.1 afla|EED49881.1 afum|EBA27410.1 agla|OJJ80989.1 akaw|GAA88474.1 aluc|OJZ88364.1 anid|CBF88234.1 anig|EHA18096.1 aory|EIT72461.1 ater|EAU39077.1 atub|OJI90961.1 aver|OJI97276.1 awen|OJJ35252.1 azon|OJJ49854.1 asyd|OJJ63658.1

Group755: abra|OJJ73793.1 acar|OOG00954.1 acla|EAW07685.1 afis|EAW22700.1 afla|EED49986.1 afum|EAL90492.1 agla|OJJ79009.1 akaw|GAA89412.1 aluc|OJZ88261.1 anid|CBF88081.1 anig|EHA18006.1 aory|EIT74617.1 asyd|OJJ63558.1 ater|EAU39002.1 atub|OJI90860.1 aver|OJI97374.1 awen|OJJ35337.1 azon|OJJ49781.1 ater|EAU30260.1 awen|OJJ38024.1

Group756: abra|OJJ73803.1 acar|OOG00958.1 acla|EAW07675.1 afis|EAW22690.1 afla|EED49983.1 afum|EAL90502.1 agla|OJJ79015.1 akaw|GAA89422.1 aluc|OJZ88250.1 anid|CBF88089.1 anig|EHA17993.1 aory|EIT74622.1 asyd|OJJ63564.1 ater|EAU39009.1 atub|OJI90849.1 aver|OJI97369.1 awen|OJJ33510.1 azon|OJJ49784.1 acar|OOG00352.1 afla|EED46078.1

Group757: abra|OJJ73866.1 acar|OOF97590.1 acla|EAW06976.1 afis|EAW16590.1 afla|EED46107.1 afum|EAL89185.1 agla|OJJ83704.1 akaw|GAA89476.1 aluc|OJZ88187.1 anid|CBF76004.1 anig|EHA17938.1 aory|EIT76398.1 asyd|OJJ57426.1 ater|EAU29326.1 atub|OJI90786.1 aver|OJJ03990.1 awen|OJJ40225.1 azon|OJJ49083.1 anid|CBF75976.1 asyd|OJJ60263.1

Group758: abra|OJJ74117.1 acar|OOG01222.1 acla|EAW07959.1 afis|EAW22994.1 afla|EED58187.1 afum|EAL90205.1 agla|OJJ83290.1 akaw|GAA87590.1 aluc|OJZ87915.1 anid|CBF87568.1 anig|EHA17710.1 aory|EIT79090.1 asyd|OJJ63243.1 ater|EAU30624.1 atub|OJI90527.1 aver|OJI97713.1 awen|OJJ38567.1 azon|OJJ50434.1 anig|EHA17709.1 afum|EAL90206.1

Group759: abra|OJJ74269.1 acar|OOF95462.1 acla|EAW07313.1 afis|EAW22333.1 afla|EED57749.1 afum|EAL90859.1 agla|OJJ87713.1 akaw|GAA86754.1 aluc|OJZ89864.1 anid|CBF78344.1 anig|EHA27064.1 aory|EIT81050.1 asyd|OJJ56131.1 ater|EAU39462.1 atub|OJI84944.1 aver|OJJ05072.1 awen|OJJ34911.1 asyd|OJJ61756.1 aver|OJI95855.1 agla|OJJ78853.1

Group760: abra|OJJ74757.1 acar|OOF95750.1 acla|EAW10654.1 afis|EAW24913.1 afla|EED46998.1 afum|EAL89567.1 agla|OJJ82924.1 akaw|GAA83654.1 aluc|OJZ92341.1 anid|CBF75675.1 anig|EHA26633.1 aory|EIT72680.1 asyd|OJJ57485.1 ater|EAU36385.1 atub|OJI86767.1 aver|OJJ05695.1 awen|OJJ40509.1 azon|OJJ49368.1 afla|EED48195.1 aory|EIT72956.1

Group761: abra|OJJ74761.1 acar|OOF95754.1 acla|EAW10650.1 afis|EAW24917.1 afla|EED47002.1 afum|EAL89572.2 agla|OJJ82145.1 akaw|GAA83650.1 aluc|OJZ92345.1 anid|CBF75683.1 anig|EHA26629.1 aory|EIT72569.1 asyd|OJJ57489.1 ater|EAU36381.1 atub|OJI86771.1 aver|OJJ05691.1 awen|OJJ40513.1 azon|OJJ49372.1 anid|CBF75685.1 atub|OJI86772.1

Group762: abra|OJJ74833.1 acar|OOF95823.1 acla|EAW11247.1 afis|EAW18253.1 afla|EED56199.1 afum|EAL87616.1 agla|OJJ88191.1 akaw|GAA83587.1 aluc|OJZ92410.1 anid|CBF86203.1 anig|EHA26555.1 aory|EIT73440.1 asyd|OJJ58331.1 ater|EAU33725.1 atub|OJI86840.1 aver|OJJ02080.1 awen|OJJ30627.1 azon|OJJ47986.1 aver|OJI98640.1 asyd|OJJ64544.1

Group763: abra|OJJ74891.1 acar|OOF95894.1 acla|EAW12408.1 afis|EAW17228.1 afla|EED45013.1 agla|OJJ86664.1 akaw|GAA83528.1 aluc|OJZ92468.1 anig|EHA26500.1 aory|EIT73647.1 asyd|OJJ62261.1 ater|EAU35311.1 atub|OJI86897.1 aver|OJI96370.1 awen|OJJ39852.1 afla|EED54064.1 aory|EIT76126.1 asyd|OJJ56095.1 aver|OJJ05039.1 afla|EED46550.1

Group764: abra|OJJ74964.1 acar|OOF97350.1 acla|EAW08410.1 afis|EAW23459.1 afla|EED57342.1 afum|EAL88007.2 agla|OJJ86690.1 akaw|GAA83462.1 aluc|OJZ92534.1 anid|CBF89818.1 anig|EHA26441.1 aory|EIT80102.1 asyd|OJJ62188.1 ater|EAU37472.1 atub|OJI86970.1 aver|OJI96297.1 awen|OJJ39771.1 azon|OJJ47905.1 afla|EED57341.1 aory|EIT80055.1

Group765: abra|OJJ75326.1 acar|OOF90139.1 acla|EAW12017.1 afis|EAW19657.1 afla|EED57008.1 afum|EAL86866.1 agla|OJJ80802.1 akaw|GAA92021.1 aluc|OJZ92889.1 anid|CBF82008.1 anig|EHA26074.1

aory|EIT82264.1 asyd|OJJ55074.1 asyd|OJJ65337.1 ater|EAU32873.1 atub|OJI87347.1 aver|OJI99483.1 aver|OJJ07134.1 awen|OJJ41915.1 agla|OJJ85377.1

Group766: abra|OJJ75618.1 acar|OOG00217.1 acla|EAW09356.1 afis|EAW21782.1 afla|EED46350.1 afum|EAL92810.1 agla|OJJ84341.1 akaw|GAA84367.1 aluc|OJZ89071.1 anid|CBF76611.1 anig|EHA23465.1 aory|EIT81207.1 asyd|OJJ61187.1 ater|EAU38459.1 atub|OJI85717.1 aver|OJJ00510.1 awen|OJJ34070.1 azon|OJJ48159.1 afum|EAL85431.1 anid|CBF76609.1

Group767: abra|OJJ75770.1 acla|EAW09729.1 afis|EAW21415.1 afla|EED54885.1 afum|EAL92430.1 agla|OJJ78973.1 akaw|GAA89736.1 aluc|OJZ82585.1 anid|CBF82747.1 aory|EIT79345.1 asyd|OJJ54779.1 ater|EAU34053.1 atub|OJI87188.1 awen|OJJ30924.1 azon|OJJ44473.1 afis|EAW17192.1 afum|EAL86620.1 akaw|GAA92574.1 anid|CBF80434.1 agla|OJJ79605.1

Group768: abra|OJJ75774.1 acla|EAW12650.1 afis|EAW18035.1 afla|EED45683.1 afum|EAL91846.1 agla|OJJ82507.1 anid|CBF81936.1 anig|EHA18442.1 aory|EIT76073.1 asyd|OJJ58678.1 ater|EAU31610.1 atub|OJI87184.1 aver|OJJ01290.1 awen|OJJ30920.1 acar|OOG00708.1 afla|EED54070.1 asyd|OJJ57410.1 aver|OJJ05771.1 awen|OJJ35374.1 aory|EIT75690.1

Group769: abra|OJJ76070.1 acar|OOF99859.1 acla|EAW09749.1 afis|EAW21393.1 afla|EED55810.1 afum|EAL92407.1 agla|OJJ85517.1 akaw|GAA88897.1 aluc|OJZ89524.1 anid|CBF71432.1 anig|EHA23077.1 aory|EIT76869.1 asyd|OJJ61713.1 ater|EAU35164.1 atub|OJI85279.1 aver|OJJ07221.1 awen|OJJ39311.1 azon|OJJ51541.1 asyd|OJJ54970.1 ater|EAU32747.1

Group770: abra|OJJ76073.1 acar|OOF99853.1 afis|EAW16840.1 afla|EED47451.1 afum|EAL86387.1 akaw|GAA88894.1 aluc|OJZ89527.1 anig|EHA23074.1 aory|EIT81896.1 atub|OJI85276.1 aver|OJJ06456.1 awen|OJJ36309.1 abra|OJJ76507.1 agla|OJJ80808.1 akaw|GAA92901.1 anid|CBF73893.1 anig|EHA22710.1 asyd|OJJ53660.1 ater|EAU36678.1 aver|OJJ07768.1

Group771: abra|OJJ76117.1 acar|OOF99808.1 acla|EAW09703.1 afis|EAW21437.1 afla|EED45636.1 afum|EAL92452.2 agla|OJJ85581.1 akaw|GAA88850.1 aluc|OJZ89574.1 anid|CBF76585.1 anig|EHA23033.1 aory|EIT76558.1 asyd|OJJ61172.1 ater|EAU35108.1 atub|OJI85230.1 aver|OJJ00497.1 awen|OJJ39261.1 azon|OJJ51593.1 aory|EIT75602.1 afla|EED55865.1

Group772: abra|OJJ76529.1 acar|OOF99449.1 acla|EAW14176.1 afis|EAW18891.1 afla|EED50873.1 afum|EBA27495.1 agla|OJJ81497.1 akaw|GAA82440.1 akaw|GAA82441.1 aluc|OJZ91675.1 aluc|OJZ91676.1 anid|CBF69904.1 anig|EHA22693.1 aory|EIT79015.1 asyd|OJJ58997.1 ater|EAU38063.1 atub|OJI86130.1 aver|OJJ01619.1 awen|OJJ32450.1 azon|OJJ47208.1

Group773: abra|OJJ77055.1 acar|OOF92945.1 acla|EAW13514.1 afis|EAW19907.1 afla|EED54205.1 afum|EAL84999.1 agla|OJJ88625.1 akaw|GAA86997.1 aluc|OJZ84460.1 anid|CBF86958.1 anig|EHA27775.1 aory|EIT76071.1 asyd|OJJ55597.1 ater|EAU35255.1 atub|OJI89615.1 aver|OJJ06677.1 awen|OJJ33456.1 azon|OJJ50712.1 afis|EAW20612.1 acla|EAW13697.1

Group774: abra|OJJ77217.1 acar|OOF93104.1 acla|EAW09073.1 afis|EAW16463.1 afla|EED45621.1 afum|EAL89059.1 akaw|GAA90721.1 aluc|OJZ84628.1 anid|CBF82099.1 anig|EHA27915.1 aory|EIT75489.1 ater|EAU35691.1 atub|OJI89451.1 azon|OJJ51362.1 awen|OJJ34525.1 acla|EAW06842.1 afis|EAW25646.1 asyd|OJJ54269.1 agla|OJJ79388.1 afum|EAL84332.1

Group775: abra|OJJ77859.1 acar|OOF98914.1 acla|EAW11267.1 afis|EAW18231.1 afla|EED52136.1 afum|EAL87599.1 agla|OJJ85846.1 akaw|GAA83790.1 aluc|OJZ90933.1 anid|CBF86176.1 anig|EHA19643.1 aory|EIT73335.1 asyd|OJJ64527.1 ater|EAU33760.1 atub|OJI82925.1 aver|OJI98625.1 awen|OJJ30611.1 azon|OJJ48005.1 azon|OJJ42776.1 awen|OJJ36209.1

Group776: abra|OJJ77974.1 acar|OOF98840.1 acla|EAW06664.1 afis|EAW16101.1 afla|EED48636.1 afum|EAL88733.1 agla|OJJ86121.1 akaw|GAA83686.1 aluc|OJZ91047.1 anid|CBF85446.1 anig|EHA19744.1 aory|EIT73060.1 asyd|OJJ64094.1 ater|EAU33175.1 aver|OJI98176.1 awen|OJJ30331.1 azon|OJJ45359.1 anid|CBF85444.1 asyd|OJJ64092.1 atub|OJI83043.1

Group777: abra|OJJ78228.1 acar|OOF98631.1 afis|EAW21866.1 afla|EED46443.1 afum|EAL92880.2 agla|OJJ84269.1 akaw|GAA85796.1 aluc|OJZ91303.1 anid|CBF76759.1 anig|EHA19985.1 aory|EIT72387.1 asyd|OJJ61264.1 ater|EAU30863.1 atub|OJI83294.1 aver|OJJ00590.1 awen|OJJ34155.1 azon|OJJ43474.1 afla|EED54738.1 anid|CBF76761.1 aver|OJJ00591.1

Group778: abra|OJJ78259.1 acar|OOF98601.1 acla|EAW09251.1 afis|EAW21896.1 afla|EED46412.1 afum|EBA27479.1 agla|OJJ84236.1 akaw|GAA85766.1 aluc|OJZ91333.1 anig|EHA20015.1 aory|EIT72351.1 asyd|OJJ61292.1 ater|EAU30891.1 atub|OJI83326.1 aver|OJJ00618.1 awen|OJJ34185.1 azon|OJJ43448.1 aluc|OJZ91334.1 akaw|GAA85767.1 anid|CBF76812.1

Group779: abra|OJJ66219.1 abra|OJJ77447.1 awen|OJJ34603.1 awen|OJJ39635.1 acar|OOF90664.1 afla|EED48277.1 afla|EED48278.1 akaw|GAA89067.1 aluc|OJZ80525.1 anig|EHA25670.1 anig|EHA25671.1 aory|EIT72908.1 aory|EIT72950.1 ater|EAU37355.1 atub|OJI88405.1 anid|CBF74667.1 acar|OOF90665.1 anid|CBF74669.1 awen|OJJ34600.1

Group780: abra|OJJ67019.1 abra|OJJ74812.1 akaw|GAA83601.1 akaw|GAA93299.1 anig|EHA25612.1 anig|EHA26582.1 afis|EAW20313.1 afla|EED56766.1 afum|EAL85436.1 agla|OJJ83095.1 aluc|OJZ92393.1 ater|EAU31040.1 atub|OJI86823.1 awen|OJJ33515.1 acla|EAW07069.1 aory|EIT73952.1 asyd|OJJ56123.1 aver|OJJ05065.1 azon|OJJ42468.1

Group781: abra|OJJ67077.1 abra|OJJ70209.1 akaw|GAA86567.1 akaw|GAA91080.1 aluc|OJZ85118.1 aluc|OJZ87895.1 anig|EHA20194.1 anig|EHA25338.1 aory|EIT75167.1 aory|EIT80564.1 atub|OJI80475.1 atub|OJI80757.1 acar|OOF90366.1 afis|EAW16753.1 afla|EED51374.1 afum|EAL86325.1 agla|OJJ78769.1 aver|OJJ05553.1 afla|EED53531.1

Group782: abra|OJJ68020.1 abra|OJJ77410.1 acar|OOF92063.1 acar|OOF93277.1 akaw|GAA90931.1 akaw|GAA91354.1 anig|EHA21318.1 anig|EHA28081.1 atub|OJI89119.1 atub|OJI89267.1 acla|EAW09048.1 afla|EED46361.1 agla|OJJ81295.1 aluc|OJZ82894.1 aory|EIT81187.1 asyd|OJJ63773.1 aver|OJI97164.1 awen|OJJ30865.1 aluc|OJZ84818.1

Group783: abra|OJJ68371.1 abra|OJJ72091.1 acar|OOF96736.1 acla|EAW09472.1 afis|EAW21661.1 afla|EED57928.1 afum|EAL92696.1 agla|OJJ79735.1 akaw|GAA84829.1 aluc|OJZ86846.1 anid|CBF83566.1 anig|EHA21792.1 aory|EIT82318.1 asyd|OJJ60745.1 ater|EAU38349.1 atub|OJI83673.1 aver|OJJ00077.1 awen|OJJ33927.1 azon|OJJ48635.1

Group784: acar|OOF90452.1 acar|OOF90459.1 abra|OJJ71882.1 acla|EAW13911.1 afis|EAW18623.1 afla|EED52721.1 afum|EAL93333.1 agla|OJJ81237.1 akaw|GAA85034.1 aluc|OJZ86620.1 anid|CBF70493.1 anig|EHA22046.1 aory|EIT78225.1 asyd|OJJ58583.1 ater|EAU34923.1 atub|OJI83905.1 aver|OJJ01187.1 awen|OJJ32086.1 azon|OJJ46665.1

Group785: acar|OOF90454.1 acar|OOF90462.1 abra|OJJ71884.1 acla|EAW09637.1 afis|EAW21503.1 afla|EED55939.1 afum|EAL92522.1 agla|OJJ85666.1 akaw|GAA85032.1 aluc|OJZ86622.1 anid|CBF76453.1 anig|EHA22044.1 aory|EIT76619.1 asyd|OJJ61107.1 ater|EAU35035.1 atub|OJI83903.1 aver|OJJ00424.1 awen|OJJ39192.1 azon|OJJ51508.1

Group786: acar|OOF90455.1 acar|OOF90461.1 abra|OJJ71885.1 acla|EAW09636.1 afis|EAW21504.1 afla|EED55940.1 afum|EAL92523.1 agla|OJJ85667.1 akaw|GAA85031.1 aluc|OJZ86623.1 anid|CBF76451.1 anig|EHA22043.1 aory|EIT76886.1 asyd|OJJ61106.1 ater|EAU35034.1 atub|OJI83902.1 aver|OJJ00423.1 awen|OJJ39191.1 azon|OJJ51507.1

Group787: acar|OOF90456.1 acar|OOF90463.1 abra|OJJ71886.1 acla|EAW09635.1 afis|EAW21505.1 afla|EED55941.1 afum|EAL92524.1 agla|OJJ85668.1 akaw|GAA85030.1 aluc|OJZ86624.1 anid|CBF76449.1 anig|EHA22042.1 aory|EIT76597.1 asyd|OJJ61105.1 ater|EAU35033.1 atub|OJI83901.1 aver|OJJ00422.1 awen|OJJ39190.1 azon|OJJ51506.1

Group788: acar|OOF90909.1 acar|OOF90910.1 abra|OJJ67526.1 afla|EED49685.1 anig|EHA18706.1 aory|EIT81018.1 asyd|OJJ57249.1 ater|EAU38522.1 aver|OJJ03045.1 awen|OJJ31267.1 abra|OJJ75516.1 acar|OOF90843.1 afla|EED46523.1 akaw|GAA84280.1 aluc|OJZ88973.1 anig|EHA23552.1 atub|OJI85817.1 aory|EIT72379.1 agla|OJJ87127.1

Group789: acar|OOF92001.1 acar|OOF92003.1 abra|OJJ67845.1 acla|EAW11054.1 afis|EAW20822.1 afla|EED52866.1 afum|EAL84807.1 agla|OJJ88409.1 akaw|GAA91851.1 aluc|OJZ82047.1 anid|CBF78348.1 anig|EHA22211.1 aory|EIT78637.1 asyd|OJJ56141.1 atub|OJI84605.1 aver|OJJ05080.1 awen|OJJ29709.1 azon|OJJ45742.1 awen|OJJ34808.1

Group790: acar|OOF92286.1 acar|OOF92327.1 abra|OJJ67516.1 acla|EAW06255.1 afis|EAW18362.1 afla|EED53043.1 afum|EAL93033.1 agla|OJJ84926.1 akaw|GAA86324.1 aluc|OJZ84848.1 anid|CBF70825.1 anig|EHA25082.1 aory|EIT77408.1 asyd|OJJ58399.1 ater|EAU32230.1 atub|OJI80748.1 aver|OJJ01000.1 awen|OJJ31788.1 azon|OJJ50438.1

Group791: acar|OOF92287.1 acar|OOF92328.1 abra|OJJ67515.1 acla|EAW06254.1 afis|EAW18361.1 afla|EED53044.1 afum|EAL93032.2 agla|OJJ84925.1 akaw|GAA86325.1 aluc|OJZ84849.1 anid|CBF70827.1 anig|EHA25083.1 aory|EIT77438.1 asyd|OJJ58398.1 ater|EAU32229.1 atub|OJI80747.1 aver|OJJ00999.1 awen|OJJ31789.1 azon|OJJ42001.1

Group792: acar|OOF92288.1 acar|OOF92329.1 abra|OJJ67514.1 acla|EAW06260.1 afis|EAW18367.1 afla|EED53038.1 afum|EAL93041.1 agla|OJJ84931.1 akaw|GAA86326.1 aluc|OJZ84850.1 anid|CBF70815.1 anig|EHA25084.1 aory|EIT77399.1 asyd|OJJ58404.1 ater|EAU32236.1 atub|OJI80746.1 aver|OJJ01005.1 awen|OJJ31783.1 azon|OJJ50443.1

Group793: acar|OOF92289.1 acar|OOF92330.1 abra|OJJ67513.1 acla|EAW06259.1 afis|EAW18366.1 afla|EED53039.1 afum|EAL93040.1 agla|OJJ84930.1 akaw|GAA86327.1 aluc|OJZ84851.1 anid|CBF70817.1 anig|EHA25085.1 aory|EIT77396.1 asyd|OJJ58403.1 ater|EAU32235.1 atub|OJI80745.1 aver|OJJ01004.1 awen|OJJ31784.1 azon|OJJ50442.1

Group794: acar|OOF92291.1 acar|OOF92332.1 abra|OJJ67510.1 afis|EAW18368.1 afla|EED53037.1 afum|EAL93043.1 agla|OJJ84933.1 akaw|GAA86329.1 aluc|OJZ84854.1 anid|CBF70814.1 anig|EHA25087.1

aory|EIT77401.1 asyd|OJJ58405.1 ater|EAU32238.1 atub|OJI80742.1 aver|OJJ01006.1 awen|OJJ31781.1 azon|OJJ43320.1 acla|EAW06262.1

Group795: acar|OOF92293.1 acar|OOF92334.1 abra|OJJ67507.1 acla|EAW06264.1 afis|EAW18370.1 afla|EED53036.1 afum|EAL93045.1 agla|OJJ84935.1 akaw|GAA86331.1 aluc|OJZ84856.1 anid|CBF70808.1 anig|EHA25089.1 aory|EIT77405.1 asyd|OJJ58408.1 ater|EAU32240.1 atub|OJI80740.1 aver|OJJ01010.1 awen|OJJ31779.1 azon|OJJ50445.1

Group796: acar|OOF92297.1 acar|OOF92338.1 abra|OJJ67503.1 acla|EAW06267.1 afis|EAW18373.1 afla|EED53033.1 afum|EAL93048.1 agla|OJJ84937.1 akaw|GAA86335.1 aluc|OJZ84860.1 anid|CBF70800.1 anig|EHA25093.1 aory|EIT77403.1 asyd|OJJ58411.1 ater|EAU32243.1 atub|OJI80736.1 aver|OJJ01013.1 awen|OJJ31777.1 azon|OJJ50448.1

Group797: acar|OOF92298.1 acar|OOF92339.1 abra|OJJ67502.1 acla|EAW06268.1 afis|EAW18374.1 afla|EED53032.1 afum|EAL93049.1 agla|OJJ84938.1 akaw|GAA86336.1 aluc|OJZ84861.1 anid|CBF70798.1 anig|EHA25094.1 aory|EIT77421.1 asyd|OJJ58412.1 ater|EAU32244.1 atub|OJI80735.1 aver|OJJ01014.1 awen|OJJ31766.1 azon|OJJ50449.1

Group798: acar|OOF92299.1 acar|OOF92340.1 abra|OJJ67501.1 acla|EAW06269.1 afis|EAW18375.1 afla|EED53031.1 afum|EAL93050.1 agla|OJJ84939.1 akaw|GAA86337.1 aluc|OJZ84862.1 anid|CBF70797.1 anig|EHA25095.1 aory|EIT77415.1 asyd|OJJ58413.1 ater|EAU32245.1 atub|OJI80734.1 aver|OJJ01015.1 awen|OJJ31767.1 azon|OJJ50450.1

Group799: acar|OOF92301.1 acar|OOF92342.1 abra|OJJ67499.1 acla|EAW06271.1 afis|EAW18377.1 afla|EED53029.1 afum|EAL93052.1 agla|OJJ84941.1 akaw|GAA86339.1 aluc|OJZ84864.1 anid|CBF70793.1 anig|EHA25096.1 aory|EIT77426.1 asyd|OJJ58415.1 ater|EAU32247.1 atub|OJI80732.1 aver|OJJ01017.1 awen|OJJ31769.1 azon|OJJ50452.1

Group800: acar|OOF92303.1 acar|OOF92343.1 abra|OJJ67498.1 acla|EAW06272.1 afis|EAW18378.1 afla|EED53028.1 afum|EAL93053.1 agla|OJJ84942.1 akaw|GAA86340.1 aluc|OJZ84865.1 anid|CBF70791.1 anig|EHA25097.1 aory|EIT77397.1 asyd|OJJ58416.1 ater|EAU32248.1 atub|OJI80731.1 aver|OJJ01018.1 awen|OJJ31770.1 azon|OJJ50453.1

Group801: acar|OOF92304.1 acar|OOF92345.1 abra|OJJ67496.1 acla|EAW06274.1 afis|EAW18380.1 afla|EED53026.1 afum|EAL93055.1 agla|OJJ84944.1 akaw|GAA86342.1 aluc|OJZ84867.1 anid|CBF70787.1 anig|EHA25099.1 aory|EIT77406.1 asyd|OJJ58418.1 ater|EAU32249.1 atub|OJI80729.1 aver|OJJ01020.1 awen|OJJ31772.1 azon|OJJ50455.1

Group802: acar|OOF92305.1 acar|OOF92346.1 abra|OJJ67495.1 acla|EAW06275.1 afis|EAW18381.1 afla|EED53025.1 afum|EAL93056.1 agla|OJJ84945.1 akaw|GAA86343.1 aluc|OJZ84869.1 anid|CBF70785.1 anig|EHA25100.1 aory|EIT77407.1 asyd|OJJ58419.1 ater|EAU32250.1 atub|OJI80728.1 aver|OJJ01021.1 awen|OJJ31773.1 azon|OJJ50456.1

Group803: acar|OOF92306.1 acar|OOF92347.1 abra|OJJ67494.1 acla|EAW06276.1 afis|EAW18382.1 afla|EED53024.1 afum|EAL93057.1 agla|OJJ84946.1 akaw|GAA86344.1 aluc|OJZ84870.1 anid|CBF70783.1 anig|EHA25101.1 aory|EIT77418.1 asyd|OJJ58420.1 ater|EAU32251.1 atub|OJI80727.1 aver|OJJ01022.1 awen|OJJ31774.1 azon|OJJ50457.1

Group804: acar|OOF92307.1 acar|OOF92348.1 abra|OJJ67492.1 acla|EAW06278.1 afis|EAW18384.1 afla|EED53022.1 afum|EAL93059.1 agla|OJJ84948.1 akaw|GAA86346.1 aluc|OJZ84872.1 anid|CBF70781.1 anig|EHA25103.1 aory|EIT77395.1 asyd|OJJ58422.1 ater|EAU32253.1 atub|OJI80726.1 aver|OJJ01023.1 awen|OJJ31775.1 azon|OJJ50458.1

Group805: acar|OOF92308.1 acar|OOF92349.1 abra|OJJ67491.1 acla|EAW06279.1 afis|EAW18385.1 afla|EED53021.1 afum|EAL93060.1 agla|OJJ84949.1 akaw|GAA86347.1 aluc|OJZ84874.1 anid|CBF70779.1 anig|EHA25104.1 aory|EIT77414.1 asyd|OJJ58423.1 ater|EAU32254.1 atub|OJI80725.1 aver|OJJ01024.1 awen|OJJ31776.1 azon|OJJ45926.1

Group806: acar|OOF92310.1 acar|OOF92351.1 abra|OJJ67490.1 acla|EAW06282.1 afis|EAW18388.1 afla|EED53019.1 afum|EAL93063.1 agla|OJJ84951.1 akaw|GAA86348.1 aluc|OJZ84875.1 anid|CBF70772.1 anig|EHA25106.1 aory|EIT77433.1 asyd|OJJ58427.1 ater|EAU32261.1 atub|OJI80723.1 aver|OJJ01028.1 awen|OJJ31763.1 azon|OJJ50461.1

Group807: acar|OOF92311.1 acar|OOF92352.1 abra|OJJ67489.1 acla|EAW06284.1 afis|EAW18390.1 afla|EED53017.1 afum|EAL93065.1 agla|OJJ84953.1 akaw|GAA86350.1 aluc|OJZ84878.1 anid|CBF70776.1 anig|EHA25107.1 aory|EIT77439.1 asyd|OJJ58425.1 ater|EAU32263.1 atub|OJI80721.1 aver|OJJ01026.1 awen|OJJ31761.1 azon|OJJ50464.1

Group808: acar|OOF92312.1 acar|OOF92353.1 abra|OJJ67488.1 acla|EAW06286.1 afis|EAW18391.1 afla|EED53016.1 afum|EAL93066.1 agla|OJJ84955.1 akaw|GAA86351.1 aluc|OJZ84880.1 anid|CBF70777.1 anig|EHA25110.1 aory|EIT77398.1 asyd|OJJ58424.1 ater|EAU32278.1 atub|OJI80719.1 aver|OJJ01025.1 awen|OJJ31758.1 azon|OJJ50466.1

Group809: acar|OOF92314.1 acar|OOF92355.1 abra|OJJ67485.1 acla|EAW06289.1 afis|EAW18394.1 afla|EED53013.1 afum|EAL93070.1 agla|OJJ84966.1 akaw|GAA86354.1 aluc|OJZ84883.1 anid|CBF70762.1 anig|EHA25112.1 aory|EIT77419.1 asyd|OJJ58430.1 ater|EAU32282.1 atub|OJI80717.1 aver|OJJ01032.1 awen|OJJ31756.1 azon|OJJ50472.1

Group810: acar|OOF92317.1 acar|OOF92359.1 abra|OJJ67482.1 acla|EAW06291.1 afis|EAW18397.1 afla|EED53009.1 afum|EAL93074.1 agla|OJJ84968.1 akaw|GAA86357.1 aluc|OJZ84886.1 anid|CBF70756.1 anig|EHA25115.1 aory|EIT77428.1 asyd|OJJ58434.1 ater|EAU32285.1 atub|OJI80714.1 aver|OJJ01035.1 awen|OJJ31754.1 azon|OJJ50470.1

Group811: acar|OOF92318.1 acar|OOF92360.1 afum|EAL85117.1 afum|EAL93075.1 abra|OJJ67480.1 acla|EAW06292.1 afis|EAW18398.1 afla|EED53008.1 akaw|GAA86358.1 aluc|OJZ84887.1 anid|CBF70755.1 anig|EHA25116.1 aory|EIT77431.1 asyd|OJJ58436.1 ater|EAU29814.1 atub|OJI80713.1 aver|OJJ01036.1 awen|OJJ31752.1 azon|OJJ50468.1

Group812: acar|OOF92320.1 acar|OOF92362.1 abra|OJJ67474.1 acla|EAW06298.1 afis|EAW18405.1 afla|EED53003.1 afum|EAL93085.1 agla|OJJ84956.1 akaw|GAA86363.1 aluc|OJZ84891.1 anid|CBF69754.1 anig|EHA25120.1 aory|EIT77409.1 asyd|OJJ58942.1 ater|EAU29822.1 atub|OJI80707.1 aver|OJJ01538.1 awen|OJJ31745.1 azon|OJJ50505.1

Group813: acar|OOF92596.1 acar|OOF92617.1 abra|OJJ68969.1 acla|EAW14218.1 afis|EAW19063.1 afla|EED54429.1 afum|EAL93792.1 agla|OJJ84544.1 akaw|GAA90496.1 aluc|OJZ85956.1 anid|CBF79645.1 anig|EHA24645.1 aory|EIT75848.1 asyd|OJJ58020.1 ater|EAU32106.1 atub|OJI79744.1 aver|OJJ04379.1 awen|OJJ31494.1 azon|OJJ46207.1

Group814: acar|OOF92598.1 acar|OOF92619.1 abra|OJJ68487.1 acla|EAW06807.1 afis|EAW16356.1 afla|EED53514.1 afum|EAL88981.1 agla|OJJ88237.1 akaw|GAA92784.1 aluc|OJZ82699.1 anid|CBF89230.1 anig|EHA21155.1 aory|EIT74940.1 asyd|OJJ62515.1 ater|EAU34103.1 atub|OJI88931.1 aver|OJI96634.1 awen|OJJ37760.1 azon|OJJ45058.1

Group815: acar|OOF92601.1 acar|OOF92622.1 abra|OJJ77122.1 acla|EAW12935.1 afis|EAW17711.1 afla|EED45713.1 afum|EAL91526.1 agla|OJJ81737.1 akaw|GAA90806.1 aluc|OJZ84532.1 anid|CBF76922.1 anig|EHA27830.1 aory|EIT75682.1 asyd|OJJ57071.1 ater|EAU31890.1 atub|OJI89548.1 aver|OJJ03227.1 awen|OJJ37125.1 azon|OJJ50824.1

Group816: acar|OOF92602.1 acar|OOF92623.1 abra|OJJ75110.1 acla|EAW08316.1 afis|EAW23324.1 afla|EED55642.1 afum|EAL88155.1 agla|OJJ86519.1 akaw|GAA88719.1 aluc|OJZ92676.1 anid|CBF89451.1 anig|EHA26322.1 aory|EIT81779.1 asyd|OJJ62432.1 ater|EAU35572.1 atub|OJI87104.1 aver|OJI96568.1 awen|OJJ39570.1 azon|OJJ47763.1

Group817: acar|OOF92604.1 acar|OOF92625.1 abra|OJJ78212.1 acla|EAW11239.1 afis|EAW18266.1 afla|EED48732.1 afum|EAL87631.1 agla|OJJ88160.1 akaw|GAA85815.1 aluc|OJZ91284.1 anid|CBF79520.1 anig|EHA19969.1 aory|EIT73261.1 asyd|OJJ57890.1 ater|EAU33273.1 atub|OJI83274.1 aver|OJJ04504.1 awen|OJJ30640.1 azon|OJJ47975.1

Group818: acar|OOF92606.1 acar|OOF92627.1 abra|OJJ72009.1 acla|EAW09531.1 afis|EAW21601.1 afla|EED57828.1 afum|EAL92620.1 agla|OJJ85796.1 akaw|GAA84924.1 aluc|OJZ86745.1 anid|CBF83460.1 anig|EHA21934.1 aory|EIT81025.1 asyd|OJJ60683.1 ater|EAU38291.1 atub|OJI83782.1 aver|OJJ00008.1 awen|OJJ39081.1 azon|OJJ51413.1

Group819: acar|OOF92607.1 acar|OOF92628.1 abra|OJJ71278.1 acla|EAW10260.1 afis|EAW25333.1 afla|EED53813.1 afum|EAL89996.1 agla|OJJ83874.1 akaw|GAA82710.1 aluc|OJZ87265.1 anid|CBF75238.1 anig|EHA20507.1 aory|EIT78421.1 asyd|OJJ59912.1 ater|EAU34479.1 atub|OJI81377.1 aver|OJJ02374.1 awen|OJJ41027.1 azon|OJJ44637.1

Group820: acar|OOF92609.1 acar|OOF92630.1 abra|OJJ74612.1 acla|EAW10770.1 afis|EAW24806.1 afla|EED47177.1 afum|EAL89448.1 agla|OJJ82901.1 akaw|GAA81996.1 aluc|OJZ92192.1 anid|CBF85894.1 anig|EHA26762.1 aory|EIT79234.1 asyd|OJJ64389.1 ater|EAU36500.1 atub|OJI86621.1 aver|OJI98483.1 awen|OJJ40390.1 azon|OJJ49240.1

Group821: acar|OOF92610.1 acar|OOF92631.1 abra|OJJ71181.1 acla|EAW10323.1 afis|EAW25269.1 afla|EED53734.1 afum|EAL89932.1 agla|OJJ89237.1 akaw|GAA82804.1 aluc|OJZ87362.1 anid|CBF85380.1 anig|EHA20597.1 aory|EIT75356.1 asyd|OJJ64050.1 ater|EAU34412.1 atub|OJI81283.1 aver|OJI98136.1 awen|OJJ40983.1 azon|OJJ44569.1

Group822: acar|OOF92611.1 acar|OOF92632.1 abra|OJJ72794.1 acla|EAW11478.1 afis|EAW23975.1 afla|EED50533.1 afum|EAL87405.1 agla|OJJ86905.1 akaw|GAA85397.1 aluc|OJZ83290.1 anid|CBF77343.1 anig|EHA24175.1 aory|EIT82080.1 asyd|OJJ53273.1 ater|EAU29777.1 atub|OJI82192.1 aver|OJJ03576.1 awen|OJJ35440.1 azon|OJJ51303.1

Group823: acar|OOF92612.1 acar|OOF92633.1 abra|OJJ66525.1 acla|EAW10966.1 afis|EAW20894.1 afla|EED50243.1 afum|EAL84739.1 agla|OJJ84001.1 akaw|GAA91188.1 aluc|OJZ81515.1 anid|CBF82444.1

anig|EHA25913.1 aory|EIT77371.1 asyd|OJJ54119.1 ater|EAU38528.1 atub|OJI88664.1 aver|OJJ08617.1 awen|OJJ34454.1 azon|OJJ51741.1

Group824: acar|OOF92616.1 acar|OOF92637.1 abra|OJJ76860.1 acla|EAW13421.1 afis|EAW20532.1 afla|EED52482.1 afum|EAL84525.1 agla|OJJ84699.1 akaw|GAA87162.1 aluc|OJZ84272.1 anid|CBF79000.1 anig|EHA27614.1 aory|EIT82763.1 asyd|OJJ55688.1 ater|EAU37152.1 atub|OJI89811.1 aver|OJJ06772.1 awen|OJJ33239.1 azon|OJJ50618.1

Group825: acar|OOF93657.1 acar|OOF93741.1 abra|OJJ66636.1 acla|EAW11779.1 afis|EAW19389.1 afla|EED50347.1 afum|EAL87106.1 agla|OJJ81100.1 akaw|GAA89589.1 aluc|OJZ80813.1 anid|CBF74291.1 anig|EHA27221.1 aory|EIT81936.1 asyd|OJJ51912.1 ater|EAU36052.1 atub|OJI87662.1 aver|OJJ05353.1 awen|OJJ36782.1 azon|OJJ43016.1

Group826: acar|OOF93659.1 acar|OOF93743.1 abra|OJJ66639.1 acla|EAW11782.1 afis|EAW19392.1 afla|EED50350.1 afum|EAL87102.1 agla|OJJ81103.1 akaw|GAA89592.1 aluc|OJZ80810.1 anid|CBF74285.1 anig|EHA27224.1 aory|EIT81937.1 asyd|OJJ51914.1 ater|EAU36055.1 atub|OJI87658.1 aver|OJJ05356.1 awen|OJJ36785.1 azon|OJJ43013.1

Group827: acar|OOF93660.1 acar|OOF93744.1 abra|OJJ66640.1 acla|EAW11783.1 afis|EAW19393.1 afla|EED50351.1 agla|OJJ81104.1 akaw|GAA89593.1 aluc|OJZ80809.1 anig|EHA27225.1 aory|EIT81941.1 asyd|OJJ51863.1 ater|EAU36056.1 atub|OJI87657.1 aver|OJJ05357.1 awen|OJJ36787.1 azon|OJJ43012.1 afum|EAL87101.1 anid|CBF74283.1

Group828: acar|OOF93663.1 acar|OOF93747.1 abra|OJJ66643.1 acla|EAW11786.1 afis|EAW19396.1 afla|EED50352.1 afum|EAL87098.1 agla|OJJ81107.1 akaw|GAA89596.1 aluc|OJZ80806.1 anid|CBF74278.1 anig|EHA27228.1 aory|EIT83119.1 asyd|OJJ51859.1 ater|EAU36122.1 atub|OJI87654.1 aver|OJJ05360.1 awen|OJJ36791.1 azon|OJJ43008.1

Group829: acar|OOF93667.1 acar|OOF93751.1 abra|OJJ66647.1 acla|EAW11790.1 afis|EAW19400.1 afla|EED50355.1 afum|EAL87094.1 agla|OJJ81111.1 akaw|GAA89600.1 aluc|OJZ80802.1 anid|CBF74336.1 anig|EHA27232.1 aory|EIT83130.1 asyd|OJJ51906.1 ater|EAU36126.1 atub|OJI87650.1 aver|OJJ05363.1 awen|OJJ36795.1 azon|OJJ43004.1

Group830: acar|OOF93669.1 acar|OOF93753.1 abra|OJJ66649.1 acla|EAW11792.1 afis|EAW19403.1 afla|EED50358.1 afum|EAL87092.1 agla|OJJ81117.1 akaw|GAA89602.1 aluc|OJZ80800.1 anid|CBF74298.1 anig|EHA27234.1 aory|EIT83146.1 asyd|OJJ51902.1 ater|EAU36143.1 atub|OJI87648.1 aver|OJJ05365.1 awen|OJJ36798.1 azon|OJJ43001.1

Group831: acar|OOF93671.1 acar|OOF93755.1 abra|OJJ66653.1 acla|EAW11795.1 afis|EAW19405.1 afla|EED50361.1 afum|EAL87090.1 agla|OJJ81119.1 akaw|GAA89604.1 aluc|OJZ80794.1 anid|CBF74345.1 anig|EHA27237.1 aory|EIT83126.1 asyd|OJJ57796.1 ater|EAU36140.1 atub|OJI87645.1 aver|OJJ05368.1 awen|OJJ36801.1 azon|OJJ42997.1

Group832: acar|OOF93672.1 acar|OOF93756.1 abra|OJJ66655.1 acla|EAW11796.1 afis|EAW19406.1 afla|EED50362.1 afum|EAL87088.1 agla|OJJ81120.1 akaw|GAA89605.1 aluc|OJZ80793.1 anid|CBF74347.1 anig|EHA27238.1 aory|EIT83144.1 asyd|OJJ57795.1 ater|EAU36139.1 atub|OJI87644.1 aver|OJJ05369.1 awen|OJJ36802.1 azon|OJJ42996.1

Group833: acar|OOF93673.1 acar|OOF93757.1 abra|OJJ66656.1 acla|EAW11797.1 afis|EAW19407.1 afla|EED50363.1 afum|EAL87087.1 agla|OJJ81121.1 akaw|GAA89606.1 aluc|OJZ80792.1 anid|CBF74349.1 anig|EHA27239.1 aory|EIT83134.1 asyd|OJJ57794.1 ater|EAU36138.1 atub|OJI87643.1 aver|OJJ05370.1 awen|OJJ36803.1 azon|OJJ42995.1

Group834: acar|OOF93674.1 acar|OOF93758.1 abra|OJJ66657.1 acla|EAW11798.1 afis|EAW19408.1 afla|EED50364.1 afum|EAL87086.1 agla|OJJ81122.1 akaw|GAA89607.1 aluc|OJZ80791.1 anid|CBF74356.1 anig|EHA27240.1 aory|EIT83115.1 asyd|OJJ57793.1 ater|EAU34715.1 atub|OJI87642.1 aver|OJJ05371.1 awen|OJJ36804.1 azon|OJJ42994.1

Group835: acar|OOF93676.1 acar|OOF93761.1 abra|OJJ66660.1 acla|EAW11802.1 afis|EAW19412.1 afla|EED50367.1 afum|EAL87082.1 agla|OJJ81126.1 akaw|GAA89610.1 aluc|OJZ80788.1 anid|CBF74358.1 anig|EHA27242.1 aory|EIT83138.1 asyd|OJJ57789.1 ater|EAU34718.1 atub|OJI87639.1 aver|OJJ05375.1 awen|OJJ36808.1 azon|OJJ42992.1

Group836: acar|OOF93677.1 acar|OOF93762.1 abra|OJJ66661.1 acla|EAW11803.1 afis|EAW19413.1 afla|EED50368.1 afum|EAL87081.1 agla|OJJ81127.1 akaw|GAA89611.1 aluc|OJZ80787.1 anid|CBF74360.1 anig|EHA27243.1 aory|EIT83132.1 asyd|OJJ57788.1 ater|EAU34719.1 atub|OJI87638.1 aver|OJJ05376.1 awen|OJJ36809.1 azon|OJJ42991.1

Group837: acar|OOF93678.1 acar|OOF93763.1 abra|OJJ66662.1 acla|EAW11804.1 afis|EAW19414.1 afla|EED50369.1 afum|EAL87080.2 agla|OJJ81128.1 akaw|GAA89612.1 aluc|OJZ80786.1 anid|CBF74362.1 anig|EHA27244.1 aory|EIT83123.1 asyd|OJJ57787.1 ater|EAU34720.1 atub|OJI87636.1 aver|OJJ05377.1 awen|OJJ36810.1 azon|OJJ42990.1

Group838: acar|OOF93679.1 acar|OOF93765.1 abra|OJJ66664.1 acla|EAW11805.1 afis|EAW19415.1 afla|EED50371.1 afum|EAL87079.1 agla|OJJ81129.1 akaw|GAA89613.1 aluc|OJZ80783.1 anid|CBF74364.1 anig|EHA27246.1 aory|EIT83116.1 asyd|OJJ57785.1 ater|EAU34721.1 atub|OJI87635.1 aver|OJJ05379.1 awen|OJJ36812.1 azon|OJJ42989.1

Group839: acar|OOF93681.1 acar|OOF93764.1 abra|OJJ66666.1 acla|EAW11807.1 afis|EAW19417.1 afla|EED50373.1 afum|EAL87077.1 agla|OJJ81130.1 akaw|GAA89615.1 aluc|OJZ80781.1 anid|CBF86348.1 anig|EHA27248.1 aory|EIT83140.1 asyd|OJJ64978.1 ater|EAU34723.1 atub|OJI87633.1 aver|OJI99100.1 awen|OJJ36815.1 azon|OJJ42987.1

Group840: acar|OOF93685.1 acar|OOF93770.1 abra|OJJ66671.1 acla|EAW11812.1 afis|EAW19422.1 afla|EED50379.1 afum|EAL87072.1 agla|OJJ81134.1 akaw|GAA89620.1 aluc|OJZ80776.1 anid|CBF74373.1 anig|EHA27253.1 aory|EIT83120.1 asyd|OJJ57781.1 ater|EAU34726.1 atub|OJI87628.1 aver|OJJ05384.1 awen|OJJ36820.1 azon|OJJ42982.1

Group841: acar|OOF93686.1 acar|OOF93771.1 abra|OJJ66672.1 acla|EAW11813.1 afis|EAW19423.1 afla|EED50380.1 afum|EAL87071.1 agla|OJJ81135.1 akaw|GAA89621.1 aluc|OJZ80775.1 anid|CBF74375.1 anig|EHA27254.1 aory|EIT83122.1 asyd|OJJ57780.1 ater|EAU34727.1 atub|OJI87627.1 aver|OJJ05385.1 awen|OJJ36821.1 azon|OJJ42981.1

Group842: acar|OOF93690.1 acar|OOF93776.1 abra|OJJ66680.1 acla|EAW11817.1 afis|EAW19428.1 afla|EED56632.1 afum|EAL87066.1 agla|OJJ81140.1 akaw|GAA89625.1 aluc|OJZ80769.1 anid|CBF74382.1 anig|EHA27259.1 aory|EIT79645.1 asyd|OJJ57775.1 ater|EAU34731.1 atub|OJI87622.1 aver|OJJ05390.1 awen|OJJ36826.1 azon|OJJ42977.1

Group843: acar|OOF93692.1 acar|OOF93778.1 abra|OJJ66683.1 acla|EAW11820.1 afis|EAW19431.1 afla|EED56635.1 afum|EAL87063.1 agla|OJJ81143.1 akaw|GAA89628.1 aluc|OJZ80766.1 anid|CBF81374.1 anig|EHA27262.1 aory|EIT79669.1 asyd|OJJ59388.1 ater|EAU34734.1 atub|OJI87619.1 aver|OJJ02885.1 awen|OJJ36832.1 azon|OJJ42974.1

Group844: acar|OOF93693.1 acar|OOF93777.1 abra|OJJ66682.1 acla|EAW11819.1 afis|EAW19430.1 afla|EED56634.1 afum|EAL87064.1 agla|OJJ81142.1 akaw|GAA89627.1 aluc|OJZ80767.1 anid|CBF74386.1 anig|EHA27261.1 aory|EIT79632.1 asyd|OJJ57773.1 ater|EAU34733.1 atub|OJI87620.1 aver|OJJ05393.1 awen|OJJ36829.1 azon|OJJ42975.1

Group845: acar|OOF93696.1 acar|OOF93781.1 abra|OJJ66687.1 acla|EAW11822.1 afis|EAW19433.1 afla|EED56638.1 afum|EAL87062.1 agla|OJJ81145.1 akaw|GAA89631.1 aluc|OJZ80762.1 anid|CBF81380.1 anig|EHA27265.1 aory|EIT79671.1 asyd|OJJ59391.1 ater|EAU34735.1 atub|OJI87616.1 aver|OJJ02881.1 awen|OJJ36833.1 azon|OJJ42972.1

Group846: acar|OOF93699.1 acar|OOF93784.1 abra|OJJ66690.1 acla|EAW11825.1 afis|EAW19436.1 afla|EED56641.1 afum|EAL87059.1 agla|OJJ81148.1 akaw|GAA89634.1 aluc|OJZ80759.1 anid|CBF81383.1 anig|EHA27268.1 aory|EIT79678.1 asyd|OJJ59395.1 ater|EAU34738.1 atub|OJI87613.1 aver|OJJ02877.1 awen|OJJ36834.1 azon|OJJ42969.1

Group847: acar|OOF93701.1 acar|OOF93787.1 abra|OJJ66693.1 acla|EAW11828.1 afis|EAW19440.1 afla|EED56647.1 afum|EAL87056.1 agla|OJJ81155.1 akaw|GAA89637.1 aluc|OJZ80752.1 anid|CBF79760.1 anig|EHA27271.1 aory|EIT79660.1 asyd|OJJ58101.1 ater|EAU34741.1 atub|OJI87609.1 aver|OJJ04303.1 awen|OJJ36840.1 azon|OJJ42961.1

Group848: acar|OOF93703.1 acar|OOF93790.1 abra|OJJ66695.1 acla|EAW11830.1 afis|EAW19442.1 afla|EED56649.1 afum|EAL87054.1 agla|OJJ81153.1 akaw|GAA89639.1 aluc|OJZ80750.1 anid|CBF81395.1 anig|EHA27272.1 aory|EIT79630.1 asyd|OJJ59401.1 ater|EAU34743.1 atub|OJI87607.1 aver|OJJ02872.1 awen|OJJ36842.1 azon|OJJ42958.1

Group849: acar|OOF93707.1 acar|OOF93793.1 abra|OJJ66703.1 acla|EAW11834.1 afis|EAW19445.1 afla|EED56653.1 afum|EAL87050.1 agla|OJJ81160.1 akaw|GAA89645.1 aluc|OJZ80744.1 anid|CBF81404.1 anig|EHA27278.1 aory|EIT79624.1 asyd|OJJ59405.1 ater|EAU34747.1 atub|OJI87601.1 aver|OJJ02868.1 awen|OJJ36846.1 azon|OJJ42954.1

Group850: acar|OOF93708.1 acar|OOF93794.1 abra|OJJ66704.1 acla|EAW11835.1 afis|EAW19446.1 afla|EED56654.1 afum|EAL87049.1 agla|OJJ81161.1 akaw|GAA89646.1 aluc|OJZ80743.1 anid|CBF81405.1 anig|EHA27279.1 aory|EIT79662.1 asyd|OJJ59406.1 ater|EAU34748.1 atub|OJI87600.1 aver|OJJ02867.1 awen|OJJ36847.1 azon|OJJ42953.1

Group851: acar|OOF93709.1 acar|OOF93795.1 abra|OJJ66706.1 acla|EAW11837.1 afis|EAW19448.1 afla|EED56656.1 afum|EBA27299.1 agla|OJJ81163.1 akaw|GAA89648.1 aluc|OJZ80741.1 anid|CBF81409.1 anig|EHA27281.1 aory|EIT79680.1 asyd|OJJ59408.1 ater|EAU34750.1 atub|OJI87598.1 aver|OJJ02865.1 awen|OJJ36849.1 azon|OJJ42951.1

Group852: acar|OOF93710.1 acar|OOF93796.1 abra|OJJ66708.1 acla|EAW11839.1 afis|EAW19450.1 afla|EED56658.1 afum|EAL87046.1 agla|OJJ81165.1 akaw|GAA89650.1 aluc|OJZ80739.1 anid|CBF81412.1

anig|EHA27283.1 aory|EIT79655.1 asyd|OJJ59410.1 ater|EAU34752.1 atub|OJI87596.1 aver|OJJ02863.1 awen|OJJ36851.1 azon|OJJ42949.1

Group853: acar|OOF93711.1 acar|OOF93797.1 abra|OJJ66709.1 acla|EAW11840.1 afis|EAW19451.1 afla|EED56659.1 afum|EAL87045.1 agla|OJJ81166.1 akaw|GAA89651.1 aluc|OJZ80738.1 anid|CBF81414.1 anig|EHA27284.1 aory|EIT79675.1 asyd|OJJ59411.1 ater|EAU34753.1 atub|OJI87595.1 aver|OJJ02862.1 awen|OJJ36852.1 azon|OJJ42948.1

Group854: acar|OOF93715.1 acar|OOF93802.1 abra|OJJ66713.1 acla|EAW11844.1 afis|EAW19455.1 afla|EED56663.1 afum|EAL87041.1 agla|OJJ81171.1 akaw|GAA89655.1 aluc|OJZ80734.1 anid|CBF81421.1 anig|EHA27287.1 aory|EIT79656.1 asyd|OJJ59415.1 ater|EAU34758.1 atub|OJI87591.1 aver|OJJ02858.1 awen|OJJ36856.1 azon|OJJ42944.1

Group855: acar|OOF93716.1 acar|OOF93803.1 abra|OJJ66714.1 acla|EAW11845.1 afis|EAW19456.1 afla|EED56664.1 afum|EAL87040.1 agla|OJJ81173.1 akaw|GAA89656.1 aluc|OJZ80733.1 anid|CBF81423.1 anig|EHA27288.1 aory|EIT79663.1 asyd|OJJ59416.1 ater|EAU34759.1 atub|OJI87590.1 aver|OJJ02857.1 awen|OJJ36858.1 azon|OJJ42943.1

Group856: acar|OOF93717.1 acar|OOF93804.1 abra|OJJ66715.1 acla|EAW11846.1 afis|EAW19457.1 afla|EED56665.1 afum|EAL87039.1 agla|OJJ81174.1 akaw|GAA89657.1 aluc|OJZ80732.1 anid|CBF81425.1 anig|EHA27289.1 aory|EIT79668.1 asyd|OJJ59417.1 ater|EAU34760.1 atub|OJI87589.1 aver|OJJ02856.1 awen|OJJ36859.1 azon|OJJ42942.1

Group857: acar|OOF93718.1 acar|OOF93805.1 abra|OJJ66716.1 acla|EAW11847.1 afis|EAW19458.1 afla|EED56669.1 afum|EAL87038.1 agla|OJJ81175.1 akaw|GAA89658.1 aluc|OJZ80731.1 anid|CBF81427.1 anig|EHA27290.1 aory|EIT79631.1 asyd|OJJ59418.1 ater|EAU34761.1 atub|OJI87588.1 aver|OJJ02851.1 awen|OJJ36860.1 azon|OJJ42941.1

Group858: acar|OOF93721.1 acar|OOF93808.1 abra|OJJ66719.1 acla|EAW11850.1 afis|EAW19461.1 afla|EED56672.1 afum|EAL87035.1 agla|OJJ81178.1 akaw|GAA89661.1 aluc|OJZ80728.1 anid|CBF74529.1 anig|EHA27293.1 aory|EIT82995.1 asyd|OJJ57737.1 ater|EAU34764.1 atub|OJI87584.1 aver|OJJ05430.1 awen|OJJ36863.1 azon|OJJ42938.1

Group859: acar|OOF93723.1 acar|OOF93810.1 abra|OJJ66721.1 acla|EAW11852.1 afis|EAW19463.1 afla|EED56674.1 afum|EAL87033.1 agla|OJJ81180.1 akaw|GAA89663.1 aluc|OJZ80726.1 anid|CBF75491.1 anig|EHA27294.1 aory|EIT73836.1 asyd|OJJ59753.1 ater|EAU34766.1 atub|OJI87582.1 aver|OJJ02535.1 awen|OJJ36865.1 azon|OJJ42936.1

Group860: acar|OOF93724.1 acar|OOF93811.1 abra|OJJ66722.1 acla|EAW11853.1 afis|EAW19464.1 afla|EED56675.1 afum|EAL87032.1 agla|OJJ81181.1 akaw|GAA89664.1 aluc|OJZ80725.1 anid|CBF75490.1 anig|EHA27295.1 aory|EIT73882.1 asyd|OJJ59754.1 ater|EAU34768.1 atub|OJI87581.1 aver|OJJ02534.1 awen|OJJ36866.1 azon|OJJ42935.1

Group861: acar|OOF93726.1 acar|OOF93814.1 abra|OJJ66726.1 acla|EAW11856.1 afis|EAW19467.1 afla|EED56677.1 afum|EAL87029.1 agla|OJJ81187.1 akaw|GAA89667.1 aluc|OJZ80722.1 anid|CBF75486.1 anig|EHA27298.1 aory|EIT73931.1 asyd|OJJ59756.1 ater|EAU34777.1 atub|OJI87578.1 aver|OJJ02532.1 awen|OJJ36872.1 azon|OJJ42932.1

Group862: acar|OOF93727.1 acar|OOF93815.1 abra|OJJ66727.1 acla|EAW11857.1 afis|EAW19468.1 afla|EED56678.1 afum|EAL87028.1 agla|OJJ81188.1 akaw|GAA89668.1 aluc|OJZ80721.1 anid|CBF75484.1 anig|EHA27299.1 aory|EIT73958.1 asyd|OJJ59757.1 ater|EAU34778.1 atub|OJI87577.1 aver|OJJ02531.1 awen|OJJ36873.1 azon|OJJ42931.1

Group863: acar|OOF93728.1 acar|OOF93816.1 abra|OJJ66728.1 acla|EAW11858.1 afis|EAW19469.1 afla|EED56679.1 afum|EAL87027.1 agla|OJJ81190.1 akaw|GAA89669.1 aluc|OJZ80720.1 anid|CBF75482.1 anig|EHA27300.1 aory|EIT73843.1 asyd|OJJ59758.1 ater|EAU34779.1 atub|OJI87576.1 aver|OJJ02530.1 awen|OJJ36874.1 azon|OJJ42930.1

Group864: acar|OOF93729.1 acar|OOF93817.1 abra|OJJ66731.1 acla|EAW11859.1 afis|EAW19470.1 afla|EED56680.1 afum|EAL87026.1 agla|OJJ81191.1 akaw|GAA89670.1 aluc|OJZ80718.1 anid|CBF75480.1 anig|EHA27301.1 aory|EIT73854.1 asyd|OJJ59759.1 ater|EAU34780.1 atub|OJI87574.1 aver|OJJ02529.1 awen|OJJ36875.1 azon|OJJ42929.1

Group865: acar|OOF93731.1 acar|OOF93819.1 abra|OJJ66733.1 acla|EAW11861.1 afis|EAW19472.1 afla|EED56682.1 afum|EAL87024.1 agla|OJJ81193.1 akaw|GAA89672.1 aluc|OJZ80716.1 anid|CBF75474.1 anig|EHA27303.1 aory|EIT73961.1 asyd|OJJ59761.1 ater|EAU34782.1 atub|OJI87572.1 aver|OJJ02527.1 awen|OJJ36877.1 azon|OJJ42927.1

Group866: acar|OOF93734.1 acar|OOF93822.1 abra|OJJ66736.1 acla|EAW11864.1 afis|EAW19475.1 afla|EED56683.1 afum|EAL87021.1 agla|OJJ81198.1 akaw|GAA89675.1 aluc|OJZ80711.1 anid|CBF75470.1 anig|EHA27306.1 aory|EIT73904.1 asyd|OJJ59764.1 ater|EAU34787.1 atub|OJI87569.1 aver|OJJ02524.1 awen|OJJ36880.1 azon|OJJ42925.1

Group867: acar|OOF93736.1 acar|OOF93825.1 abra|OJJ66739.1 acla|EAW11866.1 afis|EAW19477.1 afla|EED56685.1 afum|EAL87019.1 agla|OJJ81207.1 akaw|GAA89677.1 aluc|OJZ80709.1 anid|CBF71519.1 anig|EHA27308.1 aory|EIT73831.1 asyd|OJJ54916.1 ater|EAU34789.1 atub|OJI87567.1 aver|OJJ07287.1 awen|OJJ36882.1 azon|OJJ42923.1

Group868: acar|OOF93739.1 acar|OOF93829.1 abra|OJJ66743.1 acla|EAW11872.1 afis|EAW19483.1 afla|EED56701.1 afum|EAL87012.1 agla|OJJ81208.1 akaw|GAA89681.1 aluc|OJZ80704.1 anid|CBF75464.1 anig|EHA27313.1 aory|EIT73864.1 asyd|OJJ59767.1 ater|EAU34796.1 atub|OJI87562.1 aver|OJJ02520.1 awen|OJJ36895.1 azon|OJJ42918.1

Group869: acar|OOF94046.1 acar|OOF94116.1 agla|OJJ78942.1 agla|OJJ84364.1 agla|OJJ88800.1 asyd|OJJ55478.1 asyd|OJJ56071.1 aver|OJJ05014.1 aver|OJJ06558.1 akaw|GAA90264.1 aluc|OJZ83077.1 anig|EHA28141.1 ater|EAU36656.1 atub|OJI87869.1 awen|OJJ30395.1 aory|EIT78619.1 azon|OJJ48036.1 anig|EHA28514.1 awen|OJJ34834.1

Group870: acar|OOF94064.1 acar|OOF94134.1 abra|OJJ66360.1 acla|EAW10009.1 afis|EAW21047.1 afla|EED51623.1 afum|EAL92083.1 agla|OJJ82365.1 akaw|GAA90233.1 aluc|OJZ83108.1 anid|CBF87099.1 anig|EHA28171.1 aory|EIT80605.1 asyd|OJJ60391.1 ater|EAU36819.1 atub|OJI87826.1 aver|OJI99720.1 awen|OJJ32175.1 azon|OJJ46754.1

Group871: acar|OOF94087.1 acar|OOF94157.1 abra|OJJ66351.1 acla|EAW06467.1 afis|EAW15962.1 afla|EED58095.1 afum|EAL88595.1 agla|OJJ89350.1 akaw|GAA90224.1 aluc|OJZ83117.1 anid|CBF81081.1 anig|EHA28180.1 aory|EIT79147.1 asyd|OJJ56464.1 ater|EAU35492.1 atub|OJI87817.1 aver|OJJ06256.1 awen|OJJ35612.1 azon|OJJ46912.1

Group872: acar|OOF94090.1 acar|OOF94160.1 abra|OJJ66347.1 acla|EAW09988.1 afis|EAW21105.1 afla|EED51687.1 afum|EAL92126.1 agla|OJJ80110.1 akaw|GAA90221.1 aluc|OJZ83120.1 anid|CBF87045.1 anig|EHA28184.1 aory|EIT80607.1 asyd|OJJ60445.1 ater|EAU36794.1 atub|OJI87813.1 aver|OJI99763.1 awen|OJJ36366.1 azon|OJJ43506.1

Group873: acar|OOF94095.1 acar|OOF94164.1 abra|OJJ66343.1 acla|EAW09991.1 afis|EAW21101.1 afla|EED48394.1 afum|EAL92122.1 agla|OJJ80116.1 akaw|GAA90217.1 aluc|OJZ83126.1 anid|CBF87046.1 atub|OJI87807.1 aver|OJI99762.1 awen|OJJ37901.1 azon|OJJ44446.1 acla|EAW09992.1 awen|OJJ36357.1 afla|EED51683.1 asyd|OJJ60444.1

Group874: acar|OOF95064.1 acar|OOF95130.1 abra|OJJ74649.1 acla|EAW10737.1 afis|EAW24838.1 afla|EED47138.1 afum|EAL89484.1 agla|OJJ82029.1 akaw|GAA81961.1 aluc|OJZ92227.1 anid|CBF74603.1 anig|EHA26729.1 aory|EIT79201.1 asyd|OJJ57657.1 ater|EAU36468.1 atub|OJI86657.1 aver|OJJ05518.1 awen|OJJ40424.1 azon|OJJ49282.1

Group875: acar|OOF95067.1 acar|OOF95133.1 abra|OJJ74647.1 acla|EAW10740.1 afis|EAW24834.1 afla|EED47141.1 afum|EAL89479.1 agla|OJJ82027.1 akaw|GAA81964.1 aluc|OJZ92225.1 anid|CBF74582.1 anig|EHA26731.1 aory|EIT79244.1 asyd|OJJ57655.1 ater|EAU36471.1 atub|OJI86655.1 aver|OJJ05515.1 awen|OJJ40421.1 azon|OJJ49279.1

Group876: acar|OOF95068.1 acar|OOF95134.1 abra|OJJ74646.1 acla|EAW10741.1 afis|EAW24833.1 afla|EED47142.1 afum|EAL89478.1 agla|OJJ82026.1 akaw|GAA81965.1 aluc|OJZ92224.1 anid|CBF74580.1 anig|EHA26732.1 aory|EIT79261.1 asyd|OJJ57654.1 ater|EAU36472.1 atub|OJI86654.1 aver|OJJ05514.1 awen|OJJ40420.1 azon|OJJ49278.1

Group877: acar|OOF95069.1 acar|OOF95135.1 abra|OJJ68490.1 acla|EAW10742.1 afis|EAW24832.1 afla|EED47143.1 afum|EAL89477.1 agla|OJJ82025.1 akaw|GAA92780.1 aluc|OJZ82703.1 anid|CBF74578.1 anig|EHA21158.1 aory|EIT79304.1 asyd|OJJ57653.1 ater|EAU36473.1 atub|OJI88936.1 aver|OJJ05513.1 awen|OJJ40419.1 azon|OJJ49277.1

Group878: acar|OOF95070.1 acar|OOF95136.1 abra|OJJ74645.1 acla|EAW10743.1 afis|EAW24831.1 afla|EED47144.1 afum|EAL89476.1 agla|OJJ82019.1 akaw|GAA81966.1 aluc|OJZ92223.1 anid|CBF74576.1 anig|EHA26733.1 aory|EIT79280.1 asyd|OJJ57652.1 ater|EAU36474.1 atub|OJI86653.1 aver|OJJ05512.1 awen|OJJ40418.1 azon|OJJ49276.1

Group879: acar|OOF95076.1 acar|OOF95140.1 abra|OJJ74640.1 acla|EAW10748.1 afis|EAW24826.1 afla|EED47149.1 afum|EAL89470.1 agla|OJJ82015.1 akaw|GAA81971.1 aluc|OJZ92219.1 anid|CBF85858.1 anig|EHA26739.1 aory|EIT79249.1 asyd|OJJ64367.1 ater|EAU36479.1 atub|OJI86650.1 aver|OJI98462.1 awen|OJJ40412.1 azon|OJJ49262.1

Group880: acar|OOF95080.1 acar|OOF95145.1 abra|OJJ74631.1 acla|EAW10753.1 afis|EAW24822.1 afla|EED47153.1 afum|EAL89466.1 agla|OJJ82920.1 akaw|GAA81978.1 aluc|OJZ92212.1 anid|CBF85851.1 anig|EHA26746.1 aory|EIT79200.1 asyd|OJJ64363.1 ater|EAU36483.1 atub|OJI86641.1 aver|OJI98458.1 awen|OJJ40408.1 azon|OJJ49257.1

Group881: acar|OOF95083.1 acar|OOF95148.1 abra|OJJ74626.1 acla|EAW10756.1 afis|EAW24818.1 afla|EED47162.1 afum|EAL91338.1 akaw|GAA81982.1 aluc|OJZ92208.1 anid|CBF85876.1 anig|EHA26750.1

aory|EIT79206.1 asyd|OJJ64375.1 ater|EAU36488.1 atub|OJI86637.1 aver|OJI98471.1 awen|OJJ40404.1 afum|EAL89462.1 afis|EAW17523.1

Group882: acar|OOF95084.1 acar|OOF95149.1 abra|OJJ74624.1 acla|EAW10760.1 afis|EAW24816.1 afla|EED47167.1 afum|EAL89460.2 agla|OJJ82910.1 akaw|GAA81984.1 aluc|OJZ92206.1 anid|CBF85879.1 anig|EHA26752.1 aory|EIT79187.1 asyd|OJJ64377.1 ater|EAU39452.1 atub|OJI86635.1 aver|OJI98473.1 awen|OJJ40402.1 azon|OJJ43613.1

Group883: acar|OOF95086.1 acar|OOF95152.1 abra|OJJ74618.1 acla|EAW10764.1 afis|EAW24812.1 afla|EED47171.1 afum|EAL89455.1 agla|OJJ82907.1 akaw|GAA81988.1 aluc|OJZ92201.1 anid|CBF85885.1 anig|EHA26756.1 aory|EIT79186.1 asyd|OJJ64381.1 ater|EAU36495.1 atub|OJI86629.1 aver|OJI98477.1 awen|OJJ40396.1 azon|OJJ49249.1

Group884: acar|OOF95087.1 acar|OOF95153.1 abra|OJJ74617.1 acla|EAW10765.1 afis|EAW24811.1 afla|EED47172.1 afum|EAL89454.1 agla|OJJ82906.1 akaw|GAA81989.1 aluc|OJZ92200.1 anid|CBF85886.1 anig|EHA26757.1 aory|EIT79291.1 asyd|OJJ64382.1 ater|EAU36496.1 atub|OJI86627.1 aver|OJI98478.1 awen|OJJ40395.1 azon|OJJ49248.1

Group885: acar|OOF95089.1 acar|OOF95155.1 abra|OJJ74615.1 acla|EAW10767.1 afis|EAW24809.1 afla|EED47174.1 afum|EAL89452.1 agla|OJJ82904.1 akaw|GAA81991.1 aluc|OJZ92198.1 anid|CBF85888.1 anig|EHA26759.1 aory|EIT79300.1 asyd|OJJ64384.1 ater|EAU36497.1 atub|OJI86625.1 aver|OJI98480.1 awen|OJJ40393.1 azon|OJJ49246.1

Group886: acar|OOF95090.1 acar|OOF95156.1 abra|OJJ74614.1 acla|EAW10768.1 afis|EAW24808.1 afla|EED47175.1 afum|EAL89450.2 agla|OJJ82903.1 akaw|GAA81992.1 aluc|OJZ92197.1 anid|CBF85890.1 anig|EHA26760.1 aory|EIT79254.1 asyd|OJJ64385.1 ater|EAU36498.1 atub|OJI86624.1 aver|OJI98481.1 awen|OJJ40392.1 azon|OJJ49245.1

Group887: acar|OOF95091.1 acar|OOF95157.1 abra|OJJ74613.1 acla|EAW10769.1 afis|EAW24807.1 afla|EED47176.1 afum|EAL89449.1 agla|OJJ82902.1 akaw|GAA81995.1 aluc|OJZ92193.1 anid|CBF85892.1 anig|EHA26761.1 aory|EIT79197.1 asyd|OJJ64387.1 ater|EAU36499.1 atub|OJI86622.1 aver|OJI98482.1 awen|OJJ40391.1 azon|OJJ49241.1

Group888: acar|OOF95092.1 acar|OOF95158.1 abra|OJJ74611.1 acla|EAW10771.1 afis|EAW24805.1 afla|EED47180.1 afum|EAL89447.1 agla|OJJ82900.1 akaw|GAA81997.1 aluc|OJZ92191.1 anid|CBF81492.1 anig|EHA26763.1 aory|EIT79217.1 asyd|OJJ59459.1 ater|EAU36502.1 atub|OJI86620.1 aver|OJJ02814.1 awen|OJJ40389.1 azon|OJJ49239.1

Group889: acar|OOF95093.1 acar|OOF95159.1 abra|OJJ74610.1 acla|EAW10772.1 afis|EAW24804.1 afla|EED47181.1 afum|EAL89446.1 agla|OJJ82899.1 akaw|GAA81998.1 aluc|OJZ92190.1 anid|CBF81493.1 anig|EHA26764.1 aory|EIT79264.1 asyd|OJJ59460.1 ater|EAU36503.1 atub|OJI86619.1 aver|OJJ02813.1 awen|OJJ40388.1 azon|OJJ49238.1

Group890: acar|OOF95095.1 acar|OOF95162.1 abra|OJJ74608.1 acla|EAW10773.1 afis|EAW24803.1 afla|EED47183.1 afum|EAL89444.1 agla|OJJ82897.1 akaw|GAA82000.1 aluc|OJZ92188.1 anid|CBF81495.1 anig|EHA26765.1 aory|EIT79220.1 asyd|OJJ59463.1 ater|EAU36504.1 atub|OJI86617.1 aver|OJJ02811.1 azon|OJJ49236.1 awen|OJJ40386.1

Group891: acar|OOF95101.1 acar|OOF95167.1 abra|OJJ74603.1 acla|EAW10776.1 afis|EAW24798.1 afla|EED47189.1 afum|EAL89440.1 agla|OJJ82894.1 akaw|GAA82005.1 aluc|OJZ92182.1 anid|CBF81508.1 anig|EHA26771.1 aory|EIT79258.1 asyd|OJJ59468.1 ater|EAU36507.1 atub|OJI86610.1 aver|OJJ02806.1 awen|OJJ40383.1 azon|OJJ49232.1

Group892: acar|OOF95102.1 acar|OOF95168.1 afis|EAW19312.1 afis|EAW24797.1 awen|OJJ34628.1 awen|OJJ40382.1 abra|OJJ74602.1 acla|EAW10777.1 afla|EED47188.1 afum|EAL89439.1 akaw|GAA82006.1 aluc|OJZ92181.1 anid|CBF81506.1 anig|EHA26772.1 asyd|OJJ60343.1 ater|EAU36508.1 atub|OJI86609.1 aver|OJI99684.1 aory|EIT79270.1

Group893: acar|OOF95104.1 acar|OOF95170.1 abra|OJJ74600.1 acla|EAW10779.1 afis|EAW24795.1 afla|EED47187.1 afum|EAL89437.1 agla|OJJ82891.1 akaw|GAA82008.1 aluc|OJZ92179.1 anid|CBF81503.1 anig|EHA26774.1 aory|EIT79230.1 asyd|OJJ59466.1 ater|EAU36510.1 atub|OJI86607.1 aver|OJJ02808.1 awen|OJJ40380.1 azon|OJJ49230.1

Group894: acar|OOF95106.1 acar|OOF95172.1 abra|OJJ74598.1 acla|EAW10781.1 afis|EAW24788.1 afla|EED47191.1 afum|EAL89427.1 agla|OJJ82890.1 akaw|GAA82010.1 aluc|OJZ92176.1 anid|CBF81466.1 anig|EHA26776.1 aory|EIT79298.1 asyd|OJJ59443.1 ater|EAU36513.1 atub|OJI86605.1 aver|OJJ02830.1 awen|OJJ40378.1 azon|OJJ49229.1

Group895: acar|OOF95629.1 acar|OOF95680.1 abra|OJJ74664.1 acla|EAW10731.1 afis|EAW24844.1 afla|EED47127.1 afum|EAL89490.1 agla|OJJ82044.1 akaw|GAA81947.1 aluc|OJZ92241.1 anid|CBF75849.1 anig|EHA26713.1 aory|EIT79274.1 asyd|OJJ57571.1 ater|EAU36460.1 atub|OJI86675.1 aver|OJJ05608.1 awen|OJJ40429.1 azon|OJJ49290.1

Group896: acar|OOF95630.1 acar|OOF95681.1 abra|OJJ74665.1 acla|EAW10730.1 afis|EAW24845.1 afla|EED47126.1 afum|EAL89491.1 agla|OJJ82045.1 akaw|GAA81946.1 aluc|OJZ92242.1 anid|CBF75847.1 anig|EHA26712.1 aory|EIT79168.1 asyd|OJJ57562.1 ater|EAU36459.1 atub|OJI86676.1 aver|OJJ05616.1 awen|OJJ40430.1 azon|OJJ49291.1

Group897: acar|OOF95631.1 acar|OOF95683.1 abra|OJJ74666.1 acla|EAW10729.1 afis|EAW24846.1 afla|EED47125.1 afum|EAL89492.1 agla|OJJ82058.1 akaw|GAA81945.1 aluc|OJZ92244.1 anid|CBF75845.1 anig|EHA26711.1 aory|EIT79177.1 asyd|OJJ57561.1 ater|EAU36457.1 atub|OJI86678.1 aver|OJJ05617.1 awen|OJJ40431.1 azon|OJJ49292.1

Group898: acar|OOF95632.1 acar|OOF95684.1 abra|OJJ74671.1 acla|EAW10726.1 afis|EAW24849.1 afla|EED47122.1 afum|EAL89495.1 agla|OJJ82061.1 akaw|GAA81942.1 aluc|OJZ92248.1 anid|CBF75839.1 anig|EHA26708.1 aory|EIT79246.1 asyd|OJJ57557.1 ater|EAU36454.1 atub|OJI86681.1 aver|OJJ05621.1 awen|OJJ40434.1 azon|OJJ49295.1

Group899: acar|OOF95633.1 acar|OOF95685.1 abra|OJJ74668.1 acla|EAW10728.1 afis|EAW24847.1 afla|EED47124.1 afum|EAL89493.1 agla|OJJ82059.1 akaw|GAA81944.1 aluc|OJZ92246.1 anid|CBF75843.1 anig|EHA26710.1 aory|EIT79296.1 asyd|OJJ57560.1 ater|EAU36456.1 atub|OJI86679.1 aver|OJJ05618.1 awen|OJJ40432.1 azon|OJJ49293.1

Group900: acar|OOF95638.1 acar|OOF95689.1 abra|OJJ74675.1 acla|EAW10722.1 afis|EAW24851.1 afla|EED47120.1 afum|EAL89498.1 agla|OJJ82065.1 akaw|GAA81938.1 aluc|OJZ92252.1 anid|CBF75835.1 anig|EHA26704.1 aory|EIT79290.1 asyd|OJJ57555.1 ater|EAU36452.1 atub|OJI86685.1 aver|OJJ05623.1 awen|OJJ40436.1 azon|OJJ49297.1

Group901: acar|OOF95639.1 acar|OOF95690.1 abra|OJJ74677.1 acla|EAW10720.1 afis|EAW24853.1 afla|EED47118.1 afum|EAL89500.1 agla|OJJ82067.1 akaw|GAA81937.1 aluc|OJZ92254.1 anid|CBF75831.1 anig|EHA26703.1 aory|EIT79297.1 asyd|OJJ57553.1 ater|EAU36449.1 atub|OJI86687.1 aver|OJJ05625.1 awen|OJJ40438.1 azon|OJJ49299.1

Group902: acar|OOF95640.1 acar|OOF95691.1 abra|OJJ74678.1 acla|EAW10719.1 afis|EAW24854.1 afla|EED47117.1 afum|EAL89501.1 agla|OJJ82068.1 akaw|GAA81936.1 aluc|OJZ92255.1 anid|CBF90310.1 anig|EHA26702.1 aory|EIT79215.1 asyd|OJJ61915.1 ater|EAU36448.1 atub|OJI86688.1 aver|OJI96008.1 awen|OJJ40439.1 azon|OJJ46990.1

Group903: acar|OOF95641.1 acar|OOF95692.1 abra|OJJ74679.1 acla|EAW10716.1 afis|EAW24855.1 afla|EED47094.1 afum|EAL89503.2 agla|OJJ82069.1 akaw|GAA81935.1 aluc|OJZ92256.1 anid|CBF75829.1 anig|EHA26701.1 aory|EIT72488.1 asyd|OJJ57552.1 ater|EAU36447.1 atub|OJI86690.1 aver|OJJ05626.1 awen|OJJ40443.1 azon|OJJ49301.1

Group904: acar|OOF95643.1 acar|OOF95694.1 abra|OJJ74681.1 acla|EAW10714.1 afis|EAW24856.1 afla|EED47096.1 afum|EAL89504.1 agla|OJJ82071.1 akaw|GAA81933.1 aluc|OJZ92258.1 anid|CBF75827.1 anig|EHA26700.1 aory|EIT72489.1 asyd|OJJ57550.1 ater|EAU36446.1 atub|OJI86692.1 aver|OJJ05628.1 awen|OJJ40445.1 azon|OJJ49302.1

Group905: acar|OOF95644.1 acar|OOF95695.1 abra|OJJ74683.1 acla|EAW10713.1 afis|EAW24857.1 afla|EED47097.1 afum|EAL89505.2 agla|OJJ82072.1 akaw|GAA81931.1 aluc|OJZ92260.1 anid|CBF75825.1 anig|EHA26699.1 aory|EIT72494.1 asyd|OJJ57549.1 ater|EAU36443.1 atub|OJI86694.1 aver|OJJ05629.1 awen|OJJ40446.1 azon|OJJ49303.1

Group906: acar|OOF95645.1 acar|OOF95696.1 abra|OJJ74684.1 acla|EAW10712.1 afis|EAW24858.1 afla|EED47098.1 afum|EAL89506.2 agla|OJJ82073.1 akaw|GAA81930.1 aluc|OJZ92261.1 anid|CBF75823.1 anig|EHA26698.1 aory|EIT72492.1 asyd|OJJ57548.1 ater|EAU36442.1 atub|OJI86695.1 aver|OJJ05630.1 awen|OJJ40447.1 azon|OJJ49304.1

Group907: acar|OOF95647.1 acar|OOF95698.1 abra|OJJ74686.1 acla|EAW10710.1 afis|EAW24860.1 afla|EED47100.1 afum|EAL89509.1 agla|OJJ82078.1 akaw|GAA81928.1 aluc|OJZ92263.1 anid|CBF75819.1 anig|EHA26696.1 aory|EIT72779.1 asyd|OJJ57546.1 ater|EAU36440.1 atub|OJI86697.1 aver|OJJ05632.1 awen|OJJ40450.1 azon|OJJ49306.1

Group908: acar|OOF95648.1 acar|OOF95699.1 abra|OJJ74687.1 acla|EAW10709.1 afis|EAW24861.1 afla|EED47101.1 afum|EAL89510.1 agla|OJJ82079.1 akaw|GAA81927.1 aluc|OJZ92264.1 anid|CBF75817.1 anig|EHA26695.1 aory|EIT72784.1 asyd|OJJ57545.1 ater|EAU36439.1 atub|OJI86698.1 aver|OJJ05633.1 awen|OJJ40451.1 azon|OJJ49307.1

Group909: acar|OOF95649.1 acar|OOF95700.1 abra|OJJ74688.1 acla|EAW10708.1 afis|EAW24862.1 afla|EED47102.1 afum|EAL89511.1 agla|OJJ82080.1 akaw|GAA81926.1 aluc|OJZ92265.1 anid|CBF75815.1 anig|EHA26694.1 aory|EIT72778.1 asyd|OJJ57544.1 ater|EAU36438.1 atub|OJI86699.1 aver|OJJ05634.1 awen|OJJ40452.1 azon|OJJ49308.1

Group910: acar|OOF95650.1 acar|OOF95701.1 abra|OJJ74689.1 acla|EAW10707.1 afis|EAW24863.1 afla|EED47103.1 afum|EAL89513.1 agla|OJJ82081.1 akaw|GAA81925.1 aluc|OJZ92266.1 anid|CBF75813.1

anig|EHA26693.1 aory|EIT72782.1 asyd|OJJ57543.1 ater|EAU36437.1 atub|OJI86700.1 aver|OJJ05635.1 awen|OJJ40453.1 azon|OJJ49309.1

Group911: acar|OOF95651.1 acar|OOF95702.1 abra|OJJ74690.1 acla|EAW10706.1 afis|EAW24864.1 afla|EED47104.1 afum|EAL89514.1 agla|OJJ82082.1 akaw|GAA81924.1 aluc|OJZ92267.1 anid|CBF75811.1 anig|EHA26692.1 aory|EIT72780.1 asyd|OJJ57542.1 ater|EAU36436.1 atub|OJI86701.1 aver|OJJ05636.1 awen|OJJ40454.1 azon|OJJ49310.1

Group912: acar|OOF95653.1 acar|OOF95704.1 abra|OJJ74693.1 acla|EAW10705.1 afis|EAW24865.1 afla|EED47106.1 afum|EAL89515.1 agla|OJJ82083.1 akaw|GAA81923.1 aluc|OJZ92270.1 anid|CBF75809.1 anig|EHA26691.1 aory|EIT72781.1 asyd|OJJ57541.1 ater|EAU36435.1 atub|OJI86704.1 aver|OJJ05637.1 awen|OJJ40455.1 azon|OJJ49311.1

Group913: acar|OOF95660.1 acar|OOF95711.1 abra|OJJ68465.1 acla|EAW10698.1 afis|EAW24872.1 afla|EED47112.1 afum|EAL89522.1 agla|OJJ82093.1 akaw|GAA92385.1 aluc|OJZ83244.1 anid|CBF75794.1 anig|EHA21099.1 aory|EIT79170.1 asyd|OJJ57530.1 ater|EAU36429.1 atub|OJI87692.1 aver|OJJ05648.1 awen|OJJ40463.1 azon|OJJ49323.1

Group914: acar|OOF95661.1 acar|OOF95712.1 abra|OJJ68466.1 acla|EAW10697.1 afis|EAW24873.1 afla|EED47113.1 afum|EAL89523.1 agla|OJJ82094.1 akaw|GAA92386.1 aluc|OJZ83245.1 anid|CBF75792.1 anig|EHA21100.1 aory|EIT79294.1 asyd|OJJ57531.1 ater|EAU36428.1 atub|OJI87691.1 aver|OJJ05647.1 awen|OJJ40464.1 azon|OJJ49324.1

Group915: acar|OOF95662.1 acar|OOF95713.1 abra|OJJ68467.1 acla|EAW10696.1 afis|EAW24874.1 afla|EED47114.1 afum|EAL89524.1 agla|OJJ82095.1 akaw|GAA92388.1 aluc|OJZ83248.1 anid|CBF75790.1 anig|EHA21101.1 aory|EIT79175.1 asyd|OJJ57532.1 ater|EAU36427.1 atub|OJI87689.1 aver|OJJ05646.1 awen|OJJ40465.1 azon|OJJ49325.1

Group916: acar|OOF95663.1 acar|OOF95714.1 abra|OJJ74702.1 acla|EAW10695.1 afis|EAW24875.1 afla|EED47115.1 afum|EAL89525.1 agla|OJJ82096.1 akaw|GAA81917.1 aluc|OJZ92279.1 anid|CBF75789.1 anig|EHA26685.1 aory|EIT79273.1 asyd|OJJ57533.1 ater|EAU36426.1 atub|OJI86712.1 aver|OJJ05645.1 awen|OJJ40466.1 azon|OJJ49328.1

Group917: acar|OOF95665.1 acar|OOF95716.1 abra|OJJ74704.1 acla|EAW10693.1 afis|EAW24877.1 afla|EED47089.1 afum|EAL89527.1 agla|OJJ82098.1 akaw|GAA81914.1 aluc|OJZ92284.1 anid|CBF75758.1 anig|EHA26684.1 aory|EIT72484.1 asyd|OJJ57527.1 ater|EAU36424.1 atub|OJI86715.1 aver|OJJ05651.1 awen|OJJ40467.1 azon|OJJ49330.1

Group918: acar|OOF96224.1 acar|OOF96262.1 abra|OJJ71508.1 acla|EAW15066.1 afis|EAW20038.1 afla|EED45342.1 afum|EAL85159.1 agla|OJJ88925.1 akaw|GAA89405.1 aluc|OJZ86247.1 anid|CBF84777.1 anig|EHA21617.1 aory|EIT73643.1 asyd|OJJ63237.1 ater|EAU38653.1 atub|OJI84295.1 aver|OJI97719.1 awen|OJJ31793.1 azon|OJJ43703.1

Group919: acar|OOF96229.1 acar|OOF96267.1 abra|OJJ71515.1 acla|EAW15072.1 afis|EAW20044.1 afla|EED45336.1 afum|EAL85164.2 agla|OJJ88918.1 akaw|GAA89401.1 aluc|OJZ86253.1 anid|CBF84789.1 anig|EHA21612.1 aory|EIT73570.1 asyd|OJJ63230.1 ater|EAU38648.1 atub|OJI84290.1 aver|OJI97726.1 awen|OJJ31800.1 azon|OJJ46321.1

Group920: acar|OOF96233.1 acar|OOF96270.1 abra|OJJ71517.1 acla|EAW15075.1 afis|EAW20046.1 afla|EED45334.1 afum|EAL85167.1 agla|OJJ88914.1 akaw|GAA89400.1 aluc|OJZ86256.1 anid|CBF84795.1 anig|EHA21610.1 aory|EIT73786.1 asyd|OJJ63227.1 ater|EAU38656.1 atub|OJI84287.1 aver|OJI97728.1 awen|OJJ31804.1 azon|OJJ46318.1

Group921: acar|OOF96234.1 acar|OOF96271.1 abra|OJJ71518.1 acla|EAW15076.1 afis|EAW20047.1 afla|EED45333.1 afum|EAL85168.2 agla|OJJ88913.1 akaw|GAA89399.1 aluc|OJZ86257.1 anid|CBF84797.1 anig|EHA21609.1 aory|EIT73741.1 asyd|OJJ63226.1 ater|EAU38657.1 atub|OJI84286.1 aver|OJI97729.1 awen|OJJ31805.1 azon|OJJ46317.1

Group922: acar|OOF96235.1 acar|OOF96272.1 abra|OJJ71519.1 acla|EAW15077.1 afis|EAW20048.1 afla|EED45332.1 afum|EAL85169.1 agla|OJJ88912.1 akaw|GAA89398.1 aluc|OJZ86258.1 anid|CBF84799.1 anig|EHA21608.1 aory|EIT73661.1 asyd|OJJ63225.1 ater|EAU38658.1 atub|OJI84285.1 aver|OJI97730.1 awen|OJJ31806.1 azon|OJJ46316.1

Group923: acar|OOF96236.1 acar|OOF96273.1 abra|OJJ71520.1 acla|EAW15078.1 afis|EAW20049.1 afum|EAL85170.2 agla|OJJ88911.1 akaw|GAA89397.1 aluc|OJZ86259.1 anid|CBF84801.1 anig|EHA21607.1 aory|EIT73827.1 asyd|OJJ63224.1 ater|EAU38659.1 atub|OJI84284.1 aver|OJI97731.1 awen|OJJ31807.1 azon|OJJ46315.1 afla|EED45331.1

Group924: acar|OOF96239.1 acar|OOF96276.1 abra|OJJ71523.1 acla|EAW15080.1 afis|EAW20052.1 afla|EED45328.1 afum|EAL85172.1 agla|OJJ88908.1 akaw|GAA89394.1 aluc|OJZ86262.1 anid|CBF84806.1 anig|EHA21605.1 aory|EIT73685.1 asyd|OJJ63222.1 ater|EAU38661.1 atub|OJI84281.1 aver|OJI97733.1 awen|OJJ31810.1 azon|OJJ46312.1

Group925: acar|OOF96240.1 acar|OOF96277.1 abra|OJJ71524.1 acla|EAW15081.1 afis|EAW20053.1 afla|EED45327.1 afum|EAL85173.2 agla|OJJ88907.1 akaw|GAA89393.1 aluc|OJZ86263.1 anid|CBF84808.1 anig|EHA21604.1 aory|EIT73613.1 asyd|OJJ63221.1 ater|EAU38662.1 atub|OJI84280.1 aver|OJI97734.1 awen|OJJ31811.1 azon|OJJ46311.1

Group926: acar|OOF96242.1 acar|OOF96279.1 abra|OJJ71527.1 acla|EAW15083.1 afis|EAW20055.1 afla|EED45325.1 afum|EAL85175.2 agla|OJJ88905.1 akaw|GAA89391.1 aluc|OJZ86266.1 anid|CBF84813.1 anig|EHA21602.1 aory|EIT73716.1 asyd|OJJ63218.1 ater|EAU38664.1 atub|OJI84275.1 aver|OJI97737.1 awen|OJJ31814.1 azon|OJJ46309.1

Group927: acar|OOF96243.1 acar|OOF96280.1 afum|EAL85176.2 afum|EAL87762.1 abra|OJJ71528.1 acla|EAW15084.1 afis|EAW20056.1 afla|EED45324.1 agla|OJJ88904.1 akaw|GAA89390.1 aluc|OJZ86268.1 anid|CBF84817.1 aory|EIT73595.1 asyd|OJJ63217.1 ater|EAU38665.1 atub|OJI84274.1 aver|OJI97738.1 awen|OJJ31815.1 azon|OJJ46308.1

Group928: acar|OOF96245.1 acar|OOF96282.1 abra|OJJ71531.1 acla|EAW15086.1 afis|EAW20058.1 afla|EED45322.1 afum|EAL85178.1 agla|OJJ88902.1 akaw|GAA89388.1 aluc|OJZ86270.1 anid|CBF84840.1 anig|EHA21599.1 aory|EIT73696.1 asyd|OJJ63215.1 ater|EAU38667.1 atub|OJI84271.1 aver|OJI97740.1 awen|OJJ31817.1 azon|OJJ46306.1

Group929: acar|OOF96248.1 acar|OOF96285.1 afum|EAL85181.2 afum|EAL87756.2 abra|OJJ71535.1 acla|EAW15089.1 afis|EAW20061.1 afla|EED45318.1 agla|OJJ88898.1 akaw|GAA89385.1 aluc|OJZ86273.1 anid|CBF84834.1 anig|EHA21596.1 aory|EIT73796.1 asyd|OJJ63212.1 ater|EAU38670.1 atub|OJI84267.1 aver|OJI97743.1 awen|OJJ31821.1

Group930: acar|OOF96249.1 acar|OOF96286.1 abra|OJJ71537.1 acla|EAW15090.1 afis|EAW20062.1 afla|EED45317.1 afum|EAL85182.2 agla|OJJ88897.1 akaw|GAA89384.1 aluc|OJZ86274.1 anid|CBF84832.1 anig|EHA21595.1 aory|EIT73715.1 asyd|OJJ63211.1 ater|EAU38671.1 atub|OJI84266.1 aver|OJI97744.1 awen|OJJ31822.1 azon|OJJ46303.1

Group931: acar|OOF96250.1 acar|OOF96287.1 abra|OJJ71540.1 acla|EAW15095.1 afis|EAW20064.1 afla|EED45313.1 afum|EAL85184.1 agla|OJJ88893.1 akaw|GAA89381.1 aluc|OJZ86278.1 anid|CBF84828.1 anig|EHA21593.1 aory|EIT73604.1 asyd|OJJ63209.1 ater|EAU38673.1 atub|OJI84261.1 aver|OJI97746.1 awen|OJJ31825.1 azon|OJJ46300.1

Group932: acar|OOF96251.1 acar|OOF96290.1 abra|OJJ71543.1 acla|EAW15097.1 afis|EAW20069.1 afla|EED45310.1 afum|EAL85187.1 agla|OJJ88891.1 akaw|GAA89379.1 aluc|OJZ86281.1 anid|CBF84823.1 anig|EHA21590.1 aory|EIT73765.1 asyd|OJJ63206.1 ater|EAU38675.1 atub|OJI84258.1 aver|OJI97749.1 awen|OJJ31828.1 azon|OJJ46296.1

Group933: acar|OOF96252.1 acar|OOF96291.1 abra|OJJ71545.1 acla|EAW15099.1 afis|EAW20071.1 afla|EED45308.1 afum|EAL85189.1 agla|OJJ88889.1 akaw|GAA89377.1 aluc|OJZ86283.1 anid|CBF84845.1 anig|EHA21588.1 aory|EIT73815.1 asyd|OJJ63204.1 ater|EAU38676.1 atub|OJI84255.1 aver|OJI97751.1 awen|OJJ31830.1 azon|OJJ46294.1

Group934: acar|OOF96254.1 acar|OOF96293.1 abra|OJJ71549.1 acla|EAW15103.1 afis|EAW20075.1 afla|EED45304.1 afum|EAL85193.2 agla|OJJ88885.1 akaw|GAA89373.1 aluc|OJZ86287.1 anid|CBF84853.1 anig|EHA21584.1 aory|EIT73551.1 asyd|OJJ63200.1 ater|EAU38683.1 atub|OJI84251.1 aver|OJI97755.1 awen|OJJ31834.1 azon|OJJ46290.1

Group935: acar|OOF96255.1 acar|OOF96294.1 abra|OJJ71550.1 acla|EAW15104.1 afis|EAW20076.1 afla|EED45302.1 afum|EAL85194.1 agla|OJJ88884.1 akaw|GAA89372.1 aluc|OJZ86288.1 anid|CBF84855.1 anig|EHA21583.1 aory|EIT73645.1 asyd|OJJ63199.1 ater|EAU38684.1 atub|OJI84250.1 aver|OJI97756.1 awen|OJJ31835.1 azon|OJJ46289.1

Group936: acar|OOF96256.1 acar|OOF96295.1 abra|OJJ71551.1 acla|EAW15105.1 afis|EAW20077.1 afla|EED45298.1 afum|EAL85195.1 agla|OJJ88883.1 akaw|GAA89371.1 aluc|OJZ86289.1 anid|CBF84867.1 anig|EHA21582.1 aory|EIT73681.1 asyd|OJJ63198.1 ater|EAU38685.1 atub|OJI84249.1 aver|OJI97757.1 awen|OJJ31836.1 azon|OJJ46288.1

Group937: acar|OOF96257.1 acar|OOF96296.1 abra|OJJ71554.1 acla|EAW15108.1 afis|EAW20079.1 afla|EED45296.1 afum|EAL85198.1 agla|OJJ88880.1 akaw|GAA89368.1 aluc|OJZ86292.1 anid|CBF84858.1 anig|EHA21579.1 aory|EIT73808.1 asyd|OJJ63195.1 ater|EAU38687.1 atub|OJI84246.1 aver|OJI97760.1 awen|OJJ31839.1 azon|OJJ46284.1

Group938: acar|OOF96906.1 acar|OOF96942.1 abra|OJJ70803.1 acla|EAW11121.1 afis|EAW15850.1 afla|EED56523.1 afum|EAL88486.1 agla|OJJ87258.1 akaw|GAA90119.1 aluc|OJZ84149.1 anid|CBF81174.1 anig|EHA19253.1 aory|EIT72791.1 asyd|OJJ59291.1 ater|EAU31665.1 atub|OJI90521.1 aver|OJJ02984.1 awen|OJJ36777.1 azon|OJJ47421.1

Group939: acar|OOF96913.1 acar|OOF96948.1 abra|OJJ70797.1 acla|EAW11126.1 afis|EAW15855.1 afla|EED56517.1 afum|EAL88491.1 agla|OJJ87252.1 akaw|GAA90113.1 aluc|OJZ84142.1 anid|CBF81185.1

anig|EHA19250.1 aory|EIT72794.1 asyd|OJJ59297.1 ater|EAU31670.1 atub|OJI90516.1 aver|OJJ02978.1 awen|OJJ36772.1 azon|OJJ47426.1

Group940: acar|OOF96914.1 acar|OOF96949.1 abra|OJJ70796.1 acla|EAW11127.1 afis|EAW15856.1 afla|EED56516.1 afum|EAL88492.1 agla|OJJ87251.1 akaw|GAA90112.1 aluc|OJZ84141.1 anid|CBF81187.1 anig|EHA19249.1 aory|EIT72847.1 asyd|OJJ59298.1 ater|EAU31671.1 atub|OJI90515.1 aver|OJJ02977.1 awen|OJJ36771.1 azon|OJJ47427.1

Group941: acar|OOF96916.1 acar|OOF96951.1 abra|OJJ70794.1 acla|EAW11129.1 afis|EAW15858.1 afla|EED56514.1 afum|EAL88494.1 agla|OJJ87249.1 akaw|GAA90110.1 aluc|OJZ84139.1 anid|CBF81191.1 anig|EHA19247.1 aory|EIT72852.1 asyd|OJJ59300.1 ater|EAU31673.1 atub|OJI90513.1 aver|OJJ02975.1 awen|OJJ36769.1 azon|OJJ47429.1

Group942: acar|OOF96917.1 acar|OOF96952.1 abra|OJJ70793.1 acla|EAW11130.1 afis|EAW15861.1 afla|EED56513.1 afum|EAL88496.1 agla|OJJ87248.1 akaw|GAA90109.1 aluc|OJZ84138.1 anid|CBF81193.1 anig|EHA19246.1 aory|EIT72867.1 asyd|OJJ59301.1 ater|EAU31674.1 atub|OJI90512.1 aver|OJJ02974.1 awen|OJJ36768.1 azon|OJJ47430.1

Group943: acar|OOF96918.1 acar|OOF96953.1 abra|OJJ70792.1 acla|EAW11131.1 afis|EAW15862.1 afla|EED56512.1 afum|EAL88497.1 agla|OJJ87247.1 akaw|GAA90108.1 aluc|OJZ84137.1 anid|CBF81194.1 anig|EHA19245.1 aory|EIT72817.1 asyd|OJJ59302.1 ater|EAU31675.1 atub|OJI90511.1 aver|OJJ02973.1 awen|OJJ36767.1 azon|OJJ48009.1

Group944: acar|OOF96919.1 acar|OOF96954.1 abra|OJJ70791.1 acla|EAW11132.1 afis|EAW15863.1 afla|EED56511.1 afum|EAL88498.1 agla|OJJ87246.1 akaw|GAA90107.1 aluc|OJZ84136.1 anid|CBF81196.1 anig|EHA19244.1 aory|EIT72814.1 asyd|OJJ59303.1 ater|EAU31676.1 atub|OJI90510.1 aver|OJJ02972.1 awen|OJJ36766.1 azon|OJJ47431.1

Group945: acar|OOF96921.1 acar|OOF96956.1 abra|OJJ70789.1 acla|EAW11133.1 afis|EAW15865.1 afla|EED56510.1 afum|EAL88499.1 agla|OJJ87244.1 akaw|GAA90105.1 aluc|OJZ84134.1 anid|CBF81200.1 anig|EHA19242.1 aory|EIT72849.1 asyd|OJJ59304.1 ater|EAU31678.1 atub|OJI90508.1 aver|OJJ02971.1 awen|OJJ36765.1 azon|OJJ47433.1

Group946: acar|OOF96924.1 acar|OOF96959.1 abra|OJJ70786.1 acla|EAW11135.1 afis|EAW15867.1 afla|EED56508.1 afum|EAL88501.1 agla|OJJ87241.1 akaw|GAA90103.1 aluc|OJZ84131.1 anid|CBF81205.1 anig|EHA19239.1 aory|EIT72789.1 asyd|OJJ59307.1 ater|EAU31680.1 atub|OJI90505.1 aver|OJJ02968.1 awen|OJJ36762.1 azon|OJJ47436.1

Group947: acar|OOF96927.1 acar|OOF96962.1 abra|OJJ70781.1 acla|EAW11138.1 afis|EAW15869.1 afla|EED56505.1 afum|EAL88503.1 agla|OJJ87238.1 akaw|GAA90100.1 aluc|OJZ84128.1 anid|CBF81211.1 anig|EHA19236.1 aory|EIT72793.1 asyd|OJJ59310.1 ater|EAU31686.1 atub|OJI90502.1 aver|OJJ02965.1 awen|OJJ36760.1 azon|OJJ47439.1

Group948: acar|OOF96928.1 acar|OOF96963.1 abra|OJJ70780.1 acla|EAW11139.1 afis|EAW15870.1 afla|EED56504.1 afum|EAL88504.1 agla|OJJ87237.1 akaw|GAA90099.1 aluc|OJZ84127.1 anid|CBF81213.1 anig|EHA19235.1 aory|EIT72861.1 asyd|OJJ59311.1 ater|EAU31687.1 atub|OJI90501.1 aver|OJJ02964.1 awen|OJJ36759.1 azon|OJJ47440.1

Group949: acar|OOF96930.1 acar|OOF96965.1 abra|OJJ70778.1 acla|EAW11141.1 afis|EAW15872.1 afla|EED56503.1 afum|EAL88506.1 agla|OJJ87235.1 akaw|GAA90097.1 aluc|OJZ84125.1 anid|CBF81219.1 anig|EHA19233.1 aory|EIT72819.1 asyd|OJJ59312.1 ater|EAU31689.1 atub|OJI90499.1 aver|OJJ02963.1 awen|OJJ36758.1 azon|OJJ47442.1

Group950: acar|OOF96931.1 acar|OOF96966.1 abra|OJJ70777.1 acla|EAW11142.1 afis|EAW15873.1 afla|EED56502.1 afum|EBA27350.1 akaw|GAA90096.1 aluc|OJZ84124.1 anid|CBF81221.1 anig|EHA19232.1 aory|EIT72844.1 asyd|OJJ59313.1 ater|EAU31690.1 atub|OJI90497.1 aver|OJJ02962.1 awen|OJJ36757.1 azon|OJJ47443.1 afum|EBA27351.1

Group951: acar|OOF96932.1 acar|OOF96967.1 abra|OJJ70776.1 acla|EAW11143.1 afis|EAW15874.1 afla|EED56501.1 afum|EAL88508.1 agla|OJJ87234.1 akaw|GAA90095.1 aluc|OJZ84123.1 anid|CBF81222.1 anig|EHA19231.1 aory|EIT72827.1 asyd|OJJ59314.1 ater|EAU31691.1 atub|OJI90495.1 aver|OJJ02961.1 awen|OJJ36756.1 azon|OJJ47444.1

Group952: acar|OOF97657.1 acar|OOF97704.1 abra|OJJ71500.1 acla|EAW10048.1 afis|EAW25536.1 afla|EED44712.1 afum|EAL90200.1 agla|OJJ87267.1 akaw|GAA82520.1 aluc|OJZ87047.1 anid|CBF77819.1 anig|EHA20320.1 aory|EIT83462.1 asyd|OJJ53009.1 ater|EAU33377.1 atub|OJI81588.1 aver|OJJ03834.1 awen|OJJ41278.1 azon|OJJ49501.1

Group953: acar|OOF97658.1 acar|OOF97705.1 abra|OJJ71499.1 acla|EAW10050.1 afis|EAW25534.1 afla|EED44709.1 afum|EAL90198.1 agla|OJJ87269.1 akaw|GAA82521.1 aluc|OJZ87048.1 anid|CBF77817.1 anig|EHA20321.1 aory|EIT83458.1 asyd|OJJ53010.1 ater|EAU33376.1 atub|OJI81587.1 aver|OJJ03833.1 awen|OJJ41276.1 azon|OJJ49508.1

Group954: acar|OOF97660.1 acar|OOF97707.1 abra|OJJ71497.1 acla|EAW10052.1 afis|EAW25532.1 afla|EED54033.1 afum|EAL90196.2 agla|OJJ87270.1 akaw|GAA82523.1 aluc|OJZ87050.1 anid|CBF77813.1 anig|EHA20323.1 aory|EIT78299.1 asyd|OJJ53012.1 ater|EAU33374.1 atub|OJI81585.1 aver|OJJ03831.1 awen|OJJ41274.1 azon|OJJ49506.1

Group955: acar|OOF97661.1 acar|OOF97708.1 abra|OJJ71496.1 acla|EAW10053.1 afis|EAW25531.1 afla|EED54032.1 afum|EBA27398.1 agla|OJJ87271.1 akaw|GAA82524.1 aluc|OJZ87051.1 anid|CBF77811.1 anig|EHA20324.1 aory|EIT78333.1 asyd|OJJ53013.1 ater|EAU33373.1 atub|OJI81584.1 aver|OJJ03830.1 awen|OJJ41273.1 azon|OJJ49505.1

Group956: acar|OOF97663.1 acar|OOF97710.1 abra|OJJ71494.1 acla|EAW10055.1 afis|EAW25529.1 afla|EED54030.1 afum|EAL90194.1 agla|OJJ87273.1 akaw|GAA82526.1 aluc|OJZ87053.1 anid|CBF77821.1 anig|EHA20326.1 aory|EIT78259.1 asyd|OJJ53015.1 ater|EAU33372.1 atub|OJI81582.1 aver|OJJ03828.1 awen|OJJ41271.1 azon|OJJ49509.1

Group957: acar|OOF97664.1 acar|OOF97711.1 abra|OJJ71493.1 acla|EAW10057.1 afis|EAW25527.1 afla|EED54028.1 afum|EAL90192.1 agla|OJJ87275.1 akaw|GAA82527.1 aluc|OJZ87054.1 anid|CBF77805.1 anig|EHA20327.1 aory|EIT78292.1 asyd|OJJ53017.1 ater|EAU33370.1 atub|OJI81581.1 aver|OJJ03826.1 awen|OJJ41269.1 azon|OJJ49511.1

Group958: acar|OOF97666.1 acar|OOF97713.1 abra|OJJ71491.1 acla|EAW10059.1 afis|EAW25525.1 afla|EED55229.1 afum|EAL90190.2 agla|OJJ81067.1 akaw|GAA82529.1 aluc|OJZ87056.1 anid|CBF77802.1 anig|EHA20329.1 aory|EIT83576.1 asyd|OJJ53019.1 ater|EAU32314.1 atub|OJI81579.1 aver|OJJ03824.1 awen|OJJ40768.1 azon|OJJ44041.1

Group959: acar|OOF97669.1 acar|OOF97715.1 abra|OJJ71489.1 acla|EAW10061.1 afis|EAW25523.1 afla|EED54026.1 afum|EAL90188.2 agla|OJJ87278.1 akaw|GAA82531.1 aluc|OJZ87058.1 anid|CBF77800.1 anig|EHA20331.1 aory|EIT78257.1 asyd|OJJ53021.1 ater|EAU33368.1 atub|OJI81577.1 aver|OJJ03822.1 awen|OJJ41267.1 azon|OJJ44832.1

Group960: acar|OOF97671.1 acar|OOF97718.1 abra|OJJ71486.1 acla|EAW10064.1 afis|EAW25521.1 afla|EED54023.1 afum|EAL90186.2 agla|OJJ87281.1 akaw|GAA82534.1 aluc|OJZ87061.1 anid|CBF77796.1 anig|EHA20334.1 aory|EIT78303.1 asyd|OJJ53024.1 ater|EAU33367.1 atub|OJI81574.1 aver|OJJ03819.1 awen|OJJ41264.1 azon|OJJ44829.1

Group961: acar|OOF97672.1 acar|OOF97719.1 abra|OJJ71485.1 acla|EAW10065.1 afis|EAW25520.1 afla|EED54022.1 afum|EAL90185.1 agla|OJJ87282.1 akaw|GAA82535.1 aluc|OJZ87062.1 anid|CBF77794.1 anig|EHA20335.1 aory|EIT78334.1 asyd|OJJ53025.1 ater|EAU33366.1 atub|OJI81573.1 aver|OJJ03818.1 awen|OJJ41263.1 azon|OJJ44828.1

Group962: acar|OOF97673.1 acar|OOF97720.1 abra|OJJ71481.1 acla|EAW10068.1 afis|EAW25517.1 afla|EED54017.1 afum|EAL90182.1 agla|OJJ87286.1 akaw|GAA82538.1 aluc|OJZ87066.1 anid|CBF77788.1 anig|EHA20338.1 aory|EIT78273.1 asyd|OJJ53028.1 ater|EAU33363.1 atub|OJI81570.1 aver|OJJ03816.1 awen|OJJ41260.1 azon|OJJ44825.1

Group963: acar|OOF97674.1 acar|OOF97721.1 abra|OJJ71478.1 acla|EAW10071.1 afis|EAW25515.1 afla|EED54016.1 afum|EAL90180.1 agla|OJJ87288.1 akaw|GAA82539.1 aluc|OJZ87067.1 anid|CBF77785.1 anig|EHA20339.1 aory|EIT78267.1 asyd|OJJ53029.1 ater|EAU33361.1 atub|OJI81569.1 aver|OJJ03815.1 awen|OJJ41258.1 azon|OJJ44824.1

Group964: acar|OOF97675.1 acar|OOF97723.1 abra|OJJ71476.1 acla|EAW10073.1 afis|EAW25513.1 afla|EED54014.1 afum|EAL90178.1 agla|OJJ87291.1 akaw|GAA82540.1 aluc|OJZ87070.1 anid|CBF77781.1 anig|EHA20340.1 aory|EIT78254.1 asyd|OJJ53031.1 ater|EAU33359.1 atub|OJI81567.1 aver|OJJ03813.1 awen|OJJ41256.1 azon|OJJ44821.1

Group965: acar|OOF97678.1 acar|OOF97726.1 abra|OJJ71474.1 acla|EAW10076.1 afis|EAW25510.1 afla|EED54011.1 afum|EAL90176.2 agla|OJJ87295.1 akaw|GAA82542.1 aluc|OJZ87072.1 anid|CBF77775.1 anig|EHA20342.1 aory|EIT78253.1 asyd|OJJ53034.1 ater|EAU33357.1 atub|OJI81565.1 aver|OJJ03810.1 awen|OJJ41252.1 azon|OJJ44819.1

Group966: acar|OOF97679.1 acar|OOF97727.1 abra|OJJ71472.1 acla|EAW10078.1 afis|EAW25508.1 afla|EED54008.1 afum|EAL90174.1 agla|OJJ87297.1 akaw|GAA82544.1 aluc|OJZ87074.1 anid|CBF77772.1 anig|EHA20343.1 aory|EIT78280.1 asyd|OJJ53036.1 ater|EAU33355.1 atub|OJI81563.1 aver|OJJ03808.1 awen|OJJ41250.1 azon|OJJ44817.1

Group967: acar|OOF97680.1 acar|OOF97729.1 abra|OJJ71470.1 acla|EAW10081.1 afis|EAW25506.1 afla|EED54006.1 afum|EAL90172.1 agla|OJJ87298.1 akaw|GAA82545.1 aluc|OJZ87075.1 anid|CBF77768.1 anig|EHA20345.1 aory|EIT78338.1 asyd|OJJ53037.1 ater|EAU33354.1 atub|OJI81561.1 aver|OJJ03807.1 awen|OJJ41249.1 azon|OJJ44816.1

Group968: acar|OOF97681.1 acar|OOF97730.1 abra|OJJ71469.1 acla|EAW10082.1 afis|EAW25505.1 afla|EED54005.1 afum|EAL90171.1 agla|OJJ87299.1 akaw|GAA82546.1 aluc|OJZ87076.1 anid|CBF77766.1

anig|EHA20346.1 aory|EIT78265.1 asyd|OJJ53038.1 ater|EAU33353.1 atub|OJI81560.1 aver|OJJ03806.1 awen|OJJ41248.1 azon|OJJ44813.1

Group969: acar|OOF97682.1 acar|OOF97731.1 abra|OJJ71468.1 acla|EAW10083.1 afis|EAW25504.1 afla|EED54003.1 afum|EAL90170.1 agla|OJJ87300.1 akaw|GAA82547.1 aluc|OJZ87077.1 anid|CBF77764.1 anig|EHA20347.1 aory|EIT78261.1 asyd|OJJ53039.1 ater|EAU33352.1 atub|OJI81559.1 aver|OJJ03805.1 awen|OJJ41247.1 azon|OJJ44812.1

Group970: acar|OOF97684.1 acar|OOF97734.1 abra|OJJ71464.1 acla|EAW10086.1 afis|EAW25501.1 afla|EED54000.1 afum|EAL90167.1 agla|OJJ87303.1 akaw|GAA82550.1 aluc|OJZ87080.1 anid|CBF77756.1 anig|EHA20351.1 aory|EIT78290.1 asyd|OJJ53043.1 ater|EAU34671.1 atub|OJI81557.1 aver|OJJ03801.1 awen|OJJ41243.1 azon|OJJ44809.1

Group971: acar|OOF97685.1 acar|OOF97735.1 abra|OJJ71465.1 acla|EAW10085.1 afis|EAW25502.1 afla|EED54001.1 afum|EAL90168.1 agla|OJJ87302.1 akaw|GAA82549.1 aluc|OJZ87079.1 anid|CBF77758.1 anig|EHA20350.1 aory|EIT78293.1 asyd|OJJ53042.1 ater|EAU34672.1 atub|OJI81558.1 aver|OJJ03802.1 awen|OJJ41244.1 azon|OJJ44810.1

Group972: acar|OOF97686.1 acar|OOF97736.1 abra|OJJ71463.1 acla|EAW10088.1 afis|EAW25499.1 afla|EED53999.1 afum|EAL90165.1 agla|OJJ87305.1 akaw|GAA82551.1 aluc|OJZ87081.1 anid|CBF77754.1 anig|EHA20352.1 aory|EIT78297.1 asyd|OJJ53044.1 ater|EAU34670.1 atub|OJI81556.1 aver|OJJ03800.1 awen|OJJ41241.1 azon|OJJ44807.1

Group973: acar|OOF97690.1 acar|OOF97740.1 abra|OJJ71458.1 acla|EAW10091.1 afis|EAW25496.1 afla|EED53996.1 afum|EAL90162.1 agla|OJJ87309.1 akaw|GAA82553.1 aluc|OJZ87085.1 anid|CBF77749.1 anig|EHA20355.1 aory|EIT78288.1 asyd|OJJ53047.1 ater|EAU34667.1 atub|OJI81553.1 aver|OJJ03798.1 awen|OJJ41238.1 azon|OJJ44804.1

Group974: acar|OOF97691.1 acar|OOF97741.1 abra|OJJ71456.1 acla|EAW10093.1 afis|EAW25494.1 afla|EED53994.1 afum|EAL90160.1 agla|OJJ87311.1 akaw|GAA82555.1 aluc|OJZ87087.1 anid|CBF77745.1 anig|EHA20357.1 aory|EIT78318.1 asyd|OJJ53049.1 ater|EAU34665.1 atub|OJI81551.1 aver|OJJ03796.1 awen|OJJ41236.1 azon|OJJ44802.1

Group975: acar|OOF97692.1 acar|OOF97742.1 abra|OJJ71455.1 acla|EAW10094.1 afis|EAW25493.1 afla|EED53993.1 afum|EAL90158.1 akaw|GAA82556.1 aluc|OJZ87088.1 anig|EHA20359.1 aory|EIT78309.1 asyd|OJJ53050.1 ater|EAU34664.1 atub|OJI81550.1 aver|OJJ03795.1 awen|OJJ41235.1 azon|OJJ44801.1 afum|EAL90159.1 anig|EHA20358.1

Group976: acar|OOF97694.1 acar|OOF97743.1 abra|OJJ71454.1 acla|EAW10095.1 afis|EAW25492.1 afla|EED53992.1 afum|EAL90157.1 agla|OJJ87140.1 akaw|GAA82557.1 aluc|OJZ87089.1 anid|CBF77743.1 anig|EHA20360.1 aory|EIT78276.1 asyd|OJJ53051.1 ater|EAU34663.1 atub|OJI81549.1 aver|OJJ03794.1 awen|OJJ41234.1 azon|OJJ44799.1

Group977: acar|OOF97695.1 acar|OOF97745.1 abra|OJJ71451.1 acla|EAW10096.1 afis|EAW25491.1 afla|EED53991.1 afum|EAL90156.1 agla|OJJ87139.1 akaw|GAA82559.1 aluc|OJZ87091.1 anid|CBF77741.1 anig|EHA20362.1 aory|EIT78277.1 asyd|OJJ53052.1 ater|EAU34662.1 atub|OJI81547.1 aver|OJJ03792.1 awen|OJJ41233.1 azon|OJJ44798.1

Group978: acar|OOF97697.1 acar|OOF97747.1 abra|OJJ71447.1 acla|EAW10099.1 afis|EAW25488.1 afla|EED53986.1 afum|EAL90153.1 agla|OJJ87136.1 akaw|GAA82562.1 aluc|OJZ87094.1 anid|CBF77724.1 anig|EHA20365.1 aory|EIT78270.1 asyd|OJJ53061.1 ater|EAU34659.1 atub|OJI81544.1 aver|OJJ03783.1 awen|OJJ41229.1 azon|OJJ44796.1

Group979: acar|OOF97698.1 acar|OOF97748.1 abra|OJJ71446.1 acla|EAW10100.1 afis|EAW25487.1 afla|EED53985.1 afum|EAL90152.1 agla|OJJ87135.1 akaw|GAA82563.1 aluc|OJZ87095.1 anid|CBF77722.1 anig|EHA20366.1 aory|EIT78345.1 asyd|OJJ53062.1 ater|EAU34658.1 atub|OJI81543.1 aver|OJJ03782.1 awen|OJJ41228.1 azon|OJJ44795.1

Group980: acar|OOF97699.1 acar|OOF97749.1 abra|OJJ71444.1 acla|EAW10102.1 afis|EAW25485.1 afla|EED53983.1 afum|EAL90150.1 agla|OJJ87133.1 akaw|GAA82565.1 aluc|OJZ87097.1 anid|CBF77713.1 anig|EHA20368.1 aory|EIT78331.1 asyd|OJJ53067.1 ater|EAU34656.1 atub|OJI81541.1 aver|OJJ03778.1 awen|OJJ41226.1 azon|OJJ44781.1

Group981: acar|OOF98471.1 acar|OOF98473.1 abra|OJJ78401.1 acla|EAW09131.1 afis|EAW22015.1 afla|EED46740.1 afum|EAL93030.1 agla|OJJ84082.1 akaw|GAA85630.1 aluc|OJZ91480.1 anid|CBF82625.1 anig|EHA20142.1 aory|EIT77878.1 asyd|OJJ54012.1 ater|EAU31136.1 atub|OJI83473.1 aver|OJJ08730.1 awen|OJJ34311.1 azon|OJJ43327.1

Group982: acar|OOF98472.1 acar|OOF98486.1 abra|OJJ78402.1 acla|EAW09130.1 afis|EAW22016.1 afla|EED46741.1 afum|EAL93031.1 agla|OJJ84081.1 akaw|GAA85629.1 aluc|OJZ91481.1 anid|CBF82627.1 anig|EHA20143.1 aory|EIT77969.1 asyd|OJJ54011.1 ater|EAU31137.1 atub|OJI83474.1 aver|OJJ08731.1 awen|OJJ34312.1 azon|OJJ43328.1

Group983: acar|OOF98475.1 acar|OOF98487.1 abra|OJJ78399.1 acla|EAW09133.1 afis|EAW22013.1 afla|EED46738.1 afum|EAL93028.1 agla|OJJ84084.1 akaw|GAA85632.1 aluc|OJZ91478.1 anid|CBF82644.1 anig|EHA20140.1 aory|EIT77900.1 asyd|OJJ61676.1 ater|EAU31135.1 atub|OJI83471.1 aver|OJJ00997.1 awen|OJJ34309.1 azon|OJJ48062.1

Group984: acar|OOF98476.1 acar|OOF98489.1 abra|OJJ78398.1 acla|EAW09134.1 afis|EAW22012.1 afla|EED46737.1 afum|EAL93027.1 agla|OJJ84085.1 akaw|GAA85633.1 aluc|OJZ91477.1 anid|CBF82646.1 anig|EHA20139.1 aory|EIT77930.1 asyd|OJJ61675.1 ater|EAU31134.1 atub|OJI83470.1 aver|OJJ00996.1 awen|OJJ34308.1 azon|OJJ48061.1

Group985: acar|OOF98478.1 acar|OOF98491.1 abra|OJJ78396.1 acla|EAW09136.1 afis|EAW22011.1 afla|EED46735.1 afum|EAL93026.1 agla|OJJ84087.1 akaw|GAA85635.1 aluc|OJZ91475.1 anid|CBF82649.1 anig|EHA20137.1 aory|EIT77821.1 asyd|OJJ61673.1 ater|EAU31132.1 atub|OJI83468.1 aver|OJJ00994.1 awen|OJJ34306.1 azon|OJJ43333.1

Group986: acar|OOF98481.1 acar|OOF98494.1 abra|OJJ78393.1 acla|EAW09139.1 afis|EAW22008.1 afla|EED46732.1 afum|EAL93023.1 agla|OJJ84090.1 akaw|GAA85638.1 aluc|OJZ91472.1 anid|CBF82655.1 anig|EHA20134.1 aory|EIT77839.1 asyd|OJJ61670.1 ater|EAU31130.1 atub|OJI83465.1 aver|OJJ00991.1 awen|OJJ34303.1 azon|OJJ43335.1

Group987: acar|OOF98482.1 acar|OOF98495.1 abra|OJJ78391.1 acla|EAW09141.1 afis|EAW22006.1 afla|EED46730.1 afum|EAL93021.2 agla|OJJ84092.1 akaw|GAA85640.1 aluc|OJZ91470.1 anid|CBF82659.1 anig|EHA20132.1 aory|EIT77908.1 asyd|OJJ61668.1 ater|EAU31128.1 atub|OJI83463.1 aver|OJJ00989.1 awen|OJJ34301.1 azon|OJJ43337.1

Group988: acar|OOF98483.1 acar|OOF98496.1 abra|OJJ78392.1 acla|EAW09140.1 afis|EAW22007.1 afla|EED46731.1 afum|EAL93022.1 agla|OJJ84091.1 akaw|GAA85639.1 aluc|OJZ91471.1 anid|CBF82657.1 anig|EHA20133.1 aory|EIT77818.1 asyd|OJJ61669.1 ater|EAU31129.1 atub|OJI83464.1 aver|OJJ00990.1 awen|OJJ34302.1 azon|OJJ43336.1

Group989: acar|OOF98531.1 acar|OOF98532.1 abra|OJJ78355.1 acla|EAW09180.1 afis|EAW21966.1 afla|EED46687.1 afum|EAL92980.1 agla|OJJ84137.1 akaw|GAA85679.1 aluc|OJZ91430.1 anid|CBF82734.1 anig|EHA20099.1 aory|EIT77938.1 asyd|OJJ61626.1 ater|EAU31086.1 atub|OJI83423.1 aver|OJJ00951.1 awen|OJJ34260.1 azon|OJJ43377.1

Group990: acla|EAW12052.1 acla|EAW15326.1 afis|EAW19692.1 afis|EAW20347.1 afla|EED45214.1 afla|EED48467.1 afum|EAL85467.1 afum|EAL86834.1 aory|EIT73589.1 aory|EIT76272.1 asyd|OJJ53694.1 asyd|OJJ62962.1 aver|OJI98005.1 aver|OJJ07810.1 acar|OOF91909.1 anid|CBF75992.1 ater|EAU37786.1 awen|OJJ38808.1 azon|OJJ45237.1

Group991: afis|EAW15984.1 afis|EAW22037.1 abra|OJJ77639.1 acar|OOF99107.1 acla|EAW06489.1 afla|EED56354.1 afum|EAL88616.1 agla|OJJ85987.1 akaw|GAA83995.1 aluc|OJZ80891.1 anid|CBF85810.1 anig|EHA19447.1 aory|EIT73316.1 asyd|OJJ64340.1 ater|EAU33668.1 atub|OJI82687.1 aver|OJI98433.1 awen|OJJ30486.1 azon|OJJ45477.1

Group992: afis|EAW16169.1 afis|EAW20696.1 abra|OJJ78079.1 acar|OOF98785.1 acla|EAW06612.1 afla|EED48696.1 afum|EAL88803.1 agla|OJJ86214.1 akaw|GAA85915.1 aluc|OJZ91151.1 anid|CBF79561.1 anig|EHA19849.1 aory|EIT73253.1 asyd|OJJ57924.1 ater|EAU33245.1 atub|OJI83165.1 aver|OJJ04468.1 awen|OJJ30269.1 azon|OJJ43965.1

Group993: afis|EAW16186.1 afis|EAW21276.1 afum|EAL88817.1 afum|EAL92291.2 abra|OJJ66405.1 acla|EAW11035.1 afla|EED49635.1 agla|OJJ80815.1 akaw|GAA90273.1 aluc|OJZ83068.1 anig|EHA28132.1 aory|EIT80977.1 asyd|OJJ54301.1 ater|EAU36744.1 atub|OJI87878.1 azon|OJJ42729.1 ater|EAU31600.1 azon|OJJ45943.1 anid|CBF80686.1

Group994: afis|EAW16703.1 afis|EAW22511.1 abra|OJJ73562.1 acar|OOG00744.1 acla|EAW07479.1 afla|EED54539.1 afum|EAL90682.1 agla|OJJ80876.1 akaw|GAA88592.1 aluc|OJZ88499.1 anid|CBF88981.1 anig|EHA18208.1 aory|EIT78923.1 asyd|OJJ62789.1 ater|EAU39198.1 atub|OJI91086.1 aver|OJI96836.1 awen|OJJ35137.1 azon|OJJ49962.1

Group995: afis|EAW17302.1 afis|EAW21312.1 abra|OJJ75975.1 acar|OOF99944.1 acla|EAW09822.1 afla|EED57269.1 afum|EAL92328.1 agla|OJJ87430.1 akaw|GAA88981.1 aluc|OJZ89422.1 anid|CBF83405.1 anig|EHA23165.1 aory|EIT74144.1 asyd|OJJ60646.1 ater|EAU35941.1 atub|OJI85371.1 aver|OJI99964.1 awen|OJJ39388.1 azon|OJJ44276.1

Group996: afis|EAW20681.1 afis|EAW24898.1 abra|OJJ74730.1 acar|OOF89795.1 acla|EAW10669.1 afla|EED46977.1 afum|EAL89552.2 agla|OJJ82121.1 akaw|GAA81887.1 aluc|OJZ92314.1 anid|CBF75653.1 anig|EHA26655.1 aory|EIT72604.1 asyd|OJJ57466.1 ater|EAU36401.1 atub|OJI86743.1 aver|OJJ05715.1 awen|OJJ40490.1 azon|OJJ49353.1

Group997: afis|EAW21461.1 afis|EAW22039.1 abra|OJJ76153.1 acar|OOF89976.1 acla|EAW09678.1 afla|EED55893.1 afum|EAL92480.1 agla|OJJ85609.1 akaw|GAA82102.1 aluc|OJZ92076.1 anid|CBF76534.1

anig|EHA23002.1 aory|EIT76550.1 asyd|OJJ61148.1 ater|EAU35074.1 atub|OJI86506.1 aver|OJJ00470.1 awen|OJJ39238.1 azon|OJJ51616.1

Group998: afis|EAW24479.1 afis|EAW24670.1 abra|OJJ67630.1 acar|OOF91977.1 afis|EAW16900.1 afla|EED52360.1 afla|EED53180.1 afum|EAL86435.1 akaw|GAA89150.1 aluc|OJZ81686.1 anig|EHA25757.1 aory|EIT74904.1 atub|OJI88495.1 azon|OJJ45915.1 abra|OJJ70817.1 acla|EAW12031.1 afum|EAL86150.1 anig|EHA28156.1 awen|OJJ40789.1

Group999: afla|EED44958.1 afla|EED54307.1 aory|EIT73482.1 aory|EIT76036.1 abra|OJJ68802.1 acar|OOF91598.1 acla|EAW12204.1 afis|EAW17076.1 afum|EAL86562.1 agla|OJJ84498.1 akaw|GAA90451.1 aluc|OJZ85773.1 anid|CBF78312.1 anig|EHA24780.1 asyd|OJJ52532.1 ater|EAU37366.1 atub|OJI79558.1 aver|OJJ03869.1 awen|OJJ32505.1

Group1000: afla|EED47569.1 afla|EED49784.1 abra|OJJ77270.1 acar|OOF93140.1 acla|EAW13268.1 afis|EAW17349.1 afum|EAL91192.2 agla|OJJ88227.1 akaw|GAA90678.1 aluc|OJZ84677.1 anid|CBF84233.1 anig|EHA27955.1 aory|EIT77705.1 asyd|OJJ57184.1 ater|EAU33864.1 atub|OJI89403.1 aver|OJJ03106.1 awen|OJJ34563.1 azon|OJJ50311.1

Group1001: afla|EED51012.1 afla|EED56994.1 aory|EIT76959.1 aory|EIT77587.1 abra|OJJ72322.1 acar|OOF94890.1 acla|EAW12003.1 afis|EAW19643.1 afum|EAL86879.1 agla|OJJ80785.1 akaw|GAA86073.1 aluc|OJZ90308.1 anig|EHA23795.1 asyd|OJJ58365.1 ater|EAU32885.1 atub|OJI81732.1 aver|OJJ02041.1 awen|OJJ41894.1 azon|OJJ42706.1

Group1002: afla|EED51028.1 afla|EED52463.1 aver|OJJ01603.1 aver|OJJ05985.1 abra|OJJ72678.1 acar|OOF97379.1 acla|EAW12138.1 afis|EAW17198.1 afum|EAL86626.2 akaw|GAA85296.1 aluc|OJZ90655.1 anid|CBF69883.1 anig|EHA24091.1 aory|EIT82808.1 asyd|OJJ56742.1 asyd|OJJ59012.1 atub|OJI82085.1 afla|EED45180.1 aory|EIT73605.1

Group1003: agla|OJJ78416.1 agla|OJJ80642.1 abra|OJJ72427.1 acar|OOF94806.1 acla|EAW12660.1 afis|EAW17824.1 afla|EED46164.1 afum|EAL91634.1 akaw|GAA86168.1 aluc|OJZ90411.1 anid|CBF78007.1 anig|EHA23877.1 aory|EIT76471.1 asyd|OJJ55244.1 ater|EAU33025.1 atub|OJI81842.1 aver|OJJ06980.1 awen|OJJ41754.1 azon|OJJ48444.1

Group1004: agla|OJJ78484.1 agla|OJJ87510.1 abra|OJJ75909.1 acar|OOF99994.1 acla|EAW09880.1 afis|EAW21246.1 afla|EED54806.1 afum|EAL92259.1 akaw|GAA84615.1 aluc|OJZ89359.1 anid|CBF83238.1 anig|EHA23222.1 aory|EIT79446.1 asyd|OJJ60599.1 ater|EAU35886.1 atub|OJI85439.1 aver|OJI99922.1 awen|OJJ36559.1 azon|OJJ44332.1

Group1005: agla|OJJ78485.1 agla|OJJ87511.1 abra|OJJ75908.1 acar|OOF99995.1 acla|EAW09881.1 afis|EAW21245.1 afla|EED54805.1 afum|EAL92258.1 akaw|GAA84614.1 aluc|OJZ89358.1 anid|CBF83236.1 anig|EHA23223.1 aory|EIT79346.1 asyd|OJJ60600.1 ater|EAU35885.1 atub|OJI85440.1 aver|OJI99923.1 awen|OJJ36558.1 azon|OJJ44333.1

Group1006: agla|OJJ78486.1 agla|OJJ87512.1 abra|OJJ75907.1 acar|OOF99996.1 acla|EAW09882.1 afis|EAW21244.1 afla|EED54804.1 afum|EAL92257.1 akaw|GAA84613.1 aluc|OJZ89357.1 anid|CBF83253.1 anig|EHA23224.1 aory|EIT79338.1 asyd|OJJ60588.1 ater|EAU35884.1 atub|OJI85441.1 aver|OJI99911.1 awen|OJJ36555.1 azon|OJJ44334.1

Group1007: agla|OJJ78634.1 agla|OJJ84721.1 asyd|OJJ56854.1 asyd|OJJ56884.1 aver|OJJ03469.1 aver|OJJ07611.1 afla|EED52628.1 aory|EIT74179.1 afis|EAW23894.1 afum|EAL87322.1 acar|OOF92970.1 acla|EAW11559.1 akaw|GAA86969.1 aluc|OJZ84495.1 anid|CBF77167.1 ater|EAU33448.1 atub|OJI89585.1 awen|OJJ39055.1 azon|OJJ51386.1

Group1008: agla|OJJ80738.1 agla|OJJ86280.1 abra|OJJ77802.1 acar|OOF90102.1 agla|OJJ86304.1 akaw|GAA83847.1 aluc|OJZ90868.1 anig|EHA19588.1 aory|EIT75382.1 asyd|OJJ55038.1 atub|OJI82866.1 aver|OJJ07235.1 awen|OJJ36240.1 awen|OJJ37877.1 afla|EED45523.1 asyd|OJJ54957.1 agla|OJJ79081.1 akaw|GAA91814.1 anig|EHA23349.1

Group1009: agla|OJJ82043.1 agla|OJJ82886.1 abra|OJJ74586.1 acar|OOF95180.1 acla|EAW10792.1 afis|EAW24784.1 afla|EED47201.1 afum|EAL89423.1 akaw|GAA82019.1 aluc|OJZ92164.1 anid|CBF81451.1 anig|EHA26785.1 aory|EIT79216.1 asyd|OJJ59433.1 ater|EAU36525.1 atub|OJI86593.1 aver|OJJ02838.1 awen|OJJ40369.1 azon|OJJ49218.1

Group1010: agla|OJJ82106.1 agla|OJJ85287.1 abra|OJJ74712.1 acar|OOF95721.1 acla|EAW10686.1 afis|EAW24884.1 afla|EED47082.1 afum|EAL89534.1 akaw|GAA81905.1 aluc|OJZ92293.1 anid|CBF75745.1 anig|EHA26676.1 aory|EIT72490.1 asyd|OJJ57520.1 ater|EAU36417.1 atub|OJI86723.1 aver|OJJ05658.1 awen|OJJ40474.1 azon|OJJ49338.1

Group1011: agla|OJJ82204.1 agla|OJJ88613.1 abra|OJJ66850.1 acar|OOF91626.1 acla|EAW14428.1 afis|EAW19135.1 afla|EED54279.1 afum|EAL93867.1 akaw|GAA91556.1 aluc|OJZ85734.1 anid|CBF86332.1 anig|EHA24816.1 aory|EIT76149.1 asyd|OJJ64990.1 ater|EAU37406.1 atub|OJI79519.1 aver|OJI99110.1 awen|OJJ37471.1 azon|OJJ51151.1

Group1012: agla|OJJ87407.1 agla|OJJ87526.1 abra|OJJ76001.1 acar|OOF99924.1 acla|EAW09804.1 afis|EAW21332.1 afla|EED57290.1 afum|EAL92350.1 akaw|GAA88959.1 aluc|OJZ89446.1 anid|CBF83435.1 anig|EHA23140.1 aory|EIT74092.1 asyd|OJJ60664.1 ater|EAU35958.1 atub|OJI85347.1 aver|OJI99987.1 awen|OJJ39366.1 azon|OJJ51705.1

Group1013: akaw|GAA87623.1 akaw|GAA91524.1 abra|OJJ66888.1 acar|OOF91659.1 acla|EAW14460.1 afis|EAW19168.1 afla|EED54229.1 afum|EAL93898.1 agla|OJJ88556.1 aluc|OJZ85694.1 anid|CBF86275.1 anig|EHA24847.1 aory|EIT75898.1 asyd|OJJ65023.1 ater|EAU37305.1 atub|OJI79484.1 aver|OJI99142.1 awen|OJJ37521.1 azon|OJJ51187.1

Group1014: akaw|GAA90564.1 akaw|GAA91190.1 abra|OJJ66522.1 acar|OOF90976.1 acla|EAW10968.1 afis|EAW20892.1 afla|EED45587.1 afum|EAL84741.1 agla|OJJ84000.1 aluc|OJZ81513.1 anid|CBF82751.1 anig|EHA25911.1 aory|EIT75801.1 asyd|OJJ61613.1 ater|EAU30831.1 atub|OJI88662.1 aver|OJJ00936.1 awen|OJJ34455.1 azon|OJJ51742.1

Group1015: akaw|GAA91161.1 akaw|GAA92850.1 atub|OJI87463.1 atub|OJI88691.1 abra|OJJ66554.1 acar|OOF90999.1 acla|EAW10940.1 afla|EED50275.1 afum|EAL84713.1 agla|OJJ83968.1 aluc|OJZ81489.1 anid|CBF82446.1 ater|EAU38568.1 aver|OJJ08627.1 awen|OJJ34399.1 azon|OJJ48135.1 afis|EAW20921.1 aory|EIT77308.1 aluc|OJZ79652.1

Group1016: aluc|OJZ80175.1 aluc|OJZ91283.1 abra|OJJ78210.1 acla|EAW11240.1 afis|EAW18265.1 afum|EAL87630.1 agla|OJJ88161.1 akaw|GAA85816.1 anid|CBF79522.1 anig|EHA19968.1 aory|EIT73099.1 asyd|OJJ57891.1 ater|EAU33272.1 atub|OJI83273.1 aver|OJJ04503.1 awen|OJJ30639.1 azon|OJJ47977.1 afla|EED48730.1 afla|EED48731.1

Group1017: anid|CBF75896.1 anid|CBF87037.1 abra|OJJ68334.1 acar|OOF97603.1 acla|EAW09978.1 afis|EAW21111.1 afla|EED51763.1 afum|EAL92134.1 agla|OJJ80102.1 akaw|GAA92087.1 aluc|OJZ83637.1 anig|EHA18789.1 aory|EIT79989.1 asyd|OJJ60451.1 ater|EAU36787.1 atub|OJI90006.1 aver|OJI99769.1 awen|OJJ36440.1 azon|OJJ44433.1

Group1018: anid|CBF76123.1 anid|CBF81569.1 abra|OJJ71017.1 acar|OOF98123.1 acla|EAW10567.1 afis|EAW25033.1 afla|EED55458.1 afum|EAL89661.1 agla|OJJ82245.1 akaw|GAA82938.1 aluc|OJZ87515.1 anig|EHA20726.1 aory|EIT80849.1 asyd|OJJ59506.1 ater|EAU35752.1 atub|OJI81135.1 aver|OJJ02768.1 awen|OJJ40606.1 azon|OJJ49455.1

Group1019: anid|CBF84679.1 anid|CBF86911.1 asyd|OJJ53342.1 asyd|OJJ64609.1 awen|OJJ34698.1 awen|OJJ40005.1 abra|OJJ69889.1 acar|OOF93409.1 afla|EED48936.1 agla|OJJ87937.1 akaw|GAA85843.1 akaw|GAA87761.1 aluc|OJZ85395.1 anig|EHA25579.1 aory|EIT80037.1 ater|EAU30714.1 atub|OJI80176.1 aver|OJJ08403.1 aver|OJI98705.1

Group1020: anid|CBF85624.1 anid|CBF85626.1 abra|OJJ69051.1 acar|OOF91239.1 afis|EAW20343.1 afla|EED45065.1 afum|EAL85460.1 akaw|GAA90583.1 aluc|OJZ86041.1 anig|EHA24570.1 aory|EIT73625.1 asyd|OJJ57131.1 ater|EAU37680.1 atub|OJI79828.1 aver|OJJ03173.1 awen|OJJ38367.1 azon|OJJ45226.1 awen|OJJ38122.1 agla|OJJ87449.1

Group1021: aory|EIT72521.1 aory|EIT77023.1 abra|OJJ69072.1 acar|OOF91232.1 acla|EAW08768.1 afis|EAW15662.1 afla|EED46885.1 afum|EAL85686.1 agla|OJJ81784.1 akaw|GAA90603.1 aluc|OJZ86061.1 anid|CBF71182.1 anig|EHA24552.1 asyd|OJJ55861.1 atub|OJI79858.1 aver|OJJ04802.1 awen|OJJ38338.1 azon|OJJ43025.1 afla|EED51086.1

Group1022: aory|EIT72608.1 aory|EIT74906.1 asyd|OJJ54447.1 asyd|OJJ56044.1 aver|OJJ04989.1 aver|OJJ07712.1 abra|OJJ68724.1 acar|OOF92223.1 afla|EED46807.1 akaw|GAA89875.1 aluc|OJZ81804.1 anig|EHA22412.1 asyd|OJJ56930.1 atub|OJI84376.1 awen|OJJ34986.1 azon|OJJ48830.1 acla|EAW12522.1 afla|EED53315.1 ater|EAU31747.1

Group1023: aory|EIT76145.1 aory|EIT78526.1 abra|OJJ72823.1 acar|OOF96028.1 acla|EAW11415.1 afis|EAW24006.1 afla|EED49155.1 afum|EAL87436.1 agla|OJJ86937.1 akaw|GAA85424.1 aluc|OJZ83325.1 anid|CBF77396.1 anig|EHA24203.1 asyd|OJJ53244.1 ater|EAU29745.1 atub|OJI82222.1 aver|OJJ03606.1 awen|OJJ35478.1 azon|OJJ51270.1

Group1024: aory|EIT76394.1 aory|EIT77963.1 abra|OJJ78294.1 acar|OOF98573.1 acla|EAW09222.1 afis|EAW21926.1 afla|EED46618.1 afum|EAL92939.1 agla|OJJ84196.1 akaw|GAA85733.1 aluc|OJZ91368.1 anid|CBF76880.1 anig|EHA20046.1 asyd|OJJ61326.1 ater|EAU30932.1 atub|OJI83361.1 aver|OJJ00651.1 awen|OJJ34216.1 azon|OJJ43418.1

Group1025: aory|EIT81453.1 aory|EIT82110.1 abra|OJJ76662.1 acar|OOF99341.1 afis|EAW20834.1 afla|EED52049.1 afum|EAL84798.1 agla|OJJ80143.1 akaw|GAA93003.1 aluc|OJZ91540.1 anid|CBF88060.1 anig|EHA22577.1 asyd|OJJ54521.1 atub|OJI85994.1 aver|OJJ07664.1 awen|OJJ36032.1 afla|EED50429.1 aver|OJI97388.1 asyd|OJJ63546.1

Group1026: asyd|OJJ51877.1 asyd|OJJ54109.1 abra|OJJ66556.1 acar|OOF91001.1 acla|EAW10938.1 afis|EAW20923.1 afla|EED50277.1 afum|EAL84711.1 agla|OJJ83966.1 akaw|GAA91159.1 aluc|OJZ81487.1

anid|CBF82451.1 anig|EHA25940.1 aory|EIT77347.1 ater|EAU38570.1 atub|OJI88693.1 aver|OJJ08625.1 awen|OJJ34397.1 azon|OJJ48133.1

Group1027: asyd|OJJ51975.1 asyd|OJJ60994.1 abra|OJJ71988.1 acar|OOF96641.1 acla|EAW09550.1 afis|EAW21586.1 afla|EED57807.1 afum|EAL92604.1 agla|OJJ85779.1 akaw|GAA84941.1 aluc|OJZ86725.1 anid|CBF76287.1 anig|EHA21954.1 aory|EIT81038.1 ater|EAU34945.1 atub|OJI83803.1 aver|OJJ00314.1 awen|OJJ39095.1 azon|OJJ51428.1

Group1028: asyd|OJJ52256.1 asyd|OJJ61135.1 abra|OJJ76154.1 acar|OOF99776.1 acla|EAW09663.1 afis|EAW21474.1 afla|EED46116.1 afum|EAL92493.1 agla|OJJ85624.1 akaw|GAA82103.1 aluc|OJZ92074.1 anid|CBF76511.1 anig|EHA23001.1 aory|EIT76380.1 ater|EAU35059.1 atub|OJI86505.1 aver|OJJ00457.1 awen|OJJ39223.1 azon|OJJ51633.1

Group1029: asyd|OJJ52749.1 asyd|OJJ64102.1 abra|OJJ77967.1 acar|OOF98848.1 acla|EAW06673.1 afis|EAW16092.1 afla|EED48628.1 afum|EAL88724.1 agla|OJJ86129.1 akaw|GAA83695.1 aluc|OJZ91037.1 anid|CBF85462.1 anig|EHA19736.1 aory|EIT73247.1 ater|EAU33164.1 atub|OJI83033.1 aver|OJI98185.1 awen|OJJ30338.1 azon|OJJ45366.1

Group1030: asyd|OJJ53223.1 asyd|OJJ57663.1 aver|OJJ03629.1 aver|OJJ05525.1 abra|OJJ71299.1 acar|OOF97871.1 afis|EAW25353.1 afla|EED53834.1 afum|EAL90015.1 agla|OJJ83901.1 akaw|GAA82690.1 aluc|OJZ87244.1 anid|CBF77438.1 anig|EHA20489.1 aory|EIT78444.1 ater|EAU34501.1 atub|OJI81398.1 awen|OJJ41076.1 azon|OJJ44658.1

Group1031: asyd|OJJ53278.1 asyd|OJJ56762.1 abra|OJJ72786.1 acar|OOF96003.1 acla|EAW11483.1 afis|EAW23970.1 afla|EED50538.1 afum|EAL87400.1 agla|OJJ86899.1 akaw|GAA85393.1 aluc|OJZ83284.1 anid|CBF77331.1 anig|EHA24170.1 aory|EIT82142.1 ater|EAU29782.1 atub|OJI82187.1 aver|OJJ03570.1 awen|OJJ38973.1 azon|OJJ51308.1

Group1032: asyd|OJJ53282.1 asyd|OJJ56766.1 abra|OJJ72783.1 acar|OOF96001.1 acla|EAW11484.1 afis|EAW23969.1 afla|EED50540.1 afum|EAL87399.1 agla|OJJ86898.1 akaw|GAA85391.1 aluc|OJZ83282.1 anid|CBF77330.1 anig|EHA24169.1 aory|EIT82164.1 ater|EAU29783.1 atub|OJI82186.1 aver|OJJ03568.1 awen|OJJ38974.1 azon|OJJ51309.1

Group1033: asyd|OJJ53391.1 asyd|OJJ55131.1 abra|OJJ72369.1 acar|OOF94851.1 acla|EAW11966.1 afis|EAW19602.1 afla|EED56958.1 afum|EAL86916.1 agla|OJJ80744.1 akaw|GAA86119.1 aluc|OJZ90357.1 anid|CBF71283.1 anig|EHA23833.1 aory|EIT77537.1 ater|EAU32923.1 atub|OJI81785.1 aver|OJJ07072.1 awen|OJJ41838.1 azon|OJJ47049.1

Group1034: asyd|OJJ53445.1 asyd|OJJ58376.1 abra|OJJ68456.1 acar|OOF93941.1 afla|EED51144.1 akaw|GAA86918.1 aluc|OJZ90041.1 anid|CBF87350.1 anig|EHA21029.1 aory|EIT81354.1 asyd|OJJ61782.1 ater|EAU30292.1 atub|OJI84765.1 aver|OJI95882.1 awen|OJJ41192.1 azon|OJJ51544.1 asyd|OJJ60364.1 aver|OJI99700.1 aver|OJJ08306.1

Group1035: asyd|OJJ53802.1 asyd|OJJ61125.1 abra|OJJ76171.1 acar|OOF99768.1 acla|EAW09654.1 afis|EAW21485.1 afla|EED55918.1 afum|EAL92505.1 agla|OJJ85644.1 akaw|GAA82117.1 aluc|OJZ92059.1 anid|CBF76492.1 anig|EHA22988.1 aory|EIT76579.1 ater|EAU35053.1 atub|OJI86491.1 aver|OJJ00446.1 awen|OJJ39212.1 azon|OJJ51642.1

Group1036: asyd|OJJ55564.1 asyd|OJJ65204.1 aver|OJI99353.1 aver|OJJ06645.1 abra|OJJ77036.1 acar|OOF92935.1 acla|EAW13557.1 afis|EAW20633.1 afla|EED49759.1 afum|EAL84435.1 agla|OJJ86284.1 akaw|GAA87015.1 aluc|OJZ84439.1 anid|CBF83215.1 anig|EHA27758.1 aory|EIT77675.1 ater|EAU32168.1 atub|OJI89634.1 awen|OJJ33604.1

Group1037: asyd|OJJ55692.1 asyd|OJJ57944.1 aver|OJJ04452.1 aver|OJJ06777.1 abra|OJJ76856.1 acar|OOF91342.1 acla|EAW13417.1 afis|EAW20528.1 afla|EED52477.1 afum|EAL84529.1 agla|OJJ84707.1 akaw|GAA87166.1 aluc|OJZ84268.1 anid|CBF79013.1 anig|EHA27610.1 aory|EIT82744.1 ater|EAU37160.1 atub|OJI89815.1 awen|OJJ33235.1

Group1038: asyd|OJJ57870.1 asyd|OJJ61020.1 abra|OJJ75464.1 acar|OOF90276.1 acla|EAW13831.1 afis|EAW18300.1 afla|EED48769.1 afum|EAL87666.1 agla|OJJ88123.1 akaw|GAA84237.1 aluc|OJZ88920.1 anid|CBF79478.1 anig|EHA23592.1 aory|EIT73032.1 ater|EAU33308.1 atub|OJI85872.1 aver|OJJ04524.1 awen|OJJ30675.1 azon|OJJ43275.1

Group1039: ater|EAU29462.1 ater|EAU34080.1 ater|EAU38788.1 abra|OJJ74396.1 acar|OOF95340.1 acla|EAW06820.1 afis|EAW16368.1 afla|EED53468.1 afum|EAL88993.1 agla|OJJ78589.1 akaw|GAA87294.1 aluc|OJZ89724.1 anid|CBF89352.1 anig|EHA26952.1 aory|EIT74981.1 asyd|OJJ62501.1 atub|OJI85076.1 aver|OJI96622.1 awen|OJJ37773.1

Group1040: ater|EAU30138.1 ater|EAU38860.1 abra|OJJ73936.1 acar|OOG01067.1 acla|EAW07813.1 afis|EAW22849.1 afla|EED50124.1 afum|EAL90351.1 agla|OJJ83475.1 akaw|GAA89542.1 aluc|OJZ88108.1 anid|CBF87853.1 anig|EHA17877.1 aory|EIT74737.1 asyd|OJJ63417.1 atub|OJI90715.1 aver|OJI97521.1 awen|OJJ38872.1 azon|OJJ49662.1

Group1041: ater|EAU30572.1 ater|EAU36084.1 abra|OJJ68744.1 acar|OOF90309.1 acla|EAW14290.1 afis|EAW18993.1 afla|EED46791.1 afum|EAL93728.1 agla|OJJ82981.1 akaw|GAA89891.1 aluc|OJZ81782.1 anid|CBF77932.1 anig|EHA22433.1 aory|EIT72766.1 asyd|OJJ52417.1 atub|OJI84356.1 aver|OJJ03362.1 awen|OJJ31615.1 azon|OJJ47315.1

Group1042: ater|EAU30835.1 ater|EAU31368.1 abra|OJJ68417.1 acar|OOF91799.1 acla|EAW08827.1 afis|EAW15570.1 afla|EED51627.1 afum|EAL85763.1 agla|OJJ85441.1 akaw|GAA92531.1 aluc|OJZ90080.1 anig|EHA20993.1 aory|EIT80506.1 asyd|OJJ57633.1 atub|OJI84726.1 aver|OJJ04438.1 awen|OJJ38058.1 awen|OJJ38229.1 aver|OJI95759.1

Group1043: atub|OJI80331.1 atub|OJI90561.1 abra|OJJ74085.1 acar|OOG01195.1 acla|EAW07929.1 afis|EAW22967.1 afla|EED54404.1 afum|EAL90230.1 agla|OJJ83320.1 akaw|GAA87565.1 aluc|OJZ87950.1 anid|CBF87610.1 anig|EHA17736.1 aory|EIT75873.1 asyd|OJJ63271.1 ater|EAU30655.1 aver|OJI97686.1 awen|OJJ38596.1 azon|OJJ47331.1

Group1044: aver|OJI96628.1 aver|OJJ08269.1 abra|OJJ73489.1 acar|OOG00683.1 acla|EAW06813.1 afis|EAW16362.1 afla|EED53506.1 afum|EAL88987.1 agla|OJJ79111.1 akaw|GAA87438.1 aluc|OJZ88559.1 anid|CBF89241.1 anig|EHA18256.1 aory|EIT75032.1 asyd|OJJ62508.1 ater|EAU34090.1 atub|OJI91144.1 awen|OJJ37766.1 asyd|OJJ55388.1

Group1045: aver|OJI96684.1 aver|OJJ05918.1 abra|OJJ73439.1 acar|OOG00645.1 acla|EAW06773.1 afis|EAW16324.1 afla|EED53558.1 afum|EAL88947.1 agla|OJJ88286.1 akaw|GAA83054.1 aluc|OJZ88601.1 anid|CBF89160.1 anig|EHA18295.1 aory|EIT74902.1 asyd|OJJ62559.1 ater|EAU34133.1 atub|OJI91186.1 awen|OJJ37724.1 azon|OJJ44983.1

Group1046: aver|OJI98887.1 aver|OJJ06757.1 abra|OJJ77001.1 acar|OOF91446.1 acla|EAW13450.1 afis|EAW20561.1 afla|EED52343.1 afum|EAL84500.1 agla|OJJ88729.1 akaw|GAA87047.1 aluc|OJZ84400.1 anid|CBF78975.1 anig|EHA27730.1 aory|EIT74333.1 asyd|OJJ55674.1 ater|EAU37119.1 atub|OJI89672.1 awen|OJJ33268.1 azon|OJJ50642.1

Group1047: aver|OJI98940.1 aver|OJI99461.1 abra|OJJ69903.1 acar|OOF93421.1 acla|EAW08920.1 afis|EAW15474.1 afla|EED46925.1 afum|EAL85847.1 agla|OJJ89052.1 akaw|GAA87749.1 aluc|OJZ85382.1 anid|CBF86601.1 anig|EHA25565.1 aory|EIT72600.1 asyd|OJJ64827.1 ater|EAU32654.1 atub|OJI80188.1 awen|OJJ36395.1 azon|OJJ44109.1

Group1048: aver|OJJ00758.1 aver|OJJ05227.1 abra|OJJ75869.1 acar|OOG00025.1 acla|EAW09931.1 afis|EAW21178.1 afla|EED54765.1 afum|EAL92198.1 agla|OJJ79995.1 akaw|GAA84575.1 aluc|OJZ89312.1 anid|CBF82104.1 anig|EHA23255.1 aory|EIT79387.1 asyd|OJJ54271.1 ater|EAU35844.1 atub|OJI85487.1 awen|OJJ36517.1 afla|EED54766.1

Group1049: aver|OJJ01895.1 aver|OJJ04283.1 abra|OJJ68598.1 acar|OOF92108.1 acla|EAW12641.1 afis|EAW18044.1 afla|EED51502.1 afum|EAL91855.2 agla|OJJ85415.1 akaw|GAA91922.1 aluc|OJZ81958.1 anid|CBF79781.1 anig|EHA22289.1 aory|EIT80736.1 asyd|OJJ58122.1 ater|EAU31279.1 atub|OJI84509.1 awen|OJJ41490.1 azon|OJJ48220.1

Group1050: aver|OJJ05206.1 aver|OJJ06610.1 aluc|OJZ88621.1 anig|EHA18312.1 atub|OJI91206.1 abra|OJJ73422.1 acar|OOG00625.1 acla|EAW07037.1 afis|EAW16663.1 afla|EED44906.1 afum|EAL89267.1 agla|OJJ78706.1 akaw|GAA83070.1 anid|CBF82078.1 aory|EIT73489.1 asyd|OJJ54252.1 ater|EAU39585.1 awen|OJJ40050.1 azon|OJJ50117.1

Group1051: aver|OJJ07706.1 aver|OJJ08297.1 abra|OJJ76161.1 acar|OOF95039.1 afis|EAW19929.1 afum|EAL84979.1 agla|OJJ80644.1 akaw|GAA82108.1 aluc|OJZ92068.1 anid|CBF84561.1 anig|EHA22995.1 aory|EIT79289.1 asyd|OJJ53459.1 ater|EAU31756.1 atub|OJI86499.1 awen|OJJ33359.1 awen|OJJ33575.1 acla|EAW13677.1 afla|EED47286.1

Group1052: awen|OJJ29714.1 awen|OJJ31500.1 abra|OJJ72682.1 acar|OOF95931.1 acla|EAW12316.1 afis|EAW16904.1 afla|EED45262.1 afum|EAL86440.1 agla|OJJ84538.1 akaw|GAA85300.1 aluc|OJZ90660.1 anid|CBF80774.1 anig|EHA24095.1 aory|EIT73651.1 asyd|OJJ54502.1 ater|EAU30211.1 atub|OJI82089.1 aver|OJJ07679.1 azon|OJJ46214.1

Group1053: awen|OJJ30059.1 awen|OJJ32960.1 abra|OJJ69424.1 acar|OOF94312.1 acla|EAW14803.1 afis|EAW24334.1 afla|EED47930.1 afum|EAL86004.1 agla|OJJ83203.1 akaw|GAA88290.1 aluc|OJZ82260.1 anig|EHA28482.1 aory|EIT79819.1 ater|EAU30414.1 atub|OJI88178.1 anid|CBF74037.1 aver|OJJ07954.1 azon|OJJ48958.1 asyd|OJJ53852.1

Group1054: awen|OJJ30747.1 awen|OJJ31734.1 abra|OJJ67459.1 acar|OOF92371.1 acla|EAW06307.1 afis|EAW18416.1 afla|EED52993.1 afum|EAL93098.1 agla|OJJ84982.1 akaw|GAA86374.1 aluc|OJZ84905.1 anid|CBF70716.1 anig|EHA25129.1 aory|EIT77436.1 asyd|OJJ58446.1 ater|EAU29831.1 atub|OJI80693.1 aver|OJJ01046.1 azon|OJJ50492.1

Group1055: awen|OJJ30760.1 awen|OJJ31721.1 abra|OJJ67449.1 acar|OOF90341.1 acla|EAW06315.1 afis|EAW18429.1 afla|EED52984.1 afum|EAL93109.1 agla|OJJ84994.1 akaw|GAA86381.1 aluc|OJZ84915.1

anid|CBF70683.1 anig|EHA25136.1 aory|EIT78705.1 asyd|OJJ58466.1 ater|EAU29845.1 atub|OJI80683.1 aver|OJJ01065.1 azon|OJJ50482.1

Group1056: awen|OJJ30764.1 awen|OJJ31717.1 abra|OJJ67446.1 acar|OOF90343.1 acla|EAW06317.1 afis|EAW18431.1 afla|EED52982.1 afum|EAL93111.2 agla|OJJ84997.1 akaw|GAA86383.1 aluc|OJZ84917.1 anid|CBF70025.1 anig|EHA25137.1 aory|EIT78725.1 asyd|OJJ58872.1 ater|EAU29847.1 atub|OJI80681.1 aver|OJJ01475.1 azon|OJJ50481.1

Group1057: awen|OJJ30768.1 awen|OJJ31713.1 abra|OJJ67442.1 acar|OOF92386.1 acla|EAW06321.1 afis|EAW18435.1 afla|EED52980.1 afum|EAL93116.1 agla|OJJ85002.1 akaw|GAA86387.1 aluc|OJZ84922.1 anid|CBF70033.1 anig|EHA25140.1 aory|EIT78731.1 asyd|OJJ58868.1 ater|EAU29851.1 atub|OJI80677.1 aver|OJJ01470.1 azon|OJJ50478.1

Group1058: awen|OJJ30787.1 awen|OJJ31694.1 abra|OJJ75327.1 acar|OOF90141.1 acla|EAW15368.1 afla|EED45606.1 akaw|GAA92022.1 aluc|OJZ92890.1 anig|EHA26073.1 aory|EIT75814.1 ater|EAU36093.1 atub|OJI87348.1 aver|OJI99803.1 akaw|GAA86921.1 aluc|OJZ90045.1 anig|EHA21025.1 ater|EAU38089.1 atub|OJI84761.1 asyd|OJJ52000.1

Group1059: awen|OJJ31367.1 awen|OJJ39791.1 awen|OJJ33381.1 awen|OJJ35034.1 azon|OJJ43925.1 azon|OJJ44485.1 abra|OJJ70024.1 acar|OOF93532.1 afla|EED45807.1 agla|OJJ82719.1 akaw|GAA87637.1 aluc|OJZ85252.1 anig|EHA25453.1 atub|OJI80316.1 aver|OJJ07005.1 acar|OOF93530.1 agla|OJJ78997.1 aver|OJJ04358.1 asyd|OJJ58045.1

Group1060: awen|OJJ32955.1 awen|OJJ34548.1 abra|OJJ69452.1 acar|OOF94289.1 acla|EAW14816.1 afis|EAW24346.1 afla|EED47917.1 afum|EAL86016.1 agla|OJJ83207.1 akaw|GAA88367.1 aluc|OJZ82288.1 anid|CBF74019.1 anig|EHA28460.1 aory|EIT83370.1 asyd|OJJ53841.1 ater|EAU30400.1 atub|OJI88151.1 aver|OJJ07944.1 azon|OJJ48966.1

Group1061: awen|OJJ35632.1 awen|OJJ41410.1 abra|OJJ68673.1 acar|OOF92174.1 acla|EAW12575.1 afis|EAW24073.1 afla|EED51400.1 afum|EAL91929.1 agla|OJJ87142.1 akaw|GAA89826.1 aluc|OJZ81862.1 anid|CBF86467.1 anig|EHA22364.1 aory|EIT80717.1 asyd|OJJ64908.1 ater|EAU30495.1 atub|OJI84429.1 aver|OJI99030.1 azon|OJJ48882.1

Group1062: awen|OJJ35956.1 awen|OJJ39245.1 abra|OJJ76134.1 acar|OOF99789.1 acla|EAW09684.1 afis|EAW21455.1 afla|EED55885.1 afum|EAL92474.1 agla|OJJ85603.1 akaw|GAA88833.1 aluc|OJZ89596.1 anid|CBF76546.1 anig|EHA23016.1 aory|EIT76856.1 asyd|OJJ61154.1 ater|EAU35080.1 atub|OJI85207.1 aver|OJJ00476.1 azon|OJJ51610.1

Group1063: awen|OJJ35989.1 awen|OJJ38023.1 abra|OJJ75270.1 acar|OOF94533.1 acla|EAW12238.1 afis|EAW16989.1 afla|EED48968.1 afum|EAL86513.1 agla|OJJ79827.1 akaw|GAA91808.1 aluc|OJZ92830.1 anid|CBF87435.1 anig|EHA26179.1 aory|EIT79996.1 asyd|OJJ52914.1 ater|EAU31738.1 atub|OJI87287.1 aver|OJJ08884.1 azon|OJJ50274.1

Group1064: awen|OJJ36336.1 awen|OJJ40727.1 abra|OJJ70865.1 acar|OOF98251.1 acla|EAW10457.1 afis|EAW25110.1 afla|EED55332.1 afum|EAL89781.1 agla|OJJ89071.1 akaw|GAA91732.1 aluc|OJZ87654.1 anid|CBF86071.1 anig|EHA20848.1 aory|EIT76213.1 asyd|OJJ64460.1 ater|EAU39380.1 atub|OJI80988.1 aver|OJI98563.1 azon|OJJ44099.1

Group1065: awen|OJJ38099.1 awen|OJJ40555.1 abra|OJJ74809.1 acar|OOF95801.1 acla|EAW10605.1 afis|EAW24962.1 afla|EED55500.1 afum|EAL89620.1 agla|OJJ82197.1 akaw|GAA83603.1 aluc|OJZ92391.1 anid|CBF81656.1 anig|EHA26584.1 aory|EIT80890.1 asyd|OJJ59547.1 ater|EAU35693.1 atub|OJI86821.1 aver|OJJ02721.1 azon|OJJ49417.1

Group1066: awen|OJJ40158.1 awen|OJJ41199.1 abra|OJJ71411.1 acar|OOF97761.1 acla|EAW10126.1 afis|EAW25462.1 afla|EED53951.1 afum|EAL90125.1 agla|OJJ87114.1 akaw|GAA82589.1 aluc|OJZ87129.1 anid|CBF77649.1 anig|EHA20391.1 aory|EIT78320.1 asyd|OJJ53118.1 ater|EAU34631.1 atub|OJI81511.1 aver|OJJ03736.1 azon|OJJ44770.1

Group1067: azon|OJJ42233.1 azon|OJJ45236.1 acla|EAW12053.1 afis|EAW19693.1 afla|EED48466.1 afum|EAL86833.1 aory|EIT76269.1 asyd|OJJ62963.1 aver|OJI98004.1 acla|EAW15332.1 afis|EAW20372.1 afla|EED45213.1 afum|EAL85486.1 anid|CBF75994.1 aory|EIT73663.1 asyd|OJJ53695.1 ater|EAU37785.1 aver|OJJ07811.1 awen|OJJ38807.1

Group1068: azon|OJJ43179.1 azon|OJJ47539.1 abra|OJJ70678.1 acar|OOF97049.1 acla|EAW08086.1 afis|EAW23122.1 afla|EED48531.1 afum|EAL88358.1 agla|OJJ89617.1 akaw|GAA90010.1 aluc|OJZ84026.1 anid|CBF74402.1 anig|EHA19147.1 aory|EIT73068.1 asyd|OJJ57764.1 ater|EAU36250.1 atub|OJI90398.1 aver|OJJ05402.1 awen|OJJ36659.1

Group1069: azon|OJJ44484.1 azon|OJJ48556.1 abra|OJJ75695.1 acar|OOG00157.1 acla|EAW09413.1 afis|EAW21723.1 afla|EED57992.1 afum|EAL92756.1 agla|OJJ79861.1 akaw|GAA84431.1 aluc|OJZ89146.1 anid|CBF83716.1 anig|EHA23402.1 aory|EIT79772.1 asyd|OJJ60837.1 ater|EAU38407.1 atub|OJI85642.1 aver|OJJ00161.1 awen|OJJ33996.1

Group1070: azon|OJJ45306.1 azon|OJJ46721.1 abra|OJJ76474.1 acar|OOF99711.1 acla|EAW13959.1 afis|EAW18668.1 afla|EED52660.1 afum|EAL93386.1 agla|OJJ82407.1 akaw|GAA82386.1 aluc|OJZ91735.1 anid|CBF70575.1 anig|EHA22741.1 aory|EIT74271.1 asyd|OJJ58530.1 ater|EAU38243.1 atub|OJI86193.1 aver|OJJ01132.1 awen|OJJ32140.1

Group1071: azon|OJJ45770.1 azon|OJJ47370.1 abra|OJJ72219.1 agla|OJJ78976.1 anig|EHA21679.1 awen|OJJ32400.1 abra|OJJ74055.1 acar|OOG01168.1 acla|EAW13644.1 afla|EED58134.1 afum|EAL84922.1 akaw|GAA87537.1 aluc|OJZ87980.1 anig|EHA17763.1 aory|EIT79161.1 asyd|OJJ57210.1 ater|EAU30685.1 atub|OJI90590.1 aver|OJJ03079.1

Group1072: azon|OJJ45841.1 azon|OJJ45942.1 abra|OJJ69362.1 acar|OOF94363.1 acla|EAW14752.1 afis|EAW24283.1 afla|EED47993.1 afum|EAL85952.1 agla|OJJ83145.1 akaw|GAA88231.1 aluc|OJZ82195.1 anid|CBF74136.1 anig|EHA28542.1 aory|EIT79842.1 asyd|OJJ53916.1 ater|EAU31231.1 atub|OJI88240.1 aver|OJJ08014.1 awen|OJJ33013.1

Group1073: azon|OJJ50179.1 azon|OJJ50395.1 abra|OJJ68426.1 acar|OOF93927.1 acla|EAW11928.1 afis|EAW19562.1 afla|EED50583.1 afum|EAL86950.1 agla|OJJ81713.1 akaw|GAA86946.1 aluc|OJZ90071.1 anid|CBF89380.1 anig|EHA21002.1 aory|EIT82053.1 asyd|OJJ62464.1 ater|EAU31050.1 atub|OJI84735.1 aver|OJI96599.1 awen|OJJ35963.1

Group1074: abra|OJJ65489.1 acar|OOF90657.1 afis|EAW25622.1 afla|EED47479.1 afum|EAL85425.1 agla|OJJ86171.1 akaw|GAA88141.1 aluc|OJZ85402.1 anig|EHA18427.1 aory|EIT79955.1 asyd|OJJ55499.1 ater|EAU36065.1 atub|OJI91619.1 aver|OJJ06577.1 afla|EED49747.1 afum|EAL84310.1 akaw|GAA87767.1 aory|EIT77702.1 aluc|OJZ79947.1

Group1075: abra|OJJ65876.1 aory|EIT76450.1 abra|OJJ68863.1 acar|OOF90526.1 afis|EAW20215.1 akaw|GAA90390.1 aluc|OJZ85841.1 anig|EHA24723.1 atub|OJI79629.1 awen|OJJ31221.1 abra|OJJ77712.1 acar|OOF99028.1 agla|OJJ85976.1 aluc|OJZ90777.1 asyd|OJJ59565.1 atub|OJI82764.1 awen|OJJ41677.1 aver|OJJ02704.1 azon|OJJ46135.1

Group1076: afla|EED47310.1 aory|EIT82826.1 abra|OJJ77715.1 acar|OOF99024.1 acla|EAW06603.1 afis|EAW16220.1 afum|EAL88848.1 agla|OJJ86232.1 akaw|GAA83917.1 aluc|OJZ90780.1 anig|EHA19513.1 asyd|OJJ59566.1 atub|OJI82767.1 aver|OJJ02703.1 awen|OJJ29818.1 azon|OJJ42618.1 acar|OOF98224.1 ater|EAU33263.1 awen|OJJ30252.1

Group1077: abra|OJJ65944.1 acar|OOF92929.1 acla|EAW13567.1 afis|EAW20033.1 afla|EED46044.1 afum|EAL84883.1 agla|OJJ88021.1 akaw|GAA90822.1 aluc|OJZ83013.1 anid|CBF84765.1 anig|EHA21428.1 aory|EIT75479.1 asyd|OJJ53284.1 ater|EAU29399.1 atub|OJI89248.1 aver|OJJ08457.1 awen|OJJ30056.1 azon|OJJ46327.1 afla|EED46043.1

Group1078: abra|OJJ65999.1 acar|OOF92101.1 afla|EED56561.1 agla|OJJ88417.1 akaw|GAA90871.1 aluc|OJZ82962.1 anig|EHA21378.1 asyd|OJJ57315.1 atub|OJI89194.1 aver|OJJ05846.1 awen|OJJ33380.1 azon|OJJ44468.1 anid|CBF79162.1 akaw|GAA92926.1 aluc|OJZ82517.1 anig|EHA20902.1 ater|EAU38011.1 asyd|OJJ62803.1 aver|OJI96849.1

Group1079: abra|OJJ66158.1 acar|OOG00538.1 acla|EAW08523.1 afis|EAW23562.1 afum|EAL87906.1 agla|OJJ80848.1 asyd|OJJ55543.1 aver|OJJ06621.1 abra|OJJ67247.1 acar|OOF89887.1 afla|EED52167.1 akaw|GAA91667.1 aluc|OJZ87730.1 anig|EHA20222.1 aory|EIT74456.1 ater|EAU35672.1 atub|OJI80910.1 afla|EED47300.1 aory|EIT82893.1

Group1080: abra|OJJ66244.1 acla|EAW14177.1 afis|EAW16160.1 afla|EED50871.1 afum|EBA27496.1 agla|OJJ81498.1 akaw|GAA89047.1 aluc|OJZ80549.1 anid|CBF69906.1 anig|EHA25647.1 aory|EIT79021.1 asyd|OJJ58996.1 ater|EAU33887.1 atub|OJI88382.1 aver|OJJ01620.1 azon|OJJ47209.1 acar|OOF99450.1 afis|EAW18892.1 afla|EED48908.1

Group1081: abra|OJJ66266.1 acar|OOF97511.1 akaw|GAA89028.1 aluc|OJZ80576.1 anig|EHA25621.1 atub|OJI88355.1 abra|OJJ69860.1 afla|EED45622.1 agla|OJJ84525.1 akaw|GAA87796.1 aluc|OJZ85431.1 anid|CBF86345.1 anig|EHA25610.1 aory|EIT75614.1 asyd|OJJ64976.1 ater|EAU32692.1 atub|OJI80144.1 aver|OJI99098.1 awen|OJJ32475.1

Group1082: abra|OJJ66514.1 acar|OOF90929.1 acla|EAW15358.1 afis|EAW20353.1 afla|EED45813.1 afum|EAL85471.1 agla|OJJ82840.1 akaw|GAA91197.1 aluc|OJZ81532.1 anid|CBF78206.1 anig|EHA25904.1 aory|EIT75838.1 atub|OJI88655.1 aver|OJJ04934.1 awen|OJJ30867.1 azon|OJJ42291.1 afla|EED50237.1 aluc|OJZ81531.1 anig|EHA25905.1

Group1083: abra|OJJ66594.1 acar|OOF91029.1 acla|EAW10897.1 afis|EAW20961.1 afla|EED50316.1 afum|EAL84672.1 agla|OJJ83917.1 akaw|GAA91123.1 aluc|OJZ81453.1 anid|CBF82569.1 anig|EHA25975.1 aory|EIT77366.1 asyd|OJJ54049.1 ater|EAU38611.1 atub|OJI88728.1 aver|OJJ08694.1 awen|OJJ34356.1 azon|OJJ48092.1 aory|EIT77330.1

Group1084: abra|OJJ66648.1 acar|OOF93752.1 acla|EAW11791.1 afis|EAW19402.1 afla|EED50356.1 afum|EAL87093.1 agla|OJJ81115.1 akaw|GAA89601.1 aluc|OJZ80801.1 anid|CBF74340.1 anig|EHA27233.1

aory|EIT83133.1 asyd|OJJ51904.1 ater|EAU36127.1 atub|OJI87649.1 aver|OJJ05364.1 awen|OJJ36797.1 azon|OJJ43002.1 acar|OOF93668.1

Group1085: abra|OJJ66851.1 acar|OOF91627.1 acla|EAW14429.1 afis|EAW19136.1 afla|EED54277.1 afum|EAL93868.1 agla|OJJ88615.1 akaw|GAA91555.1 aluc|OJZ85733.1 anid|CBF86327.1 anig|EHA24817.1 aory|EIT75982.1 asyd|OJJ64992.1 ater|EAU37407.1 atub|OJI79518.1 aver|OJI99111.1 awen|OJJ37473.1 azon|OJJ51152.1 anid|CBF86329.1

Group1086: abra|OJJ66857.1 acar|OOF91634.1 acla|EAW14436.1 afis|EAW19144.1 afla|EED54269.1 afum|EAL93875.1 agla|OJJ88587.1 akaw|GAA91548.1 aluc|OJZ85726.1 anid|CBF86314.1 anig|EHA24822.1 aory|EIT75975.1 asyd|OJJ64999.1 ater|EAU37416.1 atub|OJI79512.1 aver|OJI99119.1 awen|OJJ37483.1 azon|OJJ51160.1 aory|EIT76087.1

Group1087: abra|OJJ66865.1 acar|OOF91641.1 acla|EAW14441.1 afis|EAW19149.1 afla|EED54264.1 afum|EAL93880.1 agla|OJJ88575.1 akaw|GAA91543.1 aluc|OJZ85717.1 anid|CBF86306.1 anig|EHA24827.1 aory|EIT76024.1 asyd|OJJ65005.1 ater|EAU37422.1 atub|OJI79507.1 aver|OJI99124.1 awen|OJJ37502.1 azon|OJJ51167.1 afla|EED54263.1

Group1088: abra|OJJ67116.1 acar|OOF98436.1 acla|EAW13746.1 afis|EAW19825.1 afla|EED56208.1 afum|EAL85067.1 agla|OJJ83144.1 akaw|GAA91049.1 aluc|OJZ87859.1 anid|CBF83120.1 anig|EHA20165.1 aory|EIT73304.1 asyd|OJJ61473.1 ater|EAU31280.1 atub|OJI80788.1 aver|OJJ00814.1 awen|OJJ36528.1 azon|OJJ47562.1 agla|OJJ79980.1

Group1089: abra|OJJ67209.1 acar|OOF98351.1 afis|EAW19743.1 afum|EAL86783.1 akaw|GAA90968.1 aluc|OJZ87765.1 anig|EHA20249.1 ater|EAU29472.1 atub|OJI80880.1 abra|OJJ75764.1 afla|EED56526.1 aory|EIT82926.1 asyd|OJJ58672.1 atub|OJI87194.1 aver|OJJ01283.1 awen|OJJ30928.1 azon|OJJ42672.1 awen|OJJ34642.1 aver|OJJ07429.1

Group1090: abra|OJJ67296.1 acar|OOF92515.1 afla|EED45654.1 agla|OJJ79150.1 akaw|GAA86517.1 aluc|OJZ85063.1 anid|CBF81698.1 anig|EHA25277.1 aory|EIT75443.1 asyd|OJJ59570.1 atub|OJI80535.1 aver|OJJ02697.1 acar|OOF90954.1 afla|EED50408.1 aory|EIT82054.1 asyd|OJJ59578.1 ater|EAU32270.1 aver|OJJ02691.1 awen|OJJ36407.1

Group1091: abra|OJJ67369.1 acla|EAW10405.1 afis|EAW25166.1 afla|EED44928.1 afum|EAL89839.1 anid|CBF85620.1 aory|EIT77682.1 asyd|OJJ64202.1 ater|EAU31038.1 aver|OJI98291.1 abra|OJJ68396.1 akaw|GAA92552.1 aluc|OJZ90100.1 anig|EHA20975.1 aory|EIT83236.1 atub|OJI84706.1 awen|OJJ34638.1 aory|EIT73481.1 afla|EED49793.1

Group1092: abra|OJJ67422.1 acar|OOF91907.1 acla|EAW06342.1 afis|EAW18454.1 afla|EED52954.1 afum|EAL93139.1 agla|OJJ84146.1 akaw|GAA86407.1 anig|EHA25162.1 aory|EIT78718.1 asyd|OJJ58854.1 ater|EAU29871.1 atub|OJI80655.1 aver|OJJ01455.1 azon|OJJ50520.1 anid|CBF71560.1 asyd|OJJ54214.1 aver|OJJ08517.1 agla|OJJ85037.1

Group1093: abra|OJJ67523.1 awen|OJJ30978.1 abra|OJJ69776.1 acar|OOF93595.1 acla|EAW11058.1 afis|EAW20815.1 afla|EED53102.1 afum|EBA27207.1 agla|OJJ81282.1 akaw|GAA87877.1 aluc|OJZ85518.1 anid|CBF86687.1 anig|EHA25051.1 aory|EIT74763.1 asyd|OJJ64752.1 ater|EAU38934.1 atub|OJI80048.1 aver|OJI98853.1 azon|OJJ45137.1

Group1094: abra|OJJ67596.1 acla|EAW12346.1 afis|EAW20358.1 afla|EED57868.1 afum|EAL90941.1 agla|OJJ88440.1 akaw|GAA89115.1 aluc|OJZ81724.1 anid|CBF77124.1 anig|EHA25715.1 aory|EIT81103.1 asyd|OJJ62720.1 ater|EAU34084.1 atub|OJI88457.1 aver|OJI99735.1 awen|OJJ36944.1 afum|EAL85475.1 afum|EAL91116.1 asyd|OJJ60405.1

Group1095: abra|OJJ67622.1 acar|OOF91970.1 acla|EAW12407.1 afis|EAW16866.1 afla|EED45897.1 afum|EAL86405.2 akaw|GAA89142.1 aluc|OJZ81694.1 anig|EHA25737.1 aory|EIT75474.1 asyd|OJJ56695.1 atub|OJI88486.1 aver|OJJ06029.1 awen|OJJ33543.1 azon|OJJ42803.1 acar|OOF91765.1 awen|OJJ34441.1 azon|OJJ50737.1 afla|EED52164.1

Group1096: abra|OJJ67694.1 acar|OOF90173.1 acla|EAW12072.1 afis|EAW19713.1 afla|EED56920.1 afum|EAL86814.1 akaw|GAA89203.1 aluc|OJZ81627.1 anid|CBF82745.1 anig|EHA25816.1 aory|EIT72578.1 asyd|OJJ61617.1 atub|OJI88553.1 aver|OJJ00940.1 awen|OJJ31364.1 azon|OJJ47340.1 acar|OOF91834.1 afla|EED46824.1 aory|EIT77570.1

Group1097: abra|OJJ67728.1 acar|OOF89960.1 afis|EAW20574.1 afla|EED52326.1 afum|EAL84491.1 agla|OJJ89291.1 akaw|GAA89222.1 aluc|OJZ81606.1 anid|CBF87431.1 anig|EHA25839.1 aory|EIT74400.1 asyd|OJJ52907.1 atub|OJI88579.1 aver|OJJ08876.1 awen|OJJ30548.1 awen|OJJ29983.1 akaw|GAA85320.1 aluc|OJZ90681.1 anig|EHA27494.1

Group1098: abra|OJJ67796.1 agla|OJJ84477.1 abra|OJJ75071.1 acar|OOF97263.1 acla|EAW08252.1 afis|EAW23286.1 afla|EED55688.1 afum|EAL88193.1 akaw|GAA88683.1 aluc|OJZ92637.1 anid|CBF89516.1 anig|EHA26354.1 aory|EIT77084.1 asyd|OJJ62394.1 ater|EAU37586.1 atub|OJI87068.1 aver|OJI96527.1 awen|OJJ39670.1 azon|OJJ47800.1

Group1099: aory|EIT77379.1 abra|OJJ71274.1 acar|OOF97900.1 acla|EAW10254.1 afis|EAW25337.1 afla|EED53807.1 afum|EAL90000.1 agla|OJJ83868.1 akaw|GAA82716.1 aluc|OJZ87270.1 anid|CBF75246.1 anig|EHA20511.1 asyd|OJJ59907.1 ater|EAU34477.1 atub|OJI81372.1 aver|OJJ02378.1 awen|OJJ41034.1 azon|OJJ44631.1 aory|EIT78377.1

Group1100: abra|OJJ67904.1 acar|OOF92874.1 acla|EAW11719.1 afis|EAW23740.1 afla|EED51530.1 afum|EAL87177.1 agla|OJJ78966.1 akaw|GAA91909.1 aluc|OJZ81983.1 anid|CBF73900.1 anig|EHA22271.1 aory|EIT80572.1 asyd|OJJ65360.1 atub|OJI84535.1 aver|OJI99509.1 awen|OJJ33676.1 azon|OJJ44443.1 afla|EED57578.1 aory|EIT82975.1

Group1101: ater|EAU31288.1 awen|OJJ33664.1 abra|OJJ75236.1 acar|OOF94556.1 acla|EAW11702.1 afis|EAW23723.1 afla|EED53191.1 afum|EAL87163.1 akaw|GAA91779.1 aluc|OJZ92797.1 anid|CBF86826.1 anig|EHA26211.1 aory|EIT74820.1 asyd|OJJ56249.1 atub|OJI87241.1 aver|OJJ08511.1 azon|OJJ44225.1 afla|EED51505.1 aory|EIT80632.1

Group1102: abra|OJJ67986.1 acar|OOF92028.1 acla|EAW13238.1 afis|EAW21052.1 afla|EED48858.1 akaw|GAA91216.1 aluc|OJZ82854.1 anid|CBF73882.1 anig|EHA21290.1 aory|EIT79551.1 asyd|OJJ57026.1 ater|EAU38426.1 atub|OJI89084.1 aver|OJJ06477.1 afum|EAL92088.2 asyd|OJJ55377.1 aory|EIT81277.1 anid|CBF77000.1 aver|OJJ03264.1

Group1103: abra|OJJ68002.1 akaw|GAA90948.1 aluc|OJZ82876.1 anig|EHA21302.1 atub|OJI89100.1 abra|OJJ77430.1 afis|EAW24679.1 afum|EAL89347.1 anid|CBF90095.1 asyd|OJJ62032.1 awen|OJJ40790.1 azon|OJJ48192.1 afla|EED49910.1 aory|EIT72471.1 asyd|OJJ57385.1 ater|EAU31630.1 anid|CBF88170.1 aver|OJI97314.1 awen|OJJ32480.1

Group1104: abra|OJJ68219.1 acar|OOF97509.1 acla|EAW09056.1 afis|EAW25630.1 afla|EED55187.1 afum|EAL84318.1 agla|OJJ78900.1 akaw|GAA83227.1 aluc|OJZ83727.1 anid|CBF83164.1 anig|EHA18875.1 aory|EIT83560.1 asyd|OJJ55506.1 ater|EAU32707.1 atub|OJI90103.1 aver|OJJ06581.1 awen|OJJ36050.1 azon|OJJ50396.1 acla|EAW09057.1

Group1105: abra|OJJ68227.1 afla|EED48923.1 anid|CBF73570.1 aory|EIT80015.1 asyd|OJJ56694.1 aver|OJJ06030.1 abra|OJJ72608.1 acar|OOF94647.1 afis|EAW20314.1 afla|EED52548.1 afum|EAL85437.1 akaw|GAA85234.1 aluc|OJZ90590.1 anig|EHA24029.1 ater|EAU33505.1 atub|OJI82013.1 awen|OJJ33813.1 azon|OJJ48723.1 aory|EIT74186.1

Group1106: abra|OJJ68422.1 acar|OOF97621.1 acla|EAW10999.1 afis|EAW20862.1 afla|EED45444.1 afum|EAL84773.1 agla|OJJ83749.1 akaw|GAA92526.1 aluc|OJZ90075.1 anid|CBF71444.1 anig|EHA20998.1 aory|EIT82447.1 asyd|OJJ60710.1 ater|EAU30805.1 atub|OJI84731.1 aver|OJJ00036.1 awen|OJJ34508.1 azon|OJJ51786.1 agla|OJJ89303.1

Group1107: abra|OJJ68428.1 acar|OOF94918.1 afis|EAW23747.1 afla|EED51539.1 afum|EAL87182.1 agla|OJJ79306.1 akaw|GAA86943.1 aluc|OJZ90068.1 anid|CBF81672.1 anig|EHA21006.1 aory|EIT80703.1 asyd|OJJ59555.1 ater|EAU31300.1 atub|OJI84739.1 aver|OJI97564.1 awen|OJJ33683.1 azon|OJJ44480.1 anig|EHA25691.1 aver|OJJ02713.1

Group1108: abra|OJJ68521.1 acar|OOF92726.1 acla|EAW12843.1 afis|EAW18006.1 afla|EED51889.1 afum|EAL91818.1 agla|OJJ80269.1 akaw|GAA91328.1 aluc|OJZ82735.1 anid|CBF80030.1 anig|EHA21186.1 aory|EIT82919.1 asyd|OJJ58269.1 ater|EAU31522.1 atub|OJI88969.1 aver|OJJ02154.1 awen|OJJ41536.1 azon|OJJ48254.1 agla|OJJ80268.1

Group1109: abra|OJJ68529.1 acar|OOF92733.1 acla|EAW12835.1 afis|EAW18000.1 afla|EED51896.1 afum|EAL91811.1 agla|OJJ80263.1 akaw|GAA91321.1 aluc|OJZ82742.1 anid|CBF80021.1 anig|EHA21193.1 aory|EIT76177.1 asyd|OJJ58263.1 ater|EAU31512.1 atub|OJI88977.1 aver|OJJ02161.1 awen|OJJ41542.1 azon|OJJ48260.1 aory|EIT76174.1

Group1110: abra|OJJ68552.1 acar|OOF92747.1 acla|EAW12815.1 afis|EAW17980.1 afla|EED51925.1 afum|EAL91789.1 agla|OJJ80237.1 akaw|GAA91301.1 aluc|OJZ82769.1 anid|CBF79979.1 anig|EHA21211.1 aory|EIT81371.1 asyd|OJJ58235.1 ater|EAU31490.1 atub|OJI89000.1 aver|OJJ02190.1 awen|OJJ41565.1 azon|OJJ48280.1 akaw|GAA92005.1

Group1111: abra|OJJ68565.1 acar|OOF92758.1 acla|EAW12801.1 afis|EAW17967.1 afla|EED51939.1 afum|EAL91776.1 agla|OJJ80213.1 akaw|GAA91289.1 aluc|OJZ82780.1 anid|CBF79959.1 anig|EHA21223.1 aory|EIT81465.1 asyd|OJJ58221.1 ater|EAU31477.1 atub|OJI89013.1 aver|OJJ02201.1 awen|OJJ41577.1 azon|OJJ48290.1 akaw|GAA91288.1

Group1112: abra|OJJ68634.1 acar|OOF92138.1 acla|EAW12618.1 afis|EAW18066.1 afla|EED51450.1 afum|EBA27443.1 agla|OJJ87353.1 akaw|GAA89773.1 aluc|OJZ81921.1 anid|CBF86420.1 anig|EHA22326.1 anig|EHA22327.1 aory|EIT80631.1 asyd|OJJ64939.1 ater|EAU30462.1 atub|OJI84473.1 aver|OJI99061.1 awen|OJJ41453.1 azon|OJJ48911.1

Group1113: abra|OJJ68759.1 acar|OOF92246.1 acla|EAW12492.1 afis|EAW24153.1 afla|EED46778.1 afum|EAL92008.1 agla|OJJ89544.1 akaw|GAA89903.1 aluc|OJZ81767.1 anid|CBF77909.1 anig|EHA22447.1

aory|EIT72606.1 asyd|OJJ55299.1 ater|EAU30585.1 atub|OJI84340.1 aver|OJJ06926.1 awen|OJJ41324.1 azon|OJJ48798.1 ater|EAU30584.1

Group1114: abra|OJJ68992.1 acar|OOF92704.1 acla|EAW12644.1 afis|EAW18041.1 afla|EED51855.1 afla|EED51856.1 afum|EAL91852.1 agla|OJJ85413.1 akaw|GAA90512.1 aluc|OJZ85977.1 anid|CBF80101.1 anig|EHA24629.1 aory|EIT81757.1 asyd|OJJ58297.1 ater|EAU31556.1 atub|OJI79767.1 aver|OJJ02127.1 awen|OJJ41496.1 azon|OJJ48224.1

Group1115: abra|OJJ69111.1 acar|OOF91183.1 acla|EAW08721.1 afis|EAW15726.1 afla|EED51338.1 afum|EAL85621.1 agla|OJJ81865.1 akaw|GAA90636.1 aluc|OJZ86100.1 anid|CBF71089.1 anig|EHA24519.1 aory|EIT81534.1 asyd|OJJ52700.1 ater|EAU32431.1 atub|OJI79895.1 aver|OJJ04728.1 awen|OJJ38410.1 azon|OJJ43832.1 afla|EED51337.1

Group1116: abra|OJJ69231.1 acar|OOF91089.1 acla|EAW08618.1 afis|EAW15816.1 afla|EED51231.1 afum|EAL85535.1 agla|OJJ81980.1 akaw|GAA92239.1 aluc|OJZ86211.1 anid|CBF70897.1 anig|EHA24415.1 aory|EIT83178.1 asyd|OJJ52594.1 ater|EAU33432.1 atub|OJI80005.1 aver|OJJ04621.1 awen|OJJ38530.1 azon|OJJ43732.1 atub|OJI80006.1

Group1117: abra|OJJ69301.1 acar|OOF94424.1 acla|EAW14687.1 afis|EAW24218.1 afla|EED48053.1 afum|EAL85887.1 agla|OJJ79652.1 akaw|GAA88170.1 aluc|OJZ82129.1 anid|CBF74250.1 anig|EHA28602.1 aory|EIT83328.1 asyd|OJJ53976.1 ater|EAU31168.1 atub|OJI88307.1 aver|OJJ08075.1 awen|OJJ33078.1 azon|OJJ44868.1 aory|EIT83336.1

Group1118: abra|OJJ69328.1 acar|OOF94399.1 acla|EAW14712.1 afis|EAW24247.1 afla|EED48026.1 afum|EAL85915.1 agla|OJJ79626.1 akaw|GAA88199.1 aluc|OJZ82160.1 anid|CBF74202.1 anig|EHA28572.1 aory|EIT83332.1 asyd|OJJ53955.1 ater|EAU31188.1 atub|OJI88277.1 aver|OJJ08051.1 awen|OJJ33059.1 azon|OJJ48467.1 afla|EED48027.1

Group1119: abra|OJJ69488.1 acar|OOF94260.1 acla|EAW09905.1 afis|EAW21154.1 afla|EED54658.1 afum|EAL92172.1 agla|OJJ80039.1 akaw|GAA88332.1 aluc|OJZ82327.1 anid|CBF86989.1 anig|EHA28431.1 aory|EIT78787.1 asyd|OJJ60510.1 ater|EAU39556.1 atub|OJI88111.1 aver|OJI99836.1 awen|OJJ36482.1 azon|OJJ44363.1 anig|EHA21818.1

Group1120: abra|OJJ69514.1 acar|OOF94239.1 acla|EAW09954.1 afis|EAW21142.1 afla|EED46141.1 afum|EAL92161.1 agla|OJJ80069.1 akaw|GAA88310.1 aluc|OJZ82352.1 anid|CBF87008.1 anig|EHA28408.1 aory|EIT76511.1 asyd|OJJ60471.1 ater|EAU36763.1 atub|OJI88088.1 aver|OJI99791.1 awen|OJJ36466.1 azon|OJJ44412.1 azon|OJJ42893.1

Group1121: abra|OJJ69523.1 acar|OOF94231.1 acla|EAW09971.1 afis|EAW21117.1 afla|EED51790.1 afum|EAL92141.1 agla|OJJ80094.1 akaw|GAA88302.1 aluc|OJZ82363.1 anid|CBF87028.1 anig|EHA28402.1 aory|EIT81753.1 asyd|OJJ60456.1 ater|EAU36782.1 atub|OJI88079.1 aver|OJJ06754.1 awen|OJJ36447.1 azon|OJJ44428.1 aver|OJI99774.1

Group1122: abra|OJJ69615.1 acar|OOF92691.1 acla|EAW12075.1 afis|EAW19716.1 afla|EED53109.1 afum|EAL86811.1 agla|OJJ80862.1 akaw|GAA91599.1 aluc|OJZ82423.1 anid|CBF80080.1 anig|EHA28346.1 aory|EIT74768.1 asyd|OJJ58309.1 ater|EAU37777.1 atub|OJI88017.1 aver|OJJ02103.1 awen|OJJ35106.1 azon|OJJ45858.1 awen|OJJ34696.1

Group1123: abra|OJJ69668.1 acar|OOF95768.1 acla|EAW10632.1 afis|EAW24935.1 afla|EED55537.1 afum|EAL87763.1 agla|OJJ82162.1 akaw|GAA91646.1 aluc|OJZ82473.1 anid|CBF75718.1 anig|EHA28300.1 aory|EIT80819.1 asyd|OJJ57503.1 ater|EAU35664.1 atub|OJI87964.1 aver|OJJ05675.1 awen|OJJ40530.1 azon|OJJ49390.1 afum|EAL89592.1

Group1124: abra|OJJ69841.1 acar|OOF93364.1 acla|EAW13293.1 afis|EAW24568.1 afla|EED56610.1 afum|EAL91152.1 akaw|GAA87819.1 aluc|OJZ85455.1 anid|CBF82896.1 anig|EHA24997.1 aory|EIT81324.1 asyd|OJJ62102.1 ater|EAU39297.1 atub|OJI80109.1 aver|OJI96208.1 afis|EAW17316.1 aory|EIT82251.1 ater|EAU30304.1 aory|EIT79627.1

Group1125: abra|OJJ69857.1 acar|OOF93382.1 acla|EAW08537.1 afis|EAW23575.1 afla|EED57541.1 afum|EAL87890.1 agla|OJJ85306.1 akaw|GAA87799.1 aluc|OJZ85434.1 anig|EHA24980.1 aory|EIT81841.1 asyd|OJJ56731.1 atub|OJI80134.1 awen|OJJ38171.1 azon|OJJ45131.1 agla|OJJ86836.1 awen|OJJ39903.1 azon|OJJ45879.1 afla|EED54164.1

Group1126: abra|OJJ70048.1 acar|OOF93540.1 afla|EED47673.1 agla|OJJ79802.1 akaw|GAA87627.1 aluc|OJZ85243.1 anid|CBF84432.1 anig|EHA25443.1 aory|EIT80242.1 asyd|OJJ61842.1 ater|EAU34191.1 atub|OJI80328.1 aver|OJJ05342.1 awen|OJJ36937.1 aver|OJI95942.1 agla|OJJ80121.1 asyd|OJJ53636.1 aver|OJJ01170.1 ater|EAU29232.1

Group1127: abra|OJJ70093.1 acla|EAW12322.1 afis|EAW17167.1 afla|EED51057.1 afum|EAL89052.1 agla|OJJ78763.1 akaw|GAA87602.1 aluc|OJZ85222.1 anig|EHA25426.1 aory|EIT76987.1 ater|EAU34026.1 atub|OJI80361.1 azon|OJJ42125.1 akaw|GAA87480.1 aluc|OJZ88046.1 anig|EHA17818.1 ater|EAU29611.1 atub|OJI90654.1 awen|OJJ36983.1

Group1128: abra|OJJ70121.1 acar|OOF92449.1 acla|EAW11044.1 afis|EAW16194.1 afla|EED48259.1 afum|EAL88825.1 akaw|GAA86648.1 aluc|OJZ85206.1 anid|CBF74996.1 anig|EHA25410.1 aory|EIT72896.1 ater|EAU34204.1 atub|OJI80396.1 aver|OJJ05082.1 azon|OJJ46585.1 afla|EED53310.1 aory|EIT74931.1 azon|OJJ45951.1 afla|EED49627.1

Group1129: abra|OJJ70144.1 acar|OOF96811.1 afla|EED52394.1 akaw|GAA90567.1 akaw|GAA90568.1 aluc|OJZ86936.1 anig|EHA25649.1 aory|EIT83282.1 asyd|OJJ53406.1 atub|OJI88384.1 aver|OJJ04195.1 awen|OJJ35567.1 azon|OJJ42608.1 akaw|GAA84747.1 anig|EHA21708.1 atub|OJI83585.1 afla|EED52395.1 anig|EHA25751.1 akaw|GAA91340.1

Group1130: abra|OJJ70231.1 acar|OOF91736.1 acla|EAW11658.1 afis|EAW20417.1 afla|EED53976.1 agla|OJJ82616.1 aluc|OJZ90859.1 aver|OJJ06455.1 awen|OJJ40076.1 azon|OJJ45549.1 abra|OJJ73408.1 acar|OOG00605.1 akaw|GAA83083.1 aluc|OJZ88630.1 anig|EHA18320.1 aory|EIT74373.1 atub|OJI91214.1 afla|EED52225.1 agla|OJJ88203.1

Group1131: abra|OJJ70466.1 acar|OOF97458.1 acla|EAW13048.1 afis|EAW17619.1 afla|EED49199.1 afla|EED49200.1 afum|EAL91432.1 agla|OJJ81306.1 akaw|GAA83315.1 aluc|OJZ83810.1 anid|CBF90163.1 anig|EHA18955.1 aory|EIT78513.1 asyd|OJJ61994.1 ater|EAU37053.1 atub|OJI90177.1 aver|OJI96086.1 awen|OJJ37225.1 azon|OJJ50928.1

Group1132: abra|OJJ70467.1 acar|OOF97457.1 acla|EAW13047.1 afis|EAW17620.1 afla|EED49197.1 afla|EED49198.1 afum|EAL91433.2 agla|OJJ81305.1 akaw|GAA83316.1 aluc|OJZ83811.1 anid|CBF90161.1 anig|EHA18956.1 aory|EIT78563.1 asyd|OJJ61995.1 ater|EAU37054.1 atub|OJI90178.1 aver|OJI96087.1 awen|OJJ37224.1 azon|OJJ50927.1

Group1133: abra|OJJ70612.1 acar|OOF97110.1 acla|EAW13015.1 afis|EAW17652.1 afla|EED50506.1 afum|EAL91467.1 agla|OJJ79552.1 akaw|GAA89951.1 aluc|OJZ83958.1 anid|CBF90075.1 anig|EHA19086.1 aory|EIT82132.1 asyd|OJJ62041.1 ater|EAU37088.1 atub|OJI90330.1 aver|OJI96147.1 awen|OJJ37193.1 azon|OJJ50892.1 aver|OJJ07605.1

Group1134: abra|OJJ70647.1 acar|OOF97074.1 acla|EAW08113.1 afis|EAW23138.1 afla|EED48501.1 afum|EAL88343.1 agla|OJJ89662.1 akaw|GAA89983.1 aluc|OJZ83997.1 anid|CBF74448.1 anig|EHA19120.1 aory|EIT73103.1 asyd|OJJ57741.1 ater|EAU36279.1 atub|OJI90366.1 aver|OJJ05426.1 awen|OJJ36635.1 azon|OJJ47581.1 awen|OJJ36634.1

Group1135: abra|OJJ70656.1 acar|OOF97066.1 acla|EAW08103.1 afis|EAW23148.1 afla|EED48511.1 afum|EAL88333.1 agla|OJJ89654.1 akaw|GAA89992.1 aluc|OJZ84006.1 anid|CBF74430.1 anig|EHA19128.1 aory|EIT73155.1 asyd|OJJ57750.1 ater|EAU32975.1 atub|OJI90375.1 aver|OJJ05417.1 awen|OJJ36643.1 azon|OJJ47571.1 acar|OOG00309.1

Group1136: abra|OJJ70745.1 acar|OOF96999.1 acla|EAW11175.1 afis|EAW15906.1 afla|EED56463.1 afum|EAL88541.1 agla|OJJ89566.1 akaw|GAA90066.1 aluc|OJZ84090.1 anid|CBF81270.1 anig|EHA19203.1 aory|EIT72857.1 asyd|OJJ59333.1 ater|EAU36183.1 atub|OJI90461.1 aver|OJJ02941.1 awen|OJJ36725.1 azon|OJJ47484.1 ater|EAU30153.1

Group1137: abra|OJJ70747.1 acar|OOF96998.1 acla|EAW11174.1 afis|EAW15905.1 afla|EED56464.1 afum|EAL88540.1 agla|OJJ89565.1 akaw|GAA90067.1 aluc|OJZ84091.1 anid|CBF81274.1 anig|EHA19204.1 aory|EIT72825.1 asyd|OJJ59334.1 ater|EAU36182.1 atub|OJI90462.1 aver|OJJ02940.1 awen|OJJ36726.1 azon|OJJ47471.1 aory|EIT72838.1

Group1138: abra|OJJ70748.1 acar|OOF96997.1 acla|EAW11173.1 afis|EAW15904.1 afla|EED56466.1 afum|EAL88539.1 agla|OJJ89564.1 akaw|GAA90068.1 aluc|OJZ84092.1 anid|CBF81276.1 anig|EHA19205.1 aory|EIT72390.1 asyd|OJJ59335.1 ater|EAU36181.1 atub|OJI90463.1 aver|OJJ02939.1 awen|OJJ36727.1 azon|OJJ47470.1 afla|EED46475.1

Group1139: abra|OJJ70830.1 afis|EAW19787.1 afla|EED45955.1 afum|EAL85107.2 agla|OJJ88990.1 anid|CBF74550.1 anig|EHA20974.1 aory|EIT75768.1 asyd|OJJ57685.1 ater|EAU34824.1 aver|OJJ04314.1 awen|OJJ33838.1 abra|OJJ76587.1 acar|OOF99400.1 akaw|GAA82491.1 aluc|OJZ91619.1 anig|EHA22646.1 atub|OJI86072.1 asyd|OJJ52721.1

Group1140: abra|OJJ70840.1 acar|OOF98274.1 acla|EAW13281.1 afis|EAW17336.1 afla|EED46105.1 afum|EAL91180.1 agla|OJJ82057.1 akaw|GAA91708.1 aluc|OJZ87681.1 anid|CBF85633.1 anig|EHA20871.1 aory|EIT76521.1 asyd|OJJ56056.1 ater|EAU33888.1 atub|OJI80962.1 aver|OJJ04998.1 awen|OJJ33506.1 azon|OJJ50300.1 azon|OJJ51817.1

Group1141: abra|OJJ70845.1 acar|OOF98270.1 acla|EAW10436.1 afis|EAW25131.1 afla|EED55314.1 afum|EAL89802.1 agla|OJJ89035.1 akaw|GAA91713.1 aluc|OJZ87676.1 anid|CBF86816.1 anig|EHA20866.1 aory|EIT83519.1 asyd|OJJ64657.1 ater|EAU39403.1 atub|OJI80967.1 aver|OJI98760.1 awen|OJJ40756.1 azon|OJJ44121.1 aver|OJJ02113.1

Group1142: abra|OJJ70882.1 acar|OOF98239.1 acla|EAW10470.1 afis|EAW25097.1 afla|EED55351.1 afum|EAL89768.1 agla|OJJ85476.1 akaw|GAA91744.1 aluc|OJZ87640.1 anid|CBF86010.1 anig|EHA20834.1

aory|EIT80916.1 asyd|OJJ64447.1 ater|EAU39355.1 atub|OJI81005.1 aver|OJI98550.1 awen|OJJ40711.1 azon|OJJ44087.1 ater|EAU29372.1

Group1143: abra|OJJ70985.1 acar|OOF98146.1 acla|EAW10545.1 afis|EAW25013.1 afla|EED55432.1 afum|EAL89683.1 agla|OJJ89177.1 akaw|GAA82964.1 aluc|OJZ87545.1 aluc|OJZ87546.1 anid|CBF81523.1 anig|EHA20749.1 aory|EIT80770.1 asyd|OJJ59478.1 ater|EAU35773.1 atub|OJI81100.1 aver|OJJ02795.1 awen|OJJ40627.1 azon|OJJ49056.1

Group1144: abra|OJJ71009.1 acar|OOF98133.1 acla|EAW10561.1 afis|EAW25028.1 afla|EED55451.1 afum|EAL89667.1 agla|OJJ79156.1 agla|OJJ82560.1 akaw|GAA82944.1 aluc|OJZ87522.1 anid|CBF81555.1 anig|EHA20732.1 aory|EIT80765.1 asyd|OJJ59497.1 ater|EAU35759.1 atub|OJI81127.1 aver|OJJ02777.1 awen|OJJ40611.1 azon|OJJ49460.1

Group1145: abra|OJJ71029.1 acar|OOF98110.1 acla|EAW10582.1 afis|EAW24987.1 afla|EED55471.1 afum|EAL89647.1 agla|OJJ82275.1 akaw|GAA82926.1 aluc|OJZ87501.1 anid|CBF81601.1 anig|EHA20716.1 aory|EIT80792.1 asyd|OJJ59520.1 ater|EAU35738.1 atub|OJI81148.1 aver|OJJ02753.1 awen|OJJ40583.1 azon|OJJ41984.1 azon|OJJ49442.1

Group1146: abra|OJJ71043.1 acar|OOF98096.1 acla|EAW10591.1 afis|EAW24977.1 afla|EED55482.1 afum|EAL89637.1 agla|OJJ79140.1 akaw|GAA82913.1 aluc|OJZ87485.1 anid|CBF81627.1 anig|EHA20706.1 aory|EIT80881.1 asyd|OJJ59529.1 ater|EAU35726.1 atub|OJI81163.1 aver|OJJ02741.1 awen|OJJ40574.1 azon|OJJ49436.1 afla|EED55483.1

Group1147: abra|OJJ71134.1 acar|OOF98018.1 acla|EAW10363.1 afis|EAW25229.1 afla|EED53673.1 afum|EAL89890.2 agla|OJJ79179.1 akaw|GAA82840.1 aluc|OJZ87401.1 anid|CBF85338.1 anig|EHA20630.1 aory|EIT75324.1 asyd|OJJ64023.1 ater|EAU34387.1 atub|OJI81245.1 aver|OJI98109.1 awen|OJJ40894.1 azon|OJJ44523.1 aory|EIT75321.1

Group1148: abra|OJJ71194.1 acar|OOF97972.1 acla|EAW10310.1 afis|EAW25283.1 afla|EED53749.1 afum|EAL89946.1 agla|OJJ89257.1 akaw|GAA82789.1 aluc|OJZ87348.1 anid|CBF85422.1 anig|EHA20584.1 aory|EIT78394.1 asyd|OJJ64075.1 ater|EAU34425.1 atub|OJI81295.1 aver|OJI98162.1 awen|OJJ40996.1 azon|OJJ44586.1 asyd|OJJ60350.1

Group1149: abra|OJJ71328.1 acar|OOF97846.1 acla|EAW10211.1 afis|EAW25383.1 afla|EED53860.1 afum|EAL90041.1 agla|OJJ83775.1 akaw|GAA82664.1 aluc|OJZ87214.1 anid|CBF77488.1 anig|EHA20465.1 aory|EIT78406.1 asyd|OJJ53195.1 ater|EAU34529.1 atub|OJI81425.1 aver|OJJ03655.1 awen|OJJ41109.1 azon|OJJ44687.1 aory|EIT78415.1

Group1150: abra|OJJ71401.1 acar|OOF97769.1 acla|EAW10133.1 afis|EAW25454.1 afla|EED53942.1 afla|EED53943.1 afum|EAL90113.1 agla|OJJ87107.1 akaw|GAA82596.1 aluc|OJZ87141.1 anid|CBF77635.1 anig|EHA20399.1 aory|EIT78263.1 asyd|OJJ53125.1 ater|EAU34624.1 atub|OJI81501.1 aver|OJJ03729.1 awen|OJJ41186.1 azon|OJJ44762.1

Group1151: abra|OJJ71618.1 acar|OOF96335.1 acla|EAW13285.1 afis|EAW17333.1 afla|EED57246.1 afum|EAL91170.2 akaw|GAA89309.1 aluc|OJZ86359.1 anid|CBF84952.1 anig|EHA21531.1 aory|EIT74025.1 asyd|OJJ63145.1 ater|EAU38742.1 atub|OJI84175.1 aver|OJI97809.1 azon|OJJ50869.1 acla|EAW10844.1 afla|EED47443.1 aory|EIT81909.1

Group1152: abra|OJJ71994.1 acar|OOF96648.1 acla|EAW09538.1 afis|EAW21594.1 afla|EED57816.1 afum|EAL92612.1 agla|OJJ85787.1 akaw|GAA84934.1 aluc|OJZ86732.1 anid|CBF83446.1 anig|EHA21947.1 aory|EIT81030.1 asyd|OJJ60671.1 ater|EAU34930.1 atub|OJI83796.1 aver|OJI99994.1 awen|OJJ39087.1 azon|OJJ51418.1 ater|EAU32744.1

Group1153: abra|OJJ72045.1 acar|OOF96703.1 acla|EAW09501.1 afis|EAW21635.1 afla|EED48487.1 afum|EAL92669.1 agla|OJJ79700.1 akaw|GAA84889.1 aluc|OJZ86782.1 anid|CBF83498.1 anig|EHA21900.1 aory|EIT83266.1 asyd|OJJ60712.1 ater|EAU38321.1 atub|OJI83738.1 aver|OJJ00045.1 awen|OJJ33884.1 azon|OJJ48669.1 aory|EIT73220.1

Group1154: abra|OJJ72230.1 acar|OOF96853.1 acla|EAW14990.1 afis|EAW25547.1 afla|EED47847.1 afum|EAL86183.1 agla|OJJ80332.1 akaw|GAA84707.1 aluc|OJZ86980.1 anid|CBF74013.1 anig|EHA21669.1 aory|EIT77206.1 asyd|OJJ56425.1 atub|OJI83540.1 aver|OJJ06292.1 awen|OJJ32605.1 afis|EAW24517.1 asyd|OJJ53838.1 aver|OJJ07941.1

Group1155: abra|OJJ72280.1 acar|OOF94941.1 afis|EAW20800.1 afla|EED49545.1 afum|EAL84829.1 agla|OJJ85012.1 akaw|GAA86032.1 aluc|OJZ90259.1 anid|CBF71374.1 anig|EHA23754.1 aory|EIT82577.1 asyd|OJJ55077.1 ater|EAU32870.1 atub|OJI81685.1 aver|OJJ07131.1 awen|OJJ41912.1 azon|OJJ47358.1 anig|EHA23755.1 afum|EAL84830.1

Group1156: abra|OJJ72423.1 acar|OOF90372.1 acla|EAW12860.1 afis|EAW17809.1 afla|EED46159.1 afum|EAL91617.1 agla|OJJ80653.1 akaw|GAA86165.1 aluc|OJZ90406.1 anid|CBF78020.1 anig|EHA23874.1 aory|EIT76396.1 asyd|OJJ55235.1 ater|EAU33015.1 atub|OJI81837.1 aver|OJJ06988.1 awen|OJJ41763.1 azon|OJJ44903.1 afla|EED46160.1

Group1157: abra|OJJ72434.1 acar|OOF94801.1 acla|EAW12667.1 afis|EAW17831.1 afla|EED46173.1 afum|EAL91641.1 agla|OJJ80640.1 akaw|GAA86174.1 aluc|OJZ90417.1 anid|CBF77994.1 anig|EHA23885.1 aory|EIT76438.1 asyd|OJJ55252.1 ater|EAU33034.1 atub|OJI81850.1 aver|OJI99643.1 awen|OJJ41750.1 azon|OJJ48442.1 aver|OJJ06972.1

Group1158: abra|OJJ72484.1 acar|OOF94751.1 acla|EAW12715.1 afis|EAW17877.1 afla|EED46293.1 afum|EAL91685.1 agla|OJJ83069.1 akaw|GAA86219.1 aluc|OJZ90471.1 anig|EHA23930.1 aory|EIT76328.1 asyd|OJJ61521.1 ater|EAU33097.1 atub|OJI81895.1 aver|OJJ05549.1 awen|OJJ41687.1 asyd|OJJ57661.1 aver|OJJ05523.1 aver|OJJ01273.1

Group1159: abra|OJJ72549.1 acar|OOF94703.1 acla|EAW12754.1 afis|EAW17917.1 afla|EED46320.1 afum|EBA27439.1 agla|OJJ83026.1 akaw|GAA85181.1 aluc|OJZ90537.1 anid|CBF76959.1 anig|EHA23985.1 aory|EIT81197.1 atub|OJI81958.1 awen|OJJ41628.1 asyd|OJJ57052.1 aver|OJJ03244.1 azon|OJJ48353.1 ater|EAU33139.1 asyd|OJJ57051.1

Group1160: acla|EAW12414.1 acar|OOF98070.1 afla|EED52231.1 agla|OJJ84011.1 anid|CBF69489.1 aory|EIT74442.1 asyd|OJJ59240.1 ater|EAU37441.1 awen|OJJ35818.1 azon|OJJ49326.1 acar|OOG00738.1 asyd|OJJ60713.1 ater|EAU30842.1 aver|OJJ00046.1 afla|EED47470.1 aory|EIT79973.1 ater|EAU31019.1 azon|OJJ48614.1 aver|OJJ01850.1

Group1161: abra|OJJ72629.1 acar|OOF94635.1 acla|EAW11593.1 afis|EAW23864.1 afla|EED52589.1 afum|EAL87292.1 agla|OJJ82532.1 akaw|GAA85249.1 aluc|OJZ90606.1 anid|CBF77222.1 anig|EHA24044.1 aory|EIT74309.1 asyd|OJJ56820.1 ater|EAU33487.1 atub|OJI82030.1 aver|OJJ03515.1 awen|OJJ33839.1 azon|OJJ48708.1 awen|OJJ33432.1

Group1162: abra|OJJ72716.1 acar|OOF95949.1 acla|EAW11552.1 afis|EAW23901.1 afla|EED52636.1 afum|EAL87328.1 agla|OJJ79367.1 akaw|GAA85324.1 aluc|OJZ90685.1 anid|CBF77143.1 anig|EHA24113.1 aory|EIT74168.1 asyd|OJJ56912.1 ater|EAU32337.1 atub|OJI82111.1 aver|OJJ03380.1 awen|OJJ39048.1 azon|OJJ51378.1 afis|EAW25771.1

Group1163: abra|OJJ72751.1 acar|OOF95974.1 acla|EAW11510.1 afis|EAW23943.1 afla|EED50573.1 afum|EAL87368.1 agla|OJJ79431.1 akaw|GAA85363.1 aluc|OJZ90725.1 anid|CBF77256.1 anig|EHA24143.1 aory|EIT82138.1 asyd|OJJ56806.1 ater|EAU36036.1 atub|OJI82153.1 aver|OJJ03528.1 awen|OJJ39012.1 azon|OJJ51337.1 awen|OJJ40567.1

Group1164: abra|OJJ72900.1 acar|OOF89981.1 acla|EAW11351.1 afis|EAW18143.1 afla|EED58069.1 afum|EAL87500.1 agla|OJJ80189.1 akaw|GAA85487.1 aluc|OJZ83409.1 anid|CBF81125.1 anig|EHA24260.1 aory|EIT79709.1 asyd|OJJ56489.1 atub|OJI82297.1 aver|OJJ06229.1 awen|OJJ35585.1 awen|OJJ38003.1 aver|OJJ03876.1 ater|EAU30131.1

Group1165: abra|OJJ72905.1 acar|OOF89821.1 acla|EAW11349.1 afis|EAW18146.1 afla|EED58074.1 afum|EAL87503.1 agla|OJJ83830.1 akaw|GAA85490.1 aluc|OJZ83415.1 anid|CBF81118.1 anig|EHA24264.1 asyd|OJJ56487.1 ater|EAU35469.1 atub|OJI82302.1 aver|OJJ06232.1 awen|OJJ35589.1 azon|OJJ46936.1 afla|EED58073.1 afum|EAL87504.1

Group1166: abra|OJJ72907.1 acar|OOF96090.1 acla|EAW11346.1 afis|EAW18148.1 afla|EED58076.1 afum|EAL87506.1 agla|OJJ83833.1 akaw|GAA85492.1 aluc|OJZ83417.1 anid|CBF81114.1 anig|EHA24266.1 aory|EIT79127.1 ater|EAU35470.1 atub|OJI82304.1 aver|OJJ06234.1 awen|OJJ35591.1 azon|OJJ46913.1 asyd|OJJ56485.1 asyd|OJJ57077.1

Group1167: abra|OJJ73268.1 acar|OOG00508.1 acla|EAW06945.1 afis|EAW16559.1 afla|EED56102.1 afum|EAL89157.1 agla|OJJ82763.1 akaw|GAA87914.1 aluc|OJZ81161.1 anid|CBF81806.1 anig|EHA18478.1 aory|EIT76895.1 asyd|OJJ59644.1 ater|EAU36627.1 atub|OJI91356.1 aver|OJJ02638.1 awen|OJJ40257.1 azon|OJJ49121.1 acar|OOF91712.1

Group1168: abra|OJJ73284.1 acar|OOG00520.1 acla|EAW06959.1 afis|EAW16574.1 afla|EED56090.1 afum|EAL89171.2 agla|OJJ82706.1 akaw|GAA92721.1 aluc|OJZ88765.1 anid|CBF81830.1 anig|EHA18461.1 aory|EIT76622.1 asyd|OJJ59660.1 ater|EAU36642.1 atub|OJI91338.1 aver|OJJ02622.1 awen|OJJ40244.1 azon|OJJ49105.1 asyd|OJJ52085.1

Group1169: abra|OJJ73346.1 acar|OOG00556.1 acla|EAW06973.1 afis|EAW16587.1 afla|EED56078.1 afum|EAL89182.1 agla|OJJ82679.1 akaw|GAA83136.1 aluc|OJZ88693.1 anid|CBF81890.1 anig|EHA18373.1 aory|EIT76830.1 asyd|OJJ59676.1 ater|EAU36664.1 ater|EAU39117.1 atub|OJI91273.1 aver|OJJ02610.1 awen|OJJ40228.1 azon|OJJ49093.1

Group1170: abra|OJJ73352.1 acar|OOG00563.1 acla|EAW12784.1 afis|EAW19851.1 afla|EED52646.1 afum|EAL85042.1 agla|OJJ81301.1 akaw|GAA83131.1 aluc|OJZ88687.1 anid|CBF73979.1 anig|EHA18367.1 aory|EIT74299.1 asyd|OJJ54702.1 ater|EAU32479.1 atub|OJI91267.1 aver|OJJ07471.1 awen|OJJ32014.1 azon|OJJ46075.1 afis|EAW17946.1

Group1171: abra|OJJ73409.1 acar|OOG00606.1 acla|EAW07013.1 afis|EAW16640.1 afla|EED44962.1 afum|EAL89242.1 agla|OJJ82610.1 akaw|GAA83082.1 aluc|OJZ88629.1 anid|CBF82018.1 anig|EHA18319.1

aory|EIT73512.1 asyd|OJJ56191.1 ater|EAU36751.1 atub|OJI91213.1 aver|OJJ05120.1 awen|OJJ40074.1 azon|OJJ45550.1 anid|CBF76269.1

Group1172: abra|OJJ73438.1 acar|OOG00644.1 acla|EAW06774.1 afis|EAW16325.1 afla|EED53556.1 afum|EAL88949.1 agla|OJJ88288.1 akaw|GAA83055.1 aluc|OJZ88602.1 anid|CBF89162.1 anig|EHA18296.1 aory|EIT75182.1 asyd|OJJ62547.1 ater|EAU34132.1 atub|OJI91187.1 aver|OJI96673.1 awen|OJJ37725.1 azon|OJJ44985.1 afla|EED53557.1

Group1173: abra|OJJ73449.1 acar|OOG00652.1 acla|EAW11065.1 afis|EAW20809.1 afla|EED50135.1 afum|EAL84819.1 akaw|GAA83047.1 aluc|OJZ88592.1 anid|CBF69930.1 anig|EHA18288.1 aory|EIT74700.1 asyd|OJJ58924.1 ater|EAU34826.1 atub|OJI91178.1 aver|OJJ01520.1 awen|OJJ29971.1 azon|OJJ46931.1 asyd|OJJ52486.1 aver|OJJ03920.1

Group1174: abra|OJJ73545.1 acar|OOG00719.1 acla|EAW07464.1 afis|EAW22495.1 afla|EED54524.1 afum|EAL90698.1 agla|OJJ82639.1 akaw|GAA88607.1 aluc|OJZ88515.1 anid|CBF78415.1 anig|EHA18221.1 aory|EIT78926.1 asyd|OJJ58988.1 atub|OJI91100.1 aver|OJJ01628.1 afis|EAW23820.1 afla|EED45632.1 afum|EAL87255.1 aory|EIT75782.1

Group1175: abra|OJJ73649.1 acar|OOG00830.1 acla|EAW07562.1 afis|EAW22596.1 afla|EED54646.1 afum|EAL90602.1 agla|OJJ80960.1 akaw|GAA88506.1 aluc|OJZ88401.1 anid|CBF88373.1 anig|EHA18129.1 aory|EIT78830.1 asyd|OJJ63733.1 ater|EAU39107.1 atub|OJI90997.1 aver|OJI97208.1 awen|OJJ35224.1 azon|OJJ49885.1 afla|EED54647.1

Group1176: abra|OJJ73717.1 acla|EAW14401.1 afis|EAW16390.1 afla|EED52126.1 afum|EAL89016.2 agla|OJJ84496.1 akaw|GAA88446.1 aluc|OJZ88333.1 anid|CBF79713.1 anig|EHA18069.1 aory|EIT74447.1 asyd|OJJ58060.1 ater|EAU37368.1 atub|OJI90929.1 aver|OJJ04343.1 awen|OJJ32507.1 azon|OJJ45230.1 afis|EAW19108.1 afum|EAL93839.1

Group1177: abra|OJJ73743.1 acar|OOG00906.1 acla|EAW07637.1 afis|EAW22657.1 afla|EED49925.1 afum|EAL90535.1 agla|OJJ81040.1 akaw|GAA88422.1 aluc|OJZ88309.1 anid|CBF80225.1 anig|EHA18046.1 aory|EIT72274.1 asyd|OJJ54399.1 ater|EAU39050.1 atub|OJI90904.1 aver|OJJ05335.1 awen|OJJ35266.1 azon|OJJ49829.1 ater|EAU30036.1

Group1178: abra|OJJ73792.1 acar|OOG00955.1 afis|EAW22699.1 afla|EED49985.1 afum|EAL90493.1 agla|OJJ79011.1 akaw|GAA89411.1 aluc|OJZ88262.1 anid|CBF88085.1 anig|EHA18007.1 aory|EIT74716.1 asyd|OJJ63561.1 ater|EAU39007.1 atub|OJI90861.1 aver|OJI97372.1 awen|OJJ35335.1 acar|OOF98064.1 anid|CBF78395.1 acla|EAW07684.1

Group1179: abra|OJJ73822.1 acar|OOG00974.1 acla|EAW07711.1 afis|EAW22727.1 afla|EED50013.1 afum|EAL90468.2 agla|OJJ83675.1 akaw|GAA89441.1 aluc|OJZ88226.1 anid|CBF88026.1 anig|EHA17974.1 aory|EIT74607.1 asyd|OJJ63528.1 ater|EAU38970.1 atub|OJI90827.1 aver|OJI97403.1 awen|OJJ35422.1 azon|OJJ49754.1 aory|EIT74669.1

Group1180: abra|OJJ73906.1 acar|OOG01039.1 acla|EAW07784.1 afis|EAW22820.1 afla|EED50095.1 afum|EBA27405.1 agla|OJJ83518.1 akaw|GAA89513.1 aluc|OJZ88141.1 anid|CBF87897.1 anig|EHA17904.1 aory|EIT74756.1 asyd|OJJ63443.1 ater|EAU38890.1 atub|OJI90744.1 aver|OJI97493.1 awen|OJJ38907.1 azon|OJJ49688.1 afla|EED50094.1

Group1181: abra|OJJ73972.1 acar|OOG01099.1 acla|EAW07843.1 afis|EAW22879.1 afla|EED50182.1 afum|EAL90320.1 akaw|GAA89575.1 aluc|OJZ88072.1 anid|CBF87810.1 anig|EHA17845.1 aory|EIT83503.1 asyd|OJJ63389.1 ater|EAU38843.1 atub|OJI90681.1 aver|OJI97546.1 awen|OJJ38843.1 azon|OJJ49640.1 afla|EED46055.1 azon|OJJ45935.1

Group1182: abra|OJJ73974.1 acar|OOG01101.1 acla|EAW07845.1 afis|EAW22881.1 afla|EED50179.1 afum|EAL90318.1 agla|OJJ83449.1 akaw|GAA89577.1 aluc|OJZ88070.1 anid|CBF87807.1 anig|EHA17843.1 aory|EIT77377.1 asyd|OJJ63387.1 ater|EAU38845.1 atub|OJI90679.1 aver|OJI97548.1 awen|OJJ38841.1 azon|OJJ49638.1 agla|OJJ83448.1

Group1183: abra|OJJ73990.1 acar|OOG01116.1 acla|EAW07861.1 afis|EAW22896.1 afla|EED54867.1 afum|EAL90302.1 agla|OJJ83416.1 akaw|GAA87473.1 aluc|OJZ88055.1 anid|CBF87777.1 anig|EHA17827.1 aory|EIT79357.1 asyd|OJJ63370.1 atub|OJI90663.1 aver|OJI97576.1 awen|OJJ38822.1 azon|OJJ49621.1 ater|EAU35980.1 awen|OJJ38821.1

Group1184: abra|OJJ74134.1 acar|OOF95597.1 acla|EAW08432.1 afis|EAW23475.1 afla|EED57371.1 afum|EAL87990.1 agla|OJJ86714.1 akaw|GAA89752.1 aluc|OJZ80631.1 anid|CBF89773.1 anig|EHA27200.1 aory|EIT80150.1 asyd|OJJ62211.1 ater|EAU36905.1 atub|OJI87487.1 aver|OJI96322.1 awen|OJJ37876.1 asyd|OJJ52090.1 aver|OJJ03003.1

Group1185: abra|OJJ74469.1 acar|OOF95278.1 acla|EAW07228.1 afis|EAW22242.1 afla|EED57635.1 afum|EAL90951.1 agla|OJJ87809.1 akaw|GAA87362.1 aluc|OJZ89651.1 anid|CBF88489.1 anig|EHA26889.1 aory|EIT82991.1 asyd|OJJ63790.1 ater|EAU34249.1 atub|OJI85153.1 aver|OJI97147.1 awen|OJJ34820.1 azon|OJJ45730.1 aver|OJJ06299.1

Group1186: acar|OOF99264.1 awen|OJJ30905.1 agla|OJJ80074.1 akaw|GAA89762.1 aluc|OJZ80621.1 anid|CBF86745.1 anig|EHA27210.1 aory|EIT75558.1 asyd|OJJ64702.1 ater|EAU30235.1 atub|OJI87475.1 aver|OJI98806.1 acar|OOF99265.1 afla|EED49606.1 aory|EIT80966.1 azon|OJJ42634.1 asyd|OJJ53572.1 aory|EIT80991.1 aver|OJI99007.1

Group1187: abra|OJJ74764.1 acar|OOF95757.1 acla|EAW10646.1 afis|EAW24920.1 afla|EED55553.1 afum|EAL89575.1 agla|OJJ82149.1 akaw|GAA83647.1 aluc|OJZ92348.1 anig|EHA26626.1 aory|EIT82708.1 asyd|OJJ57492.1 asyd|OJJ62481.1 ater|EAU35652.1 atub|OJI86775.1 aver|OJJ05688.1 awen|OJJ40517.1 azon|OJJ49376.1 anid|CBF75691.1

Group1188: abra|OJJ74777.1 acar|OOF95766.1 acla|EAW10634.1 afis|EAW24932.1 afla|EED55541.1 afum|EAL89588.1 agla|OJJ82160.1 akaw|GAA83635.1 aluc|OJZ92358.1 anid|CBF75714.1 anig|EHA26615.1 aory|EIT82707.1 asyd|OJJ57501.1 ater|EAU35662.1 atub|OJI86788.1 aver|OJJ05677.1 awen|OJJ40528.1 azon|OJJ49389.1 afum|EAL87767.1

Group1189: abra|OJJ74785.1 acar|OOF95775.1 acla|EAW10626.1 afis|EAW24941.1 afla|EED55524.1 afum|EAL89598.1 agla|OJJ82168.1 akaw|GAA83628.1 aluc|OJZ92365.1 anig|EHA26608.1 aory|EIT80862.1 asyd|OJJ57516.1 ater|EAU35668.1 atub|OJI86795.1 aver|OJJ05663.1 awen|OJJ40535.1 azon|OJJ46849.1 azon|OJJ49396.1 afla|EED47694.1

Group1190: abra|OJJ74880.1 acar|OOF95883.1 acla|EAW08490.1 afis|EAW23536.1 afla|EED57434.1 afum|EAL87930.1 agla|OJJ86791.1 akaw|GAA83540.1 aluc|OJZ92457.1 anid|CBF89670.1 anig|EHA26510.1 aory|EIT80126.1 asyd|OJJ62272.1 ater|EAU35322.1 atub|OJI86886.1 aver|OJI96381.1 awen|OJJ39866.1 azon|OJJ43234.1 afla|EED57433.1

Group1191: abra|OJJ74903.1 acar|OOF95901.1 acla|EAW08469.1 afis|EAW23516.1 afla|EED57417.1 afum|EAL87947.2 agla|OJJ86764.1 akaw|GAA83519.1 aluc|OJZ92476.1 anid|CBF89704.1 anig|EHA26494.1 aory|EIT80064.1 asyd|OJJ62253.1 ater|EAU35305.1 atub|OJI86907.1 aver|OJI96363.1 awen|OJJ39841.1 azon|OJJ43251.1 aory|EIT80158.1

Group1192: abra|OJJ74942.1 acar|OOF97373.1 acla|EAW08428.1 afis|EAW23472.1 afla|EED57368.1 afum|EAL87994.1 agla|OJJ86711.1 akaw|GAA83479.1 aluc|OJZ92516.1 anid|CBF89779.1 anig|EHA26459.1 aory|EIT80072.1 asyd|OJJ62207.1 ater|EAU36908.1 atub|OJI86949.1 aver|OJI96319.1 awen|OJJ39798.1 azon|OJJ47919.1 awen|OJJ40002.1

Group1193: abra|OJJ75019.1 acar|OOF89964.1 akaw|GAA88640.1 aluc|OJZ92586.1 anid|CBF84112.1 anig|EHA26399.1 atub|OJI87018.1 abra|OJJ75020.1 acla|EAW08212.1 afis|EAW23246.1 afla|EED55727.1 afum|EAL88235.1 agla|OJJ86614.1 aory|EIT83220.1 asyd|OJJ57103.1 ater|EAU37528.1 aver|OJJ03196.1 awen|OJJ39724.1 azon|OJJ47850.1

Group1194: abra|OJJ75054.1 acar|OOF97283.1 acla|EAW08239.1 afis|EAW23273.1 afla|EED51546.1 afum|EAL88206.1 agla|OJJ86575.1 akaw|GAA88667.1 aluc|OJZ92619.1 anid|CBF89542.1 anig|EHA26369.1 aory|EIT77094.1 asyd|OJJ62380.1 ater|EAU37558.1 atub|OJI87053.1 aver|OJI96514.1 awen|OJJ39684.1 azon|OJJ47821.1 aory|EIT80554.1

Group1195: abra|OJJ75063.1 acar|OOF97271.1 acla|EAW08246.1 afis|EAW23280.1 afla|EED55696.1 afum|EAL88199.1 agla|OJJ86567.1 akaw|GAA88675.1 aluc|OJZ92629.1 anid|CBF89529.1 anig|EHA26361.1 aory|EIT77085.1 asyd|OJJ62387.1 ater|EAU37578.1 atub|OJI87059.1 aver|OJI96520.1 awen|OJJ39677.1 azon|OJJ43147.1 awen|OJJ31335.1

Group1196: abra|OJJ75119.1 acar|OOF97214.1 acla|EAW09724.1 afis|EAW21417.1 afum|EAL92432.2 aluc|OJZ92686.1 anig|EHA26314.1 atub|OJI87114.1 azon|OJJ50750.1 abra|OJJ75768.1 afla|EED52344.1 agla|OJJ78972.1 aory|EIT74507.1 asyd|OJJ58674.1 atub|OJI87190.1 aver|OJJ01285.1 awen|OJJ30922.1 asyd|OJJ52504.1 aver|OJJ03875.1

Group1197: aver|OJJ04160.1 afis|EAW19670.1 afla|EED46493.1 afum|EAL92627.1 agla|OJJ86969.1 anid|CBF74912.1 anig|EHA22175.1 aory|EIT72425.1 asyd|OJJ60375.1 ater|EAU35595.1 atub|OJI91472.1 aver|OJJ05939.1 awen|OJJ39537.1 azon|OJJ51156.1 agla|OJJ86474.1 asyd|OJJ53618.1 awen|OJJ30584.1 asyd|OJJ60102.1 asyd|OJJ53622.1

Group1198: abra|OJJ75142.1 acar|OOF97194.1 anid|CBF74890.1 anig|EHA26291.1 asyd|OJJ60105.1 ater|EAU35601.1 aver|OJJ04157.1 awen|OJJ39534.1 abra|OJJ76897.1 afla|EED44885.1 agla|OJJ86440.1 akaw|GAA87147.1 aluc|OJZ84290.1 anig|EHA27630.1 aory|EIT83262.1 asyd|OJJ57332.1 atub|OJI89779.1 aver|OJJ05829.1 awen|OJJ40829.1

Group1199: abra|OJJ75173.1 acar|OOF94605.1 acla|EAW13003.1 afis|EAW17665.1 afla|EED50492.1 afum|EAL91479.1 agla|OJJ79538.1 akaw|GAA88780.1 aluc|OJZ92741.1 anid|CBF90049.1 anig|EHA26262.1 aory|EIT82090.1 asyd|OJJ62054.1 ater|EAU31988.1 atub|OJI87166.1 aver|OJI96160.1 awen|OJJ37176.1 azon|OJJ50876.1 asyd|OJJ61465.1

Group1200: abra|OJJ75218.1 acar|OOF94566.1 acla|EAW12260.1 afis|EAW16969.1 afla|EED48292.1 afum|EAL86497.1 agla|OJJ79785.1 akaw|GAA91768.1 aluc|OJZ92782.1 anig|EHA26228.1 aory|EIT76281.1

asyd|OJJ64772.1 atub|OJI87224.1 aver|OJI98872.1 awen|OJJ35998.1 azon|OJJ50271.1 abra|OJJ76878.1 afum|EAL87809.1 ater|EAU38553.1

Group1201: abra|OJJ75219.1 acar|OOF94565.1 acla|EAW12259.1 afis|EAW16978.1 afla|EED48290.1 afum|EAL86504.1 agla|OJJ79786.1 akaw|GAA91769.1 aluc|OJZ92783.1 anig|EHA26227.1 aory|EIT76317.1 asyd|OJJ64771.1 atub|OJI87225.1 aver|OJI98871.1 awen|OJJ35997.1 azon|OJJ50269.1 anig|EHA26226.1 afum|EAL86503.1 afla|EED48291.1

Group1202: abra|OJJ75302.1 acar|OOF92541.1 acla|EAW09084.1 afis|EAW25675.1 afla|EED47643.1 afum|EAL84345.1 agla|OJJ86330.1 akaw|GAA91835.1 aluc|OJZ92857.1 anid|CBF78280.1 anig|EHA26149.1 aory|EIT76311.1 asyd|OJJ52225.1 ater|EAU36081.1 atub|OJI87314.1 aver|OJJ05016.1 awen|OJJ35856.1 azon|OJJ42600.1 asyd|OJJ56073.1

Group1203: abra|OJJ75308.1 acar|OOF94521.1 acla|EAW12232.1 afis|EAW16996.1 afla|EED48959.1 afum|EAL86520.1 agla|OJJ79823.1 akaw|GAA91992.1 aluc|OJZ92862.1 anid|CBF87466.1 anig|EHA26103.1 aory|EIT80011.1 asyd|OJJ52936.1 ater|EAU31732.1 atub|OJI87320.1 aver|OJJ08903.1 awen|OJJ35985.1 azon|OJJ47407.1 azon|OJJ50280.1

Group1204: abra|OJJ75752.1 acar|OOG00116.1 acla|EAW09398.1 afis|EAW16786.1 afla|EED47412.1 afum|EAL86356.1 agla|OJJ78549.1 akaw|GAA84485.1 aluc|OJZ89206.1 anid|CBF84608.1 anig|EHA23353.1 aory|EIT81898.1 asyd|OJJ60434.1 ater|EAU37659.1 atub|OJI85586.1 aver|OJI99756.1 awen|OJJ40856.1 azon|OJJ42408.1 ater|EAU30953.1

Group1205: abra|OJJ75970.1 acar|OOF99950.1 acla|EAW09828.1 afis|EAW21305.1 afla|EED57265.1 afum|EAL92321.1 akaw|GAA88986.1 aluc|OJZ89417.1 anid|CBF71434.1 anig|EHA23171.1 aory|EIT73994.1 asyd|OJJ65347.1 ater|EAU35936.1 atub|OJI85377.1 aver|OJJ07224.1 awen|OJJ39393.1 azon|OJJ50301.1 asyd|OJJ54967.1 ater|EAU32173.1

Group1206: abra|OJJ75996.1 acar|OOF99929.1 acla|EAW09809.1 afis|EAW21327.1 afla|EED57285.1 afum|EAL92345.1 agla|OJJ87412.1 akaw|GAA88963.1 aluc|OJZ89442.1 anid|CBF83428.1 anig|EHA23144.1 aory|EIT74010.1 asyd|OJJ60659.1 ater|EAU35952.1 atub|OJI85351.1 aver|OJI99982.1 awen|OJJ39371.1 azon|OJJ51711.1 afla|EED57284.1

Group1207: abra|OJJ76229.1 acar|OOF99477.1 acla|EAW07053.1 afis|EAW16679.1 afla|EED45240.1 afum|EAL89283.1 akaw|GAA82167.1 aluc|OJZ92000.1 anid|CBF82050.1 aory|EIT73588.1 asyd|OJJ54232.1 ater|EAU29467.1 atub|OJI86434.1 aver|OJJ05185.1 awen|OJJ36977.1 azon|OJJ42100.1 akaw|GAA92942.1 atub|OJI86436.1 akaw|GAA82166.1

Group1208: abra|OJJ76593.1 acar|OOF99394.1 acla|EAW14308.1 afis|EAW18977.1 afla|EED53193.1 afum|EAL93713.2 agla|OJJ82935.1 akaw|GAA82497.1 aluc|OJZ91613.1 anid|CBF69648.1 anig|EHA22642.1 aory|EIT74974.1 asyd|OJJ59078.1 ater|EAU38168.1 atub|OJI86066.1 aver|OJJ01671.1 awen|OJJ31583.1 azon|OJJ47298.1 afla|EED53194.1

Group1209: abra|OJJ76704.1 acar|OOF89909.1 acla|EAW07574.1 afis|EAW22608.1 afla|EED49806.1 afum|EAL90590.1 agla|OJJ87452.1 akaw|GAA92495.1 aluc|OJZ91507.1 anid|CBF80167.1 aory|EIT77615.1 asyd|OJJ63679.1 atub|OJI85959.1 aver|OJI97260.1 awen|OJJ35236.1 azon|OJJ49875.1 agla|OJJ80978.1 anid|CBF88267.1 aver|OJI99521.1

Group1210: abra|OJJ76824.1 acar|OOF91318.1 acla|EAW13384.1 afis|EAW20498.1 afla|EED44740.1 afum|EAL84556.1 akaw|GAA87198.1 aluc|OJZ84235.1 anid|CBF79206.1 anig|EHA27582.1 aory|EIT77799.1 asyd|OJJ55728.1 ater|EAU37192.1 atub|OJI89850.1 aver|OJJ06814.1 awen|OJJ33198.1 azon|OJJ46398.1 aory|EIT77801.1 afla|EED44741.1

Group1211: abra|OJJ76859.1 acar|OOF91347.1 acla|EAW13420.1 afis|EAW20531.1 afla|EED52481.1 afum|EAL84526.1 agla|OJJ84700.1 akaw|GAA87163.1 aluc|OJZ84271.1 anid|CBF79002.1 anig|EHA27613.1 aory|EIT82813.1 asyd|OJJ55689.1 ater|EAU32506.1 atub|OJI89812.1 aver|OJJ06773.1 awen|OJJ33238.1 azon|OJJ50610.1 afis|EAW15383.1

Group1212: abra|OJJ76952.1 acar|OOF91399.1 acla|EAW13478.1 afis|EAW20585.1 afla|EED52259.1 afum|EAL84480.1 agla|OJJ80829.1 akaw|GAA87090.1 aluc|OJZ84348.1 anid|CBF78926.1 anig|EHA27685.1 aory|EIT76185.1 asyd|OJJ55629.1 ater|EAU37094.1 atub|OJI89720.1 aver|OJJ06706.1 awen|OJJ33298.1 azon|OJJ50675.1 afis|EAW16394.1

Group1213: abra|OJJ76962.1 acla|EAW13481.1 afis|EAW20589.1 afla|EED52239.1 afum|EAL84472.1 akaw|GAA87081.1 aluc|OJZ84357.1 anid|CBF80645.1 anig|EHA27694.1 aory|EIT74320.1 asyd|OJJ54615.1 ater|EAU35228.1 atub|OJI89710.1 aver|OJJ07544.1 azon|OJJ44240.1 acla|EAW11256.1 afis|EAW18243.1 afum|EAL87607.1 ater|EAU38946.1

Group1214: abra|OJJ77019.1 acla|EAW12022.1 afis|EAW20396.1 afla|EED51074.1 afum|EAL86857.1 akaw|GAA87033.1 aluc|OJZ84419.1 anig|EHA27741.1 aory|EIT83235.1 asyd|OJJ61467.1 ater|EAU34588.1 atub|OJI89655.1 aver|OJJ07304.1 awen|OJJ32287.1 afis|EAW19666.1 aory|EIT77057.1 ater|EAU30085.1 ater|EAU31682.1 aver|OJJ00806.1

Group1215: abra|OJJ77072.1 acar|OOF96452.1 acla|EAW11107.1 afis|EAW23663.1 afla|EED48431.1 afum|EAL85154.2 agla|OJJ81757.1 aluc|OJZ84480.1 anid|CBF75932.1 aory|EIT81669.1 atub|OJI89599.1 aver|OJI99663.1 awen|OJJ32900.1 afla|EED51081.1 aory|EIT77059.1 agla|OJJ80484.1 awen|OJJ40921.1 awen|OJJ40116.1 awen|OJJ32943.1

Group1216: akaw|GAA86981.1 anig|EHA27470.1 azon|OJJ46863.1 acla|EAW08585.1 afis|EAW16399.1 afum|EAL89027.1 azon|OJJ47065.1 acla|EAW11106.1 afla|EED48430.1 agla|OJJ80486.1 aory|EIT76978.1 atub|OJI88846.1 awen|OJJ32901.1 afla|EED51080.1 agla|OJJ88498.1 akaw|GAA92594.1 aluc|OJZ82611.1 afla|EED54189.1 awen|OJJ34609.1

Group1217: abra|OJJ77092.1 acar|OOG00596.1 afis|EAW17058.1 afla|EED52120.1 afum|EAL86554.1 akaw|GAA86963.1 aluc|OJZ84501.1 anid|CBF88306.1 anig|EHA27801.1 asyd|OJJ62781.1 ater|EAU31929.1 atub|OJI89579.1 aver|OJI96826.1 azon|OJJ45055.1 acar|OOF90672.1 aory|EIT74489.1 azon|OJJ43507.1 afla|EED52121.1 anid|CBF88998.1

Group1218: abra|OJJ77232.1 acar|OOF93114.1 afla|EED48701.1 agla|OJJ83280.1 akaw|GAA90710.1 aluc|OJZ84642.1 anid|CBF78734.1 anig|EHA27928.1 aory|EIT73111.1 asyd|OJJ55609.1 atub|OJI89439.1 aver|OJJ06684.1 awen|OJJ30084.1 abra|OJJ77423.1 acla|EAW13221.1 afis|EAW20821.1 afum|EAL84808.1 ater|EAU31194.1 awen|OJJ31931.1

Group1219: abra|OJJ77364.1 acar|OOF93233.1 acla|EAW13112.1 afis|EAW17552.1 afla|EED49273.1 afum|EAL91366.1 agla|OJJ81416.1 akaw|GAA91398.1 aluc|OJZ84773.1 anid|CBF90298.1 anig|EHA28041.1 aory|EIT82235.1 asyd|OJJ61925.1 ater|EAU36982.1 atub|OJI89308.1 aver|OJI96017.1 awen|OJJ37301.1 azon|OJJ50994.1 anid|CBF87295.1

Group1220: abra|OJJ77453.1 acar|OOF99273.1 acla|EAW12649.1 afis|EAW18036.1 afla|EED54071.1 afum|EAL91847.1 agla|OJJ78571.1 akaw|GAA84155.1 aluc|OJZ81076.1 anid|CBF81938.1 anig|EHA19286.1 aory|EIT76067.1 asyd|OJJ57409.1 ater|EAU29318.1 ater|EAU31609.1 atub|OJI82509.1 aver|OJJ05772.1 awen|OJJ34693.1 azon|OJJ48230.1

Group1221: abra|OJJ77467.1 acar|OOF99267.1 acla|EAW07127.1 afla|EED54176.1 agla|OJJ87943.1 akaw|GAA84150.1 aluc|OJZ81071.1 anig|EHA19298.1 aory|EIT75990.1 asyd|OJJ52794.1 ater|EAU29321.1 atub|OJI82515.1 aver|OJJ08772.1 awen|OJJ34711.1 akaw|GAA91805.1 atub|OJI87284.1 asyd|OJJ64953.1 aver|OJI99075.1 aluc|OJZ92827.1

Group1222: abra|OJJ77471.1 acar|OOF99253.1 acla|EAW06761.1 afis|EAW16311.1 afla|EED54184.1 afum|EAL88933.1 agla|OJJ88270.1 akaw|GAA84146.1 aluc|OJZ81067.1 anid|CBF69560.1 anig|EHA19302.1 asyd|OJJ59156.1 ater|EAU29331.1 atub|OJI88508.1 aver|OJJ01746.1 awen|OJJ37711.1 azon|OJJ44970.1 aluc|OJZ81673.1 anig|EHA25767.1

Group1223: abra|OJJ77555.1 acar|OOF99172.1 acla|EAW06522.1 afis|EAW16238.1 afla|EED53339.1 afum|EAL88859.1 agla|OJJ86247.1 akaw|GAA84068.1 aluc|OJZ80982.1 anid|CBF78861.1 anig|EHA19378.1 aory|EIT78064.1 asyd|OJJ64575.1 atub|OJI82604.1 aver|OJI98672.1 awen|OJJ30219.1 azon|OJJ43941.1 afla|EED50785.1 agla|OJJ79233.1

Group1224: abra|OJJ77564.1 acar|OOF99168.1 acla|EAW06526.1 afis|EAW16070.1 afla|EED48589.1 afum|EAL88702.1 agla|OJJ86098.1 akaw|GAA84062.1 aluc|OJZ80974.1 anid|CBF75103.1 anig|EHA19385.1 aory|EIT83270.1 asyd|OJJ60005.1 ater|EAU33561.1 atub|OJI82611.1 aver|OJJ02281.1 awen|OJJ30365.1 azon|OJJ45384.1 afla|EED57884.1

Group1225: abra|OJJ77597.1 acar|OOF99129.1 acla|EAW06561.1 afis|EAW16035.1 afla|EED56421.1 afum|EAL88666.2 agla|OJJ86065.1 akaw|GAA84031.1 aluc|OJZ80937.1 anig|EHA19414.1 aory|EIT72879.1 ater|EAU33605.1 atub|OJI82644.1 awen|OJJ34579.1 azon|OJJ45420.1 aluc|OJZ83622.1 akaw|GAA92103.1 anig|EHA27514.1 atub|OJI87849.1

Group1226: abra|OJJ77599.1 acar|OOF99127.1 acla|EAW06563.1 afis|EAW16033.1 afla|EED56415.1 afla|EED56416.1 afum|EAL88664.1 agla|OJJ86061.1 akaw|GAA84029.1 aluc|OJZ80934.1 anid|CBF75034.1 anig|EHA19416.1 aory|EIT72886.1 asyd|OJJ60050.1 ater|EAU33608.1 atub|OJI82646.1 aver|OJJ02239.1 awen|OJJ30414.1 azon|OJJ45422.1

Group1227: abra|OJJ77664.1 acar|OOF99071.1 acla|EAW06508.1 afis|EAW24531.1 afla|EED56377.1 agla|OJJ85724.1 akaw|GAA83971.1 aluc|OJZ80864.1 anig|EHA19471.1 aory|EIT73456.1 ater|EAU33642.1 atub|OJI82713.1 aver|OJJ02022.1 awen|OJJ30465.1 azon|OJJ45455.1 afum|EAL86197.1 asyd|OJJ54667.1 afum|EAL86196.1 agla|OJJ86406.1

Group1228: abra|OJJ77796.1 acar|OOF90711.1 akaw|GAA89038.1 aluc|OJZ80557.1 anig|EHA25640.1 atub|OJI88374.1 aver|OJJ02063.1 awen|OJJ38007.1 abra|OJJ77916.1 afla|EED51608.1 agla|OJJ82626.1 anid|CBF73906.1 anig|EHA18406.1 aory|EIT80458.1 asyd|OJJ53666.1 ater|EAU37641.1 aver|OJJ07773.1 awen|OJJ40190.1 agla|OJJ83009.1

Group1229: abra|OJJ77806.1 acar|OOF98961.1 afis|EAW18189.1 afla|EED56286.1 afum|EAL87553.1 agla|OJJ86044.1 akaw|GAA83843.1 aluc|OJZ90873.1 anid|CBF86100.1 anig|EHA19593.1 aory|EIT73443.1

asyd|OJJ61783.1 ater|EAU33804.1 atub|OJI82872.1 aver|OJI95883.1 awen|OJJ30553.1 azon|OJJ45513.1 aver|OJI98581.1 asyd|OJJ64479.1

Group1230: abra|OJJ77818.1 acar|OOF98950.1 acla|EAW11305.1 afis|EAW18192.1 afla|EED56282.1 afum|EAL87560.1 agla|OJJ85885.1 akaw|GAA83828.1 aluc|OJZ90890.1 anid|CBF86109.1 anig|EHA19609.1 aory|EIT73431.1 asyd|OJJ64488.1 ater|EAU33797.1 atub|OJI82884.1 aver|OJI98589.1 awen|OJJ30564.1 azon|OJJ45507.1 aory|EIT73332.1

Group1231: abra|OJJ77831.1 acar|OOF98939.1 acla|EAW11295.1 afis|EAW18207.1 afla|EED56264.1 afum|EAL87572.1 agla|OJJ85875.1 akaw|GAA83816.1 aluc|OJZ90905.1 anid|CBF86133.1 anig|EHA19620.1 aory|EIT73347.1 asyd|OJJ64503.1 ater|EAU33782.1 atub|OJI82898.1 aver|OJI98602.1 awen|OJJ30579.1 azon|OJJ45497.1 afla|EED56263.1

Group1232: abra|OJJ77887.1 acar|OOF90364.1 afis|EAW17355.1 afla|EED49462.1 agla|OJJ82474.1 akaw|GAA82230.1 aluc|OJZ91918.1 anig|EHA19662.1 aory|EIT79498.1 asyd|OJJ56938.1 ater|EAU29950.1 aver|OJJ03347.1 awen|OJJ32673.1 azon|OJJ46453.1 asyd|OJJ57438.1 aver|OJJ05740.1 awen|OJJ31966.1 asyd|OJJ57599.1 anig|EHA23994.1

Group1233: abra|OJJ77901.1 acar|OOF98882.1 acla|EAW06511.1 afis|EAW16229.1 afla|EED53352.1 afum|EAL88852.1 agla|OJJ86237.1 akaw|GAA83761.1 aluc|OJZ90970.1 anid|CBF86221.1 anig|EHA19672.1 aory|EIT75062.1 asyd|OJJ64562.1 ater|EAU33715.1 atub|OJI82961.1 aver|OJI98659.1 awen|OJJ30233.1 azon|OJJ43950.1 anid|CBF86220.1

Group1234: abra|OJJ78153.1 acar|OOF98713.1 acla|EAW12149.1 afis|EAW17172.1 afla|EED45420.1 afum|EAL86603.1 akaw|GAA85873.1 aluc|OJZ91205.1 anig|EHA19910.1 aory|EIT82401.1 ater|EAU31964.1 atub|OJI83222.1 aver|OJJ03361.1 awen|OJJ31986.1 azon|OJJ49155.1 akaw|GAA92449.1 atub|OJI88616.1 aluc|OJZ81569.1 anig|EHA25894.1

Group1235: abra|OJJ78155.1 acar|OOF90381.1 acla|EAW11243.1 afis|EAW18260.1 afla|EED48719.1 afum|EAL87623.1 agla|OJJ88167.1 akaw|GAA85871.1 aluc|OJZ91207.1 anid|CBF79523.1 anig|EHA19911.1 aory|EIT73142.1 asyd|OJJ57893.1 ater|EAU33268.1 atub|OJI83223.1 aver|OJJ04502.1 awen|OJJ30632.1 awen|OJJ30633.1 azon|OJJ47984.1

Group1236: abra|OJJ78254.1 acar|OOF98608.1 acla|EAW09255.1 afis|EAW21892.1 afla|EED46416.1 afum|EAL92906.1 agla|OJJ84240.1 akaw|GAA85772.1 aluc|OJZ91328.1 anid|CBF76804.1 anig|EHA20010.1 aory|EIT72374.1 asyd|OJJ61288.1 atub|OJI83321.1 aver|OJJ00614.1 awen|OJJ34181.1 azon|OJJ43451.1 acar|OOF99280.1 ater|EAU36657.1

Group1237: abra|OJJ78362.1 acar|OOF98525.1 acla|EAW09173.1 afis|EAW21973.1 afla|EED46696.1 afum|EAL92988.1 agla|OJJ84129.1 akaw|GAA85671.1 aluc|OJZ91436.1 anid|CBF82714.1 anig|EHA20102.1 aory|EIT77832.1 asyd|OJJ61638.1 ater|EAU31091.1 atub|OJI83430.1 aver|OJJ00961.1 awen|OJJ34266.1 azon|OJJ43370.1 aory|EIT77819.1

Group1238: abra|OJJ68129.1 abra|OJJ70102.1 acla|EAW12416.1 akaw|GAA87598.1 akaw|GAA86909.1 aluc|OJZ85218.1 aluc|OJZ90032.1 anig|EHA18784.1 anig|EHA27408.1 anig|EHA25422.1 atub|OJI80367.1 atub|OJI84774.1 awen|OJJ39118.1 anid|CBF75781.1 ater|EAU32451.1 azon|OJJ42520.1 afla|EED47323.1 aory|EIT82878.1

Group1239: abra|OJJ73597.1 abra|OJJ74454.1 acar|OOF95298.1 acar|OOG00783.1 akaw|GAA87348.1 akaw|GAA88555.1 aluc|OJZ88453.1 aluc|OJZ89666.1 anig|EHA18174.1 anig|EHA26903.1 atub|OJI85134.1 atub|OJI91051.1 afla|EED49261.1 aory|EIT82199.1 asyd|OJJ62434.1 ater|EAU29287.1 aver|OJI96570.1 aver|OJI98026.1

Group1240: acar|OOF90451.1 acar|OOF90458.1 abra|OJJ71881.1 acla|EAW13910.1 afis|EAW18622.1 afla|EED52720.1 afum|EAL93332.1 agla|OJJ81238.1 akaw|GAA85035.1 aluc|OJZ86619.1 anid|CBF70495.1 anig|EHA22047.1 asyd|OJJ58582.1 ater|EAU34922.1 atub|OJI83907.1 aver|OJJ01186.1 awen|OJJ32085.1 azon|OJJ46664.1

Group1241: acar|OOF90710.1 acar|OOF98737.1 atub|OJI84596.1 atub|OJI89205.1 abra|OJJ65987.1 afis|EAW19527.1 afla|EED49678.1 agla|OJJ87966.1 akaw|GAA90861.1 aluc|OJZ82972.1 anid|CBF78948.1 anig|EHA21389.1 aory|EIT80959.1 asyd|OJJ55653.1 ater|EAU29344.1 aver|OJJ06737.1 awen|OJJ30016.1 azon|OJJ43646.1

Group1242: acar|OOF91721.1 acar|OOF91725.1 abra|OJJ73107.1 afis|EAW19523.1 afla|EED54039.1 afum|EAL84403.1 akaw|GAA88059.1 aluc|OJZ81321.1 anid|CBF87177.1 anig|EHA18627.1 aory|EIT74598.1 asyd|OJJ63343.1 ater|EAU32279.1 atub|OJI91524.1 aver|OJI97618.1 azon|OJJ45982.1 afis|EAW25732.1 ater|EAU37760.1

Group1243: acar|OOF92284.1 acar|OOF92325.1 abra|OJJ67518.1 acla|EAW06257.1 afis|EAW18364.1 afla|EED53041.1 afum|EAL93037.1 agla|OJJ84927.1 aluc|OJZ84846.1 anid|CBF70821.1 anig|EHA25081.1 aory|EIT77411.1 asyd|OJJ58401.1 ater|EAU32232.1 atub|OJI80749.1 aver|OJJ01002.1 awen|OJJ31787.1 azon|OJJ42024.1

Group1244: acar|OOF92296.1 acar|OOF92337.1 abra|OJJ67504.1 acla|EAW06266.1 afis|EAW18372.1 afla|EED53034.1 afum|EAL93047.1 agla|OJJ84936.1 akaw|GAA86334.1 aluc|OJZ84859.1 anid|CBF70802.1 anig|EHA25092.1 asyd|OJJ58410.1 ater|EAU32242.1 atub|OJI80737.1 aver|OJJ01012.1 awen|OJJ31778.1 azon|OJJ50447.1

Group1245: acar|OOF92300.1 acar|OOF92341.1 abra|OJJ67500.1 acla|EAW06270.1 afis|EAW18376.1 afla|EED53030.1 afum|EAL93051.1 agla|OJJ84940.1 akaw|GAA86338.1 aluc|OJZ84863.1 anid|CBF70795.1 aory|EIT77422.1 asyd|OJJ58414.1 ater|EAU32246.1 atub|OJI80733.1 aver|OJJ01016.1 awen|OJJ31768.1 azon|OJJ50451.1

Group1246: acar|OOF92302.1 acar|OOF92344.1 abra|OJJ67497.1 acla|EAW06273.1 afis|EAW18379.1 afla|EED53027.1 afum|EAL93054.1 agla|OJJ84943.1 akaw|GAA86341.1 aluc|OJZ84866.1 anid|CBF70789.1 anig|EHA25098.1 aory|EIT77417.1 asyd|OJJ58417.1 atub|OJI80730.1 aver|OJJ01019.1 awen|OJJ31771.1 azon|OJJ50454.1

Group1247: acar|OOF92313.1 acar|OOF92354.1 abra|OJJ67486.1 acla|EAW06288.1 afis|EAW18393.1 afla|EED53014.1 afum|EAL93069.1 agla|OJJ84960.1 akaw|GAA86353.1 aluc|OJZ84882.1 anid|CBF70764.1 anig|EHA25111.1 asyd|OJJ58429.1 ater|EAU32281.1 atub|OJI80718.1 aver|OJJ01031.1 azon|OJJ50467.1 awen|OJJ31757.1

Group1248: acar|OOF92613.1 acar|OOF92634.1 abra|OJJ68141.1 acla|EAW07634.1 afis|EAW22654.1 afla|EED49922.1 afum|EAL90538.1 agla|OJJ81037.1 akaw|GAA92377.1 aluc|OJZ83236.1 anid|CBF88199.1 anig|EHA21093.1 asyd|OJJ63635.1 ater|EAU39052.1 atub|OJI87700.1 aver|OJI97301.1 awen|OJJ35269.1 azon|OJJ49838.1

Group1249: acar|OOF93658.1 acar|OOF93742.1 abra|OJJ66637.1 acla|EAW11780.1 afis|EAW19390.1 afla|EED50348.1 afum|EAL87105.1 agla|OJJ81101.1 akaw|GAA89590.1 aluc|OJZ80812.1 anig|EHA27222.1 aory|EIT81938.1 asyd|OJJ51911.1 ater|EAU36053.1 atub|OJI87661.1 aver|OJJ05352.1 awen|OJJ36783.1 azon|OJJ43015.1

Group1250: acar|OOF93662.1 acar|OOF93746.1 abra|OJJ66642.1 acla|EAW11785.1 afis|EAW19395.1 afum|EAL87099.1 agla|OJJ81106.1 akaw|GAA89595.1 aluc|OJZ80807.1 anid|CBF74279.1 anig|EHA27227.1 aory|EIT83136.1 asyd|OJJ51860.1 ater|EAU36121.1 atub|OJI87655.1 aver|OJJ05359.1 awen|OJJ36789.1 azon|OJJ43009.1

Group1251: acar|OOF93664.1 acar|OOF93748.1 abra|OJJ66644.1 acla|EAW11787.1 afis|EAW19397.1 afla|EED50353.1 afum|EAL87097.1 agla|OJJ81108.1 akaw|GAA89597.1 aluc|OJZ80805.1 anid|CBF74276.1 anig|EHA27229.1 aory|EIT83127.1 ater|EAU36123.1 atub|OJI87653.1 aver|OJJ05361.1 awen|OJJ36792.1 azon|OJJ43007.1

Group1252: acar|OOF93684.1 acar|OOF93769.1 abra|OJJ66669.1 acla|EAW11810.1 afis|EAW19420.1 afla|EED50377.1 afum|EAL87074.1 agla|OJJ81132.1 akaw|GAA89618.1 aluc|OJZ80778.1 anid|CBF74369.1 anig|EHA27251.1 aory|EIT83135.1 asyd|OJJ57783.1 atub|OJI87630.1 aver|OJJ05382.1 awen|OJJ36818.1 azon|OJJ42984.1

Group1253: acar|OOF93697.1 acar|OOF93782.1 abra|OJJ66688.1 acla|EAW11823.1 afis|EAW19434.1 afla|EED56639.1 afum|EAL87061.1 agla|OJJ81146.1 akaw|GAA89632.1 aluc|OJZ80761.1 anid|CBF81381.1 anig|EHA27266.1 aory|EIT79620.1 asyd|OJJ59392.1 ater|EAU34736.1 atub|OJI87615.1 aver|OJJ02880.1 azon|OJJ42971.1

Group1254: acar|OOF93702.1 acar|OOF93788.1 abra|OJJ66694.1 acla|EAW11829.1 afis|EAW19441.1 afla|EED56648.1 afum|EAL87055.1 agla|OJJ81154.1 akaw|GAA89638.1 aluc|OJZ80751.1 anid|CBF81393.1 aory|EIT79649.1 asyd|OJJ59399.1 ater|EAU34742.1 atub|OJI87608.1 aver|OJJ02873.1 awen|OJJ36841.1 azon|OJJ42959.1

Group1255: acar|OOF93712.1 acar|OOF93798.1 abra|OJJ66710.1 acla|EAW11841.1 afis|EAW19452.1 afla|EED56660.1 afum|EAL87044.1 agla|OJJ81167.1 akaw|GAA89652.1 aluc|OJZ80737.1 anid|CBF81416.1 anig|EHA27285.1 asyd|OJJ59412.1 ater|EAU34754.1 atub|OJI87594.1 aver|OJJ02861.1 azon|OJJ42947.1 awen|OJJ36853.1

Group1256: acar|OOF93719.1 acar|OOF93806.1 abra|OJJ66717.1 acla|EAW11848.1 afis|EAW19459.1 afla|EED56670.1 afum|EAL87037.1 agla|OJJ81176.1 akaw|GAA89659.1 aluc|OJZ80730.1 anig|EHA27291.1 aory|EIT79651.1 asyd|OJJ59419.1 ater|EAU34762.1 atub|OJI87586.1 aver|OJJ02850.1 awen|OJJ36861.1 azon|OJJ42940.1

Group1257: acar|OOF93720.1 acar|OOF93807.1 abra|OJJ66718.1 acla|EAW11849.1 afis|EAW19460.1 afla|EED56671.1 afum|EAL87036.1 agla|OJJ81177.1 akaw|GAA89660.1 aluc|OJZ80729.1 anid|CBF81429.1 anig|EHA27292.1 asyd|OJJ59420.1 ater|EAU34763.1 atub|OJI87585.1 aver|OJJ02849.1 awen|OJJ36862.1 azon|OJJ42939.1

Group1258: acar|OOF93730.1 acar|OOF93818.1 abra|OJJ66732.1 acla|EAW11860.1 afis|EAW19471.1 afla|EED56681.1 afum|EAL87025.1 agla|OJJ81192.1 akaw|GAA89671.1 aluc|OJZ80717.1 anid|CBF75472.1

anig|EHA27302.1 asyd|OJJ59760.1 ater|EAU34781.1 atub|OJI87573.1 aver|OJJ02528.1 awen|OJJ36876.1 azon|OJJ42928.1

Group1259: acar|OOF93732.1 acar|OOF93820.1 abra|OJJ66734.1 acla|EAW11862.1 afis|EAW19473.1 afla|EED57650.1 afum|EAL87023.1 agla|OJJ81195.1 akaw|GAA89673.1 aluc|OJZ80715.1 anid|CBF71137.1 anig|EHA27304.1 asyd|OJJ55833.1 ater|EAU34783.1 atub|OJI87571.1 aver|OJJ04775.1 awen|OJJ36878.1 azon|OJJ48920.1

Group1260: acar|OOF93733.1 acar|OOF93821.1 abra|OJJ66735.1 acla|EAW11863.1 afis|EAW19474.1 afum|EAL87022.1 agla|OJJ81194.1 akaw|GAA89674.1 aluc|OJZ80713.1 anid|CBF75476.1 anig|EHA27305.1 aory|EIT73892.1 asyd|OJJ59763.1 ater|EAU34784.1 atub|OJI87570.1 aver|OJJ02525.1 awen|OJJ36879.1 azon|OJJ42926.1

Group1261: acar|OOF93738.1 acar|OOF93827.1 abra|OJJ66742.1 acla|EAW11870.1 afis|EAW19481.1 afla|EED56691.1 afum|EAL87014.1 akaw|GAA89679.1 aluc|OJZ80707.1 anid|CBF75468.1 anig|EHA27311.1 aory|EIT73840.1 asyd|OJJ59765.1 ater|EAU34793.1 atub|OJI87564.1 aver|OJJ02523.1 awen|OJJ36887.1 azon|OJJ42920.1

Group1262: acar|OOF94028.1 acar|OOF94098.1 abra|OJJ66425.1 acla|EAW06805.1 afis|EAW16354.1 afla|EED53518.1 afum|EAL88979.1 akaw|GAA90289.1 aluc|OJZ83051.1 anid|CBF89224.1 anig|EHA28115.1 aory|EIT74854.1 asyd|OJJ62518.1 ater|EAU34107.1 atub|OJI87897.1 aver|OJI96637.1 awen|OJJ37758.1 azon|OJJ47345.1

Group1263: acar|OOF94037.1 acar|OOF94107.1 abra|OJJ71135.1 afla|EED48894.1 akaw|GAA82839.1 aluc|OJZ87400.1 anig|EHA20629.1 aory|EIT81248.1 atub|OJI81246.1 abra|OJJ74706.1 acar|OOF89904.1 akaw|GAA81911.1 aluc|OJZ92286.1 anig|EHA26682.1 atub|OJI86717.1 awen|OJJ33632.1 abra|OJJ76740.1 asyd|OJJ65386.1

Group1264: acar|OOF94049.1 acar|OOF94119.1 akaw|GAA85929.1 akaw|GAA90360.1 aluc|OJZ85872.1 aluc|OJZ91136.1 atub|OJI79658.1 atub|OJI83147.1 abra|OJJ78065.1 acla|EAW14269.1 afis|EAW19008.1 afla|EED49855.1 afum|EAL93742.1 anid|CBF71642.1 anig|EHA24713.1 asyd|OJJ61653.1 anid|CBF82888.1 awen|OJJ32641.1

Group1265: acar|OOF94065.1 acar|OOF94135.1 abra|OJJ66358.1 acla|EAW10008.1 afis|EAW21048.1 afla|EED51622.1 afum|EAL92084.1 akaw|GAA90232.1 aluc|OJZ83109.1 anid|CBF87100.1 anig|EHA28172.1 aory|EIT80748.1 asyd|OJJ60390.1 ater|EAU36820.1 atub|OJI87825.1 aver|OJI99719.1 awen|OJJ32174.1 azon|OJJ46753.1

Group1266: acar|OOF94088.1 acar|OOF94158.1 abra|OJJ66350.1 acla|EAW09997.1 afis|EAW21076.1 afla|EED51677.1 afum|EAL92099.1 akaw|GAA90223.1 aluc|OJZ83118.1 anid|CBF87050.1 anig|EHA28181.1 aory|EIT80671.1 asyd|OJJ60442.1 ater|EAU36800.1 atub|OJI87816.1 aver|OJI99760.1 awen|OJJ36350.1 azon|OJJ45249.1

Group1267: acar|OOF95043.1 acar|OOF95110.1 abra|OJJ70456.1 awen|OJJ40126.1 abra|OJJ75241.1 acla|EAW14126.1 afis|EAW18829.1 afla|EED52811.1 afum|EAL93563.1 akaw|GAA91784.1 aluc|OJZ92803.1 anig|EHA26206.1 aory|EIT77227.1 ater|EAU33497.1 atub|OJI87246.1 awen|OJJ39969.1 aory|EIT73400.1 awen|OJJ40159.1

Group1268: acar|OOF95074.1 acar|OOF95139.1 abra|OJJ74641.1 acla|EAW10747.1 afis|EAW24827.1 afla|EED47148.1 afum|EAL89472.1 agla|OJJ82016.1 akaw|GAA81970.1 aluc|OJZ92220.1 anid|CBF85861.1 anig|EHA26738.1 asyd|OJJ64368.1 ater|EAU36478.1 atub|OJI86651.1 aver|OJI98463.1 awen|OJJ40413.1 azon|OJJ49263.1

Group1269: acar|OOF95075.1 acar|OOF95141.1 abra|OJJ74639.1 acla|EAW10749.1 afis|EAW24825.1 afla|EED47150.1 afum|EAL89469.1 agla|OJJ82013.1 akaw|GAA81972.1 aluc|OJZ92218.1 anid|CBF85857.1 anig|EHA26740.1 asyd|OJJ64366.1 ater|EAU36480.1 atub|OJI86649.1 aver|OJI98461.1 awen|OJJ40411.1 azon|OJJ49260.1

Group1270: acar|OOF95077.1 acar|OOF95142.1 abra|OJJ74637.1 acla|EAW10754.1 afis|EAW24823.1 afla|EED47152.1 afum|EAL89467.1 agla|OJJ82011.1 akaw|GAA81974.1 aluc|OJZ92216.1 anid|CBF85853.1 anig|EHA26742.1 aory|EIT79248.1 asyd|OJJ64364.1 atub|OJI86647.1 aver|OJI98459.1 awen|OJJ40409.1 azon|OJJ49258.1

Group1271: acar|OOF95081.1 acar|OOF95146.1 abra|OJJ74628.1 acla|EAW10751.1 afis|EAW24820.1 afla|EED47156.1 afum|EAL89464.1 agla|OJJ82918.1 akaw|GAA81980.1 aluc|OJZ92210.1 anid|CBF85872.1 anig|EHA26748.1 asyd|OJJ64373.1 ater|EAU36485.1 atub|OJI86639.1 aver|OJI98469.1 awen|OJJ40406.1 azon|OJJ49255.1

Group1272: acar|OOF95103.1 acar|OOF95169.1 abra|OJJ74601.1 acla|EAW10778.1 afis|EAW24796.1 afum|EAL89438.1 agla|OJJ82892.1 akaw|GAA82007.1 aluc|OJZ92180.1 anid|CBF81504.1 anig|EHA26773.1 aory|EIT79228.1 asyd|OJJ59467.1 ater|EAU36509.1 atub|OJI86608.1 aver|OJJ02807.1 awen|OJJ40381.1 azon|OJJ49231.1

Group1273: acar|OOF95628.1 acar|OOF95679.1 abra|OJJ74663.1 acla|EAW10732.1 afis|EAW24843.1 afla|EED47128.1 afum|EAL89489.1 agla|OJJ82033.1 akaw|GAA81948.1 aluc|OJZ92240.1 anid|CBF75851.1 anig|EHA26714.1 aory|EIT79238.1 asyd|OJJ57572.1 ater|EAU36461.1 atub|OJI86674.1 aver|OJJ05607.1 awen|OJJ40428.1

Group1274: acar|OOF95634.1 acar|OOF95682.1 abra|OJJ74669.1 acla|EAW10727.1 afis|EAW24848.1 afla|EED47123.1 afum|EAL89494.1 agla|OJJ82060.1 akaw|GAA81943.1 aluc|OJZ92247.1 anid|CBF75841.1 anig|EHA26709.1 aory|EIT79325.1 asyd|OJJ57559.1 ater|EAU36455.1 atub|OJI86680.1 aver|OJJ05619.1 awen|OJJ40433.1

Group1275: acar|OOF95654.1 acar|OOF95705.1 abra|OJJ74694.1 acla|EAW10704.1 afis|EAW24866.1 afla|EED47107.1 afum|EAL89516.1 agla|OJJ82084.1 akaw|GAA81922.1 aluc|OJZ92271.1 anid|CBF75806.1 anig|EHA26690.1 asyd|OJJ57539.1 ater|EAU36434.1 atub|OJI86706.1 aver|OJJ05639.1 awen|OJJ40457.1 azon|OJJ49316.1

Group1276: acar|OOF95656.1 acar|OOF95707.1 abra|OJJ74696.1 acla|EAW10702.1 afis|EAW24868.1 afla|EED47108.1 afum|EAL89518.1 agla|OJJ82086.1 akaw|GAA81921.1 aluc|OJZ92273.1 anid|CBF75804.1 anig|EHA26689.1 asyd|OJJ57537.1 ater|EAU36432.1 atub|OJI86708.1 aver|OJJ05641.1 awen|OJJ40459.1 azon|OJJ49318.1

Group1277: acar|OOF95657.1 acar|OOF95708.1 abra|OJJ74697.1 acla|EAW10701.1 afis|EAW24869.1 afla|EED47109.1 afum|EAL89519.1 agla|OJJ82088.1 akaw|GAA81920.1 aluc|OJZ92276.1 anid|CBF75800.1 anig|EHA26688.1 asyd|OJJ57528.1 ater|EAU36431.1 atub|OJI86709.1 aver|OJJ05650.1 awen|OJJ40460.1 azon|OJJ49320.1

Group1278: acar|OOF95658.1 acar|OOF95709.1 abra|OJJ74699.1 acla|EAW10700.1 afis|EAW24870.1 afla|EED47110.1 afum|EAL89520.1 agla|OJJ82091.1 akaw|GAA81919.1 aluc|OJZ92277.1 anid|CBF75798.1 anig|EHA26687.1 asyd|OJJ57529.1 ater|EAU36430.1 atub|OJI86710.1 aver|OJJ05649.1 awen|OJJ40461.1 azon|OJJ49322.1

Group1279: acar|OOF96222.1 acar|OOF96260.1 abra|OJJ71506.1 acla|EAW15064.1 afis|EAW20036.1 afum|EAL85157.1 agla|OJJ88927.1 akaw|GAA89407.1 aluc|OJZ86245.1 anid|CBF84773.1 anig|EHA21619.1 aory|EIT73713.1 asyd|OJJ63239.1 ater|EAU38654.1 atub|OJI84297.1 aver|OJI97717.1 awen|OJJ31791.1 azon|OJJ46325.1

Group1280: acar|OOF96232.1 acar|OOF96269.1 abra|OJJ71516.1 acla|EAW15074.1 afis|EAW20045.1 afla|EED45335.1 afum|EAL85166.1 agla|OJJ88916.1 aluc|OJZ86255.1 anid|CBF84793.1 anig|EHA21611.1 aory|EIT73562.1 asyd|OJJ63228.1 ater|EAU38655.1 atub|OJI84288.1 aver|OJI97727.1 awen|OJJ31802.1 azon|OJJ46323.1

Group1281: acar|OOF96253.1 acar|OOF96292.1 abra|OJJ71548.1 acla|EAW15102.1 afis|EAW20074.1 afla|EED45305.1 afum|EAL85192.1 agla|OJJ88886.1 akaw|GAA89374.1 aluc|OJZ86286.1 anig|EHA21585.1 aory|EIT73597.1 asyd|OJJ63201.1 ater|EAU38682.1 atub|OJI84252.1 aver|OJI97754.1 awen|OJJ31833.1 azon|OJJ46291.1

Group1282: acar|OOF96903.1 acar|OOF96938.1 abra|OJJ70807.1 acla|EAW10044.1 afis|EAW25540.1 afum|EAL90203.1 agla|OJJ87262.1 akaw|GAA90121.1 aluc|OJZ84153.1 anid|CBF77829.1 anig|EHA19257.1 aory|EIT82996.1 asyd|OJJ53004.1 ater|EAU31663.1 atub|OJI90525.1 aver|OJJ03839.1 awen|OJJ36781.1 azon|OJJ43018.1

Group1283: acar|OOF96912.1 acar|OOF96947.1 abra|OJJ70798.1 acla|EAW11125.1 afis|EAW15854.1 afla|EED56518.1 afum|EAL88490.1 agla|OJJ87253.1 akaw|GAA90114.1 aluc|OJZ84143.1 anid|CBF81183.1 anig|EHA19251.1 asyd|OJJ59296.1 ater|EAU31669.1 atub|OJI90517.1 aver|OJJ02979.1 awen|OJJ36773.1 azon|OJJ47425.1

Group1284: acar|OOF96922.1 acar|OOF96957.1 abra|OJJ70788.1 acla|EAW11134.1 afis|EAW15866.1 afla|EED56509.1 afum|EAL88500.1 agla|OJJ87243.1 akaw|GAA90104.1 aluc|OJZ84133.1 anid|CBF81202.1 anig|EHA19241.1 asyd|OJJ59305.1 ater|EAU31679.1 atub|OJI90507.1 aver|OJJ02970.1 awen|OJJ36764.1 azon|OJJ47434.1

Group1285: acar|OOF96933.1 acar|OOF96968.1 asyd|OJJ59315.1 abra|OJJ70775.1 acla|EAW11144.1 afis|EAW15875.1 afla|EED56500.1 afum|EAL88509.2 agla|OJJ87233.1 aluc|OJZ84122.1 anid|CBF81224.1 anig|EHA19230.1 aory|EIT72804.1 ater|EAU31692.1 atub|OJI90494.1 aver|OJJ02960.1 awen|OJJ36755.1 azon|OJJ47445.1

Group1286: acar|OOF96934.1 acar|OOF96969.1 azon|OJJ42010.1 azon|OJJ45917.1 abra|OJJ68484.1 acla|EAW11145.1 afis|EAW15876.1 afla|EED56499.1 afum|EAL88510.1 agla|OJJ87232.1 akaw|GAA91928.1 aluc|OJZ82694.1 anid|CBF81226.1 anig|EHA21141.1 aory|EIT72869.1 atub|OJI88926.1 aver|OJJ02959.1 awen|OJJ36754.1

Group1287: acar|OOF96935.1 acar|OOF96970.1 abra|OJJ68483.1 acla|EAW11146.1 afis|EAW15877.1 afla|EED56498.1 afum|EAL88511.1 agla|OJJ87231.1 akaw|GAA91929.1 aluc|OJZ82693.1 anid|CBF81228.1

anig|EHA21140.1 aory|EIT72870.1 ater|EAU31697.1 atub|OJI88925.1 aver|OJJ02958.1 awen|OJJ36753.1 azon|OJJ47446.1

Group1288: acar|OOF96937.1 acar|OOF96973.1 abra|OJJ70773.1 acla|EAW11149.1 afis|EAW15880.1 afla|EED56492.1 afum|EAL88514.1 agla|OJJ89541.1 akaw|GAA90092.1 aluc|OJZ84119.1 anid|CBF81234.1 anig|EHA19228.1 aory|EIT72835.1 asyd|OJJ59317.1 atub|OJI90492.1 aver|OJJ02956.1 awen|OJJ36751.1 azon|OJJ47448.1

Group1289: acar|OOF97655.1 acar|OOF97702.1 abra|OJJ71502.1 acla|EAW10046.1 afis|EAW25538.1 afla|EED44714.1 afum|EAL90201.1 akaw|GAA82518.1 aluc|OJZ87045.1 anid|CBF77822.1 anig|EHA20318.1 aory|EIT83463.1 asyd|OJJ53007.1 ater|EAU33379.1 atub|OJI81590.1 aver|OJJ03836.1 awen|OJJ41280.1 azon|OJJ49503.1

Group1290: acar|OOF97659.1 acar|OOF97706.1 abra|OJJ71498.1 acla|EAW10051.1 afis|EAW25533.1 afla|EED44708.1 afum|EAL90197.1 akaw|GAA82522.1 aluc|OJZ87049.1 anid|CBF77815.1 anig|EHA20322.1 aory|EIT83464.1 asyd|OJJ53011.1 ater|EAU33375.1 atub|OJI81586.1 aver|OJJ03832.1 awen|OJJ41275.1 azon|OJJ49507.1

Group1291: acar|OOF97662.1 acar|OOF97709.1 abra|OJJ71495.1 acla|EAW10054.1 afis|EAW25530.1 afla|EED54031.1 afum|EBA27397.1 agla|OJJ87272.1 akaw|GAA82525.1 aluc|OJZ87052.1 anid|CBF77809.1 anig|EHA20325.1 aory|EIT78310.1 asyd|OJJ53014.1 atub|OJI81583.1 aver|OJJ03829.1 awen|OJJ41272.1 azon|OJJ49504.1

Group1292: acar|OOF97667.1 acar|OOF97714.1 abra|OJJ71490.1 acla|EAW10060.1 afis|EAW25524.1 afla|EED54027.1 afum|EAL90189.1 agla|OJJ87277.1 akaw|GAA82530.1 aluc|OJZ87057.1 anig|EHA20330.1 aory|EIT78322.1 asyd|OJJ53020.1 ater|EAU33369.1 atub|OJI81578.1 aver|OJJ03823.1 awen|OJJ41268.1 azon|OJJ49513.1

Group1293: acar|OOF97670.1 acar|OOF97717.1 abra|OJJ71487.1 acla|EAW10063.1 afis|EAW25522.1 afla|EED54024.1 afum|EAL90187.1 agla|OJJ87280.1 akaw|GAA82533.1 aluc|OJZ87060.1 anid|CBF77798.1 anig|EHA20333.1 aory|EIT78336.1 asyd|OJJ53023.1 atub|OJI81575.1 aver|OJJ03820.1 awen|OJJ41265.1 azon|OJJ44830.1

Group1294: acar|OOF97677.1 acar|OOF97725.1 abra|OJJ71475.1 acla|EAW10075.1 afis|EAW25511.1 afla|EED54012.1 afum|EAL90177.1 agla|OJJ87294.1 akaw|GAA82541.1 aluc|OJZ87071.1 anid|CBF77777.1 aory|EIT78286.1 asyd|OJJ53033.1 ater|EAU33358.1 atub|OJI81566.1 aver|OJJ03811.1 awen|OJJ41253.1 azon|OJJ44820.1

Group1295: acar|OOF97683.1 acar|OOF97732.1 abra|OJJ71466.1 acla|EAW10084.1 afis|EAW25503.1 afla|EED54002.1 afum|EAL90169.2 agla|OJJ87301.1 akaw|GAA82548.1 aluc|OJZ87078.1 anid|CBF77762.1 anig|EHA20348.1 aory|EIT78296.1 asyd|OJJ53041.1 ater|EAU33351.1 aver|OJJ03803.1 awen|OJJ41245.1 azon|OJJ44811.1

Group1296: acar|OOF97696.1 acar|OOF97746.1 ater|EAU30101.1 ater|EAU30103.1 abra|OJJ71449.1 afla|EED53988.1 agla|OJJ87138.1 akaw|GAA82561.1 aluc|OJZ87092.1 anid|CBF77728.1 anig|EHA20364.1 aory|EIT78348.1 asyd|OJJ53059.1 atub|OJI81546.1 aver|OJJ03785.1 awen|OJJ41230.1 acla|EAW14376.1 aver|OJI99265.1

Group1297: acar|OOF98470.1 acar|OOF98485.1 abra|OJJ78403.1 acla|EAW14650.1 afis|EAW17292.1 afla|EED46742.1 agla|OJJ84080.1 akaw|GAA85628.1 aluc|OJZ91482.1 anid|CBF82629.1 anig|EHA20144.1 aory|EIT77955.1 asyd|OJJ54010.1 ater|EAU31138.1 atub|OJI83475.1 aver|OJJ08732.1 awen|OJJ34313.1 azon|OJJ43329.1

Group1298: acar|OOF98474.1 acar|OOF98488.1 abra|OJJ78400.1 acla|EAW09132.1 afis|EAW22014.1 afla|EED46739.1 afum|EAL93029.1 agla|OJJ84083.1 akaw|GAA85631.1 aluc|OJZ91479.1 anid|CBF82642.1 anig|EHA20141.1 aory|EIT77943.1 asyd|OJJ61677.1 atub|OJI83472.1 aver|OJJ00998.1 awen|OJJ34310.1 azon|OJJ48063.1

Group1299: acar|OOF98480.1 acar|OOF98493.1 abra|OJJ78394.1 acla|EAW09138.1 afis|EAW22009.1 afla|EED46733.1 afum|EAL93024.1 agla|OJJ84089.1 akaw|GAA85637.1 aluc|OJZ91473.1 anid|CBF82653.1 anig|EHA20135.1 aory|EIT77921.1 asyd|OJJ61671.1 atub|OJI83466.1 aver|OJJ00992.1 awen|OJJ34304.1 azon|OJJ48064.1

Group1300: acar|OOG00033.1 acar|OOG00123.1 abra|OJJ75863.1 acla|EAW14825.1 afis|EAW24354.1 afla|EED47909.1 afum|EAL86025.2 agla|OJJ83234.1 akaw|GAA84569.1 aluc|OJZ89305.1 anig|EHA23261.1 aory|EIT83374.1 asyd|OJJ53696.1 ater|EAU30389.1 atub|OJI85494.1 aver|OJJ07812.1 awen|OJJ32871.1 azon|OJJ48979.1

Group1301: acla|EAW14649.1 afis|EAW22112.1 afis|EAW24562.1 afum|EAL86223.1 asyd|OJJ54831.1 asyd|OJJ65331.1 aver|OJJ07476.1 afla|EED54620.1 agla|OJJ78532.1 anid|CBF80350.1 aory|EIT78818.1 ater|EAU31010.1 ater|EAU33401.1 ater|EAU36517.1 aver|OJJ07330.1 acla|EAW15031.1 aver|OJI99476.1 afis|EAW21218.1

Group1302: acla|EAW08761.1 acla|EAW12263.1 abra|OJJ69061.1 acar|OOF91222.1 afis|EAW15671.1 afla|EED46862.1 afum|EAL85676.2 akaw|GAA90591.1 aluc|OJZ86049.1 anid|CBF71172.1 anig|EHA24562.1 aory|EIT72618.1 asyd|OJJ55852.1 ater|EAU32490.1 atub|OJI79846.1 aver|OJJ04795.1 awen|OJJ38348.1 azon|OJJ43869.1

Group1303: acla|EAW09069.1 acla|EAW11073.1 afla|EED55166.1 afla|EED57249.1 anid|CBF83136.1 anid|CBF84254.1 aory|EIT74130.1 aory|EIT81645.1 asyd|OJJ55425.1 asyd|OJJ57203.1 ater|EAU32710.1 ater|EAU33927.1 aver|OJJ03086.1 aver|OJJ06504.1 afis|EAW25639.1 afum|EAL84326.1 agla|OJJ78892.1 awen|OJJ36107.1

Group1304: acla|EAW09129.1 acla|EAW11451.1 aory|EIT74374.1 abra|OJJ77018.1 acar|OOF95335.1 afla|EED56545.1 akaw|GAA90906.1 aluc|OJZ82920.1 atub|OJI89146.1 awen|OJJ36105.1 acar|OOF91778.1 afla|EED52330.1 agla|OJJ85629.1 ater|EAU39199.1 awen|OJJ32501.1 azon|OJJ46534.1 aory|EIT79688.1 anid|CBF87732.1

Group1305: afis|EAW16696.1 afis|EAW24556.1 afum|EAL86220.1 afum|EAL89293.1 abra|OJJ70132.1 acar|OOF96170.1 afla|EED46901.1 agla|OJJ88675.1 akaw|GAA86632.1 aluc|OJZ85189.1 anid|CBF81791.1 anig|EHA25402.1 aory|EIT72585.1 asyd|OJJ60097.1 ater|EAU32525.1 atub|OJI80408.1 aver|OJJ04183.1 awen|OJJ36331.1

Group1306: afis|EAW17245.1 afis|EAW25661.1 abra|OJJ68041.1 acar|OOF90403.1 acla|EAW12126.1 afla|EED56768.1 afum|EAL86664.2 akaw|GAA90912.1 aluc|OJZ82914.1 anid|CBF84467.1 anig|EHA21337.1 aory|EIT73930.1 asyd|OJJ53543.1 atub|OJI89140.1 aver|OJJ08210.1 awen|OJJ40087.1 ater|EAU38594.1 aver|OJJ03052.1

Group1307: afla|EED44734.1 afla|EED49486.1 agla|OJJ78680.1 agla|OJJ85924.1 abra|OJJ66288.1 aory|EIT79948.1 aver|OJJ07505.1 abra|OJJ77218.1 akaw|GAA90720.1 aluc|OJZ84629.1 atub|OJI89450.1 afis|EAW23523.1 afla|EED47473.1 azon|OJJ42569.1 aory|EIT77798.1 ater|EAU36203.1 aver|OJJ07255.1 anid|CBF78331.1

Group1308: afla|EED47371.1 afla|EED50262.1 afla|EED50950.1 ater|EAU30176.1 ater|EAU32383.1 ater|EAU38563.1 afis|EAW17332.1 agla|OJJ84032.1 anid|CBF85356.1 afum|EAL91169.1 aory|EIT76964.1 asyd|OJJ62637.1 asyd|OJJ62661.1 aver|OJI96759.1 aver|OJI99292.1 awen|OJJ34422.1 aory|EIT82881.1 anid|CBF89127.1

Group1309: afla|EED50259.1 afla|EED52529.1 aory|EIT74308.1 aory|EIT77339.1 abra|OJJ66540.1 acar|OOF90993.1 acla|EAW10950.1 afis|EAW20911.1 afum|EAL84723.1 akaw|GAA91175.1 aluc|OJZ81504.1 anid|CBF82490.1 anig|EHA25926.1 asyd|OJJ54107.1 ater|EAU38544.1 atub|OJI88677.1 aver|OJJ08628.1 awen|OJJ34431.1

Group1310: agla|OJJ78539.1 agla|OJJ78727.1 abra|OJJ72757.1 acar|OOF95978.1 acla|EAW11507.1 afla|EED52511.1 afum|EAL91104.2 akaw|GAA85367.1 aluc|OJZ90731.1 anid|CBF77292.1 anig|EHA24147.1 aory|EIT82146.1 asyd|OJJ56788.1 ater|EAU31605.1 atub|OJI82157.1 aver|OJJ03548.1 awen|OJJ39008.1 aory|EIT82761.1

Group1311: agla|OJJ78573.1 agla|OJJ78791.1 asyd|OJJ53623.1 asyd|OJJ55815.1 awen|OJJ34401.1 awen|OJJ38082.1 afla|EED55668.1 aory|EIT77088.1 asyd|OJJ64785.1 aver|OJI98897.1 anid|CBF79150.1 akaw|GAA92796.1 aluc|OJZ88717.1 anig|EHA28200.1 anid|CBF85279.1 asyd|OJJ62636.1 aver|OJJ05940.1 asyd|OJJ61766.1

Group1312: agla|OJJ78989.1 agla|OJJ83687.1 abra|OJJ73813.1 acar|OOG00968.1 acla|EAW07700.1 afis|EAW22717.1 afla|EED50003.1 afum|EAL90477.1 akaw|GAA89432.1 aluc|OJZ88237.1 anid|CBF88050.1 anig|EHA17984.1 asyd|OJJ63539.1 ater|EAU38986.1 atub|OJI90839.1 aver|OJI97394.1 awen|OJJ35411.1 azon|OJJ49765.1

Group1313: agla|OJJ80769.1 agla|OJJ80770.1 abra|OJJ72336.1 acar|OOF94879.1 acla|EAW11986.1 afis|EAW19626.1 afla|EED56978.1 afum|EAL86895.1 akaw|GAA86088.1 aluc|OJZ90317.1 anid|CBF71317.1 asyd|OJJ55112.1 ater|EAU32900.1 atub|OJI81745.1 aver|OJJ07094.1 awen|OJJ41873.1 azon|OJJ47068.1 aory|EIT77552.1

Group1314: agla|OJJ80830.1 agla|OJJ84729.1 awen|OJJ32928.1 awen|OJJ33337.1 abra|OJJ74236.1 afla|EED48423.1 aluc|OJZ89898.1 anig|EHA27098.1 aory|EIT80749.1 atub|OJI84909.1 acla|EAW13274.1 afis|EAW17343.1 afum|EBA27425.1 anid|CBF71489.1 asyd|OJJ51916.1 afla|EED51567.1 aory|EIT83078.1 ater|EAU30102.1

Group1315: agla|OJJ81338.1 agla|OJJ84780.1 awen|OJJ36016.1 awen|OJJ38019.1 abra|OJJ73446.1 acar|OOF98836.1 afis|EAW24692.1 afla|EED45076.1 afum|EAL89334.1 akaw|GAA89024.1 aluc|OJZ80580.1 anid|CBF76032.1 anig|EHA25618.1 aory|EIT73772.1 asyd|OJJ52453.1 ater|EAU33313.1 atub|OJI88350.1 aver|OJJ03963.1

Group1316: agla|OJJ83725.1 agla|OJJ88209.1 awen|OJJ30544.1 awen|OJJ37831.1 azon|OJJ42347.1 azon|OJJ45910.1 ater|EAU34086.1 acar|OOF92105.1 aver|OJJ07715.1 acar|OOF99481.1 afis|EAW19566.1

afis|EAW19382.1 aluc|OJZ85537.1 ater|EAU36043.1 afis|EAW19567.1 anig|EHA22173.1 aver|OJJ07008.1 asyd|OJJ55209.1

Group1317: agla|OJJ84608.1 agla|OJJ88488.1 abra|OJJ68915.1 acar|OOF91503.1 acla|EAW14256.1 afis|EAW19024.1 afla|EED56627.1 afum|EAL93755.1 akaw|GAA90334.1 aluc|OJZ85898.1 anid|CBF73887.1 anig|EHA24694.1 aory|EIT79613.1 asyd|OJJ53658.1 ater|EAU32066.1 atub|OJI79683.1 aver|OJJ07766.1 awen|OJJ31397.1

Group1318: akaw|GAA84492.1 akaw|GAA92643.1 abra|OJJ68086.1 acar|OOF93885.1 afis|EAW23619.1 afum|EAL87843.1 akaw|GAA86871.1 aluc|OJZ89995.1 anig|EHA27365.1 asyd|OJJ55804.1 atub|OJI84813.1 aver|OJJ03908.1 awen|OJJ33610.1 abra|OJJ75773.1 afla|EED51751.1 aory|EIT80721.1 ater|EAU34085.1 azon|OJJ50739.1

Group1319: akaw|GAA88994.1 akaw|GAA93237.1 aluc|OJZ79544.1 aluc|OJZ80344.1 abra|OJJ70263.1 acar|OOF91460.1 afla|EED57905.1 agla|OJJ83807.1 akaw|GAA86534.1 aluc|OJZ85082.1 anid|CBF78690.1 aory|EIT82332.1 asyd|OJJ53892.1 ater|EAU33679.1 atub|OJI80517.1 aver|OJJ07990.1 afla|EED57907.1 aory|EIT82357.1

Group1320: akaw|GAA89097.1 akaw|GAA93124.1 abra|OJJ70832.1 acla|EAW13725.1 afla|EED45954.1 agla|OJJ89628.1 aluc|OJZ83078.1 anig|EHA20972.1 aory|EIT73606.1 asyd|OJJ52723.1 atub|OJI87867.1 aver|OJJ04315.1 awen|OJJ32035.1 azon|OJJ46438.1 awen|OJJ34430.1 akaw|GAA90262.1 aory|EIT75383.1 awen|OJJ34591.1

Group1321: aluc|OJZ85535.1 aluc|OJZ86787.1 anig|EHA19890.1 anig|EHA21895.1 atub|OJI83732.1 atub|OJI89790.1 abra|OJJ69763.1 ater|EAU29245.1 abra|OJJ72050.1 acar|OOF96707.1 afla|EED57904.1 agla|OJJ78987.1 akaw|GAA84884.1 aory|EIT82360.1 asyd|OJJ61024.1 aver|OJJ01910.1 awen|OJJ33896.1 azon|OJJ45975.1

Group1322: anid|CBF69554.1 anid|CBF74620.1 asyd|OJJ57601.1 asyd|OJJ59165.1 aver|OJJ01755.1 aver|OJJ05583.1 abra|OJJ77468.1 acar|OOF99266.1 acla|EAW07128.1 afis|EAW22137.1 afla|EED54177.1 afum|EAL91053.1 akaw|GAA84149.1 aluc|OJZ81070.1 anig|EHA19299.1 ater|EAU29324.1 atub|OJI82516.1 awen|OJJ34710.1

Group1323: anid|CBF71205.1 anid|CBF74966.1 anid|CBF89272.1 abra|OJJ69173.1 acar|OOF91131.1 afla|EED55558.1 agla|OJJ84719.1 akaw|GAA86289.1 aluc|OJZ86160.1 anig|EHA24464.1 asyd|OJJ61621.1 ater|EAU32677.1 aver|OJJ00945.1 awen|OJJ38097.1 azon|OJJ42541.1 aory|EIT82704.1 atub|OJI79952.1 ater|EAU34906.1

Group1324: anid|CBF77312.1 anid|CBF84491.1 abra|OJJ72772.1 acar|OOF95991.1 afis|EAW23959.1 afla|EED50553.1 afum|EAL87388.1 agla|OJJ86884.1 akaw|GAA85381.1 aluc|OJZ90749.1 anig|EHA24160.1 aory|EIT82046.1 asyd|OJJ56774.1 ater|EAU29793.1 atub|OJI82175.1 aver|OJJ03559.1 awen|OJJ38988.1 azon|OJJ51320.1

Group1325: anid|CBF87056.1 anid|CBF87058.1 abra|OJJ66247.1 acar|OOF90705.1 acla|EAW07050.1 afis|EAW17102.1 afla|EED51639.1 afum|EAL89282.1 akaw|GAA89043.1 aluc|OJZ80553.1 anig|EHA25644.1 aory|EIT80540.1 atub|OJI88378.1 aver|OJI99751.1 awen|OJJ37983.1 afis|EAW16677.1 afla|EED47132.1 aory|EIT79173.1

Group1326: anig|EHA19581.1 anig|EHA25318.1 awen|OJJ33562.1 awen|OJJ34654.1 abra|OJJ70237.1 acar|OOF92557.1 afis|EAW21623.1 afla|EED53598.1 afum|EAL92659.1 agla|OJJ88653.1 akaw|GAA86548.1 aluc|OJZ85096.1 anid|CBF79555.1 aory|EIT74955.1 asyd|OJJ57931.1 atub|OJI80502.1 aver|OJJ04466.1 azon|OJJ45043.1

Group1327: anig|EHA20767.1 anig|EHA21813.1 atub|OJI81082.1 abra|OJJ70967.1 acar|OOF98164.1 acla|EAW10527.1 afis|EAW25039.1 afla|EED55413.1 afum|EAL89707.1 agla|OJJ89154.1 akaw|GAA82981.1 aluc|OJZ87565.1 anid|CBF85903.1 aory|EIT80777.1 asyd|OJJ64393.1 ater|EAU35789.1 aver|OJI98488.1 awen|OJJ40645.1

Group1328: anig|EHA20833.1 afum|EAL89767.1 abra|OJJ70883.1 acar|OOF98234.1 acla|EAW10471.1 afis|EAW25096.1 afla|EED55352.1 agla|OJJ89081.1 akaw|GAA87402.1 aluc|OJZ87638.1 anid|CBF86008.1 aory|EIT80853.1 asyd|OJJ64446.1 ater|EAU39354.1 atub|OJI81009.1 aver|OJI98549.1 awen|OJJ40710.1 azon|OJJ44086.1

Group1329: anig|EHA21819.1 anig|EHA24599.1 abra|OJJ69025.1 acar|OOF92661.1 acla|EAW07121.1 afis|EAW22131.1 afla|EED51689.1 afum|EAL91059.1 akaw|GAA90542.1 aluc|OJZ86012.1 aory|EIT80445.1 ater|EAU33620.1 atub|OJI79801.1 awen|OJJ32638.1 azon|OJJ42095.1 afla|EED56398.1 aory|EIT83004.1 aver|OJJ06436.1

Group1330: anig|EHA25931.1 anig|EHA28231.1 abra|OJJ66546.1 acla|EAW07078.1 afla|EED45458.1 akaw|GAA91171.1 aluc|OJZ81499.1 aory|EIT82415.1 ater|EAU30796.1 atub|OJI88682.1 aver|OJJ06470.1 akaw|GAA92427.1 anig|EHA25846.1 atub|OJI88588.1 acar|OOF90934.1 anid|CBF82815.1 asyd|OJJ61574.1 aver|OJJ00895.1

Group1331: aory|EIT72308.1 aory|EIT72311.1 abra|OJJ69636.1 acar|OOF92673.1 acla|EAW10864.1 afis|EAW20994.1 afum|EAL84634.1 agla|OJJ84072.1 akaw|GAA91616.1 aluc|OJZ82442.1 anid|CBF82637.1 anig|EHA28331.1 asyd|OJJ54015.1 ater|EAU38643.1 atub|OJI87998.1 aver|OJJ08726.1 awen|OJJ34316.1 azon|OJJ48058.1

Group1332: aory|EIT72309.1 aory|EIT72310.1 abra|OJJ66632.1 acar|OOF91054.1 acla|EAW10863.1 afis|EAW20995.1 afum|EAL84633.1 agla|OJJ84073.1 akaw|GAA91095.1 aluc|OJZ81416.1 anid|CBF82633.1 anig|EHA26004.1 asyd|OJJ54013.1 ater|EAU38644.1 atub|OJI88764.1 aver|OJJ08728.1 awen|OJJ34315.1 azon|OJJ48060.1

Group1333: asyd|OJJ52021.1 asyd|OJJ52275.1 abra|OJJ73341.1 acar|OOG00528.1 acla|EAW13716.1 afis|EAW19870.1 afla|EED53094.1 afum|EAL85027.1 akaw|GAA83141.1 aluc|OJZ88698.1 anid|CBF81882.1 anig|EHA18378.1 aory|EIT74789.1 asyd|OJJ59668.1 ater|EAU30732.1 atub|OJI91279.1 aver|OJJ02615.1 ater|EAU36648.1

Group1334: asyd|OJJ52762.1 asyd|OJJ62749.1 abra|OJJ73605.1 acar|OOG00789.1 acla|EAW07522.1 afis|EAW22552.1 afla|EED54584.1 afum|EAL90643.1 agla|OJJ80914.1 akaw|GAA88548.1 aluc|OJZ88444.1 anid|CBF89060.1 anig|EHA18168.1 aory|EIT78947.1 ater|EAU39152.1 atub|OJI91042.1 aver|OJI96789.1 awen|OJJ35183.1

Group1335: asyd|OJJ54165.1 asyd|OJJ59819.1 aver|OJJ02469.1 aver|OJJ08576.1 abra|OJJ76687.1 acar|OOF99325.1 acla|EAW12040.1 afis|EAW19689.1 afla|EED56767.1 afum|EAL86837.2 akaw|GAA92507.1 aluc|OJZ91520.1 anid|CBF75398.1 anig|EHA22558.1 aory|EIT73885.1 ater|EAU37774.1 atub|OJI85975.1 awen|OJJ31435.1

Group1336: asyd|OJJ54481.1 asyd|OJJ64266.1 abra|OJJ70844.1 acar|OOF98271.1 acla|EAW10435.1 afis|EAW25133.1 afla|EED55313.1 afum|EAL89804.1 agla|OJJ89034.1 akaw|GAA91712.1 aluc|OJZ87677.1 anid|CBF85703.1 anig|EHA20867.1 aory|EIT83632.1 ater|EAU39404.1 atub|OJI80966.1 aver|OJI98358.1 awen|OJJ40757.1

Group1337: asyd|OJJ55684.1 asyd|OJJ56117.1 aver|OJJ05058.1 aver|OJJ06768.1 abra|OJJ76907.1 acar|OOF91355.1 afis|EAW20540.1 afla|EED52439.1 afum|EAL84518.2 akaw|GAA87131.1 aluc|OJZ84306.1 anid|CBF78991.1 anig|EHA27642.1 aory|EIT82783.1 ater|EAU37140.1 atub|OJI89762.1 awen|OJJ33246.1 azon|OJJ50626.1

Group1338: asyd|OJJ56868.1 asyd|OJJ60506.1 aver|OJI99831.1 aver|OJJ03458.1 aver|OJJ05527.1 abra|OJJ65536.1 afis|EAW19680.1 afum|EAL86843.1 aluc|OJZ79939.1 anig|EHA19938.1 abra|OJJ65772.1 atub|OJI89174.1 awen|OJJ39572.1 awen|OJJ39573.1 ater|EAU37595.1 anid|CBF80809.1 aory|EIT76448.1 ater|EAU39331.1

Group1339: asyd|OJJ58286.1 asyd|OJJ62494.1 abra|OJJ69000.1 acar|OOF92712.1 afis|EAW18028.1 afla|EED51869.1 afum|EAL91839.1 agla|OJJ85399.1 akaw|GAA90519.1 aluc|OJZ85984.1 anid|CBF80060.1 anig|EHA24622.1 aory|EIT81740.1 ater|EAU31540.1 atub|OJI79774.1 aver|OJJ02138.1 awen|OJJ41513.1 azon|OJJ48929.1

Group1340: asyd|OJJ60628.1 abra|OJJ75954.1 acar|OOF99958.1 acla|EAW09842.1 afis|EAW21293.1 afla|EED54845.1 afum|EAL92310.1 agla|OJJ87460.1 akaw|GAA84657.1 aluc|OJZ89403.1 anid|CBF83378.1 anig|EHA23184.1 aory|EIT79413.1 ater|EAU35926.1 atub|OJI85394.1 aver|OJI99952.1 awen|OJJ39406.1 azon|OJJ44289.1

Group1341: ater|EAU29424.1 ater|EAU33846.1 abra|OJJ77281.1 acar|OOF93155.1 acla|EAW13280.1 afis|EAW17337.1 afla|EED51045.1 afum|EAL91181.1 agla|OJJ79349.1 akaw|GAA90668.1 aluc|OJZ84691.1 anid|CBF87334.1 anig|EHA27966.1 aory|EIT77013.1 asyd|OJJ56362.1 atub|OJI89391.1 aver|OJJ06352.1 azon|OJJ44714.1

Group1342: ater|EAU29807.1 ater|EAU31871.1 abra|OJJ77150.1 acar|OOF93036.1 acla|EAW12919.1 afis|EAW17736.1 afla|EED45737.1 afum|EAL91551.1 agla|OJJ81714.1 akaw|GAA90780.1 aluc|OJZ84564.1 anid|CBF86592.1 anig|EHA27858.1 asyd|OJJ64830.1 atub|OJI89518.1 aver|OJI98943.1 awen|OJJ37099.1 azon|OJJ50800.1

Group1343: ater|EAU31940.1 ater|EAU38199.1 abra|OJJ76424.1 acar|OOF99668.1 acla|EAW14001.1 afis|EAW18709.1 afla|EED50658.1 afum|EAL93432.1 akaw|GAA82339.1 aluc|OJZ91788.1 anid|CBF70654.1 anig|EHA22785.1 aory|EIT78023.1 asyd|OJJ58483.1 atub|OJI86244.1 aver|OJJ01086.1 awen|OJJ32192.1 azon|OJJ46757.1

Group1344: ater|EAU35144.1 ater|EAU37867.1 abra|OJJ77032.1 acar|OOF91470.1 acla|EAW13563.1 afis|EAW20685.1 afla|EED49742.1 afum|EAL84413.1 agla|OJJ80325.1 akaw|GAA87019.1 aluc|OJZ84435.1 anid|CBF83195.1 anig|EHA27754.1 aory|EIT77691.1 asyd|OJJ55559.1 atub|OJI89638.1 aver|OJJ06640.1 awen|OJJ34542.1

Group1345: ater|EAU35442.1 ater|EAU37095.1 abra|OJJ76968.1 acar|OOF91413.1 acla|EAW13484.1 afis|EAW20592.1 afla|EED52233.1 afum|EAL84469.1 agla|OJJ79954.1 akaw|GAA87076.1 aluc|OJZ84364.1

anid|CBF78915.1 anig|EHA27699.1 asyd|OJJ55617.1 ater|EAU35238.1 atub|OJI89704.1 aver|OJJ06692.1 awen|OJJ33552.1

Group1346: ater|EAU35971.1 ater|EAU38852.1 abra|OJJ76019.1 acar|OOF99906.1 acla|EAW09787.1 afis|EAW21351.1 afla|EED55763.1 afum|EAL92366.1 agla|OJJ80812.1 akaw|GAA88941.1 aluc|OJZ89466.1 anid|CBF76199.1 anig|EHA23122.1 aory|EIT76697.1 asyd|OJJ60381.1 atub|OJI85329.1 aver|OJI99710.1 awen|OJJ39349.1

Group1347: aver|OJI97203.1 aver|OJJ08497.1 abra|OJJ74529.1 acar|OOF95222.1 acla|EAW07163.1 afis|EAW22178.1 afla|EED57557.1 afum|EAL91011.1 agla|OJJ87877.1 akaw|GAA82071.1 aluc|OJZ92111.1 anid|CBF88382.1 aory|EIT81848.1 asyd|OJJ63737.1 ater|EAU34183.1 atub|OJI86544.1 awen|OJJ34758.1 azon|OJJ45803.1

Group1348: awen|OJJ29894.1 awen|OJJ38081.1 abra|OJJ76942.1 akaw|GAA87098.1 aluc|OJZ84340.1 anig|EHA27674.1 atub|OJI89728.1 acla|EAW12080.1 afis|EAW19721.1 agla|OJJ86006.1 aver|OJI96480.1 acar|OOF91391.1 afla|EED57523.1 afum|EAL86805.2 anig|EHA19572.1 ater|EAU30973.1 atub|OJI83020.1 ater|EAU34067.1

Group1349: awen|OJJ30757.1 awen|OJJ31724.1 abra|OJJ67453.1 acar|OOF92380.1 acla|EAW06312.1 afis|EAW18421.1 afla|EED52987.1 afum|EAL93105.1 akaw|GAA86378.1 aluc|OJZ84912.1 anid|CBF70698.1 anig|EHA25133.1 aory|EIT78706.1 asyd|OJJ58462.1 ater|EAU29839.1 atub|OJI80686.1 aver|OJJ01062.1 azon|OJJ50486.1

Group1350: awen|OJJ30769.1 awen|OJJ31712.1 abra|OJJ67441.1 acar|OOF92385.1 acla|EAW06322.1 afis|EAW18436.1 afla|EED52979.1 afum|EAL93117.1 agla|OJJ85003.1 akaw|GAA86388.1 aluc|OJZ84923.1 anid|CBF70035.1 anig|EHA25141.1 asyd|OJJ58867.1 ater|EAU29852.1 atub|OJI80676.1 aver|OJJ01469.1 azon|OJJ50477.1

Group1351: awen|OJJ30784.1 awen|OJJ31697.1 abra|OJJ77412.1 acar|OOF93279.1 acla|EAW08581.1 afis|EAW17309.1 afla|EED48984.1 afum|EAL91144.1 agla|OJJ85394.1 akaw|GAA91352.1 aluc|OJZ84821.1 anid|CBF89993.1 anig|EHA28083.1 asyd|OJJ62080.1 ater|EAU35447.1 atub|OJI89265.1 aver|OJI96188.1 azon|OJJ44474.1

Group1352: awen|OJJ30785.1 awen|OJJ31696.1 abra|OJJ67989.1 acar|OOF92031.1 afis|EAW24766.1 afla|EED47575.1 afum|EAL89408.2 agla|OJJ88067.1 akaw|GAA90959.1 aluc|OJZ82863.1 anid|CBF90079.1 anig|EHA21293.1 aory|EIT74521.1 asyd|OJJ62039.1 ater|EAU29463.1 atub|OJI89088.1 aver|OJI96145.1 azon|OJJ46302.1

Group1353: awen|OJJ32597.1 awen|OJJ38114.1 abra|OJJ77103.1 acar|OOF92988.1 afla|EED45185.1 akaw|GAA86953.1 aluc|OJZ84512.1 anid|CBF81557.1 anig|EHA27810.1 aory|EIT73710.1 asyd|OJJ59498.1 ater|EAU38093.1 atub|OJI89569.1 aver|OJJ02776.1 azon|OJJ42741.1 afla|EED47336.1 aory|EIT82859.1 aver|OJJ03900.1

Group1354: awen|OJJ32803.1 awen|OJJ35367.1 abra|OJJ75800.1 acar|OOG00076.1 acla|EAW14886.1 afis|EAW24405.1 afla|EED47807.1 afum|EAL86076.1 agla|OJJ78882.1 akaw|GAA84516.1 aluc|OJZ89248.1 anid|CBF75173.1 anig|EHA23319.1 aory|EIT77184.1 asyd|OJJ59956.1 ater|EAU30320.1 atub|OJI85550.1 aver|OJJ02333.1

Group1355: awen|OJJ34458.1 awen|OJJ34686.1 abra|OJJ66071.1 acar|OOG00583.1 acla|EAW08522.1 afis|EAW16392.1 afla|EED52127.1 afum|EAL89031.1 akaw|GAA84173.1 aluc|OJZ81098.1 anid|CBF81685.1 anig|EHA19273.1 aory|EIT74371.1 asyd|OJJ62625.1 ater|EAU29596.1 atub|OJI82482.1 aver|OJI96704.1 azon|OJJ43880.1

Group1356: azon|OJJ42761.1 azon|OJJ42890.1 azon|OJJ42564.1 azon|OJJ50002.1 azon|OJJ44488.1 azon|OJJ42513.1 azon|OJJ43493.1 azon|OJJ42351.1 azon|OJJ42537.1 azon|OJJ48473.1 azon|OJJ43882.1 azon|OJJ44466.1 azon|OJJ51813.1 azon|OJJ42495.1 azon|OJJ46515.1 afla|EED48610.1 afla|EED57034.1 aory|EIT82256.1

Group1357: azon|OJJ42597.1 azon|OJJ49266.1 abra|OJJ70118.1 acla|EAW12241.1 afis|EAW21131.1 agla|OJJ80450.1 asyd|OJJ55221.1 atub|OJI80395.1 aver|OJI97990.1 awen|OJJ37684.1 asyd|OJJ61887.1 afum|EAL84504.1 agla|OJJ80675.1 afis|EAW20557.1 awen|OJJ35176.1 azon|OJJ42354.1 asyd|OJJ59262.1 anig|EHA25411.1

Group1358: azon|OJJ43518.1 azon|OJJ49486.1 abra|OJJ66404.1 acla|EAW12367.1 afis|EAW20845.1 agla|OJJ86411.1 akaw|GAA90272.1 aluc|OJZ83069.1 anid|CBF78174.1 anig|EHA28133.1 asyd|OJJ55965.1 atub|OJI87877.1 aver|OJJ04911.1 awen|OJJ36259.1 abra|OJJ68228.1 afum|EAL84788.1 acar|OOG00612.1 azon|OJJ45925.1

Group1359: azon|OJJ44012.1 azon|OJJ45468.1 abra|OJJ69814.1 acar|OOF93561.1 acla|EAW11673.1 afis|EAW23690.1 afla|EED51830.1 afum|EAL87135.1 agla|OJJ79265.1 akaw|GAA87843.1 aluc|OJZ85481.1 anid|CBF80383.1 anig|EHA25021.1 aory|EIT81761.1 asyd|OJJ54786.1 atub|OJI80083.1 aver|OJJ07379.1 awen|OJJ38091.1

Group1360: abra|OJJ65585.1 afum|EBA27422.1 abra|OJJ65589.1 asyd|OJJ55957.1 abra|OJJ68807.1 afla|EED46563.1 aluc|OJZ85779.1 anid|CBF70722.1 aory|EIT77899.1 atub|OJI79564.1 awen|OJJ35847.1 acla|EAW13291.1 afis|EAW17318.1 afum|EAL91154.1 azon|OJJ50647.1 aver|OJJ04903.1 afum|EAL89333.1 azon|OJJ50595.1

Group1361: abra|OJJ65623.1 acar|OOF97131.1 acla|EAW12840.1 afum|EAL87789.1 agla|OJJ86050.1 akaw|GAA92893.1 anid|CBF78703.1 asyd|OJJ62680.1 ater|EAU36754.1 awen|OJJ36388.1 azon|OJJ43355.1 afla|EED54881.1 aory|EIT79373.1 acar|OOF98798.1 acla|EAW11601.1 aluc|OJZ85600.1 atub|OJI79395.1 anig|EHA24924.1

Group1362: abra|OJJ65672.1 afis|EAW16875.1 afum|EAL86412.1 aluc|OJZ79539.1 ater|EAU30978.1 aver|OJJ03435.1 awen|OJJ36144.1 azon|OJJ46815.1 abra|OJJ67798.1 aluc|OJZ90858.1 atub|OJI82852.1 abra|OJJ68250.1 acar|OOF97529.1 akaw|GAA83211.1 aluc|OJZ83713.1 anig|EHA18864.1 atub|OJI90088.1 ater|EAU37972.1

Group1363: abra|OJJ65773.1 acar|OOF92085.1 agla|OJJ81015.1 akaw|GAA90888.1 aluc|OJZ82942.1 anid|CBF86755.1 anig|EHA21361.1 asyd|OJJ61469.1 ater|EAU35421.1 atub|OJI89175.1 aver|OJJ00810.1 awen|OJJ34702.1 afla|EED45830.1 akaw|GAA85168.1 aluc|OJZ86468.1 anig|EHA21442.1 aory|EIT75507.1 azon|OJJ48930.1

Group1364: abra|OJJ65864.1 acar|OOF95036.1 akaw|GAA92281.1 aluc|OJZ90178.1 anid|CBF80753.1 anig|EHA23677.1 asyd|OJJ54523.1 atub|OJI81605.1 aver|OJJ07660.1 abra|OJJ69850.1 acar|OOF93373.1 afla|EED55058.1 akaw|GAA87808.1 aluc|OJZ85443.1 anig|EHA24987.1 aory|EIT81967.1 atub|OJI80124.1 azon|OJJ46475.1

Group1365: abra|OJJ65896.1 acar|OOF94956.1 acla|EAW11696.1 afis|EAW23713.1 afla|EED44871.1 afum|EAL87157.1 agla|OJJ81717.1 akaw|GAA86009.1 aluc|OJZ90235.1 anid|CBF83004.1 anig|EHA23735.1 aory|EIT82717.1 asyd|OJJ61502.1 ater|EAU31796.1 atub|OJI81665.1 aver|OJJ00840.1 awen|OJJ37102.1 azon|OJJ50803.1

Group1366: abra|OJJ65913.1 acar|OOF94964.1 acla|EAW12019.1 afis|EAW19660.1 afla|EED57030.1 afum|EAL86863.1 agla|OJJ80822.1 akaw|GAA92319.1 aluc|OJZ90217.1 anid|CBF71390.1 anig|EHA23715.1 aory|EIT82277.1 asyd|OJJ55042.1 ater|EAU32852.1 atub|OJI81649.1 aver|OJJ07166.1 awen|OJJ41922.1 azon|OJJ42684.1

Group1367: abra|OJJ65945.1 acar|OOF92928.1 acla|EAW13569.1 afis|EAW20032.1 afla|EED46042.1 afum|EAL84884.1 agla|OJJ88019.1 akaw|GAA90823.1 aluc|OJZ83012.1 anid|CBF84764.1 anig|EHA21427.1 aory|EIT75566.1 asyd|OJJ53285.1 ater|EAU29398.1 atub|OJI89247.1 aver|OJJ08456.1 awen|OJJ30055.1 azon|OJJ43701.1

Group1368: abra|OJJ65946.1 acar|OOF92925.1 acla|EAW13570.1 afis|EAW20031.1 afla|EED46041.1 afum|EAL84885.1 agla|OJJ88018.1 akaw|GAA90824.1 aluc|OJZ83011.1 anid|CBF84760.1 anig|EHA21426.1 aory|EIT75583.1 asyd|OJJ53286.1 ater|EAU29395.1 atub|OJI89246.1 aver|OJJ08455.1 awen|OJJ30054.1 azon|OJJ43702.1

Group1369: abra|OJJ65948.1 acar|OOF92924.1 acla|EAW13571.1 afis|EAW20030.1 afla|EED46039.1 afum|EAL84887.1 agla|OJJ88016.1 akaw|GAA90826.1 aluc|OJZ83009.1 anid|CBF84756.1 anig|EHA21424.1 aory|EIT75781.1 asyd|OJJ53288.1 ater|EAU29393.1 atub|OJI89244.1 aver|OJJ08453.1 awen|OJJ30052.1 azon|OJJ43700.1

Group1370: abra|OJJ65949.1 acar|OOF92923.1 acla|EAW13572.1 afis|EAW20029.1 afla|EED46038.1 afum|EAL84888.1 agla|OJJ88015.1 akaw|GAA90827.1 aluc|OJZ83008.1 anid|CBF84754.1 anig|EHA21423.1 aory|EIT75542.1 asyd|OJJ53289.1 ater|EAU29392.1 atub|OJI89243.1 aver|OJJ08452.1 awen|OJJ30051.1 azon|OJJ43698.1

Group1371: abra|OJJ65951.1 acar|OOF92921.1 acla|EAW13573.1 afis|EAW20028.1 afla|EED46037.1 afum|EAL84889.1 agla|OJJ88000.1 akaw|GAA90828.1 aluc|OJZ83006.1 anid|CBF84753.1 anig|EHA21422.1 aory|EIT75829.1 asyd|OJJ53290.1 ater|EAU29391.1 atub|OJI89241.1 aver|OJJ08450.1 awen|OJJ30042.1 azon|OJJ43688.1

Group1372: abra|OJJ65952.1 acar|OOF92920.1 acla|EAW13574.1 afis|EAW20027.1 afla|EED46036.1 afum|EAL84890.1 agla|OJJ88001.1 akaw|GAA90829.1 aluc|OJZ83005.1 anid|CBF84751.1 anig|EHA21421.1 aory|EIT75375.1 asyd|OJJ53291.1 ater|EAU29390.1 atub|OJI89240.1 aver|OJJ08449.1 awen|OJJ30043.1 azon|OJJ43689.1

Group1373: abra|OJJ65954.1 acar|OOF92918.1 acla|EAW13575.1 afis|EAW20026.1 afla|EED46034.1 afum|EAL84891.1 agla|OJJ88004.1 akaw|GAA90831.1 aluc|OJZ83003.1 anid|CBF84749.1 anig|EHA21419.1 aory|EIT75468.1 asyd|OJJ53293.1 ater|EAU29388.1 atub|OJI89237.1 aver|OJJ08447.1 awen|OJJ30045.1 azon|OJJ43691.1

Group1374: abra|OJJ65956.1 acar|OOF92916.1 acla|EAW13577.1 afis|EAW20024.1 afla|EED46032.1 afum|EAL84893.1 agla|OJJ88006.1 akaw|GAA90833.1 aluc|OJZ83001.1 anid|CBF84745.1 anig|EHA21417.1

aory|EIT75584.1 asyd|OJJ53295.1 ater|EAU29386.1 atub|OJI89235.1 aver|OJJ08445.1 awen|OJJ30046.1 azon|OJJ43693.1

Group1375: abra|OJJ65957.1 acar|OOF92915.1 acla|EAW13578.1 afis|EAW20023.1 afla|EED46031.1 afum|EAL84894.1 agla|OJJ88007.1 akaw|GAA90834.1 aluc|OJZ83000.1 anid|CBF84743.1 anig|EHA21416.1 aory|EIT75718.1 asyd|OJJ53296.1 ater|EAU29385.1 atub|OJI89234.1 aver|OJJ08444.1 awen|OJJ30047.1 azon|OJJ43694.1

Group1376: abra|OJJ65958.1 acar|OOF92914.1 acla|EAW13579.1 afis|EAW20022.1 afla|EED46030.1 afum|EAL84895.1 agla|OJJ88011.1 akaw|GAA90835.1 aluc|OJZ82999.1 anid|CBF84741.1 anig|EHA21415.1 aory|EIT75399.1 asyd|OJJ53297.1 ater|EAU29384.1 atub|OJI89233.1 aver|OJJ08443.1 awen|OJJ30048.1 azon|OJJ43695.1

Group1377: abra|OJJ65959.1 acar|OOF92913.1 acla|EAW13580.1 afis|EAW20021.1 afla|EED46029.1 afum|EAL84896.2 agla|OJJ88012.1 akaw|GAA90836.1 aluc|OJZ82998.1 anid|CBF84739.1 anig|EHA21414.1 aory|EIT75790.1 asyd|OJJ53298.1 ater|EAU29383.1 atub|OJI89232.1 aver|OJJ08442.1 awen|OJJ30049.1 azon|OJJ43696.1

Group1378: abra|OJJ65963.1 acar|OOF92911.1 acla|EAW13582.1 afis|EAW20019.1 afla|EED46027.1 afum|EAL84898.1 agla|OJJ87997.1 akaw|GAA90838.1 aluc|OJZ82995.1 anid|CBF84733.1 anig|EHA21410.1 aory|EIT75391.1 asyd|OJJ53302.1 ater|EAU29380.1 atub|OJI89229.1 aver|OJJ08439.1 awen|OJJ30040.1 azon|OJJ43684.1

Group1379: abra|OJJ65969.1 acar|OOF92904.1 acla|EAW13590.1 afis|EAW20013.1 afla|EED46020.1 afum|EAL84905.1 agla|OJJ87978.1 akaw|GAA90844.1 aluc|OJZ82989.1 anid|CBF84715.1 anig|EHA21404.1 aory|EIT75372.1 asyd|OJJ53326.1 ater|EAU29355.1 atub|OJI89223.1 aver|OJJ08417.1 awen|OJJ30027.1 azon|OJJ43661.1

Group1380: abra|OJJ65976.1 acar|OOF92896.1 acla|EAW13597.1 afis|EAW20007.1 afla|EED46013.1 afum|EAL84911.1 agla|OJJ87972.1 akaw|GAA90850.1 aluc|OJZ82983.1 anid|CBF84706.1 anig|EHA21397.1 aory|EIT75456.1 asyd|OJJ53321.1 ater|EAU29349.1 atub|OJI89217.1 aver|OJJ08422.1 awen|OJJ30020.1 azon|OJJ49472.1

Group1381: abra|OJJ65993.1 acar|OOF92879.1 acla|EAW13603.1 afis|EAW15644.1 afla|EED45983.1 agla|OJJ87961.1 akaw|GAA90867.1 aluc|OJZ82966.1 anid|CBF80346.1 anig|EHA21382.1 aory|EIT75657.1 asyd|OJJ54833.1 atub|OJI89199.1 aver|OJJ07328.1 awen|OJJ30009.1 azon|OJJ46985.1 asyd|OJJ52075.1 aver|OJJ03028.1

Group1382: abra|OJJ65998.1 acar|OOF92102.1 akaw|GAA90870.1 aluc|OJZ82963.1 anig|EHA21379.1 atub|OJI89195.1 abra|OJJ66040.1 acla|EAW10004.1 afis|EAW21054.1 afum|EAL92090.1 agla|OJJ81308.1 anid|CBF83224.1 aory|EIT77099.1 asyd|OJJ55572.1 ater|EAU37572.1 aver|OJJ06651.1 awen|OJJ33474.1 afla|EED55697.1

Group1383: abra|OJJ66217.1 acar|OOF89906.1 acla|EAW11713.1 afis|EAW19322.1 afla|EED48416.1 afum|EAL94040.1 agla|OJJ88754.1 akaw|GAA89069.1 aluc|OJZ80523.1 anid|CBF74663.1 anig|EHA25672.1 aory|EIT83055.1 asyd|OJJ54973.1 atub|OJI88407.1 aver|OJJ07218.1 awen|OJJ33251.1 azon|OJJ50629.1 awen|OJJ34601.1

Group1384: abra|OJJ66228.1 acar|OOF90678.1 acla|EAW07009.1 afis|EAW16625.1 afla|EED44976.1 afum|EAL89212.2 akaw|GAA89061.1 aluc|OJZ80533.1 anig|EHA25662.1 aory|EIT73723.1 asyd|OJJ56749.1 ater|EAU36726.1 atub|OJI88398.1 aver|OJJ05982.1 awen|OJJ40148.1 azon|OJJ42882.1 acar|OOG00595.1 azon|OJJ42590.1

Group1385: abra|OJJ66483.1 acar|OOF90960.1 acla|EAW10986.1 afis|EAW20874.1 afla|EED45556.1 afum|EAL84760.1 agla|OJJ83981.1 akaw|GAA92468.1 aluc|OJZ81551.1 anid|CBF82851.1 anig|EHA25883.1 aory|EIT75688.1 asyd|OJJ61594.1 ater|EAU30815.1 atub|OJI88634.1 aver|OJJ00916.1 awen|OJJ34486.1 azon|OJJ51763.1

Group1386: akaw|GAA92469.1 aluc|OJZ81550.1 atub|OJI88635.1 abra|OJJ66485.1 acar|OOF90962.1 acla|EAW10984.1 afis|EAW20876.1 afla|EED45558.1 afum|EAL84757.1 agla|OJJ83983.1 anid|CBF82855.1 anig|EHA25885.1 aory|EIT75434.1 asyd|OJJ61595.1 ater|EAU30813.1 aver|OJJ00918.1 awen|OJJ34484.1 azon|OJJ51761.1

Group1387: abra|OJJ66488.1 acar|OOF90964.1 acla|EAW10981.1 afis|EAW20878.1 afla|EED45563.1 afum|EAL84754.1 agla|OJJ83988.1 akaw|GAA92471.1 aluc|OJZ81547.1 anid|CBF82860.1 anig|EHA25888.1 aory|EIT75724.1 asyd|OJJ61597.1 ater|EAU38706.1 atub|OJI88637.1 aver|OJJ00921.1 awen|OJJ34481.1 azon|OJJ51758.1

Group1388: abra|OJJ66491.1 acar|OOF90967.1 acla|EAW10978.1 afis|EAW20881.1 afla|EED45568.1 afum|EAL84751.1 agla|OJJ83990.1 akaw|GAA91209.1 aluc|OJZ81544.1 anid|CBF82866.1 anig|EHA25891.1 aory|EIT75376.1 asyd|OJJ61599.1 ater|EAU30823.1 atub|OJI88640.1 aver|OJJ00923.1 awen|OJJ34479.1 azon|OJJ51756.1

Group1389: abra|OJJ66504.1 acar|OOF90968.1 acla|EAW10977.1 afis|EAW20882.1 afla|EED45567.1 afum|EAL84750.1 agla|OJJ83991.1 akaw|GAA91206.1 aluc|OJZ81541.1 anid|CBF82764.1 anig|EHA25895.1 aory|EIT75654.1 asyd|OJJ61604.1 ater|EAU30824.1 atub|OJI88645.1 aver|OJJ00927.1 awen|OJJ34478.1 azon|OJJ51755.1

Group1390: abra|OJJ66506.1 acar|OOF90970.1 acla|EAW10975.1 afis|EAW20885.1 afla|EED45570.1 afum|EAL84748.1 agla|OJJ83992.1 akaw|GAA91204.1 aluc|OJZ81539.1 anid|CBF82758.1 anig|EHA25897.1 aory|EIT75723.1 asyd|OJJ61607.1 ater|EAU30825.1 atub|OJI88647.1 aver|OJJ00930.1 awen|OJJ34462.1 azon|OJJ51752.1

Group1391: abra|OJJ66507.1 acar|OOF90971.1 acla|EAW10974.1 afis|EAW20886.1 afla|EED45571.1 afum|EAL84747.1 agla|OJJ83993.1 akaw|GAA91203.1 aluc|OJZ81538.1 anid|CBF82760.1 anig|EHA25898.1 aory|EIT75837.1 asyd|OJJ61606.1 ater|EAU30826.1 atub|OJI88648.1 aver|OJJ00929.1 awen|OJJ34461.1 azon|OJJ51750.1

Group1392: abra|OJJ66508.1 acar|OOF90972.1 acla|EAW10973.1 afis|EAW20887.1 afla|EED45572.1 afum|EAL84746.1 agla|OJJ83994.1 akaw|GAA91202.1 aluc|OJZ81537.1 anid|CBF82762.1 anig|EHA25899.1 aory|EIT75742.1 asyd|OJJ61605.1 ater|EAU30827.1 atub|OJI88649.1 aver|OJJ00928.1 awen|OJJ34460.1 azon|OJJ51749.1

Group1393: abra|OJJ66518.1 acar|OOF90973.1 acla|EAW10971.1 afis|EAW20888.1 afla|EED45576.1 afum|EAL84745.1 agla|OJJ83996.1 akaw|GAA91193.1 aluc|OJZ81526.1 anid|CBF82768.1 anig|EHA25908.1 aory|EIT75514.1 asyd|OJJ61601.1 ater|EAU30828.1 atub|OJI88659.1 aver|OJJ00925.1 awen|OJJ34459.1 azon|OJJ51748.1

Group1394: abra|OJJ66532.1 acar|OOF90983.1 acla|EAW10960.1 afis|EAW20899.1 afla|EED50249.1 afum|EAL84733.1 agla|OJJ84012.1 akaw|GAA91183.1 aluc|OJZ81520.1 anid|CBF82455.1 anig|EHA25917.1 aory|EIT77292.1 asyd|OJJ54114.1 ater|EAU38532.1 atub|OJI88669.1 aver|OJJ08621.1 awen|OJJ34449.1 azon|OJJ51735.1

Group1395: abra|OJJ66534.1 acar|OOF90986.1 acla|EAW10958.1 afis|EAW20901.1 afla|EED50251.1 afum|EAL84730.1 agla|OJJ84015.1 akaw|GAA91181.1 aluc|OJZ81522.1 anid|CBF82453.1 anig|EHA25920.1 aory|EIT77336.1 asyd|OJJ54112.1 ater|EAU38534.1 atub|OJI88671.1 aver|OJJ08623.1 awen|OJJ34447.1 azon|OJJ51733.1

Group1396: abra|OJJ66536.1 acar|OOF90987.1 acla|EAW10955.1 afis|EAW20906.1 afla|EED50254.1 afum|EAL84727.1 agla|OJJ84022.1 akaw|GAA91179.1 aluc|OJZ81524.1 anid|CBF82467.1 anig|EHA25922.1 aory|EIT77306.1 asyd|OJJ54098.1 ater|EAU38536.1 atub|OJI88673.1 aver|OJJ08640.1 awen|OJJ34445.1 azon|OJJ51728.1

Group1397: abra|OJJ66538.1 acar|OOF90989.1 acla|EAW10953.1 afis|EAW20908.1 afla|EED50256.1 afum|EAL84725.1 agla|OJJ84024.1 akaw|GAA91177.1 aluc|OJZ81509.1 anid|CBF82471.1 anig|EHA25924.1 aory|EIT77316.1 asyd|OJJ54100.1 ater|EAU38538.1 atub|OJI88675.1 aver|OJJ08638.1 awen|OJJ34443.1 azon|OJJ51720.1

Group1398: abra|OJJ66542.1 acar|OOF90994.1 acla|EAW09002.1 afis|EAW25543.1 afla|EED50261.1 afum|EAL84261.1 agla|OJJ84031.1 akaw|GAA91167.1 aluc|OJZ81495.1 anid|CBF80805.1 anig|EHA25935.1 aory|EIT77304.1 asyd|OJJ54485.1 ater|EAU38562.1 atub|OJI88686.1 aver|OJJ07694.1 awen|OJJ34423.1 azon|OJJ49761.1

Group1399: abra|OJJ66544.1 acar|OOF90996.1 acla|EAW10945.1 afis|EAW20913.1 afla|EED50263.1 afum|EAL84721.1 agla|OJJ79574.1 akaw|GAA91169.1 aluc|OJZ81497.1 anid|CBF84410.1 anig|EHA25933.1 aory|EIT77352.1 asyd|OJJ53562.1 ater|EAU38564.1 atub|OJI88684.1 aver|OJJ08190.1 awen|OJJ34421.1 azon|OJJ48150.1

Group1400: abra|OJJ66552.1 acar|OOF89951.1 acla|EAW10941.1 afis|EAW20919.1 afla|EED50273.1 afum|EAL84715.1 agla|OJJ80887.1 akaw|GAA91165.1 aluc|OJZ81492.1 anid|CBF82484.1 anig|EHA25937.1 aory|EIT77325.1 asyd|OJJ54104.1 atub|OJI88688.1 aver|OJJ08631.1 awen|OJJ34402.1 azon|OJJ49948.1 awen|OJJ35153.1

Group1401: abra|OJJ66558.1 acar|OOF90013.1 acla|EAW10937.1 afis|EAW20924.1 afla|EED50278.1 afum|EAL84710.1 agla|OJJ83965.1 akaw|GAA91158.1 aluc|OJZ81486.1 anid|CBF82449.1 anig|EHA25941.1 aory|EIT77317.1 asyd|OJJ54111.1 ater|EAU38571.1 atub|OJI88694.1 aver|OJJ08624.1 awen|OJJ34396.1 azon|OJJ48132.1

Group1402: abra|OJJ66562.1 acar|OOF91003.1 acla|EAW10934.1 afis|EAW20927.1 afla|EED50281.1 afum|EAL84707.1 agla|OJJ83961.1 akaw|GAA91155.1 aluc|OJZ81483.1 anid|CBF82497.1 anig|EHA25944.1 aory|EIT77267.1 asyd|OJJ54088.1 ater|EAU38573.1 atub|OJI88698.1 aver|OJJ08650.1 awen|OJJ34392.1 azon|OJJ48123.1

Group1403: abra|OJJ66563.1 acar|OOF91004.1 acla|EAW10933.1 afis|EAW20928.1 afla|EED50282.1 afum|EAL84706.1 agla|OJJ83960.1 akaw|GAA91154.1 aluc|OJZ81482.1 anid|CBF82515.1 anig|EHA25945.1

aory|EIT77363.1 asyd|OJJ54084.1 ater|EAU38574.1 atub|OJI88699.1 aver|OJJ08654.1 awen|OJJ34390.1 azon|OJJ48122.1

Group1404: abra|OJJ66564.1 acar|OOF91005.1 acla|EAW10932.1 afis|EAW20929.1 afla|EED50283.1 afum|EAL84705.1 agla|OJJ83959.1 akaw|GAA91153.1 aluc|OJZ81481.1 anid|CBF82513.1 anig|EHA25946.1 aory|EIT77279.1 asyd|OJJ54083.1 ater|EAU38575.1 atub|OJI88700.1 aver|OJJ08655.1 awen|OJJ34389.1 azon|OJJ48121.1

Group1405: abra|OJJ66568.1 acar|OOF91009.1 acla|EAW10927.1 afis|EAW20402.1 afla|EED50288.1 afum|EAL85501.1 agla|OJJ83952.1 akaw|GAA91149.1 aluc|OJZ81476.1 anid|CBF82504.1 anig|EHA25950.1 aory|EIT77254.1 asyd|OJJ54078.1 ater|EAU38579.1 atub|OJI88703.1 aver|OJJ08660.1 awen|OJJ34384.1 azon|OJJ48115.1

Group1406: abra|OJJ66570.1 acar|OOF91010.1 acla|EAW10924.1 afis|EAW20934.1 afla|EED50291.1 afum|EAL84700.1 agla|OJJ83951.1 akaw|GAA91148.1 aluc|OJZ81474.1 anid|CBF82532.1 anig|EHA25951.1 aory|EIT77301.1 asyd|OJJ54076.1 ater|EAU38580.1 atub|OJI88704.1 aver|OJJ08663.1 awen|OJJ34382.1 azon|OJJ48114.1

Group1407: abra|OJJ66573.1 acar|OOF91014.1 acla|EAW10917.1 afis|EAW20941.1 afla|EED50296.1 afum|EAL84693.1 agla|OJJ83946.1 akaw|GAA91145.1 aluc|OJZ81471.1 anid|CBF82534.1 anig|EHA25956.1 aory|EIT77257.1 asyd|OJJ54069.1 ater|EAU38586.1 atub|OJI88707.1 aver|OJJ08673.1 awen|OJJ34376.1 azon|OJJ50768.1

Group1408: abra|OJJ66576.1 acar|OOF91017.1 acla|EAW10914.1 afis|EAW20944.1 afla|EED50298.1 afum|EAL84690.2 agla|OJJ83943.1 akaw|GAA91142.1 aluc|OJZ81468.1 anid|CBF82539.1 anig|EHA25959.1 aory|EIT77376.1 asyd|OJJ54066.1 ater|EAU38588.1 atub|OJI88710.1 aver|OJJ08676.1 awen|OJJ34372.1 azon|OJJ48108.1

Group1409: abra|OJJ66577.1 acar|OOF91018.1 acla|EAW10913.1 afis|EAW20945.1 afla|EED50299.1 afum|EAL84689.2 agla|OJJ83941.1 akaw|GAA91141.1 aluc|OJZ81467.1 anid|CBF82541.1 anig|EHA25960.1 aory|EIT77303.1 asyd|OJJ54065.1 ater|EAU38596.1 atub|OJI88711.1 aver|OJJ08677.1 awen|OJJ34371.1 azon|OJJ48107.1

Group1410: abra|OJJ66579.1 acar|OOF91019.1 acla|EAW10911.1 afis|EAW20947.1 afla|EED50301.1 afum|EAL84687.1 agla|OJJ83939.1 akaw|GAA91139.1 aluc|OJZ81465.1 anid|CBF82545.1 anig|EHA25962.1 aory|EIT77341.1 asyd|OJJ54063.1 ater|EAU38598.1 atub|OJI88713.1 aver|OJJ08679.1 awen|OJJ34369.1 azon|OJJ48105.1

Group1411: abra|OJJ66580.1 acar|OOF91020.1 acla|EAW10910.1 afis|EAW20948.1 afla|EED50302.1 afum|EAL84686.1 agla|OJJ83938.1 akaw|GAA91138.1 aluc|OJZ81464.1 anid|CBF82547.1 anig|EHA25963.1 aory|EIT77278.1 asyd|OJJ54062.1 ater|EAU38599.1 atub|OJI88714.1 aver|OJJ08680.1 awen|OJJ34368.1 azon|OJJ48104.1

Group1412: abra|OJJ66581.1 acar|OOF89819.1 acla|EAW10909.1 afis|EAW20949.1 afla|EED50303.1 afum|EAL84685.1 agla|OJJ83936.1 akaw|GAA91137.1 aluc|OJZ81463.1 anid|CBF82549.1 anig|EHA25964.1 aory|EIT77276.1 asyd|OJJ54061.1 ater|EAU38600.1 atub|OJI88715.1 aver|OJJ08681.1 awen|OJJ34367.1 azon|OJJ48103.1

Group1413: abra|OJJ66583.1 acar|OOF91021.1 acla|EAW10908.1 afis|EAW20950.1 afla|EED50305.1 afum|EAL84684.1 agla|OJJ83930.1 akaw|GAA91135.1 aluc|OJZ81462.1 anid|CBF82550.1 anig|EHA25966.1 aory|EIT77342.1 asyd|OJJ54060.1 ater|EAU38601.1 atub|OJI88716.1 aver|OJJ08682.1 awen|OJJ34366.1 azon|OJJ48101.1

Group1414: abra|OJJ66587.1 acar|OOF91024.1 acla|EAW10904.1 afis|EAW20954.1 afla|EED50310.1 afum|EAL84680.2 agla|OJJ83925.1 akaw|GAA91130.1 aluc|OJZ81459.1 anid|CBF82558.1 anig|EHA25970.1 aory|EIT77289.1 asyd|OJJ54056.1 ater|EAU38605.1 atub|OJI88720.1 aver|OJJ08685.1 awen|OJJ34362.1 azon|OJJ48098.1

Group1415: abra|OJJ66588.1 acar|OOF91025.1 acla|EAW10903.1 afis|EAW20955.1 afla|EED50311.1 afum|EAL84679.1 agla|OJJ83924.1 akaw|GAA91129.1 aluc|OJZ81458.1 anid|CBF82560.1 anig|EHA25971.1 aory|EIT77266.1 asyd|OJJ54054.1 ater|EAU38606.1 atub|OJI88721.1 aver|OJJ08687.1 awen|OJJ34361.1 azon|OJJ48097.1

Group1416: abra|OJJ66589.1 acar|OOF91026.1 acla|EAW10902.1 afis|EAW20956.1 afla|EED50312.1 afum|EAL84677.1 agla|OJJ83922.1 akaw|GAA91128.1 aluc|OJZ81457.1 anid|CBF82562.1 anig|EHA25972.1 aory|EIT77295.1 asyd|OJJ54052.1 ater|EAU38607.1 atub|OJI88722.1 aver|OJJ08688.1 awen|OJJ34360.1 azon|OJJ48096.1

Group1417: abra|OJJ66590.1 acar|OOF91027.1 acla|EAW10901.1 afis|EAW20957.1 afla|EED50313.1 afum|EAL84676.1 agla|OJJ83920.1 akaw|GAA91127.1 aluc|OJZ81456.1 anid|CBF82564.1 anig|EHA25973.1 aory|EIT77362.1 asyd|OJJ54051.1 ater|EAU38608.1 atub|OJI88723.1 aver|OJJ08689.1 awen|OJJ34359.1 azon|OJJ48095.1

Group1418: abra|OJJ66596.1 acar|OOF91030.1 acla|EAW10896.1 afis|EAW20962.1 afla|EED50317.1 afum|EAL84671.1 agla|OJJ83916.1 akaw|GAA91122.1 aluc|OJZ81450.1 anid|CBF82571.1 anig|EHA25976.1 aory|EIT77307.1 asyd|OJJ54048.1 ater|EAU38612.1 atub|OJI88729.1 aver|OJJ08695.1 awen|OJJ34354.1 azon|OJJ48091.1

Group1419: abra|OJJ66598.1 acar|OOF91031.1 acla|EAW10895.1 afis|EAW20963.1 afla|EED50318.1 afum|EAL84670.1 agla|OJJ83915.1 akaw|GAA91121.1 aluc|OJZ81449.1 anid|CBF82572.1 anig|EHA25977.1 aory|EIT77348.1 asyd|OJJ54047.1 ater|EAU38613.1 atub|OJI88730.1 aver|OJJ08696.1 awen|OJJ34352.1 azon|OJJ48089.1

Group1420: abra|OJJ66600.1 acar|OOF91033.1 acla|EAW10892.1 afis|EAW20966.1 afla|EED50320.1 afum|EAL84667.1 agla|OJJ84043.1 akaw|GAA91119.1 aluc|OJZ81446.1 anid|CBF82578.1 anig|EHA25979.1 aory|EIT77255.1 asyd|OJJ54042.1 ater|EAU38616.1 atub|OJI88732.1 aver|OJJ08699.1 awen|OJJ34349.1 azon|OJJ48086.1

Group1421: abra|OJJ66601.1 acar|OOF91034.1 acla|EAW10891.1 afis|EAW20967.1 afla|EED50321.1 afum|EAL84666.1 agla|OJJ84044.1 akaw|GAA91118.1 aluc|OJZ81445.1 anid|CBF82580.1 anig|EHA25980.1 aory|EIT77332.1 asyd|OJJ54041.1 ater|EAU38617.1 atub|OJI88733.1 aver|OJJ08700.1 awen|OJJ34348.1 azon|OJJ48085.1

Group1422: abra|OJJ66604.1 acar|OOF90114.1 acla|EAW10889.1 afis|EAW20969.1 afla|EED50323.1 afum|EAL84665.1 agla|OJJ84046.1 akaw|GAA91116.1 aluc|OJZ81442.1 anid|CBF82584.1 anig|EHA25982.1 aory|EIT77349.1 asyd|OJJ54039.1 ater|EAU38619.1 atub|OJI88736.1 aver|OJJ08701.1 awen|OJJ34346.1 azon|OJJ48084.1

Group1423: abra|OJJ66606.1 acar|OOF90112.1 acla|EAW10887.1 afis|EAW20971.1 afla|EED50325.1 afum|EAL84663.1 agla|OJJ84048.1 akaw|GAA91114.1 aluc|OJZ81440.1 anid|CBF82585.1 anig|EHA25984.1 aory|EIT77291.1 asyd|OJJ54037.1 ater|EAU38620.1 atub|OJI88738.1 aver|OJJ08703.1 awen|OJJ34344.1 azon|OJJ48083.1

Group1424: abra|OJJ66610.1 acar|OOF91035.1 acla|EAW10886.1 afis|EAW20972.1 afla|EED50326.1 afum|EAL84661.1 agla|OJJ84050.1 akaw|GAA91113.1 aluc|OJZ81439.1 anid|CBF82587.1 anig|EHA25985.1 aory|EIT77361.1 asyd|OJJ54036.1 ater|EAU38622.1 atub|OJI88742.1 aver|OJJ08704.1 awen|OJJ34341.1 azon|OJJ48081.1

Group1425: abra|OJJ66612.1 acar|OOF91036.1 acla|EAW10884.1 afis|EAW20974.1 afla|EED50329.1 afum|EAL84659.1 agla|OJJ84052.1 akaw|GAA91111.1 aluc|OJZ81437.1 anid|CBF82593.1 anig|EHA25987.1 aory|EIT77331.1 asyd|OJJ54033.1 ater|EAU38624.1 atub|OJI88744.1 aver|OJJ08707.1 awen|OJJ34339.1 azon|OJJ48079.1

Group1426: abra|OJJ66613.1 acar|OOF91038.1 acla|EAW10883.1 afis|EAW20975.1 afla|EED50330.1 afum|EAL84658.1 agla|OJJ84053.1 akaw|GAA91110.1 aluc|OJZ81436.1 anid|CBF82595.1 anig|EHA25988.1 aory|EIT77270.1 asyd|OJJ54032.1 ater|EAU38625.1 atub|OJI88745.1 aver|OJJ08708.1 awen|OJJ34337.1 azon|OJJ48078.1

Group1427: abra|OJJ66617.1 acar|OOF91040.1 acla|EAW10880.1 afis|EAW20978.1 afla|EED50333.1 afum|EAL84655.1 agla|OJJ84055.1 akaw|GAA91108.1 aluc|OJZ81432.1 anid|CBF82598.1 anig|EHA25990.1 aory|EIT77374.1 asyd|OJJ54030.1 ater|EAU38627.1 atub|OJI88748.1 aver|OJJ08710.1 awen|OJJ34334.1 azon|OJJ48076.1

Group1428: abra|OJJ66619.1 acar|OOF91042.1 acla|EAW10878.1 afis|EAW20980.1 afla|EED50335.1 afum|EAL84651.2 agla|OJJ84057.1 akaw|GAA91106.1 aluc|OJZ81430.1 anid|CBF71772.1 anig|EHA25992.1 aory|EIT77286.1 asyd|OJJ61720.1 ater|EAU38628.1 atub|OJI88750.1 aver|OJI95814.1 awen|OJJ34332.1 azon|OJJ48074.1

Group1429: abra|OJJ66624.1 acar|OOF91048.1 acla|EAW10872.1 afis|EAW20986.1 afla|EED50341.1 afum|EAL84644.1 agla|OJJ84065.1 akaw|GAA91101.1 aluc|OJZ81425.1 anid|CBF82611.1 anig|EHA25997.1 aory|EIT77327.1 asyd|OJJ54022.1 ater|EAU38635.1 atub|OJI88756.1 aver|OJJ08718.1 awen|OJJ34325.1 azon|OJJ48071.1

Group1430: abra|OJJ66627.1 acar|OOF91052.1 acla|EAW10869.1 afis|EAW20989.1 afla|EED50343.1 afum|EAL84640.2 agla|OJJ84067.1 akaw|GAA91098.1 aluc|OJZ81421.1 anid|CBF82605.1 anig|EHA26000.1 aory|EIT77281.1 asyd|OJJ54026.1 ater|EAU38638.1 atub|OJI88760.1 aver|OJJ08714.1 awen|OJJ34321.1 azon|OJJ48066.1

Group1431: abra|OJJ66749.1 acar|OOF93832.1 acla|EAW11875.1 afis|EAW19486.1 afla|EED56704.1 afum|EAL87008.1 agla|OJJ81211.1 akaw|GAA89685.1 aluc|OJZ80700.1 anid|CBF75460.1 anig|EHA27316.1 aory|EIT73946.1 asyd|OJJ59769.1 ater|EAU34799.1 atub|OJI87559.1 aver|OJJ02516.1 awen|OJJ36902.1 azon|OJJ42915.1

Group1432: abra|OJJ66751.1 acar|OOF93833.1 acla|EAW11876.1 afis|EAW19487.1 afla|EED56705.1 afum|EAL87007.1 agla|OJJ81213.1 akaw|GAA89686.1 aluc|OJZ80699.1 anid|CBF75458.1 anig|EHA27317.1

aory|EIT73903.1 asyd|OJJ59771.1 ater|EAU34800.1 atub|OJI87558.1 aver|OJJ02515.1 awen|OJJ36903.1 azon|OJJ42913.1

Group1433: abra|OJJ66752.1 acar|OOF93834.1 acla|EAW11877.1 afis|EAW19488.1 afla|EED56706.1 afum|EAL87006.1 agla|OJJ81214.1 akaw|GAA89687.1 aluc|OJZ80698.1 anid|CBF75456.1 anig|EHA27318.1 aory|EIT73834.1 asyd|OJJ59772.1 ater|EAU34801.1 atub|OJI87557.1 aver|OJJ02514.1 awen|OJJ36904.1 azon|OJJ42912.1

Group1434: abra|OJJ66753.1 acar|OOF93835.1 acla|EAW11880.1 afis|EAW19491.1 afla|EED56710.1 afum|EAL87003.1 agla|OJJ81220.1 akaw|GAA89688.1 aluc|OJZ80697.1 anid|CBF75452.1 anig|EHA27319.1 aory|EIT73847.1 asyd|OJJ59774.1 ater|EAU34803.1 atub|OJI87556.1 aver|OJJ02512.1 awen|OJJ36907.1 azon|OJJ42909.1

Group1435: abra|OJJ66755.1 acar|OOF93836.1 acla|EAW11881.1 afis|EAW19492.1 afla|EED56711.1 afum|EAL87002.1 agla|OJJ81221.1 akaw|GAA89689.1 aluc|OJZ80696.1 anid|CBF75450.1 anig|EHA27320.1 aory|EIT73907.1 asyd|OJJ59775.1 ater|EAU34804.1 atub|OJI87555.1 aver|OJJ02511.1 awen|OJJ36908.1 azon|OJJ42908.1

Group1436: abra|OJJ66773.1 acar|OOF91583.1 acla|EAW15030.1 afis|EAW20305.1 agla|OJJ85746.1 akaw|GAA87178.1 aluc|OJZ84255.1 asyd|OJJ60493.1 atub|OJI89828.1 awen|OJJ36138.1 acla|EAW10581.1 afla|EED52812.1 aory|EIT78206.1 awen|OJJ37047.1 afis|EAW17210.1 azon|OJJ49313.1 afum|EAL85433.1 awen|OJJ32513.1

Group1437: abra|OJJ66819.1 acar|OOF91602.1 acla|EAW14405.1 afis|EAW19113.1 afla|EED54301.1 afum|EAL93845.1 agla|OJJ84489.1 akaw|GAA90457.1 aluc|OJZ85767.1 anid|CBF79734.1 anig|EHA24786.1 aory|EIT76106.1 asyd|OJJ58069.1 ater|EAU37372.1 atub|OJI79552.1 aver|OJJ04338.1 awen|OJJ32523.1 azon|OJJ50791.1

Group1438: abra|OJJ66828.1 acar|OOF91606.1 acla|EAW14411.1 afis|EAW19119.1 afla|EED54295.1 afum|EAL93851.1 agla|OJJ80434.1 akaw|GAA90464.1 aluc|OJZ85759.1 anid|CBF79724.1 anig|EHA24797.1 aory|EIT76143.1 asyd|OJJ58075.1 ater|EAU37382.1 atub|OJI79544.1 aver|OJJ04332.1 awen|OJJ37450.1 azon|OJJ51127.1

Group1439: abra|OJJ66830.1 acar|OOF91608.1 acla|EAW14413.1 afis|EAW19121.1 afla|EED54293.1 afum|EAL93853.1 agla|OJJ80436.1 akaw|GAA90467.1 aluc|OJZ85757.1 anid|CBF79722.1 anig|EHA24799.1 aory|EIT76028.1 asyd|OJJ58077.1 ater|EAU37384.1 atub|OJI79542.1 aver|OJJ04330.1 awen|OJJ37452.1 azon|OJJ51129.1

Group1440: abra|OJJ66832.1 acar|OOF91609.1 acla|EAW14414.1 afis|EAW19122.1 afla|EED54292.1 afum|EAL93854.1 agla|OJJ80437.1 akaw|GAA90468.1 aluc|OJZ85756.1 anid|CBF79720.1 anig|EHA24800.1 aory|EIT76096.1 asyd|OJJ58078.1 ater|EAU37385.1 atub|OJI79541.1 aver|OJJ04329.1 awen|OJJ37453.1 azon|OJJ51130.1

Group1441: abra|OJJ66833.1 acar|OOF91610.1 acla|EAW14415.1 afis|EAW19123.1 afla|EED54291.1 afum|EAL93855.1 agla|OJJ80438.1 akaw|GAA90469.1 aluc|OJZ85755.1 anid|CBF79718.1 anig|EHA24801.1 aory|EIT76111.1 asyd|OJJ58079.1 ater|EAU37386.1 atub|OJI79540.1 aver|OJJ04328.1 awen|OJJ37454.1 azon|OJJ51131.1

Group1442: abra|OJJ66834.1 acar|OOF91611.1 acla|EAW14416.1 afis|EAW19124.1 afla|EED54290.1 afum|EAL93856.1 agla|OJJ80439.1 akaw|GAA90470.1 aluc|OJZ85754.1 anid|CBF79717.1 anig|EHA24802.1 aory|EIT76047.1 asyd|OJJ58080.1 ater|EAU37387.1 atub|OJI79538.1 aver|OJJ04327.1 awen|OJJ37455.1 azon|OJJ51134.1

Group1443: abra|OJJ66835.1 acar|OOF91613.1 acla|EAW14419.1 afis|EAW19126.1 afla|EED54288.1 afum|EAL93858.1 agla|OJJ80441.1 akaw|GAA90472.1 aluc|OJZ85752.1 anid|CBF79737.1 anig|EHA24804.1 aory|EIT75920.1 asyd|OJJ58083.1 ater|EAU37393.1 atub|OJI79535.1 aver|OJJ04325.1 awen|OJJ37457.1 azon|OJJ51137.1

Group1444: abra|OJJ66836.1 acar|OOF91614.1 acla|EAW14420.1 afis|EAW19127.1 afla|EED54287.1 afum|EAL93859.1 agla|OJJ88596.1 akaw|GAA90473.1 aluc|OJZ85751.1 anid|CBF79739.1 anig|EHA24805.1 aory|EIT75974.1 asyd|OJJ58084.1 ater|EAU37394.1 atub|OJI79534.1 aver|OJJ04324.1 awen|OJJ37459.1 azon|OJJ51138.1

Group1445: abra|OJJ66840.1 acar|OOF91617.1 agla|OJJ85152.1 akaw|GAA90476.1 aluc|OJZ85747.1 anig|EHA24808.1 atub|OJI79532.1 aver|OJI98647.1 awen|OJJ33714.1 asyd|OJJ59197.1 azon|OJJ44469.1 acla|EAW12945.1 afla|EED50142.1 aluc|OJZ85744.1 aory|EIT74655.1 asyd|OJJ64551.1 atub|OJI79529.1 azon|OJJ49271.1

Group1446: abra|OJJ66843.1 acar|OOF91620.1 acla|EAW14417.1 afis|EAW19130.1 afla|EED54284.1 afum|EAL93862.1 agla|OJJ88602.1 akaw|GAA90479.1 aluc|OJZ85740.1 anid|CBF79758.1 anig|EHA24811.1 aory|EIT76041.1 asyd|OJJ58100.1 ater|EAU37398.1 atub|OJI79525.1 aver|OJJ04304.1 awen|OJJ37465.1 azon|OJJ51143.1

Group1447: abra|OJJ66846.1 acar|OOF91624.1 acla|EAW14425.1 afis|EAW19132.1 afla|EED54281.1 afum|EAL93864.1 agla|OJJ88607.1 akaw|GAA91559.1 aluc|OJZ85737.1 anid|CBF86338.1 anig|EHA24813.1 aory|EIT76060.1 asyd|OJJ64988.1 ater|EAU37405.1 atub|OJI79522.1 aver|OJI99108.1 awen|OJJ37468.1 azon|OJJ51146.1

Group1448: abra|OJJ66847.1 acar|OOF91625.1 acla|EAW14426.1 afis|EAW19133.1 afla|EED54280.1 afum|EAL93865.1 agla|OJJ88608.1 akaw|GAA91558.1 aluc|OJZ85736.1 anid|CBF86336.1 anig|EHA24814.1 aory|EIT76085.1 asyd|OJJ64989.1 ater|EAU37401.1 atub|OJI79521.1 aver|OJI99109.1 awen|OJJ37469.1 azon|OJJ51147.1

Group1449: abra|OJJ66853.1 acar|OOF91629.1 acla|EAW14431.1 afis|EAW19138.1 afla|EED54275.1 afum|EAL93870.1 agla|OJJ88617.1 akaw|GAA91553.1 aluc|OJZ85731.1 anid|CBF86324.1 anig|EHA24818.1 aory|EIT75921.1 asyd|OJJ64993.1 ater|EAU37408.1 atub|OJI79516.1 aver|OJI99112.1 awen|OJJ37475.1 azon|OJJ51155.1

Group1450: abra|OJJ66854.1 acar|OOF91630.1 acla|EAW14432.1 afis|EAW19139.1 afla|EED54274.1 afum|EAL93871.2 agla|OJJ88591.1 akaw|GAA91552.1 aluc|OJZ85730.1 anid|CBF86322.1 anig|EHA24819.1 aory|EIT75977.1 asyd|OJJ64994.1 ater|EAU37410.1 atub|OJI79515.1 aver|OJI99113.1 awen|OJJ37476.1 azon|OJJ51157.1

Group1451: abra|OJJ66855.1 acar|OOF91632.1 acla|EAW14433.1 afis|EAW19141.1 afla|EED54272.1 afum|EAL93872.1 agla|OJJ88589.1 akaw|GAA91551.1 aluc|OJZ85728.1 anid|CBF86321.1 anig|EHA24820.1 aory|EIT76058.1 asyd|OJJ64995.1 ater|EAU37412.1 atub|OJI79514.1 aver|OJI99115.1 awen|OJJ37481.1 azon|OJJ51158.1

Group1452: abra|OJJ66856.1 acar|OOF91633.1 acla|EAW14434.1 afis|EAW19142.1 afla|EED54271.1 afum|EAL93873.1 agla|OJJ88588.1 akaw|GAA91550.1 aluc|OJZ85727.1 anid|CBF86317.1 anig|EHA24821.1 aory|EIT76153.1 asyd|OJJ64997.1 ater|EAU37413.1 atub|OJI79513.1 aver|OJI99117.1 awen|OJJ37482.1 azon|OJJ51159.1

Group1453: abra|OJJ66858.1 acar|OOF91636.1 acla|EAW14437.1 afis|EAW19145.1 afla|EED54268.1 afum|EAL93876.1 agla|OJJ88586.1 akaw|GAA91547.1 aluc|OJZ85725.1 anid|CBF86313.1 anig|EHA24823.1 aory|EIT76154.1 asyd|OJJ65000.1 ater|EAU37417.1 atub|OJI79511.1 aver|OJI99120.1 awen|OJJ37484.1 azon|OJJ51161.1

Group1454: abra|OJJ66861.1 acar|OOF91638.1 acla|EAW14438.1 afis|EAW19146.1 afla|EED54267.1 afum|EAL93877.1 agla|OJJ88584.1 akaw|GAA91546.1 aluc|OJZ85721.1 anid|CBF86311.1 anig|EHA24824.1 aory|EIT76119.1 asyd|OJJ65001.1 ater|EAU37420.1 atub|OJI79510.1 aver|OJI99121.1 awen|OJJ37485.1 azon|OJJ51163.1

Group1455: abra|OJJ66864.1 acar|OOF91640.1 acla|EAW14440.1 afis|EAW19148.1 afla|EED54265.1 afum|EAL93879.1 agla|OJJ88576.1 akaw|GAA91544.1 aluc|OJZ85719.1 anid|CBF86308.1 anig|EHA24826.1 aory|EIT75979.1 asyd|OJJ65004.1 ater|EAU37421.1 atub|OJI79508.1 aver|OJI99123.1 awen|OJJ37501.1 azon|OJJ51166.1

Group1456: abra|OJJ66866.1 acar|OOF91643.1 acla|EAW14442.1 afis|EAW19150.1 afla|EED54257.1 afum|EAL93881.1 agla|OJJ88572.1 akaw|GAA91542.1 aluc|OJZ85716.1 anid|CBF86305.1 anig|EHA24828.1 aory|EIT76051.1 asyd|OJJ65006.1 ater|EAU37424.1 atub|OJI79506.1 aver|OJI99125.1 awen|OJJ37503.1 azon|OJJ51168.1

Group1457: abra|OJJ66869.1 acar|OOF91646.1 acla|EAW14445.1 afis|EAW19153.1 afla|EED54252.1 afum|EAL93884.1 agla|OJJ88569.1 akaw|GAA91539.1 aluc|OJZ85712.1 anid|CBF86299.1 anig|EHA24831.1 aory|EIT76023.1 asyd|OJJ65009.1 ater|EAU37426.1 atub|OJI79503.1 aver|OJI99128.1 awen|OJJ37506.1 azon|OJJ51172.1

Group1458: abra|OJJ66871.1 acar|OOF91647.1 acla|EAW14446.1 afis|EAW19154.1 afla|EED54251.1 afum|EAL93885.1 agla|OJJ88568.1 akaw|GAA91538.1 aluc|OJZ85711.1 anid|CBF86297.1 anig|EHA24832.1 aory|EIT75939.1 asyd|OJJ65010.1 ater|EAU37427.1 atub|OJI79502.1 aver|OJI99129.1 awen|OJJ37507.1 azon|OJJ51173.1

Group1459: abra|OJJ66873.1 acar|OOF91649.1 acla|EAW14448.1 afis|EAW19156.1 afla|EED54246.1 afum|EAL93887.1 agla|OJJ88566.1 akaw|GAA91536.1 aluc|OJZ85708.1 anid|CBF86294.1 anig|EHA24834.1 aory|EIT75988.1 asyd|OJJ65012.1 ater|EAU37429.1 atub|OJI79499.1 aver|OJI99131.1 awen|OJJ37509.1 azon|OJJ51174.1

Group1460: abra|OJJ66874.1 acar|OOF91650.1 acla|EAW14449.1 afis|EAW19157.1 afla|EED54245.1 afum|EBA27505.1 agla|OJJ88565.1 akaw|GAA91535.1 aluc|OJZ85707.1 anid|CBF86292.1 anig|EHA24835.1 aory|EIT75937.1 asyd|OJJ65013.1 ater|EAU37430.1 atub|OJI79498.1 aver|OJI99132.1 awen|OJJ37510.1 azon|OJJ51175.1

Group1461: abra|OJJ66875.1 acar|OOF91651.1 acla|EAW14450.1 afis|EAW19158.1 afla|EED54244.1 afum|EBA27506.1 agla|OJJ88564.1 akaw|GAA91534.1 aluc|OJZ85706.1 anid|CBF86291.1 anig|EHA24836.1

aory|EIT76133.1 asyd|OJJ65014.1 ater|EAU37431.1 atub|OJI79497.1 aver|OJI99133.1 awen|OJJ37511.1 azon|OJJ51176.1

Group1462: abra|OJJ66879.1 acar|OOF91654.1 acla|EAW14454.1 afis|EAW19162.1 afla|EED54240.1 afum|EAL93892.2 agla|OJJ88562.1 akaw|GAA91530.1 aluc|OJZ85703.1 anid|CBF86285.1 anig|EHA24840.1 aory|EIT76157.1 asyd|OJJ65017.1 ater|EAU37311.1 atub|OJI79493.1 aver|OJI99135.1 awen|OJJ37515.1 azon|OJJ51178.1

Group1463: abra|OJJ66880.1 acar|OOF91655.1 acla|EAW14455.1 afis|EAW19163.1 afla|EED54239.1 afum|EAL93893.1 agla|OJJ88561.1 akaw|GAA91529.1 aluc|OJZ85702.1 anid|CBF86282.1 anig|EHA24841.1 aory|EIT76014.1 asyd|OJJ65018.1 ater|EAU37310.1 atub|OJI79492.1 aver|OJI99136.1 awen|OJJ37516.1 azon|OJJ51181.1

Group1464: abra|OJJ66883.1 acar|OOF91657.1 acla|EAW14457.1 afis|EAW19165.1 afla|EED54232.1 afum|EAL93895.1 agla|OJJ88559.1 akaw|GAA91527.1 aluc|OJZ85699.1 anid|CBF86279.1 anig|EHA24843.1 aory|EIT75967.1 asyd|OJJ65020.1 ater|EAU37308.1 atub|OJI79489.1 aver|OJI99138.1 awen|OJJ37518.1 azon|OJJ51185.1

Group1465: abra|OJJ66887.1 acar|OOF91660.1 acla|EAW14459.1 afis|EAW19167.1 afla|EED54230.1 afum|EAL93897.1 agla|OJJ88557.1 akaw|GAA91525.1 aluc|OJZ85696.1 anid|CBF86276.1 anig|EHA24846.1 aory|EIT75971.1 asyd|OJJ65022.1 ater|EAU37306.1 atub|OJI79485.1 aver|OJI99141.1 awen|OJJ37520.1 azon|OJJ51186.1

Group1466: abra|OJJ66889.1 acar|OOF91661.1 acla|EAW14461.1 afis|EAW19169.1 afla|EED54228.1 afum|EAL93899.1 agla|OJJ88555.1 akaw|GAA91523.1 aluc|OJZ85693.1 anid|CBF86273.1 anig|EHA24848.1 aory|EIT76026.1 asyd|OJJ65024.1 ater|EAU37304.1 atub|OJI79483.1 aver|OJI99143.1 awen|OJJ37522.1 azon|OJJ51188.1

Group1467: abra|OJJ66890.1 acar|OOF91662.1 acla|EAW14462.1 afis|EAW19170.1 afla|EED54226.1 afum|EAL93900.1 agla|OJJ88554.1 akaw|GAA91522.1 aluc|OJZ85692.1 anid|CBF86271.1 anig|EHA24849.1 aory|EIT75952.1 asyd|OJJ65025.1 ater|EAU37303.1 atub|OJI79482.1 aver|OJI99144.1 awen|OJJ37523.1 azon|OJJ51189.1

Group1468: abra|OJJ66891.1 acar|OOF91663.1 acla|EAW14463.1 afis|EAW19171.1 afla|EED54224.1 afum|EAL93901.1 agla|OJJ88553.1 akaw|GAA91521.1 aluc|OJZ85691.1 anid|CBF86270.1 anig|EHA24850.1 aory|EIT75965.1 asyd|OJJ65026.1 ater|EAU37302.1 atub|OJI79481.1 aver|OJI99145.1 awen|OJJ37524.1 azon|OJJ51190.1

Group1469: abra|OJJ66893.1 acar|OOF91664.1 acla|EAW14465.1 afis|EAW19173.1 afla|EED54222.1 afum|EAL93903.1 agla|OJJ88551.1 akaw|GAA91519.1 aluc|OJZ85689.1 anid|CBF86266.1 anig|EHA24852.1 aory|EIT76009.1 asyd|OJJ65029.1 ater|EAU37300.1 atub|OJI79479.1 aver|OJI99148.1 awen|OJJ37526.1 azon|OJJ51193.1

Group1470: abra|OJJ66894.1 acar|OOF91665.1 acla|EAW14466.1 afis|EAW19174.1 afla|EED54221.1 afum|EAL93904.1 agla|OJJ88550.1 akaw|GAA91518.1 aluc|OJZ85688.1 anid|CBF86265.1 anig|EHA24853.1 aory|EIT76027.1 asyd|OJJ65030.1 ater|EAU37299.1 atub|OJI79478.1 aver|OJI99149.1 awen|OJJ37527.1 azon|OJJ51194.1

Group1471: abra|OJJ66895.1 acar|OOF91666.1 acla|EAW14467.1 afis|EAW19175.1 afla|EED54220.1 afum|EAL93905.1 agla|OJJ88548.1 akaw|GAA91517.1 aluc|OJZ85687.1 anid|CBF86263.1 anig|EHA24854.1 aory|EIT75931.1 asyd|OJJ65031.1 ater|EAU37298.1 atub|OJI79477.1 aver|OJI99150.1 awen|OJJ37528.1 azon|OJJ51195.1

Group1472: abra|OJJ66896.1 acar|OOF91667.1 acla|EAW14468.1 afis|EAW19176.1 afla|EED54219.1 afum|EAL93906.1 agla|OJJ88547.1 akaw|GAA91516.1 aluc|OJZ85686.1 anid|CBF86262.1 anig|EHA24855.1 aory|EIT76150.1 asyd|OJJ65032.1 ater|EAU37297.1 atub|OJI79476.1 aver|OJI99151.1 awen|OJJ37530.1 azon|OJJ51196.1

Group1473: abra|OJJ66897.1 acar|OOF91668.1 acla|EAW14469.1 afis|EAW19177.1 afla|EED54218.1 afum|EAL93907.1 agla|OJJ88546.1 akaw|GAA91515.1 aluc|OJZ85685.1 anid|CBF86260.1 anig|EHA24856.1 aory|EIT75968.1 asyd|OJJ65033.1 ater|EAU37296.1 atub|OJI79475.1 aver|OJI99152.1 awen|OJJ37531.1 azon|OJJ51197.1

Group1474: abra|OJJ66899.1 acar|OOF91671.1 acla|EAW14470.1 afis|EAW19179.1 afla|EED54217.1 afum|EAL93909.1 agla|OJJ88544.1 akaw|GAA91513.1 aluc|OJZ85683.1 anid|CBF86259.1 anig|EHA24858.1 aory|EIT76147.1 asyd|OJJ65035.1 ater|EAU37294.1 atub|OJI79473.1 aver|OJI99154.1 awen|OJJ37533.1 azon|OJJ51199.1

Group1475: abra|OJJ66900.1 acar|OOF91672.1 acla|EAW14471.1 afis|EAW19180.1 afla|EED54216.1 afum|EAL93910.1 agla|OJJ88543.1 akaw|GAA91512.1 aluc|OJZ85682.1 anid|CBF86257.1 anig|EHA24859.1 aory|EIT76034.1 asyd|OJJ65036.1 ater|EAU37293.1 atub|OJI79472.1 aver|OJI99155.1 awen|OJJ37534.1 azon|OJJ51200.1

Group1476: abra|OJJ66903.1 acar|OOF91673.1 acla|EAW14473.1 afis|EAW19182.1 afla|EED54213.1 afum|EAL93912.1 agla|OJJ88540.1 akaw|GAA91510.1 aluc|OJZ85680.1 anid|CBF86253.1 anig|EHA24861.1 aory|EIT76125.1 asyd|OJJ65038.1 ater|EAU37291.1 atub|OJI79470.1 aver|OJI99157.1 awen|OJJ37536.1 azon|OJJ51202.1

Group1477: abra|OJJ66905.1 acar|OOF91675.1 acla|EAW14474.1 afis|EAW19183.1 afla|EED54212.1 afum|EAL93913.1 agla|OJJ88539.1 akaw|GAA91509.1 aluc|OJZ85676.1 anid|CBF86252.1 anig|EHA24864.1 aory|EIT75985.1 asyd|OJJ65041.1 ater|EAU37290.1 atub|OJI79467.1 aver|OJI99159.1 awen|OJJ37538.1 azon|OJJ51203.1

Group1478: abra|OJJ66906.1 acar|OOF91676.1 acla|EAW14475.1 afis|EAW19184.1 afla|EED54211.1 afum|EAL93915.1 akaw|GAA91508.1 aluc|OJZ85675.1 anid|CBF86250.1 anig|EHA24865.1 aory|EIT76099.1 asyd|OJJ65042.1 ater|EAU37289.1 atub|OJI79466.1 aver|OJI99160.1 awen|OJJ37539.1 azon|OJJ51204.1 agla|OJJ88538.1

Group1479: abra|OJJ66907.1 acar|OOF91677.1 acla|EAW14476.1 afla|EED54210.1 afum|EAL93916.1 agla|OJJ88537.1 akaw|GAA91507.1 aluc|OJZ85673.1 anid|CBF86248.1 anig|EHA24866.1 aory|EIT76017.1 asyd|OJJ65043.1 ater|EAU37287.1 atub|OJI79465.1 aver|OJI99162.1 awen|OJJ37542.1 afis|EAW19185.1 azon|OJJ51206.1

Group1480: abra|OJJ66909.1 acar|OOF91679.1 acla|EAW14478.1 afis|EAW19187.1 afla|EED47724.1 afum|EAL93918.1 agla|OJJ88534.1 akaw|GAA91505.1 aluc|OJZ85671.1 anid|CBF86246.1 anig|EHA24868.1 aory|EIT77160.1 asyd|OJJ65044.1 ater|EAU37286.1 atub|OJI79463.1 aver|OJI99165.1 awen|OJJ37545.1 azon|OJJ51210.1

Group1481: abra|OJJ66911.1 acar|OOF91680.1 acla|EAW14480.1 afis|EAW19189.1 afla|EED47722.1 afum|EAL93921.1 agla|OJJ88532.1 akaw|GAA91504.1 aluc|OJZ85670.1 anid|CBF86243.1 anig|EHA24869.1 aory|EIT77116.1 asyd|OJJ65046.1 ater|EAU37285.1 atub|OJI79462.1 aver|OJI99167.1 awen|OJJ37549.1 azon|OJJ51211.1

Group1482: abra|OJJ66915.1 acar|OOF91682.1 acla|EAW14482.1 afis|EAW19191.1 afla|EED47718.1 afum|EAL93922.1 agla|OJJ88518.1 akaw|GAA91503.1 aluc|OJZ85665.1 anid|CBF86239.1 anig|EHA24870.1 aory|EIT80246.1 asyd|OJJ65049.1 ater|EAU37284.1 atub|OJI79458.1 aver|OJI99171.1 awen|OJJ37551.1 azon|OJJ51212.1

Group1483: abra|OJJ66918.1 acar|OOF91685.1 acla|EAW14485.1 afis|EAW19194.1 afla|EED47714.1 afum|EAL93925.2 agla|OJJ88515.1 akaw|GAA91500.1 aluc|OJZ85661.1 anid|CBF78544.1 anig|EHA24872.1 aory|EIT80273.1 asyd|OJJ65054.1 ater|EAU37281.1 atub|OJI79455.1 aver|OJI99177.1 awen|OJJ37552.1 azon|OJJ51213.1

Group1484: abra|OJJ66919.1 acar|OOF91687.1 acla|EAW14486.1 afis|EAW19195.1 afla|EED47713.1 afum|EAL93927.2 agla|OJJ88514.1 akaw|GAA91499.1 aluc|OJZ85660.1 anid|CBF78546.1 anig|EHA24873.1 aory|EIT80279.1 asyd|OJJ65055.1 ater|EAU37280.1 atub|OJI79454.1 aver|OJI99178.1 awen|OJJ37553.1 azon|OJJ51214.1

Group1485: abra|OJJ66923.1 acar|OOF91690.1 acla|EAW14489.1 afis|EAW19203.1 afla|EED47709.1 afum|EAL93933.1 agla|OJJ88509.1 akaw|GAA91496.1 aluc|OJZ85657.1 anid|CBF78554.1 anig|EHA24876.1 aory|EIT80207.1 asyd|OJJ65059.1 ater|EAU37277.1 atub|OJI79449.1 aver|OJI99184.1 awen|OJJ37556.1 azon|OJJ51219.1

Group1486: abra|OJJ66924.1 acar|OOF91692.1 acla|EAW14490.1 afis|EAW19204.1 afla|EED47708.1 afum|EAL93934.1 agla|OJJ88508.1 akaw|GAA91493.1 aluc|OJZ85654.1 anid|CBF78558.1 anig|EHA24878.1 aory|EIT80227.1 asyd|OJJ65061.1 ater|EAU37268.1 atub|OJI79446.1 aver|OJI99185.1 awen|OJJ37557.1 azon|OJJ51220.1

Group1487: abra|OJJ66928.1 acar|OOF91696.1 acla|EAW14493.1 afis|EAW19207.1 afla|EED47705.1 afum|EAL93937.1 agla|OJJ88504.1 akaw|GAA91489.1 aluc|OJZ85651.1 anid|CBF78563.1 anig|EHA24882.1 aory|EIT80245.1 asyd|OJJ65064.1 ater|EAU37265.1 atub|OJI79442.1 aver|OJI99188.1 awen|OJJ37560.1 azon|OJJ51222.1

Group1488: abra|OJJ66931.1 acar|OOF91698.1 acla|EAW14494.1 afis|EAW19208.1 afla|EED47703.1 afum|EAL93938.2 agla|OJJ88501.1 akaw|GAA91487.1 aluc|OJZ85649.1 anid|CBF78566.1 anig|EHA24884.1 aory|EIT80217.1 asyd|OJJ65067.1 ater|EAU37260.1 atub|OJI79440.1 aver|OJI99191.1 awen|OJJ37562.1 azon|OJJ51225.1

Group1489: abra|OJJ66934.1 acar|OOF91701.1 acla|EAW14496.1 afis|EAW19210.1 afla|EED47702.1 afum|EAL93940.1 agla|OJJ88490.1 akaw|GAA91485.1 aluc|OJZ85647.1 anid|CBF78570.1 anig|EHA24887.1 aory|EIT80269.1 asyd|OJJ65075.1 ater|EAU37258.1 atub|OJI79438.1 aver|OJI99200.1 awen|OJJ37564.1 azon|OJJ51227.1

Group1490: afum|EAL92928.1 abra|OJJ78283.1 acar|OOF98585.1 acla|EAW09232.1 afis|EAW21916.1 afla|EED46633.1 agla|OJJ84207.1 akaw|GAA85744.1 aluc|OJZ91357.1 anig|EHA20035.1 aory|EIT77912.1

asyd|OJJ61312.1 ater|EAU30918.1 atub|OJI83350.1 aver|OJJ00637.1 awen|OJJ34206.1 azon|OJJ43428.1 anid|CBF76852.1

Group1491: abra|OJJ66937.1 acar|OOF91703.1 acla|EAW15005.1 afis|EAW24537.1 afla|EED47700.1 afum|EAL86201.1 agla|OJJ86874.1 akaw|GAA91483.1 aluc|OJZ85645.1 anig|EHA24890.1 aory|EIT80258.1 asyd|OJJ63536.1 ater|EAU39600.1 atub|OJI79436.1 aver|OJI96723.1 awen|OJJ38055.1 azon|OJJ42331.1 agla|OJJ81013.1

Group1492: abra|OJJ66938.1 acar|OOF91704.1 acla|EAW14497.1 afis|EAW19211.1 afla|EED47699.1 afum|EAL93941.1 agla|OJJ88486.1 akaw|GAA91482.1 aluc|OJZ85644.1 anid|CBF78583.1 anig|EHA24891.1 aory|EIT80265.1 asyd|OJJ65078.1 ater|EAU37256.1 atub|OJI79435.1 aver|OJI99203.1 awen|OJJ37565.1 azon|OJJ51228.1

Group1493: abra|OJJ66940.1 acar|OOF91705.1 acla|EAW14498.1 afis|EAW19212.1 afla|EED47698.1 afum|EBA27508.1 agla|OJJ88485.1 akaw|GAA91481.1 aluc|OJZ85642.1 anid|CBF78581.1 anig|EHA24892.1 aory|EIT80243.1 asyd|OJJ65076.1 ater|EAU37255.1 atub|OJI79433.1 aver|OJI99201.1 awen|OJJ37568.1 azon|OJJ51229.1

Group1494: abra|OJJ66948.1 acar|OOF91714.1 acla|EAW14503.1 afis|EAW19217.1 afla|EED47682.1 afum|EAL93950.1 agla|OJJ88478.1 akaw|GAA91472.1 aluc|OJZ85632.1 anid|CBF78594.1 anig|EHA24899.1 aory|EIT80231.1 asyd|OJJ65091.1 ater|EAU37251.1 atub|OJI79422.1 aver|OJI99216.1 awen|OJJ37574.1 azon|OJJ51234.1

Group1495: abra|OJJ66952.1 acar|OOF93656.1 acla|EAW14505.1 afis|EAW19218.1 afla|EED47681.1 afum|EAL93951.2 akaw|GAA91468.1 aluc|OJZ85626.1 anid|CBF78597.1 anig|EHA24902.1 aory|EIT80228.1 asyd|OJJ65093.1 ater|EAU37250.1 atub|OJI79419.1 aver|OJI99217.1 azon|OJJ51235.1 agla|OJJ88477.1 awen|OJJ37576.1

Group1496: abra|OJJ66955.1 acar|OOF93653.1 acla|EAW14506.1 afis|EAW19219.1 afla|EED47670.1 afum|EAL93952.1 agla|OJJ88476.1 akaw|GAA92206.1 aluc|OJZ85621.1 anid|CBF78603.1 anig|EHA24905.1 aory|EIT80271.1 asyd|OJJ65097.1 ater|EAU37238.1 atub|OJI79414.1 aver|OJI99223.1 awen|OJJ37577.1 azon|OJJ51236.1

Group1497: abra|OJJ66956.1 acar|OOF93652.1 acla|EAW14507.1 afis|EAW19220.1 afla|EED47669.1 afum|EAL93953.1 agla|OJJ88464.1 akaw|GAA92205.1 aluc|OJZ85620.1 anid|CBF78605.1 anig|EHA24906.1 aory|EIT80172.1 asyd|OJJ65098.1 ater|EAU37237.1 atub|OJI79413.1 aver|OJI99224.1 awen|OJJ37583.1 azon|OJJ51237.1

Group1498: abra|OJJ66962.1 acar|OOF93645.1 acla|EAW14512.1 afis|EAW19230.1 afla|EED47658.1 afum|EAL93959.1 agla|OJJ88473.1 akaw|GAA92199.1 aluc|OJZ85614.1 anid|CBF78614.1 anig|EHA24912.1 aory|EIT80215.1 asyd|OJJ65178.1 ater|EAU37233.1 atub|OJI79407.1 aver|OJI99331.1 awen|OJJ37588.1 azon|OJJ51242.1

Group1499: abra|OJJ66980.1 acar|OOF93631.1 acla|EAW11770.1 afis|EAW23678.1 afla|EED52197.1 afum|EAL87121.1 agla|OJJ78538.1 akaw|GAA92184.1 aluc|OJZ85594.1 anid|CBF89572.1 anig|EHA24928.1 aory|EIT74397.1 asyd|OJJ62335.1 ater|EAU35375.1 atub|OJI79390.1 aver|OJI96441.1 awen|OJJ35575.1 azon|OJJ43197.1

Group1500: abra|OJJ66988.1 acar|OOF93624.1 acla|EAW09074.1 afis|EAW25663.1 afla|EED46073.1 afum|EAL84335.1 akaw|GAA92175.1 aluc|OJZ85585.1 anid|CBF84674.1 anig|EHA24936.1 aory|EIT83504.1 asyd|OJJ53347.1 atub|OJI79380.1 aver|OJJ08397.1 awen|OJJ35373.1 azon|OJJ45069.1 afla|EED55027.1 aory|EIT81986.1

Group1501: abra|OJJ66992.1 acar|OOF93622.1 acla|EAW15343.1 afis|EAW20364.1 afla|EED51949.1 afum|EAL85480.2 agla|OJJ81790.1 akaw|GAA92172.1 aluc|OJZ85580.1 anid|CBF69463.1 anig|EHA24939.1 aory|EIT81480.1 asyd|OJJ59276.1 ater|EAU36758.1 atub|OJI79376.1 aver|OJJ01891.1 awen|OJJ32524.1 azon|OJJ50243.1

Group1502: abra|OJJ67094.1 acla|EAW11007.1 akaw|GAA91067.1 aluc|OJZ87881.1 anig|EHA20184.1 atub|OJI80770.1 abra|OJJ69053.1 acar|OOF91237.1 afum|EAL86660.1 abra|OJJ72679.1 acar|OOF95928.1 afis|EAW19541.1 agla|OJJ88408.1 anig|EHA24092.1 ater|EAU39521.1 atub|OJI82086.1 acar|OOF91465.1 afum|EAL86967.1

Group1503: abra|OJJ67118.1 acar|OOF98434.1 acla|EAW11710.1 afis|EAW23729.1 afla|EED47523.1 afum|EAL87167.1 agla|OJJ88177.1 akaw|GAA91047.1 aluc|OJZ87857.1 anid|CBF69394.1 anig|EHA20163.1 aory|EIT74567.1 asyd|OJJ52918.1 ater|EAU30755.1 atub|OJI80790.1 aver|OJJ08888.1 awen|OJJ32546.1 azon|OJJ51795.1

Group1504: abra|OJJ67150.1 acar|OOF98408.1 acla|EAW14575.1 afis|EAW19299.1 afla|EED47588.1 afum|EAL94018.1 agla|OJJ88383.1 akaw|GAA91023.1 aluc|OJZ87826.1 anid|CBF78774.1 anig|EHA20305.1 aory|EIT74538.1 asyd|OJJ65240.1 ater|EAU29474.1 atub|OJI80817.1 aver|OJI99390.1 awen|OJJ37648.1 azon|OJJ44913.1

Group1505: abra|OJJ67151.1 acar|OOF98407.1 acla|EAW14574.1 afis|EAW19297.1 afla|EED47589.1 afum|EAL94016.1 agla|OJJ88384.1 akaw|GAA91022.1 aluc|OJZ87825.1 anid|CBF78772.1 anig|EHA20304.1 aory|EIT74535.1 asyd|OJJ65239.1 ater|EAU29475.1 atub|OJI80819.1 aver|OJI99389.1 awen|OJJ37647.1 azon|OJJ44912.1

Group1506: abra|OJJ67153.1 acar|OOF98405.1 acla|EAW14571.1 afis|EAW19294.1 afla|EED47592.1 afum|EAL94013.1 agla|OJJ88386.1 akaw|GAA91019.1 aluc|OJZ87821.1 anid|CBF78767.1 anig|EHA20302.1 aory|EIT74534.1 asyd|OJJ65237.1 ater|EAU29478.1 atub|OJI80823.1 aver|OJI99386.1 awen|OJJ37645.1 azon|OJJ44910.1

Group1507: abra|OJJ67154.1 acar|OOF98404.1 acla|EAW14570.1 afis|EAW19283.1 afla|EED47593.1 afum|EAL94009.1 agla|OJJ88395.1 akaw|GAA91018.1 aluc|OJZ87820.1 anid|CBF78763.1 anig|EHA20301.1 aory|EIT80189.1 asyd|OJJ65235.1 ater|EAU29479.1 atub|OJI80824.1 aver|OJI99384.1 awen|OJJ37644.1 azon|OJJ44909.1

Group1508: abra|OJJ67157.1 acar|OOF98402.1 acla|EAW14568.1 afis|EAW19281.1 afla|EED47596.1 afum|EAL94007.1 agla|OJJ88393.1 akaw|GAA91015.1 aluc|OJZ87816.1 anid|CBF78760.1 anig|EHA20297.1 aory|EIT80256.1 asyd|OJJ65233.1 ater|EAU29482.1 atub|OJI80828.1 aver|OJI99382.1 awen|OJJ37642.1 azon|OJJ44906.1

Group1509: abra|OJJ67159.1 acar|OOF98400.1 acla|EAW14567.1 afis|EAW19280.1 afla|EED47598.1 afum|EAL94006.1 agla|OJJ88391.1 akaw|GAA91013.1 aluc|OJZ87814.1 anid|CBF78756.1 anig|EHA20295.1 aory|EIT80259.1 asyd|OJJ65231.1 ater|EAU29483.1 atub|OJI80830.1 aver|OJI99380.1 awen|OJJ37640.1 azon|OJJ44904.1

Group1510: abra|OJJ67163.1 acar|OOF98397.1 acla|EAW14565.1 afis|EAW19278.1 afla|EED47600.1 afum|EAL94004.1 agla|OJJ88389.1 akaw|GAA91011.1 aluc|OJZ87812.1 anid|CBF78752.1 anig|EHA20294.1 aory|EIT80253.1 asyd|OJJ65229.1 ater|EAU29485.1 atub|OJI80832.1 aver|OJI99378.1 awen|OJJ37638.1 azon|OJJ44882.1

Group1511: abra|OJJ67164.1 acar|OOF98398.1 acla|EAW14564.1 afis|EAW19277.1 afla|EED47601.1 afum|EAL94003.1 agla|OJJ88396.1 akaw|GAA91010.1 aluc|OJZ87811.1 anid|CBF78750.1 anig|EHA20293.1 aory|EIT80205.1 asyd|OJJ65228.1 ater|EAU29486.1 atub|OJI80833.1 aver|OJI99377.1 awen|OJJ37637.1 azon|OJJ44883.1

Group1512: abra|OJJ67173.1 acar|OOF98390.1 acla|EAW14550.1 afis|EAW19267.1 afla|EED47608.1 afum|EAL93994.1 agla|OJJ88426.1 akaw|GAA91001.1 aluc|OJZ87802.1 anid|CBF78695.1 anig|EHA20283.1 aory|EIT80277.1 asyd|OJJ65218.1 ater|EAU29548.1 atub|OJI80845.1 aver|OJI99368.1 awen|OJJ37628.1 azon|OJJ47016.1

Group1513: abra|OJJ67175.1 acar|OOF98388.1 acla|EAW14548.1 afis|EAW19265.1 afla|EED47609.1 afum|EAL93992.1 agla|OJJ88424.1 akaw|GAA90999.1 aluc|OJZ87800.1 anid|CBF78691.1 anig|EHA20281.1 aory|EIT80181.1 asyd|OJJ65216.1 ater|EAU29546.1 atub|OJI80847.1 aver|OJI99366.1 awen|OJJ37626.1 azon|OJJ47015.1

Group1514: abra|OJJ67176.1 acar|OOF98386.1 acla|EAW14547.1 afis|EAW19264.1 afla|EED47610.1 afum|EAL93991.1 agla|OJJ88423.1 akaw|GAA90998.1 aluc|OJZ87799.1 anid|CBF78688.1 anig|EHA20280.1 aory|EIT80241.1 asyd|OJJ65214.1 ater|EAU29545.1 atub|OJI80848.1 aver|OJI99364.1 awen|OJJ37625.1 azon|OJJ47014.1

Group1515: abra|OJJ67178.1 acar|OOF98385.1 acla|EAW14545.1 afis|EAW19263.1 afla|EED47612.1 afum|EAL93990.1 agla|OJJ88421.1 aluc|OJZ87797.1 anid|CBF78684.1 anig|EHA20278.1 aory|EIT80200.1 asyd|OJJ65212.1 ater|EAU29543.1 atub|OJI80850.1 aver|OJI99362.1 awen|OJJ37623.1 azon|OJJ47012.1 aory|EIT80261.1

Group1516: abra|OJJ67179.1 acar|OOF98384.1 acla|EAW14544.1 afis|EAW19262.1 afla|EED47613.1 afum|EAL93989.1 agla|OJJ88420.1 akaw|GAA90997.1 aluc|OJZ87796.1 anid|CBF78682.1 anig|EHA20277.1 aory|EIT80281.1 asyd|OJJ65211.1 ater|EAU29542.1 atub|OJI80851.1 aver|OJI99361.1 awen|OJJ37622.1 azon|OJJ47011.1

Group1517: abra|OJJ67180.1 acar|OOF98382.1 acla|EAW14543.1 afis|EAW19261.1 afla|EED47614.1 afum|EAL93988.1 agla|OJJ88433.1 akaw|GAA90996.1 aluc|OJZ87795.1 anid|CBF78680.1 anig|EHA20276.1 aory|EIT80274.1 asyd|OJJ65210.1 ater|EAU29514.1 atub|OJI80852.1 aver|OJI99360.1 awen|OJJ37621.1 azon|OJJ47010.1

Group1518: abra|OJJ67182.1 acar|OOF98380.1 acla|EAW14541.1 afis|EAW19259.1 afla|EED47616.1 afum|EAL93986.1 agla|OJJ88436.1 akaw|GAA90994.1 aluc|OJZ87793.1 anid|CBF78678.1 anig|EHA20274.1 aory|EIT80183.1 asyd|OJJ65209.1 ater|EAU29516.1 atub|OJI80854.1 aver|OJI99358.1 awen|OJJ37620.1 azon|OJJ47009.1

Group1519: abra|OJJ67183.1 acar|OOF98379.1 acla|EAW14540.1 afis|EAW19258.1 afla|EED47618.1 afum|EAL93985.1 agla|OJJ88437.1 akaw|GAA90993.1 aluc|OJZ87792.1 anid|CBF78676.1 anig|EHA20273.1

aory|EIT80276.1 asyd|OJJ65208.1 ater|EAU29517.1 atub|OJI80855.1 aver|OJI99357.1 awen|OJJ37619.1 azon|OJJ47008.1

Group1520: abra|OJJ67187.1 acar|OOF98377.1 acla|EAW14537.1 afis|EAW19253.1 afla|EED47621.1 afum|EAL93982.1 agla|OJJ88444.1 akaw|GAA90990.1 aluc|OJZ87788.1 anid|CBF78671.1 anig|EHA20270.1 aory|EIT80226.1 asyd|OJJ65205.1 ater|EAU29510.1 atub|OJI80859.1 aver|OJI99354.1 awen|OJJ37616.1 azon|OJJ47005.1

Group1521: abra|OJJ67189.1 acar|OOF98374.1 acla|EAW14534.1 afis|EAW19252.1 afla|EED47626.1 afum|EAL93981.1 agla|OJJ88445.1 akaw|GAA90988.1 aluc|OJZ87786.1 anid|CBF78669.1 anig|EHA20266.1 aory|EIT80202.1 asyd|OJJ65202.1 ater|EAU29506.1 atub|OJI80861.1 aver|OJI99351.1 awen|OJJ37615.1 azon|OJJ47004.1

Group1522: abra|OJJ67190.1 acar|OOF98373.1 acla|EAW14533.1 afis|EAW19251.1 afla|EED47627.1 afum|EAL93980.1 agla|OJJ88446.1 akaw|GAA90987.1 aluc|OJZ87785.1 anid|CBF78667.1 anig|EHA20265.1 aory|EIT80224.1 asyd|OJJ65201.1 ater|EAU29505.1 atub|OJI80862.1 aver|OJI99350.1 awen|OJJ37614.1 azon|OJJ51262.1

Group1523: abra|OJJ67192.1 acar|OOF98372.1 acla|EAW14532.1 afis|EAW19250.1 afla|EED47628.1 afum|EAL93979.1 agla|OJJ88447.1 akaw|GAA90986.1 aluc|OJZ87784.1 anid|CBF78663.1 anig|EHA20264.1 aory|EIT80188.1 asyd|OJJ65199.1 ater|EAU29504.1 atub|OJI80863.1 aver|OJI99348.1 awen|OJJ37612.1 azon|OJJ51261.1

Group1524: abra|OJJ67193.1 acar|OOF98371.1 acla|EAW14531.1 afis|EAW19249.1 afla|EED47629.1 afum|EAL93978.1 agla|OJJ88448.1 akaw|GAA90985.1 aluc|OJZ87782.1 anid|CBF78665.1 anig|EHA20263.1 aory|EIT80199.1 asyd|OJJ65200.1 ater|EAU29503.1 atub|OJI80865.1 aver|OJI99349.1 awen|OJJ37611.1 azon|OJJ51260.1

Group1525: abra|OJJ67195.1 acar|OOF98369.1 acla|EAW14529.1 afis|EAW19247.1 afla|EED47631.1 afum|EAL93976.1 agla|OJJ88450.1 akaw|GAA90983.1 aluc|OJZ87780.1 anid|CBF78658.1 anig|EHA20262.1 aory|EIT80177.1 asyd|OJJ65197.1 ater|EAU29501.1 atub|OJI80867.1 aver|OJI99346.1 awen|OJJ37604.1 azon|OJJ51257.1

Group1526: abra|OJJ67200.1 acar|OOF98364.1 acla|EAW14524.1 afis|EAW19242.1 afla|EED47635.1 afum|EAL93971.2 agla|OJJ88455.1 akaw|GAA90978.1 aluc|OJZ87774.1 anid|CBF78650.1 anig|EHA20258.1 aory|EIT80223.1 asyd|OJJ65194.1 ater|EAU29498.1 atub|OJI80871.1 aver|OJI99343.1 awen|OJJ37598.1 azon|OJJ51253.1

Group1527: abra|OJJ67201.1 acar|OOF98362.1 acla|EAW14523.1 afis|EAW19241.1 afla|EED47636.1 afum|EAL93970.2 agla|OJJ88456.1 akaw|GAA90977.1 aluc|OJZ87773.1 anid|CBF78648.1 anig|EHA20257.1 aory|EIT80218.1 asyd|OJJ65193.1 ater|EAU29497.1 atub|OJI80872.1 aver|OJI99342.1 awen|OJJ37597.1 azon|OJJ51252.1

Group1528: abra|OJJ67202.1 acar|OOF98361.1 acla|EAW14522.1 afis|EAW19240.1 afla|EED47637.1 afum|EAL93969.1 agla|OJJ88458.1 akaw|GAA90976.1 aluc|OJZ87772.1 anid|CBF78646.1 anig|EHA20256.1 aory|EIT80185.1 asyd|OJJ65192.1 ater|EAU37223.1 atub|OJI80873.1 aver|OJI99341.1 awen|OJJ37596.1 azon|OJJ51251.1

Group1529: abra|OJJ67203.1 acar|OOF98360.1 acla|EAW14521.1 afis|EAW19239.1 afla|EED47638.1 afum|EBA27509.1 agla|OJJ88459.1 akaw|GAA90975.1 aluc|OJZ87771.1 anid|CBF78644.1 anig|EHA20255.1 aory|EIT80222.1 asyd|OJJ65191.1 ater|EAU37224.1 atub|OJI80874.1 aver|OJI99340.1 awen|OJJ37595.1 azon|OJJ51250.1

Group1530: abra|OJJ67204.1 acar|OOF98359.1 acla|EAW14520.1 afis|EAW19238.1 afla|EED47639.1 afum|EAL93966.1 agla|OJJ88461.1 akaw|GAA90974.1 aluc|OJZ87770.1 anid|CBF78642.1 anig|EHA20254.1 aory|EIT80175.1 asyd|OJJ65190.1 ater|EAU37225.1 atub|OJI80875.1 aver|OJI99339.1 awen|OJJ37594.1 azon|OJJ51249.1

Group1531: abra|OJJ67213.1 acar|OOF98346.1 acla|EAW08553.1 afis|EAW23595.1 afla|EED47505.1 afum|EAL87872.1 agla|OJJ86855.1 akaw|GAA90964.1 aluc|OJZ87760.1 anid|CBF89585.1 anig|EHA20245.1 aory|EIT74581.1 asyd|OJJ62318.1 ater|EAU35355.1 atub|OJI80884.1 aver|OJI96426.1 awen|OJJ39917.1 azon|OJJ45891.1

Group1532: abra|OJJ67215.1 acar|OOF98343.1 acla|EAW10167.1 afis|EAW25427.1 afla|EED53915.1 afum|EAL90086.1 agla|OJJ87071.1 akaw|GAA90962.1 aluc|OJZ87757.1 anid|CBF77551.1 anig|EHA20243.1 aory|EIT78350.1 asyd|OJJ53152.1 ater|EAU34597.1 atub|OJI80886.1 aver|OJJ03699.1 awen|OJJ41156.1 azon|OJJ44734.1

Group1533: abra|OJJ67225.1 acar|OOF98326.1 acla|EAW10408.1 afis|EAW25160.1 afla|EED53622.1 afum|EAL89835.1 agla|OJJ88637.1 akaw|GAA91652.1 aluc|OJZ87749.1 anid|CBF80654.1 anig|EHA20237.1 aory|EIT75132.1 asyd|OJJ54613.1 ater|EAU34333.1 atub|OJI80894.1 aver|OJJ07545.1 awen|OJJ33447.1 azon|OJJ50704.1

Group1534: abra|OJJ67239.1 acar|OOF89889.1 acla|EAW13993.1 afis|EAW18701.1 afla|EED50650.1 afum|EAL93424.1 agla|OJJ82360.1 akaw|GAA91664.1 aluc|OJZ87736.1 anid|CBF70640.1 anig|EHA20228.1 aory|EIT78150.1 asyd|OJJ58491.1 ater|EAU38205.1 atub|OJI80904.1 aver|OJJ01094.1 awen|OJJ32184.1 azon|OJJ48705.1

Group1535: abra|OJJ67298.1 acar|OOF92514.1 acla|EAW15241.1 afis|EAW20208.1 afla|EED57135.1 afum|EAL85325.1 agla|OJJ84656.1 akaw|GAA86516.1 aluc|OJZ85061.1 anid|CBF85098.1 anig|EHA25276.1 aory|EIT74134.1 asyd|OJJ63059.1 ater|EAU32018.1 atub|OJI80536.1 aver|OJI97904.1 awen|OJJ31212.1 azon|OJJ46148.1

Group1536: abra|OJJ67306.1 acar|OOF92507.1 acla|EAW06427.1 afis|EAW18531.1 afla|EED52870.1 afum|EAL93228.1 agla|OJJ85144.1 akaw|GAA86509.1 aluc|OJZ85052.1 anid|CBF70204.1 anig|EHA25269.1 aory|EIT78588.1 asyd|OJJ58784.1 ater|EAU30039.1 atub|OJI80546.1 aver|OJJ01392.1 awen|OJJ31089.1 azon|OJJ50594.1

Group1537: abra|OJJ67308.1 acar|OOF92504.1 acla|EAW06424.1 afis|EAW18528.1 afla|EED52873.1 afum|EAL93225.1 agla|OJJ85141.1 akaw|GAA86507.1 aluc|OJZ85050.1 anid|CBF70208.1 anig|EHA25267.1 aory|EIT78611.1 asyd|OJJ58781.1 ater|EAU30041.1 atub|OJI80548.1 aver|OJJ01389.1 awen|OJJ31086.1 azon|OJJ50591.1

Group1538: abra|OJJ67310.1 acar|OOF92502.1 acla|EAW06422.1 afis|EAW18526.1 afla|EED52875.1 afum|EAL93221.1 agla|OJJ85140.1 akaw|GAA86505.1 aluc|OJZ85048.1 anid|CBF70211.1 anig|EHA25265.1 aory|EIT78615.1 asyd|OJJ58779.1 ater|EAU30043.1 atub|OJI80550.1 aver|OJJ01388.1 awen|OJJ31080.1 azon|OJJ50588.1

Group1539: abra|OJJ67311.1 acar|OOF92501.1 acla|EAW06421.1 afis|EAW18525.1 afla|EED52876.1 afum|EAL93220.1 agla|OJJ85139.1 akaw|GAA86504.1 aluc|OJZ85047.1 anid|CBF70213.1 anig|EHA25264.1 aory|EIT78600.1 asyd|OJJ58778.1 ater|EAU30044.1 atub|OJI80551.1 aver|OJJ01387.1 awen|OJJ31079.1 azon|OJJ50587.1

Group1540: abra|OJJ67319.1 acar|OOF92496.1 acla|EAW06417.1 afis|EAW18521.1 afla|EED52877.1 afum|EAL93216.1 agla|OJJ85133.1 akaw|GAA86499.1 aluc|OJZ85041.1 anid|CBF70223.1 anig|EHA25259.1 aory|EIT78623.1 asyd|OJJ58774.1 ater|EAU30049.1 atub|OJI80556.1 aver|OJJ01383.1 awen|OJJ31073.1 azon|OJJ49993.1

Group1541: abra|OJJ67321.1 acar|OOF92493.1 acla|EAW06415.1 afis|EAW18519.1 afla|EED52879.1 afum|EAL93214.1 agla|OJJ85131.1 akaw|GAA86497.1 aluc|OJZ85039.1 anid|CBF70226.1 anig|EHA25257.1 aory|EIT78673.1 asyd|OJJ58773.1 ater|EAU30051.1 atub|OJI80558.1 aver|OJJ01382.1 awen|OJJ31071.1 azon|OJJ50581.1

Group1542: abra|OJJ67323.1 acar|OOF92490.1 acla|EAW06412.1 afis|EAW18516.1 afla|EED52882.1 afum|EAL93211.1 agla|OJJ85129.1 akaw|GAA86493.1 aluc|OJZ85035.1 anid|CBF70232.1 anig|EHA25255.1 aory|EIT78656.1 asyd|OJJ58771.1 ater|EAU30054.1 atub|OJI80562.1 aver|OJJ01380.1 awen|OJJ31592.1 azon|OJJ50579.1

Group1543: abra|OJJ67325.1 acar|OOF92488.1 acla|EAW06410.1 afis|EAW18514.1 afla|EED52884.1 afum|EAL93209.1 agla|OJJ85107.1 akaw|GAA86491.1 aluc|OJZ85033.1 anid|CBF70234.1 anig|EHA25253.1 aory|EIT78594.1 asyd|OJJ58770.1 ater|EAU30055.1 atub|OJI80564.1 aver|OJJ01379.1 awen|OJJ31068.1 azon|OJJ50577.1

Group1544: abra|OJJ67326.1 acar|OOF92487.1 acla|EAW06409.1 afis|EAW18513.1 afla|EED52887.1 afum|EAL93208.1 agla|OJJ85108.1 akaw|GAA86490.1 aluc|OJZ85032.1 anid|CBF70236.1 anig|EHA25252.1 aory|EIT78631.1 asyd|OJJ58769.1 ater|EAU30057.1 atub|OJI80565.1 aver|OJJ01378.1 awen|OJJ31067.1 azon|OJJ50576.1

Group1545: abra|OJJ67327.1 acar|OOF92486.1 acla|EAW06407.1 afis|EAW18511.1 afla|EED52889.1 afum|EAL93206.1 agla|OJJ85109.1 akaw|GAA86489.1 aluc|OJZ85030.1 anid|CBF70239.1 anig|EHA25251.1 aory|EIT78676.1 asyd|OJJ58767.1 ater|EAU30058.1 atub|OJI80567.1 aver|OJJ01376.1 awen|OJJ31065.1 azon|OJJ50575.1

Group1546: abra|OJJ67328.1 acar|OOF92485.1 acla|EAW06406.1 afis|EAW18510.1 afla|EED52890.1 afum|EAL93205.1 agla|OJJ85110.1 akaw|GAA86488.1 aluc|OJZ85029.1 anid|CBF70241.1 anig|EHA25250.1 aory|EIT78606.1 asyd|OJJ58766.1 ater|EAU30059.1 atub|OJI80568.1 aver|OJJ01375.1 awen|OJJ31064.1 azon|OJJ50574.1

Group1547: abra|OJJ67329.1 acar|OOF92483.1 acla|EAW06405.1 afis|EAW18509.1 afla|EED52891.1 afum|EAL93204.1 agla|OJJ85111.1 akaw|GAA86487.1 aluc|OJZ85028.1 anid|CBF70243.1 anig|EHA25249.1 aory|EIT78599.1 asyd|OJJ58765.1 ater|EAU30060.1 atub|OJI80569.1 aver|OJJ01374.1 awen|OJJ31063.1 azon|OJJ50573.1

Group1548: abra|OJJ67330.1 acar|OOF92484.1 acla|EAW06404.1 afis|EAW18508.1 afla|EED52894.1 afum|EAL93203.1 agla|OJJ85112.1 akaw|GAA86486.1 aluc|OJZ85027.1 anid|CBF70245.1 anig|EHA25248.1

aory|EIT78590.1 asyd|OJJ58762.1 ater|EAU30063.1 atub|OJI80570.1 aver|OJJ01372.1 awen|OJJ31061.1 azon|OJJ50572.1

Group1549: abra|OJJ67334.1 acar|OOF92479.1 acla|EAW06400.1 afis|EAW18504.1 afla|EED52897.1 afum|EAL93197.1 agla|OJJ85119.1 akaw|GAA86482.1 aluc|OJZ85023.1 anid|CBF70249.1 anig|EHA25244.1 aory|EIT78613.1 asyd|OJJ58759.1 ater|EAU30067.1 atub|OJI80574.1 aver|OJJ01367.1 awen|OJJ31058.1 azon|OJJ50567.1

Group1550: abra|OJJ67335.1 acar|OOF92478.1 acla|EAW06399.1 afis|EAW18503.1 afla|EED52898.1 afum|EAL93196.2 agla|OJJ85118.1 akaw|GAA86481.1 aluc|OJZ85022.1 anid|CBF70251.1 anig|EHA25243.1 aory|EIT78654.1 asyd|OJJ58758.1 ater|EAU30068.1 atub|OJI80575.1 aver|OJJ01366.1 awen|OJJ31057.1 azon|OJJ50568.1

Group1551: abra|OJJ67336.1 acar|OOF92477.1 acla|EAW06398.1 afis|EAW18502.1 afla|EED52899.1 afum|EAL93195.1 agla|OJJ85123.1 akaw|GAA86480.1 aluc|OJZ85021.1 anid|CBF70253.1 anig|EHA25242.1 aory|EIT78660.1 asyd|OJJ58757.1 ater|EAU30069.1 atub|OJI80576.1 aver|OJJ01365.1 awen|OJJ31056.1 azon|OJJ50566.1

Group1552: abra|OJJ67339.1 acar|OOF92474.1 acla|EAW06395.1 afis|EAW18499.1 afla|EED52902.1 afum|EAL93190.1 agla|OJJ85128.1 akaw|GAA86477.1 aluc|OJZ85018.1 anid|CBF70257.1 anig|EHA25239.1 aory|EIT78634.1 asyd|OJJ58755.1 ater|EAU30071.1 atub|OJI80579.1 aver|OJJ01363.1 awen|OJJ31053.1 azon|OJJ50563.1

Group1553: abra|OJJ67340.1 acar|OOF92473.1 acla|EAW06394.1 afis|EAW18498.1 afla|EED52903.1 afum|EAL93188.1 agla|OJJ85105.1 akaw|GAA86476.1 aluc|OJZ85017.1 anid|CBF70259.1 anig|EHA25238.1 aory|EIT78622.1 asyd|OJJ58754.1 ater|EAU30072.1 atub|OJI80580.1 aver|OJJ01362.1 awen|OJJ31052.1 azon|OJJ50564.1

Group1554: abra|OJJ67346.1 acar|OOF92469.1 acla|EAW06390.1 afis|EAW18496.1 afla|EED52907.1 afum|EAL93186.1 agla|OJJ85091.1 akaw|GAA86472.1 aluc|OJZ85013.1 anid|CBF70170.1 anig|EHA25234.1 aory|EIT78612.1 asyd|OJJ58795.1 ater|EAU30073.1 atub|OJI80584.1 aver|OJJ01400.1 awen|OJJ31049.1 azon|OJJ50560.1

Group1555: abra|OJJ67347.1 acar|OOF92468.1 acla|EAW06389.1 afis|EAW18495.1 afla|EED52908.1 afum|EAL93185.1 agla|OJJ85090.1 akaw|GAA86471.1 aluc|OJZ85012.1 anid|CBF70168.1 anig|EHA25233.1 aory|EIT78663.1 asyd|OJJ58797.1 ater|EAU29921.1 atub|OJI80585.1 aver|OJJ01401.1 awen|OJJ31048.1 azon|OJJ50559.1

Group1556: abra|OJJ67350.1 acar|OOF92466.1 acla|EAW06386.1 afis|EAW18492.1 afla|EED52912.1 afum|EAL93182.1 agla|OJJ85087.1 akaw|GAA86468.1 aluc|OJZ85009.1 anid|CBF70162.1 anig|EHA25230.1 aory|EIT78650.1 asyd|OJJ58800.1 ater|EAU29918.1 atub|OJI80588.1 aver|OJJ01404.1 awen|OJJ31045.1 azon|OJJ50556.1

Group1557: abra|OJJ67375.1 acar|OOF92436.1 acla|EAW06380.1 afis|EAW18486.1 afla|EED52922.1 afum|EAL93175.1 agla|OJJ85081.1 akaw|GAA86447.1 aluc|OJZ84990.1 anid|CBF70147.1 anig|EHA25202.1 aory|EIT78638.1 asyd|OJJ58808.1 ater|EAU29911.1 atub|OJI80610.1 aver|OJJ01411.1 awen|OJJ31033.1 azon|OJJ50552.1

Group1558: abra|OJJ67377.1 acar|OOF92435.1 acla|EAW06379.1 afis|EAW18485.1 afla|EED52923.1 afum|EBA27486.1 agla|OJJ85074.1 akaw|GAA86446.1 aluc|OJZ84988.1 anid|CBF70145.1 anig|EHA25201.1 aory|EIT78668.1 asyd|OJJ58809.1 ater|EAU29910.1 atub|OJI80611.1 aver|OJJ01412.1 awen|OJJ31035.1 azon|OJJ50550.1

Group1559: abra|OJJ67380.1 acar|OOF92432.1 acla|EAW06377.1 afis|EAW18483.1 afla|EED52925.1 afum|EAL93172.1 agla|OJJ85072.1 akaw|GAA86444.1 aluc|OJZ84986.1 anid|CBF86341.1 anig|EHA25199.1 aory|EIT78658.1 asyd|OJJ64985.1 ater|EAU29909.1 atub|OJI80614.1 aver|OJI99105.1 awen|OJJ31037.1 azon|OJJ50548.1

Group1560: abra|OJJ67381.1 acar|OOF92433.1 acla|EAW06376.1 afis|EAW18482.1 afla|EED52926.1 afum|EAL93171.1 agla|OJJ85063.1 akaw|GAA86443.1 aluc|OJZ84985.1 anid|CBF86343.1 anig|EHA25198.1 aory|EIT78671.1 asyd|OJJ64984.1 ater|EAU29908.1 atub|OJI80615.1 aver|OJI99104.1 awen|OJJ31038.1 azon|OJJ50547.1

Group1561: abra|OJJ67385.1 acar|OOF92428.1 acla|EAW06374.1 afis|EAW18480.1 afla|EED52929.1 afum|EAL93169.1 agla|OJJ85066.1 akaw|GAA86439.1 aluc|OJZ84981.1 anid|CBF70139.1 anig|EHA25194.1 aory|EIT78702.1 asyd|OJJ58812.1 ater|EAU29896.1 atub|OJI80620.1 aver|OJJ01415.1 awen|OJJ31040.1 azon|OJJ50545.1

Group1562: abra|OJJ67387.1 acar|OOF92427.1 acla|EAW06372.1 afis|EAW18478.1 afla|EED52932.1 afum|EAL93166.1 agla|OJJ85068.1 akaw|GAA86437.1 aluc|OJZ84980.1 anid|CBF70136.1 anig|EHA25192.1 aory|EIT78730.1 asyd|OJJ58814.1 ater|EAU29895.1 atub|OJI80622.1 aver|OJJ01417.1 awen|OJJ31031.1 azon|OJJ50544.1

Group1563: abra|OJJ67393.1 acar|OOF92423.1 acla|EAW06370.1 afis|EAW18476.1 afla|EED52934.1 afum|EAL93163.1 agla|OJJ85071.1 akaw|GAA86434.1 aluc|OJZ84976.1 anid|CBF70132.1 anig|EHA25188.1 aory|EIT78693.1 asyd|OJJ58817.1 ater|EAU29893.1 atub|OJI80625.1 aver|OJJ01421.1 awen|OJJ31029.1 azon|OJJ50542.1

Group1564: abra|OJJ67394.1 acar|OOF92422.1 acla|EAW06369.1 afis|EAW18475.1 afla|EED52935.1 afum|EAL93162.1 agla|OJJ85062.1 akaw|GAA86433.1 aluc|OJZ84975.1 anid|CBF70116.1 anig|EHA25187.1 aory|EIT78723.1 asyd|OJJ58819.1 ater|EAU29892.1 atub|OJI80626.1 aver|OJJ01423.1 awen|OJJ31028.1 azon|OJJ50541.1

Group1565: abra|OJJ67395.1 acar|OOF92421.1 acla|EAW06368.1 afis|EAW18474.1 afla|EED52936.1 afum|EAL93161.1 agla|OJJ85061.1 akaw|GAA86432.1 aluc|OJZ84974.1 anid|CBF70108.1 anig|EHA25186.1 aory|EIT78688.1 asyd|OJJ58823.1 ater|EAU29891.1 atub|OJI80627.1 aver|OJJ01427.1 awen|OJJ31026.1 azon|OJJ50540.1

Group1566: abra|OJJ67396.1 acar|OOF92419.1 acla|EAW06364.1 afis|EAW18473.1 afla|EED52937.1 afum|EAL93160.2 agla|OJJ85060.1 akaw|GAA86431.1 aluc|OJZ84973.1 anid|CBF70107.1 anig|EHA25185.1 aory|EIT78722.1 asyd|OJJ58827.1 ater|EAU29890.1 atub|OJI80628.1 aver|OJJ01430.1 awen|OJJ31025.1 azon|OJJ50539.1

Group1567: abra|OJJ67398.1 acar|OOF92418.1 acla|EAW06362.1 afis|EAW18471.1 afla|EED52939.1 afum|EAL93158.1 agla|OJJ85056.1 akaw|GAA86429.1 aluc|OJZ84971.1 anid|CBF70105.1 anig|EHA25183.1 aory|EIT78698.1 asyd|OJJ58829.1 ater|EAU29888.1 atub|OJI80630.1 aver|OJJ01432.1 awen|OJJ31023.1 azon|OJJ50537.1

Group1568: abra|OJJ67399.1 acar|OOF92417.1 acla|EAW06359.1 afis|EAW18470.1 afla|EED52940.1 afum|EAL93156.1 agla|OJJ85055.1 akaw|GAA86428.1 aluc|OJZ84969.1 anid|CBF70103.1 anig|EHA25182.1 aory|EIT78679.1 asyd|OJJ58830.1 ater|EAU29887.1 atub|OJI80631.1 aver|OJJ01433.1 awen|OJJ31022.1 azon|OJJ50536.1

Group1569: abra|OJJ67400.1 acar|OOF92416.1 acla|EAW06358.1 afis|EAW18469.1 afla|EED52941.1 afum|EAL93155.1 agla|OJJ85054.1 akaw|GAA86427.1 aluc|OJZ84968.1 anid|CBF70101.1 anig|EHA25181.1 aory|EIT78712.1 asyd|OJJ58831.1 ater|EAU29886.1 atub|OJI80632.1 aver|OJJ01434.1 awen|OJJ31021.1 azon|OJJ50535.1

Group1570: abra|OJJ67401.1 acar|OOF92415.1 acla|EAW06357.1 afis|EAW18468.1 afla|EED52942.1 afum|EAL93154.1 agla|OJJ85053.1 akaw|GAA86426.1 aluc|OJZ84967.1 anid|CBF70100.1 anig|EHA25180.1 aory|EIT78694.1 asyd|OJJ58832.1 ater|EAU29885.1 atub|OJI80633.1 aver|OJJ01435.1 awen|OJJ31020.1 azon|OJJ50534.1

Group1571: abra|OJJ67403.1 acar|OOF92413.1 acla|EAW06355.1 afis|EAW18466.1 afla|EED52944.1 afum|EAL93152.1 agla|OJJ85051.1 akaw|GAA86424.1 aluc|OJZ84965.1 anid|CBF70096.1 anig|EHA25178.1 aory|EIT78707.1 asyd|OJJ58834.1 ater|EAU29883.1 atub|OJI80635.1 aver|OJJ01437.1 awen|OJJ31018.1 azon|OJJ50532.1

Group1572: abra|OJJ67411.1 acar|OOF89994.1 acla|EAW06348.1 afis|EAW18459.1 afla|EED52949.1 afum|EAL93145.1 agla|OJJ85042.1 akaw|GAA86416.1 aluc|OJZ84957.1 anid|CBF70085.1 anig|EHA25171.1 aory|EIT78721.1 asyd|OJJ58842.1 ater|EAU29877.1 atub|OJI80643.1 aver|OJJ01444.1 awen|OJJ31011.1 azon|OJJ50525.1

Group1573: abra|OJJ67412.1 acar|OOF89992.1 acla|EAW06347.1 afis|EAW18458.1 afla|EED52950.1 afum|EAL93143.1 agla|OJJ85041.1 akaw|GAA86415.1 aluc|OJZ84956.1 anid|CBF70084.1 anig|EHA25170.1 aory|EIT78692.1 asyd|OJJ58843.1 ater|EAU29876.1 atub|OJI80644.1 aver|OJJ01445.1 awen|OJJ31010.1 azon|OJJ50524.1

Group1574: abra|OJJ67419.1 acar|OOF92402.1 acla|EAW06345.1 afis|EAW18456.1 afla|EED52952.1 afum|EAL93141.1 agla|OJJ85039.1 akaw|GAA86409.1 aluc|OJZ84949.1 anid|CBF70053.1 anig|EHA25164.1 aory|EIT78736.1 asyd|OJJ58857.1 ater|EAU29873.1 atub|OJI80653.1 aver|OJJ01458.1 awen|OJJ31008.1 azon|OJJ50522.1

Group1575: abra|OJJ67421.1 acar|OOF92401.1 acla|EAW06344.1 afis|EAW18455.1 afla|EED52953.1 afum|EAL93140.1 agla|OJJ85038.1 akaw|GAA86408.1 aluc|OJZ84947.1 anid|CBF70057.1 anig|EHA25163.1 aory|EIT78696.1 asyd|OJJ58855.1 ater|EAU29872.1 atub|OJI80654.1 aver|OJJ01456.1 awen|OJJ31007.1 azon|OJJ50521.1

Group1576: abra|OJJ67423.1 acar|OOF89873.1 acla|EAW06341.1 afis|EAW18453.1 afla|EED52955.1 afum|EAL93138.1 agla|OJJ85032.1 akaw|GAA86406.1 aluc|OJZ84944.1 anid|CBF70061.1 anig|EHA25161.1 aory|EIT78714.1 asyd|OJJ58853.1 ater|EAU29870.1 atub|OJI80656.1 aver|OJJ01454.1 awen|OJJ31005.1 azon|OJJ50519.1

Group1577: abra|OJJ67424.1 acar|OOF89874.1 acla|EAW06340.1 afis|EAW18452.1 afla|EED52956.1 afum|EAL93137.1 agla|OJJ85030.1 akaw|GAA86405.1 aluc|OJZ84943.1 anid|CBF70062.1 anig|EHA25160.1

aory|EIT78680.1 asyd|OJJ58852.1 ater|EAU29869.1 atub|OJI80657.1 aver|OJJ01453.1 awen|OJJ31004.1 azon|OJJ50517.1

Group1578: abra|OJJ67426.1 acar|OOF92399.1 acla|EAW06338.1 afis|EAW18450.1 afla|EED52958.1 afum|EAL93135.1 agla|OJJ85028.1 akaw|GAA86404.1 aluc|OJZ84941.1 anid|CBF70064.1 anig|EHA25158.1 aory|EIT78699.1 asyd|OJJ58850.1 ater|EAU29868.1 atub|OJI80659.1 aver|OJJ01451.1 awen|OJJ31002.1 azon|OJJ50516.1

Group1579: abra|OJJ67429.1 acar|OOF92397.1 acla|EAW06335.1 afis|EAW18447.1 afla|EED52963.1 afum|EAL93132.1 agla|OJJ85024.1 akaw|GAA86401.1 aluc|OJZ84937.1 anid|CBF70070.1 anig|EHA25155.1 aory|EIT78728.1 asyd|OJJ58847.1 ater|EAU29866.1 atub|OJI80662.1 aver|OJJ01448.1 awen|OJJ30999.1 azon|OJJ50513.1

Group1580: abra|OJJ67431.1 anig|EHA25151.1 atub|OJI80666.1 abra|OJJ69856.1 acar|OOF97498.1 akaw|GAA87800.1 aluc|OJZ85435.1 anig|EHA24981.1 atub|OJI80133.1 abra|OJJ75776.1 acar|OOF95028.1 acla|EAW07821.1 afis|EAW22857.1 afla|EED47892.1 afum|EAL90343.1 akaw|GAA86398.1 aory|EIT77247.1 aluc|OJZ84933.1

Group1581: abra|OJJ67434.1 acar|OOF92394.1 acla|EAW06332.1 afis|EAW18445.1 afla|EED52967.1 afum|EAL93130.1 agla|OJJ85021.1 akaw|GAA86396.1 aluc|OJZ84930.1 anid|CBF70041.1 anig|EHA25149.1 aory|EIT78729.1 asyd|OJJ58865.1 ater|EAU29864.1 atub|OJI80669.1 aver|OJJ01466.1 awen|OJJ30993.1 azon|OJJ50510.1

Group1582: abra|OJJ67435.1 acar|OOF92393.1 acla|EAW06331.1 afis|EAW18444.1 afla|EED52968.1 afum|EAL93128.1 agla|OJJ85020.1 akaw|GAA86395.1 aluc|OJZ84929.1 anid|CBF70043.1 anig|EHA25148.1 aory|EIT78686.1 asyd|OJJ58864.1 ater|EAU29863.1 atub|OJI80670.1 aver|OJJ01465.1 awen|OJJ30992.1 azon|OJJ50511.1

Group1583: abra|OJJ67437.1 acar|OOF92391.1 acla|EAW06327.1 afis|EAW18441.1 afla|EED52972.1 afum|EAL93123.1 agla|OJJ85006.1 akaw|GAA86393.1 aluc|OJZ84927.1 anid|CBF70049.1 anig|EHA25146.1 aory|EIT78690.1 asyd|OJJ58861.1 ater|EAU29860.1 atub|OJI80672.1 aver|OJJ01462.1 awen|OJJ30989.1 azon|OJJ50507.1

Group1584: abra|OJJ67438.1 acar|OOF92389.1 acla|EAW06328.1 afis|EAW18442.1 afla|EED52971.1 afum|EAL93125.1 agla|OJJ85008.1 akaw|GAA86392.1 aluc|OJZ84926.1 anid|CBF70047.1 anig|EHA25145.1 aory|EIT78682.1 asyd|OJJ58862.1 ater|EAU29859.1 atub|OJI80673.1 aver|OJJ01463.1 awen|OJJ30990.1 azon|OJJ50508.1

Group1585: abra|OJJ67439.1 acar|OOF92390.1 acla|EAW06329.1 afis|EAW18443.1 afla|EED52970.1 afum|EAL93126.1 agla|OJJ85009.1 akaw|GAA86391.1 aluc|OJZ84925.1 anid|CBF70045.1 anig|EHA25144.1 aory|EIT78727.1 asyd|OJJ58863.1 ater|EAU29858.1 atub|OJI80674.1 aver|OJJ01464.1 awen|OJJ30991.1 azon|OJJ50509.1

Group1586: abra|OJJ67452.1 acla|EAW06313.1 afla|EED52986.1 akaw|GAA86379.1 aluc|OJZ84913.1 anid|CBF70694.1 anig|EHA25134.1 aory|EIT78726.1 ater|EAU29843.1 atub|OJI80685.1 azon|OJJ50483.1 afis|EAW18422.1 afum|EAL93106.1 asyd|OJJ58464.1 aver|OJJ01063.1 awen|OJJ31723.1 agla|OJJ84990.1 awen|OJJ30758.1

Group1587: abra|OJJ67471.1 acar|OOF89932.1 acla|EAW06300.1 afis|EAW18407.1 afla|EED52997.1 afum|EAL93087.1 agla|OJJ84959.1 akaw|GAA86365.1 aluc|OJZ84895.1 anid|CBF70741.1 anig|EHA25122.1 aory|EIT77423.1 asyd|OJJ58439.1 ater|EAU29825.1 atub|OJI80705.1 aver|OJJ01039.1 awen|OJJ31743.1 azon|OJJ50501.1

Group1588: abra|OJJ67609.1 acar|OOF91956.1 acla|EAW11006.1 afis|EAW20847.1 afla|EED45466.1 afum|EAL84786.1 akaw|GAA89125.1 aluc|OJZ81713.1 anid|CBF82875.1 anig|EHA25726.1 aory|EIT75524.1 asyd|OJJ61570.1 ater|EAU30786.1 atub|OJI88471.1 aver|OJJ00890.1 awen|OJJ34544.1 azon|OJJ42460.1 ater|EAU37631.1

Group1589: abra|OJJ67742.1 acar|OOF90949.1 acla|EAW10991.1 afis|EAW20869.1 afla|EED45539.1 afum|EAL84765.1 agla|OJJ83977.1 akaw|GAA92433.1 aluc|OJZ81588.1 anid|CBF82842.1 anig|EHA25852.1 aory|EIT75440.1 asyd|OJJ61589.1 ater|EAU30819.1 atub|OJI88594.1 aver|OJJ00911.1 awen|OJJ34495.1 azon|OJJ51769.1

Group1590: abra|OJJ67743.1 acar|OOF90948.1 acla|EAW10992.1 afis|EAW20868.1 afla|EED45543.1 afum|EAL84766.2 agla|OJJ83976.1 akaw|GAA92434.1 aluc|OJZ81586.1 anid|CBF82840.1 anig|EHA25853.1 aory|EIT75711.1 asyd|OJJ61588.1 ater|EAU30820.1 atub|OJI88595.1 aver|OJJ00910.1 awen|OJJ34498.1 azon|OJJ51773.1

Group1591: abra|OJJ67744.1 acar|OOF90947.1 acla|EAW10993.1 afis|EAW20867.1 afla|EED45544.1 afum|EAL84767.1 agla|OJJ83975.1 akaw|GAA92435.1 aluc|OJZ81585.1 anid|CBF82838.1 anig|EHA25854.1 aory|EIT75422.1 asyd|OJJ61587.1 ater|EAU30821.1 atub|OJI88596.1 aver|OJJ00909.1 awen|OJJ34499.1 azon|OJJ51774.1

Group1592: abra|OJJ67747.1 acar|OOF90944.1 acla|EAW10997.1 afis|EAW20864.1 afla|EED45548.1 afum|EAL84771.1 agla|OJJ83751.1 akaw|GAA92438.1 aluc|OJZ81582.1 anid|CBF82835.1 anig|EHA25857.1 aory|EIT75692.1 asyd|OJJ61584.1 ater|EAU30807.1 atub|OJI88599.1 aver|OJJ00906.1 awen|OJJ34506.1 azon|OJJ51784.1

Group1593: abra|OJJ67748.1 acar|OOF90943.1 acla|EAW10998.1 afis|EAW20863.1 afla|EED45549.1 afum|EAL84772.1 agla|OJJ83750.1 akaw|GAA92439.1 aluc|OJZ81581.1 anid|CBF82833.1 anig|EHA25858.1 aory|EIT75610.1 asyd|OJJ61583.1 ater|EAU30806.1 atub|OJI88600.1 aver|OJJ00905.1 awen|OJJ34507.1 azon|OJJ51785.1

Group1594: abra|OJJ67749.1 acar|OOF90950.1 acla|EAW10990.1 afis|EAW20870.1 afla|EED45552.1 afum|EAL84764.1 agla|OJJ83978.1 akaw|GAA92440.1 aluc|OJZ81580.1 anid|CBF82844.1 anig|EHA25859.1 aory|EIT75541.1 asyd|OJJ61590.1 ater|EAU30818.1 atub|OJI88601.1 aver|OJJ00912.1 awen|OJJ34494.1 azon|OJJ51768.1

Group1595: abra|OJJ67799.1 acar|OOF94206.1 agla|OJJ79105.1 anid|CBF81915.1 asyd|OJJ59697.1 ater|EAU35300.1 aver|OJJ04270.1 awen|OJJ41729.1 azon|OJJ43893.1 asyd|OJJ65109.1 aver|OJI99236.1 asyd|OJJ55389.1 aver|OJJ08268.1 aver|OJJ02590.1 awen|OJJ34589.1 asyd|OJJ54350.1 aver|OJJ05302.1 asyd|OJJ58135.1

Group1596: abra|OJJ67847.1 acar|OOF92828.1 acla|EAW11752.1 afis|EAW23785.1 afla|EED51594.1 afum|EAL87219.2 agla|OJJ78938.1 akaw|GAA91855.1 aluc|OJZ82044.1 anid|CBF79845.1 anig|EHA22213.1 aory|EIT80570.1 asyd|OJJ58153.1 ater|EAU31326.1 atub|OJI84603.1 aver|OJJ04251.1 awen|OJJ33730.1 azon|OJJ42852.1

Group1597: abra|OJJ67848.1 acar|OOF92829.1 acla|EAW11751.1 afis|EAW23784.1 afla|EED51593.1 afum|EAL87218.2 agla|OJJ78937.1 akaw|GAA91856.1 aluc|OJZ82043.1 anid|CBF79841.1 anig|EHA22214.1 aory|EIT80650.1 asyd|OJJ58152.1 ater|EAU31325.1 atub|OJI84602.1 aver|OJJ04252.1 awen|OJJ33729.1 azon|OJJ42853.1

Group1598: abra|OJJ67851.1 acar|OOF92832.1 acla|EAW11748.1 afis|EAW23781.1 afla|EED51585.1 afum|EAL87215.1 agla|OJJ78933.1 akaw|GAA91859.1 aluc|OJZ82040.1 anid|CBF79839.1 anig|EHA22216.1 aory|EIT80522.1 asyd|OJJ58148.1 ater|EAU31322.1 atub|OJI84599.1 aver|OJJ04256.1 awen|OJJ33724.1 azon|OJJ42856.1

Group1599: abra|OJJ67859.1 acar|OOF92840.1 acla|EAW11739.1 afis|EAW23772.1 afla|EED51573.1 afum|EAL87206.1 agla|OJJ78947.1 akaw|GAA91866.1 aluc|OJZ82033.1 anid|CBF79823.1 anig|EHA22223.1 aory|EIT80715.1 asyd|OJJ58141.1 ater|EAU31316.1 atub|OJI84588.1 aver|OJJ04264.1 awen|OJJ33703.1 azon|OJJ42837.1

Group1600: abra|OJJ67861.1 acar|OOF92839.1 acla|EAW11737.1 afis|EAW23770.1 afla|EED51571.1 afum|EAL87204.1 agla|OJJ78949.1 anid|CBF79821.1 anig|EHA22225.1 aory|EIT80633.1 asyd|OJJ58138.1 ater|EAU31314.1 aver|OJJ04267.1 awen|OJJ33705.1 azon|OJJ42836.1 akaw|GAA91868.1 aluc|OJZ82031.1 atub|OJI84586.1

Group1601: abra|OJJ67865.1 acar|OOF92845.1 acla|EAW11734.1 afis|EAW23766.1 afla|EED51552.1 afum|EAL87199.1 agla|OJJ78956.1 akaw|GAA91872.1 aluc|OJZ82027.1 anid|CBF79770.1 anig|EHA22229.1 aory|EIT80651.1 asyd|OJJ58112.1 ater|EAU31310.1 atub|OJI84581.1 aver|OJJ04293.1 awen|OJJ33692.1 azon|OJJ42832.1

Group1602: abra|OJJ67866.1 acar|OOF92846.1 acla|EAW11733.1 afis|EAW23765.1 afla|EED51550.1 afum|EAL87198.1 agla|OJJ78957.1 akaw|GAA91873.1 aluc|OJZ82026.1 anid|CBF79772.1 anig|EHA22230.1 aory|EIT80718.1 asyd|OJJ58110.1 ater|EAU31311.1 atub|OJI84579.1 aver|OJJ04294.1 awen|OJJ33689.1 azon|OJJ42830.1

Group1603: abra|OJJ67881.1 acar|OOF92865.1 acla|EAW11722.1 afis|EAW23744.1 afla|EED51536.1 afum|EAL87180.1 agla|OJJ78963.1 akaw|GAA91888.1 aluc|OJZ82009.1 anid|CBF81714.1 anig|EHA22248.1 aory|EIT80594.1 asyd|OJJ59582.1 ater|EAU31299.1 atub|OJI84558.1 aver|OJJ02687.1 awen|OJJ33679.1 azon|OJJ42820.1

Group1604: abra|OJJ67913.1 acar|OOF92766.1 acla|EAW12796.1 afis|EAW17959.1 afla|EED51948.1 afum|EAL91770.1 agla|OJJ80207.1 akaw|GAA91283.1 aluc|OJZ82786.1 anid|CBF79950.1 anig|EHA21227.1 aory|EIT81390.1 asyd|OJJ58216.1 ater|EAU31448.1 atub|OJI89018.1 aver|OJJ02206.1 awen|OJJ41583.1 azon|OJJ48295.1

Group1605: abra|OJJ67915.1 acar|OOF92767.1 acla|EAW12794.1 afis|EAW17957.1 afla|EED51950.1 afum|EAL91768.1 agla|OJJ80204.1 akaw|GAA91281.1 aluc|OJZ82788.1 anid|CBF79947.1 anig|EHA21229.1 aory|EIT81406.1 asyd|OJJ58214.1 ater|EAU31445.1 atub|OJI89020.1 aver|OJJ02208.1 awen|OJJ41585.1 azon|OJJ48297.1

Group1606: abra|OJJ67917.1 acar|OOF92769.1 acla|EAW12792.1 afis|EAW17955.1 afla|EED51952.1 afum|EAL91766.1 agla|OJJ80202.1 akaw|GAA91279.1 aluc|OJZ82790.1 anid|CBF79943.1 anig|EHA21231.1

aory|EIT81414.1 asyd|OJJ58212.1 ater|EAU31443.1 atub|OJI89021.1 aver|OJJ02210.1 awen|OJJ41587.1 azon|OJJ48302.1

Group1607: abra|OJJ67918.1 acar|OOF92770.1 acla|EAW12791.1 afis|EAW17951.1 afla|EED51953.1 afum|EAL91761.1 agla|OJJ80201.1 akaw|GAA91278.1 aluc|OJZ82791.1 anid|CBF79941.1 anig|EHA21232.1 aory|EIT81400.1 asyd|OJJ58211.1 ater|EAU31442.1 atub|OJI89022.1 aver|OJJ02211.1 awen|OJJ41588.1 azon|OJJ48303.1

Group1608: abra|OJJ67919.1 acar|OOF92771.1 acla|EAW12790.1 afis|EAW17952.1 afla|EED51954.1 afum|EAL91762.1 agla|OJJ80200.1 akaw|GAA91277.1 aluc|OJZ82792.1 anid|CBF79939.1 anig|EHA21233.1 aory|EIT81379.1 asyd|OJJ58210.1 ater|EAU31441.1 atub|OJI89023.1 aver|OJJ02212.1 awen|OJJ41589.1 azon|OJJ48304.1

Group1609: abra|OJJ67920.1 acar|OOF92772.1 acla|EAW12789.1 afis|EAW17953.1 afla|EED51955.1 afum|EAL91763.1 agla|OJJ80199.1 akaw|GAA91276.1 aluc|OJZ82793.1 anid|CBF79934.1 anig|EHA21234.1 aory|EIT81461.1 asyd|OJJ58207.1 ater|EAU31440.1 atub|OJI89024.1 aver|OJJ02215.1 awen|OJJ41590.1 azon|OJJ48305.1

Group1610: abra|OJJ67922.1 acar|OOF92773.1 acla|EAW12788.1 afis|EAW17954.1 afla|EED51956.1 afum|EAL91765.1 agla|OJJ80198.1 akaw|GAA91275.1 aluc|OJZ82794.1 anid|CBF79932.1 anig|EHA21235.1 aory|EIT81403.1 asyd|OJJ58206.1 ater|EAU31439.1 atub|OJI89025.1 aver|OJJ02216.1 awen|OJJ41591.1 azon|OJJ48306.1

Group1611: abra|OJJ67923.1 acar|OOF92774.1 acla|EAW12787.1 afis|EAW17950.1 afla|EED51957.1 afum|EAL91760.1 agla|OJJ80197.1 akaw|GAA91274.1 aluc|OJZ82795.1 anid|CBF79931.1 anig|EHA21236.1 aory|EIT81374.1 asyd|OJJ58205.1 ater|EAU31438.1 atub|OJI89026.1 aver|OJJ02217.1 awen|OJJ41592.1 azon|OJJ48308.1

Group1612: abra|OJJ67926.1 acar|OOF92775.1 acla|EAW12785.1 afis|EAW17947.1 afla|EED51959.1 afum|EAL91757.1 agla|OJJ80195.1 akaw|GAA91271.1 aluc|OJZ82798.1 anid|CBF79929.1 anig|EHA21239.1 aory|EIT81382.1 asyd|OJJ58204.1 ater|EAU31431.1 atub|OJI89029.1 aver|OJJ04202.1 awen|OJJ41596.1 azon|OJJ48312.1

Group1613: abra|OJJ67928.1 acar|OOF92777.1 acla|EAW12781.1 afis|EAW17943.1 afla|EED51961.1 afum|EAL91754.1 agla|OJJ82989.1 akaw|GAA91269.1 aluc|OJZ82800.1 anid|CBF79925.1 anig|EHA21240.1 aory|EIT81385.1 asyd|OJJ58202.1 ater|EAU31396.1 atub|OJI89031.1 aver|OJJ04207.1 awen|OJJ41600.1 azon|OJJ48323.1

Group1614: abra|OJJ67929.1 acar|OOF92779.1 acla|EAW11622.1 afis|EAW23836.1 afla|EED51962.1 afum|EAL87267.1 agla|OJJ82498.1 akaw|GAA91268.1 aluc|OJZ82801.1 anid|CBF79923.1 anig|EHA21242.1 aory|EIT81370.1 asyd|OJJ58200.1 ater|EAU31395.1 atub|OJI89032.1 aver|OJJ04208.1 awen|OJJ33798.1 azon|OJJ48745.1

Group1615: abra|OJJ67936.1 acar|OOF92785.1 acla|EAW11625.1 afis|EAW23831.1 afla|EED51974.1 afum|EAL87263.1 agla|OJJ82481.1 akaw|GAA91260.1 aluc|OJZ82808.1 anid|CBF79904.1 anig|EHA21248.1 aory|EIT81407.1 asyd|OJJ58187.1 ater|EAU31385.1 atub|OJI89039.1 aver|OJJ04218.1 awen|OJJ33789.1 azon|OJJ48757.1

Group1616: abra|OJJ67938.1 acar|OOF92787.1 acla|EAW11628.1 afis|EAW23828.1 afla|EED51977.1 afum|EAL87260.1 agla|OJJ82484.1 akaw|GAA91258.1 aluc|OJZ82810.1 anid|CBF79908.1 anig|EHA21249.1 aory|EIT81387.1 asyd|OJJ58189.1 ater|EAU31383.1 atub|OJI89040.1 aver|OJJ04216.1 awen|OJJ33787.1 azon|OJJ48759.1

Group1617: abra|OJJ67940.1 acar|OOF92789.1 acla|EAW11630.1 afis|EAW23826.1 afla|EED51980.1 afum|EAL87258.1 agla|OJJ82486.1 akaw|GAA91256.1 aluc|OJZ82812.1 anid|CBF79911.1 anig|EHA21252.1 aory|EIT81452.1 asyd|OJJ58190.1 ater|EAU31381.1 atub|OJI89043.1 aver|OJJ04215.1 awen|OJJ33784.1 azon|OJJ42886.1

Group1618: abra|OJJ67942.1 acar|OOF92791.1 acla|EAW11636.1 afis|EAW23821.1 afla|EED51983.1 afum|EAL87256.1 agla|OJJ82468.1 akaw|GAA91254.1 aluc|OJZ82814.1 anid|CBF79894.1 anig|EHA21254.1 aory|EIT81391.1 asyd|OJJ58182.1 ater|EAU31373.1 atub|OJI89045.1 aver|OJJ04224.1 awen|OJJ33777.1 azon|OJJ42883.1

Group1619: abra|OJJ67951.1 acar|OOF92798.1 acla|EAW11641.1 afis|EAW23816.1 afla|EED51989.1 afum|EAL87251.1 agla|OJJ82462.1 akaw|GAA91246.1 aluc|OJZ82824.1 anid|CBF79887.1 anig|EHA21262.1 aory|EIT81439.1 asyd|OJJ58175.1 ater|EAU31371.1 atub|OJI89053.1 aver|OJJ04229.1 awen|OJJ33773.1 azon|OJJ42877.1

Group1620: abra|OJJ67957.1 acar|OOF92802.1 acla|EAW11646.1 afis|EAW23810.1 afla|EED52005.1 afum|EAL87244.1 agla|OJJ82450.1 akaw|GAA91239.1 aluc|OJZ82830.1 anid|CBF79876.1 anig|EHA21268.1 aory|EIT81434.1 asyd|OJJ58169.1 ater|EAU31363.1 atub|OJI89060.1 aver|OJJ04234.1 awen|OJJ33766.1 azon|OJJ42870.1

Group1621: abra|OJJ67958.1 acar|OOF92803.1 acla|EAW11647.1 afis|EAW23809.1 afla|EED52006.1 afum|EAL87243.1 agla|OJJ82449.1 akaw|GAA91238.1 aluc|OJZ82831.1 anid|CBF79874.1 anig|EHA21269.1 aory|EIT81404.1 asyd|OJJ58168.1 ater|EAU31362.1 atub|OJI89061.1 aver|OJJ04235.1 awen|OJJ33765.1 azon|OJJ42868.1

Group1622: abra|OJJ67977.1 acar|OOF92819.1 acla|EAW11758.1 afis|EAW23791.1 afla|EED51599.1 afum|EAL87225.1 agla|OJJ78819.1 akaw|GAA91226.1 aluc|OJZ82844.1 anid|CBF79853.1 anig|EHA21282.1 aory|EIT80507.1 asyd|OJJ58157.1 ater|EAU31330.1 atub|OJI89074.1 aver|OJJ04246.1 awen|OJJ33746.1 azon|OJJ42843.1

Group1623: abra|OJJ67978.1 acar|OOF92818.1 acla|EAW11757.1 afis|EAW23790.1 afla|EED51598.1 afum|EAL87224.1 agla|OJJ78817.1 akaw|GAA91225.1 aluc|OJZ82845.1 anid|CBF79851.1 anig|EHA21283.1 aory|EIT80526.1 asyd|OJJ58156.1 ater|EAU31329.1 atub|OJI89075.1 aver|OJJ04247.1 awen|OJJ33745.1 azon|OJJ42844.1

Group1624: abra|OJJ68015.1 acar|OOF92057.1 acla|EAW08795.1 afis|EAW15605.1 afla|EED55861.1 afum|EAL85728.1 agla|OJJ79064.1 akaw|GAA90936.1 aluc|OJZ82888.1 anid|CBF69625.1 anig|EHA21314.1 aory|EIT76549.1 asyd|OJJ59120.1 ater|EAU37248.1 atub|OJI89113.1 aver|OJJ01709.1 awen|OJJ32398.1 azon|OJJ43874.1

Group1625: abra|OJJ68016.1 acar|OOF92058.1 afla|EED54104.1 agla|OJJ80413.1 akaw|GAA90935.1 aluc|OJZ82889.1 anid|CBF83706.1 anig|EHA21315.1 aory|EIT77159.1 asyd|OJJ53740.1 atub|OJI89114.1 aver|OJJ07853.1 awen|OJJ35049.1 azon|OJJ42272.1 acla|EAW14887.1 awen|OJJ34541.1 aory|EIT75976.1 awen|OJJ30142.1

Group1626: abra|OJJ68060.1 acar|OOF92081.1 acla|EAW13655.1 afis|EAW19935.1 afla|EED45904.1 afum|EAL84975.2 agla|OJJ79327.1 akaw|GAA90896.1 aluc|OJZ82932.1 anid|CBF84602.1 anig|EHA21353.1 aory|EIT75822.1 asyd|OJJ53375.1 ater|EAU36898.1 atub|OJI89161.1 aver|OJJ08369.1 awen|OJJ29978.1 azon|OJJ50215.1

Group1627: abra|OJJ68075.1 acar|OOF94470.1 acla|EAW12117.1 afis|EAW17265.1 afla|EED48857.1 afum|EAL86682.1 agla|OJJ85378.1 akaw|GAA92736.1 aluc|OJZ92944.1 anid|CBF87528.1 anig|EHA26028.1 aory|EIT81286.1 asyd|OJJ52984.1 ater|EAU36128.1 atub|OJI87403.1 aver|OJJ08953.1 awen|OJJ35769.1 azon|OJJ50248.1

Group1628: abra|OJJ68089.1 acar|OOF93893.1 acla|EAW15283.1 afis|EAW17127.1 afla|EED48286.1 afum|EAL86587.1 agla|OJJ78597.1 akaw|GAA86874.1 aluc|OJZ89998.1 anid|CBF80813.1 anig|EHA27373.1 aory|EIT72919.1 asyd|OJJ54451.1 ater|EAU34073.1 atub|OJI84810.1 aver|OJJ07703.1 awen|OJJ29885.1 azon|OJJ50148.1

Group1629: abra|OJJ68125.1 acar|OOF93919.1 acla|EAW12869.1 afis|EAW17817.1 afla|EED45601.1 afum|EAL91627.1 agla|OJJ81689.1 akaw|GAA86906.1 aluc|OJZ90029.1 anid|CBF80748.1 anig|EHA27405.1 aory|EIT75765.1 asyd|OJJ54539.1 ater|EAU30285.1 atub|OJI84778.1 aver|OJJ07642.1 awen|OJJ36934.1 azon|OJJ42822.1

Group1630: abra|OJJ68126.1 acar|OOF93920.1 acla|EAW12870.1 afis|EAW17818.1 afla|EED45602.1 afum|EAL91628.1 agla|OJJ85762.1 akaw|GAA86907.1 aluc|OJZ90030.1 anid|CBF80750.1 anig|EHA27406.1 asyd|OJJ62953.1 atub|OJI84777.1 aver|OJJ07643.1 awen|OJJ31294.1 azon|OJJ50609.1 aver|OJI98031.1 asyd|OJJ54538.1

Group1631: abra|OJJ68139.1 acar|OOF98356.1 acla|EAW14517.1 afis|EAW19235.1 afla|EED47647.1 afum|EAL93963.1 agla|OJJ88463.1 akaw|GAA92378.1 aluc|OJZ83237.1 anid|CBF78634.1 anig|EHA21094.1 aory|EIT80221.1 asyd|OJJ65123.1 ater|EAU37228.1 atub|OJI87699.1 aver|OJI99251.1 awen|OJJ37592.1 azon|OJJ51245.1

Group1632: abra|OJJ68145.1 acar|OOF89988.1 acla|EAW07390.1 afis|EAW22416.1 afla|EED52494.1 afum|EAL90776.1 agla|OJJ87680.1 akaw|GAA92373.1 aluc|OJZ83231.1 anid|CBF88053.1 anig|EHA21089.1 aory|EIT82805.1 asyd|OJJ63540.1 ater|EAU39291.1 atub|OJI87705.1 aver|OJI97393.1 awen|OJJ34995.1 azon|OJJ45638.1

Group1633: agla|OJJ86892.1 akaw|GAA88060.1 aluc|OJZ81322.1 abra|OJJ68375.1 agla|OJJ79451.1 aluc|OJZ90115.1 anig|EHA18628.1 ater|EAU37955.1 atub|OJI84684.1 awen|OJJ35546.1 azon|OJJ50297.1 acar|OOF90164.1 atub|OJI82870.1 awen|OJJ29729.1 acar|OOF90659.1 afla|EED57502.1 ater|EAU37868.1 atub|OJI91525.1

Group1634: abra|OJJ68168.1 acar|OOF97466.1 acla|EAW13054.1 afis|EAW17612.1 afla|EED49203.1 afum|EAL91425.1 agla|OJJ81325.1 akaw|GAA83275.1 aluc|OJZ83781.1 anid|CBF90174.1 anig|EHA18921.1 aory|EIT78579.1 asyd|OJJ61990.1 ater|EAU37047.1 atub|OJI90151.1 aver|OJI96082.1 awen|OJJ37230.1 azon|OJJ50933.1

Group1635: abra|OJJ68169.1 acar|OOF97467.1 acla|EAW13055.1 afis|EAW17611.1 afla|EED49202.1 afum|EAL91424.1 agla|OJJ81326.1 akaw|GAA83274.1 aluc|OJZ83780.1 anid|CBF90175.1 anig|EHA18920.1

aory|EIT78562.1 asyd|OJJ61989.1 ater|EAU37046.1 atub|OJI90150.1 aver|OJI96081.1 awen|OJJ37231.1 azon|OJJ50934.1

Group1636: abra|OJJ68171.1 acar|OOF97468.1 acla|EAW13056.1 afis|EAW17610.1 afla|EED49201.1 afum|EAL91423.1 agla|OJJ81331.1 akaw|GAA83273.1 aluc|OJZ83779.1 anid|CBF90177.1 anig|EHA18919.1 aory|EIT78514.1 asyd|OJJ61988.1 ater|EAU37045.1 atub|OJI90149.1 aver|OJI96080.1 awen|OJJ37232.1 azon|OJJ50935.1

Group1637: abra|OJJ68172.1 acar|OOF97470.1 acla|EAW13057.1 afis|EAW17609.1 afla|EED49213.1 afum|EAL91422.1 agla|OJJ81356.1 akaw|GAA83272.1 aluc|OJZ83778.1 anid|CBF90179.1 anig|EHA18918.1 aory|EIT83168.1 asyd|OJJ61987.1 ater|EAU37044.1 atub|OJI90148.1 aver|OJI96079.1 awen|OJJ37233.1 azon|OJJ50936.1

Group1638: abra|OJJ68173.1 acar|OOF97471.1 acla|EAW13058.1 afis|EAW17608.1 afla|EED49214.1 afum|EAL91421.1 agla|OJJ81355.1 akaw|GAA83271.1 aluc|OJZ83777.1 anid|CBF90181.1 anig|EHA18917.1 aory|EIT83162.1 asyd|OJJ61986.1 ater|EAU37043.1 atub|OJI90147.1 aver|OJI96078.1 awen|OJJ37234.1 azon|OJJ50937.1

Group1639: abra|OJJ68176.1 acar|OOF97473.1 acla|EAW13060.1 afis|EAW17604.1 afla|EED49217.1 afum|EAL91417.1 agla|OJJ81352.1 akaw|GAA83268.1 aluc|OJZ83774.1 anid|CBF90186.1 anig|EHA18914.1 aory|EIT83159.1 asyd|OJJ61983.1 ater|EAU37041.1 atub|OJI90145.1 aver|OJI96075.1 awen|OJJ37237.1 azon|OJJ50939.1

Group1640: abra|OJJ68177.1 acar|OOF97474.1 acla|EAW13061.1 afis|EAW17603.1 afla|EED49218.1 afum|EAL91416.1 agla|OJJ81351.1 akaw|GAA83267.1 aluc|OJZ83773.1 anid|CBF90188.1 anig|EHA18913.1 aory|EIT83167.1 asyd|OJJ61982.1 ater|EAU37040.1 atub|OJI90144.1 aver|OJI96074.1 awen|OJJ37238.1 azon|OJJ50940.1

Group1641: abra|OJJ68181.1 acar|OOF97477.1 acla|EAW13063.1 afis|EAW17601.1 afla|EED49220.1 afum|EBA27434.1 agla|OJJ81348.1 akaw|GAA83265.1 aluc|OJZ83771.1 anid|CBF90190.1 anig|EHA18911.1 aory|EIT83161.1 asyd|OJJ61981.1 ater|EAU37038.1 atub|OJI90142.1 aver|OJI96073.1 awen|OJJ37244.1 azon|OJJ50942.1

Group1642: abra|OJJ68182.1 acar|OOF97476.1 acla|EAW13064.1 afis|EAW17600.1 afla|EED49221.1 afum|EAL91414.1 agla|OJJ81347.1 akaw|GAA83264.1 aluc|OJZ83770.1 anid|CBF90192.1 anig|EHA18910.1 aory|EIT83165.1 asyd|OJJ61980.1 ater|EAU37037.1 atub|OJI90141.1 aver|OJI96072.1 awen|OJJ37245.1 azon|OJJ50943.1

Group1643: abra|OJJ68184.1 acar|OOF97479.1 acla|EAW13065.1 afis|EAW17599.1 afla|EED49222.1 afum|EAL91412.2 agla|OJJ81345.1 akaw|GAA83262.1 aluc|OJZ83768.1 anig|EHA18908.1 aory|EIT83170.1 asyd|OJJ61978.1 ater|EAU37036.1 atub|OJI90139.1 aver|OJI96070.1 awen|OJJ37247.1 azon|OJJ50945.1 anid|CBF90194.1

Group1644: abra|OJJ68190.1 acar|OOF97486.1 acla|EAW13072.1 afis|EAW17592.1 afla|EED49230.1 afum|EAL91405.1 agla|OJJ81357.1 akaw|GAA83255.1 aluc|OJZ83756.1 anid|CBF90212.1 anig|EHA18903.1 aory|EIT83166.1 asyd|OJJ61969.1 ater|EAU37029.1 atub|OJI90131.1 aver|OJI96062.1 awen|OJJ37254.1 azon|OJJ50949.1

Group1645: abra|OJJ68193.1 acar|OOF97489.1 acla|EAW13076.1 afis|EAW17588.1 afla|EED49234.1 afum|EAL91401.1 agla|OJJ81364.1 akaw|GAA83252.1 aluc|OJZ83753.1 anid|CBF90220.1 anig|EHA18900.1 aory|EIT83171.1 asyd|OJJ61966.1 ater|EAU37023.1 atub|OJI90128.1 aver|OJI96058.1 awen|OJJ37258.1 azon|OJJ50953.1

Group1646: abra|OJJ68194.1 acar|OOF97490.1 acla|EAW13077.1 afis|EAW17587.1 afla|EED49235.1 afum|EAL91400.1 agla|OJJ81365.1 akaw|GAA83251.1 aluc|OJZ83752.1 anid|CBF90222.1 anig|EHA18899.1 aory|EIT83154.1 asyd|OJJ61965.1 ater|EAU37022.1 atub|OJI90127.1 aver|OJI96057.1 awen|OJJ37259.1 azon|OJJ50954.1

Group1647: abra|OJJ68196.1 acar|OOF97491.1 acla|EAW13079.1 afis|EAW17585.1 afla|EED49237.1 afum|EAL91398.2 agla|OJJ81368.1 akaw|GAA83249.1 aluc|OJZ83750.1 anid|CBF90225.1 anig|EHA18897.1 aory|EIT83169.1 asyd|OJJ61963.1 ater|EAU37020.1 atub|OJI90125.1 aver|OJI96055.1 awen|OJJ37261.1 azon|OJJ50956.1

Group1648: abra|OJJ68197.1 acar|OOF89886.1 acla|EAW13080.1 afis|EAW17584.1 afla|EED49239.1 afum|EAL91397.1 agla|OJJ81369.1 akaw|GAA83248.1 aluc|OJZ83749.1 anid|CBF90227.1 anig|EHA18896.1 aory|EIT82222.1 asyd|OJJ61962.1 ater|EAU37019.1 atub|OJI90124.1 aver|OJI96054.1 awen|OJJ37262.1 azon|OJJ50957.1

Group1649: abra|OJJ68199.1 acar|OOF90119.1 acla|EAW13081.1 afis|EAW17583.1 afla|EED49240.1 afum|EAL91396.1 agla|OJJ81370.1 akaw|GAA83247.1 aluc|OJZ83748.1 anid|CBF90229.1 anig|EHA18895.1 aory|EIT82239.1 asyd|OJJ61961.1 ater|EAU37018.1 atub|OJI90123.1 aver|OJI96053.1 awen|OJJ37265.1 azon|OJJ50959.1

Group1650: abra|OJJ68200.1 acar|OOF90117.1 acla|EAW13083.1 afis|EAW17581.1 afla|EED49241.1 agla|OJJ81372.1 akaw|GAA83245.1 aluc|OJZ83747.1 anid|CBF90232.1 anig|EHA18893.1 aory|EIT82216.1 asyd|OJJ61959.1 ater|EAU37016.1 atub|OJI90122.1 aver|OJI96051.1 awen|OJJ37267.1 azon|OJJ50960.1 afum|EAL91394.1

Group1651: abra|OJJ68201.1 acar|OOF90116.1 acla|EAW13084.1 afis|EAW17580.1 afla|EED49242.1 afum|EAL91393.1 agla|OJJ81379.1 akaw|GAA83244.1 aluc|OJZ83746.1 anid|CBF90234.1 anig|EHA18892.1 aory|EIT82232.1 asyd|OJJ61958.1 ater|EAU37015.1 atub|OJI90121.1 aver|OJI96050.1 awen|OJJ37268.1 azon|OJJ50961.1

Group1652: abra|OJJ68203.1 acar|OOF90234.1 acla|EAW13086.1 afis|EAW17579.1 afla|EED49243.1 afum|EAL91392.1 agla|OJJ81378.1 akaw|GAA83243.1 aluc|OJZ83744.1 anid|CBF90238.1 anig|EHA18891.1 aory|EIT82211.1 asyd|OJJ61957.1 ater|EAU37014.1 atub|OJI90120.1 aver|OJI96049.1 awen|OJJ37269.1 azon|OJJ50962.1

Group1653: abra|OJJ68204.1 acar|OOF90236.1 acla|EAW13087.1 afis|EAW17577.1 afla|EED49244.1 afum|EAL91390.1 agla|OJJ81377.1 akaw|GAA83242.1 aluc|OJZ83741.1 anid|CBF90245.1 anig|EHA18890.1 aory|EIT82198.1 asyd|OJJ61951.1 ater|EAU37013.1 atub|OJI90119.1 aver|OJI96042.1 awen|OJJ37270.1 azon|OJJ50969.1

Group1654: abra|OJJ68205.1 acar|OOF90235.1 acla|EAW13088.1 afis|EAW17576.1 afla|EED49245.1 afum|EAL91389.1 agla|OJJ81381.1 akaw|GAA83241.1 aluc|OJZ83740.1 anid|CBF90247.1 anig|EHA18889.1 aory|EIT82237.1 asyd|OJJ61950.1 ater|EAU37012.1 atub|OJI90118.1 aver|OJI96041.1 awen|OJJ37271.1 azon|OJJ50971.1

Group1655: abra|OJJ68207.1 acar|OOF97493.1 acla|EAW13090.1 afis|EAW17574.1 afla|EED49247.1 afum|EAL91387.1 agla|OJJ81384.1 akaw|GAA83239.1 aluc|OJZ83738.1 anid|CBF90250.1 anig|EHA18887.1 aory|EIT82189.1 asyd|OJJ61948.1 ater|EAU37010.1 atub|OJI90116.1 aver|OJI96040.1 awen|OJJ37273.1 azon|OJJ50973.1

Group1656: abra|OJJ68211.1 acar|OOF97495.1 acla|EAW12973.1 afis|EAW17691.1 afla|EED50453.1 afum|EAL91505.1 agla|OJJ79488.1 akaw|GAA83235.1 aluc|OJZ83734.1 anid|CBF89980.1 anig|EHA18883.1 aory|EIT82051.1 asyd|OJJ62090.1 ater|EAU31953.1 atub|OJI90112.1 aver|OJI96196.1 awen|OJJ37145.1 azon|OJJ50845.1

Group1657: abra|OJJ68212.1 acar|OOF97496.1 acla|EAW12972.1 afis|EAW17692.1 afla|EED50452.1 afum|EAL91506.1 agla|OJJ79487.1 akaw|GAA83234.1 aluc|OJZ83733.1 anid|CBF89978.1 anig|EHA18882.1 aory|EIT82145.1 asyd|OJJ62091.1 ater|EAU31958.1 atub|OJI90111.1 aver|OJI96197.1 awen|OJJ37144.1 azon|OJJ50844.1

Group1658: abra|OJJ68249.1 acar|OOF97528.1 acla|EAW09061.1 afis|EAW25634.1 afla|EED55178.1 afum|EAL84322.1 agla|OJJ78898.1 akaw|GAA83212.1 aluc|OJZ83714.1 anid|CBF83155.1 anig|EHA18865.1 aory|EIT81648.1 asyd|OJJ55514.1 ater|EAU32715.1 atub|OJI90089.1 aver|OJJ06591.1 awen|OJJ36053.1 azon|OJJ50759.1

Group1659: abra|OJJ68257.1 acar|OOF97534.1 acla|EAW09064.1 afis|EAW25636.1 afla|EED55176.1 afum|EAL84324.1 agla|OJJ78896.1 akaw|GAA83205.1 aluc|OJZ83707.1 anid|CBF83151.1 anig|EHA18858.1 aory|EIT81641.1 asyd|OJJ55516.1 ater|EAU32717.1 atub|OJI90079.1 aver|OJJ06593.1 awen|OJJ36093.1 azon|OJJ50756.1

Group1660: abra|OJJ68259.1 acar|OOF97536.1 acla|EAW14109.1 afis|EAW18811.1 afla|EED50761.1 afum|EAL93547.1 agla|OJJ80544.1 akaw|GAA92415.1 aluc|OJZ83273.1 anid|CBF69795.1 anig|EHA18856.1 aory|EIT78026.1 asyd|OJJ58964.1 ater|EAU37966.1 atub|OJI87668.1 aver|OJJ01558.1 awen|OJJ32387.1 azon|OJJ47147.1

Group1661: abra|OJJ68274.1 acar|OOF97552.1 acla|EAW09071.1 agla|OJJ78841.1 akaw|GAA83195.1 aluc|OJZ83697.1 anid|CBF78459.1 anig|EHA18848.1 asyd|OJJ64190.1 ater|EAU32708.1 atub|OJI90068.1 aver|OJI98267.1 awen|OJJ35541.1 azon|OJJ44995.1 acar|OOF92523.1 azon|OJJ42182.1 afla|EED55164.1 azon|OJJ45941.1

Group1662: abra|OJJ68281.1 acar|OOF97555.1 acla|EAW09093.1 afis|EAW25687.1 afla|EED55157.1 afum|EAL84356.1 agla|OJJ78886.1 akaw|GAA83191.1 aluc|OJZ83690.1 anid|CBF83933.1 anig|EHA18844.1 aory|EIT81633.1 asyd|OJJ55435.1 ater|EAU32721.1 atub|OJI90062.1 aver|OJJ06512.1 awen|OJJ36120.1 azon|OJJ50742.1

Group1663: abra|OJJ68301.1 acar|OOF96686.1 acla|EAW12631.1 afis|EAW18053.1 afla|EED56195.1 afum|EAL91864.1 agla|OJJ87336.1 akaw|GAA83173.1 aluc|OJZ83672.1 anid|CBF83350.1 anig|EHA18827.1 aory|EIT73338.1 asyd|OJJ60615.1 ater|EAU34830.1 atub|OJI90043.1 aver|OJI99939.1 awen|OJJ41435.1 azon|OJJ48899.1

Group1664: abra|OJJ68398.1 acar|OOF93975.1 acla|EAW08574.1 afis|EAW23634.1 afla|EED47506.1 afum|EAL87826.1 akaw|GAA92549.1 aluc|OJZ90098.1 anig|EHA20978.1 aory|EIT74517.1 asyd|OJJ54836.1

atub|OJI84708.1 aver|OJJ07325.1 azon|OJJ50231.1 aory|EIT74543.1 asyd|OJJ53612.1 aver|OJJ07720.1 awen|OJJ29655.1

Group1665: abra|OJJ68415.1 acar|OOG00093.1 acla|EAW11055.1 afis|EAW20382.1 afum|EAL85495.1 agla|OJJ83841.1 akaw|GAA92533.1 aluc|OJZ90082.1 anid|CBF70730.1 anig|EHA20991.1 asyd|OJJ56111.1 ater|EAU30781.1 atub|OJI84724.1 aver|OJJ05054.1 awen|OJJ30077.1 azon|OJJ48238.1 anid|CBF78288.1 acla|EAW15020.1

Group1666: abra|OJJ68449.1 acar|OOF93946.1 acla|EAW11772.1 afis|EAW23677.1 afla|EED54146.1 afum|EAL87120.1 agla|OJJ78443.1 akaw|GAA86924.1 aluc|OJZ90048.1 anid|CBF75094.1 anig|EHA21022.1 aory|EIT75945.1 asyd|OJJ60010.1 ater|EAU29439.1 atub|OJI84758.1 aver|OJJ02275.1 awen|OJJ38063.1 azon|OJJ50775.1

Group1667: abra|OJJ68459.1 acar|OOF92643.1 acla|EAW08639.1 afis|EAW15792.1 afla|EED51253.1 afum|EAL85558.1 agla|OJJ81955.1 akaw|GAA92421.1 aluc|OJZ80815.1 anid|CBF70968.1 anig|EHA21125.1 aory|EIT81555.1 asyd|OJJ52614.1 ater|EAU32350.1 atub|OJI87664.1 aver|OJJ04641.1 awen|OJJ38500.1 azon|OJJ43801.1

Group1668: abra|OJJ68464.1 acar|OOF94169.1 acla|EAW10056.1 afis|EAW25528.1 afla|EED54029.1 afum|EAL90193.2 agla|OJJ87274.1 akaw|GAA92384.1 aluc|OJZ83243.1 anid|CBF77807.1 anig|EHA21098.1 aory|EIT78344.1 asyd|OJJ53016.1 ater|EAU33371.1 atub|OJI87693.1 aver|OJJ03827.1 awen|OJJ41270.1 azon|OJJ49510.1

Group1669: abra|OJJ68468.1 acar|OOF89893.1 acla|EAW10429.1 afis|EAW25140.1 afla|EED55305.1 afum|EAL89812.1 agla|OJJ89027.1 akaw|GAA92389.1 aluc|OJZ83249.1 anid|CBF86095.1 anig|EHA21102.1 aory|EIT83530.1 asyd|OJJ64474.1 ater|EAU39409.1 atub|OJI87688.1 aver|OJI98578.1 awen|OJJ40764.1 azon|OJJ44129.1

Group1670: abra|OJJ68475.1 acar|OOF92975.1 acla|EAW06477.1 afis|EAW15972.1 afla|EED58084.1 afum|EAL88604.1 agla|OJJ83843.1 akaw|GAA92396.1 aluc|OJZ83256.1 anid|CBF81100.1 anig|EHA21110.1 aory|EIT79134.1 asyd|OJJ56477.1 ater|EAU35476.1 atub|OJI87682.1 aver|OJJ06241.1 awen|OJJ35600.1 azon|OJJ46920.1

Group1671: abra|OJJ68476.1 acar|OOF99927.1 acla|EAW09807.1 afis|EAW21329.1 afla|EED57287.1 afum|EAL92347.1 agla|OJJ87410.1 akaw|GAA92397.1 aluc|OJZ83258.1 anid|CBF83431.1 anig|EHA21111.1 aory|EIT74125.1 asyd|OJJ60661.1 ater|EAU35955.1 atub|OJI87681.1 aver|OJI99984.1 awen|OJJ39369.1 azon|OJJ51709.1

Group1672: abra|OJJ68478.1 acar|OOF96519.1 acla|EAW13881.1 afis|EAW18593.1 afla|EED52754.1 afum|EAL93301.1 agla|OJJ81645.1 akaw|GAA92401.1 aluc|OJZ83262.1 anid|CBF70381.1 anig|EHA21115.1 aory|EIT78198.1 asyd|OJJ58642.1 ater|EAU34887.1 atub|OJI87675.1 aver|OJJ01248.1 awen|OJJ31156.1 azon|OJJ46631.1

Group1673: abra|OJJ68485.1 acar|OOG00353.1 acla|EAW07017.1 afis|EAW16643.1 afla|EED44966.1 afum|EAL89246.1 agla|OJJ82604.1 akaw|GAA92786.1 aluc|OJZ82697.1 anid|CBF82045.1 anig|EHA21146.1 aory|EIT73490.1 asyd|OJJ54225.1 ater|EAU39568.1 atub|OJI88929.1 aver|OJJ05178.1 awen|OJJ40071.1 azon|OJJ45553.1

Group1674: abra|OJJ68486.1 acar|OOF95442.1 acla|EAW07333.1 afis|EAW22365.1 afla|EED57773.1 afum|EBA27416.1 agla|OJJ87616.1 akaw|GAA92785.1 aluc|OJZ82698.1 anid|CBF88690.1 anig|EHA21153.1 aory|EIT81113.1 asyd|OJJ63908.1 ater|EAU39499.1 atub|OJI88930.1 aver|OJI97033.1 awen|OJJ34939.1 azon|OJJ45591.1

Group1675: abra|OJJ68497.1 acar|OOF99362.1 acla|EAW14277.1 afis|EAW18997.1 afla|EED53139.1 afum|EAL93732.1 agla|OJJ80191.1 akaw|GAA92773.1 aluc|OJZ82710.1 anid|CBF86779.1 anig|EHA21165.1 aory|EIT74918.1 asyd|OJJ64682.1 ater|EAU37806.1 atub|OJI88943.1 aver|OJI98784.1 awen|OJJ31625.1 azon|OJJ47325.1

Group1676: abra|OJJ68499.1 acar|OOF93648.1 acla|EAW14509.1 afis|EAW19222.1 afla|EED47663.1 afum|EAL93954.1 agla|OJJ88466.1 akaw|GAA92772.1 aluc|OJZ82712.1 anid|CBF78608.1 anig|EHA21167.1 aory|EIT80278.1 asyd|OJJ65103.1 ater|EAU37236.1 atub|OJI88945.1 aver|OJI99230.1 awen|OJJ37585.1 azon|OJJ51239.1

Group1677: abra|OJJ68500.1 acar|OOF92674.1 acla|EAW08059.1 afis|EAW23095.1 afla|EED48553.1 afum|EAL88383.1 agla|OJJ89607.1 akaw|GAA92771.1 aluc|OJZ82713.1 anid|CBF75180.1 anig|EHA21168.1 aory|EIT73191.1 asyd|OJJ59952.1 ater|EAU36223.1 atub|OJI88946.1 aver|OJJ02337.1 awen|OJJ36686.1 azon|OJJ47510.1

Group1678: abra|OJJ68501.1 acar|OOF89894.1 acla|EAW11963.1 afis|EAW19600.1 afla|EED56956.1 afum|EAL86918.1 agla|OJJ80742.1 akaw|GAA92770.1 aluc|OJZ82714.1 anid|CBF71291.1 anig|EHA21169.1 aory|EIT77492.1 asyd|OJJ55128.1 ater|EAU32925.1 atub|OJI88947.1 aver|OJJ07076.1 awen|OJJ41841.1 azon|OJJ47038.1

Group1679: abra|OJJ68507.1 acar|OOF92954.1 acla|EAW07233.1 afis|EAW22254.1 afla|EED57640.1 afum|EAL90924.1 agla|OJJ87802.1 akaw|GAA92766.1 aluc|OJZ82719.1 anid|CBF88500.1 anig|EHA21173.1 aory|EIT82931.1 asyd|OJJ63795.1 ater|EAU34255.1 atub|OJI88953.1 aver|OJI97142.1 awen|OJJ34826.1 azon|OJJ45723.1

Group1680: abra|OJJ68510.1 acar|OOF92672.1 acla|EAW07690.1 afis|EAW22705.1 afla|EED49991.1 afum|EAL90487.1 agla|OJJ79004.1 akaw|GAA92761.1 aluc|OJZ82724.1 anid|CBF88071.1 anig|EHA21178.1 aory|EIT74613.1 asyd|OJJ63553.1 ater|EAU38996.1 atub|OJI88958.1 aver|OJI97381.1 awen|OJJ35342.1 azon|OJJ49776.1

Group1681: abra|OJJ68511.1 acar|OOF92678.1 acla|EAW14940.1 afis|EAW24455.1 afla|EED55219.1 afum|EAL86124.1 agla|OJJ80302.1 akaw|GAA91335.1 aluc|OJZ82725.1 anid|CBF75392.1 anig|EHA21179.1 aory|EIT83615.1 asyd|OJJ59834.1 ater|EAU29438.1 atub|OJI88959.1 aver|OJJ02454.1 awen|OJJ32742.1 azon|OJJ45292.1

Group1682: abra|OJJ68517.1 acar|OOF99154.1 acla|EAW06536.1 afis|EAW16059.1 afla|EED48573.1 afum|EAL88692.1 agla|OJJ86106.1 akaw|GAA91331.1 aluc|OJZ82731.1 anid|CBF75082.1 anig|EHA21183.1 aory|EIT73064.1 asyd|OJJ60019.1 ater|EAU33575.1 atub|OJI88964.1 aver|OJJ02266.1 awen|OJJ30380.1 azon|OJJ45396.1

Group1683: abra|OJJ68524.1 acar|OOF92729.1 acla|EAW12839.1 afis|EAW18004.1 afla|EED51892.1 afum|EAL91815.2 agla|OJJ80272.1 akaw|GAA91325.1 aluc|OJZ82738.1 anid|CBF80032.1 anig|EHA21189.1 aory|EIT76182.1 asyd|OJJ58271.1 ater|EAU31516.1 atub|OJI88973.1 aver|OJJ02153.1 awen|OJJ41538.1 azon|OJJ48256.1

Group1684: abra|OJJ68526.1 acar|OOF92730.1 acla|EAW12838.1 afis|EAW18003.1 afla|EED51893.1 afum|EAL91814.2 agla|OJJ80266.1 akaw|GAA91324.1 aluc|OJZ82739.1 anid|CBF80026.1 anig|EHA21190.1 aory|EIT76172.1 asyd|OJJ58266.1 ater|EAU31515.1 atub|OJI88974.1 aver|OJJ02157.1 awen|OJJ41539.1 azon|OJJ48257.1

Group1685: abra|OJJ68527.1 acar|OOF92731.1 acla|EAW12837.1 afis|EAW18002.1 afla|EED51894.1 afum|EAL91813.1 agla|OJJ80265.1 akaw|GAA91323.1 aluc|OJZ82740.1 anid|CBF80025.1 anig|EHA21191.1 aory|EIT76169.1 asyd|OJJ58265.1 ater|EAU31514.1 atub|OJI88975.1 aver|OJJ02158.1 awen|OJJ41540.1 azon|OJJ48258.1

Group1686: abra|OJJ68528.1 acar|OOF92732.1 acla|EAW12836.1 afis|EAW18001.1 afla|EED51895.1 afum|EAL91812.1 agla|OJJ80264.1 akaw|GAA91322.1 aluc|OJZ82741.1 anid|CBF80023.1 anig|EHA21192.1 aory|EIT76176.1 asyd|OJJ58264.1 ater|EAU31513.1 atub|OJI88976.1 aver|OJJ02160.1 awen|OJJ41541.1 azon|OJJ48259.1

Group1687: abra|OJJ68531.1 acar|OOF92734.1 acla|EAW12834.1 afis|EAW17999.1 afla|EED51899.1 afum|EAL91810.1 agla|OJJ80262.1 akaw|GAA91320.1 aluc|OJZ82743.1 anid|CBF80019.1 anig|EHA21194.1 aory|EIT76173.1 asyd|OJJ58262.1 ater|EAU31511.1 atub|OJI88978.1 aver|OJJ02162.1 awen|OJJ41543.1 azon|OJJ48261.1

Group1688: abra|OJJ68532.1 acar|OOF92735.1 acla|EAW12833.1 afis|EAW17998.1 afla|EED51900.1 afum|EAL91809.2 agla|OJJ80261.1 akaw|GAA91319.1 aluc|OJZ82744.1 anid|CBF80017.1 anig|EHA21195.1 aory|EIT76171.1 asyd|OJJ58261.1 ater|EAU31510.1 atub|OJI88979.1 aver|OJJ02163.1 awen|OJJ41544.1 azon|OJJ48262.1

Group1689: abra|OJJ68533.1 acar|OOF92736.1 afis|EAW17997.1 afla|EED51903.1 afum|EAL91807.2 akaw|GAA91318.1 aluc|OJZ82746.1 anig|EHA21196.1 aory|EIT76183.1 asyd|OJJ58260.1 atub|OJI88980.1 aver|OJJ02164.1 awen|OJJ41545.1 acla|EAW12832.1 agla|OJJ80257.1 ater|EAU31509.1 azon|OJJ48263.1 anid|CBF80015.1

Group1690: abra|OJJ68537.1 acar|OOF90151.1 acla|EAW12827.1 afis|EAW17992.1 afla|EED51911.1 afum|EAL91802.1 agla|OJJ80253.1 akaw|GAA91314.1 aluc|OJZ82751.1 anid|CBF80004.1 anig|EHA21200.1 aory|EIT76179.1 asyd|OJJ58250.1 ater|EAU31503.1 atub|OJI88987.1 aver|OJJ02174.1 awen|OJJ41554.1 azon|OJJ48269.1

Group1691: abra|OJJ68538.1 acar|OOF90152.1 acla|EAW12828.1 afis|EAW17993.1 afla|EED51908.1 afum|EAL91803.1 agla|OJJ80254.1 akaw|GAA91313.1 aluc|OJZ82752.1 anid|CBF80005.1 anig|EHA21201.1 aory|EIT76170.1 asyd|OJJ58251.1 ater|EAU31504.1 atub|OJI88988.1 aver|OJJ02173.1 awen|OJJ41553.1 azon|OJJ48268.1

Group1692: abra|OJJ68541.1 acar|OOF92739.1 acla|EAW12829.1 afis|EAW17994.1 afla|EED51907.1 afum|EAL91804.1 agla|OJJ80255.1 akaw|GAA91312.1 aluc|OJZ82753.1 anid|CBF80009.1 anig|EHA21202.1 aory|EIT76184.1 asyd|OJJ58256.1 ater|EAU31505.1 atub|OJI88990.1 aver|OJJ02167.1 awen|OJJ41551.1 azon|OJJ48267.1

Group1693: abra|OJJ68542.1 acar|OOF92740.1 acla|EAW12830.1 afis|EAW17995.1 afla|EED51906.1 afum|EAL91805.1 agla|OJJ80259.1 akaw|GAA91311.1 aluc|OJZ82755.1 anid|CBF80011.1 anig|EHA21203.1

aory|EIT76180.1 asyd|OJJ58257.1 ater|EAU31507.1 atub|OJI88991.1 aver|OJJ02166.1 awen|OJJ41550.1 azon|OJJ48266.1

Group1694: abra|OJJ68544.1 acar|OOF92742.1 acla|EAW12823.1 afis|EAW17988.1 afla|EED51915.1 afum|EAL91797.1 agla|OJJ80248.1 akaw|GAA91309.1 aluc|OJZ82759.1 anid|CBF79996.1 anig|EHA21205.1 aory|EIT81365.1 asyd|OJJ58247.1 ater|EAU31498.1 atub|OJI88993.1 aver|OJJ02179.1 awen|OJJ41558.1 azon|OJJ48273.1

Group1695: abra|OJJ68546.1 acar|OOF92743.1 acla|EAW12822.1 afis|EAW17987.1 afla|EED51916.1 afum|EAL91796.1 agla|OJJ80242.1 akaw|GAA91308.1 aluc|OJZ82762.1 anid|CBF80000.1 anig|EHA21206.1 aory|EIT81367.1 asyd|OJJ58248.1 ater|EAU31497.1 atub|OJI88995.1 aver|OJJ02176.1 awen|OJJ41559.1 azon|OJJ48274.1

Group1696: abra|OJJ68549.1 acar|OOF92745.1 acla|EAW12818.1 afis|EAW17983.1 afla|EED51922.1 afum|EAL91792.1 agla|OJJ80240.1 akaw|GAA91304.1 aluc|OJZ82766.1 anid|CBF79992.1 anig|EHA21209.1 aory|EIT81388.1 asyd|OJJ58238.1 ater|EAU31493.1 atub|OJI88998.1 aver|OJJ02187.1 awen|OJJ41562.1 azon|OJJ48277.1

Group1697: abra|OJJ68554.1 acar|OOF92749.1 acla|EAW12813.1 afis|EAW17978.1 afla|EED51927.1 afum|EAL91787.1 agla|OJJ80235.1 akaw|GAA91299.1 aluc|OJZ82771.1 anid|CBF79976.1 anig|EHA21213.1 aory|EIT81454.1 asyd|OJJ58232.1 ater|EAU31489.1 atub|OJI89002.1 aver|OJJ02192.1 awen|OJJ41567.1 azon|OJJ44836.1

Group1698: abra|OJJ68555.1 acar|OOF92750.1 acla|EAW12812.1 afis|EAW17977.1 afla|EED51928.1 afum|EAL91786.1 agla|OJJ80234.1 akaw|GAA91298.1 aluc|OJZ82772.1 anid|CBF79975.1 anig|EHA21214.1 aory|EIT81416.1 asyd|OJJ58231.1 ater|EAU31488.1 atub|OJI89003.1 aver|OJJ02193.1 awen|OJJ41568.1 azon|OJJ44837.1

Group1699: abra|OJJ68556.1 acar|OOF92751.1 acla|EAW12811.1 afis|EAW17976.1 afla|EED51929.1 afum|EAL91785.1 agla|OJJ80233.1 akaw|GAA91297.1 aluc|OJZ82773.1 anid|CBF79973.1 anig|EHA21215.1 aory|EIT81423.1 asyd|OJJ58230.1 ater|EAU31487.1 atub|OJI89004.1 aver|OJJ02194.1 awen|OJJ41569.1 azon|OJJ44838.1

Group1700: abra|OJJ68557.1 acar|OOF92752.1 acla|EAW12810.1 afis|EAW17975.1 afla|EED51930.1 afum|EAL91784.1 agla|OJJ80232.1 akaw|GAA91296.1 aluc|OJZ82774.1 anid|CBF79972.1 anig|EHA21216.1 aory|EIT81446.1 asyd|OJJ58229.1 ater|EAU31486.1 atub|OJI89005.1 aver|OJJ02195.1 awen|OJJ41570.1 azon|OJJ48283.1

Group1701: abra|OJJ68559.1 acar|OOF92754.1 acla|EAW12806.1 afis|EAW17972.1 afla|EED51932.1 afum|EAL91781.1 agla|OJJ80230.1 akaw|GAA91294.1 aluc|OJZ82775.1 anid|CBF79968.1 anig|EHA21218.1 aory|EIT81462.1 asyd|OJJ58227.1 ater|EAU31482.1 atub|OJI89007.1 aver|OJJ02197.1 awen|OJJ41572.1 azon|OJJ48285.1

Group1702: abra|OJJ68561.1 acar|OOF92755.1 acla|EAW12804.1 afis|EAW17970.1 afla|EED51934.1 afum|EAL91779.1 agla|OJJ80228.1 akaw|GAA91292.1 aluc|OJZ82777.1 anid|CBF79964.1 anig|EHA21220.1 aory|EIT81427.1 asyd|OJJ58224.1 ater|EAU31480.1 atub|OJI89009.1 aver|OJJ02198.1 awen|OJJ41574.1 azon|OJJ48287.1

Group1703: abra|OJJ68564.1 acar|OOF92757.1 acla|EAW12802.1 afis|EAW17968.1 afla|EED51937.1 afum|EAL91777.1 agla|OJJ80226.1 akaw|GAA91290.1 aluc|OJZ82779.1 anid|CBF79961.1 anig|EHA21222.1 aory|EIT81398.1 asyd|OJJ58222.1 ater|EAU31478.1 atub|OJI89012.1 aver|OJJ02200.1 awen|OJJ41576.1 azon|OJJ48289.1

Group1704: abra|OJJ68567.1 acar|OOF92761.1 acla|EAW12798.1 afis|EAW17964.1 afla|EED51946.1 afum|EAL91774.1 agla|OJJ80209.1 akaw|GAA91286.1 aluc|OJZ82782.1 anid|CBF79954.1 anig|EHA21225.1 aory|EIT81431.1 asyd|OJJ58218.1 ater|EAU31450.1 atub|OJI89015.1 aver|OJJ02204.1 awen|OJJ41580.1 azon|OJJ48293.1

Group1705: abra|OJJ68599.1 acar|OOF92109.1 acla|EAW12640.1 afis|EAW18045.1 afla|EED51501.1 afum|EAL91857.1 agla|OJJ85416.1 akaw|GAA91923.1 aluc|OJZ81957.1 anid|CBF79779.1 anig|EHA22290.1 aory|EIT80534.1 asyd|OJJ58121.1 ater|EAU31278.1 atub|OJI84508.1 aver|OJJ04284.1 awen|OJJ41489.1 azon|OJJ48219.1

Group1706: abra|OJJ68601.1 acar|OOF92111.1 acla|EAW12638.1 afis|EAW18047.1 afla|EED51499.1 afum|EAL91859.1 agla|OJJ85418.1 akaw|GAA91925.1 aluc|OJZ81955.1 anid|CBF79776.1 anig|EHA22292.1 aory|EIT80455.1 asyd|OJJ58119.1 ater|EAU31276.1 atub|OJI84506.1 aver|OJJ04286.1 awen|OJJ41487.1 azon|OJJ48217.1

Group1707: abra|OJJ68605.1 acar|OOF92114.1 acla|EAW12588.1 afis|EAW18087.1 afla|EED51483.1 afum|EAL91899.1 agla|OJJ85421.1 akaw|GAA92916.1 aluc|OJZ81952.1 anid|CBF79764.1 anig|EHA22301.1 aory|EIT80442.1 asyd|OJJ58103.1 ater|EAU30435.1 atub|OJI84502.1 aver|OJJ04301.1 awen|OJJ41480.1 azon|OJJ48212.1

Group1708: abra|OJJ68606.1 acar|OOF92117.1 acla|EAW12587.1 afis|EAW18086.1 afla|EED51480.1 afum|EAL91898.1 agla|OJJ85422.1 akaw|GAA92915.1 aluc|OJZ81948.1 anid|CBF79762.1 anig|EHA22302.1 aory|EIT80466.1 asyd|OJJ58102.1 ater|EAU30436.1 atub|OJI84501.1 aver|OJJ04302.1 awen|OJJ41479.1 azon|OJJ48211.1

Group1709: abra|OJJ68607.1 acar|OOF92118.1 acla|EAW12593.1 afis|EAW18085.1 afla|EED51479.1 afum|EAL91897.1 agla|OJJ85423.1 akaw|GAA92914.1 aluc|OJZ81947.1 anid|CBF86384.1 aory|EIT80735.1 asyd|OJJ64960.1 ater|EAU30437.1 atub|OJI84500.1 aver|OJI99082.1 awen|OJJ41478.1 azon|OJJ48210.1 aory|EIT81994.1

Group1710: abra|OJJ68608.1 acar|OOF92119.1 acla|EAW12594.1 afis|EAW18084.1 afla|EED51478.1 afum|EAL91896.1 agla|OJJ85424.1 akaw|GAA92913.1 aluc|OJZ81946.1 anid|CBF86386.1 anig|EHA22303.1 aory|EIT80678.1 asyd|OJJ64959.1 ater|EAU30438.1 atub|OJI84499.1 aver|OJI99081.1 awen|OJJ41477.1 azon|OJJ48209.1

Group1711: abra|OJJ68610.1 acar|OOF92120.1 acla|EAW12601.1 afis|EAW18083.1 afla|EED51477.1 afum|EAL91895.1 agla|OJJ85446.1 akaw|GAA92912.1 aluc|OJZ81945.1 anid|CBF86388.1 anig|EHA22304.1 aory|EIT80541.1 asyd|OJJ64958.1 ater|EAU30439.1 atub|OJI84498.1 aver|OJI99080.1 awen|OJJ41472.1 azon|OJJ48931.1

Group1712: abra|OJJ68611.1 acar|OOF92121.1 acla|EAW12602.1 afis|EAW18082.1 afla|EED51476.1 afum|EAL91894.1 agla|OJJ85447.1 akaw|GAA92911.1 aluc|OJZ81944.1 anid|CBF86390.1 anig|EHA22305.1 aory|EIT80460.1 asyd|OJJ64957.1 ater|EAU30440.1 atub|OJI84497.1 aver|OJI99079.1 awen|OJJ41470.1 azon|OJJ48927.1

Group1713: abra|OJJ68612.1 acar|OOF92122.1 acla|EAW12603.1 afis|EAW18081.1 afla|EED51475.1 afum|EAL91893.1 agla|OJJ87374.1 akaw|GAA92910.1 aluc|OJZ81943.1 anid|CBF86391.1 anig|EHA22306.1 aory|EIT80723.1 asyd|OJJ64956.1 ater|EAU30441.1 atub|OJI84496.1 aver|OJI99078.1 awen|OJJ41469.1 azon|OJJ48926.1

Group1714: abra|OJJ68613.1 acar|OOF92123.1 acla|EAW12604.1 afis|EAW18080.1 afla|EED51474.1 afum|EAL91892.1 agla|OJJ87373.1 akaw|GAA92909.1 aluc|OJZ81942.1 anid|CBF86393.1 anig|EHA22307.1 aory|EIT80697.1 asyd|OJJ64955.1 ater|EAU30442.1 atub|OJI84495.1 aver|OJI99077.1 awen|OJJ41468.1 azon|OJJ48925.1

Group1715: abra|OJJ68615.1 acar|OOF92126.1 acla|EAW12607.1 afis|EAW18077.1 afla|EED51471.1 afum|EAL91889.1 agla|OJJ87370.1 akaw|GAA92906.1 aluc|OJZ81939.1 anid|CBF86404.1 anig|EHA22309.1 aory|EIT80562.1 asyd|OJJ64951.1 ater|EAU30446.1 atub|OJI84492.1 aver|OJI99073.1 awen|OJJ41466.1 azon|OJJ48923.1

Group1716: abra|OJJ68617.1 acar|OOF92128.1 acla|EAW12609.1 afis|EAW18075.1 afla|EED51467.1 afum|EAL91887.1 agla|OJJ87367.1 akaw|GAA92904.1 aluc|OJZ81937.1 anid|CBF86400.1 anig|EHA22311.1 aory|EIT80468.1 asyd|OJJ64949.1 ater|EAU30452.1 atub|OJI84490.1 aver|OJI99071.1 awen|OJJ41464.1 azon|OJJ48919.1

Group1717: abra|OJJ68618.1 acar|OOF92129.1 acla|EAW12610.1 afis|EAW18074.1 afla|EED51464.1 afum|EAL91886.1 agla|OJJ87366.1 akaw|GAA92903.1 aluc|OJZ81936.1 anid|CBF86398.1 anig|EHA22312.1 aory|EIT80711.1 asyd|OJJ64948.1 ater|EAU30454.1 atub|OJI84489.1 aver|OJI99070.1 awen|OJJ41463.1 azon|OJJ48918.1

Group1718: abra|OJJ68619.1 acar|OOF92130.1 acla|EAW12611.1 afis|EAW18073.1 afla|EED51463.1 afum|EAL91885.1 agla|OJJ87365.1 akaw|GAA92899.1 aluc|OJZ81935.1 anid|CBF86406.1 anig|EHA22313.1 aory|EIT80619.1 asyd|OJJ64947.1 ater|EAU30455.1 atub|OJI84488.1 aver|OJI99069.1 awen|OJJ41462.1 azon|OJJ48917.1

Group1719: abra|OJJ68620.1 acar|OOF92131.1 acla|EAW13703.1 afis|EAW19885.1 afla|EED57031.1 afum|EAL85012.1 agla|OJJ89079.1 akaw|GAA92898.1 aluc|OJZ81934.1 anid|CBF82756.1 anig|EHA22314.1 aory|EIT82313.1 asyd|OJJ61608.1 ater|EAU33697.1 atub|OJI84487.1 aver|OJJ00931.1 awen|OJJ36996.1 azon|OJJ42189.1

Group1720: abra|OJJ68623.1 acar|OOF92133.1 acla|EAW12612.1 afis|EAW18072.1 afla|EED51461.1 afum|EAL91884.1 agla|OJJ87364.1 akaw|GAA92897.1 aluc|OJZ81932.1 anid|CBF86411.1 anig|EHA22316.1 aory|EIT80603.1 asyd|OJJ64944.1 ater|EAU30457.1 atub|OJI84485.1 aver|OJI99066.1 awen|OJJ41459.1 azon|OJJ48916.1

Group1721: abra|OJJ68626.1 acar|OOF92136.1 acla|EAW12613.1 afis|EAW18071.1 afla|EED51460.1 afum|EAL91883.1 agla|OJJ87363.1 akaw|GAA92895.1 aluc|OJZ81929.1 anid|CBF86413.1 anig|EHA22318.1 aory|EIT80647.1 asyd|OJJ64943.1 ater|EAU30458.1 atub|OJI84481.1 aver|OJI99065.1 awen|OJJ41458.1 azon|OJJ48915.1

Group1722: abra|OJJ68636.1 acar|OOF92140.1 acla|EAW12615.1 afis|EAW18069.1 afla|EED51458.1 afum|EAL91881.2 agla|OJJ87356.1 akaw|GAA89775.1 aluc|OJZ81919.1 anid|CBF86416.1 anig|EHA22328.1

aory|EIT80491.1 asyd|OJJ64941.1 ater|EAU30460.1 atub|OJI84471.1 aver|OJI99063.1 awen|OJJ41456.1 azon|OJJ48913.1

Group1723: abra|OJJ68637.1 acar|OOF92141.1 acla|EAW12614.1 afis|EAW18070.1 afla|EED51459.1 afum|EAL91882.1 agla|OJJ87357.1 akaw|GAA89776.1 aluc|OJZ81918.1 anid|CBF86415.1 anig|EHA22329.1 aory|EIT80742.1 asyd|OJJ64942.1 ater|EAU30459.1 atub|OJI84470.1 aver|OJI99064.1 awen|OJJ41457.1 azon|OJJ48914.1

Group1724: abra|OJJ68642.1 acar|OOF92146.1 acla|EAW12620.1 afis|EAW18064.1 afla|EED51433.1 afum|EAL91876.1 akaw|GAA89782.1 aluc|OJZ81910.1 anid|CBF86424.1 anig|EHA22334.1 aory|EIT80728.1 asyd|OJJ64937.1 ater|EAU30467.1 atub|OJI84463.1 aver|OJI99058.1 awen|OJJ41451.1 azon|OJJ48909.1 agla|OJJ87350.1

Group1725: abra|OJJ68643.1 acar|OOF92145.1 acla|EAW12621.1 afis|EAW18063.1 afla|EED51432.1 afum|EAL91875.1 agla|OJJ87349.1 akaw|GAA89783.1 aluc|OJZ81908.1 anid|CBF86426.1 anig|EHA22335.1 aory|EIT80621.1 asyd|OJJ64936.1 ater|EAU30468.1 atub|OJI84461.1 aver|OJI99057.1 awen|OJJ41449.1 azon|OJJ48908.1

Group1726: abra|OJJ68647.1 acar|OOF92149.1 acla|EAW12623.1 afis|EAW18061.1 afla|EED51429.1 afum|EAL91872.1 agla|OJJ87325.1 akaw|GAA89787.1 aluc|OJZ81904.1 anid|CBF86430.1 anig|EHA22339.1 aory|EIT80637.1 asyd|OJJ64934.1 ater|EAU30473.1 atub|OJI84457.1 aver|OJI99054.1 awen|OJJ41445.1 azon|OJJ48907.1

Group1727: abra|OJJ68648.1 acar|OOF92150.1 acla|EAW12624.1 afis|EAW18060.1 afla|EED51428.1 afum|EAL91871.1 agla|OJJ87326.1 akaw|GAA89788.1 aluc|OJZ81903.1 anid|CBF86432.1 anig|EHA22340.1 aory|EIT80552.1 asyd|OJJ64933.1 ater|EAU30474.1 atub|OJI84456.1 aver|OJI99053.1 awen|OJJ41444.1 azon|OJJ48905.1

Group1728: abra|OJJ68649.1 acar|OOF92151.1 acla|EAW12625.1 afis|EAW18059.1 afla|EED51427.1 afum|EAL91870.1 agla|OJJ87327.1 akaw|GAA89789.1 aluc|OJZ81902.1 anid|CBF86434.1 anig|EHA22341.1 aory|EIT80495.1 asyd|OJJ64932.1 ater|EAU30475.1 atub|OJI84455.1 aver|OJI99051.1 awen|OJJ41442.1 azon|OJJ48904.1

Group1729: abra|OJJ68650.1 acar|OOF92152.1 acla|EAW12626.1 afis|EAW18058.1 afla|EED51426.1 afum|EBA27442.1 agla|OJJ87328.1 akaw|GAA89790.1 aluc|OJZ81901.1 anid|CBF86435.1 anig|EHA22342.1 aory|EIT80761.1 asyd|OJJ64931.1 ater|EAU30476.1 atub|OJI84454.1 aver|OJI99050.1 awen|OJJ41441.1 azon|OJJ48903.1

Group1730: abra|OJJ68652.1 acar|OOF92153.1 acla|EAW12628.1 afis|EAW18056.1 afla|EED51423.1 afum|EAL91867.1 agla|OJJ87330.1 akaw|GAA89792.1 aluc|OJZ81899.1 anid|CBF86443.1 anig|EHA22344.1 aory|EIT80509.1 asyd|OJJ64928.1 ater|EAU30478.1 atub|OJI84452.1 aver|OJI99047.1 awen|OJJ41439.1 azon|OJJ48901.1

Group1731: abra|OJJ68655.1 acar|OOF92155.1 acla|EAW12630.1 afis|EAW18054.1 afla|EED51421.1 afum|EAL91865.1 agla|OJJ87335.1 akaw|GAA89794.1 aluc|OJZ81897.1 anid|CBF86438.1 anig|EHA22345.1 aory|EIT80746.1 asyd|OJJ64927.1 ater|EAU30479.1 atub|OJI84450.1 aver|OJI99045.1 awen|OJJ41436.1 azon|OJJ48900.1

Group1732: abra|OJJ68656.1 acar|OOF92156.1 acla|EAW12632.1 afis|EAW18052.1 afla|EED51418.1 afum|EAL91863.1 agla|OJJ87339.1 akaw|GAA89795.1 aluc|OJZ81896.1 anid|CBF86439.1 anig|EHA22346.1 aory|EIT80553.1 asyd|OJJ64925.1 ater|EAU30480.1 atub|OJI84449.1 aver|OJI99044.1 awen|OJJ41433.1 azon|OJJ48898.1

Group1733: abra|OJJ68657.1 acar|OOF92157.1 acla|EAW12633.1 afis|EAW18051.1 afla|EED51417.1 afum|EAL91862.1 agla|OJJ87340.1 akaw|GAA89796.1 aluc|OJZ81895.1 anid|CBF86440.1 anig|EHA22347.1 aory|EIT80630.1 asyd|OJJ64924.1 ater|EAU30481.1 atub|OJI84448.1 aver|OJI99043.1 awen|OJJ41432.1 azon|OJJ48897.1

Group1734: abra|OJJ68659.1 acar|OOF92159.1 acla|EAW12590.1 afis|EAW24059.1 afla|EED51416.1 afum|EAL91914.1 agla|OJJ87341.1 akaw|GAA89797.1 aluc|OJZ81894.1 anid|CBF86444.1 anig|EHA22348.1 aory|EIT80652.1 asyd|OJJ64923.1 ater|EAU30482.1 atub|OJI84447.1 aver|OJI99042.1 awen|OJJ41431.1 azon|OJJ48896.1

Group1735: abra|OJJ68660.1 acar|OOF92158.1 acla|EAW12591.1 afis|EAW24060.1 afla|EED51415.1 afum|EAL91915.1 agla|OJJ87342.1 akaw|GAA89798.1 aluc|OJZ81893.1 anid|CBF86446.1 anig|EHA22349.1 aory|EIT80731.1 asyd|OJJ64922.1 ater|EAU30483.1 atub|OJI84446.1 aver|OJI99041.1 awen|OJJ41430.1 azon|OJJ48895.1

Group1736: abra|OJJ68662.1 acar|OOF92162.1 acla|EAW12586.1 afis|EAW24062.1 afla|EED51412.1 afum|EAL91917.1 agla|OJJ87344.1 akaw|GAA89802.1 aluc|OJZ81889.1 anid|CBF86449.1 anig|EHA22352.1 aory|EIT80533.1 asyd|OJJ64920.1 ater|EAU30484.1 atub|OJI84442.1 aver|OJI99040.1 awen|OJJ41428.1 azon|OJJ48893.1

Group1737: abra|OJJ68664.1 acar|OOF92165.1 acla|EAW12585.1 afis|EAW24063.1 afla|EED51411.1 afum|EAL91918.1 agla|OJJ87345.1 akaw|GAA89805.1 aluc|OJZ81886.1 anid|CBF86451.1 anig|EHA22354.1 aory|EIT80561.1 asyd|OJJ64919.1 ater|EAU30485.1 atub|OJI84439.1 aver|OJI99039.1 awen|OJJ41426.1 azon|OJJ48891.1

Group1738: abra|OJJ68665.1 acar|OOF92167.1 acla|EAW12584.1 afis|EAW24064.1 afla|EED51410.1 afum|EAL91919.1 agla|OJJ87346.1 akaw|GAA89806.1 aluc|OJZ81885.1 anid|CBF86452.1 anig|EHA22355.1 aory|EIT80617.1 asyd|OJJ64918.1 ater|EAU30486.1 atub|OJI84438.1 aver|OJI99038.1 awen|OJJ41425.1 azon|OJJ48890.1

Group1739: abra|OJJ68666.1 acar|OOF92166.1 acla|EAW12583.1 afis|EAW24065.1 afla|EED51409.1 afum|EAL91920.1 agla|OJJ87347.1 akaw|GAA89807.1 aluc|OJZ81884.1 anid|CBF86454.1 anig|EHA22356.1 aory|EIT80471.1 asyd|OJJ64917.1 ater|EAU30487.1 atub|OJI84437.1 aver|OJI99037.1 awen|OJJ41424.1 azon|OJJ48889.1

Group1740: abra|OJJ68670.1 acar|OOF92170.1 acla|EAW12577.1 afis|EAW24071.1 afla|EED51404.1 afum|EAL91927.1 agla|OJJ87312.1 akaw|GAA89811.1 aluc|OJZ81879.1 anid|CBF86462.1 anig|EHA22359.1 aory|EIT80762.1 asyd|OJJ64912.1 ater|EAU30492.1 atub|OJI84433.1 aver|OJI99033.1 awen|OJJ41419.1 azon|OJJ48884.1

Group1741: abra|OJJ68677.1 acar|OOF92179.1 acla|EAW12570.1 afis|EAW24080.1 afla|EED51393.1 afum|EAL91935.1 agla|OJJ87151.1 akaw|GAA89830.1 aluc|OJZ81857.1 anid|CBF86478.1 anig|EHA22367.1 aory|EIT80640.1 asyd|OJJ64902.1 ater|EAU30501.1 atub|OJI84425.1 aver|OJI99024.1 awen|OJJ41418.1 azon|OJJ48875.1

Group1742: abra|OJJ68678.1 acar|OOF92180.1 acla|EAW12569.1 afis|EAW24081.1 afla|EED51392.1 afum|EAL91936.1 agla|OJJ87152.1 akaw|GAA89831.1 aluc|OJZ81856.1 anid|CBF86480.1 anig|EHA22368.1 aory|EIT80524.1 asyd|OJJ64901.1 ater|EAU30504.1 atub|OJI84424.1 aver|OJI99022.1 awen|OJJ41407.1 azon|OJJ48873.1

Group1743: abra|OJJ68681.1 acar|OOF92183.1 acla|EAW12566.1 afis|EAW24084.1 afla|EED51389.1 afum|EAL91939.1 agla|OJJ87154.1 akaw|GAA89834.1 aluc|OJZ81852.1 anid|CBF86484.1 anig|EHA22370.1 aory|EIT80477.1 asyd|OJJ64899.1 ater|EAU30507.1 atub|OJI84421.1 aver|OJI99020.1 awen|OJJ41405.1 azon|OJJ48871.1

Group1744: abra|OJJ68682.1 acar|OOF92184.1 acla|EAW12565.1 afis|EAW24085.1 afla|EED51388.1 afum|EAL91940.1 agla|OJJ87155.1 akaw|GAA89835.1 aluc|OJZ81851.1 anid|CBF86486.1 anig|EHA22371.1 aory|EIT80535.1 asyd|OJJ64898.1 ater|EAU30508.1 atub|OJI84420.1 aver|OJI99019.1 awen|OJJ41404.1 azon|OJJ48870.1

Group1745: abra|OJJ68684.1 acar|OOF92186.1 acla|EAW12563.1 afis|EAW24087.1 afla|EED51386.1 afum|EAL91942.1 agla|OJJ87157.1 akaw|GAA89837.1 aluc|OJZ81849.1 anid|CBF86489.1 anig|EHA22373.1 aory|EIT80476.1 asyd|OJJ64896.1 ater|EAU30511.1 atub|OJI84418.1 aver|OJI99017.1 awen|OJJ41402.1 azon|OJJ48868.1

Group1746: abra|OJJ68685.1 acar|OOF92187.1 acla|EAW12562.1 afis|EAW24088.1 afla|EED46851.1 afum|EAL91943.1 agla|OJJ87158.1 akaw|GAA89838.1 aluc|OJZ81848.1 anid|CBF86491.1 anig|EHA22374.1 aory|EIT72584.1 asyd|OJJ64895.1 ater|EAU30512.1 atub|OJI84417.1 aver|OJI99016.1 awen|OJJ41401.1 azon|OJJ48867.1

Group1747: abra|OJJ68686.1 acar|OOF92188.1 acla|EAW12561.1 afis|EAW24089.1 afla|EED46850.1 afum|EAL91944.1 agla|OJJ87159.1 akaw|GAA89839.1 aluc|OJZ81847.1 anid|CBF86493.1 anig|EHA22375.1 aory|EIT72520.1 asyd|OJJ64894.1 ater|EAU30513.1 atub|OJI84416.1 aver|OJI99015.1 awen|OJJ41400.1 azon|OJJ48866.1

Group1748: abra|OJJ68689.1 acar|OOF92193.1 acla|EAW12555.1 afis|EAW24096.1 afla|EED46838.1 afum|EAL91950.1 agla|OJJ87169.1 akaw|GAA89844.1 aluc|OJZ81843.1 anid|CBF86498.1 anig|EHA22379.1 aory|EIT72590.1 asyd|OJJ64889.1 ater|EAU30523.1 atub|OJI84411.1 aver|OJI99003.1 awen|OJJ41394.1 azon|OJJ48862.1

Group1749: abra|OJJ68690.1 acar|OOF92194.1 acla|EAW12554.1 afis|EAW24097.1 afla|EED46837.1 afum|EAL91951.2 agla|OJJ87171.1 akaw|GAA89845.1 aluc|OJZ81842.1 anid|CBF86500.1 anig|EHA22380.1 aory|EIT72693.1 asyd|OJJ64888.1 ater|EAU30524.1 atub|OJI84410.1 aver|OJI99002.1 awen|OJJ41392.1 azon|OJJ48861.1

Group1750: abra|OJJ68691.1 acar|OOF92195.1 acla|EAW12553.1 afis|EAW24098.1 afla|EED46836.1 afum|EAL91952.1 agla|OJJ87172.1 akaw|GAA89846.1 aluc|OJZ81841.1 anid|CBF86501.1 anig|EHA22381.1 aory|EIT72579.1 asyd|OJJ64887.1 ater|EAU30525.1 atub|OJI84409.1 aver|OJI99001.1 awen|OJJ41391.1 azon|OJJ48860.1

Group1751: abra|OJJ68692.1 acar|OOF92196.1 acla|EAW12552.1 afis|EAW24099.1 afla|EED46835.1 afum|EAL91953.2 agla|OJJ87173.1 akaw|GAA89847.1 aluc|OJZ81840.1 anid|CBF86503.1 anig|EHA22382.1

aory|EIT72533.1 asyd|OJJ64886.1 ater|EAU30526.1 atub|OJI84408.1 aver|OJI99000.1 awen|OJJ41390.1 azon|OJJ48859.1

Group1752: abra|OJJ68693.1 acar|OOF92197.1 acla|EAW12551.1 afis|EAW24100.1 afla|EED46834.1 afum|EAL91954.1 agla|OJJ87174.1 akaw|GAA89848.1 aluc|OJZ81838.1 anid|CBF86505.1 anig|EHA22383.1 aory|EIT72706.1 asyd|OJJ64885.1 ater|EAU30527.1 atub|OJI84407.1 aver|OJI98999.1 awen|OJJ41389.1 azon|OJJ48858.1

Group1753: abra|OJJ68695.1 acar|OOF92199.1 acla|EAW12549.1 afis|EAW24101.1 afla|EED46832.1 afum|EAL91955.1 agla|OJJ87176.1 akaw|GAA89850.1 aluc|OJZ81836.1 anid|CBF86507.1 anig|EHA22385.1 aory|EIT72575.1 asyd|OJJ64883.1 ater|EAU30529.1 atub|OJI84405.1 aver|OJI98997.1 awen|OJJ41387.1 azon|OJJ48856.1

Group1754: abra|OJJ68696.1 acar|OOF92200.1 acla|EAW12548.1 afis|EAW24102.1 afla|EED46831.1 afum|EAL91956.1 agla|OJJ87177.1 akaw|GAA89851.1 aluc|OJZ81835.1 anid|CBF86509.1 anig|EHA22386.1 aory|EIT72546.1 asyd|OJJ64882.1 ater|EAU30530.1 atub|OJI84404.1 aver|OJI98996.1 awen|OJJ41386.1 azon|OJJ48855.1

Group1755: abra|OJJ68697.1 acar|OOF92201.1 acla|EAW12547.1 afis|EAW24103.1 afla|EED46830.1 afum|EAL91957.1 agla|OJJ87178.1 akaw|GAA89852.1 aluc|OJZ81834.1 anid|CBF86511.1 anig|EHA22387.1 aory|EIT72771.1 asyd|OJJ64881.1 ater|EAU30531.1 atub|OJI84403.1 aver|OJI98995.1 awen|OJJ41385.1 azon|OJJ48854.1

Group1756: abra|OJJ68698.1 acar|OOF92202.1 acla|EAW12546.1 afis|EAW24104.1 afla|EED46829.1 afum|EAL91958.1 agla|OJJ87179.1 akaw|GAA89853.1 aluc|OJZ81833.1 anid|CBF86513.1 anig|EHA22388.1 aory|EIT72709.1 asyd|OJJ64880.1 ater|EAU30532.1 atub|OJI84402.1 aver|OJI98994.1 awen|OJJ41384.1 azon|OJJ48853.1

Group1757: abra|OJJ68703.1 acar|OOF92205.1 acla|EAW12542.1 afis|EAW24107.1 afla|EED46826.1 afum|EAL91961.1 agla|OJJ87182.1 akaw|GAA89857.1 aluc|OJZ81828.1 anid|CBF86518.1 anig|EHA22391.1 aory|EIT72757.1 asyd|OJJ64877.1 ater|EAU30535.1 atub|OJI84398.1 aver|OJI98990.1 awen|OJJ41381.1 azon|OJJ48850.1

Group1758: abra|OJJ68704.1 acar|OOF92206.1 acla|EAW12541.1 afis|EAW24108.1 afla|EED46825.1 afum|EAL91962.1 agla|OJJ87183.1 akaw|GAA89858.1 aluc|OJZ81826.1 anid|CBF86520.1 anig|EHA22392.1 aory|EIT72547.1 asyd|OJJ64876.1 ater|EAU30536.1 atub|OJI84396.1 aver|OJI98989.1 awen|OJJ41380.1 azon|OJJ48848.1

Group1759: abra|OJJ68706.1 acar|OOF92208.1 acla|EAW12539.1 afis|EAW24110.1 afla|EED46822.1 afum|EAL91964.1 agla|OJJ87185.1 akaw|GAA89860.1 aluc|OJZ81824.1 anid|CBF86523.1 anig|EHA22394.1 aory|EIT72674.1 asyd|OJJ64874.1 ater|EAU30538.1 atub|OJI84394.1 aver|OJI98987.1 awen|OJJ41378.1 azon|OJJ48846.1

Group1760: abra|OJJ68709.1 acar|OOF92211.1 acla|EAW12536.1 afis|EAW24113.1 afla|EED46820.1 afum|EAL91967.1 agla|OJJ87190.1 akaw|GAA89863.1 aluc|OJZ81818.1 anid|CBF86529.1 anig|EHA22397.1 aory|EIT72599.1 asyd|OJJ64871.1 ater|EAU30541.1 atub|OJI84391.1 aver|OJI98983.1 awen|OJJ41375.1 azon|OJJ48843.1

Group1761: abra|OJJ68710.1 acar|OOF92212.1 acla|EAW12535.1 afis|EAW24114.1 afla|EED46819.1 afum|EAL91968.1 agla|OJJ87191.1 akaw|GAA89864.1 aluc|OJZ81817.1 anid|CBF86531.1 anig|EHA22398.1 aory|EIT72684.1 asyd|OJJ64870.1 ater|EAU30542.1 atub|OJI84390.1 aver|OJI98982.1 awen|OJJ41374.1 azon|OJJ48842.1

Group1762: abra|OJJ68717.1 acar|OOF92217.1 acla|EAW12529.1 afis|EAW24120.1 afla|EED46812.1 afum|EAL91975.1 agla|OJJ87194.1 akaw|GAA89869.1 aluc|OJZ81812.1 anid|CBF86542.1 anig|EHA22405.1 aory|EIT72532.1 asyd|OJJ64862.1 ater|EAU30548.1 atub|OJI84384.1 aver|OJI98973.1 awen|OJJ41369.1 azon|OJJ48836.1

Group1763: abra|OJJ68719.1 acar|OOF92218.1 acla|EAW12528.1 afis|EAW24121.1 afla|EED46810.1 afum|EAL91977.1 agla|OJJ87196.1 akaw|GAA89870.1 aluc|OJZ81810.1 anid|CBF86546.1 anig|EHA22407.1 aory|EIT72617.1 asyd|OJJ64860.1 ater|EAU30549.1 atub|OJI84382.1 aver|OJI98972.1 awen|OJJ41368.1 azon|OJJ48835.1

Group1764: abra|OJJ68722.1 acar|OOF92221.1 acla|EAW12524.1 afis|EAW24124.1 afla|EED46809.1 afum|EAL91980.1 agla|OJJ87199.1 akaw|GAA89872.1 aluc|OJZ81807.1 anid|CBF86551.1 anig|EHA22410.1 aory|EIT72759.1 asyd|OJJ64857.1 ater|EAU30551.1 atub|OJI84379.1 aver|OJI98969.1 awen|OJJ41365.1 azon|OJJ48832.1

Group1765: abra|OJJ68723.1 acar|OOF92222.1 acla|EAW12523.1 afis|EAW24125.1 afla|EED46808.1 afum|EAL91981.1 agla|OJJ87200.1 akaw|GAA89873.1 aluc|OJZ81806.1 anid|CBF86553.1 anig|EHA22411.1 aory|EIT72530.1 asyd|OJJ64856.1 ater|EAU30552.1 atub|OJI84378.1 aver|OJI98968.1 awen|OJJ41364.1 azon|OJJ48831.1

Group1766: abra|OJJ68725.1 acar|OOF92224.1 acla|EAW12521.1 afis|EAW24126.1 afla|EED46806.1 afum|EAL91982.1 agla|OJJ87202.1 akaw|GAA89876.1 aluc|OJZ81803.1 anid|CBF86555.1 anig|EHA22413.1 aory|EIT72636.1 asyd|OJJ64855.1 ater|EAU30553.1 atub|OJI84375.1 aver|OJI98967.1 awen|OJJ41363.1 azon|OJJ48829.1

Group1767: abra|OJJ68728.1 acar|OOF92225.1 acla|EAW12520.1 afis|EAW24127.1 afla|EED46805.1 afum|EAL91983.1 agla|OJJ87203.1 akaw|GAA89877.1 aluc|OJZ81802.1 anid|CBF86557.1 anig|EHA22414.1 aory|EIT72714.1 asyd|OJJ64854.1 ater|EAU30557.1 atub|OJI84374.1 aver|OJI98966.1 awen|OJJ41362.1 azon|OJJ48827.1

Group1768: abra|OJJ68729.1 acar|OOF92226.1 acla|EAW12519.1 afis|EAW24128.1 afla|EED46804.1 afum|EAL91984.1 agla|OJJ87204.1 akaw|GAA89878.1 aluc|OJZ81801.1 anid|CBF86559.1 anig|EHA22415.1 aory|EIT72675.1 asyd|OJJ64853.1 ater|EAU30558.1 atub|OJI84372.1 aver|OJI98965.1 awen|OJJ41361.1 azon|OJJ48825.1

Group1769: abra|OJJ68730.1 acar|OOF92228.1 acla|EAW12518.1 afis|EAW24129.1 afla|EED46803.1 afum|EAL91985.1 agla|OJJ87205.1 akaw|GAA89879.1 aluc|OJZ81800.1 anid|CBF86560.1 anig|EHA22416.1 aory|EIT72669.1 asyd|OJJ64852.1 ater|EAU30559.1 atub|OJI84371.1 aver|OJI98964.1 awen|OJJ41360.1 azon|OJJ48824.1

Group1770: abra|OJJ68734.1 acar|OOF92230.1 acla|EAW12513.1 afis|EAW24133.1 afla|EED46800.1 afum|EAL91988.1 agla|OJJ87210.1 akaw|GAA89882.1 aluc|OJZ81796.1 anid|CBF86566.1 anig|EHA22420.1 aory|EIT72682.1 asyd|OJJ64849.1 ater|EAU30562.1 atub|OJI84367.1 aver|OJI98961.1 awen|OJJ41356.1 azon|OJJ48819.1

Group1771: abra|OJJ68735.1 acar|OOF92231.1 acla|EAW12512.1 afis|EAW24134.1 afla|EED46799.1 afum|EAL91989.2 agla|OJJ87211.1 akaw|GAA89883.1 aluc|OJZ81794.1 anid|CBF86568.1 anig|EHA22421.1 aory|EIT72755.1 asyd|OJJ64848.1 ater|EAU30563.1 atub|OJI84366.1 aver|OJI98960.1 awen|OJJ41355.1 azon|OJJ48818.1

Group1772: abra|OJJ68736.1 acar|OOF92232.1 acla|EAW12511.1 afis|EAW24135.1 afla|EED46798.1 afum|EAL91990.1 agla|OJJ87212.1 akaw|GAA89884.1 aluc|OJZ81793.1 anid|CBF86570.1 anig|EHA22422.1 aory|EIT72542.1 asyd|OJJ64847.1 ater|EAU30564.1 atub|OJI84365.1 aver|OJI98959.1 awen|OJJ41354.1 azon|OJJ48817.1

Group1773: abra|OJJ68738.1 acar|OOF92234.1 afis|EAW24137.1 afla|EED46797.1 afum|EAL91992.1 agla|OJJ87215.1 akaw|GAA89886.1 aluc|OJZ81789.1 anid|CBF77943.1 anig|EHA22426.1 aory|EIT72631.1 asyd|OJJ55282.1 ater|EAU30566.1 atub|OJI84363.1 aver|OJJ06946.1 awen|OJJ41352.1 azon|OJJ48815.1 acla|EAW12509.1

Group1774: abra|OJJ68739.1 acar|OOF92235.1 acla|EAW12508.1 afis|EAW24138.1 afla|EED46796.1 afum|EAL91993.1 agla|OJJ87216.1 akaw|GAA89887.1 aluc|OJZ81788.1 anid|CBF77941.1 anig|EHA22427.1 aory|EIT72772.1 asyd|OJJ55283.1 ater|EAU30567.1 atub|OJI84362.1 aver|OJJ06945.1 awen|OJJ41351.1 azon|OJJ48814.1

Group1775: abra|OJJ68742.1 acar|OOF90307.1 acla|EAW12506.1 afis|EAW24141.1 afla|EED46793.1 afum|EAL91996.1 agla|OJJ87220.1 akaw|GAA89889.1 aluc|OJZ81784.1 anid|CBF77936.1 anig|EHA22431.1 aory|EIT72574.1 asyd|OJJ55286.1 ater|EAU30570.1 atub|OJI84358.1 aver|OJJ06942.1 awen|OJJ41347.1 azon|OJJ48811.1

Group1776: abra|OJJ68743.1 acar|OOF90308.1 acla|EAW12505.1 afis|EAW24142.1 afla|EED46792.1 afum|EAL91997.1 agla|OJJ87221.1 akaw|GAA89890.1 aluc|OJZ81783.1 anid|CBF77934.1 anig|EHA22432.1 aory|EIT72571.1 asyd|OJJ55287.1 ater|EAU30571.1 atub|OJI84357.1 aver|OJJ06941.1 awen|OJJ41346.1 azon|OJJ48810.1

Group1777: abra|OJJ68748.1 acar|OOF92236.1 acla|EAW12503.1 afis|EAW24143.1 afla|EED46789.1 afum|EAL91998.1 agla|OJJ87222.1 akaw|GAA89892.1 aluc|OJZ81779.1 anid|CBF77930.1 anig|EHA22436.1 aory|EIT72633.1 asyd|OJJ55290.1 ater|EAU30574.1 atub|OJI84353.1 aver|OJJ06939.1 awen|OJJ41345.1 azon|OJJ48809.1

Group1778: abra|OJJ68749.1 acar|OOF92237.1 acla|EAW12502.1 afis|EAW24144.1 afla|EED46788.1 afum|EAL91999.1 agla|OJJ87223.1 akaw|GAA89893.1 aluc|OJZ81778.1 anid|CBF77928.1 anig|EHA22438.1 aory|EIT72562.1 asyd|OJJ55291.1 ater|EAU30575.1 atub|OJI84351.1 aver|OJJ06936.1 awen|OJJ41336.1 azon|OJJ48806.1

Group1779: abra|OJJ68750.1 acar|OOF92238.1 acla|EAW12501.1 afis|EAW24145.1 afla|EED46787.1 afum|EAL92000.1 agla|OJJ87224.1 akaw|GAA89894.1 aluc|OJZ81777.1 anid|CBF77926.1 anig|EHA22439.1 aory|EIT72718.1 asyd|OJJ55292.1 ater|EAU30576.1 atub|OJI84350.1 aver|OJJ06935.1 awen|OJJ41335.1 azon|OJJ48804.1

Group1780: abra|OJJ68752.1 acar|OOF92239.1 acla|EAW12499.1 afis|EAW24146.1 afla|EED46783.1 afum|EAL92001.1 agla|OJJ87226.1 akaw|GAA89896.1 aluc|OJZ81775.1 anid|CBF77923.1 anig|EHA22441.1

aory|EIT72696.1 asyd|OJJ55294.1 ater|EAU30578.1 atub|OJI84348.1 aver|OJJ06932.1 awen|OJJ41333.1 azon|OJJ48803.1

Group1781: abra|OJJ68756.1 acar|OOF92244.1 acla|EAW12495.1 afis|EAW24150.1 afla|EED46781.1 afum|EAL92005.1 agla|OJJ89542.1 akaw|GAA89900.1 aluc|OJZ81771.1 anid|CBF77915.1 anig|EHA22445.1 aory|EIT72518.1 asyd|OJJ55297.1 ater|EAU30582.1 atub|OJI84344.1 aver|OJJ06929.1 awen|OJJ41328.1 azon|OJJ48801.1

Group1782: abra|OJJ68763.1 acar|OOF92249.1 acla|EAW12488.1 afis|EAW24157.1 afla|EED46776.1 afum|EAL92012.2 agla|OJJ89524.1 akaw|GAA89907.1 aluc|OJZ81762.1 anid|CBF77900.1 anig|EHA22451.1 aory|EIT72774.1 asyd|OJJ55303.1 ater|EAU30588.1 atub|OJI84334.1 aver|OJJ06923.1 awen|OJJ41321.1 azon|OJJ48794.1

Group1783: abra|OJJ68766.1 acar|OOF92252.1 acla|EAW12485.1 afis|EAW24160.1 afla|EED46773.1 afum|EAL92016.1 agla|OJJ89521.1 akaw|GAA89910.1 aluc|OJZ81759.1 anid|CBF77894.1 anig|EHA22453.1 aory|EIT72644.1 asyd|OJJ55306.1 ater|EAU30591.1 atub|OJI84331.1 aver|OJJ06920.1 awen|OJJ41318.1 azon|OJJ48791.1

Group1784: abra|OJJ68768.1 acar|OOF92253.1 acla|EAW12484.1 afis|EAW24161.1 afla|EED46772.1 afum|EAL92017.1 agla|OJJ89519.1 akaw|GAA89911.1 aluc|OJZ81758.1 anid|CBF77892.1 anig|EHA22454.1 aory|EIT72707.1 asyd|OJJ55307.1 ater|EAU30592.1 atub|OJI84329.1 aver|OJJ06919.1 awen|OJJ41316.1 azon|OJJ48790.1

Group1785: abra|OJJ68769.1 acar|OOF92254.1 acla|EAW12483.1 afis|EAW24162.1 afla|EED46771.1 afum|EAL92018.1 agla|OJJ89518.1 akaw|GAA89912.1 aluc|OJZ81757.1 anid|CBF77890.1 anig|EHA22455.1 aory|EIT72701.1 asyd|OJJ55308.1 ater|EAU30593.1 atub|OJI84328.1 aver|OJJ06918.1 awen|OJJ41315.1 azon|OJJ48789.1

Group1786: abra|OJJ68770.1 acar|OOF92255.1 acla|EAW12482.1 afis|EAW24163.1 afla|EED46770.1 afum|EAL92020.1 agla|OJJ89517.1 akaw|GAA89913.1 aluc|OJZ81756.1 anid|CBF77888.1 anig|EHA22456.1 aory|EIT72763.1 asyd|OJJ55309.1 ater|EAU30594.1 atub|OJI84327.1 aver|OJJ06917.1 awen|OJJ41314.1 azon|OJJ48788.1

Group1787: abra|OJJ68785.1 acar|OOF92269.1 acla|EAW12469.1 afis|EAW24172.1 afla|EED46757.1 afum|EAL92034.1 agla|OJJ89503.1 akaw|GAA89927.1 aluc|OJZ81743.1 anid|CBF77861.1 anig|EHA22469.1 aory|EIT72255.1 asyd|OJJ55323.1 ater|EAU30607.1 atub|OJI84314.1 aver|OJJ06905.1 awen|OJJ41300.1 azon|OJJ48774.1

Group1788: abra|OJJ68788.1 acar|OOF92272.1 acla|EAW12466.1 afis|EAW24175.1 afla|EED46754.1 afum|EAL92037.1 agla|OJJ89499.1 akaw|GAA89929.1 aluc|OJZ81740.1 anid|CBF77855.1 anig|EHA22472.1 aory|EIT72259.1 asyd|OJJ55326.1 ater|EAU30610.1 atub|OJI84311.1 aver|OJJ06902.1 awen|OJJ41295.1 azon|OJJ48771.1

Group1789: abra|OJJ68790.1 acar|OOF92274.1 acla|EAW12463.1 afis|EAW24178.1 afla|EED46751.1 afum|EAL92040.1 agla|OJJ89496.1 akaw|GAA89932.1 aluc|OJZ81736.1 anid|CBF77849.1 anig|EHA22475.1 aory|EIT72252.1 asyd|OJJ55329.1 ater|EAU30613.1 atub|OJI84308.1 aver|OJJ06899.1 awen|OJJ41291.1 azon|OJJ48765.1

Group1790: abra|OJJ68793.1 acar|OOF92278.1 acla|EAW12460.1 afis|EAW24182.1 afla|EED46749.1 afum|EAL92042.1 agla|OJJ89493.1 akaw|GAA89933.1 aluc|OJZ81734.1 anid|CBF77844.1 anig|EHA22478.1 aory|EIT72258.1 asyd|OJJ55332.1 ater|EAU30615.1 atub|OJI84305.1 aver|OJJ06896.1 awen|OJJ41288.1 azon|OJJ48762.1

Group1791: abra|OJJ68794.1 acar|OOF92277.1 acla|EAW12459.1 afis|EAW24183.1 afla|EED46748.1 afum|EAL92043.1 agla|OJJ89492.1 akaw|GAA89934.1 aluc|OJZ81733.1 anid|CBF77842.1 anig|EHA22479.1 aory|EIT72260.1 asyd|OJJ55333.1 ater|EAU30616.1 atub|OJI84304.1 aver|OJJ06895.1 awen|OJJ41287.1 azon|OJJ48769.1

Group1792: abra|OJJ68797.1 acar|OOF92281.1 acla|EAW14657.1 afis|EAW24186.1 afla|EED46746.1 afum|EAL92046.1 agla|OJJ89489.1 akaw|GAA89936.1 aluc|OJZ81730.1 anid|CBF77836.1 anig|EHA22482.1 aory|EIT72257.1 asyd|OJJ55336.1 ater|EAU30619.1 atub|OJI84301.1 aver|OJJ06891.1 awen|OJJ41284.1 azon|OJJ43704.1

Group1793: abra|OJJ68827.1 acla|EAW12865.1 afis|EAW17814.1 afla|EED45598.1 afum|EAL91623.1 agla|OJJ81685.1 akaw|GAA90426.1 aluc|OJZ85803.1 anid|CBF79113.1 anig|EHA24752.1 aory|EIT75707.1 asyd|OJJ54395.1 atub|OJI79588.1 aver|OJJ07596.1 awen|OJJ31341.1 anid|CBF80733.1 awen|OJJ36931.1 asyd|OJJ54560.1

Group1794: abra|OJJ68855.1 acar|OOF90533.1 acla|EAW15256.1 afis|EAW20230.1 afla|EED57124.1 afum|EAL85347.2 agla|OJJ84632.1 akaw|GAA90398.1 aluc|OJZ85833.1 anid|CBF85122.1 anig|EHA24730.1 aory|EIT74034.1 asyd|OJJ63044.1 ater|EAU32032.1 atub|OJI79621.1 aver|OJI97919.1 awen|OJJ31233.1 azon|OJJ46156.1

Group1795: abra|OJJ68856.1 acar|OOF90532.1 afla|EED57125.1 afum|EAL85346.1 agla|OJJ84633.1 akaw|GAA90397.1 aluc|OJZ85834.1 anid|CBF85120.1 anig|EHA24729.1 aory|EIT74077.1 ater|EAU32031.1 atub|OJI79622.1 aver|OJI97918.1 awen|OJJ31232.1 azon|OJJ46155.1 acla|EAW15255.1 afis|EAW20229.1 asyd|OJJ63045.1

Group1796: abra|OJJ68859.1 acar|OOF90530.1 acla|EAW15250.1 afis|EAW20218.1 afla|EED57129.1 afum|EAL85333.1 agla|OJJ84638.1 akaw|GAA90394.1 aluc|OJZ85837.1 anid|CBF85115.1 anig|EHA24726.1 aory|EIT74090.1 asyd|OJJ63049.1 ater|EAU32027.1 atub|OJI79625.1 aver|OJI97914.1 awen|OJJ31228.1 azon|OJJ46151.1

Group1797: abra|OJJ68871.1 acar|OOF89965.1 acla|EAW15248.1 afis|EAW20214.1 afla|EED57130.1 afum|EAL85331.1 agla|OJJ84640.1 akaw|GAA90383.1 aluc|OJZ85848.1 anid|CBF85111.1 anig|EHA28098.1 aory|EIT74018.1 asyd|OJJ63051.1 ater|EAU32024.1 atub|OJI79636.1 aver|OJI97912.1 awen|OJJ31220.1 azon|OJJ46137.1

Group1798: abra|OJJ68913.1 acar|OOF91500.1 acla|EAW15323.1 afis|EAW20327.1 afla|EED57093.1 afum|EAL85453.1 agla|OJJ86344.1 akaw|GAA90336.1 aluc|OJZ85896.1 anid|CBF87781.1 anig|EHA24696.1 aory|EIT82282.1 asyd|OJJ63373.1 ater|EAU32055.1 atub|OJI79681.1 aver|OJI97573.1 awen|OJJ39915.1 azon|OJJ45889.1

Group1799: abra|OJJ68918.1 acar|OOF91506.1 acla|EAW14259.1 afis|EAW19021.1 afla|EED56623.1 afum|EAL93752.1 agla|OJJ84614.1 akaw|GAA90331.1 aluc|OJZ85901.1 anid|CBF85579.1 anig|EHA24691.1 aory|EIT79672.1 asyd|OJJ64177.1 ater|EAU32063.1 atub|OJI79686.1 aver|OJI98253.1 awen|OJJ31311.1 azon|OJJ46160.1

Group1800: abra|OJJ68919.1 acar|OOF91507.1 acla|EAW14260.1 afis|EAW19020.1 afla|EED56622.1 afum|EAL93751.1 agla|OJJ84615.1 akaw|GAA90330.1 aluc|OJZ85902.1 anid|CBF85577.1 anig|EHA24690.1 aory|EIT79600.1 asyd|OJJ64178.1 ater|EAU32062.1 atub|OJI79687.1 aver|OJI98254.1 awen|OJJ31310.1 azon|OJJ46161.1

Group1801: abra|OJJ68926.1 acar|OOF91512.1 acla|EAW14248.1 afis|EAW19032.1 afla|EED50385.1 afum|EAL93761.1 agla|OJJ84590.1 akaw|GAA90322.1 aluc|OJZ85911.1 anid|CBF79596.1 anig|EHA24681.1 aory|EIT83141.1 asyd|OJJ57985.1 ater|EAU32071.1 atub|OJI79701.1 aver|OJJ04415.1 awen|OJJ31461.1 azon|OJJ46163.1

Group1802: abra|OJJ68929.1 acar|OOF90676.1 afis|EAW19225.1 afum|EAL93956.1 agla|OJJ89343.1 akaw|GAA90320.1 aluc|OJZ85913.1 anid|CBF90358.1 anig|EHA24677.1 aory|EIT72912.1 asyd|OJJ55570.1 ater|EAU37576.1 atub|OJI79703.1 aver|OJJ06649.1 awen|OJJ29830.1 afla|EED51132.1 aory|EIT81305.1 agla|OJJ81307.1

Group1803: abra|OJJ68932.1 acar|OOF91515.1 acla|EAW14247.1 afis|EAW19033.1 afla|EED50387.1 afum|EAL93762.2 agla|OJJ84588.1 akaw|GAA90317.1 aluc|OJZ85916.1 anid|CBF79600.1 anig|EHA24675.1 aory|EIT83129.1 asyd|OJJ57990.1 ater|EAU32073.1 atub|OJI79707.1 aver|OJJ04412.1 awen|OJJ31463.1 azon|OJJ46164.1

Group1804: abra|OJJ68933.1 acar|OOF91516.1 acla|EAW14246.1 afla|EED50389.1 agla|OJJ84587.1 akaw|GAA90316.1 aluc|OJZ85917.1 anig|EHA24674.1 aory|EIT83117.1 asyd|OJJ57991.1 ater|EAU32074.1 atub|OJI79710.1 aver|OJJ04411.1 awen|OJJ31464.1 azon|OJJ46166.1 afum|EAL93763.1 anid|CBF79601.1 afis|EAW19034.1

Group1805: abra|OJJ68937.1 acar|OOF91518.1 acla|EAW14243.1 afis|EAW19037.1 afla|EED50391.1 afum|EAL93766.1 agla|OJJ84584.1 akaw|GAA90314.1 aluc|OJZ85922.1 anid|CBF79605.1 anig|EHA24672.1 aory|EIT82128.1 asyd|OJJ57993.1 ater|EAU32076.1 atub|OJI79712.1 aver|OJJ04409.1 awen|OJJ31466.1 azon|OJJ46169.1

Group1806: abra|OJJ68941.1 acar|OOF91521.1 acla|EAW14242.1 afis|EAW19038.1 afla|EED50393.1 afum|EAL93767.1 agla|OJJ84582.1 akaw|GAA90312.1 aluc|OJZ85927.1 anid|CBF79609.1 anig|EHA24670.1 aory|EIT82042.1 asyd|OJJ57994.1 ater|EAU32077.1 atub|OJI79716.1 aver|OJJ04408.1 awen|OJJ31468.1 azon|OJJ46172.1

Group1807: abra|OJJ68943.1 acar|OOF91522.1 acla|EAW14241.1 afis|EAW19039.1 afla|EED50394.1 afum|EAL93768.1 agla|OJJ84573.1 akaw|GAA90310.1 aluc|OJZ85929.1 anid|CBF79610.1 anig|EHA24669.1 aory|EIT82167.1 asyd|OJJ57995.1 ater|EAU32078.1 atub|OJI79717.1 aver|OJJ04407.1 awen|OJJ31469.1 azon|OJJ46173.1

Group1808: abra|OJJ68944.1 acar|OOF91524.1 acla|EAW14239.1 afis|EAW19041.1 afla|EED50396.1 afum|EAL93770.1 agla|OJJ84564.1 akaw|GAA90309.1 aluc|OJZ85930.1 anid|CBF79612.1 anig|EHA24667.1 aory|EIT82062.1 asyd|OJJ57999.1 ater|EAU32080.1 atub|OJI79719.1 aver|OJJ04403.1 awen|OJJ31470.1 azon|OJJ46179.1

Group1809: abra|OJJ68945.1 acar|OOF91525.1 acla|EAW14238.1 afis|EAW19042.1 afla|EED50397.1 afum|EAL93771.1 agla|OJJ84563.1 akaw|GAA90308.1 aluc|OJZ85931.1 anid|CBF79613.1 anig|EHA24666.1

aory|EIT82121.1 asyd|OJJ57998.1 ater|EAU32081.1 atub|OJI79720.1 aver|OJJ04404.1 awen|OJJ31471.1 azon|OJJ46181.1

Group1810: abra|OJJ68948.1 acar|OOF91526.1 acla|EAW14236.1 afis|EAW19044.1 afla|EED50401.1 afum|EAL93773.1 agla|OJJ84561.1 akaw|GAA90306.1 aluc|OJZ85934.1 anig|EHA24664.1 aory|EIT82075.1 ater|EAU32083.1 atub|OJI79723.1 azon|OJJ46184.1 anid|CBF79617.1 asyd|OJJ58000.1 aver|OJJ04402.1 awen|OJJ31473.1

Group1811: abra|OJJ68949.1 acar|OOF91527.1 acla|EAW14235.1 afis|EAW19045.1 afla|EED50402.1 afum|EAL93774.1 agla|OJJ84560.1 akaw|GAA90305.1 aluc|OJZ85935.1 anid|CBF79619.1 anig|EHA24663.1 aory|EIT82000.1 asyd|OJJ58001.1 ater|EAU32084.1 atub|OJI79724.1 aver|OJJ04400.1 awen|OJJ31474.1 azon|OJJ46188.1

Group1812: abra|OJJ68952.1 acar|OOF91530.1 acla|EAW14234.1 afis|EAW19046.1 afla|EED50405.1 afum|EAL93775.1 agla|OJJ84559.1 akaw|GAA90481.1 aluc|OJZ85938.1 anid|CBF79621.1 anig|EHA24661.1 aory|EIT82119.1 asyd|OJJ58003.1 ater|EAU32085.1 atub|OJI79726.1 aver|OJJ04398.1 awen|OJJ31475.1 azon|OJJ46189.1

Group1813: abra|OJJ68953.1 acar|OOF91531.1 acla|EAW14233.1 afis|EAW19047.1 afla|EED50406.1 afum|EAL93776.1 agla|OJJ84558.1 akaw|GAA90482.1 aluc|OJZ85939.1 anid|CBF79622.1 anig|EHA24660.1 aory|EIT82158.1 asyd|OJJ58004.1 ater|EAU32086.1 atub|OJI79727.1 aver|OJJ04397.1 awen|OJJ31477.1 azon|OJJ46190.1

Group1814: abra|OJJ68956.1 acar|OOF91535.1 acla|EAW14229.1 afis|EAW19052.1 afla|EED54409.1 afum|EAL93781.1 agla|OJJ84555.1 akaw|GAA90484.1 aluc|OJZ85942.1 anid|CBF79627.1 anig|EHA24657.1 aory|EIT75857.1 asyd|OJJ58009.1 ater|EAU32089.1 atub|OJI79730.1 aver|OJJ04391.1 awen|OJJ31483.1 azon|OJJ46193.1

Group1815: abra|OJJ68957.1 acar|OOF91534.1 acla|EAW14228.1 afis|EAW19053.1 afla|EED54411.1 afum|EAL93782.1 agla|OJJ84554.1 akaw|GAA90485.1 aluc|OJZ85943.1 anid|CBF79628.1 anig|EHA24656.1 aory|EIT75871.1 asyd|OJJ58010.1 ater|EAU32090.1 atub|OJI79731.1 aver|OJJ04390.1 awen|OJJ31484.1 azon|OJJ46194.1

Group1816: abra|OJJ68959.1 acar|OOF91537.1 acla|EAW14226.1 afis|EAW19055.1 afla|EED54413.1 afum|EAL93784.1 agla|OJJ84552.1 akaw|GAA90487.1 aluc|OJZ85945.1 anid|CBF79630.1 anig|EHA24654.1 aory|EIT75877.1 asyd|OJJ58012.1 ater|EAU32091.1 atub|OJI79733.1 aver|OJJ04388.1 awen|OJJ31486.1 azon|OJJ46196.1

Group1817: abra|OJJ68960.1 acar|OOF91538.1 acla|EAW14225.1 afis|EAW19056.1 afla|EED54414.1 afum|EAL93785.1 agla|OJJ84551.1 akaw|GAA90488.1 aluc|OJZ85946.1 anid|CBF79632.1 anig|EHA24653.1 aory|EIT75852.1 asyd|OJJ58013.1 ater|EAU32092.1 atub|OJI79734.1 aver|OJJ04387.1 awen|OJJ31487.1 azon|OJJ46197.1

Group1818: abra|OJJ68961.1 acar|OOF91539.1 acla|EAW14224.1 afis|EAW19057.1 afla|EED54415.1 afum|EAL93786.1 agla|OJJ84550.1 akaw|GAA90489.1 aluc|OJZ85947.1 anid|CBF79634.1 anig|EHA24652.1 aory|EIT75893.1 asyd|OJJ58014.1 ater|EAU32093.1 atub|OJI79735.1 aver|OJJ04386.1 awen|OJJ31488.1 azon|OJJ46199.1

Group1819: abra|OJJ68963.1 acar|OOF91541.1 acla|EAW14223.1 afis|EAW19058.1 afla|EED54418.1 afum|EAL93787.1 agla|OJJ84549.1 akaw|GAA90491.1 aluc|OJZ85950.1 anid|CBF79638.1 anig|EHA24650.1 aory|EIT75892.1 asyd|OJJ58015.1 ater|EAU32094.1 atub|OJI79738.1 aver|OJJ04385.1 awen|OJJ31489.1 azon|OJJ46201.1

Group1820: abra|OJJ68965.1 acar|OOF91542.1 acla|EAW14222.1 afis|EAW19059.1 afla|EED54421.1 afum|EAL93788.1 agla|OJJ84548.1 akaw|GAA90492.1 aluc|OJZ85952.1 anid|CBF79639.1 anig|EHA24649.1 aory|EIT75888.1 asyd|OJJ58016.1 ater|EAU32095.1 atub|OJI79740.1 aver|OJJ04384.1 awen|OJJ31490.1 azon|OJJ46202.1

Group1821: abra|OJJ68966.1 acar|OOF91544.1 acla|EAW14221.1 afis|EAW19060.1 afla|EED54422.1 afum|EAL93789.1 agla|OJJ84547.1 akaw|GAA90493.1 aluc|OJZ85953.1 anid|CBF79640.1 anig|EHA24648.1 aory|EIT75846.1 asyd|OJJ58017.1 ater|EAU32098.1 atub|OJI79741.1 aver|OJJ04382.1 awen|OJJ31491.1 azon|OJJ46203.1

Group1822: abra|OJJ68967.1 acar|OOF91545.1 acla|EAW14220.1 afis|EAW19061.1 afla|EED54423.1 afum|EAL93790.1 agla|OJJ84546.1 akaw|GAA90494.1 aluc|OJZ85954.1 anid|CBF79641.1 anig|EHA24647.1 aory|EIT75876.1 asyd|OJJ58018.1 ater|EAU32099.1 atub|OJI79742.1 aver|OJJ04381.1 awen|OJJ31492.1 azon|OJJ46204.1

Group1823: abra|OJJ68968.1 acar|OOF91546.1 acla|EAW14219.1 afis|EAW19062.1 afla|EED54428.1 afum|EAL93791.1 agla|OJJ84545.1 akaw|GAA90495.1 aluc|OJZ85955.1 anid|CBF79643.1 anig|EHA24646.1 aory|EIT75878.1 asyd|OJJ58019.1 ater|EAU32105.1 atub|OJI79743.1 aver|OJJ04380.1 awen|OJJ31493.1 azon|OJJ46206.1

Group1824: abra|OJJ68970.1 acar|OOF91547.1 acla|EAW14217.1 afis|EAW19064.1 afla|EED54430.1 afum|EAL93793.2 agla|OJJ84543.1 akaw|GAA90497.1 aluc|OJZ85957.1 anid|CBF79646.1 anig|EHA24644.1 aory|EIT75886.1 asyd|OJJ58021.1 ater|EAU32107.1 atub|OJI79745.1 aver|OJJ04378.1 awen|OJJ31496.1 azon|OJJ46208.1

Group1825: abra|OJJ68973.1 acar|OOF91550.1 acla|EAW14214.1 afis|EAW19067.1 afla|EED54433.1 afum|EAL93796.1 agla|OJJ84539.1 akaw|GAA90500.1 aluc|OJZ85962.1 anid|CBF79652.1 anig|EHA24641.1 aory|EIT75842.1 asyd|OJJ58025.1 ater|EAU32115.1 atub|OJI79749.1 aver|OJJ04374.1 awen|OJJ31499.1 azon|OJJ46213.1

Group1826: abra|OJJ68978.1 acar|OOF91553.1 acla|EAW14210.1 afis|EAW19071.1 afla|EED54436.1 afum|EAL93799.1 agla|OJJ84534.1 akaw|GAA90503.1 aluc|OJZ85966.1 anid|CBF79663.1 anig|EHA24638.1 aory|EIT75872.1 asyd|OJJ58028.1 ater|EAU32119.1 atub|OJI79754.1 aver|OJJ04371.1 awen|OJJ31503.1 azon|OJJ46218.1

Group1827: abra|OJJ68982.1 acar|OOF91554.1 acla|EAW14209.1 afis|EAW19072.1 afla|EED54437.1 afum|EAL93800.1 agla|OJJ84532.1 akaw|GAA90504.1 aluc|OJZ85968.1 anid|CBF79661.1 anig|EHA24637.1 aory|EIT75860.1 asyd|OJJ58029.1 ater|EAU32120.1 atub|OJI79756.1 aver|OJJ04370.1 awen|OJJ32466.1 azon|OJJ46219.1

Group1828: abra|OJJ68984.1 acar|OOF91555.1 acla|EAW14207.1 afis|EAW19074.1 afla|EED54439.1 afum|EAL93802.1 agla|OJJ84529.1 akaw|GAA90506.1 aluc|OJZ85970.1 anid|CBF79667.1 anig|EHA24635.1 aory|EIT75870.1 asyd|OJJ58031.1 ater|EAU32122.1 atub|OJI79758.1 aver|OJJ04368.1 awen|OJJ32468.1 azon|OJJ46238.1

Group1829: abra|OJJ68991.1 acar|OOF92703.1 acla|EAW12643.1 afis|EAW18042.1 afla|EED51854.1 afum|EAL91853.1 agla|OJJ85414.1 akaw|GAA90511.1 aluc|OJZ85976.1 anid|CBF80097.1 anig|EHA24630.1 aory|EIT81766.1 asyd|OJJ58299.1 ater|EAU31557.1 atub|OJI79766.1 aver|OJJ02125.1 awen|OJJ41492.1 azon|OJJ48223.1

Group1830: abra|OJJ68994.1 acar|OOF92705.1 acla|EAW12645.1 afis|EAW18040.1 afla|EED51857.1 afum|EAL91851.1 agla|OJJ85412.1 akaw|GAA90513.1 aluc|OJZ85978.1 anid|CBF80103.1 anig|EHA24628.1 aory|EIT81744.1 asyd|OJJ58296.1 ater|EAU31555.1 atub|OJI79768.1 aver|OJJ02128.1 awen|OJJ41497.1 azon|OJJ48225.1

Group1831: abra|OJJ68996.1 acar|OOF92707.1 acla|EAW12647.1 afis|EAW18038.1 afla|EED51860.1 afum|EAL91849.1 agla|OJJ85410.1 akaw|GAA90515.1 aluc|OJZ85980.1 anid|CBF80073.1 anig|EHA24626.1 aory|EIT81689.1 asyd|OJJ58294.1 ater|EAU31552.1 atub|OJI79770.1 aver|OJJ02130.1 awen|OJJ41499.1 azon|OJJ48227.1

Group1832: abra|OJJ68997.1 acar|OOF92708.1 acla|EAW12648.1 afis|EAW18037.1 afla|EED51861.1 afum|EAL91848.1 agla|OJJ85409.1 akaw|GAA90516.1 aluc|OJZ85981.1 anid|CBF80074.1 anig|EHA24625.1 aory|EIT81730.1 asyd|OJJ58293.1 ater|EAU31551.1 atub|OJI79771.1 aver|OJJ02131.1 awen|OJJ41500.1 azon|OJJ48228.1

Group1833: abra|OJJ69004.1 acar|OOF92717.1 acla|EAW12855.1 afis|EAW18018.1 afla|EED51873.1 afum|EAL91830.1 agla|OJJ85372.1 akaw|GAA90522.1 aluc|OJZ85990.1 anid|CBF80052.1 anig|EHA24620.1 aory|EIT81725.1 asyd|OJJ58280.1 ater|EAU31534.1 atub|OJI79779.1 aver|OJJ02143.1 awen|OJJ41521.1 azon|OJJ48243.1

Group1834: abra|OJJ69005.1 acar|OOF92718.1 acla|EAW12854.1 afla|EED51874.1 afum|EAL91829.1 akaw|GAA90523.1 aluc|OJZ85991.1 anig|EHA24619.1 aory|EIT81686.1 ater|EAU31533.1 atub|OJI79780.1 afis|EAW18017.1 asyd|OJJ58279.1 aver|OJJ02144.1 awen|OJJ41522.1 agla|OJJ85371.1 anid|CBF80050.1 azon|OJJ48244.1

Group1835: abra|OJJ69008.1 acar|OOF92719.1 acla|EAW12850.1 afis|EAW18013.1 afla|EED51883.1 afum|EAL91825.1 agla|OJJ80286.1 akaw|GAA90525.1 aluc|OJZ85994.1 anid|CBF80040.1 anig|EHA24616.1 aory|EIT81726.1 asyd|OJJ58275.1 ater|EAU31529.1 atub|OJI79782.1 aver|OJJ02149.1 awen|OJJ41528.1 azon|OJJ48248.1

Group1836: abra|OJJ69010.1 acar|OOF92721.1 acla|EAW12848.1 afis|EAW18011.1 afla|EED51885.1 afum|EAL91823.1 agla|OJJ80283.1 akaw|GAA90527.1 aluc|OJZ85996.1 anid|CBF80037.1 anig|EHA24614.1 aory|EIT81760.1 asyd|OJJ58273.1 ater|EAU31527.1 atub|OJI79784.1 aver|OJJ02151.1 awen|OJJ41530.1 azon|OJJ48250.1

Group1837: azon|OJJ51098.1 abra|OJJ74349.1 acar|OOF95381.1 acla|EAW07403.1 afis|EAW22429.1 afla|EED52507.1 afum|EAL90762.1 agla|OJJ87594.1 akaw|GAA86681.1 aluc|OJZ89781.1 anid|CBF88824.1 anig|EHA26996.1 aory|EIT82792.1 asyd|OJJ62884.1 ater|EAU39278.1 atub|OJI85025.1 aver|OJI96936.1 awen|OJJ35008.1

Group1838: abra|OJJ69024.1 acar|OOF92985.1 acla|EAW06466.1 afis|EAW15961.1 afla|EED58096.1 afum|EAL88594.1 agla|OJJ89351.1 akaw|GAA90541.1 aluc|OJZ86011.1 anid|CBF81079.1 anig|EHA24600.1

aory|EIT79116.1 asyd|OJJ56463.1 ater|EAU35493.1 atub|OJI79800.1 aver|OJJ06258.1 awen|OJJ35613.1 azon|OJJ46911.1

Group1839: abra|OJJ69026.1 acar|OOF92660.1 acla|EAW12556.1 afis|EAW24095.1 afla|EED46839.1 afum|EAL91949.1 agla|OJJ87168.1 akaw|GAA92787.1 aluc|OJZ82696.1 anid|CBF75138.1 anig|EHA21145.1 aory|EIT72775.1 asyd|OJJ59978.1 ater|EAU30520.1 atub|OJI88928.1 aver|OJJ02313.1 awen|OJJ41395.1 azon|OJJ48863.1

Group1840: abra|OJJ69029.1 acar|OOF92658.1 acla|EAW10072.1 afis|EAW25514.1 afla|EED54015.1 afum|EAL90179.1 agla|OJJ87289.1 akaw|GAA90545.1 aluc|OJZ86015.1 anid|CBF77783.1 anig|EHA24596.1 aory|EIT78264.1 asyd|OJJ53030.1 ater|EAU33360.1 atub|OJI79804.1 aver|OJJ03814.1 awen|OJJ41257.1 azon|OJJ44822.1

Group1841: abra|OJJ69034.1 acar|OOF89828.1 acla|EAW08636.1 afis|EAW15795.1 afla|EED51250.1 afum|EAL85555.1 agla|OJJ81958.1 akaw|GAA90549.1 aluc|OJZ86019.1 anid|CBF70929.1 anig|EHA24592.1 aory|EIT81525.1 asyd|OJJ52611.1 ater|EAU32347.1 atub|OJI79808.1 aver|OJJ04638.1 awen|OJJ38503.1 azon|OJJ43748.1

Group1842: abra|OJJ69036.1 acar|OOF92649.1 acla|EAW12687.1 afis|EAW17850.1 afla|EED46204.1 afum|EAL91659.1 agla|OJJ83119.1 akaw|GAA90551.1 aluc|OJZ86021.1 anid|CBF77960.1 anig|EHA24590.1 aory|EIT76404.1 asyd|OJJ55272.1 ater|EAU33052.1 atub|OJI79810.1 aver|OJJ06955.1 awen|OJJ41723.1 azon|OJJ48421.1

Group1843: abra|OJJ69037.1 acar|OOF92647.1 acla|EAW14104.1 afis|EAW18805.1 afla|EED50771.1 afum|EAL93541.1 agla|OJJ80535.1 akaw|GAA90552.1 aluc|OJZ86022.1 anid|CBF88044.1 anig|EHA24589.1 aory|EIT78003.1 asyd|OJJ63545.1 ater|EAU37943.1 atub|OJI79811.1 aver|OJI97389.1 awen|OJJ32380.1 azon|OJJ47142.1

Group1844: abra|OJJ69038.1 acar|OOF92646.1 acla|EAW06768.1 afis|EAW16318.1 afla|EED53563.1 afum|EAL88942.1 agla|OJJ88279.1 akaw|GAA90553.1 aluc|OJZ86023.1 anid|CBF89147.1 anig|EHA24588.1 aory|EIT74896.1 asyd|OJJ62568.1 ater|EAU34139.1 atub|OJI79812.1 aver|OJI96692.1 awen|OJJ37718.1 azon|OJJ44975.1

Group1845: abra|OJJ69039.1 acar|OOF92645.1 acla|EAW11330.1 afis|EAW18165.1 afla|EED53435.1 afum|EAL87525.2 agla|OJJ85953.1 akaw|GAA90554.1 aluc|OJZ86024.1 anid|CBF85751.1 anig|EHA24587.1 aory|EIT74836.1 asyd|OJJ64296.1 ater|EAU36013.1 atub|OJI79813.1 aver|OJI98389.1 awen|OJJ30512.1 azon|OJJ50291.1

Group1846: abra|OJJ69057.1 acar|OOF91226.1 acla|EAW08757.1 afis|EAW15674.1 afla|EED46856.1 afum|EAL85673.1 agla|OJJ81821.1 akaw|GAA90587.1 aluc|OJZ86045.1 anid|CBF71161.1 anig|EHA24566.1 aory|EIT72754.1 asyd|OJJ55844.1 ater|EAU32486.1 atub|OJI79842.1 aver|OJJ04787.1 awen|OJJ38352.1 azon|OJJ42790.1

Group1847: abra|OJJ69058.1 acar|OOF91225.1 acla|EAW08756.1 afis|EAW15673.1 afla|EED46857.1 afum|EAL85675.1 agla|OJJ81820.1 akaw|GAA90588.1 aluc|OJZ86046.1 anid|CBF71163.1 anig|EHA24565.1 aory|EIT72609.1 asyd|OJJ55845.1 ater|EAU32487.1 atub|OJI79843.1 aver|OJJ04788.1 awen|OJJ38351.1 azon|OJJ42791.1

Group1848: abra|OJJ69064.1 acar|OOF91219.1 acla|EAW08760.1 afis|EAW15668.1 afla|EED46876.1 afum|EAL85680.1 agla|OJJ81806.1 akaw|GAA90594.1 aluc|OJZ86052.1 anid|CBF71174.1 anig|EHA24559.1 aory|EIT72756.1 asyd|OJJ55853.1 ater|EAU32503.1 atub|OJI79849.1 aver|OJJ04796.1 awen|OJJ38344.1 azon|OJJ43043.1

Group1849: abra|OJJ69065.1 acar|OOF91218.1 acla|EAW08759.1 afis|EAW15667.1 afla|EED46877.1 afum|EAL85681.1 agla|OJJ81805.1 akaw|GAA90595.1 aluc|OJZ86053.1 anid|CBF71176.1 anig|EHA24558.1 aory|EIT72761.1 asyd|OJJ55854.1 ater|EAU32504.1 atub|OJI79850.1 aver|OJJ04797.1 awen|OJJ38343.1 azon|OJJ43042.1

Group1850: abra|OJJ69071.1 acar|OOF91231.1 acla|EAW08767.1 afis|EAW15663.1 afla|EED46886.1 afum|EAL85685.1 agla|OJJ81785.1 akaw|GAA90602.1 aluc|OJZ86060.1 anid|CBF71180.1 anig|EHA24553.1 aory|EIT72594.1 asyd|OJJ55862.1 ater|EAU32510.1 atub|OJI79857.1 aver|OJJ04803.1 awen|OJJ38339.1 azon|OJJ43024.1

Group1851: abra|OJJ69078.1 acar|OOF91213.1 afis|EAW15680.1 afla|EED46852.1 afum|EAL85668.1 akaw|GAA90607.1 aluc|OJZ86066.1 anid|CBF71154.1 anig|EHA24548.1 aory|EIT72663.1 asyd|OJJ59218.1 ater|EAU32482.1 atub|OJI79862.1 aver|OJJ01828.1 awen|OJJ38357.1 azon|OJJ48601.1 asyd|OJJ55840.1 aver|OJJ04783.1

Group1852: abra|OJJ69080.1 acar|OOF91211.1 acla|EAW08754.1 afis|EAW15682.1 afla|EED51372.1 afum|EAL85666.1 agla|OJJ81831.1 akaw|GAA90609.1 aluc|OJZ86068.1 anid|CBF71152.1 anig|EHA24546.1 aory|EIT80462.1 asyd|OJJ55839.1 ater|EAU32475.1 atub|OJI79864.1 aver|OJJ04782.1 awen|OJJ38374.1 azon|OJJ43858.1

Group1853: abra|OJJ69081.1 acar|OOF91210.1 acla|EAW08753.1 afis|EAW15683.1 afla|EED51371.1 afum|EAL85665.1 agla|OJJ81832.1 akaw|GAA90610.1 aluc|OJZ86069.1 anid|CBF71150.1 anig|EHA24545.1 aory|EIT80441.1 asyd|OJJ55838.1 ater|EAU32474.1 atub|OJI79865.1 aver|OJJ04781.1 awen|OJJ38375.1 azon|OJJ43859.1

Group1854: abra|OJJ69084.1 acar|OOF91208.1 acla|EAW08750.1 afis|EAW15686.1 afla|EED51368.1 afum|EAL85662.1 agla|OJJ81834.1 akaw|GAA90613.1 aluc|OJZ86071.1 anid|CBF71144.1 anig|EHA24543.1 aory|EIT80616.1 asyd|OJJ55836.1 ater|EAU32467.1 atub|OJI79868.1 aver|OJJ04779.1 awen|OJJ38377.1 azon|OJJ43861.1

Group1855: abra|OJJ69089.1 acar|OOF91204.1 acla|EAW08743.1 afis|EAW15693.1 afla|EED51360.1 afum|EAL85655.1 agla|OJJ81840.1 akaw|GAA90618.1 aluc|OJZ86078.1 anid|CBF71129.1 anig|EHA24538.1 aory|EIT80527.1 asyd|OJJ55829.1 ater|EAU32462.1 atub|OJI79875.1 aver|OJJ04771.1 awen|OJJ38384.1 azon|OJJ43853.1

Group1856: abra|OJJ69090.1 acar|OOF91203.1 acla|EAW08742.1 afis|EAW15694.1 afla|EED51359.1 afum|EAL85654.1 agla|OJJ81841.1 akaw|GAA90619.1 aluc|OJZ86079.1 anid|CBF71127.1 anig|EHA24537.1 aory|EIT80431.1 asyd|OJJ55828.1 ater|EAU32461.1 atub|OJI79876.1 aver|OJJ04770.1 awen|OJJ38385.1 azon|OJJ43852.1

Group1857: abra|OJJ69091.1 acar|OOF91202.1 acla|EAW08741.1 afis|EAW15695.1 afla|EED51358.1 afum|EAL85653.1 agla|OJJ81842.1 akaw|GAA90620.1 aluc|OJZ86080.1 anid|CBF71119.1 anig|EHA24535.1 aory|EIT80729.1 asyd|OJJ55827.1 ater|EAU32460.1 atub|OJI79877.1 aver|OJJ04769.1 awen|OJJ38386.1 azon|OJJ43850.1

Group1858: abra|OJJ69093.1 acar|OOF91201.1 acla|EAW08740.1 afis|EAW15696.1 afla|EED51357.1 afum|EAL85652.2 agla|OJJ81850.1 akaw|GAA90621.1 aluc|OJZ86081.1 anid|CBF71121.1 anig|EHA24534.1 aory|EIT80641.1 asyd|OJJ55826.1 ater|EAU32455.1 atub|OJI79878.1 aver|OJJ04768.1 awen|OJJ38389.1 azon|OJJ43849.1

Group1859: abra|OJJ69094.1 acar|OOF91200.1 acla|EAW08737.1 afis|EAW15699.1 afla|EED51353.1 afum|EAL85649.1 agla|OJJ81849.1 akaw|GAA90622.1 aluc|OJZ86083.1 anid|CBF71123.1 anig|EHA24532.1 aory|EIT80448.1 asyd|OJJ55825.1 ater|EAU32452.1 atub|OJI79879.1 aver|OJJ04767.1 awen|OJJ38391.1 azon|OJJ43847.1

Group1860: abra|OJJ69099.1 acar|OOF91197.1 acla|EAW08734.1 afis|EAW15702.1 afla|EED51349.1 afum|EAL85645.1 agla|OJJ81844.1 akaw|GAA90625.1 aluc|OJZ86088.1 anid|CBF71108.1 anig|EHA24529.1 aory|EIT80559.1 asyd|OJJ52712.1 ater|EAU32442.1 atub|OJI79884.1 aver|OJJ04741.1 awen|OJJ38396.1 azon|OJJ43845.1

Group1861: abra|OJJ69100.1 acar|OOF91196.1 acla|EAW08733.1 afis|EAW15707.1 afla|EED51348.1 afum|EAL85640.1 agla|OJJ81851.1 akaw|GAA90626.1 aluc|OJZ86089.1 anid|CBF71106.1 anig|EHA24528.1 aory|EIT80499.1 asyd|OJJ52711.1 atub|OJI79885.1 aver|OJJ04740.1 awen|OJJ38397.1 ater|EAU32441.1 azon|OJJ43844.1

Group1862: abra|OJJ69104.1 acar|OOF91194.1 acla|EAW08728.1 afis|EAW15703.1 afla|EED51345.1 afum|EAL85644.1 agla|OJJ81855.1 akaw|GAA90629.1 aluc|OJZ86092.1 anid|CBF71100.1 anig|EHA24525.1 aory|EIT80418.1 asyd|OJJ52707.1 ater|EAU32438.1 atub|OJI79888.1 aver|OJJ04736.1 awen|OJJ38402.1 azon|OJJ43841.1

Group1863: abra|OJJ69108.1 acar|OOF91181.1 acla|EAW08724.1 afis|EAW15732.1 afla|EED51341.1 afum|EAL85615.2 agla|OJJ81861.1 akaw|GAA90633.1 aluc|OJZ86096.1 anid|CBF71094.1 anig|EHA24522.1 aory|EIT81523.1 asyd|OJJ52704.1 ater|EAU32434.1 atub|OJI79892.1 aver|OJJ04733.1 awen|OJJ38406.1 azon|OJJ43836.1

Group1864: abra|OJJ69110.1 acar|OOF91184.1 acla|EAW08722.1 afis|EAW15730.1 afla|EED51339.1 afum|EAL85617.1 agla|OJJ81864.1 akaw|GAA90635.1 aluc|OJZ86098.1 anid|CBF71090.1 anig|EHA24520.1 aory|EIT81546.1 asyd|OJJ52701.1 ater|EAU32432.1 atub|OJI79894.1 aver|OJJ04729.1 awen|OJJ38409.1 azon|OJJ43833.1

Group1865: abra|OJJ69113.1 acar|OOF91185.1 acla|EAW08719.1 afis|EAW15728.1 afla|EED51335.1 afum|EAL85619.1 agla|OJJ81867.1 akaw|GAA90638.1 aluc|OJZ86102.1 anid|CBF71085.1 anig|EHA24517.1 aory|EIT81524.1 asyd|OJJ52698.1 ater|EAU32430.1 atub|OJI79897.1 aver|OJJ04726.1 awen|OJJ38412.1 azon|OJJ43830.1

Group1866: abra|OJJ69116.1 acar|OOF91188.1 acla|EAW08716.1 afis|EAW15724.1 afla|EED51332.1 afum|EAL85623.1 agla|OJJ81870.1 akaw|GAA90641.1 aluc|OJZ86105.1 anid|CBF71079.1 anig|EHA24515.1 aory|EIT81503.1 asyd|OJJ52695.1 ater|EAU32427.1 atub|OJI79900.1 aver|OJJ04723.1 awen|OJJ38415.1 azon|OJJ43827.1

Group1867: abra|OJJ69119.1 acar|OOF91191.1 acla|EAW08713.1 afis|EAW15721.1 afla|EED51329.1 afum|EAL85626.1 agla|OJJ81873.1 akaw|GAA90644.1 aluc|OJZ86108.1 anid|CBF71075.1 anig|EHA24513.1

aory|EIT81515.1 asyd|OJJ52692.1 ater|EAU32424.1 atub|OJI79903.1 aver|OJJ04720.1 awen|OJJ38418.1 azon|OJJ43824.1

Group1868: abra|OJJ69120.1 acar|OOF91192.1 acla|EAW08712.1 afis|EAW15720.1 afla|EED51328.1 afum|EAL85628.1 agla|OJJ81874.1 akaw|GAA90645.1 aluc|OJZ86109.1 anid|CBF71073.1 anig|EHA24512.1 aory|EIT81554.1 asyd|OJJ52691.1 ater|EAU32423.1 atub|OJI79904.1 aver|OJJ04719.1 awen|OJJ38419.1 azon|OJJ43823.1

Group1869: abra|OJJ69121.1 acar|OOF91193.1 acla|EAW08711.1 afis|EAW15719.1 afla|EED51327.1 afum|EAL85629.2 agla|OJJ81875.1 akaw|GAA90646.1 aluc|OJZ86110.1 anid|CBF71071.1 anig|EHA24511.1 aory|EIT81517.1 asyd|OJJ52690.1 ater|EAU32420.1 atub|OJI79905.1 aver|OJJ04718.1 awen|OJJ38420.1 azon|OJJ43822.1

Group1870: abra|OJJ69122.1 acar|OOF91178.1 acla|EAW08710.1 afis|EAW15718.1 afla|EED51326.1 afum|EAL85630.2 agla|OJJ81877.1 akaw|GAA90647.1 aluc|OJZ86111.1 anid|CBF71070.1 anig|EHA24510.1 aory|EIT81526.1 asyd|OJJ52689.1 ater|EAU32421.1 atub|OJI79906.1 aver|OJJ04717.1 awen|OJJ38421.1 azon|OJJ43821.1

Group1871: abra|OJJ69123.1 acar|OOF91177.1 acla|EAW08709.1 afis|EAW15717.1 afla|EED51325.1 afum|EAL85631.1 agla|OJJ81878.1 akaw|GAA90648.1 aluc|OJZ86112.1 anid|CBF71068.1 anig|EHA24509.1 aory|EIT81505.1 asyd|OJJ52688.1 ater|EAU32422.1 atub|OJI79907.1 aver|OJJ04716.1 awen|OJJ38422.1 azon|OJJ43820.1

Group1872: abra|OJJ69125.1 acar|OOF91175.1 acla|EAW08707.1 afis|EAW15715.1 afla|EED51323.1 afum|EAL85633.1 agla|OJJ81886.1 akaw|GAA90650.1 aluc|OJZ86114.1 anid|CBF71064.1 anig|EHA24507.1 aory|EIT81539.1 asyd|OJJ52685.1 ater|EAU32419.1 atub|OJI79909.1 aver|OJJ04713.1 awen|OJJ38425.1 azon|OJJ43818.1

Group1873: abra|OJJ69126.1 acar|OOF91174.1 acla|EAW08706.1 afis|EAW15714.1 afla|EED51322.1 afum|EAL85634.1 agla|OJJ81887.1 akaw|GAA90651.1 aluc|OJZ86115.1 anid|CBF71058.1 anig|EHA24506.1 aory|EIT81529.1 asyd|OJJ52680.1 ater|EAU32418.1 atub|OJI79910.1 aver|OJJ04706.1 awen|OJJ38426.1 azon|OJJ43817.1

Group1874: abra|OJJ69127.1 acar|OOF91173.1 acla|EAW08705.1 afis|EAW15713.1 afla|EED51321.1 afum|EAL85635.1 agla|OJJ81888.1 akaw|GAA90652.1 aluc|OJZ86116.1 anid|CBF71060.1 anig|EHA24505.1 aory|EIT81530.1 asyd|OJJ52679.1 ater|EAU32417.1 atub|OJI79911.1 aver|OJJ04705.1 awen|OJJ38427.1 azon|OJJ43816.1

Group1875: abra|OJJ69128.1 acar|OOF91172.1 acla|EAW08704.1 afis|EAW15712.1 afla|EED51320.1 afum|EAL85636.1 agla|OJJ81889.1 akaw|GAA90653.1 aluc|OJZ86117.1 anid|CBF71057.1 anig|EHA24504.1 aory|EIT81550.1 asyd|OJJ52678.1 ater|EAU32416.1 atub|OJI79912.1 aver|OJJ04704.1 awen|OJJ38428.1 azon|OJJ43815.1

Group1876: abra|OJJ69131.1 acar|OOF91169.1 acla|EAW08701.1 afis|EAW15709.1 afla|EED51317.1 afum|EAL85638.1 agla|OJJ81892.1 akaw|GAA90656.1 aluc|OJZ86120.1 anid|CBF71053.1 anig|EHA24501.1 aory|EIT81516.1 asyd|OJJ52675.1 ater|EAU32414.1 atub|OJI79916.1 aver|OJJ04701.1 awen|OJJ38431.1 azon|OJJ43812.1

Group1877: abra|OJJ69132.1 acar|OOF91168.1 acla|EAW08700.1 afis|EAW15708.1 afla|EED51316.1 afum|EAL85639.1 agla|OJJ81893.1 akaw|GAA90657.1 aluc|OJZ86121.1 anid|CBF71051.1 anig|EHA24500.1 aory|EIT81557.1 asyd|OJJ52674.1 ater|EAU32413.1 atub|OJI79917.1 aver|OJJ04700.1 awen|OJJ38432.1 azon|OJJ43811.1

Group1878: abra|OJJ69136.1 acar|OOF91164.1 acla|EAW08697.1 afis|EAW15738.1 afla|EED51314.1 afum|EAL85610.1 agla|OJJ81895.1 akaw|GAA86320.1 aluc|OJZ86124.1 anid|CBF71047.1 anig|EHA24497.1 aory|EIT81566.1 asyd|OJJ52672.1 ater|EAU32406.1 atub|OJI79920.1 aver|OJJ04698.1 awen|OJJ38434.1 azon|OJJ43808.1

Group1879: abra|OJJ69138.1 acar|OOF91163.1 acla|EAW08696.1 afis|EAW15739.1 afla|EED51313.1 afum|EAL85609.1 agla|OJJ81900.1 akaw|GAA86319.1 aluc|OJZ86125.1 anid|CBF71045.1 anig|EHA24496.1 aory|EIT81532.1 asyd|OJJ52671.1 ater|EAU32408.1 atub|OJI79921.1 aver|OJJ04697.1 awen|OJJ38438.1 azon|OJJ43807.1

Group1880: abra|OJJ69143.1 acar|OOF91159.1 acla|EAW08666.1 afis|EAW15743.1 afla|EED51309.1 afum|EAL85605.1 agla|OJJ81904.1 akaw|GAA86314.1 aluc|OJZ86129.1 anid|CBF71038.1 anig|EHA24492.1 aory|EIT81535.1 asyd|OJJ52666.1 ater|EAU32402.1 atub|OJI79925.1 aver|OJJ04693.1 awen|OJJ38443.1 azon|OJJ43749.1

Group1881: abra|OJJ69147.1 acar|OOF91156.1 acla|EAW08669.1 afis|EAW15757.1 afla|EED51302.1 afum|EAL85591.1 agla|OJJ81905.1 akaw|GAA86311.1 aluc|OJZ86133.1 anid|CBF71025.1 anig|EHA24488.1 aory|EIT81545.1 asyd|OJJ52660.1 ater|EAU32400.1 atub|OJI79929.1 aver|OJJ04688.1 awen|OJJ38445.1 azon|OJJ43751.1

Group1882: abra|OJJ69154.1 acar|OOF91149.1 acla|EAW08671.1 afis|EAW15753.1 afla|EED51298.1 afum|EAL85596.1 agla|OJJ81909.1 akaw|GAA86308.1 aluc|OJZ86141.1 anid|CBF71023.1 anig|EHA24483.1 aory|EIT81538.1 asyd|OJJ52659.1 ater|EAU32397.1 atub|OJI79933.1 aver|OJJ04687.1 awen|OJJ38453.1 azon|OJJ43753.1

Group1883: abra|OJJ69155.1 acar|OOF91148.1 acla|EAW08672.1 afis|EAW15752.1 afla|EED51297.1 afum|EAL85597.1 agla|OJJ81910.1 akaw|GAA86307.1 aluc|OJZ86142.1 anid|CBF71021.1 anig|EHA24482.1 aory|EIT81540.1 asyd|OJJ52658.1 ater|EAU32396.1 atub|OJI79934.1 aver|OJJ04686.1 awen|OJJ38454.1 azon|OJJ43754.1

Group1884: abra|OJJ69156.1 acar|OOF91147.1 acla|EAW08673.1 afis|EAW15751.1 afla|EED51296.1 afum|EAL85598.1 agla|OJJ81911.1 akaw|GAA86306.1 aluc|OJZ86143.1 anid|CBF71019.1 anig|EHA24481.1 aory|EIT81520.1 asyd|OJJ52657.1 ater|EAU32395.1 atub|OJI79935.1 aver|OJJ04685.1 awen|OJJ38455.1 azon|OJJ43755.1

Group1885: abra|OJJ69157.1 acar|OOF91146.1 acla|EAW08674.1 afis|EAW15750.1 afum|EAL85599.1 agla|OJJ81913.1 akaw|GAA86305.1 aluc|OJZ86144.1 anid|CBF71017.1 anig|EHA24480.1 aory|EIT81492.1 asyd|OJJ52656.1 ater|EAU32394.1 atub|OJI79936.1 aver|OJJ04684.1 awen|OJJ38456.1 azon|OJJ43756.1 afla|EED51295.1

Group1886: abra|OJJ69158.1 acar|OOF91145.1 acla|EAW08675.1 afis|EAW15749.1 afla|EED51294.1 afum|EAL85600.1 agla|OJJ81914.1 akaw|GAA86304.1 aluc|OJZ86145.1 anid|CBF71015.1 anig|EHA24479.1 aory|EIT81541.1 asyd|OJJ52655.1 ater|EAU32393.1 atub|OJI79937.1 aver|OJJ04683.1 awen|OJJ38457.1 azon|OJJ43757.1

Group1887: abra|OJJ69159.1 acar|OOF91144.1 acla|EAW08676.1 afis|EAW15748.1 afla|EED51293.1 afum|EAL85601.1 agla|OJJ81915.1 akaw|GAA86303.1 aluc|OJZ86146.1 anid|CBF71013.1 anig|EHA24478.1 aory|EIT81547.1 asyd|OJJ52654.1 ater|EAU32392.1 atub|OJI79938.1 aver|OJJ04682.1 awen|OJJ38458.1 azon|OJJ43759.1

Group1888: abra|OJJ69161.1 acar|OOF91141.1 acla|EAW08678.1 afis|EAW15746.1 afla|EED51291.1 afum|EAL85602.1 agla|OJJ81917.1 akaw|GAA86301.1 aluc|OJZ86148.1 anid|CBF71009.1 anig|EHA24476.1 aory|EIT81488.1 asyd|OJJ52651.1 ater|EAU32390.1 atub|OJI79940.1 aver|OJJ04680.1 awen|OJJ38460.1 azon|OJJ43761.1

Group1889: abra|OJJ69162.1 acar|OOF91140.1 acla|EAW08679.1 afis|EAW15754.1 afla|EED51290.1 afum|EAL85595.1 agla|OJJ81918.1 akaw|GAA86300.1 aluc|OJZ86149.1 anid|CBF71007.1 anig|EHA24475.1 aory|EIT81493.1 asyd|OJJ52650.1 ater|EAU32389.1 atub|OJI79941.1 aver|OJJ04679.1 awen|OJJ38462.1 azon|OJJ43762.1

Group1890: abra|OJJ69164.1 acar|OOF91139.1 acla|EAW08680.1 afis|EAW15758.1 afla|EED51289.1 afum|EAL85590.1 agla|OJJ81920.1 akaw|GAA86299.1 aluc|OJZ86151.1 anid|CBF71006.1 anig|EHA24474.1 aory|EIT81496.1 asyd|OJJ52648.1 ater|EAU32388.1 atub|OJI79943.1 aver|OJJ04677.1 awen|OJJ38466.1 azon|OJJ43764.1

Group1891: abra|OJJ69165.1 acar|OOF91138.1 acla|EAW08681.1 afis|EAW15759.1 afla|EED51288.1 afum|EAL85589.1 agla|OJJ81921.1 akaw|GAA86298.1 aluc|OJZ86152.1 anid|CBF71004.1 anig|EHA24473.1 aory|EIT81511.1 asyd|OJJ52647.1 ater|EAU32387.1 atub|OJI79944.1 aver|OJJ04676.1 awen|OJJ38467.1 azon|OJJ43765.1

Group1892: abra|OJJ69166.1 acar|OOF91137.1 acla|EAW08682.1 afis|EAW15760.1 afla|EED51287.1 afum|EAL85588.1 agla|OJJ81922.1 akaw|GAA86297.1 aluc|OJZ86153.1 anid|CBF71002.1 anig|EHA24472.1 aory|EIT81510.1 asyd|OJJ52646.1 ater|EAU32386.1 atub|OJI79945.1 aver|OJJ04675.1 awen|OJJ38468.1 azon|OJJ43766.1

Group1893: abra|OJJ69167.1 acar|OOF91136.1 acla|EAW08683.1 afis|EAW15761.1 afla|EED51286.1 afum|EAL85587.1 agla|OJJ81923.1 akaw|GAA86296.1 aluc|OJZ86154.1 anid|CBF71000.1 anig|EHA24471.1 aory|EIT81497.1 asyd|OJJ52645.1 ater|EAU32385.1 atub|OJI79946.1 aver|OJJ04674.1 awen|OJJ38469.1 azon|OJJ43767.1

Group1894: abra|OJJ69168.1 acar|OOF91135.1 acla|EAW08684.1 afis|EAW15762.1 afla|EED51285.1 afum|EAL85586.1 agla|OJJ81924.1 akaw|GAA86295.1 aluc|OJZ86155.1 anid|CBF70998.1 anig|EHA24470.1 aory|EIT81522.1 asyd|OJJ52644.1 ater|EAU32384.1 atub|OJI79947.1 aver|OJJ04673.1 awen|OJJ38470.1 azon|OJJ43769.1

Group1895: abra|OJJ69177.1 acar|OOF91129.1 acla|EAW08686.1 afis|EAW15763.1 afla|EED51284.1 afum|EAL85585.1 agla|OJJ81925.1 akaw|GAA86287.1 aluc|OJZ86163.1 anid|CBF70996.1 anig|EHA24461.1 aory|EIT81489.1 asyd|OJJ52643.1 ater|EAU32378.1 atub|OJI79955.1 aver|OJJ04672.1 awen|OJJ38471.1 azon|OJJ43770.1

Group1896: abra|OJJ69178.1 acar|OOF90317.1 acla|EAW08687.1 afis|EAW15764.1 afla|EED51283.1 afum|EAL85584.1 agla|OJJ81927.1 akaw|GAA86286.1 aluc|OJZ86164.1 anid|CBF70994.1 anig|EHA24460.1

aory|EIT81513.1 asyd|OJJ52642.1 ater|EAU32377.1 atub|OJI79956.1 aver|OJJ04670.1 awen|OJJ38472.1 azon|OJJ43772.1

Group1897: abra|OJJ69179.1 acar|OOF90318.1 acla|EAW08688.1 afis|EAW15765.1 afla|EED51282.1 afum|EAL85583.1 agla|OJJ81928.1 akaw|GAA86285.1 aluc|OJZ86165.1 anid|CBF70993.1 anig|EHA24459.1 aory|EIT81558.1 asyd|OJJ52641.1 ater|EAU32376.1 atub|OJI79957.1 aver|OJJ04669.1 awen|OJJ38473.1 azon|OJJ43773.1

Group1898: abra|OJJ69180.1 acar|OOF90319.1 acla|EAW08689.1 afis|EAW15766.1 afla|EED51281.1 afum|EAL85582.1 agla|OJJ81930.1 akaw|GAA86284.1 aluc|OJZ86166.1 anig|EHA24458.1 aory|EIT81544.1 asyd|OJJ52640.1 ater|EAU32375.1 atub|OJI79958.1 awen|OJJ38474.1 anid|CBF70991.1 aver|OJJ04668.1 azon|OJJ43774.1

Group1899: abra|OJJ69181.1 acar|OOF90321.1 acla|EAW08690.1 afis|EAW15767.1 afla|EED51280.1 afum|EAL85581.1 agla|OJJ81931.1 akaw|GAA86283.1 aluc|OJZ86167.1 anid|CBF70989.1 anig|EHA24457.1 aory|EIT81536.1 asyd|OJJ52639.1 ater|EAU32374.1 atub|OJI79959.1 aver|OJJ04667.1 awen|OJJ38475.1 azon|OJJ43775.1

Group1900: abra|OJJ69183.1 acar|OOF90320.1 acla|EAW08691.1 afis|EAW15768.1 afla|EED51279.1 afum|EAL85580.1 agla|OJJ81932.1 akaw|GAA86282.1 aluc|OJZ86168.1 anid|CBF70987.1 anig|EHA24456.1 aory|EIT81531.1 asyd|OJJ52638.1 ater|EAU32373.1 atub|OJI79960.1 aver|OJJ04666.1 awen|OJJ38476.1 azon|OJJ43776.1

Group1901: abra|OJJ69184.1 acar|OOF91128.1 acla|EAW08662.1 afis|EAW15769.1 afla|EED51278.1 afum|EAL85579.1 agla|OJJ81933.1 akaw|GAA86281.1 aluc|OJZ86170.1 anid|CBF70983.1 anig|EHA24455.1 aory|EIT81490.1 asyd|OJJ52636.1 ater|EAU32372.1 atub|OJI79962.1 aver|OJJ04664.1 awen|OJJ38477.1 azon|OJJ43777.1

Group1902: abra|OJJ69186.1 acar|OOF91126.1 acla|EAW08660.1 afis|EAW15771.1 afla|EED51276.1 afum|EAL85577.1 agla|OJJ81935.1 akaw|GAA86279.1 aluc|OJZ86172.1 anid|CBF70979.1 anig|EHA24453.1 aory|EIT81507.1 asyd|OJJ52634.1 ater|EAU32370.1 atub|OJI79964.1 aver|OJJ04662.1 awen|OJJ38479.1 azon|OJJ43779.1

Group1903: abra|OJJ69187.1 acar|OOF91125.1 acla|EAW08659.1 afis|EAW15772.1 afla|EED51275.1 afum|EAL85576.1 agla|OJJ81936.1 akaw|GAA86278.1 aluc|OJZ86173.1 anid|CBF70977.1 anig|EHA24452.1 aory|EIT81560.1 asyd|OJJ52633.1 ater|EAU32369.1 atub|OJI79965.1 aver|OJJ04661.1 awen|OJJ38480.1 azon|OJJ43780.1

Group1904: abra|OJJ69189.1 acar|OOF91122.1 acla|EAW08656.1 afis|EAW15775.1 afla|EED51271.1 afum|EAL85574.1 agla|OJJ81938.1 akaw|GAA86275.1 aluc|OJZ86175.1 anid|CBF70976.1 anig|EHA24449.1 aory|EIT81533.1 asyd|OJJ52631.1 ater|EAU32366.1 atub|OJI79967.1 aver|OJJ04659.1 awen|OJJ38482.1 azon|OJJ43783.1

Group1905: abra|OJJ69191.1 acar|OOF91120.1 acla|EAW08654.1 afis|EAW15777.1 afla|EED51268.1 afum|EAL85572.1 agla|OJJ81940.1 akaw|GAA86273.1 aluc|OJZ86177.1 anid|CBF70933.1 anig|EHA24447.1 aory|EIT81563.1 asyd|OJJ52629.1 ater|EAU32364.1 atub|OJI79969.1 aver|OJJ04657.1 awen|OJJ38484.1 azon|OJJ43785.1

Group1906: abra|OJJ69192.1 acar|OOF91121.1 acla|EAW08653.1 afis|EAW15778.1 afla|EED51266.1 afum|EAL85571.1 agla|OJJ81941.1 akaw|GAA86272.1 aluc|OJZ86178.1 anid|CBF70935.1 anig|EHA24446.1 aory|EIT81565.1 asyd|OJJ52628.1 ater|EAU32363.1 atub|OJI79970.1 aver|OJJ04656.1 awen|OJJ38485.1 azon|OJJ43786.1

Group1907: abra|OJJ69193.1 acar|OOF91119.1 acla|EAW08652.1 afis|EAW15779.1 afla|EED51265.1 afum|EAL85570.1 agla|OJJ81942.1 akaw|GAA86271.1 aluc|OJZ86179.1 anid|CBF70937.1 anig|EHA24445.1 aory|EIT81542.1 asyd|OJJ52627.1 ater|EAU32362.1 atub|OJI79971.1 aver|OJJ04655.1 awen|OJJ38486.1 azon|OJJ43787.1

Group1908: abra|OJJ69194.1 acar|OOF91118.1 acla|EAW08651.1 afis|EAW15780.1 afla|EED51264.1 afum|EBA27235.1 agla|OJJ81943.1 akaw|GAA86270.1 aluc|OJZ86180.1 anid|CBF70939.1 anig|EHA24444.1 aory|EIT81528.1 asyd|OJJ52626.1 ater|EAU32361.1 atub|OJI79972.1 aver|OJJ04654.1 awen|OJJ38487.1 azon|OJJ43788.1

Group1909: abra|OJJ69196.1 acar|OOF90027.1 acla|EAW08650.1 afis|EAW15781.1 afla|EED51263.1 afum|EAL85569.1 agla|OJJ81944.1 akaw|GAA92209.1 aluc|OJZ86181.1 anid|CBF70941.1 anig|EHA24443.1 aory|EIT81567.1 asyd|OJJ52625.1 ater|EAU32360.1 atub|OJI79973.1 aver|OJJ04652.1 awen|OJJ38488.1 azon|OJJ43789.1

Group1910: abra|OJJ69197.1 acar|OOF90026.1 acla|EAW08649.1 afis|EAW15782.1 afla|EED51262.1 afum|EAL85568.1 agla|OJJ81945.1 akaw|GAA92210.1 aluc|OJZ86182.1 anid|CBF70945.1 anig|EHA24442.1 aory|EIT81562.1 asyd|OJJ52624.1 ater|EAU32359.1 atub|OJI79974.1 aver|OJJ04651.1 awen|OJJ38489.1 azon|OJJ43790.1

Group1911: abra|OJJ69198.1 acar|OOF91117.1 acla|EAW08648.1 afis|EAW15783.1 afla|EED51261.1 afum|EAL85567.1 agla|OJJ81946.1 akaw|GAA92211.1 aluc|OJZ86183.1 anid|CBF70946.1 anig|EHA24441.1 aory|EIT81552.1 asyd|OJJ52623.1 ater|EAU32358.1 atub|OJI79975.1 aver|OJJ04650.1 awen|OJJ38490.1 azon|OJJ43791.1

Group1912: abra|OJJ69200.1 acar|OOF91115.1 acla|EAW08646.1 afis|EAW15785.1 afla|EED51259.1 afum|EAL85565.1 agla|OJJ81948.1 akaw|GAA92213.1 aluc|OJZ86185.1 anid|CBF70954.1 anig|EHA24439.1 aory|EIT81568.1 asyd|OJJ52621.1 ater|EAU32356.1 atub|OJI79977.1 aver|OJJ04648.1 awen|OJJ38492.1 azon|OJJ43794.1

Group1913: abra|OJJ69204.1 acar|OOF91112.1 acla|EAW08643.1 afis|EAW15788.1 afla|EED51257.1 afum|EAL85562.1 agla|OJJ81951.1 akaw|GAA92216.1 aluc|OJZ86188.1 anid|CBF70960.1 anig|EHA24435.1 aory|EIT81501.1 asyd|OJJ52618.1 ater|EAU32353.1 atub|OJI79980.1 aver|OJJ04645.1 awen|OJJ38496.1 azon|OJJ43797.1

Group1914: abra|OJJ69205.1 acar|OOF91109.1 acla|EAW08642.1 afis|EAW15789.1 afla|EED51256.1 afum|EAL85561.1 agla|OJJ81952.1 akaw|GAA92217.1 aluc|OJZ86189.1 anid|CBF70962.1 anig|EHA24434.1 aory|EIT81537.1 asyd|OJJ52617.1 ater|EAU32352.1 atub|OJI79981.1 aver|OJJ04644.1 awen|OJJ38497.1 azon|OJJ43798.1

Group1915: abra|OJJ69206.1 acar|OOF91111.1 acla|EAW08641.1 afis|EAW15790.1 afla|EED51255.1 afum|EAL85560.1 agla|OJJ81953.1 akaw|GAA92218.1 aluc|OJZ86190.1 anid|CBF70964.1 anig|EHA24433.1 aory|EIT81514.1 asyd|OJJ52616.1 ater|EAU32351.1 atub|OJI79982.1 aver|OJJ04643.1 awen|OJJ38498.1 azon|OJJ43799.1

Group1916: abra|OJJ69208.1 acar|OOF91108.1 acla|EAW08638.1 afis|EAW15793.1 afla|EED51252.1 afum|EAL85557.1 agla|OJJ81956.1 akaw|GAA92219.1 aluc|OJZ86191.1 anid|CBF70970.1 anig|EHA24431.1 aory|EIT81504.1 asyd|OJJ52613.1 ater|EAU32349.1 atub|OJI79983.1 aver|OJJ04640.1 awen|OJJ38501.1 azon|OJJ43802.1

Group1917: abra|OJJ69209.1 acar|OOF91107.1 acla|EAW08637.1 afis|EAW15794.1 afla|EED51251.1 afum|EAL85556.1 agla|OJJ81957.1 akaw|GAA92220.1 aluc|OJZ86192.1 anid|CBF70931.1 anig|EHA24430.1 aory|EIT81500.1 asyd|OJJ52612.1 ater|EAU32348.1 atub|OJI79984.1 aver|OJJ04639.1 awen|OJJ38502.1 azon|OJJ43803.1

Group1918: abra|OJJ69210.1 acar|OOF91106.1 acla|EAW08635.1 afis|EAW15796.1 afla|EED51249.1 afum|EAL85554.1 agla|OJJ81960.1 akaw|GAA92221.1 aluc|OJZ86193.1 anid|CBF70927.1 anig|EHA24429.1 aory|EIT81508.1 asyd|OJJ52610.1 ater|EAU32346.1 atub|OJI79985.1 aver|OJJ04637.1 awen|OJJ38504.1 azon|OJJ43747.1

Group1919: abra|OJJ69211.1 acar|OOF91105.1 acla|EAW08634.1 afis|EAW15797.1 afla|EED51248.1 afum|EAL85553.2 agla|OJJ81961.1 akaw|GAA92222.1 aluc|OJZ86194.1 anid|CBF70925.1 anig|EHA24428.1 aory|EIT81561.1 asyd|OJJ52609.1 ater|EAU32345.1 atub|OJI79986.1 aver|OJJ04636.1 awen|OJJ38505.1 azon|OJJ43746.1

Group1920: abra|OJJ69212.1 acar|OOF91104.1 acla|EAW08633.1 afis|EAW15798.1 afla|EED51247.1 afum|EAL85552.1 agla|OJJ81962.1 akaw|GAA92223.1 aluc|OJZ86195.1 anid|CBF70923.1 anig|EHA24427.1 aory|EIT81502.1 asyd|OJJ52608.1 ater|EAU32344.1 atub|OJI79988.1 aver|OJJ04635.1 awen|OJJ38506.1 azon|OJJ43745.1

Group1921: abra|OJJ69215.1 acar|OOF91103.1 acla|EAW08630.1 afis|EAW15801.1 afla|EED51244.1 afum|EAL85549.2 agla|OJJ81968.1 akaw|GAA92224.1 aluc|OJZ86196.1 anid|CBF70918.1 anig|EHA24426.1 aory|EIT83186.1 asyd|OJJ52605.1 ater|EAU33447.1 atub|OJI79989.1 aver|OJJ04632.1 awen|OJJ38510.1 azon|OJJ43742.1

Group1922: abra|OJJ69217.1 acar|OOF91101.1 acla|EAW08628.1 afis|EAW15803.1 afla|EED51242.1 afum|EAL85547.1 agla|OJJ81970.1 akaw|GAA92226.1 aluc|OJZ86199.1 anid|CBF70914.1 anig|EHA24424.1 aory|EIT83176.1 asyd|OJJ52603.1 ater|EAU33445.1 atub|OJI79991.1 aver|OJJ04629.1 awen|OJJ38513.1 azon|OJJ43739.1

Group1923: abra|OJJ69222.1 acar|OOF91098.1 acla|EAW12739.1 afis|EAW17902.1 afla|EED51238.1 afum|EAL91710.1 agla|OJJ81973.1 akaw|GAA92230.1 aluc|OJZ86202.1 anid|CBF70908.1 anig|EHA24420.1 aory|EIT83180.1 asyd|OJJ52599.1 ater|EAU33438.1 atub|OJI79996.1 aver|OJJ04626.1 awen|OJJ38518.1 azon|OJJ43736.1

Group1924: abra|OJJ69230.1 acar|OOF91090.1 acla|EAW08619.1 afis|EAW15815.1 afla|EED51232.1 afum|EAL85536.1 agla|OJJ81979.1 akaw|GAA92238.1 aluc|OJZ86210.1 anid|CBF70899.1 anig|EHA24416.1 aory|EIT83190.1 asyd|OJJ52595.1 ater|EAU33433.1 atub|OJI80004.1 aver|OJJ04622.1 awen|OJJ38528.1 azon|OJJ43733.1

Group1925: abra|OJJ69232.1 acar|OOF91088.1 acla|EAW08617.1 afis|EAW15817.1 afla|EED51230.1 afum|EAL85534.1 agla|OJJ81981.1 akaw|GAA92240.1 aluc|OJZ86212.1 anid|CBF70895.1 anig|EHA24414.1

aory|EIT83179.1 asyd|OJJ52593.1 ater|EAU33431.1 atub|OJI80007.1 aver|OJJ04620.1 awen|OJJ38531.1 azon|OJJ43731.1

Group1926: abra|OJJ69234.1 acar|OOF91086.1 acla|EAW08615.1 afis|EAW15819.1 afla|EED51228.1 afum|EAL85532.1 agla|OJJ81983.1 akaw|GAA92242.1 aluc|OJZ86214.1 anid|CBF70891.1 anig|EHA24412.1 aory|EIT83174.1 asyd|OJJ52591.1 ater|EAU33429.1 atub|OJI80009.1 aver|OJJ04618.1 awen|OJJ38533.1 azon|OJJ43729.1

Group1927: abra|OJJ69235.1 acar|OOF91085.1 acla|EAW08614.1 afis|EAW15820.1 afla|EED51227.1 afum|EAL85531.1 agla|OJJ81984.1 akaw|GAA92243.1 aluc|OJZ86215.1 anid|CBF70889.1 anig|EHA24411.1 aory|EIT83196.1 asyd|OJJ52590.1 ater|EAU33428.1 atub|OJI80010.1 aver|OJJ04617.1 awen|OJJ38534.1 azon|OJJ43728.1

Group1928: abra|OJJ69237.1 acar|OOF91081.1 acla|EAW08610.1 afis|EAW15823.1 afla|EED51223.1 afum|EAL85528.1 agla|OJJ81986.1 akaw|GAA92247.1 aluc|OJZ86217.1 anid|CBF70884.1 anig|EHA24409.1 aory|EIT83191.1 asyd|OJJ52588.1 ater|EAU33425.1 atub|OJI80012.1 aver|OJJ04615.1 awen|OJJ38536.1 azon|OJJ43726.1

Group1929: abra|OJJ69238.1 acar|OOF91080.1 acla|EAW08609.1 afis|EAW15824.1 afla|EED51221.1 afum|EAL85527.1 agla|OJJ81987.1 akaw|GAA92248.1 aluc|OJZ86218.1 anid|CBF70878.1 anig|EHA24408.1 aory|EIT80400.1 asyd|OJJ52586.1 ater|EAU33424.1 atub|OJI80013.1 aver|OJJ04613.1 awen|OJJ38538.1 azon|OJJ43725.1

Group1930: abra|OJJ69239.1 acar|OOF91079.1 acla|EAW08608.1 afis|EAW15825.1 afla|EED51220.1 afum|EAL85526.1 agla|OJJ81988.1 akaw|GAA92249.1 aluc|OJZ86220.1 anid|CBF70876.1 anig|EHA24407.1 aory|EIT80394.1 asyd|OJJ52584.1 ater|EAU33423.1 atub|OJI80014.1 aver|OJJ04612.1 awen|OJJ38539.1 azon|OJJ43724.1

Group1931: abra|OJJ69240.1 acar|OOF91078.1 acla|EAW08607.1 afis|EAW15826.1 afla|EED51219.1 afum|EAL85525.1 agla|OJJ81989.1 akaw|GAA92250.1 aluc|OJZ86221.1 anid|CBF70874.1 anig|EHA24406.1 aory|EIT80397.1 asyd|OJJ52583.1 ater|EAU33422.1 atub|OJI80015.1 aver|OJJ04611.1 awen|OJJ38540.1 azon|OJJ43723.1

Group1932: abra|OJJ69244.1 acar|OOF91076.1 acla|EAW08605.1 afis|EAW15828.1 afla|EED51217.1 afum|EAL85523.1 agla|OJJ81992.1 akaw|GAA92252.1 aluc|OJZ86225.1 anid|CBF70869.1 anig|EHA24403.1 aory|EIT80398.1 asyd|OJJ52580.1 ater|EAU33419.1 atub|OJI80018.1 aver|OJJ04608.1 awen|OJJ38542.1 azon|OJJ43721.1

Group1933: abra|OJJ69247.1 acla|EAW08602.1 afis|EAW15831.1 afla|EED51215.1 afum|EAL85521.1 agla|OJJ81995.1 akaw|GAA92254.1 aluc|OJZ86228.1 anid|CBF70863.1 anig|EHA24401.1 aory|EIT80403.1 asyd|OJJ52577.1 ater|EAU33417.1 atub|OJI80021.1 aver|OJJ04606.1 awen|OJJ38544.1 azon|OJJ43718.1 aory|EIT80399.1

Group1934: abra|OJJ69248.1 acar|OOF91073.1 acla|EAW08601.1 afis|EAW15832.1 afla|EED51214.1 afum|EAL85520.1 agla|OJJ81996.1 akaw|GAA92255.1 aluc|OJZ86229.1 anid|CBF70861.1 anig|EHA24400.1 aory|EIT80409.1 asyd|OJJ52576.1 ater|EAU33416.1 atub|OJI80022.1 aver|OJJ04605.1 awen|OJJ38545.1 azon|OJJ43717.1

Group1935: abra|OJJ69249.1 acar|OOF91072.1 acla|EAW08600.1 afis|EAW15833.1 afla|EED51213.1 afum|EAL85519.1 agla|OJJ81997.1 akaw|GAA92256.1 aluc|OJZ86230.1 anid|CBF70859.1 anig|EHA24399.1 aory|EIT80405.1 asyd|OJJ52575.1 ater|EAU33415.1 atub|OJI80023.1 aver|OJJ04604.1 awen|OJJ38546.1 azon|OJJ43716.1

Group1936: abra|OJJ69250.1 acar|OOF91071.1 acla|EAW08599.1 afis|EAW15834.1 afla|EED51212.1 afum|EAL85518.1 agla|OJJ81998.1 akaw|GAA92257.1 aluc|OJZ86231.1 anid|CBF70858.1 anig|EHA24398.1 aory|EIT80395.1 asyd|OJJ52574.1 ater|EAU33414.1 atub|OJI80024.1 aver|OJJ04603.1 awen|OJJ38547.1 azon|OJJ43715.1

Group1937: abra|OJJ69252.1 acar|OOF91069.1 acla|EAW08597.1 afis|EAW15836.1 afla|EED51210.1 afum|EAL85515.1 agla|OJJ82000.1 akaw|GAA92259.1 aluc|OJZ86232.1 anid|CBF70854.1 anig|EHA24397.1 aory|EIT80401.1 asyd|OJJ52573.1 ater|EAU33412.1 atub|OJI80026.1 aver|OJJ04602.1 awen|OJJ38550.1 azon|OJJ43713.1

Group1938: atub|OJI80028.1 abra|OJJ69256.1 acar|OOF91065.1 acla|EAW08593.1 afis|EAW15841.1 afla|EED51208.1 afum|EAL85510.1 agla|OJJ84917.1 akaw|GAA92262.1 aluc|OJZ86236.1 anid|CBF70845.1 anig|EHA24392.1 aory|EIT80413.1 asyd|OJJ52568.1 ater|EAU33394.1 aver|OJJ04597.1 awen|OJJ38555.1 azon|OJJ43711.1

Group1939: abra|OJJ69257.1 acar|OOF91064.1 acla|EAW08592.1 afis|EAW15842.1 afla|EED51207.1 afum|EAL85509.1 agla|OJJ84916.1 akaw|GAA92263.1 aluc|OJZ86237.1 anid|CBF70843.1 anig|EHA24391.1 aory|EIT80396.1 asyd|OJJ52567.1 ater|EAU33393.1 atub|OJI80029.1 aver|OJJ04596.1 awen|OJJ38556.1 azon|OJJ43710.1

Group1940: abra|OJJ69259.1 acar|OOF91063.1 acla|EAW08590.1 afis|EAW15844.1 afla|EED51205.1 afum|EAL85507.1 agla|OJJ84915.1 akaw|GAA92265.1 aluc|OJZ86239.1 anid|CBF70839.1 anig|EHA24390.1 asyd|OJJ52565.1 ater|EAU33391.1 atub|OJI80031.1 aver|OJJ04594.1 awen|OJJ38559.1 azon|OJJ43708.1 awen|OJJ38558.1

Group1941: abra|OJJ69262.1 acar|OOF91060.1 acla|EAW08589.1 afis|EAW15846.1 afla|EED51204.1 afum|EAL85505.1 agla|OJJ84913.1 akaw|GAA92267.1 aluc|OJZ86241.1 anid|CBF70835.1 anig|EHA24388.1 aory|EIT80414.1 asyd|OJJ52564.1 ater|EAU33390.1 atub|OJI80033.1 aver|OJJ04592.1 awen|OJJ38561.1 azon|OJJ48761.1

Group1942: abra|OJJ69263.1 acar|OOF91059.1 acla|EAW08588.1 afis|EAW15847.1 afla|EED51203.1 afum|EAL85504.1 agla|OJJ84911.1 akaw|GAA92268.1 aluc|OJZ86242.1 anid|CBF70833.1 anig|EHA24387.1 aory|EIT80410.1 asyd|OJJ52563.1 ater|EAU33389.1 atub|OJI80034.1 aver|OJJ04591.1 awen|OJJ38562.1 azon|OJJ43615.1

Group1943: abra|OJJ69268.1 acar|OOF89861.1 acla|EAW14653.1 afis|EAW24188.1 afla|EED48081.1 afum|EAL85861.1 agla|OJJ79691.1 akaw|GAA88143.1 aluc|OJZ82097.1 anid|CBF74306.1 anig|EHA28632.1 aory|EIT82476.1 asyd|OJJ54008.1 ater|EAU31140.1 atub|OJI88340.1 aver|OJJ08103.1 awen|OJJ33111.1 azon|OJJ41999.1

Group1944: abra|OJJ69275.1 acar|OOF94447.1 acla|EAW14663.1 afis|EAW24194.1 afla|EED48077.1 afum|EAL85866.1 agla|OJJ79684.1 akaw|GAA88148.1 aluc|OJZ82102.1 anid|CBF74313.1 anig|EHA28627.1 aory|EIT82481.1 asyd|OJJ54002.1 ater|EAU31145.1 atub|OJI88333.1 aver|OJJ08099.1 awen|OJJ33105.1 azon|OJJ44844.1

Group1945: abra|OJJ69276.1 acar|OOF94446.1 acla|EAW14664.1 afis|EAW24195.1 afla|EED48076.1 afum|EAL85867.1 agla|OJJ79683.1 akaw|GAA88149.1 aluc|OJZ82103.1 anid|CBF74315.1 anig|EHA28626.1 aory|EIT82479.1 asyd|OJJ54001.1 ater|EAU31146.1 atub|OJI88332.1 aver|OJJ08098.1 awen|OJJ33104.1 azon|OJJ44845.1

Group1946: abra|OJJ69277.1 acar|OOF94445.1 acla|EAW14665.1 afis|EAW24196.1 afla|EED48075.1 afum|EAL85868.1 agla|OJJ79682.1 akaw|GAA88150.1 aluc|OJZ82104.1 anid|CBF74317.1 anig|EHA28625.1 aory|EIT82482.1 asyd|OJJ54000.1 ater|EAU31147.1 atub|OJI88331.1 aver|OJJ08097.1 awen|OJJ33103.1 azon|OJJ44846.1

Group1947: abra|OJJ69278.1 acar|OOF94444.1 acla|EAW14666.1 afis|EAW24197.1 afla|EED48074.1 afum|EAL85869.1 agla|OJJ79681.1 akaw|GAA88151.1 aluc|OJZ82105.1 anid|CBF74319.1 anig|EHA28624.1 aory|EIT82477.1 asyd|OJJ53999.1 ater|EAU31148.1 atub|OJI88330.1 aver|OJJ08096.1 awen|OJJ33102.1 azon|OJJ44847.1

Group1948: abra|OJJ69279.1 acar|OOF94443.1 acla|EAW14668.1 afis|EAW24199.1 afla|EED48073.1 afum|EAL85871.1 agla|OJJ79678.1 akaw|GAA88152.1 aluc|OJZ82106.1 anid|CBF74323.1 anig|EHA28623.1 aory|EIT83321.1 asyd|OJJ53996.1 ater|EAU31150.1 atub|OJI88329.1 aver|OJJ08094.1 awen|OJJ33099.1 azon|OJJ44848.1

Group1949: abra|OJJ69281.1 acar|OOF94441.1 acla|EAW14670.1 afis|EAW24201.1 afla|EED48071.1 afum|EBA27248.1 agla|OJJ79676.1 akaw|GAA88153.1 aluc|OJZ82109.1 anid|CBF74326.1 anig|EHA28621.1 aory|EIT83320.1 asyd|OJJ53994.1 ater|EAU31152.1 atub|OJI88327.1 aver|OJJ08092.1 awen|OJJ33097.1 azon|OJJ44850.1

Group1950: abra|OJJ69283.1 acar|OOF94439.1 acla|EAW14672.1 afis|EAW24203.1 afla|EED48069.1 afum|EAL85873.2 agla|OJJ79674.1 akaw|GAA88155.1 aluc|OJZ82112.1 anid|CBF74330.1 anig|EHA28619.1 aory|EIT83340.1 asyd|OJJ53992.1 ater|EAU31154.1 atub|OJI88325.1 aver|OJJ08090.1 awen|OJJ33095.1 azon|OJJ44852.1

Group1951: abra|OJJ69284.1 acar|OOF94438.1 acla|EAW14673.1 afis|EAW24204.1 afla|EED48067.1 afum|EAL85874.2 agla|OJJ79673.1 akaw|GAA88156.1 aluc|OJZ82113.1 anid|CBF74332.1 anig|EHA28618.1 aory|EIT83335.1 asyd|OJJ53991.1 ater|EAU31155.1 atub|OJI88324.1 aver|OJJ08089.1 awen|OJJ33094.1 azon|OJJ44854.1

Group1952: abra|OJJ69285.1 acar|OOF94436.1 acla|EAW14674.1 afis|EAW24205.1 afla|EED48066.1 afum|EAL85875.1 agla|OJJ79672.1 akaw|GAA88157.1 aluc|OJZ82114.1 anid|CBF74272.1 anig|EHA28617.1 aory|EIT83356.1 asyd|OJJ53990.1 ater|EAU31156.1 atub|OJI88323.1 aver|OJJ08088.1 awen|OJJ33093.1 azon|OJJ44855.1

Group1953: abra|OJJ69286.1 acar|OOF94437.1 acla|EAW14675.1 afis|EAW24206.1 afla|EED48065.1 afum|EAL85876.1 agla|OJJ79671.1 akaw|GAA88158.1 aluc|OJZ82115.1 anid|CBF74271.1 anig|EHA28616.1 aory|EIT83359.1 asyd|OJJ53989.1 ater|EAU31157.1 atub|OJI88322.1 aver|OJJ08087.1 awen|OJJ33092.1 azon|OJJ44856.1

Group1954: abra|OJJ69288.1 acar|OOF94434.1 acla|EAW14677.1 afis|EAW24208.1 afla|EED48063.1 afum|EAL85878.1 agla|OJJ79669.1 akaw|GAA88160.1 aluc|OJZ82117.1 anid|CBF74269.1 anig|EHA28614.1

aory|EIT83351.1 asyd|OJJ53987.1 ater|EAU31158.1 atub|OJI88320.1 aver|OJJ08085.1 awen|OJJ33089.1 azon|OJJ44858.1

Group1955: abra|OJJ69292.1 acar|OOF94431.1 acla|EAW14680.1 afis|EAW24211.1 afla|EED48060.1 afum|EAL85880.1 agla|OJJ79654.1 akaw|GAA88163.1 aluc|OJZ82121.1 anid|CBF74263.1 anig|EHA28611.1 aory|EIT83350.1 asyd|OJJ53983.1 ater|EAU31161.1 atub|OJI88317.1 aver|OJJ08082.1 awen|OJJ33085.1 azon|OJJ44861.1

Group1956: abra|OJJ69293.1 acar|OOF94430.1 acla|EAW14681.1 afis|EAW24212.1 afla|EED48059.1 afum|EAL85881.1 agla|OJJ79663.1 akaw|GAA88164.1 aluc|OJZ82122.1 anid|CBF74261.1 anig|EHA28610.1 aory|EIT83323.1 asyd|OJJ53982.1 ater|EAU31162.1 atub|OJI88316.1 aver|OJJ08081.1 awen|OJJ33084.1 azon|OJJ44862.1

Group1957: abra|OJJ69294.1 acar|OOF94429.1 acla|EAW14682.1 afis|EAW24213.1 afla|EED48058.1 afum|EAL85882.1 agla|OJJ79665.1 akaw|GAA88165.1 aluc|OJZ82123.1 anid|CBF74260.1 anig|EHA28609.1 aory|EIT83345.1 asyd|OJJ53981.1 ater|EAU31163.1 atub|OJI88315.1 aver|OJJ08080.1 awen|OJJ33083.1 azon|OJJ44863.1

Group1958: abra|OJJ69296.1 acar|OOF94428.1 acla|EAW14683.1 afis|EAW24214.1 afla|EED48057.1 afum|EAL85883.1 agla|OJJ79666.1 akaw|GAA88166.1 aluc|OJZ82125.1 anid|CBF74258.1 anig|EHA28607.1 aory|EIT83357.1 asyd|OJJ53980.1 ater|EAU31164.1 atub|OJI88313.1 aver|OJJ08079.1 awen|OJJ33082.1 azon|OJJ44864.1

Group1959: abra|OJJ69297.1 acar|OOF94427.1 acla|EAW14684.1 afis|EAW24215.1 afla|EED48056.1 afum|EAL85884.1 agla|OJJ79667.1 akaw|GAA88167.1 aluc|OJZ82126.1 anid|CBF74256.1 anig|EHA28606.1 aory|EIT83352.1 asyd|OJJ53979.1 ater|EAU31165.1 atub|OJI88312.1 aver|OJJ08078.1 awen|OJJ33081.1 azon|OJJ44865.1

Group1960: abra|OJJ69298.1 acar|OOF94425.1 acla|EAW14685.1 afis|EAW24216.1 afla|EED48055.1 afum|EAL85885.1 agla|OJJ79668.1 akaw|GAA88168.1 aluc|OJZ82127.1 anid|CBF74254.1 anig|EHA28605.1 aory|EIT83339.1 asyd|OJJ53978.1 ater|EAU31166.1 atub|OJI88311.1 aver|OJJ08077.1 awen|OJJ33080.1 azon|OJJ44866.1

Group1961: abra|OJJ69299.1 acar|OOF94426.1 acla|EAW14686.1 afis|EAW24217.1 afla|EED48054.1 afum|EAL85886.1 agla|OJJ79653.1 akaw|GAA88169.1 aluc|OJZ82128.1 anid|CBF74252.1 anig|EHA28603.1 aory|EIT83360.1 asyd|OJJ53977.1 ater|EAU31167.1 atub|OJI88310.1 aver|OJJ08076.1 awen|OJJ33079.1 azon|OJJ44867.1

Group1962: abra|OJJ69302.1 acar|OOF94423.1 acla|EAW14688.1 afis|EAW24219.1 afla|EED48052.1 afum|EAL85888.1 agla|OJJ79651.1 akaw|GAA88171.1 aluc|OJZ82130.1 anid|CBF74248.1 anig|EHA28601.1 aory|EIT83354.1 asyd|OJJ53975.1 ater|EAU31169.1 atub|OJI88306.1 aver|OJJ08074.1 awen|OJJ33077.1 azon|OJJ44869.1

Group1963: abra|OJJ69305.1 acar|OOF94420.1 acla|EAW14691.1 afis|EAW24222.1 afla|EED48050.1 afum|EAL85891.2 agla|OJJ79648.1 akaw|GAA88174.1 aluc|OJZ82134.1 anid|CBF74239.1 anig|EHA28597.1 aory|EIT83349.1 asyd|OJJ53971.1 ater|EAU31172.1 atub|OJI88303.1 aver|OJJ08070.1 awen|OJJ33074.1 azon|OJJ44873.1

Group1964: abra|OJJ69307.1 acar|OOF94419.1 acla|EAW14694.1 afis|EAW24225.1 afla|EED48046.1 afum|EAL85893.1 agla|OJJ79646.1 akaw|GAA88176.1 aluc|OJZ82136.1 anid|CBF74234.1 anig|EHA28595.1 aory|EIT83343.1 asyd|OJJ53969.1 ater|EAU31174.1 atub|OJI88301.1 aver|OJJ08067.1 awen|OJJ33071.1 azon|OJJ44875.1

Group1965: abra|OJJ69308.1 acar|OOF94418.1 acla|EAW14695.1 afis|EAW24226.1 afla|EED48045.1 afum|EAL85894.1 agla|OJJ79645.1 akaw|GAA88177.1 aluc|OJZ82137.1 anid|CBF74232.1 anig|EHA28594.1 aory|EIT83341.1 asyd|OJJ53968.1 ater|EAU31175.1 atub|OJI88300.1 aver|OJJ08066.1 awen|OJJ33070.1 azon|OJJ44877.1

Group1966: abra|OJJ69312.1 acar|OOF94415.1 acla|EAW14738.1 afis|EAW24269.1 afla|EED48008.1 afum|EAL85937.1 agla|OJJ79596.1 akaw|GAA88181.1 aluc|OJZ82141.1 anid|CBF74167.1 anig|EHA28591.1 aory|EIT82915.1 asyd|OJJ53936.1 ater|EAU31214.1 atub|OJI88296.1 aver|OJJ08034.1 awen|OJJ33031.1 azon|OJJ48488.1

Group1967: abra|OJJ69313.1 acar|OOF94413.1 acla|EAW14737.1 afis|EAW24268.1 afla|EED48009.1 afum|EAL85936.1 agla|OJJ79597.1 akaw|GAA88182.1 aluc|OJZ82142.1 anid|CBF74169.1 anig|EHA28589.1 aory|EIT82903.1 asyd|OJJ53937.1 ater|EAU31213.1 atub|OJI88295.1 aver|OJJ08035.1 awen|OJJ33032.1 azon|OJJ48487.1

Group1968: abra|OJJ69314.1 acar|OOF94412.1 acla|EAW14736.1 afis|EAW24267.1 afla|EED48010.1 afum|EAL85935.1 agla|OJJ79598.1 akaw|GAA88183.1 aluc|OJZ82143.1 anid|CBF74171.1 anig|EHA28588.1 aory|EIT82909.1 asyd|OJJ53938.1 ater|EAU31212.1 atub|OJI88294.1 aver|OJJ08036.1 awen|OJJ33033.1 azon|OJJ48486.1

Group1969: abra|OJJ69315.1 acar|OOF90302.1 acla|EAW14734.1 afis|EAW24266.1 afla|EED48011.1 afum|EAL85934.1 agla|OJJ79606.1 akaw|GAA88184.1 aluc|OJZ82144.1 anid|CBF74173.1 anig|EHA28586.1 aory|EIT82916.1 asyd|OJJ53940.1 ater|EAU31210.1 atub|OJI88292.1 aver|OJJ08037.1 awen|OJJ33034.1 azon|OJJ48485.1

Group1970: abra|OJJ69316.1 acar|OOF90303.1 acla|EAW14732.1 afis|EAW24265.1 afla|EED48012.1 afum|EAL85933.1 agla|OJJ79607.1 akaw|GAA88185.1 aluc|OJZ82145.1 anid|CBF74175.1 anig|EHA28585.1 aory|EIT82905.1 asyd|OJJ53941.1 ater|EAU31209.1 atub|OJI88291.1 aver|OJJ08038.1 awen|OJJ33035.1 azon|OJJ48484.1

Group1971: abra|OJJ69319.1 acar|OOF94411.1 acla|EAW14726.1 afis|EAW24262.1 akaw|GAA88188.1 aluc|OJZ82148.1 ater|EAU31207.1 atub|OJI88287.1 aver|OJJ08043.1 awen|OJJ33039.1 azon|OJJ48479.1 afum|EAL85930.1 agla|OJJ79610.1 anid|CBF74186.1 anig|EHA28582.1 aory|EIT82904.1 asyd|OJJ53946.1 afla|EED48015.1

Group1972: abra|OJJ69329.1 acar|OOF94398.1 acla|EAW14711.1 afis|EAW24246.1 afla|EED48028.1 afum|EAL85914.1 agla|OJJ79627.1 akaw|GAA88200.1 aluc|OJZ82161.1 anid|CBF74210.1 anig|EHA28571.1 aory|EIT83355.1 asyd|OJJ53956.1 ater|EAU31187.1 atub|OJI88276.1 aver|OJJ08052.1 awen|OJJ33058.1 azon|OJJ48466.1

Group1973: abra|OJJ69332.1 acar|OOF94395.1 acla|EAW14708.1 afis|EAW24239.1 afla|EED48031.1 afum|EAL85907.1 agla|OJJ79630.1 akaw|GAA88203.1 aluc|OJZ82164.1 anid|CBF74213.1 anig|EHA28568.1 aory|EIT83322.1 asyd|OJJ53957.1 ater|EAU31186.1 atub|OJI88273.1 aver|OJJ08053.1 awen|OJJ33054.1 azon|OJJ48465.1

Group1974: anig|EHA28560.1 abra|OJJ69342.1 acar|OOF94385.1 acla|EAW14704.1 afis|EAW24235.1 afla|EED48036.1 afum|EAL85904.1 agla|OJJ79636.1 akaw|GAA88211.1 aluc|OJZ82174.1 anid|CBF74219.1 aory|EIT83346.1 asyd|OJJ53961.1 ater|EAU31183.1 atub|OJI88263.1 aver|OJJ08057.1 awen|OJJ33063.1 azon|OJJ48461.1

Group1975: abra|OJJ69343.1 acar|OOF94386.1 acla|EAW14703.1 afis|EAW24234.1 afla|EED48037.1 afum|EAL85903.1 agla|OJJ79637.1 akaw|GAA88212.1 aluc|OJZ82175.1 anid|CBF74221.1 anig|EHA28559.1 aory|EIT83326.1 asyd|OJJ53962.1 ater|EAU31182.1 atub|OJI88262.1 aver|OJJ08058.1 awen|OJJ33064.1 azon|OJJ48460.1

Group1976: abra|OJJ69345.1 acar|OOF94379.1 acla|EAW14701.1 afis|EAW24232.1 afla|EED48039.1 afum|EAL85901.2 agla|OJJ79639.1 akaw|GAA88214.1 aluc|OJZ82177.1 anid|CBF74223.1 anig|EHA28557.1 aory|EIT83353.1 asyd|OJJ53964.1 ater|EAU31180.1 atub|OJI88260.1 aver|OJJ08060.1 awen|OJJ33065.1 azon|OJJ48458.1

Group1977: abra|OJJ69346.1 acar|OOF94378.1 acla|EAW14700.1 afis|EAW24231.1 afla|EED48040.1 afum|EAL85900.1 agla|OJJ79640.1 akaw|GAA88215.1 aluc|OJZ82178.1 anid|CBF74224.1 anig|EHA28556.1 aory|EIT83342.1 asyd|OJJ53965.1 ater|EAU31179.1 atub|OJI88259.1 aver|OJJ08061.1 awen|OJJ33066.1 azon|OJJ48457.1

Group1978: abra|OJJ69349.1 acar|OOF94376.1 acla|EAW14739.1 afis|EAW24270.1 afla|EED48007.1 afum|EAL85938.1 agla|OJJ79595.1 akaw|GAA88219.1 aluc|OJZ82181.1 anid|CBF74165.1 anig|EHA28553.1 aory|EIT82896.1 asyd|OJJ53935.1 ater|EAU31215.1 atub|OJI88254.1 aver|OJJ08033.1 awen|OJJ33030.1 azon|OJJ48489.1

Group1979: abra|OJJ69351.1 acar|OOF94375.1 acla|EAW14740.1 afis|EAW24271.1 afla|EED48006.1 afum|EAL85939.1 agla|OJJ79594.1 akaw|GAA88220.1 aluc|OJZ82183.1 anid|CBF74163.1 anig|EHA28552.1 aory|EIT82918.1 asyd|OJJ53933.1 ater|EAU31216.1 atub|OJI88253.1 aver|OJJ08031.1 awen|OJJ33028.1 azon|OJJ48490.1

Group1980: abra|OJJ69353.1 acar|OOF94372.1 acla|EAW14743.1 afis|EAW24274.1 afla|EED48002.1 afum|EAL85942.1 agla|OJJ79587.1 akaw|GAA88222.1 aluc|OJZ82186.1 anid|CBF74158.1 anig|EHA28550.1 aory|EIT82912.1 asyd|OJJ53927.1 ater|EAU31221.1 atub|OJI88251.1 aver|OJJ08026.1 awen|OJJ33023.1 azon|OJJ48494.1

Group1981: abra|OJJ69355.1 acar|OOF94370.1 acla|EAW14745.1 afis|EAW24276.1 afla|EED48000.1 afum|EAL85945.1 agla|OJJ79585.1 akaw|GAA88224.1 aluc|OJZ82187.1 anid|CBF74156.1 anig|EHA28548.1 aory|EIT82907.1 asyd|OJJ53925.1 ater|EAU31219.1 atub|OJI88249.1 aver|OJJ08024.1 awen|OJJ33021.1 azon|OJJ48496.1

Group1982: abra|OJJ69357.1 acar|OOF94367.1 acla|EAW14748.1 afis|EAW24279.1 afla|EED47997.1 afum|EAL85948.1 agla|OJJ80633.1 akaw|GAA88227.1 aluc|OJZ82189.1 anid|CBF74151.1 anig|EHA28546.1 aory|EIT82902.1 asyd|OJJ53923.1 ater|EAU31228.1 atub|OJI88246.1 aver|OJJ08021.1 awen|OJJ33018.1 azon|OJJ48499.1

Group1983: abra|OJJ69366.1 acar|OOF94359.1 acla|EAW14756.1 afis|EAW24287.1 afla|EED47989.1 afum|EAL85956.1 agla|OJJ83152.1 akaw|GAA88235.1 aluc|OJZ82200.1 anid|CBF74129.1 anig|EHA28539.1

aory|EIT79810.1 asyd|OJJ53912.1 ater|EAU31238.1 atub|OJI88236.1 aver|OJJ08010.1 awen|OJJ33009.1 azon|OJJ48506.1

Group1984: abra|OJJ69367.1 acar|OOF94358.1 acla|EAW14757.1 afis|EAW24288.1 afla|EED47988.1 afum|EAL85957.1 agla|OJJ83153.1 akaw|GAA88236.1 aluc|OJZ82201.1 anid|CBF74127.1 anig|EHA28538.1 aory|EIT79844.1 asyd|OJJ53911.1 ater|EAU31239.1 atub|OJI88235.1 aver|OJJ08009.1 awen|OJJ33008.1 azon|OJJ48507.1

Group1985: abra|OJJ69369.1 acar|OOF89837.1 acla|EAW14759.1 afis|EAW24290.1 afla|EED47986.1 afum|EAL85958.1 agla|OJJ83155.1 akaw|GAA88238.1 aluc|OJZ82203.1 anid|CBF74125.1 anig|EHA28536.1 aory|EIT79849.1 asyd|OJJ53909.1 ater|EAU31240.1 atub|OJI88233.1 aver|OJJ08007.1 awen|OJJ33006.1 azon|OJJ48509.1

Group1986: abra|OJJ69370.1 acar|OOF94357.1 acla|EAW14760.1 afis|EAW24291.1 afla|EED47985.1 afum|EAL85959.1 agla|OJJ83156.1 akaw|GAA88239.1 aluc|OJZ82204.1 anid|CBF74123.1 anig|EHA28535.1 aory|EIT79854.1 asyd|OJJ53908.1 ater|EAU31241.1 atub|OJI88232.1 aver|OJJ08006.1 awen|OJJ33005.1 azon|OJJ48510.1

Group1987: abra|OJJ69371.1 acar|OOF94355.1 acla|EAW14761.1 afis|EAW24292.1 afla|EED47984.1 afum|EAL85960.1 agla|OJJ83157.1 akaw|GAA88240.1 aluc|OJZ82205.1 anid|CBF74121.1 anig|EHA28534.1 aory|EIT79808.1 asyd|OJJ53907.1 ater|EAU31242.1 atub|OJI88231.1 aver|OJJ08005.1 awen|OJJ33004.1 azon|OJJ48511.1

Group1988: abra|OJJ69373.1 acar|OOF94354.1 acla|EAW14763.1 afis|EAW24294.1 afla|EED47982.1 afum|EAL85962.1 agla|OJJ83159.1 akaw|GAA88242.1 aluc|OJZ82208.1 anid|CBF74118.1 anig|EHA28532.1 aory|EIT79845.1 asyd|OJJ53905.1 ater|EAU31244.1 atub|OJI88228.1 aver|OJJ08003.1 awen|OJJ33001.1 azon|OJJ48513.1

Group1989: abra|OJJ69375.1 acar|OOF94351.1 acla|EAW14765.1 afis|EAW24296.1 afla|EED47980.1 afum|EAL85964.1 agla|OJJ83161.1 akaw|GAA88244.1 aluc|OJZ82210.1 anid|CBF75402.1 anig|EHA28530.1 aory|EIT79827.1 asyd|OJJ59822.1 ater|EAU31246.1 atub|OJI88226.1 aver|OJJ02465.1 awen|OJJ32998.1 azon|OJJ48514.1

Group1990: abra|OJJ69376.1 acar|OOF94349.1 acla|EAW14766.1 afis|EAW24297.1 afla|EED47979.1 afum|EAL85965.1 agla|OJJ83162.1 akaw|GAA88245.1 aluc|OJZ82211.1 anid|CBF74116.1 anig|EHA28529.1 aory|EIT79833.1 asyd|OJJ53904.1 ater|EAU31248.1 atub|OJI88225.1 aver|OJJ08001.1 awen|OJJ32997.1 azon|OJJ48515.1

Group1991: abra|OJJ69381.1 acar|OOF94344.1 acla|EAW14768.1 afis|EAW24299.1 afla|EED47973.1 afum|EAL85967.1 agla|OJJ83167.1 akaw|GAA88249.1 aluc|OJZ82216.1 anid|CBF74108.1 anig|EHA28524.1 aory|EIT79831.1 asyd|OJJ53901.1 ater|EAU31250.1 atub|OJI88219.1 aver|OJJ07998.1 awen|OJJ32993.1 azon|OJJ48523.1

Group1992: abra|OJJ69383.1 acar|OOF94343.1 acla|EAW14770.1 afis|EAW24301.1 afla|EED47971.1 afum|EAL85969.1 agla|OJJ83169.1 akaw|GAA88251.1 aluc|OJZ82217.1 anid|CBF74104.1 anig|EHA28523.1 aory|EIT79840.1 asyd|OJJ53899.1 ater|EAU31252.1 atub|OJI88218.1 aver|OJJ07996.1 awen|OJJ32991.1 azon|OJJ48525.1

Group1993: abra|OJJ69384.1 acar|OOF94342.1 acla|EAW14771.1 afis|EAW24302.1 afla|EED47970.1 afum|EAL85970.1 agla|OJJ83170.1 akaw|GAA88252.1 aluc|OJZ82218.1 anid|CBF74102.1 anig|EHA28522.1 aory|EIT79832.1 asyd|OJJ53898.1 ater|EAU31253.1 atub|OJI88217.1 aver|OJJ07995.1 awen|OJJ32990.1 azon|OJJ45840.1

Group1994: abra|OJJ69385.1 acar|OOF94341.1 acla|EAW14772.1 afis|EAW24303.1 afla|EED47969.1 afum|EAL85971.1 agla|OJJ83171.1 akaw|GAA88253.1 aluc|OJZ82219.1 anid|CBF74100.1 anig|EHA28521.1 aory|EIT79817.1 asyd|OJJ53897.1 ater|EAU31256.1 atub|OJI88216.1 aver|OJJ07994.1 awen|OJJ32989.1 azon|OJJ48526.1

Group1995: abra|OJJ69386.1 acar|OOF94340.1 acla|EAW14773.1 afis|EAW24304.1 afla|EED47968.1 afum|EAL85972.1 agla|OJJ83172.1 akaw|GAA88254.1 aluc|OJZ82220.1 anid|CBF79548.1 anig|EHA28520.1 aory|EIT79824.1 asyd|OJJ62162.1 ater|EAU31257.1 atub|OJI88215.1 aver|OJI96272.1 awen|OJJ32988.1 azon|OJJ48527.1

Group1996: abra|OJJ69387.1 acar|OOF94339.1 acla|EAW14774.1 afis|EAW24305.1 afla|EED47965.1 afum|EAL85973.1 agla|OJJ83173.1 akaw|GAA88255.1 aluc|OJZ82221.1 anid|CBF74098.1 anig|EHA28519.1 aory|EIT79856.1 asyd|OJJ53896.1 ater|EAU31258.1 atub|OJI88214.1 aver|OJJ07993.1 awen|OJJ32987.1 azon|OJJ48932.1

Group1997: abra|OJJ69398.1 acar|OOF94332.1 acla|EAW14780.1 afis|EAW24311.1 afla|EED47956.1 afum|EAL85979.1 agla|OJJ83179.1 akaw|GAA88264.1 aluc|OJZ82234.1 anid|CBF74081.1 anig|EHA28507.1 aory|EIT79851.1 asyd|OJJ53882.1 ater|EAU31264.1 atub|OJI88204.1 aver|OJJ07980.1 awen|OJJ32981.1 azon|OJJ48936.1

Group1998: abra|OJJ69400.1 acar|OOF94330.1 acla|EAW14782.1 afis|EAW24313.1 afla|EED47954.1 afum|EAL85981.1 agla|OJJ83181.1 akaw|GAA88266.1 aluc|OJZ82236.1 anid|CBF74079.1 anig|EHA28505.1 aory|EIT79862.1 asyd|OJJ53880.1 ater|EAU31266.1 atub|OJI88202.1 aver|OJJ07978.1 awen|OJJ32979.1 azon|OJJ48938.1

Group1999: abra|OJJ69402.1 acar|OOF94329.1 acla|EAW14784.1 afis|EAW24315.1 afla|EED47952.1 afum|EAL85983.1 agla|OJJ83183.1 akaw|GAA88267.1 aluc|OJZ82238.1 anid|CBF74078.1 anig|EHA28503.1 aory|EIT79841.1 asyd|OJJ53878.1 ater|EAU31267.1 atub|OJI88200.1 aver|OJJ07976.1 awen|OJJ32977.1 azon|OJJ48940.1

Group2000: abra|OJJ69404.1 acar|OOF94327.1 acla|EAW14786.1 afis|EAW24317.1 afla|EED47950.1 afum|EAL85985.1 agla|OJJ83185.1 akaw|GAA88269.1 aluc|OJZ82240.1 anid|CBF74074.1 anig|EHA28501.1 aory|EIT79859.1 asyd|OJJ53876.1 ater|EAU31270.1 atub|OJI88198.1 aver|OJJ07974.1 awen|OJJ32975.1 azon|OJJ48942.1

Group2001: abra|OJJ69405.1 acar|OOF94326.1 acla|EAW14787.1 afis|EAW24318.1 afla|EED47949.1 afum|EAL85987.1 agla|OJJ83186.1 akaw|GAA88270.1 aluc|OJZ82242.1 anid|CBF74073.1 anig|EHA28500.1 aory|EIT79816.1 asyd|OJJ53875.1 ater|EAU31271.1 atub|OJI88196.1 aver|OJJ07973.1 awen|OJJ32974.1 azon|OJJ48943.1

Group2002: abra|OJJ69410.1 acar|OOF94323.1 afla|EED47946.1 afum|EAL85990.1 akaw|GAA88277.1 aluc|OJZ82247.1 anid|CBF74069.1 anig|EHA28494.1 aory|EIT79850.1 asyd|OJJ53873.1 ater|EAU31273.1 atub|OJI88191.1 aver|OJJ07971.1 azon|OJJ48945.1 acla|EAW14789.1 afis|EAW24320.1 agla|OJJ83189.1 awen|OJJ32972.1

Group2003: abra|OJJ69411.1 acar|OOF94322.1 acla|EAW14790.1 afis|EAW24321.1 afla|EED47945.1 afum|EAL85991.1 agla|OJJ83190.1 akaw|GAA88278.1 aluc|OJZ82248.1 anid|CBF74068.1 anig|EHA28493.1 aory|EIT79836.1 asyd|OJJ53872.1 ater|EAU31274.1 atub|OJI88190.1 aver|OJJ07970.1 awen|OJJ32971.1 azon|OJJ48946.1

Group2004: abra|OJJ69412.1 acar|OOF94321.1 acla|EAW14791.1 afis|EAW24322.1 afla|EED47944.1 afum|EAL85992.1 agla|OJJ83191.1 akaw|GAA88279.1 aluc|OJZ82249.1 anid|CBF74066.1 anig|EHA28492.1 aory|EIT79820.1 asyd|OJJ53871.1 ater|EAU30430.1 atub|OJI88189.1 aver|OJJ07969.1 awen|OJJ32970.1 azon|OJJ48947.1

Group2005: abra|OJJ69413.1 acar|OOF94320.1 acla|EAW14793.1 afis|EAW24324.1 afla|EED47942.1 afum|EAL85994.1 akaw|GAA88281.1 aluc|OJZ82250.1 anid|CBF74060.1 anig|EHA28491.1 aory|EIT79823.1 asyd|OJJ53870.1 atub|OJI88187.1 aver|OJJ07968.1 awen|OJJ33577.1 azon|OJJ42851.1 afla|EED51658.1 aory|EIT80622.1

Group2006: abra|OJJ69414.1 acar|OOF94318.1 acla|EAW14795.1 afis|EAW24326.1 afla|EED47940.1 afum|EAL85996.1 agla|OJJ83193.1 akaw|GAA88282.1 aluc|OJZ82251.1 anid|CBF74056.1 anig|EHA28490.1 aory|EIT79834.1 asyd|OJJ53868.1 ater|EAU30427.1 atub|OJI88186.1 aver|OJJ07966.1 awen|OJJ32968.1 azon|OJJ48949.1

Group2007: abra|OJJ69415.1 acar|OOF94317.1 acla|EAW14796.1 afis|EAW24327.1 afla|EED47939.1 afum|EAL85997.1 agla|OJJ83194.1 akaw|GAA88283.1 aluc|OJZ82252.1 anid|CBF74054.1 anig|EHA28489.1 aory|EIT79852.1 asyd|OJJ53867.1 ater|EAU30426.1 atub|OJI88185.1 aver|OJJ07965.1 awen|OJJ32967.1 azon|OJJ48950.1

Group2008: abra|OJJ69417.1 acar|OOF94315.1 acla|EAW14798.1 afis|EAW24329.1 afla|EED47937.1 afum|EAL85999.1 agla|OJJ83196.1 akaw|GAA88285.1 aluc|OJZ82254.1 anid|CBF74052.1 anig|EHA28487.1 aory|EIT79861.1 asyd|OJJ53865.1 atub|OJI88183.1 aver|OJJ07963.1 awen|OJJ32965.1 azon|OJJ48953.1 ater|EAU30424.1

Group2009: abra|OJJ69419.1 acar|OOF90227.1 acla|EAW14799.1 afis|EAW24330.1 afla|EED47935.1 afum|EAL86000.1 agla|OJJ83197.1 akaw|GAA88286.1 aluc|OJZ82255.1 anid|CBF74050.1 anig|EHA28486.1 aory|EIT79863.1 asyd|OJJ53863.1 ater|EAU30422.1 atub|OJI88182.1 aver|OJJ07962.1 awen|OJJ32964.1 azon|OJJ48954.1

Group2010: abra|OJJ69420.1 acar|OOF90228.1 acla|EAW14800.1 afis|EAW24331.1 afla|EED47933.1 afum|EAL86001.1 agla|OJJ83198.1 akaw|GAA88287.1 aluc|OJZ82256.1 anid|CBF74048.1 anig|EHA28485.1 aory|EIT79848.1 asyd|OJJ53862.1 ater|EAU30418.1 atub|OJI88181.1 aver|OJJ07961.1 awen|OJJ32963.1 azon|OJJ48955.1

Group2011: abra|OJJ69421.1 acar|OOF90226.1 acla|EAW14801.1 afis|EAW24332.1 afla|EED47932.1 afum|EAL86002.1 agla|OJJ83199.1 akaw|GAA88288.1 aluc|OJZ82258.1 anid|CBF74045.1 anig|EHA28484.1 aory|EIT79839.1 asyd|OJJ53857.1 ater|EAU30417.1 atub|OJI88180.1 aver|OJJ07958.1 awen|OJJ32962.1 azon|OJJ48960.1

Group2012: abra|OJJ69423.1 acar|OOF94313.1 acla|EAW14802.1 afis|EAW24333.1 afla|EED47931.1 afum|EAL86003.1 agla|OJJ83202.1 akaw|GAA88289.1 aluc|OJZ82259.1 anid|CBF74039.1 anig|EHA28483.1

aory|EIT79843.1 asyd|OJJ53859.1 ater|EAU30416.1 atub|OJI88179.1 aver|OJJ07959.1 awen|OJJ32961.1 azon|OJJ48959.1

Group2013: abra|OJJ69446.1 acar|OOF94294.1 acla|EAW14811.1 afis|EAW24341.1 afla|EED47922.1 afum|EAL86012.1 agla|OJJ83224.1 akaw|GAA88372.1 aluc|OJZ82282.1 anid|CBF74027.1 anig|EHA28465.1 aory|EIT83372.1 asyd|OJJ53846.1 ater|EAU30406.1 atub|OJI88157.1 aver|OJJ07948.1 awen|OJJ32950.1 azon|OJJ48970.1

Group2014: abra|OJJ69448.1 acar|OOF94293.1 acla|EAW14812.1 afis|EAW24342.1 afla|EED47920.1 afum|EAL86013.1 agla|OJJ83223.1 akaw|GAA88371.1 aluc|OJZ82283.1 anid|CBF74025.1 anig|EHA28464.1 aory|EIT83362.1 asyd|OJJ53845.1 ater|EAU30404.1 atub|OJI88155.1 aver|OJJ07947.1 awen|OJJ32951.1 azon|OJJ48969.1

Group2015: abra|OJJ69449.1 acar|OOF94292.1 acla|EAW14813.1 afis|EAW24343.1 afla|EED47919.1 afum|EAL86014.1 agla|OJJ83210.1 akaw|GAA88370.1 aluc|OJZ82285.1 anid|CBF74028.1 anig|EHA28463.1 aory|EIT83364.1 asyd|OJJ53847.1 ater|EAU30403.1 atub|OJI88154.1 aver|OJJ07949.1 awen|OJJ32952.1 azon|OJJ48968.1

Group2016: abra|OJJ69453.1 acar|OOF94288.1 acla|EAW14817.1 afis|EAW24347.1 afla|EED47916.1 afum|EAL86017.1 agla|OJJ83206.1 akaw|GAA88359.1 aluc|OJZ82296.1 anid|CBF74017.1 anig|EHA28459.1 aory|EIT83378.1 asyd|OJJ53840.1 ater|EAU30399.1 atub|OJI88143.1 aver|OJJ07943.1 awen|OJJ32956.1 azon|OJJ48964.1

Group2017: abra|OJJ69456.1 acar|OOF94285.1 acla|EAW14821.1 afis|EAW24349.1 afla|EED47914.1 afum|EAL86019.2 agla|OJJ81762.1 akaw|GAA88356.1 aluc|OJZ82299.1 anid|CBF76155.1 anig|EHA28456.1 aory|EIT83376.1 asyd|OJJ52360.1 ater|EAU30395.1 atub|OJI88140.1 aver|OJJ01988.1 awen|OJJ32875.1 azon|OJJ48976.1

Group2018: abra|OJJ69457.1 acar|OOF94284.1 acla|EAW14820.1 afis|EAW24350.1 afla|EED47913.1 afum|EAL86020.2 agla|OJJ81761.1 akaw|GAA88355.1 aluc|OJZ82300.1 anid|CBF76157.1 anig|EHA28455.1 aory|EIT83375.1 asyd|OJJ52359.1 ater|EAU30394.1 atub|OJI88139.1 aver|OJJ01990.1 awen|OJJ32874.1 azon|OJJ48975.1

Group2019: abra|OJJ69458.1 acar|OOF94283.1 acla|EAW14819.1 afis|EAW24351.1 afla|EED47912.1 afum|EAL86021.1 agla|OJJ81760.1 akaw|GAA88354.1 aluc|OJZ82301.1 anid|CBF76159.1 anig|EHA28454.1 aory|EIT83371.1 asyd|OJJ52358.1 ater|EAU30393.1 atub|OJI88137.1 aver|OJJ01991.1 awen|OJJ32873.1 azon|OJJ48974.1

Group2020: abra|OJJ69459.1 acar|OOF94282.1 acla|EAW09925.1 afis|EAW21173.1 afla|EED54758.1 afum|EAL92193.1 agla|OJJ80005.1 akaw|GAA88353.1 aluc|OJZ82302.1 anid|CBF83317.1 anig|EHA28453.1 aory|EIT79382.1 asyd|OJJ60559.1 ater|EAU35840.1 atub|OJI88135.1 aver|OJI99885.1 awen|OJJ36511.1 azon|OJJ44387.1

Group2021: abra|OJJ69463.1 acar|OOF94278.1 acla|EAW09922.1 afis|EAW21170.1 afla|EED54735.1 afum|EAL92189.2 agla|OJJ80010.1 akaw|GAA88349.1 aluc|OJZ82306.1 anid|CBF83232.1 anig|EHA28450.1 aory|EIT78745.1 asyd|OJJ60541.1 ater|EAU35837.1 atub|OJI88132.1 aver|OJI99868.1 awen|OJJ36507.1 azon|OJJ44379.1

Group2022: abra|OJJ69467.1 acar|OOF94275.1 acla|EAW09920.1 afis|EAW21168.1 afla|EED54732.1 afum|EAL92187.1 agla|OJJ80012.1 akaw|GAA88346.1 aluc|OJZ82311.1 anid|CBF86960.1 anig|EHA28447.1 aory|EIT78891.1 asyd|OJJ60539.1 ater|EAU35835.1 atub|OJI88129.1 aver|OJI99866.1 awen|OJJ36505.1 azon|OJJ44377.1

Group2023: abra|OJJ69468.1 acar|OOF94274.1 acla|EAW09919.1 afis|EAW21167.1 afla|EED54731.1 afum|EAL92186.1 agla|OJJ80013.1 akaw|GAA88345.1 aluc|OJZ82312.1 anid|CBF86961.1 anig|EHA28446.1 aory|EIT78775.1 asyd|OJJ60537.1 ater|EAU35834.1 atub|OJI88128.1 aver|OJI99864.1 awen|OJJ36504.1 azon|OJJ44376.1

Group2024: abra|OJJ69469.1 acar|OOF94273.1 acla|EAW09918.1 afis|EAW21166.1 afla|EED54730.1 afum|EAL92185.1 agla|OJJ80015.1 akaw|GAA88344.1 aluc|OJZ82313.1 anid|CBF86963.1 anig|EHA28445.1 aory|EIT78843.1 asyd|OJJ60536.1 ater|EAU35833.1 atub|OJI88127.1 aver|OJI99863.1 awen|OJJ36503.1 azon|OJJ44375.1

Group2025: abra|OJJ69471.1 acar|OOF94272.1 acla|EAW09917.1 afis|EAW21165.1 afla|EED54729.1 afum|EAL92184.1 agla|OJJ80016.1 akaw|GAA88343.1 aluc|OJZ82314.1 anid|CBF86964.1 anig|EHA28444.1 aory|EIT78934.1 asyd|OJJ60535.1 ater|EAU35832.1 atub|OJI88126.1 aver|OJI99862.1 awen|OJJ36500.1 azon|OJJ44374.1

Group2026: abra|OJJ69473.1 acar|OOF94270.1 acla|EAW09915.1 afis|EAW21163.1 afla|EED54726.1 afum|EAL92182.1 agla|OJJ80020.1 akaw|GAA88342.1 aluc|OJZ82316.1 anid|CBF86968.1 anig|EHA28442.1 aory|EIT78844.1 asyd|OJJ60532.1 ater|EAU35829.1 atub|OJI88125.1 aver|OJI99859.1 awen|OJJ36497.1 azon|OJJ44372.1

Group2027: abra|OJJ69474.1 acar|OOF94268.1 acla|EAW09914.1 afis|EAW21162.1 afla|EED54725.1 afum|EAL92181.1 agla|OJJ80021.1 akaw|GAA88341.1 aluc|OJZ82317.1 anid|CBF86970.1 anig|EHA28441.1 aory|EIT78833.1 asyd|OJJ60531.1 ater|EAU35828.1 atub|OJI88124.1 aver|OJI99858.1 awen|OJJ36496.1 azon|OJJ44371.1

Group2028: abra|OJJ69475.1 acar|OOF94269.1 acla|EAW09913.1 afis|EAW21161.1 afla|EED54724.1 afum|EAL92180.1 agla|OJJ80022.1 akaw|GAA88340.1 aluc|OJZ82318.1 anid|CBF86972.1 anig|EHA28440.1 aory|EIT78951.1 asyd|OJJ60530.1 ater|EAU35827.1 atub|OJI88123.1 aver|OJI99857.1 awen|OJJ36495.1 azon|OJJ44370.1

Group2029: abra|OJJ69477.1 acar|OOF94265.1 acla|EAW09911.1 afis|EAW21160.1 afla|EED54649.1 afum|EAL92178.1 agla|OJJ80028.1 akaw|GAA88338.1 aluc|OJZ82320.1 anid|CBF86977.1 anig|EHA28438.1 aory|EIT78896.1 asyd|OJJ60527.1 ater|EAU35822.1 atub|OJI88120.1 aver|OJI99855.1 awen|OJJ36492.1 azon|OJJ44365.1

Group2030: abra|OJJ69481.1 acar|OOF94263.1 acla|EAW09908.1 akaw|GAA88335.1 aluc|OJZ82323.1 anig|EHA28435.1 atub|OJI88117.1 awen|OJJ36488.1 agla|OJJ80036.1 afis|EAW21157.1 afum|EAL92175.1 afla|EED54653.1 aory|EIT78820.1 ater|EAU35819.1 anid|CBF86982.1 asyd|OJJ60523.1 aver|OJI99847.1 azon|OJJ44359.1

Group2031: abra|OJJ69483.1 acar|OOF94262.1 acla|EAW09907.1 afis|EAW21156.1 afla|EED54652.1 afum|EAL92174.1 agla|OJJ80037.1 akaw|GAA88334.1 aluc|OJZ82324.1 anid|CBF86984.1 anig|EHA28434.1 aory|EIT78871.1 asyd|OJJ60518.1 ater|EAU35818.1 atub|OJI88116.1 aver|OJI99845.1 awen|OJJ36487.1 azon|OJJ44360.1

Group2032: abra|OJJ69485.1 acar|OOF94261.1 acla|EAW09906.1 afis|EAW21155.1 afla|EED54661.1 afum|EAL92173.1 agla|OJJ80038.1 akaw|GAA88333.1 aluc|OJZ82326.1 anid|CBF86986.1 anig|EHA28432.1 aory|EIT78925.1 asyd|OJJ60520.1 ater|EAU35817.1 atub|OJI88114.1 aver|OJI99846.1 awen|OJJ36486.1 azon|OJJ44362.1

Group2033: abra|OJJ69492.1 acar|OOF94251.1 acla|EAW09902.1 afis|EAW21150.1 afla|EED54662.1 afum|EAL92169.1 agla|OJJ78417.1 akaw|GAA88328.1 aluc|OJZ82332.1 anid|CBF75144.1 anig|EHA28428.1 aory|EIT78748.1 asyd|OJJ59971.1 ater|EAU36738.1 atub|OJI88108.1 aver|OJJ02320.1 awen|OJJ36477.1 asyd|OJJ54978.1

Group2034: abra|OJJ69496.1 acar|OOF94254.1 acla|EAW09950.1 afis|EAW21146.1 afla|EED46146.1 afum|EAL92165.1 agla|OJJ80070.1 akaw|GAA88325.1 aluc|OJZ82336.1 anid|CBF87010.1 anig|EHA28424.1 aory|EIT76496.1 asyd|OJJ60472.1 ater|EAU36761.1 atub|OJI88104.1 aver|OJI99792.1 awen|OJJ36469.1 azon|OJJ44354.1

Group2035: abra|OJJ69497.1 acar|OOF94256.1 acla|EAW09951.1 afis|EAW21145.1 afla|EED46147.1 afum|EAL92164.1 agla|OJJ80071.1 akaw|GAA88324.1 aluc|OJZ82337.1 anid|CBF86995.1 anig|EHA28423.1 aory|EIT76462.1 asyd|OJJ60473.1 ater|EAU36757.1 atub|OJI88103.1 aver|OJI99793.1 awen|OJJ36470.1 azon|OJJ44355.1

Group2036: abra|OJJ69498.1 acar|OOF94257.1 acla|EAW09952.1 afis|EAW21144.1 afla|EED46148.1 afum|EAL92163.1 agla|OJJ80072.1 akaw|GAA88323.1 aluc|OJZ82338.1 anid|CBF86997.1 anig|EHA28422.1 aory|EIT76514.1 asyd|OJJ60474.1 ater|EAU36756.1 atub|OJI88102.1 aver|OJI99794.1 awen|OJJ36471.1 azon|OJJ44356.1

Group2037: abra|OJJ69505.1 acar|OOF94247.1 acla|EAW09957.1 afis|EAW21136.1 afla|EED51811.1 afum|EAL92155.1 agla|OJJ80076.1 akaw|GAA88318.1 aluc|OJZ82344.1 anid|CBF87016.1 anig|EHA28416.1 aory|EIT81758.1 asyd|OJJ60463.1 ater|EAU36774.1 atub|OJI88096.1 aver|OJI99783.1 awen|OJJ36456.1 azon|OJJ44420.1

Group2038: abra|OJJ69506.1 acar|OOF94246.1 acla|EAW09958.1 afis|EAW21137.1 afla|EED51812.1 afum|EAL92156.1 agla|OJJ80081.1 akaw|GAA88317.1 aluc|OJZ82345.1 anid|CBF87014.1 anig|EHA28415.1 aory|EIT81705.1 asyd|OJJ60464.1 ater|EAU36773.1 atub|OJI88095.1 aver|OJI99784.1 awen|OJJ36457.1 azon|OJJ44418.1

Group2039: abra|OJJ69508.1 acar|OOF94245.1 acla|EAW09959.1 afis|EAW21138.1 afla|EED51813.1 afum|EAL92157.2 agla|OJJ80080.1 akaw|GAA88316.1 aluc|OJZ82346.1 anid|CBF87012.1 anig|EHA28414.1 aory|EIT81694.1 asyd|OJJ60465.1 ater|EAU36772.1 atub|OJI88094.1 aver|OJI99785.1 awen|OJJ36458.1 azon|OJJ44419.1

Group2040: abra|OJJ69510.1 acar|OOF94244.1 acla|EAW09960.1 afis|EAW21135.1 afla|EED46138.1 afum|EAL92154.1 agla|OJJ80078.1 akaw|GAA88314.1 aluc|OJZ82348.1 anid|CBF87000.1 anig|EHA28412.1 aory|EIT76494.1 asyd|OJJ60467.1 ater|EAU36767.1 atub|OJI88092.1 aver|OJI99787.1 awen|OJJ36461.1 azon|OJJ44416.1

Group2041: abra|OJJ69513.1 acar|OOF94240.1 acla|EAW09955.1 afis|EAW21141.1 afla|EED46142.1 afum|EAL92160.1 agla|OJJ80068.1 akaw|GAA88311.1 aluc|OJZ82351.1 anid|CBF87007.1 anig|EHA28409.1

aory|EIT76415.1 asyd|OJJ60470.1 ater|EAU36762.1 atub|OJI88089.1 aver|OJI99790.1 awen|OJJ36465.1 azon|OJJ44413.1

Group2042: abra|OJJ69516.1 acar|OOF94238.1 acla|EAW09964.1 afis|EAW21124.1 afla|EED51799.1 afum|EAL92147.1 agla|OJJ80086.1 akaw|GAA88308.1 aluc|OJZ82354.1 anid|CBF87018.1 anig|EHA28406.1 aory|EIT81724.1 asyd|OJJ60461.1 ater|EAU36776.1 atub|OJI88086.1 aver|OJI99781.1 awen|OJJ36454.1 azon|OJJ44423.1

Group2043: abra|OJJ69520.1 acar|OOF94237.1 acla|EAW09966.1 afis|EAW21122.1 afla|EED51795.1 afum|EAL92145.1 agla|OJJ80089.1 akaw|GAA88306.1 aluc|OJZ82359.1 anid|CBF87021.1 anig|EHA28404.1 aory|EIT81713.1 asyd|OJJ60459.1 ater|EAU36778.1 atub|OJI88082.1 aver|OJI99779.1 awen|OJJ36452.1 azon|OJJ44425.1

Group2044: abra|OJJ69522.1 acar|OOF94233.1 acla|EAW09968.1 afis|EAW21120.1 afla|EED51793.1 afum|EBA27448.1 agla|OJJ80091.1 akaw|GAA88305.1 aluc|OJZ82362.1 anid|CBF87025.1 anig|EHA28403.1 aory|EIT81754.1 asyd|OJJ60457.1 ater|EAU36779.1 atub|OJI88080.1 aver|OJI99777.1 awen|OJJ36450.1 azon|OJJ44427.1

Group2045: abra|OJJ69525.1 acar|OOF94229.1 acla|EAW09972.1 afis|EAW21116.1 afla|EED51767.1 afum|EAL92139.1 agla|OJJ80095.1 akaw|GAA88300.1 aluc|OJZ82365.1 anid|CBF87030.1 anig|EHA28400.1 aory|EIT79993.1 asyd|OJJ60455.1 ater|EAU36783.1 atub|OJI88077.1 aver|OJI99773.1 awen|OJJ36446.1 azon|OJJ44429.1

Group2046: abra|OJJ69529.1 afis|EAW22089.1 afla|EED53382.1 aory|EIT74986.1 asyd|OJJ58665.1 ater|EAU39308.1 azon|OJJ42020.1 abra|OJJ77940.1 acar|OOF93336.1 acla|EAW10026.1 akaw|GAA83724.1 aluc|OJZ91007.1 asyd|OJJ53344.1 atub|OJI82999.1 aver|OJJ08400.1 afla|EED53383.1 azon|OJJ45239.1 anid|CBF70508.1

Group2047: abra|OJJ69535.1 acar|OOF95965.1 acla|EAW11570.1 afis|EAW23883.1 afla|EED52612.1 afum|EAL87311.1 agla|OJJ82570.1 akaw|GAA88385.1 aluc|OJZ82373.1 anid|CBF77187.1 anig|EHA28391.1 aory|EIT74250.1 asyd|OJJ56841.1 ater|EAU33463.1 atub|OJI88069.1 aver|OJJ03497.1 awen|OJJ33865.1 azon|OJJ48688.1

Group2048: abra|OJJ69536.1 acar|OOF91604.1 acla|EAW14409.1 afis|EAW19117.1 afla|EED54297.1 afum|EAL93849.1 agla|OJJ80432.1 akaw|GAA91927.1 aluc|OJZ82695.1 anid|CBF79728.1 anig|EHA21143.1 aory|EIT75966.1 asyd|OJJ58073.1 ater|EAU37379.1 atub|OJI88927.1 aver|OJJ04334.1 awen|OJJ37447.1 azon|OJJ51125.1

Group2049: abra|OJJ69575.1 acar|OOF91580.1 acla|EAW14394.1 afis|EAW19102.1 afla|EED54308.1 afum|EAL93834.1 agla|OJJ84500.1 akaw|GAA91572.1 aluc|OJZ82392.1 anid|CBF79696.1 anig|EHA28372.1 aory|EIT75942.1 asyd|OJJ58050.1 ater|EAU32151.1 atub|OJI88049.1 aver|OJJ04353.1 awen|OJJ32495.1 azon|OJJ46034.1

Group2050: abra|OJJ69580.1 acar|OOF91573.1 acla|EAW14388.1 afis|EAW19095.1 afla|EED54328.1 afum|EAL93826.1 agla|OJJ84506.1 akaw|GAA91576.1 aluc|OJZ82398.1 anid|CBF79705.1 anig|EHA28367.1 aory|EIT75940.1 asyd|OJJ58055.1 ater|EAU32143.1 atub|OJI88044.1 aver|OJJ04348.1 awen|OJJ32488.1 azon|OJJ46043.1

Group2051: abra|OJJ69583.1 acar|OOF91571.1 acla|EAW14386.1 afis|EAW19092.1 afla|EED54327.1 afum|EAL93823.1 agla|OJJ84509.1 akaw|GAA91578.1 aluc|OJZ82400.1 anid|CBF79709.1 anig|EHA28365.1 asyd|OJJ58057.1 ater|EAU32141.1 atub|OJI88041.1 aver|OJJ04346.1 awen|OJJ32486.1 azon|OJJ46045.1 afum|EAL93822.1

Group2052: abra|OJJ69584.1 acar|OOF91570.1 acla|EAW14385.1 afis|EAW19091.1 afla|EED54326.1 afum|EAL93821.1 agla|OJJ84512.1 akaw|GAA91579.1 aluc|OJZ82401.1 anid|CBF79711.1 anig|EHA28364.1 aory|EIT76092.1 asyd|OJJ58058.1 ater|EAU32140.1 atub|OJI88040.1 aver|OJJ04345.1 awen|OJJ32485.1 azon|OJJ46046.1

Group2053: abra|OJJ69587.1 acar|OOF91568.1 acla|EAW14380.1 afis|EAW19086.1 afla|EED54318.1 afum|EAL93816.1 agla|OJJ84518.1 akaw|GAA91584.1 aluc|OJZ82404.1 anid|CBF79684.1 anig|EHA28362.1 aory|EIT76159.1 asyd|OJJ58047.1 ater|EAU32134.1 atub|OJI88037.1 aver|OJJ04356.1 awen|OJJ32483.1 azon|OJJ46227.1

Group2054: abra|OJJ69588.1 acar|OOF91567.1 acla|EAW14379.1 afis|EAW19085.1 afla|EED54317.1 afum|EAL93815.1 agla|OJJ84521.1 akaw|GAA91585.1 aluc|OJZ82406.1 anid|CBF79683.1 anig|EHA28361.1 aory|EIT75897.1 asyd|OJJ58046.1 ater|EAU32133.1 atub|OJI88036.1 aver|OJJ04357.1 awen|OJJ32482.1 azon|OJJ46226.1

Group2055: abra|OJJ69590.1 acar|OOF91565.1 acla|EAW14378.1 afis|EAW19084.1 afla|EED54316.1 afum|EAL93814.1 agla|OJJ84522.1 akaw|GAA91586.1 aluc|OJZ82408.1 anid|CBF79676.1 anig|EHA28360.1 aory|EIT75902.1 asyd|OJJ58038.1 ater|EAU32132.1 atub|OJI88034.1 aver|OJJ04362.1 awen|OJJ32481.1 azon|OJJ46224.1

Group2056: abra|OJJ69592.1 anid|CBF82180.1 anig|EHA28357.1 ater|EAU30193.1 abra|OJJ73493.1 acar|OOG00685.1 afis|EAW20408.1 afum|EAL89236.1 agla|OJJ88380.1 akaw|GAA87442.1 aluc|OJZ88555.1 anig|EHA18253.1 atub|OJI91140.1 awen|OJJ31333.1 azon|OJJ49482.1 afis|EAW19984.1 afum|EAL84932.1 anid|CBF84172.1

Group2057: abra|OJJ69602.1 acar|OOF92702.1 acla|EAW10013.1 afis|EAW21041.1 afla|EED51373.1 afum|EAL92080.1 agla|OJJ84213.1 akaw|GAA91590.1 aluc|OJZ82413.1 anid|CBF80091.1 anig|EHA28354.1 aory|EIT80745.1 asyd|OJJ58302.1 ater|EAU31725.1 atub|OJI88028.1 aver|OJJ02110.1 awen|OJJ32696.1 azon|OJJ50700.1

Group2058: abra|OJJ69616.1 acar|OOF92690.1 acla|EAW12076.1 afis|EAW19717.1 afla|EED53108.1 afum|EAL86810.1 agla|OJJ80861.1 akaw|GAA91600.1 aluc|OJZ82424.1 anid|CBF80078.1 anig|EHA28345.1 aory|EIT74760.1 asyd|OJJ58310.1 ater|EAU37776.1 atub|OJI88016.1 aver|OJJ02101.1 awen|OJJ35107.1 azon|OJJ49987.1

Group2059: abra|OJJ69628.1 afla|EED55079.1 agla|OJJ86032.1 aluc|OJZ82433.1 anid|CBF82983.1 anig|EHA28338.1 asyd|OJJ64730.1 ater|EAU30171.1 atub|OJI88008.1 aver|OJI98834.1 awen|OJJ30457.1 azon|OJJ45448.1 acar|OOF92682.1 afis|EAW16821.1 afum|EAL86371.1 acla|EAW12375.1 aory|EIT81573.1 akaw|GAA91608.1

Group2060: abra|OJJ69635.1 acar|OOF93616.1 acla|EAW15185.1 afis|EAW20155.1 afla|EED55122.1 afum|EAL85270.1 agla|OJJ89111.1 akaw|GAA91615.1 aluc|OJZ82441.1 anid|CBF69484.1 anig|EHA28332.1 aory|EIT79219.1 asyd|OJJ59246.1 atub|OJI87999.1 aver|OJJ01857.1 awen|OJJ36185.1 aory|EIT81639.1 afla|EED47294.1

Group2061: abra|OJJ69637.1 acar|OOF92675.1 acla|EAW12571.1 afis|EAW24079.1 afla|EED51394.1 afum|EAL91934.1 agla|OJJ87150.1 akaw|GAA91617.1 aluc|OJZ82443.1 anid|CBF86476.1 anig|EHA28330.1 aory|EIT80502.1 asyd|OJJ64903.1 ater|EAU30500.1 atub|OJI87997.1 aver|OJI99025.1 awen|OJJ41417.1 azon|OJJ48876.1

Group2062: azon|OJJ44974.1 abra|OJJ73161.1 acar|OOG00404.1 acla|EAW06767.1 afis|EAW16317.1 afla|EED53564.1 afum|EAL88940.1 agla|OJJ88278.1 akaw|GAA88011.1 aluc|OJZ81269.1 anid|CBF89145.1 anig|EHA18569.1 aory|EIT75175.1 asyd|OJJ62569.1 ater|EAU34140.1 atub|OJI91476.1 aver|OJI96693.1 awen|OJJ37717.1

Group2063: abra|OJJ69655.1 acar|OOG00750.1 acla|EAW07485.1 afis|EAW22518.1 afla|EED54547.1 afum|EAL90675.1 agla|OJJ80878.1 akaw|GAA91634.1 aluc|OJZ82461.1 anid|CBF88995.1 anig|EHA28314.1 aory|EIT78864.1 asyd|OJJ62783.1 ater|EAU39191.1 atub|OJI87977.1 aver|OJI96828.1 awen|OJJ35145.1 azon|OJJ49955.1

Group2064: abra|OJJ69657.1 acar|OOF95821.1 acla|EAW11286.1 afis|EAW18213.1 afla|EED56258.1 afum|EAL87579.1 agla|OJJ85869.1 akaw|GAA91636.1 aluc|OJZ82463.1 anid|CBF75160.1 anig|EHA28312.1 aory|EIT73422.1 asyd|OJJ59962.1 ater|EAU33776.1 atub|OJI87975.1 aver|OJJ02328.1 awen|OJJ30591.1 azon|OJJ50185.1

Group2065: abra|OJJ69663.1 acar|OOF98113.1 acla|EAW10577.1 afis|EAW24990.1 afla|EED55469.1 afum|EAL89650.1 agla|OJJ82264.1 akaw|GAA91641.1 aluc|OJZ82468.1 anid|CBF81596.1 anig|EHA28307.1 aory|EIT80882.1 asyd|OJJ59517.1 ater|EAU35741.1 atub|OJI87970.1 aver|OJJ02756.1 awen|OJJ40595.1 azon|OJJ49446.1

Group2066: abra|OJJ69664.1 acar|OOG00819.1 acla|EAW07551.1 afis|EAW22585.1 afla|EED54630.1 afum|EAL90614.1 agla|OJJ80945.1 akaw|GAA91642.1 aluc|OJZ82469.1 anid|CBF88352.1 anig|EHA28306.1 aory|EIT78765.1 asyd|OJJ63721.1 ater|EAU39114.1 atub|OJI87968.1 aver|OJI97219.1 awen|OJJ35213.1 azon|OJJ49896.1

Group2067: abra|OJJ69665.1 acar|OOG00018.1 acla|EAW09937.1 afis|EAW21182.1 afla|EED54777.1 afum|EAL92203.1 agla|OJJ79988.1 akaw|GAA91643.1 aluc|OJZ82470.1 anid|CBF83310.1 anig|EHA28303.1 aory|EIT79439.1 asyd|OJJ60549.1 ater|EAU35848.1 atub|OJI87967.1 aver|OJI99875.1 awen|OJJ36521.1 azon|OJJ44397.1

Group2068: abra|OJJ69666.1 acar|OOG00019.1 acla|EAW09936.1 afis|EAW21181.1 afla|EED54778.1 afum|EAL92202.1 agla|OJJ79998.1 akaw|GAA91644.1 aluc|OJZ82471.1 anid|CBF83308.1 anig|EHA28302.1 aory|EIT79454.1 asyd|OJJ60550.1 ater|EAU35847.1 atub|OJI87966.1 aver|OJI99876.1 awen|OJJ36520.1 azon|OJJ44396.1

Group2069: abra|OJJ69667.1 acar|OOF97488.1 acla|EAW13075.1 afis|EAW17589.1 afla|EED49233.1 afum|EAL91402.1 agla|OJJ81360.1 akaw|GAA91645.1 aluc|OJZ82472.1 anid|CBF90218.1 anig|EHA28301.1 aory|EIT83160.1 asyd|OJJ61967.1 ater|EAU37024.1 atub|OJI87965.1 aver|OJI96059.1 awen|OJJ37257.1 azon|OJJ50952.1

Group2070: abra|OJJ69669.1 acar|OOF95822.1 acla|EAW06663.1 afis|EAW16102.1 afla|EED48637.1 afum|EAL88734.1 agla|OJJ86131.1 akaw|GAA91647.1 aluc|OJZ82474.1 anid|CBF85442.1 anig|EHA28299.1

aory|EIT73069.1 asyd|OJJ64091.1 ater|EAU33179.1 atub|OJI87963.1 aver|OJI98175.1 awen|OJJ30330.1 azon|OJJ45358.1

Group2071: abra|OJJ69677.1 acar|OOF92983.1 acla|EAW14808.1 afis|EAW24337.1 afla|EED47927.1 afum|EAL86008.1 agla|OJJ83205.1 akaw|GAA88802.1 aluc|OJZ82485.1 anid|CBF74030.1 anig|EHA28289.1 aory|EIT79853.1 asyd|OJJ53849.1 ater|EAU30411.1 atub|OJI87947.1 aver|OJJ07950.1 awen|OJJ32957.1 azon|OJJ48963.1

Group2072: abra|OJJ69682.1 acar|OOF93221.1 acla|EAW13124.1 afis|EAW17540.1 afla|EED49287.1 afum|EAL91354.1 agla|OJJ81429.1 akaw|GAA92416.1 aluc|OJZ83274.1 anid|CBF90315.1 anig|EHA28284.1 aory|EIT82215.1 asyd|OJJ61912.1 ater|EAU36970.1 atub|OJI87666.1 aver|OJI96005.1 awen|OJJ37313.1 azon|OJJ51005.1

Group2073: abra|OJJ69687.1 acar|OOF92648.1 acla|EAW10319.1 afis|EAW25273.1 afla|EED53738.1 afum|EAL89936.1 agla|OJJ89247.1 akaw|GAA88811.1 aluc|OJZ82494.1 anid|CBF85387.1 anig|EHA28278.1 aory|EIT75306.1 asyd|OJJ64055.1 ater|EAU34416.1 atub|OJI87938.1 aver|OJI98140.1 awen|OJJ40987.1 azon|OJJ44574.1

Group2074: abra|OJJ69698.1 acar|OOF90521.1 acla|EAW15242.1 afis|EAW20209.1 afla|EED57134.1 afum|EAL85326.1 agla|OJJ84655.1 akaw|GAA88816.1 aluc|OJZ82500.1 anid|CBF85100.1 anig|EHA28270.1 aory|EIT74032.1 asyd|OJJ63057.1 ater|EAU32019.1 atub|OJI87931.1 aver|OJI97905.1 awen|OJJ31213.1 azon|OJJ46146.1

Group2075: abra|OJJ69699.1 acar|OOF95410.1 acla|EAW07370.1 afis|EAW22395.1 afla|EED56059.1 afum|EAL90796.1 agla|OJJ87658.1 akaw|GAA88818.1 aluc|OJZ82502.1 anid|CBF88747.1 anig|EHA28269.1 aory|EIT76693.1 asyd|OJJ63956.1 ater|EAU39322.1 atub|OJI87929.1 aver|OJI96985.1 awen|OJJ34973.1 azon|OJJ45622.1

Group2076: abra|OJJ69701.1 acar|OOF93222.1 acla|EAW13123.1 afis|EAW17541.1 afla|EED49286.1 afum|EAL91355.1 agla|OJJ81428.1 akaw|GAA88820.1 aluc|OJZ82504.1 anid|CBF90313.1 anig|EHA28266.1 aory|EIT82202.1 asyd|OJJ61913.1 ater|EAU36971.1 atub|OJI87927.1 aver|OJI96006.1 awen|OJJ37312.1 azon|OJJ51004.1

Group2077: abra|OJJ69703.1 acar|OOF98863.1 acla|EAW06689.1 afis|EAW16076.1 afla|EED48596.1 afum|EAL88708.1 agla|OJJ86093.1 akaw|GAA88822.1 aluc|OJZ82506.1 anid|CBF75115.1 anig|EHA28263.1 aory|EIT73056.1 asyd|OJJ59995.1 ater|EAU33555.1 atub|OJI87925.1 aver|OJJ02296.1 awen|OJJ30357.1 azon|OJJ45379.1

Group2078: abra|OJJ69718.1 acar|OOF94196.1 acla|EAW09976.1 afis|EAW21113.1 afla|EED51764.1 afum|EAL92136.1 agla|OJJ80098.1 akaw|GAA90171.1 aluc|OJZ83179.1 anid|CBF87036.1 anig|EHA28253.1 aory|EIT80019.1 asyd|OJJ60452.1 ater|EAU36786.1 atub|OJI87758.1 aver|OJI99770.1 awen|OJJ36441.1 azon|OJJ44432.1

Group2079: abra|OJJ69746.1 acar|OOF94176.1 acla|EAW09985.1 afis|EAW21108.1 afla|EED51696.1 afum|EAL92129.1 agla|OJJ80107.1 akaw|GAA90198.1 aluc|OJZ83150.1 anid|CBF87041.1 anig|EHA28216.1 aory|EIT80588.1 asyd|OJJ60448.1 ater|EAU36792.1 atub|OJI87787.1 aver|OJI99766.1 awen|OJJ36382.1 azon|OJJ51534.1

Group2080: aver|OJJ05453.1 abra|OJJ70544.1 acar|OOF94607.1 acla|EAW08135.1 afis|EAW23171.1 afla|EED46949.1 afum|EAL88312.2 agla|OJJ89683.1 akaw|GAA83390.1 aluc|OJZ83889.1 anid|CBF74485.1 anig|EHA19026.1 aory|EIT72627.1 asyd|OJJ57712.1 ater|EAU36300.1 atub|OJI90256.1 awen|OJJ36606.1 azon|OJJ47601.1

Group2081: abra|OJJ69784.1 acar|OOF95612.1 afum|EAL89018.1 anid|CBF84864.1 anig|EHA25046.1 asyd|OJJ56179.1 atub|OJI80053.1 aver|OJJ05815.1 abra|OJJ77383.1 acar|OOF93253.1 agla|OJJ82715.1 akaw|GAA91378.1 aluc|OJZ84791.1 anig|EHA28059.1 atub|OJI89291.1 awen|OJJ36484.1 aver|OJI99823.1 asyd|OJJ57350.1

Group2082: abra|OJJ69822.1 acar|OOF93341.1 acla|EAW08969.1 afis|EAW15448.1 afla|EED56825.1 afum|EAL84198.2 agla|OJJ86271.1 akaw|GAA87838.1 aluc|OJZ85474.1 anid|CBF78305.1 anig|EHA25015.1 aory|EIT73942.1 asyd|OJJ56090.1 ater|EAU30838.1 atub|OJI80089.1 aver|OJJ05033.1 awen|OJJ31223.1 azon|OJJ45071.1

Group2083: abra|OJJ69827.1 acar|OOF93352.1 afla|EED55192.1 akaw|GAA87833.1 aluc|OJZ85469.1 anid|CBF78301.1 anig|EHA25010.1 aory|EIT83545.1 asyd|OJJ56100.1 atub|OJI80094.1 aver|OJJ05043.1 acla|EAW08979.1 afis|EAW15430.1 afum|EAL84213.1 ater|EAU32701.1 awen|OJJ33446.1 afla|EED47528.1 aory|EIT74571.1

Group2084: abra|OJJ69888.1 acar|OOF93408.1 acla|EAW10806.1 afis|EAW24768.1 afla|EED49741.1 afum|EAL89409.1 agla|OJJ82608.1 akaw|GAA87764.1 aluc|OJZ85399.1 anid|CBF69441.1 anig|EHA25581.1 aory|EIT77620.1 ater|EAU36289.1 atub|OJI80172.1 awen|OJJ39939.1 acar|OOF92530.1 asyd|OJJ60681.1 aver|OJJ00006.1

Group2085: abra|OJJ69898.1 acar|OOF93417.1 acla|EAW08922.1 afis|EAW15471.1 afla|EED46922.1 afum|EAL85850.1 agla|OJJ80151.1 akaw|GAA87752.1 aluc|OJZ85385.1 anid|CBF78234.1 anig|EHA25569.1 aory|EIT72668.1 asyd|OJJ56036.1 ater|EAU32657.1 atub|OJI80185.1 aver|OJJ04982.1 awen|OJJ38121.1 azon|OJJ48022.1

Group2086: abra|OJJ69901.1 acar|OOF93419.1 acla|EAW08921.1 afis|EAW15473.1 afla|EED46923.1 afum|EAL85848.1 akaw|GAA87750.1 aluc|OJZ85383.1 anid|CBF78228.1 anig|EHA25567.1 aory|EIT72551.1 asyd|OJJ56034.1 ater|EAU32656.1 atub|OJI80187.1 aver|OJJ04980.1 awen|OJJ38126.1 azon|OJJ43160.1 agla|OJJ80154.1

Group2087: abra|OJJ69906.1 acar|OOF93423.1 acla|EAW08918.1 afis|EAW15476.1 afla|EED46939.1 afum|EAL85845.1 agla|OJJ80155.1 akaw|GAA87747.1 aluc|OJZ85380.1 anid|CBF78226.1 anig|EHA25563.1 aory|EIT72564.1 asyd|OJJ56033.1 ater|EAU32651.1 atub|OJI80190.1 aver|OJJ04979.1 awen|OJJ38133.1 azon|OJJ43159.1

Group2088: abra|OJJ69914.1 acar|OOF93426.1 acla|EAW08915.1 afis|EAW15479.1 afla|EED46934.1 afum|EAL85842.1 agla|OJJ80158.1 akaw|GAA87742.1 aluc|OJZ85374.1 anid|CBF78221.1 anig|EHA25558.1 aory|EIT72728.1 asyd|OJJ56028.1 ater|EAU32648.1 atub|OJI80195.1 aver|OJJ04975.1 awen|OJJ38138.1 azon|OJJ43152.1

Group2089: abra|OJJ69916.1 acar|OOF93428.1 acla|EAW08912.1 afis|EAW15483.1 afla|EED46930.1 afla|EED48092.1 afum|EAL85839.1 agla|OJJ80162.1 akaw|GAA87740.1 aluc|OJZ85372.1 anig|EHA25556.1 aory|EIT72736.1 asyd|OJJ56026.1 ater|EAU32647.1 atub|OJI80198.1 aver|OJJ04973.1 awen|OJJ38142.1 azon|OJJ43148.1

Group2090: abra|OJJ69940.1 acar|OOF93449.1 acla|EAW08883.1 afis|EAW15506.1 afla|EED49019.1 afum|EAL85818.1 agla|OJJ80183.1 akaw|GAA87719.1 aluc|OJZ85347.1 anid|CBF84473.1 anig|EHA25534.1 aory|EIT82494.1 asyd|OJJ53540.1 ater|EAU32612.1 atub|OJI80223.1 aver|OJJ08213.1 awen|OJJ38166.1 azon|OJJ45800.1

Group2091: abra|OJJ69952.1 acar|OOF93462.1 acla|EAW08870.1 afis|EAW15524.1 afla|EED49034.1 afum|EAL85804.1 agla|OJJ85222.1 akaw|GAA87707.1 aluc|OJZ85330.1 anig|EHA25519.1 aory|EIT82486.1 asyd|OJJ55992.1 ater|EAU32599.1 atub|OJI80242.1 aver|OJJ04940.1 awen|OJJ38188.1 azon|OJJ43107.1 afla|EED49033.1

Group2092: abra|OJJ69953.1 acar|OOF93463.1 acla|EAW08869.1 afis|EAW15525.1 afla|EED49036.1 afum|EAL85803.1 agla|OJJ85221.1 akaw|GAA87706.1 aluc|OJZ85329.1 anid|CBF78217.1 anig|EHA25518.1 aory|EIT82537.1 asyd|OJJ55991.1 ater|EAU32598.1 atub|OJI80243.1 aver|OJJ04939.1 awen|OJJ38189.1 azon|OJJ43106.1

Group2093: abra|OJJ69955.1 acar|OOF93465.1 acla|EAW08867.1 afis|EAW15527.1 afla|EED49038.1 afum|EAL85801.1 agla|OJJ85219.1 akaw|GAA87704.1 aluc|OJZ85327.1 anid|CBF78212.1 anig|EHA25517.1 aory|EIT82564.1 asyd|OJJ55989.1 ater|EAU32597.1 atub|OJI80245.1 aver|OJJ04937.1 awen|OJJ38191.1 azon|OJJ43104.1

Group2094: abra|OJJ69962.1 acar|OOF93470.1 acla|EAW08834.1 afis|EAW15566.1 afla|EED49075.1 afum|EAL85767.2 agla|OJJ85185.1 akaw|GAA87697.1 aluc|OJZ85321.1 anid|CBF78116.1 anig|EHA25511.1 aory|EIT82536.1 asyd|OJJ55900.1 ater|EAU32567.1 atub|OJI80252.1 aver|OJJ04845.1 awen|OJJ38226.1 azon|OJJ43069.1

Group2095: abra|OJJ69963.1 acar|OOF93471.1 acla|EAW08835.1 afis|EAW15565.1 afla|EED49074.1 agla|OJJ85186.1 akaw|GAA87696.1 aluc|OJZ85320.1 anid|CBF78118.1 anig|EHA25510.1 aory|EIT82524.1 asyd|OJJ55901.1 ater|EAU32568.1 atub|OJI80253.1 aver|OJJ04846.1 awen|OJJ38225.1 azon|OJJ43070.1 afum|EAL85768.1

Group2096: abra|OJJ69971.1 acar|OOF93475.1 acla|EAW08842.1 afis|EAW15559.1 afla|EED49063.1 afum|EAL85773.1 agla|OJJ85195.1 akaw|GAA87690.1 aluc|OJZ85311.1 anid|CBF78159.1 anig|EHA25506.1 aory|EIT82560.1 asyd|OJJ55960.1 ater|EAU32575.1 atub|OJI80260.1 aver|OJJ04906.1 awen|OJJ38217.1 azon|OJJ43077.1

Group2097: abra|OJJ69974.1 acar|OOF93479.1 acla|EAW08844.1 afis|EAW15556.1 afla|EED49061.1 afum|EAL85776.1 agla|OJJ85198.1 akaw|GAA87687.1 aluc|OJZ85307.1 anid|CBF78163.1 anig|EHA25503.1 aory|EIT82517.1 asyd|OJJ55964.1 ater|EAU32577.1 atub|OJI80264.1 aver|OJJ04910.1 awen|OJJ38214.1 azon|OJJ43081.1

Group2098: abra|OJJ69975.1 acar|OOF93481.1 acla|EAW08846.1 afis|EAW15554.1 afla|EED49057.1 afum|EAL85779.1 agla|OJJ85201.1 akaw|GAA87686.1 aluc|OJZ85306.1 anid|CBF78167.1 anig|EHA25502.1 aory|EIT82497.1 asyd|OJJ55968.1 ater|EAU32580.1 atub|OJI80265.1 aver|OJJ04915.1 awen|OJJ38210.1 azon|OJJ43084.1

Group2099: abra|OJJ69978.1 acar|OOF93484.1 acla|EAW08847.1 afis|EAW15549.1 afla|EED49055.1 afum|EAL85783.1 agla|OJJ85202.1 akaw|GAA87683.1 aluc|OJZ85302.1 anid|CBF78165.1 anig|EHA25499.1

aory|EIT82558.1 asyd|OJJ55969.1 ater|EAU32583.1 atub|OJI80268.1 aver|OJJ04916.1 awen|OJJ38209.1 azon|OJJ43086.1

Group2100: abra|OJJ69979.1 acar|OOF93483.1 acla|EAW08849.1 afis|EAW15547.1 afla|EED49054.1 afum|EAL85785.1 agla|OJJ85205.1 akaw|GAA87682.1 aluc|OJZ85301.1 anid|CBF78176.1 anig|EHA25498.1 aory|EIT82504.1 asyd|OJJ55970.1 ater|EAU32582.1 atub|OJI80269.1 aver|OJJ04917.1 awen|OJJ38208.1 azon|OJJ43087.1

Group2101: abra|OJJ69982.1 acar|OOF93486.1 acla|EAW08852.1 afis|EAW15544.1 afla|EED49051.1 afum|EAL85787.1 agla|OJJ85207.1 akaw|GAA87680.1 aluc|OJZ85298.1 anid|CBF78182.1 anig|EHA25496.1 aory|EIT82527.1 asyd|OJJ55973.1 ater|EAU32584.1 atub|OJI80272.1 aver|OJJ04921.1 awen|OJJ38205.1 azon|OJJ43090.1

Group2102: abra|OJJ69983.1 acar|OOF93487.1 acla|EAW08853.1 afis|EAW15543.1 afla|EED49050.1 afum|EAL85788.1 agla|OJJ85208.1 akaw|GAA87679.1 aluc|OJZ85297.1 anid|CBF78183.1 anig|EHA25495.1 aory|EIT82528.1 asyd|OJJ55974.1 ater|EAU32585.1 atub|OJI80273.1 aver|OJJ04922.1 awen|OJJ38204.1 azon|OJJ43091.1

Group2103: abra|OJJ69984.1 acar|OOF93489.1 acla|EAW08855.1 afis|EAW15541.1 afla|EED49049.1 afum|EAL85789.1 agla|OJJ85209.1 akaw|GAA87677.1 aluc|OJZ85295.1 anid|CBF78187.1 anig|EHA25493.1 aory|EIT82548.1 asyd|OJJ55976.1 ater|EAU32586.1 atub|OJI80275.1 aver|OJJ04924.1 awen|OJJ38202.1 azon|OJJ43093.1

Group2104: abra|OJJ69985.1 acar|OOF93490.1 acla|EAW08856.1 afis|EAW15540.1 afla|EED49048.1 afum|EAL85790.1 agla|OJJ85210.1 akaw|GAA87676.1 aluc|OJZ85294.1 anid|CBF78189.1 anig|EHA25492.1 aory|EIT82530.1 asyd|OJJ55977.1 ater|EAU32587.1 atub|OJI80276.1 aver|OJJ04925.1 awen|OJJ38201.1 azon|OJJ43094.1

Group2105: abra|OJJ69987.1 acar|OOF93491.1 acla|EAW08858.1 afis|EAW15538.1 afla|EED49046.1 afum|EAL85792.1 agla|OJJ85211.1 akaw|GAA87674.1 aluc|OJZ85292.1 anid|CBF78193.1 anig|EHA25490.1 aory|EIT82570.1 asyd|OJJ55979.1 ater|EAU32589.1 atub|OJI80278.1 aver|OJJ04927.1 awen|OJJ38200.1 azon|OJJ43095.1

Group2106: abra|OJJ69989.1 acar|OOF93494.1 acla|EAW08861.1 afis|EAW15533.1 afla|EED49044.1 afum|EAL85795.1 agla|OJJ85214.1 akaw|GAA87672.1 aluc|OJZ85290.1 anid|CBF78199.1 anig|EHA25488.1 aory|EIT82552.1 asyd|OJJ55983.1 ater|EAU32592.1 atub|OJI80280.1 aver|OJJ04930.1 awen|OJJ38197.1 azon|OJJ43096.1

Group2107: abra|OJJ69990.1 acar|OOF93496.1 acla|EAW08863.1 afis|EAW15531.1 afla|EED49042.1 afum|EAL85797.1 agla|OJJ85216.1 akaw|GAA87671.1 aluc|OJZ85289.1 anid|CBF78202.1 anig|EHA25486.1 aory|EIT82550.1 asyd|OJJ55985.1 ater|EAU32594.1 atub|OJI80281.1 aver|OJJ04932.1 awen|OJJ38195.1 azon|OJJ43099.1

Group2108: abra|OJJ69991.1 acar|OOF93497.1 acla|EAW08864.1 afis|EAW15530.1 afla|EED49041.1 afum|EAL85798.1 agla|OJJ85217.1 akaw|GAA87670.1 aluc|OJZ85288.1 anid|CBF78204.1 anig|EHA25485.1 aory|EIT82567.1 asyd|OJJ55986.1 ater|EAU32595.1 atub|OJI80282.1 aver|OJJ04933.1 awen|OJJ38194.1 azon|OJJ43100.1

Group2109: abra|OJJ69995.1 acar|OOF93502.1 acla|EAW08824.1 afis|EAW15573.1 afla|EED49082.1 afum|EAL85761.1 agla|OJJ85179.1 akaw|GAA87666.1 aluc|OJZ85284.1 anid|CBF78105.1 anig|EHA25481.1 aory|EIT82549.1 asyd|OJJ55895.1 ater|EAU32558.1 atub|OJI80286.1 aver|OJJ04840.1 awen|OJJ38232.1 azon|OJJ43064.1

Group2110: abra|OJJ70001.1 acar|OOF93507.1 acla|EAW08818.1 afis|EAW15579.1 afla|EED49093.1 afum|EAL85755.1 agla|OJJ85173.1 akaw|GAA87660.1 aluc|OJZ85278.1 anid|CBF78100.1 anig|EHA25475.1 aory|EIT82531.1 asyd|OJJ55889.1 ater|EAU32551.1 atub|OJI80292.1 aver|OJJ04834.1 awen|OJJ38241.1 azon|OJJ43058.1

Group2111: abra|OJJ70003.1 acar|OOF93509.1 acla|EAW08816.1 afis|EAW15581.1 afla|EED49090.1 afum|EAL85753.2 agla|OJJ85170.1 akaw|GAA87657.1 aluc|OJZ85276.1 anid|CBF78096.1 anig|EHA25473.1 aory|EIT82539.1 asyd|OJJ55886.1 ater|EAU32549.1 atub|OJI80294.1 aver|OJJ04831.1 awen|OJJ38243.1 azon|OJJ43055.1

Group2112: abra|OJJ70008.1 acar|OOF93514.1 acla|EAW08815.1 afis|EAW15582.1 afla|EED49088.1 afum|EAL85752.1 agla|OJJ85169.1 akaw|GAA87654.1 aluc|OJZ85271.1 anid|CBF78090.1 anig|EHA25470.1 aory|EIT82507.1 asyd|OJJ55883.1 ater|EAU32547.1 atub|OJI80300.1 aver|OJJ04828.1 awen|OJJ38246.1 azon|OJJ43052.1

Group2113: abra|OJJ70011.1 acar|OOF93517.1 acla|EAW08813.1 afis|EAW15584.1 afla|EED54445.1 afum|EAL85748.2 agla|OJJ85162.1 akaw|GAA87651.1 aluc|OJZ85267.1 anid|CBF78060.1 anig|EHA25466.1 aory|EIT81147.1 ater|EAU32545.1 atub|OJI80303.1 aver|OJJ04813.1 awen|OJJ38249.1 azon|OJJ43030.1 anig|EHA25467.1

Group2114: abra|OJJ70013.1 acar|OOF93519.1 acla|EAW08811.1 afis|EAW15586.1 afla|EED54443.1 afum|EAL85746.1 agla|OJJ85166.1 akaw|GAA87649.1 aluc|OJZ85264.1 anid|CBF78063.1 anig|EHA25464.1 aory|EIT81154.1 asyd|OJJ55870.1 ater|EAU32543.1 atub|OJI80305.1 aver|OJJ04815.1 awen|OJJ38252.1 azon|OJJ43032.1

Group2115: abra|OJJ70014.1 acar|OOF93520.1 acla|EAW08809.1 afis|EAW15587.1 afla|EED50410.1 afum|EAL85745.1 agla|OJJ85161.1 akaw|GAA87648.1 aluc|OJZ85263.1 anid|CBF78069.1 anig|EHA25463.1 aory|EIT82131.1 asyd|OJJ55872.1 ater|EAU32541.1 atub|OJI80306.1 aver|OJJ04817.1 awen|OJJ38254.1 azon|OJJ43033.1

Group2116: abra|OJJ70015.1 acar|OOF93521.1 acla|EAW08808.1 afis|EAW15588.1 afla|EED50411.1 afum|EAL85744.1 agla|OJJ85160.1 akaw|GAA87646.1 aluc|OJZ85261.1 anid|CBF78071.1 anig|EHA25462.1 aory|EIT82015.1 asyd|OJJ55873.1 ater|EAU32539.1 atub|OJI80307.1 aver|OJJ04818.1 awen|OJJ38255.1 azon|OJJ43038.1

Group2117: abra|OJJ70016.1 acar|OOF93522.1 acla|EAW08807.1 afis|EAW15589.1 afla|EED50412.1 afum|EAL85743.1 agla|OJJ85159.1 akaw|GAA87645.1 aluc|OJZ85260.1 anid|CBF78072.1 anig|EHA25461.1 aory|EIT82097.1 asyd|OJJ55874.1 ater|EAU32538.1 atub|OJI80308.1 aver|OJJ04819.1 awen|OJJ38262.1 azon|OJJ43023.1

Group2118: abra|OJJ70018.1 acar|OOF93524.1 acla|EAW08805.1 afis|EAW15591.1 afla|EED50414.1 afum|EAL85741.1 agla|OJJ85157.1 akaw|GAA87643.1 aluc|OJZ85258.1 anid|CBF78076.1 anig|EHA25459.1 aory|EIT82095.1 asyd|OJJ55876.1 ater|EAU32537.1 atub|OJI80310.1 aver|OJJ04821.1 awen|OJJ38264.1 azon|OJJ43021.1

Group2119: abra|OJJ70026.1 acar|OOF93536.1 acla|EAW08802.1 afis|EAW15595.1 afla|EED50418.1 afum|EAL85736.2 agla|OJJ85151.1 akaw|GAA87634.1 aluc|OJZ85250.1 anid|CBF78087.1 anig|EHA25450.1 aory|EIT82070.1 asyd|OJJ55881.1 ater|EAU32532.1 atub|OJI80319.1 aver|OJJ04826.1 awen|OJJ38271.1 azon|OJJ43910.1

Group2120: abra|OJJ70042.1 acar|OOF93537.1 acla|EAW08803.1 afis|EAW15594.1 afla|EED50417.1 afum|EAL85737.2 agla|OJJ85153.1 akaw|GAA87633.1 aluc|OJZ85249.1 anid|CBF78085.1 anig|EHA25449.1 aory|EIT82162.1 asyd|OJJ55880.1 ater|EAU32533.1 atub|OJI80320.1 aver|OJJ04825.1 awen|OJJ38270.1 azon|OJJ43912.1

Group2121: abra|OJJ70083.1 acla|EAW11426.1 afis|EAW20385.1 afla|EED45578.1 afum|EAL85496.1 agla|OJJ83253.1 akaw|GAA87604.1 aluc|OJZ85224.1 anid|CBF86057.1 anig|EHA25428.1 aory|EIT75627.1 asyd|OJJ54466.1 ater|EAU32965.1 atub|OJI80357.1 aver|OJJ07783.1 awen|OJJ30093.1 afis|EAW16167.1 aory|EIT75578.1

Group2122: abra|OJJ70153.1 acar|OOF93582.1 afis|EAW17230.1 agla|OJJ86975.1 akaw|GAA84496.1 aluc|OJZ89221.1 anig|EHA23341.1 asyd|OJJ53738.1 ater|EAU34915.1 atub|OJI85571.1 aver|OJI98051.1 afis|EAW19779.1 afla|EED45371.1 afum|EAL85119.2 anid|CBF71521.1 aory|EIT82393.1 asyd|OJJ62940.1 afis|EAW20309.1

Group2123: abra|OJJ70159.1 acar|OOF93314.1 acla|EAW06525.1 afis|EAW16241.1 afla|EED57104.1 afum|EAL88863.1 agla|OJJ88977.1 akaw|GAA86606.1 aluc|OJZ85156.1 anid|CBF78253.1 anig|EHA25372.1 aory|EIT73977.1 asyd|OJJ56050.1 ater|EAU34336.1 atub|OJI80433.1 aver|OJJ04993.1 awen|OJJ36019.1 azon|OJJ42535.1

Group2124: abra|OJJ70295.1 acar|OOF91749.1 acla|EAW10279.1 afis|EAW25315.1 afla|EED49708.1 afum|EAL89976.1 agla|OJJ88748.1 aluc|OJZ82560.1 anig|EHA22521.1 aory|EIT77651.1 asyd|OJJ62816.1 ater|EAU32274.1 atub|OJI88802.1 aver|OJI96860.1 awen|OJJ40751.1 azon|OJJ44610.1 awen|OJJ41048.1 agla|OJJ80540.1

Group2125: abra|OJJ70321.1 acar|OOF91776.1 acla|EAW12313.1 afis|EAW16939.1 afla|EED45249.1 afum|EAL86466.1 agla|OJJ79973.1 akaw|GAA92577.1 aluc|OJZ82590.1 anid|CBF75409.1 anig|EHA27485.1 aory|EIT73687.1 asyd|OJJ59815.1 ater|EAU30208.1 atub|OJI88825.1 aver|OJJ02473.1 awen|OJJ29713.1 azon|OJJ45106.1

Group2126: abra|OJJ70342.1 acar|OOF91814.1 abra|OJJ74452.1 acla|EAW12256.1 afla|EED48966.1 afum|EAL86656.1 agla|OJJ87924.1 akaw|GAA87346.1 aluc|OJZ89668.1 anig|EHA26906.1 aory|EIT79988.1 asyd|OJJ52239.1 atub|OJI85132.1 awen|OJJ31495.1 afla|EED48965.1 anig|EHA26905.1 aver|OJJ02177.1 aory|EIT83308.1

Group2127: abra|OJJ70367.1 acar|OOF91904.1 acla|EAW12332.1 afis|EAW16890.1 afla|EED53384.1 afum|EAL86428.2 agla|OJJ79944.1 akaw|GAA91984.1 aluc|OJZ82629.1 anid|CBF80798.1 anig|EHA27448.1 aory|EIT75200.1 asyd|OJJ54488.1 ater|EAU30224.1 atub|OJI88862.1 aver|OJJ07691.1 awen|OJJ29748.1 azon|OJJ50203.1

Group2128: abra|OJJ70389.1 acar|OOF90063.1 acla|EAW12390.1 afis|EAW16837.1 afla|EED47319.1 afum|EAL86385.1 agla|OJJ83707.1 akaw|GAA91969.1 aluc|OJZ82647.1 anid|CBF80639.1 anig|EHA27425.1

aory|EIT82834.1 asyd|OJJ54621.1 ater|EAU30244.1 atub|OJI88879.1 aver|OJJ07537.1 awen|OJJ33871.1 azon|OJJ43960.1

Group2129: abra|OJJ70449.1 acar|OOF91831.1 acla|EAW09124.1 afis|EAW24708.1 afla|EED52336.1 afum|EAL89350.1 agla|OJJ82814.1 akaw|GAA90132.1 aluc|OJZ83215.1 anid|CBF83955.1 anig|EHA21075.1 aory|EIT74337.1 asyd|OJJ61431.1 ater|EAU37947.1 atub|OJI87721.1 aver|OJJ00766.1 awen|OJJ39949.1 azon|OJJ51122.1

Group2130: abra|OJJ70463.1 acar|OOF97461.1 acla|EAW13050.1 afis|EAW17617.1 afla|EED49208.1 afum|EAL91430.1 agla|OJJ81323.1 akaw|GAA83312.1 aluc|OJZ83806.1 anid|CBF90167.1 anig|EHA18953.1 aory|EIT83163.1 asyd|OJJ61992.1 ater|EAU37051.1 atub|OJI90173.1 aver|OJI96084.1 awen|OJJ37228.1 azon|OJJ50931.1

Group2131: abra|OJJ70464.1 acar|OOF97459.1 acla|EAW13049.1 afis|EAW17618.1 afla|EED49209.1 afum|EAL91431.1 agla|OJJ81322.1 akaw|GAA83313.1 aluc|OJZ83807.1 anid|CBF90165.1 anig|EHA18954.1 aory|EIT83149.1 asyd|OJJ61993.1 ater|EAU37052.1 atub|OJI90174.1 aver|OJI96085.1 awen|OJJ37227.1 azon|OJJ50930.1

Group2132: abra|OJJ70468.1 acar|OOF97456.1 acla|EAW13046.1 afis|EAW17621.1 afla|EED49196.1 afum|EAL91434.1 agla|OJJ81304.1 akaw|GAA83317.1 aluc|OJZ83812.1 anid|CBF90159.1 anig|EHA18957.1 aory|EIT78509.1 asyd|OJJ61996.1 ater|EAU37055.1 atub|OJI90179.1 aver|OJI96088.1 awen|OJJ37223.1 azon|OJJ50926.1

Group2133: abra|OJJ70469.1 acar|OOF97455.1 acla|EAW13045.1 afis|EAW17622.1 afla|EED49195.1 afum|EAL91435.1 agla|OJJ81303.1 akaw|GAA83318.1 aluc|OJZ83813.1 anid|CBF90157.1 anig|EHA18958.1 aory|EIT78576.1 asyd|OJJ61997.1 ater|EAU37056.1 atub|OJI90180.1 aver|OJI96089.1 awen|OJJ37222.1 azon|OJJ50925.1

Group2134: abra|OJJ70470.1 acar|OOF97453.1 acla|EAW13044.1 afis|EAW17623.1 afla|EED49194.1 afum|EAL91437.1 agla|OJJ81302.1 akaw|GAA83319.1 aluc|OJZ83814.1 anid|CBF90156.1 anig|EHA18959.1 aory|EIT78552.1 asyd|OJJ61998.1 ater|EAU37057.1 atub|OJI90181.1 aver|OJI96090.1 awen|OJJ37221.1 azon|OJJ50924.1

Group2135: abra|OJJ70473.1 acar|OOF97452.1 acla|EAW13041.1 afis|EAW17626.1 afla|EED49192.1 afum|EAL91440.1 agla|OJJ81276.1 akaw|GAA83322.1 aluc|OJZ83817.1 anid|CBF90150.1 anig|EHA18962.1 aory|EIT78505.1 asyd|OJJ62001.1 ater|EAU37060.1 atub|OJI90184.1 aver|OJI96093.1 awen|OJJ37218.1 azon|OJJ50921.1

Group2136: abra|OJJ70475.1 acar|OOF97450.1 acla|EAW13039.1 afis|EAW17628.1 afla|EED49190.1 afum|EAL91442.1 agla|OJJ81275.1 akaw|GAA83324.1 aluc|OJZ83819.1 anid|CBF90147.1 anig|EHA18964.1 aory|EIT78550.1 asyd|OJJ62002.1 ater|EAU37062.1 atub|OJI90186.1 aver|OJI96094.1 awen|OJJ37216.1 azon|OJJ50919.1

Group2137: abra|OJJ70476.1 acar|OOF97449.1 acla|EAW13038.1 afis|EAW17629.1 afla|EED49189.1 afum|EAL91443.1 agla|OJJ81272.1 akaw|GAA83325.1 aluc|OJZ83820.1 anid|CBF90141.1 anig|EHA18965.1 aory|EIT78529.1 asyd|OJJ62006.1 ater|EAU37063.1 atub|OJI90187.1 aver|OJI96098.1 awen|OJJ37215.1 azon|OJJ50916.1

Group2138: abra|OJJ70477.1 acar|OOF97448.1 acla|EAW13037.1 afis|EAW17630.1 afla|EED49188.1 afum|EAL91445.1 agla|OJJ81271.1 akaw|GAA83326.1 aluc|OJZ83822.1 anid|CBF90143.1 anig|EHA18966.1 aory|EIT78536.1 asyd|OJJ62005.1 ater|EAU37065.1 atub|OJI90189.1 aver|OJI96096.1 awen|OJJ37214.1 azon|OJJ50914.1

Group2139: abra|OJJ70478.1 acar|OOF97447.1 acla|EAW13036.1 afis|EAW17631.1 afla|EED49187.1 afum|EAL91446.1 agla|OJJ81270.1 akaw|GAA83327.1 aluc|OJZ83823.1 anid|CBF90145.1 anig|EHA18967.1 aory|EIT78534.1 asyd|OJJ62004.1 ater|EAU37066.1 atub|OJI90190.1 aver|OJI96095.1 awen|OJJ37213.1 azon|OJJ50913.1

Group2140: abra|OJJ70481.1 acar|OOF97444.1 acla|EAW13035.1 afis|EAW17632.1 afla|EED49186.1 afum|EAL91447.1 agla|OJJ81269.1 akaw|GAA83329.1 aluc|OJZ83825.1 anid|CBF90139.1 anig|EHA18969.1 aory|EIT78523.1 asyd|OJJ62011.1 ater|EAU37067.1 atub|OJI90192.1 aver|OJI96103.1 awen|OJJ37212.1 azon|OJJ50912.1

Group2141: abra|OJJ70482.1 acar|OOF97443.1 acla|EAW13034.1 afis|EAW17633.1 afla|EED50531.1 afum|EAL91448.1 agla|OJJ81266.1 akaw|GAA83330.1 aluc|OJZ83826.1 anid|CBF90137.1 anig|EHA18970.1 aory|EIT82093.1 asyd|OJJ62012.1 ater|EAU37069.1 atub|OJI90193.1 aver|OJI96104.1 awen|OJJ37211.1 azon|OJJ50911.1

Group2142: abra|OJJ70483.1 acar|OOF97442.1 acla|EAW13033.1 afis|EAW17634.1 afla|EED50530.1 afum|EAL91449.1 agla|OJJ81265.1 akaw|GAA83331.1 aluc|OJZ83827.1 anid|CBF90135.1 anig|EHA18971.1 aory|EIT82030.1 asyd|OJJ62013.1 ater|EAU37070.1 atub|OJI90194.1 aver|OJI96105.1 awen|OJJ37210.1 azon|OJJ50910.1

Group2143: abra|OJJ70484.1 acar|OOF97441.1 acla|EAW13031.1 afis|EAW17636.1 afla|EED50528.1 afum|EAL91451.2 agla|OJJ81263.1 akaw|GAA83333.1 aluc|OJZ83829.1 anid|CBF90132.1 anig|EHA18972.1 aory|EIT82038.1 asyd|OJJ62015.1 ater|EAU37072.1 atub|OJI90196.1 aver|OJI96106.1 awen|OJJ37208.1 azon|OJJ50908.1

Group2144: abra|OJJ70488.1 acar|OOF97438.1 acla|EAW13028.1 afis|EAW17639.1 afla|EED50524.1 afum|EAL91454.1 agla|OJJ79567.1 akaw|GAA83336.1 aluc|OJZ83832.1 anid|CBF90125.1 anig|EHA18975.1 aory|EIT82049.1 asyd|OJJ62018.1 ater|EAU37075.1 atub|OJI90199.1 aver|OJI96109.1 awen|OJJ37206.1 azon|OJJ50905.1

Group2145: abra|OJJ70489.1 acar|OOF97437.1 acla|EAW13027.1 afis|EAW17640.1 afla|EED50523.1 afum|EAL91455.1 agla|OJJ79568.1 akaw|GAA83337.1 aluc|OJZ83833.1 anig|EHA18976.1 aory|EIT82102.1 asyd|OJJ62019.1 ater|EAU37076.1 atub|OJI90200.1 aver|OJI96110.1 awen|OJJ37205.1 azon|OJJ50904.1 anid|CBF90123.1

Group2146: abra|OJJ70490.1 acar|OOF97436.1 acla|EAW13026.1 afis|EAW17641.1 afla|EED50522.1 afum|EAL91456.1 agla|OJJ79569.1 akaw|GAA83338.1 aluc|OJZ83834.1 anid|CBF90121.1 anig|EHA18977.1 aory|EIT82016.1 asyd|OJJ62020.1 ater|EAU37077.1 atub|OJI90201.1 aver|OJI96111.1 awen|OJJ37204.1 azon|OJJ50903.1

Group2147: abra|OJJ70492.1 acar|OOF97435.1 acla|EAW13025.1 afis|EAW17642.1 afla|EED50518.1 afum|EAL91457.1 agla|OJJ79562.1 akaw|GAA83340.1 aluc|OJZ83836.1 anid|CBF90119.1 anig|EHA18979.1 aory|EIT82059.1 asyd|OJJ62021.1 ater|EAU37078.1 atub|OJI90203.1 aver|OJI96112.1 awen|OJJ37203.1 azon|OJJ50902.1

Group2148: abra|OJJ70493.1 acar|OOF97432.1 acla|EAW13024.1 afis|EAW17643.1 afla|EED50517.1 afum|EAL91458.1 agla|OJJ79561.1 akaw|GAA83341.1 aluc|OJZ83837.1 anid|CBF90118.1 anig|EHA18980.1 aory|EIT82149.1 asyd|OJJ62022.1 ater|EAU37079.1 atub|OJI90204.1 aver|OJI96113.1 awen|OJJ37202.1 azon|OJJ50901.1

Group2149: abra|OJJ70495.1 acar|OOF97431.1 acla|EAW13022.1 afis|EAW17645.1 afla|EED50512.1 afum|EAL91460.1 agla|OJJ79559.1 akaw|GAA83343.1 aluc|OJZ83840.1 anid|CBF90114.1 anig|EHA18982.1 aory|EIT82064.1 asyd|OJJ62024.1 ater|EAU37081.1 atub|OJI90206.1 aver|OJI96115.1 awen|OJJ37200.1 azon|OJJ50899.1

Group2150: abra|OJJ70498.1 acar|OOF97428.1 acla|EAW08176.1 afis|EAW23212.1 afla|EED47039.1 afum|EAL88272.1 agla|OJJ78733.1 akaw|GAA83346.1 aluc|OJZ83843.1 anid|CBF74740.1 anig|EHA18985.1 aory|EIT72595.1 asyd|OJJ60184.1 ater|EAU36340.1 atub|OJI90209.1 aver|OJJ04080.1 awen|OJJ39445.1 azon|OJJ47647.1

Group2151: abra|OJJ70499.1 acar|OOF97427.1 acla|EAW08175.1 afis|EAW23211.1 afla|EED47040.1 afum|EAL88273.1 agla|OJJ78734.1 akaw|GAA83347.1 aluc|OJZ83844.1 anid|CBF74738.1 anig|EHA18986.1 aory|EIT72653.1 asyd|OJJ60185.1 ater|EAU36339.1 atub|OJI90210.1 aver|OJJ04079.1 awen|OJJ39444.1 azon|OJJ47646.1

Group2152: abra|OJJ70500.1 acar|OOF97425.1 acla|EAW08174.1 afis|EAW23210.1 afla|EED47041.1 afum|EAL88274.1 agla|OJJ78735.1 akaw|GAA83348.1 aluc|OJZ83845.1 anid|CBF74736.1 anig|EHA18987.1 aory|EIT72592.1 asyd|OJJ60186.1 ater|EAU36338.1 atub|OJI90211.1 aver|OJJ04078.1 awen|OJJ39443.1 azon|OJJ47645.1

Group2153: abra|OJJ70504.1 acar|OOF97421.1 acla|EAW08170.1 afis|EAW23206.1 afla|EED47047.1 afum|EAL88277.1 agla|OJJ78739.1 akaw|GAA83353.1 aluc|OJZ83849.1 anid|CBF74734.1 anig|EHA18991.1 aory|EIT72747.1 asyd|OJJ60189.1 ater|EAU36334.1 atub|OJI90215.1 aver|OJJ04075.1 awen|OJJ39438.1 azon|OJJ47641.1

Group2154: abra|OJJ70509.1 acar|OOF97420.1 acla|EAW08166.1 afis|EAW23204.1 afla|EED47050.1 afum|EAL88279.1 agla|OJJ78743.1 akaw|GAA83356.1 aluc|OJZ83854.1 anid|CBF74730.1 anig|EHA18995.1 aory|EIT72509.1 asyd|OJJ60194.1 ater|EAU36332.1 atub|OJI90221.1 aver|OJJ04071.1 awen|OJJ39434.1 azon|OJJ47638.1

Group2155: abra|OJJ70510.1 acar|OOF97419.1 acla|EAW08165.1 afis|EAW23203.1 afla|EED47051.1 afum|EAL88280.1 agla|OJJ78745.1 akaw|GAA83357.1 aluc|OJZ83855.1 anid|CBF74728.1 anig|EHA18996.1 aory|EIT72511.1 asyd|OJJ60195.1 ater|EAU36331.1 atub|OJI90222.1 aver|OJJ04070.1 awen|OJJ39433.1 azon|OJJ47637.1

Group2156: abra|OJJ70512.1 acar|OOF97417.1 acla|EAW08163.1 afis|EAW23201.1 afla|EED47053.1 afum|EAL88282.1 agla|OJJ78747.1 akaw|GAA83359.1 aluc|OJZ83857.1 anid|CBF74721.1 anig|EHA18998.1 aory|EIT72498.1 asyd|OJJ60201.1 ater|EAU36329.1 atub|OJI90224.1 aver|OJJ04065.1 awen|OJJ39431.1 azon|OJJ47635.1

Group2157: abra|OJJ70514.1 acar|OOF97415.1 acla|EAW08161.1 afis|EAW23199.1 afla|EED47055.1 afum|EAL88284.1 agla|OJJ78749.1 akaw|GAA83361.1 aluc|OJZ83859.1 anid|CBF74717.1 anig|EHA19000.1

aory|EIT72508.1 asyd|OJJ60203.1 ater|EAU36327.1 atub|OJI90226.1 aver|OJJ04063.1 awen|OJJ39429.1 azon|OJJ47633.1

Group2158: abra|OJJ70515.1 acar|OOF97414.1 acla|EAW08160.1 afis|EAW23198.1 afla|EED47056.1 afum|EAL88285.1 agla|OJJ86331.1 akaw|GAA83362.1 aluc|OJZ83860.1 anid|CBF74715.1 anig|EHA19001.1 aory|EIT72476.1 asyd|OJJ60205.1 ater|EAU36325.1 atub|OJI90228.1 aver|OJJ04062.1 awen|OJJ39428.1 azon|OJJ47632.1

Group2159: abra|OJJ70521.1 acar|OOF97407.1 acla|EAW08154.1 afis|EAW23185.1 afla|EED47062.1 afum|EAL88298.1 agla|OJJ86360.1 akaw|GAA83368.1 aluc|OJZ83866.1 anid|CBF74698.1 anig|EHA19007.1 aory|EIT72505.1 asyd|OJJ60215.1 ater|EAU36320.1 atub|OJI90234.1 aver|OJJ04053.1 awen|OJJ36587.1 azon|OJJ47619.1

Group2160: abra|OJJ70522.1 acar|OOF97406.1 acla|EAW08153.1 afis|EAW23186.1 afla|EED47064.1 afum|EAL88297.1 agla|OJJ86361.1 akaw|GAA83369.1 aluc|OJZ83867.1 anid|CBF74696.1 anig|EHA19008.1 aory|EIT72495.1 asyd|OJJ60214.1 ater|EAU36319.1 atub|OJI90235.1 aver|OJJ04054.1 awen|OJJ36588.1 azon|OJJ47618.1

Group2161: abra|OJJ70529.1 acar|OOF97402.1 acla|EAW08142.1 afis|EAW23178.1 afla|EED49000.1 afum|EAL88305.1 agla|OJJ89690.1 akaw|GAA83374.1 aluc|OJZ83872.1 anid|CBF74512.1 anig|EHA19013.1 aory|EIT80028.1 asyd|OJJ57730.1 ater|EAU36311.1 atub|OJI90240.1 aver|OJJ05439.1 awen|OJJ36601.1 azon|OJJ47607.1

Group2162: abra|OJJ70532.1 acar|OOF97400.1 acla|EAW08145.1 afis|EAW23181.1 afla|EED47073.1 afum|EAL88302.1 akaw|GAA83377.1 aluc|OJZ83875.1 anid|CBF74516.1 anig|EHA19015.1 aory|EIT72487.1 asyd|OJJ57732.1 ater|EAU36314.1 atub|OJI90243.1 aver|OJJ05437.1 awen|OJJ36597.1 azon|OJJ47611.1 agla|OJJ86370.1

Group2163: abra|OJJ70536.1 acar|OOF97397.1 acla|EAW08148.1 afis|EAW23191.1 afla|EED47070.1 afum|EAL88292.1 agla|OJJ86367.1 akaw|GAA83381.1 aluc|OJZ83880.1 anid|CBF74521.1 anig|EHA19018.1 aory|EIT72473.1 asyd|OJJ57733.1 ater|EAU36315.1 atub|OJI90247.1 aver|OJJ05434.1 awen|OJJ36593.1 azon|OJJ47612.1

Group2164: abra|OJJ70537.1 acar|OOF97396.1 acla|EAW08149.1 afis|EAW23190.1 afla|EED47069.1 afum|EAL88293.1 agla|OJJ86365.1 akaw|GAA83382.1 aluc|OJZ83881.1 anid|CBF74523.1 anig|EHA19019.1 aory|EIT72486.1 asyd|OJJ57734.1 ater|EAU36309.1 atub|OJI90249.1 aver|OJJ05433.1 awen|OJJ36592.1 azon|OJJ47614.1

Group2165: abra|OJJ70539.1 acar|OOF97394.1 acla|EAW08151.1 afis|EAW23188.1 afla|EED47067.1 afum|EAL88295.1 agla|OJJ86363.1 akaw|GAA83384.1 aluc|OJZ83884.1 anid|CBF74527.1 anig|EHA19021.1 aory|EIT72500.1 asyd|OJJ57736.1 ater|EAU36310.1 atub|OJI90251.1 aver|OJJ05431.1 awen|OJJ36590.1 azon|OJJ47616.1

Group2166: abra|OJJ70541.1 acar|OOF97392.1 acla|EAW08137.1 afis|EAW23173.1 afla|EED46946.1 afum|EAL88310.1 agla|OJJ89685.1 akaw|GAA83387.1 aluc|OJZ83886.1 anid|CBF74495.1 anig|EHA19023.1 aory|EIT72576.1 asyd|OJJ57718.1 ater|EAU36302.1 atub|OJI90253.1 aver|OJJ05449.1 awen|OJJ36604.1 azon|OJJ47602.1

Group2167: abra|OJJ70552.1 acar|OOF97168.1 acla|EAW08344.1 afis|EAW23379.1 afla|EED55578.1 afum|EAL88092.1 agla|OJJ86444.1 akaw|GAA83396.1 aluc|OJZ83895.1 anid|CBF74849.1 anig|EHA19031.1 aory|EIT82685.1 asyd|OJJ60128.1 ater|EAU35633.1 atub|OJI90263.1 aver|OJJ04133.1 awen|OJJ39503.1 azon|OJJ47704.1

Group2168: abra|OJJ70554.1 acar|OOF97165.1 acla|EAW08350.1 afis|EAW23383.1 afla|EED55571.1 afum|EAL88088.1 agla|OJJ86437.1 akaw|GAA83399.1 aluc|OJZ83897.1 anid|CBF74854.1 anig|EHA19034.1 aory|EIT82660.1 asyd|OJJ60126.1 ater|EAU35636.1 atub|OJI90267.1 aver|OJJ04136.1 awen|OJJ39498.1 azon|OJJ47700.1

Group2169: abra|OJJ70555.1 acar|OOF97164.1 acla|EAW08353.1 afis|EAW23384.1 afla|EED55572.1 afum|EAL88087.1 agla|OJJ86436.1 akaw|GAA83400.1 aluc|OJZ83898.1 anid|CBF74856.1 anig|EHA19035.1 aory|EIT82663.1 asyd|OJJ60125.1 ater|EAU35637.1 atub|OJI90268.1 aver|OJJ04137.1 awen|OJJ39497.1 azon|OJJ47699.1

Group2170: abra|OJJ70556.1 acar|OOF97163.1 acla|EAW08354.1 afis|EAW23385.1 afla|EED55573.1 afum|EAL88086.2 agla|OJJ86435.1 akaw|GAA83401.1 aluc|OJZ83899.1 anid|CBF74875.1 anig|EHA19036.1 aory|EIT82690.1 asyd|OJJ60114.1 ater|EAU35638.1 atub|OJI90269.1 aver|OJJ04148.1 awen|OJJ39496.1 azon|OJJ47698.1

Group2171: abra|OJJ70557.1 acar|OOF97162.1 acla|EAW08355.1 afis|EAW23386.1 afla|EED55574.1 afum|EAL88085.1 agla|OJJ86432.1 akaw|GAA83402.1 aluc|OJZ83900.1 anid|CBF74873.1 anig|EHA19037.1 aory|EIT82673.1 asyd|OJJ60115.1 ater|EAU35639.1 atub|OJI90270.1 aver|OJJ04147.1 awen|OJJ39495.1 azon|OJJ47696.1

Group2172: abra|OJJ70559.1 acar|OOF97161.1 acla|EAW08357.1 afis|EAW23388.1 afla|EED55576.1 afum|EAL88083.1 agla|OJJ86430.1 akaw|GAA83404.1 aluc|OJZ83903.1 anid|CBF74870.1 anig|EHA19038.1 aory|EIT82706.1 asyd|OJJ60117.1 ater|EAU35640.1 atub|OJI90272.1 aver|OJJ04145.1 awen|OJJ39493.1 azon|OJJ47694.1

Group2173: abra|OJJ70560.1 acar|OOF97160.1 acla|EAW08358.1 afis|EAW23389.1 afla|EED55568.1 afum|EAL88082.1 agla|OJJ86428.1 akaw|GAA83405.1 aluc|OJZ83904.1 anid|CBF74868.1 anig|EHA19039.1 aory|EIT82664.1 asyd|OJJ60118.1 ater|EAU35642.1 atub|OJI90273.1 aver|OJJ04144.1 awen|OJJ39492.1 azon|OJJ47693.1

Group2174: abra|OJJ70561.1 acar|OOF97158.1 acla|EAW08360.1 afis|EAW23391.1 afla|EED55566.1 afum|EAL88080.1 agla|OJJ86426.1 akaw|GAA83406.1 aluc|OJZ83905.1 anid|CBF74864.1 anig|EHA19040.1 aory|EIT82703.1 asyd|OJJ60121.1 ater|EAU35644.1 atub|OJI90276.1 aver|OJJ04141.1 awen|OJJ39488.1 azon|OJJ47691.1

Group2175: abra|OJJ70564.1 acar|OOF97156.1 acla|EAW08362.1 afis|EAW23393.1 afla|EED55564.1 afum|EAL88077.1 agla|OJJ86420.1 akaw|GAA83408.1 aluc|OJZ83907.1 anid|CBF74860.1 anig|EHA19042.1 aory|EIT82692.1 asyd|OJJ60123.1 ater|EAU35646.1 atub|OJI90278.1 aver|OJJ04139.1 awen|OJJ39486.1 azon|OJJ47689.1

Group2176: abra|OJJ70567.1 acar|OOF97155.1 acla|EAW08366.1 afis|EAW23396.1 afla|EED55560.1 afum|EAL88074.1 agla|OJJ86410.1 akaw|GAA83411.1 aluc|OJZ83910.1 anid|CBF74807.1 anig|EHA19044.1 aory|EIT82680.1 asyd|OJJ60148.1 ater|EAU35649.1 atub|OJI90281.1 aver|OJJ04115.1 awen|OJJ39483.1 azon|OJJ48236.1

Group2177: abra|OJJ70568.1 acar|OOF97154.1 acla|EAW08367.1 afis|EAW23397.1 afla|EED55559.1 afum|EAL88073.1 agla|OJJ86409.1 akaw|GAA83412.1 aluc|OJZ83911.1 anid|CBF74805.1 anig|EHA19045.1 aory|EIT82705.1 asyd|OJJ60149.1 ater|EAU35650.1 atub|OJI90282.1 aver|OJJ04114.1 awen|OJJ39482.1 azon|OJJ47684.1

Group2178: abra|OJJ70569.1 acar|OOF97152.1 acla|EAW08371.1 afis|EAW23398.1 afla|EED55554.1 afum|EAL88072.1 agla|OJJ86403.1 akaw|GAA83413.1 aluc|OJZ83913.1 anid|CBF74803.1 anig|EHA19046.1 aory|EIT82712.1 asyd|OJJ60150.1 ater|EAU35651.1 atub|OJI90283.1 aver|OJJ04113.1 awen|OJJ39481.1 azon|OJJ47683.1

Group2179: abra|OJJ70570.1 acar|OOF97151.1 acla|EAW08370.1 afis|EAW23399.1 afla|EED47005.1 afum|EAL88071.1 agla|OJJ86402.1 akaw|GAA83414.1 aluc|OJZ83914.1 anid|CBF74801.1 anig|EHA19047.1 aory|EIT72537.1 asyd|OJJ60152.1 ater|EAU36378.1 atub|OJI90284.1 aver|OJJ04112.1 awen|OJJ39480.1 azon|OJJ47682.1

Group2180: abra|OJJ70571.1 acar|OOF97150.1 acla|EAW08369.1 afis|EAW23400.1 afla|EED47006.1 afum|EAL88070.1 agla|OJJ86401.1 akaw|GAA83415.1 aluc|OJZ83915.1 anid|CBF73601.1 anig|EHA19048.1 aory|EIT72638.1 asyd|OJJ56680.1 ater|EAU36377.1 atub|OJI90285.1 aver|OJJ06043.1 awen|OJJ39479.1 azon|OJJ47681.1

Group2181: abra|OJJ70574.1 acar|OOF97147.1 acla|EAW08373.1 afis|EAW23402.1 afla|EED47009.1 afum|EAL88068.1 agla|OJJ86389.1 akaw|GAA83418.1 aluc|OJZ83918.1 anid|CBF74795.1 anig|EHA19051.1 aory|EIT72635.1 asyd|OJJ60155.1 ater|EAU36373.1 atub|OJI90289.1 aver|OJJ04109.1 awen|OJJ39476.1 azon|OJJ47679.1

Group2182: abra|OJJ70576.1 acar|OOF97145.1 acla|EAW08375.1 afis|EAW23404.1 afla|EED47011.1 afum|EAL88066.1 agla|OJJ86392.1 akaw|GAA83420.1 aluc|OJZ83920.1 anid|CBF74793.1 anig|EHA19053.1 aory|EIT72673.1 asyd|OJJ60156.1 ater|EAU36371.1 atub|OJI90292.1 aver|OJJ04108.1 awen|OJJ39473.1 azon|OJJ47675.1

Group2183: abra|OJJ70577.1 acar|OOF97144.1 acla|EAW08203.1 afis|EAW23237.1 afla|EED47012.1 afum|EAL88245.1 agla|OJJ86393.1 akaw|GAA83421.1 aluc|OJZ83921.1 anid|CBF74787.1 anig|EHA19054.1 aory|EIT72690.1 asyd|OJJ60159.1 ater|EAU36370.1 atub|OJI90293.1 aver|OJJ04105.1 awen|OJJ39472.1 azon|OJJ47674.1

Group2184: abra|OJJ70578.1 acar|OOF97143.1 acla|EAW08202.1 afis|EAW23236.1 afla|EED47013.1 afum|EAL88246.1 agla|OJJ86394.1 akaw|GAA83422.1 aluc|OJZ83922.1 anid|CBF74785.1 anig|EHA19055.1 aory|EIT72768.1 asyd|OJJ60160.1 ater|EAU36369.1 atub|OJI90294.1 aver|OJJ04104.1 awen|OJJ39471.1 azon|OJJ47673.1

Group2185: abra|OJJ70580.1 acar|OOF97138.1 acla|EAW08200.1 afis|EAW23234.1 afla|EED47015.1 afum|EAL88248.1 agla|OJJ86396.1 akaw|GAA83424.1 aluc|OJZ83925.1 anid|CBF74783.1 anig|EHA19057.1 aory|EIT72553.1 asyd|OJJ60161.1 ater|EAU36367.1 atub|OJI90297.1 aver|OJJ04103.1 awen|OJJ39469.1 azon|OJJ47669.1

Group2186: abra|OJJ70582.1 acar|OOF97137.1 acla|EAW08198.1 afis|EAW23232.1 afla|EED47016.1 afum|EAL88250.1 agla|OJJ86398.1 akaw|GAA83426.1 aluc|OJZ83927.1 anid|CBF74779.1 anig|EHA19059.1

aory|EIT72651.1 asyd|OJJ60163.1 ater|EAU36361.1 atub|OJI90299.1 aver|OJJ04101.1 awen|OJJ39467.1 azon|OJJ47667.1

Group2187: abra|OJJ70584.1 acar|OOF97136.1 acla|EAW08197.1 afis|EAW23231.1 afla|EED47019.1 afum|EAL88252.1 agla|OJJ86399.1 akaw|GAA83427.1 aluc|OJZ83928.1 anid|CBF74777.1 anig|EHA19060.1 aory|EIT72752.1 asyd|OJJ60164.1 ater|EAU36360.1 atub|OJI90300.1 aver|OJJ04100.1 awen|OJJ39466.1 azon|OJJ47666.1

Group2188: abra|OJJ70585.1 acar|OOF97135.1 acla|EAW08196.1 afis|EAW23230.1 afla|EED47020.1 afum|EAL88253.1 agla|OJJ86400.1 akaw|GAA83428.1 aluc|OJZ83929.1 anid|CBF74775.1 anig|EHA19061.1 aory|EIT72543.1 asyd|OJJ60165.1 ater|EAU36359.1 atub|OJI90301.1 aver|OJJ04099.1 awen|OJJ39465.1 azon|OJJ47665.1

Group2189: abra|OJJ70589.1 acar|OOF97128.1 acla|EAW08192.1 afis|EAW23226.1 afla|EED47024.1 afum|EAL88257.1 agla|OJJ86383.1 akaw|GAA83433.1 aluc|OJZ83934.1 anid|CBF74767.1 anig|EHA19065.1 aory|EIT72765.1 asyd|OJJ60169.1 ater|EAU36356.1 atub|OJI90306.1 aver|OJJ04095.1 awen|OJJ39461.1 azon|OJJ47660.1

Group2190: abra|OJJ70592.1 acar|OOF97126.1 acla|EAW08188.1 afis|EAW23222.1 afla|EED47028.1 afum|EAL88262.1 agla|OJJ86379.1 akaw|GAA83437.1 aluc|OJZ83938.1 anid|CBF74759.1 anig|EHA19068.1 aory|EIT72692.1 asyd|OJJ60173.1 ater|EAU36353.1 atub|OJI90311.1 aver|OJJ04091.1 awen|OJJ39458.1 azon|OJJ47656.1

Group2191: abra|OJJ70594.1 acar|OOF97124.1 acla|EAW08186.1 afis|EAW23220.1 afla|EED47030.1 afum|EAL88264.1 agla|OJJ86377.1 akaw|GAA83439.1 aluc|OJZ83940.1 anid|CBF74757.1 anig|EHA19070.1 aory|EIT72550.1 asyd|OJJ60174.1 ater|EAU36351.1 atub|OJI90313.1 aver|OJJ04089.1 awen|OJJ39456.1 azon|OJJ47654.1

Group2192: abra|OJJ70595.1 acar|OOF97123.1 acla|EAW08185.1 afis|EAW23219.1 afla|EED47031.1 afum|EAL88265.1 agla|OJJ86376.1 akaw|GAA83440.1 aluc|OJZ83941.1 anid|CBF74755.1 anig|EHA19071.1 aory|EIT72573.1 asyd|OJJ60175.1 ater|EAU36350.1 atub|OJI90314.1 aver|OJJ04088.1 awen|OJJ39455.1 azon|OJJ47653.1

Group2193: abra|OJJ70600.1 acar|OOF97120.1 acla|EAW08182.1 afis|EAW23217.1 afla|EED47035.1 afum|EAL88267.1 agla|OJJ86373.1 akaw|GAA89940.1 aluc|OJZ83945.1 anid|CBF74749.1 anig|EHA19074.1 aory|EIT72655.1 asyd|OJJ60178.1 ater|EAU36347.1 atub|OJI90316.1 aver|OJJ04084.1 awen|OJJ39451.1 azon|OJJ47651.1

Group2194: abra|OJJ70606.1 acar|OOF97115.1 acla|EAW13020.1 afis|EAW17647.1 afla|EED50511.1 afum|EAL91462.1 agla|OJJ79557.1 akaw|GAA89946.1 aluc|OJZ83952.1 anid|CBF90111.1 anig|EHA19080.1 aory|EIT82166.1 asyd|OJJ62026.1 ater|EAU37083.1 atub|OJI90323.1 aver|OJI96117.1 awen|OJJ37198.1 azon|OJJ50897.1

Group2195: abra|OJJ70607.1 acar|OOF97114.1 acla|EAW13019.1 afis|EAW17648.1 afla|EED50510.1 afum|EAL91464.1 agla|OJJ79556.1 akaw|GAA89947.1 aluc|OJZ83954.1 anid|CBF90087.1 anig|EHA19081.1 aory|EIT82125.1 asyd|OJJ62036.1 ater|EAU37084.1 atub|OJI90324.1 aver|OJI96142.1 awen|OJJ37197.1 azon|OJJ50896.1

Group2196: abra|OJJ70608.1 acar|OOF97113.1 acla|EAW13018.1 afis|EAW17649.1 afla|EED50509.1 afum|EAL91465.1 agla|OJJ79555.1 akaw|GAA89948.1 aluc|OJZ83955.1 anid|CBF90085.1 anig|EHA19082.1 aory|EIT82043.1 asyd|OJJ62037.1 ater|EAU37085.1 atub|OJI90325.1 aver|OJI96143.1 awen|OJJ37196.1 azon|OJJ50895.1

Group2197: abra|OJJ70611.1 acar|OOF97111.1 acla|EAW13016.1 afis|EAW17651.1 afla|EED50508.1 afum|EAL91466.1 agla|OJJ79553.1 akaw|GAA89950.1 aluc|OJZ83957.1 anid|CBF90077.1 anig|EHA19084.1 aory|EIT82060.1 asyd|OJJ62040.1 ater|EAU37087.1 atub|OJI90327.1 aver|OJI96146.1 awen|OJJ37194.1 azon|OJJ50893.1

Group2198: abra|OJJ70613.1 acar|OOF97109.1 acla|EAW13014.1 afis|EAW17653.1 afla|EED50505.1 afum|EAL91468.1 agla|OJJ79551.1 akaw|GAA89952.1 aluc|OJZ83959.1 anid|CBF90074.1 anig|EHA19087.1 aory|EIT82010.1 asyd|OJJ62042.1 ater|EAU37089.1 atub|OJI90331.1 aver|OJI96148.1 awen|OJJ37192.1 azon|OJJ50891.1

Group2199: abra|OJJ70616.1 acar|OOF97105.1 acla|EAW13011.1 afis|EAW17656.1 afla|EED50502.1 afum|EAL91471.1 agla|OJJ79549.1 akaw|GAA89955.1 aluc|OJZ83962.1 anid|CBF90066.1 anig|EHA19090.1 aory|EIT82103.1 asyd|OJJ62045.1 ater|EAU31997.1 atub|OJI90334.1 aver|OJI96151.1 awen|OJJ37187.1 azon|OJJ50889.1

Group2200: abra|OJJ70617.1 acar|OOF97104.1 acla|EAW13010.1 afis|EAW17657.1 afla|EED50501.1 afum|EAL91472.1 agla|OJJ79548.1 akaw|GAA89956.1 aluc|OJZ83963.1 anid|CBF90065.1 anig|EHA19091.1 aory|EIT82023.1 asyd|OJJ62046.1 ater|EAU31996.1 atub|OJI90335.1 aver|OJI96152.1 awen|OJJ37186.1 azon|OJJ50888.1

Group2201: abra|OJJ70618.1 acar|OOF97103.1 acla|EAW13009.1 afis|EAW17658.1 afla|EED50500.1 afum|EAL91473.1 agla|OJJ79547.1 akaw|GAA89957.1 aluc|OJZ83964.1 anid|CBF90063.1 anig|EHA19092.1 aory|EIT82147.1 asyd|OJJ62048.1 ater|EAU31994.1 atub|OJI90337.1 aver|OJI96153.1 awen|OJJ37185.1 azon|OJJ50886.1

Group2202: abra|OJJ70619.1 acar|OOF97102.1 acla|EAW13008.1 afis|EAW17659.1 afla|EED50499.1 afum|EAL91474.1 agla|OJJ79546.1 akaw|GAA89958.1 aluc|OJZ83965.1 anid|CBF90061.1 anig|EHA19093.1 aory|EIT82055.1 asyd|OJJ62049.1 ater|EAU31993.1 atub|OJI90338.1 aver|OJI96154.1 awen|OJJ37184.1 azon|OJJ50885.1

Group2203: abra|OJJ70620.1 acar|OOF97101.1 acla|EAW13007.1 afis|EAW17660.1 afla|EED50497.1 afum|EAL91475.1 agla|OJJ79529.1 akaw|GAA89959.1 aluc|OJZ83966.1 anid|CBF90059.1 anig|EHA19094.1 aory|EIT82172.1 asyd|OJJ62050.1 ater|EAU31992.1 atub|OJI90339.1 aver|OJI96155.1 awen|OJJ37182.1 azon|OJJ50884.1

Group2204: abra|OJJ70625.1 acar|OOF97099.1 acla|EAW13005.1 afis|EAW17663.1 afla|EED50495.1 afum|EAL91477.2 agla|OJJ79532.1 akaw|GAA89963.1 aluc|OJZ83970.1 anid|CBF90056.1 anig|EHA19099.1 aory|EIT82160.1 asyd|OJJ62052.1 ater|EAU31990.1 atub|OJI90343.1 aver|OJI96158.1 awen|OJJ37179.1 azon|OJJ50881.1

Group2205: abra|OJJ70626.1 acar|OOF97098.1 acla|EAW08131.1 afis|EAW23168.1 afla|EED46953.1 afum|EAL88316.1 agla|OJJ89679.1 akaw|GAA89964.1 aluc|OJZ83971.1 anid|CBF74478.1 anig|EHA19100.1 aory|EIT72624.1 asyd|OJJ57707.1 ater|EAU36297.1 atub|OJI90344.1 aver|OJJ05457.1 awen|OJJ36615.1 azon|OJJ47598.1

Group2206: abra|OJJ70627.1 acar|OOF97097.1 acla|EAW08130.1 afis|EAW23167.1 afla|EED46954.1 afum|EAL88317.2 agla|OJJ89678.1 akaw|GAA89965.1 aluc|OJZ83972.1 anid|CBF74476.1 anig|EHA19101.1 aory|EIT72656.1 asyd|OJJ57706.1 ater|EAU36296.1 atub|OJI90345.1 aver|OJJ05458.1 awen|OJJ36616.1 azon|OJJ41979.1

Group2207: abra|OJJ70628.1 acar|OOF97096.1 acla|EAW08129.1 afis|EAW23166.1 afla|EED46955.1 afum|EAL88318.1 agla|OJJ89677.1 akaw|GAA89966.1 aluc|OJZ83974.1 anid|CBF74475.1 anig|EHA19102.1 aory|EIT72623.1 asyd|OJJ57705.1 ater|EAU36295.1 atub|OJI90347.1 aver|OJJ05459.1 awen|OJJ36617.1 azon|OJJ47597.1

Group2208: abra|OJJ70629.1 acar|OOF97095.1 acla|EAW08128.1 afis|EAW23165.1 afla|EED46957.1 afum|EBA27343.1 agla|OJJ89676.1 akaw|GAA89967.1 aluc|OJZ83976.1 anid|CBF74473.1 anig|EHA19103.1 aory|EIT72540.1 asyd|OJJ57704.1 ater|EAU36294.1 atub|OJI90349.1 aver|OJJ05460.1 awen|OJJ36618.1 azon|OJJ47595.1

Group2209: abra|OJJ70630.1 acar|OOF97093.1 acla|EAW08126.1 afis|EAW23163.1 afla|EED46958.1 afum|EAL88320.1 agla|OJJ89674.1 akaw|GAA89969.1 aluc|OJZ83978.1 anid|CBF74467.1 anig|EHA19105.1 aory|EIT72637.1 asyd|OJJ57700.1 ater|EAU36293.1 atub|OJI90351.1 aver|OJJ05464.1 awen|OJJ36620.1 azon|OJJ47593.1

Group2210: abra|OJJ70639.1 acar|OOF97080.1 acla|EAW08118.1 afis|EAW23133.1 afla|EED48995.1 afum|EAL88348.1 agla|OJJ89666.1 akaw|GAA89975.1 aluc|OJZ83986.1 anid|CBF74456.1 anig|EHA19112.1 aory|EIT80032.1 asyd|OJJ57677.1 ater|EAU36286.1 atub|OJI90358.1 aver|OJJ05483.1 awen|OJJ36627.1 azon|OJJ47586.1

Group2211: abra|OJJ70640.1 acar|OOF97081.1 acla|EAW08117.1 afis|EAW23134.1 afla|EED48497.1 afum|EAL88347.1 agla|OJJ89665.1 akaw|GAA89976.1 aluc|OJZ83987.1 anid|CBF74455.1 anig|EHA19113.1 aory|EIT73203.1 asyd|OJJ57676.1 ater|EAU36282.1 atub|OJI90359.1 aver|OJJ05482.1 awen|OJJ36630.1 azon|OJJ47584.1

Group2212: abra|OJJ70646.1 acar|OOF97075.1 acla|EAW08114.1 afis|EAW23137.1 afla|EED48500.1 afum|EAL88344.1 agla|OJJ89663.1 akaw|GAA89982.1 aluc|OJZ83996.1 anid|CBF74450.1 anig|EHA19119.1 aory|EIT73193.1 asyd|OJJ57740.1 ater|EAU36280.1 atub|OJI90365.1 aver|OJJ05427.1 awen|OJJ36633.1 azon|OJJ47582.1

Group2213: abra|OJJ70653.1 acar|OOF97070.1 acla|EAW08106.1 afis|EAW23145.1 afla|EED48508.1 afum|EAL88336.1 agla|OJJ89657.1 akaw|GAA89989.1 aluc|OJZ84003.1 anid|CBF74436.1 anig|EHA19125.1 aory|EIT73083.1 asyd|OJJ57747.1 ater|EAU36266.1 atub|OJI90372.1 aver|OJJ05420.1 awen|OJJ36640.1 azon|OJJ47574.1

Group2214: abra|OJJ70654.1 acar|OOF97068.1 acla|EAW08105.1 afis|EAW23146.1 afla|EED48509.1 afum|EAL88335.1 agla|OJJ89656.1 akaw|GAA89990.1 aluc|OJZ84004.1 anid|CBF74434.1 anig|EHA19126.1 aory|EIT73165.1 asyd|OJJ57748.1 ater|EAU36265.1 atub|OJI90373.1 aver|OJJ05419.1 awen|OJJ36641.1 azon|OJJ47573.1

Group2215: abra|OJJ70657.1 acar|OOF97065.1 acla|EAW08102.1 afis|EAW23149.1 afla|EED48512.1 afum|EAL88332.1 agla|OJJ89653.1 akaw|GAA89993.1 aluc|OJZ84007.1 anid|CBF74428.1 anig|EHA19129.1

aory|EIT73157.1 asyd|OJJ57751.1 ater|EAU36263.1 atub|OJI90376.1 aver|OJJ05416.1 awen|OJJ36645.1 azon|OJJ47570.1

Group2216: abra|OJJ70659.1 acar|OOF97063.1 acla|EAW08100.1 afis|EAW23151.1 afla|EED48514.1 afum|EAL88330.2 agla|OJJ89651.1 akaw|GAA89995.1 aluc|OJZ84009.1 anid|CBF74424.1 anig|EHA19131.1 aory|EIT73110.1 asyd|OJJ57753.1 ater|EAU36261.1 atub|OJI90378.1 aver|OJJ05414.1 awen|OJJ36647.1 azon|OJJ47568.1

Group2217: abra|OJJ70661.1 acar|OOF97060.1 acla|EAW08097.1 afis|EAW23153.1 afla|EED48516.1 afum|EAL88328.1 agla|OJJ89648.1 akaw|GAA89997.1 aluc|OJZ84011.1 anid|CBF74421.1 anig|EHA19134.1 aory|EIT73108.1 asyd|OJJ57755.1 ater|EAU36258.1 atub|OJI90380.1 aver|OJJ05412.1 awen|OJJ36649.1 azon|OJJ47553.1

Group2218: abra|OJJ70666.1 acar|OOF97058.1 acla|EAW08096.1 afis|EAW23154.1 afla|EED48519.1 afum|EAL88327.2 agla|OJJ89646.1 akaw|GAA90001.1 aluc|OJZ84016.1 anid|CBF74419.1 anig|EHA19138.1 aory|EIT73174.1 asyd|OJJ57756.1 ater|EAU36257.1 atub|OJI90387.1 aver|OJJ05411.1 awen|OJJ36650.1 azon|OJJ47545.1

Group2219: abra|OJJ70669.1 acar|OOF97057.1 acla|EAW08094.1 afis|EAW23129.1 afla|EED48521.1 afum|EAL88351.1 agla|OJJ89645.1 akaw|GAA90002.1 aluc|OJZ84017.1 anid|CBF74417.1 anig|EHA19139.1 aory|EIT73127.1 asyd|OJJ57757.1 ater|EAU36256.1 atub|OJI90389.1 aver|OJJ05410.1 awen|OJJ36651.1 azon|OJJ47547.1

Group2220: abra|OJJ70671.1 acar|OOF97055.1 acla|EAW08093.1 afis|EAW23128.1 afla|EED48522.1 afum|EAL88352.1 agla|OJJ89644.1 akaw|GAA90003.1 aluc|OJZ84019.1 anid|CBF74413.1 anig|EHA19140.1 aory|EIT73195.1 asyd|OJJ57758.1 ater|EAU36255.1 atub|OJI90390.1 aver|OJJ05408.1 awen|OJJ36652.1 azon|OJJ47548.1

Group2221: abra|OJJ70672.1 acar|OOF97054.1 acla|EAW08092.1 afis|EAW23127.1 afla|EED48523.1 afum|EAL88353.1 agla|OJJ89643.1 akaw|GAA90004.1 aluc|OJZ84020.1 anid|CBF74412.1 anig|EHA19141.1 aory|EIT73139.1 asyd|OJJ57759.1 ater|EAU36254.1 atub|OJI90391.1 aver|OJJ05407.1 awen|OJJ36653.1 azon|OJJ47549.1

Group2222: abra|OJJ70675.1 acar|OOF97052.1 acla|EAW08089.1 afis|EAW23124.1 afla|EED48528.1 afum|EAL88356.1 agla|OJJ89641.1 akaw|GAA90007.1 aluc|OJZ84023.1 anid|CBF74406.1 anig|EHA19144.1 aory|EIT73244.1 asyd|OJJ57762.1 ater|EAU36252.1 atub|OJI90395.1 aver|OJJ05404.1 awen|OJJ36656.1 azon|OJJ47552.1

Group2223: abra|OJJ70677.1 acar|OOF97050.1 acla|EAW08087.1 afis|EAW23123.1 afla|EED48530.1 afum|EAL88357.1 agla|OJJ89616.1 akaw|GAA90009.1 aluc|OJZ84025.1 anid|CBF74404.1 anig|EHA19146.1 aory|EIT73116.1 asyd|OJJ57763.1 ater|EAU36251.1 atub|OJI90397.1 aver|OJJ05403.1 awen|OJJ36658.1 azon|OJJ47540.1

Group2224: abra|OJJ70680.1 acar|OOF97047.1 acla|EAW08084.1 afis|EAW23120.1 afla|EED48533.1 afum|EAL88360.1 agla|OJJ89619.1 akaw|GAA90012.1 aluc|OJZ84028.1 anid|CBF74398.1 anig|EHA19149.1 aory|EIT73210.1 asyd|OJJ57766.1 ater|EAU36249.1 atub|OJI90400.1 aver|OJJ05400.1 awen|OJJ36661.1 azon|OJJ47537.1

Group2225: abra|OJJ70681.1 acar|OOF97046.1 acla|EAW08083.1 afis|EAW23119.1 afla|EED48534.1 afum|EAL88361.1 agla|OJJ89621.1 akaw|GAA90013.1 aluc|OJZ84029.1 anid|CBF74396.1 anig|EHA19150.1 aory|EIT73258.1 asyd|OJJ57767.1 ater|EAU36248.1 atub|OJI90401.1 aver|OJJ05399.1 awen|OJJ36662.1 azon|OJJ47536.1

Group2226: abra|OJJ70684.1 acar|OOF97043.1 acla|EAW08078.1 afis|EAW23117.1 afla|EED48535.1 afum|EAL88363.1 agla|OJJ89624.1 akaw|GAA90016.1 aluc|OJZ84032.1 anid|CBF74393.1 anig|EHA19153.1 aory|EIT73171.1 asyd|OJJ57769.1 ater|EAU36245.1 atub|OJI90404.1 aver|OJJ05397.1 awen|OJJ36665.1 azon|OJJ47534.1

Group2227: abra|OJJ70685.1 acar|OOF97042.1 acla|EAW08077.1 afis|EAW23116.1 afla|EED48536.1 afum|EAL88364.1 agla|OJJ89625.1 akaw|GAA90017.1 aluc|OJZ84033.1 anid|CBF74391.1 anig|EHA19154.1 aory|EIT73186.1 asyd|OJJ57770.1 ater|EAU36244.1 atub|OJI90405.1 aver|OJJ05396.1 awen|OJJ36666.1 azon|OJJ47533.1

Group2228: abra|OJJ70686.1 acar|OOF97041.1 afis|EAW23115.1 afla|EED53328.1 afum|EAL88365.1 agla|OJJ89626.1 akaw|GAA90018.1 aluc|OJZ84034.1 anid|CBF76024.1 anig|EHA19155.1 aory|EIT74989.1 asyd|OJJ52450.1 ater|EAU36243.1 atub|OJI90406.1 aver|OJJ03966.1 awen|OJJ36667.1 azon|OJJ47532.1 aory|EIT74930.1

Group2229: abra|OJJ70689.1 acar|OOF97038.1 acla|EAW08076.1 afis|EAW23111.1 afla|EED48537.1 afum|EAL88368.1 agla|OJJ89630.1 akaw|GAA90021.1 aluc|OJZ84037.1 anid|CBF74389.1 anig|EHA19158.1 aory|EIT73070.1 asyd|OJJ57771.1 ater|EAU36239.1 atub|OJI90409.1 aver|OJJ05395.1 awen|OJJ36670.1 azon|OJJ47529.1

Group2230: abra|OJJ70694.1 acar|OOF97035.1 acla|EAW08071.1 afis|EAW23107.1 afla|EED48541.1 afum|EAL88372.1 agla|OJJ89636.1 akaw|GAA90025.1 aluc|OJZ84041.1 anid|CBF81370.1 anig|EHA19163.1 aory|EIT73129.1 asyd|OJJ59386.1 ater|EAU36235.1 atub|OJI90413.1 aver|OJJ02887.1 awen|OJJ36674.1 azon|OJJ47524.1

Group2231: abra|OJJ70696.1 acar|OOF97033.1 acla|EAW08069.1 afis|EAW23105.1 afla|EED48543.1 afum|EAL88374.1 agla|OJJ89638.1 akaw|GAA90027.1 aluc|OJZ84043.1 anid|CBF81367.1 anig|EHA19165.1 aory|EIT73169.1 asyd|OJJ59384.1 ater|EAU36233.1 atub|OJI90415.1 aver|OJJ02889.1 awen|OJJ36676.1 azon|OJJ47522.1

Group2232: abra|OJJ70700.1 acar|OOF90048.1 acla|EAW08065.1 afis|EAW23101.1 afla|EED48549.1 afum|EAL88377.1 agla|OJJ89613.1 akaw|GAA90031.1 aluc|OJZ84046.1 anid|CBF81359.1 anig|EHA19167.1 aory|EIT73156.1 asyd|OJJ59381.1 ater|EAU36227.1 atub|OJI90418.1 aver|OJJ02892.1 awen|OJJ36680.1 azon|OJJ47517.1

Group2233: abra|OJJ70701.1 acar|OOF89978.1 acla|EAW08064.1 afis|EAW23100.1 afla|EED48548.1 afum|EAL88378.2 agla|OJJ89612.1 akaw|GAA90032.1 aluc|OJZ84047.1 anig|EHA19168.1 aory|EIT73049.1 asyd|OJJ59380.1 ater|EAU36228.1 atub|OJI90419.1 aver|OJJ02893.1 awen|OJJ36681.1 azon|OJJ47516.1 anid|CBF81358.1

Group2234: abra|OJJ70702.1 acar|OOF89980.1 acla|EAW08063.1 afis|EAW23099.1 afla|EED48547.1 afum|EAL88379.1 agla|OJJ89611.1 akaw|GAA90033.1 aluc|OJZ84049.1 anid|CBF81356.1 anig|EHA19169.1 aory|EIT73063.1 asyd|OJJ59379.1 ater|EAU36230.1 atub|OJI90420.1 aver|OJJ02894.1 awen|OJJ36682.1 azon|OJJ47515.1

Group2235: abra|OJJ70704.1 acar|OOF97032.1 acla|EAW08061.1 afis|EAW23097.1 afla|EED48551.1 afum|EAL88381.2 agla|OJJ89609.1 akaw|GAA90035.1 aluc|OJZ84051.1 anid|CBF81352.1 anig|EHA19171.1 aory|EIT73218.1 asyd|OJJ59377.1 ater|EAU36225.1 atub|OJI90422.1 aver|OJJ02896.1 awen|OJJ36684.1 azon|OJJ47512.1

Group2236: abra|OJJ70705.1 acar|OOF97031.1 acla|EAW08060.1 afis|EAW23096.1 afla|EED48552.1 afum|EAL88382.2 agla|OJJ89608.1 akaw|GAA90036.1 aluc|OJZ84052.1 anid|CBF81350.1 anig|EHA19172.1 aory|EIT73272.1 asyd|OJJ59376.1 ater|EAU36224.1 atub|OJI90423.1 aver|OJJ02897.1 awen|OJJ36685.1 azon|OJJ47511.1

Group2237: abra|OJJ70706.1 acar|OOF97029.1 acla|EAW08058.1 afis|EAW23094.1 afla|EED48554.1 afum|EAL88384.2 agla|OJJ89606.1 akaw|GAA90037.1 aluc|OJZ84053.1 anid|CBF87089.1 anig|EHA19173.1 aory|EIT73275.1 asyd|OJJ60399.1 atub|OJI90424.1 aver|OJI99728.1 awen|OJJ36688.1 azon|OJJ47509.1 aory|EIT73221.1

Group2238: abra|OJJ70708.1 acar|OOF97028.1 acla|EAW08056.1 afis|EAW23092.1 afla|EED48556.1 afum|EBA27345.1 agla|OJJ89604.1 akaw|GAA90039.1 aluc|OJZ84055.1 anid|CBF81348.1 anig|EHA19176.1 aory|EIT73227.1 asyd|OJJ59374.1 ater|EAU36215.1 atub|OJI90426.1 aver|OJJ02900.1 awen|OJJ36690.1 azon|OJJ47506.1

Group2239: abra|OJJ70710.1 acar|OOF97026.1 acla|EAW08054.1 afis|EAW23090.1 afla|EED48558.1 afum|EAL88389.1 agla|OJJ89602.1 akaw|GAA90041.1 aluc|OJZ84057.1 anid|CBF81345.1 anig|EHA19177.1 aory|EIT73059.1 asyd|OJJ59372.1 ater|EAU36213.1 atub|OJI90428.1 aver|OJJ02902.1 awen|OJJ36692.1 azon|OJJ47504.1

Group2240: abra|OJJ70711.1 acar|OOF97025.1 acla|EAW08053.1 afis|EAW23089.1 afla|EED48559.1 afum|EAL88390.1 agla|OJJ89601.1 akaw|GAA90042.1 aluc|OJZ84058.1 anid|CBF81343.1 anig|EHA19178.1 aory|EIT73173.1 asyd|OJJ59371.1 ater|EAU36212.1 atub|OJI90429.1 aver|OJJ02903.1 awen|OJJ36694.1 azon|OJJ47503.1

Group2241: abra|OJJ70714.1 acar|OOF97022.1 acla|EAW08049.1 afis|EAW23085.1 afla|EED48563.1 afum|EAL88394.1 agla|OJJ89595.1 akaw|GAA90045.1 aluc|OJZ84061.1 anid|CBF81339.1 anig|EHA19181.1 aory|EIT73232.1 asyd|OJJ59369.1 ater|EAU36209.1 atub|OJI90432.1 aver|OJJ02905.1 awen|OJJ36699.1 azon|OJJ47498.1

Group2242: abra|OJJ70715.1 acar|OOF97021.1 acla|EAW08048.1 afis|EAW23084.1 afla|EED48564.1 afum|EAL88395.1 agla|OJJ89594.1 akaw|GAA90046.1 aluc|OJZ84062.1 anid|CBF81337.1 anig|EHA19182.1 aory|EIT73208.1 asyd|OJJ59368.1 ater|EAU36208.1 atub|OJI90433.1 aver|OJJ02906.1 awen|OJJ36700.1 azon|OJJ47497.1

Group2243: abra|OJJ70716.1 acar|OOF97020.1 acla|EAW08047.1 afis|EAW23083.1 afla|EED48565.1 afum|EAL88396.1 agla|OJJ89592.1 akaw|GAA90047.1 aluc|OJZ84063.1 anid|CBF81335.1 anig|EHA19183.1 aory|EIT73176.1 asyd|OJJ59367.1 ater|EAU36207.1 atub|OJI90434.1 aver|OJJ02907.1 awen|OJJ36701.1 azon|OJJ47496.1

Group2244: abra|OJJ70717.1 acar|OOF97019.1 acla|EAW08046.1 afis|EAW23082.1 afla|EED48566.1 afum|EAL88397.1 agla|OJJ89591.1 akaw|GAA90048.1 aluc|OJZ84064.1 anid|CBF81333.1 anig|EHA19184.1

aory|EIT73236.1 asyd|OJJ59366.1 ater|EAU36206.1 atub|OJI90435.1 aver|OJJ02908.1 awen|OJJ36702.1 azon|OJJ47495.1

Group2245: abra|OJJ70718.1 acar|OOF97018.1 acla|EAW08044.1 afis|EAW23080.1 afla|EED48567.1 afum|EAL88399.1 agla|OJJ89588.1 akaw|GAA90049.1 aluc|OJZ84065.1 anid|CBF81330.1 anig|EHA19185.1 aory|EIT73202.1 asyd|OJJ59364.1 ater|EAU36205.1 atub|OJI90436.1 aver|OJJ02910.1 awen|OJJ36704.1 azon|OJJ47494.1

Group2246: abra|OJJ70719.1 acar|OOF97017.1 acla|EAW08043.1 afis|EAW23079.1 afla|EED48568.1 afum|EAL88400.1 agla|OJJ89587.1 akaw|GAA90050.1 aluc|OJZ84066.1 anid|CBF81328.1 anig|EHA19186.1 aory|EIT73095.1 asyd|OJJ59363.1 ater|EAU36204.1 atub|OJI90437.1 aver|OJJ02911.1 awen|OJJ36705.1 azon|OJJ47493.1

Group2247: abra|OJJ70720.1 acar|OOF97016.1 acla|EAW08042.1 afis|EAW23078.1 afla|EED48569.1 afum|EAL88401.1 agla|OJJ89586.1 akaw|GAA90051.1 aluc|OJZ84067.1 anid|CBF81326.1 anig|EHA19187.1 aory|EIT73149.1 asyd|OJJ59362.1 ater|EAU36202.1 atub|OJI90438.1 aver|OJJ02912.1 awen|OJJ36706.1 azon|OJJ47489.1

Group2248: abra|OJJ70722.1 acar|OOF97015.1 afis|EAW23077.1 afla|EED48570.1 afum|EAL88402.1 agla|OJJ89585.1 akaw|GAA90052.1 aluc|OJZ84068.1 anid|CBF81324.1 anig|EHA19188.1 aory|EIT73161.1 asyd|OJJ59361.1 ater|EAU36201.1 atub|OJI90440.1 aver|OJJ02913.1 awen|OJJ36707.1 azon|OJJ47490.1 aver|OJJ06633.1

Group2249: abra|OJJ70726.1 acar|OOF97013.1 acla|EAW08040.1 afis|EAW23075.1 afla|EED56447.1 afum|EAL88404.1 agla|OJJ89582.1 akaw|GAA90054.1 aluc|OJZ84071.1 anid|CBF81318.1 anig|EHA19190.1 aory|EIT72820.1 asyd|OJJ59359.1 ater|EAU36199.1 atub|OJI90443.1 aver|OJJ02915.1 awen|OJJ36709.1 azon|OJJ47492.1

Group2250: abra|OJJ70727.1 acar|OOF97012.1 acla|EAW08039.1 afis|EAW23074.1 afla|EED56449.1 afum|EAL88405.1 agla|OJJ89581.1 akaw|GAA90055.1 aluc|OJZ84072.1 anid|CBF81316.1 anig|EHA19191.1 aory|EIT72790.1 asyd|OJJ59358.1 ater|EAU36198.1 atub|OJI90444.1 aver|OJJ02916.1 awen|OJJ36710.1 azon|OJJ47487.1

Group2251: abra|OJJ70728.1 acar|OOF97011.1 acla|EAW08038.1 afis|EAW23073.1 afla|EED56450.1 afum|EAL88406.1 agla|OJJ89580.1 akaw|GAA90056.1 aluc|OJZ84073.1 anid|CBF81314.1 anig|EHA19192.1 aory|EIT72843.1 asyd|OJJ59357.1 ater|EAU36197.1 atub|OJI90445.1 aver|OJJ02917.1 awen|OJJ36711.1 azon|OJJ47488.1

Group2252: abra|OJJ70729.1 acar|OOF97009.1 acla|EAW11184.1 afis|EAW15915.1 afla|EED56455.1 afum|EAL88550.2 agla|OJJ89579.1 akaw|GAA90057.1 aluc|OJZ84074.1 anid|CBF81309.1 anig|EHA19193.1 aory|EIT72851.1 asyd|OJJ59354.1 ater|EAU36193.1 atub|OJI90446.1 aver|OJJ02920.1 awen|OJJ36715.1 azon|OJJ47474.1

Group2253: abra|OJJ70733.1 acar|OOF97005.1 acla|EAW11180.1 afis|EAW15911.1 afla|EED56457.1 afum|EAL88546.2 agla|OJJ89575.1 akaw|GAA90061.1 aluc|OJZ84078.1 anid|CBF81301.1 anig|EHA19197.1 aory|EIT72816.1 asyd|OJJ59350.1 ater|EAU36191.1 atub|OJI90450.1 aver|OJJ02924.1 awen|OJJ36719.1 azon|OJJ47478.1

Group2254: abra|OJJ70735.1 acar|OOF97004.1 acla|EAW11179.1 afis|EAW15910.1 afla|EED56458.1 afum|EAL88545.1 agla|OJJ89574.1 akaw|GAA90062.1 aluc|OJZ84080.1 anid|CBF81299.1 anig|EHA19198.1 aory|EIT72821.1 asyd|OJJ59349.1 ater|EAU36189.1 atub|OJI90451.1 aver|OJJ02925.1 awen|OJJ36720.1 azon|OJJ47479.1

Group2255: abra|OJJ70738.1 acar|OOF97003.1 acla|EAW11178.1 afis|EAW15909.1 afla|EED56459.1 afum|EAL88544.1 agla|OJJ89573.1 akaw|GAA90063.1 aluc|OJZ84083.1 anid|CBF81298.1 anig|EHA19199.1 aory|EIT72803.1 asyd|OJJ59348.1 ater|EAU36188.1 atub|OJI90453.1 aver|OJJ02926.1 awen|OJJ36721.1 azon|OJJ47480.1

Group2256: abra|OJJ70741.1 acar|OOF97002.1 acla|EAW11176.1 afis|EAW15907.1 afla|EED56461.1 afum|EAL88542.1 agla|OJJ78421.1 akaw|GAA90065.1 aluc|OJZ84086.1 anid|CBF81263.1 anig|EHA19202.1 aory|EIT72874.1 asyd|OJJ59332.1 ater|EAU36186.1 atub|OJI90457.1 aver|OJJ02942.1 awen|OJJ36723.1 azon|OJJ47482.1

Group2257: abra|OJJ70751.1 acar|OOF96994.1 acla|EAW11170.1 afis|EAW15901.1 afla|EED56470.1 afum|EAL88536.1 agla|OJJ89561.1 akaw|GAA90071.1 aluc|OJZ84095.1 anid|CBF81281.1 anig|EHA19208.1 aory|EIT72864.1 asyd|OJJ59338.1 ater|EAU36180.1 atub|OJI90468.1 aver|OJJ02936.1 awen|OJJ36730.1 azon|OJJ47467.1

Group2258: abra|OJJ70752.1 acar|OOF96992.1 acla|EAW11169.1 afis|EAW15900.1 afla|EED56471.1 afum|EAL88535.1 agla|OJJ89560.1 akaw|GAA90072.1 aluc|OJZ84097.1 anid|CBF81283.1 anig|EHA19209.1 aory|EIT72802.1 asyd|OJJ59340.1 ater|EAU36179.1 atub|OJI90470.1 aver|OJJ02935.1 awen|OJJ36731.1 azon|OJJ47466.1

Group2259: abra|OJJ70753.1 acar|OOF96991.1 acla|EAW11168.1 afis|EAW15899.1 afla|EED56472.1 afum|EAL88534.1 agla|OJJ89553.1 akaw|GAA90073.1 aluc|OJZ84098.1 anid|CBF81285.1 anig|EHA19210.1 aory|EIT72807.1 asyd|OJJ59341.1 ater|EAU36178.1 atub|OJI90471.1 aver|OJJ02934.1 awen|OJJ36732.1 azon|OJJ47465.1

Group2260: abra|OJJ70754.1 acar|OOF96990.1 acla|EAW11167.1 afis|EAW15898.1 afla|EED56473.1 afum|EAL88533.1 agla|OJJ89552.1 akaw|GAA90074.1 aluc|OJZ84099.1 anid|CBF81287.1 anig|EHA19211.1 aory|EIT72796.1 asyd|OJJ59342.1 ater|EAU36177.1 atub|OJI90472.1 aver|OJJ02933.1 awen|OJJ36733.1 azon|OJJ47464.1

Group2261: abra|OJJ70755.1 acar|OOF96989.1 acla|EAW11166.1 afis|EAW15897.1 afla|EED56474.1 afum|EAL88532.1 agla|OJJ89551.1 akaw|GAA90075.1 aluc|OJZ84100.1 anid|CBF81288.1 anig|EHA19212.1 aory|EIT72834.1 asyd|OJJ59343.1 ater|EAU36176.1 atub|OJI90473.1 aver|OJJ02931.1 awen|OJJ36734.1 azon|OJJ47463.1

Group2262: abra|OJJ70756.1 acar|OOF96988.1 acla|EAW11165.1 afis|EAW15896.1 afla|EED56475.1 afum|EAL88531.1 agla|OJJ89550.1 akaw|GAA90076.1 aluc|OJZ84101.1 anid|CBF81290.1 anig|EHA19213.1 aory|EIT72841.1 asyd|OJJ59344.1 ater|EAU36175.1 atub|OJI90474.1 aver|OJJ02930.1 awen|OJJ36735.1 azon|OJJ47462.1

Group2263: abra|OJJ70758.1 acar|OOF96986.1 acla|EAW11163.1 afis|EAW15894.1 afla|EED56477.1 afum|EAL88529.1 agla|OJJ89548.1 akaw|GAA90078.1 aluc|OJZ84103.1 anid|CBF81294.1 anig|EHA19215.1 aory|EIT72822.1 asyd|OJJ59346.1 ater|EAU36173.1 atub|OJI90476.1 aver|OJJ02928.1 awen|OJJ36737.1 azon|OJJ47460.1

Group2264: abra|OJJ70760.1 acar|OOF96984.1 acla|EAW11161.1 afis|EAW15892.1 afla|EED56479.1 afum|EAL88527.1 agla|OJJ89530.1 akaw|GAA90080.1 aluc|OJZ84105.1 anid|CBF81259.1 anig|EHA19217.1 aory|EIT72795.1 asyd|OJJ59330.1 ater|EAU31714.1 atub|OJI90479.1 aver|OJJ02944.1 awen|OJJ36739.1 azon|OJJ47459.1

Group2265: abra|OJJ70762.1 acar|OOF96982.1 acla|EAW11159.1 afis|EAW15890.1 afla|EED56482.1 afum|EAL88525.2 agla|OJJ89532.1 akaw|GAA90082.1 aluc|OJZ84107.1 anid|CBF81256.1 anig|EHA19219.1 aory|EIT72811.1 asyd|OJJ59328.1 ater|EAU31712.1 atub|OJI90481.1 aver|OJJ02946.1 awen|OJJ36740.1 azon|OJJ47457.1

Group2266: abra|OJJ70766.1 acar|OOF96979.1 acla|EAW11156.1 afis|EAW15887.1 afla|EED56485.1 afum|EAL88521.2 agla|OJJ89534.1 akaw|GAA90086.1 aluc|OJZ84112.1 anid|CBF81250.1 anig|EHA19222.1 aory|EIT72797.1 asyd|OJJ59325.1 ater|EAU31709.1 atub|OJI90485.1 aver|OJJ02949.1 awen|OJJ36743.1 azon|OJJ47454.1

Group2267: abra|OJJ70767.1 acar|OOF96977.1 acla|EAW11154.1 afis|EAW15885.1 afla|EED56487.1 afum|EAL88519.1 agla|OJJ89536.1 akaw|GAA90087.1 aluc|OJZ84113.1 anid|CBF81243.1 anig|EHA19223.1 aory|EIT72824.1 asyd|OJJ59321.1 ater|EAU31705.1 atub|OJI90486.1 aver|OJJ02952.1 awen|OJJ36746.1 azon|OJJ47453.1

Group2268: abra|OJJ70768.1 acar|OOF96978.1 acla|EAW11153.1 afis|EAW15884.1 afla|EED56488.1 afum|EAL88518.1 agla|OJJ89537.1 akaw|GAA90088.1 aluc|OJZ84114.1 anid|CBF81241.1 anig|EHA19224.1 aory|EIT72873.1 asyd|OJJ59320.1 ater|EAU31704.1 atub|OJI90487.1 aver|OJJ02953.1 awen|OJJ36747.1 azon|OJJ47452.1

Group2269: abra|OJJ70770.1 acar|OOF96976.1 acla|EAW11151.1 afis|EAW15882.1 afla|EED56489.1 afum|EAL88516.1 agla|OJJ89539.1 akaw|GAA90090.1 aluc|OJZ84116.1 anid|CBF81237.1 anig|EHA19226.1 aory|EIT72859.1 asyd|OJJ59319.1 ater|EAU31703.1 atub|OJI90489.1 aver|OJJ02954.1 awen|OJJ36749.1 azon|OJJ47450.1

Group2270: abra|OJJ70774.1 acar|OOF96936.1 acla|EAW11147.1 afis|EAW15878.1 afla|EED56497.1 afum|EAL88512.1 agla|OJJ87230.1 akaw|GAA90094.1 aluc|OJZ84121.1 anid|CBF81230.1 anig|EHA19229.1 aory|EIT72868.1 asyd|OJJ59316.1 ater|EAU31699.1 atub|OJI90493.1 aver|OJJ02957.1 awen|OJJ36752.1 azon|OJJ47447.1

Group2271: abra|OJJ70795.1 acar|OOF96950.1 acla|EAW11128.1 afis|EAW15857.1 afla|EED56515.1 afum|EAL88493.1 agla|OJJ87250.1 akaw|GAA90111.1 aluc|OJZ84140.1 anid|CBF81189.1 anig|EHA19248.1 aory|EIT72805.1 asyd|OJJ59299.1 ater|EAU31672.1 atub|OJI90514.1 aver|OJJ02976.1 awen|OJJ36770.1 azon|OJJ47428.1

Group2272: abra|OJJ70809.1 acar|OOF98293.1 acla|EAW10418.1 afis|EAW25151.1 afla|EED55282.1 afum|EAL89826.2 agla|OJJ89004.1 akaw|GAA91689.1 aluc|OJZ87705.1 anid|CBF81138.1 anig|EHA20888.1 aory|EIT83553.1 asyd|OJJ56507.1 ater|EAU39430.1 atub|OJI80938.1 aver|OJJ06212.1 awen|OJJ40780.1 azon|OJJ46872.1

Group2273: abra|OJJ70815.1 acar|OOF98285.1 acla|EAW10424.1 afis|EAW25145.1 afla|EED55294.1 afum|EAL89818.1 agla|OJJ89008.1 akaw|GAA91695.1 aluc|OJZ87698.1 anid|CBF85679.1 anig|EHA20885.1

aory|EIT83533.1 asyd|OJJ64254.1 ater|EAU39423.1 atub|OJI80945.1 aver|OJI98344.1 awen|OJJ40770.1 azon|OJJ46878.1

Group2274: abra|OJJ70833.1 acar|OOF98281.1 acla|EAW10427.1 afis|EAW25142.1 afla|EED55303.1 afum|EAL89814.1 agla|OJJ89025.1 akaw|GAA91701.1 aluc|OJZ87689.1 anid|CBF85686.1 anig|EHA20878.1 aory|EIT83517.1 asyd|OJJ64258.1 ater|EAU39411.1 atub|OJI80951.1 aver|OJI98350.1 awen|OJJ40766.1 azon|OJJ44131.1

Group2275: abra|OJJ70834.1 acar|OOF98280.1 acla|EAW10428.1 afis|EAW25141.1 afla|EED55304.1 afum|EAL89813.1 agla|OJJ89026.1 akaw|GAA91702.1 aluc|OJZ87688.1 anid|CBF86093.1 anig|EHA20877.1 aory|EIT83571.1 asyd|OJJ64473.1 ater|EAU39410.1 atub|OJI80952.1 aver|OJI98577.1 awen|OJJ40765.1 azon|OJJ44130.1

Group2276: abra|OJJ70837.1 acar|OOF98277.1 acla|EAW10432.1 afis|EAW25137.1 afla|EED55309.1 afum|EAL89808.2 agla|OJJ89030.1 akaw|GAA91705.1 aluc|OJZ87684.1 anid|CBF85710.1 anig|EHA20874.1 aory|EIT83613.1 asyd|OJJ64270.1 ater|EAU39407.1 atub|OJI80959.1 aver|OJI98362.1 awen|OJJ40761.1 azon|OJJ44125.1

Group2277: abra|OJJ70839.1 acar|OOF98275.1 acla|EAW10433.1 afis|EAW25135.1 afla|EED55311.1 afum|EAL89806.2 agla|OJJ89032.1 akaw|GAA91707.1 aluc|OJZ87682.1 anid|CBF85706.1 anig|EHA20872.1 aory|EIT83546.1 asyd|OJJ64268.1 ater|EAU39406.1 atub|OJI80961.1 aver|OJI98360.1 awen|OJJ40759.1 azon|OJJ44123.1

Group2278: abra|OJJ70843.1 acar|OOF98272.1 acla|EAW10434.1 afis|EAW25134.1 afla|EED55312.1 afum|EAL89805.1 agla|OJJ89033.1 akaw|GAA91711.1 aluc|OJZ87678.1 anid|CBF85705.1 anig|EHA20868.1 aory|EIT83614.1 asyd|OJJ64267.1 ater|EAU39405.1 atub|OJI80965.1 aver|OJI98359.1 awen|OJJ40758.1 azon|OJJ44122.1

Group2279: abra|OJJ70848.1 acar|OOF98266.1 acla|EAW10439.1 afis|EAW25128.1 afla|EED55316.1 afum|EAL89799.1 agla|OJJ89042.1 akaw|GAA91716.1 aluc|OJZ87672.1 anid|CBF85697.1 anig|EHA20863.1 aory|EIT83568.1 asyd|OJJ64263.1 ater|EAU39401.1 atub|OJI80970.1 aver|OJI98355.1 awen|OJJ40753.1 azon|OJJ44117.1

Group2280: abra|OJJ70851.1 acar|OOF98265.1 acla|EAW10441.1 afis|EAW25126.1 afla|EED55317.1 afum|EAL89797.1 agla|OJJ89044.1 akaw|GAA91718.1 aluc|OJZ87670.1 anid|CBF85694.1 anig|EHA20861.1 aory|EIT83542.1 asyd|OJJ64261.1 ater|EAU39399.1 atub|OJI80972.1 aver|OJI98353.1 awen|OJJ40750.1 azon|OJJ44115.1

Group2281: abra|OJJ70854.1 acar|OOF98262.1 acla|EAW10447.1 afis|EAW25120.1 afla|EED55322.1 afum|EAL89791.1 agla|OJJ89053.1 akaw|GAA91721.1 aluc|OJZ87667.1 anid|CBF86087.1 anig|EHA20858.1 aory|EIT76263.1 asyd|OJJ64469.1 ater|EAU39392.1 atub|OJI80976.1 aver|OJI98573.1 awen|OJJ40739.1 azon|OJJ44108.1

Group2282: abra|OJJ70855.1 acar|OOF98261.1 acla|EAW10446.1 afis|EAW25121.1 afla|EED55320.1 afum|EAL89792.1 agla|OJJ89049.1 akaw|GAA91722.1 aluc|OJZ87665.1 anid|CBF86089.1 anig|EHA20857.1 aory|EIT76239.1 asyd|OJJ64471.1 ater|EAU39393.1 atub|OJI80978.1 aver|OJI98575.1 awen|OJJ40740.1 azon|OJJ44110.1

Group2283: abra|OJJ70856.1 acar|OOF98260.1 acla|EAW10445.1 afis|EAW25122.1 afla|EED55319.1 afum|EAL89793.1 agla|OJJ89048.1 akaw|GAA91723.1 aluc|OJZ87664.1 anid|CBF86091.1 anig|EHA20856.1 aory|EIT76220.1 asyd|OJJ64472.1 ater|EAU39394.1 atub|OJI80979.1 aver|OJI98576.1 awen|OJJ40741.1 azon|OJJ44112.1

Group2284: abra|OJJ70857.1 acar|OOF98259.1 acla|EAW10449.1 afis|EAW25118.1 afla|EED55324.1 afum|EAL89789.1 agla|OJJ89059.1 akaw|GAA91724.1 aluc|OJZ87663.1 anid|CBF86086.1 anig|EHA20855.1 aory|EIT76226.1 asyd|OJJ64468.1 ater|EAU39386.1 atub|OJI80980.1 aver|OJI98572.1 awen|OJJ40737.1 azon|OJJ44107.1

Group2285: abra|OJJ70858.1 acar|OOF98258.1 acla|EAW10450.1 afis|EAW25117.1 afla|EED55325.1 afum|EAL89788.1 agla|OJJ89063.1 akaw|GAA91725.1 aluc|OJZ87662.1 anid|CBF86084.1 anig|EHA20854.1 aory|EIT76188.1 asyd|OJJ64467.1 ater|EAU39387.1 atub|OJI80981.1 aver|OJI98571.1 awen|OJJ40734.1 azon|OJJ44106.1

Group2286: abra|OJJ70859.1 acar|OOF98257.1 acla|EAW10451.1 afis|EAW25116.1 afla|EED55326.1 afum|EAL89787.1 agla|OJJ89064.1 akaw|GAA91726.1 aluc|OJZ87661.1 anid|CBF86082.1 anig|EHA20853.1 aory|EIT76238.1 asyd|OJJ64466.1 ater|EAU39388.1 atub|OJI80982.1 aver|OJI98570.1 awen|OJJ40733.1 azon|OJJ44105.1

Group2287: abra|OJJ70860.1 acar|OOF98256.1 acla|EAW10452.1 afis|EAW25115.1 afla|EED55327.1 afum|EAL89786.1 agla|OJJ89065.1 akaw|GAA91727.1 aluc|OJZ87660.1 anid|CBF86080.1 anig|EHA20852.1 aory|EIT76212.1 asyd|OJJ64465.1 ater|EAU39389.1 atub|OJI80983.1 aver|OJI98569.1 awen|OJJ40732.1 azon|OJJ44104.1

Group2288: abra|OJJ70861.1 acar|OOF98255.1 acla|EAW10453.1 afis|EAW25114.1 afla|EED55328.1 afum|EAL89785.1 agla|OJJ89066.1 akaw|GAA91728.1 aluc|OJZ87659.1 anid|CBF86079.1 anig|EHA20851.1 aory|EIT76255.1 asyd|OJJ64464.1 ater|EAU39390.1 atub|OJI80984.1 aver|OJI98568.1 awen|OJJ40731.1 azon|OJJ44103.1

Group2289: abra|OJJ70862.1 acar|OOF98254.1 acla|EAW10454.1 afis|EAW25113.1 afla|EED55329.1 afum|EAL89784.1 agla|OJJ89067.1 akaw|GAA91729.1 aluc|OJZ87658.1 anid|CBF86077.1 anig|EHA20850.1 aory|EIT76257.1 asyd|OJJ64463.1 ater|EAU39383.1 atub|OJI80985.1 aver|OJI98567.1 awen|OJJ40730.1 azon|OJJ44102.1

Group2290: abra|OJJ70864.1 acar|OOF98252.1 acla|EAW10456.1 afis|EAW25111.1 afla|EED55331.1 afum|EAL89782.2 agla|OJJ89070.1 akaw|GAA91731.1 aluc|OJZ87656.1 anid|CBF86073.1 anig|EHA20849.1 aory|EIT76253.1 asyd|OJJ64461.1 ater|EAU39381.1 atub|OJI80987.1 aver|OJI98564.1 awen|OJJ40728.1 azon|OJJ44100.1

Group2291: abra|OJJ70878.1 acar|OOF98242.1 acla|EAW10467.1 afis|EAW25100.1 afla|EED55345.1 afum|EAL89771.1 agla|OJJ85468.1 akaw|GAA91741.1 aluc|OJZ87644.1 anid|CBF86015.1 anig|EHA20837.1 aory|EIT80879.1 asyd|OJJ64450.1 ater|EAU39362.1 atub|OJI81001.1 aver|OJI98553.1 awen|OJJ40714.1 azon|OJJ44091.1

Group2292: abra|OJJ70880.1 acar|OOF98241.1 acla|EAW10468.1 afis|EAW25099.1 afla|EED55347.1 afum|EAL89770.1 agla|OJJ85472.1 akaw|GAA91742.1 aluc|OJZ87643.1 anid|CBF86013.1 anig|EHA20836.1 aory|EIT80895.1 asyd|OJJ64449.1 ater|EAU39361.1 atub|OJI81002.1 aver|OJI98552.1 awen|OJJ40713.1 azon|OJJ44090.1

Group2293: abra|OJJ70890.1 acar|OOF98227.1 acla|EAW10472.1 afis|EAW25095.1 afla|EED55353.1 afum|EAL89766.2 agla|OJJ89082.1 akaw|GAA87406.1 aluc|OJZ87634.1 anid|CBF86004.1 anig|EHA20829.1 aory|EIT80918.1 asyd|OJJ64445.1 ater|EAU39353.1 atub|OJI81015.1 aver|OJI98548.1 awen|OJJ40709.1 azon|OJJ44085.1

Group2294: abra|OJJ70892.1 acar|OOF90217.1 acla|EAW10474.1 afis|EAW25093.1 afla|EED55354.1 afum|EAL89764.1 agla|OJJ89084.1 akaw|GAA87408.1 aluc|OJZ87631.1 anid|CBF86001.1 anig|EHA20827.1 aory|EIT80886.1 asyd|OJJ64443.1 ater|EAU39351.1 atub|OJI81018.1 aver|OJI98545.1 awen|OJJ40707.1 azon|OJJ44083.1

Group2295: abra|OJJ70895.1 acar|OOF90219.1 acla|EAW10482.1 afis|EAW25085.1 afla|EED55362.1 afum|EAL89754.1 agla|OJJ89097.1 akaw|GAA87411.1 aluc|OJZ87628.1 anid|CBF85988.1 anig|EHA20824.1 aory|EIT80873.1 asyd|OJJ64434.1 ater|EAU39341.1 atub|OJI81022.1 aver|OJI98535.1 awen|OJJ40697.1 azon|OJJ44075.1

Group2296: abra|OJJ70897.1 acar|OOF90220.1 acla|EAW10481.1 afis|EAW25086.1 afla|EED55361.1 afum|EAL89755.1 agla|OJJ89096.1 akaw|GAA87412.1 aluc|OJZ87626.1 anid|CBF85990.1 anig|EHA20823.1 aory|EIT80880.1 asyd|OJJ64435.1 ater|EAU39342.1 atub|OJI81023.1 aver|OJI98536.1 awen|OJJ40698.1 azon|OJJ44076.1

Group2297: abra|OJJ70898.1 acar|OOF98228.1 acla|EAW10480.1 afis|EAW25087.1 afla|EED55360.1 afum|EAL89756.1 agla|OJJ89095.1 akaw|GAA87413.1 aluc|OJZ87625.1 anid|CBF85992.1 anig|EHA20822.1 aory|EIT80799.1 asyd|OJJ64436.1 ater|EAU39343.1 atub|OJI81024.1 aver|OJI98537.1 awen|OJJ40700.1 azon|OJJ44077.1

Group2298: abra|OJJ70899.1 acar|OOF98221.1 acla|EAW10479.1 afis|EAW25088.1 afla|EED55359.1 afum|EAL89757.1 agla|OJJ89094.1 akaw|GAA87414.1 aluc|OJZ87624.1 anid|CBF85994.1 anig|EHA20821.1 aory|EIT80835.1 asyd|OJJ64437.1 ater|EAU39344.1 atub|OJI81025.1 aver|OJI98538.1 awen|OJJ40701.1 azon|OJJ44078.1

Group2299: abra|OJJ70901.1 acar|OOF98218.1 acla|EAW10477.1 afis|EAW25090.1 afla|EED55357.1 afum|EAL89760.2 agla|OJJ89087.1 akaw|GAA87416.1 aluc|OJZ87622.1 anid|CBF85997.1 anig|EHA20819.1 aory|EIT80906.1 asyd|OJJ64440.1 ater|EAU39345.1 atub|OJI81027.1 aver|OJI98542.1 awen|OJJ40704.1 azon|OJJ44080.1

Group2300: abra|OJJ70906.1 acar|OOF98214.1 acla|EAW10484.1 afis|EAW25083.1 afla|EED55367.1 afum|EAL89752.1 agla|OJJ89102.1 akaw|GAA87420.1 aluc|OJZ87618.1 anid|CBF85985.1 anig|EHA20816.1 aory|EIT80861.1 asyd|OJJ64432.1 ater|EAU39339.1 atub|OJI81031.1 aver|OJI98533.1 awen|OJJ40695.1 azon|OJJ44072.1

Group2301: abra|OJJ70911.1 acar|OOF98211.1 acla|EAW10486.1 afis|EAW25081.1 afla|EED55369.1 afum|EAL89750.1 agla|OJJ89105.1 akaw|GAA87422.1 aluc|OJZ87614.1 anid|CBF85977.1 anig|EHA20811.1 aory|EIT80845.1 asyd|OJJ64429.1 ater|EAU39519.1 atub|OJI81035.1 aver|OJI98529.1 awen|OJJ40693.1 azon|OJJ44069.1

Group2302: abra|OJJ70912.1 acar|OOF98210.1 acla|EAW10487.1 afis|EAW25080.1 afla|EED55370.1 afum|EAL89749.1 agla|OJJ89106.1 akaw|GAA87423.1 aluc|OJZ87613.1 anid|CBF85975.1 anig|EHA20810.1

aory|EIT80866.1 asyd|OJJ64428.1 ater|EAU39539.1 atub|OJI81036.1 aver|OJI98528.1 awen|OJJ40692.1 azon|OJJ44068.1

Group2303: abra|OJJ70913.1 acar|OOF89971.1 acla|EAW10488.1 afis|EAW25079.1 afla|EED55371.1 afum|EAL89748.1 agla|OJJ89107.1 akaw|GAA87424.1 aluc|OJZ87612.1 anid|CBF85973.1 anig|EHA20809.1 aory|EIT80872.1 asyd|OJJ64427.1 ater|EAU39540.1 atub|OJI81037.1 aver|OJI98527.1 awen|OJJ40691.1 azon|OJJ44067.1

Group2304: abra|OJJ70915.1 acar|OOF98209.1 acla|EAW10489.1 afis|EAW25078.1 afla|EED55375.1 afum|EAL89747.1 agla|OJJ89115.1 akaw|GAA87425.1 aluc|OJZ87610.1 anid|CBF85970.1 anig|EHA20808.1 aory|EIT80925.1 asyd|OJJ64426.1 ater|EAU39545.1 atub|OJI81038.1 aver|OJI98526.1 awen|OJJ40689.1 azon|OJJ44066.1

Group2305: abra|OJJ70916.1 acar|OOF98208.1 acla|EAW10490.1 afis|EAW25077.1 afla|EED55376.1 afum|EAL89746.1 agla|OJJ89116.1 akaw|GAA87426.1 aluc|OJZ87609.1 anid|CBF85968.1 anig|EHA20807.1 aory|EIT80812.1 asyd|OJJ64425.1 ater|EAU39546.1 atub|OJI81039.1 aver|OJI98524.1 awen|OJJ40688.1 azon|OJJ44065.1

Group2306: abra|OJJ70918.1 acar|OOF98206.1 acla|EAW10492.1 afis|EAW25075.1 afla|EED55378.1 afum|EAL89744.1 agla|OJJ89117.1 akaw|GAA87428.1 aluc|OJZ87608.1 anid|CBF85964.1 anig|EHA20806.1 aory|EIT80830.1 asyd|OJJ64423.1 ater|EAU39550.1 atub|OJI81040.1 aver|OJI98522.1 awen|OJJ40686.1 azon|OJJ44063.1

Group2307: abra|OJJ70931.1 acar|OOF98195.1 acla|EAW10497.1 afis|EAW25070.1 afla|EED55384.1 afum|EAL89739.1 agla|OJJ89124.1 akaw|GAA83011.1 aluc|OJZ87596.1 anid|CBF85958.1 anig|EHA20796.1 aory|EIT80805.1 asyd|OJJ64418.1 ater|EAU39555.1 atub|OJI81051.1 aver|OJI98517.1 awen|OJJ40681.1 azon|OJJ44058.1

Group2308: abra|OJJ70933.1 acar|OOF89985.1 acla|EAW10499.1 afis|EAW25068.1 afla|EED55386.1 afum|EAL89737.2 agla|OJJ89126.1 akaw|GAA83009.1 aluc|OJZ87594.1 anid|CBF85954.1 anig|EHA20794.1 aory|EIT80870.1 asyd|OJJ64416.1 ater|EAU35815.1 atub|OJI81053.1 aver|OJI98515.1 awen|OJJ40675.1 azon|OJJ44056.1

Group2309: abra|OJJ70940.1 acar|OOF98192.1 acla|EAW10505.1 afis|EAW25062.1 afla|EED55391.1 afum|EAL89731.2 agla|OJJ89133.1 akaw|GAA83004.1 aluc|OJZ87588.1 anid|CBF85943.1 anig|EHA20789.1 aory|EIT80778.1 asyd|OJJ64412.1 ater|EAU35810.1 atub|OJI81060.1 aver|OJI98511.1 awen|OJJ40669.1 azon|OJJ44050.1

Group2310: abra|OJJ70941.1 acar|OOF98191.1 acla|EAW10506.1 afis|EAW25061.1 afla|EED55392.1 afum|EAL89729.1 agla|OJJ89134.1 akaw|GAA83003.1 aluc|OJZ87587.1 anid|CBF85941.1 anig|EHA20788.1 aory|EIT80784.1 asyd|OJJ64411.1 ater|EAU35809.1 atub|OJI81061.1 aver|OJI98510.1 awen|OJJ40668.1 azon|OJJ44049.1

Group2311: abra|OJJ70942.1 acar|OOF98190.1 acla|EAW10507.1 afis|EAW25060.1 afla|EED55393.1 afum|EAL89728.1 agla|OJJ89135.1 akaw|GAA83002.1 aluc|OJZ87586.1 anid|CBF85940.1 anig|EHA20787.1 aory|EIT80892.1 asyd|OJJ64410.1 ater|EAU35808.1 atub|OJI81062.1 aver|OJI98509.1 awen|OJJ40667.1 azon|OJJ44048.1

Group2312: abra|OJJ70944.1 acar|OOF98187.1 acla|EAW10512.1 afis|EAW25055.1 afla|EED55396.1 afum|EAL89723.2 agla|OJJ89137.1 akaw|GAA82999.1 aluc|OJZ87584.1 anid|CBF85933.1 anig|EHA20785.1 aory|EIT80802.1 asyd|OJJ64408.1 ater|EAU35805.1 atub|OJI81064.1 aver|OJI98506.1 awen|OJJ40665.1 azon|OJJ44044.1

Group2313: abra|OJJ70945.1 acar|OOF98186.1 acla|EAW10513.1 afis|EAW25054.1 afla|EED55397.1 afum|EAL89722.1 agla|OJJ89138.1 akaw|GAA82998.1 aluc|OJZ87583.1 anid|CBF85931.1 anig|EHA20784.1 aory|EIT80901.1 asyd|OJJ64407.1 ater|EAU35804.1 atub|OJI81065.1 aver|OJI98505.1 awen|OJJ40664.1 azon|OJJ44045.1

Group2314: abra|OJJ70946.1 acar|OOF98185.1 acla|EAW10514.1 afis|EAW25053.1 afla|EED55398.1 afum|EAL89721.1 agla|OJJ89139.1 akaw|GAA82997.1 aluc|OJZ87582.1 anid|CBF85929.1 anig|EHA20783.1 aory|EIT80773.1 asyd|OJJ64406.1 ater|EAU35803.1 atub|OJI81066.1 aver|OJI98504.1 awen|OJJ40663.1 azon|OJJ44043.1

Group2315: abra|OJJ70954.1 acar|OOF98176.1 acla|EAW10515.1 afis|EAW25051.1 afla|EED55400.1 afum|EAL89719.1 agla|OJJ89141.1 akaw|GAA82993.1 aluc|OJZ87578.1 anid|CBF85925.1 anig|EHA20780.1 aory|EIT80833.1 asyd|OJJ64405.1 ater|EAU35801.1 atub|OJI81071.1 aver|OJI98501.1 awen|OJJ40661.1 azon|OJJ44040.1

Group2316: abra|OJJ70955.1 acar|OOF98175.1 acla|EAW10516.1 afis|EAW25050.1 afla|EED55401.1 afum|EAL89718.1 agla|OJJ89142.1 akaw|GAA82992.1 aluc|OJZ87577.1 anid|CBF85923.1 anig|EHA20779.1 aory|EIT80841.1 asyd|OJJ64404.1 ater|EAU35800.1 atub|OJI81072.1 aver|OJI98500.1 awen|OJJ40660.1 azon|OJJ44039.1

Group2317: abra|OJJ70957.1 acar|OOF98173.1 acla|EAW10518.1 afis|EAW25048.1 afla|EED55403.1 afum|EAL89716.1 agla|OJJ89144.1 akaw|GAA82990.1 aluc|OJZ87575.1 anid|CBF85920.1 anig|EHA20777.1 aory|EIT80783.1 asyd|OJJ64402.1 ater|EAU35798.1 atub|OJI81074.1 aver|OJI98498.1 awen|OJJ40659.1 azon|OJJ44037.1

Group2318: abra|OJJ70958.1 acar|OOF98172.1 acla|EAW10519.1 afis|EAW25047.1 afla|EED55404.1 afum|EAL89715.1 agla|OJJ89145.1 akaw|GAA82989.1 aluc|OJZ87574.1 anid|CBF85918.1 anig|EHA20776.1 aory|EIT80839.1 asyd|OJJ64401.1 ater|EAU35797.1 atub|OJI81075.1 aver|OJI98497.1 awen|OJJ40658.1 azon|OJJ44036.1

Group2319: abra|OJJ70959.1 acar|OOF98171.1 acla|EAW10520.1 afis|EAW25046.1 afla|EED55405.1 afum|EAL89714.1 agla|OJJ89148.1 akaw|GAA82988.1 aluc|OJZ87573.1 anid|CBF85916.1 anig|EHA20775.1 aory|EIT80804.1 asyd|OJJ64400.1 ater|EAU35796.1 atub|OJI81076.1 aver|OJI98496.1 awen|OJJ40657.1 azon|OJJ44035.1

Group2320: abra|OJJ70963.1 acar|OOF98167.1 acla|EAW10524.1 afis|EAW25042.1 afla|EED55410.1 afum|EAL89710.1 agla|OJJ89152.1 akaw|GAA82984.1 aluc|OJZ87569.1 anid|CBF85908.1 anig|EHA20771.1 aory|EIT80900.1 asyd|OJJ64396.1 ater|EAU35792.1 atub|OJI81080.1 aver|OJI98492.1 awen|OJJ40647.1 azon|OJJ44032.1

Group2321: abra|OJJ70964.1 acar|OOF98166.1 acla|EAW10525.1 afis|EAW25041.1 afla|EED55411.1 afum|EAL89709.1 agla|OJJ89153.1 akaw|GAA82983.1 aluc|OJZ87568.1 anid|CBF85907.1 anig|EHA20770.1 aory|EIT80795.1 asyd|OJJ64395.1 ater|EAU35791.1 atub|OJI81081.1 aver|OJI98490.1 awen|OJJ40646.1 azon|OJJ44031.1

Group2322: abra|OJJ70968.1 acar|OOF98162.1 acla|EAW10528.1 afis|EAW25038.1 afla|EED55415.1 afum|EAL89706.1 agla|OJJ89155.1 akaw|GAA82980.1 aluc|OJZ87564.1 anid|CBF85901.1 anig|EHA20766.1 aory|EIT80889.1 asyd|OJJ64392.1 ater|EAU35788.1 atub|OJI81083.1 aver|OJI98487.1 awen|OJJ40644.1 azon|OJJ44028.1

Group2323: abra|OJJ70971.1 acar|OOF98159.1 acla|EAW10531.1 afis|EAW24998.1 afla|EED55418.1 afum|EAL89703.1 agla|OJJ89158.1 akaw|GAA82977.1 aluc|OJZ87561.1 anid|CBF85898.1 anig|EHA20763.1 aory|EIT80823.1 asyd|OJJ64390.1 ater|EAU35786.1 atub|OJI81086.1 aver|OJI98485.1 awen|OJJ40641.1 azon|OJJ44026.1

Group2324: abra|OJJ70973.1 acar|OOF98158.1 acla|EAW10532.1 afis|EAW24999.1 afla|EED55419.1 afum|EAL89699.1 agla|OJJ89161.1 akaw|GAA82976.1 aluc|OJZ87559.1 anid|CBF81499.1 anig|EHA20762.1 aory|EIT80834.1 asyd|OJJ59465.1 ater|EAU35785.1 atub|OJI81088.1 aver|OJJ02809.1 awen|OJJ40640.1 azon|OJJ44024.1

Group2325: abra|OJJ70976.1 acar|OOF98154.1 acla|EAW10535.1 afis|EAW25002.1 afla|EED55422.1 afum|EAL89696.1 agla|OJJ89165.1 akaw|GAA82973.1 aluc|OJZ87556.1 anid|CBF81486.1 anig|EHA20759.1 aory|EIT80883.1 ater|EAU35783.1 atub|OJI81091.1 awen|OJJ40637.1 azon|OJJ49046.1 asyd|OJJ59457.1 aver|OJJ02816.1

Group2326: abra|OJJ70977.1 acar|OOF98155.1 acla|EAW10536.1 afis|EAW25003.1 afla|EED55423.1 afum|EAL89695.1 agla|OJJ89166.1 akaw|GAA82972.1 aluc|OJZ87555.1 anid|CBF81484.1 anig|EHA20758.1 aory|EIT80840.1 asyd|OJJ59456.1 ater|EAU35782.1 atub|OJI81092.1 aver|OJJ02817.1 awen|OJJ40636.1 azon|OJJ49047.1

Group2327: abra|OJJ70979.1 acar|OOF98153.1 acla|EAW10538.1 afis|EAW25005.1 afla|EED55425.1 afum|EAL89693.1 agla|OJJ89168.1 akaw|GAA82970.1 aluc|OJZ87553.1 anid|CBF81481.1 anig|EHA20756.1 aory|EIT80871.1 asyd|OJJ59454.1 ater|EAU35780.1 atub|OJI81093.1 aver|OJJ02819.1 awen|OJJ40634.1 azon|OJJ49049.1

Group2328: abra|OJJ70980.1 acar|OOF98151.1 acla|EAW10539.1 afis|EAW25006.1 afla|EED55426.1 afum|EAL89691.2 agla|OJJ89169.1 akaw|GAA82969.1 aluc|OJZ87551.1 anid|CBF81512.1 anig|EHA20754.1 aory|EIT80772.1 asyd|OJJ59470.1 ater|EAU35779.1 atub|OJI81094.1 aver|OJJ02804.1 awen|OJJ40633.1 azon|OJJ49050.1

Group2329: abra|OJJ70981.1 acar|OOF98150.1 acla|EAW10540.1 afis|EAW25007.1 afla|EED55427.1 afum|EAL89690.1 agla|OJJ89170.1 akaw|GAA82968.1 aluc|OJZ87550.1 anid|CBF81514.1 anig|EHA20753.1 aory|EIT80826.1 asyd|OJJ59472.1 ater|EAU35778.1 atub|OJI81095.1 aver|OJJ02802.1 awen|OJJ40632.1 azon|OJJ49051.1

Group2330: abra|OJJ70982.1 acar|OOF98148.1 acla|EAW10541.1 afis|EAW25008.1 afla|EED55428.1 afum|EBA27387.1 agla|OJJ89173.1 akaw|GAA82967.1 aluc|OJZ87549.1 anid|CBF81516.1 anig|EHA20752.1 aory|EIT80794.1 asyd|OJJ59473.1 ater|EAU35777.1 atub|OJI81097.1 aver|OJJ02801.1 awen|OJJ40631.1 azon|OJJ49052.1

Group2331: abra|OJJ70983.1 acar|OOF98149.1 acla|EAW10542.1 afis|EAW25010.1 afla|EED55430.1 afum|EAL89686.1 agla|OJJ89174.1 akaw|GAA82966.1 aluc|OJZ87548.1 anid|CBF81518.1 anig|EHA20751.1

aory|EIT80803.1 asyd|OJJ59474.1 ater|EAU35776.1 atub|OJI81098.1 aver|OJJ02799.1 awen|OJJ40630.1 azon|OJJ49053.1

Group2332: abra|OJJ70984.1 acar|OOF98147.1 acla|EAW10543.1 afis|EAW25011.1 afla|EED55431.1 afum|EAL89685.1 agla|OJJ89175.1 akaw|GAA82965.1 aluc|OJZ87547.1 anid|CBF81519.1 anig|EHA20750.1 aory|EIT80824.1 asyd|OJJ59475.1 ater|EAU35775.1 atub|OJI81099.1 aver|OJJ02798.1 awen|OJJ40629.1 azon|OJJ49054.1

Group2333: abra|OJJ70989.1 acar|OOF98144.1 acla|EAW10547.1 afis|EAW25015.1 afla|EED55437.1 afum|EAL89681.1 agla|OJJ89181.1 akaw|GAA82962.1 aluc|OJZ87543.1 anid|CBF81527.1 anig|EHA20747.1 aory|EIT80927.1 asyd|OJJ59481.1 ater|EAU35771.1 atub|OJI81104.1 aver|OJJ02793.1 awen|OJJ40625.1 azon|OJJ49058.1

Group2334: abra|OJJ70991.1 acar|OOF98143.1 acla|EAW10548.1 afis|EAW25016.1 afla|EED55439.1 afum|EAL89680.1 agla|OJJ89182.1 akaw|GAA82960.1 aluc|OJZ87541.1 anid|CBF81529.1 anig|EHA20746.1 aory|EIT80844.1 asyd|OJJ59483.1 ater|EAU35770.1 atub|OJI81106.1 aver|OJJ02791.1 awen|OJJ40624.1 azon|OJJ49059.1

Group2335: abra|OJJ70996.1 acar|OOF90034.1 acla|EAW10552.1 afis|EAW25019.1 afla|EED55442.1 afum|EAL89676.1 agla|OJJ89187.1 akaw|GAA82952.1 aluc|OJZ87532.1 anid|CBF81538.1 anig|EHA20741.1 aory|EIT80858.1 asyd|OJJ59488.1 ater|EAU35767.1 atub|OJI81117.1 aver|OJJ02786.1 awen|OJJ40621.1 azon|OJJ49469.1

Group2336: abra|OJJ70998.1 acar|OOF98142.1 acla|EAW10555.1 afis|EAW25022.1 afla|EED55444.1 afum|EAL89673.1 agla|OJJ89190.1 akaw|GAA82950.1 aluc|OJZ87529.1 anid|CBF81541.1 anig|EHA20739.1 aory|EIT80864.1 asyd|OJJ59490.1 ater|EAU35765.1 atub|OJI81119.1 aver|OJJ02784.1 awen|OJJ40618.1 azon|OJJ49466.1

Group2337: abra|OJJ71001.1 acar|OOF98139.1 acla|EAW10558.1 afis|EAW25025.1 afla|EED55447.1 afum|EAL89670.1 agla|OJJ79159.1 akaw|GAA82947.1 aluc|OJZ87525.1 anid|CBF81547.1 anig|EHA20736.1 aory|EIT80809.1 asyd|OJJ59493.1 ater|EAU35762.1 atub|OJI81123.1 aver|OJJ02781.1 awen|OJJ40614.1 azon|OJJ49463.1

Group2338: abra|OJJ71008.1 acar|OOF98134.1 acla|EAW10560.1 afis|EAW25027.1 afla|EED55449.1 afum|EAL89668.1 agla|OJJ79157.1 akaw|GAA82945.1 aluc|OJZ87523.1 anid|CBF81551.1 anig|EHA20733.1 aory|EIT80929.1 asyd|OJJ59495.1 ater|EAU35760.1 atub|OJI81125.1 aver|OJJ02779.1 awen|OJJ40612.1 azon|OJJ49461.1

Group2339: abra|OJJ71013.1 acar|OOF98126.1 acla|EAW10564.1 afis|EAW25031.1 afla|EED55454.1 afum|EAL89664.1 agla|OJJ82242.1 akaw|GAA82941.1 aluc|OJZ87518.1 anid|CBF81564.1 anig|EHA20729.1 aory|EIT80774.1 asyd|OJJ59502.1 ater|EAU35756.1 atub|OJI81131.1 aver|OJJ02772.1 awen|OJJ40608.1 azon|OJJ49458.1

Group2340: abra|OJJ71016.1 acar|OOF98124.1 acla|EAW10566.1 afis|EAW25032.1 afla|EED55457.1 afum|EAL89662.1 agla|OJJ82244.1 akaw|GAA82939.1 aluc|OJZ87516.1 anid|CBF81567.1 anig|EHA20727.1 aory|EIT80779.1 asyd|OJJ59504.1 ater|EAU35755.1 atub|OJI81134.1 aver|OJJ02770.1 awen|OJJ40607.1 azon|OJJ49456.1

Group2341: abra|OJJ71019.1 acar|OOF98122.1 acla|EAW10568.1 afis|EAW25034.1 afla|EED55459.1 afum|EAL89660.1 agla|OJJ82255.1 akaw|GAA82936.1 aluc|OJZ87513.1 anid|CBF81574.1 anig|EHA20725.1 aory|EIT80788.1 asyd|OJJ59507.1 ater|EAU35751.1 atub|OJI81138.1 aver|OJJ02767.1 awen|OJJ40604.1 azon|OJJ49454.1

Group2342: abra|OJJ71021.1 acar|OOF98119.1 acla|EAW10570.1 afis|EAW25036.1 afla|EED55462.1 afum|EAL89658.1 agla|OJJ82257.1 akaw|GAA82934.1 aluc|OJZ87511.1 anid|CBF81583.1 anig|EHA20723.1 aory|EIT80855.1 asyd|OJJ59510.1 ater|EAU35747.1 atub|OJI81140.1 aver|OJJ02764.1 awen|OJJ40602.1 azon|OJJ49452.1

Group2343: abra|OJJ71022.1 acar|OOF98117.1 acla|EAW10571.1 afis|EAW25037.1 afla|EED55463.1 afum|EAL89657.1 agla|OJJ82258.1 akaw|GAA82933.1 aluc|OJZ87510.1 anid|CBF81585.1 anig|EHA20722.1 aory|EIT80922.1 asyd|OJJ59511.1 ater|EAU38720.1 atub|OJI81141.1 aver|OJJ02762.1 awen|OJJ40601.1 azon|OJJ49451.1

Group2344: abra|OJJ71023.1 acar|OOF98118.1 acla|EAW10572.1 afis|EAW24995.1 afla|EED55464.1 afum|EAL89656.1 agla|OJJ82259.1 akaw|GAA82932.1 aluc|OJZ87509.1 anid|CBF81586.1 anig|EHA20721.1 aory|EIT80875.1 asyd|OJJ59512.1 ater|EAU35746.1 atub|OJI81142.1 aver|OJJ02761.1 awen|OJJ40600.1 azon|OJJ49450.1

Group2345: abra|OJJ71028.1 acar|OOF98111.1 acla|EAW10579.1 afis|EAW24988.1 afla|EED55470.1 afum|EAL89648.1 agla|OJJ82274.1 akaw|GAA82927.1 aluc|OJZ87502.1 anid|CBF81599.1 anig|EHA20717.1 aory|EIT80811.1 asyd|OJJ59519.1 ater|EAU35739.1 atub|OJI81147.1 aver|OJJ02754.1 awen|OJJ40593.1 azon|OJJ49443.1

Group2346: abra|OJJ71030.1 acar|OOF98109.1 acla|EAW10583.1 afis|EAW24986.1 afla|EED55472.1 afum|EAL89646.1 agla|OJJ82276.1 akaw|GAA82925.1 aluc|OJZ87498.1 anid|CBF81603.1 anig|EHA20715.1 aory|EIT80838.1 asyd|OJJ59521.1 ater|EAU35737.1 atub|OJI81149.1 aver|OJJ02752.1 awen|OJJ40582.1 azon|OJJ49441.1

Group2347: abra|OJJ71036.1 acar|OOF98101.1 acla|EAW10586.1 afis|EAW24982.1 afla|EED55477.1 afum|EAL89642.1 agla|OJJ82279.1 akaw|GAA82919.1 aluc|OJZ87491.1 anid|CBF81614.1 anig|EHA20711.1 aory|EIT80831.1 asyd|OJJ59526.1 ater|EAU35734.1 atub|OJI81157.1 aver|OJJ02746.1 awen|OJJ40579.1 azon|OJJ49430.1

Group2348: abra|OJJ71038.1 acar|OOF98100.1 acla|EAW10587.1 afis|EAW24981.1 afla|EED55478.1 afum|EAL89641.1 agla|OJJ79136.1 akaw|GAA82918.1 aluc|OJZ87490.1 anid|CBF81612.1 anig|EHA20710.1 aory|EIT80854.1 asyd|OJJ59525.1 ater|EAU35733.1 atub|OJI81158.1 aver|OJJ02747.1 awen|OJJ40578.1 azon|OJJ49431.1

Group2349: abra|OJJ71040.1 acar|OOF98098.1 acla|EAW10589.1 afis|EAW24979.1 afla|EED55480.1 afum|EAL89639.1 agla|OJJ79138.1 akaw|GAA82916.1 aluc|OJZ87488.1 anid|CBF81608.1 anig|EHA20708.1 aory|EIT80915.1 asyd|OJJ59523.1 ater|EAU35729.1 atub|OJI81160.1 aver|OJJ02749.1 awen|OJJ40576.1 azon|OJJ49434.1

Group2350: abra|OJJ71042.1 acar|OOF98097.1 acla|EAW10592.1 afis|EAW24976.1 afla|EED55484.1 afum|EAL89636.1 agla|OJJ79141.1 akaw|GAA82914.1 aluc|OJZ87486.1 anid|CBF81629.1 anig|EHA20707.1 aory|EIT80868.1 asyd|OJJ59530.1 ater|EAU35725.1 atub|OJI81162.1 aver|OJJ02740.1 awen|OJJ40573.1 azon|OJJ49438.1

Group2351: abra|OJJ71045.1 acar|OOF98094.1 acla|EAW10593.1 afis|EAW24975.1 afla|EED55485.1 afum|EAL89635.1 agla|OJJ82241.1 akaw|GAA82911.1 aluc|OJZ87481.1 anid|CBF81630.1 anig|EHA20703.1 aory|EIT80859.1 asyd|OJJ59531.1 ater|EAU35723.1 atub|OJI81167.1 aver|OJJ02739.1 awen|OJJ40572.1 azon|OJJ49429.1

Group2352: abra|OJJ71047.1 acar|OOF98092.1 acla|EAW10595.1 afis|EAW24973.1 afla|EED55486.1 afum|EAL89633.1 agla|OJJ82239.1 akaw|GAA82909.1 aluc|OJZ87479.1 anid|CBF81634.1 anig|EHA20701.1 aory|EIT80885.1 asyd|OJJ59533.1 ater|EAU35721.1 atub|OJI81169.1 aver|OJJ02737.1 awen|OJJ40570.1 azon|OJJ49427.1

Group2353: abra|OJJ71048.1 acar|OOF98091.1 acla|EAW10596.1 afis|EAW24972.1 afla|EED55487.1 afum|EBA27384.1 agla|OJJ82238.1 akaw|GAA82908.1 aluc|OJZ87478.1 anid|CBF81636.1 anig|EHA20700.1 aory|EIT80911.1 asyd|OJJ59534.1 ater|EAU35720.1 atub|OJI81170.1 aver|OJJ02736.1 awen|OJJ40569.1 azon|OJJ49426.1

Group2354: abra|OJJ71049.1 acar|OOF98090.1 acla|EAW10597.1 afis|EAW24971.1 afla|EED55490.1 afum|EAL89630.2 agla|OJJ82237.1 akaw|GAA82907.1 aluc|OJZ87477.1 anid|CBF81638.1 anig|EHA20699.1 aory|EIT80796.1 asyd|OJJ59535.1 ater|EAU35719.1 atub|OJI81171.1 aver|OJJ02735.1 awen|OJJ40568.1 azon|OJJ49425.1

Group2355: abra|OJJ71052.1 acar|OOF98087.1 acla|EAW10603.1 afis|EAW24965.1 afla|EED55495.1 afum|EAL89623.1 agla|OJJ82224.1 akaw|GAA82905.1 aluc|OJZ87475.1 anid|CBF81647.1 anig|EHA20695.1 aory|EIT80847.1 asyd|OJJ59541.1 ater|EAU35709.1 atub|OJI81173.1 aver|OJJ02727.1 awen|OJJ40558.1 azon|OJJ49419.1

Group2356: abra|OJJ71054.1 acar|OOF98085.1 acla|EAW14735.1 afis|EAW19536.1 afla|EED54353.1 afum|EAL86970.2 agla|OJJ85406.1 akaw|GAA82902.1 aluc|OJZ87473.1 anid|CBF76891.1 anig|EHA20693.1 aory|EIT78008.1 asyd|OJJ61335.1 ater|EAU31542.1 atub|OJI81175.1 aver|OJJ00659.1 awen|OJJ34757.1 aory|EIT75941.1

Group2357: abra|OJJ71061.1 acar|OOF98082.1 acla|EAW10598.1 afis|EAW24970.1 afla|EED55491.1 afum|EAL89629.1 agla|OJJ82236.1 akaw|GAA82898.1 aluc|OJZ87467.1 anid|CBF81640.1 anig|EHA20689.1 aory|EIT80891.1 asyd|OJJ59536.1 ater|EAU35715.1 atub|OJI81181.1 aver|OJJ02733.1 awen|OJJ40564.1 azon|OJJ49424.1

Group2358: abra|OJJ71075.1 acar|OOF98069.1 acla|EAW10604.1 afis|EAW24964.1 afla|EED55497.1 afum|EAL89622.1 agla|OJJ82226.1 akaw|GAA82887.1 aluc|OJZ87455.1 anid|CBF81649.1 anig|EHA20676.1 aory|EIT80869.1 asyd|OJJ59542.1 ater|EAU35707.1 atub|OJI81193.1 aver|OJJ02726.1 awen|OJJ40556.1 azon|OJJ49418.1

Group2359: abra|OJJ71103.1 acar|OOF90221.1 acla|EAW10388.1 afis|EAW25205.1 afla|EED53664.1 afum|EAL89866.2 agla|OJJ88951.1 akaw|GAA82865.1 aluc|OJZ87432.1 anid|CBF85221.1 anig|EHA20653.1 aory|EIT75288.1 asyd|OJJ62997.1 ater|EAU34349.1 atub|OJI81216.1 aver|OJI97970.1 awen|OJJ40840.1 azon|OJJ46822.1

Group2360: abra|OJJ71104.1 acar|OOF90222.1 acla|EAW10387.1 afis|EAW25206.1 afla|EED53662.1 afum|EAL89867.1 agla|OJJ88952.1 akaw|GAA82864.1 aluc|OJZ87431.1 anid|CBF85223.1 anig|EHA20652.1

aory|EIT75326.1 asyd|OJJ62996.1 ater|EAU34348.1 atub|OJI81217.1 aver|OJI97971.1 awen|OJJ40841.1 azon|OJJ46823.1

Group2361: abra|OJJ71105.1 acar|OOF90223.1 acla|EAW10386.1 afis|EAW25207.1 afla|EED53660.1 afum|EAL89868.1 agla|OJJ79791.1 akaw|GAA82863.1 aluc|OJZ87430.1 anid|CBF85225.1 anig|EHA20651.1 aory|EIT75316.1 asyd|OJJ62995.1 ater|EAU34347.1 atub|OJI81218.1 aver|OJI97972.1 awen|OJJ35846.1 azon|OJJ45132.1

Group2362: abra|OJJ71112.1 acar|OOF98038.1 acla|EAW10380.1 afis|EAW25213.1 afla|EED53667.1 afum|EAL89874.1 agla|OJJ88948.1 akaw|GAA82857.1 aluc|OJZ87422.1 anid|CBF85606.1 anig|EHA20645.1 aory|EIT75347.1 asyd|OJJ64197.1 ater|EAU34370.1 atub|OJI81226.1 aver|OJI98286.1 awen|OJJ40875.1 azon|OJJ46812.1

Group2363: abra|OJJ71117.1 acar|OOF98033.1 acla|EAW10378.1 afis|EAW25215.1 afla|EED53692.1 afum|EAL89876.1 agla|OJJ88944.1 akaw|GAA82853.1 aluc|OJZ87418.1 anid|CBF85254.1 anig|EHA20642.1 aory|EIT75323.1 asyd|OJJ62921.1 ater|EAU34372.1 atub|OJI81230.1 aver|OJI98068.1 awen|OJJ40878.1 azon|OJJ44506.1

Group2364: abra|OJJ71119.1 acar|OOF98031.1 acla|EAW10376.1 afis|EAW25217.1 afla|EED53690.1 afum|EAL89878.1 agla|OJJ79184.1 akaw|GAA82851.1 aluc|OJZ87417.1 anid|CBF85273.1 anig|EHA20641.1 aory|EIT75328.1 asyd|OJJ62920.1 ater|EAU34374.1 atub|OJI81231.1 aver|OJI98070.1 awen|OJJ40880.1 azon|OJJ44508.1

Group2365: abra|OJJ71125.1 acar|OOF98028.1 acla|EAW10374.1 afis|EAW25218.1 afla|EED53688.1 afum|EAL89879.1 agla|OJJ79186.1 akaw|GAA82850.1 aluc|OJZ87410.1 anid|CBF85299.1 anig|EHA20640.1 aory|EIT75354.1 asyd|OJJ62917.1 ater|EAU34377.1 atub|OJI81235.1 aver|OJI98073.1 awen|OJJ40883.1 azon|OJJ44512.1

Group2366: abra|OJJ71128.1 acar|OOF98024.1 acla|EAW10370.1 afis|EAW25222.1 afla|EED53684.1 afum|EAL89883.1 agla|OJJ79189.1 akaw|GAA82846.1 aluc|OJZ87407.1 anid|CBF85258.1 anig|EHA20637.1 aory|EIT75281.1 asyd|OJJ62911.1 ater|EAU34381.1 atub|OJI81239.1 aver|OJI98078.1 awen|OJJ40888.1 azon|OJJ44517.1

Group2367: abra|OJJ71132.1 acar|OOF98020.1 acla|EAW10365.1 afis|EAW25227.1 afla|EED53671.1 afum|EAL89888.1 agla|OJJ79181.1 akaw|GAA82842.1 aluc|OJZ87403.1 anid|CBF85269.1 anig|EHA20632.1 aory|EIT75290.1 asyd|OJJ62908.1 ater|EAU34385.1 atub|OJI81243.1 aver|OJI98080.1 awen|OJJ40892.1 azon|OJJ44521.1

Group2368: abra|OJJ71133.1 acar|OOF98019.1 acla|EAW10364.1 afis|EAW25228.1 afla|EED53672.1 afum|EAL89889.1 agla|OJJ79180.1 akaw|GAA82841.1 aluc|OJZ87402.1 anid|CBF85262.1 anig|EHA20631.1 aory|EIT75302.1 asyd|OJJ62905.1 ater|EAU34386.1 atub|OJI81244.1 aver|OJI98084.1 awen|OJJ40893.1 azon|OJJ44522.1

Group2369: abra|OJJ71142.1 acar|OOF98014.1 acla|EAW10359.1 afis|EAW25234.1 afla|EED53675.1 afum|EAL89895.1 agla|OJJ89203.1 akaw|GAA82835.1 aluc|OJZ87396.1 anid|CBF85334.1 anig|EHA20626.1 aory|EIT75320.1 asyd|OJJ64021.1 ater|EAU34389.1 atub|OJI81250.1 aver|OJI98107.1 awen|OJJ40946.1 azon|OJJ44527.1

Group2370: abra|OJJ71143.1 acar|OOF98013.1 acla|EAW10358.1 afis|EAW25235.1 afla|EED53676.1 afum|EAL89896.2 agla|OJJ89204.1 akaw|GAA82834.1 aluc|OJZ87395.1 anid|CBF85332.1 anig|EHA20625.1 aory|EIT75309.1 asyd|OJJ64020.1 ater|EAU34390.1 atub|OJI81251.1 aver|OJI98106.1 awen|OJJ40947.1 azon|OJJ44528.1

Group2371: abra|OJJ71145.1 acar|OOF98011.1 acla|EAW10361.1 afis|EAW25232.1 afla|EED53678.1 afum|EAL89893.1 agla|OJJ79175.1 akaw|GAA82832.1 aluc|OJZ87393.1 anid|CBF85329.1 anig|EHA20623.1 aory|EIT75284.1 asyd|OJJ64018.1 ater|EAU34391.1 atub|OJI81253.1 aver|OJI98104.1 awen|OJJ40898.1 azon|OJJ44525.1

Group2372: abra|OJJ71150.1 acar|OOF98007.1 acla|EAW10352.1 afis|EAW25242.1 afla|EED53702.1 afum|EAL89904.1 agla|OJJ89208.1 akaw|GAA82827.1 aluc|OJZ87388.1 anid|CBF85319.1 anig|EHA20617.1 aory|EIT75296.1 asyd|OJJ64011.1 ater|EAU34367.1 atub|OJI81259.1 aver|OJI98099.1 awen|OJJ40955.1 azon|OJJ44538.1

Group2373: abra|OJJ71151.1 acar|OOF98006.1 acla|EAW10346.1 afis|EAW25243.1 afla|EED53700.1 afum|EAL89905.1 agla|OJJ89209.1 akaw|GAA82826.1 aluc|OJZ87387.1 anid|CBF85321.1 anig|EHA20616.1 aory|EIT75325.1 asyd|OJJ64012.1 ater|EAU34360.1 atub|OJI81260.1 aver|OJI98100.1 awen|OJJ40956.1 azon|OJJ44537.1

Group2374: abra|OJJ71158.1 acar|OOF98000.1 acla|EAW10339.1 afis|EAW25248.1 afla|EED53716.1 afum|EAL89910.1 agla|OJJ89222.1 akaw|GAA82820.1 aluc|OJZ87381.1 anid|CBF85341.1 anig|EHA20612.1 aory|EIT75359.1 asyd|OJJ64025.1 ater|EAU34355.1 atub|OJI81265.1 aver|OJI98111.1 awen|OJJ40964.1 azon|OJJ44545.1

Group2375: abra|OJJ71167.1 acar|OOF97996.1 acla|EAW10336.1 afis|EAW25253.1 afla|EED53722.1 afum|EAL89916.1 agla|OJJ89227.1 akaw|GAA82814.1 aluc|OJZ87374.1 anid|CBF85349.1 anig|EHA20607.1 aory|EIT75275.1 asyd|OJJ64033.1 ater|EAU34398.1 atub|OJI81272.1 aver|OJI98117.1 awen|OJJ40967.1 azon|OJJ44551.1

Group2376: abra|OJJ71169.1 acar|OOF97994.1 acla|EAW10333.1 afis|EAW25259.1 afla|EED53724.1 afum|EAL89921.1 agla|OJJ89230.1 akaw|GAA82812.1 aluc|OJZ87372.1 anid|CBF85360.1 anig|EHA20605.1 aory|EIT75308.1 asyd|OJJ64039.1 ater|EAU34400.1 atub|OJI81274.1 aver|OJI98123.1 awen|OJJ40972.1 azon|OJJ44558.1

Group2377: abra|OJJ71170.1 acar|OOF97993.1 acla|EAW10332.1 afla|EED53725.1 agla|OJJ89231.1 akaw|GAA82811.1 aluc|OJZ87371.1 anid|CBF85362.1 anig|EHA20604.1 aory|EIT75319.1 asyd|OJJ64040.1 ater|EAU34401.1 atub|OJI81275.1 aver|OJI98124.1 awen|OJJ40973.1 afis|EAW25260.1 afum|EAL89922.1 azon|OJJ44559.1

Group2378: abra|OJJ71173.1 acar|OOF97992.1 acla|EAW10331.1 afis|EAW25263.1 afla|EED53727.1 afum|EAL89926.2 agla|OJJ89233.1 akaw|GAA82810.1 aluc|OJZ87369.1 anid|CBF85365.1 anig|EHA20603.1 aory|EIT75277.1 asyd|OJJ64043.1 ater|EAU34402.1 atub|OJI81276.1 aver|OJI98127.1 awen|OJJ40975.1 azon|OJJ47355.1

Group2379: abra|OJJ71174.1 acar|OOF97991.1 acla|EAW10330.1 afis|EAW25264.1 afla|EED53728.1 afum|EAL89927.1 agla|OJJ89234.1 akaw|GAA82809.1 aluc|OJZ87368.1 anid|CBF85367.1 anig|EHA20602.1 aory|EIT75295.1 asyd|OJJ64045.1 ater|EAU34403.1 atub|OJI81277.1 aver|OJI98128.1 awen|OJJ40976.1 azon|OJJ44563.1

Group2380: abra|OJJ71179.1 acar|OOF97987.1 acla|EAW10327.1 afis|EAW25267.1 afla|EED53731.1 afum|EAL89930.1 agla|OJJ89241.1 akaw|GAA82806.1 aluc|OJZ87364.1 anid|CBF85374.1 anig|EHA20599.1 aory|EIT75331.1 asyd|OJJ64048.1 ater|EAU34408.1 atub|OJI81280.1 aver|OJI98133.1 awen|OJJ40980.1 azon|OJJ44567.1

Group2381: abra|OJJ71182.1 acar|OOF97984.1 acla|EAW10322.1 afis|EAW25270.1 afla|EED53735.1 afum|EAL89933.1 agla|OJJ89243.1 akaw|GAA82803.1 aluc|OJZ87361.1 anid|CBF85382.1 anig|EHA20596.1 aory|EIT75338.1 asyd|OJJ64051.1 ater|EAU34413.1 atub|OJI81284.1 aver|OJI98137.1 awen|OJJ40984.1 azon|OJJ44570.1

Group2382: abra|OJJ71183.1 acar|OOF97983.1 acla|EAW10321.1 afis|EAW25271.1 afla|EED53736.1 afum|EAL89934.1 agla|OJJ89244.1 akaw|GAA82802.1 aluc|OJZ87360.1 anid|CBF85384.1 anig|EHA20595.1 aory|EIT75335.1 asyd|OJJ64053.1 ater|EAU34414.1 atub|OJI81285.1 aver|OJI98138.1 awen|OJJ40985.1 azon|OJJ44572.1

Group2383: abra|OJJ71184.1 acar|OOF97982.1 acla|EAW10320.1 afis|EAW25272.1 afla|EED53737.1 afum|EAL89935.1 agla|OJJ89245.1 akaw|GAA82801.1 aluc|OJZ87359.1 anid|CBF85385.1 anig|EHA20594.1 aory|EIT75311.1 asyd|OJJ64054.1 ater|EAU34415.1 atub|OJI81286.1 aver|OJI98139.1 awen|OJJ40986.1 azon|OJJ44573.1

Group2384: abra|OJJ71185.1 acar|OOF97981.1 acla|EAW10318.1 afis|EAW25274.1 afla|EED53739.1 afum|EAL89937.1 agla|OJJ89248.1 akaw|GAA82800.1 aluc|OJZ87358.1 anid|CBF85389.1 anig|EHA20593.1 aory|EIT75291.1 asyd|OJJ64056.1 ater|EAU34417.1 atub|OJI81287.1 aver|OJI98141.1 awen|OJJ40988.1 azon|OJJ44575.1

Group2385: abra|OJJ71187.1 acar|OOF97980.1 acla|EAW10316.1 afis|EAW25276.1 afla|EED53742.1 afum|EAL89939.1 agla|OJJ89250.1 akaw|GAA82798.1 aluc|OJZ87357.1 anid|CBF85392.1 anig|EHA20591.1 aory|EIT75317.1 asyd|OJJ64058.1 ater|EAU34419.1 atub|OJI81288.1 aver|OJI98143.1 awen|OJJ40990.1 azon|OJJ44578.1

Group2386: abra|OJJ71188.1 acar|OOF97979.1 acla|EAW10315.1 afis|EAW25277.1 afla|EED53744.1 afum|EAL89940.1 agla|OJJ89251.1 akaw|GAA82797.1 aluc|OJZ87356.1 anid|CBF85394.1 anig|EHA20590.1 aory|EIT75351.1 asyd|OJJ64059.1 ater|EAU34420.1 atub|OJI81289.1 aver|OJI98145.1 awen|OJJ40991.1 azon|OJJ44580.1

Group2387: abra|OJJ71189.1 acar|OOF97978.1 acla|EAW10314.1 afis|EAW25278.1 afla|EED53745.1 afum|EAL89941.1 agla|OJJ89252.1 akaw|GAA82796.1 aluc|OJZ87355.1 anid|CBF85396.1 anig|EHA20589.1 aory|EIT75313.1 asyd|OJJ64060.1 ater|EAU34421.1 atub|OJI81290.1 aver|OJI98146.1 awen|OJJ40992.1 azon|OJJ44581.1

Group2388: abra|OJJ71192.1 acar|OOF97975.1 acla|EAW10312.1 afis|EAW25281.1 afla|EED53747.1 afum|EAL89944.1 agla|OJJ89255.1 akaw|GAA82793.1 aluc|OJZ87352.1 anid|CBF85418.1 anig|EHA20586.1 aory|EIT75318.1 asyd|OJJ64073.1 ater|EAU34422.1 atub|OJI81293.1 aver|OJI98160.1 awen|OJJ40994.1 azon|OJJ44583.1

Group2389: abra|OJJ71198.1 acar|OOF97970.1 acla|EAW10305.1 afis|EAW25287.1 afla|EED53752.1 afum|EAL89950.1 agla|OJJ89260.1 akaw|GAA82785.1 aluc|OJZ87344.1 anid|CBF85429.1 anig|EHA20581.1

asyd|OJJ64083.1 ater|EAU34428.1 atub|OJI81300.1 aver|OJI98168.1 awen|OJJ40999.1 azon|OJJ44591.1 aory|EIT78420.1

Group2390: abra|OJJ71203.1 acar|OOF97968.1 acla|EAW10304.1 afis|EAW25288.1 afla|EED53753.1 afum|EAL89951.1 agla|OJJ89261.1 akaw|GAA82781.1 aluc|OJZ87340.1 anid|CBF85431.1 anig|EHA20577.1 aory|EIT78400.1 asyd|OJJ64084.1 ater|EAU34429.1 atub|OJI81304.1 aver|OJI98169.1 awen|OJJ41000.1 azon|OJJ44594.1

Group2391: abra|OJJ71204.1 acar|OOF97967.1 acla|EAW10303.1 afis|EAW25289.1 afla|EED53754.1 afum|EAL89952.1 agla|OJJ89262.1 akaw|GAA82780.1 aluc|OJZ87339.1 anid|CBF85433.1 anig|EHA20576.1 aory|EIT78451.1 asyd|OJJ64085.1 ater|EAU34430.1 atub|OJI81305.1 aver|OJI98170.1 awen|OJJ41001.1 azon|OJJ44593.1

Group2392: abra|OJJ71205.1 acar|OOF97965.1 acla|EAW10302.1 afis|EAW25290.1 afla|EED53755.1 afum|EAL89953.1 agla|OJJ89263.1 akaw|GAA82779.1 aluc|OJZ87338.1 anid|CBF85435.1 anig|EHA20575.1 aory|EIT78386.1 asyd|OJJ64086.1 ater|EAU34431.1 atub|OJI81306.1 aver|OJI98171.1 awen|OJJ41002.1 azon|OJJ44592.1

Group2393: abra|OJJ71206.1 acar|OOF97966.1 acla|EAW10301.1 afis|EAW25291.1 afla|EED53756.1 afum|EAL89954.1 agla|OJJ89265.1 akaw|GAA82778.1 aluc|OJZ87337.1 anid|CBF85413.1 anig|EHA20574.1 aory|EIT78379.1 asyd|OJJ64069.1 ater|EAU34432.1 atub|OJI81307.1 aver|OJI98156.1 awen|OJJ41003.1 azon|OJJ44595.1

Group2394: abra|OJJ71210.1 acar|OOF97956.1 acla|EAW10300.1 afis|EAW25292.1 afla|EED53757.1 afum|EAL89955.1 agla|OJJ89269.1 akaw|GAA82774.1 aluc|OJZ87333.1 anid|CBF85411.1 anig|EHA20569.1 aory|EIT78389.1 asyd|OJJ64068.1 ater|EAU34433.1 atub|OJI81311.1 aver|OJI98155.1 awen|OJJ41006.1 azon|OJJ44602.1

Group2395: abra|OJJ71211.1 acar|OOF97955.1 acla|EAW10298.1 afis|EAW25294.1 afla|EED53759.1 afum|EAL89957.1 agla|OJJ89270.1 akaw|GAA82773.1 aluc|OJZ87332.1 anid|CBF85409.1 anig|EHA20568.1 aory|EIT78363.1 asyd|OJJ64067.1 ater|EAU34434.1 atub|OJI81312.1 aver|OJI98154.1 awen|OJJ41008.1 azon|OJJ44601.1

Group2396: abra|OJJ71227.1 acar|OOF97952.1 acla|EAW10295.1 afis|EAW25297.1 afla|EED53763.1 afum|EAL89960.1 agla|OJJ89273.1 akaw|GAA82762.1 aluc|OJZ87320.1 anid|CBF85405.1 anig|EHA20554.1 aory|EIT78455.1 asyd|OJJ64064.1 ater|EAU34438.1 atub|OJI81325.1 aver|OJI98151.1 awen|OJJ41011.1 azon|OJJ44598.1

Group2397: abra|OJJ71241.1 acar|OOF97926.1 acla|EAW09108.1 afis|EAW25717.1 afla|EED53769.1 afum|EAL84392.2 agla|OJJ85442.1 akaw|GAA82746.1 aluc|OJZ87301.1 anid|CBF77088.1 anig|EHA20541.1 aory|EIT78470.1 asyd|OJJ56975.1 ater|EAU34440.1 atub|OJI81339.1 aver|OJJ03313.1 awen|OJJ33601.1 azon|OJJ44472.1

Group2398: abra|OJJ71244.1 acar|OOF97924.1 acla|EAW10291.1 afis|EAW25300.1 afla|EED53774.1 afum|EAL89963.2 agla|OJJ89280.1 akaw|GAA82743.1 aluc|OJZ87298.1 anid|CBF75193.1 anig|EHA20539.1 aory|EIT78388.1 asyd|OJJ59938.1 ater|EAU34448.1 atub|OJI81342.1 aver|OJJ02349.1 awen|OJJ41015.1 azon|OJJ44603.1

Group2399: abra|OJJ71245.1 acar|OOF97923.1 acla|EAW10290.1 afis|EAW25301.1 afla|EED53776.1 afum|EAL89964.1 agla|OJJ89281.1 akaw|GAA82742.1 aluc|OJZ87297.1 anid|CBF75195.1 anig|EHA20538.1 aory|EIT78454.1 asyd|OJJ59937.1 ater|EAU34449.1 atub|OJI81343.1 aver|OJJ02350.1 awen|OJJ41016.1 azon|OJJ42625.1

Group2400: abra|OJJ71248.1 acar|OOF97920.1 acla|EAW10287.1 afis|EAW25305.1 afla|EED53783.1 afum|EAL89968.2 agla|OJJ89284.1 akaw|GAA82739.1 aluc|OJZ87294.1 anid|CBF75296.1 anig|EHA20535.1 aory|EIT78359.1 asyd|OJJ59884.1 ater|EAU34453.1 atub|OJI81346.1 aver|OJJ02402.1 awen|OJJ41019.1 azon|OJJ44606.1

Group2401: abra|OJJ71255.1 acar|OOF97916.1 acla|EAW10281.1 afis|EAW25313.1 afla|EED53788.1 afum|EAL89974.1 agla|OJJ83848.1 akaw|GAA82733.1 aluc|OJZ87288.1 anid|CBF75287.1 anig|EHA20529.1 aory|EIT78401.1 asyd|OJJ59889.1 ater|EAU34458.1 atub|OJI81353.1 aver|OJJ02397.1 awen|OJJ41053.1 azon|OJJ44614.1

Group2402: abra|OJJ71256.1 acar|OOF97915.1 acla|EAW10280.1 afis|EAW25314.1 afla|EED53789.1 afum|EAL89975.1 agla|OJJ83849.1 akaw|GAA82732.1 aluc|OJZ87287.1 anid|CBF75285.1 anig|EHA20528.1 aory|EIT78413.1 asyd|OJJ59890.1 ater|EAU34459.1 atub|OJI81354.1 aver|OJJ02396.1 awen|OJJ41050.1 azon|OJJ44612.1

Group2403: abra|OJJ71258.1 acar|OOF97914.1 acla|EAW10278.1 afis|EAW25316.1 afla|EED53791.1 afum|EAL89977.2 agla|OJJ83854.1 akaw|GAA82731.1 aluc|OJZ87285.1 anid|CBF75283.1 anig|EHA20527.1 aory|EIT78447.1 asyd|OJJ59892.1 ater|EAU34460.1 atub|OJI81356.1 aver|OJJ02394.1 awen|OJJ41047.1 azon|OJJ44616.1

Group2404: abra|OJJ71259.1 acar|OOF97913.1 acla|EAW10277.1 afis|EAW25317.1 afla|EED53792.1 afum|EAL89978.2 agla|OJJ83855.1 akaw|GAA82730.1 aluc|OJZ87284.1 anid|CBF75281.1 anig|EHA20526.1 aory|EIT78371.1 asyd|OJJ59893.1 ater|EAU34464.1 atub|OJI81357.1 aver|OJJ02393.1 awen|OJJ41046.1 azon|OJJ44617.1

Group2405: abra|OJJ71262.1 acar|OOF97912.1 acla|EAW10275.1 afis|EAW25319.1 afla|EED53794.1 afum|EAL89980.2 agla|OJJ83857.1 akaw|GAA82727.1 aluc|OJZ87281.1 anid|CBF75269.1 anig|EHA20523.1 aory|EIT78456.1 asyd|OJJ59895.1 ater|EAU34465.1 atub|OJI81360.1 aver|OJJ02391.1 awen|OJJ41044.1 azon|OJJ44619.1

Group2406: abra|OJJ71263.1 acar|OOF97911.1 acla|EAW10274.1 afis|EAW25320.1 afla|EED53795.1 afum|EAL89981.1 agla|OJJ83858.1 akaw|GAA82726.1 aluc|OJZ87280.1 anid|CBF75267.1 anig|EHA20522.1 aory|EIT78395.1 asyd|OJJ59896.1 ater|EAU34466.1 atub|OJI81361.1 aver|OJJ02390.1 awen|OJJ41043.1 azon|OJJ44620.1

Group2407: abra|OJJ71264.1 acar|OOF97910.1 acla|EAW10273.1 afis|EAW25321.1 afla|EED53796.1 afum|EAL89982.1 agla|OJJ83859.1 akaw|GAA82725.1 aluc|OJZ87279.1 anid|CBF75265.1 anig|EHA20521.1 aory|EIT78365.1 asyd|OJJ59897.1 ater|EAU34467.1 atub|OJI81362.1 aver|OJJ02389.1 awen|OJJ41042.1 azon|OJJ44621.1

Group2408: abra|OJJ71265.1 acar|OOF97909.1 acla|EAW10272.1 afis|EAW25322.1 afla|EED53797.1 afum|EAL89983.1 agla|OJJ83860.1 akaw|GAA82724.1 aluc|OJZ87278.1 anid|CBF75263.1 anig|EHA20520.1 aory|EIT78429.1 asyd|OJJ59898.1 ater|EAU34468.1 atub|OJI81363.1 aver|OJJ02388.1 awen|OJJ41041.1 azon|OJJ44624.1

Group2409: abra|OJJ71267.1 acar|OOF97908.1 acla|EAW10271.1 afis|EAW25323.1 afla|EED53798.1 afum|EAL89984.1 agla|OJJ83861.1 akaw|GAA82723.1 aluc|OJZ87277.1 anid|CBF75261.1 anig|EHA20519.1 aory|EIT78438.1 asyd|OJJ59899.1 ater|EAU34469.1 atub|OJI81364.1 aver|OJJ02387.1 awen|OJJ41040.1 azon|OJJ44626.1

Group2410: abra|OJJ71270.1 acar|OOF97902.1 acla|EAW10268.1 afis|EAW25326.1 afla|EED53803.1 afum|EAL89987.1 agla|OJJ83870.1 akaw|GAA82720.1 aluc|OJZ87274.1 anid|CBF75254.1 anig|EHA20515.1 aory|EIT78467.1 asyd|OJJ59904.1 ater|EAU34473.1 atub|OJI81368.1 aver|OJJ02381.1 awen|OJJ41031.1 azon|OJJ44634.1

Group2411: abra|OJJ71271.1 acar|OOF97903.1 acla|EAW10267.1 afis|EAW25327.1 afla|EED53804.1 afum|EAL89988.1 agla|OJJ83869.1 akaw|GAA82719.1 aluc|OJZ87273.1 anid|CBF75252.1 anig|EHA20514.1 aory|EIT78360.1 asyd|OJJ59905.1 ater|EAU34474.1 atub|OJI81369.1 aver|OJJ02380.1 awen|OJJ41032.1 azon|OJJ44633.1

Group2412: abra|OJJ71276.1 acar|OOF97897.1 acla|EAW10257.1 afis|EAW25336.1 afla|EED53810.1 afum|EAL89999.1 agla|OJJ83871.1 akaw|GAA82713.1 aluc|OJZ87267.1 anid|CBF75242.1 anig|EHA20509.1 aory|EIT78463.1 asyd|OJJ59910.1 atub|OJI81375.1 aver|OJJ02376.1 awen|OJJ41030.1 azon|OJJ44635.1 afla|EED53809.1

Group2413: abra|OJJ71277.1 acar|OOF97896.1 acla|EAW10258.1 afis|EAW25335.1 afla|EED53811.1 afum|EAL89998.1 agla|OJJ83872.1 akaw|GAA82712.1 aluc|OJZ87266.1 anid|CBF75240.1 anig|EHA20508.1 aory|EIT78414.1 asyd|OJJ59911.1 ater|EAU34478.1 atub|OJI81376.1 aver|OJJ02375.1 awen|OJJ41029.1 azon|OJJ44636.1

Group2414: abra|OJJ71279.1 acar|OOF97892.1 acla|EAW10261.1 afis|EAW25332.1 afla|EED53814.1 afum|EAL89994.1 agla|OJJ83875.1 akaw|GAA82709.1 aluc|OJZ87264.1 anid|CBF75236.1 anig|EHA20506.1 aory|EIT78424.1 asyd|OJJ59913.1 ater|EAU34480.1 atub|OJI81378.1 aver|OJJ02373.1 awen|OJJ41026.1 azon|OJJ44638.1

Group2415: abra|OJJ71280.1 acar|OOF97893.1 acla|EAW10262.1 afis|EAW25331.1 afla|EED53815.1 afum|EAL89993.1 agla|OJJ83877.1 akaw|GAA82708.1 aluc|OJZ87263.1 anid|CBF75234.1 anig|EHA20505.1 aory|EIT78448.1 asyd|OJJ59914.1 ater|EAU34481.1 atub|OJI81379.1 aver|OJJ02372.1 awen|OJJ41025.1 azon|OJJ44639.1

Group2416: abra|OJJ71286.1 acar|OOF97886.1 acla|EAW10251.1 afis|EAW25341.1 afla|EED53822.1 afum|EBA27392.1 agla|OJJ83884.1 akaw|GAA82703.1 aluc|OJZ87257.1 anid|CBF75213.1 anig|EHA20502.1 aory|EIT78427.1 asyd|OJJ59928.1 ater|EAU34487.1 atub|OJI81385.1 aver|OJJ02358.1 awen|OJJ41062.1 azon|OJJ44645.1

Group2417: abra|OJJ71287.1 acar|OOF97884.1 acla|EAW10250.1 afis|EAW25342.1 afla|EED53823.1 afum|EAL90005.2 agla|OJJ83885.1 akaw|GAA82702.1 aluc|OJZ87256.1 anid|CBF75211.1 anig|EHA20501.1 aory|EIT78440.1 asyd|OJJ59927.1 ater|EAU34488.1 atub|OJI81386.1 aver|OJJ02359.1 awen|OJJ41063.1 azon|OJJ44646.1

Group2418: abra|OJJ71288.1 acar|OOF97885.1 acla|EAW10249.1 afis|EAW25343.1 afla|EED53824.1 afum|EAL90006.1 agla|OJJ83886.1 akaw|GAA82701.1 aluc|OJZ87255.1 anid|CBF75209.1 anig|EHA20500.1

aory|EIT78410.1 asyd|OJJ59926.1 ater|EAU34489.1 atub|OJI81387.1 aver|OJJ02360.1 awen|OJJ41065.1 azon|OJJ44647.1

Group2419: abra|OJJ71289.1 acar|OOF97883.1 acla|EAW10248.1 afis|EAW25344.1 afla|EED53825.1 afum|EAL90007.1 agla|OJJ83887.1 akaw|GAA82700.1 aluc|OJZ87254.1 anid|CBF75207.1 anig|EHA20499.1 aory|EIT78419.1 asyd|OJJ59925.1 ater|EAU34490.1 atub|OJI81388.1 aver|OJJ02361.1 awen|OJJ41066.1 azon|OJJ44648.1

Group2420: abra|OJJ71290.1 acar|OOF97882.1 acla|EAW10247.1 afis|EAW25345.1 afla|EED53826.1 afum|EAL90008.2 agla|OJJ83891.1 akaw|GAA82699.1 aluc|OJZ87253.1 anid|CBF75205.1 anig|EHA20498.1 aory|EIT78441.1 asyd|OJJ59924.1 ater|EAU34491.1 atub|OJI81389.1 aver|OJJ02362.1 awen|OJJ41068.1 azon|OJJ44650.1

Group2421: abra|OJJ71291.1 acar|OOF97881.1 acla|EAW10246.1 afis|EAW25346.1 afla|EED53827.1 afum|EAL90009.1 agla|OJJ83892.1 akaw|GAA82698.1 aluc|OJZ87252.1 anid|CBF75203.1 anig|EHA20497.1 aory|EIT78374.1 asyd|OJJ59923.1 ater|EAU34492.1 atub|OJI81390.1 aver|OJJ02363.1 awen|OJJ41069.1 azon|OJJ44651.1

Group2422: abra|OJJ71293.1 acar|OOF97877.1 acla|EAW10245.1 afis|EAW25347.1 afla|EED53828.1 afum|EAL90010.2 agla|OJJ83893.1 akaw|GAA82696.1 aluc|OJZ87250.1 anid|CBF77426.1 anig|EHA20495.1 aory|EIT78392.1 asyd|OJJ53229.1 ater|EAU34495.1 atub|OJI81392.1 aver|OJJ03623.1 awen|OJJ41070.1 azon|OJJ44652.1

Group2423: abra|OJJ71294.1 acar|OOF97876.1 acla|EAW10244.1 afis|EAW25348.1 afla|EED53829.1 afum|EAL90011.1 agla|OJJ83894.1 akaw|GAA82695.1 aluc|OJZ87249.1 anid|CBF77428.1 anig|EHA20494.1 aory|EIT78450.1 asyd|OJJ53228.1 ater|EAU34496.1 atub|OJI81393.1 aver|OJJ03624.1 awen|OJJ41071.1 azon|OJJ44653.1

Group2424: abra|OJJ71295.1 acar|OOF97875.1 acla|EAW10243.1 afis|EAW25349.1 afla|EED53830.1 afum|EAL90012.1 agla|OJJ83895.1 akaw|GAA82694.1 aluc|OJZ87248.1 anid|CBF77430.1 anig|EHA20493.1 asyd|OJJ53227.1 ater|EAU34497.1 atub|OJI81394.1 aver|OJJ03625.1 awen|OJJ41072.1 azon|OJJ44654.1 aory|EIT78356.1

Group2425: abra|OJJ71296.1 acar|OOF97874.1 acla|EAW10242.1 afis|EAW25350.1 afla|EED53831.1 afum|EAL90013.1 agla|OJJ83896.1 akaw|GAA82693.1 aluc|OJZ87247.1 anid|CBF77432.1 anig|EHA20492.1 aory|EIT78425.1 asyd|OJJ53226.1 ater|EAU34498.1 atub|OJI81395.1 aver|OJJ03626.1 awen|OJJ41073.1 azon|OJJ44655.1

Group2426: abra|OJJ71297.1 acar|OOF97873.1 acla|EAW10241.1 afis|EAW25351.1 afla|EED53832.1 afum|EAL90014.1 agla|OJJ83897.1 akaw|GAA82692.1 aluc|OJZ87246.1 anid|CBF77434.1 anig|EHA20491.1 aory|EIT78366.1 asyd|OJJ53225.1 ater|EAU34499.1 atub|OJI81396.1 aver|OJJ03627.1 awen|OJJ41074.1 azon|OJJ44656.1

Group2427: abra|OJJ71300.1 acar|OOF97870.1 acla|EAW10238.1 afis|EAW25354.1 afla|EED53835.1 afum|EAL90016.1 agla|OJJ83902.1 akaw|GAA82689.1 aluc|OJZ87243.1 anid|CBF77471.1 anig|EHA20488.1 aory|EIT78370.1 asyd|OJJ53222.1 ater|EAU34502.1 atub|OJI81399.1 aver|OJJ03630.1 awen|OJJ41077.1 azon|OJJ44659.1

Group2428: abra|OJJ71302.1 acar|OOF97869.1 acla|EAW10237.1 afis|EAW25355.1 afla|EED53836.1 afum|EAL90017.1 agla|OJJ83903.1 akaw|GAA82688.1 aluc|OJZ87241.1 anid|CBF77469.1 anig|EHA20487.1 aory|EIT78369.1 asyd|OJJ53221.1 ater|EAU34503.1 atub|OJI81401.1 aver|OJJ03631.1 awen|OJJ41078.1 azon|OJJ44660.1

Group2429: abra|OJJ71303.1 acar|OOF97868.1 acla|EAW10236.1 afis|EAW25356.1 afla|EED53838.1 afum|EAL90018.1 agla|OJJ83905.1 akaw|GAA82687.1 aluc|OJZ87240.1 anid|CBF77467.1 anig|EHA20486.1 aory|EIT78449.1 asyd|OJJ53220.1 ater|EAU34504.1 atub|OJI81402.1 aver|OJJ03632.1 awen|OJJ41081.1 azon|OJJ44662.1

Group2430: abra|OJJ71304.1 acar|OOF97867.1 acla|EAW10235.1 afis|EAW25357.1 afla|EED53839.1 afum|EAL90019.1 agla|OJJ83906.1 akaw|GAA82686.1 aluc|OJZ87239.1 anid|CBF77465.1 anig|EHA20485.1 aory|EIT78442.1 asyd|OJJ53219.1 ater|EAU34505.1 atub|OJI81403.1 aver|OJJ03633.1 awen|OJJ41082.1 azon|OJJ44663.1

Group2431: abra|OJJ71308.1 acar|OOF97863.1 acla|EAW10232.1 afis|EAW25360.1 afum|EAL90022.1 agla|OJJ83910.1 akaw|GAA82683.1 aluc|OJZ87236.1 anid|CBF77459.1 anig|EHA20483.1 aory|EIT78368.1 asyd|OJJ53216.1 ater|EAU34508.1 atub|OJI81406.1 aver|OJJ03635.1 awen|OJJ41086.1 azon|OJJ44666.1 afla|EED53842.1

Group2432: abra|OJJ71310.1 acar|OOF97862.1 acla|EAW10230.1 afis|EAW25362.1 afla|EED53844.1 afum|EAL90024.1 agla|OJJ83912.1 akaw|GAA82681.1 aluc|OJZ87234.1 anid|CBF77455.1 anig|EHA20481.1 aory|EIT78471.1 asyd|OJJ53214.1 ater|EAU34509.1 atub|OJI81408.1 aver|OJJ03637.1 awen|OJJ41088.1 azon|OJJ44668.1

Group2433: abra|OJJ71311.1 acar|OOF97861.1 acla|EAW10229.1 afis|EAW25363.1 afla|EED53845.1 afum|EAL90025.1 agla|OJJ83913.1 akaw|GAA82680.1 aluc|OJZ87233.1 anid|CBF77453.1 anig|EHA20480.1 aory|EIT78383.1 asyd|OJJ53213.1 ater|EAU34510.1 atub|OJI81409.1 aver|OJJ03638.1 awen|OJJ41089.1 azon|OJJ44669.1

Group2434: abra|OJJ71312.1 acar|OOF97860.1 acla|EAW10228.1 afis|EAW25364.1 afla|EED53846.1 afum|EAL90026.1 agla|OJJ83752.1 akaw|GAA82679.1 aluc|OJZ87232.1 anid|CBF77452.1 anig|EHA20479.1 aory|EIT78459.1 asyd|OJJ53211.1 ater|EAU34511.1 atub|OJI81410.1 aver|OJJ03639.1 awen|OJJ41091.1 azon|OJJ44670.1

Group2435: abra|OJJ71315.1 acar|OOF97857.1 acla|EAW10224.1 afis|EAW25368.1 afla|EED53849.1 afum|EAL90028.2 agla|OJJ83755.1 akaw|GAA82676.1 aluc|OJZ87229.1 anid|CBF77446.1 anig|EHA20476.1 aory|EIT78461.1 asyd|OJJ53208.1 ater|EAU34513.1 atub|OJI81413.1 aver|OJJ03642.1 awen|OJJ41094.1 azon|OJJ44673.1

Group2436: abra|OJJ71316.1 acar|OOF97856.1 acla|EAW10223.1 afis|EAW25369.1 afla|EED53850.1 afum|EAL90029.2 agla|OJJ83756.1 akaw|GAA82675.1 aluc|OJZ87228.1 anid|CBF77444.1 anig|EHA20475.1 aory|EIT78384.1 asyd|OJJ53207.1 ater|EAU34514.1 atub|OJI81414.1 aver|OJJ03643.1 awen|OJJ41095.1 azon|OJJ44674.1

Group2437: abra|OJJ71317.1 acar|OOF97855.1 acla|EAW10222.1 afis|EAW25370.1 afla|EED53851.1 afum|EAL90030.1 agla|OJJ83757.1 akaw|GAA82674.1 aluc|OJZ87227.1 anid|CBF77442.1 anig|EHA20474.1 aory|EIT78393.1 asyd|OJJ53206.1 ater|EAU34515.1 atub|OJI81415.1 aver|OJJ03644.1 awen|OJJ41096.1 azon|OJJ44675.1

Group2438: abra|OJJ71320.1 acar|OOF97854.1 acla|EAW10219.1 afis|EAW25373.1 afla|EED53854.1 afum|EAL90033.1 agla|OJJ83760.1 akaw|GAA82671.1 aluc|OJZ87224.1 anid|CBF77475.1 anig|EHA20471.1 aory|EIT78376.1 asyd|OJJ53203.1 ater|EAU34518.1 atub|OJI81418.1 aver|OJJ03647.1 awen|OJJ41099.1 azon|OJJ44678.1

Group2439: abra|OJJ71321.1 acar|OOF97852.1 acla|EAW10218.1 afis|EAW25375.1 afla|EED53855.1 afum|EAL90034.1 agla|OJJ83761.1 akaw|GAA82670.1 aluc|OJZ87223.1 anid|CBF77477.1 anig|EHA20470.1 aory|EIT78390.1 asyd|OJJ53202.1 ater|EAU34519.1 atub|OJI81419.1 aver|OJJ03648.1 awen|OJJ41100.1 azon|OJJ44679.1

Group2440: abra|OJJ71322.1 acar|OOF97853.1 acla|EAW10217.1 afis|EAW25376.1 afla|EED53856.1 afum|EAL90035.1 agla|OJJ83765.1 akaw|GAA82669.1 aluc|OJZ87222.1 anid|CBF77479.1 anig|EHA20469.1 aory|EIT78453.1 asyd|OJJ53201.1 ater|EAU34520.1 atub|OJI81420.1 aver|OJJ03649.1 awen|OJJ41103.1 azon|OJJ44680.1

Group2441: abra|OJJ71324.1 acar|OOF97850.1 acla|EAW10215.1 afis|EAW25378.1 afla|EED53858.1 afum|EAL90037.1 agla|OJJ83767.1 akaw|GAA82667.1 aluc|OJZ87220.1 anid|CBF77483.1 anig|EHA20468.1 aory|EIT78426.1 asyd|OJJ53199.1 ater|EAU34522.1 atub|OJI81421.1 aver|OJJ03651.1 awen|OJJ41105.1 azon|OJJ44682.1

Group2442: abra|OJJ71325.1 acar|OOF97849.1 acla|EAW10214.1 afis|EAW25379.1 afla|EED53859.1 afum|EAL90038.1 agla|OJJ83772.1 akaw|GAA82666.1 aluc|OJZ87219.1 anid|CBF77485.1 anig|EHA20467.1 aory|EIT78378.1 asyd|OJJ53197.1 ater|EAU34527.1 atub|OJI81422.1 aver|OJJ03653.1 awen|OJJ41106.1 azon|OJJ44683.1

Group2443: abra|OJJ71329.1 acla|EAW10210.1 afis|EAW25384.1 afla|EED54692.1 afum|EAL90043.1 agla|OJJ83776.1 akaw|GAA82663.1 aluc|OJZ87213.1 anid|CBF77490.1 anig|EHA20464.1 asyd|OJJ53194.1 ater|EAU34530.1 atub|OJI81426.1 aver|OJJ03656.1 awen|OJJ41111.1 azon|OJJ44688.1 acar|OOF97845.1 afla|EED53861.1

Group2444: abra|OJJ71333.1 acar|OOF97842.1 acla|EAW10206.1 afis|EAW25389.1 afla|EED53866.1 afum|EAL90048.2 agla|OJJ83792.1 akaw|GAA82659.1 aluc|OJZ87209.1 anid|CBF77500.1 anig|EHA20461.1 aory|EIT78380.1 asyd|OJJ53190.1 ater|EAU34537.1 atub|OJI81430.1 aver|OJJ03660.1 awen|OJJ41116.1 azon|OJJ44692.1

Group2445: abra|OJJ71334.1 acar|OOF97838.1 acla|EAW10205.1 afis|EAW25390.1 afla|EED53867.1 afum|EAL90049.1 agla|OJJ87028.1 akaw|GAA82658.1 aluc|OJZ87208.1 anid|CBF77502.1 anig|EHA20459.1 aory|EIT78439.1 asyd|OJJ53189.1 ater|EAU34538.1 atub|OJI81431.1 aver|OJJ03661.1 awen|OJJ41117.1 azon|OJJ44693.1

Group2446: abra|OJJ71337.1 acar|OOF97835.1 acla|EAW10202.1 afis|EAW25393.1 afla|EED53870.1 afum|EAL90052.1 agla|OJJ87031.1 akaw|GAA82655.1 aluc|OJZ87205.1 anid|CBF77508.1 anig|EHA20458.1 aory|EIT78391.1 asyd|OJJ53186.1 ater|EAU34541.1 atub|OJI81435.1 aver|OJJ03665.1 awen|OJJ41119.1 azon|OJJ43212.1

Group2447: abra|OJJ71339.1 acar|OOF97833.1 acla|EAW10200.1 afis|EAW25395.1 afla|EED53872.1 afum|EAL90054.1 agla|OJJ87033.1 akaw|GAA82653.1 aluc|OJZ87203.1 anid|CBF77512.1 anig|EHA20456.1

aory|EIT78468.1 asyd|OJJ53185.1 ater|EAU34543.1 atub|OJI81437.1 aver|OJJ03666.1 awen|OJJ41121.1 azon|OJJ44696.1

Group2448: abra|OJJ71340.1 acar|OOF97832.1 acla|EAW10199.1 afis|EAW25396.1 afla|EED53873.1 afum|EAL90055.1 agla|OJJ87034.1 akaw|GAA82652.1 aluc|OJZ87202.1 anid|CBF77514.1 anig|EHA20455.1 aory|EIT78362.1 asyd|OJJ53184.1 ater|EAU34544.1 atub|OJI81438.1 aver|OJJ03667.1 awen|OJJ41122.1 azon|OJJ44697.1

Group2449: abra|OJJ71341.1 acar|OOF97831.1 acla|EAW10198.1 afis|EAW25397.1 afla|EED53874.1 afum|EAL90056.1 agla|OJJ87035.1 akaw|GAA82651.1 aluc|OJZ87201.1 anid|CBF77516.1 anig|EHA20454.1 aory|EIT78399.1 asyd|OJJ53183.1 ater|EAU34545.1 atub|OJI81439.1 aver|OJJ03668.1 awen|OJJ41123.1 azon|OJJ44698.1

Group2450: abra|OJJ71342.1 acar|OOF97830.1 acla|EAW10197.1 afis|EAW25398.1 afla|EED53875.1 afum|EAL90057.1 agla|OJJ87036.1 akaw|GAA82650.1 aluc|OJZ87200.1 anid|CBF77518.1 anig|EHA20453.1 aory|EIT78445.1 asyd|OJJ53182.1 ater|EAU34549.1 atub|OJI81440.1 aver|OJJ03669.1 awen|OJJ41124.1 azon|OJJ44699.1

Group2451: abra|OJJ71345.1 acar|OOF97827.1 acla|EAW10194.1 afis|EAW25401.1 afla|EED53879.1 afum|EAL90060.1 agla|OJJ87042.1 akaw|GAA82647.1 aluc|OJZ87196.1 anid|CBF77524.1 anig|EHA20451.1 aory|EIT78402.1 asyd|OJJ53180.1 ater|EAU34552.1 atub|OJI81443.1 aver|OJJ03671.1 awen|OJJ41128.1 azon|OJJ44702.1

Group2452: abra|OJJ71346.1 acar|OOF97824.1 acla|EAW10192.1 afis|EAW25403.1 afla|EED53882.1 afum|EAL90062.1 agla|OJJ87044.1 akaw|GAA82646.1 aluc|OJZ87193.1 anid|CBF77530.1 anig|EHA20450.1 aory|EIT78396.1 asyd|OJJ53177.1 ater|EAU34554.1 atub|OJI81445.1 aver|OJJ03675.1 awen|OJJ41130.1 azon|OJJ44704.1

Group2453: abra|OJJ71347.1 acar|OOF97823.1 acla|EAW10191.1 afis|EAW25404.1 afla|EED53883.1 afum|EAL90063.1 agla|OJJ87045.1 akaw|GAA82645.1 aluc|OJZ87192.1 anid|CBF77532.1 anig|EHA20449.1 aory|EIT78372.1 asyd|OJJ53176.1 ater|EAU34555.1 atub|OJI81446.1 aver|OJJ03676.1 awen|OJJ41131.1 azon|OJJ44705.1

Group2454: abra|OJJ71349.1 acar|OOF97821.1 acla|EAW10189.1 afis|EAW25406.1 afla|EED53885.1 afum|EAL90065.1 agla|OJJ87047.1 akaw|GAA82643.1 aluc|OJZ87190.1 anid|CBF77536.1 anig|EHA20447.1 aory|EIT78483.1 asyd|OJJ53174.1 ater|EAU34557.1 atub|OJI81448.1 aver|OJJ03678.1 awen|OJJ41133.1 azon|OJJ44707.1

Group2455: abra|OJJ71351.1 acar|OOF97818.1 acla|EAW10187.1 afis|EAW25408.1 afla|EED53887.1 afum|EAL90067.1 agla|OJJ87049.1 akaw|GAA82641.1 aluc|OJZ87188.1 anid|CBF77540.1 anig|EHA20445.1 aory|EIT78477.1 asyd|OJJ53172.1 ater|EAU34559.1 atub|OJI81450.1 aver|OJJ03680.1 awen|OJJ41135.1 azon|OJJ44709.1

Group2456: abra|OJJ71352.1 acar|OOF97819.1 acla|EAW10186.1 afis|EAW25409.1 afla|EED53888.1 afum|EAL90068.1 agla|OJJ87050.1 akaw|GAA82640.1 aluc|OJZ87187.1 anid|CBF77588.1 anig|EHA20444.1 aory|EIT78490.1 asyd|OJJ53171.1 ater|EAU34560.1 atub|OJI81451.1 aver|OJJ03681.1 awen|OJJ41136.1 azon|OJJ44715.1

Group2457: abra|OJJ71353.1 acar|OOF97817.1 acla|EAW10183.1 afis|EAW25411.1 afla|EED53890.1 afum|EAL90070.1 agla|OJJ87052.1 akaw|GAA82639.1 aluc|OJZ87186.1 anid|CBF77584.1 anig|EHA20443.1 aory|EIT78494.1 asyd|OJJ53169.1 ater|EAU34562.1 atub|OJI81452.1 aver|OJJ03683.1 awen|OJJ41138.1 azon|OJJ44717.1

Group2458: abra|OJJ71354.1 acar|OOF97816.1 acla|EAW10182.1 afis|EAW25412.1 afla|EED53891.1 afum|EAL90071.1 agla|OJJ87054.1 akaw|GAA82638.1 aluc|OJZ87185.1 anid|CBF77582.1 anig|EHA20442.1 aory|EIT78487.1 asyd|OJJ53168.1 ater|EAU34563.1 atub|OJI81453.1 aver|OJJ03684.1 awen|OJJ41139.1 azon|OJJ44719.1

Group2459: abra|OJJ71355.1 acar|OOF97815.1 acla|EAW10181.1 afis|EAW25413.1 afla|EED53892.1 afum|EAL90072.1 agla|OJJ87055.1 akaw|GAA82637.1 aluc|OJZ87184.1 anid|CBF77580.1 anig|EHA20441.1 aory|EIT78502.1 asyd|OJJ53167.1 ater|EAU34564.1 atub|OJI81455.1 aver|OJJ03685.1 awen|OJJ41140.1 azon|OJJ44720.1

Group2460: abra|OJJ71357.1 acar|OOF97813.1 acla|EAW10179.1 afis|EAW25415.1 afla|EED53894.1 afum|EAL90074.1 agla|OJJ87057.1 akaw|GAA82635.1 aluc|OJZ87182.1 anid|CBF77576.1 anig|EHA20440.1 aory|EIT78485.1 asyd|OJJ53165.1 ater|EAU34566.1 atub|OJI81457.1 aver|OJJ03687.1 awen|OJJ41142.1 azon|OJJ44722.1

Group2461: abra|OJJ71358.1 acar|OOF97812.1 acla|EAW10178.1 afis|EAW25416.1 afla|EED53895.1 afum|EAL90075.1 agla|OJJ87058.1 akaw|GAA82634.1 aluc|OJZ87181.1 anid|CBF77574.1 anig|EHA20439.1 aory|EIT78501.1 asyd|OJJ53164.1 ater|EAU34567.1 atub|OJI81458.1 aver|OJJ03688.1 awen|OJJ41144.1 azon|OJJ44723.1

Group2462: abra|OJJ71359.1 acar|OOF97811.1 acla|EAW10177.1 afis|EAW25417.1 afla|EED53896.1 afum|EAL90076.1 agla|OJJ87059.1 akaw|GAA82633.1 aluc|OJZ87180.1 anid|CBF77573.1 anig|EHA20438.1 aory|EIT78493.1 asyd|OJJ53163.1 ater|EAU34568.1 atub|OJI81459.1 aver|OJJ03689.1 awen|OJJ41145.1 azon|OJJ44724.1

Group2463: abra|OJJ71360.1 acar|OOF97810.1 acla|EAW10176.1 afis|EAW25418.1 afla|EED53897.1 afum|EAL90077.1 agla|OJJ87060.1 akaw|GAA82632.1 aluc|OJZ87179.1 anid|CBF77571.1 anig|EHA20437.1 aory|EIT78486.1 asyd|OJJ53162.1 ater|EAU34569.1 atub|OJI81460.1 aver|OJJ03690.1 awen|OJJ41146.1 azon|OJJ44725.1

Group2464: abra|OJJ71361.1 acar|OOF97809.1 acla|EAW10175.1 afis|EAW25419.1 afla|EED53898.1 afum|EAL90078.1 agla|OJJ87061.1 akaw|GAA82631.1 aluc|OJZ87178.1 anid|CBF77569.1 anig|EHA20436.1 aory|EIT78503.1 asyd|OJJ53161.1 ater|EAU34570.1 atub|OJI81461.1 aver|OJJ03691.1 awen|OJJ41147.1 azon|OJJ44726.1

Group2465: abra|OJJ71363.1 acar|OOF97808.1 acla|EAW10174.1 afis|EAW25420.1 afla|EED53899.1 afum|EAL90079.1 agla|OJJ87064.1 akaw|GAA82630.1 aluc|OJZ87177.1 anid|CBF77567.1 anig|EHA20435.1 aory|EIT78497.1 asyd|OJJ53160.1 ater|EAU34571.1 atub|OJI81462.1 aver|OJJ03692.1 awen|OJJ41148.1 azon|OJJ44727.1

Group2466: abra|OJJ71365.1 acar|OOF97807.1 acla|EAW10173.1 afis|EAW25421.1 afla|EED53900.1 afum|EAL90080.2 agla|OJJ87063.1 akaw|GAA82629.1 aluc|OJZ87175.1 anid|CBF77563.1 anig|EHA20433.1 aory|EIT78488.1 asyd|OJJ53158.1 ater|EAU34572.1 atub|OJI81463.1 aver|OJJ03693.1 awen|OJJ41149.1 azon|OJJ44728.1

Group2467: abra|OJJ71366.1 acar|OOF97806.1 acla|EAW10172.1 afis|EAW25422.1 afla|EED53901.1 afum|EAL90081.1 agla|OJJ87062.1 akaw|GAA82628.1 aluc|OJZ87174.1 anid|CBF77561.1 anig|EHA20432.1 aory|EIT78499.1 asyd|OJJ53157.1 ater|EAU34573.1 atub|OJI81464.1 aver|OJJ03694.1 awen|OJJ41150.1 azon|OJJ44729.1

Group2468: abra|OJJ71367.1 acar|OOF97805.1 acla|EAW10171.1 afis|EAW25423.1 afla|EED53902.1 afum|EAL90082.1 agla|OJJ87065.1 akaw|GAA82627.1 aluc|OJZ87173.1 anid|CBF77559.1 anig|EHA20431.1 aory|EIT78495.1 asyd|OJJ53156.1 ater|EAU34577.1 atub|OJI81465.1 aver|OJJ03695.1 awen|OJJ41151.1 azon|OJJ44730.1

Group2469: abra|OJJ71368.1 acar|OOF97804.1 acla|EAW10170.1 afis|EAW25424.1 afla|EED53903.1 afum|EAL90083.1 agla|OJJ87066.1 akaw|GAA82626.1 aluc|OJZ87172.1 anid|CBF77557.1 anig|EHA20430.1 aory|EIT78480.1 asyd|OJJ53155.1 ater|EAU34578.1 atub|OJI81466.1 aver|OJJ03696.1 awen|OJJ41152.1 azon|OJJ44731.1

Group2470: abra|OJJ71370.1 acar|OOF97798.1 acla|EAW10168.1 afis|EAW25426.1 afla|EED53914.1 afum|EAL90085.1 agla|OJJ87070.1 akaw|GAA82624.1 aluc|OJZ87170.1 anid|CBF77553.1 anig|EHA20428.1 aory|EIT78484.1 asyd|OJJ53153.1 ater|EAU34591.1 atub|OJI81469.1 aver|OJJ03698.1 awen|OJJ41155.1 azon|OJJ44733.1

Group2471: abra|OJJ71371.1 acar|OOF97797.1 acla|EAW10166.1 afis|EAW25428.1 afla|EED53916.1 afum|EAL90087.1 agla|OJJ87073.1 akaw|GAA82623.1 aluc|OJZ87169.1 anid|CBF77549.1 anig|EHA20427.1 aory|EIT78314.1 asyd|OJJ53151.1 ater|EAU34598.1 atub|OJI81470.1 aver|OJJ03700.1 awen|OJJ41157.1 azon|OJJ44735.1

Group2472: abra|OJJ71372.1 acar|OOF97796.1 acla|EAW10165.1 afis|EAW25429.1 afla|EED53917.1 afum|EAL90088.1 agla|OJJ87074.1 akaw|GAA82622.1 aluc|OJZ87168.1 anid|CBF77547.1 anig|EHA20426.1 aory|EIT78324.1 asyd|OJJ53150.1 ater|EAU34600.1 atub|OJI81471.1 aver|OJJ03701.1 awen|OJJ41158.1 azon|OJJ44736.1

Group2473: abra|OJJ71374.1 acar|OOF97794.1 acla|EAW10163.1 afis|EAW25431.1 afla|EED53919.1 afum|EAL90089.1 agla|OJJ87076.1 akaw|GAA82620.1 aluc|OJZ87166.1 anid|CBF77543.1 anig|EHA20425.1 aory|EIT78269.1 asyd|OJJ53148.1 ater|EAU34603.1 atub|OJI81473.1 aver|OJJ03703.1 awen|OJJ41160.1 azon|OJJ44738.1

Group2474: abra|OJJ71376.1 acar|OOF97792.1 acla|EAW10148.1 afis|EAW25445.1 afla|EED53931.1 afum|EAL90103.1 agla|OJJ87089.1 akaw|GAA82618.1 aluc|OJZ87164.1 anid|CBF77610.1 anig|EHA20423.1 aory|EIT78328.1 asyd|OJJ53135.1 ater|EAU34615.1 atub|OJI81475.1 aver|OJJ03717.1 awen|OJJ41174.1 azon|OJJ44750.1

Group2475: abra|OJJ71378.1 acar|OOF97791.1 acla|EAW10146.1 afis|EAW25447.1 afla|EED53929.1 afum|EAL90105.1 agla|OJJ87086.1 akaw|GAA82616.1 aluc|OJZ87162.1 anid|CBF77606.1 anig|EHA20421.1 aory|EIT78352.1 asyd|OJJ53138.1 ater|EAU34613.1 atub|OJI81477.1 aver|OJJ03714.1 awen|OJJ41169.1 azon|OJJ44747.1

Group2476: abra|OJJ71379.1 acar|OOF97790.1 acla|EAW10154.1 afis|EAW25440.1 afla|EED53928.1 afum|EAL90098.1 agla|OJJ87085.1 akaw|GAA82615.1 aluc|OJZ87161.1 anid|CBF77602.1 anig|EHA20420.1

aory|EIT78271.1 asyd|OJJ53139.1 ater|EAU34612.1 atub|OJI81478.1 aver|OJJ03713.1 awen|OJJ41168.1 azon|OJJ44746.1

Group2477: abra|OJJ71380.1 acar|OOF97789.1 acla|EAW10155.1 afis|EAW25439.1 afla|EED53927.1 afum|EAL90097.1 agla|OJJ87084.1 akaw|GAA82614.1 aluc|OJZ87160.1 anid|CBF77600.1 anig|EHA20419.1 aory|EIT78298.1 asyd|OJJ53140.1 ater|EAU34611.1 atub|OJI81480.1 aver|OJJ03712.1 awen|OJJ41167.1 azon|OJJ44745.1

Group2478: abra|OJJ71384.1 acar|OOF97785.1 acla|EAW10159.1 afis|EAW25435.1 afla|EED53923.1 afum|EAL90093.1 agla|OJJ87080.1 akaw|GAA82610.1 aluc|OJZ87156.1 anid|CBF77592.1 anig|EHA20415.1 aory|EIT78272.1 asyd|OJJ53144.1 ater|EAU34607.1 atub|OJI81484.1 aver|OJJ03708.1 awen|OJJ41164.1 azon|OJJ44742.1

Group2479: abra|OJJ71386.1 acar|OOF97783.1 acla|EAW10161.1 afis|EAW25433.1 afla|EED53921.1 afum|EAL90091.1 agla|OJJ87078.1 akaw|GAA82608.1 aluc|OJZ87154.1 anid|CBF77590.1 anig|EHA20413.1 aory|EIT78355.1 asyd|OJJ53146.1 ater|EAU34605.1 atub|OJI81486.1 aver|OJJ03706.1 awen|OJJ41162.1 azon|OJJ44740.1

Group2480: abra|OJJ71389.1 acar|OOF97779.1 acla|EAW10150.1 afis|EAW25443.1 afla|EED53933.1 afum|EAL90101.1 agla|OJJ87092.1 akaw|GAA82605.1 aluc|OJZ87151.1 anid|CBF77614.1 anig|EHA20410.1 aory|EIT78302.1 asyd|OJJ53133.1 ater|EAU34616.1 atub|OJI81489.1 aver|OJJ03719.1 awen|OJJ41176.1 azon|OJJ44752.1

Group2481: abra|OJJ71390.1 acar|OOF97778.1 acla|EAW10151.1 afis|EAW25442.1 afla|EED53934.1 afum|EAL90100.1 agla|OJJ87094.1 akaw|GAA82604.1 aluc|OJZ87150.1 anid|CBF77616.1 anig|EHA20409.1 aory|EIT78339.1 asyd|OJJ53132.1 ater|EAU34617.1 atub|OJI81490.1 aver|OJJ03720.1 awen|OJJ41177.1 azon|OJJ44753.1

Group2482: abra|OJJ71393.1 acar|OOF97775.1 acla|EAW10145.1 afis|EAW25448.1 afla|EED53935.1 afum|EAL90106.1 agla|OJJ87095.1 akaw|GAA82603.1 aluc|OJZ87148.1 anid|CBF77624.1 anig|EHA20407.1 aory|EIT78256.1 asyd|OJJ53131.1 ater|EAU34618.1 atub|OJI81492.1 aver|OJJ03723.1 awen|OJJ41179.1 azon|OJJ44755.1

Group2483: abra|OJJ71395.1 acar|OOF97774.1 acla|EAW10144.1 afis|EAW25449.1 afla|EED53936.1 afum|EAL90108.1 agla|OJJ87101.1 akaw|GAA82602.1 aluc|OJZ87147.1 anid|CBF77626.1 anig|EHA20406.1 aory|EIT78279.1 asyd|OJJ53130.1 ater|EAU34619.1 atub|OJI81494.1 aver|OJJ03724.1 awen|OJJ41180.1 azon|OJJ44756.1

Group2484: abra|OJJ71397.1 acar|OOF97772.1 acla|EAW10142.1 afis|EAW25451.1 afla|EED53938.1 afum|EAL90110.1 agla|OJJ87103.1 akaw|GAA82600.1 aluc|OJZ87145.1 anid|CBF77629.1 anig|EHA20404.1 aory|EIT78274.1 asyd|OJJ53128.1 ater|EAU34621.1 atub|OJI81496.1 aver|OJJ03726.1 awen|OJJ41182.1 azon|OJJ44758.1

Group2485: abra|OJJ71399.1 acar|OOF97771.1 acla|EAW10135.1 afis|EAW25452.1 afla|EED53940.1 afum|EAL90111.1 agla|OJJ87105.1 akaw|GAA82598.1 aluc|OJZ87143.1 anid|CBF77056.1 anig|EHA20403.1 aory|EIT78282.1 asyd|OJJ56993.1 ater|EAU34622.1 atub|OJI81498.1 aver|OJJ03294.1 awen|OJJ41184.1 azon|OJJ44760.1

Group2486: abra|OJJ71400.1 acar|OOF97770.1 acla|EAW10134.1 afis|EAW25453.1 afla|EED53941.1 afum|EAL90112.1 agla|OJJ87106.1 akaw|GAA82597.1 aluc|OJZ87142.1 anid|CBF77633.1 anig|EHA20402.1 aory|EIT78319.1 asyd|OJJ53126.1 ater|EAU34623.1 atub|OJI81500.1 aver|OJJ03728.1 awen|OJJ41185.1 azon|OJJ44761.1

Group2487: abra|OJJ71402.1 acar|OOF97768.1 acla|EAW10132.1 afis|EAW25455.1 afla|EED53944.1 afum|EAL90114.1 agla|OJJ87108.1 akaw|GAA82595.1 aluc|OJZ87139.1 anid|CBF77637.1 anig|EHA20398.1 aory|EIT78284.1 asyd|OJJ53124.1 ater|EAU34625.1 atub|OJI81502.1 aver|OJJ03730.1 awen|OJJ41187.1 azon|OJJ44763.1

Group2488: abra|OJJ71404.1 acar|OOF97767.1 acla|EAW10131.1 afis|EAW25456.1 afla|EED53945.1 afum|EAL90115.2 agla|OJJ87109.1 akaw|GAA82594.1 aluc|OJZ87138.1 anid|CBF77639.1 anig|EHA20397.1 aory|EIT78325.1 asyd|OJJ53123.1 ater|EAU34626.1 atub|OJI81503.1 aver|OJJ03731.1 awen|OJJ41188.1 azon|OJJ44764.1

Group2489: abra|OJJ71405.1 acar|OOF97766.1 acla|EAW10130.1 afis|EAW25457.1 afla|EED53946.1 afum|EAL90117.1 agla|OJJ87110.1 akaw|GAA82593.1 aluc|OJZ87137.1 anid|CBF77641.1 anig|EHA20396.1 aory|EIT78306.1 asyd|OJJ53122.1 ater|EAU34627.1 atub|OJI81504.1 aver|OJJ03732.1 awen|OJJ41189.1 azon|OJJ44767.1

Group2490: abra|OJJ71407.1 acar|OOF97764.1 acla|EAW10128.1 afis|EAW25459.1 afla|EED53948.1 afum|EAL90119.1 agla|OJJ87112.1 akaw|GAA82592.1 aluc|OJZ87135.1 anid|CBF77645.1 anig|EHA20394.1 aory|EIT78349.1 asyd|OJJ53120.1 ater|EAU34629.1 atub|OJI81507.1 aver|OJJ03734.1 awen|OJJ41191.1 azon|OJJ44768.1

Group2491: abra|OJJ71409.1 acla|EAW10127.1 afis|EAW25460.1 afla|EED53949.1 afum|EAL90121.1 agla|OJJ87113.1 akaw|GAA82591.1 aluc|OJZ87131.1 anid|CBF77647.1 anig|EHA20393.1 aory|EIT78304.1 asyd|OJJ53119.1 ater|EAU34630.1 atub|OJI81509.1 aver|OJJ03735.1 awen|OJJ41195.1 azon|OJJ44769.1 afum|EAL87795.1

Group2492: abra|OJJ71413.1 acar|OOF90159.1 acla|EAW10125.1 afis|EAW25463.1 afla|EED53955.1 afum|EAL90126.1 agla|OJJ87115.1 akaw|GAA82588.1 aluc|OJZ87128.1 anid|CBF77653.1 anig|EHA20389.1 aory|EIT78291.1 asyd|OJJ53114.1 ater|EAU34632.1 atub|OJI81512.1 aver|OJJ03741.1 awen|OJJ41200.1 azon|OJJ44771.1

Group2493: abra|OJJ71416.1 acar|OOF90160.1 acla|EAW10123.1 afis|EAW25464.1 afla|EED53958.1 afum|EAL90127.1 agla|OJJ87116.1 akaw|GAA82586.1 aluc|OJZ87124.1 anid|CBF77655.1 anig|EHA20387.1 aory|EIT78307.1 asyd|OJJ53115.1 ater|EAU34634.1 atub|OJI81516.1 aver|OJJ03740.1 awen|OJJ41201.1 azon|OJJ44774.1

Group2494: abra|OJJ71418.1 acar|OOF97758.1 acla|EAW10122.1 afis|EAW25465.1 afla|EED53960.1 afum|EAL90128.2 agla|OJJ87117.1 akaw|GAA82585.1 aluc|OJZ87122.1 anid|CBF77651.1 anig|EHA20386.1 aory|EIT78354.1 asyd|OJJ53117.1 ater|EAU34635.1 atub|OJI81519.1 aver|OJJ03737.1 awen|OJJ41205.1 azon|OJJ44775.1

Group2495: abra|OJJ71420.1 acar|OOF97757.1 acla|EAW10120.1 afis|EAW25467.1 afla|EED53963.1 afum|EAL90131.1 agla|OJJ87118.1 akaw|GAA82582.1 aluc|OJZ87118.1 anid|CBF77666.1 anig|EHA20384.1 aory|EIT78330.1 asyd|OJJ53102.1 ater|EAU34636.1 atub|OJI81522.1 aver|OJJ03751.1 awen|OJJ41206.1 azon|OJJ44777.1

Group2496: abra|OJJ71421.1 acar|OOF97756.1 acla|EAW10111.1 afis|EAW25476.1 afla|EED53964.1 afum|EAL90139.1 agla|OJJ87120.1 akaw|GAA82581.1 aluc|OJZ87117.1 anid|CBF77674.1 anig|EHA20383.1 aory|EIT78326.1 asyd|OJJ53091.1 ater|EAU34640.1 atub|OJI81523.1 aver|OJJ03755.1 awen|OJJ41207.1 azon|OJJ44779.1

Group2497: abra|OJJ71423.1 acar|OOF97755.1 acla|EAW10116.1 afis|EAW25471.1 afla|EED53968.1 afum|EAL90135.1 agla|OJJ87122.1 akaw|GAA82579.1 aluc|OJZ87115.1 anid|CBF77682.1 anig|EHA20381.1 aory|EIT78300.1 asyd|OJJ53088.1 ater|EAU34644.1 atub|OJI81525.1 aver|OJJ03758.1 awen|OJJ41212.1 azon|OJJ44793.1

Group2498: abra|OJJ71424.1 acar|OOF97754.1 acla|EAW10119.1 afis|EAW25470.1 afla|EED53969.1 afum|EAL90134.1 agla|OJJ87123.1 akaw|GAA82578.1 aluc|OJZ87114.1 anid|CBF77684.1 anig|EHA20380.1 aory|EIT78285.1 asyd|OJJ53087.1 ater|EAU34646.1 atub|OJI81526.1 aver|OJJ03759.1 awen|OJJ41213.1 azon|OJJ44792.1

Group2499: abra|OJJ71425.1 acar|OOF97753.1 acla|EAW10118.1 afis|EAW25469.1 afla|EED53970.1 afum|EAL90133.1 agla|OJJ87124.1 akaw|GAA82577.1 aluc|OJZ87113.1 anid|CBF77686.1 anig|EHA20379.1 aory|EIT78275.1 asyd|OJJ53086.1 ater|EAU34647.1 atub|OJI81527.1 aver|OJJ03760.1 awen|OJJ41214.1 azon|OJJ44791.1

Group2500: abra|OJJ71426.1 acar|OOF97752.1 acla|EAW10117.1 afis|EAW25468.1 afla|EED53971.1 afum|EAL90132.1 agla|OJJ87125.1 akaw|GAA82576.1 aluc|OJZ87112.1 anid|CBF77688.1 anig|EHA20378.1 aory|EIT78308.1 asyd|OJJ53085.1 ater|EAU34648.1 atub|OJI81528.1 aver|OJJ03761.1 awen|OJJ41215.1 azon|OJJ44790.1

Group2501: abra|OJJ71428.1 acar|OOF97750.1 acla|EAW10110.1 afis|EAW25477.1 afla|EED53972.1 afum|EAL90140.1 agla|OJJ87126.1 akaw|GAA82575.1 aluc|OJZ87110.1 anid|CBF77709.1 anig|EHA20377.1 aory|EIT78305.1 asyd|OJJ53077.1 ater|EAU34649.1 atub|OJI81529.1 aver|OJJ03771.1 awen|OJJ41216.1 azon|OJJ44789.1

Group2502: abra|OJJ71432.1 acar|OOF90401.1 acla|EAW10106.1 afis|EAW25481.1 afla|EED53979.1 afum|EAL90146.1 agla|OJJ87129.1 akaw|GAA82571.1 aluc|OJZ87106.1 anid|CBF77701.1 anig|EHA20374.1 aory|EIT78311.1 asyd|OJJ53072.1 ater|EAU34652.1 atub|OJI81532.1 aver|OJJ03773.1 awen|OJJ41222.1 azon|OJJ44785.1

Group2503: abra|OJJ71435.1 acar|OOF90399.1 acla|EAW10104.1 afis|EAW25483.1 afla|EED53981.1 afum|EAL90148.1 agla|OJJ87131.1 akaw|GAA82569.1 aluc|OJZ87103.1 anid|CBF77698.1 anig|EHA20372.1 aory|EIT78335.1 asyd|OJJ53070.1 ater|EAU34654.1 atub|OJI81535.1 aver|OJJ03775.1 awen|OJJ41224.1 azon|OJJ44783.1

Group2504: abra|OJJ71507.1 acar|OOF96261.1 acla|EAW15065.1 afis|EAW20037.1 afla|EED45343.1 afum|EAL85158.1 agla|OJJ88926.1 akaw|GAA89406.1 aluc|OJZ86246.1 anid|CBF84775.1 anig|EHA21618.1 aory|EIT73818.1 asyd|OJJ63238.1 ater|EAU38647.1 atub|OJI84296.1 aver|OJI97718.1 awen|OJJ31792.1 azon|OJJ46326.1

Group2505: abra|OJJ71542.1 acar|OOF96289.1 acla|EAW15096.1 afis|EAW20068.1 afla|EED45311.1 afum|EAL85186.1 agla|OJJ88892.1 akaw|GAA89380.1 aluc|OJZ86280.1 anid|CBF84825.1 anig|EHA21591.1

aory|EIT73564.1 asyd|OJJ63207.1 ater|EAU38674.1 atub|OJI84259.1 aver|OJI97748.1 awen|OJJ31827.1 azon|OJJ46297.1

Group2506: abra|OJJ71556.1 acar|OOF90418.1 acla|EAW15109.1 afis|EAW20080.1 afla|EED45295.1 afum|EAL85199.1 agla|OJJ88879.1 akaw|GAA89367.1 aluc|OJZ86293.1 anid|CBF84857.1 anig|EHA21578.1 aory|EIT73533.1 asyd|OJJ63194.1 ater|EAU38688.1 atub|OJI84243.1 aver|OJI97761.1 awen|OJJ31840.1 azon|OJJ46283.1

Group2507: abra|OJJ71560.1 acar|OOF90420.1 acla|EAW15112.1 afis|EAW20083.1 afla|EED45291.1 afum|EAL85202.1 agla|OJJ88875.1 akaw|GAA89364.1 aluc|OJZ86297.1 anid|CBF84871.1 anig|EHA21575.1 aory|EIT73626.1 asyd|OJJ63191.1 ater|EAU38691.1 atub|OJI84240.1 aver|OJI97765.1 awen|OJJ31843.1 azon|OJJ46280.1

Group2508: abra|OJJ71561.1 acar|OOF90421.1 acla|EAW15113.1 afis|EAW20084.1 afla|EED45290.1 afum|EAL85203.1 agla|OJJ88874.1 akaw|GAA89363.1 aluc|OJZ86298.1 anid|CBF84873.1 anig|EHA21574.1 aory|EIT73721.1 asyd|OJJ63190.1 ater|EAU38692.1 atub|OJI84239.1 aver|OJI97766.1 awen|OJJ31844.1 azon|OJJ46279.1

Group2509: abra|OJJ71562.1 acar|OOF96298.1 acla|EAW15114.1 afis|EAW20085.1 afla|EED45289.1 afum|EAL85204.1 agla|OJJ88873.1 akaw|GAA89362.1 aluc|OJZ86299.1 anid|CBF84875.1 anig|EHA21573.1 aory|EIT73546.1 asyd|OJJ63189.1 ater|EAU38693.1 atub|OJI84237.1 aver|OJI97767.1 awen|OJJ31845.1 azon|OJJ46278.1

Group2510: abra|OJJ71563.1 acar|OOF96299.1 acla|EAW15115.1 afis|EAW20086.1 afla|EED45288.1 afum|EAL85205.1 agla|OJJ88872.1 akaw|GAA89361.1 aluc|OJZ86300.1 anid|CBF84877.1 anig|EHA21572.1 aory|EIT73633.1 asyd|OJJ63188.1 ater|EAU38694.1 atub|OJI84236.1 aver|OJI97768.1 awen|OJJ31846.1 azon|OJJ46277.1

Group2511: abra|OJJ71568.1 acar|OOF96302.1 acla|EAW15118.1 afis|EAW20088.1 afla|EED45282.1 afum|EAL85207.1 agla|OJJ88868.1 akaw|GAA89356.1 aluc|OJZ86305.1 anid|CBF84880.1 anig|EHA21567.1 aory|EIT73561.1 asyd|OJJ63185.1 ater|EAU38697.1 atub|OJI84231.1 aver|OJI97770.1 awen|OJJ31849.1 azon|OJJ46274.1

Group2512: abra|OJJ71570.1 acar|OOF96303.1 acla|EAW15119.1 afis|EAW20089.1 afla|EED45281.1 afum|EAL85208.1 agla|OJJ88867.1 akaw|GAA89355.1 aluc|OJZ86307.1 anid|CBF84882.1 anig|EHA21566.1 aory|EIT73628.1 asyd|OJJ63184.1 ater|EAU38698.1 atub|OJI84229.1 aver|OJI97771.1 awen|OJJ31851.1 azon|OJJ46273.1

Group2513: abra|OJJ71573.1 acar|OOF96305.1 acla|EAW15122.1 afis|EAW20092.1 afla|EED45278.1 afum|EAL85211.1 agla|OJJ88862.1 akaw|GAA89351.1 aluc|OJZ86310.1 anid|CBF84897.1 anig|EHA21563.1 aory|EIT73632.1 asyd|OJJ63175.1 ater|EAU38701.1 atub|OJI84225.1 aver|OJI97779.1 awen|OJJ31855.1 azon|OJJ46270.1

Group2514: abra|OJJ71577.1 acar|OOF96306.1 acla|EAW15124.1 afis|EAW20094.1 afla|EED45276.1 afum|EAL85212.1 agla|OJJ88860.1 akaw|GAA89347.1 aluc|OJZ86314.1 anid|CBF84893.1 anig|EHA21561.1 aory|EIT73817.1 asyd|OJJ63177.1 ater|EAU38704.1 atub|OJI84221.1 aver|OJI97777.1 awen|OJJ31857.1 azon|OJJ46268.1

Group2515: abra|OJJ71580.1 acar|OOF96310.1 acla|EAW15127.1 afis|EAW20097.1 afla|EED45273.1 afum|EAL85215.1 agla|OJJ88857.1 akaw|GAA89344.1 aluc|OJZ86317.1 anid|CBF84888.1 anig|EHA21558.1 aory|EIT73668.1 asyd|OJJ63180.1 ater|EAU38705.1 atub|OJI84218.1 aver|OJI97774.1 awen|OJJ31860.1 azon|OJJ46265.1

Group2516: abra|OJJ71584.1 acar|OOF96314.1 acla|EAW15129.1 afis|EAW20101.1 afla|EED53372.1 afum|EAL85218.1 agla|OJJ88854.1 akaw|GAA89339.1 aluc|OJZ86324.1 anid|CBF84901.1 anig|EHA21554.1 aory|EIT74990.1 asyd|OJJ63172.1 ater|EAU38721.1 atub|OJI84210.1 aver|OJI97782.1 awen|OJJ31871.1 azon|OJJ46263.1

Group2517: abra|OJJ71585.1 acar|OOF96315.1 acla|EAW15130.1 afis|EAW20102.1 afla|EED53371.1 afum|EAL85219.1 agla|OJJ88853.1 akaw|GAA89338.1 aluc|OJZ86325.1 anid|CBF84903.1 anig|EHA21553.1 aory|EIT75070.1 asyd|OJJ63171.1 ater|EAU38722.1 atub|OJI84209.1 aver|OJI97783.1 awen|OJJ31872.1 azon|OJJ46262.1

Group2518: abra|OJJ71586.1 acar|OOF96316.1 acla|EAW15131.1 afis|EAW20103.1 afla|EED53370.1 afum|EAL85220.1 agla|OJJ88851.1 akaw|GAA89337.1 aluc|OJZ86326.1 anid|CBF84905.1 anig|EHA21552.1 aory|EIT75110.1 asyd|OJJ63170.1 ater|EAU38723.1 atub|OJI84208.1 aver|OJI97784.1 awen|OJJ31875.1 azon|OJJ46260.1

Group2519: abra|OJJ71593.1 acar|OOF96321.1 acla|EAW15138.1 afis|EAW20110.1 afla|EED53362.1 afum|EAL85227.1 agla|OJJ88842.1 akaw|GAA89330.1 aluc|OJZ86335.1 anid|CBF84919.1 anig|EHA21546.1 aory|EIT74851.1 asyd|OJJ63164.1 ater|EAU38728.1 atub|OJI84200.1 aver|OJI97791.1 awen|OJJ31881.1 azon|OJJ46253.1

Group2520: abra|OJJ71594.1 acar|OOF96322.1 acla|EAW15139.1 afis|EAW20111.1 afla|EED53361.1 afum|EAL85228.2 agla|OJJ88841.1 akaw|GAA89329.1 aluc|OJZ86336.1 anid|CBF84921.1 anig|EHA21545.1 aory|EIT75176.1 asyd|OJJ63163.1 ater|EAU38729.1 atub|OJI84199.1 aver|OJI97792.1 awen|OJJ31882.1 azon|OJJ46252.1

Group2521: abra|OJJ71595.1 acar|OOF96323.1 acla|EAW15140.1 afis|EAW20112.1 afla|EED53360.1 afum|EAL85229.1 agla|OJJ88840.1 akaw|GAA89328.1 aluc|OJZ86337.1 anid|CBF84923.1 anig|EHA21544.1 aory|EIT75201.1 asyd|OJJ63162.1 ater|EAU38730.1 atub|OJI84198.1 aver|OJI97793.1 awen|OJJ31883.1 azon|OJJ46251.1

Group2522: abra|OJJ71596.1 acar|OOF96324.1 acla|EAW15141.1 afis|EAW20113.1 afla|EED53357.1 afum|EAL85230.1 agla|OJJ82514.1 akaw|GAA89327.1 aluc|OJZ86338.1 anid|CBF84924.1 anig|EHA21543.1 aory|EIT75098.1 asyd|OJJ63161.1 ater|EAU38732.1 atub|OJI84197.1 aver|OJI97794.1 azon|OJJ46250.1 awen|OJJ31884.1

Group2523: abra|OJJ71615.1 acar|OOF96331.1 acla|EAW15157.1 afis|EAW20125.1 afla|EED57251.1 afum|EAL85243.1 agla|OJJ88822.1 akaw|GAA89312.1 aluc|OJZ86356.1 anid|CBF84949.1 anig|EHA21534.1 aory|EIT74081.1 asyd|OJJ63147.1 ater|EAU38741.1 atub|OJI84178.1 aver|OJI97807.1 awen|OJJ31894.1 azon|OJJ46242.1

Group2524: abra|OJJ71621.1 acar|OOF96338.1 acla|EAW15161.1 afis|EAW20130.1 afla|EED57222.1 afum|EAL85246.1 agla|OJJ88805.1 akaw|GAA89305.1 aluc|OJZ86364.1 anid|CBF84956.1 anig|EHA21528.1 aory|EIT74137.1 asyd|OJJ63143.1 ater|EAU38747.1 atub|OJI84170.1 aver|OJI97811.1 awen|OJJ31996.1 azon|OJJ46051.1

Group2525: abra|OJJ71623.1 acar|OOF96340.1 acla|EAW15164.1 afis|EAW20133.1 afla|EED57219.1 afum|EAL85249.1 agla|OJJ88802.1 akaw|GAA89302.1 aluc|OJZ86366.1 anid|CBF84962.1 anig|EHA21526.1 aory|EIT74020.1 asyd|OJJ63140.1 ater|EAU38749.1 atub|OJI84168.1 aver|OJI97814.1 awen|OJJ32001.1 azon|OJJ46054.1

Group2526: abra|OJJ71624.1 acar|OOF96341.1 acla|EAW15165.1 afis|EAW20134.1 afla|EED57218.1 afum|EAL85250.1 agla|OJJ88801.1 akaw|GAA89301.1 aluc|OJZ86367.1 anid|CBF84964.1 anig|EHA21525.1 aory|EIT74013.1 asyd|OJJ63139.1 ater|EAU38750.1 atub|OJI84167.1 aver|OJI97815.1 awen|OJJ32002.1 azon|OJJ46055.1

Group2527: abra|OJJ71626.1 acar|OOF96343.1 acla|EAW15166.1 afis|EAW20135.1 afla|EED57217.1 afum|EBA27225.1 agla|OJJ88799.1 akaw|GAA89300.1 aluc|OJZ86368.1 anid|CBF84965.1 anig|EHA21524.1 aory|EIT73997.1 asyd|OJJ63138.1 ater|EAU38758.1 atub|OJI84166.1 aver|OJI97816.1 awen|OJJ32003.1 azon|OJJ46056.1

Group2528: abra|OJJ71627.1 acar|OOF96345.1 acla|EAW15168.1 afis|EAW20137.1 afla|EED57212.1 afum|EAL85252.1 agla|OJJ88798.1 akaw|GAA89298.1 aluc|OJZ86369.1 anid|CBF84968.1 anig|EHA21523.1 aory|EIT74094.1 asyd|OJJ63136.1 ater|EAU38756.1 atub|OJI84165.1 aver|OJI97818.1 awen|OJJ32006.1 azon|OJJ46058.1

Group2529: abra|OJJ71628.1 acar|OOF96346.1 acla|EAW15169.1 afis|EAW20138.1 afla|EED57210.1 afum|EAL85253.1 agla|OJJ88797.1 akaw|GAA89297.1 aluc|OJZ86370.1 anid|CBF84970.1 anig|EHA21522.1 aory|EIT74150.1 asyd|OJJ63135.1 ater|EAU38755.1 atub|OJI84164.1 aver|OJI97819.1 awen|OJJ32007.1 azon|OJJ46063.1

Group2530: abra|OJJ71630.1 acar|OOF96347.1 acla|EAW15172.1 afis|EAW20141.1 afla|EED57209.1 afum|EAL85256.1 agla|OJJ88791.1 akaw|GAA89295.1 aluc|OJZ86372.1 anid|CBF84973.1 anig|EHA21520.1 aory|EIT74038.1 asyd|OJJ63133.1 ater|EAU38753.1 atub|OJI84162.1 aver|OJI97821.1 awen|OJJ32010.1 azon|OJJ46079.1

Group2531: abra|OJJ71633.1 acar|OOF96350.1 acla|EAW15174.1 afis|EAW20143.1 afla|EED57205.1 afum|EAL85258.1 agla|OJJ88789.1 akaw|GAA89293.1 aluc|OJZ86376.1 anid|CBF84978.1 anig|EHA21518.1 aory|EIT74107.1 asyd|OJJ63130.1 atub|OJI84158.1 aver|OJI97824.1 awen|OJJ32013.1 azon|OJJ46076.1 ater|EAU38760.1

Group2532: abra|OJJ71637.1 acar|OOF96354.1 acla|EAW15177.1 afis|EAW20147.1 afla|EED57202.1 afum|EAL85262.1 agla|OJJ88786.1 akaw|GAA89289.1 aluc|OJZ86380.1 anid|CBF84980.1 anig|EHA21513.1 aory|EIT73995.1 asyd|OJJ63127.1 ater|EAU38763.1 atub|OJI84153.1 aver|OJI97828.1 awen|OJJ32021.1 azon|OJJ46069.1

Group2533: abra|OJJ71643.1 acar|OOF96359.1 acla|EAW15183.1 afis|EAW20153.1 afla|EED57197.1 afum|EAL85268.1 agla|OJJ88781.1 akaw|GAA89283.1 aluc|OJZ86386.1 anid|CBF84993.1 anig|EHA21509.1 aory|EIT74014.1 asyd|OJJ63121.1 ater|EAU38767.1 atub|OJI84147.1 aver|OJI97837.1 awen|OJJ32026.1 azon|OJJ46080.1

Group2534: abra|OJJ71649.1 acar|OOF96363.1 acla|EAW15186.1 afis|EAW20156.1 afla|EED57194.1 afum|EAL85271.1 agla|OJJ88774.1 akaw|GAA89280.1 aluc|OJZ86393.1 anid|CBF84998.1 anig|EHA21505.1

aory|EIT74147.1 asyd|OJJ63118.1 ater|EAU38770.1 atub|OJI84140.1 aver|OJI97841.1 awen|OJJ32031.1 azon|OJJ46083.1

Group2535: abra|OJJ71655.1 acar|OOF96369.1 acla|EAW15192.1 afis|EAW20161.1 afla|EED57187.1 afum|EAL85279.1 agla|OJJ88769.1 akaw|GAA89274.1 aluc|OJZ86401.1 anid|CBF85009.1 anig|EHA21499.1 aory|EIT74084.1 asyd|OJJ63112.1 ater|EAU38778.1 atub|OJI84134.1 aver|OJI97847.1 awen|OJJ32056.1 azon|OJJ46090.1

Group2536: abra|OJJ71656.1 acar|OOF96370.1 acla|EAW15194.1 afis|EAW20163.1 afla|EED57186.1 afum|EAL85281.1 agla|OJJ88767.1 akaw|GAA89273.1 aluc|OJZ86402.1 anid|CBF85011.1 anig|EHA21498.1 aory|EIT74088.1 asyd|OJJ63111.1 ater|EAU38794.1 atub|OJI84133.1 aver|OJI97849.1 awen|OJJ32058.1 azon|OJJ46092.1

Group2537: abra|OJJ71663.1 acar|OOF96373.1 acla|EAW15199.1 afis|EAW20165.1 afla|EED57183.1 afum|EAL85283.1 agla|OJJ88764.1 akaw|GAA89270.1 aluc|OJZ86405.1 anid|CBF85022.1 anig|EHA21494.1 aory|EIT74158.1 asyd|OJJ63107.1 ater|EAU38797.1 atub|OJI84126.1 aver|OJI97854.1 awen|OJJ32062.1 azon|OJJ46095.1

Group2538: abra|OJJ71665.1 acar|OOF96375.1 acla|EAW15202.1 afis|EAW20167.1 afla|EED57181.1 afum|EAL85285.1 agla|OJJ88762.1 akaw|GAA89268.1 aluc|OJZ86407.1 anid|CBF85019.1 anig|EHA21492.1 aory|EIT74007.1 asyd|OJJ63105.1 ater|EAU38799.1 atub|OJI84124.1 aver|OJI97856.1 awen|OJJ32064.1 azon|OJJ46097.1

Group2539: abra|OJJ71668.1 acar|OOF96380.1 acla|EAW15208.1 afis|EAW20173.1 afla|EED57175.1 afum|EAL85291.1 agla|OJJ88757.1 akaw|GAA89263.1 aluc|OJZ86411.1 anid|CBF85031.1 anig|EHA21488.1 aory|EIT74021.1 asyd|OJJ63101.1 ater|EAU35209.1 atub|OJI84120.1 aver|OJI97861.1 awen|OJJ32068.1 azon|OJJ46101.1

Group2540: abra|OJJ71670.1 acar|OOF96382.1 acla|EAW15210.1 afis|EAW20175.1 afla|EED57173.1 afum|EAL85293.1 agla|OJJ84691.1 akaw|GAA89261.1 aluc|OJZ86413.1 anid|CBF85035.1 anig|EHA21486.1 aory|EIT73987.1 asyd|OJJ63099.1 ater|EAU35211.1 atub|OJI84118.1 aver|OJI97863.1 awen|OJJ31174.1 azon|OJJ46104.1

Group2541: abra|OJJ71671.1 acar|OOF96383.1 acla|EAW15211.1 afis|EAW20176.1 afla|EED57172.1 afum|EAL85294.1 agla|OJJ84690.1 akaw|GAA89260.1 aluc|OJZ86414.1 anid|CBF85037.1 anig|EHA21485.1 aory|EIT73964.1 asyd|OJJ63098.1 ater|EAU35212.1 atub|OJI84117.1 aver|OJI97864.1 awen|OJJ31175.1 azon|OJJ46105.1

Group2542: abra|OJJ71672.1 acar|OOF96384.1 acla|EAW15212.1 afis|EAW20177.1 afla|EED57171.1 afum|EAL85295.1 agla|OJJ84689.1 akaw|GAA89259.1 aluc|OJZ86415.1 anid|CBF85039.1 anig|EHA21484.1 aory|EIT74066.1 asyd|OJJ63097.1 ater|EAU35213.1 atub|OJI84116.1 aver|OJI97865.1 awen|OJJ31176.1 azon|OJJ46106.1

Group2543: abra|OJJ71675.1 acar|OOF96386.1 acla|EAW15214.1 afis|EAW20179.1 afla|EED57169.1 afum|EAL85297.1 agla|OJJ84687.1 akaw|GAA89257.1 aluc|OJZ86417.1 anid|CBF85042.1 anig|EHA21482.1 aory|EIT74151.1 asyd|OJJ63095.1 ater|EAU35215.1 atub|OJI84114.1 aver|OJI97867.1 awen|OJJ31178.1 azon|OJJ46109.1

Group2544: abra|OJJ71676.1 acar|OOF96387.1 acla|EAW15215.1 afis|EAW20180.1 afla|EED57168.1 afum|EAL85298.1 agla|OJJ84686.1 akaw|GAA89256.1 aluc|OJZ86418.1 anid|CBF85044.1 anig|EHA21481.1 aory|EIT73991.1 asyd|OJJ63094.1 ater|EAU35216.1 atub|OJI84113.1 aver|OJI97868.1 awen|OJJ31179.1 azon|OJJ46110.1

Group2545: abra|OJJ71677.1 acar|OOF96388.1 acla|EAW15216.1 afis|EAW20181.1 afla|EED57167.1 afum|EAL85299.1 agla|OJJ84685.1 akaw|GAA89255.1 aluc|OJZ86419.1 anid|CBF85046.1 anig|EHA21480.1 aory|EIT74127.1 asyd|OJJ63093.1 ater|EAU35217.1 atub|OJI84112.1 aver|OJI97869.1 awen|OJJ31180.1 azon|OJJ46111.1

Group2546: abra|OJJ71678.1 acar|OOF96389.1 acla|EAW15217.1 afis|EAW20182.1 afla|EED57166.1 afum|EAL85300.1 agla|OJJ84684.1 akaw|GAA89254.1 aluc|OJZ86420.1 anid|CBF85048.1 anig|EHA21479.1 aory|EIT74003.1 asyd|OJJ63092.1 ater|EAU35218.1 atub|OJI84111.1 aver|OJI97870.1 awen|OJJ31181.1 azon|OJJ46112.1

Group2547: abra|OJJ71679.1 acar|OOF96390.1 acla|EAW15218.1 afis|EAW20183.1 afla|EED57165.1 afum|EAL85301.1 agla|OJJ84683.1 akaw|GAA89253.1 aluc|OJZ86421.1 anid|CBF85050.1 anig|EHA21478.1 aory|EIT74002.1 asyd|OJJ63091.1 ater|EAU35219.1 atub|OJI84110.1 aver|OJI97871.1 awen|OJJ31182.1 azon|OJJ46113.1

Group2548: abra|OJJ71682.1 acar|OOF96393.1 acla|EAW15221.1 afis|EAW20186.1 afla|EED57164.1 afum|EAL85305.1 agla|OJJ84680.1 akaw|GAA89250.1 aluc|OJZ86424.1 anid|CBF85054.1 anig|EHA21474.1 aory|EIT74048.1 asyd|OJJ63088.1 ater|EAU35222.1 atub|OJI84107.1 aver|OJI97874.1 awen|OJJ31186.1 azon|OJJ46116.1

Group2549: abra|OJJ71685.1 acar|OOF96395.1 acla|EAW15223.1 afis|EAW20188.1 afla|EED57158.1 afum|EAL85307.1 agla|OJJ84678.1 akaw|GAA89248.1 aluc|OJZ86427.1 anid|CBF85058.1 anig|EHA21472.1 aory|EIT73974.1 asyd|OJJ63086.1 ater|EAU35223.1 atub|OJI84104.1 aver|OJI97876.1 awen|OJJ31188.1 azon|OJJ46118.1

Group2550: abra|OJJ71686.1 acar|OOF96396.1 acla|EAW15224.1 afis|EAW20189.1 afla|EED57157.1 afum|EAL85308.1 agla|OJJ84677.1 akaw|GAA89247.1 aluc|OJZ86428.1 anid|CBF85060.1 anig|EHA21471.1 aory|EIT74135.1 asyd|OJJ63085.1 ater|EAU35224.1 atub|OJI84103.1 aver|OJI97877.1 awen|OJJ31189.1 azon|OJJ46119.1

Group2551: abra|OJJ71687.1 acar|OOF96397.1 acla|EAW15225.1 afis|EAW20190.1 afla|EED57156.1 afum|EAL85309.1 agla|OJJ84676.1 akaw|GAA89246.1 aluc|OJZ86429.1 anid|CBF85062.1 anig|EHA21470.1 aory|EIT74033.1 asyd|OJJ63084.1 ater|EAU35225.1 atub|OJI84102.1 aver|OJI97879.1 awen|OJJ31190.1 azon|OJJ46124.1

Group2552: abra|OJJ71689.1 acar|OOF96398.1 acla|EAW15226.1 afis|EAW20191.1 afla|EED57155.1 afum|EAL85310.1 agla|OJJ84675.1 akaw|GAA89245.1 aluc|OJZ86431.1 anid|CBF85064.1 anig|EHA21469.1 aory|EIT74046.1 asyd|OJJ63083.1 ater|EAU35226.1 atub|OJI84100.1 aver|OJI97881.1 awen|OJJ31196.1 azon|OJJ46120.1

Group2553: abra|OJJ71691.1 acar|OOF96399.1 acla|EAW15228.1 afis|EAW20192.1 afla|EED57152.1 afum|EAL85312.2 agla|OJJ84673.1 akaw|GAA89244.1 aluc|OJZ86435.1 anid|CBF85065.1 anig|EHA21468.1 aory|EIT74165.1 asyd|OJJ63082.1 ater|EAU32000.1 atub|OJI84095.1 aver|OJI97882.1 awen|OJJ31197.1 azon|OJJ46125.1

Group2554: abra|OJJ71692.1 acar|OOF96400.1 acla|EAW15229.1 afis|EAW20193.1 afla|EED57151.1 afum|EAL85313.1 agla|OJJ84672.1 akaw|GAA89243.1 aluc|OJZ86436.1 anid|CBF85067.1 anig|EHA21467.1 aory|EIT74001.1 asyd|OJJ63081.1 ater|EAU32001.1 atub|OJI84094.1 aver|OJI97883.1 awen|OJJ31198.1 azon|OJJ46126.1

Group2555: abra|OJJ71693.1 acar|OOF96402.1 acla|EAW15230.1 afis|EAW20194.1 afla|EED57150.1 afum|EAL85314.1 agla|OJJ84671.1 akaw|GAA89242.1 aluc|OJZ86437.1 anid|CBF85069.1 anig|EHA21466.1 aory|EIT74019.1 asyd|OJJ63080.1 ater|EAU32002.1 atub|OJI84093.1 aver|OJI97884.1 awen|OJJ31199.1 azon|OJJ46127.1

Group2556: abra|OJJ71697.1 acar|OOF96405.1 acla|EAW15233.1 afis|EAW20197.1 afla|EED57147.1 afum|EAL85317.1 agla|OJJ84668.1 akaw|GAA89238.1 aluc|OJZ86440.1 anid|CBF85078.1 anig|EHA21461.1 aory|EIT74146.1 asyd|OJJ63076.1 ater|EAU32005.1 atub|OJI84088.1 aver|OJI97888.1 awen|OJJ31203.1 azon|OJJ46128.1

Group2557: abra|OJJ71699.1 acar|OOF96407.1 acla|EAW15235.1 afis|EAW20199.1 afla|EED57145.1 afum|EAL85319.1 agla|OJJ84666.1 akaw|GAA89236.1 aluc|OJZ86442.1 anid|CBF85082.1 anig|EHA21459.1 aory|EIT74005.1 asyd|OJJ63073.1 ater|EAU32007.1 atub|OJI84086.1 aver|OJI97890.1 awen|OJJ31205.1 azon|OJJ41971.1

Group2558: abra|OJJ71700.1 acar|OOF96408.1 acla|EAW15236.1 afis|EAW20200.1 afla|EED57144.1 afum|EAL85320.1 agla|OJJ84665.1 akaw|GAA89235.1 aluc|OJZ86443.1 anid|CBF85084.1 anig|EHA21458.1 aory|EIT74097.1 asyd|OJJ63072.1 ater|EAU32008.1 atub|OJI84085.1 aver|OJI97891.1 awen|OJJ31206.1 azon|OJJ46130.1

Group2559: abra|OJJ71701.1 acar|OOF96409.1 acla|EAW15237.1 afis|EAW20201.1 afla|EED57143.1 afum|EAL85321.1 agla|OJJ84663.1 akaw|GAA89234.1 aluc|OJZ86444.1 anid|CBF85085.1 anig|EHA21457.1 aory|EIT73998.1 asyd|OJJ63070.1 ater|EAU32009.1 atub|OJI84084.1 aver|OJI97892.1 awen|OJJ31207.1 azon|OJJ46131.1

Group2560: abra|OJJ71705.1 acar|OOF96415.1 afis|EAW20206.1 afla|EED57137.1 afum|EAL85324.1 agla|OJJ84661.1 akaw|GAA89232.1 aluc|OJZ86447.1 anig|EHA21454.1 aory|EIT74126.1 asyd|OJJ63064.1 ater|EAU32017.1 atub|OJI84081.1 aver|OJI97897.1 awen|OJJ31210.1 acla|EAW15239.1 anid|CBF85093.1 azon|OJJ46134.1

Group2561: abra|OJJ71714.1 acar|OOF96419.1 acla|EAW06442.1 afis|EAW18547.1 afla|EED52833.1 afum|EAL93247.1 agla|OJJ81584.1 akaw|GAA85175.1 aluc|OJZ86459.1 anid|CBF70177.1 anig|EHA21449.1 aory|EIT78161.1 asyd|OJJ58793.1 ater|EAU30022.1 atub|OJI84068.1 aver|OJJ01397.1 awen|OJJ31099.1 azon|OJJ46565.1

Group2562: abra|OJJ71715.1 acar|OOF96420.1 acla|EAW06441.1 afis|EAW18546.1 afla|EED52832.1 afum|EAL93246.1 agla|OJJ81587.1 akaw|GAA85174.1 aluc|OJZ86461.1 anid|CBF70265.1 anig|EHA21448.1 aory|EIT78207.1 asyd|OJJ58750.1 ater|EAU34832.1 atub|OJI84067.1 aver|OJJ01360.1 awen|OJJ31100.1 azon|OJJ46562.1

Group2563: abra|OJJ71716.1 acar|OOF96421.1 acla|EAW06440.1 afis|EAW18545.1 afla|EED52831.1 afum|EAL93245.1 agla|OJJ81589.1 akaw|GAA85173.1 aluc|OJZ86462.1 anid|CBF70267.1 anig|EHA21447.1

aory|EIT78194.1 asyd|OJJ58749.1 ater|EAU34833.1 atub|OJI84066.1 aver|OJJ01359.1 awen|OJJ31101.1 azon|OJJ46561.1

Group2564: abra|OJJ71719.1 acar|OOF96423.1 acla|EAW06439.1 afis|EAW18543.1 afla|EED52829.1 afum|EAL93243.1 agla|OJJ81591.1 akaw|GAA85171.1 aluc|OJZ86465.1 anid|CBF70269.1 anig|EHA21445.1 aory|EIT78205.1 asyd|OJJ58748.1 ater|EAU34834.1 atub|OJI84064.1 aver|OJJ01358.1 awen|OJJ31103.1 azon|OJJ46558.1

Group2565: abra|OJJ71720.1 acar|OOF96425.1 acla|EAW06444.1 afis|EAW18549.1 afla|EED52828.1 afum|EAL93249.1 agla|OJJ81592.1 akaw|GAA85170.1 aluc|OJZ86466.1 anid|CBF70271.1 anig|EHA21444.1 aory|EIT78197.1 asyd|OJJ58747.1 ater|EAU34836.1 atub|OJI84063.1 aver|OJJ01357.1 awen|OJJ31105.1 azon|OJJ46557.1

Group2566: abra|OJJ71742.1 acar|OOF96433.1 acla|EAW06446.1 afis|EAW18551.1 afla|EED52823.1 afum|EAL93251.1 agla|OJJ81605.1 akaw|GAA85156.1 aluc|OJZ86481.1 anid|CBF70277.1 anig|EHA22164.1 aory|EIT78237.1 asyd|OJJ58744.1 ater|EAU34839.1 atub|OJI84049.1 aver|OJJ01354.1 awen|OJJ31111.1 azon|OJJ46587.1

Group2567: abra|OJJ71745.1 acar|OOF96436.1 acla|EAW06449.1 afis|EAW18553.1 afla|EED52822.1 afum|EAL93253.1 agla|OJJ81603.1 akaw|GAA85159.1 aluc|OJZ86478.1 anid|CBF70280.1 anig|EHA22162.1 aory|EIT78230.1 asyd|OJJ58741.1 ater|EAU34841.1 atub|OJI84052.1 aver|OJJ01351.1 awen|OJJ31114.1 azon|OJJ46590.1

Group2568: abra|OJJ71803.1 acar|OOF96483.1 acla|EAW13844.1 afis|EAW18558.1 afla|EED52808.1 afum|EAL93258.1 agla|OJJ81601.1 akaw|GAA85107.1 aluc|OJZ86533.1 anid|CBF70286.1 anig|EHA22113.1 aory|EIT78195.1 asyd|OJJ58737.1 ater|EAU34847.1 atub|OJI83988.1 aver|OJJ01348.1 awen|OJJ31116.1 azon|OJJ46596.1

Group2569: abra|OJJ71806.1 acar|OOF96484.1 acla|EAW13845.1 afis|EAW18559.1 afla|EED52807.1 afum|EAL93259.1 agla|OJJ81600.1 akaw|GAA85106.1 aluc|OJZ86536.1 anid|CBF70288.1 anig|EHA22112.1 aory|EIT78169.1 asyd|OJJ58735.1 ater|EAU34848.1 atub|OJI83987.1 aver|OJJ01347.1 awen|OJJ31117.1 azon|OJJ46595.1

Group2570: abra|OJJ71809.1 acar|OOF96487.1 acla|EAW13848.1 afis|EAW18562.1 afla|EED52792.1 afum|EAL93263.1 agla|OJJ81609.1 akaw|GAA85103.1 aluc|OJZ86539.1 anid|CBF70294.1 anig|EHA22110.1 aory|EIT78177.1 asyd|OJJ58732.1 ater|EAU34850.1 atub|OJI83984.1 aver|OJJ01344.1 awen|OJJ31120.1 azon|OJJ46597.1

Group2571: abra|OJJ71810.1 acar|OOF96488.1 acla|EAW13849.1 afis|EAW18563.1 afla|EED52791.1 afum|EAL93265.2 agla|OJJ81612.1 akaw|GAA85102.1 aluc|OJZ86541.1 anid|CBF70298.1 anig|EHA22109.1 aory|EIT78239.1 asyd|OJJ58730.1 ater|EAU34851.1 atub|OJI83982.1 aver|OJJ01343.1 awen|OJJ31121.1 azon|OJJ46602.1

Group2572: abra|OJJ71811.1 acar|OOF96489.1 acla|EAW13850.1 afis|EAW18564.1 afla|EED52790.1 afum|EAL93266.1 agla|OJJ81613.1 akaw|GAA85101.1 aluc|OJZ86542.1 anid|CBF70300.1 anig|EHA22108.1 aory|EIT78209.1 asyd|OJJ58729.1 ater|EAU34852.1 atub|OJI83981.1 aver|OJJ01342.1 awen|OJJ31122.1 azon|OJJ46603.1

Group2573: abra|OJJ71813.1 acar|OOF96491.1 acla|EAW13853.1 afis|EAW18567.1 afla|EED52786.1 afum|EAL93269.2 agla|OJJ81616.1 akaw|GAA85097.1 aluc|OJZ86546.1 anid|CBF70305.1 anig|EHA22104.1 aory|EIT78200.1 asyd|OJJ58726.1 ater|EAU34854.1 atub|OJI83977.1 aver|OJJ01339.1 awen|OJJ31124.1 azon|OJJ46605.1

Group2574: abra|OJJ71816.1 acar|OOF96493.1 acla|EAW13856.1 afis|EAW18570.1 afla|EED52781.1 afum|EAL93273.1 agla|OJJ81621.1 akaw|GAA85094.1 aluc|OJZ86548.1 anid|CBF70311.1 anig|EHA22101.1 aory|EIT78224.1 asyd|OJJ58723.1 ater|EAU34855.1 atub|OJI83974.1 aver|OJJ01336.1 awen|OJJ31127.1 azon|OJJ46608.1

Group2575: abra|OJJ71817.1 acar|OOF96494.1 acla|EAW13857.1 afis|EAW18571.1 afla|EED52780.1 afum|EAL93274.1 agla|OJJ81622.1 akaw|GAA85093.1 aluc|OJZ86549.1 anid|CBF70313.1 anig|EHA22100.1 aory|EIT78222.1 asyd|OJJ58722.1 ater|EAU34856.1 atub|OJI83973.1 aver|OJJ01335.1 awen|OJJ31128.1 azon|OJJ46609.1

Group2576: abra|OJJ71818.1 acar|OOF96495.1 acla|EAW13860.1 afis|EAW18572.1 afla|EED52776.1 afum|EAL93275.1 agla|OJJ81623.1 akaw|GAA85092.1 aluc|OJZ86550.1 anid|CBF70315.1 anig|EHA22099.1 aory|EIT78170.1 asyd|OJJ58721.1 ater|EAU34857.1 atub|OJI83972.1 aver|OJJ01334.1 awen|OJJ31129.1 azon|OJJ46610.1

Group2577: abra|OJJ71819.1 acar|OOF96496.1 acla|EAW13861.1 afis|EAW18573.1 afla|EED52775.1 afum|EAL93276.1 agla|OJJ81624.1 akaw|GAA85091.1 aluc|OJZ86551.1 anid|CBF70317.1 anig|EHA22098.1 aory|EIT78219.1 asyd|OJJ58720.1 ater|EAU34858.1 atub|OJI83971.1 aver|OJJ01333.1 awen|OJJ31130.1 azon|OJJ46611.1

Group2578: abra|OJJ71820.1 acar|OOF96497.1 acla|EAW13862.1 afis|EAW18574.1 afla|EED52774.1 afum|EAL93277.1 agla|OJJ81625.1 akaw|GAA85090.1 aluc|OJZ86552.1 anid|CBF70319.1 anig|EHA22097.1 aory|EIT78236.1 asyd|OJJ58719.1 ater|EAU34869.1 atub|OJI83970.1 aver|OJJ01332.1 awen|OJJ31131.1 azon|OJJ46612.1

Group2579: abra|OJJ71821.1 acar|OOF96498.1 acla|EAW13863.1 afis|EAW18575.1 afla|EED52773.1 afum|EAL93278.1 agla|OJJ81626.1 akaw|GAA85089.1 aluc|OJZ86553.1 anid|CBF70320.1 anig|EHA22096.1 aory|EIT78159.1 asyd|OJJ58718.1 ater|EAU34868.1 atub|OJI83969.1 aver|OJJ01331.1 awen|OJJ31132.1 azon|OJJ46613.1

Group2580: abra|OJJ71823.1 acar|OOF96499.1 acla|EAW13864.1 afis|EAW18576.1 afla|EED52772.1 afum|EAL93279.1 agla|OJJ81627.1 akaw|GAA85087.1 aluc|OJZ86555.1 anid|CBF70322.1 anig|EHA22094.1 aory|EIT78160.1 asyd|OJJ58717.1 ater|EAU34867.1 atub|OJI83967.1 aver|OJJ01330.1 awen|OJJ31133.1 azon|OJJ46614.1

Group2581: abra|OJJ71824.1 acar|OOF96500.1 acla|EAW13865.1 afis|EAW18577.1 afla|EED52771.1 afum|EAL93280.1 agla|OJJ81628.1 akaw|GAA85086.1 aluc|OJZ86556.1 anid|CBF70324.1 anig|EHA22093.1 aory|EIT78235.1 asyd|OJJ58716.1 ater|EAU34866.1 atub|OJI83966.1 aver|OJJ01329.1 awen|OJJ31134.1 azon|OJJ46615.1

Group2582: abra|OJJ71829.1 acar|OOF96505.1 acla|EAW13866.1 afis|EAW18578.1 afla|EED52770.1 afum|EAL93281.1 agla|OJJ81629.1 akaw|GAA85082.1 aluc|OJZ86561.1 anid|CBF70354.1 anig|EHA22089.1 aory|EIT78184.1 asyd|OJJ58657.1 ater|EAU34861.1 atub|OJI83960.1 aver|OJJ01265.1 awen|OJJ31135.1 azon|OJJ46616.1

Group2583: abra|OJJ71837.1 acar|OOF90146.1 acla|EAW13868.1 afis|EAW18580.1 afla|EED52766.1 afum|EAL93284.1 agla|OJJ81631.1 akaw|GAA85078.1 aluc|OJZ86568.1 anid|CBF70360.1 anig|EHA22086.1 aory|EIT78154.1 asyd|OJJ58653.1 ater|EAU34873.1 atub|OJI83953.1 aver|OJJ01262.1 awen|OJJ31143.1 azon|OJJ46619.1

Group2584: abra|OJJ71840.1 acar|OOF90149.1 acla|EAW13871.1 afis|EAW18582.1 afla|EED52762.1 afum|EAL93286.1 agla|OJJ81634.1 akaw|GAA85077.1 aluc|OJZ86573.1 anid|CBF70362.1 anig|EHA22084.1 aory|EIT78249.1 asyd|OJJ58652.1 ater|EAU34876.1 atub|OJI83950.1 aver|OJJ01258.1 awen|OJJ31146.1 azon|OJJ46621.1

Group2585: abra|OJJ71842.1 acar|OOF96511.1 acla|EAW13873.1 afis|EAW18585.1 afla|EED52761.1 afum|EAL93289.1 agla|OJJ81636.1 akaw|GAA85075.1 aluc|OJZ86575.1 anid|CBF70366.1 anig|EHA22082.1 aory|EIT78178.1 asyd|OJJ58650.1 ater|EAU34878.1 atub|OJI83948.1 aver|OJJ01256.1 awen|OJJ31148.1 azon|OJJ46623.1

Group2586: abra|OJJ71843.1 acar|OOF96512.1 acla|EAW13874.1 afis|EAW18586.1 afla|EED52760.1 afum|EAL93290.1 agla|OJJ81637.1 akaw|GAA85074.1 aluc|OJZ86576.1 anid|CBF70370.1 anig|EHA22081.1 aory|EIT78214.1 asyd|OJJ58649.1 ater|EAU34879.1 atub|OJI83946.1 aver|OJJ01255.1 awen|OJJ31149.1 azon|OJJ46624.1

Group2587: abra|OJJ71844.1 acar|OOF96513.1 acla|EAW13875.1 afis|EAW18587.1 afla|EED52759.1 afum|EAL93294.2 agla|OJJ81638.1 akaw|GAA85073.1 aluc|OJZ86577.1 anid|CBF70372.1 anig|EHA22080.1 aory|EIT78199.1 asyd|OJJ58648.1 ater|EAU34880.1 atub|OJI83945.1 aver|OJJ01254.1 awen|OJJ31150.1 azon|OJJ46625.1

Group2588: abra|OJJ71845.1 acar|OOF96514.1 acla|EAW13876.1 afis|EAW18588.1 afla|EED52758.1 afum|EAL93295.2 agla|OJJ81640.1 akaw|GAA85072.1 aluc|OJZ86578.1 anid|CBF70374.1 anig|EHA22079.1 aory|EIT78218.1 asyd|OJJ58647.1 ater|EAU34881.1 atub|OJI83944.1 aver|OJJ01253.1 awen|OJJ31151.1 azon|OJJ46626.1

Group2589: abra|OJJ71846.1 acar|OOF96515.1 acla|EAW13877.1 afis|EAW18589.1 afla|EED52757.1 afum|EAL93296.2 agla|OJJ81641.1 akaw|GAA85071.1 aluc|OJZ86579.1 anid|CBF70375.1 anig|EHA22078.1 aory|EIT78231.1 asyd|OJJ58646.1 ater|EAU34882.1 atub|OJI83943.1 aver|OJJ01252.1 awen|OJJ31152.1 azon|OJJ46627.1

Group2590: abra|OJJ71851.1 acar|OOF96521.1 acla|EAW13883.1 afis|EAW18595.1 afla|EED52752.1 afum|EAL93303.1 agla|OJJ81647.1 akaw|GAA85066.1 aluc|OJZ86585.1 anid|CBF70391.1 anig|EHA22075.1 aory|EIT78172.1 asyd|OJJ58636.1 ater|EAU34889.1 atub|OJI83938.1 aver|OJJ01242.1 awen|OJJ31158.1 azon|OJJ46633.1

Group2591: abra|OJJ71854.1 acar|OOF96524.1 acla|EAW13886.1 afis|EAW18598.1 afla|EED52749.1 afum|EAL93306.1 agla|OJJ81651.1 akaw|GAA85063.1 aluc|OJZ86588.1 anid|CBF70397.1 anig|EHA22072.1 aory|EIT78201.1 asyd|OJJ58633.1 ater|EAU34892.1 atub|OJI83935.1 aver|OJJ01239.1 awen|OJJ31161.1 azon|OJJ46637.1

Group2592: abra|OJJ71855.1 acar|OOF96525.1 acla|EAW13887.1 afis|EAW18599.1 afla|EED52748.1 afum|EAL93307.1 agla|OJJ81652.1 akaw|GAA85062.1 aluc|OJZ86589.1 anid|CBF70399.1 anig|EHA22071.1

aory|EIT78171.1 asyd|OJJ58632.1 ater|EAU34893.1 atub|OJI83934.1 aver|OJJ01238.1 awen|OJJ31162.1 azon|OJJ46638.1

Group2593: abra|OJJ71857.1 acar|OOF96527.1 acla|EAW13889.1 afis|EAW18601.1 afla|EED52746.1 afum|EAL93309.1 agla|OJJ81653.1 akaw|GAA85060.1 aluc|OJZ86591.1 anid|CBF70403.1 anig|EHA22069.1 aory|EIT78188.1 asyd|OJJ58630.1 ater|EAU34895.1 atub|OJI83932.1 aver|OJJ01236.1 awen|OJJ31166.1 azon|OJJ46640.1

Group2594: abra|OJJ71858.1 acar|OOF96528.1 acla|EAW13890.1 afis|EAW18602.1 afla|EED52745.1 afum|EAL93310.1 agla|OJJ81654.1 akaw|GAA85059.1 aluc|OJZ86592.1 anid|CBF70405.1 anig|EHA22068.1 aory|EIT78217.1 asyd|OJJ58629.1 ater|EAU34896.1 atub|OJI83931.1 aver|OJJ01235.1 awen|OJJ31167.1 azon|OJJ46641.1

Group2595: abra|OJJ71861.1 acar|OOF96533.1 acla|EAW13892.1 afis|EAW18604.1 afla|EED52743.1 afum|EAL93312.1 agla|OJJ81656.1 akaw|GAA85054.1 aluc|OJZ86598.1 anid|CBF70409.1 anig|EHA22064.1 aory|EIT78243.1 asyd|OJJ58627.1 ater|EAU34899.1 atub|OJI83925.1 aver|OJJ01233.1 awen|OJJ31170.1 azon|OJJ46647.1

Group2596: abra|OJJ71864.1 acar|OOF96534.1 acla|EAW13894.1 afis|EAW18606.1 afla|EED52740.1 afum|EBA27488.1 agla|OJJ81660.1 akaw|GAA85052.1 aluc|OJZ86601.1 anig|EHA22062.1 aory|EIT78163.1 ater|EAU34901.1 atub|OJI83922.1 awen|OJJ31172.1 azon|OJJ46649.1 anid|CBF70412.1 asyd|OJJ58625.1 aver|OJJ01231.1

Group2597: abra|OJJ71865.1 acar|OOF96535.1 acla|EAW13895.1 afis|EAW18607.1 afla|EED52739.1 afum|EAL93317.1 agla|OJJ81666.1 akaw|GAA85051.1 aluc|OJZ86602.1 anid|CBF70414.1 anig|EHA22061.1 aory|EIT78220.1 asyd|OJJ58624.1 ater|EAU34902.1 atub|OJI83921.1 aver|OJJ01230.1 awen|OJJ31173.1 azon|OJJ46650.1

Group2598: abra|OJJ71866.1 acar|OOF96536.1 acla|EAW13896.1 afis|EAW18608.1 afla|EED52738.1 afum|EAL93318.1 agla|OJJ81665.1 akaw|GAA85049.1 aluc|OJZ86603.1 anid|CBF70416.1 anig|EHA22060.1 aory|EIT78223.1 asyd|OJJ58623.1 ater|EAU34903.1 atub|OJI83920.1 aver|OJJ01229.1 awen|OJJ32069.1 azon|OJJ46651.1

Group2599: abra|OJJ71869.1 acar|OOF96540.1 acla|EAW13899.1 afis|EAW18611.1 afla|EED52736.1 afum|EAL93321.1 agla|OJJ81234.1 akaw|GAA85046.1 aluc|OJZ86608.1 anid|CBF70420.1 anig|EHA22058.1 aory|EIT78190.1 asyd|OJJ58620.1 ater|EAU34908.1 atub|OJI83918.1 aver|OJJ01227.1 awen|OJJ32072.1 azon|OJJ46652.1

Group2600: abra|OJJ71873.1 acar|OOF96544.1 acla|EAW13903.1 afis|EAW18615.1 afla|EED48366.1 afum|EAL93325.1 agla|OJJ81229.1 akaw|GAA85042.1 aluc|OJZ86612.1 anid|CBF70428.1 anig|EHA22054.1 aory|EIT83110.1 asyd|OJJ58616.1 ater|EAU34912.1 atub|OJI83914.1 aver|OJJ01223.1 awen|OJJ32076.1 azon|OJJ46656.1

Group2601: abra|OJJ71874.1 acar|OOF96545.1 acla|EAW13904.1 afis|EAW18616.1 afla|EED52730.1 afum|EAL93326.1 agla|OJJ81228.1 akaw|GAA85041.1 aluc|OJZ86613.1 anid|CBF70426.1 anig|EHA22053.1 aory|EIT78192.1 asyd|OJJ58617.1 ater|EAU34913.1 atub|OJI83913.1 aver|OJJ01224.1 awen|OJJ32077.1 azon|OJJ46657.1

Group2602: abra|OJJ71876.1 acla|EAW13905.1 afis|EAW18617.1 afla|EED52728.1 afum|EAL93327.1 agla|OJJ81227.1 akaw|GAA85040.1 aluc|OJZ86614.1 anid|CBF70424.1 anig|EHA22052.1 aory|EIT78248.1 asyd|OJJ58618.1 ater|EAU34917.1 atub|OJI83912.1 aver|OJJ01225.1 awen|OJJ32078.1 azon|OJJ46658.1 afla|EED52729.1

Group2603: abra|OJJ71878.1 acar|OOF96546.1 acla|EAW13907.1 afis|EAW18619.1 afla|EED52726.1 afum|EAL93329.1 agla|OJJ81241.1 akaw|GAA85038.1 aluc|OJZ86616.1 anid|CBF70501.1 anig|EHA22050.1 aory|EIT78153.1 asyd|OJJ58579.1 ater|EAU34919.1 atub|OJI83910.1 aver|OJJ01183.1 awen|OJJ32081.1 azon|OJJ46660.1

Group2604: abra|OJJ71879.1 acar|OOF96547.1 acla|EAW13908.1 afis|EAW18620.1 afla|EED52725.1 afum|EAL93330.1 agla|OJJ81240.1 akaw|GAA85037.1 aluc|OJZ86617.1 anid|CBF70499.1 anig|EHA22049.1 aory|EIT78189.1 asyd|OJJ58580.1 ater|EAU34920.1 atub|OJI83909.1 aver|OJJ01184.1 awen|OJJ32082.1 azon|OJJ46661.1

Group2605: abra|OJJ71880.1 acar|OOF96548.1 acla|EAW13909.1 afis|EAW18621.1 afla|EED52724.1 afum|EAL93331.1 agla|OJJ81239.1 akaw|GAA85036.1 aluc|OJZ86618.1 anid|CBF70497.1 anig|EHA22048.1 aory|EIT78181.1 asyd|OJJ58581.1 ater|EAU34921.1 atub|OJI83908.1 aver|OJJ01185.1 awen|OJJ32084.1 azon|OJJ46663.1

Group2606: abra|OJJ71889.1 acar|OOF96551.1 acla|EAW09632.1 afis|EAW21508.1 afla|EED55945.1 afum|EAL92528.1 agla|OJJ85671.1 akaw|GAA85028.1 aluc|OJZ86626.1 anid|CBF76443.1 anig|EHA22039.1 aory|EIT76729.1 asyd|OJJ61102.1 ater|EAU35030.1 atub|OJI83899.1 aver|OJJ00419.1 awen|OJJ39187.1 azon|OJJ51504.1

Group2607: abra|OJJ71891.1 acar|OOF96552.1 acla|EAW09631.1 afis|EAW21509.1 afla|EED55947.1 afum|EAL92529.1 agla|OJJ85673.1 akaw|GAA85027.1 aluc|OJZ86630.1 anid|CBF76441.1 anig|EHA22038.1 aory|EIT76718.1 asyd|OJJ61101.1 ater|EAU35029.1 atub|OJI83898.1 aver|OJJ00416.1 awen|OJJ39185.1 azon|OJJ51503.1

Group2608: abra|OJJ71895.1 acar|OOF96556.1 acla|EAW09627.1 afis|EAW21513.1 afla|EED55951.1 afum|EAL92533.1 agla|OJJ85677.1 akaw|GAA85023.1 aluc|OJZ86634.1 anid|CBF76433.1 anig|EHA22035.1 aory|EIT76705.1 asyd|OJJ61096.1 ater|EAU35027.1 atub|OJI83894.1 aver|OJJ00411.1 awen|OJJ39181.1 azon|OJJ51512.1

Group2609: abra|OJJ71896.1 acar|OOF96557.1 acla|EAW09626.1 afis|EAW21514.1 afla|EED55952.1 afum|EAL92534.1 agla|OJJ85678.1 akaw|GAA85022.1 aluc|OJZ86635.1 anid|CBF76431.1 anig|EHA22034.1 aory|EIT76554.1 asyd|OJJ61095.1 ater|EAU35026.1 atub|OJI83893.1 aver|OJJ00410.1 awen|OJJ39180.1 azon|OJJ51513.1

Group2610: abra|OJJ71898.1 acar|OOF96559.1 acla|EAW09624.1 afis|EAW21516.1 afla|EED55954.1 afum|EAL92536.1 agla|OJJ85680.1 akaw|GAA85020.1 aluc|OJZ86637.1 anid|CBF76427.1 anig|EHA22032.1 aory|EIT76837.1 asyd|OJJ61093.1 ater|EAU35024.1 atub|OJI83891.1 aver|OJJ00408.1 awen|OJJ39178.1 azon|OJJ51516.1

Group2611: abra|OJJ71902.1 acar|OOF96560.1 acla|EAW09621.1 afis|EAW21518.1 afla|EED55956.1 afum|EAL92539.1 agla|OJJ85681.1 akaw|GAA85018.1 aluc|OJZ86639.1 anid|CBF76423.1 anig|EHA22030.1 aory|EIT76726.1 asyd|OJJ61091.1 ater|EAU35022.1 atub|OJI83888.1 aver|OJJ00406.1 awen|OJJ39175.1 azon|OJJ51515.1

Group2612: abra|OJJ71903.1 acar|OOF96561.1 acla|EAW09620.1 afis|EAW21519.1 afla|EED55957.1 afum|EAL92540.1 agla|OJJ85682.1 akaw|GAA85017.1 aluc|OJZ86640.1 anid|CBF76419.1 anig|EHA22029.1 aory|EIT76756.1 asyd|OJJ61090.1 ater|EAU35021.1 atub|OJI83887.1 aver|OJJ00405.1 awen|OJJ39174.1 azon|OJJ51514.1

Group2613: abra|OJJ71905.1 acar|OOF96562.1 acla|EAW09618.1 afis|EAW21521.1 afla|EED55959.1 afum|EAL92542.1 agla|OJJ85684.1 akaw|GAA85015.1 aluc|OJZ86642.1 anid|CBF76416.1 anig|EHA22027.1 aory|EIT76533.1 asyd|OJJ61088.1 ater|EAU35020.1 atub|OJI83885.1 aver|OJJ00403.1 awen|OJJ39172.1 azon|OJJ51501.1

Group2614: abra|OJJ71908.1 acar|OOF96568.1 acla|EAW09615.1 afis|EAW21524.1 afla|EED55962.1 afum|EAL92545.1 agla|OJJ85687.1 akaw|GAA85011.1 aluc|OJZ86646.1 anid|CBF76410.1 anig|EHA22024.1 aory|EIT76688.1 asyd|OJJ61085.1 ater|EAU35017.1 atub|OJI83881.1 aver|OJJ00400.1 awen|OJJ39169.1 azon|OJJ51498.1

Group2615: abra|OJJ71917.1 acar|OOF96574.1 acla|EAW09613.1 afis|EAW21526.1 afla|EED55965.1 afum|EAL92547.1 agla|OJJ85689.1 akaw|GAA85005.1 aluc|OJZ86654.1 anid|CBF76406.1 anig|EHA22017.1 aory|EIT76778.1 asyd|OJJ61082.1 ater|EAU35015.1 atub|OJI83873.1 aver|OJJ00398.1 awen|OJJ39167.1 azon|OJJ51496.1

Group2616: abra|OJJ71918.1 acar|OOF96575.1 acla|EAW09612.1 afis|EAW21527.1 afla|EED55966.1 afum|EAL92548.1 agla|OJJ85690.1 akaw|GAA85004.1 aluc|OJZ86655.1 anid|CBF76404.1 anig|EHA22016.1 aory|EIT76686.1 asyd|OJJ61081.1 ater|EAU35014.1 atub|OJI83872.1 aver|OJJ00397.1 awen|OJJ39166.1 azon|OJJ51495.1

Group2617: abra|OJJ71921.1 acar|OOF96578.1 acla|EAW09607.1 afis|EAW21530.1 afla|EED55979.1 afum|EAL92551.1 agla|OJJ85694.1 akaw|GAA85001.1 aluc|OJZ86658.1 anid|CBF76398.1 anig|EHA22013.1 aory|EIT76728.1 asyd|OJJ61078.1 ater|EAU35009.1 atub|OJI83869.1 aver|OJJ00394.1 awen|OJJ39162.1 azon|OJJ51492.1

Group2618: abra|OJJ71922.1 acar|OOF96579.1 acla|EAW09606.1 afis|EAW21531.1 afla|EED55980.1 afum|EAL92552.1 agla|OJJ85695.1 akaw|GAA85000.1 aluc|OJZ86659.1 anid|CBF76396.1 anig|EHA22012.1 aory|EIT76765.1 asyd|OJJ61077.1 ater|EAU35008.1 atub|OJI83868.1 aver|OJJ00393.1 awen|OJJ39161.1 azon|OJJ51491.1

Group2619: abra|OJJ71923.1 acar|OOF96580.1 acla|EAW09605.1 afis|EAW21532.1 afla|EED55981.1 afum|EAL92553.1 agla|OJJ85696.1 akaw|GAA84999.1 aluc|OJZ86660.1 anid|CBF76394.1 anig|EHA22011.1 aory|EIT76691.1 asyd|OJJ61076.1 ater|EAU35007.1 atub|OJI83867.1 aver|OJJ00392.1 awen|OJJ39160.1 azon|OJJ51490.1

Group2620: abra|OJJ71924.1 acar|OOF96581.1 acla|EAW09604.1 afis|EAW21533.1 afla|EED55982.1 afum|EAL92554.1 agla|OJJ85697.1 akaw|GAA84998.1 aluc|OJZ86661.1 anid|CBF76392.1 anig|EHA22010.1 aory|EIT76892.1 asyd|OJJ61073.1 ater|EAU35006.1 atub|OJI83864.1 aver|OJJ00390.1 awen|OJJ39159.1 azon|OJJ51489.1

Group2621: abra|OJJ71928.1 acar|OOF96585.1 afis|EAW21536.1 afla|EED55986.1 afum|EAL92556.2 agla|OJJ85700.1 anid|CBF76386.1 aory|EIT76707.1 aver|OJJ00386.1 acla|EAW09601.1 akaw|GAA84995.1

aluc|OJZ86665.1 anig|EHA22008.1 asyd|OJJ61070.1 ater|EAU35003.1 atub|OJI83861.1 awen|OJJ39155.1 azon|OJJ51486.1

Group2622: abra|OJJ71929.1 acar|OOF96586.1 acla|EAW09600.1 afis|EAW21537.1 afla|EED55989.1 afum|EAL92557.1 agla|OJJ85701.1 akaw|GAA84994.1 aluc|OJZ86666.1 anid|CBF76382.1 anig|EHA22007.1 aory|EIT76583.1 asyd|OJJ61068.1 ater|EAU35002.1 atub|OJI83860.1 aver|OJJ00384.1 awen|OJJ39154.1 azon|OJJ51485.1

Group2623: abra|OJJ71931.1 acar|OOF96588.1 acla|EAW09598.1 afis|EAW21539.1 afla|EED55991.1 afum|EAL92559.2 agla|OJJ85703.1 akaw|GAA84992.1 aluc|OJZ86668.1 anid|CBF76378.1 anig|EHA22005.1 aory|EIT76546.1 asyd|OJJ61066.1 ater|EAU35001.1 atub|OJI83859.1 aver|OJJ00382.1 awen|OJJ39152.1 azon|OJJ51478.1

Group2624: abra|OJJ71935.1 acar|OOF96591.1 acla|EAW09595.1 afis|EAW21542.1 afla|EED55993.1 afum|EAL92562.1 agla|OJJ85705.1 akaw|GAA84989.1 aluc|OJZ86672.1 anid|CBF76374.1 anig|EHA22002.1 aory|EIT76774.1 asyd|OJJ61064.1 ater|EAU34997.1 atub|OJI83856.1 aver|OJJ00381.1 awen|OJJ39148.1 azon|OJJ51482.1

Group2625: abra|OJJ71938.1 acar|OOF96596.1 acla|EAW09591.1 afis|EAW21547.1 afla|EED55999.1 afum|EAL92567.1 agla|OJJ85710.1 akaw|GAA84986.1 aluc|OJZ86675.1 anid|CBF76364.1 anig|EHA21998.1 aory|EIT76832.1 asyd|OJJ61060.1 ater|EAU34992.1 atub|OJI83853.1 aver|OJJ00377.1 awen|OJJ39143.1 azon|OJJ51464.1

Group2626: abra|OJJ71940.1 acar|OOF96598.1 acla|EAW09590.1 afis|EAW21548.1 afla|EED56001.1 afum|EAL92568.1 agla|OJJ85726.1 akaw|GAA84985.1 aluc|OJZ86676.1 anid|CBF76362.1 anig|EHA21997.1 aory|EIT76581.1 asyd|OJJ61059.1 ater|EAU34991.1 atub|OJI83852.1 aver|OJJ00376.1 awen|OJJ39142.1 azon|OJJ51465.1

Group2627: abra|OJJ71942.1 acar|OOF96599.1 acla|EAW09588.1 afis|EAW21550.1 afla|EED56002.1 afum|EAL92570.1 agla|OJJ85728.1 akaw|GAA84983.1 aluc|OJZ86678.1 anid|CBF76358.1 anig|EHA21995.1 aory|EIT76540.1 asyd|OJJ61057.1 ater|EAU34989.1 atub|OJI83849.1 aver|OJJ00374.1 awen|OJJ39140.1 azon|OJJ51467.1

Group2628: abra|OJJ71946.1 acar|OOF96600.1 acla|EAW09587.1 afis|EAW21551.1 afla|EED56003.1 afum|EAL92571.1 agla|OJJ85729.1 akaw|GAA84980.1 aluc|OJZ86682.1 anid|CBF76356.1 anig|EHA21992.1 aory|EIT76839.1 asyd|OJJ61056.1 ater|EAU34988.1 atub|OJI83845.1 aver|OJJ00373.1 awen|OJJ39139.1 azon|OJJ51470.1

Group2629: abra|OJJ71951.1 acar|OOF96602.1 acla|EAW09583.1 afis|EAW21554.1 afla|EED56006.1 afum|EAL92574.1 agla|OJJ85731.1 akaw|GAA84977.1 aluc|OJZ86685.1 anid|CBF76350.1 anig|EHA21988.1 aory|EIT76802.1 asyd|OJJ61053.1 ater|EAU34980.1 atub|OJI83841.1 aver|OJJ00370.1 awen|OJJ39136.1 azon|OJJ51474.1

Group2630: abra|OJJ71956.1 acar|OOF96615.1 acla|EAW09579.1 afis|EAW21558.1 afla|EED56010.1 afum|EAL92577.1 agla|OJJ85736.1 akaw|GAA84971.1 aluc|OJZ86691.1 anid|CBF76344.1 anig|EHA21982.1 aory|EIT76530.1 asyd|OJJ61049.1 ater|EAU34976.1 atub|OJI83833.1 aver|OJJ00365.1 awen|OJJ39132.1 azon|OJJ51460.1

Group2631: abra|OJJ71957.1 acar|OOF96613.1 acla|EAW09578.1 afis|EAW21559.1 afla|EED56011.1 afum|EAL92578.1 agla|OJJ85737.1 akaw|GAA84970.1 aluc|OJZ86692.1 anid|CBF76342.1 anig|EHA21981.1 aory|EIT76831.1 asyd|OJJ61048.1 ater|EAU34974.1 atub|OJI83832.1 aver|OJJ00364.1 awen|OJJ39131.1 azon|OJJ51459.1

Group2632: abra|OJJ71959.1 acar|OOF96611.1 acla|EAW09577.1 afis|EAW21560.1 afla|EED56012.1 afum|EAL92579.1 agla|OJJ85739.1 akaw|GAA84969.1 aluc|OJZ86693.1 anid|CBF76340.1 anig|EHA21980.1 aory|EIT76670.1 asyd|OJJ61046.1 atub|OJI83831.1 aver|OJJ00363.1 awen|OJJ39129.1 azon|OJJ51458.1 ater|EAU34973.1

Group2633: abra|OJJ71960.1 acar|OOF96610.1 acla|EAW09576.1 afis|EAW21561.1 afla|EED56014.1 afum|EAL92580.1 agla|OJJ85740.1 akaw|GAA84968.1 aluc|OJZ86694.1 anid|CBF76338.1 anig|EHA21979.1 aory|EIT76536.1 asyd|OJJ61045.1 ater|EAU34972.1 atub|OJI83830.1 aver|OJJ00362.1 awen|OJJ39128.1 azon|OJJ51456.1

Group2634: abra|OJJ71961.1 acar|OOF96609.1 acla|EAW09575.1 afis|EAW21562.1 afla|EED56015.1 afum|EAL92581.1 agla|OJJ85741.1 akaw|GAA84967.1 aluc|OJZ86696.1 anid|CBF76336.1 anig|EHA21978.1 aory|EIT76848.1 asyd|OJJ61044.1 ater|EAU34971.1 atub|OJI83829.1 aver|OJJ00361.1 awen|OJJ39127.1 azon|OJJ51455.1

Group2635: abra|OJJ71966.1 acar|OOF89890.1 acla|EAW09571.1 afis|EAW21565.1 afum|EAL92584.1 agla|OJJ85750.1 akaw|GAA84962.1 aluc|OJZ86702.1 anid|CBF76330.1 anig|EHA21974.1 aory|EIT76870.1 asyd|OJJ61038.1 ater|EAU34967.1 atub|OJI83824.1 aver|OJJ00356.1 awen|OJJ39120.1 azon|OJJ51450.1 afla|EED56022.1

Group2636: abra|OJJ71969.1 acar|OOF96604.1 acla|EAW09570.1 afis|EAW21566.1 afla|EED56031.1 afum|EAL92585.1 agla|OJJ85751.1 akaw|GAA84961.1 aluc|OJZ86704.1 anid|CBF76328.1 anig|EHA21972.1 aory|EIT76642.1 asyd|OJJ61016.1 ater|EAU34965.1 atub|OJI83823.1 aver|OJJ00336.1 awen|OJJ39115.1 azon|OJJ51449.1

Group2637: abra|OJJ71970.1 acar|OOF96621.1 acla|EAW09569.1 afis|EAW21567.1 afla|EED56034.1 afum|EAL92586.1 agla|OJJ85752.1 akaw|GAA84960.1 aluc|OJZ86705.1 anid|CBF76326.1 anig|EHA21971.1 aory|EIT76629.1 asyd|OJJ61015.1 ater|EAU34964.1 atub|OJI83822.1 aver|OJJ00334.1 awen|OJJ39113.1 azon|OJJ51448.1

Group2638: abra|OJJ71971.1 acar|OOF96622.1 acla|EAW09568.1 afis|EAW21568.1 afla|EED56035.1 afum|EAL92587.1 agla|OJJ85753.1 akaw|GAA84959.1 aluc|OJZ86706.1 anid|CBF76324.1 anig|EHA21970.1 aory|EIT76766.1 asyd|OJJ61014.1 ater|EAU34963.1 atub|OJI83821.1 aver|OJJ00333.1 awen|OJJ39112.1 azon|OJJ51447.1

Group2639: abra|OJJ71972.1 acar|OOF96623.1 acla|EAW09567.1 afis|EAW21569.1 afla|EED56036.1 afum|EBA27467.1 agla|OJJ85754.1 akaw|GAA84958.1 aluc|OJZ86707.1 anid|CBF76322.1 anig|EHA21969.1 aory|EIT76617.1 asyd|OJJ61013.1 ater|EAU34962.1 atub|OJI83820.1 aver|OJJ00332.1 awen|OJJ39111.1 azon|OJJ51446.1

Group2640: abra|OJJ71973.1 acar|OOF96625.1 acla|EAW09565.1 afis|EAW21571.1 afla|EED56037.1 afum|EAL92589.1 agla|OJJ85771.1 akaw|GAA84957.1 aluc|OJZ86708.1 anid|CBF76318.1 anig|EHA21968.1 aory|EIT76745.1 asyd|OJJ61011.1 ater|EAU34961.1 atub|OJI83819.1 aver|OJJ00330.1 awen|OJJ39110.1 azon|OJJ51445.1

Group2641: abra|OJJ71974.1 acar|OOF96624.1 acla|EAW09564.1 afis|EAW21572.1 afla|EED56038.1 afum|EAL92590.1 agla|OJJ85768.1 akaw|GAA84956.1 aluc|OJZ86709.1 anid|CBF76316.1 anig|EHA21967.1 aory|EIT76889.1 asyd|OJJ61010.1 ater|EAU34960.1 atub|OJI83818.1 aver|OJJ00329.1 awen|OJJ39109.1 azon|OJJ51444.1

Group2642: abra|OJJ71975.1 acar|OOF96626.1 acla|EAW09563.1 afis|EAW21573.1 afla|EED57792.1 afum|EAL92591.2 agla|OJJ85766.1 akaw|GAA84955.1 aluc|OJZ86710.1 anid|CBF76314.1 anig|EHA21966.1 aory|EIT81122.1 asyd|OJJ61009.1 ater|EAU34959.1 atub|OJI83817.1 aver|OJJ00328.1 awen|OJJ39108.1 azon|OJJ51443.1

Group2643: abra|OJJ71978.1 acar|OOF96628.1 acla|EAW09562.1 afis|EAW21575.1 afla|EED57793.1 afum|EAL92593.1 agla|OJJ85764.1 akaw|GAA84952.1 aluc|OJZ86715.1 anid|CBF76306.1 anig|EHA21963.1 aory|EIT81108.1 asyd|OJJ61006.1 ater|EAU34957.1 atub|OJI83814.1 aver|OJJ00324.1 awen|OJJ39105.1 azon|OJJ51438.1

Group2644: abra|OJJ71983.1 acar|OOF96634.1 acla|EAW09556.1 afis|EAW21581.1 afla|EED57799.1 afum|EAL92599.2 agla|OJJ85757.1 akaw|GAA84946.1 aluc|OJZ86720.1 anid|CBF76300.1 anig|EHA21959.1 aory|EIT81064.1 asyd|OJJ61001.1 ater|EAU34953.1 atub|OJI83809.1 aver|OJJ00319.1 awen|OJJ39100.1 azon|OJJ51434.1

Group2645: abra|OJJ71987.1 acar|OOF96642.1 acla|EAW09551.1 afis|EAW21585.1 afla|EED57806.1 afum|EAL92603.2 agla|OJJ85777.1 akaw|GAA84942.1 aluc|OJZ86724.1 anid|CBF76289.1 anig|EHA21955.1 aory|EIT81058.1 asyd|OJJ60995.1 ater|EAU34946.1 atub|OJI83804.1 aver|OJJ00315.1 awen|OJJ39096.1 azon|OJJ51429.1

Group2646: abra|OJJ71990.1 acar|OOF96644.1 acla|EAW09545.1 afis|EAW21588.1 afla|EED57809.1 afum|EAL92606.1 agla|OJJ85781.1 akaw|GAA84939.1 aluc|OJZ86727.1 anid|CBF76285.1 anig|EHA21952.1 aory|EIT81040.1 asyd|OJJ60992.1 ater|EAU34943.1 atub|OJI83801.1 aver|OJJ00312.1 awen|OJJ39093.1 azon|OJJ51426.1

Group2647: abra|OJJ71991.1 acar|OOF96645.1 acla|EAW09544.1 afis|EAW21589.1 afla|EED57811.1 afum|EAL92607.1 agla|OJJ85782.1 akaw|GAA84938.1 aluc|OJZ86728.1 anid|CBF76283.1 anig|EHA21951.1 aory|EIT81035.1 asyd|OJJ60991.1 ater|EAU34942.1 atub|OJI83800.1 aver|OJJ00311.1 awen|OJJ39092.1 azon|OJJ51425.1

Group2648: abra|OJJ71993.1 acar|OOF96647.1 acla|EAW09542.1 afis|EAW21591.1 afla|EED57813.1 afum|EAL92609.1 agla|OJJ85784.1 akaw|GAA84935.1 aluc|OJZ86731.1 anid|CBF83440.1 anig|EHA21948.1 aory|EIT81099.1 asyd|OJJ60668.1 ater|EAU34940.1 atub|OJI83797.1 aver|OJI99991.1 awen|OJJ39090.1 azon|OJJ51421.1

Group2649: abra|OJJ71995.1 acar|OOF96649.1 acla|EAW09540.1 afis|EAW21593.1 afla|EED57815.1 afum|EAL92611.1 agla|OJJ85788.1 akaw|GAA84933.1 aluc|OJZ86733.1 anid|CBF83444.1 anig|EHA21946.1 aory|EIT81101.1 asyd|OJJ60670.1 ater|EAU34931.1 atub|OJI83795.1 aver|OJI99993.1 awen|OJJ39088.1 azon|OJJ51419.1

Group2650: abra|OJJ71997.1 acar|OOF96651.1 acla|EAW09541.1 afis|EAW21592.1 afla|EED57814.1 afum|EAL92610.1 agla|OJJ85789.1 akaw|GAA84932.1 aluc|OJZ86735.1 anid|CBF83442.1 anig|EHA21944.1

aory|EIT81111.1 asyd|OJJ60669.1 ater|EAU34932.1 atub|OJI83793.1 aver|OJI99992.1 awen|OJJ39089.1 azon|OJJ51420.1

Group2651: abra|OJJ71998.1 acar|OOF96653.1 acla|EAW09536.1 afis|EAW21596.1 afla|EED57819.1 afum|EAL92614.1 agla|OJJ85790.1 akaw|GAA84931.1 aluc|OJZ86736.1 anid|CBF83453.1 anig|EHA21943.1 aory|EIT81091.1 asyd|OJJ60672.1 ater|EAU34928.1 atub|OJI83792.1 aver|OJI99995.1 awen|OJJ39086.1 azon|OJJ51417.1

Group2652: abra|OJJ72007.1 acar|OOF96657.1 acla|EAW09534.1 afis|EAW21599.1 afla|EED57824.1 afum|EAL92618.1 agla|OJJ85794.1 akaw|GAA84926.1 aluc|OJZ86743.1 anid|CBF83457.1 anig|EHA21936.1 aory|EIT81107.1 asyd|OJJ60679.1 ater|EAU38289.1 atub|OJI83784.1 aver|OJJ00004.1 awen|OJJ39083.1 azon|OJJ51415.1

Group2653: abra|OJJ72008.1 acla|EAW09533.1 anid|CBF83458.1 asyd|OJJ60682.1 ater|EAU38290.1 aver|OJJ00007.1 azon|OJJ51414.1 acla|EAW09532.1 afis|EAW21600.1 afla|EED57827.1 afum|EAL92619.1 agla|OJJ85795.1 akaw|GAA84925.1 aluc|OJZ86744.1 anig|EHA21935.1 aory|EIT81065.1 atub|OJI83783.1 awen|OJJ39082.1

Group2654: abra|OJJ72010.1 acar|OOF96658.1 acla|EAW09529.1 afis|EAW21603.1 afla|EED57829.1 afum|EAL92622.1 agla|OJJ85797.1 akaw|GAA84923.1 aluc|OJZ86746.1 anid|CBF83462.1 anig|EHA21933.1 aory|EIT81061.1 asyd|OJJ60684.1 ater|EAU38292.1 atub|OJI83781.1 aver|OJJ00009.1 awen|OJJ39080.1 azon|OJJ51412.1

Group2655: abra|OJJ72014.1 acar|OOF96663.1 acla|EAW09526.1 afis|EAW21606.1 afla|EED57833.1 afum|EAL92625.1 agla|OJJ85801.1 akaw|GAA84919.1 aluc|OJZ86750.1 anid|CBF83469.1 anig|EHA21929.1 aory|EIT81024.1 asyd|OJJ60688.1 ater|EAU38295.1 atub|OJI83777.1 aver|OJJ00013.1 awen|OJJ39076.1 azon|OJJ51408.1

Group2656: abra|OJJ72015.1 acar|OOF96664.1 acla|EAW09525.1 afis|EAW21607.1 afla|EED57834.1 afum|EAL92626.1 agla|OJJ85802.1 akaw|GAA84918.1 aluc|OJZ86751.1 anid|CBF83473.1 anig|EHA21928.1 aory|EIT81105.1 asyd|OJJ60690.1 ater|EAU38296.1 atub|OJI83775.1 aver|OJJ00015.1 awen|OJJ39075.1 azon|OJJ51407.1

Group2657: abra|OJJ72016.1 acar|OOF96666.1 acla|EAW09524.1 afis|EAW21608.1 afla|EED57837.1 afum|EAL92642.1 agla|OJJ85803.1 akaw|GAA84917.1 aluc|OJZ86752.1 anid|CBF83475.1 anig|EHA21927.1 aory|EIT81080.1 asyd|OJJ60691.1 ater|EAU38297.1 atub|OJI83774.1 aver|OJJ00016.1 awen|OJJ39074.1 azon|OJJ51406.1

Group2658: abra|OJJ72017.1 acar|OOF96665.1 acla|EAW09523.1 afis|EAW21609.1 afla|EED57838.1 afum|EAL92643.1 agla|OJJ85804.1 akaw|GAA84916.1 aluc|OJZ86753.1 anid|CBF83477.1 anig|EHA21926.1 aory|EIT81100.1 asyd|OJJ60692.1 ater|EAU38298.1 atub|OJI83773.1 aver|OJJ00017.1 awen|OJJ39073.1 azon|OJJ51405.1

Group2659: abra|OJJ72018.1 acar|OOF96667.1 acla|EAW09521.1 afis|EAW21611.1 afla|EED57840.1 afum|EAL92645.1 agla|OJJ78616.1 akaw|GAA84914.1 aluc|OJZ86755.1 anid|CBF83480.1 anig|EHA21924.1 aory|EIT81096.1 asyd|OJJ60694.1 ater|EAU38302.1 atub|OJI83772.1 aver|OJJ00020.1 awen|OJJ39071.1 azon|OJJ51402.1

Group2660: abra|OJJ72019.1 acar|OOF96669.1 acla|EAW09520.1 afis|EAW21612.1 afla|EED57841.1 afum|EAL92646.1 agla|OJJ78617.1 akaw|GAA84913.1 aluc|OJZ86756.1 anid|CBF83482.1 anig|EHA21923.1 aory|EIT81062.1 asyd|OJJ60695.1 ater|EAU38303.1 atub|OJI83771.1 aver|OJJ00021.1 awen|OJJ39070.1 azon|OJJ51401.1

Group2661: abra|OJJ72020.1 acar|OOF96668.1 acla|EAW09515.1 afis|EAW21617.1 afla|EED57842.1 afum|EAL92652.1 agla|OJJ78622.1 akaw|GAA84912.1 aluc|OJZ86757.1 anid|CBF83484.1 anig|EHA21922.1 aory|EIT81032.1 asyd|OJJ60696.1 ater|EAU38304.1 atub|OJI83770.1 aver|OJJ00022.1 awen|OJJ39069.1 azon|OJJ51400.1

Group2662: abra|OJJ72022.1 acar|OOF96671.1 acla|EAW09517.1 afis|EAW21615.1 afla|EED57844.1 afum|EAL92649.1 agla|OJJ78624.1 akaw|GAA84910.1 aluc|OJZ86759.1 anid|CBF83485.1 anig|EHA21920.1 aory|EIT81023.1 asyd|OJJ60698.1 ater|EAU38305.1 atub|OJI83768.1 aver|OJJ00024.1 awen|OJJ39067.1 azon|OJJ51398.1

Group2663: abra|OJJ72024.1 acar|OOF96673.1 acla|EAW09519.1 afis|EAW21613.1 afla|EED57846.1 afum|EAL92647.1 agla|OJJ78626.1 akaw|GAA84908.1 aluc|OJZ86761.1 anid|CBF83489.1 anig|EHA21918.1 aory|EIT81029.1 asyd|OJJ60700.1 ater|EAU38307.1 atub|OJI83766.1 aver|OJJ00026.1 awen|OJJ39065.1 azon|OJJ51396.1

Group2664: abra|OJJ72032.1 acar|OOF96682.1 acla|EAW09511.1 afis|EAW21621.1 afla|EED57850.1 afum|EAL92657.2 agla|OJJ82578.1 akaw|GAA84900.1 aluc|OJZ86770.1 anid|CBF83495.1 anig|EHA21910.1 aory|EIT81046.1 asyd|OJJ60704.1 ater|EAU38310.1 atub|OJI83757.1 aver|OJJ00029.1 awen|OJJ33873.1 azon|OJJ48677.1

Group2665: abra|OJJ72044.1 acar|OOF96702.1 acla|EAW09502.1 afis|EAW21634.1 afla|EED57876.1 afum|EAL92667.1 agla|OJJ79699.1 akaw|GAA84890.1 aluc|OJZ86781.1 anid|CBF83500.1 anig|EHA21901.1 aory|EIT83264.1 asyd|OJJ60711.1 ater|EAU38320.1 atub|OJI83739.1 aver|OJJ00038.1 awen|OJJ33883.1 azon|OJJ48670.1

Group2666: abra|OJJ72046.1 acar|OOF96704.1 acla|EAW09500.1 afis|EAW21636.1 afla|EED57879.1 afum|EAL92670.1 agla|OJJ79701.1 akaw|GAA84888.1 aluc|OJZ86783.1 anid|CBF83517.1 anig|EHA21899.1 aory|EIT83275.1 asyd|OJJ60714.1 ater|EAU38322.1 atub|OJI83737.1 aver|OJJ00047.1 awen|OJJ33885.1 azon|OJJ48668.1

Group2667: abra|OJJ72048.1 acar|OOF96706.1 acla|EAW09498.1 afis|EAW21638.1 afla|EED57881.1 afum|EAL92672.2 agla|OJJ79703.1 akaw|GAA84886.1 aluc|OJZ86785.1 anid|CBF83521.1 anig|EHA21897.1 aory|EIT83269.1 asyd|OJJ60716.1 ater|EAU38323.1 atub|OJI83735.1 aver|OJJ00049.1 awen|OJJ33887.1 azon|OJJ48666.1

Group2668: abra|OJJ72052.1 acar|OOF96709.1 acla|EAW09495.1 afis|EAW21641.1 afla|EED57902.1 afum|EAL92675.1 agla|OJJ79708.1 akaw|GAA84882.1 aluc|OJZ86789.1 anid|CBF83526.1 anig|EHA21894.1 aory|EIT82320.1 asyd|OJJ60718.1 ater|EAU38326.1 atub|OJI83730.1 aver|OJJ00051.1 awen|OJJ33898.1 azon|OJJ48662.1

Group2669: abra|OJJ72054.1 acar|OOF96711.1 acla|EAW09493.1 afis|EAW21643.1 afla|EED57900.1 afum|EAL92677.1 agla|OJJ79710.1 akaw|GAA84880.1 aluc|OJZ86791.1 anid|CBF83528.1 anig|EHA21892.1 aory|EIT82343.1 asyd|OJJ60720.1 ater|EAU38328.1 atub|OJI83728.1 aver|OJJ00053.1 awen|OJJ33900.1 azon|OJJ48660.1

Group2670: abra|OJJ72056.1 acar|OOF96713.1 acla|EAW09492.1 afis|EAW21644.1 afla|EED57899.1 afum|EAL92678.1 agla|OJJ79711.1 akaw|GAA84878.1 aluc|OJZ86793.1 anid|CBF83530.1 anig|EHA21890.1 aory|EIT82331.1 asyd|OJJ60721.1 ater|EAU38329.1 atub|OJI83726.1 aver|OJJ00054.1 awen|OJJ33901.1 azon|OJJ48658.1

Group2671: abra|OJJ72062.1 acar|OOF96716.1 acla|EAW09489.1 afis|EAW21647.1 afla|EED57896.1 afum|EAL92681.1 agla|OJJ79718.1 akaw|GAA84873.1 aluc|OJZ86798.1 anid|CBF83537.1 anig|EHA21885.1 aory|EIT82735.1 asyd|OJJ60725.1 ater|EAU38332.1 atub|OJI83720.1 aver|OJJ00058.1 awen|OJJ33907.1 azon|OJJ48655.1

Group2672: abra|OJJ72063.1 acar|OOF96717.1 acla|EAW09479.1 afis|EAW21657.1 afla|EED57895.1 afum|EAL92692.1 agla|OJJ79719.1 akaw|GAA84872.1 aluc|OJZ86799.1 anid|CBF83539.1 anig|EHA21884.1 aory|EIT82741.1 asyd|OJJ60727.1 ater|EAU38333.1 atub|OJI83719.1 aver|OJJ00059.1 awen|OJJ33908.1 azon|OJJ48653.1

Group2673: abra|OJJ72064.1 acar|OOF96718.1 acla|EAW09478.1 afis|EAW21658.1 afla|EED57894.1 afum|EAL92693.1 agla|OJJ79720.1 akaw|GAA84871.1 aluc|OJZ86800.1 anid|CBF83541.1 anig|EHA21883.1 aory|EIT82736.1 asyd|OJJ60728.1 ater|EAU38334.1 atub|OJI83718.1 aver|OJJ00060.1 awen|OJJ33909.1 azon|OJJ48652.1

Group2674: abra|OJJ72065.1 acar|OOF96719.1 acla|EAW09477.1 afis|EAW21659.1 afla|EED57893.1 afum|EAL92694.1 agla|OJJ79721.1 akaw|GAA84870.1 aluc|OJZ86801.1 anid|CBF83542.1 anig|EHA21882.1 aory|EIT82740.1 asyd|OJJ60729.1 ater|EAU38335.1 atub|OJI83717.1 aver|OJJ00061.1 awen|OJJ33910.1 azon|OJJ48651.1

Group2675: abra|OJJ72068.1 acar|OOF96724.1 acla|EAW09483.1 afis|EAW21653.1 afla|EED57886.1 afum|EAL92688.1 agla|OJJ79724.1 akaw|GAA84867.1 aluc|OJZ86803.1 anid|CBF83550.1 anig|EHA21879.1 aory|EIT83265.1 asyd|OJJ60733.1 ater|EAU38339.1 atub|OJI83715.1 aver|OJJ00065.1 awen|OJJ33914.1 azon|OJJ48647.1

Group2676: abra|OJJ72075.1 acar|OOF96727.1 acla|EAW09485.1 afis|EAW21651.1 afla|EED57918.1 afum|EAL92685.2 agla|OJJ79727.1 akaw|GAA84842.1 aluc|OJZ86829.1 anid|CBF83553.1 anig|EHA21806.1 aory|EIT82364.1 asyd|OJJ60735.1 ater|EAU38343.1 atub|OJI83689.1 aver|OJJ00067.1 awen|OJJ33917.1 azon|OJJ48643.1

Group2677: abra|OJJ72085.1 acar|OOF96730.1 acla|EAW09488.1 afis|EAW21648.1 afla|EED57923.1 afum|EAL92682.2 agla|OJJ79730.1 akaw|GAA84835.1 aluc|OJZ86839.1 anid|CBF83559.1 anig|EHA21798.1 aory|EIT82355.1 asyd|OJJ60738.1 ater|EAU38345.1 atub|OJI83679.1 aver|OJJ00070.1 awen|OJJ33919.1 azon|OJJ48645.1

Group2678: abra|OJJ72086.1 acar|OOF96732.1 acla|EAW09467.1 afis|EAW21669.1 afla|EED57933.1 afum|EAL92704.1 agla|OJJ79740.1 akaw|GAA84834.1 aluc|OJZ86840.1 anid|CBF83582.1 anig|EHA21797.1 aory|EIT82344.1 asyd|OJJ60781.1 ater|EAU38353.1 atub|OJI83678.1 aver|OJJ00114.1 awen|OJJ33932.1 azon|OJJ48640.1

Group2679: abra|OJJ72087.1 acar|OOF96731.1 acla|EAW09468.1 afis|EAW21668.1 afla|EED57932.1 afum|EAL92703.1 agla|OJJ79739.1 akaw|GAA84833.1 aluc|OJZ86841.1 anid|CBF83580.1 anig|EHA21796.1

aory|EIT82317.1 asyd|OJJ60749.1 ater|EAU38352.1 atub|OJI83677.1 aver|OJJ00081.1 awen|OJJ33931.1 azon|OJJ48639.1

Group2680: abra|OJJ72089.1 acar|OOF96735.1 acla|EAW09470.1 afis|EAW21666.1 afla|EED57930.1 afum|EAL92701.1 agla|OJJ79737.1 akaw|GAA84831.1 aluc|OJZ86843.1 anid|CBF83569.1 anig|EHA21794.1 aory|EIT82323.1 asyd|OJJ60747.1 ater|EAU38351.1 atub|OJI83675.1 aver|OJJ00079.1 awen|OJJ33930.1 azon|OJJ48637.1

Group2681: abra|OJJ72090.1 acar|OOF96734.1 acla|EAW09471.1 afis|EAW21665.1 afla|EED57929.1 afum|EAL92700.1 agla|OJJ79736.1 akaw|GAA84830.1 aluc|OJZ86844.1 anid|CBF83568.1 anig|EHA21793.1 aory|EIT82319.1 asyd|OJJ60746.1 ater|EAU38350.1 atub|OJI83674.1 aver|OJJ00078.1 awen|OJJ33929.1 azon|OJJ48636.1

Group2682: abra|OJJ72092.1 acar|OOF96737.1 acla|EAW09473.1 afis|EAW21662.1 afla|EED57927.1 afum|EAL92697.1 agla|OJJ79734.1 akaw|GAA84828.1 aluc|OJZ86847.1 anid|CBF83564.1 anig|EHA21791.1 aory|EIT82336.1 asyd|OJJ60744.1 ater|EAU38348.1 atub|OJI83672.1 aver|OJJ00076.1 awen|OJJ33926.1 azon|OJJ48634.1

Group2683: abra|OJJ72094.1 acar|OOF96739.1 acla|EAW09475.1 afis|EAW21664.1 afla|EED57925.1 afum|EAL92699.1 agla|OJJ79732.1 akaw|GAA84826.1 aluc|OJZ86849.1 anid|CBF83562.1 anig|EHA21789.1 aory|EIT82328.1 asyd|OJJ60742.1 ater|EAU38347.1 atub|OJI83670.1 aver|OJJ00074.1 awen|OJJ33924.1 azon|OJJ48632.1

Group2684: abra|OJJ72095.1 acar|OOF96740.1 acla|EAW09476.1 afis|EAW21660.1 afla|EED57924.1 afum|EAL92695.1 agla|OJJ79731.1 akaw|GAA84825.1 aluc|OJZ86850.1 anid|CBF83560.1 anig|EHA21788.1 aory|EIT82356.1 asyd|OJJ60741.1 ater|EAU38346.1 atub|OJI83669.1 aver|OJJ00073.1 awen|OJJ33923.1 azon|OJJ48631.1

Group2685: abra|OJJ72096.1 acar|OOF96741.1 acla|EAW09391.1 afis|EAW21742.1 afla|EED58036.1 afum|EAL92775.1 agla|OJJ84431.1 akaw|GAA84824.1 aluc|OJZ86851.1 anid|CBF83748.1 anig|EHA21787.1 aory|EIT79774.1 asyd|OJJ60855.1 ater|EAU38446.1 atub|OJI83668.1 aver|OJJ00179.1 awen|OJJ34024.1 azon|OJJ48534.1

Group2686: abra|OJJ72099.1 acar|OOF96744.1 acla|EAW09390.1 afis|EAW21743.1 afla|EED58034.1 afum|EAL92776.1 agla|OJJ84427.1 akaw|GAA84822.1 aluc|OJZ86854.1 anid|CBF83738.1 anig|EHA21785.1 aory|EIT79762.1 asyd|OJJ60853.1 ater|EAU38445.1 atub|OJI83665.1 aver|OJJ00177.1 awen|OJJ34026.1 azon|OJJ48530.1

Group2687: abra|OJJ72101.1 acar|OOF96746.1 acla|EAW09388.1 afis|EAW21746.1 afla|EED58032.1 afum|EAL92778.1 agla|OJJ84430.1 akaw|GAA84820.1 aluc|OJZ86856.1 anid|CBF83662.1 anig|EHA21783.1 aory|EIT79789.1 asyd|OJJ60804.1 ater|EAU38443.1 atub|OJI83663.1 aver|OJJ00133.1 awen|OJJ34028.1 azon|OJJ48201.1

Group2688: abra|OJJ72105.1 acar|OOF96748.1 acla|EAW09386.1 afis|EAW21749.1 afla|EED58029.1 afum|EAL92780.2 agla|OJJ84425.1 akaw|GAA84817.1 aluc|OJZ86860.1 anid|CBF83665.1 anig|EHA21780.1 aory|EIT79704.1 asyd|OJJ60806.1 ater|EAU38441.1 atub|OJI83660.1 aver|OJJ00135.1 awen|OJJ34030.1 azon|OJJ48203.1

Group2689: abra|OJJ72110.1 acar|OOF90022.1 acla|EAW14910.1 afis|EAW24431.1 afla|EED55244.1 afum|EAL86099.1 agla|OJJ79245.1 akaw|GAA84812.1 aluc|OJZ86865.1 anid|CBF73773.1 anig|EHA21776.1 aory|EIT83529.1 asyd|OJJ56593.1 ater|EAU30014.1 atub|OJI83655.1 aver|OJJ06132.1 awen|OJJ32771.1 azon|OJJ44001.1

Group2690: abra|OJJ72112.1 acar|OOF96752.1 acla|EAW14912.1 afis|EAW24433.1 afla|EED55247.1 afum|EAL86101.1 agla|OJJ79248.1 akaw|GAA84810.1 aluc|OJZ86867.1 anid|CBF73767.1 anig|EHA21774.1 aory|EIT83512.1 asyd|OJJ56591.1 ater|EAU30012.1 atub|OJI83653.1 aver|OJJ06134.1 awen|OJJ32768.1 azon|OJJ43999.1

Group2691: abra|OJJ72115.1 acar|OOF96755.1 acla|EAW14916.1 afis|EAW24438.1 afla|EED55241.1 afum|EAL86106.1 agla|OJJ78777.1 akaw|GAA84807.1 aluc|OJZ86870.1 anid|CBF73762.1 anig|EHA21771.1 aory|EIT83515.1 asyd|OJJ56588.1 ater|EAU30009.1 atub|OJI83649.1 aver|OJJ06137.1 awen|OJJ32761.1 azon|OJJ43996.1

Group2692: abra|OJJ72116.1 acar|OOF96756.1 acla|EAW14917.1 afis|EAW24439.1 afla|EED55239.1 afum|EAL86107.1 agla|OJJ78778.1 akaw|GAA84806.1 aluc|OJZ86871.1 anid|CBF73760.1 anig|EHA21770.1 aory|EIT83598.1 asyd|OJJ56587.1 ater|EAU30008.1 atub|OJI83648.1 aver|OJJ06138.1 awen|OJJ32760.1 azon|OJJ43995.1

Group2693: abra|OJJ72117.1 acar|OOF96758.1 acla|EAW14918.1 afis|EAW24440.1 afla|EED55238.1 afum|EAL86108.1 agla|OJJ78779.1 akaw|GAA84805.1 aluc|OJZ86872.1 anid|CBF73758.1 anig|EHA21769.1 aory|EIT83628.1 asyd|OJJ56586.1 ater|EAU30007.1 atub|OJI83647.1 aver|OJJ06139.1 awen|OJJ32759.1 azon|OJJ43994.1

Group2694: abra|OJJ72118.1 acar|OOF96759.1 acla|EAW14919.1 afis|EAW24441.1 afla|EED55237.1 afum|EAL86109.1 agla|OJJ78780.1 akaw|GAA84804.1 aluc|OJZ86873.1 anid|CBF73756.1 anig|EHA21768.1 aory|EIT83564.1 asyd|OJJ56585.1 ater|EAU30006.1 atub|OJI83645.1 aver|OJJ06140.1 awen|OJJ32758.1 azon|OJJ43993.1

Group2695: abra|OJJ72119.1 acar|OOF96760.1 acla|EAW14920.1 afis|EAW24442.1 afla|EED55236.1 afum|EAL86110.1 agla|OJJ78783.1 akaw|GAA84803.1 aluc|OJZ86875.1 anid|CBF73754.1 anig|EHA21767.1 aory|EIT83587.1 asyd|OJJ56584.1 ater|EAU30005.1 atub|OJI83643.1 aver|OJJ06141.1 awen|OJJ32757.1 azon|OJJ43991.1

Group2696: abra|OJJ72123.1 acar|OOF96763.1 acla|EAW14923.1 afis|EAW24445.1 afla|EED55232.1 afum|EAL86114.1 agla|OJJ80290.1 akaw|GAA84800.1 aluc|OJZ86880.1 anid|CBF73749.1 anig|EHA21764.1 aory|EIT83556.1 asyd|OJJ56580.1 ater|EAU30002.1 atub|OJI83639.1 aver|OJJ06144.1 awen|OJJ32754.1 azon|OJJ43989.1

Group2697: abra|OJJ72124.1 acar|OOF96764.1 acla|EAW14930.1 afis|EAW24446.1 afla|EED55231.1 afum|EAL86115.1 agla|OJJ80291.1 akaw|GAA84799.1 aluc|OJZ86881.1 anid|CBF73806.1 anig|EHA21763.1 aory|EIT83582.1 asyd|OJJ56579.1 ater|EAU30001.1 atub|OJI83638.1 aver|OJJ06145.1 awen|OJJ32753.1 azon|OJJ43992.1

Group2698: abra|OJJ72129.1 acar|OOF93633.1 acla|EAW13225.1 afis|EAW17419.1 afla|EED53977.1 agla|OJJ86132.1 akaw|GAA84793.1 aluc|OJZ86887.1 anid|CBF71783.1 aory|EIT78268.1 atub|OJI83632.1 aver|OJI95842.1 awen|OJJ40006.1 azon|OJJ48326.1 asyd|OJJ53805.1 ater|EAU32275.1 afis|EAW19998.1 ater|EAU38678.1

Group2699: abra|OJJ72139.1 acar|OOF96777.1 acla|EAW14933.1 afis|EAW24448.1 afla|EED55225.1 afum|EAL86117.1 agla|OJJ80294.1 akaw|GAA84783.1 aluc|OJZ86897.1 anid|CBF73811.1 anig|EHA21746.1 aory|EIT83551.1 asyd|OJJ56576.1 ater|EAU29998.1 atub|OJI83622.1 aver|OJJ06147.1 awen|OJJ32749.1 azon|OJJ45300.1

Group2700: abra|OJJ72140.1 acar|OOF96778.1 acla|EAW14934.1 afis|EAW24449.1 afla|EED55224.1 afum|EAL86118.1 agla|OJJ80295.1 akaw|GAA84784.1 aluc|OJZ86896.1 anid|CBF73813.1 anig|EHA21745.1 aory|EIT83509.1 asyd|OJJ56575.1 ater|EAU29997.1 atub|OJI83623.1 aver|OJJ06148.1 awen|OJJ32748.1 azon|OJJ45301.1

Group2701: abra|OJJ72141.1 acar|OOF96779.1 acla|EAW14935.1 afis|EAW24450.1 afla|EED55223.1 afum|EAL86119.1 agla|OJJ80296.1 akaw|GAA84782.1 aluc|OJZ86898.1 anid|CBF73815.1 anig|EHA21744.1 aory|EIT83569.1 asyd|OJJ56574.1 ater|EAU29996.1 atub|OJI83621.1 aver|OJJ06149.1 awen|OJJ32747.1 azon|OJJ45299.1

Group2702: abra|OJJ72145.1 acar|OOF96782.1 acla|EAW14939.1 afis|EAW24454.1 afla|EED55220.1 afum|EAL86123.1 agla|OJJ80301.1 akaw|GAA84779.1 aluc|OJZ86902.1 anid|CBF73822.1 anig|EHA21741.1 aory|EIT83594.1 asyd|OJJ56567.1 ater|EAU29991.1 atub|OJI83618.1 aver|OJJ06154.1 awen|OJJ32743.1 azon|OJJ45293.1

Group2703: abra|OJJ72146.1 acar|OOF96783.1 acla|EAW14941.1 afis|EAW24456.1 afla|EED55218.1 afum|EAL86126.1 agla|OJJ80303.1 akaw|GAA84778.1 aluc|OJZ86903.1 anid|CBF73824.1 anig|EHA21740.1 aory|EIT83584.1 asyd|OJJ56566.1 ater|EAU29988.1 atub|OJI83617.1 aver|OJJ06155.1 awen|OJJ32741.1 azon|OJJ45291.1

Group2704: abra|OJJ72147.1 acar|OOF96784.1 acla|EAW14942.1 afis|EAW24457.1 afla|EED55217.1 afum|EAL86127.1 agla|OJJ80304.1 akaw|GAA84777.1 aluc|OJZ86904.1 anid|CBF73826.1 anig|EHA21739.1 aory|EIT83603.1 asyd|OJJ56564.1 ater|EAU29985.1 atub|OJI83616.1 aver|OJJ06157.1 awen|OJJ32740.1 azon|OJJ45290.1

Group2705: abra|OJJ72148.1 acar|OOF96785.1 acla|EAW14943.1 afis|EAW24458.1 afla|EED55216.1 afum|EAL86128.1 agla|OJJ80305.1 akaw|GAA84776.1 aluc|OJZ86905.1 anid|CBF73828.1 anig|EHA21738.1 aory|EIT83601.1 asyd|OJJ56563.1 ater|EAU29984.1 atub|OJI83615.1 aver|OJJ06158.1 awen|OJJ32739.1 azon|OJJ45289.1

Group2706: abra|OJJ72149.1 acar|OOF96786.1 acla|EAW14944.1 afis|EAW24459.1 afla|EED55215.1 afum|EAL86129.1 agla|OJJ80306.1 akaw|GAA84775.1 aluc|OJZ86906.1 anid|CBF73830.1 anig|EHA21737.1 aory|EIT83574.1 asyd|OJJ56562.1 ater|EAU29983.1 atub|OJI83614.1 aver|OJJ06159.1 awen|OJJ32738.1 azon|OJJ45288.1

Group2707: abra|OJJ72150.1 acar|OOF96787.1 acla|EAW14945.1 afis|EAW24460.1 afla|EED55214.1 afum|EAL86130.1 agla|OJJ80308.1 akaw|GAA84774.1 aluc|OJZ86909.1 anid|CBF73832.1 anig|EHA21736.1 aory|EIT83521.1 asyd|OJJ56561.1 ater|EAU29982.1 atub|OJI83613.1 aver|OJJ06160.1 awen|OJJ32736.1 azon|OJJ45287.1

Group2708: abra|OJJ72209.1 acar|OOF96830.1 acla|EAW14967.1 afis|EAW24490.1 afla|EED54675.1 afum|EAL86159.1 agla|OJJ81567.1 akaw|GAA84725.1 aluc|OJZ86961.1 anid|CBF89709.1 anig|EHA21687.1

aory|EIT78852.1 asyd|OJJ62250.1 ater|EAU29966.1 atub|OJI83562.1 aver|OJI96360.1 awen|OJJ32706.1 azon|OJJ45248.1

Group2709: abra|OJJ72241.1 acar|OOF96866.1 acla|EAW07579.1 afis|EAW22615.1 afla|EED47379.1 afum|EAL90583.1 akaw|GAA84698.1 aluc|OJZ86989.1 anid|CBF84548.1 anig|EHA21657.1 aory|EIT82884.1 asyd|OJJ53472.1 ater|EAU39426.1 atub|OJI83530.1 aver|OJJ08284.1 awen|OJJ36695.1 azon|OJJ47502.1 aver|OJJ03406.1

Group2710: abra|OJJ72282.1 acar|OOF94939.1 afis|EAW20798.1 afla|EED49549.1 afum|EAL84831.1 akaw|GAA86034.1 aluc|OJZ90261.1 anig|EHA23757.1 asyd|OJJ55079.1 ater|EAU30148.1 atub|OJI81687.1 aver|OJJ07129.1 awen|OJJ41910.1 azon|OJJ47366.1 acar|OOF97630.1 afla|EED56695.1 aory|EIT73901.1 asyd|OJJ57120.1

Group2711: abra|OJJ72290.1 acar|OOF94933.1 acla|EAW12012.1 afis|EAW19652.1 afla|EED57003.1 afum|EAL86870.1 agla|OJJ80798.1 akaw|GAA86042.1 aluc|OJZ90271.1 anid|CBF71361.1 anig|EHA23763.1 aory|EIT77490.1 asyd|OJJ55084.1 ater|EAU32876.1 atub|OJI81695.1 aver|OJJ07123.1 awen|OJJ41903.1 azon|OJJ42697.1

Group2712: abra|OJJ72296.1 acar|OOF94928.1 acla|EAW12007.1 afis|EAW19647.1 afla|EED56998.1 afum|EAL86875.1 agla|OJJ80789.1 akaw|GAA86047.1 aluc|OJZ90276.1 anid|CBF71354.1 anig|EHA23768.1 aory|EIT77515.1 asyd|OJJ55089.1 ater|EAU32881.1 atub|OJI81700.1 aver|OJJ07118.1 awen|OJJ41898.1 azon|OJJ42702.1

Group2713: abra|OJJ72298.1 acar|OOF94926.1 acla|EAW12006.1 afis|EAW19646.1 afla|EED56997.1 afum|EAL86876.1 agla|OJJ80788.1 akaw|GAA86049.1 aluc|OJZ90278.1 anid|CBF71352.1 anig|EHA23769.1 aory|EIT77535.1 asyd|OJJ55091.1 ater|EAU32883.1 atub|OJI81703.1 aver|OJJ07117.1 awen|OJJ41897.1 azon|OJJ42703.1

Group2714: abra|OJJ72299.1 acar|OOF94924.1 acla|EAW12005.1 afis|EAW19645.1 afla|EED56996.1 afum|EAL86877.1 agla|OJJ80787.1 akaw|GAA86050.1 aluc|OJZ90280.1 anid|CBF71350.1 anig|EHA23770.1 aory|EIT77477.1 asyd|OJJ55092.1 ater|EAU32884.1 atub|OJI81704.1 aver|OJJ07116.1 awen|OJJ41896.1 azon|OJJ42704.1

Group2715: abra|OJJ72318.1 acar|OOF94893.1 acla|EAW12000.1 afis|EAW19640.1 afla|EED56991.1 afum|EAL86882.1 agla|OJJ80782.1 akaw|GAA86067.1 aluc|OJZ90301.1 anid|CBF71343.1 anig|EHA23789.1 aory|EIT77491.1 asyd|OJJ55098.1 ater|EAU32889.1 atub|OJI81727.1 aver|OJJ07108.1 awen|OJJ41885.1 azon|OJJ42710.1

Group2716: abra|OJJ72324.1 acar|OOF89799.1 acla|EAW11999.1 afis|EAW19639.1 afla|EED56990.1 afum|EAL86883.1 agla|OJJ80781.1 akaw|GAA86074.1 aluc|OJZ90309.1 anid|CBF71341.1 anig|EHA23796.1 aory|EIT77478.1 asyd|OJJ55099.1 ater|EAU32890.1 atub|OJI81734.1 aver|OJJ07107.1 awen|OJJ41884.1 azon|OJJ42711.1

Group2717: abra|OJJ72327.1 acar|OOF94887.1 acla|EAW11996.1 afis|EAW19636.1 afla|EED56988.1 afum|EAL86886.1 agla|OJJ80778.1 akaw|GAA86077.1 aluc|OJZ90312.1 anid|CBF71335.1 anig|EHA23799.1 aory|EIT77538.1 asyd|OJJ55103.1 ater|EAU32892.1 atub|OJI81737.1 aver|OJJ07104.1 awen|OJJ41881.1 azon|OJJ47079.1

Group2718: abra|OJJ72328.1 acar|OOF94886.1 acla|EAW11995.1 afis|EAW19635.1 afla|EED56986.1 afum|EAL86887.1 agla|OJJ80777.1 akaw|GAA86078.1 aluc|OJZ90313.1 anid|CBF71333.1 anig|EHA23800.1 aory|EIT77532.1 asyd|OJJ55104.1 atub|OJI81738.1 aver|OJJ07103.1 awen|OJJ41880.1 azon|OJJ47078.1 ater|EAU32893.1

Group2719: abra|OJJ72338.1 acar|OOF94878.1 acla|EAW11985.1 afis|EAW19625.1 afla|EED56977.1 afum|EBA27293.1 agla|OJJ80768.1 akaw|GAA86089.1 aluc|OJZ90318.1 anid|CBF71315.1 anig|EHA23804.1 aory|EIT77446.1 asyd|OJJ55113.1 ater|EAU32903.1 atub|OJI81747.1 aver|OJJ07093.1 awen|OJJ41872.1 azon|OJJ47067.1

Group2720: abra|OJJ72339.1 acar|OOF94877.1 acla|EAW11984.1 afis|EAW19624.1 afla|EED56976.1 afum|EAL86898.1 agla|OJJ80767.1 akaw|GAA86090.1 aluc|OJZ90319.1 anid|CBF71313.1 anig|EHA23805.1 aory|EIT77523.1 asyd|OJJ55114.1 ater|EAU32902.1 atub|OJI81748.1 aver|OJJ07092.1 awen|OJJ41871.1 azon|OJJ47066.1

Group2721: abra|OJJ72343.1 acar|OOF94874.1 acla|EAW11983.1 afis|EAW19621.1 afla|EED56973.1 afum|EAL86900.1 agla|OJJ80766.1 akaw|GAA86092.1 aluc|OJZ90322.1 anid|CBF71293.1 anig|EHA23808.1 aory|EIT77575.1 asyd|OJJ55117.1 ater|EAU32905.1 atub|OJI81749.1 aver|OJJ07089.1 awen|OJJ41868.1 azon|OJJ47061.1

Group2722: abra|OJJ72344.1 acar|OOF94873.1 acla|EAW11982.1 afis|EAW19620.1 afla|EED56972.1 afum|EAL86902.1 agla|OJJ80765.1 akaw|GAA86093.1 aluc|OJZ90323.1 anid|CBF71296.1 anig|EHA23809.1 aory|EIT77533.1 asyd|OJJ55118.1 ater|EAU32906.1 atub|OJI81750.1 aver|OJJ07088.1 awen|OJJ41867.1 azon|OJJ47060.1

Group2723: abra|OJJ72345.1 acar|OOF94872.1 acla|EAW11981.1 afis|EAW19619.1 afla|EED56967.1 afum|EAL86903.1 agla|OJJ80764.1 akaw|GAA86094.1 aluc|OJZ90324.1 anid|CBF71298.1 anig|EHA23810.1 aory|EIT77589.1 asyd|OJJ55119.1 ater|EAU32907.1 atub|OJI81751.1 aver|OJJ07087.1 awen|OJJ41866.1 azon|OJJ47059.1

Group2724: abra|OJJ72346.1 acar|OOF94871.1 acla|EAW11980.1 afis|EAW19618.1 afla|EED56968.1 afum|EAL86904.1 agla|OJJ80763.1 akaw|GAA86095.1 aluc|OJZ90325.1 anid|CBF71300.1 anig|EHA23811.1 aory|EIT77479.1 asyd|OJJ55120.1 ater|EAU32908.1 atub|OJI81752.1 aver|OJJ07086.1 awen|OJJ41865.1 azon|OJJ47058.1

Group2725: abra|OJJ72349.1 acar|OOF94869.1 acla|EAW11977.1 afis|EAW19609.1 afla|EED56969.1 afum|EAL86913.1 agla|OJJ80761.1 akaw|GAA86096.1 aluc|OJZ90328.1 anid|CBF71302.1 anig|EHA23812.1 aory|EIT77540.1 asyd|OJJ55121.1 ater|EAU32910.1 atub|OJI81755.1 aver|OJJ07083.1 awen|OJJ41862.1 azon|OJJ47055.1

Group2726: abra|OJJ72351.1 acar|OOF94867.1 acla|EAW11975.1 afis|EAW19611.1 afla|EED56966.1 afum|EAL86911.1 agla|OJJ80759.1 akaw|GAA86098.1 aluc|OJZ90330.1 anid|CBF71306.1 anig|EHA23814.1 aory|EIT77494.1 asyd|OJJ55123.1 ater|EAU32921.1 atub|OJI81757.1 aver|OJJ07080.1 awen|OJJ41859.1 azon|OJJ47053.1

Group2727: abra|OJJ72352.1 acar|OOF94866.1 acla|EAW11973.1 afis|EAW19613.1 afla|EED56961.1 afum|EAL86909.1 agla|OJJ80758.1 akaw|GAA86099.1 aluc|OJZ90331.1 anid|CBF71308.1 anig|EHA23815.1 aory|EIT77512.1 asyd|OJJ55126.1 ater|EAU32914.1 atub|OJI81758.1 aver|OJJ07078.1 awen|OJJ41857.1 azon|OJJ47051.1

Group2728: abra|OJJ72353.1 acar|OOF94865.1 acla|EAW11972.1 afis|EAW19614.1 afla|EED56962.1 afum|EAL86908.1 agla|OJJ80757.1 akaw|GAA86100.1 aluc|OJZ90332.1 anid|CBF71310.1 anig|EHA23816.1 aory|EIT77559.1 asyd|OJJ55127.1 ater|EAU32915.1 atub|OJI81759.1 aver|OJJ07077.1 awen|OJJ41856.1 azon|OJJ47050.1

Group2729: abra|OJJ72355.1 acar|OOF94863.1 acla|EAW11961.1 afis|EAW19598.1 afla|EED56954.1 afum|EAL86920.1 agla|OJJ80740.1 akaw|GAA86102.1 aluc|OJZ90334.1 anid|CBF71287.1 anig|EHA23818.1 aory|EIT77544.1 asyd|OJJ55129.1 ater|EAU32927.1 atub|OJI81761.1 aver|OJJ07074.1 awen|OJJ41844.1 azon|OJJ44888.1

Group2730: abra|OJJ72368.1 acar|OOF94849.1 acla|EAW11964.1 afis|EAW19601.1 afla|EED56957.1 afum|EAL86917.1 agla|OJJ80743.1 akaw|GAA86118.1 aluc|OJZ90356.1 anid|CBF71281.1 anig|EHA23834.1 aory|EIT77555.1 asyd|OJJ55133.1 ater|EAU32924.1 atub|OJI81783.1 aver|OJJ07071.1 awen|OJJ41839.1 azon|OJJ47044.1

Group2731: abra|OJJ72370.1 acar|OOF94852.1 afis|EAW25546.1 afla|EED51182.1 agla|OJJ80811.1 akaw|GAA86120.1 aluc|OJZ90359.1 anid|CBF80641.1 anig|EHA23832.1 aory|EIT81311.1 asyd|OJJ65320.1 ater|EAU36020.1 atub|OJI81786.1 aver|OJI99465.1 anid|CBF82274.1 asyd|OJJ59966.1 anid|CBF86654.1 aver|OJJ02324.1

Group2732: abra|OJJ72373.1 acar|OOF94855.1 acla|EAW11967.1 afis|EAW19617.1 afla|EED56965.1 afum|EAL86905.1 agla|OJJ80746.1 akaw|GAA86123.1 aluc|OJZ90362.1 anid|CBF71277.1 anig|EHA23829.1 aory|EIT77547.1 asyd|OJJ55139.1 ater|EAU32917.1 atub|OJI81789.1 aver|OJJ07066.1 awen|OJJ41850.1 azon|OJJ47046.1

Group2733: abra|OJJ72377.1 acar|OOF94841.1 acla|EAW11958.1 afis|EAW19595.1 afla|EED56951.1 afum|EAL86923.1 agla|OJJ80736.1 akaw|GAA86125.1 aluc|OJZ90364.1 anid|CBF71262.1 anig|EHA23839.1 aory|EIT77501.1 asyd|OJJ55150.1 ater|EAU32929.1 atub|OJI81791.1 aver|OJJ07057.1 awen|OJJ41849.1 azon|OJJ47020.1

Group2734: abra|OJJ72379.1 acar|OOF94840.1 acla|EAW11957.1 afis|EAW19594.1 afla|EED56950.1 afum|EAL86924.1 agla|OJJ80735.1 akaw|GAA86127.1 aluc|OJZ90366.1 anid|CBF71275.1 anig|EHA23841.1 aory|EIT77449.1 asyd|OJJ55149.1 ater|EAU32930.1 atub|OJI81793.1 aver|OJJ07058.1 awen|OJJ41811.1 azon|OJJ47021.1

Group2735: abra|OJJ72381.1 acar|OOF94838.1 acla|EAW11955.1 afis|EAW19592.1 afla|EED56948.1 afum|EAL86926.1 agla|OJJ80733.1 akaw|GAA86128.1 aluc|OJZ90368.1 anid|CBF71268.1 anig|EHA23842.1 aory|EIT77465.1 asyd|OJJ55147.1 ater|EAU32933.1 atub|OJI81794.1 aver|OJJ07060.1 awen|OJJ41809.1 azon|OJJ47023.1

Group2736: abra|OJJ72388.1 acar|OOF94835.1 acla|EAW11949.1 afis|EAW19586.1 afla|EED56914.1 afum|EAL86932.1 agla|OJJ80723.1 akaw|GAA86136.1 aluc|OJZ90374.1 anid|CBF71254.1 anig|EHA23848.1 aory|EIT77498.1 asyd|OJJ55153.1 ater|EAU32962.1 atub|OJI81800.1 aver|OJJ07054.1 awen|OJJ41800.1 azon|OJJ47031.1

Group2737: abra|OJJ72389.1 acar|OOF94834.1 acla|EAW11948.1 afis|EAW19585.1 afla|EED56913.1 afum|EAL86933.1 agla|OJJ80722.1 akaw|GAA86137.1 aluc|OJZ90376.1 anid|CBF71253.1 anig|EHA23849.1

aory|EIT77525.1 asyd|OJJ55155.1 ater|EAU32963.1 atub|OJI81801.1 aver|OJJ07053.1 awen|OJJ41799.1 azon|OJJ47032.1

Group2738: abra|OJJ72391.1 acar|OOF94833.1 acla|EAW11947.1 afis|EAW19584.1 afla|EED56912.1 afum|EAL86934.1 agla|OJJ80712.1 akaw|GAA86138.1 aluc|OJZ90378.1 anid|CBF71251.1 anig|EHA23850.1 aory|EIT77463.1 asyd|OJJ55156.1 ater|EAU32967.1 atub|OJI81803.1 aver|OJJ07052.1 awen|OJJ41798.1 azon|OJJ47033.1

Group2739: abra|OJJ72394.1 acar|OOF94832.1 acla|EAW11946.1 afis|EAW19583.1 afla|EED56911.1 afum|EAL86935.2 agla|OJJ80711.1 akaw|GAA86139.1 aluc|OJZ90379.1 anid|CBF71249.1 anig|EHA23851.1 aory|EIT77567.1 asyd|OJJ55157.1 ater|EAU32968.1 atub|OJI81804.1 aver|OJJ07051.1 awen|OJJ41797.1 azon|OJJ47034.1

Group2740: abra|OJJ72395.1 acar|OOF94831.1 acla|EAW11945.1 afis|EAW19581.1 afla|EED56910.1 afum|EAL86937.1 agla|OJJ80710.1 akaw|GAA86140.1 aluc|OJZ90380.1 anid|CBF71245.1 anig|EHA23852.1 aory|EIT77560.1 asyd|OJJ55159.1 ater|EAU32972.1 atub|OJI81806.1 aver|OJJ07049.1 awen|OJJ41795.1 azon|OJJ47036.1

Group2741: abra|OJJ72396.1 acar|OOF94830.1 acla|EAW11944.1 afis|EAW19580.1 afla|EED56909.1 afum|EAL86938.1 agla|OJJ80709.1 akaw|GAA86141.1 aluc|OJZ90381.1 anid|CBF71243.1 anig|EHA23853.1 aory|EIT77483.1 asyd|OJJ55160.1 ater|EAU32973.1 atub|OJI81807.1 aver|OJJ07047.1 awen|OJJ41794.1 azon|OJJ47037.1

Group2742: abra|OJJ72397.1 acar|OOF94829.1 acla|EAW11943.1 afis|EAW19579.1 afla|EED56804.1 afum|EAL86939.1 agla|OJJ80708.1 akaw|GAA86142.1 aluc|OJZ90382.1 anid|CBF71239.1 anig|EHA23854.1 aory|EIT73874.1 asyd|OJJ55163.1 ater|EAU32976.1 atub|OJI81808.1 aver|OJJ07044.1 awen|OJJ41792.1 azon|OJJ47041.1

Group2743: abra|OJJ72399.1 acar|OOF94828.1 acla|EAW11941.1 afis|EAW19577.1 afla|EED56805.1 afum|EAL86941.2 agla|OJJ80706.1 akaw|GAA86144.1 aluc|OJZ90384.1 anid|CBF71236.1 anig|EHA23856.1 aory|EIT73858.1 asyd|OJJ55165.1 ater|EAU33003.1 atub|OJI81811.1 aver|OJJ07042.1 awen|OJJ41790.1 azon|OJJ47039.1

Group2744: abra|OJJ72401.1 acar|OOF94827.1 acla|EAW11940.1 afis|EAW19575.1 afla|EED56808.1 afum|EAL86942.1 agla|OJJ80704.1 akaw|GAA86145.1 aluc|OJZ90386.1 anid|CBF71234.1 anig|EHA23857.1 aory|EIT73879.1 asyd|OJJ55166.1 ater|EAU33002.1 atub|OJI81814.1 aver|OJJ07040.1 awen|OJJ41788.1 azon|OJJ44890.1

Group2745: abra|OJJ72402.1 acar|OOF94826.1 acla|EAW11939.1 afis|EAW19574.1 afla|EED56809.1 afum|EAL86943.1 agla|OJJ80702.1 akaw|GAA86146.1 aluc|OJZ90387.1 anid|CBF71232.1 anig|EHA23858.1 aory|EIT73889.1 asyd|OJJ55167.1 ater|EAU33001.1 atub|OJI81815.1 aver|OJJ07039.1 awen|OJJ41787.1 azon|OJJ44891.1

Group2746: abra|OJJ72404.1 acar|OOF94824.1 acla|EAW11937.1 afis|EAW19572.1 afla|EED56811.1 afum|EAL86945.1 agla|OJJ80700.1 akaw|GAA86148.1 aluc|OJZ90389.1 anid|CBF71228.1 anig|EHA23859.1 aory|EIT73949.1 asyd|OJJ55170.1 ater|EAU32995.1 atub|OJI81818.1 aver|OJJ07037.1 awen|OJJ41785.1 azon|OJJ44893.1

Group2747: abra|OJJ72405.1 acar|OOF94823.1 acla|EAW11936.1 afis|EAW19571.1 afla|EED56812.1 afum|EAL86946.1 agla|OJJ80699.1 akaw|GAA86149.1 aluc|OJZ90390.1 anid|CBF71226.1 anig|EHA23860.1 aory|EIT73876.1 asyd|OJJ55171.1 ater|EAU32994.1 atub|OJI81819.1 aver|OJJ07036.1 awen|OJJ41784.1 azon|OJJ44894.1

Group2748: abra|OJJ72408.1 acar|OOF94821.1 acla|EAW11933.1 afis|EAW19569.1 afla|EED56814.1 afum|EAL86947.1 agla|OJJ80697.1 akaw|GAA86151.1 aluc|OJZ90392.1 anid|CBF78033.1 anig|EHA23862.1 aory|EIT73916.1 asyd|OJJ55227.1 ater|EAU32992.1 atub|OJI81821.1 aver|OJJ06994.1 awen|OJJ41782.1 azon|OJJ44896.1

Group2749: abra|OJJ72412.1 acar|OOF94817.1 acla|EAW11929.1 afis|EAW19563.1 afla|EED56817.1 afum|EAL86949.1 agla|OJJ80692.1 akaw|GAA86155.1 aluc|OJZ90396.1 anid|CBF78024.1 anig|EHA23866.1 aory|EIT73850.1 asyd|OJJ55233.1 ater|EAU32986.1 atub|OJI81827.1 aver|OJJ06990.1 awen|OJJ41778.1 azon|OJJ44899.1

Group2750: abra|OJJ72413.1 acar|OOF94816.1 acla|EAW12857.1 afis|EAW17821.1 afla|EED56818.1 afum|EAL91631.1 agla|OJJ80690.1 akaw|GAA86156.1 aluc|OJZ90397.1 anid|CBF78026.1 anig|EHA23867.1 aory|EIT73888.1 asyd|OJJ55232.1 ater|EAU32985.1 atub|OJI81828.1 aver|OJJ06991.1 awen|OJJ41777.1 azon|OJJ44900.1

Group2751: abra|OJJ72414.1 acar|OOF94815.1 acla|EAW12858.1 afis|EAW17820.1 afla|EED56819.1 afum|EAL91630.1 agla|OJJ80689.1 akaw|GAA86157.1 aluc|OJZ90398.1 anid|CBF78027.1 anig|EHA23868.1 aory|EIT73947.1 asyd|OJJ55231.1 ater|EAU32984.1 atub|OJI81829.1 aver|OJJ06992.1 awen|OJJ41776.1 azon|OJJ44901.1

Group2752: abra|OJJ72415.1 acar|OOF94814.1 acla|EAW12859.1 afis|EAW17808.1 afla|EED56820.1 afum|EAL91616.1 agla|OJJ80688.1 akaw|GAA86158.1 aluc|OJZ90399.1 anid|CBF78029.1 anig|EHA23869.1 aory|EIT73877.1 asyd|OJJ55230.1 ater|EAU32982.1 atub|OJI81830.1 aver|OJJ06993.1 awen|OJJ41775.1 azon|OJJ44902.1

Group2753: abra|OJJ72417.1 acar|OOF90199.1 acla|EAW13737.1 afis|EAW17067.1 afla|EED52009.1 afum|EAL87736.1 agla|OJJ83275.1 akaw|GAA86160.1 aluc|OJZ90401.1 aory|EIT81373.1 awen|OJJ36556.1 azon|OJJ46436.1 abra|OJJ73401.1 acar|OOF98722.1 anid|CBF84645.1 anig|EHA18326.1 ater|EAU36994.1 aver|OJJ04312.1

Group2754: abra|OJJ72421.1 acar|OOF90370.1 acla|EAW12862.1 afis|EAW17811.1 afla|EED46162.1 afum|EAL91619.1 agla|OJJ80655.1 akaw|GAA86163.1 aluc|OJZ90404.1 anid|CBF78013.1 anig|EHA23872.1 aory|EIT76383.1 asyd|OJJ55240.1 ater|EAU33017.1 atub|OJI81835.1 aver|OJJ06983.1 awen|OJJ41761.1 azon|OJJ44878.1

Group2755: abra|OJJ72422.1 acar|OOF90371.1 acla|EAW12861.1 afis|EAW17810.1 afla|EED46161.1 afum|EAL91618.1 agla|OJJ80654.1 akaw|GAA86164.1 aluc|OJZ90405.1 anid|CBF78014.1 anig|EHA23873.1 aory|EIT76437.1 asyd|OJJ55239.1 ater|EAU33016.1 atub|OJI81836.1 aver|OJJ06984.1 awen|OJJ41762.1 azon|OJJ44879.1

Group2756: abra|OJJ72424.1 acar|OOF90373.1 afis|EAW17822.1 afla|EED46167.1 agla|OJJ80652.1 akaw|GAA86166.1 aluc|OJZ90407.1 anid|CBF78011.1 anig|EHA23875.1 aory|EIT76519.1 asyd|OJJ63016.1 ater|EAU33023.1 atub|OJI81838.1 aver|OJI97948.1 awen|OJJ41760.1 azon|OJJ48452.1 acar|OOF98065.1 anid|CBF85197.1

Group2757: abra|OJJ72425.1 acar|OOF94810.1 acla|EAW12659.1 afis|EAW17823.1 afla|EED46166.1 afum|EAL91633.1 agla|OJJ80641.1 akaw|GAA86167.1 aluc|OJZ90408.1 anid|CBF78009.1 anig|EHA23876.1 aory|EIT76477.1 asyd|OJJ55242.1 ater|EAU33024.1 atub|OJI81840.1 aver|OJJ06982.1 awen|OJJ41751.1 azon|OJJ48443.1

Group2758: abra|OJJ72428.1 acar|OOF94804.1 acla|EAW12661.1 afis|EAW17825.1 afla|EED46163.1 afum|EAL91635.1 agla|OJJ80646.1 akaw|GAA86169.1 aluc|OJZ90412.1 anid|CBF78005.1 anig|EHA23878.1 aory|EIT76524.1 asyd|OJJ55245.1 ater|EAU33026.1 atub|OJI81843.1 aver|OJJ06979.1 awen|OJJ41755.1 azon|OJJ48445.1

Group2759: abra|OJJ72429.1 acar|OOF94805.1 acla|EAW12662.1 afis|EAW17826.1 afla|EED46168.1 afum|EAL91636.1 agla|OJJ80647.1 akaw|GAA86170.1 aluc|OJZ90413.1 anid|CBF78003.1 anig|EHA23879.1 aory|EIT76408.1 asyd|OJJ55246.1 ater|EAU33027.1 atub|OJI81845.1 aver|OJJ06978.1 awen|OJJ41756.1 azon|OJJ48446.1

Group2760: abra|OJJ72430.1 acar|OOF94803.1 acla|EAW12663.1 afis|EAW17827.1 afla|EED46169.1 afum|EAL91637.1 agla|OJJ80648.1 akaw|GAA86171.1 aluc|OJZ90414.1 anid|CBF78001.1 anig|EHA23880.1 aory|EIT76516.1 asyd|OJJ55247.1 ater|EAU33031.1 atub|OJI81846.1 aver|OJJ06977.1 awen|OJJ41757.1 azon|OJJ48447.1

Group2761: abra|OJJ72439.1 acar|OOF94798.1 acla|EAW12672.1 afis|EAW17835.1 afla|EED46180.1 afum|EAL91645.1 agla|OJJ83131.1 akaw|GAA86178.1 aluc|OJZ90423.1 anid|CBF77987.1 anig|EHA23892.1 aory|EIT76377.1 asyd|OJJ55256.1 ater|EAU33039.1 atub|OJI81855.1 aver|OJJ06968.1 awen|OJJ41741.1 azon|OJJ48437.1

Group2762: abra|OJJ72440.1 acar|OOF94797.1 acla|EAW12673.1 afis|EAW17836.1 afla|EED46181.1 afum|EAL91646.1 agla|OJJ83130.1 akaw|GAA86179.1 aluc|OJZ90424.1 anid|CBF77985.1 anig|EHA23893.1 aory|EIT76515.1 asyd|OJJ55257.1 ater|EAU33040.1 atub|OJI81856.1 aver|OJJ06967.1 awen|OJJ41740.1 azon|OJJ48436.1

Group2763: abra|OJJ72443.1 acar|OOF94793.1 acla|EAW12676.1 afis|EAW17839.1 afla|EED46184.1 afum|EAL91649.1 agla|OJJ83128.1 akaw|GAA86182.1 aluc|OJZ90427.1 anid|CBF77979.1 anig|EHA23897.1 aory|EIT76493.1 asyd|OJJ55260.1 ater|EAU33042.1 atub|OJI81859.1 aver|OJJ06965.1 awen|OJJ41737.1 azon|OJJ48434.1

Group2764: abra|OJJ72445.1 acar|OOF94791.1 acla|EAW12678.1 afis|EAW17841.1 afla|EED46186.1 afum|EAL91650.1 agla|OJJ83126.1 akaw|GAA86183.1 aluc|OJZ90429.1 anid|CBF77975.1 anig|EHA23898.1 aory|EIT76513.1 asyd|OJJ55262.1 ater|EAU33043.1 atub|OJI81861.1 aver|OJJ06963.1 awen|OJJ41735.1 azon|OJJ48432.1

Group2765: abra|OJJ72446.1 acar|OOF94790.1 acla|EAW12679.1 afis|EAW17842.1 afla|EED46187.1 afum|EAL91651.1 agla|OJJ83125.1 akaw|GAA86184.1 aluc|OJZ90430.1 anid|CBF77973.1 anig|EHA23899.1 aory|EIT76407.1 asyd|OJJ55263.1 ater|EAU33044.1 atub|OJI81862.1 aver|OJJ06962.1 awen|OJJ41734.1 azon|OJJ48431.1

Group2766: abra|OJJ72450.1 acar|OOF94786.1 acla|EAW12684.1 afis|EAW17847.1 afla|EED46201.1 afum|EAL91656.1 agla|OJJ83122.1 akaw|GAA86187.1 aluc|OJZ90435.1 anid|CBF77964.1 anig|EHA23902.1

aory|EIT76452.1 asyd|OJJ55269.1 ater|EAU33049.1 atub|OJI81866.1 aver|OJJ06958.1 awen|OJJ41726.1 azon|OJJ48425.1

Group2767: abra|OJJ72458.1 acar|OOF94779.1 acla|EAW12690.1 afis|EAW17854.1 afla|EED46208.1 afum|EAL91662.1 agla|OJJ83116.1 akaw|GAA86194.1 aluc|OJZ90445.1 anid|CBF77955.1 anig|EHA23908.1 aory|EIT76378.1 asyd|OJJ55275.1 ater|EAU33056.1 atub|OJI81873.1 aver|OJJ06952.1 awen|OJJ41719.1 azon|OJJ48416.1

Group2768: abra|OJJ72459.1 acar|OOF94780.1 acla|EAW12691.1 afis|EAW17855.1 afla|EED46209.1 afum|EAL91663.1 agla|OJJ83115.1 akaw|GAA86195.1 aluc|OJZ90446.1 anid|CBF77953.1 anig|EHA23909.1 aory|EIT76395.1 asyd|OJJ55276.1 ater|EAU33057.1 atub|OJI81874.1 aver|OJJ06951.1 awen|OJJ41718.1 azon|OJJ48415.1

Group2769: abra|OJJ72460.1 acar|OOF94778.1 acla|EAW12692.1 afis|EAW17856.1 afla|EED46210.1 afum|EAL91664.1 agla|OJJ83113.1 akaw|GAA86196.1 aluc|OJZ90447.1 anid|CBF77951.1 anig|EHA23910.1 aory|EIT76442.1 asyd|OJJ55277.1 ater|EAU33058.1 atub|OJI81875.1 aver|OJJ06950.1 awen|OJJ41716.1 azon|OJJ48411.1

Group2770: abra|OJJ72461.1 acar|OOF94777.1 acla|EAW12693.1 afis|EAW17857.1 afla|EED46212.1 afum|EAL91665.1 agla|OJJ83112.1 akaw|GAA86197.1 aluc|OJZ90448.1 anid|CBF77949.1 anig|EHA23911.1 aory|EIT76364.1 asyd|OJJ55278.1 ater|EAU33059.1 atub|OJI81876.1 aver|OJJ06949.1 awen|OJJ41715.1 azon|OJJ48410.1

Group2771: abra|OJJ72462.1 acar|OOF94775.1 acla|EAW12694.1 afis|EAW17858.1 afla|EED46213.1 afum|EAL91666.1 agla|OJJ83111.1 akaw|GAA86198.1 aluc|OJZ90449.1 anid|CBF77947.1 anig|EHA23912.1 aory|EIT76487.1 asyd|OJJ55279.1 ater|EAU33064.1 atub|OJI81877.1 aver|OJJ06948.1 awen|OJJ41714.1 azon|OJJ48409.1

Group2772: abra|OJJ72467.1 acar|OOF94773.1 acla|EAW12699.1 afis|EAW17862.1 afla|EED46225.1 afum|EAL91670.2 agla|OJJ83101.1 akaw|GAA86203.1 aluc|OJZ90454.1 anid|CBF88078.1 anig|EHA23916.1 aory|EIT76473.1 asyd|OJJ57087.1 ater|EAU33070.1 atub|OJI81881.1 aver|OJJ03214.1 awen|OJJ41709.1 azon|OJJ48404.1

Group2773: abra|OJJ72468.1 acar|OOF94772.1 acla|EAW12700.1 afis|EAW17863.1 afla|EED46226.1 afum|EAL91671.1 agla|OJJ83100.1 akaw|GAA86204.1 aluc|OJZ90455.1 anid|CBF84085.1 anig|EHA23917.1 aory|EIT76453.1 asyd|OJJ57088.1 ater|EAU33078.1 atub|OJI81882.1 aver|OJJ03213.1 awen|OJJ41707.1 azon|OJJ48403.1

Group2774: abra|OJJ72471.1 acar|OOF94764.1 acla|EAW12704.1 afis|EAW17867.1 afla|EED46236.1 afum|EAL91675.1 agla|OJJ83087.1 akaw|GAA86209.1 aluc|OJZ90458.1 anid|CBF89893.1 anig|EHA23920.1 aory|EIT76466.1 asyd|OJJ62139.1 ater|EAU33082.1 atub|OJI81885.1 aver|OJI96249.1 awen|OJJ41704.1 azon|OJJ48400.1

Group2775: abra|OJJ72476.1 acar|OOF94760.1 acla|EAW12707.1 afis|EAW17870.1 afla|EED46238.1 afum|EAL91678.1 agla|OJJ83090.1 akaw|GAA86212.1 aluc|OJZ90462.1 anid|CBF89899.1 anig|EHA23923.1 aory|EIT76429.1 asyd|OJJ62136.1 ater|EAU33088.1 atub|OJI81888.1 aver|OJI96246.1 awen|OJJ41700.1 azon|OJJ48397.1

Group2776: abra|OJJ72478.1 acar|OOF94756.1 acla|EAW12709.1 afis|EAW17872.1 afla|EED46239.1 afum|EAL91680.1 agla|OJJ83092.1 akaw|GAA86214.1 aluc|OJZ90465.1 anid|CBF89903.1 anig|EHA23925.1 aory|EIT76379.1 asyd|OJJ62134.1 ater|EAU33090.1 atub|OJI81890.1 aver|OJI96244.1 awen|OJJ41698.1 azon|OJJ48395.1

Group2777: abra|OJJ72482.1 acar|OOF94754.1 acla|EAW12712.1 afis|EAW17875.1 afla|EED46242.1 afum|EAL91683.1 agla|OJJ83071.1 akaw|GAA86217.1 aluc|OJZ90469.1 anid|CBF89904.1 anig|EHA23928.1 aory|EIT76495.1 asyd|OJJ59281.1 ater|EAU33095.1 atub|OJI81893.1 aver|OJJ01894.1 awen|OJJ41689.1 aver|OJI96243.1

Group2778: abra|OJJ72488.1 acar|OOF94747.1 acla|EAW12719.1 afis|EAW17882.1 afla|EED46269.1 afum|EAL91690.1 agla|OJJ83065.1 akaw|GAA86223.1 aluc|OJZ90475.1 anig|EHA23934.1 aory|EIT76427.1 asyd|OJJ57008.1 ater|EAU33102.1 atub|OJI81900.1 aver|OJJ03279.1 awen|OJJ41684.1 azon|OJJ48392.1 anid|CBF77035.1

Group2779: abra|OJJ72489.1 acar|OOF92642.1 acla|EAW12720.1 afis|EAW17883.1 afla|EED46270.1 afum|EAL91691.1 agla|OJJ83064.1 akaw|GAA86224.1 aluc|OJZ90476.1 anid|CBF77033.1 anig|EHA23935.1 aory|EIT76482.1 asyd|OJJ57009.1 ater|EAU33103.1 atub|OJI81901.1 aver|OJJ03278.1 awen|OJJ41682.1 azon|OJJ48391.1

Group2780: abra|OJJ72493.1 acar|OOF94745.1 acla|EAW12722.1 afis|EAW17885.1 afla|EED46272.1 afum|EAL91693.1 agla|OJJ83062.1 akaw|GAA86227.1 aluc|OJZ90479.1 anid|CBF77029.1 anig|EHA23938.1 aory|EIT76321.1 asyd|OJJ57011.1 ater|EAU33105.1 atub|OJI81904.1 aver|OJJ03276.1 awen|OJJ41674.1 azon|OJJ48389.1

Group2781: abra|OJJ72494.1 acar|OOF94744.1 acla|EAW12723.1 afis|EAW17886.1 afla|EED46273.1 afum|EAL91694.1 agla|OJJ83061.1 akaw|GAA86228.1 aluc|OJZ90480.1 anid|CBF77027.1 anig|EHA23939.1 aory|EIT76334.1 asyd|OJJ57012.1 ater|EAU33106.1 atub|OJI81905.1 aver|OJJ03275.1 awen|OJJ41673.1 azon|OJJ48388.1

Group2782: abra|OJJ72497.1 acar|OOF94742.1 acla|EAW12725.1 afis|EAW17888.1 afla|EED46275.1 afum|EAL91697.1 agla|OJJ83059.1 akaw|GAA86229.1 aluc|OJZ90481.1 anid|CBF77026.1 anig|EHA23940.1 aory|EIT76333.1 asyd|OJJ57013.1 ater|EAU33107.1 atub|OJI81906.1 aver|OJJ03274.1 awen|OJJ41671.1 azon|OJJ48386.1

Group2783: abra|OJJ72503.1 acar|OOF94738.1 acla|EAW12727.1 afis|EAW17890.1 afla|EED46282.1 afum|EAL91699.1 agla|OJJ83055.1 akaw|GAA86231.1 aluc|OJZ90485.1 anid|CBF77016.1 anig|EHA23943.1 aory|EIT76352.1 asyd|OJJ57017.1 ater|EAU33110.1 atub|OJI81910.1 aver|OJJ03272.1 awen|OJJ41667.1 azon|OJJ48384.1

Group2784: abra|OJJ72507.1 acar|OOF94735.1 acla|EAW12730.1 afis|EAW17893.1 afla|EED46286.1 afum|EAL91702.1 agla|OJJ83049.1 akaw|GAA86235.1 aluc|OJZ90490.1 anid|CBF77006.1 anig|EHA23948.1 aory|EIT76341.1 asyd|OJJ57021.1 ater|EAU33114.1 atub|OJI81914.1 aver|OJJ03268.1 awen|OJJ41659.1 azon|OJJ48379.1

Group2785: abra|OJJ72509.1 acar|OOF94733.1 acla|EAW12732.1 afis|EAW17895.1 afla|EED46267.1 afum|EAL91703.1 agla|OJJ83047.1 akaw|GAA86237.1 aluc|OJZ90492.1 anid|CBF77004.1 anig|EHA23950.1 aory|EIT76414.1 asyd|OJJ57023.1 ater|EAU33115.1 atub|OJI81916.1 aver|OJJ03266.1 awen|OJJ41657.1 azon|OJJ48377.1

Group2786: abra|OJJ72511.1 acar|OOF94732.1 acla|EAW12733.1 afis|EAW17896.1 afla|EED46266.1 afum|EAL91704.1 agla|OJJ83046.1 akaw|GAA86238.1 aluc|OJZ90493.1 anid|CBF77002.1 anig|EHA23951.1 aory|EIT76411.1 asyd|OJJ57025.1 ater|EAU33116.1 atub|OJI81917.1 aver|OJJ03265.1 awen|OJJ41655.1 azon|OJJ48375.1

Group2787: abra|OJJ72513.1 acar|OOF94730.1 acla|EAW12734.1 afis|EAW17897.1 afla|EED46265.1 afum|EAL91705.1 agla|OJJ83045.1 akaw|GAA86240.1 aluc|OJZ90495.1 anid|CBF76998.1 anig|EHA23954.1 aory|EIT76381.1 asyd|OJJ57027.1 ater|EAU33117.1 atub|OJI81920.1 aver|OJJ03263.1 awen|OJJ41654.1 azon|OJJ48374.1

Group2788: abra|OJJ72514.1 acar|OOF94729.1 acla|EAW12735.1 afis|EAW17898.1 afla|EED46264.1 afum|EAL91706.1 agla|OJJ83044.1 akaw|GAA86241.1 aluc|OJZ90496.1 anid|CBF76996.1 anig|EHA23955.1 aory|EIT76461.1 asyd|OJJ57028.1 ater|EAU33118.1 atub|OJI81921.1 aver|OJJ03262.1 awen|OJJ41653.1 azon|OJJ48373.1

Group2789: abra|OJJ72515.1 acar|OOF94728.1 afis|EAW17899.1 afla|EED46263.1 agla|OJJ83043.1 akaw|GAA86242.1 aluc|OJZ90497.1 anid|CBF76994.1 anig|EHA23956.1 aory|EIT76422.1 asyd|OJJ57029.1 atub|OJI81922.1 aver|OJJ03261.1 awen|OJJ41652.1 azon|OJJ48372.1 acla|EAW12736.1 afum|EAL91707.1 ater|EAU33119.1

Group2790: abra|OJJ72516.1 acar|OOF94727.1 acla|EAW12737.1 afis|EAW17900.1 afla|EED46262.1 afum|EAL91708.1 agla|OJJ83042.1 akaw|GAA86243.1 aluc|OJZ90498.1 anid|CBF76992.1 anig|EHA23957.1 aory|EIT76428.1 asyd|OJJ57030.1 ater|EAU33120.1 atub|OJI81923.1 aver|OJJ03260.1 awen|OJJ41651.1 azon|OJJ48371.1

Group2791: abra|OJJ72518.1 acar|OOF94726.1 acla|EAW12738.1 afis|EAW17901.1 afla|EED46261.1 afum|EAL91709.1 agla|OJJ83041.1 akaw|GAA86244.1 aluc|OJZ90499.1 anid|CBF76990.1 anig|EHA23958.1 aory|EIT76470.1 asyd|OJJ57032.1 ater|EAU33121.1 atub|OJI81924.1 aver|OJJ03259.1 awen|OJJ41648.1 azon|OJJ48370.1

Group2792: abra|OJJ72538.1 acar|OOF89947.1 acla|EAW12744.1 afis|EAW17907.1 afla|EED46301.1 afum|EAL91716.2 agla|OJJ83035.1 akaw|GAA86262.1 aluc|OJZ90520.1 anid|CBF76981.1 anig|EHA23974.1 aory|EIT76350.1 asyd|OJJ57037.1 ater|EAU33125.1 atub|OJI81944.1 aver|OJJ03255.1 awen|OJJ41643.1 azon|OJJ48364.1

Group2793: abra|OJJ72539.1 acar|OOF89942.1 acla|EAW12745.1 afis|EAW17908.1 afla|EED46302.1 afum|EAL91717.2 agla|OJJ83034.1 akaw|GAA86263.1 aluc|OJZ90522.1 anid|CBF76979.1 anig|EHA23975.1 aory|EIT76327.1 asyd|OJJ57038.1 ater|EAU33126.1 atub|OJI81945.1 aver|OJJ03253.1 awen|OJJ41641.1 azon|OJJ48363.1

Group2794: abra|OJJ72543.1 acar|OOF94712.1 acla|EAW12747.1 afis|EAW17911.1 afla|EED46313.1 afum|EAL91720.1 agla|OJJ83032.1 akaw|GAA86267.1 aluc|OJZ90527.1 anid|CBF76967.1 anig|EHA23979.1 aory|EIT76323.1 asyd|OJJ57041.1 ater|EAU33131.1 atub|OJI81949.1 aver|OJJ03252.1 awen|OJJ41638.1 azon|OJJ48360.1

Group2795: abra|OJJ72544.1 acar|OOF94711.1 acla|EAW12748.1 afis|EAW17912.1 afla|EED46314.1 afum|EAL91721.1 agla|OJJ83031.1 akaw|GAA86268.1 aluc|OJZ90528.1 anid|CBF76969.1 anig|EHA23980.1

aory|EIT76335.1 asyd|OJJ57042.1 ater|EAU33132.1 atub|OJI81950.1 aver|OJJ03251.1 awen|OJJ41637.1 azon|OJJ48359.1

Group2796: abra|OJJ72545.1 acar|OOF94710.1 acla|EAW12749.1 afis|EAW17913.1 afla|EED46315.1 afum|EAL91722.1 agla|OJJ83030.1 akaw|GAA86269.1 aluc|OJZ90529.1 anid|CBF76971.1 anig|EHA23981.1 aory|EIT76319.1 asyd|OJJ57043.1 ater|EAU33133.1 atub|OJI81951.1 aver|OJJ03250.1 awen|OJJ41636.1 azon|OJJ48356.1

Group2797: abra|OJJ72551.1 acar|OOF94702.1 acla|EAW12756.1 afis|EAW17919.1 afla|EED46322.1 afum|EAL91730.1 agla|OJJ83023.1 akaw|GAA85183.1 aluc|OJZ90538.1 anid|CBF76955.1 anig|EHA23986.1 aory|EIT81188.1 asyd|OJJ57054.1 ater|EAU33554.1 atub|OJI81959.1 aver|OJJ03243.1 awen|OJJ41627.1 azon|OJJ48350.1

Group2798: abra|OJJ72554.1 acar|OOF94700.1 acla|EAW12759.1 afis|EAW17921.1 afla|EED46324.1 afum|EAL91732.1 agla|OJJ83020.1 akaw|GAA85186.1 aluc|OJZ90541.1 anid|CBF76951.1 anig|EHA23989.1 aory|EIT81201.1 asyd|OJJ57057.1 ater|EAU33550.1 atub|OJI81963.1 aver|OJJ03241.1 awen|OJJ41624.1 azon|OJJ48347.1

Group2799: abra|OJJ72555.1 acar|OOF94698.1 acla|EAW12760.1 afis|EAW17922.1 afla|EED46325.1 afum|EAL91733.1 agla|OJJ83019.1 akaw|GAA85187.1 aluc|OJZ90542.1 anid|CBF76949.1 anig|EHA23990.1 aory|EIT81261.1 asyd|OJJ57058.1 ater|EAU33549.1 atub|OJI81964.1 aver|OJJ03240.1 awen|OJJ41623.1 azon|OJJ48346.1

Group2800: abra|OJJ72562.1 acar|OOF94694.1 acla|EAW11409.1 afis|EAW24012.1 afla|EED49148.1 afum|EAL87442.1 agla|OJJ86944.1 akaw|GAA85191.1 aluc|OJZ90546.1 anid|CBF77408.1 aory|EIT78554.1 asyd|OJJ53238.1 ater|EAU29735.1 atub|OJI81968.1 aver|OJJ03612.1 awen|OJJ35484.1 azon|OJJ51264.1 awen|OJJ37387.1

Group2801: abra|OJJ72564.1 acar|OOF94690.1 acla|EAW12764.1 afis|EAW17926.1 afla|EED46328.1 afum|EAL91737.1 agla|OJJ83013.1 akaw|GAA92409.1 aluc|OJZ83268.1 anid|CBF76942.1 anig|EHA21121.1 aory|EIT81217.1 asyd|OJJ57062.1 ater|EAU33545.1 atub|OJI87669.1 aver|OJJ03236.1 awen|OJJ41620.1 azon|OJJ48343.1

Group2802: abra|OJJ72565.1 acar|OOF94689.1 acla|EAW12765.1 afis|EAW17927.1 afla|EED58037.1 afum|EAL91738.1 agla|OJJ83007.1 akaw|GAA92406.1 aluc|OJZ83267.1 anid|CBF76940.1 anig|EHA21120.1 aory|EIT79801.1 asyd|OJJ57063.1 ater|EAU33544.1 atub|OJI87670.1 aver|OJJ03235.1 awen|OJJ41619.1 azon|OJJ48342.1

Group2803: abra|OJJ72567.1 acar|OOF94687.1 acla|EAW12767.1 afis|EAW17930.1 afla|EED49111.1 afum|EAL91741.1 agla|OJJ83003.1 akaw|GAA85194.1 aluc|OJZ90549.1 anid|CBF76936.1 anig|EHA23997.1 aory|EIT78530.1 asyd|OJJ57064.1 ater|EAU33540.1 atub|OJI81971.1 aver|OJJ03234.1 awen|OJJ41616.1 azon|OJJ48337.1

Group2804: abra|OJJ72568.1 acar|OOF97957.1 acla|EAW14929.1 afla|EED48920.1 afum|EAL85693.1 akaw|GAA85196.1 aluc|OJZ90551.1 anig|EHA23998.1 aory|EIT81168.1 ater|EAU36518.1 atub|OJI81973.1 aver|OJJ07138.1 afis|EAW22084.1 acla|EAW14284.1 afla|EED49819.1 aory|EIT77604.1 afla|EED55283.1 aory|EIT83580.1

Group2805: abra|OJJ72581.1 acar|OOF94671.1 acla|EAW12768.1 afis|EAW17931.1 afla|EED49110.1 afum|EAL91742.1 agla|OJJ83002.1 akaw|GAA85207.1 aluc|OJZ90562.1 anid|CBF76934.1 anig|EHA24008.1 aory|EIT78507.1 asyd|OJJ57065.1 ater|EAU33539.1 atub|OJI81985.1 aver|OJJ03233.1 awen|OJJ41615.1 azon|OJJ48336.1

Group2806: abra|OJJ72582.1 acar|OOF94670.1 acla|EAW12769.1 afis|EAW17932.1 afla|EED49109.1 afum|EAL91743.1 agla|OJJ83001.1 akaw|GAA85208.1 aluc|OJZ90563.1 anid|CBF76932.1 anig|EHA24009.1 aory|EIT78546.1 asyd|OJJ57066.1 ater|EAU33538.1 atub|OJI81986.1 aver|OJJ03232.1 awen|OJJ41614.1 azon|OJJ48335.1

Group2807: abra|OJJ72583.1 acar|OOF94669.1 acla|EAW12770.1 afis|EAW17933.1 afla|EED49108.1 afum|EAL91744.1 agla|OJJ83000.1 akaw|GAA85209.1 aluc|OJZ90564.1 anid|CBF76930.1 anig|EHA24010.1 aory|EIT78564.1 asyd|OJJ57067.1 ater|EAU33537.1 atub|OJI81987.1 aver|OJJ03231.1 awen|OJJ41613.1 azon|OJJ48333.1

Group2808: abra|OJJ72585.1 acar|OOF94667.1 acla|EAW12771.1 afis|EAW17934.1 afla|EED49107.1 afum|EAL91745.1 agla|OJJ82999.1 akaw|GAA85211.1 aluc|OJZ90566.1 anid|CBF77039.1 anig|EHA24012.1 aory|EIT78519.1 asyd|OJJ57002.1 ater|EAU33536.1 atub|OJI81989.1 aver|OJJ03285.1 awen|OJJ41612.1 azon|OJJ48331.1

Group2809: abra|OJJ72588.1 acar|OOF94662.1 acla|EAW12774.1 afis|EAW17937.1 afla|EED49105.1 afum|EAL91748.1 agla|OJJ82995.1 akaw|GAA85214.1 aluc|OJZ90569.1 anid|CBF77044.1 anig|EHA24014.1 aory|EIT78518.1 asyd|OJJ56999.1 ater|EAU33533.1 atub|OJI81992.1 aver|OJJ03288.1 awen|OJJ41609.1 azon|OJJ48328.1

Group2810: abra|OJJ72589.1 acar|OOF94661.1 acla|EAW12775.1 afis|EAW17938.1 afla|EED49103.1 afum|EAL91749.1 agla|OJJ82994.1 akaw|GAA85215.1 aluc|OJZ90570.1 anid|CBF77046.1 anig|EHA24015.1 aory|EIT82547.1 asyd|OJJ56998.1 ater|EAU33530.1 atub|OJI81993.1 aver|OJJ03289.1 awen|OJJ41607.1 azon|OJJ48325.1

Group2811: abra|OJJ72592.1 acar|OOF94658.1 acla|EAW12778.1 afis|EAW17941.1 afla|EED52574.1 afum|EAL91752.1 agla|OJJ82991.1 akaw|GAA85219.1 aluc|OJZ90573.1 anid|CBF77049.1 anig|EHA24018.1 aory|EIT74256.1 asyd|OJJ56996.1 ater|EAU33524.1 atub|OJI81997.1 aver|OJJ03291.1 awen|OJJ41602.1 azon|OJJ48320.1

Group2812: abra|OJJ72596.1 acar|OOF94657.1 acla|EAW11620.1 afla|EED52571.1 akaw|GAA85224.1 aluc|OJZ90577.1 anig|EHA24021.1 aory|EIT74280.1 ater|EAU33521.1 atub|OJI82002.1 azon|OJJ48744.1 afis|EAW23838.1 afum|EAL87269.1 asyd|OJJ56992.1 aver|OJJ03295.1 anid|CBF77058.1 agla|OJJ82497.1 awen|OJJ33801.1

Group2813: abra|OJJ72598.1 acar|OOF94656.1 acla|EAW11619.1 afis|EAW23853.1 afla|EED52570.1 afum|EAL87281.1 agla|OJJ82496.1 akaw|GAA85225.1 aluc|OJZ90579.1 anid|CBF77060.1 anig|EHA24022.1 aory|EIT74300.1 asyd|OJJ56991.1 ater|EAU33520.1 atub|OJI82004.1 aver|OJJ03296.1 awen|OJJ33802.1 azon|OJJ48743.1

Group2814: abra|OJJ72599.1 acar|OOF94655.1 acla|EAW11618.1 afis|EAW23852.1 afla|EED52569.1 afum|EAL87280.1 agla|OJJ82495.1 akaw|GAA85226.1 aluc|OJZ90580.1 anid|CBF77062.1 anig|EHA24023.1 aory|EIT74211.1 asyd|OJJ56990.1 ater|EAU33519.1 atub|OJI82005.1 aver|OJJ03297.1 awen|OJJ33803.1 azon|OJJ48742.1

Group2815: abra|OJJ72600.1 acar|OOF94653.1 acla|EAW11617.1 afis|EAW23851.1 afla|EED52568.1 afum|EAL87279.1 agla|OJJ82494.1 akaw|GAA85228.1 aluc|OJZ90583.1 anid|CBF77063.1 anig|EHA24024.1 aory|EIT74296.1 asyd|OJJ56989.1 ater|EAU33518.1 atub|OJI82006.1 aver|OJJ03298.1 awen|OJJ33804.1 azon|OJJ48740.1

Group2816: abra|OJJ72603.1 acar|OOF94650.1 acla|EAW11611.1 afis|EAW23844.1 afla|EED52553.1 afum|EAL87275.1 agla|OJJ82490.1 akaw|GAA85231.1 aluc|OJZ90586.1 anid|CBF77071.1 anig|EHA24026.1 aory|EIT74313.1 asyd|OJJ56986.1 ater|EAU33511.1 atub|OJI82009.1 aver|OJJ03301.1 awen|OJJ33807.1 azon|OJJ48737.1

Group2817: abra|OJJ72605.1 acar|OOF94649.1 afis|EAW23848.1 afla|EED52565.1 agla|OJJ82491.1 akaw|GAA85232.1 aluc|OJZ90588.1 anig|EHA24027.1 aory|EIT74169.1 atub|OJI82011.1 aver|OJJ03300.1 acla|EAW11614.1 afum|EAL87276.2 anid|CBF77067.1 asyd|OJJ56987.1 ater|EAU33515.1 awen|OJJ33806.1 azon|OJJ48727.1

Group2818: abra|OJJ72625.1 acar|OOF94639.1 acla|EAW11599.1 afis|EAW23858.1 afla|EED52583.1 afum|EAL87286.1 agla|OJJ82525.1 akaw|GAA85245.1 aluc|OJZ90602.1 anid|CBF77236.1 anig|EHA24039.1 aory|EIT74247.1 asyd|OJJ56814.1 ater|EAU33493.1 atub|OJI82026.1 aver|OJJ03521.1 awen|OJJ33819.1 azon|OJJ48716.1

Group2819: abra|OJJ72626.1 acar|OOF94638.1 acla|EAW11598.1 afis|EAW23859.1 afla|EED52584.1 afum|EAL87287.1 agla|OJJ82526.1 akaw|GAA85246.1 aluc|OJZ90603.1 anid|CBF77234.1 anig|EHA24040.1 aory|EIT74284.1 asyd|OJJ56815.1 ater|EAU33492.1 atub|OJI82027.1 aver|OJJ03520.1 awen|OJJ33820.1 azon|OJJ48715.1

Group2820: abra|OJJ72627.1 acar|OOF94637.1 acla|EAW11596.1 afis|EAW23860.1 afla|EED52585.1 afum|EAL87288.1 agla|OJJ82527.1 akaw|GAA85247.1 aluc|OJZ90604.1 anid|CBF77232.1 anig|EHA24041.1 aory|EIT74206.1 asyd|OJJ56816.1 ater|EAU33490.1 atub|OJI82028.1 awen|OJJ33821.1 azon|OJJ48714.1 aver|OJJ03519.1

Group2821: abra|OJJ72635.1 acar|OOF94633.1 acla|EAW11594.1 afis|EAW23862.1 afla|EED52586.1 agla|OJJ82528.1 akaw|GAA85256.1 aluc|OJZ90614.1 anid|CBF77230.1 anig|EHA24052.1 aory|EIT74306.1 asyd|OJJ56817.1 ater|EAU33489.1 atub|OJI82040.1 aver|OJJ03518.1 awen|OJJ33822.1 azon|OJJ48713.1 aluc|OJZ91238.1

Group2822: abra|OJJ72640.1 acar|OOF94631.1 acla|EAW11584.1 afis|EAW23869.1 afla|EED52597.1 afum|EAL87297.1 agla|OJJ82538.1 akaw|GAA85261.1 aluc|OJZ90619.1 anid|CBF77217.1 anig|EHA24056.1 aory|EIT74235.1 asyd|OJJ56823.1 ater|EAU33478.1 atub|OJI82045.1 aver|OJJ03512.1 awen|OJJ33845.1 azon|OJJ48702.1

Group2823: abra|OJJ72641.1 acar|OOF94630.1 acla|EAW11583.1 afis|EAW23870.1 afla|EED52598.1 afum|EAL87298.1 agla|OJJ82539.1 akaw|GAA85262.1 aluc|OJZ90620.1 anid|CBF77219.1 anig|EHA24057.1 aory|EIT74294.1 asyd|OJJ56822.1 ater|EAU33479.1 atub|OJI82046.1 aver|OJJ03513.1 awen|OJJ33846.1 azon|OJJ48701.1

Group2824: abra|OJJ72642.1 acar|OOF94629.1 acla|EAW11582.1 afis|EAW23871.1 afla|EED52599.1 afum|EAL87299.1 agla|OJJ82540.1 akaw|GAA85263.1 aluc|OJZ90621.1 anid|CBF77220.1 anig|EHA24058.1

aory|EIT74246.1 asyd|OJJ56821.1 ater|EAU33475.1 atub|OJI82047.1 aver|OJJ03514.1 awen|OJJ33847.1 azon|OJJ48700.1

Group2825: abra|OJJ72647.1 acar|OOF94623.1 acla|EAW11581.1 afis|EAW23872.1 afla|EED52600.1 afum|EAL87300.1 agla|OJJ82542.1 akaw|GAA85267.1 aluc|OJZ90625.1 anid|CBF84521.1 anig|EHA24062.1 aory|EIT74293.1 asyd|OJJ60278.1 ater|EAU33474.1 atub|OJI82052.1 aver|OJJ03978.1 awen|OJJ33848.1 azon|OJJ48699.1

Group2826: abra|OJJ72648.1 acar|OOF94622.1 acla|EAW11580.1 afis|EAW23874.1 afla|EED52602.1 afum|EAL87302.1 agla|OJJ82544.1 akaw|GAA85269.1 aluc|OJZ90627.1 anid|CBF77209.1 anig|EHA24063.1 aory|EIT74307.1 asyd|OJJ56829.1 ater|EAU33472.1 atub|OJI82053.1 aver|OJJ03507.1 awen|OJJ33850.1 azon|OJJ48698.1

Group2827: abra|OJJ72653.1 acar|OOF94620.1 afis|EAW23877.1 afla|EED52605.1 afum|EAL87305.1 agla|OJJ82547.1 akaw|GAA85272.1 aluc|OJZ90631.1 anid|CBF77201.1 anig|EHA24068.1 aory|EIT74181.1 asyd|OJJ56833.1 ater|EAU33469.1 atub|OJI82057.1 aver|OJJ03504.1 awen|OJJ33854.1 azon|OJJ48695.1 acla|EAW11577.1

Group2828: abra|OJJ72654.1 acar|OOF94619.1 acla|EAW11576.1 afis|EAW23878.1 afla|EED52606.1 afum|EAL87306.1 agla|OJJ82548.1 akaw|GAA85273.1 aluc|OJZ90632.1 anid|CBF77199.1 anig|EHA24069.1 aory|EIT74260.1 asyd|OJJ56834.1 ater|EAU33468.1 atub|OJI82059.1 aver|OJJ03503.1 awen|OJJ33858.1 azon|OJJ48694.1

Group2829: abra|OJJ72658.1 acar|OOF94615.1 acla|EAW11522.1 afis|EAW23931.1 afla|EED50587.1 afum|EAL87356.1 agla|OJJ79411.1 akaw|GAA85281.1 aluc|OJZ90639.1 anid|CBF77275.1 anig|EHA24074.1 aory|EIT82143.1 asyd|OJJ56796.1 ater|EAU32296.1 atub|OJI82067.1 aver|OJJ03538.1 awen|OJJ39023.1 azon|OJJ51349.1

Group2830: abra|OJJ72659.1 acar|OOF94614.1 acla|EAW11523.1 afis|EAW23930.1 afla|EED50588.1 afum|EAL87355.1 agla|OJJ79407.1 akaw|GAA85282.1 aluc|OJZ90640.1 anid|CBF77277.1 anig|EHA24075.1 aory|EIT82106.1 asyd|OJJ56795.1 ater|EAU32306.1 atub|OJI82068.1 aver|OJJ03539.1 awen|OJJ39024.1 azon|OJJ51350.1

Group2831: abra|OJJ72661.1 acar|OOF94612.1 acla|EAW11526.1 afis|EAW23928.1 afla|EED50590.1 afum|EAL87353.1 agla|OJJ79406.1 akaw|GAA85284.1 aluc|OJZ90642.1 anid|CBF77281.1 anig|EHA24077.1 aory|EIT82136.1 asyd|OJJ56793.1 ater|EAU32307.1 atub|OJI82070.1 aver|OJJ03541.1 awen|OJJ39026.1 azon|OJJ51352.1

Group2832: abra|OJJ72662.1 acar|OOF94611.1 acla|EAW11527.1 afis|EAW23927.1 afla|EED50591.1 afum|EAL87352.1 agla|OJJ79405.1 akaw|GAA85285.1 aluc|OJZ90643.1 anid|CBF77254.1 anig|EHA24078.1 aory|EIT82117.1 asyd|OJJ56807.1 ater|EAU32308.1 atub|OJI82071.1 aver|OJJ03527.1 awen|OJJ39027.1 azon|OJJ51353.1

Group2833: abra|OJJ72663.1 acar|OOF94610.1 acla|EAW11528.1 afis|EAW23926.1 afla|EED50594.1 afum|EAL87351.1 agla|OJJ79404.1 akaw|GAA85286.1 aluc|OJZ90644.1 anid|CBF77252.1 anig|EHA24079.1 aory|EIT82151.1 asyd|OJJ56808.1 ater|EAU32309.1 atub|OJI82072.1 aver|OJJ03526.1 awen|OJJ39028.1 azon|OJJ51354.1

Group2834: abra|OJJ72669.1 acar|OOF97389.1 acla|EAW11531.1 afis|EAW23923.1 afla|EED50599.1 afum|EAL87348.1 agla|OJJ79397.1 akaw|GAA85289.1 aluc|OJZ90647.1 anid|CBF77090.1 anig|EHA24082.1 aory|EIT78106.1 asyd|OJJ56959.1 ater|EAU32313.1 atub|OJI82077.1 aver|OJJ03326.1 awen|OJJ39032.1 azon|OJJ51360.1

Group2835: abra|OJJ72670.1 acar|OOF97388.1 acla|EAW11537.1 afis|EAW23919.1 afla|EED50608.1 afum|EAL87345.1 agla|OJJ79386.1 akaw|GAA85290.1 aluc|OJZ90648.1 anid|CBF77096.1 anig|EHA24083.1 aory|EIT78069.1 asyd|OJJ56955.1 ater|EAU32318.1 atub|OJI82078.1 aver|OJJ03330.1 awen|OJJ39036.1 azon|OJJ51364.1

Group2836: abra|OJJ72675.1 acar|OOF97380.1 acla|EAW11538.1 afis|EAW23913.1 afla|EED50618.1 afum|EAL87342.1 agla|OJJ79382.1 akaw|GAA85294.1 aluc|OJZ90653.1 anid|CBF77098.1 anig|EHA24088.1 aory|EIT78036.1 asyd|OJJ56954.1 ater|EAU32319.1 atub|OJI82082.1 aver|OJJ03331.1 awen|OJJ39038.1 azon|OJJ51365.1

Group2837: abra|OJJ72683.1 acar|OOF95932.1 acla|EAW13159.1 afis|EAW17487.1 afla|EED49329.1 afum|EAL91310.1 agla|OJJ81475.1 akaw|GAA85301.1 aluc|OJZ90661.1 anid|CBF71613.1 anig|EHA24096.1 aory|EIT79552.1 asyd|OJJ61862.1 ater|EAU36932.1 atub|OJI82090.1 aver|OJI95960.1 awen|OJJ37355.1 azon|OJJ51040.1

Group2838: abra|OJJ72684.1 acar|OOF95933.1 acla|EAW11540.1 afis|EAW23911.1 afla|EED52657.1 afum|EAL87340.1 agla|OJJ79380.1 akaw|GAA85302.1 aluc|OJZ90662.1 anid|CBF77104.1 anig|EHA24097.1 aory|EIT74175.1 asyd|OJJ56950.1 ater|EAU32321.1 atub|OJI82091.1 aver|OJJ03335.1 awen|OJJ39040.1 azon|OJJ51367.1

Group2839: abra|OJJ72685.1 acar|OOF95934.1 acla|EAW11541.1 afis|EAW23910.1 afla|EED52655.1 afum|EAL87339.1 agla|OJJ79379.1 akaw|GAA85303.1 aluc|OJZ90663.1 anid|CBF77106.1 anig|EHA24098.1 aory|EIT74315.1 asyd|OJJ56949.1 ater|EAU32322.1 atub|OJI82092.1 aver|OJJ03336.1 awen|OJJ39041.1 azon|OJJ51368.1

Group2840: abra|OJJ72687.1 acar|OOF95936.1 acla|EAW11542.1 afis|EAW23909.1 afla|EED52654.1 afum|EAL87337.1 agla|OJJ79378.1 akaw|GAA85305.1 aluc|OJZ90665.1 anid|CBF77108.1 anig|EHA24100.1 aory|EIT74231.1 asyd|OJJ56948.1 ater|EAU32324.1 atub|OJI82094.1 aver|OJJ03337.1 awen|OJJ39042.1 azon|OJJ51370.1

Group2841: abra|OJJ72706.1 acar|OOF95943.1 acla|EAW11546.1 afis|EAW23907.1 afla|EED52642.1 afum|EAL87335.1 agla|OJJ79369.1 akaw|GAA85310.1 aluc|OJZ90671.1 anid|CBF77127.1 anig|EHA24104.1 aory|EIT74245.1 asyd|OJJ56941.1 ater|EAU32331.1 atub|OJI82101.1 aver|OJJ03344.1 awen|OJJ39043.1 azon|OJJ51372.1

Group2842: abra|OJJ72707.1 acar|OOF95944.1 acla|EAW11548.1 afis|EAW23905.1 afla|EED52640.1 afum|EAL87333.1 agla|OJJ79370.1 akaw|GAA85311.1 aluc|OJZ90672.1 anid|CBF77125.1 anig|EHA24105.1 aory|EIT74243.1 asyd|OJJ56940.1 ater|EAU32332.1 atub|OJI82102.1 aver|OJJ03345.1 awen|OJJ39044.1 azon|OJJ51373.1

Group2843: abra|OJJ72714.1 acar|OOF95946.1 acla|EAW11550.1 afis|EAW23903.1 afla|EED52638.1 afum|EAL87331.1 agla|OJJ79372.1 akaw|GAA85322.1 aluc|OJZ90683.1 anid|CBF77139.1 anig|EHA24111.1 aory|EIT74295.1 asyd|OJJ56914.1 ater|EAU32335.1 atub|OJI82109.1 aver|OJJ03378.1 awen|OJJ39046.1 azon|OJJ51375.1

Group2844: abra|OJJ72715.1 acar|OOF95947.1 acla|EAW11551.1 afis|EAW23902.1 afla|EED52637.1 afum|EAL87329.1 agla|OJJ79373.1 akaw|GAA85323.1 aluc|OJZ90684.1 anid|CBF77141.1 anig|EHA24112.1 aory|EIT74272.1 asyd|OJJ56913.1 ater|EAU32336.1 atub|OJI82110.1 aver|OJJ03379.1 awen|OJJ39047.1 azon|OJJ51376.1

Group2845: abra|OJJ72719.1 acar|OOF95952.1 acla|EAW11555.1 afis|EAW23898.1 afla|EED52632.1 afum|EAL87325.1 agla|OJJ78640.1 akaw|GAA85326.1 aluc|OJZ90688.1 anid|CBF77148.1 anig|EHA24116.1 aory|EIT74173.1 asyd|OJJ56910.1 ater|EAU32339.1 atub|OJI82113.1 aver|OJJ03383.1 awen|OJJ39050.1 azon|OJJ51382.1

Group2846: abra|OJJ72721.1 acar|OOF95954.1 acla|EAW11557.1 afis|EAW23896.1 afla|EED52630.1 afum|EAL87324.1 agla|OJJ78637.1 akaw|GAA85327.1 aluc|OJZ90690.1 anid|CBF77152.1 anig|EHA24117.1 aory|EIT74255.1 asyd|OJJ56908.1 ater|EAU32340.1 atub|OJI82115.1 aver|OJJ03385.1 awen|OJJ39052.1 azon|OJJ51384.1

Group2847: abra|OJJ72722.1 acar|OOF95955.1 acla|EAW11558.1 afis|EAW23895.1 afla|EED52629.1 afum|EAL87323.1 agla|OJJ78636.1 akaw|GAA85328.1 aluc|OJZ90691.1 anid|CBF77154.1 anig|EHA24118.1 aory|EIT74200.1 asyd|OJJ56907.1 ater|EAU32341.1 atub|OJI82116.1 aver|OJJ03386.1 awen|OJJ39053.1 azon|OJJ51385.1

Group2848: abra|OJJ72725.1 acar|OOF95958.1 acla|EAW11562.1 afis|EAW23891.1 afla|EED52621.1 afum|EAL87319.1 agla|OJJ78631.1 akaw|GAA85331.1 aluc|OJZ90694.1 anid|CBF77171.1 anig|EHA24119.1 aory|EIT74302.1 asyd|OJJ56851.1 ater|EAU33451.1 atub|OJI82119.1 aver|OJJ03466.1 awen|OJJ39058.1 azon|OJJ51389.1

Group2849: abra|OJJ72727.1 acar|OOF95960.1 acla|EAW11564.1 afis|EAW23889.1 afla|EED52619.1 afum|EBA27309.1 agla|OJJ78629.1 akaw|GAA85333.1 aluc|OJZ90696.1 anid|CBF77175.1 anig|EHA24121.1 aory|EIT74266.1 asyd|OJJ56848.1 ater|EAU33454.1 atub|OJI82122.1 aver|OJJ03491.1 awen|OJJ39061.1 azon|OJJ51391.1

Group2850: abra|OJJ72729.1 acar|OOF95961.1 acla|EAW11565.1 afis|EAW23888.1 afla|EED52617.1 afum|EAL87315.1 agla|OJJ78628.1 akaw|GAA85334.1 aluc|OJZ90697.1 anid|CBF77177.1 anig|EHA24123.1 aory|EIT74304.1 asyd|OJJ56847.1 ater|EAU33456.1 atub|OJI82123.1 aver|OJJ03492.1 awen|OJJ39063.1 azon|OJJ51392.1

Group2851: abra|OJJ72733.1 acar|OOF95963.1 acla|EAW11568.1 afis|EAW23885.1 afla|EED52614.1 afum|EBA27307.1 agla|OJJ82572.1 akaw|GAA85339.1 aluc|OJZ90701.1 anid|CBF77183.1 anig|EHA24127.1 aory|EIT74261.1 asyd|OJJ56843.1 ater|EAU33461.1 atub|OJI82128.1 aver|OJJ03495.1 awen|OJJ33867.1 azon|OJJ48687.1

Group2852: abra|OJJ72736.1 acar|OOF95967.1 acla|EAW11572.1 afis|EAW23881.1 afla|EED52610.1 afum|EAL87309.1 agla|OJJ82558.1 akaw|GAA85342.1 aluc|OJZ90704.1 anid|CBF77191.1 anig|EHA24130.1 aory|EIT74269.1 asyd|OJJ56839.1 ater|EAU33465.1 atub|OJI82131.1 aver|OJJ03499.1 awen|OJJ33863.1 azon|OJJ48690.1

Group2853: abra|OJJ72748.1 acar|OOF95971.1 acla|EAW11515.1 afis|EAW23938.1 afla|EED50579.1 afum|EAL87363.1 agla|OJJ79426.1 akaw|GAA85360.1 aluc|OJZ90722.1 anid|CBF77265.1 anig|EHA24140.1

aory|EIT82037.1 asyd|OJJ56801.1 ater|EAU32295.1 atub|OJI82150.1 aver|OJJ03533.1 awen|OJJ39017.1 azon|OJJ51342.1

Group2854: abra|OJJ72749.1 acar|OOF95972.1 acla|EAW11514.1 afis|EAW23939.1 afla|EED50578.1 afum|EAL87364.1 agla|OJJ79427.1 akaw|GAA85361.1 aluc|OJZ90723.1 anid|CBF77263.1 anig|EHA24141.1 aory|EIT82108.1 asyd|OJJ56802.1 ater|EAU32294.1 atub|OJI82151.1 aver|OJJ03532.1 awen|OJJ39016.1 azon|OJJ51341.1

Group2855: abra|OJJ72750.1 acar|OOF95973.1 acla|EAW11513.1 afis|EAW23940.1 afla|EED50577.1 afum|EAL87365.1 agla|OJJ79428.1 akaw|GAA85362.1 aluc|OJZ90724.1 anid|CBF77262.1 anig|EHA24142.1 aory|EIT82017.1 asyd|OJJ56803.1 ater|EAU32293.1 atub|OJI82152.1 aver|OJJ03531.1 awen|OJJ39015.1 azon|OJJ51340.1

Group2856: abra|OJJ72755.1 acar|OOF95976.1 acla|EAW11512.1 afis|EAW23941.1 afla|EED50576.1 afum|EAL87366.1 agla|OJJ79429.1 akaw|GAA85365.1 aluc|OJZ90728.1 anid|CBF77260.1 anig|EHA24145.1 aory|EIT82066.1 asyd|OJJ56804.1 ater|EAU32292.1 atub|OJI82155.1 aver|OJJ03530.1 awen|OJJ39014.1 azon|OJJ51339.1

Group2857: abra|OJJ72756.1 acar|OOF95977.1 acla|EAW11508.1 afis|EAW23945.1 afla|EED50571.1 afum|EAL87370.1 agla|OJJ79436.1 akaw|GAA85366.1 aluc|OJZ90729.1 anid|CBF77290.1 anig|EHA24146.1 aory|EIT82165.1 asyd|OJJ56784.1 ater|EAU32289.1 atub|OJI82156.1 aver|OJJ03549.1 awen|OJJ39009.1 azon|OJJ51335.1

Group2858: abra|OJJ72761.1 acar|OOF95980.1 acla|EAW11503.1 afis|EAW23949.1 afla|EED50567.1 afum|EAL87375.1 agla|OJJ79442.1 akaw|GAA85371.1 aluc|OJZ90735.1 anid|CBF77283.1 anig|EHA24151.1 aory|EIT82002.1 asyd|OJJ56792.1 ater|EAU29803.1 atub|OJI82163.1 aver|OJJ03542.1 awen|OJJ39000.1 azon|OJJ51331.1

Group2859: abra|OJJ72763.1 acar|OOF95983.1 acla|EAW11501.1 afis|EAW23951.1 afla|EED50565.1 afum|EAL87379.1 agla|OJJ79444.1 akaw|GAA85373.1 aluc|OJZ90738.1 anid|CBF77296.1 anig|EHA24153.1 aory|EIT82176.1 asyd|OJJ56782.1 ater|EAU29801.1 atub|OJI82165.1 aver|OJJ03551.1 awen|OJJ38997.1 azon|OJJ51329.1

Group2860: abra|OJJ72764.1 acar|OOF95984.1 acla|EAW11500.1 afis|EAW23952.1 afla|EED50564.1 afum|EAL87380.1 agla|OJJ79445.1 akaw|GAA85374.1 aluc|OJZ90739.1 anid|CBF77298.1 anig|EHA24154.1 aory|EIT82174.1 asyd|OJJ56781.1 ater|EAU29800.1 atub|OJI82166.1 aver|OJJ03552.1 awen|OJJ38996.1 azon|OJJ51328.1

Group2861: abra|OJJ72765.1 acar|OOF95985.1 acla|EAW11499.1 afis|EAW23953.1 afla|EED50562.1 afum|EAL87381.1 agla|OJJ79446.1 akaw|GAA85375.1 aluc|OJZ90740.1 anid|CBF77300.1 anig|EHA24155.1 aory|EIT82008.1 asyd|OJJ56780.1 ater|EAU29799.1 atub|OJI82167.1 aver|OJJ03553.1 awen|OJJ38995.1 azon|OJJ51327.1

Group2862: abra|OJJ72767.1 acar|OOF95986.1 acla|EAW11498.1 afis|EAW23954.1 afla|EED50561.1 afum|EAL87382.1 agla|OJJ79447.1 akaw|GAA85376.1 aluc|OJZ90743.1 anid|CBF77302.1 anig|EHA24156.1 aory|EIT82091.1 asyd|OJJ56779.1 ater|EAU29798.1 atub|OJI82170.1 aver|OJJ03554.1 awen|OJJ38994.1 azon|OJJ51326.1

Group2863: abra|OJJ72768.1 acar|OOF95987.1 acla|EAW11497.1 afis|EAW23955.1 afla|EED50557.1 afum|EAL87383.1 agla|OJJ86878.1 akaw|GAA85377.1 aluc|OJZ90745.1 anid|CBF77304.1 anig|EHA24157.1 aory|EIT82133.1 asyd|OJJ56778.1 ater|EAU29797.1 atub|OJI82171.1 aver|OJJ03555.1 awen|OJJ38993.1 azon|OJJ51324.1

Group2864: abra|OJJ72769.1 acar|OOF95988.1 acla|EAW11496.1 afis|EAW23956.1 afla|EED50556.1 afum|EAL87384.1 agla|OJJ86879.1 akaw|GAA85378.1 aluc|OJZ90746.1 anid|CBF77306.1 anig|EHA24158.1 aory|EIT82152.1 asyd|OJJ56777.1 ater|EAU29796.1 atub|OJI82172.1 aver|OJJ03556.1 awen|OJJ38991.1 azon|OJJ51323.1

Group2865: abra|OJJ72770.1 acar|OOF95989.1 acla|EAW11495.1 afis|EAW23957.1 afla|EED50555.1 afum|EAL87385.1 agla|OJJ86880.1 akaw|GAA85379.1 aluc|OJZ90747.1 anid|CBF77308.1 anig|EHA24159.1 aory|EIT82065.1 asyd|OJJ56776.1 ater|EAU29795.1 atub|OJI82173.1 aver|OJJ03557.1 awen|OJJ38990.1 azon|OJJ51322.1

Group2866: abra|OJJ72773.1 acar|OOF95992.1 acla|EAW11493.1 afis|EAW23960.1 afla|EED50552.1 afum|EAL87389.1 agla|OJJ86885.1 akaw|GAA85382.1 aluc|OJZ90750.1 anid|CBF77314.1 anig|EHA24161.1 aory|EIT82096.1 asyd|OJJ56773.1 ater|EAU29792.1 atub|OJI82176.1 aver|OJJ03560.1 awen|OJJ38987.1 azon|OJJ51319.1

Group2867: abra|OJJ72775.1 acar|OOF95993.1 acla|EAW11491.1 afis|EAW23962.1 afla|EED50548.1 afum|EAL87391.2 agla|OJJ86887.1 akaw|GAA85384.1 anig|EHA24162.1 aory|EIT82083.1 ater|EAU29790.1 atub|OJI82177.1 awen|OJJ38985.1 azon|OJJ51317.1 anid|CBF83986.1 asyd|OJJ61403.1 aver|OJJ00731.1 aluc|OJZ90752.1

Group2868: abra|OJJ72776.1 acar|OOF95994.1 acla|EAW11490.1 afis|EAW23963.1 afla|EED50547.1 afum|EAL87393.1 agla|OJJ86888.1 akaw|GAA85385.1 aluc|OJZ90754.1 anid|CBF77316.1 anig|EHA24163.1 aory|EIT82035.1 asyd|OJJ56772.1 ater|EAU29789.1 atub|OJI82178.1 aver|OJJ03561.1 awen|OJJ38984.1 azon|OJJ51316.1

Group2869: abra|OJJ72779.1 acar|OOF95996.1 acla|EAW11488.1 afis|EAW23965.1 afla|EED50545.1 afum|EAL87395.1 agla|OJJ86894.1 akaw|GAA85387.1 aluc|OJZ83277.1 anid|CBF77322.1 anig|EHA24165.1 aory|EIT82007.1 asyd|OJJ56770.1 ater|EAU29787.1 atub|OJI82181.1 aver|OJJ03563.1 awen|OJJ38978.1 azon|OJJ51314.1

Group2870: abra|OJJ72780.1 acar|OOF95999.1 acla|EAW11487.1 afis|EAW23966.1 afla|EED50544.1 afum|EAL87396.1 agla|OJJ86895.1 akaw|GAA85388.1 aluc|OJZ83279.1 anid|CBF77324.1 anig|EHA24166.1 aory|EIT82088.1 asyd|OJJ56769.1 ater|EAU29786.1 atub|OJI82183.1 aver|OJJ03565.1 awen|OJJ38977.1 azon|OJJ51313.1

Group2871: abra|OJJ72781.1 acar|OOF96000.1 acla|EAW11486.1 afis|EAW23967.1 afla|EED50542.1 afum|EAL87397.1 agla|OJJ86896.1 akaw|GAA85389.1 aluc|OJZ83280.1 anid|CBF77326.1 anig|EHA24167.1 aory|EIT82086.1 asyd|OJJ56768.1 ater|EAU29785.1 atub|OJI82184.1 aver|OJJ03566.1 awen|OJJ38976.1 azon|OJJ51312.1

Group2872: abra|OJJ72782.1 acar|OOF96002.1 acla|EAW11485.1 afis|EAW23968.1 afla|EED50541.1 afum|EAL87398.2 agla|OJJ86897.1 akaw|GAA85390.1 aluc|OJZ83281.1 anid|CBF77328.1 anig|EHA24168.1 aory|EIT82024.1 asyd|OJJ56767.1 ater|EAU29784.1 atub|OJI82185.1 aver|OJJ03567.1 awen|OJJ38975.1 azon|OJJ51310.1

Group2873: abra|OJJ72792.1 acar|OOF90123.1 acla|EAW11480.1 afis|EAW23973.1 afla|EED50535.1 afum|EAL87403.1 agla|OJJ86902.1 akaw|GAA85395.1 aluc|OJZ83288.1 anid|CBF77339.1 anig|EHA24173.1 aory|EIT82084.1 asyd|OJJ53275.1 ater|EAU29779.1 atub|OJI82190.1 aver|OJJ03574.1 awen|OJJ35438.1 azon|OJJ51305.1

Group2874: abra|OJJ72797.1 acar|OOF96005.1 acla|EAW11475.1 afis|EAW23978.1 afla|EED49183.1 afum|EAL87408.1 agla|OJJ86908.1 akaw|GAA85400.1 aluc|OJZ83294.1 anid|CBF77349.1 anig|EHA24178.1 aory|EIT78581.1 asyd|OJJ53271.1 ater|EAU29772.1 atub|OJI82195.1 aver|OJJ03579.1 awen|OJJ35446.1 azon|OJJ51300.1

Group2875: abra|OJJ72798.1 acar|OOF96006.1 acla|EAW11474.1 afis|EAW23979.1 afla|EED49182.1 afum|EAL87409.1 agla|OJJ86910.1 akaw|GAA85401.1 aluc|OJZ83295.1 anid|CBF77351.1 anig|EHA24179.1 aory|EIT78531.1 asyd|OJJ53270.1 ater|EAU29771.1 atub|OJI82196.1 aver|OJJ03580.1 awen|OJJ35447.1 azon|OJJ51299.1

Group2876: abra|OJJ72800.1 acar|OOF96008.1 acla|EAW11444.1 afis|EAW23981.1 afla|EED49178.1 afum|EAL87411.1 agla|OJJ86914.1 akaw|GAA85403.1 aluc|OJZ83298.1 anid|CBF77356.1 anig|EHA24181.1 aory|EIT78527.1 asyd|OJJ53268.1 ater|EAU29770.1 atub|OJI82198.1 aver|OJJ03583.1 awen|OJJ35449.1 azon|OJJ51297.1

Group2877: abra|OJJ72801.1 acar|OOF96009.1 acla|EAW11443.1 afis|EAW23982.1 afla|EED49177.1 afum|EAL87412.1 agla|OJJ86916.1 akaw|GAA85404.1 aluc|OJZ83299.1 anid|CBF77358.1 anig|EHA24182.1 aory|EIT78537.1 asyd|OJJ53267.1 ater|EAU29769.1 atub|OJI82199.1 aver|OJJ03584.1 awen|OJJ35450.1 azon|OJJ51293.1

Group2878: abra|OJJ72803.1 acar|OOF96011.1 acla|EAW11441.1 afis|EAW23984.1 afla|EED49175.1 afum|EAL87413.1 agla|OJJ86918.1 akaw|GAA85406.1 aluc|OJZ83301.1 anid|CBF77362.1 anig|EHA24183.1 aory|EIT78558.1 asyd|OJJ53265.1 ater|EAU29767.1 atub|OJI82201.1 aver|OJJ03586.1 awen|OJJ35452.1 azon|OJJ51291.1

Group2879: abra|OJJ72804.1 acar|OOF96012.1 acla|EAW11440.1 afis|EAW23985.1 afla|EED49173.1 afum|EAL87414.1 agla|OJJ86919.1 akaw|GAA85407.1 aluc|OJZ83302.1 anid|CBF77364.1 anig|EHA24184.1 aory|EIT78557.1 asyd|OJJ53264.1 ater|EAU29766.1 atub|OJI82202.1 aver|OJJ03587.1 awen|OJJ35453.1 azon|OJJ51290.1

Group2880: abra|OJJ72808.1 acar|OOF96014.1 acla|EAW11433.1 afis|EAW23989.1 afla|EED49169.1 afum|EAL87419.1 agla|OJJ86923.1 akaw|GAA85411.1 aluc|OJZ83306.1 anid|CBF77367.1 anig|EHA24189.1 aory|EIT78567.1 asyd|OJJ53261.1 ater|EAU29761.1 atub|OJI82206.1 aver|OJJ03590.1 awen|OJJ35457.1 azon|OJJ51286.1

Group2881: abra|OJJ72810.1 acar|OOF96017.1 acla|EAW11430.1 afis|EAW23993.1 afla|EED49167.1 afum|EAL87423.1 agla|OJJ86926.1 akaw|GAA85413.1 aluc|OJZ83308.1 anid|CBF77373.1 anig|EHA24191.1 aory|EIT78512.1 asyd|OJJ53258.1 ater|EAU29758.1 atub|OJI82208.1 aver|OJJ03593.1 awen|OJJ35462.1 azon|OJJ51281.1

Group2882: abra|OJJ72811.1 acar|OOF96018.1 acla|EAW11429.1 afis|EAW23994.1 afla|EED49166.1 afum|EAL87424.1 agla|OJJ86927.1 akaw|GAA85414.1 aluc|OJZ83309.1 anid|CBF77375.1 anig|EHA24192.1

aory|EIT78551.1 asyd|OJJ53257.1 ater|EAU29757.1 atub|OJI82209.1 aver|OJJ03594.1 awen|OJJ35463.1 azon|OJJ51280.1

Group2883: abra|OJJ72814.1 acar|OOF96020.1 acla|EAW11428.1 afis|EAW23995.1 afla|EED49165.1 afum|EAL87425.1 agla|OJJ86928.1 akaw|GAA85415.1 aluc|OJZ83312.1 anid|CBF77378.1 anig|EHA24194.1 aory|EIT78566.1 asyd|OJJ53255.1 ater|EAU29756.1 atub|OJI82212.1 aver|OJJ03597.1 awen|OJJ35465.1 azon|OJJ51278.1

Group2884: abra|OJJ72815.1 acar|OOF96021.1 acla|EAW11427.1 afis|EAW23996.1 afla|EED49164.1 afum|EAL87426.1 agla|OJJ86929.1 akaw|GAA85416.1 aluc|OJZ83314.1 anid|CBF77379.1 anig|EHA24195.1 aory|EIT78572.1 asyd|OJJ53254.1 ater|EAU29755.1 atub|OJI82213.1 aver|OJJ03599.1 awen|OJJ35467.1 azon|OJJ51277.1

Group2885: abra|OJJ72818.1 acar|OOF96024.1 acla|EAW11422.1 afis|EAW23999.1 afla|EED49162.1 afum|EAL87429.1 agla|OJJ86933.1 akaw|GAA85419.1 aluc|OJZ83320.1 anid|CBF77385.1 anig|EHA24198.1 aory|EIT78542.1 asyd|OJJ53250.1 ater|EAU29750.1 atub|OJI82217.1 awen|OJJ35470.1 azon|OJJ51274.1 aver|OJJ03602.1

Group2886: abra|OJJ72820.1 acar|OOF96025.1 acla|EAW11419.1 afis|EAW24003.1 afla|EED49158.1 afum|EAL87433.1 agla|OJJ86935.1 akaw|GAA85421.1 aluc|OJZ83322.1 anid|CBF77390.1 anig|EHA24200.1 aory|EIT78547.1 asyd|OJJ53247.1 ater|EAU29748.1 atub|OJI82219.1 aver|OJJ03604.1 awen|OJJ35474.1 azon|OJJ51272.1

Group2887: abra|OJJ72822.1 acar|OOF96027.1 acla|EAW11417.1 afis|EAW24005.1 afla|EED49156.1 afum|EAL87435.1 agla|OJJ86936.1 akaw|GAA85423.1 aluc|OJZ83324.1 anid|CBF77394.1 anig|EHA24202.1 aory|EIT78532.1 asyd|OJJ53245.1 ater|EAU29746.1 atub|OJI82221.1 aver|OJJ03605.1 awen|OJJ35477.1 azon|OJJ51271.1

Group2888: abra|OJJ72824.1 acar|OOF96029.1 acla|EAW11414.1 afis|EAW24007.1 afla|EED49154.1 afum|EAL87437.1 agla|OJJ86938.1 akaw|GAA85425.1 aluc|OJZ83326.1 anid|CBF77398.1 anig|EHA24204.1 aory|EIT78570.1 asyd|OJJ53243.1 ater|EAU29744.1 atub|OJI82223.1 aver|OJJ03607.1 awen|OJJ35479.1 azon|OJJ51269.1

Group2889: abra|OJJ72825.1 acar|OOF96030.1 acla|EAW11413.1 afis|EAW24008.1 afla|EED49153.1 afum|EAL87438.1 agla|OJJ86939.1 akaw|GAA85426.1 aluc|OJZ83327.1 anid|CBF77400.1 anig|EHA24205.1 aory|EIT78506.1 asyd|OJJ53242.1 ater|EAU29743.1 atub|OJI82224.1 aver|OJJ03608.1 awen|OJJ35480.1 azon|OJJ51268.1

Group2890: abra|OJJ72826.1 acar|OOF96031.1 acla|EAW11412.1 afis|EAW24009.1 afla|EED49152.1 afum|EAL87439.1 agla|OJJ86940.1 akaw|GAA85427.1 aluc|OJZ83328.1 anid|CBF77402.1 anig|EHA24206.1 aory|EIT78555.1 asyd|OJJ53241.1 ater|EAU29742.1 atub|OJI82225.1 aver|OJJ03609.1 awen|OJJ35481.1 azon|OJJ51267.1

Group2891: abra|OJJ72827.1 acar|OOF96033.1 acla|EAW11410.1 afis|EAW24011.1 afla|EED49150.1 afum|EAL87441.1 agla|OJJ86943.1 akaw|GAA85428.1 aluc|OJZ83330.1 anid|CBF77406.1 anig|EHA24207.1 aory|EIT78559.1 asyd|OJJ53239.1 ater|EAU29740.1 atub|OJI82226.1 aver|OJJ03611.1 awen|OJJ35483.1 azon|OJJ51265.1

Group2892: abra|OJJ72831.1 acar|OOF96035.1 acla|EAW11408.1 afis|EAW24013.1 afla|EED49147.1 afum|EAL87443.1 agla|OJJ86945.1 akaw|GAA85430.1 aluc|OJZ83332.1 anid|CBF77410.1 anig|EHA24209.1 aory|EIT78522.1 asyd|OJJ53237.1 ater|EAU29734.1 atub|OJI82228.1 aver|OJJ03614.1 awen|OJJ35485.1 azon|OJJ51263.1

Group2893: abra|OJJ72833.1 acar|OOF96036.1 acla|EAW11406.1 afis|EAW24015.1 afla|EED49144.1 afum|EAL87445.1 agla|OJJ86949.1 akaw|GAA85431.1 aluc|OJZ83334.1 anid|CBF77414.1 anig|EHA24210.1 aory|EIT78508.1 asyd|OJJ53235.1 ater|EAU29731.1 atub|OJI82230.1 aver|OJJ03616.1 awen|OJJ35487.1 azon|OJJ47003.1

Group2894: abra|OJJ72835.1 acar|OOF96037.1 acla|EAW11405.1 afis|EAW24016.1 afla|EED49143.1 afum|EAL87446.1 agla|OJJ86950.1 akaw|GAA85432.1 aluc|OJZ83335.1 anid|CBF77416.1 anig|EHA24211.1 aory|EIT78565.1 asyd|OJJ53234.1 ater|EAU29730.1 atub|OJI82232.1 aver|OJJ03617.1 awen|OJJ35488.1 azon|OJJ47001.1

Group2895: abra|OJJ72837.1 acar|OOF96039.1 acla|EAW06923.1 afis|EAW16540.1 afla|EED53743.1 afum|EAL89137.1 agla|OJJ85576.1 akaw|GAA85434.1 aluc|OJZ83337.1 anid|CBF69989.1 anig|EHA24213.1 aory|EIT75293.1 asyd|OJJ58892.1 ater|EAU39054.1 atub|OJI82234.1 aver|OJJ01493.1 awen|OJJ40280.1 azon|OJJ49143.1

Group2896: abra|OJJ72840.1 acar|OOF96042.1 acla|EAW11402.1 afis|EAW24019.1 afla|EED49137.1 afum|EAL87449.1 agla|OJJ86952.1 akaw|GAA85442.1 aluc|OJZ83347.1 anid|CBF77420.1 anig|EHA24215.1 aory|EIT78560.1 asyd|OJJ53232.1 ater|EAU29727.1 atub|OJI82241.1 aver|OJJ03619.1 awen|OJJ35492.1 azon|OJJ46998.1

Group2897: abra|OJJ72844.1 acar|OOF96045.1 acla|EAW11399.1 afis|EAW24022.1 afla|EED49132.1 afum|EAL87453.1 agla|OJJ86955.1 akaw|GAA85445.1 aluc|OJZ83352.1 anid|CBF75300.1 anig|EHA24218.1 aory|EIT78561.1 asyd|OJJ59881.1 ater|EAU29724.1 atub|OJI82244.1 aver|OJJ02405.1 awen|OJJ35495.1 azon|OJJ46994.1

Group2898: abra|OJJ72847.1 acar|OOF96048.1 acla|EAW11396.1 afis|EAW24025.1 afla|EED49129.1 afum|EAL87456.1 agla|OJJ86958.1 akaw|GAA85448.1 aluc|OJZ83355.1 anid|CBF75305.1 anig|EHA24221.1 aory|EIT78574.1 asyd|OJJ59876.1 ater|EAU29719.1 atub|OJI82247.1 aver|OJJ02410.1 awen|OJJ35498.1 azon|OJJ46991.1

Group2899: abra|OJJ72850.1 acar|OOF96051.1 acla|EAW11393.1 afis|EAW24028.1 afla|EED49126.1 afum|EAL87459.1 agla|OJJ86961.1 akaw|GAA85451.1 aluc|OJZ83359.1 anid|CBF75310.1 anig|EHA24224.1 aory|EIT78543.1 asyd|OJJ59874.1 ater|EAU29716.1 atub|OJI82250.1 aver|OJJ02413.1 awen|OJJ35501.1 azon|OJJ46988.1

Group2900: abra|OJJ72853.1 acar|OOF96052.1 acla|EAW11391.1 afis|EAW24031.1 afla|EED49123.1 afum|EAL87462.1 agla|OJJ87001.1 akaw|GAA85454.1 aluc|OJZ83362.1 anid|CBF75316.1 anig|EHA24227.1 aory|EIT78584.1 asyd|OJJ59871.1 ater|EAU29713.1 atub|OJI82253.1 aver|OJJ02416.1 awen|OJJ35503.1 azon|OJJ46983.1

Group2901: abra|OJJ72854.1 acar|OOF96053.1 acla|EAW11390.1 afis|EAW24032.1 afla|EED49121.1 afum|EAL87463.1 agla|OJJ87000.1 akaw|GAA85455.1 aluc|OJZ83363.1 anid|CBF75318.1 anig|EHA24228.1 aory|EIT78515.1 asyd|OJJ59870.1 ater|EAU29712.1 atub|OJI82254.1 aver|OJJ02417.1 awen|OJJ35504.1 azon|OJJ46981.1

Group2902: abra|OJJ72858.1 acar|OOF96055.1 acla|EAW11388.1 afis|EAW24034.1 afla|EED49119.1 afum|EAL87465.1 agla|OJJ86998.1 akaw|GAA85457.1 aluc|OJZ83365.1 anid|CBF75324.1 anig|EHA24230.1 aory|EIT78541.1 asyd|OJJ59868.1 ater|EAU29710.1 atub|OJI82256.1 aver|OJJ02419.1 awen|OJJ35506.1 azon|OJJ46978.1

Group2903: abra|OJJ72860.1 acar|OOF96057.1 acla|EAW11386.1 afis|EAW18110.1 afla|EED49117.1 afum|EAL87467.1 agla|OJJ86996.1 akaw|GAA85459.1 aluc|OJZ83367.1 anid|CBF75328.1 anig|EHA24232.1 aory|EIT78582.1 asyd|OJJ59865.1 ater|EAU29708.1 atub|OJI82259.1 aver|OJJ02422.1 awen|OJJ35508.1 azon|OJJ47630.1

Group2904: abra|OJJ72861.1 acar|OOF96058.1 acla|EAW11385.1 afis|EAW18111.1 afla|EED49114.1 afum|EAL87468.1 agla|OJJ86995.1 akaw|GAA85460.1 aluc|OJZ83368.1 anid|CBF75330.1 anig|EHA24233.1 aory|EIT78525.1 asyd|OJJ59864.1 ater|EAU29707.1 atub|OJI82260.1 aver|OJJ02423.1 awen|OJJ35509.1 azon|OJJ46976.1

Group2905: abra|OJJ72862.1 acar|OOF96060.1 acla|EAW11384.1 afis|EAW18112.1 afla|EED49113.1 afum|EAL87469.1 agla|OJJ86994.1 akaw|GAA85461.1 aluc|OJZ83369.1 anid|CBF75332.1 anig|EHA24234.1 aory|EIT78586.1 asyd|OJJ59863.1 ater|EAU29706.1 atub|OJI82261.1 aver|OJJ02424.1 awen|OJJ35510.1 azon|OJJ46975.1

Group2906: abra|OJJ72863.1 acar|OOF96062.1 acla|EAW11379.1 afis|EAW18116.1 afla|EED58041.1 afum|EAL87473.1 agla|OJJ86980.1 akaw|GAA85462.1 aluc|OJZ83376.1 anid|CBF75340.1 anig|EHA24235.1 aory|EIT79770.1 asyd|OJJ59859.1 ater|EAU29701.1 atub|OJI82262.1 aver|OJJ02428.1 awen|OJJ35515.1 azon|OJJ46970.1

Group2907: abra|OJJ72865.1 acar|OOF96064.1 acla|EAW11382.1 afis|EAW18114.1 afla|EED58039.1 afum|EAL87471.1 agla|OJJ86982.1 akaw|GAA85464.1 aluc|OJZ83378.1 anid|CBF75336.1 anig|EHA24237.1 aory|EIT79795.1 asyd|OJJ59861.1 ater|EAU29703.1 atub|OJI82264.1 aver|OJJ02426.1 awen|OJJ35512.1 azon|OJJ46973.1

Group2908: abra|OJJ72866.1 acar|OOF96065.1 acla|EAW11383.1 afis|EAW18113.1 afla|EED58038.1 afum|EAL87470.1 agla|OJJ86991.1 akaw|GAA85465.1 aluc|OJZ83379.1 anid|CBF75334.1 anig|EHA24238.1 aory|EIT79794.1 asyd|OJJ59862.1 ater|EAU29705.1 atub|OJI82265.1 aver|OJJ02425.1 awen|OJJ35511.1 azon|OJJ46974.1

Group2909: abra|OJJ72869.1 acar|OOF96068.1 acla|EAW11376.1 afis|EAW18119.1 afla|EED58044.1 afum|EAL87477.1 agla|OJJ87011.1 akaw|GAA85468.1 aluc|OJZ83382.1 anid|CBF75346.1 anig|EHA24241.1 aory|EIT79722.1 asyd|OJJ59854.1 ater|EAU29698.1 atub|OJI82268.1 aver|OJJ02431.1 awen|OJJ35518.1 azon|OJJ46966.1

Group2910: abra|OJJ72871.1 acar|OOF96069.1 acla|EAW11375.1 afis|EAW18120.1 afla|EED58045.1 afum|EAL87478.1 agla|OJJ87014.1 akaw|GAA85469.1 aluc|OJZ83385.1 anid|CBF75348.1 anig|EHA24243.1 aory|EIT79798.1 asyd|OJJ59853.1 ater|EAU29697.1 atub|OJI82271.1 aver|OJJ02432.1 awen|OJJ35521.1 azon|OJJ46965.1

Group2911: abra|OJJ72874.1 acar|OOF96071.1 acla|EAW11374.1 afis|EAW18121.1 afla|EED58046.1 afum|EAL87479.1 agla|OJJ87015.1 akaw|GAA85470.1 aluc|OJZ83386.1 anid|CBF75352.1 anig|EHA24244.1

aory|EIT79741.1 asyd|OJJ59852.1 ater|EAU29695.1 atub|OJI82272.1 aver|OJJ02435.1 awen|OJJ35523.1 azon|OJJ46963.1

Group2912: abra|OJJ72880.1 acar|OOF96073.1 acla|EAW11366.1 afis|EAW18129.1 afla|EED58053.1 afum|EAL87486.1 agla|OJJ83795.1 akaw|GAA85472.1 aluc|OJZ83391.1 anid|CBF75366.1 anig|EHA24246.1 aory|EIT79707.1 asyd|OJJ59848.1 ater|EAU35448.1 atub|OJI82277.1 aver|OJJ02440.1 awen|OJJ35557.1 azon|OJJ46953.1

Group2913: abra|OJJ72886.1 acar|OOF96076.1 acla|EAW11364.1 afis|EAW18130.1 afla|EED58054.1 afum|EAL87487.1 agla|OJJ83802.1 akaw|GAA85473.1 aluc|OJZ83394.1 anid|CBF75370.1 anig|EHA24247.1 aory|EIT79721.1 asyd|OJJ59847.1 ater|EAU35452.1 atub|OJI82280.1 aver|OJJ02441.1 awen|OJJ35561.1 azon|OJJ46951.1

Group2914: abra|OJJ72887.1 acar|OOF96077.1 acla|EAW11363.1 afis|EAW18132.1 afla|EED58056.1 afum|EAL87489.1 agla|OJJ83814.1 akaw|GAA85474.1 aluc|OJZ83395.1 anid|CBF75372.1 anig|EHA24248.1 aory|EIT79771.1 asyd|OJJ59846.1 ater|EAU35454.1 atub|OJI82281.1 aver|OJJ02442.1 awen|OJJ35571.1 azon|OJJ46949.1

Group2915: abra|OJJ72890.1 acar|OOF96080.1 acla|EAW11360.1 afis|EAW18134.1 afla|EED58059.1 afum|EAL87491.1 agla|OJJ83818.1 akaw|GAA85477.1 aluc|OJZ83398.1 anid|CBF75380.1 anig|EHA24251.1 aory|EIT79745.1 asyd|OJJ59843.1 ater|EAU35457.1 atub|OJI82284.1 aver|OJJ02446.1 awen|OJJ35576.1 azon|OJJ46946.1

Group2916: abra|OJJ72892.1 acar|OOF96083.1 acla|EAW11358.1 afis|EAW18136.1 afla|EED58061.1 afum|EAL87493.1 agla|OJJ83819.1 akaw|GAA85479.1 aluc|OJZ83400.1 anid|CBF75376.1 anig|EHA24252.1 aory|EIT79756.1 asyd|OJJ59840.1 ater|EAU35459.1 atub|OJI82288.1 aver|OJJ02449.1 awen|OJJ35578.1 azon|OJJ46945.1

Group2917: abra|OJJ72894.1 acar|OOF96085.1 acla|EAW11357.1 afis|EAW18137.1 afla|EED58062.1 afum|EAL87494.1 agla|OJJ83820.1 akaw|GAA85481.1 aluc|OJZ83403.1 anid|CBF75384.1 anig|EHA24254.1 aory|EIT79803.1 asyd|OJJ59838.1 ater|EAU35460.1 atub|OJI82291.1 aver|OJJ02450.1 awen|OJJ35579.1 azon|OJJ46944.1

Group2918: abra|OJJ72898.1 acar|OOF96088.1 acla|EAW11353.1 afis|EAW18141.1 afla|EED58067.1 afum|EAL87498.1 agla|OJJ83826.1 akaw|GAA85485.1 aluc|OJZ83407.1 anid|CBF81129.1 anig|EHA24258.1 aory|EIT79748.1 asyd|OJJ56491.1 ater|EAU35464.1 atub|OJI82296.1 aver|OJJ06227.1 awen|OJJ35583.1 azon|OJJ46939.1

Group2919: abra|OJJ72904.1 acar|OOF89820.1 acla|EAW11350.1 afis|EAW18145.1 afla|EED58072.1 afum|EAL87502.1 agla|OJJ83828.1 akaw|GAA85489.1 aluc|OJZ83414.1 anid|CBF81120.1 anig|EHA24263.1 aory|EIT79155.1 asyd|OJJ56488.1 ater|EAU35468.1 atub|OJI82301.1 aver|OJJ06231.1 awen|OJJ35588.1 azon|OJJ46937.1

Group2920: abra|OJJ72906.1 acar|OOF96089.1 acla|EAW11348.1 afis|EAW18147.1 afla|EED58075.1 afum|EAL87505.1 agla|OJJ83832.1 akaw|GAA85491.1 aluc|OJZ83416.1 anid|CBF81116.1 anig|EHA24265.1 aory|EIT79152.1 asyd|OJJ56486.1 ater|EAU35446.1 atub|OJI82303.1 aver|OJJ06233.1 awen|OJJ35590.1 azon|OJJ46935.1

Group2921: abra|OJJ72909.1 acar|OOF96092.1 acla|EAW11344.1 afis|EAW18150.1 afla|EED58078.1 afum|EAL87508.1 agla|OJJ83835.1 akaw|GAA85494.1 aluc|OJZ83419.1 anid|CBF81110.1 anig|EHA24268.1 aory|EIT79111.1 asyd|OJJ56483.1 ater|EAU35472.1 atub|OJI82306.1 aver|OJJ06236.1 awen|OJJ35593.1 azon|OJJ46915.1

Group2922: abra|OJJ72910.1 acar|OOF96093.1 acla|EAW11343.1 afis|EAW18151.1 afla|EED58080.1 afum|EAL87509.1 agla|OJJ83836.1 akaw|GAA85495.1 aluc|OJZ83421.1 anid|CBF81108.1 anig|EHA24269.1 aory|EIT79142.1 asyd|OJJ56481.1 ater|EAU35473.1 atub|OJI82307.1 aver|OJJ06238.1 awen|OJJ35594.1 azon|OJJ46916.1

Group2923: abra|OJJ72912.1 acar|OOF96094.1 acla|EAW06479.1 afis|EAW15974.1 afla|EED58082.1 afum|EAL88606.1 agla|OJJ83838.1 akaw|GAA85496.1 aluc|OJZ83422.1 anid|CBF81105.1 anig|EHA24270.1 aory|EIT79133.1 asyd|OJJ56479.1 ater|EAU35474.1 atub|OJI82308.1 aver|OJJ06239.1 awen|OJJ35597.1 azon|OJJ46918.1

Group2924: abra|OJJ72913.1 acar|OOF96095.1 acla|EAW06478.1 afis|EAW15973.1 afla|EED58083.1 afum|EAL88605.1 agla|OJJ83842.1 akaw|GAA85497.1 aluc|OJZ83423.1 anid|CBF81101.1 anig|EHA24271.1 aory|EIT79117.1 asyd|OJJ56478.1 ater|EAU35475.1 atub|OJI82309.1 aver|OJJ06240.1 awen|OJJ35599.1 azon|OJJ46919.1

Group2925: abra|OJJ72914.1 acar|OOF96096.1 acla|EAW06476.1 afis|EAW15971.1 afla|EED58085.1 afum|EAL88603.1 agla|OJJ83844.1 akaw|GAA85498.1 aluc|OJZ83424.1 anid|CBF81098.1 anig|EHA24272.1 aory|EIT79112.1 asyd|OJJ56476.1 ater|EAU35477.1 atub|OJI82310.1 aver|OJJ06242.1 awen|OJJ35601.1 azon|OJJ46921.1

Group2926: abra|OJJ72917.1 acar|OOF96099.1 acla|EAW06474.1 afis|EAW15968.1 afla|EED58089.1 afum|EAL88601.1 agla|OJJ89290.1 akaw|GAA85500.1 aluc|OJZ83427.1 anid|CBF81094.1 anig|EHA24279.1 aory|EIT79146.1 asyd|OJJ56470.1 ater|EAU35480.1 atub|OJI82315.1 aver|OJJ06249.1 awen|OJJ35605.1 azon|OJJ46925.1

Group2927: abra|OJJ72920.1 acar|OOF96102.1 acla|EAW06471.1 afis|EAW15965.1 afla|EED58091.1 afum|EAL88598.1 agla|OJJ89346.1 akaw|GAA85503.1 aluc|OJZ83430.1 anid|CBF81089.1 anig|EHA24281.1 aory|EIT79110.1 asyd|OJJ56467.1 ater|EAU35487.1 atub|OJI82318.1 aver|OJJ06252.1 awen|OJJ35608.1 azon|OJJ46928.1

Group2928: abra|OJJ72922.1 acar|OOF96103.1 acla|EAW06469.1 afis|EAW15964.1 afla|EED58093.1 afum|EAL88597.1 agla|OJJ89348.1 akaw|GAA85504.1 aluc|OJZ83432.1 anid|CBF81085.1 anig|EHA24282.1 aory|EIT79125.1 asyd|OJJ56465.1 ater|EAU35488.1 atub|OJI82321.1 aver|OJJ06254.1 awen|OJJ35610.1 azon|OJJ46930.1

Group2929: abra|OJJ72930.1 acar|OOF96110.1 acla|EAW06460.1 afis|EAW15956.1 afla|EED58102.1 afum|EAL88589.1 agla|OJJ89357.1 akaw|GAA85512.1 aluc|OJZ83440.1 anid|CBF81067.1 anig|EHA24286.1 aory|EIT79135.1 asyd|OJJ56458.1 ater|EAU35501.1 atub|OJI82331.1 aver|OJJ06264.1 awen|OJJ35618.1 azon|OJJ46904.1

Group2930: abra|OJJ72931.1 acar|OOF96111.1 acla|EAW06459.1 afis|EAW15955.1 afla|EED58103.1 afum|EAL88588.1 agla|OJJ89358.1 akaw|GAA85513.1 aluc|OJZ83441.1 anid|CBF81065.1 anig|EHA24287.1 aory|EIT79109.1 asyd|OJJ56457.1 ater|EAU35502.1 atub|OJI82332.1 aver|OJJ06265.1 awen|OJJ35619.1 azon|OJJ46903.1

Group2931: abra|OJJ72935.1 acar|OOF96116.1 acla|EAW06456.1 afis|EAW15952.1 afla|EED58106.1 afum|EAL88585.1 agla|OJJ89362.1 akaw|GAA85516.1 aluc|OJZ83444.1 anid|CBF81059.1 anig|EHA24290.1 aory|EIT79114.1 asyd|OJJ56454.1 ater|EAU35505.1 atub|OJI82335.1 aver|OJJ06268.1 awen|OJJ35624.1 azon|OJJ46901.1

Group2932: abra|OJJ72936.1 acar|OOF96117.1 acla|EAW06455.1 afis|EAW15951.1 afla|EED58107.1 afum|EAL88584.1 agla|OJJ89363.1 akaw|GAA85517.1 aluc|OJZ83445.1 anid|CBF81057.1 anig|EHA24291.1 aory|EIT79166.1 asyd|OJJ56453.1 ater|EAU35506.1 atub|OJI82336.1 aver|OJJ06269.1 awen|OJJ35625.1 azon|OJJ46900.1

Group2933: abra|OJJ72937.1 acar|OOF96119.1 acla|EAW06454.1 afis|EAW15950.1 afla|EED58108.1 afum|EAL88583.1 agla|OJJ89364.1 akaw|GAA85518.1 aluc|OJZ83446.1 anid|CBF81056.1 anig|EHA24292.1 aory|EIT79143.1 asyd|OJJ56451.1 ater|EAU35507.1 atub|OJI82337.1 aver|OJJ06270.1 awen|OJJ35626.1 azon|OJJ46899.1

Group2934: abra|OJJ72942.1 acar|OOF96123.1 acla|EAW11209.1 afis|EAW15942.1 afla|EED58116.1 afum|EAL88576.1 agla|OJJ89368.1 akaw|GAA85524.1 aluc|OJZ83452.1 anid|CBF80959.1 anig|EHA24298.1 aory|EIT79144.1 asyd|OJJ56346.1 ater|EAU35514.1 atub|OJI82344.1 aver|OJJ06367.1 awen|OJJ35640.1 azon|OJJ46895.1

Group2935: abra|OJJ72945.1 acar|OOF96126.1 acla|EAW11205.1 afis|EAW15935.1 afla|EED58120.1 afum|EAL88571.1 agla|OJJ89371.1 akaw|GAA85527.1 aluc|OJZ83455.1 anid|CBF80966.1 anig|EHA24301.1 aory|EIT79156.1 asyd|OJJ56349.1 ater|EAU35517.1 atub|OJI82347.1 aver|OJJ06364.1 awen|OJJ35644.1 azon|OJJ46892.1

Group2936: abra|OJJ72949.1 acar|OOF96130.1 acla|EAW11195.1 afis|EAW15925.1 afla|EED54936.1 afum|EAL88561.1 agla|OJJ89380.1 akaw|GAA85530.1 aluc|OJZ83459.1 anid|CBF81019.1 anig|EHA24303.1 aory|EIT80380.1 asyd|OJJ56382.1 ater|EAU35530.1 atub|OJI82351.1 aver|OJJ06331.1 awen|OJJ35655.1 azon|OJJ46884.1

Group2937: abra|OJJ72956.1 acar|OOF96137.1 acla|EAW11201.1 afis|EAW15931.1 afla|EED54930.1 afum|EAL88567.1 agla|OJJ89375.1 akaw|GAA85537.1 aluc|OJZ83465.1 anid|CBF81029.1 anig|EHA24309.1 aory|EIT80323.1 asyd|OJJ56388.1 ater|EAU35521.1 atub|OJI82358.1 aver|OJJ06325.1 awen|OJJ35648.1 azon|OJJ46889.1

Group2938: abra|OJJ72960.1 acar|OOF96142.1 acla|EAW11192.1 afis|EAW15922.1 afla|EED54938.1 afum|EAL88559.1 agla|OJJ89386.1 akaw|GAA85540.1 aluc|OJZ83468.1 anid|CBF81013.1 anig|EHA24312.1 aory|EIT80338.1 asyd|OJJ56379.1 ater|EAU35533.1 atub|OJI82362.1 aver|OJJ06334.1 awen|OJJ35658.1 azon|OJJ46881.1

Group2939: abra|OJJ72967.1 acar|OOF96149.1 acla|EAW11187.1 afis|EAW15918.1 afla|EED54943.1 afum|EAL88554.1 agla|OJJ89393.1 akaw|GAA85546.1 aluc|OJZ83478.1 anid|CBF81002.1 anig|EHA24315.1 aory|EIT80355.1 asyd|OJJ56373.1 ater|EAU35538.1 atub|OJI82368.1 aver|OJJ06340.1 awen|OJJ35661.1 azon|OJJ44132.1

Group2940: abra|OJJ72971.1 acar|OOF96150.1 acla|EAW08037.1 afis|EAW23072.1 afla|EED54944.1 afum|EAL88407.1 agla|OJJ89394.1 akaw|GAA85547.1 aluc|OJZ83482.1 anid|CBF80992.1 anig|EHA24317.1

aory|EIT80389.1 asyd|OJJ56368.1 ater|EAU35539.1 atub|OJI82372.1 aver|OJJ06346.1 awen|OJJ35663.1 azon|OJJ44133.1

Group2941: abra|OJJ72972.1 acar|OOF96151.1 acla|EAW08036.1 afis|EAW23071.1 afla|EED54945.1 afum|EAL88408.1 agla|OJJ89395.1 akaw|GAA85548.1 aluc|OJZ83483.1 anid|CBF80994.1 anig|EHA24318.1 aory|EIT80359.1 asyd|OJJ56369.1 ater|EAU35540.1 atub|OJI82373.1 aver|OJJ06345.1 awen|OJJ35664.1 azon|OJJ44134.1

Group2942: abra|OJJ72973.1 acar|OOF96152.1 acla|EAW08035.1 afis|EAW23070.1 afla|EED54946.1 afum|EAL88409.1 agla|OJJ89396.1 akaw|GAA85549.1 aluc|OJZ83484.1 anid|CBF80996.1 anig|EHA24319.1 aory|EIT80382.1 asyd|OJJ56370.1 ater|EAU37593.1 atub|OJI82374.1 aver|OJJ06344.1 awen|OJJ35665.1 azon|OJJ44135.1

Group2943: abra|OJJ72975.1 acar|OOF96153.1 acla|EAW08034.1 afis|EAW23069.1 afla|EED54947.1 afum|EAL88410.1 agla|OJJ89397.1 akaw|GAA85550.1 aluc|OJZ83485.1 anid|CBF80998.1 anig|EHA24320.1 aory|EIT80368.1 asyd|OJJ56371.1 ater|EAU37596.1 atub|OJI82376.1 aver|OJJ06343.1 awen|OJJ35666.1 azon|OJJ44137.1

Group2944: abra|OJJ72976.1 acar|OOF96154.1 acla|EAW08033.1 afis|EAW23068.1 afla|EED54948.1 afum|EAL88411.1 agla|OJJ89398.1 akaw|GAA85551.1 aluc|OJZ83486.1 anid|CBF81000.1 anig|EHA24321.1 aory|EIT80295.1 asyd|OJJ56372.1 ater|EAU37597.1 atub|OJI82377.1 aver|OJJ06342.1 awen|OJJ35667.1 azon|OJJ44138.1

Group2945: abra|OJJ72979.1 acar|OOF96158.1 acla|EAW08031.1 afis|EAW23065.1 afla|EED54952.1 afum|EAL88414.1 agla|OJJ89409.1 akaw|GAA85554.1 aluc|OJZ83489.1 anid|CBF80981.1 anig|EHA24324.1 aory|EIT80321.1 asyd|OJJ56358.1 ater|EAU37601.1 atub|OJI82381.1 aver|OJJ06356.1 awen|OJJ35670.1 azon|OJJ44140.1

Group2946: abra|OJJ72980.1 acar|OOF96156.1 acla|EAW08030.1 afis|EAW23064.1 afla|EED54953.1 afum|EAL88415.1 agla|OJJ89410.1 akaw|GAA85555.1 aluc|OJZ83490.1 anid|CBF80979.1 anig|EHA24325.1 aory|EIT80361.1 asyd|OJJ56357.1 ater|EAU37602.1 atub|OJI82382.1 aver|OJJ06357.1 awen|OJJ35671.1 azon|OJJ44141.1

Group2947: abra|OJJ72982.1 acar|OOF96159.1 acla|EAW08028.1 afis|EAW23062.1 afla|EED54955.1 afum|EAL88417.1 agla|OJJ89412.1 akaw|GAA85557.1 aluc|OJZ83492.1 anid|CBF80975.1 anig|EHA24327.1 aory|EIT80363.1 asyd|OJJ56355.1 ater|EAU37603.1 atub|OJI82386.1 aver|OJJ06359.1 awen|OJJ35673.1 azon|OJJ44142.1

Group2948: abra|OJJ72983.1 acar|OOF96160.1 acla|EAW08027.1 afis|EAW23061.1 afla|EED54957.1 afum|EAL88418.1 agla|OJJ89414.1 akaw|GAA85558.1 aluc|OJZ83493.1 anid|CBF80973.1 anig|EHA24328.1 aory|EIT80353.1 asyd|OJJ56354.1 ater|EAU37604.1 atub|OJI82387.1 aver|OJJ06360.1 awen|OJJ35676.1 azon|OJJ44143.1

Group2949: abra|OJJ72984.1 acar|OOF96161.1 acla|EAW08026.1 afis|EAW23060.1 afla|EED54958.1 afum|EAL88419.1 agla|OJJ89415.1 akaw|GAA85559.1 aluc|OJZ83494.1 anid|CBF80972.1 anig|EHA24329.1 aory|EIT80387.1 asyd|OJJ56353.1 ater|EAU37605.1 atub|OJI82388.1 aver|OJJ06361.1 awen|OJJ35677.1 azon|OJJ44144.1

Group2950: abra|OJJ72985.1 acar|OOF96162.1 acla|EAW08025.1 afis|EAW23059.1 afla|EED54959.1 afum|EAL88420.1 agla|OJJ89417.1 akaw|GAA85560.1 aluc|OJZ83495.1 anid|CBF80970.1 anig|EHA24330.1 aory|EIT80388.1 asyd|OJJ56352.1 ater|EAU37606.1 atub|OJI82389.1 aver|OJJ06362.1 awen|OJJ35678.1 azon|OJJ44146.1

Group2951: abra|OJJ72989.1 acar|OOF96165.1 acla|EAW08022.1 afis|EAW23056.1 afla|EED54961.1 afum|EAL88424.1 agla|OJJ89419.1 akaw|GAA85563.1 aluc|OJZ83497.1 anid|CBF81039.1 anig|EHA24332.1 aory|EIT80362.1 asyd|OJJ56392.1 ater|EAU37608.1 atub|OJI82391.1 aver|OJJ06321.1 awen|OJJ35680.1 azon|OJJ44148.1

Group2952: abra|OJJ72990.1 acar|OOF96166.1 acla|EAW08021.1 afis|EAW23055.1 afla|EED54962.1 afum|EAL88425.1 agla|OJJ89420.1 akaw|GAA85564.1 aluc|OJZ83498.1 anid|CBF81041.1 anig|EHA24333.1 aory|EIT80303.1 asyd|OJJ56393.1 ater|EAU37609.1 atub|OJI82392.1 aver|OJJ06320.1 awen|OJJ35681.1 azon|OJJ44149.1

Group2953: abra|OJJ72991.1 acar|OOF96167.1 acla|EAW08020.1 afis|EAW23054.1 afla|EED54964.1 afum|EAL88426.1 agla|OJJ89422.1 akaw|GAA85565.1 aluc|OJZ83499.1 anid|CBF81043.1 anig|EHA24334.1 aory|EIT80378.1 asyd|OJJ56441.1 ater|EAU37610.1 atub|OJI82393.1 aver|OJJ06280.1 awen|OJJ35682.1 azon|OJJ44150.1

Group2954: abra|OJJ72997.1 acar|OOF96173.1 acla|EAW08016.1 afis|EAW23050.1 afla|EED54968.1 afum|EAL88430.1 agla|OJJ89429.1 akaw|GAA85571.1 aluc|OJZ83505.1 anid|CBF80953.1 anig|EHA24338.1 aory|EIT80340.1 asyd|OJJ56343.1 ater|EAU29602.1 atub|OJI82399.1 aver|OJJ06370.1 awen|OJJ35689.1 azon|OJJ44154.1

Group2955: abra|OJJ72998.1 acar|OOF89938.1 acla|EAW08015.1 afis|EAW23049.1 afla|EED54969.1 afum|EAL88431.1 agla|OJJ89430.1 akaw|GAA85572.1 aluc|OJZ83506.1 anid|CBF80951.1 anig|EHA24339.1 aory|EIT80346.1 asyd|OJJ56342.1 ater|EAU29601.1 atub|OJI82400.1 aver|OJJ06371.1 awen|OJJ35690.1 azon|OJJ44155.1

Group2956: abra|OJJ72999.1 acar|OOF89939.1 acla|EAW08014.1 afis|EAW23048.1 afla|EED54970.1 afum|EAL88432.1 agla|OJJ89431.1 akaw|GAA85573.1 aluc|OJZ83509.1 anid|CBF80949.1 anig|EHA24340.1 aory|EIT80379.1 asyd|OJJ56341.1 ater|EAU29600.1 atub|OJI82402.1 aver|OJJ06372.1 awen|OJJ35691.1 azon|OJJ44157.1

Group2957: abra|OJJ73002.1 acar|OOF96175.1 acla|EAW08011.1 afis|EAW23046.1 afla|EED54973.1 afum|EAL88435.1 agla|OJJ89432.1 akaw|GAA85580.1 aluc|OJZ83516.1 anid|CBF80946.1 anig|EHA24341.1 aory|EIT80343.1 asyd|OJJ56339.1 ater|EAU29614.1 atub|OJI82407.1 aver|OJJ06374.1 awen|OJJ35694.1 azon|OJJ44158.1

Group2958: abra|OJJ73003.1 acar|OOF96176.1 acla|EAW08010.1 afis|EAW23045.1 afla|EED54974.1 afum|EAL88436.1 agla|OJJ89433.1 akaw|GAA85581.1 aluc|OJZ83517.1 anid|CBF80944.1 anig|EHA24342.1 aory|EIT80339.1 asyd|OJJ56338.1 ater|EAU29613.1 atub|OJI82408.1 aver|OJJ06375.1 awen|OJJ35695.1 azon|OJJ44159.1

Group2959: abra|OJJ73006.1 acar|OOF96178.1 acla|EAW08007.1 afis|EAW23042.1 afla|EED54976.1 afum|EAL88439.1 agla|OJJ89436.1 akaw|GAA85584.1 aluc|OJZ83521.1 anid|CBF80938.1 anig|EHA24344.1 aory|EIT80316.1 asyd|OJJ56312.1 ater|EAU29617.1 atub|OJI82410.1 aver|OJJ06402.1 awen|OJJ35700.1 azon|OJJ44162.1

Group2960: abra|OJJ73008.1 acar|OOF96180.1 acla|EAW08005.1 afis|EAW23040.1 afla|EED54978.1 afum|EAL88441.1 agla|OJJ89438.1 akaw|GAA85586.1 aluc|OJZ83523.1 anid|CBF80935.1 anig|EHA24346.1 aory|EIT80392.1 asyd|OJJ56314.1 ater|EAU29603.1 atub|OJI82412.1 aver|OJJ06400.1 awen|OJJ35702.1 azon|OJJ44164.1

Group2961: abra|OJJ73016.1 acar|OOF96186.1 acla|EAW07997.1 afis|EAW23032.1 afla|EED54984.1 afum|EAL88450.1 agla|OJJ89445.1 akaw|GAA85594.1 aluc|OJZ83531.1 anid|CBF80910.1 anig|EHA24353.1 aory|EIT80327.1 asyd|OJJ56328.1 ater|EAU29625.1 atub|OJI82421.1 aver|OJJ06385.1 awen|OJJ35713.1 azon|OJJ44172.1

Group2962: abra|OJJ73022.1 acar|OOF96190.1 acla|EAW07993.1 afis|EAW23028.1 afla|EED54987.1 afum|EAL88453.2 agla|OJJ89451.1 akaw|GAA85597.1 aluc|OJZ83535.1 anid|CBF80920.1 anig|EHA24356.1 aory|EIT80371.1 asyd|OJJ56322.1 ater|EAU29630.1 atub|OJI82426.1 aver|OJJ06391.1 awen|OJJ35717.1 azon|OJJ44176.1

Group2963: abra|OJJ73023.1 acar|OOF96191.1 acla|EAW07992.1 afis|EAW23027.1 afla|EED54988.1 afum|EAL88455.1 agla|OJJ89452.1 akaw|GAA85598.1 aluc|OJZ83536.1 anid|CBF80918.1 anig|EHA24357.1 aory|EIT80332.1 asyd|OJJ56323.1 ater|EAU29629.1 atub|OJI82427.1 aver|OJJ06390.1 awen|OJJ35718.1 azon|OJJ44177.1

Group2964: abra|OJJ73025.1 acar|OOF96192.1 acla|EAW07991.1 afis|EAW23026.1 afla|EED54989.1 afum|EAL88456.1 agla|OJJ89453.1 akaw|GAA85599.1 aluc|OJZ83537.1 anid|CBF80916.1 anig|EHA24358.1 aory|EIT80337.1 asyd|OJJ56324.1 ater|EAU29628.1 atub|OJI82428.1 aver|OJJ06389.1 awen|OJJ35719.1 azon|OJJ44178.1

Group2965: abra|OJJ73029.1 acar|OOF96195.1 acla|EAW07988.1 afis|EAW23023.1 afla|EED54993.1 afum|EAL88459.1 agla|OJJ89456.1 akaw|GAA85602.1 aluc|OJZ83540.1 anid|CBF80905.1 anig|EHA24360.1 aory|EIT80347.1 asyd|OJJ56332.1 ater|EAU29651.1 atub|OJI82432.1 aver|OJJ06382.1 awen|OJJ30720.1 azon|OJJ44181.1

Group2966: abra|OJJ73030.1 acar|OOF96196.1 acla|EAW07987.1 afis|EAW23022.1 afla|EED54994.1 afum|EAL88460.1 agla|OJJ89457.1 akaw|GAA85603.1 aluc|OJZ83541.1 anid|CBF80903.1 anig|EHA24361.1 aory|EIT80374.1 asyd|OJJ56333.1 ater|EAU29253.1 atub|OJI82433.1 aver|OJJ06381.1 awen|OJJ30721.1 azon|OJJ44182.1

Group2967: abra|OJJ73031.1 acar|OOF96197.1 acla|EAW07986.1 afis|EAW23021.1 afla|EED54995.1 afum|EAL88461.1 agla|OJJ89458.1 akaw|GAA85604.1 aluc|OJZ83542.1 anid|CBF80901.1 anig|EHA24362.1 aory|EIT80385.1 asyd|OJJ56334.1 ater|EAU29652.1 atub|OJI82434.1 aver|OJJ06380.1 awen|OJJ30722.1 azon|OJJ44183.1

Group2968: abra|OJJ73032.1 acar|OOF96198.1 acla|EAW07985.1 afis|EAW23020.1 afla|EED54996.1 afum|EAL88462.1 agla|OJJ89459.1 akaw|GAA85605.1 aluc|OJZ83543.1 anid|CBF80900.1 anig|EHA24363.1 aory|EIT80298.1 asyd|OJJ56335.1 ater|EAU29653.1 atub|OJI82435.1 aver|OJJ06379.1 awen|OJJ30723.1 azon|OJJ44184.1

Group2969: abra|OJJ73037.1 acar|OOF96200.1 acla|EAW07982.1 afis|EAW23017.1 afla|EED54999.1 afum|EAL88464.1 agla|OJJ89463.1 akaw|GAA85608.1 aluc|OJZ83548.1 anig|EHA24366.1 aory|EIT80350.1

asyd|OJJ56309.1 ater|EAU29657.1 atub|OJI82439.1 aver|OJJ06405.1 awen|OJJ29535.1 azon|OJJ44187.1 anid|CBF80894.1

Group2970: abra|OJJ73038.1 acar|OOF96201.1 acla|EAW07981.1 afis|EAW23016.1 afla|EED55000.1 afum|EAL88465.1 agla|OJJ89464.1 akaw|GAA85609.1 aluc|OJZ83549.1 anid|CBF80896.1 anig|EHA24367.1 aory|EIT80294.1 asyd|OJJ56310.1 ater|EAU29658.1 atub|OJI82440.1 aver|OJJ06404.1 awen|OJJ29534.1 azon|OJJ44188.1

Group2971: abra|OJJ73039.1 acar|OOF96204.1 acla|EAW07980.1 afis|EAW23015.1 afla|EED55001.1 afum|EAL88466.1 agla|OJJ89465.1 akaw|GAA85610.1 aluc|OJZ83550.1 anid|CBF80890.1 anig|EHA24368.1 aory|EIT80296.1 asyd|OJJ56307.1 ater|EAU29659.1 atub|OJI82441.1 aver|OJJ06408.1 awen|OJJ30728.1 azon|OJJ44189.1

Group2972: abra|OJJ73040.1 acar|OOF96203.1 acla|EAW07979.1 afis|EAW23014.1 afla|EED55002.1 afum|EAL88467.2 agla|OJJ89466.1 akaw|GAA85611.1 aluc|OJZ83551.1 anid|CBF80888.1 anig|EHA24369.1 aory|EIT80286.1 asyd|OJJ56306.1 ater|EAU29660.1 atub|OJI82442.1 aver|OJJ06409.1 awen|OJJ30729.1 azon|OJJ44190.1

Group2973: abra|OJJ73041.1 acar|OOF96202.1 acla|EAW07978.1 afis|EAW23013.1 afla|EED55003.1 afum|EAL88468.1 agla|OJJ89467.1 akaw|GAA85612.1 aluc|OJZ83552.1 anid|CBF80886.1 anig|EHA24370.1 aory|EIT80391.1 asyd|OJJ56305.1 ater|EAU29661.1 atub|OJI82443.1 aver|OJJ06410.1 awen|OJJ30730.1 azon|OJJ44191.1

Group2974: abra|OJJ73042.1 acar|OOF96205.1 acla|EAW07977.1 afis|EAW23012.1 afla|EED55004.1 afum|EAL88469.1 agla|OJJ89468.1 akaw|GAA85613.1 aluc|OJZ83553.1 anid|CBF80885.1 anig|EHA24371.1 aory|EIT80335.1 asyd|OJJ56304.1 ater|EAU29662.1 atub|OJI82444.1 aver|OJJ06411.1 awen|OJJ30731.1 azon|OJJ44192.1

Group2975: abra|OJJ73046.1 acar|OOF96208.1 acla|EAW07973.1 afis|EAW23008.1 afla|EED55007.1 afum|EAL88472.1 agla|OJJ89472.1 akaw|GAA85617.1 aluc|OJZ83557.1 anid|CBF80879.1 anig|EHA24375.1 aory|EIT80310.1 asyd|OJJ56302.1 ater|EAU29665.1 atub|OJI82447.1 aver|OJJ06415.1 awen|OJJ30733.1 azon|OJJ44196.1

Group2976: abra|OJJ73048.1 acar|OOF96211.1 acla|EAW07971.1 afis|EAW23006.1 afla|EED55009.1 afum|EAL88474.1 agla|OJJ89473.1 akaw|GAA85619.1 aluc|OJZ83559.1 anid|CBF80875.1 anig|EHA24377.1 aory|EIT80349.1 asyd|OJJ56300.1 ater|EAU29667.1 atub|OJI82449.1 aver|OJJ06417.1 awen|OJJ30735.1 azon|OJJ44197.1

Group2977: abra|OJJ73049.1 acar|OOF96212.1 acla|EAW07970.1 afis|EAW23005.1 afla|EED55010.1 afum|EAL88475.1 agla|OJJ89474.1 akaw|GAA85620.1 aluc|OJZ83560.1 anid|CBF80873.1 anig|EHA24378.1 aory|EIT80326.1 asyd|OJJ56299.1 ater|EAU29668.1 atub|OJI82450.1 aver|OJJ06418.1 awen|OJJ30736.1 azon|OJJ44198.1

Group2978: abra|OJJ73050.1 acar|OOF96213.1 acla|EAW07969.1 afis|EAW23004.1 afla|EED55011.1 afum|EAL88476.2 agla|OJJ89475.1 akaw|GAA85621.1 aluc|OJZ83561.1 anid|CBF80871.1 anig|EHA24379.1 aory|EIT80312.1 asyd|OJJ56298.1 ater|EAU29669.1 atub|OJI82451.1 aver|OJJ06419.1 awen|OJJ30737.1 azon|OJJ44199.1

Group2979: abra|OJJ73051.1 acar|OOF96214.1 acla|EAW07968.1 afis|EAW23003.1 afla|EED55012.1 afum|EAL88477.1 agla|OJJ89476.1 akaw|GAA85622.1 aluc|OJZ83562.1 anid|CBF80869.1 anig|EHA24380.1 aory|EIT80369.1 asyd|OJJ56297.1 ater|EAU29670.1 atub|OJI82452.1 aver|OJJ06420.1 awen|OJJ30738.1 azon|OJJ44200.1

Group2980: abra|OJJ73052.1 acar|OOF96215.1 acla|EAW07967.1 afis|EAW23002.1 afla|EED55013.1 afum|EAL88478.1 agla|OJJ89477.1 akaw|GAA85623.1 aluc|OJZ83564.1 anid|CBF80868.1 anig|EHA24381.1 aory|EIT80329.1 asyd|OJJ56296.1 ater|EAU29671.1 atub|OJI82454.1 aver|OJJ06421.1 awen|OJJ30739.1 azon|OJJ44201.1

Group2981: abra|OJJ73056.1 acar|OOF96217.1 acla|EAW07965.1 afis|EAW23000.1 afla|EED55015.1 afum|EAL88480.1 agla|OJJ89479.1 akaw|GAA85624.1 aluc|OJZ83567.1 anid|CBF80864.1 anig|EHA24383.1 aory|EIT80314.1 asyd|OJJ56294.1 ater|EAU29673.1 atub|OJI82456.1 aver|OJJ06423.1 awen|OJJ30741.1 azon|OJJ44204.1

Group2982: abra|OJJ73058.1 acar|OOF96218.1 acla|EAW07963.1 afis|EAW22998.1 afla|EED55017.1 afum|EAL88482.1 agla|OJJ89481.1 akaw|GAA85626.1 aluc|OJZ83569.1 anid|CBF80860.1 anig|EHA24385.1 aory|EIT80381.1 asyd|OJJ56292.1 ater|EAU29674.1 atub|OJI82458.1 aver|OJJ06425.1 awen|OJJ30743.1 azon|OJJ44202.1

Group2983: abra|OJJ73059.1 acar|OOF96220.1 acla|EAW07962.1 afis|EAW22997.1 afla|EED55018.1 afum|EAL88483.1 agla|OJJ89482.1 akaw|GAA85627.1 aluc|OJZ83570.1 anid|CBF80856.1 anig|EHA24386.1 aory|EIT80291.1 asyd|OJJ56291.1 ater|EAU29675.1 atub|OJI82459.1 aver|OJJ06426.1 awen|OJJ30744.1 azon|OJJ44205.1

Group2984: abra|OJJ73062.1 acar|OOG00286.1 afla|EED50225.1 agla|OJJ79957.1 akaw|GAA88097.1 aluc|OJZ81364.1 anid|CBF85609.1 anig|EHA18664.1 aory|EIT76192.1 asyd|OJJ64207.1 ater|EAU30207.1 atub|OJI91568.1 aver|OJI98295.1 awen|OJJ39951.1 afla|EED45252.1 aory|EIT73530.1 agla|OJJ80099.1 aory|EIT77256.1

Group2985: abra|OJJ73142.1 acar|OOG00354.1 acla|EAW07018.1 afis|EAW16644.1 afla|EED44956.1 afum|EAL89248.1 agla|OJJ82603.1 akaw|GAA88030.1 aluc|OJZ81289.1 anid|CBF82053.1 anig|EHA18588.1 aory|EIT73503.1 asyd|OJJ54235.1 ater|EAU39569.1 atub|OJI91497.1 aver|OJJ05187.1 awen|OJJ40070.1 azon|OJJ45554.1

Group2986: abra|OJJ73143.1 acar|OOG00355.1 acla|EAW07019.1 afis|EAW16645.1 afla|EED44955.1 afum|EAL89249.1 agla|OJJ82602.1 akaw|GAA88029.1 aluc|OJZ81288.1 anid|CBF82055.1 anig|EHA18587.1 aory|EIT73491.1 asyd|OJJ54236.1 ater|EAU39570.1 atub|OJI91496.1 aver|OJJ05188.1 awen|OJJ40069.1 azon|OJJ45555.1

Group2987: abra|OJJ73147.1 acar|OOG00358.1 acla|EAW07023.1 afis|EAW16649.1 afla|EED44951.1 afum|EAL89253.1 agla|OJJ78458.1 akaw|GAA88025.1 aluc|OJZ81284.1 anid|CBF82061.1 anig|EHA18583.1 aory|EIT73464.1 asyd|OJJ54240.1 ater|EAU39573.1 atub|OJI91491.1 aver|OJJ05192.1 awen|OJJ40065.1 azon|OJJ45561.1

Group2988: abra|OJJ73148.1 acar|OOG00360.1 acla|EAW07024.1 afis|EAW16650.1 afla|EED44950.1 afum|EAL89254.1 agla|OJJ78459.1 akaw|GAA88024.1 aluc|OJZ81282.1 anid|CBF82063.1 anig|EHA18582.1 aory|EIT73478.1 asyd|OJJ54241.1 ater|EAU39574.1 atub|OJI91490.1 aver|OJJ05193.1 awen|OJJ40064.1 azon|OJJ45562.1

Group2989: abra|OJJ73149.1 acar|OOG00361.1 acla|EAW07025.1 afis|EAW16651.1 afla|EED44949.1 afum|EAL89255.1 agla|OJJ78460.1 akaw|GAA88023.1 aluc|OJZ81281.1 anid|CBF82064.1 anig|EHA18581.1 aory|EIT73505.1 asyd|OJJ54242.1 ater|EAU39575.1 atub|OJI91489.1 aver|OJJ05194.1 awen|OJJ40063.1 azon|OJJ45563.1

Group2990: abra|OJJ73151.1 acar|OOG00363.1 acla|EAW07028.1 afis|EAW16654.1 afla|EED44946.1 afum|EAL89258.1 agla|OJJ78687.1 akaw|GAA88021.1 aluc|OJZ81279.1 anid|CBF82067.1 anig|EHA18579.1 aory|EIT73521.1 asyd|OJJ54244.1 ater|EAU39578.1 atub|OJI91487.1 aver|OJJ05196.1 awen|OJJ40060.1 azon|OJJ45567.1

Group2991: abra|OJJ73156.1 acar|OOG00371.1 acla|EAW07030.1 afis|EAW16656.1 afla|EED44941.1 afum|EAL89260.1 agla|OJJ78716.1 akaw|GAA88016.1 aluc|OJZ81274.1 anid|CBF82070.1 anig|EHA18574.1 aory|EIT73470.1 asyd|OJJ54246.1 ater|EAU39579.1 atub|OJI91481.1 aver|OJJ05199.1 awen|OJJ40058.1 azon|OJJ50081.1

Group2992: abra|OJJ73158.1 acar|OOG00369.1 acla|EAW07032.1 afis|EAW16658.1 afla|EED44944.1 afum|EAL89262.1 agla|OJJ78714.1 akaw|GAA88014.1 aluc|OJZ81272.1 anid|CBF82072.1 anig|EHA18572.1 aory|EIT73479.1 asyd|OJJ54248.1 ater|EAU39581.1 atub|OJI91479.1 aver|OJJ05202.1 awen|OJJ40056.1 azon|OJJ50105.1

Group2993: abra|OJJ73159.1 acar|OOG00368.1 acla|EAW07033.1 afis|EAW16659.1 afla|EED44936.1 afum|EAL89263.1 agla|OJJ78713.1 akaw|GAA88013.1 aluc|OJZ81271.1 anid|CBF82073.1 anig|EHA18571.1 aory|EIT73463.1 asyd|OJJ54249.1 ater|EAU39582.1 atub|OJI91478.1 aver|OJJ05203.1 awen|OJJ40055.1 azon|OJJ50106.1

Group2994: abra|OJJ73160.1 acar|OOG00405.1 acla|EAW06769.1 afis|EAW16319.1 afla|EED53562.1 afum|EAL88943.1 agla|OJJ88280.1 akaw|GAA88012.1 aluc|OJZ81270.1 anid|CBF89151.1 anig|EHA18570.1 aory|EIT74869.1 asyd|OJJ62566.1 ater|EAU34138.1 atub|OJI91477.1 aver|OJI96690.1 awen|OJJ37720.1 azon|OJJ44978.1

Group2995: abra|OJJ73168.1 acar|OOG00409.1 acla|EAW07130.1 afis|EAW22141.1 afla|EED53567.1 afum|EAL91049.1 agla|OJJ87934.1 akaw|GAA88003.1 aluc|OJZ81259.1 anid|CBF89137.1 anig|EHA18566.1 aory|EIT75049.1 asyd|OJJ62649.1 ater|EAU34143.1 atub|OJI91455.1 aver|OJI96749.1 awen|OJJ34715.1 azon|OJJ45847.1

Group2996: abra|OJJ73170.1 acar|OOG00410.1 acla|EAW10796.1 afis|EAW24780.1 afla|EED47205.1 afum|EAL89419.1 agla|OJJ82876.1 akaw|GAA88001.1 aluc|OJZ81257.1 anid|CBF81439.1 anig|EHA18564.1 aory|EIT79182.1 asyd|OJJ59428.1 ater|EAU36528.1 atub|OJI91453.1 aver|OJJ02843.1 awen|OJJ40366.1 azon|OJJ49215.1

Group2997: abra|OJJ73173.1 acar|OOG00413.1 acla|EAW06856.1 afis|EAW16473.1 afla|EED47208.1 afum|EAL89067.1 agla|OJJ82873.1 akaw|GAA87998.1 aluc|OJZ81254.1 anid|CBF81435.1 anig|EHA18562.1 aory|EIT79309.1 asyd|OJJ59424.1 ater|EAU36569.1 atub|OJI91450.1 aver|OJJ02846.1 awen|OJJ40363.1 azon|OJJ49211.1

Group2998: abra|OJJ73176.1 acar|OOG00416.1 acla|EAW06858.1 afis|EAW16475.1 afla|EED47210.1 afum|EAL89069.1 agla|OJJ82871.1 akaw|GAA87996.1 aluc|OJZ81251.1 anid|CBF81431.1 anig|EHA18560.1

aory|EIT79330.1 asyd|OJJ59422.1 ater|EAU36562.1 atub|OJI91448.1 aver|OJJ02848.1 awen|OJJ40361.1 azon|OJJ49209.1

Group2999: abra|OJJ73183.1 acar|OOG00420.1 acla|EAW06861.1 afis|EAW16478.1 afla|EED47215.1 afum|EAL89072.1 agla|OJJ82867.1 akaw|GAA87992.1 aluc|OJZ81246.1 anid|CBF75501.1 anig|EHA18555.1 aory|EIT79241.1 asyd|OJJ59747.1 ater|EAU36560.1 atub|OJI91442.1 aver|OJJ02541.1 awen|OJJ40357.1 azon|OJJ49206.1

Group3000: abra|OJJ73184.1 acar|OOG00421.1 acla|EAW06862.1 afis|EAW16479.1 afla|EED47216.1 afum|EAL89073.1 agla|OJJ82866.1 akaw|GAA87991.1 aluc|OJZ81245.1 anid|CBF75499.1 anig|EHA18554.1 aory|EIT79319.1 asyd|OJJ59748.1 ater|EAU36559.1 atub|OJI91441.1 aver|OJJ02540.1 awen|OJJ40356.1 azon|OJJ49205.1

Group3001: abra|OJJ73185.1 acar|OOG00422.1 acla|EAW06863.1 afis|EAW16480.1 afla|EED47217.1 afum|EAL89074.1 agla|OJJ82865.1 akaw|GAA87990.1 aluc|OJZ81244.1 anid|CBF75503.1 anig|EHA18553.1 aory|EIT79172.1 asyd|OJJ59746.1 ater|EAU36558.1 atub|OJI91440.1 aver|OJJ02542.1 awen|OJJ40354.1 azon|OJJ49204.1

Group3002: abra|OJJ73189.1 acar|OOG00425.1 acla|EAW06865.1 afis|EAW16482.1 afla|EED47222.1 afum|EAL89076.1 agla|OJJ82862.1 akaw|GAA87988.1 aluc|OJZ81241.1 anid|CBF75506.1 anig|EHA18551.1 aory|EIT79303.1 asyd|OJJ59744.1 ater|EAU36556.1 atub|OJI91438.1 aver|OJJ02544.1 awen|OJJ40350.1 azon|OJJ49202.1

Group3003: abra|OJJ73190.1 acar|OOG00426.1 acla|EAW06866.1 afis|EAW16483.1 afla|EED47223.1 afum|EAL89077.1 agla|OJJ82861.1 akaw|GAA87987.1 aluc|OJZ81240.1 anid|CBF75508.1 anig|EHA18550.1 aory|EIT79190.1 asyd|OJJ59743.1 ater|EAU36555.1 atub|OJI91437.1 aver|OJJ02545.1 awen|OJJ40348.1 azon|OJJ49201.1

Group3004: abra|OJJ73191.1 acar|OOG00428.1 acla|EAW06867.1 afis|EAW16484.1 afla|EED47224.1 afum|EAL89078.1 agla|OJJ82860.1 akaw|GAA87986.1 aluc|OJZ81239.1 anid|CBF75520.1 anig|EHA18549.1 aory|EIT79323.1 asyd|OJJ59736.1 ater|EAU36554.1 atub|OJI91436.1 aver|OJJ02551.1 awen|OJJ40343.1 azon|OJJ49200.1

Group3005: abra|OJJ73193.1 acar|OOG00429.1 acla|EAW06869.1 afis|EAW16486.1 afla|EED47226.1 afum|EAL89080.1 agla|OJJ82858.1 akaw|GAA87984.1 aluc|OJZ81236.1 anid|CBF75516.1 anig|EHA18547.1 aory|EIT79231.1 asyd|OJJ59738.1 ater|EAU36552.1 atub|OJI91434.1 aver|OJJ02549.1 awen|OJJ40345.1 azon|OJJ49198.1

Group3006: abra|OJJ73194.1 acar|OOG00431.1 acla|EAW06871.1 afis|EAW16488.1 afla|EED47229.1 afum|EAL89082.1 agla|OJJ82852.1 akaw|GAA87983.1 aluc|OJZ81235.1 anid|CBF75510.1 anig|EHA18546.1 aory|EIT79266.1 asyd|OJJ59742.1 ater|EAU36548.1 atub|OJI91432.1 aver|OJJ02546.1 awen|OJJ40342.1 azon|OJJ49196.1

Group3007: abra|OJJ73196.1 acar|OOG00434.1 acla|EAW06873.1 afis|EAW16490.1 afla|EED47231.1 afum|EAL89084.1 agla|OJJ82829.1 akaw|GAA87981.1 aluc|OJZ81233.1 anid|CBF75522.1 anig|EHA18544.1 aory|EIT79209.1 asyd|OJJ59735.1 ater|EAU36547.1 atub|OJI91430.1 aver|OJJ02552.1 awen|OJJ40339.1 azon|OJJ49193.1

Group3008: abra|OJJ73199.1 acar|OOG00438.1 acla|EAW06876.1 afis|EAW16493.1 afla|EED47237.1 afum|EAL89087.1 agla|OJJ82832.1 akaw|GAA87978.1 aluc|OJZ81230.1 anid|CBF75527.1 anig|EHA18541.1 aory|EIT79313.1 asyd|OJJ59732.1 ater|EAU36545.1 atub|OJI91427.1 aver|OJJ02555.1 awen|OJJ40336.1 azon|OJJ49190.1

Group3009: abra|OJJ73202.1 acar|OOG00441.1 acla|EAW06879.1 afis|EAW16496.1 afla|EED47251.1 afum|EAL89090.1 agla|OJJ82835.1 akaw|GAA87975.1 aluc|OJZ81227.1 anid|CBF75606.1 anig|EHA18538.1 aory|EIT79275.1 asyd|OJJ59727.1 ater|EAU36543.1 atub|OJI91424.1 aver|OJJ02559.1 awen|OJJ40333.1 azon|OJJ49187.1

Group3010: abra|OJJ73203.1 acar|OOG00442.1 acla|EAW06880.1 afis|EAW16497.1 afla|EED56182.1 afum|EBA27367.1 agla|OJJ82836.1 akaw|GAA87974.1 aluc|OJZ81224.1 anid|CBF75605.1 anig|EHA18537.1 aory|EIT73365.1 asyd|OJJ59726.1 ater|EAU36542.1 atub|OJI91422.1 aver|OJJ02560.1 awen|OJJ40332.1 azon|OJJ49186.1

Group3011: abra|OJJ73204.1 acar|OOG00443.1 acla|EAW06881.1 afis|EAW16498.1 afla|EED56181.1 afum|EAL89093.1 agla|OJJ82838.1 akaw|GAA87973.1 aluc|OJZ81222.1 anid|CBF75604.1 anig|EHA18536.1 aory|EIT73379.1 asyd|OJJ59725.1 ater|EAU36541.1 atub|OJI91420.1 aver|OJJ02561.1 awen|OJJ40331.1 azon|OJJ49185.1

Group3012: abra|OJJ73205.1 acar|OOG00444.1 acla|EAW06882.1 afis|EAW16499.1 afla|EED56180.1 afum|EAL89094.1 agla|OJJ82826.1 akaw|GAA87972.1 aluc|OJZ81221.1 anid|CBF75602.1 anig|EHA18535.1 aory|EIT76650.1 asyd|OJJ59724.1 ater|EAU36540.1 atub|OJI91419.1 aver|OJJ02562.1 awen|OJJ40330.1 azon|OJJ49184.1

Group3013: abra|OJJ73206.1 acar|OOG00445.1 acla|EAW06883.1 afis|EAW16500.1 afla|EED56179.1 afum|EAL89095.1 agla|OJJ82827.1 akaw|GAA87971.1 aluc|OJZ81220.1 anid|CBF75600.1 anig|EHA18534.1 aory|EIT76763.1 asyd|OJJ59723.1 ater|EAU36539.1 atub|OJI91418.1 aver|OJJ02563.1 awen|OJJ40328.1 azon|OJJ49183.1

Group3014: abra|OJJ73207.1 acar|OOG00446.1 acla|EAW06884.1 afis|EAW16501.1 afla|EED56178.1 afum|EAL89096.1 agla|OJJ82828.1 akaw|GAA87970.1 aluc|OJZ81219.1 anid|CBF75598.1 anig|EHA18533.1 aory|EIT76626.1 asyd|OJJ59722.1 ater|EAU36538.1 atub|OJI91417.1 aver|OJJ02564.1 awen|OJJ40327.1 azon|OJJ49182.1

Group3015: abra|OJJ73209.1 acar|OOG00447.1 acla|EAW06885.1 afis|EAW16502.1 afla|EED56177.1 afum|EAL89097.1 agla|OJJ82815.1 akaw|GAA87969.1 aluc|OJZ81218.1 anid|CBF75589.1 anig|EHA18532.1 aory|EIT76868.1 asyd|OJJ59721.1 ater|EAU36537.1 atub|OJI91416.1 aver|OJJ02565.1 awen|OJJ40326.1 azon|OJJ49181.1

Group3016: abra|OJJ73210.1 acar|OOG00449.1 acla|EAW06886.1 afis|EAW16503.1 afla|EED56175.1 afum|EAL89098.1 agla|OJJ82816.1 akaw|GAA87968.1 aluc|OJZ81217.1 anid|CBF75591.1 anig|EHA18531.1 aory|EIT76792.1 asyd|OJJ59720.1 ater|EAU36536.1 atub|OJI91415.1 aver|OJJ02566.1 awen|OJJ40324.1 azon|OJJ49180.1

Group3017: abra|OJJ73212.1 acar|OOG00448.1 acla|EAW06888.1 afis|EAW16505.1 afla|EED56173.1 afum|EAL89100.1 agla|OJJ82818.1 akaw|GAA87966.1 aluc|OJZ81215.1 anid|CBF75594.1 anig|EHA18529.1 aory|EIT76612.1 asyd|OJJ59718.1 ater|EAU36535.1 atub|OJI91412.1 aver|OJJ02568.1 awen|OJJ40321.1 azon|OJJ49178.1

Group3018: abra|OJJ73216.1 acar|OOG00453.1 acla|EAW06891.1 afis|EAW16508.1 afla|EED56170.1 afum|EAL89103.1 agla|OJJ82822.1 akaw|GAA87963.1 aluc|OJZ81212.1 anid|CBF75578.1 anig|EHA18526.1 aory|EIT76719.1 asyd|OJJ59714.1 ater|EAU36572.1 atub|OJI91407.1 aver|OJJ02572.1 awen|OJJ40317.1 azon|OJJ47186.1

Group3019: abra|OJJ73218.1 acar|OOG00455.1 acla|EAW06892.1 afis|EAW16509.1 afla|EED56164.1 afum|EAL89104.2 agla|OJJ82812.1 akaw|GAA87961.1 aluc|OJZ81210.1 anid|CBF75572.1 anig|EHA18524.1 aory|EIT76824.1 asyd|OJJ59711.1 ater|EAU36575.1 atub|OJI91405.1 aver|OJJ02575.1 awen|OJJ40315.1 azon|OJJ49175.1

Group3020: abra|OJJ73219.1 acar|OOG00457.1 acla|EAW06894.1 afis|EAW16511.1 afla|EED56162.1 afum|EAL89106.1 agla|OJJ82811.1 akaw|GAA87959.1 aluc|OJZ81208.1 anid|CBF75568.1 anig|EHA18523.1 aory|EIT76692.1 asyd|OJJ59709.1 ater|EAU36577.1 atub|OJI91404.1 aver|OJJ02576.1 awen|OJJ40313.1 azon|OJJ49173.1

Group3021: abra|OJJ73220.1 acar|OOG00458.1 acla|EAW06895.1 afis|EAW16512.1 afla|EED56161.1 afum|EAL89107.2 agla|OJJ82810.1 akaw|GAA87958.1 aluc|OJZ81207.1 anid|CBF75566.1 anig|EHA18522.1 aory|EIT76654.1 asyd|OJJ59708.1 ater|EAU36578.1 atub|OJI91403.1 aver|OJJ02577.1 awen|OJJ40312.1 azon|OJJ49172.1

Group3022: abra|OJJ73222.1 acar|OOG00460.1 acla|EAW06897.1 afis|EAW16514.1 afla|EED56159.1 afum|EAL89109.1 agla|OJJ82808.1 akaw|GAA87956.1 aluc|OJZ81205.1 anid|CBF75562.1 anig|EHA18520.1 aory|EIT76696.1 asyd|OJJ59706.1 ater|EAU36580.1 atub|OJI91401.1 aver|OJJ02579.1 awen|OJJ40309.1 azon|OJJ49170.1

Group3023: abra|OJJ73223.1 acar|OOG00461.1 acla|EAW06898.1 afis|EAW16515.1 afla|EED56158.1 afum|EAL89110.2 agla|OJJ82807.1 akaw|GAA87955.1 aluc|OJZ81204.1 anid|CBF75560.1 anig|EHA18519.1 aory|EIT76553.1 asyd|OJJ59705.1 ater|EAU36581.1 atub|OJI91400.1 aver|OJJ02580.1 awen|OJJ40308.1 azon|OJJ49169.1

Group3024: abra|OJJ73224.1 acar|OOG00462.1 acla|EAW06899.1 afis|EAW16516.1 afla|EED56157.1 afum|EAL89111.2 agla|OJJ82806.1 akaw|GAA87954.1 aluc|OJZ81203.1 anid|CBF75558.1 anig|EHA18518.1 aory|EIT76604.1 asyd|OJJ59704.1 ater|EAU36582.1 atub|OJI91398.1 aver|OJJ02582.1 awen|OJJ40307.1 azon|OJJ49168.1

Group3025: abra|OJJ73228.1 acar|OOG00466.1 acla|EAW06902.1 afis|EAW16519.1 afla|EED56152.1 afum|EAL89114.1 agla|OJJ82801.1 akaw|GAA87952.1 aluc|OJZ81200.1 anid|CBF75555.1 anig|EHA18515.1 aory|EIT76840.1 asyd|OJJ57431.1 ater|EAU36585.1 atub|OJI91395.1 aver|OJJ05749.1 awen|OJJ40303.1 azon|OJJ49165.1

Group3026: abra|OJJ73229.1 acar|OOG00468.1 acla|EAW06904.1 afis|EAW16521.1 afla|EED56150.1 afum|EAL89116.1 agla|OJJ82800.1 akaw|GAA87951.1 aluc|OJZ81199.1 anid|CBF75547.1 anig|EHA18514.1 aory|EIT76779.1 asyd|OJJ57441.1 ater|EAU36586.1 atub|OJI91394.1 aver|OJJ05737.1 awen|OJJ40302.1 azon|OJJ49164.1

Group3027: abra|OJJ73230.1 acar|OOG00469.1 acla|EAW06905.1 afis|EAW16522.1 afla|EED56149.1 afum|EAL89117.1 agla|OJJ82799.1 akaw|GAA87950.1 aluc|OJZ81198.1 anid|CBF75545.1 anig|EHA18513.1

aory|EIT76528.1 asyd|OJJ57442.1 ater|EAU36587.1 atub|OJI91393.1 aver|OJJ05736.1 awen|OJJ40301.1 azon|OJJ49163.1

Group3028: abra|OJJ73231.1 acar|OOG00470.1 acla|EAW06906.1 afis|EAW16523.1 afla|EED56148.1 afum|EAL89118.1 agla|OJJ82798.1 akaw|GAA87949.1 aluc|OJZ81197.1 anid|CBF75543.1 anig|EHA18512.1 aory|EIT76854.1 asyd|OJJ57443.1 ater|EAU36588.1 atub|OJI91392.1 aver|OJJ05735.1 awen|OJJ40300.1 azon|OJJ49162.1

Group3029: abra|OJJ73232.1 acar|OOG00471.1 acla|EAW06907.1 afis|EAW16524.1 afla|EED56147.1 afum|EAL89119.1 agla|OJJ82797.1 akaw|GAA87948.1 aluc|OJZ81196.1 anid|CBF75541.1 anig|EHA18511.1 aory|EIT76769.1 asyd|OJJ57446.1 ater|EAU36589.1 atub|OJI91391.1 aver|OJJ05734.1 awen|OJJ40299.1 azon|OJJ49161.1

Group3030: abra|OJJ73234.1 acar|OOG00472.1 acla|EAW06909.1 afis|EAW16526.1 afla|EED56145.1 afum|EAL89122.1 agla|OJJ82796.1 akaw|GAA87946.1 aluc|OJZ81194.1 anid|CBF75537.1 anig|EHA18509.1 aory|EIT76537.1 asyd|OJJ57447.1 ater|EAU36590.1 atub|OJI91389.1 aver|OJJ05732.1 awen|OJJ40297.1 azon|OJJ49159.1

Group3031: abra|OJJ73236.1 acar|OOG00473.1 acla|EAW06910.1 afis|EAW16527.1 afla|EED56144.1 afum|EAL89123.2 agla|OJJ82795.1 akaw|GAA87945.1 aluc|OJZ81193.1 anid|CBF75535.1 anig|EHA18508.1 aory|EIT76806.1 asyd|OJJ57448.1 ater|EAU36591.1 atub|OJI91388.1 aver|OJJ05731.1 awen|OJJ40296.1 azon|OJJ49158.1

Group3032: abra|OJJ73237.1 acar|OOG00474.1 acla|EAW06911.1 afis|EAW16528.1 afla|EED56143.1 afum|EAL89124.1 agla|OJJ82793.1 akaw|GAA87944.1 aluc|OJZ81191.1 anid|CBF75533.1 anig|EHA18507.1 aory|EIT76880.1 asyd|OJJ57449.1 ater|EAU36592.1 atub|OJI91387.1 aver|OJJ05730.1 awen|OJJ40295.1 azon|OJJ49157.1

Group3033: abra|OJJ73239.1 acar|OOG00476.1 acla|EAW06912.1 afis|EAW16529.1 afla|EED56141.1 afum|EAL89125.1 agla|OJJ82791.1 akaw|GAA87942.1 aluc|OJZ81189.1 anid|CBF75529.1 anig|EHA18505.1 aory|EIT76699.1 asyd|OJJ57450.1 ater|EAU36593.1 atub|OJI91385.1 aver|OJJ05728.1 awen|OJJ40293.1 azon|OJJ49154.1

Group3034: abra|OJJ73240.1 acar|OOG00477.1 acla|EAW06913.1 afis|EAW16530.1 afla|EED56138.1 afum|EAL89127.1 agla|OJJ82790.1 akaw|GAA87941.1 aluc|OJZ81188.1 anid|CBF81731.1 anig|EHA18504.1 aory|EIT76798.1 asyd|OJJ59592.1 ater|EAU36594.1 atub|OJI91384.1 aver|OJJ02677.1 awen|OJJ40291.1 azon|OJJ49153.1

Group3035: abra|OJJ73246.1 acar|OOG00485.1 acla|EAW06920.1 afis|EAW16537.1 afla|EED56132.1 afum|EAL89134.1 agla|OJJ82785.1 akaw|GAA87934.1 aluc|OJZ81181.1 anid|CBF81743.1 anig|EHA18499.1 aory|EIT76834.1 asyd|OJJ59598.1 ater|EAU36600.1 atub|OJI91378.1 aver|OJJ02670.1 awen|OJJ40285.1 azon|OJJ49146.1

Group3036: abra|OJJ73248.1 acar|OOG00488.1 acla|EAW06922.1 afis|EAW16539.1 afla|EED56130.1 afum|EAL89136.1 agla|OJJ82783.1 akaw|GAA87932.1 aluc|OJZ81179.1 anid|CBF81746.1 anig|EHA18497.1 aory|EIT76822.1 asyd|OJJ59600.1 ater|EAU36602.1 atub|OJI91375.1 aver|OJJ02668.1 awen|OJJ40282.1 azon|OJJ49144.1

Group3037: abra|OJJ73249.1 acar|OOG00491.1 acla|EAW06924.1 afis|EAW16541.1 afla|EED56127.1 afum|EAL89138.1 agla|OJJ82782.1 akaw|GAA87931.1 aluc|OJZ81178.1 anid|CBF81748.1 anig|EHA18496.1 aory|EIT76785.1 asyd|OJJ59601.1 ater|EAU36603.1 atub|OJI91374.1 aver|OJJ02667.1 awen|OJJ40279.1 azon|OJJ49142.1

Group3038: abra|OJJ73251.1 acar|OOG00495.1 acla|EAW06929.1 afis|EAW16544.1 afla|EED56121.1 afum|EAL89142.2 agla|OJJ82779.1 akaw|GAA87929.1 aluc|OJZ81176.1 anid|CBF81754.1 anig|EHA18494.1 aory|EIT76628.1 asyd|OJJ59604.1 ater|EAU36607.1 atub|OJI91372.1 aver|OJJ02665.1 awen|OJJ40276.1 azon|OJJ49139.1

Group3039: abra|OJJ73252.1 acar|OOG00494.1 acla|EAW06930.1 afis|EAW16545.1 afla|EED56120.1 afum|EAL89143.1 agla|OJJ82778.1 akaw|GAA87928.1 aluc|OJZ81175.1 anid|CBF81755.1 anig|EHA18493.1 aory|EIT76556.1 asyd|OJJ59605.1 ater|EAU36608.1 atub|OJI91371.1 aver|OJJ02664.1 awen|OJJ40275.1 azon|OJJ49138.1

Group3040: abra|OJJ73253.1 acar|OOG00496.1 acla|EAW06931.1 afis|EAW16546.1 afum|EAL89144.1 agla|OJJ82777.1 akaw|GAA87927.1 aluc|OJZ81174.1 anid|CBF81757.1 anig|EHA18492.1 aory|EIT76862.1 asyd|OJJ59606.1 ater|EAU36609.1 atub|OJI91370.1 aver|OJJ02663.1 awen|OJJ40271.1 azon|OJJ49137.1 afla|EED56118.1

Group3041: abra|OJJ73256.1 acar|OOG00499.1 acla|EAW06933.1 afis|EAW16548.1 afla|EED56116.1 afum|EAL89146.1 agla|OJJ82775.1 akaw|GAA87925.1 aluc|OJZ81172.1 anid|CBF81761.1 anig|EHA18490.1 aory|EIT76624.1 asyd|OJJ59607.1 ater|EAU36611.1 atub|OJI91367.1 aver|OJJ02662.1 awen|OJJ40269.1 azon|OJJ49133.1

Group3042: abra|OJJ73257.1 acar|OOG00498.1 acla|EAW06934.1 afis|EAW16549.1 afla|EED56115.1 afum|EAL89147.1 agla|OJJ82774.1 akaw|GAA87924.1 aluc|OJZ81171.1 anid|CBF81763.1 anig|EHA18489.1 aory|EIT76873.1 asyd|OJJ59608.1 ater|EAU36612.1 atub|OJI91366.1 aver|OJJ02661.1 awen|OJJ40268.1 azon|OJJ49132.1

Group3043: abra|OJJ73258.1 acar|OOG00500.1 acla|EAW06935.1 afis|EAW16550.1 afla|EED56112.1 afum|EAL89148.1 agla|OJJ82773.1 akaw|GAA87923.1 aluc|OJZ81170.1 anid|CBF81765.1 anig|EHA18488.1 aory|EIT76657.1 ater|EAU36613.1 atub|OJI91365.1 awen|OJJ40267.1 azon|OJJ49131.1 asyd|OJJ59609.1 aver|OJJ02660.1

Group3044: abra|OJJ73259.1 acar|OOG00501.1 acla|EAW06936.1 afis|EAW16551.1 afla|EED56111.1 afum|EAL89149.1 agla|OJJ82772.1 akaw|GAA87922.1 aluc|OJZ81169.1 anid|CBF81766.1 anig|EHA18487.1 aory|EIT76585.1 asyd|OJJ59610.1 ater|EAU36617.1 atub|OJI91364.1 aver|OJJ02659.1 awen|OJJ40266.1 azon|OJJ49130.1

Group3045: abra|OJJ73262.1 acar|OOG00502.1 acla|EAW06939.1 afis|EAW16554.1 afla|EED56108.1 afum|EAL89152.1 agla|OJJ82769.1 akaw|GAA87920.1 aluc|OJZ81167.1 anid|CBF81795.1 anig|EHA18484.1 aory|EIT76711.1 asyd|OJJ59637.1 ater|EAU36619.1 atub|OJI91362.1 aver|OJJ02644.1 awen|OJJ40264.1 azon|OJJ49127.1

Group3046: abra|OJJ73263.1 acar|OOG00503.1 acla|EAW06940.1 afis|EAW16555.1 afla|EED56107.1 afum|EAL89153.1 agla|OJJ82768.1 akaw|GAA87919.1 aluc|OJZ81166.1 anid|CBF81796.1 anig|EHA18483.1 aory|EIT76566.1 asyd|OJJ59638.1 ater|EAU36620.1 atub|OJI91361.1 aver|OJJ02643.1 awen|OJJ40263.1 azon|OJJ49126.1

Group3047: abra|OJJ73264.1 acar|OOG00504.1 acla|EAW06941.1 afis|EAW16556.1 afla|EED56106.1 afum|EAL89154.1 agla|OJJ82767.1 akaw|GAA87918.1 aluc|OJZ81165.1 anid|CBF81798.1 anig|EHA18482.1 aory|EIT76736.1 asyd|OJJ59639.1 ater|EAU36621.1 atub|OJI91360.1 aver|OJJ02642.1 awen|OJJ40262.1 azon|OJJ49125.1

Group3048: abra|OJJ73265.1 acar|OOG00505.1 acla|EAW06942.1 afis|EAW16557.1 afla|EED56105.1 afum|EAL89155.1 agla|OJJ82766.1 akaw|GAA87917.1 aluc|OJZ81164.1 anid|CBF81802.1 anig|EHA18481.1 aory|EIT76677.1 asyd|OJJ59642.1 ater|EAU36624.1 atub|OJI91359.1 aver|OJJ02640.1 awen|OJJ40260.1 azon|OJJ49122.1

Group3049: abra|OJJ73266.1 acar|OOG00506.1 acla|EAW06943.1 afis|EAW16558.1 afla|EED56104.1 afum|EAL89156.1 agla|OJJ82765.1 akaw|GAA87916.1 aluc|OJZ81163.1 anid|CBF81800.1 anig|EHA18480.1 aory|EIT76573.1 asyd|OJJ59640.1 ater|EAU36625.1 atub|OJI91358.1 aver|OJJ02641.1 awen|OJJ40259.1 azon|OJJ49123.1

Group3050: abra|OJJ73269.1 acar|OOG00509.1 acla|EAW06946.1 afis|EAW16561.1 afla|EED56101.1 afum|EAL89159.1 agla|OJJ82762.1 akaw|GAA87913.1 aluc|OJZ81160.1 anid|CBF81808.1 anig|EHA18477.1 aory|EIT76636.1 asyd|OJJ59645.1 ater|EAU36628.1 atub|OJI91355.1 aver|OJJ02637.1 awen|OJJ40256.1 azon|OJJ49120.1

Group3051: abra|OJJ73275.1 acar|OOG00514.1 acla|EAW06950.1 afis|EAW16565.1 afla|EED56098.1 afum|EAL89162.1 agla|OJJ82756.1 akaw|GAA92730.1 aluc|OJZ88774.1 anid|CBF81817.1 anig|EHA18468.1 aory|EIT76722.1 asyd|OJJ59651.1 ater|EAU36634.1 atub|OJI91345.1 aver|OJJ02631.1 awen|OJJ40253.1 azon|OJJ49115.1

Group3052: abra|OJJ73278.1 acar|OOG00515.1 acla|EAW06954.1 afis|EAW16569.1 afla|EED56095.1 afum|EAL89166.1 agla|OJJ82751.1 akaw|GAA92727.1 aluc|OJZ88771.1 anid|CBF81822.1 anig|EHA18467.1 aory|EIT82359.1 asyd|OJJ59654.1 ater|EAU36637.1 atub|OJI91344.1 aver|OJJ02628.1 awen|OJJ40250.1 azon|OJJ41980.1

Group3053: abra|OJJ73281.1 acar|OOG00517.1 acla|EAW06956.1 afis|EAW16571.1 afla|EED56093.1 afum|EAL89168.1 agla|OJJ82703.1 akaw|GAA92724.1 aluc|OJZ88768.1 anid|CBF81826.1 anig|EHA18464.1 aory|EIT76754.1 asyd|OJJ59657.1 ater|EAU36639.1 atub|OJI91341.1 aver|OJJ02625.1 awen|OJJ40247.1 azon|OJJ49108.1

Group3054: abra|OJJ73283.1 acar|OOG00519.1 acla|EAW06958.1 afis|EAW16573.1 afla|EED56091.1 afum|EAL89170.2 agla|OJJ82705.1 akaw|GAA92722.1 aluc|OJZ88766.1 anid|CBF81828.1 anig|EHA18462.1 aory|EIT76851.1 asyd|OJJ59659.1 ater|EAU36641.1 atub|OJI91339.1 aver|OJJ02623.1 awen|OJJ40245.1 azon|OJJ49106.1

Group3055: abra|OJJ73287.1 acar|OOG00522.1 acla|EAW06962.1 afis|EAW16577.1 afla|EED56087.1 afum|EAL89173.1 agla|OJJ82700.1 akaw|GAA92718.1 aluc|OJZ88763.1 anid|CBF81876.1 anig|EHA18459.1 aory|EIT76884.1 asyd|OJJ59665.1 ater|EAU36645.1 atub|OJI91335.1 aver|OJJ02618.1 awen|OJJ40240.1 azon|OJJ49103.1

Group3056: abra|OJJ73331.1 acar|OOF91495.1 agla|OJJ78824.1 anig|EHA18400.1 abra|OJJ78190.1 acar|OOF98667.1 acla|EAW12173.1 akaw|GAA85839.1 aluc|OJZ91261.1 anig|EHA19949.1 asyd|OJJ52476.1

ater|EAU36116.1 atub|OJI83250.1 aver|OJJ03936.1 awen|OJJ39598.1 azon|OJJ42251.1 afla|EED54616.1 aory|EIT78824.1

Group3057: abra|OJJ73338.1 acar|OOG00523.1 acla|EAW06963.1 afis|EAW16578.1 afla|EED56086.1 afum|EAL89174.1 agla|OJJ82699.1 akaw|GAA83146.1 aluc|OJZ88703.1 anid|CBF81878.1 anig|EHA18387.1 aory|EIT76746.1 asyd|OJJ59666.1 ater|EAU36646.1 atub|OJI91284.1 aver|OJJ02617.1 awen|OJJ40239.1 azon|OJJ49102.1

Group3058: abra|OJJ73339.1 acar|OOG00524.1 acla|EAW06964.1 afis|EAW16579.1 afla|EED56085.1 afum|EAL89175.1 agla|OJJ82698.1 akaw|GAA83145.1 aluc|OJZ88702.1 anid|CBF81880.1 anig|EHA18386.1 aory|EIT76894.1 asyd|OJJ59667.1 ater|EAU36647.1 atub|OJI91283.1 aver|OJJ02616.1 awen|OJJ40238.1 azon|OJJ49101.1

Group3059: abra|OJJ73340.1 acar|OOG00527.1 acla|EAW06965.1 afis|EAW16580.1 afla|EED56082.1 afum|EAL89176.1 agla|OJJ82695.1 akaw|GAA83142.1 aluc|OJZ88699.1 anid|CBF81883.1 anig|EHA18379.1 aory|EIT76744.1 asyd|OJJ59669.1 ater|EAU36649.1 atub|OJI91280.1 aver|OJJ02614.1 awen|OJJ40234.1 azon|OJJ49100.1

Group3060: abra|OJJ73343.1 acar|OOG00553.1 acla|EAW06970.1 afis|EAW16584.1 afla|EED56081.1 afum|EAL89179.1 agla|OJJ82694.1 akaw|GAA83139.1 aluc|OJZ88696.1 anid|CBF81885.1 anig|EHA18376.1 aory|EIT76749.1 asyd|OJJ59671.1 ater|EAU36661.1 atub|OJI91277.1 aver|OJJ02613.1 awen|OJJ40232.1 azon|OJJ49096.1

Group3061: abra|OJJ73344.1 acar|OOG00554.1 acla|EAW06971.1 afis|EAW16585.1 afla|EED56080.1 afum|EAL89180.1 agla|OJJ82693.1 akaw|GAA83138.1 aluc|OJZ88695.1 anid|CBF81887.1 anig|EHA18375.1 aory|EIT76713.1 asyd|OJJ59672.1 ater|EAU36662.1 atub|OJI91275.1 aver|OJJ02612.1 awen|OJJ40231.1 azon|OJJ49095.1

Group3062: abra|OJJ73345.1 acar|OOG00555.1 acla|EAW06972.1 afis|EAW16586.1 afla|EED56079.1 afum|EAL89181.2 agla|OJJ82692.1 akaw|GAA83137.1 aluc|OJZ88694.1 anid|CBF81889.1 anig|EHA18374.1 aory|EIT76590.1 asyd|OJJ59673.1 ater|EAU36663.1 atub|OJI91274.1 aver|OJJ02611.1 awen|OJJ40230.1 azon|OJJ49094.1

Group3063: abra|OJJ73359.1 acar|OOG00567.1 acla|EAW06979.1 afis|EAW16593.1 afla|EED45008.1 afum|EAL89189.1 agla|OJJ82660.1 akaw|GAA83125.1 aluc|OJZ88681.1 anid|CBF81897.1 anig|EHA18362.1 aory|EIT73639.1 asyd|OJJ59682.1 ater|EAU36673.1 atub|OJI91262.1 aver|OJJ02606.1 awen|OJJ40214.1 azon|OJJ49080.1

Group3064: abra|OJJ73361.1 acar|OOG00568.1 acla|EAW06981.1 afis|EAW16595.1 afla|EED45014.1 afum|EAL89191.1 agla|OJJ82659.1 akaw|GAA83123.1 aluc|OJZ88679.1 anid|CBF81901.1 anig|EHA18360.1 aory|EIT73637.1 asyd|OJJ59685.1 ater|EAU36674.1 atub|OJI91260.1 aver|OJJ02602.1 awen|OJJ40213.1 azon|OJJ49079.1

Group3065: abra|OJJ73362.1 acar|OOG00569.1 acla|EAW06982.1 afis|EAW16596.1 afla|EED45015.1 afum|EAL89192.1 agla|OJJ82658.1 akaw|GAA83122.1 aluc|OJZ88678.1 anid|CBF81902.1 anig|EHA18359.1 aory|EIT73689.1 asyd|OJJ59686.1 ater|EAU36675.1 atub|OJI91259.1 aver|OJJ02601.1 awen|OJJ40212.1 azon|OJJ49078.1

Group3066: abra|OJJ73365.1 acar|OOG00572.1 acla|EAW06985.1 afis|EAW16599.1 afla|EED45018.1 afum|EAL89195.1 agla|OJJ82655.1 akaw|GAA83119.1 aluc|OJZ88675.1 anid|CBF81905.1 anig|EHA18357.1 aory|EIT73617.1 asyd|OJJ59688.1 ater|EAU36680.1 atub|OJI91256.1 aver|OJJ02599.1 awen|OJJ40209.1 azon|OJJ49075.1

Group3067: abra|OJJ73368.1 acar|OOG00574.1 acla|EAW06988.1 afis|EAW16602.1 afla|EED45021.1 afum|EAL89198.1 agla|OJJ82650.1 akaw|GAA83116.1 aluc|OJZ88672.1 anid|CBF81909.1 anig|EHA18354.1 aory|EIT73783.1 asyd|OJJ59690.1 ater|EAU36683.1 atub|OJI91253.1 aver|OJJ02597.1 awen|OJJ40204.1 azon|OJJ49071.1

Group3068: abra|OJJ73378.1 acar|OOF90413.1 acla|EAW06994.1 afis|EAW16609.1 afla|EED45027.1 afum|EAL89205.1 agla|OJJ82638.1 akaw|GAA83111.1 aluc|OJZ88665.1 anid|CBF81965.1 anig|EHA18350.1 aory|EIT73642.1 asyd|OJJ57355.1 ater|EAU36693.1 atub|OJI91247.1 aver|OJJ05810.1 awen|OJJ40198.1 azon|OJJ49069.1

Group3069: abra|OJJ73382.1 acar|OOG00581.1 acla|EAW06997.1 afis|EAW16611.1 afla|EED45000.1 afum|EAL89207.1 agla|OJJ82634.1 akaw|GAA83107.1 aluc|OJZ88656.1 anid|CBF81963.1 anig|EHA18347.1 aory|EIT73611.1 asyd|OJJ57358.1 ater|EAU36696.1 atub|OJI91242.1 aver|OJJ05808.1 awen|OJJ40193.1 azon|OJJ49061.1

Group3070: abra|OJJ73383.1 acar|OOG00582.1 acla|EAW06998.1 afis|EAW16613.1 afla|EED44999.1 afum|EAL89223.1 agla|OJJ82633.1 akaw|GAA83106.1 aluc|OJZ88654.1 anid|CBF81961.1 anig|EHA18346.1 aory|EIT73720.1 asyd|OJJ57359.1 ater|EAU36697.1 atub|OJI91241.1 aver|OJJ05807.1 awen|OJJ40192.1 azon|OJJ49060.1

Group3071: abra|OJJ73402.1 acar|OOG00600.1 acla|EAW07005.1 afis|EAW16622.1 afla|EED44970.1 afum|EAL89215.1 agla|OJJ82622.1 akaw|GAA83088.1 aluc|OJZ88635.1 anid|CBF81987.1 anig|EHA18325.1 aory|EIT73499.1 asyd|OJJ56156.1 ater|EAU36736.1 atub|OJI91219.1 aver|OJJ05096.1 awen|OJJ40086.1 azon|OJJ49496.1

Group3072: abra|OJJ73424.1 acar|OOG00628.1 acla|EAW07034.1 afis|EAW16660.1 afla|EED44893.1 afum|EAL89264.1 agla|OJJ78709.1 akaw|GAA83068.1 aluc|OJZ88619.1 anid|CBF82075.1 anig|EHA18310.1 aory|EIT83240.1 asyd|OJJ54250.1 ater|EAU39583.1 atub|OJI91204.1 aver|OJJ05204.1 awen|OJJ40053.1 azon|OJJ50109.1

Group3073: abra|OJJ73425.1 acar|OOG00629.1 acla|EAW07035.1 afis|EAW16661.1 afla|EED44894.1 afum|EAL89265.1 agla|OJJ78708.1 akaw|GAA83067.1 aluc|OJZ88618.1 anid|CBF82076.1 anig|EHA18309.1 aory|EIT83251.1 asyd|OJJ54251.1 ater|EAU39584.1 atub|OJI91203.1 aver|OJJ05205.1 awen|OJJ40052.1 azon|OJJ50116.1

Group3074: abra|OJJ73432.1 acar|OOG00639.1 acla|EAW06771.1 afis|EAW16322.1 afla|EED53560.1 afum|EAL88945.1 agla|OJJ88284.1 akaw|GAA83060.1 aluc|OJZ88609.1 anid|CBF89157.1 anig|EHA18302.1 aory|EIT75216.1 asyd|OJJ62563.1 ater|EAU34135.1 atub|OJI91194.1 aver|OJI96687.1 awen|OJJ37722.1 azon|OJJ44981.1

Group3075: abra|OJJ73437.1 acar|OOG00643.1 acla|EAW06775.1 afis|EAW16326.1 afla|EED53555.1 afum|EAL88950.1 agla|OJJ88266.1 akaw|GAA83056.1 aluc|OJZ88604.1 anid|CBF89164.1 anig|EHA18297.1 aory|EIT75038.1 asyd|OJJ62546.1 ater|EAU34131.1 atub|OJI91188.1 aver|OJI96672.1 awen|OJJ37726.1 azon|OJJ44986.1

Group3076: abra|OJJ73440.1 acar|OOG00646.1 acla|EAW06772.1 afis|EAW16323.1 afla|EED53559.1 afum|EAL88946.1 agla|OJJ88285.1 akaw|GAA83053.1 aluc|OJZ88600.1 anid|CBF89158.1 anig|EHA18294.1 aory|EIT74917.1 asyd|OJJ62560.1 ater|EAU34134.1 atub|OJI91185.1 aver|OJI96685.1 awen|OJJ37723.1 azon|OJJ44982.1

Group3077: abra|OJJ73442.1 acar|OOG00648.1 acla|EAW06777.1 afis|EAW16328.1 afla|EED53553.1 afum|EAL88952.1 agla|OJJ88264.1 akaw|GAA83052.1 aluc|OJZ88598.1 anid|CBF89168.1 anig|EHA18292.1 aory|EIT74949.1 asyd|OJJ62543.1 ater|EAU34129.1 atub|OJI91183.1 aver|OJI96670.1 awen|OJJ37729.1 azon|OJJ44988.1

Group3078: abra|OJJ73451.1 acar|OOG00654.1 acla|EAW06782.1 afis|EAW16332.1 afla|EED53545.1 afum|EAL88957.1 agla|OJJ88260.1 akaw|GAA83045.1 aluc|OJZ88590.1 anid|CBF89183.1 anig|EHA18285.1 aory|EIT75091.1 asyd|OJJ62537.1 ater|EAU34126.1 atub|OJI91176.1 aver|OJI96663.1 awen|OJJ37733.1 azon|OJJ44990.1

Group3079: abra|OJJ73456.1 acar|OOG00656.1 acla|EAW06783.1 afis|EAW16333.1 afla|EED53543.1 afum|EAL88958.1 agla|OJJ88259.1 akaw|GAA83042.1 aluc|OJZ88587.1 anid|CBF89188.1 anig|EHA18281.1 aory|EIT75083.1 asyd|OJJ62536.1 ater|EAU34125.1 atub|OJI91173.1 aver|OJI96661.1 awen|OJJ37734.1 azon|OJJ44993.1

Group3080: abra|OJJ73458.1 acar|OOG00659.1 acla|EAW06785.1 afis|EAW16335.1 afla|EED53541.1 afum|EAL88960.1 agla|OJJ88251.1 akaw|GAA83040.1 aluc|OJZ88585.1 anid|CBF89192.1 anig|EHA18280.1 aory|EIT74882.1 asyd|OJJ62534.1 ater|EAU34123.1 atub|OJI91171.1 aver|OJI96659.1 awen|OJJ37737.1 azon|OJJ44997.1

Group3081: abra|OJJ73460.1 acar|OOG00661.1 acla|EAW06787.1 afis|EAW16336.1 afla|EED53539.1 afum|EAL88962.1 agla|OJJ88254.1 akaw|GAA83038.1 aluc|OJZ88583.1 anid|CBF89196.1 anig|EHA18278.1 aory|EIT75013.1 asyd|OJJ62532.1 ater|EAU34121.1 atub|OJI91169.1 aver|OJI96657.1 awen|OJJ37739.1 azon|OJJ45000.1

Group3082: abra|OJJ73463.1 acar|OOG00664.1 acla|EAW06789.1 afis|EAW16339.1 afla|EED53536.1 afum|EAL88964.1 agla|OJJ88256.1 akaw|GAA83035.1 aluc|OJZ88581.1 anid|CBF89199.1 anig|EHA18277.1 aory|EIT74845.1 asyd|OJJ62530.1 ater|EAU34119.1 atub|OJI91167.1 aver|OJI96655.1 awen|OJJ37741.1 azon|OJJ45002.1

Group3083: abra|OJJ73464.1 acar|OOG00665.1 acla|EAW06790.1 afis|EAW16340.1 afla|EED53535.1 afum|EAL88965.1 agla|OJJ88257.1 akaw|GAA83034.1 aluc|OJZ88580.1 anid|CBF89201.1 anig|EHA18276.1 aory|EIT75162.1 asyd|OJJ62529.1 ater|EAU34117.1 atub|OJI91166.1 aver|OJI96654.1 awen|OJJ37742.1 azon|OJJ45003.1

Group3084: abra|OJJ73465.1 acar|OOG00666.1 acla|EAW06792.1 afis|EAW16341.1 afla|EED53534.1 afum|EAL88966.1 agla|OJJ88247.1 akaw|GAA83033.1 aluc|OJZ88579.1 anid|CBF89203.1 anig|EHA18275.1 aory|EIT75067.1 asyd|OJJ62528.1 ater|EAU34116.1 atub|OJI91165.1 aver|OJI96651.1 awen|OJJ37743.1 azon|OJJ45004.1

Group3085: abra|OJJ73466.1 acar|OOG00667.1 acla|EAW06793.1 afis|EAW16342.1 afla|EED53533.1 afum|EAL88967.1 agla|OJJ88246.1 akaw|GAA83032.1 aluc|OJZ88578.1 anid|CBF89205.1 anig|EHA18274.1

aory|EIT75133.1 asyd|OJJ62527.1 ater|EAU34115.1 atub|OJI91164.1 aver|OJI96650.1 awen|OJJ37744.1 azon|OJJ45005.1

Group3086: abra|OJJ73467.1 acar|OOG00668.1 acla|EAW06794.1 afis|EAW16343.1 afla|EED53532.1 afum|EAL88968.1 agla|OJJ88245.1 akaw|GAA83031.1 aluc|OJZ88577.1 anid|CBF89206.1 anig|EHA18273.1 aory|EIT74961.1 asyd|OJJ62526.1 ater|EAU34114.1 atub|OJI91163.1 aver|OJI96649.1 awen|OJJ37745.1 azon|OJJ45006.1

Group3087: abra|OJJ73470.1 acar|OOG00669.1 acla|EAW06795.1 afis|EAW16344.1 afla|EED53526.1 afum|EAL88969.1 agla|OJJ88244.1 akaw|GAA83030.1 aluc|OJZ88576.1 anid|CBF89217.1 anig|EHA18272.1 aory|EIT74890.1 asyd|OJJ62524.1 ater|EAU34113.1 atub|OJI91162.1 aver|OJI96645.1 awen|OJJ37747.1 azon|OJJ45008.1

Group3088: abra|OJJ73474.1 acar|OOG00670.1 acla|EAW06799.1 afis|EAW16348.1 afla|EED53523.1 afum|EAL88973.1 agla|OJJ88239.1 akaw|GAA83027.1 aluc|OJZ88573.1 anid|CBF89210.1 anig|EHA18271.1 aory|EIT75044.1 asyd|OJJ62522.1 ater|EAU34111.1 atub|OJI91159.1 aver|OJI96641.1 awen|OJJ37751.1 azon|OJJ45010.1

Group3089: abra|OJJ73476.1 acar|OOG00673.1 acla|EAW06802.1 afis|EAW16351.1 afla|EED53521.1 afum|EAL88976.1 agla|OJJ88249.1 akaw|GAA83025.1 aluc|OJZ88571.1 anid|CBF89221.1 anig|EHA18269.1 aory|EIT75100.1 asyd|OJJ62520.1 ater|EAU34110.1 atub|OJI91157.1 aver|OJI96639.1 awen|OJJ37754.1 azon|OJJ45013.1

Group3090: abra|OJJ73484.1 acar|OOG00678.1 acla|EAW06808.1 afis|EAW16357.1 afla|EED53513.1 afum|EAL88982.1 agla|OJJ88236.1 akaw|GAA87433.1 aluc|OJZ88564.1 anid|CBF89232.1 anig|EHA18261.1 aory|EIT75027.1 asyd|OJJ62514.1 ater|EAU34102.1 atub|OJI91150.1 aver|OJI96633.1 awen|OJJ37761.1 azon|OJJ45061.1

Group3091: abra|OJJ73485.1 acar|OOG00680.1 acla|EAW06809.1 afis|EAW16358.1 afla|EED53512.1 afum|EAL88983.1 agla|OJJ88235.1 akaw|GAA87434.1 aluc|OJZ88563.1 anid|CBF89234.1 anig|EHA18260.1 aory|EIT75048.1 asyd|OJJ62513.1 ater|EAU34101.1 atub|OJI91149.1 aver|OJI96632.1 awen|OJJ37762.1 azon|OJJ45062.1

Group3092: abra|OJJ73486.1 acar|OOG00679.1 acla|EAW06810.1 afis|EAW16359.1 afla|EED53510.1 afum|EAL88984.1 agla|OJJ88233.1 akaw|GAA87435.1 aluc|OJZ88562.1 anid|CBF89236.1 anig|EHA18259.1 aory|EIT75112.1 asyd|OJJ62512.1 ater|EAU34100.1 atub|OJI91147.1 aver|OJI96631.1 awen|OJJ37763.1 azon|OJJ45063.1

Group3093: abra|OJJ73487.1 acar|OOG00681.1 acla|EAW06811.1 afis|EAW16360.1 afla|EED53508.1 afum|EAL88985.1 agla|OJJ88232.1 akaw|GAA87436.1 aluc|OJZ88561.1 anid|CBF89237.1 anig|EHA18258.1 aory|EIT75230.1 asyd|OJJ62510.1 ater|EAU34092.1 atub|OJI91146.1 aver|OJI96630.1 awen|OJJ37764.1 azon|OJJ45064.1

Group3094: abra|OJJ73488.1 acar|OOG00682.1 acla|EAW06812.1 afis|EAW16361.1 afla|EED53507.1 afum|EAL88986.1 agla|OJJ88231.1 akaw|GAA87437.1 aluc|OJZ88560.1 anid|CBF89239.1 anig|EHA18257.1 aory|EIT75024.1 asyd|OJJ62509.1 ater|EAU34091.1 atub|OJI91145.1 aver|OJI96629.1 awen|OJJ37765.1 azon|OJJ45065.1

Group3095: abra|OJJ73496.1 acar|OOG00694.1 acla|EAW07430.1 afis|EAW22456.1 afla|EED54488.1 afum|EAL90735.1 agla|OJJ87568.1 akaw|GAA87445.1 aluc|OJZ88552.1 anid|CBF88921.1 anig|EHA18250.1 aory|EIT78863.1 asyd|OJJ62846.1 ater|EAU39249.1 atub|OJI91137.1 aver|OJI96890.1 awen|OJJ35081.1 azon|OJJ50026.1

Group3096: abra|OJJ73497.1 acar|OOG00693.1 acla|EAW07431.1 afis|EAW22457.1 afla|EED54487.1 afum|EAL90734.1 agla|OJJ87567.1 akaw|GAA87446.1 aluc|OJZ88551.1 anid|CBF88920.1 anig|EHA18249.1 aory|EIT78747.1 asyd|OJJ62845.1 ater|EAU39248.1 atub|OJI91136.1 aver|OJI96889.1 awen|OJJ35082.1 azon|OJJ50025.1

Group3097: abra|OJJ73507.1 acar|OOG00697.1 acla|EAW07440.1 afis|EAW22467.1 afla|EED54499.1 afum|EAL90724.1 agla|OJJ87557.1 akaw|GAA87455.1 aluc|OJZ88540.1 anid|CBF88903.1 anig|EHA18241.1 aory|EIT78913.1 asyd|OJJ62837.1 ater|EAU39241.1 atub|OJI91125.1 aver|OJI96880.1 awen|OJJ35093.1 azon|OJJ50016.1

Group3098: abra|OJJ73508.1 acar|OOG00698.1 acla|EAW07441.1 afis|EAW22468.1 afla|EED54500.1 afum|EAL90723.1 agla|OJJ87556.1 akaw|GAA87456.1 aluc|OJZ88539.1 anid|CBF88902.1 anig|EHA18240.1 aory|EIT78813.1 asyd|OJJ62836.1 ater|EAU39240.1 atub|OJI91124.1 aver|OJI96879.1 awen|OJJ35094.1 azon|OJJ50015.1

Group3099: abra|OJJ73510.1 acar|OOG00699.1 acla|EAW07443.1 afis|EAW22470.1 afla|EED54502.1 afum|EAL90721.1 agla|OJJ87554.1 akaw|GAA87458.1 aluc|OJZ88537.1 anid|CBF88900.1 anig|EHA18238.1 aory|EIT78910.1 asyd|OJJ62834.1 ater|EAU39238.1 atub|OJI91122.1 aver|OJI96877.1 awen|OJJ35096.1 azon|OJJ50013.1

Group3100: abra|OJJ73512.1 acar|OOG00701.1 acla|EAW07445.1 afis|EAW22472.1 afla|EED54504.1 afum|EAL90719.1 agla|OJJ87552.1 akaw|GAA87460.1 aluc|OJZ88535.1 anid|CBF88896.1 anig|EHA18236.1 aory|EIT78949.1 asyd|OJJ62832.1 ater|EAU39236.1 atub|OJI91120.1 aver|OJI96875.1 awen|OJJ35098.1 azon|OJJ50011.1

Group3101: abra|OJJ73513.1 acar|OOG00700.1 acla|EAW07447.1 afis|EAW22474.1 afla|EED54506.1 afum|EAL90717.1 agla|OJJ87550.1 akaw|GAA87462.1 aluc|OJZ88534.1 anid|CBF88892.1 anig|EHA18235.1 aory|EIT78850.1 asyd|OJJ62831.1 ater|EAU39235.1 atub|OJI91119.1 aver|OJI96874.1 awen|OJJ35099.1 azon|OJJ50010.1

Group3102: abra|OJJ73516.1 acar|OOG00706.1 acla|EAW07451.1 afis|EAW22483.1 afla|EED54509.1 afum|EAL90711.1 agla|OJJ87543.1 akaw|GAA87465.1 aluc|OJZ88531.1 anid|CBF88887.1 anig|EHA18232.1 aory|EIT78939.1 asyd|OJJ62826.1 ater|EAU39232.1 atub|OJI91116.1 aver|OJI96871.1 awen|OJJ35102.1 azon|OJJ50006.1

Group3103: abra|OJJ73529.1 acar|OOG00710.1 acla|EAW07454.1 afis|EAW22486.1 afla|EED54512.1 afum|EAL90707.1 agla|OJJ80863.1 akaw|GAA88617.1 aluc|OJZ88526.1 anid|CBF88942.1 anig|EHA18228.1 aory|EIT78750.1 asyd|OJJ62815.1 ater|EAU39228.1 atub|OJI91111.1 aver|OJI96859.1 awen|OJJ35105.1 azon|OJJ49989.1

Group3104: abra|OJJ73534.1 acar|OOG00712.1 acla|EAW07456.1 afis|EAW22487.1 afla|EED54516.1 afum|EAL90706.1 agla|OJJ80860.1 akaw|GAA88616.1 aluc|OJZ88525.1 anid|CBF88944.1 anig|EHA18227.1 aory|EIT78867.1 asyd|OJJ62814.1 ater|EAU39227.1 atub|OJI91110.1 aver|OJI96858.1 awen|OJJ35108.1 azon|OJJ49986.1

Group3105: abra|OJJ73537.1 acar|OOG00713.1 acla|EAW07459.1 afis|EAW22490.1 afla|EED54519.1 afum|EAL90703.1 agla|OJJ80856.1 akaw|GAA88613.1 aluc|OJZ88522.1 anid|CBF88950.1 anig|EHA18225.1 aory|EIT78823.1 asyd|OJJ62811.1 ater|EAU39225.1 atub|OJI91107.1 aver|OJI96857.1 awen|OJJ35110.1 azon|OJJ49983.1

Group3106: abra|OJJ73539.1 acar|OOG00715.1 acla|EAW07460.1 afis|EAW22491.1 afla|EED54520.1 afum|EAL90702.1 agla|OJJ80854.1 akaw|GAA88612.1 aluc|OJZ88520.1 anid|CBF88951.1 anig|EHA18224.1 aory|EIT78935.1 asyd|OJJ62809.1 ater|EAU39224.1 atub|OJI91105.1 aver|OJI96855.1 awen|OJJ35112.1 azon|OJJ49981.1

Group3107: abra|OJJ73541.1 acar|OOG00716.1 acla|EAW07461.1 afis|EAW22492.1 afla|EED54521.1 afum|EAL90701.1 agla|OJJ80853.1 akaw|GAA88610.1 aluc|OJZ88518.1 anid|CBF88953.1 anig|EHA18223.1 aory|EIT78920.1 asyd|OJJ62808.1 ater|EAU39223.1 atub|OJI91103.1 aver|OJI96854.1 awen|OJJ35113.1 azon|OJJ49980.1

Group3108: abra|OJJ73547.1 acar|OOG00721.1 acla|EAW07466.1 afis|EAW22499.1 afla|EED54526.1 afum|EAL90694.1 agla|OJJ80847.1 akaw|GAA88605.1 aluc|OJZ88513.1 anid|CBF88960.1 anig|EHA18219.1 aory|EIT78800.1 asyd|OJJ62804.1 ater|EAU39219.1 atub|OJI91098.1 aver|OJI96850.1 awen|OJJ35122.1 azon|OJJ49976.1

Group3109: abra|OJJ73549.1 acar|OOG00723.1 acla|EAW07468.1 afis|EAW22501.1 afla|EED54529.1 afum|EAL90692.1 agla|OJJ80851.1 akaw|GAA88603.1 aluc|OJZ88511.1 anid|CBF88962.1 anig|EHA18217.1 aory|EIT78773.1 asyd|OJJ62799.1 ater|EAU39217.1 atub|OJI91096.1 aver|OJI96846.1 awen|OJJ35124.1 azon|OJJ49974.1

Group3110: abra|OJJ73550.1 acar|OOG00724.1 acla|EAW07469.1 afis|EAW22502.1 afla|EED54530.1 afum|EAL90691.1 agla|OJJ80852.1 akaw|GAA88602.1 aluc|OJZ88510.1 anid|CBF88965.1 anig|EHA18216.1 aory|EIT78738.1 asyd|OJJ62798.1 ater|EAU39216.1 atub|OJI91095.1 aver|OJI96845.1 awen|OJJ35125.1 azon|OJJ49973.1

Group3111: abra|OJJ73555.1 acar|OOG00726.1 acla|EAW07473.1 afis|EAW22505.1 afla|EED54533.1 afum|EAL90688.1 agla|OJJ87536.1 akaw|GAA88598.1 aluc|OJZ88506.1 anid|CBF88970.1 anig|EHA18213.1 aory|EIT78812.1 asyd|OJJ62795.1 ater|EAU39210.1 atub|OJI91092.1 aver|OJI96842.1 awen|OJJ35130.1 azon|OJJ49969.1

Group3112: abra|OJJ73556.1 acar|OOG00728.1 acla|EAW07474.1 afis|EAW22506.1 afla|EED54534.1 afum|EAL90687.1 agla|OJJ87537.1 akaw|GAA88597.1 aluc|OJZ88505.1 anid|CBF88972.1 anig|EHA18212.1 aory|EIT78875.1 asyd|OJJ62794.1 ater|EAU39209.1 atub|OJI91091.1 aver|OJI96841.1 awen|OJJ35131.1 azon|OJJ49968.1

Group3113: abra|OJJ73560.1 acar|OOG00731.1 acla|EAW07477.1 afis|EAW22509.1 afla|EED54537.1 afum|EAL90684.1 agla|OJJ80867.1 akaw|GAA88594.1 aluc|OJZ88502.1 anid|CBF88977.1 anig|EHA18210.1 aory|EIT78879.1 asyd|OJJ62791.1 ater|EAU39205.1 atub|OJI91088.1 aver|OJI96838.1 awen|OJJ35134.1 azon|OJJ49964.1

Group3114: abra|OJJ73563.1 acar|OOG00746.1 acla|EAW07480.1 afis|EAW22512.1 afla|EED54540.1 afum|EAL90681.1 agla|OJJ80875.1 akaw|GAA88591.1 aluc|OJZ88498.1 anid|CBF88983.1 anig|EHA18207.1

aory|EIT78924.1 asyd|OJJ62788.1 ater|EAU39197.1 atub|OJI91085.1 aver|OJI96835.1 awen|OJJ35138.1 azon|OJJ49961.1

Group3115: abra|OJJ73566.1 acar|OOG00748.1 acla|EAW07482.1 afis|EAW22514.1 afla|EED54544.1 afum|EAL90679.1 agla|OJJ80873.1 akaw|GAA88589.1 aluc|OJZ88496.1 anid|CBF88984.1 anig|EHA18205.1 aory|EIT78876.1 asyd|OJJ62786.1 ater|EAU39195.1 atub|OJI91083.1 aver|OJI96833.1 awen|OJJ35140.1 azon|OJJ49959.1

Group3116: abra|OJJ73567.1 acar|OOG00745.1 acla|EAW07483.1 afis|EAW22515.1 afla|EED54545.1 afum|EAL90678.1 agla|OJJ80872.1 akaw|GAA88588.1 aluc|OJZ88495.1 anid|CBF88986.1 anig|EHA18204.1 aory|EIT78884.1 asyd|OJJ62785.1 ater|EAU39194.1 atub|OJI91082.1 aver|OJI96831.1 awen|OJJ35141.1 azon|OJJ49958.1

Group3117: abra|OJJ73568.1 acar|OOG00749.1 acla|EAW07484.1 afis|EAW22516.1 afla|EED54546.1 afum|EAL90677.1 agla|OJJ80877.1 akaw|GAA88587.1 aluc|OJZ88494.1 anid|CBF88988.1 anig|EHA18203.1 aory|EIT78756.1 asyd|OJJ62784.1 ater|EAU39193.1 atub|OJI91081.1 aver|OJI96830.1 awen|OJJ35142.1 azon|OJJ49957.1

Group3118: abra|OJJ73573.1 acar|OOG00754.1 acla|EAW07490.1 afis|EAW22523.1 afla|EED54548.1 afum|EAL90671.1 agla|OJJ80884.1 akaw|GAA88577.1 aluc|OJZ88480.1 anid|CBF89007.1 anig|EHA18196.1 aory|EIT78743.1 asyd|OJJ62776.1 ater|EAU39187.1 atub|OJI91074.1 aver|OJI96821.1 awen|OJJ35150.1 azon|OJJ49951.1

Group3119: abra|OJJ73574.1 acar|OOG00755.1 acla|EAW07491.1 afis|EAW22524.1 afla|EED54549.1 afum|EAL90670.1 agla|OJJ80885.1 akaw|GAA88576.1 aluc|OJZ88479.1 anid|CBF89009.1 anig|EHA18195.1 aory|EIT78837.1 asyd|OJJ62775.1 ater|EAU39186.1 atub|OJI91073.1 aver|OJI96820.1 awen|OJJ35151.1 azon|OJJ49950.1

Group3120: abra|OJJ73575.1 acar|OOG00756.1 acla|EAW07492.1 afis|EAW22525.1 afla|EED54553.1 afum|EAL90669.1 agla|OJJ80886.1 akaw|GAA88575.1 aluc|OJZ88477.1 anid|CBF89011.1 anig|EHA18194.1 aory|EIT78752.1 asyd|OJJ62774.1 ater|EAU39185.1 atub|OJI91072.1 aver|OJI96819.1 awen|OJJ35152.1 azon|OJJ49949.1

Group3121: abra|OJJ73578.1 acar|OOG00759.1 acla|EAW07496.1 afis|EAW22529.1 afla|EED54557.1 afum|EAL90665.1 agla|OJJ80890.1 akaw|GAA88573.1 aluc|OJZ88474.1 anid|CBF89014.1 anig|EHA18191.1 aory|EIT78796.1 asyd|OJJ62772.1 ater|EAU39182.1 atub|OJI91069.1 aver|OJI96817.1 awen|OJJ35156.1 azon|OJJ49945.1

Group3122: abra|OJJ73580.1 acar|OOG00761.1 acla|EAW07498.1 afis|EAW22531.1 afla|EED54559.1 afum|EAL90663.1 agla|OJJ80892.1 akaw|GAA88571.1 aluc|OJZ88472.1 anid|CBF89018.1 anig|EHA18189.1 aory|EIT78759.1 asyd|OJJ62770.1 ater|EAU39178.1 atub|OJI91067.1 aver|OJI96815.1 awen|OJJ35158.1 azon|OJJ49944.1

Group3123: abra|OJJ73581.1 acar|OOG00762.1 acla|EAW07499.1 afis|EAW22532.1 afla|EED54560.1 afum|EAL90662.1 agla|OJJ80893.1 akaw|GAA88570.1 aluc|OJZ88471.1 anid|CBF89020.1 anig|EHA18188.1 aory|EIT78827.1 asyd|OJJ62769.1 ater|EAU39177.1 atub|OJI91066.1 aver|OJI96814.1 awen|OJJ35159.1 azon|OJJ49943.1

Group3124: abra|OJJ73582.1 acar|OOG00763.1 acla|EAW07500.1 afis|EAW22533.1 afla|EED54561.1 afum|EAL90661.1 agla|OJJ80894.1 akaw|GAA88569.1 aluc|OJZ88470.1 anid|CBF89023.1 anig|EHA18187.1 aory|EIT78937.1 asyd|OJJ62768.1 ater|EAU39176.1 atub|OJI91065.1 aver|OJI96813.1 awen|OJJ35160.1 azon|OJJ49942.1

Group3125: abra|OJJ73583.1 acar|OOG00764.1 acla|EAW07501.1 afis|EAW22534.1 afla|EED54562.1 afum|EAL90660.1 agla|OJJ80895.1 akaw|GAA88568.1 aluc|OJZ88469.1 anid|CBF89025.1 anig|EHA18186.1 aory|EIT78784.1 asyd|OJJ62767.1 ater|EAU39175.1 atub|OJI91064.1 aver|OJI96812.1 awen|OJJ35161.1 azon|OJJ49941.1

Group3126: abra|OJJ73585.1 acar|OOG00766.1 acla|EAW07503.1 afis|EAW22536.1 afla|EED54564.1 afum|EAL90658.1 agla|OJJ80897.1 akaw|GAA88566.1 aluc|OJZ88467.1 anid|CBF89029.1 anig|EHA18184.1 aory|EIT78903.1 asyd|OJJ62765.1 ater|EAU39173.1 atub|OJI91062.1 aver|OJI96810.1 awen|OJJ35163.1 azon|OJJ49940.1

Group3127: abra|OJJ73586.1 acar|OOG00767.1 acla|EAW07504.1 afis|EAW22537.1 afla|EED54565.1 afum|EAL90657.1 agla|OJJ80898.1 akaw|GAA88565.1 aluc|OJZ88466.1 anid|CBF89031.1 anig|EHA18183.1 aory|EIT78873.1 asyd|OJJ62764.1 ater|EAU39172.1 atub|OJI91061.1 aver|OJI96809.1 awen|OJJ35165.1 azon|OJJ49939.1

Group3128: abra|OJJ73589.1 acar|OOG00771.1 acla|EAW07507.1 afis|EAW22539.1 afla|EED54568.1 afum|EAL90655.1 agla|OJJ80899.1 akaw|GAA88563.1 aluc|OJZ88461.1 anid|CBF89036.1 anig|EHA18181.1 aory|EIT78782.1 asyd|OJJ62762.1 ater|EAU39170.1 atub|OJI91059.1 aver|OJI96805.1 awen|OJJ35168.1 azon|OJJ49938.1

Group3129: abra|OJJ73592.1 acar|OOG00777.1 acla|EAW07511.1 afis|EAW22542.1 afla|EED54573.1 afum|EAL90652.1 agla|OJJ80904.1 akaw|GAA88560.1 aluc|OJZ88458.1 anid|CBF89040.1 anig|EHA18178.1 aory|EIT78776.1 asyd|OJJ62760.1 ater|EAU39166.1 atub|OJI91056.1 aver|OJI96803.1 awen|OJJ35171.1 azon|OJJ49936.1

Group3130: abra|OJJ73596.1 acar|OOG00782.1 akaw|GAA88556.1 aluc|OJZ88454.1 anig|EHA18175.1 atub|OJI91052.1 abra|OJJ74453.1 acar|OOF95299.1 afla|EED49262.1 akaw|GAA87347.1 aluc|OJZ89667.1 anig|EHA26904.1 aory|EIT82192.1 asyd|OJJ62435.1 ater|EAU29288.1 atub|OJI85133.1 aver|OJI96571.1 aver|OJI98027.1

Group3131: abra|OJJ73598.1 acar|OOG00784.1 acla|EAW07516.1 afis|EAW22546.1 afla|EED54578.1 afum|EAL90649.1 agla|OJJ80908.1 akaw|GAA88554.1 aluc|OJZ88452.1 anid|CBF89049.1 anig|EHA18173.1 aory|EIT78955.1 asyd|OJJ62755.1 ater|EAU39159.1 atub|OJI91050.1 aver|OJI96797.1 awen|OJJ35177.1 azon|OJJ49931.1

Group3132: abra|OJJ73599.1 acar|OOG00785.1 acla|EAW07517.1 afis|EAW22547.1 afla|EED54579.1 afum|EAL90648.1 agla|OJJ80909.1 akaw|GAA88553.1 aluc|OJZ88451.1 anid|CBF89051.1 anig|EHA18172.1 aory|EIT78753.1 asyd|OJJ62754.1 ater|EAU39158.1 atub|OJI91049.1 aver|OJI96796.1 awen|OJJ35178.1 azon|OJJ49930.1

Group3133: abra|OJJ73601.1 acar|OOG00786.1 acla|EAW07519.1 afis|EAW22549.1 afla|EED54582.1 afum|EAL90646.1 agla|OJJ80911.1 akaw|GAA88551.1 aluc|OJZ88447.1 anid|CBF89057.1 anig|EHA18170.1 aory|EIT78932.1 asyd|OJJ62752.1 ater|EAU39155.1 atub|OJI91046.1 aver|OJI96794.1 awen|OJJ35180.1 azon|OJJ49927.1

Group3134: abra|OJJ73606.1 acar|OOG00790.1 acla|EAW07523.1 afis|EAW22553.1 afla|EED54585.1 afum|EAL90642.1 agla|OJJ80915.1 akaw|GAA88547.1 aluc|OJZ88443.1 anid|CBF89062.1 anig|EHA18167.1 aory|EIT78786.1 asyd|OJJ62748.1 ater|EAU39151.1 atub|OJI91041.1 aver|OJI96788.1 awen|OJJ35184.1 azon|OJJ49924.1

Group3135: abra|OJJ73607.1 acar|OOG00791.1 acla|EAW07524.1 afis|EAW22554.1 afla|EED54586.1 afum|EAL90641.1 agla|OJJ80916.1 akaw|GAA88546.1 aluc|OJZ88442.1 anid|CBF89063.1 anig|EHA18166.1 aory|EIT78927.1 asyd|OJJ62747.1 ater|EAU39150.1 atub|OJI91040.1 aver|OJI96787.1 awen|OJJ35185.1 azon|OJJ49923.1

Group3136: abra|OJJ73609.1 acar|OOG00793.1 acla|EAW07526.1 afis|EAW22556.1 afla|EED54587.1 afum|EAL90639.1 agla|OJJ80917.1 akaw|GAA88544.1 aluc|OJZ88440.1 anid|CBF89065.1 anig|EHA18164.1 aory|EIT78856.1 asyd|OJJ62745.1 ater|EAU39148.1 atub|OJI91038.1 aver|OJI96785.1 awen|OJJ35187.1 azon|OJJ49921.1

Group3137: abra|OJJ73611.1 acar|OOG00794.1 acla|EAW07528.1 afis|EAW22558.1 afla|EED54589.1 afum|EAL90637.1 agla|OJJ80918.1 akaw|GAA88542.1 aluc|OJZ88439.1 anid|CBF89071.1 anig|EHA18162.1 aory|EIT78846.1 asyd|OJJ62743.1 ater|EAU39146.1 atub|OJI91036.1 aver|OJI96784.1 awen|OJJ35188.1 azon|OJJ49918.1

Group3138: abra|OJJ73612.1 acar|OOG00795.1 acla|EAW07529.1 afis|EAW22559.1 afla|EED54590.1 afum|EAL90636.1 agla|OJJ80919.1 akaw|GAA88541.1 aluc|OJZ88437.1 anid|CBF88377.1 anig|EHA18161.1 aory|EIT78952.1 asyd|OJJ63734.1 ater|EAU39145.1 atub|OJI91034.1 aver|OJI97206.1 awen|OJJ35189.1 azon|OJJ49917.1

Group3139: abra|OJJ73616.1 acar|OOG00800.1 acla|EAW07531.1 afis|EAW22562.1 afla|EED54592.1 afum|EAL90633.1 agla|OJJ80921.1 akaw|GAA88537.1 aluc|OJZ88433.1 anid|CBF88314.1 anig|EHA18157.1 aory|EIT78915.1 asyd|OJJ63700.1 ater|EAU39142.1 atub|OJI91030.1 aver|OJI97239.1 awen|OJJ35191.1 azon|OJJ49915.1

Group3140: abra|OJJ73617.1 acar|OOG00799.1 acla|EAW07532.1 afis|EAW22561.1 afla|EED54596.1 afum|EAL90634.1 agla|OJJ80922.1 akaw|GAA88536.1 aluc|OJZ88432.1 anid|CBF88316.1 anig|EHA18156.1 aory|EIT78857.1 asyd|OJJ63701.1 ater|EAU39141.1 atub|OJI91029.1 aver|OJI97238.1 awen|OJJ35192.1 azon|OJJ49914.1

Group3141: abra|OJJ73618.1 acar|OOG00801.1 acla|EAW07533.1 afis|EAW22567.1 afla|EED54597.1 afum|EAL90631.1 agla|OJJ80923.1 akaw|GAA88535.1 aluc|OJZ88431.1 anid|CBF88318.1 anig|EHA18155.1 aory|EIT78761.1 asyd|OJJ63702.1 ater|EAU39140.1 atub|OJI91028.1 aver|OJI97237.1 awen|OJJ35193.1 azon|OJJ49913.1

Group3142: abra|OJJ73619.1 acar|OOG00802.1 acla|EAW07534.1 afis|EAW22568.1 afla|EED54598.1 afum|EAL90630.1 agla|OJJ80924.1 akaw|GAA88534.1 aluc|OJZ88430.1 anid|CBF88320.1 anig|EHA18154.1 aory|EIT78762.1 asyd|OJJ63703.1 ater|EAU39139.1 atub|OJI91027.1 aver|OJI97236.1 awen|OJJ35194.1 azon|OJJ49912.1

Group3143: abra|OJJ73621.1 acar|OOG00805.1 acla|EAW07536.1 afis|EAW22570.1 afla|EED54600.1 afum|EAL90628.1 agla|OJJ80926.1 akaw|GAA88532.1 aluc|OJZ88427.1 anid|CBF88323.1 anig|EHA18152.1

aory|EIT78841.1 asyd|OJJ63705.1 ater|EAU39137.1 atub|OJI91025.1 aver|OJI97234.1 awen|OJJ35196.1 azon|OJJ49911.1

Group3144: abra|OJJ73622.1 acar|OOG00804.1 acla|EAW07537.1 afis|EAW22571.1 afla|EED54601.1 afum|EAL90627.1 agla|OJJ80928.1 akaw|GAA88531.1 aluc|OJZ88426.1 anid|CBF88325.1 anig|EHA18151.1 aory|EIT78890.1 asyd|OJJ63706.1 ater|EAU39136.1 atub|OJI91024.1 aver|OJI97233.1 awen|OJJ35198.1 azon|OJJ49910.1

Group3145: abra|OJJ73624.1 acar|OOG00807.1 acla|EAW07539.1 afis|EAW22573.1 afla|EED54602.1 afum|EAL90625.1 agla|OJJ80930.1 akaw|GAA88529.1 aluc|OJZ88424.1 anid|CBF88328.1 anig|EHA18149.1 aory|EIT78905.1 asyd|OJJ63708.1 ater|EAU39134.1 atub|OJI91020.1 aver|OJI97230.1 awen|OJJ35200.1 azon|OJJ49907.1

Group3146: abra|OJJ73627.1 acar|OOG00809.1 acla|EAW07541.1 afis|EAW22575.1 afla|EED54604.1 afum|EAL90623.1 agla|OJJ80931.1 akaw|GAA88526.1 aluc|OJZ88422.1 anid|CBF88332.1 anig|EHA18147.1 aory|EIT78895.1 asyd|OJJ63711.1 ater|EAU39129.1 atub|OJI91018.1 aver|OJI97228.1 awen|OJJ35203.1 azon|OJJ49905.1

Group3147: abra|OJJ73632.1 acar|OOG00814.1 acla|EAW07546.1 afis|EAW22580.1 afla|EED54625.1 afum|EAL90619.2 agla|OJJ80940.1 akaw|GAA88521.1 aluc|OJZ88417.1 anid|CBF88341.1 anig|EHA18143.1 aory|EIT78840.1 asyd|OJJ63716.1 ater|EAU39124.1 atub|OJI91013.1 aver|OJI97224.1 awen|OJJ35208.1 azon|OJJ49901.1

Group3148: abra|OJJ73633.1 acar|OOG00815.1 acla|EAW07547.1 afis|EAW22581.1 afla|EED54626.1 afum|EAL90618.1 agla|OJJ80941.1 akaw|GAA88520.1 aluc|OJZ88416.1 anid|CBF88343.1 anig|EHA18142.1 aory|EIT78811.1 asyd|OJJ63717.1 ater|EAU39123.1 atub|OJI91012.1 aver|OJI97223.1 awen|OJJ35209.1 azon|OJJ49900.1

Group3149: abra|OJJ73637.1 acar|OOG00817.1 acla|EAW07549.1 afis|EAW22583.1 afla|EED54628.1 afum|EAL90616.1 agla|OJJ80943.1 akaw|GAA88518.1 aluc|OJZ88413.1 anid|CBF88349.1 anig|EHA18140.1 aory|EIT78801.1 asyd|OJJ63719.1 ater|EAU39121.1 atub|OJI91009.1 aver|OJI97221.1 awen|OJJ35211.1 azon|OJJ49898.1

Group3150: abra|OJJ73638.1 acar|OOG00818.1 acla|EAW07550.1 afis|EAW22584.1 afla|EED54629.1 afum|EAL90615.1 agla|OJJ80944.1 akaw|GAA88517.1 aluc|OJZ88412.1 anid|CBF88350.1 anig|EHA18139.1 aory|EIT78791.1 asyd|OJJ63720.1 ater|EAU39120.1 atub|OJI91007.1 aver|OJI97220.1 awen|OJJ35212.1 azon|OJJ49897.1

Group3151: abra|OJJ73640.1 acar|OOG00820.1 acla|EAW07553.1 afis|EAW22587.1 afla|EED54633.1 afum|EAL90612.1 agla|OJJ80947.1 akaw|GAA88515.1 aluc|OJZ88410.1 anid|CBF88356.1 anig|EHA18137.1 aory|EIT78894.1 asyd|OJJ63723.1 ater|EAU39113.1 atub|OJI91006.1 aver|OJI97217.1 awen|OJJ35215.1 azon|OJJ49894.1

Group3152: abra|OJJ73641.1 acar|OOG00822.1 acla|EAW07554.1 afis|EAW22588.1 afla|EED54634.1 afum|EAL90611.1 agla|OJJ80948.1 akaw|GAA88514.1 aluc|OJZ88409.1 anid|CBF88358.1 anig|EHA18136.1 aory|EIT78862.1 asyd|OJJ63724.1 ater|EAU39112.1 atub|OJI91005.1 aver|OJI97216.1 awen|OJJ35216.1 azon|OJJ49893.1

Group3153: abra|OJJ73643.1 acar|OOG00824.1 acla|EAW07556.1 afis|EAW22590.1 afla|EED54636.1 afum|EAL90609.1 agla|OJJ80950.1 akaw|GAA88512.1 aluc|OJZ88407.1 anid|CBF88360.1 anig|EHA18134.1 aory|EIT78889.1 asyd|OJJ63726.1 ater|EAU39119.1 atub|OJI91003.1 aver|OJI97214.1 awen|OJJ35218.1 azon|OJJ49890.1

Group3154: abra|OJJ73644.1 acar|OOG00825.1 acla|EAW07557.1 afis|EAW22591.1 afla|EED54641.1 afum|EAL90608.1 agla|OJJ80951.1 akaw|GAA88511.1 aluc|OJZ88406.1 anid|CBF88364.1 anig|EHA18133.1 aory|EIT78790.1 asyd|OJJ63727.1 ater|EAU39111.1 atub|OJI91002.1 aver|OJI97213.1 awen|OJJ35219.1 azon|OJJ49889.1

Group3155: abra|OJJ73645.1 acar|OOG00826.1 acla|EAW07558.1 afis|EAW22592.1 afla|EED54642.1 afum|EAL90607.1 agla|OJJ80952.1 akaw|GAA88510.1 aluc|OJZ88405.1 anid|CBF88365.1 anig|EHA18132.1 aory|EIT78880.1 asyd|OJJ63728.1 ater|EAU39110.1 atub|OJI91001.1 aver|OJI97212.1 awen|OJJ35220.1 azon|OJJ49888.1

Group3156: abra|OJJ73647.1 acar|OOG00829.1 acla|EAW07560.1 afis|EAW22594.1 afla|EED54644.1 afum|EAL90605.1 agla|OJJ80954.1 akaw|GAA88508.1 aluc|OJZ88403.1 anid|CBF88369.1 anig|EHA18131.1 aory|EIT78768.1 asyd|OJJ63730.1 ater|EAU39109.1 atub|OJI90999.1 aver|OJI97210.1 awen|OJJ35222.1 azon|OJJ49886.1

Group3157: abra|OJJ73654.1 acar|OOG00832.1 acla|EAW07564.1 afis|EAW22598.1 afla|EED54720.1 afum|EAL90600.1 agla|OJJ80966.1 akaw|GAA88504.1 aluc|OJZ88399.1 anid|CBF88312.1 anig|EHA18127.1 aory|EIT78821.1 asyd|OJJ63698.1 ater|EAU39105.1 atub|OJI90994.1 aver|OJI97241.1 awen|OJJ35226.1 azon|OJJ49884.1

Group3158: abra|OJJ73655.1 acar|OOG00833.1 acla|EAW07565.1 afis|EAW22599.1 afla|EED54719.1 afum|EAL90599.1 agla|OJJ80967.1 akaw|GAA88503.1 aluc|OJZ88398.1 anid|CBF88310.1 anig|EHA18126.1 aory|EIT78916.1 asyd|OJJ63697.1 ater|EAU39104.1 atub|OJI90993.1 aver|OJI97242.1 awen|OJJ35228.1 azon|OJJ49883.1

Group3159: abra|OJJ73656.1 acar|OOG00834.1 acla|EAW07566.1 afis|EAW22600.1 afla|EED54718.1 afum|EAL90598.1 agla|OJJ80968.1 akaw|GAA88502.1 aluc|OJZ88397.1 anid|CBF88308.1 anig|EHA18125.1 aory|EIT78770.1 asyd|OJJ63696.1 ater|EAU39103.1 atub|OJI90992.1 aver|OJI97243.1 awen|OJJ35229.1 azon|OJJ49882.1

Group3160: abra|OJJ73657.1 acar|OOG00835.1 acla|EAW07567.1 afis|EAW22601.1 afla|EED54717.1 afum|EAL90597.1 agla|OJJ80971.1 akaw|GAA88501.1 aluc|OJZ88396.1 anid|CBF88256.1 anig|EHA18124.1 aory|EIT78942.1 asyd|OJJ63672.1 ater|EAU39102.1 atub|OJI90991.1 aver|OJI97266.1 awen|OJJ35230.1 azon|OJJ49881.1

Group3161: abra|OJJ73658.1 acar|OOG00836.1 acla|EAW07568.1 afis|EAW22602.1 afla|EED54716.1 afum|EAL90596.1 agla|OJJ80972.1 akaw|GAA88500.1 aluc|OJZ88395.1 anid|CBF88257.1 anig|EHA18123.1 aory|EIT78742.1 asyd|OJJ63673.1 ater|EAU39101.1 atub|OJI90990.1 aver|OJI97265.1 awen|OJJ35231.1 azon|OJJ49880.1

Group3162: abra|OJJ73665.1 acar|OOG00843.1 acla|EAW07572.1 afis|EAW22606.1 afla|EED49514.1 afum|EAL90592.1 agla|OJJ80975.1 akaw|GAA88491.1 aluc|OJZ88387.1 anid|CBF88263.1 anig|EHA18114.1 aory|EIT82633.1 asyd|OJJ63677.1 ater|EAU39097.1 atub|OJI90982.1 aver|OJI97262.1 awen|OJJ35234.1 azon|OJJ49877.1

Group3163: abra|OJJ73667.1 acar|OOG00844.1 acla|EAW07573.1 afis|EAW22607.1 afla|EED49805.1 afum|EAL90591.1 agla|OJJ80977.1 akaw|GAA88490.1 aluc|OJZ88385.1 anid|CBF88265.1 anig|EHA18113.1 aory|EIT77683.1 asyd|OJJ63678.1 ater|EAU39096.1 atub|OJI90980.1 aver|OJI97261.1 awen|OJJ35235.1 azon|OJJ49876.1

Group3164: abra|OJJ73668.1 acar|OOG00845.1 acla|EAW07575.1 afis|EAW22609.1 afla|EED49807.1 afum|EAL90589.1 agla|OJJ81009.1 akaw|GAA88489.1 aluc|OJZ88384.1 anid|CBF88269.1 anig|EHA18112.1 aory|EIT77641.1 asyd|OJJ63680.1 ater|EAU39095.1 atub|OJI90979.1 aver|OJI97259.1 awen|OJJ35237.1 azon|OJJ49870.1

Group3165: abra|OJJ73671.1 acar|OOG00846.1 acla|EAW07583.1 afis|EAW22611.1 afla|EED49809.1 afum|EAL90587.1 agla|OJJ81005.1 akaw|GAA88486.1 aluc|OJZ88382.1 anid|CBF88281.1 anig|EHA18109.1 aory|EIT77633.1 asyd|OJJ63684.1 ater|EAU39092.1 atub|OJI90976.1 aver|OJI97255.1 awen|OJJ35239.1 azon|OJJ49868.1

Group3166: abra|OJJ73672.1 acar|OOG00847.1 acla|EAW07582.1 afis|EAW22612.1 afla|EED49810.1 afum|EAL90586.1 agla|OJJ81004.1 akaw|GAA88485.1 aluc|OJZ88381.1 anid|CBF88283.1 anig|EHA18108.1 aory|EIT77693.1 asyd|OJJ63685.1 ater|EAU39091.1 atub|OJI90975.1 aver|OJI97254.1 awen|OJJ35240.1 azon|OJJ49867.1

Group3167: abra|OJJ73673.1 acar|OOG00848.1 acla|EAW07581.1 afis|EAW22613.1 afla|EED49811.1 afum|EAL90585.1 agla|OJJ81003.1 akaw|GAA88484.1 aluc|OJZ88379.1 anid|CBF88287.1 anig|EHA18106.1 aory|EIT77666.1 asyd|OJJ63686.1 ater|EAU39090.1 atub|OJI90973.1 aver|OJI97253.1 awen|OJJ35241.1 azon|OJJ49866.1

Group3168: abra|OJJ73681.1 acar|OOG00853.1 acla|EAW07596.1 afis|EAW22627.1 afla|EED49827.1 afum|EAL90569.1 agla|OJJ80994.1 akaw|GAA88478.1 aluc|OJZ88368.1 anid|CBF88241.1 anig|EHA18101.1 aory|EIT77681.1 asyd|OJJ63662.1 ater|EAU39082.1 atub|OJI90966.1 aver|OJI97272.1 awen|OJJ35248.1 azon|OJJ49858.1

Group3169: abra|OJJ73684.1 acar|OOG00854.1 acla|EAW07599.1 afis|EAW22630.1 afla|EED49879.1 afum|EAL90565.1 agla|OJJ80990.1 akaw|GAA88475.1 aluc|OJZ88365.1 anid|CBF88236.1 anig|EHA18097.1 aory|EIT72464.1 asyd|OJJ63659.1 ater|EAU39078.1 atub|OJI90963.1 aver|OJI97275.1 awen|OJJ35251.1 azon|OJJ49855.1

Group3170: abra|OJJ73688.1 acar|OOG00860.1 acla|EAW07603.1 afis|EAW22634.1 afla|EED49884.1 afum|EAL90559.1 agla|OJJ80985.1 akaw|GAA88471.1 aluc|OJZ88361.1 anid|CBF88229.1 anig|EHA18093.1 aory|EIT72459.1 asyd|OJJ63655.1 ater|EAU39074.1 atub|OJI90957.1 aver|OJI97279.1 awen|OJJ35256.1 azon|OJJ49851.1

Group3171: abra|OJJ73689.1 acar|OOG00861.1 acla|EAW07604.1 afis|EAW22635.1 afum|EAL90558.2 akaw|GAA88470.1 aluc|OJZ88360.1 anid|CBF88227.1 anig|EHA18092.1 asyd|OJJ63654.1 ater|EAU39071.1 atub|OJI90956.1 aver|OJI97280.1 awen|OJJ35257.1 azon|OJJ49850.1 afla|EED49885.1 aory|EIT72467.1 agla|OJJ80984.1

Group3172: abra|OJJ73692.1 acar|OOG00863.1 acla|EAW07607.1 afis|EAW22637.1 afla|EED49888.1 afum|EAL90555.1 agla|OJJ80981.1 akaw|GAA88468.1 aluc|OJZ88357.1 anid|CBF88221.1 anig|EHA18090.1

aory|EIT72465.1 asyd|OJJ63652.1 ater|EAU39069.1 atub|OJI90952.1 aver|OJI97282.1 awen|OJJ35260.1 azon|OJJ49847.1

Group3173: abra|OJJ73724.1 acar|OOG00893.1 acla|EAW07613.1 afis|EAW22643.1 afla|EED49912.1 afum|EAL90549.2 agla|OJJ81025.1 akaw|GAA88438.1 aluc|OJZ88326.1 anid|CBF88175.1 anig|EHA18062.1 aory|EIT72277.1 asyd|OJJ63625.1 ater|EAU39064.1 atub|OJI90921.1 aver|OJI97311.1 awen|OJJ35281.1 azon|OJJ49845.1

Group3174: abra|OJJ73731.1 acar|OOG00897.1 acla|EAW07616.1 afis|EAW22645.1 afla|EED49914.1 afum|EAL90547.1 agla|OJJ81028.1 akaw|GAA88432.1 aluc|OJZ88319.1 anid|CBF88179.1 anig|EHA18055.1 aory|EIT72268.1 asyd|OJJ63627.1 ater|EAU39062.1 atub|OJI90914.1 aver|OJI97309.1 awen|OJJ35278.1 azon|OJJ49841.1

Group3175: abra|OJJ73733.1 acar|OOG00898.1 acla|EAW07617.1 afis|EAW22646.1 afla|EED49915.1 afum|EAL90546.1 agla|OJJ81029.1 akaw|GAA88431.1 aluc|OJZ88318.1 anid|CBF88180.1 anig|EHA18054.1 aory|EIT72267.1 asyd|OJJ63628.1 ater|EAU39061.1 atub|OJI90913.1 aver|OJI97308.1 awen|OJJ35277.1 azon|OJJ49840.1

Group3176: abra|OJJ73734.1 acar|OOG00899.1 acla|EAW07618.1 afis|EAW22647.1 afla|EED49916.1 afum|EAL90545.1 agla|OJJ81030.1 akaw|GAA88430.1 aluc|OJZ88317.1 anid|CBF88182.1 anig|EHA18053.1 aory|EIT72271.1 asyd|OJJ63629.1 ater|EAU39060.1 atub|OJI90912.1 aver|OJI97307.1 awen|OJJ35276.1 azon|OJJ49839.1

Group3177: abra|OJJ73735.1 acar|OOG00900.1 acla|EAW07619.1 afis|EAW22648.1 afla|EED49917.1 afum|EAL90544.1 agla|OJJ81031.1 akaw|GAA88429.1 aluc|OJZ88316.1 anid|CBF88184.1 anig|EHA18052.1 aory|EIT72269.1 asyd|OJJ63630.1 ater|EAU39059.1 atub|OJI90911.1 aver|OJI97306.1 awen|OJJ35275.1 azon|OJJ49832.1

Group3178: abra|OJJ73736.1 acar|OOG00901.1 acla|EAW07620.1 afis|EAW22649.1 afla|EED49918.1 afum|EAL90543.1 agla|OJJ81032.1 akaw|GAA88428.1 aluc|OJZ88315.1 anid|CBF88186.1 anig|EHA18051.1 aory|EIT72278.1 asyd|OJJ63631.1 ater|EAU39058.1 atub|OJI90910.1 aver|OJI97305.1 awen|OJJ35274.1 azon|OJJ49833.1

Group3179: abra|OJJ73739.1 acar|OOG00902.1 acla|EAW07633.1 afis|EAW22653.1 afla|EED49921.1 afum|EAL90539.1 agla|OJJ81036.1 akaw|GAA88425.1 aluc|OJZ88312.1 anid|CBF88197.1 anig|EHA18049.1 aory|EIT72265.1 asyd|OJJ63634.1 ater|EAU39053.1 atub|OJI90907.1 aver|OJI97302.1 awen|OJJ35270.1 azon|OJJ49837.1

Group3180: abra|OJJ73741.1 acar|OOG00904.1 acla|EAW07635.1 afis|EAW22655.1 afla|EED49923.1 afum|EAL90537.1 agla|OJJ81038.1 akaw|GAA88424.1 aluc|OJZ88311.1 anid|CBF88201.1 anig|EHA18048.1 aory|EIT72272.1 asyd|OJJ63637.1 ater|EAU39051.1 atub|OJI90906.1 aver|OJI97299.1 awen|OJJ35268.1 azon|OJJ49831.1

Group3181: abra|OJJ73744.1 acar|OOG00908.1 acla|EAW07640.1 afis|EAW22660.1 afla|EED49930.1 afum|EAL90532.1 agla|OJJ81041.1 akaw|GAA88421.1 aluc|OJZ88308.1 anid|CBF88218.1 anig|EHA18045.1 aory|EIT72279.1 asyd|OJJ63649.1 ater|EAU39048.1 atub|OJI90903.1 aver|OJI97284.1 awen|OJJ35265.1 azon|OJJ49828.1

Group3182: abra|OJJ73745.1 acar|OOG00909.1 acla|EAW07639.1 afis|EAW22659.1 afla|EED49931.1 afum|EAL90533.1 agla|OJJ81042.1 akaw|GAA88420.1 aluc|OJZ88307.1 anid|CBF88215.1 anig|EHA18044.1 aory|EIT72275.1 asyd|OJJ63648.1 ater|EAU39049.1 atub|OJI90902.1 aver|OJI97285.1 awen|OJJ35283.1 azon|OJJ49827.1

Group3183: abra|OJJ73751.1 acar|OOG00914.1 acla|EAW07642.1 afis|EAW22662.1 afla|EED49935.1 afum|EAL90530.1 agla|OJJ79062.1 akaw|GAA88416.1 aluc|OJZ88301.1 anid|CBF88168.1 anig|EHA18040.1 aory|EIT83423.1 asyd|OJJ63622.1 ater|EAU39046.1 atub|OJI90896.1 aver|OJI97315.1 awen|OJJ35287.1 azon|OJJ49826.1

Group3184: abra|OJJ73752.1 acar|OOG00915.1 acla|EAW07643.1 afis|EAW22663.1 afla|EED49937.1 afum|EAL90529.1 akaw|GAA88415.1 aluc|OJZ88300.1 anid|CBF88166.1 anig|EHA18039.1 aory|EIT83433.1 asyd|OJJ63621.1 ater|EAU39045.1 atub|OJI90894.1 aver|OJI97316.1 awen|OJJ35288.1 azon|OJJ49825.1 agla|OJJ79061.1

Group3185: abra|OJJ73753.1 acar|OOG00916.1 acla|EAW07644.1 afis|EAW22664.1 afla|EED49938.1 afum|EAL90528.1 agla|OJJ79060.1 akaw|GAA88414.1 aluc|OJZ88298.1 anid|CBF88164.1 anig|EHA18038.1 aory|EIT83434.1 asyd|OJJ63620.1 ater|EAU39044.1 atub|OJI90893.1 aver|OJI97318.1 awen|OJJ35290.1 azon|OJJ49824.1

Group3186: abra|OJJ73756.1 acar|OOG00918.1 acla|EAW07646.1 afis|EAW22666.1 afla|EED49940.1 afum|EAL90526.1 agla|OJJ79058.1 akaw|GAA88412.1 aluc|OJZ88296.1 anid|CBF88161.1 anig|EHA18036.1 aory|EIT83451.1 asyd|OJJ63619.1 ater|EAU39042.1 atub|OJI90892.1 aver|OJI97320.1 awen|OJJ35292.1 azon|OJJ49822.1

Group3187: abra|OJJ73757.1 acar|OOG00919.1 acla|EAW07647.1 afis|EAW22667.1 afla|EED49941.1 afum|EAL90525.1 agla|OJJ79057.1 akaw|GAA88411.1 aluc|OJZ88295.1 anid|CBF88159.1 anig|EHA18035.1 aory|EIT83456.1 asyd|OJJ63618.1 ater|EAU39041.1 atub|OJI90891.1 aver|OJI97321.1 awen|OJJ35293.1 azon|OJJ49821.1

Group3188: abra|OJJ73758.1 acar|OOG00920.1 acla|EAW07648.1 afis|EAW22668.1 afla|EED49942.1 afum|EAL90524.1 agla|OJJ79056.1 akaw|GAA88410.1 aluc|OJZ88294.1 anid|CBF88157.1 anig|EHA18034.1 aory|EIT83429.1 asyd|OJJ63617.1 ater|EAU39040.1 atub|OJI90890.1 aver|OJI97322.1 awen|OJJ35294.1 azon|OJJ49820.1

Group3189: abra|OJJ73766.1 acar|OOG00925.1 acla|EAW07652.1 afis|EAW22670.1 afla|EED49947.1 afum|EAL90522.1 agla|OJJ79049.1 akaw|GAA88408.1 aluc|OJZ88290.1 anid|CBF88155.1 anig|EHA18031.1 aory|EIT83455.1 asyd|OJJ63608.1 ater|EAU39038.1 atub|OJI90887.1 aver|OJI97329.1 awen|OJJ35298.1 azon|OJJ49817.1

Group3190: abra|OJJ73768.1 acar|OOG00927.1 acla|EAW07653.1 afis|EAW22671.1 afla|EED49949.1 afum|EAL90521.1 agla|OJJ79048.1 akaw|GAA88407.1 aluc|OJZ88289.1 anid|CBF88151.1 anig|EHA18030.1 aory|EIT83435.1 asyd|OJJ63607.1 ater|EAU39037.1 atub|OJI90886.1 aver|OJI97330.1 awen|OJJ35299.1 azon|OJJ49816.1

Group3191: abra|OJJ73769.1 acar|OOG00926.1 acla|EAW07654.1 afis|EAW22672.1 afla|EED49951.1 afum|EAL90520.1 agla|OJJ79046.1 akaw|GAA88406.1 aluc|OJZ88288.1 anid|CBF88145.1 anig|EHA18029.1 aory|EIT83454.1 asyd|OJJ63604.1 ater|EAU39036.1 atub|OJI90885.1 aver|OJI97332.1 awen|OJJ35300.1 azon|OJJ49815.1

Group3192: abra|OJJ73776.1 acar|OOG00933.1 acla|EAW07661.1 afis|EAW22678.1 afla|EED49959.1 afum|EAL90514.1 agla|OJJ79038.1 akaw|GAA88400.1 aluc|OJZ88278.1 anid|CBF88130.1 anig|EHA18023.1 aory|EIT83426.1 asyd|OJJ63588.1 ater|EAU39031.1 atub|OJI90878.1 aver|OJI97345.1 awen|OJJ35306.1 azon|OJJ49809.1

Group3193: abra|OJJ73778.1 acar|OOG00934.1 acla|EAW07662.1 afis|EAW22679.1 afla|EED49960.1 afum|EAL90513.1 agla|OJJ79037.1 akaw|GAA88399.1 aluc|OJZ88277.1 anid|CBF88128.1 anig|EHA18022.1 aory|EIT83445.1 asyd|OJJ63587.1 ater|EAU39030.1 atub|OJI90877.1 aver|OJI97347.1 awen|OJJ35307.1 azon|OJJ49806.1

Group3194: abra|OJJ73781.1 acar|OOG00937.1 acla|EAW07666.1 afis|EAW22683.1 afla|EED49967.1 afum|EAL90509.1 agla|OJJ79035.1 akaw|GAA88396.1 aluc|OJZ88274.1 anid|CBF88119.1 anig|EHA18019.1 aory|EIT83437.1 asyd|OJJ63583.1 ater|EAU39026.1 atub|OJI90874.1 aver|OJI97351.1 awen|OJJ35317.1 azon|OJJ49799.1

Group3195: abra|OJJ73783.1 acar|OOG00939.1 acla|EAW07668.1 afis|EAW22685.1 afla|EED49969.1 afum|EAL90508.1 agla|OJJ79032.1 akaw|GAA88394.1 aluc|OJZ88272.1 anid|CBF88111.1 anig|EHA18017.1 aory|EIT83432.1 asyd|OJJ63580.1 ater|EAU39019.1 atub|OJI90872.1 aver|OJI97356.1 awen|OJJ35318.1 azon|OJJ49798.1

Group3196: abra|OJJ73784.1 acar|OOG00940.1 acla|EAW07671.1 afis|EAW22686.1 afla|EED49971.1 afum|EAL90507.1 agla|OJJ79031.1 akaw|GAA88393.1 aluc|OJZ88271.1 anid|CBF88109.1 anig|EHA18016.1 aory|EIT74696.1 asyd|OJJ63578.1 ater|EAU39021.1 atub|OJI90871.1 aver|OJI97357.1 awen|OJJ35320.1 azon|OJJ49796.1

Group3197: abra|OJJ73785.1 acar|OOG00941.1 acla|EAW07672.1 afis|EAW22687.1 afla|EED49972.1 afum|EAL90506.1 agla|OJJ79029.1 akaw|GAA88392.1 aluc|OJZ88270.1 anid|CBF88107.1 anig|EHA18014.1 aory|EIT74698.1 asyd|OJJ63577.1 ater|EAU39022.1 atub|OJI90870.1 aver|OJI97359.1 awen|OJJ35321.1 azon|OJJ49794.1

Group3198: abra|OJJ73786.1 acar|OOG00942.1 acla|EAW07673.1 afis|EAW22688.1 afla|EED49973.1 afum|EAL90505.1 agla|OJJ79028.1 akaw|GAA88391.1 aluc|OJZ88269.1 anid|CBF88106.1 anig|EHA18013.1 aory|EIT74642.1 asyd|OJJ63576.1 ater|EAU39023.1 atub|OJI90869.1 aver|OJI97360.1 awen|OJJ35322.1 azon|OJJ49793.1

Group3199: abra|OJJ73787.1 acar|OOF90345.1 acla|EAW07674.1 afis|EAW22689.1 afla|EED49974.1 afum|EAL90504.1 agla|OJJ79024.1 akaw|GAA88390.1 aluc|OJZ88267.1 anid|CBF88104.1 anig|EHA18012.1 aory|EIT74677.1 asyd|OJJ63575.1 ater|EAU39024.1 atub|OJI90867.1 aver|OJI97361.1 awen|OJJ35323.1 azon|OJJ49792.1

Group3200: abra|OJJ73791.1 acar|OOF90347.1 acla|EAW07680.1 afis|EAW22695.1 afla|EED49977.1 afum|EAL90498.1 agla|OJJ79021.1 akaw|GAA89410.1 aluc|OJZ88263.1 anid|CBF88098.1 anig|EHA18009.1 aory|EIT74603.1 asyd|OJJ63570.1 ater|EAU39015.1 atub|OJI90862.1 aver|OJI97364.1 awen|OJJ35325.1 azon|OJJ49789.1

Group3201: abra|OJJ73801.1 acar|OOG00957.1 acla|EAW07683.1 afis|EAW22698.1 afla|EED49984.1 afum|EAL90494.1 agla|OJJ79012.1 akaw|GAA89421.1 aluc|OJZ88252.1 anid|CBF88087.1 anig|EHA17994.1

aory|EIT74667.1 asyd|OJJ63563.1 ater|EAU39008.1 atub|OJI90850.1 aver|OJI97370.1 awen|OJJ35334.1 azon|OJJ49783.1

Group3202: abra|OJJ73804.1 acar|OOG00959.1 acla|EAW07676.1 afis|EAW22691.1 afla|EED49982.1 afum|EAL90501.1 agla|OJJ79016.1 akaw|GAA89423.1 aluc|OJZ88249.1 anid|CBF88090.1 anig|EHA17992.1 aory|EIT74721.1 asyd|OJJ63565.1 ater|EAU39010.1 atub|OJI90848.1 aver|OJI97368.1 awen|OJJ35333.1 azon|OJJ49785.1

Group3203: abra|OJJ73805.1 acar|OOG00960.1 acla|EAW07677.1 afis|EAW22692.1 afla|EED49980.1 afum|EBA27409.1 agla|OJJ79018.1 akaw|GAA89425.1 aluc|OJZ88247.1 anid|CBF88095.1 anig|EHA17991.1 aory|EIT74734.1 asyd|OJJ63566.1 ater|EAU39012.1 atub|OJI90847.1 aver|OJI97367.1 awen|OJJ35332.1 azon|OJJ49786.1

Group3204: abra|OJJ73806.1 acar|OOG00961.1 acla|EAW07694.1 afis|EAW22709.1 afla|EED49996.1 afum|EAL90483.1 agla|OJJ79001.1 akaw|GAA89426.1 aluc|OJZ88246.1 anid|CBF88064.1 anig|EHA17990.1 aory|EIT74673.1 asyd|OJJ63549.1 ater|EAU38992.1 atub|OJI90846.1 aver|OJI97385.1 awen|OJJ35346.1 azon|OJJ49771.1

Group3205: abra|OJJ73807.1 acar|OOG00963.1 acla|EAW07695.1 afis|EAW22712.1 afla|EED49997.1 afum|EAL90482.1 agla|OJJ79000.1 akaw|GAA89427.1 aluc|OJZ88243.1 anid|CBF88062.1 anig|EHA17989.1 aory|EIT74610.1 asyd|OJJ63548.1 ater|EAU38993.1 atub|OJI90845.1 aver|OJI97386.1 awen|OJJ35347.1 azon|OJJ49770.1

Group3206: abra|OJJ73816.1 acar|OOG00970.1 acla|EAW07703.1 afis|EAW22721.1 afla|EED50006.1 afum|EAL90474.1 agla|OJJ83682.1 akaw|GAA89435.1 aluc|OJZ88234.1 anid|CBF88036.1 anig|EHA17980.1 aory|EIT74720.1 asyd|OJJ63535.1 ater|EAU38983.1 atub|OJI90836.1 aver|OJI97397.1 awen|OJJ35415.1 azon|OJJ49760.1

Group3207: abra|OJJ73821.1 acar|OOG00973.1 acla|EAW07710.1 afis|EAW22726.1 afla|EED50012.1 afum|EAL90469.1 agla|OJJ83676.1 akaw|GAA89440.1 aluc|OJZ88227.1 anid|CBF88028.1 anig|EHA17975.1 aory|EIT74707.1 asyd|OJJ63529.1 ater|EAU38978.1 atub|OJI90828.1 aver|OJI97402.1 awen|OJJ35421.1 azon|OJJ49755.1

Group3208: abra|OJJ73830.1 acar|OOG00981.1 acla|EAW07725.1 afis|EAW22757.1 afla|EED50018.1 afum|EAL90442.2 agla|OJJ83666.1 akaw|GAA89448.1 aluc|OJZ88219.1 anid|CBF88015.1 anig|EHA17966.1 aory|EIT74656.1 asyd|OJJ63519.1 ater|EAU38962.1 atub|OJI90819.1 aver|OJI97411.1 awen|OJJ35429.1 azon|OJJ49747.1

Group3209: abra|OJJ73831.1 acar|OOG00982.1 acla|EAW07726.1 afis|EAW22758.1 afla|EED50019.1 afum|EAL90441.1 agla|OJJ83662.1 akaw|GAA89449.1 aluc|OJZ88218.1 anig|EHA17965.1 aory|EIT74708.1 asyd|OJJ63518.1 ater|EAU38961.1 atub|OJI90818.1 aver|OJI97412.1 awen|OJJ35430.1 azon|OJJ49746.1 aory|EIT74649.1

Group3210: abra|OJJ73832.1 acar|OOG00983.1 acla|EAW07727.1 afis|EAW22759.1 afla|EED50020.1 afum|EAL90440.1 agla|OJJ83661.1 akaw|GAA89450.1 aluc|OJZ88217.1 anid|CBF88013.1 anig|EHA17964.1 aory|EIT74627.1 asyd|OJJ63517.1 ater|EAU38960.1 atub|OJI90817.1 aver|OJI97413.1 awen|OJJ35431.1 azon|OJJ49745.1

Group3211: abra|OJJ73833.1 acar|OOG00984.1 acla|EAW07728.1 afis|EAW22760.1 afla|EED50021.1 afum|EAL90439.1 agla|OJJ83660.1 akaw|GAA89451.1 aluc|OJZ88216.1 anid|CBF88011.1 anig|EHA17963.1 aory|EIT74690.1 asyd|OJJ63516.1 ater|EAU38959.1 atub|OJI90816.1 aver|OJI97414.1 awen|OJJ35433.1 azon|OJJ49744.1

Group3212: abra|OJJ73834.1 acar|OOG00985.1 acla|EAW07729.1 afis|EAW22761.1 afla|EED50022.1 afum|EAL90438.1 agla|OJJ83659.1 akaw|GAA89452.1 aluc|OJZ88215.1 anid|CBF88009.1 anig|EHA17962.1 aory|EIT74638.1 asyd|OJJ63515.1 ater|EAU38958.1 atub|OJI90815.1 aver|OJI97415.1 awen|OJJ35434.1 azon|OJJ49743.1

Group3213: abra|OJJ73836.1 acar|OOG00987.1 acla|EAW07731.1 afis|EAW22763.1 afla|EED50025.1 afum|EAL90436.2 agla|OJJ83657.1 akaw|GAA89454.1 aluc|OJZ88213.1 anid|CBF88005.1 anig|EHA17961.1 aory|EIT74679.1 asyd|OJJ63513.1 ater|EAU38956.1 atub|OJI90813.1 aver|OJI97417.1 awen|OJJ35436.1 azon|OJJ49741.1

Group3214: abra|OJJ73837.1 acar|OOG00988.1 acla|EAW07732.1 afis|EAW22764.1 afla|EED50026.1 afum|EAL90435.1 agla|OJJ83656.1 akaw|GAA89455.1 aluc|OJZ88212.1 anid|CBF88003.1 anig|EHA17960.1 aory|EIT74726.1 asyd|OJJ63509.1 ater|EAU38953.1 atub|OJI90812.1 aver|OJI97421.1 awen|OJJ38969.1 azon|OJJ49740.1

Group3215: abra|OJJ73838.1 acar|OOG00989.1 acla|EAW07733.1 afis|EAW22765.1 afla|EED50027.1 afum|EAL90434.1 agla|OJJ83655.1 akaw|GAA89456.1 aluc|OJZ88211.1 anid|CBF88001.1 anig|EHA17958.1 aory|EIT74637.1 asyd|OJJ63508.1 ater|EAU38952.1 atub|OJI90811.1 aver|OJI97422.1 awen|OJJ38968.1 azon|OJJ49738.1

Group3216: abra|OJJ73841.1 acar|OOG00992.1 acla|EAW07736.1 afis|EAW22772.1 afla|EED50031.1 afum|EAL90426.1 agla|OJJ83602.1 akaw|GAA89459.1 aluc|OJZ88207.1 anid|CBF87996.1 anig|EHA17955.1 aory|EIT74633.1 asyd|OJJ63505.1 ater|EAU38941.1 atub|OJI90808.1 aver|OJI97425.1 awen|OJJ38965.1 azon|OJJ49736.1

Group3217: abra|OJJ73842.1 acar|OOG00993.1 acla|EAW07737.1 afis|EAW22773.1 afla|EED50032.1 afum|EAL90425.1 agla|OJJ83603.1 akaw|GAA89460.1 aluc|OJZ88206.1 anid|CBF87994.1 anig|EHA17954.1 aory|EIT74732.1 asyd|OJJ63504.1 ater|EAU38940.1 atub|OJI90807.1 aver|OJI97426.1 awen|OJJ38964.1 azon|OJJ49735.1

Group3218: abra|OJJ73843.1 acar|OOG00994.1 acla|EAW07738.1 afis|EAW22774.1 afla|EED50033.1 afum|EAL90424.1 agla|OJJ83604.1 akaw|GAA89461.1 aluc|OJZ88205.1 anid|CBF87992.1 anig|EHA17953.1 aory|EIT74665.1 asyd|OJJ63503.1 ater|EAU38939.1 atub|OJI90806.1 aver|OJI97427.1 awen|OJJ38963.1 azon|OJJ49734.1

Group3219: abra|OJJ73844.1 acar|OOG00995.1 acla|EAW07739.1 afis|EAW22775.1 afla|EED50034.1 afum|EAL90423.1 agla|OJJ83605.1 akaw|GAA89462.1 aluc|OJZ88204.1 anid|CBF87991.1 anig|EHA17952.1 aory|EIT74686.1 asyd|OJJ63502.1 ater|EAU38938.1 atub|OJI90805.1 aver|OJI97428.1 awen|OJJ38962.1 azon|OJJ49733.1

Group3220: abra|OJJ73846.1 acar|OOG00997.1 acla|EAW07741.1 afis|EAW22777.1 afla|EED50037.1 afum|EAL90421.1 agla|OJJ83628.1 akaw|GAA89464.1 aluc|OJZ88202.1 anid|CBF87987.1 anig|EHA17950.1 aory|EIT74713.1 asyd|OJJ63500.1 ater|EAU38937.1 atub|OJI90802.1 aver|OJI97430.1 awen|OJJ38960.1 azon|OJJ49731.1

Group3221: abra|OJJ73848.1 acar|OOG00998.1 acla|EAW07742.1 afis|EAW22778.1 afla|EED50038.1 afum|EAL90420.1 agla|OJJ83632.1 akaw|GAA89465.1 aluc|OJZ88201.1 anid|CBF87983.1 anig|EHA17949.1 aory|EIT74616.1 asyd|OJJ63497.1 ater|EAU38936.1 atub|OJI90799.1 aver|OJI97432.1 awen|OJJ38959.1 azon|OJJ49730.1

Group3222: abra|OJJ73850.1 acar|OOG01000.1 acla|EAW07743.1 afis|EAW22780.1 afla|EED50041.1 afum|EAL90419.1 agla|OJJ83633.1 akaw|GAA89467.1 aluc|OJZ88199.1 anid|CBF87970.1 anig|EHA17947.1 aory|EIT74714.1 asyd|OJJ63494.1 ater|EAU38932.1 atub|OJI90797.1 aver|OJI97435.1 awen|OJJ38957.1 azon|OJJ49729.1

Group3223: abra|OJJ73851.1 acar|OOG01001.1 acla|EAW07745.1 afis|EAW22782.1 afla|EED50044.1 afum|EAL90417.1 agla|OJJ83644.1 akaw|GAA89468.1 aluc|OJZ88197.1 anid|CBF87972.1 anig|EHA17946.1 aory|EIT74643.1 asyd|OJJ63493.1 ater|EAU38930.1 atub|OJI90796.1 aver|OJI97436.1 awen|OJJ38955.1 azon|OJJ49727.1

Group3224: abra|OJJ73854.1 acar|OOG01002.1 acla|EAW07746.1 afis|EAW22783.1 afla|EED50045.1 afum|EAL90416.1 agla|OJJ83653.1 akaw|GAA89470.1 aluc|OJZ88194.1 anid|CBF87974.1 anig|EHA17945.1 aory|EIT74701.1 asyd|OJJ63492.1 ater|EAU38926.1 atub|OJI90793.1 aver|OJI97437.1 awen|OJJ38954.1 azon|OJJ49726.1

Group3225: abra|OJJ73861.1 acar|OOG01007.1 acla|EAW07751.1 afis|EAW22788.1 afla|EED50052.1 afum|EAL90411.1 agla|OJJ83588.1 akaw|GAA89481.1 aluc|OJZ88182.1 anid|CBF87963.1 anig|EHA17934.1 aory|EIT74670.1 asyd|OJJ63484.1 ater|EAU38922.1 atub|OJI90781.1 aver|OJI97445.1 awen|OJJ38946.1 azon|OJJ49718.1

Group3226: abra|OJJ73864.1 acar|OOF89914.1 acla|EAW07754.1 afis|EAW22791.1 afla|EED50058.1 afum|EAL90408.1 agla|OJJ83567.1 akaw|GAA89478.1 aluc|OJZ88185.1 anid|CBF87957.1 anig|EHA17936.1 aory|EIT74695.1 asyd|OJJ63481.1 ater|EAU38920.1 atub|OJI90784.1 aver|OJI97449.1 awen|OJJ38943.1 azon|OJJ49716.1

Group3227: abra|OJJ73867.1 acar|OOF89912.1 acla|EAW07755.1 afis|EAW22792.1 afla|EED50059.1 afum|EAL90407.2 agla|OJJ83568.1 akaw|GAA89475.1 aluc|OJZ88188.1 anid|CBF87955.1 anig|EHA17939.1 aory|EIT74728.1 asyd|OJJ63480.1 ater|EAU38919.1 atub|OJI90787.1 aver|OJI97450.1 awen|OJJ38941.1 azon|OJJ49715.1

Group3228: abra|OJJ73869.1 acar|OOG01012.1 acla|EAW07757.1 afis|EAW22794.1 afla|EED50062.1 afum|EAL90405.1 agla|OJJ83570.1 akaw|GAA89473.1 aluc|OJZ88190.1 anid|CBF87952.1 anig|EHA17941.1 aory|EIT74606.1 asyd|OJJ63478.1 ater|EAU38917.1 atub|OJI90789.1 aver|OJI97452.1 awen|OJJ38939.1 azon|OJJ49713.1

Group3229: abra|OJJ73874.1 acar|OOG01014.1 acla|EAW07759.1 afis|EAW22795.1 afla|EED50063.1 afum|EAL90404.1 agla|OJJ83571.1 akaw|GAA89486.1 aluc|OJZ88176.1 anid|CBF87950.1 anig|EHA17928.1 aory|EIT74699.1 asyd|OJJ63477.1 ater|EAU38915.1 atub|OJI90774.1 aver|OJI97453.1 awen|OJJ38938.1 azon|OJJ49712.1

Group3230: abra|OJJ73876.1 acar|OOG01016.1 acla|EAW07761.1 afis|EAW22797.1 afla|EED50066.1 afum|EAL90402.1 agla|OJJ83574.1 akaw|GAA89488.1 aluc|OJZ88173.1 anid|CBF87946.1 anig|EHA17926.1

aory|EIT74703.1 asyd|OJJ63475.1 ater|EAU38913.1 atub|OJI90771.1 aver|OJI97455.1 awen|OJJ38932.1 azon|OJJ49708.1

Group3231: abra|OJJ73881.1 acar|OOG01020.1 acla|EAW07763.1 afis|EAW22799.1 afla|EED50069.1 afum|EAL90400.1 agla|OJJ83576.1 akaw|GAA89492.1 aluc|OJZ88169.1 anid|CBF87936.1 anig|EHA17923.1 aory|EIT74640.1 asyd|OJJ63466.1 ater|EAU38910.1 atub|OJI90767.1 aver|OJI97466.1 awen|OJJ38930.1 azon|OJJ49706.1

Group3232: abra|OJJ73882.1 acar|OOG01021.1 acla|EAW07764.1 afis|EAW22800.1 afla|EED50070.1 afum|EAL90399.1 agla|OJJ83577.1 akaw|GAA89493.1 aluc|OJZ88168.1 anid|CBF87934.1 anig|EHA17922.1 aory|EIT74735.1 asyd|OJJ63465.1 ater|EAU38909.1 atub|OJI90766.1 aver|OJI97467.1 awen|OJJ38929.1 azon|OJJ49705.1

Group3233: abra|OJJ73883.1 acar|OOG01022.1 acla|EAW07765.1 afis|EAW22801.1 afla|EED50071.1 afum|EAL90398.1 agla|OJJ83578.1 akaw|GAA89494.1 aluc|OJZ88167.1 anid|CBF87932.1 anig|EHA17921.1 aory|EIT74619.1 asyd|OJJ63464.1 ater|EAU38908.1 atub|OJI90765.1 aver|OJI97468.1 awen|OJJ38928.1 azon|OJJ49704.1

Group3234: abra|OJJ73884.1 acar|OOG01023.1 acla|EAW07766.1 afis|EAW22802.1 afla|EED50072.1 afum|EAL90397.1 agla|OJJ83579.1 akaw|GAA89495.1 aluc|OJZ88166.1 anid|CBF87930.1 anig|EHA17920.1 aory|EIT74719.1 asyd|OJJ63463.1 ater|EAU38907.1 atub|OJI90764.1 aver|OJI97469.1 awen|OJJ38927.1 azon|OJJ49703.1

Group3235: abra|OJJ73886.1 acar|OOG01024.1 acla|EAW07768.1 afis|EAW22804.1 afla|EED50074.1 afum|EAL90395.1 agla|OJJ83581.1 akaw|GAA89497.1 aluc|OJZ88164.1 anid|CBF87926.1 anig|EHA17918.1 aory|EIT74660.1 asyd|OJJ63461.1 ater|EAU38905.1 atub|OJI90762.1 aver|OJI97471.1 awen|OJJ38924.1 azon|OJJ49701.1

Group3236: abra|OJJ73889.1 acar|OOG01027.1 acla|EAW07773.1 afis|EAW22809.1 afla|EED50078.1 afum|EAL90392.1 agla|OJJ83584.1 akaw|GAA89501.1 aluc|OJZ88160.1 anid|CBF87904.1 anig|EHA17915.1 aory|EIT74748.1 asyd|OJJ63457.1 ater|EAU38902.1 atub|OJI90759.1 aver|OJI97475.1 awen|OJJ38921.1 azon|OJJ49698.1

Group3237: abra|OJJ73890.1 acar|OOG01030.1 acla|EAW07774.1 afis|EAW22810.1 afla|EED50079.1 afum|EAL90391.1 agla|OJJ83585.1 akaw|GAA89502.1 aluc|OJZ88159.1 anid|CBF87906.1 anig|EHA17914.1 aory|EIT74744.1 asyd|OJJ63456.1 ater|EAU38901.1 atub|OJI90758.1 aver|OJI97476.1 awen|OJJ38920.1 azon|OJJ49697.1

Group3238: abra|OJJ73891.1 acar|OOG01029.1 acla|EAW07775.1 afis|EAW22811.1 afla|EED50080.1 afum|EAL90390.1 agla|OJJ83566.1 akaw|GAA89503.1 aluc|OJZ88158.1 anid|CBF87908.1 anig|EHA17913.1 aory|EIT74652.1 asyd|OJJ63455.1 ater|EAU38900.1 atub|OJI90757.1 aver|OJI97477.1 awen|OJJ38915.1 azon|OJJ49696.1

Group3239: abra|OJJ73897.1 acar|OOG01034.1 acla|EAW07778.1 afis|EAW22815.1 afla|EED50088.1 afum|EAL90386.1 agla|OJJ83527.1 akaw|GAA89507.1 aluc|OJZ88149.1 anid|CBF87915.1 anig|EHA17910.1 aory|EIT74671.1 asyd|OJJ63448.1 ater|EAU38896.1 atub|OJI90752.1 aver|OJI97486.1 awen|OJJ38913.1 azon|OJJ49693.1

Group3240: abra|OJJ73901.1 acar|OOG01036.1 acla|EAW07780.1 afis|EAW22816.1 afla|EED50089.1 afum|EAL90385.1 agla|OJJ83524.1 akaw|GAA89508.1 aluc|OJZ88145.1 anid|CBF87918.1 anig|EHA17908.1 aory|EIT74605.1 asyd|OJJ63447.1 ater|EAU38895.1 atub|OJI90749.1 aver|OJI97489.1 awen|OJJ38911.1 azon|OJJ49692.1

Group3241: abra|OJJ73902.1 acar|OOG01037.1 acla|EAW07781.1 afis|EAW22817.1 afla|EED50090.1 afum|EAL90384.1 agla|OJJ83521.1 akaw|GAA89509.1 aluc|OJZ88144.1 anid|CBF87920.1 anig|EHA17907.1 aory|EIT74746.1 asyd|OJJ63446.1 ater|EAU38894.1 atub|OJI90748.1 aver|OJI97490.1 awen|OJJ38910.1 azon|OJJ49691.1

Group3242: abra|OJJ73904.1 acar|OOG01038.1 acla|EAW07783.1 afis|EAW22819.1 afla|EED50092.1 afum|EAL90382.1 agla|OJJ83519.1 akaw|GAA89511.1 aluc|OJZ88142.1 anid|CBF87899.1 anig|EHA17905.1 aory|EIT74751.1 asyd|OJJ63444.1 ater|EAU38891.1 atub|OJI90746.1 aver|OJI97492.1 awen|OJJ38908.1 azon|OJJ49689.1

Group3243: abra|OJJ73908.1 acar|OOG01041.1 acla|EAW07786.1 afis|EAW22822.1 afla|EED50097.1 afum|EAL90378.2 agla|OJJ83516.1 akaw|GAA89515.1 aluc|OJZ88139.1 anid|CBF87894.1 anig|EHA17903.1 aory|EIT74681.1 asyd|OJJ63441.1 ater|EAU38888.1 atub|OJI90742.1 aver|OJI97495.1 awen|OJJ38905.1 azon|OJJ49686.1

Group3244: abra|OJJ73909.1 acar|OOG01042.1 acla|EAW07787.1 afis|EAW22823.1 afla|EED50098.1 afum|EAL90377.1 agla|OJJ83515.1 akaw|GAA89516.1 aluc|OJZ88138.1 anid|CBF87892.1 anig|EHA17902.1 aory|EIT74745.1 asyd|OJJ63440.1 ater|EAU38887.1 atub|OJI90741.1 aver|OJI97496.1 awen|OJJ38904.1 azon|OJJ49685.1

Group3245: abra|OJJ73910.1 acar|OOG01043.1 acla|EAW07788.1 afis|EAW22824.1 afla|EED50099.1 afum|EAL90376.1 agla|OJJ83514.1 akaw|GAA89517.1 aluc|OJZ88137.1 anid|CBF87890.1 anig|EHA17901.1 aory|EIT74620.1 asyd|OJJ63439.1 ater|EAU38886.1 atub|OJI90740.1 aver|OJI97497.1 awen|OJJ38903.1 azon|OJJ49684.1

Group3246: abra|OJJ73912.1 acar|OOG01044.1 acla|EAW07789.1 afis|EAW22825.1 afla|EED50100.1 afum|EAL90375.2 agla|OJJ83513.1 akaw|GAA89518.1 aluc|OJZ88136.1 anid|CBF87888.1 anig|EHA17899.1 aory|EIT74685.1 asyd|OJJ63438.1 ater|EAU38885.1 atub|OJI90739.1 aver|OJI97499.1 awen|OJJ38902.1 azon|OJJ49683.1

Group3247: abra|OJJ73915.1 acar|OOG01047.1 acla|EAW07791.1 afis|EAW22829.1 afla|EED50104.1 afum|EAL90373.2 agla|OJJ83510.1 akaw|GAA89521.1 aluc|OJZ88133.1 anid|CBF87886.1 anig|EHA17897.1 aory|EIT74692.1 asyd|OJJ63437.1 ater|EAU38882.1 atub|OJI90737.1 aver|OJI97500.1 awen|OJJ38899.1 azon|OJJ49680.1

Group3248: abra|OJJ73916.1 acar|OOG01048.1 acla|EAW07792.1 afis|EAW22830.1 afla|EED50105.1 afum|EAL90372.2 agla|OJJ83509.1 akaw|GAA89522.1 aluc|OJZ88131.1 anid|CBF87883.1 anig|EHA17896.1 aory|EIT74639.1 asyd|OJJ63435.1 ater|EAU38881.1 atub|OJI90736.1 aver|OJI97502.1 awen|OJJ38898.1 azon|OJJ49679.1

Group3249: abra|OJJ73920.1 acar|OOG01051.1 acla|EAW07797.1 afis|EAW22834.1 afla|EED50109.1 afum|EAL90368.1 agla|OJJ83505.1 akaw|GAA89527.1 aluc|OJZ88126.1 anid|CBF87876.1 anig|EHA17892.1 aory|EIT74609.1 asyd|OJJ63432.1 ater|EAU38875.1 atub|OJI90733.1 aver|OJI97505.1 awen|OJJ38890.1 azon|OJJ49676.1

Group3250: abra|OJJ73923.1 acar|OOG01056.1 acla|EAW07800.1 afis|EAW22837.1 afla|EED50112.1 afum|EAL90365.2 agla|OJJ83504.1 akaw|GAA89530.1 aluc|OJZ88120.1 anid|CBF87867.1 anig|EHA17889.1 aory|EIT74684.1 asyd|OJJ63428.1 ater|EAU38873.1 atub|OJI90730.1 aver|OJI97509.1 awen|OJJ38887.1 azon|OJJ49675.1

Group3251: abra|OJJ73924.1 acar|OOG01057.1 acla|EAW07801.1 afis|EAW22838.1 afla|EED50113.1 afum|EAL90364.2 agla|OJJ83503.1 akaw|GAA89531.1 aluc|OJZ88119.1 anid|CBF87865.1 anig|EHA17888.1 aory|EIT74621.1 asyd|OJJ63427.1 ater|EAU38872.1 atub|OJI90729.1 aver|OJI97510.1 awen|OJJ38885.1 azon|OJJ49674.1

Group3252: abra|OJJ73926.1 acar|OOG01058.1 acla|EAW07803.1 afis|EAW22840.1 afla|EED50115.1 afum|EAL90363.1 agla|OJJ83501.1 akaw|GAA89533.1 aluc|OJZ88117.1 anid|CBF87861.1 anig|EHA17887.1 aory|EIT74702.1 asyd|OJJ63425.1 ater|EAU38870.1 atub|OJI90727.1 aver|OJI97512.1 awen|OJJ38883.1 azon|OJJ49672.1

Group3253: abra|OJJ73927.1 acar|OOG01060.1 acla|EAW07804.1 afis|EAW22841.1 afla|EED50116.1 afum|EAL90362.2 agla|OJJ83500.1 akaw|GAA89534.1 aluc|OJZ88116.1 anid|CBF87860.1 anig|EHA17886.1 aory|EIT74608.1 asyd|OJJ63424.1 ater|EAU38869.1 atub|OJI90725.1 aver|OJI97513.1 awen|OJJ38882.1 azon|OJJ49671.1

Group3254: abra|OJJ73928.1 acar|OOG01061.1 acla|EAW07805.1 afis|EAW22842.1 afla|EED50117.1 afum|EAL90361.1 agla|OJJ83496.1 akaw|GAA89535.1 aluc|OJZ88115.1 anid|CBF87858.1 anig|EHA17885.1 aory|EIT74650.1 asyd|OJJ63423.1 ater|EAU38868.1 atub|OJI90724.1 aver|OJI97514.1 awen|OJJ38881.1 azon|OJJ49670.1

Group3255: abra|OJJ73930.1 acar|OOG01062.1 acla|EAW07808.1 afis|EAW22845.1 afla|EED50120.1 afum|EAL90357.1 agla|OJJ83492.1 akaw|GAA89538.1 aluc|OJZ88112.1 anid|CBF87846.1 anig|EHA17882.1 aory|EIT74689.1 asyd|OJJ63421.1 ater|EAU38865.1 atub|OJI90720.1 aver|OJI97517.1 awen|OJJ38878.1 azon|OJJ49667.1

Group3256: abra|OJJ73931.1 acar|OOG01063.1 acla|EAW07809.1 afis|EAW22846.1 afla|EED50121.1 afum|EAL90356.1 agla|OJJ83485.1 akaw|GAA89539.1 aluc|OJZ88111.1 anid|CBF87848.1 anig|EHA17881.1 aory|EIT74731.1 asyd|OJJ63420.1 ater|EAU38864.1 atub|OJI90719.1 aver|OJI97518.1 awen|OJJ38877.1 azon|OJJ49666.1

Group3257: abra|OJJ73938.1 acar|OOG01069.1 afis|EAW22850.1 afla|EED50125.1 afum|EAL90350.1 agla|OJJ83473.1 akaw|GAA89543.1 aluc|OJZ88106.1 anid|CBF87855.1 anig|EHA17875.1 aory|EIT74705.1 asyd|OJJ63416.1 ater|EAU38859.1 atub|OJI90714.1 aver|OJI97522.1 awen|OJJ38871.1 azon|OJJ49663.1 acla|EAW07814.1

Group3258: abra|OJJ73939.1 acar|OOG01068.1 acla|EAW07816.1 afis|EAW22851.1 afla|EED50126.1 afum|EAL90349.1 agla|OJJ83471.1 akaw|GAA89544.1 aluc|OJZ88105.1 anid|CBF87841.1 anig|EHA17874.1 aory|EIT74661.1 asyd|OJJ63414.1 ater|EAU38858.1 atub|OJI90713.1 aver|OJI97524.1 awen|OJJ38867.1 azon|OJJ49661.1

Group3259: abra|OJJ73944.1 acar|OOG01072.1 acla|EAW07817.1 afis|EAW22852.1 afla|EED50132.1 afum|EAL90348.1 agla|OJJ83469.1 akaw|GAA89549.1 aluc|OJZ88100.1 anid|CBF87839.1 anig|EHA17869.1

aory|EIT74662.1 asyd|OJJ63413.1 ater|EAU38857.1 atub|OJI90708.1 aver|OJI97525.1 awen|OJJ38866.1 azon|OJJ49660.1

Group3260: abra|OJJ73945.1 acar|OOG01073.1 acla|EAW07818.1 afis|EAW22853.1 afla|EED50133.1 afum|EAL90347.1 agla|OJJ83468.1 akaw|GAA89550.1 aluc|OJZ88099.1 anid|CBF87838.1 anig|EHA17868.1 aory|EIT74757.1 asyd|OJJ63412.1 ater|EAU38856.1 atub|OJI90707.1 aver|OJI97526.1 awen|OJJ38865.1 azon|OJJ49659.1

Group3261: abra|OJJ73946.1 acar|OOG01074.1 acla|EAW07819.1 afis|EAW22854.1 afla|EED50134.1 afum|EAL90346.1 agla|OJJ83467.1 akaw|GAA89551.1 aluc|OJZ88098.1 anid|CBF87836.1 anig|EHA17867.1 aory|EIT74630.1 asyd|OJJ63410.1 atub|OJI90706.1 aver|OJI97527.1 awen|OJJ38864.1 azon|OJJ49658.1 ater|EAU38807.1

Group3262: abra|OJJ73949.1 acar|OOG01077.1 acla|EAW07823.1 afis|EAW22859.1 afla|EED50137.1 afum|EAL90341.1 agla|OJJ83465.1 akaw|GAA89554.1 aluc|OJZ88095.1 anid|CBF87833.1 anig|EHA17865.1 aory|EIT74739.1 asyd|OJJ63408.1 ater|EAU38809.1 atub|OJI90703.1 aver|OJI97529.1 awen|OJJ38861.1 azon|OJJ49655.1

Group3263: abra|OJJ73952.1 acar|OOG01080.1 acla|EAW07826.1 afis|EAW22862.1 afla|EED50146.1 afum|EAL90338.1 agla|OJJ83462.1 akaw|GAA89557.1 aluc|OJZ88092.1 anid|CBF87832.1 anig|EHA17862.1 aory|EIT74749.1 asyd|OJJ63407.1 ater|EAU38816.1 atub|OJI90700.1 aver|OJI97530.1 awen|OJJ38858.1 azon|OJJ49656.1

Group3264: abra|OJJ73959.1 acar|OOG01086.1 acla|EAW07828.1 afis|EAW22864.1 afla|EED50148.1 afum|EAL90336.1 agla|OJJ83460.1 akaw|GAA89562.1 aluc|OJZ88086.1 anid|CBF87829.1 anig|EHA17857.1 aory|EIT74755.1 asyd|OJJ63405.1 ater|EAU38824.1 atub|OJI90695.1 aver|OJI97531.1 awen|OJJ38856.1 azon|OJJ49654.1

Group3265: abra|OJJ73961.1 acar|OOG01087.1 acla|EAW07830.1 afis|EAW22866.1 afla|EED50150.1 afum|EAL90333.1 agla|OJJ83458.1 akaw|GAA89564.1 aluc|OJZ88084.1 anid|CBF87827.1 anig|EHA17855.1 aory|EIT74659.1 asyd|OJJ63403.1 ater|EAU38827.1 atub|OJI90693.1 aver|OJI97533.1 awen|OJJ38854.1 azon|OJJ49652.1

Group3266: abra|OJJ73962.1 acar|OOG01089.1 acla|EAW07831.1 afis|EAW22867.1 afla|EED50151.1 afum|EAL90332.1 agla|OJJ83457.1 akaw|GAA89565.1 aluc|OJZ88083.1 anid|CBF87826.1 anig|EHA17854.1 aory|EIT74722.1 asyd|OJJ63402.1 ater|EAU38828.1 atub|OJI90692.1 aver|OJI97534.1 awen|OJJ38853.1 azon|OJJ49651.1

Group3267: abra|OJJ73964.1 acar|OOG01090.1 acla|EAW07834.1 afis|EAW22871.1 afla|EED50154.1 afum|EAL90327.1 agla|OJJ83452.1 akaw|GAA89567.1 aluc|OJZ88080.1 anid|CBF87822.1 anig|EHA17852.1 aory|EIT76938.1 asyd|OJJ63400.1 ater|EAU38835.1 atub|OJI90689.1 aver|OJI97537.1 awen|OJJ38851.1 azon|OJJ49647.1

Group3268: abra|OJJ73967.1 acar|OOG01093.1 acla|EAW07837.1 afis|EAW22874.1 afla|EED50157.1 afum|EAL90324.1 agla|OJJ83443.1 akaw|GAA89570.1 aluc|OJZ88077.1 anid|CBF87816.1 anig|EHA17849.1 aory|EIT76898.1 asyd|OJJ63395.1 ater|EAU38838.1 atub|OJI90686.1 aver|OJI97541.1 awen|OJJ38848.1 azon|OJJ49644.1

Group3269: abra|OJJ73971.1 acar|OOG01097.1 acla|EAW07842.1 afis|EAW22878.1 afla|EED50183.1 afum|EAL90321.1 agla|OJJ83446.1 akaw|GAA89574.1 aluc|OJZ88073.1 anid|CBF87811.1 anig|EHA17846.1 aory|EIT77380.1 asyd|OJJ63390.1 ater|EAU38842.1 atub|OJI90682.1 aver|OJI97545.1 awen|OJJ38844.1 azon|OJJ49641.1

Group3270: abra|OJJ73976.1 acar|OOG01103.1 acla|EAW07847.1 afis|EAW22883.1 afla|EED50176.1 afum|EAL90316.1 agla|OJJ83441.1 akaw|GAA89579.1 aluc|OJZ88068.1 anid|CBF87803.1 anig|EHA17841.1 aory|EIT72282.1 asyd|OJJ63384.1 ater|EAU38846.1 atub|OJI90677.1 aver|OJI97551.1 awen|OJJ38839.1 azon|OJJ49636.1

Group3271: abra|OJJ73978.1 acar|OOG01104.1 acla|EAW07849.1 afis|EAW22885.1 afla|EED50174.1 afum|EAL90313.1 agla|OJJ83438.1 akaw|GAA89581.1 aluc|OJZ88066.1 anid|CBF87801.1 anig|EHA17839.1 aory|EIT72285.1 asyd|OJJ63382.1 ater|EAU35978.1 atub|OJI90675.1 aver|OJI97553.1 awen|OJJ38837.1 azon|OJJ49634.1

Group3272: abra|OJJ73980.1 acar|OOG01107.1 acla|EAW07851.1 afis|EAW22887.1 afla|EED50172.1 afum|EAL90311.1 agla|OJJ83436.1 akaw|GAA89583.1 aluc|OJZ88064.1 anid|CBF87798.1 anig|EHA17837.1 aory|EIT72283.1 asyd|OJJ63380.1 ater|EAU35985.1 atub|OJI90673.1 aver|OJI97555.1 awen|OJJ38835.1 azon|OJJ49632.1

Group3273: abra|OJJ73982.1 acar|OOG01109.1 acla|EAW07854.1 afis|EAW22890.1 afla|EED54859.1 afum|EAL90308.1 agla|OJJ83420.1 akaw|GAA89586.1 aluc|OJZ88062.1 anid|CBF87791.1 anig|EHA17835.1 aory|EIT79448.1 asyd|OJJ63377.1 ater|EAU35982.1 atub|OJI90671.1 aver|OJI97569.1 awen|OJJ38831.1 azon|OJJ49630.1

Group3274: abra|OJJ73983.1 acar|OOG01110.1 acla|EAW07855.1 afis|EAW22891.1 afla|EED54860.1 afum|EAL90307.1 agla|OJJ83407.1 akaw|GAA89587.1 aluc|OJZ88061.1 anid|CBF87790.1 anig|EHA17834.1 aory|EIT79392.1 asyd|OJJ63376.1 ater|EAU36000.1 atub|OJI90670.1 aver|OJI97570.1 awen|OJJ38830.1 azon|OJJ49629.1

Group3275: abra|OJJ73986.1 acar|OOG01111.1 acla|EAW07857.1 afis|EAW22893.1 afla|EED54862.1 afum|EAL90305.2 agla|OJJ83410.1 akaw|GAA87469.1 aluc|OJZ88059.1 anid|CBF87783.1 anig|EHA17831.1 aory|EIT79335.1 asyd|OJJ63374.1 ater|EAU35990.1 atub|OJI90667.1 aver|OJI97572.1 awen|OJJ38827.1 azon|OJJ49627.1

Group3276: abra|OJJ73989.1 acar|OOG01115.1 acla|EAW07860.1 afis|EAW22895.1 afla|EED54864.1 afum|EAL90303.1 agla|OJJ83415.1 akaw|GAA87472.1 aluc|OJZ88056.1 anid|CBF74977.1 anig|EHA17828.1 aory|EIT79412.1 asyd|OJJ60090.1 ater|EAU35987.1 atub|OJI90664.1 aver|OJJ04177.1 awen|OJJ38823.1 azon|OJJ49622.1

Group3277: abra|OJJ73991.1 acar|OOG01117.1 acla|EAW07862.1 afis|EAW22897.1 afla|EED54868.1 afum|EAL90301.1 agla|OJJ83417.1 akaw|GAA87474.1 aluc|OJZ88054.1 anid|CBF87775.1 anig|EHA17826.1 aory|EIT79359.1 asyd|OJJ63369.1 ater|EAU35981.1 atub|OJI90662.1 aver|OJI97577.1 awen|OJJ38820.1 azon|OJJ49620.1

Group3278: abra|OJJ73992.1 acar|OOG01119.1 acla|EAW07863.1 afis|EAW22898.1 afla|EED54869.1 afum|EAL90299.1 agla|OJJ83393.1 akaw|GAA87475.1 aluc|OJZ88053.1 anid|CBF87773.1 anig|EHA17825.1 asyd|OJJ63368.1 ater|EAU36001.1 atub|OJI90661.1 aver|OJI97578.1 awen|OJJ38732.1 azon|OJJ49619.1 agla|OJJ81800.1

Group3279: abra|OJJ73993.1 acar|OOG01118.1 acla|EAW07864.1 afis|EAW22899.1 afla|EED54870.1 afum|EAL90298.1 agla|OJJ83392.1 akaw|GAA87476.1 aluc|OJZ88052.1 anid|CBF87771.1 anig|EHA17824.1 aory|EIT79377.1 asyd|OJJ63367.1 ater|EAU36002.1 atub|OJI90660.1 aver|OJI97579.1 awen|OJJ38731.1 azon|OJJ49618.1

Group3280: abra|OJJ74000.1 acar|OOG01121.1 acla|EAW07866.1 afis|EAW22901.1 afla|EED54872.1 afum|EAL90296.1 agla|OJJ83389.1 akaw|GAA87482.1 aluc|OJZ88043.1 anid|CBF87767.1 anig|EHA17816.1 aory|EIT79383.1 asyd|OJJ63365.1 ater|EAU36006.1 atub|OJI90650.1 aver|OJI97582.1 awen|OJJ38729.1 azon|OJJ49615.1

Group3281: abra|OJJ74002.1 acar|OOG01122.1 acla|EAW07867.1 afis|EAW22902.1 afla|EED54873.1 afum|EAL90295.1 agla|OJJ83388.1 akaw|GAA87483.1 aluc|OJZ88042.1 anid|CBF87766.1 anig|EHA17815.1 aory|EIT79350.1 asyd|OJJ63364.1 ater|EAU36007.1 atub|OJI90649.1 aver|OJI97583.1 awen|OJJ38728.1 azon|OJJ49614.1

Group3282: abra|OJJ74003.1 acar|OOG01124.1 acla|EAW07868.1 afis|EAW22903.1 afla|EED54874.1 afum|EAL90294.1 agla|OJJ83387.1 akaw|GAA87484.1 aluc|OJZ88041.1 anid|CBF87764.1 anig|EHA17814.1 aory|EIT79433.1 asyd|OJJ63363.1 ater|EAU36010.1 atub|OJI90648.1 aver|OJI97584.1 awen|OJJ38727.1 azon|OJJ49613.1

Group3283: abra|OJJ74016.1 acar|OOG01137.1 acla|EAW07870.1 afis|EAW22905.1 afla|EED54897.1 afum|EAL90292.1 agla|OJJ83384.1 akaw|GAA87498.1 aluc|OJZ88024.1 anid|CBF87713.1 anig|EHA17801.1 aory|EIT80309.1 asyd|OJJ63338.1 ater|EAU36034.1 atub|OJI90632.1 aver|OJI97623.1 awen|OJJ38725.1 azon|OJJ49609.1

Group3284: abra|OJJ74017.1 acar|OOG01138.1 acla|EAW07871.1 afis|EAW22906.1 afla|EED54898.1 afum|EAL90291.1 agla|OJJ83383.1 akaw|GAA87499.1 aluc|OJZ88022.1 anid|CBF87712.1 anig|EHA17800.1 aory|EIT80293.1 asyd|OJJ63337.1 ater|EAU36033.1 atub|OJI90631.1 aver|OJI97625.1 awen|OJJ38724.1 azon|OJJ49608.1

Group3285: abra|OJJ74020.1 acar|OOG01139.1 acla|EAW07874.1 afis|EAW22909.1 afla|EED54901.1 afum|EAL90288.1 agla|OJJ83380.1 akaw|GAA87502.1 aluc|OJZ88019.1 anid|CBF87705.1 anig|EHA17798.1 aory|EIT80370.1 asyd|OJJ63332.1 ater|EAU36030.1 atub|OJI90628.1 aver|OJI97630.1 awen|OJJ38656.1 azon|OJJ49605.1

Group3286: abra|OJJ74021.1 acar|OOG01140.1 acla|EAW07875.1 afis|EAW22910.1 afla|EED54902.1 afum|EAL90287.1 agla|OJJ83379.1 akaw|GAA87503.1 aluc|OJZ88018.1 anid|CBF87703.1 anig|EHA17797.1 aory|EIT80365.1 asyd|OJJ63331.1 ater|EAU36029.1 atub|OJI90627.1 aver|OJI97631.1 awen|OJJ38655.1 azon|OJJ49604.1

Group3287: abra|OJJ74022.1 acar|OOG01141.1 acla|EAW07876.1 afis|EAW22911.1 afla|EED54904.1 afum|EAL90286.1 agla|OJJ83378.1 akaw|GAA87505.1 aluc|OJZ88015.1 anid|CBF87694.1 anig|EHA17796.1 aory|EIT80313.1 asyd|OJJ63326.1 ater|EAU36028.1 atub|OJI90625.1 aver|OJI97636.1 awen|OJJ38654.1 azon|OJJ49603.1

Group3288: abra|OJJ74023.1 acar|OOG01142.1 acla|EAW07877.1 afis|EAW22912.1 afla|EED54905.1 afum|EAL90285.1 agla|OJJ83377.1 akaw|GAA87504.1 aluc|OJZ88017.1 anid|CBF87695.1 anig|EHA17795.1

aory|EIT80317.1 asyd|OJJ63327.1 ater|EAU36027.1 atub|OJI90626.1 aver|OJI97635.1 awen|OJJ38653.1 azon|OJJ49602.1

Group3289: abra|OJJ74024.1 acar|OOG01143.1 acla|EAW07878.1 afis|EAW22913.1 afla|EED54906.1 afum|EAL90284.1 agla|OJJ83376.1 akaw|GAA87506.1 aluc|OJZ88014.1 anid|CBF87699.1 anig|EHA17794.1 aory|EIT80324.1 asyd|OJJ63329.1 ater|EAU36026.1 atub|OJI90623.1 aver|OJI97633.1 awen|OJJ38652.1 azon|OJJ49601.1

Group3290: abra|OJJ74027.1 acar|OOG01146.1 acla|EAW07879.1 afis|EAW22914.1 afla|EED54907.1 afum|EAL90283.1 agla|OJJ83375.1 akaw|GAA87510.1 aluc|OJZ88010.1 anid|CBF87697.1 anig|EHA17790.1 aory|EIT80315.1 asyd|OJJ63328.1 ater|EAU30708.1 atub|OJI90619.1 aver|OJI97634.1 awen|OJJ38651.1 azon|OJJ49599.1

Group3291: abra|OJJ74029.1 acar|OOG01147.1 acla|EAW07880.1 afis|EAW22915.1 afla|EED54911.1 afum|EAL90282.1 agla|OJJ83373.1 akaw|GAA87512.1 aluc|OJZ88008.1 anid|CBF87690.1 anig|EHA17788.1 aory|EIT80322.1 asyd|OJJ63325.1 ater|EAU30707.1 atub|OJI90617.1 aver|OJI97637.1 awen|OJJ38649.1 azon|OJJ49595.1

Group3292: abra|OJJ74032.1 acar|OOG01151.1 acla|EAW07883.1 afis|EAW22918.1 afla|EED54915.1 afum|EAL90279.1 agla|OJJ83371.1 akaw|GAA87516.1 aluc|OJZ88005.1 anid|CBF87687.1 anig|EHA17786.1 aory|EIT80289.1 asyd|OJJ63320.1 ater|EAU30705.1 atub|OJI90613.1 aver|OJI97639.1 awen|OJJ38646.1 azon|OJJ49588.1

Group3293: abra|OJJ74033.1 acar|OOG01152.1 acla|EAW07884.1 afis|EAW22919.1 afla|EED54916.1 afum|EAL90278.1 agla|OJJ83369.1 akaw|GAA87517.1 aluc|OJZ88004.1 anid|CBF87685.1 anig|EHA17785.1 aory|EIT80336.1 asyd|OJJ63319.1 ater|EAU30704.1 atub|OJI90612.1 aver|OJI97640.1 awen|OJJ38645.1 azon|OJJ49587.1

Group3294: abra|OJJ74035.1 acla|EAW07885.1 afis|EAW22920.1 afla|EED54917.1 afum|EAL90277.1 agla|OJJ83368.1 akaw|GAA87518.1 anid|CBF87683.1 aory|EIT80285.1 asyd|OJJ63318.1 ater|EAU30703.1 aver|OJI97641.1 awen|OJJ38644.1 azon|OJJ49586.1 acar|OOG01153.1 aluc|OJZ88003.1 anig|EHA17784.1 atub|OJI90611.1

Group3295: abra|OJJ74036.1 acar|OOG01155.1 acla|EAW07886.1 afis|EAW22921.1 afla|EED54918.1 afum|EAL90276.1 agla|OJJ83367.1 akaw|GAA87519.1 aluc|OJZ88000.1 anid|CBF87681.1 anig|EHA17782.1 aory|EIT80300.1 asyd|OJJ63317.1 ater|EAU30702.1 atub|OJI90609.1 aver|OJI97642.1 awen|OJJ38643.1 azon|OJJ49585.1

Group3296: abra|OJJ74037.1 acar|OOG01156.1 acla|EAW07887.1 afis|EAW22922.1 afla|EED54919.1 afum|EAL90275.1 agla|OJJ83366.1 akaw|GAA87520.1 aluc|OJZ87999.1 anid|CBF87680.1 anig|EHA17781.1 aory|EIT80390.1 asyd|OJJ63316.1 ater|EAU30701.1 atub|OJI90608.1 aver|OJI97643.1 awen|OJJ38642.1 azon|OJJ49584.1

Group3297: abra|OJJ74038.1 acar|OOG01157.1 acla|EAW07888.1 afis|EAW22923.1 afla|EED54920.1 afum|EAL90274.1 agla|OJJ83365.1 akaw|GAA87521.1 aluc|OJZ87998.1 anid|CBF87678.1 anig|EHA17780.1 aory|EIT80297.1 asyd|OJJ63315.1 ater|EAU30700.1 atub|OJI90607.1 aver|OJI97644.1 awen|OJJ38641.1 azon|OJJ49583.1

Group3298: abra|OJJ74039.1 acar|OOG01158.1 acla|EAW07889.1 afis|EAW22924.1 afla|EED54921.1 afum|EAL90273.1 agla|OJJ83364.1 akaw|GAA87522.1 aluc|OJZ87997.1 anid|CBF87676.1 anig|EHA17779.1 aory|EIT80292.1 asyd|OJJ63314.1 ater|EAU30699.1 atub|OJI90606.1 aver|OJI97645.1 awen|OJJ38640.1 azon|OJJ49582.1

Group3299: abra|OJJ74040.1 acar|OOG01159.1 acla|EAW07890.1 afis|EAW22925.1 afla|EED54922.1 afum|EAL90272.1 agla|OJJ83363.1 akaw|GAA87523.1 aluc|OJZ87996.1 anid|CBF87674.1 anig|EHA17778.1 aory|EIT80341.1 asyd|OJJ63313.1 ater|EAU30698.1 atub|OJI90605.1 aver|OJI97646.1 awen|OJJ38639.1 azon|OJJ49581.1

Group3300: abra|OJJ74043.1 acar|OOG01161.1 acla|EAW07892.1 afis|EAW22927.1 afla|EED54924.1 afum|EAL90270.1 agla|OJJ83361.1 akaw|GAA87525.1 aluc|OJZ87994.1 anid|CBF87671.1 anig|EHA17776.1 aory|EIT80287.1 asyd|OJJ63311.1 ater|EAU30696.1 atub|OJI90603.1 aver|OJI97648.1 awen|OJJ38637.1 azon|OJJ49579.1

Group3301: abra|OJJ74045.1 acar|OOG01164.1 acla|EAW07895.1 afis|EAW22930.1 afla|EED58123.1 afum|EAL90267.1 agla|OJJ83358.1 akaw|GAA87528.1 aluc|OJZ87990.1 anid|CBF87667.1 anig|EHA17773.1 aory|EIT79163.1 asyd|OJJ63308.1 ater|EAU30694.1 atub|OJI90600.1 aver|OJI97651.1 awen|OJJ38634.1 azon|OJJ49576.1

Group3302: abra|OJJ74048.1 acar|OOF89921.1 acla|EAW07897.1 afis|EAW22932.1 afla|EED58126.1 afum|EAL90264.1 agla|OJJ83355.1 akaw|GAA87530.1 aluc|OJZ87987.1 anid|CBF87662.1 anig|EHA17770.1 aory|EIT79157.1 asyd|OJJ63305.1 ater|EAU30692.1 atub|OJI90597.1 aver|OJI97654.1 awen|OJJ38631.1 azon|OJJ49573.1

Group3303: abra|OJJ74049.1 acar|OOF89922.1 acla|EAW07898.1 afis|EAW22934.1 afla|EED58127.1 afum|EAL90262.1 agla|OJJ83354.1 akaw|GAA87531.1 aluc|OJZ87986.1 anid|CBF87661.1 anig|EHA17769.1 aory|EIT79153.1 asyd|OJJ63304.1 ater|EAU30691.1 atub|OJI90596.1 aver|OJI97655.1 awen|OJJ38630.1 azon|OJJ49572.1

Group3304: abra|OJJ74050.1 acar|OOF89923.1 acla|EAW07899.1 afis|EAW22935.1 afla|EED58128.1 afum|EAL90261.1 agla|OJJ83353.1 akaw|GAA87532.1 aluc|OJZ87985.1 anid|CBF87659.1 anig|EHA17768.1 aory|EIT79137.1 asyd|OJJ63303.1 ater|EAU30690.1 atub|OJI90595.1 aver|OJI97656.1 awen|OJJ38629.1 azon|OJJ49571.1

Group3305: abra|OJJ74053.1 acar|OOG01166.1 acla|EAW07901.1 afis|EAW22938.1 afla|EED58131.1 afum|EAL90258.1 agla|OJJ83351.1 akaw|GAA87535.1 aluc|OJZ87982.1 anid|CBF87654.1 anig|EHA17765.1 aory|EIT79120.1 asyd|OJJ63299.1 ater|EAU30687.1 atub|OJI90592.1 aver|OJI97659.1 awen|OJJ38626.1 azon|OJJ49568.1

Group3306: abra|OJJ74054.1 acar|OOG01167.1 acla|EAW07902.1 afis|EAW22939.1 afla|EED58133.1 afum|EAL90257.1 agla|OJJ83349.1 akaw|GAA87536.1 aluc|OJZ87981.1 anid|CBF87652.1 anig|EHA17764.1 aory|EIT79138.1 asyd|OJJ63298.1 ater|EAU30686.1 atub|OJI90591.1 aver|OJI97660.1 awen|OJJ38625.1 azon|OJJ49567.1

Group3307: abra|OJJ74056.1 acar|OOG01169.1 acla|EAW07903.1 afis|EAW22940.1 afla|EED58154.1 afum|EAL90256.1 agla|OJJ83348.1 akaw|GAA87538.1 aluc|OJZ87979.1 anid|CBF87650.1 anig|EHA17762.1 aory|EIT79084.1 asyd|OJJ63297.1 ater|EAU30684.1 atub|OJI90589.1 aver|OJI97661.1 awen|OJJ38624.1 azon|OJJ49565.1

Group3308: abra|OJJ74060.1 acar|OOG01172.1 acla|EAW07905.1 afis|EAW22943.1 afla|EED58153.1 afum|EAL90254.1 agla|OJJ83345.1 akaw|GAA87541.1 aluc|OJZ87976.1 anid|CBF87646.1 anig|EHA17760.1 aory|EIT79073.1 asyd|OJJ63295.1 ater|EAU30681.1 atub|OJI90586.1 aver|OJI97663.1 awen|OJJ38621.1 azon|OJJ49562.1

Group3309: abra|OJJ74061.1 acar|OOG01173.1 acla|EAW07906.1 afis|EAW22944.1 afla|EED58151.1 afum|EAL90253.1 agla|OJJ83344.1 akaw|GAA87542.1 aluc|OJZ87975.1 anid|CBF87644.1 anig|EHA17759.1 aory|EIT79080.1 asyd|OJJ63294.1 ater|EAU30680.1 atub|OJI90585.1 aver|OJI97664.1 awen|OJJ38620.1 azon|OJJ49561.1

Group3310: abra|OJJ74062.1 acar|OOG01175.1 acla|EAW07907.1 afis|EAW22945.1 afla|EED58150.1 afum|EAL90252.1 agla|OJJ83343.1 akaw|GAA87543.1 aluc|OJZ87974.1 anid|CBF87642.1 anig|EHA17758.1 aory|EIT79097.1 asyd|OJJ63293.1 ater|EAU30679.1 atub|OJI90584.1 aver|OJI97665.1 awen|OJJ38619.1 azon|OJJ49560.1

Group3311: abra|OJJ74063.1 acar|OOG01176.1 acla|EAW07908.1 afis|EAW22946.1 afla|EED58149.1 afum|EAL90251.1 agla|OJJ83342.1 akaw|GAA87544.1 aluc|OJZ87973.1 anid|CBF87640.1 anig|EHA17757.1 aory|EIT79094.1 asyd|OJJ63292.1 ater|EAU30678.1 atub|OJI90583.1 aver|OJI97666.1 awen|OJJ38618.1 azon|OJJ49559.1

Group3312: abra|OJJ74064.1 acar|OOG01174.1 acla|EAW07909.1 afis|EAW22947.1 afla|EED58148.1 afum|EAL90250.1 agla|OJJ83341.1 akaw|GAA87545.1 aluc|OJZ87972.1 anid|CBF87639.1 anig|EHA17756.1 aory|EIT79089.1 asyd|OJJ63291.1 ater|EAU30677.1 atub|OJI90582.1 aver|OJI97667.1 awen|OJJ38617.1 azon|OJJ49558.1

Group3313: abra|OJJ74065.1 acar|OOG01177.1 acla|EAW07910.1 afis|EAW22948.1 afla|EED58147.1 afum|EAL90249.1 agla|OJJ83339.1 akaw|GAA87546.1 aluc|OJZ87971.1 anid|CBF87637.1 anig|EHA17755.1 aory|EIT79088.1 asyd|OJJ63290.1 ater|EAU30676.1 atub|OJI90581.1 aver|OJI97668.1 awen|OJJ38616.1 azon|OJJ49557.1

Group3314: abra|OJJ74066.1 acar|OOG01179.1 acla|EAW07911.1 afis|EAW22949.1 afla|EED58146.1 afum|EAL90248.1 agla|OJJ83338.1 akaw|GAA87547.1 aluc|OJZ87970.1 anid|CBF87636.1 anig|EHA17754.1 aory|EIT79070.1 asyd|OJJ63289.1 ater|EAU30675.1 atub|OJI90580.1 aver|OJI97669.1 awen|OJJ38615.1 azon|OJJ49556.1

Group3315: abra|OJJ74067.1 acar|OOG01178.1 acla|EAW07912.1 afis|EAW22950.1 afla|EED58145.1 afum|EAL90247.1 agla|OJJ83337.1 akaw|GAA87548.1 aluc|OJZ87969.1 anid|CBF87634.1 anig|EHA17753.1 aory|EIT79067.1 asyd|OJJ63288.1 ater|EAU30674.1 atub|OJI90579.1 aver|OJI97670.1 awen|OJJ38614.1 azon|OJJ49555.1

Group3316: abra|OJJ74068.1 acar|OOG01180.1 acla|EAW07913.1 afis|EAW22951.1 afla|EED58144.1 afum|EAL90246.1 agla|OJJ83336.1 akaw|GAA87549.1 aluc|OJZ87968.1 anid|CBF87632.1 anig|EHA17752.1 aory|EIT79092.1 asyd|OJJ63287.1 ater|EAU30673.1 atub|OJI90578.1 aver|OJI97671.1 awen|OJJ38613.1 azon|OJJ49554.1

Group3317: abra|OJJ74069.1 acar|OOG01181.1 acla|EAW07914.1 afis|EAW22952.1 afla|EED58143.1 afum|EAL90245.1 agla|OJJ83335.1 akaw|GAA87550.1 aluc|OJZ87967.1 anid|CBF87630.1 anig|EHA17751.1

aory|EIT79082.1 asyd|OJJ63286.1 ater|EAU30672.1 atub|OJI90577.1 aver|OJI97672.1 awen|OJJ38612.1 azon|OJJ49553.1

Group3318: abra|OJJ74070.1 acar|OOG01182.1 acla|EAW07915.1 afis|EAW22953.1 afla|EED58142.1 afum|EAL90244.1 agla|OJJ83334.1 akaw|GAA87551.1 aluc|OJZ87966.1 anid|CBF87629.1 anig|EHA17750.1 aory|EIT79065.1 asyd|OJJ63285.1 ater|EAU30671.1 atub|OJI90576.1 aver|OJI97673.1 awen|OJJ38611.1 azon|OJJ49552.1

Group3319: abra|OJJ74071.1 acar|OOG01183.1 acla|EAW07916.1 afis|EAW22954.1 afla|EED58141.1 afum|EAL90243.1 agla|OJJ83333.1 akaw|GAA87552.1 aluc|OJZ87965.1 anid|CBF87627.1 anig|EHA17749.1 aory|EIT79103.1 asyd|OJJ63284.1 ater|EAU30670.1 atub|OJI90575.1 aver|OJI97674.1 awen|OJJ38610.1 azon|OJJ49551.1

Group3320: abra|OJJ74072.1 acar|OOG01184.1 acla|EAW07917.1 afis|EAW22955.1 afla|EED58140.1 afum|EAL90242.1 agla|OJJ83332.1 akaw|GAA87553.1 aluc|OJZ87964.1 anid|CBF87625.1 anig|EHA17748.1 aory|EIT79149.1 asyd|OJJ63283.1 ater|EAU30669.1 atub|OJI90574.1 aver|OJI97675.1 awen|OJJ38608.1 azon|OJJ49550.1

Group3321: abra|OJJ74075.1 acar|OOG01187.1 acla|EAW07920.1 afis|EAW22958.1 afla|EED58138.1 afum|EAL90239.1 agla|OJJ83330.1 akaw|GAA87556.1 aluc|OJZ87961.1 anid|CBF87624.1 anig|EHA17745.1 aory|EIT79160.1 asyd|OJJ63281.1 ater|EAU30667.1 atub|OJI90571.1 aver|OJI97677.1 awen|OJJ38605.1 azon|OJJ49547.1

Group3322: abra|OJJ74076.1 acar|OOG01188.1 acla|EAW07921.1 afis|EAW22959.1 afla|EED58137.1 afum|EAL90238.1 agla|OJJ83329.1 akaw|GAA87557.1 aluc|OJZ87960.1 anid|CBF87622.1 anig|EHA17744.1 aory|EIT79140.1 asyd|OJJ63280.1 ater|EAU30666.1 atub|OJI90570.1 aver|OJI97678.1 awen|OJJ38604.1 azon|OJJ49546.1

Group3323: abra|OJJ74078.1 acar|OOG01190.1 acla|EAW07923.1 afis|EAW22961.1 afla|EED58135.1 afum|EAL90236.1 agla|OJJ83327.1 akaw|GAA87559.1 aluc|OJZ87957.1 anid|CBF87621.1 anig|EHA17742.1 aory|EIT79121.1 asyd|OJJ63278.1 ater|EAU30664.1 atub|OJI90568.1 aver|OJI97680.1 awen|OJJ38602.1 azon|OJJ49544.1

Group3324: abra|OJJ74079.1 acar|OOG01191.1 acla|EAW07924.1 afis|EAW22962.1 afla|EED58155.1 afum|EAL90235.1 agla|OJJ83326.1 akaw|GAA87560.1 aluc|OJZ87956.1 anid|CBF87619.1 anig|EHA17741.1 aory|EIT79083.1 asyd|OJJ63277.1 ater|EAU30663.1 atub|OJI90567.1 aver|OJI97681.1 awen|OJJ38601.1 azon|OJJ49543.1

Group3325: abra|OJJ74080.1 acar|OOG01192.1 acla|EAW07925.1 afis|EAW22963.1 afla|EED58156.1 afum|EAL90234.1 agla|OJJ83324.1 akaw|GAA87561.1 aluc|OJZ87955.1 anid|CBF87617.1 anig|EHA17740.1 aory|EIT79101.1 asyd|OJJ63276.1 ater|EAU30662.1 atub|OJI90566.1 aver|OJI97682.1 awen|OJJ38600.1 azon|OJJ49542.1

Group3326: abra|OJJ74081.1 acar|OOG01193.1 acla|EAW07926.1 afis|EAW22964.1 afla|EED58157.1 afum|EAL90233.1 agla|OJJ83323.1 akaw|GAA87562.1 aluc|OJZ87954.1 anid|CBF87615.1 anig|EHA17739.1 aory|EIT79095.1 asyd|OJJ63275.1 ater|EAU30661.1 atub|OJI90565.1 aver|OJI97683.1 awen|OJJ38599.1 azon|OJJ49541.1

Group3327: abra|OJJ74082.1 acar|OOG01194.1 acla|EAW07927.1 afis|EAW22965.1 afla|EED58158.1 afum|EAL90232.1 agla|OJJ83322.1 akaw|GAA87563.1 aluc|OJZ87953.1 anid|CBF87613.1 anig|EHA17738.1 aory|EIT79104.1 asyd|OJJ63274.1 ater|EAU30653.1 atub|OJI90564.1 aver|OJI97684.1 awen|OJJ38598.1 azon|OJJ49540.1

Group3328: abra|OJJ74084.1 acar|OOG01196.1 acla|EAW07928.1 afis|EAW22966.1 afla|EED58160.1 afum|EAL90231.1 agla|OJJ83321.1 akaw|GAA87564.1 aluc|OJZ87951.1 anid|CBF87612.1 anig|EHA17737.1 aory|EIT79091.1 asyd|OJJ63272.1 ater|EAU30654.1 atub|OJI90563.1 aver|OJI97685.1 awen|OJJ38597.1 azon|OJJ49538.1

Group3329: abra|OJJ74086.1 acar|OOG01197.1 acla|EAW07930.1 afis|EAW22968.1 afla|EED58161.1 afum|EAL90229.1 agla|OJJ83319.1 akaw|GAA87566.1 aluc|OJZ87949.1 anid|CBF87609.1 anig|EHA17735.1 aory|EIT79100.1 asyd|OJJ63270.1 ater|EAU30659.1 atub|OJI90560.1 aver|OJI97687.1 awen|OJJ38595.1 azon|OJJ49537.1

Group3330: abra|OJJ74089.1 acar|OOG01198.1 acla|EAW07932.1 afis|EAW22970.1 afla|EED58163.1 afum|EAL90227.2 agla|OJJ83317.1 akaw|GAA87568.1 aluc|OJZ87947.1 anid|CBF74548.1 anig|EHA17733.1 aory|EIT79078.1 asyd|OJJ57686.1 ater|EAU30652.1 atub|OJI90558.1 aver|OJJ05492.1 awen|OJJ38593.1 azon|OJJ49536.1

Group3331: abra|OJJ74090.1 acar|OOG01199.1 acla|EAW07933.1 afis|EAW22971.1 afla|EED58164.1 afum|EAL90226.1 agla|OJJ83316.1 akaw|GAA87569.1 aluc|OJZ87946.1 anid|CBF87607.1 anig|EHA17732.1 aory|EIT79076.1 asyd|OJJ63269.1 ater|EAU30651.1 atub|OJI90557.1 aver|OJI97688.1 awen|OJJ38591.1 azon|OJJ49535.1

Group3332: abra|OJJ74091.1 acar|OOG01200.1 acla|EAW07934.1 afis|EAW22972.1 afla|EED58166.1 afum|EAL90225.1 agla|OJJ83315.1 akaw|GAA87570.1 aluc|OJZ87945.1 anid|CBF87606.1 anig|EHA17731.1 aory|EIT79075.1 asyd|OJJ63268.1 ater|EAU30650.1 atub|OJI90556.1 aver|OJI97689.1 awen|OJJ38590.1 azon|OJJ49533.1

Group3333: abra|OJJ74093.1 acar|OOG01202.1 acla|EAW07936.1 afis|EAW22974.1 afla|EED58168.1 afum|EAL90223.1 agla|OJJ83313.1 akaw|GAA87572.1 aluc|OJZ87943.1 anid|CBF87602.1 anig|EHA17730.1 aory|EIT79071.1 asyd|OJJ63266.1 ater|EAU30647.1 atub|OJI90554.1 aver|OJI97691.1 awen|OJJ38589.1 azon|OJJ49532.1

Group3334: abra|OJJ74094.1 acar|OOG01204.1 acla|EAW07937.1 afis|EAW22975.1 afla|EED58169.1 afum|EAL90222.1 agla|OJJ83312.1 akaw|GAA87573.1 aluc|OJZ87942.1 anid|CBF87600.1 anig|EHA17729.1 aory|EIT79096.1 asyd|OJJ63265.1 ater|EAU30646.1 atub|OJI90553.1 aver|OJI97692.1 awen|OJJ38588.1 azon|OJJ49531.1

Group3335: abra|OJJ74095.1 acar|OOG01203.1 acla|EAW07938.1 afis|EAW22976.1 afla|EED58170.1 afum|EAL90221.1 agla|OJJ83311.1 akaw|GAA87574.1 aluc|OJZ87941.1 anid|CBF87598.1 anig|EHA17728.1 aory|EIT79085.1 asyd|OJJ63264.1 ater|EAU30645.1 atub|OJI90552.1 aver|OJI97693.1 awen|OJJ38587.1 azon|OJJ49530.1

Group3336: abra|OJJ74096.1 acar|OOG01205.1 acla|EAW07940.1 afis|EAW22978.1 afla|EED58171.1 afum|EAL90219.1 agla|OJJ83310.1 akaw|GAA87575.1 aluc|OJZ87940.1 anid|CBF87596.1 anig|EHA17727.1 aory|EIT79081.1 asyd|OJJ63262.1 ater|EAU30644.1 atub|OJI90551.1 aver|OJI97695.1 awen|OJJ38586.1 azon|OJJ49528.1

Group3337: abra|OJJ74100.1 acar|OOG01209.1 acla|EAW07943.1 afis|EAW22981.1 afla|EED58174.1 afum|EAL90216.1 agla|OJJ83306.1 akaw|GAA87578.1 aluc|OJZ87936.1 anid|CBF87589.1 anig|EHA17724.1 aory|EIT79069.1 asyd|OJJ63258.1 ater|EAU30640.1 atub|OJI90548.1 aver|OJI97699.1 awen|OJJ38582.1 azon|OJJ49524.1

Group3338: abra|OJJ74102.1 acar|OOG01211.1 acla|EAW07945.1 afis|EAW22983.1 afla|EED58176.1 afum|EAL90215.1 agla|OJJ83304.1 akaw|GAA87580.1 aluc|OJZ87934.1 anid|CBF87597.1 anig|EHA17723.1 aory|EIT79086.1 asyd|OJJ63263.1 ater|EAU30638.1 atub|OJI90546.1 aver|OJI97694.1 awen|OJJ38580.1 azon|OJJ49522.1

Group3339: abra|OJJ74103.1 acar|OOG01213.1 acla|EAW07946.1 afis|EAW22985.1 afla|EED58177.1 afum|EAL90214.1 agla|OJJ83302.1 akaw|GAA87581.1 aluc|OJZ87933.1 anid|CBF87584.1 anig|EHA17721.1 aory|EIT79072.1 asyd|OJJ63255.1 ater|EAU30637.1 atub|OJI90545.1 aver|OJI97702.1 awen|OJJ38579.1 azon|OJJ49520.1

Group3340: abra|OJJ74109.1 acar|OOG01217.1 acla|EAW07953.1 afis|EAW22990.1 afla|EED58182.1 afum|EAL90210.1 agla|OJJ83296.1 akaw|GAA87585.1 aluc|OJZ87925.1 anid|CBF87579.1 anig|EHA17717.1 aory|EIT79105.1 asyd|OJJ63251.1 ater|EAU30632.1 atub|OJI90537.1 aver|OJI97707.1 awen|OJJ38574.1 azon|OJJ49515.1

Group3341: abra|OJJ74116.1 acar|OOG01220.1 acla|EAW07958.1 afis|EAW22993.1 afla|EED58186.1 afum|EAL90208.1 agla|OJJ83291.1 akaw|GAA87589.1 aluc|OJZ87918.1 anid|CBF87570.1 anig|EHA17712.1 aory|EIT79074.1 asyd|OJJ63244.1 ater|EAU30627.1 atub|OJI90529.1 aver|OJI97712.1 awen|OJJ38568.1 azon|OJJ44834.1

Group3342: abra|OJJ74118.1 acar|OOG01223.1 acla|EAW07960.1 afis|EAW22995.1 afla|EED58188.1 afum|EAL90204.1 agla|OJJ83289.1 akaw|GAA87591.1 aluc|OJZ87914.1 anid|CBF87567.1 anig|EHA17708.1 aory|EIT79079.1 asyd|OJJ63242.1 ater|EAU30623.1 atub|OJI90526.1 aver|OJI97714.1 awen|OJJ38566.1 azon|OJJ50435.1

Group3343: abra|OJJ74159.1 acar|OOF95567.1 afum|EAL91052.1 akaw|GAA89725.1 aluc|OJZ80658.1 anid|CBF80314.1 anig|EHA27174.1 asyd|OJJ58662.1 atub|OJI87513.1 aver|OJJ01268.1 azon|OJJ43927.1 acar|OOF96898.1 akaw|GAA89030.1 aluc|OJZ80574.1 anig|EHA25625.1 atub|OJI88358.1 afis|EAW22138.1 azon|OJJ42294.1

Group3344: abra|OJJ74170.1 acar|OOF95550.1 acla|EAW07066.1 afis|EAW19330.1 afla|EED56451.1 afum|EAL94047.1 agla|OJJ84974.1 akaw|GAA86846.1 aluc|OJZ89965.1 anid|CBF78050.1 anig|EHA27165.1 asyd|OJJ55213.1 ater|EAU36486.1 atub|OJI84840.1 aver|OJJ07002.1 awen|OJJ31742.1 azon|OJJ42370.1 asyd|OJJ57144.1

Group3345: abra|OJJ74188.1 acar|OOF95533.1 acla|EAW07238.1 afis|EAW22261.1 afla|EED57649.1 afum|EAL90919.1 agla|OJJ87796.1 akaw|GAA86829.1 aluc|OJZ89947.1 anid|CBF88508.1 anig|EHA27148.1 aory|EIT79898.1 asyd|OJJ63801.1 ater|EAU34268.1 atub|OJI84859.1 aver|OJI97138.1 awen|OJJ34833.1 azon|OJJ45713.1

Group3346: abra|OJJ74189.1 acar|OOF95532.1 acla|EAW07241.1 afis|EAW22262.1 afla|EED57651.1 afum|EAL90918.1 agla|OJJ87794.1 akaw|GAA86828.1 aluc|OJZ89946.1 anid|CBF88511.1 anig|EHA27145.1

aory|EIT79914.1 asyd|OJJ63803.1 ater|EAU34269.1 atub|OJI84860.1 aver|OJI97135.1 awen|OJJ34835.1 azon|OJJ45712.1

Group3347: abra|OJJ74190.1 acar|OOF95531.1 acla|EAW07242.1 afis|EAW22263.1 afla|EED57655.1 afum|EAL90917.1 agla|OJJ87792.1 akaw|GAA86827.1 aluc|OJZ89945.1 anid|CBF88513.1 anig|EHA27144.1 aory|EIT79925.1 asyd|OJJ63804.1 ater|EAU34273.1 atub|OJI84861.1 aver|OJI97134.1 awen|OJJ34836.1 azon|OJJ45711.1

Group3348: abra|OJJ74193.1 acar|OOF95529.1 acla|EAW07245.1 afis|EAW22266.1 afla|EED57658.1 afum|EAL90914.1 agla|OJJ87789.1 akaw|GAA86824.1 aluc|OJZ89942.1 anid|CBF88519.1 anig|EHA27141.1 aory|EIT79939.1 asyd|OJJ63807.1 ater|EAU34277.1 atub|OJI84864.1 aver|OJI97131.1 awen|OJJ34838.1 azon|OJJ45708.1

Group3349: abra|OJJ74194.1 acar|OOF95527.1 acla|EAW07247.1 afis|EAW22268.1 afla|EED57660.1 afum|EAL90912.1 agla|OJJ87787.1 akaw|GAA86823.1 aluc|OJZ89941.1 anid|CBF88523.1 anig|EHA27140.1 aory|EIT79938.1 asyd|OJJ63808.1 ater|EAU34279.1 atub|OJI84865.1 aver|OJI97129.1 awen|OJJ34841.1 azon|OJJ45706.1

Group3350: abra|OJJ74195.1 acar|OOF95526.1 acla|EAW07248.1 afis|EAW22269.1 afla|EED57661.1 afum|EAL90911.1 agla|OJJ87786.1 akaw|GAA86822.1 aluc|OJZ89939.1 anid|CBF88527.1 anig|EHA27139.1 aory|EIT79909.1 asyd|OJJ63810.1 ater|EAU34280.1 atub|OJI84866.1 aver|OJI97127.1 awen|OJJ34842.1 azon|OJJ45705.1

Group3351: abra|OJJ74196.1 acar|OOF95524.1 acla|EAW07249.1 afis|EAW22270.1 afla|EED57662.1 afum|EAL90910.1 agla|OJJ87785.1 akaw|GAA86821.1 aluc|OJZ89938.1 anid|CBF88528.1 anig|EHA27138.1 aory|EIT79867.1 asyd|OJJ63811.1 ater|EAU34281.1 atub|OJI84868.1 aver|OJI97126.1 awen|OJJ34843.1 azon|OJJ45704.1

Group3352: abra|OJJ74197.1 acar|OOF95523.1 acla|EAW07250.1 afis|EAW22271.1 afla|EED57663.1 afum|EAL90909.1 agla|OJJ87784.1 akaw|GAA86820.1 aluc|OJZ89937.1 anid|CBF88530.1 anig|EHA27137.1 aory|EIT79903.1 asyd|OJJ63812.1 ater|EAU34282.1 atub|OJI84869.1 aver|OJI97125.1 awen|OJJ34844.1 azon|OJJ45703.1

Group3353: abra|OJJ74198.1 acar|OOF95521.1 acla|EAW07252.1 afis|EAW22273.1 afla|EED57665.1 afum|EAL90908.1 agla|OJJ87781.1 akaw|GAA86819.1 aluc|OJZ89936.1 anid|CBF88536.1 anig|EHA27136.1 aory|EIT79943.1 asyd|OJJ63820.1 ater|EAU34283.1 atub|OJI84870.1 aver|OJI97118.1 awen|OJJ34846.1 azon|OJJ45702.1

Group3354: abra|OJJ74199.1 acar|OOF95520.1 acla|EAW07253.1 afis|EAW22274.1 afla|EED57666.1 afum|EAL90907.1 agla|OJJ87780.1 akaw|GAA86818.1 aluc|OJZ89935.1 anid|CBF88537.1 anig|EHA27135.1 aory|EIT79937.1 asyd|OJJ63821.1 ater|EAU34284.1 atub|OJI84871.1 aver|OJI97117.1 awen|OJJ34847.1 azon|OJJ45700.1

Group3355: abra|OJJ74201.1 acar|OOF95519.1 acla|EAW07254.1 afis|EAW22275.1 afla|EED57667.1 afum|EAL90906.1 agla|OJJ87779.1 akaw|GAA86817.1 aluc|OJZ89934.1 anid|CBF88539.1 anig|EHA27134.1 aory|EIT79875.1 asyd|OJJ63822.1 ater|EAU34285.1 atub|OJI84872.1 aver|OJI97116.1 awen|OJJ34848.1 azon|OJJ45699.1

Group3356: abra|OJJ74202.1 acar|OOF95518.1 afla|EED57668.1 afum|EAL90905.1 agla|OJJ87777.1 akaw|GAA86816.1 aluc|OJZ89933.1 anid|CBF88541.1 anig|EHA27133.1 aory|EIT79884.1 ater|EAU34286.1 atub|OJI84873.1 aver|OJI97115.1 azon|OJJ45698.1 acla|EAW07255.1 afis|EAW22276.1 asyd|OJJ63823.1 awen|OJJ34849.1

Group3357: abra|OJJ74203.1 acar|OOF95517.1 acla|EAW07256.1 afis|EAW22277.1 afla|EED57669.1 afum|EAL90904.1 agla|OJJ87776.1 akaw|GAA86815.1 aluc|OJZ89932.1 anid|CBF88543.1 anig|EHA27132.1 aory|EIT79926.1 asyd|OJJ63824.1 ater|EAU34287.1 atub|OJI84874.1 aver|OJI97114.1 awen|OJJ34850.1 azon|OJJ45697.1

Group3358: abra|OJJ74204.1 acar|OOF95516.1 acla|EAW07257.1 afis|EAW22278.1 afla|EED57671.1 afum|EAL90903.1 agla|OJJ87775.1 akaw|GAA86814.1 aluc|OJZ89931.1 anid|CBF88545.1 anig|EHA27131.1 aory|EIT79927.1 asyd|OJJ63825.1 ater|EAU34288.1 atub|OJI84875.1 aver|OJI97113.1 awen|OJJ34851.1 azon|OJJ45696.1

Group3359: abra|OJJ74206.1 acar|OOF95514.1 acla|EAW07259.1 afis|EAW22280.1 afla|EED57677.1 afum|EAL90901.1 agla|OJJ87773.1 akaw|GAA86812.1 aluc|OJZ89929.1 anid|CBF88548.1 anig|EHA27129.1 aory|EIT79874.1 asyd|OJJ63827.1 ater|EAU34290.1 atub|OJI84877.1 aver|OJI97111.1 awen|OJJ34853.1 azon|OJJ45677.1

Group3360: abra|OJJ74207.1 acar|OOF95513.1 acla|EAW07260.1 afis|EAW22281.1 afla|EED57678.1 afum|EAL90900.1 agla|OJJ87768.1 akaw|GAA86811.1 aluc|OJZ89928.1 anid|CBF88550.1 anig|EHA27128.1 aory|EIT79890.1 asyd|OJJ63828.1 ater|EAU34291.1 atub|OJI84878.1 aver|OJI97110.1 awen|OJJ34854.1 azon|OJJ45676.1

Group3361: abra|OJJ74208.1 acar|OOF95512.1 acla|EAW07261.1 afis|EAW22282.1 afla|EED57679.1 afum|EAL90899.1 agla|OJJ87767.1 akaw|GAA86810.1 aluc|OJZ89926.1 anid|CBF88552.1 anig|EHA27127.1 aory|EIT79907.1 asyd|OJJ63829.1 ater|EAU34292.1 atub|OJI84879.1 aver|OJI97109.1 awen|OJJ34855.1 azon|OJJ45674.1

Group3362: abra|OJJ74209.1 acar|OOF95511.1 acla|EAW07262.1 afis|EAW22283.1 afla|EED57680.1 afum|EAL90898.1 agla|OJJ87766.1 akaw|GAA86809.1 aluc|OJZ89925.1 anid|CBF88554.1 anig|EHA27126.1 aory|EIT79921.1 asyd|OJJ63830.1 ater|EAU34293.1 atub|OJI84880.1 aver|OJI97108.1 awen|OJJ34856.1 azon|OJJ45673.1

Group3363: abra|OJJ74210.1 acar|OOF95510.1 acla|EAW07263.1 afis|EAW22284.1 afla|EED57682.1 afum|EAL90897.1 agla|OJJ87764.1 akaw|GAA86808.1 aluc|OJZ89924.1 anid|CBF88556.1 anig|EHA27124.1 aory|EIT79901.1 asyd|OJJ63831.1 ater|EAU34296.1 atub|OJI84881.1 aver|OJI97107.1 awen|OJJ34857.1 azon|OJJ45671.1

Group3364: abra|OJJ74211.1 acar|OOF95509.1 acla|EAW07264.1 afis|EAW22285.1 afla|EED57685.1 afum|EAL90896.1 agla|OJJ87763.1 akaw|GAA86807.1 aluc|OJZ89923.1 anid|CBF88558.1 anig|EHA27123.1 aory|EIT79887.1 asyd|OJJ63832.1 ater|EAU34297.1 atub|OJI84882.1 aver|OJI97106.1 awen|OJJ34858.1 azon|OJJ45670.1

Group3365: abra|OJJ74218.1 acar|OOF95503.1 acla|EAW07273.1 afis|EAW22294.1 afla|EED57690.1 afum|EAL90887.1 agla|OJJ87758.1 akaw|GAA86800.1 aluc|OJZ89916.1 anid|CBF88569.1 anig|EHA27116.1 aory|EIT79904.1 asyd|OJJ63837.1 ater|EAU34303.1 atub|OJI84889.1 aver|OJI97100.1 awen|OJJ34864.1 azon|OJJ45665.1

Group3366: abra|OJJ74220.1 acar|OOF90433.1 acla|EAW07284.1 afis|EAW22315.1 afla|EED57695.1 afum|EAL90875.1 agla|OJJ87735.1 akaw|GAA86798.1 aluc|OJZ89914.1 anid|CBF88577.1 anig|EHA27114.1 aory|EIT79936.1 asyd|OJJ63841.1 ater|EAU34308.1 atub|OJI84891.1 aver|OJI97096.1 awen|OJJ34868.1 azon|OJJ50045.1

Group3367: abra|OJJ74223.1 acar|OOF90434.1 acla|EAW07282.1 afis|EAW22313.1 afla|EED57697.1 afum|EAL90877.1 agla|OJJ87737.1 akaw|GAA86796.1 aluc|OJZ89911.1 anid|CBF88580.1 anig|EHA27111.1 aory|EIT79934.1 asyd|OJJ63843.1 ater|EAU34310.1 atub|OJI84895.1 aver|OJI97094.1 awen|OJJ34872.1 azon|OJJ50047.1

Group3368: abra|OJJ74224.1 acar|OOF90435.1 acla|EAW07281.1 afis|EAW22312.1 afla|EED57698.1 afum|EAL90878.1 agla|OJJ87738.1 akaw|GAA86795.1 aluc|OJZ89910.1 anid|CBF88582.1 anig|EHA27110.1 aory|EIT79916.1 asyd|OJJ63844.1 ater|EAU34311.1 atub|OJI84896.1 aver|OJI97093.1 awen|OJJ34873.1 azon|OJJ50048.1

Group3369: abra|OJJ74225.1 acar|OOF95502.1 acla|EAW07280.1 afis|EAW22311.1 afla|EED57701.1 afum|EAL90879.1 agla|OJJ87739.1 akaw|GAA86794.1 aluc|OJZ89908.1 anid|CBF88584.1 anig|EHA27108.1 aory|EIT79931.1 asyd|OJJ63846.1 ater|EAU34313.1 atub|OJI84898.1 aver|OJI97090.1 awen|OJJ34876.1 azon|OJJ50049.1

Group3370: abra|OJJ74228.1 acar|OOF95498.1 acla|EAW07276.1 afis|EAW22297.1 afla|EED57705.1 afum|EAL90884.1 agla|OJJ87748.1 akaw|GAA86791.1 aluc|OJZ89905.1 anid|CBF88592.1 anig|EHA27105.1 aory|EIT79881.1 asyd|OJJ63850.1 ater|EAU34317.1 atub|OJI84901.1 aver|OJI97087.1 awen|OJJ34881.1 azon|OJJ50050.1

Group3371: abra|OJJ74231.1 acar|OOF95495.1 acla|EAW07286.1 afis|EAW22319.1 afla|EED57710.1 afum|EAL90871.1 agla|OJJ87745.1 akaw|GAA86788.1 aluc|OJZ89902.1 anid|CBF88599.1 anig|EHA27102.1 aory|EIT79923.1 asyd|OJJ63854.1 ater|EAU34320.1 atub|OJI84905.1 aver|OJI97084.1 awen|OJJ34885.1 azon|OJJ50053.1

Group3372: abra|OJJ74237.1 acar|OOF95492.1 acla|EAW07288.1 afis|EAW22321.1 afla|EED57718.1 afum|EAL90870.1 agla|OJJ87749.1 akaw|GAA86783.1 aluc|OJZ89895.1 anid|CBF88602.1 anig|EHA27094.1 aory|EIT79929.1 asyd|OJJ63855.1 ater|EAU34321.1 atub|OJI84912.1 aver|OJI97083.1 awen|OJJ34888.1 azon|OJJ50055.1

Group3373: abra|OJJ74242.1 acar|OOF95488.1 acla|EAW07291.1 afis|EAW22325.1 afla|EED57723.1 afum|EAL90867.1 agla|OJJ87721.1 akaw|GAA86778.1 aluc|OJZ89890.1 anid|CBF88609.1 anig|EHA27089.1 aory|EIT79878.1 asyd|OJJ63859.1 ater|EAU34324.1 atub|OJI84917.1 aver|OJI97080.1 awen|OJJ34896.1 azon|OJJ50065.1

Group3374: abra|OJJ74246.1 acar|OOF95484.1 acla|EAW07298.1 afis|EAW22347.1 afla|EED57735.1 afum|EAL90845.1 agla|OJJ87687.1 akaw|GAA86774.1 aluc|OJZ89886.1 anid|CBF88623.1 anig|EHA27085.1 aory|EIT79913.1 asyd|OJJ63868.1 ater|EAU39483.1 atub|OJI84921.1 aver|OJI97071.1 awen|OJJ34928.1 azon|OJJ50070.1

Group3375: abra|OJJ74249.1 acar|OOF95479.1 acla|EAW07293.1 afis|EAW22327.1 afla|EED57726.1 afum|EAL90865.1 agla|OJJ87723.1 akaw|GAA86772.1 aluc|OJZ89883.1 anid|CBF88613.1 anig|EHA27082.1

aory|EIT79866.1 asyd|OJJ63861.1 ater|EAU34326.1 atub|OJI84924.1 aver|OJI97078.1 awen|OJJ34894.1 azon|OJJ50063.1

Group3376: abra|OJJ74250.1 acar|OOF95480.1 acla|EAW07294.1 afis|EAW22328.1 afla|EED57727.1 afum|EAL90864.1 agla|OJJ87724.1 akaw|GAA86771.1 aluc|OJZ89882.1 anid|CBF88615.1 anig|EHA27081.1 aory|EIT79906.1 asyd|OJJ63862.1 ater|EAU34327.1 atub|OJI84925.1 aver|OJI97077.1 awen|OJJ34893.1 azon|OJJ50062.1

Group3377: abra|OJJ74252.1 acar|OOF95477.1 acla|EAW07296.1 afis|EAW22330.1 afla|EED57729.1 afum|EAL90862.1 agla|OJJ87752.1 akaw|GAA86769.1 aluc|OJZ89880.1 anid|CBF88619.1 anig|EHA27079.1 aory|EIT79894.1 asyd|OJJ63864.1 ater|EAU34331.1 atub|OJI84927.1 aver|OJI97075.1 awen|OJJ34891.1 azon|OJJ50060.1

Group3378: abra|OJJ74256.1 acar|OOF95472.1 acla|EAW07302.1 afis|EAW22343.1 afla|EED57738.1 afum|EAL90848.1 agla|OJJ87691.1 akaw|GAA86765.1 aluc|OJZ89876.1 anid|CBF88627.1 anig|EHA27075.1 aory|EIT79915.1 asyd|OJJ63870.1 ater|EAU39481.1 atub|OJI84931.1 aver|OJI97069.1 awen|OJJ34922.1 azon|OJJ50072.1

Group3379: abra|OJJ74257.1 acar|OOF95471.1 acla|EAW07303.1 afis|EAW22342.1 afla|EED57739.1 afum|EAL90849.1 agla|OJJ87692.1 akaw|GAA86764.1 aluc|OJZ89875.1 anid|CBF88628.1 anig|EHA27074.1 aory|EIT79892.1 asyd|OJJ63871.1 ater|EAU39480.1 atub|OJI84932.1 aver|OJI97068.1 awen|OJJ34921.1 azon|OJJ50073.1

Group3380: abra|OJJ74260.1 acar|OOF89900.1 acla|EAW07305.1 afis|EAW22340.1 afla|EED57741.1 afum|EAL90852.1 agla|OJJ87698.1 akaw|GAA86761.1 aluc|OJZ89871.1 anid|CBF88632.1 anig|EHA27071.1 aory|EIT79893.1 asyd|OJJ63874.1 ater|EAU39478.1 atub|OJI84936.1 aver|OJI97064.1 awen|OJJ34919.1 azon|OJJ50075.1

Group3381: abra|OJJ74261.1 acar|OOF89901.1 acla|EAW07306.1 afis|EAW22339.1 afla|EED57742.1 afum|EAL90853.1 agla|OJJ87699.1 akaw|GAA86760.1 aluc|OJZ89870.1 anid|CBF88634.1 anig|EHA27070.1 aory|EIT79922.1 asyd|OJJ63875.1 ater|EAU39477.1 atub|OJI84937.1 aver|OJI97063.1 awen|OJJ34918.1 azon|OJJ50076.1

Group3382: abra|OJJ74264.1 acar|OOF95466.1 acla|EAW07308.1 afis|EAW22338.1 afla|EED57743.1 afum|EAL90854.1 agla|OJJ87700.1 akaw|GAA86759.1 aluc|OJZ89869.1 anid|CBF88636.1 anig|EHA27069.1 aory|EIT81083.1 asyd|OJJ63877.1 ater|EAU39475.1 atub|OJI84939.1 aver|OJI97062.1 awen|OJJ34916.1 azon|OJJ50079.1

Group3383: abra|OJJ74265.1 acar|OOF95465.1 acla|EAW07309.1 afis|EAW22337.1 afla|EED57744.1 afum|EAL90855.1 agla|OJJ87701.1 akaw|GAA86758.1 aluc|OJZ89868.1 anid|CBF88638.1 anig|EHA27068.1 aory|EIT81047.1 asyd|OJJ63878.1 ater|EAU39474.1 atub|OJI84940.1 aver|OJI97061.1 awen|OJJ34915.1 azon|OJJ45569.1

Group3384: abra|OJJ74266.1 acar|OOF95464.1 acla|EAW07310.1 afis|EAW22336.1 afla|EED57746.1 afum|EAL90856.1 agla|OJJ87702.1 akaw|GAA86757.1 aluc|OJZ89867.1 anid|CBF88640.1 anig|EHA27067.1 aory|EIT81092.1 asyd|OJJ63879.1 ater|EAU39473.1 atub|OJI84941.1 aver|OJI97060.1 awen|OJJ34914.1 azon|OJJ45570.1

Group3385: abra|OJJ74267.1 acar|OOF89814.1 acla|EAW07311.1 afis|EAW22335.1 afla|EED57747.1 afum|EAL90857.2 agla|OJJ87715.1 akaw|GAA86756.1 aluc|OJZ89866.1 anid|CBF88641.1 anig|EHA27066.1 aory|EIT81022.1 asyd|OJJ63880.1 ater|EAU39472.1 atub|OJI84942.1 aver|OJI97059.1 awen|OJJ34913.1 azon|OJJ45571.1

Group3386: abra|OJJ74270.1 acar|OOF95460.1 acla|EAW07314.1 afis|EAW22332.1 afla|EED57751.1 afum|EAL90860.1 agla|OJJ87712.1 akaw|GAA86753.1 aluc|OJZ89863.1 anid|CBF88647.1 anig|EHA27063.1 aory|EIT81116.1 asyd|OJJ63882.1 ater|EAU39461.1 atub|OJI84945.1 aver|OJI97057.1 awen|OJJ34910.1 azon|OJJ45575.1

Group3387: abra|OJJ74272.1 acar|OOF95458.1 acla|EAW07317.1 afis|EAW22349.1 afla|EED57753.1 afum|EAL90843.1 agla|OJJ87710.1 akaw|GAA86751.1 aluc|OJZ89861.1 anid|CBF88651.1 anig|EHA27061.1 aory|EIT81051.1 asyd|OJJ63885.1 ater|EAU39459.1 atub|OJI84947.1 aver|OJI97055.1 awen|OJJ34905.1 azon|OJJ45577.1

Group3388: abra|OJJ74276.1 acar|OOF95455.1 acla|EAW07320.1 afis|EAW22352.1 afla|EED57756.1 afum|EAL90840.1 agla|OJJ87707.1 akaw|GAA86748.1 aluc|OJZ89857.1 anid|CBF88661.1 anig|EHA27058.1 aory|EIT81039.1 asyd|OJJ63889.1 ater|EAU39488.1 atub|OJI84949.1 aver|OJI97050.1 awen|OJJ34903.1 azon|OJJ45579.1

Group3389: abra|OJJ74277.1 acar|OOF95454.1 acla|EAW07321.1 afis|EAW22353.1 afla|EED57759.1 afum|EAL90839.1 agla|OJJ87716.1 akaw|GAA86747.1 aluc|OJZ89856.1 anid|CBF88662.1 anig|EHA27057.1 aory|EIT81042.1 asyd|OJJ63890.1 ater|EAU39487.1 atub|OJI84950.1 aver|OJI97049.1 awen|OJJ34902.1 azon|OJJ45580.1

Group3390: abra|OJJ74278.1 acar|OOF95453.1 acla|EAW07322.1 afis|EAW22354.1 afla|EED57760.1 afum|EAL90838.1 agla|OJJ87717.1 akaw|GAA86746.1 aluc|OJZ89855.1 anid|CBF88664.1 anig|EHA27056.1 aory|EIT81028.1 asyd|OJJ63891.1 ater|EAU39486.1 atub|OJI84951.1 aver|OJI97048.1 awen|OJJ34901.1 azon|OJJ45581.1

Group3391: abra|OJJ74280.1 acar|OOF95451.1 acla|EAW07324.1 afis|EAW22356.1 afla|EED57762.1 afum|EAL90835.1 agla|OJJ87598.1 akaw|GAA86744.1 aluc|OJZ89853.1 anid|CBF88668.1 anig|EHA27054.1 aory|EIT81074.1 asyd|OJJ63893.1 ater|EAU39490.1 atub|OJI84953.1 aver|OJI97046.1 awen|OJJ34930.1 azon|OJJ45583.1

Group3392: abra|OJJ74281.1 acar|OOF95450.1 acla|EAW07325.1 afis|EAW22357.1 afla|EED57763.1 afum|EAL90834.1 agla|OJJ87682.1 akaw|GAA86743.1 aluc|OJZ89852.1 anid|CBF88672.1 anig|EHA27053.1 aory|EIT81043.1 asyd|OJJ63895.1 ater|EAU39491.1 atub|OJI84954.1 aver|OJI97044.1 awen|OJJ34931.1 azon|OJJ45584.1

Group3393: abra|OJJ74282.1 acar|OOF95449.1 acla|EAW07326.1 afis|EAW22358.1 afla|EED57764.1 afum|EAL90833.1 agla|OJJ87683.1 akaw|GAA86742.1 aluc|OJZ89851.1 anid|CBF88674.1 anig|EHA27052.1 aory|EIT81053.1 asyd|OJJ63896.1 ater|EAU39492.1 atub|OJI84955.1 aver|OJI97043.1 awen|OJJ34932.1 azon|OJJ45585.1

Group3394: abra|OJJ74283.1 acar|OOF95447.1 acla|EAW07327.1 afis|EAW22359.1 afla|EED57765.1 afum|EAL90832.1 agla|OJJ87684.1 akaw|GAA86741.1 aluc|OJZ89850.1 anid|CBF88676.1 anig|EHA27051.1 aory|EIT81093.1 asyd|OJJ63897.1 ater|EAU39493.1 atub|OJI84956.1 aver|OJI97042.1 awen|OJJ34933.1 azon|OJJ45586.1

Group3395: abra|OJJ74290.1 acar|OOF95441.1 acla|EAW07334.1 afis|EAW22366.1 afla|EED57774.1 afum|EBA27415.1 agla|OJJ87624.1 akaw|GAA86734.1 aluc|OJZ89844.1 anid|CBF88692.1 anig|EHA27045.1 aory|EIT81109.1 asyd|OJJ63910.1 ater|EAU39501.1 atub|OJI84964.1 aver|OJI97031.1 awen|OJJ34940.1 azon|OJJ45592.1

Group3396: abra|OJJ74291.1 acar|OOF95440.1 acla|EAW07335.1 afis|EAW22367.1 afla|EED57775.1 afum|EAL90825.1 agla|OJJ87622.1 akaw|GAA86733.1 aluc|OJZ89843.1 anid|CBF88694.1 anig|EHA27044.1 aory|EIT81063.1 asyd|OJJ63911.1 ater|EAU39502.1 atub|OJI84965.1 aver|OJI97030.1 awen|OJJ34941.1 azon|OJJ45593.1

Group3397: abra|OJJ74292.1 acar|OOF95439.1 acla|EAW07336.1 afis|EAW22368.1 afla|EED57776.1 afum|EAL90824.1 akaw|GAA86732.1 aluc|OJZ89842.1 anid|CBF88695.1 anig|EHA27043.1 aory|EIT81045.1 asyd|OJJ63913.1 ater|EAU39503.1 atub|OJI84966.1 aver|OJI97029.1 awen|OJJ34942.1 azon|OJJ45594.1 agla|OJJ87621.1

Group3398: abra|OJJ74293.1 acar|OOF95438.1 acla|EAW07337.1 afis|EAW22369.1 afla|EED57777.1 afum|EAL90823.1 agla|OJJ87620.1 akaw|GAA86731.1 aluc|OJZ89841.1 anid|CBF88697.1 anig|EHA27042.1 aory|EIT81034.1 asyd|OJJ63914.1 ater|EAU39504.1 atub|OJI84967.1 aver|OJI97028.1 awen|OJJ34944.1 azon|OJJ45595.1

Group3399: abra|OJJ74294.1 acar|OOF95437.1 acla|EAW07339.1 afis|EAW22370.1 afla|EED57778.1 afum|EAL90822.2 agla|OJJ87619.1 akaw|GAA86730.1 aluc|OJZ89840.1 anid|CBF88699.1 anig|EHA27041.1 aory|EIT81037.1 asyd|OJJ63915.1 ater|EAU39505.1 atub|OJI84968.1 aver|OJI97027.1 awen|OJJ34945.1 azon|OJJ45596.1

Group3400: abra|OJJ74295.1 acar|OOF95436.1 acla|EAW07340.1 afis|EAW22372.1 afla|EED57779.1 afum|EAL90820.2 agla|OJJ87617.1 akaw|GAA86729.1 aluc|OJZ89839.1 anid|CBF88703.1 anig|EHA27040.1 aory|EIT81082.1 asyd|OJJ63919.1 ater|EAU39506.1 atub|OJI84969.1 aver|OJI97024.1 awen|OJJ34947.1 azon|OJJ45597.1

Group3401: abra|OJJ74296.1 acar|OOF95435.1 acla|EAW07341.1 afis|EAW22373.1 afla|EED57780.1 afum|EAL90819.1 agla|OJJ87626.1 akaw|GAA86728.1 aluc|OJZ89838.1 anid|CBF88705.1 anig|EHA27039.1 aory|EIT81056.1 asyd|OJJ63920.1 ater|EAU39507.1 atub|OJI84970.1 aver|OJI97023.1 awen|OJJ34948.1 azon|OJJ45598.1

Group3402: abra|OJJ74297.1 acar|OOF95434.1 acla|EAW07342.1 afis|EAW22374.1 afla|EED57781.1 afum|EAL90818.1 agla|OJJ87627.1 akaw|GAA86727.1 aluc|OJZ89837.1 anid|CBF88706.1 anig|EHA27038.1 aory|EIT81052.1 asyd|OJJ63923.1 ater|EAU39508.1 atub|OJI84971.1 aver|OJI97022.1 awen|OJJ34949.1 azon|OJJ45599.1

Group3403: abra|OJJ74298.1 acar|OOF95433.1 acla|EAW07343.1 afis|EAW22375.1 afum|EAL90817.1 agla|OJJ87628.1 akaw|GAA86726.1 aluc|OJZ89835.1 anid|CBF88710.1 anig|EHA27037.1 aory|EIT81071.1 asyd|OJJ63924.1 ater|EAU39509.1 atub|OJI84972.1 aver|OJI97020.1 awen|OJJ34950.1 azon|OJJ45600.1 afla|EED57783.1

Group3404: abra|OJJ74299.1 acar|OOF95431.1 acla|EAW07344.1 afis|EAW22376.1 afla|EED57784.1 afum|EAL90816.1 agla|OJJ87629.1 akaw|GAA86725.1 aluc|OJZ89834.1 anid|CBF88712.1 anig|EHA27036.1

aory|EIT81070.1 asyd|OJJ63925.1 ater|EAU39510.1 atub|OJI84973.1 aver|OJI97019.1 awen|OJJ34951.1 azon|OJJ45601.1

Group3405: abra|OJJ74301.1 acar|OOF95429.1 acla|EAW07346.1 afis|EAW22378.1 afla|EED57786.1 afum|EAL90814.1 agla|OJJ87631.1 akaw|GAA86724.1 aluc|OJZ89832.1 anid|CBF88714.1 anig|EHA27034.1 aory|EIT81069.1 asyd|OJJ63926.1 ater|EAU39512.1 atub|OJI84974.1 aver|OJI97018.1 awen|OJJ34952.1 azon|OJJ45602.1

Group3406: abra|OJJ74302.1 acar|OOF95428.1 acla|EAW07349.1 afis|EAW22379.1 afla|EED57787.1 afum|EAL90812.1 agla|OJJ87635.1 akaw|GAA86723.1 aluc|OJZ89830.1 anid|CBF88716.1 anig|EHA27033.1 aory|EIT81084.1 asyd|OJJ63927.1 ater|EAU39513.1 atub|OJI84975.1 aver|OJI97017.1 awen|OJJ34953.1 azon|OJJ45603.1

Group3407: abra|OJJ74304.1 acar|OOF95424.1 acla|EAW07352.1 afis|EAW22381.1 afla|EED57790.1 afum|EAL90810.1 agla|OJJ87640.1 akaw|GAA86720.1 aluc|OJZ89824.1 anid|CBF88718.1 anig|EHA27031.1 aory|EIT81089.1 asyd|OJJ63928.1 ater|EAU39515.1 atub|OJI84982.1 aver|OJI97016.1 awen|OJJ34956.1 azon|OJJ45608.1

Group3408: abra|OJJ74305.1 acar|OOF95423.1 acla|EAW07353.1 afis|EAW22382.1 afla|EED56040.1 afum|EAL90809.1 agla|OJJ87641.1 akaw|GAA86719.1 aluc|OJZ89822.1 anid|CBF88721.1 anig|EHA27030.1 aory|EIT76582.1 asyd|OJJ63929.1 ater|EAU39516.1 atub|OJI84983.1 aver|OJI97014.1 awen|OJJ34957.1 azon|OJJ45609.1

Group3409: abra|OJJ74306.1 acar|OOF95422.1 acla|EAW07354.1 afis|EAW22383.1 afla|EED56041.1 afum|EAL90808.1 agla|OJJ87642.1 akaw|GAA86718.1 aluc|OJZ89821.1 anid|CBF88723.1 anig|EHA27029.1 aory|EIT76563.1 asyd|OJJ63930.1 ater|EAU39517.1 atub|OJI84984.1 aver|OJI97013.1 awen|OJJ34958.1 azon|OJJ45610.1

Group3410: abra|OJJ74307.1 acar|OOF95420.1 acla|EAW07355.1 afis|EAW22384.1 afla|EED56044.1 afum|EAL90806.1 agla|OJJ87644.1 akaw|GAA86717.1 aluc|OJZ89820.1 anid|CBF88725.1 anig|EHA27028.1 aory|EIT76594.1 asyd|OJJ63931.1 ater|EAU39337.1 atub|OJI84985.1 aver|OJI97012.1 awen|OJJ34959.1 azon|OJJ45611.1

Group3411: abra|OJJ74310.1 acar|OOF95419.1 acla|EAW07357.1 afis|EAW22386.1 afla|EED56046.1 afum|EAL90804.1 agla|OJJ87647.1 akaw|GAA86714.1 aluc|OJZ89816.1 anid|CBF88730.1 anig|EHA27025.1 aory|EIT76610.1 asyd|OJJ63934.1 ater|EAU39335.1 atub|OJI84989.1 aver|OJI97008.1 awen|OJJ34961.1 azon|OJJ45613.1

Group3412: abra|OJJ74311.1 acar|OOF95417.1 acla|EAW07358.1 afis|EAW22387.1 afla|EED56047.1 afum|EAL90803.1 agla|OJJ87648.1 akaw|GAA86713.1 aluc|OJZ89815.1 anid|CBF88732.1 anig|EHA27024.1 aory|EIT76591.1 asyd|OJJ63935.1 ater|EAU39334.1 atub|OJI84990.1 aver|OJI97007.1 awen|OJJ34962.1 azon|OJJ45614.1

Group3413: abra|OJJ74315.1 acar|OOF95412.1 acla|EAW07366.1 afis|EAW22393.1 afla|EED56056.1 afum|EAL90798.1 agla|OJJ87655.1 akaw|GAA86710.1 aluc|OJZ89811.1 anid|CBF88743.1 anig|EHA27021.1 aory|EIT76567.1 asyd|OJJ63954.1 ater|EAU39325.1 atub|OJI84994.1 aver|OJI96987.1 awen|OJJ34970.1 azon|OJJ45619.1

Group3414: abra|OJJ74316.1 acar|OOF95411.1 acla|EAW07367.1 afis|EAW22394.1 afla|EED56057.1 afum|EAL90797.1 agla|OJJ87657.1 akaw|GAA86709.1 aluc|OJZ89810.1 anid|CBF88745.1 anig|EHA27020.1 aory|EIT76865.1 asyd|OJJ63955.1 ater|EAU39324.1 atub|OJI84995.1 aver|OJI96986.1 awen|OJJ34971.1 azon|OJJ45620.1

Group3415: abra|OJJ74320.1 acar|OOF95409.1 acla|EAW07372.1 afis|EAW22397.1 afla|EED56061.1 afum|EAL90794.1 agla|OJJ87660.1 akaw|GAA86706.1 aluc|OJZ89808.1 anid|CBF88750.1 anig|EHA27018.1 aory|EIT76888.1 asyd|OJJ63958.1 ater|EAU39320.1 atub|OJI84998.1 aver|OJI96983.1 awen|OJJ34975.1 azon|OJJ45624.1

Group3416: abra|OJJ74325.1 acar|OOF95406.1 acla|EAW07377.1 afis|EAW22402.1 afla|EED56066.1 afum|EAL90789.1 agla|OJJ87665.1 akaw|GAA86701.1 aluc|OJZ89803.1 anid|CBF88765.1 anig|EHA27016.1 aory|EIT76819.1 asyd|OJJ63981.1 ater|EAU39306.1 atub|OJI85003.1 aver|OJI96972.1 awen|OJJ34981.1 azon|OJJ45629.1

Group3417: abra|OJJ74326.1 acar|OOF95405.1 acla|EAW07378.1 afis|EAW22403.1 afla|EED56067.1 afum|EAL90788.1 agla|OJJ87666.1 akaw|GAA86700.1 aluc|OJZ89802.1 anid|CBF88763.1 anig|EHA27015.1 aory|EIT76544.1 asyd|OJJ63982.1 ater|EAU39305.1 atub|OJI85004.1 aver|OJI96971.1 awen|OJJ34982.1 azon|OJJ45631.1

Group3418: abra|OJJ74329.1 acar|OOF95402.1 acla|EAW07379.1 afis|EAW22404.1 afla|EED56068.1 afum|EAL90787.1 agla|OJJ87667.1 akaw|GAA86699.1 aluc|OJZ89801.1 anid|CBF88785.1 anig|EHA27014.1 aory|EIT76781.1 asyd|OJJ62903.1 ater|EAU39304.1 atub|OJI85005.1 aver|OJI96957.1 awen|OJJ34983.1 azon|OJJ45633.1

Group3419: abra|OJJ74336.1 acar|OOF95391.1 acla|EAW07386.1 afis|EAW22411.1 afla|EED52491.1 afum|EAL90781.1 agla|OJJ87678.1 akaw|GAA86693.1 aluc|OJZ89794.1 anid|CBF88796.1 anig|EHA27007.1 aory|EIT82814.1 asyd|OJJ62897.1 ater|EAU39294.1 atub|OJI85012.1 aver|OJI96950.1 awen|OJJ34992.1 azon|OJJ45635.1

Group3420: abra|OJJ74340.1 acar|OOF89991.1 acla|EAW07391.1 afis|EAW22417.1 afla|EED52495.1 afum|EAL90775.1 agla|OJJ87599.1 akaw|GAA86690.1 aluc|OJZ89791.1 anid|CBF88802.1 anig|EHA27004.1 aory|EIT82790.1 asyd|OJJ62895.1 ater|EAU39289.1 atub|OJI85015.1 aver|OJI96947.1 awen|OJJ34996.1 azon|OJJ45639.1

Group3421: abra|OJJ74341.1 acar|OOF95389.1 acla|EAW07392.1 afis|EAW22418.1 afla|EED52496.1 afum|EAL90774.1 agla|OJJ87600.1 akaw|GAA86689.1 aluc|OJZ89790.1 anid|CBF88804.1 anig|EHA27003.1 aory|EIT82800.1 asyd|OJJ62894.1 ater|EAU39288.1 atub|OJI85016.1 aver|OJI96946.1 awen|OJJ34997.1 azon|OJJ45646.1

Group3422: abra|OJJ74343.1 acar|OOF95387.1 acla|EAW07397.1 afis|EAW22423.1 afla|EED52501.1 afum|EAL90768.2 agla|OJJ87605.1 akaw|GAA86687.1 aluc|OJZ89788.1 anid|CBF88813.1 anig|EHA27002.1 aory|EIT82762.1 asyd|OJJ62889.1 ater|EAU39284.1 atub|OJI85018.1 aver|OJI96941.1 awen|OJJ35002.1 azon|OJJ45642.1

Group3423: abra|OJJ74344.1 acar|OOF95386.1 acla|EAW07398.1 afis|EAW22424.1 afla|EED52502.1 afum|EAL90767.1 agla|OJJ87606.1 akaw|GAA86686.1 aluc|OJZ89787.1 anid|CBF75162.1 anig|EHA27001.1 aory|EIT82765.1 asyd|OJJ59961.1 ater|EAU39283.1 atub|OJI85019.1 aver|OJJ02329.1 awen|OJJ35003.1 azon|OJJ45641.1

Group3424: abra|OJJ74347.1 acar|OOF95382.1 acla|EAW07401.1 afis|EAW22427.1 afla|EED52505.1 afum|EAL90764.1 agla|OJJ87596.1 akaw|GAA86683.1 aluc|OJZ89783.1 anid|CBF88819.1 anig|EHA26998.1 aory|EIT82749.1 asyd|OJJ62886.1 ater|EAU39280.1 atub|OJI85022.1 aver|OJI96938.1 awen|OJJ35006.1 azon|OJJ45648.1

Group3425: abra|OJJ74348.1 acar|OOF95380.1 acla|EAW07402.1 afis|EAW22428.1 afla|EED52506.1 afum|EAL90763.1 agla|OJJ87595.1 akaw|GAA86682.1 aluc|OJZ89782.1 anid|CBF88822.1 anig|EHA26997.1 aory|EIT82757.1 asyd|OJJ62885.1 ater|EAU39279.1 atub|OJI85024.1 aver|OJI96937.1 awen|OJJ35007.1 azon|OJJ45649.1

Group3426: abra|OJJ74352.1 acar|OOF95378.1 acla|EAW07405.1 afis|EAW22431.1 afla|EED52510.1 afum|EAL90760.1 agla|OJJ87592.1 akaw|GAA86679.1 aluc|OJZ89776.1 anid|CBF88828.1 anig|EHA26994.1 aory|EIT82794.1 asyd|OJJ62882.1 ater|EAU39276.1 atub|OJI85027.1 aver|OJI96934.1 awen|OJJ35010.1 azon|OJJ45651.1

Group3427: abra|OJJ74353.1 acar|OOF95377.1 acla|EAW07406.1 afis|EAW22432.1 afla|EED52513.1 afum|EAL90759.1 agla|OJJ87590.1 akaw|GAA86678.1 aluc|OJZ89775.1 anid|CBF88832.1 anig|EHA26993.1 aory|EIT82776.1 asyd|OJJ62880.1 ater|EAU39275.1 atub|OJI85028.1 aver|OJI96932.1 awen|OJJ35011.1 azon|OJJ45652.1

Group3428: abra|OJJ74355.1 acar|OOF95375.1 acla|EAW07409.1 afis|EAW22435.1 afla|EED52515.1 afum|EAL90756.1 agla|OJJ87587.1 akaw|GAA86676.1 aluc|OJZ89773.1 anid|CBF88835.1 anig|EHA26991.1 aory|EIT82785.1 asyd|OJJ62878.1 ater|EAU39273.1 atub|OJI85030.1 aver|OJI96930.1 awen|OJJ35013.1 azon|OJJ45654.1

Group3429: abra|OJJ74359.1 acar|OOF95370.1 acla|EAW07410.1 afis|EAW22436.1 afla|EED54446.1 afum|EAL90755.1 agla|OJJ87586.1 akaw|GAA86672.1 aluc|OJZ89769.1 anid|CBF88882.1 anig|EHA26989.1 asyd|OJJ62877.1 ater|EAU39272.1 atub|OJI85034.1 aver|OJI96929.1 awen|OJJ35014.1 azon|OJJ45656.1 aory|EIT81137.1

Group3430: abra|OJJ74361.1 acar|OOF95368.1 acla|EAW07413.1 afis|EAW22439.1 afla|EED54449.1 afum|EAL90752.1 agla|OJJ87584.1 akaw|GAA86669.1 aluc|OJZ89766.1 anid|CBF88862.1 anig|EHA26986.1 aory|EIT81135.1 asyd|OJJ62874.1 ater|EAU39269.1 atub|OJI85036.1 aver|OJI96927.1 awen|OJJ35016.1 azon|OJJ45661.1

Group3431: abra|OJJ74364.1 acar|OOF95366.1 acla|EAW07414.1 afis|EAW22441.1 afla|EED54457.1 afum|EAL90750.1 agla|OJJ87583.1 akaw|GAA86666.1 aluc|OJZ89763.1 anid|CBF88845.1 anig|EHA26983.1 aory|EIT81148.1 asyd|OJJ62867.1 ater|EAU39263.1 atub|OJI85039.1 aver|OJI96920.1 awen|OJJ35018.1 azon|OJJ50043.1

Group3432: abra|OJJ74386.1 acar|OOF95352.1 acla|EAW07427.1 afis|EAW22453.1 afla|EED54469.1 afum|EAL90738.1 agla|OJJ87570.1 akaw|GAA87287.1 aluc|OJZ89732.1 anid|CBF88931.1 anig|EHA26958.1 aory|EIT81158.1 asyd|OJJ62850.1 ater|EAU39253.1 atub|OJI85069.1 aver|OJI96903.1 awen|OJJ35078.1 azon|OJJ50029.1

Group3433: abra|OJJ74395.1 acar|OOF95341.1 acla|EAW06819.1 afis|EAW16367.1 afla|EED53469.1 afum|EAL88992.2 agla|OJJ78588.1 akaw|GAA87293.1 aluc|OJZ89725.1 anid|CBF89350.1 anig|EHA26953.1

aory|EIT75003.1 asyd|OJJ62502.1 ater|EAU34081.1 atub|OJI85075.1 aver|OJI96623.1 awen|OJJ37772.1 azon|OJJ45083.1

Group3434: abra|OJJ74414.1 acar|OOF95329.1 acla|EAW06828.1 afis|EAW16378.1 afla|EED53458.1 afum|EAL89003.1 agla|OJJ78596.1 akaw|GAA87305.1 aluc|OJZ89712.1 anid|CBF88042.1 anig|EHA26943.1 aory|EIT75071.1 asyd|OJJ63543.1 ater|EAU34074.1 atub|OJI85090.1 aver|OJI97391.1 awen|OJJ37912.1 azon|OJJ45091.1

Group3435: abra|OJJ74417.1 acar|OOF95326.1 acla|EAW06832.1 afis|EAW16382.1 afla|EED53452.1 afum|EAL89007.1 agla|OJJ88205.1 akaw|GAA87308.1 aluc|OJZ89709.1 anid|CBF89369.1 anig|EHA26940.1 aory|EIT75129.1 asyd|OJJ62475.1 ater|EAU34070.1 atub|OJI85093.1 aver|OJI96611.1 awen|OJJ37915.1 azon|OJJ46177.1

Group3436: abra|OJJ74438.1 acar|OOF95308.1 acla|EAW12299.1 afis|EAW23841.1 afla|EED49145.1 afum|EAL87272.1 agla|OJJ79053.1 akaw|GAA87331.1 aluc|OJZ89683.1 anid|CBF89626.1 anig|EHA26919.1 aory|EIT78517.1 asyd|OJJ62298.1 ater|EAU29595.1 atub|OJI85117.1 aver|OJI96406.1 awen|OJJ35935.1 azon|OJJ49112.1

Group3437: abra|OJJ74456.1 acar|OOF95294.1 acla|EAW07237.1 afis|EAW22260.1 afla|EED57647.1 afum|EAL90920.1 agla|OJJ87795.1 akaw|GAA87350.1 aluc|OJZ89664.1 anid|CBF88509.1 anig|EHA26901.1 aory|EIT79886.1 asyd|OJJ63802.1 ater|EAU34263.1 atub|OJI85136.1 aver|OJI97136.1 awen|OJJ34832.1 azon|OJJ45714.1

Group3438: abra|OJJ74457.1 acar|OOF95293.1 acla|EAW07236.1 afis|EAW22259.1 afla|EED57646.1 afum|EAL90921.1 agla|OJJ87799.1 akaw|GAA87351.1 aluc|OJZ89663.1 anid|CBF88506.1 anig|EHA26900.1 aory|EIT79868.1 asyd|OJJ63799.1 ater|EAU34262.1 atub|OJI85137.1 aver|OJI97139.1 awen|OJJ34831.1 azon|OJJ45715.1

Group3439: abra|OJJ74463.1 acar|OOF95288.1 acla|EAW07235.1 afis|EAW22256.1 afla|EED57643.1 afum|EAL90922.1 agla|OJJ87800.1 akaw|GAA87355.1 aluc|OJZ89659.1 anid|CBF88504.1 anig|EHA26896.1 aory|EIT79873.1 asyd|OJJ63798.1 ater|EAU34257.1 atub|OJI85141.1 aver|OJI97140.1 awen|OJJ34828.1 azon|OJJ45720.1

Group3440: abra|OJJ74464.1 acar|OOF95286.1 acla|EAW07234.1 afis|EAW22255.1 afla|EED57641.1 afum|EAL90923.1 agla|OJJ87801.1 akaw|GAA87356.1 aluc|OJZ89658.1 anid|CBF88502.1 anig|EHA26895.1 aory|EIT82944.1 asyd|OJJ63796.1 ater|EAU34256.1 atub|OJI85143.1 aver|OJI97141.1 awen|OJJ34827.1 azon|OJJ45722.1

Group3441: abra|OJJ74466.1 acar|OOF95281.1 acla|EAW07231.1 afis|EAW22245.1 afla|EED57638.1 afum|EAL90948.1 agla|OJJ87805.1 akaw|GAA87359.1 aluc|OJZ89654.1 anid|CBF88496.1 anig|EHA26892.1 aory|EIT82948.1 asyd|OJJ63793.1 ater|EAU34253.1 atub|OJI85150.1 aver|OJI97144.1 awen|OJJ34823.1 azon|OJJ45727.1

Group3442: abra|OJJ74467.1 acar|OOF95280.1 acla|EAW07230.1 afis|EAW22244.1 afla|EED57637.1 afum|EAL90949.1 agla|OJJ87807.1 akaw|GAA87360.1 aluc|OJZ89653.1 anid|CBF88493.1 anig|EHA26891.1 aory|EIT82977.1 asyd|OJJ63792.1 ater|EAU34252.1 atub|OJI85151.1 aver|OJI97145.1 awen|OJJ34822.1 azon|OJJ45728.1

Group3443: abra|OJJ74468.1 acar|OOF95279.1 acla|EAW07229.1 afis|EAW22243.1 afla|EED57636.1 afum|EAL90950.1 agla|OJJ87808.1 akaw|GAA87361.1 aluc|OJZ89652.1 anid|CBF88491.1 anig|EHA26890.1 aory|EIT82978.1 asyd|OJJ63791.1 ater|EAU34251.1 atub|OJI85152.1 aver|OJI97146.1 awen|OJJ34821.1 azon|OJJ45729.1

Group3444: abra|OJJ74473.1 acar|OOF95271.1 acla|EAW07224.1 afis|EAW22238.1 afla|EED57627.1 afum|EAL90955.1 agla|OJJ87813.1 akaw|GAA87367.1 aluc|OJZ89645.1 anid|CBF88481.1 anig|EHA26884.1 aory|EIT82957.1 asyd|OJJ63785.1 ater|EAU34245.1 atub|OJI85158.1 aver|OJI97152.1 awen|OJJ34816.1 azon|OJJ45733.1

Group3445: abra|OJJ74477.1 acar|OOF95269.1 acla|EAW07223.1 afis|EAW22237.1 afla|EED57626.1 afum|EAL90956.1 agla|OJJ87814.1 akaw|GAA87370.1 aluc|OJZ89642.1 anid|CBF88479.1 anig|EHA26881.1 aory|EIT82989.1 asyd|OJJ63784.1 ater|EAU34244.1 atub|OJI85161.1 aver|OJI97153.1 awen|OJJ34815.1 azon|OJJ45734.1

Group3446: abra|OJJ74478.1 acar|OOF95268.1 acla|EAW07222.1 afis|EAW22236.1 afla|EED57625.1 afum|EAL90957.1 agla|OJJ87815.1 akaw|GAA87371.1 aluc|OJZ89641.1 anid|CBF88477.1 anig|EHA26880.1 aory|EIT82990.1 asyd|OJJ63783.1 ater|EAU34243.1 atub|OJI85162.1 aver|OJI97154.1 awen|OJJ34814.1 azon|OJJ45735.1

Group3447: abra|OJJ74481.1 acar|OOF95267.1 acla|EAW07220.1 afis|EAW22234.1 afla|EED57623.1 afum|EAL90959.1 agla|OJJ87817.1 akaw|GAA87374.1 aluc|OJZ89638.1 anid|CBF88474.1 anig|EHA26879.1 aory|EIT82951.1 asyd|OJJ63782.1 ater|EAU34237.1 atub|OJI85165.1 aver|OJI97155.1 awen|OJJ34813.1 azon|OJJ45736.1

Group3448: abra|OJJ74484.1 acar|OOF95266.1 acla|EAW07217.1 afis|EAW22231.1 afla|EED57620.1 afum|EAL90962.1 agla|OJJ87820.1 akaw|GAA87377.1 aluc|OJZ89635.1 anid|CBF88469.1 anig|EHA26877.1 aory|EIT82992.1 asyd|OJJ63780.1 ater|EAU34234.1 atub|OJI85168.1 aver|OJI97157.1 awen|OJJ34811.1 azon|OJJ45738.1

Group3449: abra|OJJ74489.1 acar|OOF95260.1 acla|EAW07206.1 afis|EAW22218.1 afla|EED57611.1 afum|EAL90976.1 agla|OJJ87826.1 akaw|GAA87383.1 aluc|OJZ89629.1 anid|CBF88456.1 anig|EHA26872.1 aory|EIT82939.1 asyd|OJJ63771.1 ater|EAU34228.1 atub|OJI85173.1 aver|OJI97167.1 awen|OJJ34804.1 azon|OJJ45749.1

Group3450: abra|OJJ74490.1 acar|OOF95259.1 acla|EAW07204.1 afis|EAW22216.1 afla|EED57610.1 afum|EAL90978.1 agla|OJJ87830.1 akaw|GAA87384.1 aluc|OJZ89628.1 anid|CBF88454.1 anig|EHA26871.1 aory|EIT82985.1 asyd|OJJ63770.1 ater|EAU34224.1 atub|OJI85174.1 aver|OJI97168.1 awen|OJJ34801.1 azon|OJJ45748.1

Group3451: abra|OJJ74491.1 acar|OOF95258.1 acla|EAW07203.1 afis|EAW22215.1 afla|EED57609.1 afum|EAL90979.1 agla|OJJ87829.1 akaw|GAA87385.1 aluc|OJZ89627.1 anid|CBF88452.1 anig|EHA26870.1 aory|EIT82971.1 asyd|OJJ63769.1 ater|EAU34223.1 atub|OJI85175.1 aver|OJI97169.1 awen|OJJ34800.1 azon|OJJ45747.1

Group3452: abra|OJJ74493.1 acar|OOF95256.1 acla|EAW07201.1 afis|EAW22213.1 afla|EED57607.1 afum|EAL90981.2 agla|OJJ87831.1 akaw|GAA87386.1 aluc|OJZ89625.1 anid|CBF88448.1 anig|EHA26869.1 aory|EIT82952.1 asyd|OJJ63768.1 ater|EAU34222.1 atub|OJI85177.1 aver|OJI97170.1 awen|OJJ34798.1 azon|OJJ45745.1

Group3453: abra|OJJ74494.1 acar|OOF95255.1 acla|EAW07200.1 afis|EAW22212.1 afla|EED57606.1 afum|EAL90982.1 agla|OJJ87832.1 akaw|GAA87387.1 aluc|OJZ89624.1 anid|CBF88446.1 anig|EHA26868.1 aory|EIT82953.1 asyd|OJJ63767.1 ater|EAU34221.1 atub|OJI85178.1 aver|OJI97171.1 awen|OJJ34797.1 azon|OJJ45744.1

Group3454: abra|OJJ74496.1 acar|OOF95254.1 acla|EAW07197.1 afis|EAW22209.1 afla|EED57603.1 afum|EAL90985.1 agla|OJJ87835.1 akaw|GAA87390.1 aluc|OJZ89622.1 anig|EHA26866.1 asyd|OJJ63765.1 ater|EAU34219.1 atub|OJI85180.1 aver|OJI97174.1 awen|OJJ34794.1 anid|CBF88442.1 aory|EIT82943.1 azon|OJJ45750.1

Group3455: abra|OJJ74497.1 acar|OOF95253.1 acla|EAW07195.1 afis|EAW22207.1 afla|EED57602.1 afum|EAL90986.2 agla|OJJ87837.1 akaw|GAA87392.1 aluc|OJZ89621.1 anid|CBF88439.1 anig|EHA26865.1 aory|EIT82955.1 asyd|OJJ63764.1 ater|EAU34218.1 atub|OJI85181.1 aver|OJI97176.1 awen|OJJ34793.1 azon|OJJ45753.1

Group3456: abra|OJJ74498.1 acar|OOF95252.1 acla|EAW13756.1 afis|EAW19810.1 afla|EED57601.1 afum|EAL85082.1 agla|OJJ87838.1 akaw|GAA87393.1 aluc|OJZ89620.1 anid|CBF88437.1 anig|EHA26864.1 aory|EIT82959.1 asyd|OJJ63763.1 ater|EAU34217.1 atub|OJI85182.1 aver|OJI97177.1 awen|OJJ34792.1 azon|OJJ45754.1

Group3457: abra|OJJ74503.1 acar|OOF95241.1 acla|EAW07189.1 afis|EAW22202.1 afla|EED57593.1 afum|EAL90990.1 agla|OJJ87842.1 akaw|GAA87397.1 aluc|OJZ89615.1 anid|CBF88430.1 anig|EHA26860.1 aory|EIT82963.1 asyd|OJJ63760.1 ater|EAU34212.1 atub|OJI85188.1 aver|OJI97180.1 awen|OJJ34787.1 azon|OJJ45771.1

Group3458: abra|OJJ74506.1 acar|OOF89839.1 acla|EAW07192.1 afis|EAW22205.1 afla|EED57588.1 afum|EAL90988.1 agla|OJJ87845.1 akaw|GAA88823.1 aluc|OJZ89611.1 anid|CBF88417.1 anig|EHA26856.1 aory|EIT82968.1 asyd|OJJ63756.1 ater|EAU34209.1 atub|OJI85192.1 aver|OJI97184.1 awen|OJJ34786.1 azon|OJJ45766.1

Group3459: abra|OJJ74508.1 acar|OOF95234.1 acla|EAW07184.1 afis|EAW22197.1 afla|EED57584.1 afum|EAL90994.1 agla|OJJ87848.1 akaw|GAA82091.1 aluc|OJZ92089.1 anid|CBF88409.1 anig|EHA26852.1 aory|EIT82932.1 asyd|OJJ63754.1 ater|EAU34207.1 atub|OJI86519.1 aver|OJI97187.1 awen|OJJ34780.1 azon|OJJ45761.1

Group3460: abra|OJJ74513.1 acar|OOF95230.1 acla|EAW07177.1 afis|EAW22191.1 afla|EED57575.1 afum|EAL91000.1 agla|OJJ87856.1 akaw|GAA82086.1 aluc|OJZ92094.1 anid|CBF88400.1 anig|EHA26847.1 aory|EIT82946.1 asyd|OJJ63748.1 ater|EAU34199.1 atub|OJI86527.1 aver|OJI97192.1 awen|OJJ34771.1 azon|OJJ45775.1

Group3461: abra|OJJ74519.1 acar|OOF95229.1 acla|EAW07174.1 afis|EAW22188.1 afla|EED57572.1 afum|EAL91002.1 agla|OJJ87861.1 akaw|GAA82081.1 aluc|OJZ92098.1 anid|CBF88395.1 anig|EHA26844.1 aory|EIT82984.1 asyd|OJJ63745.1 ater|EAU34195.1 atub|OJI86531.1 aver|OJI97195.1 awen|OJJ34768.1 azon|OJJ45778.1

Group3462: abra|OJJ74523.1 acar|OOF95226.1 acla|EAW07170.1 afis|EAW22185.1 afla|EED57563.1 afum|EAL91004.1 agla|OJJ87870.1 akaw|GAA82078.1 aluc|OJZ92102.1 anid|CBF88391.1 anig|EHA26841.1

aory|EIT81827.1 asyd|OJJ63742.1 ater|EAU34193.1 atub|OJI86536.1 aver|OJI97198.1 awen|OJJ34765.1 azon|OJJ45780.1

Group3463: abra|OJJ74524.1 acar|OOF95225.1 acla|EAW07169.1 afis|EAW22184.1 afla|EED57562.1 afum|EAL91005.1 agla|OJJ87869.1 akaw|GAA82077.1 aluc|OJZ92104.1 anid|CBF75419.1 anig|EHA26840.1 aory|EIT81846.1 asyd|OJJ59792.1 ater|EAU34188.1 atub|OJI86538.1 aver|OJJ02495.1 awen|OJJ34764.1 azon|OJJ45781.1

Group3464: abra|OJJ74527.1 acar|OOF95223.1 acla|EAW07164.1 afis|EAW22179.1 afla|EED57558.1 afum|EAL91010.1 agla|OJJ87876.1 akaw|GAA82074.1 aluc|OJZ92108.1 anid|CBF88384.1 anig|EHA26837.1 aory|EIT81836.1 asyd|OJJ63738.1 ater|EAU34184.1 atub|OJI86542.1 aver|OJI97202.1 awen|OJJ34760.1 azon|OJJ45791.1

Group3465: abra|OJJ74532.1 acar|OOF95220.1 acla|EAW07160.1 afis|EAW22175.1 afla|EED57554.1 afum|EAL91014.1 agla|OJJ87879.1 akaw|GAA82068.1 aluc|OJZ92114.1 anid|CBF89077.1 anig|EHA26831.1 aory|EIT81832.1 asyd|OJJ62739.1 ater|EAU34180.1 atub|OJI86547.1 aver|OJI96782.1 awen|OJJ39689.1 azon|OJJ45808.1

Group3466: abra|OJJ74533.1 acar|OOF95219.1 acla|EAW07159.1 afis|EAW22174.1 afla|EED57553.1 afum|EAL91015.1 agla|OJJ87880.1 akaw|GAA82067.1 aluc|OJZ92115.1 anid|CBF89079.1 anig|EHA26830.1 aory|EIT81853.1 asyd|OJJ62738.1 ater|EAU34179.1 atub|OJI86548.1 aver|OJI96781.1 awen|OJJ34749.1 azon|OJJ45810.1

Group3467: abra|OJJ74546.1 acar|OOF95210.1 acla|EAW07152.1 afis|EAW22166.1 afla|EED57536.1 afum|EAL91025.1 agla|OJJ87891.1 akaw|GAA82052.1 aluc|OJZ92128.1 anid|CBF89087.1 anig|EHA26820.1 aory|EIT81873.1 asyd|OJJ62734.1 ater|EAU34173.1 atub|OJI86560.1 aver|OJI96777.1 awen|OJJ34740.1 azon|OJJ45819.1

Group3468: abra|OJJ74548.1 acar|OOF95209.1 acla|EAW07150.1 afis|EAW22164.1 afla|EED57535.1 afum|EAL91027.1 agla|OJJ87893.1 akaw|GAA82050.1 aluc|OJZ92130.1 anid|CBF89090.1 anig|EHA26818.1 aory|EIT81851.1 asyd|OJJ62732.1 ater|EAU34171.1 atub|OJI86562.1 aver|OJI96775.1 awen|OJJ34738.1 azon|OJJ45821.1

Group3469: abra|OJJ74549.1 acar|OOF95208.1 acla|EAW07149.1 afis|EAW22163.1 afla|EED57534.1 afum|EAL91028.1 agla|OJJ87894.1 akaw|GAA82049.1 aluc|OJZ92131.1 anid|CBF89094.1 anig|EHA26817.1 aory|EIT81858.1 asyd|OJJ62731.1 ater|EAU34166.1 atub|OJI86563.1 aver|OJI96774.1 awen|OJJ34737.1 azon|OJJ45822.1

Group3470: abra|OJJ74553.1 acar|OOF95202.1 acla|EAW07145.1 afis|EAW22159.1 afla|EED57531.1 afum|EAL91031.1 agla|OJJ87905.1 akaw|GAA82044.1 aluc|OJZ92136.1 anid|CBF89101.1 anig|EHA26813.1 aory|EIT81829.1 asyd|OJJ62727.1 ater|EAU34162.1 atub|OJI86568.1 aver|OJI96770.1 awen|OJJ34733.1 azon|OJJ45826.1

Group3471: abra|OJJ74569.1 acar|OOF95192.1 acla|EAW07140.1 afis|EAW22153.1 afla|EED53614.1 afum|EAL91037.1 agla|OJJ87909.1 akaw|GAA82036.1 aluc|OJZ92147.1 anid|CBF89105.1 anig|EHA26802.1 aory|EIT75057.1 asyd|OJJ62725.1 ater|EAU34157.1 atub|OJI86575.1 aver|OJI96768.1 awen|OJJ34729.1 azon|OJJ45834.1

Group3472: abra|OJJ74571.1 acar|OOF95191.1 acla|EAW07138.1 afis|EAW22151.1 afla|EED53613.1 afum|EAL91039.1 agla|OJJ87911.1 akaw|GAA82034.1 aluc|OJZ92149.1 anid|CBF89108.1 anig|EHA26800.1 aory|EIT75195.1 asyd|OJJ62667.1 ater|EAU34156.1 atub|OJI86577.1 aver|OJI96765.1 awen|OJJ34727.1 azon|OJJ45835.1

Group3473: abra|OJJ74573.1 acar|OOF95189.1 acla|EAW07136.1 afis|EAW22149.1 afla|EED53610.1 afum|EAL91041.1 agla|OJJ87914.1 akaw|GAA82032.1 aluc|OJZ92151.1 anid|CBF89112.1 anig|EHA26798.1 aory|EIT74840.1 asyd|OJJ62665.1 ater|EAU34155.1 atub|OJI86579.1 aver|OJI96763.1 awen|OJJ34725.1 azon|OJJ45839.1

Group3474: abra|OJJ74577.1 acar|OOF95187.1 acla|EAW07133.1 afis|EAW22146.1 afla|EED45848.1 afum|EAL91044.1 agla|OJJ87922.1 akaw|GAA82028.1 aluc|OJZ92155.1 anid|CBF78365.1 anig|EHA26794.1 aory|EIT75625.1 asyd|OJJ57324.1 ater|EAU34149.1 atub|OJI86583.1 aver|OJJ05836.1 awen|OJJ34721.1 azon|OJJ46642.1

Group3475: abra|OJJ74578.1 acar|OOF95186.1 acla|EAW07132.1 afis|EAW22145.1 afla|EED45849.1 afum|EAL91045.1 agla|OJJ87923.1 akaw|GAA82027.1 aluc|OJZ92156.1 anid|CBF78367.1 anig|EHA26793.1 aory|EIT75756.1 asyd|OJJ57325.1 ater|EAU34148.1 atub|OJI86584.1 aver|OJJ05835.1 awen|OJJ34720.1 azon|OJJ46643.1

Group3476: abra|OJJ74581.1 acar|OOF95183.1 acla|EAW07131.1 afis|EAW22142.1 afla|EED53568.1 afum|EAL91048.2 agla|OJJ87932.1 akaw|GAA82024.1 aluc|OJZ92159.1 anid|CBF89555.1 anig|EHA26790.1 aory|EIT75148.1 asyd|OJJ62347.1 ater|EAU31752.1 atub|OJI86587.1 aver|OJI96453.1 awen|OJJ34717.1 azon|OJJ45846.1

Group3477: abra|OJJ74584.1 acar|OOF95182.1 acla|EAW10794.1 afis|EAW24782.1 afla|EED47203.1 afum|EAL89421.1 agla|OJJ82878.1 akaw|GAA82021.1 aluc|OJZ92162.1 anid|CBF81444.1 anig|EHA26787.1 aory|EIT79171.1 asyd|OJJ59430.1 ater|EAU36526.1 atub|OJI86590.1 aver|OJJ02841.1 awen|OJJ40368.1 azon|OJJ49217.1

Group3478: abra|OJJ74585.1 acar|OOF95181.1 acla|EAW10793.1 afis|EAW24783.1 afla|EED45489.1 afum|EAL89422.1 akaw|GAA82020.1 aluc|OJZ92163.1 anid|CBF81449.1 anig|EHA26786.1 aory|EIT79242.1 asyd|OJJ59432.1 atub|OJI86592.1 aver|OJJ02839.1 awen|OJJ36318.1 azon|OJJ43590.1 aory|EIT75730.1 afla|EED47202.1

Group3479: abra|OJJ74588.1 acar|OOF95178.1 acla|EAW10790.1 afis|EAW24786.1 afla|EED47199.1 afum|EAL89425.1 agla|OJJ82884.1 akaw|GAA82017.1 aluc|OJZ92165.1 anid|CBF81455.1 anig|EHA26784.1 aory|EIT79195.1 asyd|OJJ59435.1 ater|EAU36523.1 atub|OJI86595.1 aver|OJJ02836.1 awen|OJJ40370.1 azon|OJJ49220.1

Group3480: abra|OJJ74589.1 acar|OOF95177.1 acla|EAW10789.1 afis|EAW24787.1 afla|EED47197.1 afum|EAL89426.1 agla|OJJ82883.1 akaw|GAA82016.1 aluc|OJZ92167.1 anid|CBF81457.1 anig|EHA26782.1 aory|EIT79260.1 asyd|OJJ59437.1 ater|EAU36522.1 atub|OJI86598.1 aver|OJJ02834.1 awen|OJJ40371.1 azon|OJJ49221.1

Group3481: abra|OJJ74594.1 acar|OOF95175.1 acla|EAW10788.1 afis|EAW24791.1 afla|EED47195.1 afum|EAL89433.1 agla|OJJ82881.1 akaw|GAA82014.1 aluc|OJZ92169.1 anid|CBF81460.1 anig|EHA26780.1 aory|EIT79181.1 asyd|OJJ59439.1 ater|EAU36521.1 atub|OJI86601.1 aver|OJJ02833.1 awen|OJJ40372.1 azon|OJJ49223.1

Group3482: abra|OJJ74595.1 acar|OOF95174.1 acla|EAW10786.1 afis|EAW24792.1 afla|EED47194.1 afum|EAL89434.1 agla|OJJ82879.1 akaw|GAA82013.1 aluc|OJZ92170.1 anid|CBF81464.1 anig|EHA26779.1 aory|EIT79314.1 asyd|OJJ59442.1 ater|EAU36516.1 atub|OJI86602.1 aver|OJJ02831.1 awen|OJJ40373.1 azon|OJJ49224.1

Group3483: abra|OJJ74629.1 acla|EAW10752.1 afis|EAW24821.1 afla|EED47154.1 afum|EAL89465.1 agla|OJJ82919.1 akaw|GAA81979.1 aluc|OJZ92211.1 anid|CBF85870.1 anig|EHA26747.1 aory|EIT79196.1 asyd|OJJ64372.1 ater|EAU36484.1 atub|OJI86640.1 aver|OJI98468.1 awen|OJJ40407.1 azon|OJJ49256.1 afla|EED47155.1

Group3484: abra|OJJ74705.1 acar|OOF89903.1 acla|EAW10692.1 afis|EAW24878.1 afla|EED47088.1 afum|EAL89528.1 agla|OJJ82100.1 akaw|GAA81912.1 aluc|OJZ92285.1 anid|CBF75756.1 anig|EHA26683.1 aory|EIT72477.1 asyd|OJJ57526.1 ater|EAU36423.1 atub|OJI86716.1 aver|OJJ05652.1 awen|OJJ40468.1 azon|OJJ49332.1

Group3485: abra|OJJ74707.1 acar|OOF89905.1 acla|EAW10691.1 afis|EAW24879.1 afla|EED47087.1 afum|EAL89529.1 agla|OJJ82101.1 akaw|GAA81910.1 aluc|OJZ92288.1 anid|CBF75754.1 anig|EHA26681.1 aory|EIT72507.1 asyd|OJJ57525.1 ater|EAU36422.1 atub|OJI86718.1 aver|OJJ05653.1 awen|OJJ40469.1 azon|OJJ49333.1

Group3486: abra|OJJ74708.1 acar|OOF95718.1 acla|EAW10690.1 afis|EAW24880.1 afla|EED47086.1 afum|EAL89530.1 agla|OJJ82102.1 akaw|GAA81909.1 aluc|OJZ92289.1 anid|CBF75752.1 anig|EHA26680.1 aory|EIT72478.1 asyd|OJJ57524.1 ater|EAU36421.1 atub|OJI86719.1 aver|OJJ05654.1 awen|OJJ40470.1 azon|OJJ49334.1

Group3487: abra|OJJ74709.1 acar|OOF95719.1 acla|EAW10689.1 afis|EAW24881.1 afla|EED47085.1 afum|EAL89531.1 agla|OJJ82103.1 akaw|GAA81908.1 aluc|OJZ92290.1 anid|CBF75750.1 anig|EHA26679.1 aory|EIT72499.1 asyd|OJJ57523.1 ater|EAU36420.1 atub|OJI86720.1 aver|OJJ05655.1 awen|OJJ40471.1 azon|OJJ49335.1

Group3488: abra|OJJ74711.1 acar|OOF95722.1 acla|EAW10687.1 afis|EAW24883.1 afla|EED47083.1 afum|EAL89533.1 agla|OJJ82105.1 akaw|GAA81906.1 aluc|OJZ92292.1 anid|CBF75747.1 anig|EHA26677.1 aory|EIT72512.1 asyd|OJJ57521.1 ater|EAU36418.1 atub|OJI86722.1 aver|OJJ05657.1 awen|OJJ40473.1 azon|OJJ49337.1

Group3489: abra|OJJ74714.1 acar|OOF95723.1 acla|EAW10685.1 afis|EAW24885.1 afla|EED47081.1 afum|EAL89535.2 agla|OJJ82108.1 akaw|GAA81904.1 aluc|OJZ92294.1 anid|CBF75617.1 anig|EHA26675.1 aory|EIT72482.1 asyd|OJJ57452.1 ater|EAU36416.1 atub|OJI86724.1 aver|OJJ05726.1 awen|OJJ40475.1 azon|OJJ49339.1

Group3490: abra|OJJ74715.1 acar|OOF95724.1 acla|EAW10684.1 afis|EAW24886.1 afla|EED47080.1 afum|EAL89537.1 agla|OJJ82109.1 akaw|GAA81903.1 aluc|OJZ92295.1 anid|CBF75619.1 anig|EHA26674.1 aory|EIT72515.1 asyd|OJJ57453.1 ater|EAU36415.1 atub|OJI86725.1 aver|OJJ05725.1 awen|OJJ40476.1 azon|OJJ49340.1

Group3491: abra|OJJ74717.1 acar|OOF95728.1 acla|EAW10681.1 afis|EAW24889.1 afla|EED46965.1 afum|EAL89540.1 agla|OJJ82112.1 akaw|GAA81899.1 aluc|OJZ92299.1 anid|CBF75625.1 anig|EHA26672.1

aory|EIT72622.1 asyd|OJJ57456.1 ater|EAU36412.1 atub|OJI86728.1 aver|OJJ05723.1 awen|OJJ40479.1 azon|OJJ49343.1

Group3492: abra|OJJ74722.1 acar|OOF95731.1 acla|EAW10675.1 afis|EAW24891.1 afla|EED46970.1 afum|EAL89544.1 agla|OJJ82115.1 akaw|GAA81894.1 aluc|OJZ92306.1 anid|CBF75630.1 anig|EHA26662.1 aory|EIT72703.1 asyd|OJJ57459.1 ater|EAU36407.1 atub|OJI86736.1 aver|OJJ05721.1 awen|OJJ40484.1 azon|OJJ49346.1

Group3493: abra|OJJ74723.1 acar|OOF95732.1 acla|EAW10674.1 afis|EAW24892.1 afla|EED46971.1 afum|EAL89545.1 agla|OJJ82116.1 akaw|GAA81893.1 aluc|OJZ92307.1 anid|CBF75632.1 anig|EHA26661.1 aory|EIT72525.1 asyd|OJJ57460.1 ater|EAU36406.1 atub|OJI86737.1 aver|OJJ05720.1 awen|OJJ40485.1 azon|OJJ49347.1

Group3494: abra|OJJ74724.1 acar|OOF95733.1 acla|EAW10673.1 afis|EAW24893.1 afla|EED46972.1 afum|EAL89546.1 agla|OJJ82117.1 akaw|GAA81892.1 aluc|OJZ92308.1 anid|CBF75634.1 anig|EHA26660.1 aory|EIT72626.1 asyd|OJJ57461.1 ater|EAU36405.1 atub|OJI86738.1 aver|OJJ05719.1 awen|OJJ40486.1 azon|OJJ49348.1

Group3495: abra|OJJ74727.1 acar|OOF90030.1 acla|EAW10671.1 afis|EAW24895.1 afla|EED46975.1 afum|EAL89549.1 agla|OJJ82119.1 akaw|GAA81890.1 aluc|OJZ92311.1 anid|CBF75657.1 anig|EHA26658.1 aory|EIT72598.1 asyd|OJJ57463.1 ater|EAU36403.1 atub|OJI86740.1 aver|OJJ05717.1 awen|OJJ40488.1 azon|OJJ49351.1

Group3496: abra|OJJ74735.1 acar|OOF95734.1 acla|EAW10667.1 afis|EAW24899.1 afla|EED46978.1 afum|EAL89553.1 agla|OJJ82124.1 akaw|GAA83674.1 aluc|OJZ92317.1 anid|CBF75649.1 anig|EHA26654.1 aory|EIT72632.1 asyd|OJJ57470.1 ater|EAU36400.1 atub|OJI86746.1 aver|OJJ05712.1 awen|OJJ40493.1 azon|OJJ49355.1

Group3497: abra|OJJ74736.1 acar|OOF95735.1 acla|EAW10666.1 afis|EAW24900.1 afla|EED46980.1 afum|EAL89554.1 agla|OJJ82125.1 akaw|GAA83673.1 aluc|OJZ92318.1 anid|CBF75648.1 anig|EHA26653.1 aory|EIT72726.1 asyd|OJJ57472.1 ater|EAU36399.1 atub|OJI86747.1 aver|OJJ05711.1 awen|OJJ40494.1 azon|OJJ49356.1

Group3498: abra|OJJ74738.1 acar|OOF95736.1 acla|EAW10665.1 afis|EAW24901.1 afla|EED46981.1 afum|EAL89555.1 agla|OJJ82126.1 akaw|GAA83672.1 aluc|OJZ92319.1 anid|CBF75646.1 anig|EHA26651.1 aory|EIT72528.1 asyd|OJJ57473.1 ater|EAU36398.1 atub|OJI86748.1 aver|OJJ05710.1 awen|OJJ40495.1 azon|OJJ49357.1

Group3499: abra|OJJ74739.1 acar|OOF95737.1 acla|EAW10664.1 afis|EAW24902.1 afla|EED46982.1 afum|EAL89556.1 agla|OJJ82127.1 akaw|GAA83671.1 aluc|OJZ92320.1 anid|CBF75644.1 anig|EHA26650.1 aory|EIT72548.1 asyd|OJJ57474.1 ater|EAU36397.1 atub|OJI86749.1 aver|OJJ05707.1 awen|OJJ40496.1 azon|OJJ49358.1

Group3500: abra|OJJ74749.1 acar|OOF95742.1 acla|EAW10661.1 afis|EAW24906.1 afla|EED46989.1 afum|EAL89559.1 agla|OJJ82130.1 akaw|GAA83662.1 aluc|OJZ92330.1 anid|CBF75661.1 anig|EHA26641.1 aory|EIT72764.1 asyd|OJJ57478.1 ater|EAU36392.1 atub|OJI86759.1 aver|OJJ05703.1 awen|OJJ40499.1 azon|OJJ49361.1

Group3501: abra|OJJ74750.1 acar|OOF95743.1 acla|EAW10660.1 afis|EAW24907.1 afla|EED46991.1 afum|EAL89560.1 agla|OJJ82131.1 akaw|GAA83661.1 aluc|OJZ92331.1 anid|CBF75665.1 anig|EHA26640.1 aory|EIT72700.1 asyd|OJJ57479.1 ater|EAU36391.1 atub|OJI86760.1 aver|OJJ05702.1 awen|OJJ40500.1 azon|OJJ49362.1

Group3502: abra|OJJ74751.1 acar|OOF95744.1 acla|EAW10659.1 afis|EAW24908.1 afla|EED46992.1 afum|EAL89561.1 agla|OJJ82132.1 akaw|GAA83660.1 aluc|OJZ92332.1 anid|CBF75667.1 anig|EHA26639.1 aory|EIT72681.1 asyd|OJJ57480.1 ater|EAU36390.1 atub|OJI86761.1 aver|OJJ05701.1 awen|OJJ40501.1 azon|OJJ49363.1

Group3503: abra|OJJ74752.1 acar|OOF95745.1 acla|EAW10658.1 afis|EAW24909.1 afla|EED46994.1 afum|EAL89562.1 agla|OJJ82133.1 akaw|GAA83659.1 aluc|OJZ92333.1 anid|CBF75669.1 anig|EHA26638.1 aory|EIT72691.1 asyd|OJJ57481.1 ater|EAU36389.1 atub|OJI86762.1 aver|OJJ05700.1 awen|OJJ40503.1 azon|OJJ49364.1

Group3504: abra|OJJ74754.1 acar|OOF95747.1 acla|EAW10656.1 afis|EAW24911.1 afla|EED46996.1 afum|EAL89565.1 agla|OJJ82135.1 akaw|GAA83657.1 aluc|OJZ92335.1 anid|CBF75671.1 anig|EHA26636.1 aory|EIT72517.1 asyd|OJJ57483.1 ater|EAU36387.1 atub|OJI86764.1 aver|OJJ05698.1 awen|OJJ40506.1 azon|OJJ49366.1

Group3505: abra|OJJ74756.1 acar|OOF95749.1 acla|EAW10655.1 afis|EAW24912.1 afla|EED46997.1 afum|EAL89566.2 agla|OJJ82141.1 akaw|GAA83655.1 aluc|OJZ92338.1 anid|CBF75673.1 anig|EHA26634.1 aory|EIT72758.1 asyd|OJJ57484.1 ater|EAU36386.1 atub|OJI86766.1 aver|OJJ05697.1 awen|OJJ40508.1 azon|OJJ49367.1

Group3506: abra|OJJ74758.1 acar|OOF95751.1 acla|EAW10653.1 afis|EAW24914.1 afla|EED46999.1 afum|EAL89568.1 agla|OJJ82142.1 akaw|GAA83653.1 aluc|OJZ92342.1 anid|CBF75677.1 anig|EHA26632.1 aory|EIT72541.1 asyd|OJJ57486.1 ater|EAU36384.1 atub|OJI86768.1 aver|OJJ05694.1 awen|OJJ40510.1 azon|OJJ49369.1

Group3507: abra|OJJ74759.1 acar|OOF95752.1 acla|EAW10652.1 afis|EAW24915.1 afla|EED47000.1 afum|EAL89569.1 agla|OJJ82143.1 akaw|GAA83652.1 aluc|OJZ92343.1 anid|CBF75679.1 anig|EHA26631.1 aory|EIT72710.1 asyd|OJJ57487.1 ater|EAU36383.1 atub|OJI86769.1 aver|OJJ05693.1 awen|OJJ40511.1 azon|OJJ49370.1

Group3508: abra|OJJ74760.1 acar|OOF95753.1 acla|EAW10651.1 afis|EAW24916.1 afla|EED47001.1 afum|EAL89571.1 agla|OJJ82144.1 akaw|GAA83651.1 aluc|OJZ92344.1 anid|CBF75681.1 anig|EHA26630.1 aory|EIT72621.1 asyd|OJJ57488.1 ater|EAU36382.1 atub|OJI86770.1 aver|OJJ05692.1 awen|OJJ40512.1 azon|OJJ49371.1

Group3509: abra|OJJ74762.1 acar|OOF95755.1 acla|EAW10649.1 afis|EAW24918.1 afla|EED47003.1 afum|EAL89573.1 agla|OJJ82147.1 akaw|GAA83649.1 aluc|OJZ92346.1 anid|CBF75687.1 anig|EHA26628.1 aory|EIT72583.1 asyd|OJJ57490.1 ater|EAU36380.1 atub|OJI86773.1 aver|OJJ05690.1 awen|OJJ40515.1 azon|OJJ49374.1

Group3510: abra|OJJ74763.1 acar|OOF95756.1 acla|EAW10647.1 afis|EAW24919.1 afla|EED47004.1 afum|EAL89574.1 agla|OJJ82148.1 akaw|GAA83648.1 aluc|OJZ92347.1 anid|CBF75689.1 anig|EHA26627.1 aory|EIT72654.1 asyd|OJJ57491.1 ater|EAU36379.1 atub|OJI86774.1 aver|OJJ05689.1 awen|OJJ40516.1 azon|OJJ49375.1

Group3511: abra|OJJ74766.1 acar|OOF95758.1 acla|EAW10645.1 afis|EAW24921.1 afla|EED55552.1 afum|EAL89576.1 agla|OJJ82150.1 akaw|GAA83646.1 aluc|OJZ92349.1 anid|CBF75693.1 anig|EHA26625.1 aory|EIT82689.1 asyd|OJJ57493.1 ater|EAU35653.1 atub|OJI86777.1 aver|OJJ05687.1 awen|OJJ40518.1 azon|OJJ49377.1

Group3512: abra|OJJ74767.1 acar|OOF95759.1 acla|EAW10644.1 afis|EAW24922.1 afla|EED55551.1 afum|EAL89577.2 agla|OJJ82151.1 akaw|GAA83645.1 aluc|OJZ92350.1 anid|CBF75695.1 anig|EHA26624.1 aory|EIT82687.1 asyd|OJJ57494.1 ater|EAU35654.1 atub|OJI86778.1 aver|OJJ05686.1 awen|OJJ40519.1 azon|OJJ49378.1

Group3513: abra|OJJ74772.1 acar|OOF95762.1 acla|EAW10639.1 afis|EAW24927.1 afla|EED55546.1 afum|EAL89583.1 agla|OJJ82155.1 akaw|GAA83640.1 aluc|OJZ92353.1 anid|CBF75704.1 anig|EHA26621.1 aory|EIT82681.1 asyd|OJJ57497.1 ater|EAU35657.1 atub|OJI86783.1 aver|OJJ05681.1 awen|OJJ40523.1 azon|OJJ49383.1

Group3514: abra|OJJ74774.1 acar|OOF95763.1 acla|EAW10637.1 afis|EAW24929.1 afla|EED55544.1 afum|EAL89585.1 agla|OJJ82157.1 akaw|GAA83638.1 aluc|OJZ92355.1 anid|CBF75708.1 anig|EHA26619.1 aory|EIT82710.1 asyd|OJJ57499.1 ater|EAU35659.1 atub|OJI86785.1 aver|OJJ05679.1 awen|OJJ40525.1 azon|OJJ49385.1

Group3515: abra|OJJ74775.1 acar|OOF95764.1 acla|EAW10636.1 afis|EAW24930.1 afla|EED55543.1 afum|EAL89586.1 agla|OJJ82158.1 akaw|GAA83637.1 aluc|OJZ92356.1 anid|CBF75710.1 anig|EHA26618.1 aory|EIT82674.1 asyd|OJJ57500.1 ater|EAU35660.1 atub|OJI86786.1 aver|OJJ05678.1 awen|OJJ40526.1 azon|OJJ49386.1

Group3516: abra|OJJ74776.1 acar|OOF95765.1 acla|EAW10635.1 afis|EAW24931.1 afla|EED55542.1 afum|EAL89587.1 agla|OJJ82159.1 akaw|GAA83636.1 aluc|OJZ92357.1 anid|CBF75712.1 anig|EHA26617.1 aory|EIT82695.1 asyd|OJJ57502.1 ater|EAU35661.1 atub|OJI86787.1 aver|OJJ05676.1 awen|OJJ40527.1 azon|OJJ49387.1

Group3517: abra|OJJ74779.1 acar|OOF95769.1 acla|EAW10631.1 afis|EAW24936.1 afla|EED55536.1 afum|EAL89593.1 agla|OJJ82163.1 akaw|GAA83633.1 aluc|OJZ92360.1 anid|CBF75723.1 anig|EHA26613.1 aory|EIT80806.1 asyd|OJJ57506.1 ater|EAU35665.1 atub|OJI86790.1 aver|OJJ05672.1 awen|OJJ40531.1 azon|OJJ49391.1

Group3518: abra|OJJ74780.1 acar|OOF95771.1 acla|EAW10630.1 afis|EAW24937.1 afla|EED55535.1 afum|EAL89594.1 agla|OJJ82164.1 akaw|GAA83632.1 aluc|OJZ92361.1 anid|CBF75725.1 anig|EHA26612.1 aory|EIT80813.1 asyd|OJJ57507.1 ater|EAU35666.1 atub|OJI86791.1 aver|OJJ05671.1 awen|OJJ40532.1 azon|OJJ49392.1

Group3519: abra|OJJ74781.1 acar|OOF95772.1 acla|EAW10629.1 afis|EAW24938.1 afla|EED55534.1 afum|EAL89595.1 agla|OJJ82165.1 akaw|GAA83631.1 aluc|OJZ92362.1 anid|CBF75727.1 anig|EHA26611.1 aory|EIT80903.1 asyd|OJJ57508.1 ater|EAU35667.1 atub|OJI86792.1 aver|OJJ05670.1 awen|OJJ40533.1 azon|OJJ49393.1

Group3520: abra|OJJ74784.1 acar|OOF95774.1 acla|EAW10627.1 afis|EAW24940.1 afla|EED55525.1 afum|EAL89597.1 agla|OJJ82167.1 akaw|GAA83629.1 aluc|OJZ92364.1 anid|CBF75735.1 anig|EHA26609.1

aory|EIT80775.1 asyd|OJJ57512.1 ater|EAU35669.1 atub|OJI86794.1 aver|OJJ05667.1 awen|OJJ40536.1 azon|OJJ49395.1

Group3521: abra|OJJ74786.1 acar|OOF95776.1 acla|EAW10625.1 afis|EAW24942.1 afla|EED55523.1 afum|EAL89599.1 agla|OJJ82169.1 akaw|GAA83627.1 aluc|OJZ92366.1 anid|CBF75741.1 anig|EHA26607.1 aory|EIT80928.1 asyd|OJJ57517.1 ater|EAU35674.1 atub|OJI86796.1 aver|OJJ05662.1 awen|OJJ40537.1 azon|OJJ49397.1

Group3522: abra|OJJ74788.1 acar|OOF95777.1 acla|EAW10623.1 afis|EAW24944.1 afla|EED55521.1 afum|EAL89601.1 agla|OJJ82171.1 akaw|GAA83625.1 aluc|OJZ92368.1 anid|CBF75743.1 anig|EHA26605.1 aory|EIT80857.1 asyd|OJJ57519.1 ater|EAU35675.1 atub|OJI86798.1 aver|OJJ05659.1 awen|OJJ40539.1 azon|OJJ49399.1

Group3523: abra|OJJ74789.1 acar|OOF95779.1 acla|EAW10622.1 afis|EAW24945.1 afla|EED55520.1 afum|EAL89602.1 agla|OJJ82173.1 akaw|GAA83624.1 aluc|OJZ92369.1 anid|CBF75615.1 anig|EHA26604.1 aory|EIT80768.1 asyd|OJJ57451.1 ater|EAU35676.1 atub|OJI86799.1 aver|OJJ05727.1 awen|OJJ40540.1 azon|OJJ49400.1

Group3524: abra|OJJ74790.1 acar|OOF95780.1 acla|EAW10621.1 afis|EAW24946.1 afla|EED55519.1 afum|EAL89603.1 agla|OJJ82174.1 akaw|GAA83623.1 aluc|OJZ92370.1 anid|CBF81729.1 anig|EHA26603.1 aory|EIT80898.1 asyd|OJJ59591.1 ater|EAU35678.1 atub|OJI86800.1 aver|OJJ02678.1 awen|OJJ40541.1 azon|OJJ49401.1

Group3525: abra|OJJ74791.1 acar|OOF95781.1 acla|EAW10620.1 afis|EAW24947.1 afla|EED55518.1 afum|EAL89604.1 agla|OJJ82175.1 akaw|GAA83622.1 aluc|OJZ92371.1 anid|CBF81728.1 anig|EHA26602.1 aory|EIT80815.1 asyd|OJJ59590.1 ater|EAU35679.1 atub|OJI86801.1 aver|OJJ02679.1 awen|OJJ40542.1 azon|OJJ49402.1

Group3526: abra|OJJ74792.1 acar|OOF95782.1 acla|EAW10619.1 afis|EAW24948.1 afla|EED55517.1 afum|EAL89605.1 agla|OJJ82176.1 akaw|GAA83621.1 aluc|OJZ92372.1 anid|CBF81726.1 anig|EHA26601.1 aory|EIT80913.1 asyd|OJJ59589.1 ater|EAU35680.1 atub|OJI86802.1 aver|OJJ02680.1 awen|OJJ40543.1 azon|OJJ49403.1

Group3527: abra|OJJ74797.1 acar|OOF95789.1 acla|EAW10609.1 afis|EAW24958.1 afla|EED55505.1 afum|EAL89616.1 agla|OJJ82189.1 akaw|GAA83615.1 aluc|OJZ92378.1 anid|CBF81663.1 anig|EHA26595.1 aory|EIT80867.1 asyd|OJJ59551.1 ater|EAU35697.1 atub|OJI86808.1 aver|OJJ02717.1 awen|OJJ40552.1 azon|OJJ49411.1

Group3528: abra|OJJ74802.1 acar|OOF95795.1 acla|EAW10614.1 afis|EAW24953.1 afla|EED55511.1 afum|EAL89610.1 agla|OJJ82184.1 akaw|GAA83611.1 aluc|OJZ92383.1 anid|CBF81717.1 anig|EHA26592.1 aory|EIT80894.1 asyd|OJJ59584.1 ater|EAU35685.1 atub|OJI86812.1 aver|OJJ02686.1 awen|OJJ40548.1 azon|OJJ49408.1

Group3529: abra|OJJ74804.1 acar|OOF95796.1 acla|EAW10616.1 afis|EAW24951.1 afla|EED55513.1 afum|EAL89608.1 agla|OJJ82187.1 akaw|GAA83609.1 aluc|OJZ92385.1 anid|CBF81721.1 anig|EHA26590.1 aory|EIT80836.1 asyd|OJJ59586.1 atub|OJI86814.1 aver|OJJ02683.1 awen|OJJ40546.1 azon|OJJ49406.1 ater|EAU35683.1

Group3530: abra|OJJ74805.1 acar|OOF95797.1 acla|EAW10617.1 afis|EAW24950.1 afla|EED55514.1 afum|EAL89607.1 agla|OJJ82179.1 akaw|GAA83608.1 aluc|OJZ92386.1 anid|CBF81722.1 anig|EHA26589.1 aory|EIT80921.1 asyd|OJJ59587.1 ater|EAU35682.1 atub|OJI86815.1 aver|OJJ02682.1 awen|OJJ40545.1 azon|OJJ49405.1

Group3531: abra|OJJ74806.1 acar|OOF95798.1 acla|EAW10618.1 afis|EAW24949.1 afla|EED55516.1 afum|EAL89606.1 agla|OJJ82177.1 akaw|GAA83606.1 aluc|OJZ92387.1 anid|CBF81724.1 anig|EHA26588.1 aory|EIT80820.1 asyd|OJJ59588.1 ater|EAU35681.1 atub|OJI86817.1 aver|OJJ02681.1 awen|OJJ40544.1 azon|OJJ49404.1

Group3532: abra|OJJ74808.1 acar|OOF95800.1 acla|EAW10606.1 afis|EAW24961.1 afla|EED55501.1 afum|EAL89619.1 agla|OJJ82196.1 akaw|GAA83604.1 aluc|OJZ92389.1 anid|CBF81658.1 anig|EHA26585.1 aory|EIT80766.1 asyd|OJJ59548.1 ater|EAU35694.1 atub|OJI86819.1 aver|OJJ02720.1 awen|OJJ40554.1 azon|OJJ49416.1

Group3533: abra|OJJ74830.1 acar|OOF95817.1 acla|EAW11373.1 afis|EAW18122.1 afla|EED58048.1 afum|EAL87480.1 agla|OJJ87016.1 akaw|GAA83590.1 aluc|OJZ92407.1 anid|CBF75358.1 anig|EHA26558.1 aory|EIT79720.1 asyd|OJJ59851.1 ater|EAU29689.1 atub|OJI86835.1 aver|OJJ02436.1 awen|OJJ35524.1 azon|OJJ46961.1

Group3534: abra|OJJ74831.1 acar|OOF95819.1 acla|EAW11372.1 afis|EAW18123.1 afla|EED58049.1 afum|EAL87481.1 agla|OJJ87017.1 akaw|GAA83589.1 aluc|OJZ92408.1 anid|CBF80348.1 anig|EHA26557.1 aory|EIT79773.1 asyd|OJJ54832.1 ater|EAU29688.1 atub|OJI86836.1 aver|OJJ07329.1 awen|OJJ35525.1 azon|OJJ46959.1

Group3535: abra|OJJ74840.1 acar|OOF95843.1 acla|EAW09369.1 afis|EAW21766.1 afla|EED46332.1 afum|EAL92796.1 agla|OJJ84385.1 akaw|GAA83581.1 aluc|OJZ92416.1 anid|CBF83756.1 anig|EHA26550.1 aory|EIT81192.1 asyd|OJJ60860.1 ater|EAU38449.1 atub|OJI86846.1 aver|OJJ00185.1 awen|OJJ34049.1 azon|OJJ48182.1

Group3536: abra|OJJ74841.1 acar|OOF94168.1 acla|EAW08544.1 afis|EAW23581.1 afla|EED57489.1 afum|EAL87884.1 agla|OJJ86848.1 akaw|GAA83580.1 aluc|OJZ92417.1 anid|CBF89912.1 anig|EHA26549.1 aory|EIT81889.1 asyd|OJJ62129.1 ater|EAU35371.1 atub|OJI86847.1 aver|OJI96238.1 awen|OJJ39909.1 azon|OJJ45885.1

Group3537: abra|OJJ74847.1 acar|OOF95848.1 acla|EAW08541.1 afis|EAW23578.1 afla|EED57477.1 afum|EAL87887.1 agla|OJJ86843.1 akaw|GAA83575.1 aluc|OJZ92422.1 anid|CBF89598.1 anig|EHA26545.1 aory|EIT80110.1 asyd|OJJ62311.1 ater|EAU35366.1 atub|OJI86852.1 aver|OJI96419.1 awen|OJJ39906.1 azon|OJJ45883.1

Group3538: abra|OJJ74848.1 acar|OOF95849.1 acla|EAW08540.1 afis|EAW23577.1 afla|EED57476.1 afum|EAL87888.1 agla|OJJ86838.1 akaw|GAA83574.1 aluc|OJZ92423.1 anid|CBF89600.1 anig|EHA26544.1 aory|EIT80147.1 asyd|OJJ62310.1 ater|EAU35367.1 atub|OJI86853.1 aver|OJI96418.1 awen|OJJ39905.1 azon|OJJ45882.1

Group3539: abra|OJJ74852.1 acar|OOF95851.1 acla|EAW08535.1 afis|EAW23573.1 afla|EED50962.1 afum|EAL87892.2 agla|OJJ86833.1 akaw|GAA83570.1 aluc|OJZ92428.1 anid|CBF89610.1 anig|EHA26539.1 aory|EIT77015.1 asyd|OJJ62305.1 ater|EAU35343.1 atub|OJI86858.1 aver|OJI96413.1 awen|OJJ39901.1 azon|OJJ42316.1

Group3540: abra|OJJ74854.1 acar|OOF95854.1 acla|EAW08531.1 afis|EAW23570.1 afla|EED57463.1 afum|EAL87897.2 agla|OJJ86831.1 akaw|GAA83568.1 aluc|OJZ92430.1 anid|CBF89617.1 anig|EHA26536.1 aory|EIT80101.1 asyd|OJJ62302.1 ater|EAU35346.1 atub|OJI86860.1 aver|OJI96411.1 awen|OJJ39899.1 azon|OJJ45876.1

Group3541: abra|OJJ74856.1 acar|OOF95857.1 acla|EAW08528.1 afis|EAW23568.1 afla|EED57459.1 afum|EAL87899.1 agla|OJJ86829.1 akaw|GAA83566.1 aluc|OJZ92432.1 anid|CBF89621.1 anig|EHA26534.1 aory|EIT80063.1 asyd|OJJ62300.1 ater|EAU35349.1 atub|OJI86862.1 aver|OJI96408.1 awen|OJJ39896.1 azon|OJJ45874.1

Group3542: abra|OJJ74859.1 acar|OOF95859.1 acla|EAW08521.1 afis|EAW23559.1 afla|EED57454.1 afum|EAL87907.1 agla|OJJ86825.1 akaw|GAA83561.1 aluc|OJZ92437.1 anid|CBF89634.1 anig|EHA26531.1 aory|EIT80133.1 asyd|OJJ62295.1 ater|EAU35342.1 atub|OJI86866.1 aver|OJI96402.1 awen|OJJ39893.1 azon|OJJ43217.1

Group3543: abra|OJJ74860.1 acar|OOF95860.1 acla|EAW08520.1 afis|EAW23558.1 afla|EED57453.1 afum|EAL87908.1 agla|OJJ86826.1 akaw|GAA83560.1 aluc|OJZ92438.1 anid|CBF89636.1 anig|EHA26530.1 aory|EIT80071.1 asyd|OJJ62294.1 ater|EAU35341.1 atub|OJI86867.1 aver|OJI96401.1 awen|OJJ39891.1 azon|OJJ43218.1

Group3544: abra|OJJ74862.1 acar|OOF95862.1 acla|EAW08519.1 afis|EAW23557.1 afla|EED57452.1 afum|EAL87909.1 agla|OJJ86819.1 akaw|GAA83558.1 aluc|OJZ92440.1 anid|CBF89638.1 anig|EHA26527.1 aory|EIT80146.1 asyd|OJJ62292.1 ater|EAU35340.1 atub|OJI86869.1 aver|OJI96400.1 awen|OJJ39888.1 azon|OJJ43219.1

Group3545: abra|OJJ74867.1 acar|OOF95868.1 acla|EAW08513.1 afis|EAW23551.1 afla|EED57446.1 afum|EAL87915.1 agla|OJJ86807.1 akaw|GAA83553.1 aluc|OJZ92445.1 anid|CBF89646.1 anig|EHA26523.1 aory|EIT80111.1 asyd|OJJ62286.1 ater|EAU35335.1 atub|OJI86873.1 aver|OJI96394.1 awen|OJJ39882.1 azon|OJJ43223.1

Group3546: abra|OJJ74869.1 acar|OOF95870.1 acla|EAW08511.1 afis|EAW23549.1 afla|EED57444.1 afum|EAL87917.2 agla|OJJ86805.1 akaw|GAA83551.1 aluc|OJZ92447.1 anid|CBF89650.1 anig|EHA26521.1 aory|EIT80076.1 asyd|OJJ62284.1 ater|EAU35334.1 atub|OJI86875.1 aver|OJI96392.1 awen|OJJ39880.1 azon|OJJ43224.1

Group3547: abra|OJJ74872.1 acar|OOF95873.1 acla|EAW08508.1 afis|EAW23546.1 afla|EED57441.1 afum|EAL87920.1 agla|OJJ86800.1 akaw|GAA83548.1 aluc|OJZ92450.1 anid|CBF89654.1 anig|EHA26518.1 aory|EIT80061.1 asyd|OJJ62282.1 ater|EAU35331.1 atub|OJI86878.1 aver|OJI96390.1 awen|OJJ39877.1 azon|OJJ43226.1

Group3548: abra|OJJ74874.1 acar|OOF95875.1 acla|EAW08498.1 afis|EAW23543.1 afla|EED57440.1 afum|EAL87923.2 agla|OJJ86797.1 akaw|GAA83546.1 aluc|OJZ92452.1 anid|CBF89657.1 anig|EHA26516.1 aory|EIT80096.1 asyd|OJJ62279.1 ater|EAU35329.1 atub|OJI86880.1 aver|OJI96388.1 awen|OJJ39875.1 azon|OJJ43227.1

Group3549: abra|OJJ74877.1 acar|OOF95880.1 acla|EAW08493.1 afis|EAW23539.1 afla|EED57437.1 afum|EAL87927.1 agla|OJJ86794.1 akaw|GAA83543.1 aluc|OJZ92454.1 anid|CBF89665.1 anig|EHA26513.1

aory|EIT80117.1 asyd|OJJ62275.1 ater|EAU35325.1 atub|OJI86883.1 aver|OJI96384.1 awen|OJJ39870.1 azon|OJJ43231.1

Group3550: abra|OJJ74879.1 acar|OOF95882.1 acla|EAW08491.1 afis|EAW23537.1 afla|EED57435.1 afum|EAL87929.1 agla|OJJ86792.1 akaw|GAA83541.1 aluc|OJZ92456.1 anid|CBF89668.1 anig|EHA26511.1 aory|EIT80166.1 asyd|OJJ62273.1 ater|EAU35323.1 atub|OJI86885.1 aver|OJI96382.1 awen|OJJ39867.1 azon|OJJ43233.1

Group3551: abra|OJJ74881.1 acar|OOF95884.1 acla|EAW08489.1 afis|EAW23535.1 afla|EED57432.1 afum|EAL87931.1 agla|OJJ86790.1 akaw|GAA83539.1 aluc|OJZ92458.1 anid|CBF89672.1 anig|EHA26509.1 aory|EIT80162.1 asyd|OJJ62271.1 ater|EAU35321.1 atub|OJI86887.1 aver|OJI96380.1 awen|OJJ39865.1 azon|OJJ43235.1

Group3552: abra|OJJ74882.1 acar|OOF95886.1 acla|EAW08487.1 afis|EAW23533.1 afla|EED57430.1 afum|EAL87933.1 agla|OJJ86782.1 akaw|GAA83537.1 aluc|OJZ92459.1 anid|CBF89678.1 anig|EHA26508.1 aory|EIT80077.1 asyd|OJJ62269.1 ater|EAU35319.1 atub|OJI86888.1 aver|OJI96378.1 awen|OJJ39862.1 azon|OJJ43236.1

Group3553: abra|OJJ74884.1 akaw|GAA83535.1 aluc|OJZ92461.1 anig|EHA26506.1 atub|OJI86890.1 acar|OOF95889.1 acla|EAW08483.1 afis|EAW23529.1 afla|EED57428.1 afum|EAL87937.1 agla|OJJ86778.1 anid|CBF89683.1 asyd|OJJ62266.1 ater|EAU35317.1 aver|OJI96375.1 awen|OJJ39858.1 azon|OJJ43239.1 aory|EIT80152.1

Group3554: abra|OJJ74888.1 acar|OOF95893.1 acla|EAW08479.1 afis|EAW23525.1 afla|EED57424.1 afum|EAL87940.1 agla|OJJ86774.1 akaw|GAA83531.1 aluc|OJZ92465.1 anid|CBF89691.1 anig|EHA26502.1 aory|EIT80080.1 asyd|OJJ62262.1 ater|EAU35313.1 atub|OJI86894.1 aver|OJI96371.1 awen|OJJ39854.1 azon|OJJ43245.1

Group3555: abra|OJJ74889.1 acar|OOF95892.1 acla|EAW08478.1 afis|EAW23524.1 afla|EED50993.1 afum|EAL87941.1 agla|OJJ86773.1 akaw|GAA83530.1 aluc|OJZ92466.1 anid|CBF76066.1 anig|EHA26501.1 aory|EIT76998.1 asyd|OJJ60396.1 ater|EAU31002.1 atub|OJI86895.1 aver|OJI99725.1 awen|OJJ39853.1 azon|OJJ50688.1

Group3556: abra|OJJ74897.1 acar|OOF95897.1 acla|EAW08475.1 afis|EAW23521.1 afum|EAL87943.1 agla|OJJ86768.1 akaw|GAA83524.1 aluc|OJZ92471.1 anig|EHA26497.1 ater|EAU35309.1 atub|OJI86901.1 awen|OJJ39846.1 afla|EED57421.1 aory|EIT80149.1 aver|OJI96367.1 azon|OJJ43248.1 anid|CBF89696.1 asyd|OJJ62258.1

Group3557: abra|OJJ74901.1 acar|OOF95900.1 acla|EAW08472.1 afis|EAW23518.1 afla|EED57418.1 afum|EAL87946.1 agla|OJJ86765.1 akaw|GAA83521.1 aluc|OJZ92474.1 anid|CBF89702.1 anig|EHA26495.1 aory|EIT80062.1 asyd|OJJ62254.1 atub|OJI86905.1 aver|OJI96364.1 awen|OJJ39843.1 azon|OJJ43250.1 ater|EAU35306.1

Group3558: abra|OJJ74905.1 acar|OOF90301.1 acla|EAW08467.1 afis|EAW23514.1 afla|EED57413.1 afum|EAL87949.1 agla|OJJ86762.1 akaw|GAA83517.1 aluc|OJZ92478.1 anig|EHA26492.1 aory|EIT80167.1 asyd|OJJ59973.1 ater|EAU35303.1 atub|OJI86909.1 aver|OJJ02318.1 awen|OJJ39839.1 azon|OJJ43253.1 anid|CBF75148.1

Group3559: abra|OJJ74907.1 acar|OOF90299.1 acla|EAW08465.1 afis|EAW23510.1 afla|EED57410.1 afum|EAL87951.1 agla|OJJ86760.1 akaw|GAA83515.1 aluc|OJZ92480.1 anid|CBF89718.1 anig|EHA26490.1 aory|EIT80124.1 asyd|OJJ62245.1 ater|EAU35298.1 atub|OJI86911.1 aver|OJI96355.1 awen|OJJ39836.1 azon|OJJ43256.1

Group3560: abra|OJJ74909.1 acar|OOF90294.1 acla|EAW08463.1 afis|EAW23508.1 afla|EED57408.1 afum|EAL87953.1 agla|OJJ86758.1 akaw|GAA83513.1 aluc|OJZ92482.1 anid|CBF89714.1 anig|EHA26489.1 aory|EIT80104.1 asyd|OJJ62247.1 ater|EAU35296.1 atub|OJI86913.1 aver|OJI96357.1 awen|OJJ39834.1 azon|OJJ43258.1

Group3561: abra|OJJ74910.1 acar|OOF90297.1 acla|EAW08462.1 afis|EAW23507.1 afla|EED57407.1 afum|EAL87954.1 agla|OJJ86757.1 akaw|GAA83512.1 aluc|OJZ92483.1 anid|CBF89712.1 anig|EHA26488.1 aory|EIT80089.1 asyd|OJJ62248.1 ater|EAU35295.1 atub|OJI86914.1 aver|OJI96358.1 awen|OJJ39833.1 azon|OJJ43259.1

Group3562: abra|OJJ74916.1 acar|OOF95908.1 acla|EAW08454.1 afis|EAW23501.1 afla|EED57399.1 afum|EAL87961.1 agla|OJJ86746.1 akaw|GAA83506.1 aluc|OJZ92488.1 anid|CBF89733.1 anig|EHA26484.1 aory|EIT80085.1 asyd|OJJ62233.1 ater|EAU35288.1 atub|OJI86921.1 aver|OJI96342.1 awen|OJJ39824.1 azon|OJJ47948.1

Group3563: abra|OJJ74919.1 acar|OOF95910.1 afis|EAW23497.1 afla|EED57397.1 afum|EAL87966.1 agla|OJJ86744.1 akaw|GAA83504.1 aluc|OJZ92490.1 anid|CBF89737.1 anig|EHA26482.1 aory|EIT80105.1 asyd|OJJ62231.1 ater|EAU35286.1 atub|OJI86923.1 aver|OJI96340.1 awen|OJJ39822.1 azon|OJJ47946.1 acla|EAW08452.1

Group3564: abra|OJJ74922.1 acar|OOF95912.1 acla|EAW08449.1 afis|EAW23494.1 afla|EED57394.1 afum|EAL87969.1 agla|OJJ86741.1 akaw|GAA83501.1 aluc|OJZ92493.1 anid|CBF89742.1 anig|EHA26479.1 aory|EIT80159.1 asyd|OJJ62228.1 ater|EAU35283.1 atub|OJI86926.1 aver|OJI96337.1 awen|OJJ39819.1 azon|OJJ47943.1

Group3565: abra|OJJ74923.1 acar|OOF95914.1 acla|EAW08448.1 afis|EAW23493.1 afla|EED57393.1 afum|EAL87970.1 agla|OJJ86740.1 akaw|GAA83500.1 aluc|OJZ92494.1 anid|CBF89744.1 anig|EHA26478.1 aory|EIT80132.1 asyd|OJJ62227.1 ater|EAU35282.1 atub|OJI86927.1 aver|OJI96336.1 awen|OJJ39818.1 azon|OJJ47941.1

Group3566: abra|OJJ74924.1 acar|OOF95915.1 acla|EAW08447.1 afis|EAW23492.1 afla|EED57391.1 afum|EAL87971.1 agla|OJJ86737.1 akaw|GAA83499.1 aluc|OJZ92495.1 anid|CBF89746.1 anig|EHA26477.1 aory|EIT80165.1 asyd|OJJ62225.1 ater|EAU35279.1 atub|OJI86928.1 aver|OJI96334.1 awen|OJJ39816.1 azon|OJJ47939.1

Group3567: abra|OJJ74925.1 acar|OOF95916.1 acla|EAW08446.1 afis|EAW23491.1 afla|EED57390.1 afum|EAL87972.1 agla|OJJ86736.1 akaw|GAA83498.1 aluc|OJZ92496.1 anid|CBF89747.1 anig|EHA26476.1 aory|EIT80058.1 asyd|OJJ62224.1 ater|EAU35278.1 atub|OJI86929.1 aver|OJI96333.1 awen|OJJ39815.1 azon|OJJ47938.1

Group3568: abra|OJJ74928.1 acar|OOF95917.1 acla|EAW08442.1 afis|EAW23487.1 afla|EED57386.1 afum|EAL87976.1 agla|OJJ86732.1 akaw|GAA83494.1 aluc|OJZ92499.1 anid|CBF89755.1 anig|EHA26473.1 aory|EIT80123.1 asyd|OJJ62220.1 ater|EAU35274.1 atub|OJI86932.1 aver|OJI96330.1 awen|OJJ39810.1 azon|OJJ47934.1

Group3569: abra|OJJ74930.1 acar|OOF95920.1 acla|EAW08439.1 afis|EAW23484.1 afla|EED57379.1 afum|EAL87979.1 agla|OJJ86720.1 akaw|GAA83491.1 aluc|OJZ92502.1 anid|CBF89760.1 anig|EHA26471.1 aory|EIT80056.1 asyd|OJJ62217.1 ater|EAU35271.1 atub|OJI86934.1 aver|OJI96328.1 awen|OJJ39808.1 azon|OJJ47929.1

Group3570: abra|OJJ74931.1 acar|OOF95921.1 acla|EAW08438.1 afis|EAW23483.1 afla|EED57378.1 afum|EAL87980.1 agla|OJJ86719.1 akaw|GAA83490.1 aluc|OJZ92503.1 anid|CBF89762.1 anig|EHA26470.1 aory|EIT80160.1 asyd|OJJ62216.1 ater|EAU36899.1 atub|OJI86935.1 aver|OJI96327.1 awen|OJJ39807.1 azon|OJJ47928.1

Group3571: abra|OJJ74932.1 acar|OOF95922.1 acla|EAW08437.1 afis|EAW23482.1 afla|EED57377.1 afum|EAL87982.1 agla|OJJ86718.1 akaw|GAA83488.1 aluc|OJZ92505.1 anid|CBF89764.1 anig|EHA26468.1 aory|EIT80168.1 asyd|OJJ62215.1 ater|EAU36900.1 atub|OJI86937.1 aver|OJI96326.1 awen|OJJ39806.1 azon|OJJ47927.1

Group3572: abra|OJJ74935.1 acar|OOF97377.1 acla|EAW08436.1 afis|EAW23481.1 afla|EED57376.1 afum|EAL87983.1 agla|OJJ86717.1 akaw|GAA83485.1 aluc|OJZ92508.1 anid|CBF89766.1 anig|EHA26465.1 aory|EIT80075.1 asyd|OJJ62214.1 ater|EAU36901.1 atub|OJI86941.1 aver|OJI96325.1 awen|OJJ39805.1 azon|OJJ47926.1

Group3573: abra|OJJ74938.1 acar|OOF97376.1 acla|EAW08433.1 afis|EAW23476.1 afla|EED57372.1 afum|EAL87989.2 agla|OJJ86715.1 akaw|GAA83482.1 aluc|OJZ92513.1 anid|CBF89771.1 anig|EHA26462.1 aory|EIT80114.1 asyd|OJJ62212.1 ater|EAU36904.1 atub|OJI86944.1 aver|OJI96323.1 awen|OJJ39802.1 azon|OJJ47923.1

Group3574: abra|OJJ74939.1 acar|OOF97375.1 acla|EAW08431.1 afis|EAW23474.1 afla|EED57370.1 afum|EAL87991.2 agla|OJJ86713.1 akaw|GAA83481.1 aluc|OJZ92514.1 anid|CBF89775.1 anig|EHA26461.1 aory|EIT80156.1 asyd|OJJ62209.1 ater|EAU36906.1 atub|OJI86945.1 aver|OJI96321.1 awen|OJJ39800.1 azon|OJJ47922.1

Group3575: abra|OJJ74941.1 acar|OOF97374.1 acla|EAW08429.1 afis|EAW23473.1 afla|EED57369.1 afum|EAL87993.1 agla|OJJ86712.1 akaw|GAA83480.1 aluc|OJZ92515.1 anid|CBF89777.1 anig|EHA26460.1 aory|EIT80066.1 asyd|OJJ62208.1 ater|EAU36907.1 atub|OJI86948.1 aver|OJI96320.1 awen|OJJ39799.1 azon|OJJ47920.1

Group3576: abra|OJJ74943.1 acar|OOF97372.1 acla|EAW08427.1 afis|EAW23471.1 afla|EED57367.1 afum|EAL87995.1 agla|OJJ86710.1 akaw|GAA83478.1 aluc|OJZ92517.1 anid|CBF89782.1 anig|EHA26458.1 aory|EIT80170.1 asyd|OJJ62205.1 ater|EAU36909.1 atub|OJI86950.1 aver|OJI96317.1 awen|OJJ39796.1 azon|OJJ47918.1

Group3577: abra|OJJ74945.1 acar|OOF97371.1 acla|EAW08426.1 afis|EAW23442.1 afla|EED57366.1 afum|EAL88021.1 agla|OJJ86709.1 akaw|GAA83477.1 aluc|OJZ92518.1 anid|CBF89784.1 anig|EHA26457.1 aory|EIT80053.1 asyd|OJJ62204.1 ater|EAU36910.1 atub|OJI86952.1 aver|OJI96316.1 awen|OJJ39790.1 azon|OJJ47917.1

Group3578: abra|OJJ74946.1 acar|OOF97370.1 acla|EAW08425.1 afis|EAW23444.1 afla|EED57365.1 afum|EAL88020.1 agla|OJJ86707.1 akaw|GAA83476.1 aluc|OJZ92519.1 anid|CBF89786.1 anig|EHA26456.1

aory|EIT80115.1 asyd|OJJ62203.1 ater|EAU36911.1 atub|OJI86953.1 aver|OJI96315.1 awen|OJJ39786.1 azon|OJJ47916.1

Group3579: abra|OJJ74947.1 acar|OOF97369.1 acla|EAW08424.1 afis|EAW23445.1 afla|EED57364.1 afum|EAL88019.1 agla|OJJ86706.1 akaw|GAA83475.1 aluc|OJZ92520.1 anid|CBF89788.1 anig|EHA26455.1 aory|EIT80057.1 asyd|OJJ62202.1 ater|EAU36912.1 atub|OJI86954.1 aver|OJI96314.1 awen|OJJ39785.1 azon|OJJ47915.1

Group3580: abra|OJJ74949.1 acar|OOF97367.1 acla|EAW08422.1 afis|EAW23447.1 afla|EED57362.1 afum|EAL88018.1 agla|OJJ86702.1 akaw|GAA83473.1 aluc|OJZ92522.1 anid|CBF89791.1 anig|EHA26453.1 aory|EIT80100.1 asyd|OJJ62200.1 ater|EAU36913.1 atub|OJI86956.1 aver|OJI96312.1 awen|OJJ39782.1 azon|OJJ47913.1

Group3581: abra|OJJ74952.1 acar|OOF97362.1 acla|EAW08421.1 afis|EAW23448.1 afla|EED57359.1 afum|EAL88017.1 agla|OJJ86701.1 akaw|GAA83471.1 aluc|OJZ92524.1 anid|CBF89793.1 anig|EHA26450.1 aory|EIT80142.1 asyd|OJJ62199.1 ater|EAU36914.1 atub|OJI86959.1 aver|OJI96311.1 awen|OJJ39781.1 azon|OJJ47912.1

Group3582: abra|OJJ74953.1 acar|OOF97360.1 acla|EAW08420.1 afis|EAW23449.1 afla|EED57358.1 afum|EBA27332.1 agla|OJJ86700.1 akaw|GAA83470.1 aluc|OJZ92525.1 anid|CBF89795.1 anig|EHA26449.1 aory|EIT80082.1 asyd|OJJ62198.1 ater|EAU36916.1 atub|OJI86961.1 awen|OJJ39780.1 azon|OJJ47911.1 aver|OJI96310.1

Group3583: abra|OJJ74954.1 acar|OOF97359.1 acla|EAW08418.1 afis|EAW23451.1 afla|EED57352.1 afum|EAL88015.1 agla|OJJ86699.1 akaw|GAA83469.1 aluc|OJZ92526.1 anid|CBF89799.1 anig|EHA26448.1 aory|EIT80134.1 asyd|OJJ62196.1 ater|EAU36917.1 atub|OJI86962.1 aver|OJI96308.1 awen|OJJ39778.1 azon|OJJ47908.1

Group3584: abra|OJJ74956.1 acar|OOF97357.1 acla|EAW08416.1 afis|EAW23453.1 afla|EED57350.1 afum|EAL88013.1 agla|OJJ86697.1 akaw|GAA83467.1 aluc|OJZ92528.1 anid|CBF89802.1 anig|EHA26447.1 aory|EIT80106.1 asyd|OJJ62194.1 ater|EAU36919.1 atub|OJI86964.1 aver|OJI96306.1 awen|OJJ39776.1 azon|OJJ47906.1

Group3585: abra|OJJ74958.1 acar|OOF97355.1 acla|EAW08413.1 afis|EAW23456.1 afla|EED57345.1 afum|EBA27330.1 akaw|GAA83464.1 aluc|OJZ92530.1 anid|CBF89808.1 anig|EHA26444.1 aory|EIT80113.1 asyd|OJJ62191.1 ater|EAU36922.1 atub|OJI86967.1 aver|OJI96301.1 awen|OJJ39773.1 azon|OJJ47902.1 agla|OJJ86693.1

Group3586: abra|OJJ74959.1 acar|OOF97354.1 acla|EAW08412.1 afis|EAW23457.1 afla|EED57344.1 afum|EBA27329.1 agla|OJJ86692.1 akaw|GAA83463.1 aluc|OJZ92531.1 anid|CBF89810.1 anig|EHA26443.1 aory|EIT80054.1 asyd|OJJ62190.1 ater|EAU36923.1 atub|OJI86968.1 aver|OJI96300.1 awen|OJJ39772.1 azon|OJJ47903.1

Group3587: abra|OJJ74965.1 acar|OOF97349.1 acla|EAW08409.1 afis|EAW23460.1 afla|EED57340.1 afum|EAL88006.1 agla|OJJ86689.1 akaw|GAA83461.1 aluc|OJZ92535.1 anid|CBF89820.1 anig|EHA26440.1 aory|EIT74102.1 asyd|OJJ62187.1 ater|EAU37473.1 atub|OJI86971.1 aver|OJI96296.1 awen|OJJ39770.1 azon|OJJ47900.1

Group3588: abra|OJJ74966.1 acar|OOF97348.1 acla|EAW08408.1 afis|EAW23461.1 afla|EED57339.1 afum|EAL88005.1 agla|OJJ86688.1 akaw|GAA83460.1 aluc|OJZ92536.1 anid|CBF89850.1 anig|EHA26439.1 aory|EIT74042.1 asyd|OJJ62186.1 ater|EAU37474.1 atub|OJI86972.1 aver|OJI96295.1 awen|OJJ39769.1 azon|OJJ47899.1

Group3589: abra|OJJ74967.1 acar|OOF97346.1 acla|EAW08407.1 afis|EAW23462.1 afla|EED57338.1 afum|EAL88004.1 agla|OJJ86687.1 akaw|GAA83459.1 aluc|OJZ92537.1 anid|CBF89848.1 anig|EHA26438.1 aory|EIT74136.1 asyd|OJJ62185.1 ater|EAU37475.1 atub|OJI86973.1 aver|OJI96294.1 awen|OJJ39768.1 azon|OJJ47898.1

Group3590: abra|OJJ74969.1 acar|OOF97345.1 acla|EAW08405.1 afis|EAW23464.1 afla|EED57335.1 afum|EAL88002.1 agla|OJJ86685.1 akaw|GAA83457.1 aluc|OJZ92539.1 anid|CBF89843.1 anig|EHA26437.1 aory|EIT74036.1 asyd|OJJ62183.1 ater|EAU37480.1 atub|OJI86975.1 aver|OJI96292.1 awen|OJJ39766.1 azon|OJJ47896.1

Group3591: abra|OJJ74975.1 acar|OOF97344.1 acla|EAW08404.1 afis|EAW23465.1 afla|EED57334.1 afum|EAL88001.1 agla|OJJ86684.1 akaw|GAA83456.1 aluc|OJZ92540.1 anid|CBF89841.1 anig|EHA26436.1 aory|EIT73981.1 asyd|OJJ62182.1 ater|EAU37481.1 atub|OJI86976.1 aver|OJI96291.1 awen|OJJ39765.1 azon|OJJ47895.1

Group3592: abra|OJJ74976.1 acar|OOF97343.1 acla|EAW08403.1 afis|EAW23466.1 afla|EED57333.1 afum|EAL88000.1 agla|OJJ86683.1 akaw|GAA83455.1 aluc|OJZ92541.1 anid|CBF89839.1 anig|EHA26435.1 aory|EIT74149.1 asyd|OJJ62181.1 ater|EAU37482.1 atub|OJI86977.1 aver|OJI96290.1 awen|OJJ39764.1 azon|OJJ47894.1

Group3593: abra|OJJ74980.1 acar|OOF97340.1 acla|EAW08400.1 afis|EAW23469.1 afla|EED57331.1 afum|EAL87997.1 agla|OJJ86680.1 akaw|GAA83452.1 aluc|OJZ92544.1 anid|CBF89833.1 anig|EHA26432.1 aory|EIT74122.1 asyd|OJJ62178.1 ater|EAU37485.1 atub|OJI86980.1 aver|OJI96287.1 awen|OJJ39760.1 azon|OJJ47889.1

Group3594: abra|OJJ74981.1 acar|OOF97339.1 acla|EAW08399.1 afis|EAW23470.1 afla|EED57330.1 afum|EAL87996.1 agla|OJJ86679.1 akaw|GAA83451.1 aluc|OJZ92545.1 anid|CBF89831.1 anig|EHA26431.1 aory|EIT74162.1 asyd|OJJ62177.1 ater|EAU37486.1 atub|OJI86981.1 aver|OJI96286.1 awen|OJJ39759.1 azon|OJJ47888.1

Group3595: abra|OJJ74983.1 acar|OOF97338.1 acla|EAW08397.1 afis|EAW23440.1 afla|EED57329.1 afum|EAL88022.1 agla|OJJ86677.1 akaw|GAA83449.1 aluc|OJZ92547.1 anid|CBF89828.1 anig|EHA26429.1 asyd|OJJ62169.1 ater|EAU37487.1 atub|OJI86983.1 aver|OJI96281.1 awen|OJJ39757.1 azon|OJJ47885.1 anid|CBF89830.1

Group3596: abra|OJJ74988.1 acar|OOF97333.1 acla|EAW08394.1 afis|EAW23437.1 afla|EED57327.1 afum|EAL88025.1 agla|OJJ86674.1 akaw|GAA83446.1 aluc|OJZ92550.1 anid|CBF89852.1 anig|EHA26427.1 aory|EIT74016.1 asyd|OJJ62176.1 ater|EAU37489.1 atub|OJI86987.1 aver|OJI96285.1 awen|OJJ39754.1 azon|OJJ47881.1

Group3597: abra|OJJ74991.1 acar|OOF97329.1 acla|EAW08392.1 afis|EAW23423.1 afla|EED57322.1 afum|EAL88033.1 agla|OJJ86673.1 akaw|GAA83444.1 aluc|OJZ92555.1 anid|CBF89824.1 anig|EHA26424.1 aory|EIT74106.1 asyd|OJJ62171.1 ater|EAU37494.1 atub|OJI86991.1 aver|OJI96283.1 awen|OJJ39753.1 azon|OJJ47880.1

Group3598: abra|OJJ74993.1 acar|OOF97326.1 acla|EAW08390.1 afis|EAW23419.1 afla|EED57321.1 afum|EAL88050.1 agla|OJJ86644.1 akaw|GAA83442.1 aluc|OJZ92559.1 anid|CBF89854.1 anig|EHA26421.1 aory|EIT74073.1 asyd|OJJ62161.1 ater|EAU37503.1 atub|OJI86995.1 aver|OJI96271.1 awen|OJJ39752.1 azon|OJJ47878.1

Group3599: abra|OJJ74996.1 acar|OOF97324.1 acla|EAW08388.1 afis|EAW23417.1 afla|EED57318.1 afum|EAL88052.2 agla|OJJ86634.1 akaw|GAA88620.1 aluc|OJZ92562.1 anid|CBF89861.1 anig|EHA26419.1 aory|EIT73973.1 asyd|OJJ62158.1 ater|EAU37505.1 atub|OJI86998.1 aver|OJI96268.1 awen|OJJ39750.1 azon|OJJ47876.1

Group3600: abra|OJJ74997.1 acar|OOF97323.1 acla|EAW08387.1 afis|EAW23416.1 afla|EED57317.1 afum|EAL88053.2 agla|OJJ86633.1 akaw|GAA88621.1 aluc|OJZ92563.1 anid|CBF89862.1 anig|EHA26418.1 aory|EIT74065.1 asyd|OJJ62157.1 ater|EAU37506.1 atub|OJI86999.1 aver|OJI96267.1 awen|OJJ39749.1 azon|OJJ47875.1

Group3601: abra|OJJ74999.1 acar|OOF97322.1 acla|EAW08386.1 afis|EAW23415.1 afla|EED57316.1 afum|EAL88054.1 agla|OJJ86632.1 akaw|GAA88622.1 aluc|OJZ92564.1 anid|CBF89864.1 anig|EHA26417.1 aory|EIT73976.1 asyd|OJJ62156.1 ater|EAU37507.1 atub|OJI87000.1 aver|OJI96266.1 awen|OJJ39748.1 azon|OJJ47874.1

Group3602: abra|OJJ75000.1 acar|OOF97321.1 acla|EAW08385.1 afis|EAW23414.1 afla|EED57315.1 afum|EAL88055.1 agla|OJJ86631.1 akaw|GAA88623.1 aluc|OJZ92565.1 anid|CBF89865.1 anig|EHA26416.1 aory|EIT74071.1 asyd|OJJ62155.1 ater|EAU37508.1 atub|OJI87001.1 aver|OJI96265.1 awen|OJJ39747.1 azon|OJJ47873.1

Group3603: abra|OJJ75002.1 acar|OOF90037.1 acla|EAW08383.1 afis|EAW23412.1 afla|EED57313.1 afum|EAL88057.1 agla|OJJ86629.1 akaw|GAA88625.1 aluc|OJZ92567.1 anid|CBF89869.1 anig|EHA26414.1 aory|EIT74133.1 asyd|OJJ62153.1 ater|EAU37510.1 atub|OJI87003.1 aver|OJI96263.1 awen|OJJ39745.1 azon|OJJ47871.1

Group3604: abra|OJJ75003.1 acar|OOF90036.1 acla|EAW08382.1 afis|EAW23411.1 afla|EED57312.1 afum|EAL88058.1 agla|OJJ86628.1 akaw|GAA88626.1 aluc|OJZ92568.1 anid|CBF89871.1 anig|EHA26413.1 aory|EIT74029.1 asyd|OJJ62152.1 ater|EAU37511.1 atub|OJI87004.1 aver|OJI96262.1 awen|OJJ39744.1 azon|OJJ47870.1

Group3605: abra|OJJ75004.1 acar|OOF90035.1 acla|EAW08381.1 afis|EAW23410.1 afla|EED57310.1 afum|EAL88060.1 agla|OJJ86627.1 akaw|GAA88627.1 aluc|OJZ92569.1 anig|EHA26412.1 aory|EIT74104.1 asyd|OJJ62151.1 ater|EAU37516.1 atub|OJI87005.1 aver|OJI96261.1 awen|OJJ39743.1 azon|OJJ47869.1 agla|OJJ86626.1

Group3606: abra|OJJ75005.1 acar|OOF97320.1 acla|EAW08380.1 afis|EAW23409.1 afla|EED57309.1 afum|EAL88061.2 agla|OJJ86625.1 akaw|GAA88628.1 aluc|OJZ92570.1 anid|CBF89873.1 anig|EHA26411.1 aory|EIT74087.1 asyd|OJJ62150.1 ater|EAU37515.1 atub|OJI87006.1 aver|OJI96260.1 awen|OJJ39742.1 azon|OJJ47868.1

Group3607: abra|OJJ75006.1 acar|OOF97319.1 acla|EAW08379.1 afis|EAW23408.1 afla|EED57308.1 afum|EAL88062.1 agla|OJJ86624.1 akaw|GAA88629.1 aluc|OJZ92571.1 anid|CBF89874.1 anig|EHA26410.1

aory|EIT74117.1 asyd|OJJ62149.1 ater|EAU37512.1 atub|OJI87007.1 aver|OJI96259.1 awen|OJJ39735.1 azon|OJJ47867.1

Group3608: abra|OJJ75007.1 acar|OOF97318.1 acla|EAW08378.1 afis|EAW23407.1 afla|EED57307.1 afum|EAL88063.2 agla|OJJ86623.1 akaw|GAA88630.1 aluc|OJZ92572.1 anid|CBF89876.1 anig|EHA26409.1 aory|EIT74022.1 asyd|OJJ62148.1 ater|EAU37513.1 atub|OJI87008.1 aver|OJI96258.1 awen|OJJ39734.1 azon|OJJ47866.1

Group3609: abra|OJJ75009.1 acar|OOF97317.1 acla|EAW08204.1 afis|EAW23238.1 afla|EED55718.1 afum|EAL88244.1 agla|OJJ86622.1 akaw|GAA88632.1 aluc|OJZ92574.1 anid|CBF89882.1 anig|EHA26407.1 aory|EIT83207.1 asyd|OJJ62146.1 ater|EAU37519.1 atub|OJI87010.1 aver|OJI96256.1 awen|OJJ39732.1 azon|OJJ47864.1

Group3610: abra|OJJ75010.1 acar|OOF97316.1 acla|EAW08205.1 afis|EAW23239.1 afla|EED55719.1 afum|EAL88243.1 agla|OJJ86620.1 akaw|GAA88633.1 aluc|OJZ92575.1 anid|CBF89884.1 anig|EHA26406.1 aory|EIT83209.1 asyd|OJJ62145.1 ater|EAU37520.1 atub|OJI87011.1 aver|OJI96255.1 awen|OJJ39731.1 azon|OJJ47863.1

Group3611: abra|OJJ75011.1 acar|OOF97315.1 acla|EAW08206.1 afis|EAW23240.1 afla|EED55720.1 afum|EAL88242.1 agla|OJJ86619.1 akaw|GAA88634.1 aluc|OJZ92576.1 anid|CBF89885.1 anig|EHA26405.1 aory|EIT83217.1 asyd|OJJ62144.1 ater|EAU37521.1 atub|OJI87012.1 aver|OJI96254.1 awen|OJJ39730.1 azon|OJJ47862.1

Group3612: abra|OJJ75012.1 acar|OOF97314.1 acla|EAW08207.1 afis|EAW23241.1 afla|EED55722.1 afum|EAL88241.1 agla|OJJ86618.1 akaw|GAA88635.1 aluc|OJZ92577.1 anid|CBF89887.1 anig|EHA26404.1 aory|EIT83231.1 asyd|OJJ62143.1 ater|EAU37522.1 atub|OJI87013.1 aver|OJI96253.1 awen|OJJ39729.1 azon|OJJ47861.1

Group3613: abra|OJJ75014.1 acar|OOF97312.1 acla|EAW08209.1 afis|EAW23243.1 afla|EED55724.1 afum|EAL88239.1 agla|OJJ86617.1 akaw|GAA88637.1 aluc|OJZ92579.1 anid|CBF89891.1 anig|EHA26402.1 aory|EIT83225.1 asyd|OJJ62140.1 ater|EAU37524.1 atub|OJI87015.1 aver|OJI96250.1 awen|OJJ39727.1 azon|OJJ47858.1

Group3614: abra|OJJ75022.1 acar|OOF89975.1 acla|EAW08214.1 afis|EAW23248.1 afla|EED55729.1 afum|EAL88233.1 agla|OJJ86609.1 akaw|GAA88642.1 aluc|OJZ92589.1 anid|CBF84108.1 anig|EHA26397.1 aory|EIT83221.1 asyd|OJJ57101.1 ater|EAU37530.1 atub|OJI87021.1 aver|OJJ03198.1 awen|OJJ39716.1 azon|OJJ47847.1

Group3615: abra|OJJ75025.1 acar|OOF97307.1 acla|EAW08217.1 afis|EAW23251.1 afla|EED55732.1 afum|EAL88230.1 agla|OJJ86603.1 akaw|GAA88645.1 aluc|OJZ92592.1 anid|CBF84103.1 anig|EHA26394.1 aory|EIT83210.1 asyd|OJJ57097.1 ater|EAU37549.1 atub|OJI87025.1 aver|OJJ03202.1 awen|OJJ39713.1 azon|OJJ47844.1

Group3616: abra|OJJ75026.1 acar|OOF97306.1 acla|EAW08218.1 afis|EAW23252.1 afla|EED55733.1 afum|EAL88229.1 agla|OJJ86602.1 akaw|GAA88646.1 aluc|OJZ92593.1 anid|CBF84101.1 anig|EHA26393.1 aory|EIT83216.1 asyd|OJJ57096.1 ater|EAU37548.1 atub|OJI87027.1 aver|OJJ03203.1 awen|OJJ39712.1 azon|OJJ47843.1

Group3617: abra|OJJ75027.1 acar|OOF97305.1 acla|EAW08219.1 afis|EAW23253.1 afla|EED55734.1 afum|EAL88228.1 agla|OJJ86601.1 akaw|GAA88647.1 aluc|OJZ92594.1 anid|CBF84100.1 anig|EHA26392.1 aory|EIT83226.1 asyd|OJJ57095.1 ater|EAU37547.1 atub|OJI87028.1 aver|OJJ03204.1 awen|OJJ39711.1 azon|OJJ47842.1

Group3618: abra|OJJ75030.1 acar|OOF97303.1 acla|EAW08221.1 afis|EAW23255.1 afla|EED55736.1 afum|EAL88226.1 agla|OJJ86599.1 akaw|GAA88649.1 aluc|OJZ92597.1 anid|CBF84096.1 anig|EHA26390.1 aory|EIT83204.1 asyd|OJJ57093.1 ater|EAU37544.1 atub|OJI87031.1 aver|OJJ03206.1 awen|OJJ39709.1 azon|OJJ47840.1

Group3619: abra|OJJ75031.1 acar|OOF97302.1 acla|EAW08222.1 afis|EAW23256.1 afla|EED55737.1 afum|EAL88225.1 agla|OJJ86598.1 akaw|GAA88650.1 aluc|OJZ92598.1 anid|CBF84092.1 anig|EHA26389.1 aory|EIT83229.1 asyd|OJJ57092.1 ater|EAU37543.1 atub|OJI87032.1 aver|OJJ03208.1 awen|OJJ39708.1 azon|OJJ47839.1

Group3620: abra|OJJ75032.1 acar|OOF97301.1 acla|EAW08223.1 afis|EAW23257.1 afla|EED55738.1 afum|EAL88224.1 agla|OJJ86597.1 akaw|GAA88651.1 aluc|OJZ92599.1 anid|CBF84094.1 anig|EHA26388.1 aory|EIT83202.1 asyd|OJJ57091.1 ater|EAU37542.1 atub|OJI87034.1 aver|OJJ03209.1 awen|OJJ39707.1 azon|OJJ47838.1

Group3621: abra|OJJ75033.1 acar|OOF97300.1 acla|EAW08224.1 afis|EAW23258.1 afla|EED55739.1 afum|EAL88223.1 agla|OJJ86596.1 akaw|GAA88652.1 aluc|OJZ92600.1 anid|CBF84116.1 anig|EHA26387.1 aory|EIT83200.1 asyd|OJJ57105.1 ater|EAU37541.1 atub|OJI87035.1 aver|OJJ03194.1 awen|OJJ39706.1 azon|OJJ47837.1

Group3622: abra|OJJ75034.1 acar|OOF97299.1 acla|EAW08225.1 afis|EAW23259.1 afla|EED55740.1 afum|EAL88222.1 agla|OJJ86595.1 akaw|GAA88653.1 aluc|OJZ92602.1 anid|CBF84117.1 anig|EHA26386.1 aory|EIT83218.1 asyd|OJJ57106.1 ater|EAU37540.1 atub|OJI87036.1 aver|OJJ03193.1 awen|OJJ39705.1 azon|OJJ47835.1

Group3623: abra|OJJ75035.1 acar|OOF97298.1 acla|EAW08226.1 afis|EAW23260.1 afla|EED55741.1 afum|EAL88221.1 agla|OJJ86594.1 akaw|GAA88654.1 aluc|OJZ92603.1 anid|CBF84119.1 anig|EHA26385.1 aory|EIT83223.1 asyd|OJJ57107.1 ater|EAU37539.1 atub|OJI87037.1 aver|OJJ03192.1 awen|OJJ39704.1 azon|OJJ47834.1

Group3624: abra|OJJ75036.1 acar|OOF97297.1 acla|EAW08231.1 afis|EAW23265.1 afla|EED55746.1 afum|EAL88215.1 agla|OJJ86587.1 akaw|GAA88655.1 aluc|OJZ92604.1 anid|CBF84128.1 anig|EHA26384.1 aory|EIT83198.1 asyd|OJJ57112.1 ater|EAU37536.1 atub|OJI87038.1 aver|OJJ03187.1 awen|OJJ39699.1 azon|OJJ47829.1

Group3625: abra|OJJ75038.1 acar|OOF97294.1 acla|EAW08229.1 afis|EAW23263.1 afla|EED55744.1 afum|EAL88217.1 agla|OJJ86589.1 akaw|GAA88657.1 aluc|OJZ92606.1 anid|CBF84124.1 anig|EHA26382.1 aory|EIT83214.1 asyd|OJJ57110.1 ater|EAU37534.1 atub|OJI87040.1 aver|OJJ03189.1 awen|OJJ39701.1 azon|OJJ47831.1

Group3626: abra|OJJ75039.1 acar|OOF97293.1 acla|EAW08227.1 afis|EAW23261.1 afla|EED55742.1 afum|EAL88219.1 agla|OJJ86591.1 akaw|GAA88658.1 aluc|OJZ92607.1 anid|CBF84123.1 anig|EHA26381.1 aory|EIT83212.1 asyd|OJJ57109.1 ater|EAU37538.1 atub|OJI87041.1 aver|OJJ03190.1 awen|OJJ39703.1 azon|OJJ47833.1

Group3627: abra|OJJ75040.1 acar|OOF97292.1 acla|EAW08228.1 afis|EAW23262.1 afla|EED55743.1 afum|EAL88218.1 agla|OJJ86590.1 akaw|GAA88659.1 aluc|OJZ92608.1 anid|CBF84121.1 anig|EHA26380.1 aory|EIT83233.1 asyd|OJJ57108.1 ater|EAU37537.1 atub|OJI87042.1 aver|OJJ03191.1 awen|OJJ39702.1 azon|OJJ47832.1

Group3628: abra|OJJ75046.1 acar|OOF89831.1 acla|EAW08235.1 afis|EAW23269.1 afla|EED55710.1 afum|EAL88210.2 agla|OJJ86583.1 akaw|GAA88663.1 aluc|OJZ92612.1 anid|CBF89550.1 anig|EHA26375.1 aory|EIT83205.1 asyd|OJJ62375.1 ater|EAU37553.1 atub|OJI87046.1 aver|OJI96509.1 awen|OJJ39692.1 azon|OJJ47825.1

Group3629: abra|OJJ75047.1 acar|OOF97288.1 acla|EAW08236.1 afis|EAW23270.1 afla|EED55709.1 afum|EAL88209.1 agla|OJJ86582.1 akaw|GAA88664.1 aluc|OJZ92613.1 anid|CBF89548.1 anig|EHA26374.1 aory|EIT83232.1 asyd|OJJ62376.1 ater|EAU37554.1 atub|OJI87047.1 aver|OJI96510.1 awen|OJJ39691.1 azon|OJJ47824.1

Group3630: abra|OJJ75048.1 acar|OOF97286.1 acla|EAW08237.1 afis|EAW23271.1 afla|EED55706.1 afum|EAL88208.1 agla|OJJ86578.1 akaw|GAA88665.1 aluc|OJZ92615.1 anid|CBF89546.1 anig|EHA26373.1 aory|EIT83206.1 asyd|OJJ62377.1 ater|EAU37556.1 atub|OJI87049.1 aver|OJI96511.1 awen|OJJ39687.1 azon|OJJ47823.1

Group3631: abra|OJJ75052.1 acar|OOF97284.1 acla|EAW08238.1 afis|EAW23272.1 afla|EED55705.1 afum|EAL88207.1 agla|OJJ86577.1 akaw|GAA88666.1 aluc|OJZ92618.1 anid|CBF89544.1 anig|EHA26370.1 aory|EIT77089.1 asyd|OJJ62379.1 ater|EAU37557.1 atub|OJI87052.1 aver|OJI96513.1 awen|OJJ39685.1 azon|OJJ47822.1

Group3632: abra|OJJ75056.1 acar|OOF97281.1 acla|EAW08241.1 afis|EAW23275.1 afla|EED55703.1 afum|EAL88204.1 agla|OJJ86573.1 akaw|GAA88669.1 aluc|OJZ92622.1 anid|CBF89539.1 anig|EHA26367.1 aory|EIT77072.1 asyd|OJJ62382.1 ater|EAU37560.1 atub|OJI87055.1 aver|OJI96516.1 awen|OJJ39682.1 azon|OJJ47819.1

Group3633: abra|OJJ75057.1 acar|OOF97279.1 acla|EAW08242.1 afis|EAW23276.1 afla|EED55701.1 afum|EAL88203.1 agla|OJJ86571.1 akaw|GAA88670.1 aluc|OJZ92626.1 anid|CBF89533.1 anig|EHA26364.1 aory|EIT77071.1 asyd|OJJ62384.1 ater|EAU37561.1 atub|OJI87056.1 aver|OJI96518.1 awen|OJJ39680.1 azon|OJJ47818.1

Group3634: abra|OJJ75064.1 acar|OOF97270.1 acla|EAW08245.1 afis|EAW23279.1 afla|EED55695.1 afum|EAL88200.1 agla|OJJ86566.1 akaw|GAA88676.1 aluc|OJZ92630.1 anid|CBF89527.1 anig|EHA26360.1 aory|EIT77073.1 asyd|OJJ62388.1 ater|EAU37592.1 atub|OJI87061.1 aver|OJI96521.1 awen|OJJ39676.1 azon|OJJ47816.1

Group3635: abra|OJJ75066.1 acar|OOF97268.1 acla|EAW08247.1 afis|EAW23281.1 afla|EED55694.1 afum|EAL88198.2 agla|OJJ86565.1 akaw|GAA88678.1 aluc|OJZ92632.1 anid|CBF89525.1 anig|EHA26359.1 aory|EIT77087.1 asyd|OJJ62389.1 ater|EAU37591.1 atub|OJI87063.1 aver|OJI96522.1 awen|OJJ39675.1 azon|OJJ47805.1

Group3636: abra|OJJ75069.1 acar|OOF97264.1 acla|EAW08250.1 afis|EAW23284.1 afla|EED55691.1 afum|EAL88195.1 agla|OJJ86562.1 akaw|GAA88681.1 aluc|OJZ92635.1 anid|CBF89520.1 anig|EHA26356.1

aory|EIT77082.1 asyd|OJJ62392.1 ater|EAU37588.1 atub|OJI87066.1 aver|OJI96525.1 awen|OJJ39672.1 azon|OJJ47802.1

Group3637: abra|OJJ75070.1 acar|OOF97265.1 acla|EAW08251.1 afis|EAW23285.1 afla|EED55690.1 afum|EAL88194.1 agla|OJJ86561.1 akaw|GAA88682.1 aluc|OJZ92636.1 anid|CBF89518.1 anig|EHA26355.1 aory|EIT77093.1 asyd|OJJ62393.1 ater|EAU37587.1 atub|OJI87067.1 aver|OJI96526.1 awen|OJJ39671.1 azon|OJJ47801.1

Group3638: abra|OJJ75077.1 acar|OOF97256.1 acla|EAW08287.1 afis|EAW23320.1 afla|EED55678.1 afum|EAL88156.2 agla|OJJ86545.1 akaw|GAA88690.1 aluc|OJZ92643.1 anid|CBF89504.1 anig|EHA26349.1 aory|EIT77070.1 asyd|OJJ62400.1 ater|EAU37581.1 atub|OJI87074.1 aver|OJI96533.1 awen|OJJ39665.1 azon|OJJ47793.1

Group3639: abra|OJJ75080.1 acar|OOF97253.1 acla|EAW08284.1 afis|EAW23317.1 afla|EED55676.1 afum|EBA27338.1 agla|OJJ86542.1 akaw|GAA88693.1 aluc|OJZ92646.1 anid|CBF89487.1 anig|EHA26346.1 aory|EIT77097.1 asyd|OJJ62404.1 ater|EAU35544.1 atub|OJI87077.1 aver|OJI96537.1 awen|OJJ39662.1 azon|OJJ47791.1

Group3640: abra|OJJ75081.1 acar|OOF97252.1 acla|EAW08283.1 afis|EAW23316.1 afla|EED55675.1 afum|EAL88161.2 agla|OJJ86541.1 akaw|GAA88694.1 aluc|OJZ92647.1 anid|CBF89489.1 anig|EHA26345.1 aory|EIT77095.1 asyd|OJJ62405.1 ater|EAU35545.1 atub|OJI87078.1 aver|OJI96538.1 awen|OJJ39661.1 azon|OJJ47789.1

Group3641: abra|OJJ75082.1 acar|OOF97251.1 acla|EAW08282.1 afis|EAW23315.1 afla|EED55674.1 afum|EAL88162.1 agla|OJJ86540.1 akaw|GAA88695.1 aluc|OJZ92648.1 anid|CBF89491.1 anig|EHA26344.1 aory|EIT77078.1 asyd|OJJ62406.1 ater|EAU35546.1 atub|OJI87079.1 aver|OJI96539.1 awen|OJJ39660.1 azon|OJJ47788.1

Group3642: abra|OJJ75084.1 acar|OOF97249.1 acla|EAW08280.1 afis|EAW23313.1 afla|EED55672.1 afum|EAL88164.1 agla|OJJ86538.1 akaw|GAA88697.1 aluc|OJZ92650.1 anid|CBF89495.1 anig|EHA26342.1 aory|EIT77083.1 asyd|OJJ62408.1 ater|EAU35548.1 atub|OJI87081.1 aver|OJI96541.1 awen|OJJ39658.1 azon|OJJ47786.1

Group3643: abra|OJJ75087.1 acar|OOF97246.1 acla|EAW08277.1 afis|EAW23310.1 afla|EED55665.1 afum|EAL88167.1 akaw|GAA88699.1 aluc|OJZ92653.1 anid|CBF89485.1 anig|EHA26341.1 asyd|OJJ62412.1 ater|EAU35551.1 atub|OJI87084.1 aver|OJI96546.1 awen|OJJ39655.1 azon|OJJ47783.1 agla|OJJ86535.1 aory|EIT81799.1

Group3644: abra|OJJ75089.1 acar|OOF97244.1 acla|EAW08275.1 afis|EAW23308.1 afla|EED55663.1 afum|EAL88169.1 agla|OJJ86533.1 akaw|GAA88701.1 aluc|OJZ92655.1 anid|CBF89482.1 anig|EHA26339.1 aory|EIT81804.1 asyd|OJJ62413.1 ater|EAU35553.1 atub|OJI87086.1 aver|OJI96548.1 awen|OJJ39653.1 azon|OJJ47781.1

Group3645: abra|OJJ75090.1 acar|OOF97243.1 acla|EAW08274.1 afis|EAW23307.1 afla|EED55662.1 afum|EAL88170.1 agla|OJJ86532.1 akaw|GAA88702.1 aluc|OJZ92656.1 anid|CBF89480.1 anig|EHA26338.1 aory|EIT81820.1 asyd|OJJ62414.1 ater|EAU35554.1 atub|OJI87087.1 aver|OJI96549.1 awen|OJJ39652.1 azon|OJJ47780.1

Group3646: abra|OJJ75091.1 acar|OOF97241.1 acla|EAW08273.1 afis|EAW23306.1 afla|EED55661.1 afum|EAL88171.1 agla|OJJ86531.1 akaw|GAA88703.1 aluc|OJZ92657.1 anid|CBF89478.1 anig|EHA26337.1 aory|EIT81795.1 asyd|OJJ62415.1 ater|EAU35569.1 atub|OJI87088.1 aver|OJI96550.1 awen|OJJ39590.1 azon|OJJ47779.1

Group3647: abra|OJJ75093.1 acar|OOF97239.1 acla|EAW08271.1 afis|EAW23304.1 afla|EED55659.1 afum|EAL88173.1 agla|OJJ86529.1 akaw|GAA88705.1 anid|CBF89474.1 anig|EHA26335.1 aory|EIT81791.1 asyd|OJJ62418.1 ater|EAU35568.1 atub|OJI87090.1 aver|OJI96553.1 awen|OJJ39586.1 azon|OJJ47766.1 aluc|OJZ92659.1

Group3648: abra|OJJ75094.1 acar|OOF97238.1 acla|EAW08270.1 afis|EAW23303.1 afla|EED55656.1 afum|EAL88175.1 agla|OJJ86528.1 akaw|GAA88706.1 aluc|OJZ92660.1 anid|CBF89472.1 anig|EHA26334.1 aory|EIT81787.1 asyd|OJJ62419.1 ater|EAU35567.1 atub|OJI87091.1 aver|OJI96555.1 awen|OJJ39585.1 azon|OJJ47767.1

Group3649: abra|OJJ75096.1 acar|OOF97236.1 acla|EAW08268.1 afis|EAW23301.1 afla|EED55653.1 afum|EAL88178.1 agla|OJJ86507.1 akaw|GAA88708.1 aluc|OJZ92662.1 anid|CBF89468.1 anig|EHA26332.1 aory|EIT81800.1 asyd|OJJ62421.1 ater|EAU35565.1 atub|OJI87093.1 aver|OJI96556.1 awen|OJJ39583.1 azon|OJJ47769.1

Group3650: abra|OJJ75102.1 acar|OOF97232.1 acla|EAW08266.1 afis|EAW23299.1 afla|EED55649.1 afum|EAL88180.1 agla|OJJ86511.1 akaw|GAA88711.1 aluc|OJZ92666.1 anid|CBF89465.1 anig|EHA26329.1 aory|EIT81776.1 asyd|OJJ62424.1 ater|EAU35561.1 atub|OJI87097.1 aver|OJI96561.1 awen|OJJ39581.1 azon|OJJ47771.1

Group3651: abra|OJJ75103.1 acar|OOF97231.1 acla|EAW08265.1 afis|EAW23298.1 afla|EED55648.1 afum|EAL88181.2 agla|OJJ86513.1 akaw|GAA88712.1 aluc|OJZ92668.1 anid|CBF89463.1 anig|EHA26328.1 aory|EIT81789.1 asyd|OJJ62425.1 ater|EAU35559.1 atub|OJI87098.1 aver|OJI96562.1 awen|OJJ39579.1 azon|OJJ47772.1

Group3652: abra|OJJ75106.1 acar|OOF97228.1 acla|EAW08262.1 afis|EAW23295.1 afla|EED55645.1 afum|EAL88183.1 agla|OJJ86516.1 akaw|GAA88715.1 aluc|OJZ92671.1 anid|CBF89459.1 anig|EHA26326.1 aory|EIT81783.1 asyd|OJJ62427.1 ater|EAU35558.1 atub|OJI87100.1 aver|OJI96564.1 awen|OJJ39576.1 azon|OJJ47775.1

Group3653: abra|OJJ75107.1 acar|OOF97227.1 acla|EAW08261.1 afis|EAW23294.1 afla|EED55644.1 afum|EAL88184.1 agla|OJJ86517.1 akaw|GAA88716.1 aluc|OJZ92672.1 anid|CBF89457.1 anig|EHA26325.1 aory|EIT81790.1 asyd|OJJ62428.1 ater|EAU35557.1 atub|OJI87101.1 aver|OJI96565.1 awen|OJJ39575.1 azon|OJJ47776.1

Group3654: abra|OJJ75109.1 acar|OOF97223.1 acla|EAW08315.1 afis|EAW23325.1 afla|EED55641.1 afum|EAL88154.1 agla|OJJ86520.1 akaw|GAA88718.1 aluc|OJZ92675.1 anid|CBF89453.1 anig|EHA26323.1 aory|EIT81818.1 asyd|OJJ62431.1 ater|EAU35573.1 atub|OJI87103.1 aver|OJI96567.1 awen|OJJ39569.1 azon|OJJ47762.1

Group3655: abra|OJJ75114.1 acar|OOF97217.1 acla|EAW08309.1 afis|EAW23330.1 afla|EED55637.1 afum|EAL88149.2 agla|OJJ86525.1 akaw|GAA88724.1 aluc|OJZ92681.1 anid|CBF89441.1 anig|EHA26318.1 aory|EIT81798.1 asyd|OJJ62439.1 ater|EAU35577.1 atub|OJI87109.1 aver|OJI96575.1 awen|OJJ39564.1 azon|OJJ47757.1

Group3656: abra|OJJ75115.1 acar|OOF97216.1 acla|EAW08308.1 afis|EAW23331.1 afla|EED55636.1 afum|EAL88148.1 agla|OJJ86503.1 akaw|GAA88725.1 aluc|OJZ92682.1 anid|CBF89439.1 anig|EHA26317.1 aory|EIT81819.1 asyd|OJJ62440.1 ater|EAU35578.1 atub|OJI87110.1 aver|OJI96576.1 awen|OJJ39563.1 azon|OJJ47756.1

Group3657: abra|OJJ75120.1 acar|OOF97213.1 acla|EAW08306.1 afis|EAW23332.1 afla|EED55634.1 afum|EAL88146.2 agla|OJJ86500.1 akaw|GAA88729.1 aluc|OJZ92687.1 anid|CBF89437.1 anig|EHA26313.1 aory|EIT81801.1 asyd|OJJ62442.1 ater|EAU35580.1 atub|OJI87115.1 aver|OJI96578.1 awen|OJJ39561.1 azon|OJJ47754.1

Group3658: abra|OJJ75122.1 acar|OOF97212.1 acla|EAW08305.1 afis|EAW23334.1 afla|EED55632.1 afum|EAL88144.1 agla|OJJ86499.1 akaw|GAA88730.1 aluc|OJZ92688.1 anid|CBF89435.1 anig|EHA26312.1 aory|EIT81824.1 asyd|OJJ62443.1 ater|EAU35581.1 atub|OJI87116.1 aver|OJI96579.1 awen|OJJ39560.1 azon|OJJ47753.1

Group3659: abra|OJJ75124.1 acar|OOF97210.1 acla|EAW08304.1 afis|EAW23336.1 afla|EED55630.1 afum|EAL88142.1 agla|OJJ86494.1 akaw|GAA88732.1 aluc|OJZ92690.1 anid|CBF89432.1 anig|EHA26310.1 aory|EIT81797.1 asyd|OJJ62444.1 ater|EAU35582.1 atub|OJI87118.1 aver|OJI96581.1 awen|OJJ39558.1 azon|OJJ47749.1

Group3660: abra|OJJ75126.1 acar|OOF97209.1 acla|EAW08303.1 afis|EAW23337.1 afla|EED55629.1 afum|EAL88141.1 agla|OJJ86493.1 akaw|GAA88733.1 aluc|OJZ92691.1 anid|CBF89430.1 anig|EHA26309.1 aory|EIT81788.1 asyd|OJJ62445.1 ater|EAU35583.1 atub|OJI87119.1 aver|OJI96582.1 awen|OJJ39557.1 azon|OJJ47750.1

Group3661: abra|OJJ75129.1 acar|OOF97207.1 acla|EAW08300.1 afis|EAW23340.1 afla|EED55626.1 afum|EAL88138.1 agla|OJJ86490.1 akaw|GAA88736.1 aluc|OJZ92694.1 anid|CBF89425.1 anig|EHA26306.1 aory|EIT81810.1 asyd|OJJ62448.1 ater|EAU35585.1 atub|OJI87122.1 aver|OJI96585.1 awen|OJJ39554.1 azon|OJJ47748.1

Group3662: abra|OJJ75130.1 acar|OOF97205.1 acla|EAW08298.1 afis|EAW23342.1 afla|EED55624.1 afum|EAL88136.2 agla|OJJ86486.1 akaw|GAA88737.1 aluc|OJZ92695.1 anid|CBF89422.1 anig|EHA26304.1 aory|EIT81811.1 asyd|OJJ62451.1 ater|EAU35586.1 atub|OJI87123.1 aver|OJI96586.1 awen|OJJ39552.1 azon|OJJ47747.1

Group3663: abra|OJJ75131.1 acar|OOF97204.1 acla|EAW08297.1 afis|EAW23343.1 afla|EED55623.1 afum|EAL88135.1 agla|OJJ86485.1 akaw|GAA88738.1 aluc|OJZ92696.1 anid|CBF89420.1 anig|EHA26302.1 aory|EIT81805.1 asyd|OJJ62452.1 ater|EAU35587.1 atub|OJI87124.1 aver|OJI96587.1 awen|OJJ39550.1 azon|OJJ47746.1

Group3664: abra|OJJ75133.1 acar|OOF97203.1 acla|EAW08294.1 afis|EAW23345.1 afla|EED55621.1 afum|EAL88132.1 agla|OJJ86482.1 akaw|GAA88740.1 aluc|OJZ92699.1 anid|CBF89412.1 anig|EHA26300.1 aory|EIT81825.1 asyd|OJJ62455.1 ater|EAU35589.1 atub|OJI87127.1 aver|OJI96590.1 awen|OJJ39547.1 azon|OJJ47744.1

Group3665: abra|OJJ75134.1 acar|OOF97202.1 acla|EAW08293.1 afis|EAW23346.1 afla|EED55620.1 afum|EAL88130.1 agla|OJJ86481.1 akaw|GAA88741.1 aluc|OJZ92700.1 anid|CBF74918.1 anig|EHA26299.1

aory|EIT81822.1 asyd|OJJ60098.1 ater|EAU35590.1 atub|OJI87128.1 aver|OJJ04164.1 awen|OJJ39545.1 azon|OJJ47743.1

Group3666: abra|OJJ75136.1 acar|OOF97201.1 acla|EAW08292.1 afis|EAW23347.1 afla|EED55619.1 afum|EAL88129.1 agla|OJJ86480.1 akaw|GAA88742.1 aluc|OJZ92702.1 anid|CBF74916.1 anig|EHA26298.1 aory|EIT81794.1 asyd|OJJ60099.1 ater|EAU35591.1 atub|OJI87129.1 aver|OJJ04163.1 awen|OJJ39544.1 azon|OJJ47742.1

Group3667: abra|OJJ75146.1 acar|OOF97192.1 acla|EAW08319.1 afis|EAW23354.1 afla|EED55604.1 afum|EAL88122.2 agla|OJJ86471.1 akaw|GAA88751.1 aluc|OJZ92711.1 anid|CBF74886.1 anig|EHA26288.1 aory|EIT82711.1 asyd|OJJ60107.1 ater|EAU35606.1 atub|OJI87137.1 aver|OJJ04155.1 awen|OJJ39532.1 azon|OJJ47729.1

Group3668: abra|OJJ75149.1 acar|OOF97188.1 acla|EAW08323.1 afis|EAW23358.1 afla|EED55608.1 afum|EAL88118.1 agla|OJJ86468.1 akaw|GAA88755.1 aluc|OJZ92715.1 anid|CBF74882.1 anig|EHA26285.1 aory|EIT81782.1 asyd|OJJ60110.1 ater|EAU35609.1 atub|OJI87141.1 aver|OJJ04152.1 awen|OJJ39529.1 azon|OJJ47725.1

Group3669: abra|OJJ75150.1 acar|OOF97187.1 acla|EAW08324.1 afis|EAW23359.1 afla|EED55610.1 afum|EBA27336.1 agla|OJJ86467.1 akaw|GAA88756.1 aluc|OJZ92716.1 anid|CBF74880.1 anig|EHA26283.1 aory|EIT81806.1 asyd|OJJ60111.1 ater|EAU35610.1 atub|OJI87142.1 aver|OJJ04151.1 awen|OJJ39528.1 azon|OJJ47724.1

Group3670: abra|OJJ75152.1 acar|OOF97185.1 acla|EAW08327.1 afis|EAW23362.1 afla|EED55596.1 afum|EAL88112.1 agla|OJJ86464.1 akaw|GAA88757.1 aluc|OJZ92720.1 anid|CBF74837.1 anig|EHA26280.1 aory|EIT82684.1 asyd|OJJ60134.1 ater|EAU35613.1 atub|OJI87145.1 aver|OJJ04128.1 awen|OJJ39525.1 azon|OJJ47721.1

Group3671: abra|OJJ75153.1 acar|OOF97184.1 acla|EAW08328.1 afis|EAW23363.1 afla|EED55595.1 afum|EBA27334.1 agla|OJJ86463.1 akaw|GAA88758.1 aluc|OJZ92721.1 anid|CBF74835.1 anig|EHA26279.1 aory|EIT82682.1 asyd|OJJ60135.1 ater|EAU35614.1 atub|OJI87146.1 aver|OJJ04127.1 awen|OJJ39524.1 azon|OJJ47720.1

Group3672: abra|OJJ75157.1 acar|OOF97182.1 acla|EAW08332.1 afis|EAW23367.1 afla|EED55590.1 afum|EAL88106.1 agla|OJJ86457.1 akaw|GAA88763.1 aluc|OJZ92727.1 anid|CBF74823.1 anig|EHA26274.1 aory|EIT82667.1 asyd|OJJ60140.1 ater|EAU35618.1 atub|OJI87151.1 aver|OJJ04122.1 awen|OJJ39520.1 azon|OJJ47716.1

Group3673: abra|OJJ75159.1 acar|OOF97179.1 acla|EAW08335.1 afis|EAW23370.1 afla|EED55587.1 afum|EAL88103.2 agla|OJJ86453.1 akaw|GAA88770.1 aluc|OJZ92729.1 anid|CBF74817.1 anig|EHA26272.1 aory|EIT82666.1 asyd|OJJ60143.1 ater|EAU35621.1 atub|OJI87153.1 aver|OJJ04120.1 awen|OJJ39513.1 azon|OJJ47712.1

Group3674: abra|OJJ75160.1 acar|OOF97178.1 acla|EAW08336.1 afis|EAW23371.1 afla|EED55586.1 afum|EAL88101.1 agla|OJJ86452.1 akaw|GAA88771.1 aluc|OJZ92730.1 anid|CBF74815.1 anig|EHA26271.1 aory|EIT82693.1 asyd|OJJ60144.1 ater|EAU35622.1 atub|OJI87154.1 aver|OJJ04119.1 awen|OJJ39512.1 azon|OJJ47711.1

Group3675: abra|OJJ75162.1 acar|OOF97176.1 acla|EAW08338.1 afis|EAW23373.1 afla|EED55585.1 afum|EAL88099.1 agla|OJJ86450.1 akaw|GAA88773.1 aluc|OJZ92732.1 anid|CBF74811.1 anig|EHA26269.1 aory|EIT82709.1 asyd|OJJ60146.1 ater|EAU35624.1 atub|OJI87156.1 aver|OJJ04117.1 awen|OJJ39510.1 azon|OJJ47709.1

Group3676: abra|OJJ75165.1 acar|OOF97173.1 acla|EAW08339.1 afis|EAW23374.1 afla|EED55584.1 afum|EAL88097.1 agla|OJJ86449.1 akaw|GAA88776.1 aluc|OJZ92736.1 anid|CBF74839.1 anig|EHA26266.1 aory|EIT82678.1 asyd|OJJ60133.1 ater|EAU35628.1 atub|OJI87160.1 aver|OJJ04129.1 awen|OJJ39509.1 azon|OJJ47708.1

Group3677: abra|OJJ75166.1 acar|OOF97172.1 acla|EAW08340.1 afis|EAW23375.1 afla|EED55583.1 afum|EAL88096.1 agla|OJJ86448.1 akaw|GAA88777.1 aluc|OJZ92737.1 anid|CBF74841.1 anig|EHA26265.1 aory|EIT82676.1 asyd|OJJ60132.1 ater|EAU35629.1 atub|OJI87161.1 aver|OJJ04130.1 awen|OJJ39508.1 azon|OJJ47707.1

Group3678: abra|OJJ75168.1 acar|OOF94606.1 acla|EAW08132.1 afis|EAW23169.1 afla|EED46952.1 afum|EAL88315.1 agla|OJJ89680.1 akaw|GAA88778.1 aluc|OJZ92738.1 anid|CBF74480.1 anig|EHA26264.1 aory|EIT72535.1 asyd|OJJ57708.1 ater|EAU36298.1 atub|OJI87163.1 aver|OJJ05456.1 awen|OJJ36614.1 azon|OJJ47599.1

Group3679: abra|OJJ75172.1 acar|OOF94604.1 acla|EAW13004.1 afis|EAW17664.1 afla|EED50493.1 afum|EAL91478.1 agla|OJJ79537.1 akaw|GAA88779.1 aluc|OJZ92740.1 anid|CBF90052.1 anig|EHA26263.1 aory|EIT82027.1 asyd|OJJ62053.1 ater|EAU31989.1 atub|OJI87165.1 aver|OJI96159.1 awen|OJJ37177.1 azon|OJJ50877.1

Group3680: abra|OJJ75177.1 acar|OOF94602.1 acla|EAW13001.1 afis|EAW17667.1 afla|EED50488.1 afum|EAL91481.1 agla|OJJ79539.1 akaw|GAA88782.1 aluc|OJZ92743.1 anid|CBF90043.1 anig|EHA26260.1 aory|EIT82124.1 asyd|OJJ62058.1 ater|EAU31980.1 atub|OJI87168.1 aver|OJI96164.1 awen|OJJ37174.1 azon|OJJ50873.1

Group3681: abra|OJJ75178.1 acar|OOF94601.1 acla|EAW13000.1 afis|EAW17668.1 afla|EED50487.1 afum|EAL91482.1 agla|OJJ79540.1 akaw|GAA88783.1 aluc|OJZ92744.1 anid|CBF90041.1 anig|EHA26259.1 aory|EIT82171.1 asyd|OJJ62059.1 ater|EAU31979.1 atub|OJI87169.1 aver|OJI96165.1 awen|OJJ37173.1 azon|OJJ50872.1

Group3682: abra|OJJ75179.1 acar|OOF94600.1 acla|EAW12999.1 afis|EAW17669.1 afla|EED50486.1 afum|EAL91483.1 agla|OJJ79541.1 akaw|GAA88784.1 aluc|OJZ92745.1 anid|CBF90039.1 anig|EHA26258.1 aory|EIT82129.1 asyd|OJJ62060.1 ater|EAU31978.1 atub|OJI87170.1 aver|OJI96166.1 awen|OJJ37172.1 azon|OJJ50871.1

Group3683: abra|OJJ75182.1 acar|OOF94598.1 acla|EAW12995.1 afis|EAW17671.1 afla|EED50482.1 afum|EAL91485.1 agla|OJJ79543.1 akaw|GAA88786.1 aluc|OJZ92746.1 anid|CBF90036.1 anig|EHA26257.1 aory|EIT82081.1 asyd|OJJ62061.1 ater|EAU31976.1 atub|OJI87173.1 aver|OJI96167.1 awen|OJJ37171.1 azon|OJJ50867.1

Group3684: abra|OJJ75186.1 acar|OOF94596.1 acla|EAW12991.1 afis|EAW17674.1 afla|EED50477.1 afum|EAL91487.1 agla|OJJ79515.1 akaw|GAA88788.1 aluc|OJZ92749.1 anid|CBF90029.1 anig|EHA26254.1 aory|EIT82056.1 asyd|OJJ62064.1 ater|EAU31972.1 atub|OJI87175.1 aver|OJI96170.1 awen|OJJ37167.1 azon|OJJ50863.1

Group3685: abra|OJJ75189.1 acar|OOF94594.1 acla|EAW12990.1 afis|EAW17675.1 afla|EED50476.1 afum|EAL91488.1 agla|OJJ79514.1 akaw|GAA88790.1 aluc|OJZ92752.1 anid|CBF90024.1 anig|EHA26252.1 aory|EIT82044.1 asyd|OJJ62067.1 ater|EAU31971.1 atub|OJI87177.1 aver|OJI96173.1 awen|OJJ37165.1 azon|OJJ50862.1

Group3686: abra|OJJ75190.1 acar|OOF94593.1 acla|EAW12989.1 afis|EAW17676.1 afla|EED50475.1 afum|EAL91489.1 agla|OJJ79500.1 akaw|GAA88791.1 aluc|OJZ92754.1 anid|CBF90022.1 anig|EHA26251.1 aory|EIT82153.1 asyd|OJJ62068.1 ater|EAU31970.1 atub|OJI87179.1 aver|OJI96174.1 awen|OJJ37164.1 azon|OJJ50860.1

Group3687: abra|OJJ75198.1 acar|OOF94588.1 acla|EAW12983.1 afis|EAW17681.1 afla|EED50470.1 afum|EAL91495.1 agla|OJJ79499.1 akaw|GAA91750.1 aluc|OJZ92761.1 anid|CBF90009.1 anig|EHA26245.1 aory|EIT82018.1 asyd|OJJ62073.1 ater|EAU31944.1 atub|OJI87205.1 aver|OJI96180.1 awen|OJJ37158.1 azon|OJJ50856.1

Group3688: abra|OJJ75199.1 acar|OOF94589.1 acla|EAW12982.1 afis|EAW17682.1 afla|EED50469.1 afum|EAL91496.1 agla|OJJ79498.1 akaw|GAA91751.1 aluc|OJZ92762.1 anid|CBF90007.1 anig|EHA26244.1 aory|EIT82107.1 asyd|OJJ62074.1 ater|EAU31945.1 atub|OJI87206.1 aver|OJI96181.1 awen|OJJ37157.1 azon|OJJ50855.1

Group3689: abra|OJJ75201.1 acar|OOF94587.1 acla|EAW12981.1 afis|EAW17683.1 afla|EED50468.1 afum|EAL91497.1 agla|OJJ79496.1 akaw|GAA91752.1 aluc|OJZ92763.1 anid|CBF90005.1 anig|EHA26243.1 aory|EIT82022.1 asyd|OJJ62075.1 ater|EAU31946.1 atub|OJI87207.1 aver|OJI96182.1 awen|OJJ37156.1 azon|OJJ50854.1

Group3690: abra|OJJ75203.1 acar|OOF94585.1 acla|EAW12979.1 afis|EAW17685.1 afla|EED50466.1 afum|EAL91499.1 agla|OJJ79494.1 akaw|GAA91754.1 aluc|OJZ92766.1 anid|CBF90002.1 anig|EHA26241.1 aory|EIT82013.1 asyd|OJJ62078.1 ater|EAU31948.1 atub|OJI87210.1 aver|OJI96185.1 awen|OJJ37154.1 azon|OJJ50852.1

Group3691: abra|OJJ75206.1 acar|OOF94583.1 acla|EAW12977.1 afis|EAW17687.1 afla|EED50464.1 afum|EAL91501.1 agla|OJJ79492.1 akaw|GAA91756.1 aluc|OJZ92768.1 anid|CBF89987.1 anig|EHA26239.1 asyd|OJJ56120.1 ater|EAU31950.1 atub|OJI87212.1 aver|OJJ05061.1 awen|OJJ37152.1 asyd|OJJ62085.1 aver|OJI96192.1

Group3692: abra|OJJ75207.1 acar|OOF94582.1 acla|EAW12976.1 afis|EAW17688.1 afla|EED50463.1 afum|EAL91502.1 agla|OJJ79491.1 akaw|GAA91757.1 aluc|OJZ92769.1 anid|CBF89985.1 anig|EHA26238.1 aory|EIT82159.1 asyd|OJJ62086.1 ater|EAU31951.1 atub|OJI87213.1 aver|OJI96193.1 awen|OJJ37151.1 azon|OJJ50848.1

Group3693: abra|OJJ75208.1 acar|OOF94581.1 acla|EAW12975.1 afis|EAW17689.1 afla|EED50455.1 afum|EAL91503.1 agla|OJJ79490.1 akaw|GAA91758.1 aluc|OJZ92770.1 anid|CBF89984.1 anig|EHA26237.1 aory|EIT82021.1 asyd|OJJ62087.1 ater|EAU31952.1 atub|OJI87214.1 aver|OJI96194.1 awen|OJJ37147.1 azon|OJJ50847.1

Group3694: abra|OJJ75212.1 acar|OOF94572.1 acla|EAW13091.1 afis|EAW17572.1 afla|EED49249.1 afum|EAL91386.1 agla|OJJ81386.1 akaw|GAA91762.1 aluc|OJZ92776.1 anid|CBF90252.1 anig|EHA26234.1

aory|EIT82180.1 asyd|OJJ61946.1 ater|EAU37009.1 atub|OJI87218.1 aver|OJI96038.1 awen|OJJ37275.1 azon|OJJ50975.1

Group3695: abra|OJJ75213.1 acar|OOF94571.1 acla|EAW13092.1 afis|EAW17571.1 afla|EED49250.1 afum|EBA27432.1 agla|OJJ81387.1 akaw|GAA91763.1 aluc|OJZ92777.1 anid|CBF90254.1 anig|EHA26233.1 aory|EIT82196.1 asyd|OJJ61945.1 ater|EAU37008.1 atub|OJI87219.1 aver|OJI96037.1 awen|OJJ37278.1 azon|OJJ50976.1

Group3696: abra|OJJ75214.1 acar|OOF94570.1 acla|EAW13093.1 afis|EAW17570.1 afla|EED49251.1 afum|EAL91383.1 agla|OJJ81390.1 akaw|GAA91764.1 aluc|OJZ92778.1 anid|CBF90256.1 anig|EHA26232.1 aory|EIT82218.1 asyd|OJJ61944.1 ater|EAU37007.1 atub|OJI87220.1 aver|OJI96036.1 awen|OJJ37281.1 azon|OJJ50977.1

Group3697: abra|OJJ75215.1 acar|OOF94569.1 acla|EAW13094.1 afis|EAW17569.1 afla|EED49252.1 afum|EAL91382.1 agla|OJJ81395.1 akaw|GAA91765.1 aluc|OJZ92779.1 anid|CBF90258.1 anig|EHA26231.1 aory|EIT82201.1 asyd|OJJ61943.1 ater|EAU37006.1 atub|OJI87221.1 awen|OJJ37282.1 azon|OJJ50978.1 aver|OJI96035.1

Group3698: abra|OJJ75227.1 acar|OOF94562.1 acla|EAW12254.1 afis|EAW16982.1 afla|EED48301.1 afum|EAL86509.1 agla|OJJ79789.1 akaw|GAA91773.1 aluc|OJZ92788.1 anid|CBF86682.1 anig|EHA26219.1 aory|EIT76292.1 asyd|OJJ64757.1 ater|EAU31794.1 atub|OJI87231.1 aver|OJI98859.1 awen|OJJ35994.1 azon|OJJ50263.1

Group3699: abra|OJJ75272.1 acar|OOF94531.1 acla|EAW12236.1 afis|EAW16991.1 afla|EED48964.1 afum|EAL86515.1 agla|OJJ81058.1 akaw|GAA91810.1 aluc|OJZ92832.1 anid|CBF87436.1 anig|EHA26177.1 asyd|OJJ59098.1 ater|EAU31737.1 atub|OJI87289.1 aver|OJJ01689.1 awen|OJJ35979.1 azon|OJJ50286.1 aory|EIT80025.1

Group3700: abra|OJJ75274.1 acar|OOF94529.1 acla|EAW12235.1 afis|EAW16993.1 afla|EED48956.1 afum|EAL86517.1 agla|OJJ79819.1 akaw|GAA91812.1 aluc|OJZ92834.1 anid|CBF87472.1 anig|EHA26175.1 aory|EIT80048.1 asyd|OJJ52939.1 ater|EAU31735.1 atub|OJI87291.1 aver|OJJ08906.1 awen|OJJ35982.1 azon|OJJ50283.1

Group3701: abra|OJJ75279.1 afis|EAW25673.1 afla|EED49857.1 akaw|GAA91817.1 aluc|OJZ92840.1 anid|CBF82114.1 anig|EHA26171.1 aory|EIT83389.1 asyd|OJJ54289.1 ater|EAU31659.1 atub|OJI87296.1 aver|OJJ05243.1 awen|OJJ40812.1 afla|EED48617.1 ater|EAU33458.1 aory|EIT73274.1 ater|EAU35345.1 ater|EAU35445.1

Group3702: abra|OJJ75284.1 acar|OOF94523.1 acla|EAW12234.1 afis|EAW16994.1 afla|EED48957.1 afum|EAL86518.2 agla|OJJ79820.1 akaw|GAA91822.1 aluc|OJZ92845.1 anid|CBF87470.1 anig|EHA26165.1 aory|EIT79980.1 asyd|OJJ52938.1 ater|EAU31734.1 atub|OJI87301.1 aver|OJJ08905.1 awen|OJJ35983.1 azon|OJJ50282.1

Group3703: abra|OJJ75307.1 acar|OOF94522.1 acla|EAW12233.1 afis|EAW16995.1 afla|EED48958.1 afum|EAL86519.2 agla|OJJ79822.1 akaw|GAA91991.1 aluc|OJZ92861.1 anid|CBF87468.1 anig|EHA26104.1 aory|EIT79977.1 asyd|OJJ52937.1 ater|EAU31733.1 atub|OJI87319.1 aver|OJJ08904.1 awen|OJJ35984.1 azon|OJJ50281.1

Group3704: abra|OJJ75309.1 acar|OOF94520.1 acla|EAW12231.1 afis|EAW16997.1 afla|EED48962.1 afum|EAL86522.2 agla|OJJ79824.1 akaw|GAA91993.1 aluc|OJZ92863.1 anid|CBF87465.1 anig|EHA26102.1 aory|EIT79990.1 asyd|OJJ52935.1 ater|EAU31731.1 atub|OJI87321.1 aver|OJJ08902.1 awen|OJJ35986.1 azon|OJJ42560.1

Group3705: abra|OJJ75310.1 acar|OOF94519.1 acla|EAW12230.1 afis|EAW16998.1 afla|EED48952.1 afum|EAL86523.1 agla|OJJ79825.1 akaw|GAA91994.1 aluc|OJZ92864.1 anid|CBF87476.1 anig|EHA26101.1 aory|EIT79981.1 asyd|OJJ52941.1 ater|EAU31729.1 atub|OJI87322.1 aver|OJJ08908.1 awen|OJJ35987.1 azon|OJJ50276.1

Group3706: abra|OJJ75314.1 acar|OOF94515.1 acla|EAW10816.1 afis|EAW24758.1 afla|EED56728.1 afum|EAL89400.1 agla|OJJ78831.1 akaw|GAA91998.1 aluc|OJZ92868.1 anid|CBF89399.1 anig|EHA26097.1 aory|EIT73833.1 asyd|OJJ62458.1 ater|EAU34827.1 atub|OJI87326.1 aver|OJI96593.1 awen|OJJ33340.1 azon|OJJ42602.1

Group3707: abra|OJJ75322.1 acar|OOF94504.1 acla|EAW12347.1 afis|EAW16741.1 afla|EED48943.1 afum|EAL86316.1 akaw|GAA92017.1 aluc|OJZ92883.1 anid|CBF87481.1 anig|EHA26077.1 aory|EIT80047.1 asyd|OJJ64592.1 ater|EAU31723.1 atub|OJI87342.1 aver|OJI98689.1 awen|OJJ34684.1 azon|OJJ47373.1 anid|CBF75776.1

Group3708: abra|OJJ75342.1 acar|OOF89833.1 afla|EED48926.1 agla|OJJ81063.1 akaw|GAA92035.1 aluc|OJZ92905.1 anid|CBF87500.1 anig|EHA26058.1 atub|OJI87364.1 aver|OJJ08935.1 awen|OJJ35965.1 azon|OJJ50308.1 acla|EAW12182.1 afis|EAW17116.1 afum|EAL86578.1 asyd|OJJ52969.1 ater|EAU36171.1 aory|EIT80020.1

Group3709: abra|OJJ75368.1 acar|OOF94179.1 afla|EED48878.1 agla|OJJ83621.1 akaw|GAA92062.1 aluc|OJZ92937.1 anig|EHA26035.1 aory|EIT81224.1 asyd|OJJ57425.1 ater|EAU36149.1 atub|OJI87397.1 aver|OJJ04755.1 awen|OJJ31289.1 azon|OJJ50359.1 asyd|OJJ55807.1 awen|OJJ38783.1 azon|OJJ42402.1 awen|OJJ29661.1

Group3710: abra|OJJ75381.1 acar|OOF94466.1 acla|EAW12111.1 afis|EAW17274.1 afla|EED48845.1 afum|EAL86692.1 agla|OJJ81078.1 akaw|GAA92743.1 aluc|OJZ92951.1 anid|CBF87538.1 anig|EHA26024.1 aory|EIT81278.1 asyd|OJJ52990.1 ater|EAU34713.1 atub|OJI87410.1 aver|OJJ08959.1 awen|OJJ35760.1 azon|OJJ45524.1

Group3711: abra|OJJ75383.1 acar|OOF94464.1 acla|EAW12109.1 afis|EAW17276.1 afla|EED48843.1 afum|EAL86694.1 agla|OJJ81080.1 akaw|GAA92745.1 aluc|OJZ92953.1 anid|CBF87542.1 anig|EHA26022.1 aory|EIT81280.1 asyd|OJJ52992.1 ater|EAU34711.1 atub|OJI87412.1 aver|OJJ08961.1 awen|OJJ35758.1 azon|OJJ45526.1

Group3712: abra|OJJ75384.1 acar|OOF94463.1 acla|EAW12108.1 afis|EAW17277.1 afla|EED48842.1 afum|EAL86695.1 agla|OJJ81081.1 akaw|GAA92746.1 aluc|OJZ92954.1 anid|CBF87544.1 anig|EHA26021.1 aory|EIT81283.1 asyd|OJJ52993.1 ater|EAU34710.1 atub|OJI87413.1 aver|OJJ08962.1 awen|OJJ35757.1 azon|OJJ45527.1

Group3713: abra|OJJ75385.1 acar|OOF94461.1 acla|EAW12107.1 afis|EAW17278.1 afla|EED48841.1 afum|EAL86696.1 agla|OJJ81082.1 akaw|GAA92747.1 aluc|OJZ92955.1 anid|CBF87545.1 anig|EHA26020.1 aory|EIT81279.1 asyd|OJJ52994.1 ater|EAU34709.1 atub|OJI87414.1 aver|OJJ08963.1 awen|OJJ35756.1 azon|OJJ45528.1

Group3714: abra|OJJ75391.1 acar|OOF94456.1 acla|EAW12101.1 afis|EAW17287.1 afla|EED48833.1 afum|EAL86705.1 agla|OJJ81090.1 akaw|GAA92754.1 aluc|OJZ92962.1 anid|CBF87556.1 anig|EHA26013.1 aory|EIT81270.1 asyd|OJJ53001.1 ater|EAU34702.1 atub|OJI87420.1 aver|OJJ08970.1 awen|OJJ35750.1 azon|OJJ45536.1

Group3715: abra|OJJ75394.1 acar|OOF94455.1 acla|EAW12099.1 afis|EAW17285.1 afla|EED48831.1 afum|EAL86703.1 agla|OJJ81092.1 akaw|GAA92756.1 aluc|OJZ92965.1 anid|CBF87558.1 anig|EHA26010.1 aory|EIT81288.1 asyd|OJJ53003.1 ater|EAU34700.1 atub|OJI87423.1 aver|OJJ08971.1 awen|OJJ35749.1 azon|OJJ45542.1

Group3716: abra|OJJ75396.1 acar|OOF94453.1 acla|EAW12097.1 afis|EAW17289.1 afla|EED48829.1 afum|EAL86709.1 agla|OJJ81094.1 akaw|GAA92758.1 aluc|OJZ92968.1 anid|CBF81166.1 anig|EHA26008.1 aory|EIT81272.1 asyd|OJJ59287.1 ater|EAU34699.1 atub|OJI87425.1 aver|OJJ02988.1 awen|OJJ35747.1 azon|OJJ45540.1

Group3717: abra|OJJ75397.1 acar|OOF94451.1 acla|EAW12096.1 afis|EAW17290.1 afla|EED48828.1 afum|EAL86710.1 agla|OJJ81095.1 akaw|GAA92759.1 aluc|OJZ92969.1 anid|CBF74295.1 anig|EHA26007.1 aory|EIT81266.1 asyd|OJJ51909.1 ater|EAU34698.1 atub|OJI87426.1 aver|OJJ05350.1 awen|OJJ35746.1 azon|OJJ45539.1

Group3718: abra|OJJ75399.1 acar|OOF90746.1 acla|EAW13772.1 afis|EAW18360.1 afla|EED48826.1 afum|EAL87724.1 agla|OJJ88025.1 akaw|GAA84178.1 aluc|OJZ88851.1 anid|CBF79349.1 anig|EHA23652.1 aory|EIT72979.1 asyd|OJJ57798.1 ater|EAU34692.1 atub|OJI85939.1 aver|OJJ04589.1 awen|OJJ35744.1 azon|OJJ49497.1

Group3719: abra|OJJ75400.1 acar|OOF90747.1 acla|EAW13773.1 afis|EAW18359.1 afla|EED48825.1 afum|EAL87723.1 agla|OJJ88026.1 akaw|GAA84179.1 aluc|OJZ88852.1 anid|CBF79351.1 anig|EHA23651.1 aory|EIT73008.1 asyd|OJJ57799.1 ater|EAU34691.1 atub|OJI85938.1 aver|OJJ04588.1 awen|OJJ35743.1 azon|OJJ42023.1

Group3720: abra|OJJ75401.1 acar|OOF90748.1 acla|EAW08507.1 afis|EAW23545.1 afla|EED48824.1 afum|EAL87921.2 agla|OJJ79762.1 akaw|GAA84180.1 aluc|OJZ88853.1 anid|CBF78889.1 anig|EHA23650.1 aory|EIT73018.1 asyd|OJJ64596.1 ater|EAU31465.1 atub|OJI85937.1 aver|OJI98693.1 awen|OJJ36969.1 anig|EHA19941.1

Group3721: abra|OJJ75402.1 acar|OOF90749.1 acla|EAW13774.1 afis|EAW18358.1 afla|EED48823.1 afum|EAL87722.1 agla|OJJ88027.1 akaw|GAA84181.1 aluc|OJZ88854.1 anid|CBF79353.1 anig|EHA23649.1 aory|EIT72976.1 asyd|OJJ57800.1 ater|EAU34690.1 atub|OJI85936.1 aver|OJJ04587.1 awen|OJJ35742.1 azon|OJJ43326.1

Group3722: abra|OJJ75404.1 acar|OOF90751.1 acla|EAW13776.1 afis|EAW18356.1 afum|EAL87719.1 agla|OJJ88029.1 akaw|GAA84182.1 aluc|OJZ88856.1 anid|CBF79356.1 anig|EHA23647.1 aory|EIT72985.1 asyd|OJJ57802.1 ater|EAU34689.1 atub|OJI85934.1 aver|OJJ04585.1 awen|OJJ35740.1 azon|OJJ43323.1 afla|EED48821.1

Group3723: abra|OJJ75408.1 acar|OOF90754.1 acla|EAW13781.1 afis|EAW18351.1 afla|EED48816.1 afum|EAL87714.1 agla|OJJ88033.1 akaw|GAA84184.1 aluc|OJZ88859.1 anid|CBF79365.1 anig|EHA23644.1

aory|EIT72998.1 asyd|OJJ57807.1 ater|EAU34685.1 atub|OJI85931.1 aver|OJJ04581.1 awen|OJJ35735.1 azon|OJJ43321.1

Group3724: abra|OJJ75409.1 acar|OOF90755.1 acla|EAW13780.1 afis|EAW18352.1 afla|EED48817.1 afum|EAL87715.1 agla|OJJ88032.1 akaw|GAA84185.1 aluc|OJZ88860.1 anid|CBF79364.1 anig|EHA23643.1 aory|EIT73011.1 asyd|OJJ57806.1 ater|EAU34686.1 atub|OJI85930.1 aver|OJJ04582.1 awen|OJJ35736.1 azon|OJJ43322.1

Group3725: abra|OJJ75410.1 acar|OOF90756.1 acla|EAW13782.1 afis|EAW18350.1 afla|EED48815.1 afum|EAL87713.1 agla|OJJ88034.1 akaw|GAA84186.1 aluc|OJZ88861.1 anid|CBF79367.1 anig|EHA23642.1 aory|EIT73016.1 asyd|OJJ57808.1 ater|EAU34684.1 atub|OJI85929.1 aver|OJJ04580.1 awen|OJJ35734.1 azon|OJJ43319.1

Group3726: abra|OJJ75413.1 acar|OOF90757.1 acla|EAW13785.1 afis|EAW18347.1 afla|EED48812.1 afum|EAL87711.1 agla|OJJ88037.1 akaw|GAA84188.1 aluc|OJZ88864.1 anid|CBF79386.1 anig|EHA23639.1 aory|EIT73027.1 asyd|OJJ57811.1 ater|EAU34682.1 atub|OJI85926.1 aver|OJJ04577.1 awen|OJJ35731.1 azon|OJJ43318.1

Group3727: abra|OJJ75414.1 acar|OOF90760.1 acla|EAW13786.1 afis|EAW18346.1 afla|EED48811.1 afum|EAL87710.1 agla|OJJ88038.1 akaw|GAA84189.1 aluc|OJZ88865.1 anid|CBF79384.1 anig|EHA23638.1 aory|EIT73037.1 asyd|OJJ57812.1 ater|EAU34681.1 atub|OJI85925.1 aver|OJJ04576.1 awen|OJJ35730.1 azon|OJJ43317.1

Group3728: abra|OJJ75415.1 acar|OOF90759.1 acla|EAW13787.1 afis|EAW18345.1 afla|EED48810.1 afum|EAL87709.1 agla|OJJ88039.1 akaw|GAA84190.1 aluc|OJZ88866.1 anid|CBF79382.1 anig|EHA23637.1 aory|EIT72992.1 asyd|OJJ57813.1 ater|EAU34680.1 atub|OJI85924.1 aver|OJJ04575.1 awen|OJJ35729.1 azon|OJJ43316.1

Group3729: abra|OJJ75417.1 acar|OOF90762.1 acla|EAW13789.1 afis|EAW18343.1 afla|EED48808.1 afum|EAL87706.1 agla|OJJ88041.1 akaw|GAA84192.1 aluc|OJZ88868.1 anid|CBF79378.1 anig|EHA23635.1 aory|EIT73022.1 asyd|OJJ57815.1 ater|EAU34678.1 atub|OJI85922.1 aver|OJJ04573.1 awen|OJJ35727.1 azon|OJJ43315.1

Group3730: abra|OJJ75421.1 acar|OOF90765.1 acla|EAW13793.1 afis|EAW18339.1 afla|EED48803.1 afum|EAL87702.1 agla|OJJ88045.1 akaw|GAA84196.1 aluc|OJZ88873.1 anid|CBF79387.1 anig|EHA23632.1 aory|EIT73019.1 asyd|OJJ57819.1 ater|EAU34674.1 atub|OJI85918.1 aver|OJJ04572.1 awen|OJJ35722.1 azon|OJJ43312.1

Group3731: abra|OJJ75425.1 acar|OOF90770.1 acla|EAW13795.1 afis|EAW18337.1 afla|EED48801.1 afum|EAL87701.1 agla|OJJ88047.1 akaw|GAA84200.1 aluc|OJZ88877.1 anid|CBF79391.1 anig|EHA23628.1 aory|EIT73024.1 asyd|OJJ57821.1 ater|EAU33350.1 atub|OJI85914.1 aver|OJJ04570.1 awen|OJJ35720.1 azon|OJJ43310.1

Group3732: abra|OJJ75426.1 acar|OOF90771.1 acla|EAW13796.1 afis|EAW18336.1 afla|EED48800.1 afum|EAL87700.1 agla|OJJ88049.1 akaw|GAA84201.1 aluc|OJZ88878.1 anid|CBF79395.1 anig|EHA23627.1 aory|EIT73005.1 asyd|OJJ57825.1 ater|EAU33349.1 atub|OJI85913.1 aver|OJJ04567.1 awen|OJJ30717.1 azon|OJJ43309.1

Group3733: abra|OJJ75427.1 acar|OOF90773.1 acla|EAW13798.1 afis|EAW18334.1 afla|EED48798.1 afum|EAL87699.1 agla|OJJ88051.1 akaw|GAA84203.1 aluc|OJZ88880.1 anid|CBF79393.1 anig|EHA23626.1 aory|EIT73035.1 asyd|OJJ57822.1 ater|EAU33347.1 atub|OJI85912.1 aver|OJJ04569.1 awen|OJJ30715.1 azon|OJJ43307.1

Group3734: abra|OJJ75428.1 acar|OOF90774.1 acla|EAW11898.1 afis|EAW19508.1 afla|EED56888.1 afum|EAL86986.1 agla|OJJ88052.1 akaw|GAA84204.1 aluc|OJZ88881.1 anid|CBF82976.1 anig|EHA23625.1 aory|EIT77571.1 asyd|OJJ61561.1 ater|EAU33346.1 atub|OJI85911.1 aver|OJJ00884.1 awen|OJJ30714.1 azon|OJJ45886.1

Group3735: abra|OJJ75429.1 acar|OOF90775.1 acla|EAW13799.1 afis|EAW18333.1 afla|EED48797.1 afum|EAL87698.1 agla|OJJ88053.1 akaw|GAA84205.1 aluc|OJZ88882.1 anid|CBF79397.1 anig|EHA23624.1 aory|EIT73004.1 asyd|OJJ57826.1 ater|EAU33345.1 atub|OJI85910.1 aver|OJJ04566.1 awen|OJJ30713.1 azon|OJJ43306.1

Group3736: abra|OJJ75432.1 acar|OOF90777.1 acla|EAW13804.1 afis|EAW18327.1 afla|EED48793.1 afum|EAL87693.1 agla|OJJ88056.1 akaw|GAA84208.1 aluc|OJZ88885.1 anid|CBF79406.1 anig|EHA23621.1 aory|EIT72996.1 asyd|OJJ57829.1 ater|EAU33336.1 atub|OJI85905.1 aver|OJJ04563.1 awen|OJJ30711.1 azon|OJJ43303.1

Group3737: abra|OJJ75433.1 acar|OOF90778.1 acla|EAW13805.1 afis|EAW18325.1 afla|EED48792.1 afum|EAL87691.1 agla|OJJ88066.1 akaw|GAA84209.1 aluc|OJZ88886.1 anid|CBF79408.1 anig|EHA23620.1 aory|EIT72982.1 asyd|OJJ57830.1 ater|EAU33334.1 atub|OJI85904.1 aver|OJJ04562.1 awen|OJJ30707.1 azon|OJJ43302.1

Group3738: abra|OJJ75435.1 acar|OOF90779.1 acla|EAW13827.1 afis|EAW18304.1 afla|EED48773.1 afum|EAL87670.1 agla|OJJ88119.1 akaw|GAA84210.1 aluc|OJZ88888.1 anid|CBF79447.1 anig|EHA23619.1 aory|EIT73020.1 asyd|OJJ57851.1 ater|EAU33311.1 atub|OJI85901.1 aver|OJJ04541.1 awen|OJJ30678.1 azon|OJJ43279.1

Group3739: abra|OJJ75436.1 acar|OOF90780.1 acla|EAW13826.1 afis|EAW18305.1 afla|EED48774.1 afum|EAL87671.1 agla|OJJ88118.1 akaw|GAA84211.1 aluc|OJZ88889.1 anid|CBF79445.1 anig|EHA23618.1 aory|EIT73030.1 asyd|OJJ57850.1 ater|EAU33312.1 atub|OJI85900.1 aver|OJJ04542.1 awen|OJJ30679.1 azon|OJJ43280.1

Group3740: abra|OJJ75438.1 acar|OOF90783.1 acla|EAW12400.1 afis|EAW16848.1 afla|EED51031.1 afum|EAL86394.1 agla|OJJ81486.1 akaw|GAA84213.1 aluc|OJZ88891.1 anid|CBF81652.1 anig|EHA23615.1 aory|EIT77036.1 asyd|OJJ59545.1 ater|EAU30241.1 atub|OJI85898.1 aver|OJJ02722.1 awen|OJJ30081.1 azon|OJJ44766.1

Group3741: abra|OJJ75442.1 acar|OOF90787.1 acla|EAW13821.1 afis|EAW18310.1 afla|EED48779.1 afum|EAL87676.1 agla|OJJ88079.1 akaw|GAA84217.1 aluc|OJZ88895.1 anid|CBF79434.1 anig|EHA23611.1 aory|EIT73023.1 asyd|OJJ57844.1 ater|EAU33320.1 atub|OJI85894.1 aver|OJJ04548.1 awen|OJJ30685.1 azon|OJJ43284.1

Group3742: abra|OJJ75443.1 acar|OOF90788.1 acla|EAW13820.1 afis|EAW18311.1 afla|EED48780.1 afum|EAL87677.1 agla|OJJ88080.1 akaw|GAA84218.1 aluc|OJZ88896.1 anid|CBF79432.1 anig|EHA23610.1 aory|EIT72999.1 asyd|OJJ57845.1 ater|EAU33321.1 atub|OJI85893.1 aver|OJJ04547.1 awen|OJJ30686.1 azon|OJJ43285.1

Group3743: abra|OJJ75445.1 acar|OOF90789.1 acla|EAW13818.1 afis|EAW18312.1 afla|EED48781.1 afum|EAL87679.1 agla|OJJ88081.1 akaw|GAA84220.1 aluc|OJZ88900.1 anid|CBF79430.1 anig|EHA23608.1 aory|EIT73002.1 asyd|OJJ57841.1 ater|EAU33322.1 atub|OJI85891.1 aver|OJJ04549.1 awen|OJJ30688.1 azon|OJJ43286.1

Group3744: abra|OJJ75446.1 acar|OOF90790.1 acla|EAW13817.1 afis|EAW18313.1 afla|EED48782.1 afum|EAL87680.1 agla|OJJ88082.1 akaw|GAA84221.1 aluc|OJZ88901.1 anid|CBF79428.1 anig|EHA23607.1 aory|EIT73015.1 asyd|OJJ57840.1 ater|EAU33323.1 atub|OJI85890.1 aver|OJJ04550.1 awen|OJJ30689.1 azon|OJJ43287.1

Group3745: abra|OJJ75447.1 acar|OOF90791.1 acla|EAW13816.1 afis|EAW18314.1 afla|EED48783.1 afum|EAL87681.1 agla|OJJ88083.1 akaw|GAA84222.1 aluc|OJZ88902.1 anid|CBF79427.1 anig|EHA23606.1 aory|EIT73014.1 asyd|OJJ57839.1 ater|EAU33324.1 atub|OJI85889.1 aver|OJJ04551.1 awen|OJJ30690.1 azon|OJJ43288.1

Group3746: abra|OJJ75448.1 acar|OOF90792.1 acla|EAW13815.1 afis|EAW18315.1 afla|EED48784.1 afum|EAL87682.1 agla|OJJ88084.1 akaw|GAA84223.1 aluc|OJZ88903.1 anid|CBF79423.1 anig|EHA23605.1 aory|EIT72980.1 asyd|OJJ57837.1 ater|EAU33325.1 atub|OJI85888.1 aver|OJJ04553.1 awen|OJJ30691.1 azon|OJJ43289.1

Group3747: abra|OJJ75450.1 acar|OOF90794.1 acla|EAW13813.1 afis|EAW18317.1 afla|EED48785.1 afum|EAL87684.1 agla|OJJ88101.1 akaw|GAA84225.1 aluc|OJZ88905.1 anid|CBF79421.1 anig|EHA23603.1 aory|EIT73000.1 asyd|OJJ57836.1 ater|EAU33326.1 atub|OJI85886.1 aver|OJJ04554.1 awen|OJJ30699.1 azon|OJJ43292.1

Group3748: abra|OJJ75452.1 acar|OOF90796.1 acla|EAW13811.1 afis|EAW18319.1 afla|EED48787.1 afum|EAL87686.1 agla|OJJ88073.1 akaw|GAA84227.1 aluc|OJZ88907.1 anid|CBF87726.1 anig|EHA23601.1 aory|EIT72981.1 asyd|OJJ63342.1 ater|EAU33329.1 atub|OJI85884.1 aver|OJI97619.1 awen|OJJ30701.1 azon|OJJ50771.1

Group3749: abra|OJJ75454.1 acar|OOF90798.1 acla|EAW13809.1 afis|EAW18321.1 afla|EED48789.1 afum|EAL87688.1 agla|OJJ88070.1 akaw|GAA84229.1 aluc|OJZ88909.1 anid|CBF79416.1 anig|EHA23599.1 aory|EIT73021.1 asyd|OJJ57833.1 ater|EAU33331.1 atub|OJI85882.1 aver|OJJ04557.1 awen|OJJ30703.1 azon|OJJ43296.1

Group3750: abra|OJJ75457.1 acar|OOF90802.1 acla|EAW13808.1 afis|EAW18322.1 afla|EED48790.1 afum|EAL87689.2 agla|OJJ88069.1 akaw|GAA84232.1 aluc|OJZ88913.1 anid|CBF79414.1 anig|EHA23597.1 aory|EIT73033.1 asyd|OJJ57832.1 ater|EAU33332.1 atub|OJI85878.1 aver|OJJ04558.1 awen|OJJ30704.1 azon|OJJ43297.1

Group3751: abra|OJJ75460.1 acar|OOF90279.1 acla|EAW13828.1 afis|EAW18303.1 afla|EED48772.1 afum|EAL87669.1 agla|OJJ88120.1 akaw|GAA84234.1 aluc|OJZ88917.1 anid|CBF79449.1 anig|EHA23595.1 aory|EIT72974.1 asyd|OJJ57852.1 ater|EAU33310.1 atub|OJI85875.1 aver|OJJ04540.1 awen|OJJ30677.1 azon|OJJ43278.1

Group3752: abra|OJJ75463.1 acar|OOF90277.1 acla|EAW13830.1 afis|EAW18301.1 afla|EED48770.1 afum|EAL87667.2 agla|OJJ88122.1 akaw|GAA84236.1 aluc|OJZ88919.1 anid|CBF79480.1 anig|EHA23593.1

aory|EIT72978.1 asyd|OJJ57871.1 ater|EAU33309.1 atub|OJI85873.1 aver|OJJ04523.1 awen|OJJ30676.1 azon|OJJ43276.1

Group3753: abra|OJJ75465.1 acar|OOF90275.1 acla|EAW13832.1 afis|EAW18299.1 afla|EED48768.1 afum|EAL87665.1 agla|OJJ88124.1 akaw|GAA84238.1 aluc|OJZ88921.1 anid|CBF79476.1 anig|EHA23591.1 aory|EIT73017.1 asyd|OJJ57869.1 ater|EAU33307.1 atub|OJI85871.1 aver|OJJ04525.1 awen|OJJ30673.1 azon|OJJ43274.1

Group3754: abra|OJJ75466.1 acar|OOF90803.1 acla|EAW13833.1 afis|EAW18298.1 afla|EED48767.1 afum|EAL87664.2 agla|OJJ88125.1 akaw|GAA84239.1 aluc|OJZ88922.1 anid|CBF79472.1 anig|EHA23590.1 aory|EIT73036.1 asyd|OJJ57867.1 ater|EAU33306.1 atub|OJI85870.1 aver|OJJ04526.1 awen|OJJ30672.1 azon|OJJ43272.1

Group3755: abra|OJJ75468.1 acar|OOF90805.1 acla|EAW13834.1 afis|EAW18297.1 afla|EED48763.1 afum|EAL87663.1 agla|OJJ88126.1 akaw|GAA84240.1 aluc|OJZ88925.1 anid|CBF79470.1 anig|EHA23588.1 aory|EIT73013.1 asyd|OJJ57865.1 ater|EAU33305.1 atub|OJI85867.1 aver|OJJ04528.1 awen|OJJ30671.1 azon|OJJ43271.1

Group3756: abra|OJJ75472.1 acar|OOF90809.1 acla|EAW13837.1 afis|EAW18294.1 afla|EED48761.1 afum|EAL87660.1 agla|OJJ88129.1 akaw|GAA84243.1 aluc|OJZ88931.1 anid|CBF79465.1 anig|EHA23585.1 aory|EIT73038.1 asyd|OJJ57861.1 ater|EAU33303.1 atub|OJI85861.1 aver|OJJ04531.1 awen|OJJ30669.1 azon|OJJ43268.1

Group3757: abra|OJJ75473.1 acar|OOF90810.1 acla|EAW13838.1 afis|EAW18293.1 afla|EED48760.1 afum|EAL87659.1 agla|OJJ88132.1 akaw|GAA84244.1 aluc|OJZ88932.1 anid|CBF79463.1 anig|EHA23584.1 aory|EIT72991.1 asyd|OJJ57860.1 ater|EAU33302.1 atub|OJI85860.1 aver|OJJ04532.1 awen|OJJ30668.1 azon|OJJ43267.1

Group3758: abra|OJJ75474.1 acar|OOF90811.1 acla|EAW13839.1 afis|EAW18292.1 afla|EED48759.1 afum|EAL87658.1 agla|OJJ88133.1 akaw|GAA84245.1 aluc|OJZ88933.1 anid|CBF79461.1 anig|EHA23583.1 aory|EIT72984.1 asyd|OJJ57859.1 ater|EAU33301.1 atub|OJI85859.1 aver|OJJ04534.1 awen|OJJ30667.1 azon|OJJ43265.1

Group3759: abra|OJJ75476.1 acar|OOF90812.1 acla|EAW13841.1 afis|EAW18291.1 afla|EED48758.1 afum|EAL87656.1 agla|OJJ88134.1 akaw|GAA84246.1 aluc|OJZ88935.1 anid|CBF79459.1 anig|EHA23582.1 aory|EIT73034.1 asyd|OJJ57858.1 ater|EAU33299.1 atub|OJI85857.1 aver|OJJ04535.1 awen|OJJ30666.1 azon|OJJ43263.1

Group3760: abra|OJJ75480.1 acar|OOF90816.1 acla|EAW11214.1 afis|EAW18288.1 afla|EED48756.1 afum|EAL87654.2 agla|OJJ88136.1 akaw|GAA84249.1 aluc|OJZ88939.1 anid|CBF79454.1 anig|EHA23579.1 aory|EIT73128.1 asyd|OJJ57856.1 ater|EAU33296.1 atub|OJI85851.1 aver|OJJ04538.1 awen|OJJ30661.1 azon|OJJ47954.1

Group3761: abra|OJJ75486.1 acar|OOF90818.1 acla|EAW11217.1 afis|EAW18286.1 afla|EED48753.1 afum|EAL87652.1 agla|OJJ88138.1 akaw|GAA84252.1 aluc|OJZ88945.1 anid|CBF79450.1 anig|EHA23576.1 aory|EIT73238.1 asyd|OJJ57853.1 ater|EAU33292.1 atub|OJI85844.1 aver|OJJ04539.1 awen|OJJ30658.1 azon|OJJ47956.1

Group3762: abra|OJJ75487.1 acar|OOF90820.1 acla|EAW11218.1 afis|EAW18285.1 afla|EED48752.1 afum|EAL87651.1 agla|OJJ88139.1 akaw|GAA84253.1 aluc|OJZ88946.1 anid|CBF79482.1 anig|EHA23575.1 aory|EIT73219.1 asyd|OJJ57872.1 ater|EAU33291.1 atub|OJI85843.1 aver|OJJ04522.1 awen|OJJ30657.1 azon|OJJ47957.1

Group3763: abra|OJJ75488.1 acar|OOF90819.1 acla|EAW11219.1 afis|EAW18284.1 afla|EED48751.1 afum|EAL87650.1 agla|OJJ88140.1 akaw|GAA84254.1 aluc|OJZ88947.1 anid|CBF79483.1 anig|EHA23574.1 aory|EIT73141.1 asyd|OJJ57873.1 ater|EAU33290.1 atub|OJI85842.1 aver|OJJ04521.1 awen|OJJ30655.1 azon|OJJ47959.1

Group3764: abra|OJJ75491.1 acar|OOF90823.1 acla|EAW11223.1 afis|EAW18280.1 afla|EED48747.1 afum|EAL87646.1 agla|OJJ88146.1 akaw|GAA84256.1 aluc|OJZ88950.1 anid|CBF79491.1 anig|EHA23571.1 aory|EIT73260.1 asyd|OJJ57876.1 ater|EAU33286.1 atub|OJI85839.1 aver|OJJ04518.1 awen|OJJ30652.1 azon|OJJ47963.1

Group3765: abra|OJJ75493.1 acar|OOF90826.1 acla|EAW11226.1 afis|EAW18277.1 afla|EED48744.1 afum|EAL87643.1 agla|OJJ88149.1 akaw|GAA84259.1 aluc|OJZ88952.1 anid|CBF79496.1 anig|EHA23568.1 aory|EIT73150.1 asyd|OJJ57878.1 ater|EAU33283.1 atub|OJI85837.1 aver|OJJ04516.1 awen|OJJ30650.1 azon|OJJ47965.1

Group3766: abra|OJJ75494.1 acar|OOF90827.1 acla|EAW09288.1 afis|EAW21859.1 afla|EED46451.1 afum|EAL92872.1 agla|OJJ84276.1 akaw|GAA84260.1 aluc|OJZ88953.1 anid|CBF76751.1 anig|EHA23567.1 aory|EIT72375.1 asyd|OJJ61260.1 ater|EAU30858.1 atub|OJI85836.1 aver|OJJ00583.1 awen|OJJ34143.1 azon|OJJ43483.1

Group3767: abra|OJJ75495.1 acar|OOF90828.1 acla|EAW09289.1 afis|EAW21858.1 afla|EED46452.1 afum|EAL92871.1 agla|OJJ84277.1 akaw|GAA84261.1 aluc|OJZ88954.1 anid|CBF76749.1 anig|EHA23566.1 aory|EIT72324.1 asyd|OJJ61259.1 ater|EAU30857.1 atub|OJI85835.1 aver|OJJ00582.1 awen|OJJ34142.1 azon|OJJ43484.1

Group3768: abra|OJJ75496.1 acar|OOF90829.1 acla|EAW09290.1 afis|EAW21857.1 afla|EED46453.1 afum|EAL92870.1 agla|OJJ84278.1 akaw|GAA84262.1 aluc|OJZ88955.1 anid|CBF76747.1 anig|EHA23565.1 aory|EIT72382.1 asyd|OJJ61258.1 ater|EAU30856.1 atub|OJI85834.1 aver|OJJ00581.1 awen|OJJ34141.1 azon|OJJ43485.1

Group3769: abra|OJJ75497.1 acar|OOF90830.1 acla|EAW09291.1 afis|EAW21856.1 afla|EED46454.1 afum|EAL92869.1 agla|OJJ84279.1 akaw|GAA84263.1 aluc|OJZ88956.1 anid|CBF76745.1 aory|EIT72446.1 asyd|OJJ61257.1 ater|EAU30855.1 atub|OJI85833.1 aver|OJJ00580.1 awen|OJJ34140.1 azon|OJJ43486.1 anig|EHA23564.1

Group3770: abra|OJJ75506.1 acar|OOF89956.1 acla|EAW09296.1 afis|EAW21851.1 afla|EED46459.1 afum|EAL92864.1 agla|OJJ84284.1 akaw|GAA84269.1 aluc|OJZ88962.1 anid|CBF76735.1 anig|EHA23559.1 aory|EIT72419.1 asyd|OJJ61252.1 ater|EAU30850.1 atub|OJI85828.1 aver|OJJ00576.1 awen|OJJ34134.1 azon|OJJ44210.1

Group3771: abra|OJJ75507.1 acar|OOF89955.1 acla|EAW09297.1 afis|EAW21850.1 afla|EED46460.1 afum|EAL92863.2 agla|OJJ84285.1 akaw|GAA84270.1 aluc|OJZ88963.1 anid|CBF76733.1 anig|EHA23558.1 aory|EIT72388.1 asyd|OJJ61251.1 ater|EAU30849.1 atub|OJI85827.1 aver|OJJ00575.1 awen|OJJ34133.1 azon|OJJ46409.1

Group3772: abra|OJJ75511.1 acar|OOF90837.1 acla|EAW09299.1 afis|EAW21848.1 afum|EAL92861.2 agla|OJJ84287.1 akaw|GAA84275.1 aluc|OJZ88968.1 anid|CBF76727.1 anig|EHA23555.1 asyd|OJJ61248.1 ater|EAU30847.1 atub|OJI85822.1 aver|OJJ00572.1 awen|OJJ34131.1 azon|OJJ44212.1 afla|EED46464.1 aory|EIT72335.1

Group3773: abra|OJJ75512.1 acar|OOF90839.1 acla|EAW09300.1 afis|EAW21847.1 afla|EED46468.1 afum|EAL92860.1 agla|OJJ84288.1 akaw|GAA84276.1 aluc|OJZ88969.1 anid|CBF76725.1 anig|EHA23554.1 aory|EIT72394.1 asyd|OJJ61246.1 ater|EAU30846.1 atub|OJI85821.1 aver|OJJ00570.1 awen|OJJ34130.1 azon|OJJ44213.1

Group3774: abra|OJJ75518.1 acar|OOF90845.1 acla|EAW09302.1 afis|EAW21844.1 afla|EED46526.1 afum|EAL92856.1 agla|OJJ84292.1 akaw|GAA84282.1 aluc|OJZ88975.1 anid|CBF76717.1 anig|EHA23550.1 aory|EIT72426.1 asyd|OJJ61242.1 ater|EAU38521.1 atub|OJI85814.1 aver|OJJ00565.1 awen|OJJ34128.1 azon|OJJ44215.1

Group3775: abra|OJJ75521.1 acar|OOF90848.1 acla|EAW09308.1 afis|EAW21837.1 afla|EED46538.1 afum|EAL92851.1 agla|OJJ84305.1 akaw|GAA84285.1 aluc|OJZ88978.1 anid|CBF76701.1 anig|EHA23547.1 aory|EIT72362.1 asyd|OJJ61233.1 ater|EAU38512.1 atub|OJI85811.1 aver|OJJ00556.1 awen|OJJ34113.1 azon|OJJ44227.1

Group3776: abra|OJJ75523.1 acar|OOF90850.1 acla|EAW09310.1 afis|EAW21835.1 afla|EED46540.1 afum|EAL92850.1 agla|OJJ84309.1 akaw|GAA84286.1 aluc|OJZ88980.1 anid|CBF76699.1 anig|EHA23546.1 aory|EIT72364.1 asyd|OJJ61232.1 ater|EAU38510.1 atub|OJI85809.1 aver|OJJ00555.1 awen|OJJ34109.1 azon|OJJ44232.1

Group3777: abra|OJJ75527.1 acar|OOF90854.1 acla|EAW09313.1 afis|EAW21832.1 afla|EED46544.1 afum|EAL92847.1 agla|OJJ84312.1 akaw|GAA84289.1 aluc|OJZ88983.1 anid|CBF76694.1 anig|EHA23543.1 aory|EIT77942.1 asyd|OJJ61229.1 ater|EAU38508.1 atub|OJI85806.1 aver|OJJ00552.1 awen|OJJ34105.1 azon|OJJ44236.1

Group3778: abra|OJJ75528.1 acar|OOF90855.1 acla|EAW09314.1 afis|EAW21831.1 afla|EED46545.1 afum|EAL92846.1 agla|OJJ84313.1 akaw|GAA84290.1 aluc|OJZ88984.1 anid|CBF76680.1 anig|EHA23542.1 aory|EIT77881.1 asyd|OJJ61223.1 ater|EAU38507.1 atub|OJI85804.1 aver|OJJ00546.1 awen|OJJ34104.1 azon|OJJ44238.1

Group3779: abra|OJJ75539.1 acar|OOF90858.1 acla|EAW09315.1 afis|EAW21830.1 afla|EED46546.1 afum|EAL92845.1 agla|OJJ84314.1 akaw|GAA84293.1 aluc|OJZ88987.1 anid|CBF76676.1 anig|EHA23539.1 aory|EIT77971.1 asyd|OJJ61221.1 ater|EAU38506.1 atub|OJI85801.1 aver|OJJ00545.1 awen|OJJ34103.1 azon|OJJ44239.1

Group3780: abra|OJJ75544.1 acar|OOF90864.1 acla|EAW09316.1 afis|EAW21829.1 afla|EED46547.1 afum|EAL92844.1 agla|OJJ84315.1 akaw|GAA84300.1 aluc|OJZ88994.1 anid|CBF76674.1 anig|EHA23533.1 aory|EIT77834.1 asyd|OJJ61220.1 ater|EAU38505.1 atub|OJI85794.1 aver|OJJ00544.1 awen|OJJ34102.1 azon|OJJ44242.1

Group3781: abra|OJJ75545.1 acar|OOF90866.1 acla|EAW09317.1 afis|EAW21828.1 afla|EED46548.1 afum|EAL92843.1 agla|OJJ84316.1 akaw|GAA84301.1 aluc|OJZ88996.1 anid|CBF76670.1 anig|EHA23532.1

aory|EIT77919.1 asyd|OJJ61219.1 ater|EAU38504.1 atub|OJI85793.1 aver|OJJ00543.1 awen|OJJ34101.1 azon|OJJ44243.1

Group3782: abra|OJJ75548.1 acar|OOF90868.1 acla|EAW09319.1 afis|EAW21826.1 afla|EED46553.1 afum|EAL92841.1 agla|OJJ84318.1 akaw|GAA84303.1 aluc|OJZ88998.1 anid|CBF76666.1 anig|EHA23530.1 aory|EIT77928.1 asyd|OJJ61217.1 ater|EAU38502.1 atub|OJI85791.1 aver|OJJ00541.1 awen|OJJ34099.1 azon|OJJ44245.1

Group3783: abra|OJJ75552.1 acar|OOF90870.1 acla|EAW09324.1 afis|EAW21821.1 afla|EED46558.1 afum|EAL92837.1 agla|OJJ84321.1 akaw|GAA84307.1 aluc|OJZ89002.1 anid|CBF76660.1 anig|EHA23526.1 aory|EIT77966.1 asyd|OJJ61212.1 ater|EAU38495.1 atub|OJI85787.1 aver|OJJ00537.1 awen|OJJ34095.1 azon|OJJ44249.1

Group3784: abra|OJJ75553.1 acar|OOF90872.1 acla|EAW09355.1 afis|EAW21820.1 afla|EED46559.1 afum|EAL92836.1 agla|OJJ84322.1 akaw|GAA84308.1 aluc|OJZ89003.1 anid|CBF76658.1 anig|EHA23525.1 aory|EIT77877.1 asyd|OJJ61211.1 ater|EAU38494.1 atub|OJI85786.1 aver|OJJ00535.1 awen|OJJ34094.1 azon|OJJ44252.1

Group3785: abra|OJJ75554.1 acar|OOF90873.1 acla|EAW09354.1 afis|EAW21819.1 afla|EED46564.1 afum|EAL92835.1 agla|OJJ84327.1 akaw|GAA84309.1 aluc|OJZ89004.1 anid|CBF76654.1 anig|EHA23524.1 aory|EIT77873.1 asyd|OJJ61210.1 ater|EAU38490.1 atub|OJI85785.1 aver|OJJ00534.1 awen|OJJ34093.1 azon|OJJ44253.1

Group3786: abra|OJJ75574.1 acar|OOF90890.1 acla|EAW09346.1 afis|EAW21799.1 afla|EED46590.1 afum|EAL92824.2 agla|OJJ84324.1 akaw|GAA84325.1 aluc|OJZ89022.1 anid|CBF76633.1 anig|EHA23509.1 aory|EIT77934.1 asyd|OJJ61197.1 ater|EAU38481.1 atub|OJI85767.1 aver|OJJ00521.1 awen|OJJ34086.1 azon|OJJ44256.1

Group3787: abra|OJJ75575.1 acar|OOF90893.1 acla|EAW09343.1 afis|EAW21797.1 afla|EED46598.1 afum|EAL92822.2 agla|OJJ84331.1 akaw|GAA84327.1 aluc|OJZ89024.1 anid|CBF76629.1 anig|EHA23508.1 aory|EIT77937.1 asyd|OJJ61196.1 ater|EAU38479.1 atub|OJI85766.1 aver|OJJ00520.1 awen|OJJ34082.1 azon|OJJ44260.1

Group3788: akaw|GAA84326.1 aluc|OJZ89023.1 acar|OOF90891.1 acla|EAW09345.1 afis|EAW21798.1 afla|EED46597.1 afum|EAL92823.2 agla|OJJ84323.1 aory|EIT77884.1 awen|OJJ34085.1 acar|OOF90892.1 acla|EAW09344.1 agla|OJJ84329.1 anid|CBF76631.1 aory|EIT77853.1 ater|EAU38480.1 awen|OJJ34084.1 azon|OJJ44257.1

Group3789: abra|OJJ75579.1 acar|OOF90896.1 acla|EAW09340.1 afis|EAW21795.1 afla|EED46600.1 afum|EAL92820.1 agla|OJJ84333.1 akaw|GAA84331.1 aluc|OJZ89030.1 anid|CBF76625.1 anig|EHA23504.1 aory|EIT77931.1 asyd|OJJ61194.1 ater|EAU38477.1 atub|OJI85761.1 aver|OJJ00518.1 azon|OJJ44262.1 awen|OJJ34079.1

Group3790: abra|OJJ75581.1 acar|OOF90899.1 acla|EAW09337.1 afis|EAW21791.1 afla|EED46604.1 afum|EAL92816.1 agla|OJJ88519.1 akaw|GAA84333.1 aluc|OJZ89032.1 anid|CBF76623.1 anig|EHA23502.1 aory|EIT77970.1 asyd|OJJ61193.1 ater|EAU38475.1 atub|OJI85758.1 aver|OJJ00517.1 awen|OJJ37963.1 azon|OJJ46824.1

Group3791: abra|OJJ75592.1 acar|OOG00236.1 acla|EAW09330.1 afis|EAW21786.1 afla|EED46610.1 afum|EAL92813.1 agla|OJJ84337.1 akaw|GAA84344.1 aluc|OJZ89044.1 anid|CBF76617.1 anig|EHA23491.1 aory|EIT77835.1 asyd|OJJ61189.1 ater|EAU38469.1 atub|OJI85743.1 aver|OJJ00513.1 awen|OJJ34072.1 azon|OJJ48155.1

Group3792: abra|OJJ75602.1 acar|OOG00235.1 acla|EAW09327.1 afis|EAW21784.1 afla|EED46611.1 afum|EAL92811.1 agla|OJJ84339.1 akaw|GAA84352.1 aluc|OJZ89052.1 anid|CBF76613.1 anig|EHA23486.1 aory|EIT77805.1 asyd|OJJ61188.1 ater|EAU38467.1 atub|OJI85735.1 aver|OJJ00511.1 awen|OJJ34071.1 azon|OJJ48157.1

Group3793: abra|OJJ75605.1 acar|OOG00232.1 acla|EAW09367.1 afis|EAW21770.1 afla|EED46330.1 afum|EAL92799.1 agla|OJJ84378.1 akaw|GAA84356.1 aluc|OJZ89058.1 anid|CBF83758.1 anig|EHA23484.1 aory|EIT81243.1 asyd|OJJ60861.1 ater|EAU38448.1 atub|OJI85728.1 aver|OJJ00187.1 awen|OJJ34057.1 azon|OJJ48175.1

Group3794: abra|OJJ75610.1 acar|OOG00228.1 acla|EAW09372.1 afis|EAW21763.1 afla|EED46336.1 afum|EAL92793.1 agla|OJJ84388.1 akaw|GAA84360.1 aluc|OJZ89062.1 anid|CBF83751.1 anig|EHA23476.1 aory|EIT81185.1 asyd|OJJ60857.1 ater|EAU38452.1 atub|OJI85724.1 aver|OJJ00181.1 awen|OJJ34046.1 azon|OJJ48185.1

Group3795: abra|OJJ75611.1 acar|OOG00227.1 acla|EAW09373.1 afis|EAW21762.1 afla|EED46337.1 afum|EAL92792.1 agla|OJJ84389.1 akaw|GAA84361.1 aluc|OJZ89063.1 anid|CBF83749.1 anig|EHA23475.1 aory|EIT81205.1 asyd|OJJ60856.1 ater|EAU38453.1 atub|OJI85723.1 aver|OJJ00180.1 awen|OJJ34045.1 azon|OJJ48186.1

Group3796: abra|OJJ75615.1 acar|OOG00215.1 acla|EAW09363.1 afis|EAW21774.1 afla|EED46340.1 afum|EAL92803.1 agla|OJJ84350.1 akaw|GAA84364.1 aluc|OJZ89068.1 anid|CBF76597.1 anig|EHA23468.1 aory|EIT81181.1 asyd|OJJ61178.1 ater|EAU38464.1 atub|OJI85720.1 aver|OJJ00504.1 awen|OJJ34062.1 azon|OJJ48170.1

Group3797: abra|OJJ75625.1 acar|OOG00211.1 acla|EAW09360.1 afis|EAW21777.1 afla|EED46344.1 afum|EAL92806.1 agla|OJJ84342.1 akaw|GAA84372.1 aluc|OJZ89076.1 anid|CBF76603.1 anig|EHA23461.1 aory|EIT81165.1 asyd|OJJ61182.1 ater|EAU38462.1 atub|OJI85709.1 aver|OJJ00507.1 awen|OJJ34066.1 azon|OJJ48165.1

Group3798: abra|OJJ75628.1 acar|OOG00210.1 acla|EAW09465.1 afis|EAW21671.1 afla|EED57935.1 afum|EAL92706.1 agla|OJJ79745.1 akaw|GAA84375.1 aluc|OJZ89079.1 anid|CBF83585.1 anig|EHA23458.1 aory|EIT82333.1 asyd|OJJ60779.1 ater|EAU38355.1 atub|OJI85706.1 aver|OJJ00112.1 awen|OJJ33935.1 azon|OJJ48642.1

Group3799: abra|OJJ75630.1 acar|OOG00205.1 acla|EAW09463.1 afis|EAW21674.1 afla|EED57936.1 afum|EAL92708.1 agla|OJJ79747.1 akaw|GAA84377.1 aluc|OJZ89081.1 anid|CBF83589.1 anig|EHA23456.1 aory|EIT82341.1 asyd|OJJ60776.1 ater|EAU38357.1 atub|OJI85703.1 aver|OJJ00110.1 awen|OJJ33940.1 azon|OJJ48629.1

Group3800: abra|OJJ75633.1 acar|OOG00202.1 acla|EAW09461.1 agla|OJJ79927.1 akaw|GAA84380.1 aluc|OJZ89084.1 anig|EHA23453.1 asyd|OJJ60773.1 ater|EAU38359.1 atub|OJI85700.1 aver|OJJ00107.1 awen|OJJ33943.1 afla|EED57941.1 anid|CBF83591.1 afis|EAW21676.1 afum|EAL92710.1 aory|EIT82327.1 azon|OJJ48627.1

Group3801: abra|OJJ75634.1 acar|OOG00201.1 acla|EAW09460.1 afis|EAW21677.1 afla|EED57942.1 afum|EAL92711.1 agla|OJJ79926.1 akaw|GAA84381.1 aluc|OJZ89085.1 anid|CBF83593.1 anig|EHA23452.1 aory|EIT82349.1 asyd|OJJ60772.1 ater|EAU38360.1 atub|OJI85699.1 aver|OJJ00106.1 awen|OJJ33944.1 azon|OJJ48626.1

Group3802: abra|OJJ75636.1 acar|OOG00200.1 acla|EAW09459.1 afis|EAW21678.1 afla|EED57943.1 afum|EAL92712.1 agla|OJJ79925.1 akaw|GAA84382.1 aluc|OJZ89086.1 anid|CBF83595.1 anig|EHA23451.1 aory|EIT82316.1 asyd|OJJ60771.1 ater|EAU38361.1 atub|OJI85698.1 aver|OJJ00105.1 awen|OJJ33945.1 azon|OJJ48625.1

Group3803: abra|OJJ75637.1 acar|OOG00199.1 acla|EAW09458.1 afis|EAW21679.1 afla|EED57944.1 afum|EAL92713.1 agla|OJJ79924.1 akaw|GAA84383.1 aluc|OJZ89087.1 anid|CBF83597.1 anig|EHA23450.1 aory|EIT82325.1 asyd|OJJ60770.1 ater|EAU38362.1 atub|OJI85697.1 aver|OJJ00104.1 awen|OJJ33946.1 azon|OJJ48624.1

Group3804: abra|OJJ75639.1 acar|OOG00198.1 acla|EAW09457.1 afis|EAW21680.1 afla|EED57945.1 afum|EAL92714.1 agla|OJJ79923.1 akaw|GAA84384.1 aluc|OJZ89088.1 anid|CBF83598.1 anig|EHA23449.1 aory|EIT82335.1 asyd|OJJ60769.1 ater|EAU38363.1 atub|OJI85694.1 aver|OJJ00103.1 awen|OJJ33947.1 azon|OJJ48623.1

Group3805: abra|OJJ75642.1 acar|OOG00196.1 acla|EAW09456.1 afis|EAW21681.1 afla|EED57946.1 afum|EAL92715.1 agla|OJJ79921.1 akaw|GAA84386.1 aluc|OJZ89091.1 anid|CBF83600.1 anig|EHA23447.1 aory|EIT82326.1 asyd|OJJ60768.1 ater|EAU38364.1 atub|OJI85692.1 aver|OJJ00102.1 awen|OJJ33948.1 azon|OJJ48622.1

Group3806: abra|OJJ75644.1 acar|OOG00195.1 acla|EAW09454.1 afis|EAW21683.1 afla|EED57950.1 afum|EAL92717.1 agla|OJJ79918.1 akaw|GAA84389.1 aluc|OJZ89093.1 anid|CBF83605.1 anig|EHA23444.1 aory|EIT82322.1 asyd|OJJ60766.1 ater|EAU38367.1 atub|OJI85688.1 aver|OJJ00100.1 awen|OJJ33950.1 azon|OJJ48620.1

Group3807: abra|OJJ75645.1 acar|OOG00194.1 acla|EAW09453.1 afis|EAW21684.1 afla|EED57951.1 afum|EAL92718.2 agla|OJJ79917.1 akaw|GAA84390.1 aluc|OJZ89094.1 anid|CBF83607.1 anig|EHA23443.1 aory|EIT82348.1 asyd|OJJ60765.1 ater|EAU38368.1 atub|OJI85687.1 aver|OJJ00099.1 awen|OJJ33951.1 azon|OJJ48619.1

Group3808: abra|OJJ75650.1 acar|OOG00190.1 acla|EAW09452.1 afis|EAW21685.1 afla|EED57956.1 afum|EAL92719.1 agla|OJJ79914.1 akaw|GAA84393.1 aluc|OJZ89098.1 anid|CBF83611.1 anig|EHA23440.1 aory|EIT79731.1 asyd|OJJ60762.1 ater|EAU38371.1 atub|OJI85683.1 aver|OJJ00096.1 awen|OJJ33955.1 azon|OJJ48615.1

Group3809: abra|OJJ75651.1 acar|OOG00189.1 acla|EAW09449.1 afis|EAW21687.1 afla|EED57957.1 afum|EAL92721.2 agla|OJJ79913.1 akaw|GAA84394.1 aluc|OJZ89099.1 anid|CBF83612.1 anig|EHA23439.1 aory|EIT79765.1 asyd|OJJ60761.1 ater|EAU38372.1 atub|OJI85682.1 aver|OJJ00095.1 awen|OJJ33956.1 azon|OJJ48612.1

Group3810: abra|OJJ75652.1 acar|OOG00188.1 acla|EAW09448.1 afis|EAW21688.1 afla|EED57958.1 afum|EAL92722.1 agla|OJJ79912.1 akaw|GAA84395.1 aluc|OJZ89100.1 anid|CBF83614.1 anig|EHA23438.1

aory|EIT79734.1 asyd|OJJ60760.1 ater|EAU38373.1 atub|OJI85681.1 aver|OJJ00094.1 awen|OJJ33957.1 azon|OJJ48611.1

Group3811: abra|OJJ75654.1 acar|OOG00187.1 acla|EAW09446.1 afis|EAW21690.1 afla|EED57960.1 afum|EAL92724.1 agla|OJJ79910.1 akaw|GAA84397.1 aluc|OJZ89103.1 anid|CBF83617.1 anig|EHA23436.1 aory|EIT79781.1 asyd|OJJ60758.1 ater|EAU38375.1 atub|OJI85678.1 aver|OJJ00092.1 awen|OJJ33959.1 azon|OJJ48609.1

Group3812: abra|OJJ75656.1 acar|OOG00186.1 acla|EAW09444.1 afis|EAW21692.1 afla|EED57962.1 afum|EAL92726.1 agla|OJJ79907.1 akaw|GAA84399.1 aluc|OJZ89106.1 anid|CBF83621.1 anig|EHA23434.1 aory|EIT79782.1 asyd|OJJ60756.1 ater|EAU38377.1 atub|OJI85676.1 aver|OJJ00090.1 awen|OJJ33961.1 azon|OJJ48607.1

Group3813: abra|OJJ75657.1 acar|OOG00185.1 acla|EAW09442.1 afis|EAW21694.1 afla|EED57963.1 afum|EAL92728.1 agla|OJJ79905.1 akaw|GAA84400.1 aluc|OJZ89107.1 anid|CBF83623.1 anig|EHA23433.1 aory|EIT79708.1 asyd|OJJ60755.1 ater|EAU38379.1 atub|OJI85675.1 aver|OJJ00089.1 awen|OJJ33963.1 azon|OJJ48605.1

Group3814: abra|OJJ75658.1 acar|OOG00184.1 acla|EAW09441.1 afis|EAW21695.1 afla|EED57964.1 afum|EAL92729.1 agla|OJJ79904.1 akaw|GAA84401.1 aluc|OJZ89109.1 anid|CBF83624.1 anig|EHA23432.1 aory|EIT79788.1 asyd|OJJ60754.1 ater|EAU38380.1 atub|OJI85674.1 aver|OJJ00088.1 awen|OJJ33964.1 azon|OJJ48603.1

Group3815: abra|OJJ75662.1 acar|OOG00181.1 acla|EAW09439.1 afis|EAW21697.1 afla|EED57966.1 afum|EAL92731.1 agla|OJJ79901.1 akaw|GAA84403.1 aluc|OJZ89113.1 anid|CBF83628.1 anig|EHA23430.1 aory|EIT79705.1 asyd|OJJ60752.1 ater|EAU38382.1 atub|OJI85671.1 aver|OJJ00085.1 awen|OJJ33966.1 azon|OJJ48600.1

Group3816: abra|OJJ75664.1 acar|OOG00180.1 acla|EAW09437.1 afis|EAW21699.1 afla|EED57968.1 afum|EAL92733.1 agla|OJJ79899.1 akaw|GAA84405.1 aluc|OJZ89115.1 anid|CBF83632.1 anig|EHA23428.1 aory|EIT79768.1 asyd|OJJ60750.1 ater|EAU38384.1 atub|OJI85669.1 aver|OJJ00083.1 awen|OJJ33968.1 azon|OJJ48598.1

Group3817: abra|OJJ75665.1 acar|OOG00179.1 acla|EAW09436.1 afis|EAW21700.1 afla|EED57969.1 afum|EAL92734.1 agla|OJJ79898.1 akaw|GAA84406.1 aluc|OJZ89116.1 anid|CBF83633.1 anig|EHA23427.1 aory|EIT79802.1 asyd|OJJ60782.1 ater|EAU38385.1 atub|OJI85668.1 aver|OJJ00115.1 awen|OJJ33970.1 azon|OJJ48589.1

Group3818: abra|OJJ75667.1 acar|OOG00177.1 acla|EAW09434.1 afis|EAW21702.1 afla|EED57971.1 afum|EAL92736.1 agla|OJJ79896.1 akaw|GAA84408.1 aluc|OJZ89118.1 anid|CBF83639.1 anig|EHA23425.1 aory|EIT79763.1 asyd|OJJ60785.1 ater|EAU38387.1 atub|OJI85666.1 aver|OJJ00118.1 awen|OJJ33972.1 azon|OJJ48587.1

Group3819: abra|OJJ75668.1 acar|OOG00176.1 acla|EAW09433.1 afis|EAW21703.1 afla|EED57972.1 afum|EAL92737.1 agla|OJJ84485.1 akaw|GAA84409.1 aluc|OJZ89119.1 anid|CBF83649.1 anig|EHA23424.1 aory|EIT79804.1 asyd|OJJ60792.1 ater|EAU38388.1 atub|OJI85665.1 aver|OJJ00125.1 awen|OJJ33973.1 azon|OJJ48586.1

Group3820: abra|OJJ75670.1 acar|OOG00175.1 acla|EAW09432.1 afis|EAW21704.1 afla|EED57973.1 afum|EAL92738.1 agla|OJJ79888.1 akaw|GAA84410.1 aluc|OJZ89120.1 anid|CBF83648.1 anig|EHA23423.1 aory|EIT79806.1 asyd|OJJ60791.1 ater|EAU38389.1 atub|OJI85664.1 aver|OJJ00124.1 awen|OJJ33975.1 azon|OJJ48581.1

Group3821: abra|OJJ75672.1 acar|OOG00173.1 acla|EAW09425.1 afis|EAW21711.1 afla|EED57975.1 afum|EAL92746.1 agla|OJJ79890.1 akaw|GAA84413.1 aluc|OJZ89122.1 anid|CBF83646.1 anig|EHA23421.1 aory|EIT79737.1 asyd|OJJ60789.1 ater|EAU38391.1 atub|OJI85662.1 aver|OJJ00122.1 awen|OJJ33977.1 azon|OJJ48583.1

Group3822: abra|OJJ75673.1 acar|OOG00172.1 acla|EAW09426.1 afis|EAW21710.1 afla|EED57976.1 afum|EAL92745.1 agla|OJJ79891.1 akaw|GAA84414.1 aluc|OJZ89123.1 anid|CBF83644.1 anig|EHA23420.1 aory|EIT79753.1 asyd|OJJ60788.1 ater|EAU38392.1 atub|OJI85661.1 aver|OJJ00121.1 awen|OJJ33980.1 azon|OJJ48575.1

Group3823: abra|OJJ75674.1 acar|OOG00171.1 acla|EAW09427.1 afis|EAW21709.1 afla|EED57977.1 afum|EAL92744.1 agla|OJJ79892.1 akaw|GAA84415.1 aluc|OJZ89124.1 anid|CBF83642.1 anig|EHA23419.1 aory|EIT79730.1 asyd|OJJ60787.1 ater|EAU38393.1 atub|OJI85660.1 aver|OJJ00120.1 awen|OJJ33981.1 azon|OJJ48574.1

Group3824: abra|OJJ75675.1 acar|OOG00170.1 acla|EAW09428.1 afis|EAW21708.1 afla|EED57978.1 afum|EAL92743.1 agla|OJJ79893.1 akaw|GAA84416.1 aluc|OJZ89125.1 anid|CBF83641.1 anig|EHA23418.1 aory|EIT79718.1 asyd|OJJ60786.1 ater|EAU38394.1 atub|OJI85659.1 aver|OJJ00119.1 awen|OJJ33982.1 azon|OJJ48573.1

Group3825: abra|OJJ75677.1 acar|OOG00169.1 acla|EAW09423.1 afis|EAW21712.1 afla|EED57980.1 afum|EAL92747.1 agla|OJJ79895.1 akaw|GAA84418.1 aluc|OJZ89127.1 anid|CBF83653.1 anig|EHA23416.1 aory|EIT79727.1 asyd|OJJ60794.1 ater|EAU38396.1 atub|OJI85657.1 aver|OJJ00127.1 awen|OJJ33984.1 azon|OJJ48570.1

Group3826: abra|OJJ75679.1 acar|OOG00168.1 acla|EAW09424.1 afis|EAW21713.1 afla|EED57981.1 afum|EAL92748.1 agla|OJJ79886.1 akaw|GAA84419.1 aluc|OJZ89129.1 anid|CBF83655.1 anig|EHA23415.1 aory|EIT79767.1 asyd|OJJ60795.1 ater|EAU38398.1 atub|OJI85656.1 aver|OJJ00128.1 awen|OJJ33985.1 azon|OJJ48568.1

Group3827: abra|OJJ75680.1 acar|OOG00166.1 acla|EAW09422.1 afis|EAW21714.1 afla|EED57984.1 afum|EAL92749.1 agla|OJJ79885.1 akaw|GAA84420.1 aluc|OJZ89130.1 anid|CBF83657.1 anig|EHA23414.1 aory|EIT79791.1 asyd|OJJ60796.1 ater|EAU38399.1 atub|OJI85655.1 aver|OJJ00129.1 awen|OJJ33986.1 azon|OJJ48567.1

Group3828: abra|OJJ75682.1 acla|EAW09420.1 afis|EAW21716.1 afla|EED57986.1 afum|EAL92750.1 agla|OJJ79882.1 agla|OJJ79883.1 akaw|GAA84422.1 aluc|OJZ89133.1 anid|CBF83658.1 anig|EHA23412.1 aory|EIT79755.1 asyd|OJJ60798.1 ater|EAU38401.1 atub|OJI85653.1 aver|OJJ00131.1 awen|OJJ33988.1 azon|OJJ48565.1

Group3829: abra|OJJ75686.1 acar|OOG00164.1 acla|EAW09419.1 afis|EAW21717.1 afla|EED57987.1 afum|EAL92751.1 agla|OJJ79880.1 akaw|GAA84424.1 aluc|OJZ89137.1 anid|CBF83708.1 anig|EHA23410.1 aory|EIT79780.1 asyd|OJJ60831.1 ater|EAU38402.1 atub|OJI85648.1 aver|OJJ00156.1 awen|OJJ33990.1 azon|OJJ48563.1

Group3830: abra|OJJ75687.1 acar|OOG00163.1 acla|EAW09417.1 afis|EAW21719.1 afla|EED57990.1 afum|EAL92752.1 agla|OJJ79865.1 akaw|GAA84425.1 aluc|OJZ89138.1 anid|CBF83710.1 anig|EHA23407.1 aory|EIT79712.1 asyd|OJJ60833.1 ater|EAU38403.1 atub|OJI85647.1 aver|OJJ00157.1 awen|OJJ33992.1 azon|OJJ48561.1

Group3831: abra|OJJ75688.1 acar|OOG00162.1 acla|EAW09416.1 afis|EAW21720.1 afla|EED57989.1 afum|EAL92753.1 agla|OJJ79864.1 akaw|GAA84426.1 aluc|OJZ89139.1 anid|CBF83711.1 anig|EHA23406.1 aory|EIT79747.1 asyd|OJJ60834.1 ater|EAU38404.1 atub|OJI85646.1 aver|OJJ00158.1 awen|OJJ33993.1 azon|OJJ48560.1

Group3832: abra|OJJ75689.1 acar|OOG00161.1 acla|EAW09415.1 afis|EAW21721.1 afla|EED57988.1 afum|EAL92754.1 agla|OJJ79863.1 akaw|GAA84427.1 aluc|OJZ89140.1 anid|CBF83713.1 anig|EHA23405.1 aory|EIT79743.1 asyd|OJJ60835.1 ater|EAU38405.1 atub|OJI85645.1 aver|OJJ00159.1 awen|OJJ33994.1 azon|OJJ48559.1

Group3833: abra|OJJ75692.1 acar|OOG00160.1 acla|EAW09414.1 afis|EAW21722.1 afla|EED57991.1 afum|EAL92755.1 agla|OJJ79862.1 akaw|GAA84429.1 aluc|OJZ89143.1 anid|CBF83715.1 anig|EHA23404.1 aory|EIT79797.1 asyd|OJJ60836.1 ater|EAU38406.1 atub|OJI85644.1 aver|OJJ00160.1 awen|OJJ33995.1 azon|OJJ48558.1

Group3834: abra|OJJ75696.1 acar|OOG00158.1 acla|EAW09412.1 afis|EAW21724.1 afla|EED57993.1 afum|EAL92757.1 agla|OJJ79860.1 akaw|GAA84432.1 aluc|OJZ89147.1 anid|CBF83718.1 anig|EHA23401.1 aory|EIT79706.1 asyd|OJJ60838.1 ater|EAU38408.1 atub|OJI85641.1 aver|OJJ00162.1 awen|OJJ33997.1 azon|OJJ48555.1

Group3835: abra|OJJ75698.1 acar|OOG00155.1 acla|EAW09410.1 afis|EAW21726.1 afla|EED57995.1 afum|EAL92759.1 agla|OJJ79858.1 akaw|GAA84434.1 aluc|OJZ89149.1 anid|CBF83722.1 anig|EHA23399.1 aory|EIT79805.1 asyd|OJJ60840.1 ater|EAU38410.1 atub|OJI85639.1 aver|OJJ00164.1 awen|OJJ33999.1 azon|OJJ48553.1

Group3836: abra|OJJ75701.1 acar|OOG00153.1 acla|EAW09407.1 afis|EAW21729.1 afla|EED57998.1 afum|EAL92762.1 agla|OJJ79855.1 akaw|GAA84437.1 aluc|OJZ89152.1 anid|CBF83727.1 anig|EHA23397.1 aory|EIT79787.1 asyd|OJJ60843.1 ater|EAU38413.1 atub|OJI85636.1 aver|OJJ00168.1 awen|OJJ34002.1 azon|OJJ48550.1

Group3837: abra|OJJ75707.1 acar|OOG00151.1 acla|EAW09404.1 afis|EAW21732.1 afla|EED58001.1 afum|EAL92765.1 agla|OJJ79853.1 akaw|GAA84442.1 aluc|OJZ89158.1 anid|CBF83732.1 anig|EHA23395.1 aory|EIT79716.1 asyd|OJJ60846.1 ater|EAU38415.1 atub|OJI85632.1 aver|OJJ00171.1 awen|OJJ34005.1 azon|OJJ48546.1

Group3838: abra|OJJ75709.1 acar|OOG00149.1 acla|EAW09403.1 afis|EAW21733.1 afla|EED58002.1 afum|EAL92766.1 agla|OJJ79850.1 akaw|GAA84443.1 aluc|OJZ89160.1 anid|CBF83734.1 anig|EHA23394.1 aory|EIT79783.1 asyd|OJJ60848.1 ater|EAU38416.1 atub|OJI85630.1 aver|OJJ00174.1 awen|OJJ34006.1 azon|OJJ48545.1

Group3839: abra|OJJ75710.1 acar|OOG00150.1 acla|EAW09402.1 afis|EAW21734.1 afla|EED58006.1 afum|EAL92767.1 agla|OJJ79849.1 akaw|GAA84444.1 aluc|OJZ89161.1 anid|CBF83736.1 anig|EHA23393.1

aory|EIT79710.1 asyd|OJJ60850.1 ater|EAU36017.1 atub|OJI85629.1 aver|OJJ00172.1 awen|OJJ34007.1 azon|OJJ48544.1

Group3840: abra|OJJ75716.1 acar|OOG00143.1 acla|EAW09397.1 afis|EAW21737.1 afla|EED58011.1 afum|EAL92771.1 agla|OJJ84451.1 akaw|GAA84450.1 aluc|OJZ89168.1 anid|CBF83703.1 anig|EHA23387.1 aory|EIT79723.1 asyd|OJJ60826.1 ater|EAU38419.1 atub|OJI85623.1 aver|OJJ00152.1 awen|OJJ34019.1 azon|OJJ48539.1

Group3841: abra|OJJ75719.1 acar|OOG00137.1 acla|EAW09375.1 afis|EAW21760.1 afla|EED58018.1 afum|EAL92790.2 agla|OJJ84390.1 akaw|GAA84455.1 aluc|OJZ89171.1 anid|CBF83695.1 anig|EHA23384.1 aory|EIT79793.1 asyd|OJJ60824.1 ater|EAU38430.1 atub|OJI85619.1 aver|OJJ00150.1 awen|OJJ34044.1 azon|OJJ48187.1

Group3842: abra|OJJ75722.1 acar|OOG00136.1 acla|EAW09378.1 afis|EAW21757.1 afla|EED58020.1 afum|EAL92787.1 agla|OJJ84393.1 akaw|GAA84458.1 aluc|OJZ89174.1 anid|CBF83682.1 anig|EHA23381.1 aory|EIT79724.1 asyd|OJJ60821.1 ater|EAU38433.1 atub|OJI85616.1 aver|OJJ00147.1 awen|OJJ34041.1 azon|OJJ48198.1

Group3843: abra|OJJ75723.1 acar|OOG00135.1 acla|EAW09379.1 afis|EAW21756.1 afla|EED58022.1 afum|EAL92786.1 agla|OJJ84394.1 akaw|GAA84459.1 aluc|OJZ89175.1 anid|CBF83684.1 anig|EHA23380.1 asyd|OJJ60820.1 ater|EAU38434.1 atub|OJI85615.1 aver|OJJ00146.1 awen|OJJ34040.1 azon|OJJ48197.1 aory|EIT79736.1

Group3844: abra|OJJ75724.1 acar|OOG00134.1 acla|EAW09380.1 afis|EAW21755.1 afla|EED58023.1 afum|EAL92785.1 agla|OJJ84398.1 akaw|GAA84460.1 aluc|OJZ89176.1 anid|CBF83685.1 anig|EHA23379.1 aory|EIT79744.1 asyd|OJJ60819.1 ater|EAU38435.1 atub|OJI85613.1 aver|OJJ00145.1 awen|OJJ34039.1 azon|OJJ48196.1

Group3845: abra|OJJ75726.1 acar|OOG00132.1 acla|EAW09383.1 afis|EAW21752.1 afla|EED58026.1 afum|EAL92782.1 agla|OJJ84401.1 akaw|GAA84462.1 aluc|OJZ89178.1 anid|CBF83693.1 anig|EHA23377.1 aory|EIT79749.1 asyd|OJJ60816.1 ater|EAU38438.1 atub|OJI85611.1 aver|OJJ00142.1 awen|OJJ34035.1 azon|OJJ48207.1

Group3846: abra|OJJ75728.1 acar|OOF90230.1 acla|EAW08986.1 afis|EAW24686.1 afla|EED54452.1 afum|EAL89327.1 akaw|GAA84463.1 aluc|OJZ89180.1 anid|CBF83674.1 anig|EHA23375.1 aory|EIT76299.1 asyd|OJJ60813.1 ater|EAU39265.1 atub|OJI85609.1 aver|OJJ00140.1 awen|OJJ29740.1 afla|EED48344.1 ater|EAU31420.1

Group3847: abra|OJJ75732.1 acar|OOF89927.1 acla|EAW14909.1 afis|EAW24430.1 afla|EED55243.1 afum|EAL86098.1 agla|OJJ79244.1 akaw|GAA84465.1 aluc|OJZ89183.1 anid|CBF73775.1 anig|EHA23373.1 aory|EIT83557.1 asyd|OJJ56594.1 ater|EAU30015.1 atub|OJI85606.1 aver|OJJ06131.1 awen|OJJ32772.1 azon|OJJ44003.1

Group3848: abra|OJJ75734.1 acar|OOF89928.1 acla|EAW14908.1 afis|EAW24429.1 afla|EED55249.1 afum|EAL86097.1 agla|OJJ79242.1 akaw|GAA84467.1 aluc|OJZ89185.1 anid|CBF73777.1 anig|EHA23372.1 aory|EIT83561.1 asyd|OJJ56596.1 ater|EAU30016.1 atub|OJI85604.1 aver|OJJ06129.1 awen|OJJ32774.1 azon|OJJ44004.1

Group3849: abra|OJJ75735.1 acar|OOF89929.1 acla|EAW14907.1 afis|EAW24428.1 afla|EED55250.1 afum|EAL86096.1 agla|OJJ79241.1 akaw|GAA84468.1 aluc|OJZ89186.1 anid|CBF73778.1 anig|EHA23371.1 aory|EIT83631.1 asyd|OJJ56597.1 ater|EAU30017.1 atub|OJI85603.1 aver|OJJ06128.1 awen|OJJ32775.1 azon|OJJ44005.1

Group3850: abra|OJJ75737.1 acar|OOF89931.1 acla|EAW14904.1 afis|EAW24425.1 afla|EED55254.1 afum|EAL86093.1 agla|OJJ79239.1 akaw|GAA84470.1 aluc|OJZ89189.1 anid|CBF73782.1 anig|EHA23368.1 aory|EIT83565.1 asyd|OJJ56599.1 ater|EAU30020.1 atub|OJI85601.1 aver|OJJ06126.1 awen|OJJ32781.1 azon|OJJ44007.1

Group3851: abra|OJJ75738.1 acar|OOG00131.1 acla|EAW14903.1 afis|EAW24424.1 afla|EED55258.1 afum|EAL86092.1 agla|OJJ79238.1 akaw|GAA84471.1 aluc|OJZ89191.1 anid|CBF73784.1 anig|EHA23367.1 aory|EIT83539.1 asyd|OJJ56600.1 ater|EAU30021.1 atub|OJI85599.1 aver|OJJ06125.1 awen|OJJ32780.1 azon|OJJ44009.1

Group3852: abra|OJJ75784.1 acar|OOG00104.1 acla|EAW14898.1 afis|EAW24420.1 afla|EED47748.1 afum|EAL86089.1 agla|OJJ79224.1 akaw|GAA84500.1 aluc|OJZ89227.1 anid|CBF73792.1 anig|EHA23335.1 aory|EIT77177.1 asyd|OJJ56604.1 ater|EAU30307.1 atub|OJI85565.1 aver|OJJ06119.1 awen|OJJ32789.1 azon|OJJ44022.1

Group3853: abra|OJJ75785.1 acar|OOG00103.1 acla|EAW14897.1 afis|EAW24419.1 afla|EED47749.1 afum|EAL86088.1 agla|OJJ79226.1 akaw|GAA84501.1 aluc|OJZ89229.1 anid|CBF73795.1 anig|EHA23334.1 aory|EIT77122.1 asyd|OJJ56605.1 ater|EAU30308.1 atub|OJI85564.1 aver|OJJ06118.1 awen|OJJ32790.1 azon|OJJ44021.1

Group3854: abra|OJJ75786.1 acar|OOG00102.1 acla|EAW14896.1 afis|EAW24418.1 afla|EED47750.1 afum|EAL86087.1 agla|OJJ79227.1 akaw|GAA84502.1 aluc|OJZ89230.1 anid|CBF73797.1 anig|EHA23333.1 aory|EIT77133.1 asyd|OJJ56607.1 ater|EAU30309.1 atub|OJI85563.1 aver|OJJ06117.1 awen|OJJ32791.1 azon|OJJ44020.1

Group3855: abra|OJJ75787.1 acar|OOG00101.1 acla|EAW14895.1 afis|EAW24417.1 afla|EED47751.1 afum|EAL86086.1 agla|OJJ79228.1 akaw|GAA84503.1 aluc|OJZ89231.1 anid|CBF73799.1 anig|EHA23332.1 aory|EIT77182.1 asyd|OJJ56608.1 ater|EAU30310.1 atub|OJI85562.1 aver|OJJ06116.1 awen|OJJ32792.1 azon|OJJ44019.1

Group3856: abra|OJJ75788.1 acar|OOG00099.1 acla|EAW14893.1 afis|EAW24415.1 afla|EED47766.1 afum|EAL86084.1 agla|OJJ79218.1 akaw|GAA84504.1 aluc|OJZ89232.1 anid|CBF73803.1 anig|EHA23331.1 aory|EIT77193.1 asyd|OJJ56615.1 ater|EAU30314.1 atub|OJI85561.1 aver|OJJ06109.1 awen|OJJ32794.1 azon|OJJ49041.1

Group3857: abra|OJJ75789.1 acar|OOG00098.1 acla|EAW14892.1 afis|EAW24414.1 afla|EED47765.1 afum|EAL86083.1 agla|OJJ79217.1 akaw|GAA84505.1 aluc|OJZ89233.1 anid|CBF73747.1 anig|EHA23330.1 aory|EIT77149.1 asyd|OJJ56622.1 ater|EAU30315.1 atub|OJI85560.1 aver|OJJ06104.1 awen|OJJ32795.1 azon|OJJ49038.1

Group3858: abra|OJJ75792.1 acar|OOG00086.1 acla|EAW14889.1 afis|EAW24411.1 afum|EAL86080.1 agla|OJJ79214.1 akaw|GAA84511.1 aluc|OJZ89240.1 anid|CBF73741.1 anig|EHA23327.1 aory|EIT77168.1 asyd|OJJ56625.1 ater|EAU30318.1 atub|OJI85558.1 aver|OJJ06101.1 awen|OJJ32799.1 azon|OJJ49033.1 afla|EED47762.1

Group3859: abra|OJJ75796.1 acar|OOG00085.1 acla|EAW14888.1 afis|EAW24410.1 afla|EED47761.1 afum|EAL86079.1 agla|OJJ79213.1 akaw|GAA84514.1 aluc|OJZ89243.1 anid|CBF73739.1 anig|EHA23322.1 aory|EIT77110.1 asyd|OJJ56626.1 ater|EAU30319.1 atub|OJI85552.1 aver|OJJ06100.1 awen|OJJ32800.1 azon|OJJ49032.1

Group3860: abra|OJJ75804.1 acar|OOG00071.1 acla|EAW14879.1 afis|EAW24396.1 afla|EED47814.1 afum|EAL86068.1 agla|OJJ78876.1 akaw|GAA84520.1 aluc|OJZ89252.1 anid|CBF73724.1 anig|EHA23315.1 aory|EIT77191.1 asyd|OJJ56638.1 ater|EAU30339.1 atub|OJI85546.1 aver|OJJ06088.1 awen|OJJ32809.1 azon|OJJ49030.1

Group3861: abra|OJJ75805.1 acar|OOG00070.1 acla|EAW14878.1 afis|EAW24395.1 afla|EED47815.1 afum|EAL86067.1 agla|OJJ78875.1 akaw|GAA84521.1 aluc|OJZ89253.1 anid|CBF73720.1 anig|EHA23314.1 aory|EIT77139.1 asyd|OJJ56639.1 ater|EAU30340.1 atub|OJI85545.1 aver|OJJ06087.1 awen|OJJ32810.1 azon|OJJ49031.1

Group3862: abra|OJJ75808.1 acar|OOG00068.1 acla|EAW14864.1 afis|EAW24392.1 afla|EED47818.1 afum|EAL86064.1 agla|OJJ78872.1 akaw|GAA84523.1 aluc|OJZ89256.1 anid|CBF73713.1 anig|EHA23312.1 aory|EIT77112.1 asyd|OJJ56642.1 ater|EAU30342.1 atub|OJI85543.1 aver|OJJ06084.1 awen|OJJ32812.1 azon|OJJ49020.1

Group3863: abra|OJJ75810.1 acar|OOG00067.1 acla|EAW14863.1 afis|EAW24391.1 afla|EED47819.1 afum|EAL86063.1 agla|OJJ78871.1 akaw|GAA84524.1 aluc|OJZ89257.1 anid|CBF73711.1 anig|EHA23311.1 aory|EIT77158.1 asyd|OJJ56643.1 ater|EAU30343.1 atub|OJI85542.1 aver|OJJ06083.1 awen|OJJ32813.1 azon|OJJ49019.1

Group3864: abra|OJJ75813.1 acar|OOG00062.1 acla|EAW14860.1 afis|EAW24388.1 afla|EED47821.1 afum|EAL86060.1 agla|OJJ83284.1 akaw|GAA84527.1 aluc|OJZ89260.1 anid|CBF73705.1 anig|EHA23308.1 aory|EIT77124.1 asyd|OJJ56646.1 ater|EAU30346.1 atub|OJI85539.1 aver|OJJ06080.1 awen|OJJ32817.1 azon|OJJ49015.1

Group3865: abra|OJJ75814.1 acar|OOG00061.1 acla|EAW14859.1 afis|EAW24387.1 afla|EED47822.1 afum|EAL86059.1 agla|OJJ83283.1 akaw|GAA84528.1 aluc|OJZ89261.1 anid|CBF73703.1 anig|EHA23307.1 aory|EIT77142.1 asyd|OJJ56647.1 ater|EAU30347.1 atub|OJI85538.1 aver|OJJ06079.1 awen|OJJ32818.1 azon|OJJ49014.1

Group3866: abra|OJJ75817.1 acar|OOG00058.1 acla|EAW14858.1 afis|EAW24385.1 afla|EED47824.1 afum|EAL86057.1 agla|OJJ83281.1 akaw|GAA84531.1 aluc|OJZ89264.1 anid|CBF73697.1 anig|EHA23304.1 aory|EIT77137.1 asyd|OJJ56650.1 ater|EAU30350.1 atub|OJI85535.1 aver|OJJ06075.1 awen|OJJ32825.1 azon|OJJ49011.1

Group3867: abra|OJJ75821.1 acar|OOG00055.1 acla|EAW14854.1 afis|EAW24381.1 afla|EED47829.1 afum|EAL86053.1 agla|OJJ83269.1 akaw|GAA84534.1 aluc|OJZ89267.1 anid|CBF73667.1 anig|EHA23301.1 aory|EIT77192.1 asyd|OJJ56666.1 ater|EAU30354.1 atub|OJI85532.1 aver|OJJ06057.1 awen|OJJ32831.1 azon|OJJ49005.1

Group3868: abra|OJJ75822.1 acar|OOG00054.1 acla|EAW14853.1 afis|EAW24380.1 afla|EED47830.1 afum|EAL86052.1 agla|OJJ83268.1 akaw|GAA84535.1 aluc|OJZ89268.1 anid|CBF73669.1 anig|EHA23300.1

aory|EIT77144.1 asyd|OJJ56665.1 ater|EAU30355.1 atub|OJI85531.1 aver|OJJ06058.1 awen|OJJ32832.1 azon|OJJ49004.1

Group3869: abra|OJJ75828.1 acar|OOG00052.1 acla|EAW14850.1 afis|EAW24377.1 afla|EED47834.1 afum|EAL86049.1 agla|OJJ83263.1 akaw|GAA84538.1 aluc|OJZ89271.1 anid|CBF73674.1 anig|EHA23298.1 aory|EIT77135.1 asyd|OJJ56662.1 ater|EAU30359.1 atub|OJI85528.1 aver|OJJ06062.1 awen|OJJ32835.1 azon|OJJ49000.1

Group3870: abra|OJJ75829.1 acar|OOG00051.1 acla|EAW14849.1 afis|EAW24376.1 afla|EED47835.1 afum|EAL86048.1 agla|OJJ83262.1 akaw|GAA84539.1 aluc|OJZ89272.1 anid|CBF73680.1 anig|EHA23297.1 aory|EIT77202.1 asyd|OJJ56659.1 ater|EAU30360.1 atub|OJI85527.1 aver|OJJ06066.1 awen|OJJ32836.1 azon|OJJ48998.1

Group3871: abra|OJJ75831.1 acar|OOG00049.1 acla|EAW14847.1 afis|EAW24374.1 afla|EED47837.1 afum|EAL86046.1 agla|OJJ83260.1 akaw|GAA84541.1 aluc|OJZ89274.1 anid|CBF73682.1 anig|EHA23295.1 aory|EIT77185.1 asyd|OJJ56657.1 ater|EAU30362.1 atub|OJI85525.1 aver|OJJ06068.1 awen|OJJ32838.1 azon|OJJ48996.1

Group3872: abra|OJJ75832.1 acar|OOG00048.1 acla|EAW14846.1 afis|EAW24373.1 afla|EED47838.1 afum|EAL86045.1 agla|OJJ83259.1 akaw|GAA84542.1 aluc|OJZ89275.1 anid|CBF73684.1 anig|EHA23294.1 aory|EIT77136.1 asyd|OJJ56656.1 ater|EAU30363.1 atub|OJI85524.1 aver|OJJ06069.1 awen|OJJ32839.1 azon|OJJ48995.1

Group3873: abra|OJJ75843.1 acar|OOG00045.1 acla|EAW14844.1 afis|EAW24371.1 afla|EED47846.1 afum|EAL86043.1 agla|OJJ83256.1 akaw|GAA84553.1 aluc|OJZ89285.1 anid|CBF73688.1 anig|EHA23281.1 aory|EIT77207.1 asyd|OJJ56654.1 ater|EAU30368.1 atub|OJI85514.1 aver|OJJ06071.1 awen|OJJ32844.1 azon|OJJ46816.1

Group3874: abra|OJJ75851.1 acar|OOG00042.1 acla|EAW14835.1 afis|EAW24364.1 afla|EED47849.1 afum|EBA27254.1 agla|OJJ83249.1 akaw|GAA84556.1 aluc|OJZ89288.1 anid|CBF73935.1 anig|EHA23273.1 aory|EIT77237.1 asyd|OJJ53672.1 ater|EAU30373.1 atub|OJI85511.1 aver|OJJ07779.1 awen|OJJ32869.1 azon|OJJ48991.1

Group3875: abra|OJJ75854.1 acar|OOG00041.1 acla|EAW14833.1 afis|EAW24362.1 afla|EED47851.1 afum|EAL86032.1 agla|OJJ83247.1 akaw|GAA84559.1 aluc|OJZ89291.1 anid|CBF73928.1 anig|EHA23271.1 aory|EIT77246.1 asyd|OJJ53677.1 ater|EAU30376.1 atub|OJI85508.1 aver|OJJ07793.1 awen|OJJ32865.1 azon|OJJ48989.1

Group3876: abra|OJJ75855.1 acar|OOG00040.1 acla|EAW14832.1 afis|EAW24361.1 afla|EED47852.1 afum|EAL86031.1 agla|OJJ83246.1 akaw|GAA84560.1 aluc|OJZ89293.1 anid|CBF73926.1 anig|EHA23270.1 aory|EIT77210.1 asyd|OJJ53678.1 ater|EAU30377.1 atub|OJI85507.1 aver|OJJ07794.1 awen|OJJ32864.1 azon|OJJ48988.1

Group3877: abra|OJJ75856.1 acar|OOG00038.1 acla|EAW14831.1 afis|EAW24360.1 afla|EED47853.1 afum|EAL86030.1 agla|OJJ83245.1 akaw|GAA84561.1 aluc|OJZ89294.1 anid|CBF73924.1 anig|EHA23269.1 aory|EIT77236.1 asyd|OJJ53679.1 ater|EAU30378.1 atub|OJI85506.1 aver|OJJ07795.1 awen|OJJ32863.1 azon|OJJ48987.1

Group3878: abra|OJJ75858.1 acar|OOG00037.1 acla|EAW14829.1 afis|EAW24358.1 afla|EED47856.1 afum|EAL86028.1 agla|OJJ83242.1 akaw|GAA84563.1 aluc|OJZ89296.1 anid|CBF73921.1 anig|EHA23267.1 aory|EIT77221.1 asyd|OJJ53681.1 ater|EAU30381.1 atub|OJI85503.1 aver|OJJ07797.1 awen|OJJ32861.1 azon|OJJ48985.1

Group3879: abra|OJJ75864.1 acar|OOG00032.1 acla|EAW14824.1 afis|EAW24353.1 afla|EED47910.1 afum|EAL86024.2 agla|OJJ83233.1 akaw|GAA84570.1 aluc|OJZ89306.1 anid|CBF73944.1 anig|EHA23260.1 aory|EIT83380.1 asyd|OJJ53698.1 ater|EAU30386.1 atub|OJI85493.1 aver|OJJ07816.1 awen|OJJ32872.1 azon|OJJ48973.1

Group3880: abra|OJJ75865.1 acar|OOG00029.1 acla|EAW09926.1 afis|EAW21174.1 afla|EED54761.1 afum|EAL92194.1 agla|OJJ80004.1 akaw|GAA84571.1 aluc|OJZ89307.1 anid|CBF83297.1 anig|EHA23259.1 aory|EIT79426.1 asyd|OJJ60558.1 ater|EAU35841.1 atub|OJI85491.1 aver|OJI99884.1 awen|OJJ36513.1 azon|OJJ44389.1

Group3881: abra|OJJ75867.1 acar|OOG00027.1 acla|EAW09928.1 afis|EAW21176.1 afla|EED54763.1 afum|EAL92196.1 agla|OJJ80001.1 akaw|GAA84573.1 aluc|OJZ89309.1 anid|CBF83306.1 anig|EHA23257.1 aory|EIT79399.1 asyd|OJJ60555.1 ater|EAU35843.1 atub|OJI85490.1 aver|OJI99881.1 awen|OJJ36515.1 azon|OJJ44391.1

Group3882: abra|OJJ75873.1 acar|OOG00022.1 acla|EAW09932.1 afis|EAW21179.1 afla|EED54769.1 afum|EAL92199.1 agla|OJJ79993.1 akaw|GAA84576.1 aluc|OJZ89317.1 anid|CBF83303.1 anig|EHA23254.1 aory|EIT79449.1 asyd|OJJ60552.1 ater|EAU35845.1 atub|OJI85480.1 aver|OJI99878.1 awen|OJJ36518.1 azon|OJJ44393.1

Group3883: abra|OJJ75875.1 acar|OOG00020.1 acla|EAW09935.1 afis|EAW21180.1 afla|EED54785.1 afum|EAL92201.1 agla|OJJ79997.1 akaw|GAA84577.1 aluc|OJZ89319.1 anid|CBF83301.1 anig|EHA23253.1 aory|EIT79362.1 asyd|OJJ60551.1 ater|EAU35846.1 atub|OJI85479.1 aver|OJI99877.1 awen|OJJ36519.1 azon|OJJ44395.1

Group3884: abra|OJJ75876.1 acar|OOG00017.1 acla|EAW09938.1 afis|EAW21183.1 afla|EED54776.1 afum|EAL92204.1 agla|OJJ79987.1 akaw|GAA84578.1 aluc|OJZ89320.1 anid|CBF83312.1 anig|EHA23252.1 aory|EIT79349.1 asyd|OJJ60548.1 ater|EAU35849.1 atub|OJI85478.1 aver|OJI99874.1 awen|OJJ36522.1 azon|OJJ44398.1

Group3885: abra|OJJ75879.1 acar|OOG00014.1 acla|EAW09940.1 afis|EAW21185.1 afla|EED54774.1 afum|EAL92207.1 agla|OJJ79982.1 akaw|GAA84582.1 aluc|OJZ89325.1 anid|CBF83315.1 anig|EHA23249.1 aory|EIT79429.1 asyd|OJJ60547.1 ater|EAU35850.1 atub|OJI85474.1 aver|OJI99873.1 awen|OJJ36526.1 azon|OJJ44399.1

Group3886: abra|OJJ75880.1 acar|OOG00013.1 acla|EAW09942.1 afis|EAW21187.1 afla|EED54773.1 afum|EAL92209.1 agla|OJJ79979.1 akaw|GAA84583.1 aluc|OJZ89326.1 anid|CBF83319.1 anig|EHA23248.1 aory|EIT79437.1 asyd|OJJ60546.1 ater|EAU35851.1 atub|OJI85473.1 aver|OJI99872.1 awen|OJJ36529.1 azon|OJJ44401.1

Group3887: abra|OJJ75884.1 acar|OOF90248.1 acla|EAW09944.1 afis|EAW21222.1 afla|EED54784.1 afum|EBA27454.1 agla|OJJ78510.1 akaw|GAA84592.1 aluc|OJZ89337.1 anid|CBF83295.1 anig|EHA23244.1 aory|EIT79434.1 asyd|OJJ60560.1 ater|EAU35852.1 atub|OJI85462.1 aver|OJI99886.1 awen|OJJ36530.1 azon|OJJ44404.1

Group3888: abra|OJJ75886.1 acar|OOF90247.1 acla|EAW09945.1 afis|EAW21223.1 afla|EED54783.1 afum|EAL92236.1 agla|OJJ78507.1 akaw|GAA84593.1 aluc|OJZ89338.1 anid|CBF83293.1 anig|EHA23243.1 aory|EIT79443.1 asyd|OJJ60561.1 ater|EAU35853.1 atub|OJI85461.1 aver|OJI99887.1 awen|OJJ36531.1 azon|OJJ44405.1

Group3889: abra|OJJ75888.1 acar|OOF90245.1 acla|EAW09946.1 afis|EAW21224.1 afla|EED54782.1 afum|EAL92237.1 agla|OJJ78506.1 akaw|GAA84594.1 aluc|OJZ89340.1 anid|CBF83291.1 anig|EHA23242.1 aory|EIT79409.1 asyd|OJJ60562.1 ater|EAU35854.1 atub|OJI85459.1 aver|OJI99888.1 awen|OJJ36532.1 azon|OJJ44406.1

Group3890: abra|OJJ75889.1 acar|OOF90244.1 acla|EAW09948.1 afis|EAW21226.1 afla|EED54779.1 afum|EBA27456.1 agla|OJJ78504.1 akaw|GAA84596.1 aluc|OJZ89341.1 anid|CBF83289.1 anig|EHA23241.1 aory|EIT79431.1 asyd|OJJ60563.1 ater|EAU35857.1 atub|OJI85458.1 aver|OJI99890.1 awen|OJJ36534.1 azon|OJJ44408.1

Group3891: abra|OJJ75890.1 acar|OOG00012.1 acla|EAW09898.1 afis|EAW21228.1 afla|EED54786.1 afum|EAL92240.1 agla|OJJ78502.1 akaw|GAA84597.1 aluc|OJZ89342.1 anid|CBF83280.1 anig|EHA23240.1 aory|EIT79332.1 asyd|OJJ60568.1 ater|EAU35859.1 atub|OJI85457.1 aver|OJI99893.1 awen|OJJ36537.1 azon|OJJ44351.1

Group3892: abra|OJJ75891.1 acar|OOG00011.1 acla|EAW09897.1 afis|EAW21229.1 afla|EED54787.1 afum|EAL92241.2 agla|OJJ78501.1 akaw|GAA84598.1 aluc|OJZ89343.1 anid|CBF83278.1 anig|EHA23239.1 aory|EIT79422.1 asyd|OJJ60569.1 ater|EAU35860.1 atub|OJI85456.1 aver|OJI99894.1 awen|OJJ36538.1 azon|OJJ44349.1

Group3893: abra|OJJ75892.1 acar|OOG00010.1 acla|EAW09896.1 afis|EAW21230.1 afla|EED54788.1 afum|EAL92242.1 agla|OJJ78492.1 akaw|GAA84599.1 aluc|OJZ89344.1 anid|CBF83277.1 anig|EHA23238.1 aory|EIT79453.1 asyd|OJJ60570.1 ater|EAU35863.1 atub|OJI85455.1 aver|OJI99895.1 awen|OJJ36539.1 azon|OJJ44348.1

Group3894: abra|OJJ75897.1 acar|OOG00007.1 acla|EAW09894.1 afis|EAW21232.1 afla|EED54793.1 afum|EAL92244.1 agla|OJJ78494.1 akaw|GAA84603.1 aluc|OJZ89347.1 anid|CBF83273.1 anig|EHA23234.1 aory|EIT79342.1 asyd|OJJ60573.1 ater|EAU35865.1 atub|OJI85451.1 aver|OJI99898.1 awen|OJJ36542.1 azon|OJJ44346.1

Group3895: abra|OJJ75898.1 acar|OOG00006.1 acla|EAW09893.1 afis|EAW21233.1 afla|EED54794.1 afum|EAL92245.1 agla|OJJ78495.1 akaw|GAA84604.1 aluc|OJZ89348.1 anid|CBF83271.1 anig|EHA23233.1 aory|EIT79361.1 asyd|OJJ60574.1 ater|EAU35866.1 atub|OJI85450.1 aver|OJI99899.1 awen|OJJ36543.1 azon|OJJ44345.1

Group3896: abra|OJJ75899.1 acar|OOG00005.1 acla|EAW09892.1 afis|EAW21234.1 afla|EED54795.1 afum|EAL92246.1 agla|OJJ78496.1 akaw|GAA84605.1 aluc|OJZ89349.1 anid|CBF83270.1 anig|EHA23232.1 aory|EIT79442.1 asyd|OJJ60575.1 ater|EAU35867.1 atub|OJI85449.1 aver|OJI99900.1 awen|OJJ36544.1 azon|OJJ44344.1

Group3897: abra|OJJ75902.1 acar|OOG00002.1 acla|EAW09888.1 afis|EAW21238.1 afla|EED54798.1 afum|EAL92250.1 agla|OJJ78500.1 akaw|GAA84608.1 aluc|OJZ89352.1 anid|CBF83266.1 anig|EHA23229.1

aory|EIT79394.1 asyd|OJJ60579.1 ater|EAU35879.1 atub|OJI85446.1 aver|OJI99904.1 awen|OJJ36549.1 azon|OJJ44340.1

Group3898: abra|OJJ75903.1 acar|OOG00001.1 acla|EAW09887.1 afis|EAW21239.1 afla|EED54799.1 afum|EAL92252.1 agla|OJJ78491.1 akaw|GAA84609.1 aluc|OJZ89353.1 anid|CBF83264.1 anig|EHA23228.1 aory|EIT79376.1 asyd|OJJ60580.1 ater|EAU35880.1 atub|OJI85445.1 aver|OJI99905.1 awen|OJJ36550.1 azon|OJJ44339.1

Group3899: abra|OJJ75904.1 acar|OOF99999.1 acla|EAW09886.1 afis|EAW21240.1 afla|EED54800.1 afum|EAL92253.1 agla|OJJ78490.1 akaw|GAA84610.1 aluc|OJZ89354.1 anid|CBF83262.1 anig|EHA23227.1 aory|EIT79341.1 asyd|OJJ60581.1 ater|EAU35881.1 atub|OJI85444.1 aver|OJI99906.1 awen|OJJ36551.1 azon|OJJ44338.1

Group3900: abra|OJJ75905.1 acar|OOF99998.1 acla|EAW09885.1 afis|EAW21241.1 afla|EED54802.1 afum|EAL92254.1 agla|OJJ78489.1 akaw|GAA84611.1 aluc|OJZ89355.1 anid|CBF83259.1 anig|EHA23226.1 aory|EIT79395.1 asyd|OJJ60584.1 ater|EAU35882.1 atub|OJI85443.1 aver|OJI99907.1 awen|OJJ36552.1 azon|OJJ44337.1

Group3901: abra|OJJ75906.1 acar|OOF99997.1 acla|EAW09883.1 afis|EAW21243.1 afla|EED54803.1 afum|EAL92256.1 agla|OJJ78487.1 akaw|GAA84612.1 aluc|OJZ89356.1 anid|CBF83255.1 anig|EHA23225.1 aory|EIT79348.1 asyd|OJJ60587.1 ater|EAU35883.1 atub|OJI85442.1 aver|OJI99910.1 awen|OJJ36554.1 azon|OJJ44335.1

Group3902: abra|OJJ75910.1 acar|OOF99993.1 acla|EAW09879.1 afis|EAW21247.1 afla|EED54807.1 afum|EAL92260.1 agla|OJJ87509.1 akaw|GAA84616.1 aluc|OJZ89360.1 anid|CBF83240.1 anig|EHA23221.1 aory|EIT79440.1 asyd|OJJ60598.1 ater|EAU35887.1 atub|OJI85438.1 aver|OJI99921.1 awen|OJJ36560.1 azon|OJJ44331.1

Group3903: abra|OJJ75911.1 acar|OOF99992.1 acla|EAW09878.1 afis|EAW21248.1 afla|EED54808.1 afum|EAL92261.1 agla|OJJ87508.1 akaw|GAA84617.1 aluc|OJZ89361.1 anid|CBF83242.1 anig|EHA23220.1 aory|EIT79438.1 asyd|OJJ60597.1 ater|EAU35888.1 atub|OJI85437.1 aver|OJI99920.1 awen|OJJ36561.1 azon|OJJ44327.1

Group3904: abra|OJJ75914.1 acar|OOF99989.1 acla|EAW09876.1 afis|EAW21250.1 afla|EED54810.1 afum|EAL92263.1 agla|OJJ87501.1 akaw|GAA84619.1 aluc|OJZ89363.1 anid|CBF83245.1 anig|EHA23219.1 aory|EIT79398.1 asyd|OJJ60594.1 ater|EAU35890.1 atub|OJI85435.1 aver|OJI99918.1 awen|OJJ36564.1 azon|OJJ44324.1

Group3905: abra|OJJ75916.1 acar|OOF90029.1 acla|EAW09874.1 afis|EAW21252.1 afla|EED54812.1 afum|EAL92266.1 agla|OJJ87495.1 akaw|GAA84621.1 aluc|OJZ89365.1 anid|CBF83247.1 anig|EHA23217.1 aory|EIT79364.1 asyd|OJJ60592.1 ater|EAU35892.1 atub|OJI85433.1 aver|OJI99916.1 awen|OJJ36567.1 azon|OJJ44321.1

Group3906: abra|OJJ75919.1 acar|OOF90028.1 acla|EAW09873.1 afis|EAW21253.1 afla|EED54815.1 afum|EAL92268.1 agla|OJJ87497.1 akaw|GAA84622.1 aluc|OJZ89368.1 anid|CBF83249.1 anig|EHA23215.1 aory|EIT79356.1 asyd|OJJ60590.1 ater|EAU35893.1 atub|OJI85430.1 aver|OJI99913.1 awen|OJJ36568.1 azon|OJJ44319.1

Group3907: abra|OJJ75920.1 acar|OOF99987.1 acla|EAW09872.1 afis|EAW21254.1 afla|EED54816.1 afum|EAL92269.1 agla|OJJ87498.1 akaw|GAA84623.1 aluc|OJZ89369.1 anid|CBF83251.1 anig|EHA23214.1 aory|EIT79384.1 asyd|OJJ60589.1 ater|EAU35894.1 atub|OJI85429.1 aver|OJI99912.1 awen|OJJ36569.1 azon|OJJ44318.1

Group3908: abra|OJJ75922.1 acar|OOF99986.1 acla|EAW09866.1 afis|EAW21259.1 afla|EED54818.1 afum|EAL92274.1 agla|OJJ87491.1 akaw|GAA84626.1 aluc|OJZ89372.1 anid|CBF83325.1 anig|EHA23212.1 aory|EIT79360.1 asyd|OJJ60601.1 ater|EAU35896.1 atub|OJI85426.1 aver|OJI99924.1 awen|OJJ36574.1 azon|OJJ44315.1

Group3909: abra|OJJ75924.1 acar|OOF99984.1 acla|EAW09865.1 afis|EAW21260.1 afla|EED54819.1 afum|EAL92275.1 agla|OJJ87490.1 akaw|GAA84627.1 aluc|OJZ89373.1 anid|CBF83329.1 anig|EHA23210.1 aory|EIT79435.1 asyd|OJJ60605.1 ater|EAU35897.1 atub|OJI85425.1 aver|OJI99929.1 awen|OJJ36575.1 azon|OJJ44314.1

Group3910: abra|OJJ75931.1 acar|OOF99979.1 acla|EAW09862.1 afis|EAW21264.1 afla|EED54823.1 afum|EAL92279.1 agla|OJJ87471.1 akaw|GAA84632.1 aluc|OJZ89378.1 anid|CBF83340.1 anig|EHA23205.1 aory|EIT79396.1 asyd|OJJ60609.1 ater|EAU35901.1 atub|OJI85420.1 aver|OJI99933.1 awen|OJJ36581.1 azon|OJJ44309.1

Group3911: abra|OJJ75932.1 acar|OOF99978.1 acla|EAW09861.1 afis|EAW21265.1 afla|EED54824.1 afum|EAL92280.1 agla|OJJ87472.1 akaw|GAA84633.1 aluc|OJZ89379.1 anid|CBF83342.1 anig|EHA23204.1 aory|EIT79447.1 asyd|OJJ60610.1 ater|EAU35902.1 atub|OJI85419.1 aver|OJI99934.1 awen|OJJ36582.1 azon|OJJ44308.1

Group3912: abra|OJJ75934.1 acar|OOF99976.1 acla|EAW09859.1 afis|EAW21267.1 afla|EED54826.1 afum|EAL92282.1 agla|OJJ87474.1 akaw|GAA84635.1 aluc|OJZ89381.1 anid|CBF83345.1 anig|EHA23202.1 aory|EIT79337.1 asyd|OJJ60612.1 ater|EAU35903.1 atub|OJI85417.1 aver|OJI99936.1 awen|OJJ36584.1 azon|OJJ47371.1

Group3913: abra|OJJ75935.1 acar|OOF99975.1 acla|EAW09858.1 afis|EAW21268.1 afla|EED54827.1 afum|EAL92283.1 agla|OJJ87475.1 akaw|GAA84636.1 aluc|OJZ89382.1 anid|CBF83347.1 anig|EHA23201.1 aory|EIT79343.1 asyd|OJJ60613.1 ater|EAU35904.1 atub|OJI85416.1 aver|OJI99937.1 awen|OJJ39424.1 azon|OJJ44306.1

Group3914: abra|OJJ75936.1 acar|OOF99974.1 acla|EAW09857.1 afis|EAW21269.1 afla|EED54829.1 afum|EBA27457.1 agla|OJJ87476.1 akaw|GAA84637.1 aluc|OJZ89383.1 anid|CBF83349.1 anig|EHA23200.1 aory|EIT79419.1 asyd|OJJ60614.1 ater|EAU35905.1 atub|OJI85415.1 aver|OJI99938.1 awen|OJJ39423.1 azon|OJJ44305.1

Group3915: abra|OJJ75942.1 acar|OOF99968.1 acla|EAW09851.1 afis|EAW21284.1 afla|EED54835.1 afum|EAL92301.1 agla|OJJ87479.1 akaw|GAA84644.1 aluc|OJZ89389.1 anid|CBF83363.1 anig|EHA23195.1 aory|EIT79451.1 asyd|OJJ60620.1 ater|EAU35913.1 atub|OJI85408.1 aver|OJI99944.1 awen|OJJ39415.1 azon|OJJ44298.1

Group3916: abra|OJJ75944.1 acar|OOF99967.1 acla|EAW09849.1 afis|EAW21286.1 afla|EED54837.1 afum|EAL92303.1 agla|OJJ87468.1 akaw|GAA84646.1 aluc|OJZ89391.1 anid|CBF83367.1 anig|EHA23193.1 aory|EIT79347.1 asyd|OJJ60622.1 ater|EAU35918.1 atub|OJI85406.1 aver|OJI99946.1 awen|OJJ39413.1 azon|OJJ44296.1

Group3917: abra|OJJ75949.1 acar|OOF99964.1 acla|EAW09847.1 afis|EAW21288.1 afla|EED54839.1 afum|EAL92305.1 agla|OJJ87466.1 akaw|GAA84652.1 aluc|OJZ89398.1 anid|CBF83371.1 anig|EHA23189.1 aory|EIT79388.1 asyd|OJJ60624.1 ater|EAU35920.1 atub|OJI85399.1 aver|OJI99948.1 awen|OJJ39411.1 azon|OJJ44294.1

Group3918: abra|OJJ75950.1 acar|OOF99962.1 acla|EAW09846.1 afis|EAW21289.1 afla|EED54840.1 afum|EAL92306.1 agla|OJJ87464.1 akaw|GAA84653.1 aluc|OJZ89399.1 anid|CBF83372.1 anig|EHA23188.1 aory|EIT79351.1 asyd|OJJ60625.1 ater|EAU35921.1 atub|OJI85398.1 aver|OJI99949.1 awen|OJJ39410.1 azon|OJJ44293.1

Group3919: abra|OJJ75951.1 acar|OOF99961.1 acla|EAW09845.1 afis|EAW21290.1 afla|EED54842.1 afum|EAL92307.1 agla|OJJ87463.1 akaw|GAA84654.1 aluc|OJZ89400.1 anid|CBF85245.1 anig|EHA23187.1 aory|EIT79441.1 asyd|OJJ62975.1 ater|EAU35922.1 atub|OJI85397.1 aver|OJI97994.1 awen|OJJ35914.1 azon|OJJ44292.1

Group3920: abra|OJJ75952.1 acar|OOF99960.1 acla|EAW09844.1 afis|EAW21291.1 afla|EED54843.1 afum|EAL92308.1 agla|OJJ87462.1 akaw|GAA84655.1 aluc|OJZ89401.1 anid|CBF83374.1 anig|EHA23186.1 aory|EIT79331.1 asyd|OJJ60626.1 ater|EAU35923.1 atub|OJI85396.1 aver|OJI99950.1 awen|OJJ39408.1 azon|OJJ44291.1

Group3921: abra|OJJ75958.1 acar|OOF99956.1 acla|EAW09840.1 afis|EAW21295.1 afla|EED54846.1 afum|EAL92312.1 agla|OJJ87444.1 akaw|GAA84659.1 aluc|OJZ89406.1 anid|CBF83381.1 anig|EHA23181.1 aory|EIT79417.1 asyd|OJJ60629.1 ater|EAU35928.1 atub|OJI85391.1 aver|OJI99953.1 awen|OJJ39404.1 azon|OJJ44288.1

Group3922: abra|OJJ75959.1 acar|OOF99955.1 acla|EAW09839.1 afis|EAW21296.1 afla|EED54847.1 afum|EAL92313.1 agla|OJJ87443.1 akaw|GAA84660.1 aluc|OJZ89407.1 anid|CBF83383.1 anig|EHA23180.1 aory|EIT79405.1 asyd|OJJ60630.1 ater|EAU35929.1 atub|OJI85388.1 aver|OJI99954.1 awen|OJJ39403.1 azon|OJJ44287.1

Group3923: abra|OJJ75963.1 acar|OOF90049.1 acla|EAW09834.1 afis|EAW21299.1 afla|EED54851.1 afum|EAL92316.1 agla|OJJ87439.1 akaw|GAA84663.1 aluc|OJZ89411.1 anid|CBF83388.1 anig|EHA23176.1 aory|EIT79436.1 asyd|OJJ60634.1 ater|EAU35932.1 atub|OJI85385.1 aver|OJI99957.1 awen|OJJ39399.1 azon|OJJ44283.1

Group3924: abra|OJJ75965.1 acar|OOF90050.1 acla|EAW09833.1 afis|EAW21300.1 afla|EED54852.1 afum|EAL92317.1 agla|OJJ87438.1 akaw|GAA84664.1 aluc|OJZ89412.1 anid|CBF83392.1 anig|EHA23175.1 aory|EIT79390.1 asyd|OJJ60635.1 ater|EAU35933.1 atub|OJI85384.1 aver|OJI99958.1 awen|OJJ39398.1 azon|OJJ44282.1

Group3925: abra|OJJ75966.1 acar|OOF90052.1 acla|EAW09832.1 afis|EAW21301.1 afla|EED57261.1 afum|EAL92318.1 agla|OJJ87436.1 akaw|GAA84665.1 aluc|OJZ89413.1 anid|CBF83394.1 anig|EHA23174.1 aory|EIT73984.1 asyd|OJJ60636.1 ater|EAU35934.1 atub|OJI85382.1 aver|OJI99959.1 awen|OJJ39397.1 azon|OJJ44281.1

Group3926: abra|OJJ75973.1 acar|OOF99946.1 acla|EAW09823.1 afis|EAW21310.1 afla|EED57266.1 afum|EAL92326.1 agla|OJJ87432.1 akaw|GAA88983.1 aluc|OJZ89420.1 anid|CBF83404.1 anig|EHA23167.1

aory|EIT74110.1 asyd|OJJ60640.1 ater|EAU35940.1 atub|OJI85373.1 aver|OJI99961.1 awen|OJJ39390.1 azon|OJJ44278.1

Group3927: abra|OJJ75977.1 acar|OOF99943.1 acla|EAW09821.1 afis|EAW21313.1 afla|EED57270.1 afum|EAL92330.1 agla|OJJ87429.1 akaw|GAA88980.1 aluc|OJZ89423.1 anid|CBF83407.1 anig|EHA23164.1 aory|EIT74037.1 asyd|OJJ60647.1 ater|EAU35942.1 atub|OJI85370.1 aver|OJI99965.1 awen|OJJ39386.1 azon|OJJ44275.1

Group3928: abra|OJJ75978.1 acar|OOF99941.1 acla|EAW09820.1 afis|EAW21314.1 afla|EED57271.1 afum|EAL92331.1 agla|OJJ87426.1 akaw|GAA88979.1 aluc|OJZ89424.1 anid|CBF83408.1 anig|EHA23163.1 aory|EIT74076.1 asyd|OJJ60648.1 ater|EAU35943.1 atub|OJI85369.1 aver|OJI99968.1 awen|OJJ39383.1 azon|OJJ44273.1

Group3929: abra|OJJ75980.1 acar|OOF99939.1 acla|EAW09819.1 afis|EAW21315.1 afla|EED57273.1 afum|EAL92332.1 agla|OJJ87424.1 akaw|GAA88977.1 aluc|OJZ89426.1 anid|CBF83412.1 anig|EHA23160.1 aory|EIT74008.1 asyd|OJJ60650.1 ater|EAU35944.1 atub|OJI85367.1 aver|OJI99970.1 awen|OJJ39381.1 azon|OJJ44271.1

Group3930: abra|OJJ75982.1 acar|OOF99937.1 acla|EAW09817.1 afis|EAW21317.1 afla|EED57275.1 afum|EAL92334.1 agla|OJJ87422.1 akaw|GAA88975.1 aluc|OJZ89428.1 anid|CBF83416.1 anig|EHA23159.1 aory|EIT74079.1 asyd|OJJ60652.1 ater|EAU35945.1 atub|OJI85365.1 aver|OJI99972.1 awen|OJJ39379.1 azon|OJJ44269.1

Group3931: abra|OJJ75983.1 acar|OOF99936.1 acla|EAW09816.1 afis|EAW21318.1 afla|EED57276.1 afum|EAL92335.1 agla|OJJ87421.1 akaw|GAA88974.1 aluc|OJZ89429.1 anid|CBF83417.1 anig|EHA23158.1 aory|EIT74114.1 asyd|OJJ60653.1 ater|EAU35946.1 atub|OJI85364.1 aver|OJI99973.1 awen|OJJ39378.1 azon|OJJ44268.1

Group3932: abra|OJJ75984.1 acar|OOF99935.1 acla|EAW09815.1 afis|EAW21319.1 afla|EED57277.1 afum|EAL92336.1 agla|OJJ87420.1 akaw|GAA88973.1 aluc|OJZ89430.1 anid|CBF83419.1 anig|EHA23157.1 aory|EIT74055.1 asyd|OJJ60654.1 ater|EAU35947.1 atub|OJI85363.1 aver|OJI99974.1 awen|OJJ39377.1 azon|OJJ44267.1

Group3933: abra|OJJ75987.1 acar|OOF99934.1 acla|EAW09813.1 afis|EAW21321.1 afla|EED57279.1 afum|EAL92338.1 agla|OJJ87416.1 akaw|GAA88971.1 aluc|OJZ89432.1 anid|CBF83421.1 anig|EHA23155.1 aory|EIT74031.1 asyd|OJJ60655.1 ater|EAU35948.1 atub|OJI85361.1 aver|OJI99976.1 awen|OJJ39375.1 azon|OJJ44265.1

Group3934: abra|OJJ75988.1 acar|OOF99933.1 acla|EAW09812.1 afis|EAW21323.1 afla|EED57280.1 afum|EAL92340.1 agla|OJJ87415.1 akaw|GAA88970.1 aluc|OJZ89433.1 anid|CBF83423.1 anig|EHA23154.1 aory|EIT74009.1 asyd|OJJ60656.1 ater|EAU35949.1 atub|OJI85360.1 aver|OJI99977.1 awen|OJJ39374.1 azon|OJJ44264.1

Group3935: abra|OJJ75995.1 acar|OOF99930.1 acla|EAW09810.1 afis|EAW21325.1 afla|EED57283.1 afum|EAL92343.1 agla|OJJ87413.1 akaw|GAA88964.1 aluc|OJZ89441.1 anid|CBF83426.1 anig|EHA23145.1 aory|EIT74153.1 asyd|OJJ60658.1 ater|EAU35951.1 atub|OJI85352.1 aver|OJI99981.1 awen|OJJ39372.1 azon|OJJ51712.1

Group3936: abra|OJJ76003.1 acar|OOF99921.1 acla|EAW09802.1 afis|EAW21334.1 afla|EED57292.1 afum|EAL92352.1 agla|OJJ87399.1 akaw|GAA88957.1 aluc|OJZ89448.1 anid|CBF76279.1 anig|EHA23138.1 aory|EIT74027.1 asyd|OJJ60988.1 ater|EAU35959.1 atub|OJI85345.1 aver|OJJ00308.1 awen|OJJ39364.1 azon|OJJ51703.1

Group3937: abra|OJJ76004.1 acar|OOF99922.1 acla|EAW09801.1 afis|EAW21335.1 afla|EED57293.1 afum|EAL92353.1 agla|OJJ87398.1 akaw|GAA88956.1 aluc|OJZ89449.1 anid|CBF76277.1 anig|EHA23137.1 aory|EIT73971.1 asyd|OJJ60987.1 ater|EAU35960.1 atub|OJI85344.1 aver|OJJ00307.1 awen|OJJ39363.1 azon|OJJ51702.1

Group3938: abra|OJJ76005.1 acar|OOF99920.1 acla|EAW09800.1 afis|EAW21336.1 afla|EED57294.1 afum|EAL92354.1 agla|OJJ82541.1 akaw|GAA88955.1 aluc|OJZ89451.1 anid|CBF83881.1 anig|EHA23136.1 aory|EIT74083.1 asyd|OJJ60940.1 ater|EAU35961.1 atub|OJI85343.1 aver|OJJ00266.1 awen|OJJ39362.1 azon|OJJ51701.1

Group3939: abra|OJJ76007.1 acar|OOF99919.1 acla|EAW09798.1 afis|EAW21338.1 afla|EED57296.1 afum|EAL92356.1 agla|OJJ87396.1 akaw|GAA88953.1 aluc|OJZ89453.1 anid|CBF83884.1 anig|EHA23134.1 aory|EIT74155.1 asyd|OJJ60942.1 ater|EAU35963.1 atub|OJI85341.1 aver|OJJ00268.1 awen|OJJ39360.1 azon|OJJ51699.1

Group3940: abra|OJJ76008.1 acar|OOF99917.1 acla|EAW09797.1 afis|EAW21339.1 afla|EED55754.1 afum|EBA27459.1 agla|OJJ87395.1 akaw|GAA88952.1 aluc|OJZ89454.1 anid|CBF83886.1 anig|EHA23133.1 aory|EIT76828.1 asyd|OJJ60943.1 ater|EAU35964.1 atub|OJI85340.1 aver|OJJ00269.1 awen|OJJ39359.1 azon|OJJ51698.1

Group3941: abra|OJJ76010.1 acar|OOF99914.1 acla|EAW09795.1 afis|EAW21341.1 afla|EED55756.1 afum|EAL92358.1 agla|OJJ87391.1 akaw|GAA88950.1 aluc|OJZ89456.1 anid|CBF76183.1 anig|EHA23131.1 aory|EIT76602.1 asyd|OJJ60962.1 ater|EAU35965.1 atub|OJI85338.1 aver|OJJ00287.1 awen|OJJ39356.1 azon|OJJ51697.1

Group3942: abra|OJJ76023.1 acar|OOF99901.1 acla|EAW09785.1 afis|EAW21353.1 afla|EED55768.1 afum|EAL92368.1 agla|OJJ87381.1 akaw|GAA88934.1 aluc|OJZ89473.1 anid|CBF76255.1 anig|EHA23118.1 aory|EIT76752.1 asyd|OJJ60965.1 ater|EAU35974.1 atub|OJI85325.1 aver|OJJ00290.1 awen|OJJ39347.1 azon|OJJ51691.1

Group3943: abra|OJJ76025.1 acar|OOF99902.1 acla|EAW09784.1 afis|EAW21354.1 afla|EED55769.1 afum|EAL92369.1 agla|OJJ87375.1 akaw|GAA88933.1 aluc|OJZ89475.1 anid|CBF76253.1 anig|EHA23117.1 aory|EIT76709.1 asyd|OJJ60966.1 ater|EAU35204.1 atub|OJI85324.1 aver|OJJ00291.1 awen|OJJ39346.1 azon|OJJ51690.1

Group3944: abra|OJJ76035.1 acar|OOF99893.1 acla|EAW09768.1 afis|EAW21370.1 afla|EED55788.1 afum|EAL92387.1 agla|OJJ85492.1 akaw|GAA88929.1 aluc|OJZ89490.1 anid|CBF83869.1 anig|EHA23108.1 aory|EIT76876.1 asyd|OJJ60933.1 ater|EAU35186.1 atub|OJI85313.1 aver|OJJ00259.1 awen|OJJ39327.1 azon|OJJ51672.1

Group3945: abra|OJJ76037.1 acar|OOF99892.1 acla|EAW09769.1 afis|EAW21369.1 afla|EED55786.1 afum|EAL92386.1 agla|OJJ85493.1 akaw|GAA88928.1 aluc|OJZ89492.1 anid|CBF83867.1 anig|EHA23107.1 aory|EIT76715.1 asyd|OJJ60932.1 ater|EAU35187.1 atub|OJI85311.1 aver|OJJ00258.1 awen|OJJ39328.1 azon|OJJ51673.1

Group3946: abra|OJJ76039.1 acar|OOF99890.1 acla|EAW09770.1 afis|EAW21368.1 afla|EED55782.1 afum|EAL92385.2 agla|OJJ85494.1 akaw|GAA88926.1 aluc|OJZ89495.1 anid|CBF83874.1 anig|EHA23105.1 aory|EIT76864.1 asyd|OJJ60937.1 ater|EAU35189.1 atub|OJI85308.1 aver|OJJ00263.1 awen|OJJ39329.1 azon|OJJ51674.1

Group3947: abra|OJJ76040.1 acar|OOF99889.1 acla|EAW09771.1 afis|EAW21367.1 afla|EED55781.1 afum|EAL92384.1 agla|OJJ85495.1 akaw|GAA88925.1 aluc|OJZ89496.1 anid|CBF83872.1 anig|EHA23104.1 aory|EIT76541.1 asyd|OJJ60936.1 ater|EAU35190.1 atub|OJI85307.1 aver|OJJ00262.1 awen|OJJ39330.1 azon|OJJ51675.1

Group3948: abra|OJJ76044.1 acar|OOF99886.1 acla|EAW09774.1 afis|EAW21364.1 afla|EED55778.1 afum|EAL92380.1 agla|OJJ85485.1 akaw|GAA88921.1 aluc|OJZ89500.1 anid|CBF83875.1 anig|EHA23101.1 aory|EIT76770.1 asyd|OJJ60938.1 ater|EAU35196.1 atub|OJI85303.1 aver|OJJ00264.1 awen|OJJ39334.1 azon|OJJ51679.1

Group3949: abra|OJJ76046.1 acar|OOF99884.1 acla|EAW09777.1 afis|EAW21361.1 afla|EED55775.1 afum|EAL92377.1 agla|OJJ85480.1 akaw|GAA88919.1 aluc|OJZ89502.1 anid|CBF76267.1 anig|EHA23098.1 aory|EIT76605.1 asyd|OJJ60973.1 ater|EAU35199.1 atub|OJI85301.1 aver|OJJ00298.1 awen|OJJ39337.1 azon|OJJ51682.1

Group3950: abra|OJJ76048.1 acar|OOF99882.1 acla|EAW09779.1 afis|EAW21359.1 afla|EED55774.1 afum|EAL92375.1 agla|OJJ85478.1 akaw|GAA88917.1 aluc|OJZ89504.1 anid|CBF76265.1 anig|EHA23096.1 aory|EIT76598.1 asyd|OJJ60972.1 ater|EAU35200.1 atub|OJI85299.1 aver|OJJ00297.1 awen|OJJ39339.1 azon|OJJ51684.1

Group3951: abra|OJJ76056.1 acar|OOF99874.1 acla|EAW09762.1 afis|EAW21375.1 afla|EED55793.1 afum|EAL92392.1 agla|OJJ85508.1 akaw|GAA88910.1 aluc|OJZ89511.1 anid|CBF83860.1 anig|EHA23088.1 aory|EIT76584.1 asyd|OJJ60926.1 ater|EAU35182.1 atub|OJI85292.1 aver|OJJ00252.1 awen|OJJ39322.1 azon|OJJ51667.1

Group3952: abra|OJJ76057.1 acar|OOF99873.1 acla|EAW09763.1 afis|EAW21374.1 afla|EED55792.1 afum|EAL92391.1 agla|OJJ85500.1 akaw|GAA88909.1 aluc|OJZ89512.1 anid|CBF83862.1 anig|EHA23087.1 aory|EIT76633.1 asyd|OJJ60927.1 ater|EAU35183.1 atub|OJI85291.1 aver|OJJ00253.1 awen|OJJ39323.1 azon|OJJ51668.1

Group3953: abra|OJJ76058.1 acar|OOF99872.1 acla|EAW09760.1 afis|EAW21381.1 afla|EED55795.1 afum|EAL92396.1 agla|OJJ85505.1 akaw|GAA88908.1 aluc|OJZ89513.1 anid|CBF83856.1 anig|EHA23086.1 aory|EIT76717.1 asyd|OJJ60924.1 ater|EAU35180.1 atub|OJI85290.1 aver|OJJ00250.1 awen|OJJ39320.1 azon|OJJ51665.1

Group3954: abra|OJJ76059.1 acar|OOF99871.1 acla|EAW09759.1 afis|EAW21382.1 afla|EED55796.1 afum|EAL92397.1 agla|OJJ85504.1 akaw|GAA88907.1 aluc|OJZ89514.1 anid|CBF83854.1 anig|EHA23085.1 aory|EIT76703.1 asyd|OJJ60923.1 ater|EAU35179.1 atub|OJI85289.1 aver|OJJ00249.1 awen|OJJ39319.1 azon|OJJ51664.1

Group3955: abra|OJJ76061.1 acar|OOF99870.1 acla|EAW09757.1 afis|EAW21384.1 afla|EED55798.1 afum|EAL92399.1 agla|OJJ85509.1 akaw|GAA88905.1 aluc|OJZ89516.1 anid|CBF83847.1 anig|EHA23083.1

aory|EIT76813.1 asyd|OJJ60919.1 ater|EAU35178.1 atub|OJI85287.1 aver|OJJ00245.1 awen|OJJ39317.1 azon|OJJ51530.1

Group3956: abra|OJJ76062.1 acar|OOF99869.1 acla|EAW09756.1 afis|EAW21385.1 afla|EED55799.1 afum|EAL92400.1 agla|OJJ85510.1 akaw|GAA88904.1 aluc|OJZ89517.1 anid|CBF83845.1 anig|EHA23082.1 aory|EIT76797.1 asyd|OJJ60918.1 ater|EAU35171.1 atub|OJI85286.1 aver|OJJ00244.1 awen|OJJ39316.1 azon|OJJ51531.1

Group3957: abra|OJJ76064.1 acar|OOF99865.1 acla|EAW09754.1 afis|EAW21387.1 afla|EED55801.1 afum|EAL92402.1 agla|OJJ85512.1 akaw|GAA88902.1 aluc|OJZ89519.1 anid|CBF83842.1 anig|EHA23081.1 aory|EIT76678.1 asyd|OJJ60916.1 ater|EAU35173.1 atub|OJI85284.1 aver|OJJ00243.1 awen|OJJ39314.1 azon|OJJ51532.1

Group3958: abra|OJJ76069.1 acar|OOF99862.1 acla|EAW09751.1 afis|EAW21391.1 afla|EED55805.1 afum|EAL92405.1 agla|OJJ85515.1 akaw|GAA88898.1 aluc|OJZ89523.1 anid|CBF83838.1 anig|EHA23078.1 aory|EIT76825.1 asyd|OJJ60913.1 ater|EAU35176.1 atub|OJI85280.1 aver|OJJ00240.1 awen|OJJ39312.1 azon|OJJ51538.1

Group3959: abra|OJJ76071.1 acar|OOF99857.1 acla|EAW09748.1 afis|EAW21394.1 afla|EED55811.1 afum|EAL92408.1 agla|OJJ85518.1 akaw|GAA88896.1 aluc|OJZ89525.1 anid|CBF83826.1 anig|EHA23076.1 aory|EIT76796.1 asyd|OJJ60904.1 ater|EAU35162.1 atub|OJI85278.1 aver|OJJ00230.1 awen|OJJ39310.1 azon|OJJ51542.1

Group3960: abra|OJJ76079.1 acar|OOF99846.1 acla|EAW09742.1 afis|EAW21400.1 afla|EED55821.1 afum|EAL92414.1 agla|OJJ85525.1 akaw|GAA88887.1 aluc|OJZ89536.1 anid|CBF83829.1 anig|EHA23068.1 aory|EIT76664.1 asyd|OJJ60906.1 ater|EAU35150.1 atub|OJI85270.1 aver|OJJ00232.1 awen|OJJ39303.1 azon|OJJ51549.1

Group3961: abra|OJJ76080.1 acar|OOF99845.1 acla|EAW09741.1 afis|EAW21401.1 afla|EED55822.1 afum|EAL92415.1 agla|OJJ85528.1 akaw|GAA88886.1 aluc|OJZ89538.1 anid|CBF83820.1 anig|EHA23067.1 aory|EIT76768.1 asyd|OJJ60901.1 ater|EAU35149.1 atub|OJI85269.1 aver|OJJ00227.1 awen|OJJ39302.1 azon|OJJ51550.1

Group3962: abra|OJJ76082.1 acar|OOF99844.1 acla|EAW09739.1 afis|EAW21402.1 afla|EED55824.1 afum|EAL92416.1 agla|OJJ85530.1 akaw|GAA88884.1 aluc|OJZ89540.1 anid|CBF83819.1 anig|EHA23066.1 aory|EIT76826.1 asyd|OJJ60900.1 ater|EAU29588.1 atub|OJI85267.1 aver|OJJ00225.1 awen|OJJ30539.1 azon|OJJ51552.1

Group3963: abra|OJJ76083.1 acar|OOF99843.1 acla|EAW09738.1 afis|EAW21403.1 afla|EED55825.1 afum|EAL92417.1 agla|OJJ85531.1 akaw|GAA88883.1 aluc|OJZ89541.1 anid|CBF83817.1 anig|EHA23065.1 aory|EIT76589.1 asyd|OJJ60899.1 ater|EAU35147.1 atub|OJI85266.1 aver|OJJ00224.1 awen|OJJ39300.1 azon|OJJ51553.1

Group3964: abra|OJJ76084.1 acar|OOF99842.1 acla|EAW09737.1 afis|EAW21404.1 afla|EED55826.1 afum|EAL92418.1 agla|OJJ85533.1 akaw|GAA88882.1 aluc|OJZ89542.1 anid|CBF83815.1 anig|EHA23064.1 aory|EIT76656.1 asyd|OJJ60898.1 ater|EAU35146.1 atub|OJI85265.1 aver|OJJ00223.1 awen|OJJ39299.1 azon|OJJ51554.1

Group3965: abra|OJJ76088.1 acar|OOF99837.1 acla|EAW09734.1 afis|EAW21407.1 afla|EED55829.1 afum|EAL92421.1 agla|OJJ85537.1 akaw|GAA88879.1 aluc|OJZ89545.1 anid|CBF83810.1 anig|EHA23061.1 aory|EIT76811.1 asyd|OJJ60895.1 ater|EAU35141.1 atub|OJI85261.1 aver|OJJ00220.1 awen|OJJ39294.1 azon|OJJ51560.1

Group3966: abra|OJJ76090.1 acar|OOF99835.1 acla|EAW09731.1 afla|EED55831.1 agla|OJJ85541.1 akaw|GAA88877.1 aluc|OJZ89547.1 anig|EHA23059.1 aory|EIT76755.1 atub|OJI85259.1 awen|OJJ39291.1 azon|OJJ51563.1 afis|EAW21410.1 afum|EAL92424.1 anid|CBF83805.1 asyd|OJJ60892.1 ater|EAU35136.1 aver|OJJ00216.1

Group3967: abra|OJJ76092.1 acar|OOF99833.1 acla|EAW09730.1 afis|EAW21411.1 afla|EED55834.1 afum|EAL92425.1 agla|OJJ85542.1 akaw|GAA88875.1 aluc|OJZ89548.1 anid|CBF83803.1 anig|EHA23058.1 aory|EIT76568.1 asyd|OJJ60891.1 ater|EAU35135.1 atub|OJI85258.1 aver|OJJ00215.1 awen|OJJ39290.1 azon|OJJ51564.1

Group3968: abra|OJJ76095.1 acar|OOF99829.1 acla|EAW09723.1 afis|EAW21418.1 afla|EED55843.1 afum|EAL92433.1 agla|OJJ85551.1 akaw|GAA88871.1 aluc|OJZ89551.1 anid|CBF83792.1 anig|EHA23055.1 aory|EIT76877.1 asyd|OJJ60881.1 ater|EAU35131.1 atub|OJI85254.1 aver|OJJ00207.1 awen|OJJ39285.1 azon|OJJ51569.1

Group3969: abra|OJJ76096.1 acar|OOF99828.1 acla|EAW09722.1 afis|EAW21419.1 afla|EED55844.1 afum|EAL92434.1 agla|OJJ85553.1 akaw|GAA88870.1 aluc|OJZ89552.1 anid|CBF83790.1 anig|EHA23054.1 aory|EIT76586.1 asyd|OJJ60880.1 ater|EAU35130.1 atub|OJI85253.1 aver|OJJ00206.1 awen|OJJ39284.1 azon|OJJ51570.1

Group3970: abra|OJJ76098.1 acar|OOF99827.1 acla|EAW09720.1 afis|EAW21421.1 afla|EED55846.1 afum|EAL92436.1 agla|OJJ85557.1 akaw|GAA88868.1 aluc|OJZ89554.1 anid|CBF83787.1 anig|EHA23052.1 aory|EIT76632.1 asyd|OJJ60878.1 ater|EAU35128.1 atub|OJI85251.1 aver|OJJ00203.1 awen|OJJ39280.1 azon|OJJ51572.1

Group3971: abra|OJJ76099.1 acar|OOF99825.1 acla|EAW09719.1 afis|EAW21422.1 afla|EED55847.1 afum|EAL92437.1 agla|OJJ85558.1 akaw|GAA88867.1 aluc|OJZ89555.1 anid|CBF83785.1 anig|EHA23051.1 aory|EIT76561.1 asyd|OJJ60877.1 ater|EAU35127.1 atub|OJI85249.1 aver|OJJ00202.1 awen|OJJ39279.1 azon|OJJ51573.1

Group3972: abra|OJJ76100.1 acar|OOF99824.1 acla|EAW09718.1 afis|EAW21423.1 afla|EED55848.1 afum|EAL92438.1 agla|OJJ85559.1 akaw|GAA88866.1 aluc|OJZ89556.1 anid|CBF83783.1 anig|EHA23050.1 aory|EIT76818.1 asyd|OJJ60876.1 ater|EAU35126.1 atub|OJI85248.1 aver|OJJ00201.1 awen|OJJ39278.1 azon|OJJ51574.1

Group3973: abra|OJJ76102.1 acar|OOF99823.1 acla|EAW09716.1 afis|EAW21425.1 afla|EED55850.1 afum|EAL92440.1 agla|OJJ85562.1 akaw|GAA88864.1 aluc|OJZ89558.1 anid|CBF83781.1 anig|EHA23048.1 aory|EIT76846.1 asyd|OJJ60874.1 ater|EAU35124.1 atub|OJI85246.1 aver|OJJ00199.1 awen|OJJ39276.1 azon|OJJ51576.1

Group3974: abra|OJJ76103.1 acar|OOF99822.1 acla|EAW09715.1 afis|EAW21426.1 afla|EED55853.1 afum|EAL92441.1 agla|OJJ85563.1 akaw|GAA88863.1 aluc|OJZ89559.1 anid|CBF83780.1 anig|EHA23047.1 aory|EIT76704.1 asyd|OJJ60873.1 ater|EAU35123.1 atub|OJI85245.1 aver|OJJ00198.1 awen|OJJ39275.1 azon|OJJ51577.1

Group3975: abra|OJJ76104.1 acar|OOF99821.1 acla|EAW09714.1 afis|EAW21427.1 afla|EED55854.1 afum|EAL92442.1 agla|OJJ85564.1 akaw|GAA88862.1 aluc|OJZ89560.1 anid|CBF83778.1 anig|EHA23046.1 aory|EIT76600.1 asyd|OJJ60871.1 ater|EAU35121.1 atub|OJI85244.1 aver|OJJ00197.1 awen|OJJ39274.1 azon|OJJ51579.1

Group3976: abra|OJJ76107.1 acar|OOF99819.1 acla|EAW09713.1 afis|EAW21428.1 afla|EED55855.1 afum|EAL92443.1 agla|OJJ85566.1 akaw|GAA88859.1 aluc|OJZ89563.1 anid|CBF83776.1 anig|EHA23043.1 aory|EIT76883.1 asyd|OJJ60870.1 ater|EAU35120.1 atub|OJI85241.1 aver|OJJ00195.1 awen|OJJ39273.1 azon|OJJ51582.1

Group3977: abra|OJJ76108.1 acar|OOF99818.1 acla|EAW09712.1 afis|EAW21429.1 afla|EED55856.1 afum|EAL92444.1 agla|OJJ85567.1 akaw|GAA88858.1 aluc|OJZ89564.1 anid|CBF83774.1 anig|EHA23042.1 aory|EIT76794.1 asyd|OJJ60869.1 ater|EAU35119.1 atub|OJI85240.1 aver|OJJ00194.1 awen|OJJ39271.1 azon|OJJ51583.1

Group3978: abra|OJJ76109.1 acar|OOF99817.1 acla|EAW09711.1 afis|EAW21430.1 afla|EED55857.1 afum|EAL92445.1 agla|OJJ85568.1 akaw|GAA88857.1 aluc|OJZ89565.1 anid|CBF83772.1 anig|EHA23041.1 aory|EIT76635.1 asyd|OJJ60868.1 ater|EAU35118.1 atub|OJI85239.1 aver|OJJ00193.1 awen|OJJ39270.1 azon|OJJ51584.1

Group3979: abra|OJJ76111.1 acar|OOF99815.1 acla|EAW09709.1 afis|EAW21431.1 afla|EED55859.1 afum|EAL92446.2 agla|OJJ85572.1 akaw|GAA88856.1 aluc|OJZ89567.1 anid|CBF83769.1 anig|EHA23040.1 aory|EIT76616.1 asyd|OJJ60865.1 ater|EAU35117.1 atub|OJI85237.1 aver|OJJ00191.1 awen|OJJ39269.1 azon|OJJ51587.1

Group3980: abra|OJJ76112.1 acar|OOF99813.1 acla|EAW09708.1 afis|EAW21432.1 afla|EED55860.1 afum|EAL92447.1 agla|OJJ85573.1 akaw|GAA88855.1 aluc|OJZ89568.1 anid|CBF83767.1 anig|EHA23039.1 aory|EIT76829.1 asyd|OJJ60864.1 ater|EAU35116.1 atub|OJI85236.1 aver|OJJ00190.1 awen|OJJ39267.1 azon|OJJ51588.1

Group3981: abra|OJJ76113.1 acar|OOF99812.1 acla|EAW09707.1 afis|EAW21433.1 afla|EED55862.1 afum|EAL92448.2 agla|OJJ85574.1 akaw|GAA88854.1 aluc|OJZ89569.1 anid|CBF83765.1 anig|EHA23037.1 aory|EIT76807.1 asyd|OJJ60863.1 ater|EAU35111.1 atub|OJI85234.1 aver|OJJ00189.1 awen|OJJ39266.1 azon|OJJ51589.1

Group3982: abra|OJJ76114.1 acar|OOF99811.1 acla|EAW09706.1 afis|EAW21434.1 afla|EED55863.1 afum|EAL92449.1 agla|OJJ85575.1 akaw|GAA88853.1 aluc|OJZ89571.1 anid|CBF76591.1 anig|EHA23036.1 aory|EIT76762.1 asyd|OJJ61175.1 ater|EAU35110.1 atub|OJI85233.1 aver|OJJ00501.1 awen|OJJ39265.1 azon|OJJ51590.1

Group3983: abra|OJJ76116.1 acar|OOF99810.1 acla|EAW09704.1 afis|EAW21436.1 afla|EED55864.1 afum|EAL92451.1 agla|OJJ85579.1 akaw|GAA88851.1 aluc|OJZ89573.1 anid|CBF76587.1 anig|EHA23034.1 aory|EIT76708.1 asyd|OJJ61173.1 ater|EAU35109.1 atub|OJI85231.1 aver|OJJ00499.1 awen|OJJ39263.1 azon|OJJ51592.1

Group3984: abra|OJJ76118.1 acar|OOF99807.1 acla|EAW09701.1 afis|EAW21438.1 afla|EED55866.1 afum|EAL92453.2 agla|OJJ85583.1 akaw|GAA88849.1 aluc|OJZ89575.1 anid|CBF76583.1 anig|EHA23032.1

aory|EIT76853.1 asyd|OJJ61171.1 ater|EAU35107.1 atub|OJI85229.1 aver|OJJ00496.1 awen|OJJ39260.1 azon|OJJ51595.1

Group3985: abra|OJJ76120.1 acar|OOF99805.1 acla|EAW09699.1 afis|EAW21440.1 afla|EED55868.1 afum|EAL92456.1 agla|OJJ85587.1 akaw|GAA88847.1 aluc|OJZ89577.1 anid|CBF76579.1 anig|EHA23031.1 aory|EIT76773.1 asyd|OJJ61169.1 ater|EAU35105.1 atub|OJI85227.1 aver|OJJ00494.1 awen|OJJ39258.1 azon|OJJ51598.1

Group3986: abra|OJJ76121.1 acar|OOF99804.1 acla|EAW09698.1 afis|EAW21441.1 afla|EED55869.1 afum|EAL92458.2 agla|OJJ85588.1 akaw|GAA88846.1 aluc|OJZ89578.1 anid|CBF76577.1 anig|EHA23030.1 aory|EIT76534.1 asyd|OJJ61168.1 ater|EAU35104.1 atub|OJI85226.1 aver|OJJ00493.1 awen|OJJ39257.1 azon|OJJ51599.1

Group3987: abra|OJJ76122.1 acar|OOF99803.1 acla|EAW09697.1 afis|EAW21442.1 afla|EED55870.1 afum|EAL92460.1 agla|OJJ85589.1 akaw|GAA88845.1 aluc|OJZ89579.1 anid|CBF76575.1 anig|EHA23029.1 aory|EIT76841.1 asyd|OJJ61167.1 ater|EAU35103.1 atub|OJI85225.1 aver|OJJ00492.1 awen|OJJ39256.1 azon|OJJ51600.1

Group3988: abra|OJJ76123.1 acar|OOF99802.1 acla|EAW09696.1 afis|EAW21443.1 afla|EED55871.1 afum|EAL92461.2 agla|OJJ85590.1 akaw|GAA88844.1 aluc|OJZ89580.1 anid|CBF76573.1 anig|EHA23028.1 aory|EIT76804.1 asyd|OJJ61166.1 ater|EAU35102.1 atub|OJI85224.1 aver|OJJ00491.1 awen|OJJ39255.1 azon|OJJ51601.1

Group3989: abra|OJJ76125.1 acar|OOF99800.1 acla|EAW09694.1 afis|EAW21445.1 afla|EED55873.1 afum|EAL92464.2 agla|OJJ85593.1 akaw|GAA88842.1 aluc|OJZ89583.1 anid|CBF76569.1 anig|EHA23026.1 aory|EIT76681.1 asyd|OJJ61164.1 ater|EAU35099.1 atub|OJI85220.1 aver|OJJ00488.1 awen|OJJ39253.1 azon|OJJ51602.1

Group3990: abra|OJJ76126.1 acar|OOF99798.1 acla|EAW09693.1 afis|EAW21446.1 afla|EED55874.1 afum|EAL92465.2 agla|OJJ85594.1 akaw|GAA88841.1 aluc|OJZ89587.1 anid|CBF76565.1 anig|EHA23024.1 aory|EIT76673.1 asyd|OJJ61162.1 ater|EAU35097.1 atub|OJI85216.1 aver|OJJ00486.1 awen|OJJ39252.1 azon|OJJ51603.1

Group3991: abra|OJJ76127.1 acar|OOF99796.1 acla|EAW09692.1 afis|EAW21447.1 afla|EED55876.1 afum|EAL92466.1 agla|OJJ85595.1 akaw|GAA88840.1 aluc|OJZ89588.1 anid|CBF76563.1 anig|EHA23023.1 aory|EIT76593.1 asyd|OJJ61161.1 ater|EAU35096.1 atub|OJI85214.1 aver|OJJ00483.1 awen|OJJ39251.1 azon|OJJ51604.1

Group3992: abra|OJJ76128.1 acar|OOF99795.1 acla|EAW09691.1 afis|EAW21448.1 afla|EED55877.1 afum|EAL92467.1 agla|OJJ85596.1 akaw|GAA88839.1 aluc|OJZ89589.1 anid|CBF76559.1 anig|EHA23022.1 aory|EIT76881.1 asyd|OJJ61159.1 ater|EAU35095.1 atub|OJI85213.1 aver|OJJ00481.1 awen|OJJ39250.1 azon|OJJ51605.1

Group3993: abra|OJJ76129.1 acar|OOF99794.1 acla|EAW09690.1 afis|EAW21449.1 afla|EED55878.1 afum|EAL92468.1 agla|OJJ85597.1 akaw|GAA88838.1 aluc|OJZ89590.1 anid|CBF76557.1 anig|EHA23021.1 aory|EIT76638.1 asyd|OJJ61158.1 ater|EAU35094.1 atub|OJI85212.1 aver|OJJ00480.1 awen|OJJ39249.1 azon|OJJ51606.1

Group3994: abra|OJJ76133.1 acar|OOF99790.1 acla|EAW09685.1 afis|EAW21454.1 afla|EED55884.1 afum|EAL92473.1 agla|OJJ85602.1 akaw|GAA88834.1 aluc|OJZ89594.1 anid|CBF76548.1 anig|EHA23017.1 aory|EIT76723.1 asyd|OJJ61155.1 ater|EAU35081.1 atub|OJI85208.1 aver|OJJ00477.1 awen|OJJ39246.1 azon|OJJ51609.1

Group3995: abra|OJJ76138.1 acar|OOF99786.1 acla|EAW09681.1 afis|EAW21458.1 afla|EED55888.1 afum|EAL92477.1 agla|OJJ85605.1 akaw|GAA88830.1 aluc|OJZ89601.1 anid|CBF76540.1 anig|EHA23014.1 aory|EIT76702.1 asyd|OJJ61151.1 ater|EAU35077.1 atub|OJI85203.1 aver|OJJ00473.1 awen|OJJ39242.1 azon|OJJ51613.1

Group3996: abra|OJJ76140.1 acar|OOF99785.1 acla|EAW09679.1 afis|EAW21460.1 afla|EED55891.1 afum|EAL92479.1 agla|OJJ85608.1 akaw|GAA88828.1 aluc|OJZ89603.1 anid|CBF76536.1 anig|EHA23012.1 aory|EIT76836.1 asyd|OJJ61149.1 ater|EAU35075.1 atub|OJI85201.1 aver|OJJ00471.1 awen|OJJ39239.1 azon|OJJ51615.1

Group3997: abra|OJJ76141.1 acar|OOF99784.1 acla|EAW09664.1 afis|EAW21473.1 afla|EED55908.1 afum|EAL92492.1 agla|OJJ85622.1 akaw|GAA88826.1 aluc|OJZ89605.1 anid|CBF76513.1 anig|EHA23011.1 aory|EIT76607.1 asyd|OJJ61136.1 ater|EAU35060.1 atub|OJI85198.1 aver|OJJ00458.1 awen|OJJ39224.1 azon|OJJ51630.1

Group3998: abra|OJJ76142.1 acar|OOF99783.1 acla|EAW09665.1 afis|EAW21472.1 afla|EED55902.1 afum|EAL92491.1 agla|OJJ85621.1 akaw|GAA88825.1 aluc|OJZ89606.1 anid|CBF76515.1 anig|EHA23010.1 aory|EIT76799.1 asyd|OJJ61137.1 ater|EAU35061.1 atub|OJI85197.1 aver|OJJ00459.1 awen|OJJ39225.1 azon|OJJ51629.1

Group3999: abra|OJJ76143.1 acar|OOF99782.1 acla|EAW09666.1 afis|EAW21471.1 afla|EED55904.1 afum|EAL92490.1 agla|OJJ85620.1 akaw|GAA88824.1 aluc|OJZ89607.1 anid|CBF76517.1 anig|EHA23009.1 aory|EIT76648.1 asyd|OJJ61138.1 ater|EAU35062.1 atub|OJI85196.1 aver|OJJ00460.1 awen|OJJ39227.1 azon|OJJ51628.1

Group4000: abra|OJJ76145.1 acar|OOF99781.1 acla|EAW09668.1 afis|EAW21469.1 afla|EED55907.1 afum|EAL92488.1 agla|OJJ85618.1 akaw|GAA82094.1 aluc|OJZ92086.1 anid|CBF76521.1 anig|EHA23008.1 aory|EIT76871.1 asyd|OJJ61140.1 ater|EAU35064.1 atub|OJI86515.1 aver|OJJ00462.1 awen|OJJ39229.1 azon|OJJ51626.1

Group4001: abra|OJJ76146.1 acar|OOF99780.1 acla|EAW09671.1 afis|EAW21467.1 afla|EED55900.1 afum|EAL92487.1 agla|OJJ85617.1 akaw|GAA82095.1 aluc|OJZ92084.1 anid|CBF76523.1 anig|EHA23007.1 aory|EIT76694.1 asyd|OJJ61141.1 ater|EAU35066.1 atub|OJI86514.1 aver|OJJ00463.1 awen|OJJ39230.1 azon|OJJ51625.1

Group4002: abra|OJJ76150.1 acar|OOF99777.1 acla|EAW09675.1 afis|EAW21464.1 afla|EED55896.1 afum|EAL92484.1 agla|OJJ85612.1 akaw|GAA82099.1 aluc|OJZ92079.1 anid|CBF76529.1 anig|EHA23005.1 aory|EIT76592.1 asyd|OJJ61145.1 ater|EAU35071.1 atub|OJI86509.1 aver|OJJ00467.1 awen|OJJ39235.1 azon|OJJ51620.1

Group4003: abra|OJJ76151.1 acar|OOF89898.1 acla|EAW09676.1 afis|EAW21463.1 afla|EED55895.1 afum|EAL92483.1 agla|OJJ85611.1 akaw|GAA82100.1 aluc|OJZ92078.1 anid|CBF76530.1 anig|EHA23004.1 aory|EIT76817.1 asyd|OJJ61146.1 ater|EAU35072.1 atub|OJI86508.1 aver|OJJ00468.1 awen|OJJ39236.1 azon|OJJ51619.1

Group4004: abra|OJJ76152.1 acar|OOF89899.1 acla|EAW09677.1 afis|EAW21462.1 afla|EED55894.1 afum|EAL92481.1 agla|OJJ85610.1 akaw|GAA82101.1 aluc|OJZ92077.1 anid|CBF76532.1 anig|EHA23003.1 aory|EIT76700.1 asyd|OJJ61147.1 ater|EAU35073.1 atub|OJI86507.1 aver|OJJ00469.1 awen|OJJ39237.1 azon|OJJ51618.1

Group4005: abra|OJJ76157.1 acar|OOF99775.1 acla|EAW09661.1 afis|EAW21478.1 afla|EED55910.1 afum|EAL92498.1 agla|OJJ85630.1 akaw|GAA82105.1 aluc|OJZ92071.1 anid|CBF76508.1 anig|EHA23000.1 aory|EIT76734.1 asyd|OJJ61133.1 ater|EAU35058.1 atub|OJI86503.1 aver|OJJ00454.1 awen|OJJ39221.1 azon|OJJ51635.1

Group4006: abra|OJJ76160.1 acar|OOF99772.1 acla|EAW09658.1 afis|EAW21481.1 afla|EED55913.1 afum|EAL92501.1 agla|OJJ85635.1 akaw|GAA82107.1 aluc|OJZ92069.1 anid|CBF76502.1 anig|EHA22997.1 aory|EIT76621.1 asyd|OJJ61130.1 ater|EAU35056.1 atub|OJI86500.1 aver|OJJ00451.1 awen|OJJ39216.1 azon|OJJ51638.1

Group4007: abra|OJJ76166.1 acar|OOF99771.1 acla|EAW09657.1 afis|EAW21482.1 afla|EED55914.1 afum|EAL92502.1 agla|OJJ85636.1 akaw|GAA82114.1 aluc|OJZ92062.1 anid|CBF76498.1 anig|EHA22991.1 aory|EIT76649.1 asyd|OJJ61128.1 ater|EAU35055.1 atub|OJI86494.1 aver|OJJ00449.1 awen|OJJ39215.1 azon|OJJ51640.1

Group4008: abra|OJJ76172.1 acar|OOF99767.1 acla|EAW09653.1 afis|EAW21486.1 afla|EED55919.1 afum|EAL92506.1 agla|OJJ85645.1 akaw|GAA82118.1 aluc|OJZ92058.1 anid|CBF76490.1 anig|EHA22987.1 aory|EIT76891.1 asyd|OJJ61124.1 ater|EAU35051.1 atub|OJI86490.1 aver|OJJ00445.1 awen|OJJ39211.1 azon|OJJ51643.1

Group4009: abra|OJJ76174.1 acar|OOF99766.1 acla|EAW09652.1 afis|EAW21488.1 afla|EED55923.1 afum|EAL92507.1 agla|OJJ85648.1 akaw|GAA82119.1 aluc|OJZ92056.1 anid|CBF76488.1 anig|EHA22985.1 aory|EIT76645.1 asyd|OJJ61123.1 ater|EAU35050.1 atub|OJI86487.1 aver|OJJ00441.1 awen|OJJ39207.1 azon|OJJ51645.1

Group4010: abra|OJJ76175.1 acar|OOF99765.1 acla|EAW09651.1 afis|EAW21489.1 afla|EED55924.1 afum|EAL92508.1 agla|OJJ85649.1 akaw|GAA82120.1 aluc|OJZ92055.1 anid|CBF76486.1 anig|EHA22984.1 aory|EIT76531.1 asyd|OJJ61122.1 ater|EAU35049.1 atub|OJI86486.1 aver|OJJ00440.1 awen|OJJ39206.1 azon|OJJ51646.1

Group4011: abra|OJJ76176.1 acar|OOF99764.1 acla|EAW09650.1 afis|EAW21490.1 afla|EED55925.1 afum|EAL92509.1 agla|OJJ85652.1 akaw|GAA82121.1 aluc|OJZ92054.1 anid|CBF76484.1 anig|EHA22983.1 aory|EIT76596.1 asyd|OJJ61121.1 ater|EAU35048.1 atub|OJI86485.1 aver|OJJ00439.1 awen|OJJ39205.1 azon|OJJ51647.1

Group4012: abra|OJJ76177.1 acar|OOF99762.1 acla|EAW09649.1 afis|EAW21491.1 afla|EED55926.1 afum|EAL92510.1 agla|OJJ85653.1 akaw|GAA82122.1 aluc|OJZ92053.1 anid|CBF76482.1 anig|EHA22982.1 aory|EIT76879.1 asyd|OJJ61120.1 ater|EAU35047.1 atub|OJI86484.1 aver|OJJ00438.1 awen|OJJ39204.1 azon|OJJ51648.1

Group4013: abra|OJJ76178.1 acar|OOF99763.1 acla|EAW09648.1 afis|EAW21492.1 afla|EED55927.1 afum|EAL92511.1 agla|OJJ85654.1 akaw|GAA82123.1 aluc|OJZ92052.1 anid|CBF76480.1 anig|EHA22981.1

aory|EIT76815.1 asyd|OJJ61119.1 ater|EAU35046.1 atub|OJI86483.1 aver|OJJ00437.1 awen|OJJ39203.1 azon|OJJ51649.1

Group4014: abra|OJJ76180.1 acar|OOF99760.1 acla|EAW09646.1 afis|EAW21494.1 afla|EED55929.1 afum|EAL92513.1 agla|OJJ85656.1 akaw|GAA82125.1 aluc|OJZ92049.1 anid|CBF76476.1 anig|EHA22979.1 aory|EIT76689.1 asyd|OJJ61117.1 ater|EAU35044.1 atub|OJI86481.1 aver|OJJ00435.1 awen|OJJ39201.1 azon|OJJ51526.1

Group4015: abra|OJJ76181.1 acar|OOF99759.1 acla|EAW09645.1 afis|EAW21495.1 afla|EED55930.1 afum|EAL92514.1 agla|OJJ85657.1 akaw|GAA82126.1 aluc|OJZ92048.1 anid|CBF76474.1 anig|EHA22978.1 aory|EIT76659.1 asyd|OJJ61116.1 ater|EAU35043.1 atub|OJI86480.1 aver|OJJ00434.1 awen|OJJ39200.1 azon|OJJ51525.1

Group4016: abra|OJJ76182.1 acar|OOF99758.1 acla|EAW09644.1 afis|EAW21496.1 afla|EED55931.1 afum|EAL92515.1 agla|OJJ85658.1 akaw|GAA82127.1 aluc|OJZ92047.1 anid|CBF76472.1 anig|EHA22977.1 aory|EIT76569.1 asyd|OJJ61115.1 ater|EAU35042.1 atub|OJI86479.1 aver|OJJ00433.1 awen|OJJ39199.1 azon|OJJ51524.1

Group4017: abra|OJJ76183.1 acar|OOF99757.1 acla|EAW09643.1 afis|EAW21497.1 afla|EED55933.1 afum|EAL92516.1 agla|OJJ85660.1 akaw|GAA82128.1 aluc|OJZ92046.1 anid|CBF76468.1 anig|EHA22976.1 aory|EIT76844.1 asyd|OJJ61114.1 ater|EAU35041.1 atub|OJI86478.1 aver|OJJ00430.1 awen|OJJ39198.1 azon|OJJ51523.1

Group4018: abra|OJJ76187.1 acar|OOF99755.1 acla|EAW09640.1 afis|EAW21500.1 afla|EED55936.1 afum|EAL92519.1 agla|OJJ85663.1 akaw|GAA82131.1 aluc|OJZ92042.1 anid|CBF76459.1 anig|EHA22974.1 aory|EIT76547.1 asyd|OJJ61111.1 ater|EAU35038.1 atub|OJI86475.1 aver|OJJ00427.1 awen|OJJ39195.1 azon|OJJ51522.1

Group4019: abra|OJJ76188.1 acar|OOF99754.1 acla|EAW09639.1 afis|EAW21501.1 afla|EED55937.1 afum|EAL92520.1 agla|OJJ85664.1 akaw|GAA82132.1 aluc|OJZ92041.1 anid|CBF76457.1 anig|EHA22973.1 aory|EIT76679.1 asyd|OJJ61110.1 ater|EAU35037.1 atub|OJI86474.1 aver|OJJ00426.1 awen|OJJ39194.1 azon|OJJ51519.1

Group4020: abra|OJJ76191.1 acar|OOF99751.1 acla|EAW13914.1 afis|EAW18625.1 afla|EED52717.1 afum|EAL93337.1 agla|OJJ81242.1 akaw|GAA82134.1 aluc|OJZ92038.1 anid|CBF70486.1 anig|EHA22970.1 aory|EIT78208.1 asyd|OJJ58585.1 ater|EAU34925.1 atub|OJI86472.1 aver|OJJ01189.1 awen|OJJ32090.1 azon|OJJ46667.1

Group4021: abra|OJJ76192.1 acar|OOF99752.1 acla|EAW13915.1 afis|EAW18626.1 afla|EED52716.1 afum|EAL93338.1 agla|OJJ81246.1 akaw|GAA82135.1 aluc|OJZ92037.1 anid|CBF70484.1 anig|EHA22969.1 aory|EIT78180.1 asyd|OJJ58586.1 ater|EAU34926.1 atub|OJI86470.1 aver|OJJ01190.1 awen|OJJ32092.1 azon|OJJ46669.1

Group4022: abra|OJJ76193.1 acar|OOF99750.1 acla|EAW13916.1 afis|EAW18627.1 afla|EED52715.1 afum|EAL93339.1 agla|OJJ81245.1 akaw|GAA82136.1 aluc|OJZ92036.1 anid|CBF70478.1 anig|EHA22968.1 aory|EIT78215.1 asyd|OJJ58587.1 ater|EAU38285.1 atub|OJI86469.1 aver|OJJ01191.1 awen|OJJ32093.1 azon|OJJ46670.1

Group4023: abra|OJJ76194.1 acar|OOF99749.1 acla|EAW13917.1 afis|EAW18628.1 afla|EED52714.1 afum|EAL93340.1 agla|OJJ81244.1 akaw|GAA82137.1 aluc|OJZ92035.1 anid|CBF70480.1 anig|EHA22967.1 aory|EIT78226.1 asyd|OJJ58588.1 ater|EAU38284.1 atub|OJI86468.1 aver|OJJ01192.1 awen|OJJ32094.1 azon|OJJ46671.1

Group4024: abra|OJJ76197.1 acar|OOF99747.1 acla|EAW13918.1 afis|EAW18629.1 afla|EED52712.1 afum|EAL93341.1 agla|OJJ81251.1 akaw|GAA82139.1 aluc|OJZ92032.1 anid|CBF70482.1 anig|EHA22965.1 aory|EIT78174.1 asyd|OJJ58592.1 ater|EAU38282.1 atub|OJI86465.1 aver|OJJ01194.1 awen|OJJ32096.1 azon|OJJ46675.1

Group4025: abra|OJJ76200.1 acar|OOF99744.1 acla|EAW13921.1 afis|EAW18632.1 afla|EED52709.1 afum|EAL93345.1 agla|OJJ82445.1 akaw|GAA82143.1 aluc|OJZ92028.1 anid|CBF70472.1 anig|EHA22962.1 aory|EIT74199.1 asyd|OJJ58595.1 ater|EAU38279.1 atub|OJI86462.1 aver|OJJ01197.1 awen|OJJ32099.1 azon|OJJ46681.1

Group4026: abra|OJJ76201.1 acar|OOF99743.1 acla|EAW13922.1 afis|EAW18633.1 afla|EED52708.1 afum|EAL93346.1 agla|OJJ82444.1 akaw|GAA82144.1 aluc|OJZ92027.1 anid|CBF70470.1 anig|EHA22961.1 aory|EIT74198.1 asyd|OJJ58596.1 ater|EAU38278.1 atub|OJI86461.1 aver|OJJ01198.1 awen|OJJ32100.1 azon|OJJ46682.1

Group4027: abra|OJJ76202.1 acar|OOF99742.1 acla|EAW13923.1 afis|EAW18634.1 afla|EED52707.1 afum|EAL93347.1 agla|OJJ82443.1 akaw|GAA82145.1 aluc|OJZ92026.1 anid|CBF70468.1 anig|EHA22960.1 aory|EIT74275.1 asyd|OJJ58597.1 ater|EAU38277.1 atub|OJI86460.1 aver|OJJ01199.1 awen|OJJ32101.1 azon|OJJ46683.1

Group4028: abra|OJJ76204.1 acar|OOF99741.1 acla|EAW13925.1 afis|EAW18636.1 afla|EED52705.1 afum|EAL93349.1 akaw|GAA82147.1 aluc|OJZ92024.1 anid|CBF70461.1 anig|EHA22958.1 aory|EIT74316.1 asyd|OJJ58600.1 atub|OJI86458.1 aver|OJJ01202.1 awen|OJJ32103.1 azon|OJJ46685.1 agla|OJJ82439.1 ater|EAU38275.1

Group4029: abra|OJJ76210.1 acar|OOF99739.1 acla|EAW13927.1 afis|EAW18638.1 afla|EED52702.1 afum|EAL93351.1 agla|OJJ82436.1 akaw|GAA82150.1 aluc|OJZ92018.1 anid|CBF70453.1 anig|EHA22956.1 aory|EIT74209.1 asyd|OJJ58604.1 ater|EAU38273.1 atub|OJI86453.1 aver|OJJ01207.1 awen|OJJ32106.1 azon|OJJ46687.1

Group4030: abra|OJJ76213.1 acar|OOF99737.1 acla|EAW13929.1 afis|EAW18640.1 afla|EED52700.1 afum|EAL93354.2 agla|OJJ82434.1 akaw|GAA82152.1 aluc|OJZ92016.1 anid|CBF70449.1 anig|EHA22955.1 aory|EIT74286.1 asyd|OJJ58606.1 ater|EAU38271.1 atub|OJI86451.1 aver|OJJ01210.1 awen|OJJ32108.1 azon|OJJ46689.1

Group4031: abra|OJJ76214.1 acar|OOF99736.1 acla|EAW13930.1 afis|EAW18641.1 afla|EED52699.1 afum|EAL93355.1 agla|OJJ82433.1 akaw|GAA82153.1 aluc|OJZ92015.1 anid|CBF70447.1 anig|EHA22954.1 aory|EIT74167.1 asyd|OJJ58607.1 ater|EAU38270.1 atub|OJI86450.1 aver|OJJ01211.1 awen|OJJ32109.1 azon|OJJ46690.1

Group4032: abra|OJJ76215.1 acar|OOF99735.1 acla|EAW13931.1 afis|EAW18642.1 afla|EED52698.1 afum|EAL93356.1 agla|OJJ82432.1 akaw|GAA82154.1 aluc|OJZ92014.1 anid|CBF70445.1 anig|EHA22953.1 aory|EIT74249.1 asyd|OJJ58608.1 ater|EAU38269.1 atub|OJI86449.1 aver|OJJ01212.1 awen|OJJ32110.1 azon|OJJ46691.1

Group4033: abra|OJJ76220.1 acar|OOF99731.1 acla|EAW13935.1 afis|EAW18646.1 afla|EED52696.1 afum|EAL93361.1 agla|OJJ82429.1 akaw|GAA82158.1 aluc|OJZ92010.1 anid|CBF70437.1 anig|EHA22950.1 aory|EIT74305.1 asyd|OJJ58611.1 ater|EAU38265.1 atub|OJI86445.1 aver|OJJ01217.1 awen|OJJ32114.1 azon|OJJ46695.1

Group4034: abra|OJJ76225.1 acar|OOF99472.1 acla|EAW14365.1 afis|EAW18915.1 afla|EED50899.1 afum|EBA27498.1 agla|OJJ81529.1 akaw|GAA82161.1 aluc|OJZ92006.1 anid|CBF69691.1 anig|EHA22946.1 aory|EIT78987.1 asyd|OJJ59055.1 ater|EAU38082.1 atub|OJI86441.1 aver|OJJ01649.1 awen|OJJ31508.1 azon|OJJ47227.1

Group4035: abra|OJJ76226.1 acar|OOF99473.1 acla|EAW14366.1 afis|EAW18916.1 afla|EED50900.1 afum|EAL93647.1 agla|OJJ81530.1 akaw|GAA82162.1 aluc|OJZ92005.1 anid|CBF69689.1 anig|EHA22945.1 aory|EIT79047.1 asyd|OJJ59056.1 ater|EAU38083.1 atub|OJI86440.1 aver|OJJ01650.1 awen|OJJ31509.1 azon|OJJ47228.1

Group4036: abra|OJJ76227.1 acar|OOF99474.1 acla|EAW14367.1 afis|EAW18917.1 afla|EED50901.1 afum|EAL93648.1 agla|OJJ81531.1 akaw|GAA82164.1 aluc|OJZ92002.1 anid|CBF69688.1 anig|EHA22943.1 aory|EIT79014.1 asyd|OJJ59057.1 ater|EAU38084.1 atub|OJI86438.1 aver|OJJ01651.1 awen|OJJ31510.1 azon|OJJ47229.1

Group4037: abra|OJJ76228.1 acar|OOF99475.1 acla|EAW14368.1 afis|EAW18918.1 afla|EED50902.1 afum|EAL93649.2 agla|OJJ81532.1 akaw|GAA82165.1 aluc|OJZ92001.1 anid|CBF69686.1 anig|EHA22942.1 aory|EIT78973.1 asyd|OJJ59058.1 ater|EAU38085.1 atub|OJI86437.1 aver|OJJ01652.1 awen|OJJ31511.1 azon|OJJ47230.1

Group4038: abra|OJJ76242.1 acar|OOF99487.1 acla|EAW14197.1 afis|EAW18925.1 afla|EED50914.1 afum|EBA27500.1 agla|OJJ85252.1 akaw|GAA82175.1 aluc|OJZ91989.1 anid|CBF81679.1 anig|EHA22936.1 aory|EIT79011.1 asyd|OJJ59560.1 ater|EAU38100.1 atub|OJI86424.1 aver|OJJ02708.1 awen|OJJ31519.1 azon|OJJ47235.1

Group4039: abra|OJJ76245.1 acar|OOF99491.1 acla|EAW14362.1 afis|EAW18927.1 afla|EED50920.1 afum|EAL93658.1 agla|OJJ85256.1 akaw|GAA82178.1 aluc|OJZ91986.1 anid|CBF69671.1 anig|EHA22932.1 aory|EIT78966.1 asyd|OJJ59063.1 ater|EAU38106.1 atub|OJI86421.1 aver|OJJ01657.1 awen|OJJ31521.1 azon|OJJ47237.1

Group4040: abra|OJJ76246.1 acar|OOF99492.1 acla|EAW14361.1 afis|EAW18928.1 afla|EED50921.1 afum|EAL93659.1 agla|OJJ85257.1 akaw|GAA82179.1 aluc|OJZ91985.1 anid|CBF69669.1 anig|EHA22931.1 aory|EIT79013.1 asyd|OJJ59062.1 ater|EAU38107.1 atub|OJI86420.1 aver|OJJ01656.1 awen|OJJ31522.1 azon|OJJ47238.1

Group4041: abra|OJJ76248.1 acar|OOF89844.1 acla|EAW14359.1 afis|EAW18931.1 afla|EED50936.1 afum|EAL93663.1 agla|OJJ85260.1 akaw|GAA82181.1 aluc|OJZ91982.1 anid|CBF86796.1 anig|EHA22929.1 aory|EIT78972.1 asyd|OJJ64670.1 ater|EAU38110.1 atub|OJI86418.1 aver|OJI98772.1 awen|OJJ31525.1 azon|OJJ47240.1

Group4042: abra|OJJ76250.1 acar|OOF99495.1 acla|EAW14357.1 afis|EAW18933.1 afla|EED50938.1 afum|EAL93666.2 agla|OJJ85262.1 akaw|GAA82183.1 aluc|OJZ91977.1 anid|CBF86800.1 anig|EHA22927.1

aory|EIT77014.1 asyd|OJJ64668.1 ater|EAU38112.1 atub|OJI86416.1 aver|OJI98770.1 awen|OJJ31527.1 azon|OJJ47242.1

Group4043: abra|OJJ76251.1 acar|OOF99496.1 acla|EAW14356.1 afis|EAW18934.1 afla|EED50939.1 afum|EAL93667.2 agla|OJJ85263.1 akaw|GAA82184.1 aluc|OJZ91976.1 anid|CBF86802.1 anig|EHA22926.1 aory|EIT77060.1 asyd|OJJ64667.1 ater|EAU38113.1 atub|OJI86415.1 aver|OJI98769.1 awen|OJJ31528.1 azon|OJJ47243.1

Group4044: abra|OJJ76252.1 acar|OOF99497.1 acla|EAW14355.1 afis|EAW18935.1 afla|EED50940.1 afum|EAL93668.2 agla|OJJ85264.1 akaw|GAA82185.1 aluc|OJZ91975.1 anid|CBF86804.1 anig|EHA22925.1 aory|EIT76996.1 asyd|OJJ64666.1 ater|EAU38114.1 atub|OJI86414.1 aver|OJI98768.1 awen|OJJ31529.1 azon|OJJ47244.1

Group4045: abra|OJJ76253.1 acar|OOF99498.1 acla|EAW14354.1 afis|EAW18936.1 afla|EED50941.1 afum|EAL93669.2 agla|OJJ85265.1 akaw|GAA82186.1 aluc|OJZ91974.1 anid|CBF86805.1 anig|EHA22924.1 aory|EIT77063.1 asyd|OJJ64665.1 ater|EAU38115.1 atub|OJI86413.1 aver|OJI98767.1 awen|OJJ31530.1 azon|OJJ47245.1

Group4046: abra|OJJ76254.1 acar|OOF99499.1 acla|EAW14353.1 afis|EAW18937.1 afla|EED50942.1 afum|EAL93670.1 agla|OJJ85270.1 akaw|GAA82188.1 aluc|OJZ91972.1 anid|CBF86807.1 anig|EHA22918.1 aory|EIT77016.1 asyd|OJJ64664.1 ater|EAU38116.1 atub|OJI86411.1 aver|OJI98766.1 awen|OJJ31532.1 azon|OJJ47246.1

Group4047: abra|OJJ76256.1 acar|OOF99501.1 acla|EAW14350.1 afis|EAW18940.1 afla|EED50966.1 afum|EAL93673.1 agla|OJJ85271.1 akaw|GAA82190.1 aluc|OJZ91970.1 anid|CBF86811.1 anig|EHA22920.1 aory|EIT76988.1 asyd|OJJ64661.1 ater|EAU38119.1 atub|OJI86409.1 aver|OJI98763.1 awen|OJJ31534.1 azon|OJJ47250.1

Group4048: abra|OJJ76259.1 acar|OOF99504.1 acla|EAW14351.1 afis|EAW18939.1 afla|EED50969.1 afum|EAL93672.1 agla|OJJ85268.1 akaw|GAA82192.1 aluc|OJZ91964.1 anid|CBF86815.1 anig|EHA22923.1 aory|EIT77055.1 asyd|OJJ64658.1 ater|EAU38120.1 atub|OJI86407.1 aver|OJI98761.1 awen|OJJ31535.1 azon|OJJ47249.1

Group4049: abra|OJJ76262.1 acar|OOF99506.1 acla|EAW14346.1 afis|EAW18944.1 afla|EED50978.1 afum|EAL93677.1 agla|OJJ85278.1 akaw|GAA82194.1 aluc|OJZ91960.1 anid|CBF86822.1 anig|EHA22916.1 aory|EIT77009.1 asyd|OJJ64655.1 ater|EAU38124.1 atub|OJI86404.1 aver|OJI98757.1 awen|OJJ31540.1 azon|OJJ47257.1

Group4050: abra|OJJ76266.1 afla|EED50981.1 afum|EAL93681.2 anid|CBF86829.1 aory|EIT76942.1 asyd|OJJ64651.1 atub|OJI86400.1 aver|OJI98752.1 acar|OOF99509.1 acla|EAW14343.1 afis|EAW18947.1 agla|OJJ85308.1 akaw|GAA82198.1 aluc|OJZ91954.1 anig|EHA22913.1 ater|EAU38127.1 awen|OJJ31543.1 azon|OJJ47260.1

Group4051: abra|OJJ76267.1 acar|OOF99511.1 acla|EAW14341.1 afis|EAW18949.1 afla|EED50985.1 afum|EAL93683.1 agla|OJJ85320.1 akaw|GAA82200.1 aluc|OJZ91953.1 anid|CBF86833.1 anig|EHA22912.1 aory|EIT77049.1 asyd|OJJ64649.1 ater|EAU38135.1 atub|OJI86398.1 aver|OJI98749.1 awen|OJJ31547.1 azon|OJJ47263.1

Group4052: abra|OJJ76269.1 acar|OOF99517.1 acla|EAW12851.1 afis|EAW18014.1 afla|EED51880.1 afum|EAL91826.1 agla|OJJ85368.1 akaw|GAA82202.1 aluc|OJZ91951.1 anid|CBF80042.1 anig|EHA22910.1 aory|EIT81710.1 asyd|OJJ58276.1 ater|EAU31530.1 atub|OJI86396.1 aver|OJJ02148.1 awen|OJJ41526.1 azon|OJJ48247.1

Group4053: abra|OJJ76288.1 acar|OOF99529.1 acla|EAW14328.1 afis|EAW18960.1 afla|EED51006.1 afum|EAL93693.1 agla|OJJ85342.1 akaw|GAA82217.1 aluc|OJZ91934.1 anid|CBF69600.1 anig|EHA22894.1 aory|EIT77056.1 asyd|OJJ59135.1 ater|EAU38143.1 atub|OJI86376.1 aver|OJJ01724.1 awen|OJJ31559.1 azon|OJJ47276.1

Group4054: abra|OJJ76290.1 acar|OOF99535.1 acla|EAW14325.1 afis|EAW18963.1 afla|EED51010.1 afum|EAL93695.1 agla|OJJ85344.1 akaw|GAA82219.1 aluc|OJZ91932.1 anid|CBF69605.1 anig|EHA22893.1 aory|EIT76961.1 asyd|OJJ59132.1 ater|EAU38148.1 atub|OJI86374.1 aver|OJJ01721.1 awen|OJJ31562.1 azon|OJJ47283.1

Group4055: abra|OJJ76292.1 acar|OOF99537.1 acla|EAW14324.1 afis|EAW18964.1 afla|EED51011.1 afum|EAL93696.1 agla|OJJ85346.1 akaw|GAA82220.1 aluc|OJZ91930.1 anid|CBF69607.1 anig|EHA22892.1 aory|EIT77048.1 asyd|OJJ59130.1 atub|OJI86373.1 aver|OJJ01720.1 awen|OJJ31564.1 azon|OJJ47282.1 ater|EAU38149.1

Group4056: abra|OJJ76296.1 acar|OOF99542.1 acla|EAW14322.1 afis|EAW18966.1 afla|EED51032.1 afum|EAL93698.2 agla|OJJ85352.1 akaw|GAA82223.1 aluc|OJZ91926.1 anid|CBF69611.1 anig|EHA22889.1 aory|EIT76971.1 asyd|OJJ59128.1 ater|EAU38151.1 atub|OJI86370.1 aver|OJJ01716.1 awen|OJJ31566.1 azon|OJJ47284.1

Group4057: abra|OJJ76297.1 acar|OOF99545.1 acla|EAW14321.1 afis|EAW18967.1 afla|EED51033.1 afum|EAL93699.1 agla|OJJ85353.1 akaw|GAA82224.1 aluc|OJZ91925.1 anid|CBF69613.1 anig|EHA22888.1 aory|EIT76951.1 asyd|OJJ59127.1 ater|EAU38153.1 atub|OJI86369.1 aver|OJJ01715.1 awen|OJJ31567.1 azon|OJJ47285.1

Group4058: abra|OJJ76299.1 acar|OOF99546.1 acla|EAW14319.1 afis|EAW18969.1 afla|EED51035.1 afum|EAL93702.1 agla|OJJ85355.1 akaw|GAA82226.1 aluc|OJZ91923.1 anid|CBF69617.1 anig|EHA22887.1 aory|EIT77006.1 asyd|OJJ59124.1 ater|EAU38155.1 atub|OJI86367.1 aver|OJJ01713.1 awen|OJJ31569.1 azon|OJJ47287.1

Group4059: abra|OJJ76300.1 acar|OOF99544.1 acla|EAW14318.1 afis|EAW18970.1 afla|EED51036.1 afum|EAL93703.1 agla|OJJ85356.1 akaw|GAA82227.1 aluc|OJZ91922.1 anid|CBF69619.1 anig|EHA22886.1 aory|EIT77012.1 asyd|OJJ59123.1 ater|EAU38156.1 atub|OJI86366.1 aver|OJJ01712.1 awen|OJJ31570.1 azon|OJJ47288.1

Group4060: abra|OJJ76301.1 acar|OOF99547.1 acla|EAW14317.1 afis|EAW18971.1 afla|EED51037.1 afum|EAL93704.1 agla|OJJ85357.1 akaw|GAA82228.1 aluc|OJZ91921.1 anid|CBF69621.1 anig|EHA22885.1 aory|EIT77040.1 asyd|OJJ59122.1 ater|EAU38157.1 atub|OJI86365.1 aver|OJJ01711.1 awen|OJJ31571.1 azon|OJJ47289.1

Group4061: abra|OJJ76302.1 acar|OOF99548.1 acla|EAW14316.1 afis|EAW18972.1 afla|EED51038.1 afum|EAL93705.1 agla|OJJ85360.1 akaw|GAA82232.1 aluc|OJZ91916.1 anid|CBF69627.1 anig|EHA22884.1 aory|EIT77044.1 asyd|OJJ59119.1 ater|EAU38158.1 atub|OJI86364.1 aver|OJJ01708.1 awen|OJJ31572.1 azon|OJJ47291.1

Group4062: abra|OJJ76303.1 acar|OOF99549.1 acla|EAW14315.1 afis|EAW18973.1 afla|EED51039.1 afum|EAL93706.1 agla|OJJ85361.1 akaw|GAA82233.1 aluc|OJZ91914.1 anid|CBF69629.1 anig|EHA22883.1 aory|EIT77038.1 asyd|OJJ59117.1 ater|EAU38159.1 atub|OJI86363.1 aver|OJJ01707.1 awen|OJJ31573.1 azon|OJJ47290.1

Group4063: abra|OJJ76307.1 acar|OOF99552.1 acla|EAW14108.1 afis|EAW18810.1 afla|EED50762.1 afum|EAL93546.1 agla|OJJ80543.1 akaw|GAA82238.1 aluc|OJZ91908.1 anid|CBF69793.1 anig|EHA22882.1 aory|EIT78035.1 asyd|OJJ58963.1 ater|EAU37962.1 atub|OJI86358.1 aver|OJJ01557.1 awen|OJJ32385.1 azon|OJJ47146.1

Group4064: abra|OJJ76314.1 acar|OOF99566.1 acla|EAW14101.1 afis|EAW18802.1 afla|EED50774.1 afum|EAL93538.1 agla|OJJ80532.1 akaw|GAA82245.1 aluc|OJZ91900.1 anid|CBF69784.1 anig|EHA22879.1 aory|EIT77990.1 asyd|OJJ58960.1 ater|EAU37940.1 atub|OJI86353.1 aver|OJJ01554.1 awen|OJJ32376.1 azon|OJJ47139.1

Group4065: abra|OJJ76323.1 acar|OOF99575.1 acla|EAW14092.1 afis|EAW18798.1 afla|EED50791.1 afum|EAL93534.1 agla|OJJ80527.1 akaw|GAA82252.1 aluc|OJZ91892.1 anid|CBF69777.1 anig|EHA22871.1 aory|EIT78043.1 asyd|OJJ58956.1 ater|EAU37930.1 atub|OJI86344.1 aver|OJJ01550.1 awen|OJJ32372.1 azon|OJJ47135.1

Group4066: abra|OJJ76325.1 acar|OOF99577.1 acla|EAW14090.1 afis|EAW18796.1 afla|EED50793.1 afum|EAL93532.1 agla|OJJ80525.1 akaw|GAA82254.1 aluc|OJZ91890.1 anid|CBF69773.1 anig|EHA22870.1 aory|EIT78128.1 asyd|OJJ58954.1 ater|EAU37927.1 atub|OJI86342.1 aver|OJJ01548.1 awen|OJJ32370.1 azon|OJJ47133.1

Group4067: abra|OJJ76326.1 acar|OOF99578.1 afis|EAW18795.1 afla|EED50794.1 afum|EAL93531.1 agla|OJJ80524.1 akaw|GAA82255.1 aluc|OJZ91889.1 anid|CBF69771.1 anig|EHA22869.1 aory|EIT78000.1 asyd|OJJ58953.1 ater|EAU37926.1 atub|OJI86341.1 aver|OJJ01547.1 awen|OJJ32369.1 azon|OJJ47132.1 acla|EAW14089.1

Group4068: abra|OJJ76327.1 acar|OOF99580.1 acla|EAW14088.1 afis|EAW18794.1 afla|EED50795.1 afum|EAL93530.1 agla|OJJ80523.1 akaw|GAA82256.1 aluc|OJZ91888.1 anid|CBF69769.1 anig|EHA22868.1 aory|EIT78079.1 asyd|OJJ58952.1 ater|EAU37925.1 atub|OJI86340.1 aver|OJJ01546.1 awen|OJJ32367.1 azon|OJJ47131.1

Group4069: abra|OJJ76332.1 acar|OOF99584.1 acla|EAW14083.1 afis|EAW18790.1 afla|EED50748.1 afum|EAL93525.1 agla|OJJ80519.1 akaw|GAA82260.1 aluc|OJZ91881.1 anid|CBF69761.1 anig|EHA22859.1 aory|EIT78056.1 asyd|OJJ58947.1 ater|EAU37921.1 atub|OJI86332.1 aver|OJJ01542.1 awen|OJJ32363.1 azon|OJJ47126.1

Group4070: abra|OJJ76334.1 acar|OOF99587.1 acla|EAW14079.1 afis|EAW18786.1 afla|EED50745.1 afum|EAL93519.1 agla|OJJ80509.1 akaw|GAA82262.1 aluc|OJZ91878.1 anid|CBF69752.1 anig|EHA22856.1 aory|EIT78009.1 asyd|OJJ58941.1 ater|EAU37918.1 atub|OJI86329.1 aver|OJJ01537.1 awen|OJJ32360.1 azon|OJJ47123.1

Group4071: abra|OJJ76336.1 acar|OOF99588.1 acla|EAW14078.1 afis|EAW18785.1 afla|EED50744.1 afum|EAL93517.2 agla|OJJ80507.1 akaw|GAA82264.1 aluc|OJZ91876.1 anid|CBF69748.1 anig|EHA22855.1

aory|EIT78137.1 asyd|OJJ58938.1 ater|EAU37917.1 atub|OJI86327.1 aver|OJJ01534.1 awen|OJJ32358.1 azon|OJJ47121.1

Group4072: abra|OJJ76337.1 acar|OOF99589.1 acla|EAW14077.1 afis|EAW18784.1 afla|EED50743.1 afum|EAL93516.2 agla|OJJ80506.1 akaw|GAA82265.1 aluc|OJZ91875.1 anid|CBF69746.1 anig|EHA22854.1 aory|EIT78027.1 asyd|OJJ58937.1 ater|EAU37916.1 atub|OJI86326.1 aver|OJJ01533.1 awen|OJJ32357.1 azon|OJJ47120.1

Group4073: abra|OJJ76338.1 acar|OOF99590.1 acla|EAW14076.1 afis|EAW18783.1 afla|EED50742.1 afum|EAL93515.1 agla|OJJ80504.1 akaw|GAA82266.1 aluc|OJZ91874.1 anid|CBF69744.1 anig|EHA22853.1 aory|EIT78012.1 asyd|OJJ58936.1 ater|EAU37915.1 atub|OJI86325.1 aver|OJJ01532.1 awen|OJJ32356.1 azon|OJJ47119.1

Group4074: abra|OJJ76340.1 acar|OOF99591.1 acla|EAW14075.1 afis|EAW18782.1 afla|EED50741.1 afum|EAL93514.1 agla|OJJ80502.1 akaw|GAA82267.1 aluc|OJZ91873.1 anid|CBF69742.1 anig|EHA22852.1 aory|EIT78041.1 asyd|OJJ58935.1 ater|EAU37914.1 atub|OJI86324.1 aver|OJJ01531.1 awen|OJJ32354.1 azon|OJJ47118.1

Group4075: abra|OJJ76342.1 acar|OOF99593.1 acla|EAW14073.1 afis|EAW18780.1 afla|EED50739.1 afum|EAL93512.1 agla|OJJ80500.1 akaw|GAA82269.1 aluc|OJZ91871.1 anid|CBF69738.1 anig|EHA22850.1 aory|EIT77981.1 asyd|OJJ58933.1 ater|EAU37912.1 atub|OJI86322.1 aver|OJJ01529.1 awen|OJJ32352.1 azon|OJJ47116.1

Group4076: abra|OJJ76344.1 acar|OOF99595.1 acla|EAW14071.1 afis|EAW18778.1 afla|EED50737.1 afum|EAL93510.1 agla|OJJ80495.1 akaw|GAA82271.1 aluc|OJZ91869.1 anid|CBF69734.1 anig|EHA22849.1 aory|EIT78123.1 asyd|OJJ58931.1 ater|EAU37910.1 atub|OJI86320.1 aver|OJJ01527.1 awen|OJJ32349.1 azon|OJJ47114.1

Group4077: abra|OJJ76345.1 acar|OOF99596.1 acla|EAW14070.1 afis|EAW18777.1 afla|EED50736.1 afum|EAL93509.1 agla|OJJ80494.1 akaw|GAA82272.1 aluc|OJZ91868.1 anid|CBF69732.1 anig|EHA22848.1 aory|EIT78071.1 asyd|OJJ58930.1 ater|EAU37909.1 atub|OJI86319.1 aver|OJJ01526.1 awen|OJJ32348.1 azon|OJJ47113.1

Group4078: abra|OJJ76346.1 acar|OOF99597.1 acla|EAW14069.1 afis|EAW18776.1 afla|EED50735.1 afum|EAL93508.1 agla|OJJ80493.1 akaw|GAA82273.1 aluc|OJZ91867.1 anid|CBF69730.1 anig|EHA22847.1 aory|EIT78130.1 asyd|OJJ58929.1 ater|EAU37908.1 atub|OJI86318.1 aver|OJJ01525.1 awen|OJJ32347.1 azon|OJJ47112.1

Group4079: abra|OJJ76347.1 acar|OOF99598.1 acla|EAW14068.1 afis|EAW18775.1 afla|EED50734.1 afum|EAL93507.1 agla|OJJ80491.1 akaw|GAA82274.1 aluc|OJZ91866.1 anid|CBF69728.1 anig|EHA22846.1 aory|EIT78021.1 asyd|OJJ58928.1 ater|EAU37906.1 atub|OJI86317.1 aver|OJJ01524.1 awen|OJJ32346.1 azon|OJJ47111.1

Group4080: abra|OJJ76349.1 acar|OOF99600.1 acla|EAW14066.1 afis|EAW18773.1 afla|EED50732.1 afum|EAL93505.1 agla|OJJ80489.1 akaw|GAA82276.1 aluc|OJZ91864.1 anid|CBF69724.1 anig|EHA22844.1 aory|EIT78029.1 asyd|OJJ58927.1 ater|EAU37904.1 atub|OJI86315.1 aver|OJJ01523.1 awen|OJJ32344.1 azon|OJJ47110.1

Group4081: abra|OJJ76351.1 acar|OOF99603.1 acla|EAW14064.1 afis|EAW18771.1 afla|EED50730.1 afum|EAL93502.1 agla|OJJ80467.1 akaw|GAA82278.1 aluc|OJZ91862.1 anid|CBF69934.1 anig|EHA22842.1 aory|EIT78074.1 asyd|OJJ58922.1 ater|EAU37902.1 atub|OJI86314.1 aver|OJJ01519.1 awen|OJJ32340.1 azon|OJJ47106.1

Group4082: abra|OJJ76353.1 acar|OOF99604.1 acla|EAW14062.1 afis|EAW18769.1 afla|EED50728.1 afum|EAL93500.2 agla|OJJ82285.1 akaw|GAA82279.1 aluc|OJZ91860.1 anid|CBF69938.1 anig|EHA22840.1 aory|EIT78133.1 asyd|OJJ58920.1 ater|EAU37900.1 atub|OJI86311.1 aver|OJJ01517.1 awen|OJJ32338.1 azon|OJJ47103.1

Group4083: abra|OJJ76354.1 acar|OOF99605.1 acla|EAW14061.1 afis|EAW18768.1 afla|EED50727.1 afum|EAL93499.1 agla|OJJ82284.1 akaw|GAA82280.1 aluc|OJZ91859.1 anid|CBF69940.1 anig|EHA22839.1 aory|EIT78038.1 asyd|OJJ58919.1 ater|EAU37899.1 atub|OJI86310.1 aver|OJJ01516.1 awen|OJJ32337.1 azon|OJJ47102.1

Group4084: abra|OJJ76356.1 acar|OOF99607.1 acla|EAW14059.1 afis|EAW18765.1 afla|EED50725.1 afum|EAL93497.1 agla|OJJ80456.1 akaw|GAA82282.1 aluc|OJZ91857.1 anid|CBF69944.1 anig|EHA22837.1 aory|EIT78144.1 asyd|OJJ58917.1 ater|EAU37895.1 atub|OJI86308.1 aver|OJJ01514.1 awen|OJJ32335.1 azon|OJJ47101.1

Group4085: abra|OJJ76358.1 acar|OOF99609.1 acla|EAW14058.1 afis|EAW18764.1 afla|EED50724.1 afum|EAL93496.2 agla|OJJ80457.1 akaw|GAA82284.1 aluc|OJZ91855.1 anid|CBF69945.1 anig|EHA22836.1 aory|EIT78031.1 asyd|OJJ58915.1 ater|EAU37894.1 atub|OJI86306.1 aver|OJJ01512.1 awen|OJJ32333.1 azon|OJJ47099.1

Group4086: abra|OJJ76359.1 acar|OOF99610.1 acla|EAW14057.1 afis|EAW18763.1 afla|EED50723.1 afum|EAL93495.1 agla|OJJ80458.1 akaw|GAA82285.1 aluc|OJZ91854.1 anid|CBF69947.1 anig|EHA22835.1 aory|EIT78108.1 asyd|OJJ58914.1 ater|EAU37893.1 atub|OJI86305.1 aver|OJJ01511.1 awen|OJJ32332.1 azon|OJJ47098.1

Group4087: abra|OJJ76362.1 acar|OOF99613.1 acla|EAW14054.1 afis|EAW18760.1 afla|EED50720.1 afum|EAL93491.1 agla|OJJ82333.1 akaw|GAA82288.1 aluc|OJZ91850.1 anid|CBF69953.1 anig|EHA22832.1 aory|EIT78011.1 asyd|OJJ58911.1 ater|EAU37891.1 atub|OJI86302.1 aver|OJJ01508.1 awen|OJJ32330.1 azon|OJJ47095.1

Group4088: abra|OJJ76363.1 acar|OOF99614.1 acla|EAW14053.1 afis|EAW18759.1 afla|EED50719.1 afum|EAL93489.1 agla|OJJ82332.1 akaw|GAA82289.1 aluc|OJZ91849.1 anid|CBF69955.1 anig|EHA22831.1 aory|EIT78126.1 asyd|OJJ58910.1 ater|EAU37928.1 atub|OJI86301.1 aver|OJJ01507.1 awen|OJJ32329.1 azon|OJJ47094.1

Group4089: abra|OJJ76364.1 acar|OOF99615.1 acla|EAW14052.1 afis|EAW18758.1 afla|EED50718.1 afum|EAL93488.2 agla|OJJ82331.1 akaw|GAA82290.1 aluc|OJZ91848.1 anid|CBF69957.1 anig|EHA22830.1 aory|EIT78109.1 asyd|OJJ58909.1 ater|EAU37890.1 atub|OJI86300.1 aver|OJJ01506.1 awen|OJJ32328.1 azon|OJJ47093.1

Group4090: abra|OJJ76366.1 acar|OOF89987.1 acla|EAW14050.1 afis|EAW18756.1 afla|EED50716.1 afum|EAL93484.1 agla|OJJ82324.1 akaw|GAA82292.1 aluc|OJZ91846.1 anid|CBF69963.1 anig|EHA22828.1 aory|EIT78115.1 asyd|OJJ58907.1 ater|EAU37887.1 atub|OJI86298.1 aver|OJJ01504.1 awen|OJJ32325.1 azon|OJJ47092.1

Group4091: abra|OJJ76368.1 acar|OOF99617.1 acla|EAW14048.1 afis|EAW18754.1 afla|EED50714.1 afum|EAL93481.1 agla|OJJ82322.1 akaw|GAA82294.1 aluc|OJZ91844.1 anid|CBF69971.1 anig|EHA22827.1 aory|EIT77980.1 asyd|OJJ58901.1 ater|EAU37885.1 atub|OJI86296.1 aver|OJJ01501.1 awen|OJJ32323.1 azon|OJJ47090.1

Group4092: abra|OJJ76370.1 acar|OOF99619.1 acla|EAW14047.1 afis|EAW18753.1 afla|EED50712.1 afum|EAL93480.1 agla|OJJ82321.1 akaw|GAA82296.1 aluc|OJZ91842.1 anid|CBF69973.1 anig|EHA22825.1 aory|EIT78076.1 asyd|OJJ58900.1 ater|EAU37884.1 atub|OJI86294.1 aver|OJJ01500.1 awen|OJJ32322.1 azon|OJJ47089.1

Group4093: abra|OJJ76378.1 acar|OOF99623.1 acla|EAW14044.1 afis|EAW18750.1 afla|EED50709.1 afum|EAL93477.1 agla|OJJ82317.1 akaw|GAA82299.1 aluc|OJZ91836.1 anid|CBF69979.1 anig|EHA22823.1 aory|EIT78032.1 asyd|OJJ58896.1 ater|EAU37879.1 atub|OJI86290.1 aver|OJJ01497.1 awen|OJJ32317.1 azon|OJJ47085.1

Group4094: abra|OJJ76379.1 acar|OOF99624.1 acla|EAW14043.1 afis|EAW18749.1 afla|EED50708.1 afum|EAL93475.1 agla|OJJ82316.1 akaw|GAA82300.1 aluc|OJZ91835.1 anid|CBF69983.1 anig|EHA22822.1 aory|EIT77996.1 asyd|OJJ58895.1 ater|EAU37878.1 atub|OJI86289.1 aver|OJJ01496.1 awen|OJJ32316.1 azon|OJJ47084.1

Group4095: abra|OJJ76381.1 acar|OOF89925.1 acla|EAW14041.1 afis|EAW18747.1 afla|EED50706.1 afum|EAL93473.1 agla|OJJ82314.1 akaw|GAA82302.1 aluc|OJZ91833.1 anid|CBF69987.1 anig|EHA22820.1 aory|EIT78116.1 asyd|OJJ58893.1 ater|EAU37876.1 atub|OJI86287.1 aver|OJJ01494.1 awen|OJJ32313.1 azon|OJJ47082.1

Group4096: abra|OJJ76382.1 acar|OOF89924.1 acla|EAW14040.1 afis|EAW18746.1 afla|EED50705.1 afum|EAL93472.1 agla|OJJ82313.1 akaw|GAA82303.1 aluc|OJZ91832.1 anid|CBF69992.1 anig|EHA22819.1 aory|EIT78094.1 asyd|OJJ58891.1 ater|EAU37875.1 atub|OJI86286.1 aver|OJJ01492.1 awen|OJJ32312.1 azon|OJJ42712.1

Group4097: abra|OJJ76387.1 acar|OOF99628.1 acla|EAW14036.1 afis|EAW18742.1 afla|EED50698.1 afum|EAL93468.1 agla|OJJ82308.1 akaw|GAA82307.1 aluc|OJZ91826.1 anid|CBF69998.1 anig|EHA22815.1 aory|EIT78061.1 asyd|OJJ58887.1 ater|EAU37869.1 atub|OJI86281.1 aver|OJJ01488.1 awen|OJJ32307.1 azon|OJJ42719.1

Group4098: abra|OJJ76389.1 acar|OOF99630.1 acla|EAW14035.1 afis|EAW18741.1 afla|EED50696.1 afum|EAL93467.1 agla|OJJ82307.1 akaw|GAA82308.1 aluc|OJZ91824.1 anid|CBF70000.1 anig|EHA22814.1 aory|EIT78051.1 asyd|OJJ58886.1 ater|EAU37864.1 atub|OJI86279.1 aver|OJJ01487.1 awen|OJJ32306.1 azon|OJJ42720.1

Group4099: abra|OJJ76390.1 acar|OOF99631.1 acla|EAW14034.1 afis|EAW18740.1 afla|EED50695.1 afum|EAL93466.1 agla|OJJ82306.1 akaw|GAA82309.1 aluc|OJZ91823.1 anid|CBF70002.1 anig|EHA22813.1 aory|EIT78073.1 asyd|OJJ58884.1 ater|EAU37863.1 atub|OJI86278.1 aver|OJJ01486.1 awen|OJJ32305.1 azon|OJJ42721.1

Group4100: abra|OJJ76392.1 acar|OOF99632.1 acla|EAW14032.1 afis|EAW18738.1 afla|EED50693.1 afum|EAL93464.1 agla|OJJ82304.1 akaw|GAA82311.1 aluc|OJZ91821.1 anid|CBF70006.1 anig|EHA22811.1

aory|EIT78112.1 asyd|OJJ58882.1 ater|EAU37861.1 atub|OJI86276.1 aver|OJJ01484.1 awen|OJJ32302.1 azon|OJJ42723.1

Group4101: abra|OJJ76393.1 acar|OOF99633.1 acla|EAW14031.1 afis|EAW18737.1 afla|EED50692.1 afum|EAL93462.1 agla|OJJ82303.1 akaw|GAA82312.1 aluc|OJZ91820.1 anid|CBF70008.1 anig|EHA22810.1 aory|EIT78068.1 asyd|OJJ58881.1 ater|EAU37860.1 atub|OJI86275.1 aver|OJJ01483.1 awen|OJJ32301.1 azon|OJJ42724.1

Group4102: abra|OJJ76395.1 acar|OOF99635.1 acla|EAW14029.1 afis|EAW18735.1 afla|EED50690.1 afum|EAL93460.1 agla|OJJ82301.1 akaw|GAA82314.1 aluc|OJZ91818.1 anid|CBF70012.1 anig|EHA22808.1 aory|EIT78132.1 asyd|OJJ58879.1 ater|EAU37858.1 atub|OJI86273.1 aver|OJJ01481.1 awen|OJJ32298.1 azon|OJJ42734.1

Group4103: abra|OJJ76396.1 acar|OOF99637.1 acla|EAW14027.1 afis|EAW18733.1 afla|EED50688.1 afum|EAL93458.1 agla|OJJ82299.1 akaw|GAA82316.1 aluc|OJZ91816.1 anid|CBF70014.1 anig|EHA22807.1 aory|EIT78099.1 asyd|OJJ58878.1 ater|EAU37848.1 atub|OJI86272.1 aver|OJJ01480.1 awen|OJJ32296.1 azon|OJJ42736.1

Group4104: abra|OJJ76404.1 acar|OOF99647.1 acla|EAW14017.1 afis|EAW18725.1 afla|EED50682.1 afum|EAL93450.1 agla|OJJ80475.1 akaw|GAA82324.1 aluc|OJZ91808.1 anid|CBF70021.1 anig|EHA22801.1 aory|EIT77978.1 asyd|OJJ58874.1 ater|EAU38179.1 atub|OJI86263.1 aver|OJJ01477.1 awen|OJJ32229.1 azon|OJJ46783.1

Group4105: abra|OJJ76406.1 acar|OOF99649.1 acla|EAW14019.1 afis|EAW18723.1 afla|EED50680.1 afum|EAL93448.1 agla|OJJ80477.1 akaw|GAA82326.1 aluc|OJZ91806.1 anid|CBF70688.1 anig|EHA22799.1 aory|EIT78149.1 asyd|OJJ58468.1 ater|EAU38180.1 atub|OJI86261.1 aver|OJJ01067.1 awen|OJJ32231.1 azon|OJJ46781.1

Group4106: abra|OJJ76409.1 acar|OOF99651.1 acla|EAW14015.1 afis|EAW18721.1 afla|EED50678.1 afum|EAL93445.1 agla|OJJ82334.1 akaw|GAA82328.1 aluc|OJZ91802.1 anid|CBF70681.1 anig|EHA22797.1 aory|EIT78078.1 asyd|OJJ58470.1 ater|EAU38183.1 atub|OJI86257.1 aver|OJJ01071.1 awen|OJJ32227.1 azon|OJJ46778.1

Group4107: abra|OJJ76411.1 acar|OOF99652.1 acla|EAW14014.1 afis|EAW18720.1 afla|EED50677.1 afum|EAL93444.1 agla|OJJ82335.1 akaw|GAA82329.1 aluc|OJZ91801.1 anid|CBF70679.1 anig|EHA22796.1 aory|EIT78134.1 asyd|OJJ58471.1 ater|EAU38184.1 atub|OJI86256.1 aver|OJJ01072.1 awen|OJJ32226.1 azon|OJJ46777.1

Group4108: abra|OJJ76417.1 acar|OOF99659.1 acla|EAW14009.1 afis|EAW18715.1 afla|EED50669.1 afum|EAL93440.1 agla|OJJ82344.1 akaw|GAA82332.1 aluc|OJZ91795.1 anid|CBF70670.1 anig|EHA22792.1 aory|EIT78070.1 asyd|OJJ58476.1 ater|EAU38192.1 atub|OJI86251.1 aver|OJJ01079.1 awen|OJJ32202.1 azon|OJJ46771.1

Group4109: abra|OJJ76418.1 acar|OOF99660.1 acla|EAW14008.1 afis|EAW18714.1 afla|EED50667.1 afum|EAL93439.1 agla|OJJ82345.1 akaw|GAA82333.1 aluc|OJZ91794.1 anid|CBF70668.1 anig|EHA22791.1 aory|EIT78037.1 asyd|OJJ58477.1 ater|EAU38193.1 atub|OJI86250.1 aver|OJJ01080.1 awen|OJJ32201.1 azon|OJJ46770.1

Group4110: abra|OJJ76419.1 acar|OOF99661.1 acla|EAW14007.1 afis|EAW18713.1 afla|EED50666.1 afum|EAL93438.1 agla|OJJ82346.1 akaw|GAA82334.1 aluc|OJZ91793.1 anid|CBF70666.1 anig|EHA22790.1 aory|EIT78088.1 asyd|OJJ58478.1 ater|EAU38194.1 atub|OJI86249.1 aver|OJJ01081.1 awen|OJJ32200.1 azon|OJJ46769.1

Group4111: abra|OJJ76420.1 acar|OOF99662.1 acla|EAW14006.1 afis|EAW18712.1 afla|EED50665.1 afum|EAL93437.1 agla|OJJ82347.1 akaw|GAA82335.1 aluc|OJZ91792.1 anid|CBF70665.1 anig|EHA22789.1 aory|EIT77998.1 asyd|OJJ58480.1 ater|EAU38195.1 atub|OJI86248.1 aver|OJJ01082.1 awen|OJJ32199.1 azon|OJJ46768.1

Group4112: abra|OJJ76422.1 acar|OOF99665.1 acla|EAW14003.1 afis|EAW18711.1 afla|EED50662.1 afum|EAL93434.1 agla|OJJ82348.1 akaw|GAA82337.1 aluc|OJZ91790.1 anid|CBF70661.1 anig|EHA22787.1 asyd|OJJ58481.1 ater|EAU38197.1 atub|OJI86246.1 aver|OJJ01083.1 awen|OJJ32197.1 azon|OJJ46766.1 aory|EIT77986.1

Group4113: abra|OJJ76423.1 acar|OOF99667.1 acla|EAW14002.1 afis|EAW18710.1 afla|EED50660.1 afum|EAL93433.1 agla|OJJ82349.1 akaw|GAA82338.1 aluc|OJZ91789.1 anid|CBF70657.1 anig|EHA22786.1 aory|EIT78084.1 asyd|OJJ58482.1 ater|EAU38198.1 atub|OJI86245.1 aver|OJJ01085.1 awen|OJJ32196.1 azon|OJJ46765.1

Group4114: abra|OJJ76429.1 acar|OOF99673.1 acla|EAW13996.1 afis|EAW18704.1 afla|EED50653.1 afum|EAL93427.2 agla|OJJ82354.1 akaw|GAA82344.1 aluc|OJZ91783.1 anid|CBF70646.1 anig|EHA22780.1 aory|EIT78100.1 asyd|OJJ58488.1 ater|EAU38202.1 atub|OJI86239.1 aver|OJJ01091.1 awen|OJJ32187.1 azon|OJJ46760.1

Group4115: abra|OJJ76430.1 acar|OOF99674.1 acla|EAW13995.1 afis|EAW18703.1 afla|EED50652.1 afum|EAL93426.1 agla|OJJ82355.1 akaw|GAA82345.1 aluc|OJZ91782.1 anid|CBF70644.1 anig|EHA22779.1 aory|EIT78020.1 asyd|OJJ58489.1 ater|EAU38203.1 atub|OJI86238.1 aver|OJJ01092.1 awen|OJJ32186.1 azon|OJJ46761.1

Group4116: abra|OJJ76431.1 acar|OOF99675.1 acla|EAW13994.1 afis|EAW18702.1 afla|EED50651.1 afum|EAL93425.1 agla|OJJ82359.1 akaw|GAA82346.1 aluc|OJZ91781.1 anid|CBF70642.1 anig|EHA22778.1 aory|EIT78030.1 asyd|OJJ58490.1 ater|EAU38204.1 atub|OJI86237.1 aver|OJJ01093.1 awen|OJJ32185.1 azon|OJJ46762.1

Group4117: abra|OJJ76433.1 acar|OOF99677.1 acla|EAW13991.1 afis|EAW18699.1 afla|EED50647.1 afum|EAL93421.1 agla|OJJ82362.1 akaw|GAA82348.1 aluc|OJZ91779.1 anid|CBF70635.1 anig|EHA22776.1 aory|EIT78093.1 asyd|OJJ58494.1 ater|EAU38207.1 atub|OJI86235.1 aver|OJJ01097.1 awen|OJJ32181.1 azon|OJJ46756.1

Group4118: abra|OJJ76434.1 acar|OOF99679.1 acla|EAW13989.1 afis|EAW18697.1 afla|EED50645.1 afum|EAL93419.1 agla|OJJ82371.1 akaw|GAA82349.1 aluc|OJZ91778.1 anid|CBF70631.1 anig|EHA22775.1 aory|EIT78142.1 asyd|OJJ58496.1 ater|EAU38209.1 atub|OJI86234.1 aver|OJJ01099.1 awen|OJJ32168.1 azon|OJJ46752.1

Group4119: abra|OJJ76440.1 acar|OOF99685.1 acla|EAW13984.1 afis|EAW18693.1 afla|EED50642.1 afum|EAL93414.1 agla|OJJ82368.1 akaw|GAA82354.1 aluc|OJZ91769.1 anid|CBF70618.1 anig|EHA22769.1 aory|EIT78025.1 asyd|OJJ58503.1 ater|EAU38215.1 atub|OJI86227.1 aver|OJJ01104.1 awen|OJJ32164.1 azon|OJJ46745.1

Group4120: abra|OJJ76441.1 acar|OOF99686.1 acla|EAW13983.1 afis|EAW18692.1 afla|EED50641.1 afum|EAL93413.2 agla|OJJ82366.1 akaw|GAA82355.1 aluc|OJZ91768.1 anid|CBF70620.1 anig|EHA22768.1 aory|EIT78095.1 asyd|OJJ58502.1 ater|EAU38216.1 atub|OJI86226.1 aver|OJJ01103.1 awen|OJJ32163.1 azon|OJJ46746.1

Group4121: abra|OJJ76446.1 acar|OOF99689.1 acla|EAW13982.1 afis|EAW18691.1 afla|EED50638.1 afum|EAL93412.1 agla|OJJ82381.1 akaw|GAA82358.1 aluc|OJZ91764.1 anid|CBF70624.1 anig|EHA22765.1 aory|EIT78085.1 asyd|OJJ58499.1 ater|EAU38217.1 atub|OJI86222.1 aver|OJJ01102.1 awen|OJJ32162.1 azon|OJJ46748.1

Group4122: abra|OJJ76448.1 acar|OOF99690.1 acla|EAW13980.1 afis|EAW18689.1 afla|EED50637.1 afum|EAL93410.1 agla|OJJ82383.1 akaw|GAA82360.1 aluc|OJZ91762.1 anid|CBF70616.1 anig|EHA22763.1 aory|EIT78124.1 asyd|OJJ58506.1 ater|EAU38219.1 atub|OJI86220.1 aver|OJJ01107.1 awen|OJJ32160.1 azon|OJJ46744.1

Group4123: abra|OJJ76449.1 acar|OOF99692.1 acla|EAW13979.1 afis|EAW18688.1 afla|EED50636.1 afum|EAL93409.1 agla|OJJ82384.1 akaw|GAA82361.1 aluc|OJZ91761.1 anid|CBF70612.1 anig|EHA22762.1 aory|EIT78146.1 asyd|OJJ58510.1 ater|EAU38220.1 atub|OJI86219.1 aver|OJJ01111.1 awen|OJJ32159.1 azon|OJJ46743.1

Group4124: abra|OJJ76455.1 acar|OOF99696.1 acla|EAW13975.1 afis|EAW18684.1 afla|EED50622.1 afum|EAL93405.1 agla|OJJ82387.1 akaw|GAA82367.1 aluc|OJZ91755.1 anid|CBF70607.1 anig|EHA22758.1 aory|EIT78141.1 asyd|OJJ58514.1 ater|EAU38226.1 atub|OJI86213.1 aver|OJJ01115.1 awen|OJJ32155.1 azon|OJJ46739.1

Group4125: abra|OJJ76456.1 acar|OOF99695.1 acla|EAW13974.1 afis|EAW18683.1 afla|EED50627.1 afum|EAL93404.1 agla|OJJ82392.1 akaw|GAA82368.1 aluc|OJZ91754.1 anid|CBF70605.1 anig|EHA22757.1 aory|EIT77985.1 asyd|OJJ58515.1 ater|EAU38227.1 atub|OJI86212.1 aver|OJJ01116.1 awen|OJJ32154.1 azon|OJJ46738.1

Group4126: abra|OJJ76460.1 acar|OOF99699.1 acla|EAW13970.1 afis|EAW18679.1 afla|EED50631.1 afum|EAL93399.1 agla|OJJ82396.1 akaw|GAA82373.1 aluc|OJZ91749.1 anid|CBF70596.1 anig|EHA22752.1 aory|EIT78016.1 asyd|OJJ58519.1 ater|EAU38232.1 atub|OJI86207.1 aver|OJJ01120.1 awen|OJJ32151.1 azon|OJJ46735.1

Group4127: abra|OJJ76464.1 acar|OOF99702.1 acla|EAW13967.1 afis|EAW18676.1 afla|EED50634.1 afum|EAL93396.1 agla|OJJ82400.1 akaw|GAA82376.1 aluc|OJZ91746.1 anid|CBF70590.1 anig|EHA22749.1 aory|EIT78033.1 asyd|OJJ58522.1 ater|EAU38234.1 atub|OJI86204.1 aver|OJJ01123.1 awen|OJJ32148.1 azon|OJJ46732.1

Group4128: abra|OJJ76467.1 acar|OOF99705.1 acla|EAW13964.1 afis|EAW18673.1 afla|EED50626.1 afum|EAL93393.2 agla|OJJ82402.1 akaw|GAA82378.1 aluc|OJZ91743.1 anid|CBF70584.1 anig|EHA22746.1 aory|EIT78060.1 asyd|OJJ58525.1 ater|EAU38237.1 atub|OJI86201.1 aver|OJJ01126.1 awen|OJJ32145.1 azon|OJJ46729.1

Group4129: abra|OJJ76468.1 acar|OOF99706.1 acla|EAW13963.1 afis|EAW18672.1 afla|EED50621.1 afum|EAL93391.1 agla|OJJ82403.1 akaw|GAA82379.1 aluc|OJZ91742.1 anid|CBF70583.1 anig|EHA22745.1

aory|EIT78045.1 asyd|OJJ58526.1 ater|EAU38238.1 atub|OJI86200.1 aver|OJJ01127.1 awen|OJJ32144.1 azon|OJJ46728.1

Group4130: abra|OJJ76472.1 acar|OOF99709.1 acla|EAW13961.1 afis|EAW18670.1 afla|EED52658.1 afum|EAL93388.1 agla|OJJ82405.1 akaw|GAA82384.1 aluc|OJZ91737.1 anid|CBF70579.1 anig|EHA22743.1 aory|EIT74274.1 asyd|OJJ58528.1 ater|EAU38241.1 atub|OJI86195.1 aver|OJJ01130.1 awen|OJJ32142.1 azon|OJJ46723.1

Group4131: abra|OJJ76479.1 acar|OOF90192.1 acla|EAW13956.1 afis|EAW18666.1 afla|EED52663.1 afum|EAL93384.1 agla|OJJ82411.1 akaw|GAA82392.1 aluc|OJZ91728.1 anid|CBF70569.1 anig|EHA22735.1 aory|EIT74303.1 asyd|OJJ58533.1 ater|EAU38246.1 atub|OJI86186.1 aver|OJJ01135.1 awen|OJJ32134.1 azon|OJJ46717.1

Group4132: acar|OOF90191.1 aluc|OJZ91727.1 anig|EHA22734.1 atub|OJI86185.1 abra|OJJ76481.1 acla|EAW13954.1 afis|EAW18664.1 afla|EED52665.1 afum|EAL93382.1 agla|OJJ82412.1 akaw|GAA82394.1 anid|CBF70567.1 aory|EIT74310.1 asyd|OJJ58535.1 ater|EAU38248.1 aver|OJJ01136.1 awen|OJJ32132.1 azon|OJJ46715.1

Group4133: abra|OJJ76482.1 acar|OOF90190.1 acla|EAW13953.1 afis|EAW18663.1 afla|EED52666.1 afum|EBA27491.1 agla|OJJ82413.1 akaw|GAA82395.1 aluc|OJZ91726.1 anid|CBF70565.1 anig|EHA22733.1 aory|EIT74219.1 asyd|OJJ58536.1 ater|EAU38249.1 atub|OJI86184.1 aver|OJJ01137.1 awen|OJJ32131.1 azon|OJJ46714.1

Group4134: abra|OJJ76483.1 acar|OOF99714.1 acla|EAW13951.1 afis|EAW18661.1 afla|EED52669.1 afum|EAL93380.1 agla|OJJ82416.1 akaw|GAA82397.1 aluc|OJZ91725.1 anid|CBF70557.1 anig|EHA22732.1 aory|EIT74184.1 asyd|OJJ58538.1 ater|EAU38251.1 atub|OJI86182.1 aver|OJJ01138.1 awen|OJJ32128.1 azon|OJJ46711.1

Group4135: abra|OJJ76484.1 acar|OOF99715.1 acla|EAW13950.1 afis|EAW18659.1 afla|EED52670.1 afum|EAL93377.1 agla|OJJ82417.1 akaw|GAA82398.1 aluc|OJZ91724.1 anid|CBF70556.1 anig|EHA22731.1 aory|EIT74290.1 asyd|OJJ58539.1 ater|EAU38252.1 atub|OJI86181.1 aver|OJJ01139.1 awen|OJJ32127.1 azon|OJJ46710.1

Group4136: abra|OJJ76485.1 acar|OOF99718.1 acla|EAW13948.1 afis|EAW18658.1 afla|EED52672.1 afum|EAL93375.1 agla|OJJ82418.1 akaw|GAA82401.1 aluc|OJZ91721.1 anid|CBF70552.1 anig|EHA22728.1 aory|EIT74194.1 asyd|OJJ58540.1 ater|EAU38254.1 atub|OJI86178.1 aver|OJJ01141.1 awen|OJJ32125.1 azon|OJJ46709.1

Group4137: abra|OJJ76487.1 acar|OOF99720.1 acla|EAW13946.1 afis|EAW18656.1 afla|EED52674.1 afum|EAL93372.1 agla|OJJ82419.1 akaw|GAA82403.1 aluc|OJZ91718.1 anid|CBF70546.1 anig|EHA22726.1 aory|EIT74203.1 asyd|OJJ58543.1 ater|EAU38256.1 atub|OJI86175.1 aver|OJJ01143.1 awen|OJJ32123.1 azon|OJJ46708.1

Group4138: abra|OJJ76501.1 acar|OOF99466.1 acla|EAW14195.1 afis|EAW18911.1 afla|EED50838.1 afum|EAL93642.1 agla|OJJ81515.1 akaw|GAA82417.1 aluc|OJZ91701.1 anid|CBF69699.1 anig|EHA22715.1 aory|EIT79018.1 asyd|OJJ59050.1 ater|EAU38078.1 atub|OJI86160.1 aver|OJJ01646.1 awen|OJJ31504.1 azon|OJJ47224.1

Group4139: abra|OJJ76513.1 acar|OOF99459.1 acla|EAW14187.1 afis|EAW18902.1 afla|EED50846.1 afum|EAL93634.1 agla|OJJ81507.1 akaw|GAA82426.1 aluc|OJZ91692.1 anid|CBF69923.1 anig|EHA22705.1 aory|EIT79043.1 asyd|OJJ59039.1 ater|EAU38072.1 atub|OJI86145.1 aver|OJJ01634.1 awen|OJJ32459.1 azon|OJJ47217.1

Group4140: abra|OJJ76516.1 acar|OOF99457.1 acla|EAW14186.1 afis|EAW18901.1 afla|EED50847.1 afum|EAL93633.1 agla|OJJ81506.1 akaw|GAA82428.1 aluc|OJZ91690.1 anid|CBF69921.1 anig|EHA22703.1 aory|EIT79032.1 asyd|OJJ58987.1 ater|EAU38069.1 atub|OJI86143.1 aver|OJJ01629.1 awen|OJJ32458.1 azon|OJJ47216.1

Group4141: abra|OJJ76520.1 acar|OOF99453.1 acla|EAW14181.1 afis|EAW18896.1 afla|EED50867.1 afum|EAL93628.1 agla|OJJ81502.1 akaw|GAA82433.1 aluc|OJZ91688.1 anid|CBF69913.1 anig|EHA22700.1 aory|EIT79004.1 asyd|OJJ58992.1 ater|EAU38066.1 atub|OJI86139.1 aver|OJJ01623.1 awen|OJJ32454.1 azon|OJJ47211.1

Group4142: abra|OJJ76524.1 acar|OOF99445.1 acla|EAW14173.1 afis|EAW18887.1 afla|EED50878.1 afum|EAL93620.1 agla|OJJ81493.1 akaw|GAA82437.1 aluc|OJZ91684.1 anid|CBF69898.1 anig|EHA22696.1 aory|EIT79044.1 asyd|OJJ59000.1 ater|EAU38059.1 atub|OJI86136.1 aver|OJJ01615.1 awen|OJJ32445.1 azon|OJJ47203.1

Group4143: abra|OJJ76528.1 acar|OOF99447.1 acla|EAW14175.1 afis|EAW18889.1 afla|EED50874.1 afum|EAL93622.1 agla|OJJ81495.1 akaw|GAA82439.1 aluc|OJZ91677.1 anid|CBF69902.1 anig|EHA22694.1 aory|EIT78988.1 asyd|OJJ58998.1 ater|EAU38061.1 atub|OJI86132.1 aver|OJJ01617.1 awen|OJJ32448.1 azon|OJJ47206.1

Group4144: abra|OJJ76530.1 acar|OOF99443.1 acla|EAW14171.1 afis|EAW18885.1 afla|EED50880.1 afum|EAL93618.1 agla|OJJ80620.1 akaw|GAA82442.1 aluc|OJZ91674.1 anid|CBF69894.1 anig|EHA22692.1 aory|EIT78957.1 asyd|OJJ59002.1 ater|EAU38057.1 atub|OJI86129.1 aver|OJJ01613.1 awen|OJJ32443.1 azon|OJJ47201.1

Group4145: abra|OJJ76534.1 acar|OOF99440.1 acla|EAW14169.1 afis|EAW18883.1 afla|EED50882.1 afum|EAL93616.1 agla|OJJ80618.1 akaw|GAA82445.1 aluc|OJZ91671.1 anid|CBF69892.1 anig|EHA22689.1 aory|EIT79022.1 asyd|OJJ59005.1 ater|EAU38055.1 atub|OJI86126.1 aver|OJJ01611.1 awen|OJJ32440.1 azon|OJJ47199.1

Group4146: abra|OJJ76535.1 acar|OOF99439.1 acla|EAW14168.1 afis|EAW18882.1 afla|EED50883.1 afum|EAL93614.1 agla|OJJ80617.1 akaw|GAA82446.1 aluc|OJZ91670.1 anid|CBF70466.1 anig|EHA22688.1 aory|EIT78979.1 asyd|OJJ58598.1 ater|EAU38054.1 atub|OJI86125.1 aver|OJJ01200.1 awen|OJJ32441.1 azon|OJJ47198.1

Group4147: abra|OJJ76536.1 acar|OOF99432.1 acla|EAW14162.1 afis|EAW18875.1 afla|EED50892.1 afum|EAL93607.1 agla|OJJ80612.1 akaw|GAA82447.1 aluc|OJZ91669.1 anid|CBF69869.1 anig|EHA22687.1 aory|EIT79035.1 asyd|OJJ59019.1 ater|EAU38040.1 atub|OJI86124.1 aver|OJJ01597.1 awen|OJJ32434.1 azon|OJJ47192.1

Group4148: abra|OJJ76537.1 acar|OOF99433.1 acla|EAW14163.1 afis|EAW18876.1 afla|EED50891.1 afum|EAL93608.1 agla|OJJ80613.1 akaw|GAA82448.1 aluc|OJZ91667.1 anid|CBF69873.1 anig|EHA22686.1 aory|EIT79049.1 asyd|OJJ59018.1 ater|EAU38041.1 atub|OJI86122.1 aver|OJJ01598.1 awen|OJJ32435.1 azon|OJJ47191.1

Group4149: abra|OJJ76540.1 acar|OOF99435.1 acla|EAW14165.1 afis|EAW18878.1 afla|EED50888.1 afum|EAL93610.1 agla|OJJ80615.1 akaw|GAA82450.1 aluc|OJZ91663.1 anid|CBF69877.1 anig|EHA22683.1 aory|EIT79008.1 asyd|OJJ59015.1 ater|EAU38043.1 atub|OJI86120.1 aver|OJJ01600.1 awen|OJJ32437.1 azon|OJJ47196.1

Group4150: abra|OJJ76542.1 acar|OOF99437.1 acla|EAW12334.1 afis|EAW16888.1 afla|EED49676.1 afum|EAL86426.1 agla|OJJ79936.1 akaw|GAA82451.1 aluc|OJZ91661.1 anid|CBF84569.1 anig|EHA22681.1 aory|EIT80968.1 asyd|OJJ53441.1 ater|EAU39143.1 atub|OJI86117.1 aver|OJJ08310.1 awen|OJJ29756.1 azon|OJJ43187.1

Group4151: abra|OJJ76543.1 acar|OOF99431.1 acla|EAW14161.1 afis|EAW18874.1 afla|EED50893.1 afum|EAL93606.1 agla|OJJ80611.1 akaw|GAA82452.1 aluc|OJZ91660.1 anid|CBF69867.1 anig|EHA22680.1 aory|EIT79007.1 asyd|OJJ59020.1 ater|EAU38039.1 atub|OJI86116.1 aver|OJJ01596.1 awen|OJJ32433.1 azon|OJJ47193.1

Group4152: abra|OJJ76545.1 acar|OOF99429.1 acla|EAW14159.1 afis|EAW18872.1 afla|EED50895.1 afum|EAL93604.1 agla|OJJ80609.1 akaw|GAA82454.1 aluc|OJZ91658.1 anid|CBF69863.1 anig|EHA22678.1 aory|EIT79057.1 asyd|OJJ59023.1 ater|EAU38036.1 atub|OJI86114.1 aver|OJJ01594.1 awen|OJJ32431.1 azon|OJJ47188.1

Group4153: abra|OJJ76546.1 acar|OOF99428.1 acla|EAW14158.1 afis|EAW18871.1 afla|EED50831.1 afum|EAL93603.1 agla|OJJ80595.1 akaw|GAA82455.1 aluc|OJZ91657.1 anid|CBF69852.1 anig|EHA22677.1 aory|EIT79042.1 asyd|OJJ59028.1 ater|EAU38035.1 atub|OJI86112.1 aver|OJJ01588.1 awen|OJJ32430.1 azon|OJJ47185.1

Group4154: abra|OJJ76547.1 acar|OOF99427.1 acla|EAW14157.1 afis|EAW18870.1 afla|EED50832.1 afum|EAL93602.1 agla|OJJ80596.1 akaw|GAA82456.1 aluc|OJZ91656.1 anid|CBF69854.1 anig|EHA22676.1 aory|EIT79054.1 asyd|OJJ59027.1 ater|EAU38034.1 atub|OJI86111.1 aver|OJJ01589.1 awen|OJJ32429.1 azon|OJJ47184.1

Group4155: abra|OJJ76549.1 acar|OOF99426.1 acla|EAW14156.1 afis|EAW18869.1 afla|EED50833.1 afum|EAL93601.1 agla|OJJ80600.1 akaw|GAA82457.1 aluc|OJZ91654.1 anid|CBF69856.1 anig|EHA22675.1 aory|EIT78961.1 asyd|OJJ59026.1 ater|EAU38033.1 atub|OJI86108.1 aver|OJJ01591.1 awen|OJJ32428.1 azon|OJJ47183.1

Group4156: abra|OJJ76550.1 acar|OOF89946.1 acla|EAW14155.1 afis|EAW18868.1 afla|EED50834.1 afum|EAL93600.1 agla|OJJ80601.1 akaw|GAA82458.1 aluc|OJZ91653.1 anid|CBF69857.1 anig|EHA22674.1 aory|EIT78982.1 asyd|OJJ59025.1 ater|EAU38032.1 atub|OJI86107.1 aver|OJJ01592.1 awen|OJJ32427.1 azon|OJJ47182.1

Group4157: abra|OJJ76551.1 acar|OOF89945.1 acla|EAW14153.1 afis|EAW18866.1 afla|EED50897.1 afum|EAL93598.1 agla|OJJ80603.1 akaw|GAA82459.1 aluc|OJZ91652.1 anid|CBF69861.1 anig|EHA22673.1 aory|EIT79016.1 asyd|OJJ59024.1 ater|EAU38030.1 atub|OJI86106.1 aver|OJJ01593.1 awen|OJJ32425.1 azon|OJJ47180.1

Group4158: abra|OJJ76554.1 acar|OOF99424.1 acla|EAW14150.1 afis|EAW18863.1 afla|EED50830.1 afum|EAL93595.1 agla|OJJ80586.1 akaw|GAA82461.1 aluc|OJZ91650.1 anid|CBF69848.1 anig|EHA22671.1

aory|EIT79053.1 asyd|OJJ59030.1 ater|EAU38025.1 atub|OJI86104.1 aver|OJJ01586.1 awen|OJJ32419.1 azon|OJJ47176.1

Group4159: abra|OJJ76558.1 acar|OOF99421.1 acla|EAW14147.1 afis|EAW18860.1 afla|EED50827.1 afum|EAL93592.1 agla|OJJ80583.1 akaw|GAA82464.1 aluc|OJZ91647.1 anid|CBF69842.1 anig|EHA22668.1 aory|EIT78975.1 asyd|OJJ59033.1 ater|EAU38023.1 atub|OJI86101.1 aver|OJJ01583.1 awen|OJJ32416.1 azon|OJJ47173.1

Group4160: abra|OJJ76563.1 acar|OOF99419.1 acla|EAW14144.1 afis|EAW18857.1 afla|EED50824.1 afum|EAL93589.2 agla|OJJ80579.1 akaw|GAA82467.1 aluc|OJZ91643.1 anid|CBF69834.1 anig|EHA22665.1 aory|EIT78963.1 asyd|OJJ58985.1 ater|EAU38020.1 atub|OJI86097.1 aver|OJJ01580.1 awen|OJJ32413.1 azon|OJJ47170.1

Group4161: abra|OJJ76565.1 acar|OOF99418.1 acla|EAW14143.1 afis|EAW18856.1 afla|EED50823.1 afum|EAL93587.1 akaw|GAA82469.1 aluc|OJZ91641.1 anid|CBF69831.1 anig|EHA22664.1 aory|EIT79024.1 asyd|OJJ58983.1 ater|EAU38019.1 atub|OJI86095.1 aver|OJJ01578.1 awen|OJJ32411.1 azon|OJJ47168.1 agla|OJJ80577.1

Group4162: abra|OJJ76566.1 acar|OOF99355.1 acla|EAW14142.1 afis|EAW18855.1 afla|EED50822.1 afum|EAL93586.1 agla|OJJ80574.1 akaw|GAA82470.1 aluc|OJZ91640.1 anid|CBF69829.1 anig|EHA22663.1 aory|EIT78993.1 asyd|OJJ58981.1 ater|EAU38013.1 atub|OJI86094.1 aver|OJJ01576.1 awen|OJJ32410.1 azon|OJJ47167.1

Group4163: abra|OJJ76567.1 acar|OOF99417.1 acla|EAW14141.1 afis|EAW18854.1 afla|EED50821.1 afum|EAL93585.1 agla|OJJ80573.1 akaw|GAA82471.1 aluc|OJZ91639.1 anid|CBF69827.1 anig|EHA22662.1 aory|EIT78968.1 asyd|OJJ58980.1 ater|EAU38012.1 atub|OJI86093.1 aver|OJJ01575.1 awen|OJJ32409.1 azon|OJJ47166.1

Group4164: abra|OJJ76569.1 acar|OOF99416.1 acla|EAW14139.1 afis|EAW18852.1 afla|EED50819.1 afum|EAL93583.1 agla|OJJ80571.1 akaw|GAA82473.1 aluc|OJZ91636.1 anid|CBF89917.1 anig|EHA22660.1 aory|EIT79009.1 asyd|OJJ62126.1 ater|EAU38005.1 atub|OJI86089.1 aver|OJI96234.1 awen|OJJ32406.1 azon|OJJ47164.1

Group4165: abra|OJJ76570.1 acar|OOF99415.1 acla|EAW14138.1 afis|EAW18851.1 afla|EED50818.1 afum|EAL93582.1 agla|OJJ80570.1 akaw|GAA82474.1 aluc|OJZ91635.1 anid|CBF69823.1 anig|EHA22659.1 aory|EIT78969.1 asyd|OJJ58978.1 ater|EAU38004.1 atub|OJI86088.1 aver|OJJ01573.1 awen|OJJ32405.1 azon|OJJ47163.1

Group4166: abra|OJJ76571.1 acar|OOF99414.1 acla|EAW14137.1 afis|EAW18850.1 afla|EED50817.1 afum|EAL93581.1 agla|OJJ80569.1 akaw|GAA82475.1 aluc|OJZ91634.1 anid|CBF69821.1 anig|EHA22658.1 aory|EIT79031.1 asyd|OJJ58977.1 ater|EAU38003.1 atub|OJI86087.1 aver|OJJ01572.1 awen|OJJ32404.1 azon|OJJ47162.1

Group4167: abra|OJJ76572.1 acar|OOF99413.1 acla|EAW14136.1 afis|EAW18849.1 afla|EED50816.1 afum|EAL93580.1 agla|OJJ80568.1 akaw|GAA82476.1 aluc|OJZ91633.1 anid|CBF69819.1 anig|EHA22657.1 aory|EIT78989.1 asyd|OJJ58976.1 ater|EAU38002.1 atub|OJI86086.1 aver|OJJ01571.1 awen|OJJ32403.1 azon|OJJ47161.1

Group4168: abra|OJJ76573.1 acar|OOF99412.1 acla|EAW14134.1 afis|EAW18847.1 afla|EED50814.1 afum|EAL93579.1 agla|OJJ80567.1 akaw|GAA82478.1 aluc|OJZ91632.1 anid|CBF69817.1 anig|EHA22656.1 aory|EIT79029.1 asyd|OJJ58975.1 ater|EAU38001.1 atub|OJI86085.1 aver|OJJ01570.1 awen|OJJ32402.1 azon|OJJ47160.1

Group4169: abra|OJJ76576.1 acar|OOF99408.1 acla|EAW14117.1 afis|EAW18819.1 afla|EED50808.1 afum|EAL93555.1 agla|OJJ80554.1 akaw|GAA82481.1 aluc|OJZ91629.1 anid|CBF69809.1 anig|EHA22653.1 aory|EIT78964.1 asyd|OJJ58971.1 ater|EAU37998.1 atub|OJI86082.1 aver|OJJ01566.1 awen|OJJ32395.1 azon|OJJ47157.1

Group4170: abra|OJJ76577.1 acar|OOF99407.1 acla|EAW14116.1 afis|EAW18818.1 afla|EED50809.1 afum|EAL93554.1 agla|OJJ80552.1 akaw|GAA82482.1 aluc|OJZ91628.1 anid|CBF69811.1 anig|EHA22652.1 aory|EIT78995.1 asyd|OJJ58972.1 ater|EAU37997.1 atub|OJI86081.1 aver|OJJ01567.1 awen|OJJ32394.1 azon|OJJ47156.1

Group4171: abra|OJJ76579.1 acar|OOF99405.1 acla|EAW14114.1 afis|EAW18816.1 afla|EED50804.1 afum|EAL93552.1 agla|OJJ80549.1 akaw|GAA82484.1 aluc|OJZ91626.1 anid|CBF69815.1 anig|EHA22650.1 aory|EIT78104.1 asyd|OJJ58974.1 ater|EAU37991.1 atub|OJI86079.1 aver|OJJ01569.1 awen|OJJ32392.1 azon|OJJ47153.1

Group4172: abra|OJJ76581.1 acar|OOF99404.1 acla|EAW14113.1 afis|EAW18815.1 afla|EED50802.1 afum|EAL93551.1 agla|OJJ80548.1 akaw|GAA82485.1 aluc|OJZ91624.1 anid|CBF69803.1 anig|EHA22649.1 aory|EIT77988.1 asyd|OJJ58968.1 ater|EAU37982.1 atub|OJI86077.1 aver|OJJ01563.1 awen|OJJ32391.1 azon|OJJ47152.1

Group4173: abra|OJJ76582.1 acar|OOF99403.1 acla|EAW14112.1 afis|EAW18814.1 afla|EED50800.1 afum|EAL93550.2 agla|OJJ80547.1 akaw|GAA82486.1 aluc|OJZ91623.1 anid|CBF69801.1 anig|EHA22648.1 aory|EIT78081.1 asyd|OJJ58967.1 ater|EAU37981.1 atub|OJI86076.1 aver|OJJ01562.1 awen|OJJ32390.1 azon|OJJ47151.1

Group4174: abra|OJJ76588.1 acar|OOF99398.1 acla|EAW14313.1 afis|EAW18974.1 afla|EED53230.1 afum|EAL93708.1 agla|OJJ82930.1 akaw|GAA82492.1 aluc|OJZ91618.1 anid|CBF69639.1 anig|EHA22645.1 aory|EIT74874.1 asyd|OJJ59084.1 ater|EAU38166.1 atub|OJI86071.1 aver|OJJ01677.1 awen|OJJ31576.1 azon|OJJ47293.1

Group4175: abra|OJJ76590.1 acar|OOF99397.1 acla|EAW14311.1 afis|EAW18975.1 afla|EED53222.1 afum|EAL93710.1 agla|OJJ82932.1 akaw|GAA82494.1 aluc|OJZ91616.1 anid|CBF69642.1 anig|EHA22644.1 aory|EIT74886.1 asyd|OJJ59081.1 ater|EAU38164.1 atub|OJI86069.1 aver|OJJ01674.1 awen|OJJ31580.1 azon|OJJ47295.1

Group4176: abra|OJJ76591.1 acar|OOF99396.1 acla|EAW14310.1 afis|EAW18976.1 afla|EED53221.1 afum|EAL93711.2 agla|OJJ82933.1 akaw|GAA82495.1 aluc|OJZ91615.1 anid|CBF69644.1 anig|EHA22643.1 aory|EIT74842.1 asyd|OJJ59080.1 ater|EAU38163.1 atub|OJI86068.1 aver|OJJ01673.1 awen|OJJ31581.1 azon|OJJ47296.1

Group4177: abra|OJJ76594.1 acar|OOF99393.1 acla|EAW14307.1 afis|EAW18978.1 afla|EED53192.1 afum|EAL93714.1 agla|OJJ82936.1 akaw|GAA82498.1 aluc|OJZ91612.1 anid|CBF69649.1 anig|EHA22641.1 aory|EIT75152.1 asyd|OJJ59077.1 ater|EAU38169.1 atub|OJI86065.1 aver|OJJ01670.1 awen|OJJ31584.1 azon|OJJ47299.1

Group4178: abra|OJJ76601.1 acar|OOF99390.1 acla|EAW14305.1 afis|EAW18979.1 afla|EED53188.1 afum|EAL93715.1 agla|OJJ82941.1 akaw|GAA82504.1 aluc|OJZ91607.1 anid|CBF69652.1 anig|EHA22636.1 aory|EIT74907.1 asyd|OJJ59076.1 ater|EAU37838.1 atub|OJI86059.1 aver|OJJ01669.1 awen|OJJ31587.1 azon|OJJ47303.1

Group4179: abra|OJJ76605.1 acar|OOF99386.1 acla|EAW14301.1 afis|EAW18983.1 afla|EED53161.1 afum|EAL93719.1 agla|OJJ82958.1 akaw|GAA82509.1 aluc|OJZ91603.1 anid|CBF69659.1 anig|EHA22632.1 aory|EIT74944.1 asyd|OJJ59072.1 ater|EAU37822.1 atub|OJI86055.1 aver|OJJ01665.1 awen|OJJ31600.1 azon|OJJ47307.1

Group4180: abra|OJJ76606.1 acar|OOF99384.1 acla|EAW14300.1 afis|EAW18984.1 afla|EED53162.1 afum|EAL93720.1 agla|OJJ82959.1 akaw|GAA82510.1 aluc|OJZ91602.1 anid|CBF69661.1 anig|EHA22631.1 aory|EIT74985.1 asyd|OJJ59071.1 ater|EAU37821.1 atub|OJI86054.1 aver|OJJ01664.1 awen|OJJ31604.1 azon|OJJ47308.1

Group4181: abra|OJJ76609.1 acar|OOF99383.1 acla|EAW14297.1 afis|EAW18986.1 afla|EED53164.1 afum|EAL93722.1 agla|OJJ82964.1 akaw|GAA82512.1 aluc|OJZ91599.1 anid|CBF69665.1 anig|EHA22629.1 aory|EIT75120.1 asyd|OJJ59069.1 ater|EAU37819.1 atub|OJI86050.1 aver|OJJ01662.1 awen|OJJ31606.1 azon|OJJ47310.1

Group4182: abra|OJJ76624.1 acar|OOF99374.1 acla|EAW14295.1 afis|EAW18988.1 afla|EED53173.1 afum|EAL93724.1 agla|OJJ82975.1 akaw|GAA92833.1 aluc|OJZ91588.1 anid|CBF86791.1 anig|EHA22612.1 aory|EIT75118.1 asyd|OJJ64674.1 ater|EAU37816.1 atub|OJI86037.1 aver|OJI98775.1 awen|OJJ31609.1 azon|OJJ47312.1

Group4183: abra|OJJ76631.1 acar|OOF99366.1 acla|EAW08624.1 afis|EAW15808.1 afla|EED53158.1 afum|EAL85543.1 agla|OJJ81974.1 akaw|GAA92825.1 aluc|OJZ91580.1 anid|CBF86787.1 anig|EHA22607.1 aory|EIT75169.1 asyd|OJJ64676.1 ater|EAU37812.1 atub|OJI86029.1 aver|OJI98777.1 awen|OJJ38519.1 azon|OJJ50132.1

Group4184: abra|OJJ76632.1 acar|OOF99365.1 acla|EAW14288.1 afis|EAW18995.1 afla|EED53157.1 afum|EAL93730.1 agla|OJJ83149.1 akaw|GAA92824.1 aluc|OJZ91578.1 anid|CBF86656.1 anig|EHA22605.1 aory|EIT74972.1 asyd|OJJ64788.1 atub|OJI86028.1 aver|OJJ07926.1 awen|OJJ29936.1 aver|OJI98879.1 aver|OJI98900.1

Group4185: abra|OJJ76658.1 acar|OOF99343.1 acla|EAW14620.1 afis|EAW19358.1 afla|EED52157.1 afum|EAL94078.1 akaw|GAA92998.1 aluc|OJZ91547.1 anid|CBF73438.1 anig|EHA22582.1 aory|EIT74377.1 asyd|OJJ52447.1 ater|EAU36409.1 atub|OJI86000.1 aver|OJJ08112.1 azon|OJJ42907.1 azon|OJJ42486.1 aver|OJJ03969.1

Group4186: abra|OJJ76671.1 acar|OOF99339.1 acla|EAW09095.1 afis|EAW25689.1 afum|EAL84358.1 agla|OJJ78884.1 akaw|GAA92521.1 aluc|OJZ91535.1 anig|EHA22572.1 asyd|OJJ55501.1 atub|OJI85989.1 aver|OJJ06579.1 awen|OJJ38060.1 azon|OJJ45319.1 acar|OOG00611.1 anid|CBF80316.1 asyd|OJJ57586.1 aver|OJJ05596.1

Group4187: abra|OJJ76753.1 acar|OOF91249.1 acla|EAW13307.1 afis|EAW20421.1 afla|EED44850.1 afum|EAL84629.1 agla|OJJ84904.1 akaw|GAA87271.1 aluc|OJZ84156.1 anid|CBF79342.1 anig|EHA27518.1

aory|EIT77742.1 asyd|OJJ55796.1 ater|EAU37735.1 atub|OJI89928.1 aver|OJJ06885.1 awen|OJJ33114.1 azon|OJJ46330.1

Group4188: abra|OJJ76756.1 acar|OOF91251.1 acla|EAW13310.1 afis|EAW20424.1 afla|EED44846.1 afum|EAL84624.2 agla|OJJ84901.1 akaw|GAA87269.1 aluc|OJZ84159.1 anid|CBF79340.1 anig|EHA27521.1 aory|EIT77736.1 asyd|OJJ55792.1 ater|EAU37733.1 atub|OJI89925.1 aver|OJJ06882.1 awen|OJJ33117.1 azon|OJJ46333.1

Group4189: abra|OJJ76758.1 acar|OOF91253.1 acla|EAW13312.1 afis|EAW20426.1 afla|EED44842.1 afum|EAL84623.1 agla|OJJ84899.1 akaw|GAA87268.1 aluc|OJZ84161.1 anid|CBF79338.1 anig|EHA27522.1 aory|EIT77746.1 asyd|OJJ55790.1 ater|EAU37731.1 atub|OJI89923.1 aver|OJJ06880.1 awen|OJJ33119.1 azon|OJJ46335.1

Group4190: abra|OJJ76760.1 acar|OOF91255.1 acla|EAW13314.1 afis|EAW20428.1 afla|EED44840.1 afum|EAL84622.1 agla|OJJ84897.1 akaw|GAA87266.1 aluc|OJZ84163.1 anid|CBF79334.1 anig|EHA27524.1 aory|EIT77728.1 asyd|OJJ55788.1 ater|EAU37729.1 atub|OJI89921.1 aver|OJJ06878.1 awen|OJJ33121.1 azon|OJJ46337.1

Group4191: abra|OJJ76761.1 acar|OOF91256.1 acla|EAW13315.1 afis|EAW20429.1 afla|EED44839.1 afum|EAL84621.1 agla|OJJ84896.1 akaw|GAA87265.1 aluc|OJZ84164.1 anid|CBF79332.1 anig|EHA27525.1 aory|EIT77744.1 asyd|OJJ55787.1 ater|EAU37728.1 atub|OJI89920.1 aver|OJJ06877.1 awen|OJJ33122.1 azon|OJJ46338.1

Group4192: abra|OJJ76762.1 acar|OOF91257.1 acla|EAW13316.1 afis|EAW20430.1 afla|EED44838.1 afum|EAL84620.1 agla|OJJ84895.1 akaw|GAA87264.1 aluc|OJZ84165.1 anid|CBF79330.1 anig|EHA27526.1 aory|EIT77755.1 asyd|OJJ55786.1 ater|EAU37727.1 atub|OJI89919.1 aver|OJJ06876.1 awen|OJJ33123.1 azon|OJJ46339.1

Group4193: abra|OJJ76764.1 acar|OOF91259.1 acla|EAW13318.1 afis|EAW20432.1 afla|EED44836.1 afum|EAL84619.1 agla|OJJ84893.1 akaw|GAA87262.1 aluc|OJZ84168.1 anid|CBF79325.1 anig|EHA27528.1 aory|EIT77720.1 asyd|OJJ55784.1 ater|EAU37725.1 atub|OJI89916.1 aver|OJJ06874.1 awen|OJJ33125.1 azon|OJJ46341.1

Group4194: abra|OJJ76766.1 acar|OOF91260.1 acla|EAW13320.1 afis|EAW20435.1 afla|EED44835.1 afum|EAL84617.1 agla|OJJ84891.1 akaw|GAA87260.1 aluc|OJZ84169.1 anid|CBF79321.1 anig|EHA27530.1 aory|EIT77727.1 asyd|OJJ55783.1 ater|EAU37723.1 atub|OJI89915.1 aver|OJJ06872.1 awen|OJJ33126.1 azon|OJJ46342.1

Group4195: abra|OJJ76768.1 acar|OOF91262.1 acla|EAW13322.1 afis|EAW20437.1 afla|EED44833.1 afum|EAL84615.1 agla|OJJ84886.1 akaw|GAA87258.1 aluc|OJZ84171.1 anid|CBF79317.1 anig|EHA27532.1 aory|EIT77715.1 asyd|OJJ55781.1 ater|EAU37721.1 atub|OJI89913.1 aver|OJJ06870.1 awen|OJJ33128.1 azon|OJJ46344.1

Group4196: abra|OJJ76769.1 acar|OOF91263.1 acla|EAW13323.1 afis|EAW20438.1 afla|EED44832.1 afum|EAL84614.1 agla|OJJ84885.1 akaw|GAA87257.1 aluc|OJZ84172.1 anid|CBF79312.1 anig|EHA27533.1 aory|EIT77757.1 asyd|OJJ55780.1 ater|EAU37720.1 atub|OJI89912.1 aver|OJJ06869.1 awen|OJJ33129.1 azon|OJJ46345.1

Group4197: abra|OJJ76774.1 acar|OOF91269.1 acla|EAW13331.1 afis|EAW20446.1 afla|EED44824.1 afum|EAL84607.1 agla|OJJ84875.1 akaw|GAA87250.1 aluc|OJZ84178.1 anid|CBF79292.1 anig|EHA27537.1 aory|EIT77756.1 asyd|OJJ55775.1 ater|EAU37713.1 atub|OJI89905.1 aver|OJJ06864.1 awen|OJJ33136.1 azon|OJJ46349.1

Group4198: abra|OJJ76776.1 acar|OOF91271.1 acla|EAW13335.1 afis|EAW20450.1 afla|EED44818.1 afum|EAL84603.1 agla|OJJ84872.1 akaw|GAA87248.1 aluc|OJZ84180.1 anid|CBF79297.1 anig|EHA27540.1 aory|EIT77719.1 asyd|OJJ55773.1 ater|EAU37709.1 atub|OJI89903.1 aver|OJJ06860.1 awen|OJJ33140.1 azon|OJJ46351.1

Group4199: abra|OJJ76779.1 acar|OOF91275.1 acla|EAW13338.1 afis|EAW20453.1 afla|EED44815.1 afum|EAL84600.1 agla|OJJ84869.1 akaw|GAA87245.1 aluc|OJZ84183.1 anid|CBF79284.1 anig|EHA27543.1 aory|EIT77717.1 asyd|OJJ55770.1 ater|EAU37706.1 atub|OJI89900.1 aver|OJJ06856.1 awen|OJJ33143.1 azon|OJJ46354.1

Group4200: abra|OJJ76780.1 acar|OOF91276.1 acla|EAW13339.1 afis|EAW20454.1 afla|EED44814.1 afum|EAL84599.1 agla|OJJ84868.1 akaw|GAA87244.1 aluc|OJZ84184.1 anid|CBF79286.1 anig|EHA27544.1 aory|EIT77725.1 asyd|OJJ55769.1 ater|EAU37705.1 atub|OJI89899.1 aver|OJJ06855.1 awen|OJJ33144.1 azon|OJJ46355.1

Group4201: abra|OJJ76784.1 acar|OOF91279.1 acla|EAW13342.1 afis|EAW20457.1 afla|EED44810.1 afum|EAL84595.1 agla|OJJ84862.1 akaw|GAA87241.1 aluc|OJZ84187.1 anid|CBF79283.1 anig|EHA27547.1 aory|EIT77730.1 asyd|OJJ55766.1 ater|EAU37703.1 atub|OJI89896.1 aver|OJJ06852.1 awen|OJJ33149.1 azon|OJJ46359.1

Group4202: abra|OJJ76787.1 acar|OOF91281.1 acla|EAW13345.1 afis|EAW20460.1 afla|EED44808.1 afum|EAL84593.1 agla|OJJ84858.1 akaw|GAA87238.1 aluc|OJZ84189.1 anid|CBF79277.1 anig|EHA27549.1 aory|EIT77754.1 asyd|OJJ55763.1 ater|EAU37700.1 atub|OJI89893.1 aver|OJJ06849.1 awen|OJJ33153.1 azon|OJJ46362.1

Group4203: abra|OJJ76790.1 acar|OOF91284.1 acla|EAW13348.1 afis|EAW20463.1 afla|EED44805.1 afum|EAL84590.1 agla|OJJ84854.1 akaw|GAA87235.1 aluc|OJZ84193.1 anid|CBF79267.1 anig|EHA27552.1 aory|EIT77734.1 asyd|OJJ55760.1 ater|EAU37738.1 atub|OJI89890.1 aver|OJJ06845.1 awen|OJJ33157.1 azon|OJJ46366.1

Group4204: abra|OJJ76792.1 acar|OOF91283.1 acla|EAW13351.1 afis|EAW20465.1 afla|EED44802.1 afum|EAL84588.1 agla|OJJ84852.1 akaw|GAA87233.1 aluc|OJZ84196.1 anid|CBF79269.1 anig|EHA27553.1 aory|EIT77749.1 asyd|OJJ55759.1 ater|EAU37698.1 atub|OJI89888.1 aver|OJJ06844.1 awen|OJJ33159.1 azon|OJJ46367.1

Group4205: abra|OJJ76793.1 acar|OOF91286.1 acla|EAW13352.1 afis|EAW20466.1 afla|EED44801.1 afum|EAL84586.2 agla|OJJ84851.1 akaw|GAA87232.1 aluc|OJZ84197.1 anid|CBF79271.1 anig|EHA27554.1 aory|EIT77739.1 asyd|OJJ55758.1 ater|EAU37697.1 atub|OJI89887.1 aver|OJJ06843.1 awen|OJJ33160.1 azon|OJJ46368.1

Group4206: abra|OJJ76796.1 acar|OOF91289.1 acla|EAW13355.1 afis|EAW20469.1 afla|EED44799.1 afum|EAL84583.1 agla|OJJ84849.1 akaw|GAA87227.1 aluc|OJZ84203.1 anid|CBF79257.1 anig|EHA27557.1 aory|EIT77745.1 asyd|OJJ55748.1 ater|EAU37694.1 atub|OJI89881.1 aver|OJJ06835.1 awen|OJJ33163.1 azon|OJJ46370.1

Group4207: abra|OJJ76798.1 acar|OOF91291.1 acla|EAW13357.1 afis|EAW20471.1 afla|EED44797.1 afum|EAL84582.1 agla|OJJ84847.1 akaw|GAA87225.1 aluc|OJZ84205.1 anid|CBF79258.1 anig|EHA27558.1 aory|EIT77743.1 asyd|OJJ55750.1 ater|EAU37691.1 atub|OJI89879.1 aver|OJJ06837.1 awen|OJJ33165.1 azon|OJJ46372.1

Group4208: abra|OJJ76799.1 acar|OOF91292.1 acla|EAW13358.1 afis|EAW20472.1 afla|EED44796.1 afum|EAL84580.2 agla|OJJ84846.1 akaw|GAA87224.1 aluc|OJZ84206.1 anid|CBF79260.1 anig|EHA27559.1 aory|EIT77751.1 asyd|OJJ55751.1 ater|EAU37690.1 atub|OJI89878.1 aver|OJJ06838.1 awen|OJJ33166.1 azon|OJJ46373.1

Group4209: abra|OJJ76800.1 acar|OOF91294.1 acla|EAW13359.1 afis|EAW20473.1 afla|EED44794.1 afum|EAL84579.1 agla|OJJ84845.1 akaw|GAA87223.1 aluc|OJZ84207.1 anid|CBF79262.1 anig|EHA27561.1 aory|EIT77790.1 asyd|OJJ55754.1 ater|EAU37689.1 atub|OJI89877.1 aver|OJJ06839.1 awen|OJJ33167.1 azon|OJJ46375.1

Group4210: abra|OJJ76802.1 acar|OOF91296.1 acla|EAW13361.1 afis|EAW20475.1 afla|EED44793.1 afum|EAL84578.1 agla|OJJ84842.1 akaw|GAA87221.1 aluc|OJZ84209.1 anid|CBF79255.1 anig|EHA27563.1 aory|EIT77775.1 asyd|OJJ55747.1 ater|EAU37687.1 atub|OJI89874.1 aver|OJJ06834.1 awen|OJJ33170.1 azon|OJJ46377.1

Group4211: abra|OJJ76806.1 acar|OOF91300.1 acla|EAW13365.1 afis|EAW20479.1 afla|EED44789.1 afum|EAL84575.1 agla|OJJ84831.1 akaw|GAA87217.1 aluc|OJZ84213.1 anid|CBF79213.1 anig|EHA27566.1 aory|EIT77792.1 asyd|OJJ55738.1 ater|EAU29558.1 atub|OJI89870.1 aver|OJJ06824.1 awen|OJJ33174.1 azon|OJJ46381.1

Group4212: abra|OJJ76809.1 acar|OOF91305.1 acla|EAW13370.1 afis|EAW20484.1 afla|EED44784.1 afum|EAL84571.1 agla|OJJ84813.1 akaw|GAA87212.1 aluc|OJZ84218.1 anid|CBF79235.1 anig|EHA27569.1 aory|EIT77759.1 asyd|OJJ55742.1 ater|EAU29562.1 atub|OJI89866.1 aver|OJJ06829.1 awen|OJJ33185.1 azon|OJJ46384.1

Group4213: abra|OJJ76810.1 acar|OOF91306.1 acla|EAW13371.1 afis|EAW20485.1 afla|EED44782.1 afum|EAL84570.1 agla|OJJ84822.1 akaw|GAA87211.1 aluc|OJZ84221.1 anid|CBF79237.1 anig|EHA27570.1 aory|EIT77768.1 asyd|OJJ55743.1 ater|EAU29563.1 atub|OJI89865.1 aver|OJJ06830.1 awen|OJJ33186.1 azon|OJJ46388.1

Group4214: abra|OJJ76811.1 acar|OOF91307.1 acla|EAW13372.1 afis|EAW20486.1 afla|EED44781.1 afum|EAL84569.1 agla|OJJ84825.1 akaw|GAA87210.1 aluc|OJZ84222.1 anid|CBF79215.1 anig|EHA27571.1 aory|EIT77774.1 asyd|OJJ55737.1 ater|EAU29564.1 atub|OJI89864.1 aver|OJJ06823.1 awen|OJJ33187.1 azon|OJJ46389.1

Group4215: abra|OJJ76813.1 acar|OOF91309.1 acla|EAW13374.1 afis|EAW20488.1 afla|EED44776.1 afum|EAL84567.1 agla|OJJ84826.1 akaw|GAA87208.1 aluc|OJZ84224.1 anid|CBF79219.1 anig|EHA27573.1 aory|EIT77760.1 asyd|OJJ55736.1 ater|EAU29566.1 atub|OJI89861.1 aver|OJJ06822.1 awen|OJJ33188.1 azon|OJJ46390.1

Group4216: abra|OJJ76819.1 acar|OOF91315.1 acla|EAW13379.1 afis|EAW20493.1 afla|EED44737.1 afum|EAL84561.1 agla|OJJ84833.1 akaw|GAA87203.1 aluc|OJZ84229.1 anid|CBF79226.1 anig|EHA27578.1

aory|EIT77797.1 asyd|OJJ55732.1 ater|EAU29569.1 atub|OJI89855.1 aver|OJJ06818.1 awen|OJJ33193.1 azon|OJJ46403.1

Group4217: abra|OJJ76821.1 acar|OOF91316.1 acla|EAW13381.1 afis|EAW20495.1 afla|EED44771.1 afum|EAL84559.1 agla|OJJ84807.1 akaw|GAA87201.1 aluc|OJZ84232.1 anid|CBF79222.1 anig|EHA27579.1 aory|EIT77762.1 asyd|OJJ55734.1 ater|EAU37189.1 atub|OJI89853.1 aver|OJJ06820.1 awen|OJJ33195.1 azon|OJJ46401.1

Group4218: abra|OJJ76823.1 acar|OOF91319.1 acla|EAW13383.1 afis|EAW20497.1 afla|EED44739.1 afum|EAL84557.1 agla|OJJ84805.1 akaw|GAA87199.1 aluc|OJZ84234.1 anid|CBF79208.1 anig|EHA27581.1 aory|EIT77796.1 asyd|OJJ55729.1 ater|EAU37191.1 atub|OJI89851.1 aver|OJJ06815.1 awen|OJJ33197.1 azon|OJJ46399.1

Group4219: abra|OJJ76827.1 acar|OOF91321.1 acla|EAW13387.1 afis|EAW20501.1 afla|EED44744.1 afum|EAL84554.1 agla|OJJ84797.1 akaw|GAA87195.1 aluc|OJZ84239.1 anid|CBF79200.1 anig|EHA27586.1 aory|EIT77773.1 asyd|OJJ55725.1 ater|EAU37195.1 atub|OJI89844.1 aver|OJJ06811.1 awen|OJJ33202.1 azon|OJJ46396.1

Group4220: abra|OJJ76830.1 acar|OOF91324.1 acla|EAW13392.1 afis|EAW20504.1 afla|EED44747.1 afum|EAL84552.1 agla|OJJ84800.1 akaw|GAA87192.1 aluc|OJZ84242.1 anid|CBF79195.1 anig|EHA27587.1 aory|EIT77784.1 asyd|OJJ55722.1 ater|EAU37199.1 atub|OJI89841.1 aver|OJJ06808.1 awen|OJJ33205.1 azon|OJJ46478.1

Group4221: abra|OJJ76832.1 acar|OOF91326.1 afis|EAW20506.1 afum|EAL84550.1 akaw|GAA87190.1 aluc|OJZ84244.1 anig|EHA27589.1 asyd|OJJ55720.1 ater|EAU37200.1 atub|OJI89839.1 aver|OJJ06806.1 awen|OJJ33207.1 acla|EAW13395.1 afla|EED52484.1 aory|EIT82804.1 afla|EED52486.1 anid|CBF79191.1 aory|EIT82815.1

Group4222: abra|OJJ76834.1 acar|OOF91327.1 acla|EAW13396.1 afis|EAW20507.1 afla|EED52448.1 afum|EAL84549.1 agla|OJJ84784.1 akaw|GAA87189.1 aluc|OJZ84245.1 anid|CBF79193.1 anig|EHA27590.1 aory|EIT82772.1 asyd|OJJ55719.1 ater|EAU37201.1 atub|OJI89838.1 aver|OJJ06805.1 awen|OJJ33208.1 azon|OJJ46481.1

Group4223: abra|OJJ76835.1 acar|OOF91328.1 acla|EAW13397.1 afis|EAW20508.1 afla|EED52449.1 afum|EAL84548.1 agla|OJJ84785.1 akaw|GAA87188.1 aluc|OJZ84246.1 anid|CBF79190.1 anig|EHA27591.1 aory|EIT82778.1 asyd|OJJ55718.1 ater|EAU37202.1 atub|OJI89837.1 aver|OJJ06804.1 awen|OJJ33209.1 azon|OJJ46482.1

Group4224: abra|OJJ76836.1 acar|OOF90072.1 acla|EAW13398.1 afis|EAW20509.1 afla|EED52450.1 afum|EAL84547.1 agla|OJJ84786.1 akaw|GAA87187.1 aluc|OJZ84247.1 anid|CBF79189.1 anig|EHA27592.1 aory|EIT82759.1 asyd|OJJ55717.1 ater|EAU37203.1 atub|OJI89836.1 aver|OJJ06803.1 awen|OJJ33210.1 azon|OJJ46483.1

Group4225: abra|OJJ76838.1 acar|OOF91330.1 acla|EAW13400.1 afis|EAW20511.1 afla|EED52452.1 afum|EAL84545.2 agla|OJJ84788.1 akaw|GAA87185.1 aluc|OJZ84249.1 anid|CBF79186.1 anig|EHA27594.1 aory|EIT82799.1 asyd|OJJ55715.1 ater|EAU37205.1 atub|OJI89834.1 aver|OJJ06801.1 awen|OJJ33213.1 azon|OJJ46485.1

Group4226: abra|OJJ76839.1 acar|OOF91331.1 acla|EAW13401.1 afis|EAW20512.1 afla|EED52453.1 afum|EBA27192.1 agla|OJJ84789.1 akaw|GAA87184.1 aluc|OJZ84250.1 anid|CBF79184.1 anig|EHA27595.1 aory|EIT82753.1 asyd|OJJ55714.1 ater|EAU37208.1 atub|OJI89833.1 aver|OJJ06800.1 awen|OJJ33214.1 azon|OJJ46486.1

Group4227: abra|OJJ76840.1 acar|OOF91333.1 acla|EAW13403.1 afis|EAW20514.1 afla|EED52468.1 afum|EAL84543.2 agla|OJJ84776.1 akaw|GAA87182.1 aluc|OJZ84251.1 anid|CBF79055.1 anig|EHA27596.1 aory|EIT82784.1 asyd|OJJ55710.1 ater|EAU37187.1 atub|OJI89832.1 aver|OJJ06796.1 awen|OJJ33220.1 azon|OJJ46488.1

Group4228: abra|OJJ76843.1 acar|OOF89969.1 acla|EAW13406.1 afis|EAW20517.1 afla|EED52458.1 afum|EAL84540.1 agla|OJJ84769.1 akaw|GAA87179.1 aluc|OJZ84254.1 anid|CBF88058.1 anig|EHA27599.1 aory|EIT82793.1 asyd|OJJ63541.1 ater|EAU37184.1 atub|OJI89829.1 aver|OJI97392.1 awen|OJJ33223.1 azon|OJJ46553.1

Group4229: abra|OJJ76844.1 acar|OOF89968.1 acla|EAW13407.1 afis|EAW20518.1 afla|EED52457.1 afum|EAL84539.1 agla|OJJ84768.1 akaw|GAA87177.1 aluc|OJZ84256.1 anid|CBF79061.1 anig|EHA27600.1 aory|EIT82769.1 asyd|OJJ55707.1 ater|EAU37183.1 atub|OJI89827.1 aver|OJJ06793.1 awen|OJJ33224.1 azon|OJJ46581.1

Group4230: abra|OJJ76845.1 acar|OOF91335.1 acla|EAW13408.1 afis|EAW20519.1 afla|EED52455.1 afum|EAL84538.1 agla|OJJ84767.1 akaw|GAA87176.1 aluc|OJZ84257.1 anid|CBF79067.1 anig|EHA27601.1 aory|EIT82798.1 asyd|OJJ55706.1 ater|EAU37182.1 atub|OJI89826.1 aver|OJJ06792.1 awen|OJJ33225.1 azon|OJJ46580.1

Group4231: abra|OJJ76847.1 acar|OOF91337.1 acla|EAW13410.1 afis|EAW20521.1 afla|EED52470.1 afum|EAL84536.1 agla|OJJ84765.1 akaw|GAA87174.1 aluc|OJZ84259.1 anid|CBF79070.1 anig|EHA27602.1 aory|EIT82770.1 asyd|OJJ55704.1 ater|EAU37167.1 atub|OJI89824.1 aver|OJJ06790.1 awen|OJJ33227.1 azon|OJJ46577.1

Group4232: abra|OJJ76849.1 acar|OOF91338.1 acla|EAW13412.1 afis|EAW20523.1 afla|EED52472.1 afum|EAL84534.1 agla|OJJ84740.1 akaw|GAA87172.1 aluc|OJZ84261.1 anid|CBF79074.1 anig|EHA27604.1 aory|EIT82821.1 asyd|OJJ55702.1 ater|EAU37165.1 atub|OJI89822.1 aver|OJJ06787.1 awen|OJJ33229.1 azon|OJJ46575.1

Group4233: abra|OJJ76851.1 acar|OOF91339.1 acla|EAW13413.1 afis|EAW20524.1 afla|EED52473.1 afum|EAL84533.1 agla|OJJ84742.1 akaw|GAA87171.1 aluc|OJZ84262.1 anid|CBF79076.1 anig|EHA27605.1 aory|EIT82758.1 asyd|OJJ55701.1 ater|EAU37164.1 atub|OJI89821.1 aver|OJJ06786.1 awen|OJJ33230.1 azon|OJJ46574.1

Group4234: abra|OJJ76852.1 acar|OOF91340.1 acla|EAW13414.1 afis|EAW20525.1 afla|EED52474.1 afum|EAL84532.1 agla|OJJ84743.1 akaw|GAA87170.1 aluc|OJZ84264.1 anid|CBF79078.1 anig|EHA27606.1 aory|EIT82817.1 asyd|OJJ55699.1 ater|EAU37163.1 atub|OJI89819.1 aver|OJJ06784.1 awen|OJJ33231.1 azon|OJJ46573.1

Group4235: abra|OJJ76855.1 acar|OOF91341.1 acla|EAW13416.1 afis|EAW20527.1 afla|EED52476.1 afum|EAL84530.1 agla|OJJ84745.1 akaw|GAA87167.1 aluc|OJZ84267.1 anid|CBF79081.1 anig|EHA27609.1 aory|EIT82771.1 asyd|OJJ55696.1 ater|EAU37161.1 atub|OJI89816.1 aver|OJJ06781.1 awen|OJJ33234.1 azon|OJJ46570.1

Group4236: abra|OJJ76857.1 acar|OOF91343.1 acla|EAW13418.1 afis|EAW20529.1 afla|EED52478.1 afum|EAL84528.1 agla|OJJ84706.1 akaw|GAA87165.1 aluc|OJZ84269.1 anid|CBF79006.1 anig|EHA27611.1 aory|EIT82803.1 asyd|OJJ55691.1 ater|EAU37159.1 atub|OJI89814.1 aver|OJJ06776.1 awen|OJJ33236.1 azon|OJJ50608.1

Group4237: abra|OJJ76903.1 acar|OOF91351.1 acla|EAW13425.1 afis|EAW20536.1 afla|EED52442.1 afum|EAL84522.1 agla|OJJ84695.1 akaw|GAA87135.1 aluc|OJZ84302.1 anid|CBF78995.1 anig|EHA27638.1 aory|EIT82797.1 asyd|OJJ55685.1 ater|EAU37143.1 atub|OJI89766.1 aver|OJJ06769.1 awen|OJJ33243.1 azon|OJJ50622.1

Group4238: abra|OJJ76916.1 acar|OOF91363.1 acla|EAW13428.1 afis|EAW20544.1 afla|EED52424.1 afum|EAL84514.1 agla|OJJ88753.1 akaw|GAA87122.1 aluc|OJZ84316.1 anid|CBF78988.1 anig|EHA27651.1 aory|EIT83288.1 asyd|OJJ55682.1 ater|EAU37137.1 atub|OJI89752.1 aver|OJJ06766.1 awen|OJJ33252.1 azon|OJJ50638.1

Group4239: abra|OJJ76917.1 acar|OOF91364.1 acla|EAW13429.1 afis|EAW20545.1 afla|EED52423.1 afum|EBA27188.1 agla|OJJ88752.1 akaw|GAA87121.1 aluc|OJZ84317.1 anid|CBF78986.1 anig|EHA27652.1 aory|EIT83281.1 asyd|OJJ55681.1 ater|EAU37136.1 atub|OJI89751.1 aver|OJJ06765.1 awen|OJJ33253.1 azon|OJJ50637.1

Group4240: abra|OJJ76918.1 acar|OOF91365.1 acla|EAW13430.1 afis|EAW20546.1 afla|EED52422.1 afum|EAL84513.1 agla|OJJ88751.1 akaw|GAA87120.1 aluc|OJZ84318.1 anid|CBF78985.1 anig|EHA27653.1 aory|EIT83315.1 asyd|OJJ55680.1 ater|EAU37135.1 atub|OJI89750.1 aver|OJJ06763.1 awen|OJJ33254.1 azon|OJJ50636.1

Group4241: abra|OJJ76919.1 acar|OOF91366.1 acla|EAW13431.1 afis|EAW20547.1 afla|EED52421.1 afum|EAL84512.1 agla|OJJ88750.1 akaw|GAA87119.1 aluc|OJZ84319.1 anid|CBF78983.1 anig|EHA27654.1 aory|EIT83284.1 asyd|OJJ55679.1 ater|EAU37134.1 atub|OJI89749.1 aver|OJJ06762.1 awen|OJJ33255.1 azon|OJJ50635.1

Group4242: abra|OJJ76923.1 acar|OOF91370.1 acla|EAW13432.1 afis|EAW20548.1 afla|EED52408.1 afum|EAL84511.1 agla|OJJ88739.1 akaw|GAA87115.1 aluc|OJZ84323.1 anid|CBF78977.1 anig|EHA27658.1 aory|EIT83309.1 asyd|OJJ55675.1 ater|EAU37129.1 atub|OJI89745.1 aver|OJJ06758.1 awen|OJJ33260.1 aver|OJJ07651.1

Group4243: abra|OJJ76937.1 acar|OOF91386.1 acla|EAW12145.1 afis|EAW17130.1 afla|EED55089.1 afum|EAL86590.1 agla|OJJ82232.1 akaw|GAA87102.1 aluc|OJZ84336.1 anid|CBF80327.1 anig|EHA27670.1 aory|EIT81595.1 asyd|OJJ54844.1 ater|EAU31746.1 atub|OJI89732.1 aver|OJJ07318.1 awen|OJJ31282.1 awen|OJJ38657.1

Group4244: abra|OJJ76950.1 acar|OOF91396.1 acla|EAW13470.1 afis|EAW20582.1 afla|EED52277.1 afum|EAL84482.1 agla|OJJ88700.1 akaw|GAA87091.1 aluc|OJZ84347.1 anid|CBF78946.1 anig|EHA27683.1 aory|EIT74415.1 asyd|OJJ55651.1 ater|EAU37100.1 atub|OJI89721.1 aver|OJJ06736.1 awen|OJJ33287.1 azon|OJJ50663.1

Group4245: abra|OJJ76954.1 acar|OOF91398.1 acla|EAW13476.1 afis|EAW20583.1 afla|EED52245.1 afum|EBA27180.1 agla|OJJ88686.1 akaw|GAA87088.1 aluc|OJZ84350.1 anid|CBF78930.1 anig|EHA27687.1

aory|EIT74502.1 asyd|OJJ55642.1 ater|EAU37097.1 atub|OJI89717.1 aver|OJJ06725.1 awen|OJJ33300.1 azon|OJJ50671.1

Group4246: abra|OJJ76970.1 acar|OOF91415.1 acla|EAW13495.1 afis|EAW20595.1 afla|EED52229.1 afum|EAL84466.1 agla|OJJ88661.1 akaw|GAA87074.1 aluc|OJZ84366.1 anid|CBF78911.1 anig|EHA27701.1 aory|EIT74324.1 asyd|OJJ55615.1 ater|EAU35241.1 atub|OJI89702.1 aver|OJJ06690.1 awen|OJJ33385.1 azon|OJJ50691.1

Group4247: abra|OJJ76971.1 acar|OOF91416.1 acla|EAW13496.1 afis|EAW20596.1 afla|EED52228.1 afum|EAL84465.1 agla|OJJ88660.1 akaw|GAA87073.1 aluc|OJZ84367.1 anid|CBF78909.1 anig|EHA27702.1 aory|EIT74434.1 asyd|OJJ55614.1 ater|EAU35242.1 atub|OJI89701.1 aver|OJJ06689.1 awen|OJJ33388.1 azon|OJJ50692.1

Group4248: abra|OJJ76972.1 acar|OOF91417.1 acla|EAW13498.1 afis|EAW20597.1 afla|EED52213.1 afum|EAL84464.1 agla|OJJ88659.1 akaw|GAA87072.1 aluc|OJZ84368.1 anid|CBF78908.1 anig|EHA27703.1 aory|EIT74430.1 asyd|OJJ55612.1 ater|EAU35243.1 atub|OJI89700.1 aver|OJJ06687.1 awen|OJJ33389.1 azon|OJJ50693.1

Group4249: abra|OJJ76976.1 acar|OOF91419.1 acla|EAW13502.1 afis|EAW20601.1 afla|EED47729.1 afum|EAL84460.1 agla|OJJ88650.1 akaw|GAA87069.1 aluc|OJZ84372.1 anid|CBF75136.1 anig|EHA27707.1 aory|EIT77171.1 asyd|OJJ59976.1 ater|EAU35246.1 atub|OJI89695.1 aver|OJJ02315.1 awen|OJJ33393.1 azon|OJJ50697.1

Group4250: abra|OJJ76977.1 acar|OOF91422.1 acla|EAW13504.1 afis|EAW20603.1 afla|EED47732.1 afum|EAL84458.1 agla|OJJ88646.1 akaw|GAA87068.1 aluc|OJZ84374.1 anid|CBF86930.1 anig|EHA27708.1 aory|EIT77153.1 asyd|OJJ55600.1 ater|EAU35248.1 atub|OJI89694.1 aver|OJJ06680.1 awen|OJJ33395.1 azon|OJJ50698.1

Group4251: abra|OJJ76978.1 acar|OOF91424.1 acla|EAW13505.1 afis|EAW20604.1 afla|EED47733.1 afum|EAL84457.1 agla|OJJ88645.1 akaw|GAA87067.1 aluc|OJZ84376.1 anid|CBF86928.1 anig|EHA27709.1 aory|EIT77125.1 asyd|OJJ55601.1 ater|EAU35250.1 atub|OJI89693.1 aver|OJJ06681.1 awen|OJJ33396.1 azon|OJJ50394.1

Group4252: abra|OJJ76981.1 acar|OOF91427.1 acla|EAW12964.1 afis|EAW17699.1 afla|EED45692.1 afum|EAL91514.1 agla|OJJ79475.1 akaw|GAA87064.1 aluc|OJZ84381.1 anid|CBF89963.1 anig|EHA27712.1 aory|EIT75518.1 asyd|OJJ62104.1 ater|EAU31915.1 atub|OJI89690.1 aver|OJI96210.1 awen|OJJ37134.1 azon|OJJ50834.1

Group4253: abra|OJJ76984.1 acar|OOF91429.1 acla|EAW12966.1 afis|EAW17697.1 afla|EED50445.1 afum|EAL91511.1 agla|OJJ79478.1 akaw|GAA87062.1 aluc|OJZ84383.1 anid|CBF89965.1 anig|EHA27714.1 aory|EIT82014.1 asyd|OJJ62100.1 ater|EAU31918.1 atub|OJI89688.1 aver|OJI96206.1 awen|OJJ37137.1 azon|OJJ50836.1

Group4254: abra|OJJ76985.1 acar|OOF91430.1 acla|EAW12967.1 afis|EAW17696.1 afla|EED50446.1 afum|EAL91510.1 agla|OJJ79479.1 akaw|GAA87061.1 aluc|OJZ84384.1 anid|CBF89966.1 anig|EHA27715.1 aory|EIT82098.1 asyd|OJJ62099.1 ater|EAU31919.1 atub|OJI89687.1 aver|OJI96205.1 awen|OJJ37139.1 azon|OJJ50839.1

Group4255: abra|OJJ76986.1 acar|OOF92639.1 acla|EAW12968.1 afis|EAW17695.1 afla|EED50447.1 afum|EAL91509.1 agla|OJJ79483.1 akaw|GAA87060.1 aluc|OJZ84385.1 anid|CBF89968.1 anig|EHA27716.1 aory|EIT82034.1 asyd|OJJ62098.1 ater|EAU31920.1 atub|OJI89686.1 aver|OJI96204.1 awen|OJJ37140.1 azon|OJJ50840.1

Group4256: abra|OJJ76987.1 acar|OOF91431.1 acla|EAW12969.1 afis|EAW17694.1 afla|EED50448.1 afum|EAL91508.1 agla|OJJ79485.1 akaw|GAA87059.1 aluc|OJZ84386.1 anid|CBF89970.1 anig|EHA27717.1 aory|EIT82134.1 asyd|OJJ62097.1 ater|EAU31961.1 atub|OJI89685.1 aver|OJI96203.1 awen|OJJ37141.1 azon|OJJ50841.1

Group4257: abra|OJJ76993.1 acar|OOF91438.1 acla|EAW13455.1 afis|EAW20563.1 afum|EAL84498.1 agla|OJJ88728.1 akaw|GAA87053.1 aluc|OJZ84393.1 anid|CBF78959.1 anig|EHA27722.1 aory|EIT74501.1 asyd|OJJ55658.1 ater|EAU37112.1 atub|OJI89678.1 aver|OJJ06742.1 awen|OJJ33269.1 azon|OJJ50641.1 afla|EED52341.1

Group4258: abra|OJJ76996.1 acar|OOF91443.1 acla|EAW13460.1 afis|EAW20566.1 afla|EED52324.1 afum|EAL84495.1 agla|OJJ88727.1 akaw|GAA87050.1 aluc|OJZ84396.1 anid|CBF78935.1 anig|EHA27726.1 aory|EIT74477.1 asyd|OJJ55644.1 ater|EAU37111.1 atub|OJI89676.1 aver|OJJ06728.1 awen|OJJ33271.1 azon|OJJ50646.1

Group4259: abra|OJJ77016.1 acar|OOF95265.1 acla|EAW07210.1 afis|EAW22222.1 afla|EED57616.1 afum|EAL90972.1 agla|OJJ87822.1 akaw|GAA87034.1 aluc|OJZ84418.1 anid|CBF88465.1 anig|EHA27740.1 aory|EIT82988.1 asyd|OJJ63777.1 ater|EAU34232.1 atub|OJI89656.1 aver|OJI97160.1 awen|OJJ34809.1 azon|OJJ45741.1

Group4260: abra|OJJ77024.1 acar|OOF91461.1 acla|EAW12057.1 afis|EAW19698.1 afla|EED54134.1 afum|EAL86829.1 agla|OJJ80810.1 akaw|GAA87028.1 aluc|OJZ84424.1 anid|CBF81131.1 anig|EHA27746.1 aory|EIT76042.1 asyd|OJJ56500.1 ater|EAU32171.1 atub|OJI89649.1 aver|OJJ06219.1 awen|OJJ41934.1 azon|OJJ48232.1

Group4261: abra|OJJ77043.1 acar|OOF90273.1 acla|EAW13554.1 afis|EAW20631.1 afla|EED49762.1 afum|EAL84436.1 agla|OJJ80447.1 akaw|GAA87010.1 aluc|OJZ84445.1 anid|CBF83219.1 anig|EHA27763.1 aory|EIT77598.1 asyd|OJJ55568.1 ater|EAU32166.1 atub|OJI89629.1 aver|OJJ06647.1 awen|OJJ33600.1 azon|OJJ50722.1

Group4262: abra|OJJ77049.1 acar|OOF92938.1 acla|EAW13549.1 afis|EAW20628.1 afla|EED49778.1 afum|EAL84442.1 agla|OJJ88595.1 akaw|GAA87004.1 aluc|OJZ84453.1 anid|CBF83230.1 anig|EHA27768.1 aory|EIT77696.1 asyd|OJJ55582.1 ater|EAU32163.1 atub|OJI89623.1 aver|OJJ06661.1 awen|OJJ33461.1 azon|OJJ50717.1

Group4263: abra|OJJ77050.1 acar|OOF92939.1 acla|EAW13544.1 afis|EAW20616.1 afla|EED54198.1 afum|EAL84445.1 agla|OJJ88594.1 akaw|GAA87003.1 aluc|OJZ84454.1 anid|CBF86935.1 anig|EHA27769.1 aory|EIT75987.1 asyd|OJJ55584.1 ater|EAU35262.1 atub|OJI89621.1 aver|OJJ06663.1 awen|OJJ33460.1 azon|OJJ50716.1

Group4264: abra|OJJ77064.1 acar|OOF92948.1 acla|EAW13511.1 afis|EAW20609.1 afla|EED54207.1 afum|EAL84451.1 agla|OJJ88630.1 akaw|GAA86988.1 aluc|OJZ84472.1 anid|CBF86951.1 anig|EHA27781.1 aory|EIT76020.1 asyd|OJJ55594.1 ater|EAU35257.1 atub|OJI89606.1 aver|OJJ06673.1 awen|OJJ33452.1 azon|OJJ50709.1

Group4265: abra|OJJ77068.1 acar|OOF92951.1 acla|EAW13508.1 afis|EAW20606.1 afla|EED47725.1 afum|EAL84454.1 agla|OJJ88635.1 akaw|GAA86985.1 aluc|OJZ84476.1 anid|CBF86946.1 anig|EHA27784.1 aory|EIT77120.1 asyd|OJJ55591.1 ater|EAU35260.1 atub|OJI89603.1 aver|OJJ06670.1 awen|OJJ33449.1 azon|OJJ50706.1

Group4266: abra|OJJ77076.1 acar|OOF92958.1 acla|EAW11204.1 afis|EAW15934.1 afla|EED54927.1 afum|EAL88570.1 agla|OJJ89373.1 akaw|GAA86977.1 aluc|OJZ84486.1 anid|CBF86351.1 anig|EHA27790.1 aory|EIT80330.1 asyd|OJJ64980.1 ater|EAU35519.1 atub|OJI89594.1 aver|OJI99102.1 awen|OJJ35645.1 azon|OJJ46891.1

Group4267: abra|OJJ77079.1 acar|OOF92961.1 acla|EAW12041.1 afis|EAW19690.1 afla|EED57010.1 afum|EAL86836.1 agla|OJJ80804.1 akaw|GAA86974.1 aluc|OJZ84489.1 anid|CBF75140.1 anig|EHA27793.1 aory|EIT82303.1 asyd|OJJ59969.1 atub|OJI89591.1 aver|OJJ02322.1 awen|OJJ41917.1 azon|OJJ42689.1 ater|EAU32856.1

Group4268: abra|OJJ77090.1 acar|OOF92974.1 acla|EAW08632.1 afis|EAW15799.1 afla|EED51246.1 afum|EAL85551.1 agla|OJJ81963.1 akaw|GAA86965.1 aluc|OJZ84499.1 anid|CBF70921.1 anig|EHA27799.1 aory|EIT83184.1 asyd|OJJ52607.1 ater|EAU32343.1 atub|OJI89581.1 aver|OJJ04634.1 awen|OJJ38507.1 azon|OJJ43744.1

Group4269: abra|OJJ77091.1 acar|OOF92976.1 acla|EAW10011.1 afis|EAW21045.1 afla|EED51625.1 afum|EAL92081.1 agla|OJJ82363.1 akaw|GAA86964.1 aluc|OJZ84500.1 anid|CBF87097.1 anig|EHA27800.1 aory|EIT80549.1 asyd|OJJ60392.1 ater|EAU36817.1 atub|OJI89580.1 aver|OJI99721.1 awen|OJJ32179.1 azon|OJJ46755.1

Group4270: abra|OJJ77094.1 acar|OOF92977.1 acla|EAW10918.1 afis|EAW20940.1 afla|EED50295.1 afum|EAL84694.1 agla|OJJ83947.1 akaw|GAA86961.1 aluc|OJZ84503.1 anid|CBF82521.1 anig|EHA27803.1 aory|EIT77252.1 asyd|OJJ54070.1 ater|EAU38585.1 atub|OJI89577.1 aver|OJJ08672.1 awen|OJJ34377.1 azon|OJJ48111.1

Group4271: abra|OJJ77095.1 acar|OOF92978.1 acla|EAW10919.1 afis|EAW20939.1 afla|EED50294.1 afum|EAL84695.1 agla|OJJ83948.1 akaw|GAA86960.1 aluc|OJZ84504.1 anid|CBF82523.1 anig|EHA27804.1 aory|EIT77268.1 asyd|OJJ54071.1 ater|EAU38584.1 atub|OJI89576.1 aver|OJJ08671.1 awen|OJJ34378.1 azon|OJJ48112.1

Group4272: abra|OJJ77106.1 acar|OOF92991.1 acla|EAW11608.1 afis|EAW23840.1 afla|EED52551.1 afum|EAL87271.1 agla|OJJ82489.1 akaw|GAA86950.1 aluc|OJZ84515.1 anid|CBF77077.1 anig|EHA27813.1 aory|EIT74221.1 asyd|OJJ56983.1 ater|EAU33507.1 atub|OJI89566.1 aver|OJJ03304.1 awen|OJJ33810.1 azon|OJJ48724.1

Group4273: abra|OJJ77111.1 acar|OOF93001.1 acla|EAW07270.1 afis|EAW22291.1 afla|EED57693.1 afum|EAL90890.1 agla|OJJ87755.1 akaw|GAA90817.1 aluc|OJZ84520.1 anid|CBF88575.1 anig|EHA27818.1 aory|EIT79896.1 asyd|OJJ63840.1 ater|EAU34306.1 atub|OJI89560.1 aver|OJI97097.1 awen|OJJ34867.1 azon|OJJ50044.1

Group4274: abra|OJJ77116.1 acar|OOF93005.1 acla|EAW12952.1 afis|EAW17704.1 afla|EED45696.1 afum|EAL91519.2 agla|OJJ81740.1 akaw|GAA90813.1 aluc|OJZ84526.1 anid|CBF89929.1 anig|EHA27823.1

aory|EIT75367.1 asyd|OJJ62119.1 ater|EAU31912.1 atub|OJI89554.1 aver|OJI96226.1 awen|OJJ37131.1 azon|OJJ50830.1

Group4275: abra|OJJ77120.1 acar|OOF93007.1 acla|EAW12932.1 afis|EAW17709.1 afla|EED45711.1 afum|EAL91524.2 agla|OJJ81738.1 akaw|GAA90809.1 aluc|OJZ84530.1 anid|CBF89906.1 anig|EHA27827.1 aory|EIT75650.1 asyd|OJJ62132.1 ater|EAU31903.1 atub|OJI89550.1 aver|OJI96241.1 awen|OJJ37127.1 azon|OJJ50826.1

Group4276: abra|OJJ77123.1 acar|OOF93009.1 acla|EAW12936.1 afis|EAW17712.1 afla|EED45714.1 afum|EAL91527.1 agla|OJJ81736.1 akaw|GAA90805.1 aluc|OJZ84533.1 anid|CBF76920.1 anig|EHA27831.1 aory|EIT75661.1 asyd|OJJ57072.1 ater|EAU31891.1 atub|OJI89547.1 aver|OJJ03226.1 awen|OJJ37124.1 azon|OJJ50823.1

Group4277: abra|OJJ77125.1 acar|OOF93011.1 acla|EAW12938.1 afis|EAW17714.1 afla|EED45716.1 afum|EAL91529.1 agla|OJJ81734.1 akaw|GAA90803.1 aluc|OJZ84535.1 anid|CBF76916.1 anig|EHA27833.1 aory|EIT75429.1 asyd|OJJ57079.1 ater|EAU31892.1 atub|OJI89545.1 aver|OJJ03224.1 awen|OJJ37122.1 azon|OJJ50821.1

Group4278: abra|OJJ77126.1 acar|OOF93012.1 acla|EAW12939.1 afis|EAW17715.1 afla|EED45717.1 afum|EAL91530.1 agla|OJJ81733.1 akaw|GAA90802.1 aluc|OJZ84536.1 anid|CBF76914.1 anig|EHA27834.1 aory|EIT75816.1 asyd|OJJ57080.1 ater|EAU31893.1 atub|OJI89544.1 aver|OJJ03223.1 awen|OJJ37121.1 azon|OJJ50820.1

Group4279: abra|OJJ77130.1 acar|OOF93018.1 acla|EAW12941.1 afis|EAW17717.1 afla|EED45719.1 afum|EAL91532.1 agla|OJJ81731.1 akaw|GAA90798.1 aluc|OJZ84540.1 anid|CBF84088.1 anig|EHA27838.1 aory|EIT75412.1 asyd|OJJ57082.1 ater|EAU31895.1 atub|OJI89540.1 aver|OJJ03220.1 awen|OJJ37119.1 azon|OJJ50818.1

Group4280: abra|OJJ77131.1 acar|OOF93019.1 acla|EAW12942.1 afis|EAW17718.1 afla|EED45720.1 afum|EAL91533.1 agla|OJJ81728.1 akaw|GAA90797.1 aluc|OJZ84541.1 anid|CBF76908.1 anig|EHA27839.1 aory|EIT75617.1 asyd|OJJ57083.1 ater|EAU31896.1 atub|OJI89539.1 aver|OJJ03219.1 awen|OJJ37118.1 azon|OJJ50817.1

Group4281: abra|OJJ77133.1 acar|OOF93021.1 acla|EAW12944.1 afis|EAW17720.1 afla|EED45722.1 afum|EAL91535.2 agla|OJJ81730.1 akaw|GAA90795.1 aluc|OJZ84543.1 anid|CBF76904.1 anig|EHA27841.1 aory|EIT75737.1 asyd|OJJ57085.1 ater|EAU31898.1 atub|OJI89537.1 aver|OJJ03217.1 awen|OJJ37116.1 azon|OJJ50815.1

Group4282: abra|OJJ77138.1 acar|OOF90283.1 acla|EAW12948.1 afis|EAW17723.1 afla|EED45725.1 afum|EAL91538.1 agla|OJJ81727.1 akaw|GAA90791.1 aluc|OJZ84549.1 anid|CBF86584.1 anig|EHA27845.1 aory|EIT75656.1 asyd|OJJ64839.1 ater|EAU31901.1 atub|OJI89531.1 aver|OJI98950.1 awen|OJJ37115.1 azon|OJJ50813.1

Group4283: abra|OJJ77139.1 acar|OOF90282.1 acla|EAW12950.1 afis|EAW17725.1 afla|EED45726.1 afum|EAL91540.1 agla|OJJ81726.1 akaw|GAA90790.1 aluc|OJZ84552.1 anid|CBF86583.1 anig|EHA27846.1 aory|EIT75496.1 asyd|OJJ64840.1 ater|EAU31881.1 atub|OJI89530.1 aver|OJI98951.1 awen|OJJ37113.1 azon|OJJ50812.1

Group4284: abra|OJJ77141.1 acar|OOF93027.1 acla|EAW12927.1 afis|EAW17728.1 afla|EED45728.1 afum|EAL91543.1 agla|OJJ81723.1 akaw|GAA90788.1 aluc|OJZ84555.1 anid|CBF86577.1 anig|EHA27849.1 aory|EIT75836.1 asyd|OJJ64842.1 ater|EAU31879.1 atub|OJI89527.1 aver|OJI98953.1 awen|OJJ37110.1 azon|OJJ50809.1

Group4285: abra|OJJ77142.1 acar|OOF93028.1 acla|EAW12926.1 afis|EAW17729.1 afla|EED45729.1 afum|EAL91544.1 agla|OJJ81722.1 akaw|GAA90787.1 aluc|OJZ84556.1 anid|CBF86575.1 anig|EHA27850.1 aory|EIT75504.1 asyd|OJJ64843.1 ater|EAU31878.1 atub|OJI89526.1 aver|OJI98954.1 awen|OJJ37109.1 azon|OJJ50808.1

Group4286: abra|OJJ77143.1 acar|OOF93029.1 acla|EAW12925.1 afis|EAW17730.1 afla|EED45730.1 afum|EAL91545.1 agla|OJJ81721.1 akaw|GAA90786.1 aluc|OJZ84558.1 anid|CBF86573.1 anig|EHA27851.1 aory|EIT75486.1 asyd|OJJ64844.1 ater|EAU31877.1 atub|OJI89525.1 aver|OJI98955.1 awen|OJJ37108.1 azon|OJJ50807.1

Group4287: abra|OJJ77144.1 acar|OOF93030.1 acla|EAW12924.1 afis|EAW17731.1 afla|EED45731.1 afum|EAL91546.1 agla|OJJ81720.1 akaw|GAA90785.1 aluc|OJZ84559.1 anid|CBF86588.1 anig|EHA27852.1 aory|EIT75623.1 asyd|OJJ64838.1 ater|EAU31876.1 atub|OJI89524.1 aver|OJI98948.1 awen|OJJ37107.1 azon|OJJ50806.1

Group4288: abra|OJJ77145.1 acar|OOF93031.1 acla|EAW12923.1 afis|EAW17732.1 afla|EED45732.1 afum|EAL91547.1 agla|OJJ81719.1 akaw|GAA90784.1 aluc|OJZ84560.1 anid|CBF86586.1 anig|EHA27853.1 aory|EIT75469.1 asyd|OJJ64837.1 ater|EAU31875.1 atub|OJI89523.1 aver|OJI98947.1 awen|OJJ37105.1 azon|OJJ50805.1

Group4289: abra|OJJ77146.1 acar|OOF93032.1 acla|EAW12922.1 afis|EAW17733.1 afla|EED45734.1 afum|EAL91548.1 agla|OJJ81718.1 akaw|GAA90783.1 aluc|OJZ84561.1 anid|CBF86597.1 anig|EHA27855.1 aory|EIT75719.1 asyd|OJJ64833.1 ater|EAU31874.1 atub|OJI89521.1 aver|OJI98946.1 awen|OJJ37104.1 azon|OJJ50804.1

Group4290: abra|OJJ77148.1 acar|OOF93034.1 acla|EAW12921.1 afis|EAW17734.1 afla|EED45735.1 afum|EAL91549.1 agla|OJJ81716.1 akaw|GAA90782.1 aluc|OJZ84562.1 anid|CBF86595.1 anig|EHA27856.1 aory|EIT75759.1 asyd|OJJ64832.1 ater|EAU31873.1 atub|OJI89520.1 aver|OJI98945.1 azon|OJJ50802.1 awen|OJJ37101.1

Group4291: abra|OJJ77149.1 acar|OOF93035.1 acla|EAW12920.1 afis|EAW17735.1 afla|EED45736.1 afum|EAL91550.1 agla|OJJ81715.1 akaw|GAA90781.1 aluc|OJZ84563.1 anid|CBF86593.1 anig|EHA27857.1 aory|EIT75423.1 asyd|OJJ64831.1 ater|EAU31872.1 atub|OJI89519.1 aver|OJI98944.1 awen|OJJ37100.1 azon|OJJ50801.1

Group4292: abra|OJJ77157.1 acar|OOF93044.1 acla|EAW12915.1 afis|EAW17745.1 afla|EED45745.1 afum|EAL91557.1 agla|OJJ81708.1 akaw|GAA90773.1 aluc|OJZ84571.1 anid|CBF86614.1 anig|EHA27866.1 aory|EIT75582.1 asyd|OJJ64818.1 ater|EAU31862.1 atub|OJI89511.1 aver|OJI98931.1 awen|OJJ37092.1 azon|OJJ50796.1

Group4293: abra|OJJ77164.1 acar|OOF93050.1 acla|EAW12910.1 afis|EAW17754.1 afla|EED45758.1 afum|EAL91567.1 agla|OJJ81699.1 akaw|GAA90768.1 aluc|OJZ84577.1 anid|CBF86624.1 anig|EHA27870.1 aory|EIT75538.1 asyd|OJJ64811.1 ater|EAU31854.1 atub|OJI89504.1 aver|OJI98924.1 awen|OJJ37082.1 azon|OJJ50787.1

Group4294: abra|OJJ77165.1 acar|OOF93054.1 acla|EAW12901.1 afis|EAW17764.1 afla|EED45766.1 afum|EAL91574.1 agla|OJJ81698.1 akaw|GAA90767.1 aluc|OJZ84578.1 anid|CBF86628.1 anig|EHA27871.1 aory|EIT75559.1 asyd|OJJ64809.1 ater|EAU31853.1 atub|OJI89503.1 aver|OJI98922.1 awen|OJJ37081.1 azon|OJJ50784.1

Group4295: abra|OJJ77166.1 acar|OOF93055.1 acla|EAW12900.1 afis|EAW17765.1 afla|EED45767.1 afum|EAL91575.1 agla|OJJ81697.1 akaw|GAA90766.1 aluc|OJZ84579.1 anid|CBF86630.1 anig|EHA27872.1 aory|EIT75709.1 asyd|OJJ64808.1 ater|EAU31852.1 atub|OJI89502.1 aver|OJI98921.1 awen|OJJ37080.1 azon|OJJ50783.1

Group4296: abra|OJJ77176.1 acar|OOF93064.1 acla|EAW13174.1 afis|EAW17464.1 afla|EED49356.1 afum|EAL91293.1 agla|OJJ80340.1 akaw|GAA90759.1 aluc|OJZ84587.1 anid|CBF71650.1 anig|EHA27880.1 aory|EIT79568.1 asyd|OJJ61840.1 ater|EAU33949.1 atub|OJI89491.1 aver|OJI95939.1 awen|OJJ37381.1 azon|OJJ51055.1

Group4297: abra|OJJ77177.1 acar|OOF93065.1 acla|EAW13175.1 afis|EAW17463.1 afla|EED49357.1 afum|EAL91292.1 agla|OJJ80342.1 akaw|GAA90758.1 aluc|OJZ84589.1 anid|CBF71651.1 anig|EHA27881.1 aory|EIT79484.1 asyd|OJJ61839.1 ater|EAU33948.1 atub|OJI89490.1 aver|OJI95938.1 awen|OJJ37382.1 azon|OJJ51056.1

Group4298: abra|OJJ77186.1 acar|OOF93074.1 acla|EAW13176.1 afis|EAW17462.1 afla|EED49360.1 afum|EAL91291.1 agla|OJJ80343.1 akaw|GAA90753.1 aluc|OJZ84595.1 anid|CBF71661.1 anig|EHA27884.1 aory|EIT79468.1 asyd|OJJ61834.1 ater|EAU33947.1 atub|OJI89484.1 aver|OJI95933.1 awen|OJJ37384.1 azon|OJJ51059.1

Group4299: abra|OJJ77188.1 acar|OOF92641.1 acla|EAW13178.1 afis|EAW17461.1 afla|EED49361.1 afum|EAL91289.1 agla|OJJ80344.1 akaw|GAA90752.1 aluc|OJZ84596.1 anid|CBF71663.1 anig|EHA27885.1 aory|EIT79490.1 asyd|OJJ61833.1 ater|EAU33946.1 atub|OJI89483.1 aver|OJI95932.1 awen|OJJ37385.1 azon|OJJ51060.1

Group4300: abra|OJJ77190.1 acar|OOF93076.1 acla|EAW13179.1 afis|EAW17460.1 afla|EED49362.1 afum|EAL91288.1 agla|OJJ80345.1 akaw|GAA90750.1 aluc|OJZ84598.1 anid|CBF71665.1 anig|EHA27887.1 aory|EIT79506.1 asyd|OJJ61832.1 ater|EAU33945.1 atub|OJI89481.1 aver|OJI95931.1 awen|OJJ37386.1 azon|OJJ51061.1

Group4301: abra|OJJ77193.1 acar|OOF93078.1 acla|EAW13183.1 afis|EAW17457.1 afla|EED49365.1 afum|EAL91285.1 agla|OJJ80354.1 akaw|GAA90748.1 aluc|OJZ84600.1 anid|CBF71668.1 anig|EHA27890.1 aory|EIT79576.1 asyd|OJJ61829.1 ater|EAU33944.1 atub|OJI89479.1 aver|OJI95929.1 awen|OJJ37390.1 azon|OJJ51063.1

Group4302: abra|OJJ77194.1 acar|OOF93079.1 acla|EAW13184.1 afis|EAW17456.1 afla|EED49366.1 afum|EAL91284.1 agla|OJJ80353.1 akaw|GAA90747.1 aluc|OJZ84601.1 anid|CBF71670.1 anig|EHA27891.1 aory|EIT79508.1 asyd|OJJ61828.1 ater|EAU33936.1 atub|OJI89478.1 aver|OJI95928.1 awen|OJJ37391.1 azon|OJJ51064.1

Group4303: abra|OJJ77195.1 acar|OOF93080.1 acla|EAW13186.1 afis|EAW17455.1 afla|EED49367.1 afum|EAL91283.1 agla|OJJ80352.1 akaw|GAA90746.1 aluc|OJZ84602.1 anid|CBF71671.1 anig|EHA27892.1

aory|EIT79573.1 asyd|OJJ61827.1 ater|EAU33935.1 atub|OJI89477.1 aver|OJI95927.1 awen|OJJ37392.1 azon|OJJ51065.1

Group4304: abra|OJJ77198.1 acar|OOF93081.1 acla|EAW13187.1 afis|EAW17454.1 afla|EED49368.1 afum|EAL91282.1 agla|OJJ80351.1 akaw|GAA90743.1 aluc|OJZ84605.1 anid|CBF71673.1 anig|EHA27894.1 aory|EIT79544.1 asyd|OJJ61826.1 ater|EAU33934.1 atub|OJI89474.1 aver|OJI95926.1 awen|OJJ37393.1 azon|OJJ51066.1

Group4305: abra|OJJ77201.1 acar|OOF93089.1 acla|EAW13192.1 afis|EAW17449.1 afla|EED49373.1 afum|EBA27429.1 akaw|GAA90737.1 aluc|OJZ84611.1 anid|CBF71685.1 anig|EHA27900.1 aory|EIT79520.1 asyd|OJJ61821.1 ater|EAU33929.1 atub|OJI89470.1 aver|OJI95921.1 awen|OJJ37397.1 azon|OJJ51070.1 agla|OJJ80368.1

Group4306: abra|OJJ77202.1 acar|OOF93090.1 acla|EAW13193.1 afis|EAW17448.1 afla|EED49374.1 afum|EAL91275.2 agla|OJJ80369.1 akaw|GAA90736.1 aluc|OJZ84612.1 anid|CBF71687.1 anig|EHA27901.1 aory|EIT79525.1 asyd|OJJ61820.1 ater|EAU33928.1 atub|OJI89469.1 aver|OJI95920.1 awen|OJJ37398.1 azon|OJJ51071.1

Group4307: abra|OJJ77204.1 acar|OOF93092.1 acla|EAW13196.1 akaw|GAA90734.1 aluc|OJZ84615.1 anid|CBF71688.1 anig|EHA27903.1 aory|EIT79511.1 asyd|OJJ61819.1 ater|EAU33925.1 atub|OJI89466.1 aver|OJI95919.1 awen|OJJ37400.1 afis|EAW17446.1 afla|EED49381.1 afum|EAL91272.1 agla|OJJ80371.1 azon|OJJ51074.1

Group4308: abra|OJJ77205.1 acar|OOF93094.1 acla|EAW13197.1 afis|EAW17445.1 afla|EED49382.1 afum|EAL91271.1 agla|OJJ80372.1 akaw|GAA90733.1 aluc|OJZ84616.1 anid|CBF71690.1 anig|EHA27904.1 aory|EIT79527.1 asyd|OJJ61818.1 ater|EAU33924.1 atub|OJI89465.1 aver|OJI95918.1 awen|OJJ37401.1 azon|OJJ51075.1

Group4309: abra|OJJ77206.1 acar|OOF93093.1 acla|EAW13198.1 afis|EAW17444.1 afla|EED49383.1 afum|EAL91270.1 agla|OJJ80373.1 akaw|GAA90732.1 aluc|OJZ84617.1 anid|CBF71692.1 anig|EHA27905.1 aory|EIT79499.1 asyd|OJJ61817.1 ater|EAU33923.1 atub|OJI89464.1 aver|OJI95917.1 awen|OJJ37402.1 azon|OJJ51076.1

Group4310: abra|OJJ77207.1 acar|OOF93095.1 acla|EAW13199.1 afis|EAW17443.1 afla|EED49384.1 afum|EAL91269.1 agla|OJJ80374.1 akaw|GAA90731.1 aluc|OJZ84618.1 anid|CBF71694.1 anig|EHA27906.1 aory|EIT79562.1 asyd|OJJ61816.1 ater|EAU33922.1 atub|OJI89463.1 aver|OJI95916.1 awen|OJJ37403.1 azon|OJJ51077.1

Group4311: abra|OJJ77209.1 acar|OOF93096.1 acla|EAW13201.1 afis|EAW17441.1 afla|EED49386.1 afum|EAL91267.1 agla|OJJ80376.1 akaw|GAA90729.1 aluc|OJZ84620.1 anid|CBF71698.1 anig|EHA27908.1 aory|EIT79558.1 asyd|OJJ61814.1 ater|EAU33920.1 atub|OJI89461.1 aver|OJI95913.1 awen|OJJ37405.1 azon|OJJ51079.1

Group4312: abra|OJJ77210.1 acar|OOF93098.1 acla|EAW13202.1 afis|EAW17440.1 afla|EED49387.1 afum|EAL91266.1 agla|OJJ80377.1 akaw|GAA90728.1 aluc|OJZ84621.1 anid|CBF71700.1 anig|EHA27909.1 aory|EIT79563.1 asyd|OJJ61813.1 ater|EAU33919.1 atub|OJI89460.1 aver|OJI95912.1 awen|OJJ37406.1 azon|OJJ51080.1

Group4313: abra|OJJ77211.1 acar|OOF93097.1 acla|EAW13203.1 afis|EAW17439.1 afla|EED49388.1 afum|EAL91265.1 agla|OJJ80378.1 akaw|GAA90727.1 aluc|OJZ84622.1 anid|CBF71702.1 anig|EHA27910.1 aory|EIT79503.1 asyd|OJJ61812.1 ater|EAU33918.1 atub|OJI89459.1 aver|OJI95911.1 awen|OJJ37407.1 azon|OJJ51081.1

Group4314: abra|OJJ77213.1 acar|OOF93099.1 acla|EAW13205.1 afis|EAW17437.1 afla|EED49390.1 afla|EED49391.1 afum|EAL91264.1 agla|OJJ80380.1 akaw|GAA90725.1 aluc|OJZ84624.1 anig|EHA27911.1 aory|EIT79569.1 asyd|OJJ61810.1 ater|EAU33916.1 atub|OJI89457.1 aver|OJI95909.1 awen|OJJ37409.1 azon|OJJ51083.1

Group4315: abra|OJJ77214.1 acar|OOF93101.1 acla|EAW13206.1 afis|EAW17436.1 afla|EED49392.1 afum|EAL91263.1 agla|OJJ80381.1 akaw|GAA90724.1 aluc|OJZ84625.1 anid|CBF71706.1 anig|EHA27912.1 aory|EIT79513.1 asyd|OJJ61809.1 ater|EAU33915.1 atub|OJI89456.1 aver|OJI95908.1 awen|OJJ37410.1 azon|OJJ51084.1

Group4316: abra|OJJ77215.1 acar|OOF93102.1 acla|EAW13207.1 afis|EAW17435.1 afla|EED49409.1 afum|EAL91262.1 agla|OJJ80383.1 akaw|GAA90723.1 aluc|OJZ84626.1 anid|CBF71708.1 anig|EHA27913.1 aory|EIT79458.1 asyd|OJJ61808.1 ater|EAU33914.1 atub|OJI89455.1 aver|OJI95907.1 awen|OJJ37411.1 azon|OJJ51085.1

Group4317: abra|OJJ77216.1 acar|OOF93103.1 acla|EAW13209.1 afis|EAW17432.1 afla|EED49408.1 afum|EAL91260.1 agla|OJJ80384.1 akaw|GAA90722.1 aluc|OJZ84627.1 anid|CBF71710.1 anig|EHA27914.1 aory|EIT79545.1 asyd|OJJ61807.1 ater|EAU33913.1 atub|OJI89452.1 aver|OJI95906.1 awen|OJJ37412.1 azon|OJJ51086.1

Group4318: abra|OJJ77220.1 acar|OOF93105.1 acla|EAW13210.1 afis|EAW17431.1 afla|EED49407.1 afum|EAL91259.1 agla|OJJ80387.1 akaw|GAA90719.1 aluc|OJZ84630.1 anid|CBF71712.1 anig|EHA27918.1 aory|EIT79560.1 asyd|OJJ61806.1 ater|EAU33912.1 atub|OJI89449.1 aver|OJI95905.1 awen|OJJ37413.1 azon|OJJ51087.1

Group4319: abra|OJJ77223.1 acar|OOF93108.1 acla|EAW13214.1 afis|EAW17428.1 afla|EED49402.1 afum|EAL91256.1 agla|OJJ80396.1 akaw|GAA90716.1 aluc|OJZ84633.1 anid|CBF71716.1 anig|EHA27921.1 aory|EIT79534.1 asyd|OJJ61804.1 ater|EAU33909.1 atub|OJI89446.1 aver|OJI95903.1 awen|OJJ37415.1 azon|OJJ51089.1

Group4320: abra|OJJ77225.1 acar|OOF93110.1 acla|EAW13216.1 afis|EAW17426.1 afla|EED49400.1 afum|EAL91254.1 agla|OJJ80398.1 akaw|GAA90714.1 aluc|OJZ84635.1 anid|CBF71720.1 anig|EHA27923.1 aory|EIT79524.1 asyd|OJJ61802.1 ater|EAU33908.1 atub|OJI89444.1 aver|OJI95901.1 awen|OJJ37417.1 azon|OJJ51091.1

Group4321: abra|OJJ77226.1 acar|OOF93111.1 acla|EAW13217.1 afis|EAW17425.1 afla|EED49399.1 afum|EAL91253.1 agla|OJJ80399.1 akaw|GAA90713.1 aluc|OJZ84636.1 anid|CBF71722.1 anig|EHA27924.1 aory|EIT79475.1 asyd|OJJ61801.1 ater|EAU33907.1 atub|OJI89443.1 aver|OJI95900.1 awen|OJJ37418.1 azon|OJJ51092.1

Group4322: abra|OJJ77230.1 acar|OOF93113.1 acla|EAW13219.1 afis|EAW17423.1 afla|EED49397.1 afum|EAL91251.1 agla|OJJ80401.1 akaw|GAA90711.1 aluc|OJZ84640.1 anid|CBF71729.1 anig|EHA27926.1 aory|EIT79564.1 asyd|OJJ61799.1 ater|EAU33905.1 atub|OJI89440.1 aver|OJI95898.1 awen|OJJ37421.1 azon|OJJ51094.1

Group4323: abra|OJJ77238.1 acar|OOF93119.1 acla|EAW13245.1 afis|EAW17391.1 afla|EED49477.1 afum|EAL91228.1 agla|OJJ80406.1 akaw|GAA90705.1 aluc|OJZ84648.1 anid|CBF71739.1 anig|EHA27932.1 aory|EIT79509.1 asyd|OJJ61792.1 ater|EAU33896.1 atub|OJI89432.1 aver|OJI95892.1 awen|OJJ37424.1 azon|OJJ51104.1

Group4324: abra|OJJ77240.1 acar|OOF93121.1 acla|EAW13247.1 afis|EAW17389.1 afla|EED49481.1 afum|EAL91225.1 agla|OJJ80408.1 akaw|GAA90703.1 aluc|OJZ84650.1 anid|CBF71743.1 anig|EHA27933.1 aory|EIT79553.1 asyd|OJJ61790.1 ater|EAU33894.1 atub|OJI89430.1 aver|OJI95890.1 awen|OJJ37426.1 azon|OJJ51102.1

Group4325: abra|OJJ77242.1 acar|OOF93123.1 acla|EAW14606.1 afis|EAW19335.1 afla|EED56869.1 afum|EAL94052.1 agla|OJJ79421.1 akaw|GAA90701.1 aluc|OJZ84652.1 anid|CBF82394.1 anig|EHA27935.1 aory|EIT77502.1 asyd|OJJ54156.1 atub|OJI89428.1 aver|OJJ08579.1 awen|OJJ38873.1 asyd|OJJ59232.1 aver|OJJ01842.1

Group4326: abra|OJJ77244.1 acar|OOF93125.1 acla|EAW13248.1 afis|EAW17388.1 afla|EED49482.1 afum|EAL91224.1 agla|OJJ80409.1 akaw|GAA90699.1 aluc|OJZ84654.1 anid|CBF71787.1 anig|EHA27937.1 aory|EIT79464.1 asyd|OJJ61746.1 ater|EAU33893.1 atub|OJI89426.1 aver|OJI95843.1 awen|OJJ37427.1 azon|OJJ51101.1

Group4327: abra|OJJ77247.1 acar|OOF90006.1 acla|EAW13252.1 afis|EAW17384.1 afla|EED49501.1 afum|EAL91219.1 agla|OJJ80419.1 akaw|GAA90695.1 aluc|OJZ84658.1 anid|CBF84196.1 anig|EHA27940.1 aory|EIT82601.1 asyd|OJJ57160.1 ater|EAU33890.1 atub|OJI89422.1 aver|OJJ03153.1 awen|OJJ33371.1 azon|OJJ51109.1

Group4328: abra|OJJ77250.1 acar|OOF90005.1 acla|EAW13253.1 afis|EAW17383.1 afla|EED49504.1 afum|EAL91218.1 agla|OJJ80420.1 akaw|GAA90693.1 aluc|OJZ84661.1 anid|CBF84198.1 anig|EHA27943.1 aory|EIT82610.1 asyd|OJJ57161.1 ater|EAU33880.1 atub|OJI89419.1 aver|OJJ03152.1 awen|OJJ37433.1 azon|OJJ51112.1

Group4329: abra|OJJ77260.1 acar|OOF93128.1 afla|EED49508.1 akaw|GAA90687.1 aluc|OJZ84667.1 anig|EHA27946.1 aory|EIT82587.1 asyd|OJJ57166.1 ater|EAU33878.1 atub|OJI89413.1 awen|OJJ37439.1 azon|OJJ51115.1 acla|EAW13256.1 afis|EAW17380.1 afum|EAL91215.1 agla|OJJ80422.1 anid|CBF84207.1 aver|OJJ03148.1

Group4330: abra|OJJ77261.1 acar|OOF93130.1 acla|EAW13257.1 afis|EAW17379.1 afla|EED49509.1 afum|EAL91214.1 agla|OJJ80423.1 akaw|GAA90686.1 aluc|OJZ84668.1 anid|CBF84209.1 anig|EHA27947.1 aory|EIT82656.1 asyd|OJJ57167.1 ater|EAU33877.1 atub|OJI89412.1 aver|OJJ03147.1 awen|OJJ37441.1 azon|OJJ51117.1

Group4331: abra|OJJ77262.1 acar|OOF93132.1 acla|EAW13258.1 afis|EAW17378.1 afla|EED49510.1 afum|EAL91213.1 agla|OJJ80424.1 akaw|GAA90685.1 aluc|OJZ84669.1 anid|CBF84210.1 anig|EHA27948.1 aory|EIT82657.1 asyd|OJJ57168.1 ater|EAU33876.1 atub|OJI89411.1 aver|OJJ03146.1 awen|OJJ37442.1 azon|OJJ51120.1

Group4332: abra|OJJ77263.1 acar|OOF93131.1 acla|EAW13259.1 afis|EAW17377.1 afla|EED49511.1 afum|EAL91212.1 agla|OJJ80425.1 akaw|GAA90684.1 aluc|OJZ84671.1 anid|CBF84212.1 anig|EHA27949.1

aory|EIT82654.1 asyd|OJJ57169.1 ater|EAU33875.1 atub|OJI89409.1 aver|OJJ03145.1 awen|OJJ37443.1 azon|OJJ51121.1

Group4333: abra|OJJ77275.1 acar|OOF93150.1 acla|EAW13275.1 afis|EAW17342.1 afla|EED49781.1 afum|EBA27424.1 agla|OJJ79342.1 akaw|GAA90674.1 aluc|OJZ84684.1 anid|CBF84217.1 anig|EHA27960.1 aory|EIT77695.1 asyd|OJJ57179.1 ater|EAU33855.1 atub|OJI89397.1 aver|OJJ03135.1 awen|OJJ29675.1 azon|OJJ45994.1

Group4334: abra|OJJ77289.1 acar|OOF93167.1 acla|EAW13173.1 afis|EAW17465.1 afla|EED49355.1 afum|EAL91294.1 agla|OJJ80339.1 akaw|GAA90660.1 aluc|OJZ84700.1 anid|CBF71646.1 anig|EHA27975.1 aory|EIT79489.1 asyd|OJJ61841.1 ater|EAU33950.1 atub|OJI89382.1 aver|OJI95941.1 awen|OJJ37380.1 azon|OJJ51054.1

Group4335: abra|OJJ77290.1 acar|OOF93168.1 acla|EAW13172.1 afis|EAW17466.1 afla|EED49354.1 afum|EAL91295.1 agla|OJJ80338.1 akaw|GAA90659.1 aluc|OJZ84701.1 anid|CBF71638.1 anig|EHA27976.1 aory|EIT79580.1 asyd|OJJ61844.1 ater|EAU33951.1 atub|OJI89381.1 aver|OJI95944.1 awen|OJJ37379.1 azon|OJJ51053.1

Group4336: abra|OJJ77291.1 acar|OOF93169.1 acla|EAW13171.1 afis|EAW17468.1 afla|EED49353.1 afum|EAL91297.1 agla|OJJ80336.1 akaw|GAA90658.1 aluc|OJZ84702.1 anid|CBF71640.1 anig|EHA27977.1 aory|EIT79590.1 asyd|OJJ61843.1 ater|EAU33952.1 atub|OJI89380.1 aver|OJI95943.1 awen|OJJ37378.1 azon|OJJ51052.1

Group4337: abra|OJJ77295.1 acar|OOF93172.1 acla|EAW13169.1 afis|EAW17470.1 afla|EED49348.1 afum|EAL91299.1 agla|OJJ78440.1 akaw|GAA91456.1 aluc|OJZ84707.1 anid|CBF71630.1 anig|EHA27981.1 aory|EIT79461.1 asyd|OJJ61849.1 ater|EAU33954.1 atub|OJI89376.1 aver|OJI95948.1 awen|OJJ37372.1 azon|OJJ51050.1

Group4338: abra|OJJ77297.1 acar|OOF93177.1 acla|EAW13167.1 afis|EAW17472.1 afla|EED49343.1 akaw|GAA91454.1 aluc|OJZ84709.1 anid|CBF71626.1 anig|EHA27983.1 asyd|OJJ61851.1 ater|EAU33956.1 atub|OJI89374.1 aver|OJI95950.1 afum|EAL91301.1 agla|OJJ81490.1 aory|EIT79471.1 awen|OJJ37370.1 azon|OJJ51049.1

Group4339: abra|OJJ77300.1 acar|OOF93179.1 acla|EAW13165.1 afis|EAW17474.1 afla|EED49340.1 afum|EAL91303.1 agla|OJJ81488.1 akaw|GAA91452.1 aluc|OJZ84712.1 anid|CBF71622.1 anig|EHA27985.1 aory|EIT79516.1 asyd|OJJ61854.1 ater|EAU33958.1 atub|OJI89371.1 aver|OJI95953.1 awen|OJJ37368.1 azon|OJJ51047.1

Group4340: abra|OJJ77301.1 acar|OOF93180.1 acla|EAW13164.1 afis|EAW17475.1 afla|EED49339.1 afum|EAL91304.1 agla|OJJ81487.1 akaw|GAA91451.1 aluc|OJZ84713.1 anid|CBF71620.1 anig|EHA27986.1 aory|EIT79565.1 asyd|OJJ61855.1 ater|EAU33959.1 atub|OJI89370.1 aver|OJI95954.1 awen|OJJ37367.1 azon|OJJ51046.1

Group4341: abra|OJJ77303.1 acar|OOF93183.1 acla|EAW13163.1 afis|EAW17477.1 afla|EED49337.1 afum|EAL91306.1 agla|OJJ81480.1 akaw|GAA91448.1 aluc|OJZ84716.1 anid|CBF71603.1 anig|EHA27988.1 aory|EIT79497.1 asyd|OJJ61857.1 ater|EAU33693.1 atub|OJI89367.1 aver|OJI95956.1 awen|OJJ37365.1 azon|OJJ51045.1

Group4342: abra|OJJ77304.1 acar|OOF93182.1 acla|EAW13162.1 afis|EAW17478.1 afla|EED49336.1 afum|EAL91307.1 agla|OJJ81479.1 akaw|GAA91447.1 aluc|OJZ84717.1 anid|CBF71605.1 anig|EHA27989.1 aory|EIT79535.1 asyd|OJJ61858.1 ater|EAU33694.1 atub|OJI89366.1 aver|OJI95957.1 awen|OJJ37364.1 azon|OJJ51044.1

Group4343: abra|OJJ77305.1 acar|OOF93184.1 acla|EAW13161.1 afis|EAW17479.1 afla|EED49335.1 afum|EAL91308.1 agla|OJJ81478.1 akaw|GAA91446.1 aluc|OJZ84718.1 anid|CBF71607.1 anig|EHA27990.1 aory|EIT79462.1 asyd|OJJ61859.1 ater|EAU33695.1 atub|OJI89365.1 aver|OJI95958.1 awen|OJJ37362.1 azon|OJJ51043.1

Group4344: abra|OJJ77306.1 acar|OOF93185.1 acla|EAW13160.1 afis|EAW17481.1 afla|EED49334.1 afum|EAL91309.1 agla|OJJ81477.1 akaw|GAA91445.1 aluc|OJZ84720.1 anid|CBF71609.1 anig|EHA27991.1 aory|EIT79526.1 asyd|OJJ61860.1 ater|EAU36927.1 atub|OJI89363.1 aver|OJI95959.1 awen|OJJ37357.1 azon|OJJ51041.1

Group4345: abra|OJJ77315.1 acar|OOF93191.1 acla|EAW13157.1 afis|EAW17489.1 afla|EED49327.1 afum|EAL91311.1 agla|OJJ81467.1 akaw|GAA91440.1 aluc|OJZ84725.1 anid|CBF71617.1 anig|EHA27999.1 aory|EIT79549.1 asyd|OJJ61864.1 ater|EAU36934.1 atub|OJI89357.1 aver|OJI95962.1 awen|OJJ37352.1 azon|OJJ51038.1

Group4346: abra|OJJ77318.1 acar|OOF93194.1 acla|EAW13154.1 afis|EAW17492.1 afla|EED49324.1 afum|EAL91314.1 agla|OJJ81464.1 akaw|GAA91438.1 aluc|OJZ84728.1 anid|CBF71599.1 anig|EHA28001.1 aory|EIT79543.1 asyd|OJJ61866.1 ater|EAU36936.1 atub|OJI89354.1 aver|OJI95964.1 awen|OJJ37349.1 azon|OJJ51035.1

Group4347: abra|OJJ77319.1 acar|OOF93195.1 acla|EAW13153.1 afis|EAW17493.1 afla|EED49323.1 afum|EAL91315.1 agla|OJJ81463.1 akaw|GAA91437.1 aluc|OJZ84729.1 anid|CBF71595.1 anig|EHA28002.1 aory|EIT79479.1 asyd|OJJ61868.1 ater|EAU36937.1 atub|OJI89353.1 aver|OJI95966.1 awen|OJJ37348.1 azon|OJJ51034.1

Group4348: abra|OJJ77320.1 acar|OOF93196.1 acla|EAW13152.1 afis|EAW17494.1 afla|EED49322.1 afum|EAL91316.1 agla|OJJ81460.1 akaw|GAA91436.1 aluc|OJZ84730.1 anid|CBF71589.1 anig|EHA28003.1 aory|EIT79493.1 asyd|OJJ61870.1 ater|EAU36938.1 atub|OJI89352.1 aver|OJI95968.1 awen|OJJ37347.1 azon|OJJ51033.1

Group4349: abra|OJJ77326.1 acar|OOF93202.1 acla|EAW13146.1 afis|EAW17518.1 afla|EED49315.1 afum|EAL91336.1 agla|OJJ81452.1 akaw|GAA91430.1 aluc|OJZ84736.1 anid|CBF71576.1 anig|EHA28009.1 aory|EIT82190.1 asyd|OJJ61877.1 ater|EAU36948.1 atub|OJI89345.1 aver|OJI95975.1 awen|OJJ37339.1 azon|OJJ51025.1

Group4350: abra|OJJ77328.1 acar|OOF93203.1 acla|EAW13145.1 afis|EAW17517.1 afla|EED49314.1 afum|EAL91335.1 agla|OJJ81451.1 akaw|GAA91429.1 aluc|OJZ84737.1 anid|CBF71574.1 anig|EHA28010.1 aory|EIT82219.1 asyd|OJJ61878.1 ater|EAU36949.1 atub|OJI89344.1 aver|OJI95976.1 awen|OJJ37338.1 azon|OJJ51024.1

Group4351: abra|OJJ77329.1 acar|OOF93204.1 acla|EAW13144.1 afis|EAW17516.1 afla|EED49313.1 afum|EAL91334.1 agla|OJJ81450.1 akaw|GAA91428.1 aluc|OJZ84738.1 anid|CBF71572.1 anig|EHA28011.1 aory|EIT82242.1 asyd|OJJ61879.1 ater|EAU36950.1 atub|OJI89343.1 aver|OJI95977.1 awen|OJJ37337.1 azon|OJJ51023.1

Group4352: abra|OJJ77334.1 acar|OOF89885.1 acla|EAW13138.1 afis|EAW17507.1 afla|EED49308.1 afum|EAL91329.1 agla|OJJ81445.1 akaw|GAA91424.1 aluc|OJZ84742.1 anid|CBF71533.1 anig|EHA28016.1 aory|EIT82210.1 asyd|OJJ61892.1 ater|EAU36953.1 atub|OJI89339.1 aver|OJI95988.1 awen|OJJ37332.1 azon|OJJ51019.1

Group4353: abra|OJJ77338.1 acar|OOF93209.1 acla|EAW13137.1 afis|EAW17506.1 afla|EED49306.1 afum|EAL91328.2 agla|OJJ81444.1 akaw|GAA91423.1 aluc|OJZ84747.1 anid|CBF71535.1 anig|EHA28017.1 aory|EIT82204.1 asyd|OJJ61889.1 ater|EAU36955.1 atub|OJI89335.1 aver|OJI95986.1 awen|OJJ37329.1 azon|OJJ51018.1

Group4354: abra|OJJ77339.1 acar|OOF93210.1 acla|EAW13136.1 afis|EAW17505.1 afla|EED49305.1 afum|EAL91327.1 agla|OJJ81443.1 akaw|GAA91422.1 aluc|OJZ84748.1 anid|CBF71537.1 anig|EHA28018.1 aory|EIT82207.1 asyd|OJJ61894.1 ater|EAU36956.1 atub|OJI89334.1 aver|OJI95990.1 awen|OJJ37328.1 azon|OJJ51017.1

Group4355: abra|OJJ77341.1 acar|OOF93211.1 acla|EAW13134.1 afis|EAW17503.1 afla|EED49301.1 afum|EBA27430.1 agla|OJJ81441.1 akaw|GAA91420.1 aluc|OJZ84750.1 anid|CBF71541.1 anig|EHA28020.1 aory|EIT82193.1 asyd|OJJ61896.1 ater|EAU36958.1 atub|OJI89332.1 aver|OJI95992.1 awen|OJJ37326.1 azon|OJJ51015.1

Group4356: abra|OJJ77342.1 acar|OOF93212.1 acla|EAW13133.1 afis|EAW17502.1 afla|EED49300.1 afum|EAL91325.1 agla|OJJ81440.1 akaw|GAA91419.1 aluc|OJZ84751.1 anid|CBF71543.1 anig|EHA28021.1 aory|EIT82195.1 asyd|OJJ61897.1 ater|EAU36959.1 atub|OJI89331.1 aver|OJI95993.1 awen|OJJ37325.1 azon|OJJ51014.1

Group4357: abra|OJJ77343.1 acar|OOF93213.1 acla|EAW13132.1 afis|EAW17501.1 afla|EED49299.1 afum|EAL91324.1 agla|OJJ81439.1 akaw|GAA91418.1 aluc|OJZ84753.1 anid|CBF85589.1 anig|EHA28022.1 aory|EIT82233.1 asyd|OJJ64171.1 ater|EAU36960.1 atub|OJI89330.1 aver|OJI98246.1 awen|OJJ37324.1 azon|OJJ51013.1

Group4358: abra|OJJ77344.1 acar|OOF93214.1 acla|EAW13131.1 afis|EAW17532.1 afla|EED49298.1 afum|EAL91346.1 agla|OJJ81438.1 akaw|GAA91417.1 aluc|OJZ84754.1 anid|CBF90335.1 anig|EHA28023.1 aory|EIT82183.1 asyd|OJJ61898.1 ater|EAU36961.1 atub|OJI89329.1 aver|OJI95994.1 awen|OJJ37320.1 azon|OJJ51012.1

Group4359: abra|OJJ77346.1 acar|OOF93216.1 acla|EAW13128.1 afis|EAW17535.1 afla|EED49294.1 afum|EAL91349.1 agla|OJJ81436.1 akaw|GAA91414.1 aluc|OJZ84757.1 anid|CBF90326.1 anig|EHA28026.1 aory|EIT82178.1 asyd|OJJ61907.1 ater|EAU36964.1 atub|OJI89327.1 aver|OJI96000.1 awen|OJJ37318.1 azon|OJJ51010.1

Group4360: abra|OJJ77347.1 acar|OOF93217.1 acla|EAW13127.1 afis|EAW17536.1 afla|EED49293.1 afum|EAL91350.1 agla|OJJ81433.1 akaw|GAA91413.1 aluc|OJZ84758.1 anid|CBF90324.1 anig|EHA28027.1 aory|EIT82228.1 asyd|OJJ61908.1 ater|EAU36965.1 atub|OJI89326.1 aver|OJI96001.1 awen|OJJ37317.1 azon|OJJ51009.1

Group4361: abra|OJJ77348.1 acar|OOF93218.1 acla|EAW13126.1 afis|EAW17537.1 afla|EED49290.1 afum|EAL91351.1 agla|OJJ81432.1 akaw|GAA91412.1 aluc|OJZ84759.1 anid|CBF90322.1 anig|EHA28028.1

aory|EIT82241.1 asyd|OJJ61909.1 ater|EAU36967.1 atub|OJI89325.1 aver|OJI96002.1 awen|OJJ37315.1 azon|OJJ51007.1

Group4362: abra|OJJ77350.1 acar|OOF93220.1 acla|EAW13125.1 afis|EAW17539.1 afla|EED49288.1 afum|EAL91353.1 agla|OJJ81430.1 akaw|GAA91411.1 aluc|OJZ84761.1 anid|CBF90317.1 anig|EHA28030.1 aory|EIT82231.1 asyd|OJJ61911.1 ater|EAU36969.1 atub|OJI89323.1 aver|OJI96004.1 awen|OJJ37314.1 azon|OJJ51006.1

Group4363: abra|OJJ77353.1 acar|OOF93223.1 acla|EAW13121.1 afis|EAW17543.1 afla|EED49284.1 afum|EAL91357.1 agla|OJJ81426.1 akaw|GAA91408.1 aluc|OJZ84764.1 anid|CBF90308.1 anig|EHA28033.1 aory|EIT82220.1 asyd|OJJ61932.1 ater|EAU36973.1 atub|OJI89320.1 aver|OJI96024.1 awen|OJJ37310.1 azon|OJJ51002.1

Group4364: abra|OJJ77355.1 acar|OOF93225.1 acla|EAW13119.1 afis|EAW17545.1 afla|EED49282.1 afum|EAL91359.1 agla|OJJ81424.1 akaw|GAA91406.1 aluc|OJZ84765.1 anid|CBF90305.1 anig|EHA28034.1 aory|EIT82244.1 asyd|OJJ61930.1 ater|EAU36975.1 atub|OJI89319.1 aver|OJI96022.1 awen|OJJ37309.1 azon|OJJ51001.1

Group4365: abra|OJJ77356.1 acar|OOF93226.1 acla|EAW13118.1 afis|EAW17546.1 afla|EED49281.1 afum|EAL91360.1 agla|OJJ81423.1 akaw|GAA91405.1 aluc|OJZ84766.1 anid|CBF90303.1 anig|EHA28035.1 aory|EIT82243.1 asyd|OJJ61929.1 ater|EAU36976.1 atub|OJI89318.1 aver|OJI96021.1 awen|OJJ37308.1 azon|OJJ51000.1

Group4366: abra|OJJ77359.1 acar|OOF93228.1 acla|EAW13117.1 afis|EAW17547.1 afla|EED49278.1 afum|EAL91361.1 agla|OJJ81421.1 akaw|GAA91404.1 aluc|OJZ84767.1 anid|CBF90290.1 anig|EHA28037.1 aory|EIT82197.1 asyd|OJJ61921.1 ater|EAU36977.1 atub|OJI89315.1 aver|OJI96013.1 awen|OJJ37307.1 azon|OJJ50999.1

Group4367: abra|OJJ77361.1 acar|OOF93230.1 acla|EAW13115.1 afis|EAW17549.1 afla|EED49276.1 afum|EAL91363.1 agla|OJJ81419.1 akaw|GAA91402.1 aluc|OJZ84769.1 anid|CBF90294.1 anig|EHA28038.1 aory|EIT82240.1 asyd|OJJ61923.1 ater|EAU36985.1 atub|OJI89312.1 aver|OJI96015.1 awen|OJJ37305.1 azon|OJJ50997.1

Group4368: abra|OJJ77362.1 acar|OOF93231.1 acla|EAW13114.1 afis|EAW17550.1 afla|EED49275.1 afum|EAL91364.1 agla|OJJ81418.1 akaw|GAA91400.1 aluc|OJZ84771.1 anid|CBF90296.1 anig|EHA28039.1 aory|EIT82229.1 asyd|OJJ61924.1 ater|EAU36984.1 atub|OJI89310.1 aver|OJI96016.1 awen|OJJ37303.1 azon|OJJ50996.1

Group4369: abra|OJJ77365.1 acar|OOF93235.1 acla|EAW13111.1 afis|EAW17553.1 afla|EED49272.1 afum|EAL91367.1 agla|OJJ81415.1 akaw|GAA91397.1 aluc|OJZ84774.1 anid|CBF90299.1 anig|EHA28042.1 aory|EIT82208.1 asyd|OJJ61926.1 ater|EAU36981.1 atub|OJI89307.1 aver|OJI96018.1 awen|OJJ37300.1 azon|OJJ50993.1

Group4370: abra|OJJ77366.1 acar|OOF93236.1 acla|EAW13110.1 afis|EAW17554.1 afla|EED49271.1 afum|EAL91368.1 agla|OJJ81414.1 akaw|GAA91396.1 aluc|OJZ84775.1 anid|CBF90301.1 anig|EHA28043.1 aory|EIT82221.1 asyd|OJJ61927.1 ater|EAU36980.1 atub|OJI89306.1 aver|OJI96019.1 awen|OJJ37299.1 azon|OJJ50992.1

Group4371: abra|OJJ77368.1 acar|OOF93238.1 acla|EAW13108.1 afis|EAW17556.1 afla|EED49268.1 afum|EAL91370.1 agla|OJJ81412.1 akaw|GAA91394.1 aluc|OJZ84777.1 anid|CBF90282.1 anig|EHA28045.1 aory|EIT82203.1 asyd|OJJ61918.1 ater|EAU36988.1 atub|OJI89304.1 aver|OJI96010.1 awen|OJJ37297.1 azon|OJJ50990.1

Group4372: abra|OJJ77370.1 acar|OOF93241.1 acla|EAW13105.1 afis|EAW17559.1 afla|EED49265.1 afum|EAL91373.1 agla|OJJ81409.1 akaw|GAA91391.1 aluc|OJZ84780.1 anid|CBF90277.1 anig|EHA28047.1 aory|EIT82185.1 asyd|OJJ61933.1 ater|EAU36990.1 atub|OJI89302.1 aver|OJI96025.1 awen|OJJ37295.1 azon|OJJ50988.1

Group4373: abra|OJJ77371.1 acar|OOF93240.1 acla|EAW13104.1 afis|EAW17560.1 afla|EED49264.1 afum|EAL91374.1 agla|OJJ81408.1 akaw|GAA91390.1 aluc|OJZ84781.1 anid|CBF90275.1 anig|EHA28048.1 aory|EIT82187.1 asyd|OJJ61934.1 ater|EAU36991.1 atub|OJI89301.1 aver|OJI96026.1 awen|OJJ37294.1 azon|OJJ50987.1

Group4374: abra|OJJ77372.1 acar|OOF93242.1 acla|EAW13103.1 afis|EAW17561.1 afla|EED49263.1 afum|EAL91375.1 agla|OJJ81407.1 akaw|GAA91389.1 aluc|OJZ84782.1 anid|CBF90273.1 anig|EHA28049.1 aory|EIT82191.1 asyd|OJJ61935.1 ater|EAU36992.1 atub|OJI89300.1 aver|OJI96027.1 awen|OJJ37293.1 azon|OJJ50986.1

Group4375: abra|OJJ77373.1 acar|OOF93244.1 acla|EAW13102.1 afis|EAW17562.1 afla|EED49259.1 afum|EAL91376.1 agla|OJJ81406.1 akaw|GAA91387.1 aluc|OJZ84783.1 anid|CBF90271.1 anig|EHA28050.1 aory|EIT82238.1 asyd|OJJ61936.1 ater|EAU37000.1 atub|OJI89299.1 aver|OJI96028.1 awen|OJJ37292.1 azon|OJJ50985.1

Group4376: abra|OJJ77374.1 acar|OOF93243.1 acla|EAW13101.1 afis|EAW17563.1 afla|EED49258.1 afum|EAL91377.1 agla|OJJ81405.1 akaw|GAA91386.1 aluc|OJZ84784.1 anid|CBF90269.1 anig|EHA28051.1 aory|EIT82186.1 asyd|OJJ61937.1 ater|EAU37001.1 atub|OJI89298.1 aver|OJI96029.1 awen|OJJ37291.1 azon|OJJ50984.1

Group4377: abra|OJJ77375.1 acar|OOF93245.1 acla|EAW13100.1 afis|EAW17564.1 afla|EED49257.1 afum|EAL91378.1 agla|OJJ81402.1 akaw|GAA91385.1 aluc|OJZ84785.1 anid|CBF90267.1 anig|EHA28052.1 aory|EIT82246.1 asyd|OJJ61938.1 ater|EAU37002.1 atub|OJI89297.1 aver|OJI96030.1 awen|OJJ37290.1 azon|OJJ50983.1

Group4378: abra|OJJ77376.1 acar|OOF93246.1 acla|EAW13098.1 afis|EAW17565.1 afla|EED49256.1 afum|EAL91379.1 agla|OJJ81401.1 akaw|GAA91384.1 aluc|OJZ84786.1 anid|CBF90266.1 anig|EHA28053.1 aory|EIT82227.1 asyd|OJJ61939.1 ater|EAU37003.1 atub|OJI89296.1 aver|OJI96031.1 awen|OJJ37288.1 azon|OJJ50982.1

Group4379: abra|OJJ77378.1 acar|OOF93247.1 acla|EAW13096.1 afis|EAW17567.1 afla|EED49254.1 afum|EAL91381.1 agla|OJJ81398.1 akaw|GAA91382.1 aluc|OJZ84787.1 anid|CBF90262.1 anig|EHA28055.1 aory|EIT82184.1 asyd|OJJ61941.1 ater|EAU37004.1 atub|OJI89295.1 aver|OJI96033.1 awen|OJJ37286.1 azon|OJJ50980.1

Group4380: abra|OJJ77476.1 acar|OOF99249.1 acla|EAW06738.1 afis|EAW16288.1 afla|EED53265.1 afum|EAL88910.1 agla|OJJ88310.1 akaw|GAA84142.1 aluc|OJZ81062.1 anid|CBF86860.1 anig|EHA19306.1 aory|EIT75107.1 asyd|OJJ64634.1 ater|EAU37333.1 atub|OJI82523.1 aver|OJI98733.1 awen|OJJ37692.1 azon|OJJ44952.1

Group4381: abra|OJJ77477.1 acar|OOF99248.1 acla|EAW06739.1 afis|EAW16289.1 afla|EED53264.1 afum|EAL88911.1 agla|OJJ88311.1 akaw|GAA84141.1 aluc|OJZ81061.1 anid|CBF86858.1 anig|EHA19307.1 aory|EIT74825.1 asyd|OJJ64635.1 ater|EAU37334.1 atub|OJI82524.1 aver|OJI98734.1 awen|OJJ37691.1 azon|OJJ44951.1

Group4382: abra|OJJ77478.1 acar|OOF99247.1 acla|EAW06740.1 afis|EAW16290.1 afla|EED53263.1 afum|EAL88912.1 agla|OJJ88312.1 akaw|GAA84140.1 aluc|OJZ81060.1 anid|CBF86856.1 anig|EHA19308.1 aory|EIT75015.1 asyd|OJJ64636.1 ater|EAU37335.1 atub|OJI82525.1 aver|OJI98735.1 awen|OJJ37690.1 azon|OJJ44950.1

Group4383: abra|OJJ77479.1 acar|OOF99246.1 acla|EAW06741.1 afis|EAW16291.1 afla|EED53262.1 afum|EAL88913.1 agla|OJJ88313.1 akaw|GAA84139.1 aluc|OJZ81059.1 anid|CBF86854.1 anig|EHA19309.1 aory|EIT74947.1 asyd|OJJ64637.1 ater|EAU37336.1 atub|OJI82526.1 aver|OJI98736.1 awen|OJJ37689.1 azon|OJJ44949.1

Group4384: abra|OJJ77485.1 acar|OOF99240.1 acla|EAW06746.1 afis|EAW16296.1 afla|EED53258.1 afum|EAL88918.1 agla|OJJ88303.1 akaw|GAA84133.1 aluc|OJZ81051.1 anid|CBF86847.1 anig|EHA19316.1 aory|EIT75226.1 asyd|OJJ64642.1 ater|EAU29340.1 atub|OJI82533.1 aver|OJI98742.1 awen|OJJ37695.1 azon|OJJ44956.1

Group4385: abra|OJJ77489.1 acar|OOF99236.1 acla|EAW06751.1 afis|EAW16301.1 afla|EED53253.1 afum|EAL88923.1 agla|OJJ88293.1 akaw|GAA84130.1 aluc|OJZ81047.1 anid|CBF86841.1 anig|EHA19320.1 aory|EIT74998.1 asyd|OJJ64646.1 ater|EAU37346.1 atub|OJI82537.1 aver|OJI98746.1 awen|OJJ37700.1 azon|OJJ44961.1

Group4386: abra|OJJ77490.1 acar|OOF99235.1 acla|EAW06752.1 afis|EAW16302.1 afla|EED53252.1 afum|EAL88924.1 agla|OJJ88292.1 akaw|GAA84129.1 aluc|OJZ81046.1 anid|CBF86839.1 anig|EHA19321.1 aory|EIT75063.1 asyd|OJJ64647.1 ater|EAU37345.1 atub|OJI82538.1 aver|OJI98747.1 awen|OJJ37701.1 azon|OJJ44962.1

Group4387: abra|OJJ77493.1 acar|OOF99233.1 acla|EAW06754.1 afis|EAW16304.1 afla|EED53250.1 afum|EAL88926.1 agla|OJJ88290.1 akaw|GAA84127.1 aluc|OJZ81044.1 anid|CBF69582.1 anig|EHA19323.1 aory|EIT75043.1 asyd|OJJ59144.1 ater|EAU29332.1 atub|OJI82540.1 aver|OJJ01734.1 awen|OJJ37704.1 azon|OJJ44964.1

Group4388: abra|OJJ77494.1 acar|OOF99232.1 acla|EAW06755.1 afis|EAW16305.1 afla|EED53249.1 afum|EAL88927.1 agla|OJJ88267.1 akaw|GAA84126.1 aluc|OJZ81043.1 anid|CBF69583.1 anig|EHA19324.1 aory|EIT75153.1 asyd|OJJ59145.1 ater|EAU29333.1 atub|OJI82541.1 aver|OJJ01735.1 awen|OJJ37705.1 azon|OJJ44965.1

Group4389: abra|OJJ77495.1 acla|EAW06756.1 afis|EAW16306.1 afla|EED53248.1 afum|EAL88928.1 agla|OJJ88268.1 akaw|GAA84125.1 aluc|OJZ81042.1 anid|CBF69575.1 anig|EHA19325.1 aory|EIT75004.1 asyd|OJJ59149.1 ater|EAU29334.1 atub|OJI82542.1 aver|OJJ01739.1 awen|OJJ37706.1 azon|OJJ44966.1 aver|OJJ05343.1

Group4390: abra|OJJ77499.1 acar|OOF99227.1 acla|EAW06736.1 afis|EAW16287.1 afla|EED53269.1 afum|EAL88909.1 agla|OJJ88309.1 akaw|GAA84122.1 aluc|OJZ81039.1 anid|CBF86861.1 anig|EHA19328.1

aory|EIT74855.1 asyd|OJJ64633.1 ater|EAU37332.1 atub|OJI82545.1 aver|OJI98732.1 awen|OJJ37682.1 azon|OJJ44954.1

Group4391: abra|OJJ77502.1 acar|OOF99225.1 acla|EAW06734.1 afis|EAW16285.1 afla|EED53271.1 afum|EAL88906.1 agla|OJJ88306.1 akaw|GAA84119.1 aluc|OJZ81035.1 anid|CBF86869.1 anig|EHA19331.1 aory|EIT75236.1 asyd|OJJ64631.1 ater|EAU37329.1 atub|OJI82548.1 aver|OJI98729.1 awen|OJJ37679.1 azon|OJJ44945.1

Group4392: abra|OJJ77503.1 acar|OOF99224.1 acla|EAW06732.1 afis|EAW16284.1 afla|EED53272.1 afum|EAL88905.1 agla|OJJ88305.1 akaw|GAA84118.1 aluc|OJZ81034.1 anid|CBF86871.1 anig|EHA19332.1 aory|EIT75050.1 asyd|OJJ64630.1 ater|EAU37328.1 atub|OJI82549.1 aver|OJI98728.1 awen|OJJ37677.1 azon|OJJ44942.1

Group4393: abra|OJJ77504.1 acar|OOF99222.1 acla|EAW06730.1 afis|EAW16283.1 afla|EED53273.1 afum|EAL88904.1 agla|OJJ88318.1 akaw|GAA84116.1 aluc|OJZ81032.1 anid|CBF86876.1 anig|EHA19333.1 aory|EIT75123.1 asyd|OJJ64627.1 ater|EAU37327.1 atub|OJI82551.1 aver|OJI98725.1 awen|OJJ37675.1 azon|OJJ44940.1

Group4394: abra|OJJ77507.1 acar|OOF99219.1 acla|EAW06727.1 afis|EAW16280.1 afla|EED53276.1 afum|EAL88901.1 agla|OJJ88324.1 akaw|GAA84113.1 aluc|OJZ81029.1 anid|CBF86880.1 anig|EHA19336.1 aory|EIT74958.1 asyd|OJJ64626.1 ater|EAU37325.1 atub|OJI82554.1 aver|OJI98722.1 awen|OJJ37672.1 azon|OJJ44937.1

Group4395: abra|OJJ77508.1 acar|OOF99218.1 acla|EAW06726.1 afis|EAW16279.1 afla|EED53277.1 afum|EAL88900.1 agla|OJJ88323.1 akaw|GAA84112.1 aluc|OJZ81028.1 anid|CBF86882.1 anig|EHA19337.1 aory|EIT74848.1 asyd|OJJ64625.1 ater|EAU37324.1 atub|OJI82555.1 aver|OJI98721.1 awen|OJJ37671.1 azon|OJJ44936.1

Group4396: abra|OJJ77509.1 acar|OOF99217.1 acla|EAW06725.1 afis|EAW16278.1 afla|EED53279.1 afum|EAL88899.1 agla|OJJ88321.1 akaw|GAA84111.1 aluc|OJZ81027.1 anid|CBF86883.1 anig|EHA19338.1 aory|EIT74824.1 asyd|OJJ64624.1 ater|EAU37323.1 atub|OJI82556.1 aver|OJI98720.1 awen|OJJ37670.1 azon|OJJ44935.1

Group4397: abra|OJJ77510.1 acar|OOF99216.1 acla|EAW06724.1 afis|EAW16277.1 afla|EED53280.1 afum|EAL88898.1 agla|OJJ88328.1 akaw|GAA84110.1 aluc|OJZ81026.1 anid|CBF86885.1 anig|EHA19339.1 aory|EIT75207.1 asyd|OJJ64623.1 ater|EAU37322.1 atub|OJI82557.1 aver|OJI98719.1 awen|OJJ37669.1 azon|OJJ44934.1

Group4398: abra|OJJ77511.1 acar|OOF99214.1 acla|EAW06723.1 afis|EAW16276.1 afla|EED53281.1 afum|EAL88897.1 agla|OJJ88329.1 akaw|GAA84109.1 aluc|OJZ81025.1 anid|CBF86892.1 anig|EHA18669.1 aory|EIT75064.1 asyd|OJJ64622.1 ater|EAU37321.1 atub|OJI82558.1 aver|OJI98718.1 awen|OJJ37668.1 azon|OJJ44933.1

Group4399: abra|OJJ77513.1 acar|OOF99211.1 acla|EAW06722.1 afis|EAW16265.1 afla|EED53285.1 afum|EAL88886.1 agla|OJJ88330.1 akaw|GAA84107.1 aluc|OJZ81022.1 anid|CBF86894.1 anig|EHA19341.1 aory|EIT74876.1 asyd|OJJ64621.1 ater|EAU37438.1 atub|OJI82561.1 aver|OJI98717.1 awen|OJJ37667.1 azon|OJJ44930.1

Group4400: abra|OJJ77514.1 acar|OOF99210.1 acla|EAW06721.1 afis|EAW16266.1 afla|EED53284.1 afum|EAL88887.1 agla|OJJ88331.1 akaw|GAA84106.1 aluc|OJZ81021.1 anid|CBF86895.1 anig|EHA19342.1 aory|EIT75173.1 asyd|OJJ64620.1 ater|EAU37439.1 atub|OJI82562.1 aver|OJI98716.1 awen|OJJ37666.1 azon|OJJ44931.1

Group4401: abra|OJJ77516.1 acar|OOF99207.1 acla|EAW06720.1 afis|EAW16267.1 afla|EED53282.1 afum|EAL88888.1 agla|OJJ88333.1 akaw|GAA84104.1 aluc|OJZ81019.1 anid|CBF86897.1 anig|EHA19343.1 aory|EIT75090.1 asyd|OJJ64618.1 ater|EAU37440.1 atub|OJI82564.1 aver|OJI98714.1 awen|OJJ37664.1 azon|OJJ44923.1

Group4402: abra|OJJ77517.1 acar|OOF99208.1 acla|EAW06719.1 afis|EAW16268.1 afla|EED53288.1 afum|EAL88889.1 agla|OJJ88337.1 akaw|GAA84103.1 aluc|OJZ81018.1 anid|CBF86899.1 anig|EHA19344.1 aory|EIT74865.1 asyd|OJJ64617.1 ater|EAU37443.1 atub|OJI82565.1 aver|OJI98713.1 awen|OJJ37663.1 azon|OJJ44924.1

Group4403: abra|OJJ77520.1 acar|OOF99203.1 acla|EAW06715.1 afla|EED53293.1 afum|EAL88893.1 akaw|GAA84100.1 aluc|OJZ81016.1 anid|CBF86904.1 anig|EHA19346.1 aory|EIT74861.1 asyd|OJJ64613.1 atub|OJI82568.1 aver|OJI98709.1 awen|OJJ37656.1 azon|OJJ44929.1 afis|EAW16272.1 agla|OJJ88342.1 ater|EAU37447.1

Group4404: abra|OJJ77522.1 acar|OOF99202.1 acla|EAW06717.1 afis|EAW16270.1 afla|EED53295.1 afum|EAL88891.1 agla|OJJ88339.1 akaw|GAA84098.1 aluc|OJZ81014.1 anid|CBF86903.1 anig|EHA19347.1 aory|EIT74914.1 asyd|OJJ64615.1 ater|EAU37445.1 atub|OJI82570.1 aver|OJI98711.1 awen|OJJ37660.1 azon|OJJ44927.1

Group4405: abra|OJJ77524.1 acar|OOF99200.1 acla|EAW06718.1 afis|EAW16269.1 afla|EED53289.1 afum|EAL88890.1 agla|OJJ88338.1 akaw|GAA84095.1 aluc|OJZ81011.1 anid|CBF86901.1 anig|EHA19350.1 aory|EIT74922.1 asyd|OJJ64616.1 ater|EAU37444.1 atub|OJI82573.1 aver|OJI98712.1 awen|OJJ37661.1 azon|OJJ44926.1

Group4406: abra|OJJ77526.1 acar|OOF99198.1 acla|EAW06712.1 afis|EAW16275.1 afla|EED53297.1 afum|EAL88896.1 agla|OJJ88345.1 akaw|GAA84094.1 aluc|OJZ81009.1 anid|CBF86909.1 anig|EHA19351.1 aory|EIT75079.1 asyd|OJJ64610.1 ater|EAU37450.1 atub|OJI82575.1 aver|OJI98706.1 awen|OJJ37658.1 azon|OJJ44920.1

Group4407: abra|OJJ77535.1 acar|OOF99189.1 acla|EAW06704.1 afis|EAW16249.1 afla|EED53313.1 afum|EAL88877.1 agla|OJJ86260.1 akaw|GAA84085.1 aluc|OJZ81000.1 anid|CBF78891.1 anig|EHA19359.1 aory|EIT74965.1 asyd|OJJ64597.1 ater|EAU37466.1 atub|OJI82585.1 aver|OJI98694.1 awen|OJJ30206.1 azon|OJJ43931.1

Group4408: abra|OJJ77554.1 acar|OOF99173.1 acla|EAW06523.1 afis|EAW16239.1 afla|EED53338.1 afum|EAL88860.1 agla|OJJ86246.1 akaw|GAA84069.1 aluc|OJZ80983.1 anid|CBF78863.1 anig|EHA19377.1 aory|EIT75140.1 asyd|OJJ64574.1 ater|EAU33704.1 atub|OJI82603.1 aver|OJI98671.1 awen|OJJ30218.1 azon|OJJ43940.1

Group4409: abra|OJJ77557.1 acar|OOG00298.1 afis|EAW16398.1 afla|EED45629.1 afum|EAL89029.2 agla|OJJ82194.1 anid|CBF85652.1 anig|EHA19380.1 aory|EIT75689.1 asyd|OJJ54151.1 ater|EAU33011.1 aver|OJJ08588.1 awen|OJJ38072.1 aory|EIT76357.1 azon|OJJ46582.1 asyd|OJJ56065.1 aver|OJJ05008.1 awen|OJJ31109.1

Group4410: abra|OJJ77561.1 acar|OOF99169.1 acla|EAW06694.1 afis|EAW16072.1 afla|EED48591.1 afum|EAL88704.1 agla|OJJ86096.1 akaw|GAA84064.1 aluc|OJZ80977.1 anid|CBF75113.1 anig|EHA19382.1 aory|EIT73146.1 asyd|OJJ59998.1 ater|EAU33559.1 atub|OJI82608.1 aver|OJJ02293.1 awen|OJJ30362.1 azon|OJJ45381.1

Group4411: abra|OJJ77566.1 acar|OOF99164.1 acla|EAW06531.1 afis|EAW16064.1 afla|EED48578.1 afum|EAL88696.1 agla|OJJ86101.1 akaw|GAA84060.1 aluc|OJZ80972.1 anid|CBF75092.1 anig|EHA19387.1 aory|EIT73042.1 asyd|OJJ60011.1 ater|EAU33571.1 atub|OJI82613.1 aver|OJJ02274.1 awen|OJJ30375.1 azon|OJJ45391.1

Group4412: abra|OJJ77567.1 acar|OOF99162.1 acla|EAW06532.1 afis|EAW16063.1 afla|EED48577.1 afum|EAL88695.1 agla|OJJ86102.1 akaw|GAA84059.1 aluc|OJZ80971.1 anid|CBF75090.1 anig|EHA19388.1 aory|EIT73179.1 asyd|OJJ60012.1 ater|EAU33572.1 atub|OJI82614.1 aver|OJJ02273.1 awen|OJJ30376.1 azon|OJJ45392.1

Group4413: abra|OJJ77568.1 acar|OOF99163.1 acla|EAW06533.1 afis|EAW16062.1 afla|EED48576.1 afum|EAL88694.1 agla|OJJ86103.1 akaw|GAA84058.1 aluc|OJZ80970.1 anid|CBF75088.1 anig|EHA19389.1 aory|EIT73163.1 asyd|OJJ60013.1 ater|EAU33573.1 atub|OJI82615.1 aver|OJJ02272.1 awen|OJJ30377.1 azon|OJJ45393.1

Group4414: abra|OJJ77572.1 acar|OOF99157.1 acla|EAW06539.1 afis|EAW16056.1 afla|EED56442.1 afum|EAL88688.1 agla|OJJ86089.1 akaw|GAA84054.1 aluc|OJZ80966.1 anid|CBF75076.1 anig|EHA19392.1 aory|EIT72786.1 asyd|OJJ60016.1 ater|EAU33578.1 atub|OJI82620.1 aver|OJJ02269.1 awen|OJJ30383.1 azon|OJJ45400.1

Group4415: abra|OJJ77573.1 acar|OOF99156.1 acla|EAW06538.1 afis|EAW16057.1 afla|EED56441.1 afum|EAL88689.1 agla|OJJ86090.1 akaw|GAA84053.1 aluc|OJZ80965.1 anid|CBF75078.1 anig|EHA19393.1 aory|EIT72787.1 asyd|OJJ60017.1 ater|EAU33577.1 atub|OJI82621.1 aver|OJJ02268.1 awen|OJJ30382.1 azon|OJJ45399.1

Group4416: abra|OJJ77574.1 acar|OOF99155.1 acla|EAW06537.1 afis|EAW16058.1 afla|EED48572.1 afum|EAL88690.2 agla|OJJ86107.1 akaw|GAA84051.1 aluc|OJZ80963.1 anid|CBF75080.1 anig|EHA19394.1 aory|EIT73249.1 asyd|OJJ60018.1 ater|EAU33576.1 atub|OJI82623.1 aver|OJJ02267.1 awen|OJJ30381.1 azon|OJJ45398.1

Group4417: abra|OJJ77577.1 acar|OOF99150.1 acla|EAW06543.1 afis|EAW16052.1 afla|EED56446.1 afum|EAL88684.1 agla|OJJ86086.1 akaw|GAA84048.1 aluc|OJZ80959.1 anid|CBF75068.1 anig|EHA19397.1 aory|EIT72800.1 asyd|OJJ60022.1 ater|EAU33581.1 atub|OJI82626.1 aver|OJJ02262.1 awen|OJJ30390.1 azon|OJJ45402.1

Group4418: abra|OJJ77579.1 acar|OOF99148.1 acla|EAW06545.1 afis|EAW16049.1 afla|EED56438.1 afum|EAL88682.1 agla|OJJ86083.1 akaw|GAA84046.1 aluc|OJZ80956.1 anid|CBF75064.1 anig|EHA19399.1 aory|EIT72808.1 asyd|OJJ60024.1 ater|EAU33587.1 atub|OJI82628.1 aver|OJJ02260.1 awen|OJJ30394.1 azon|OJJ45404.1

Group4419: abra|OJJ77580.1 acar|OOF99147.1 acla|EAW06546.1 afis|EAW16048.1 afla|EED56437.1 afum|EAL88681.1 agla|OJJ86082.1 akaw|GAA84045.1 aluc|OJZ80955.1 anid|CBF75062.1 anig|EHA19400.1

aory|EIT72865.1 asyd|OJJ60025.1 ater|EAU33588.1 atub|OJI82629.1 aver|OJJ02259.1 awen|OJJ30396.1 azon|OJJ45405.1

Group4420: abra|OJJ77581.1 acar|OOF99146.1 acla|EAW06547.1 afis|EAW16047.1 afla|EED56436.1 afum|EAL88680.1 agla|OJJ86081.1 akaw|GAA84044.1 aluc|OJZ80954.1 anid|CBF75060.1 anig|EHA19401.1 aory|EIT72831.1 asyd|OJJ60026.1 ater|EAU33590.1 atub|OJI82630.1 aver|OJJ02258.1 awen|OJJ30398.1 azon|OJJ45406.1

Group4421: abra|OJJ77585.1 acar|OOF92640.1 acla|EAW06551.1 afis|EAW16044.1 afla|EED56433.1 afum|EAL88676.1 agla|OJJ86077.1 akaw|GAA84041.1 aluc|OJZ80950.1 anid|CBF75054.1 anig|EHA19404.1 aory|EIT72801.1 asyd|OJJ60029.1 ater|EAU33593.1 atub|OJI82633.1 aver|OJJ02255.1 awen|OJJ30402.1 azon|OJJ45410.1

Group4422: abra|OJJ77586.1 acar|OOF99144.1 acla|EAW06552.1 afis|EAW16043.1 afla|EED56432.1 afum|EAL88675.1 agla|OJJ86076.1 akaw|GAA84040.1 aluc|OJZ80949.1 anid|CBF75052.1 anig|EHA19405.1 aory|EIT72860.1 asyd|OJJ60030.1 ater|EAU33594.1 atub|OJI82634.1 aver|OJJ02254.1 awen|OJJ30403.1 azon|OJJ45411.1

Group4423: abra|OJJ77588.1 acar|OOF99143.1 acla|EAW06554.1 afis|EAW16041.1 afla|EED56430.1 afum|EAL88673.1 agla|OJJ86074.1 akaw|GAA84038.1 aluc|OJZ80947.1 anid|CBF75050.1 anig|EHA19407.1 aory|EIT72854.1 asyd|OJJ60032.1 ater|EAU33596.1 atub|OJI82636.1 aver|OJJ02252.1 awen|OJJ30405.1 azon|OJJ45413.1

Group4424: abra|OJJ77590.1 acar|OOF99141.1 acla|EAW06556.1 afis|EAW16039.1 afla|EED56427.1 afum|EAL88671.1 agla|OJJ86072.1 akaw|GAA84036.1 aluc|OJZ80945.1 anid|CBF75046.1 anig|EHA19409.1 aory|EIT72877.1 asyd|OJJ60034.1 ater|EAU33599.1 atub|OJI82638.1 aver|OJJ02250.1 awen|OJJ30407.1 azon|OJJ45415.1

Group4425: abra|OJJ77598.1 acar|OOF99128.1 acla|EAW06562.1 afis|EAW16034.1 afla|EED56420.1 afum|EAL88665.1 agla|OJJ86066.1 akaw|GAA84030.1 aluc|OJZ80935.1 anid|CBF75036.1 anig|EHA19415.1 aory|EIT72883.1 asyd|OJJ60049.1 ater|EAU33606.1 atub|OJI82645.1 aver|OJJ02240.1 awen|OJJ30413.1 azon|OJJ45421.1

Group4426: abra|OJJ77601.1 acar|OOF99126.1 acla|EAW06565.1 afis|EAW16031.1 afla|EED56413.1 afum|EAL88662.1 agla|OJJ86059.1 akaw|GAA84027.1 aluc|OJZ80932.1 anid|CBF75030.1 anig|EHA19418.1 aory|EIT72887.1 asyd|OJJ60052.1 ater|EAU33610.1 atub|OJI82648.1 aver|OJJ02237.1 awen|OJJ30417.1 azon|OJJ45424.1

Group4427: abra|OJJ77602.1 acar|OOF99124.1 acla|EAW06567.1 afis|EAW16029.1 afla|EED56412.1 afum|EAL88660.1 agla|OJJ86057.1 akaw|GAA84026.1 aluc|OJZ80931.1 anid|CBF75016.1 anig|EHA19419.1 aory|EIT72882.1 asyd|OJJ60070.1 ater|EAU33611.1 atub|OJI82649.1 aver|OJJ04192.1 awen|OJJ30419.1 azon|OJJ45426.1

Group4428: abra|OJJ77603.1 acar|OOF99123.1 acla|EAW06568.1 afis|EAW16028.1 afla|EED56411.1 afum|EAL88658.1 agla|OJJ86056.1 akaw|GAA84025.1 aluc|OJZ80930.1 anid|CBF75018.1 anig|EHA19420.1 aory|EIT72881.1 asyd|OJJ60069.1 ater|EAU33612.1 atub|OJI82650.1 aver|OJJ02226.1 awen|OJJ30420.1 azon|OJJ45427.1

Group4429: abra|OJJ77605.1 acar|OOF99122.1 acla|EAW06569.1 afis|EAW16027.1 afla|EED56410.1 afum|EAL88657.1 agla|OJJ86055.1 akaw|GAA84024.1 aluc|OJZ80928.1 anid|CBF75020.1 anig|EHA19421.1 aory|EIT83014.1 asyd|OJJ60068.1 ater|EAU33613.1 atub|OJI82651.1 aver|OJJ02227.1 awen|OJJ30421.1 azon|OJJ45428.1

Group4430: abra|OJJ77613.1 acar|OOF99115.1 acla|EAW06571.1 afis|EAW16025.1 afla|EED56406.1 afum|EAL88654.1 agla|OJJ86053.1 akaw|GAA84018.1 aluc|OJZ80921.1 anid|CBF75014.1 anig|EHA19426.1 aory|EIT83006.1 asyd|OJJ60057.1 ater|EAU33616.1 atub|OJI82659.1 aver|OJJ02232.1 awen|OJJ30432.1 azon|OJJ45431.1

Group4431: abra|OJJ77614.1 acar|OOF90324.1 acla|EAW11334.1 afis|EAW18161.1 afla|EED56332.1 afum|EAL87521.1 agla|OJJ85965.1 akaw|GAA84017.1 aluc|OJZ80920.1 anid|CBF85769.1 anig|EHA19427.1 aory|EIT73455.1 asyd|OJJ64306.1 ater|EAU33688.1 atub|OJI82660.1 aver|OJI98399.1 awen|OJJ30506.1 azon|OJJ45495.1

Group4432: abra|OJJ77615.1 acar|OOF90323.1 acla|EAW11335.1 afis|EAW18160.1 afla|EED56333.1 afum|EBA27314.1 agla|OJJ85966.1 akaw|GAA84016.1 aluc|OJZ80919.1 anid|CBF85772.1 anig|EHA19428.1 aory|EIT73306.1 asyd|OJJ64308.1 ater|EAU33687.1 atub|OJI82661.1 aver|OJI98400.1 awen|OJJ30505.1 azon|OJJ45494.1

Group4433: abra|OJJ77616.1 acar|OOF90325.1 acla|EAW11336.1 afis|EAW18159.1 afla|EED56335.1 afum|EAL87518.1 agla|OJJ85968.1 akaw|GAA84015.1 aluc|OJZ80918.1 anid|CBF85776.1 anig|EHA19429.1 aory|EIT73326.1 asyd|OJJ64310.1 ater|EAU33686.1 atub|OJI82662.1 aver|OJI98403.1 awen|OJJ30504.1 azon|OJJ45493.1

Group4434: abra|OJJ77617.1 acar|OOF90326.1 acla|EAW11337.1 afis|EAW18158.1 afla|EED56336.1 afum|EAL87517.1 agla|OJJ85969.1 akaw|GAA84014.1 aluc|OJZ80917.1 anid|CBF85777.1 anig|EHA19430.1 aory|EIT73425.1 asyd|OJJ64311.1 ater|EAU33685.1 atub|OJI82663.1 aver|OJI98404.1 awen|OJJ30503.1 azon|OJJ45492.1

Group4435: abra|OJJ77618.1 acar|OOF90327.1 acla|EAW11338.1 afis|EAW18157.1 afla|EED56337.1 afum|EAL87516.1 agla|OJJ85970.1 akaw|GAA84013.1 aluc|OJZ80916.1 anid|CBF85779.1 anig|EHA19431.1 aory|EIT73376.1 asyd|OJJ64312.1 ater|EAU33684.1 atub|OJI82664.1 aver|OJI98405.1 awen|OJJ30502.1 azon|OJJ45491.1

Group4436: abra|OJJ77619.1 acar|OOF90328.1 acla|EAW11339.1 afis|EAW18156.1 afla|EED56338.1 afum|EAL87515.1 agla|OJJ85971.1 akaw|GAA84012.1 aluc|OJZ80915.1 anid|CBF85781.1 anig|EHA19432.1 aory|EIT73330.1 asyd|OJJ64313.1 ater|EAU33683.1 atub|OJI82665.1 aver|OJI98406.1 awen|OJJ30500.1 azon|OJJ45490.1

Group4437: abra|OJJ77620.1 acar|OOF90329.1 acla|EAW11340.1 afis|EAW18155.1 afla|EED56339.1 afum|EAL87514.1 agla|OJJ85972.1 akaw|GAA84011.1 aluc|OJZ80914.1 anid|CBF85783.1 anig|EHA19433.1 aory|EIT73446.1 asyd|OJJ64314.1 ater|EAU33682.1 atub|OJI82666.1 aver|OJI98407.1 awen|OJJ30499.1 azon|OJJ45489.1

Group4438: abra|OJJ77621.1 acar|OOF99114.1 acla|EAW11341.1 afis|EAW18154.1 afla|EED56340.1 afum|EAL87513.1 agla|OJJ85973.1 akaw|GAA84010.1 aluc|OJZ80913.1 anid|CBF85784.1 anig|EHA19434.1 aory|EIT73328.1 asyd|OJJ64316.1 ater|EAU33681.1 atub|OJI82667.1 aver|OJI98409.1 awen|OJJ30498.1 azon|OJJ45488.1

Group4439: abra|OJJ77626.1 acar|OOF99112.1 acla|EAW11342.1 afis|EAW18152.1 afla|EED56342.1 afum|EAL87511.1 agla|OJJ85975.1 akaw|GAA84007.1 aluc|OJZ80905.1 anid|CBF85790.1 anig|EHA19436.1 aory|EIT73287.1 asyd|OJJ64325.1 ater|EAU33677.1 atub|OJI82672.1 aver|OJI98419.1 awen|OJJ30495.1 azon|OJJ45487.1

Group4440: abra|OJJ77629.1 acar|OOF99097.1 acla|EAW06480.1 afis|EAW15975.1 afla|EED56345.1 afum|EAL88607.1 agla|OJJ85980.1 akaw|GAA84005.1 aluc|OJZ80901.1 anid|CBF85795.1 anig|EHA19438.1 aory|EIT73353.1 asyd|OJJ64330.1 ater|EAU33676.1 atub|OJI82678.1 aver|OJI98423.1 awen|OJJ30494.1 azon|OJJ45485.1

Group4441: abra|OJJ77630.1 acar|OOF99098.1 acla|EAW06481.1 afis|EAW15976.1 afla|EED56346.1 afum|EAL88608.1 agla|OJJ85981.1 akaw|GAA84004.1 aluc|OJZ80900.1 anid|CBF85797.1 anig|EHA19439.1 aory|EIT73314.1 asyd|OJJ64331.1 ater|EAU33675.1 atub|OJI82679.1 aver|OJI98424.1 awen|OJJ30493.1 azon|OJJ45484.1

Group4442: abra|OJJ77631.1 acar|OOF99099.1 acla|EAW06482.1 afis|EAW15977.1 afla|EED56347.1 afum|EAL88609.1 agla|OJJ85982.1 akaw|GAA84003.1 aluc|OJZ80898.1 anid|CBF85799.1 anig|EHA19440.1 aory|EIT73397.1 asyd|OJJ64332.1 ater|EAU33674.1 atub|OJI82680.1 aver|OJI98425.1 awen|OJJ30492.1 azon|OJJ45483.1

Group4443: abra|OJJ77632.1 acar|OOF99100.1 acla|EAW06484.1 afis|EAW15979.1 afla|EED56349.1 afum|EAL88611.1 agla|OJJ85984.1 akaw|GAA84001.1 aluc|OJZ80897.1 anid|CBF85803.1 anig|EHA19442.1 aory|EIT73329.1 asyd|OJJ64334.1 ater|EAU33672.1 atub|OJI82681.1 aver|OJI98427.1 awen|OJJ30491.1 azon|OJJ45482.1

Group4444: abra|OJJ77637.1 acar|OOF99105.1 acla|EAW06487.1 afis|EAW15982.1 afla|EED56352.1 afum|EAL88614.1 agla|OJJ85985.1 akaw|GAA83997.1 aluc|OJZ80893.1 anid|CBF85806.1 anig|EHA19445.1 aory|EIT73461.1 asyd|OJJ64337.1 ater|EAU33670.1 atub|OJI82685.1 aver|OJI98430.1 awen|OJJ30488.1 azon|OJJ45479.1

Group4445: abra|OJJ77638.1 acar|OOF99106.1 acla|EAW06488.1 afis|EAW15983.1 afla|EED56353.1 afum|EAL88615.1 agla|OJJ85986.1 akaw|GAA83996.1 aluc|OJZ80892.1 anid|CBF85808.1 anig|EHA19446.1 aory|EIT73319.1 asyd|OJJ64339.1 ater|EAU33669.1 atub|OJI82686.1 aver|OJI98432.1 awen|OJJ30487.1 azon|OJJ45478.1

Group4446: abra|OJJ77640.1 acar|OOF99108.1 acla|EAW06490.1 afis|EAW15985.1 afla|EED56355.1 afum|EAL88617.1 agla|OJJ85988.1 akaw|GAA83994.1 aluc|OJZ80890.1 anid|CBF85812.1 anig|EHA19448.1 aory|EIT73389.1 asyd|OJJ64341.1 ater|EAU33667.1 atub|OJI82688.1 aver|OJI98434.1 awen|OJJ30485.1 azon|OJJ45476.1

Group4447: abra|OJJ77642.1 acar|OOF99094.1 acla|EAW06492.1 afis|EAW15988.1 afla|EED56360.1 afum|EAL88620.1 agla|OJJ85990.1 akaw|GAA83992.1 aluc|OJZ80888.1 anid|CBF85815.1 anig|EHA19450.1 aory|EIT73300.1 asyd|OJJ64342.1 ater|EAU33661.1 atub|OJI82690.1 aver|OJI98435.1 awen|OJJ30482.1 azon|OJJ45473.1

Group4448: abra|OJJ77644.1 acar|OOF99091.1 acla|EAW07074.1 afis|EAW20698.1 afla|EED46367.1 afum|EAL84873.1 akaw|GAA83990.1 aluc|OJZ80885.1 anid|CBF86210.1 anig|EHA19452.1 aory|EIT81200.1

asyd|OJJ64550.1 atub|OJI82692.1 aver|OJI98646.1 awen|OJJ36028.1 acar|OOF98893.1 afis|EAW20291.1 afum|EAL85423.1

Group4449: abra|OJJ77652.1 acar|OOF99084.1 acla|EAW06494.1 afis|EAW15990.1 afla|EED56362.1 afum|EAL88622.1 agla|OJJ85992.1 akaw|GAA83983.1 aluc|OJZ80877.1 anid|CBF85819.1 anig|EHA19459.1 aory|EIT73339.1 asyd|OJJ64343.1 ater|EAU33658.1 atub|OJI82700.1 aver|OJI98440.1 awen|OJJ30480.1 azon|OJJ45471.1

Group4450: abra|OJJ77654.1 acar|OOF99083.1 acla|EAW06496.1 afis|EAW15992.1 afla|EED56364.1 afum|EAL88624.2 agla|OJJ86003.1 akaw|GAA83981.1 aluc|OJZ80875.1 anid|CBF85823.1 anig|EHA19460.1 aory|EIT73404.1 asyd|OJJ64344.1 ater|EAU33657.1 atub|OJI82702.1 aver|OJI98442.1 awen|OJJ30478.1 azon|OJJ45470.1

Group4451: abra|OJJ77655.1 acar|OOF99081.1 acla|EAW06497.1 afis|EAW15993.1 afla|EED56365.1 afum|EAL88625.1 agla|OJJ86004.1 akaw|GAA83980.1 aluc|OJZ80874.1 anid|CBF85824.1 anig|EHA19462.1 aory|EIT73307.1 asyd|OJJ64345.1 ater|EAU33656.1 atub|OJI82704.1 aver|OJI98443.1 awen|OJJ30477.1 azon|OJJ45469.1

Group4452: abra|OJJ77656.1 acar|OOF99080.1 acla|EAW06498.1 afis|EAW15994.1 afla|EED56367.1 afum|EAL88626.1 agla|OJJ86008.1 akaw|GAA83979.1 aluc|OJZ80872.1 anid|CBF85828.1 anig|EHA19463.1 asyd|OJJ64348.1 ater|EAU33654.1 atub|OJI82705.1 aver|OJI98445.1 awen|OJJ30475.1 azon|OJJ45467.1 aory|EIT73315.1

Group4453: abra|OJJ77657.1 acar|OOF99079.1 acla|EAW06499.1 afis|EAW15995.1 afla|EED56369.1 afum|EAL88627.1 agla|OJJ86009.1 akaw|GAA83978.1 aluc|OJZ80871.1 anid|CBF85830.1 anig|EHA19464.1 aory|EIT73436.1 asyd|OJJ64349.1 ater|EAU33652.1 atub|OJI82706.1 aver|OJI98446.1 awen|OJJ30474.1 azon|OJJ45466.1

Group4454: abra|OJJ77659.1 acar|OOF99076.1 acla|EAW06501.1 afis|EAW15997.1 afla|EED56371.1 afum|EAL88629.1 agla|OJJ86017.1 akaw|GAA83976.1 aluc|OJZ80869.1 anid|CBF85832.1 anig|EHA19466.1 aory|EIT73442.1 asyd|OJJ64350.1 ater|EAU33649.1 atub|OJI82708.1 aver|OJI98447.1 awen|OJJ30472.1 azon|OJJ45464.1

Group4455: abra|OJJ77660.1 acar|OOF99075.1 acla|EAW06504.1 afis|EAW15998.1 afla|EED56372.1 afum|EAL88630.1 agla|OJJ86013.1 akaw|GAA83975.1 aluc|OJZ80868.1 anid|CBF85834.1 anig|EHA19467.1 aory|EIT73360.1 asyd|OJJ64351.1 ater|EAU33648.1 atub|OJI82709.1 aver|OJI98448.1 awen|OJJ30471.1 azon|OJJ45463.1

Group4456: abra|OJJ77661.1 acar|OOF99073.1 acla|EAW06598.1 afis|EAW16006.1 afla|EED56380.1 afum|EAL88638.2 agla|OJJ86025.1 akaw|GAA83974.1 aluc|OJZ80867.1 anid|CBF85868.1 anig|EHA19468.1 aory|EIT73448.1 asyd|OJJ64371.1 ater|EAU33637.1 atub|OJI82710.1 aver|OJI98467.1 awen|OJJ30461.1 azon|OJJ45451.1

Group4457: abra|OJJ77665.1 acar|OOF99070.1 acla|EAW06507.1 afis|EAW16002.1 afla|EED56376.1 afum|EAL88634.1 agla|OJJ86024.1 akaw|GAA83970.1 aluc|OJZ80863.1 anid|CBF85844.1 anig|EHA19472.1 aory|EIT73423.1 asyd|OJJ64356.1 ater|EAU33643.1 atub|OJI82714.1 aver|OJI98452.1 awen|OJJ30466.1 azon|OJJ45457.1

Group4458: abra|OJJ77667.1 acar|OOF99068.1 acla|EAW06506.1 afis|EAW16000.1 afla|EED56375.1 afum|EAL88633.1 agla|OJJ86020.1 akaw|GAA83962.1 aluc|OJZ80850.1 anid|CBF85840.1 anig|EHA19474.1 aory|EIT73310.1 asyd|OJJ64354.1 ater|EAU33645.1 atub|OJI82718.1 aver|OJI98450.1 awen|OJJ30469.1 azon|OJJ45461.1

Group4459: abra|OJJ77671.1 acar|OOF99066.1 acla|EAW06505.1 afis|EAW15999.1 afla|EED56374.1 afum|EAL88631.1 agla|OJJ86019.1 akaw|GAA83961.1 aluc|OJZ80848.1 anid|CBF85835.1 anig|EHA19475.1 aory|EIT73377.1 asyd|OJJ64352.1 ater|EAU33647.1 atub|OJI82719.1 aver|OJI98449.1 awen|OJJ30470.1 azon|OJJ45462.1

Group4460: abra|OJJ77680.1 acar|OOF99060.1 acla|EAW06591.1 afis|EAW16014.1 afla|EED56391.1 afum|EAL88643.1 agla|OJJ86040.1 akaw|GAA83954.1 aluc|OJZ80838.1 anid|CBF74562.1 anig|EHA19482.1 aory|EIT83009.1 asyd|OJJ57647.1 ater|EAU33630.1 atub|OJI82730.1 aver|OJJ05504.1 awen|OJJ30450.1 azon|OJJ45442.1

Group4461: abra|OJJ77681.1 acar|OOF99058.1 acla|EAW06592.1 afis|EAW16015.1 afla|EED56392.1 afum|EAL88644.1 agla|OJJ86041.1 akaw|GAA83952.1 aluc|OJZ80836.1 anid|CBF74560.1 anig|EHA19483.1 aory|EIT83002.1 asyd|OJJ57646.1 ater|EAU33629.1 atub|OJI82732.1 aver|OJJ05503.1 awen|OJJ30449.1 azon|OJJ45441.1

Group4462: abra|OJJ77682.1 acar|OOF99059.1 acla|EAW06594.1 afis|EAW16017.1 afla|EED56393.1 afum|EAL88646.1 agla|OJJ86042.1 akaw|GAA83951.1 aluc|OJZ80835.1 anid|CBF74558.1 anig|EHA19484.1 aory|EIT83003.1 asyd|OJJ57645.1 ater|EAU33628.1 atub|OJI82733.1 aver|OJJ05502.1 awen|OJJ30447.1 azon|OJJ45439.1

Group4463: abra|OJJ77683.1 acar|OOF99057.1 acla|EAW06595.1 afis|EAW16018.1 afla|EED56394.1 afum|EAL88647.1 agla|OJJ86045.1 akaw|GAA83950.1 aluc|OJZ80834.1 anid|CBF74555.1 anig|EHA19485.1 aory|EIT83010.1 asyd|OJJ57644.1 ater|EAU33627.1 atub|OJI82734.1 aver|OJJ05501.1 awen|OJJ30446.1 azon|OJJ45438.1

Group4464: abra|OJJ77684.1 acar|OOF99056.1 acla|EAW06596.1 afis|EAW16019.1 afla|EED56396.1 afum|EAL88648.1 agla|OJJ86046.1 akaw|GAA83949.1 aluc|OJZ80833.1 anid|CBF74553.1 anig|EHA19486.1 aory|EIT83017.1 asyd|OJJ57642.1 ater|EAU33626.1 atub|OJI82735.1 aver|OJJ05499.1 awen|OJJ30445.1 azon|OJJ45437.1

Group4465: abra|OJJ77690.1 acar|OOF99049.1 acla|EAW06575.1 afis|EAW16021.1 afla|EED56399.1 afum|EAL88650.1 agla|OJJ86048.1 akaw|GAA83944.1 aluc|OJZ80825.1 anid|CBF74538.1 anig|EHA19490.1 aory|EIT83016.1 asyd|OJJ57695.1 ater|EAU33619.1 atub|OJI82740.1 aver|OJJ05471.1 awen|OJJ30443.1 azon|OJJ45435.1

Group4466: abra|OJJ77691.1 acar|OOF99048.1 acla|EAW06574.1 afis|EAW16022.1 afla|EED56401.1 afum|EAL88651.1 agla|OJJ86051.1 akaw|GAA83939.1 aluc|OJZ80821.1 anid|CBF74540.1 anig|EHA19491.1 aory|EIT83005.1 asyd|OJJ57694.1 ater|EAU33618.1 atub|OJI82741.1 aver|OJJ05472.1 awen|OJJ30439.1 azon|OJJ45434.1

Group4467: abra|OJJ77695.1 acar|OOF99044.1 acla|EAW11326.1 afis|EAW18169.1 afla|EED56325.1 afum|EBA27315.1 agla|OJJ85948.1 akaw|GAA83935.1 aluc|OJZ90758.1 anid|CBF85744.1 anig|EHA19495.1 aory|EIT73371.1 asyd|OJJ64290.1 ater|EAU33835.1 atub|OJI82745.1 aver|OJI98383.1 awen|OJJ30516.1 azon|OJJ50197.1

Group4468: abra|OJJ77697.1 acar|OOF99043.1 acla|EAW11327.1 afis|EAW18168.1 afla|EED56326.1 afum|EAL87529.2 agla|OJJ85949.1 akaw|GAA83934.1 aluc|OJZ90761.1 anid|CBF85746.1 anig|EHA19496.1 aory|EIT73447.1 asyd|OJJ64291.1 ater|EAU33836.1 atub|OJI82748.1 aver|OJI98384.1 awen|OJJ30514.1 azon|OJJ50195.1

Group4469: abra|OJJ77699.1 acar|OOF99040.1 acla|EAW11324.1 afis|EAW18171.1 afla|EED56322.1 afum|EAL87533.1 agla|OJJ85935.1 akaw|GAA83931.1 aluc|OJZ90764.1 anid|CBF85742.1 anig|EHA19498.1 aory|EIT73405.1 asyd|OJJ64289.1 ater|EAU33833.1 atub|OJI82752.1 aver|OJI98382.1 awen|OJJ30518.1 azon|OJJ50199.1

Group4470: abra|OJJ77704.1 acar|OOF99036.1 acla|EAW11315.1 afis|EAW18183.1 afla|EED56304.1 afum|EAL87545.1 agla|OJJ85903.1 akaw|GAA83926.1 aluc|OJZ90769.1 anid|CBF85723.1 anig|EHA19504.1 aory|EIT73331.1 asyd|OJJ64277.1 ater|EAU33819.1 atub|OJI82757.1 aver|OJI98369.1 awen|OJJ30531.1 azon|OJJ50212.1

Group4471: abra|OJJ77708.1 acar|OOF99032.1 acla|EAW11317.1 afis|EAW18179.1 afla|EED56307.1 afum|EAL87540.1 agla|OJJ85910.1 akaw|GAA83922.1 aluc|OJZ90773.1 anid|CBF85729.1 anig|EHA19507.1 aory|EIT73380.1 asyd|OJJ64280.1 ater|EAU33821.1 atub|OJI82760.1 aver|OJI98372.1 awen|OJJ30527.1 azon|OJJ50208.1

Group4472: abra|OJJ77709.1 acar|OOF99031.1 acla|EAW11318.1 afis|EAW18178.1 afla|EED56306.1 afum|EAL87539.1 agla|OJJ85912.1 akaw|GAA83921.1 aluc|OJZ90775.1 anid|CBF85732.1 anig|EHA19508.1 aory|EIT73415.1 asyd|OJJ64283.1 ater|EAU33827.1 atub|OJI82761.1 aver|OJI98375.1 awen|OJJ30526.1 azon|OJJ50207.1

Group4473: abra|OJJ77710.1 acar|OOF99030.1 acla|EAW11319.1 afis|EAW18177.1 afla|EED56312.1 afum|EAL87538.1 agla|OJJ85913.1 akaw|GAA83920.1 aluc|OJZ90776.1 anid|CBF85733.1 anig|EHA19509.1 aory|EIT73345.1 asyd|OJJ64284.1 ater|EAU33828.1 atub|OJI82762.1 aver|OJI98376.1 awen|OJJ30525.1 azon|OJJ50206.1

Group4474: abra|OJJ77717.1 acar|OOF99022.1 acla|EAW11320.1 afis|EAW18176.1 afla|EED56313.1 afum|EAL87537.1 agla|OJJ85914.1 akaw|GAA83915.1 aluc|OJZ90782.1 anid|CBF85735.1 anig|EHA19515.1 aory|EIT73299.1 asyd|OJJ64285.1 ater|EAU33829.1 atub|OJI82769.1 aver|OJI98377.1 awen|OJJ30524.1 azon|OJJ50205.1

Group4475: abra|OJJ77718.1 acar|OOF99019.1 acla|EAW11321.1 afis|EAW18175.1 afla|EED56314.1 afum|EAL87536.1 agla|OJJ85915.1 akaw|GAA83914.1 aluc|OJZ90783.1 anid|CBF85737.1 anig|EHA19516.1 aory|EIT73381.1 asyd|OJJ64286.1 ater|EAU33830.1 atub|OJI82770.1 aver|OJI98378.1 awen|OJJ30523.1 azon|OJJ50204.1

Group4476: abra|OJJ77810.1 acar|OOF98957.1 acla|EAW11307.1 afis|EAW18194.1 afla|EED56284.1 afum|EBA27318.1 agla|OJJ85895.1 akaw|GAA83839.1 aluc|OJZ90877.1 anid|CBF86104.1 anig|EHA19597.1 aory|EIT73433.1 asyd|OJJ64482.1 ater|EAU33799.1 atub|OJI82874.1 aver|OJI98583.1 awen|OJJ30558.1 azon|OJJ45509.1

Group4477: abra|OJJ77811.1 acar|OOF98956.1 acla|EAW11306.1 afis|EAW18193.1 afla|EED56283.1 afum|EAL87559.1 agla|OJJ85893.1 akaw|GAA83838.1 aluc|OJZ90878.1 anid|CBF86108.1 anig|EHA19599.1

aory|EIT73325.1 asyd|OJJ64487.1 ater|EAU33798.1 atub|OJI82875.1 aver|OJI98588.1 awen|OJJ30559.1 azon|OJJ45508.1

Group4478: abra|OJJ77819.1 acar|OOF98949.1 acla|EAW11304.1 afis|EAW18191.1 afla|EED56281.1 afum|EAL87562.2 agla|OJJ85884.1 akaw|GAA83827.1 aluc|OJZ90891.1 anid|CBF86111.1 anig|EHA19610.1 aory|EIT73396.1 asyd|OJJ64489.1 ater|EAU33796.1 atub|OJI82885.1 aver|OJI98590.1 awen|OJJ30565.1 azon|OJJ45506.1

Group4479: abra|OJJ77821.1 acar|OOF98945.1 acla|EAW11303.1 afis|EAW18199.1 afla|EED56279.1 afum|EAL87564.1 agla|OJJ85883.1 akaw|GAA83824.1 aluc|OJZ90894.1 anid|CBF86115.1 anig|EHA19613.1 aory|EIT73401.1 asyd|OJJ64491.1 ater|EAU33795.1 atub|OJI82887.1 aver|OJI98593.1 awen|OJJ30567.1 azon|OJJ45505.1

Group4480: abra|OJJ77824.1 acar|OOF98944.1 acla|EAW11301.1 afis|EAW18201.1 afla|EED56277.1 afum|EAL87566.1 agla|OJJ85881.1 akaw|GAA83822.1 aluc|OJZ90897.1 anid|CBF86122.1 anig|EHA19615.1 aory|EIT73449.1 asyd|OJJ64496.1 ater|EAU33793.1 atub|OJI82889.1 aver|OJI98598.1 awen|OJJ30569.1 azon|OJJ45503.1

Group4481: abra|OJJ77826.1 acar|OOF98942.1 acla|EAW11299.1 afis|EAW18203.1 afla|EED56269.1 afum|EAL87568.1 agla|OJJ85879.1 akaw|GAA83820.1 aluc|OJZ90900.1 anid|CBF86119.1 anig|EHA19616.1 aory|EIT73413.1 asyd|OJJ64494.1 ater|EAU33786.1 atub|OJI82893.1 aver|OJI98596.1 awen|OJJ30571.1 azon|OJJ45501.1

Group4482: abra|OJJ77827.1 acar|OOF98941.1 acla|EAW11298.1 afis|EAW18204.1 afla|EED56268.1 afum|EAL87569.1 agla|OJJ85878.1 akaw|GAA83819.1 aluc|OJZ90901.1 anid|CBF86124.1 anig|EHA19617.1 aory|EIT73341.1 asyd|OJJ64497.1 ater|EAU33785.1 atub|OJI82894.1 aver|OJI98599.1 awen|OJJ30572.1 azon|OJJ45500.1

Group4483: abra|OJJ77830.1 acar|OOF98940.1 acla|EAW11296.1 afis|EAW18206.1 afla|EED56265.1 afum|EAL87571.1 agla|OJJ85876.1 akaw|GAA83817.1 aluc|OJZ90904.1 anid|CBF86132.1 anig|EHA19619.1 aory|EIT73429.1 asyd|OJJ64502.1 ater|EAU33783.1 atub|OJI82897.1 aver|OJI98601.1 awen|OJJ30578.1 azon|OJJ45498.1

Group4484: abra|OJJ77834.1 acar|OOF98937.1 acla|EAW11289.1 afis|EAW18210.1 afla|EED56260.1 afum|EAL87576.2 agla|OJJ85872.1 akaw|GAA83813.1 aluc|OJZ90908.1 anid|CBF86139.1 anig|EHA19623.1 aory|EIT73444.1 asyd|OJJ64506.1 ater|EAU33778.1 atub|OJI82901.1 aver|OJI98605.1 awen|OJJ30588.1 azon|OJJ50189.1

Group4485: abra|OJJ77841.1 acar|OOF98930.1 acla|EAW11282.1 afis|EAW18218.1 afla|EED56252.1 afum|EAL87584.1 agla|OJJ85864.1 akaw|GAA83806.1 aluc|OJZ90916.1 anid|CBF86148.1 anig|EHA19628.1 aory|EIT73348.1 asyd|OJJ64512.1 ater|EAU33773.1 atub|OJI82908.1 aver|OJI98610.1 awen|OJJ30596.1 azon|OJJ50172.1

Group4486: abra|OJJ77844.1 acar|OOF98928.1 acla|EAW11279.1 afis|EAW18221.1 afla|EED56249.1 afum|EAL87587.1 agla|OJJ85861.1 akaw|GAA83803.1 aluc|OJZ90919.1 anid|CBF86155.1 anig|EHA19631.1 aory|EIT73362.1 asyd|OJJ64515.1 ater|EAU33770.1 atub|OJI82911.1 aver|OJI98613.1 awen|OJJ30599.1 azon|OJJ50169.1

Group4487: abra|OJJ77845.1 acar|OOF98927.1 acla|EAW11278.1 afis|EAW18222.1 afla|EED56247.1 afum|EAL87588.1 agla|OJJ85860.1 akaw|GAA83802.1 aluc|OJZ90920.1 anid|CBF86153.1 anig|EHA19632.1 aory|EIT73414.1 asyd|OJJ64516.1 ater|EAU33769.1 atub|OJI82912.1 aver|OJI98614.1 awen|OJJ30600.1 azon|OJJ50168.1

Group4488: abra|OJJ77847.1 acar|OOF98925.1 acla|EAW11276.1 afis|EAW18224.1 afla|EED56245.1 afum|EAL87590.1 agla|OJJ85858.1 akaw|GAA83800.1 aluc|OJZ90922.1 anid|CBF86159.1 anig|EHA19634.1 aory|EIT73297.1 asyd|OJJ64518.1 ater|EAU33767.1 atub|OJI82914.1 aver|OJI98616.1 awen|OJJ30602.1 azon|OJJ50166.1

Group4489: abra|OJJ77851.1 acar|OOF98922.1 acla|EAW11275.1 afis|EAW18225.1 afla|EED56244.1 afum|EAL87591.1 agla|OJJ85857.1 akaw|GAA83797.1 aluc|OJZ90925.1 anid|CBF86161.1 anig|EHA19637.1 aory|EIT73369.1 asyd|OJJ64519.1 ater|EAU33766.1 atub|OJI82917.1 aver|OJI98617.1 awen|OJJ30603.1 azon|OJJ50165.1

Group4490: abra|OJJ77852.1 acar|OOF98921.1 acla|EAW11274.1 afis|EAW18226.1 afla|EED56243.1 afum|EAL87593.2 agla|OJJ85856.1 akaw|GAA83796.1 aluc|OJZ90926.1 anid|CBF86162.1 anig|EHA19638.1 aory|EIT73293.1 asyd|OJJ64520.1 ater|EAU33765.1 atub|OJI82918.1 aver|OJI98618.1 awen|OJJ30604.1 azon|OJJ50164.1

Group4491: abra|OJJ77853.1 acar|OOF98920.1 acla|EAW11273.1 afis|EAW18227.1 afla|EED56242.1 afum|EAL87594.1 agla|OJJ85855.1 akaw|GAA83795.1 aluc|OJZ90927.1 anid|CBF86164.1 anig|EHA19639.1 aory|EIT73313.1 asyd|OJJ64521.1 ater|EAU33764.1 atub|OJI82919.1 aver|OJI98619.1 awen|OJJ30605.1 azon|OJJ50163.1

Group4492: abra|OJJ77854.1 acar|OOF98919.1 acla|EAW11272.1 afis|EAW18228.1 afla|EED56241.1 afum|EAL87595.1 agla|OJJ85854.1 akaw|GAA83794.1 aluc|OJZ90928.1 anid|CBF86166.1 anig|EHA19640.1 aory|EIT73375.1 asyd|OJJ64522.1 ater|EAU33763.1 atub|OJI82920.1 aver|OJI98620.1 awen|OJJ30606.1 azon|OJJ50162.1

Group4493: abra|OJJ77857.1 acar|OOF98917.1 acla|EAW11269.1 afis|EAW18230.1 afla|EED56238.1 afum|EAL87597.1 agla|OJJ85844.1 akaw|GAA83792.1 aluc|OJZ90931.1 anid|CBF86172.1 anig|EHA19642.1 aory|EIT73450.1 asyd|OJJ64525.1 ater|EAU33761.1 atub|OJI82923.1 aver|OJI98623.1 awen|OJJ30609.1 azon|OJJ50159.1

Group4494: abra|OJJ77862.1 acar|OOF98911.1 acla|EAW11264.1 afis|EAW18234.1 afla|EED56232.1 afum|EBA27319.1 agla|OJJ85850.1 akaw|GAA83787.1 aluc|OJZ90937.1 anid|CBF86182.1 anig|EHA19645.1 aory|EIT73318.1 asyd|OJJ64530.1 ater|EAU33752.1 atub|OJI82928.1 aver|OJI98628.1 awen|OJJ30614.1 azon|OJJ48003.1

Group4495: abra|OJJ77864.1 acar|OOF98909.1 acla|EAW11262.1 afis|EAW18236.1 afla|EED56230.1 afum|EAL87603.1 agla|OJJ85838.1 akaw|GAA83785.1 aluc|OJZ90939.1 anid|CBF86185.1 anig|EHA19647.1 aory|EIT73458.1 asyd|OJJ64532.1 ater|EAU33749.1 atub|OJI82930.1 aver|OJI98630.1 awen|OJJ30616.1 azon|OJJ48001.1

Group4496: abra|OJJ77865.1 acar|OOF98907.1 acla|EAW11261.1 afis|EAW18237.1 afla|EED56229.1 afum|EAL87604.2 agla|OJJ85837.1 akaw|GAA83784.1 aluc|OJZ90940.1 anid|CBF86187.1 anig|EHA19648.1 aory|EIT73322.1 asyd|OJJ64534.1 ater|EAU33748.1 atub|OJI82931.1 aver|OJI98631.1 awen|OJJ30617.1 azon|OJJ48000.1

Group4497: abra|OJJ77866.1 acar|OOF98906.1 acla|EAW11259.1 afis|EAW18239.1 afla|EED56227.1 afum|EBA27321.1 agla|OJJ85831.1 akaw|GAA83782.1 aluc|OJZ90942.1 anid|CBF86191.1 anig|EHA19650.1 aory|EIT73427.1 asyd|OJJ64536.1 ater|EAU33744.1 atub|OJI82933.1 aver|OJI98633.1 awen|OJJ30619.1 azon|OJJ47998.1

Group4498: abra|OJJ77876.1 acar|OOF98895.1 acla|EAW06509.1 afis|EAW16222.1 afla|EED50188.1 afum|EAL88850.1 agla|OJJ86234.1 akaw|GAA83776.1 aluc|OJZ90951.1 anid|CBF86216.1 anig|EHA19657.1 aory|EIT77385.1 asyd|OJJ64558.1 ater|EAU33719.1 atub|OJI82942.1 aver|OJI98655.1 awen|OJJ30248.1 azon|OJJ43951.1

Group4499: abra|OJJ77878.1 acar|OOF98894.1 acla|EAW06602.1 afis|EAW16221.1 afla|EED50189.1 afum|EAL88849.1 agla|OJJ86233.1 akaw|GAA83775.1 aluc|OJZ90952.1 anid|CBF86215.1 anig|EHA19658.1 aory|EIT77381.1 asyd|OJJ64557.1 ater|EAU33720.1 atub|OJI82943.1 aver|OJI98654.1 awen|OJJ30250.1 azon|OJJ43952.1

Group4500: abra|OJJ77888.1 acar|OOF90363.1 afis|EAW17356.1 afla|EED49463.1 agla|OJJ82473.1 akaw|GAA82231.1 aluc|OJZ91917.1 anig|EHA19663.1 aory|EIT79554.1 asyd|OJJ56937.1 ater|EAU32565.1 aver|OJJ05741.1 awen|OJJ32672.1 aory|EIT75760.1 asyd|OJJ57437.1 awen|OJJ31967.1 afla|EED45648.1 asyd|OJJ57600.1

Group4501: abra|OJJ77890.1 acar|OOF98890.1 acla|EAW11245.1 afis|EAW18255.1 afla|EED56196.1 afum|EAL87618.1 agla|OJJ88189.1 akaw|GAA83768.1 aluc|OJZ90960.1 anid|CBF86206.1 anig|EHA19665.1 aory|EIT73437.1 asyd|OJJ64546.1 ater|EAU33727.1 atub|OJI82951.1 aver|OJI98642.1 awen|OJJ30629.1 azon|OJJ46567.1

Group4502: abra|OJJ77893.1 acar|OOF98889.1 acla|EAW11246.1 afis|EAW18254.1 afla|EED56198.1 afum|EAL87617.1 agla|OJJ88190.1 akaw|GAA83767.1 aluc|OJZ90963.1 anid|CBF86205.1 anig|EHA19667.1 aory|EIT73303.1 asyd|OJJ64545.1 ater|EAU33726.1 atub|OJI82954.1 aver|OJI98641.1 awen|OJJ30628.1 azon|OJJ47987.1

Group4503: abra|OJJ77897.1 acar|OOF98887.1 acla|EAW11248.1 afis|EAW18249.1 afla|EED56215.1 afum|EAL87614.1 agla|OJJ85821.1 akaw|GAA83765.1 aluc|OJZ90967.1 anid|CBF86200.1 anig|EHA19669.1 aory|EIT73361.1 asyd|OJJ64542.1 ater|EAU33731.1 atub|OJI82957.1 aver|OJI98638.1 awen|OJJ30625.1 azon|OJJ47993.1

Group4504: abra|OJJ77898.1 acar|OOF98886.1 acla|EAW11249.1 afis|EAW18248.1 afla|EED56216.1 afum|EAL87613.1 agla|OJJ85822.1 akaw|GAA83764.1 aluc|OJZ90968.1 anid|CBF86198.1 anig|EHA19670.1 aory|EIT73459.1 asyd|OJJ64541.1 ater|EAU33732.1 atub|OJI82958.1 aver|OJI98637.1 awen|OJJ30624.1 azon|OJJ47994.1

Group4505: abra|OJJ77902.1 acar|OOF98881.1 acla|EAW06512.1 afis|EAW16230.1 afla|EED53351.1 afum|EAL88853.1 agla|OJJ86245.1 akaw|GAA83760.1 aluc|OJZ90971.1 anid|CBF86223.1 anig|EHA19673.1 aory|EIT75232.1 asyd|OJJ64563.1 ater|EAU33714.1 atub|OJI82962.1 aver|OJI98660.1 awen|OJJ30232.1 azon|OJJ43949.1

Group4506: abra|OJJ77903.1 acar|OOF98879.1 acla|EAW06513.1 afis|EAW16231.1 afla|EED53350.1 afum|EAL88854.1 agla|OJJ86244.1 akaw|GAA83759.1 aluc|OJZ90972.1 anid|CBF86224.1 anig|EHA19674.1

aory|EIT74926.1 asyd|OJJ64564.1 ater|EAU33713.1 atub|OJI82963.1 aver|OJI98661.1 awen|OJJ30231.1 azon|OJJ43948.1

Group4507: abra|OJJ77910.1 acar|OOF98875.1 acla|EAW06518.1 afis|EAW16236.1 afla|EED53345.1 afum|EAL88857.1 agla|OJJ86239.1 akaw|GAA83749.1 aluc|OJZ90982.1 anid|CBF86232.1 anig|EHA19687.1 aory|EIT74884.1 asyd|OJJ64566.1 ater|EAU33708.1 atub|OJI82973.1 aver|OJI98663.1 awen|OJJ30224.1 azon|OJJ43944.1

Group4508: anig|EHA19693.1 abra|OJJ77921.1 acar|OOF98864.1 acla|EAW06687.1 afis|EAW16078.1 afla|EED48598.1 afum|EAL88710.1 agla|OJJ86111.1 akaw|GAA83742.1 aluc|OJZ90989.1 anid|CBF75119.1 aory|EIT73211.1 asyd|OJJ59993.1 ater|EAU33144.1 atub|OJI82980.1 aver|OJJ02298.1 awen|OJJ30355.1 azon|OJJ45377.1

Group4509: abra|OJJ77936.1 acar|OOF98872.1 acla|EAW06678.1 afis|EAW16087.1 afla|EED48608.1 afum|EAL88719.1 agla|OJJ86119.1 akaw|GAA83727.1 aluc|OJZ91004.1 anid|CBF75125.1 anig|EHA19709.1 aory|EIT73213.1 asyd|OJJ59984.1 ater|EAU33157.1 atub|OJI82995.1 aver|OJJ02307.1 awen|OJJ30344.1 azon|OJJ45369.1

Group4510: abra|OJJ77937.1 acar|OOF98873.1 acla|EAW06677.1 afis|EAW16088.1 afla|EED48609.1 afum|EAL88720.1 agla|OJJ86120.1 akaw|GAA83726.1 aluc|OJZ91005.1 anid|CBF75127.1 anig|EHA19710.1 aory|EIT73207.1 asyd|OJJ59983.1 ater|EAU33159.1 atub|OJI82996.1 aver|OJJ02308.1 awen|OJJ30343.1 azon|OJJ45368.1

Group4511: abra|OJJ77968.1 acar|OOF98847.1 acla|EAW06672.1 afis|EAW16093.1 afla|EED48629.1 afum|EAL88725.1 akaw|GAA83694.1 aluc|OJZ91038.1 anid|CBF85460.1 anig|EHA19737.1 aory|EIT73172.1 asyd|OJJ64101.1 ater|EAU33165.1 atub|OJI83034.1 aver|OJI98184.1 awen|OJJ30337.1 azon|OJJ45367.1 agla|OJJ86128.1

Group4512: abra|OJJ77971.1 acar|OOF98843.1 acla|EAW06670.1 afis|EAW16095.1 afla|EED48631.1 afum|EAL88727.1 agla|OJJ86126.1 akaw|GAA83691.1 aluc|OJZ91041.1 anid|CBF85455.1 anig|EHA19740.1 aory|EIT73094.1 asyd|OJJ64098.1 ater|EAU33171.1 atub|OJI83037.1 aver|OJI98181.1 awen|OJJ30335.1 azon|OJJ45363.1

Group4513: abra|OJJ77972.1 acar|OOF98842.1 acla|EAW06667.1 afis|EAW16098.1 afla|EED48633.1 afum|EAL88730.1 agla|OJJ86124.1 akaw|GAA83689.1 aluc|OJZ91043.1 anid|CBF85451.1 anig|EHA19742.1 aory|EIT73279.1 asyd|OJJ64096.1 ater|EAU33173.1 atub|OJI83039.1 aver|OJI98179.1 awen|OJJ30333.1 azon|OJJ45362.1

Group4514: abra|OJJ77973.1 acar|OOF98841.1 acla|EAW06665.1 afis|EAW16100.1 afla|EED48635.1 afum|EAL88732.1 agla|OJJ86122.1 anid|CBF85447.1 anig|EHA19743.1 aory|EIT73131.1 asyd|OJJ64095.1 ater|EAU33174.1 aver|OJI98177.1 awen|OJJ30332.1 azon|OJJ45360.1 akaw|GAA83687.1 aluc|OJZ91045.1 atub|OJI83041.1

Group4515: abra|OJJ77988.1 acar|OOF98835.1 acla|EAW06661.1 afis|EAW16104.1 afla|EED48640.1 afum|EAL88736.1 agla|OJJ86133.1 akaw|GAA85992.1 aluc|OJZ91063.1 anid|CBF85480.1 anig|EHA19767.1 aory|EIT73283.1 asyd|OJJ64108.1 ater|EAU33183.1 atub|OJI83059.1 aver|OJI98190.1 awen|OJJ30328.1 azon|OJJ45345.1

Group4516: abra|OJJ77989.1 acar|OOF98834.1 acla|EAW06660.1 afis|EAW16105.1 afla|EED48641.1 afum|EAL88737.1 agla|OJJ86134.1 akaw|GAA85991.1 aluc|OJZ91064.1 anid|CBF85482.1 anig|EHA19768.1 aory|EIT73197.1 asyd|OJJ64109.1 ater|EAU33184.1 atub|OJI83060.1 aver|OJI98191.1 awen|OJJ30327.1 azon|OJJ45344.1

Group4517: abra|OJJ77990.1 acar|OOF98833.1 acla|EAW06659.1 afis|EAW16106.1 afla|EED48642.1 afum|EAL88738.1 agla|OJJ86135.1 akaw|GAA85990.1 aluc|OJZ91066.1 anid|CBF85484.1 anig|EHA19769.1 aory|EIT73122.1 asyd|OJJ64110.1 ater|EAU33185.1 atub|OJI83061.1 aver|OJI98192.1 awen|OJJ30325.1 azon|OJJ45343.1

Group4518: abra|OJJ77991.1 acar|OOF98831.1 acla|EAW06658.1 afis|EAW16107.1 afla|EED48643.1 afum|EAL88739.2 agla|OJJ86136.1 akaw|GAA85989.1 aluc|OJZ91067.1 anid|CBF85486.1 anig|EHA19770.1 aory|EIT73085.1 asyd|OJJ64111.1 ater|EAU33190.1 atub|OJI83062.1 aver|OJI98193.1 awen|OJJ30324.1 azon|OJJ45342.1

Group4519: abra|OJJ77992.1 acar|OOF98830.1 acla|EAW06657.1 afis|EAW16108.1 afla|EED48644.1 afum|EAL88740.1 agla|OJJ86137.1 akaw|GAA85988.1 aluc|OJZ91068.1 anid|CBF85487.1 anig|EHA19771.1 aory|EIT73264.1 asyd|OJJ64112.1 ater|EAU33191.1 atub|OJI83063.1 aver|OJI98194.1 awen|OJJ30323.1 azon|OJJ45341.1

Group4520: abra|OJJ77993.1 acar|OOF98829.1 acla|EAW06655.1 afis|EAW16110.1 afla|EED48650.1 afum|EAL88741.1 agla|OJJ86145.1 akaw|GAA85987.1 aluc|OJZ91069.1 anid|CBF85492.1 anig|EHA19772.1 aory|EIT73074.1 asyd|OJJ64113.1 ater|EAU33193.1 atub|OJI83064.1 aver|OJI98196.1 awen|OJJ30320.1 azon|OJJ45339.1

Group4521: abra|OJJ77994.1 acar|OOF98828.1 acla|EAW06654.1 afis|EAW16111.1 afla|EED48651.1 afum|EAL88742.1 agla|OJJ86146.1 akaw|GAA85986.1 aluc|OJZ91070.1 anid|CBF85494.1 anig|EHA19773.1 aory|EIT73265.1 asyd|OJJ64114.1 ater|EAU33194.1 atub|OJI83065.1 aver|OJI98197.1 awen|OJJ30319.1 azon|OJJ45338.1

Group4522: abra|OJJ77995.1 acar|OOF98827.1 acla|EAW06653.1 afis|EAW16112.1 afla|EED48652.1 afum|EAL88743.1 agla|OJJ86151.1 akaw|GAA85985.1 aluc|OJZ91071.1 anid|CBF85496.1 anig|EHA19774.1 aory|EIT73201.1 asyd|OJJ64115.1 ater|EAU33195.1 atub|OJI83066.1 aver|OJI98198.1 awen|OJJ30318.1 azon|OJJ45337.1

Group4523: abra|OJJ78001.1 acar|OOF98821.1 acla|EAW06649.1 afis|EAW16116.1 afla|EED48657.1 afum|EAL88747.1 agla|OJJ86155.1 akaw|GAA85982.1 aluc|OJZ91077.1 anid|CBF85505.1 anig|EHA19778.1 aory|EIT73053.1 asyd|OJJ64119.1 ater|EAU33199.1 atub|OJI83072.1 aver|OJI98202.1 awen|OJJ30314.1 azon|OJJ45331.1

Group4524: abra|OJJ78004.1 acar|OOF98819.1 acla|EAW06646.1 afis|EAW16119.1 afla|EED48660.1 afum|EAL88750.1 agla|OJJ86160.1 akaw|GAA85979.1 aluc|OJZ91080.1 anid|CBF85543.1 anig|EHA19781.1 aory|EIT73098.1 asyd|OJJ64143.1 ater|EAU33202.1 atub|OJI83075.1 aver|OJI98224.1 awen|OJJ30311.1 azon|OJJ45328.1

Group4525: abra|OJJ78005.1 acar|OOF98818.1 acla|EAW06645.1 afis|EAW16120.1 afla|EED48661.1 afum|EAL88751.1 agla|OJJ86161.1 akaw|GAA85978.1 aluc|OJZ91081.1 anid|CBF85541.1 anig|EHA19782.1 aory|EIT73067.1 asyd|OJJ64142.1 ater|EAU33203.1 atub|OJI83077.1 aver|OJI98223.1 awen|OJJ30310.1 azon|OJJ45327.1

Group4526: abra|OJJ78008.1 acar|OOF98815.1 acla|EAW06642.1 afis|EAW16123.1 afla|EED48663.1 afum|EAL88753.1 agla|OJJ86164.1 akaw|GAA85975.1 aluc|OJZ91084.1 anid|CBF85534.1 anig|EHA19785.1 aory|EIT73192.1 asyd|OJJ64136.1 ater|EAU33206.1 atub|OJI83080.1 aver|OJI98219.1 awen|OJJ30307.1 azon|OJJ45310.1

Group4527: abra|OJJ78010.1 acar|OOF98813.1 acla|EAW06639.1 afis|EAW16126.1 afla|EED48666.1 afum|EAL88756.1 agla|OJJ86168.1 akaw|GAA85973.1 aluc|OJZ91086.1 anid|CBF85529.1 anig|EHA19787.1 aory|EIT73204.1 asyd|OJJ64133.1 ater|EAU33209.1 atub|OJI83082.1 aver|OJI98216.1 awen|OJJ30303.1 azon|OJJ45304.1

Group4528: abra|OJJ78011.1 acar|OOF98812.1 acla|EAW06638.1 afis|EAW16127.1 afla|EED48667.1 afum|EAL88757.1 agla|OJJ86169.1 akaw|GAA85972.1 aluc|OJZ91087.1 anid|CBF85527.1 anig|EHA19788.1 aory|EIT73187.1 asyd|OJJ64132.1 ater|EAU33210.1 atub|OJI83083.1 aver|OJI98215.1 awen|OJJ30302.1 azon|OJJ45303.1

Group4529: abra|OJJ78012.1 acar|OOF98811.1 acla|EAW06637.1 afis|EAW16128.1 afla|EED48668.1 afum|EAL88758.1 agla|OJJ86174.1 akaw|GAA85971.1 aluc|OJZ91088.1 anid|CBF85525.1 anig|EHA19789.1 aory|EIT73230.1 asyd|OJJ64131.1 ater|EAU33211.1 atub|OJI83085.1 aver|OJI98214.1 awen|OJJ30301.1 azon|OJJ45302.1

Group4530: abra|OJJ78013.1 acar|OOF98810.1 acla|EAW06635.1 afis|EAW16130.1 afla|EED48670.1 afum|EAL88760.1 agla|OJJ86175.1 akaw|GAA85970.1 aluc|OJZ91089.1 anid|CBF85523.1 anig|EHA19790.1 aory|EIT73154.1 asyd|OJJ64130.1 ater|EAU33212.1 atub|OJI83086.1 aver|OJI98213.1 awen|OJJ30299.1 azon|OJJ43987.1

Group4531: abra|OJJ78014.1 acar|OOF98809.1 acla|EAW06634.1 afis|EAW16131.1 afla|EED48669.1 afum|EAL88761.1 agla|OJJ86176.1 akaw|GAA85969.1 aluc|OJZ91090.1 anid|CBF85521.1 anig|EHA19791.1 aory|EIT73222.1 asyd|OJJ64128.1 ater|EAU33213.1 atub|OJI83087.1 aver|OJI98211.1 awen|OJJ30298.1 azon|OJJ43986.1

Group4532: abra|OJJ78018.1 acar|OOF98805.1 acla|EAW06629.1 afis|EAW16136.1 afla|EED48676.1 afum|EAL88766.2 agla|OJJ86181.1 akaw|GAA85965.1 aluc|OJZ91094.1 anid|CBF85512.1 anig|EHA19795.1 aory|EIT73051.1 asyd|OJJ64123.1 ater|EAU33218.1 atub|OJI83091.1 aver|OJI98206.1 awen|OJJ30292.1 azon|OJJ43981.1

Group4533: abra|OJJ78021.1 acar|OOF98803.1 acla|EAW06627.1 afis|EAW16138.1 afla|EED48678.1 afum|EAL88767.1 agla|OJJ86183.1 akaw|GAA85964.1 aluc|OJZ91096.1 anid|CBF85545.1 anig|EHA19797.1 aory|EIT73188.1 asyd|OJJ64144.1 ater|EAU33219.1 atub|OJI83094.1 aver|OJI98225.1 awen|OJJ30290.1 azon|OJJ43979.1

Group4534: abra|OJJ78023.1 acar|OOF98802.1 acla|EAW06626.1 afis|EAW16139.1 afla|EED48679.1 afum|EAL88768.1 agla|OJJ86185.1 akaw|GAA85963.1 aluc|OJZ91098.1 anid|CBF85546.1 anig|EHA19798.1 aory|EIT73152.1 asyd|OJJ64148.1 ater|EAU33220.1 atub|OJI83095.1 aver|OJI98228.1 awen|OJJ30289.1 azon|OJJ43978.1

Group4535: abra|OJJ78024.1 acar|OOF98801.1 acla|EAW06625.1 afis|EAW16140.1 afla|EED48681.1 afum|EAL88769.2 agla|OJJ86189.1 akaw|GAA85962.1 aluc|OJZ91099.1 anid|CBF85548.1 anig|EHA19799.1

aory|EIT73077.1 asyd|OJJ64149.1 ater|EAU33225.1 atub|OJI83096.1 aver|OJI98229.1 awen|OJJ30288.1 azon|OJJ43977.1

Group4536: abra|OJJ78026.1 acar|OOF98799.1 acla|EAW06623.1 afis|EAW16142.1 afla|EED48683.1 afum|EAL88771.1 agla|OJJ86190.1 akaw|GAA85960.1 aluc|OJZ91101.1 anid|CBF85551.1 anig|EHA19801.1 aory|EIT73175.1 asyd|OJJ64151.1 ater|EAU33227.1 atub|OJI83098.1 aver|OJI98231.1 awen|OJJ30286.1 azon|OJJ43975.1

Group4537: abra|OJJ78027.1 acar|OOF98796.1 acla|EAW06622.1 afis|EAW16143.1 afla|EED48684.1 afum|EAL88772.1 agla|OJJ86191.1 akaw|GAA85959.1 aluc|OJZ91102.1 anid|CBF79592.1 anig|EHA19802.1 aory|EIT73194.1 asyd|OJJ57983.1 ater|EAU33228.1 atub|OJI83099.1 aver|OJJ04418.1 awen|OJJ30285.1 azon|OJJ43974.1

Group4538: abra|OJJ78060.1 acar|OOF98793.1 acla|EAW13353.1 afis|EAW20467.1 afla|EED47863.1 afum|EAL84585.1 akaw|GAA85932.1 aluc|OJZ91131.1 anid|CBF79265.1 anig|EHA19830.1 aory|EIT77212.1 asyd|OJJ55757.1 atub|OJI83142.1 aver|OJJ06842.1 awen|OJJ33161.1 azon|OJJ51457.1 acar|OOF91287.1 awen|OJJ32661.1

Group4539: abra|OJJ78062.1 acar|OOF98792.1 acla|EAW06621.1 afis|EAW16144.1 afla|EED48686.1 afum|EAL88773.1 agla|OJJ86192.1 akaw|GAA85931.1 aluc|OJZ91133.1 anid|CBF79590.1 anig|EHA19832.1 aory|EIT73255.1 asyd|OJJ57981.1 ater|EAU33229.1 atub|OJI83143.1 aver|OJJ04419.1 awen|OJJ30283.1 azon|OJJ43973.1

Group4540: abra|OJJ78073.1 acar|OOF98790.1 acla|EAW15356.1 afis|EAW20356.1 afla|EED46131.1 afum|EAL85473.1 agla|OJJ81763.1 akaw|GAA85921.1 aluc|OJZ91146.1 anid|CBF74973.1 anig|EHA19844.1 aory|EIT76365.1 asyd|OJJ60088.1 ater|EAU34035.1 atub|OJI83160.1 aver|OJJ04175.1 awen|OJJ38942.1 azon|OJJ45059.1

Group4541: abra|OJJ78074.1 acar|OOF98789.1 afis|EAW16145.1 afla|EED48687.1 agla|OJJ86198.1 akaw|GAA85920.1 aluc|OJZ91147.1 anid|CBF79583.1 anig|EHA19845.1 aory|EIT73058.1 asyd|OJJ57977.1 ater|EAU33230.1 atub|OJI83161.1 aver|OJJ04423.1 awen|OJJ30281.1 azon|OJJ43972.1 acla|EAW06620.1 afum|EAL88774.1

Group4542: abra|OJJ78078.1 acar|OOF98786.1 acla|EAW06611.1 afis|EAW16204.1 afla|EED48697.1 afum|EAL88832.1 agla|OJJ86215.1 akaw|GAA85916.1 aluc|OJZ91150.1 anid|CBF79563.1 anig|EHA19848.1 aory|EIT73216.1 asyd|OJJ57913.1 ater|EAU33246.1 atub|OJI83164.1 aver|OJJ04480.1 awen|OJJ30268.1 azon|OJJ43964.1

Group4543: abra|OJJ78083.1 acar|OOF98783.1 acla|EAW06613.1 afis|EAW16177.1 afla|EED54377.1 afum|EAL88810.1 agla|OJJ86213.1 akaw|GAA85909.1 aluc|OJZ91157.1 anid|CBF79566.1 anig|EHA19853.1 aory|EIT75854.1 asyd|OJJ57917.1 ater|EAU33243.1 atub|OJI83172.1 aver|OJJ04474.1 awen|OJJ30272.1 azon|OJJ43966.1

Group4544: abra|OJJ78105.1 acar|OOF98760.1 acla|EAW06617.1 afis|EAW16148.1 afla|EED48691.1 afum|EBA27357.1 agla|OJJ86200.1 akaw|GAA85900.1 aluc|OJZ91169.1 anig|EHA19864.1 aory|EIT73263.1 ater|EAU33231.1 atub|OJI83184.1 awen|OJJ30276.1 asyd|OJJ57971.1 aver|OJJ04431.1 anig|EHA19863.1 aver|OJJ04432.1

Group4545: abra|OJJ78110.1 acar|OOF98756.1 acla|EAW06606.1 afis|EAW16217.1 afla|EED48711.1 afum|EAL88844.1 agla|OJJ86227.1 akaw|GAA85896.1 aluc|OJZ91175.1 anid|CBF79538.1 anig|EHA19867.1 aory|EIT73084.1 asyd|OJJ57899.1 ater|EAU33261.1 atub|OJI83188.1 aver|OJJ04496.1 awen|OJJ30255.1 azon|OJJ43955.1

Group4546: abra|OJJ78112.1 acar|OOF98755.1 acla|EAW06605.1 afis|EAW16218.1 afla|EED48712.1 afum|EAL88846.1 agla|OJJ86225.1 akaw|GAA85895.1 aluc|OJZ91177.1 anid|CBF79536.1 anig|EHA19868.1 aory|EIT73123.1 asyd|OJJ57898.1 ater|EAU33262.1 atub|OJI83189.1 aver|OJJ04497.1 awen|OJJ30254.1 azon|OJJ43957.1

Group4547: abra|OJJ78113.1 acar|OOF98753.1 acla|EAW07002.1 afis|EAW16619.1 afla|EED48441.1 afum|EAL89218.1 akaw|GAA85894.1 aluc|OJZ91178.1 anig|EHA19869.1 aory|EIT81677.1 asyd|OJJ60003.1 ater|EAU30738.1 atub|OJI83190.1 aver|OJJ02288.1 awen|OJJ30079.1 azon|OJJ42185.1 aory|EIT74880.1 awen|OJJ37785.1

Group4548: abra|OJJ78189.1 acar|OOF98670.1 acla|EAW06798.1 afis|EAW16347.1 afla|EED51752.1 afum|EAL88972.1 agla|OJJ88241.1 akaw|GAA85840.1 aluc|OJZ91260.1 anid|CBF89212.1 anig|EHA19948.1 aory|EIT80660.1 asyd|OJJ62523.1 ater|EAU36051.1 atub|OJI83249.1 aver|OJI96642.1 awen|OJJ37750.1 azon|OJJ47558.1

Group4549: abra|OJJ78192.1 acar|OOF98665.1 afum|EAL85073.2 akaw|GAA85837.1 aluc|OJZ91263.1 anid|CBF87346.1 asyd|OJJ56202.1 atub|OJI83252.1 aver|OJJ06457.1 acar|OOF90595.1 afis|EAW19818.1 afla|EED53224.1 aory|EIT75036.1 aver|OJJ05129.1 azon|OJJ42171.1 afla|EED53225.1 anig|EHA19951.1 anid|CBF75950.1

Group4550: abra|OJJ78206.1 acar|OOF98648.1 acla|EAW11242.1 afis|EAW18261.1 afla|EED48720.1 afum|EAL87625.1 agla|OJJ88165.1 akaw|GAA85820.1 aluc|OJZ91279.1 anid|CBF79529.1 anig|EHA19964.1 aory|EIT73088.1 asyd|OJJ57894.1 ater|EAU33269.1 atub|OJI83269.1 aver|OJJ04501.1 awen|OJJ30635.1 azon|OJJ47983.1

Group4551: abra|OJJ78213.1 acar|OOF98642.1 acla|EAW11237.1 afis|EAW18268.1 afla|EED48733.1 afum|EAL87633.1 agla|OJJ88159.1 akaw|GAA85814.1 aluc|OJZ91285.1 anid|CBF79516.1 anig|EHA19970.1 aory|EIT73048.1 asyd|OJJ57888.1 ater|EAU33275.1 atub|OJI83275.1 aver|OJJ04506.1 awen|OJJ30642.1 azon|OJJ47973.1

Group4552: abra|OJJ78216.1 acar|OOF98640.1 acla|EAW11234.1 afis|EAW18271.1 afla|EED48737.1 afum|EAL87636.1 agla|OJJ88156.1 akaw|GAA85811.1 aluc|OJZ91288.1 anid|CBF79510.1 anig|EHA19973.1 aory|EIT73125.1 asyd|OJJ57885.1 ater|EAU33277.1 atub|OJI83278.1 aver|OJJ04509.1 awen|OJJ30644.1 azon|OJJ47971.1

Group4553: abra|OJJ78217.1 acar|OOF98639.1 acla|EAW11232.1 afis|EAW18272.1 afla|EED48738.1 afum|EAL87637.1 agla|OJJ88155.1 akaw|GAA85807.1 aluc|OJZ91291.1 anid|CBF79508.1 anig|EHA19974.1 aory|EIT73235.1 asyd|OJJ57884.1 ater|EAU33278.1 atub|OJI83282.1 aver|OJJ04510.1 awen|OJJ30645.1 azon|OJJ47970.1

Group4554: abra|OJJ78218.1 acar|OOF98638.1 acla|EAW11231.1 afis|EAW18273.1 afla|EED48739.1 afum|EAL87638.1 agla|OJJ88154.1 akaw|GAA85806.1 aluc|OJZ91292.1 anid|CBF79507.1 anig|EHA19975.1 aory|EIT73224.1 asyd|OJJ57883.1 ater|EAU33279.1 atub|OJI83283.1 aver|OJJ04511.1 awen|OJJ30646.1 azon|OJJ47969.1

Group4555: abra|OJJ78220.1 acar|OOF98636.1 acla|EAW11229.1 afis|EAW18274.1 afla|EED48741.1 afum|EAL87640.1 agla|OJJ88152.1 akaw|GAA85805.1 aluc|OJZ91294.1 anid|CBF79503.1 anig|EHA19977.1 aory|EIT73281.1 asyd|OJJ57881.1 ater|EAU33280.1 atub|OJI83285.1 aver|OJJ04513.1 awen|OJJ30647.1 azon|OJJ47968.1

Group4556: abra|OJJ78221.1 acar|OOF98635.1 acla|EAW11228.1 afis|EAW18275.1 afla|EED48742.1 afum|EAL87641.1 agla|OJJ88151.1 akaw|GAA85804.1 aluc|OJZ91296.1 anid|CBF79499.1 anig|EHA19978.1 aory|EIT73223.1 asyd|OJJ57880.1 ater|EAU33281.1 atub|OJI83286.1 aver|OJJ04514.1 awen|OJJ30648.1 azon|OJJ47967.1

Group4557: abra|OJJ78226.1 acar|OOF98632.1 acla|EAW09284.1 afis|EAW21863.1 afla|EED46448.1 afum|EAL92877.2 agla|OJJ84270.1 akaw|GAA85799.1 aluc|OJZ91301.1 anid|CBF76755.1 anig|EHA19983.1 aory|EIT72377.1 asyd|OJJ61262.1 ater|EAU30861.1 atub|OJI83292.1 aver|OJJ00588.1 awen|OJJ34153.1 azon|OJJ43475.1

Group4558: abra|OJJ78229.1 acar|OOF98630.1 acla|EAW09280.1 afis|EAW21867.1 afla|EED46442.1 afum|EAL92881.1 agla|OJJ84268.1 akaw|GAA85795.1 aluc|OJZ91304.1 anid|CBF76763.1 anig|EHA19986.1 aory|EIT72329.1 asyd|OJJ61265.1 ater|EAU30864.1 atub|OJI83295.1 aver|OJJ00592.1 awen|OJJ34156.1 azon|OJJ43473.1

Group4559: abra|OJJ78230.1 acar|OOF98629.1 acla|EAW09279.1 afis|EAW21868.1 afla|EED46441.1 afum|EAL92882.1 agla|OJJ84267.1 akaw|GAA85794.1 aluc|OJZ91305.1 anid|CBF76765.1 anig|EHA19987.1 aory|EIT72366.1 asyd|OJJ61266.1 ater|EAU30866.1 atub|OJI83296.1 aver|OJJ00593.1 awen|OJJ34157.1 azon|OJJ43472.1

Group4560: abra|OJJ78231.1 acar|OOF98628.1 acla|EAW09278.1 afis|EAW21869.1 afla|EED46440.1 afum|EAL92883.1 agla|OJJ84264.1 akaw|GAA85793.1 aluc|OJZ91306.1 anid|CBF76767.1 anig|EHA19988.1 aory|EIT72428.1 asyd|OJJ61267.1 ater|EAU30867.1 atub|OJI83297.1 aver|OJJ00594.1 awen|OJJ34158.1 azon|OJJ43471.1

Group4561: abra|OJJ78233.1 acar|OOF98627.1 acla|EAW09277.1 afis|EAW21870.1 afla|EED46438.1 afum|EAL92884.1 agla|OJJ84262.1 akaw|GAA85792.1 aluc|OJZ91307.1 anid|CBF76769.1 anig|EHA19991.1 aory|EIT72393.1 asyd|OJJ61268.1 ater|EAU30868.1 atub|OJI83300.1 aver|OJJ00597.1 awen|OJJ34159.1 azon|OJJ43470.1

Group4562: abra|OJJ78234.1 acar|OOF98626.1 acla|EAW09276.1 afis|EAW21871.1 afla|EED46437.1 afum|EAL92885.1 agla|OJJ84261.1 akaw|GAA85791.1 aluc|OJZ91308.1 anid|CBF76771.1 anig|EHA19992.1 aory|EIT72319.1 asyd|OJJ61269.1 ater|EAU30869.1 atub|OJI83301.1 aver|OJJ00598.1 awen|OJJ34160.1 azon|OJJ43469.1

Group4563: abra|OJJ78237.1 acar|OOF98623.1 acla|EAW09273.1 afis|EAW21875.1 afla|EED46433.1 afum|EAL92889.2 agla|OJJ84257.1 akaw|GAA85788.1 aluc|OJZ91311.1 anid|CBF76776.1 anig|EHA19995.1 aory|EIT72389.1 asyd|OJJ61273.1 ater|EAU30874.1 atub|OJI83304.1 aver|OJJ00601.1 awen|OJJ34164.1 azon|OJJ43466.1

Group4564: abra|OJJ78238.1 acar|OOF98622.1 acla|EAW09272.1 afis|EAW21876.1 afla|EED46432.1 afum|EAL92890.1 agla|OJJ84256.1 akaw|GAA85787.1 aluc|OJZ91312.1 anid|CBF76778.1 anig|EHA19996.1

aory|EIT72368.1 asyd|OJJ61274.1 ater|EAU30875.1 atub|OJI83305.1 aver|OJJ00602.1 awen|OJJ34165.1 azon|OJJ43465.1

Group4565: abra|OJJ78239.1 acar|OOF98621.1 acla|EAW09271.1 afis|EAW21877.1 afla|EED46431.1 afum|EAL92891.2 agla|OJJ84255.1 akaw|GAA85786.1 aluc|OJZ91313.1 anid|CBF76780.1 anig|EHA19997.1 aory|EIT72420.1 asyd|OJJ61275.1 ater|EAU30876.1 atub|OJI83306.1 aver|OJJ00603.1 awen|OJJ34166.1 azon|OJJ43464.1

Group4566: abra|OJJ78241.1 acar|OOF98619.1 acla|EAW09269.1 afis|EAW21879.1 afla|EED46429.1 afum|EAL92893.1 agla|OJJ84253.1 akaw|GAA85783.1 aluc|OJZ91315.1 anid|CBF76784.1 anig|EHA19998.1 aory|EIT72355.1 asyd|OJJ61277.1 ater|EAU30877.1 atub|OJI83309.1 aver|OJJ00605.1 awen|OJJ34168.1 azon|OJJ43462.1

Group4567: abra|OJJ78242.1 acar|OOF98618.1 acla|EAW09268.1 afis|EAW21880.1 afla|EED46428.1 afum|EAL92894.2 agla|OJJ84252.1 akaw|GAA85782.1 aluc|OJZ91316.1 anid|CBF76786.1 anig|EHA19999.1 aory|EIT72330.1 asyd|OJJ61278.1 ater|EAU30878.1 atub|OJI83310.1 aver|OJJ00606.1 awen|OJJ34169.1 azon|OJJ43461.1

Group4568: abra|OJJ78244.1 acar|OOF98616.1 acla|EAW09265.1 afis|EAW21883.1 afla|EED46424.1 afum|EAL92897.1 agla|OJJ84251.1 akaw|GAA85780.1 aluc|OJZ91318.1 anid|CBF76788.1 anig|EHA20001.1 aory|EIT72383.1 asyd|OJJ61280.1 ater|EAU30881.1 atub|OJI83312.1 aver|OJJ00607.1 awen|OJJ34172.1 azon|OJJ43460.1

Group4569: abra|OJJ78247.1 acar|OOF98615.1 acla|EAW09263.1 afis|EAW21884.1 afla|EED46423.1 afum|EAL92898.1 agla|OJJ84249.1 akaw|GAA85779.1 aluc|OJZ91321.1 anid|CBF76790.1 anig|EHA20003.1 aory|EIT72443.1 asyd|OJJ61281.1 ater|EAU30882.1 atub|OJI83314.1 aver|OJJ00608.1 awen|OJJ34173.1 azon|OJJ43459.1

Group4570: abra|OJJ78249.1 acar|OOF98614.1 acla|EAW09262.1 afis|EAW21885.1 afla|EED46422.1 afum|EAL92899.1 agla|OJJ84248.1 akaw|GAA85778.1 aluc|OJZ91322.1 anid|CBF76792.1 anig|EHA20004.1 aory|EIT72353.1 asyd|OJJ61282.1 ater|EAU30883.1 atub|OJI83315.1 aver|OJJ00609.1 awen|OJJ34174.1 azon|OJJ43458.1

Group4571: abra|OJJ78250.1 acar|OOF98612.1 acla|EAW09261.1 afis|EAW21886.1 afla|EED46421.1 afum|EAL92900.1 agla|OJJ84247.1 akaw|GAA85777.1 aluc|OJZ91323.1 anid|CBF76794.1 anig|EHA20005.1 aory|EIT72442.1 asyd|OJJ61283.1 ater|EAU30884.1 atub|OJI83316.1 aver|OJJ00610.1 awen|OJJ34175.1 azon|OJJ43457.1

Group4572: abra|OJJ78252.1 acar|OOF98611.1 acla|EAW09259.1 afis|EAW21888.1 afla|EED46419.1 afum|EAL92902.1 agla|OJJ84245.1 akaw|GAA85775.1 aluc|OJZ91325.1 anid|CBF76798.1 anig|EHA20007.1 aory|EIT72406.1 asyd|OJJ61285.1 ater|EAU30885.1 atub|OJI83318.1 aver|OJJ00611.1 awen|OJJ34177.1 azon|OJJ43455.1

Group4573: abra|OJJ78253.1 acar|OOF98610.1 acla|EAW09258.1 afis|EAW21889.1 afla|EED46418.1 afum|EAL92903.1 agla|OJJ84244.1 akaw|GAA85774.1 aluc|OJZ91326.1 anid|CBF76800.1 anig|EHA20008.1 aory|EIT72432.1 asyd|OJJ61286.1 ater|EAU30886.1 atub|OJI83319.1 aver|OJJ00612.1 awen|OJJ34178.1 azon|OJJ43454.1

Group4574: abra|OJJ78255.1 acar|OOF98607.1 acla|EAW09254.1 afis|EAW21893.1 afla|EED46415.1 afum|EAL92907.1 agla|OJJ84239.1 akaw|GAA85771.1 aluc|OJZ91329.1 anid|CBF76806.1 anig|EHA20011.1 aory|EIT72313.1 asyd|OJJ61289.1 ater|EAU30889.1 atub|OJI83322.1 aver|OJJ00615.1 awen|OJJ34182.1 azon|OJJ43450.1

Group4575: abra|OJJ78256.1 acar|OOF98606.1 acla|EAW09253.1 afis|EAW21894.1 afla|EED46414.1 afum|EAL92908.1 agla|OJJ84238.1 akaw|GAA85770.1 aluc|OJZ91330.1 anid|CBF76808.1 anig|EHA20012.1 aory|EIT72378.1 asyd|OJJ61290.1 ater|EAU30890.1 atub|OJI83323.1 aver|OJJ00616.1 awen|OJJ34183.1 azon|OJJ43449.1

Group4576: abra|OJJ78260.1 acar|OOF98600.1 acla|EAW09250.1 afis|EAW21897.1 afla|EED46411.1 afum|EAL92910.1 agla|OJJ84227.1 akaw|GAA85765.1 aluc|OJZ91335.1 anid|CBF76814.1 anig|EHA20016.1 aory|EIT72440.1 asyd|OJJ61293.1 ater|EAU30892.1 atub|OJI83327.1 aver|OJJ00619.1 awen|OJJ34186.1 azon|OJJ43447.1

Group4577: abra|OJJ78261.1 acar|OOF98599.1 acla|EAW09249.1 afis|EAW21898.1 afla|EED46408.1 afum|EAL92911.2 agla|OJJ84228.1 akaw|GAA85764.1 aluc|OJZ91336.1 anid|CBF76816.1 anig|EHA20017.1 aory|EIT72346.1 asyd|OJJ61294.1 ater|EAU30893.1 atub|OJI83328.1 aver|OJJ00620.1 awen|OJJ34187.1 azon|OJJ43446.1

Group4578: abra|OJJ78263.1 acar|OOF98598.1 acla|EAW09248.1 afis|EAW21899.1 afla|EED46407.1 afum|EAL92912.1 agla|OJJ84229.1 akaw|GAA85762.1 aluc|OJZ91338.1 anid|CBF76818.1 anig|EHA20018.1 aory|EIT72328.1 asyd|OJJ61295.1 ater|EAU30894.1 atub|OJI83330.1 aver|OJJ00621.1 awen|OJJ34188.1 azon|OJJ43445.1

Group4579: abra|OJJ78264.1 acar|OOF98597.1 acla|EAW09247.1 afis|EAW21900.1 afla|EED46406.1 afum|EAL92913.1 agla|OJJ84230.1 akaw|GAA85761.1 aluc|OJZ91339.1 anid|CBF76820.1 anig|EHA20019.1 aory|EIT72352.1 asyd|OJJ61296.1 ater|EAU30895.1 atub|OJI83331.1 aver|OJJ00622.1 awen|OJJ34189.1 azon|OJJ43444.1

Group4580: abra|OJJ78266.1 acar|OOF98596.1 acla|EAW09246.1 afis|EAW21901.1 afla|EED46405.1 afum|EAL92914.1 agla|OJJ84231.1 akaw|GAA85760.1 aluc|OJZ91340.1 anid|CBF76822.1 anig|EHA20020.1 aory|EIT72444.1 asyd|OJJ61297.1 ater|EAU30896.1 atub|OJI83332.1 aver|OJJ00623.1 awen|OJJ34190.1 azon|OJJ43443.1

Group4581: abra|OJJ78267.1 acar|OOF98595.1 acla|EAW09245.1 afis|EAW21902.1 afla|EED46403.1 afum|EAL92915.1 agla|OJJ84232.1 akaw|GAA85759.1 aluc|OJZ91341.1 anid|CBF76824.1 anig|EHA20021.1 aory|EIT72339.1 asyd|OJJ61298.1 ater|EAU30897.1 atub|OJI83333.1 aver|OJJ00624.1 awen|OJJ34191.1 azon|OJJ43442.1

Group4582: abra|OJJ78268.1 acar|OOF98594.1 acla|EAW09244.1 afis|EAW21903.1 afla|EED46402.1 afum|EAL92916.1 agla|OJJ84233.1 akaw|GAA85758.1 aluc|OJZ91342.1 anid|CBF76826.1 anig|EHA20022.1 aory|EIT72439.1 asyd|OJJ61299.1 ater|EAU30898.1 atub|OJI83334.1 aver|OJJ00625.1 awen|OJJ34192.1 azon|OJJ43441.1

Group4583: abra|OJJ78275.1 acar|OOF98592.1 acla|EAW09239.1 afis|EAW21909.1 afla|EED46641.1 afum|EAL92921.1 agla|OJJ84222.1 akaw|GAA85751.1 aluc|OJZ91350.1 anid|CBF76837.1 anig|EHA20028.1 aory|EIT77855.1 asyd|OJJ61305.1 ater|EAU30909.1 atub|OJI83342.1 aver|OJJ00630.1 awen|OJJ34199.1 azon|OJJ43435.1

Group4584: abra|OJJ78277.1 acar|OOF98590.1 acla|EAW09237.1 afis|EAW21911.1 afla|EED46639.1 afum|EAL92923.1 agla|OJJ84220.1 akaw|GAA85749.1 aluc|OJZ91352.1 anid|CBF76842.1 anig|EHA20030.1 aory|EIT77915.1 asyd|OJJ61307.1 ater|EAU30911.1 atub|OJI83344.1 aver|OJJ00632.1 awen|OJJ34201.1 azon|OJJ43433.1

Group4585: abra|OJJ78278.1 acar|OOF98588.1 acla|EAW09236.1 afis|EAW21912.1 afla|EED46638.1 afum|EAL92924.1 agla|OJJ84219.1 akaw|GAA85748.1 aluc|OJZ91353.1 anid|CBF76844.1 anig|EHA20031.1 aory|EIT77829.1 asyd|OJJ61308.1 ater|EAU30912.1 atub|OJI83345.1 aver|OJJ00633.1 awen|OJJ34202.1 azon|OJJ43432.1

Group4586: abra|OJJ78280.1 acar|OOF98589.1 acla|EAW09235.1 afis|EAW21913.1 afla|EED46637.1 afum|EAL92925.1 agla|OJJ84210.1 akaw|GAA85747.1 aluc|OJZ91354.1 anid|CBF76846.1 anig|EHA20032.1 aory|EIT77951.1 asyd|OJJ61309.1 ater|EAU30914.1 atub|OJI83347.1 aver|OJJ00634.1 awen|OJJ34203.1 azon|OJJ43431.1

Group4587: abra|OJJ78282.1 acar|OOF98586.1 acla|EAW09233.1 afis|EAW21915.1 afla|EED46634.1 afum|EAL92927.1 agla|OJJ84208.1 akaw|GAA85745.1 aluc|OJZ91356.1 anid|CBF76850.1 anig|EHA20034.1 aory|EIT77867.1 asyd|OJJ61311.1 ater|EAU30916.1 atub|OJI83349.1 aver|OJJ00636.1 awen|OJJ34205.1 azon|OJJ43429.1

Group4588: abra|OJJ78284.1 acar|OOF98584.1 acla|EAW09231.1 afis|EAW21917.1 afla|EED46632.1 afum|EAL92929.1 agla|OJJ84206.1 akaw|GAA85743.1 aluc|OJZ91358.1 anid|CBF76854.1 anig|EHA20036.1 aory|EIT77843.1 asyd|OJJ61313.1 ater|EAU30919.1 atub|OJI83351.1 aver|OJJ00638.1 awen|OJJ34207.1 azon|OJJ43427.1

Group4589: abra|OJJ78285.1 acar|OOF98583.1 acla|EAW09230.1 afis|EAW21918.1 afum|EAL92930.1 agla|OJJ84205.1 akaw|GAA85742.1 aluc|OJZ91359.1 anid|CBF76856.1 anig|EHA20037.1 asyd|OJJ61314.1 ater|EAU30920.1 atub|OJI83352.1 aver|OJJ00639.1 awen|OJJ34208.1 azon|OJJ43426.1 afla|EED46631.1 aory|EIT77816.1

Group4590: abra|OJJ78287.1 acar|OOF98581.1 acla|EAW09228.1 afis|EAW21920.1 afla|EED46630.1 afum|EAL92932.1 agla|OJJ84203.1 akaw|GAA85740.1 aluc|OJZ91361.1 anid|CBF76860.1 anig|EHA20039.1 aory|EIT77823.1 asyd|OJJ61316.1 ater|EAU30925.1 atub|OJI83354.1 aver|OJJ00641.1 awen|OJJ34210.1 azon|OJJ43424.1

Group4591: abra|OJJ78288.1 acar|OOF98579.1 acla|EAW09227.1 afis|EAW21921.1 afla|EED46629.1 afum|EAL92934.1 agla|OJJ84202.1 akaw|GAA85739.1 aluc|OJZ91362.1 anid|CBF76862.1 anig|EHA20040.1 aory|EIT77918.1 asyd|OJJ61317.1 ater|EAU30926.1 atub|OJI83355.1 aver|OJJ00642.1 awen|OJJ34211.1 azon|OJJ43423.1

Group4592: abra|OJJ78289.1 acar|OOF98580.1 acla|EAW09226.1 afis|EAW21922.1 afla|EED46628.1 afum|EAL92935.1 agla|OJJ84201.1 akaw|GAA85738.1 aluc|OJZ91363.1 anid|CBF76864.1 anig|EHA20041.1 aory|EIT77831.1 asyd|OJJ61318.1 ater|EAU30927.1 atub|OJI83356.1 aver|OJJ00643.1 awen|OJJ34212.1 azon|OJJ43422.1

Group4593: abra|OJJ78290.1 acar|OOF98578.1 acla|EAW09225.1 afis|EAW21923.1 afla|EED46627.1 afum|EAL92936.1 agla|OJJ84200.1 akaw|GAA85737.1 aluc|OJZ91364.1 anid|CBF76866.1 anig|EHA20042.1

aory|EIT77896.1 asyd|OJJ61319.1 ater|EAU30928.1 atub|OJI83357.1 aver|OJJ00644.1 awen|OJJ34213.1 azon|OJJ43421.1

Group4594: abra|OJJ78292.1 acar|OOF98576.1 afis|EAW21924.1 afla|EED46615.1 afum|EAL92937.1 akaw|GAA85735.1 aluc|OJZ91366.1 anig|EHA20044.1 aory|EIT77890.1 ater|EAU30929.1 atub|OJI83359.1 aver|OJJ00647.1 azon|OJJ43420.1 acla|EAW09224.1 agla|OJJ84199.1 anid|CBF76872.1 asyd|OJJ61322.1 awen|OJJ34214.1

Group4595: abra|OJJ78293.1 acar|OOF98575.1 acla|EAW09223.1 afis|EAW21925.1 afla|EED46616.1 afum|EAL92938.1 agla|OJJ84198.1 akaw|GAA85734.1 aluc|OJZ91367.1 anid|CBF76882.1 anig|EHA20045.1 aory|EIT77869.1 asyd|OJJ61327.1 ater|EAU30930.1 atub|OJI83360.1 aver|OJJ00652.1 awen|OJJ34215.1 azon|OJJ43419.1

Group4596: abra|OJJ78298.1 acar|OOF98571.1 acla|EAW09218.1 afis|EAW21930.1 afla|EED46623.1 afum|EAL92942.1 agla|OJJ84191.1 akaw|GAA85729.1 aluc|OJZ91372.1 anid|CBF76884.1 anig|EHA20050.1 aory|EIT77852.1 asyd|OJJ61328.1 ater|EAU30936.1 atub|OJI83365.1 aver|OJJ00654.1 awen|OJJ34220.1 azon|OJJ43414.1

Group4597: abra|OJJ78299.1 acar|OOF98567.1 acla|EAW09216.1 afis|EAW21931.1 afla|EED46626.1 afum|EAL92943.2 agla|OJJ84190.1 akaw|GAA85727.1 aluc|OJZ91376.1 anid|CBF76885.1 anig|EHA20051.1 aory|EIT77815.1 asyd|OJJ61330.1 ater|EAU30937.1 atub|OJI83366.1 aver|OJJ00655.1 awen|OJJ34222.1 azon|OJJ43413.1

Group4598: abra|OJJ78301.1 acar|OOF98565.1 acla|EAW09214.1 afis|EAW21933.1 afla|EED46353.1 afum|EAL92946.1 agla|OJJ84187.1 akaw|GAA85725.1 aluc|OJZ91378.1 anid|CBF76887.1 anig|EHA20053.1 aory|EIT81186.1 asyd|OJJ61333.1 ater|EAU30938.1 atub|OJI83371.1 aver|OJJ00657.1 awen|OJJ34224.1 azon|OJJ43411.1

Group4599: abra|OJJ78302.1 acar|OOF98564.1 acla|EAW09213.1 afis|EAW21934.1 afla|EED46354.1 afum|EAL92947.1 agla|OJJ84186.1 akaw|GAA85724.1 aluc|OJZ91379.1 anid|CBF76889.1 anig|EHA20054.1 aory|EIT81211.1 asyd|OJJ61334.1 ater|EAU30939.1 atub|OJI83372.1 aver|OJJ00658.1 awen|OJJ34225.1 azon|OJJ43410.1

Group4600: abra|OJJ78308.1 acar|OOF98558.1 acla|EAW09212.1 afis|EAW21935.1 afla|EED46652.1 afum|EAL92948.2 agla|OJJ84185.1 akaw|GAA85721.1 aluc|OJZ91383.1 anid|CBF76899.1 anig|EHA20060.1 aory|EIT77952.1 asyd|OJJ61338.1 ater|EAU30941.1 atub|OJI83376.1 aver|OJJ00662.1 awen|OJJ34226.1 azon|OJJ43409.1

Group4601: abra|OJJ78319.1 acar|OOF98556.1 acla|EAW09206.1 afis|EAW21941.1 afla|EED46658.1 afum|EAL92955.1 agla|OJJ84182.1 akaw|GAA85711.1 aluc|OJZ91392.1 anid|CBF84078.1 anig|EHA20070.1 aory|EIT77820.1 asyd|OJJ61342.1 ater|EAU30946.1 atub|OJI83385.1 aver|OJJ00666.1 awen|OJJ34232.1 azon|OJJ43403.1

Group4602: abra|OJJ78323.1 acar|OOF98551.1 acla|EAW09202.1 afis|EAW21945.1 afla|EED46662.1 afum|EAL92959.1 agla|OJJ84179.1 akaw|GAA85705.1 aluc|OJZ91398.1 anid|CBF84050.1 anig|EHA20075.1 aory|EIT77962.1 asyd|OJJ61343.1 ater|EAU30993.1 atub|OJI83390.1 aver|OJJ00667.1 awen|OJJ34236.1 azon|OJJ43400.1

Group4603: abra|OJJ78325.1 acar|OOF98550.1 acla|EAW09201.1 afis|EAW21946.1 afla|EED46663.1 afum|EAL92960.1 agla|OJJ84174.1 akaw|GAA85704.1 aluc|OJZ91399.1 anid|CBF84059.1 anig|EHA20076.1 aory|EIT77808.1 asyd|OJJ61345.1 ater|EAU30994.1 atub|OJI83391.1 aver|OJJ00670.1 awen|OJJ34237.1 azon|OJJ43399.1

Group4604: abra|OJJ78327.1 acar|OOF98548.1 acla|EAW09199.1 afis|EAW21948.1 afla|EED46666.1 afum|EAL92962.1 agla|OJJ84172.1 akaw|GAA85702.1 aluc|OJZ91401.1 anid|CBF84062.1 anig|EHA20078.1 aory|EIT77953.1 asyd|OJJ61347.1 ater|EAU30996.1 atub|OJI83393.1 aver|OJJ00674.1 awen|OJJ34240.1 azon|OJJ43397.1

Group4605: abra|OJJ78328.1 acar|OOF98547.1 acla|EAW09198.1 afis|EAW21949.1 afum|EAL92963.1 agla|OJJ84171.1 akaw|GAA85701.1 aluc|OJZ91402.1 anid|CBF84064.1 anig|EHA20079.1 aory|EIT77849.1 asyd|OJJ61348.1 ater|EAU30997.1 atub|OJI83395.1 aver|OJJ00675.1 awen|OJJ34241.1 azon|OJJ43396.1 afla|EED46667.1

Group4606: abra|OJJ78329.1 acar|OOF98546.1 acla|EAW09197.1 afis|EAW21950.1 afla|EED46668.1 afum|EAL92964.1 agla|OJJ84170.1 akaw|GAA85700.1 aluc|OJZ91403.1 anid|CBF84066.1 anig|EHA20080.1 aory|EIT77926.1 asyd|OJJ61349.1 ater|EAU30998.1 atub|OJI83396.1 aver|OJJ00676.1 awen|OJJ34242.1 azon|OJJ43395.1

Group4607: abra|OJJ78334.1 acar|OOF98544.1 acla|EAW09194.1 afis|EAW21952.1 afla|EED46669.1 afum|EAL92966.1 agla|OJJ84159.1 akaw|GAA85696.1 aluc|OJZ91407.1 anid|CBF84069.1 anig|EHA20083.1 aory|EIT77916.1 asyd|OJJ61351.1 ater|EAU31000.1 atub|OJI83400.1 aver|OJJ00678.1 awen|OJJ34244.1 azon|OJJ43394.1

Group4608: abra|OJJ78337.1 acar|OOF98543.1 acla|EAW09193.1 afis|EAW21953.1 afla|EED46671.1 afum|EAL92967.1 agla|OJJ84160.1 akaw|GAA85695.1 aluc|OJZ91409.1 anid|CBF84070.1 anig|EHA20084.1 aory|EIT77807.1 asyd|OJJ61352.1 ater|EAU31001.1 atub|OJI83403.1 aver|OJJ00679.1 awen|OJJ34245.1 azon|OJJ43393.1

Group4609: abra|OJJ78339.1 acar|OOF98541.1 acla|EAW09192.1 afis|EAW21954.1 afla|EED46672.1 afum|EAL92968.1 agla|OJJ84161.1 akaw|GAA85694.1 aluc|OJZ91411.1 anid|CBF84074.1 anig|EHA20085.1 aory|EIT77806.1 asyd|OJJ61353.1 ater|EAU31060.1 atub|OJI83404.1 aver|OJJ00680.1 awen|OJJ34246.1 azon|OJJ43392.1

Group4610: abra|OJJ78345.1 acar|OOF90269.1 acla|EAW09189.1 afis|EAW21957.1 afla|EED46676.1 afum|EAL92971.1 agla|OJJ84164.1 akaw|GAA85688.1 aluc|OJZ91418.1 anid|CBF84037.1 anig|EHA20090.1 aory|EIT77863.1 asyd|OJJ61362.1 ater|EAU31063.1 atub|OJI83412.1 aver|OJJ00689.1 awen|OJJ34249.1 azon|OJJ43389.1

Group4611: abra|OJJ78346.1 acar|OOF90268.1 acla|EAW09188.1 afis|EAW21958.1 afla|EED46678.1 afum|EAL92972.1 agla|OJJ84158.1 akaw|GAA85687.1 aluc|OJZ91420.1 anid|CBF84041.1 anig|EHA20091.1 aory|EIT77880.1 asyd|OJJ61360.1 ater|EAU31078.1 atub|OJI83413.1 aver|OJJ00687.1 awen|OJJ34251.1 azon|OJJ43387.1

Group4612: abra|OJJ78347.1 acar|OOF90267.1 acla|EAW09187.1 afis|EAW21959.1 afla|EED46679.1 afum|EAL92973.1 agla|OJJ84157.1 akaw|GAA85686.1 aluc|OJZ91421.1 anid|CBF84043.1 anig|EHA20092.1 aory|EIT77956.1 asyd|OJJ61359.1 ater|EAU31079.1 atub|OJI83414.1 aver|OJJ00686.1 awen|OJJ34252.1 azon|OJJ43386.1

Group4613: abra|OJJ78351.1 acar|OOF98536.1 acla|EAW09184.1 afis|EAW21962.1 afla|EED46683.1 afum|EAL92976.1 agla|OJJ84141.1 akaw|GAA85683.1 aluc|OJZ91425.1 anid|CBF82742.1 anig|EHA20095.1 aory|EIT77876.1 asyd|OJJ61622.1 ater|EAU31082.1 atub|OJI83418.1 aver|OJJ00946.1 awen|OJJ34256.1 azon|OJJ43383.1

Group4614: abra|OJJ78353.1 acar|OOF98535.1 acla|EAW09182.1 afis|EAW21964.1 afla|EED46685.1 afum|EAL92978.2 agla|OJJ84139.1 akaw|GAA85681.1 aluc|OJZ91428.1 anid|CBF82738.1 anig|EHA20097.1 aory|EIT77960.1 asyd|OJJ61624.1 ater|EAU31084.1 atub|OJI83421.1 aver|OJJ00949.1 awen|OJJ34258.1 azon|OJJ43381.1

Group4615: abra|OJJ78354.1 acar|OOF98534.1 acla|EAW09181.1 afis|EAW21965.1 afla|EED46686.1 afum|EAL92979.1 agla|OJJ84138.1 akaw|GAA85680.1 aluc|OJZ91429.1 anid|CBF82736.1 anig|EHA20098.1 aory|EIT77854.1 asyd|OJJ61625.1 ater|EAU31085.1 atub|OJI83422.1 aver|OJJ00950.1 awen|OJJ34259.1 azon|OJJ43378.1

Group4616: abra|OJJ78356.1 acar|OOF98533.1 acla|EAW09178.1 afis|EAW21968.1 afla|EED46689.1 afum|EAL92982.1 agla|OJJ84131.1 akaw|GAA85677.1 aluc|OJZ91431.1 anid|CBF82731.1 anig|EHA20100.1 aory|EIT77885.1 asyd|OJJ61628.1 ater|EAU31088.1 atub|OJI83424.1 aver|OJJ00952.1 awen|OJJ34261.1 azon|OJJ43376.1

Group4617: abra|OJJ78363.1 acar|OOF98524.1 acla|EAW09172.1 afis|EAW21974.1 afla|EED46697.1 afum|EAL92989.1 agla|OJJ84128.1 akaw|GAA85670.1 aluc|OJZ91438.1 anid|CBF82716.1 anig|EHA20103.1 aory|EIT77925.1 asyd|OJJ61637.1 ater|EAU31092.1 atub|OJI83432.1 aver|OJJ00960.1 awen|OJJ34267.1 azon|OJJ43369.1

Group4618: abra|OJJ78365.1 acar|OOF98521.1 acla|EAW09170.1 afis|EAW21976.1 afla|EED46699.1 afum|EAL92991.1 agla|OJJ84125.1 akaw|GAA85668.1 aluc|OJZ91442.1 anid|CBF82720.1 anig|EHA20106.1 aory|EIT77848.1 asyd|OJJ61635.1 ater|EAU31094.1 atub|OJI83436.1 aver|OJJ00958.1 awen|OJJ34269.1 azon|OJJ43368.1

Group4619: abra|OJJ78366.1 acar|OOF98520.1 acla|EAW09169.1 afis|EAW21977.1 afla|EED46700.1 afum|EAL92992.1 agla|OJJ84124.1 akaw|GAA85667.1 aluc|OJZ91443.1 anid|CBF82722.1 anig|EHA20107.1 aory|EIT77809.1 asyd|OJJ61634.1 ater|EAU31095.1 atub|OJI83437.1 aver|OJJ00957.1 awen|OJJ34270.1 azon|OJJ43366.1

Group4620: abra|OJJ78368.1 acar|OOF98518.1 acla|EAW09167.1 afis|EAW21979.1 afla|EED46702.1 afum|EAL92994.1 agla|OJJ84120.1 akaw|GAA85665.1 aluc|OJZ91445.1 anid|CBF82711.1 anig|EHA20109.1 aory|EIT77847.1 asyd|OJJ61640.1 ater|EAU31097.1 atub|OJI83439.1 aver|OJJ00963.1 awen|OJJ34272.1 azon|OJJ43364.1

Group4621: abra|OJJ78369.1 acar|OOF90130.1 acla|EAW09166.1 afis|EAW21980.1 afla|EED46704.1 afum|EAL92995.1 agla|OJJ84119.1 akaw|GAA85664.1 aluc|OJZ91446.1 anid|CBF82709.1 anig|EHA20110.1 aory|EIT77845.1 asyd|OJJ61641.1 ater|EAU31100.1 atub|OJI83440.1 aver|OJJ00964.1 awen|OJJ34274.1 azon|OJJ43363.1

Group4622: abra|OJJ78371.1 acar|OOF90128.1 acla|EAW09164.1 afis|EAW21982.1 afla|EED46706.1 afum|EAL92997.1 agla|OJJ84118.1 akaw|GAA85662.1 aluc|OJZ91448.1 anid|CBF82705.1 anig|EHA20112.1

aory|EIT77874.1 asyd|OJJ61643.1 ater|EAU31101.1 atub|OJI83441.1 aver|OJJ00965.1 awen|OJJ34276.1 azon|OJJ43361.1

Group4623: abra|OJJ78372.1 acar|OOF90125.1 acla|EAW09163.1 afis|EAW21983.1 afla|EED46707.1 afum|EAL92998.1 agla|OJJ84115.1 akaw|GAA85661.1 aluc|OJZ91449.1 anid|CBF82703.1 anig|EHA20113.1 aory|EIT77859.1 asyd|OJJ61644.1 ater|EAU31102.1 atub|OJI83442.1 aver|OJJ00966.1 awen|OJJ34280.1 azon|OJJ43360.1

Group4624: abra|OJJ78374.1 acar|OOF98515.1 acla|EAW09161.1 afis|EAW21985.1 afla|EED46709.1 afum|EAL93000.1 agla|OJJ84113.1 akaw|GAA85659.1 aluc|OJZ91451.1 anid|CBF82698.1 anig|EHA20115.1 aory|EIT77948.1 asyd|OJJ61646.1 ater|EAU31104.1 atub|OJI83444.1 aver|OJJ00968.1 awen|OJJ34282.1 azon|OJJ43358.1

Group4625: abra|OJJ78375.1 acar|OOF98514.1 acla|EAW09160.1 afis|EAW21986.1 afla|EED46710.1 afum|EAL93002.1 agla|OJJ84112.1 akaw|GAA85658.1 aluc|OJZ91452.1 anid|CBF82696.1 anig|EHA20116.1 aory|EIT77888.1 asyd|OJJ61647.1 ater|EAU31106.1 atub|OJI83445.1 aver|OJJ00969.1 awen|OJJ34283.1 azon|OJJ43357.1

Group4626: abra|OJJ78378.1 acar|OOF98510.1 acla|EAW09155.1 afis|EAW21991.1 afla|EED46715.1 afum|EAL93006.2 agla|OJJ84107.1 akaw|GAA85654.1 aluc|OJZ91457.1 anid|CBF82687.1 anig|EHA20120.1 aory|EIT77887.1 asyd|OJJ61652.1 ater|EAU31110.1 atub|OJI83450.1 aver|OJJ00973.1 awen|OJJ34287.1 azon|OJJ43351.1

Group4627: abra|OJJ78380.1 acar|OOF98508.1 acla|EAW09153.1 afis|EAW21993.1 afla|EED46717.1 afum|EAL93008.1 agla|OJJ84104.1 akaw|GAA85652.1 aluc|OJZ91459.1 anid|CBF82683.1 anig|EHA20122.1 aory|EIT77842.1 asyd|OJJ61655.1 ater|EAU31112.1 atub|OJI83452.1 aver|OJJ00976.1 awen|OJJ34289.1 azon|OJJ43349.1

Group4628: abra|OJJ78381.1 acar|OOF98507.1 acla|EAW09152.1 afis|EAW21994.1 afla|EED46718.1 afum|EAL93009.1 agla|OJJ84103.1 akaw|GAA85651.1 aluc|OJZ91460.1 anid|CBF82681.1 anig|EHA20123.1 aory|EIT77902.1 asyd|OJJ61656.1 ater|EAU31113.1 atub|OJI83453.1 aver|OJJ00977.1 awen|OJJ34290.1 azon|OJJ43348.1

Group4629: abra|OJJ78382.1 acar|OOF98506.1 acla|EAW09151.1 afis|EAW21995.1 afla|EED46719.1 afum|EAL93010.1 agla|OJJ84102.1 akaw|GAA85650.1 aluc|OJZ91461.1 anid|CBF82679.1 anig|EHA20124.1 aory|EIT77906.1 asyd|OJJ61657.1 ater|EAU31114.1 atub|OJI83454.1 aver|OJJ00978.1 awen|OJJ34291.1 azon|OJJ43347.1

Group4630: abra|OJJ78383.1 acar|OOF98505.1 acla|EAW09150.1 afis|EAW21996.1 afla|EED46720.1 afum|EAL93011.1 agla|OJJ84101.1 akaw|GAA85649.1 aluc|OJZ91462.1 anid|CBF82677.1 anig|EHA20125.1 aory|EIT77812.1 asyd|OJJ61658.1 ater|EAU31115.1 atub|OJI83455.1 aver|OJJ00979.1 awen|OJJ34292.1 azon|OJJ43346.1

Group4631: abra|OJJ78384.1 acar|OOF98504.1 acla|EAW09149.1 afis|EAW21997.1 afla|EED46721.1 afum|EAL93012.1 agla|OJJ84100.1 akaw|GAA85648.1 aluc|OJZ91463.1 anid|CBF82675.1 anig|EHA20126.1 aory|EIT77875.1 asyd|OJJ61659.1 ater|EAU31116.1 atub|OJI83456.1 aver|OJJ00980.1 awen|OJJ34293.1 azon|OJJ43345.1

Group4632: abra|OJJ78385.1 acar|OOF98503.1 acla|EAW09148.1 afis|EAW21998.1 afla|EED46722.1 afum|EAL93013.1 agla|OJJ84099.1 akaw|GAA85647.1 aluc|OJZ91464.1 anid|CBF82674.1 anig|EHA20127.1 aory|EIT77891.1 asyd|OJJ61660.1 ater|EAU31117.1 atub|OJI83457.1 aver|OJJ00981.1 awen|OJJ34294.1 azon|OJJ43344.1

Group4633: abra|OJJ78386.1 acar|OOF98502.1 acla|EAW09147.1 afis|EAW21999.1 afla|EED46723.1 afum|EAL93014.1 agla|OJJ84098.1 akaw|GAA85646.1 aluc|OJZ91465.1 anid|CBF82672.1 anig|EHA20128.1 aory|EIT77907.1 asyd|OJJ61661.1 ater|EAU31118.1 atub|OJI83458.1 aver|OJJ00982.1 awen|OJJ34295.1 azon|OJJ43343.1

Group4634: abra|OJJ78388.1 acar|OOF98500.1 acla|EAW09145.1 afis|EAW22002.1 afla|EED46727.1 afum|EAL93017.1 agla|OJJ84096.1 akaw|GAA85644.1 aluc|OJZ91467.1 anid|CBF82666.1 anig|EHA20129.1 aory|EIT77844.1 asyd|OJJ61664.1 ater|EAU31121.1 atub|OJI83460.1 aver|OJJ00985.1 awen|OJJ34297.1 azon|OJJ43341.1

Group4635: acar|OOG00510.1 afis|EAW25745.1 akaw|GAA87910.1 aluc|OJZ88780.1 anid|CBF84274.1 anig|EHA18474.1 aory|EIT75519.1 asyd|OJJ57228.1 ater|EAU32770.1 atub|OJI91352.1 aver|OJI97599.1 awen|OJJ32621.1 afis|EAW24516.1 afla|EED47665.1 aver|OJJ03061.1 aluc|OJZ85622.1 aory|EIT80232.1 atub|OJI79415.1

Group4636: acla|EAW13541.1 afis|EAW20614.1 afla|EED54203.1 afum|EAL84447.1 agla|OJJ88592.1 akaw|GAA87001.1 aluc|OJZ84456.1 anid|CBF86940.1 anig|EHA27771.1 aory|EIT75953.1 asyd|OJJ55587.1 atub|OJI89619.1 aver|OJJ06666.1 awen|OJJ33458.1 azon|OJJ50714.1 ater|EAU35253.1 asyd|OJJ55586.1 aver|OJJ06665.1

Group4637: abra|OJJ65435.1 abra|OJJ65508.1 abra|OJJ65821.1 asyd|OJJ51932.1 asyd|OJJ52321.1 asyd|OJJ51963.1 asyd|OJJ51982.1 awen|OJJ29548.1 awen|OJJ29621.1 akaw|GAA93288.1 afis|EAW20669.1 agla|OJJ83544.1 aluc|OJZ79579.1 anig|EHA17693.1 ater|EAU30109.1 akaw|GAA93088.1 aluc|OJZ80408.1

Group4638: abra|OJJ65486.1 abra|OJJ65539.1 akaw|GAA92968.1 anig|EHA20201.1 afla|EED53240.1 agla|OJJ82729.1 anid|CBF82095.1 aory|EIT74878.1 aver|OJJ05216.1 awen|OJJ30575.1 abra|OJJ75529.1 acar|OOF90856.1 akaw|GAA84291.1 aluc|OJZ88985.1 anig|EHA23541.1 atub|OJI85803.1 ater|EAU39592.1

Group4639: abra|OJJ65671.1 abra|OJJ69720.1 acar|OOF92570.1 acar|OOG00879.1 afla|EED52525.1 afla|EED53603.1 akaw|GAA90173.1 akaw|GAA93196.1 aory|EIT74948.1 aory|EIT82807.1 acla|EAW11895.1 afum|EAL84364.1 aluc|OJZ83177.1 anig|EHA28251.1 atub|OJI87760.1 awen|OJJ36063.1 azon|OJJ42163.1

Group4640: abra|OJJ68069.1 abra|OJJ68262.1 akaw|GAA83203.1 aluc|OJZ83704.1 anig|EHA18854.1 atub|OJI90076.1 acar|OOF97541.1 afla|EED45998.1 agla|OJJ80130.1 anid|CBF90047.1 aory|EIT75615.1 asyd|OJJ62055.1 ater|EAU38008.1 aver|OJI96161.1 awen|OJJ30952.1 anid|CBF79559.1 awen|OJJ36502.1

Group4641: acar|OOF90194.1 acar|OOF90196.1 abra|OJJ67673.1 afis|EAW24512.1 afum|EBA27260.1 akaw|GAA89189.1 aluc|OJZ81642.1 anid|CBF75855.1 asyd|OJJ62592.1 ater|EAU37120.1 atub|OJI88540.1 aver|OJJ04434.1 awen|OJJ36101.1 afis|EAW15651.1 aluc|OJZ79951.1 aluc|OJZ80155.1 asyd|OJJ57969.1

Group4642: acar|OOF90453.1 acar|OOF90460.1 abra|OJJ71883.1 acla|EAW09638.1 afis|EAW21502.1 afla|EED55938.1 afum|EAL92521.1 agla|OJJ85665.1 akaw|GAA85033.1 aluc|OJZ86621.1 anig|EHA22045.1 asyd|OJJ61108.1 ater|EAU35036.1 atub|OJI83904.1 aver|OJJ00425.1 awen|OJJ39193.1 azon|OJJ51518.1

Group4643: acar|OOF92292.1 acar|OOF92333.1 abra|OJJ67509.1 acla|EAW06263.1 afis|EAW18369.1 afum|EAL93044.1 agla|OJJ84934.1 akaw|GAA86330.1 aluc|OJZ84855.1 anid|CBF70810.1 anig|EHA25088.1 aory|EIT77442.1 asyd|OJJ58407.1 atub|OJI80741.1 aver|OJJ01008.1 awen|OJJ31780.1 azon|OJJ42022.1

Group4644: acar|OOF92294.1 acar|OOF92335.1 abra|OJJ67506.1 acla|EAW06265.1 afis|EAW18371.1 afla|EED53035.1 afum|EAL93046.1 akaw|GAA86332.1 aluc|OJZ84857.1 anid|CBF70806.1 anig|EHA25090.1 aory|EIT77400.1 asyd|OJJ58409.1 ater|EAU32241.1 atub|OJI80739.1 aver|OJJ01011.1 azon|OJJ50444.1

Group4645: acar|OOF92315.1 acar|OOF92357.1 abra|OJJ67484.1 acla|EAW06290.1 afis|EAW18396.1 afla|EED53012.1 afum|EAL93072.1 agla|OJJ84967.1 akaw|GAA86355.1 aluc|OJZ84884.1 anid|CBF70760.1 anig|EHA25113.1 aory|EIT77413.1 asyd|OJJ58432.1 atub|OJI80716.1 aver|OJJ01033.1 azon|OJJ50471.1

Group4646: acar|OOF93666.1 acar|OOF93750.1 abra|OJJ66646.1 acla|EAW11789.1 afis|EAW19399.1 afum|EAL87095.1 agla|OJJ81110.1 akaw|GAA89599.1 aluc|OJZ80803.1 anid|CBF74334.1 anig|EHA27231.1 aory|EIT83139.1 asyd|OJJ51907.1 ater|EAU36125.1 atub|OJI87651.1 awen|OJJ36794.1 azon|OJJ43005.1

Group4647: acar|OOF93683.1 acar|OOF93768.1 abra|OJJ66668.1 acla|EAW11809.1 afis|EAW19419.1 afla|EED50376.1 afum|EAL87075.1 akaw|GAA89617.1 aluc|OJZ80779.1 anid|CBF74367.1 anig|EHA27250.1 aory|EIT83147.1 ater|EAU34725.1 atub|OJI87631.1 aver|OJJ05381.1 awen|OJJ36817.1 azon|OJJ42985.1

Group4648: acar|OOF93688.1 acar|OOF93773.1 abra|OJJ66674.1 acla|EAW11814.1 afis|EAW19424.1 afla|EED50382.1 afum|EAL87070.1 agla|OJJ81136.1 akaw|GAA89622.1 aluc|OJZ80774.1 anig|EHA27256.1 aory|EIT83125.1 asyd|OJJ57779.1 ater|EAU34728.1 atub|OJI87626.1 aver|OJJ05386.1 awen|OJJ36822.1

Group4649: acar|OOF93689.1 acar|OOF93774.1 abra|OJJ66676.1 acla|EAW11815.1 afis|EAW19426.1 afum|EAL87068.1 agla|OJJ81138.1 akaw|GAA89624.1 aluc|OJZ80772.1 anig|EHA27258.1 aory|EIT83128.1 asyd|OJJ57777.1 ater|EAU34730.1 atub|OJI87624.1 aver|OJJ05388.1 awen|OJJ36824.1 azon|OJJ42491.1

Group4650: acar|OOF93695.1 acar|OOF93780.1 abra|OJJ66686.1 acla|EAW11821.1 afis|EAW19432.1 afla|EED56637.1 agla|OJJ81144.1 akaw|GAA89630.1 aluc|OJZ80763.1 anig|EHA27264.1 aory|EIT79667.1 asyd|OJJ59390.1 atub|OJI87617.1 aver|OJJ02882.1 awen|OJJ36830.1 azon|OJJ42973.1 anid|CBF81378.1

Group4651: acar|OOF93698.1 acar|OOF93783.1 abra|OJJ66689.1 acla|EAW11824.1 afis|EAW19435.1 afla|EED56640.1 afum|EAL87060.1 agla|OJJ81147.1 akaw|GAA89633.1 aluc|OJZ80760.1 anig|EHA27267.1 aory|EIT79665.1 asyd|OJJ59394.1 ater|EAU34737.1 atub|OJI87614.1 aver|OJJ02878.1 azon|OJJ42970.1

Group4652: acar|OOF93704.1 acar|OOF93789.1 abra|OJJ66696.1 afla|EED56650.1 afum|EAL87053.1 agla|OJJ81156.1 akaw|GAA89640.1 aluc|OJZ80749.1 anid|CBF81397.1 anig|EHA27273.1 aory|EIT79652.1 asyd|OJJ59402.1 ater|EAU34744.1 atub|OJI87606.1 aver|OJJ02871.1 awen|OJJ36843.1 azon|OJJ42957.1

Group4653: acar|OOF93713.1 acar|OOF93799.1 abra|OJJ66711.1 acla|EAW11842.1 afis|EAW19453.1 afla|EED56661.1 afum|EAL87043.1 agla|OJJ81168.1 akaw|GAA89653.1 aluc|OJZ80736.1 anid|CBF81418.1 asyd|OJJ59413.1 ater|EAU34755.1 atub|OJI87593.1 aver|OJJ02860.1 awen|OJJ36854.1 azon|OJJ42946.1

Group4654: acar|OOF93722.1 acar|OOF93809.1 abra|OJJ66720.1 acla|EAW11851.1 afis|EAW19462.1 afla|EED56673.1 afum|EAL87034.1 agla|OJJ81179.1 akaw|GAA89662.1 aluc|OJZ80727.1 anid|CBF75493.1 asyd|OJJ59751.1 ater|EAU34765.1 atub|OJI87583.1 aver|OJJ02536.1 awen|OJJ36864.1 azon|OJJ42937.1

Group4655: acar|OOF94041.1 acar|OOF94111.1 abra|OJJ66400.1 afla|EED49553.1 akaw|GAA90268.1 aluc|OJZ83073.1 anig|EHA28137.1 aory|EIT82620.1 asyd|OJJ61454.1 atub|OJI87873.1 aver|OJJ00790.1 awen|OJJ39517.1 abra|OJJ69534.1 agla|OJJ79509.1 akaw|GAA88384.1 anig|EHA28392.1 acar|OOF92662.1

Group4656: acar|OOF94050.1 acar|OOF94120.1 abra|OJJ66395.1 acla|EAW12087.1 afla|EED49519.1 akaw|GAA90260.1 aluc|OJZ83080.1 anid|CBF73558.1 anig|EHA28143.1 aory|EIT82588.1 asyd|OJJ56699.1 atub|OJI87865.1 aver|OJJ06026.1 awen|OJJ40206.1 azon|OJJ47473.1 awen|OJJ35025.1 afis|EAW19730.1

Group4657: acar|OOF94052.1 acar|OOF94122.1 abra|OJJ66392.1 acla|EAW13709.1 afis|EAW19881.1 afla|EED46102.1 afum|EAL85017.1 akaw|GAA90258.1 aluc|OJZ83082.1 anid|CBF75188.1 anig|EHA28145.1 aory|EIT76369.1 asyd|OJJ59947.1 ater|EAU35603.1 atub|OJI87863.1 aver|OJJ02342.1 azon|OJJ51816.1

Group4658: acar|OOF94076.1 acar|OOF94146.1 abra|OJJ66026.1 acla|EAW07129.1 afis|EAW22139.1 anid|CBF89247.1 atub|OJI87440.1 awen|OJJ40945.1 azon|OJJ42435.1 abra|OJJ67016.1 afum|EAL91051.1 aver|OJJ03311.1 abra|OJJ67290.1 akaw|GAA86523.1 aluc|OJZ85069.1 anig|EHA25283.1 atub|OJI80529.1

Group4659: acar|OOF95088.1 acar|OOF95154.1 abra|OJJ74616.1 acla|EAW10766.1 afis|EAW24810.1 afla|EED47173.1 afum|EAL89453.2 agla|OJJ82905.1 akaw|GAA81990.1 aluc|OJZ92199.1 anig|EHA26758.1 aory|EIT79174.1 asyd|OJJ64383.1 atub|OJI86626.1 aver|OJI98479.1 awen|OJJ40394.1 azon|OJJ49247.1

Group4660: acar|OOF95105.1 acar|OOF95171.1 abra|OJJ74599.1 acla|EAW10780.1 afis|EAW24794.1 afla|EED46333.1 afum|EAL89436.1 agla|OJJ79801.1 akaw|GAA82009.1 aluc|OJZ92178.1 anig|EHA26775.1 aory|EIT81232.1 asyd|OJJ59471.1 ater|EAU36511.1 atub|OJI86606.1 awen|OJJ40379.1 aver|OJJ02803.1

Group4661: acar|OOF96237.1 acar|OOF96274.1 afum|EAL85171.1 afum|EAL87775.1 abra|OJJ71521.1 afis|EAW20050.1 afla|EED45330.1 agla|OJJ88910.1 akaw|GAA89396.1 aluc|OJZ86260.1 anid|CBF84802.1 anig|EHA21606.1 aory|EIT73757.1 ater|EAU38660.1 atub|OJI84283.1 awen|OJJ31808.1 azon|OJJ46314.1

Group4662: acar|OOF96905.1 acar|OOF96940.1 abra|OJJ70805.1 acla|EAW11119.1 afis|EAW15849.1 afum|EBA27349.1 agla|OJJ87260.1 akaw|GAA90120.1 aluc|OJZ84151.1 anid|CBF81170.1 anig|EHA19255.1 asyd|OJJ59289.1 ater|EAU31664.1 atub|OJI90523.1 aver|OJJ02986.1 awen|OJJ36779.1 azon|OJJ43020.1

Group4663: acar|OOF96910.1 acar|OOF96944.1 abra|OJJ70800.1 acla|EAW11124.1 afis|EAW15853.1 afla|EED56520.1 afum|EAL88489.1 agla|OJJ87255.1 akaw|GAA90116.1 aluc|OJZ84145.1 anid|CBF81179.1 asyd|OJJ59294.1 ater|EAU31667.1 atub|OJI90519.1 aver|OJJ02981.1 awen|OJJ36775.1 azon|OJJ47423.1

Group4664: acar|OOF96925.1 acar|OOF96960.1 abra|OJJ70784.1 acla|EAW11136.1 afis|EAW15868.1 afum|EAL88502.1 agla|OJJ87240.1 akaw|GAA90102.1 aluc|OJZ84130.1 anid|CBF81207.1 anig|EHA19238.1 asyd|OJJ59308.1 ater|EAU31683.1 atub|OJI90504.1 aver|OJJ02967.1 awen|OJJ29537.1 azon|OJJ47437.1

Group4665: acar|OOF96926.1 acar|OOF96961.1 abra|OJJ70782.1 afla|EED56506.1 agla|OJJ87239.1 akaw|GAA90101.1 aluc|OJZ84129.1 anid|CBF81209.1 anig|EHA19237.1 aory|EIT72792.1 asyd|OJJ59309.1 atub|OJI90503.1 aver|OJJ02966.1 awen|OJJ36761.1 azon|OJJ47438.1 acla|EAW11137.1 awen|OJJ29536.1

Group4666: acar|OOF97656.1 acar|OOF97703.1 abra|OJJ71501.1 acla|EAW10047.1 afla|EED44713.1 afum|EBA27399.1 agla|OJJ81159.1 akaw|GAA82519.1 aluc|OJZ87046.1 anig|EHA20319.1 aory|EIT83457.1 asyd|OJJ53008.1 ater|EAU33378.1 atub|OJI81589.1 aver|OJJ03835.1 awen|OJJ41279.1 azon|OJJ49502.1

Group4667: acar|OOF98479.1 acar|OOF98492.1 abra|OJJ78395.1 acla|EAW09137.1 afis|EAW22010.1 afla|EED46734.1 afum|EAL93025.1 agla|OJJ84088.1 akaw|GAA85636.1 aluc|OJZ91474.1 anig|EHA20136.1 asyd|OJJ61672.1 atub|OJI83467.1 awen|OJJ34305.1 azon|OJJ43332.1 anid|CBF82651.1 aver|OJJ00993.1

Group4668: acar|OOF99310.1 acar|OOF99311.1 acla|EAW09382.1 afis|EAW21753.1 afla|EED58025.1 afum|EAL92783.1 agla|OJJ84400.1 anid|CBF87161.1 aory|EIT79777.1 asyd|OJJ58846.1 ater|EAU38437.1 aver|OJJ01447.1 awen|OJJ34037.1 azon|OJJ45317.1 anid|CBF83691.1 asyd|OJJ60817.1 aver|OJJ00143.1

Group4669: acar|OOG00248.1 acar|OOG00266.1 abra|OJJ67542.1 acla|EAW13608.1 afis|EAW20002.1 afla|EED45812.1 afum|EAL84917.1 akaw|GAA88117.1 aluc|OJZ81390.1 anid|CBF78636.1 anig|EHA18683.1 aory|EIT75677.1 asyd|OJJ65124.1 ater|EAU37945.1 atub|OJI91595.1 aver|OJI99252.1 azon|OJJ47329.1

Group4670: afla|EED49436.1 afla|EED53000.1 aory|EIT77416.1 aory|EIT79505.1 abra|OJJ67468.1 acar|OOF92366.1 afis|EAW18409.1 afum|EAL93089.1 agla|OJJ84976.1 akaw|GAA86369.1 aluc|OJZ84898.1 anid|CBF70739.1 anig|EHA25125.1 asyd|OJJ58440.1 atub|OJI80700.1 aver|OJJ01040.1 awen|OJJ31740.1

Group4671: agla|OJJ81704.1 agla|OJJ85950.1 anig|EHA24273.1 anig|EHA24275.1 asyd|OJJ53801.1 asyd|OJJ63907.1 awen|OJJ37085.1 awen|OJJ39694.1 acla|EAW12912.1 afis|EAW17749.1 afla|EED55100.1 afum|EAL91563.1 anid|CBF80577.1 ater|EAU31857.1 aver|OJI97034.1 azon|OJJ44442.1 aory|EIT81664.1

Group4672: agla|OJJ84696.1 agla|OJJ88299.1 abra|OJJ65609.1 afla|EED54385.1 aory|EIT75847.1 abra|OJJ76926.1 acar|OOF91371.1 afla|EED46863.1 akaw|GAA87114.1 aluc|OJZ84324.1 anid|CBF74601.1 anig|EHA27659.1 aory|EIT72704.1 asyd|OJJ61216.1 atub|OJI89744.1 aver|OJJ00540.1 anig|EHA18441.1

Group4673: akaw|GAA82959.1 akaw|GAA85567.1 aluc|OJZ83501.1 aluc|OJZ87540.1 anig|EHA18316.1 anig|EHA24335.1 atub|OJI81107.1 atub|OJI82395.1 abra|OJJ72993.1 acar|OOF96169.1 afla|EED49853.1 agla|OJJ88852.1 anid|CBF73514.1 aory|EIT83415.1 asyd|OJJ61476.1 aver|OJJ00818.1 awen|OJJ31873.1

Group4674: akaw|GAA87889.1 akaw|GAA92892.1 asyd|OJJ52060.1 asyd|OJJ56757.1 abra|OJJ66170.1 afla|EED52364.1 afum|EAL88779.1 agla|OJJ83554.1 anid|CBF75918.1 anig|EHA26110.1 asyd|OJJ52025.1 azon|OJJ42071.1 acla|EAW11462.1 agla|OJJ81341.1 aluc|OJZ79991.1 acla|EAW11473.1 afis|EAW17037.1

Group4675: anid|CBF79042.1 anid|CBF89822.1 abra|OJJ74989.1 acar|OOF97331.1 acla|EAW08393.1 afis|EAW23436.1 afla|EED57325.1 afum|EAL88026.1 akaw|GAA83445.1 aluc|OJZ92551.1 anig|EHA26426.1 aory|EIT74067.1 asyd|OJJ62175.1 ater|EAU37490.1 atub|OJI86989.1 aver|OJI96284.1 azon|OJJ44145.1

Group4676: anid|CBF83201.1 anid|CBF86907.1 asyd|OJJ55555.1 asyd|OJJ58381.1 asyd|OJJ64611.1 ater|EAU29240.1 ater|EAU35231.1 ater|EAU39358.1 aver|OJI98707.1 aver|OJJ06635.1 aver|OJJ06637.1 azon|OJJ46028.1 afis|EAW17327.1 afla|EED54401.1 afum|EAL91164.1 agla|OJJ79869.1 aory|EIT75849.1

Group4677: asyd|OJJ53784.1 asyd|OJJ56688.1 abra|OJJ67840.1 acar|OOF92016.1 acla|EAW12885.1 afis|EAW17792.1 afla|EED53092.1 agla|OJJ85532.1 akaw|GAA91846.1 aluc|OJZ82052.1 anid|CBF73574.1 anig|EHA22207.1 aory|EIT74818.1 ater|EAU38815.1 atub|OJI84610.1 aver|OJJ06035.1 awen|OJJ35973.1

Group4678: asyd|OJJ54404.1 asyd|OJJ54753.1 asyd|OJJ64139.1 abra|OJJ65870.1 afla|EED51457.1 agla|OJJ85481.1 akaw|GAA92276.1 aluc|OJZ90170.1 aory|EIT80565.1 ater|EAU33973.1 atub|OJI81598.1 aver|OJI99607.1 awen|OJJ36266.1 asyd|OJJ58123.1 aver|OJJ08845.1 anid|CBF71445.1 asyd|OJJ56514.1

Group4679: asyd|OJJ54407.1 asyd|OJJ56037.1 abra|OJJ69897.1 acar|OOF93416.1 acla|EAW08923.1 afis|EAW15470.1 agla|OJJ80150.1 akaw|GAA87753.1 aluc|OJZ85386.1 anid|CBF78232.1 anig|EHA25570.1 aory|EIT72697.1 ater|EAU32658.1 atub|OJI80184.1 aver|OJJ04983.1 awen|OJJ38120.1 afum|EAL85852.1

Group4680: asyd|OJJ54582.1 asyd|OJJ59187.1 awen|OJJ38449.1 awen|OJJ38819.1 abra|OJJ67702.1 akaw|GAA92941.1 abra|OJJ77957.1 afis|EAW20204.1 afla|EED45854.1 aluc|OJZ83508.1 anig|EHA19712.1 aory|EIT75426.1 ater|EAU35724.1 atub|OJI91308.1 azon|OJJ45690.1 ater|EAU31781.1 agla|OJJ83813.1

Group4681: asyd|OJJ58287.1 asyd|OJJ62495.1 abra|OJJ68999.1 acar|OOF92711.1 afis|EAW18027.1 afla|EED51868.1 afum|EAL91838.1 agla|OJJ85400.1 akaw|GAA90518.1 aluc|OJZ85983.1 anid|CBF80061.1 anig|EHA24623.1 ater|EAU31541.1 atub|OJI79773.1 aver|OJJ02137.1 awen|OJJ41512.1 azon|OJJ48928.1

Group4682: ater|EAU30451.1 ater|EAU31200.1 abra|OJJ68616.1 acar|OOF92127.1 acla|EAW12608.1 afis|EAW18076.1 afla|EED51468.1 afum|EAL91888.1 akaw|GAA92905.1 aluc|OJZ81938.1 anid|CBF86402.1 anig|EHA22310.1 aory|EIT80658.1 asyd|OJJ64950.1 atub|OJI84491.1 aver|OJI99072.1 awen|OJJ41465.1

Group4683: ater|EAU30817.1 ater|EAU34589.1 abra|OJJ67750.1 acar|OOF90955.1 acla|EAW10989.1 afis|EAW20871.1 afla|EED45553.1 afum|EAL84763.1 akaw|GAA92441.1 aluc|OJZ81579.1 anid|CBF82846.1 anig|EHA25860.1 asyd|OJJ61591.1 atub|OJI88602.1 aver|OJJ00913.1 awen|OJJ34490.1 azon|OJJ51767.1

Group4684: atub|OJI81487.1 atub|OJI81722.1 abra|OJJ71387.1 acar|OOF97781.1 afis|EAW25058.1 afum|EAL89726.1 agla|OJJ79169.1 akaw|GAA82607.1 aluc|OJZ87153.1 anig|EHA20412.1 aver|OJJ05909.1 awen|OJJ33722.1 azon|OJJ46843.1 acar|OOF98188.1 aluc|OJZ90300.1 anig|EHA23788.1 akaw|GAA86066.1

Group4685: awen|OJJ30759.1 awen|OJJ31722.1 abra|OJJ67450.1 acar|OOF90340.1 acla|EAW06314.1 afla|EED52985.1 agla|OJJ84993.1 akaw|GAA86380.1 aluc|OJZ84914.1 anid|CBF70692.1 anig|EHA25135.1 aory|EIT78708.1 asyd|OJJ58465.1 ater|EAU29844.1 atub|OJI80684.1 aver|OJJ01064.1 azon|OJJ50485.1

Group4686: awen|OJJ30765.1 awen|OJJ31716.1 abra|OJJ67445.1 acar|OOF92382.1 acla|EAW06318.1 afis|EAW18432.1 afum|EAL93113.1 agla|OJJ84998.1 akaw|GAA86384.1 aluc|OJZ84918.1 anid|CBF70027.1 anig|EHA25138.1 asyd|OJJ58871.1 ater|EAU29848.1 atub|OJI80680.1 aver|OJJ01473.1 azon|OJJ50480.1

Group4687: awen|OJJ30766.1 awen|OJJ31715.1 abra|OJJ67444.1 acar|OOF92383.1 acla|EAW06319.1 afis|EAW18433.1 afum|EAL93114.1 agla|OJJ84999.1 akaw|GAA86385.1 aluc|OJZ84920.1 anid|CBF70029.1 anig|EHA25139.1 asyd|OJJ58870.1 ater|EAU29849.1 atub|OJI80679.1 aver|OJJ01472.1 azon|OJJ50479.1

Group4688: azon|OJJ42401.1 azon|OJJ48129.1 abra|OJJ68278.1 acar|OOG00615.1 afla|EED57500.1 akaw|GAA85996.1 aluc|OJZ83693.1 anig|EHA19762.1 aory|EIT81886.1 asyd|OJJ62122.1 ater|EAU34294.1 atub|OJI90065.1 aver|OJI96230.1 acar|OOF96457.1 aluc|OJZ91059.1 atub|OJI83055.1 azon|OJJ47352.1

Group4689: azon|OJJ47108.1 azon|OJJ47396.1 abra|OJJ67013.1 acar|OOF93607.1 acla|EAW15355.1 afis|EAW20357.1 afum|EAL85474.1 agla|OJJ80638.1 akaw|GAA92160.1 aluc|OJZ85567.1 anig|EHA24951.1 aory|EIT76052.1 asyd|OJJ64186.1 ater|EAU31045.1 atub|OJI79362.1 aver|OJI98263.1 awen|OJJ33014.1

Group4690: azon|OJJ50112.1 azon|OJJ50416.1 abra|OJJ68033.1 acar|OOF91810.1 acla|EAW06966.1 afla|EED55046.1 afum|EAL86259.2 agla|OJJ83082.1 akaw|GAA90919.1 aluc|OJZ82907.1 anig|EHA21330.1 aory|EIT81961.1 asyd|OJJ53715.1 ater|EAU36650.1 atub|OJI89133.1 aver|OJJ07841.1 awen|OJJ33892.1

Group4691: azon|OJJ50963.1 azon|OJJ51217.1 abra|OJJ67715.1 afis|EAW16634.1 anig|EHA27628.1 asyd|OJJ65143.1 aver|OJI99311.1 abra|OJJ68588.1 afis|EAW25637.1 afum|EBA27174.1 aver|OJJ02989.1 awen|OJJ33493.1 akaw|GAA85860.1 aluc|OJZ91219.1 ater|EAU36712.1 aver|OJJ06469.1 ater|EAU33968.1

Group4692: acla|EAW12782.1 awen|OJJ36316.1 acar|OOF94301.1 afis|EAW24051.1 afla|EED49757.1 afum|EAL87858.1 agla|OJJ80393.1 akaw|GAA87492.1 aluc|OJZ88032.1 anig|EHA17806.1 aory|EIT77642.1 asyd|OJJ56970.1 atub|OJI90638.1 aver|OJJ03318.1 afis|EAW23607.1 awen|OJJ41819.1 asyd|OJJ51929.1

Group4693: abra|OJJ65584.1 acar|OOF94381.1 acla|EAW15324.1 afis|EAW24551.1 afla|EED49772.1 afum|EAL91136.1 anid|CBF84508.1 aory|EIT77709.1 asyd|OJJ53518.1 ater|EAU38754.1 aver|OJJ08233.1 awen|OJJ37782.1 azon|OJJ42493.1 aver|OJJ07928.1 awen|OJJ31444.1 acla|EAW15019.1 afum|EAL86216.1

Group4694: abra|OJJ65640.1 afla|EED47428.1 aluc|OJZ91197.1 anid|CBF82230.1 aory|EIT81901.1 ater|EAU35410.1 awen|OJJ40835.1 abra|OJJ65888.1 acar|OOF94950.1 acla|EAW12250.1 agla|OJJ79071.1 akaw|GAA86016.1 aluc|OJZ90244.1 anig|EHA23739.1 asyd|OJJ57337.1 atub|OJI81672.1 aver|OJJ05825.1

Group4695: abra|OJJ65778.1 agla|OJJ79806.1 aluc|OJZ82947.1 atub|OJI89179.1 aver|OJJ07886.1 awen|OJJ35386.1 abra|OJJ69436.1 acla|EAW11436.1 afis|EAW23987.1 afla|EED52190.1 afum|EAL87416.1 aluc|OJZ82272.1 anid|CBF82043.1 asyd|OJJ54224.1 atub|OJI88167.1 aver|OJJ05177.1 afla|EED57914.1

Group4696: abra|OJJ65915.1 acar|OOF94966.1 afis|EAW19972.1 afla|EED50462.1 afum|EAL84938.1 agla|OJJ80064.1 akaw|GAA92317.1 aluc|OJZ90215.1 anid|CBF84460.1 anig|EHA23713.1 aory|EIT82029.1 asyd|OJJ53547.1 ater|EAU31470.1 atub|OJI81646.1 aver|OJJ08206.1 awen|OJJ36012.1 azon|OJJ46810.1

Group4697: abra|OJJ65924.1 acar|OOF94985.1 afis|EAW19706.1 afla|EED57047.1 afum|EAL86820.1 agla|OJJ78913.1 akaw|GAA92309.1 aluc|OJZ90207.1 anid|CBF90284.1 anig|EHA23704.1 aory|EIT82250.1 asyd|OJJ61919.1 ater|EAU32843.1 atub|OJI81636.1 aver|OJI96011.1 awen|OJJ29912.1 acla|EAW12063.1

Group4698: abra|OJJ65938.1 acar|OOF91905.1 acla|EAW12331.1 afis|EAW16891.1 afla|EED46396.1 afum|EAL86429.1 agla|OJJ79945.1 akaw|GAA92298.1 aluc|OJZ90195.1 anid|CBF78493.1 anig|EHA23694.1 aory|EIT72430.1 asyd|OJJ65325.1 ater|EAU30223.1 atub|OJI81622.1 aver|OJI99470.1 awen|OJJ29747.1

Group4699: abra|OJJ65941.1 acar|OOF95011.1 acla|EAW09018.1 afis|EAW19837.1 afla|EED47289.1 afum|EAL85104.1 akaw|GAA92294.1 aluc|OJZ90191.1 anid|CBF84686.1 anig|EHA23690.1 aory|EIT79198.1 asyd|OJJ53334.1 atub|OJI81618.1 aver|OJJ08249.1 aver|OJJ08410.1 acla|EAW13741.1 asyd|OJJ53496.1

Group4700: abra|OJJ65962.1 acar|OOF92912.1 acla|EAW13581.1 afis|EAW20020.1 afla|EED46028.1 afum|EAL84897.1 agla|OJJ88014.1 akaw|GAA90837.1 aluc|OJZ82996.1 anig|EHA21411.1 aory|EIT75634.1 asyd|OJJ53300.1 ater|EAU29382.1 atub|OJI89231.1 aver|OJJ08441.1 awen|OJJ30050.1 azon|OJJ43697.1

Group4701: abra|OJJ65966.1 acar|OOF92909.1 acla|EAW13585.1 afla|EED46023.1 afum|EAL84901.1 agla|OJJ87990.1 akaw|GAA90841.1 aluc|OJZ82992.1 anid|CBF84726.1 anig|EHA21406.1 aory|EIT75803.1 asyd|OJJ53306.1 ater|EAU29377.1 atub|OJI89226.1 aver|OJJ08435.1 awen|OJJ30034.1 azon|OJJ43683.1

Group4702: abra|OJJ65974.1 acar|OOF92898.1 acla|EAW13595.1 afis|EAW20009.1 afla|EED46015.1 afum|EAL84909.1 agla|OJJ87974.1 akaw|GAA90848.1 aluc|OJZ82985.1 anid|CBF84710.1 anig|EHA21399.1 aory|EIT75417.1 asyd|OJJ53323.1 ater|EAU29351.1 atub|OJI89219.1 aver|OJJ08420.1 awen|OJJ30022.1

Group4703: abra|OJJ65975.1 acar|OOF92897.1 acla|EAW13596.1 afis|EAW20008.1 afla|EED46014.1 afum|EAL84910.1 agla|OJJ87973.1 akaw|GAA90849.1 aluc|OJZ82984.1 anid|CBF84708.1 anig|EHA21398.1 aory|EIT75605.1 asyd|OJJ53322.1 ater|EAU29350.1 atub|OJI89218.1 aver|OJJ08421.1 awen|OJJ30021.1

Group4704: abra|OJJ65988.1 acar|OOF92882.1 afis|EAW19525.1 afla|EED49677.1 agla|OJJ87965.1 akaw|GAA90862.1 aluc|OJZ82971.1 anid|CBF78949.1 anig|EHA21387.1 aory|EIT80940.1 asyd|OJJ55654.1 ater|EAU29343.1 atub|OJI89204.1 aver|OJJ06738.1 awen|OJJ30015.1 azon|OJJ43645.1 anig|EHA21388.1

Group4705: abra|OJJ66002.1 acar|OOF92096.1 acla|EAW13639.1 afis|EAW19989.1 afla|EED45931.1 afum|EAL84926.1 agla|OJJ78858.1 akaw|GAA90874.1 aluc|OJZ82958.1 anid|CBF84689.1 anig|EHA21373.1 aory|EIT75732.1 asyd|OJJ53331.1 atub|OJI89190.1 aver|OJJ08413.1 awen|OJJ30007.1 azon|OJJ43636.1

Group4706: abra|OJJ66003.1 acar|OOF92095.1 acla|EAW13640.1 afis|EAW19990.1 afla|EED45930.1 afum|EAL84925.1 agla|OJJ79320.1 akaw|GAA90875.1 aluc|OJZ82957.1 anid|CBF82047.1 anig|EHA21372.1 aory|EIT75591.1 ater|EAU32162.1 atub|OJI89189.1 awen|OJJ30006.1 asyd|OJJ54230.1 aver|OJJ05183.1

Group4707: abra|OJJ66126.1 afis|EAW15452.1 afla|EED45675.1 anid|CBF89338.1 aory|EIT75361.1 abra|OJJ77946.1 akaw|GAA83717.1 aluc|OJZ91016.1 anid|CBF71846.1 asyd|OJJ59800.1 ater|EAU29638.1 atub|OJI83007.1 afum|EAL84194.2 anid|CBF80232.1 afis|EAW22090.1 aver|OJJ02490.1 azon|OJJ46524.1

Group4708: abra|OJJ66193.1 akaw|GAA89095.1 anig|EHA25697.1 atub|OJI88434.1 abra|OJJ77442.1 acla|EAW11799.1 afis|EAW19409.1 afla|EED54078.1 afum|EAL87085.1 agla|OJJ81123.1 anid|CBF74355.1 aory|EIT76033.1 asyd|OJJ57792.1 ater|EAU35399.1 aver|OJJ05372.1 awen|OJJ36805.1 ater|EAU34716.1

Group4709: abra|OJJ66245.1 acar|OOF91971.1 acla|EAW08549.1 afis|EAW23590.1 afla|EED51817.1 afum|EAL87876.1 agla|OJJ79051.1 akaw|GAA89046.1 aluc|OJZ80550.1 anid|CBF74981.1 anig|EHA25646.1 aory|EIT81750.1 asyd|OJJ54563.1 ater|EAU37769.1 atub|OJI88381.1 aver|OJJ07593.1 awen|OJJ35894.1

Group4710: abra|OJJ66282.1 acla|EAW10347.1 afis|EAW20261.1 anig|EHA23662.1 ater|EAU32983.1 atub|OJI89941.1 abra|OJJ70126.1 afla|EED49622.1 akaw|GAA86644.1 aluc|OJZ85201.1 anig|EHA25407.1 asyd|OJJ54176.1 atub|OJI80400.1 aver|OJJ08563.1 azon|OJJ42583.1 afla|EED54705.1 aory|EIT78877.1

Group4711: abra|OJJ66374.1 acla|EAW12222.1 afis|EAW20296.1 afla|EED51865.1 agla|OJJ84731.1 akaw|GAA90242.1 aluc|OJZ83098.1 anig|EHA28162.1 aory|EIT81762.1 asyd|OJJ64162.1 ater|EAU31056.1 atub|OJI87838.1 aver|OJI98239.1 awen|OJJ35379.1 anig|EHA25212.1 aluc|OJZ87036.1 atub|OJI83479.1

Group4712: abra|OJJ66475.1 afla|EED46093.1 akaw|GAA92461.1 aluc|OJZ81556.1 anid|CBF82161.1 anig|EHA25878.1 aory|EIT74924.1 asyd|OJJ54339.1 ater|EAU37565.1 atub|OJI88629.1 aver|OJJ05291.1 awen|OJJ39935.1 aory|EIT76371.1 afla|EED53488.1 asyd|OJJ56980.1 ater|EAU30836.1 aver|OJJ03307.1

Group4713: abra|OJJ66482.1 acar|OOF90959.1 acla|EAW10987.1 afis|EAW20873.1 afla|EED45555.1 afum|EAL84761.1 agla|OJJ83980.1 akaw|GAA92467.1 aluc|OJZ81552.1 anid|CBF82849.1 anig|EHA25882.1 aory|EIT75793.1 asyd|OJJ61593.1 atub|OJI88633.1 aver|OJJ00915.1 awen|OJJ34487.1 azon|OJJ51764.1

Group4714: abra|OJJ66487.1 acar|OOF90963.1 acla|EAW10982.1 afis|EAW20877.1 afla|EED45560.1 afum|EAL84755.1 agla|OJJ83987.1 akaw|GAA92470.1 aluc|OJZ81548.1 anid|CBF82859.1 anig|EHA25887.1 asyd|OJJ61596.1 ater|EAU30812.1 atub|OJI88636.1 aver|OJJ00920.1 awen|OJJ34482.1 azon|OJJ51759.1

Group4715: abra|OJJ66520.1 acar|OOF90974.1 acla|EAW10970.1 afis|EAW20890.1 afla|EED45585.1 afum|EAL84743.1 agla|OJJ83998.1 akaw|GAA91192.1 aluc|OJZ81511.1 anig|EHA25909.1 aory|EIT75393.1 asyd|OJJ61611.1 ater|EAU30829.1 atub|OJI88660.1 aver|OJJ00934.1 awen|OJJ34457.1 azon|OJJ51744.1

Group4716: abra|OJJ66521.1 acar|OOF90975.1 acla|EAW10969.1 afis|EAW20891.1 afla|EED45586.1 afum|EAL84742.1 agla|OJJ83999.1 akaw|GAA91191.1 aluc|OJZ81512.1 anig|EHA25910.1 aory|EIT75451.1 asyd|OJJ61612.1 ater|EAU30830.1 atub|OJI88661.1 aver|OJJ00935.1 awen|OJJ34456.1 azon|OJJ51743.1

Group4717: abra|OJJ66526.1 acar|OOF90979.1 acla|EAW10964.1 afis|EAW20896.1 afla|EED50245.1 afum|EAL84737.2 agla|OJJ84003.1 akaw|GAA91187.1 aluc|OJZ81516.1 anid|CBF82464.1 anig|EHA25914.1 asyd|OJJ54117.1 ater|EAU38530.1 atub|OJI88665.1 aver|OJJ08619.1 awen|OJJ34452.1 azon|OJJ51740.1

Group4718: abra|OJJ66547.1 acar|OOF89822.1 acla|EAW10944.1 afis|EAW20914.1 afla|EED50271.1 afum|EAL84720.1 agla|OJJ84038.1 akaw|GAA91172.1 aluc|OJZ81500.1 anid|CBF82481.1 anig|EHA25930.1 aory|EIT77321.1 asyd|OJJ54102.1 atub|OJI88681.1 aver|OJJ08633.1 awen|OJJ34407.1 azon|OJJ48149.1

Group4719: asyd|OJJ54108.1 abra|OJJ66555.1 acar|OOF91000.1 acla|EAW10939.1 afis|EAW20922.1 afla|EED50276.1 afum|EAL84712.1 agla|OJJ83967.1 akaw|GAA91160.1 aluc|OJZ81488.1 anid|CBF82447.1 aory|EIT77271.1 ater|EAU38569.1 atub|OJI88692.1 aver|OJJ08626.1 awen|OJJ34398.1 azon|OJJ48134.1

Group4720: abra|OJJ66561.1 acar|OOF91002.1 acla|EAW10936.1 afis|EAW20925.1 afla|EED50280.1 afum|EAL84709.1 agla|OJJ83962.1 akaw|GAA91156.1 aluc|OJZ81484.1 anid|CBF82499.1 anig|EHA25943.1 aory|EIT77329.1 asyd|OJJ54089.1 atub|OJI88697.1 aver|OJJ08649.1 awen|OJJ34393.1 azon|OJJ48124.1

Group4721: abra|OJJ66567.1 acar|OOF91008.1 acla|EAW10928.1 afis|EAW20933.1 afla|EED50287.1 afum|EAL84701.1 akaw|GAA91150.1 aluc|OJZ81477.1 anid|CBF82506.1 anig|EHA25949.1 aory|EIT77334.1 asyd|OJJ54079.1 ater|EAU38578.1 atub|OJI88702.1 aver|OJJ08659.1 awen|OJJ34385.1 azon|OJJ48116.1

Group4722: abra|OJJ66575.1 acar|OOF91016.1 acla|EAW10915.1 afis|EAW20943.1 afla|EED50297.1 afum|EAL84691.2 agla|OJJ83944.1 akaw|GAA91143.1 aluc|OJZ81469.1 anid|CBF82538.1 anig|EHA25958.1 asyd|OJJ54067.1 ater|EAU38587.1 atub|OJI88709.1 aver|OJJ08675.1 awen|OJJ34373.1 azon|OJJ48109.1

Group4723: abra|OJJ66586.1 acar|OOF91023.1 acla|EAW10905.1 afis|EAW20953.1 afla|EED50309.1 afum|EAL84681.2 agla|OJJ83927.1 akaw|GAA91131.1 aluc|OJZ81460.1 anid|CBF82556.1 anig|EHA25969.1 asyd|OJJ54057.1 ater|EAU38604.1 atub|OJI88719.1 aver|OJJ08684.1 awen|OJJ34363.1 azon|OJJ48099.1

Group4724: abra|OJJ66599.1 acla|EAW10893.1 afis|EAW20965.1 afla|EED50319.1 afum|EAL84668.1 agla|OJJ84042.1 akaw|GAA91120.1 aluc|OJZ81448.1 anid|CBF82576.1 anig|EHA25978.1 aory|EIT77287.1 asyd|OJJ54044.1 ater|EAU38615.1 atub|OJI88731.1 aver|OJJ08698.1 awen|OJJ34350.1 azon|OJJ48087.1

Group4725: abra|OJJ66611.1 acar|OOF91037.1 acla|EAW10885.1 afis|EAW20973.1 afla|EED50328.1 afum|EAL84660.1 agla|OJJ84051.1 akaw|GAA91112.1 aluc|OJZ81438.1 anid|CBF82591.1 anig|EHA25986.1 asyd|OJJ54034.1 ater|EAU38623.1 atub|OJI88743.1 aver|OJJ08706.1 awen|OJJ34340.1 azon|OJJ48080.1

Group4726: abra|OJJ66614.1 acla|EAW10882.1 afis|EAW20976.1 afla|EED50331.1 afum|EAL84657.1 agla|OJJ84054.1 akaw|GAA91109.1 aluc|OJZ81435.1 anid|CBF82596.1 anig|EHA25989.1 aory|EIT77310.1 asyd|OJJ54031.1 ater|EAU38626.1 atub|OJI88746.1 aver|OJJ08709.1 awen|OJJ34336.1 azon|OJJ48077.1

Group4727: abra|OJJ66618.1 acar|OOF91041.1 acla|EAW10879.1 afis|EAW20979.1 afla|EED50334.1 afum|EAL84654.1 agla|OJJ84056.1 akaw|GAA91107.1 aluc|OJZ81431.1 anid|CBF82600.1 anig|EHA25991.1 aory|EIT77351.1 asyd|OJJ54029.1 atub|OJI88749.1 aver|OJJ08711.1 awen|OJJ34333.1 azon|OJJ48075.1

Group4728: abra|OJJ66620.1 acar|OOF91043.1 acla|EAW10877.1 afis|EAW20981.1 afla|EED50336.1 afum|EAL84649.2 agla|OJJ84058.1 akaw|GAA91105.1 aluc|OJZ81429.1 anid|CBF82602.1 anig|EHA25993.1 aory|EIT77328.1 asyd|OJJ54028.1 ater|EAU38629.1 atub|OJI88751.1 aver|OJJ08712.1 awen|OJJ34331.1

Group4729: abra|OJJ66625.1 acar|OOF91049.1 acla|EAW10871.1 afis|EAW20987.1 afla|EED50342.1 afum|EAL84643.1 agla|OJJ84066.1 akaw|GAA91100.1 aluc|OJZ81423.1 anid|CBF82609.1 anig|EHA25998.1 aory|EIT77368.1 asyd|OJJ54024.1 ater|EAU38636.1 atub|OJI88758.1 aver|OJJ08716.1 awen|OJJ34323.1

Group4730: abra|OJJ66659.1 acla|EAW11801.1 afis|EAW19411.1 afla|EED50366.1 afum|EAL87083.1 agla|OJJ81125.1 akaw|GAA89609.1 aluc|OJZ80789.1 anid|CBF74351.1 anig|EHA27241.1 aory|EIT83143.1 asyd|OJJ57790.1 ater|EAU34717.1 atub|OJI87640.1 aver|OJJ05374.1 awen|OJJ36807.1 azon|OJJ42993.1

Group4731: abra|OJJ66692.1 acla|EAW11827.1 afis|EAW19439.1 afla|EED56646.1 afum|EAL87057.1 agla|OJJ81151.1 akaw|GAA89636.1 aluc|OJZ80754.1 anid|CBF81391.1 anig|EHA27270.1 aory|EIT79642.1 asyd|OJJ59398.1 ater|EAU34740.1 atub|OJI87610.1 aver|OJJ02874.1 awen|OJJ36836.1 azon|OJJ42962.1

Group4732: abra|OJJ66705.1 acla|EAW11836.1 afis|EAW19447.1 afla|EED56655.1 afum|EAL87048.1 agla|OJJ81162.1 akaw|GAA89647.1 aluc|OJZ80742.1 anid|CBF81407.1 anig|EHA27280.1 aory|EIT79638.1 asyd|OJJ59407.1 ater|EAU34749.1 atub|OJI87599.1 aver|OJJ02866.1 awen|OJJ36848.1 azon|OJJ42952.1

Group4733: abra|OJJ66707.1 acla|EAW11838.1 afis|EAW19449.1 afla|EED56657.1 afum|EAL87047.1 akaw|GAA89649.1 aluc|OJZ80740.1 anid|CBF81410.1 anig|EHA27282.1 aory|EIT79686.1 asyd|OJJ59409.1 ater|EAU34751.1 atub|OJI87597.1 aver|OJJ02864.1 awen|OJJ36850.1 azon|OJJ42950.1 agla|OJJ81164.1

Group4734: abra|OJJ66724.1 acar|OOF93812.1 acla|EAW11854.1 afis|EAW19465.1 afla|EED56676.1 afum|EAL87031.2 agla|OJJ81183.1 akaw|GAA89665.1 aluc|OJZ80724.1 anid|CBF75488.1 anig|EHA27296.1 asyd|OJJ59755.1 ater|EAU34775.1 atub|OJI87580.1 aver|OJJ02533.1 awen|OJJ36868.1 azon|OJJ42934.1

Group4735: abra|OJJ66756.1 acar|OOF93837.1 acla|EAW11882.1 afis|EAW19493.1 afla|EED56712.1 afum|EAL87001.1 agla|OJJ81223.1 akaw|GAA89690.1 aluc|OJZ80695.1 anid|CBF75448.1 anig|EHA27321.1 aory|EIT73873.1 asyd|OJJ59776.1 atub|OJI87554.1 aver|OJJ02510.1 awen|OJJ36909.1 azon|OJJ42906.1

Group4736: abra|OJJ66763.1 acar|OOF93844.1 acla|EAW11889.1 afis|EAW19501.1 afla|EED56721.1 afum|EAL86992.2 agla|OJJ81672.1 akaw|GAA89699.1 aluc|OJZ80686.1 anid|CBF75440.1 anig|EHA27328.1 asyd|OJJ59782.1 ater|EAU34816.1 atub|OJI87544.1 aver|OJJ02506.1 awen|OJJ36916.1 azon|OJJ42901.1

Group4737: abra|OJJ66766.1 acar|OOF93847.1 acla|EAW11892.1 afis|EAW19503.1 afla|EED56726.1 afum|EAL86990.1 akaw|GAA89703.1 aluc|OJZ80681.1 anid|CBF75427.1 anig|EHA27331.1 aory|EIT73853.1 asyd|OJJ59788.1 ater|EAU34818.1 atub|OJI87540.1 aver|OJJ02500.1 awen|OJJ36922.1 azon|OJJ43879.1

Group4738: abra|OJJ66797.1 acar|OOF93875.1 acla|EAW14976.1 afis|EAW24501.1 afla|EED45009.1 afum|EAL86171.1 akaw|GAA86862.1 aluc|OJZ89986.1 anid|CBF82363.1 anig|EHA27357.1 aory|EIT73712.1 asyd|OJJ53691.1 ater|EAU36993.1 atub|OJI84824.1 aver|OJJ03933.1 awen|OJJ33356.1 azon|OJJ42743.1

Group4739: abra|OJJ66827.1 acar|OOF91605.1 acla|EAW14410.1 afis|EAW19118.1 afla|EED54296.1 afum|EAL93850.1 agla|OJJ80433.1 akaw|GAA90463.1 aluc|OJZ85760.1 anid|CBF79726.1 anig|EHA24796.1 aory|EIT76076.1 asyd|OJJ58074.1 atub|OJI79545.1 aver|OJJ04333.1 awen|OJJ37449.1 azon|OJJ51126.1

Group4740: abra|OJJ66837.1 acar|OOF91615.1 acla|EAW14421.1 afis|EAW19128.1 afla|EED54286.1 afum|EAL93860.1 agla|OJJ88598.1 akaw|GAA90474.1 aluc|OJZ85750.1 anid|CBF79741.1 anig|EHA24806.1 aory|EIT76040.1 asyd|OJJ58085.1 ater|EAU37395.1 aver|OJJ04323.1 awen|OJJ37461.1 azon|OJJ51139.1

Group4741: abra|OJJ66867.1 acar|OOF91645.1 acla|EAW14443.1 afis|EAW19151.1 afla|EED54254.1 afum|EAL93882.1 agla|OJJ88571.1 akaw|GAA91541.1 aluc|OJZ85715.1 anid|CBF86303.1 anig|EHA24829.1 aory|EIT76113.1 asyd|OJJ65007.1 atub|OJI79505.1 aver|OJI99126.1 awen|OJJ37504.1 azon|OJJ51169.1

Group4742: abra|OJJ66868.1 acar|OOF91644.1 acla|EAW14444.1 afis|EAW19152.1 afla|EED54253.1 afum|EAL93883.1 agla|OJJ88570.1 akaw|GAA91540.1 aluc|OJZ85714.1 anid|CBF86301.1 anig|EHA24830.1 aory|EIT76066.1 asyd|OJJ65008.1 atub|OJI79504.1 aver|OJI99127.1 awen|OJJ37505.1 azon|OJJ51171.1

Group4743: abra|OJJ66882.1 acar|OOF91656.1 acla|EAW14456.1 afis|EAW19164.1 afla|EED54233.1 afum|EAL93894.1 agla|OJJ88560.1 akaw|GAA91528.1 aluc|OJZ85701.1 anig|EHA24842.1 aory|EIT75922.1 asyd|OJJ65019.1 ater|EAU37309.1 atub|OJI79491.1 aver|OJI99137.1 awen|OJJ37517.1 azon|OJJ51184.1

Group4744: abra|OJJ66892.1 acla|EAW14464.1 afis|EAW19172.1 afla|EED54223.1 afum|EAL93902.1 agla|OJJ88552.1 akaw|GAA91520.1 aluc|OJZ85690.1 anid|CBF86268.1 anig|EHA24851.1 aory|EIT76131.1 asyd|OJJ65027.1 ater|EAU37301.1 atub|OJI79480.1 aver|OJI99146.1 awen|OJJ37525.1 azon|OJJ51191.1

Group4745: abra|OJJ66901.1 acla|EAW14472.1 afis|EAW19181.1 afla|EED54214.1 afum|EAL93911.1 agla|OJJ88542.1 akaw|GAA91511.1 aluc|OJZ85681.1 anid|CBF86255.1 anig|EHA24860.1 aory|EIT76103.1 asyd|OJJ65037.1 ater|EAU37292.1 atub|OJI79471.1 aver|OJI99156.1 awen|OJJ37535.1 azon|OJJ51201.1

Group4746: abra|OJJ66922.1 acar|OOF91689.1 acla|EAW14488.1 afis|EAW19202.1 afla|EED47710.1 afum|EAL93932.1 agla|OJJ88510.1 akaw|GAA91497.1 aluc|OJZ85658.1 anid|CBF78552.1 anig|EHA24875.1 asyd|OJJ65058.1 ater|EAU37278.1 atub|OJI79450.1 aver|OJI99183.1 awen|OJJ37555.1 azon|OJJ51216.1

Group4747: abra|OJJ66925.1 acar|OOF91693.1 acla|EAW14491.1 afis|EAW19205.1 afla|EED47707.1 afum|EAL93935.1 agla|OJJ88506.1 akaw|GAA91492.1 aluc|OJZ85653.1 anid|CBF78559.1 anig|EHA24879.1 aory|EIT80229.1 asyd|OJJ65062.1 ater|EAU37267.1 atub|OJI79445.1 aver|OJI99186.1 awen|OJJ37558.1

Group4748: abra|OJJ66941.1 acar|OOF91707.1 acla|EAW14499.1 afis|EAW19213.1 afla|EED47687.1 afum|EAL93946.1 agla|OJJ88483.1 akaw|GAA91480.1 aluc|OJZ85641.1 anid|CBF78587.1 anig|EHA24893.1 aory|EIT80252.1 asyd|OJJ65082.1 atub|OJI79432.1 aver|OJI99207.1 awen|OJJ37570.1 azon|OJJ51230.1

Group4749: abra|OJJ66943.1 acar|OOF91709.1 acla|EAW14502.1 afis|EAW19216.1 afla|EED47684.1 afum|EAL93949.1 agla|OJJ88481.1 akaw|GAA91477.1 aluc|OJZ85639.1 anid|CBF78593.1 anig|EHA24895.1 asyd|OJJ65085.1 ater|EAU37252.1 atub|OJI79430.1 aver|OJI99210.1 awen|OJJ37573.1 azon|OJJ51232.1

Group4750: acla|EAW15049.1 afis|EAW24612.1 afla|EED56125.1 afum|EAL86242.1 aory|EIT75528.1 asyd|OJJ61495.1 ater|EAU36605.1 aver|OJJ07466.1 awen|OJJ35562.1 acar|OOF93879.1 akaw|GAA86866.1 aluc|OJZ89989.1 anig|EHA27359.1 atub|OJI84820.1 afis|EAW22020.1 afla|EED45483.1 awen|OJJ30949.1

Group4751: ater|EAU37911.1 abra|OJJ76343.1 acar|OOF99594.1 acla|EAW14072.1 afis|EAW18779.1 afla|EED50738.1 afum|EAL93511.1 agla|OJJ80499.1 akaw|GAA82270.1 aluc|OJZ91870.1 anid|CBF69736.1 aory|EIT78057.1 asyd|OJJ58932.1 atub|OJI86321.1 aver|OJJ01528.1 awen|OJJ32350.1 azon|OJJ47115.1

Group4752: abra|OJJ67071.1 acla|EAW06987.1 afis|EAW16601.1 afla|EED45020.1 afum|EAL89197.1 agla|OJJ82651.1 akaw|GAA91084.1 aluc|OJZ87901.1 anid|CBF84000.1 anig|EHA20197.1 aory|EIT73543.1 asyd|OJJ64729.1 ater|EAU36681.1 atub|OJI80750.1 aver|OJI98833.1 awen|OJJ40205.1 azon|OJJ49072.1

Group4753: abra|OJJ67141.1 acar|OOF98415.1 afis|EAW15396.1 afla|EED47554.1 agla|OJJ83078.1 akaw|GAA91028.1 aluc|OJZ87831.1 anid|CBF80436.1 anig|EHA20310.1 aory|EIT74522.1 asyd|OJJ54780.1 ater|EAU29457.1 atub|OJI80810.1 aver|OJJ07385.1 awen|OJJ38130.1 agla|OJJ86276.1 aver|OJJ05913.1

Group4754: abra|OJJ67165.1 acar|OOF98396.1 acla|EAW14563.1 afis|EAW19276.1 afum|EAL94002.1 agla|OJJ88397.1 akaw|GAA91009.1 aluc|OJZ87810.1 anid|CBF80662.1 anig|EHA20292.1 aory|EIT80262.1 asyd|OJJ54608.1 ater|EAU38851.1 atub|OJI80835.1 aver|OJJ07548.1 awen|OJJ37636.1 azon|OJJ44884.1

Group4755: abra|OJJ67166.1 acar|OOF98395.1 acla|EAW14562.1 afis|EAW19275.1 afum|EAL94001.1 agla|OJJ88398.1 akaw|GAA91008.1 aluc|OJZ87809.1 anid|CBF78749.1 anig|EHA20291.1 aory|EIT80174.1 asyd|OJJ65226.1 ater|EAU29489.1 atub|OJI80836.1 aver|OJI99376.1 awen|OJJ37635.1 azon|OJJ44885.1

Group4756: abra|OJJ67170.1 acar|OOF98393.1 acla|EAW14559.1 afis|EAW19272.1 afla|EED47604.1 afum|EAL93998.1 agla|OJJ88411.1 akaw|GAA91004.1 aluc|OJZ87805.1 anid|CBF78725.1 anig|EHA20287.1 aory|EIT80192.1 asyd|OJJ65224.1 ater|EAU29492.1 atub|OJI80840.1 aver|OJI99373.1 awen|OJJ37633.1

Group4757: abra|OJJ67184.1 acla|EAW14539.1 afis|EAW19257.1 afla|EED47619.1 afum|EAL93984.1 agla|OJJ88438.1 akaw|GAA90992.1 aluc|OJZ87791.1 anid|CBF78675.1 anig|EHA20272.1 aory|EIT80266.1 asyd|OJJ65207.1 ater|EAU29518.1 atub|OJI80856.1 aver|OJI99356.1 awen|OJJ37618.1 azon|OJJ47007.1

Group4758: abra|OJJ67185.1 acar|OOF98378.1 acla|EAW14538.1 afis|EAW19254.1 afla|EED47620.1 afum|EAL93983.1 agla|OJJ88441.1 akaw|GAA90991.1 aluc|OJZ87789.1 anid|CBF78673.1 anig|EHA20271.1 asyd|OJJ65206.1 ater|EAU29513.1 atub|OJI80857.1 aver|OJI99355.1 awen|OJJ37617.1 azon|OJJ47006.1

Group4759: abra|OJJ67194.1 acar|OOF98370.1 acla|EAW14530.1 afis|EAW19248.1 afla|EED47630.1 afum|EAL93977.1 agla|OJJ88449.1 akaw|GAA90984.1 aluc|OJZ87781.1 anid|CBF78660.1 aory|EIT80194.1 asyd|OJJ65198.1 ater|EAU29502.1 atub|OJI80866.1 aver|OJI99347.1 awen|OJJ37605.1 azon|OJJ51258.1

Group4760: abra|OJJ67196.1 acar|OOF98368.1 acla|EAW14528.1 afis|EAW19246.1 afla|EED47632.1 afum|EAL93975.1 agla|OJJ88451.1 akaw|GAA90982.1 aluc|OJZ87779.1 anid|CBF78656.1 anig|EHA20261.1 aory|EIT80195.1 asyd|OJJ65196.1 atub|OJI80868.1 aver|OJI99345.1 awen|OJJ37603.1 azon|OJJ51256.1

Group4761: abra|OJJ67198.1 acar|OOF98367.1 acla|EAW14527.1 afis|EAW19245.1 afum|EAL93974.1 agla|OJJ88452.1 akaw|GAA90981.1 aluc|OJZ87776.1 anid|CBF85414.1 anig|EHA20260.1 aory|EIT80249.1 asyd|OJJ64070.1 ater|EAU36216.1 atub|OJI80869.1 aver|OJI98157.1 awen|OJJ37602.1 azon|OJJ50356.1

Group4762: abra|OJJ67214.1 acar|OOF98344.1 acla|EAW14514.1 afis|EAW19232.1 afla|EED47656.1 afum|EAL93961.1 agla|OJJ88474.1 akaw|GAA90963.1 aluc|OJZ87759.1 anid|CBF78621.1 anig|EHA20244.1 asyd|OJJ65162.1 ater|EAU37231.1 atub|OJI80885.1 aver|OJI99313.1 awen|OJJ37589.1 azon|OJJ51243.1

Group4763: agla|OJJ81007.1 abra|OJJ73670.1 acla|EAW07584.1 afis|EAW22610.1 afla|EED49808.1 afum|EAL90588.1 akaw|GAA88487.1 aluc|OJZ88383.1 anid|CBF88276.1 anig|EHA18110.1 aory|EIT77703.1 asyd|OJJ63682.1 ater|EAU39094.1 atub|OJI90977.1 awen|OJJ35238.1 azon|OJJ49869.1 aver|OJI97256.1

Group4764: abra|OJJ67253.1 acar|OOF98308.1 acla|EAW10399.1 afis|EAW25178.1 afla|EED53636.1 afum|EAL89852.1 agla|OJJ81059.1 akaw|GAA91670.1 aluc|OJZ87725.1 anid|CBF85243.1 aory|EIT75074.1 asyd|OJJ62971.1 ater|EAU34337.1 atub|OJI80918.1 aver|OJI97996.1 awen|OJJ33660.1 azon|OJJ42611.1

Group4765: abra|OJJ67256.1 acar|OOF98307.1 acla|EAW10397.1 afis|EAW25180.1 afla|EED48197.1 afum|EAL89854.1 agla|OJJ84746.1 akaw|GAA91671.1 aluc|OJZ87723.1 anig|EHA20217.1 aory|EIT74879.1 asyd|OJJ62969.1 atub|OJI80919.1 aver|OJI97998.1 azon|OJJ47985.1 aluc|OJZ87724.1 aory|EIT72909.1

Group4766: abra|OJJ67270.1 acar|OOF98295.1 acla|EAW10415.1 afis|EAW25154.1 afla|EED55279.1 afum|EAL89829.2 akaw|GAA91685.1 aluc|OJZ87709.1 anid|CBF85640.1 anig|EHA20891.1 aory|EIT83616.1 asyd|OJJ64240.1 ater|EAU39433.1 atub|OJI80933.1 aver|OJI98333.1 awen|OJJ40784.1 azon|OJJ46869.1

Group4767: abra|OJJ67317.1 acar|OOF89802.1 acla|EAW06419.1 afis|EAW18523.1 afum|EAL93218.1 agla|OJJ85135.1 akaw|GAA86501.1 aluc|OJZ85043.1 anid|CBF70217.1 anig|EHA25261.1 aory|EIT78635.1 asyd|OJJ58776.1 ater|EAU30045.1 atub|OJI80554.1 aver|OJJ01385.1 awen|OJJ31075.1 azon|OJJ50584.1

Group4768: abra|OJJ67318.1 acar|OOF92497.1 acla|EAW06418.1 afis|EAW18522.1 afum|EAL93217.1 agla|OJJ85134.1 akaw|GAA86500.1 aluc|OJZ85042.1 anid|CBF70221.1 anig|EHA25260.1 aory|EIT78625.1 asyd|OJJ58775.1 ater|EAU30046.1 atub|OJI80555.1 aver|OJJ01384.1 awen|OJJ31074.1 azon|OJJ50583.1

Group4769: abra|OJJ67348.1 acar|OOF92467.1 acla|EAW06388.1 afis|EAW18494.1 afla|EED52910.1 afum|EAL93184.2 agla|OJJ85089.1 akaw|GAA86470.1 aluc|OJZ85011.1 anid|CBF70166.1 anig|EHA25232.1 asyd|OJJ58798.1 ater|EAU29920.1 atub|OJI80586.1 aver|OJJ01402.1 awen|OJJ31047.1 azon|OJJ50558.1

Group4770: abra|OJJ67349.1 acla|EAW06387.1 afis|EAW18493.1 afla|EED52911.1 afum|EAL93183.1 agla|OJJ85088.1 akaw|GAA86469.1 aluc|OJZ85010.1 anid|CBF70164.1 anig|EHA25231.1 aory|EIT78601.1 asyd|OJJ58799.1 ater|EAU29919.1 atub|OJI80587.1 aver|OJJ01403.1 awen|OJJ31046.1 azon|OJJ50557.1

Group4771: abra|OJJ67361.1 acar|OOF92461.1 acla|EAW06382.1 afis|EAW18488.1 afla|EED52919.1 afum|EAL93178.1 agla|OJJ85084.1 akaw|GAA86459.1 aluc|OJZ85002.1 anid|CBF70153.1 anig|EHA25219.1 aory|EIT78648.1 asyd|OJJ58805.1 ater|EAU29913.1 atub|OJI80597.1 aver|OJJ01408.1 awen|OJJ31042.1

Group4772: abra|OJJ67384.1 acar|OOF92429.1 acla|EAW06375.1 afis|EAW18481.1 afla|EED52928.1 afum|EAL93170.1 agla|OJJ85064.1 akaw|GAA86440.1 aluc|OJZ84982.1 anig|EHA25195.1 aory|EIT78719.1 asyd|OJJ58811.1 ater|EAU29904.1 atub|OJI80619.1 aver|OJJ01414.1 awen|OJJ31039.1 azon|OJJ50546.1

Group4773: abra|OJJ67390.1 acla|EAW06371.1 afis|EAW18477.1 afla|EED52933.1 afum|EAL93165.1 agla|OJJ85069.1 akaw|GAA86435.1 aluc|OJZ84977.1 anid|CBF70134.1 anig|EHA25190.1 aory|EIT78710.1 asyd|OJJ58815.1 ater|EAU29894.1 atub|OJI80624.1 aver|OJJ01418.1 awen|OJJ31030.1 azon|OJJ50543.1

Group4774: abra|OJJ67402.1 acar|OOF92414.1 acla|EAW06356.1 afis|EAW18467.1 afla|EED52943.1 afum|EAL93153.1 agla|OJJ85052.1 akaw|GAA86425.1 aluc|OJZ84966.1 anid|CBF70098.1 anig|EHA25179.1 asyd|OJJ58833.1 ater|EAU29884.1 atub|OJI80634.1 aver|OJJ01436.1 awen|OJJ31019.1 azon|OJJ50533.1

Group4775: abra|OJJ67407.1 acar|OOF92409.1 acla|EAW06351.1 afis|EAW18463.1 afla|EED52946.1 afum|EAL93149.1 agla|OJJ85047.1 akaw|GAA86420.1 aluc|OJZ84961.1 anig|EHA25175.1 aory|EIT78711.1 asyd|OJJ58839.1 ater|EAU29881.1 atub|OJI80639.1 aver|OJJ01441.1 awen|OJJ31015.1 azon|OJJ50528.1

Group4776: abra|OJJ67408.1 acar|OOF92408.1 acla|EAW06350.1 afis|EAW18462.1 afum|EAL93148.2 agla|OJJ85046.1 akaw|GAA86419.1 aluc|OJZ84960.1 anid|CBF70091.1 anig|EHA25174.1 aory|EIT78697.1 asyd|OJJ58840.1 ater|EAU29880.1 atub|OJI80640.1 aver|OJJ01442.1 awen|OJJ31014.1 azon|OJJ50527.1

Group4777: abra|OJJ67427.1 acla|EAW06337.1 afis|EAW18449.1 afla|EED52959.1 afum|EAL93134.1 agla|OJJ85027.1 akaw|GAA86403.1 aluc|OJZ84940.1 anid|CBF70066.1 anig|EHA25157.1 aory|EIT78716.1 asyd|OJJ58849.1 ater|EAU29867.1 atub|OJI80660.1 aver|OJJ01450.1 awen|OJJ31001.1 azon|OJJ50515.1

Group4778: abra|OJJ67430.1 acar|OOF92396.1 acla|EAW06334.1 afis|EAW18446.1 afla|EED52965.1 afum|EAL93131.1 agla|OJJ85023.1 akaw|GAA86400.1 aluc|OJZ84936.1 anig|EHA25154.1 aory|EIT78687.1 asyd|OJJ56611.1 ater|EAU29865.1 atub|OJI80663.1 aver|OJJ06112.1 awen|OJJ30998.1 azon|OJJ50512.1

Group4779: abra|OJJ67436.1 acar|OOF92392.1 acla|EAW06326.1 afis|EAW18440.1 afla|EED52973.1 afum|EAL93122.1 agla|OJJ85005.1 akaw|GAA86394.1 aluc|OJZ84928.1 anid|CBF70051.1 anig|EHA25147.1 aory|EIT78715.1 asyd|OJJ58860.1 ater|EAU36110.1 atub|OJI80671.1 aver|OJJ01461.1 awen|OJJ30988.1

Group4780: abra|OJJ67464.1 acar|OOF92368.1 acla|EAW06304.1 afis|EAW18413.1 afum|EAL93093.2 agla|OJJ84980.1 akaw|GAA86372.1 aluc|OJZ84901.1 anid|CBF70733.1 anig|EHA25127.1 aory|EIT77394.1 asyd|OJJ58443.1 ater|EAU29828.1 atub|OJI80697.1 aver|OJJ01043.1 awen|OJJ31737.1 azon|OJJ50496.1

Group4781: abra|OJJ67573.1 acar|OOF91947.1 acla|EAW14347.1 afis|EAW18943.1 afla|EED50976.1 afum|EAL93676.1 agla|OJJ83697.1 akaw|GAA89102.1 anid|CBF79785.1 anig|EHA25704.1 aory|EIT77067.1 asyd|OJJ58115.1 ater|EAU33169.1 atub|OJI88441.1 aver|OJJ04290.1 awen|OJJ30692.1 azon|OJJ47252.1

Group4782: abra|OJJ67611.1 acar|OOF91958.1 afla|EED45464.1 akaw|GAA89128.1 aluc|OJZ81710.1 anid|CBF87139.1 anig|EHA19582.1 aory|EIT82377.1 asyd|OJJ60382.1 ater|EAU30789.1 atub|OJI88474.1 aver|OJI99711.1 awen|OJJ41962.1 azon|OJJ48033.1 anid|CBF87137.1 ater|EAU29990.1 azon|OJJ46093.1

Group4783: abra|OJJ67612.1 acar|OOF91957.1 acla|EAW11005.1 afis|EAW20848.1 afum|EAL84785.1 agla|OJJ83719.1 akaw|GAA89129.1 aluc|OJZ81709.1 anid|CBF82870.1 anig|EHA25729.1 aory|EIT82455.1 asyd|OJJ61571.1 ater|EAU30790.1 atub|OJI88475.1 aver|OJJ00891.1 awen|OJJ34515.1 azon|OJJ51801.1

Group4784: abra|OJJ67727.1 acar|OOF89959.1 acla|EAW11003.1 afis|EAW20853.1 afum|EAL84779.1 agla|OJJ83722.1 akaw|GAA89221.1 aluc|OJZ81607.1 anid|CBF82773.1 anig|EHA25838.1 aory|EIT82434.1 asyd|OJJ61572.1 ater|EAU30792.1 atub|OJI88578.1 aver|OJJ00892.1 awen|OJJ34520.1 azon|OJJ51799.1

Group4785: abra|OJJ67740.1 acar|OOF90939.1 acla|EAW11002.1 afis|EAW20858.1 afla|EED45450.1 afum|EAL84777.1 agla|OJJ83739.1 akaw|GAA92431.1 aluc|OJZ81591.1 anid|CBF82824.1 anig|EHA25850.1 aory|EIT82442.1 asyd|OJJ61579.1 ater|EAU30801.1 atub|OJI88592.1 aver|OJJ00901.1 awen|OJJ34524.1

Group4786: abra|OJJ67741.1 acar|OOF90942.1 acla|EAW11001.1 afis|EAW20860.1 afla|EED45446.1 afum|EAL84775.2 agla|OJJ83746.1 akaw|GAA92432.1 aluc|OJZ81589.1 anid|CBF84701.1 anig|EHA25851.1 aory|EIT82460.1 asyd|OJJ53318.1 ater|EAU30803.1 atub|OJI88593.1 aver|OJJ08425.1 awen|OJJ34510.1

Group4787: abra|OJJ67745.1 acar|OOF90946.1 acla|EAW10994.1 afis|EAW20866.1 afla|EED45545.1 afum|EAL84768.1 agla|OJJ83974.1 akaw|GAA92436.1 aluc|OJZ81584.1 anid|CBF82836.1 anig|EHA25855.1 aory|EIT75798.1 asyd|OJJ61586.1 ater|EAU30822.1 atub|OJI88597.1 aver|OJJ00908.1 azon|OJJ51775.1

Group4788: abra|OJJ67856.1 acar|OOF92835.1 acla|EAW11744.1 afis|EAW23777.1 afla|EED51577.1 afum|EAL87211.1 agla|OJJ78928.1 akaw|GAA91862.1 aluc|OJZ82036.1 anid|CBF79830.1 anig|EHA22220.1 aory|EIT80612.1 asyd|OJJ58143.1 atub|OJI84591.1 aver|OJJ04260.1 awen|OJJ33700.1 azon|OJJ42841.1

Group4789: abra|OJJ67858.1 acar|OOF92838.1 acla|EAW11740.1 afis|EAW23773.1 afum|EAL87207.1 agla|OJJ78925.1 akaw|GAA91865.1 aluc|OJZ82034.1 anid|CBF79824.1 anig|EHA22222.1 aory|EIT80706.1 asyd|OJJ58142.1 ater|EAU31317.1 atub|OJI84589.1 aver|OJJ04262.1 awen|OJJ33702.1 azon|OJJ42839.1

Group4790: abra|OJJ67862.1 acar|OOF92842.1 acla|EAW11736.1 afis|EAW23769.1 afla|EED51570.1 afum|EAL87203.1 agla|OJJ78951.1 akaw|GAA91869.1 aluc|OJZ82030.1 anid|CBF79819.1 anig|EHA22226.1 asyd|OJJ58139.1 ater|EAU31313.1 atub|OJI84584.1 aver|OJJ04266.1 awen|OJJ33706.1 azon|OJJ42834.1

Group4791: abra|OJJ67868.1 acar|OOF92850.1 acla|EAW11729.1 afis|EAW23761.1 afla|EED51548.1 afum|EBA27305.1 agla|OJJ78961.1 akaw|GAA91875.1 aluc|OJZ82024.1 anid|CBF79789.1 anig|EHA22231.1 aory|EIT80682.1 asyd|OJJ58124.1 atub|OJI84577.1 aver|OJJ04282.1 awen|OJJ33686.1 azon|OJJ42828.1

Group4792: acar|OOF90067.1 abra|OJJ73116.1 acla|EAW15001.1 afla|EED51114.1 agla|OJJ82961.1 akaw|GAA88053.1 aluc|OJZ81315.1 anid|CBF80452.1 anig|EHA18619.1 aory|EIT81295.1 asyd|OJJ54765.1 atub|OJI91518.1 aver|OJJ07402.1 awen|OJJ40911.1 afla|EED45581.1 agla|OJJ78689.1 aory|EIT75557.1

Group4793: abra|OJJ67907.1 acar|OOF92871.1 acla|EAW11701.1 afis|EAW23722.1 afla|EED51506.1 afum|EAL87162.1 agla|OJJ78977.1 akaw|GAA91912.1 aluc|OJZ81980.1 anid|CBF85571.1 anig|EHA22274.1 aory|EIT80734.1 asyd|OJJ58128.1 atub|OJI84532.1 aver|OJJ04277.1 afis|EAW24685.1 afum|EAL89325.1

Group4794: abra|OJJ67912.1 acar|OOF92764.1 acla|EAW12797.1 afis|EAW17960.1 afla|EED51947.1 afum|EAL91771.1 agla|OJJ80208.1 akaw|GAA91284.1 aluc|OJZ82785.1 anid|CBF79952.1 aory|EIT81471.1 asyd|OJJ58217.1 ater|EAU31449.1 atub|OJI89017.1 aver|OJJ02205.1 awen|OJJ41581.1 azon|OJJ48294.1

Group4795: abra|OJJ67914.1 acar|OOF92765.1 acla|EAW12795.1 afis|EAW17958.1 afum|EAL91769.1 agla|OJJ80206.1 akaw|GAA91282.1 aluc|OJZ82787.1 anid|CBF79949.1 anig|EHA21228.1 aory|EIT81485.1 asyd|OJJ58215.1 ater|EAU31447.1 atub|OJI89019.1 aver|OJJ02207.1 awen|OJJ41584.1 azon|OJJ48296.1

Group4796: abra|OJJ67916.1 acar|OOF92768.1 acla|EAW12793.1 afis|EAW17956.1 afla|EED51951.1 afum|EAL91767.1 agla|OJJ80203.1 akaw|GAA91280.1 aluc|OJZ82789.1 anid|CBF79945.1 anig|EHA21230.1 aory|EIT81421.1 asyd|OJJ58213.1 ater|EAU31444.1 aver|OJJ02209.1 awen|OJJ41586.1 azon|OJJ48301.1

Group4797: abra|OJJ67924.1 acla|EAW12786.1 afis|EAW17949.1 afla|EED51958.1 afum|EAL91759.1 agla|OJJ80196.1 akaw|GAA91273.1 aluc|OJZ82796.1 anid|CBF79927.1 anig|EHA21237.1 aory|EIT81486.1 asyd|OJJ58203.1 ater|EAU31432.1 atub|OJI89027.1 aver|OJJ04203.1 awen|OJJ41594.1 azon|OJJ48309.1

Group4798: abra|OJJ67932.1 acar|OOF92782.1 afis|EAW23834.1 afla|EED53910.1 afum|EAL87265.1 agla|OJJ82500.1 akaw|GAA91265.1 aluc|OJZ82804.1 anid|CBF79896.1 anig|EHA21245.1 aory|EIT78489.1 asyd|OJJ58183.1 ater|EAU31393.1 atub|OJI89035.1 aver|OJJ04222.1 awen|OJJ33792.1 azon|OJJ48752.1

Group4799: abra|OJJ67933.1 acar|OOF92783.1 afis|EAW20400.1 afla|EED51970.1 afum|EAL85499.2 agla|OJJ82479.1 akaw|GAA91264.1 aluc|OJZ82805.1 anid|CBF79898.1 anig|EHA21246.1 aory|EIT81412.1 asyd|OJJ58184.1 ater|EAU31392.1 atub|OJI89036.1 aver|OJJ04221.1 awen|OJJ33791.1 azon|OJJ48751.1

Group4800: abra|OJJ67935.1 acar|OOF92784.1 afis|EAW23832.1 afla|EED51973.1 akaw|GAA91261.1 aluc|OJZ82807.1 aory|EIT81418.1 asyd|OJJ58186.1 ater|EAU31390.1 atub|OJI89038.1 awen|OJJ33790.1 azon|OJJ48756.1 acla|EAW11624.1 afum|EAL87264.2 agla|OJJ82480.1 aver|OJJ04219.1 anid|CBF79902.1

Group4801: abra|OJJ67941.1 acar|OOF92790.1 acla|EAW11631.1 afis|EAW23825.1 afla|EED51981.1 agla|OJJ82487.1 akaw|GAA91255.1 aluc|OJZ82813.1 anig|EHA21253.1 aory|EIT81429.1 asyd|OJJ57257.1 ater|EAU31380.1 atub|OJI89044.1 aver|OJJ05344.1 awen|OJJ33783.1 azon|OJJ42885.1 ater|EAU29233.1

Group4802: abra|OJJ67948.1 acar|OOF92797.1 acla|EAW11638.1 afis|EAW23819.1 afla|EED51986.1 afum|EAL87254.1 agla|OJJ82465.1 akaw|GAA91249.1 aluc|OJZ82821.1 anid|CBF79892.1 anig|EHA21260.1 asyd|OJJ58178.1 ater|EAU31374.1 atub|OJI89050.1 aver|OJJ04226.1 awen|OJJ33776.1 azon|OJJ42881.1

Group4803: abra|OJJ67949.1 acar|OOF92796.1 acla|EAW11639.1 afis|EAW23818.1 afla|EED51987.1 afum|EAL87253.1 agla|OJJ82466.1 akaw|GAA91248.1 aluc|OJZ82822.1 anid|CBF79891.1 aory|EIT81399.1 asyd|OJJ58177.1 ater|EAU31375.1 atub|OJI89051.1 aver|OJJ04227.1 awen|OJJ33775.1 azon|OJJ42880.1

Group4804: abra|OJJ67950.1 acla|EAW11640.1 afla|EED51988.1 afum|EAL87252.1 agla|OJJ82463.1 akaw|GAA91247.1 aluc|OJZ82823.1 anid|CBF79889.1 anig|EHA21261.1 asyd|OJJ58176.1 ater|EAU31372.1 atub|OJI89052.1 aver|OJJ04228.1 awen|OJJ33774.1 azon|OJJ42878.1 afis|EAW23817.1 aory|EIT81468.1

Group4805: abra|OJJ67953.1 acla|EAW11642.1 afis|EAW23815.1 afla|EED51990.1 afum|EAL87250.1 agla|OJJ82456.1 akaw|GAA91244.1 aluc|OJZ82826.1 anid|CBF79885.1 anig|EHA21263.1 aory|EIT81442.1 asyd|OJJ58174.1 ater|EAU31370.1 atub|OJI89056.1 aver|OJJ04230.1 awen|OJJ33772.1 azon|OJJ42875.1

Group4806: abra|OJJ67956.1 acar|OOF92801.1 acla|EAW11645.1 afis|EAW23811.1 afla|EED52004.1 afum|EAL87246.1 agla|OJJ82451.1 akaw|GAA91240.1 aluc|OJZ82829.1 anid|CBF79877.1 anig|EHA21267.1 asyd|OJJ58170.1 ater|EAU31364.1 atub|OJI89059.1 aver|OJJ04233.1 awen|OJJ33767.1 azon|OJJ42871.1

Group4807: abra|OJJ67959.1 acar|OOF92805.1 acla|EAW11649.1 afis|EAW23807.1 afla|EED52008.1 afum|EAL87241.1 agla|OJJ82447.1 akaw|GAA91237.1 aluc|OJZ82832.1 anid|CBF79870.1 anig|EHA21271.1 asyd|OJJ58166.1 ater|EAU31359.1 atub|OJI89062.1 aver|OJJ04236.1 awen|OJJ33764.1 azon|OJJ42867.1

Group4808: abra|OJJ67965.1 acar|OOF92811.1 acla|EAW11655.1 afis|EAW23798.1 afum|EAL87233.1 agla|OJJ78846.1 akaw|GAA91232.1 aluc|OJZ82838.1 anid|CBF79861.1 anig|EHA21275.1 aory|EIT80592.1 asyd|OJJ58161.1 ater|EAU31336.1 atub|OJI89068.1 aver|OJJ04241.1 awen|OJJ33757.1 azon|OJJ42863.1

Group4809: abra|OJJ67967.1 acar|OOF92812.1 acla|EAW11656.1 afis|EAW23797.1 afla|EED51616.1 afum|EAL87231.2 agla|OJJ78845.1 akaw|GAA91231.1 aluc|OJZ82839.1 anid|CBF79860.1 anig|EHA21276.1 asyd|OJJ58160.1 ater|EAU31335.1 atub|OJI89069.1 aver|OJJ04242.1 awen|OJJ33755.1 azon|OJJ42862.1

Group4810: abra|OJJ67987.1 acar|OOF92029.1 acla|EAW12429.1 afis|EAW16736.1 afla|EED47284.1 afum|EAL86311.1 akaw|GAA91215.1 aluc|OJZ82856.1 anid|CBF73583.1 anig|EHA21291.1 aory|EIT79240.1 asyd|OJJ56684.1 ater|EAU29285.1 atub|OJI89086.1 aver|OJJ06039.1 awen|OJJ39986.1 azon|OJJ43671.1

Group4811: abra|OJJ68043.1 acar|OOF90405.1 acla|EAW13685.1 afis|EAW19921.1 afla|EED45891.1 afum|EAL84988.1 akaw|GAA90910.1 aluc|OJZ82916.1 anig|EHA21339.1 aory|EIT75607.1 atub|OJI89142.1 aver|OJJ07332.1 awen|OJJ29957.1 agla|OJJ79335.1 anig|EHA23723.1 atub|OJI82465.1 azon|OJJ42323.1

Group4812: abra|OJJ68119.1 acar|OOF93913.1 acla|EAW09088.1 afis|EAW25680.1 afla|EED51102.1 afum|EAL84350.1 agla|OJJ78750.1 akaw|GAA86900.1 aluc|OJZ90023.1 anid|CBF83938.1 anig|EHA27399.1 aory|EIT81294.1 asyd|OJJ61451.1 ater|EAU29935.1 atub|OJI84784.1 aver|OJJ00786.1 awen|OJJ35396.1

Group4813: abra|OJJ68122.1 acar|OOF93916.1 acla|EAW12863.1 afis|EAW17812.1 afla|EED45597.1 afum|EAL91621.1 agla|OJJ81684.1 akaw|GAA86903.1 aluc|OJZ90026.1 anid|CBF80724.1 anig|EHA27402.1 aory|EIT75545.1 asyd|OJJ54569.1 ater|EAU30289.1 atub|OJI84781.1 aver|OJJ07585.1 awen|OJJ36929.1

Group4814: abra|OJJ68124.1 acar|OOF93918.1 acla|EAW12868.1 afis|EAW17816.1 afla|EED45600.1 afum|EAL91626.1 agla|OJJ81688.1 akaw|GAA86905.1 aluc|OJZ90028.1 anid|CBF80746.1 anig|EHA27404.1 aory|EIT75820.1 asyd|OJJ54540.1 ater|EAU30286.1 atub|OJI84779.1 aver|OJJ07641.1 awen|OJJ36933.1

Group4815: abra|OJJ68159.1 acla|EAW08944.1 akaw|GAA83283.1 aluc|OJZ83793.1 anig|EHA18929.1 atub|OJI90161.1 awen|OJJ34623.1 abra|OJJ68167.1 acar|OOF93405.1 akaw|GAA83276.1 aluc|OJZ83782.1 anig|EHA22297.1 aory|EIT74145.1 asyd|OJJ63613.1 ater|EAU35823.1 atub|OJI90152.1 afum|EAL91589.1

Group4816: abra|OJJ68187.1 acar|OOF97480.1 acla|EAW13068.1 afis|EAW17596.1 afla|EED49224.1 afum|EAL91409.1 agla|OJJ81334.1 akaw|GAA83258.1 aluc|OJZ83764.1 anid|CBF90205.1 anig|EHA18905.1 aory|EIT83157.1 asyd|OJJ61975.1 ater|EAU37034.1 atub|OJI90135.1 aver|OJI96068.1 awen|OJJ37250.1

Group4817: abra|OJJ68191.1 acar|OOF97487.1 acla|EAW13073.1 afis|EAW17591.1 afla|EED49231.1 afum|EAL91404.1 agla|OJJ81358.1 akaw|GAA83254.1 aluc|OJZ83755.1 anid|CBF90214.1 anig|EHA18902.1 aory|EIT83155.1 ater|EAU37028.1 atub|OJI90130.1 aver|OJI96061.1 awen|OJJ37255.1 azon|OJJ50950.1

Group4818: abra|OJJ68192.1 acla|EAW13074.1 afis|EAW17590.1 afla|EED49232.1 afum|EAL91403.1 agla|OJJ81359.1 akaw|GAA83253.1 aluc|OJZ83754.1 anid|CBF90216.1 anig|EHA18901.1 aory|EIT83156.1 asyd|OJJ61968.1 ater|EAU37027.1 atub|OJI90129.1 aver|OJI96060.1 awen|OJJ37256.1 azon|OJJ50951.1

Group4819: abra|OJJ68195.1 acla|EAW13078.1 afis|EAW17586.1 afla|EED49236.1 afum|EAL91399.1 agla|OJJ81366.1 akaw|GAA83250.1 aluc|OJZ83751.1 anid|CBF90223.1 anig|EHA18898.1 aory|EIT83150.1 asyd|OJJ61964.1 ater|EAU37021.1 atub|OJI90126.1 aver|OJI96056.1 awen|OJJ37260.1 azon|OJJ50955.1

Group4820: abra|OJJ68214.1 acar|OOF97497.1 acla|EAW12971.1 afis|EAW17693.1 afla|EED50451.1 afum|EAL91507.1 agla|OJJ79486.1 akaw|GAA83232.1 aluc|OJZ83732.1 anid|CBF89976.1 anig|EHA18881.1 asyd|OJJ62092.1 ater|EAU31959.1 atub|OJI90109.1 aver|OJI96198.1 awen|OJJ37143.1 azon|OJJ50843.1

Group4821: abra|OJJ68216.1 acar|OOF97500.1 acla|EAW12280.1 afis|EAW16960.1 afum|EAL86488.2 agla|OJJ85938.1 akaw|GAA83230.1 aluc|OJZ83730.1 anid|CBF78343.1 anig|EHA18879.1 aory|EIT77597.1 asyd|OJJ56130.1 ater|EAU34038.1 atub|OJI90107.1 aver|OJJ05071.1 awen|OJJ35391.1 azon|OJJ49853.1

Group4822: abra|OJJ68225.1 acar|OOF97522.1 afis|EAW23333.1 afum|EAL88145.1 akaw|GAA83218.1 aluc|OJZ83720.1 anid|CBF84160.1 anig|EHA18869.1 asyd|OJJ57124.1 ater|EAU33402.1 atub|OJI90094.1 aver|OJJ03180.1 awen|OJJ38697.1 azon|OJJ47302.1 anid|CBF78048.1 atub|OJI86339.1 awen|OJJ40872.1

Group4823: abra|OJJ68270.1 acar|OOF97549.1 acla|EAW07692.1 afis|EAW22707.1 afla|EED49993.1 afum|EAL90485.1 agla|OJJ79003.1 akaw|GAA83199.1 aluc|OJZ83700.1 anid|CBF88067.1 anig|EHA18851.1 aory|EIT74717.1 asyd|OJJ63551.1 atub|OJI90072.1 aver|OJI97383.1 awen|OJJ35344.1 azon|OJJ49773.1

Group4824: abra|OJJ68273.1 acar|OOF97553.1 acla|EAW09070.1 afis|EAW25638.1 afla|EED55165.1 afum|EAL84325.1 agla|OJJ78891.1 akaw|GAA83196.1 aluc|OJZ83698.1 anid|CBF83138.1 anig|EHA18849.1 aory|EIT81612.1 asyd|OJJ55426.1 ater|EAU32709.1 atub|OJI90069.1 aver|OJJ06505.1 awen|OJJ36108.1

Group4825: abra|OJJ68286.1 acar|OOF90109.1 afis|EAW22105.1 afla|EED55143.1 afum|EAL91086.1 agla|OJJ82697.1 akaw|GAA83186.1 aluc|OJZ83685.1 anid|CBF84478.1 anig|EHA18839.1 aory|EIT81640.1 asyd|OJJ53529.1 ater|EAU32729.1 atub|OJI90056.1 aver|OJJ08221.1 awen|OJJ40237.1 azon|OJJ48799.1

Group4826: abra|OJJ68304.1 acar|OOF96684.1 acla|EAW12949.1 afis|EAW17724.1 afla|EED55147.1 afum|EAL91539.1 agla|OJJ81577.1 akaw|GAA83170.1 aluc|OJZ83669.1 anid|CBF83920.1 anig|EHA18824.1 aory|EIT81584.1 asyd|OJJ55430.1 ater|EAU32724.1 atub|OJI90040.1 aver|OJJ06509.1 awen|OJJ31293.1

Group4827: abra|OJJ68343.1 acar|OOF97641.1 acla|EAW08568.1 afis|EAW23614.1 afla|EED49585.1 afum|EAL87848.1 akaw|GAA92110.1 aluc|OJZ83615.1 anid|CBF76101.1 anig|EHA18772.1 asyd|OJJ59936.1 ater|EAU33073.1 atub|OJI89988.1 aver|OJJ02351.1 awen|OJJ39632.1 azon|OJJ50314.1 anid|CBF75197.1

Group4828: abra|OJJ68358.1 acar|OOF99377.1 afla|EED48863.1 akaw|GAA92838.1 aluc|OJZ91595.1 anig|EHA22625.1 asyd|OJJ53448.1 aver|OJJ08304.1 abra|OJJ78138.1 afla|EED53385.1 aluc|OJZ82076.1 aory|EIT75247.1 awen|OJJ29928.1 anig|EHA21837.1 aory|EIT81210.1 awen|OJJ31481.1 awen|OJJ29599.1

Group4829: abra|OJJ68384.1 acla|EAW12359.1 afis|EAW16783.1 afla|EED47253.1 afum|EAL86354.1 akaw|GAA92561.1 aluc|OJZ90107.1 anid|CBF80601.1 anig|EHA20963.1 aory|EIT79232.1 ater|EAU30268.1 atub|OJI84695.1 awen|OJJ29849.1 azon|OJJ43199.1 agla|OJJ79765.1 asyd|OJJ54699.1 aver|OJJ07475.1

Group4830: abra|OJJ68411.1 acar|OOF93966.1 acla|EAW13740.1 afis|EAW19836.1 afum|EAL85056.1 agla|OJJ86861.1 akaw|GAA92539.1 aluc|OJZ90087.1 anid|CBF81579.1 anig|EHA20987.1 aory|EIT79584.1 asyd|OJJ57609.1 atub|OJI84719.1 aver|OJJ05575.1 awen|OJJ31663.1 azon|OJJ45931.1 awen|OJJ39923.1

Group4831: abra|OJJ68416.1 acar|OOF93924.1 acla|EAW13751.1 afis|EAW19814.1 afla|EED45417.1 afum|EAL85077.1 agla|OJJ83693.1 akaw|GAA92532.1 aluc|OJZ90081.1 anid|CBF82421.1 anig|EHA20992.1 aory|EIT82441.1 asyd|OJJ54129.1 ater|EAU30780.1 atub|OJI84725.1 aver|OJJ08607.1 awen|OJJ34554.1

Group4832: abra|OJJ68446.1 acar|OOF93949.1 acla|EAW13754.1 afis|EAW19812.1 afla|EED45411.1 afum|EAL85079.1 agla|OJJ83694.1 akaw|GAA86928.1 aluc|OJZ90052.1 anid|CBF82391.1 anig|EHA21018.1 aory|EIT82394.1 asyd|OJJ54153.1 ater|EAU30741.1 atub|OJI84755.1 aver|OJJ08587.1 awen|OJJ34559.1

Group4833: abra|OJJ68461.1 acar|OOF95945.1 acla|EAW11549.1 afis|EAW23904.1 afla|EED52639.1 agla|OJJ79371.1 akaw|GAA92383.1 aluc|OJZ83242.1 anid|CBF77137.1 anig|EHA21097.1 asyd|OJJ56915.1 ater|EAU32333.1 atub|OJI87694.1 aver|OJJ03377.1 awen|OJJ39045.1 afum|EAL87332.1 azon|OJJ51374.1

Group4834: abra|OJJ68469.1 acar|OOF90193.1 acla|EAW13957.1 afis|EAW18667.1 afla|EED52662.1 afum|EAL93385.1 agla|OJJ82410.1 akaw|GAA92390.1 aluc|OJZ83250.1 anid|CBF70571.1 anig|EHA21103.1 asyd|OJJ58532.1 ater|EAU38245.1 atub|OJI87687.1 aver|OJJ01134.1 awen|OJJ32135.1 azon|OJJ46718.1

Group4835: abra|OJJ68473.1 acar|OOF94210.1 acla|EAW14616.1 afis|EAW19354.1 afla|EED46571.1 afum|EAL85102.1 akaw|GAA92394.1 aluc|OJZ83254.1 anid|CBF76650.1 anig|EHA21108.1 aory|EIT77929.1 asyd|OJJ61207.1 ater|EAU38488.1 atub|OJI87683.1 aver|OJJ00531.1 ater|EAU36160.1 afum|EAL94074.2

Group4836: abra|OJJ68481.1 acar|OOF91471.1 acla|EAW13584.1 afis|EAW20017.1 afla|EED46025.1 afum|EAL84900.1 agla|OJJ87994.1 akaw|GAA92405.1 aluc|OJZ83266.1 anid|CBF84729.1 anig|EHA21119.1 aory|EIT75379.1 asyd|OJJ53304.1 atub|OJI87671.1 aver|OJJ08437.1 awen|OJJ30036.1 azon|OJJ43680.1

Group4837: abra|OJJ68488.1 acar|OOF95813.1 acla|EAW13752.1 afis|EAW19815.1 afla|EED45414.1 afum|EAL85076.1 agla|OJJ83692.1 akaw|GAA92783.1 aluc|OJZ82700.1 anid|CBF82393.1 anig|EHA21156.1 aory|EIT82424.1 asyd|OJJ54157.1 ater|EAU30740.1 atub|OJI88932.1 aver|OJJ08580.1 awen|OJJ36130.1

Group4838: abra|OJJ68489.1 acar|OOF99836.1 acla|EAW09732.1 afis|EAW21409.1 afum|EAL92423.1 agla|OJJ85539.1 akaw|GAA92781.1 aluc|OJZ82702.1 anid|CBF83806.1 anig|EHA21157.1 aory|EIT76599.1 asyd|OJJ60893.1 ater|EAU35137.1 atub|OJI88935.1 aver|OJJ00217.1 awen|OJJ39292.1 azon|OJJ51562.1

Group4839: abra|OJJ68491.1 acar|OOF92638.1 acla|EAW08749.1 afis|EAW15687.1 afla|EED51367.1 afum|EAL85661.1 agla|OJJ81835.1 akaw|GAA92779.1 aluc|OJZ82704.1 anid|CBF71140.1 anig|EHA21159.1 aory|EIT80679.1 asyd|OJJ55835.1 atub|OJI88937.1 aver|OJJ04777.1 awen|OJJ38378.1 azon|OJJ43862.1

Group4840: abra|OJJ68506.1 acar|OOF95814.1 acla|EAW12116.1 afis|EAW17267.1 afla|EED48852.1 afum|EAL86684.1 agla|OJJ81072.1 akaw|GAA92767.1 aluc|OJZ82718.1 anig|EHA21172.1 aory|EIT81274.1 asyd|OJJ59981.1 atub|OJI88952.1 aver|OJJ02310.1 awen|OJJ35766.1 azon|OJJ45516.1 ater|EAU36132.1

Group4841: abra|OJJ68509.1 acar|OOF92671.1 acla|EAW07689.1 afis|EAW22704.1 afla|EED49990.1 afum|EAL90488.1 agla|OJJ79005.1 akaw|GAA92762.1 aluc|OJZ82723.1 anid|CBF88072.1 anig|EHA21177.1 aory|EIT74747.1 asyd|OJJ63554.1 atub|OJI88957.1 aver|OJI97380.1 awen|OJJ35341.1 azon|OJJ49777.1

Group4842: abra|OJJ68513.1 acar|OOF95416.1 acla|EAW07359.1 afis|EAW22388.1 afla|EED56048.1 afum|EAL90802.1 akaw|GAA91333.1 aluc|OJZ82728.1 anid|CBF88736.1 anig|EHA21181.1 aory|EIT76661.1 asyd|OJJ63936.1 ater|EAU39333.1 atub|OJI88961.1 aver|OJI97006.1 awen|OJJ34963.1 azon|OJJ45615.1

Group4843: abra|OJJ68520.1 acar|OOF92725.1 acla|EAW12844.1 afis|EAW18007.1 afla|EED51888.1 afum|EAL91819.1 agla|OJJ80267.1 akaw|GAA91329.1 aluc|OJZ82734.1 anid|CBF80028.1 anig|EHA21185.1 asyd|OJJ58268.1 ater|EAU31523.1 atub|OJI88967.1 aver|OJJ02156.1 awen|OJJ41534.1 azon|OJJ48253.1

Group4844: abra|OJJ68523.1 acar|OOF92728.1 acla|EAW12842.1 afis|EAW18005.1 afla|EED51890.1 afum|EAL91817.1 agla|OJJ80271.1 akaw|GAA91326.1 aluc|OJZ82737.1 anid|CBF81140.1 anig|EHA21188.1 aory|EIT82920.1 asyd|OJJ56508.1 ater|EAU31517.1 atub|OJI88972.1 aver|OJJ06211.1 awen|OJJ41537.1

Group4845: abra|OJJ68534.1 acla|EAW12824.1 afis|EAW17989.1 afla|EED51914.1 afum|EAL91798.1 agla|OJJ80249.1 akaw|GAA91317.1 aluc|OJZ82747.1 anid|CBF79981.1 anig|EHA21197.1 aory|EIT81368.1 asyd|OJJ58246.1 ater|EAU31499.1 atub|OJI88981.1 aver|OJJ02180.1 awen|OJJ41557.1 azon|OJJ48272.1

Group4846: abra|OJJ68543.1 acar|OOF92741.1 acla|EAW12831.1 afis|EAW17996.1 afla|EED51905.1 afum|EAL91806.2 akaw|GAA91310.1 aluc|OJZ82757.1 anid|CBF80013.1 anig|EHA21204.1 aory|EIT76178.1 asyd|OJJ58258.1 ater|EAU31508.1 atub|OJI88992.1 aver|OJJ02165.1 awen|OJJ41547.1 azon|OJJ48265.1

Group4847: abra|OJJ68550.1 acar|OOF92746.1 acla|EAW12817.1 afis|EAW17982.1 afla|EED51923.1 afum|EAL91791.1 agla|OJJ80239.1 akaw|GAA91303.1 aluc|OJZ82767.1 anid|CBF79994.1 anig|EHA21210.1 aory|EIT81466.1 asyd|OJJ58237.1 ater|EAU31492.1 aver|OJJ02188.1 awen|OJJ41563.1 azon|OJJ48278.1

Group4848: abra|OJJ68553.1 acar|OOF92748.1 acla|EAW12814.1 afis|EAW17979.1 afla|EED51926.1 afum|EAL91788.1 agla|OJJ80236.1 akaw|GAA91300.1 aluc|OJZ82770.1 anid|CBF79978.1 anig|EHA21212.1 aory|EIT81426.1 asyd|OJJ58233.1 atub|OJI89001.1 aver|OJJ02191.1 awen|OJJ41566.1 azon|OJJ44835.1

Group4849: abra|OJJ68558.1 acar|OOF92753.1 acla|EAW12807.1 afis|EAW17973.1 afla|EED51931.1 afum|EAL91782.1 agla|OJJ80231.1 akaw|GAA91295.1 anid|CBF79970.1 anig|EHA21217.1 aory|EIT81474.1 asyd|OJJ58228.1 ater|EAU31485.1 atub|OJI89006.1 aver|OJJ02196.1 awen|OJJ41571.1 azon|OJJ48284.1

Group4850: abra|OJJ68563.1 acar|OOF92756.1 acla|EAW12803.1 afis|EAW17969.1 afla|EED51936.1 afum|EAL91778.1 agla|OJJ80227.1 akaw|GAA91291.1 aluc|OJZ82778.1 anid|CBF79966.1 anig|EHA21221.1 aory|EIT81395.1 asyd|OJJ58226.1 ater|EAU31479.1 atub|OJI89011.1 awen|OJJ41575.1 azon|OJJ48288.1

Group4851: abra|OJJ68566.1 acar|OOF92762.1 acla|EAW12799.1 afis|EAW17965.1 afla|EED51945.1 afum|EBA27440.1 agla|OJJ80210.1 akaw|GAA91287.1 aluc|OJZ82781.1 anid|CBF79956.1 anig|EHA21224.1 aory|EIT81435.1 asyd|OJJ58219.1 atub|OJI89014.1 aver|OJJ02203.1 awen|OJJ41579.1 azon|OJJ48292.1

Group4852: abra|OJJ68569.1 acar|OOF98183.1 afla|EED57921.1 afum|EAL86294.1 agla|OJJ78785.1 aluc|OJZ81967.1 anid|CBF90243.1 anig|EHA22280.1 aory|EIT82365.1 asyd|OJJ61952.1 atub|OJI84518.1 aver|OJI96043.1 awen|OJJ35925.1 azon|OJJ42130.1 azon|OJJ47732.1 acar|OOF91241.1 aver|OJI99323.1

Group4853: abra|OJJ68600.1 acar|OOF92110.1 acla|EAW12639.1 afis|EAW18046.1 afla|EED51500.1 afum|EAL91858.1 agla|OJJ85417.1 akaw|GAA91924.1 aluc|OJZ81956.1 anid|CBF79777.1 anig|EHA22291.1 asyd|OJJ58120.1 ater|EAU31277.1 atub|OJI84507.1 aver|OJJ04285.1 awen|OJJ41488.1 azon|OJJ48218.1

Group4854: abra|OJJ68635.1 acar|OOF92139.1 acla|EAW12617.1 afis|EAW18067.1 afla|EED51451.1 afum|EBA27444.1 agla|OJJ87355.1 akaw|GAA89774.1 aluc|OJZ81920.1 anid|CBF86418.1 aory|EIT80649.1 asyd|OJJ64940.1 ater|EAU30461.1 atub|OJI84472.1 aver|OJI99062.1 awen|OJJ41454.1 azon|OJJ48912.1

Group4855: abra|OJJ68651.1 acla|EAW12627.1 afis|EAW18057.1 afla|EED51425.1 afum|EBA27441.1 agla|OJJ87329.1 akaw|GAA89791.1 aluc|OJZ81900.1 anid|CBF86437.1 anig|EHA22343.1 aory|EIT80763.1 asyd|OJJ64930.1 ater|EAU30477.1 atub|OJI84453.1 aver|OJI99049.1 awen|OJJ41440.1 azon|OJJ48902.1

Group4856: abra|OJJ68674.1 acar|OOF92175.1 acla|EAW12574.1 afis|EAW24074.1 afla|EED51399.1 afum|EAL91930.2 agla|OJJ87143.1 akaw|GAA89827.1 aluc|OJZ81861.1 anid|CBF86469.1 aory|EIT80583.1 asyd|OJJ64907.1 ater|EAU30496.1 atub|OJI84428.1 aver|OJI99029.1 awen|OJJ41411.1 azon|OJJ48881.1

Group4857: abra|OJJ68675.1 acar|OOF92176.1 acla|EAW12573.1 afis|EAW24075.1 afla|EED51398.1 afum|EBA27446.1 agla|OJJ87144.1 akaw|GAA89828.1 aluc|OJZ81860.1 anid|CBF86471.1 anig|EHA22365.1 aory|EIT80528.1 asyd|OJJ64906.1 atub|OJI84427.1 aver|OJI99028.1 awen|OJJ41412.1 azon|OJJ48880.1

Group4858: abra|OJJ68683.1 acar|OOF92185.1 acla|EAW12564.1 afis|EAW24086.1 afla|EED51387.1 afum|EAL91941.1 agla|OJJ87156.1 akaw|GAA89836.1 aluc|OJZ81850.1 anid|CBF86487.1 anig|EHA22372.1 asyd|OJJ64897.1 ater|EAU30509.1 atub|OJI84419.1 aver|OJI99018.1 awen|OJJ41403.1 azon|OJJ48869.1

Group4859: abra|OJJ68688.1 acar|OOF92191.1 acla|EAW12557.1 afis|EAW24093.1 afum|EAL91948.1 agla|OJJ87167.1 akaw|GAA89843.1 aluc|OJZ81845.1 anid|CBF86496.1 anig|EHA22377.1 aory|EIT72686.1 asyd|OJJ64892.1 ater|EAU30518.1 atub|OJI84414.1 aver|OJI99013.1 awen|OJJ41396.1 azon|OJJ48864.1

Group4860: abra|OJJ68699.1 acar|OOF92203.1 acla|EAW12545.1 afis|EAW24105.1 afla|EED46828.1 afum|EAL91959.1 agla|OJJ87180.1 akaw|GAA89854.1 aluc|OJZ81832.1 anid|CBF86515.1 anig|EHA22389.1 asyd|OJJ64879.1 ater|EAU30533.1 atub|OJI84401.1 aver|OJI98993.1 awen|OJJ41383.1 azon|OJJ48852.1

Group4861: abra|OJJ68700.1 acar|OOF92204.1 acla|EAW12544.1 afis|EAW24106.1 afla|EED46827.1 afum|EAL91960.1 akaw|GAA89855.1 aluc|OJZ81831.1 anid|CBF86516.1 anig|EHA22390.1 aory|EIT72643.1 asyd|OJJ64878.1 ater|EAU30534.1 atub|OJI84400.1 aver|OJI98992.1 awen|OJJ41382.1 azon|OJJ48851.1

Group4862: abra|OJJ68705.1 acar|OOF92207.1 acla|EAW12540.1 afis|EAW24109.1 afla|EED46823.1 afum|EAL91963.1 agla|OJJ87184.1 akaw|GAA89859.1 aluc|OJZ81825.1 anid|CBF86522.1 anig|EHA22393.1 asyd|OJJ64875.1 ater|EAU30537.1 atub|OJI84395.1 aver|OJI98988.1 awen|OJJ41379.1 azon|OJJ48847.1

Group4863: abra|OJJ68711.1 acla|EAW12534.1 afis|EAW24115.1 afla|EED46818.1 afum|EAL91969.1 agla|OJJ87192.1 akaw|GAA89865.1 aluc|OJZ81816.1 anid|CBF86533.1 anig|EHA22399.1 aory|EIT72744.1 asyd|OJJ64869.1 ater|EAU30543.1 atub|OJI84389.1 aver|OJI98981.1 awen|OJJ41373.1 azon|OJJ48841.1

Group4864: abra|OJJ68720.1 acar|OOF92219.1 acla|EAW12527.1 afis|EAW24122.1 afum|EAL91978.1 agla|OJJ87197.1 akaw|GAA89871.1 aluc|OJZ81809.1 anid|CBF86548.1 anig|EHA22408.1 aory|EIT72689.1 asyd|OJJ64859.1 ater|EAU30550.1 atub|OJI84381.1 aver|OJI98971.1 awen|OJJ41367.1 azon|OJJ48834.1

Group4865: abra|OJJ68740.1 acla|EAW12507.1 afis|EAW24139.1 afla|EED46795.1 afum|EAL91994.1 agla|OJJ87217.1 akaw|GAA89888.1 aluc|OJZ81787.1 anid|CBF77940.1 anig|EHA22428.1 aory|EIT72678.1 asyd|OJJ55284.1 ater|EAU30568.1 atub|OJI84361.1 aver|OJJ06944.1 awen|OJJ41350.1 azon|OJJ48813.1

Group4866: abra|OJJ68754.1 acar|OOF92241.1 acla|EAW12496.1 afis|EAW24149.1 afla|EED46782.1 afum|EAL92004.1 agla|OJJ87229.1 akaw|GAA89899.1 aluc|OJZ81772.1 anid|CBF77917.1 anig|EHA22444.1 aory|EIT72767.1 asyd|OJJ55296.1 ater|EAU30581.1 atub|OJI84345.1 aver|OJJ06930.1 awen|OJJ41329.1

Group4867: abra|OJJ68760.1 acar|OOF92247.1 acla|EAW12491.1 afis|EAW24154.1 afum|EAL92009.1 agla|OJJ89529.1 akaw|GAA89904.1 aluc|OJZ81766.1 anid|CBF77906.1 anig|EHA22448.1 aory|EIT72713.1 asyd|OJJ55300.1 ater|EAU30586.1 atub|OJI84338.1 aver|OJJ06925.1 awen|OJJ41323.1 azon|OJJ48796.1

Group4868: abra|OJJ68771.1 acar|OOF92256.1 acla|EAW12481.1 afis|EAW24164.1 afla|EED46769.1 afum|EAL92021.1 agla|OJJ89516.1 akaw|GAA89914.1 aluc|OJZ81755.1 anid|CBF77886.1 anig|EHA22457.1 asyd|OJJ55310.1 ater|EAU30595.1 atub|OJI84326.1 aver|OJJ06916.1 awen|OJJ41313.1 azon|OJJ48787.1

Group4869: abra|OJJ68772.1 acar|OOF92257.1 acla|EAW12480.1 afis|EAW24165.1 afla|EED46768.1 afum|EAL92022.1 agla|OJJ89515.1 akaw|GAA89915.1 aluc|OJZ81754.1 anid|CBF77885.1 anig|EHA22458.1 asyd|OJJ55311.1 ater|EAU30596.1 atub|OJI84325.1 aver|OJJ06915.1 awen|OJJ41312.1 azon|OJJ48786.1

Group4870: abra|OJJ68773.1 acar|OOF92258.1 acla|EAW12479.1 afis|EAW24166.1 afla|EED46767.1 afum|EAL92023.1 agla|OJJ89514.1 akaw|GAA89916.1 aluc|OJZ81753.1 anid|CBF77883.1 anig|EHA22459.1 asyd|OJJ55312.1 ater|EAU30597.1 atub|OJI84324.1 aver|OJJ06914.1 awen|OJJ41311.1 azon|OJJ48785.1

Group4871: abra|OJJ68775.1 acar|OOF92262.1 acla|EAW12476.1 afis|EAW24167.1 afla|EED46765.1 afum|EAL92025.1 agla|OJJ89511.1 akaw|GAA89919.1 aluc|OJZ81751.1 anid|CBF77876.1 anig|EHA22461.1 asyd|OJJ55314.1 ater|EAU30600.1 atub|OJI84322.1 aver|OJJ06912.1 awen|OJJ41309.1 azon|OJJ48782.1

Group4872: abra|OJJ68776.1 acar|OOF92260.1 acla|EAW12475.1 afis|EAW24168.1 afla|EED46764.1 afum|EAL92026.1 agla|OJJ89510.1 akaw|GAA89920.1 aluc|OJZ81750.1 anid|CBF77874.1 anig|EHA22462.1 asyd|OJJ55315.1 ater|EAU30601.1 atub|OJI84321.1 aver|OJJ06911.1 awen|OJJ41308.1 azon|OJJ48781.1

Group4873: abra|OJJ68779.1 acar|OOF92265.1 acla|EAW12472.1 afis|EAW24169.1 afla|EED46761.1 afum|EAL92031.1 agla|OJJ89507.1 akaw|GAA89923.1 aluc|OJZ81747.1 anid|CBF77869.1 anig|EHA22465.1 asyd|OJJ55319.1 ater|EAU30604.1 atub|OJI84318.1 aver|OJJ06909.1 awen|OJJ41304.1 azon|OJJ48778.1

Group4874: abra|OJJ68782.1 acar|OOF92267.1 acla|EAW12470.1 afis|EAW24171.1 afla|EED46758.1 afum|EAL92033.1 agla|OJJ89504.1 akaw|GAA89926.1 aluc|OJZ81744.1 anid|CBF77863.1 anig|EHA22468.1 asyd|OJJ55322.1 ater|EAU30606.1 atub|OJI84315.1 aver|OJJ06906.1 awen|OJJ41301.1 azon|OJJ48775.1

Group4875: abra|OJJ68786.1 acar|OOF92270.1 acla|EAW12468.1 afis|EAW24173.1 afla|EED46755.1 afum|EAL92035.1 agla|OJJ89501.1 akaw|GAA89928.1 aluc|OJZ81742.1 anid|CBF77859.1 anig|EHA22470.1 asyd|OJJ55324.1 ater|EAU30608.1 atub|OJI84313.1 aver|OJJ06904.1 awen|OJJ41297.1 azon|OJJ48773.1

Group4876: abra|OJJ68789.1 acla|EAW12464.1 afis|EAW24177.1 afla|EED46752.1 afum|EAL92039.1 agla|OJJ89497.1 akaw|GAA89931.1 aluc|OJZ81738.1 anid|CBF77851.1 anig|EHA22474.1 aory|EIT72262.1 asyd|OJJ55328.1 ater|EAU30612.1 atub|OJI84309.1 aver|OJJ06900.1 awen|OJJ41292.1 azon|OJJ48766.1

Group4877: abra|OJJ68792.1 acar|OOF92276.1 acla|EAW12461.1 afis|EAW24180.1 afla|EED46750.1 afum|EAL92041.1 agla|OJJ89494.1 aluc|OJZ81735.1 anid|CBF77846.1 anig|EHA22477.1 aory|EIT72256.1 asyd|OJJ55331.1 ater|EAU30614.1 atub|OJI84306.1 aver|OJJ06897.1 awen|OJJ41289.1 azon|OJJ48763.1

Group4878: abra|OJJ68795.1 acar|OOF92279.1 acla|EAW14659.1 afis|EAW24184.1 afla|EED46747.1 afum|EAL92044.1 agla|OJJ89491.1 aluc|OJZ81732.1 anid|CBF77840.1 anig|EHA22480.1 aory|EIT72264.1 asyd|OJJ55334.1 ater|EAU30617.1 atub|OJI84303.1 aver|OJJ06894.1 awen|OJJ41286.1 azon|OJJ42002.1

Group4879: abra|OJJ68799.1 acar|OOF92283.1 acla|EAW14654.1 afis|EAW24187.1 afla|EED46744.1 afum|EAL92047.1 agla|OJJ89487.1 akaw|GAA89938.1 aluc|OJZ81728.1 anid|CBF77833.1 aory|EIT72263.1 asyd|OJJ55338.1 ater|EAU30621.1 atub|OJI84299.1 aver|OJJ06889.1 awen|OJJ41282.1 azon|OJJ43706.1

Group4880: abra|OJJ68803.1 acar|OOF91597.1 acla|EAW14402.1 afis|EAW19109.1 afla|EED54306.1 afum|EAL93840.1 agla|OJJ84497.1 akaw|GAA90450.1 aluc|OJZ85774.1 anid|CBF79695.1 aory|EIT76105.1 asyd|OJJ58049.1 ater|EAU37367.1 atub|OJI79559.1 aver|OJJ04354.1 awen|OJJ32506.1 azon|OJJ46032.1

Group4881: abra|OJJ68849.1 acar|OOF90540.1 acla|EAW15272.1 afis|EAW20242.1 afla|EED57103.1 afum|EAL85359.1 agla|OJJ84630.1 akaw|GAA90400.1 aluc|OJZ85829.1 anid|CBF85142.1 anig|EHA24732.1 asyd|OJJ63037.1 ater|EAU32034.1 atub|OJI79617.1 aver|OJI97930.1 awen|OJJ31237.1 azon|OJJ46159.1

Group4882: abra|OJJ68857.1 acar|OOF90531.1 acla|EAW15254.1 afis|EAW20228.1 afla|EED57126.1 afum|EAL85345.2 agla|OJJ84634.1 akaw|GAA90396.1 aluc|OJZ85835.1 anid|CBF86775.1 anig|EHA24728.1 aory|EIT74040.1 asyd|OJJ64690.1 ater|EAU32030.1 atub|OJI79623.1 aver|OJI98793.1 awen|OJJ31231.1

Group4883: abra|OJJ68858.1 acla|EAW15251.1 afis|EAW20224.1 afla|EED57127.1 afum|EAL85339.1 agla|OJJ84637.1 akaw|GAA90395.1 aluc|OJZ85836.1 anid|CBF85118.1 anig|EHA24727.1 aory|EIT74129.1 asyd|OJJ63046.1 ater|EAU32029.1 atub|OJI79624.1 aver|OJI97917.1 awen|OJJ31230.1 azon|OJJ46153.1

Group4884: abra|OJJ68873.1 acar|OOF89966.1 acla|EAW15247.1 afis|EAW20213.1 afla|EED57131.1 afum|EAL85330.1 agla|OJJ84641.1 akaw|GAA90382.1 aluc|OJZ85849.1 anid|CBF85109.1 anig|EHA28097.1 aory|EIT74159.1 asyd|OJJ63052.1 ater|EAU32023.1 aver|OJI97911.1 awen|OJJ31219.1 azon|OJJ46138.1

Group4885: abra|OJJ68875.1 acla|EAW15245.1 afis|EAW20212.1 afla|EED57132.1 afum|EAL85329.1 agla|OJJ84642.1 akaw|GAA90380.1 aluc|OJZ85851.1 anid|CBF85107.1 anig|EHA28096.1 aory|EIT73979.1 asyd|OJJ63054.1 ater|EAU32021.1 atub|OJI79638.1 aver|OJI97909.1 awen|OJJ31217.1 azon|OJJ46140.1

Group4886: acar|OOF92273.1 acla|EAW12465.1 afis|EAW24176.1 afla|EED46753.1 afum|EAL92038.1 agla|OJJ89498.1 akaw|GAA89930.1 aluc|OJZ81739.1 anid|CBF77853.1 anig|EHA22473.1 aory|EIT72253.1 asyd|OJJ55327.1 ater|EAU30611.1 atub|OJI84310.1 aver|OJJ06901.1 awen|OJJ41294.1 azon|OJJ48770.1

Group4887: abra|OJJ68877.1 acar|OOF90522.1 acla|EAW15243.1 afis|EAW20210.1 afla|EED57133.1 afum|EAL85327.1 agla|OJJ84654.1 akaw|GAA90378.1 aluc|OJZ85853.1 anid|CBF85102.1 aory|EIT73969.1 asyd|OJJ63056.1 ater|EAU32020.1 atub|OJI79640.1 aver|OJI97906.1 awen|OJJ31215.1 azon|OJJ46145.1

Group4888: abra|OJJ68878.1 acar|OOF97048.1 acla|EAW07071.1 afis|EAW25746.1 afum|EBA27245.1 asyd|OJJ52302.1 ater|EAU38964.1 azon|OJJ44440.1 abra|OJJ69169.1 acla|EAW08352.1 akaw|GAA86294.1 aluc|OJZ86156.1 anid|CBF89377.1 anig|EHA24469.1 atub|OJI79948.1 awen|OJJ37486.1 acla|EAW12600.1

Group4889: abra|OJJ68916.1 acar|OOF91504.1 acla|EAW14257.1 afis|EAW19023.1 afla|EED56625.1 afum|EAL93754.1 agla|OJJ84609.1 akaw|GAA90333.1 aluc|OJZ85899.1 anid|CBF85582.1 anig|EHA24693.1 aory|EIT79644.1 asyd|OJJ64175.1 ater|EAU32065.1 atub|OJI79684.1 aver|OJI98250.1 awen|OJJ31398.1

Group4890: abra|OJJ68924.1 acar|OOF91510.1 acla|EAW13235.1 afis|EAW16374.1 afla|EED45428.1 afum|EAL88998.1 akaw|GAA90324.1 aluc|OJZ85909.1 anid|CBF83908.1 anig|EHA24683.1 aory|EIT82385.1 asyd|OJJ55419.1 atub|OJI79693.1 aver|OJJ06496.1 awen|OJJ31350.1 azon|OJJ46550.1 acar|OOF92458.1

Group4891: abra|OJJ68934.1 acar|OOF91517.1 acla|EAW14244.1 afis|EAW19036.1 afla|EED50390.1 afum|EAL93765.2 agla|OJJ84585.1 akaw|GAA90315.1 aluc|OJZ85919.1 anid|CBF79603.1 anig|EHA24673.1 asyd|OJJ57992.1 ater|EAU32075.1 atub|OJI79711.1 aver|OJJ04410.1 awen|OJJ31465.1 azon|OJJ46167.1

Group4892: abra|OJJ68955.1 acar|OOF91533.1 acla|EAW14231.1 afis|EAW19050.1 afla|EED54442.1 afum|EAL93779.1 agla|OJJ84556.1 akaw|GAA90483.1 aluc|OJZ85941.1 anig|EHA24658.1 aory|EIT81131.1 asyd|OJJ58006.1 ater|EAU32088.1 atub|OJI79729.1 aver|OJJ04394.1 awen|OJJ31479.1 azon|OJJ46192.1

Group4893: abra|OJJ68977.1 acar|OOF91552.1 acla|EAW14211.1 afis|EAW19070.1 afla|EED54435.1 afum|EAL93798.1 agla|OJJ84535.1 akaw|GAA90502.1 aluc|OJZ85965.1 anid|CBF79660.1 anig|EHA24639.1 asyd|OJJ58026.1 ater|EAU32118.1 atub|OJI79753.1 aver|OJJ04372.1 awen|OJJ31502.1 azon|OJJ46217.1

Group4894: abra|OJJ68983.1 acla|EAW14208.1 afis|EAW19073.1 afla|EED54438.1 afum|EAL93801.1 agla|OJJ84530.1 akaw|GAA90505.1 aluc|OJZ85969.1 anid|CBF79665.1 anig|EHA24636.1 aory|EIT75855.1 asyd|OJJ58030.1 ater|EAU32123.1 atub|OJI79757.1 aver|OJJ04369.1 awen|OJJ32467.1 azon|OJJ46239.1

Group4895: abra|OJJ68988.1 acar|OOF91559.1 acla|EAW14203.1 afis|EAW19079.1 afla|EED54337.1 afum|EAL93807.1 agla|OJJ84527.1 akaw|GAA90509.1 aluc|OJZ85974.1 anid|CBF79668.1 aory|EIT76081.1 asyd|OJJ58033.1 ater|EAU32125.1 atub|OJI79763.1 aver|OJJ04366.1 awen|OJJ32471.1 azon|OJJ46235.1

Group4896: abra|OJJ69001.1 acar|OOF92713.1 acla|EAW12656.1 afis|EAW18024.1 afla|EED51870.1 afum|EAL91836.1 agla|OJJ85376.1 akaw|GAA90520.1 aluc|OJZ85985.1 anid|CBF80058.1 aory|EIT81751.1 asyd|OJJ58285.1 ater|EAU31538.1 atub|OJI79775.1 aver|OJJ02139.1 awen|OJJ41516.1 azon|OJJ48239.1

Group4897: abra|OJJ69003.1 acar|OOF92716.1 acla|EAW12856.1 afis|EAW18019.1 afla|EED51872.1 afum|EAL91831.1 agla|OJJ85373.1 akaw|GAA90521.1 aluc|OJZ85989.1 anid|CBF80054.1 anig|EHA24621.1 asyd|OJJ58281.1 ater|EAU31535.1 atub|OJI79778.1 aver|OJJ02142.1 awen|OJJ41520.1 azon|OJJ48242.1

Group4898: abra|OJJ69007.1 acla|EAW12852.1 afis|EAW18015.1 afla|EED51879.1 afum|EAL91827.1 agla|OJJ85367.1 akaw|GAA90524.1 aluc|OJZ85992.1 anid|CBF80046.1 anig|EHA24618.1 aory|EIT81690.1 asyd|OJJ58277.1 ater|EAU31531.1 atub|OJI79781.1 aver|OJJ02147.1 awen|OJJ41525.1 azon|OJJ48246.1

Group4899: abra|OJJ69009.1 acar|OOF92720.1 acla|EAW12849.1 afis|EAW18012.1 afla|EED51884.1 afum|EAL91824.1 agla|OJJ80284.1 akaw|GAA90526.1 aluc|OJZ85995.1 anid|CBF80039.1 anig|EHA24615.1 asyd|OJJ58274.1 ater|EAU31528.1 atub|OJI79783.1 aver|OJJ02150.1 awen|OJJ41529.1 azon|OJJ48249.1

Group4900: abra|OJJ69033.1 acar|OOF92651.1 acla|EAW08890.1 afis|EAW15499.1 afla|EED49014.1 afum|EAL85824.1 agla|OJJ80177.1 akaw|GAA90548.1 aluc|OJZ86018.1 anig|EHA24593.1 aory|EIT82509.1 asyd|OJJ56012.1 ater|EAU32620.1 atub|OJI79807.1 aver|OJJ04957.1 awen|OJJ38158.1 azon|OJJ43131.1

Group4901: abra|OJJ69035.1 acar|OOF92650.1 acla|EAW14479.1 afis|EAW19188.1 afla|EED47723.1 afum|EAL93919.1 agla|OJJ88533.1 akaw|GAA90550.1 aluc|OJZ86020.1 anid|CBF86244.1 anig|EHA24591.1 aory|EIT77180.1 asyd|OJJ65045.1 atub|OJI79809.1 aver|OJI99166.1 awen|OJJ37546.1 azon|OJJ51209.1

Group4902: abra|OJJ69040.1 acar|OOF92967.1 acla|EAW11878.1 afis|EAW19489.1 afla|EED56707.1 afum|EAL87005.1 agla|OJJ81219.1 akaw|GAA90555.1 aluc|OJZ86025.1 anid|CBF75454.1 anig|EHA24586.1 aory|EIT73897.1 asyd|OJJ59773.1 atub|OJI79814.1 aver|OJJ02513.1 awen|OJJ36905.1 azon|OJJ42910.1

Group4903: abra|OJJ69056.1 acar|OOF91227.1 acla|EAW08758.1 afis|EAW15675.1 afla|EED46855.1 afum|EAL85672.1 agla|OJJ81822.1 akaw|GAA90586.1 aluc|OJZ86044.1 anid|CBF71159.1 anig|EHA24567.1 asyd|OJJ55843.1 ater|EAU32485.1 atub|OJI79841.1 aver|OJJ04786.1 awen|OJJ38353.1 azon|OJJ43867.1

Group4904: abra|OJJ69073.1 acar|OOF91233.1 acla|EAW08769.1 afis|EAW15661.1 afla|EED46884.1 afum|EAL85687.1 agla|OJJ81783.1 akaw|GAA90604.1 aluc|OJZ86062.1 anid|CBF71184.1 anig|EHA24551.1 aory|EIT72716.1 asyd|OJJ55860.1 atub|OJI79859.1 aver|OJJ04801.1 awen|OJJ38289.1 azon|OJJ43026.1

Group4905: abra|OJJ69086.1 acar|OOF91207.1 acla|EAW08746.1 afis|EAW15690.1 afum|EAL85658.1 agla|OJJ81837.1 akaw|GAA90615.1 aluc|OJZ86074.1 anid|CBF71135.1 anig|EHA24541.1 aory|EIT80497.1 asyd|OJJ55832.1 ater|EAU32464.1 atub|OJI79871.1 aver|OJJ04774.1 awen|OJJ38381.1 azon|OJJ43856.1

Group4906: abra|OJJ69087.1 acar|OOF91206.1 acla|EAW08745.1 afis|EAW15691.1 afla|EED51363.1 afum|EAL85657.1 agla|OJJ81838.1 akaw|GAA90616.1 aluc|OJZ86075.1 anid|CBF71133.1 anig|EHA24540.1 aory|EIT80567.1 asyd|OJJ55831.1 atub|OJI79872.1 aver|OJJ04773.1 awen|OJJ38382.1 azon|OJJ43855.1

Group4907: abra|OJJ69088.1 acar|OOF91205.1 acla|EAW08744.1 afis|EAW15692.1 afla|EED51362.1 afum|EAL85656.1 agla|OJJ81839.1 akaw|GAA90617.1 aluc|OJZ86076.1 anid|CBF71131.1 anig|EHA24539.1 asyd|OJJ55830.1 ater|EAU32463.1 atub|OJI79873.1 aver|OJJ04772.1 awen|OJJ38383.1 azon|OJJ43854.1

Group4908: abra|OJJ69095.1 acar|OOF91199.1 acla|EAW08736.1 afis|EAW15700.1 afla|EED51351.1 afum|EAL85648.1 agla|OJJ81848.1 akaw|GAA90623.1 aluc|OJZ86085.1 anid|CBF71125.1 anig|EHA24531.1 asyd|OJJ55824.1 ater|EAU32448.1 atub|OJI79881.1 aver|OJJ04766.1 awen|OJJ38392.1 azon|OJJ43846.1

Group4909: abra|OJJ69103.1 acla|EAW08730.1 afis|EAW15704.1 afla|EED51346.1 afum|EAL85643.1 agla|OJJ81854.1 akaw|GAA90628.1 aluc|OJZ86091.1 anid|CBF71102.1 anig|EHA24526.1 aory|EIT80416.1 asyd|OJJ52708.1 ater|EAU32439.1 atub|OJI79887.1 aver|OJJ04737.1 awen|OJJ38401.1 azon|OJJ43842.1

Group4910: abra|OJJ69105.1 acar|OOF91179.1 acla|EAW08727.1 afis|EAW15735.1 afla|EED51344.1 afum|EBA27236.1 akaw|GAA90630.1 aluc|OJZ86093.1 anid|CBF83999.1 anig|EHA24524.1 aory|EIT80417.1 asyd|OJJ61395.1 ater|EAU32437.1 atub|OJI79889.1 aver|OJJ00724.1 awen|OJJ38403.1 azon|OJJ43840.1

Group4911: abra|OJJ69106.1 acar|OOF91180.1 acla|EAW08726.1 afis|EAW15734.1 afla|EED51343.1 afum|EBA27237.1 agla|OJJ81859.1 akaw|GAA90631.1 aluc|OJZ86094.1 anid|CBF71098.1 aory|EIT81543.1 asyd|OJJ52706.1 ater|EAU32436.1 atub|OJI79890.1 aver|OJJ04735.1 awen|OJJ38404.1 azon|OJJ43838.1

Group4912: abra|OJJ69107.1 acla|EAW08725.1 afis|EAW15733.1 afla|EED51342.1 afum|EAL85614.1 agla|OJJ81860.1 akaw|GAA90632.1 aluc|OJZ86095.1 anid|CBF71096.1 anig|EHA24523.1 aory|EIT81551.1 asyd|OJJ52705.1 ater|EAU32435.1 atub|OJI79891.1 aver|OJJ04734.1 awen|OJJ38405.1 azon|OJJ43837.1

Group4913: abra|OJJ69109.1 acar|OOF91182.1 acla|EAW08723.1 afis|EAW15731.1 afla|EED51340.1 afum|EAL85616.1 agla|OJJ81862.1 akaw|GAA90634.1 aluc|OJZ86097.1 anid|CBF71092.1 anig|EHA24521.1 asyd|OJJ52702.1 ater|EAU32433.1 atub|OJI79893.1 aver|OJJ04730.1 awen|OJJ38407.1 azon|OJJ43835.1

Group4914: abra|OJJ69114.1 acar|OOF91187.1 acla|EAW08718.1 afis|EAW15729.1 afla|EED51334.1 afum|EAL85618.1 agla|OJJ81868.1 akaw|GAA90639.1 aluc|OJZ86103.1 anid|CBF71083.1 anig|EHA24516.1 asyd|OJJ52697.1 ater|EAU32429.1 atub|OJI79898.1 aver|OJJ04725.1 awen|OJJ38413.1 azon|OJJ43829.1

Group4915: abra|OJJ69118.1 acar|OOF91190.1 acla|EAW08714.1 afis|EAW15722.1 afla|EED51330.1 afum|EAL85625.2 agla|OJJ81872.1 akaw|GAA90643.1 aluc|OJZ86107.1 anid|CBF71077.1 anig|EHA23837.1 asyd|OJJ52693.1 ater|EAU32425.1 atub|OJI79902.1 aver|OJJ04721.1 awen|OJJ38417.1 azon|OJJ43825.1

Group4916: abra|OJJ69124.1 acar|OOF91176.1 acla|EAW08708.1 afis|EAW15716.1 afla|EED51324.1 afum|EAL85632.1 agla|OJJ81880.1 akaw|GAA90649.1 aluc|OJZ86113.1 anid|CBF71066.1 anig|EHA24508.1 aory|EIT81491.1 asyd|OJJ52686.1 atub|OJI79908.1 aver|OJJ04714.1 awen|OJJ38424.1 azon|OJJ43819.1

Group4917: abra|OJJ69146.1 acar|OOF91157.1 acla|EAW08668.1 afis|EAW15756.1 afla|EED51301.1 afum|EAL85593.1 agla|OJJ81907.1 akaw|GAA86312.1 aluc|OJZ86132.1 anid|CBF71029.1 anig|EHA24489.1 aory|EIT81548.1 asyd|OJJ52662.1 ater|EAU32399.1 atub|OJI79928.1 aver|OJJ04689.1 awen|OJJ38448.1

Group4918: abra|OJJ69153.1 acar|OOF91150.1 acla|EAW08670.1 afis|EAW15755.1 afla|EED51299.1 afum|EAL85594.1 agla|OJJ81908.1 akaw|GAA86309.1 aluc|OJZ86140.1 anid|CBF71031.1 anig|EHA24484.1 asyd|OJJ52663.1 ater|EAU32398.1 atub|OJI79932.1 aver|OJJ04690.1 awen|OJJ38452.1 azon|OJJ43752.1

Group4919: abra|OJJ69160.1 acar|OOF91143.1 acla|EAW08677.1 afis|EAW15747.1 afla|EED51292.1 agla|OJJ81916.1 akaw|GAA86302.1 aluc|OJZ86147.1 anid|CBF71011.1 anig|EHA24477.1 aory|EIT81495.1 asyd|OJJ52652.1 ater|EAU32391.1 atub|OJI79939.1 aver|OJJ04681.1 awen|OJJ38459.1 azon|OJJ43760.1

Group4920: abra|OJJ69185.1 acar|OOF91127.1 acla|EAW08661.1 afis|EAW15770.1 afla|EED51277.1 afum|EAL85578.1 agla|OJJ81934.1 akaw|GAA86280.1 aluc|OJZ86171.1 anid|CBF70981.1 anig|EHA24454.1 asyd|OJJ52635.1 ater|EAU32371.1 atub|OJI79963.1 aver|OJJ04663.1 awen|OJJ38478.1 azon|OJJ43778.1

Group4921: abra|OJJ69190.1 acar|OOF91123.1 acla|EAW08655.1 afis|EAW15776.1 afla|EED51270.1 afum|EAL85573.1 agla|OJJ81939.1 akaw|GAA86274.1 aluc|OJZ86176.1 anig|EHA24448.1 aory|EIT81527.1 asyd|OJJ52630.1 ater|EAU32365.1 atub|OJI79968.1 aver|OJJ04658.1 awen|OJJ38483.1 azon|OJJ43784.1

Group4922: abra|OJJ69202.1 acar|OOF91114.1 acla|EAW08645.1 afis|EAW15786.1 afum|EAL85564.1 agla|OJJ81949.1 akaw|GAA92214.1 aluc|OJZ86186.1 anid|CBF70956.1 anig|EHA24438.1 aory|EIT81494.1 asyd|OJJ52620.1 ater|EAU32355.1 atub|OJI79978.1 aver|OJJ04647.1 awen|OJJ38493.1 azon|OJJ43795.1

Group4923: abra|OJJ69203.1 acar|OOF91113.1 acla|EAW08644.1 afis|EAW15787.1 afla|EED51258.1 afum|EAL85563.1 agla|OJJ81950.1 akaw|GAA92215.1 aluc|OJZ86187.1 anid|CBF70958.1 anig|EHA24437.1 asyd|OJJ52619.1 ater|EAU32354.1 atub|OJI79979.1 aver|OJJ04646.1 awen|OJJ38495.1 azon|OJJ43796.1

Group4924: abra|OJJ69223.1 acar|OOF91097.1 acla|EAW08623.1 afis|EAW15809.1 afum|EAL85542.1 agla|OJJ81975.1 akaw|GAA92231.1 aluc|OJZ86203.1 anid|CBF70907.1 anig|EHA24419.1 aory|EIT83175.1 asyd|OJJ52598.1 ater|EAU33439.1 atub|OJI79997.1 aver|OJJ04625.1 awen|OJJ38520.1 azon|OJJ43735.1

Group4925: abra|OJJ69225.1 acar|OOF91095.1 afis|EAW15811.1 afum|EAL85540.1 agla|OJJ81977.1 akaw|GAA92233.1 aluc|OJZ86205.1 anid|CBF75271.1 aory|EIT83193.1 asyd|OJJ55915.1 ater|EAU33621.1 atub|OJI79999.1 aver|OJJ04855.1 awen|OJJ38522.1 abra|OJJ70041.1 anig|EHA18723.1 afla|EED51236.1

Group4926: abra|OJJ69233.1 acar|OOF91087.1 acla|EAW08616.1 afis|EAW15818.1 afla|EED51229.1 afum|EAL85533.1 agla|OJJ81982.1 akaw|GAA92241.1 aluc|OJZ86213.1 anid|CBF70893.1 anig|EHA24413.1 asyd|OJJ52592.1 ater|EAU33430.1 atub|OJI80008.1 aver|OJJ04619.1 awen|OJJ38532.1 azon|OJJ43730.1

Group4927: abra|OJJ69245.1 acar|OOF91075.1 acla|EAW08604.1 afis|EAW15829.1 afla|EED51216.1 afum|EAL85522.2 agla|OJJ81993.1 akaw|GAA92253.1 aluc|OJZ86226.1 anid|CBF70867.1 anig|EHA24402.1 aory|EIT80404.1 asyd|OJJ52579.1 atub|OJI80019.1 aver|OJJ04607.1 awen|OJJ38543.1 azon|OJJ43720.1

Group4928: abra|OJJ69266.1 acar|OOF89860.1 acla|EAW14656.1 afis|EAW24190.1 afum|EAL85863.1 agla|OJJ79689.1 akaw|GAA88142.1 aluc|OJZ82095.1 anid|CBF80855.1 anig|EHA28634.1 aory|EIT82483.1 asyd|OJJ56290.1 ater|EAU31141.1 atub|OJI88342.1 aver|OJJ06427.1 awen|OJJ33109.1 azon|OJJ44840.1

Group4929: abra|OJJ69272.1 acar|OOF94449.1 acla|EAW14661.1 afis|EAW24192.1 afla|EED48079.1 agla|OJJ79687.1 akaw|GAA88146.1 aluc|OJZ82099.1 anid|CBF74310.1 anig|EHA28630.1 aory|EIT82480.1 asyd|OJJ54006.1 ater|EAU31143.1 atub|OJI88338.1 aver|OJJ08101.1 awen|OJJ33107.1 azon|OJJ44841.1

Group4930: abra|OJJ69303.1 acla|EAW14689.1 afis|EAW24220.1 afla|EED48051.1 afum|EAL85889.1 agla|OJJ79650.1 akaw|GAA88172.1 aluc|OJZ82131.1 anid|CBF74246.1 anig|EHA28600.1 aory|EIT83327.1 asyd|OJJ53974.1 ater|EAU31170.1 atub|OJI88305.1 aver|OJJ08073.1 awen|OJJ33076.1 azon|OJJ44870.1

Group4931: abra|OJJ69304.1 acar|OOF94422.1 acla|EAW14690.1 afis|EAW24221.1 afum|EAL85890.1 agla|OJJ79649.1 akaw|GAA88173.1 aluc|OJZ82132.1 anid|CBF74244.1 anig|EHA28599.1 aory|EIT83331.1 asyd|OJJ53973.1 ater|EAU31171.1 atub|OJI88304.1 aver|OJJ08072.1 awen|OJJ33075.1 azon|OJJ44871.1

Group4932: abra|OJJ69317.1 acar|OOF90304.1 acla|EAW14731.1 afis|EAW24264.1 afla|EED48013.1 afum|EAL85932.1 agla|OJJ79608.1 akaw|GAA88186.1 aluc|OJZ82146.1 anid|CBF74177.1 anig|EHA28584.1 aory|EIT82900.1 asyd|OJJ53942.1 atub|OJI88290.1 aver|OJJ08039.1 awen|OJJ33037.1 azon|OJJ48482.1

Group4933: abra|OJJ69320.1 acar|OOF94410.1 acla|EAW14725.1 afis|EAW24261.1 afla|EED48017.1 afum|EAL85929.1 agla|OJJ79611.1 akaw|GAA88189.1 aluc|OJZ82149.1 anid|CBF74187.1 anig|EHA28581.1 asyd|OJJ53948.1 ater|EAU31206.1 atub|OJI88286.1 aver|OJJ08044.1 awen|OJJ33041.1 azon|OJJ48595.1

Group4934: abra|OJJ69324.1 acar|OOF94403.1 acla|EAW14716.1 afis|EAW24251.1 afla|EED48022.1 afum|EAL85920.2 agla|OJJ79622.1 akaw|GAA88195.1 aluc|OJZ82156.1 anid|CBF74197.1 anig|EHA28576.1 asyd|OJJ53951.1 ater|EAU31192.1 atub|OJI88281.1 aver|OJJ08047.1 awen|OJJ33048.1 azon|OJJ48471.1

Group4935: abra|OJJ69325.1 acar|OOF94402.1 acla|EAW14715.1 afis|EAW24250.1 afla|EED48023.1 afum|EAL85919.1 agla|OJJ79623.1 akaw|GAA88196.1 aluc|OJZ82157.1 anid|CBF74199.1 anig|EHA28575.1 asyd|OJJ53952.1 ater|EAU31191.1 atub|OJI88280.1 aver|OJJ08048.1 awen|OJJ33062.1 azon|OJJ48470.1

Group4936: abra|OJJ69326.1 acar|OOF94400.1 acla|EAW14714.1 afis|EAW24249.1 afla|EED48024.1 afum|EAL85917.2 agla|OJJ79624.1 akaw|GAA88197.1 aluc|OJZ82158.1 anid|CBF74201.1 anig|EHA28574.1 asyd|OJJ53953.1 ater|EAU31190.1 atub|OJI88279.1 aver|OJJ08049.1 awen|OJJ33061.1 azon|OJJ48469.1

Group4937: abra|OJJ69339.1 acla|EAW14707.1 afis|EAW24238.1 afla|EED48033.1 afum|EAL85906.1 agla|OJJ79633.1 akaw|GAA88208.1 aluc|OJZ82171.1 anid|CBF74215.1 anig|EHA28562.1 aory|EIT83347.1 asyd|OJJ53959.1 ater|EAU31184.1 atub|OJI88266.1 aver|OJJ08055.1 awen|OJJ33052.1 azon|OJJ48463.1

Group4938: abra|OJJ69347.1 acar|OOF94377.1 acla|EAW14699.1 afis|EAW24230.1 afla|EED48041.1 afum|EAL85899.2 agla|OJJ79641.1 akaw|GAA88216.1 aluc|OJZ82179.1 anid|CBF74226.1 anig|EHA28555.1 aory|EIT83348.1 asyd|OJJ53966.1 atub|OJI88258.1 aver|OJJ08062.1 awen|OJJ33067.1 azon|OJJ48456.1

Group4939: abra|OJJ69356.1 acar|OOF94368.1 acla|EAW14747.1 afis|EAW24278.1 afla|EED47998.1 afum|EAL85947.1 agla|OJJ79584.1 akaw|GAA88226.1 aluc|OJZ82188.1 anid|CBF74153.1 anig|EHA28547.1 aory|EIT82913.1 asyd|OJJ53924.1 atub|OJI88247.1 aver|OJJ08022.1 awen|OJJ33019.1 azon|OJJ48498.1

Group4940: abra|OJJ69358.1 acar|OOF94366.1 acla|EAW14749.1 afis|EAW24280.1 afla|EED47996.1 afum|EAL85949.1 agla|OJJ80634.1 akaw|GAA88228.1 aluc|OJZ82190.1 anid|CBF74149.1 anig|EHA28545.1 aory|EIT82910.1 asyd|OJJ53922.1 atub|OJI88245.1 aver|OJJ08020.1 awen|OJJ33017.1 azon|OJJ48500.1

Group4941: abra|OJJ69364.1 acar|OOF94361.1 acla|EAW14754.1 afis|EAW24285.1 afla|EED47991.1 afum|EAL85954.1 agla|OJJ83147.1 akaw|GAA88233.1 aluc|OJZ82198.1 anid|CBF74133.1 aory|EIT79826.1 asyd|OJJ53914.1 ater|EAU31236.1 atub|OJI88238.1 aver|OJJ08012.1 awen|OJJ33011.1 azon|OJJ48504.1

Group4942: abra|OJJ69365.1 acar|OOF94360.1 acla|EAW14755.1 afis|EAW24286.1 afla|EED47990.1 afum|EAL85955.1 agla|OJJ83150.1 akaw|GAA88234.1 aluc|OJZ82199.1 anig|EHA28540.1 aory|EIT79821.1 asyd|OJJ53913.1 ater|EAU31237.1 atub|OJI88237.1 aver|OJJ08011.1 awen|OJJ33010.1 azon|OJJ48505.1

Group4943: abra|OJJ69372.1 acar|OOF94356.1 acla|EAW14762.1 afis|EAW24293.1 afum|EAL85961.1 agla|OJJ83158.1 akaw|GAA88241.1 aluc|OJZ82206.1 anid|CBF74120.1 anig|EHA28533.1 aory|EIT79846.1 asyd|OJJ53906.1 ater|EAU31243.1 atub|OJI88230.1 aver|OJJ08004.1 awen|OJJ33003.1 azon|OJJ48512.1

Group4944: abra|OJJ69379.1 acar|OOF94346.1 acla|EAW14767.1 afis|EAW24298.1 afla|EED47974.1 afum|EAL85966.1 agla|OJJ83165.1 akaw|GAA88247.1 aluc|OJZ82214.1 anid|CBF74112.1 anig|EHA28526.1 asyd|OJJ53902.1 ater|EAU31249.1 atub|OJI88221.1 aver|OJJ07999.1 awen|OJJ32994.1 azon|OJJ48521.1

Group4945: abra|OJJ69392.1 acla|EAW14777.1 afis|EAW24308.1 afum|EAL85976.1 agla|OJJ83177.1 akaw|GAA88260.1 aluc|OJZ82229.1 anid|CBF74089.1 anig|EHA28510.1 aory|EIT79815.1 asyd|OJJ53886.1 ater|EAU31261.1 atub|OJI88208.1 aver|OJJ07984.1 awen|OJJ32984.1 azon|OJJ48933.1 afla|EED47961.1

Group4946: abra|OJJ69395.1 acar|OOF94334.1 afis|EAW24310.1 afla|EED47957.1 akaw|GAA88262.1 aluc|OJZ82231.1 anig|EHA28508.1 aory|EIT79855.1 asyd|OJJ53884.1 ater|EAU31263.1 atub|OJI88206.1 aver|OJJ07982.1 awen|OJJ32982.1 azon|OJJ48935.1 acla|EAW14779.1 afum|EBA27252.1 anid|CBF74085.1

Group4947: abra|OJJ69399.1 acar|OOF94331.1 acla|EAW14781.1 afis|EAW24312.1 afla|EED47955.1 afum|EAL85980.1 agla|OJJ83180.1 akaw|GAA88265.1 aluc|OJZ82235.1 anig|EHA28506.1 aory|EIT79818.1 asyd|OJJ53881.1 ater|EAU31265.1 atub|OJI88203.1 aver|OJJ07979.1 awen|OJJ32980.1 azon|OJJ48937.1

Group4948: abra|OJJ69403.1 acar|OOF94328.1 acla|EAW14785.1 afis|EAW24316.1 afla|EED47951.1 afum|EAL85984.1 agla|OJJ83184.1 akaw|GAA88268.1 aluc|OJZ82239.1 anid|CBF74076.1 anig|EHA28502.1 asyd|OJJ53877.1 ater|EAU31268.1 atub|OJI88199.1 aver|OJJ07975.1 awen|OJJ32976.1 azon|OJJ48941.1

Group4949: abra|OJJ69406.1 acla|EAW14788.1 afis|EAW24319.1 afla|EED47948.1 afum|EAL85989.1 agla|OJJ83187.1 akaw|GAA88271.1 aluc|OJZ82243.1 anig|EHA28499.1 ater|EAU31272.1 atub|OJI88195.1 aver|OJJ07972.1 awen|OJJ32973.1 anid|CBF74071.1 asyd|OJJ53874.1 azon|OJJ48944.1 aory|EIT79838.1

Group4950: abra|OJJ69416.1 acar|OOF94316.1 acla|EAW14797.1 afis|EAW24328.1 afla|EED47938.1 afum|EAL85998.1 agla|OJJ83195.1 akaw|GAA88284.1 aluc|OJZ82253.1 anig|EHA28488.1 aory|EIT79811.1 asyd|OJJ53866.1 ater|EAU30425.1 atub|OJI88184.1 aver|OJJ07964.1 awen|OJJ32966.1 azon|OJJ48951.1

Group4951: abra|OJJ69438.1 acar|OOF94302.1 acla|EAW14810.1 afis|EAW24339.1 afla|EED47924.1 afum|EAL86010.1 agla|OJJ83227.1 aluc|OJZ82274.1 anid|CBF74023.1 anig|EHA28471.1 aory|EIT83369.1 asyd|OJJ53843.1 ater|EAU30409.1 atub|OJI88165.1 aver|OJJ07946.1 awen|OJJ32883.1 azon|OJJ48972.1

Group4952: abra|OJJ69444.1 acar|OOF94295.1 acla|EAW11741.1 afis|EAW23774.1 afla|EED57694.1 afum|EAL87208.1 akaw|GAA88374.1 aluc|OJZ82280.1 anid|CBF69507.1 anig|EHA28466.1 aory|EIT79917.1 asyd|OJJ59220.1 ater|EAU31469.1 atub|OJI88159.1 aver|OJJ01831.1 awen|OJJ40003.1 azon|OJJ49871.1

Group4953: abra|OJJ69454.1 acar|OOF94287.1 acla|EAW14818.1 afis|EAW24348.1 afla|EED47915.1 afum|EAL86018.1 agla|OJJ83236.1 akaw|GAA88358.1 aluc|OJZ82297.1 anid|CBF74015.1 anig|EHA28458.1 aory|EIT83377.1 asyd|OJJ53839.1 atub|OJI88142.1 aver|OJJ07942.1 awen|OJJ32876.1 ater|EAU32154.1

Group4954: abra|OJJ69461.1 acar|OOF94279.1 acla|EAW09923.1 afis|EAW21171.1 afla|EED54736.1 afum|EBA27452.1 agla|OJJ80009.1 akaw|GAA88351.1 aluc|OJZ82305.1 anid|CBF83234.1 anig|EHA28451.1 asyd|OJJ60542.1 ater|EAU35838.1 atub|OJI88133.1 aver|OJI99869.1 awen|OJJ36509.1 azon|OJJ44380.1

Group4955: abra|OJJ69466.1 acar|OOF94276.1 acla|EAW09921.1 afis|EAW21169.1 afla|EED54734.1 afum|EAL92188.2 agla|OJJ80011.1 akaw|GAA88347.1 aluc|OJZ82309.1 anig|EHA28448.1 aory|EIT78858.1 asyd|OJJ60540.1 ater|EAU35836.1 atub|OJI88130.1 aver|OJI99867.1 awen|OJJ36506.1 azon|OJJ44378.1

Group4956: abra|OJJ69479.1 acla|EAW09910.1 afis|EAW21159.1 afla|EED54650.1 afum|EAL92177.1 agla|OJJ80029.1 akaw|GAA88337.1 aluc|OJZ82321.1 anid|CBF86979.1 anig|EHA28437.1 aory|EIT78788.1 asyd|OJJ60526.1 ater|EAU35821.1 atub|OJI88119.1 aver|OJI99854.1 awen|OJJ36491.1 azon|OJJ44357.1

Group4957: abra|OJJ69480.1 acar|OOF94264.1 acla|EAW09909.1 afis|EAW21158.1 afum|EAL92176.1 agla|OJJ80030.1 akaw|GAA88336.1 aluc|OJZ82322.1 anid|CBF86981.1 anig|EHA28436.1 aory|EIT78777.1 asyd|OJJ60525.1 ater|EAU35820.1 atub|OJI88118.1 aver|OJI99853.1 awen|OJJ36490.1 azon|OJJ44358.1

Group4958: abra|OJJ69493.1 acar|OOF94252.1 acla|EAW09901.1 afis|EAW21149.1 afla|EED54663.1 afum|EAL92168.1 agla|OJJ80052.1 akaw|GAA88327.1 aluc|OJZ82333.1 anid|CBF75142.1 anig|EHA28427.1 aory|EIT78868.1 asyd|OJJ59970.1 ater|EAU36737.1 atub|OJI88107.1 aver|OJJ02321.1 awen|OJJ36476.1

Group4959: abra|OJJ69503.1 acar|OOF94248.1 afla|EED54665.1 akaw|GAA88320.1 aluc|OJZ82342.1 anig|EHA28418.1 aory|EIT78849.1 atub|OJI88098.1 awen|OJJ36473.1 acla|EAW09899.1 afis|EAW21147.1 afum|EAL92166.1 agla|OJJ80054.1 anid|CBF83168.1 asyd|OJJ55503.1 aver|OJJ06583.1 ater|EAU39451.1

Group4960: abra|OJJ69504.1 acla|EAW09963.1 afis|EAW21132.1 afla|EED51810.1 afum|EAL92150.1 agla|OJJ80085.1 akaw|GAA88319.1 aluc|OJZ82343.1 anig|EHA28417.1 aory|EIT81769.1 asyd|OJJ60462.1 ater|EAU36775.1 atub|OJI88097.1 aver|OJI99782.1 awen|OJJ36455.1 anid|CBF87017.1 azon|OJJ44421.1

Group4961: abra|OJJ69512.1 acar|OOF94241.1 acla|EAW09956.1 afis|EAW21140.1 afla|EED46143.1 afum|EAL92159.1 agla|OJJ80067.1 akaw|GAA88312.1 aluc|OJZ82350.1 anid|CBF87005.1 anig|EHA28410.1 aory|EIT76500.1 asyd|OJJ60469.1 atub|OJI88090.1 aver|OJI99789.1 awen|OJJ36464.1 azon|OJJ44414.1

Group4962: abra|OJJ69530.1 acar|OOF92669.1 acla|EAW07395.1 afis|EAW22421.1 afla|EED52499.1 afum|EAL90771.1 agla|OJJ87603.1 akaw|GAA88381.1 aluc|OJZ82369.1 anid|CBF88809.1 anig|EHA28396.1 aory|EIT82809.1 asyd|OJJ62891.1 atub|OJI88073.1 aver|OJI96942.1 awen|OJJ35000.1 azon|OJJ45643.1

Group4963: abra|OJJ69551.1 acar|OOF95793.1 acla|EAW10612.1 afis|EAW24955.1 afla|EED55509.1 afum|EAL89613.1 agla|OJJ82182.1 akaw|GAA91563.1 aluc|OJZ82381.1 anid|CBF81670.1 anig|EHA28384.1 aory|EIT80837.1 asyd|OJJ59554.1 ater|EAU35702.1 atub|OJI88060.1 aver|OJJ02714.1 awen|OJJ40550.1

Group4964: abra|OJJ69578.1 acar|OOF91577.1 acla|EAW14392.1 afis|EAW19099.1 afla|EED54333.1 afum|EAL93831.1 agla|OJJ84502.1 akaw|GAA91574.1 aluc|OJZ82396.1 anid|CBF79698.1 aory|EIT75935.1 asyd|OJJ58052.1 ater|EAU32146.1 atub|OJI88046.1 aver|OJJ04352.1 awen|OJJ32492.1 azon|OJJ46039.1

Group4965: abra|OJJ69579.1 acar|OOF91575.1 acla|EAW14389.1 afis|EAW19096.1 afla|EED54329.1 afum|EBA27504.1 agla|OJJ84505.1 akaw|GAA91575.1 aluc|OJZ82397.1 anid|CBF79704.1 asyd|OJJ58054.1 ater|EAU32144.1 atub|OJI88045.1 aver|OJJ04349.1 awen|OJJ32489.1 azon|OJJ46042.1 aory|EIT76045.1

Group4966: abra|OJJ69596.1 acar|OOF91563.1 acla|EAW14374.1 afis|EAW19082.1 afla|EED54314.1 afum|EAL93811.1 agla|OJJ84524.1 akaw|GAA91588.1 aluc|OJZ82411.1 anid|CBF79670.1 anig|EHA28356.1 aory|EIT75963.1 asyd|OJJ58035.1 ater|EAU32129.1 atub|OJI88030.1 aver|OJJ04364.1 awen|OJJ32477.1

Group4967: abra|OJJ69613.1 acar|OOF92693.1 acla|EAW11766.1 afis|EAW23684.1 afla|EED51826.1 afum|EAL87129.1 agla|OJJ86862.1 akaw|GAA91597.1 aluc|OJZ82421.1 anid|CBF80086.1 anig|EHA28348.1 aory|EIT81697.1 asyd|OJJ58305.1 ater|EAU31583.1 atub|OJI88020.1 aver|OJJ02107.1 azon|OJJ45792.1

Group4968: abra|OJJ69651.1 acar|OOG00403.1 acla|EAW06766.1 afis|EAW16316.1 afla|EED53565.1 afum|EAL88939.1 agla|OJJ88277.1 akaw|GAA91628.1 aluc|OJZ82455.1 anid|CBF89144.1 anig|EHA28320.1 aory|EIT75184.1 asyd|OJJ62570.1 ater|EAU34141.1 atub|OJI87983.1 aver|OJI96694.1 awen|OJJ37714.1

Group4969: abra|OJJ69653.1 acar|OOF93647.1 acla|EAW14511.1 afis|EAW19229.1 afla|EED47661.1 agla|OJJ88470.1 akaw|GAA91632.1 aluc|OJZ82459.1 anid|CBF78612.1 anig|EHA28316.1 asyd|OJJ65179.1 ater|EAU37234.1 atub|OJI87979.1 aver|OJI99332.1 awen|OJJ37587.1 azon|OJJ51241.1 aory|EIT80209.1

Group4970: abra|OJJ69678.1 acar|OOF93500.1 acla|EAW08823.1 afis|EAW15574.1 afla|EED49083.1 afum|EAL85760.1 agla|OJJ85178.1 akaw|GAA88803.1 aluc|OJZ82486.1 anid|CBF78103.1 anig|EHA28288.1 aory|EIT82543.1 asyd|OJJ55894.1 ater|EAU32557.1 atub|OJI87946.1 aver|OJJ04839.1 awen|OJJ38234.1

Group4971: abra|OJJ69700.1 acar|OOF90941.1 afis|EAW20859.1 afla|EED45448.1 afum|EAL84776.1 agla|OJJ83745.1 akaw|GAA88817.1 aluc|OJZ82501.1 anid|CBF84702.1 anig|EHA28268.1 aory|EIT82427.1 asyd|OJJ53319.1 ater|EAU30802.1 atub|OJI87930.1 aver|OJJ08424.1 awen|OJJ34514.1 azon|OJJ45199.1

Group4972: abra|OJJ69708.1 acar|OOF94202.1 acla|EAW09975.1 afis|EAW21114.1 afla|EED51765.1 afum|EAL92137.1 agla|OJJ80097.1 akaw|GAA90157.1 aluc|OJZ83190.1 anid|CBF87034.1 anig|EHA28260.1 aory|EIT80039.1 asyd|OJJ60453.1 ater|EAU36785.1 atub|OJI87749.1 aver|OJI99771.1 awen|OJJ36444.1

Group4973: abra|OJJ69711.1 acar|OOF94201.1 acla|EAW09974.1 afis|EAW21115.1 afla|EED51766.1 afum|EAL92138.1 agla|OJJ80096.1 akaw|GAA90161.1 aluc|OJZ83186.1 anid|CBF87032.1 anig|EHA28258.1 asyd|OJJ60454.1 ater|EAU36784.1 atub|OJI87753.1 aver|OJI99772.1 awen|OJJ36445.1 azon|OJJ44431.1

Group4974: abra|OJJ69731.1 acar|OOF94190.1 acla|EAW11683.1 afla|EED51759.1 agla|OJJ86787.1 akaw|GAA90184.1 aluc|OJZ83165.1 anid|CBF73433.1 anig|EHA28229.1 aory|EIT80571.1 atub|OJI87772.1 aver|OJJ08109.1 awen|OJJ38074.1 azon|OJJ42488.1 acar|OOF93383.1 afum|EAL86339.2 afis|EAW16771.1

Group4975: abra|OJJ69741.1 acar|OOF94180.1 acla|EAW09983.1 afis|EAW21109.1 afla|EED51713.1 afum|EAL92131.1 akaw|GAA90193.1 aluc|OJZ83155.1 anid|CBF80441.1 anig|EHA28221.1 aory|EIT80457.1 asyd|OJJ54783.1 ater|EAU36790.1 atub|OJI87782.1 aver|OJJ07383.1 awen|OJJ36437.1 azon|OJJ45175.1

Group4976: abra|OJJ69743.1 acar|OOG00883.1 acla|EAW07453.1 afis|EAW22485.1 afla|EED56932.1 afum|EAL89359.2 akaw|GAA90196.1 aluc|OJZ83152.1 anig|EHA28218.1 aory|EIT77590.1 ater|EAU39229.1 atub|OJI87785.1 awen|OJJ35104.1 afla|EED47459.1 afum|EAL90708.1 aory|EIT79961.1 afla|EED56933.1

Group4977: abra|OJJ69747.1 acar|OOF94175.1 acla|EAW09986.1 afis|EAW21107.1 afla|EED51688.1 afum|EAL92128.1 agla|OJJ80109.1 akaw|GAA90199.1 aluc|OJZ83149.1 anig|EHA28215.1 aory|EIT80537.1 asyd|OJJ60447.1 ater|EAU36793.1 atub|OJI87788.1 aver|OJI99765.1 awen|OJJ36368.1 azon|OJJ51535.1

Group4978: abra|OJJ69772.1 agla|OJJ89535.1 akaw|GAA87882.1 aluc|OJZ85527.1 anig|EHA25058.1 asyd|OJJ59178.1 atub|OJI80041.1 aver|OJJ01798.1 abra|OJJ76053.1 afla|EED48186.1 akaw|GAA88912.1 aluc|OJZ89509.1 anig|EHA23091.1 aory|EIT72940.1 ater|EAU37178.1 atub|OJI85294.1 awen|OJJ39948.1

Group4979: acar|OOF93589.1 anig|EHA25040.1 atub|OJI80057.1 abra|OJJ69791.1 acla|EAW11764.1 afis|EAW23687.1 afla|EED57653.1 afum|EAL87132.1 agla|OJJ78642.1 akaw|GAA87867.1 aluc|OJZ85508.1 anid|CBF84203.1 aory|EIT79945.1 asyd|OJJ57163.1 ater|EAU34272.1 aver|OJJ03150.1 awen|OJJ33314.1

Group4980: abra|OJJ69803.1 acar|OOF93573.1 acla|EAW11066.1 afis|EAW20808.1 afla|EED51436.1 afum|EAL84820.1 akaw|GAA87856.1 aluc|OJZ85498.1 anid|CBF80564.1 anig|EHA25031.1 aory|EIT80626.1 asyd|OJJ54681.1 ater|EAU30269.1 atub|OJI80067.1 aver|OJJ07487.1 awen|OJJ31034.1 azon|OJJ42532.1

Group4981: abra|OJJ69845.1 acar|OOF93368.1 acla|EAW08980.1 afis|EAW15429.1 afla|EED55190.1 afum|EAL84214.1 agla|OJJ80141.1 akaw|GAA87814.1 aluc|OJZ85449.1 anid|CBF78299.1 anig|EHA24992.1 aory|EIT83595.1 asyd|OJJ56101.1 ater|EAU32702.1 atub|OJI80118.1 aver|OJJ05044.1 awen|OJJ38049.1

Group4982: anid|CBF87945.1 abra|OJJ77908.1 acla|EAW13240.1 afis|EAW17394.1 afla|EED55526.1 afum|EAL91232.1 agla|OJJ86275.1 akaw|GAA83751.1 aluc|OJZ90980.1 anig|EHA19685.1 aory|EIT80850.1 asyd|OJJ63474.1 ater|EAU38912.1 atub|OJI82971.1 aver|OJI97456.1 awen|OJJ32549.1 azon|OJJ47335.1

Group4983: abra|OJJ69868.1 acar|OOF90336.1 acla|EAW15316.1 afis|EAW20318.1 afla|EED57091.1 afum|EAL85446.1 akaw|GAA87788.1 aluc|OJZ85423.1 anig|EHA25602.1 aory|EIT82311.1 asyd|OJJ56070.1 ater|EAU32680.1 atub|OJI80152.1 aver|OJJ05013.1 awen|OJJ31345.1 asyd|OJJ52769.1 aver|OJJ08752.1

Group4984: abra|OJJ69874.1 acar|OOF93387.1 acla|EAW08929.1 afis|EAW15467.1 afla|EED46919.1 afum|EAL85855.1 agla|OJJ80148.1 akaw|GAA87783.1 aluc|OJZ85418.1 anid|CBF78284.1 anig|EHA25597.1 aory|EIT72664.1 asyd|OJJ56069.1 ater|EAU32661.1 atub|OJI80158.1 aver|OJJ05012.1 awen|OJJ38113.1

Group4985: abra|OJJ69904.1 acar|OOF97941.1 acla|EAW08919.1 afis|EAW15475.1 afla|EED47464.1 afum|EAL85846.1 agla|OJJ89051.1 akaw|GAA87748.1 aluc|OJZ85381.1 anid|CBF86603.1 anig|EHA25564.1 aory|EIT79959.1 asyd|OJJ64826.1 ater|EAU32653.1 atub|OJI80189.1 aver|OJI98939.1 awen|OJJ36394.1

Group4986: abra|OJJ69912.1 acar|OOF90389.1 acla|EAW08916.1 afis|EAW15478.1 afum|EAL85843.1 agla|OJJ78432.1 akaw|GAA87745.1 aluc|OJZ85376.1 anid|CBF78223.1 anig|EHA25559.1 aory|EIT72649.1 asyd|OJJ56031.1 ater|EAU32649.1 atub|OJI80193.1 aver|OJJ04978.1 awen|OJJ38135.1 azon|OJJ43156.1

Group4987: abra|OJJ69915.1 acar|OOF93427.1 acla|EAW08913.1 afis|EAW15482.1 afla|EED46931.1 afum|EAL85840.1 agla|OJJ80161.1 akaw|GAA87741.1 aluc|OJZ85373.1 anid|CBF78219.1 anig|EHA25557.1 aory|EIT72658.1 asyd|OJJ56027.1 atub|OJI80197.1 aver|OJJ04974.1 awen|OJJ38141.1 azon|OJJ43149.1

Group4988: abra|OJJ69919.1 acar|OOF93431.1 acla|EAW08910.1 afis|EAW15486.1 afla|EED46941.1 afum|EAL85837.1 agla|OJJ80164.1 akaw|GAA87737.1 aluc|OJZ85369.1 anig|EHA25552.1 aory|EIT72770.1 asyd|OJJ56024.1 ater|EAU32646.1 atub|OJI80201.1 aver|OJJ04971.1 awen|OJJ38143.1 azon|OJJ43145.1

Group4989: abra|OJJ69924.1 acar|OOF93435.1 acla|EAW08906.1 afis|EAW15490.1 afla|EED49004.1 afum|EAL85833.1 agla|OJJ80167.1 akaw|GAA87733.1 aluc|OJZ85364.1 anig|EHA25547.1 aory|EIT82535.1 asyd|OJJ56021.1 ater|EAU32629.1 atub|OJI80206.1 aver|OJJ04968.1 awen|OJJ38146.1 azon|OJJ43141.1

Group4990: abra|OJJ69926.1 acar|OOF93437.1 acla|EAW08904.1 afis|EAW15492.1 afla|EED49007.1 afum|EAL85831.1 agla|OJJ80169.1 akaw|GAA87731.1 aluc|OJZ85362.1 anig|EHA25545.1 aory|EIT82513.1 asyd|OJJ56019.1 ater|EAU32627.1 atub|OJI80209.1 aver|OJJ04966.1 awen|OJJ38148.1 azon|OJJ43139.1

Group4991: abra|OJJ69927.1 acar|OOF93438.1 acla|EAW08903.1 afis|EAW15493.1 afla|EED49008.1 afum|EAL85830.1 agla|OJJ80170.1 akaw|GAA87730.1 aluc|OJZ85361.1 anig|EHA25544.1 aory|EIT82514.1 asyd|OJJ56018.1 ater|EAU32626.1 atub|OJI80210.1 aver|OJJ04964.1 awen|OJJ38149.1 azon|OJJ43138.1

Group4992: abra|OJJ69928.1 acar|OOF93439.1 acla|EAW08902.1 afis|EAW15494.1 afla|EED49009.1 afum|EAL85829.1 agla|OJJ80171.1 akaw|GAA87729.1 aluc|OJZ85359.1 anig|EHA25543.1 aory|EIT82523.1 asyd|OJJ56017.1 ater|EAU32625.1 atub|OJI80212.1 aver|OJJ04963.1 awen|OJJ38150.1 azon|OJJ43137.1

Group4993: abra|OJJ69929.1 acar|OOF93440.1 acla|EAW08901.1 afis|EAW15495.1 afla|EED49010.1 afum|EAL85828.1 agla|OJJ80173.1 akaw|GAA87728.1 aluc|OJZ85358.1 anig|EHA25542.1 aory|EIT82519.1 asyd|OJJ56016.1 ater|EAU32624.1 atub|OJI80213.1 aver|OJJ04962.1 awen|OJJ38151.1 azon|OJJ43136.1

Group4994: abra|OJJ69930.1 acar|OOF93441.1 acla|EAW08893.1 afis|EAW15496.1 afla|EED49011.1 afum|EAL85827.1 agla|OJJ80175.1 akaw|GAA87727.1 aluc|OJZ85357.1 anig|EHA25541.1 aory|EIT82520.1 asyd|OJJ56015.1 ater|EAU32623.1 atub|OJI80214.1 aver|OJJ04961.1 awen|OJJ38154.1 azon|OJJ43135.1

Group4995: abra|OJJ69934.1 acar|OOF93443.1 acla|EAW08892.1 afis|EAW15497.1 afla|EED49012.1 afum|EAL85826.1 agla|OJJ80176.1 akaw|GAA87725.1 aluc|OJZ85353.1 anig|EHA25539.1 aory|EIT82506.1 asyd|OJJ56014.1 ater|EAU32622.1 atub|OJI80217.1 aver|OJJ04959.1 awen|OJJ38156.1 azon|OJJ43134.1

Group4996: abra|OJJ69937.1 acar|OOF93446.1 acla|EAW08886.1 afis|EAW15503.1 afla|EED49016.1 afum|EAL85821.1 agla|OJJ80180.1 akaw|GAA87722.1 aluc|OJZ85350.1 anig|EHA25537.1 aory|EIT82554.1 asyd|OJJ56008.1 ater|EAU32615.1 atub|OJI80220.1 aver|OJJ04953.1 awen|OJJ38163.1 azon|OJJ43124.1

Group4997: abra|OJJ69938.1 acar|OOF93447.1 acla|EAW08885.1 afis|EAW15504.1 afla|EED49017.1 afum|EAL85820.1 agla|OJJ80181.1 akaw|GAA87721.1 aluc|OJZ85349.1 anig|EHA25536.1 aory|EIT82559.1 asyd|OJJ56007.1 ater|EAU32614.1 atub|OJI80221.1 aver|OJJ04952.1 awen|OJJ38164.1 azon|OJJ43125.1

Group4998: abra|OJJ69939.1 acar|OOF93448.1 acla|EAW08884.1 afis|EAW15505.1 afla|EED49018.1 afum|EAL85819.1 agla|OJJ80182.1 akaw|GAA87720.1 aluc|OJZ85348.1 anig|EHA25535.1 aory|EIT82495.1 asyd|OJJ56006.1 ater|EAU32613.1 atub|OJI80222.1 aver|OJJ04951.1 awen|OJJ38165.1 azon|OJJ43126.1

Group4999: abra|OJJ69944.1 acar|OOF93453.1 acla|EAW08879.1 afis|EAW15516.1 afla|EED49022.1 afum|EAL85813.1 agla|OJJ80187.1 akaw|GAA87716.1 aluc|OJZ85343.1 anig|EHA25531.1 aory|EIT82503.1 asyd|OJJ56002.1 ater|EAU32608.1 atub|OJI80228.1 aver|OJJ04948.1 awen|OJJ38177.1 azon|OJJ43120.1

Group5000: abra|OJJ69945.1 acar|OOF93454.1 acla|EAW08878.1 afis|EAW15517.1 afla|EED49023.1 afum|EAL85812.1 agla|OJJ85228.1 akaw|GAA87715.1 aluc|OJZ85342.1 anig|EHA25530.1 aory|EIT82489.1 asyd|OJJ56001.1 ater|EAU32607.1 atub|OJI80229.1 aver|OJJ04947.1 awen|OJJ38178.1 azon|OJJ43119.1

Group5001: abra|OJJ69946.1 acar|OOF93455.1 acla|EAW08877.1 afis|EAW15518.1 afla|EED49025.1 afum|EAL85811.1 agla|OJJ85227.1 akaw|GAA87714.1 aluc|OJZ85341.1 anig|EHA25529.1 aory|EIT82492.1 asyd|OJJ56000.1 ater|EAU32606.1 atub|OJI80230.1 aver|OJJ04946.1 awen|OJJ38179.1 azon|OJJ43118.1

Group5002: abra|OJJ69949.1 acar|OOF93457.1 acla|EAW08875.1 afis|EAW15520.1 afla|EED49027.1 afum|EAL85809.1 agla|OJJ85226.1 akaw|GAA87711.1 aluc|OJZ85338.1 anig|EHA25526.1 aory|EIT82544.1 asyd|OJJ55997.1 ater|EAU32604.1 atub|OJI80233.1 aver|OJJ04944.1 awen|OJJ38181.1 azon|OJJ43112.1

Group5003: abra|OJJ69957.1 acar|OOF93467.1 acla|EAW08865.1 afis|EAW15529.1 afla|EED49040.1 afum|EAL85799.1 akaw|GAA87703.1 aluc|OJZ85325.1 anid|CBF78208.1 anig|EHA25515.1 aory|EIT82500.1 asyd|OJJ55987.1 ater|EAU32596.1 atub|OJI80247.1 aver|OJJ04935.1 awen|OJJ38193.1 azon|OJJ43101.1

Group5004: abra|OJJ69966.1 acla|EAW08838.1 afis|EAW15562.1 afla|EED49068.1 afum|EAL85770.1 agla|OJJ85189.1 akaw|GAA87693.1 aluc|OJZ85316.1 anid|CBF78123.1 anig|EHA25509.1 aory|EIT82555.1 asyd|OJJ55904.1 ater|EAU32570.1 atub|OJI80256.1 aver|OJJ04849.1 awen|OJJ38222.1 azon|OJJ43073.1

Group5005: abra|OJJ69969.1 acar|OOF93474.1 acla|EAW08840.1 afis|EAW15561.1 afla|EED49065.1 afum|EAL85771.1 agla|OJJ85193.1 akaw|GAA87692.1 aluc|OJZ85313.1 anig|EHA25508.1 aory|EIT82526.1 asyd|OJJ55911.1 ater|EAU32571.1 atub|OJI80258.1 aver|OJJ04851.1 awen|OJJ38219.1 azon|OJJ43074.1

Group5006: abra|OJJ69970.1 acar|OOF93476.1 acla|EAW08841.1 afis|EAW15560.1 afla|EED49064.1 afum|EAL85772.1 agla|OJJ85194.1 akaw|GAA87691.1 aluc|OJZ85312.1 anig|EHA25507.1 aory|EIT82521.1 asyd|OJJ55912.1 ater|EAU32572.1 atub|OJI80259.1 aver|OJJ04852.1 awen|OJJ38218.1 azon|OJJ43075.1

Group5007: abra|OJJ69972.1 acar|OOF93477.1 acla|EAW08843.1 afis|EAW15558.1 afla|EED49062.1 afum|EAL85774.1 agla|OJJ85196.1 akaw|GAA87689.1 aluc|OJZ85310.1 anid|CBF78161.1 anig|EHA25505.1 asyd|OJJ55962.1 ater|EAU32576.1 atub|OJI80261.1 aver|OJJ04907.1 awen|OJJ38216.1 azon|OJJ43079.1

Group5008: abra|OJJ69980.1 acla|EAW08850.1 afis|EAW15546.1 afum|EAL85786.1 agla|OJJ85206.1 akaw|GAA87681.1 aluc|OJZ85300.1 anid|CBF78178.1 anig|EHA25497.1 aory|EIT82561.1 asyd|OJJ55971.1 ater|EAU32581.1 atub|OJI80270.1 aver|OJJ04918.1 awen|OJJ38207.1 azon|OJJ43088.1 afla|EED49053.1

Group5009: abra|OJJ69988.1 acar|OOF93493.1 acla|EAW08859.1 afis|EAW15535.1 afla|EED49045.1 afum|EAL85793.1 agla|OJJ85212.1 akaw|GAA87673.1 aluc|OJZ85291.1 anid|CBF78195.1 anig|EHA25489.1 aory|EIT82566.1 asyd|OJJ55980.1 ater|EAU32590.1 atub|OJI80279.1 aver|OJJ04928.1 awen|OJJ38199.1

Group5010: abra|OJJ69998.1 acar|OOF93505.1 acla|EAW08821.1 afis|EAW15576.1 afla|EED49085.1 afum|EAL85758.1 agla|OJJ85176.1 akaw|GAA87663.1 aluc|OJZ85281.1 anig|EHA25478.1 aory|EIT82569.1 asyd|OJJ55892.1 ater|EAU32555.1 atub|OJI80289.1 aver|OJJ04837.1 awen|OJJ38236.1 azon|OJJ46061.1

Group5011: abra|OJJ70002.1 acar|OOF93508.1 acla|EAW08817.1 afis|EAW15580.1 afla|EED49092.1 afum|EAL85754.1 agla|OJJ85172.1 akaw|GAA87659.1 aluc|OJZ85277.1 anid|CBF78098.1 anig|EHA25474.1 asyd|OJJ55888.1 ater|EAU32550.1 atub|OJI80293.1 aver|OJJ04832.1 awen|OJJ38242.1 azon|OJJ43056.1

Group5012: abra|OJJ70005.1 acar|OOF93510.1 acla|EAW07277.1 afis|EAW22308.1 afla|EED57704.1 afum|EAL90882.1 agla|OJJ87742.1 akaw|GAA87656.1 aluc|OJZ85274.1 anid|CBF88590.1 anig|EHA25472.1 aory|EIT79905.1 asyd|OJJ63849.1 ater|EAU34316.1 atub|OJI80296.1 aver|OJI97088.1 awen|OJJ34879.1

Group5013: abra|OJJ70010.1 acar|OOF93516.1 acla|EAW08814.1 afis|EAW15583.1 afla|EED49087.1 afum|EAL85751.1 agla|OJJ85168.1 akaw|GAA87652.1 aluc|OJZ85269.1 anid|CBF78058.1 anig|EHA25468.1 aory|EIT82488.1 ater|EAU32546.1 atub|OJI80302.1 aver|OJJ04812.1 awen|OJJ38248.1 azon|OJJ43028.1

Group5014: abra|OJJ70012.1 acar|OOF93518.1 acla|EAW08812.1 afis|EAW15585.1 afla|EED54444.1 afum|EAL85747.1 agla|OJJ85164.1 akaw|GAA87650.1 aluc|OJZ85266.1 anid|CBF78061.1 anig|EHA25465.1 asyd|OJJ55869.1 ater|EAU32544.1 atub|OJI80304.1 aver|OJJ04814.1 awen|OJJ38251.1 azon|OJJ43031.1

Group5015: abra|OJJ70137.1 acar|OOF93594.1 acla|EAW13744.1 afis|EAW19842.1 afla|EED46533.1 afum|EAL85053.1 akaw|GAA86628.1 aluc|OJZ85183.1 anid|CBF76707.1 anig|EHA25395.1 aory|EIT72392.1 asyd|OJJ61237.1 ater|EAU38516.1 atub|OJI80414.1 aver|OJJ00560.1 awen|OJJ34118.1 azon|OJJ49476.1

Group5016: abra|OJJ70140.1 acar|OOF93597.1 acla|EAW13733.1 afis|EAW19843.1 afla|EED46534.1 afum|EAL85052.1 akaw|GAA86625.1 aluc|OJZ85180.1 anid|CBF76705.1 anig|EHA25392.1 aory|EIT72326.1 asyd|OJJ61236.1 ater|EAU38515.1 atub|OJI80417.1 aver|OJJ00559.1 awen|OJJ34117.1 azon|OJJ49477.1

Group5017: abra|OJJ70193.1 acar|OOF92582.1 acla|EAW06999.1 afis|EAW16614.1 afla|EED49260.1 afum|EAL89222.2 agla|OJJ82625.1 akaw|GAA86582.1 aluc|OJZ85133.1 anid|CBF86355.1 anig|EHA25352.1 asyd|OJJ57360.1 ater|EAU36698.1 atub|OJI80460.1 aver|OJJ05806.1 awen|OJJ40191.1 afla|EED44998.1

Group5018: abra|OJJ70232.1 acar|OOF93866.1 afla|EED56154.1 afum|EAL90745.1 agla|OJJ88092.1 akaw|GAA86552.1 aluc|OJZ85100.1 anig|EHA26109.1 asyd|OJJ59762.1 ater|EAU37896.1 aver|OJJ02526.1 awen|OJJ39210.1 azon|OJJ50036.1 anig|EHA25322.1 acla|EAW07420.1 afis|EAW22446.1 awen|OJJ35071.1

Group5019: abra|OJJ70304.1 acar|OOF91757.1 acla|EAW12288.1 afis|EAW25658.1 afla|EED45243.1 afum|EAL86477.2 agla|OJJ79117.1 akaw|GAA93018.1 aluc|OJZ82567.1 anid|CBF80474.1 anig|EHA27507.1 aory|EIT73558.1 asyd|OJJ54715.1 ater|EAU30190.1 atub|OJI88811.1 aver|OJJ07452.1 afis|EAW16950.1

Group5020: abra|OJJ70306.1 acar|OOF91761.1 acla|EAW12289.1 afis|EAW16947.1 afla|EED45244.1 afum|EAL86474.1 agla|OJJ79960.1 akaw|GAA93016.1 aluc|OJZ82569.1 anid|CBF80495.1 anig|EHA27505.1 aory|EIT73662.1 asyd|OJJ54729.1 ater|EAU30192.1 atub|OJI88813.1 aver|OJJ07439.1 awen|OJJ29711.1

Group5021: abra|OJJ70329.1 acar|OOF91784.1 acla|EAW12320.1 afis|EAW16901.1 afla|EED45272.1 afum|EAL86436.1 agla|OJJ79952.1 akaw|GAA92584.1 aluc|OJZ82598.1 anid|CBF80772.1 anig|EHA27479.1 aory|EIT73781.1 asyd|OJJ54500.1 ater|EAU30216.1 atub|OJI88834.1 aver|OJJ07680.1 awen|OJJ29730.1

Group5022: abra|OJJ70366.1 acar|OOF91906.1 acla|EAW12330.1 afis|EAW16893.1 afum|EAL86431.1 agla|OJJ79946.1 akaw|GAA91985.1 aluc|OJZ82628.1 anid|CBF80796.1 anig|EHA27449.1 aory|EIT75240.1 asyd|OJJ54490.1 ater|EAU30222.1 atub|OJI88861.1 aver|OJJ07689.1 awen|OJJ29744.1 azon|OJJ42525.1

Group5023: abra|OJJ70382.1 acar|OOF91889.1 afis|EAW16883.1 afla|EED47346.1 afum|EAL86419.1 agla|OJJ79750.1 akaw|GAA91974.1 aluc|OJZ82641.1 anid|CBF80677.1 anig|EHA27429.1 aory|EIT82848.1 asyd|OJJ54589.1 ater|EAU30232.1 atub|OJI88874.1 aver|OJJ07564.1 awen|OJJ29766.1 azon|OJJ50379.1

Group5024: abra|OJJ70471.1 acar|OOF97454.1 acla|EAW13043.1 afis|EAW17624.1 afla|EED49193.1 afum|EAL91438.1 agla|OJJ81279.1 akaw|GAA83320.1 aluc|OJZ83815.1 anid|CBF90154.1 anig|EHA18960.1 asyd|OJJ61999.1 ater|EAU37058.1 atub|OJI90182.1 aver|OJI96091.1 awen|OJJ37220.1 azon|OJJ50923.1

Group5025: abra|OJJ70486.1 acar|OOF97440.1 acla|EAW13030.1 afis|EAW17637.1 afla|EED50527.1 afum|EAL91452.1 agla|OJJ81257.1 akaw|GAA83334.1 aluc|OJZ83830.1 anid|CBF90130.1 anig|EHA18973.1 asyd|OJJ62016.1 ater|EAU37073.1 atub|OJI90197.1 aver|OJI96107.1 awen|OJJ37207.1 azon|OJJ50906.1

Group5026: abra|OJJ70494.1 acar|OOF97433.1 acla|EAW13023.1 afis|EAW17644.1 afla|EED50516.1 agla|OJJ79560.1 akaw|GAA83342.1 aluc|OJZ83838.1 anid|CBF90116.1 anig|EHA18981.1 aory|EIT82026.1 asyd|OJJ62023.1 ater|EAU37080.1 atub|OJI90205.1 aver|OJI96114.1 awen|OJJ37201.1 azon|OJJ50900.1

Group5027: abra|OJJ70501.1 acar|OOF97426.1 acla|EAW08173.1 afis|EAW23209.1 afla|EED47042.1 afum|EAL88275.1 agla|OJJ78736.1 akaw|GAA83349.1 aluc|OJZ83846.1 anig|EHA18988.1 aory|EIT72737.1 asyd|OJJ60187.1 ater|EAU36337.1 atub|OJI90212.1 aver|OJJ04077.1 awen|OJJ39442.1 azon|OJJ47644.1

Group5028: abra|OJJ70503.1 acar|OOF97423.1 acla|EAW08172.1 afis|EAW23208.1 afla|EED47046.1 afum|EBA27341.1 agla|OJJ78738.1 akaw|GAA83351.1 aluc|OJZ83848.1 anig|EHA18990.1 aory|EIT72619.1 asyd|OJJ60188.1 ater|EAU36333.1 atub|OJI90214.1 aver|OJJ04076.1 awen|OJJ39441.1 azon|OJJ47642.1

Group5029: abra|OJJ70511.1 acar|OOF97418.1 acla|EAW08164.1 afis|EAW23202.1 afla|EED47052.1 afum|EAL88281.1 agla|OJJ78746.1 akaw|GAA83358.1 aluc|OJZ83856.1 anid|CBF74726.1 anig|EHA18997.1 asyd|OJJ60196.1 ater|EAU36330.1 atub|OJI90223.1 aver|OJJ04069.1 awen|OJJ39432.1 azon|OJJ47636.1

Group5030: abra|OJJ70523.1 acla|EAW08152.1 afis|EAW23187.1 afla|EED47066.1 afum|EAL88296.1 agla|OJJ86362.1 akaw|GAA83370.1 aluc|OJZ83868.1 anid|CBF74683.1 anig|EHA19009.1 aory|EIT72474.1 asyd|OJJ60221.1 ater|EAU36318.1 atub|OJI90236.1 aver|OJJ04047.1 awen|OJJ36589.1 azon|OJJ47617.1

Group5031: abra|OJJ70527.1 acar|OOF97404.1 acla|EAW08140.1 afis|EAW23176.1 afla|EED46943.1 afum|EAL88307.2 agla|OJJ89688.1 akaw|GAA83372.1 aluc|OJZ83870.1 anid|CBF74500.1 anig|EHA19011.1 aory|EIT72572.1 asyd|OJJ57721.1 atub|OJI90238.1 aver|OJJ05447.1 awen|OJJ36602.1 azon|OJJ47606.1

Group5032: abra|OJJ70533.1 acar|OOF97399.1 afis|EAW16751.1 afla|EED47075.1 agla|OJJ86369.1 akaw|GAA83378.1 aluc|OJZ83876.1 anig|EHA19016.1 aory|EIT72481.1 atub|OJI90244.1 awen|OJJ36596.1 azon|OJJ50257.1 afis|EAW23182.1 acla|EAW12439.1 acla|EAW12440.1 afum|EBA27268.1 afum|EAL88300.1

Group5033: abra|OJJ70547.1 acla|EAW08134.1 afis|EAW23170.1 afla|EED46951.1 afum|EAL88314.1 agla|OJJ89681.1 akaw|GAA83391.1 aluc|OJZ83891.1 anid|CBF74482.1 anig|EHA19027.1 asyd|OJJ57710.1 ater|EAU36299.1 atub|OJI90258.1 aver|OJJ05455.1 awen|OJJ36613.1 azon|OJJ47600.1 agla|OJJ89682.1

Group5034: abra|OJJ70551.1 acar|OOF97169.1 acla|EAW08343.1 afis|EAW23378.1 afla|EED55579.1 afum|EAL88093.1 agla|OJJ86445.1 akaw|GAA83395.1 aluc|OJZ83894.1 anid|CBF74847.1 anig|EHA19030.1 asyd|OJJ60129.1 atub|OJI90262.1 aver|OJJ04132.1 awen|OJJ39506.1 azon|OJJ47705.1 ater|EAU35632.1

Group5035: abra|OJJ70553.1 acar|OOF97166.1 acla|EAW08348.1 afis|EAW23382.1 afla|EED55569.1 afum|EAL88090.1 agla|OJJ86441.1 akaw|GAA83398.1 aluc|OJZ83896.1 anig|EHA19032.1 aory|EIT82669.1 ater|EAU35635.1 atub|OJI90265.1 awen|OJJ39502.1 azon|OJJ47702.1 afum|EAL88089.1 anig|EHA19033.1

Group5036: abra|OJJ70562.1 acar|OOF97157.1 acla|EAW08361.1 afis|EAW23392.1 afla|EED55565.1 afum|EAL88078.1 agla|OJJ86425.1 akaw|GAA83407.1 aluc|OJZ83906.1 anid|CBF74862.1 anig|EHA19041.1 asyd|OJJ60122.1 ater|EAU35645.1 atub|OJI90277.1 aver|OJJ04140.1 awen|OJJ39487.1 azon|OJJ47690.1

Group5037: abra|OJJ70586.1 acar|OOF97134.1 acla|EAW08195.1 afis|EAW23229.1 afla|EED47021.1 afum|EAL88254.1 agla|OJJ86386.1 akaw|GAA83429.1 aluc|OJZ83930.1 anid|CBF74773.1 anig|EHA19062.1 asyd|OJJ60166.1 ater|EAU36358.1 atub|OJI90302.1 aver|OJJ04098.1 awen|OJJ39464.1 azon|OJJ47664.1

Group5038: abra|OJJ70587.1 acar|OOF97133.1 acla|EAW08194.1 afis|EAW23228.1 afla|EED47022.1 afum|EAL88255.1 agla|OJJ86385.1 akaw|GAA83430.1 aluc|OJZ83931.1 anid|CBF74771.1 anig|EHA19063.1 asyd|OJJ60167.1 ater|EAU36357.1 atub|OJI90303.1 aver|OJJ04097.1 awen|OJJ39463.1 azon|OJJ47663.1

Group5039: abra|OJJ70590.1 acar|OOF97129.1 acla|EAW08191.1 afis|EAW23225.1 afla|EED47025.1 afum|EAL88258.1 agla|OJJ86382.1 akaw|GAA83434.1 aluc|OJZ83935.1 anid|CBF74765.1 anig|EHA19066.1 aory|EIT72720.1 asyd|OJJ60170.1 atub|OJI90307.1 aver|OJJ04094.1 awen|OJJ39460.1 azon|OJJ47659.1

Group5040: abra|OJJ70591.1 afla|EED47026.1 afum|EAL88260.1 agla|OJJ86381.1 akaw|GAA83435.1 aluc|OJZ83937.1 anid|CBF74763.1 aory|EIT72526.1 azon|OJJ47658.1 awen|OJJ39459.1 acla|EAW08190.1 afis|EAW23224.1 anig|EHA19067.1 asyd|OJJ60171.1 ater|EAU36355.1 atub|OJI90309.1 aver|OJJ04093.1

Group5041: abra|OJJ70597.1 acar|OOF97122.1 afis|EAW23218.1 afla|EED47033.1 afum|EAL88266.1 agla|OJJ86374.1 akaw|GAA83441.1 aluc|OJZ83943.1 anid|CBF74754.1 anig|EHA19072.1 aory|EIT72711.1 asyd|OJJ60177.1 ater|EAU36349.1 atub|OJI90315.1 aver|OJJ04087.1 awen|OJJ39452.1 azon|OJJ47652.1

Group5042: abra|OJJ70601.1 acar|OOF97119.1 acla|EAW08181.1 afis|EAW23216.1 afum|EAL88268.1 agla|OJJ86372.1 akaw|GAA89941.1 aluc|OJZ83946.1 anid|CBF74747.1 anig|EHA19075.1 aory|EIT72596.1 asyd|OJJ60179.1 ater|EAU36346.1 atub|OJI90317.1 aver|OJJ04083.1 awen|OJJ39450.1 azon|OJJ47650.1

Group5043: abra|OJJ70614.1 acar|OOF97107.1 acla|EAW13013.1 afis|EAW17654.1 afla|EED50504.1 afum|EAL91469.1 agla|OJJ79550.1 akaw|GAA89953.1 aluc|OJZ83960.1 anig|EHA19088.1 aory|EIT82141.1 atub|OJI90332.1 aver|OJI96149.1 awen|OJJ37191.1 azon|OJJ50890.1 anid|CBF90070.1 asyd|OJJ62043.1

Group5044: abra|OJJ70615.1 acar|OOF97106.1 acla|EAW13012.1 afis|EAW17655.1 afla|EED50503.1 afum|EAL91470.1 akaw|GAA89954.1 aluc|OJZ83961.1 anid|CBF90068.1 anig|EHA19089.1 aory|EIT82061.1 asyd|OJJ62044.1 ater|EAU31998.1 atub|OJI90333.1 aver|OJI96150.1 awen|OJJ37190.1 azon|OJJ48749.1

Group5045: abra|OJJ70631.1 acar|OOF97092.1 acla|EAW08125.1 afis|EAW23162.1 afla|EED46959.1 afum|EAL88321.1 agla|OJJ89673.1 akaw|GAA89970.1 aluc|OJZ83979.1 anid|CBF74465.1 anig|EHA19106.1 aory|EIT72687.1 asyd|OJJ57699.1 atub|OJI90352.1 aver|OJJ05465.1 awen|OJJ36621.1 azon|OJJ47592.1

Group5046: abra|OJJ70636.1 acar|OOF97078.1 acla|EAW08120.1 afis|EAW23131.1 afla|EED48997.1 afum|EAL88350.2 agla|OJJ89668.1 akaw|GAA89974.1 aluc|OJZ83984.1 anid|CBF74460.1 aory|EIT80038.1 asyd|OJJ57680.1 ater|EAU36284.1 atub|OJI90355.1 aver|OJJ05485.1 awen|OJJ36629.1 azon|OJJ47588.1

Group5047: abra|OJJ70638.1 acar|OOF97079.1 acla|EAW08119.1 afis|EAW23132.1 afla|EED48996.1 afum|EAL88349.1 agla|OJJ89667.1 aluc|OJZ83985.1 anid|CBF74458.1 anig|EHA19111.1 aory|EIT80017.1 asyd|OJJ57678.1 ater|EAU36285.1 atub|OJI90356.1 aver|OJJ05484.1 awen|OJJ36628.1 azon|OJJ47587.1

Group5048: abra|OJJ70652.1 acla|EAW08107.1 afis|EAW23144.1 afla|EED48507.1 afum|EAL88337.2 agla|OJJ89658.1 akaw|GAA89988.1 aluc|OJZ84002.1 anid|CBF74437.1 anig|EHA19124.1 aory|EIT73158.1 asyd|OJJ57746.1 ater|EAU36267.1 atub|OJI90371.1 aver|OJJ05421.1 awen|OJJ36639.1 azon|OJJ47576.1

Group5049: abra|OJJ70655.1 acla|EAW08104.1 afis|EAW23147.1 afla|EED48510.1 afum|EAL88334.1 agla|OJJ89655.1 akaw|GAA89991.1 aluc|OJZ84005.1 anid|CBF74432.1 anig|EHA19127.1 aory|EIT73217.1 asyd|OJJ57749.1 ater|EAU36264.1 atub|OJI90374.1 aver|OJJ05418.1 awen|OJJ36642.1 azon|OJJ47572.1

Group5050: abra|OJJ70658.1 acar|OOF97064.1 acla|EAW08101.1 afis|EAW23150.1 afla|EED48513.1 afum|EAL88331.1 agla|OJJ89652.1 akaw|GAA89994.1 aluc|OJZ84008.1 anid|CBF74426.1 anig|EHA19130.1 asyd|OJJ57752.1 ater|EAU36262.1 atub|OJI90377.1 aver|OJJ05415.1 awen|OJJ36646.1 azon|OJJ47569.1

Group5051: abra|OJJ70687.1 acar|OOF97040.1 afis|EAW23113.1 afla|EED53331.1 afum|EAL88366.1 agla|OJJ89627.1 akaw|GAA90019.1 aluc|OJZ84035.1 anid|CBF76022.1 anig|EHA19156.1 aory|EIT75261.1 asyd|OJJ52449.1 ater|EAU36241.1 atub|OJI90407.1 aver|OJJ03967.1 awen|OJJ36668.1 azon|OJJ47531.1

Group5052: abra|OJJ70688.1 acar|OOF97039.1 afis|EAW23112.1 afla|EED53332.1 afum|EAL88367.1 agla|OJJ89629.1 akaw|GAA90020.1 aluc|OJZ84036.1 anid|CBF76020.1 anig|EHA19157.1 aory|EIT75265.1 asyd|OJJ52448.1 ater|EAU36240.1 atub|OJI90408.1 aver|OJJ03968.1 awen|OJJ36669.1 azon|OJJ47530.1

Group5053: abra|OJJ70690.1 acla|EAW08074.1 afis|EAW23110.1 afla|EED48538.1 afum|EAL88369.1 agla|OJJ89631.1 akaw|GAA90022.1 aluc|OJZ84038.1 anid|CBF74387.1 anig|EHA19159.1 aory|EIT73045.1 asyd|OJJ57772.1 ater|EAU36238.1 atub|OJI90410.1 aver|OJJ05394.1 awen|OJJ36671.1 azon|OJJ47528.1

Group5054: abra|OJJ70692.1 acla|EAW08072.1 afis|EAW23108.1 afla|EED48540.1 afum|EAL88371.1 agla|OJJ89633.1 akaw|GAA90023.1 aluc|OJZ84039.1 anid|CBF81372.1 anig|EHA19161.1 aory|EIT73071.1 asyd|OJJ59387.1 ater|EAU36236.1 atub|OJI90411.1 aver|OJJ02886.1 awen|OJJ36673.1 azon|OJJ47526.1

Group5055: abra|OJJ70695.1 acar|OOF97034.1 acla|EAW08070.1 afis|EAW23106.1 afla|EED48542.1 afum|EAL88373.1 agla|OJJ89637.1 akaw|GAA90026.1 aluc|OJZ84042.1 anid|CBF81369.1 anig|EHA19164.1 asyd|OJJ59385.1 ater|EAU36234.1 atub|OJI90414.1 aver|OJJ02888.1 awen|OJJ36675.1 azon|OJJ47523.1

Group5056: abra|OJJ70703.1 acla|EAW08062.1 afis|EAW23098.1 afla|EED48546.1 afum|EAL88380.2 agla|OJJ89610.1 akaw|GAA90034.1 aluc|OJZ84050.1 anid|CBF81354.1 anig|EHA19170.1 aory|EIT73119.1 asyd|OJJ59378.1 ater|EAU36231.1 atub|OJI90421.1 aver|OJJ02895.1 awen|OJJ36683.1 azon|OJJ47514.1

Group5057: abra|OJJ70712.1 acar|OOF97024.1 acla|EAW08052.1 afis|EAW23088.1 afla|EED48560.1 afum|EAL88391.1 agla|OJJ89598.1 akaw|GAA90043.1 aluc|OJZ84059.1 anid|CBF81137.1 anig|EHA19179.1 aory|EIT73115.1 asyd|OJJ56502.1 atub|OJI90430.1 aver|OJJ06217.1 awen|OJJ36696.1 azon|OJJ47501.1

Group5058: abra|OJJ70713.1 acar|OOF97023.1 acla|EAW08050.1 afis|EAW23086.1 afla|EED48562.1 afum|EAL88393.1 agla|OJJ89596.1 akaw|GAA90044.1 aluc|OJZ84060.1 anid|CBF81341.1 anig|EHA19180.1 asyd|OJJ59370.1 ater|EAU36210.1 atub|OJI90431.1 aver|OJJ02904.1 awen|OJJ36698.1 azon|OJJ47499.1

Group5059: abra|OJJ70724.1 acar|OOF97014.1 acla|EAW08041.1 afis|EAW23076.1 afum|EAL88403.1 agla|OJJ89583.1 akaw|GAA90053.1 aluc|OJZ84069.1 anid|CBF81322.1 anig|EHA19189.1 aory|EIT73284.1 asyd|OJJ59360.1 ater|EAU36200.1 atub|OJI90442.1 aver|OJJ02914.1 awen|OJJ36708.1 azon|OJJ47491.1

Group5060: abra|OJJ70732.1 acar|OOF97006.1 acla|EAW11181.1 afis|EAW15912.1 afla|EED56456.1 afum|EAL88547.2 agla|OJJ89576.1 akaw|GAA90060.1 aluc|OJZ84077.1 anid|CBF81303.1 anig|EHA19196.1 aory|EIT72846.1 asyd|OJJ59351.1 atub|OJI90449.1 aver|OJJ02923.1 awen|OJJ36718.1 azon|OJJ47477.1

Group5061: abra|OJJ70739.1 acla|EAW11177.1 afis|EAW15908.1 afla|EED56460.1 afum|EAL88543.1 agla|OJJ89572.1 akaw|GAA90064.1 aluc|OJZ84084.1 anid|CBF81261.1 anig|EHA19200.1 aory|EIT72862.1 asyd|OJJ59331.1 ater|EAU36187.1 atub|OJI90454.1 aver|OJJ02943.1 awen|OJJ36722.1 azon|OJJ47481.1

Group5062: abra|OJJ70749.1 acar|OOF96996.1 acla|EAW11172.1 afis|EAW15903.1 afla|EED56467.1 afum|EAL88538.1 agla|OJJ89563.1 akaw|GAA90069.1 aluc|OJZ84093.1 anid|CBF81278.1 anig|EHA19206.1 aory|EIT72833.1 asyd|OJJ59336.1 atub|OJI90465.1 aver|OJJ02938.1 awen|OJJ36728.1 azon|OJJ47469.1

Group5063: abra|OJJ70750.1 acar|OOF96995.1 acla|EAW11171.1 afis|EAW15902.1 afla|EED56468.1 afum|EAL88537.1 agla|OJJ89562.1 akaw|GAA90070.1 aluc|OJZ84094.1 anid|CBF81279.1 anig|EHA19207.1 aory|EIT72855.1 asyd|OJJ59337.1 atub|OJI90466.1 aver|OJJ02937.1 awen|OJJ36729.1 azon|OJJ47468.1

Group5064: abra|OJJ70757.1 acar|OOF96987.1 acla|EAW11164.1 afis|EAW15895.1 afum|EAL88530.1 agla|OJJ89549.1 akaw|GAA90077.1 aluc|OJZ84102.1 anid|CBF81292.1 anig|EHA19214.1 aory|EIT72812.1 asyd|OJJ59345.1 ater|EAU36174.1 atub|OJI90475.1 aver|OJJ02929.1 awen|OJJ36736.1 azon|OJJ47461.1

Group5065: abra|OJJ70761.1 acar|OOF96983.1 acla|EAW11160.1 afis|EAW15891.1 afla|EED56481.1 afum|EAL88526.2 agla|OJJ89531.1 akaw|GAA90081.1 aluc|OJZ84106.1 anid|CBF81258.1 anig|EHA19218.1 aory|EIT72809.1 asyd|OJJ59329.1 ater|EAU31713.1 atub|OJI90480.1 aver|OJJ02945.1 azon|OJJ47458.1

Group5066: abra|OJJ70763.1 afis|EAW15889.1 afla|EED56483.1 akaw|GAA90083.1 aluc|OJZ84108.1 anid|CBF81254.1 anig|EHA19220.1 aory|EIT72840.1 asyd|OJJ59327.1 atub|OJI90482.1 aver|OJJ02947.1 awen|OJJ36741.1 azon|OJJ47456.1 acla|EAW11158.1 afum|EAL88523.1 agla|OJJ89533.1 ater|EAU31711.1

Group5067: abra|OJJ70764.1 acar|OOF96981.1 acla|EAW11157.1 afis|EAW15888.1 afla|EED56484.1 afum|EAL88522.2 akaw|GAA90084.1 aluc|OJZ84109.1 anid|CBF81252.1 anig|EHA19221.1 aory|EIT72836.1 asyd|OJJ59326.1 ater|EAU31710.1 atub|OJI90483.1 aver|OJJ02948.1 awen|OJJ36742.1 azon|OJJ47455.1

Group5068: abra|OJJ70772.1 acar|OOF96974.1 acla|EAW11150.1 afis|EAW15881.1 afla|EED56491.1 afum|EAL88515.1 agla|OJJ89540.1 akaw|GAA90091.1 aluc|OJZ84118.1 anid|CBF81236.1 anig|EHA19227.1 asyd|OJJ59318.1 ater|EAU31701.1 atub|OJI90491.1 aver|OJJ02955.1 awen|OJJ36750.1 azon|OJJ47449.1

Group5069: abra|OJJ70810.1 acar|OOF98292.1 acla|EAW10419.1 afis|EAW25150.1 afla|EED55285.1 afum|EAL89825.1 agla|OJJ89005.1 akaw|GAA91690.1 aluc|OJZ87704.1 anid|CBF85670.1 anig|EHA20887.1 asyd|OJJ64248.1 ater|EAU39418.1 atub|OJI80939.1 aver|OJI98339.1 awen|OJJ40779.1 azon|OJJ46873.1

Group5070: abra|OJJ70813.1 acar|OOF98289.1 acla|EAW10422.1 afis|EAW25147.1 afla|EED55291.1 afum|EAL89822.1 agla|OJJ89010.1 akaw|GAA91693.1 aluc|OJZ87701.1 anid|CBF85675.1 aory|EIT83562.1 asyd|OJJ64252.1 ater|EAU39421.1 atub|OJI80942.1 aver|OJI98342.1 awen|OJJ40772.1 azon|OJJ46876.1

Group5071: abra|OJJ70814.1 acar|OOF98288.1 acla|EAW10423.1 afis|EAW25146.1 afla|EED55292.1 afum|EAL89821.1 agla|OJJ89009.1 akaw|GAA91694.1 aluc|OJZ87700.1 anid|CBF85677.1 aory|EIT83581.1 asyd|OJJ64253.1 ater|EAU39422.1 atub|OJI80943.1 aver|OJI98343.1 awen|OJJ40771.1 azon|OJJ46877.1

Group5072: abra|OJJ70835.1 acar|OOF98279.1 acla|EAW10430.1 afis|EAW25139.1 afla|EED55306.1 afum|EAL89811.1 agla|OJJ89028.1 akaw|GAA91703.1 aluc|OJZ87687.1 anid|CBF86097.1 anig|EHA20876.1 aory|EIT83522.1 asyd|OJJ64475.1 atub|OJI80953.1 aver|OJI98579.1 awen|OJJ40763.1 azon|OJJ44128.1

Group5073: abra|OJJ70847.1 acar|OOF98268.1 acla|EAW10438.1 afis|EAW25129.1 afla|EED55315.1 afum|EAL89800.1 agla|OJJ89037.1 akaw|GAA91715.1 aluc|OJZ87674.1 anid|CBF85699.1 anig|EHA20864.1 asyd|OJJ64264.1 ater|EAU39402.1 atub|OJI80969.1 aver|OJI98356.1 awen|OJJ40754.1 azon|OJJ44118.1

Group5074: abra|OJJ70849.1 acar|OOF98267.1 acla|EAW10440.1 afis|EAW25127.1 afum|EAL89798.1 agla|OJJ89043.1 akaw|GAA91717.1 aluc|OJZ87671.1 anid|CBF85695.1 anig|EHA20862.1 aory|EIT83507.1 asyd|OJJ64262.1 ater|EAU39400.1 atub|OJI80971.1 aver|OJI98354.1 awen|OJJ40752.1 azon|OJJ44116.1

Group5075: abra|OJJ70863.1 acar|OOF98253.1 acla|EAW10455.1 afis|EAW25112.1 afla|EED55330.1 afum|EAL89783.2 agla|OJJ89069.1 akaw|GAA91730.1 aluc|OJZ87657.1 anid|CBF86075.1 aory|EIT76201.1 asyd|OJJ64462.1 ater|EAU39382.1 atub|OJI80986.1 aver|OJI98565.1 awen|OJJ40729.1 azon|OJJ44101.1

Group5076: abra|OJJ70868.1 acar|OOF90122.1 acla|EAW10460.1 afis|EAW25107.1 afum|EAL89778.1 agla|OJJ89080.1 akaw|GAA91735.1 aluc|OJZ87651.1 anid|CBF86066.1 anig|EHA20845.1 aory|EIT76240.1 asyd|OJJ64457.1 ater|EAU39375.1 atub|OJI80992.1 aver|OJI98560.1 awen|OJJ40725.1 azon|OJJ44097.1

Group5077: abra|OJJ70870.1 acar|OOF98249.1 acla|EAW10461.1 afis|EAW25106.1 afla|EED55338.1 afum|EAL89777.1 agla|OJJ89073.1 akaw|GAA91736.1 aluc|OJZ87650.1 anid|CBF86025.1 anig|EHA20844.1 asyd|OJJ64455.1 ater|EAU39374.1 atub|OJI80993.1 aver|OJI98558.1 awen|OJJ40719.1 azon|OJJ44094.1

Group5078: abra|OJJ70877.1 acar|OOF98243.1 acla|EAW10466.1 afis|EAW25101.1 afla|EED55344.1 afum|EAL89772.1 agla|OJJ85449.1 akaw|GAA91740.1 aluc|OJZ87645.1 anid|CBF86017.1 anig|EHA20838.1 asyd|OJJ64451.1 ater|EAU39368.1 atub|OJI81000.1 aver|OJI98554.1 awen|OJJ40715.1 azon|OJJ44092.1

Group5079: abra|OJJ70881.1 acar|OOF98240.1 acla|EAW10469.1 afis|EAW25098.1 afla|EED55348.1 afum|EAL89769.1 akaw|GAA91743.1 aluc|OJZ87642.1 anid|CBF86012.1 anig|EHA20835.1 aory|EIT80829.1 asyd|OJJ64448.1 ater|EAU39356.1 atub|OJI81003.1 aver|OJI98551.1 awen|OJJ40712.1 azon|OJJ44089.1

Group5080: abra|OJJ70891.1 acar|OOF90216.1 acla|EAW10473.1 afis|EAW25094.1 afum|EAL89765.2 agla|OJJ89083.1 akaw|GAA87407.1 aluc|OJZ87632.1 anid|CBF86003.1 anig|EHA20828.1 aory|EIT80821.1 asyd|OJJ64444.1 ater|EAU39352.1 atub|OJI81017.1 aver|OJI98546.1 awen|OJJ40708.1 azon|OJJ44084.1

Group5081: abra|OJJ70893.1 acla|EAW10475.1 afis|EAW25092.1 afla|EED55355.1 afum|EAL89762.2 agla|OJJ89085.1 akaw|GAA87409.1 aluc|OJZ87630.1 anid|CBF85999.1 anig|EHA20826.1 aory|EIT80902.1 asyd|OJJ64442.1 ater|EAU39350.1 atub|OJI81019.1 aver|OJI98544.1 awen|OJJ40706.1 azon|OJJ44082.1

Group5082: abra|OJJ70905.1 acar|OOF98215.1 acla|EAW10483.1 afis|EAW25084.1 afla|EED55366.1 afum|EAL89753.1 agla|OJJ89101.1 akaw|GAA87419.1 aluc|OJZ87619.1 anid|CBF85986.1 anig|EHA20817.1 aory|EIT80910.1 asyd|OJJ64433.1 ater|EAU39340.1 atub|OJI81030.1 aver|OJI98534.1 awen|OJJ40696.1

Group5083: abra|OJJ70907.1 acar|OOF98213.1 acla|EAW10485.1 afis|EAW25082.1 afla|EED55368.1 afum|EAL89751.1 agla|OJJ89103.1 akaw|GAA87421.1 aluc|OJZ87617.1 anid|CBF85983.1 anig|EHA20815.1 aory|EIT80932.1 asyd|OJJ64431.1 atub|OJI81032.1 aver|OJI98532.1 awen|OJJ40694.1 azon|OJJ44071.1

Group5084: abra|OJJ70919.1 acar|OOF98205.1 acla|EAW10493.1 afis|EAW25074.1 afla|EED55379.1 afum|EAL89743.1 agla|OJJ89118.1 akaw|GAA83021.1 aluc|OJZ87607.1 anig|EHA20805.1 aory|EIT80877.1 asyd|OJJ64422.1 ater|EAU39551.1 atub|OJI81041.1 aver|OJI98521.1 awen|OJJ40685.1 azon|OJJ44062.1

Group5085: abra|OJJ70924.1 acla|EAW13722.1 afis|EAW21022.1 afla|EED51437.1 afum|EAL92061.1 akaw|GAA83016.1 aluc|OJZ87602.1 anid|CBF87343.1 anig|EHA20801.1 asyd|OJJ52825.1 ater|EAU33971.1 atub|OJI81046.1 aver|OJJ08801.1 ater|EAU36221.1 aver|OJJ04859.1 aory|EIT80432.1 ater|EAU31577.1

Group5086: abra|OJJ70932.1 acla|EAW10498.1 afis|EAW25069.1 afla|EED55385.1 afum|EAL89738.1 agla|OJJ89125.1 akaw|GAA83010.1 aluc|OJZ87595.1 anid|CBF85956.1 anig|EHA20795.1 aory|EIT80780.1 asyd|OJJ64417.1 ater|EAU35816.1 atub|OJI81052.1 aver|OJI98516.1 awen|OJJ40676.1 azon|OJJ44057.1

Group5087: abra|OJJ70935.1 acar|OOF89983.1 acla|EAW10501.1 afis|EAW25066.1 afla|EED55388.1 afum|EAL89735.2 agla|OJJ89127.1 aluc|OJZ87592.1 anid|CBF85951.1 anig|EHA20793.1 aory|EIT80771.1 asyd|OJJ64415.1 ater|EAU35813.1 atub|OJI81055.1 aver|OJI98514.1 awen|OJJ40674.1 azon|OJJ44055.1

Group5088: abra|OJJ70938.1 acla|EAW10503.1 afis|EAW25064.1 afla|EED55390.1 afum|EAL89733.1 agla|OJJ89129.1 akaw|GAA83006.1 aluc|OJZ87590.1 anid|CBF85945.1 anig|EHA20791.1 aory|EIT80842.1 asyd|OJJ64413.1 ater|EAU35811.1 atub|OJI81058.1 aver|OJI98512.1 awen|OJJ40671.1 azon|OJJ44052.1

Group5089: abra|OJJ70943.1 acar|OOF98189.1 acla|EAW10508.1 afis|EAW25059.1 afla|EED55394.1 afum|EAL89727.1 agla|OJJ89136.1 akaw|GAA83001.1 aluc|OJZ87585.1 anid|CBF85938.1 anig|EHA20786.1 asyd|OJJ64409.1 ater|EAU35807.1 atub|OJI81063.1 aver|OJI98507.1 awen|OJJ40666.1 azon|OJJ44047.1

Group5090: abra|OJJ70956.1 acar|OOF98174.1 acla|EAW10517.1 afis|EAW25049.1 afla|EED55402.1 afum|EAL89717.1 agla|OJJ89143.1 akaw|GAA82991.1 aluc|OJZ87576.1 anid|CBF85921.1 anig|EHA20778.1 aory|EIT80931.1 asyd|OJJ64403.1 ater|EAU35799.1 atub|OJI81073.1 aver|OJI98499.1 azon|OJJ44038.1

Group5091: abra|OJJ70962.1 acla|EAW10523.1 afis|EAW25043.1 afla|EED55409.1 afum|EAL89711.1 agla|OJJ89151.1 akaw|GAA82985.1 aluc|OJZ87570.1 anig|EHA20772.1 aory|EIT80767.1 asyd|OJJ64397.1 ater|EAU35793.1 atub|OJI81079.1 aver|OJI98493.1 awen|OJJ40648.1 azon|OJJ44033.1 anid|CBF85910.1

Group5092: abra|OJJ70970.1 acar|OOF98160.1 acla|EAW10530.1 afis|EAW24997.1 akaw|GAA82978.1 aluc|OJZ87562.1 anid|CBF85899.1 anig|EHA20764.1 asyd|OJJ64391.1 ater|EAU35787.1 atub|OJI81085.1 awen|OJJ40642.1 azon|OJJ44027.1 afla|EED55417.1 afum|EAL89704.1 aory|EIT80790.1 aver|OJI98486.1

Group5093: abra|OJJ70994.1 acar|OOF90032.1 acla|EAW10549.1 afis|EAW25017.1 afla|EED55440.1 afum|EAL89678.1 agla|OJJ89185.1 akaw|GAA82954.1 aluc|OJZ87534.1 anid|CBF81534.1 anig|EHA20743.1 asyd|OJJ59484.1 ater|EAU35769.1 atub|OJI81115.1 aver|OJJ02790.1 awen|OJJ40623.1 azon|OJJ49471.1

Group5094: abra|OJJ70995.1 acar|OOF90033.1 acla|EAW10551.1 afis|EAW25018.1 afla|EED55441.1 afum|EAL89677.1 agla|OJJ89186.1 akaw|GAA82953.1 aluc|OJZ87533.1 anid|CBF81536.1 anig|EHA20742.1 asyd|OJJ59487.1 ater|EAU35768.1 atub|OJI81116.1 aver|OJJ02787.1 awen|OJJ40622.1 azon|OJJ49470.1

Group5095: abra|OJJ70997.1 acla|EAW10553.1 afis|EAW25020.1 afla|EED55443.1 afum|EAL89675.1 agla|OJJ89188.1 akaw|GAA82951.1 aluc|OJZ87530.1 anid|CBF81540.1 anig|EHA20740.1 aory|EIT80782.1 asyd|OJJ59489.1 ater|EAU35766.1 atub|OJI81118.1 aver|OJJ02785.1 awen|OJJ40620.1 azon|OJJ49468.1

Group5096: abra|OJJ71012.1 acar|OOF98131.1 acla|EAW10563.1 afis|EAW25030.1 afla|EED55453.1 afum|EAL89665.1 agla|OJJ79154.1 akaw|GAA82942.1 aluc|OJZ87519.1 anid|CBF81562.1 anig|EHA20730.1 aory|EIT80874.1 asyd|OJJ59501.1 ater|EAU35757.1 atub|OJI81130.1 aver|OJJ02773.1 awen|OJJ40609.1

Group5097: abra|OJJ71020.1 acar|OOF98120.1 acla|EAW10569.1 afis|EAW25035.1 afla|EED55461.1 afum|EAL89659.2 agla|OJJ82256.1 akaw|GAA82935.1 aluc|OJZ87512.1 anid|CBF81576.1 anig|EHA20724.1 asyd|OJJ59508.1 ater|EAU35748.1 atub|OJI81139.1 aver|OJJ02766.1 awen|OJJ40603.1 azon|OJJ49453.1

Group5098: abra|OJJ71026.1 acar|OOF98115.1 acla|EAW10576.1 afis|EAW24991.1 afla|EED55468.1 afum|EAL89651.1 akaw|GAA82929.1 aluc|OJZ87505.1 anid|CBF81594.1 anig|EHA20718.1 aory|EIT80848.1 asyd|OJJ59516.1 ater|EAU35742.1 atub|OJI81145.1 aver|OJJ02757.1 awen|OJJ40596.1 azon|OJJ49447.1

Group5099: abra|OJJ71039.1 acar|OOF98099.1 acla|EAW10588.1 afis|EAW24980.1 afla|EED55479.1 afum|EAL89640.1 agla|OJJ79137.1 akaw|GAA82917.1 aluc|OJZ87489.1 anid|CBF81610.1 anig|EHA20709.1 asyd|OJJ59524.1 ater|EAU35732.1 atub|OJI81159.1 aver|OJJ02748.1 awen|OJJ40577.1 azon|OJJ49432.1

Group5100: abra|OJJ71046.1 acar|OOF98093.1 acla|EAW10594.1 afis|EAW24974.1 afum|EAL89634.1 agla|OJJ82240.1 akaw|GAA82910.1 aluc|OJZ87480.1 anid|CBF81632.1 anig|EHA20702.1 aory|EIT80851.1 asyd|OJJ59532.1 ater|EAU35722.1 atub|OJI81168.1 aver|OJJ02738.1 awen|OJJ40571.1 azon|OJJ49428.1

Group5101: abra|OJJ71058.1 acla|EAW10601.1 afis|EAW24967.1 afla|EED55493.1 afum|EAL89625.2 agla|OJJ82221.1 akaw|GAA82901.1 aluc|OJZ87470.1 anid|CBF81645.1 anig|EHA20692.1 aory|EIT80912.1 asyd|OJJ59539.1 ater|EAU35710.1 atub|OJI81178.1 aver|OJJ02729.1 awen|OJJ40560.1 azon|OJJ49421.1

Group5102: abra|OJJ71060.1 acar|OOF98081.1 acla|EAW10599.1 afis|EAW24969.1 afla|EED55492.1 afum|EAL89628.1 agla|OJJ82235.1 akaw|GAA82899.1 aluc|OJZ87468.1 anid|CBF81641.1 anig|EHA20690.1 asyd|OJJ59537.1 ater|EAU35714.1 atub|OJI81180.1 aver|OJJ02732.1 awen|OJJ40563.1 azon|OJJ49423.1

Group5103: abra|OJJ71065.1 acar|OOF98080.1 afis|EAW17397.1 afla|EED54883.1 afum|EAL91235.1 agla|OJJ83972.1 akaw|GAA82897.1 aluc|OJZ87466.1 anid|CBF88997.1 anig|EHA20688.1 aory|EIT79374.1 asyd|OJJ62782.1 ater|EAU29507.1 atub|OJI81182.1 aver|OJI96827.1 awen|OJJ36326.1 acla|EAW13233.1

Group5104: abra|OJJ71068.1 acar|OOF98077.1 acla|EAW13231.1 afis|EAW17399.1 afla|EED54882.1 afum|EAL91237.1 agla|OJJ83970.1 akaw|GAA82894.1 aluc|OJZ87463.1 anid|CBF83002.1 anig|EHA20685.1 aory|EIT79368.1 asyd|OJJ61518.1 ater|EAU29509.1 atub|OJI81185.1 aver|OJJ00854.1 awen|OJJ36327.1

Group5105: abra|OJJ71113.1 acar|OOF98037.1 acla|EAW10379.1 afis|EAW25214.1 afla|EED53668.1 afum|EAL89875.1 akaw|GAA82856.1 aluc|OJZ87421.1 anid|CBF85252.1 aory|EIT75344.1 asyd|OJJ62922.1 atub|OJI81227.1 aver|OJI98067.1 awen|OJJ40876.1 azon|OJJ46811.1 agla|OJJ88947.1 ater|EAU34371.1

Group5106: abra|OJJ71127.1 acla|EAW10371.1 afis|EAW25221.1 afla|EED53685.1 afum|EAL89882.1 agla|OJJ79188.1 akaw|GAA82847.1 aluc|OJZ87408.1 anid|CBF85260.1 anig|EHA20638.1 aory|EIT75267.1 asyd|OJJ62912.1 ater|EAU34380.1 atub|OJI81238.1 aver|OJI98077.1 awen|OJJ40887.1 azon|OJJ44516.1

Group5107: abra|OJJ71131.1 acar|OOF98021.1 acla|EAW10366.1 afis|EAW25226.1 afla|EED53670.1 afum|EAL89887.1 agla|OJJ79182.1 akaw|GAA82843.1 aluc|OJZ87404.1 anid|CBF85267.1 anig|EHA20633.1 aory|EIT75270.1 asyd|OJJ62907.1 atub|OJI81242.1 aver|OJI98081.1 awen|OJJ40891.1 azon|OJJ44520.1

Group5108: abra|OJJ71144.1 acar|OOF98012.1 acla|EAW10362.1 afis|EAW25231.1 afla|EED53677.1 afum|EAL89892.1 agla|OJJ79176.1 akaw|GAA82833.1 aluc|OJZ87394.1 anid|CBF85331.1 anig|EHA20624.1 aory|EIT75332.1 asyd|OJJ64019.1 atub|OJI81252.1 aver|OJI98105.1 awen|OJJ40897.1 azon|OJJ44524.1

Group5109: abra|OJJ71149.1 acla|EAW10353.1 afis|EAW25241.1 afla|EED53703.1 afum|EAL89903.1 agla|OJJ89207.1 akaw|GAA82828.1 aluc|OJZ87389.1 anid|CBF85318.1 anig|EHA20618.1 aory|EIT75342.1 asyd|OJJ64010.1 ater|EAU34366.1 atub|OJI81258.1 aver|OJI98098.1 awen|OJJ40954.1 azon|OJJ44539.1

Group5110: abra|OJJ71152.1 acar|OOF98005.1 acla|EAW10345.1 afis|EAW25244.1 afla|EED53699.1 afum|EAL89906.1 agla|OJJ89212.1 akaw|GAA82825.1 aluc|OJZ87386.1 anig|EHA20615.1 aory|EIT75333.1 asyd|OJJ64013.1 ater|EAU34359.1 atub|OJI81261.1 aver|OJI98101.1 awen|OJJ40957.1 azon|OJJ44536.1

Group5111: abra|OJJ71168.1 acla|EAW10334.1 afis|EAW25258.1 afla|EED53723.1 afum|EAL89919.1 agla|OJJ89229.1 akaw|GAA82813.1 aluc|OJZ87373.1 anid|CBF85358.1 anig|EHA20606.1 aory|EIT75289.1 asyd|OJJ64038.1 ater|EAU34399.1 atub|OJI81273.1 aver|OJI98122.1 awen|OJJ40971.1 azon|OJJ44557.1

Group5112: abra|OJJ71176.1 acar|OOF97990.1 acla|EAW10329.1 afis|EAW25265.1 afla|EED53729.1 afum|EAL89928.1 akaw|GAA82808.1 aluc|OJZ87367.1 anid|CBF85371.1 anig|EHA20601.1 aory|EIT75310.1 asyd|OJJ64046.1 ater|EAU34405.1 atub|OJI81278.1 aver|OJI98131.1 awen|OJJ40977.1 azon|OJJ44565.1

Group5113: abra|OJJ71191.1 acar|OOF97976.1 acla|EAW10313.1 afis|EAW25279.1 afla|EED53746.1 afum|EAL89942.1 agla|OJJ89253.1 akaw|GAA82794.1 aluc|OJZ87353.1 anid|CBF85416.1 anig|EHA20587.1 aory|EIT75305.1 asyd|OJJ64071.1 atub|OJI81292.1 aver|OJI98158.1 awen|OJJ40993.1 azon|OJJ44582.1

Group5114: abra|OJJ71196.1 acar|OOF97971.1 acla|EAW10306.1 afis|EAW25286.1 afum|EAL89949.1 agla|OJJ89259.1 akaw|GAA82787.1 aluc|OJZ87346.1 anid|CBF85427.1 anig|EHA20583.1 aory|EIT78443.1 asyd|OJJ64081.1 ater|EAU34427.1 atub|OJI81298.1 aver|OJI98167.1 awen|OJJ40998.1 azon|OJJ44588.1

Group5115: abra|OJJ71219.1 akaw|GAA82767.1 aluc|OJZ87326.1 anig|EHA20563.1 atub|OJI81319.1 abra|OJJ75210.1 acar|OOF94578.1 acla|EAW15349.1 afla|EED53880.1 aory|EIT80547.1 asyd|OJJ52896.1 aver|OJJ08867.1 awen|OJJ32543.1 aluc|OJZ90682.1 afla|EED51735.1 akaw|GAA85321.1 aory|EIT78423.1

Group5116: abra|OJJ71225.1 acar|OOF97954.1 acla|EAW10297.1 afis|EAW25295.1 afum|EAL89958.1 agla|OJJ89271.1 akaw|GAA82764.1 aluc|OJZ87322.1 anig|EHA20556.1 ater|EAU34436.1 atub|OJI81323.1 aver|OJI98153.1 azon|OJJ44600.1 afla|EED53761.1 anid|CBF85407.1 asyd|OJJ64066.1 awen|OJJ41009.1

Group5117: abra|OJJ71226.1 acar|OOF97953.1 acla|EAW10296.1 afis|EAW25296.1 afla|EED53762.1 afum|EAL89959.2 agla|OJJ89272.1 akaw|GAA82763.1 aluc|OJZ87321.1 anig|EHA20555.1 aory|EIT78357.1 asyd|OJJ64065.1 ater|EAU34437.1 atub|OJI81324.1 aver|OJI98152.1 awen|OJJ41010.1 azon|OJJ44599.1

Group5118: abra|OJJ71232.1 acla|EAW13299.1 afis|EAW17306.1 afla|EED54153.1 afum|EAL91142.1 agla|OJJ82653.1 akaw|GAA82756.1 aluc|OJZ87313.1 anid|CBF78484.1 anig|EHA20549.1 aory|EIT76008.1 asyd|OJJ65333.1 ater|EAU33840.1 atub|OJI81332.1 aver|OJI99478.1 awen|OJJ34703.1 azon|OJJ50430.1

Group5119: abra|OJJ71249.1 acla|EAW10286.1 afis|EAW25306.1 afla|EED53784.1 afum|EAL89969.1 agla|OJJ89285.1 akaw|GAA82738.1 aluc|OJZ87293.1 anid|CBF75294.1 anig|EHA20534.1 aory|EIT78475.1 asyd|OJJ59885.1 ater|EAU34454.1 atub|OJI81347.1 aver|OJJ02401.1 awen|OJJ41020.1 azon|OJJ44607.1

Group5120: abra|OJJ71260.1 afla|EED53793.1 acla|EAW10276.1 afis|EAW25318.1 afum|EAL89979.1 aory|EIT78464.1 asyd|OJJ59894.1 ater|EAU34463.1 awen|OJJ41045.1 azon|OJJ44618.1 agla|OJJ83856.1 akaw|GAA82729.1 aluc|OJZ87283.1 anid|CBF75279.1 anig|EHA20525.1 atub|OJI81358.1 aver|OJJ02392.1

Group5121: abra|OJJ71282.1 acar|OOF97890.1 acla|EAW10265.1 afis|EAW25329.1 afla|EED53817.1 afum|EAL89990.1 agla|OJJ83879.1 akaw|GAA82706.1 aluc|OJZ87261.1 anid|CBF75230.1 anig|EHA20504.1 asyd|OJJ59916.1 ater|EAU34482.1 atub|OJI81381.1 aver|OJJ02370.1 awen|OJJ41024.1 azon|OJJ44641.1

Group5122: abra|OJJ71283.1 acar|OOF97889.1 acla|EAW10266.1 afis|EAW25328.1 afla|EED53818.1 afum|EAL89989.1 agla|OJJ83880.1 akaw|GAA82705.1 aluc|OJZ87260.1 anid|CBF75228.1 aory|EIT78476.1 asyd|OJJ59918.1 ater|EAU34484.1 atub|OJI81382.1 aver|OJJ02368.1 awen|OJJ41023.1 azon|OJJ44642.1

Group5123: abra|OJJ71284.1 acar|OOF97888.1 acla|EAW10252.1 afis|EAW25340.1 afla|EED53821.1 afum|EAL90003.1 agla|OJJ83882.1 akaw|GAA82704.1 aluc|OJZ87259.1 anid|CBF75215.1 anig|EHA20503.1 asyd|OJJ59930.1 ater|EAU34486.1 atub|OJI81383.1 aver|OJJ02356.1 awen|OJJ41059.1 azon|OJJ44643.1

Group5124: abra|OJJ71298.1 acar|OOF97872.1 acla|EAW10240.1 afis|EAW25352.1 afla|EED53833.1 agla|OJJ83900.1 akaw|GAA82691.1 aluc|OJZ87245.1 anid|CBF77436.1 anig|EHA20490.1 aory|EIT78382.1 asyd|OJJ53224.1 ater|EAU34500.1 atub|OJI81397.1 aver|OJJ03628.1 awen|OJJ41075.1 azon|OJJ44657.1

Group5125: abra|OJJ71314.1 acar|OOF97858.1 acla|EAW10225.1 afis|EAW25367.1 afla|EED53848.1 afum|EAL90027.1 agla|OJJ83754.1 akaw|GAA82677.1 aluc|OJZ87230.1 anid|CBF77448.1 anig|EHA20477.1 asyd|OJJ53209.1 ater|EAU34512.1 atub|OJI81412.1 aver|OJJ03641.1 awen|OJJ41093.1 azon|OJJ44672.1

Group5126: abra|OJJ71318.1 acla|EAW10221.1 afis|EAW25371.1 afla|EED53852.1 afum|EAL90031.1 agla|OJJ83758.1 akaw|GAA82673.1 aluc|OJZ87226.1 anid|CBF77440.1 anig|EHA20473.1 aory|EIT78361.1 asyd|OJJ53205.1 ater|EAU34516.1 atub|OJI81416.1 aver|OJJ03645.1 awen|OJJ41097.1 azon|OJJ44676.1

Group5127: abra|OJJ71319.1 acla|EAW10220.1 afis|EAW25372.1 afla|EED53853.1 afum|EAL90032.1 agla|OJJ83759.1 akaw|GAA82672.1 aluc|OJZ87225.1 anid|CBF77473.1 anig|EHA20472.1 aory|EIT78430.1 asyd|OJJ53204.1 ater|EAU34517.1 atub|OJI81417.1 aver|OJJ03646.1 awen|OJJ41098.1 azon|OJJ44677.1

Group5128: abra|OJJ71331.1 acar|OOF97844.1 acla|EAW10209.1 afis|EAW25386.1 afla|EED53863.1 afum|EAL90045.1 agla|OJJ83787.1 akaw|GAA82661.1 aluc|OJZ87211.1 anid|CBF77494.1 anig|EHA20462.1 asyd|OJJ53193.1 ater|EAU34533.1 atub|OJI81428.1 aver|OJJ03657.1 awen|OJJ41113.1 azon|OJJ44689.1

Group5129: abra|OJJ71332.1 acar|OOF97843.1 acla|EAW10208.1 afis|EAW25387.1 afla|EED53864.1 afum|EAL90046.2 agla|OJJ83788.1 akaw|GAA82660.1 aluc|OJZ87210.1 anid|CBF77496.1 aory|EIT78422.1 asyd|OJJ53192.1 ater|EAU34534.1 atub|OJI81429.1 aver|OJJ03658.1 awen|OJJ41114.1 azon|OJJ44690.1

Group5130: abra|OJJ71335.1 acar|OOF97837.1 acla|EAW10204.1 afis|EAW25391.1 afla|EED53868.1 afum|EAL90050.1 agla|OJJ87029.1 akaw|GAA82657.1 aluc|OJZ87207.1 anid|CBF77504.1 aory|EIT78375.1 asyd|OJJ53188.1 ater|EAU34539.1 atub|OJI81432.1 aver|OJJ03662.1 awen|OJJ41118.1 azon|OJJ44694.1

Group5131: abra|OJJ71348.1 acar|OOF97822.1 acla|EAW10190.1 afis|EAW25405.1 afla|EED53884.1 afum|EAL90064.1 agla|OJJ87046.1 akaw|GAA82644.1 aluc|OJZ87191.1 anid|CBF77534.1 anig|EHA20448.1 asyd|OJJ53175.1 ater|EAU34556.1 atub|OJI81447.1 aver|OJJ03677.1 awen|OJJ41132.1 azon|OJJ44706.1

Group5132: abra|OJJ71350.1 acar|OOF97820.1 acla|EAW10188.1 afis|EAW25407.1 afla|EED53886.1 afum|EAL90066.1 agla|OJJ87048.1 akaw|GAA82642.1 aluc|OJZ87189.1 anid|CBF77538.1 anig|EHA20446.1 asyd|OJJ53173.1 ater|EAU34558.1 atub|OJI81449.1 aver|OJJ03679.1 awen|OJJ41134.1 azon|OJJ44708.1

Group5133: abra|OJJ71369.1 acar|OOF97803.1 acla|EAW10169.1 afis|EAW25425.1 afum|EAL90084.1 agla|OJJ87067.1 akaw|GAA82625.1 aluc|OJZ87171.1 anid|CBF77555.1 anig|EHA20429.1 aory|EIT78498.1 asyd|OJJ53154.1 ater|EAU34579.1 atub|OJI81467.1 aver|OJJ03697.1 awen|OJJ41153.1 azon|OJJ44732.1

Group5134: abra|OJJ71375.1 acar|OOF97793.1 afis|EAW25432.1 afum|EAL90090.1 agla|OJJ87077.1 akaw|GAA82619.1 aluc|OJZ87165.1 anig|EHA20424.1 aory|EIT78287.1 atub|OJI81474.1 aver|OJJ03704.1 awen|OJJ41161.1 azon|OJJ44739.1 acla|EAW10162.1 afla|EED53920.1 asyd|OJJ53147.1 anid|CBF77542.1

Group5135: abra|OJJ71377.1 acla|EAW10147.1 afis|EAW25446.1 afla|EED53930.1 afum|EAL90104.1 agla|OJJ87088.1 akaw|GAA82617.1 aluc|OJZ87163.1 anid|CBF77608.1 anig|EHA20422.1 aory|EIT78346.1 asyd|OJJ53136.1 ater|EAU34614.1 atub|OJI81476.1 aver|OJJ03716.1 awen|OJJ41170.1 azon|OJJ44749.1

Group5136: abra|OJJ71381.1 acar|OOF97788.1 acla|EAW10156.1 afis|EAW25438.1 afla|EED53926.1 afum|EAL90096.1 agla|OJJ87083.1 akaw|GAA82613.1 aluc|OJZ87159.1 anid|CBF77598.1 anig|EHA20418.1 asyd|OJJ53141.1 ater|EAU34610.1 atub|OJI81481.1 aver|OJJ03711.1 awen|OJJ41166.1 azon|OJJ44744.1

Group5137: abra|OJJ71385.1 acar|OOF97784.1 acla|EAW10160.1 afis|EAW25434.1 afla|EED53922.1 afum|EAL90092.1 agla|OJJ87079.1 akaw|GAA82609.1 aluc|OJZ87155.1 anig|EHA20414.1 aory|EIT78289.1 asyd|OJJ53145.1 ater|EAU34606.1 atub|OJI81485.1 aver|OJJ03707.1 awen|OJJ41163.1 azon|OJJ44741.1

Group5138: abra|OJJ71388.1 acar|OOF97780.1 acla|EAW10149.1 afis|EAW25444.1 afla|EED53932.1 afum|EAL90102.1 agla|OJJ87091.1 akaw|GAA82606.1 aluc|OJZ87152.1 anid|CBF77612.1 anig|EHA20411.1 aory|EIT78343.1 asyd|OJJ53134.1 atub|OJI81488.1 aver|OJJ03718.1 awen|OJJ41175.1 azon|OJJ44751.1

Group5139: abra|OJJ71396.1 acar|OOF97773.1 acla|EAW10143.1 afis|EAW25450.1 afla|EED53937.1 afum|EAL90109.2 agla|OJJ87102.1 akaw|GAA82601.1 aluc|OJZ87146.1 anid|CBF77627.1 anig|EHA20405.1 asyd|OJJ53129.1 ater|EAU34620.1 atub|OJI81495.1 aver|OJJ03725.1 awen|OJJ41181.1 azon|OJJ44757.1

Group5140: abra|OJJ71422.1 acla|EAW10112.1 afis|EAW25473.1 afla|EED53965.1 afum|EAL90136.2 agla|OJJ87121.1 akaw|GAA82580.1 aluc|OJZ87116.1 anid|CBF77676.1 anig|EHA20382.1 aory|EIT78353.1 asyd|OJJ53090.1 ater|EAU34642.1 atub|OJI81524.1 aver|OJJ03756.1 awen|OJJ41208.1 azon|OJJ44794.1

Group5141: abra|OJJ71433.1 acar|OOF90400.1 acla|EAW10105.1 afis|EAW25482.1 afla|EED53980.1 afum|EAL90147.1 agla|OJJ87130.1 akaw|GAA82570.1 aluc|OJZ87105.1 anid|CBF77699.1 anig|EHA20373.1 asyd|OJJ53071.1 ater|EAU34653.1 atub|OJI81533.1 aver|OJJ03774.1 awen|OJJ41223.1 azon|OJJ44784.1

Group5142: abra|OJJ71445.1 acla|EAW10101.1 afis|EAW25486.1 afla|EED53984.1 afum|EAL90151.1 agla|OJJ87134.1 akaw|GAA82564.1 aluc|OJZ87096.1 anid|CBF77715.1 anig|EHA20367.1 aory|EIT78340.1 asyd|OJJ53066.1 ater|EAU34657.1 atub|OJI81542.1 aver|OJJ03779.1 awen|OJJ41227.1 azon|OJJ44780.1

Group5143: abra|OJJ71457.1 acla|EAW10092.1 afis|EAW25495.1 afla|EED53995.1 afum|EAL90161.1 agla|OJJ87310.1 akaw|GAA82554.1 aluc|OJZ87086.1 anid|CBF77747.1 anig|EHA20356.1 aory|EIT78281.1 asyd|OJJ53048.1 ater|EAU34666.1 atub|OJI81552.1 aver|OJJ03797.1 awen|OJJ41237.1 azon|OJJ44803.1

Group5144: abra|OJJ71482.1 acla|EAW10067.1 afis|EAW25518.1 afla|EED54019.1 afum|EAL90183.1 agla|OJJ87285.1 akaw|GAA82537.1 aluc|OJZ87064.1 anid|CBF77790.1 anig|EHA20337.1 aory|EIT78295.1 asyd|OJJ53027.1 ater|EAU33364.1 atub|OJI81571.1 aver|OJJ03817.1 awen|OJJ41261.1 azon|OJJ44826.1

Group5145: abra|OJJ71539.1 acla|EAW15094.1 afis|EAW20063.1 afla|EED45314.1 afum|EAL85183.1 agla|OJJ88896.1 akaw|GAA89382.1 aluc|OJZ86277.1 anid|CBF84830.1 anig|EHA21594.1 aory|EIT73621.1 asyd|OJJ63210.1 ater|EAU38672.1 atub|OJI84262.1 aver|OJI97745.1 awen|OJJ31824.1 azon|OJJ46301.1

Group5146: abra|OJJ71546.1 acla|EAW15100.1 afis|EAW20072.1 afla|EED45307.1 afum|EAL85190.1 agla|OJJ88888.1 akaw|GAA89376.1 aluc|OJZ86284.1 anid|CBF84847.1 anig|EHA21587.1 aory|EIT73619.1 asyd|OJJ63203.1 ater|EAU38679.1 atub|OJI84254.1 aver|OJI97752.1 awen|OJJ31831.1 azon|OJJ46293.1

Group5147: abra|OJJ71547.1 acla|EAW15101.1 afis|EAW20073.1 afla|EED45306.1 afum|EAL85191.1 agla|OJJ88887.1 akaw|GAA89375.1 aluc|OJZ86285.1 anid|CBF84849.1 anig|EHA21586.1 aory|EIT73795.1 asyd|OJJ63202.1 ater|EAU38681.1 atub|OJI84253.1 aver|OJI97753.1 awen|OJJ31832.1 azon|OJJ46292.1

Group5148: abra|OJJ71552.1 acla|EAW15106.1 afis|EAW20078.1 afla|EED45297.1 afum|EAL85196.1 agla|OJJ88882.1 akaw|GAA89370.1 aluc|OJZ86290.1 anid|CBF84862.1 anig|EHA21581.1 aory|EIT73726.1 asyd|OJJ63197.1 ater|EAU38686.1 atub|OJI84248.1 aver|OJI97758.1 awen|OJJ31837.1 azon|OJJ46286.1

Group5149: abra|OJJ71557.1 acla|EAW15110.1 afis|EAW20081.1 afla|EED45293.1 afum|EAL85200.1 agla|OJJ88878.1 akaw|GAA89366.1 aluc|OJZ86295.1 anid|CBF84868.1 anig|EHA21577.1 aory|EIT73574.1 asyd|OJJ63193.1 ater|EAU38689.1 atub|OJI84242.1 aver|OJI97762.1 awen|OJJ31841.1 azon|OJJ46282.1

Group5150: abra|OJJ71559.1 acar|OOF90419.1 acla|EAW15111.1 afis|EAW20082.1 afla|EED45292.1 afum|EAL85201.1 agla|OJJ88876.1 akaw|GAA89365.1 aluc|OJZ86296.1 anid|CBF84870.1 anig|EHA21576.1 asyd|OJJ63192.1 ater|EAU38690.1 atub|OJI84241.1 aver|OJI97764.1 awen|OJJ31842.1 azon|OJJ46281.1

Group5151: abra|OJJ71564.1 acar|OOF96300.1 acla|EAW15116.1 afis|EAW20087.1 afla|EED45286.1 afum|EAL85206.1 akaw|GAA89360.1 aluc|OJZ86301.1 anid|CBF84879.1 anig|EHA21571.1 aory|EIT73732.1 asyd|OJJ63187.1 ater|EAU38696.1 atub|OJI84235.1 aver|OJI97769.1 awen|OJJ31847.1 azon|OJJ46276.1

Group5152: abra|OJJ71571.1 acla|EAW15120.1 afis|EAW20090.1 afla|EED45280.1 afum|EAL85209.1 agla|OJJ88866.1 akaw|GAA89354.1 aluc|OJZ86308.1 anid|CBF84884.1 anig|EHA21565.1 aory|EIT73541.1 asyd|OJJ63183.1 ater|EAU38699.1 atub|OJI84228.1 aver|OJI97772.1 awen|OJJ31852.1 azon|OJJ46272.1

Group5153: abra|OJJ71572.1 acar|OOF96304.1 acla|EAW15121.1 afis|EAW20091.1 afla|EED45279.1 afum|EAL85210.1 agla|OJJ88863.1 akaw|GAA89352.1 aluc|OJZ86309.1 anid|CBF84886.1 anig|EHA21564.1 asyd|OJJ63181.1 ater|EAU38700.1 atub|OJI84226.1 aver|OJI97773.1 awen|OJJ31854.1 azon|OJJ46271.1

Group5154: abra|OJJ71578.1 acar|OOF96308.1 acla|EAW15125.1 afis|EAW20095.1 afla|EED45275.1 afum|EAL85213.1 agla|OJJ88859.1 akaw|GAA89346.1 aluc|OJZ86315.1 anid|CBF84891.1 anig|EHA21560.1 aory|EIT73717.1 asyd|OJJ63178.1 atub|OJI84220.1 aver|OJI97776.1 awen|OJJ31858.1 azon|OJJ46267.1

Group5155: abra|OJJ71583.1 acar|OOF96312.1 acla|EAW15128.1 afis|EAW20098.1 afla|EED53373.1 afum|EAL85216.1 agla|OJJ88856.1 akaw|GAA89340.1 aluc|OJZ86323.1 anid|CBF84899.1 anig|EHA21555.1 aory|EIT74942.1 asyd|OJJ63174.1 atub|OJI84211.1 aver|OJI97780.1 awen|OJJ31866.1 azon|OJJ46264.1

Group5156: abra|OJJ71589.1 acar|OOF96318.1 acla|EAW15134.1 afis|EAW20106.1 afum|EAL85223.1 agla|OJJ88847.1 akaw|GAA89334.1 aluc|OJZ86331.1 anid|CBF84912.1 anig|EHA21549.1 aory|EIT74895.1 asyd|OJJ63167.1 ater|EAU38725.1 atub|OJI84205.1 aver|OJI97787.1 awen|OJJ31878.1 azon|OJJ46256.1

Group5157: abra|OJJ71591.1 acla|EAW15137.1 afis|EAW20109.1 afla|EED53363.1 afum|EAL85226.1 akaw|GAA89331.1 aluc|OJZ86334.1 anid|CBF84917.1 anig|EHA21547.1 aory|EIT75266.1 asyd|OJJ63165.1 ater|EAU38727.1 atub|OJI84201.1 aver|OJI97790.1 awen|OJJ31880.1 azon|OJJ46254.1 agla|OJJ88843.1

Group5158: abra|OJJ71598.1 acar|OOF96325.1 acla|EAW15146.1 afis|EAW20114.1 afla|EED53359.1 afum|EAL85231.1 agla|OJJ88837.1 akaw|GAA89326.1 aluc|OJZ86339.1 anid|CBF84928.1 anig|EHA21542.1 aory|EIT74994.1 asyd|OJJ63160.1 ater|EAU38731.1 atub|OJI84196.1 aver|OJI97797.1 awen|OJJ31886.1

Group5159: abra|OJJ71607.1 acla|EAW15149.1 afis|EAW20117.1 afla|EED53354.1 afum|EAL85234.1 agla|OJJ88832.1 akaw|GAA89321.1 aluc|OJZ86349.1 anid|CBF84936.1 anig|EHA21537.1 aory|EIT75234.1 asyd|OJJ63157.1 ater|EAU38735.1 atub|OJI84185.1 aver|OJI97799.1 awen|OJJ31887.1 azon|OJJ46247.1

Group5160: abra|OJJ71616.1 acar|OOF96332.1 acla|EAW15158.1 afis|EAW20126.1 afla|EED57250.1 afum|EAL85244.1 agla|OJJ88821.1 akaw|GAA89311.1 aluc|OJZ86357.1 anid|CBF84950.1 anig|EHA21533.1 aory|EIT74017.1 asyd|OJJ63146.1 atub|OJI84177.1 aver|OJI97808.1 awen|OJJ31895.1 azon|OJJ46241.1

Group5161: abra|OJJ71634.1 acar|OOF91865.1 afis|EAW20144.1 afla|EED57204.1 afum|EAL85259.1 agla|OJJ88788.1 akaw|GAA89292.1 aluc|OJZ86377.1 anid|CBF78474.1 anig|EHA21517.1 aory|EIT74128.1 asyd|OJJ58349.1 ater|EAU38761.1 atub|OJI84157.1 aver|OJJ02058.1 awen|OJJ32015.1 azon|OJJ46072.1

Group5162: abra|OJJ71636.1 acar|OOF96353.1 acla|EAW15176.1 afis|EAW20146.1 afla|EED57203.1 afum|EAL85261.1 agla|OJJ88787.1 akaw|GAA89290.1 aluc|OJZ86379.1 anig|EHA21515.1 aory|EIT74050.1 asyd|OJJ63129.1 ater|EAU38762.1 atub|OJI84155.1 aver|OJI97826.1 awen|OJJ32020.1 azon|OJJ46070.1

Group5163: abra|OJJ71638.1 acar|OOF96355.1 acla|EAW15178.1 afis|EAW20148.1 afla|EED57201.1 afum|EAL85263.1 agla|OJJ88785.1 akaw|GAA89288.1 aluc|OJZ86381.1 anid|CBF84982.1 aory|EIT74108.1 asyd|OJJ63126.1 ater|EAU38764.1 atub|OJI84152.1 aver|OJI97829.1 awen|OJJ32022.1 azon|OJJ46068.1

Group5164: abra|OJJ71650.1 acla|EAW15187.1 afis|EAW20157.1 afla|EED57193.1 afum|EAL85273.1 agla|OJJ88773.1 akaw|GAA89279.1 aluc|OJZ86394.1 anid|CBF85000.1 anig|EHA21504.1 aory|EIT74099.1 asyd|OJJ63117.1 ater|EAU38771.1 atub|OJI84139.1 aver|OJI97842.1 awen|OJJ32051.1 azon|OJJ46084.1

Group5165: abra|OJJ71651.1 acar|OOF96364.1 acla|EAW15188.1 afla|EED57190.1 afum|EAL85274.1 agla|OJJ88772.1 akaw|GAA89278.1 aluc|OJZ86395.1 anid|CBF85002.1 anig|EHA21503.1 asyd|OJJ63116.1 ater|EAU38772.1 atub|OJI84138.1 aver|OJI97843.1 awen|OJJ32052.1 azon|OJJ46085.1 agla|OJJ79968.1

Group5166: abra|OJJ71652.1 acar|OOF96365.1 acla|EAW15189.1 afis|EAW20159.1 afla|EED57189.1 afum|EAL85276.1 akaw|GAA89277.1 aluc|OJZ86398.1 anid|CBF85004.1 anig|EHA21502.1 aory|EIT74024.1 asyd|OJJ63115.1 ater|EAU38775.1 atub|OJI84137.1 aver|OJI97844.1 awen|OJJ32053.1 azon|OJJ46086.1

Group5167: abra|OJJ71653.1 akaw|GAA89276.1 aluc|OJZ86399.1 anig|EHA21501.1 ater|EAU38776.1 atub|OJI84136.1 awen|OJJ32054.1 acar|OOF96367.1 acla|EAW15191.1 afis|EAW20160.1 afla|EED57188.1 afum|EAL85277.1 anid|CBF85006.1 asyd|OJJ63114.1 aver|OJI97845.1 agla|OJJ88771.1 azon|OJJ46087.1

Group5168: abra|OJJ71658.1 acar|OOF96371.1 acla|EAW15195.1 afis|EAW20164.1 afla|EED57185.1 afum|EAL85282.1 agla|OJJ88766.1 akaw|GAA89272.1 aluc|OJZ86403.1 anid|CBF85013.1 anig|EHA21497.1 aory|EIT74112.1 asyd|OJJ63110.1 ater|EAU38795.1 atub|OJI84132.1 aver|OJI97850.1 awen|OJJ32059.1

Group5169: abra|OJJ71664.1 acar|OOF96374.1 acla|EAW15200.1 afis|EAW20166.1 afla|EED57182.1 afum|EAL85284.1 agla|OJJ88763.1 akaw|GAA89269.1 aluc|OJZ86406.1 anid|CBF85020.1 anig|EHA21493.1 asyd|OJJ63106.1 ater|EAU38798.1 atub|OJI84125.1 aver|OJI97855.1 awen|OJJ32063.1 azon|OJJ46096.1

Group5170: abra|OJJ71680.1 acar|OOF96391.1 acla|EAW15219.1 afis|EAW20184.1 afla|EED57162.1 afum|EAL85302.2 agla|OJJ84682.1 akaw|GAA89252.1 aluc|OJZ86422.1 anig|EHA21477.1 aory|EIT74012.1 asyd|OJJ63090.1 ater|EAU35220.1 atub|OJI84109.1 aver|OJI97872.1 awen|OJJ31183.1 azon|OJJ46114.1

Group5171: abra|OJJ71681.1 acar|OOF96392.1 acla|EAW15220.1 afis|EAW20185.1 afla|EED57163.1 afum|EAL85304.2 agla|OJJ84681.1 akaw|GAA89251.1 aluc|OJZ86423.1 anid|CBF85052.1 anig|EHA21475.1 asyd|OJJ63089.1 ater|EAU35221.1 atub|OJI84108.1 aver|OJI97873.1 awen|OJJ31185.1 azon|OJJ46115.1

Group5172: abra|OJJ71694.1 acar|OOF96401.1 acla|EAW15231.1 afis|EAW20195.1 afla|EED57149.1 afum|EAL85315.1 agla|OJJ84670.1 akaw|GAA89241.1 aluc|OJZ86438.1 anid|CBF85071.1 anig|EHA21465.1 aory|EIT73968.1 asyd|OJJ63079.1 ater|EAU32003.1 atub|OJI84092.1 aver|OJI97885.1 awen|OJJ31201.1

Group5173: abra|OJJ71695.1 acar|OOF96403.1 acla|EAW15232.1 afis|EAW20196.1 afla|EED57148.1 afum|EAL85316.1 agla|OJJ84669.1 akaw|GAA89240.1 aluc|OJZ86439.1 anid|CBF85073.1 anig|EHA21464.1 aory|EIT74078.1 asyd|OJJ63078.1 ater|EAU32004.1 atub|OJI84090.1 aver|OJI97886.1 awen|OJJ31202.1

Group5174: abra|OJJ71698.1 acar|OOF96406.1 acla|EAW15234.1 afis|EAW20198.1 afla|EED57146.1 afum|EAL85318.1 agla|OJJ84667.1 akaw|GAA89237.1 aluc|OJZ86441.1 anid|CBF85080.1 anig|EHA21460.1 aory|EIT74082.1 asyd|OJJ63075.1 atub|OJI84087.1 aver|OJI97889.1 awen|OJJ31204.1 azon|OJJ46129.1

Group5175: abra|OJJ71712.1 acar|OOF96417.1 acla|EAW06436.1 afis|EAW18541.1 afla|EED52854.1 afum|EAL93241.1 agla|OJJ81573.1 akaw|GAA85177.1 aluc|OJZ86456.1 anid|CBF70194.1 anig|EHA21451.1 aory|EIT78642.1 asyd|OJJ58789.1 ater|EAU30024.1 atub|OJI84071.1 aver|OJJ01396.1 awen|OJJ31097.1

Group5176: abra|OJJ71724.1 acar|OOF96428.1 acla|EAW10028.1 afla|EED54155.1 akaw|GAA85166.1 aluc|OJZ86470.1 anig|EHA21440.1 aory|EIT81946.1 asyd|OJJ53835.1 ater|EAU30727.1 atub|OJI84059.1 aver|OJI98821.1 awen|OJJ38368.1 azon|OJJ45250.1 afla|EED55034.1 asyd|OJJ64718.1 ater|EAU37147.1

Group5177: acla|EAW09043.1 afis|EAW25210.1 aory|EIT73738.1 akaw|GAA86660.1 aluc|OJZ89756.1 atub|OJI85045.1 awen|OJJ36425.1 azon|OJJ48013.1 acla|EAW09045.1 afla|EED45079.1 anid|CBF79126.1 asyd|OJJ53761.1 ater|EAU35542.1 afum|EAL89871.1 asyd|OJJ57939.1 aory|EIT73659.1 agla|OJJ78860.1

Group5178: abra|OJJ71744.1 acar|OOF96435.1 acla|EAW06448.1 afis|EAW18552.1 afum|EAL93252.1 agla|OJJ81604.1 akaw|GAA85158.1 aluc|OJZ86479.1 anid|CBF70279.1 anig|EHA22163.1 aory|EIT78165.1 asyd|OJJ58742.1 ater|EAU34840.1 atub|OJI84051.1 aver|OJJ01352.1 awen|OJJ31113.1 azon|OJJ46589.1

Group5179: abra|OJJ71802.1 acar|OOF96482.1 acla|EAW13843.1 afis|EAW18557.1 afla|EED52809.1 afum|EAL93257.1 agla|OJJ81602.1 akaw|GAA85108.1 aluc|OJZ86532.1 anid|CBF70284.1 anig|EHA22114.1 aory|EIT78228.1 asyd|OJJ58738.1 ater|EAU34846.1 atub|OJI83989.1 aver|OJJ01349.1 awen|OJJ31115.1

Group5180: abra|OJJ71807.1 acar|OOF96485.1 acla|EAW13846.1 afis|EAW18560.1 afla|EED52800.1 afum|EAL93260.1 agla|OJJ81599.1 akaw|GAA85105.1 aluc|OJZ86537.1 anid|CBF70290.1 anig|EHA22111.1 aory|EIT78240.1 asyd|OJJ58734.1 atub|OJI83986.1 aver|OJJ01346.1 awen|OJJ31118.1 azon|OJJ46591.1

Group5181: abra|OJJ71832.1 acar|OOF96508.1 acla|EAW13867.1 afis|EAW18579.1 afla|EED52769.1 afum|EAL93282.1 akaw|GAA85079.1 aluc|OJZ86564.1 anid|CBF70356.1 anig|EHA22087.1 aory|EIT78246.1 asyd|OJJ58656.1 ater|EAU34872.1 atub|OJI83957.1 aver|OJJ01264.1 awen|OJJ31138.1 azon|OJJ46618.1

Group5182: abra|OJJ71847.1 acar|OOF96516.1 acla|EAW13878.1 afis|EAW18590.1 afla|EED52756.1 afum|EAL93297.1 agla|OJJ81642.1 akaw|GAA85070.1 aluc|OJZ86580.1 anid|CBF70377.1 aory|EIT78203.1 asyd|OJJ58645.1 ater|EAU34883.1 atub|OJI83942.1 aver|OJJ01251.1 awen|OJJ31153.1 azon|OJJ46628.1

Group5183: abra|OJJ71848.1 acar|OOF96517.1 acla|EAW13879.1 afis|EAW18591.1 afum|EAL93298.1 agla|OJJ81643.1 akaw|GAA85069.1 aluc|OJZ86581.1 anid|CBF70379.1 anig|EHA22077.1 aory|EIT78204.1 asyd|OJJ58644.1 ater|EAU34884.1 atub|OJI83941.1 aver|OJJ01250.1 awen|OJJ31154.1 azon|OJJ46629.1

Group5184: abra|OJJ71852.1 acar|OOF96522.1 acla|EAW13884.1 afis|EAW18596.1 afla|EED52751.1 afum|EAL93304.2 agla|OJJ81649.1 akaw|GAA85065.1 aluc|OJZ86586.1 anid|CBF70393.1 anig|EHA22074.1 asyd|OJJ58635.1 ater|EAU34890.1 atub|OJI83937.1 aver|OJJ01241.1 awen|OJJ31159.1 azon|OJJ46635.1

Group5185: abra|OJJ71863.1 acla|EAW13893.1 afis|EAW18605.1 afla|EED52741.1 afum|EAL93314.1 agla|OJJ81657.1 akaw|GAA85053.1 aluc|OJZ86600.1 anid|CBF70410.1 anig|EHA22063.1 aory|EIT78173.1 asyd|OJJ58626.1 ater|EAU34900.1 atub|OJI83923.1 aver|OJJ01232.1 awen|OJJ31171.1 azon|OJJ46648.1

Group5186: abra|OJJ71867.1 acar|OOF96537.1 acla|EAW13897.1 afis|EAW18609.1 afla|EED52737.1 afum|EAL93319.1 agla|OJJ81664.1 akaw|GAA85048.1 aluc|OJZ86604.1 anid|CBF70418.1 anig|EHA22059.1 aory|EIT78182.1 asyd|OJJ58621.1 ater|EAU34907.1 atub|OJI83919.1 aver|OJJ01228.1 awen|OJJ32070.1

Group5187: abra|OJJ71872.1 acar|OOF96543.1 acla|EAW13902.1 afis|EAW18614.1 afla|EED52732.1 afum|EAL93324.1 agla|OJJ81230.1 akaw|GAA85043.1 aluc|OJZ86611.1 anid|CBF70430.1 anig|EHA22055.1 asyd|OJJ58615.1 ater|EAU34911.1 atub|OJI83915.1 aver|OJJ01222.1 awen|OJJ32075.1 azon|OJJ46655.1

Group5188: abra|OJJ71877.1 aluc|OJZ86615.1 atub|OJI83911.1 acla|EAW13906.1 afla|EED52727.1 afis|EAW18618.1 anig|EHA22051.1 afum|EAL93328.1 anid|CBF70503.1 aory|EIT78247.1 agla|OJJ81226.1 akaw|GAA85039.1 asyd|OJJ58578.1 ater|EAU34918.1 aver|OJJ01182.1 awen|OJJ32080.1 azon|OJJ46659.1

Group5189: abra|OJJ71893.1 acar|OOF96553.1 acla|EAW09629.1 afis|EAW21511.1 afla|EED55949.1 afum|EAL92531.1 agla|OJJ85675.1 akaw|GAA85025.1 aluc|OJZ86632.1 anid|CBF76437.1 anig|EHA22037.1 aory|EIT76814.1 asyd|OJJ61099.1 atub|OJI83896.1 aver|OJJ00414.1 awen|OJJ39183.1 azon|OJJ51510.1

Group5190: abra|OJJ71894.1 acar|OOF96555.1 acla|EAW09628.1 afis|EAW21512.1 afla|EED55950.1 afum|EAL92532.1 agla|OJJ85676.1 akaw|GAA85024.1 aluc|OJZ86633.1 anid|CBF76435.1 anig|EHA22036.1 asyd|OJJ61097.1 ater|EAU35028.1 atub|OJI83895.1 aver|OJJ00412.1 awen|OJJ39182.1 azon|OJJ51511.1

Group5191: abra|OJJ71897.1 acar|OOF96558.1 acla|EAW09625.1 afis|EAW21515.1 afla|EED55953.1 afum|EAL92535.1 agla|OJJ85679.1 akaw|GAA85021.1 aluc|OJZ86636.1 anid|CBF76429.1 anig|EHA22033.1 asyd|OJJ61094.1 ater|EAU35025.1 atub|OJI83892.1 aver|OJJ00409.1 awen|OJJ39179.1 azon|OJJ51517.1

Group5192: abra|OJJ71904.1 acar|OOF96563.1 acla|EAW09619.1 afis|EAW21520.1 afla|EED55958.1 afum|EAL92541.1 agla|OJJ85683.1 akaw|GAA85016.1 aluc|OJZ86641.1 anid|CBF76417.1 anig|EHA22028.1 aory|EIT76716.1 asyd|OJJ61089.1 atub|OJI83886.1 aver|OJJ00404.1 awen|OJJ39173.1 azon|OJJ51500.1

Group5193: abra|OJJ71906.1 acla|EAW09617.1 afis|EAW21522.1 afla|EED55960.1 afum|EAL92543.1 agla|OJJ85685.1 akaw|GAA85014.1 aluc|OJZ86643.1 anid|CBF76414.1 anig|EHA22026.1 aory|EIT76548.1 asyd|OJJ61087.1 ater|EAU35019.1 atub|OJI83884.1 aver|OJJ00402.1 awen|OJJ39171.1 azon|OJJ51502.1

Group5194: abra|OJJ71910.1 afis|EAW21525.1 afla|EED55963.1 afum|EAL92546.1 agla|OJJ85688.1 akaw|GAA85010.1 aluc|OJZ86647.1 anid|CBF76408.1 anig|EHA22023.1 aory|EIT76627.1 asyd|OJJ61084.1 ater|EAU35016.1 atub|OJI83880.1 aver|OJJ00399.1 awen|OJJ39168.1 azon|OJJ51497.1 acla|EAW09614.1

Group5195: abra|OJJ71919.1 acar|OOF96576.1 acla|EAW09611.1 afis|EAW21528.1 afla|EED55976.1 afum|EAL92549.1 agla|OJJ85692.1 akaw|GAA85003.1 aluc|OJZ86656.1 anid|CBF76402.1 anig|EHA22015.1 asyd|OJJ61080.1 ater|EAU35013.1 atub|OJI83871.1 aver|OJJ00396.1 awen|OJJ39165.1 azon|OJJ51494.1

Group5196: abra|OJJ71925.1 acar|OOF96582.1 acla|EAW09603.1 afis|EAW21534.1 afla|EED55984.1 afum|EBA27462.1 akaw|GAA84997.1 aluc|OJZ86662.1 anid|CBF76390.1 anig|EHA22009.1 aory|EIT76780.1 asyd|OJJ61072.1 ater|EAU35005.1 atub|OJI83863.1 aver|OJJ00389.1 awen|OJJ39158.1 azon|OJJ51488.1

Group5197: abra|OJJ71941.1 acar|OOF96597.1 acla|EAW09589.1 afis|EAW21549.1 afum|EAL92569.1 agla|OJJ85727.1 akaw|GAA84984.1 aluc|OJZ86677.1 anid|CBF76360.1 anig|EHA21996.1 aory|EIT76795.1 asyd|OJJ61058.1 ater|EAU34990.1 atub|OJI83850.1 aver|OJJ00375.1 awen|OJJ39141.1 azon|OJJ51466.1

Group5198: abra|OJJ71948.1 acla|EAW09586.1 afis|EAW21552.1 afla|EED56004.1 afum|EAL92572.2 agla|OJJ85730.1 akaw|GAA84979.1 aluc|OJZ86683.1 anid|CBF76354.1 anig|EHA21990.1 aory|EIT76847.1 asyd|OJJ61055.1 ater|EAU34986.1 atub|OJI83844.1 aver|OJJ00372.1 awen|OJJ39138.1 azon|OJJ51471.1

Group5199: abra|OJJ71949.1 acla|EAW09585.1 afis|EAW21553.1 afla|EED56005.1 afum|EAL92573.1 agla|OJJ85733.1 akaw|GAA84978.1 aluc|OJZ86684.1 anid|CBF76352.1 anig|EHA21989.1 aory|EIT76552.1 asyd|OJJ61054.1 ater|EAU34981.1 atub|OJI83843.1 aver|OJJ00371.1 awen|OJJ39137.1 azon|OJJ51472.1

Group5200: abra|OJJ71954.1 acla|EAW09582.1 afis|EAW21555.1 afla|EED56007.1 afum|EBA27465.1 agla|OJJ85734.1 akaw|GAA84974.1 aluc|OJZ86688.1 anid|CBF76348.1 aory|EIT76671.1 asyd|OJJ61052.1 ater|EAU34979.1 atub|OJI83837.1 aver|OJJ00368.1 awen|OJJ39135.1 azon|OJJ51463.1 anig|EHA21985.1

Group5201: abra|OJJ71955.1 acar|OOF96617.1 acla|EAW09580.1 afis|EAW21557.1 afla|EED56009.1 afum|EAL92576.1 agla|OJJ85735.1 akaw|GAA84972.1 aluc|OJZ86690.1 anig|EHA21983.1 aory|EIT76882.1 asyd|OJJ61050.1 ater|EAU34977.1 atub|OJI83834.1 aver|OJJ00366.1 awen|OJJ39133.1 azon|OJJ51461.1

Group5202: abra|OJJ71964.1 acar|OOF89892.1 acla|EAW09572.1 afis|EAW21564.1 afla|EED56020.1 afum|EAL92583.1 agla|OJJ85749.1 akaw|GAA84964.1 aluc|OJZ86700.1 anid|CBF76332.1 anig|EHA21976.1 asyd|OJJ61037.1 ater|EAU34968.1 atub|OJI83826.1 aver|OJJ00355.1 awen|OJJ39122.1 azon|OJJ51452.1

Group5203: abra|OJJ71981.1 acar|OOF96632.1 acla|EAW09558.1 afis|EAW21579.1 afla|EED57796.1 afum|EAL92597.1 agla|OJJ85760.1 akaw|GAA84948.1 aluc|OJZ86718.1 anid|CBF76296.1 anig|EHA21961.1 aory|EIT81066.1 asyd|OJJ61003.1 atub|OJI83811.1 aver|OJJ00321.1 awen|OJJ39102.1 azon|OJJ51432.1

Group5204: abra|OJJ71982.1 acar|OOF96633.1 acla|EAW09557.1 afis|EAW21580.1 afla|EED57797.1 afum|EAL92598.1 agla|OJJ85759.1 akaw|GAA84947.1 aluc|OJZ86719.1 anid|CBF76298.1 anig|EHA21960.1 asyd|OJJ61002.1 ater|EAU34954.1 atub|OJI83810.1 awen|OJJ39101.1 azon|OJJ51433.1 aver|OJJ00320.1

Group5205: abra|OJJ71984.1 acar|OOF96636.1 acla|EAW09554.1 afis|EAW21582.1 afla|EED57802.1 afum|EAL92600.1 agla|OJJ85755.1 akaw|GAA84945.1 aluc|OJZ86721.1 anig|EHA21958.1 aory|EIT81095.1 asyd|OJJ60998.1 ater|EAU34951.1 atub|OJI83807.1 aver|OJJ00318.1 awen|OJJ39099.1 azon|OJJ51435.1

Group5206: abra|OJJ71985.1 acar|OOF96639.1 acla|EAW09553.1 afis|EAW21583.1 afla|EED57804.1 afum|EAL92601.2 agla|OJJ85773.1 akaw|GAA84944.1 aluc|OJZ86722.1 anid|CBF76291.1 anig|EHA21957.1 asyd|OJJ60997.1 ater|EAU34948.1 atub|OJI83806.1 aver|OJJ00317.1 awen|OJJ39098.1 azon|OJJ51436.1

Group5207: abra|OJJ71986.1 acar|OOF96640.1 acla|EAW09552.1 afis|EAW21584.1 afla|EED57805.1 afum|EAL92602.1 agla|OJJ85776.1 akaw|GAA84943.1 aluc|OJZ86723.1 anig|EHA21956.1 aory|EIT81057.1 asyd|OJJ60996.1 ater|EAU34947.1 atub|OJI83805.1 aver|OJJ00316.1 awen|OJJ39097.1 azon|OJJ51430.1

Group5208: abra|OJJ71989.1 acar|OOF96643.1 acla|EAW09546.1 afis|EAW21587.1 afla|EED57808.1 afum|EAL92605.1 agla|OJJ85780.1 akaw|GAA84940.1 aluc|OJZ86726.1 anig|EHA21953.1 aory|EIT81079.1 asyd|OJJ60993.1 ater|EAU34944.1 atub|OJI83802.1 aver|OJJ00313.1 awen|OJJ39094.1 azon|OJJ51427.1

Group5209: abra|OJJ72011.1 acla|EAW09530.1 afis|EAW21602.1 afla|EED57830.1 afum|EAL92621.1 agla|OJJ85798.1 akaw|GAA84922.1 aluc|OJZ86747.1 anid|CBF83464.1 anig|EHA21932.1 aory|EIT81055.1 asyd|OJJ60685.1 ater|EAU38293.1 atub|OJI83780.1 aver|OJJ00010.1 awen|OJJ39079.1 azon|OJJ51411.1

Group5210: abra|OJJ72013.1 acar|OOF96662.1 acla|EAW09527.1 afis|EAW21605.1 afla|EED57832.1 afum|EAL92624.1 agla|OJJ85800.1 akaw|GAA84920.1 aluc|OJZ86749.1 anid|CBF83467.1 anig|EHA21930.1 asyd|OJJ60687.1 ater|EAU38294.1 atub|OJI83778.1 aver|OJJ00012.1 awen|OJJ39077.1 azon|OJJ51409.1

Group5211: abra|OJJ72023.1 acar|OOF96672.1 acla|EAW09518.1 afis|EAW21614.1 afla|EED57845.1 afum|EAL92648.1 agla|OJJ78625.1 akaw|GAA84909.1 aluc|OJZ86760.1 anid|CBF83487.1 anig|EHA21919.1 asyd|OJJ60699.1 ater|EAU38306.1 atub|OJI83767.1 aver|OJJ00025.1 awen|OJJ39066.1 azon|OJJ51397.1

Group5212: abra|OJJ72025.1 acar|OOF96674.1 acla|EAW09514.1 afis|EAW21618.1 afla|EED57847.1 afum|EAL92654.1 agla|OJJ78627.1 akaw|GAA84907.1 aluc|OJZ86762.1 anid|CBF83491.1 anig|EHA21917.1 asyd|OJJ60701.1 ater|EAU38308.1 atub|OJI83765.1 aver|OJJ00027.1 awen|OJJ39064.1 azon|OJJ51395.1

Group5213: abra|OJJ72051.1 acar|OOF96708.1 acla|EAW09496.1 afis|EAW21640.1 afla|EED57903.1 afum|EAL92674.1 agla|OJJ79707.1 akaw|GAA84883.1 aluc|OJZ86788.1 anid|CBF83525.1 aory|EIT82339.1 asyd|OJJ60717.1 ater|EAU38325.1 atub|OJI83731.1 aver|OJJ00050.1 awen|OJJ33897.1 azon|OJJ48663.1

Group5214: abra|OJJ72053.1 acar|OOF96710.1 acla|EAW09494.1 afis|EAW21642.1 afla|EED57901.1 afum|EAL92676.1 agla|OJJ79709.1 akaw|GAA84881.1 aluc|OJZ86790.1 anig|EHA21893.1 aory|EIT82345.1 asyd|OJJ60719.1 ater|EAU38327.1 atub|OJI83729.1 aver|OJJ00052.1 awen|OJJ33899.1 azon|OJJ48661.1

Group5215: abra|OJJ72059.1 acar|OOF89883.1 acla|EAW09490.1 afis|EAW21646.1 afla|EED48456.1 afum|EAL92680.1 agla|OJJ79714.1 akaw|GAA84876.1 aluc|OJZ86795.1 anid|CBF83533.1 anig|EHA21888.1 asyd|OJJ60723.1 ater|EAU32953.1 atub|OJI83723.1 aver|OJJ00056.1 awen|OJJ33904.1 azon|OJJ48657.1

Group5216: abra|OJJ72080.1 acar|OOF96728.1 acla|EAW09487.1 afis|EAW21649.1 afla|EED57920.1 afum|EAL92683.1 agla|OJJ79729.1 aluc|OJZ86834.1 anid|CBF83557.1 anig|EHA21801.1 aory|EIT82340.1 asyd|OJJ60737.1 ater|EAU38344.1 atub|OJI83684.1 aver|OJJ00069.1 awen|OJJ33918.1 azon|OJJ48644.1

Group5217: abra|OJJ72107.1 acar|OOF96750.1 acla|EAW09384.1 afis|EAW21751.1 afla|EED58027.1 afum|EBA27473.1 agla|OJJ84423.1 akaw|GAA84815.1 aluc|OJZ86862.1 anid|CBF83669.1 aory|EIT79785.1 asyd|OJJ60809.1 ater|EAU38439.1 atub|OJI83658.1 aver|OJJ00137.1 awen|OJJ34032.1 azon|OJJ48205.1

Group5218: abra|OJJ72114.1 acar|OOF96754.1 acla|EAW14915.1 afis|EAW24437.1 afla|EED55242.1 afum|EAL86105.1 agla|OJJ78776.1 akaw|GAA84808.1 aluc|OJZ86869.1 anid|CBF73764.1 anig|EHA21772.1 aory|EIT83629.1 asyd|OJJ56589.1 atub|OJI83650.1 aver|OJJ06136.1 awen|OJJ32762.1 azon|OJJ43997.1

Group5219: abra|OJJ72122.1 acar|OOF96762.1 acla|EAW14922.1 afis|EAW24444.1 afla|EED55233.1 afum|EAL86113.1 agla|OJJ80289.1 akaw|GAA84801.1 aluc|OJZ86879.1 anid|CBF73751.1 anig|EHA21765.1 aory|EIT83604.1 asyd|OJJ56581.1 ater|EAU30003.1 atub|OJI83640.1 aver|OJJ06143.1 awen|OJJ32755.1

Group5220: abra|OJJ72142.1 acar|OOF96780.1 acla|EAW14936.1 afis|EAW24451.1 afum|EAL86120.1 agla|OJJ80297.1 akaw|GAA84781.1 aluc|OJZ86899.1 anid|CBF73817.1 anig|EHA21743.1 aory|EIT83518.1 asyd|OJJ56573.1 ater|EAU29995.1 atub|OJI83620.1 aver|OJJ06150.1 awen|OJJ32746.1 azon|OJJ45298.1

Group5221: abra|OJJ72151.1 afis|EAW24461.1 afla|EED55212.1 agla|OJJ80309.1 akaw|GAA84773.1 aluc|OJZ86910.1 anid|CBF73833.1 anig|EHA21735.1 asyd|OJJ56559.1 atub|OJI83612.1 awen|OJJ32735.1 azon|OJJ45286.1 acla|EAW14946.1 afla|EED55213.1 afum|EAL86131.1 aory|EIT83597.1 ater|EAU29981.1

Group5222: abra|OJJ72192.1 afis|EAW21198.1 afum|EAL90047.1 akaw|GAA84741.1 aluc|OJZ86943.1 anig|EHA21701.1 aory|EIT78387.1 asyd|OJJ53191.1 atub|OJI83579.1 aver|OJI97565.1 awen|OJJ41115.1 azon|OJJ44691.1 acla|EAW10207.1 afis|EAW25388.1 afla|EED53865.1 ater|EAU34535.1 aver|OJJ03659.1

Group5223: abra|OJJ72201.1 acar|OOF96821.1 acla|EAW14948.1 afis|EAW24463.1 afla|EED55203.1 afum|EAL86134.1 agla|OJJ80321.1 akaw|GAA84735.1 aluc|OJZ86951.1 anid|CBF73839.1 anig|EHA21696.1 aory|EIT83590.1 asyd|OJJ56556.1 ater|EAU29977.1 atub|OJI83572.1 aver|OJJ06164.1 awen|OJJ32728.1

Group5224: abra|OJJ72208.1 acar|OOF96829.1 acla|EAW14968.1 afis|EAW24491.1 afum|EAL86160.1 agla|OJJ80328.1 akaw|GAA84726.1 aluc|OJZ86960.1 anid|CBF73841.1 anig|EHA21688.1 aory|EIT78779.1 asyd|OJJ56555.1 ater|EAU29943.1 atub|OJI83563.1 aver|OJJ06165.1 awen|OJJ32704.1 azon|OJJ45096.1

Group5225: abra|OJJ72253.1 acar|OOF96884.1 acla|EAW12203.1 afis|EAW17077.1 afla|EED52139.1 agla|OJJ81367.1 akaw|GAA84684.1 aluc|OJZ87004.1 anid|CBF79753.1 anig|EHA21642.1 aory|EIT74390.1 asyd|OJJ58098.1 ater|EAU30779.1 atub|OJI83515.1 aver|OJJ04306.1 awen|OJJ36962.1 azon|OJJ45322.1

Group5226: abra|OJJ72287.1 acar|OOF94936.1 acla|EAW12015.1 afis|EAW19654.1 afla|EED57006.1 afum|EAL86868.1 agla|OJJ80800.1 akaw|GAA86039.1 aluc|OJZ90266.1 anid|CBF71365.1 anig|EHA23761.1 asyd|OJJ55082.1 ater|EAU32874.1 atub|OJI81691.1 aver|OJJ07125.1 awen|OJJ41906.1 azon|OJJ42696.1

Group5227: abra|OJJ72289.1 acar|OOF94934.1 acla|EAW12013.1 afis|EAW19653.1 afla|EED57004.1 afum|EAL86869.1 agla|OJJ80799.1 akaw|GAA86041.1 aluc|OJZ90269.1 anid|CBF71363.1 anig|EHA23762.1 asyd|OJJ55083.1 ater|EAU32875.1 atub|OJI81694.1 aver|OJJ07124.1 awen|OJJ41904.1 azon|OJJ42693.1

Group5228: abra|OJJ72291.1 acar|OOF94929.1 acla|EAW12011.1 afis|EAW19651.1 afla|EED57002.1 afum|EAL86871.1 agla|OJJ80797.1 akaw|GAA86043.1 aluc|OJZ90272.1 anid|CBF71359.1 anig|EHA23764.1 asyd|OJJ55085.1 ater|EAU32877.1 atub|OJI81696.1 aver|OJJ07122.1 awen|OJJ41902.1 azon|OJJ42698.1

Group5229: abra|OJJ72292.1 acar|OOF94932.1 acla|EAW12010.1 afis|EAW19650.1 afla|EED57001.1 agla|OJJ80796.1 akaw|GAA86044.1 aluc|OJZ90273.1 anid|CBF71357.1 anig|EHA23765.1 aory|EIT77584.1 asyd|OJJ55086.1 ater|EAU32878.1 atub|OJI81697.1 aver|OJJ07121.1 awen|OJJ41901.1 azon|OJJ42699.1

Group5230: abra|OJJ72293.1 acar|OOF94930.1 acla|EAW12009.1 afis|EAW19649.1 afla|EED57000.1 afum|EAL86873.1 agla|OJJ80791.1 akaw|GAA86045.1 aluc|OJZ90274.1 anig|EHA23766.1 aory|EIT77489.1 asyd|OJJ55087.1 ater|EAU32879.1 atub|OJI81698.1 aver|OJJ07120.1 awen|OJJ41900.1 azon|OJJ42700.1

Group5231: abra|OJJ72294.1 acar|OOF94931.1 acla|EAW12008.1 afis|EAW19648.1 afla|EED56999.1 afum|EAL86874.1 agla|OJJ80790.1 akaw|GAA86046.1 aluc|OJZ90275.1 anid|CBF71356.1 anig|EHA23767.1 asyd|OJJ55088.1 ater|EAU32880.1 atub|OJI81699.1 aver|OJJ07119.1 awen|OJJ41899.1 azon|OJJ42701.1

Group5232: abra|OJJ72300.1 acar|OOF94925.1 acla|EAW12004.1 afis|EAW19644.1 afla|EED56995.1 afum|EAL86878.1 agla|OJJ80786.1 akaw|GAA86051.1 aluc|OJZ90281.1 anid|CBF71348.1 anig|EHA23771.1 aory|EIT77548.1 asyd|OJJ55093.1 atub|OJI81705.1 aver|OJJ07115.1 awen|OJJ41895.1 azon|OJJ42705.1

Group5233: abra|OJJ72308.1 acar|OOF94905.1 acla|EAW13442.1 afis|EAW20849.1 afum|EAL84784.1 aluc|OJZ90289.1 anid|CBF89941.1 anig|EHA23779.1 asyd|OJJ61203.1 ater|EAU29955.1 atub|OJI81713.1 aver|OJJ00527.1 awen|OJJ41681.1 azon|OJJ50849.1 anig|EHA21820.1 asyd|OJJ61389.1 aver|OJJ00717.1

Group5234: abra|OJJ72315.1 acar|OOF94895.1 afis|EAW19532.1 afla|EED48961.1 afum|EAL86974.1 akaw|GAA86062.1 aluc|OJZ90296.1 anig|EHA23785.1 aory|EIT79991.1 ater|EAU31962.1 atub|OJI81720.1 awen|OJJ35408.1 azon|OJJ49084.1 afla|EED56029.1 aory|EIT76743.1 atub|OJI89169.1 awen|OJJ35688.1

Group5235: abra|OJJ72326.1 acar|OOF94888.1 acla|EAW11997.1 afis|EAW19637.1 afla|EED56989.1 afum|EAL86885.1 agla|OJJ80779.1 akaw|GAA86076.1 aluc|OJZ90311.1 anid|CBF71337.1 anig|EHA23798.1 asyd|OJJ55102.1 ater|EAU32891.1 atub|OJI81736.1 aver|OJJ07105.1 awen|OJJ41882.1 azon|OJJ47080.1

Group5236: abra|OJJ72329.1 acar|OOF94885.1 acla|EAW11994.1 afis|EAW19634.1 afla|EED56985.1 afum|EAL86888.1 agla|OJJ86606.1 akaw|GAA86079.1 aluc|OJZ90314.1 anid|CBF71331.1 anig|EHA23801.1 aory|EIT77448.1 asyd|OJJ55105.1 ater|EAU32894.1 atub|OJI81739.1 aver|OJJ07102.1 awen|OJJ41879.1

Group5237: abra|OJJ72350.1 acar|OOF94868.1 acla|EAW11976.1 afis|EAW19610.1 afla|EED56970.1 afum|EAL86912.1 agla|OJJ80760.1 akaw|GAA86097.1 aluc|OJZ90329.1 anid|CBF71304.1 anig|EHA23813.1 asyd|OJJ55122.1 ater|EAU32911.1 atub|OJI81756.1 aver|OJJ07081.1 awen|OJJ41860.1 azon|OJJ47054.1

Group5238: abra|OJJ72357.1 acar|OOF94861.1 acla|EAW11962.1 afis|EAW19599.1 afla|EED56955.1 afum|EAL86919.1 agla|OJJ80741.1 akaw|GAA86104.1 aluc|OJZ90336.1 anid|CBF71289.1 anig|EHA23820.1 aory|EIT77466.1 ater|EAU32926.1 atub|OJI81763.1 aver|OJJ07075.1 awen|OJJ41842.1 azon|OJJ44889.1

Group5239: abra|OJJ72378.1 acar|OOF94839.1 acla|EAW11956.1 afis|EAW19593.1 afla|EED56949.1 afum|EAL86925.1 akaw|GAA86126.1 aluc|OJZ90365.1 anid|CBF71271.1 anig|EHA23840.1 aory|EIT77564.1 asyd|OJJ55148.1 ater|EAU32931.1 atub|OJI81792.1 aver|OJJ07059.1 awen|OJJ41810.1 azon|OJJ47022.1

Group5240: abra|OJJ72383.1 acar|OOF90287.1 acla|EAW11952.1 afis|EAW19589.1 afla|EED56943.1 afum|EAL86929.1 agla|OJJ80729.1 akaw|GAA86132.1 aluc|OJZ90370.1 anig|EHA23845.1 aory|EIT77526.1 asyd|OJJ55143.1 ater|EAU32937.1 atub|OJI81796.1 aver|OJJ07064.1 awen|OJJ41804.1 azon|OJJ47027.1

Group5241: abra|OJJ72386.1 acar|OOF90292.1 acla|EAW11950.1 afis|EAW19587.1 afla|EED56915.1 afum|EAL86931.1 agla|OJJ80724.1 akaw|GAA86135.1 aluc|OJZ90373.1 anig|EHA23847.1 aory|EIT77573.1 asyd|OJJ55152.1 ater|EAU32961.1 atub|OJI81799.1 aver|OJJ07055.1 awen|OJJ41801.1 azon|OJJ47030.1

Group5242: abra|OJJ72398.1 acla|EAW11942.1 afis|EAW19578.1 afla|EED56803.1 afum|EAL86940.2 agla|OJJ80707.1 akaw|GAA86143.1 aluc|OJZ90383.1 anid|CBF71237.1 anig|EHA23855.1 aory|EIT73849.1 asyd|OJJ55164.1 ater|EAU32977.1 atub|OJI81809.1 aver|OJJ07043.1 awen|OJJ41791.1 azon|OJJ47042.1

Group5243: abra|OJJ72407.1 acar|OOF94822.1 acla|EAW11935.1 afis|EAW19570.1 afla|EED56813.1 agla|OJJ80698.1 akaw|GAA86150.1 aluc|OJZ90391.1 anid|CBF71224.1 anig|EHA23861.1 aory|EIT73940.1 asyd|OJJ55172.1 ater|EAU32993.1 atub|OJI81820.1 aver|OJJ07035.1 awen|OJJ41783.1 azon|OJJ44895.1

Group5244: abra|OJJ72432.1 acla|EAW12664.1 afis|EAW17828.1 afla|EED46170.1 afum|EAL91638.1 agla|OJJ80649.1 akaw|GAA86172.1 aluc|OJZ90415.1 anid|CBF77999.1 anig|EHA23881.1 aory|EIT76464.1 asyd|OJJ55248.1 ater|EAU33032.1 atub|OJI81848.1 aver|OJJ06975.1 awen|OJJ41758.1 azon|OJJ48449.1

Group5245: abra|OJJ72433.1 acar|OOF94802.1 acla|EAW12665.1 afis|EAW17829.1 afla|EED46171.1 afum|EAL91639.1 agla|OJJ80650.1 akaw|GAA86173.1 aluc|OJZ90416.1 anid|CBF77998.1 anig|EHA23884.1 aory|EIT76389.1 asyd|OJJ55249.1 ater|EAU33033.1 atub|OJI81849.1 aver|OJJ06974.1 azon|OJJ48450.1

Group5246: abra|OJJ72436.1 acar|OOF94799.1 acla|EAW12669.1 afis|EAW17833.1 afla|EED46175.1 afum|EAL91643.1 akaw|GAA86176.1 aluc|OJZ90420.1 anid|CBF77990.1 anig|EHA23890.1 aory|EIT76472.1 asyd|OJJ55254.1 ater|EAU33036.1 atub|OJI81852.1 aver|OJJ06970.1 awen|OJJ41748.1 azon|OJJ48441.1

Group5247: abra|OJJ72438.1 acla|EAW12671.1 afis|EAW17834.1 afla|EED46179.1 afum|EAL91644.1 agla|OJJ83132.1 akaw|GAA86177.1 aluc|OJZ90422.1 anid|CBF77988.1 anig|EHA23891.1 aory|EIT76441.1 asyd|OJJ55255.1 ater|EAU33038.1 atub|OJI81854.1 aver|OJJ06969.1 awen|OJJ41742.1 azon|OJJ48438.1

Group5248: abra|OJJ72441.1 acar|OOF94796.1 acla|EAW12674.1 afis|EAW17837.1 afla|EED46182.1 afum|EAL91647.1 agla|OJJ83129.1 akaw|GAA86180.1 aluc|OJZ90425.1 anid|CBF77983.1 anig|EHA23894.1 aory|EIT76412.1 asyd|OJJ55258.1 ater|EAU33041.1 atub|OJI81857.1 awen|OJJ41739.1 azon|OJJ48435.1

Group5249: abra|OJJ72447.1 acar|OOF94789.1 acla|EAW12680.1 afis|EAW17843.1 afla|EED46188.1 afum|EAL91652.1 agla|OJJ83124.1 akaw|GAA86185.1 aluc|OJZ90431.1 anid|CBF77971.1 anig|EHA23900.1 aory|EIT76400.1 asyd|OJJ55264.1 ater|EAU33045.1 atub|OJI81863.1 awen|OJJ41733.1 azon|OJJ48430.1

Group5250: abra|OJJ72452.1 acar|OOF94784.1 acla|EAW12686.1 afis|EAW17849.1 afla|EED46202.1 afum|EAL91658.1 agla|OJJ83120.1 akaw|GAA86189.1 aluc|OJZ90437.1 anig|EHA23904.1 aory|EIT76465.1 asyd|OJJ55271.1 ater|EAU33051.1 atub|OJI81868.1 aver|OJJ06956.1 awen|OJJ41724.1 azon|OJJ48422.1

Group5251: abra|OJJ72463.1 acar|OOF94776.1 acla|EAW12695.1 afis|EAW17859.1 afla|EED46214.1 afum|EAL91667.1 agla|OJJ83110.1 akaw|GAA86199.1 aluc|OJZ90450.1 anid|CBF77945.1 anig|EHA23913.1 aory|EIT76391.1 asyd|OJJ55280.1 atub|OJI81878.1 aver|OJJ06947.1 awen|OJJ41713.1 azon|OJJ48408.1

Group5252: abra|OJJ72472.1 acar|OOF94763.1 acla|EAW12705.1 afis|EAW17868.1 afum|EAL91676.1 agla|OJJ83088.1 akaw|GAA86210.1 aluc|OJZ90459.1 anid|CBF89895.1 anig|EHA23921.1 aory|EIT76489.1 asyd|OJJ62138.1 ater|EAU33086.1 atub|OJI81886.1 aver|OJI96248.1 awen|OJJ41703.1 azon|OJJ48399.1

Group5253: abra|OJJ72473.1 acar|OOF94762.1 acla|EAW12706.1 afis|EAW17869.1 afla|EED46237.1 afum|EAL91677.1 agla|OJJ83089.1 akaw|GAA86211.1 aluc|OJZ90460.1 anid|CBF89897.1 anig|EHA23922.1 asyd|OJJ62137.1 ater|EAU33087.1 atub|OJI81887.1 aver|OJI96247.1 awen|OJJ41702.1 azon|OJJ48398.1

Group5254: abra|OJJ72477.1 acar|OOF94759.1 acla|EAW12708.1 afis|EAW17871.1 afum|EAL91679.1 agla|OJJ83091.1 akaw|GAA86213.1 aluc|OJZ90463.1 anid|CBF89900.1 anig|EHA23924.1 aory|EIT76455.1 asyd|OJJ62135.1 ater|EAU33089.1 atub|OJI81889.1 aver|OJI96245.1 awen|OJJ41699.1 azon|OJJ48396.1

Group5255: abra|OJJ72479.1 acar|OOF94757.1 acla|EAW12710.1 afis|EAW17873.1 afla|EED46240.1 afum|EAL91681.1 agla|OJJ83075.1 akaw|GAA86215.1 aluc|OJZ90467.1 anid|CBF89932.1 anig|EHA23926.1 aory|EIT76355.1 asyd|OJJ62115.1 ater|EAU33091.1 atub|OJI81891.1 aver|OJI96222.1 awen|OJJ41695.1

Group5256: abra|OJJ72486.1 acar|OOF94749.1 afis|EAW17880.1 afla|EED51757.1 afum|EAL91688.1 akaw|GAA86221.1 aluc|OJZ90473.1 anid|CBF88138.1 anig|EHA23932.1 aory|EIT80744.1 asyd|OJJ63602.1 ater|EAU33100.1 atub|OJI81897.1 aver|OJJ05546.1 awen|OJJ36319.1 ater|EAU29427.1 aver|OJI97334.1

Group5257: abra|OJJ72491.1 acar|OOF94746.1 acla|EAW12721.1 afis|EAW17884.1 afla|EED46271.1 afum|EAL91692.1 agla|OJJ83063.1 akaw|GAA86225.1 aluc|OJZ90477.1 anid|CBF77031.1 anig|EHA23936.1 asyd|OJJ57010.1 ater|EAU33104.1 atub|OJI81902.1 aver|OJJ03277.1 awen|OJJ41675.1 azon|OJJ48390.1

Group5258: abra|OJJ72504.1 acla|EAW12728.1 afis|EAW17891.1 afla|EED46283.1 afum|EAL91700.1 agla|OJJ83052.1 akaw|GAA86232.1 aluc|OJZ90487.1 anid|CBF77012.1 anig|EHA23945.1 aory|EIT76351.1 asyd|OJJ57018.1 ater|EAU33111.1 atub|OJI81911.1 aver|OJJ03271.1 awen|OJJ41662.1 azon|OJJ48381.1

Group5259: abra|OJJ72506.1 acar|OOF94736.1 acla|EAW12729.1 afis|EAW17892.1 afla|EED46285.1 afum|EAL91701.1 agla|OJJ83050.1 akaw|GAA86234.1 aluc|OJZ90489.1 anid|CBF77008.1 anig|EHA23947.1 asyd|OJJ57020.1 ater|EAU33113.1 atub|OJI81913.1 aver|OJJ03269.1 awen|OJJ41660.1 azon|OJJ48380.1

Group5260: abra|OJJ72533.1 acar|OOF94720.1 acla|EAW12740.1 afis|EAW17903.1 afla|EED46295.1 afum|EAL91711.1 agla|OJJ83040.1 akaw|GAA86255.1 aluc|OJZ90512.1 anid|CBF76988.1 aory|EIT76329.1 ater|EAU33122.1 atub|OJI81937.1 aver|OJJ03258.1 awen|OJJ41647.1 asyd|OJJ57034.1 azon|OJJ48369.1

Group5261: abra|OJJ72536.1 acla|EAW12743.1 afis|EAW17906.1 afla|EED46300.1 afum|EAL91715.1 agla|OJJ83036.1 akaw|GAA86259.1 aluc|OJZ90517.1 anid|CBF76983.1 anig|EHA23971.1 aory|EIT76325.1 asyd|OJJ57036.1 ater|EAU33124.1 atub|OJI81941.1 aver|OJJ03256.1 awen|OJJ41644.1 azon|OJJ48365.1

Group5262: abra|OJJ72546.1 acar|OOF94707.1 acla|EAW12750.1 afis|EAW17914.1 afla|EED46317.1 afum|EAL91724.1 agla|OJJ83029.1 akaw|GAA85179.1 aluc|OJZ90533.1 anid|CBF76973.1 anig|EHA23982.1 asyd|OJJ57046.1 ater|EAU33135.1 atub|OJI81954.1 aver|OJJ03249.1 awen|OJJ41633.1 azon|OJJ48358.1

Group5263: abra|OJJ72563.1 acar|OOF94691.1 acla|EAW12763.1 afis|EAW17925.1 afum|EAL91736.1 agla|OJJ83017.1 akaw|GAA85192.1 aluc|OJZ90547.1 anid|CBF76944.1 anig|EHA23995.1 aory|EIT81166.1 asyd|OJJ57061.1 ater|EAU33547.1 atub|OJI81969.1 aver|OJJ03237.1 awen|OJJ41621.1 azon|OJJ48344.1

Group5264: abra|OJJ72572.1 acar|OOF99282.1 akaw|GAA85198.1 aluc|OJZ90553.1 anig|EHA24000.1 asyd|OJJ56262.1 atub|OJI81976.1 abra|OJJ78185.1 acla|EAW08831.1 anig|EHA19946.1 ater|EAU38338.1 aver|OJJ08473.1 acar|OOF96678.1 afla|EED51444.1 agla|OJJ88347.1 aory|EIT80756.1 ater|EAU37977.1

Group5265: abra|OJJ72579.1 acar|OOF94673.1 afla|EED56580.1 agla|OJJ88581.1 akaw|GAA85205.1 aluc|OJZ90560.1 anig|EHA24006.1 aory|EIT79653.1 asyd|OJJ60421.1 ater|EAU30076.1 atub|OJI81983.1 aver|OJJ01323.1 awen|OJJ33716.1 azon|OJJ42313.1 asyd|OJJ58710.1 awen|OJJ40585.1 akaw|GAA87620.1

Group5266: abra|OJJ72586.1 acar|OOF94663.1 acla|EAW12772.1 afis|EAW17935.1 afum|EAL91746.1 agla|OJJ82997.1 akaw|GAA85212.1 aluc|OJZ90567.1 anid|CBF77040.1 anig|EHA24013.1 aory|EIT78504.1 asyd|OJJ57001.1 ater|EAU33535.1 atub|OJI81990.1 aver|OJJ03286.1 awen|OJJ41611.1 azon|OJJ48330.1

Group5267: abra|OJJ72587.1 acar|OOF94664.1 acla|EAW12773.1 afis|EAW17936.1 afla|EED49106.1 agla|OJJ82996.1 akaw|GAA85213.1 aluc|OJZ90568.1 anid|CBF77042.1 aory|EIT78510.1 asyd|OJJ57000.1 ater|EAU33534.1 atub|OJI81991.1 aver|OJJ03287.1 awen|OJJ41610.1 azon|OJJ48329.1 afum|EAL91747.1

Group5268: abra|OJJ72590.1 acar|OOF94660.1 acla|EAW12776.1 afis|EAW17939.1 afla|EED49102.1 afum|EAL91750.1 agla|OJJ82993.1 akaw|GAA85216.1 aluc|OJZ90571.1 anid|CBF77047.1 anig|EHA24016.1 asyd|OJJ56997.1 ater|EAU33529.1 atub|OJI81994.1 aver|OJJ03290.1 awen|OJJ41606.1 azon|OJJ48324.1

Group5269: abra|OJJ72615.1 acla|EAW07008.1 afis|EAW16624.1 afla|EED44980.1 afum|EAL89213.1 agla|OJJ82624.1 akaw|GAA85240.1 aluc|OJZ90597.1 anid|CBF83188.1 anig|EHA24036.1 aory|EIT73566.1 asyd|OJJ55547.1 ater|EAU36728.1 atub|OJI82021.1 aver|OJJ06624.1 awen|OJJ36245.1 azon|OJJ42444.1

Group5270: abra|OJJ72621.1 acar|OOF90202.1 acla|EAW11603.1 afis|EAW23855.1 afla|EED49096.1 afum|EAL87283.1 agla|OJJ82506.1 akaw|GAA85242.1 aluc|OJZ90599.1 anid|CBF77246.1 aory|EIT82505.1 asyd|OJJ56811.1 ater|EAU33500.1 atub|OJI82023.1 aver|OJJ03524.1 awen|OJJ33816.1 azon|OJJ48722.1

Group5271: abra|OJJ72624.1 acar|OOF94640.1 acla|EAW11600.1 afis|EAW23857.1 afla|EED52582.1 afum|EAL87285.1 agla|OJJ82511.1 akaw|GAA85244.1 aluc|OJZ90601.1 anid|CBF77238.1 aory|EIT74177.1 asyd|OJJ56813.1 ater|EAU33494.1 atub|OJI82025.1 aver|OJJ03522.1 awen|OJJ33818.1 azon|OJJ48717.1

Group5272: abra|OJJ72631.1 acar|OOF94634.1 afis|EAW23863.1 afla|EED52587.1 afum|EAL87291.1 agla|OJJ82529.1 akaw|GAA85251.1 aluc|OJZ90608.1 anid|CBF77226.1 anig|EHA24046.1 aory|EIT74239.1 asyd|OJJ56818.1 ater|EAU33488.1 atub|OJI82032.1 aver|OJJ03517.1 awen|OJJ33823.1 azon|OJJ48712.1

Group5273: abra|OJJ72634.1 acar|OOF93305.1 afis|EAW18838.1 afla|EED47562.1 afum|EAL93572.1 akaw|GAA85255.1 aluc|OJZ90613.1 anid|CBF74711.1 anig|EHA24051.1 aory|EIT74513.1 asyd|OJJ59476.1 ater|EAU38813.1 atub|OJI82039.1 aver|OJJ02797.1 aver|OJJ08238.1 awen|OJJ32594.1 asyd|OJJ53508.1

Group5274: abra|OJJ72637.1 acla|EAW11587.1 afis|EAW23866.1 afla|EED52594.1 afum|EAL87294.1 agla|OJJ82535.1 akaw|GAA85258.1 aluc|OJZ90616.1 anid|CBF77211.1 anig|EHA24053.1 aory|EIT74298.1 asyd|OJJ56826.1 ater|EAU33476.1 atub|OJI82042.1 aver|OJJ03509.1 awen|OJJ33842.1 azon|OJJ48707.1

Group5275: abra|OJJ72638.1 acla|EAW11586.1 afis|EAW23867.1 afla|EED52595.1 afum|EAL87295.1 agla|OJJ82536.1 akaw|GAA85259.1 aluc|OJZ90617.1 anid|CBF77213.1 anig|EHA24054.1 aory|EIT74234.1 asyd|OJJ56825.1 ater|EAU33477.1 atub|OJI82043.1 aver|OJJ03510.1 awen|OJJ33843.1 azon|OJJ48704.1

Group5276: abra|OJJ72639.1 acar|OOF94632.1 acla|EAW11585.1 afis|EAW23868.1 afla|EED52596.1 afum|EAL87296.1 agla|OJJ82537.1 akaw|GAA85260.1 aluc|OJZ90618.1 anid|CBF77215.1 anig|EHA24055.1 aory|EIT74172.1 asyd|OJJ56824.1 atub|OJI82044.1 aver|OJJ03511.1 awen|OJJ33844.1 azon|OJJ48703.1

Group5277: abra|OJJ72649.1 acar|OOF94621.1 acla|EAW11579.1 afis|EAW23875.1 afla|EED52603.1 afum|EAL87303.1 agla|OJJ82545.1 akaw|GAA85270.1 aluc|OJZ90628.1 anid|CBF77207.1 anig|EHA24064.1 asyd|OJJ56830.1 ater|EAU33471.1 atub|OJI82054.1 aver|OJJ03506.1 awen|OJJ33851.1 azon|OJJ48697.1

Group5278: abra|OJJ72651.1 acla|EAW11578.1 afis|EAW23876.1 afla|EED52604.1 afum|EAL87304.1 agla|OJJ82546.1 akaw|GAA85271.1 aluc|OJZ90629.1 anid|CBF77205.1 anig|EHA24065.1 aory|EIT74232.1 asyd|OJJ56831.1 ater|EAU33470.1 atub|OJI82055.1 aver|OJJ03505.1 awen|OJJ33852.1 azon|OJJ48696.1

Group5279: abra|OJJ72656.1 acar|OOF94618.1 acla|EAW11519.1 afis|EAW23934.1 afla|EED50584.1 afum|EAL87359.1 agla|OJJ79414.1 akaw|GAA85278.1 aluc|OJZ90637.1 anid|CBF77271.1 anig|EHA24072.1 asyd|OJJ56798.1 ater|EAU32303.1 atub|OJI82065.1 aver|OJJ03536.1 awen|OJJ39021.1 azon|OJJ51346.1

Group5280: abra|OJJ72660.1 acar|OOF94613.1 acla|EAW11524.1 afis|EAW23929.1 afla|EED50589.1 afum|EAL87354.1 agla|OJJ79408.1 akaw|GAA85283.1 aluc|OJZ90641.1 anid|CBF77279.1 anig|EHA24076.1 aory|EIT82068.1 asyd|OJJ56794.1 atub|OJI82069.1 aver|OJJ03540.1 awen|OJJ39025.1 azon|OJJ51351.1

Group5281: abra|OJJ72674.1 acar|OOF97383.1 acla|EAW11536.1 afis|EAW23918.1 afla|EED50607.1 afum|EAL87344.1 agla|OJJ79387.1 akaw|GAA85293.1 aluc|OJZ90652.1 anid|CBF77094.1 anig|EHA24087.1 asyd|OJJ56956.1 ater|EAU32317.1 atub|OJI82081.1 aver|OJJ03329.1 awen|OJJ39035.1 azon|OJJ51363.1

Group5282: abra|OJJ72718.1 acar|OOF95950.1 acla|EAW11554.1 afis|EAW23899.1 afla|EED52633.1 afum|EAL87326.1 agla|OJJ78641.1 aluc|OJZ90687.1 anid|CBF77146.1 anig|EHA24115.1 aory|EIT74241.1 asyd|OJJ56911.1 ater|EAU32338.1 atub|OJI82112.1 aver|OJJ03382.1 awen|OJJ39049.1 azon|OJJ51381.1

Group5283: abra|OJJ72724.1 acar|OOF95957.1 acla|EAW11561.1 afis|EAW23892.1 afla|EED52622.1 afum|EAL87320.1 agla|OJJ78632.1 akaw|GAA85330.1 aluc|OJZ90693.1 anid|CBF77169.1 aory|EIT74282.1 asyd|OJJ56852.1 ater|EAU33450.1 atub|OJI82118.1 aver|OJJ03467.1 awen|OJJ39057.1 azon|OJJ51388.1

Group5284: abra|OJJ72726.1 acar|OOF95959.1 acla|EAW11563.1 afis|EAW23890.1 afla|EED52620.1 afum|EAL87318.1 agla|OJJ78630.1 akaw|GAA85332.1 aluc|OJZ90695.1 anid|CBF77173.1 anig|EHA24120.1 asyd|OJJ56849.1 ater|EAU33453.1 atub|OJI82121.1 aver|OJJ03490.1 awen|OJJ39059.1 azon|OJJ51390.1

Group5285: abra|OJJ72731.1 acla|EAW11566.1 afis|EAW23887.1 afla|EED52616.1 afum|EAL87314.2 agla|OJJ82574.1 akaw|GAA85337.1 aluc|OJZ90700.1 anid|CBF77179.1 anig|EHA24126.1 aory|EIT74278.1 asyd|OJJ56845.1 ater|EAU33459.1 atub|OJI82126.1 aver|OJJ03493.1 awen|OJJ33869.1 azon|OJJ48685.1

Group5286: abra|OJJ72734.1 acar|OOF95964.1 acla|EAW11569.1 afis|EAW23884.1 afla|EED52613.1 afum|EAL87312.1 agla|OJJ82571.1 akaw|GAA85340.1 aluc|OJZ90702.1 anid|CBF77185.1 anig|EHA24128.1 aory|EIT74192.1 asyd|OJJ56842.1 ater|EAU33462.1 atub|OJI82129.1 aver|OJJ03496.1 awen|OJJ33866.1

Group5287: abra|OJJ72740.1 acar|OOF95968.1 acla|EAW11575.1 afis|EAW23879.1 afla|EED52609.1 afum|EAL87307.1 agla|OJJ82569.1 akaw|GAA85350.1 aluc|OJZ90712.1 anig|EHA24132.1 aory|EIT74197.1 asyd|OJJ56837.1 ater|EAU33466.1 atub|OJI82139.1 aver|OJJ03500.1 awen|OJJ33860.1 azon|OJJ48692.1

Group5288: abra|OJJ72752.1 acar|OOF95975.1 acla|EAW11511.1 afis|EAW23942.1 afla|EED50574.1 afum|EAL87367.1 agla|OJJ79430.1 akaw|GAA85364.1 aluc|OJZ90726.1 anid|CBF77258.1 anig|EHA24144.1 asyd|OJJ56805.1 ater|EAU32291.1 atub|OJI82154.1 aver|OJJ03529.1 awen|OJJ39013.1 azon|OJJ51338.1

Group5289: abra|OJJ72758.1 acar|OOF95979.1 acla|EAW11506.1 afis|EAW23946.1 afum|EAL87372.1 agla|OJJ79439.1 akaw|GAA85368.1 aluc|OJZ90732.1 anid|CBF77288.1 anig|EHA24148.1 aory|EIT82036.1 asyd|OJJ56789.1 ater|EAU32288.1 atub|OJI82158.1 aver|OJJ03547.1 awen|OJJ39005.1 azon|OJJ51334.1

Group5290: abra|OJJ72759.1 acla|EAW11505.1 afis|EAW23947.1 afla|EED50569.1 afum|EAL87373.1 agla|OJJ79440.1 akaw|GAA85369.1 aluc|OJZ90733.1 anid|CBF77286.1 anig|EHA24149.1 aory|EIT82150.1 asyd|OJJ56790.1 ater|EAU32287.1 atub|OJI82159.1 aver|OJJ03546.1 awen|OJJ39004.1 azon|OJJ51333.1

Group5291: abra|OJJ72760.1 acla|EAW11504.1 afis|EAW23948.1 afla|EED50568.1 afum|EAL87374.1 agla|OJJ79441.1 akaw|GAA85370.1 aluc|OJZ90734.1 anid|CBF77285.1 anig|EHA24150.1 aory|EIT82140.1 asyd|OJJ56791.1 ater|EAU29804.1 atub|OJI82161.1 aver|OJJ03543.1 awen|OJJ39002.1 azon|OJJ51332.1

Group5292: abra|OJJ72771.1 acar|OOF95990.1 acla|EAW11494.1 afis|EAW23958.1 afla|EED50554.1 afum|EAL87386.1 agla|OJJ86881.1 akaw|GAA85380.1 aluc|OJZ90748.1 anid|CBF77310.1 aory|EIT82130.1 asyd|OJJ56775.1 ater|EAU29794.1 atub|OJI82174.1 aver|OJJ03558.1 awen|OJJ38989.1 azon|OJJ51321.1

Group5293: abra|OJJ72777.1 acar|OOF95995.1 acla|EAW11489.1 afis|EAW23964.1 afla|EED50546.1 afum|EAL87394.1 agla|OJJ86893.1 akaw|GAA85386.1 aluc|OJZ90755.1 anid|CBF77318.1 aory|EIT82079.1 asyd|OJJ56771.1 ater|EAU29788.1 atub|OJI82179.1 aver|OJJ03562.1 awen|OJJ38981.1 azon|OJJ51315.1

Group5294: abra|OJJ72787.1 acar|OOF96004.1 acla|EAW11482.1 afis|EAW23971.1 afla|EED50537.1 afum|EAL87401.1 agla|OJJ86900.1 akaw|GAA85394.1 aluc|OJZ83285.1 anid|CBF77333.1 anig|EHA24171.1 asyd|OJJ53277.1 ater|EAU29781.1 atub|OJI82188.1 aver|OJJ03571.1 awen|OJJ38972.1 azon|OJJ51307.1

Group5295: abra|OJJ72790.1 acar|OOF90124.1 acla|EAW11481.1 afis|EAW23972.1 afla|EED50536.1 afum|EAL87402.1 aluc|OJZ83287.1 anid|CBF77337.1 anig|EHA24172.1 aory|EIT82163.1 ater|EAU29780.1 atub|OJI82189.1 aver|OJJ03573.1 awen|OJJ38971.1 azon|OJJ51306.1 agla|OJJ86901.1 asyd|OJJ53276.1

Group5296: abra|OJJ72793.1 acla|EAW11479.1 afis|EAW23974.1 afla|EED50534.1 afum|EAL87404.1 agla|OJJ86903.1 akaw|GAA85396.1 aluc|OJZ83289.1 anid|CBF77341.1 anig|EHA24174.1 aory|EIT82105.1 asyd|OJJ53274.1 ater|EAU29778.1 atub|OJI82191.1 aver|OJJ03575.1 awen|OJJ35439.1 azon|OJJ51304.1

Group5297: abra|OJJ72799.1 acar|OOF96007.1 acla|EAW11445.1 afis|EAW23980.1 afla|EED49179.1 afum|EAL87410.1 agla|OJJ86912.1 akaw|GAA85402.1 aluc|OJZ83297.1 anid|CBF77354.1 anig|EHA24180.1 aory|EIT78569.1 asyd|OJJ53269.1 atub|OJI82197.1 awen|OJJ35448.1 azon|OJJ51298.1 aver|OJJ03582.1

Group5298: abra|OJJ72802.1 acar|OOF96010.1 acla|EAW11442.1 afis|EAW23983.1 afla|EED49176.1 afum|EBA27313.1 agla|OJJ86917.1 akaw|GAA85405.1 aluc|OJZ83300.1 anid|CBF77360.1 aory|EIT78516.1 asyd|OJJ53266.1 ater|EAU29768.1 atub|OJI82200.1 aver|OJJ03585.1 awen|OJJ35451.1 azon|OJJ51292.1

Group5299: abra|OJJ72806.1 afla|EED49171.1 akaw|GAA85409.1 aluc|OJZ83304.1 anig|EHA24187.1 aory|EIT78556.1 ater|EAU29763.1 atub|OJI82204.1 awen|OJJ35455.1 acla|EAW11435.1 afis|EAW23988.1 afum|EAL87417.2 agla|OJJ86921.1 anid|CBF77366.1 asyd|OJJ53263.1 aver|OJJ03588.1 azon|OJJ51288.1

Group5300: abra|OJJ72816.1 acar|OOF96022.1 acla|EAW11424.1 afis|EAW23997.1 agla|OJJ86931.1 akaw|GAA85417.1 aluc|OJZ83318.1 anid|CBF77381.1 anig|EHA24196.1 aory|EIT78578.1 asyd|OJJ53252.1 ater|EAU29752.1 atub|OJI82214.1 aver|OJJ03600.1 awen|OJJ35468.1 azon|OJJ51276.1 afum|EAL87427.1

Group5301: abra|OJJ72845.1 acar|OOF96046.1 acla|EAW11398.1 afis|EAW24023.1 afla|EED49131.1 afum|EAL87454.1 agla|OJJ86956.1 akaw|GAA85446.1 aluc|OJZ83353.1 anid|CBF75302.1 anig|EHA24219.1 asyd|OJJ59880.1 ater|EAU29723.1 atub|OJI82245.1 aver|OJJ02406.1 awen|OJJ35496.1 azon|OJJ46993.1

Group5302: abra|OJJ72846.1 acar|OOF96047.1 acla|EAW11397.1 afis|EAW24024.1 afla|EED49130.1 afum|EAL87455.1 agla|OJJ86957.1 akaw|GAA85447.1 aluc|OJZ83354.1 anid|CBF75304.1 anig|EHA24220.1 asyd|OJJ59879.1 ater|EAU29722.1 atub|OJI82246.1 aver|OJJ02407.1 awen|OJJ35497.1 azon|OJJ46992.1

Group5303: abra|OJJ72849.1 acar|OOF96050.1 acla|EAW11394.1 afis|EAW24027.1 afla|EED49127.1 afum|EAL87458.1 agla|OJJ86960.1 akaw|GAA85450.1 aluc|OJZ83358.1 anid|CBF75308.1 anig|EHA24223.1 asyd|OJJ59875.1 ater|EAU29717.1 atub|OJI82249.1 aver|OJJ02411.1 awen|OJJ35500.1 azon|OJJ46989.1

Group5304: abra|OJJ72851.1 acla|EAW11392.1 afis|EAW24029.1 afla|EED49125.1 afum|EAL87460.1 agla|OJJ86963.1 akaw|GAA85452.1 aluc|OJZ83360.1 anid|CBF75312.1 anig|EHA24225.1 aory|EIT78520.1 asyd|OJJ59873.1 ater|EAU29715.1 atub|OJI82251.1 aver|OJJ02414.1 awen|OJJ35502.1 azon|OJJ46987.1

Group5305: abra|OJJ72864.1 acar|OOF96063.1 acla|EAW11381.1 afis|EAW18115.1 afla|EED58040.1 afum|EAL87472.1 agla|OJJ86981.1 akaw|GAA85463.1 aluc|OJZ83377.1 anid|CBF75338.1 anig|EHA24236.1 asyd|OJJ59860.1 ater|EAU29702.1 atub|OJI82263.1 aver|OJJ02427.1 awen|OJJ35514.1 azon|OJJ46969.1

Group5306: abra|OJJ72867.1 acar|OOF96066.1 acla|EAW11378.1 afis|EAW18117.1 afla|EED58042.1 afum|EAL87475.1 agla|OJJ86979.1 akaw|GAA85466.1 aluc|OJZ83380.1 anid|CBF75342.1 anig|EHA24239.1 asyd|OJJ59858.1 ater|EAU29700.1 atub|OJI82266.1 aver|OJJ02429.1 awen|OJJ35516.1 azon|OJJ46968.1

Group5307: abra|OJJ72868.1 acar|OOF96067.1 acla|EAW11377.1 afis|EAW18118.1 afla|EED58043.1 afum|EAL87476.1 agla|OJJ86978.1 akaw|GAA85467.1 aluc|OJZ83381.1 anid|CBF75344.1 anig|EHA24240.1 aory|EIT79729.1 asyd|OJJ59857.1 ater|EAU29699.1 atub|OJI82267.1 aver|OJJ02430.1 awen|OJJ35517.1

Group5308: abra|OJJ72897.1 acla|EAW11354.1 afis|EAW18140.1 afla|EED58066.1 afum|EAL87497.1 agla|OJJ83825.1 akaw|GAA85484.1 aluc|OJZ83406.1 anid|CBF75390.1 anig|EHA24257.1 aory|EIT79738.1 asyd|OJJ59835.1 ater|EAU35463.1 atub|OJI82295.1 aver|OJJ02453.1 awen|OJJ35582.1 azon|OJJ46940.1

Group5309: abra|OJJ72908.1 acar|OOF96091.1 acla|EAW11345.1 afis|EAW18149.1 afla|EED58077.1 afum|EAL87507.1 agla|OJJ83834.1 akaw|GAA85493.1 aluc|OJZ83418.1 anid|CBF81112.1 anig|EHA24267.1 asyd|OJJ56484.1 ater|EAU35471.1 atub|OJI82305.1 aver|OJJ06235.1 awen|OJJ35592.1 azon|OJJ46914.1

Group5310: abra|OJJ72925.1 acar|OOF96106.1 acla|EAW06464.1 afis|EAW15959.1 afla|EED58099.1 afum|EAL88592.1 agla|OJJ89353.1 akaw|GAA85508.1 aluc|OJZ83435.1 aory|EIT79128.1 ater|EAU35497.1 atub|OJI82326.1 aver|OJJ06259.1 awen|OJJ35614.1 azon|OJJ46909.1 anid|CBF81076.1 asyd|OJJ56462.1

Group5311: abra|OJJ72926.1 acar|OOF96107.1 acla|EAW06463.1 afis|EAW15958.1 afla|EED58100.1 afum|EAL88591.1 agla|OJJ89354.1 akaw|GAA85509.1 aluc|OJZ83436.1 anid|CBF81074.1 anig|EHA24284.1 aory|EIT79118.1 asyd|OJJ56461.1 atub|OJI82327.1 aver|OJJ06260.1 awen|OJJ35615.1 azon|OJJ46908.1

Group5312: abra|OJJ72943.1 acla|EAW11208.1 afis|EAW15941.1 afla|EED58117.1 afum|EAL88575.1 agla|OJJ89369.1 akaw|GAA85525.1 aluc|OJZ83453.1 anig|EHA24299.1 aory|EIT79113.1 asyd|OJJ56347.1 ater|EAU35515.1 atub|OJI82345.1 aver|OJJ06366.1 azon|OJJ46894.1 anid|CBF80960.1 awen|OJJ35641.1

Group5313: abra|OJJ72944.1 acar|OOF96125.1 acla|EAW11207.1 afis|EAW15937.1 afla|EED58118.1 afum|EAL88573.1 agla|OJJ89370.1 akaw|GAA85526.1 aluc|OJZ83454.1 anid|CBF80962.1 anig|EHA24300.1 aory|EIT79136.1 asyd|OJJ56348.1 atub|OJI82346.1 aver|OJJ06365.1 awen|OJJ35642.1 azon|OJJ46893.1

Group5314: abra|OJJ72951.1 acar|OOF96133.1 acla|EAW11197.1 afis|EAW15927.1 afla|EED54934.1 afum|EAL88563.1 agla|OJJ89378.1 akaw|GAA85532.1 aluc|OJZ83461.1 anid|CBF81022.1 aory|EIT80375.1 asyd|OJJ56384.1 ater|EAU35528.1 atub|OJI82353.1 aver|OJJ06328.1 awen|OJJ35653.1 azon|OJJ46885.1

Group5315: abra|OJJ72955.1 acar|OOF96136.1 acla|EAW11200.1 afis|EAW15930.1 afla|EED54931.1 afum|EAL88566.1 agla|OJJ89376.1 akaw|GAA85536.1 aluc|OJZ83464.1 anid|CBF81028.1 anig|EHA24308.1 asyd|OJJ56387.1 ater|EAU35522.1 atub|OJI82357.1 aver|OJJ06326.1 awen|OJJ35650.1 azon|OJJ46888.1

Group5316: abra|OJJ72964.1 acar|OOF96146.1 acla|EAW11190.1 afis|EAW15921.1 afla|EED54940.1 afum|EAL88557.2 agla|OJJ89389.1 akaw|GAA85542.1 aluc|OJZ83473.1 anid|CBF81005.1 aory|EIT80301.1 asyd|OJJ56378.1 ater|EAU35535.1 atub|OJI82363.1 aver|OJJ06335.1 awen|OJJ35660.1 azon|OJJ46880.1

Group5317: abra|OJJ72995.1 acar|OOF96172.1 acla|EAW08017.1 afis|EAW23051.1 afum|EAL88429.1 agla|OJJ89428.1 akaw|GAA85569.1 aluc|OJZ83503.1 anid|CBF89694.1 anig|EHA24337.1 aory|EIT80308.1 asyd|OJJ56447.1 ater|EAU29597.1 atub|OJI82397.1 aver|OJJ06275.1 awen|OJJ35687.1 azon|OJJ50729.1

Group5318: abra|OJJ73005.1 acla|EAW08008.1 afis|EAW23043.1 afla|EED54975.1 afum|EAL88438.1 agla|OJJ89435.1 akaw|GAA85583.1 aluc|OJZ83520.1 anid|CBF80940.1 anig|EHA24343.1 aory|EIT80357.1 asyd|OJJ56336.1 ater|EAU29604.1 atub|OJI82409.1 aver|OJJ06377.1 awen|OJJ35697.1 azon|OJJ44161.1

Group5319: abra|OJJ73007.1 acar|OOF96179.1 acla|EAW08006.1 afis|EAW23041.1 afla|EED54977.1 afum|EAL88440.1 agla|OJJ89437.1 akaw|GAA85585.1 aluc|OJZ83522.1 anid|CBF80937.1 anig|EHA24345.1 aory|EIT80307.1 asyd|OJJ56313.1 atub|OJI82411.1 aver|OJJ06401.1 awen|OJJ35701.1 azon|OJJ44163.1

Group5320: abra|OJJ73011.1 acar|OOF96184.1 acla|EAW08001.1 afis|EAW23036.1 afla|EED54981.1 afum|EAL88445.1 agla|OJJ89441.1 akaw|GAA85590.1 aluc|OJZ83526.1 anid|CBF80927.1 anig|EHA24349.1 asyd|OJJ56317.1 ater|EAU29622.1 atub|OJI82415.1 aver|OJJ06397.1 awen|OJJ35705.1 azon|OJJ44167.1

Group5321: abra|OJJ73015.1 acla|EAW07998.1 afis|EAW23033.1 afla|EED54983.1 afum|EAL88448.1 akaw|GAA85593.1 aluc|OJZ83530.1 anid|CBF80922.1 anig|EHA24352.1 aory|EIT80306.1 asyd|OJJ56321.1 ater|EAU29624.1 atub|OJI82420.1 awen|OJJ35712.1 azon|OJJ44170.1 aver|OJJ06392.1 agla|OJJ89444.1

Group5322: abra|OJJ73017.1 acar|OOF96187.1 acla|EAW07996.1 afis|EAW23031.1 afum|EAL88451.2 agla|OJJ89446.1 akaw|GAA85595.1 aluc|OJZ83532.1 anid|CBF80912.1 anig|EHA24354.1 asyd|OJJ56327.1 ater|EAU29626.1 atub|OJI82422.1 awen|OJJ35714.1 afla|EED54985.1 aory|EIT80319.1 aver|OJJ06386.1

Group5323: abra|OJJ73026.1 acar|OOF96193.1 acla|EAW07990.1 afis|EAW23025.1 afla|EED54990.1 afum|EAL88457.1 agla|OJJ89454.1 akaw|GAA85600.1 aluc|OJZ83538.1 anid|CBF80909.1 anig|EHA24359.1 aory|EIT80325.1 asyd|OJJ56330.1 ater|EAU29650.1 atub|OJI82429.1 awen|OJJ30718.1 azon|OJJ44179.1

Group5324: abra|OJJ73047.1 acla|EAW07972.1 afis|EAW23007.1 afla|EED48101.1 afum|EAL88473.1 agla|OJJ80223.1 akaw|GAA85618.1 aluc|OJZ83558.1 anid|CBF80877.1 anig|EHA24376.1 aory|EIT76233.1 asyd|OJJ56301.1 ater|EAU29666.1 atub|OJI82448.1 aver|OJJ06416.1 awen|OJJ31316.1 azon|OJJ50668.1

Group5325: abra|OJJ73054.1 acar|OOF96216.1 acla|EAW07966.1 afis|EAW23001.1 afla|EED55014.1 afum|EAL88479.2 agla|OJJ89478.1 aluc|OJZ83565.1 anid|CBF80866.1 anig|EHA24382.1 aory|EIT80356.1 asyd|OJJ56295.1 ater|EAU29672.1 atub|OJI82455.1 aver|OJJ06422.1 awen|OJJ30740.1 azon|OJJ44206.1

Group5326: abra|OJJ73057.1 acar|OOF96219.1 acla|EAW07964.1 afis|EAW22999.1 afla|EED55016.1 afum|EAL88481.1 agla|OJJ89480.1 akaw|GAA85625.1 aluc|OJZ83568.1 anid|CBF80862.1 anig|EHA24384.1 aory|EIT80386.1 asyd|OJJ56293.1 atub|OJI82457.1 aver|OJJ06424.1 awen|OJJ30742.1 azon|OJJ44203.1

Group5327: abra|OJJ73083.1 acar|OOG00317.1 acla|EAW12281.1 afis|EAW19608.1 afla|EED57664.1 afum|EAL89356.1 akaw|GAA88077.1 aluc|OJZ81340.1 anid|CBF88728.1 anig|EHA18646.1 aory|EIT79918.1 asyd|OJJ63933.1 ater|EAU35193.1 atub|OJI91545.1 aver|OJI97009.1 awen|OJJ31943.1 azon|OJJ50754.1

Group5328: abra|OJJ73108.1 acar|OOG00341.1 acla|EAW07041.1 afis|EAW16668.1 afla|EED44908.1 afum|EAL89272.1 agla|OJJ78701.1 akaw|GAA88058.1 aluc|OJZ81320.1 anid|CBF82081.1 anig|EHA18626.1 aory|EIT73518.1 asyd|OJJ54255.1 atub|OJI91523.1 aver|OJJ05208.1 awen|OJJ40045.1 azon|OJJ50124.1

Group5329: abra|OJJ73144.1 acar|OOG00356.1 acla|EAW07020.1 afis|EAW16646.1 afla|EED44954.1 afum|EAL89250.1 agla|OJJ82597.1 akaw|GAA88028.1 aluc|OJZ81287.1 anid|CBF82056.1 anig|EHA18586.1 asyd|OJJ54237.1 ater|EAU39571.1 atub|OJI91494.1 aver|OJJ05189.1 awen|OJJ40068.1 azon|OJJ45556.1

Group5330: abra|OJJ73150.1 acar|OOG00362.1 acla|EAW07027.1 afis|EAW16653.1 afla|EED44947.1 afum|EAL89257.1 agla|OJJ78686.1 akaw|GAA88022.1 aluc|OJZ81280.1 anig|EHA18580.1 aory|EIT73514.1 asyd|OJJ54243.1 ater|EAU39577.1 atub|OJI91488.1 aver|OJJ05195.1 awen|OJJ40061.1 azon|OJJ45566.1

Group5331: abra|OJJ73157.1 acar|OOG00370.1 acla|EAW07031.1 afis|EAW16657.1 afla|EED44942.1 afum|EAL89261.1 agla|OJJ78715.1 akaw|GAA88015.1 aluc|OJZ81273.1 anig|EHA18573.1 aory|EIT73488.1 asyd|OJJ54247.1 ater|EAU39580.1 atub|OJI91480.1 aver|OJJ05201.1 awen|OJJ40057.1 azon|OJJ50085.1

Group5332: abra|OJJ73169.1 acla|EAW10795.1 afis|EAW24781.1 afla|EED47204.1 afum|EAL89420.1 agla|OJJ82877.1 akaw|GAA88002.1 aluc|OJZ81258.1 anid|CBF81440.1 anig|EHA18565.1 aory|EIT79259.1 asyd|OJJ59429.1 atub|OJI91454.1 aver|OJJ02842.1 awen|OJJ40367.1 azon|OJJ49216.1 ater|EAU36527.1

Group5333: abra|OJJ73174.1 acar|OOG00414.1 acla|EAW06857.1 afis|EAW16474.1 afla|EED47209.1 afum|EAL89068.1 agla|OJJ82872.1 akaw|GAA87997.1 aluc|OJZ81253.1 anid|CBF81433.1 anig|EHA18561.1 asyd|OJJ59423.1 ater|EAU36568.1 atub|OJI91449.1 aver|OJJ02847.1 awen|OJJ40362.1 azon|OJJ49210.1

Group5334: abra|OJJ73182.1 acar|OOG00419.1 acla|EAW06860.1 afis|EAW16477.1 afla|EED47214.1 afum|EAL89071.1 agla|OJJ82868.1 akaw|GAA87993.1 aluc|OJZ81247.1 anid|CBF75497.1 anig|EHA18556.1 aory|EIT79205.1 asyd|OJJ59749.1 atub|OJI91443.1 aver|OJJ02539.1 awen|OJJ40358.1 azon|OJJ49207.1

Group5335: abra|OJJ73188.1 acar|OOG00423.1 acla|EAW06864.1 afis|EAW16481.1 afla|EED47218.1 afum|EAL89075.1 agla|OJJ82864.1 akaw|GAA87989.1 aluc|OJZ81243.1 anig|EHA18552.1 aory|EIT79179.1 asyd|OJJ59745.1 ater|EAU36557.1 atub|OJI91439.1 aver|OJJ02543.1 awen|OJJ40353.1 azon|OJJ49203.1

Group5336: abra|OJJ73192.1 acar|OOG00427.1 acla|EAW06868.1 afis|EAW16485.1 afla|EED47225.1 afum|EAL89079.1 agla|OJJ82859.1 akaw|GAA87985.1 aluc|OJZ81237.1 anid|CBF75518.1 anig|EHA18548.1 asyd|OJJ59737.1 ater|EAU36553.1 atub|OJI91435.1 aver|OJJ02550.1 awen|OJJ40344.1 azon|OJJ49199.1

Group5337: abra|OJJ73197.1 acar|OOG00436.1 acla|EAW06874.1 afis|EAW16491.1 afla|EED47232.1 afum|EAL89085.1 agla|OJJ82830.1 akaw|GAA87980.1 aluc|OJZ81232.1 anid|CBF75524.1 anig|EHA18543.1 asyd|OJJ59734.1 ater|EAU36546.1 atub|OJI91429.1 aver|OJJ02553.1 awen|OJJ40338.1 azon|OJJ49192.1

Group5338: abra|OJJ73198.1 acar|OOG00437.1 acla|EAW06875.1 afis|EAW16492.1 afla|EED47233.1 afum|EAL89086.1 agla|OJJ82831.1 akaw|GAA87979.1 aluc|OJZ81231.1 anid|CBF75525.1 anig|EHA18542.1 aory|EIT79252.1 asyd|OJJ59733.1 atub|OJI91428.1 aver|OJJ02554.1 awen|OJJ40337.1 azon|OJJ49191.1

Group5339: abra|OJJ73201.1 acar|OOG00440.1 acla|EAW06878.1 afis|EAW16495.1 afum|EAL89089.1
agla|OJJ82834.1 akaw|GAA87976.1 aluc|OJZ81228.1 anid|CBF75608.1 anig|EHA18539.1 aory|EIT79203.1
asyd|OJJ59728.1 ater|EAU36544.1 atub|OJI91425.1 aver|OJJ02558.1 awen|OJJ40334.1 azon|OJJ49188.1

Group5340: abra|OJJ73214.1 acar|OOG00451.1 acla|EAW06889.1 afis|EAW16506.1 afla|EED56172.1
afum|EAL89101.1 agla|OJJ82819.1 akaw|GAA87965.1 aluc|OJZ81214.1 anid|CBF75596.1 anig|EHA18528.1
aory|EIT76809.1 asyd|OJJ59717.1 atub|OJI91409.1 aver|OJJ02569.1 awen|OJJ40319.1 azon|OJJ49177.1

Group5341: abra|OJJ73221.1 acar|OOG00459.1 acla|EAW06896.1 afis|EAW16513.1 afla|EED56160.1
afum|EAL89108.1 agla|OJJ82809.1 akaw|GAA87957.1 aluc|OJZ81206.1 anid|CBF75564.1 anig|EHA18521.1
asyd|OJJ59707.1 ater|EAU36579.1 atub|OJI91402.1 aver|OJJ02578.1 awen|OJJ40311.1 azon|OJJ49171.1

Group5342: abra|OJJ73243.1 acar|OOG00482.1 acla|EAW06917.1 afis|EAW16534.1 afla|EED56135.1
afum|EAL89131.1 akaw|GAA87937.1 aluc|OJZ81185.1 anid|CBF81738.1 anig|EHA18502.1 aory|EIT76790.1
asyd|OJJ59595.1 ater|EAU36597.1 atub|OJI91381.1 aver|OJJ02673.1 awen|OJJ40288.1 azon|OJJ49149.1

Group5343: abra|OJJ73267.1 acar|OOG00507.1 acla|EAW06944.1 afis|EAW16560.1 afla|EED56103.1
afum|EAL89158.1 agla|OJJ82764.1 akaw|GAA87915.1 aluc|OJZ81162.1 anid|CBF81804.1 anig|EHA18479.1
aory|EIT76866.1 asyd|OJJ59643.1 ater|EAU36626.1 atub|OJI91357.1 aver|OJJ02639.1 awen|OJJ40258.1

Group5344: abra|OJJ73272.1 acar|OOG00512.1 acla|EAW06949.1 afis|EAW16564.1 afla|EED56099.1
afum|EAL89161.1 agla|OJJ82757.1 akaw|GAA87908.1 aluc|OJZ88777.1 anid|CBF81811.1 anig|EHA18471.1
aory|EIT76849.1 asyd|OJJ59647.1 atub|OJI91349.1 aver|OJJ02635.1 awen|OJJ40254.1 azon|OJJ49116.1

Group5345: abra|OJJ73282.1 acar|OOG00518.1 acla|EAW06957.1 afis|EAW16572.1 afla|EED56092.1
afum|EAL89169.2 agla|OJJ82704.1 akaw|GAA92723.1 aluc|OJZ88767.1 anig|EHA18463.1 aory|EIT76859.1
asyd|OJJ59658.1 ater|EAU36640.1 atub|OJI91340.1 aver|OJJ02624.1 awen|OJJ40246.1 azon|OJJ49107.1

Group5346: abra|OJJ73285.1 acar|OOG00521.1 acla|EAW06960.1 afis|EAW16575.1 afla|EED56089.1
afum|EBA27368.1 agla|OJJ82702.1 akaw|GAA92720.1 aluc|OJZ88764.1 anig|EHA18460.1 aory|EIT76647.1
asyd|OJJ59661.1 atub|OJI91337.1 aver|OJJ02621.1 awen|OJJ40243.1 azon|OJJ49104.1 anid|CBF81831.1

Group5347: abra|OJJ73298.1 afis|EAW19827.1 afla|EED51781.1 afum|EAL85065.1 akaw|GAA92708.1
aluc|OJZ88750.1 anig|EHA18452.1 asyd|OJJ59177.1 atub|OJI91321.1 aver|OJJ01796.1 awen|OJJ34692.1
azon|OJJ49039.1 agla|OJJ79503.1 acar|OOF93317.1 asyd|OJJ62364.1 aver|OJI96498.1 awen|OJJ30127.1

Group5348: abra|OJJ73323.1 acla|EAW15335.1 afis|EAW18094.1 afla|EED54259.1 afum|EAL91902.1
agla|OJJ78550.1 akaw|GAA86996.1 aluc|OJZ84463.1 anid|CBF75962.1 anig|EHA18708.1 aory|EIT76021.1
ater|EAU36995.1 atub|OJI89614.1 aver|OJJ00568.1 awen|OJJ32938.1 azon|OJJ42340.1 anid|CBF75964.1

Group5349: abra|OJJ73354.1 acla|EAW15249.1 afis|EAW20217.1 afla|EED44916.1 afum|EAL85332.1
agla|OJJ84639.1 akaw|GAA83130.1 aluc|OJZ88686.1 anid|CBF78511.1 anig|EHA18366.1 aory|EIT73493.1
asyd|OJJ65310.1 atub|OJI91266.1 aver|OJI99450.1 awen|OJJ31229.1 afla|EED57128.1 aory|EIT74120.1

Group5350: acla|EAW13469.1 agla|OJJ88694.1 abra|OJJ76945.1 acar|OOF91395.1 afla|EED52278.1
afum|EAL86634.1 akaw|GAA87095.1 aluc|OJZ84343.1 anid|CBF71790.1 anig|EHA27679.1 aory|EIT74372.1
asyd|OJJ61714.1 ater|EAU37102.1 atub|OJI89725.1 aver|OJI95809.1 awen|OJJ33286.1 azon|OJJ50664.1

Group5351: abra|OJJ73356.1 afla|EED45197.1 agla|OJJ82670.1 akaw|GAA83128.1 aluc|OJZ88684.1
anid|CBF81892.1 aory|EIT73648.1 asyd|OJJ59679.1 ater|EAU36665.1 atub|OJI91264.1 aver|OJJ02609.1
awen|OJJ40226.1 azon|OJJ49091.1 acla|EAW06975.1 afis|EAW16589.1 afum|EAL89184.1 anig|EHA18364.1

Group5352: abra|OJJ73363.1 acar|OOG00570.1 acla|EAW06983.1 afis|EAW16597.1 afla|EED45016.1
afum|EAL89193.1 agla|OJJ82657.1 akaw|GAA83121.1 aluc|OJZ88677.1 anid|CBF81904.1 aory|EIT73770.1
asyd|OJJ59687.1 ater|EAU36676.1 atub|OJI91258.1 aver|OJJ02600.1 awen|OJJ40211.1 azon|OJJ49077.1

Group5353: abra|OJJ73373.1 acar|OOG00578.1 acla|EAW06991.1 afis|EAW16605.1 afla|EED45024.1
afum|EAL89202.2 agla|OJJ82648.1 akaw|GAA83113.1 aluc|OJZ88668.1 anid|CBF81912.1 aory|EIT73735.1
asyd|OJJ59693.1 ater|EAU36687.1 atub|OJI91250.1 aver|OJJ02594.1 awen|OJJ40201.1 azon|OJJ49066.1

Group5354: abra|OJJ73377.1 acar|OOG00580.1 acla|EAW06990.1 afis|EAW16604.1 afla|EED45023.1
afum|EAL89200.1 akaw|GAA83112.1 aluc|OJZ88666.1 anid|CBF81911.1 anig|EHA18351.1 aory|EIT73764.1
asyd|OJJ59692.1 ater|EAU36686.1 atub|OJI91249.1 aver|OJJ02595.1 awen|OJJ40202.1 azon|OJJ49070.1

Group5355: abra|OJJ73379.1 acla|EAW06993.1 afis|EAW16608.1 afla|EED45026.1 afum|EAL89204.1
agla|OJJ82645.1 akaw|GAA83110.1 aluc|OJZ88664.1 anid|CBF81966.1 anig|EHA18349.1 aory|EIT73734.1
asyd|OJJ57354.1 ater|EAU36692.1 atub|OJI91246.1 aver|OJJ05811.1 awen|OJJ40199.1 azon|OJJ49068.1

Group5356: abra|OJJ73380.1 acar|OOF90416.1 acla|EAW06996.1 afis|EAW16610.1 afla|EED45002.1
afum|EAL89206.1 agla|OJJ82636.1 akaw|GAA83109.1 aluc|OJZ88663.1 anid|CBF81964.1 aory|EIT73646.1
asyd|OJJ57357.1 ater|EAU36695.1 atub|OJI91244.1 aver|OJJ05809.1 awen|OJJ40196.1 azon|OJJ49063.1

Group5357: abra|OJJ73386.1 acar|OOG00588.1 acla|EAW07010.1 afis|EAW16629.1 afla|EED44985.1 afum|EAL89224.2 agla|OJJ82614.1 akaw|GAA83103.1 aluc|OJZ88651.1 anid|CBF81970.1 anig|EHA18342.1 aory|EIT73790.1 asyd|OJJ57346.1 ater|EAU36722.1 atub|OJI91238.1 aver|OJJ05819.1 azon|OJJ44014.1

Group5358: abra|OJJ73387.1 acar|OOG00589.1 acla|EAW07001.1 afis|EAW16616.1 afla|EED44988.1 afum|EAL89220.1 agla|OJJ82631.1 akaw|GAA83102.1 aluc|OJZ88650.1 anid|CBF81968.1 anig|EHA18341.1 aory|EIT73725.1 asyd|OJJ57348.1 ater|EAU36704.1 atub|OJI91237.1 aver|OJJ05817.1 awen|OJJ40182.1

Group5359: abra|OJJ73404.1 acar|OOG00602.1 acla|EAW07011.1 afis|EAW16631.1 afla|EED44967.1 afum|EAL89227.1 agla|OJJ82618.1 akaw|GAA83086.1 aluc|OJZ88633.1 anid|CBF82012.1 anig|EHA18323.1 aory|EIT73524.1 asyd|OJJ56189.1 atub|OJI91217.1 aver|OJJ05117.1 awen|OJJ40078.1 azon|OJJ49493.1

Group5360: abra|OJJ73441.1 acar|OOG00647.1 acla|EAW06776.1 afis|EAW16327.1 afla|EED53554.1 afum|EAL88951.1 agla|OJJ88265.1 aluc|OJZ88599.1 anid|CBF89166.1 anig|EHA18293.1 aory|EIT74850.1 asyd|OJJ62544.1 ater|EAU34130.1 atub|OJI91184.1 aver|OJI96671.1 awen|OJJ37728.1 azon|OJJ44987.1

Group5361: abra|OJJ73444.1 acla|EAW06779.1 afis|EAW16330.1 afla|EED53548.1 afum|EAL88954.1 agla|OJJ88262.1 akaw|GAA83050.1 aluc|OJZ88596.1 anid|CBF89171.1 anig|EHA18290.1 aory|EIT75161.1 asyd|OJJ62541.1 ater|EAU34128.1 atub|OJI91181.1 aver|OJI96668.1 awen|OJJ37731.1 azon|OJJ44989.1

Group5362: abra|OJJ73457.1 acar|OOG00658.1 acla|EAW06784.1 afis|EAW16334.1 afla|EED53542.1 afum|EAL88959.1 agla|OJJ88258.1 akaw|GAA83041.1 aluc|OJZ88586.1 anid|CBF89190.1 aory|EIT75196.1 asyd|OJJ62535.1 ater|EAU34124.1 atub|OJI91172.1 aver|OJI96660.1 awen|OJJ37735.1 azon|OJJ44994.1

Group5363: abra|OJJ73459.1 acar|OOG00660.1 acla|EAW06786.1 afla|EED53540.1 afum|EAL88961.1 agla|OJJ88253.1 akaw|GAA83039.1 aluc|OJZ88584.1 anid|CBF89194.1 anig|EHA18279.1 aory|EIT74903.1 asyd|OJJ62533.1 ater|EAU34122.1 atub|OJI91170.1 aver|OJI96658.1 awen|OJJ37738.1 azon|OJJ44999.1

Group5364: abra|OJJ73461.1 acar|OOG00662.1 acla|EAW06788.1 afis|EAW16337.1 afla|EED53538.1 afum|EAL88963.1 agla|OJJ88255.1 akaw|GAA83037.1 aluc|OJZ88582.1 anid|CBF89197.1 aory|EIT75002.1 asyd|OJJ62531.1 ater|EAU34120.1 atub|OJI91168.1 aver|OJI96656.1 awen|OJJ37740.1 azon|OJJ45001.1

Group5365: abra|OJJ73499.1 acar|OOG00691.1 acla|EAW07433.1 afis|EAW22459.1 afla|EED54485.1 afum|EAL90733.1 agla|OJJ87564.1 akaw|GAA87448.1 aluc|OJZ88549.1 anid|CBF88916.1 anig|EHA18247.1 asyd|OJJ62843.1 ater|EAU39246.1 atub|OJI91134.1 aver|OJI96887.1 awen|OJJ35084.1 azon|OJJ50023.1

Group5366: abra|OJJ73501.1 acar|OOG00689.1 acla|EAW07434.1 afis|EAW22460.1 afla|EED54484.1 afum|EAL90731.1 agla|OJJ87562.1 akaw|GAA87449.1 aluc|OJZ88546.1 anid|CBF88914.1 anig|EHA18246.1 asyd|OJJ62842.1 ater|EAU39245.1 atub|OJI91131.1 aver|OJI96886.1 awen|OJJ35086.1 azon|OJJ50021.1

Group5367: abra|OJJ73504.1 acar|OOG00687.1 acla|EAW07437.1 afis|EAW22463.1 afla|EED54481.1 afum|EAL90728.1 agla|OJJ87560.1 aluc|OJZ88544.1 anid|CBF88909.1 anig|EHA18244.1 aory|EIT81141.1 asyd|OJJ62840.1 ater|EAU39243.1 atub|OJI91129.1 aver|OJI96883.1 awen|OJJ35089.1 azon|OJJ50019.1

Group5368: abra|OJJ73505.1 acla|EAW07438.1 afis|EAW22465.1 afla|EED54496.1 afum|EAL90726.1 agla|OJJ87559.1 akaw|GAA87452.1 aluc|OJZ88543.1 anid|CBF88907.1 anig|EHA18243.1 aory|EIT78760.1 asyd|OJJ62839.1 ater|EAU39242.1 atub|OJI91128.1 aver|OJI96882.1 awen|OJJ35090.1 azon|OJJ50018.1

Group5369: abra|OJJ73511.1 acla|EAW07444.1 afis|EAW22471.1 afla|EED54503.1 afum|EAL90720.1 agla|OJJ87553.1 akaw|GAA87459.1 aluc|OJZ88536.1 anid|CBF88898.1 anig|EHA18237.1 aory|EIT78805.1 asyd|OJJ62833.1 atub|OJI91121.1 aver|OJI96876.1 awen|OJJ35097.1 azon|OJJ50012.1 ater|EAU39237.1

Group5370: abra|OJJ73514.1 acar|OOG00702.1 acla|EAW07448.1 afis|EAW22475.1 afla|EED54507.1 afum|EAL90716.1 agla|OJJ87549.1 akaw|GAA87463.1 aluc|OJZ88533.1 anid|CBF88890.1 anig|EHA18234.1 asyd|OJJ62830.1 ater|EAU39234.1 atub|OJI91118.1 aver|OJI96873.1 awen|OJJ35100.1 azon|OJJ50008.1

Group5371: abra|OJJ73517.1 acar|OOG00707.1 acla|EAW07452.1 afis|EAW22484.1 afla|EED54510.1 afum|EAL90710.1 agla|OJJ87542.1 akaw|GAA87466.1 aluc|OJZ88530.1 anid|CBF88885.1 anig|EHA18231.1 asyd|OJJ62825.1 ater|EAU39233.1 atub|OJI91115.1 aver|OJI96870.1 awen|OJJ35103.1 azon|OJJ50004.1

Group5372: abra|OJJ73553.1 acar|OOG00725.1 acla|EAW07471.1 afis|EAW22504.1 afla|EED54531.1 afum|EAL90689.1 agla|OJJ80845.1 akaw|GAA88600.1 aluc|OJZ88508.1 anid|CBF88968.1 anig|EHA18215.1 asyd|OJJ62796.1 ater|EAU39214.1 atub|OJI91094.1 aver|OJI96843.1 awen|OJJ35128.1 azon|OJJ49972.1

Group5373: abra|OJJ73558.1 acar|OOG00729.1 acla|EAW07475.1 afis|EAW22507.1 afla|EED54535.1 afum|EAL90686.1 agla|OJJ87538.1 akaw|GAA88596.1 aluc|OJZ88504.1 anid|CBF88973.1 anig|EHA18211.1 asyd|OJJ62793.1 ater|EAU39206.1 atub|OJI91090.1 aver|OJI96840.1 awen|OJJ35132.1 azon|OJJ49966.1

Group5374: abra|OJJ73569.1 acar|OOG00751.1 acla|EAW07486.1 afis|EAW22519.1 afum|EAL90674.1 agla|OJJ80879.1 akaw|GAA88586.1 aluc|OJZ88491.1 anid|CBF89002.1 anig|EHA18200.1 aory|EIT78853.1 asyd|OJJ62780.1 ater|EAU39190.1 atub|OJI91078.1 aver|OJI96825.1 awen|OJJ35146.1 azon|OJJ49954.1

Group5375: abra|OJJ73577.1 acar|OOG00758.1 acla|EAW07495.1 afis|EAW22528.1 afla|EED54556.1 afum|EAL90666.2 agla|OJJ80888.1 aluc|OJZ88475.1 anid|CBF89012.1 anig|EHA18192.1 aory|EIT78898.1 asyd|OJJ62773.1 ater|EAU39183.1 atub|OJI91070.1 aver|OJI96818.1 awen|OJJ35155.1 azon|OJJ49946.1

Group5376: abra|OJJ73579.1 acar|OOG00760.1 acla|EAW07497.1 afis|EAW22530.1 afla|EED54558.1 afum|EAL90664.1 agla|OJJ80891.1 akaw|GAA88572.1 aluc|OJZ88473.1 anid|CBF89016.1 anig|EHA18190.1 aory|EIT81483.1 asyd|OJJ62771.1 ater|EAU39179.1 atub|OJI91068.1 aver|OJI96816.1 awen|OJJ35157.1

Group5377: abra|OJJ73584.1 acar|OOG00765.1 acla|EAW07502.1 afis|EAW22535.1 afla|EED54563.1 afum|EAL90659.1 agla|OJJ80896.1 akaw|GAA88567.1 aluc|OJZ88468.1 anid|CBF89027.1 anig|EHA18185.1 aory|EIT78861.1 asyd|OJJ62766.1 ater|EAU39174.1 atub|OJI91063.1 aver|OJI96811.1 awen|OJJ35162.1

Group5378: abra|OJJ73595.1 acar|OOG00780.1 acla|EAW07514.1 afis|EAW22545.1 afla|EED54576.1 afum|EAL90650.1 agla|OJJ80907.1 akaw|GAA88557.1 aluc|OJZ88455.1 anid|CBF89045.1 anig|EHA18176.1 asyd|OJJ62757.1 ater|EAU39163.1 atub|OJI91053.1 aver|OJI96799.1 awen|OJJ35174.1 azon|OJJ49933.1

Group5379: abra|OJJ73600.1 acla|EAW07518.1 afis|EAW22548.1 afla|EED54580.1 afum|EAL90647.1 agla|OJJ80910.1 akaw|GAA88552.1 aluc|OJZ88450.1 anid|CBF89053.1 anig|EHA18171.1 aory|EIT78826.1 asyd|OJJ62753.1 ater|EAU39157.1 atub|OJI91048.1 aver|OJI96795.1 awen|OJJ35179.1 azon|OJJ49929.1

Group5380: abra|OJJ73613.1 acar|OOG00796.1 afla|EED54591.1 agla|OJJ80920.1 akaw|GAA88540.1 aluc|OJZ88436.1 anid|CBF88375.1 anig|EHA18160.1 aory|EIT78809.1 atub|OJI91033.1 aver|OJI97207.1 azon|OJJ49916.1 afis|EAW22560.1 afum|EAL90635.1 awen|OJJ35190.1 acla|EAW07530.1 ater|EAU39144.1

Group5381: abra|OJJ73620.1 acar|OOG00803.1 acla|EAW07535.1 afis|EAW22569.1 afla|EED54599.1 afum|EAL90629.1 agla|OJJ80925.1 akaw|GAA88533.1 aluc|OJZ88429.1 anid|CBF88321.1 anig|EHA18153.1 aory|EIT78845.1 asyd|OJJ63704.1 ater|EAU39138.1 atub|OJI91026.1 aver|OJI97235.1 awen|OJJ35195.1

Group5382: abra|OJJ73623.1 acar|OOG00806.1 acla|EAW07538.1 afis|EAW22572.1 afum|EAL90626.1 agla|OJJ80929.1 akaw|GAA88530.1 aluc|OJZ88425.1 anid|CBF88327.1 anig|EHA18150.1 aory|EIT78792.1 asyd|OJJ63707.1 ater|EAU39135.1 atub|OJI91023.1 aver|OJI97232.1 awen|OJJ35199.1 azon|OJJ49909.1

Group5383: abra|OJJ73626.1 acar|OOG00808.1 acla|EAW07540.1 afis|EAW22574.1 afla|EED54603.1 afum|EAL90624.2 akaw|GAA88527.1 aluc|OJZ88423.1 anid|CBF88330.1 anig|EHA18148.1 aory|EIT78769.1 asyd|OJJ63710.1 ater|EAU39130.1 atub|OJI91019.1 aver|OJI97229.1 awen|OJJ35202.1 azon|OJJ49906.1

Group5384: abra|OJJ73628.1 acla|EAW07542.1 afis|EAW22576.1 afla|EED54615.1 afum|EAL90622.2 agla|OJJ80936.1 akaw|GAA88525.1 aluc|OJZ88421.1 anid|CBF88334.1 anig|EHA18146.1 aory|EIT78740.1 asyd|OJJ63712.1 ater|EAU39128.1 atub|OJI91017.1 aver|OJI97227.1 awen|OJJ35204.1 azon|OJJ49904.1

Group5385: abra|OJJ73630.1 acar|OOG00812.1 acla|EAW07544.1 afis|EAW22578.1 afla|EED54621.1 afum|EAL90620.2 agla|OJJ80938.1 akaw|GAA88523.1 aluc|OJZ88419.1 anid|CBF88337.1 anig|EHA18145.1 aory|EIT78944.1 asyd|OJJ63714.1 ater|EAU39126.1 atub|OJI91015.1 awen|OJJ35206.1 azon|OJJ49902.1

Group5386: abra|OJJ73642.1 acar|OOG00823.1 acla|EAW07555.1 afis|EAW22589.1 afla|EED54635.1 afum|EAL90610.1 agla|OJJ80949.1 akaw|GAA88513.1 aluc|OJZ88408.1 anid|CBF88362.1 anig|EHA18135.1 asyd|OJJ63725.1 ater|EAU39118.1 atub|OJI91004.1 aver|OJI97215.1 awen|OJJ35217.1 azon|OJJ49892.1

Group5387: abra|OJJ73664.1 acla|EAW07571.1 afis|EAW22605.1 afla|EED49515.1 afum|EAL90593.1 agla|OJJ80973.1 akaw|GAA88492.1 aluc|OJZ88388.1 anid|CBF88261.1 anig|EHA18115.1 aory|EIT82604.1 asyd|OJJ63675.1 ater|EAU39099.1 atub|OJI90983.1 aver|OJI97263.1 awen|OJJ35233.1 azon|OJJ49878.1

Group5388: abra|OJJ73677.1 acla|EAW07578.1 afis|EAW22616.1 afla|EED49824.1 afum|EAL90581.1 agla|OJJ80998.1 akaw|GAA88482.1 aluc|OJZ88372.1 anid|CBF88249.1 anig|EHA18105.1 aory|EIT77667.1 asyd|OJJ63668.1 ater|EAU39085.1 atub|OJI90970.1 aver|OJI97269.1 awen|OJJ35243.1 azon|OJJ49862.1

Group5389: abra|OJJ73679.1 acar|OOG00850.1 acla|EAW07594.1 afis|EAW22625.1 afla|EED49826.1 afum|EAL90571.1 agla|OJJ80996.1 akaw|GAA88480.1 aluc|OJZ88370.1 anid|CBF88245.1 anig|EHA18103.1 aory|EIT77671.1 asyd|OJJ63665.1 ater|EAU39084.1 atub|OJI90968.1 aver|OJI97270.1 awen|OJJ35245.1

Group5390: abra|OJJ73682.1 acar|OOG00852.1 acla|EAW07597.1 afis|EAW22628.1 afla|EED49877.1 afum|EAL90568.1 agla|OJJ80993.1 akaw|GAA88477.1 aluc|OJZ88367.1 anid|CBF88239.1 anig|EHA18100.1 asyd|OJJ63661.1 ater|EAU39080.1 atub|OJI90965.1 aver|OJI97273.1 awen|OJJ35249.1 azon|OJJ49857.1

Group5391: abra|OJJ73683.1 afis|EAW22629.1 afla|EED49878.1 afum|EAL90566.1 agla|OJJ80991.1 akaw|GAA88476.1 aluc|OJZ88366.1 anid|CBF88238.1 aory|EIT72447.1 asyd|OJJ63660.1 ater|EAU39079.1 atub|OJI90964.1 aver|OJI97274.1 awen|OJJ35250.1 azon|OJJ49856.1 acla|EAW07598.1 anig|EHA18098.1

Group5392: abra|OJJ73687.1 acar|OOG00859.1 afis|EAW22633.1 afla|EED49883.1 afum|EAL90560.1 agla|OJJ80986.1 akaw|GAA88472.1 aluc|OJZ88362.1 anid|CBF88230.1 anig|EHA18094.1 aory|EIT72448.1 asyd|OJJ63656.1 ater|EAU39075.1 atub|OJI90958.1 aver|OJI97278.1 awen|OJJ35255.1 acla|EAW07602.1

Group5393: abra|OJJ73691.1 acla|EAW07606.1 afis|EAW22636.1 afla|EED49887.1 afum|EAL90556.2 agla|OJJ80982.1 akaw|GAA88469.1 aluc|OJZ88358.1 anid|CBF88223.1 anig|EHA18091.1 aory|EIT72468.1 asyd|OJJ63653.1 ater|EAU39070.1 atub|OJI90953.1 aver|OJI97281.1 awen|OJJ35259.1 azon|OJJ49848.1

Group5394: abra|OJJ73721.1 acar|OOG00887.1 acla|EAW12122.1 afis|EAW17258.1 afla|EED45960.1 afum|EAL86675.1 agla|OJJ83737.1 akaw|GAA88441.1 aluc|OJZ88329.1 anid|CBF88172.1 anig|EHA18064.1 aory|EIT75555.1 asyd|OJJ63623.1 ater|EAU32152.1 atub|OJI90924.1 aver|OJI97313.1 awen|OJJ37054.1

Group5395: abra|OJJ73730.1 acar|OOG00895.1 acla|EAW07614.1 afis|EAW22644.1 afla|EED49913.1 afum|EAL90548.1 agla|OJJ81026.1 aluc|OJZ88320.1 anid|CBF88177.1 anig|EHA18056.1 aory|EIT72270.1 asyd|OJJ63626.1 ater|EAU39063.1 atub|OJI90915.1 aver|OJI97310.1 awen|OJJ35280.1 azon|OJJ49844.1

Group5396: abra|OJJ73738.1 acar|OOG00903.1 acla|EAW07622.1 afis|EAW22651.1 afla|EED49920.1 afum|EAL90541.1 agla|OJJ81034.1 akaw|GAA88426.1 aluc|OJZ88313.1 anid|CBF88192.1 anig|EHA18050.1 asyd|OJJ63633.1 ater|EAU39056.1 atub|OJI90908.1 aver|OJI97303.1 awen|OJJ35272.1 azon|OJJ49835.1

Group5397: abra|OJJ73765.1 acla|EAW07651.1 afis|EAW22669.1 afla|EED49946.1 afum|EAL90523.1 agla|OJJ79052.1 akaw|GAA88409.1 aluc|OJZ88291.1 anid|CBF88153.1 anig|EHA18032.1 aory|EIT83448.1 asyd|OJJ63614.1 ater|EAU39039.1 atub|OJI90888.1 aver|OJI97324.1 awen|OJJ35297.1 azon|OJJ49819.1

Group5398: abra|OJJ73772.1 acar|OOG00930.1 acla|EAW07656.1 afis|EAW22674.1 afla|EED49955.1 afum|EAL90518.1 agla|OJJ79043.1 akaw|GAA88404.1 aluc|OJZ88284.1 anid|CBF88144.1 anig|EHA18027.1 aory|EIT83450.1 asyd|OJJ63600.1 atub|OJI90882.1 aver|OJI97337.1 awen|OJJ35303.1 azon|OJJ49813.1

Group5399: abra|OJJ73780.1 acar|OOG00936.1 acla|EAW07664.1 afis|EAW22681.1 afla|EED49962.1 afum|EAL90511.1 akaw|GAA88397.1 aluc|OJZ88275.1 anid|CBF88122.1 anig|EHA18020.1 aory|EIT83441.1 asyd|OJJ63586.1 ater|EAU39028.1 atub|OJI90875.1 aver|OJI97348.1 awen|OJJ35309.1 azon|OJJ49802.1

Group5400: abra|OJJ73796.1 acar|OOG00951.1 acla|EAW07687.1 afis|EAW22702.1 afum|EAL90490.1 agla|OJJ79007.1 akaw|GAA89414.1 aluc|OJZ88259.1 anid|CBF88076.1 anig|EHA18004.1 aory|EIT74750.1 asyd|OJJ63556.1 ater|EAU39000.1 atub|OJI90858.1 aver|OJI97378.1 awen|OJJ35339.1 azon|OJJ49779.1

Group5401: abra|OJJ73799.1 acar|OOG00948.1 acla|EAW07693.1 afis|EAW22708.1 afla|EED49994.1 afum|EAL90484.1 agla|OJJ79002.1 akaw|GAA89417.1 aluc|OJZ88256.1 anid|CBF88066.1 aory|EIT74634.1 asyd|OJJ63550.1 ater|EAU38994.1 atub|OJI90854.1 aver|OJI97384.1 awen|OJJ35345.1 azon|OJJ49772.1

Group5402: abra|OJJ73814.1 acla|EAW07701.1 afis|EAW22718.1 afla|EED50004.1 afum|EAL90476.1 agla|OJJ83685.1 akaw|GAA89433.1 aluc|OJZ88236.1 anid|CBF88048.1 anig|EHA17983.1 aory|EIT74712.1 asyd|OJJ63538.1 ater|EAU38985.1 atub|OJI90838.1 aver|OJI97395.1 awen|OJJ35412.1 azon|OJJ49764.1

Group5403: abra|OJJ73815.1 acar|OOG00969.1 acla|EAW07702.1 afis|EAW22719.1 afla|EED50005.1 afum|EAL90475.1 agla|OJJ83684.1 akaw|GAA89434.1 aluc|OJZ88235.1 anid|CBF88040.1 anig|EHA17982.1 asyd|OJJ63537.1 ater|EAU38984.1 atub|OJI90837.1 aver|OJI97396.1 awen|OJJ35413.1 azon|OJJ49763.1

Group5404: abra|OJJ73817.1 acar|OOG00971.1 acla|EAW07706.1 afis|EAW22723.1 afla|EED50008.1 afum|EAL90473.1 agla|OJJ83680.1 akaw|GAA89436.1 aluc|OJZ88233.1 anid|CBF88032.1 anig|EHA17979.1 aory|EIT74623.1 asyd|OJJ63533.1 ater|EAU38982.1 atub|OJI90834.1 aver|OJI97399.1 awen|OJJ35417.1

Group5405: abra|OJJ73818.1 acla|EAW07707.1 afis|EAW22724.1 afla|EED50009.1 afum|EAL90472.1 agla|OJJ83679.1 akaw|GAA89437.1 aluc|OJZ88232.1 anid|CBF88031.1 anig|EHA17978.1 aory|EIT74693.1 ater|EAU38981.1 atub|OJI90833.1 awen|OJJ35418.1 azon|OJJ49759.1 asyd|OJJ63532.1 aver|OJI97400.1

Group5406: abra|OJJ73820.1 acla|EAW07709.1 afis|EAW22725.1 afla|EED50011.1 afum|EAL90471.1 agla|OJJ83677.1 akaw|GAA89439.1 aluc|OJZ88229.1 anid|CBF88029.1 anig|EHA17976.1 aory|EIT74724.1 asyd|OJJ63531.1 ater|EAU38979.1 atub|OJI90831.1 aver|OJI97401.1 awen|OJJ35420.1 azon|OJJ49757.1

Group5407: abra|OJJ73826.1 acar|OOG00978.1 acla|EAW07721.1 afis|EAW22753.1 afum|EAL90446.2 agla|OJJ83672.1 akaw|GAA89444.1 aluc|OJZ88223.1 anid|CBF88020.1 anig|EHA17970.1 aory|EIT74674.1 asyd|OJJ63523.1 ater|EAU38967.1 atub|OJI90823.1 aver|OJI97407.1 awen|OJJ35425.1 azon|OJJ49751.1

Group5408: abra|OJJ73828.1 acar|OOG00980.1 acla|EAW07723.1 afis|EAW22755.1 afla|EED50016.1 afum|EAL90444.2 agla|OJJ83670.1 akaw|GAA89446.1 aluc|OJZ88221.1 anig|EHA17968.1 aory|EIT74646.1 asyd|OJJ63521.1 ater|EAU38965.1 atub|OJI90821.1 aver|OJI97409.1 awen|OJJ35427.1 azon|OJJ49749.1

Group5409: abra|OJJ73829.1 acla|EAW07724.1 afis|EAW22756.1 afla|EED50017.1 afum|EAL90443.1 agla|OJJ83667.1 akaw|GAA89447.1 aluc|OJZ88220.1 anid|CBF88017.1 aory|EIT74727.1 asyd|OJJ63520.1 ater|EAU38963.1 atub|OJI90820.1 aver|OJI97410.1 awen|OJJ35428.1 azon|OJJ49748.1 anig|EHA17967.1

Group5410: abra|OJJ73845.1 acar|OOG00996.1 acla|EAW07740.1 afis|EAW22776.1 afla|EED50036.1 afum|EAL90422.1 agla|OJJ83612.1 akaw|GAA89463.1 aluc|OJZ88203.1 anid|CBF87989.1 anig|EHA17951.1 aory|EIT74654.1 asyd|OJJ63501.1 atub|OJI90803.1 aver|OJI97429.1 awen|OJJ38961.1 azon|OJJ49732.1

Group5411: abra|OJJ73858.1 acla|EAW07749.1 afis|EAW22786.1 afla|EED50049.1 afum|EAL90413.1 agla|OJJ83591.1 akaw|GAA89483.1 aluc|OJZ88179.1 anid|CBF87967.1 anig|EHA17931.1 aory|EIT74754.1 asyd|OJJ63487.1 ater|EAU38924.1 atub|OJI90778.1 aver|OJI97442.1 awen|OJJ38949.1 azon|OJJ49721.1

Group5412: abra|OJJ73860.1 acar|OOG01008.1 acla|EAW07750.1 afis|EAW22787.1 afla|EED50051.1 afum|EAL90412.1 agla|OJJ83589.1 akaw|GAA89482.1 aluc|OJZ88181.1 anid|CBF87965.1 anig|EHA17933.1 asyd|OJJ63485.1 ater|EAU38923.1 atub|OJI90780.1 aver|OJI97444.1 awen|OJJ38947.1 azon|OJJ49719.1

Group5413: abra|OJJ73863.1 acar|OOF89915.1 acla|EAW07753.1 afis|EAW22790.1 afla|EED50057.1 afum|EAL90409.1 agla|OJJ83586.1 akaw|GAA89479.1 aluc|OJZ88184.1 anid|CBF87959.1 anig|EHA17935.1 aory|EIT74648.1 asyd|OJJ63482.1 atub|OJI90783.1 aver|OJI97448.1 awen|OJJ38944.1 azon|OJJ49717.1

Group5414: abra|OJJ73875.1 acla|EAW07760.1 afis|EAW22796.1 afla|EED50065.1 afum|EAL90403.1 agla|OJJ83573.1 akaw|GAA89487.1 aluc|OJZ88174.1 anid|CBF87948.1 anig|EHA17927.1 aory|EIT74629.1 asyd|OJJ63476.1 ater|EAU38914.1 atub|OJI90772.1 aver|OJI97454.1 awen|OJJ38933.1 azon|OJJ49709.1

Group5415: abra|OJJ73879.1 acar|OOG01019.1 acla|EAW07762.1 afis|EAW22798.1 afla|EED50068.1 afum|EAL90401.1 agla|OJJ83575.1 akaw|GAA89491.1 aluc|OJZ88170.1 anid|CBF87937.1 anig|EHA17924.1 aory|EIT74758.1 asyd|OJJ63467.1 atub|OJI90768.1 aver|OJI97465.1 awen|OJJ38931.1 azon|OJJ49707.1

Group5416: abra|OJJ73885.1 acla|EAW07767.1 afis|EAW22803.1 afla|EED50073.1 afum|EAL90396.1 agla|OJJ83580.1 akaw|GAA89496.1 aluc|OJZ88165.1 anid|CBF87928.1 anig|EHA17919.1 aory|EIT74628.1 asyd|OJJ63462.1 ater|EAU38906.1 atub|OJI90763.1 aver|OJI97470.1 awen|OJJ38925.1 azon|OJJ49702.1

Group5417: abra|OJJ73895.1 acla|EAW07777.1 afis|EAW22813.1 afla|EED50086.1 afum|EAL90388.2 agla|OJJ83556.1 akaw|GAA89505.1 aluc|OJZ88152.1 anid|CBF87911.1 anig|EHA17912.1 aory|EIT74666.1 asyd|OJJ63450.1 ater|EAU38897.1 atub|OJI90754.1 aver|OJI97484.1 awen|OJJ38918.1 azon|OJJ49695.1

Group5418: abra|OJJ73903.1 acla|EAW07782.1 afis|EAW22818.1 afla|EED50091.1 afum|EAL90383.1 akaw|GAA89510.1 aluc|OJZ88143.1 anig|EHA17906.1 aory|EIT74676.1 ater|EAU38892.1 atub|OJI90747.1 awen|OJJ38909.1 azon|OJJ49690.1 anid|CBF87900.1 asyd|OJJ63445.1 aver|OJI97491.1 agla|OJJ83520.1

Group5419: abra|OJJ73914.1 acar|OOG01046.1 acla|EAW07790.1 afis|EAW22826.1 afla|EED50102.1 afum|EAL90374.2 agla|OJJ83511.1 akaw|GAA89520.1 aluc|OJZ88134.1 anid|CBF87885.1 anig|EHA17898.1 aory|EIT74725.1 asyd|OJJ63436.1 ater|EAU38883.1 aver|OJI97501.1 awen|OJJ38900.1 azon|OJJ49681.1

Group5420: abra|OJJ73929.1 acla|EAW07806.1 afis|EAW22843.1 afla|EED50118.1 afum|EAL90360.1 agla|OJJ83495.1 akaw|GAA89536.1 aluc|OJZ88114.1 anid|CBF87856.1 anig|EHA17884.1 aory|EIT74743.1 asyd|OJJ63422.1 ater|EAU38867.1 atub|OJI90723.1 aver|OJI97515.1 awen|OJJ38880.1 azon|OJJ49669.1

Group5421: abra|OJJ73950.1 acar|OOG01078.1 acla|EAW07825.1 afis|EAW22861.1 afla|EED50144.1 afum|EAL90339.1 agla|OJJ83463.1 akaw|GAA89555.1 aluc|OJZ88094.1 anid|CBF84626.1 anig|EHA17864.1 aory|EIT74618.1 asyd|OJJ60002.1 atub|OJI90702.1 aver|OJJ02289.1 awen|OJJ38859.1 agla|OJJ78756.1

Group5422: abra|OJJ73951.1 acar|OOG01079.1 acla|EAW07824.1 afis|EAW22860.1 afla|EED50141.1 afum|EAL90340.1 agla|OJJ83464.1 akaw|GAA89556.1 aluc|OJZ88093.1 anid|CBF84628.1 anig|EHA17863.1 aory|EIT74753.1 asyd|OJJ60001.1 atub|OJI90701.1 aver|OJJ02290.1 awen|OJJ38860.1 agla|OJJ78752.1

Group5423: abra|OJJ73955.1 acar|OOG01082.1 acla|EAW14167.1 afis|EAW18881.1 afla|EED50885.1 afum|EAL93613.1 agla|OJJ88981.1 akaw|GAA89560.1 aluc|OJZ88088.1 anid|CBF80630.1 anig|EHA17859.1 aory|EIT77121.1 asyd|OJJ54635.1 ater|EAU38049.1 atub|OJI90697.1 aver|OJJ07521.1 afla|EED47797.1

Group5424: abra|OJJ73958.1 acar|OOG01085.1 acla|EAW07827.1 afis|EAW22863.1 afla|EED50147.1 afum|EAL90337.1 agla|OJJ83461.1 akaw|GAA89561.1 aluc|OJZ88087.1 anid|CBF87830.1 anig|EHA17858.1 aory|EIT74683.1 asyd|OJJ63406.1 ater|EAU38823.1 atub|OJI90696.1 awen|OJJ38857.1 azon|OJJ49657.1

Group5425: abra|OJJ73960.1 acar|OOG01088.1 acla|EAW07829.1 afis|EAW22865.1 afla|EED50149.1 afum|EAL90334.1 agla|OJJ83459.1 akaw|GAA89563.1 aluc|OJZ88085.1 anig|EHA17856.1 aory|EIT74687.1 asyd|OJJ63404.1 ater|EAU38825.1 atub|OJI90694.1 aver|OJI97532.1 awen|OJJ38855.1 azon|OJJ49653.1

Group5426: abra|OJJ73965.1 acar|OOG01091.1 acla|EAW07835.1 afis|EAW22872.1 afla|EED50155.1 afum|EAL90326.1 agla|OJJ83451.1 akaw|GAA89568.1 aluc|OJZ88079.1 anid|CBF87819.1 anig|EHA17851.1 asyd|OJJ63398.1 ater|EAU38836.1 atub|OJI90688.1 aver|OJI97538.1 awen|OJJ38850.1 azon|OJJ49646.1

Group5427: abra|OJJ73966.1 acar|OOG01092.1 acla|EAW07836.1 afis|EAW22873.1 afla|EED50156.1 afum|EAL90325.2 agla|OJJ83450.1 akaw|GAA89569.1 aluc|OJZ88078.1 anid|CBF87817.1 anig|EHA17850.1 asyd|OJJ63396.1 ater|EAU38837.1 atub|OJI90687.1 aver|OJI97540.1 awen|OJJ38849.1 azon|OJJ49645.1

Group5428: abra|OJJ73981.1 acar|OOG01108.1 acla|EAW07853.1 afis|EAW22889.1 afla|EED50170.1 afum|EAL90309.1 agla|OJJ83421.1 akaw|GAA89585.1 aluc|OJZ88063.1 anid|CBF87794.1 anig|EHA17836.1 asyd|OJJ63379.1 ater|EAU35984.1 atub|OJI90672.1 aver|OJI97556.1 awen|OJJ38833.1 azon|OJJ49631.1

Group5429: abra|OJJ73984.1 acla|EAW07856.1 afis|EAW22892.1 afla|EED54861.1 afum|EAL90306.1 agla|OJJ83409.1 akaw|GAA89588.1 aluc|OJZ88060.1 anid|CBF87788.1 anig|EHA17833.1 aory|EIT79389.1 asyd|OJJ63375.1 ater|EAU35991.1 atub|OJI90668.1 aver|OJI97571.1 awen|OJJ38828.1 azon|OJJ49626.1

Group5430: abra|OJJ74014.1 acla|EAW07869.1 afis|EAW22904.1 afla|EED54896.1 afum|EAL90293.1 agla|OJJ83386.1 akaw|GAA87497.1 aluc|OJZ88025.1 anid|CBF87715.1 anig|EHA17802.1 aory|EIT79432.1 asyd|OJJ63339.1 ater|EAU36035.1 atub|OJI90633.1 aver|OJI97622.1 awen|OJJ38726.1 azon|OJJ49612.1

Group5431: abra|OJJ74042.1 acar|OOG01160.1 acla|EAW07891.1 afis|EAW22926.1 afla|EED54923.1 afum|EAL90271.1 agla|OJJ83362.1 akaw|GAA87524.1 aluc|OJZ87995.1 anid|CBF87673.1 anig|EHA17777.1 asyd|OJJ63312.1 ater|EAU30697.1 atub|OJI90604.1 aver|OJI97647.1 awen|OJJ38638.1 azon|OJJ49580.1

Group5432: abra|OJJ74046.1 acar|OOG01163.1 acla|EAW07896.1 afis|EAW22931.1 afla|EED58124.1 afum|EAL90266.1 agla|OJJ83357.1 akaw|GAA87529.1 aluc|OJZ87989.1 anid|CBF87666.1 anig|EHA17772.1 asyd|OJJ63307.1 ater|EAU30693.1 atub|OJI90599.1 aver|OJI97652.1 awen|OJJ38633.1 azon|OJJ49575.1

Group5433: abra|OJJ74051.1 acar|OOF89807.1 acla|EAW07900.1 afis|EAW22936.1 afla|EED58129.1 afum|EAL90260.1 agla|OJJ83352.1 akaw|GAA87533.1 aluc|OJZ87984.1 anid|CBF87657.1 anig|EHA17767.1 aory|EIT79148.1 asyd|OJJ63302.1 atub|OJI90594.1 aver|OJI97657.1 awen|OJJ38628.1 azon|OJJ49570.1

Group5434: abra|OJJ74074.1 acar|OOG01186.1 acla|EAW07919.1 afis|EAW22957.1 afla|EED58139.1 afum|EAL90240.1 agla|OJJ83331.1 akaw|GAA87555.1 aluc|OJZ87962.1 anig|EHA17746.1 aory|EIT79129.1 asyd|OJJ63282.1 ater|EAU30668.1 atub|OJI90572.1 aver|OJI97676.1 awen|OJJ38606.1 azon|OJJ49548.1

Group5435: abra|OJJ74077.1 acar|OOG01189.1 acla|EAW07922.1 afis|EAW22960.1 afla|EED58136.1 afum|EAL90237.1 agla|OJJ83328.1 akaw|GAA87558.1 aluc|OJZ87959.1 anig|EHA17743.1 aory|EIT79122.1 asyd|OJJ63279.1 ater|EAU30665.1 atub|OJI90569.1 aver|OJI97679.1 awen|OJJ38603.1 azon|OJJ49545.1

Group5436: abra|OJJ74098.1 acar|OOG01207.1 acla|EAW07941.1 afis|EAW22979.1 afla|EED58172.1 agla|OJJ83308.1 akaw|GAA87576.1 aluc|OJZ87938.1 anid|CBF87592.1 anig|EHA17726.1 aory|EIT79098.1 asyd|OJJ63260.1 ater|EAU30642.1 atub|OJI90550.1 aver|OJI97697.1 awen|OJJ38584.1 azon|OJJ49526.1

Group5437: abra|OJJ74099.1 acar|OOG01208.1 acla|EAW07942.1 afis|EAW22980.1 afla|EED58173.1 afum|EAL90217.1 agla|OJJ83307.1 akaw|GAA87577.1 aluc|OJZ87937.1 anid|CBF87590.1 anig|EHA17725.1 asyd|OJJ63259.1 ater|EAU30641.1 atub|OJI90549.1 aver|OJI97698.1 awen|OJJ38583.1 azon|OJJ49525.1

Group5438: abra|OJJ74105.1 acla|EAW07950.1 afis|EAW22987.1 afla|EED58179.1 afum|EAL90212.1 agla|OJJ83300.1 akaw|GAA87583.1 aluc|OJZ87931.1 anid|CBF87581.1 anig|EHA17720.1 aory|EIT79066.1 asyd|OJJ63253.1 ater|EAU30636.1 atub|OJI90543.1 aver|OJI97705.1 awen|OJJ38577.1 azon|OJJ49519.1

Group5439: abra|OJJ74106.1 acar|OOG01215.1 acla|EAW07951.1 afis|EAW22988.1 afla|EED58180.1 afum|EAL90211.1 agla|OJJ83298.1 aluc|OJZ87930.1 anid|CBF75423.1 anig|EHA17719.1 aory|EIT79102.1 asyd|OJJ59794.1 ater|EAU30635.1 atub|OJI90542.1 aver|OJJ02493.1 awen|OJJ38576.1 azon|OJJ49518.1

Group5440: abra|OJJ74111.1 acar|OOF89999.1 acla|EAW07954.1 afis|EAW22991.1 afla|EED58183.1 afum|EAL90209.1 agla|OJJ83295.1 akaw|GAA87586.1 aluc|OJZ87924.1 anid|CBF87578.1 anig|EHA17716.1 aory|EIT79107.1 asyd|OJJ63249.1 ater|EAU30631.1 atub|OJI90536.1 aver|OJI97708.1 awen|OJJ38573.1

Group5441: abra|OJJ74114.1 acar|OOG01219.1 acla|EAW07956.1 afis|EAW22992.1 afla|EED58184.1 afum|EBA27400.1 agla|OJJ83293.1 akaw|GAA87588.1 aluc|OJZ87920.1 anid|CBF87574.1 anig|EHA17714.1 asyd|OJJ63246.1 ater|EAU30628.1 atub|OJI90531.1 aver|OJI97710.1 awen|OJJ38570.1 azon|OJJ42000.1

Group5442: abra|OJJ74131.1 acar|OOF95603.1 acla|EAW11690.1 afis|EAW23706.1 afla|EED56137.1 afum|EAL87150.1 agla|OJJ78978.1 akaw|GAA89755.1 aluc|OJZ80628.1 anid|CBF84383.1 anig|EHA27203.1 aory|EIT76571.1 asyd|OJJ58682.1 ater|EAU36843.1 atub|OJI87484.1 aver|OJJ01294.1 awen|OJJ30914.1

Group5443: abra|OJJ74154.1 acar|OOF95571.1 acla|EAW12278.1 afis|EAW16962.1 afla|EED54137.1 afum|EAL86489.1 agla|OJJ88175.1 akaw|GAA89729.1 aluc|OJZ80654.1 anid|CBF89392.1 anig|EHA27178.1 aory|EIT75949.1 asyd|OJJ62463.1 ater|EAU34050.1 atub|OJI87509.1 aver|OJI96598.1 awen|OJJ40679.1

Group5444: abra|OJJ74174.1 acar|OOF95554.1 acla|EAW10815.1 afis|EAW24759.1 afla|EED48096.1 afum|EAL89401.1 agla|OJJ79193.1 akaw|GAA86842.1 aluc|OJZ89961.1 anid|CBF89394.1 anig|EHA27161.1 aory|EIT76187.1 asyd|OJJ62462.1 ater|EAU34013.1 atub|OJI84844.1 aver|OJI96597.1 awen|OJJ30980.1

Group5445: abra|OJJ74205.1 acar|OOF95515.1 acla|EAW07258.1 afis|EAW22279.1 afla|EED57672.1 afum|EAL90902.1 agla|OJJ87774.1 akaw|GAA86813.1 aluc|OJZ89930.1 anid|CBF88546.1 anig|EHA27130.1 asyd|OJJ63826.1 ater|EAU34289.1 atub|OJI84876.1 aver|OJI97112.1 awen|OJJ34852.1 azon|OJJ45695.1

Group5446: abra|OJJ74216.1 acar|OOF95505.1 acla|EAW07268.1 afis|EAW22289.1 afla|EED57688.1 afum|EAL90892.1 agla|OJJ87760.1 akaw|GAA86802.1 aluc|OJZ89918.1 anid|CBF88566.1 anig|EHA27118.1 asyd|OJJ63836.1 ater|EAU34301.1 atub|OJI84887.1 aver|OJI97101.1 awen|OJJ34862.1 azon|OJJ45666.1

Group5447: abra|OJJ74219.1 acar|OOF90432.1 acla|EAW07272.1 afis|EAW22293.1 afla|EED57691.1 afum|EAL90888.1 agla|OJJ87757.1 akaw|GAA86799.1 aluc|OJZ89915.1 anid|CBF88571.1 anig|EHA27115.1 asyd|OJJ63838.1 ater|EAU34304.1 atub|OJI84890.1 aver|OJI97099.1 awen|OJJ34865.1 azon|OJJ45664.1

Group5448: abra|OJJ74222.1 acla|EAW07283.1 afis|EAW22314.1 afla|EED57696.1 afum|EAL90876.1 agla|OJJ87736.1 akaw|GAA86797.1 aluc|OJZ89912.1 anid|CBF88579.1 anig|EHA27112.1 aory|EIT79872.1 asyd|OJJ63842.1 ater|EAU34309.1 atub|OJI84893.1 aver|OJI97095.1 awen|OJJ34870.1 azon|OJJ50046.1

Group5449: abra|OJJ74227.1 acar|OOF95499.1 acla|EAW07278.1 afis|EAW22309.1 afla|EED57703.1 afum|EAL90881.1 agla|OJJ87741.1 akaw|GAA86792.1 aluc|OJZ89906.1 anid|CBF88588.1 anig|EHA27106.1 aory|EIT79911.1 asyd|OJJ63847.1 ater|EAU34315.1 atub|OJI84900.1 aver|OJI97089.1 awen|OJJ34878.1

Group5450: abra|OJJ74230.1 acar|OOF95496.1 acla|EAW07274.1 afis|EAW22295.1 afla|EED57708.1 afum|EAL90886.1 agla|OJJ87747.1 akaw|GAA86789.1 aluc|OJZ89903.1 anid|CBF88596.1 anig|EHA27103.1 asyd|OJJ63852.1 ater|EAU34319.1 atub|OJI84903.1 aver|OJI97085.1 awen|OJJ34883.1 azon|OJJ50051.1

Group5451: abra|OJJ74238.1 acar|OOF95493.1 acla|EAW07289.1 afis|EAW22322.1 afla|EED57719.1 afum|EAL90869.1 agla|OJJ87750.1 akaw|GAA86782.1 aluc|OJZ89894.1 anid|CBF88603.1 anig|EHA27093.1 aory|EIT79885.1 asyd|OJJ63856.1 atub|OJI84913.1 aver|OJI97082.1 awen|OJJ34889.1 azon|OJJ50056.1

Group5452: abra|OJJ74243.1 acar|OOF95487.1 acla|EAW07292.1 afis|EAW22326.1 afla|EED57725.1 afum|EAL90866.1 agla|OJJ87722.1 akaw|GAA86777.1 aluc|OJZ89889.1 anid|CBF88611.1 anig|EHA27088.1 aory|EIT79882.1 asyd|OJJ63860.1 ater|EAU34325.1 atub|OJI84918.1 aver|OJI97079.1 awen|OJJ34895.1

Group5453: abra|OJJ74247.1 acar|OOF95482.1 acla|EAW07301.1 afis|EAW22344.1 afla|EED57737.1 afum|EAL90847.1 agla|OJJ87690.1 aluc|OJZ89885.1 anid|CBF88625.1 anig|EHA27084.1 aory|EIT79933.1 asyd|OJJ63869.1 ater|EAU39482.1 atub|OJI84922.1 aver|OJI97070.1 awen|OJJ34923.1 azon|OJJ50071.1

Group5454: abra|OJJ74251.1 acar|OOF95478.1 acla|EAW07295.1 afis|EAW22329.1 afla|EED57728.1 afum|EAL90863.1 agla|OJJ87754.1 akaw|GAA86770.1 aluc|OJZ89881.1 anid|CBF88617.1 anig|EHA27080.1 aory|EIT79902.1 asyd|OJJ63863.1 atub|OJI84926.1 aver|OJI97076.1 awen|OJJ34892.1 azon|OJJ50061.1

Group5455: abra|OJJ74253.1 acar|OOF95476.1 acla|EAW07297.1 afis|EAW22348.1 afla|EED57730.1 afum|EAL90844.1 agla|OJJ87751.1 akaw|GAA86768.1 aluc|OJZ89879.1 anid|CBF88621.1 anig|EHA27078.1 aory|EIT79888.1 asyd|OJJ63865.1 ater|EAU39456.1 atub|OJI84928.1 aver|OJI97074.1 azon|OJJ50059.1

Group5456: abra|OJJ74259.1 acla|EAW07304.1 afis|EAW22341.1 afla|EED57740.1 afum|EAL90851.1 agla|OJJ87694.1 akaw|GAA86762.1 aluc|OJZ89872.1 anid|CBF88630.1 anig|EHA27072.1 aory|EIT79897.1 asyd|OJJ63873.1 ater|EAU39479.1 atub|OJI84935.1 aver|OJI97065.1 awen|OJJ34920.1 azon|OJJ50074.1

Group5457: abra|OJJ74287.1 acar|OOF95444.1 acla|EAW07331.1 afis|EAW22363.1 afla|EED57771.1 afum|EAL90828.1 agla|OJJ87612.1 akaw|GAA86737.1 aluc|OJZ89846.1 anig|EHA27047.1 aory|EIT81068.1 asyd|OJJ63905.1 ater|EAU39497.1 atub|OJI84960.1 aver|OJI97036.1 awen|OJJ34937.1 azon|OJJ45589.1

Group5458: abra|OJJ74288.1 acar|OOF95443.1 acla|EAW07332.1 afis|EAW22364.1 afla|EED57772.1 afum|EAL90827.1 akaw|GAA86735.1 aluc|OJZ89845.1 anid|CBF88688.1 anig|EHA27046.1 aory|EIT81112.1 asyd|OJJ63906.1 ater|EAU39498.1 atub|OJI84961.1 aver|OJI97035.1 awen|OJJ34938.1 azon|OJJ45590.1

Group5459: abra|OJJ74308.1 acla|EAW07356.1 afis|EAW22385.1 afla|EED56045.1 afum|EAL90805.1 agla|OJJ87646.1 akaw|GAA86716.1 aluc|OJZ89819.1 anid|CBF88727.1 anig|EHA27027.1 aory|EIT76587.1 asyd|OJJ63932.1 ater|EAU39336.1 atub|OJI84987.1 aver|OJI97011.1 awen|OJJ34960.1 azon|OJJ45612.1

Group5460: abra|OJJ74313.1 acar|OOF95415.1 acla|EAW07364.1 afis|EAW22391.1 afla|EED56054.1 afum|EBA27413.1 agla|OJJ87653.1 aluc|OJZ89813.1 anid|CBF88739.1 anig|EHA27023.1 aory|EIT76735.1 asyd|OJJ63952.1 ater|EAU39327.1 atub|OJI84992.1 aver|OJI96989.1 awen|OJJ34968.1 azon|OJJ45617.1

Group5461: abra|OJJ74321.1 acla|EAW07373.1 afis|EAW22398.1 afla|EED56062.1 afum|EAL90793.1 agla|OJJ87661.1 akaw|GAA86705.1 aluc|OJZ89807.1 anid|CBF88752.1 anig|EHA27017.1 aory|EIT76767.1 asyd|OJJ63959.1 ater|EAU39315.1 atub|OJI84999.1 aver|OJI96982.1 awen|OJJ34976.1 azon|OJJ45625.1

Group5462: abra|OJJ74323.1 acar|OOF95407.1 acla|EAW07375.1 afis|EAW22400.1 afla|EED56064.1 afum|EAL90791.1 agla|OJJ87663.1 akaw|GAA86703.1 aluc|OJZ89805.1 anid|CBF88768.1 aory|EIT76742.1 asyd|OJJ63979.1 ater|EAU39307.1 atub|OJI85001.1 aver|OJI96974.1 awen|OJJ34979.1 azon|OJJ45627.1

Group5463: abra|OJJ74330.1 acar|OOF95401.1 acla|EAW07381.1 afis|EAW22406.1 afla|EED56070.1 afum|EAL90786.1 agla|OJJ87669.1 akaw|GAA86698.1 aluc|OJZ89799.1 anid|CBF88789.1 anig|EHA27012.1 asyd|OJJ62901.1 ater|EAU39303.1 atub|OJI85006.1 aver|OJI96955.1 awen|OJJ34985.1 azon|OJJ42755.1

Group5464: abra|OJJ74334.1 acar|OOF95398.1 acla|EAW07384.1 afis|EAW22409.1 afla|EED52489.1 afum|EAL90783.1 agla|OJJ87677.1 akaw|GAA86695.1 aluc|OJZ89795.1 anid|CBF82129.1 anig|EHA27009.1 aory|EIT82766.1 asyd|OJJ54297.1 ater|EAU39299.1 atub|OJI85010.1 aver|OJJ05252.1 awen|OJJ34989.1

Group5465: abra|OJJ74350.1 acar|OOF95379.1 acla|EAW07404.1 afis|EAW22430.1 afla|EED52508.1 agla|OJJ87593.1 akaw|GAA86680.1 aluc|OJZ89780.1 anid|CBF88826.1 anig|EHA26995.1 asyd|OJJ62883.1 ater|EAU39277.1 atub|OJI85026.1 aver|OJI96935.1 awen|OJJ35009.1 azon|OJJ45650.1 afum|EAL90761.1

Group5466: abra|OJJ74354.1 acar|OOF95376.1 acla|EAW07407.1 afis|EAW22433.1 afla|EED52514.1 afum|EAL90758.1 agla|OJJ87588.1 akaw|GAA86677.1 aluc|OJZ89774.1 anid|CBF88834.1 anig|EHA26992.1 asyd|OJJ62879.1 ater|EAU39274.1 atub|OJI85029.1 aver|OJI96931.1 awen|OJJ35012.1 azon|OJJ45653.1

Group5467: abra|OJJ74360.1 acla|EAW07411.1 afis|EAW22437.1 afla|EED54447.1 afum|EAL90754.1 agla|OJJ87585.1 akaw|GAA86671.1 aluc|OJZ89768.1 anid|CBF88866.1 anig|EHA26988.1 aory|EIT81146.1 asyd|OJJ62876.1 ater|EAU39271.1 atub|OJI85035.1 aver|OJI96928.1 awen|OJJ35015.1 azon|OJJ45660.1

Group5468: abra|OJJ74380.1 acar|OOF95357.1 acla|EAW07421.1 afis|EAW22447.1 afla|EED54461.1 afum|EAL90744.1 agla|OJJ87577.1 akaw|GAA87280.1 aluc|OJZ89740.1 anid|CBF88852.1 anig|EHA26964.1 asyd|OJJ62863.1 ater|EAU39260.1 atub|OJI85061.1 aver|OJI96916.1 awen|OJJ35072.1 azon|OJJ50035.1

Group5469: abra|OJJ74381.1 acar|OOF95356.1 acla|EAW07422.1 afis|EAW22448.1 afla|EED54463.1 afum|EAL90743.1 agla|OJJ87576.1 akaw|GAA87281.1 aluc|OJZ89739.1 anid|CBF88854.1 anig|EHA26963.1 asyd|OJJ62862.1 ater|EAU39259.1 atub|OJI85062.1 aver|OJI96915.1 awen|OJJ35073.1 azon|OJJ50034.1

Group5470: abra|OJJ74382.1 acar|OOF95355.1 acla|EAW07425.1 afis|EAW22451.1 afla|EED54464.1 afum|EAL90740.1 akaw|GAA87283.1 aluc|OJZ89736.1 anid|CBF88856.1 anig|EHA26962.1 aory|EIT81161.1 asyd|OJJ62870.1 ater|EAU39258.1 atub|OJI85065.1 aver|OJI96923.1 awen|OJJ35074.1 azon|OJJ50033.1

Group5471: abra|OJJ74385.1 afis|EAW22452.1 agla|OJJ87571.1 akaw|GAA87286.1 aluc|OJZ89733.1 anid|CBF88929.1 anig|EHA26959.1 ater|EAU39254.1 atub|OJI85068.1 aver|OJI96904.1 awen|OJJ35077.1 azon|OJJ50030.1 acla|EAW07426.1 afum|EAL90739.1 asyd|OJJ62851.1 afla|EED54468.1 aory|EIT81152.1

Group5472: abra|OJJ74390.1 acar|OOF95346.1 acla|EAW06816.1 afis|EAW16365.1 afum|EAL88990.1 agla|OJJ78586.1 akaw|GAA87290.1 aluc|OJZ89729.1 anid|CBF89345.1 anig|EHA26956.1 aory|EIT78416.1 asyd|OJJ62506.1 ater|EAU34087.1 atub|OJI85072.1 aver|OJI96626.1 awen|OJJ37769.1 afla|EED53778.1

Group5473: abra|OJJ74394.1 acla|EAW06818.1 afis|EAW16366.1 afla|EED53470.1 afum|EAL88991.1 agla|OJJ78587.1 akaw|GAA87292.1 aluc|OJZ89726.1 anid|CBF89348.1 anig|EHA26954.1 aory|EIT75141.1 asyd|OJJ62503.1 ater|EAU34082.1 atub|OJI85074.1 aver|OJI96624.1 awen|OJJ37771.1 azon|OJJ45082.1

Group5474: abra|OJJ74398.1 acar|OOF95339.1 acla|EAW06821.1 afis|EAW16369.1 afla|EED53466.1 afum|EAL88994.1 agla|OJJ78590.1 akaw|GAA87296.1 aluc|OJZ89722.1 anid|CBF89353.1 anig|EHA26950.1 aory|EIT74909.1 atub|OJI85078.1 aver|OJJ03881.1 awen|OJJ37906.1 azon|OJJ45084.1 aver|OJI96621.1

Group5475: abra|OJJ74409.1 acar|OOF95333.1 acla|EAW06823.1 afis|EAW16371.1 afla|EED53464.1 afum|EAL88996.1 agla|OJJ78592.1 akaw|GAA87302.1 aluc|OJZ89716.1 anid|CBF89355.1 aory|EIT75209.1 asyd|OJJ62500.1 ater|EAU34078.1 atub|OJI85086.1 aver|OJI96620.1 awen|OJJ37908.1 azon|OJJ45086.1

Group5476: abra|OJJ74413.1 acar|OOF95330.1 acla|EAW06825.1 afis|EAW16376.1 afla|EED53461.1 afum|EAL89001.1 agla|OJJ78594.1 akaw|GAA87304.1 aluc|OJZ89713.1 anid|CBF84606.1 anig|EHA26944.1 aory|EIT74960.1 asyd|OJJ53373.1 ater|EAU34076.1 atub|OJI85089.1 aver|OJJ08371.1 awen|OJJ37910.1

Group5477: abra|OJJ74419.1 acar|OOF95321.1 acla|EAW06835.1 afis|EAW16383.1 afla|EED53449.1 afum|EAL89011.1 agla|OJJ88210.1 akaw|GAA87313.1 aluc|OJZ89704.1 anid|CBF69425.1 anig|EHA26938.1 aory|EIT75059.1 asyd|OJJ54913.1 ater|EAU34063.1 atub|OJI85095.1 aver|OJJ07290.1 awen|OJJ37921.1

Group5478: abra|OJJ74424.1 acar|OOF95316.1 acla|EAW06836.1 afis|EAW16384.1 afla|EED54095.1 afum|EAL89012.1 akaw|GAA87317.1 aluc|OJZ89700.1 anid|CBF74710.1 anig|EHA26934.1 aory|EIT75993.1 asyd|OJJ60207.1 ater|EAU39463.1 atub|OJI85101.1 aver|OJJ04060.1 awen|OJJ37924.1 azon|OJJ45093.1

Group5479: abra|OJJ74470.1 acar|OOF95277.1 acla|EAW07227.1 afis|EAW22241.1 afla|EED57634.1 afum|EAL90952.1 agla|OJJ87810.1 akaw|GAA87363.1 aluc|OJZ89650.1 anid|CBF88487.1 anig|EHA26888.1 aory|EIT82970.1 asyd|OJJ63789.1 ater|EAU34248.1 atub|OJI85154.1 aver|OJI97149.1 awen|OJJ34819.1

Group5480: abra|OJJ74483.1 acla|EAW07218.1 afis|EAW22232.1 afla|EED57621.1 afum|EAL90961.1 agla|OJJ87819.1 akaw|GAA87376.1 aluc|OJZ89636.1 anid|CBF88470.1 anig|EHA26878.1 aory|EIT82969.1 asyd|OJJ63781.1 ater|EAU34235.1 atub|OJI85167.1 aver|OJI97156.1 awen|OJJ34812.1 azon|OJJ45737.1

Group5481: abra|OJJ74485.1 acar|OOF95264.1 acla|EAW09107.1 afis|EAW25711.1 afla|EED54262.1 afum|EAL84384.1 agla|OJJ84991.1 akaw|GAA87378.1 aluc|OJZ89634.1 anid|CBF88463.1 anig|EHA26876.1 aory|EIT76037.1 asyd|OJJ63776.1 ater|EAU37423.1 atub|OJI85169.1 aver|OJI97161.1 awen|OJJ38524.1

Group5482: abra|OJJ74501.1 acar|OOF95243.1 acla|EAW07185.1 afis|EAW22198.1 afla|EED57599.1 afum|EBA27419.1 akaw|GAA87395.1 aluc|OJZ89617.1 anid|CBF88433.1 anig|EHA26862.1 aory|EIT82949.1 asyd|OJJ63761.1 ater|EAU34216.1 atub|OJI85186.1 aver|OJI97179.1 awen|OJJ34791.1 azon|OJJ45755.1

Group5483: abra|OJJ74504.1 acar|OOF95240.1 acla|EAW07190.1 afis|EAW22203.1 afla|EED57592.1 afum|EAL90989.1 agla|OJJ87843.1 akaw|GAA87399.1 aluc|OJZ89613.1 anig|EHA26859.1 aory|EIT82935.1 asyd|OJJ63759.1 ater|EAU34211.1 atub|OJI85189.1 aver|OJI97181.1 awen|OJJ32000.1 azon|OJJ50253.1

Group5484: abra|OJJ74507.1 acla|EAW07194.1 afis|EAW22206.1 afla|EED57585.1 afum|EAL90987.2 agla|OJJ87847.1 akaw|GAA82092.1 aluc|OJZ92088.1 anid|CBF88411.1 anig|EHA26853.1 aory|EIT82967.1 asyd|OJJ63755.1 ater|EAU34208.1 atub|OJI86517.1 aver|OJI97186.1 awen|OJJ34781.1 azon|OJJ45763.1

Group5485: abra|OJJ74515.1 acla|EAW07176.1 afis|EAW22190.1 afla|EED57574.1 afum|EAL91001.1 agla|OJJ87857.1 akaw|GAA82084.1 aluc|OJZ92096.1 anid|CBF88398.1 anig|EHA26845.1 aory|EIT82936.1 asyd|OJJ63747.1 ater|EAU34198.1 atub|OJI86529.1 aver|OJI97193.1 awen|OJJ34770.1 azon|OJJ45776.1

Group5486: abra|OJJ74525.1 acla|EAW07166.1 afis|EAW22181.1 afla|EED57560.1 afum|EAL91008.1 agla|OJJ87874.1 akaw|GAA82076.1 aluc|OJZ92105.1 anid|CBF88387.1 anig|EHA26839.1 aory|EIT81854.1 asyd|OJJ63740.1 ater|EAU34186.1 atub|OJI86539.1 aver|OJI97200.1 awen|OJJ34762.1 azon|OJJ45788.1

Group5487: abra|OJJ74526.1 acla|EAW07165.1 afis|EAW22180.1 afla|EED57559.1 afum|EAL91009.1 agla|OJJ87875.1 akaw|GAA82075.1 aluc|OJZ92107.1 anid|CBF88385.1 anig|EHA26838.1 aory|EIT81828.1 asyd|OJJ63739.1 ater|EAU34185.1 atub|OJI86541.1 aver|OJI97201.1 awen|OJJ34761.1 azon|OJJ45789.1

Group5488: abra|OJJ74550.1 acla|EAW07148.1 afis|EAW22162.1 afla|EED57533.1 afum|EAL91029.1 agla|OJJ87902.1 akaw|GAA82047.1 aluc|OJZ92133.1 anid|CBF89096.1 anig|EHA26816.1 aory|EIT81833.1 asyd|OJJ62730.1 ater|EAU34164.1 atub|OJI86565.1 aver|OJI96773.1 awen|OJJ34736.1 azon|OJJ45823.1

Group5489: abra|OJJ74551.1 acar|OOF95204.1 acla|EAW07147.1 afis|EAW22161.1 afla|EED57532.1 afum|EAL91030.1 agla|OJJ87903.1 akaw|GAA82046.1 aluc|OJZ92134.1 anid|CBF89097.1 anig|EHA26815.1 aory|EIT81868.1 asyd|OJJ62729.1 atub|OJI86566.1 aver|OJI96772.1 awen|OJJ34735.1 azon|OJJ45824.1

Group5490: abra|OJJ74572.1 acar|OOF95190.1 acla|EAW07137.1 afis|EAW22150.1 afla|EED53612.1 afum|EAL91040.1 agla|OJJ87912.1 akaw|GAA82033.1 aluc|OJZ92150.1 anid|CBF89110.1 anig|EHA26799.1 aory|EIT75168.1 asyd|OJJ62666.1 atub|OJI86578.1 aver|OJI96764.1 awen|OJJ34726.1 azon|OJJ45836.1

Group5491: abra|OJJ74574.1 acar|OOF95188.1 acla|EAW07135.1 afis|EAW22148.1 afla|EED53609.1 afum|EAL91042.1 agla|OJJ87913.1 akaw|GAA82031.1 aluc|OJZ92152.1 anid|CBF87368.1 anig|EHA26797.1 aory|EIT74984.1 asyd|OJJ52838.1 ater|EAU34154.1 atub|OJI86580.1 aver|OJJ08809.1 awen|OJJ34724.1

Group5492: abra|OJJ74580.1 acar|OOF95184.1 afis|EAW22143.1 afla|EED53569.1 afum|EAL91047.1 agla|OJJ87931.1 akaw|GAA82025.1 aluc|OJZ92158.1 anid|CBF78371.1 anig|EHA26791.1 aory|EIT75000.1 asyd|OJJ57327.1 ater|EAU34146.1 atub|OJI86586.1 aver|OJJ05833.1 awen|OJJ34718.1 azon|OJJ46644.1

Group5493: abra|OJJ74625.1 acla|EAW10757.1 afis|EAW24817.1 afla|EED47165.1 afum|EAL89461.1 agla|OJJ82916.1 akaw|GAA81983.1 aluc|OJZ92207.1 anid|CBF85877.1 anig|EHA26751.1 aory|EIT79320.1 asyd|OJJ64376.1 ater|EAU36489.1 atub|OJI86636.1 aver|OJI98472.1 awen|OJJ40403.1 azon|OJJ49252.1

Group5494: abra|OJJ74638.1 acla|EAW10750.1 afis|EAW24824.1 afla|EED47151.1 afum|EAL89468.1 agla|OJJ82012.1 akaw|GAA81973.1 aluc|OJZ92217.1 anid|CBF85855.1 anig|EHA26741.1 aory|EIT79315.1 asyd|OJJ64365.1 ater|EAU36481.1 atub|OJI86648.1 aver|OJI98460.1 awen|OJJ40410.1 azon|OJJ49259.1

Group5495: abra|OJJ74642.1 acla|EAW10746.1 afis|EAW24828.1 afla|EED47147.1 agla|OJJ82018.1 akaw|GAA81969.1 aluc|OJZ92222.1 anid|CBF85863.1 anig|EHA26736.1 aory|EIT79265.1 asyd|OJJ64369.1 ater|EAU36477.1 atub|OJI86652.1 aver|OJI98465.1 awen|OJJ40414.1 afum|EAL89473.1 azon|OJJ49264.1

Group5496: abra|OJJ74710.1 acar|OOF95720.1 acla|EAW10688.1 afis|EAW24882.1 afla|EED47084.1 afum|EAL89532.1 agla|OJJ82104.1 akaw|GAA81907.1 aluc|OJZ92291.1 anid|CBF75748.1 anig|EHA26678.1 asyd|OJJ57522.1 ater|EAU36419.1 atub|OJI86721.1 aver|OJJ05656.1 awen|OJJ40472.1 azon|OJJ49336.1

Group5497: abra|OJJ74716.1 acar|OOF95725.1 acla|EAW10683.1 afis|EAW24887.1 afum|EAL89538.2 agla|OJJ82110.1 akaw|GAA81902.1 aluc|OJZ92296.1 anid|CBF75621.1 anig|EHA26673.1 aory|EIT72493.1 asyd|OJJ57454.1 ater|EAU36413.1 atub|OJI86727.1 aver|OJJ05724.1 awen|OJJ40477.1 azon|OJJ49341.1

Group5498: abra|OJJ74728.1 acar|OOF90031.1 acla|EAW10670.1 afis|EAW24896.1 afum|EAL89550.1 agla|OJJ82120.1 akaw|GAA81889.1 aluc|OJZ92312.1 anid|CBF75655.1 anig|EHA26657.1 aory|EIT72662.1 asyd|OJJ57464.1 ater|EAU36402.1 atub|OJI86741.1 aver|OJJ05716.1 awen|OJJ40489.1 azon|OJJ49352.1

Group5499: abra|OJJ74748.1 acla|EAW10662.1 afis|EAW24905.1 afla|EED46988.1 afum|EAL89558.1 agla|OJJ82129.1 akaw|GAA83663.1 aluc|OJZ92329.1 anid|CBF75659.1 anig|EHA26642.1 asyd|OJJ57477.1 ater|EAU36393.1 atub|OJI86758.1 aver|OJJ05704.1 awen|OJJ40498.1 azon|OJJ49360.1 aory|EIT72554.1

Group5500: abra|OJJ74753.1 acar|OOF95746.1 acla|EAW10657.1 afis|EAW24910.1 afla|EED46995.1 afum|EAL89564.1 agla|OJJ82134.1 akaw|GAA83658.1 aluc|OJZ92334.1 anig|EHA26637.1 aory|EIT72538.1 asyd|OJJ57482.1 ater|EAU36388.1 atub|OJI86763.1 aver|OJJ05699.1 awen|OJJ40505.1 azon|OJJ49365.1

Group5501: akaw|GAA83616.1 aluc|OJZ92377.1 acar|OOF95790.1 acla|EAW10610.1 afis|EAW24957.1 afla|EED55506.1 afum|EAL89615.2 agla|OJJ82188.1 anid|CBF81665.1 anig|EHA26596.1 aory|EIT80801.1 asyd|OJJ59552.1 ater|EAU35698.1 atub|OJI86807.1 aver|OJJ02716.1 awen|OJJ40551.1 azon|OJJ49410.1

Group5502: abra|OJJ74801.1 acar|OOF95794.1 acla|EAW10613.1 afis|EAW24954.1 afla|EED55510.1 afum|EAL89611.1 agla|OJJ82183.1 akaw|GAA83612.1 aluc|OJZ92382.1 anid|CBF81715.1 anig|EHA26593.1 aory|EIT80800.1 asyd|OJJ59583.1 ater|EAU35704.1 atub|OJI86811.1 awen|OJJ40549.1 azon|OJJ49409.1

Group5503: abra|OJJ74803.1 acla|EAW10615.1 afis|EAW24952.1 afla|EED55512.1 afum|EAL89609.1 agla|OJJ82185.1 akaw|GAA83610.1 aluc|OJZ92384.1 anid|CBF81719.1 anig|EHA26591.1 aory|EIT80787.1 asyd|OJJ59585.1 ater|EAU35684.1 atub|OJI86813.1 aver|OJJ02685.1 awen|OJJ40547.1 azon|OJJ49407.1

Group5504: abra|OJJ74832.1 acar|OOF95820.1 acla|EAW11371.1 afis|EAW18124.1 afla|EED58050.1 afum|EAL87482.1 akaw|GAA83588.1 aluc|OJZ92409.1 anid|CBF75360.1 anig|EHA26556.1 aory|EIT79713.1 asyd|OJJ59850.1 atub|OJI86837.1 aver|OJJ02437.1 awen|OJJ35526.1 azon|OJJ46958.1 agla|OJJ87018.1

Group5505: abra|OJJ74845.1 acar|OOF95846.1 acla|EAW08543.1 afis|EAW23580.1 afla|EED57485.1 afum|EAL87885.1 agla|OJJ86846.1 akaw|GAA83577.1 aluc|OJZ92420.1 anig|EHA26546.1 aory|EIT81877.1 asyd|OJJ62315.1 ater|EAU35364.1 atub|OJI86850.1 aver|OJI96422.1 awen|OJJ39908.1 azon|OJJ50702.1

Group5506: abra|OJJ74846.1 acar|OOF95847.1 acla|EAW08542.1 afis|EAW23579.1 afla|EED57478.1 afum|EAL87886.1 agla|OJJ86844.1 akaw|GAA83576.1 aluc|OJZ92421.1 anid|CBF89596.1 aory|EIT80108.1 asyd|OJJ62312.1 ater|EAU35365.1 atub|OJI86851.1 aver|OJI96420.1 awen|OJJ39907.1 azon|OJJ45884.1

Group5507: abra|OJJ74851.1 acar|OOF95852.1 acla|EAW08536.1 afis|EAW23574.1 afla|EED57465.1 afum|EAL87891.1 agla|OJJ86834.1 akaw|GAA83571.1 aluc|OJZ92427.1 anid|CBF89608.1 anig|EHA26540.1 aory|EIT80065.1 asyd|OJJ62306.1 atub|OJI86857.1 aver|OJI96414.1 awen|OJJ39902.1 azon|OJJ45878.1

Group5508: abra|OJJ74871.1 acar|OOF95872.1 acla|EAW08509.1 afis|EAW23547.1 afla|EED57442.1 afum|EAL87919.1 akaw|GAA83549.1 aluc|OJZ92449.1 anid|CBF89652.1 anig|EHA26519.1 aory|EIT80070.1 asyd|OJJ62283.1 ater|EAU35332.1 atub|OJI86877.1 aver|OJI96391.1 awen|OJJ39878.1 azon|OJJ43225.1

Group5509: abra|OJJ74878.1 acar|OOF95881.1 acla|EAW08492.1 afis|EAW23538.1 afla|EED57436.1 afum|EAL87928.1 agla|OJJ86793.1 akaw|GAA83542.1 aluc|OJZ92455.1 anid|CBF89666.1 anig|EHA26512.1 asyd|OJJ62274.1 ater|EAU35324.1 atub|OJI86884.1 aver|OJI96383.1 awen|OJJ39869.1 azon|OJJ43232.1

Group5510: abra|OJJ74885.1 acar|OOF95890.1 acla|EAW08482.1 afis|EAW23528.1 afla|EED57427.1 afum|EBA27327.1 akaw|GAA83534.1 aluc|OJZ92462.1 anig|EHA26505.1 aory|EIT80107.1 asyd|OJJ62265.1 ater|EAU35316.1 atub|OJI86891.1 aver|OJI96374.1 awen|OJJ39857.1 azon|OJJ43241.1 agla|OJJ86777.1

Group5511: abra|OJJ74887.1 acar|OOF95891.1 acla|EAW08480.1 afis|EAW23526.1 afla|EED57425.1 afum|EAL87939.1 agla|OJJ86775.1 akaw|GAA83532.1 aluc|OJZ92464.1 anid|CBF89689.1 anig|EHA26503.1 asyd|OJJ62263.1 ater|EAU35314.1 atub|OJI86893.1 aver|OJI96372.1 awen|OJJ39855.1 azon|OJJ43246.1

Group5512: abra|OJJ74899.1 acar|OOF95899.1 acla|EAW08473.1 afis|EAW23519.1 afla|EED57419.1 afum|EAL87945.2 agla|OJJ86766.1 akaw|GAA83522.1 aluc|OJZ92473.1 anid|CBF89700.1 anig|EHA26496.1 aory|EIT80138.1 asyd|OJJ62256.1 ater|EAU35307.1 atub|OJI86904.1 aver|OJI96365.1 awen|OJJ39844.1

Group5513: abra|OJJ74906.1 acar|OOF90300.1 acla|EAW08466.1 afis|EAW23513.1 afla|EED57411.1 afum|EAL87950.1 agla|OJJ86761.1 akaw|GAA83516.1 aluc|OJZ92479.1 anid|CBF89707.1 anig|EHA26491.1 asyd|OJJ62251.1 ater|EAU35302.1 atub|OJI86910.1 aver|OJI96361.1 awen|OJJ39837.1 azon|OJJ43254.1

Group5514: abra|OJJ74908.1 acar|OOF90298.1 acla|EAW08464.1 afis|EAW23509.1 afla|EED57409.1 afum|EAL87952.1 agla|OJJ86759.1 akaw|GAA83514.1 aluc|OJZ92481.1 aory|EIT80131.1 asyd|OJJ62246.1 ater|EAU35297.1 atub|OJI86912.1 aver|OJI96356.1 awen|OJJ39835.1 azon|OJJ43257.1 anid|CBF89716.1

Group5515: abra|OJJ74911.1 acar|OOF90295.1 acla|EAW08461.1 afis|EAW23506.1 afla|EED57406.1 afum|EAL87955.1 agla|OJJ86756.1 akaw|GAA83511.1 aluc|OJZ92484.1 anid|CBF89710.1 aory|EIT80118.1 asyd|OJJ62249.1 ater|EAU35294.1 atub|OJI86915.1 aver|OJI96359.1 awen|OJJ39832.1 azon|OJJ43260.1

Group5516: abra|OJJ74912.1 acar|OOF90296.1 acla|EAW08460.1 afis|EAW23505.1 afla|EED57405.1 afum|EAL87956.1 agla|OJJ86752.1 akaw|GAA83510.1 aluc|OJZ92485.1 anid|CBF89721.1 aory|EIT80097.1 asyd|OJJ62237.1 ater|EAU35293.1 atub|OJI86916.1 aver|OJI96346.1 awen|OJJ39831.1 azon|OJJ46407.1

Group5517: abra|OJJ74921.1 acar|OOF95913.1 acla|EAW08450.1 afis|EAW23495.1 afla|EED57395.1 afum|EAL87968.1 agla|OJJ86742.1 akaw|GAA83502.1 aluc|OJZ92492.1 anid|CBF89740.1 anig|EHA26480.1 asyd|OJJ62229.1 ater|EAU35284.1 atub|OJI86925.1 aver|OJI96338.1 awen|OJJ39820.1 azon|OJJ47944.1

Group5518: abra|OJJ74926.1 acla|EAW08443.1 afis|EAW23488.1 afla|EED57389.1 afum|EAL87975.1 agla|OJJ86735.1 akaw|GAA83497.1 aluc|OJZ92497.1 anid|CBF89749.1 anig|EHA26475.1 aory|EIT80081.1 asyd|OJJ62222.1 ater|EAU35277.1 atub|OJI86930.1 aver|OJI96332.1 awen|OJJ39811.1 azon|OJJ47937.1

Group5519: abra|OJJ74929.1 acar|OOF95919.1 acla|EAW08440.1 afis|EAW23485.1 afla|EED57384.1 afum|EAL87978.2 akaw|GAA83492.1 aluc|OJZ92500.1 anid|CBF89759.1 anig|EHA26472.1 aory|EIT80161.1 asyd|OJJ62219.1 ater|EAU35273.1 atub|OJI86933.1 aver|OJI96329.1 awen|OJJ39809.1 azon|OJJ47931.1

Group5520: abra|OJJ74937.1 acla|EAW08434.1 afis|EAW23477.1 afla|EED57374.1 afum|EAL87988.1 agla|OJJ86716.1 akaw|GAA83483.1 aluc|OJZ92512.1 anid|CBF89769.1 anig|EHA26463.1 aory|EIT80086.1 asyd|OJJ62213.1 ater|EAU36903.1 atub|OJI86943.1 aver|OJI96324.1 awen|OJJ39803.1 azon|OJJ47924.1

Group5521: abra|OJJ74968.1 acar|OOF97347.1 acla|EAW08406.1 afis|EAW23463.1 afla|EED57337.1 afum|EAL88003.1 agla|OJJ86686.1 akaw|GAA83458.1 aluc|OJZ92538.1 anid|CBF89845.1 aory|EIT74160.1 asyd|OJJ62184.1 ater|EAU37478.1 atub|OJI86974.1 aver|OJI96293.1 awen|OJJ39767.1 azon|OJJ47897.1

Group5522: abra|OJJ74978.1 acar|OOF97342.1 acla|EAW08402.1 afis|EAW23467.1 afum|EAL87999.1 agla|OJJ86681.1 akaw|GAA83454.1 aluc|OJZ92542.1 anid|CBF89837.1 anig|EHA26434.1 aory|EIT74085.1 asyd|OJJ62180.1 ater|EAU37483.1 atub|OJI86978.1 aver|OJI96289.1 awen|OJJ39762.1 azon|OJJ47891.1

Group5523: abra|OJJ75001.1 acar|OOF90038.1 acla|EAW08384.1 afis|EAW23413.1 afla|EED57314.1 afum|EAL88056.1 agla|OJJ86630.1 akaw|GAA88624.1 aluc|OJZ92566.1 anid|CBF89867.1 anig|EHA26415.1 asyd|OJJ62154.1 ater|EAU37509.1 atub|OJI87002.1 aver|OJI96264.1 awen|OJJ39746.1 azon|OJJ47872.1

Group5524: abra|OJJ75013.1 acar|OOF97313.1 acla|EAW08208.1 afis|EAW23242.1 afla|EED55723.1 afum|EAL88240.1 akaw|GAA88636.1 aluc|OJZ92578.1 anid|CBF89889.1 anig|EHA26403.1 aory|EIT83219.1 asyd|OJJ62141.1 ater|EAU37523.1 atub|OJI87014.1 aver|OJI96251.1 awen|OJJ39728.1 azon|OJJ47859.1

Group5525: abra|OJJ75016.1 acar|OOF97310.1 acla|EAW08211.1 afis|EAW23245.1 afla|EED55726.1 afum|EAL88237.1 agla|OJJ86615.1 akaw|GAA88639.1 aluc|OJZ92582.1 anid|CBF84114.1 anig|EHA26400.1 aory|EIT83230.1 asyd|OJJ57104.1 ater|EAU37526.1 atub|OJI87017.1 aver|OJJ03195.1 awen|OJJ39725.1

Group5526: abra|OJJ75021.1 acar|OOF89973.1 acla|EAW08213.1 afis|EAW23247.1 afla|EED55728.1 afum|EAL88234.2 akaw|GAA88641.1 aluc|OJZ92588.1 anid|CBF84110.1 anig|EHA26398.1 aory|EIT83211.1 asyd|OJJ57102.1 ater|EAU37529.1 atub|OJI87020.1 aver|OJJ03197.1 awen|OJJ39722.1 azon|OJJ47848.1

Group5527: abra|OJJ75023.1 acar|OOF97309.1 acla|EAW08215.1 afis|EAW23249.1 afla|EED55730.1 afum|EAL88232.2 agla|OJJ86607.1 akaw|GAA88643.1 aluc|OJZ92590.1 anid|CBF84107.1 anig|EHA26396.1 asyd|OJJ57099.1 ater|EAU37551.1 atub|OJI87022.1 aver|OJJ03200.1 awen|OJJ39715.1 azon|OJJ47846.1

Group5528: abra|OJJ75024.1 acla|EAW08216.1 afis|EAW23250.1 afla|EED55731.1 afum|EAL88231.2 agla|OJJ86604.1 akaw|GAA88644.1 aluc|OJZ92591.1 anid|CBF84105.1 anig|EHA26395.1 aory|EIT83234.1 asyd|OJJ57098.1 ater|EAU37550.1 atub|OJI87024.1 aver|OJJ03201.1 awen|OJJ39714.1 azon|OJJ47845.1

Group5529: abra|OJJ75029.1 acar|OOF97304.1 acla|EAW08220.1 afis|EAW23254.1 afla|EED55735.1 afum|EAL88227.1 agla|OJJ86600.1 akaw|GAA88648.1 aluc|OJZ92596.1 anid|CBF84098.1 anig|EHA26391.1 asyd|OJJ57094.1 ater|EAU37545.1 atub|OJI87030.1 aver|OJJ03205.1 awen|OJJ39710.1 azon|OJJ47841.1

Group5530: abra|OJJ75037.1 acar|OOF97295.1 acla|EAW08230.1 afis|EAW23264.1 afla|EED55745.1 afum|EAL88216.1 agla|OJJ86588.1 akaw|GAA88656.1 aluc|OJZ92605.1 anig|EHA26383.1 aory|EIT83208.1 asyd|OJJ57111.1 ater|EAU37535.1 atub|OJI87039.1 aver|OJJ03188.1 awen|OJJ39700.1 azon|OJJ47830.1

Group5531: abra|OJJ75045.1 acar|OOF89832.1 acla|EAW08234.1 afis|EAW23268.1 afla|EED55714.1 afum|EAL88211.1 agla|OJJ86584.1 akaw|GAA88662.1 aluc|OJZ92611.1 anid|CBF89552.1 anig|EHA26376.1 asyd|OJJ62374.1 ater|EAU37531.1 atub|OJI87045.1 aver|OJI96508.1 awen|OJJ39693.1 azon|OJJ47826.1

Group5532: abra|OJJ75055.1 acar|OOF97282.1 acla|EAW08240.1 afis|EAW23274.1 afla|EED55704.1 afum|EAL88205.1 agla|OJJ86574.1 akaw|GAA88668.1 aluc|OJZ92620.1 anid|CBF89540.1 anig|EHA26368.1 asyd|OJJ62381.1 ater|EAU37559.1 atub|OJI87054.1 aver|OJI96515.1 awen|OJJ39683.1 azon|OJJ47820.1

Group5533: abra|OJJ75058.1 acar|OOF97276.1 acla|EAW08243.1 afis|EAW23277.1 afla|EED55699.1 afum|EAL88202.1 agla|OJJ86569.1 akaw|GAA88671.1 aluc|OJZ92627.1 anid|CBF89532.1 anig|EHA26363.1 asyd|OJJ62385.1 ater|EAU37562.1 atub|OJI87057.1 aver|OJI96519.1 awen|OJJ39679.1 azon|OJJ47817.1

Group5534: abra|OJJ75067.1 acar|OOF97267.1 acla|EAW08248.1 afis|EAW23282.1 afla|EED55693.1 afum|EAL88197.2 agla|OJJ86564.1 akaw|GAA88679.1 aluc|OJZ92633.1 anid|CBF89523.1 anig|EHA26358.1 asyd|OJJ62390.1 ater|EAU37590.1 atub|OJI87064.1 aver|OJI96523.1 awen|OJJ39674.1 azon|OJJ47804.1

Group5535: abra|OJJ75068.1 acar|OOF97266.1 acla|EAW08249.1 afis|EAW23283.1 afla|EED55692.1 afum|EAL88196.2 agla|OJJ86563.1 akaw|GAA88680.1 aluc|OJZ92634.1 anid|CBF89522.1 anig|EHA26357.1 asyd|OJJ62391.1 ater|EAU37589.1 atub|OJI87065.1 aver|OJI96524.1 awen|OJJ39673.1 azon|OJJ47803.1

Group5536: abra|OJJ75073.1 acar|OOF97260.1 acla|EAW08255.1 afis|EAW23289.1 afla|EED55685.1 afum|EAL88190.1 agla|OJJ86556.1 akaw|GAA88686.1 aluc|OJZ92639.1 anid|CBF89511.1 anig|EHA26352.1 aory|EIT77081.1 asyd|OJJ62396.1 ater|EAU37584.1 atub|OJI87070.1 aver|OJI96530.1 awen|OJJ39668.1

Group5537: abra|OJJ75078.1 acar|OOF97255.1 acla|EAW08286.1 afis|EAW23319.1 afla|EED55677.1 afum|EAL88157.1 agla|OJJ86544.1 akaw|GAA88691.1 aluc|OJZ92644.1 anid|CBF89502.1 anig|EHA26348.1 asyd|OJJ62401.1 ater|EAU37580.1 atub|OJI87075.1 aver|OJI96534.1 awen|OJJ39664.1 azon|OJJ47794.1

Group5538: abra|OJJ75079.1 acar|OOF97254.1 acla|EAW08285.1 afis|EAW23318.1 afum|EAL88158.1 agla|OJJ86543.1 akaw|GAA88692.1 aluc|OJZ92645.1 anid|CBF89500.1 anig|EHA26347.1 aory|EIT77080.1 asyd|OJJ62402.1 ater|EAU37579.1 atub|OJI87076.1 aver|OJI96535.1 awen|OJJ39663.1 azon|OJJ47795.1

Group5539: abra|OJJ75083.1 acar|OOF97250.1 acla|EAW08281.1 afis|EAW23314.1 afla|EED55673.1 afum|EAL88163.1 agla|OJJ86539.1 akaw|GAA88696.1 aluc|OJZ92649.1 anid|CBF89493.1 anig|EHA26343.1 asyd|OJJ62407.1 ater|EAU35547.1 atub|OJI87080.1 aver|OJI96540.1 awen|OJJ39659.1 azon|OJJ47787.1

Group5540: abra|OJJ75088.1 acar|OOF97245.1 acla|EAW08276.1 afis|EAW23309.1 afla|EED55664.1 afum|EAL88168.1 agla|OJJ86534.1 akaw|GAA88700.1 aluc|OJZ92654.1 anid|CBF89483.1 anig|EHA26340.1 aory|EIT81812.1 ater|EAU35552.1 atub|OJI87085.1 aver|OJI96547.1 awen|OJJ39654.1 azon|OJJ47782.1

Group5541: abra|OJJ75092.1 acar|OOF97240.1 acla|EAW08272.1 afis|EAW23305.1 afla|EED55660.1 afum|EAL88172.1 agla|OJJ86530.1 akaw|GAA88704.1 aluc|OJZ92658.1 anid|CBF89476.1 anig|EHA26336.1 aory|EIT81785.1 asyd|OJJ62417.1 atub|OJI87089.1 aver|OJI96552.1 awen|OJJ39587.1 azon|OJJ47778.1

Group5542: abra|OJJ75095.1 acar|OOF97237.1 acla|EAW08269.1 afis|EAW23302.1 afla|EED55654.1 afum|EAL88177.1 agla|OJJ86527.1 akaw|GAA88707.1 aluc|OJZ92661.1 anid|CBF89470.1 anig|EHA26333.1 aory|EIT81821.1 asyd|OJJ62420.1 ater|EAU35566.1 atub|OJI87092.1 awen|OJJ39584.1 azon|OJJ47768.1

Group5543: abra|OJJ75100.1 acar|OOF97234.1 acla|EAW08267.1 afis|EAW23300.1 afla|EED55652.1 afum|EAL88179.1 agla|OJJ86509.1 akaw|GAA88709.1 aluc|OJZ92664.1 anid|CBF89467.1 anig|EHA26330.1 asyd|OJJ62422.1 ater|EAU35563.1 atub|OJI87095.1 aver|OJI96559.1 awen|OJJ39582.1 azon|OJJ47770.1

Group5544: abra|OJJ75105.1 acar|OOF97229.1 acla|EAW08263.1 afis|EAW23296.1 afla|EED55646.1 afum|EAL88182.1 agla|OJJ86515.1 akaw|GAA88714.1 aluc|OJZ92670.1 anid|CBF89461.1 anig|EHA26327.1 aory|EIT81781.1 asyd|OJJ62426.1 atub|OJI87099.1 aver|OJI96563.1 awen|OJJ39577.1 azon|OJJ47774.1

Group5545: abra|OJJ75108.1 acar|OOF97225.1 acla|EAW08260.1 afis|EAW23293.1 afla|EED55643.1 afum|EAL88186.1 agla|OJJ86518.1 akaw|GAA88717.1 aluc|OJZ92674.1 anid|CBF89455.1 anig|EHA26324.1 asyd|OJJ62430.1 ater|EAU35556.1 atub|OJI87102.1 aver|OJI96566.1 awen|OJJ39574.1 azon|OJJ47764.1

Group5546: abra|OJJ75112.1 acar|OOF97219.1 acla|EAW08312.1 afis|EAW23328.1 afla|EED55639.1 afum|EAL88151.1 agla|OJJ86523.1 akaw|GAA88722.1 aluc|OJZ92679.1 anid|CBF89444.1 anig|EHA26320.1 asyd|OJJ62437.1 ater|EAU35575.1 atub|OJI87107.1 aver|OJI96573.1 awen|OJJ39566.1 azon|OJJ47759.1

Group5547: abra|OJJ75113.1 acar|OOF97218.1 acla|EAW08311.1 afis|EAW23329.1 afla|EED55638.1 afum|EAL88150.1 agla|OJJ86524.1 akaw|GAA88723.1 aluc|OJZ92680.1 anid|CBF89443.1 anig|EHA26319.1 asyd|OJJ62438.1 ater|EAU35576.1 atub|OJI87108.1 awen|OJJ39565.1 azon|OJJ47758.1 aver|OJI96574.1

Group5548: abra|OJJ75128.1 akaw|GAA88735.1 anig|EHA26307.1 atub|OJI87121.1 asyd|OJJ62447.1 aver|OJI96584.1 acla|EAW08301.1 afla|EED55627.1 afum|EAL88139.1 aory|EIT81809.1 afis|EAW23339.1 anid|CBF89427.1 ater|EAU35584.1 agla|OJJ86491.1 awen|OJJ39555.1 azon|OJJ47752.1 aluc|OJZ92693.1

Group5549: abra|OJJ75132.1 acla|EAW08295.1 afis|EAW23344.1 afla|EED55622.1 afum|EAL88133.1 agla|OJJ86483.1 akaw|GAA88739.1 aluc|OJZ92698.1 anid|CBF89414.1 anig|EHA26301.1 aory|EIT81796.1 asyd|OJJ62454.1 ater|EAU35588.1 atub|OJI87126.1 aver|OJI96589.1 awen|OJJ39548.1 azon|OJJ47745.1

Group5550: abra|OJJ75151.1 acar|OOF97186.1 acla|EAW08326.1 afis|EAW23361.1 afla|EED55597.1 afum|EAL88113.1 agla|OJJ86465.1 aluc|OJZ92719.1 anid|CBF74877.1 anig|EHA26281.1 aory|EIT82694.1 asyd|OJJ60113.1 ater|EAU35612.1 atub|OJI87144.1 aver|OJJ04149.1 awen|OJJ39526.1 azon|OJJ47722.1

Group5551: abra|OJJ75154.1 acla|EAW08329.1 afis|EAW23364.1 afla|EED55594.1 afum|EAL88109.1 agla|OJJ86462.1 akaw|GAA88759.1 aluc|OJZ92722.1 anid|CBF74833.1 anig|EHA26278.1 aory|EIT82699.1 asyd|OJJ60136.1 ater|EAU35615.1 atub|OJI87147.1 aver|OJJ04126.1 awen|OJJ39523.1 azon|OJJ47719.1

Group5552: abra|OJJ75176.1 acar|OOF94603.1 acla|EAW13002.1 afis|EAW17666.1 afla|EED50489.1 afum|EAL91480.2 akaw|GAA88781.1 aluc|OJZ92742.1 anid|CBF90045.1 anig|EHA26261.1 aory|EIT82041.1 asyd|OJJ62057.1 ater|EAU31987.1 atub|OJI87167.1 aver|OJI96163.1 awen|OJJ37175.1 azon|OJJ50874.1

Group5553: abra|OJJ75191.1 acar|OOF94592.1 acla|EAW12988.1 afis|EAW17677.1 afla|EED50474.1 afum|EAL91490.1 agla|OJJ79501.1 akaw|GAA88792.1 aluc|OJZ92755.1 anid|CBF90020.1 anig|EHA26250.1 asyd|OJJ62069.1 ater|EAU31969.1 atub|OJI87181.1 aver|OJI96175.1 awen|OJJ37163.1 azon|OJJ50859.1

Group5554: abra|OJJ75192.1 acar|OOF94591.1 acla|EAW12987.1 afis|EAW17678.1 afla|EED50472.1 afum|EAL91491.1 agla|OJJ79502.1 akaw|GAA91745.1 aluc|OJZ92756.1 anid|CBF90017.1 anig|EHA26249.1 asyd|OJJ62071.1 ater|EAU31968.1 atub|OJI87183.1 aver|OJI96176.1 awen|OJJ37161.1 azon|OJJ50858.1

Group5555: abra|OJJ75202.1 acar|OOF94586.1 acla|EAW12980.1 afis|EAW17684.1 afla|EED50467.1 afum|EAL91498.1 agla|OJJ79495.1 akaw|GAA91753.1 aluc|OJZ92765.1 anid|CBF90004.1 anig|EHA26242.1 asyd|OJJ62077.1 ater|EAU31947.1 atub|OJI87209.1 aver|OJI96184.1 awen|OJJ37155.1 azon|OJJ50853.1

Group5556: abra|OJJ75204.1 acar|OOF94584.1 acla|EAW12978.1 afis|EAW17686.1 afla|EED50465.1 afum|EAL91500.1 agla|OJJ79493.1 akaw|GAA91755.1 aluc|OJZ92767.1 anid|CBF89989.1 anig|EHA26240.1 asyd|OJJ62084.1 ater|EAU31949.1 atub|OJI87211.1 aver|OJI96191.1 awen|OJJ37153.1 azon|OJJ50851.1

Group5557: abra|OJJ75216.1 acar|OOF94568.1 acla|EAW13095.1 afis|EAW17568.1 afla|EED49253.1 afum|EBA27431.1 agla|OJJ81397.1 akaw|GAA91766.1 aluc|OJZ92780.1 anid|CBF90260.1 anig|EHA26230.1 asyd|OJJ61942.1 ater|EAU37005.1 atub|OJI87222.1 aver|OJI96034.1 awen|OJJ37283.1 azon|OJJ50979.1

Group5558: abra|OJJ75243.1 acar|OOF94550.1 acla|EAW13700.1 afis|EAW19906.1 afla|EED45982.1 afum|EAL85001.1 akaw|GAA91786.1 aluc|OJZ92805.1 anid|CBF78482.1 anig|EHA26204.1 aory|EIT75561.1 asyd|OJJ65334.1 ater|EAU31595.1 atub|OJI87248.1 aver|OJI99479.1 awen|OJJ30802.1 azon|OJJ47379.1

Group5559: awen|OJJ30208.1 abra|OJJ77534.1 acar|OOF99192.1 afis|EAW16248.1 afla|EED53312.1 afum|EAL88876.1 agla|OJJ86258.1 akaw|GAA84087.1 aluc|OJZ81002.1 anid|CBF78877.1 anig|EHA19358.1 aory|EIT74927.1 asyd|OJJ64593.1 ater|EAU37458.1 atub|OJI82583.1 aver|OJI98690.1 azon|OJJ43932.1

Group5560: abra|OJJ75273.1 acar|OOF94530.1 afis|EAW16992.1 afla|EED48963.1 afum|EAL86516.1 akaw|GAA91811.1 aluc|OJZ92833.1 anid|CBF77694.1 anig|EHA26176.1 aory|EIT80034.1 asyd|OJJ53081.1 ater|EAU31736.1 atub|OJI87290.1 aver|OJJ03766.1 awen|OJJ35980.1 azon|OJJ50285.1 agla|OJJ80492.1

Group5561: abra|OJJ75340.1 acar|OOF89855.1 acla|EAW07715.1 afum|EAL86289.1 akaw|GAA92033.1 aluc|OJZ92903.1 anid|CBF81870.1 anig|EHA26060.1 asyd|OJJ53488.1 atub|OJI87362.1 aver|OJJ00349.1 awen|OJJ40854.1 azon|OJJ43191.1 azon|OJJ50965.1 ater|EAU31350.1 azon|OJJ42649.1 azon|OJJ45030.1

Group5562: abra|OJJ75378.1 acar|OOF94469.1 acla|EAW12114.1 afis|EAW17271.1 afla|EED48848.1 afum|EAL86688.1 agla|OJJ81075.1 akaw|GAA92740.1 aluc|OJZ92948.1 anid|CBF87533.1 aory|EIT81268.1 asyd|OJJ52987.1 ater|EAU36135.1 atub|OJI87407.1 aver|OJJ08956.1 awen|OJJ35763.1 azon|OJJ45521.1

Group5563: abra|OJJ75379.1 acar|OOF94468.1 acla|EAW12113.1 afis|EAW17272.1 afla|EED48847.1 afum|EAL86689.1 agla|OJJ81076.1 akaw|GAA92741.1 aluc|OJZ92949.1 anid|CBF87535.1 aory|EIT81287.1 asyd|OJJ52988.1 ater|EAU36136.1 atub|OJI87408.1 aver|OJJ08957.1 awen|OJJ35762.1 azon|OJJ45522.1

Group5564: abra|OJJ75380.1 acar|OOF94467.1 acla|EAW12112.1 afis|EAW17273.1 afla|EED48846.1 afum|EAL86691.1 agla|OJJ81077.1 akaw|GAA92742.1 aluc|OJZ92950.1 anid|CBF87537.1 aory|EIT81267.1 asyd|OJJ52989.1 ater|EAU34714.1 atub|OJI87409.1 aver|OJJ08958.1 awen|OJJ35761.1 azon|OJJ45523.1

Group5565: abra|OJJ75386.1 acar|OOF94462.1 acla|EAW12106.1 afis|EAW17279.1 afla|EED48840.1 afum|EAL86697.1 agla|OJJ81083.1 akaw|GAA92748.1 aluc|OJZ92956.1 anid|CBF87547.1 anig|EHA26019.1 asyd|OJJ52995.1 ater|EAU34708.1 atub|OJI87415.1 aver|OJJ08964.1 awen|OJJ35755.1 azon|OJJ45529.1

Group5566: abra|OJJ75387.1 acar|OOF94459.1 acla|EAW12105.1 afis|EAW17281.1 afla|EED48838.1 afum|EAL86699.1 agla|OJJ81084.1 akaw|GAA92750.1 aluc|OJZ92958.1 anid|CBF87551.1 anig|EHA26017.1 asyd|OJJ52997.1 ater|EAU34707.1 atub|OJI87416.1 aver|OJJ08966.1 awen|OJJ35754.1 azon|OJJ45530.1

Group5567: abra|OJJ75403.1 acar|OOF90750.1 acla|EAW13775.1 afis|EAW18357.1 afla|EED48822.1 afum|EAL87721.1 agla|OJJ88028.1 aluc|OJZ88855.1 anid|CBF79354.1 anig|EHA23648.1 aory|EIT73028.1 asyd|OJJ57801.1 atub|OJI85935.1 aver|OJJ04586.1 awen|OJJ35741.1 azon|OJJ43325.1 afum|EAL87720.1

Group5568: abra|OJJ75407.1 acar|OOF90752.1 acla|EAW13779.1 afis|EAW18353.1 afla|EED48818.1 afum|EAL87716.1 agla|OJJ88031.1 aluc|OJZ88858.1 anid|CBF79362.1 anig|EHA23645.1 aory|EIT73012.1 asyd|OJJ57805.1 ater|EAU34687.1 atub|OJI85932.1 aver|OJJ04583.1 awen|OJJ35737.1 azon|OJJ50439.1

Group5569: abra|OJJ75416.1 acar|OOF90761.1 acla|EAW13788.1 afis|EAW18344.1 afla|EED48809.1 afum|EAL87708.1 agla|OJJ88040.1 akaw|GAA84191.1 aluc|OJZ88867.1 anid|CBF79380.1 anig|EHA23636.1 aory|EIT73003.1 asyd|OJJ57814.1 ater|EAU34679.1 atub|OJI85923.1 aver|OJJ04574.1 awen|OJJ35728.1

Group5570: abra|OJJ75418.1 acar|OOF90763.1 acla|EAW13790.1 afis|EAW18342.1 afla|EED48807.1 afum|EAL87705.1 agla|OJJ88042.1 akaw|GAA84193.1 aluc|OJZ88869.1 anid|CBF79376.1 anig|EHA23634.1 aory|EIT73006.1 asyd|OJJ57816.1 ater|EAU34677.1 atub|OJI85921.1 awen|OJJ35726.1 azon|OJJ43314.1

Group5571: abra|OJJ75422.1 acar|OOF90767.1 acla|EAW13794.1 afis|EAW18338.1 afla|EED48802.1 agla|OJJ88046.1 akaw|GAA84197.1 aluc|OJZ88874.1 anid|CBF79389.1 anig|EHA23631.1 aory|EIT72987.1 asyd|OJJ57820.1 ater|EAU34673.1 atub|OJI85917.1 aver|OJJ04571.1 awen|OJJ35721.1 azon|OJJ43311.1

Group5572: abra|OJJ75437.1 acar|OOF90781.1 acla|EAW13825.1 afis|EAW18306.1 afla|EED48775.1 afum|EAL87672.1 agla|OJJ88117.1 akaw|GAA84212.1 aluc|OJZ88890.1 anid|CBF79443.1 anig|EHA23617.1 aory|EIT72975.1 asyd|OJJ57849.1 ater|EAU33316.1 atub|OJI85899.1 aver|OJJ04543.1 awen|OJJ30680.1

Group5573: abra|OJJ75440.1 acar|OOF90784.1 acla|EAW13823.1 afis|EAW18308.1 afum|EAL87674.1 agla|OJJ88074.1 akaw|GAA84215.1 aluc|OJZ88893.1 anid|CBF79440.1 anig|EHA23613.1 aory|EIT72977.1 asyd|OJJ57847.1 ater|EAU33318.1 atub|OJI85896.1 aver|OJJ04545.1 awen|OJJ30682.1 azon|OJJ43282.1

Group5574: abra|OJJ75451.1 acar|OOF90795.1 acla|EAW13812.1 afis|EAW18318.1 afla|EED48786.1 afum|EAL87685.1 agla|OJJ88100.1 akaw|GAA84226.1 aluc|OJZ88906.1 anid|CBF79419.1 anig|EHA23602.1 asyd|OJJ57835.1 ater|EAU33328.1 atub|OJI85885.1 aver|OJJ04555.1 awen|OJJ30700.1 azon|OJJ43293.1

Group5575: abra|OJJ75453.1 acar|OOF90797.1 acla|EAW13810.1 afis|EAW18320.1 afla|EED48788.1 agla|OJJ88071.1 akaw|GAA84228.1 aluc|OJZ88908.1 anid|CBF79417.1 anig|EHA23600.1 aory|EIT73031.1 asyd|OJJ57834.1 ater|EAU33330.1 atub|OJI85883.1 aver|OJJ04556.1 awen|OJJ30702.1 azon|OJJ43294.1

Group5576: abra|OJJ75458.1 acar|OOF90280.1 acla|EAW13807.1 afis|EAW18323.1 afla|EED48791.1 afum|EAL87690.2 agla|OJJ88068.1 akaw|GAA84233.1 aluc|OJZ88915.1 anid|CBF79412.1 anig|EHA23596.1 asyd|OJJ57831.1 ater|EAU33333.1 atub|OJI85877.1 aver|OJJ04559.1 awen|OJJ30705.1 azon|OJJ43299.1

Group5577: abra|OJJ75469.1 acar|OOF90804.1 acla|EAW13835.1 afis|EAW18296.1 afla|EED48762.1 afum|EAL87662.1 agla|OJJ88127.1 akaw|GAA84241.1 aluc|OJZ88926.1 anid|CBF79468.1 anig|EHA23587.1 asyd|OJJ57864.1 ater|EAU33304.1 atub|OJI85866.1 aver|OJJ04529.1 awen|OJJ30670.1 azon|OJJ43270.1

Group5578: abra|OJJ75489.1 acar|OOF90821.1 acla|EAW11220.1 afis|EAW18283.1 afla|EED48750.1 afum|EAL87649.1 agla|OJJ88144.1 aluc|OJZ88948.1 anid|CBF79485.1 anig|EHA23573.1 aory|EIT73079.1 asyd|OJJ57874.1 ater|EAU33289.1 atub|OJI85841.1 aver|OJJ04520.1 awen|OJJ30654.1 azon|OJJ47960.1

Group5579: abra|OJJ75524.1 acar|OOF90851.1 acla|EAW09311.1 afis|EAW21834.1 afla|EED46541.1 afum|EAL92849.1 agla|OJJ84310.1 akaw|GAA84287.1 aluc|OJZ88981.1 anig|EHA23545.1 aory|EIT77894.1 asyd|OJJ61231.1 ater|EAU38509.1 atub|OJI85808.1 aver|OJJ00554.1 awen|OJJ34107.1 azon|OJJ44234.1

Group5580: abra|OJJ75547.1 acar|OOF90867.1 acla|EAW09318.1 afis|EAW21827.1 afla|EED46549.1 afum|EAL92842.1 agla|OJJ84317.1 akaw|GAA84302.1 aluc|OJZ88997.1 anid|CBF76668.1 anig|EHA23531.1 asyd|OJJ61218.1 ater|EAU38503.1 atub|OJI85792.1 aver|OJJ00542.1 awen|OJJ34100.1 azon|OJJ44244.1

Group5581: abra|OJJ75550.1 acar|OOF90869.1 afis|EAW21823.1 afla|EED46555.1 afum|EAL92839.1 agla|OJJ84319.1 akaw|GAA84305.1 aluc|OJZ89000.1 anid|CBF86661.1 anig|EHA23528.1 aory|EIT77858.1 asyd|OJJ61620.1 ater|EAU38497.1 atub|OJI85789.1 aver|OJJ00944.1 awen|OJJ34097.1 azon|OJJ44247.1

Group5582: abra|OJJ75551.1 acar|OOF90871.1 acla|EAW09323.1 afis|EAW21822.1 afla|EED46557.1 afum|EAL92838.1 agla|OJJ84320.1 akaw|GAA84306.1 aluc|OJZ89001.1 anid|CBF76662.1 anig|EHA23527.1 asyd|OJJ61214.1 ater|EAU38496.1 atub|OJI85788.1 aver|OJJ00538.1 awen|OJJ34096.1 azon|OJJ44248.1

Group5583: abra|OJJ75580.1 acar|OOF90898.1 acla|EAW09338.1 afis|EAW21793.1 afla|EED46606.1 afum|EAL92818.1 agla|OJJ83098.1 akaw|GAA84332.1 aluc|OJZ89031.1 anid|CBF76621.1 anig|EHA23503.1 aory|EIT77822.1 asyd|OJJ61191.1 ater|EAU38472.1 atub|OJI85760.1 aver|OJJ00515.1 awen|OJJ34077.1

Group5584: abra|OJJ75591.1 acar|OOG00237.1 acla|EAW09331.1 afis|EAW21787.1 afla|EED46609.1 afum|EAL92814.1 agla|OJJ84336.1 akaw|GAA84343.1 aluc|OJZ89043.1 anid|CBF76619.1 anig|EHA23492.1 asyd|OJJ61190.1 ater|EAU38470.1 atub|OJI85744.1 aver|OJJ00514.1 awen|OJJ34073.1 azon|OJJ48156.1

Group5585: abra|OJJ75604.1 acar|OOG00233.1 acla|EAW09366.1 afis|EAW21771.1 afla|EED46329.1 afum|EAL92800.1 agla|OJJ84379.1 akaw|GAA84355.1 aluc|OJZ89056.1 anid|CBF83760.1 anig|EHA23485.1 asyd|OJJ60862.1 ater|EAU38447.1 atub|OJI85731.1 aver|OJJ00188.1 awen|OJJ34058.1 azon|OJJ48174.1

Group5586: abra|OJJ75613.1 acar|OOG00223.1 acla|EAW09365.1 afis|EAW21772.1 afla|EED46338.1 afum|EAL92801.1 agla|OJJ84352.1 akaw|GAA84362.1 aluc|OJZ89066.1 anid|CBF76593.1 anig|EHA23470.1 aory|EIT81227.1 asyd|OJJ61176.1 atub|OJI85722.1 aver|OJJ00502.1 awen|OJJ34059.1 azon|OJJ48173.1

Group5587: abra|OJJ75617.1 acla|EAW09361.1 afis|EAW21776.1 afla|EED46342.1 afum|EAL92805.1 agla|OJJ84348.1 akaw|GAA84366.1 aluc|OJZ89070.1 anid|CBF76601.1 anig|EHA23466.1 aory|EIT81196.1 asyd|OJJ61181.1 ater|EAU38463.1 atub|OJI85718.1 aver|OJJ00506.1 awen|OJJ34064.1 azon|OJJ48168.1

Group5588: abra|OJJ75623.1 acar|OOG00219.1 acla|EAW09358.1 afis|EAW21779.1 afla|EED46346.1 afum|EAL92808.1 akaw|GAA84370.1 aluc|OJZ89075.1 anid|CBF76605.1 anig|EHA23463.1 aory|EIT81237.1 asyd|OJJ61184.1 ater|EAU38461.1 atub|OJI85711.1 aver|OJJ00508.1 awen|OJJ34068.1 azon|OJJ48161.1

Group5589: abra|OJJ75627.1 acla|EAW09466.1 afis|EAW21670.1 afla|EED57934.1 afum|EAL92705.1 agla|OJJ79744.1 akaw|GAA84374.1 aluc|OJZ89078.1 anid|CBF83584.1 anig|EHA23459.1 aory|EIT82346.1 asyd|OJJ60780.1 ater|EAU38354.1 atub|OJI85707.1 aver|OJJ00113.1 awen|OJJ33934.1 azon|OJJ48641.1

Group5590: abra|OJJ75629.1 acar|OOG00206.1 acla|EAW09464.1 afis|EAW21672.1 afum|EAL92707.1 agla|OJJ79746.1 akaw|GAA84376.1 aluc|OJZ89080.1 anid|CBF83587.1 anig|EHA23457.1 aory|EIT82330.1 asyd|OJJ60778.1 ater|EAU38356.1 atub|OJI85705.1 aver|OJJ00111.1 awen|OJJ33937.1 azon|OJJ48630.1

Group5591: abra|OJJ75643.1 acla|EAW09455.1 afis|EAW21682.1 afla|EED57949.1 afum|EAL92716.2 agla|OJJ79919.1 akaw|GAA84388.1 aluc|OJZ89092.1 anid|CBF83604.1 anig|EHA23445.1 aory|EIT82329.1 asyd|OJJ60767.1 ater|EAU38366.1 atub|OJI85689.1 aver|OJJ00101.1 awen|OJJ33949.1 azon|OJJ48621.1

Group5592: abra|OJJ75653.1 acla|EAW09447.1 afis|EAW21689.1 afla|EED57959.1 afum|EAL92723.2 agla|OJJ79911.1 akaw|GAA84396.1 aluc|OJZ89102.1 anid|CBF83616.1 anig|EHA23437.1 aory|EIT79725.1 asyd|OJJ60759.1 ater|EAU38374.1 atub|OJI85679.1 aver|OJJ00093.1 awen|OJJ33958.1 azon|OJJ48610.1

Group5593: abra|OJJ75655.1 acla|EAW09445.1 afis|EAW21691.1 afla|EED57961.1 afum|EAL92725.1 agla|OJJ79909.1 akaw|GAA84398.1 aluc|OJZ89104.1 anid|CBF83619.1 anig|EHA23435.1 aory|EIT79711.1 asyd|OJJ60757.1 ater|EAU38376.1 atub|OJI85677.1 aver|OJJ00091.1 awen|OJJ33960.1 azon|OJJ48608.1

Group5594: abra|OJJ75660.1 acar|OOG00183.1 acla|EAW09440.1 afis|EAW21696.1 afla|EED57965.1 afum|EAL92730.1 agla|OJJ79903.1 akaw|GAA84402.1 aluc|OJZ89110.1 anid|CBF83626.1 anig|EHA23431.1 asyd|OJJ60753.1 ater|EAU38381.1 atub|OJI85673.1 aver|OJJ00087.1 awen|OJJ33965.1 azon|OJJ48604.1

Group5595: abra|OJJ75663.1 acar|OOG00182.1 acla|EAW09438.1 afis|EAW21698.1 afla|EED57967.1 afum|EAL92732.1 agla|OJJ79900.1 akaw|GAA84404.1 aluc|OJZ89114.1 anid|CBF83630.1 anig|EHA23429.1 asyd|OJJ60751.1 ater|EAU38383.1 atub|OJI85670.1 aver|OJJ00084.1 awen|OJJ33967.1 azon|OJJ48599.1

Group5596: abra|OJJ75666.1 acar|OOG00178.1 acla|EAW09435.1 afis|EAW21701.1 afla|EED57970.1 afum|EAL92735.1 agla|OJJ79897.1 akaw|GAA84407.1 aluc|OJZ89117.1 anid|CBF83635.1 anig|EHA23426.1 asyd|OJJ60783.1 ater|EAU38386.1 atub|OJI85667.1 aver|OJJ00116.1 awen|OJJ33971.1 azon|OJJ48588.1

Group5597: abra|OJJ75676.1 acla|EAW09429.1 afis|EAW21707.1 afla|EED57979.1 afum|EAL92742.1 agla|OJJ79894.1 akaw|GAA84417.1 aluc|OJZ89126.1 anid|CBF83651.1 anig|EHA23417.1 aory|EIT79779.1 asyd|OJJ60793.1 ater|EAU38395.1 atub|OJI85658.1 aver|OJJ00126.1 awen|OJJ33983.1 azon|OJJ48576.1

Group5598: abra|OJJ75681.1 acar|OOG00167.1 acla|EAW09421.1 afis|EAW21715.1 afla|EED57985.1 afum|EBA27470.1 agla|OJJ79884.1 akaw|GAA84421.1 aluc|OJZ89132.1 anig|EHA23413.1 aory|EIT79799.1 asyd|OJJ60797.1 ater|EAU38400.1 atub|OJI85654.1 aver|OJJ00130.1 awen|OJJ33987.1 azon|OJJ48566.1

Group5599: abra|OJJ75693.1 acar|OOG00159.1 acla|EAW07961.1 afis|EAW22996.1 afla|EED49502.1 agla|OJJ83288.1 akaw|GAA84430.1 aluc|OJZ89145.1 anid|CBF80648.1 anig|EHA23403.1 aory|EIT82603.1 asyd|OJJ54616.1 ater|EAU29307.1 atub|OJI85643.1 aver|OJJ07543.1 awen|OJJ38565.1 azon|OJJ43626.1

Group5600: abra|OJJ75725.1 acar|OOG00133.1 acla|EAW09381.1 afis|EAW21754.1 afla|EED58024.1 afum|EAL92784.1 agla|OJJ84399.1 akaw|GAA84461.1 aluc|OJZ89177.1 anid|CBF83689.1 anig|EHA23378.1 asyd|OJJ60818.1 ater|EAU38436.1 atub|OJI85612.1 aver|OJJ00144.1 awen|OJJ34038.1 azon|OJJ48208.1

Group5601: abra|OJJ75782.1 acla|EAW14900.1 afis|EAW24422.1 afla|EED47746.1 afum|EAL86090.1 agla|OJJ79222.1 akaw|GAA84499.1 aluc|OJZ89224.1 anid|CBF73788.1 anig|EHA23337.1 aory|EIT77143.1 asyd|OJJ56602.1 ater|EAU30306.1 atub|OJI85567.1 aver|OJJ06122.1 awen|OJJ32787.1 azon|OJJ49043.1

Group5602: abra|OJJ75803.1 acar|OOG00073.1 acla|EAW14880.1 afis|EAW24398.1 afla|EED47813.1 afum|EAL86069.1 agla|OJJ78879.1 akaw|GAA84519.1 aluc|OJZ89251.1 anid|CBF73726.1 anig|EHA23316.1 aory|EIT77134.1 asyd|OJJ56635.1 atub|OJI85547.1 aver|OJJ06091.1 awen|OJJ32807.1 azon|OJJ49029.1

Group5603: abra|OJJ75818.1 acar|OOG00057.1 acla|EAW14857.1 afis|EAW24384.1 afla|EED47825.1 afum|EAL86056.1 akaw|GAA84532.1 aluc|OJZ89265.1 anid|CBF73699.1 anig|EHA23303.1 aory|EIT77173.1 asyd|OJJ56649.1 ater|EAU30351.1 atub|OJI85534.1 aver|OJJ06077.1 awen|OJJ32827.1 azon|OJJ49010.1

Group5604: abra|OJJ75826.1 acar|OOG00053.1 acla|EAW14851.1 afis|EAW24378.1 afla|EED47833.1 afum|EAL86050.1 agla|OJJ83264.1 akaw|GAA84537.1 aluc|OJZ89270.1 anid|CBF73672.1 anig|EHA23299.1 aory|EIT77204.1 asyd|OJJ56663.1 atub|OJI85529.1 aver|OJJ06061.1 awen|OJJ32834.1 azon|OJJ49001.1

Group5605: abra|OJJ75830.1 acar|OOG00050.1 acla|EAW14848.1 afis|EAW24375.1 afla|EED47836.1 afum|EAL86047.1 agla|OJJ83261.1 akaw|GAA84540.1 aluc|OJZ89273.1 anig|EHA23296.1 aory|EIT77176.1 asyd|OJJ56658.1 ater|EAU30361.1 atub|OJI85526.1 aver|OJJ06067.1 awen|OJJ32837.1 azon|OJJ48997.1

Group5606: abra|OJJ75842.1 acar|OOG00046.1 acla|EAW14845.1 afis|EAW24372.1 afla|EED47842.1 afum|EAL86044.1 agla|OJJ83257.1 akaw|GAA84552.1 aluc|OJZ89284.1 anid|CBF73686.1 anig|EHA23282.1 aory|EIT77226.1 asyd|OJJ56655.1 ater|EAU30365.1 atub|OJI85515.1 aver|OJJ06070.1 awen|OJJ32842.1

Group5607: abra|OJJ75853.1 acla|EAW14834.1 afis|EAW24363.1 afla|EED47850.1 afum|EAL86034.1 agla|OJJ83248.1 akaw|GAA84558.1 aluc|OJZ89290.1 anid|CBF73930.1 anig|EHA23272.1 aory|EIT77222.1 asyd|OJJ53676.1 ater|EAU30375.1 atub|OJI85509.1 aver|OJJ07792.1 awen|OJJ32868.1 azon|OJJ48990.1

Group5608: abra|OJJ75857.1 acar|OOG00039.1 acla|EAW14830.1 afis|EAW24359.1 afla|EED47854.1 afum|EAL86029.1 agla|OJJ83244.1 akaw|GAA84562.1 aluc|OJZ89295.1 anid|CBF73923.1 anig|EHA23268.1 asyd|OJJ53680.1 ater|EAU30379.1 atub|OJI85505.1 aver|OJJ07796.1 awen|OJJ32862.1 azon|OJJ48986.1

Group5609: abra|OJJ75861.1 acar|OOG00035.1 acla|EAW14828.1 afis|EAW24357.1 afla|EED47857.1 afum|EAL86027.1 agla|OJJ83239.1 akaw|GAA84566.1 aluc|OJZ89299.1 anid|CBF73919.1 anig|EHA23264.1 aory|EIT77243.1 asyd|OJJ53682.1 ater|EAU30382.1 atub|OJI85499.1 aver|OJJ07799.1 awen|OJJ32860.1

Group5610: abra|OJJ75862.1 acar|OOG00034.1 acla|EAW14827.1 afis|EAW24356.1 afla|EED47906.1 afum|EAL86026.2 akaw|GAA84568.1 aluc|OJZ89303.1 anid|CBF73945.1 anig|EHA23263.1 aory|EIT83365.1 asyd|OJJ53697.1 ater|EAU30390.1 atub|OJI85496.1 aver|OJJ07813.1 awen|OJJ32870.1 azon|OJJ48983.1

Group5611: abra|OJJ75868.1 acar|OOG00026.1 acla|EAW09929.1 afis|EAW21177.1 afla|EED54764.1 afum|EAL92197.1 agla|OJJ80000.1 akaw|GAA84574.1 aluc|OJZ89310.1 anid|CBF83304.1 anig|EHA23256.1 aory|EIT79450.1 asyd|OJJ60554.1 atub|OJI85489.1 aver|OJI99880.1 awen|OJJ36516.1 azon|OJJ44392.1

Group5612: abra|OJJ75894.1 acar|OOG00009.1 acla|EAW09895.1 afis|EAW21231.1 afum|EAL92243.1 agla|OJJ78493.1 akaw|GAA84600.1 aluc|OJZ89345.1 anid|CBF83275.1 anig|EHA23237.1 aory|EIT79428.1 asyd|OJJ60571.1 ater|EAU35864.1 atub|OJI85454.1 aver|OJI99896.1 awen|OJJ36541.1 azon|OJJ44347.1

Group5613: abra|OJJ75901.1 acar|OOG00004.1 acla|EAW09889.1 afis|EAW21237.1 afla|EED54797.1 afum|EAL92249.1 agla|OJJ78499.1 akaw|GAA84607.1 aluc|OJZ89351.1 anid|CBF83268.1 anig|EHA23230.1 asyd|OJJ60577.1 ater|EAU35878.1 atub|OJI85447.1 aver|OJI99903.1 awen|OJJ36548.1 azon|OJJ44341.1

Group5614: abra|OJJ75913.1 acar|OOF99991.1 afla|EED54809.1 akaw|GAA84618.1 aluc|OJZ89362.1 aory|EIT79401.1 ater|EAU35889.1 atub|OJI85436.1 awen|OJJ36562.1 azon|OJJ44326.1 acla|EAW09877.1 afis|EAW21249.1 agla|OJJ87507.1 anid|CBF83244.1 asyd|OJJ60596.1 aver|OJI99919.1 afum|EAL92262.1

Group5615: abra|OJJ75930.1 acar|OOF99980.1 acla|EAW09863.1 afis|EAW21263.1 afla|EED54822.1 afum|EAL92278.1 akaw|GAA84631.1 aluc|OJZ89377.1 anid|CBF83338.1 anig|EHA23206.1 aory|EIT79385.1 asyd|OJJ60608.1 ater|EAU35900.1 atub|OJI85421.1 aver|OJI99932.1 awen|OJJ36580.1 azon|OJJ44310.1

Group5616: abra|OJJ75943.1 acla|EAW09850.1 afis|EAW21285.1 afla|EED54836.1 afum|EAL92302.1 agla|OJJ87469.1 akaw|GAA84645.1 aluc|OJZ89390.1 anid|CBF83365.1 anig|EHA23194.1 aory|EIT79369.1 asyd|OJJ60621.1 ater|EAU35917.1 atub|OJI85407.1 aver|OJI99945.1 awen|OJJ39414.1 azon|OJJ44297.1

Group5617: abra|OJJ75999.1 acar|OOF99925.1 acla|EAW09805.1 afis|EAW21331.1 afla|EED57289.1 afum|EAL92349.1 agla|OJJ87408.1 akaw|GAA88960.1 aluc|OJZ89445.1 anid|CBF83433.1 anig|EHA23141.1 asyd|OJJ60663.1 ater|EAU35957.1 atub|OJI85348.1 aver|OJI99986.1 awen|OJJ39367.1 azon|OJJ51707.1

Group5618: abra|OJJ76002.1 acar|OOF99923.1 acla|EAW09803.1 afis|EAW21333.1 afla|EED57291.1 afum|EAL92351.1 agla|OJJ87401.1 akaw|GAA88958.1 aluc|OJZ89447.1 anid|CBF83437.1 anig|EHA23139.1 aory|EIT73967.1 asyd|OJJ60665.1 atub|OJI85346.1 aver|OJI99988.1 awen|OJJ39365.1 azon|OJJ51704.1

Group5619: abra|OJJ76006.1 acar|OOF99918.1 acla|EAW09799.1 afis|EAW21337.1 afla|EED57295.1 afum|EAL92355.2 agla|OJJ87397.1 akaw|GAA88954.1 aluc|OJZ89452.1 anid|CBF83883.1 anig|EHA23135.1 asyd|OJJ60941.1 ater|EAU35962.1 atub|OJI85342.1 aver|OJJ00267.1 awen|OJJ39361.1 azon|OJJ51700.1

Group5620: abra|OJJ76050.1 acar|OOF99881.1 acla|EAW09781.1 afis|EAW21357.1 afla|EED55772.1 afum|EAL92372.1 agla|OJJ87379.1 akaw|GAA88915.1 aluc|OJZ89506.1 anid|CBF76261.1 anig|EHA23094.1 aory|EIT76646.1 asyd|OJJ60970.1 atub|OJI85297.1 aver|OJJ00295.1 awen|OJJ39341.1 azon|OJJ51687.1

Group5621: abra|OJJ76052.1 acar|OOF99875.1 acla|EAW09765.1 afis|EAW21372.1 afla|EED55790.1 afum|EAL92389.1 agla|OJJ85497.1 akaw|GAA88913.1 aluc|OJZ89508.1 anid|CBF83865.1 anig|EHA23092.1 aory|EIT76543.1 asyd|OJJ60931.1 atub|OJI85295.1 aver|OJJ00257.1 awen|OJJ39325.1 azon|OJJ51670.1

Group5622: abra|OJJ76054.1 acla|EAW09764.1 afis|EAW21373.1 afla|EED55791.1 afum|EAL92390.1 agla|OJJ85498.1 akaw|GAA88911.1 aluc|OJZ89510.1 anid|CBF83863.1 anig|EHA23090.1 aory|EIT76741.1 asyd|OJJ60929.1 ater|EAU35184.1 atub|OJI85293.1 aver|OJJ00255.1 awen|OJJ39324.1 azon|OJJ51669.1

Group5623: abra|OJJ76075.1 acar|OOF99850.1 acla|EAW09746.1 afis|EAW21396.1 afla|EED55815.1 afum|EAL92410.1 agla|OJJ85521.1 akaw|GAA88892.1 aluc|OJZ89531.1 anid|CBF83835.1 anig|EHA23071.1 aory|EIT76634.1 asyd|OJJ60911.1 atub|OJI85274.1 aver|OJJ00238.1 awen|OJJ39307.1 azon|OJJ51547.1

Group5624: abra|OJJ76085.1 acar|OOF99840.1 acla|EAW09736.1 afis|EAW21405.1 afla|EED55827.1 afum|EAL92419.1 agla|OJJ85534.1 akaw|GAA88881.1 aluc|OJZ89543.1 anid|CBF83813.1 anig|EHA23063.1 asyd|OJJ60897.1 ater|EAU35145.1 atub|OJI85264.1 aver|OJJ00222.1 awen|OJJ39298.1 azon|OJJ51555.1

Group5625: abra|OJJ76086.1 acar|OOF99838.1 acla|EAW09735.1 afis|EAW21406.1 afla|EED55828.1 afum|EAL92420.1 agla|OJJ85535.1 akaw|GAA88880.1 aluc|OJZ89544.1 anid|CBF83812.1 anig|EHA23062.1 asyd|OJJ60896.1 ater|EAU35142.1 atub|OJI85262.1 aver|OJJ00221.1 awen|OJJ39297.1 azon|OJJ51558.1

Group5626: abra|OJJ76089.1 acla|EAW09733.1 afis|EAW21408.1 afla|EED55830.1 afum|EAL92422.1 agla|OJJ85538.1 akaw|GAA88878.1 aluc|OJZ89546.1 anid|CBF83808.1 anig|EHA23060.1 aory|EIT76672.1 asyd|OJJ60894.1 ater|EAU35140.1 atub|OJI85260.1 aver|OJJ00219.1 awen|OJJ39293.1 azon|OJJ51561.1

Group5627: abra|OJJ76093.1 acar|OOF99832.1 acla|EAW09726.1 afis|EAW21412.1 afla|EED55837.1 afum|EAL92426.1 agla|OJJ85545.1 akaw|GAA88874.1 aluc|OJZ89549.1 anid|CBF83796.1 anig|EHA23057.1 aory|EIT76701.1 asyd|OJJ60886.1 atub|OJI85257.1 aver|OJJ00210.1 awen|OJJ39287.1 azon|OJJ51567.1

Group5628: abra|OJJ76097.1 acla|EAW09721.1 afis|EAW21420.1 afla|EED55845.1 afum|EAL92435.1 agla|OJJ85554.1 akaw|GAA88869.1 aluc|OJZ89553.1 anid|CBF83789.1 anig|EHA23053.1 aory|EIT76872.1 asyd|OJJ60879.1 ater|EAU35129.1 atub|OJI85252.1 aver|OJJ00205.1 awen|OJJ39283.1 azon|OJJ51571.1

Group5629: abra|OJJ76101.1 acar|OOF99826.1 acla|EAW09717.1 afis|EAW21424.1 afla|EED55849.1 afum|EAL92439.1 agla|OJJ85561.1 akaw|GAA88865.1 aluc|OJZ89557.1 anig|EHA23049.1 aory|EIT76560.1 asyd|OJJ60875.1 ater|EAU35125.1 atub|OJI85247.1 aver|OJJ00200.1 awen|OJJ39277.1 azon|OJJ51575.1

Group5630: abra|OJJ76119.1 acar|OOF99806.1 acla|EAW09700.1 afis|EAW21439.1 afla|EED55867.1 afum|EAL92455.1 agla|OJJ85584.1 akaw|GAA88848.1 aluc|OJZ89576.1 anid|CBF76581.1 aory|EIT76843.1 asyd|OJJ61170.1 ater|EAU35106.1 atub|OJI85228.1 aver|OJJ00495.1 awen|OJJ39259.1 azon|OJJ51596.1

Group5631: abra|OJJ76135.1 acar|OOF99788.1 acla|EAW09683.1 afis|EAW21456.1 afla|EED55886.1 afum|EAL92475.1 agla|OJJ85604.1 akaw|GAA88832.1 aluc|OJZ89598.1 anid|CBF76544.1 anig|EHA23015.1 asyd|OJJ61153.1 ater|EAU35079.1 atub|OJI85206.1 aver|OJJ00475.1 awen|OJJ39244.1 azon|OJJ51611.1

Group5632: abra|OJJ76139.1 acla|EAW09680.1 afis|EAW21459.1 afla|EED55890.1 afum|EAL92478.1 agla|OJJ85607.1 akaw|GAA88829.1 aluc|OJZ89602.1 anid|CBF76538.1 anig|EHA23013.1 aory|EIT76890.1 asyd|OJJ61150.1 ater|EAU35076.1 atub|OJI85202.1 aver|OJJ00472.1 awen|OJJ39240.1 azon|OJJ51614.1

Group5633: abra|OJJ76147.1 acar|OOF99779.1 acla|EAW09672.1 afis|EAW21466.1 afla|EED55899.1 afum|EAL92486.1 agla|OJJ85616.1 akaw|GAA82097.1 aluc|OJZ92082.1 anid|CBF76525.1 aory|EIT76748.1 asyd|OJJ61142.1 atub|OJI86512.1 aver|OJJ00464.1 awen|OJJ39231.1 ater|EAU35067.1 azon|OJJ51624.1

Group5634: abra|OJJ76159.1 acar|OOF99773.1 acla|EAW09659.1 afis|EAW21480.1 afla|EED55912.1 afum|EAL92500.1 agla|OJJ85634.1 akaw|GAA92403.1 aluc|OJZ83264.1 anid|CBF76504.1 anig|EHA21117.1 asyd|OJJ61131.1 ater|EAU35057.1 atub|OJI87673.1 aver|OJJ00452.1 awen|OJJ39217.1 azon|OJJ51637.1

Group5635: abra|OJJ76184.1 acla|EAW09642.1 afis|EAW21498.1 afla|EED55934.1 afum|EAL92517.1 agla|OJJ85661.1 akaw|GAA82129.1 aluc|OJZ92045.1 anid|CBF76464.1 anig|EHA22975.1 aory|EIT76682.1 asyd|OJJ61113.1 ater|EAU35040.1 atub|OJI86477.1 aver|OJJ00429.1 awen|OJJ39197.1 azon|OJJ51520.1

Group5636: abra|OJJ76186.1 acar|OOF99756.1 acla|EAW09641.1 afis|EAW21499.1 afla|EED55935.1 afum|EAL92518.1 agla|OJJ85662.1 akaw|GAA82130.1 aluc|OJZ92043.1 anid|CBF76460.1 aory|EIT76668.1 asyd|OJJ61112.1 ater|EAU35039.1 atub|OJI86476.1 aver|OJJ00428.1 awen|OJJ39196.1 azon|OJJ51521.1

Group5637: abra|OJJ76198.1 acar|OOF99746.1 acla|EAW13919.1 afis|EAW18630.1 afla|EED52710.1 afum|EAL93342.1 agla|OJJ81255.1 akaw|GAA82140.1 aluc|OJZ92031.1 anid|CBF70476.1 anig|EHA22964.1 asyd|OJJ58593.1 ater|EAU38281.1 atub|OJI86464.1 aver|OJJ01195.1 awen|OJJ32097.1 azon|OJJ46676.1

Group5638: abra|OJJ76199.1 acar|OOF99745.1 acla|EAW13920.1 afis|EAW18631.1 afla|EED52711.1 afum|EAL93343.2 agla|OJJ82446.1 akaw|GAA82142.1 aluc|OJZ92029.1 anid|CBF70475.1 anig|EHA22963.1 asyd|OJJ58594.1 ater|EAU38280.1 atub|OJI86463.1 aver|OJJ01196.1 awen|OJJ32098.1 azon|OJJ46678.1

Group5639: abra|OJJ76203.1 acla|EAW13924.1 afis|EAW18635.1 afla|EED52706.1 afum|EAL93348.2 agla|OJJ82441.1 akaw|GAA82146.1 aluc|OJZ92025.1 anid|CBF70464.1 anig|EHA22959.1 aory|EIT74268.1 asyd|OJJ58599.1 ater|EAU38276.1 atub|OJI86459.1 aver|OJJ01201.1 awen|OJJ32102.1 azon|OJJ46684.1

Group5640: abra|OJJ76205.1 acla|EAW13926.1 afis|EAW18637.1 afla|EED52704.1 afum|EAL93350.1 agla|OJJ82438.1 akaw|GAA82148.1 aluc|OJZ92023.1 anid|CBF70459.1 anig|EHA22957.1 aory|EIT74180.1 asyd|OJJ58601.1 ater|EAU38274.1 atub|OJI86456.1 aver|OJJ01203.1 awen|OJJ32105.1 azon|OJJ46686.1

Group5641: abra|OJJ76216.1 acar|OOF99734.1 acla|EAW13932.1 afis|EAW18643.1 afla|EED52693.1 afum|EAL93357.1 agla|OJJ82431.1 akaw|GAA82155.1 aluc|OJZ92013.1 anid|CBF70443.1 anig|EHA22952.1 asyd|OJJ58609.1 ater|EAU38268.1 atub|OJI86448.1 aver|OJJ01214.1 awen|OJJ32111.1 azon|OJJ46692.1

Group5642: abra|OJJ76218.1 acar|OOF99733.1 acla|EAW13933.1 afis|EAW18644.1 afla|EED52694.1 afum|EAL93359.1 agla|OJJ82430.1 akaw|GAA82156.1 aluc|OJZ92012.1 anid|CBF70441.1 anig|EHA22951.1 asyd|OJJ58610.1 ater|EAU38267.1 atub|OJI86447.1 aver|OJJ01215.1 awen|OJJ32112.1 azon|OJJ46693.1

Group5643: abra|OJJ76224.1 acar|OOF99471.1 acla|EAW14364.1 afis|EAW18914.1 afla|EED50898.1 afum|EBA27497.1 agla|OJJ81518.1 akaw|GAA82160.1 aluc|OJZ92007.1 anid|CBF69693.1 aory|EIT79056.1 asyd|OJJ59054.1 ater|EAU38081.1 atub|OJI86442.1 aver|OJJ01648.1 awen|OJJ31507.1 azon|OJJ47226.1

Group5644: abra|OJJ76235.1 acla|EAW14372.1 afis|EAW18921.1 afla|EED50907.1 afum|EAL93652.1 agla|OJJ81522.1 akaw|GAA82170.1 aluc|OJZ91995.1 anid|CBF69680.1 anig|EHA22940.1 aory|EIT78977.1 asyd|OJJ59061.1 ater|EAU38094.1 atub|OJI86430.1 aver|OJJ01655.1 awen|OJJ31514.1 azon|OJJ47232.1

Group5645: abra|OJJ76236.1 acar|OOF99480.1 acla|EAW14373.1 afis|EAW18922.1 afla|EED50908.1 afum|EAL93653.1 agla|OJJ81521.1 akaw|GAA82171.1 aluc|OJZ91994.1 anid|CBF69678.1 anig|EHA22939.1 asyd|OJJ59066.1 ater|EAU38095.1 atub|OJI86429.1 aver|OJJ01660.1 awen|OJJ31515.1 azon|OJJ47233.1

Group5646: abra|OJJ76247.1 acar|OOF99493.1 acla|EAW14360.1 afis|EAW18930.1 afla|EED50923.1 afum|EAL93661.1 agla|OJJ85259.1 akaw|GAA82180.1 aluc|OJZ91984.1 anig|EHA22930.1 aory|EIT78976.1 asyd|OJJ64673.1 atub|OJI86419.1 aver|OJI98774.1 awen|OJJ31524.1 azon|OJJ47239.1 anid|CBF86792.1

Group5647: abra|OJJ76249.1 acar|OOF99494.1 acla|EAW14358.1 afis|EAW18932.1 afla|EED50937.1 afum|EAL93664.1 agla|OJJ85261.1 akaw|GAA82182.1 aluc|OJZ91981.1 anid|CBF86798.1 anig|EHA22928.1 asyd|OJJ64669.1 ater|EAU38111.1 atub|OJI86417.1 aver|OJI98771.1 awen|OJJ31526.1 azon|OJJ47241.1

Group5648: abra|OJJ76255.1 acar|OOF99500.1 acla|EAW14352.1 afis|EAW18938.1 afla|EED50943.1 afum|EAL93671.1 agla|OJJ85269.1 akaw|GAA82189.1 aluc|OJZ91971.1 anid|CBF86809.1 anig|EHA22919.1 asyd|OJJ64663.1 ater|EAU38117.1 atub|OJI86410.1 aver|OJI98765.1 awen|OJJ31533.1 azon|OJJ47247.1

Group5649: abra|OJJ76261.1 acar|OOF99505.1 afis|EAW18942.1 afla|EED50975.1 afum|EAL93675.1 akaw|GAA82193.1 aluc|OJZ91961.1 anid|CBF86818.1 anig|EHA22917.1 aory|EIT76997.1 asyd|OJJ64656.1 ater|EAU38123.1 atub|OJI86405.1 aver|OJI98759.1 awen|OJJ31539.1 azon|OJJ47251.1 acla|EAW14349.1

Group5650: abra|OJJ76263.1 acar|OOF99507.1 acla|EAW14345.1 afis|EAW18945.1 afla|EED50979.1 afum|EAL93678.1 agla|OJJ85272.1 akaw|GAA82195.1 aluc|OJZ91959.1 anid|CBF86824.1 anig|EHA22915.1 aory|EIT76970.1 asyd|OJJ64653.1 ater|EAU38125.1 atub|OJI86402.1 aver|OJI98755.1 awen|OJJ31541.1

Group5651: abra|OJJ76265.1 acar|OOF99508.1 acla|EAW14344.1 afis|EAW18946.1 afla|EED50982.1 afum|EAL93680.1 akaw|GAA82197.1 aluc|OJZ91956.1 anid|CBF86827.1 anig|EHA22914.1 aory|EIT77065.1 asyd|OJJ64652.1 ater|EAU38128.1 atub|OJI86401.1 aver|OJI98753.1 awen|OJJ31544.1 azon|OJJ47261.1

Group5652: abra|OJJ76273.1 acar|OOF99521.1 acla|EAW14339.1 afis|EAW18951.1 afla|EED50988.1 afum|EAL93686.1 agla|OJJ85322.1 akaw|GAA82206.1 aluc|OJZ91947.1 anid|CBF69580.1 anig|EHA22906.1 aory|EIT77062.1 asyd|OJJ59143.1 ater|EAU38130.1 atub|OJI86392.1 aver|OJJ01733.1 azon|OJJ47265.1

Group5653: abra|OJJ76280.1 acar|OOF99525.1 acla|EAW14335.1 afis|EAW18954.1 afla|EED51001.1 agla|OJJ85329.1 akaw|GAA82212.1 aluc|OJZ91940.1 anid|CBF69592.1 anig|EHA22899.1 aory|EIT77031.1 asyd|OJJ59140.1 ater|EAU38138.1 atub|OJI86382.1 aver|OJJ01729.1 awen|OJJ31552.1 azon|OJJ47269.1

Group5654: abra|OJJ76284.1 acar|OOF99526.1 acla|EAW14332.1 afis|EAW18956.1 afla|EED51003.1 agla|OJJ85334.1 akaw|GAA82213.1 aluc|OJZ91937.1 anid|CBF69594.1 anig|EHA22897.1 asyd|OJJ59139.1 ater|EAU38139.1 atub|OJI86379.1 aver|OJJ01728.1 awen|OJJ31555.1 azon|OJJ47272.1 afum|EAL93690.1

Group5655: abra|OJJ76285.1 acar|OOF99528.1 acla|EAW14331.1 afis|EAW18957.1 afla|EED51004.1 agla|OJJ85335.1 akaw|GAA82214.1 aluc|OJZ91936.1 anid|CBF69596.1 anig|EHA22896.1 aory|EIT77061.1 asyd|OJJ59137.1 ater|EAU38140.1 atub|OJI86378.1 aver|OJJ01727.1 awen|OJJ31556.1 azon|OJJ47273.1

Group5656: abra|OJJ76315.1 acla|EAW14100.1 afis|EAW18801.1 afla|EED50775.1 agla|OJJ80531.1 akaw|GAA82246.1 aluc|OJZ91899.1 anid|CBF69782.1 anig|EHA22878.1 aory|EIT78048.1 asyd|OJJ58959.1 ater|EAU37933.1 atub|OJI86352.1 aver|OJJ01553.1 awen|OJJ32375.1 afum|EAL93537.1 azon|OJJ47138.1

Group5657: abra|OJJ76319.1 acar|OOF99572.1 acla|EAW14098.1 afis|EAW18800.1 afla|EED50776.1 afum|EAL93536.1 agla|OJJ80529.1 akaw|GAA82249.1 aluc|OJZ91895.1 anid|CBF69780.1 anig|EHA22874.1 asyd|OJJ58958.1 ater|EAU37932.1 atub|OJI86348.1 aver|OJJ01552.1 awen|OJJ32374.1 azon|OJJ47137.1

Group5658: abra|OJJ76321.1 acar|OOF99574.1 acla|EAW14093.1 afis|EAW18799.1 afla|EED50790.1 afum|EAL93535.1 akaw|GAA82250.1 aluc|OJZ91894.1 anid|CBF69778.1 anig|EHA22873.1 aory|EIT78129.1 asyd|OJJ58957.1 ater|EAU37931.1 atub|OJI86346.1 aver|OJJ01551.1 awen|OJJ32373.1 azon|OJJ47136.1

Group5659: abra|OJJ76324.1 acar|OOF99576.1 acla|EAW14091.1 afis|EAW18797.1 afla|EED50792.1 afum|EAL93533.1 agla|OJJ80526.1 akaw|GAA82253.1 aluc|OJZ91891.1 anid|CBF69775.1 aory|EIT78127.1 asyd|OJJ58955.1 ater|EAU37929.1 atub|OJI86343.1 aver|OJJ01549.1 awen|OJJ32371.1 azon|OJJ47134.1

Group5660: abra|OJJ76330.1 acar|OOF99582.1 acla|EAW14085.1 afis|EAW18792.1 afla|EED50750.1 afum|EAL93527.1 agla|OJJ80521.1 aluc|OJZ91883.1 anid|CBF69765.1 anig|EHA22861.1 aory|EIT77994.1 asyd|OJJ58949.1 ater|EAU37923.1 atub|OJI86334.1 aver|OJJ01544.1 awen|OJJ32365.1 azon|OJJ47129.1

Group5661: abra|OJJ76331.1 acar|OOF99583.1 acla|EAW14084.1 afis|EAW18791.1 afla|EED50749.1 afum|EAL93526.1 agla|OJJ80520.1 akaw|GAA82259.1 aluc|OJZ91882.1 anid|CBF69763.1 anig|EHA22860.1 asyd|OJJ58948.1 ater|EAU37922.1 atub|OJI86333.1 aver|OJJ01543.1 awen|OJJ32364.1 azon|OJJ47127.1

Group5662: abra|OJJ76333.1 acar|OOF99585.1 acla|EAW14082.1 afis|EAW18789.1 afla|EED50747.1 afum|EAL93523.1 agla|OJJ80518.1 aluc|OJZ91880.1 anid|CBF69759.1 anig|EHA22858.1 aory|EIT78001.1 asyd|OJJ58945.1 ater|EAU37920.1 atub|OJI86331.1 aver|OJJ01541.1 awen|OJJ32362.1 azon|OJJ47125.1

Group5663: abra|OJJ76341.1 acar|OOF99592.1 acla|EAW14074.1 afis|EAW18781.1 afla|EED50740.1 afum|EAL93513.1 agla|OJJ80501.1 akaw|GAA82268.1 aluc|OJZ91872.1 anid|CBF69740.1 anig|EHA22851.1 asyd|OJJ58934.1 ater|EAU37913.1 atub|OJI86323.1 aver|OJJ01530.1 awen|OJJ32353.1 azon|OJJ47117.1

Group5664: abra|OJJ76352.1 acar|OOF99602.1 acla|EAW14063.1 afis|EAW18770.1 afum|EAL93501.2 agla|OJJ80472.1 aluc|OJZ91861.1 anid|CBF69936.1 anig|EHA22841.1 aory|EIT78053.1 asyd|OJJ58921.1 ater|EAU37901.1 atub|OJI86312.1 aver|OJJ01518.1 awen|OJJ32339.1 azon|OJJ47105.1 afla|EED50729.1

Group5665: abra|OJJ76361.1 acar|OOF99612.1 acla|EAW14055.1 afis|EAW18761.1 afla|EED50721.1 afum|EAL93492.2 agla|OJJ80460.1 akaw|GAA82287.1 aluc|OJZ91851.1 anid|CBF69951.1 anig|EHA22833.1 asyd|OJJ58912.1 ater|EAU37892.1 atub|OJI86303.1 aver|OJJ01509.1 awen|OJJ32331.1 azon|OJJ47096.1

Group5666: abra|OJJ76365.1 acar|OOF99616.1 acla|EAW14051.1 afis|EAW18757.1 afla|EED50717.1 afum|EAL93486.1 agla|OJJ82325.1 akaw|GAA82291.1 aluc|OJZ91847.1 anid|CBF69959.1 anig|EHA22829.1 aory|EIT78006.1 asyd|OJJ58908.1 ater|EAU37889.1 atub|OJI86299.1 aver|OJJ01505.1 awen|OJJ32327.1

Group5667: abra|OJJ76367.1 acar|OOF89986.1 acla|EAW14049.1 afis|EAW18755.1 afla|EED50715.1 afum|EAL93482.1 akaw|GAA82293.1 aluc|OJZ91845.1 aory|EIT78024.1 asyd|OJJ58906.1 atub|OJI86297.1 aver|OJJ01503.1 awen|OJJ32324.1 azon|OJJ47091.1 anid|CBF69965.1 ater|EAU37886.1 agla|OJJ82323.1

Group5668: abra|OJJ76372.1 acar|OOF99620.1 acla|EAW14046.1 afis|EAW18752.1 afla|EED50711.1 afum|EAL93479.1 agla|OJJ82320.1 akaw|GAA82297.1 aluc|OJZ91841.1 anid|CBF69975.1 aory|EIT77974.1 asyd|OJJ58899.1 ater|EAU37883.1 atub|OJI86293.1 aver|OJJ01499.1 awen|OJJ32321.1 azon|OJJ47088.1

Group5669: abra|OJJ76373.1 acar|OOF99621.1 acla|EAW14045.1 afis|EAW18751.1 afla|EED50710.1 afum|EAL93478.1 agla|OJJ82319.1 akaw|GAA82298.1 aluc|OJZ91840.1 anig|EHA22824.1 aory|EIT78136.1 asyd|OJJ58898.1 ater|EAU37882.1 atub|OJI86292.1 aver|OJJ01498.1 awen|OJJ32320.1 azon|OJJ47087.1

Group5670: abra|OJJ76380.1 acar|OOF89926.1 acla|EAW14042.1 afis|EAW18748.1 afla|EED50707.1 afum|EAL93474.1 agla|OJJ82315.1 akaw|GAA82301.1 aluc|OJZ91834.1 anid|CBF69985.1 anig|EHA22821.1 asyd|OJJ58894.1 ater|EAU37877.1 atub|OJI86288.1 aver|OJJ01495.1 awen|OJJ32315.1 azon|OJJ47083.1

Group5671: abra|OJJ76385.1 acar|OOF99626.1 acla|EAW14039.1 afis|EAW18745.1 afla|EED50701.1 afum|EAL93471.1 agla|OJJ82310.1 akaw|GAA82304.1 aluc|OJZ91830.1 anid|CBF69991.1 anig|EHA22817.1 aory|EIT78013.1 asyd|OJJ58890.1 atub|OJI86284.1 aver|OJJ01490.1 awen|OJJ32310.1 azon|OJJ42714.1

Group5672: abra|OJJ76391.1 acla|EAW14033.1 afis|EAW18739.1 afla|EED50694.1 afum|EAL93465.1 agla|OJJ82305.1 akaw|GAA82310.1 aluc|OJZ91822.1 anid|CBF70004.1 anig|EHA22812.1 aory|EIT78125.1 asyd|OJJ58883.1 ater|EAU37862.1 atub|OJI86277.1 aver|OJJ01485.1 awen|OJJ32304.1 azon|OJJ42722.1

Group5673: abra|OJJ76394.1 acar|OOF99634.1 acla|EAW14030.1 afis|EAW18736.1 afla|EED50691.1 afum|EAL93461.1 agla|OJJ82302.1 akaw|GAA82313.1 aluc|OJZ91819.1 anid|CBF70010.1 anig|EHA22809.1 asyd|OJJ58880.1 ater|EAU37859.1 atub|OJI86274.1 aver|OJJ01482.1 awen|OJJ32300.1 azon|OJJ42733.1

Group5674: abra|OJJ76403.1 acar|OOF99646.1 acla|EAW14016.1 afis|EAW18726.1 afla|EED50683.1 afum|EAL93451.1 agla|OJJ80473.1 akaw|GAA82323.1 aluc|OJZ91809.1 anid|CBF70019.1 anig|EHA22802.1 asyd|OJJ58876.1 ater|EAU38178.1 atub|OJI86264.1 aver|OJJ01478.1 awen|OJJ32228.1 azon|OJJ46784.1

Group5675: abra|OJJ76405.1 acar|OOF99648.1 acla|EAW14018.1 afis|EAW18724.1 afla|EED50681.1 afum|EAL93449.1 agla|OJJ80476.1 akaw|GAA82325.1 aluc|OJZ91807.1 anid|CBF70686.1 anig|EHA22800.1 aory|EIT78066.1 asyd|OJJ58467.1 atub|OJI86262.1 aver|OJJ01066.1 awen|OJJ32230.1 azon|OJJ46782.1

Group5676: abra|OJJ76416.1 acar|OOF99656.1 acla|EAW14011.1 afis|EAW18717.1 afla|EED50671.1 afum|EBA27493.1 agla|OJJ82342.1 akaw|GAA82331.1 aluc|OJZ91797.1 anid|CBF70674.1 anig|EHA22793.1 asyd|OJJ58474.1 ater|EAU38188.1 atub|OJI86253.1 aver|OJJ01076.1 awen|OJJ32211.1 azon|OJJ46772.1

Group5677: abra|OJJ76425.1 acar|OOF99669.1 acla|EAW14000.1 afis|EAW18708.1 afla|EED50657.1 afum|EAL93431.1 agla|OJJ82350.1 akaw|GAA82340.1 aluc|OJZ91787.1 anid|CBF70652.1 anig|EHA22784.1 aory|EIT78022.1 asyd|OJJ58484.1 ater|EAU38200.1 atub|OJI86243.1 aver|OJJ01087.1 awen|OJJ32191.1

Group5678: abra|OJJ76426.1 acar|OOF99670.1 acla|EAW13999.1 afis|EAW18707.1 afla|EED50656.1 afum|EAL93430.1 agla|OJJ82351.1 akaw|GAA82341.1 aluc|OJZ91786.1 anid|CBF70650.1 anig|EHA22783.1 aory|EIT78121.1 asyd|OJJ58485.1 ater|EAU38201.1 atub|OJI86242.1 aver|OJJ01088.1 awen|OJJ32190.1

Group5679: abra|OJJ76427.1 acar|OOF99672.1 acla|EAW13998.1 afis|EAW18706.1 afla|EED50655.1 afum|EAL93429.1 agla|OJJ82352.1 akaw|GAA82342.1 aluc|OJZ91785.1 anid|CBF70649.1 anig|EHA22782.1 aory|EIT78135.1 asyd|OJJ58486.1 atub|OJI86241.1 aver|OJJ01089.1 awen|OJJ32189.1 azon|OJJ46758.1

Group5680: abra|OJJ76437.1 acla|EAW13986.1 afis|EAW18694.1 afla|EED50643.1 afum|EAL93416.1 agla|OJJ82369.1 akaw|GAA82352.1 aluc|OJZ91773.1 anid|CBF70626.1 anig|EHA22773.1 aory|EIT78019.1 asyd|OJJ58498.1 ater|EAU38212.1 atub|OJI86230.1 aver|OJJ01101.1 awen|OJJ32165.1 azon|OJJ46750.1

Group5681: abra|OJJ76450.1 acar|OOF99693.1 acla|EAW13978.1 afis|EAW18687.1 afla|EED50635.1 afum|EAL93408.2 agla|OJJ82385.1 akaw|GAA82362.1 aluc|OJZ91760.1 anid|CBF70611.1 aory|EIT78005.1 asyd|OJJ58511.1 ater|EAU38221.1 atub|OJI86218.1 aver|OJJ01112.1 awen|OJJ32158.1 azon|OJJ46742.1

Group5682: abra|OJJ76454.1 acla|EAW13976.1 afis|EAW18685.1 afla|EED50623.1 afum|EAL93406.1 agla|OJJ82386.1 akaw|GAA82366.1 aluc|OJZ91756.1 anid|CBF70609.1 anig|EHA22759.1 aory|EIT78050.1 asyd|OJJ58513.1 ater|EAU38223.1 atub|OJI86214.1 aver|OJJ01114.1 awen|OJJ32156.1 azon|OJJ46740.1

Group5683: abra|OJJ76458.1 acar|OOF99698.1 acla|EAW13971.1 afis|EAW18680.1 afla|EED50630.1 afum|EAL93401.1 agla|OJJ82395.1 akaw|GAA82371.1 aluc|OJZ91751.1 anid|CBF70599.1 anig|EHA22754.1 asyd|OJJ58518.1 ater|EAU38229.1 atub|OJI86209.1 aver|OJJ01119.1 awen|OJJ32152.1 azon|OJJ46736.1

Group5684: abra|OJJ76461.1 acar|OOF99700.1 acla|EAW13969.1 afis|EAW18678.1 afla|EED50632.1 agla|OJJ82398.1 akaw|GAA82374.1 aluc|OJZ91748.1 anid|CBF70594.1 anig|EHA22751.1 aory|EIT78090.1 asyd|OJJ58520.1 ater|EAU38233.1 atub|OJI86206.1 aver|OJJ01121.1 awen|OJJ32150.1 azon|OJJ46734.1

Group5685: abra|OJJ76463.1 acar|OOF99701.1 acla|EAW13968.1 afis|EAW18677.1 afla|EED50633.1 afum|EAL93397.1 agla|OJJ82399.1 akaw|GAA82375.1 aluc|OJZ91747.1 anid|CBF70592.1 anig|EHA22750.1 aory|EIT78096.1 asyd|OJJ58521.1 atub|OJI86205.1 aver|OJJ01122.1 awen|OJJ32149.1 azon|OJJ46733.1

Group5686: abra|OJJ76473.1 acar|OOF99710.1 acla|EAW13960.1 afis|EAW18669.1 afla|EED52659.1 afum|EAL93387.1 agla|OJJ82406.1 akaw|GAA82385.1 aluc|OJZ91736.1 anid|CBF70577.1 anig|EHA22742.1 asyd|OJJ58529.1 ater|EAU38242.1 atub|OJI86194.1 aver|OJJ01131.1 awen|OJJ32141.1 azon|OJJ46722.1

Group5687: abra|OJJ76490.1 acla|EAW13945.1 afis|EAW18654.1 afla|EED52682.1 afum|EAL93370.1 agla|OJJ82421.1 akaw|GAA82405.1 aluc|OJZ91715.1 anid|CBF70542.1 anig|EHA22725.1 aory|EIT74201.1 asyd|OJJ58548.1 ater|EAU38258.1 atub|OJI86173.1 aver|OJJ01146.1 awen|OJJ32121.1 azon|OJJ46707.1

Group5688: abra|OJJ76492.1 acar|OOF99724.1 acla|EAW13940.1 afis|EAW18651.1 afla|EED52688.1 afum|EAL93367.1 agla|OJJ82423.1 akaw|GAA82408.1 aluc|OJZ91712.1 anid|CBF70537.1 anig|EHA22723.1 asyd|OJJ58550.1 ater|EAU38261.1 atub|OJI86171.1 aver|OJJ01148.1 awen|OJJ32119.1 azon|OJJ46705.1

Group5689: abra|OJJ76494.1 acar|OOF99726.1 acla|EAW13938.1 afis|EAW18649.1 afla|EED52690.1 afum|EAL93365.1 agla|OJJ82425.1 akaw|GAA82410.1 aluc|OJZ91710.1 anid|CBF70535.1 anig|EHA22721.1 aory|EIT74193.1 asyd|OJJ58552.1 atub|OJI86169.1 aver|OJJ01150.1 awen|OJJ32117.1 azon|OJJ46703.1

Group5690: azon|OJJ47218.1 abra|OJJ76512.1 acar|OOF99460.1 acla|EAW14188.1 afis|EAW18903.1 afla|EED50845.1 afum|EAL93635.1 agla|OJJ81508.1 akaw|GAA82425.1 aluc|OJZ91693.1 anid|CBF69928.1 anig|EHA22706.1 asyd|OJJ59040.1 ater|EAU38071.1 atub|OJI86147.1 aver|OJJ01635.1 awen|OJJ32460.1

Group5691: abra|OJJ76522.1 acar|OOF99451.1 acla|EAW14179.1 afis|EAW18894.1 afla|EED50869.1 afum|EAL93626.1 agla|OJJ81500.1 akaw|GAA82435.1 aluc|OJZ91686.1 anid|CBF69910.1 anig|EHA22698.1 aory|EIT78965.1 asyd|OJJ58994.1 ater|EAU38065.1 atub|OJI86138.1 awen|OJJ32452.1 azon|OJJ47210.1

Group5692: abra|OJJ76532.1 acar|OOF99441.1 acla|EAW14170.1 afis|EAW18884.1 afla|EED50881.1 afum|EAL93617.1 agla|OJJ80619.1 akaw|GAA82444.1 aluc|OJZ91672.1 anig|EHA22690.1 aory|EIT78974.1 asyd|OJJ59003.1 ater|EAU38056.1 atub|OJI86127.1 aver|OJJ01612.1 azon|OJJ47200.1 awen|OJJ32439.1

Group5693: abra|OJJ76544.1 acar|OOF99430.1 acla|EAW14160.1 afis|EAW18873.1 afla|EED50894.1 afum|EAL93605.1 agla|OJJ80610.1 akaw|GAA82453.1 aluc|OJZ91659.1 anig|EHA22679.1 aory|EIT78984.1 asyd|OJJ59022.1 ater|EAU38037.1 atub|OJI86115.1 aver|OJJ01595.1 awen|OJJ32432.1 azon|OJJ47190.1

Group5694: abra|OJJ76553.1 acar|OOF89944.1 acla|EAW14152.1 afis|EAW18865.1 afla|EED50896.1 afum|EAL93597.1 agla|OJJ80587.1 akaw|GAA82460.1 aluc|OJZ91651.1 anid|CBF69850.1 anig|EHA22672.1 aory|EIT79012.1 asyd|OJJ59029.1 atub|OJI86105.1 aver|OJJ01587.1 awen|OJJ32424.1 azon|OJJ47179.1

Group5695: abra|OJJ76556.1 acar|OOF99423.1 acla|EAW14149.1 afis|EAW18862.1 afla|EED50829.1 afum|EAL93594.1 agla|OJJ80585.1 akaw|GAA82462.1 aluc|OJZ91649.1 anid|CBF69846.1 anig|EHA22670.1 asyd|OJJ59031.1 ater|EAU38024.1 atub|OJI86103.1 aver|OJJ01585.1 awen|OJJ32418.1 azon|OJJ47175.1

Group5696: abra|OJJ76559.1 acla|EAW14146.1 afis|EAW18859.1 afla|EED50826.1 afum|EAL93591.1 agla|OJJ80582.1 akaw|GAA82465.1 aluc|OJZ91646.1 anid|CBF69840.1 anig|EHA22667.1 aory|EIT78992.1 asyd|OJJ59034.1 ater|EAU38022.1 atub|OJI86100.1 aver|OJJ01582.1 awen|OJJ32415.1 azon|OJJ47172.1

Group5697: abra|OJJ76560.1 acla|EAW14145.1 afis|EAW18858.1 afla|EED50825.1 afum|EAL93590.2 agla|OJJ80580.1 akaw|GAA82466.1 aluc|OJZ91644.1 anid|CBF69838.1 anig|EHA22666.1 aory|EIT78960.1 asyd|OJJ59035.1 ater|EAU38021.1 atub|OJI86099.1 aver|OJJ01581.1 awen|OJJ32414.1 azon|OJJ47171.1

Group5698: abra|OJJ76568.1 afis|EAW18853.1 afum|EAL93584.1 agla|OJJ80572.1 akaw|GAA82472.1 aluc|OJZ91638.1 anid|CBF69825.1 anig|EHA22661.1 asyd|OJJ58979.1 atub|OJI86091.1 aver|OJJ01574.1 awen|OJJ32407.1 azon|OJJ47165.1 acla|EAW14140.1 afla|EED50820.1 aory|EIT79040.1 ater|EAU38009.1

Group5699: abra|OJJ76574.1 acar|OOF99410.1 afis|EAW18821.1 afla|EED50805.1 afum|EAL93558.1 agla|OJJ80565.1 akaw|GAA82479.1 aluc|OJZ91631.1 anid|CBF82810.1 anig|EHA22654.1 aory|EIT78039.1 asyd|OJJ60269.1 ater|EAU38000.1 atub|OJI86084.1 aver|OJJ03985.1 awen|OJJ32399.1 azon|OJJ47159.1

Group5700: abra|OJJ76575.1 acar|OOF99409.1 acla|EAW14118.1 afis|EAW18820.1 afla|EED50807.1 afum|EAL93557.1 agla|OJJ80555.1 akaw|GAA82480.1 aluc|OJZ91630.1 anid|CBF69807.1 aory|EIT79005.1 asyd|OJJ58970.1 ater|EAU37999.1 atub|OJI86083.1 aver|OJJ01565.1 awen|OJJ32396.1 azon|OJJ47158.1

Group5701: abra|OJJ76578.1 acar|OOF99406.1 acla|EAW14115.1 afis|EAW18817.1 afla|EED50803.1 afum|EAL93553.1 agla|OJJ80550.1 akaw|GAA82483.1 aluc|OJZ91627.1 anid|CBF69813.1 anig|EHA22651.1 asyd|OJJ58973.1 ater|EAU37992.1 atub|OJI86080.1 aver|OJJ01568.1 awen|OJJ32393.1 azon|OJJ47154.1

Group5702: abra|OJJ76602.1 acar|OOF99389.1 acla|EAW14302.1 afis|EAW18982.1 afla|EED52436.1 afum|EAL93718.1 agla|OJJ82953.1 akaw|GAA82505.1 aluc|OJZ91606.1 anid|CBF69657.1 anig|EHA22635.1 asyd|OJJ59073.1 ater|EAU37829.1 atub|OJI86058.1 aver|OJJ01666.1 awen|OJJ31597.1 azon|OJJ47306.1

Group5703: abra|OJJ76607.1 acla|EAW14299.1 afis|EAW18985.1 afla|EED53163.1 afum|EAL93721.1 agla|OJJ82960.1 akaw|GAA82511.1 aluc|OJZ91600.1 anid|CBF69663.1 anig|EHA22630.1 aory|EIT75021.1 asyd|OJJ59070.1 ater|EAU37820.1 atub|OJI86051.1 aver|OJJ01663.1 awen|OJJ31605.1 azon|OJJ47309.1

Group5704: abra|OJJ76610.1 agla|OJJ82965.1 akaw|GAA82513.1 aluc|OJZ91598.1 anig|EHA22628.1 atub|OJI86049.1 acla|EAW14296.1 afis|EAW18987.1 afla|EED53166.1 afum|EAL93723.1 anid|CBF69667.1 aory|EIT75116.1 asyd|OJJ59068.1 ater|EAU37818.1 aver|OJJ01661.1 awen|OJJ31607.1 azon|OJJ47311.1

Group5705: abra|OJJ76625.1 acar|OOF99373.1 acla|EAW14294.1 afis|EAW18989.1 afla|EED53174.1 afum|EAL93725.1 agla|OJJ82976.1 akaw|GAA92832.1 aluc|OJZ91587.1 anid|CBF75890.1 anig|EHA22611.1 aory|EIT75047.1 asyd|OJJ60261.1 atub|OJI86036.1 aver|OJJ03992.1 awen|OJJ31610.1 anid|CBF82973.1

Group5706: abra|OJJ76683.1 acar|OOF99328.1 acla|EAW12908.1 afis|EAW17784.1 afla|EED57245.1 afum|EAL91592.1 agla|OJJ78673.1 akaw|GAA92511.1 aluc|OJZ91524.1 anid|CBF82109.1 aory|EIT74118.1 asyd|OJJ54283.1 ater|EAU37365.1 atub|OJI85979.1 aver|OJJ05237.1 awen|OJJ33522.1 azon|OJJ50362.1

Group5707: abra|OJJ76685.1 acar|OOF99326.1 acla|EAW07047.1 afis|EAW16672.1 afla|EED52488.1 afum|EAL89277.1 agla|OJJ78694.1 akaw|GAA92509.1 aluc|OJZ91522.1 anig|EHA22559.1 aory|EIT82788.1 asyd|OJJ56612.1 ater|EAU33886.1 atub|OJI85977.1 aver|OJJ06111.1 awen|OJJ40037.1 azon|OJJ45870.1

Group5708: abra|OJJ76694.1 acla|EAW11086.1 afis|EAW20772.1 afla|EED45937.1 afum|EAL84847.1 akaw|GAA92502.1 aluc|OJZ91515.1 anid|CBF71393.1 anig|EHA22553.1 aory|EIT75761.1 asyd|OJJ55030.1 ater|EAU36978.1 atub|OJI85967.1 aver|OJJ07173.1 awen|OJJ29779.1 anid|CBF69396.1 awen|OJJ41548.1

Group5709: abra|OJJ76751.1 acar|OOF91247.1 acla|EAW13770.1 afis|EAW20419.1 afum|EAL84630.1 agla|OJJ84906.1 akaw|GAA87273.1 aluc|OJZ84154.1 anid|CBF79344.1 aory|EIT77737.1 asyd|OJJ55798.1 ater|EAU37737.1 atub|OJI89930.1 aver|OJI98814.1 awen|OJJ33112.1 azon|OJJ45537.1 agla|OJJ83727.1

Group5710: abra|OJJ76754.1 acar|OOF91250.1 acla|EAW13308.1 afis|EAW20422.1 afla|EED44849.1 afum|EAL84628.1 agla|OJJ84903.1 akaw|GAA87270.1 aluc|OJZ84157.1 anig|EHA27519.1 aory|EIT77733.1 asyd|OJJ55795.1 ater|EAU37734.1 atub|OJI89927.1 aver|OJJ06884.1 awen|OJJ33115.1 azon|OJJ46331.1

Group5711: abra|OJJ76763.1 acar|OOF91258.1 acla|EAW13317.1 afis|EAW20431.1 afla|EED44837.1 agla|OJJ84894.1 akaw|GAA87263.1 aluc|OJZ84166.1 anid|CBF79329.1 anig|EHA27527.1 aory|EIT77758.1 asyd|OJJ55785.1 atub|OJI89918.1 aver|OJJ06875.1 awen|OJJ33124.1 azon|OJJ46340.1 ater|EAU37726.1

Group5712: abra|OJJ76770.1 acar|OOF91265.1 acla|EAW13324.1 afis|EAW20439.1 afla|EED44831.1 afum|EAL84613.1 agla|OJJ84884.1 akaw|GAA87256.1 aluc|OJZ84174.1 anid|CBF79313.1 anig|EHA27534.1 aory|EIT77752.1 asyd|OJJ55779.1 ater|EAU37719.1 atub|OJI89911.1 aver|OJJ06867.1 awen|OJJ33130.1

Group5713: abra|OJJ76773.1 acar|OOF91268.1 acla|EAW13330.1 afis|EAW20445.1 afla|EED44825.1 afum|EBA27197.1 agla|OJJ84876.1 akaw|GAA87251.1 aluc|OJZ84177.1 anid|CBF79305.1 anig|EHA27536.1 aory|EIT77731.1 ater|EAU37714.1 atub|OJI89906.1 aver|OJJ06865.1 awen|OJJ33135.1 azon|OJJ46348.1

Group5714: abra|OJJ76777.1 acar|OOF91273.1 acla|EAW13336.1 afis|EAW20451.1 afla|EED44817.1 afum|EAL84602.1 agla|OJJ84871.1 akaw|GAA87247.1 aluc|OJZ84181.1 anig|EHA27541.1 aory|EIT77722.1 asyd|OJJ52097.1 ater|EAU37708.1 atub|OJI89902.1 aver|OJJ03020.1 awen|OJJ33141.1 azon|OJJ46352.1

Group5715: abra|OJJ76782.1 acla|EAW13340.1 afis|EAW20455.1 afla|EED44813.1 afum|EBA27196.1 agla|OJJ84867.1 akaw|GAA87243.1 aluc|OJZ84185.1 anid|CBF79288.1 anig|EHA27545.1 aory|EIT77735.1 asyd|OJJ55768.1 ater|EAU37704.1 atub|OJI89898.1 aver|OJJ06854.1 awen|OJJ33145.1 azon|OJJ46357.1

Group5716: abra|OJJ76783.1 acar|OOF91277.1 acla|EAW13341.1 afis|EAW20456.1 afla|EED44812.1 afum|EAL84596.1 agla|OJJ84866.1 akaw|GAA87242.1 aluc|OJZ84186.1 anid|CBF79290.1 anig|EHA27546.1 aory|EIT77713.1 asyd|OJJ55767.1 atub|OJI89897.1 aver|OJJ06853.1 awen|OJJ33146.1 azon|OJJ46358.1

Group5717: abra|OJJ76788.1 acla|EAW13346.1 afis|EAW20461.1 afla|EED44807.1 afum|EAL84592.1 agla|OJJ84856.1 akaw|GAA87237.1 aluc|OJZ84190.1 anid|CBF79275.1 anig|EHA27550.1 aory|EIT77750.1 asyd|OJJ55762.1 ater|EAU37699.1 atub|OJI89892.1 aver|OJJ06847.1 awen|OJJ33154.1 azon|OJJ46363.1

Group5718: abra|OJJ76789.1 acar|OOF91282.1 acla|EAW13347.1 afis|EAW20462.1 afla|EED44806.1 afum|EAL84591.1 agla|OJJ84855.1 akaw|GAA87236.1 aluc|OJZ84191.1 anid|CBF79273.1 anig|EHA27551.1 aory|EIT77738.1 asyd|OJJ55761.1 atub|OJI89891.1 aver|OJJ06846.1 awen|OJJ33155.1 azon|OJJ46364.1

Group5719: abra|OJJ76805.1 acla|EAW13364.1 afis|EAW20478.1 afla|EED44790.1 afum|EAL84576.1 agla|OJJ84839.1 akaw|GAA87218.1 aluc|OJZ84212.1 anid|CBF79250.1 anig|EHA27565.1 aory|EIT77766.1 asyd|OJJ55744.1 ater|EAU29556.1 atub|OJI89871.1 aver|OJJ06831.1 awen|OJJ33173.1 azon|OJJ46380.1

Group5720: abra|OJJ76820.1 acar|OOF91314.1 acla|EAW13380.1 afis|EAW20494.1 afla|EED44770.1 afum|EAL84560.2 agla|OJJ84808.1 akaw|GAA87202.1 aluc|OJZ84230.1 anid|CBF79224.1 aory|EIT77791.1 asyd|OJJ55733.1 ater|EAU29570.1 atub|OJI89854.1 aver|OJJ06819.1 awen|OJJ33194.1 azon|OJJ46402.1

Group5721: abra|OJJ76841.1 acar|OOF91334.1 acla|EAW13404.1 afis|EAW20515.1 afla|EED52467.1 afum|EAL84542.1 agla|OJJ84775.1 akaw|GAA87181.1 aluc|OJZ84252.1 anid|CBF79057.1 anig|EHA27597.1 aory|EIT82760.1 asyd|OJJ55709.1 ater|EAU37186.1 atub|OJI89831.1 aver|OJJ06795.1 awen|OJJ33221.1

Group5722: abra|OJJ76846.1 acar|OOF91336.1 acla|EAW13409.1 afis|EAW20520.1 afla|EED52469.1 afum|EAL84537.1 agla|OJJ84766.1 akaw|GAA87175.1 aluc|OJZ84258.1 anid|CBF79069.1 aory|EIT82811.1 asyd|OJJ55705.1 ater|EAU37168.1 atub|OJI89825.1 aver|OJJ06791.1 awen|OJJ33226.1 azon|OJJ46578.1

Group5723: abra|OJJ76848.1 acla|EAW13411.1 afis|EAW20522.1 afla|EED52471.1 afum|EAL84535.1 agla|OJJ84739.1 akaw|GAA87173.1 aluc|OJZ84260.1 anid|CBF79072.1 anig|EHA27603.1 aory|EIT82780.1 asyd|OJJ55703.1 ater|EAU37166.1 atub|OJI89823.1 aver|OJJ06789.1 awen|OJJ33228.1 azon|OJJ46576.1

Group5724: abra|OJJ76853.1 acla|EAW13415.1 afis|EAW20526.1 afla|EED52475.1 afum|EAL84531.1 agla|OJJ84744.1 akaw|GAA87169.1 aluc|OJZ84265.1 anid|CBF79080.1 anig|EHA27607.1 aory|EIT82795.1 asyd|OJJ55698.1 ater|EAU37162.1 atub|OJI89818.1 aver|OJJ06783.1 awen|OJJ33232.1 azon|OJJ46572.1

Group5725: abra|OJJ76858.1 acar|OOF91346.1 acla|EAW13419.1 afis|EAW20530.1 afla|EED52480.1 afum|EAL84527.1 agla|OJJ84704.1 akaw|GAA87164.1 aluc|OJZ84270.1 anid|CBF79004.1 anig|EHA27612.1 aory|EIT82752.1 asyd|OJJ55690.1 ater|EAU37155.1 atub|OJI89813.1 aver|OJJ06774.1 awen|OJJ33237.1

Group5726: abra|OJJ76902.1 acar|OOF91350.1 acla|EAW13423.1 afis|EAW20535.1 afla|EED54118.1 afum|EAL84523.1 akaw|GAA87136.1 aluc|OJZ84301.1 anid|CBF78997.1 anig|EHA27637.1 aory|EIT76011.1 asyd|OJJ55686.1 ater|EAU37148.1 atub|OJI89767.1 aver|OJJ06770.1 awen|OJJ33242.1 azon|OJJ50621.1

Group5727: abra|OJJ76915.1 acar|OOF91362.1 afis|EAW20543.1 afla|EED52430.1 afum|EAL84515.1 agla|OJJ88755.1 akaw|GAA87123.1 aluc|OJZ84315.1 anid|CBF78989.1 anig|EHA27650.1 aory|EIT83314.1 asyd|OJJ55683.1 ater|EAU37138.1 atub|OJI89753.1 aver|OJJ06767.1 awen|OJJ33250.1 azon|OJJ42414.1

Group5728: abra|OJJ76974.1 acar|OOF91418.1 acla|EAW13500.1 afis|EAW20599.1 afla|EED52205.1 afum|EAL84462.1 agla|OJJ88656.1 akaw|GAA87071.1 aluc|OJZ84370.1 anid|CBF78906.1 anig|EHA27705.1 asyd|OJJ55610.1 ater|EAU35244.1 atub|OJI89697.1 aver|OJJ06685.1 awen|OJJ33391.1 azon|OJJ50695.1

Group5729: abra|OJJ76975.1 acar|OOF91420.1 acla|EAW13501.1 afis|EAW20600.1 afla|EED47728.1 afum|EAL84461.1 agla|OJJ88657.1 akaw|GAA87070.1 aluc|OJZ84371.1 anid|CBF86933.1 anig|EHA27706.1 aory|EIT77127.1 asyd|OJJ55598.1 atub|OJI89696.1 aver|OJJ06678.1 awen|OJJ33392.1 azon|OJJ50696.1

Group5730: abra|OJJ76999.1 acar|OOF91445.1 acla|EAW13462.1 afis|EAW20568.1 afla|EED52322.1 afum|EAL84494.1 agla|OJJ88725.1 akaw|GAA87049.1 aluc|OJZ84397.1 anid|CBF87400.1 anig|EHA27728.1 aory|EIT74352.1 asyd|OJJ52861.1 ater|EAU37115.1 atub|OJI89675.1 aver|OJJ08839.1 awen|OJJ33272.1

Group5731: abra|OJJ77006.1 acar|OOF91451.1 afis|EAW25624.1 afla|EED54122.1 afum|EAL84312.1 agla|OJJ78904.1 akaw|GAA87042.1 aluc|OJZ84405.1 anid|CBF83175.1 anig|EHA27735.1 aory|EIT76161.1 asyd|OJJ55524.1 ater|EAU32184.1 atub|OJI89667.1 aver|OJJ06602.1 awen|OJJ36043.1 azon|OJJ42823.1

Group5732: abra|OJJ77035.1 acla|EAW06829.1 afis|EAW16379.1 afla|EED54135.1 afum|EAL89004.1 agla|OJJ88206.1 akaw|GAA87016.1 aluc|OJZ84438.1 anid|CBF81133.1 anig|EHA27757.1 aory|EIT76057.1 asyd|OJJ56501.1 ater|EAU32642.1 atub|OJI89635.1 aver|OJJ06218.1 awen|OJJ37913.1 azon|OJJ45092.1

Group5733: abra|OJJ77047.1 acar|OOF92936.1 acla|EAW13551.1 afis|EAW20626.1 afla|EED49776.1 afum|EAL84440.1 agla|OJJ80442.1 akaw|GAA87006.1 aluc|OJZ84451.1 anid|CBF83226.1 anig|EHA27766.1 aory|EIT77694.1 asyd|OJJ55580.1 atub|OJI89625.1 aver|OJJ06659.1 awen|OJJ33463.1 azon|OJJ50719.1

Group5734: abra|OJJ77051.1 acar|OOF92940.1 acla|EAW13542.1 afis|EAW20615.1 afla|EED54202.1 afum|EAL84446.1 agla|OJJ88593.1 akaw|GAA87002.1 aluc|OJZ84455.1 anid|CBF86938.1 anig|EHA27770.1 asyd|OJJ55588.1 ater|EAU35252.1 atub|OJI89620.1 aver|OJJ06667.1 awen|OJJ33459.1 azon|OJJ45125.1

Group5735: abra|OJJ77052.1 acar|OOF92941.1 acla|EAW13540.1 afis|EAW20613.1 afum|EAL84448.1 agla|OJJ88622.1 akaw|GAA87000.1 aluc|OJZ84457.1 anid|CBF86943.1 anig|EHA27772.1 aory|EIT76152.1 asyd|OJJ55585.1 ater|EAU35254.1 atub|OJI89618.1 aver|OJJ06664.1 awen|OJJ33457.1 azon|OJJ50713.1

Group5736: abra|OJJ77062.1 acla|EAW13513.1 afis|EAW20611.1 afla|EED54206.1 afum|EAL84450.1 agla|OJJ88628.1 akaw|GAA86990.1 aluc|OJZ84470.1 anid|CBF86957.1 anig|EHA27780.1 aory|EIT75918.1 asyd|OJJ55596.1 ater|EAU35256.1 atub|OJI89608.1 aver|OJJ06675.1 awen|OJJ33454.1 azon|OJJ50711.1

Group5737: abra|OJJ77066.1 acar|OOF92950.1 acla|EAW13509.1 afis|EAW20607.1 afla|EED54208.1 afum|EAL84453.1 agla|OJJ88632.1 akaw|GAA86986.1 aluc|OJZ84475.1 anid|CBF86948.1 anig|EHA27783.1 asyd|OJJ55592.1 ater|EAU35259.1 atub|OJI89604.1 aver|OJJ06671.1 awen|OJJ33450.1 azon|OJJ50707.1

Group5738: abra|OJJ77070.1 acla|EAW09727.1 afis|EAW21413.1 afla|EED55836.1 afum|EAL92427.1 agla|OJJ85544.1 akaw|GAA86983.1 aluc|OJZ84478.1 anid|CBF83797.1 anig|EHA27786.1 aory|EIT76757.1 asyd|OJJ60887.1 ater|EAU35133.1 atub|OJI89601.1 aver|OJJ00211.1 awen|OJJ39288.1 azon|OJJ51566.1

Group5739: abra|OJJ77071.1 acar|OOF92953.1 acla|EAW09728.1 afis|EAW21414.1 afla|EED55835.1 afum|EAL92428.1 agla|OJJ85543.1 akaw|GAA86982.1 aluc|OJZ84479.1 anig|EHA27787.1 aory|EIT76805.1 asyd|OJJ60888.1 ater|EAU35134.1 atub|OJI89600.1 aver|OJJ00212.1 awen|OJJ39289.1 azon|OJJ51565.1

Group5740: abra|OJJ77074.1 acar|OOF92955.1 acla|EAW12062.1 afis|EAW19705.1 afla|EED57046.1 afum|EAL86821.1 agla|OJJ78914.1 akaw|GAA86979.1 aluc|OJZ84484.1 anid|CBF71407.1 anig|EHA27788.1 aory|EIT82262.1 asyd|OJJ55014.1 ater|EAU32844.1 atub|OJI89596.1 aver|OJJ07197.1 awen|OJJ29913.1

Group5741: abra|OJJ77080.1 acar|OOF92963.1 acla|EAW06701.1 afis|EAW16246.1 afla|EED53307.1 afum|EAL88874.1 agla|OJJ86255.1 akaw|GAA86973.1 aluc|OJZ84490.1 anid|CBF78902.1 anig|EHA27794.1 aory|EIT75199.1 asyd|OJJ64604.1 ater|EAU37456.1 atub|OJI89589.1 aver|OJI98701.1 awen|OJJ30210.1

Group5742: abra|OJJ77087.1 acar|OOF92971.1 afis|EAW21842.1 afla|EED46528.1 afum|EAL92854.1 agla|OJJ84295.1 akaw|GAA86968.1 aluc|OJZ84496.1 anid|CBF76713.1 anig|EHA27798.1 aory|EIT72340.1 asyd|OJJ61240.1 ater|EAU38519.1 atub|OJI89584.1 aver|OJJ00563.1 awen|OJJ34126.1 azon|OJJ44217.1

Group5743: abra|OJJ77088.1 acar|OOF92972.1 acla|EAW10185.1 afis|EAW25410.1 afla|EED53889.1 afum|EAL90069.1 agla|OJJ87051.1 akaw|GAA86967.1 aluc|OJZ84497.1 anid|CBF77586.1 aory|EIT78478.1 asyd|OJJ53170.1 ater|EAU34561.1 atub|OJI89583.1 aver|OJJ03682.1 awen|OJJ41137.1 azon|OJJ44716.1

Group5744: abra|OJJ77089.1 acar|OOF92973.1 acla|EAW08631.1 afis|EAW15800.1 afla|EED51245.1 afum|EAL85550.2 agla|OJJ81967.1 akaw|GAA86966.1 aluc|OJZ84498.1 anid|CBF70920.1 aory|EIT83182.1 asyd|OJJ52606.1 ater|EAU32342.1 atub|OJI89582.1 aver|OJJ04633.1 awen|OJJ38509.1 azon|OJJ43743.1

Group5745: abra|OJJ77099.1 acar|OOF92981.1 afis|EAW16895.1 afla|EED53380.1 afum|EAL86432.1 agla|OJJ79067.1 akaw|GAA86957.1 aluc|OJZ84507.1 anid|CBF80785.1 anig|EHA27806.1 aory|EIT75077.1 asyd|OJJ54496.1 ater|EAU30219.1 atub|OJI89573.1 aver|OJJ07684.1 awen|OJJ29738.1 agla|OJJ79950.1

Group5746: abra|OJJ77115.1 acar|OOF93004.1 acla|EAW12953.1 afis|EAW17703.1 afla|EED45697.1 afum|EAL91518.1 agla|OJJ79466.1 akaw|GAA90814.1 aluc|OJZ84525.1 anid|CBF89930.1 anig|EHA27822.1 aory|EIT75749.1 asyd|OJJ62118.1 ater|EAU31911.1 atub|OJI89555.1 aver|OJI96225.1 awen|OJJ37132.1

Group5747: abra|OJJ77117.1 acar|OOF93234.1 acla|EAW12934.1 afis|EAW17706.1 afla|EED45708.1 afum|EAL91520.2 akaw|GAA90812.1 aluc|OJZ84527.1 anig|EHA27824.1 aory|EIT75608.1 asyd|OJJ62133.1 ater|EAU31913.1 atub|OJI89553.1 aver|OJI96242.1 awen|OJJ37130.1 azon|OJJ50829.1 afis|EAW17705.1

Group5748: abra|OJJ77119.1 acla|EAW12931.1 afis|EAW17708.1 afla|EED45710.1 afum|EAL91522.2 agla|OJJ81739.1 akaw|GAA90810.1 aluc|OJZ84529.1 anid|CBF89908.1 anig|EHA27826.1 aory|EIT75384.1 asyd|OJJ62131.1 ater|EAU31904.1 atub|OJI89551.1 aver|OJI96240.1 awen|OJJ37128.1 azon|OJJ50827.1

Group5749: abra|OJJ77124.1 acar|OOF93010.1 acla|EAW12937.1 afis|EAW17713.1 afla|EED45715.1 afum|EAL91528.1 agla|OJJ81735.1 akaw|GAA90804.1 aluc|OJZ84534.1 anid|CBF76918.1 anig|EHA27832.1 aory|EIT75535.1 asyd|OJJ57073.1 atub|OJI89546.1 aver|OJJ03225.1 awen|OJJ37123.1 azon|OJJ50822.1

Group5750: abra|OJJ77129.1 acla|EAW12940.1 afis|EAW17716.1 afla|EED45718.1 afum|EAL91531.1 agla|OJJ81732.1 akaw|GAA90799.1 aluc|OJZ84539.1 anid|CBF84090.1 anig|EHA27837.1 aory|EIT75537.1 asyd|OJJ57081.1 ater|EAU31894.1 atub|OJI89541.1 aver|OJJ03221.1 awen|OJJ37120.1 azon|OJJ50819.1

Group5751: abra|OJJ77132.1 acar|OOF93020.1 acla|EAW12943.1 afis|EAW17719.1 afla|EED45721.1 afum|EAL91534.2 agla|OJJ81729.1 akaw|GAA90796.1 aluc|OJZ84542.1 anid|CBF76906.1 anig|EHA27840.1 asyd|OJJ57084.1 ater|EAU31897.1 atub|OJI89538.1 aver|OJJ03218.1 awen|OJJ37117.1 azon|OJJ50816.1

Group5752: abra|OJJ77137.1 acar|OOF90284.1 afis|EAW16801.1 afla|EED47170.1 afum|EAL84245.1 agla|OJJ88968.1 akaw|GAA90792.1 aluc|OJZ84548.1 anid|CBF78601.1 anig|EHA27844.1 asyd|OJJ59267.1 atub|OJI89532.1 aver|OJJ01879.1 awen|OJJ32033.1 acla|EAW12954.1 afis|EAW15402.1 aory|EIT79292.1

Group5753: abra|OJJ77151.1 acar|OOF93037.1 acla|EAW12918.1 afis|EAW17737.1 afla|EED45738.1 afum|EAL91552.1 akaw|GAA90779.1 aluc|OJZ84565.1 anid|CBF86590.1 anig|EHA27859.1 aory|EIT75449.1 asyd|OJJ64829.1 ater|EAU31870.1 atub|OJI89517.1 aver|OJI98942.1 awen|OJJ37098.1 azon|OJJ50799.1

Group5754: abra|OJJ77169.1 acar|OOF93060.1 acla|EAW12897.1 afis|EAW17772.1 afla|EED45786.1 afum|EAL91581.1 agla|OJJ81695.1 akaw|GAA90764.1 aluc|OJZ84582.1 anig|EHA27875.1 aory|EIT75547.1 ater|EAU31841.1 atub|OJI89498.1 awen|OJJ37074.1 acla|EAW15270.1 afis|EAW20239.1 afum|EAL85357.1

Group5755: abra|OJJ77208.1 acla|EAW13200.1 afis|EAW17442.1 afla|EED49385.1 afum|EAL91268.1 agla|OJJ80375.1 akaw|GAA90730.1 aluc|OJZ84619.1 anid|CBF71696.1 anig|EHA27907.1 aory|EIT79456.1 asyd|OJJ61815.1 ater|EAU33921.1 atub|OJI89462.1 aver|OJI95914.1 awen|OJJ37404.1 azon|OJJ51078.1

Group5756: abra|OJJ77224.1 acar|OOF93109.1 acla|EAW13215.1 afis|EAW17427.1 afla|EED49401.1 afum|EAL91255.1 agla|OJJ80397.1 akaw|GAA90715.1 aluc|OJZ84634.1 anid|CBF71718.1 anig|EHA27922.1 aory|EIT79578.1 asyd|OJJ61803.1 atub|OJI89445.1 aver|OJI95902.1 awen|OJJ37416.1 azon|OJJ51090.1

Group5757: abra|OJJ77227.1 acar|OOF93112.1 acla|EAW13218.1 afis|EAW17424.1 afla|EED49398.1 afum|EAL91252.1 agla|OJJ80400.1 akaw|GAA90712.1 aluc|OJZ84637.1 anid|CBF71724.1 anig|EHA27925.1 asyd|OJJ61800.1 ater|EAU33906.1 atub|OJI89442.1 aver|OJI95899.1 awen|OJJ37419.1 azon|OJJ51093.1

Group5758: abra|OJJ77237.1 acar|OOF93118.1 acla|EAW13244.1 afis|EAW17392.1 afla|EED49476.1 afum|EAL91230.1 agla|OJJ80402.1 akaw|GAA90706.1 aluc|OJZ84647.1 anid|CBF71737.1 anig|EHA27931.1 asyd|OJJ61793.1 ater|EAU33897.1 atub|OJI89433.1 aver|OJI95893.1 awen|OJJ37423.1 azon|OJJ51106.1

Group5759: abra|OJJ77239.1 acar|OOF93120.1 acla|EAW13246.1 afis|EAW17390.1 afla|EED49478.1 afum|EAL91226.1 agla|OJJ80407.1 akaw|GAA90704.1 aluc|OJZ84649.1 anid|CBF71741.1 aory|EIT79473.1 asyd|OJJ61791.1 ater|EAU33895.1 atub|OJI89431.1 aver|OJI95891.1 awen|OJJ37425.1 azon|OJJ51103.1

Group5760: abra|OJJ77245.1 acla|EAW13249.1 afis|EAW17387.1 afla|EED49496.1 afum|EAL91221.2 agla|OJJ80412.1 akaw|GAA90697.1 aluc|OJZ84656.1 anid|CBF84185.1 anig|EHA27938.1 aory|EIT82598.1 asyd|OJJ57142.1 ater|EAU33892.1 atub|OJI89424.1 aver|OJJ03162.1 awen|OJJ37429.1 azon|OJJ51100.1

Group5761: abra|OJJ77246.1 acar|OOF93127.1 acla|EAW13250.1 afis|EAW17386.1 afla|EED49497.1 afum|EBA27427.1 agla|OJJ80416.1 akaw|GAA90696.1 aluc|OJZ84657.1 anid|CBF84181.1 anig|EHA27939.1 aory|EIT82594.1 asyd|OJJ57158.1 atub|OJI89423.1 aver|OJJ03155.1 awen|OJJ37430.1 azon|OJJ51107.1

Group5762: abra|OJJ77251.1 acar|OOF90004.1 acla|EAW13254.1 afis|EAW17382.1 afla|EED49505.1 afum|EAL91217.1 akaw|GAA90692.1 aluc|OJZ84662.1 anid|CBF84200.1 anig|EHA27944.1 aory|EIT82574.1 asyd|OJJ57162.1 ater|EAU33879.1 atub|OJI89418.1 aver|OJJ03151.1 awen|OJJ37435.1 azon|OJJ51113.1

Group5763: abra|OJJ77259.1 acar|OOF90003.1 acla|EAW13255.1 afis|EAW17381.1 afla|EED49507.1 afum|EAL91216.1 agla|OJJ80421.1 akaw|GAA90688.1 aluc|OJZ84666.1 anid|CBF84205.1 anig|EHA27945.1 aory|EIT82652.1 asyd|OJJ57165.1 atub|OJI89415.1 aver|OJJ03149.1 awen|OJJ37437.1 azon|OJJ51114.1

Group5764: abra|OJJ77276.1 acar|OOF93151.1 acla|EAW13276.1 afis|EAW17341.1 afla|EED49780.1 afum|EAL91186.1 agla|OJJ79343.1 akaw|GAA90673.1 aluc|OJZ84686.1 anid|CBF84219.1 anig|EHA27961.1 aory|EIT77670.1 asyd|OJJ57177.1 atub|OJI89396.1 aver|OJJ03137.1 awen|OJJ36045.1 azon|OJJ45995.1

Group5765: abra|OJJ77280.1 acar|OOF93154.1 acla|EAW13279.1 afis|EAW17338.1 afla|EED51043.1 afum|EAL91182.1 agla|OJJ79348.1 akaw|GAA90669.1 aluc|OJZ84690.1 anig|EHA27965.1 aory|EIT76982.1 asyd|OJJ57176.1 ater|EAU33848.1 atub|OJI89392.1 aver|OJJ03138.1 awen|OJJ40817.1 azon|OJJ50225.1

Group5766: abra|OJJ77294.1 acar|OOF93171.1 acla|EAW13170.1 afis|EAW17469.1 afla|EED49347.1 afum|EAL91298.1 agla|OJJ78441.1 akaw|GAA91457.1 aluc|OJZ84706.1 anid|CBF71628.1 anig|EHA27980.1 aory|EIT79559.1 asyd|OJJ61848.1 ater|EAU33953.1 atub|OJI89377.1 aver|OJI95947.1 awen|OJJ37373.1

Group5767: abra|OJJ77296.1 acar|OOF93173.1 acla|EAW13168.1 afis|EAW17471.1 afla|EED49349.1 afum|EAL91300.1 agla|OJJ81491.1 akaw|GAA91455.1 aluc|OJZ84708.1 anid|CBF71632.1 anig|EHA27982.1 aory|EIT79572.1 asyd|OJJ61850.1 ater|EAU33955.1 atub|OJI89375.1 aver|OJI95949.1 awen|OJJ37371.1

Group5768: abra|OJJ77299.1 acar|OOF93178.1 acla|EAW13166.1 afis|EAW17473.1 afla|EED49342.1 afum|EAL91302.1 agla|OJJ81489.1 akaw|GAA91453.1 aluc|OJZ84711.1 anid|CBF71624.1 anig|EHA27984.1 asyd|OJJ61852.1 ater|EAU33957.1 atub|OJI89372.1 aver|OJI95952.1 awen|OJJ37369.1 azon|OJJ51048.1

Group5769: abra|OJJ77302.1 acar|OOF93181.1 afis|EAW17476.1 afla|EED49338.1 afum|EAL91305.1 agla|OJJ81485.1 akaw|GAA91449.1 aluc|OJZ84715.1 anid|CBF71601.1 anig|EHA27987.1 aory|EIT79492.1 asyd|OJJ61856.1 ater|EAU33692.1 atub|OJI89368.1 aver|OJI95955.1 awen|OJJ37366.1 azon|OJJ45454.1

Group5770: abra|OJJ77316.1 acar|OOF93192.1 acla|EAW13156.1 afis|EAW17490.1 afla|EED49326.1 afum|EAL91312.1 agla|OJJ81466.1 aluc|OJZ84726.1 anid|CBF71619.1 anig|EHA28000.1 aory|EIT79591.1 asyd|OJJ61865.1 ater|EAU36935.1 atub|OJI89356.1 aver|OJI95963.1 awen|OJJ37351.1 azon|OJJ51037.1

Group5771: abra|OJJ77323.1 acar|OOF93199.1 afis|EAW17497.1 afla|EED49319.1 afum|EAL91320.1 agla|OJJ81458.1 akaw|GAA91433.1 aluc|OJZ84733.1 anid|CBF71584.1 anig|EHA28006.1 asyd|OJJ61873.1 ater|EAU36941.1 atub|OJI89348.1 aver|OJI95971.1 awen|OJJ37344.1 azon|OJJ51030.1 ater|EAU38793.1

Group5772: abra|OJJ77324.1 acar|OOF93200.1 acla|EAW13148.1 afis|EAW17499.1 afla|EED49317.1 afum|EAL91322.1 agla|OJJ81454.1 akaw|GAA91432.1 aluc|OJZ84734.1 anid|CBF71580.1 anig|EHA28007.1 asyd|OJJ61875.1 ater|EAU36944.1 atub|OJI89347.1 aver|OJI95973.1 awen|OJJ37342.1 azon|OJJ51027.1

Group5773: abra|OJJ77330.1 acar|OOF93205.1 acla|EAW13143.1 afis|EAW17515.1 afla|EED49312.1 afum|EAL91333.1 agla|OJJ81449.1 akaw|GAA91427.1 aluc|OJZ84739.1 anid|CBF71570.1 anig|EHA28012.1 aory|EIT82234.1 asyd|OJJ61880.1 atub|OJI89342.1 aver|OJI95978.1 awen|OJJ37336.1 azon|OJJ51022.1

Group5774: abra|OJJ77332.1 acar|OOF93206.1 acla|EAW13142.1 afis|EAW17514.1 afla|EED49311.1 afum|EAL91332.1 agla|OJJ81448.1 akaw|GAA91426.1 aluc|OJZ84740.1 anid|CBF73599.1 anig|EHA28013.1 aory|EIT82247.1 asyd|OJJ56681.1 ater|EAU36951.1 atub|OJI89341.1 aver|OJJ06042.1 awen|OJJ37335.1

Group5775: abra|OJJ77340.1 acla|EAW13135.1 afis|EAW17504.1 afla|EED49304.1 afum|EAL91326.1 agla|OJJ81442.1 akaw|GAA91421.1 aluc|OJZ84749.1 anid|CBF71539.1 anig|EHA28019.1 aory|EIT82224.1 asyd|OJJ61895.1 ater|EAU36957.1 atub|OJI89333.1 aver|OJI95991.1 awen|OJJ37327.1 azon|OJJ51016.1

Group5776: abra|OJJ77360.1 acar|OOF93229.1 acla|EAW13116.1 afis|EAW17548.1 afla|EED49277.1 afum|EAL91362.1 agla|OJJ81420.1 akaw|GAA91403.1 aluc|OJZ84768.1 anid|CBF90292.1 aory|EIT82223.1 asyd|OJJ61922.1 ater|EAU36987.1 atub|OJI89314.1 aver|OJI96014.1 awen|OJJ37306.1 azon|OJJ50998.1

Group5777: abra|OJJ77389.1 acar|OOF93258.1 acla|EAW08796.1 afis|EAW15599.1 afla|EED45975.1 afum|EAL85733.1 akaw|GAA91372.1 aluc|OJZ84797.1 anid|CBF78871.1 anig|EHA28064.1 aory|EIT75420.1 asyd|OJJ64587.1 ater|EAU30348.1 atub|OJI89285.1 aver|OJI98684.1 awen|OJJ37986.1 azon|OJJ43887.1

Group5778: abra|OJJ77395.1 acar|OOF93263.1 afis|EAW17805.1 afla|EED45800.1 afum|EAL91613.1 agla|OJJ78527.1 akaw|GAA91367.1 aluc|OJZ84803.1 anid|CBF86646.1 anig|EHA28068.1 aory|EIT75621.1 asyd|OJJ64799.1 ater|EAU31836.1 atub|OJI89280.1 aver|OJI98913.1 awen|OJJ41514.1 agla|OJJ80100.1

Group5779: abra|OJJ77466.1 acar|OOF99268.1 acla|EAW07126.1 afis|EAW22136.1 afla|EED54175.1 afum|EAL91054.1 akaw|GAA84151.1 aluc|OJZ81072.1 anid|CBF69552.1 anig|EHA19297.1 aory|EIT75896.1 asyd|OJJ59166.1 ater|EAU29320.1 atub|OJI82514.1 aver|OJJ01756.1 awen|OJJ34713.1 aory|EIT76090.1

Group5780: abra|OJJ77469.1 acar|OOF99255.1 acla|EAW06765.1 afis|EAW16315.1 afla|EED54179.1 afum|EAL88938.1 agla|OJJ88273.1 akaw|GAA84148.1 aluc|OJZ81069.1 anid|CBF69555.1 anig|EHA19300.1 aory|EIT75912.1 asyd|OJJ59164.1 ater|EAU29325.1 atub|OJI82517.1 aver|OJJ01754.1 awen|OJJ37713.1

Group5781: abra|OJJ77470.1 acar|OOF99254.1 acla|EAW06762.1 afis|EAW16312.1 afla|EED54183.1 afum|EAL88934.1 agla|OJJ88271.1 akaw|GAA84147.1 aluc|OJZ81068.1 anig|EHA19301.1 aory|EIT76029.1 asyd|OJJ59157.1 ater|EAU29330.1 atub|OJI82518.1 aver|OJJ01747.1 awen|OJJ37712.1 azon|OJJ44971.1

Group5782: abra|OJJ77472.1 acar|OOF99252.1 acla|EAW06759.1 afis|EAW16309.1 afla|EED54185.1 afum|EAL88931.1 agla|OJJ86289.1 akaw|GAA84145.1 aluc|OJZ81065.1 anid|CBF69562.1 anig|EHA19303.1 aory|EIT75996.1 asyd|OJJ59155.1 ater|EAU29338.1 atub|OJI82520.1 aver|OJJ01745.1 awen|OJJ37708.1

Group5783: abra|OJJ77473.1 acar|OOF99251.1 acla|EAW06758.1 afis|EAW16308.1 afla|EED53241.1 afum|EAL88930.1 agla|OJJ86288.1 akaw|GAA84144.1 aluc|OJZ81064.1 anid|CBF69564.1 anig|EHA19304.1 aory|EIT75223.1 asyd|OJJ59154.1 ater|EAU29337.1 atub|OJI82521.1 aver|OJJ01744.1 awen|OJJ37707.1

Group5784: abra|OJJ77481.1 acar|OOF99244.1 acla|EAW06743.1 afis|EAW16293.1 afum|EAL88915.1 agla|OJJ88315.1 akaw|GAA84137.1 aluc|OJZ81056.1 anid|CBF86850.1 anig|EHA19311.1 aory|EIT75158.1 asyd|OJJ64639.1 ater|EAU37337.1 atub|OJI82528.1 aver|OJI98738.1 awen|OJJ37687.1 azon|OJJ44947.1

Group5785: abra|OJJ77483.1 acar|OOF99243.1 acla|EAW06744.1 afis|EAW16294.1 afla|EED53260.1 afum|EAL88916.1 agla|OJJ88316.1 akaw|GAA84136.1 aluc|OJZ81054.1 anid|CBF86848.1 anig|EHA19313.1 aory|EIT74888.1 asyd|OJJ64640.1 atub|OJI82530.1 aver|OJI98740.1 awen|OJJ37685.1 azon|OJJ44946.1

Group5786: abra|OJJ77484.1 acar|OOF99241.1 acla|EAW06747.1 afis|EAW16297.1 afla|EED53257.1 afum|EAL88919.1 agla|OJJ88298.1 akaw|GAA84134.1 aluc|OJZ81052.1 anid|CBF86845.1 anig|EHA19315.1 asyd|OJJ64643.1 ater|EAU29339.1 atub|OJI82532.1 aver|OJI98743.1 awen|OJJ37696.1 azon|OJJ44957.1

Group5787: abra|OJJ77487.1 acar|OOF99238.1 acla|EAW06749.1 afis|EAW16299.1 afla|EED53255.1 afum|EAL88921.1 agla|OJJ88296.1 akaw|GAA84132.1 aluc|OJZ81049.1 anig|EHA19318.1 aory|EIT75108.1 asyd|OJJ64645.1 ater|EAU37341.1 atub|OJI82535.1 aver|OJI98745.1 awen|OJJ37698.1 azon|OJJ44959.1

Group5788: abra|OJJ77519.1 acar|OOF99205.1 acla|EAW06713.1 afis|EAW16274.1 afla|EED53296.1 afum|EAL88895.1 agla|OJJ78577.1 akaw|GAA84102.1 aluc|OJZ81017.1 anid|CBF78275.1 anig|EHA19345.1 aory|EIT75252.1 asyd|OJJ56075.1 ater|EAU37449.1 atub|OJI82566.1 aver|OJJ05018.1 azon|OJJ50409.1

Group5789: abra|OJJ77531.1 acla|EAW06706.1 afis|EAW16263.1 afla|EED53301.1 afum|EAL88884.1 agla|OJJ88379.1 akaw|GAA84089.1 aluc|OJZ81004.1 anid|CBF86916.1 anig|EHA19356.1 aory|EIT75165.1 asyd|OJJ64606.1 ater|EAU37452.1 atub|OJI82581.1 aver|OJI98703.1 awen|OJJ37653.1 azon|OJJ44917.1

Group5790: abra|OJJ77536.1 acar|OOF99190.1 acla|EAW06711.1 afis|EAW16257.1 afla|EED53323.1 afum|EAL88878.1 akaw|GAA84084.1 aluc|OJZ80998.1 anid|CBF78893.1 anig|EHA19362.1 aory|EIT75253.1 asyd|OJJ64598.1 ater|EAU37468.1 atub|OJI82586.1 aver|OJI98695.1 awen|OJJ30202.1 azon|OJJ50681.1

Group5791: abra|OJJ77546.1 acar|OOF99177.1 acla|EAW06708.1 afis|EAW16261.1 afla|EED53325.1 afum|EAL88882.1 agla|OJJ88382.1 akaw|GAA84077.1 aluc|OJZ80991.1 anid|CBF78870.1 anig|EHA19371.1 asyd|OJJ64588.1 ater|EAU33700.1 atub|OJI82594.1 aver|OJI98685.1 awen|OJJ37651.1 azon|OJJ44915.1

Group5792: abra|OJJ77547.1 acar|OOF99176.1 acla|EAW06707.1 afis|EAW16262.1 afla|EED53326.1 afum|EAL88883.1 agla|OJJ88381.1 akaw|GAA84076.1 aluc|OJZ80990.1 anid|CBF78868.1 aory|EIT74871.1 asyd|OJJ64589.1 ater|EAU33701.1 atub|OJI82595.1 aver|OJI98686.1 awen|OJJ37652.1 azon|OJJ44916.1

Group5793: abra|OJJ77549.1 acar|OOF95414.1 acla|EAW08956.1 afla|EED49536.1 afum|EAL88030.1 agla|OJJ78723.1 akaw|GAA84074.1 aluc|OJZ80988.1 anid|CBF70524.1 anig|EHA19372.1 asyd|OJJ57565.1 atub|OJI82598.1 aver|OJJ05614.1 awen|OJJ32036.1 azon|OJJ46060.1 asyd|OJJ56222.1 aver|OJJ05154.1

Group5794: abra|OJJ77558.1 acar|OOG00297.1 afis|EAW16397.1 afla|EED51760.1 afum|EAL89030.2 agla|OJJ88351.1 akaw|GAA84066.1 aluc|OJZ80980.1 anid|CBF85653.1 anig|EHA19381.1 aory|EIT80739.1 asyd|OJJ62365.1 ater|EAU33012.1 atub|OJI82606.1 aver|OJI96499.1 awen|OJJ30119.1 agla|OJJ82192.1

Group5795: abra|OJJ77562.1 acla|EAW06695.1 afis|EAW16071.1 afla|EED48590.1 afum|EAL88703.2 agla|OJJ86097.1 akaw|GAA84063.1 aluc|OJZ80976.1 anid|CBF75105.1 anig|EHA19383.1 aory|EIT73151.1 asyd|OJJ59999.1 ater|EAU33560.1 atub|OJI82609.1 aver|OJJ02292.1 awen|OJJ30363.1 azon|OJJ45382.1

Group5796: abra|OJJ77565.1 acar|OOF99165.1 acla|EAW06530.1 afis|EAW16065.1 afla|EED48586.1 afum|EAL88697.1 agla|OJJ86100.1 akaw|GAA84061.1 aluc|OJZ80973.1 anid|CBF75096.1 anig|EHA19386.1 asyd|OJJ60008.1 ater|EAU33568.1 atub|OJI82612.1 aver|OJJ02277.1 awen|OJJ30370.1 azon|OJJ45390.1

Group5797: abra|OJJ77570.1 acla|EAW06535.1 afis|EAW16060.1 afla|EED48574.1 afum|EAL88693.1 akaw|GAA84056.1 aluc|OJZ80968.1 anid|CBF75084.1 anig|EHA19390.1 aory|EIT73112.1 asyd|OJJ60015.1 atub|OJI82618.1 aver|OJJ02270.1 awen|OJJ30379.1 azon|OJJ45395.1 agla|OJJ86105.1 ater|EAU33574.1

Group5798: abra|OJJ77571.1 acar|OOF99158.1 acla|EAW06540.1 afis|EAW16055.1 afla|EED56443.1 afum|EAL88687.1 agla|OJJ86088.1 akaw|GAA84055.1 aluc|OJZ80967.1 anid|CBF75074.1 anig|EHA19391.1 aory|EIT72848.1 asyd|OJJ60020.1 ater|EAU33579.1 atub|OJI82619.1 aver|OJJ02265.1 awen|OJJ30384.1

Group5799: abra|OJJ77578.1 acar|OOF99149.1 acla|EAW06544.1 afis|EAW16050.1 afla|EED56439.1 afum|EAL88683.1 agla|OJJ86084.1 akaw|GAA84047.1 aluc|OJZ80957.1 anid|CBF75066.1 anig|EHA19398.1 asyd|OJJ60023.1 atub|OJI82627.1 aver|OJJ02261.1 awen|OJJ30393.1 azon|OJJ45403.1 ater|EAU33586.1

Group5800: abra|OJJ77594.1 acla|EAW06558.1 afis|EAW16038.1 afla|EED56426.1 afum|EAL88669.1 agla|OJJ86062.1 akaw|GAA84034.1 aluc|OJZ80941.1 anid|CBF75038.1 anig|EHA19412.1 aory|EIT72878.1 asyd|OJJ60036.1 ater|EAU33602.1 atub|OJI82641.1 aver|OJJ02249.1 awen|OJJ30408.1 azon|OJJ45416.1

Group5801: abra|OJJ77595.1 acar|OOF99132.1 acla|EAW06559.1 afis|EAW16037.1 afla|EED56425.1 afum|EBA27355.1 agla|OJJ86063.1 akaw|GAA84033.1 aluc|OJZ80940.1 anid|CBF75042.1 aory|EIT72875.1 asyd|OJJ60037.1 ater|EAU33603.1 atub|OJI82642.1 aver|OJJ02248.1 awen|OJJ30409.1 azon|OJJ45417.1

Group5802: abra|OJJ77596.1 acar|OOF99130.1 acla|EAW06560.1 afis|EAW16036.1 afla|EED56424.1 afum|EBA27354.1 agla|OJJ86064.1 akaw|GAA84032.1 aluc|OJZ80939.1 anid|CBF75040.1 anig|EHA19413.1 asyd|OJJ60038.1 ater|EAU33604.1 atub|OJI82643.1 aver|OJJ02247.1 awen|OJJ30410.1 azon|OJJ45419.1

Group5803: abra|OJJ77600.1 acar|OOF99125.1 acla|EAW06564.1 afis|EAW16032.1 afla|EED56414.1 afum|EAL88663.1 agla|OJJ86060.1 akaw|GAA84028.1 aluc|OJZ80933.1 anid|CBF75032.1 anig|EHA19417.1 asyd|OJJ60051.1 ater|EAU33609.1 atub|OJI82647.1 aver|OJJ02238.1 awen|OJJ30415.1 azon|OJJ45423.1

Group5804: abra|OJJ77647.1 acar|OOF99089.1 acla|EAW06493.1 afis|EAW15989.1 afla|EED56361.1 afum|EAL88621.1 agla|OJJ85991.1 akaw|GAA83988.1 aluc|OJZ80882.1 anid|CBF85817.1 anig|EHA19454.1 aory|EIT73312.1 ater|EAU33660.1 atub|OJI82695.1 aver|OJI98436.1 awen|OJJ30481.1 azon|OJJ45472.1

Group5805: abra|OJJ77649.1 acar|OOF99087.1 akaw|GAA83986.1 aluc|OJZ80880.1 anig|EHA19456.1 atub|OJI82697.1 aver|OJI98438.1 abra|OJJ78132.1 afis|EAW20412.1 afla|EED52849.1 afum|EAL89232.1 aluc|OJZ91194.1 aory|EIT78655.1 ater|EAU29526.1 atub|OJI83213.1 awen|OJJ38076.1 anid|CBF87263.1

Group5806: abra|OJJ77662.1 acar|OOF99072.1 acla|EAW06599.1 afis|EAW16005.1 afla|EED56379.1 afum|EAL88637.2 agla|OJJ86029.1 akaw|GAA83973.1 aluc|OJZ80866.1 anid|CBF85850.1 anig|EHA19469.1 asyd|OJJ64361.1 ater|EAU33638.1 atub|OJI82711.1 aver|OJI98457.1 awen|OJJ30462.1 azon|OJJ45452.1

Group5807: abra|OJJ77663.1 acla|EAW06600.1 afis|EAW16004.1 afum|EAL88636.1 agla|OJJ86028.1 akaw|GAA83972.1 aluc|OJZ80865.1 anid|CBF85848.1 anig|EHA19470.1 aory|EIT73333.1 asyd|OJJ64358.1 ater|EAU33639.1 atub|OJI82712.1 aver|OJI98454.1 awen|OJJ30463.1 azon|OJJ45453.1 afla|EED56378.1

Group5808: abra|OJJ77676.1 acla|EAW06588.1 afis|EAW16011.1 afla|EED56388.1 afum|EAL88641.1 agla|OJJ86036.1 akaw|GAA83956.1 aluc|OJZ80843.1 anid|CBF74567.1 anig|EHA19480.1 aory|EIT83019.1 asyd|OJJ57650.1 ater|EAU33632.1 atub|OJI82724.1 aver|OJJ05509.1 awen|OJJ30454.1 azon|OJJ45445.1

Group5809: abra|OJJ77677.1 acla|EAW06589.1 afis|EAW16012.1 afla|EED56389.1 afum|EAL88642.1 agla|OJJ86037.1 akaw|GAA83955.1 aluc|OJZ80842.1 anid|CBF74566.1 anig|EHA19481.1 aory|EIT83001.1 asyd|OJJ57649.1 ater|EAU33631.1 atub|OJI82725.1 aver|OJJ05508.1 awen|OJJ30453.1 azon|OJJ45444.1

Group5810: abra|OJJ77692.1 acar|OOF99047.1 acla|EAW06573.1 afis|EAW16023.1 afla|EED56402.1 afum|EAL88652.1 agla|OJJ86052.1 akaw|GAA83938.1 aluc|OJZ80820.1 anid|CBF75011.1 anig|EHA19492.1 aory|EIT82997.1 asyd|OJJ60055.1 atub|OJI82742.1 aver|OJJ02234.1 awen|OJJ30438.1 azon|OJJ45433.1

Group5811: abra|OJJ77698.1 acar|OOF99042.1 acla|EAW11328.1 afis|EAW18167.1 afla|EED56328.1 afum|EAL87527.2 agla|OJJ85954.1 akaw|GAA83932.1 aluc|OJZ90763.1 anid|CBF85748.1 anig|EHA19497.1 aory|EIT73301.1 asyd|OJJ64294.1 ater|EAU33837.1 atub|OJI82751.1 aver|OJI98386.1 awen|OJJ30513.1

Group5812: abra|OJJ77705.1 acar|OOF99035.1 acla|EAW11316.1 afis|EAW18180.1 afla|EED56308.1 afum|EAL87541.1 akaw|GAA83925.1 aluc|OJZ90770.1 anid|CBF85727.1 anig|EHA19505.1 aory|EIT73291.1 asyd|OJJ64279.1 ater|EAU33820.1 atub|OJI82758.1 aver|OJI98371.1 awen|OJJ30528.1 azon|OJJ50210.1

Group5813: abra|OJJ77739.1 acar|OOF98987.1 afis|EAW21097.1 afla|EED49614.1 afum|EAL92120.1 agla|OJJ80115.1 akaw|GAA83890.1 aluc|OJZ90805.1 anid|CBF84486.1 anig|EHA19532.1 aory|EIT80984.1 asyd|OJJ53525.1 ater|EAU37778.1 atub|OJI82794.1 aver|OJJ08225.1 awen|OJJ36023.1 acla|EAW09993.1

Group5814: abra|OJJ77772.1 acar|OOF98962.1 acla|EAW11312.1 afis|EAW18186.1 afla|EED56292.1 afum|EAL87549.1 agla|OJJ85898.1 akaw|GAA83874.1 aluc|OJZ90822.1 anid|CBF85717.1 anig|EHA19548.1 aory|EIT73285.1 asyd|OJJ64274.1 ater|EAU33808.1 atub|OJI82810.1 aver|OJI98366.1 awen|OJJ30541.1

Group5815: abra|OJJ77773.1 acar|OOF98964.1 acla|EAW08571.1 afis|EAW23629.1 afla|EED46113.1 afum|EAL87831.1 akaw|GAA83873.1 aluc|OJZ90825.1 anid|CBF84280.1 anig|EHA19549.1 aory|EIT76433.1 asyd|OJJ57232.1 ater|EAU38029.1 atub|OJI82812.1 aver|OJJ03057.1 awen|OJJ36003.1 azon|OJJ51529.1

Group5816: abra|OJJ77774.1 acar|OOF98965.1 acla|EAW08572.1 afis|EAW23630.1 afla|EED46115.1 afum|EBA27325.1 akaw|GAA83872.1 aluc|OJZ90826.1 anid|CBF84282.1 anig|EHA19550.1 aory|EIT76372.1 asyd|OJJ57233.1 ater|EAU38028.1 atub|OJI82813.1 aver|OJJ03056.1 awen|OJJ36002.1 azon|OJJ51527.1

Group5817: abra|OJJ77776.1 acar|OOF98966.1 acla|EAW11313.1 afis|EAW18184.1 afla|EED56301.1 afum|EBA27317.1 agla|OJJ85900.1 akaw|GAA83871.1 aluc|OJZ90829.1 anid|CBF85715.1 anig|EHA19552.1 aory|EIT73399.1 asyd|OJJ64273.1 ater|EAU33816.1 atub|OJI82815.1 aver|OJI98365.1 awen|OJJ30532.1

Group5818: abra|OJJ77780.1 acar|OOF98970.1 afla|EED53399.1 akaw|GAA83867.1 aluc|OJZ90833.1 anid|CBF80617.1 anig|EHA19556.1 aory|EIT74939.1 asyd|OJJ54627.1 atub|OJI82819.1 aver|OJJ07529.1 awen|OJJ34502.1 afla|EED44856.1 aory|EIT82733.1 aver|OJI99413.1 anid|CBF78813.1 asyd|OJJ65262.1

Group5819: abra|OJJ77823.1 acar|OOF98946.1 acla|EAW11302.1 afis|EAW18200.1 afla|EED56278.1 afum|EAL87565.1 agla|OJJ85882.1 akaw|GAA83823.1 aluc|OJZ90895.1 anid|CBF86117.1 anig|EHA19614.1 aory|EIT73354.1 ater|EAU33794.1 atub|OJI82888.1 aver|OJI98595.1 awen|OJJ30568.1 azon|OJJ45504.1

Group5820: abra|OJJ77829.1 acla|EAW11297.1 afis|EAW18205.1 afla|EED56267.1 afum|EAL87570.1 agla|OJJ85877.1 akaw|GAA83818.1 aluc|OJZ90903.1 anid|CBF86130.1 anig|EHA19618.1 aory|EIT73457.1 asyd|OJJ64500.1 ater|EAU33784.1 atub|OJI82896.1 aver|OJI98600.1 awen|OJJ30576.1 azon|OJJ45499.1

Group5821: abra|OJJ77832.1 acar|OOF98938.1 acla|EAW11294.1 afis|EAW18208.1 afla|EED56262.1 afum|EAL87574.1 agla|OJJ85874.1 akaw|GAA83815.1 aluc|OJZ90906.1 anid|CBF86135.1 anig|EHA19621.1 asyd|OJJ64504.1 ater|EAU33780.1 atub|OJI82899.1 aver|OJI98603.1 awen|OJJ30580.1 azon|OJJ50191.1

Group5822: abra|OJJ77833.1 acla|EAW11293.1 afis|EAW18209.1 afla|EED56261.1 afum|EAL87575.2 agla|OJJ85873.1 akaw|GAA83814.1 aluc|OJZ90907.1 anid|CBF86137.1 anig|EHA19622.1 aory|EIT73295.1 asyd|OJJ64505.1 ater|EAU33779.1 atub|OJI82900.1 aver|OJI98604.1 awen|OJJ30581.1 azon|OJJ50190.1

Group5823: abra|OJJ77835.1 acar|OOF98936.1 acla|EAW11288.1 afis|EAW18211.1 afum|EAL87577.1 agla|OJJ85871.1 akaw|GAA83812.1 aluc|OJZ90909.1 anid|CBF86140.1 anig|EHA19624.1 aory|EIT73309.1 asyd|OJJ64507.1 ater|EAU33777.1 atub|OJI82902.1 aver|OJI98606.1 awen|OJJ30589.1 azon|OJJ50187.1

Group5824: abra|OJJ77842.1 acla|EAW11281.1 afis|EAW18219.1 afla|EED56251.1 afum|EAL87585.1 agla|OJJ85863.1 akaw|GAA83805.1 aluc|OJZ90917.1 anid|CBF86150.1 anig|EHA19629.1 aory|EIT73410.1 asyd|OJJ64513.1 ater|EAU33772.1 atub|OJI82909.1 aver|OJI98611.1 awen|OJJ30597.1 azon|OJJ50171.1

Group5825: abra|OJJ77843.1 acar|OOF98929.1 acla|EAW11280.1 afis|EAW18220.1 afla|EED56250.1 afum|EAL87586.1 agla|OJJ85862.1 akaw|GAA83804.1 aluc|OJZ90918.1 anid|CBF86157.1 anig|EHA19630.1 asyd|OJJ64514.1 ater|EAU33771.1 atub|OJI82910.1 aver|OJI98612.1 awen|OJJ30598.1 azon|OJJ50170.1

Group5826: abra|OJJ77856.1 acar|OOF98918.1 acla|EAW11270.1 afis|EAW18229.1 afla|EED56239.1 afum|EAL87596.1 agla|OJJ85852.1 akaw|GAA83793.1 aluc|OJZ90930.1 anid|CBF86170.1 anig|EHA19641.1 asyd|OJJ64524.1 ater|EAU33762.1 atub|OJI82922.1 aver|OJI98622.1 awen|OJJ30608.1 azon|OJJ50160.1

Group5827: abra|OJJ77863.1 acar|OOF98910.1 acla|EAW11263.1 afis|EAW18235.1 afla|EED56231.1 afum|EAL87602.2 agla|OJJ85839.1 akaw|GAA83786.1 aluc|OJZ90938.1 anid|CBF86184.1 anig|EHA19646.1 asyd|OJJ64531.1 ater|EAU33750.1 atub|OJI82929.1 aver|OJI98629.1 awen|OJJ30615.1 azon|OJJ48002.1

Group5828: abra|OJJ77909.1 acar|OOF98876.1 acla|EAW06517.1 afis|EAW16235.1 afla|EED53346.1 agla|OJJ86240.1 akaw|GAA83750.1 aluc|OJZ90981.1 anid|CBF86229.1 anig|EHA19686.1 aory|EIT75244.1 asyd|OJJ64565.1 ater|EAU33709.1 atub|OJI82972.1 aver|OJI98662.1 awen|OJJ30225.1 azon|OJJ43945.1

Group5829: abra|OJJ77922.1 acla|EAW06686.1 afis|EAW16079.1 afla|EED48599.1 afum|EAL88711.1 agla|OJJ86112.1 akaw|GAA83741.1 aluc|OJZ90990.1 anid|CBF75121.1 anig|EHA19694.1 aory|EIT73245.1 asyd|OJJ59992.1 ater|EAU33145.1 atub|OJI82981.1 aver|OJJ02299.1 awen|OJJ30354.1 azon|OJJ45376.1

Group5830: abra|OJJ77927.1 acar|OOF98865.1 acla|EAW06685.1 afis|EAW16080.1 afla|EED48600.1 afum|EAL88712.1 agla|OJJ86113.1 akaw|GAA83737.1 aluc|OJZ90995.1 anig|EHA19702.1 aory|EIT73241.1 asyd|OJJ59991.1 ater|EAU33146.1 atub|OJI82985.1 aver|OJJ02300.1 awen|OJJ30353.1 azon|OJJ45375.1

Group5831: abra|OJJ77928.1 acar|OOF98866.1 acla|EAW06684.1 afis|EAW16081.1 afla|EED48601.1 afum|EAL88713.1 agla|OJJ86114.1 akaw|GAA83736.1 aluc|OJZ90996.1 anig|EHA19703.1 aory|EIT73043.1 asyd|OJJ59990.1 ater|EAU33147.1 atub|OJI82986.1 aver|OJJ02301.1 awen|OJJ30352.1 azon|OJJ45374.1

Group5832: abra|OJJ77931.1 acar|OOF98868.1 acla|EAW06683.1 afis|EAW16082.1 afla|EED48602.1 afum|EAL88714.1 agla|OJJ86116.1 akaw|GAA83732.1 aluc|OJZ90999.1 anig|EHA19706.1 aory|EIT73073.1 asyd|OJJ59989.1 ater|EAU33148.1 atub|OJI82989.1 aver|OJJ02302.1 awen|OJJ30351.1 azon|OJJ45373.1

Group5833: abra|OJJ77932.1 acar|OOF98869.1 acla|EAW06682.1 afis|EAW16083.1 afla|EED48603.1 afum|EAL88715.1 agla|OJJ86115.1 akaw|GAA83731.1 aluc|OJZ91000.1 anig|EHA19707.1 aory|EIT73153.1 asyd|OJJ59988.1 ater|EAU33149.1 atub|OJI82990.1 aver|OJJ02303.1 awen|OJJ30350.1 azon|OJJ45372.1

Group5834: abra|OJJ77934.1 acla|EAW06680.1 afis|EAW16085.1 afla|EED48605.1 afum|EAL88717.1 agla|OJJ86117.1 akaw|GAA83729.1 aluc|OJZ91002.1 anid|CBF75123.1 anig|EHA19708.1 aory|EIT73254.1 asyd|OJJ59986.1 ater|EAU33151.1 atub|OJI82992.1 aver|OJJ02305.1 awen|OJJ30347.1 azon|OJJ45371.1

Group5835: abra|OJJ77953.1 afla|EED49771.1 agla|OJJ89120.1 akaw|GAA83710.1 aluc|OJZ91024.1 anid|CBF87975.1 anig|EHA19724.1 aory|EIT77708.1 asyd|OJJ63489.1 ater|EAU33503.1 atub|OJI83016.1 aver|OJI97440.1 awen|OJJ30074.1 azon|OJJ42166.1 atub|OJI83019.1 agla|OJJ84720.1 agla|OJJ88987.1

Group5836: abra|OJJ77986.1 acar|OOF98837.1 acla|EAW06662.1 afis|EAW16103.1 afla|EED48638.1 afum|EAL88735.1 akaw|GAA85994.1 aluc|OJZ91061.1 anid|CBF85479.1 anig|EHA19764.1 aory|EIT73148.1 asyd|OJJ64107.1 ater|EAU33180.1 atub|OJI83057.1 aver|OJI98189.1 awen|OJJ30329.1 azon|OJJ45346.1

Group5837: abra|OJJ77996.1 acar|OOF98825.1 acla|EAW06652.1 afis|EAW16113.1 afla|EED48653.1 afum|EAL88744.1 agla|OJJ86152.1 akaw|GAA85984.1 aluc|OJZ91072.1 anid|CBF85498.1 anig|EHA19775.1 aory|EIT73209.1 asyd|OJJ64116.1 ater|EAU33196.1 atub|OJI83067.1 aver|OJI98199.1 awen|OJJ30317.1

Group5838: abra|OJJ78002.1 acla|EAW06648.1 afis|EAW16117.1 afla|EED48658.1 afum|EAL88748.1 agla|OJJ86156.1 akaw|GAA85981.1 aluc|OJZ91078.1 anid|CBF85507.1 anig|EHA19779.1 aory|EIT73114.1 asyd|OJJ64120.1 ater|EAU33200.1 atub|OJI83073.1 aver|OJI98203.1 awen|OJJ30313.1 azon|OJJ45330.1

Group5839: abra|OJJ78003.1 acar|OOF98820.1 acla|EAW06647.1 afis|EAW16118.1 afla|EED48659.1 afum|EAL88749.1 agla|OJJ86159.1 akaw|GAA85980.1 aluc|OJZ91079.1 anid|CBF85509.1 anig|EHA19780.1 asyd|OJJ64121.1 ater|EAU33201.1 atub|OJI83074.1 aver|OJI98204.1 awen|OJJ30312.1 azon|OJJ45329.1

Group5840: abra|OJJ78006.1 acar|OOF98816.1 acla|EAW06644.1 afis|EAW16121.1 afum|EAL88752.1 agla|OJJ86162.1 akaw|GAA85977.1 aluc|OJZ91082.1 anid|CBF85539.1 anig|EHA19783.1 aory|EIT73243.1 asyd|OJJ64141.1 ater|EAU33204.1 atub|OJI83078.1 aver|OJI98222.1 awen|OJJ30309.1 azon|OJJ45312.1

Group5841: abra|OJJ78009.1 acar|OOF98814.1 acla|EAW06640.1 afis|EAW16125.1 afla|EED48665.1 afum|EAL88755.1 agla|OJJ86167.1 akaw|GAA85974.1 aluc|OJZ91085.1 anid|CBF85530.1 anig|EHA19786.1 asyd|OJJ64134.1 ater|EAU33208.1 atub|OJI83081.1 aver|OJI98217.1 awen|OJJ30304.1 azon|OJJ45305.1

Group5842: abra|OJJ78016.1 acar|OOF98808.1 acla|EAW06632.1 afis|EAW16133.1 afla|EED48672.1 afum|EAL88763.1 agla|OJJ86178.1 akaw|GAA85967.1 aluc|OJZ91092.1 anid|CBF85518.1 anig|EHA19793.1 asyd|OJJ64126.1 ater|EAU33215.1 atub|OJI83089.1 aver|OJI98209.1 awen|OJJ30295.1 azon|OJJ43985.1

Group5843: abra|OJJ78017.1 acar|OOF98806.1 acla|EAW06630.1 afis|EAW16135.1 afla|EED48674.1 afum|EAL88765.1 agla|OJJ86180.1 akaw|GAA85966.1 aluc|OJZ91093.1 anid|CBF85514.1 anig|EHA19794.1 asyd|OJJ64124.1 ater|EAU33217.1 atub|OJI83090.1 aver|OJI98207.1 awen|OJJ30293.1 azon|OJJ43982.1

Group5844: abra|OJJ78025.1 acar|OOF98800.1 acla|EAW06624.1 afis|EAW16141.1 afla|EED48682.1 afum|EAL88770.2 akaw|GAA85961.1 aluc|OJZ91100.1 anid|CBF85550.1 anig|EHA19800.1 aory|EIT73246.1 asyd|OJJ64150.1 ater|EAU33226.1 atub|OJI83097.1 aver|OJI98230.1 awen|OJJ30287.1 azon|OJJ43976.1

Group5845: abra|OJJ78056.1 afis|EAW20703.1 afla|EED51737.1 afum|EAL84878.1 agla|OJJ85717.1 akaw|GAA85936.1 aluc|OJZ91126.1 anid|CBF70385.1 anig|EHA19827.1 aory|EIT80428.1 asyd|OJJ58639.1 atub|OJI83137.1 aver|OJJ01246.1 asyd|OJJ56271.1 aver|OJJ08494.1 anid|CBF79145.1 atub|OJI79837.1

Group5846: afla|EED48481.1 ater|EAU29541.1 acar|OOF98852.1 acla|EAW09008.1 akaw|GAA93107.1 aluc|OJZ80597.1 anig|EHA22185.1 asyd|OJJ64213.1 awen|OJJ29864.1 anid|CBF80392.1 aory|EIT73087.1 asyd|OJJ54790.1 aver|OJJ07373.1 azon|OJJ43202.1 afis|EAW17320.1 aver|OJJ06502.1 anid|CBF89303.1

Group5847: abra|OJJ78077.1 acla|EAW06610.1 afis|EAW16205.1 afla|EED48698.1 agla|OJJ86216.1 akaw|GAA85917.1 aluc|OJZ91149.1 anid|CBF79565.1 anig|EHA19847.1 aory|EIT73041.1 asyd|OJJ57914.1 ater|EAU33247.1 atub|OJI83163.1 aver|OJJ04479.1 awen|OJJ30267.1 azon|OJJ43963.1 afum|EAL88833.1

Group5848: abra|OJJ78095.1 acar|OOF98778.1 acla|EAW11017.1 afis|EAW20831.1 afla|EED57883.1 afum|EAL84801.1 akaw|GAA85903.1 aluc|OJZ91164.1 anid|CBF69443.1 anig|EHA19859.1 aory|EIT83273.1 asyd|OJJ52396.1 ater|EAU34241.1 atub|OJI83180.1 aver|OJJ01927.1 azon|OJJ48593.1 azon|OJJ42147.1

Group5849: abra|OJJ78102.1 acar|OOF90265.1 acla|EAW06615.1 afis|EAW16152.1 afla|EED48694.1 agla|OJJ86204.1 akaw|GAA85901.1 aluc|OJZ91167.1 anid|CBF79575.1 anig|EHA19860.1 aory|EIT73256.1 asyd|OJJ57908.1 ater|EAU33236.1 atub|OJI83182.1 aver|OJJ04484.1 awen|OJJ30273.1 azon|OJJ43967.1

Group5850: abra|OJJ78123.1 acar|OOF97938.1 acla|EAW13664.1 afis|EAW24511.1 afla|EED51090.1 afum|EBA27259.1 akaw|GAA85885.1 aluc|OJZ91185.1 anid|CBF83112.1 anig|EHA19879.1 asyd|OJJ54499.1 ater|EAU31566.1 atub|OJI83204.1 aver|OJJ07681.1 awen|OJJ38687.1 acar|OOF98765.1 anid|CBF80779.1

Group5851: abra|OJJ78208.1 acar|OOF98646.1 afis|EAW18263.1 afla|EED48722.1 afum|EAL87627.1 agla|OJJ88163.1 akaw|GAA85818.1 aluc|OJZ91281.1 anid|CBF79532.1 anig|EHA19966.1 aory|EIT73066.1 asyd|OJJ57896.1 ater|EAU29421.1 atub|OJI83271.1 aver|OJJ04499.1 awen|OJJ30637.1 azon|OJJ47979.1

Group5852: abra|OJJ78222.1 acar|OOF98634.1 acla|EAW11227.1 afis|EAW18276.1 afla|EED48743.1 afum|EAL87642.1 agla|OJJ88150.1 akaw|GAA85803.1 aluc|OJZ91297.1 anid|CBF79497.1 anig|EHA19979.1 asyd|OJJ57879.1 ater|EAU33282.1 atub|OJI83287.1 aver|OJJ04515.1 awen|OJJ30649.1 azon|OJJ47966.1

Group5853: abra|OJJ78227.1 acla|EAW09282.1 afis|EAW21865.1 afla|EED46446.1 afum|EAL92879.1 agla|OJJ84272.1 akaw|GAA85797.1 aluc|OJZ91302.1 anid|CBF76757.1 anig|EHA19984.1 aory|EIT72438.1 asyd|OJJ61263.1 ater|EAU30862.1 atub|OJI83293.1 aver|OJJ00589.1 awen|OJJ34151.1 azon|OJJ43478.1

Group5854: abra|OJJ78271.1 acar|OOF98593.1 acla|EAW09242.1 afis|EAW21905.1 afla|EED46646.1 afum|EBA27480.1 agla|OJJ84225.1 akaw|GAA85755.1 aluc|OJZ91345.1 anid|CBF76830.1 anig|EHA20024.1 asyd|OJJ61301.1 ater|EAU30900.1 atub|OJI83337.1 aver|OJJ00627.1 awen|OJJ34195.1 azon|OJJ43439.1

Group5855: abra|OJJ78273.1 acla|EAW09241.1 afis|EAW21907.1 afum|EAL92919.2 akaw|GAA85753.1 aluc|OJZ91347.1 anig|EHA20026.1 asyd|OJJ61303.1 ater|EAU30901.1 atub|OJI83339.1 aver|OJJ00628.1 awen|OJJ34197.1 afla|EED46644.1 agla|OJJ84224.1 aory|EIT77882.1 anid|CBF76833.1 azon|OJJ43437.1

Group5856: abra|OJJ78274.1 acla|EAW09240.1 afis|EAW21908.1 afla|EED46643.1 afum|EAL92920.1 agla|OJJ84223.1 akaw|GAA85752.1 aluc|OJZ91349.1 anid|CBF76835.1 anig|EHA20027.1 aory|EIT77824.1 asyd|OJJ61304.1 ater|EAU30908.1 atub|OJI83340.1 aver|OJJ00629.1 awen|OJJ34198.1 azon|OJJ43436.1

Group5857: abra|OJJ78276.1 acar|OOF98591.1 acla|EAW09238.1 afis|EAW21910.1 afla|EED46640.1 afum|EAL92922.2 agla|OJJ84221.1 akaw|GAA85750.1 aluc|OJZ91351.1 anid|CBF76839.1 anig|EHA20029.1 asyd|OJJ61306.1 ater|EAU30910.1 atub|OJI83343.1 aver|OJJ00631.1 awen|OJJ34200.1 azon|OJJ43434.1

Group5858: abra|OJJ78281.1 acar|OOF98587.1 acla|EAW09234.1 afis|EAW21914.1 afla|EED46636.1 afum|EBA27482.1 agla|OJJ84209.1 akaw|GAA85746.1 aluc|OJZ91355.1 anid|CBF76848.1 anig|EHA20033.1 asyd|OJJ61310.1 ater|EAU30915.1 atub|OJI83348.1 aver|OJJ00635.1 awen|OJJ34204.1 azon|OJJ43430.1

Group5859: abra|OJJ78286.1 acar|OOF98582.1 acla|EAW09229.1 afis|EAW21919.1 afum|EAL92931.1 agla|OJJ84204.1 akaw|GAA85741.1 aluc|OJZ91360.1 anid|CBF76858.1 anig|EHA20038.1 aory|EIT77872.1 asyd|OJJ61315.1 ater|EAU30924.1 atub|OJI83353.1 aver|OJJ00640.1 awen|OJJ34209.1 azon|OJJ43425.1

Group5860: abra|OJJ78295.1 acla|EAW09221.1 afis|EAW21927.1 afla|EED46620.1 afum|EAL92940.1 agla|OJJ84194.1 akaw|GAA85732.1 aluc|OJZ91369.1 anid|CBF76878.1 anig|EHA20047.1 aory|EIT77851.1 asyd|OJJ61325.1 ater|EAU30933.1 atub|OJI83362.1 aver|OJJ00650.1 awen|OJJ34217.1 azon|OJJ43417.1

Group5861: abra|OJJ78296.1 acar|OOF98572.1 acla|EAW09220.1 afis|EAW21928.1 afla|EED46621.1 afum|EAL92941.1 agla|OJJ84193.1 akaw|GAA85731.1 aluc|OJZ91370.1 anid|CBF76876.1 anig|EHA20048.1 asyd|OJJ61324.1 ater|EAU30934.1 atub|OJI83363.1 aver|OJJ00649.1 awen|OJJ34218.1 azon|OJJ43416.1

Group5862: abra|OJJ78306.1 acar|OOF98560.1 acla|EAW09210.1 afis|EAW21937.1 afla|EED46654.1 afum|EAL92950.2 akaw|GAA85719.1 aluc|OJZ91385.1 anid|CBF76897.1 anig|EHA20058.1 aory|EIT77879.1 asyd|OJJ61337.1 ater|EAU30943.1 atub|OJI83378.1 aver|OJJ00661.1 awen|OJJ34228.1 azon|OJJ43407.1

Group5863: abra|OJJ78315.1 acar|OOF90168.1 acla|EAW12152.1 afis|EAW17176.1 afla|EED54162.1 afum|EAL86606.1 akaw|GAA85715.1 aluc|OJZ91388.1 anid|CBF76640.1 anig|EHA20066.1 aory|EIT76137.1 asyd|OJJ61201.1 ater|EAU35069.1 atub|OJI83381.1 aver|OJJ00526.1 awen|OJJ37819.1 azon|OJJ42628.1

Group5864: abra|OJJ78318.1 acla|EAW09207.1 afis|EAW21940.1 afla|EED46657.1 afum|EAL92953.1 agla|OJJ84183.1 akaw|GAA85712.1 aluc|OJZ91391.1 anid|CBF84079.1 anig|EHA20069.1 aory|EIT77958.1 asyd|OJJ61341.1 ater|EAU30945.1 atub|OJI83384.1 aver|OJJ00665.1 awen|OJJ34231.1 azon|OJJ43404.1

Group5865: abra|OJJ78322.1 acar|OOF98552.1 acla|EAW09203.1 afis|EAW21944.1 afla|EED46661.1 afum|EAL92958.1 agla|OJJ84180.1 akaw|GAA85706.1 aluc|OJZ91397.1 anid|CBF84051.1 anig|EHA20074.1 aory|EIT77927.1 ater|EAU30992.1 atub|OJI83389.1 aver|OJJ00668.1 awen|OJJ34235.1 azon|OJJ43401.1

Group5866: abra|OJJ78326.1 afla|EED46664.1 acar|OOF98549.1 akaw|GAA85703.1 aluc|OJZ91400.1 anid|CBF84060.1 anig|EHA20077.1 asyd|OJJ61346.1 atub|OJI83392.1 aver|OJJ00671.1 azon|OJJ43398.1 acla|EAW09200.1 afis|EAW21947.1 afum|EAL92961.1 ater|EAU30995.1 agla|OJJ84173.1 awen|OJJ34238.1

Group5867: abra|OJJ78344.1 acar|OOF90270.1 acla|EAW09190.1 afis|EAW21956.1 afla|EED46675.1 afum|EAL92970.2 agla|OJJ84163.1 akaw|GAA85689.1 aluc|OJZ91417.1 anid|CBF84036.1 anig|EHA20089.1 aory|EIT77945.1 asyd|OJJ61363.1 atub|OJI83411.1 aver|OJJ00690.1 awen|OJJ34248.1 azon|OJJ43390.1

Group5868: abra|OJJ78349.1 afis|EAW21961.1 afla|EED46682.1 afum|EAL92975.1 agla|OJJ84155.1 akaw|GAA85684.1 aluc|OJZ91424.1 anid|CBF84046.1 anig|EHA20094.1 aory|EIT77949.1 asyd|OJJ61357.1 ater|EAU31081.1 atub|OJI83417.1 aver|OJJ00684.1 awen|OJJ34255.1 acla|EAW09185.1 azon|OJJ43384.1

Group5869: abra|OJJ78352.1 acla|EAW09183.1 afis|EAW21963.1 afla|EED46684.1 afum|EAL92977.1 agla|OJJ84140.1 akaw|GAA85682.1 aluc|OJZ91427.1 anid|CBF82740.1 anig|EHA20096.1 aory|EIT77811.1 asyd|OJJ61623.1 ater|EAU31083.1 atub|OJI83420.1 aver|OJJ00948.1 awen|OJJ34257.1 azon|OJJ43382.1

Group5870: aver|OJJ00953.1 abra|OJJ78358.1 acar|OOF98528.1 acla|EAW09176.1 afis|EAW21970.1 afla|EED46692.1 afum|EAL92984.1 agla|OJJ84133.1 akaw|GAA85675.1 aluc|OJZ91433.1 anid|CBF82727.1 aory|EIT77959.1 asyd|OJJ61630.1 ater|EAU31090.1 atub|OJI83426.1 awen|OJJ34263.1 azon|OJJ43374.1

Group5871: abra|OJJ78367.1 acla|EAW09168.1 afis|EAW21978.1 afla|EED46701.1 afum|EAL92993.1 agla|OJJ84122.1 akaw|GAA85666.1 aluc|OJZ91444.1 anid|CBF82712.1 anig|EHA20108.1 aory|EIT77893.1 asyd|OJJ61639.1 ater|EAU31096.1 atub|OJI83438.1 aver|OJJ00962.1 awen|OJJ34271.1 azon|OJJ43365.1

Group5872: abra|OJJ78373.1 acar|OOF98516.1 acla|EAW09162.1 afis|EAW21984.1 afla|EED46708.1 afum|EAL92999.1 agla|OJJ84114.1 akaw|GAA85660.1 aluc|OJZ91450.1 anid|CBF82701.1 anig|EHA20114.1 asyd|OJJ61645.1 ater|EAU31103.1 atub|OJI83443.1 aver|OJJ00967.1 awen|OJJ34281.1 azon|OJJ43359.1

Group5873: abra|OJJ78376.1 acar|OOF98512.1 acla|EAW09158.1 afis|EAW21988.1 afla|EED46712.1 afum|EAL93004.1 agla|OJJ84110.1 akaw|GAA85657.1 aluc|OJZ91454.1 anid|CBF82692.1 anig|EHA20117.1 aory|EIT77910.1 asyd|OJJ61649.1 atub|OJI83447.1 aver|OJJ00970.1 awen|OJJ34284.1 azon|OJJ43354.1

Group5874: abra|OJJ78377.1 acla|EAW09157.1 afis|EAW21989.1 afla|EED46713.1 afum|EAL93005.2 agla|OJJ84109.1 akaw|GAA85656.1 aluc|OJZ91455.1 anid|CBF82690.1 anig|EHA20118.1 aory|EIT77905.1 asyd|OJJ61650.1 ater|EAU31108.1 atub|OJI83448.1 aver|OJJ00971.1 awen|OJJ34285.1 azon|OJJ43353.1

Group5875: abra|OJJ78379.1 acar|OOF98509.1 acla|EAW09154.1 afis|EAW21992.1 afla|EED46716.1 afum|EAL93007.1 agla|OJJ84105.1 akaw|GAA85653.1 aluc|OJZ91458.1 anid|CBF82685.1 anig|EHA20121.1 asyd|OJJ61654.1 ater|EAU31111.1 atub|OJI83451.1 aver|OJJ00975.1 awen|OJJ34288.1 azon|OJJ43350.1

Group5876: abra|OJJ78389.1 acar|OOF98499.1 acla|EAW09144.1 afis|EAW22003.1 afla|EED46728.1 afum|EAL93018.1 agla|OJJ84095.1 akaw|GAA85643.1 aluc|OJZ91468.1 anid|CBF82664.1 anig|EHA20130.1 asyd|OJJ61665.1 ater|EAU31122.1 atub|OJI83461.1 aver|OJJ00986.1 awen|OJJ34298.1 azon|OJJ43340.1

Group5877: acar|OOF90682.1 acla|EAW14843.1 afis|EAW24370.1 afla|EED49848.1 afum|EAL86042.1 anid|CBF87190.1 awen|OJJ30943.1 acar|OOG00289.1 acla|EAW12454.1 afla|EED51634.1 anid|CBF84176.1 aory|EIT80587.1 asyd|OJJ57129.1 aver|OJJ03175.1 azon|OJJ46526.1 awen|OJJ32845.1 awen|OJJ36074.1

Group5878: acar|OOF91612.1 acla|EAW14418.1 afis|EAW19125.1 afla|EED54289.1 afum|EAL93857.1 agla|OJJ80440.1 akaw|GAA90471.1 aluc|OJZ85753.1 anid|CBF79735.1 anig|EHA24803.1 aory|EIT76095.1 asyd|OJJ58082.1 ater|EAU37392.1 atub|OJI79537.1 aver|OJJ04326.1 awen|OJJ37456.1 azon|OJJ51135.1

Group5879: acar|OOF93414.1 acla|EAW08926.1 afis|EAW15468.1 afla|EED46920.1 afum|EAL85854.1 agla|OJJ80149.1 akaw|GAA87755.1 aluc|OJZ85388.1 anid|CBF78258.1 anig|EHA25572.1 aory|EIT72527.1 asyd|OJJ56063.1 ater|EAU32660.1 atub|OJI80182.1 aver|OJJ05006.1 awen|OJJ38118.1 azon|OJJ51809.1

Group5880: acar|OOF98511.1 acla|EAW09156.1 afis|EAW21990.1 afla|EED46714.1 afum|EBA27484.1 agla|OJJ84108.1 akaw|GAA85655.1 aluc|OJZ91456.1 anid|CBF82688.1 anig|EHA20119.1 aory|EIT77857.1 asyd|OJJ61651.1 ater|EAU31109.1 atub|OJI83449.1 aver|OJJ00972.1 awen|OJJ34286.1 azon|OJJ43352.1

Group5881: acar|OOG01162.1 acla|EAW07893.1 afis|EAW22928.1 afla|EED58121.1 afum|EAL90269.1 agla|OJJ83360.1 akaw|GAA87526.1 aluc|OJZ87993.1 anid|CBF87669.1 anig|EHA17775.1 aory|EIT79165.1 asyd|OJJ63310.1 ater|EAU30695.1 atub|OJI90602.1 aver|OJI97649.1 awen|OJJ38636.1 azon|OJJ49578.1

Group5882: acla|EAW09522.1 afis|EAW21610.1 afla|EED57839.1 afum|EAL92644.1 agla|OJJ85805.1 akaw|GAA84915.1 aluc|OJZ86754.1 anid|CBF83478.1 anig|EHA21925.1 aory|EIT81104.1 asyd|OJJ60693.1 ater|EAU38299.1 aver|OJJ00018.1 awen|OJJ39072.1 azon|OJJ51403.1 azon|OJJ51404.1 aver|OJJ00019.1

Group5883: acla|EAW14913.1 afis|EAW24434.1 afla|EED45083.1 afum|EAL86102.1 akaw|GAA88070.1 aluc|OJZ81335.1 aory|EIT73616.1 asyd|OJJ56673.1 ater|EAU29576.1 atub|OJI91538.1 aver|OJJ06051.1 awen|OJJ38123.1 azon|OJJ48557.1 agla|OJJ87162.1 ater|EAU29898.1 awen|OJJ30898.1 aory|EIT74968.1

Group5884: abra|OJJ66154.1 abra|OJJ75559.1 asyd|OJJ58905.1 asyd|OJJ59701.1 aver|OJJ01502.1 aver|OJJ02586.1 atub|OJI85780.1 awen|OJJ38282.1 acar|OOF90877.1 agla|OJJ88520.1 akaw|GAA84314.1 aluc|OJZ89009.1 anid|CBF69967.1 anig|EHA23519.1 aory|EIT75405.1 ater|EAU30290.1

Group5885: abra|OJJ67080.1 abra|OJJ68912.1 acar|OOF91499.1 afis|EAW20326.1 afla|EED57092.1 agla|OJJ78923.1 akaw|GAA90337.1 aluc|OJZ85895.1 anid|CBF81708.1 anig|EHA24697.1 aory|EIT82304.1 asyd|OJJ59577.1 ater|EAU32053.1 atub|OJI79680.1 aver|OJJ02692.1 awen|OJJ38052.1

Group5886: acar|OOF91513.1 acar|OOF98315.1 abra|OJJ67236.1 afis|EAW24732.1 afla|EED46087.1 afum|EAL89379.1 agla|OJJ86786.1 akaw|GAA91660.1 aluc|OJZ87739.1 anid|CBF69482.1 anig|EHA20231.1 aory|EIT76445.1 asyd|OJJ59273.1 atub|OJI80901.1 aver|OJJ01886.1 awen|OJJ40220.1

Group5887: acar|OOF92290.1 acar|OOF92331.1 abra|OJJ67511.1 acla|EAW06261.1 afla|EED57038.1 afum|EAL93042.1 agla|OJJ84932.1 aluc|OJZ84853.1 anid|CBF70812.1 aory|EIT77427.1 asyd|OJJ58406.1 ater|EAU32237.1 atub|OJI80743.1 aver|OJJ01007.1 awen|OJJ31782.1 asyd|OJJ61026.1

Group5888: acar|OOF92605.1 acar|OOF92626.1 abra|OJJ69658.1 acla|EAW13777.1 afis|EAW18355.1 afla|EED48820.1 akaw|GAA91637.1 aluc|OJZ82464.1 anid|CBF79358.1 anig|EHA28311.1 aory|EIT73001.1 asyd|OJJ57803.1 ater|EAU34688.1 atub|OJI87974.1 awen|OJJ35739.1 azon|OJJ43324.1

Group5889: acar|OOF92826.1 acar|OOF98200.1 acla|EAW12158.1 acla|EAW13719.1 afis|EAW17190.1 afis|EAW21021.1 afum|EAL86616.2 afum|EAL92059.1 ater|EAU31621.1 ater|EAU36218.1 abra|OJJ70923.1 akaw|GAA83017.1 aluc|OJZ87603.1 anig|EHA20802.1 atub|OJI81045.1 aver|OJJ05979.1

Group5890: acar|OOF93694.1 acar|OOF93779.1 abra|OJJ66685.1 acla|EAW09000.1 afla|EED45351.1 agla|OJJ85436.1 akaw|GAA89629.1 aluc|OJZ80764.1 anid|CBF81376.1 anig|EHA27263.1 aory|EIT82467.1 asyd|OJJ59389.1 atub|OJI87618.1 aver|OJJ02883.1 awen|OJJ39926.1 azon|OJJ50230.1

Group5891: acar|OOF94035.1 acar|OOF94105.1 abra|OJJ66415.1 afis|EAW20234.1 afla|EED55132.1 afum|EAL85351.1 akaw|GAA90282.1 aluc|OJZ83058.1 anid|CBF73520.1 anig|EHA28124.1 aory|EIT81578.1 asyd|OJJ56746.1 ater|EAU37765.1 atub|OJI87888.1 aver|OJJ05984.1 azon|OJJ47372.1

Group5892: acar|OOF94043.1 acar|OOF94113.1 abra|OJJ66398.1 afis|EAW16735.1 afla|EED49556.1 afum|EAL86309.1 agla|OJJ79510.1 akaw|GAA90266.1 aluc|OJZ83075.1 anig|EHA28139.1 asyd|OJJ61452.1 atub|OJI87871.1 aver|OJJ00787.1 awen|OJJ39514.1 azon|OJJ51715.1 anid|CBF83128.1

Group5893: acar|OOF94086.1 acar|OOF94156.1 abra|OJJ66352.1 afis|EAW17344.1 afla|EED46461.1 afum|EAL91188.1 agla|OJJ88648.1 akaw|GAA90225.1 aluc|OJZ83116.1 anid|CBF82993.1 anig|EHA28179.1 aory|EIT72314.1 asyd|OJJ54261.1 atub|OJI87818.1 aver|OJJ05214.1 awen|OJJ40134.1

Group5894: acar|OOF94093.1 acar|OOF94163.1 abra|OJJ66344.1 acla|EAW09990.1 afis|EAW21102.1 afla|EED51684.1 afum|EAL92123.1 akaw|GAA90218.1 aluc|OJZ83123.1 anid|CBF86612.1 anig|EHA28187.1 aory|EIT80601.1 asyd|OJJ64820.1 ater|EAU36797.1 atub|OJI87809.1 aver|OJI98933.1

Group5895: acar|OOF94752.1 acar|OOF95317.1 abra|OJJ72483.1 acla|EAW12714.1 afis|EAW17876.1 afla|EED46294.1 afum|EAL91684.1 agla|OJJ83070.1 akaw|GAA86218.1 aluc|OJZ90470.1 anig|EHA23929.1 asyd|OJJ52333.1 ater|EAU33096.1 atub|OJI81894.1 aver|OJJ05550.1 awen|OJJ41688.1

Group5896: acar|OOF95617.1 acar|OOF95669.1 abra|OJJ67062.1 acla|EAW12224.1 afla|EED54494.1 agla|OJJ85767.1 akaw|GAA82365.1 aluc|OJZ91758.1 anid|CBF81941.1 anig|EHA18944.1 aory|EIT78917.1 asyd|OJJ56618.1 ater|EAU31797.1 atub|OJI86216.1 aver|OJJ04440.1 anig|EHA26144.1

Group5897: acar|OOF96227.1 acar|OOF96265.1 abra|OJJ71512.1 acla|EAW15069.1 afis|EAW20041.1 afum|EAL85161.2 agla|OJJ88921.1 aluc|OJZ86250.1 anid|CBF84782.1 anig|EHA21615.1 aory|EIT73556.1 asyd|OJJ63233.1 atub|OJI84292.1 aver|OJI97723.1 awen|OJJ31797.1 azon|OJJ41993.1

Group5898: acar|OOF96911.1 acar|OOF96946.1 abra|OJJ70799.1 afla|EED56519.1 agla|OJJ87254.1 akaw|GAA90115.1 aluc|OJZ84144.1 anid|CBF81181.1 anig|EHA19252.1 aory|EIT72828.1 asyd|OJJ59295.1 ater|EAU31668.1 atub|OJI90518.1 aver|OJJ02980.1 awen|OJJ36774.1 azon|OJJ47424.1

Group5899: acar|OOF97665.1 acar|OOF97712.1 abra|OJJ71492.1 acla|EAW10058.1 afis|EAW25526.1 afum|EAL90191.2 agla|OJJ87276.1 akaw|GAA82528.1 aluc|OJZ87055.1 anid|CBF77803.1 anig|EHA20328.1 aory|EIT78255.1 asyd|OJJ53018.1 atub|OJI81580.1 aver|OJJ03825.1 azon|OJJ49512.1

Group5900: acar|OOF98477.1 acar|OOF98490.1 abra|OJJ78397.1 acla|EAW09135.1 afla|EED46736.1 agla|OJJ84086.1 akaw|GAA85634.1 aluc|OJZ91476.1 anid|CBF82648.1 anig|EHA20138.1 asyd|OJJ61674.1 ater|EAU31133.1 atub|OJI83469.1 aver|OJJ00995.1 awen|OJJ34307.1 azon|OJJ43334.1

Group5901: acar|OOG00239.1 acar|OOG00258.1 abra|OJJ67535.1 acla|EAW11018.1 afis|EAW20832.1 afla|EED45130.1 afum|EAL84800.1 akaw|GAA88124.1 aluc|OJZ81397.1 anid|CBF80709.1 anig|EHA18689.1 aory|EIT73782.1 asyd|OJJ62619.1 atub|OJI91602.1 aver|OJI96745.1 azon|OJJ42683.1

Group5902: acar|OOG00251.1 acar|OOG00269.1 abra|OJJ67545.1 acla|EAW12867.1 afla|EED45590.1 agla|OJJ81687.1 akaw|GAA88115.1 aluc|OJZ81387.1 anid|CBF82370.1 anig|EHA18680.1 aory|EIT75758.1 asyd|OJJ54193.1 ater|EAU37669.1 atub|OJI91592.1 aver|OJJ08533.1 awen|OJJ35116.1

Group5903: afis|EAW17151.1 afis|EAW18536.1 agla|OJJ85246.1 agla|OJJ86248.1 abra|OJJ71801.1 afla|EED49211.1 akaw|GAA85109.1 aluc|OJZ86531.1 anid|CBF80198.1 anig|EHA22115.1 aory|EIT83151.1 asyd|OJJ62941.1 ater|EAU39447.1 atub|OJI83990.1 aver|OJI98049.1 awen|OJJ30934.1

Group5904: afis|EAW22768.1 afis|EAW24693.1 abra|OJJ73447.1 afla|EED45075.1 afum|EAL89335.1 agla|OJJ81339.1 akaw|GAA89025.1 aluc|OJZ80579.1 anid|CBF76030.1 anig|EHA25619.1 aory|EIT73722.1 asyd|OJJ52452.1 ater|EAU33314.1 atub|OJI88351.1 aver|OJJ03964.1 awen|OJJ38020.1

Group5905: afla|EED45997.1 afla|EED55181.1 aory|EIT75482.1 aory|EIT81589.1 asyd|OJJ55511.1 asyd|OJJ55533.1 asyd|OJJ57370.1 aver|OJJ05798.1 aver|OJJ06588.1 acla|EAW09058.1 afis|EAW25631.1 afum|EAL84319.1 anid|CBF83157.1 ater|EAU32719.1 awen|OJJ36051.1 aver|OJJ06611.1

Group5906: afla|EED49561.1 afla|EED49753.1 abra|OJJ67096.1 acar|OOF98458.1 acla|EAW08935.1 afis|EAW15463.1 afum|EAL85858.1 akaw|GAA91065.1 aluc|OJZ87878.1 anid|CBF74671.1 anig|EHA20182.1 aory|EIT82641.1 ater|EAU39081.1 atub|OJI80772.1 awen|OJJ37034.1 azon|OJJ44465.1

Group5907: afum|EAL86255.1 afum|EAL89177.1 abra|OJJ68037.1 acar|OOF91806.1 acla|EAW06968.1 afis|EAW16582.1 afla|EED55050.1 akaw|GAA90915.1 aluc|OJZ82911.1 anig|EHA21334.1 aory|EIT81979.1 asyd|OJJ53719.1 ater|EAU36654.1 atub|OJI89137.1 aver|OJJ07845.1 awen|OJJ33891.1

Group5908: agla|OJJ78990.1 agla|OJJ83688.1 abra|OJJ73812.1 acar|OOG00967.1 acla|EAW07699.1 afis|EAW22716.1 afla|EED50000.1 afum|EAL90478.1 akaw|GAA89431.1 aluc|OJZ88238.1 anid|CBF88051.1 anig|EHA17985.1 aory|EIT74675.1 atub|OJI90840.1 awen|OJJ35410.1 azon|OJJ49766.1

Group5909: agla|OJJ83138.1 agla|OJJ86319.1 abra|OJJ68823.1 afum|EAL86208.1 akaw|GAA90430.1 aluc|OJZ85799.1 anid|CBF78855.1 anig|EHA24758.1 asyd|OJJ55033.1 atub|OJI79584.1 aver|OJJ07170.1 awen|OJJ30830.1 afla|EED50787.1 aory|EIT77976.1 afla|EED57506.1 aory|EIT81869.1

Group5910: agla|OJJ85816.1 agla|OJJ85832.1 agla|OJJ85818.1 acar|OOF98908.1 acla|EAW11260.1 akaw|GAA83783.1 aluc|OJZ90941.1 anid|CBF86189.1 anig|EHA19649.1 asyd|OJJ64535.1 ater|EAU33747.1 atub|OJI82932.1 aver|OJI98632.1 awen|OJJ30618.1 azon|OJJ47999.1 afla|EED56228.1

Group5911: agla|OJJ86769.1 agla|OJJ86770.1 abra|OJJ74895.1 acar|OOF95896.1 acla|EAW08476.1 afis|EAW23522.1 afla|EED57422.1 akaw|GAA83525.1 aluc|OJZ92470.1 anig|EHA26498.1 aory|EIT80073.1 asyd|OJJ62259.1 atub|OJI86900.1 aver|OJI96368.1 awen|OJJ39847.1 afum|EAL87942.1

Group5912: anid|CBF71552.1 anid|CBF84169.1 acla|EAW08561.1 afis|EAW23603.1 afla|EED55065.1 afum|EAL87863.2 aory|EIT77141.1 asyd|OJJ56282.1 ater|EAU35362.1 aver|OJJ08479.1 awen|OJJ32575.1 acla|EAW13726.1 afla|EED50166.1 aory|EIT76902.1 afla|EED47738.1 anid|CBF71809.1

Group5913: aory|EIT75268.1 aory|EIT75646.1 abra|OJJ66105.1 acar|OOF93992.1 afis|EAW16816.1 afla|EED53712.1 afum|EAL86367.1 akaw|GAA92674.1 aluc|OJZ90129.1 anid|CBF78556.1 anig|EHA20942.1 asyd|OJJ65060.1 atub|OJI84667.1 azon|OJJ46860.1 acar|OOF91691.1 aory|EIT80433.1

Group5914: asyd|OJJ54204.1 asyd|OJJ54927.1 aver|OJJ01905.1 aver|OJJ07268.1 awen|OJJ35311.1 awen|OJJ36432.1 acla|EAW07665.1 afis|EAW22682.1 afla|EED49966.1 afum|EAL90510.1 agla|OJJ79757.1 anid|CBF88120.1 aory|EIT83446.1 asyd|OJJ63584.1 ater|EAU39027.1 aver|OJI97350.1

Group5915: asyd|OJJ54824.1 asyd|OJJ57889.1 ater|EAU33274.1 ater|EAU38853.1 acar|OOF98643.1 acla|EAW11238.1 afis|EAW18267.1 afum|EAL87632.1 anid|CBF79518.1 aory|EIT73252.1 atub|OJI88770.1 aver|OJJ04505.1 awen|OJJ30641.1 azon|OJJ47974.1 ater|EAU34238.1 aver|OJJ07336.1

Group5916: asyd|OJJ56712.1 asyd|OJJ57429.1 aver|OJJ05750.1 aver|OJJ06013.1 abra|OJJ67120.1 acar|OOF98431.1 acla|EAW11078.1 afla|EED52028.1 agla|OJJ86869.1 akaw|GAA91045.1 aluc|OJZ87855.1 anig|EHA20160.1 aory|EIT81420.1 atub|OJI80792.1 awen|OJJ33344.1 ater|EAU37270.1

Group5917: asyd|OJJ57667.1 asyd|OJJ64961.1 atub|OJI90353.1 abra|OJJ70633.1 acar|OOF97091.1 afis|EAW23159.1 afla|EED46961.1 afum|EAL88324.1 akaw|GAA89972.1 aluc|OJZ83981.1 anid|CBF86381.1 anig|EHA19108.1 aory|EIT72519.1 ater|EAU36292.1 aver|OJI99083.1 awen|OJJ36624.1

Group5918: asyd|OJJ60004.1 asyd|OJJ62600.1 awen|OJJ32651.1 awen|OJJ37094.1 abra|OJJ77155.1 acar|OOF93042.1 afla|EED45743.1 agla|OJJ81794.1 akaw|GAA90775.1 aluc|OJZ84569.1 anid|CBF79863.1 anig|EHA27864.1 aory|EIT75727.1 ater|EAU31865.1 atub|OJI89513.1 aver|OJJ02282.1

Group5919: asyd|OJJ60338.1 asyd|OJJ65170.1 abra|OJJ69828.1 acar|OOF93353.1 afum|EAL86293.1 agla|OJJ79198.1 akaw|GAA87832.1 aluc|OJZ85468.1 anid|CBF87111.1 anig|EHA25009.1 ater|EAU37987.1 atub|OJI80095.1 aver|OJI99322.1 awen|OJJ32858.1 azon|OJJ47733.1 azon|OJJ45214.1

Group5920: ater|EAU32685.1 ater|EAU32837.1 abra|OJJ69617.1 acar|OOF92689.1 akaw|GAA91601.1 aluc|OJZ82425.1 anid|CBF82164.1 anig|EHA28344.1 asyd|OJJ54340.1 atub|OJI88015.1 aver|OJJ05292.1 awen|OJJ36838.1 azon|OJJ43517.1 anig|EHA28204.1 akaw|GAA92799.1 aluc|OJZ88721.1

Group5921: awen|OJJ29955.1 awen|OJJ35055.1 afla|EED47573.1 abra|OJJ72994.1 acar|OOF96171.1 afis|EAW16182.1 afla|EED52079.1 akaw|GAA85568.1 aluc|OJZ83502.1 anig|EHA24336.1 aory|EIT76901.1 atub|OJI82396.1 awen|OJJ30004.1 afum|EAL88813.1 ater|EAU29301.1 aluc|OJZ80354.1

Group5922: awen|OJJ30767.1 awen|OJJ31714.1 abra|OJJ67443.1 acar|OOF92384.1 afis|EAW18434.1 afla|EED52981.1 afum|EAL93115.1 agla|OJJ85001.1 akaw|GAA86386.1 aluc|OJZ84921.1 anid|CBF70031.1 aory|EIT78703.1 asyd|OJJ58869.1 ater|EAU29850.1 atub|OJI80678.1 aver|OJJ01471.1

Group5923: awen|OJJ30908.1 awen|OJJ38004.1 abra|OJJ73700.1 acar|OOF90210.1 akaw|GAA88464.1 aluc|OJZ88353.1 anig|EHA18085.1 aory|EIT81772.1 atub|OJI90948.1 abra|OJJ75777.1 acar|OOF91917.1 acla|EAW07822.1 afis|EAW22858.1 afum|EAL90342.1 akaw|GAA87320.1 afla|EED51804.1

Group5924: awen|OJJ30944.1 awen|OJJ32848.1 abra|OJJ75187.1 acar|OOF94595.1 acla|EAW14838.1 afis|EAW24366.1 afla|EED46591.1 afum|EAL86038.2 agla|OJJ82520.1 akaw|GAA88789.1 aluc|OJZ92750.1 anid|CBF87187.1 anig|EHA26253.1 aory|EIT77903.1 atub|OJI87176.1 acar|OOF90681.1

Group5925: awen|OJJ32657.1 awen|OJJ33840.1 abra|OJJ66139.1 acar|OOF95826.1 acla|EAW11590.1 afla|EED52591.1 agla|OJJ88404.1 akaw|GAA92695.1 aluc|OJZ90153.1 anid|CBF83901.1 anig|EHA20916.1 aory|EIT73374.1 ater|EAU33482.1 atub|OJI84646.1 agla|OJJ82534.1 aory|EIT74204.1

Group5926: awen|OJJ33832.1 awen|OJJ36387.1 abra|OJJ72946.1 acar|OOF96127.1 afla|EED48176.1 akaw|GAA85528.1 aluc|OJZ83456.1 anid|CBF83118.1 anig|EHA24302.1 aory|EIT83024.1 asyd|OJJ53474.1 ater|EAU31957.1 atub|OJI82348.1 aver|OJJ03999.1 azon|OJJ42763.1 asyd|OJJ60256.1

Group5927: azon|OJJ42111.1 azon|OJJ45189.1 azon|OJJ44136.1 azon|OJJ49610.1 abra|OJJ73127.1 acar|OOG00350.1 afla|EED48144.1 akaw|GAA88037.1 aluc|OJZ81296.1 anig|EHA18604.1 aory|EIT76194.1 asyd|OJJ56200.1 ater|EAU39558.1 atub|OJI91505.1 aver|OJJ05127.1 awen|OJJ36253.1

Group5928: azon|OJJ44493.1 azon|OJJ50272.1 abra|OJJ71252.1 acar|OOF97918.1 acla|EAW10283.1 afis|EAW25309.1 akaw|GAA82735.1 aluc|OJZ87290.1 anig|EHA20531.1 atub|OJI81351.1 awen|OJJ41057.1 acar|OOG00279.1 akaw|GAA88103.1 aluc|OJZ81370.1 anig|EHA18670.1 atub|OJI91575.1

Group5929: acla|EAW11715.1 agla|OJJ78979.1 acar|OOF97633.1 akaw|GAA92095.1 aluc|OJZ83630.1 anig|EHA18783.1 aory|EIT80453.1 atub|OJI89999.1 afis|EAW23734.1 afla|EED51512.1 afum|EAL87172.1 anid|CBF87375.1 asyd|OJJ52839.1 ater|EAU31294.1 aver|OJJ08808.1 awen|OJJ33670.1

Group5930: abra|OJJ65588.1 acla|EAW13295.1 afis|EAW17315.1 asyd|OJJ60476.1 abra|OJJ68806.1 acar|OOF91595.1 afum|EAL91135.2 akaw|GAA90447.1 aluc|OJZ85778.1 anid|CBF74648.1 anig|EHA24776.1 ater|EAU39376.1 atub|OJI79563.1 aver|OJI99796.1 awen|OJJ35960.1 afum|EAL91151.1

Group5931: abra|OJJ65608.1 acar|OOF92455.1 acla|EAW12305.1 afla|EED51167.1 anid|CBF81654.1 aory|EIT81330.1 asyd|OJJ54344.1 ater|EAU31008.1 aver|OJJ05296.1 abra|OJJ77464.1 anig|EHA19296.1 asyd|OJJ56405.1 ater|EAU39560.1 aver|OJJ06307.1 anid|CBF80165.1 aver|OJI99520.1

Group5932: abra|OJJ65769.1 afum|EAL87801.2 anig|EHA21875.1 aver|OJI99576.1 awen|OJJ29597.1 acar|OOF90907.1 acla|EAW09702.1 agla|OJJ85451.1 anig|EHA21874.1 asyd|OJJ52319.1 acla|EAW12595.1 acla|EAW12596.1 aory|EIT75343.1 afis|EAW16225.1 afum|EAL87819.1 aory|EIT79546.1

Group5933: afis|EAW19924.1 acar|OOF93587.1 acla|EAW13682.1 abra|OJJ75101.1 acar|OOF97233.1 afla|EED55651.1 afum|EBA27214.1 agla|OJJ86510.1 akaw|GAA88710.1 aluc|OJZ92665.1 aory|EIT81813.1 asyd|OJJ62423.1 atub|OJI87096.1 aver|OJI96560.1 awen|OJJ39580.1 azon|OJJ50107.1

Group5934: afla|EED49755.1 afum|EAL84309.2 acar|OOF98690.1 acla|EAW13141.1 afis|EAW25621.1 agla|OJJ86173.1 aluc|OJZ88033.1 aory|EIT77706.1 asyd|OJJ56973.1 atub|OJI90639.1 aver|OJJ03316.1 awen|OJJ36298.1 afis|EAW17513.1 afla|EED52201.1 aluc|OJZ79945.1 aory|EIT74476.1

Group5935: abra|OJJ65917.1 acar|OOF94967.1 afla|EED50458.1 afum|EAL84940.1 agla|OJJ80059.1 akaw|GAA92315.1 aluc|OJZ90213.1 anid|CBF84461.1 anig|EHA23711.1 aory|EIT82170.1 asyd|OJJ53548.1 ater|EAU31471.1 atub|OJI81644.1 aver|OJJ08205.1 awen|OJJ31371.1 asyd|OJJ54265.1

Group5936: abra|OJJ65943.1 acar|OOF92930.1 acla|EAW13568.1 afis|EAW20034.1 afum|EAL84882.1 agla|OJJ88020.1 akaw|GAA90821.1 aluc|OJZ83014.1 anid|CBF84767.1 anig|EHA21429.1 asyd|OJJ53283.1 ater|EAU29400.1 atub|OJI89249.1 aver|OJJ08459.1 awen|OJJ30057.1 azon|OJJ46328.1

Group5937: abra|OJJ65947.1 acar|OOF92926.1 afla|EED46040.1 afum|EAL84886.1 agla|OJJ88017.1 akaw|GAA90825.1 aluc|OJZ83010.1 anid|CBF84758.1 anig|EHA21425.1 aory|EIT75670.1 asyd|OJJ53287.1 ater|EAU29394.1 atub|OJI89245.1 aver|OJJ08454.1 awen|OJJ30053.1 azon|OJJ43699.1

Group5938: abra|OJJ65955.1 acar|OOF92917.1 acla|EAW13576.1 afis|EAW20025.1 afla|EED46033.1 afum|EAL84892.1 agla|OJJ88005.1 akaw|GAA90832.1 aluc|OJZ83002.1 anid|CBF84747.1 anig|EHA21418.1 asyd|OJJ53294.1 ater|EAU29387.1 atub|OJI89236.1 aver|OJJ08446.1 azon|OJJ43692.1

Group5939: abra|OJJ65970.1 acar|OOF92903.1 acla|EAW13591.1 afla|EED46019.1 agla|OJJ87977.1 akaw|GAA90845.1 aluc|OJZ82988.1 anid|CBF87092.1 anig|EHA21403.1 aory|EIT75531.1 asyd|OJJ60402.1 atub|OJI89222.1 aver|OJI99731.1 awen|OJJ30026.1 afla|EED54119.1 awen|OJJ36029.1

Group5940: abra|OJJ66005.1 acar|OOF92092.1 acla|EAW13642.1 afis|EAW19993.1 afla|EED45929.1 afum|EAL84924.1 akaw|GAA90878.1 aluc|OJZ82955.1 anid|CBF84688.1 anig|EHA21370.1 aory|EIT75499.1 asyd|OJJ53332.1 ater|EAU35263.1 atub|OJI89187.1 aver|OJJ08412.1 azon|OJJ43627.1

Group5941: afis|EAW17530.1 agla|OJJ79940.1 anid|CBF76169.1 abra|OJJ69571.1 afla|EED57484.1 akaw|GAA92494.1 aluc|OJZ91506.1 anig|EHA28383.1 aory|EIT80112.1 asyd|OJJ64206.1 ater|EAU32458.1 atub|OJI82869.1 aver|OJI98294.1 awen|OJJ29896.1 agla|OJJ82855.1 agla|OJJ86642.1

Group5942: abra|OJJ66098.1 acar|OOF90927.1 acla|EAW14985.1 afis|EAW24507.1 afla|EED45890.1 afum|EAL86175.1 akaw|GAA93286.1 aluc|OJZ90158.1 anid|CBF89388.1 anig|EHA20914.1 aory|EIT75697.1 asyd|OJJ56086.1 ater|EAU35712.1 atub|OJI84641.1 aver|OJJ05029.1 awen|OJJ38109.1

Group5943: abra|OJJ66100.1 akaw|GAA92666.1 aluc|OJZ90120.1 anig|EHA20949.1 atub|OJI84675.1 abra|OJJ66420.1 acar|OOG00283.1 afla|EED54053.1 afum|EAL87184.1 anid|CBF86035.1 anig|EHA28120.1 aory|EIT76094.1 aver|OJJ07930.1 azon|OJJ48538.1 ater|EAU37533.1 awen|OJJ41204.1

Group5944: abra|OJJ66195.1 acar|OOF91936.1 acla|EAW12300.1 afis|EAW16934.1 afla|EED53214.1 afum|EAL86459.1 agla|OJJ86546.1 akaw|GAA89093.1 anid|CBF82438.1 anig|EHA25695.1 aory|EIT74921.1 asyd|OJJ54125.1 ater|EAU37364.1 atub|OJI88432.1 aver|OJJ08611.1 awen|OJJ30070.1

Group5945: abra|OJJ66233.1 acar|OOF90691.1 afla|EED47090.1 agla|OJJ84413.1 akaw|GAA89056.1 aluc|OJZ80538.1 anid|CBF85759.1 anig|EHA25657.1 aory|EIT72497.1 asyd|OJJ64301.1 atub|OJI88393.1 aver|OJJ07894.1 awen|OJJ31375.1 agla|OJJ80120.1 aver|OJI98394.1 awen|OJJ36365.1

Group5946: abra|OJJ66246.1 acar|OOF90704.1 afis|EAW24436.1 afla|EED55245.1 afum|EAL86104.1 agla|OJJ79251.1 akaw|GAA89044.1 aluc|OJZ80552.1 anid|CBF86218.1 anig|EHA25645.1 asyd|OJJ64561.1 ater|EAU30010.1 atub|OJI88379.1 aver|OJI98658.1 awen|OJJ32763.1 azon|OJJ42168.1

Group5947: abra|OJJ66261.1 acar|OOF90732.1 acla|EAW09306.1 afis|EAW21840.1 afla|EED46532.1 aluc|OJZ80569.1 anig|EHA25629.1 aory|EIT72369.1 asyd|OJJ59252.1 ater|EAU38517.1 atub|OJI88362.1 aver|OJJ01861.1 awen|OJJ34122.1 azon|OJJ48038.1 aory|EIT82363.1 afla|EED57910.1

Group5948: abra|OJJ66429.1 acar|OOF92895.1 afla|EED53122.1 akaw|GAA90297.1 aluc|OJZ83040.1 anid|CBF88837.1 anig|EHA28111.1 aory|EIT72885.1 asyd|OJJ62821.1 atub|OJI87909.1 azon|OJJ51632.1 akaw|GAA84751.1 aluc|OJZ86932.1 anig|EHA21714.1 atub|OJI83589.1 afla|EED56423.1

Group5949: abra|OJJ66471.1 afis|EAW16806.1 afla|EED53911.1 agla|OJJ89421.1 akaw|GAA92130.1 aluc|OJZ83592.1 anid|CBF80483.1 asyd|OJJ55016.1 ater|EAU31818.1 atub|OJI89964.1 aver|OJI99385.1 awen|OJJ37491.1 acar|OOF94407.1 awen|OJJ36143.1 anid|CBF78765.1 asyd|OJJ65236.1

Group5950: abra|OJJ66505.1 acar|OOF90969.1 acla|EAW10976.1 afis|EAW20883.1 afla|EED57731.1 afum|EAL84749.1 agla|OJJ85833.1 akaw|GAA91205.1 aluc|OJZ81540.1 anid|CBF85489.1 anig|EHA25896.1 aory|EIT79869.1 asyd|OJJ54961.1 atub|OJI88646.1 aver|OJJ07230.1 awen|OJJ34477.1

Group5951: abra|OJJ66533.1 acar|OOF90984.1 acla|EAW10959.1 afis|EAW20900.1 afla|EED50250.1 afum|EBA27203.1 agla|OJJ84013.1 akaw|GAA91182.1 aluc|OJZ81521.1 anig|EHA25919.1 asyd|OJJ54113.1 ater|EAU38533.1 atub|OJI88670.1 aver|OJJ08622.1 awen|OJJ34448.1 azon|OJJ51734.1

Group5952: abra|OJJ66537.1 acar|OOF90988.1 acla|EAW10954.1 afis|EAW20907.1 afla|EED50255.1 afum|EAL84726.1 agla|OJJ84023.1 akaw|GAA91178.1 aluc|OJZ81510.1 anig|EHA25923.1 aory|EIT77300.1 asyd|OJJ54099.1 ater|EAU38537.1 atub|OJI88674.1 aver|OJJ08639.1 awen|OJJ34444.1

Group5953: abra|OJJ66539.1 acar|OOF90992.1 acla|EAW10951.1 afis|EAW20910.1 afla|EED50258.1 afum|EAL84724.2 agla|OJJ84026.1 akaw|GAA91176.1 aluc|OJZ81505.1 anid|CBF82479.1 anig|EHA25925.1 aory|EIT77280.1 ater|EAU38540.1 atub|OJI88676.1 awen|OJJ34436.1 azon|OJJ51718.1

Group5954: abra|OJJ66549.1 acar|OOF89953.1 acla|EAW10943.1 afis|EAW20915.1 afla|EED48929.1 afum|EAL84719.1 agla|OJJ78905.1 akaw|GAA91174.1 aluc|OJZ81502.1 anid|CBF87874.1 anig|EHA25928.1 aory|EIT80003.1 asyd|OJJ65183.1 atub|OJI88679.1 aver|OJI99336.1 awen|OJJ30158.1

Group5955: abra|OJJ66560.1 acla|EAW10935.1 afis|EAW20926.1 afla|EED50279.1 afum|EAL84708.1 agla|OJJ83963.1 akaw|GAA91157.1 aluc|OJZ81485.1 anid|CBF82501.1 anig|EHA25942.1 aory|EIT77314.1 ater|EAU38572.1 atub|OJI88696.1 aver|OJJ08648.1 awen|OJJ34394.1 azon|OJJ48125.1

Group5956: abra|OJJ66571.1 acar|OOF91011.1 acla|EAW10922.1 afis|EAW20936.1 afum|EAL84698.1 agla|OJJ83950.1 akaw|GAA91147.1 aluc|OJZ81473.1 anid|CBF82528.1 anig|EHA25953.1 asyd|OJJ54074.1 ater|EAU38582.1 atub|OJI88705.1 aver|OJJ08665.1 awen|OJJ34381.1 azon|OJJ48113.1

Group5957: abra|OJJ66603.1 acar|OOF90115.1 acla|EAW10890.1 afis|EAW20968.1 afla|EED50322.1 afum|EBA27201.1 agla|OJJ84045.1 akaw|GAA91117.1 aluc|OJZ81443.1 anid|CBF82582.1 anig|EHA25981.1 aory|EIT77258.1 asyd|OJJ54040.1 ater|EAU38618.1 atub|OJI88735.1 awen|OJJ34347.1

Group5958: abra|OJJ66605.1 acar|OOF90113.1 acla|EAW10888.1 afis|EAW20970.1 afla|EED50324.1 afum|EAL84664.1 agla|OJJ84047.1 akaw|GAA91115.1 aluc|OJZ81441.1 anig|EHA25983.1 aory|EIT77253.1 asyd|OJJ54038.1 atub|OJI88737.1 aver|OJJ08702.1 awen|OJJ34345.1 azon|OJJ48082.1

Group5959: abra|OJJ66658.1 acar|OOF93675.1 acla|EAW11800.1 afis|EAW19410.1 afla|EED50365.1 afum|EAL87084.2 agla|OJJ81124.1 akaw|GAA89608.1 aluc|OJZ80790.1 anid|CBF74353.1 aory|EIT83118.1 asyd|OJJ57791.1 atub|OJI87641.1 aver|OJJ05373.1 awen|OJJ36806.1 acar|OOF93760.1

Group5960: abra|OJJ66762.1 acla|EAW11888.1 afis|EAW19500.1 afla|EED56719.1 afum|EBA27297.1 agla|OJJ81669.1 akaw|GAA89698.1 aluc|OJZ80687.1 anid|CBF75438.1 anig|EHA27327.1 asyd|OJJ59784.1 ater|EAU34815.1 atub|OJI87546.1 aver|OJJ02504.1 awen|OJJ36915.1 azon|OJJ42900.1

Group5961: abra|OJJ66768.1 acar|OOF93849.1 acla|EAW11605.1 afla|EED49852.1 agla|OJJ83400.1 akaw|GAA89705.1 aluc|OJZ80679.1 anid|CBF82408.1 anig|EHA27333.1 aory|EIT83408.1 asyd|OJJ54169.1 atub|OJI87538.1 aver|OJJ08570.1 awen|OJJ40864.1 azon|OJJ49073.1 awen|OJJ40207.1

Group5962: abra|OJJ66778.1 acar|OOF93857.1 afis|EAW16744.1 afla|EED45880.1 afum|EAL86319.2 agla|OJJ80280.1 akaw|GAA89713.1 aluc|OJZ80671.1 anid|CBF75770.1 anig|EHA27340.1 asyd|OJJ62106.1 ater|EAU36169.1 atub|OJI87525.1 aver|OJI96212.1 awen|OJJ29904.1 azon|OJJ42471.1

Group5963: abra|OJJ66785.1 acar|OOF96742.1 acla|EAW12028.1 afis|EAW19677.1 afum|EAL86845.1 akaw|GAA89719.1 aluc|OJZ80665.1 anid|CBF81955.1 anig|EHA27345.1 asyd|OJJ52953.1 atub|OJI87520.1 aver|OJJ08919.1 awen|OJJ37896.1 afla|EED47776.1 agla|OJJ81344.1 aory|EIT77196.1

Group5964: abra|OJJ66826.1 acla|EAW14408.1 afis|EAW19116.1 afla|EED54298.1 afum|EAL93848.2 agla|OJJ80431.1 akaw|GAA90462.1 aluc|OJZ85761.1 anid|CBF79730.1 anig|EHA24795.1 asyd|OJJ58072.1 ater|EAU37378.1 atub|OJI79546.1 aver|OJJ04335.1 awen|OJJ37446.1 azon|OJJ51124.1

Group5965: abra|OJJ66841.1 acar|OOF91618.1 acla|EAW14422.1 afis|EAW19129.1 afla|EED54285.1 afum|EAL93861.1 agla|OJJ88601.1 akaw|GAA90477.1 aluc|OJZ85743.1 anid|CBF79747.1 anig|EHA24809.1 asyd|OJJ58089.1 atub|OJI79528.1 aver|OJJ04311.1 awen|OJJ37462.1 azon|OJJ51140.1

Group5966: abra|OJJ66872.1 acar|OOF91648.1 acla|EAW14447.1 afis|EAW19155.1 afla|EED54247.1 afum|EAL93886.1 akaw|GAA91537.1 aluc|OJZ85709.1 anid|CBF86296.1 anig|EHA24833.1 aory|EIT76022.1 asyd|OJJ65011.1 ater|EAU37428.1 atub|OJI79500.1 aver|OJI99130.1 awen|OJJ37508.1

Group5967: abra|OJJ66877.1 acar|OOF91652.1 acla|EAW14452.1 afis|EAW19160.1 afla|EED54242.1 afum|EAL93890.2 akaw|GAA91532.1 aluc|OJZ85705.1 anid|CBF86287.1 anig|EHA24837.1 aory|EIT76015.1 asyd|OJJ65016.1 ater|EAU37433.1 atub|OJI79495.1 aver|OJI99134.1 awen|OJJ37513.1

Group5968: abra|OJJ66878.1 acar|OOF91653.1 acla|EAW14453.1 afis|EAW19161.1 afla|EED54241.1 afum|EAL93891.1 agla|OJJ88563.1 akaw|GAA91531.1 aluc|OJZ85704.1 anid|CBF75184.1 anig|EHA24838.1 aory|EIT76132.1 ater|EAU37319.1 atub|OJI79494.1 awen|OJJ37514.1 azon|OJJ51177.1

Group5969: abra|OJJ66959.1 acar|OOF93649.1 acla|EAW14961.1 afis|EAW24484.1 afla|EED52058.1 afum|EAL86153.1 agla|OJJ88957.1 akaw|GAA92202.1 aluc|OJZ85617.1 anig|EHA24909.1 aory|EIT81432.1 asyd|OJJ55928.1 ater|EAU37957.1 atub|OJI79410.1 aver|OJJ04878.1 azon|OJJ51805.1

Group5970: abra|OJJ66989.1 acar|OOF93623.1 acla|EAW12086.1 afis|EAW19729.1 afla|EED52168.1 afum|EAL86798.1 akaw|GAA92174.1 aluc|OJZ85584.1 anid|CBF87096.1 anig|EHA24937.1 aory|EIT74461.1 asyd|OJJ60395.1 atub|OJI79379.1 aver|OJI99724.1 awen|OJJ29795.1 azon|OJJ50255.1

Group5971: abra|OJJ67092.1 acar|OOF97365.1 acla|EAW12323.1 afis|EAW25701.1 afum|EAL84374.1 agla|OJJ86316.1 akaw|GAA91069.1 aluc|OJZ87883.1 anid|CBF82025.1 anig|EHA20186.1 asyd|OJJ58346.1 ater|EAU39413.1 atub|OJI80768.1 aver|OJJ02067.1 afis|EAW23748.1 anid|CBF80131.1

Group5972: abra|OJJ67100.1 acar|OOF98452.1 acla|EAW13713.1 afis|EAW20273.1 afla|EED48132.1 afum|EAL85407.1 agla|OJJ87505.1 akaw|GAA91061.1 aluc|OJZ87873.1 anid|CBF78240.1 anig|EHA20179.1 aory|EIT76216.1 ater|EAU31788.1 atub|OJI80776.1 awen|OJJ35880.1 afis|EAW23647.1

Group5973: abra|OJJ67109.1 acar|OOF98443.1 afis|EAW24483.1 afla|EED52545.1 afum|EAL86152.1 agla|OJJ85836.1 akaw|GAA91055.1 aluc|OJZ87866.1 anig|EHA20172.1 aory|EIT74263.1 ater|EAU30095.1 atub|OJI80782.1 awen|OJJ30838.1 ater|EAU33502.1 afum|EAL92050.2 asyd|OJJ52467.1

Group5974: abra|OJJ67132.1 acar|OOF99014.1 afla|EED53374.1 anid|CBF81678.1 aory|EIT74911.1 asyd|OJJ59559.1 aver|OJI98274.1 awen|OJJ38788.1 azon|OJJ42559.1 abra|OJJ70398.1 acar|OOF91875.1 akaw|GAA91960.1 aluc|OJZ82657.1 anig|EHA27416.1 atub|OJI88889.1 aver|OJJ02709.1

Group5975: abra|OJJ67156.1 acar|OOF98403.1 acla|EAW14569.1 afis|EAW19282.1 afla|EED47595.1 agla|OJJ88394.1 akaw|GAA91016.1 aluc|OJZ87817.1 anid|CBF78761.1 anig|EHA20298.1 aory|EIT80196.1 asyd|OJJ65234.1 atub|OJI80827.1 aver|OJI99383.1 awen|OJJ37643.1 azon|OJJ44907.1

Group5976: abra|OJJ67161.1 acar|OOF98399.1 afis|EAW19279.1 afla|EED47599.1 akaw|GAA91012.1 aluc|OJZ87813.1 anid|CBF78754.1 aory|EIT80238.1 asyd|OJJ65230.1 ater|EAU29484.1 atub|OJI80831.1 aver|OJI99379.1 awen|OJJ37639.1 acla|EAW14566.1 afum|EAL94005.1 azon|OJJ44880.1

Group5977: abra|OJJ67167.1 acar|OOF98394.1 acla|EAW14561.1 afis|EAW19274.1 afum|EAL94000.1 akaw|GAA91007.1 aluc|OJZ87808.1 anid|CBF78747.1 anig|EHA20290.1 asyd|OJJ65225.1 ater|EAU29490.1 atub|OJI80837.1 aver|OJI99375.1 afla|EED47602.1 agla|OJJ88399.1 aory|EIT80191.1

Group5978: abra|OJJ67230.1 acar|OOF98323.1 acla|EAW10403.1 afis|EAW25169.1 afla|EED57527.1 afum|EAL89842.1 agla|OJJ88986.1 akaw|GAA91655.1 aluc|OJZ87744.1 anid|CBF85618.1 asyd|OJJ64203.1 ater|EAU39453.1 atub|OJI80897.1 aver|OJI98292.1 awen|OJJ40820.1 azon|OJJ46864.1

Group5979: abra|OJJ67233.1 asyd|OJJ53601.1 aver|OJJ08147.1 awen|OJJ35947.1 azon|OJJ42412.1 abra|OJJ77556.1 acar|OOF99171.1 afla|EED46976.1 akaw|GAA84067.1 aluc|OJZ80981.1 anig|EHA19379.1 aory|EIT72531.1 ater|EAU33717.1 atub|OJI82605.1 ater|EAU31461.1 awen|OJJ34676.1

Group5980: abra|OJJ67269.1 acar|OOF98297.1 afis|EAW17110.1 afla|EED55274.1 afum|EAL86574.1 agla|OJJ88999.1 akaw|GAA91684.1 aluc|OJZ87710.1 anid|CBF85646.1 anig|EHA20204.1 aory|EIT83570.1 asyd|OJJ64237.1 ater|EAU39436.1 atub|OJI80932.1 aver|OJI98330.1 awen|OJJ40797.1

Group5981: abra|OJJ67271.1 acla|EAW10416.1 afis|EAW25153.1 afla|EED55280.1 afum|EAL89828.1 akaw|GAA91686.1 aluc|OJZ87708.1 anid|CBF85639.1 anig|EHA20890.1 aory|EIT83511.1 asyd|OJJ64241.1 atub|OJI80934.1 aver|OJI98334.1 awen|OJJ40782.1 azon|OJJ46870.1 agla|OJJ89001.1

Group5982: akaw|GAA86510.1 aluc|OJZ85054.1 anig|EHA25270.1 acar|OOF92509.1 acla|EAW06428.1 afis|EAW18532.1 afla|EED52868.1 agla|OJJ85145.1 anid|CBF70202.1 asyd|OJJ58785.1 ater|EAU30038.1 aver|OJJ01393.1 awen|OJJ31090.1 azon|OJJ50596.1 afum|EAL93230.1 aory|EIT78632.1

Group5983: abra|OJJ67307.1 acar|OOF92506.1 acla|EAW06426.1 afis|EAW18530.1 afla|EED52871.1 afum|EAL93227.1 agla|OJJ85143.1 akaw|GAA86508.1 aluc|OJZ85051.1 anid|CBF70206.1 anig|EHA25268.1 asyd|OJJ58783.1 atub|OJI80547.1 aver|OJJ01391.1 awen|OJJ31088.1 azon|OJJ50593.1

Group5984: abra|OJJ67312.1 acla|EAW06420.1 afis|EAW18524.1 afum|EAL93219.1 agla|OJJ85136.1 akaw|GAA86503.1 aluc|OJZ85046.1 anid|CBF70215.1 anig|EHA25263.1 aory|EIT78618.1 asyd|OJJ58777.1 ater|EAU30048.1 atub|OJI80552.1 aver|OJJ01386.1 awen|OJJ31078.1 azon|OJJ50586.1

Group5985: abra|OJJ67320.1 acar|OOF92494.1 acla|EAW06416.1 afis|EAW18520.1 afla|EED52878.1 afum|EAL93215.1 agla|OJJ85132.1 akaw|GAA86498.1 aluc|OJZ85040.1 anid|CBF70224.1 anig|EHA25258.1 aory|EIT78617.1 ater|EAU30050.1 atub|OJI80557.1 awen|OJJ31072.1 azon|OJJ50582.1

Group5986: abra|OJJ67333.1 acar|OOF92480.1 acla|EAW06401.1 afis|EAW18505.1 afla|EED52896.1 afum|EAL93199.1 agla|OJJ85117.1 akaw|GAA86483.1 aluc|OJZ85024.1 anid|CBF87122.1 anig|EHA25245.1 aory|EIT78621.1 asyd|OJJ60361.1 ater|EAU30066.1 atub|OJI80573.1 aver|OJI99698.1

Group5987: afla|EED54124.1 aory|EIT76004.1 abra|OJJ77008.1 afis|EAW25620.1 agla|OJJ78907.1 akaw|GAA87040.1 aluc|OJZ84407.1 anig|EHA27737.1 asyd|OJJ55522.1 ater|EAU32182.1 atub|OJI89665.1 aver|OJJ06600.1 awen|OJJ36039.1 azon|OJJ45113.1 acar|OOF91454.1 anid|CBF83171.1

Group5988: abra|OJJ67397.1 acar|OOF92420.1 acla|EAW06363.1 afis|EAW18472.1 afla|EED52938.1 afum|EAL93159.2 agla|OJJ85059.1 akaw|GAA86430.1 aluc|OJZ84972.1 anig|EHA25184.1 asyd|OJJ58828.1 ater|EAU29889.1 atub|OJI80629.1 aver|OJJ01431.1 awen|OJJ31024.1 azon|OJJ50538.1

Group5989: abra|OJJ67406.1 acar|OOF92410.1 acla|EAW06352.1 afis|EAW18464.1 afum|EAL93151.2 agla|OJJ85048.1 akaw|GAA86421.1 aluc|OJZ84962.1 anid|CBF70093.1 aory|EIT78734.1 asyd|OJJ58838.1 ater|EAU29882.1 atub|OJI80638.1 aver|OJJ01440.1 awen|OJJ31016.1 azon|OJJ50529.1

Group5990: abra|OJJ67428.1 acar|OOF92398.1 acla|EAW06336.1 afis|EAW18448.1 afla|EED52960.1 afum|EAL93133.2 agla|OJJ85025.1 akaw|GAA86402.1 aluc|OJZ84938.1 anid|CBF70068.1 anig|EHA25156.1 asyd|OJJ58848.1 atub|OJI80661.1 aver|OJJ01449.1 awen|OJJ31000.1 azon|OJJ50514.1

Group5991: abra|OJJ67532.1 acar|OOG01225.1 acla|EAW14583.1 afis|EAW20812.1 afla|EED55975.1 afum|EAL84816.1 akaw|GAA88126.1 anid|CBF77711.1 anig|EHA18691.1 asyd|OJJ53078.1 ater|EAU32035.1 atub|OJI91604.1 aver|OJJ03769.1 awen|OJJ32225.1 acla|EAW11063.1 awen|OJJ30947.1

Group5992: abra|OJJ67550.1 acar|OOF90250.1 acla|EAW13271.1 afis|EAW17346.1 afla|EED45503.1 afum|EAL91190.1 akaw|GAA88110.1 aluc|OJZ81380.1 anig|EHA18675.1 aory|EIT75626.1 asyd|OJJ56676.1 ater|EAU29418.1 atub|OJI91586.1 aver|OJJ06048.1 acar|OOF94391.1 ater|EAU35969.1

Group5993: abra|OJJ67617.1 afla|EED45202.1 akaw|GAA89134.1 aluc|OJZ81702.1 anig|EHA19895.1 aory|EIT73724.1 atub|OJI81348.1 abra|OJJ73693.1 acar|OOG00866.1 agla|OJJ78660.1 akaw|GAA88467.1 aluc|OJZ88356.1 anig|EHA18089.1 ater|EAU39318.1 atub|OJI90951.1 awen|OJJ33717.1

Group5994: abra|OJJ67624.1 acar|OOF91972.1 afis|EAW20775.1 afla|EED45948.1 afum|EAL84845.1 agla|OJJ79050.1 akaw|GAA89144.1 aluc|OJZ81692.1 anid|CBF74983.1 anig|EHA25752.1 aory|EIT75387.1 asyd|OJJ54562.1 atub|OJI88488.1 aver|OJJ07594.1 awen|OJJ35893.1 azon|OJJ42145.1

Group5995: abra|OJJ67669.1 acar|OOF90912.1 acla|EAW12248.1 afis|EAW16768.1 akaw|GAA89185.1 aluc|OJZ81646.1 anid|CBF84388.1 anig|EHA25797.1 asyd|OJJ54909.1 ater|EAU34859.1 atub|OJI88536.1 aver|OJJ07293.1 azon|OJJ43892.1 anid|CBF78140.1 asyd|OJJ53585.1 aver|OJJ08163.1

Group5996: abra|OJJ67692.1 acar|OOF97519.1 acla|EAW14628.1 afis|EAW17251.1 afla|EED53073.1 afum|EAL86670.1 akaw|GAA89201.1 aluc|OJZ81629.1 anid|CBF88038.1 anig|EHA25814.1 aory|EIT74791.1 asyd|OJJ58695.1 ater|EAU30968.1 atub|OJI88551.1 aver|OJJ01307.1 awen|OJJ32905.1

Group5997: abra|OJJ67729.1 acar|OOF90931.1 afis|EAW20573.1 afla|EED52328.1 afum|EAL84492.1 agla|OJJ88971.1 akaw|GAA89223.1 aluc|OJZ81605.1 anid|CBF74531.1 anig|EHA25840.1 aory|EIT74343.1 atub|OJI88580.1 aver|OJJ06519.1 awen|OJJ29984.1 asyd|OJJ55446.1 awen|OJJ36164.1

Group5998: abra|OJJ67829.1 acar|OOG00541.1 acla|EAW09505.1 afum|EAL84476.1 akaw|GAA91840.1 aluc|OJZ82058.1 anid|CBF78144.1 anig|EHA22199.1 ater|EAU32305.1 atub|OJI84617.1 aver|OJI99602.1 awen|OJJ31459.1 azon|OJJ43641.1 acar|OOF92586.1 azon|OJJ44381.1 aver|OJI99600.1

Group5999: abra|OJJ67857.1 acar|OOF92837.1 acla|EAW11743.1 afis|EAW23776.1 afla|EED51576.1 afum|EAL87210.1 agla|OJJ78927.1 akaw|GAA91863.1 aluc|OJZ82035.1 anid|CBF79828.1 anig|EHA22221.1 aory|EIT80538.1 atub|OJI84590.1 aver|OJJ04261.1 awen|OJJ33701.1 azon|OJJ42840.1

Group6000: abra|OJJ67860.1 acar|OOF92841.1 acla|EAW11738.1 afis|EAW23771.1 afla|EED51572.1 afum|EAL87205.1 agla|OJJ78948.1 akaw|GAA91867.1 aluc|OJZ82032.1 anig|EHA22224.1 aory|EIT80473.1 asyd|OJJ58140.1 ater|EAU31315.1 atub|OJI84587.1 aver|OJJ04265.1 awen|OJJ33704.1

Group6001: abra|OJJ67864.1 acar|OOF92844.1 afis|EAW23767.1 afla|EED51553.1 afum|EAL87201.1 agla|OJJ78955.1 akaw|GAA91871.1 aluc|OJZ82028.1 anid|CBF84704.1 anig|EHA22228.1 aory|EIT80560.1 asyd|OJJ53320.1 atub|OJI84582.1 aver|OJJ08423.1 awen|OJJ33696.1 azon|OJJ42545.1

Group6002: abra|OJJ67880.1 acar|OOF92856.1 acla|EAW11723.1 afis|EAW23746.1 afla|EED51538.1 afum|EAL87181.1 akaw|GAA91887.1 aluc|OJZ82010.1 anid|CBF87336.1 anig|EHA22247.1 aory|EIT80493.1 asyd|OJJ56363.1 atub|OJI84559.1 aver|OJJ06351.1 awen|OJJ33681.1 azon|OJJ44710.1

Group6003: abra|OJJ67882.1 acar|OOF92863.1 acla|EAW11720.1 afis|EAW23741.1 afla|EED51535.1 afum|EAL87178.1 agla|OJJ78964.1 akaw|GAA91889.1 aluc|OJZ82008.1 anid|CBF79813.1 anig|EHA22249.1 aory|EIT80693.1 ater|EAU31298.1 atub|OJI84557.1 awen|OJJ33678.1 azon|OJJ42819.1

Group6004: abra|OJJ67888.1 acla|EAW11717.1 afis|EAW23736.1 afla|EED51513.1 afum|EAL87174.1 agla|OJJ78970.1 akaw|GAA91894.1 aluc|OJZ82002.1 anid|CBF84245.1 anig|EHA22254.1 asyd|OJJ57198.1 ater|EAU31295.1 atub|OJI84552.1 aver|OJJ03093.1 awen|OJJ33671.1 azon|OJJ42814.1

Group6005: abra|OJJ67889.1 acar|OOF92859.1 afis|EAW23738.1 afla|EED51514.1 afum|EAL87175.1 agla|OJJ78967.1 akaw|GAA91895.1 aluc|OJZ82001.1 anid|CBF79809.1 anig|EHA22255.1 aory|EIT80532.1 asyd|OJJ58130.1 ater|EAU31296.1 atub|OJI84551.1 aver|OJJ04275.1 awen|OJJ33674.1

Group6006: abra|OJJ67890.1 acar|OOF92858.1 acla|EAW11060.1 afis|EAW20814.1 afla|EED47512.1 afum|EAL84814.1 akaw|GAA91896.1 aluc|OJZ82000.1 anid|CBF78358.1 anig|EHA22256.1 aory|EIT74562.1 asyd|OJJ56145.1 ater|EAU30844.1 atub|OJI84550.1 aver|OJJ05086.1 awen|OJJ35834.1

Group6007: ater|EAU35708.1 anid|CBF69522.1 afis|EAW21018.1 afla|EED56839.1 afum|EAL92056.1 agla|OJJ80564.1 aory|EIT77488.1 asyd|OJJ59206.1 aver|OJJ01817.1 awen|OJJ37826.1 afum|EAL85152.1 agla|OJJ88496.1 atub|OJI81729.1 akaw|GAA86070.1 aluc|OJZ90305.1 awen|OJJ31968.1

Group6008: acla|EAW12904.1 abra|OJJ78317.1 acar|OOF90170.1 akaw|GAA85713.1 aluc|OJZ91390.1 anid|CBF75868.1 anig|EHA20068.1 asyd|OJJ60241.1 atub|OJI83383.1 aver|OJJ04025.1 afis|EAW17776.1 afla|EED56744.1 afum|EAL91584.2 aory|EIT73880.1 ater|EAU31816.1 asyd|OJJ52432.1

Group6009: abra|OJJ67939.1 acar|OOF92788.1 acla|EAW11629.1 afis|EAW23827.1 afla|EED51979.1 afum|EAL87259.1 agla|OJJ82485.1 akaw|GAA91257.1 aluc|OJZ82811.1 anid|CBF79909.1 anig|EHA21250.1 aory|EIT81475.1 ater|EAU31382.1 atub|OJI89041.1 awen|OJJ33785.1 azon|OJJ42887.1

Group6010: abra|OJJ67972.1 acar|OOF92813.1 acla|EAW11762.1 afis|EAW23795.1 afla|EED51602.1 afum|EAL87229.2 agla|OJJ78844.1 akaw|GAA91230.1 aluc|OJZ82840.1 anid|CBF79856.1 anig|EHA21277.1 aory|EIT80530.1 atub|OJI89070.1 aver|OJJ04244.1 awen|OJJ33751.1 azon|OJJ42860.1

Group6011: abra|OJJ67984.1 acar|OOF92026.1 afis|EAW25692.1 afla|EED46905.1 afum|EAL84362.1 akaw|GAA91218.1 aluc|OJZ82852.1 anid|CBF78965.1 anig|EHA21288.1 aory|EIT72639.1 asyd|OJJ55667.1 ater|EAU35088.1 atub|OJI89082.1 aver|OJJ06748.1 awen|OJJ36205.1 azon|OJJ50743.1

Group6012: abra|OJJ68114.1 acar|OOF93908.1 afis|EAW15405.1 afla|EED44866.1 afum|EAL84241.1 agla|OJJ86207.1 akaw|GAA86895.1 aluc|OJZ90018.1 anid|CBF86936.1 anig|EHA27394.1 aory|EIT82714.1 asyd|OJJ55589.1 atub|OJI84789.1 aver|OJJ06668.1 awen|OJJ41765.1 agla|OJJ83696.1

Group6013: abra|OJJ68115.1 acar|OOF93909.1 afis|EAW16723.1 afla|EED45933.1 afum|EAL86297.2 agla|OJJ88975.1 akaw|GAA86896.1 aluc|OJZ90019.1 anig|EHA27395.1 aory|EIT75655.1 asyd|OJJ56047.1 atub|OJI84788.1 aver|OJJ04990.1 awen|OJJ36020.1 azon|OJJ45077.1 anid|CBF78249.1

Group6014: abra|OJJ68116.1 acar|OOF93910.1 afis|EAW16724.1 afla|EED45934.1 afum|EAL86298.1 agla|OJJ88976.1 akaw|GAA86897.1 aluc|OJZ90020.1 anid|CBF78251.1 anig|EHA27396.1 aory|EIT75493.1 asyd|OJJ56049.1 atub|OJI84787.1 aver|OJJ04992.1 acla|EAW13226.1 acar|OOF94484.1

Group6015: agla|OJJ87804.1 abra|OJJ74465.1 acla|EAW07232.1 afis|EAW22246.1 afum|EAL90947.1 akaw|GAA87358.1 aluc|OJZ89655.1 anid|CBF88498.1 anig|EHA26893.1 asyd|OJJ63794.1 ater|EAU34254.1 atub|OJI85149.1 aver|OJI97143.1 awen|OJJ34824.1 afla|EED57639.1 aory|EIT82987.1

Group6016: abra|OJJ68174.1 acar|OOF97472.1 acla|EAW13059.1 afis|EAW17606.1 afla|EED49216.1 afum|EAL91419.1 agla|OJJ81353.1 akaw|GAA83270.1 aluc|OJZ83776.1 anid|CBF90185.1 anig|EHA18915.1 asyd|OJJ61984.1 atub|OJI90146.1 aver|OJI96076.1 awen|OJJ37236.1 azon|OJJ50938.1

Group6017: abra|OJJ68180.1 acar|OOF97475.1 acla|EAW13062.1 afis|EAW17602.1 afla|EED49219.1 afum|EAL91415.1 akaw|GAA83266.1 aluc|OJZ83772.1 anig|EHA18912.1 aory|EIT83164.1 asyd|OJJ60509.1 ater|EAU37039.1 atub|OJI90143.1 aver|OJI99834.1 awen|OJJ37243.1 azon|OJJ50941.1

Group6018: abra|OJJ68188.1 acar|OOF97482.1 acla|EAW13070.1 afis|EAW17594.1 afla|EED49226.1 afum|EAL91407.1 akaw|GAA83257.1 aluc|OJZ83760.1 anid|CBF90208.1 anig|EHA18904.1 asyd|OJJ61971.1 ater|EAU37032.1 atub|OJI90133.1 aver|OJI96064.1 awen|OJJ37252.1 azon|OJJ50947.1

Group6019: abra|OJJ68189.1 acar|OOF97485.1 acla|EAW13071.1 afis|EAW17593.1 afla|EED49228.1 afum|EAL91406.1 agla|OJJ81332.1 akaw|GAA83256.1 aluc|OJZ83759.1 asyd|OJJ61970.1 ater|EAU37030.1 atub|OJI90132.1 aver|OJI96063.1 awen|OJJ37253.1 azon|OJJ50948.1 anid|CBF90210.1

Group6020: abra|OJJ68210.1 acar|OOF97494.1 acla|EAW12974.1 afis|EAW17690.1 afla|EED50454.1 afum|EAL91504.1 agla|OJJ79489.1 akaw|GAA83236.1 aluc|OJZ83735.1 anid|CBF89982.1 anig|EHA18884.1 asyd|OJJ62089.1 atub|OJI90113.1 aver|OJI96195.1 awen|OJJ37146.1 azon|OJJ50846.1

Group6021: abra|OJJ68215.1 acla|EAW12970.1 afla|EED50449.1 akaw|GAA83231.1 aluc|OJZ83731.1 anid|CBF89974.1 anig|EHA18880.1 aory|EIT82144.1 asyd|OJJ58334.1 ater|EAU29907.1 atub|OJI90108.1 aver|OJJ02076.1 awen|OJJ37142.1 agla|OJJ80592.1 asyd|OJJ62093.1 aver|OJI96199.1

Group6022: abra|OJJ68236.1 acla|EAW12223.1 afla|EED56299.1 anid|CBF87461.1 anig|EHA18314.1 aory|EIT73296.1 asyd|OJJ52778.1 ater|EAU35485.1 atub|OJI89935.1 aver|OJJ08340.1 awen|OJJ36976.1 anid|CBF87418.1 aver|OJJ03445.1 afla|EED56300.1 asyd|OJJ53411.1 aver|OJJ03428.1

Group6023: abra|OJJ68253.1 acar|OOF97532.1 afis|EAW24691.1 afla|EED46562.1 afum|EAL89332.1 akaw|GAA83208.1 aluc|OJZ83710.1 anid|CBF70720.1 anig|EHA18861.1 aory|EIT77828.1 asyd|OJJ61535.1 ater|EAU29994.1 atub|OJI90082.1 aver|OJJ00863.1 aver|OJJ07656.1 asyd|OJJ54532.1

Group6024: abra|OJJ68295.1 acar|OOF97562.1 acla|EAW08965.1 afis|EAW15446.1 afla|EED46465.1 afum|EAL84200.1 akaw|GAA83178.1 aluc|OJZ83677.1 anig|EHA18833.1 aory|EIT72318.1 atub|OJI90048.1 ater|EAU36715.1 asyd|OJJ53380.1 aver|OJJ08363.1 anid|CBF73480.1 anid|CBF87739.1

Group6025: abra|OJJ68325.1 acar|OOF97617.1 afla|EED57063.1 agla|OJJ78511.1 akaw|GAA92076.1 aluc|OJZ83648.1 anig|EHA18800.1 aory|EIT83411.1 asyd|OJJ56739.1 atub|OJI90017.1 awen|OJJ38714.1 asyd|OJJ52101.1 afla|EED49837.1 akaw|GAA93219.1 anid|CBF89124.1 anig|EHA22004.1

Group6026: abra|OJJ68348.1 acar|OOF94844.1 afis|EAW17519.1 afla|EED45478.1 afum|EAL91337.1 agla|OJJ78830.1 akaw|GAA92115.1 aluc|OJZ83610.1 anid|CBF80533.1 anig|EHA18769.1 aory|EIT75491.1 asyd|OJJ54654.1 ater|EAU36947.1 atub|OJI89983.1 aver|OJJ07507.1 awen|OJJ37340.1

Group6027: abra|OJJ68355.1 afla|EED53490.1 agla|OJJ82739.1 akaw|GAA92127.1 aluc|OJZ83595.1 anig|EHA18755.1 aory|EIT72952.1 asyd|OJJ56978.1 ater|EAU39005.1 atub|OJI89967.1 aver|OJJ03309.1 awen|OJJ36414.1 acar|OOF91796.1 afla|EED48206.1 ater|EAU30833.1 awen|OJJ37862.1

Group6028: abra|OJJ68383.1 acar|OOF93985.1 afis|EAW16782.1 afla|EED56189.1 afum|EAL86353.1 agla|OJJ79766.1 akaw|GAA92562.1 aluc|OJZ90109.1 anig|EHA20962.1 aory|EIT73337.1 asyd|OJJ59099.1 ater|EAU30270.1 atub|OJI84694.1 aver|OJJ01690.1 awen|OJJ29848.1 azon|OJJ50360.1

Group6029: abra|OJJ68391.1 afis|EAW19987.1 afum|EAL84928.1 akaw|GAA92556.1 aluc|OJZ90103.1 anig|EHA20967.1 aory|EIT82613.1 atub|OJI84702.1 acla|EAW13638.1 akaw|GAA92555.1 anid|CBF73971.1 anig|EHA20968.1 asyd|OJJ62368.1 aver|OJI96502.1 afla|EED53131.1 aory|EIT74881.1

Group6030: abra|OJJ68402.1 acar|OOF93970.1 afis|EAW17326.1 afla|EED54080.1 afum|EAL91163.1 akaw|GAA92545.1 aluc|OJZ90094.1 anid|CBF82407.1 anig|EHA20982.1 aory|EIT75924.1 asyd|OJJ54167.1 ater|EAU29239.1 atub|OJI84712.1 aver|OJJ08575.1 awen|OJJ29674.1 azon|OJJ46027.1

Group6031: abra|OJJ68447.1 acar|OOF93948.1 acla|EAW15354.1 afis|EAW20359.1 afla|EED47313.1 afum|EAL85476.1 akaw|GAA86927.1 aluc|OJZ90051.1 anid|CBF71653.1 anig|EHA21019.1 aory|EIT82832.1 asyd|OJJ61838.1 ater|EAU30275.1 atub|OJI84756.1 aver|OJI95937.1 azon|OJJ50770.1

Group6032: abra|OJJ68454.1 afla|EED56946.1 agla|OJJ87926.1 akaw|GAA86920.1 aluc|OJZ90044.1 anid|CBF70196.1 anig|EHA21027.1 aory|EIT77506.1 asyd|OJJ58788.1 ater|EAU30025.1 atub|OJI84763.1 aver|OJJ01395.1 awen|OJJ37051.1 azon|OJJ46448.1 afla|EED48364.1 agla|OJJ81574.1

Group6033: abra|OJJ68480.1 acar|OOF91469.1 acla|EAW06709.1 afis|EAW16259.1 afla|EED48914.1 afum|EAL88880.1 akaw|GAA92404.1 aluc|OJZ83265.1 anid|CBF80822.1 anig|EHA21118.1 asyd|OJJ54455.1 ater|EAU32190.1 atub|OJI87672.1 aver|OJJ07700.1 awen|OJJ38053.1 azon|OJJ43162.1

Group6034: abra|OJJ68493.1 acar|OOF90054.1 acla|EAW12143.1 afis|EAW17193.1 afum|EAL86621.1 agla|OJJ86661.1 akaw|GAA92777.1 aluc|OJZ82706.1 anid|CBF69881.1 anig|EHA21161.1 aory|EIT73563.1 asyd|OJJ59013.1 ater|EAU37674.1 atub|OJI88939.1 aver|OJJ01602.1 azon|OJJ42624.1

Group6035: abra|OJJ68547.1 afis|EAW17986.1 afla|EED51918.1 afum|EAL91795.1 akaw|GAA91307.1 aluc|OJZ82763.1 anid|CBF80002.1 anig|EHA21207.1 aory|EIT81366.1 asyd|OJJ58249.1 ater|EAU31496.1 atub|OJI88996.1 aver|OJJ02175.1 azon|OJJ48275.1 acla|EAW12821.1 awen|OJJ41560.1

Group6036: abra|OJJ68579.1 afla|EED56440.1 anid|CBF75415.1 aory|EIT72799.1 asyd|OJJ56522.1 atub|OJI91463.1 aver|OJJ06200.1 awen|OJJ37869.1 abra|OJJ68930.1 acar|OOF90673.1 afis|EAW19226.1 afum|EAL93957.1 akaw|GAA90321.1 aluc|OJZ85912.1 anig|EHA24676.1 atub|OJI79702.1

Group6037: anid|CBF78851.1 abra|OJJ74275.1 acar|OOF95456.1 acla|EAW07319.1 afis|EAW22351.1 afla|EED57755.1 afum|EAL90841.1 agla|OJJ87708.1 akaw|GAA86749.1 aluc|OJZ89858.1 anig|EHA27059.1 aory|EIT81036.1 asyd|OJJ63888.1 atub|OJI84948.1 aver|OJI97051.1 anid|CBF88659.1

Group6038: abra|OJJ68644.1 acar|OOF92147.1 acla|EAW07072.1 afis|EAW24963.1 afum|EAL89621.1 akaw|GAA89784.1 aluc|OJZ81907.1 anig|EHA22336.1 asyd|OJJ55374.1 ater|EAU30793.1 atub|OJI84460.1 aver|OJJ06453.1 awen|OJJ33779.1 azon|OJJ50607.1 anid|CBF82913.1 ater|EAU34596.1

Group6039: abra|OJJ68669.1 acar|OOF92169.1 acla|EAW12578.1 afis|EAW24070.1 afla|EED51405.1 afum|EAL91926.1 agla|OJJ87313.1 akaw|GAA89810.1 aluc|OJZ81880.1 anid|CBF86460.1 asyd|OJJ64913.1 ater|EAU30491.1 atub|OJI84434.1 aver|OJI99034.1 awen|OJJ41420.1 azon|OJJ48885.1

Group6040: abra|OJJ68671.1 acar|OOF92172.1 acla|EAW12576.1 afis|EAW24072.1 afum|EAL91928.1 agla|OJJ87141.1 akaw|GAA89824.1 aluc|OJZ81866.1 anid|CBF86464.1 anig|EHA22361.1 aory|EIT80514.1 asyd|OJJ64911.1 ater|EAU30493.1 atub|OJI84432.1 aver|OJI99032.1 awen|OJJ41408.1

Group6041: abra|OJJ68680.1 acar|OOF92182.1 acla|EAW12567.1 afis|EAW24083.1 afla|EED51390.1 afum|EAL91938.1 agla|OJJ87153.1 akaw|GAA89833.1 aluc|OJZ81853.1 anid|CBF86482.1 asyd|OJJ64900.1 ater|EAU30506.1 atub|OJI84422.1 aver|OJI99021.1 awen|OJJ41406.1 azon|OJJ48872.1

Group6042: abra|OJJ68737.1 acar|OOF92233.1 acla|EAW12510.1 afis|EAW24136.1 afum|EAL91991.1 agla|OJJ87213.1 akaw|GAA89885.1 aluc|OJZ81791.1 anid|CBF86571.1 anig|EHA22424.1 asyd|OJJ64846.1 ater|EAU30565.1 atub|OJI84364.1 aver|OJI98958.1 awen|OJJ41353.1 azon|OJJ48816.1

Group6043: abra|OJJ68764.1 acar|OOF92250.1 acla|EAW12487.1 afis|EAW24158.1 afum|EAL92014.2 agla|OJJ89523.1 akaw|GAA89908.1 aluc|OJZ81761.1 anid|CBF77898.1 anig|EHA22452.1 asyd|OJJ55304.1 ater|EAU30589.1 atub|OJI84333.1 aver|OJJ06922.1 awen|OJJ41320.1 azon|OJJ48793.1

Group6044: abra|OJJ68765.1 acar|OOF92251.1 acla|EAW12486.1 afis|EAW24159.1 afla|EED46774.1 afum|EAL92015.1 agla|OJJ89522.1 akaw|GAA89909.1 aluc|OJZ81760.1 anid|CBF77896.1 aory|EIT72588.1 asyd|OJJ55305.1 atub|OJI84332.1 aver|OJJ06921.1 awen|OJJ41319.1 azon|OJJ48792.1

Group6045: abra|OJJ68778.1 acar|OOF92264.1 acla|EAW12473.1 afla|EED46762.1 afum|EAL92028.1 agla|OJJ89508.1 akaw|GAA89922.1 aluc|OJZ81748.1 anid|CBF77870.1 anig|EHA22464.1 asyd|OJJ55318.1 ater|EAU30603.1 atub|OJI84319.1 aver|OJJ06910.1 awen|OJJ41305.1 azon|OJJ48779.1

Group6046: abra|OJJ68781.1 acar|OOF92268.1 acla|EAW12471.1 afla|EED46759.1 afum|EAL92032.1 agla|OJJ89505.1 akaw|GAA89925.1 aluc|OJZ81745.1 anid|CBF77865.1 anig|EHA22467.1 asyd|OJJ55321.1 ater|EAU30605.1 atub|OJI84316.1 aver|OJJ06907.1 awen|OJJ41302.1 azon|OJJ48776.1

Group6047: abra|OJJ68787.1 acar|OOF92271.1 acla|EAW12467.1 afis|EAW24174.1 afum|EAL92036.2 agla|OJJ89500.1 aluc|OJZ81741.1 anid|CBF77857.1 anig|EHA22471.1 aory|EIT72261.1 asyd|OJJ55325.1 ater|EAU30609.1 atub|OJI84312.1 aver|OJJ06903.1 awen|OJJ41296.1 azon|OJJ48772.1

Group6048: abra|OJJ68796.1 acar|OOF92280.1 acla|EAW14658.1 afis|EAW24185.1 afum|EAL92045.1 agla|OJJ89490.1 akaw|GAA89935.1 aluc|OJZ81731.1 anid|CBF77838.1 anig|EHA22481.1 asyd|OJJ55335.1 ater|EAU30618.1 atub|OJI84302.1 aver|OJJ06892.1 awen|OJJ41285.1 azon|OJJ48768.1

Group6049: acar|OOF97439.1 acla|EAW13029.1 afis|EAW17638.1 afum|EAL91453.1 agla|OJJ81258.1 anid|CBF90127.1 aory|EIT82094.1 asyd|OJJ62017.1 ater|EAU37074.1 aver|OJI96108.1 azon|OJJ50907.1 afla|EED50525.1 akaw|GAA83335.1 aluc|OJZ83831.1 anig|EHA18974.1 atub|OJI90198.1

Group6050: acla|EAW15285.1 afum|EAL85363.2 agla|OJJ84627.1 acar|OOF90548.1 afis|EAW20280.1 afla|EED57098.1 akaw|GAA90407.1 aluc|OJZ85821.1 anid|CBF85204.1 asyd|OJJ63028.1 atub|OJI79608.1 aver|OJI97940.1 awen|OJJ31242.1 azon|OJJ50674.1 acla|EAW15275.1 afis|EAW20245.1

Group6051: abra|OJJ68843.1 acar|OOF90546.1 acla|EAW11068.1 afis|EAW20806.1 afla|EED47530.1 afum|EAL84822.1 akaw|GAA90405.1 aluc|OJZ85823.1 anid|CBF85130.1 anig|EHA24738.1 aory|EIT74519.1 asyd|OJJ63043.1 ater|EAU37215.1 atub|OJI79610.1 aver|OJI97921.1 azon|OJJ44328.1

Group6052: abra|OJJ68845.1 acar|OOF90544.1 acla|EAW15280.1 afis|EAW20249.1 afla|EED57096.1 afum|EAL85367.1 akaw|GAA90403.1 aluc|OJZ85825.1 anid|CBF85206.1 anig|EHA24736.1 aory|EIT82292.1 asyd|OJJ63030.1 ater|EAU32040.1 atub|OJI79613.1 aver|OJI97938.1 azon|OJJ50397.1

Group6053: abra|OJJ68847.1 acla|EAW15274.1 afis|EAW20244.1 afla|EED57100.1 afum|EAL85361.1 agla|OJJ84629.1 aluc|OJZ85827.1 anid|CBF85138.1 anig|EHA24734.1 aory|EIT82275.1 asyd|OJJ63035.1 ater|EAU30255.1 atub|OJI79615.1 aver|OJI97932.1 awen|OJJ31241.1 akaw|GAA92649.1

Group6054: abra|OJJ68925.1 acar|OOF91511.1 acla|EAW14249.1 afis|EAW19031.1 afla|EED50384.1 afum|EAL93760.2 agla|OJJ84591.1 akaw|GAA90323.1 aluc|OJZ85910.1 anid|CBF79594.1 anig|EHA24682.1 asyd|OJJ57984.1 ater|EAU32070.1 atub|OJI79699.1 aver|OJJ04417.1 awen|OJJ31460.1

Group6055: abra|OJJ68928.1 afis|EAW16673.1 afum|EAL89278.1 akaw|GAA90319.1 aluc|OJZ85914.1 anig|EHA24678.1 atub|OJI79704.1 afla|EED48203.1 acar|OOF90674.1 agla|OJJ79205.1 anid|CBF83970.1 aory|EIT72893.1 asyd|OJJ61414.1 aver|OJJ00750.1 ater|EAU34353.1 awen|OJJ31870.1

Group6056: abra|OJJ68986.1 acar|OOF91557.1 afis|EAW19076.1 afla|EED54441.1 agla|OJJ83213.1 akaw|GAA90507.1 aluc|OJZ85972.1 anig|EHA24633.1 aory|EIT81163.1 asyd|OJJ58661.1 ater|EAU32124.1 atub|OJI79760.1 aver|OJJ01267.1 awen|OJJ32470.1 azon|OJJ46236.1 acar|OOF91848.1

Group6057: abra|OJJ69019.1 acar|OOF98449.1 afis|EAW20276.1 afla|EED53202.1 afum|EAL85410.1 agla|OJJ87502.1 akaw|GAA90536.1 aluc|OJZ86005.1 anid|CBF78245.1 anig|EHA24605.1 aory|EIT75242.1 asyd|OJJ56192.1 ater|EAU31765.1 atub|OJI79794.1 aver|OJJ05121.1 awen|OJJ35878.1

Group6058: acla|EAW14513.1 azon|OJJ44015.1 acar|OOF98345.1 afis|EAW19231.1 afla|EED47657.1 afum|EAL93960.1 akaw|GAA88443.1 aluc|OJZ88331.1 anid|CBF78616.1 anig|EHA18066.1 aory|EIT80280.1 asyd|OJJ65166.1 ater|EAU37232.1 atub|OJI90926.1 aver|OJI99318.1 afis|EAW25599.1

Group6059: abra|OJJ69042.1 acar|OOF89866.1 acla|EAW11011.1 afis|EAW16707.1 afla|EED53056.1 afum|EAL89298.1 akaw|GAA90571.1 aluc|OJZ86027.1 anid|CBF85134.1 anig|EHA24585.1 aory|EIT74798.1 asyd|OJJ63344.1 ater|EAU32201.1 atub|OJI79816.1 aver|OJI97617.1 azon|OJJ45979.1

Group6060: abra|OJJ69085.1 acla|EAW08747.1 afis|EAW15689.1 afla|EED51364.1 afum|EAL85659.1 akaw|GAA90614.1 aluc|OJZ86073.1 anid|CBF86339.1 anig|EHA24542.1 aory|EIT80722.1 asyd|OJJ64986.1 ater|EAU32465.1 atub|OJI79870.1 aver|OJI99106.1 awen|OJJ38380.1 azon|OJJ43864.1

Group6061: abra|OJJ69101.1 acar|OOF91195.1 acla|EAW08731.1 afis|EAW15705.1 afla|EED51347.1 afum|EAL85642.1 akaw|GAA90627.1 aluc|OJZ86090.1 anid|CBF71104.1 anig|EHA24527.1 asyd|OJJ52709.1 ater|EAU32440.1 atub|OJI79886.1 aver|OJJ04738.1 azon|OJJ43843.1 agla|OJJ81853.1

Group6062: abra|OJJ69112.1 acla|EAW08720.1 afum|EAL85620.1 agla|OJJ81866.1 akaw|GAA90637.1 aluc|OJZ86101.1 anid|CBF71087.1 anig|EHA24518.1 asyd|OJJ52699.1 aver|OJJ04727.1 awen|OJJ38411.1 azon|OJJ43831.1 acar|OOF91186.1 atub|OJI79896.1 afis|EAW15727.1 afla|EED51336.1

Group6063: abra|OJJ69117.1 acar|OOF91189.1 acla|EAW08715.1 afis|EAW15723.1 afla|EED51331.1 afum|EAL85624.1 agla|OJJ81871.1 akaw|GAA90642.1 aluc|OJZ86106.1 anig|EHA24514.1 asyd|OJJ52694.1 ater|EAU32426.1 atub|OJI79901.1 aver|OJJ04722.1 awen|OJJ38416.1 azon|OJJ43826.1

Group6064: abra|OJJ69129.1 acar|OOF91171.1 acla|EAW08703.1 afis|EAW15711.1 afla|EED51319.1 akaw|GAA90654.1 aluc|OJZ86118.1 anid|CBF71055.1 anig|EHA24503.1 aory|EIT81518.1 asyd|OJJ52677.1 ater|EAU32415.1 atub|OJI79914.1 aver|OJJ04703.1 awen|OJJ38429.1 azon|OJJ43814.1

Group6065: abra|OJJ69139.1 acla|EAW08695.1 afis|EAW15740.1 afla|EED51312.1 afum|EAL85608.1 agla|OJJ81901.1 akaw|GAA86318.1 aluc|OJZ86126.1 anig|EHA24495.1 aory|EIT81512.1 atub|OJI79922.1 awen|OJJ38439.1 azon|OJJ43806.1 anid|CBF71043.1 asyd|OJJ52670.1 aver|OJJ04696.1

Group6066: abra|OJJ69227.1 acar|OOF91093.1 acla|EAW08621.1 afis|EAW15812.1 afla|EED51234.1 afum|EAL85539.1 agla|OJJ81978.1 akaw|GAA92235.1 aluc|OJZ86207.1 anid|CBF75275.1 aory|EIT83185.1 asyd|OJJ55913.1 ater|EAU33442.1 atub|OJI80001.1 aver|OJJ04853.1 awen|OJJ38525.1

Group6067: abra|OJJ69258.1 acar|OOF91062.1 acla|EAW08591.1 afis|EAW15843.1 afla|EED51206.1 afum|EAL85508.1 agla|OJJ84914.1 akaw|GAA92264.1 aluc|OJZ86238.1 anid|CBF70841.1 asyd|OJJ52566.1 ater|EAU33392.1 atub|OJI80030.1 aver|OJJ04595.1 awen|OJJ38557.1 azon|OJJ43709.1

Group6068: abra|OJJ69270.1 acar|OOF94450.1 acla|EAW14660.1 afis|EAW24191.1 afum|EAL85864.1 agla|OJJ79688.1 akaw|GAA88145.1 aluc|OJZ82098.1 anid|CBF74308.1 anig|EHA28631.1 asyd|OJJ54007.1 ater|EAU31142.1 atub|OJI88339.1 aver|OJJ08102.1 awen|OJJ33108.1 azon|OJJ44839.1

Group6069: abra|OJJ69287.1 acar|OOF94435.1 acla|EAW14676.1 afis|EAW24207.1 afla|EED48064.1 afum|EAL85877.1 agla|OJJ79670.1 akaw|GAA88159.1 aluc|OJZ82116.1 anig|EHA28615.1 aory|EIT83334.1 asyd|OJJ53988.1 atub|OJI88321.1 aver|OJJ08086.1 awen|OJJ33091.1 azon|OJJ44857.1

Group6070: abra|OJJ69290.1 acar|OOF94432.1 acla|EAW14679.1 afis|EAW24210.1 afla|EED48061.1 agla|OJJ79656.1 akaw|GAA88162.1 aluc|OJZ82120.1 anid|CBF74265.1 anig|EHA28612.1 asyd|OJJ53985.1 ater|EAU31160.1 atub|OJI88318.1 aver|OJJ08083.1 awen|OJJ33086.1 azon|OJJ44860.1

Group6071: abra|OJJ69323.1 acar|OOF94405.1 acla|EAW14721.1 afis|EAW24257.1 afum|EAL85925.1 agla|OJJ79616.1 akaw|GAA88192.1 aluc|OJZ82153.1 anid|CBF74191.1 anig|EHA28578.1 asyd|OJJ53950.1 ater|EAU31203.1 atub|OJI88283.1 aver|OJJ08046.1 awen|OJJ33043.1 azon|OJJ48476.1

Group6072: abra|OJJ69327.1 acar|OOF94401.1 acla|EAW14713.1 afis|EAW24248.1 afla|EED48025.1 afum|EAL85916.1 agla|OJJ79625.1 akaw|GAA88198.1 aluc|OJZ82159.1 anig|EHA28573.1 asyd|OJJ53954.1 ater|EAU31189.1 atub|OJI88278.1 aver|OJJ08050.1 awen|OJJ33060.1 azon|OJJ48468.1

Group6073: abra|OJJ69344.1 acar|OOF94384.1 acla|EAW14702.1 afis|EAW24233.1 afla|EED48038.1 afum|EAL85902.1 agla|OJJ79638.1 akaw|GAA88213.1 aluc|OJZ82176.1 anig|EHA28558.1 aory|EIT83330.1 asyd|OJJ53963.1 ater|EAU31181.1 atub|OJI88261.1 aver|OJJ08059.1 azon|OJJ48459.1

Group6074: abra|OJJ69354.1 acar|OOF94371.1 acla|EAW14744.1 afis|EAW24275.1 afla|EED48001.1 afum|EAL85943.1 agla|OJJ79586.1 akaw|GAA88223.1 anig|EHA28549.1 aory|EIT82906.1 asyd|OJJ53926.1 ater|EAU31220.1 atub|OJI88250.1 aver|OJJ08025.1 awen|OJJ33022.1 azon|OJJ48495.1

Group6075: abra|OJJ69388.1 acar|OOF94338.1 acla|EAW14775.1 afis|EAW24306.1 afla|EED47964.1 afum|EAL85974.1 agla|OJJ83174.1 akaw|GAA88256.1 aluc|OJZ82222.1 anid|CBF74097.1 anig|EHA28518.1 asyd|OJJ53895.1 ater|EAU31259.1 atub|OJI88213.1 aver|OJJ07992.1 awen|OJJ32986.1

Group6076: abra|OJJ69431.1 acar|OOF94305.1 acla|EAW14807.1 afis|EAW24336.1 afla|EED47928.1 afum|EAL86006.1 akaw|GAA88296.1 aluc|OJZ82268.1 anid|CBF74032.1 anig|EHA28475.1 aory|EIT79835.1 asyd|OJJ53850.1 ater|EAU30412.1 atub|OJI88170.1 aver|OJJ07951.1 azon|OJJ48962.1

Group6077: abra|OJJ69451.1 acar|OOF94290.1 acla|EAW14815.1 afis|EAW24345.1 afla|EED47918.1 agla|OJJ83208.1 akaw|GAA88368.1 aluc|OJZ82287.1 anid|CBF74021.1 anig|EHA28461.1 aory|EIT83361.1 asyd|OJJ53842.1 ater|EAU30401.1 atub|OJI88152.1 aver|OJJ07945.1 awen|OJJ32954.1

Group6078: abra|OJJ69455.1 acar|OOF94286.1 acla|EAW07408.1 afis|EAW22434.1 afla|EED53608.1 afum|EAL90757.1 akaw|GAA88357.1 aluc|OJZ82298.1 anig|EHA28457.1 aory|EIT75075.1 asyd|OJJ57319.1 ater|EAU34153.1 atub|OJI88141.1 aver|OJJ05842.1 awen|OJJ36494.1 azon|OJJ44369.1

Group6079: abra|OJJ69500.1 aluc|OJZ82340.1 anig|EHA28420.1 atub|OJI88100.1 awen|OJJ30574.1 azon|OJJ45335.1 abra|OJJ78072.1 acar|OOF91543.1 acla|EAW15328.1 afum|EAL85362.2 aory|EIT73816.1 asyd|OJJ63986.1 aver|OJI96967.1 awen|OJJ40088.1 azon|OJJ46957.1 afis|EAW21468.1

Group6080: abra|OJJ69531.1 acar|OOF92664.1 afis|EAW18043.1 afla|EED51503.1 akaw|GAA88382.1 aluc|OJZ82370.1 anid|CBF75024.1 anig|EHA28394.1 asyd|OJJ60073.1 ater|EAU31283.1 atub|OJI88072.1 aver|OJJ04188.1 awen|OJJ41491.1 azon|OJJ48222.1 afum|EAL91854.1 aory|EIT80430.1

Group6081: akaw|GAA82187.1 abra|OJJ71154.1 acar|OOF93867.1 acla|EAW11932.1 afum|EAL92066.1 agla|OJJ88460.1 aluc|OJZ84300.1 asyd|OJJ52830.1 ater|EAU33249.1 atub|OJI89769.1 ater|EAU31455.1 acar|OOF98994.1 afis|EAW21129.1 aluc|OJZ91973.1 atub|OJI86412.1 akaw|GAA87137.1

Group6082: abra|OJJ69576.1 acar|OOF91579.1 acla|EAW14393.1 afis|EAW19101.1 afum|EAL93833.1 agla|OJJ84501.1 akaw|GAA91573.1 aluc|OJZ82394.1 anid|CBF79700.1 anig|EHA28371.1 aory|EIT75927.1 ater|EAU32147.1 atub|OJI88048.1 awen|OJJ32494.1 azon|OJJ46037.1 afla|EED54334.1

Group6083: abra|OJJ69597.1 acar|OOF91562.1 acla|EAW14201.1 afis|EAW19081.1 afla|EED54313.1 afum|EAL93810.1 akaw|GAA91589.1 aluc|OJZ82412.1 anid|CBF75163.1 anig|EHA28355.1 aory|EIT76019.1 asyd|OJJ59960.1 ater|EAU32128.1 atub|OJI88029.1 aver|OJJ02330.1 awen|OJJ32476.1

Group6084: abra|OJJ69674.1 afla|EED49568.1 anid|CBF90241.1 anig|EHA28292.1 aory|EIT82591.1 abra|OJJ69681.1 acar|OOF93865.1 afis|EAW19351.1 afum|EAL94070.1 akaw|GAA88806.1 aluc|OJZ82489.1 anig|EHA28285.1 ater|EAU37356.1 atub|OJI87943.1 awen|OJJ41942.1 atub|OJI85778.1

Group6085: abra|OJJ69689.1 acar|OOF93402.1 acla|EAW11116.1 afla|EED56732.1 agla|OJJ88353.1 akaw|GAA88813.1 aluc|OJZ82496.1 anid|CBF69500.1 anig|EHA28276.1 aory|EIT73912.1 asyd|OJJ59234.1 ater|EAU33166.1 atub|OJI87935.1 aver|OJJ01844.1 awen|OJJ29946.1 azon|OJJ50326.1

Group6086: abra|OJJ69739.1 acar|OOF94181.1 acla|EAW09980.1 afis|EAW21110.1 afla|EED51761.1 afum|EAL92132.1 akaw|GAA90192.1 aluc|OJZ83157.1 anid|CBF87039.1 anig|EHA28222.1 aory|EIT80519.1 asyd|OJJ60449.1 ater|EAU36788.1 atub|OJI87780.1 aver|OJI99767.1 azon|OJJ44437.1

Group6087: abra|OJJ69810.1 acar|OOF93567.1 afis|EAW25601.1 afla|EED57247.1 afum|EBA27172.1 akaw|GAA87850.1 aluc|OJZ85490.1 anid|CBF81957.1 anig|EHA25026.1 aory|EIT74113.1 asyd|OJJ54537.1 ater|EAU34250.1 atub|OJI80075.1 aver|OJJ07644.1 awen|OJJ35936.1 azon|OJJ43522.1

Group6088: abra|OJJ69818.1 acar|OOF93345.1 acla|EAW12083.1 afis|EAW19726.1 afla|EED51052.1 agla|OJJ78576.1 akaw|GAA87842.1 aluc|OJZ85478.1 anid|CBF78377.1 anig|EHA25019.1 aory|EIT76968.1 asyd|OJJ57329.1 ater|EAU33553.1 atub|OJI80085.1 aver|OJJ05832.1 awen|OJJ40132.1

Group6089: abra|OJJ69861.1 afis|EAW15433.1 afla|EED45625.1 afum|EAL84211.1 agla|OJJ86265.1 akaw|GAA87795.1 aluc|OJZ85430.1 anig|EHA25609.1 aory|EIT75726.1 asyd|OJJ64001.1 ater|EAU31307.1 atub|OJI80145.1 aver|OJI98090.1 awen|OJJ32639.1 aluc|OJZ83144.1 anig|EHA23287.1

Group6090: abra|OJJ69871.1 acar|OOF93385.1 acla|EAW08953.1 afis|EAW20571.1 afla|EED54199.1 afum|EBA27182.1 agla|OJJ80145.1 akaw|GAA87785.1 aluc|OJZ85420.1 anid|CBF78263.1 anig|EHA25599.1 asyd|OJJ56066.1 ater|EAU32669.1 atub|OJI80155.1 aver|OJJ05009.1 awen|OJJ38089.1

Group6091: abra|OJJ69922.1 acar|OOF93434.1 acla|EAW08907.1 afis|EAW15489.1 afla|EED49003.1 afum|EAL85834.1 akaw|GAA87734.1 aluc|OJZ85366.1 anig|EHA25548.1 asyd|OJJ56022.1 ater|EAU32631.1 atub|OJI80205.1 aver|OJJ04969.1 azon|OJJ43142.1 awen|OJJ38145.1 agla|OJJ80166.1

Group6092: abra|OJJ69936.1 acar|OOF93445.1 acla|EAW08889.1 afis|EAW15500.1 afum|EAL85823.1 agla|OJJ80178.1 akaw|GAA87723.1 aluc|OJZ85351.1 anig|EHA25538.1 aory|EIT82557.1 asyd|OJJ56011.1 ater|EAU32619.1 atub|OJI80219.1 aver|OJJ04956.1 awen|OJJ38159.1 azon|OJJ43130.1

Group6093: abra|OJJ69942.1 acar|OOF93452.1 acla|EAW08880.1 afis|EAW15515.1 afum|EAL85816.1 agla|OJJ80186.1 akaw|GAA87717.1 aluc|OJZ85344.1 anig|EHA25532.1 aory|EIT82510.1 asyd|OJJ56004.1 ater|EAU32609.1 atub|OJI80226.1 aver|OJJ04949.1 awen|OJJ38176.1 azon|OJJ43121.1

Group6094: abra|OJJ69950.1 acla|EAW08874.1 afis|EAW15522.1 afla|EED49028.1 afum|EBA27244.1 agla|OJJ85224.1 akaw|GAA87710.1 aluc|OJZ85336.1 anig|EHA25525.1 aory|EIT82532.1 asyd|OJJ55995.1 ater|EAU32603.1 atub|OJI80234.1 aver|OJJ04943.1 awen|OJJ38182.1 azon|OJJ43111.1

Group6095: abra|OJJ69954.1 acar|OOF93464.1 acla|EAW08868.1 afis|EAW15526.1 afla|EED49037.1 afum|EAL85802.1 agla|OJJ85220.1 akaw|GAA87705.1 aluc|OJZ85328.1 anid|CBF78213.1 aory|EIT82546.1 asyd|OJJ55990.1 atub|OJI80244.1 aver|OJJ04938.1 awen|OJJ38190.1 azon|OJJ43105.1

Group6096: abra|OJJ69956.1 acar|OOF93466.1 acla|EAW08866.1 afis|EAW15528.1 afla|EED49039.1 afum|EAL85800.1 agla|OJJ85218.1 aluc|OJZ85326.1 anid|CBF78210.1 anig|EHA25516.1 aory|EIT82502.1 asyd|OJJ55988.1 atub|OJI80246.1 aver|OJJ04936.1 awen|OJJ38192.1 azon|OJJ43102.1

Group6097: abra|OJJ69965.1 acar|OOF93473.1 acla|EAW08837.1 afis|EAW15563.1 afum|EAL85769.1 agla|OJJ85188.1 akaw|GAA87694.1 aluc|OJZ85318.1 anid|CBF78122.1 aory|EIT82485.1 asyd|OJJ55903.1 ater|EAU32569.1 atub|OJI80255.1 aver|OJJ04848.1 awen|OJJ38223.1 azon|OJJ43072.1

Group6098: abra|OJJ69977.1 acla|EAW08845.1 afis|EAW15555.1 afla|EED49059.1 afum|EAL85777.1 agla|OJJ85199.1 akaw|GAA87684.1 aluc|OJZ85303.1 anid|CBF78170.1 anig|EHA25500.1 aory|EIT82563.1 asyd|OJJ55966.1 ater|EAU32578.1 atub|OJI80267.1 aver|OJJ04913.1 awen|OJJ38213.1

Group6099: abra|OJJ69992.1 acar|OOF93498.1 acla|EAW08828.1 afis|EAW15569.1 afla|EED49079.1 afum|EAL85764.1 agla|OJJ85183.1 akaw|GAA87669.1 aluc|OJZ85287.1 anig|EHA25484.1 asyd|OJJ55898.1 ater|EAU32561.1 atub|OJI80283.1 aver|OJJ04843.1 awen|OJJ38228.1 azon|OJJ43067.1

Group6100: abra|OJJ69993.1 acar|OOF93499.1 acla|EAW08826.1 afis|EAW15571.1 afla|EED49080.1 afum|EAL85762.1 agla|OJJ85181.1 akaw|GAA87668.1 aluc|OJZ85286.1 anid|CBF74534.1 anig|EHA25483.1 aory|EIT82491.1 ater|EAU32560.1 atub|OJI80284.1 awen|OJJ38230.1 azon|OJJ43066.1

Group6101: abra|OJJ69994.1 acar|OOF93501.1 acla|EAW08825.1 afis|EAW15572.1 afla|EED49081.1 agla|OJJ85180.1 akaw|GAA87667.1 aluc|OJZ85285.1 anid|CBF78107.1 anig|EHA25482.1 aory|EIT82508.1 asyd|OJJ55896.1 ater|EAU32559.1 atub|OJI80285.1 aver|OJJ04841.1 awen|OJJ38231.1

Group6102: abra|OJJ69996.1 acar|OOF93503.1 acla|EAW08822.1 afis|EAW15575.1 afla|EED49084.1 afum|EAL85759.1 agla|OJJ85177.1 akaw|GAA87665.1 aluc|OJZ85283.1 anig|EHA25480.1 aory|EIT82540.1 asyd|OJJ55893.1 ater|EAU32556.1 atub|OJI80287.1 aver|OJJ04838.1 awen|OJJ38235.1

Group6103: abra|OJJ69999.1 acar|OOF93506.1 acla|EAW08820.1 afis|EAW15577.1 afla|EED49086.1 afum|EAL85757.1 agla|OJJ85175.1 akaw|GAA87662.1 aluc|OJZ85280.1 anig|EHA25477.1 aory|EIT82487.1 asyd|OJJ55891.1 ater|EAU32554.1 atub|OJI80290.1 aver|OJJ04836.1 awen|OJJ38238.1

Group6104: abra|OJJ70000.1 acla|EAW08819.1 afis|EAW15578.1 afla|EED49094.1 afum|EAL85756.1 agla|OJJ85174.1 akaw|GAA87661.1 aluc|OJZ85279.1 anid|CBF78102.1 anig|EHA25476.1 aory|EIT82512.1 asyd|OJJ55890.1 atub|OJI80291.1 aver|OJJ04835.1 awen|OJJ38240.1 azon|OJJ43059.1

Group6105: abra|OJJ70017.1 acar|OOF93523.1 acla|EAW08806.1 afis|EAW15590.1 afla|EED50413.1 afum|EAL85742.1 agla|OJJ85158.1 akaw|GAA87644.1 aluc|OJZ85259.1 anid|CBF78074.1 anig|EHA25460.1 asyd|OJJ55875.1 atub|OJI80309.1 aver|OJJ04820.1 awen|OJJ38263.1 azon|OJJ43022.1

Group6106: abra|OJJ70019.1 acar|OOF93525.1 afis|EAW15592.1 afla|EED50415.1 afum|EAL85740.1 akaw|GAA87642.1 aluc|OJZ85257.1 anid|CBF78078.1 anig|EHA25458.1 aory|EIT82135.1 asyd|OJJ55877.1 ater|EAU32536.1 atub|OJI80311.1 aver|OJJ04822.1 awen|OJJ38265.1 azon|OJJ43913.1

Group6107: abra|OJJ70033.1 afis|EAW17103.1 anig|EHA18718.1 atub|OJI79322.1 awen|OJJ29677.1 abra|OJJ72237.1 acar|OOF96859.1 afla|EED54714.1 akaw|GAA84702.1 aluc|OJZ86985.1 anid|CBF76384.1 anig|EHA21662.1 asyd|OJJ61069.1 ater|EAU29940.1 atub|OJI83534.1 aver|OJJ00385.1

Group6108: abra|OJJ70131.1 acar|OOF93325.1 acla|EAW09068.1 afis|EAW25640.1 afla|EED55189.1 afum|EAL84327.2 agla|OJJ80957.1 akaw|GAA86633.1 aluc|OJZ85190.1 anid|CBF86061.1 anig|EHA25404.1 aory|EIT83514.1 ater|EAU32713.1 atub|OJI80407.1 awen|OJJ36096.1 agla|OJJ78893.1

Group6109: abra|OJJ70178.1 acar|OOF91400.1 acla|EAW13479.1 afis|EAW20586.1 afum|EAL84479.1 agla|OJJ80828.1 akaw|GAA86595.1 aluc|OJZ85142.1 anig|EHA25358.1 asyd|OJJ55630.1 ater|EAU37093.1 atub|OJI80450.1 aver|OJJ03121.1 awen|OJJ33297.1 anig|EHA27684.1 aver|OJJ06707.1

Group6110: abra|OJJ70197.1 acar|OOF90057.1 acla|EAW14580.1 afis|EAW19314.1 afla|EED47568.1 afum|EAL94030.1 akaw|GAA86579.1 aluc|OJZ85130.1 anid|CBF78805.1 anig|EHA25349.1 aory|EIT74569.1 asyd|OJJ65257.1 ater|EAU29461.1 atub|OJI80463.1 aver|OJI99408.1 awen|OJJ35850.1

Group6111: abra|OJJ70293.1 acar|OOF91747.1 afis|EAW22073.1 afum|EAL91097.1 akaw|GAA92855.1 aluc|OJZ82558.1 anid|CBF82784.1 anig|EHA22520.1 aory|EIT73943.1 asyd|OJJ57122.1 ater|EAU30151.1 atub|OJI88800.1 aver|OJJ02365.1 awen|OJJ35472.1 azon|OJJ47361.1 awen|OJJ33482.1

Group6112: abra|OJJ70368.1 acar|OOF91902.1 afis|EAW16892.1 afla|EED48420.1 afum|EAL86430.2 agla|OJJ79761.1 akaw|GAA91983.1 aluc|OJZ82630.1 anig|EHA27447.1 aory|EIT83082.1 atub|OJI88863.1 awen|OJJ31356.1 azon|OJJ49611.1 afla|EED51051.1 aory|EIT77047.1 awen|OJJ35872.1

Group6113: abra|OJJ70384.1 acar|OOF91887.1 acla|EAW12345.1 afis|EAW16882.1 afum|EAL86418.1 agla|OJJ79752.1 akaw|GAA91973.1 aluc|OJZ82642.1 anid|CBF80675.1 anig|EHA27428.1 asyd|OJJ54588.1 ater|EAU30233.1 atub|OJI88875.1 aver|OJJ07566.1 awen|OJJ29769.1 azon|OJJ50378.1

Group6114: abra|OJJ70385.1 acar|OOF91886.1 acla|EAW12343.1 afis|EAW16879.1 afla|EED47344.1 afum|EAL86416.1 akaw|GAA91972.1 aluc|OJZ82643.1 anid|CBF80671.1 anig|EHA27427.1 aory|EIT82869.1 asyd|OJJ54586.1 ater|EAU30234.1 atub|OJI88876.1 aver|OJJ07568.1 awen|OJJ29771.1

Group6115: abra|OJJ70391.1 acar|OOF90062.1 acla|EAW12389.1 afis|EAW16836.1 afla|EED47326.1 afum|EAL86384.1 agla|OJJ89425.1 akaw|GAA91968.1 aluc|OJZ82648.1 anid|CBF74685.1 anig|EHA27424.1 asyd|OJJ60209.1 ater|EAU30243.1 atub|OJI88880.1 aver|OJJ04059.1 awen|OJJ35684.1

Group6116: abra|OJJ70472.1 acla|EAW13042.1 afis|EAW17625.1 afum|EAL91439.1 agla|OJJ81278.1 akaw|GAA83321.1 aluc|OJZ83816.1 anid|CBF90152.1 anig|EHA18961.1 aory|EIT78528.1 asyd|OJJ62000.1 ater|EAU37059.1 atub|OJI90183.1 aver|OJI96092.1 awen|OJJ37219.1 azon|OJJ50922.1

Group6117: abra|OJJ70506.1 afis|EAW19833.1 afla|EED46252.1 agla|OJJ88103.1 akaw|GAA83355.1 aluc|OJZ83851.1 anig|EHA18993.1 aory|EIT76504.1 asyd|OJJ56258.1 atub|OJI90217.1 aver|OJJ08506.1 awen|OJJ32240.1 afla|EED57615.1 afum|EAL85059.1 aory|EIT82986.1 ater|EAU31581.1

Group6118: abra|OJJ70520.1 acar|OOF97408.1 acla|EAW08155.1 afis|EAW23184.1 afla|EED47061.1 afum|EAL88299.1 akaw|GAA83367.1 aluc|OJZ83865.1 anid|CBF74700.1 anig|EHA19006.1 aory|EIT72501.1 asyd|OJJ60216.1 atub|OJI90233.1 aver|OJJ04052.1 awen|OJJ36586.1 azon|OJJ47621.1

Group6119: abra|OJJ70538.1 acar|OOF97395.1 acla|EAW08150.1 afis|EAW23189.1 afla|EED47068.1 afum|EAL88294.2 agla|OJJ86364.1 akaw|GAA83383.1 aluc|OJZ83882.1 anid|CBF74525.1 anig|EHA19020.1 asyd|OJJ57735.1 atub|OJI90250.1 aver|OJJ05432.1 awen|OJJ36591.1 azon|OJJ47615.1

Group6120: abra|OJJ70542.1 acar|OOF97391.1 acla|EAW08136.1 afis|EAW23172.1 afla|EED46947.1 afum|EAL88311.2 agla|OJJ89684.1 akaw|GAA83388.1 aluc|OJZ83887.1 anid|CBF74493.1 anig|EHA19024.1 aory|EIT72523.1 asyd|OJJ57717.1 atub|OJI90254.1 aver|OJJ05450.1 awen|OJJ36605.1

Group6121: abra|OJJ70573.1 acar|OOF97148.1 acla|EAW08372.1 afis|EAW23401.1 afla|EED47008.1 afum|EAL88069.1 akaw|GAA83417.1 aluc|OJZ83917.1 anid|CBF74797.1 anig|EHA19050.1 aory|EIT72568.1 asyd|OJJ60154.1 atub|OJI90287.1 aver|OJJ04110.1 awen|OJJ39477.1 azon|OJJ47680.1

Group6122: abra|OJJ70588.1 acar|OOF97132.1 acla|EAW08193.1 afis|EAW23227.1 afla|EED47023.1 afum|EAL88256.1 agla|OJJ86384.1 akaw|GAA83431.1 aluc|OJZ83932.1 anid|CBF74769.1 anig|EHA19064.1 asyd|OJJ60168.1 atub|OJI90304.1 aver|OJJ04096.1 awen|OJJ39462.1 azon|OJJ47662.1

Group6123: abra|OJJ70624.1 acla|EAW13006.1 afis|EAW17662.1 afla|EED50496.1 afum|EAL91476.2 agla|OJJ79531.1 akaw|GAA89962.1 aluc|OJZ83969.1 anid|CBF90058.1 anig|EHA19098.1 aory|EIT82078.1 ater|EAU31991.1 atub|OJI90342.1 aver|OJI96157.1 awen|OJJ37180.1 azon|OJJ50882.1

Group6124: abra|OJJ70645.1 acar|OOF97076.1 acla|EAW08115.1 afis|EAW23136.1 afla|EED48499.1 afum|EAL88345.2 agla|OJJ89664.1 akaw|GAA89981.1 aluc|OJZ83995.1 anid|CBF74452.1 asyd|OJJ57739.1 ater|EAU36281.1 atub|OJI90364.1 aver|OJJ05428.1 awen|OJJ36632.1 azon|OJJ47583.1

Group6125: abra|OJJ70649.1 acar|OOF97071.1 acla|EAW08110.1 afis|EAW23141.1 afum|EAL88340.1 agla|OJJ89660.1 akaw|GAA89985.1 aluc|OJZ83999.1 anid|CBF74442.1 aory|EIT73199.1 asyd|OJJ57743.1 ater|EAU36276.1 atub|OJI90368.1 aver|OJJ05424.1 awen|OJJ36637.1 azon|OJJ47579.1

Group6126: abra|OJJ70651.1 acar|OOF97069.1 acla|EAW08108.1 afis|EAW23143.1 afla|EED48506.1 afum|EAL88338.1 akaw|GAA89987.1 aluc|OJZ84001.1 anid|CBF74438.1 anig|EHA19123.1 aory|EIT73205.1 asyd|OJJ57745.1 ater|EAU36268.1 atub|OJI90370.1 aver|OJJ05422.1 azon|OJJ47577.1

Group6127: abra|OJJ70682.1 acar|OOF97045.1 acla|EAW13290.1 afis|EAW17319.1 afum|EAL91155.1 agla|OJJ89622.1 akaw|GAA90014.1 aluc|OJZ84030.1 anid|CBF86689.1 anig|EHA19151.1 asyd|OJJ64751.1 ater|EAU36247.1 atub|OJI90402.1 aver|OJI98852.1 awen|OJJ36663.1 acla|EAW08082.1

Group6128: abra|OJJ70683.1 acar|OOF97044.1 acla|EAW08079.1 afis|EAW23118.1 afum|EAL88362.1 agla|OJJ89623.1 akaw|GAA90015.1 aluc|OJZ84031.1 anid|CBF74395.1 anig|EHA19152.1 asyd|OJJ57768.1 ater|EAU36246.1 atub|OJI90403.1 aver|OJJ05398.1 awen|OJJ36664.1 azon|OJJ47535.1

Group6129: abra|OJJ70707.1 acar|OOF97030.1 acla|EAW08057.1 afis|EAW23093.1 afla|EED48555.1 afum|EAL88385.1 agla|OJJ89605.1 akaw|GAA90038.1 aluc|OJZ84054.1 anig|EHA19174.1 asyd|OJJ59375.1 ater|EAU36217.1 atub|OJI90425.1 aver|OJJ02898.1 awen|OJJ36689.1 azon|OJJ47508.1

Group6130: abra|OJJ70709.1 acar|OOF97027.1 acla|EAW08055.1 afis|EAW23091.1 afla|EED48557.1 afum|EAL88388.1 agla|OJJ89603.1 akaw|GAA90040.1 aluc|OJZ84056.1 anid|CBF81347.1 asyd|OJJ59373.1 ater|EAU36214.1 atub|OJI90427.1 aver|OJJ02901.1 awen|OJJ36691.1 azon|OJJ47505.1

Group6131: abra|OJJ70759.1 acar|OOF96985.1 acla|EAW11162.1 afis|EAW15893.1 afla|EED56478.1 afum|EAL88528.1 akaw|GAA90079.1 aluc|OJZ84104.1 anid|CBF81296.1 anig|EHA19216.1 aory|EIT72872.1 asyd|OJJ59347.1 ater|EAU36172.1 atub|OJI90478.1 aver|OJJ02927.1 awen|OJJ36738.1

Group6132: abra|OJJ70808.1 acar|OOF98294.1 acla|EAW10417.1 afis|EAW25152.1 afla|EED55281.1 afum|EAL89827.1 agla|OJJ89002.1 akaw|GAA91688.1 aluc|OJZ87706.1 anid|CBF89561.1 aory|EIT83566.1 ater|EAU39431.1 atub|OJI80937.1 aver|OJI96448.1 awen|OJJ40781.1 azon|OJJ46871.1

Group6133: abra|OJJ70811.1 acar|OOF98290.1 acla|EAW10420.1 afis|EAW25149.1 afla|EED55287.1 afum|EAL89824.1 agla|OJJ89006.1 akaw|GAA91691.1 aluc|OJZ87703.1 anid|CBF85672.1 anig|EHA20886.1 asyd|OJJ64249.1 atub|OJI80940.1 aver|OJI98340.1 awen|OJJ40777.1 azon|OJJ46874.1

Group6134: abra|OJJ70812.1 acar|OOF98286.1 acla|EAW10421.1 afis|EAW25148.1 afla|EED55288.1 afum|EAL89823.1 agla|OJJ89007.1 akaw|GAA91692.1 aluc|OJZ87702.1 anid|CBF85673.1 asyd|OJJ64251.1 ater|EAU39420.1 atub|OJI80941.1 aver|OJI98341.1 awen|OJJ40773.1 azon|OJJ46875.1

Group6135: abra|OJJ70819.1 acar|OOF98283.1 acla|EAW10426.1 afis|EAW25143.1 afla|EED55302.1 afum|EAL89815.2 agla|OJJ89024.1 akaw|GAA91698.1 aluc|OJZ87692.1 anid|CBF85685.1 anig|EHA20881.1 aory|EIT83611.1 ater|EAU39412.1 atub|OJI80948.1 aver|OJI98348.1 awen|OJJ40767.1

Group6136: abra|OJJ70836.1 acar|OOF98278.1 acla|EAW10431.1 afis|EAW25138.1 afla|EED55308.1 afum|EAL89809.2 akaw|GAA91704.1 aluc|OJZ87685.1 anid|CBF85712.1 anig|EHA20875.1 asyd|OJJ64271.1 ater|EAU39408.1 atub|OJI80958.1 aver|OJI98363.1 awen|OJJ40762.1 azon|OJJ44127.1

Group6137: abra|OJJ70838.1 acar|OOF98276.1 afis|EAW25136.1 afla|EED55310.1 afum|EAL89807.2 agla|OJJ89031.1 akaw|GAA91706.1 aluc|OJZ87683.1 anid|CBF85708.1 anig|EHA20873.1 aory|EIT83586.1 asyd|OJJ64269.1 atub|OJI80960.1 aver|OJI98361.1 awen|OJJ40760.1 azon|OJJ44124.1

Group6138: abra|OJJ70841.1 acar|OOF98273.1 acla|EAW13282.1 afis|EAW17335.1 afla|EED46106.1 afum|EAL91178.1 akaw|GAA91709.1 aluc|OJZ87680.1 anig|EHA20870.1 aory|EIT76506.1 asyd|OJJ56055.1 ater|EAU33889.1 atub|OJI80963.1 aver|OJJ04997.1 awen|OJJ33505.1 azon|OJJ50299.1

Group6139: acar|OOF98702.1 afis|EAW20762.1 afum|EAL84857.1 anid|CBF85637.1 aver|OJJ01161.1 azon|OJJ46003.1 acla|EAW11101.1 afla|EED53201.1 agla|OJJ86968.1 akaw|GAA92918.1 anig|EHA23722.1 aory|EIT75034.1 asyd|OJJ58562.1 ater|EAU29736.1 atub|OJI82462.1 awen|OJJ37494.1

Group6140: abra|OJJ70852.1 acar|OOF98264.1 acla|EAW10442.1 afis|EAW25125.1 afum|EAL89796.1 agla|OJJ89045.1 akaw|GAA91719.1 aluc|OJZ87669.1 anid|CBF85690.1 anig|EHA20860.1 asyd|OJJ64260.1 ater|EAU39398.1 atub|OJI80973.1 aver|OJI98352.1 awen|OJJ40743.1 azon|OJJ44114.1

Group6141: abra|OJJ70894.1 acar|OOF90218.1 acla|EAW10476.1 afis|EAW25091.1 afla|EED55356.1 afum|EAL89761.2 agla|OJJ89086.1 akaw|GAA87410.1 aluc|OJZ87629.1 anig|EHA20825.1 aory|EIT80828.1 asyd|OJJ64441.1 atub|OJI81020.1 aver|OJI98543.1 awen|OJJ40705.1 azon|OJJ44081.1

Group6142: abra|OJJ70900.1 acar|OOF98220.1 acla|EAW10478.1 afis|EAW25089.1 afla|EED55358.1 afum|EAL89758.2 agla|OJJ89088.1 akaw|GAA87415.1 aluc|OJZ87623.1 anid|CBF85995.1 anig|EHA20820.1 asyd|OJJ64439.1 atub|OJI81026.1 aver|OJI98540.1 awen|OJJ40702.1 azon|OJJ44079.1

Group6143: abra|OJJ70930.1 acar|OOF98197.1 acla|EAW10496.1 afis|EAW25071.1 afla|EED55383.1 afum|EAL89740.1 agla|OJJ89123.1 akaw|GAA83012.1 aluc|OJZ87597.1 anig|EHA20797.1 asyd|OJJ64419.1 ater|EAU39554.1 atub|OJI81050.1 aver|OJI98518.1 awen|OJJ40682.1 azon|OJJ44059.1

Group6144: abra|OJJ70936.1 acla|EAW10502.1 afis|EAW25065.1 afla|EED55389.1 afum|EAL89734.1 agla|OJJ89128.1 akaw|GAA83007.1 anid|CBF85947.1 anig|EHA20792.1 aory|EIT80920.1 asyd|OJJ64414.1 ater|EAU35812.1 atub|OJI81056.1 aver|OJI98513.1 awen|OJJ40673.1 azon|OJJ44053.1

Group6145: abra|OJJ70974.1 acar|OOF98157.1 acla|EAW10533.1 afis|EAW25000.1 afla|EED55420.1 afum|EAL89698.1 agla|OJJ89162.1 akaw|GAA82975.1 aluc|OJZ87558.1 anid|CBF81497.1 anig|EHA20761.1 asyd|OJJ59464.1 atub|OJI81089.1 aver|OJJ02810.1 awen|OJJ40639.1 azon|OJJ44023.1

Group6146: abra|OJJ70978.1 acar|OOF98152.1 afis|EAW25004.1 afla|EED55424.1 afum|EAL89694.1 agla|OJJ89167.1 akaw|GAA82971.1 aluc|OJZ87554.1 anid|CBF81483.1 anig|EHA20757.1 asyd|OJJ59455.1 ater|EAU35781.1 aver|OJJ02818.1 awen|OJJ40635.1 azon|OJJ49048.1 acla|EAW10537.1

Group6147: abra|OJJ71000.1 acar|OOF98140.1 afla|EED55446.1 akaw|GAA82948.1 aluc|OJZ87527.1 anig|EHA20737.1 aory|EIT80919.1 asyd|OJJ59492.1 atub|OJI81121.1 aver|OJJ02782.1 awen|OJJ40616.1 acla|EAW10557.1 afis|EAW25024.1 afum|EAL89671.1 anid|CBF81545.1 ater|EAU35763.1

Group6148: abra|OJJ71024.1 acar|OOF98116.1 acla|EAW10574.1 afis|EAW24993.1 afum|EAL89653.1 agla|OJJ82262.1 akaw|GAA82931.1 aluc|OJZ87507.1 anid|CBF81590.1 anig|EHA20720.1 asyd|OJJ59514.1 ater|EAU35744.1 atub|OJI81143.1 aver|OJJ02759.1 awen|OJJ40598.1 azon|OJJ49448.1

Group6149: abra|OJJ71025.1 acla|EAW10575.1 afis|EAW24992.1 afla|EED55467.1 afum|EAL89652.1 agla|OJJ82263.1 akaw|GAA82930.1 aluc|OJZ87506.1 anid|CBF81592.1 anig|EHA20719.1 aory|EIT80781.1 asyd|OJJ59515.1 ater|EAU35743.1 atub|OJI81144.1 awen|OJJ40597.1 aver|OJJ02758.1

Group6150: abra|OJJ71076.1 acar|OOF98068.1 acla|EAW11904.1 afis|EAW19816.1 afla|EED46069.1 afum|EAL85075.1 akaw|GAA82886.1 aluc|OJZ87454.1 anig|EHA20675.1 aory|EIT83499.1 atub|OJI81194.1 awen|OJJ34557.1 afis|EAW25580.1 afla|EED52141.1 afum|EAL84291.1 ater|EAU36155.1

Group6151: abra|OJJ71095.1 afla|EED48308.1 akaw|GAA90277.1 aluc|OJZ83064.1 anig|EHA28129.1 aory|EIT76315.1 atub|OJI87881.1 abra|OJJ71758.1 afis|EAW17369.1 afum|EAL91206.2 agla|OJJ79798.1 aluc|OJZ84837.1 anig|EHA25747.1 asyd|OJJ61751.1 aver|OJI95848.1 awen|OJJ37888.1

Group6152: abra|OJJ71098.1 acla|EAW14582.1 afis|EAW19316.1 afla|EED48318.1 afum|EAL94033.1 akaw|GAA83836.1 aluc|OJZ90881.1 anig|EHA28512.1 aory|EIT76307.1 atub|OJI83754.1 azon|OJJ46593.1 afis|EAW17411.1 afla|EED55973.1 aory|EIT76588.1 aver|OJJ06557.1 acla|EAW15051.1

Group6153: abra|OJJ71101.1 acar|OOF98042.1 afis|EAW25201.1 afum|EAL89864.1 akaw|GAA82867.1 aluc|OJZ87434.1 anig|EHA20656.1 atub|OJI81214.1 afla|EED53648.1 anid|CBF85217.1 aory|EIT75339.1 aver|OJI97967.1 awen|OJJ40836.1 acla|EAW10390.1 agla|OJJ88974.1 asyd|OJJ62999.1

Group6154: abra|OJJ71153.1 acar|OOF98004.1 acla|EAW10344.1 afis|EAW25245.1 afum|EAL89907.2 akaw|GAA82824.1 aluc|OJZ87385.1 anid|CBF85325.1 anig|EHA20614.1 asyd|OJJ64014.1 ater|EAU34358.1 atub|OJI81262.1 aver|OJI98102.1 awen|OJJ40958.1 azon|OJJ43036.1 anid|CBF82000.1

Group6155: abra|OJJ71159.1 acar|OOF98001.1 acla|EAW10340.1 afis|EAW25249.1 afla|EED53715.1 afum|EAL89911.1 agla|OJJ89221.1 akaw|GAA82819.1 aluc|OJZ87380.1 anid|CBF85303.1 anig|EHA20611.1 asyd|OJJ63997.1 atub|OJI81266.1 aver|OJI98086.1 awen|OJJ40963.1 azon|OJJ44544.1

Group6156: abra|OJJ71199.1 acar|OOF97969.1 afis|EAW20331.1 afla|EED48992.1 afum|EAL85457.1 agla|OJJ88970.1 akaw|GAA82784.1 aluc|OJZ87343.1 anid|CBF82369.1 anig|EHA20580.1 asyd|OJJ54197.1 ater|EAU37619.1 atub|OJI81301.1 aver|OJJ08527.1 awen|OJJ31947.1 akaw|GAA90166.1

Group6157: abra|OJJ71228.1 acar|OOF97951.1 acla|EAW10294.1 afis|EAW25298.1 afla|EED53764.1 afum|EAL89961.1 agla|OJJ89277.1 akaw|GAA82761.1 aluc|OJZ87319.1 anig|EHA20553.1 aory|EIT78432.1 ater|EAU34439.1 atub|OJI81326.1 aver|OJI98150.1 awen|OJJ41012.1 azon|OJJ44597.1

Group6158: abra|OJJ71247.1 acar|OOF97921.1 acla|EAW10288.1 afis|EAW25304.1 afum|EAL89967.2 agla|OJJ89283.1 akaw|GAA82740.1 aluc|OJZ87295.1 anid|CBF75298.1 anig|EHA20536.1 asyd|OJJ59883.1 ater|EAU34452.1 atub|OJI81345.1 aver|OJJ02403.1 awen|OJJ41018.1 azon|OJJ44605.1

Group6159: abra|OJJ71250.1 acla|EAW10285.1 afis|EAW25307.1 afum|EAL89970.1 agla|OJJ89286.1 akaw|GAA82737.1 aluc|OJZ87292.1 anid|CBF75293.1 anig|EHA20533.1 aory|EIT78358.1 asyd|OJJ59886.1 ater|EAU34455.1 atub|OJI81349.1 aver|OJJ02400.1 awen|OJJ41021.1 azon|OJJ44608.1

Group6160: abra|OJJ71313.1 acar|OOF97859.1 acla|EAW10226.1 afis|EAW25366.1 afla|EED53847.1 afum|EBA27393.1 akaw|GAA82678.1 aluc|OJZ87231.1 anid|CBF77450.1 aory|EIT78418.1 asyd|OJJ53210.1 atub|OJI81411.1 aver|OJJ03640.1 awen|OJJ41092.1 agla|OJJ83753.1 azon|OJJ44671.1

Group6161: abra|OJJ71323.1 acar|OOF97851.1 acla|EAW10216.1 afis|EAW25377.1 afla|EED53857.1 afum|EAL90036.1 agla|OJJ83766.1 akaw|GAA82668.1 aluc|OJZ87221.1 anid|CBF77481.1 aory|EIT78408.1 asyd|OJJ53200.1 ater|EAU34521.1 aver|OJJ03650.1 awen|OJJ41104.1 azon|OJJ44681.1

Group6162: abra|OJJ71338.1 acar|OOF97834.1 acla|EAW10201.1 afis|EAW25394.1 afla|EED53871.1 afum|EAL90053.1 agla|OJJ87032.1 akaw|GAA82654.1 aluc|OJZ87204.1 anid|CBF77510.1 anig|EHA20457.1 aory|EIT78385.1 ater|EAU34542.1 atub|OJI81436.1 awen|OJJ41120.1 azon|OJJ44695.1

Group6163: abra|OJJ71356.1 acar|OOF97814.1 acla|EAW10180.1 afis|EAW25414.1 afla|EED53893.1 afum|EAL90073.1 agla|OJJ87056.1 akaw|GAA82636.1 aluc|OJZ87183.1 asyd|OJJ53166.1 ater|EAU34565.1 atub|OJI81456.1 aver|OJJ03686.1 awen|OJJ41141.1 azon|OJJ44721.1 anid|CBF77578.1

Group6164: abra|OJJ71473.1 acla|EAW10077.1 afis|EAW25509.1 afla|EED54010.1 afum|EAL90175.2 agla|OJJ87296.1 akaw|GAA82543.1 aluc|OJZ87073.1 aory|EIT78294.1 asyd|OJJ53035.1 ater|EAU33356.1 atub|OJI81564.1 aver|OJJ03809.1 awen|OJJ41251.1 azon|OJJ44818.1 anid|CBF77773.1

Group6165: abra|OJJ71590.1 acar|OOF96319.1 acla|EAW15136.1 afis|EAW20108.1 afum|EAL85225.1 agla|OJJ88846.1 akaw|GAA89332.1 aluc|OJZ86333.1 anid|CBF84915.1 anig|EHA21548.1 asyd|OJJ63166.1 ater|EAU38726.1 atub|OJI84202.1 aver|OJI97789.1 awen|OJJ31879.1 azon|OJJ46255.1

Group6166: abra|OJJ71632.1 acar|OOF96349.1 acla|EAW15173.1 afis|EAW20142.1 afla|EED57207.1 afum|EAL85257.1 aluc|OJZ86375.1 anid|CBF84976.1 anig|EHA21519.1 aory|EIT74096.1 asyd|OJJ63131.1 ater|EAU38759.1 atub|OJI84159.1 aver|OJI97823.1 awen|OJJ32012.1 azon|OJJ46077.1

Group6167: abra|OJJ71642.1 acar|OOF96358.1 acla|EAW15182.1 afis|EAW20152.1 afum|EAL85267.1 agla|OJJ88782.1 akaw|GAA89284.1 aluc|OJZ86385.1 anid|CBF84989.1 anig|EHA21510.1 aory|EIT74139.1 asyd|OJJ63122.1 atub|OJI84148.1 aver|OJI97832.1 awen|OJJ32025.1 azon|OJJ46065.1

Group6168: abra|OJJ71703.1 acar|OOF96414.1 acla|EAW15238.1 afis|EAW20203.1 afla|EED57138.1 afum|EAL85323.1 agla|OJJ84662.1 aluc|OJZ86446.1 anid|CBF85089.1 anig|EHA21455.1 aory|EIT74000.1 asyd|OJJ63068.1 atub|OJI84082.1 aver|OJI97894.1 awen|OJJ31209.1 azon|OJJ46132.1

Group6169: abra|OJJ71814.1 acla|EAW13854.1 afis|EAW18568.1 afla|EED52783.1 afum|EAL93270.1 agla|OJJ81619.1 akaw|GAA85096.1 aluc|OJZ86547.1 anid|CBF70307.1 anig|EHA22103.1 aory|EIT78241.1 asyd|OJJ58725.1 atub|OJI83976.1 aver|OJJ01338.1 awen|OJJ31125.1 azon|OJJ46606.1

Group6170: abra|OJJ71841.1 acar|OOF96510.1 acla|EAW13872.1 afis|EAW18583.1 afum|EAL93288.1 agla|OJJ81635.1 akaw|GAA85076.1 aluc|OJZ86574.1 anid|CBF70364.1 anig|EHA22083.1 asyd|OJJ58651.1 ater|EAU34877.1 atub|OJI83949.1 aver|OJJ01257.1 awen|OJJ31147.1 azon|OJJ46622.1

Group6171: abra|OJJ71871.1 acar|OOF96542.1 acla|EAW13901.1 afis|EAW18613.1 afla|EED52731.1 afum|EAL93323.1 akaw|GAA85044.1 aluc|OJZ86610.1 anid|CBF70432.1 anig|EHA22056.1 aory|EIT78245.1 asyd|OJJ58614.1 ater|EAU34910.1 atub|OJI83916.1 aver|OJJ01221.1 awen|OJJ32074.1

Group6172: abra|OJJ71916.1 acar|OOF96573.1 afis|EAW20251.1 afla|EED46917.1 afum|EAL85369.1 agla|OJJ80821.1 akaw|GAA85006.1 aluc|OJZ86652.1 anid|CBF80720.1 anig|EHA22018.1 aory|EIT72602.1 asyd|OJJ54567.1 ater|EAU31463.1 atub|OJI83875.1 aver|OJJ07587.1 awen|OJJ33373.1

Group6173: abra|OJJ71926.1 acar|OOF96583.1 acla|EAW09602.1 afis|EAW21535.1 afla|EED55985.1 afum|EBA27463.1 agla|OJJ85698.1 akaw|GAA84996.1 aluc|OJZ86663.1 anid|CBF76388.1 asyd|OJJ61071.1 ater|EAU35004.1 atub|OJI83862.1 aver|OJJ00388.1 awen|OJJ39157.1 azon|OJJ51487.1

Group6174: abra|OJJ71930.1 acar|OOF96587.1 acla|EAW09599.1 afis|EAW21538.1 afla|EED55990.1 afum|EAL92558.2 agla|OJJ85702.1 akaw|GAA84993.1 aluc|OJZ86667.1 anid|CBF76380.1 anig|EHA22006.1 aory|EIT76800.1 asyd|OJJ61067.1 aver|OJJ00383.1 awen|OJJ39153.1 azon|OJJ51484.1

Group6175: abra|OJJ71936.1 acar|OOF96592.1 acla|EAW09594.1 afis|EAW21543.1 afla|EED55994.1 afum|EAL92563.1 agla|OJJ85706.1 akaw|GAA84988.1 aluc|OJZ86673.1 anid|CBF76372.1 anig|EHA22001.1 aory|EIT76857.1 ater|EAU34996.1 atub|OJI83855.1 awen|OJJ39147.1 azon|OJJ51483.1

Group6176: acar|OOF96616.1 anig|EHA21984.1 atub|OJI83835.1 acla|EAW09581.1 afis|EAW21556.1 afla|EED56008.1 afum|EBA27466.1 akaw|GAA84973.1 aluc|OJZ86689.1 anid|CBF76346.1 aory|EIT76788.1 asyd|OJJ61051.1 ater|EAU34978.1 aver|OJJ00367.1 awen|OJJ39134.1 azon|OJJ51462.1

Group6177: abra|OJJ72004.1 acla|EAW09535.1 afis|EAW21598.1 afla|EED57822.1 afum|EAL92617.1 agla|OJJ85793.1 akaw|GAA84928.1 aluc|OJZ86740.1 anid|CBF83455.1 anig|EHA21939.1 asyd|OJJ60678.1 ater|EAU38287.1 atub|OJI83788.1 aver|OJJ00001.1 awen|OJJ39084.1 azon|OJJ51416.1

Group6178: abra|OJJ72006.1 acar|OOF96656.1 afis|EAW17213.1 afla|EED56213.1 afum|EAL86640.1 akaw|GAA84927.1 aluc|OJZ86742.1 anid|CBF83257.1 anig|EHA21937.1 aory|EIT73317.1 asyd|OJJ60586.1 ater|EAU38288.1 atub|OJI83785.1 aver|OJI99909.1 awen|OJJ41450.1 azon|OJJ50967.1

Group6179: abra|OJJ72012.1 acar|OOF96661.1 acla|EAW09528.1 afis|EAW21604.1 afla|EED57831.1 afum|EAL92623.1 agla|OJJ85799.1 akaw|GAA84921.1 aluc|OJZ86748.1 anid|CBF83465.1 anig|EHA21931.1 asyd|OJJ60686.1 atub|OJI83779.1 aver|OJJ00011.1 awen|OJJ39078.1 azon|OJJ51410.1

Group6180: abra|OJJ72021.1 acar|OOF96670.1 acla|EAW09516.1 afis|EAW21616.1 afum|EAL92650.1 agla|OJJ78623.1 akaw|GAA84911.1 aluc|OJZ86758.1 anig|EHA21921.1 asyd|OJJ60697.1 atub|OJI83769.1 aver|OJJ00023.1 awen|OJJ39068.1 azon|OJJ51399.1 afla|EED57843.1 aory|EIT81106.1

Group6181: abra|OJJ72041.1 acar|OOF96700.1 afis|EAW21632.1 afla|EED57860.1 afum|EAL92665.1 akaw|GAA84892.1 aluc|OJZ86779.1 anig|EHA21903.1 aory|EIT81090.1 asyd|OJJ54353.1 ater|EAU38318.1 atub|OJI83741.1 aver|OJJ05307.1 awen|OJJ33881.1 azon|OJJ42153.1 afis|EAW19761.1

Group6182: abra|OJJ72047.1 acar|OOF96705.1 acla|EAW09499.1 afis|EAW21637.1 afla|EED57880.1 afum|EAL92671.1 agla|OJJ79702.1 akaw|GAA84887.1 aluc|OJZ86784.1 anid|CBF83519.1 anig|EHA21898.1 asyd|OJJ60715.1 atub|OJI83736.1 aver|OJJ00048.1 awen|OJJ33886.1 azon|OJJ48667.1

Group6183: abra|OJJ72066.1 acar|OOF96720.1 acla|EAW09481.1 afis|EAW21655.1 afla|EED57890.1 afum|EAL92690.1 akaw|GAA84868.1 aluc|OJZ86802.1 anid|CBF83546.1 anig|EHA21881.1 aory|EIT82739.1 asyd|OJJ60731.1 atub|OJI83716.1 aver|OJJ00063.1 awen|OJJ33912.1 azon|OJJ48649.1

Group6184: abra|OJJ72069.1 acar|OOF96725.1 acla|EAW09484.1 afis|EAW21652.1 afla|EED57885.1 afum|EBA27469.1 agla|OJJ79725.1 akaw|GAA84866.1 aluc|OJZ86804.1 anid|CBF83552.1 anig|EHA21878.1 aory|EIT83276.1 asyd|OJJ60734.1 atub|OJI83714.1 aver|OJJ00066.1 awen|OJJ33916.1

Group6185: abra|OJJ72088.1 acar|OOF96733.1 acla|EAW09469.1 afis|EAW21667.1 afla|EED57931.1 afum|EAL92702.1 agla|OJJ79738.1 akaw|GAA84832.1 aluc|OJZ86842.1 anid|CBF83571.1 anig|EHA21795.1 aory|EIT82338.1 asyd|OJJ60748.1 atub|OJI83676.1 aver|OJJ00080.1 azon|OJJ48638.1
[truncated: 743,394 more chars]
